# Characterizing Hotspots and Frontier Landscapes of Diabetes-Specific Distress from 2000 to 2018: A Bibliometric Study

**DOI:** 10.1155/2020/8691451

**Published:** 2020-01-15

**Authors:** Dan Li, Fu-Min Dai, Juan-Juan Xu, Meng-Die Jiang

**Affiliations:** ^1^College of Nursing, Zhengzhou University, Zhengzhou, Henan, China; ^2^Department of Gerontology, Henan Provincial People's Hospital, Zhengzhou, Henan, China; ^3^College of Nursing & Allied Health Sciences, Henan University, Kaifeng, Henan, China

## Abstract

**Objectives:**

This work aims to comprehensively characterize hotspots and frontier landscapes concerning diabetes-specific distress from 2000 to 2018.

**Materials and Methods:**

Firstly, diabetes-specific distress-related literature was retrieved and downloaded from the Web of Science Core Collection (WoSCC). Secondly, WoSCC self-contained toolkits and GraphPad Prism7 were conducted to analyze general characteristics, including literature products, countries, institutes, authors, and journal resource. Finally, CiteSpace V Toolkits was put forward to implement advanced analysis, consisting of keyword-term frequency and co-occurrence, references-cited frequency and co-occurrence, and burst detection for keyword terms and references cited, which uncovers the hotspots and frontiers of diabetes-specific distress.

**Results:**

After preprocessing, our study included a total of 1051 papers concerning diabetes-specific distress. Publication outputs increased smoothly year by year. Compared with other journals, *diabetic medicine* delivered the largest number of documents. The United States occupied the leading positions, and the most productive institution was the University of California System in terms of literature products. Fisher L. has the highest references-cited frequency. Prevalence of diabetes-specific distress, diabetes-specific distress and glycemic control, diabetes-specific distress and depression comorbidity, and diabetes-specific distress and risk factors were the research hotspots, whereas the measure of diabetes-specific distress and latent and serious/severe diabetes-specific distress was the research frontiers.

**Conclusions:**

Overall, our study may inspire researchers to show great interest in diabetes-specific distress in the next few years.

## 1. Introduction

An overwhelmingly increasing number of publications over the past decades have suggested that diabetes mellitus is one of the most frequently diagnosed noninfectious chronic diseases and ranks first on the incidence of complications in noncommunicable chronic disorders [1–4]. Most strikingly, prevalence of diabetes mellitus has significantly increased with the aging of the population, especially type 2 diabetes [5–7]. Furthermore, there is a large volume of published studies suggesting that diabetes mellitus poses a serious burden on the public health services and social economy, whether the developed or developing countries [8, 9].

Fortunately, the association between diabetes-related psychological disorders and prognosis of diabetes has been widely studied during the past 30 years [10–12]. However, research on diabetes-related psychological disorders has been mostly restricted to anxiety and depression [13–15]. Previous studies mostly defined “diabetes-specific distress” as a diabetes-specific psychological dysfunction, which covered much wider and deeper psychological and emotional experience than depression comorbidity for those who suffered from progressive diabetes and serious diabetic complications [16, 17]. For example, a large-sample retrospective study has demonstrated that 36% of patients with type 2 diabetes suffered from diabetes-specific distress, which uncovered that diabetes-specific distress is not uncommon nowadays. Moreover, an association analysis has indicated that diabetes-specific distress frequently occurred in diabetes mellitus with a higher incidence of depressive symptoms [18]. Interestingly, many studies have shown that diabetes-specific distress and depression comorbidity do not work alone but influence each other and interweave each other, which explain in a certain extent the reasons why the effect of diabetes-specific distress was left out [19, 20]. However, diabetes-specific distress is more common and widespread than depression in patients with diabetes mellitus [21, 22]. In view of this, the American Diabetes Association (ADA) proposes regular evaluations for patients with diabetes-specific distress for the management and prevention of severe diabetic complications [23]. However, the lack of systematic research on diabetes-specific distress and the lack of a specific definition and grading system are badly restricting further research.

Recently, bibliometrics relying on literature databases has been widely applied in medical research [24]. Bibliometrics enables researchers to extract essential literature information to capture research hotspots and frontier based on mathematical and statistical methods [25–27], thereby assisting researchers to work on its main areas of interest in a short time. Meanwhile, the theoretical framework and analytical tools of bibliometrics are developing vigorously [28]. To the best of our knowledge, a number of bibliometrics software, especially CiteSpace Toolkits, which was designed and freely supported by Chen et al., covered all sorts of things from management and conversion of data to construction of matrices and visualization [29, 30]. However, the bibliometric strategies have not yet been applied to the research field of diabetes-specific distress so far.

In the present study, we retrieved diabetes-specific distress-related publications from 2000 to 2018 and explored the research hotspots and frontiers by means of CiteSpace V Toolkits. Taken together, we aimed to identify hotspots and frontiers by time and warrants further studies in the diabetes-specific distress filed.

## 2. Materials and Methods

### 2.1. Data Sources and Retrieval Strategy

To avoid the discrepancies resulted from database daily updates, diabetes-specific distress-related publication was retrieved through the Science Citation Index-Expanded (SCI-E) of the Web of Science Core Collection (WoSCC) (Clarivate Analytics) on January 6, 2019. The search expressions were constructed as follows: TS = ((“diabetes distress”) OR (“diabetes” AND “distress”) OR (“diabetes” AND “psychological distress”) OR (“diabetes-related distress”) OR (“diabetes-specific distress”) OR (“diabetes” AND “emotional distress”) OR (“diabetes-specific emotional distress”) OR (“diabetes-related emotional distress”) AND Language = English AND Time range = 2000–2018). In this study, only the original articles and reviews were included.

### 2.2. Data Download and Data Clean

Two authors (Dan Li and Fu-Min Dai) were independently in charge of literature retrieval, download, extraction, and verification. Any controversies were discussed, standardized, and unified by all authors. The literature contents including full records and references cited from WoSCC were downloaded in a text plain format, compliant with software input format. Then, literature profiling was imported into CiteSpace V (Drexel University, Philadelphia, United States) for data preprocessing, including deduplication and sorting.

### 2.3. Statistical Methods

The WoSCC self-contained toolkits were used to analyze literature's general characteristics, including the number of annual publications, countries, institutes, author's information, and journal sources. GraphPad Prism7.0 (GraphPad Prism Software, Inc., San Diego, California) was conducted to depict and visualize publication outputs and growth trends by means of a polynomial regression model.

CiteSpace V was carried out to decode keyword-term frequency and co-occurrence, references-cited frequency and co-occurrence, detect keyword terms with the strongest citation burst, and construct visualization maps, thereby uncovering diabetes-specific distress-related hotspot and frontiers. Generally speaking, nodes standing for keyword terms and references cited with high frequency and centrality are identified as hotspots, while nodes with strong citation burst are considered as frontiers [31, 32].

## 3. Results

### 3.1. Flow Chart for Selection Criteria

**Figure 1:**
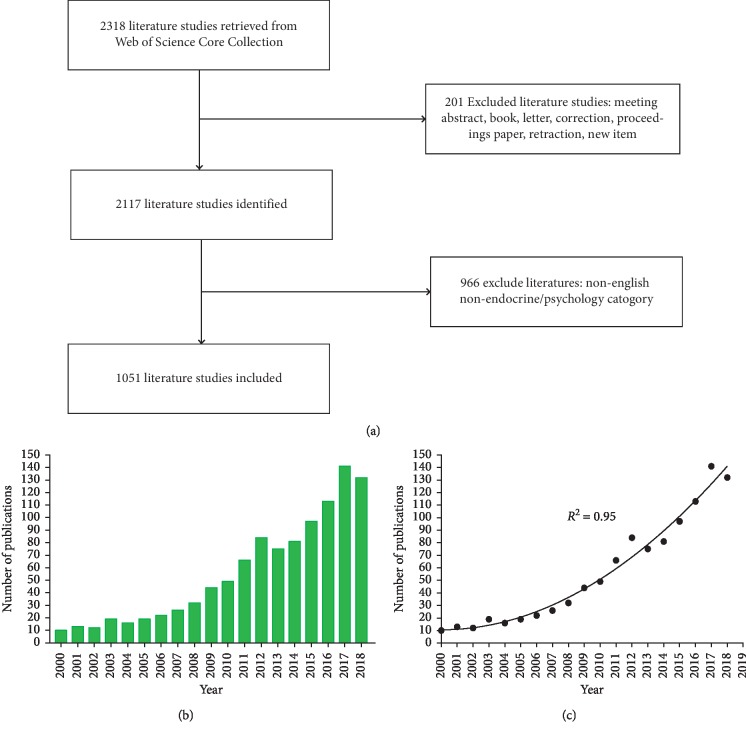
(a) Flow diagram of diabetes-specific distress-related literature inclusion and exclusion criteria. (b) The annual quantities of diabetes-specific distress-related literature from 2000 to 2018. (c) The polynomial fitting curve of growth trends on diabetes-specific distress-related literature.

The criteria for selecting publications are shown as follows (Figure 1(a)): (1) time interval covering 2000 to 2018; (2) publications indexed in WoSCC; (3) documents published on endocrine and psychology category; (4) articles and reviews. In addition, this study excluded the following documents: (1) meeting, corrected, and retracted articles; (2) articles with incomplete and biased information; (3) non-English language.

### 3.2. Annual Publications and Growth Forecast

According to the inclusion and exclusion criteria, a total of 1,051 publications were included in this study ([Supplementary-material supplementary-material-1]). As was presented in Figure 1(b), the number of documents maintained a growing trend from 2000 to 2018 per year ([Supplementary-material supplementary-material-1]). Moreover, as shown in Figure 1(c), the polynomial curve fitting of document growth presented a strongly positive correlation (the coefficient of determination (*R*^2^) equals 0.95) by the year of publication. It is estimated from curve fitting that document circulations will reach approximately 213 in 2019. In light of the above, diabetes-specific distress is a relatively new field and is still in the developing phase at home and abroad. Therefore, there is also a need to pay more attention to diabetes-specific distress over the days ahead.

### 3.3. Countries and Institute Distribution

**Figure 2:**
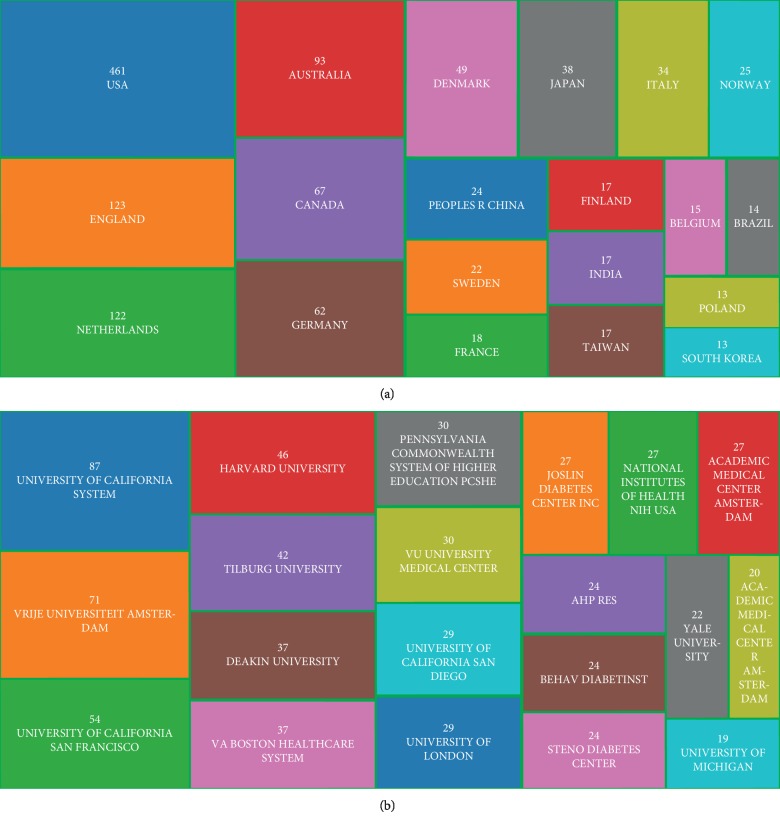
(a) The treemap of top 20 countries that published literature about diabetes-specific distress. (b) The treemap of top 20 institutes that published literature about diabetes-specific distress.

The 1,051 publications on diabetes-specific distress were contributed by a total of 68 countries ([Supplementary-material supplementary-material-1]). According to the treemap of top 20 countries (Figure 2(a)), the United States contributed most publications (461), followed by England (123), the Netherlands (122), Australia (93), and Canada (67). In addition, a total of 1,527 institutes published 1,051 diabetes-specific distress-related papers ([Supplementary-material supplementary-material-1]). As was illustrated in the treemap of the top 20 institutes (Figure 2(b)), the University of California System contributed the most publications (87), followed by Vrije University Amsterdam (71), Harvard University (46), and Tilburg University (42). Taking the ranking of countries and institution's publication number into account, the United States and the University of California System maintained their leading position in the field of diabetes-specific distress.

### 3.4. Authors and Journal Distribution

**Figure 3:**
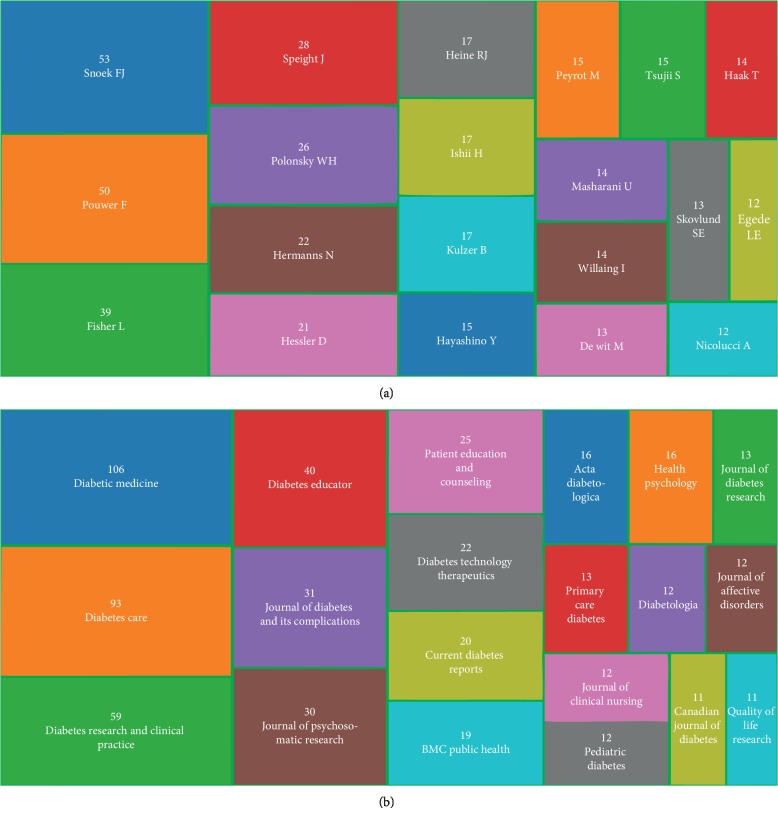
(a) The treemap of the top 20 authors that published literature about diabetes-specific distress. (b) The treemap of top 20 journals that published literature about diabetes-specific distress.

The 1,051 articles were contributed by more than 4,073 authors about diabetes-specific distress research ([Supplementary-material supplementary-material-1]). The top 20 authors publishing articles were listed in Figure 3(a). Among the top 20 contributing authors, Snoek F. J. (53 publications) was ranked first, followed by Pouwer F. (50 publications), Fisher L. (39 publications), Speight J. (28 publications), and Polonsky W. H. (26 publications). In view of the quantity and quality of the publication, Snoek F. J. is regarded as a prolific author in the field of diabetes-specific distress.

Despite the disadvantages, the impact factor (IF) is also a relatively objective index to evaluate the journal's quality and can reflect the academic level of journals at a certain degree at the present time [33]. In total, 236 academic journals have published papers in the diabetes-specific distress research category ([Supplementary-material supplementary-material-1]).  Figure 3(b) maps the top 20 journals publishing diabetes distress research. Moreover, it is very clearly seen that *diabetic medicine* (IF = 3.132) published the most papers, followed by *diabetes care* (IF = 13.397), *diabetes research and clinical practice* (IF = 2.548), and *diabetes educator* (IF = 1.736).

### 3.5. Analysis of Keyword-Term Frequency Distribution and Co-Occurrence

The premise that keywords can reflect research hotspots is that researchers carefully and accurately select professional terms. This team is inclined to integrate keywords and terms as bibliometrics research objects, which ensured that subsequent analysis maximizes the coverage degree of major points of a diabetes-specific distress domain. In this part, the keyword-term cluster view, timeline view, and frequency distribution were drawn/plotted via CiteSpace V. The parameters of CiteSpace V were configured as follows: time slicing (from 2000 to 2018, years per slice = 3), node types (term and keyword), selection criteria (top 30), and visualization (cluster view-static, show merged network).

As was presented in Table 1, the top 5 keyword terms on diabetes-specific distress were listed in order as follows: “depression,” “glycemic control,” “mellitus,” “distress,” and “adult.”

**Figure 4:**
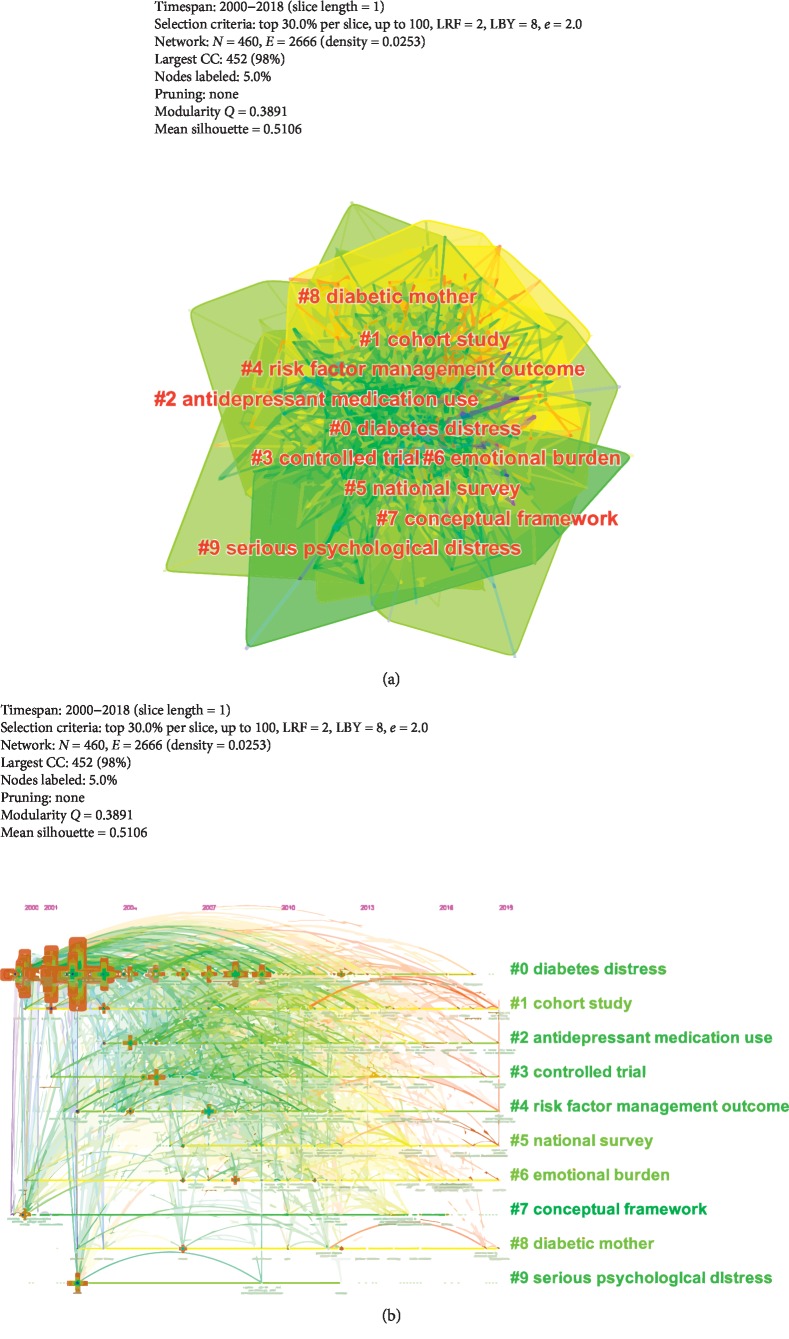
(a) Keyword-term co-occurrence cluster map related to diabetes-specific distress from 2000 to 2018. (b) Keywords/terms co-occurrence timeline map related to diabetes-specific distress from 2000 to 2018. Notes: rings represent the keyword-term history of keyword term; the color of ring denotes the time of corresponding frequency; the thickness of a ring is proportional to the number of keyword term in a given time slice.

The keyword-term cluster view (Figure 4(a)) and timeline view (Figure 4(b)) consist of 153 nodes and 893 links, and the values of Modularity *Q* and Mean Silhouette were 0.3891 and 0.5106, which demonstrated that clustering quality was acceptable. Top 5 keyword-term clusters were illustrated as follows: “diabetes distress,” “cohort study,” “anti-depression medication use,” “controlled trial,” and “risk factor management outcome.”

### 3.6. Analysis of References-Cited Frequency Distribution and Co-Occurrence

References cited have always been considered as a core component of the bibliometrics research. Meanwhile, the CiteSpace toolkit has an absolute advantage in references-cited analysis. Therefore, CiteSpace V was performed to analyze references cited frequently and drawing reference-cited cluster view and timeline view. The parameters of CiteSpace V were configured as follows: node types (references cited). Other parameters are set as default.

**Figure 5:**
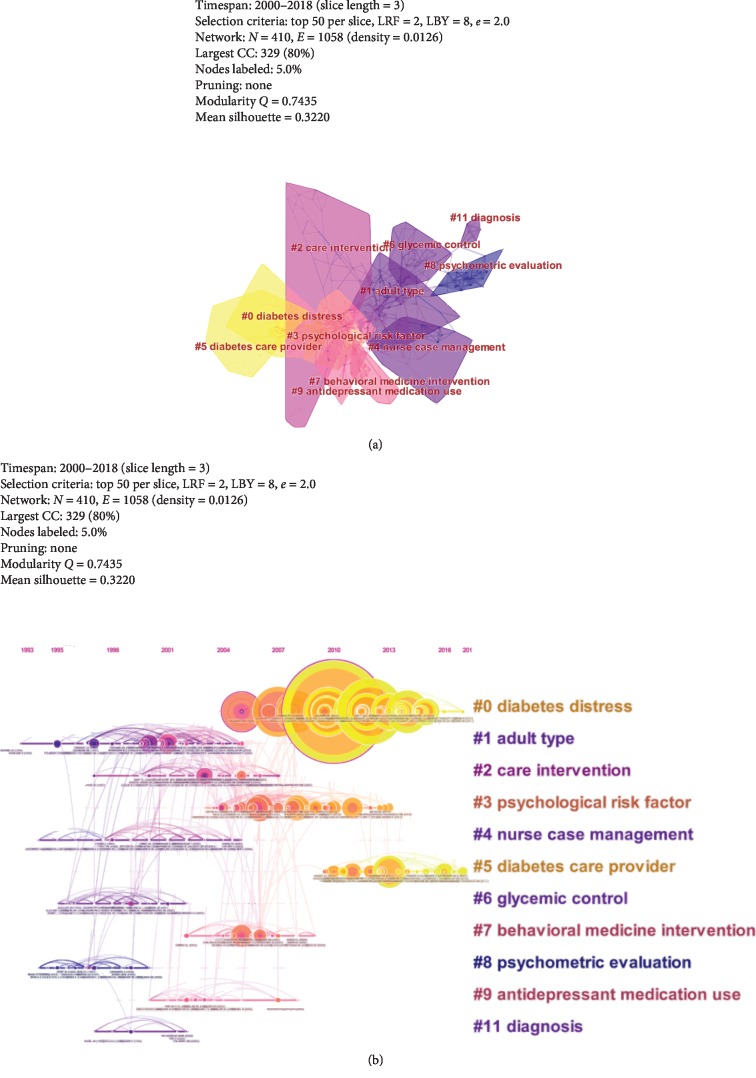
(a) References-cited cluster map related to diabetes-specific distress from 2000 to 2018. (b) References-cited timeline map related to diabetes-specific distress from 2000 to 2018. Notes: citation rings represent the citation history of an article; the color of a citation ring denotes the time of corresponding citations; the thickness of a ring is proportional to the number of citations in a given time slice.

As shown in Table 2, the top 5 references cited on diabetes-specific distress were listed in the following order: “Fisher L. (2010),” “Fisher L. (2012),” “Fisher L. (2007),” “Fisher L. (2010),” and “Fisher L. (2018).” References-cited cluster view (Figure 5(a)) and timeline view (Figure 5(b)) contain 410 nodes and 1,058 links, and values of Modularity *Q* and Mean Silhouette were 0.7435 and 0.322, which indicated that cluster quality was reasonable. Top 5 references-cited clusters were presented as follows: “diabetes distress,” “adult type,” “care intervention,” “psychological risk factor,” and “nurse case management.”

### 3.7. Analysis of Burst Detection for Keyword Terms and References Cited

**Figure 6:**
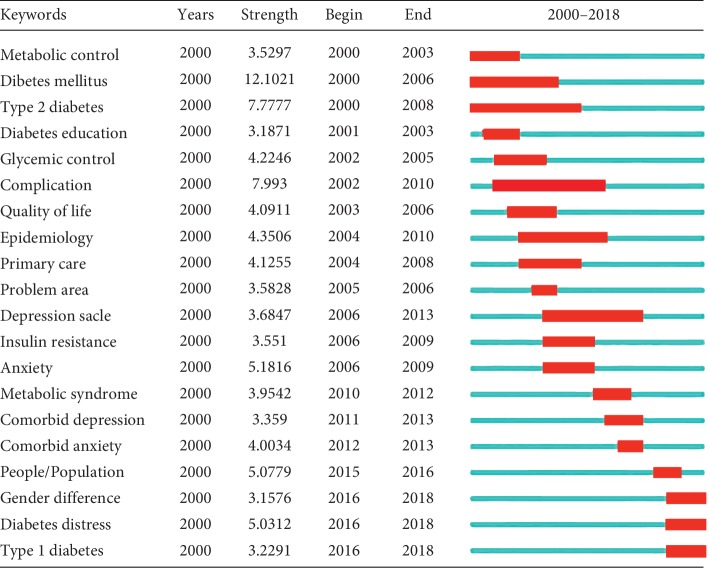
Top 20 keyword terms with the strongest citation bursts related to diabetes-specific distress from 2000 to 2018. Notes: the red bars mean keywords cited frequently; the green bars were keywords cited infrequently.

**Figure 7:**
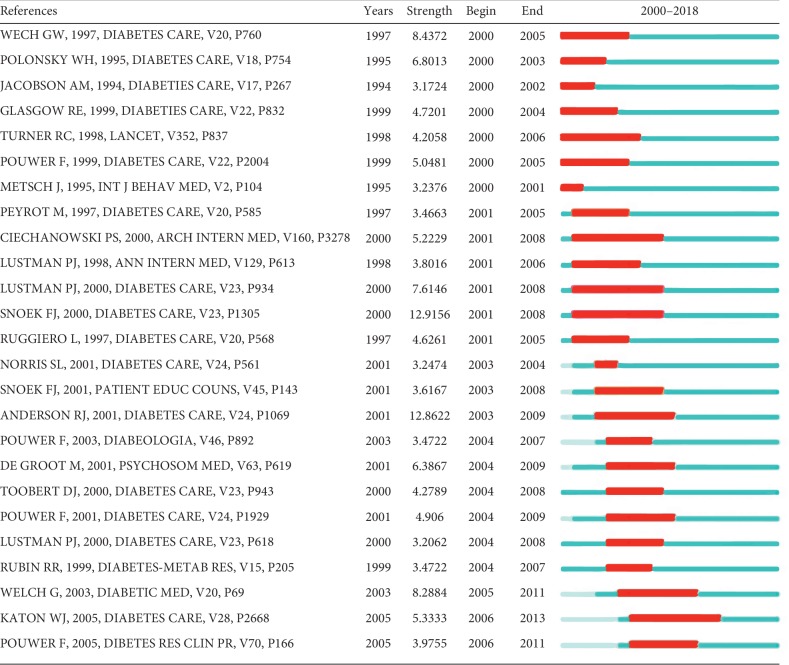
Top 25 references cited with the strongest citation bursts related to diabetes-specific distress from 2000 to 2018. Notes: the red bars mean keywords cited frequently; the green bars were keywords cited infrequently.

Keyword-term burst detection and references-cited burst detection were considered as indicators of research frontiers or emerging trends over time.  Figure 6 shows the top 25 keyword terms with the strongest frequency bursts. The strongest ones include “Mellitus (12.1021),” “complication (7.993),” “diabetes mellitus (7.7777),” and “anxiety (5.1816).” As shown in Figure 7, the top 25 references cited with the strongest citation bursts include Snoek F. J. 2000 (12.9156), Anderson R. J. 2001 (12.8622), Welch G. W. 1997 (8.4372), and Welch G. 2003 (8.2884).

## 4. Discussion

In our study, we analyzed 1,051 publications that originated from the WoSCC database and diabetes-specific distress-related publication outputs maintained a steady overall growth trend from 2000 to 2018. Among countries, the United States had an absolute advantage in the number of paper outputs. And the top ten institutes were all from the United States. Remarkably, China was the only one from the developing country, showing its vast progress in diabetes-specific distress over the past decade. Among academic journals, *diabetes care* (IF = 13.397) had an IF higher than 10. Moreover, among the top ten authors, each person has published at least 17 papers; they were regarded as prolific authors, especially Snoek F. J. In addition, Fisher L. owned the highest references-cited frequency.

Herein, considering the findings from keyword-term frequency distribution and co-occurrence and references-cited frequency distribution and co-occurrence, we summarized the following diabetes-specific distress-related research hotspots:

- *Prevalence of Diabetes-Specific Distress*. More and more attention has been paid to explore the prevalence of diabetes-specific distress in recent years. A meta-analysis including fifty-five original articles suggested that 36% people with type 2 diabetes suffer from diabetes-specific distress [18]. Furthermore, Fisher et al. reported that almost 60% of patients with type 2 diabetes experience diabetes-specific distress [34]. More strikingly, it was reported that diabetes-specific distress frequently occurred in the younger age group, not limited to elder population [35]. Taken together, diabetes-specific distress may have an even bigger impact in the wider population, which motivates researchers to pay greater attention to diabetes-specific distress.
- *Diabetes-Specific Distress and Glycemic Control*. Glycemic control has always been an essential indicator of the treatment and prognosis of diabetes. Long-term controllable glycemic control can greatly improve patient's quality of life. Data from several sources have identified that diabetes-specific distress has a strong negative correlation with glycemic control [36–38]. Moreover, a cross-sectional study demonstrated that diabetes-specific not only directly affects blood sugar but also indirectly affects blood sugar through adherence to insulin and type 2 diabetes treatment [39,40]. Based on the published works of the literature, we have described the landscapes of diabetes-specific distress on glycemic control and quality of life in diabetic patients, thereby attracting the attention of clinicians.
- *Diabetes-Specific Distress and Depression Comorbidity*. Common psychological comorbidities, including depression, anxiety, and diabetes-specific distress, are widely screened and are closely associated with poor outcomes in patients with diabetes [19]. For example, a study from Australia suggested that diabetes-specific distress was more closely associated with HbA1c level than depressive comorbidity in adolescents with type 1 diabetes [22]. Moreover, Tsujii et al. found that diabetes-specific distress, but not depressive symptoms, was associated with glycemic control among Japanese patients with type 2 diabetes [21]. However, it is not easy to distinguish the manifestations of diabetes distress and depression. Therefore, it is crucial to clarify the difference between diabetes-specific distress and depression in patients with diabetes.
- *Diabetes-Specific Distress and Risk Factors*. A number of studies have suggested that the prevalence of diabetes-specific distress is caused by many risk factors. In terms of gender, the females are more likely to suffer from diabetes-specific distress than male peers [18]. Moreover, Fisher et al. presented that the prevalence of diabetes-specific distress was closely associated with patient demographic background and disease-related patient characteristics [41]. In addition, diabetes-specific distress was significantly related to physical exercise, education level, monthly income, frequency of administration of medication, adherence to medical treatment, and the number of complications [42]. Therefore, we propose that diabetes-specific distress should be recommended as routine screening in clinical practice, thereby assisting primary prevention for diabetes-specific distress.
- *Diabetes-Specific Distress and Management and Intervention*. Precise management and the personalized intervention of diabetes-specific distress play a vital role in controlling blood sugar and preventing complications in patients with diabetes [43]. A systematic review indicated that an efficient intervention project can significantly reduce both diabetes-specific distress and HbA1c level by means of motivational interviewing and personalized interventions [44]. Remarkably, a program including 30 RCTs with 9177 participants showed the elevated self-efficacy and downregulated HbA1c after a diabetes-specific distress intervention [45]. However, the specific projects and objectives of intervention need further study.

With views of burst detection for keyword terms and references cited, the research frontier can be shown as follows:

- *Measurement of Diabetes Distress*. The vigorous advance of diabetes distress benefits from the development, evaluation, and validation of the corresponding diabetes-specific distress scale. To the best of our knowledge, diabetes-specific distress has been measured in diverse scales so far. Polonsky et al. first described the diabetes-specific distress measurement and introduced the Problem Areas in Diabetes Scale (PAID) in 1995 [16]. Subsequently, Polonsky et al. further improved the diabetes-specific distress and invented the 17-item Diabetes Distress Scale (DDS-17) in 2005 [46]. Although the PAID and DDS remain the same scale construct, they have significant differences in problem entry [17]. Moreover, Fisher et al. developed the Type 1 Diabetes Distress Scale (T1-DDS) to elucidate specific sources of diabetes distress for type 1 diabetes [41]. Although more and more diabetes distress scales are being developed, the measurement of diabetes distress still faces great challenges. The following reasons can account for the great challenges: (1) complex diabetes classification system; (2) fuzzy definition of diabetes distress; (3) nonuniversal diabetes distress scale. Therefore, a justified choice for assessing diabetes distress greatly depends on the clinical practice or scientific purpose.
- *Latent and Serious/Severe Diabetes-Specific Distress*. Thus far, there have been no studies focusing on the analysis of the latent diabetes-specific distress based on the existing literature. The current domestic academic research on severe diabetes-specific distress is only sporadic discussion [47,48]. In addition, the symptoms of latent diabetes-specific distress are difficult to measure quickly and accurately because of lacking specific index. Moreover, we are lacking effective preventive intervention measures for severe diabetes-specific distress. Our team found that patients with diabetes may be unknowingly afflicted with diabetes-specific distress based on daily clinical practice. Therefore, the identification of diabetes-specific distress to reduce the occurrence of multiple complications as well as improving the prognosis of patients is particularly important in patients with diabetes.

To the best of our knowledge, this study is one of the first attempts to comprehensively draw the outline of diabetes-specific distress-related hotspots and frontiers based on a bibliometric method. Our findings will provide a new direction for research on diabetes-specific distress from multiple dimension.

However, the generalizability of findings above is subject to certain limitations, including several biases: (1) Language bias: all publications included in this study were written in English. Therefore, there are many shortcomings such as much relevant information included other languages. (2) Literature-type bias: this study only includes the original articles and reviews published between 2000 and 2018, which may not be enough to represent all diabetes-specific distress literature. (3) Database selection bias: the literature included in this study was downloaded from WoSCC. However, there is no doubt that other databases such as PubMed, Google Scholar, Baidu Scholar, Scopus, and EMBASE may include a broader and deeper range of documentary coverage. (5) Literature-cited bias: document-cited counts need to be excluded from man-made malicious manipulation. (6) Bibliometrics and corresponding software bias: owing to limitations of the software, there is no uniform standard for setting parameters in CiteSpace. In spite of its limitations, this study certainly adds to our understanding of the research hotspots and frontiers. However, this study finding still needs further confirmation.

## 5. Conclusions

The present study suggested that publication outputs of diabetes-specific distress have been rising over the past quarter century. Although diabetes-specific distress develops very late, we can foresee that the quantity and quality of publication have been expanded and promoted in recent years. More and more researchers show their interest in this fresh and promising discipline. Besides, our findings have a number of important implications for future clinical practice.

## Figures and Tables

**Table 1:** Top 20 keyword terms shown in terms of frequency and centrality for diabetes-specific distress.

Ranking	Cocitation counts	Centrality	Keywords/terms
1	332	0.06	Depression
2	261	0.05	Glycemic control
3	229	0.04	Mellitus
4	211	0.02	Distress
5	200	0.02	Adult
6	190	0.03	Quality of life
7	169	0.04	Diabetes
8	158	0.05	Prevalence
9	141	0.07	Care
10	136	0.02	Meta-analysis
11	123	0.01	Scale
12	120	0.07	Health
13	104	0.02	Association
14	104	0.04	Type 2 diabetes
15	103	0.03	Management
16	99	0.05	Anxiety
17	93	0.04	Outcome
18	92	0.03	Depressive symptom
19	91	0.03	Psychological distress
20	86	0.02	Randomized controlled trial

**Table 2:** Top 20 references cited shown in terms of frequency and centrality for diabetes-specific distress.

Ranking	Cocitation counts	Centrality	Representative author (publication year)	Journal	Vol	Page
1	125	0.17	Fisher L. (2010)	Diabetes care	33	23
2	80	0.05	Fisher L. (2012)	Diabetes care	35	259
3	61	0.09	Fisher L. (2007)	Diabetes care	30	542
4	59	0.04	Fisher L. (2010)	Diabetes care	33	1034
5	55	0.02	Fisher L. (2008)	Diabetic med	25	1096
6	53	0.02	Fisher L. (2014)	Diabetic med	31	764
7	49	0.11	Polonsky W. H. (2005)	Diabetes care	28	626
8	49	0.06	Gonzalez J. S. (2011)	Diabetes care	34	236
9	46	0.02	Aikens J. E. (2012)	Diabetes care	35	2472
10	43	0.03	Fisher L. (2013)	Diabetes care	36	2551
11	41	0.03	Fisher L. (2008)	Ann fam med	6	246
12	40	0.03	Nicolucci A. (2013)	Diabetic med	30	767
13	40	0.01	Mcguire B. E. (2010)	Diabetologia	53	66
14	37	0.07	Hermanns N. (2006)	Diabetologia	49	469
15	36	0.06	van Bastelaar K. (2010)	Diabetic med	27	798
16	35	0.02	Gonzalez J. S. (2008)	Diabetes care	31	2398
17	35	0.01	Fisher L. (2009)	Diabetic med	26	622
18	34	0.01	Fisher L. (2015)	J. diabetes comp	29	572
19	31	0.08	Peyrot M. (2005)	Diabetic med	22	1379
20	29	0.02	Snoek F. J. (2015)	Lancet diabetes	3	450

## Data Availability

Firstly, raw literature data that support the findings of this study have been downloaded from the Science Citation Index-Expanded (SCI-E) of the Web of Science Core Collection (WoSCC) (Thomson Reuters Company) on January 6, 2019. The search expressions were constructed as follows: TS = ((“diabetes distress”) OR (“diabetes” AND “distress”) OR (“diabetes” AND “psychological distress”) OR (“diabetes-related distress”) OR (“diabetes-specific distress”) OR (“diabetes” AND “emotional distress”) OR (“diabetes-specific emotional distress”) OR (“diabetes-related emotional distress”) AND Language = English AND Time range = 2000–2018). In our study, only original article and review papers were included. Secondly, supplementary data that support the findings of this study have been deposited in Figshare database, which is a repository where users can make all of their research outputs available in a citable, shareable, and discoverable manner. Our supplementary materials are available at https://figshare.com/articles/Supplementary_Materials/8224685.

## Supplementary Materials

Supplementary Materials S1-1: raw citing literature and cited references. S1-2: raw citing literature and cited references (1). S1-3: raw citing literatures and cited references. S2: the number of publications by year. S3: country/region distribution. S4: institutes distribution. S5: authors distribution. S6: journals distribution.
