# Supplementary material for: Characterizing Hotspots and Frontier Landscapes of Diabetes-Specific Distress from 2000 to 2018: A Bibliometric Study
Source: Biomed Res Int. 2020 Jan 15;2020:8691451. doi: 10.1155/2020/8691451 (PMC6985931; doi:10.1155/2020/8691451)
Supplement: Supplementary Materials — S1-1: raw citing literature and cited references. S1-2: raw citing literature and cited references (1). S1-3: raw citing literatures and cited references. S2: the number of publications by year. S3: country/region distribution. S4: institutes distribution. S5: authors distribution. S6: journals distribution. [file 8691451.f1.zip › 8691451.f1/S1-1 Raw citing literatures and cited references.doc]

FN Clarivate Analytics Web of Science

VR 1.0

PT J

AU Teasdale, A

Limbers, C

AF Teasdale, Ashley

Limbers, Christine

TI Avoidant coping moderates the relationship between paternal involvement

in the child's type 1 diabetes (T1D) care and parenting stress

SO JOURNAL OF CHILD HEALTH CARE

LA English

DT Article

DE Chronic illness; diabetes; father; parenting; psychology

ID YOUNG-CHILDREN; MOTHERS; FATHERS; STRATEGIES; INTERVENTIONS;

ADOLESCENTS; INVENTORY; DISTRESS; SUPPORT

AB Fathers may experience greater parenting stress and anxiety when they are more involved in their child's type 1 diabetes (T1D) care. The present study evaluated whether seeking social support and avoidant coping strategies moderate the relationship between paternal involvement in the child's T1D care and parenting stress in an international sample. Two hundred forty-nine fathers of young children with T1D completed the Parenting Stress Index (PSI), Pediatric Inventory for Parents (PIP), Dads' Active Disease Support scale (DADS), COPE Inventory, Self-Care Inventory (SCI-R), and a demographic questionnaire online. Pearson's product moment correlations were computed, and multiple linear regression analysis was conducted with three separate models in which the PSI Child Domain, PIP Frequency, and PIP Difficulty scores represented different parenting stress outcomes. The interaction between use of denial coping and DADS Involvement was significantly correlated with general parenting stress (p < .05). There were no significant interactions between instrumental social support and DADS Involvement; however, use of instrumental social support coping was significantly correlated with difficulty of pediatric parenting stress (p < .05), DADS Involvement (p < .001), and SCI-R better adherence to the child diabetes treatment regimen (p < .001). Avoidant coping strategies are associated with more general parenting stress, especially when fathers are more involved in T1D management.

C1 [Teasdale, Ashley; Limbers, Christine] Baylor Univ, Dept Psychol & Neurosci, One Bear Pl 97334, Waco, TX 76798 USA.

RP Teasdale, A (reprint author), Baylor Univ, Dept Psychol & Neurosci, One Bear Pl 97334, Waco, TX 76798 USA.

EM ashley_teasdale@baylor.edu

FU College of Arts and Sciences at Baylor University

FX The author(s) disclosed receipt of the following financial support for

the research, authorship, and/or publication of this article: This work

was supported by internal funding from the College of Arts and Sciences

at Baylor University.

CR Abidin R. R., 2012, PARENTING STRESS IND

Azar R, 2001, J Pediatr Nurs, V16, P418, DOI 10.1053/jpdn.2001.27878

Beran D, 2013, DIABETES CARE, V36, pE144, DOI 10.2337/dc13-0744

Berinsky AJ, 2012, POLIT ANAL, V20, P351, DOI 10.1093/pan/mpr057

Blankfeld DF, 1996, J FAM PSYCHOL, V10, P173

Breevaart K, 2011, ANXIETY STRESS COPIN, V24, P239, DOI 10.1080/10615806.2010.527958

CARVER CS, 1989, J PERS SOC PSYCHOL, V56, P267, DOI 10.1037/0022-3514.56.2.267

Casler K, 2013, COMPUT HUM BEHAV, V29, P2156, DOI 10.1016/j.chb.2013.05.009

Chandler J, 2016, ANNU REV CLIN PSYCHO, V12, P53, DOI 10.1146/annurev-clinpsy-021815-093623

Dardas LA, 2015, STRESS HEALTH, V31, P5, DOI 10.1002/smi.2513

Dashiff C J, 1993, J Pediatr Nurs, V8, P361

Davis CL, 2001, J PEDIATR PSYCHOL, V26, P123, DOI 10.1093/jpepsy/26.2.123

FOLKMAN S, 1988, J PERS SOC PSYCHOL, V54, P466, DOI 10.1037//0022-3514.54.3.466

Grover Sandeep, 2016, Perspect Clin Res, V7, P32, DOI 10.4103/2229-3485.173776

Hansen JA, 2012, FAM SYST HEALTH, V30, P47, DOI 10.1037/a0027519

Hayes SC, 2006, BEHAV RES THER, V44, P1, DOI 10.1016/j.brat.2005.06.006

Hessler D, 2016, J PEDIATR PSYCHOL, V41, P750, DOI 10.1093/jpepsy/jsw002

Jaser SS, 2014, MATERN CHILD HLTH J, V18, P101, DOI 10.1007/s10995-013-1239-4

Kalra Sanjay, 2013, J Midlife Health, V4, P176, DOI 10.4103/0976-7800.118998

Konradsdottir E, 2011, J SPEC PEDIATR NURS, V16, P295, DOI 10.1111/j.1744-6155.2011.00297.x

Law EF, 2014, J PEDIATR PSYCHOL, V39, P866, DOI 10.1093/jpepsy/jsu032

Lazarus R. S, 1984, STRESS APPRAISAL COP

Lewin A. B., 2005, FAMILIES SYSTEMS HLT, V23, P56, DOI DOI 10.1037/1091-7527.23.1.56

Lohan A, 2015, CHILD CARE HLTH DEV, V41, P803, DOI 10.1111/cch.12278

Lyne K, 2000, PERS INDIV DIFFER, V29, P321, DOI 10.1016/S0191-8869(99)00196-8

Mitchell SJ, 2009, FAM SYST HEALTH, V27, P314, DOI 10.1037/a0018191

Nieuwesteeg A, 2017, DIABETIC MED, V34, P821, DOI 10.1111/dme.13300

Nunnally JC, 1994, PSYCHOMETRIC THEORY

Palermo Tonya M, 2014, Clin Pract Pediatr Psychol, V2, P212

Pottie CG, 2008, J FAM PSYCHOL, V22, P855, DOI 10.1037/a0013604

Ryan JL, 2011, CHILD HEALTH CARE, V40, P297, DOI 10.1080/02739615.2011.617237

Sahler OJZ, 2005, J CONSULT CLIN PSYCH, V73, P272, DOI 10.1037/0022-006X.73.2.272

Schleider JL, 2015, J CHILD FAM STUD, V24, P3235, DOI 10.1007/s10826-015-0126-6

Seppanen S M, 1999, Nurs Health Sci, V1, P63, DOI 10.1046/j.1442-2018.1999.00009.x

Sparud-Lundin C, 2013, J FAM NURS, V19, P249, DOI 10.1177/1074840713484386

Streisand R, 2001, J PEDIATR PSYCHOL, V26, P155, DOI 10.1093/jpepsy/26.3.155

Thorsteinsson EB, 2017, PEER J PREPRINTS

WALLANDER JL, 1989, J PEDIATR PSYCHOL, V14, P371, DOI 10.1093/jpepsy/14.3.371

Weinger K, 2005, DIABETES CARE, V28, P1346, DOI 10.2337/diacare.28.6.1346

Wysocki T, 2004, J PEDIATR PSYCHOL, V29, P231, DOI 10.1093/jpepsy/jsh024

NR 40

TC 0

Z9 0

U1 0

U2 0

PU SAGE PUBLICATIONS LTD

PI LONDON

PA 1 OLIVERS YARD, 55 CITY ROAD, LONDON EC1Y 1SP, ENGLAND

SN 1367-4935

EI 1741-2889

J9 J CHILD HEALTH CARE

JI J. Child Health Care

PD DEC

PY 2018

VL 22

IS 4

BP 606

EP 618

DI 10.1177/1367493518767068

PG 13

WC Nursing; Pediatrics

SC Nursing; Pediatrics

GA HE5TT

UT WOS:000453452600008

PM 29606015

OA Bronze

DA 2019-01-08

ER

PT J

AU Vigen, CLP

Carandang, K

Blanchard, J

Sequeira, PA

Wood, JR

Spruijt-Metz, D

Whittemore, R

Peters, AL

Pyatak, EA

AF Vigen, Cheryl L. P.

Carandang, Kristine

Blanchard, Jeanine

Sequeira, Paola A.

Wood, Jamie R.

Spruijt-Metz, Donna

Whittemore, Robin

Peters, Anne L.

Pyatak, Elizabeth A.

TI Psychosocial and Behavioral Correlates of A1C and Quality of Life Among

Young Adults With Diabetes

SO DIABETES EDUCATOR

LA English

DT Article

ID SELF-MANAGEMENT; GLYCEMIC CONTROL; DEPRESSIVE SYMPTOMS; CHILDHOOD

ADVERSITY; BLOOD-GLUCOSE; MENTAL-HEALTH; PARTICIPATION; IMPACT; CARE;

ASSOCIATION

AB Purpose The purpose of this study was to evaluate relationships between behavioral and psychosocial constructs, A1C, and diabetes-dependent quality of life (DQoL) among low-socioeconomic status, ethnically diverse young adults with diabetes. Methods Using baseline data of 81 participants in the Resilient, Empowered, Active Living (REAL) randomized controlled trial, behavioral, cognitive, affective, and experiential variables were correlated with A1C and DQoL while adjusting for demographic characteristics, and these relationships were examined for potential effect modification. Results The data indicate that depressive symptoms and satisfaction with daily activities are associated with both A1C and DQoL, while diabetes knowledge and participation in daily activities are associated with neither A1C nor DQoL. Two constructs, diabetes distress and life satisfaction, were associated with DQoL and were unrelated to A1C, while 2 constructs, self-monitoring of blood glucose and medication adherence, were associated with A1C but unrelated to DQoL. These relationships were largely unchanged by adjusting for demographic characteristics, while numerous effect modifications were found. Conclusion The data suggest that when tailoring interventions, depressive symptoms and satisfaction with daily activities may be particularly fruitful intervention targets, as they represent modifiable risk factors that are associated with both A1C and DQoL.

C1 [Vigen, Cheryl L. P.; Carandang, Kristine; Blanchard, Jeanine; Pyatak, Elizabeth A.] Univ Southern Calif, Div Occupat Sci & Occupat Therapy, 1540 Alcazar St,CHP-133, Los Angeles, CA 90089 USA.

[Sequeira, Paola A.] Univ Southern Calif, Dept Pediat, Los Angeles, CA 90089 USA.

[Wood, Jamie R.] Childrens Hosp Los Angeles, Los Angeles, CA 90027 USA.

[Spruijt-Metz, Donna] Univ Southern Calif, Dornsife Ctr Econ & Social Res, Los Angeles, CA USA.

[Whittemore, Robin] Yale Sch Nursing, New Haven, CT USA.

[Peters, Anne L.] Univ Southern Calif, Div Endocrinol, Beverly Hills, CA USA.

RP Vigen, CLP (reprint author), Univ Southern Calif, Div Occupat Sci & Occupat Therapy, 1540 Alcazar St,CHP-133, Los Angeles, CA 90089 USA.

EM vigen@usc.edu

OI Pyatak, Elizabeth/0000-0003-3280-8912

FU National Institute of Diabetes and Digestive and Kidney Diseases at the

National Institutes of Health (NIH/NIDDK) [1K01 DK099202-01A1]

FX This work was supported by the National Institute of Diabetes and

Digestive and Kidney Diseases at the National Institutes of Health

(NIH/NIDDK 1K01 DK099202-01A1). The content is solely the responsibility

of the authors and does not represent the official view of the National

Institutes of Health. The study is registered at ClinicalTrials.gov

(identifier number NCT02214641).

CR American Diabetes Association, 2016, DIABETES, V39, pS13, DOI DOI 10.2337/DC16-S005

Anderson RM, 2003, DIABETES CARE, V26, P1641, DOI 10.2337/diacare.26.5.1641-a

[Anonymous], 2000, Diabetes Care, V23, P1619

Arora S, 2012, DIABETES TECHNOL THE, V14, P492, DOI 10.1089/dia.2011.0252

Arora S, 2011, ETHNIC DIS, V21, P1

Bains SS, 2011, DIABETES TECHNOL THE, V13, P335, DOI 10.1089/dia.2010.0160

Benka J, 2016, DISABIL REHABIL, V38, P1172, DOI 10.3109/09638288.2015.1076071

Berg C, 2013, OTJR-OCCUP PART HEAL, V33, P21, DOI 10.3928/15394492-20120607-01

Bernstein CM, 2013, CLIN PEDIATR, V52, P10, DOI 10.1177/0009922812459950

Bot M, 2013, DIABETIC MED, V30, pE115, DOI 10.1111/dme.12082

Bradley C, 2002, DIABETES METAB RES, V18, pS64, DOI 10.1002/dmrr.279

Bradley C, 1999, QUAL LIFE RES, V8, P79, DOI 10.1023/A:1026485130100

Brown M, 2004, J HEAD TRAUMA REHAB, V19, P459, DOI 10.1097/00001199-200411000-00004

Browne JL, 2015, DIABETIC MED, V32, P133, DOI 10.1111/dme.12566

Dabelea D, 2017, JAMA-J AM MED ASSOC, V317, P825, DOI 10.1001/jama.2017.0686

DIENER E, 1985, J PERS ASSESS, V49, P71, DOI 10.1207/s15327752jpa4901_13

Elhai JD, 2013, J TRAUMA STRESS, V26, P10, DOI 10.1002/jts.21755

Fisher L, 2016, J DIABETES COMPLICAT, V30, P1123, DOI 10.1016/j.jdiacomp.2016.03.032

Garcia AA, 2001, DIABETES CARE, V24, P16, DOI 10.2337/diacare.24.1.16

Glasgow RE, 2007, DIABETES CARE, V30, P33, DOI 10.2337/dc06-1390

Hamman RF, 2014, DIABETES CARE, V37, P3336, DOI 10.2337/dc14-0574

Hill-Briggs F, 2011, J GEN INTERN MED, V26, P972, DOI 10.1007/s11606-011-1689-6

Hislop AL, 2008, DIABETIC MED, V25, P91, DOI 10.1111/j.1464-5491.2007.02310.x

Hood KK, 2009, PEDIATRICS, V124, pE1171, DOI 10.1542/peds.2009-0207

Johnson B, 2014, DIABETIC MED, V31, P227, DOI 10.1111/dme.12305

King DK, 2010, DIABETES CARE, V33, P751, DOI 10.2337/dc09-1746

Kroenke K, 2009, J AFFECT DISORDERS, V114, P163, DOI 10.1016/j.jad.2008.06.026

Lane JT, 2007, DIABETES RES CLIN PR, V78, P385, DOI 10.1016/j.diabres.2007.04.014

Law M, 2002, AM J OCCUP THER, V56, P640, DOI 10.5014/ajot.56.6.640

McGuire BE, 2010, DIABETOLOGIA, V53, P66, DOI 10.1007/s00125-009-1559-5

Miller KM, 2015, DIABETES CARE, V38, P971, DOI 10.2337/dc15-0078

Miller KM, 2013, DIABETES CARE, V36, P2009, DOI 10.2337/dc12-1770

Nagrebetsky A, 2012, DIABETES RES CLIN PR, V96, P119, DOI 10.1016/j.diabres.2011.12.003

Polonsky WH, 2011, DIABETES CARE, V34, P262, DOI 10.2337/dc10-1732

Pyatak EA, 2014, DIABETIC MED, V31, P1615, DOI 10.1111/dme.12485

Pyatak EA, 2013, DIABETIC MED, V30, P1140, DOI 10.1111/dme.12248

Pyatak E, 2011, AM J OCCUP THER, V65, P462, DOI 10.5014/ajot.2011.001453

Pyatak EA, 2017, CONTEMP CLIN TRIALS, V54, P8, DOI 10.1016/j.cct.2016.12.025

Sarkar U, 2006, DIABETES CARE, V29, P823, DOI 10.2337/diacare.29.04.06.dc05-1615

Schilling EA, 2008, SOC SCI MED, V66, P1140, DOI 10.1016/j.socscimed.2007.11.023

Sequeira PA, 2015, DIABETES CARE, V38, P1412, DOI 10.2337/dc14-2577

Strandberg RB, 2014, J PSYCHOSOM RES, V77, P174, DOI 10.1016/j.jpsychores.2014.06.015

Stratton IM, 2000, BRIT MED J, V321, P405, DOI 10.1136/bmj.321.7258.405

Toobert DJ, 2000, DIABETES CARE, V23, P943, DOI 10.2337/diacare.23.7.943

Turner HA, 2003, J YOUTH ADOLESCENCE, V32, P89, DOI 10.1023/A:1021853600645

van Tol E, 2011, BRAIN INJURY, V25, P1279, DOI 10.3109/02699052.2011.613089

Walders-Abramson N, 2014, J PEDIAT, V165

Wood Jamie R, 2012, J Diabetes Sci Technol, V6, P380

NR 48

TC 0

Z9 0

U1 0

U2 0

PU SAGE PUBLICATIONS INC

PI THOUSAND OAKS

PA 2455 TELLER RD, THOUSAND OAKS, CA 91320 USA

SN 0145-7217

EI 1554-6063

J9 DIABETES EDUCATOR

JI Diabetes Educ.

PD DEC

PY 2018

VL 44

IS 6

BP 489

EP 500

DI 10.1177/0145721718804170

PG 12

WC Endocrinology & Metabolism; Public, Environmental & Occupational Health

SC Endocrinology & Metabolism; Public, Environmental & Occupational Health

GA HD7KN

UT WOS:000452731000001

PM 30295170

DA 2019-01-08

ER

PT J

AU Jeong, M

Reifsnider, E

AF Jeong, Mihyun

Reifsnider, Elizabeth

TI Associations of Diabetes-Related Distress and Depressive Symptoms With

Glycemic Control in Korean Americans With Type 2 Diabetes

SO DIABETES EDUCATOR

LA English

DT Article

ID EMOTIONAL DISTRESS; GLUCOSE CONTROL; CUT POINTS; ADULTS; CARE;

PREDICTORS; OUTCOMES; HEALTH; PREVALENCE; IMMIGRANTS

AB Purpose The purpose of this study was to investigate the associations of diabetes-related distress and depressive symptoms with glycemic control in Korean Americans (KAs) with type 2 diabetes mellitus (T2DM). Methods The cross-sectional, correlational descriptive study explored glycemic control in KA. A total of 119 KA adults with T2DM were recruited from Korean communities in Arizona between July and November 2016. Data were collected by a finger-stick glucose test for A1C, the Diabetes Distress Scale for diabetes-related distress, the Center for Epidemiological Studies Depression for depressive symptoms, and sociodemographics. Height and weight were measured to calculate body mass index. Descriptive statistics, bivariate correlation, and multiple linear regression analyses were performed. Results The total score for diabetes-related distress was positively associated with A1C in both unadjusted and adjusted models, while depressive symptoms were not associated with glycemic control regardless of analysis methods. In the further analysis of the 4 subscales of diabetes-related distress, only regimen-related distress was a significant predictor of poor glycemic control after adjusting for covariates. Emotional burden was not associated with glycemic control, even though there was a weak but significant correlation between emotional burden and glycemic control. Conclusions Diabetes-related distress and depressive symptoms should be recognized as different psychological aspects in T2DM, and individual-centered approaches should be used for diabetes self-management education and support. In addition, diabetes-focused psychological interventions and culturally tailored diabetes self-management programs should be included in comprehensive education and support for KAs with T2D to achieve good glycemic control.

C1 [Jeong, Mihyun] Gyeongju Univ, Dept Nursing, 188 Taejong Ro, Gyeongju 38065, Gyeongsangbuk D, South Korea.

[Reifsnider, Elizabeth] Arizona State Univ, Coll Nursing & Hlth Innovat, Phoenix, AZ USA.

RP Jeong, M (reprint author), Gyeongju Univ, Dept Nursing, 188 Taejong Ro, Gyeongju 38065, Gyeongsangbuk D, South Korea.

EM mihyeon@naver.com

CR Aikens JE, 2012, DIABETES CARE, V35, P2472, DOI 10.2337/dc12-0181

Amer Diabet Assoc, 2018, DIABETES CARE, V41, pS55, DOI 10.2337/dc18-S006

American Diabetes Association, 2017, Diabetes Care, V40, pS33

Anderson RJ, 2001, DIABETES CARE, V24, P1069, DOI 10.2337/diacare.24.6.1069

Arrendale Justin R, 2008, J Diabetes Sci Technol, V2, P822

Asuzu CC, 2017, J DIABETES COMPLICAT, V31, P169, DOI 10.1016/j.jdiacomp.2016.09.013

Benoit SR, 2005, BMC PUBLIC HEALTH, V5, DOI 10.1186/1471-2458-5-36

Burns RJ, 2015, DIABETIC MED, V32, P1272, DOI 10.1111/dme.12860

Campbell JA, 2012, ENDOCRINE, V42, P471, DOI 10.1007/s12020-012-9744-6

Centers for Disease Control and Prevention, 2017, NAT DIAB STAT REP 20

Choi E, 2007, THESIS

Choi S, 2009, WESTERN J NURS RES, V31, P347, DOI 10.1177/0193945908328472

Choi SE, 2013, NURS RES, V62, P115, DOI 10.1097/NNR.0b013e31827aec29

Chung J, 2018, INT J OLDER PEOPLE N, V13, DOI 10.1111/opn.12179

Cummings DM, 2014, ANN PHARMACOTHER, V48, P970, DOI 10.1177/1060028014536532

Delahanty LM, 2007, DIABETIC MED, V24, P48, DOI 10.1111/j.1464-5491.2007.02028.x

Fisher L, 2008, DIABETIC MED, V25, P1096, DOI 10.1111/j.1464-5491.2008.02533.x

Fisher L, 2014, DIABETIC MED, V31, P764, DOI 10.1111/dme.12428

Fisher L, 2012, DIABETES CARE, V35, P259, DOI 10.2337/dc11-1572

Fisher L, 2010, DIABETES CARE, V33, P23, DOI 10.2337/dc09-1238

Hagger V, 2017, DIABETES CARE, V40, P1462, DOI 10.2337/dc17-0441

Hessler D, 2014, DIABETES CARE, V37, P617, DOI 10.2337/dc13-0762

Karter AJ, 2013, DIABETES CARE, V36, P574, DOI 10.2337/dc12-0722

Kendzor DE, 2014, BMC PUBLIC HEALTH, V14, DOI 10.1186/1471-2458-14-176

Kim MT, 2015, AM J GERIAT PSYCHIAT, V23, P671, DOI 10.1016/j.jagp.2014.11.003

Kim Miyong To, 2017, BMJ Open Diabetes Res Care, V5, pe000337, DOI 10.1136/bmjdrc-2016-000337

Koro CE, 2004, DIABETES CARE, V27, P17, DOI 10.2337/diacare.27.1.17

Lin KK, 2017, DIABETES RES CLIN PR, V131, P179, DOI 10.1016/j.diabres.2017.03.013

Lustman PJ, 2000, DIABETES CARE, V23, P934, DOI 10.2337/diacare.23.7.934

Meng RW, 2018, J AFFECT DISORDERS, V234, P59, DOI 10.1016/j.jad.2018.02.052

Nichols GA, 2000, DIABETES CARE, V23, P273, DOI 10.2337/diacare.23.3.273

Noh S, 1998, CROSS CULTURAL RES J, V32, P358, DOI DOI 10.1177/106939719803200403

Oh KM, 2014, HEALTH EDUC BEHAV, V41, P173, DOI 10.1177/1090198113496789

Ozcan B, 2018, DIABETES CARE, V41, P1854, DOI 10.2337/dc17-2181

Pandit AU, 2014, J EPIDEMIOL COMMUN H, V68, P557, DOI 10.1136/jech-2013-203063

PARIKH RM, 1988, INT J PSYCHIAT MED, V18, P169, DOI 10.2190/BH75-EUYA-4FM1-J7QA

Polonsky WH, 2005, DIABETES CARE, V28, P626, DOI 10.2337/diacare.28.3.626

Pouwer F, 2013, ENDOCRIN METAB CLIN, V42, P529, DOI 10.1016/j.ecl.2013.05.002

RADLOFF L S, 1977, Applied Psychological Measurement, V1, P385, DOI 10.1177/014662167700100306

Roy T, 2012, J AFFECT DISORDERS, V142, pS8, DOI 10.1016/S0165-0327(12)70004-6

Sidhu R, 2017, CAN J DIABETES, V41, P69, DOI 10.1016/j.jcjd.2016.07.008

Strandberg RB, 2014, J PSYCHOSOM RES, V77, P174, DOI 10.1016/j.jpsychores.2014.06.015

Ting RZW, 2011, DIABETES CARE, V34, P1094, DOI 10.2337/dc10-1612

Tsujii S, 2012, DIABETIC MED, V29, P1451, DOI 10.1111/j.1464-5491.2012.03647.x

UK Prospective Diabetes Study Grp, 1999, DIABETES CARE, V22, P1125

Winchester RJ, 2016, J DIABETES COMPLICAT, V30, P312, DOI 10.1016/j.jdiacomp.2015.11.010

Wong EM, 2017, CAN J DIABETES, V41, P362, DOI 10.1016/j.jcjd.2016.11.006

Zurita-Cruz JN, 2018, HEALTH QUAL LIFE OUT, V16, DOI 10.1186/s12955-018-0906-y

NR 48

TC 0

Z9 0

U1 0

U2 0

PU SAGE PUBLICATIONS INC

PI THOUSAND OAKS

PA 2455 TELLER RD, THOUSAND OAKS, CA 91320 USA

SN 0145-7217

EI 1554-6063

J9 DIABETES EDUCATOR

JI Diabetes Educ.

PD DEC

PY 2018

VL 44

IS 6

BP 531

EP 540

DI 10.1177/0145721718807443

PG 10

WC Endocrinology & Metabolism; Public, Environmental & Occupational Health

SC Endocrinology & Metabolism; Public, Environmental & Occupational Health

GA HD7KN

UT WOS:000452731000005

PM 30319059

DA 2019-01-08

ER

PT J

AU Kontopantelis, E

Buchan, I

Webb, RT

Ashcroft, DM

Mamas, MA

Doran, T

AF Kontopantelis, Evangelos

Buchan, Iain

Webb, Roger T.

Ashcroft, Darren M.

Mamas, Mamas A.

Doran, Tim

TI Disparities in mortality among 25-44-year-olds in England: a

longitudinal, population-based study

SO LANCET PUBLIC HEALTH

LA English

DT Article

ID ALCOHOL-RELATED MORTALITY; TRAFFIC ACCIDENTS; LIFE EXPECTANCY;

GREAT-BRITAIN; SOCIAL-CLASS; HEALTH; INEQUALITIES; DEPRIVATION; SUICIDE;

TRENDS

AB Background Since the mid-1990s, excess mortality has increased markedly for adults aged 25-44 years in the north compared with the south of England. We examined the underlying causes of this excess mortality and the contribution of socioeconomic deprivation.

Methods Mortality data from the Office of National Statistics for adults aged 25-44 years were aggregated and compared between England's five most northern versus five most southern government office regions between Jan 1,1981, and Dec 31,2016. Poisson regression models, adjusted for age and sex, were used to quantify excess mortality in the north compared with the south by underlying cause of death (accidents, alcohol related, cardiovascular disease and diabetes, drug related, suicide, cancer, and other causes). The role of socioeconomic deprivation, as measured by the 2015 Index of Multiple Deprivation, in explaining the excess and regional variability was also explored.

Findings A mortality divide between the north and south appeared in the mid-1990s and rapidly expanded thereafter for deaths attributed to accidents, alcohol misuse, and drug misuse. In the 2014-16 period, the northern excess was incidence rate ratio (IRR) 1.47 (95% CI 1.39-1.54) for cardiovascular reasons, 2.09 (1.94-2.25) for alcohol misuse, and 1.60 (1.51-1.70) for drug misuse, across both men and women aged 25-44 years. National mortality rates for cardiovascular deaths declined over the study period but a longstanding gap between north and south persisted (from 33.3 [95% CI 31.8-34.8] in 1981 to 15.0 [14.0-15.9] in 2016 in the north vs from 23.5 [22.3-24.8] to 9.9 [9.2-10.5] in the south). Between 2014 and 2016, estimated excess numbers of death in the north versus the south for those aged 25-44 years were 1881 (95% CI 726-2627) for women and 3530 (2216-4511) for men. Socioeconomic deprivation explained up to two-thirds of the excess mortality in the north (IRR for northern effect reduced from 1.15 [95% CI 1.14-1.15; unadjusted] to 1.05 [1.04-1.05; adjusted for Index of Multiple Deprivation]). By 2016, in addition to the persistent north-south gap, mortality rates in London were lower than in all other regions, with IRRs ranging from IRR 1.13 (95% CI 1.12-1.15) for the East England to 1.22 (1.20-1.24) for the North East, even after adjusting for deprivation.

Interpretation Sharp relative rises in deaths from cardiovascular reasons, alcohol misuse and drug misuse in the north compared with the south seem to have created new health divisions between England's regions. This gap might be due to exacerbation of existing social and health inequalities that have been experienced for many years. These divisions might suggest increasing psychological distress, despair, and risk taking among young and middle-aged adults, particularly outside of London. Copyright (C) 2018 The Author(s). Published by Elsevier Ltd.

C1 [Kontopantelis, Evangelos; Buchan, Iain] Univ Manchester, Fac Biol Med & Hlth, Div Informat Imaging & Data Sci, Manchester, Lancs, England.

[Webb, Roger T.] Univ Manchester, Fac Biol Med & Hlth, Div Psychol & Mental Hlth, Manchester, Lancs, England.

[Ashcroft, Darren M.] Univ Manchester, Fac Biol Med & Hlth, Div Pharm & Optometry, Manchester, Lancs, England.

[Buchan, Iain] Univ Liverpool, Fac Hlth & Life Sci, Dept Publ Hlth & Policy, Publ Hlth & Clin Informat, Liverpool, Merseyside, England.

[Kontopantelis, Evangelos] Univ Manchester, Natl Inst Hlth Res, Sch Primary Care Res, Manchester, Lancs, England.

[Webb, Roger T.; Ashcroft, Darren M.] Univ Manchester, NIHR Greater Manchester Patient Safety Translat R, Manchester, Lancs, England.

[Webb, Roger T.] Manchester Acad Hlth Sci Ctr, Manchester, Lancs, England.

[Mamas, Mamas A.] Keele Univ, Ctr Prognosis Res, Inst Primary Care & Hlth Sci, Keele Cardiovasc Res Grp, Keele, Staffs, England.

[Doran, Tim] Univ York, Dept Hlth Sci, Seebohm Rowntree Bldg, York, N Yorkshire, England.

RP Kontopantelis, E (reprint author), Univ Manchester, Fac Biol Med & Hlth, Manchester M13 9PL, Lancs, England.

EM e.kontopantelis@manchester.ac.uk

OI Kontopantelis, Evangelos/0000-0001-6450-5815; Ashcroft,

Darren/0000-0002-2958-915X

FU Medical Research Council; Wellcome Trust

FX Medical Research Council and Wellcome Trust.

CR Asaria P, 2012, INT J EPIDEMIOL, V41, P1737, DOI 10.1093/ije/dys151

Barbieri M, 2013, POPULATION, V68, P433, DOI 10.3917/popu.1303.0433

Barr B, 2012, BRIT MED J, V345, DOI 10.1136/bmj.e5142

Blakely TA, 2003, J EPIDEMIOL COMMUN H, V57, P594, DOI 10.1136/jech.57.8.594

Bosch FX, 2002, J CLIN PATHOL, V55, P244, DOI 10.1136/jcp.55.4.244

Buchan IE, 2017, J EPIDEMIOL COMMUN H, V71, P928, DOI 10.1136/jech-2017-209195

Cancer Research UK, 2018, DEPR GRAD CANC MORT

Cancer Research UK, DEPR GRAD CANC MORT

Case A, 2017, BROOKINGS PAP ECO AC, P397

Chang MH, 2013, MMWR-MORBID MORTAL W, V62, P561

Clarke DD, 2010, BEH RES ROAD SAF 200, P81

Coleman MP, 2004, BRIT J CANCER, V90, P1367, DOI 10.1038/sj.bjc.6601696

Communities and Local Government, 2015, ENGL IND DEPR 2015 T

Connolly S, 2007, SOC SCI MED, V64, P2008, DOI 10.1016/j.socscimed.2007.02.021

Cox E, 2011, WRONG TRACK ANAL AUT

Davies AR, 2007, EUR HEART J, V28, P2142, DOI [10.1093/eurheartj/ehm272, 10.1093/eurheartj/ehm323]

Doran T, 2004, BRIT MED J, V328, P1043, DOI 10.1136/bmj.328.7447.1043

Erskine S, 2010, BMC PUBLIC HEALTH, V10, DOI 10.1186/1471-2458-10-99

Gutierrez-Fisac JL, 2000, J EPIDEMIOL COMMUN H, V54, P451, DOI 10.1136/jech.54.6.451

Hacking JM, 2011, BRIT MED J, V342, DOI 10.1136/bmj.d508

Harrison L, 1999, ADDICTION, V94, P1871, DOI 10.1046/j.1360-0443.1999.9412187112.x

Health and Safety Executive, 2015, STAND IND FAT INJ RA, P11

Holborn PG, 2003, FIRE SAFETY J, V38, P1, DOI 10.1016/S0379-7112(02)00049-8

Jones AP, 2008, HEALTH PLACE, V14, P519, DOI 10.1016/j.healthplace.2007.10.001

Kontopantelis E, 2018, J EPIDEMIOL COMMUN H, V72, P140, DOI 10.1136/jech-2017-209999

Law E., 2018, DOES ITALYS N S DIVI

Leon DA, 2006, LANCET, V367, P52, DOI 10.1016/S0140-6736(06)67924-5

Lewis G, 1998, BMJ-BRIT MED J, V317, P1283, DOI 10.1136/bmj.317.7168.1283

Li ZY, 2011, SOC SCI MED, V72, P608, DOI 10.1016/j.socscimed.2010.11.008

Mackenbach JP, 2011, J EPIDEMIOL COMMUN H, V65, P568, DOI 10.1136/jech.2010.128280

Makela P, 1999, ADDICTION, V94, P867, DOI 10.1046/j.1360-0443.1999.94686710.x

Marmot M, 2012, PUBLIC HEALTH, V126, pS4, DOI 10.1016/j.puhe.2012.05.014

Mensah GA, 2017, CIRC RES, V120, P366, DOI 10.1161/CIRCRESAHA.116.309115

National Health Service, 2000, MOD STAND SERV MOD C

Nutt DJ, 2010, LANCET, V376, P1558, DOI 10.1016/S0140-6736(10)61462-6

Office for National Statistics, METH US GUID MORT ST

Organization for Economic Co-operation Development [OECD], 2018, OECD EMPL OUTL 2018

Parkinson J, 2018, BMC PUBLIC HEALTH, V18, DOI 10.1186/s12889-018-5267-2

Rechel B, 2013, LANCET, V381, P1235, DOI 10.1016/S0140-6736(12)62086-8

Road Safety Foundation, 2017, CUTT COST DANG ROADS

Scantlebury R, 2015, BRIT J GEN PRACT, V65, pE649, DOI 10.3399/bjgp15X686893

Skog OJ, 2001, ADDICTION, V96, pS49

Social Mobility Commission, 2017, STAT NAT 2017 SOC MO

Wardle J, 2003, PREV MED, V36, P721, DOI 10.1016/S0091-7435(03)00047-1

Webb RT, 2012, ARCH GEN PSYCHIAT, V69, P256, DOI 10.1001/archgenpsychiatry.2011.1561

Whitehead M, 2014, DUE N REPORT INQUIRY

NR 46

TC 1

Z9 1

U1 0

U2 0

PU ELSEVIER SCI LTD

PI OXFORD

PA THE BOULEVARD, LANGFORD LANE, KIDLINGTON, OXFORD OX5 1GB, OXON, ENGLAND

SN 2468-2667

J9 LANCET PUBLIC HEALTH

JI Lancet Public Health

PD DEC

PY 2018

VL 3

IS 12

BP E567

EP E575

DI 10.1016/S2468-2667(18)30177-4

PG 9

WC Public, Environmental & Occupational Health

SC Public, Environmental & Occupational Health

GA HC8CP

UT WOS:000452029600010

PM 30389570

OA DOAJ Gold, Green Published

DA 2019-01-08

ER

PT J

AU Kane, NS

Hoogendoorn, CJ

Tanenbaum, ML

Gonzalez, JS

AF Kane, N. S.

Hoogendoorn, C. J.

Tanenbaum, M. L.

Gonzalez, J. S.

TI Physical symptom complaints, cognitive emotion regulation strategies,

self-compassion and diabetes distress among adults with Type 2 diabetes

SO DIABETIC MEDICINE

LA English

DT Article

ID TREATMENT ADHERENCE; GLYCEMIC CONTROL; DEPRESSION; ASSOCIATION;

PREVALENCE; TRIAL; LIFE

AB Aims Diabetes-related distress is common among adults with Type 2 diabetes and is consistently associated with poorer self-management and treatment outcomes. However, little is known about the psychological factors that may contribute to or protect against diabetes distress. This study examined illness burden, and positive and negative ways of thinking and relating to oneself in times of stress, as independent correlates of diabetes distress, cross sectionally and longitudinally. Method A total of 120 adults treated for Type 2 diabetes reported their physical symptom complaints, cognitive emotion regulation, self-compassion and diabetes distress at baseline; 110 completed a 3-month follow-up assessment of diabetes distress. Pearson correlations and multivariable linear regression tested baseline and longitudinal relationships. Results Baseline diabetes distress was associated with greater use of negative cognitive emotion regulation strategies (r = 0.43, P < 0.01), greater tendency towards self-criticism, self-judgement and over-identification (r = 0.37, P < 0.01), and greater physical symptom burden (r = 0.50, P < 0.01). Baseline physical symptoms and negative cognitive emotion regulation were independently associated with baseline diabetes distress. Baseline physical symptoms and negative aspects of self-compassion significantly predicted diabetes distress over 3 months. Positive aspects of cognitive emotion regulation and self-compassion were not independently associated with diabetes distress cross sectionally or longitudinally. Conclusion What's new? ? Greater symptom burden along with the use of negative cognitive emotion regulation and negative aspects of self-compassion were independently associated with diabetes distress. If these relations are explained by causal influence, these modifiable factors could be fruitful targets for intervention research.

C1 [Kane, N. S.; Hoogendoorn, C. J.; Gonzalez, J. S.] Yeshiva Univ, Ferkauf Grad Sch Psychol, New York, NY 10033 USA.

[Kane, N. S.] VA New Jersey Hlth Care Syst, War Related Injury & Illness Study Ctr, E Orange, NJ USA.

[Tanenbaum, M. L.] Stanford Univ, Dept Pediat, Sch Med, Stanford, CA 94305 USA.

[Gonzalez, J. S.] Albert Einstein Coll Med, Dept Med, Bronx, NY 10461 USA.

[Gonzalez, J. S.] Albert Einstein Coll Med, Dept Epidemiol & Populat Hlth, Bronx, NY 10461 USA.

[Gonzalez, J. S.] New York Reg Ctr Diabet Translat Res, New York, NY 10461 USA.

RP Gonzalez, JS (reprint author), Yeshiva Univ, Ferkauf Grad Sch Psychol, New York, NY 10033 USA.; Gonzalez, JS (reprint author), Albert Einstein Coll Med, Dept Med, Bronx, NY 10461 USA.; Gonzalez, JS (reprint author), Albert Einstein Coll Med, Dept Epidemiol & Populat Hlth, Bronx, NY 10461 USA.; Gonzalez, JS (reprint author), New York Reg Ctr Diabet Translat Res, New York, NY 10461 USA.

EM jeffrey.gonzalez@einstein.yu.edu

FU Einstein-Mount Sinai Diabetes Research Center [P30 DK020541]; New York

Regional Center for Diabetes Translation Research [P30 DK111022];

National Institutes of Health [R01 DK104845, R18 DK098742]; Drs David

and Jane Willner Bloomgarden Family Fellowship Fund

FX This study was partially supported by the Einstein-Mount Sinai Diabetes

Research Center (P30 DK020541) and the New York Regional Center for

Diabetes Translation Research (P30 DK111022). Dr. Gonzalez is also

supported by grants R01 DK104845 and R18 DK098742 from the National

Institutes of Health. Support was also provided by the Drs David and

Jane Willner Bloomgarden Family Fellowship Fund.

CR Baek RN, 2014, ANN BEHAV MED, V48, P145, DOI 10.1007/s12160-013-9585-4

Bair MJ, 2010, DIABETIC MED, V27, P578, DOI 10.1111/j.1464-5491.2010.02971.x

Coroiu A, 2018, PLOS ONE, V13, DOI 10.1371/journal.pone.0190771

Delahanty LM, 2007, DIABETIC MED, V24, P48, DOI 10.1111/j.1464-5491.2007.02028.x

Fisher L, 2018, DIABETES CARE, V41, P1862, DOI 10.2337/dc18-0391

Fisher L, 2018, PATIENT EDUC COUNS, V101, P124, DOI 10.1016/j.pec.2017.06.036

Fisher L, 2013, DIABETES CARE, V36, P2551, DOI 10.2337/dc12-2493

Fisher L, 2010, DIABETES CARE, V33, P1034, DOI 10.2337/dc09-2175

Friis AM, 2016, DIABETES CARE, V39, P1963, DOI 10.2337/dc16-0416

Friis Anna M, 2015, Diabetes Spectr, V28, P252, DOI 10.2337/diaspect.28.4.252

Garnefski N, 2006, PERS INDIV DIFFER, V40, P1659, DOI 10.1016/j.paid.2005.12.009

Garnefski N, 2001, PERS INDIV DIFFER, V30, P1311, DOI 10.1016/S0191-8869(00)00113-6

Gonzalez JS, 2016, DIABETES CARE, V39, P2182, DOI 10.2337/dc16-1657

Gonzalez JS, 2015, HEALTH PSYCHOL, V34, P505, DOI 10.1037/hea0000131

Grierson AB, 2016, PSYCHOL MED, V46, P2467, DOI 10.1017/S0033291716001392

GROOTENHUIS PA, 1994, DIABETIC MED, V11, P253, DOI 10.1111/j.1464-5491.1994.tb00268.x

Gross JJ, 2003, J PERS SOC PSYCHOL, V85, P348, DOI 10.1037/0022-3514.85.2.348

Huffman JC, 2015, J DIABETES RES, DOI 10.1155/2015/428349

Iturralde E, 2017, HEALTH PSYCHOL, V36, P236, DOI 10.1037/hea0000445

Kasteleyn MJ, 2015, DIABETIC MED, V32, P1617, DOI 10.1111/dme.12743

Katon W, 2007, GEN HOSP PSYCHIAT, V29, P147, DOI 10.1016/j.genhosppsych.2006.11.005

Leventhal H, 2016, J BEHAV MED, V39, P935, DOI 10.1007/s10865-016-9782-2

Montero-Marin J, 2016, FRONT PSYCHOL, V7, P1

Moss-Morris R, 2002, PSYCHOL HEALTH, V17, P1, DOI 10.1080/08870440290001494

Moulton CD, 2015, LANCET DIABETES ENDO, V3, P461, DOI 10.1016/S2213-8587(15)00134-5

Muris P, 2017, CLIN PSYCHOL PSYCHOT, V24, P373, DOI 10.1002/cpp.2005

Neff KD, 2003, SELF IDENTITY, V2, P223, DOI 10.1080/15298860390209035

Perrin NE, 2017, DIABETIC MED, V34, P1508, DOI 10.1111/dme.13448

Wadsworth LP, 2018, COGNITIVE THER RES, V42, P236, DOI 10.1007/s10608-017-9887-0

WATSON D, 1989, PSYCHOL REV, V96, P234, DOI 10.1037//0033-295X.96.2.234

NR 30

TC 1

Z9 1

U1 2

U2 2

PU WILEY

PI HOBOKEN

PA 111 RIVER ST, HOBOKEN 07030-5774, NJ USA

SN 0742-3071

EI 1464-5491

J9 DIABETIC MED

JI Diabetic Med.

PD DEC

PY 2018

VL 35

IS 12

BP 1671

EP 1677

DI 10.1111/dme.13830

PG 7

WC Endocrinology & Metabolism

SC Endocrinology & Metabolism

GA HB9SK

UT WOS:000451432800009

PM 30264898

DA 2019-01-08

ER

PT J

AU Pintaudi, B

Fresa, R

Dalfra, M

Dodesini, AR

Vitacolonna, E

Tumminia, A

Sciacca, L

Lencioni, C

Marcone, T

Lucisano, G

Nicolucci, A

Bonomo, M

Napoli, A

AF Pintaudi, Basilio

Fresa, Raffaella

Dalfra, Mariagrazia

Dodesini, Alessandro Roberto

Vitacolonna, Ester

Tumminia, Andrea

Sciacca, Laura

Lencioni, Cristina

Marcone, Teresa

Lucisano, Giuseppe

Nicolucci, Antonio

Bonomo, Matteo

Napoli, Angela

CA STRONG Study Collaborators

TI The risk stratification of adverse neonatal outcomes in women with

gestational diabetes (STRONG) study

SO ACTA DIABETOLOGICA

LA English

DT Article

DE Gestational diabetes; Neonatal outcomes; Risk stratification; Obesity

ID MATERNAL OBESITY; WEIGHT-GAIN; PREGNANCY; MELLITUS; HYPERGLYCEMIA;

IMPACT; CLASSIFICATION; EPIDEMIOLOGY; ASSOCIATIONS; IMPROVEMENT

AB AimsTo assess the risk of adverse neonatal outcomes in women with gestational diabetes (GDM) by identifying subgroups of women at higher risk to recognize the characteristics most associated with an excess of risk.MethodsObservational, retrospective, multicenter study involving consecutive women with GDM. To identify distinct and homogeneous subgroups of women at a higher risk, the RECursive Partitioning and AMalgamation (RECPAM) method was used. Overall, 2736 pregnancies complicated by GDM were analyzed. The main outcome measure was the occurrence of adverse neonatal outcomes in pregnancies complicated by GDM.ResultsAmong study participants (median age 36.8 years, pre-gestational BMI 24.8kg/m(2)), six miscarriages, one neonatal death, but no maternal death was recorded. The occurrence of the cumulative adverse outcome (OR 2.48, 95% CI 1.59-3.87), large for gestational age (OR 3.99, 95% CI 2.40-6.63), fetal malformation (OR 2.66, 95% CI 1.00-7.18), and respiratory distress (OR 4.33, 95% CI 1.33-14.12) was associated with previous macrosomia. Large for gestational age was also associated with obesity (OR 1.46, 95% CI 1.00-2.15). Small for gestational age was associated with first trimester glucose levels (OR 1.96, 95% CI 1.04-3.69). Neonatal hypoglycemia was associated with overweight (OR 1.52, 95% CI 1.02-2.27) and obesity (OR 1.62, 95% CI 1.04-2.51). The RECPAM analysis identified high-risk subgroups mainly characterized by high pre-pregnancy BMI (OR 1.68, 95% CI 1.21-2.33 for obese; OR 1.38 95% CI 1.03-1.87 for overweight).ConclusionsA deep investigation on the factors associated with adverse neonatal outcomes requires a risk stratification. In particular, great attention must be paid to the prevention and treatment of obesity.

C1 [Pintaudi, Basilio; Bonomo, Matteo] Osped Niguarda Ca Granda, SSD Diabet Unit, I-20162 Milan, Italy.

[Fresa, Raffaella] ASL Salerno, Endocrinol & Diabet Unit, Salerno, Italy.

[Dalfra, Mariagrazia] Colli ULSS16, Diabetol, Padua, Italy.

[Dodesini, Alessandro Roberto] Papa Giovanni XXIII Hosp, Bergamo, Italy.

[Vitacolonna, Ester] DAnnunzio Univ, Dept Med & Aging, Chieti, Italy.

[Tumminia, Andrea; Sciacca, Laura] Univ Catania, Endocrinol Sect, Dept Clin & Expt Med, Catania, Italy.

[Lencioni, Cristina] Usl Nord Ovest Toscana, Diabet Unit, Lucca, Italy.

[Marcone, Teresa] Univ Hosp, SSD Diabetol, Foggia, Italy.

[Lucisano, Giuseppe; Nicolucci, Antonio] COREsearch, Pescara, Italy.

[Napoli, Angela] Sapienza Univ, S Andrea Hosp, Rome, Italy.

RP Pintaudi, B (reprint author), Osped Niguarda Ca Granda, SSD Diabet Unit, I-20162 Milan, Italy.

EM basilio.pintaudi@ospedaleniguarda.it

OI Bianchi, Cristina/0000-0003-2799-2380

FU Italian Diabetes and Pregnancy AMD-SID study group

FX The authors want to thank all the participating local study

investigators. We thank the Italian Diabetes and Pregnancy AMD-SID study

group for its support.

CR A merican Diabetes Association, 2015, DIABETES CARE S1, V38, P13

[Anonymous], 2001, ACOG PRACTICE B, V30

Bellamy L, 2009, LANCET, V373, P1773, DOI 10.1016/S0140-6736(09)60731-5

Beyerlein A, 2010, DIABETIC MED, V27, P1379, DOI 10.1111/j.1464-5491.2010.03109.x

Black MH, 2010, DIABETES CARE, V33, P2524, DOI 10.2337/dc10.1445

Buckley BS, 2012, DIABETIC MED, V29, P844, DOI 10.1111/j.1464-5491.2011.03541.x

Burlina S, 2017, ACTA DIABETOL, V54, P881, DOI 10.1007/s00592-017-1003-0

Casey BM, 1997, OBSTET GYNECOL, V90, P869, DOI 10.1016/S0029-7844(97)00542-5

Catalano PM, 2012, DIABETES CARE, V35, P780, DOI 10.2337/dc11-1790

Ciampi A, 1992, P PRAG 1991 SUMM SCH, P165

Corrado F, 2014, J MATERN-FETAL NEO M, V27, P1445, DOI 10.3109/14767058.2013.860961

Crowther CA, 2005, NEW ENGL J MED, V352, P2477, DOI 10.1056/NEJMoa042973

Devlieger R, 2016, EUR J OBSTET GYN R B, V201, P203, DOI 10.1016/j.ejogrb.2016.04.005

Fadl HE, 2010, DIABETIC MED, V27, P436, DOI 10.1111/j.1464-5491.2010.02978.x

Feig DS, 2014, DIABETES CARE, V37, P1590, DOI 10.2337/dc13-2717

Franciosi M, 2005, DIABETIC MED, V22, P900, DOI 10.1111/j.1464-5491.2005.01546.x

Fuchs F, 2013, BMC PREGNANCY CHILDB, V13, DOI 10.1186/1471-2393-13-90

Gaillard R, 2013, OBESITY, V21, P1046, DOI 10.1002/oby.20088

Goldstein RF, 2017, JAMA-J AM MED ASSOC, V317, P2207, DOI 10.1001/jama.2017.3635

Goueslard K, 2017, ACTA DIABETOL, V54, P645, DOI 10.1007/s00592-017-0986-x

HJALMARSON O, 1981, ACTA PAEDIATR SCAND, V70, P773, DOI 10.1111/j.1651-2227.1981.tb06228.x

Lai FY, 2016, J DIABETES, V8, P45, DOI 10.1111/1753-0407.12255

Landon MB, 2009, NEW ENGL J MED, V361, P1339, DOI 10.1056/NEJMoa0902430

Lapolla A, 2004, DIABETES NUTR METAB, V17, P358

MCFARLAND LV, 1986, OBSTET GYNECOL, V68, P784

McKenzie-Sampson S, 2018, ACTA DIABETOL, V55, P315, DOI 10.1007/s00592-017-1099-2

Metzger BE, 2008, NEW ENGL J MED, V358, P1991, DOI 10.1056/NEJMoa0707943

Metzger BE, 2010, DIABETES CARE, V33, P676, DOI 10.2337/dc09-1848

MILLS JL, 1979, DIABETES, V28, P292, DOI 10.2337/diabetes.28.4.292

Mokkala K, 2017, ACTA DIABETOL, V54, P1147, DOI 10.1007/s00592-017-1056-0

Much D, 2016, HORM METAB RES, V48, P16, DOI 10.1055/s-0035-1565084

O'Sullivan EP, 2011, DIABETOLOGIA, V54, P1670, DOI 10.1007/s00125-011-2150-4

Parnell AS, 2017, MATERN CHILD HLTH J, V21, P1105, DOI 10.1007/s10995-016-2209-4

Persson B, 1998, DIABETES CARE, V21, pB79

Pintaudi B, 2014, EUR J ENDOCRINOL, V170, P87, DOI 10.1530/EJE-13-0759

Reece EA, 2009, LANCET, V373, P1789, DOI 10.1016/S0140-6736(09)60515-8

Santangeli L, 2015, BEST PRACT RES CL OB, V29, P438, DOI 10.1016/j.bpobgyn.2014.10.009

Shand AW, 2008, DIABETIC MED, V25, P708, DOI 10.1111/j.1464-5491.2008.02431.x

Watson D, 2003, AUST NZ J OBSTET GYN, V43, P429, DOI 10.1046/j.0004-8666.2003.00116.x

NR 39

TC 0

Z9 0

U1 0

U2 0

PU SPRINGER-VERLAG ITALIA SRL

PI MILAN

PA VIA DECEMBRIO, 28, MILAN, 20137, ITALY

SN 0940-5429

EI 1432-5233

J9 ACTA DIABETOL

JI Acta Diabetol.

PD DEC

PY 2018

VL 55

IS 12

BP 1261

EP 1273

DI 10.1007/s00592-018-1208-x

PG 13

WC Endocrinology & Metabolism

SC Endocrinology & Metabolism

GA HA7KK

UT WOS:000450459800008

PM 30221320

DA 2019-01-08

ER

PT J

AU Ehrmann, D

Kulzer, B

Schipfer, M

Lippmann-Grob, B

Haak, T

Hermanns, N

AF Ehrmann, Dominic

Kulzer, Bernhard

Schipfer, Melanie

Lippmann-Grob, Bernhard

Haak, Thomas

Hermanns, Norbert

TI Efficacy of an Education Program for People With Diabetes and Insulin

Pump Treatment (INPUT): Results From a Randomized Controlled Trial

SO DIABETES CARE

LA English

DT Article

ID THERAPY; DISTRESS; MELLITUS; ADULTS; ADOLESCENTS; SYMPTOMS; DELIVERY;

INFUSION

AB OBJECTIVE

Continuous subcutaneous insulin infusion (CSII) is the most advanced form of insulin delivery, but it requires structured education to provide users with the necessary knowledge/skills and to support their motivation. Currently, no structured education program designed to provide this training has been evaluated. We developed a CSII-specific, structured education program (Insulin Pump Treatment [INPUT]) and evaluated its impact on glycemic control, behavior, and psychosocial status.

RESEARCH DESIGN AND METHODS

This was a multicenter, randomized, parallel trial with a 6-month follow-up. Eligible participants (age 16-75 years) currently were treated with insulin pump therapy. Participants were randomly assigned (1:1) to the INPUT program or to usual care using a computer-generated algorithm, with study center as the stratification factor. The primary outcome was HbA(1c) change from baseline to 6 months. Secondary outcomes were incidence of severe hypoglycemia and changes in behavioral and psychosocial measures.

RESULTS

Between 1 April 2016 and 26 April 2016, 268 people with diabetes and a mean duration of CSII therapy of 9.5 years were randomly assigned to the INPUT group (n = 135) or control group (n = 133). At 6 months, HbA(1c) improved in the INPUT group (8.33 +/- 0.8 vs. 8.04 +/- 0.9; P < 0.0001) but not in the control group (8.33 +/- 1.0 vs. 8.27 +/- 1.0; P = 0.11). The between-group difference in HbA(1c) reduction was significant, favoring INPUT (-0.28% vs. -0.06%, Delta -0.22%, 95% CI -0.38 to -0.06; P = 0.0029). The incidence rate ratio of severe hypoglycemia was 3.55 times higher for participants in the control group than for those in the INPUT group (95% CI 1.50-8.43; P = 0.0041).

CONCLUSIONS

The INPUT education program led to a significant improvement in glycemic control and incidence of severe hypoglycemia in insulin pump users.

C1 [Ehrmann, Dominic; Kulzer, Bernhard; Schipfer, Melanie; Hermanns, Norbert] Res Inst Diabet Acad Mergentheim, Bad Mergentheim, Germany.

[Ehrmann, Dominic; Kulzer, Bernhard; Hermanns, Norbert] Univ Bamberg, Dept Clin Psychol & Psychotherapy, Bamberg, Germany.

[Kulzer, Bernhard; Lippmann-Grob, Bernhard; Haak, Thomas; Hermanns, Norbert] Diabet Clin Mergentheim, Bad Mergentheim, Germany.

RP Hermanns, N (reprint author), Res Inst Diabet Acad Mergentheim, Bad Mergentheim, Germany.; Hermanns, N (reprint author), Univ Bamberg, Dept Clin Psychol & Psychotherapy, Bamberg, Germany.; Hermanns, N (reprint author), Diabet Clin Mergentheim, Bad Mergentheim, Germany.

EM hermanns@fidam.de

OI Hermanns, Norbert/0000-0002-2903-2677; Ehrmann,

Dominic/0000-0002-5794-5596

FU Research Institute Diabetes Academy Mergentheim

FX The authors thank all participating study centers for their effort in

recruiting and conducting this study: Diabeteszentrum am

Sophie-Charlotte-Platz, Dr. Kristina Pralle (Berlin, Germany);

Diabetes-und Stoffwechselpraxis Bochum, Stephan Bonnermann (Bochum,

Germany); Die Zuckerpraxis, Dr. Ewald Jammers (Bramsche, Germany);

Diabetologische Schwerpunktpraxis Dr. Golz, Dr. Stefan Golz (Esslingen,

Germany); Praxis Dres. Sammler/Denger, Dr. Armin Sammler

(Friedrichsthal, Germany); Zentrum fur Diabetologie Bergedorf, Dr. Jens

Kr6ger (Hamburg, Germany); Diabetologische Schwerpunktpraxis Dr. Milek,

Dr. Karsten Milek (Hohenmolsen, Germany); Gemeinschaftspraxis Dres.

Puth/Konig/Brockmann, Dr. Kerstin Konig (Kamen, Germany); Hormonzentrum

Karlsruhe, Sebastian Zink (Karlsruhe, Germany); Diabetologische

Schwerpunktpraxis Dres. Closs/Brahimi, Dr. Beqir Brahimi (Kempen,

Germany); Gemein-schaftspraxis Dres.

Schlotmann/Hochlehnert/Zavaleta/Birgel, Dr. Michael Birgel (Koln,

Germany); Praxis Dres. Reichert/Hinck, Dr. Dorothea Reichert (Landau,

Germany); Diabetologische Schwerpunktpraxis Dr. Lang, Dr. Vera Lang

(Lauf, Germany); Praxis Dr. GlaB, Dr. Florian GlaB (Magdeburg, Germany);

Diabeteszentrum Neckar-Odenwald, Dr. Carsten lannello (Mosbach,

Germany); Schwerpunktpraxis fur Diabetes und Ernahrungsmedizin Dr.

Keuthage, Dr. Winfried Keuthage (Munster, Germany); Zentrum fur Diabetes

und Gefasserkrankungen Munster, Dr. Ludger Rose (Munster, Germany);

Praxis Marck-Linn-Pickel, Dr. Cornelia Marck (Pohlheim, Germany); Praxis

Dr. Lange, Dr. Martina Lange (Rheinbach, Germany); Diabetologische

Schwerpunktpraxis Dr. Dietlein, Dr. Michael Dietlein (Stadtbergen,

Germany); Diabetologische Schwerpunktpraxis Dr. Schreiber, Dr. Anne

Schreiber (Stuttgart, Germany); Praxis Dres. Etzrodt/Alexopoulos, Dr.

Gwendolin Etzrodt-Walter (Ulm, Germany); Gemeinschaftspraxis Dr.

Schreiber/Werkmeister, Petra Werkmeister (Volkertshausen, Germany);

Praxis Dr. Sturmer, Dr. Annette Klupfel (Wurzburg, Germany); Diabendo

Praxisgemeinschaft, Dr. Stephan Arndt (Rostock, Germany); and

Diabetologische Schwerpunktpraxis Galatea-Anlage, Dr. Dorothea Herber

(Wiesbaden, Germany). The authors also thank Chris Parkin for assistance

with editing the final version of the manuscript (sponsored by the

Research Institute Diabetes Academy Mergentheim).

CR American Diabetes Association, 2004, Diabetes Care, V27 Suppl 1, pS110

Benkhadra K, 2017, ENDOCRINE, V55, P86, DOI 10.1007/s12020-016-1039-x

Bergis N, 2012, DIABETOL STOFFWECHS, V7, P9

CLARKE WL, 1995, DIABETES CARE, V18, P517, DOI 10.2337/diacare.18.4.517

Deeb A, 2015, DIABETES TECHNOL THE, V17, P166, DOI 10.1089/dia.2014.0224

Ehrmann D, 2015, DIABETIC MED, V32, P1264, DOI 10.1111/dme.12861

Fisher L, 2015, J DIABETES COMPLICAT, V29, P572, DOI 10.1016/j.jdiacomp.2015.01.012

Fisher L, 2010, DIABETES CARE, V33, P23, DOI 10.2337/dc09-1238

Garmo A, 2013, DIABETIC MED, V30, P717, DOI 10.1111/dme.12155

Gonder-Frederick Linda, 2011, J Diabetes Sci Technol, V5, P1387

Gonder-Frederick LA, 2016, AM PSYCHOL, V71, P577, DOI 10.1037/a0040383

Greiner W, 2005, Eur J Health Econ, V6, P124, DOI 10.1007/s10198-004-0264-z

Hautzinger M, 1993, ALLGEMEINE DEPRESSIO

Heller S, 2017, BMJ-BRIT MED J, V356, DOI 10.1136/bmj.j1285

Hermanns N, 2017, DIABETOL STOFFWECHS, V12, P224

Hislop AL, 2008, DIABETIC MED, V25, P91, DOI 10.1111/j.1464-5491.2007.02310.x

Kalyani RR, 2018, DIABETES CARE, V41, pS3, DOI [10.2337/dc18-Sppc01, 10.2337/dc18-SPPC01]

Katon W, 2008, J GEN INTERN MED, V23, P1571, DOI 10.1007/s11606-008-0731-9

KULZER B, 1995, VERHALTENSTHERAPIE, V5, pA72

Kulzer B, 2016, DIABETES, V65, pA178

Misso ML, 2010, COCHRANE DB SYST REV, DOI 10.1002/14651858.CD005103.pub2

Muhlhauser I, 2002, DIABETOLOGIA, V45, P1723, DOI 10.1007/s00125-002-0987-2

National Institute for Health and Care Excellence, 2008, CONT SUBC INS INF TR

Patton Susana R, 2017, J Diabetes Sci Technol, V11, P87, DOI 10.1177/1932296816658901

Payk M, 2018, J ADV NURS, V74, P528, DOI 10.1111/jan.13463

PICKUP JC, 1978, BRIT MED J, V1, P204, DOI 10.1136/bmj.1.6107.204

Pickup JC, 2012, NEW ENGL J MED, V366, P1616, DOI 10.1056/NEJMct1113948

POLONSKY WH, 1995, DIABETES CARE, V18, P754, DOI 10.2337/diacare.18.6.754

Reichert D, 2013, DIABETES STOFFWECH H, V22, P367

Reimer A, 2017, PLOS ONE, V12, DOI 10.1371/journal.pone.0181218

Ritholz MD, 2007, DIABETES CARE, V30, P549, DOI 10.2337/dc06-1755

Schmitt A, 2013, HEALTH QUAL LIFE OUT, V11, DOI 10.1186/1477-7525-11-138

Seereiner S, 2010, DIABETES TECHNOL THE, V12, P89, DOI 10.1089/dia.2009.0080

Weisman A, 2017, LANCET DIABETES ENDO, V5, P501, DOI 10.1016/S2213-8587(17)30167-5

Wong JC, 2015, PEDIATR DIABETES, V16, P592, DOI 10.1111/pedi.12221

Wood JR, 2006, DIABETES CARE, V29, P2355, DOI 10.2337/dc06-1141

Yeh HC, 2012, ANN INTERN MED, V157, P336, DOI 10.7326/0003-4819-157-5-201209040-00508

Ziegler R, 2016, PEDIATR DIABETES, V17, P311, DOI 10.1111/pedi.12290

NR 38

TC 0

Z9 0

U1 3

U2 3

PU AMER DIABETES ASSOC

PI ALEXANDRIA

PA 1701 N BEAUREGARD ST, ALEXANDRIA, VA 22311-1717 USA

SN 0149-5992

EI 1935-5548

J9 DIABETES CARE

JI Diabetes Care

PD DEC 1

PY 2018

VL 41

IS 12

BP 2453

EP 2462

DI 10.2337/dc18-0917

PG 10

WC Endocrinology & Metabolism

SC Endocrinology & Metabolism

GA HA8RI

UT WOS:000450560000010

PM 30305343

DA 2019-01-08

ER

PT J

AU Rassart, J

Oris, L

Prikken, S

Weets, I

Moons, P

Luyckx, K

AF Rassart, Jessica

Oris, Leen

Prikken, Sofie

Weets, Ilse

Moons, Philip

Luyckx, Koen

TI Personality Functioning in Adolescents and Emerging Adults With Type 1

Diabetes

SO JOURNAL OF ADOLESCENT HEALTH

LA English

DT Article

DE Personality; the Big Five; Treatment adherence; Diabetes-specific

distress; Glycemic control; Type 1 diabetes; Adolescents; Emerging

adults; Longitudinal; Cross-lagged analysis; Latent growth curve

modeling

ID GLYCEMIC CONTROL; SELF-CARE; MEASUREMENT INVARIANCE; EMOTIONAL DISTRESS;

YOUNG ADULTHOOD; LIFE-COURSE; MEAN-LEVEL; METAANALYSIS; TRAITS;

CONSCIENTIOUSNESS

AB Purpose: Although prior research has stressed the role of personality in adjusting to type 1 diabetes, longitudinal research is lacking. The objectives of the present study were twofold: (1) to chart the development of patients' personality over a 2-year period; and (2) to examine prospective associations among personality, treatment adherence, glycemic control, and diabetes-specific distress.

Methods: Adolescents and emerging adults with type 1 diabetes, aged 14-25 years (M-age = 18.86 years, 54% female), participated in a three-wave longitudinal study spanning 2 years (n = 560 at Time 1). Patients filled out questionnaires on Big Five personality traits, treatment adherence, and diabetes-specific distress. HbA(1c) values were obtained from treating physicians. We used latent growth curve modeling to examine the development of patients' personality. Cross-lagged path analysis was performed to examine prospective associations among the study variables.

Results: First, we observed mean-level increases in extraversion, agreeableness, and conscientiousness over the course of the study. Second, we uncovered bidirectional associations between personality and several important indicators of adjustment. Lower conscientiousness and higher extraversion predicted a relative decrease in treatment adherence 1 year later. Poorer treatment adherence, in turn, predicted relative decreases in conscientiousness and agreeableness over time. Furthermore, lower emotional stability predicted a relative increase in distress 1 year later. Higher distress, in turn, predicted a relative decrease in agreeableness over time. Finally, lower conscientiousness predicted poorer glycemic control 1 year later.

Conclusions: This study found young patients to move toward a more mature personality and stressed the importance of personality in adjusting to type 1 diabetes. (C) 2018 Society for Adolescent Health and Medicine. All rights reserved.

C1 [Rassart, Jessica; Oris, Leen; Prikken, Sofie; Luyckx, Koen] Univ Leuven, Fac Psychol & Educ Sci, Leuven, Belgium.

[Rassart, Jessica; Oris, Leen; Prikken, Sofie] Res Fdn Flanders, Brussels, Belgium.

[Weets, Ilse] Free Univ Brussels, Univ Hosp Brussels, Diabet Res Ctr Clin Biol, Brussels, Belgium.

[Moons, Philip] Univ Leuven, Dept Publ Hlth & Primary Care, Leuven, Belgium.

[Moons, Philip] Univ Gothenburg, Inst Hlth & Care Sci, Gothenburg, Sweden.

RP Rassart, J (reprint author), Katholieke Univ Leuven, Fac Psychol & Educ Sci, Tiensestr 102,Box 3717, B-3000 Leuven, Belgium.

EM Jessica.Rassart@kuleuven.be

FU FWO Flanders [G.0B35.14N]

FX Funding was provided through research project G.0B35.14N granted by FWO

Flanders to K.L.

CR Amer Diabet Assoc, 2016, DIABETES CARE, V39, pS86, DOI 10.2337/dc16-S014

Arnett JJ, 1999, AM PSYCHOL, V54, P317, DOI 10.1037/0003-066X.54.5.317

Berg CA, 2017, DEV REV, V46, P1, DOI 10.1016/j.dr.2017.09.001

Bogg T, 2004, PSYCHOL BULL, V130, P887, DOI 10.1037/0033-2909-130.6.887

Brickman AL, 1996, PSYCHOSOMATICS, V37, P459, DOI 10.1016/S0033-3182(96)71534-7

Caspi A, 2005, ANNU REV PSYCHOL, V56, P453, DOI 10.1146/annurev.psych.55.090902.141913

Chapman BP, 2014, DEV PSYCHOL, V50, P1426, DOI 10.1037/a0034135

Cheung GW, 2002, STRUCT EQU MODELING, V9, P233, DOI 10.1207/S15328007SEM0902_5

Cole DA, 2003, J ABNORM PSYCHOL, V112, P558, DOI 10.1037/0021-843X.112.4.558

Connor-Smith JK, 2007, J PERS SOC PSYCHOL, V93, P1080, DOI 10.1037/0022-3514.93.6.1080

Denissen JJA, 2008, J PERS ASSESS, V90, P152, DOI 10.1080/00223890701845229

Drew LM, 2010, J FAM PSYCHOL, V24, P299, DOI 10.1037/a0019352

Gerlitz JY, 2005, DIW RES NOTES, P4

Hilliard ME, 2013, J ADOLESCENT HEALTH, V52, P28, DOI 10.1016/j.jadohealth.2012.05.009

Jeronimus BF, 2014, J PERS SOC PSYCHOL, V107, P751, DOI 10.1037/a0037009

Klimstra TA, 2014, SOC PSYCH PSYCH EPID, V49, P1319, DOI 10.1007/s00127-014-0827-y

Klimstra TA, 2009, J PERS SOC PSYCHOL, V96, P898, DOI 10.1037/a0014746

Kline RB, PRINCIPLES PRACTICE

KOVACS M, 1992, J AM ACAD CHILD PSY, V31, P1112, DOI 10.1097/00004583-199211000-00020

Lane JD, 2000, DIABETES CARE, V23, P1321, DOI 10.2337/diacare.23.9.1321

Lewin AB, 2009, J PEDIATR PSYCHOL, V34, P999, DOI 10.1093/jpepsy/jsp032

LITTLE RJA, 1988, J AM STAT ASSOC, V83, P1198, DOI 10.2307/2290157

Luan Z, 2017, J RES PERS, V67, P44, DOI 10.1016/j.jrp.2016.03.004

POLONSKY WH, 1995, DIABETES CARE, V18, P754, DOI 10.2337/diacare.18.6.754

Rassart J, 2014, J CLIN PSYCHOL MED S, V21, P41, DOI 10.1007/s10880-014-9387-2

Rassart J, 2014, J PSYCHOSOM RES, V76, P139, DOI 10.1016/j.jpsychores.2013.11.015

Roberts BW, 2006, PSYCHOL BULL, V132, P1, DOI 10.1037/0033-2909.132.1.1

Shiner RL, 2017, J RES PERS, V67, P171, DOI 10.1016/j.jrp.2016.10.002

Skinner TC, 2002, HEALTH PSYCHOL, V21, P61, DOI 10.1037//0278-6133.21.1.61

Snoek FJ, 2000, DIABETES CARE, V23, P1305, DOI 10.2337/diacare.23.9.1305

Specht J, 2011, J PERS SOC PSYCHOL, V101, P862, DOI 10.1037/a0024950

Strandberg RB, 2014, J PSYCHOSOM RES, V77, P174, DOI 10.1016/j.jpsychores.2014.06.015

Tackett JL, 2006, CLIN PSYCHOL REV, V26, P584, DOI 10.1016/j.cpr.2006.04.003

Taylor MD, 2003, DIABETIC MED, V20, P135, DOI 10.1046/j.1464-5491.2003.00887.x

Vandenberg RJ, 2000, ORGAN RES METHODS, V3, P4, DOI 10.1177/109442810031002

Vollrath ME, 2007, DIABETIC MED, V24, P1028, DOI 10.1111/j.1464-5491.2007.02215.x

Waller D, 2013, DIABETES CARE, V36, P2968, DOI 10.2337/dc12-1743

Weinger K, 2005, DIABETES CARE, V28, P1346, DOI 10.2337/diacare.28.6.1346

Weissberg-Benchell J, 2007, DIABETES CARE, V30, P2441, DOI 10.2337/dc07-1249

Wheeler K, 2012, J CHILD ADOL PS NURS, V25, P66, DOI 10.1111/j.1744-6171.2012.00329.x

NR 40

TC 0

Z9 0

U1 1

U2 1

PU ELSEVIER SCIENCE INC

PI NEW YORK

PA 360 PARK AVE SOUTH, NEW YORK, NY 10010-1710 USA

SN 1054-139X

EI 1879-1972

J9 J ADOLESCENT HEALTH

JI J. Adolesc. Health

PD DEC

PY 2018

VL 63

IS 6

BP 792

EP 798

DI 10.1016/j.jadohealth.2018.06.019

PG 7

WC Psychology, Developmental; Public, Environmental & Occupational Health;

Pediatrics

SC Psychology; Public, Environmental & Occupational Health; Pediatrics

GA HA5FQ

UT WOS:000450295000023

PM 30266568

DA 2019-01-08

ER

PT J

AU Del Valle, KL

McDonnell, ME

AF Del Valle, Kayla L.

McDonnell, Marie E.

TI Chronic Care Management Services for Complex Diabetes Management: a

Practical Overview

SO CURRENT DIABETES REPORTS

LA English

DT Review

DE Diabetes; Chronic care management; Chronic care model; Type 1 diabetes;

Type 2 diabetes; Complex diabetes; Virtual care

ID IMPROVING PRIMARY-CARE; REGRESSION-ANALYSIS; EMOTIONAL DISTRESS;

GLYCEMIC CONTROL; CHRONIC ILLNESS; INTERVENTIONS; COMPLICATIONS;

OUTCOMES

AB Purpose of ReviewFormalized chronic care management has the potential to improve the quality and cost-effectiveness of complex diabetes management in adults, but has historically not been sustainably supported by health care systems. This review discusses the application of the chronic care model in the care of complex diabetes and its translation in the current reimbursement structure designed by Centers for Medicare and Medicaid Services (CMS).Recent FindingsFollowing the introduction of Wagner's Chronic Care Model (CCM) in the late 1990s, evidence gathered over the past 2 decades has supported the shift in focus of health care systems from acute to chronic disease management and proactive care. Acknowledging evidence and potential for improved cost-effectiveness, in 2015, Medicare began reimbursing for chronic care management services (CCMS) for patients with multiple chronic conditions. The CCMS billing codes allow a program to be reimbursed for up to 90min per month spent by clinical staff performing interim care within a comprehensive care plan. Recent data from local and global programs support the application of formalized CCM in diabetes management.SummaryAlthough reimbursement models for CCM have been designed for use in primary care, the challenges of the reimbursement model has opened the door for specialty areas focused on multimorbidity care such as diabetes care to explore this approach. With the broader availability of remote glucose monitoring and telemedicine, a strategy that combines goal-oriented care and telehealth solutions appears to be most effective in diabetes CCM care. Despite widespread acceptance of the chronic care model of care, there remain significant barriers to its incorporation into standard practice.

C1 [Del Valle, Kayla L.; McDonnell, Marie E.] Harvard Med Sch, Brigham & Womens Hosp, Div Endocrinol, 221 Longwood Ave,Suite 381, Boston, MA 02115 USA.

RP McDonnell, ME (reprint author), Harvard Med Sch, Brigham & Womens Hosp, Div Endocrinol, 221 Longwood Ave,Suite 381, Boston, MA 02115 USA.

EM kdelvalle@bwh.harvard.edu; MMCDONNELL@BWH.HARVARD.EDU

CR Aikens JE, 2012, DIABETES CARE, V35, P2472, DOI 10.2337/dc12-0181

Amer Diabet Assoc, 2018, DIABETES CARE, V41, pS73, DOI 10.2337/dc18-S008

Amer Diabet Assoc, 2018, DIABETES CARE, V41, pS38, DOI 10.2337/dc18-S004

Amer Diabet Assoc, 2018, DIABETES CARE, V41, pS86, DOI 10.2337/dc18-S009

Amer Diabet Assoc, 2018, DIABETES CARE, V41, pS105, DOI 10.2337/dc18-S010

Beck J, 2017, DIABETES CARE, V40, P1409, DOI 10.2337/dci17-0025

Bodenheimer T, 2002, JAMA-J AM MED ASSOC, V288, P1775, DOI 10.1001/jama.288.14.1775

Bodenheimer T, 2002, JAMA-J AM MED ASSOC, V288, P1909, DOI 10.1001/jama.288.15.1909

Boehmer KR, 2018, PLOS ONE, V13, DOI 10.1371/journal.pone.0190852

Davy C, 2015, BMC HEALTH SERV RES, V15, DOI 10.1186/s12913-015-0854-8

Dwyer-Lindgren L, 2016, DIABETES CARE, V39, P1556, DOI 10.2337/dc16-0678

Fisher L, 2018, DIABETES CARE, V41, P1862, DOI 10.2337/dc18-0391

Fremont Allen, 2018, Health Aff (Millwood), V37, P1457, DOI 10.1377/hlthaff.2018.0443

Gregg EW, 2014, NEW ENGL J MED, V370, P1514, DOI 10.1056/NEJMoa1310799

Huynh P, 2018, ENDOCR PRACT, V24, P489, DOI 10.4158/EP-2017-0267

Mendola N, 2018, PREVALENCE TOTAL DIA

Moher D, 2009, ANN INTERN MED, V151, P264, DOI 10.7326/0003-4819-151-4-200908180-00135

Nefs G, 2015, DIABETES RES CLIN PR, V108, P94, DOI 10.1016/j.diabres.2015.01.015

Nuckols TK, 2018, DIABETES CARE, V41, P985, DOI 10.2337/dc17-1495

Panagioti M, 2018, JAMA INTERN MED, V178, P1317, DOI 10.1001/jamainternmed.2018.3713

Rein R, 2017, AM DIAB ASS SCI SESS

Sadhu AR, 2017, CURR DIABETES REP, V17, DOI 10.1007/s11892-017-0936-6

Serrano V, 2017, CURR DIABETES REP, V17, DOI 10.1007/s11892-017-0935-7

Shojania KG, 2006, JAMA-J AM MED ASSOC, V296, P427, DOI 10.1001/jama.296.4.427

Stellefson M, 2013, PREV CHRONIC DIS, V10, DOI 10.5888/pcd10.120180

The Centers for Medicare & Medicaid Services (CMS), 2018, CHRON CAR MAN SERV

Tsai AC, 2005, AM J MANAG CARE, V11, P478

Wan EYF, 2018, DIABETES CARE, V41, P49, DOI [10.2337/dc17-0426, 10.2337/dc17-1149]

Yang WY, 2018, DIABETES CARE, V41, P917, DOI 10.2337/dci18-0007

NR 29

TC 0

Z9 0

U1 16

U2 16

PU CURRENT MEDICINE GROUP

PI PHILADELPHIA

PA 400 MARKET STREET, STE 700, PHILADELPHIA, PA 19106 USA

SN 1534-4827

EI 1539-0829

J9 CURR DIABETES REP

JI Curr. Diabetes Rep.

PD DEC

PY 2018

VL 18

IS 12

AR 135

DI 10.1007/s11892-018-1118-x

PG 8

WC Endocrinology & Metabolism

SC Endocrinology & Metabolism

GA GX5PV

UT WOS:000447802900009

PM 30343337

DA 2019-01-08

ER

PT J

AU Sheikh, MA

AF Sheikh, Mashhood Ahmed

TI Child maltreatment, psychopathological symptoms, and onset of diabetes

mellitus, hypothyroidism and COPD in adulthood

SO JOURNAL OF AFFECTIVE DISORDERS

LA English

DT Article

DE Diabetes mellitus; Hypothyroidism; Low metabolism; Chronic bronchitis;

Emphysema; COPD; Childhood maltreatment; Social epidemiology; Social

causation; Life course

ID ACUTE MYOCARDIAL-INFARCTION; EARLY-LIFE ADVERSITY; PSYCHOLOGICAL

DISTRESS; BIPOLAR DISORDER; INFLAMMATORY MARKERS; METABOLIC SYNDROME;

ALLOSTATIC LOAD; RISK-FACTOR; ABUSE; DEPRESSION

AB Background: The aim of this study was to assess the associations between child maltreatment (CM), psychopathological symptoms, and onset of diabetes mellitus, hypothyroidism (i.e., low metabolism), and chronic bronchitis/emphysema/COPD in adulthood.

Methods: The present analysis used cross-sectional data collected in 2007-2008 within the Troms phi Study, Norway (N = 12,981). CM was measured with a single item, and self-reported information on psychopathological symptoms and physical health outcomes was used. The associations between CM, psychopathological symptoms, and physical health outcomes were assessed with linear and Poisson regression models. Mediation was assessed with difference-in-coefficients method.

Results: In the fully-adjusted models, CM was associated with higher levels of anxiety and depression, psychological distress, difficulty in sleeping, insomnia, and use of sleeping pills and antidepressants in adulthood (p < 0.05). Moreover, CM was associated with a more than two-folds increased risk of consultation with psychiatrist (p < 0.001), a 26% increased risk of forgetfulness (p < 0.001), a 15% increased risk of decline in memory (p < 0.001), and a 96% increased risk of psychiatric problems (p < 0.001) over the course of life. In the fully-adjusted models, CM was associated with a 27-82% increased risk of physical health outcomes in adulthood (p < 0.05). Indicators of psychopathological symptoms significantly (p < 0.05) mediate the associations between CM and physical health outcomes.

Limitations: The design of this study is cross-sectional, and all measures are self-reported.

Conclusion: The associations between retrospectively-reported CM and physical health outcomes in adulthood are partially driven by psychopathological symptoms in adulthood.

C1 [Sheikh, Mashhood Ahmed] Univ Tromso, Dept Community Med, N-9037 Tromso, Norway.

RP Sheikh, MA (reprint author), Univ Tromso, Dept Community Med, N-9037 Tromso, Norway.

EM mashhood.a.sheikh@uit.no

OI Sheikh, Mashhood Ahmed/0000-0002-3212-592X

CR Anda R. F., 2006, EUR ARCH PSYCHIAT CL, V256

Baumeister D, 2016, MOL PSYCHIATR, V21, P642, DOI 10.1038/mp.2015.67

Baumeister D, 2014, SOC PSYCH PSYCH EPID, V49, P841, DOI 10.1007/s00127-014-0887-z

Blanchette CM, 2008, ANN EPIDEMIOL, V18, P316, DOI 10.1016/j.annepidem.2007.11.004

Cerda M, 2012, DEPRESS ANXIETY, V29, P359, DOI 10.1002/da.21947

Cigolle C. T., 2016, J GERONTOL B-PSYCHOL

Coates D, 2010, AUST SOC WORK, V63, P391, DOI 10.1080/0312407X.2010.508533

Coccaro EF, 2014, JAMA PSYCHIAT, V71, P158, DOI 10.1001/jamapsychiatry.2013.3297

Coelho R, 2014, ACTA PSYCHIAT SCAND, V129, P180, DOI 10.1111/acps.12217

Cohen HW, 2000, AM J MED, V108, P2, DOI 10.1016/S0002-9343(99)00301-0

Correll CU, 2017, WORLD PSYCHIATRY, V16, P163, DOI 10.1002/wps.20420

Correll CU, 2015, WORLD PSYCHIATRY, V14, P119, DOI 10.1002/wps.20204

COUPLAND C, 2011, BMJ, V343

Danese A, 2012, PHYSIOL BEHAV, V106, P29, DOI 10.1016/j.physbeh.2011.08.019

De Bellis MD, 2014, CHILD ADOL PSYCH CL, V23, P185, DOI 10.1016/j.chc.2014.01.002

Dortland AKBV, 2010, ACTA PSYCHIAT SCAND, V122, P30, DOI 10.1111/j.1600-0447.2010.01565.x

Duncan AE, 2015, PREV CHRONIC DIS, V12, DOI 10.5888/pcd12.140434

Fagundes CP, 2013, BRAIN BEHAV IMMUN, V27, P8, DOI 10.1016/j.bbi.2012.06.014

Felitti VJ, 1998, AM J PREV MED, V14, P245, DOI 10.1016/S0749-3797(98)00017-8

Fosgate GT, 2006, EMERG THEMES EPIDEMI, V3, DOI 10.1186/1742-7622-3-7

Gee DG, 2015, NEUROBIOL STRESS, V1, P184, DOI 10.1016/j.ynstr.2015.02.001

Heffner KL, 2011, IMMUNOL ALLERGY CLIN, V31, P95, DOI 10.1016/j.iac.2010.09.005

HELLHAMMER DH, 1993, PSYCHOTHER PSYCHOSOM, V60, P8, DOI 10.1159/000288675

Hippisley-Cox J, 2001, BRIT MED J, V323, P666, DOI 10.1136/bmj.323.7314.666

Hostinar CE, 2017, PSYCHONEUROENDOCRINO, V75, P152, DOI 10.1016/j.psyneuen.2016.10.026

Huang KL, 2017, J PSYCHOPHARMACOL, V31, P1544, DOI 10.1177/0269881117714047

Jacobsen BK, 2012, INT J EPIDEMIOL, V41, P961, DOI 10.1093/ije/dyr049

Joung KE, 2014, J CLIN ENDOCR METAB, V99, pE1055, DOI 10.1210/jc.2013-3669

Kalmakis KA, 2015, J AM ASSOC NURSE PRA, V27, P457, DOI 10.1002/2327-6924.12215

Kaufman Jay S, 2004, Epidemiol Perspect Innov, V1, P4, DOI 10.1186/1742-5573-1-4

Kaufman JS, 2009, INT J EPIDEMIOL, V38, P845, DOI 10.1093/ije/dyp163

Lorton D, 2006, NEUROIMMUNOMODULAT, V13, P357, DOI 10.1159/000104864

McEwen BS, 2000, NEUROPSYCHOPHARMACOL, V22, P108, DOI 10.1016/S0893-133X(99)00129-3

Mock S. E., 2011, FRONT PSYCHOL, V1

Naimi AI, 2014, INT J EPIDEMIOL, V43, P1656, DOI 10.1093/ije/dyu107

Nusslock R, 2016, BIOL PSYCHIAT, V80, P23, DOI 10.1016/j.biopsych.2015.05.017

Pan A, 2010, ARCH INTERN MED, V170, P1884, DOI 10.1001/archinternmed.2010.356

Plaza A, 2012, PSYCHIAT RES, V200, P329, DOI 10.1016/j.psychres.2012.06.032

Post RM, 2013, J AFFECT DISORDERS, V147, P288, DOI 10.1016/j.jad.2012.11.020

Procaccini C, 2014, FRONT IMMUNOL, V5, DOI 10.3389/fimmu.2014.00143

Rich-Edwards JW, 2010, AM J PREV MED, V39, P529, DOI 10.1016/j.amepre.2010.09.007

Rubin RR, 2008, DIABETES CARE, V31, P420, DOI 10.2337/dc07-182T

Scott KM, 2011, ARCH GEN PSYCHIAT, V68, P838, DOI 10.1001/archgenpsychiatry.2011.77

Sheikh MA, 2018, J PSYCHIATR RES, V103, P173, DOI 10.1016/j.jpsychires.2018.05.027

Sheikh MA, 2018, EUR CHILD ADOLES PSY, V27, P481, DOI 10.1007/s00787-017-1090-z

Sheikh MA, 2018, J PSYCHOSOM RES, V108, P78, DOI 10.1016/j.jpsychores.2018.03.001

Sheikh MA, 2018, J PSYCHIATR RES, V101, P50, DOI 10.1016/j.jpsychires.2018.02.025

Sheikh MA, 2018, INT J PUBLIC HEALTH, V63, P181, DOI 10.1007/s00038-017-1021-2

Sheikh MA, 2018, J AFFECT DISORDERS, V229, P206, DOI 10.1016/j.jad.2017.12.051

Sheikh MA, 2018, J AFFECT DISORDERS, V226, P21, DOI 10.1016/j.jad.2017.09.015

Sheikh MA, 2014, LIPIDS HEALTH DIS, V13, DOI 10.1186/1476-511X-13-164

Shields ME, 2016, BMC PUBLIC HEALTH, V16, DOI 10.1186/s12889-016-3491-1

Slavich GM, 2010, NEUROSCI BIOBEHAV R, V35, P39, DOI 10.1016/j.neubiorev.2010.01.003

Smith Sean M, 2006, Dialogues Clin Neurosci, V8, P383

Springer KW, 2003, J GEN INTERN MED, V18, P864, DOI 10.1046/j.1525-1497.2003.20918.x

Tata LJ, 2005, HEART, V91, P465, DOI 10.1136/hrt.2004.037457

Teicher MH, 2013, AM J PSYCHIAT, V170, P1114, DOI 10.1176/appi.ajp.2013.12070957

Teicher MH, 2002, PSYCHIAT CLIN N AM, V25, P397, DOI 10.1016/S0193-953X(01)00003-X

Vancampfort D, 2015, WORLD PSYCHIATRY, V14, P339, DOI 10.1002/wps.20252

Vogelzangs N, 2013, TRANSL PSYCHIAT, V3, DOI 10.1038/tp.2013.27

Weis J. G., 1989, CRIME JUSTICE ANN RE, V11, P117

Yang YC, 2017, BIODEMOGR SOC BIOL, V63, P87, DOI 10.1080/19485565.2017.1279536

Yoon JM, 2013, KOREAN J FAM MED, V34, P228, DOI 10.4082/kjfm.2013.34.4.228

NR 63

TC 1

Z9 1

U1 105

U2 105

PU ELSEVIER SCIENCE BV

PI AMSTERDAM

PA PO BOX 211, 1000 AE AMSTERDAM, NETHERLANDS

SN 0165-0327

EI 1573-2517

J9 J AFFECT DISORDERS

JI J. Affect. Disord.

PD DEC 1

PY 2018

VL 241

BP 80

EP 85

DI 10.1016/j.jad.2018.07.085

PG 6

WC Clinical Neurology; Psychiatry

SC Neurosciences & Neurology; Psychiatry

GA GS6OK

UT WOS:000443816800011

PM 30099267

DA 2019-01-08

ER

PT J

AU Noser, AE

Majidi, S

Finch, J

Clements, MA

Youngkin, EM

Patton, SR

AF Noser, Amy E.

Majidi, Shideh

Finch, Jonathan

Clements, Mark A.

Youngkin, Erin M.

Patton, Susana R.

TI Authoritarian parenting style predicts poorer glycemic control in

children with new-onset type 1 diabetes

SO PEDIATRIC DIABETES

LA English

DT Article

DE child; hemoglobin A1c; new onset; parenting; type 1 diabetes mellitus

ID ASSOCIATION; ADOLESCENTS; YOUTH; STATEMENT; DISTRESS; PATTERNS;

BEHAVIOR; PROGRAM; SUPPORT; MOTHERS

AB Objective: To examine cross-sectional and longitudinal associations among parenting styles (ie, authoritative, authoritarian, and permissive) and youth glycated hemoglobin (HbA1c) in a cohort of families of children with new-onset type 1 diabetes (T1D).

Methods: One-hundred two parents completed a baseline measure of parenting style, and we collected child HbA1c values at baseline and at three- and six-month follow-ups. We examined correlations among use of different parenting strategies and child HbA1cs. We conducted multiple regressions to assess the impact of these strategies on child HbA1c at three-month and six-month follow-ups, while controlling for baseline HbA1c, family income, and T1D duration.

Results: Correlational analyses showed negative associations between authoritative strategies and child HbA1c at baseline, three-month, and six-month assessments and positive associations between authoritarian strategies and child HbA1c at three-month and six-month assessments. Regression analyses found use of authoritarian-like strategies were the only parenting strategies associated with child HbA1c at three-month and six-month follow-ups, while controlling for baseline HbA1c, family income, and T1D duration.

Conclusion: Parents' use of authoritarian-like strategies may negatively impact glycemic control over the course of six-month in children with new-onset T1D.

C1 [Noser, Amy E.] Univ Kansas, Clin Child Psychol Program, Lawrence, KS 66045 USA.

[Noser, Amy E.; Finch, Jonathan; Clements, Mark A.; Patton, Susana R.] Childrens Mercy Kansas City, Ctr Childrens Hlth Lifestyles & Nutr, Kansas City, MO USA.

[Majidi, Shideh; Youngkin, Erin M.] Univ Colorado, Sch Med, Barbara Davis Ctr Childhood Diabet, Div Endocrinol, Aurora, CO USA.

[Finch, Jonathan; Patton, Susana R.] Univ Kansas, Med Ctr, Dept Pediat, MS 4004,3901 Rainbow Blvd, Kansas City, KS 66160 USA.

[Clements, Mark A.] Childrens Mercy Kansas City, Div Endocrinol & Diabet, Dept Pediat, Kansas City, MO USA.

RP Patton, SR (reprint author), Univ Kansas, Med Ctr, Dept Pediat, MS 4004,3901 Rainbow Blvd, Kansas City, KS 66160 USA.

EM spatton2@kumc.edu

FU National Institute of Diabetes and Digestive and Kidney Diseases [R01

DK100779]

FX National Institute of Diabetes and Digestive and Kidney Diseases,

Grant/Award Number: R01 DK100779

CR Anderson BJ, 2011, DIABETES CARE, V34, P1885, DOI 10.2337/dc11-1028

BAUMRIND D, 1966, CHILD DEV, V37, P887, DOI 10.2307/1126611

BAUMRIND D, 1971, DEV PSYCHOL, V4, P1, DOI 10.1037/h0030372

BAUMRIND D, 1965, CHILDREN, V12, P230

Baumrind D, 2005, NEW DIR CHILD ADOLES, V108, P61, DOI 10.1002/cd.128

Butler DA, 2008, PEDIATR DIABETES, V9, P373, DOI 10.1111/j.1399-5448.2008.00370.x

Chiang JL, 2014, DIABETES CARE, V37, P2034, DOI 10.2337/dc14-1140

Davis CL, 2001, J PEDIATR PSYCHOL, V26, P123, DOI 10.1093/jpepsy/26.2.123

Delamater AM, 2009, PEDIATR DIABETES, V10, P175, DOI 10.1111/j.1399-5448.2009.00580.x

Duke DC, 2008, J PEDIATR PSYCHOL, V33, P719, DOI 10.1093/jpepsy/jsn012

Forgatch MS, 1999, J CONSULT CLIN PSYCH, V67, P711, DOI 10.1037/0022-006X.67.5.711

Gallegos-Macias Angela R, 2003, Pediatr Diabetes, V4, P19, DOI 10.1034/j.1399-5448.2003.00020.x

Gandhi KK, 2016, PEDIATR RES, V80, P347, DOI 10.1038/pr.2016.87

Greene MS, 2010, DIABETES EDUCATOR, V36, P326, DOI 10.1177/0145721710361270

Gruhn MA, 2016, HEALTH PSYCHOL, V35, P652, DOI 10.1037/hea0000349

Harris MA, 2001, DIABETES SPECTRUM, V14, P182

Haugstvedt A, 2011, PEDIATR DIABETES, V12, P107, DOI 10.1111/j.1399-5448.2010.00661.x

Hessler D, 2016, J PEDIATR PSYCHOL, V41, P750, DOI 10.1093/jpepsy/jsw002

Jaser S, 2010, J PEDIATR PSYCHOL, V35, P977

Langer SL, 2017, APPETITE, V110, P72, DOI 10.1016/j.appet.2016.12.011

Langer SL, 2014, J PEDIATR PSYCHOL, V39, P633, DOI 10.1093/jpepsy/jsu021

Mlynarczyk Susan M, 2013, Pediatr Nurs, V39, P181

Monaghan M, 2012, J CLIN PSYCHOL MED S, V19, P255, DOI 10.1007/s10880-011-9284-x

Pierce JS, 2017, PEDIATR DIABETES, V18, P17, DOI 10.1111/pedi.12351

Radcliff Z, 2018, J PEDIATR PSYCHOL, V43, P185, DOI 10.1093/jpepsy/jsx107

Redondo MJ, 2014, PEDIATR DIABETES, V15, P294, DOI 10.1111/pedi.12061

Roberts L, 2018, APPETITE, V120, P318, DOI 10.1016/j.appet.2017.09.024

Robinson C. C., 2001, HDB FAMILY MEASUREME, V3, P319

Rodriguez MMD, 2009, FAM PROCESS, V48, P195

Sanders MR, 2014, CLIN PSYCHOL REV, V34, P337, DOI 10.1016/j.cpr.2014.04.003

Shah R, 2016, PEDIATRICS, V137, DOI 10.1542/peds.2015-3393

Sherifali D, 2009, DIABETES EDUCATOR, V35, P476, DOI 10.1177/0145721709333268

Silverstein J, 2005, DIABETES CARE, V28, P186, DOI 10.2337/diacare.28.1.186

Snorer M, 2011, DIABETES CARE, V34, P1735, DOI 10.2337/dc10-1602

Sweenie R, 2014, FAM SYST HEALTH, V32, P31, DOI 10.1037/fsh0000001

Topham GL, 2010, PUBLIC HEALTH NUTR, V13, P1237, DOI 10.1017/S1368980009992163

Weissberg-Benchell J, 2009, J PEDIATR PSYCHOL, V34, P977, DOI 10.1093/jpepsy/jsp003

Zhang HY, 2015, J CHILD FAM STUD, V24, P1886, DOI 10.1007/s10826-014-9990-8

NR 38

TC 0

Z9 0

U1 7

U2 7

PU WILEY

PI HOBOKEN

PA 111 RIVER ST, HOBOKEN 07030-5774, NJ USA

SN 1399-543X

EI 1399-5448

J9 PEDIATR DIABETES

JI Pediatr. Diabetes

PD NOV

PY 2018

VL 19

IS 7

BP 1315

EP 1321

DI 10.1111/pedi.12726

PG 7

WC Endocrinology & Metabolism; Pediatrics

SC Endocrinology & Metabolism; Pediatrics

GA GW0NS

UT WOS:000446564900024

PM 30014608

DA 2019-01-08

ER

PT J

AU Forlenza, GP

Messer, LH

Berget, C

Wadwa, RP

Driscoll, KA

AF Forlenza, Gregory P.

Messer, Laurel H.

Berget, Cari

Wadwa, R. Paul

Driscoll, Kimberly A.

TI Biopsychosocial Factors Associated With Satisfaction and Sustained Use

of Artificial Pancreas Technology and Its Components: a Call to the

Technology Field

SO CURRENT DIABETES REPORTS

LA English

DT Review

DE Type 1 diabetes; Artificial pancreas; Insulin pumps; Continuous glucose

monitoring; Behavioral health

ID INSULIN PUMP THERAPY; QUALITY-OF-LIFE; CONTINUOUS GLUCOSE MONITORS;

RANDOMIZED CONTROLLED-TRIAL; ADVERSE EVENTS; CLINICAL-TRIAL; GLYCEMIC

CONTROL; DELIVERY-SYSTEM; PREGNANT-WOMEN; ADULTS

AB Purpose of Review Summarize biopsychosocial factors associated with using continuous glucose monitors (CGMs), insulin pumps, and artificial pancreas (AP) systems and provide a "call to the field" about their importance to technology uptake and maintained use.

Recent Findings Insulin pumps and CGMs are becoming standard of care for individuals with type 1 diabetes (T1D). AP systems combining a CGM, insulin pump, and automated dosing algorithm are available for commercial use. Despite improved glycemic control with AP system use, numerous barriers exist which may limit their benefit. Studies on components of AP systems (pumps, CGMs) are limited and demonstrate mixed results of their impact on fear of hypoglycemia, adherence, quality of life, depression and anxiety, and diabetes distress. Studies examining biopsychological factors associated specifically with sustained use of AP systems are also sparse.

Summary Biological, psychological and social impacts of AP systems have been understudied and the information they provide has not been capitalized upon.

C1 [Forlenza, Gregory P.; Messer, Laurel H.; Berget, Cari; Wadwa, R. Paul; Driscoll, Kimberly A.] Univ Colorado Denver, Barbara Davis Ctr, 1775 Aurora CT MS A140, Aurora, CO 80045 USA.

RP Driscoll, KA (reprint author), Univ Colorado Denver, Barbara Davis Ctr, 1775 Aurora CT MS A140, Aurora, CO 80045 USA.

EM gregory.forlenza@ucdenver.edu; laurel.messer@ucdnver.edu;

cari.berget@ucdenver.edu; paul.wadwa@ucdenver.edu;

kimberly.driscoll@ucdenver.edu

FU NIH K12 grant [K12DK094712-06]

FX Dr. Forlenza's work on this project was funded in part by an NIH K12

grant (K12DK094712-06).

CR Barnard KD, 2007, DIABETIC MED, V24, P607, DOI 10.1111/j.1464-5491.2007.02120.x

Barnard Katharine D, 2015, J Diabetes Sci Technol, V9, P925, DOI 10.1177/1932296815588332

Battelino T, 2012, DIABETOLOGIA, V55, P3155, DOI 10.1007/s00125-012-2708-9

Beck RW, 2017, ANN INTERN MED, V167, P365, DOI 10.7326/M16-2855

Beck RW, 2017, LANCET DIABETES ENDO, V5, P700, DOI 10.1016/S2213-8587(17)30217-6

Beck RW, 2017, JAMA-J AM MED ASSOC, V317, P371, DOI 10.1001/jama.2016.19975

Beck RW, 2010, DIABETES CARE, V33, P2175, DOI 10.2337/dc10-0331

Beck RW, 2009, DIABETES CARE, V32, P1947, DOI 10.2337/dc09-0889

Bekiari E, 2018, BMJ-BRIT MED J, V361, DOI 10.1136/bmj.k1310

Bequette B Wayne, 2013, J Diabetes Sci Technol, V7, P1632

Berg AK, 2018, PEDIATR DIABETES, V19, P733, DOI 10.1111/pedi.12652

Bergenstal RM, 2016, JAMA-J AM MED ASSOC, V316, P1407, DOI 10.1001/jama.2016.11708

Bergenstal RM, 2014, DIABETES CARE, V37, P659, DOI 10.2337/dc12-2621

Berget C, 2018, DIABETES, V67, pA254

Binder E, 2015, EXP CLIN ENDOCR DIAB, V123, P260, DOI 10.1055/s-0034-1394381

Cemeroglu AP, 2010, PEDIATR DIABETES, V11, P182, DOI 10.1111/j.1399-5448.2009.00549.x

Chase HP, 2010, DIABETES TECHNOL THE, V12, P507, DOI 10.1089/dia.2010.0021

Christiansen M, 2013, DIABETES TECHNOL THE, V15, P881, DOI 10.1089/dia.2013.0077

Cope Judith U, 2012, J Diabetes Sci Technol, V6, P1053

Datye Karishma A, 2018, J Diabetes Sci Technol, V12, P349, DOI 10.1177/1932296817728525

DeSalvo DJ, 2018, PEDIAT DIABETES

Doyle FJ, 2014, DIABETES CARE, V37, P1191, DOI 10.2337/dc13-2108

Driscoll KA, 2017, J PEDIAT PSYCHOL

Driscoll Kimberly A, 2016, J Diabetes Sci Technol, V10, P724, DOI 10.1177/1932296815623568

Driscoll Kimberly A, 2013, J Diabetes Sci Technol, V7, P898

Driscoll Kimberly A, 2013, J Diabetes Sci Technol, V7, P646

Driscoll KA, 2011, DIABETES CARE, V34, P2170, DOI 10.2337/dc11-0388

Englert Kimberly, 2014, J Diabetes Sci Technol, V8, P745, DOI 10.1177/1932296814529893

Facchinetti A, 2016, SENSORS-BASEL, V16, DOI 10.3390/s16122093

Faccioli Simone, 2017, J Diabetes Sci Technol, V11, P1147, DOI 10.1177/1932296817706377

Farrington C, 2017, LANCET DIABETES ENDO, V5, P332, DOI 10.1016/S2213-8587(16)30397-7

Feig DS, 2017, LANCET, V390, P2347, DOI 10.1016/S0140-6736(17)32400-5

Fisher L, 2018, DIABETES CARE, V41, P1862, DOI 10.2337/dc18-0391

Forlenza GP, 2018, DIABETES, V67, pW204

Forlenza GP, 2018, DIABETES TECHNOL THE, V20, P335, DOI 10.1089/dia.2017.0424

Forlenza GP, 2016, J PEDIATR-US, V169, P13, DOI 10.1016/j.jpeds.2015.10.015

Garg SK, 2017, DIABETES TECHNOL THE, V19, P155, DOI 10.1089/dia.2016.0421

Garmo A, 2013, DIABETIC MED, V30, P717, DOI 10.1111/dme.12155

Giani Elisa, 2017, J Diabetes Sci Technol, V11, P476, DOI 10.1177/1932296816676280

Heinemann Lutz, 2012, J Diabetes Sci Technol, V6, P954

Heller S, 2017, HEALTH TECHNOL ASSES, V21, P1, DOI 10.3310/hta21200

Hermanides Jeroen, 2011, Evid Based Med, V16, P46, DOI 10.1136/ebm1159

Hilliard ME, 2009, J PEDIATR PSYCHOL, V34, P137, DOI 10.1093/jpepsy/jsn062

Hirose M, 2012, CURR DIABETES REP, V12, P711, DOI 10.1007/s11892-012-0313-4

Hirsch IB, 2008, DIABETES TECHNOL THE, V10, P377, DOI 10.1089/dia.2008.0068

Hirsch IB, 2009, J CLIN ENDOCR METAB, V94, P2232, DOI 10.1210/jc.2008-2625

Hommel E, 2014, ACTA DIABETOL, V51, P845, DOI 10.1007/s00592-014-0598-7

Iturralde E, 2017, DIABETES EDUCATOR, V43, P223, DOI 10.1177/0145721717697244

Ives B, 2010, DIABETES EDUCATOR, V36, P53, DOI 10.1177/0145721709352010

James Steven, 2016, J Diabetes Sci Technol, V10, P1115, DOI 10.1177/1932296816660326

Kerr David, 2013, J Diabetes Sci Technol, V7, P1595

Kovatchev B, 2008, DIABETES CARE, V31, P1160, DOI 10.2337/dc07-2401

Kropff J, 2017, DIABETIC MED, V34, P262, DOI 10.1111/dme.13268

Laffel L, 2016, DIABETES TECHNOL THE, V18, P23, DOI 10.1089/dia.2015.0380

Lind M, 2017, JAMA-J AM MED ASSOC, V317, P379, DOI 10.1001/jama.2016.19976

Markowitz JT, 2012, DIABETES TECHNOL THE, V14, P523, DOI 10.1089/dia.2011.0201

Mauras N, 2012, DIABETES CARE, V35, P204, DOI 10.2337/dc11-1746

Messer LH, 2018, DIABETIC MED, V35, P409, DOI 10.1111/dme.13568

Messer L, 2009, DIABETES EDUCATOR, V35, P124, DOI 10.1177/0145721708325157

Messer LH, 2018, DIABETES TECHNOL THE, V20, P54, DOI 10.1089/dia.2018.0080

Messer LH, 2018, DIABETES CARE, V41, P789, DOI 10.2337/dc17-1682

Messer LH, 2018, PEDIATR DIABETES, V19, P14, DOI 10.1111/pedi.12535

Miller KM, 2015, DIABETES CARE, V38, P971, DOI 10.2337/dc15-0078

Nimri R, 2006, PEDIATRICS, V117, P2126, DOI 10.1542/peds.2005-2621

Nuboer R, 2008, PEDIATR DIABETES, V9, P291, DOI 10.1111/j.1399-5448.2008.00396.x

O'Connell MA, 2011, PEDIATR DIABETES, V12, P556, DOI 10.1111/j.1399-5448.2010.00740.x

Patton Susana R, 2017, J Diabetes Sci Technol, V11, P87, DOI 10.1177/1932296816658901

Patton Susana R, 2016, J Diabetes Sci Technol, V10, P656, DOI 10.1177/1932296816638109

Petrie JR, 2017, DIABETOLOGIA, V60, P2319, DOI 10.1007/s00125-017-4463-4

Pickup JC, 2015, DIABETES CARE, V38, P544, DOI 10.2337/dc14-1855

Polonsky WH, 2017, DIABETES CARE, V40, P736, DOI 10.2337/dc17-0133

Polonsky WH, 2017, DIABETES TECHNOL THE, V19, P131, DOI 10.1089/dia.2016.0366

Polonsky WH, 2013, DIABETES TECHNOL THE, V15, P295, DOI 10.1089/dia.2012.0298

Rashotte J, 2014, CAN J DIABETES, V38, P256, DOI 10.1016/j.jcjd.2014.02.002

Riddlesworth T, 2017, DIABETES THER, V8, P947, DOI 10.1007/s13300-017-0281-4

Ritholz MD, 2010, DIABETIC MED, V27, P1060, DOI 10.1111/j.1464-5491.2010.03061.x

Rodbard D, 2016, DIABETES TECHNOL THE, V18, P3, DOI 10.1089/dia.2015.0417

Ross PL, 2015, ACTA DIABETOL, V52, P1017, DOI 10.1007/s00592-015-0784-2

Rubin RR, 2012, DIABETES TECHNOL THE, V14, P143, DOI 10.1089/dia.2011.0162

Rubin Richard R, 2009, J Diabetes Sci Technol, V3, P1402

Ruedy Katrina J, 2017, J Diabetes Sci Technol, V11, P1138, DOI 10.1177/1932296817704445

Ruiz Jessica L, 2012, J Diabetes Sci Technol, V6, P1123

Secher AL, 2014, DIABETIC MED, V31, P352, DOI 10.1111/dme.12383

Seereiner S, 2010, DIABETES TECHNOL THE, V12, P89, DOI 10.1089/dia.2009.0080

Shah VN, 2018, DIABETES TECHNOL THE, V20, P428, DOI 10.1089/dia.2018.0143

Sherr JL, 2016, DIABETOLOGIA, V59, P87, DOI 10.1007/s00125-015-3790-6

Shivers Joseph P, 2013, J Diabetes Sci Technol, V7, P789

Steil Garry M, 2013, J Diabetes Sci Technol, V7, P1621

Tanenbaum Molly L, 2017, J Diabetes Sci Technol, V11, P484, DOI 10.1177/1932296816688010

Tanenbaum ML, 2017, DIABETES CARE, V40, P181, DOI 10.2337/dc16-1536

Vloemans AF, 2017, DIABETIC MED, V34, P1470, DOI 10.1111/dme.13429

Wadwa RP, 2018, DIABETES TECHNOL THE, V20, P395, DOI 10.1089/dia.2018.0150

Wadwa RP, 2009, DIABETES TECHNOL THE, V11, pS83, DOI 10.1089/dia.2008.0122

Weinzimer S, 2010, DIABETES CARE, V33, P17, DOI 10.2337/dc09-1502

Weisman A, 2017, LANCET DIABETES ENDO, V5, P501, DOI 10.1016/S2213-8587(17)30167-5

Weissberg-Benchell J, 2003, DIABETES CARE, V26, P1079, DOI 10.2337/diacare.26.4.1079

Weissberg-Benchell Jill, 2016, J Diabetes Sci Technol, V10, P799, DOI 10.1177/1932296815619637

Wheeler BJ, 2014, DIABETES TECHNOL THE, V16, P558, DOI 10.1089/dia.2013.0388

Wheeler BJ, 2014, DIABETES TECHNOL THE, V16, P204, DOI 10.1089/dia.2013.0315

Wong Jenise C, 2017, J Diabetes Sci Technol, V11, P224, DOI 10.1177/1932296816663963

Wong JC, 2015, PEDIATR DIABETES, V16, P592, DOI 10.1111/pedi.12221

Wysocki T, 2016, DIABETES EDUCATOR, V42, P395, DOI 10.1177/0145721716647492

NR 102

TC 0

Z9 0

U1 4

U2 4

PU CURRENT MEDICINE GROUP

PI PHILADELPHIA

PA 400 MARKET STREET, STE 700, PHILADELPHIA, PA 19106 USA

SN 1534-4827

EI 1539-0829

J9 CURR DIABETES REP

JI Curr. Diabetes Rep.

PD NOV

PY 2018

VL 18

IS 11

AR 114

DI 10.1007/s11892-018-1078-1

PG 10

WC Endocrinology & Metabolism

SC Endocrinology & Metabolism

GA GV5IZ

UT WOS:000446136300002

PM 30259309

DA 2019-01-08

ER

PT J

AU Hosseini, E

Janghorbani, M

Shahshahan, Z

AF Hosseini, Elham

Janghorbani, Mohsen

Shahshahan, Zahra

TI Comparison of risk factors and pregnancy outcomes of gestational

diabetes mellitus diagnosed during early and late pregnancy

SO MIDWIFERY

LA English

DT Article

DE Early screening; Gestational diabetes; Pregnancy outcomes

ID FASTING PLASMA-GLUCOSE; 1ST PRENATAL VISIT; HYPERGLYCEMIA; IRAN

AB Objective: To compare risk factors and pregnancy outcomes of gestational diabetes mellitus (GDM) diagnosed during early and late pregnancy.

Design: Prospective population-based cohort study.

Setting: Community health care centers of Isfahan, Iran.

Participants and measurements: 1000 pregnant women who were eligible and consented to participate underwent fasting plasma glucose testing at the first prenatal visit (6-14 weeks). The women free from GDM or overt diabetes were screened at 24-28 weeks of gestation using a 75-g, 2-hour oral glucose tolerance test. The diagnosis of GDM was reached through the International Association of the Diabetes and Pregnancy Study Groups. Early-onset GDM was defined as the diagnosis of GDM at the first prenatal visit. Late-onset GDM was defined as the diagnosis of GDM later at 24-28 weeks.

Findings: Prevalence of GDM was 10% (95% CI: 8.1-11.9) at the first prenatal visit. GDM incidence was 9.3% (95% CI: 7.4-11.2) at 24-28 weeks of gestation. Family history of diabetes, and previous gestational diabetes and maternal age were the independent risk factors for GDM during early and late diagnosis. GDM was associated with increased risk of macrosomia, large for gestational age, and cesarean section in both periods while, neonates of women with early-onset GDM were more likely to have an apgar score at 1-min < 7, and neonatal respiratory distress syndrome and were more admitted to the neonatal intensive care unit.

Key conclusion and implication for practice: Despite early screening and current practice management, early-onset GDM was associated with poorer pregnancy outcomes compared to the late-onset group. Women with early-onset GDM would benefit from more strict surveillance and management strategies to improve pregnancy outcomes. Further studies are needed to evaluate the efficacy of alternative management approaches in these high risk women.

C1 [Hosseini, Elham; Janghorbani, Mohsen] Isfahan Univ Med Sci, Isfahan Endocrine & Metab Res Ctr, Esfahan, Iran.

[Shahshahan, Zahra] Isfahan Univ Med Sci, Sch Med, Dept Obstet & Gynecol, Esfahan, Iran.

RP Janghorbani, M (reprint author), Isfahan Univ Med Sci, Isfahan Endocrine & Metab Res Ctr, Esfahan, Iran.

EM janghorbani@hlth.mui.ac.ir; shahshahan@med.mui.ac.ir

FU Elite Researcher Grant Committee from the National Institutes of Medical

Research Development (NIMAD), Tehran, Iran [963232]

FX This study was supported by the Elite Researcher Grant Committee under

award number 963232 from the National Institutes of Medical Research

Development (NIMAD), Tehran, Iran.

CR Agarwal MM, 2016, WORLD J DIABETES, V7, P279, DOI 10.4239/wjd.v7.i14.279

Ainsworth BE, 2000, MED SCI SPORT EXER, V32, pS498, DOI 10.1097/00005768-200009001-00009

Amer Diabet Assoc, 2018, DIABETES CARE, V41, pS13, DOI 10.2337/dc18-S002

Bartha JL, 2000, AM J OBSTET GYNECOL, V182, P346, DOI 10.1016/S0002-9378(00)70222-5

Bartha JL, 2003, EUR J OBSTET GYN R B, V109, P41, DOI 10.1016/S0301-2115(02)00480-3

Bunthalarath Sittiwate, 2004, Journal of the Medical Association of Thailand, V87, pS50

Fenton TR, 2013, BMC PEDIATR, V13, DOI 10.1186/1471-2431-13-59

Hawkins J. S, 2008, AM J OBSTET GYNECOL, V198

Hollander MH, 2007, OBSTET GYNECOL SURV, V62, P125, DOI 10.1097/01.ogx.0000253303.92229.59

Jafari-Shobeiri M, 2015, IRAN J PUBLIC HEALTH, V44, P1036

Jain R, 2014, INT J MED SCI PUBLIC, V3, P1283

Keshavarz M, 2005, DIABETES RES CLIN PR, V69, P279, DOI 10.1016/j.diabres.2005.01.011

McIntyre HD, 2016, DIABETES CARE, V39, P53, DOI 10.2337/dc15-1887

Meek CL, 2015, DIABETOLOGIA, V58, P2003, DOI 10.1007/s00125-015-3647-z

Metzger BE, 2008, NEW ENGL J MED, V358, P1991, DOI 10.1056/NEJMoa0707943

Metzger BE, 2007, DIABETES CARE, V30, pS251, DOI 10.2337/dc07-s225

Metzger BE, 2010, DIABETES CARE, V33, P676, DOI 10.2337/dc09-1848

Moosazadeh M, 2017, DIABETES METAB SYND, V11, pS99, DOI 10.1016/j.dsx.2016.12.016

Oster RT, 2014, BMC PREGNANCY CHILDB, V14, DOI 10.1186/1471-2393-14-136

Page RCL, 1996, DIABETIC MED, V13, P170, DOI 10.1002/(SICI)1096-9136(199602)13:2<170::AID-DIA8>3.0.CO;2-4

Riskin-Mashiah S, 2009, DIABETES CARE, V32, P1639, DOI 10.2337/dc09-0688

Sacks DA, 2003, OBSTET GYNECOL, V101, P1197, DOI 10.1016/S0029-7844(03)00049-8

Sweeting AN, 2016, DIABETES CARE, V39, P75, DOI 10.2337/dc15-0433

The Population Office FamilyHealth, 2016, NAT PROT PREGN

Vignoles P, 2011, ARCH GYNECOL OBSTET, V284, P1099, DOI 10.1007/s00404-010-1810-9

Wong SF, 2002, DIABETES CARE, V25, P1681, DOI 10.2337/diacare.25.10.1681

Zhu WW, 2013, DIABETES CARE, V36, P586, DOI 10.2337/dc12-1157

NR 27

TC 0

Z9 0

U1 25

U2 25

PU ELSEVIER SCI LTD

PI OXFORD

PA THE BOULEVARD, LANGFORD LANE, KIDLINGTON, OXFORD OX5 1GB, OXON, ENGLAND

SN 0266-6138

EI 1532-3099

J9 MIDWIFERY

JI Midwifery

PD NOV

PY 2018

VL 66

BP 64

EP 69

DI 10.1016/j.midw.2018.07.017

PG 6

WC Nursing

SC Nursing

GA GT3NC

UT WOS:000444409000009

PM 30130677

DA 2019-01-08

ER

PT J

AU Jeong, YM

Quinn, L

Kim, N

Martyn-Nemeth, P

AF Jeong, Yoo Mi

Quinn, Laurie

Kim, Nahyun

Martyn-Nemeth, Pamela

TI Health-Related Stigma in Young Adults With Type 1 Diabetes Mellitus

SO JOURNAL OF PSYCHOSOCIAL NURSING AND MENTAL HEALTH SERVICES

LA English

DT Article

ID LIFE-STYLE; CARE; RECOMMENDATIONS; TRANSITION; PERSPECTIVES;

ADOLESCENCE; CHALLENGES; EPIDEMIC; PEOPLE

AB Young adults with type 1 diabetes mellitus (T1DM) often exhibit poor treatment adherence and suboptimal glucose control. Health-related stigma may adversely influence diabetes self-management behavior. The aim of the current study was to explore health-related stigma among young adults with T1DM using qualitative descriptive methods in focus groups. Data were collected from a purposive sample of 14 young adults ages 20 to 34 with T1DM. Using content analysis, transcripts from two focus groups were analyzed, and five main themes were identified: (a) Desire to be Seen as a Person, Not a Disease; (b) Wanting to be "Normal"; (c) Feeling Ashamed Managing Diabetes in Public; (d) Struggling to Overcome Anger and Distress; and (e) Feeling Distrusted by Others to Manage Their Condition. Young adults with T1DM reported experiencing a high degree of stigma in their daily lives, which negatively influenced their self-management and psychological well-being. Stigma led to delaying blood glucose monitoring and insulin dosing and feelings of anger and distress. Young adults with T1DM require strategies to help cope with stigma and the anger and distress they experience.

C1 [Jeong, Yoo Mi] Konyang Univ, Coll Nursing, Daejun Si, South Korea.

[Kim, Nahyun] Keimyung Univ, Coll Nursing, Daegu Si, South Korea.

[Quinn, Laurie; Martyn-Nemeth, Pamela] Univ Illinois, Coll Nursing, Biobehav Hlth Sci, Chicago, IL USA.

RP Jeong, YM (reprint author), Konyang Univ, Coll Nursing 209, Gwanjeodonglo 158, Daejun Si 35365, Chungnam Do, South Korea.

EM yjeong20@konyang.ac.kr

FU University of Illinois at Chicago College of Nursing

FX The authors have disclosed no potential conflicts of interest, financial

or otherwise. Financial support was provided by internal funding

supplied by the University of Illinois at Chicago College of Nursing.

The authors thank the study participants for sharing their experience of

health-related stigma associated with type 1 diabetes mellitus. The

authors also acknowledge the time and expertise devoted to review by

Kevin Grandfield, Publication Manager of the University of Illinois at

Chicago Department of Biobehavioral Health Science.

CR Abdoli S, 2017, DIABETES EDUCATOR, V43, P206, DOI 10.1177/0145721717697245

Abdoli Samereh, 2013, Iran J Nurs Midwifery Res, V18, P65

Anderson BJ, 2004, PATIENT EDUC COUNS, V53, P347, DOI 10.1016/j.pec.2003.03.001

Arnett JJ, 2001, J ADULT DEV, V8, P133, DOI 10.1023/A:1026450103225

Balfe M, 2013, BMC ENDOCR DISORD, V13, DOI 10.1186/1472-6823-13-25

Berger BE, 2001, RES NURS HEALTH, V24, P518, DOI 10.1002/nur.10011

Beverly E. A., 2016, J CLIN OUTCOMES MANA, V23, P509

Browne JL, 2014, BMJ OPEN, V4, DOI 10.1136/bmjopen-2014-005625

Bryden KS, 2001, DIABETES CARE, V24, P1536, DOI 10.2337/diacare.24.9.1536

Celik Selda, 2015, Int J Community Based Nurs Midwifery, V3, P84

Dashiff C, 2009, CHILD CARE HLTH DEV, V35, P79, DOI 10.1111/j.1365-2214.2008.00892.x

Dashiff C, 2008, J ADV NURS, V62, P140, DOI 10.1111/j.1365-2648.2007.04549.x

DCCT Res Grp, 1994, J PEDIATR-US, V125, P177, DOI 10.1016/S0022-3476(94)70190-3

Dudley J., 1983, LIVING STIGMA PLIGHT

Freeborn D, 2013, J CLIN NURS, V22, P1890, DOI 10.1111/jocn.12046

Garvey K, 2011, DIABETES SPECTRUM, V24, P22, DOI [10.2337/diaspect.24.1.22, DOI 10.2337/DIASPECT.24.1.22]

Goffman E, 1963, STIGMA NOTES MANAGEM

Hapunda G, 2015, BMC ENDOCR DISORD, V15, DOI 10.1186/s12902-015-0013-6

Horowitz AD, 2007, YOUTH SOC, V39, P209, DOI 10.1177/0044118X06296692

Hsieh HF, 2005, QUAL HEALTH RES, V15, P1277, DOI 10.1177/1049732305276687

Lawrence SE, 2015, PAED CHILD HEALT-CAN, V20, P35, DOI 10.1093/pch/20.1.35

Leroux C, 2014, CAN J DIABETES, V38, P62, DOI 10.1016/j.jcjd.2013.08.268

Mahajan AP, 2008, AIDS, V22, pS67, DOI 10.1097/01.aids.0000327438.13291.62

Miller TA, 2013, DIABETES METAB SYNDR, V6, P421, DOI 10.2147/DMSO.S36368

Monaghan M, 2015, CURR DIABETES REV, V11, P239, DOI 10.2174/1573399811666150421114957

Nielsen SJ, 2004, AM J PREV MED, V27, P205, DOI 10.1016/j.amepre.2004.05.005

Onyeka TC, 2010, INDIAN J PALLIAT CAR, V16, P123, DOI 10.4103/0973-1075.73642

Paterson B, 1999, QUAL HEALTH RES, V9, P786, DOI 10.1177/104973299129122289

Pescosolido BA, 2015, ANNU REV SOCIOL, V41, P87, DOI 10.1146/annurev-soc-071312-145702

Peters A, 2011, DIABETES CARE, V34, P2477, DOI 10.2337/dc11-1723

Pitacinski S, 2014, ARCH MED SCI, V10, P124, DOI 10.5114/aoms.2014.40739

Pyatak EA, 2013, DIABETIC MED, V30, P1140, DOI 10.1111/dme.12248

Saarinen T, 2014, EUROPEAN DIABETES NU, V11, P38, DOI [10.1002/edn.246, DOI 10.1002/EDN.246, 10.1002/edn.246.]

Schabert J, 2013, PATIENT, V6, P1, DOI 10.1007/s40271-012-0001-0

Sparud-Lundin C, 2010, J ADV NURS, V66, P128, DOI 10.1111/j.1365-2648.2009.05166.x

Wahowiak L., 2015, DIABETES FORECAST, V68, P30

Young-Hyman D, 2016, DIABETES CARE, V39, P2126, DOI 10.2337/dc16-2053

NR 37

TC 0

Z9 0

U1 3

U2 3

PU SLACK INC

PI THOROFARE

PA 6900 GROVE RD, THOROFARE, NJ 08086 USA

SN 0279-3695

EI 1938-2413

J9 J PSYCHOSOC NURS MEN

JI J. Psychosoc. Nurs. Ment. Health Serv.

PD OCT

PY 2018

VL 56

IS 10

BP 44

EP 51

DI 10.3928/02793695-20180503-01

PG 8

WC Nursing

SC Nursing

GA GW1YX

UT WOS:000446679900008

PM 29741747

DA 2019-01-08

ER

PT J

AU Adams, RN

Tanenbaum, ML

Hanes, SJ

Ambrosino, JM

Ly, TT

Maahs, DM

Naranjo, D

Walders-Abramson, N

Weinzimer, SA

Buckingham, BA

Hood, KK

AF Adams, Rebecca N.

Tanenbaum, Molly L.

Hanes, Sarah J.

Ambrosino, Jodie M.

Ly, Trang T.

Maahs, David M.

Naranjo, Diana

Walders-Abramson, Natalie

Weinzimer, Stuart A.

Buckingham, Bruce A.

Hood, Korey K.

TI Psychosocial and Human Factors During a Trial of a Hybrid Closed Loop

System for Type 1 Diabetes Management

SO DIABETES TECHNOLOGY & THERAPEUTICS

LA English

DT Article

DE Type 1 diabetes; Artificial pancreas; Human factors

ID INSULIN DELIVERY-SYSTEM; GLYCEMIC CONTROL; ADULTS; ADOLESCENTS;

DEPRESSION; HYPOGLYCEMIA; DISTRESS; CHILDREN; FEAR

AB Background: Hybrid closed loop (HCL) systems are designed to automate insulin delivery to improve type 1 diabetes (T1D) outcomes and reduce user burden and distress. Because the systems only automate some aspects of diabetes care, psychosocial and human factors remain an important consideration in their use. Thus, we examined whether psychosocial and human factors (i.e., distress related to diabetes management, fear of hypoglycemia, and technology attitudes) would (1) change after using the system and (2) predict glycemic outcomes during the trial. Subjects and Methods: Fourteen adults and 15 adolescents with T1D participated in a multisite clinical trial of an investigational version of the MiniMed 670G system (Medtronic, Northridge, CA) over 4 to 5 days in a semisupervised outpatient setting. Users completed surveys assessing psychosocial and human factors before beginning the HCL system and at the conclusion of the study. t-Tests and regression analyses were conducted to examine whether these factors changed following trial exposure to the HCL system and predicted glycemic outcomes during the trial. Results: Diabetes management distress decreased and diabetes technology attitudes became more positive over the trial period. Fear of hypoglycemia did not change over the trial period. There was a trend toward greater pretrial management distress predicting less time in range during the trial, controlling for time in range before the trial. Conclusions: Results suggest that this system is promising for enhancing technology attitudes and reducing management distress. Psychosocial factors, such as management distress, may negatively impact glycemic outcomes and should be a priority area for further investigation.

C1 [Adams, Rebecca N.; Tanenbaum, Molly L.; Hanes, Sarah J.; Ly, Trang T.; Maahs, David M.; Naranjo, Diana; Buckingham, Bruce A.; Hood, Korey K.] Stanford Univ, Sch Med, Dept Pediat, 780 Welch Rd,MC 5776, Palo Alto, CA 94304 USA.

[Ambrosino, Jodie M.; Weinzimer, Stuart A.] Yale Univ, Sch Med, New Haven, CT USA.

[Ly, Trang T.] Univ Western Australia, Sch Paediat & Child Hlth, Crawley, WA, Australia.

[Walders-Abramson, Natalie] Univ Colorado, Barbara Davis Ctr Childhood Diabet, Aurora, CO USA.

RP Hood, KK (reprint author), Stanford Univ, Sch Med, Dept Pediat, 780 Welch Rd,MC 5776, Palo Alto, CA 94304 USA.

EM kkhood@stanford.edu

FU JDRF; Michael D. Ryan and Rosemary McNicholas Ryan Pediatric Diabetes

Research Fund

FX This work was supported by a grant from JDRF. Researchers at Yale were

also supported by the Michael D. Ryan and Rosemary McNicholas Ryan

Pediatric Diabetes Research Fund.

CR Barnard KD, 2015, DIABETIC MED, V32, P601, DOI 10.1111/dme.12706

Barnard Katharine D, 2017, J Diabetes Sci Technol, V11, P1080, DOI 10.1177/1932296817702656

Barnard KD, 2014, BMJ OPEN DIAB RES CA, V2, DOI 10.1136/bmjdrc-2014-000025

Bergenstal RM, 2016, JAMA-J AM MED ASSOC, V316, P1407, DOI 10.1001/jama.2016.11708

Farrington C, 2018, DIABETIC MED, V35, P436, DOI 10.1111/dme.13567

Fisher L, 2015, J DIABETES COMPLICAT, V29, P572, DOI 10.1016/j.jdiacomp.2015.01.012

Fisher L, 2013, DIABETES CARE, V36, P2551, DOI 10.2337/dc12-2493

Garg SK, 2017, DIABETES TECHNOL THE, V19, P155, DOI 10.1089/dia.2016.0421

Gonder-Frederick LA, 2011, DIABETES CARE, V34, P801, DOI 10.2337/dc10-1343

Gonzalez JS, 2008, DIABETES CARE, V31, P2398, DOI 10.2337/dc08-1341

Hilliard ME, 2018, PEDIATR DIABETES, V19, P534, DOI 10.1111/pedi.12575

Hood KK, 2006, DIABETES CARE, V29, P1389, DOI 10.2337/dc06-0087

Iturralde E, 2017, DIABETES EDUCATOR, V43, P223, DOI 10.1177/0145721717697244

Lustman PJ, 2000, DIABETES CARE, V23, P934, DOI 10.2337/diacare.23.7.934

Ly TT, 2017, PEDIATR DIABETES, V18, P348, DOI 10.1111/pedi.12399

Messer LH, 2018, DIABETES CARE, V41, P789, DOI 10.2337/dc17-1682

Naranjo Diana, 2016, J Diabetes Sci Technol, V10, P852, DOI 10.1177/1932296816650900

Pickup JC, 2015, DIABETES CARE, V38, P544, DOI 10.2337/dc14-1855

Quintal A, 2018, Diabetes Metab, DOI 10.1016/j.diabet.2018.04.003

Tanenbaum Molly L, 2017, J Health Psychol, p1359105317718615, DOI 10.1177/1359105317718615

Tanenbaum ML, 2017, DIABETES CARE, V40, P181, DOI 10.2337/dc16-1536

Trief PM, 2014, DIABETES CARE, V37, P1563, DOI 10.2337/dc13-1867

Vallis M, 2014, CURR DIABETES REV, V10, P364, DOI 10.2174/1573399810666141113115026

Weissberg-Benchell J, 2017, DIABETES TECHNOL THE, V19, P723, DOI 10.1089/dia.2017.0174

Wheeler BJ, 2014, DIABETES TECHNOL THE, V16, P558, DOI 10.1089/dia.2013.0388

Wild D, 2007, PATIENT EDUC COUNS, V68, P10, DOI 10.1016/j.pec.2007.05.003

Wong JC, 2015, PEDIATR DIABETES, V16, P592, DOI 10.1111/pedi.12221

Ziegler C, 2015, J DIABETES RES, V2015

NR 28

TC 0

Z9 0

U1 0

U2 0

PU MARY ANN LIEBERT, INC

PI NEW ROCHELLE

PA 140 HUGUENOT STREET, 3RD FL, NEW ROCHELLE, NY 10801 USA

SN 1520-9156

EI 1557-8593

J9 DIABETES TECHNOL THE

JI Diabetes Technol. Ther.

PD OCT

PY 2018

VL 20

IS 10

BP 648

EP 653

DI 10.1089/dia.2018.0174

EA SEP

EY 2018

PG 6

WC Endocrinology & Metabolism

SC Endocrinology & Metabolism

GA GV3NX

UT WOS:000445347300001

PM 30239219

DA 2019-01-08

ER

PT J

AU Staite, E

Zaremba, N

Macdonald, P

Allan, J

Treasure, J

Ismail, K

Stadler, M

AF Staite, E.

Zaremba, N.

Macdonald, P.

Allan, J.

Treasure, J.

Ismail, K.

Stadler, M.

TI "Diabulima' through the lens of social media: a qualitative review and

analysis of online blogs by people with Type 1 diabetes mellitus and

eating disorders

SO DIABETIC MEDICINE

LA English

DT Review

ID INSULIN RESTRICTION; MORTALITY; INFORMATION; OUTCOMES; COHORT; ADULTS

AB AimsTo perform a qualitative review of online blogs authored by people self-identifying as having Type 1 diabetes and an eating disorder or diabulimia', a term used by people with Type 1 diabetes to describe an eating disorder that is characterized by deliberate restriction of insulin to control weight.

MethodsWe conducted a structured qualitative review of online blogs published between 2012 and 2017 authored by people who report having Type 1 diabetes and an eating disorder or diabulimia. The subsequent thematic analysis followed a six-phase process and was conducted by two independent researchers.

ResultsFrom 147 000 search results, 11 blogs (304 posts) matched criteria for further analyses. Three key themes and 18 subthemes emerged: 1) different aspects of bloggers' relationship with insulin, including motives for omitting insulin, secrecy of insulin omission and perception of control; 2) bloggers' experiences of diabetes complications, and diabetes ketoacidosis in particular, as well as their worries about future complications; 3) strategies for recovery and triggers for relapse, which involved diabetes self-management and setting up a support system.

ConclusionsQualitative analyses of blogs authored by people with Type 1 diabetes and an eating disorder or diabulimia have identified high levels of diabetes distress and provided insight into different motives for insulin omission and strategies for recovery. Considering the limited evidence for effective interventions, these findings may help the development of complex interventions to improve biomedical and psychological outcomes in this group.

C1 [Staite, E.; Zaremba, N.; Ismail, K.] Kings Coll London, Dept Psychol Med, Weston Educ Ctr, London, England.

[Macdonald, P.; Treasure, J.] Kings Coll London, Inst Psychiat Psychol & Neurosci, Dept Psychol Med, London, England.

[Allan, J.] Birkbeck Univ London, London, England.

[Stadler, M.] Kings Coll London, Weston Educ Ctr, Diabet Res Grp, London, England.

RP Stadler, M (reprint author), Kings Coll London, Weston Educ Ctr, Diabet Res Grp, London, England.

EM Marietta.stadler@kcl.ac.uk

OI Zaremba, Natalie/0000-0002-1720-1621; ismail,

khalida/0000-0001-6084-449X; Staite, Emily Rose/0000-0003-0414-9892

FU King's Health Partners; King's College London; National Institute of

Health Research (NIHR) Mental Health Biomedical Research Centre at South

London and Maudsley NHS Foundation Trust and King's College London;

NIHR; Academy of Medical Sciences

FX E.S.'s salary was funded by King's Health Partners. N.Z.'s salary was

part funded by King's College London. Parenting leave funds awarded to

M.S., J.T. and K.I. are part funded by the National Institute of Health

Research (NIHR) Mental Health Biomedical Research Centre at South London

and Maudsley NHS Foundation Trust and King's College London. M.S. was

funded through an NIHR Academic Clinical Lecturership, NIHR Clinician

Scientist Fellowship and Academy of Medical Sciences Starter Grant for

Clinical Lecturers (2017). The views expressed are those of the

author(s) and not necessarily those of the NHS, the NIHR or the

Department of Health.

CR Allan JA, 2015, J DIABETES NURS, V19, P99

Anderson JG, 2017, J FAM NURS, V23, P34, DOI 10.1177/1074840716681289

[Anonymous], 1996, DIABETES, V45, P1289

[Anonymous], 2013, DIAGN STAT MAN MENT

Avena NM, 2012, NEUROPHARMACOLOGY, V63, P87, DOI 10.1016/j.neuropharm.2011.11.010

Braun V., 2008, QUALITATIVE RES PSYC, V3, P77, DOI DOI 10.1191/1478088706QP0630A

Clery P, 2017, DIABETIC MED, V34, P1667, DOI 10.1111/dme.13509

Davidson Jennifer, 2014, Nurs Stand, V29, P44, DOI 10.7748/ns.29.2.44.e7877

de Groot M, 2016, AM PSYCHOL, V71, P552, DOI 10.1037/a0040408

Dickinson JK, 2017, DIABETES CARE, V40, P1790, DOI 10.2337/dci17-0041

Eynon R., 2009, TWENTY FIRST CENTURY, V4, P187, DOI DOI 10.1080/17450140903000308

Feltbower RG, 2008, DIABETES CARE, V31, P922, DOI 10.2337/dc07-2029

Garbett K, 2017, J HEALTH PSYCHOL, V22, P1755, DOI 10.1177/1359105316638549

Goebel-Fabbri AE, 2008, DIABETES CARE, V31, P415, DOI 10.2337/dc07-2026

Goebel-Fabbri AE, 2011, DIABETES CARE, V34, P545, DOI 10.2337/dc10-1547

Griffiths S, 2015, INT J EAT DISORDER, V48, P775, DOI 10.1002/eat.22413

Hanna K M, 2000, Issues Compr Pediatr Nurs, V23, P193

Hookway N, 2008, QUAL RES, V8, P91, DOI 10.1177/1468794107085298

Iturralde E, 2017, HEALTH PSYCHOL, V36, P236, DOI 10.1037/hea0000445

Jones JM, 2000, BMJ-BRIT MED J, V320, P1563, DOI 10.1136/bmj.320.7249.1563

Merwin RM, 2015, DIABETES CARE, V38, P2025, DOI 10.2337/dc15-0753

Micali N, 2013, BMJ OPEN, V3, DOI 10.1136/bmjopen-2013-002646

Miura A, 2007, J COMPUT-MEDIAT COMM, V12, P1452, DOI 10.1111/j.1083-6101.2007.00381.x

Scheuing N, 2014, DIABETES CARE, V37, P1581, DOI 10.2337/dc13-2156

Schulte EM, 2015, PLOS ONE, V10, DOI 10.1371/journal.pone.0117959

Skrivarhaug T, 2006, DIABETOLOGIA, V49, P298, DOI 10.1007/s00125-005-0082-6

Stadler M, 2014, J CLIN ENDOCR METAB, V99, P4523, DOI 10.1210/jc.2014-2701

Treasure J, 2015, DIABETIC MED, V32, P1541, DOI 10.1111/dme.12839

Watson A, 2018, CANCER NURS, V41, P117, DOI 10.1097/NCC.0000000000000460

Wilkinson D, 2011, SOC SCI COMPUT REV, V29, P387, DOI 10.1177/0894439310378979

NR 30

TC 0

Z9 0

U1 7

U2 7

PU WILEY

PI HOBOKEN

PA 111 RIVER ST, HOBOKEN 07030-5774, NJ USA

SN 0742-3071

EI 1464-5491

J9 DIABETIC MED

JI Diabetic Med.

PD OCT

PY 2018

VL 35

IS 10

BP 1329

EP 1336

DI 10.1111/dme.13700

PG 8

WC Endocrinology & Metabolism

SC Endocrinology & Metabolism

GA GU5ME

UT WOS:000445328800004

PM 29855073

DA 2019-01-08

ER

PT J

AU Ochoa, CY

Haardorfer, R

Escoffery, C

Stein, K

Alcaraz, KI

AF Ochoa, Carol Y.

Haardoerfer, Regine

Escoffery, Cam

Stein, Kevin

Alcaraz, Kassandra I.

TI Examining the role of social support and spirituality on the general

health perceptions of Hispanic cancer survivors

SO PSYCHO-ONCOLOGY

LA English

DT Article

DE cancer; cancer survivors; general health perception; health disparities;

Hispanics; HRQOL; oncology; quality of life; social support;

spirituality

ID QUALITY-OF-LIFE; BREAST-CANCER; INTERVIEW SURVEY; UNITED-STATES;

DISPARITIES; SOCIETY; LATINA; SCALE; CARE; SATISFACTION

AB Objective: Although cancer survival rates continue to improve, overall disparities persist for ethnic minority survivors, who have a disproportionately high risk of experiencing poor quality of life, despite documented higher levels of self-reported spirituality. Yet little is known about the relationship between spirituality and social support and health outcomes among Hispanic survivors. This study examined (1) differences between Hispanic and non-Hispanic white survivors on health, social support, and spirituality and (2) the potential mediating roles of mental health and emotional distress on general health perceptions.

Methods: We analyzed data (N = 7778) from the American Cancer Society's Study of Cancer Survivors-II, a national cross-sectional study of adult cancer survivors. Preliminary analysis compared sociodemographic and medical characteristics between the 2 groups to identify significant covariates. Structural equation modeling assessed whether mental health and emotional distress mediate the impact of social support and spirituality on Hispanics' general health perceptions.

Results: Overall, 693 survivors were Hispanic and 7085 were non-Hispanic whites. Hispanics reported poorer health and were more likely to have comorbid conditions such as diabetes and depression compared with non-Hispanic whites. Structural equation modeling indicated that the impact of spirituality on general health perceptions was fully mediated through mental health. Emotional distress did not have a direct effect on general health perceptions nor did it mediate effects of spirituality and social support and on general health perceptions. There was a mediated effect of social support on general health perceptions.

Conclusions: Spirituality is an important factor in the health of Hispanic survivors. Future studies should explore the impact and effectiveness of spiritual interventions and the beneficial effect for mental health on general health perceptions.

C1 [Ochoa, Carol Y.; Haardoerfer, Regine; Escoffery, Cam; Stein, Kevin] Univ Southern Calif, Keck Sch Med, Dept Prevent Med, 2001 N Soto St, Los Angeles, CA 90033 USA.

[Ochoa, Carol Y.] Univ Southern Calif, Keck Sch Med, Dept Prevent Med, Los Angeles, CA 90033 USA.

[Alcaraz, Kassandra I.] Amer Canc Soc, Behav Res Ctr, Atlanta, GA 30329 USA.

RP Ochoa, CY (reprint author), Univ Southern Calif, Keck Sch Med, Dept Prevent Med, 2001 N Soto St, Los Angeles, CA 90033 USA.

EM caroloch@usc.edu

FU American Cancer Society (ACS) Studies of Cancer Survivors (SCS)

FX The American Cancer Society (ACS) Studies of Cancer Survivors (SCS) were

funded as an intramural program of research conducted by the ACS

Behavioral Research. Overall approval for SCS was obtained from the

Institutional Review Board (IRB) of Emory University and from the IRBs

and/or regulatory boards for each participating cancer registry. We wish

to acknowledge the cooperation and efforts of the cancer registries and

public health departments from the states of Arizona, California

(Regions 2-6), Colorado, Delaware, Illinois, Iowa, Maine, Massachusetts,

Michigan, Nebraska, New Jersey, Pennsylvania, Washington, and Wyoming.

We also thank the staff of the hundreds of hospitals contributing cases

to the participating cancer registries; their data made this research

possible. Lastly, we salute the thousands of cancer survivors, their

physicians, and their loved ones who contributed to the collection of

these data. The authors assume full responsibility for analyses and

interpretation of these data.

CR American Cancer Society, 2015, CANC FACTS FIG HISP

Bai M, 2015, J PALLIAT MED, V18, P286, DOI 10.1089/jpm.2014.0189

Brady MJ, 1999, PSYCHO-ONCOLOGY, V8, P417, DOI 10.1002/(SICI)1099-1611(199909/10)8:5<417::AID-PON398>3.3.CO;2-W

Canada AL, 2013, J BEHAV MED, V36, P441, DOI 10.1007/s10865-012-9439-8

Clarke TC, 2015, ANN EPIDEMIOL, V25, P832, DOI 10.1016/j.annepidem.2015.07.011

COHEN S, 1985, PSYCHOL BULL, V98, P310, DOI 10.1037//0033-2909.98.2.310

Culliford L, 2002, BRIT MED J, V325, P1434, DOI 10.1136/bmj.325.7378.1434

Eversley R, 2005, ONCOL NURS FORUM, V32, P250, DOI 10.1188/05.ONF.250-256

Healthy People, 2020, HLTH REL QUAL LIF WE

IBM Corp., 2015, IBM SPSS STAT WIND V

Kline R. B., 2015, PRINCIPLES PRACTICE

Lazarus RS, 1984, FS STRESS APPRAISAL

Lopez-Class M, 2012, PSYCHO-ONCOLOGY, V21, P115, DOI 10.1002/pon.1998

Lopez-Class M, 2011, J CANCER EDUC, V26, P724, DOI 10.1007/s13187-011-0249-4

Luckett T, 2011, LANCET ONCOL, V12, P1240, DOI 10.1016/S1470-2045(11)70212-1

Miller WR, 2003, AM PSYCHOL, V58, P24, DOI 10.1037/0003-066X.58.1.24

Mukwato K. P., 2010, MED J ZAMBIA, V37, P40

Muthen L.K., 2012, MPLUS VERSION 7 USER

Napoles AM, 2017, J COMMUNITY SUPPORT, V15, P20, DOI 10.12788/jcso.0325

Nelson C, 2009, ANN BEHAV MED, V38, P105, DOI 10.1007/s12160-009-9139-y

Niu XL, 2013, CANCER MED-US, V2, P403, DOI 10.1002/cam4.84

Prince P, 2015, J PSYCHOSOC ONCOL, V33, P635, DOI 10.1080/07347332.2015.1082167

Rim SH, 2016, EXPERT REV PHARM OUT, V16, P579, DOI 10.1080/14737167.2016.1239533

Ross LE, 2008, J ALTERN COMPLEM MED, V14, P931, DOI 10.1089/acm.2007.0788

Sammarco A, 2008, ONCOL NURS FORUM, V35, P844, DOI 10.1188/08.ONF.844-849

Sammarco A, 2010, ONCOL NURS FORUM, V37, P93, DOI 10.1188/10.ONF.93-99

Short PF, 2006, MED CARE, V44, P16, DOI 10.1097/01.mlr.0000188986.84819.3a

Smith T, 2007, CANCER, V109, P1, DOI 10.1002/cncr.22387

Stephens C, 2010, PSYCHO-ONCOLOGY, V19, P376, DOI 10.1002/pon.1566

WARE JE, 1992, MED CARE, V30, P473, DOI 10.1097/00005650-199206000-00002

Ware JE, 1993, SF 36 HLTH SURVEY MA

Weaver KE, 2012, CANCER EPIDEM BIOMAR, V21, P2108, DOI 10.1158/1055-9965.EPI-12-0740

Weaver KE, 2010, CANCER-AM CANCER SOC, V116, P3493, DOI 10.1002/cncr.25209

Wildes KA, 2009, PSYCHO-ONCOLOGY, V18, P831, DOI 10.1002/pon.1475

Zhao LH, 2009, J PAIN SYMPTOM MANAG, V37, P676, DOI 10.1016/j.jpainsymman.2008.04.011

ZIMET GD, 1990, J PERS ASSESS, V55, P610, DOI 10.1207/s15327752jpa5503&4_17

Zlatar ZZ, 2015, PSYCHO-ONCOLOGY, V24, P241, DOI 10.1002/pon.3600

NR 37

TC 0

Z9 0

U1 3

U2 3

PU WILEY

PI HOBOKEN

PA 111 RIVER ST, HOBOKEN 07030-5774, NJ USA

SN 1057-9249

EI 1099-1611

J9 PSYCHO-ONCOLOGY

JI Psycho-Oncol.

PD SEP

PY 2018

VL 27

IS 9

BP 2189

EP 2197

DI 10.1002/pon.4795

PG 9

WC Oncology; Psychology; Psychology, Multidisciplinary; Social Sciences,

Biomedical

SC Oncology; Psychology; Biomedical Social Sciences

GA GV5WH

UT WOS:000446175500016

PM 29900621

DA 2019-01-08

ER

PT J

AU Roy, M

Sengupta, N

Sahana, PK

Das, C

Talukdar, P

Baidya, A

Goswami, S

AF Roy, Mukut

Sengupta, Nilanjan

Sahana, Pranab Kumar

Das, Chanchal

Talukdar, Payel

Baidya, Arjun

Goswami, Soumik

TI Type 2 diabetes and influence of diabetes-specific distress on

depression

SO DIABETES RESEARCH AND CLINICAL PRACTICE

LA English

DT Article

ID CLINICAL DEPRESSION; GLYCEMIC CONTROL; METAANALYSIS; INVENTORY;

PREVALENCE; ADULTS; CARE

AB AIMS: Common psycho-social emotional reactions of patients with diabetes may be termed as diabetes-specific distress which is conceptually distinctive from depression. In patients with type 2 diabetes, different screening methods for depression may get influenced by simultaneous presence of diabetes distress. This study was planned to assess magnitude and relationship of depression and diabetes specific distress in patients with type 2 diabetes.

Methods: Two hundred and fifty (250) adult patients with type 2 diabetes (T2DM) were assessed for depression based on Beck Depression Inventory (BDI) and Diagnostic and Statistical Manual, Fourth edition (DSM IV) criteria. Diabetes specific distress was assessed as per Diabetes Distress Scale (DDS) score.

Results: Among study population of 250 adult T2DM patients, based on BDI score, 97 (38.8%) patients were found to suffer from depression and based on DSM IV criteria, prevalence of depression was 29.2%. A total of 62 (24.8%) patients were found to suffer from diabetes specific distress based on DDS score. Patients with severe diabetes specific distress had associated matching of symptoms with mild depression based on BDI score which was also statistically significant (p < 0.0001). However, these same individuals were non-depressed as per DSM-IV criteria.

Conclusion: Recognizing depression with self-administered questionnaires may be influenced by concomitant presence of symptoms due to diabetes specific distress. Therefore, proper diagnosis of depression may be established by structured clinical interview and psycho-social management of type 2 diabetes should possibly include both assessment of depression and diabetes specific distress. (C) 2018 Elsevier B.V. All rights reserved.

C1 [Roy, Mukut] Tripura Med Coll, Dept Med, Agartala 799014, Tripura, India.

[Roy, Mukut] DRBRAM Teaching Hosp, Agartala 799014, Tripura, India.

[Roy, Mukut; Sengupta, Nilanjan; Sahana, Pranab Kumar; Baidya, Arjun; Goswami, Soumik] Nilratan Sircar Med Coll, Dept Endocrinol, Kolkata, W Bengal, India.

[Das, Chanchal] West Bengal Hlth Serv, Kolkata, India.

[Das, Chanchal; Talukdar, Payel] Nilratan Sircar Med Coll, Dept Psychiat, Kolkata, W Bengal, India.

RP Roy, M (reprint author), Tripura Med Coll, Dept Med, Agartala 799014, Tripura, India.; Roy, M (reprint author), DRBRAM Teaching Hosp, Agartala 799014, Tripura, India.

EM mukutdoc@gmail.com

CR Amer Diabet Assoc, 2012, DIABETES CARE, V35, pS64, DOI [10.2337/dc12-s064, 10.2337/dc12-S064]

American Psychiatric Association, 1994, DIAGN STAT MAN MENT

Anderson RJ, 2001, DIABETES CARE, V24, P1069, DOI 10.2337/diacare.24.6.1069

Bajaj Sarita, 2012, Indian J Endocrinol Metab, V16, P759, DOI 10.4103/2230-8210.100670

Beck AT, 1996, J PERS ASSESS, V67, P588, DOI 10.1207/s15327752jpa6703_13

BECK AT, 1961, ARCH GEN PSYCHIAT, V4, P561

First MB, 1995, J PERSONALITY DISORD, V9, P2

Fisher L, 2001, DIABETES CARE, V24, P1751, DOI 10.2337/diacare.24.10.1751

Fisher L, 2007, DIABETES CARE, V30, P542, DOI 10.2337/dc06-1614

Fisher L, 2010, DIABETES CARE, V33, P1034, DOI 10.2337/dc09-2175

Fournier JC, 2010, JAMA-J AM MED ASSOC, V303, P47, DOI 10.1001/jama.2009.1943

Gask L, 2011, CHRONIC ILLN, V7, P239, DOI 10.1177/1742395311403636

Gonzalez JS, 2008, DIABETOLOGIA, V51, P1822, DOI 10.1007/s00125-008-1113-x

Joseph Nitin, 2013, Indian J Endocrinol Metab, V17, P681, DOI 10.4103/2230-8210.113761

Lustman PJ, 1997, PSYCHOSOM MED, V59, P24, DOI 10.1097/00006842-199701000-00004

Lustman PJ, 2000, DIABETES CARE, V23, P934, DOI 10.2337/diacare.23.7.934

Mathew Cynthia Susan, 2012, Indian J Endocrinol Metab, V16, P764, DOI 10.4103/2230-8210.100671

Mezuk B, 2008, DIABETES CARE, V31, P2383, DOI 10.2337/dc08-0985

Polonsky WH, 2005, DIABETES CARE, V28, P626, DOI 10.2337/diacare.28.3.626

Raval A, 2010, INDIAN J MED RES, V132, P195

Roy T, 2012, DIABETIC MED, V29, P164, DOI 10.1111/j.1464-5491.2011.03401.x

Talbot F, 2000, DIABETES CARE, V23, P1556, DOI 10.2337/diacare.23.10.1556

Zhang J, 2013, NEUROPSYCH DIS TREAT, V9, P1289, DOI 10.2147/NDT.S49798

NR 23

TC 0

Z9 0

U1 3

U2 3

PU ELSEVIER IRELAND LTD

PI CLARE

PA ELSEVIER HOUSE, BROOKVALE PLAZA, EAST PARK SHANNON, CO, CLARE, 00000,

IRELAND

SN 0168-8227

EI 1872-8227

J9 DIABETES RES CLIN PR

JI Diabetes Res. Clin. Pract.

PD SEP

PY 2018

VL 143

BP 194

EP 198

DI 10.1016/j.diabres.2018.07.006

PG 5

WC Endocrinology & Metabolism

SC Endocrinology & Metabolism

GA GU2VR

UT WOS:000445129900023

PM 30009934

DA 2019-01-08

ER

PT J

AU Humayun, MA

Jenkins, E

Knott, J

Ryder, J

Shaban, C

Weiss, M

Charman, J

Cavan, D

Brooks, A

AF Humayun, Malik Asif

Jenkins, Emma

Knott, Julia

Ryder, Jacky

Shaban, Clare

Weiss, Melanie

Charman, Julie

Cavan, David

Brooks, Augustin

TI Intensive structured education for type 1 diabetes management using

BERTIE: Long-term follow-up to assess impact on glycaemic control and

quality of life indices

SO DIABETES RESEARCH AND CLINICAL PRACTICE

LA English

DT Article

DE Type 1 diabetes; Structured education; BERTIE

ID ROUTINE CARE; PROGRAM; DISTRESS

AB Aims: Bournemouth Type 1 Intensive Education (BERTIE) is a structured education course delivered 1 day a week for 4 weeks for self-management of type 1 diabetes. BERTIE outcomes were analysed to assess long-term effectiveness: primary outcome assessed impact of BERTIE on glycaemic control, secondary outcomes assessed impact on Problem Area in Diabetes (PAID) scale, severe hypoglycaemia and diabetic ketoacidosis incidence (DKA).

Methods: Prospectively collected outcome data from attendees included glycated haemoglobin (HbA1c), PAID, severe hypoglycaemia and DKA incidence recorded pre-course, 6 months and 1 year post-attendance, with HbA1c assessed annually at subsequent clinic visits.

Results: Between 1999 and 2015, 524 people attended BERTIE with 5 year follow-up in 316 (60.3%) attendees. HbA1c was reduced from 74 +/- 17 mmol/mol (8.9 +/- 1.6%) at baseline to 71 +/- 15 mmol/mol (8.6 +/- 1.4%) at 1 year and 70 +/- 15 mmol/mol (8.6 +/- 1.3%) at 5 years (p < 0.0001); severe hypoglycaemia incidence reduced from 0.8 +/- 2.1 to 0.4 +/- 2.2 episodes/person/year at 1 year (p < 0.0001); PAID scale reduced from 23 +/- 16 to 15 +/- 12 (p < 0.0001) at 1 year; DKA incidence was 0.06 +/- 0.34 episodes/person/year pre-course and 0.03 +/- 0.21 at 1 year (p = 0.5271).

Conclusions: BERTIE outcome data demonstrate favorable biochemical and psychological outcomes supporting recommendations that structured education be provided to adults with type 1 diabetes. (C) 2018 Elsevier B.V. All rights reserved.

C1 [Humayun, Malik Asif; Jenkins, Emma; Knott, Julia; Ryder, Jacky; Shaban, Clare; Weiss, Melanie; Charman, Julie; Cavan, David; Brooks, Augustin] Royal Bournemouth Hosp, Bournemouth Diabet & Endocrine Ctr, Castle Lane East, Bournemouth BH7 7DW, Dorset, England.

RP Brooks, A (reprint author), Royal Bournemouth Hosp, Bournemouth Diabet & Endocrine Ctr, Castle Lane East, Bournemouth BH7 7DW, Dorset, England.

EM Augustin.Brooks@rbch.nhs.uk

OI Humayun, Malik Asif/0000-0003-3313-303X

CR Amiel S, 2002, BRIT MED J, V325, P746

Bandura A., 1977, SOCIAL LEARNING THEO

Cooke D, 2013, DIABETES CARE, V36, P270, DOI 10.2337/dc12-0080

Elliott J, 2014, DIABETIC MED, V31, P847, DOI 10.1111/dme.12441

Gunn D, 2012, DIABETIC MED, V29, P807, DOI 10.1111/j.1464-5491.2011.03525.x

Hopkins D, 2012, DIABETES CARE, V35, P1638, DOI 10.2337/dc11-1579

Lowe J, 2008, DIABETES RES CLIN PR, V80, P439, DOI 10.1016/j.diabres.2008.02.003

MUHLHAUSER I, 1983, DIABETOLOGIA, V25, P470

MUHLHAUSER I, 1987, DIABETOLOGIA, V30, P681, DOI 10.1007/BF00296989

POLONSKY WH, 1995, DIABETES CARE, V18, P754, DOI 10.2337/diacare.18.6.754

Reas DL, 2007, BMC PUBLIC HEALTH, V7, DOI 10.1186/1471-2458-7-269

SHAMOON H, 1993, NEW ENGL J MED, V329, P977

Speight J, 2010, DIABETES RES CLIN PR, V89, P22, DOI 10.1016/j.diabres.2010.03.017

Speight J, 2016, DIABETES RES CLIN PR, V112, P65, DOI 10.1016/j.diabres.2015.11.002

NR 14

TC 0

Z9 0

U1 3

U2 3

PU ELSEVIER IRELAND LTD

PI CLARE

PA ELSEVIER HOUSE, BROOKVALE PLAZA, EAST PARK SHANNON, CO, CLARE, 00000,

IRELAND

SN 0168-8227

EI 1872-8227

J9 DIABETES RES CLIN PR

JI Diabetes Res. Clin. Pract.

PD SEP

PY 2018

VL 143

BP 275

EP 281

DI 10.1016/j.diabres.2018.07.034

PG 7

WC Endocrinology & Metabolism

SC Endocrinology & Metabolism

GA GU2VR

UT WOS:000445129900034

PM 30076871

DA 2019-01-08

ER

PT J

AU Aghili, R

Ridderstrale, M

Farshchi, A

Valojerdi, AE

Banazadeh, Z

Malek, M

Kia, M

Khamseh, ME

AF Aghili, Rokhsareh

Ridderstrale, Martin

Farshchi, Amir

Valojerdi, Ameneh Ebrahim

Banazadeh, Zahra

Malek, Mojtaba

Kia, Maryam

Khamseh, Mohammad Ebrahim

TI Psychosocial factors and glycemic control in insulin-naive and

insulin-experienced people with type 2 diabetes: a path analysis model

SO INTERNATIONAL JOURNAL OF DIABETES IN DEVELOPING COUNTRIES

LA English

DT Article

DE Diabetes; type 2; Psychosocial; Glycemic control

ID CLINICAL INERTIA; DEPRESSION; BARRIERS; CARE; PREVALENCE; MANAGEMENT;

KNOWLEDGE; DISTRESS; THERAPY; METAANALYSIS

AB The purpose of this study was to compare the status of psychosocial factors and glycemic control in insulin-naive and insulin-experienced people with type 2 diabetes (T2D). In this observational study on people with T2D, demographic, self-care behavior, resources, and affective variables as well as health-related quality of life were assessed and compared in insulin-naive and insulin-experienced considering the number of oral glucose-lowering drugs (OGLDs). Measured variable path analysis was used to test the association among variables and their effect on HbA1c in both groups. In total, 215 insulin-naive and 165 insulin-experienced patients were recruited in this study. The mean duration of diabetes was 11.7 +/- 7.0years in insulin-experienced and 6.8 +/- 5.4years in insulin-naive (p<0.001). The mean hemoglobin A1c (HbA1c) was significantly higher in insulin-experienced subjects irrespective of the number of OGLDs [68 +/- 20mmol/mol (8.4 +/- 1.8%) vs. 56 +/- 16mmol/mol (7.3 +/- 1.4%); p<0.001]. Moreover, insulin-experienced subjects had significantly higher level of diabetes-related distress (2.2 +/- 0.9 vs. 1.9 +/- 0.8), depression (9.5 +/- 5.5 vs. 8.1 +/- 5.1), anxiety (18.3 +/- 12.0 vs. 15.1 +/- 10.5), and lower knowledge of insulin use considering the results of 9-item insulin-use subscale of Michigan diabetes knowledge test (mean 3.9 +/- 1.8) compared to insulin-naive subjects (p<0.05). Higher levels of distress, depression, and anxiety are found in insulin-experienced people with T2D. Therefore, one should be aware that, at the time of insulin need/initiation, people with T2D have reached a more vulnerable state and this should be taken into consideration when implementing a complex insulin initiation plan.

C1 [Aghili, Rokhsareh; Farshchi, Amir; Valojerdi, Ameneh Ebrahim; Khamseh, Mohammad Ebrahim] Iran Univ Med Sci, Inst Endocrinol & Metab, Endocrine Res Ctr, 10 Firoozeh St,South Vali Asr Ave,Vali Asr Sq, Tehran, Iran.

[Ridderstrale, Martin] Lund Univ, Skane Univ Hosp Malmo, Dept Clin Sci, Clin Obes, S-20502 Malmo, Sweden.

[Banazadeh, Zahra] Iran Univ Med Sci, Lolagar Hosp, Tehran, Iran.

[Malek, Mojtaba] Iran Univ Med Sci, Inst Endocrinol & Metab, Res Ctr Prevent Cardiovasc Dis, Tehran, Iran.

[Kia, Maryam] Univ Tehran Med Sci, Ziaeian Hosp, Dept Internal Med, Tehran, Iran.

RP Khamseh, ME (reprint author), Iran Univ Med Sci, Inst Endocrinol & Metab, Endocrine Res Ctr, 10 Firoozeh St,South Vali Asr Ave,Vali Asr Sq, Tehran, Iran.

EM aghili.r@tak.iums.ac.ir; martin.ridderstrale@med.lu.se;

farshchi_a@razi.tums.ac.ir; ebrahimv86@gmail.com;

zahrabanazadeh@yahoo.com; malek.m@iums.ac.ir; mkia220@gmail.com;

khamseh.m@iums.ac.ir

RI Khamseh, Mohammad Ebrahim/L-9391-2018

FU Iran University of Medical Sciences [93-02-122-24800]

FX This study was funded and supported by the Iran University of Medical

Sciences, grant number: 93-02-122-24800. The authors wish to thank all

people who participated in this study.

CR Abu Hassan H, 2013, BMC FAM PRACT, V14, DOI 10.1186/1471-2296-14-164

Aghili R, 2016, CAN J DIABETES, V40, P424, DOI 10.1016/j.jcjd.2016.02.014

American Diabetes A. 6, 2017, DIABETES CARE S1, V40, pS48, DOI DOI 10.2337/DC17-S009

Anderson RJ, 2001, DIABETES CARE, V24, P1069, DOI 10.2337/diacare.24.6.1069

BROWNE MW, 1992, SOCIOL METHOD RES, V21, P230, DOI 10.1177/0049124192021002005

Byrne B. M., 2013, STRUCTURAL EQUATION

Camara A, 2015, DIABETES METAB, V41, P62, DOI 10.1016/j.diabet.2014.04.007

Casagrande SS, 2013, DIABETES CARE, V36, P2271, DOI 10.2337/dc12-2258

Ciechanowski PS, 2000, ARCH INTERN MED, V160, P3278, DOI 10.1001/archinte.160.21.3278

Dalal MR, 2017, ADV THER, V34, P2083, DOI 10.1007/s12325-017-0592-x

de Groot M, 2001, PSYCHOSOM MED, V63, P619, DOI 10.1097/00006842-200107000-00015

Fisher L, 2012, DIABETES CARE, V35, P259, DOI 10.2337/dc11-1572

Fitzgerald JT, 1998, DIABETES CARE, V21, P706, DOI 10.2337/diacare.21.5.706

Glasgow RE, 2005, DIABETES CARE, V28, P2655, DOI 10.2337/diacare.28.11.2655

Harris SB, 2010, CAN FAM PHYSICIAN, V56, pE418

Holmes-Truscott E, 2016, PRIM CARE DIABETES, V10, P75, DOI 10.1016/j.pcd.2015.06.006

Home P, 2011, DIABETES RES CLIN PR, V94, P352, DOI 10.1016/j.diabres.2011.10.021

Janes R, 2013, J PRIM HEALTH CARE, V5, P114, DOI 10.1071/HC13114

Jasper US, 2014, J DIABETES METAB DIS, V13, DOI 10.1186/2251-6581-13-10

Katon W, 2004, DIABETES CARE, V27, P914, DOI 10.2337/diacare.27.4.914

Kaviani H, 2008, TEHRAN U MED J, V66, P136

Khamseh ME, 2011, BMC PSYCHIATRY, V11, DOI 10.1186/1471-244X-11-61

Khodapanahi MK, 2009, J FAMILY RES, V5, P423

Larkin ME, 2008, DIABETES EDUCATOR, V34, P511, DOI 10.1177/0145721708317869

Limaye TY, 2016, INT J DIABETES DEV C, V36, P263, DOI 10.1007/s13410-015-0367-3

Lustman PJ, 2000, DIABETES CARE, V23, P934, DOI 10.2337/diacare.23.7.934

Makine C, 2009, DIABETIC MED, V26, P28, DOI 10.1111/j.1464-5491.2008.02606.x

Murata GH, 2003, DIABETOLOGIA, V46, P1170, DOI 10.1007/s00125-003-1161-1

Peyrot M, 2005, DIABETIC MED, V22, P1379, DOI 10.1111/j.1464-5491.2005.01644.x

Peyrot M, 2012, HEALTH QUAL LIFE OUT, V10, DOI 10.1186/1477-7525-10-125

Peyrot M, 2010, DIABETES CARE, V33, P240, DOI 10.2337/dc09-1348

Pieber TR, 2000, DIABETES CARE, V23, P157, DOI 10.2337/diacare.23.2.157

Reach G, 2017, DIABETES METAB, V43, P501, DOI 10.1016/j.diabet.2017.06.003

Reaney M, 2013, DIABETES SPECTR, V26, P236

Ross SA, 2011, CURR MED RES OPIN, V27, P13, DOI 10.1185/03007995.2011.621416

Rushforth B, 2016, BRIT J GEN PRACT, V66, pE114, DOI 10.3399/bjgp16X683509

Russell-Jones D, 2007, DIABETES OBES METAB, V9, P799, DOI 10.1111/j.1463-1326.2006.00686.x

Tong WT, 2015, BMJ OPEN, V5, DOI 10.1136/bmjopen-2014-006407

van Raalte DH, 2017, DIABETES OBES METAB, V19, P1205, DOI 10.1111/dom.12935

Vanderlee L, 2016, INT J DIABETES DEV C, V36, P320, DOI 10.1007/s13410-015-0460-7

Vasheghani-Farahani Ali, 2011, Asian J Sports Med, V2, P106

Wallace TM, 2000, QJM-MON J ASSOC PHYS, V93, P369, DOI 10.1093/qjmed/93.6.369

Weinger K, 2010, DIABETES CARE, V33, P450, DOI 10.2337/dc09-2132

NR 43

TC 1

Z9 1

U1 0

U2 0

PU SPRINGER INDIA

PI NEW DELHI

PA 7TH FLOOR, VIJAYA BUILDING, 17, BARAKHAMBA ROAD, NEW DELHI, 110 001,

INDIA

SN 0973-3930

EI 1998-3832

J9 INT J DIABETES DEV C

JI Int. Diabetes Dev. Ctries.

PD SEP

PY 2018

VL 38

IS 3

BP 289

EP 297

DI 10.1007/s13410-017-0581-2

PG 9

WC Endocrinology & Metabolism

SC Endocrinology & Metabolism

GA GS8ZZ

UT WOS:000444005700004

DA 2019-01-08

ER

PT J

AU Kilpatrick, M

Nelson, M

Palmer, A

Jose, K

Venn, A

AF Kilpatrick, Michelle

Nelson, Mark

Palmer, Andrew

Jose, Kim

Venn, Alison

TI Who discusses reaching a healthy weight with a general practitioner?

Findings from the 2014-15 Australian National Health Survey

SO OBESITY RESEARCH & CLINICAL PRACTICE

LA English

DT Article

DE Primary care; Obesity; Weight management

ID PRIMARY-CARE; BRIEF INTERVENTION; YOUNG-ADULTS; MANAGEMENT; OBESITY;

OVERWEIGHT; BEHAVIORS; FUTURE; TRIAL

AB Objective: General practitioners (GPs) can positively impact upon patient intentions to lose weight and weight management, and are important in the referral pathway to specialist weight-loss programs and surgical interventions. The aim of this study was to investigate the characteristics and proportions of Australians who report talking to a GP about weight management.

Methods: Cross-sectional data from 15,329 participants aged 15 years and over in the 2014-15 Australian National Health Survey were used. Proportions (with 95% confidence intervals (95%CI)) of respondents who reported discussing reaching a healthy weight with a GP in the previous 12 months were estimated, categorised by demographic, social and health characteristics.

Results: We found that 10.8% (95%CI:9.8-11.8) of overweight participants, 24.4% (95%CI:22.7-26.4) with Class 1 obesity (30 <= BMI < 35 kg/m(2)) and 41.8% (95%CI:38.3-45.3) with Classes II/III obesity (BMI >= 35 kg/m(2)) reported discussing weight with a GP. Higher proportions of respondents with Class II/Class III obesity and poor/fair self-reported health (50.2%, 95%CI:43.3-57.0), or high/very high levels of psychological distress (53.3%, 95%CI:43.7-61.4), or diabetes ( 64.8%, 95%CI:51.9-77.3) reported discussing weight. As age, number of GP visits, or comorbid conditions increased, the proportions of people who discussed their weight with a GP also increased, across all weight classes.

Conclusions: While discussions are more likely with increasing BMI and comorbidities, most Australians with overweight and obesity appear to be missing opportunities to discuss reaching a healthy weight with their GP. Policies, training and education programs to encourage this dialogue could lead to earlier and more beneficial weight-related interventions. (C) 2018 Asia Oceania Association for the Study of Obesity. Published by Elsevier Ltd. All rights reserved.

C1 [Kilpatrick, Michelle; Nelson, Mark; Palmer, Andrew; Jose, Kim; Venn, Alison] Univ Tasmania, Menzies Inst Med Res, Med Sci 1 Bldg,Private Bag 23, Hobart, Tas 7000, Australia.

RP Venn, A (reprint author), Univ Tasmania, Menzies Inst Med Res, Med Sci 1 Bldg,Private Bag 23, Hobart, Tas 7000, Australia.

EM Alison.Venn@utas.edu.au

OI , Mark/0000-0001-9941-7161

FU National Health and Medical Research Council [APP1076899]

FX The research has been supported by a Partnership Project grant

(APP1076899) from the National Health and Medical Research Council.

CR Afshin A, 2017, NEW ENGL J MED, V377, P13, DOI 10.1056/NEJMoa1614362

[Anonymous], 2013, SUMM GUID MAN OV OB

Ashman Freya, 2016, Int J Family Med, V2016, P8212837, DOI 10.1155/2016/8212837

Australian Bureau of Statistics, 2015, NAT HLTH SURV 1 RES

Australian Bureau of Statistics, 2017, NAT HLTH SURV US GUI

Australian Institute of Health and Welfare, 2017, AUS BURD DIS STUD SE

Aveyard P, 2016, LANCET, V388, P2492, DOI 10.1016/S0140-6736(16)31893-1

Beeken RJ, 2017, INT J OBESITY, V41, P246, DOI 10.1038/ijo.2016.206

Bonney A, 2017, AUST FAM PHYSICIAN, V46, P928

Burke MA, 2010, OBESITY, V18, P1226, DOI 10.1038/oby.2009.369

Chauhan BF, 2017, IMPLEMENT SCI, V12, DOI 10.1186/s13012-017-0568-x

Daly M, 2017, PSYCHOL SCI, V28, P872, DOI 10.1177/0956797617696311

Dewhurst A, 2017, PATIENT EDUC COUNS, V100, P897, DOI 10.1016/j.pec.2016.12.017

Finucane MM, 2011, LANCET, V377, P557, DOI 10.1016/S0140-6736(10)62037-5

Galuska DA, 1999, JAMA, V282

Hayden MJ, 2008, AUST J PRIM HEALTH, V14, P9, DOI 10.1071/PY08031

Hayes AJ, 2017, INT J OBESITY, V41, P178, DOI 10.1038/ijo.2016.165

Haynes A, 2018, OBES REV, V19, P347, DOI 10.1111/obr.12634

Hemmes RA, 2016, CLIN OBES, V6, P202, DOI 10.1111/cob.12143

Herbert J, 2017, OBES RES CLIN PRACT, V11, P414, DOI 10.1016/j.orcp.2016.12.009

Jackson SE, 2013, BMJ OPEN, V3, DOI 10.1136/bmjopen-2013-003693

Lanoye A, 2017, CURR DIABETES REP, V17, DOI 10.1007/s11892-017-0938-4

LaRose JG, 2013, OBESITY, V21, P449, DOI 10.1002/oby.20053

Laska MN, 2012, J ADOLESCENT HEALTH, V50, P324, DOI 10.1016/j.jadohealth.2012.01.016

Lewis AL, 2013, CURR OBES REP, V2, P341, DOI 10.1007/s13679-013-0073-8

McDonald J, 2008, MED J AUSTRALIA, V188, pS69

Michie Susan, 2007, Psychol Health Med, V12, P521, DOI 10.1080/13548500701203441

Rose SA, 2013, INT J OBESITY, V37, P118, DOI 10.1038/ijo.2012.24

Sheehan TJ, 2003, INT J OBESITY, V27, P498, DOI 10.1038/sj.ijo.0802263

Sturgiss E, 2017, BMJ OPEN, V7, DOI 10.1136/bmjopen-2016-014314

Tan D, 2006, MED J AUSTRALIA, V185, P73

Tang JW, 2010, BMC FAM PRACT, V11, DOI 10.1186/1471-2296-11-31

Truesdale KP, 2006, INT J OBESITY, V30, P1397, DOI 10.1038/sj.ijo.0803307

Turner LR, 2015, MED J AUSTRALIA, V202, P370, DOI 10.5694/mja14.00998

NR 34

TC 0

Z9 0

U1 0

U2 0

PU ELSEVIER SCI LTD

PI OXFORD

PA THE BOULEVARD, LANGFORD LANE, KIDLINGTON, OXFORD OX5 1GB, OXON, ENGLAND

SN 1871-403X

EI 1878-0318

J9 OBES RES CLIN PRACT

JI Obes. Res. Clin. Pract.

PD SEP-OCT

PY 2018

VL 12

IS 5

BP 459

EP 464

DI 10.1016/j.orcp.2018.07.003

PG 6

WC Endocrinology & Metabolism; Nutrition & Dietetics

SC Endocrinology & Metabolism; Nutrition & Dietetics

GA GT4CD

UT WOS:000444450500010

PM 30033355

DA 2019-01-08

ER

PT J

AU Vesco, AT

Feldman, MA

Evans, MA

Weissberg-Benchell, J

AF Vesco, Anthony T.

Feldman, Marissa A.

Evans, Meredyth A.

Weissberg-Benchell, Jill

TI Parent-Adolescent Dyadic Diabetes Distress: Associations With A1c and

Diabetes-Related Strengths

SO FAMILIES SYSTEMS & HEALTH

LA English

DT Article

DE adolescent; diabetes mellitus; type 1; emotional stress; family systems

ID QUALITY-OF-LIFE; RESPONSE-SURFACE ANALYSIS; FAMILY SYSTEMS THERAPY;

GLYCEMIC CONTROL; METABOLIC-CONTROL; SELF-EFFICACY; TYPE-1; PERCEPTIONS;

MANAGEMENT; CONFLICT

AB Introduction: This study examined concordance and discordance between parent-adolescent report of diabetes-specific emotional distress and associations with A1c and diabetes-related strengths. Diabetes strengths refer to adaptive behaviors and attitudes that enhance resilience in living with diabetes. Method: One thousand two hundred sixteen adolescent-parent dyads completed measures of diabetes distress and adolescent-reported diabetes strengths. Polynomial regression with response surface analysis assessed concordant and discordant reports of adolescent-parent distress and independent associations of each reporter's distress on measures of diabetes strengths and A1c. Results: A1c was lower in concordantly lower distress dyads compared to concordantly higher distress dyads. For discordant dyadic reports, A1c was higher when distress was higher for parents and lower for adolescents compared to low-parent-high-adolescent distress. Greater diabetes-related strengths were reported when distress was concordantly low among dyads and fewer strengths were reported when distress was concordantly higher. Greater strengths were reported when distress was lower for adolescents and higher for parents compared to high-adolescent/low-parent distress. Dyadic distress for both A1c and diabetes strengths were robust when adjusted for significant demographic predictors of outcome including age, race, income, pump use, and continuous glucose monitoring use. Discussion: A1c and diabetes strengths are associated with degree of concordant and discordant reports of diabetes distress among adolescent-parent dyads. Programmatic interventions should target parents and adolescents, particularly when diabetes distress is higher among both adolescents and their parents.

C1 [Vesco, Anthony T.; Feldman, Marissa A.; Evans, Meredyth A.; Weissberg-Benchell, Jill] Ann & Robert H Lurie Childrens Hosp Chicago, Dept Psychiat & Behav Hlth, Chicago, IL 60611 USA.

[Feldman, Marissa A.; Evans, Meredyth A.; Weissberg-Benchell, Jill] Northwestern Univ, Feinberg Sch Med, Evanston, IL 60208 USA.

RP Vesco, AT (reprint author), Ann & Robert H Lurie Childrens Hosp Chicago, Dept Psychiat, 225 East Chicago Ave,Mailbox 10A, Chicago, IL 60611 USA.

EM avesco@luriechildrens.org

FU Helmsley Charitable Trust Foundation

FX This research was supported in part by a grant from the Helmsley

Charitable Trust Foundation.

CR Anderson BJ, 2009, FAM SYST HEALTH, V27, P141, DOI 10.1037/a0015759

Anderson BJ, 1999, DIABETES CARE, V22, P713, DOI 10.2337/diacare.22.5.713

Bogenschneider K, 2008, J MARRIAGE FAM, V70, P1015, DOI 10.1111/j.1741-3737.2008.00543.x

Cook S, 2002, DIABETES EDUCATOR, V28, P115, DOI 10.1177/014572170202800113

De Los Reyes A, 2015, PSYCHOL BULL, V141, P858, DOI 10.1037/a0038498

Delamater AM, 2014, PEDIATR DIABETES, V15, P232, DOI 10.1111/pedi.12191

Fisher L, 2010, DIABETES CARE, V33, P23, DOI 10.2337/dc09-1238

Grey M, 2000, J PEDIATR-US, V137, P107, DOI 10.1067/mpd.2000.106568

Hagger V, 2016, CURR DIABETES REP, V16, DOI 10.1007/s11892-015-0694-2

Hains AA, 2007, J PEDIATR PSYCHOL, V32, P561, DOI 10.1093/jpepsy/jsl040

Helgeson VS, 2009, J PEDIATR PSYCHOL, V34, P254, DOI 10.1093/jpepsy/jsn079

Hilliard ME, 2017, J PEDIATR PSYCHOL, V42, P995, DOI 10.1093/jpepsy/jsx086

Hilliard ME, 2016, AM PSYCHOL, V71, P590, DOI 10.1037/a0040359

Hilliard ME, 2015, J PEDIATR PSYCHOL, V40, P835, DOI 10.1093/jpepsy/jsv072

Hilliard ME, 2013, HEALTH PSYCHOL, V32, P388, DOI 10.1037/a0027811

Hilliard ME, 2012, CURR DIABETES REP, V12, P739, DOI 10.1007/s11892-012-0314-3

Human LJ, 2016, J YOUTH ADOLESCENCE, V45, P2022, DOI 10.1007/s10964-016-0517-z

Jaser SS, 2017, ANN BEHAV MED, V51, P30, DOI 10.1007/s12160-016-9825-5

Jaser SS, 2014, MATERN CHILD HLTH J, V18, P101, DOI 10.1007/s10995-013-1239-4

Laffel LMB, 2003, DIABETES CARE, V26, P3067, DOI 10.2337/diacare.26.11.3067

Law GU, 2013, J PSYCHOSOM RES, V74, P334, DOI 10.1016/j.jpsychores.2012.12.009

Miller VA, 2003, J PEDIATR PSYCHOL, V28, P265, DOI 10.1093/jpepsy/jsg014

Nouwen A, 2009, PSYCHOL HEALTH, V24, P1071, DOI 10.1080/08870440802254597

Ohannessian CM, 2000, INT J BEHAV DEV, V24, P362, DOI 10.1080/01650250050118358

Rybak TM, 2017, J PEDIATR PSYCHOL, V42, P40, DOI 10.1093/jpepsy/jsw071

Shanock LR, 2010, J BUS PSYCHOL, V25, P543, DOI 10.1007/s10869-010-9183-4

Vesco AT, 2010, J PEDIATR PSYCHOL, V35, P1168, DOI 10.1093/jpepsy/jsq038

Weissberg-Benchell J, 2014, AM DIAB ASS 74 SCI S

Weissberg-Benchell J, 2017, PEDIATR DIABETES, V18, P853, DOI 10.1111/pedi.12499

Weissberg-Benchell J, 2011, PEDIATR DIABETES, V12, P341, DOI 10.1111/j.1399-5448.2010.00720.x

Wysocki T, 1996, DIABETES CARE, V19, P119, DOI 10.2337/diacare.19.2.119

Wysocki T, 2008, BEHAV THER, V39, P33, DOI 10.1016/j.beth.2007.04.001

Wysocki T, 2006, J PEDIATR PSYCHOL, V31, P928, DOI 10.1093/jpepsy/jsj098

NR 33

TC 0

Z9 0

U1 2

U2 2

PU EDUCATIONAL PUBLISHING FOUNDATION-AMERICAN PSYCHOLOGICAL ASSOC

PI WASHINGTON

PA 750 FIRST ST, NE, WASHINGTON, DC 20002-4242 USA

SN 1091-7527

EI 1939-0602

J9 FAM SYST HEALTH

JI Fam. Syst. Health

PD SEP

PY 2018

VL 36

IS 3

BP 357

EP 367

DI 10.1037/fsh0000358

PG 11

WC Health Care Sciences & Services; Family Studies; Public, Environmental &

Occupational Health

SC Health Care Sciences & Services; Family Studies; Public, Environmental &

Occupational Health

GA GT0LJ

UT WOS:000444131900010

PM 29999340

DA 2019-01-08

ER

PT J

AU Tanaka, N

Yabe, D

Murotani, K

Ueno, S

Kuwata, H

Hamamoto, Y

Kurose, T

Takahashi, N

Akashi, T

Matsuoka, T

Osonoi, T

Minami, M

Shimono, D

Seino, Y

AF Tanaka, Nagaaki

Yabe, Daisuke

Murotani, Kenta

Ueno, Shinji

Kuwata, Hitoshi

Hamamoto, Yoshiyuki

Kurose, Takeshi

Takahashi, Nobuo

Akashi, Tomoyuki

Matsuoka, Takashi

Osonoi, Takeshi

Minami, Masae

Shimono, Dai

Seino, Yutaka

CA Positive SMBG Study Grp

TI Mental distress and health-related quality of life among type 1 and type

2 diabetes patients using self-monitoring of blood glucose: A

cross-sectional questionnaire study in Japan

SO JOURNAL OF DIABETES INVESTIGATION

LA English

DT Article

DE Education; Health-related quality of life; Self-monitoring of blood

glucose

ID RANDOMIZED CONTROLLED-TRIAL; IMPACT

AB Aims/IntroductionThe present multicenter, cross-sectional survey was initiated to evaluate self-monitoring of blood glucose (SMBG)-associated mental distress among patients with diabetes.

Materials and MethodsThe survey was carried out in patients with type 1 diabetes and type 2 diabetes using SMBG recruited from 42 medical institutions. Profiles of Mood States 2 and diabetes therapy-related quality of life questionnaires were used to evaluate mood status and health-related quality of life. Two original questionnaires were also developed to evaluate SMBG importance,' painfulness' and confidence' among patients, and to evaluate physician attitudes to SMBG use.

ResultsQuestionnaires from 517 type 1 diabetes and 1,648 type 2 diabetes patients showed that 46.0% of type 1 diabetes and 37.5% of type 2 diabetes patients reported painfulness,' and that these patients reporting painfulness' showed significantly higher Profiles of Mood States 2 scores, lower diabetes therapy-related quality of life scores and higher glycated hemoglobin compared with those not reporting painfulness,' whereas the number of their daily SMBG tests were comparable. Patients reporting painfulness' also reported that SMBG use was significantly less important. Whether or not patients recognized the importance of SMBG use was well correlated with the frequency of physicians checking patient diaries.

ConclusionsType 1 diabetes and type 2 diabetes patients reporting painfulness' in SMBG use had more mental distress, lower health-related quality of life and higher glycated hemoglobin regardless of their number of daily SMBG tests. The importance of SMBG use was recognized less by patients experiencing pain, and the importance of SMBG use was recognized more in medical institutions in which physicians regularly checked SMBG diaries to provide meaningful feedback to patients in clinical settings.

C1 [Tanaka, Nagaaki; Yabe, Daisuke; Ueno, Shinji; Kuwata, Hitoshi; Hamamoto, Yoshiyuki; Kurose, Takeshi; Seino, Yutaka] Kansai Elect Power Med Res Inst, Yutaka Seino Distinguished Ctr Diabet Res, Kobe, Hyogo, Japan.

[Tanaka, Nagaaki; Ueno, Shinji; Kuwata, Hitoshi; Hamamoto, Yoshiyuki; Kurose, Takeshi; Seino, Yutaka] Kansai Elect Power Hosp, Ctr Diabet Endocrinol & Metab, Osaka, Japan.

[Murotani, Kenta] Aichi Med Univ Hosp, Div Biostat, Clin Res Ctr, Nagakute, Aichi, Japan.

[Hamamoto, Yoshiyuki] Kansai Elect Power Hosp, Ctr Metab & Clin Nutr, Osaka, Japan.

[Takahashi, Nobuo] Takahashi Family Clin, Nagoya, Aichi, Japan.

[Akashi, Tomoyuki] Shimada Hosp, Ogori, Japan.

[Matsuoka, Takashi] Kurashiki Cent Hosp, Kurashiki, Okayama, Japan.

[Osonoi, Takeshi] Nakakinen Clin, Naka, Ibaraki, Japan.

[Minami, Masae] Masae Minami Clin, Fukuoka, Japan.

[Shimono, Dai] Futata Tetsuhiro Clin, Fukuoka, Japan.

RP Seino, Y (reprint author), Kansai Elect Power Med Res Inst, Yutaka Seino Distinguished Ctr Diabet Res, Kobe, Hyogo, Japan.; Seino, Y (reprint author), Kansai Elect Power Hosp, Ctr Diabet Endocrinol & Metab, Osaka, Japan.

EM seino.yutaka@e2.kepco.co.jp

OI Yabe, Daisuke/0000-0002-5334-7687

FU Terumo Corporation

FX The authors are grateful to H Abe of Kansai Electric Power Medical

Research Institute and M Yamane of Kansai Electric Power Hospital for

secretarial assistance. The authors also deeply thank the patients and

colleagues who contributed to this study. This study was funded by

Terumo Corporation.

CR Al Hayek AA, 2017, DIABETES THER, V8, P75, DOI 10.1007/s13300-016-0221-8

American Diabetes Association, 2017, Diabetes Care, V40, pS33

Educators AAoD, 2008, DIABETES ED, V2008, P445

Farmer A, 2007, BRIT MED J, V335, P132, DOI 10.1136/bmj.39247.447431.BE

Franciosi M, 2001, DIABETES CARE, V24, P1870, DOI 10.2337/diacare.24.11.1870

Heinemann Lutz, 2008, J Diabetes Sci Technol, V2, P919

Heuchert J. P, 2012, PROFILE MOOD STATES

Iga R, 2017, DIABETES THER, V8, P783, DOI 10.1007/s13300-017-0269-0

International Diabetes Federation, 2015, IDF DIABETES ATLAS

Ishii Hitoshi, 2012, J Med Econ, V15, P556, DOI 10.3111/13696998.2012.665111

Malanda UL, 2012, COCHRANE DB SYST REV, DOI 10.1002/14651858.CD005060.pub3

Miller KM, 2013, DIABETES CARE, V36, P2009, DOI 10.2337/dc12-1770

O'Kane MJ, 2008, BRIT MED J, V336, P1174, DOI 10.1136/bmj.39534.571644.BE

Okada M, 2015, J PHARM HEALTH CARE, V1, DOI 10.1186/s40780-015-0027-2

Peel E, 2007, BRIT MED J, V335, P493, DOI 10.1136/bmj.39302.444572.DE

Polonsky WH, 2013, DIABETES CARE, V36, P179, DOI 10.2337/dc12-0731

Polonsky WH, 2011, DIABETES CARE, V34, P262, DOI 10.2337/dc10-1732

Russo GT, 2016, DIABETES TECHNOL THE, V18, P421, DOI 10.1089/dia.2015.0358

SHAMOON H, 1993, NEW ENGL J MED, V329, P977

Statement AP Education DS, 2014, DIABETES ED, V33, P45

NR 20

TC 0

Z9 0

U1 0

U2 0

PU WILEY

PI HOBOKEN

PA 111 RIVER ST, HOBOKEN 07030-5774, NJ USA

SN 2040-1124

J9 J DIABETES INVEST

JI J. Diabetes Investig.

PD SEP

PY 2018

VL 9

IS 5

BP 1203

EP 1211

DI 10.1111/jdi.12827

PG 9

WC Endocrinology & Metabolism

SC Endocrinology & Metabolism

GA GS3YI

UT WOS:000443564400029

PM 29493881

OA DOAJ Gold

DA 2019-01-08

ER

PT J

AU Ikeda, K

Fujimoto, S

Morling, B

Ayano-Takahara, S

Harashima, S

Uchida, Y

Inagaki, N

AF Ikeda, Kaori

Fujimoto, Shimpei

Morling, Beth

Ayano-Takahara, Shiho

Harashima, Shin-ichi

Uchida, Yukiko

Inagaki, Nobuya

TI Cross-cultural comparison of predictors for self-care behaviors in

patients with type 2 diabetes

SO JOURNAL OF DIABETES INVESTIGATION

LA English

DT Article

DE Cultural psychology; Diabetes self-care; Social psychology

ID SUPPORT; SCALE; INTERVENTIONS; STRATEGIES; AMERICAN; DISTRESS; JAPAN

AB The aim of the present study was to evaluate how culture moderates the behavioral and psychosocial predictors of diabetes self-care activities. Patients with type 2 diabetes were recruited in the outpatient department at two sites: Kyoto University hospital in Japan and the Christiana Care Health System in the USA. The data were collected by survey using questionnaires including questions on the frequency of self-care activities, behavioral and psychosocial predictors, and other background information from 149 Japanese patients and 48 American patients. The cultural impact was observed by multiple regression analyses with interaction terms on the association between emotional support and self-care activities in diet in female patients. The findings of the present study serve as an example of how cultural context can affect patients with diabetes, and lead to a better understanding of culturally sensitive behavioral intervention.

C1 [Ikeda, Kaori; Fujimoto, Shimpei; Ayano-Takahara, Shiho; Harashima, Shin-ichi; Inagaki, Nobuya] Kyoto Univ, Grad Sch Med, Dept Diabet Endocrinol & Nutr, Kyoto, Japan.

[Fujimoto, Shimpei] Kochi Univ, Kochi Med Sch, Dept Endocrinol Metab & Nephrol, Kochi, Japan.

[Morling, Beth] Univ Delaware, Dept Psychol & Brain Sci, Newark, DE USA.

[Uchida, Yukiko] Kyoto Univ, Kokoro Res Ctr, Kyoto, Japan.

RP Ikeda, K (reprint author), Kyoto Univ, Grad Sch Med, Dept Diabet Endocrinol & Nutr, Kyoto, Japan.

EM krikeda@kuhp.kyoto-u.ac.jp

OI Ikeda, Kaori/0000-0003-2273-9693

FU Kokoro Research Center Kyoto University; JSPS KAKENHI [15K20698]

FX This study was supported by Kokoro Research Center Kyoto University and

JSPS KAKENHI Grant Number 15K20698.

CR [Anonymous], 2015, DIABETES CARE, V38, pS20, DOI 10.2337/dc15-S007

Daitoku Masuko, 2006, Journal of the Japan Diabetes Society, V49, P1

Ikeda K, 2014, PLOS ONE, V9, DOI 10.1371/journal.pone.0109323

Ishii H, 1999, DIABETES, V48, pA319

Kashiwagi A, 2012, J DIABETES INVEST, V3, P39, DOI 10.1111/j.2040-1124.2012.00207.x

Kitayama S, 2006, HDB CULTURAL PSYCHOL, P136

Lirussi F, 2010, DIABETES-METAB RES, V26, P421, DOI 10.1002/dmrr.1105

MARKUS HR, 1991, PSYCHOL REV, V98, P224, DOI 10.1037/0033-295X.98.2.224

Morling B, 2003, PERS SOC PSYCHOL B, V29, P1533, DOI 10.1177/0146167203256878

Morling B, 2015, PLOS ONE, V10, DOI 10.1371/journal.pone.0127737

Peyrot M, 2007, DIABETES CARE, V30, P2433, DOI 10.2337/dc07-1222

POLONSKY WH, 1995, DIABETES CARE, V18, P754, DOI 10.2337/diacare.18.6.754

Powers MA, 2015, DIABETES CARE, V38, P1372, DOI 10.2337/dc15-0730

Snoek FJ, 2000, DIABETES CARE, V23, P1305, DOI 10.2337/diacare.23.9.1305

Tafarodi RW, 2011, J CROSS CULT PSYCHOL, V42, P155, DOI 10.1177/0022022110386373

Taylor SE, 2007, PSYCHOL SCI, V18, P831, DOI 10.1111/j.1467-9280.2007.01987.x

Toobert DJ, 2000, DIABETES CARE, V23, P943, DOI 10.2337/diacare.23.7.943

Torenholt R, 2014, DIABETIC MED, V31, P15, DOI 10.1111/dme.12290

Uchida Y, 2008, PERS SOC PSYCHOL B, V34, P741, DOI 10.1177/0146167208315157

NR 19

TC 0

Z9 0

U1 0

U2 0

PU WILEY

PI HOBOKEN

PA 111 RIVER ST, HOBOKEN 07030-5774, NJ USA

SN 2040-1124

J9 J DIABETES INVEST

JI J. Diabetes Investig.

PD SEP

PY 2018

VL 9

IS 5

BP 1212

EP 1215

DI 10.1111/jdi.12822

PG 4

WC Endocrinology & Metabolism

SC Endocrinology & Metabolism

GA GS3YI

UT WOS:000443564400030

PM 29453793

OA DOAJ Gold

DA 2019-01-08

ER

PT J

AU Ozcan, B

Rutters, F

Snoek, FJ

Roosendaal, M

Sijbrands, EJ

Elders, PJM

Holleman, F

Pijl, H

Tack, CJ

Abbink, EJ

de Valk, HW

Wolffenbuttel, BHR

Stehouwer, CDA

Schaper, NC

Dekker, JM

Schram, MT

AF Ozcan, Behiye

Rutters, Femke

Snoek, Frank J.

Roosendaal, Mandy

Sijbrands, Eric J.

Elders, Petra J. M.

Holleman, Frits

Pijl, Hanno

Tack, Cees J.

Abbink, Evertine J.

de Valk, Harold W.

Wolffenbuttel, Bruce H. R.

Stehouwer, Coen D. A.

Schaper, Nicholas C.

Dekker, Jacqueline M.

Schram, Miranda T.

CA Diabet Peart Parelsnoer Initiative

TI High Diabetes Distress Among Ethnic Minorities Is Not Explained by

Metabolic, Cardiovascular, or Lifestyle Factors: Findings From the Dutch

Diabetes Pearl Cohort

SO DIABETES CARE

LA English

DT Article

ID EMOTIONAL DISTRESS; SELF-MANAGEMENT; CONTROLLED-TRIAL; OLDER-ADULTS;

DEPRESSION; EFFICACY; PEOPLE; HEALTH; RECOGNITION; HISPANICS

AB OBJECTIVEDiabetes distress among patients from ethnic minorities is still poorly understood. We investigated the association between ethnicity and diabetes distress among ethnic minority groups of people with type 2 diabetes in the Netherlands, focusing on the possible effects of glycemic control, lifestyle factors, cardiovascular risk factors, and diabetes complications.RESEARCH DESIGN AND METHODSCross-sectional data from the Dutch Diabetes Pearl cohort included people with type 2 diabetes from primary, secondary, and tertiary diabetes care programs. We used the 20-item Problem Areas in Diabetes Survey (PAID) scale to assess diabetes distress; a score 40 is considered to represent high distress. Ethnicity was estimated on the basis of country of birth. Sociodemographic and lifestyle data were self-reported; cardiovascular and metabolic data were retrieved from medical charts. Logistic regression analysis determined the association between ethnicity and diabetes distress, with Caucasians as the reference group.RESULTSDiabetes distress scores and ethnicity were available for 4,191 people with type 2 diabetes: 3,684 were Caucasian, 83 were Asian, 51 were Moroccan, 92 were African, 134 were Latin American, 46 were Turkish, and 101 were Hindustani-Surinamese. Overall, participants in minority groups had worse health outcomes than those of Caucasian descent, and diabetes distress was more prevalent (ranging from 9.6 to 31.7%, compared with 5.8% among Caucasians), even after adjusting for age, sex, education level, alcohol use, smoking, BMI, lipid profile, HbA(1c), medication use, and the presence of diabetes complications.CONCLUSIONSAmong people with type 2 diabetes in the Netherlands, ethnicity is independently associated with high diabetes distress. Further research is warranted to explain the higher prevalence of diabetes distress in minority groups and to develop effective interventions.

C1 [Ozcan, Behiye; Sijbrands, Eric J.] Erasmus MC, Rotterdam, Netherlands.

[Rutters, Femke; Snoek, Frank J.; Elders, Petra J. M.; Dekker, Jacqueline M.] Vrije Univ Amsterdam, Med Ctr, Amsterdam, Netherlands.

[Snoek, Frank J.; Holleman, Frits] Acad Med Ctr, Amsterdam, Netherlands.

[Roosendaal, Mandy; Stehouwer, Coen D. A.; Schaper, Nicholas C.; Schram, Miranda T.] CARIM, Cardiovasc Res Inst Maastricht CARIM, Maastricht, Netherlands.

[Pijl, Hanno] Leiden Univ, Med Ctr, Leiden, Netherlands.

[Tack, Cees J.; Abbink, Evertine J.] Radboud Univ Nijmegen, Med Ctr, Nijmegen, Netherlands.

[de Valk, Harold W.] Univ Med Ctr Utrecht, Utrecht, Netherlands.

[Wolffenbuttel, Bruce H. R.] Univ Med Ctr Groningen, Groningen, Netherlands.

RP Ozcan, B (reprint author), Erasmus MC, Rotterdam, Netherlands.

EM b.ozcan@erasmusmc.nl

OI Sijbrands, Eric/0000-0001-8857-7389; Pijl, Hanno/0000-0002-3076-1551;

Schram, Miranda/0000-0001-8113-7604

FU Dutch Federation of University Medical Centres; Dutch Government

FX The work described in this study was carried out in the context of the

Parelsnoer Initiative (PSI). PSI is part of and is funded by the Dutch

Federation of University Medical Centres and from 2007 to 2011 received

initial funding from the Dutch Government.

CR Bindraban NR, 2008, BMC PUBLIC HEALTH, V8, DOI 10.1186/1471-2458-8-271

Cinnirella M, 1999, BRIT J MED PSYCHOL, V72, P505, DOI 10.1348/000711299160202

Department of Health, 2016, UK CHIEF MED OFF ALC

Fisher L, 2014, DIABETIC MED, V31, P764, DOI 10.1111/dme.12428

Fisher L, 2014, DIABETIC MED, V31, P739, DOI 10.1111/dme.12403

Fisher L, 2013, DIABETES CARE, V36, P2551, DOI 10.2337/dc12-2493

Fisher L, 2012, DIABETES CARE, V35, P259, DOI 10.2337/dc11-1572

Fredrickson BL, 2005, COGNITION EMOTION, V19, P313, DOI 10.1080/02699930441000238

Glasgow RE, 2012, PATIENT EDUC COUNS, V87, P81, DOI 10.1016/j.pec.2011.07.024

Grzywacz JG, 2012, BEHAV MED, V38, P115, DOI 10.1080/08964289.2012.693976

Huang ES, 2009, DIABETES CARE, V32, P311, DOI 10.2337/dc08-1307

Hudson DL, 2013, AM J MANAG CARE, V19, P344

Ince BU, 2014, BMC PSYCHIATRY, V14, DOI 10.1186/s12888-014-0252-5

Jansen YFM, 2011, NED TIJDSCHR GENEES, V155, pA3117

Joensen LE, 2013, DIABETIC MED, V30, P1132, DOI 10.1111/dme.12241

KDOQI, 2007, Am J Kidney Dis, V49, pS12

Kim G, 2015, J AGING HEALTH, V27, P320, DOI 10.1177/0898264314549662

Kohinor MJE, 2011, BMC PUBLIC HEALTH, V11, DOI 10.1186/1471-2458-11-399

Lavery LA, 1996, DIABETES CARE, V19, P48, DOI 10.2337/diacare.19.1.48

Lazarus R. S, 1984, STRESS APPRAISAL COP

Loewenthal KM, 2001, BRIT J MED PSYCHOL, V74, P293, DOI 10.1348/000711201160993

Lustman PJ, 1998, ANN INTERN MED, V129, P613, DOI 10.7326/0003-4819-129-8-199810150-00005

Nouwen A, 2015, DIABETIC MED, V32, P1261, DOI 10.1111/dme.12863

Peyrot M, 2014, CURR MED RES OPIN, V30, P2241, DOI 10.1185/03007995.2014.947023

Pouwer F, 2006, PATIENT EDUC COUNS, V60, P235, DOI 10.1016/j.pec.2005.01.009

Pouwer F, 2013, DIABETIC MED, V30, pE25, DOI 10.1111/dme.12031

Rosal MC, 2003, DIABETES EDUCATOR, V29, P1006, DOI 10.1177/014572170302900611

Schmidt CB, 2017, J DIABETES RES, DOI 10.1155/2017/1204237

Snoek FJ, 2000, DIABETES CARE, V23, P1305, DOI 10.2337/diacare.23.9.1305

Spencer MS, 2006, J HEALTH CARE POOR U, V17, P88, DOI 10.1353/hpu.2006.0095

Stoop CH, 2014, DIABETIC MED, V31, P1252, DOI 10.1111/dme.12472

Stronks K, 2009, J PUBLIC HEALTH-UK, V31, P324, DOI 10.1093/pubmed/fdp070

Tang TS, 2014, DIABETES CARE, V37, P1525, DOI 10.2337/dc13-2161

Trief PM, 2009, AGE AGEING, V38, P219, DOI 10.1093/ageing/afn299

Ujcic-Voortman JK, 2009, EUR J PUBLIC HEALTH, V19, P511, DOI 10.1093/eurpub/ckp096

van't Riet E, 2012, BMC PUBLIC HLTH, V12, P949

Weng C, 2000, DIABETIC MED, V17, P612, DOI 10.1046/j.1464-5491.2000.00352.x

World Health Organization, 2016, GLOB REP DIAB

YOUNG MJ, 1993, DIABETES RES CLIN PR, V20, P129, DOI 10.1016/0168-8227(93)90006-Q

Young-Hyman D, 2016, DIABETES CARE, V39, P2126, DOI 10.2337/dc16-2053

NR 40

TC 1

Z9 1

U1 2

U2 2

PU AMER DIABETES ASSOC

PI ALEXANDRIA

PA 1701 N BEAUREGARD ST, ALEXANDRIA, VA 22311-1717 USA

SN 0149-5992

EI 1935-5548

J9 DIABETES CARE

JI Diabetes Care

PD SEP 1

PY 2018

VL 41

IS 9

BP 1854

EP 1861

DI 10.2337/dc17-2181

PG 8

WC Endocrinology & Metabolism

SC Endocrinology & Metabolism

GA GR1UJ

UT WOS:000442337700009

PM 29945936

DA 2019-01-08

ER

PT J

AU Fisher, L

Hessler, D

Polonsky, WH

Masharani, U

Guzman, S

Bowyer, V

Strycker, L

Ahmann, A

Basina, M

Blumer, I

Chloe, C

Kim, S

Peters, AL

Shumway, M

Weihs, K

Wu, P

AF Fisher, Lawrence

Hessler, Danielle

Polonsky, William H.

Masharani, Umesh

Guzman, Susan

Bowyer, Vicky

Strycker, Lisa

Ahmann, Andrew

Basina, Marina

Blumer, Ian

Chloe, Charles

Kim, Sarah

Peters, Anne L.

Shumway, Martha

Weihs, Karen

Wu, Patricia

TI T1-REDEEM: A Randomized Controlled Trial to Reduce Diabetes Distress

Among Adults With Type 1 Diabetes

SO DIABETES CARE

LA English

DT Article

ID SELF-REGULATION; REGIMEN DISTRESS; TIME; DEPRESSION; MANAGEMENT

AB OBJECTIVETo compare the effectiveness of two interventions to reduce diabetes distress (DD) and improve glycemic control among adults with type 1 diabetes (T1D).RESEARCH DESIGN AND METHODSIndividuals with T1D (n = 301) with elevated DD and HbA(1c) were recruited from multiple settings and randomly assigned to OnTrack, an emotion-focused intervention, or to KnowIt, an educational/behavioral intervention. Each group attended a full-day workshop plus four online meetings over 3 months. Assessments occurred at baseline and 3 and 9 months. Primary and secondary outcomes were change in DD and change in HbA(1c), respectively.RESULTSWith 12% attrition, both groups demonstrated dramatic reductions in DD (effect size d = 1.06; 78.4% demonstrated a reduction of at least one minimal clinically important difference). There were, however, no significant differences in DD reduction between OnTrack and KnowIt. Moderator analyses indicated that OnTrack provided greater DD reduction to those with initially poorer cognitive or emotion regulation skills, higher baseline DD, or greater initial diabetes knowledge than those in KnowIt. Significant but modest reductions in HbA(1c) occurred with no between-group differences. Change in DD was modestly associated with change in HbA(1c) (r = 0.14, P = 0.01), with no significant between-group differences.CONCLUSIONSDD can be successfully reduced among distressed individuals with T1D with elevated HbA(1c) using both education/behavioral and emotion-focused approaches. Reductions in DD are only modestly associated with reductions in HbA(1c). These findings point to the importance of tailoring interventions to address affective, knowledge, and cognitive skills when intervening to reduce DD and improve glycemic control.

C1 [Fisher, Lawrence; Hessler, Danielle; Masharani, Umesh; Bowyer, Vicky; Kim, Sarah; Shumway, Martha] Univ Calif San Francisco, San Francisco, CA 94143 USA.

[Polonsky, William H.] Univ Calif San Diego, San Diego, CA 92103 USA.

[Guzman, Susan] Behav Diabet Inst, San Diego, CA USA.

[Strycker, Lisa] Oregon Res Inst, Eugene, OR 97403 USA.

[Ahmann, Andrew] Oregon Hlth & Sci Univ, Portland, OR 97201 USA.

[Basina, Marina] Stanford Univ, Stanford, CA 94305 USA.

[Blumer, Ian] Univ Toronto, Toronto, ON, Canada.

[Chloe, Charles] Univ Calif San Diego, Vet Affairs San Diego Healthcare Syst, San Diego, CA 92103 USA.

[Peters, Anne L.] Univ Southern Calif, Keck Sch Med, Los Angeles, CA 90033 USA.

[Weihs, Karen] Univ Arizona, Coll Med, Tucson, AZ USA.

[Wu, Patricia] Kaiser Permanente Med Grp, San Diego, CA USA.

RP Fisher, L (reprint author), Univ Calif San Francisco, San Francisco, CA 94143 USA.

EM larry.fisher@ucsf.edu

FU National Institute of Diabetes and Digestive and Kidney Diseases [R01

DK-094863]

FX This research was supported by the National Institute of Diabetes and

Digestive and Kidney Diseases (R01 DK-094863).

CR Baer RA, 2006, ASSESSMENT, V13, P27, DOI 10.1177/1073191105283504

Blair C, 2008, DEV PSYCHOPATHOL, V20, P899, DOI 10.1017/S0954579408000436

Cohen J, 1988, STAT POWER ANAL BEHA

Delahanty LM, 2007, DIABETIC MED, V24, P48, DOI 10.1111/j.1464-5491.2007.02028.x

Fisher L, 2014, DIABETIC MED, V31, P764, DOI 10.1111/dme.12428

Fisher L, 2018, PATIENT EDUC COUNS, V101, P124, DOI 10.1016/j.pec.2017.06.036

Fisher L, 2016, J DIABETES COMPLICAT, V30, P1123, DOI 10.1016/j.jdiacomp.2016.03.032

Fisher L, 2015, J DIABETES COMPLICAT, V29, P572, DOI 10.1016/j.jdiacomp.2015.01.012

Fisher L, 2013, DIABETES CARE, V36, P2551, DOI 10.2337/dc12-2493

Fisher L, 2012, PATIENT EDUC COUNS, V86, P372, DOI 10.1016/j.pec.2011.07.002

Fisher L, 2012, DIABETES CARE, V35, P259, DOI 10.2337/dc11-1572

Fitzgerald JT, 2016, DIABETES EDUCATOR, V42, P178, DOI 10.1177/0145721715624968

Fredrickson BL, 2005, COGNITION EMOTION, V19, P313, DOI 10.1080/02699930441000238

Funnell MM, 2004, CLIN DIABETES, V22, P123, DOI DOI 10.2337/DIACLIN.22.3.123

Gonzalez JS, 2011, DIABETES CARE, V34, P236, DOI 10.2337/dc10-1970

Guendelman S, 2017, FRONT PSYCHOL, V8, P1, DOI 10.3389/fpsyg.2017.00220

Hessler DM, 2017, DIABETIC MED, V34, P1228, DOI 10.1111/dme.13381

Hessler D, 2015, DIABETES RES CLIN PR, V108, P360, DOI 10.1016/j.diabres.2014.12.017

Hessler D, 2014, DIABETES CARE, V37, P617, DOI 10.2337/dc13-0762

Hilliard ME, 2016, CURR DIABETES REP, V16, DOI 10.1007/s11892-016-0761-3

IBM Corp, 2010, IBM SPSS STAT WIND V

Lansing AH, 2014, J PEDIATR PSYCHOL, V39, P1091, DOI 10.1093/jpepsy/jsu067

Leventhal H., 2003, SELF REGULATION HLTH, V1, P42

Lloyd C, 2005, DIABETES SPECTR, V18, P121, DOI [DOI 10.2337/DIASPECT.18.2.121, 10.2337/diaspect.18.2.121]

Miller W. R., 2013, MOTIVATIONAL INTERVI

Moss-Morris R, 2002, PSYCHOL HEALTH, V17, P1, DOI 10.1080/08870440290001494

Nigg JT, 2017, J CHILD PSYCHOL PSYC, V58, P361, DOI 10.1111/jcpp.12675

Polonsky WH, 2017, DIABETES CARE, V40, P736, DOI 10.2337/dc17-0133

Quoidbach J, 2015, PSYCHOL BULL, V141, P655, DOI 10.1037/a0038648

Sturt J, 2015, INT DIAB NURS, V12, P40, DOI DOI 10.1179/2057332415Y.0000000004

Zwarenstein M, 2008, BRIT MED J, V337, DOI 10.1136/bmj.a2390

NR 31

TC 3

Z9 3

U1 0

U2 0

PU AMER DIABETES ASSOC

PI ALEXANDRIA

PA 1701 N BEAUREGARD ST, ALEXANDRIA, VA 22311-1717 USA

SN 0149-5992

EI 1935-5548

J9 DIABETES CARE

JI Diabetes Care

PD SEP 1

PY 2018

VL 41

IS 9

BP 1862

EP 1869

DI 10.2337/dc18-0391

PG 8

WC Endocrinology & Metabolism

SC Endocrinology & Metabolism

GA GR1UJ

UT WOS:000442337700010

PM 29976567

DA 2019-01-08

ER

PT J

AU Danne, T

Cariou, B

Banks, P

Brandle, M

Brath, H

Franek, E

Kushner, JA

Lapuerta, P

McGuire, DK

Peters, AL

Sawhney, S

Strumph, P

AF Danne, Thomas

Cariou, Bertrand

Banks, Phillip

Brandle, Michael

Brath, Helmut

Franek, Edward

Kushner, Jake A.

Lapuerta, Pablo

McGuire, Darren K.

Peters, Anne L.

Sawhney, Sangeeta

Strumph, Paul

TI HbA(1c) and Hypoglycemia Reductions at 24 and 52 Weeks With

Sotagliflozin in Combination With Insulin in Adults With Type 1

Diabetes: The European inTandem2 Study

SO DIABETES CARE

LA English

DT Article

ID EXCHANGE CLINIC REGISTRY; PLACEBO-CONTROLLED TRIAL; T1D EXCHANGE;

DOUBLE-BLIND; AMERICAN ASSOCIATION; GLYCEMIC CONTROL; COMPLICATIONS;

KETOACIDOSIS; INHIBITORS; MELLITUS

AB OBJECTIVEThe objective of this study was to evaluate the efficacy and safety of the dual sodium-glucose cotransporter 1 and 2 inhibitor sotagliflozin compared with placebo when combined with optimized insulin in adults with type 1 diabetes (T1D).RESEARCH DESIGN AND METHODSIn a double-blind, 52-week, international phase 3 trial, adults with T1D were randomized to placebo (n = 258) or once-daily oral sotagliflozin 200 mg (n = 261) or 400 mg (n = 263) after 6 weeks of insulin optimization. The primary outcome was change in HbA(1c) from baseline to 24 weeks. The first secondary end point was a composite of the proportion of patients with HbA(1c) <7.0%, no episode of severe hypoglycemia, and no episode of diabetic ketoacidosis (DKA) at week 24. Fasting glucose, weight, insulin dose, and safety end points were assessed through 52 weeks.RESULTSAt 24 weeks, placebo-adjusted changes in HbA(1c) from baseline (7.8%) were -0.37% and -0.35% with sotagliflozin 200 and 400 mg, respectively (P < 0.001), and differences were maintained at 52 weeks. At 52 weeks, greater proportions of sotagliflozin-treated patients (200 mg: 25.67%; 400 mg: 26.62%) than placebo-treated patients (14.34%; P 0.001) met the composite end point, and sotagliflozin 400 mg reduced fasting plasma glucose (-0.87 mmol/L; P = 0.008), weight (-2.92 kg; P < 0.001), and total daily insulin dose (-8.2%; P = 0.001). In a 24-week continuous glucose monitoring (CGM) substudy, postprandial glucose decreased (P 0.009) and CGM demonstrated up to 3 h more time in the target range of 3.9-10.0 mmol/L with sotagliflozin. Treatment satisfaction increased and diabetes distress decreased with sotagliflozin (P < 0.05 vs. placebo). The frequency of documented hypoglycemia was lower with sotagliflozin, and severe hypoglycemia occurred by week 52 in 13 patients (5.0%), 13 patients (5.0%), and 6 patients (2.3%) treated with placebo and sotagliflozin 200 and 400 mg, respectively. DKA occurred in 0 of 258 patients, 6 of 261 patients (2.3%), and 9 of 263 patients (3.4%) in these respective groups.CONCLUSIONSIn a 1-year study, sotagliflozin was associated with statistically significant HbA(1c) reductions. More episodes of DKA and fewer episodes of documented and severe hypoglycemia were observed in patients using sotagliflozin relative to those receiving placebo (ClinicalTrials.gov, NCT02421510).

C1 [Danne, Thomas] Hannover Med Sch, Childrens & Youth Hosp Bult, Dept Diabet Endocrinol & Clin Res, Hannover, Germany.

[Cariou, Bertrand] CHU Nantes, Dept Endocrinol, Inst Thorax, INSERM,CIC 1413, Nantes, France.

[Banks, Phillip; Lapuerta, Pablo; Sawhney, Sangeeta; Strumph, Paul] Lexicon Pharmaceut Inc, The Woodlands, TX USA.

[Brandle, Michael] Kantonsspital St Gallen, Dept Internal Med, St Gallen, Switzerland.

[Brath, Helmut] Hlth Ctr South, Diabet Outpatient Clin, Vienna, Austria.

[Franek, Edward] Polish Acad Sci, Mossakowski Med Res Ctr, Warsaw, Poland.

[Kushner, Jake A.] Baylor Coll Med, Dept Pediat, Houston, TX 77030 USA.

[Kushner, Jake A.] Texas Childrens Hosp, Houston, TX 77030 USA.

[McGuire, Darren K.] Univ Texas Southwestern Med Ctr Dallas, Dept Internal Med, Div Cardiol, Dallas, TX USA.

[Peters, Anne L.] Univ Southern Calif, Keck Sch Med, Los Angeles, CA USA.

RP Danne, T (reprint author), Hannover Med Sch, Childrens & Youth Hosp Bult, Dept Diabet Endocrinol & Clin Res, Hannover, Germany.

EM danne@hka.de

OI Kushner, Jake A/0000-0002-3646-1744

FU Lexicon Pharmaceuticals, Inc.; Sanofi

FX This study was supported and conducted by Lexicon Pharmaceuticals, Inc.

Lexicon Pharmaceuticals, Inc., and Sanofi entered a license agreement

effective November 2015 and are collaborating on the development and

commercialization of sotagliflozin.

CR Agiostratidou G, 2017, DIABETES CARE, V40, P1622, DOI 10.2337/dc17-1624

Amer Diabet Assoc, 2018, DIABETES CARE, V41, pS144, DOI 10.2337/dc18-S014

Bode BW, 2016, ENDOCR PRACT, V22, P220, DOI 10.4158/EP15869.RA

Buse JB, 2018, DIABETES CARE, V41, P1970, DOI [10.2337/dc17-2212, 10.2337/dc18-0343]

Cariou B, 2015, DIABETES METAB, V41, P116, DOI 10.1016/j.diabet.2014.10.007

Cariou B, 2015, EXPERT OPIN INV DRUG, V24, P1647, DOI 10.1517/13543784.2015.1100361

Dandona P, 2017, LANCET DIABETES ENDO, V5, P864, DOI 10.1016/S2213-8587(17)30308-X

Dobbins RL, 2015, AM J PHYSIOL-GASTR L, V308, pG946, DOI 10.1152/ajpgi.00286.2014

Ellis SL, 2011, DIABETIC MED, V28, P1176, DOI 10.1111/j.1464-5491.2011.03331.x

Farsani SF, 2017, BMJ OPEN, V7, DOI 10.1136/bmjopen-2017-016587

Garg SK, 2018, NEW ENGL J MED, V378, P967, DOI 10.1056/NEJMc1800394

Garg SK, 2017, NEW ENGL J MED, V377, P2337, DOI 10.1056/NEJMoa1708337

Garg SK, 2017, DIABETES TECHNOL THE, V19, P155, DOI 10.1089/dia.2016.0421

Garg SK, 2013, ENDOCR PRACT, V19, P19, DOI 10.4158/EP12100.OR

Handelsman Y, 2016, ENDOCR PRACT, V22, P753, DOI 10.4158/EP161292.PS

Henry RR, 2015, DIABETES CARE, V38, P2258, DOI 10.2337/dc15-1730

Jonasson JM, 2008, DIABETES CARE, V31, P1536, DOI 10.2337/dc08-0344

Khunti K, 2016, DIABETES OBES METAB, V18, P907, DOI 10.1111/dom.12689

Lapuerta P, 2015, DIABETES VASC DIS RE, V12, P101, DOI 10.1177/1479164114563304

Lyons SK, 2017, DIABETES CARE, V40, pE139, DOI 10.2337/dc17-0403

Mathieu C, 2016, DIABETES CARE, V39, P1702, DOI 10.2337/dc16-0691

McKnight JA, 2015, DIABETIC MED, V32, P1036, DOI 10.1111/dme.12676

Miller KM, 2015, DIABETES CARE, V38, P971, DOI 10.2337/dc15-0078

Nathan DM, 2001, DIABETES CARE, V24, P1711

Neal B, 2017, NEW ENGL J MED, V377, P644, DOI 10.1056/NEJMoa1611925

Petrie JR, 2017, LANCET DIABETES ENDO, V5, P597, DOI 10.1016/S2213-8587(17)30194-8

Pieber T R, 2015, Diabetes Obes Metab, V17, P928, DOI 10.1111/dom.12494

Purnell JQ, 2013, CIRCULATION, V127, P180, DOI 10.1161/CIRCULATIONAHA.111.077487

Russell SJ, 2014, NEW ENGL J MED, V371, P313, DOI 10.1056/NEJMoa1314474

Sands AT, 2015, DIABETES CARE, V38, P1181, DOI 10.2337/dc14-2806

Taylor SI, 2015, J CLIN ENDOCR METAB, V100, P2849, DOI 10.1210/jc.2015-1884

Weinstock RS, 2016, DIABETES RES CLIN PR, V122, P28, DOI 10.1016/j.diabres.2016.09.024

Weinstock RS, 2013, J CLIN ENDOCR METAB, V98, P3411, DOI 10.1210/jc.2013-1589

Zambrowicz B, 2013, CLIN THER, V35

NR 34

TC 6

Z9 6

U1 3

U2 3

PU AMER DIABETES ASSOC

PI ALEXANDRIA

PA 1701 N BEAUREGARD ST, ALEXANDRIA, VA 22311-1717 USA

SN 0149-5992

EI 1935-5548

J9 DIABETES CARE

JI Diabetes Care

PD SEP 1

PY 2018

VL 41

IS 9

BP 1981

EP 1990

DI 10.2337/dc18-0342

PG 10

WC Endocrinology & Metabolism

SC Endocrinology & Metabolism

GA GR1UJ

UT WOS:000442337700024

PM 29937431

DA 2019-01-08

ER

PT J

AU Teufel-Shone, NI

Jiang, LH

Rockell, J

Chang, J

Beals, J

Bullock, A

Manson, SM

AF Teufel-Shone, Nicolette, I

Jiang, Luohua

Rockell, Jennifer

Chang, Jennifer

Beals, Janette

Bullock, Ann

Manson, Spero M.

TI Food choices and distress in reservation-based American Indians and

Alaska Natives with type 2 diabetes

SO PUBLIC HEALTH NUTRITION

LA English

DT Article

DE American Indians/Alaska Natives; Type 2 diabetes; Food choice; Diet;

Distress

ID NONSPECIFIC PSYCHOLOGICAL DISTRESS; DEMONSTRATION PROJECT;

GENERAL-POPULATION; PHYSICAL-ACTIVITY; SCREENING SCALES;

NATIONAL-SURVEY; HEART-DISEASE; US ADULTS; STRESS; HEALTH

AB Objective: To examine the association between food choice and distress in a large national sample of American Indians/Alaska Natives (AI/AN) with type 2 diabetes.

Design: Participants completed a sociodemographic survey, an FFQ and the Kessler-6 Distress Scale. Foods were identified as 'healthy' or 'unhealthy' using a classification grounded in the health education provided by the programme case managers; healthy and unhealthy food scores were calculated using reported intake frequencies. Pearson's correlation coefficients for distress and food scores were calculated for all participants and by gender. Multiple linear regression models stratified by gender assessed the association between distress and food scores, controlling for sociodemogaphics and duration of type 2 diabetes.

Setting: Rural AI reservations and AN villages.

Subjects: AI/AN (n 2484) with type 2 diabetes.

Results: Both males (34.9%) and females (65.1%) had higher healthy food scores than unhealthy scores. In bivariate analysis, distress level had a significant negative correlation with healthy food scores among female participants, but the association was not significant among males. Significant positive correlations between distress and unhealthy food scores were found in both genders. In the final multivariate models, healthy food scores were not significantly related to distress; however, unhealthy food scores showed significant positive relationships with distress for both genders (females: beta = 0.078, P = 0.0007; males: beta = 0.139, P < 0.0001).

Conclusions: Health professionals working with AI/AN diagnosed with type 2 diabetes should offer food choice strategies during difficult times and recognize that males may be more likely than females to select unhealthy foods when distressed.

C1 [Teufel-Shone, Nicolette, I] No Arizona Univ, Dept Hlth Sci, POB 4065,ARD Bldg,Suite 120, Flagstaff, AZ 86011 USA.

[Teufel-Shone, Nicolette, I] No Arizona Univ, Ctr Hlth Equ Res, POB 4065,ARD Bldg,Suite 120, Flagstaff, AZ 86011 USA.

[Jiang, Luohua; Chang, Jennifer] Univ Calif Irvine, Dept Epidemiol, Irvine, CA USA.

[Rockell, Jennifer; Beals, Janette; Manson, Spero M.] Univ Colorado, Ctr Amer Indian & Alaska Nat Hlth, Anschutz Med Ctr, Aurora, CO USA.

[Bullock, Ann] Indian Hlth Serv, Div Diabet Treatment & Prevent, Rockville, MD USA.

RP Teufel-Shone, NI (reprint author), No Arizona Univ, Dept Hlth Sci, POB 4065,ARD Bldg,Suite 120, Flagstaff, AZ 86011 USA.; Teufel-Shone, NI (reprint author), No Arizona Univ, Ctr Hlth Equ Res, POB 4065,ARD Bldg,Suite 120, Flagstaff, AZ 86011 USA.

EM nicky.teufel@nau.edu

FU Indian Health Service [HHSI242200400049C]; American Diabetes Association

(ADA) [7-12-CT-36]; National Institute of Diabetes and Digestive and

Kidney Diseases (NIDDK) [1P30DK092923, R21DK108187]

FX Funding for the SDPI-HH project was provided by the Indian Health

Service (HHSI242200400049C, S.M. Manson). Manuscript preparation was

supported in part by American Diabetes Association (ADA #7-12-CT-36, L.

Jiang), and National Institute of Diabetes and Digestive and Kidney

Diseases (NIDDK) (1P30DK092923, S.M. Manson and R21DK108187, L. Jiang).

The IHS contributed to the study design, interpretation of findings and

the preparation of the manuscript. The ADA had no role in the design,

analysis or writing of this manuscript.

CR Best LG, 2015, CANCER CAUSE CONTROL, V26, P1551, DOI 10.1007/s10552-015-0648-7

Brand-Miller J, 2003, DIABETES CARE, V26, P2261, DOI 10.2337/diacare.26.8.2261

Brewerton TD, 2011, J WOMENS HEALTH, V20, P1133, DOI 10.1089/jwh.2011.3050

Bryant AN, 2012, AGING MENT HEALTH, V16, P208, DOI 10.1080/13607863.2011.615735

Centers for Disease Control and Prevention, 2014, NAT DIAB STAT REP ES

Choudhary P, 2004, DIABETES RES CLIN PR, V65, pS9, DOI 10.1016/j.diabres.2004.07.003

Cota D, 2006, SCIENCE, V312, P927, DOI 10.1126/science.1124147

Dohle S, 2014, PSYCHOL HEALTH, V29, P1062, DOI 10.1080/08870446.2014.909042

DOHRENWEND BP, 1980, ARCH GEN PSYCHIAT, V37, P1229

Eisenberg DM, 2015, ACAD MED, V90, P854, DOI 10.1097/ACM.0000000000000682

Furukawa TA, 2003, PSYCHOL MED, V33, P357, DOI 10.1017/S0033291702006700

Gibson EL, 2006, PHYSIOL BEHAV, V89, P53, DOI 10.1016/j.physbeh.2006.01.024

Hickman NJ, 2014, TOB CONTROL, V23, P7, DOI 10.1136/tobaccocontrol-2012-050479

Huyser KR, 2015, ETHNIC DIS, V25, P145

Jenkins DJA, 2011, DIABETOLOGIA, V54, P271, DOI 10.1007/s00125-010-1927-1

Kattelmann KK, 2009, J AM DIET ASSOC, V109, P1532, DOI 10.1016/j.jada.2009.06.362

Kessler RC, 2003, ARCH GEN PSYCHIAT, V60, P184, DOI 10.1001/archpsyc.60.2.184

Kessler RC, 2002, PSYCHOL MED, V32, P959, DOI 10.1017/S0033291702006074

King DE, 2009, AM J MED, V122, P528, DOI 10.1016/j.amjmed.2008.11.013

Li Y, 2017, PSYCHIAT RES, V253, P373, DOI 10.1016/j.psychres.2017.04.020

Lloyd-Jones DM, 2010, CIRCULATION, V121, P586, DOI 10.1161/CIRCULATIONAHA.109.192703

Mendenhall TJ, 2010, NURS INQ, V17, P359, DOI 10.1111/j.1440-1800.2010.00508.x

MICHAUD C, 1990, STRESS MEDICINE, V6, P57, DOI 10.1002/smi.2460060112

Moore K, 2014, AM J PUBLIC HEALTH, V104, pE158, DOI 10.2105/AJPH.2014.302108

Morton LW, 2007, RURAL REALITIES, V1, P4

Novotny J, 2003, J AM DIET ASSOC, V103, P1146, DOI [10.1016/S0002-8223(03)00975-1, 10.1053/jada.2003.50568]

Raghunathan TES, 2002, IVEWARE IMPUTATION V

Richardson T, 2015, INT J EAT DISORDER, V48, P517, DOI 10.1002/eat.22392

Rubin RR, 2001, J CLIN PSYCHOL, V57, P457, DOI 10.1002/jclp.1041

Sangha O, 2003, ARTHRIT RHEUM-ARTHR, V49, P156, DOI 10.1002/art.10993

Saylors K, 2005, J PSYCHOACTIVE DRUGS, V37, P273, DOI 10.1080/02791072.2005.10400520

Schuz B, 2015, APPETITE, V87, P310, DOI 10.1016/j.appet.2015.01.002

Teufel-Shone NI, 2015, PREV CHRONIC DIS, V12, DOI 10.5888/pcd12.150266

Teufel-Shone NI, 2015, ETHNIC HEALTH, V20, P327, DOI 10.1080/13557858.2014.921890

Teufel-Shone NI, 2009, AM J HEALTH PROMOT, V23, pS8, DOI 10.4278/ajhp.07053151

Ulrich-Lai YM, 2010, P NATL ACAD SCI USA, V107, P20529, DOI 10.1073/pnas.1007740107

Veazie M, 2014, AM J PUBLIC HEALTH, V104, pS359, DOI 10.2105/AJPH.2013.301715

Verhoeven AAC, 2015, APPETITE, V84, P20, DOI 10.1016/j.appet.2014.09.013

Wallis DJ, 2009, APPETITE, V52, P355, DOI 10.1016/j.appet.2008.11.007

Wansink B, 2003, PHYSIOL BEHAV, V79, P739, DOI 10.1016/S0031-9384(03)00203-8

Weidner G, 1996, ANXIETY STRESS COPIN, V9, P123, DOI 10.1080/10615809608249396

Wethington H, 2014, J PUBLIC HEALTH-UK, V36, P490, DOI 10.1093/pubmed/fdt109

Yellow Horse Brave Heart M, 2008, J HLTH SOC POLICY, V10, P1

Zellner DA, 2006, PHYSIOL BEHAV, V87, P789, DOI 10.1016/j.physbeh.2006.01.014

NR 44

TC 0

Z9 0

U1 9

U2 9

PU CAMBRIDGE UNIV PRESS

PI CAMBRIDGE

PA EDINBURGH BLDG, SHAFTESBURY RD, CB2 8RU CAMBRIDGE, ENGLAND

SN 1368-9800

EI 1475-2727

J9 PUBLIC HEALTH NUTR

JI Public Health Nutr.

PD SEP

PY 2018

VL 21

IS 13

BP 2367

EP 2375

DI 10.1017/S136898018000897

PG 9

WC Public, Environmental & Occupational Health; Nutrition & Dietetics

SC Public, Environmental & Occupational Health; Nutrition & Dietetics

GA GP4SM

UT WOS:000440860200003

PM 29681247

DA 2019-01-08

ER

PT J

AU Seligman, HK

Smith, M

Rosenmoss, S

Marshall, MB

Waxman, E

AF Seligman, Hilary K.

Smith, Morgan

Rosenmoss, Sophie

Marshall, Michelle Berger

Waxman, Elaine

TI Comprehensive Diabetes Self-Management Support From Food Banks: A

Randomized Controlled Trial

SO AMERICAN JOURNAL OF PUBLIC HEALTH

LA English

DT Article

ID LOW-INCOME PATIENTS; GLYCEMIC CONTROL; HOSPITAL-ADMISSIONS; REGRESSION

APPROACH; UNITED-STATES; INSECURITY; HYPOGLYCEMIA; EFFICACY;

INTERVENTION; DISPARITIES

AB Objectives. To determine whether food bank provision of self-management support and diabetes-appropriate food improves glycemic control among clients with diabetes.

Methods. We screened 5329 adults for diabetes at food pantries (n = 27) affiliated with food banks in Oakland, California; Detroit, Michigan; and Houston, Texas, between October 2015 and September 2016. We individually randomized 568 participants with hemoglobin A1c (HbA1c) 7.5% or greater to waitlist control or 6-month intervention including food, diabetes education, health care referral, and glucose monitoring. The primary outcome was HbA1c at 6 months.

Results. Food security (relative risk [RR] = 0.85; 95% confidence interval [CI] = 0.73, 0.98), food stability (RR = 0.77; 95% CI = 0.64, 0.93), and fruit and vegetable intake (risk difference [RD] = 0.34; 95% CI = 0.34, 0.34) significantly improved among intervention participants. There were no differences in self-management (depressive symptoms, diabetes distress, self-care, hypoglycemia, self-efficacy) or HbA1c (RD = 0.24; 95% CI = -0.09, 0.58).

Conclusions. Food banks are ideally situated to provide diabetes-appropriate food to food-insecure households. Effective strategies for food banks to support improvements in diabetes clinical outcomes require additional study.

Public Health Implications. Moving chronic disease support from clinics into communities expands reach into vulnerable populations. However, it is unclear how community interventions should be integrated with clinical care to improve disease outcomes.

C1 [Seligman, Hilary K.; Rosenmoss, Sophie] Univ Calif San Francisco, Div Gen Internal Med, San Francisco, CA 94143 USA.

[Seligman, Hilary K.; Rosenmoss, Sophie] Univ Calif San Francisco, Ctr Vulnerable Populat, San Francisco, CA 94143 USA.

[Seligman, Hilary K.; Smith, Morgan; Marshall, Michelle Berger; Waxman, Elaine] Urban Inst, Washington, DC 20037 USA.

RP Seligman, HK (reprint author), Univ Calif San Francisco, POB 1364, San Francisco, CA 94143 USA.

EM hilary.seligman@ucsf.edu

FU Feeding America; Laura and John Arnold Foundation; Urban Institute via a

Robert Wood Johnson Foundation grant; National Institute of Diabetes and

Digestive and Kidney Diseases of the National Institutes of Health

[P30DK092924]; Centers for Disease Control and Prevention

[3U48DP004998-01S1]

FX Funding for the Feeding America Intervention Trial for Health-Diabetes

Mellitus (FAITH-DM) was provided by Feeding America, the Laura and John

Arnold Foundation, the Urban Institute via a Robert Wood Johnson

Foundation grant, National Institute of Diabetes and Digestive and

Kidney Diseases of the National Institutes of Health under award

P30DK092924, and Centers for Disease Control and Prevention under award

3U48DP004998-01S1.

CR American Association of Diabetes Educators, 2018, AADE7 SELF CAR BEH

[Anonymous], 2014, TYP 2 DIAB BASICS CU

Basu S, 2017, MED CARE, V55, P639, DOI 10.1097/MLR.0000000000000728

Berkowitz Seth A, 2018, Health Aff (Millwood), V37, P535, DOI 10.1377/hlthaff.2017.0999

Berkowitz SA, 2015, JAMA INTERN MED, V175, P257, DOI 10.1001/jamainternmed.2014.6888

Berkowitz SA, 2014, AM J MED, V127, P303, DOI 10.1016/j.amjmed.2014.01.002

Chetty R, 2016, JAMA-J AM MED ASSOC, V315, P1750, DOI 10.1001/jama.2016.4226

Cheung YB, 2007, AM J EPIDEMIOL, V166, P1337, DOI 10.1093/aje/kwm223

Coleman-Jensen A, 2016, STAT HOUSEHOLD FOO S, V72

Epidemiology and Genomics Research Program National Cancer Institute Division of Cancer Control and Population Sciences, 2015, DIET SCREEN 2005 CHI

Heisler M, 2002, J GEN INTERN MED, V17, P243, DOI 10.1046/j.1525-1497.2002.10905.x

Ippolito MM, 2017, PUBLIC HEALTH NUTR, V20, P183, DOI 10.1017/S1368980016001786

Johnston-Brooks CH, 2002, PSYCHOSOM MED, V64, P43, DOI 10.1097/00006842-200201000-00007

KAVANAGH DJ, 1993, J BEHAV MED, V16, P509, DOI 10.1007/BF00844820

Kroenke K, 2009, J AFFECT DISORDERS, V114, P163, DOI 10.1016/j.jad.2008.06.026

Lyles CR, 2013, DIABETES CARE, V36, P1448, DOI 10.2337/dc12-1961

Menke A, 2015, JAMA-J AM MED ASSOC, V314, P1021, DOI 10.1001/jama.2015.10029

MORISKY DE, 1986, MED CARE, V24, P67, DOI 10.1097/00005650-198601000-00007

Peek ME, 2012, HEALTH AFFAIR, V31, P177, DOI 10.1377/hlthaff.2011.1058

Sarkar U, 2006, DIABETES CARE, V29, P823, DOI 10.2337/diacare.29.04.06.dc05-1615

Seligman HK, 2015, HEALTH AFFAIR, V34, P1956, DOI 10.1377/hlthaff.2015.0641

Seligman HK, 2014, HEALTH AFFAIR, V33, P116, DOI 10.1377/hlthaff.2013.0096

Seligman HK, 2012, DIABETES CARE, V35, P233, DOI 10.2337/dc11-1627

Seligman HK, 2011, ARCH INTERN MED, V171, P1204, DOI 10.1001/archinternmed.2011.287

Seligman HK, 2010, NEW ENGL J MED, V363, P6, DOI 10.1056/NEJMp1000072

Smith S, 2018, SOC TEACH FAM MED AN

Talbot F, 1997, J BEHAV MED, V20, P291, DOI 10.1023/A:1025508928696

Thom DH, 2013, ANN FAM MED, V11, P137, DOI 10.1370/afm.1443

UCLA Center for Health Policy Research California Department of Health Services Public Health Institute., 2010, CHIS 2005 AD QUEST V

United States Department of Agriculture Economic Research Service, 2012, US HOUS FOOD SEC SUR

University of Chicago, 2018, IMPR DIAB CAR OUTC S

Weinfield NS, 2014, FEEDING AM HUNGER AM

Zou GY, 2004, AM J EPIDEMIOL, V159, P702, DOI 10.1093/aje/kwh090

NR 33

TC 0

Z9 0

U1 6

U2 6

PU AMER PUBLIC HEALTH ASSOC INC

PI WASHINGTON

PA 800 I STREET, NW, WASHINGTON, DC 20001-3710 USA

SN 0090-0036

EI 1541-0048

J9 AM J PUBLIC HEALTH

JI Am. J. Public Health

PD SEP

PY 2018

VL 108

IS 9

BP 1227

EP 1234

DI 10.2105/AJPH.2018.304528

PG 8

WC Public, Environmental & Occupational Health

SC Public, Environmental & Occupational Health

GA GP7DZ

UT WOS:000441054400049

PM 30024798

DA 2019-01-08

ER

PT J

AU Zhu, BQ

Quinn, L

Kapella, MC

Bronas, UG

Collins, EG

Ruggiero, L

Park, CG

Fritschi, C

AF Zhu, Bingqian

Quinn, Laurie

Kapella, Mary C.

Bronas, Ulf G.

Collins, Eileen G.

Ruggiero, Laurie

Park, Chang G.

Fritschi, Cynthia

TI Relationship between sleep disturbance and self-care in adults with type

2 diabetes

SO ACTA DIABETOLOGICA

LA English

DT Article

DE Actigraphy; Nocturnal awakening; Self-care; Sleep; Symptom; Type 2

diabetes

ID PHYSICAL-ACTIVITY; DAYTIME SLEEPINESS; DISTRESS; MELLITUS; SCALE;

FRAGMENTATION; MANAGEMENT; BEHAVIORS; OUTCOMES; QUALITY

AB Type 2 diabetes (T2D) is a metabolic disorder requiring intensive self-care to maintain optimal glycemic control. Sleep disturbance is common in T2D patients and likely impairs glycemic control. Our aim was to examine the relationship between sleep disturbance and self-care in adults with T2D.

This study used a correlational design. Sixty-four adults with T2D were recruited. Validated instruments were used to measure self-care, subjective sleep disturbance, and covariates (e.g., diabetes distress, self-efficacy, fatigue, and daytime sleepiness). Over an 8-day period, the ActiGraph-wGT3X was used to measure objective sleep outcomes (e.g., total sleep time and number of awakenings). Bivariate correlation and multiple linear regression analyses were conducted.

The mean age of the participants was 60.6 (SD 6.8) years (range 50-78), and 51.6% were women. Controlling for covariates, subjective sleep disturbance (beta = - 0.26), diabetes distress (beta = - 0.39), and daytime sleepiness (beta = - 0.21) were strong predictors of diabetes self-care (R (2) = 0.51, p < 0.001). When objective sleep parameters were used, the number of awakenings (beta = - 0.23) also predicted self-care along with diabetes distress, fatigue, and daytime sleepiness (R (2) = 0.57, p < 0.001).

Subjective sleep disturbance and frequent nocturnal awakenings are associated with worse diabetes self-care in adults with T2D. Healthcare providers are recommended to include comprehensive sleep assessment at every clinical visit. Diabetes educators may consider including sleep-related education in their diabetes self-management classes and/or counseling sessions.

C1 [Zhu, Bingqian; Quinn, Laurie; Kapella, Mary C.; Bronas, Ulf G.; Collins, Eileen G.; Park, Chang G.; Fritschi, Cynthia] Univ Illinois, Coll Nursing, Dept Biobehav Hlth Sci, 845 S Damen Ave,M-C 802, Chicago, IL 60612 USA.

[Ruggiero, Laurie] Univ Illinois, Sch Publ Hlth, Inst Hlth Res & Policy, Chicago, IL 60612 USA.

[Ruggiero, Laurie] Univ Delaware, Sch Nursing, Newark, DE USA.

[Zhu, Bingqian] Shanghai Jiao Tong Univ, Sch Nursing, Shanghai, Peoples R China.

RP Fritschi, C (reprint author), Univ Illinois, Coll Nursing, Dept Biobehav Hlth Sci, 845 S Damen Ave,M-C 802, Chicago, IL 60612 USA.

EM fritschi@uic.edu

OI Fritschi, Cynthia/0000-0001-5447-8315

FU Midwest Roybal Center for Health Promotion and Translation from the

National Institute on Aging [P30AG022849]; Institute for Health Research

and Policy Grant support [Center for Clinical and Translational Science

(CCTS)] [UL1RR029879]; Chancellor's Graduate Research Award of the

University of Illinois at Chicago; Provost Award for Graduate Research

of the University of Illinois at Chicago.; National Institutes of

Health/National Institute for Nursing Research and Department of

Veterans Affairs, Research Career Scientist Award [K99 R00 NR012219]

FX This research was supported by the Midwest Roybal Center for Health

Promotion and Translation from the National Institute on Aging (Grant#

P30AG022849). This research was also supported by the Institute for

Health Research and Policy Grant support [Center for Clinical and

Translational Science (CCTS) UL1RR029879], the Chancellor's Graduate

Research Award, and the Provost Award for Graduate Research of the

University of Illinois at Chicago. This research was also supported by

the National Institutes of Health/National Institute for Nursing

Research and Department of Veterans Affairs, Research Career Scientist

Award (EC) [K99 R00 NR012219 (CF)].

CR Amer Diabet Assoc, 2018, DIABETES CARE, V41, pS28, DOI 10.2337/dc18-S003

American Diabetes Association, 2017, Diabetes Care, V40, pS48

American Diabetes Association, 2015, Clin Diabetes, V33, P97, DOI 10.2337/diaclin.33.2.97

Ancoli-Israel S, 2015, BEHAV SLEEP MED, V13, pS4, DOI 10.1080/15402002.2015.1046356

Ancoli-Israel S, 2009, SLEEP MED, V10, pS7, DOI 10.1016/j.sleep.2009.07.004

Anderson RM, 2003, DIABETES CARE, V26, P1641, DOI 10.2337/diacare.26.5.1641-a

Arbuckle RA, 2009, VALUE HEALTH, V12, P1168, DOI 10.1111/j.1524-4733.2009.00571.x

Beverly EA, 2016, CURR DIABETES REP, V16, DOI 10.1007/s11892-016-0741-7

Bode Bruce W, 2007, J Diabetes Sci Technol, V1, P405

BUYSSE DJ, 1991, SLEEP, V14, P331

Cellini N, 2016, PHYSIOL BEHAV, V157, P79, DOI 10.1016/j.physbeh.2016.01.034

Chasens ER, 2013, DIABETES EDUCATOR, V39, P74, DOI 10.1177/0145721712467683

Chasens Eileen R, 2008, Issues Ment Health Nurs, V29, P1134, DOI 10.1080/01612840802319878

COLE RJ, 1992, SLEEP, V15, P461, DOI 10.1093/sleep/15.5.461

Crowley K, 2011, NEUROPSYCHOL REV, V21, P41, DOI 10.1007/s11065-010-9154-6

Grandner MA, 2016, CURR DIABETES REP, V16, DOI 10.1007/s11892-016-0805-8

Gupta S, 2016, DIABETES METAB SYND, V10, P213, DOI 10.1016/j.dsx.2016.06.009

Harris PA, 2009, J BIOMED INFORM, V42, P377, DOI 10.1016/j.jbi.2008.08.010

Hursel R, 2011, AM J CLIN NUTR, V94, P804, DOI 10.3945/ajcn.111.017632

JOHNS MW, 1991, SLEEP, V14, P540, DOI 10.1093/sleep/14.6.540

Kirk JK, 2015, DIABETES RES CLIN PR, V107, P54, DOI 10.1016/j.diabres.2014.10.005

Kontopantelis E, 2016, J STAT SOFTW, V74, P1, DOI 10.18637/jss.v074.i12

Larcher S, 2015, DIABETES METAB, V41, P263, DOI 10.1016/j.diabet.2014.12.004

Lee SWH, 2017, SLEEP MED REV, V31, P91, DOI 10.1016/j.smrv.2016.02.001

Lee YJ, 2016, PATIENT EDUC COUNS, V99, P287, DOI 10.1016/j.pec.2015.08.021

Libman E, 2016, SLEEP DISORD, V2016, P1

Martins RC, 2008, BRAZ J MED BIOL RES, V41, P180, DOI 10.1590/S0100-879X2006005000194

Nefs G, 2015, DIABETES RES CLIN PR, V109, P466, DOI 10.1016/j.diabres.2015.07.008

Ohayon MM, 2010, J PSYCHOSOM RES, V69, P565, DOI 10.1016/j.jpsychores.2010.03.010

Pandit AU, 2014, J EPIDEMIOL COMMUN H, V68, P557, DOI 10.1136/jech-2013-203063

Pintaudi B, 2015, J PSYCHOSOM RES, V79, P348, DOI 10.1016/j.jpsychores.2015.08.010

Polonsky WH, 2005, DIABETES CARE, V28, P626, DOI 10.2337/diacare.28.3.626

Redeker NS, 2008, J CARDIOVASC NURS, V23, P231, DOI 10.1097/01.JCN.0000305094.20012.76

Reutrakul S, 2014, ANN NY ACAD SCI, V1311, P151, DOI 10.1111/nyas.12355

Riegel B, 2009, EUR J CARDIOVASC NUR, V8, P337, DOI 10.1016/j.ejcnurse.2009.06.003

Rosenberger ME, 2016, MED SCI SPORT EXER, V48, P457, DOI 10.1249/MSS.0000000000000778

Sadeh A, 2011, SLEEP MED REV, V15, P259, DOI 10.1016/j.smrv.2010.10.001

Schmitt A, 2013, HEALTH QUAL LIFE OUT, V11, DOI 10.1186/1477-7525-11-138

Shen JH, 2006, SLEEP MED REV, V10, P63, DOI 10.1016/j.smrv.2005.05.004

Short MA, 2014, SLEEP DEPRIVATION DI, P13

Slater JA, 2015, SLEEP BIOL RHYTHMS, V13, P172, DOI 10.1111/sbr.12103

Stepanski EJ, 2002, SLEEP, V25, P268, DOI 10.1093/sleep/25.3.268

Weinger K, 2005, DIABETES CARE, V28, P1346, DOI 10.2337/diacare.28.6.1346

Zhuo XH, 2013, AM J PREV MED, V45, P253, DOI 10.1016/j.amepre.2013.04.017

NR 44

TC 0

Z9 0

U1 9

U2 9

PU SPRINGER-VERLAG ITALIA SRL

PI MILAN

PA VIA DECEMBRIO, 28, MILAN, 20137, ITALY

SN 0940-5429

EI 1432-5233

J9 ACTA DIABETOL

JI Acta Diabetol.

PD SEP

PY 2018

VL 55

IS 9

BP 963

EP 970

DI 10.1007/s00592-018-1181-4

PG 8

WC Endocrinology & Metabolism

SC Endocrinology & Metabolism

GA GQ2AC

UT WOS:000441445300010

PM 29931420

DA 2019-01-08

ER

PT J

AU Schmidt, CB

van Loon, BJP

Vergouwen, ACM

Snoek, FJ

Honig, A

AF Schmidt, C. B.

van Loon, B. J. Potter

Vergouwen, A. C. M.

Snoek, F. J.

Honig, A.

TI Systematic review and meta-analysis of psychological interventions in

people with diabetes and elevated diabetes-distress

SO DIABETIC MEDICINE

LA English

DT Review

ID RANDOMIZED CONTROLLED-TRIAL; QUALITY-OF-LIFE; GLYCEMIC CONTROL;

EMOTIONAL DISTRESS; DEPRESSION; ASSOCIATION; TYPE-1; HBA(1C); HEALTH

AB AimsThe clinical relevance of diabetes-distress is increasingly recognized, but little is known about the efficacy of interventions specifically targeted to treat elevated diabetes-distress. Therefore, this systematic review sought to determine the efficacy of psychological interventions aimed at treating elevated diabetes-distress in people with Type 1 or Type 2 diabetes.

MethodsWe systematically searched literature from five databases. Randomized controlled trials (RCTs) with an English abstract, describing the results of a psychological intervention in adults with diabetes were included. Articles were eligible for inclusion if the primary outcome was diabetes-distress measured by the Problem Areas in Diabetes Scale (PAID-5/PAID-20) or the Diabetes Distress Scale (DDS-17). Only mean group diabetes-distress values above cut-off at baseline or the results of a subgroup above cut-off (PAID-5 8, PAID-20 40 or DDS-17 3) were included.

ResultsThe search yielded 8907 articles. After removing 2800 duplicates, 6107 articles remained. Titles and abstracts were screened, leaving 394 potential articles of interest, nine of which were RCTs. In a random-effects meta-analysis, the pooled effect size for diabetes-distress was 0.48 (Cohen's d), Z=3.91, P<0.0001. Statistical heterogeneity was I-2=46.67% (confidence intervals 45.06% to 48.28%). Diabetes-tailored psychological interventions reduced HbA(1c) (Cohen's d=0.57), whereas mindfulness-based interventions did not (Cohen's d=0.11).

ConclusionsThis systematic review shows that specifically diabetes-tailored psychological interventions are effective in reducing elevated diabetes-distress and HbA(1c). More rigorous studies are warranted to establish the full potential of these interventions. PROSPERO database registration ID: CRD42017075290.

C1 [Schmidt, C. B.; Vergouwen, A. C. M.; Honig, A.] Dept Psychiat, Amsterdam, Netherlands.

[van Loon, B. J. Potter] OLVG, Dept Internal Med, Amsterdam, Netherlands.

[Schmidt, C. B.; Snoek, F. J.; Honig, A.] Dept Amsterdam Publ Hlth Res Inst, Amsterdam, Netherlands.

[Snoek, F. J.] Acad Med Ctr AMC, Dept Med Psychol, Amsterdam, Netherlands.

[Snoek, F. J.] Dept Med Psychol, Amsterdam, Netherlands.

[Honig, A.] Vrije Univ Amsterdam Med Ctr, Dept Psychiat, Amsterdam, Netherlands.

RP Schmidt, CB (reprint author), Dept Psychiat, Amsterdam, Netherlands.; Schmidt, CB (reprint author), Dept Amsterdam Publ Hlth Res Inst, Amsterdam, Netherlands.

EM c.schmidt@olvg.nl

OI Schmidt, Charlotte/0000-0002-7852-4264

CR Baumeister H, 2011, PSYCHOTHER PSYCHOSOM, V80, P275, DOI 10.1159/000323404

Becker L. A., 2000, EFFECT SIZE ES

Berry E, 2015, POSTGRAD MED J, V91, P278, DOI 10.1136/postgradmedj-2014-133017

Browne JL, 2016, AUST J PRIM HEALTH, V22, P409, DOI 10.1071/PY15020

Button KS, 2013, NAT REV NEUROSCI, V14, P365, DOI 10.1038/nrn3475

Chaimani Anna, 2015, Evid Based Ment Health, V18, P23, DOI 10.1136/eb-2014-102035

Chew BH, 2017, COCHRANE DB SYST REV, V9

Elmagarmid A, 2014, EVIDENCE INFORM PUBL

Fisher L, 2015, J DIABETES COMPLICAT, V29, P572, DOI 10.1016/j.jdiacomp.2015.01.012

Fisher L, 2013, DIABETES CARE, V36, P2551, DOI 10.2337/dc12-2493

Fisher L, 2012, DIABETES CARE, V35, P259, DOI 10.2337/dc11-1572

Fisher L, 2010, DIABETES CARE, V33, P23, DOI 10.2337/dc09-1238

Friis AM, 2016, DIABETES CARE, V39, P1963, DOI 10.2337/dc16-0416

Hansen BP, 2017, FAG FORSKNING, V1, P64

Harkness E, 2010, DIABETES CARE, V33, P926, DOI 10.2337/dc09-1519

Hermanns N, 2006, DIABETOLOGIA, V49, P469, DOI 10.1007/s00125-005-0094-2

Higgins JPT, 2002, STAT MED, V21, P1539, DOI 10.1002/sim.1186

Jung HY, 2015, NURS HEALTH SCI, V17, P516, DOI 10.1111/nhs.12229

Kasteleyn MJ, 2016, DIABETIC MED, V33, P125, DOI 10.1111/dme.12816

Lamers F, 2011, J ADV NURS, V67, P788, DOI 10.1111/j.1365-2648.2010.05540.x

Imazu MFM, 2015, REV LAT-AM ENFERM, V23, P200, DOI 10.1590/0104-1169.0247.2543

McGuire BE, 2010, DIABETOLOGIA, V53, P66, DOI 10.1007/s00125-009-1559-5

Nakaya N, 2014, EUR J PUBLIC HEALTH, V24, P45, DOI 10.1093/eurpub/ckt017

Newby Jill, 2017, J Med Internet Res, V19, pe157, DOI 10.2196/jmir.7274

Perrin NE, 2017, DIABETIC MED, V34, P1508, DOI 10.1111/dme.13448

Rees G, 2017, BMJ OPEN DIABETES RE, V5

Roy T, 2012, J AFFECT DISORDERS, V142, pS8, DOI 10.1016/S0165-0327(12)70004-6

Schroevers MJ, 2015, MINDFULNESS, V6, P99, DOI 10.1007/s12671-013-0235-5

Smith KJ, 2013, J PSYCHOSOM RES, V74, P89, DOI 10.1016/j.jpsychores.2012.11.013

Snoek FJ, 2015, LANCET DIABETES ENDO, V3, P450, DOI 10.1016/S2213-8587(15)00135-7

Snoek FJ, 2012, DIABETES CARE, V35, P2128, DOI 10.2337/dc11-1326

Sturt J, 2015, INT DIAB NURS, V12, P40, DOI DOI 10.1179/2057332415Y.0000000004

Thorlund K, 2012, PLOS ONE, V7, DOI 10.1371/journal.pone.0039471

van Bastelaar KMP, 2010, DIABETIC MED, V27, P798, DOI 10.1111/j.1464-5491.2010.03025.x

van der Ven NCW, 2005, DIABETIC MED, V22, P1619

van Son J, 2013, DIABETES CARE, V36, P823, DOI 10.2337/dc12-1477

von Hippel PT, 2015, BMC MED RES METHODOL, V15, DOI 10.1186/s12874-015-0024-z

Wagner JA, 2016, DIABETES RES CLIN PR, V120, P162, DOI 10.1016/j.diabres.2016.07.022

Wardian JL, 2018, PATIENT ED COUNS, V23

Whittemore R, 2004, DIABETES EDUCATOR, V30, P795, DOI 10.1177/014572170403000515

Wilson AM, 2014, PLOS ONE, V9, DOI 10.1371/journal.pone.0094175

NR 41

TC 0

Z9 0

U1 8

U2 8

PU WILEY

PI HOBOKEN

PA 111 RIVER ST, HOBOKEN 07030-5774, NJ USA

SN 0742-3071

EI 1464-5491

J9 DIABETIC MED

JI Diabetic Med.

PD SEP

PY 2018

VL 35

IS 9

BP 1157

EP 1172

DI 10.1111/dme.13709

PG 16

WC Endocrinology & Metabolism

SC Endocrinology & Metabolism

GA GP6KX

UT WOS:000440990200003

PM 29896760

DA 2019-01-08

ER

PT J

AU Hayashino, Y

Okarnura, S

Tsujii, S

Ishii, H

AF Hayashino, Yasuaki

Okarnura, Shintaro

Tsujii, Satoru

Ishii, Hitoshi

CA Diabet Distress Care Registry Tenr

TI Association between diabetes distress and all-cause mortality in

Japanese individuals with type 2 diabetes: a prospective cohort study

(Diabetes Distress and Care Registry in Tenri [DDCRT 18])

SO DIABETOLOGIA

LA English

DT Article

DE Diabetes distress; Epidemiology; Human; Mortality; Problem Areas in

Diabetes survey; Type 2 diabetes

ID GLYCEMIC CONTROL; PROBLEM AREAS; DEPRESSION; COMPLICATIONS;

BENCHMARKING; OUTCOMES; PEOPLE; TRIAL; SCALE

AB Aims/hypothesis The absence of data on the direct association between diabetes-specific distress and all-cause mortality in individuals with diabetes prompted us to examine the temporal association between Problem Areas in Diabetes (PAID) survey scores and the subsequent risk of all-cause mortality in a cohort of individuals with type 2 diabetes.

Methods Longitudinal data from 3305 individuals with diabetes were obtained from a large Japanese diabetes registry. Independent correlations between quintiles of PAID total scores or PAID scores of >= 40 and all-cause mortality (median follow-up of 6.1 years) were examined using Cox proportional hazards models with adjustment for potential confounders.

Results The study population included 1280 women and 2025 men with a mean age of 64.9 years, BMI of 24.6 kg/m(2) and HbA(1c) level of 58.7 mmol/mol (7.5%). In the multivariable-adjusted model, compared with the first quintile of PAID scores, the multivariable-adjusted HRs (95% CIs) for all-muse mortality for the second to fifth quintiles were 1.11 (0.77, 1.60; p = 0.56), 0.87 (0.56, 1.35;p = 0.524), 0.95 (0.63, 1.46;p = 0.802) and 1.60 (1.09, 2.36;p = 0.016), respectively. Compared with a PAID score of <40, the multivariable-adjusted HR for all-cause mortality of those with a score of >= 10 was 1.56 (95% CI 1.17, 2.08; p = 0.002). In subgroup analyses, the association between PAID score and all-cause mortality was found in men (HR 1.76; 95% CI 1.26, 2.46) but not in women (HR 1.09; 95% CI 0.60, 2.00), with a significant interaction between diabetes distress and sex (p = 0.0336).

Conclusions/interpretation We observed a significant positive association between high diabetes distress and all-cause mortality in men with diabetes.

C1 [Hayashino, Yasuaki; Okarnura, Shintaro; Tsujii, Satoru] Tenri Hosp, Dept Endocrinol, 200 Mishima Cho, Tenri, Nara 6328552, Japan.

[Ishii, Hitoshi] Nara Med Univ, Dept Diabetol, Kashihara, Nara, Japan.

RP Hayashino, Y (reprint author), Tenri Hosp, Dept Endocrinol, 200 Mishima Cho, Tenri, Nara 6328552, Japan.

EM hayasino-y@umin.net

OI Hayashino, Yasuaki/0000-0002-2932-8478

FU Manpei Suzuki Diabetes Foundation; Japan Society for the Promotion of

Science (JSPS) KAKENHI [25460641]

FX This study was partially supported by the Manpei Suzuki Diabetes

Foundation and the Japan Society for the Promotion of Science (JSPS)

KAKENHI (grant number 25460641). These organisations played no role in

the study design or conduct, data collection, analysis or

interpretation, or preparation, review or approval of the manuscript.

CR Buse JB, 2007, AM J CARDIOL, V99, p21I, DOI 10.1016/j.amjcard.2007.03.003

Craig CL, 2003, MED SCI SPORT EXER, V35, P1381, DOI 10.1249/01.MSS.0000078924.61453.FB

Cuijpers P, 2014, J AFFECT DISORDERS, V161, P47, DOI 10.1016/j.jad.2014.03.003

Dalsgaard EM, 2014, DIABETOLOGIA, V57, P710, DOI 10.1007/s00125-014-3165-4

Fisher L, 2010, DIABETES CARE, V33, P1034, DOI 10.2337/dc09-2175

Gonzalez JS, 2011, DIABETES CARE, V34, P236, DOI 10.2337/dc10-1970

Hay SI, 2017, LANCET, V390, P1260, DOI 10.1016/S0140-6736(17)32130-X

Hayashino Y, 2013, EXP CLIN ENDOCR DIAB, V121, P628, DOI 10.1055/s-0033-1355424

Hayashino Y, 2012, DIABETES RES CLIN PR, V97, P405, DOI 10.1016/j.diabres.2012.04.005

International Federation of Clinical Chemistry (IFCC) Working Group, 2010, STAND HBA1C

Ishii H, 2000, TONYOBYO J JAPAN DIA, V43, P13

Ismail K, 2017, DIABETOLOGIA, V60, P2092, DOI 10.1007/s00125-017-4367-3

Kahn SE, 2014, LANCET, V383, P1068, DOI 10.1016/S0140-6736(13)62154-6

Nakamura J, 2017, J DIABETES INVEST, V8, P397, DOI 10.1111/jdi.12645

Nicolucci A, 2013, DIABETIC MED, V30, P767, DOI 10.1111/dme.12245

Pintaudi B, 2015, J PSYCHOSOM RES, V79, P348, DOI 10.1016/j.jpsychores.2015.08.010

POLONSKY WH, 1995, DIABETES CARE, V18, P754, DOI 10.2337/diacare.18.6.754

Schmitt A, 2014, DIABETIC MED, V31, P1446, DOI 10.1111/dme.12553

Schmitt A, 2015, J BEHAV MED, V38, P556, DOI 10.1007/s10865-014-9604-3

Seino Y, 2010, J DIABETES INVEST, V1, P1, DOI 10.1111/j.2040-1124.2010.00005.x

Ware JE, 2001, SCORE INTERPRET SING

Welch GW, 1997, DIABETES CARE, V20, P760, DOI 10.2337/diacare.20.5.760

Whiting DR, 2011, DIABETES RES CLIN PR, V94, P311, DOI 10.1016/j.diabres.2011.10.029

NR 23

TC 1

Z9 1

U1 2

U2 2

PU SPRINGER

PI NEW YORK

PA 233 SPRING ST, NEW YORK, NY 10013 USA

SN 0012-186X

EI 1432-0428

J9 DIABETOLOGIA

JI Diabetologia

PD SEP

PY 2018

VL 61

IS 9

BP 1978

EP 1984

DI 10.1007/s00125-018-4657-4

PG 7

WC Endocrinology & Metabolism

SC Endocrinology & Metabolism

GA GO9EP

UT WOS:000440408500010

PM 29947921

OA Bronze

DA 2019-01-08

ER

PT J

AU Caballero, AE

AF Caballero, A. Enrique

TI The "A to Z" of Managing Type 2 Diabetes in Culturally Diverse

Populations

SO FRONTIERS IN ENDOCRINOLOGY

LA English

DT Review

DE type 2 diabetes; race; ethnicity; culture; health care disparities;

social medicine; treatment; vulnerable populations

ID SELF-MANAGEMENT EDUCATION; IMPAIRED GLUCOSE-TOLERANCE; HEALTH-CARE

PROFESSIONALS; PREDOMINANTLY LOW-INCOME; UNITED-STATES;

AFRICAN-AMERICAN; GLYCEMIC CONTROL; MEDICATION ADHERENCE;

INSULIN-RESISTANCE; PHYSICAL-ACTIVITY

AB Type 2 diabetes affects racial/ethnic minorities at an alarming rate in the US and in many countries around the world. The quality of health care provided to these groups is often suboptimal, resulting in worse patient-related outcomes when compared to those in mainstream populations. Understanding the complex biological elements that influence the development and course of the disease in high-risk populations is extremely important but often insufficient to implement effective prevention and treatment plans. Multiple factors must be addressed in routine diabetes clinical care. This paper discusses various key factors, organized in alphabetical order. These are acculturation, biology, clinician's cultural awareness, depression and diabetes-specific emotional distress, educational level, fears, group integration, health literacy, intimacy and sexual dysfunction, judging, knowledge of the disease, language, medication adherence, nutritional preferences, other forms of medicine (alternative), perception of body image, quality of life, religion and faith, socio-economic status, technology, unconscious bias, vulnerable groups, asking why?, exercise, "you are in charge" and zip it! Considering these factors in the development of type 2 diabetes prevention and treatment programs will help improve diabetes-related outcomes in culturally diverse populations and reduce health care disparities.

C1 [Caballero, A. Enrique] Harvard Med Sch, Off External Educ, Boston, MA 02115 USA.

RP Caballero, AE (reprint author), Harvard Med Sch, Off External Educ, Boston, MA 02115 USA.

EM enrique_caballero@hms.harvard.edu

CR Al Sayah F, 2015, J HEALTH COMMUN, V20, P581, DOI 10.1080/10810730.2015.1012235

Al-Dahir S, 2013, J HEALTH CARE POOR U, V24, P47, DOI 10.1353/hpu.2013.0038

Ali MK, 2013, NEW ENGL J MED, V368, P1613, DOI 10.1056/NEJMsa1213829

American Diabetes Association, 2017, Diabetes Care, V40, pS6

Angstman KB, 2015, MED CARE, V53, P32, DOI 10.1097/MLR.0000000000000280

[Anonymous], 2002, UNEQUAL TREATMENT CO

Bacha F, 2003, J CLIN ENDOCR METAB, V88, P2534, DOI 10.1210/jc.2002-021267

Baig AA, 2014, J HEALTH CARE POOR U, V25, P527, DOI 10.1353/hpu.2014.0086

Baig AA, 2014, ETHNIC DIS, V24, P28

Beck J, 2017, DIABETES CARE, V40, P1409, DOI 10.2337/dci17-0025

Betancourt Joseph R, 2006, Consult Pharm, V21, P988, DOI 10.4140/TCP.n.2006.988

Betancourt JR, 2004, NEW ENGL J MED, V351, P953, DOI 10.1056/NEJMp048033

Bijlani RL, 2005, J ALTERN COMPLEM MED, V11, P267, DOI 10.1089/acm.2005.11.267

Black SA, 2003, DIABETES CARE, V26, P2822, DOI 10.2337/diacare.26.10.2822

Blomster JI, 2017, DIABETES RES CLIN PR, V127, P212, DOI 10.1016/j.diabres.2017.03.015

Brown SA, 2005, DIABETES CARE, V28, P527, DOI 10.2337/diacare.28.3.527

Brown-Riggs C, 2016, INT J ENV RES PUB HE, V13, DOI 10.3390/ijerph13010028

Brunton SA, 2017, J FAM PRACTICE, V66, pS46

Butler M, 2016, 16EHC006EF AHRQ COMP

Caballero A Enrique, 2007, Curr Opin Endocrinol Diabetes Obes, V14, P151, DOI 10.1097/MED.0b013e32809f9531

Caballero AE, 2008, DIABETES CARE, V31, P576, DOI 10.2337/dc07-1540

Caballero A Enrique, 2005, Curr Diab Rep, V5, P217, DOI 10.1007/s11892-005-0012-5

Caballero A Enrique, 2004, Curr Diab Rep, V4, P237, DOI 10.1007/s11892-004-0074-9

Caballero AE, 2011, AM J MED, V124, pS10, DOI 10.1016/j.amjmed.2011.07.018

Caballero A Enrique, 2007, Curr Diab Rep, V7, P321, DOI 10.1007/s11892-007-0052-0

Caballero AE, 2006, ETHNIC DIS, V16, P559

Caballero AE, 2007, INSULIN, V2, P81, DOI [10.1016/S1557-0843(07)80019-4, DOI 10.1016/S1557-0843(07)80019-4]

Caballero AE, 2016, SCI SESS AM DIAB ASS

Caballero AE, 2005, JOSLINS DIABETES MEL, P505

Caballero AE, 2017, PRINCIPLES DIABETES, P159

Caballero E, 2004, DIABETES, V53, pA514

Capoccia K, 2016, DIABETES EDUCATOR, V42, P34, DOI 10.1177/0145721715619038

Centers for Disease Control and Prevention (CDC), 2005, MMWR Morb Mortal Wkly Rep, V54, P991

Chatterjee S, 2018, LANCET DIABETES ENDO, V6, P130, DOI 10.1016/S2213-8587(17)30239-5

Chesla CA, 2004, DIABETES CARE, V27, P2850, DOI 10.2337/diacare.27.12.2850

Chiu KC, 2000, DIABETES CARE, V23, P1353, DOI 10.2337/diacare.23.9.1353

Clark DO, 1997, DIABETES CARE, V20, P1176, DOI 10.2337/diacare.20.7.1176

Cortes DE, 2013, AM J PREV MED, V44, pS267, DOI 10.1016/j.amepre.2012.11.012

Coughlin SS, 2018, J HOSP MANAG HLTH PO, V2, DOI [10.21037/jhmhp.2017.12.04, DOI 10.21037/JHMHP.2017.12.04]

Dai SF, 2015, J PHYS ACT HEALTH, V12, pS128, DOI 10.1123/jpah.2015-0038

DeFronzo RA, 2009, DIABETES, V58, P773, DOI 10.2337/db09-9028

Devlin H, 2006, HEALTH PROMOT PRACT, V7, P47, DOI 10.1177/1524839905275395

Dham Shefali, 2006, Curr Diab Rep, V6, P251, DOI 10.1007/s11892-006-0042-7

Doshi T, 2016, AM J MED SCI, V352, P36, DOI 10.1016/j.amjms.2016.03.020

Ferdinand KC, 2014, CARDIORENAL MED, V4, P1, DOI 10.1159/000357236

Fernandez A, 2011, J GEN INTERN MED, V26, P170, DOI 10.1007/s11606-010-1507-6

Fisher L, 2000, DIABETES CARE, V23, P267, DOI 10.2337/diacare.23.3.267

FitzGerald C, 2017, BMC MED ETHICS, V18, DOI 10.1186/s12910-017-0179-8

Fox M, 2017, SOC SCI MED, V176, P123, DOI 10.1016/j.socscimed.2017.01.029

Garrow D, 2006, DIABETES CARE, V29, P15, DOI 10.2337/diacare.29.01.06.dc05-1448

Glasgow RE, 1997, DIABETES CARE, V20, P556, DOI 10.2337/diacare.20.4.556

Goran MI, 2002, DIABETES CARE, V25, P2184, DOI 10.2337/diacare.25.12.2184

Gurka MJ, 2006, OBESITY, V14, P1085, DOI 10.1038/oby.2006.124

Hahn EA, 2015, J HEALTH COMMUN, V20, P4, DOI [10.1080/10810730.2015.1061071, 10.1097/MLR.0B013E31817A835D]

Hall WJ, 2015, AM J PUBLIC HEALTH, V105, pE60, DOI 10.2105/AJPH.2015.302903

Han BH, 2016, J NURS SCHOLARSHIP, V48, P48, DOI 10.1111/jnu.12180

Hansel B, 2017, J MED INTERNET RES, V19, DOI 10.2196/jmir.7947

Hassanein M, 2017, DIABETES RES CLIN PR, V126, P303, DOI 10.1016/j.diabres.2017.03.003

Hasson BR, 2015, CURR OBES REP, V4, P241, DOI 10.1007/s13679-015-0150-2

Heisler M, 2014, ANN INTERN MED, V161, pS13, DOI 10.7326/M13-3012

Hite RW, 2000, NEW ENGL J MED, V343, P1340

Hornberger JC, 1996, MED CARE, V34, P845, DOI 10.1097/00005650-199608000-00011

Horvat L, 2014, COCHRANE DB SYST REV, DOI 10.1002/14651858.CD009405.pub2

Hsu WC, 2015, DIABETES CARE, V38, P150, DOI 10.2337/dc14-2391

Inzucchi SE, 2015, DIABETES CARE, V38, P140, DOI 10.2337/dc14-2441

Jaam M, 2017, DIABETES RES CLIN PR, V129, P1, DOI 10.1016/j.diabres.2017.04.015

Jin K, 2017, EUR J PREV CARDIOL, V24, P2000, DOI 10.1177/2047487317736828

Joo JY, 2014, DIABETES EDUCATOR, V40, P605, DOI 10.1177/0145721714534994

Gagliardino JJ, 2013, DIABETES-METAB RES, V29, P152, DOI 10.1002/dmrr.2368

Kandula NR, 2008, DIABETES CARE, V31, P1621, DOI 10.2337/dc07-2182

Kennedy-Martin T, 2017, PATIENT PREFER ADHER, V11, P1103, DOI 10.2147/PPA.S136639

Khunti K, 2017, DIABETES CARE, V40, P1588, DOI 10.2337/dc16-1925

Kim S, 2004, DIABETES CARE, V27, P2980, DOI 10.2337/diacare.27.12.2980

Kizilay Fuat, 2017, Sex Med Rev, V5, P45, DOI 10.1016/j.sxmr.2016.07.002

Kouidrat Y, 2017, DIABETIC MED, V34, P1185, DOI 10.1111/dme.13403

Krass I, 2015, DIABETIC MED, V32, P725, DOI 10.1111/dme.12651

Kutob RM, 2013, J CONTIN EDUC HEALTH, V33, P164, DOI 10.1002/chp.21181

Lara M, 2005, ANNU REV PUBL HEALTH, V26, P367, DOI 10.1146/annurev.publhealth.26.021304.144615

Lee EH, 2017, J ADV NURS, V73, P2035, DOI 10.1111/jan.13256

Leonetti Donna L., 1992, Ethnicity and Disease, V2, P326

Lerman I, 2004, BIOMED PHARMACOTHER, V58, P566, DOI 10.1016/j.biopha.2004.09.003

Liburd LC, 1999, DIABETES EDUCATOR, V25, P382, DOI 10.1177/014572179902500309

Lopez L, 2016, J DIABETES COMPLICAT, V30, P554, DOI 10.1016/j.jdiacomp.2015.12.003

Luscombe F A, 2000, Value Health, V3 Suppl 1, P15

Marvel MK, 1999, JAMA-J AM MED ASSOC, V281, P283, DOI 10.1001/jama.281.3.283

Maty SC, 2005, INT J EPIDEMIOL, V34, P1274, DOI 10.1093/ije/dyi167

Mayberry LS, 2016, CURR DIABETES REP, V16, DOI 10.1007/s11892-016-0796-5

McBean AM, 2004, DIABETES CARE, V27, P2317, DOI 10.2337/diacare.27.10.2317

McCabe MP, 2016, J SEX MED, V13, P153, DOI 10.1016/j.jsxm.2015.12.015

McCabe M, 2006, DIABETES EDUCATOR, V32, P714, DOI 10.1177/0145721706292101

Meece J, 2006, DIABETES EDUCATOR, V32, p9S, DOI 10.1177/014572170585638

Mendivil CO, 2013, DIABETOL METAB SYNDR, V5, DOI 10.1186/1758-5996-5-37

Menke A, 2015, JAMA-J AM MED ASSOC, V314, P1021, DOI 10.1001/jama.2015.10029

Menon K, 2017, FRONT ENDOCRINOL, V8, DOI 10.3389/fendo.2017.00263

Merino J, 2018, ANN NY ACAD SCI, V1411, P140, DOI 10.1111/nyas.13588

Millan-Ferro Andreina, 2007, Curr Diab Rep, V7, P391, DOI 10.1007/s11892-007-0064-9

Mitchell MF, 2010, FAM PRACT S1, V27, pi17, DOI 10.1093/fampra/cmp027

Montori VM, 2006, HEALTH EXPECT, V9, P25, DOI 10.1111/j.1369-7625.2006.00359.x

Moussa M, 2013, INT J NURS PRACT, V19, P36, DOI 10.1111/ijn.12167

National Diabetes Statistics, 2005, NAT DIAB INF CLEAR H

NEEL JV, 1962, AM J HUM GENET, V14, P353

O'Brien MJ, 2014, PREV CHRONIC DIS, V11, DOI 10.5888/pcd11.140142

Odgers-Jewell K, 2017, DIABETIC MED, V34, P1027, DOI 10.1111/dme.13340

Ogden CL, 2014, JAMA-J AM MED ASSOC, V311, P806, DOI 10.1001/jama.2014.732

Osborn CY, 2013, J NATL MED ASSOC, V105, P51, DOI 10.1016/S0027-9684(15)30085-7

Osei K, 2004, DIABETES CARE, V27, P1439, DOI 10.2337/diacare.27.6.1439

Perrin NE, 2017, DIABETIC MED, V34, P1508, DOI 10.1111/dme.13448

Polonsky WH, 2005, DIABETES CARE, V28, P626, DOI 10.2337/diacare.28.3.626

Puckrein GA, 2015, ETHNIC DIS, V25, P521, DOI 10.18865/ed.25.4.521

Qi L, 2015, BMC PUBLIC HEALTH, V15, DOI 10.1186/s12889-015-1798-y

Raji A, 2001, J CLIN ENDOCR METAB, V86, P5366, DOI 10.1210/jc.86.11.5366

Rasouli N, 2007, J CLIN ENDOCR METAB, V92, P4656, DOI 10.1210/jc.2007-0919

Reynolds DB, 2015, DIABETES TECHNOL THE, V17, P243, DOI 10.1089/dia.2014.0285

Robbins JM, 2005, DIABETES RES CLIN PR, V68, P230, DOI 10.1016/j.diabres.2004.09.007

Rodriguez-Gutierrez R, 2015, J DIABETES METAB, V6, DOI 10.4172/2155-6156.1000482

Rosal MC, 2004, HEALTH EDUC BEHAV, V31, P388, DOI 10.1177/1090198104263360

Roux AVD, 2005, CIRCULATION, V112, P1557, DOI 10.1161/CIRCULATIONAHA.104.530147

Santiago-Torres M, 2016, AM J CLIN NUTR, V103, P366, DOI 10.3945/ajcn.115.119016

Sarkar U, 2006, DIABETES CARE, V29, P823, DOI 10.2337/diacare.29.04.06.dc05-1615

Saver BG, 2007, J AM BOARD FAM MED, V20, P28, DOI 10.3122/jabfm.2007.01.060026

Schillinger D, 2002, JAMA-J AM MED ASSOC, V288, P475, DOI 10.1001/jama.288.4.475

Seligman HK, 2010, NEW ENGL J MED, V363, P6, DOI 10.1056/NEJMp1000072

Shai I, 2006, DIABETES CARE, V29, P1585, DOI 10.2337/dc06-0057

Shane-McWhorter Laura, 2005, Curr Diab Rep, V5, P391, DOI 10.1007/s11892-005-0099-8

Singh GK, 2006, INT J EPIDEMIOL, V35, P903, DOI 10.1093/ije/dyl089

Snehalatha C, 2003, DIABETES-METAB RES, V19, P329, DOI 10.1002/dmrr.388

Sohal T, 2015, PLOS ONE, V10, DOI 10.1371/journal.pone.0136202

Sorkin DH, 2015, J AM GERIATR SOC, V63, P2158, DOI 10.1111/jgs.13655

Stringer Jaime D, 2016, FP Essent, V449, P18

Strizich G, 2016, DIABETES RES CLIN PR, V117, P64, DOI 10.1016/j.diabres.2016.04.052

Stuckey HL, 2015, CLIN THER, V37, P1986, DOI 10.1016/j.clinthera.2015.06.010

Tamhane S, 2015, CURR DIABETES REP, V15, DOI 10.1007/s11892-015-0688-0

Tang TS, 2014, DIABETES CARE, V37, P1525, DOI 10.2337/dc13-2161

Teal CR, 2012, MED EDUC, V46, P80, DOI 10.1111/j.1365-2923.2011.04101.x

Thackeray R, 2004, DIABETES EDUCATOR, V30, P665, DOI 10.1177/014572170403000418

Trikkalinou A, 2017, WORLD J DIABETES, V8, P120, DOI 10.4239/wjd.v8.i4.120

Tucker Katherine L, 2005, J Med Invest, V52 Suppl, P252, DOI 10.2152/jmi.52.252

US Census Bureau, 2014, US POP PROJ RAC HISP

van Dijk SEM, 2018, QUAL LIFE RES, V27, P1415, DOI 10.1007/s11136-018-1782-y

Vongmany J, 2018, DIABETIC MED, V35, P184, DOI 10.1111/dme.13547

Wachtel MS, 2005, J NATL MED ASSOC, V97, P334

Walker RJ, 2014, ETHNIC DIS, V24, P349

Watson KB, 2016, MMWR-MORBID MORTAL W, V65, P954, DOI 10.15585/mmwr.mm6536a3

Wee HL, 2006, HEALTH QUAL LIFE OUT, V4, DOI 10.1186/1477-7525-4-93

Weiss R, 2006, DIABETOLOGIA, V49, P571, DOI 10.1007/s00125-005-0109-z

Weitzman PF, 2013, DIABETES EDUCATOR, V39, P856, DOI 10.1177/0145721713507113

Welch G, 2006, DIABETES SPECTR, V19, P5, DOI DOI 10.2337/diaspect.19.1.5

Wen LK, 2004, FAM MED, V36, P423

Williams JW, 1999, AM J MED, V106, P36, DOI 10.1016/S0002-9343(98)00371-4

Wood FG, 2004, J ADV NURS, V45, P190, DOI 10.1046/j.1365-2648.2003.02880.x

Yazla E, 2018, J RELIG HEALTH, V57, P1010, DOI 10.1007/s10943-017-0504-z

Zanoveli JM, 2016, CURR DIABETES REV, V12, P165, DOI 10.2174/1573399811666150515125349

Zhang XX, 2016, PATIENT EDUC COUNS, V99, P186, DOI 10.1016/j.pec.2015.09.006

NR 153

TC 0

Z9 0

U1 6

U2 6

PU FRONTIERS MEDIA SA

PI LAUSANNE

PA AVENUE DU TRIBUNAL FEDERAL 34, LAUSANNE, CH-1015, SWITZERLAND

SN 1664-2392

J9 FRONT ENDOCRINOL

JI Front. Endocrinol.

PD AUG 28

PY 2018

VL 9

AR 479

DI 10.3389/fendo.2018.00479

PG 15

WC Endocrinology & Metabolism

SC Endocrinology & Metabolism

GA GR7IE

UT WOS:000442861700001

PM 30233490

OA DOAJ Gold

DA 2019-01-08

ER

PT J

AU Ponizovsky, AM

Haklai, Z

Goldberger, N

AF Ponizovsky, Alexander Michael

Haklai, Ziona

Goldberger, Nehama

TI Association between psychological distress and mortality: the case of

Israel

SO JOURNAL OF EPIDEMIOLOGY AND COMMUNITY HEALTH

LA English

DT Article

ID NATIONAL-HEALTH SURVEY; CORONARY-HEART-DISEASE; PSYCHOSOCIAL FACTORS;

MISSILE ATTACKS; MENTAL-HEALTH; GULF-WAR; POPULATION; RISK; CARE;

DEPRESSION

AB Background Previous studies have demonstrated the association between psychological distress (measured by the 12-item General Health Questionnaire, GHQ-12) and risks of all-cause mortality and deaths from cardiovascular, cancer and other causes. We hypothesised that in the Israeli population permanently exposed to war/terror stressors, this relationship is absent.

Methods We performed an analysis of data from participants in the Israel National Health Survey conducted in 2003-2004, who died during the follow-up decade. Subjects were assigned to groups based on GHQ-12 score: 0-10 (asymptomatic), 11-14 (subclinically symptomatic), 15-19 (symptomatic) and 20-36 (highly symptomatic). Data were weighted to the total population.

Results We analysed data from 4843 individuals, median age 40.5 (IQR 28-54); 473 participants died during the follow-up. We found a significant increase in total mortality, cardiovascular and other causes of mortality (but not cancer deaths) with increase in GHQ-12 score (P for linear trend of ungrouped GHQ scores < 0.0001, 0.0015 and < 0.0001, respectively). The age-sex-adjusted HR for the highest GHQ-12 compared with the lowest asymptomatic category was 2.1 (95% CI 1.6 to 2.7) for all-cause deaths, 2.3 (95% CI 1.3 to 4.1) for cardiovascular disease deaths and 2.7 (95% CI 1.9 to 3.9) for other deaths. The HR remained significant after adjustment for education, smoking, alcohol consumption and diabetes. The HR even increased after excluding participants with baseline cardiovascular diseases and cancer.

Conclusion Contrary to our hypothesis, psychological distress was associated with all-cause and cardiovascular but not cancer mortality. The absence of reverse causality provides evidence for the direct deleterious effects of psychological distress on mortality outcomes.

C1 [Ponizovsky, Alexander Michael] Minist Hlth, Mental Hlth Serv, IL-91000 Jerusalem, Israel.

[Haklai, Ziona; Goldberger, Nehama] Minist Hlth, Dept Hlth Informat, Jerusalem, Israel.

RP Ponizovsky, AM (reprint author), Minist Hlth, Mental Hlth Serv, IL-91000 Jerusalem, Israel.

EM alexpon8@gmail.com

CR [Anonymous], 1993, ICD 10 CLASS MENT BE

Batty GD, 2017, BMJ-BRIT MED J, V356, DOI 10.1136/bmj.j108

Batty GD, 2014, ATHEROSCLEROSIS, V236, P385, DOI 10.1016/j.atherosclerosis.2014.06.025

Brotman DJ, 2007, LANCET, V370, P1089, DOI 10.1016/S0140-6736(07)61305-1

Chida Y, 2008, NAT CLIN PRACT ONCOL, V5, P466, DOI 10.1038/ncponc1134

COX DR, 1972, J R STAT SOC B, V34, P187

COYNE JC, 1994, PSYCHOL BULL, V116, P29, DOI 10.1037/0033-2909.116.1.29

EASTON JA, 1991, BRIT MED J, V303, P1231, DOI 10.1136/bmj.303.6812.1231

Goldberg D., 1978, MANUAL GEN HLTH QUES

Goldberg DP, 1997, PSYCHOL MED, V27, P191, DOI 10.1017/S0033291796004242

Goldberger N, LEADING CAUSES DEATH

Gulsvik AK, 2011, EUR J EPIDEMIOL, V26, P221, DOI 10.1007/s10654-010-9535-4

Hamer M, 2008, J AM COLL CARDIOL, V52, P2156, DOI 10.1016/j.jacc.2008.08.057

Hamer M, 2009, J PSYCHOSOM RES, V66, P255, DOI 10.1016/j.jpsychores.2008.11.002

HARRIS MD, 1991, J CLIN PATHOL, V44, P862, DOI 10.1136/jcp.44.10.862

Hemingway H, 1999, BMJ-BRIT MED J, V318, P1460, DOI 10.1136/bmj.318.7196.1460

HUPPERT FA, 1995, PSYCHOL MED, V25, P1073, DOI 10.1017/S0033291700037569

Kahan NR, 2018, INT CLIN PSYCHOPHARM, V33, P73, DOI 10.1097/YIC.0000000000000197

KARK JD, 1995, JAMA-J AM MED ASSOC, V273, P1208, DOI 10.1001/jama.273.15.1208

Kessler RC, 2004, INT J METH PSYCH RES, V13, P93, DOI 10.1002/mpr.168

Kessler RC, 2017, EUR J PSYCHOTRAUMATO, V8, DOI 10.1080/20008198.2017.1353383

Lerner Y, 1996, PSYCHOL MED, V26, P503, DOI 10.1017/S0033291700035583

Levav I, 2009, PSYCHIAT BEHAV DISOR

Levav I, 2006, AM J PSYCHIAT, V163, P1355, DOI 10.1176/appi.ajp.163.8.1355

Leventer-Roberts M, 2017, STUD HEALTH TECHNOL, V244, P107, DOI 10.3233/978-1-61499-824-2-107

Levinson D, 2007, ISR J PSYCHIATR REL, V44, P85

Levinson D, 2007, ISR J PSYCHIATR REL, V44, P79

Lubin G, 2016, ISR J PSYCHIATR REL, V53, P3

Neumark YD, 2007, ISR J PSYCHIATR REL, V44, P126

Nicholson A, 2006, EUR HEART J, V27, P2763, DOI 10.1093/eurheartj/ehl338

Niskar A, 2009, PREV CHRONIC DIS, V6

Ponizovsky AM, 2009, CAN J PSYCHIAT, V54, P409, DOI 10.1177/070674370905400608

Puustinen PJ, 2011, SCAND J PUBLIC HEALT, V39, P577, DOI 10.1177/1403494811414244

Rasul F, 2005, J EPIDEMIOL COMMUN H, V59, P140, DOI 10.1136/jech.2004.019786

Robinson KL, 2004, ANN EPIDEMIOL, V14, P467, DOI 10.1016/j.annepidem.2003.11.007

RUSS TC, 2012, BRIT MED J, V345, DOI [10.1136/bmj.e4933, DOI 10.1136/BMJ.E4933]

Schmitz N, 1999, SOC PSYCH PSYCH EPID, V34, P360, DOI 10.1007/s001270050156

Soskolne V, 1996, SOC SCI MED, V42, P1039, DOI 10.1016/0277-9536(95)00215-4

NR 38

TC 0

Z9 0

U1 0

U2 0

PU BMJ PUBLISHING GROUP

PI LONDON

PA BRITISH MED ASSOC HOUSE, TAVISTOCK SQUARE, LONDON WC1H 9JR, ENGLAND

SN 0143-005X

EI 1470-2738

J9 J EPIDEMIOL COMMUN H

JI J. Epidemiol. Community Health

PD AUG

PY 2018

VL 72

IS 8

BP 726

EP 732

DI 10.1136/jech-2017-210356

PG 7

WC Public, Environmental & Occupational Health

SC Public, Environmental & Occupational Health

GA GR3DP

UT WOS:000442469800012

PM 29599386

DA 2019-01-08

ER

PT J

AU Braizat, O

Feinn, R

Abbott, G

Wagner, J

AF Braizat, Omar

Feinn, Richard

Abbott, Gina

Wagner, Julie

TI Relationship style and glycaemic control in women with type 2 diabetes:

The mediating role of psychological distress

SO STRESS AND HEALTH

LA English

DT Article

DE attachment; continuous glucose monitoring; depression; diabetes;

diabetes distress; mediation

ID ATTACHMENT STYLE; SOCIAL SUPPORT; DEPRESSION; ADULTS; CARE; ASSOCIATION;

MORTALITY; OUTCOMES; DISEASE; SCALE

AB This study examined whether depressive symptoms and/or diabetes distress mediate the association between relationship style and glycaemic control in women with diabetes. Seventy-five women with type 2 diabetes completed the Relationship Questionnaire. Participants endorsing secure or preoccupied adult attachment were combined into the interactive relationship style and dismissing/avoidant or fearful adult attachment were combined into the independent relationship style. Glycaemic control was a latent variable composed of A1c and 48-hr continuously measured glucose. Diabetes distress was assessed with the Problem Areas in Diabetes scale and depressive symptoms with the Center for Epidemiological Studies Depression scale. A parallel multiple mediation model with relationship style as the independent variable, glycaemic control as the dependent variable, and Problem Areas in Diabetes and Center for Epidemiological Studies Depression scale as mediators, tested indirect effects. Bias-corrected bootstrap with 10,000 replications was used to construct 95% confidence intervals. The indirect association of relationship style with glycaemic control through diabetes distress was significant (effect=-0.09, p=.036, 95CI=-0.19-0.01), but through depressive symptoms was not. A model testing the indirect association of relationship style with diabetes distress through glycaemic control was not significant. Results suggest that relationship style is associated with glycaemic control through diabetes distress in women with type 2 diabetes.

C1 [Braizat, Omar] Univ Connecticut, Storrs, CT USA.

[Feinn, Richard] Quinnipiac Univ, Frank Netter Sch Med, North Haven, CT USA.

[Abbott, Gina] Quinnipiac Univ, Hamden, CT USA.

[Wagner, Julie] UConn Hlth, Farmington, CT USA.

RP Wagner, J (reprint author), UConn Hlth, Behav Sci & Community Hlth, 263 Farmington Ave, Farmington, CT 06030 USA.

EM juwagner@uchc.edu

FU Foundation for the National Institutes of Health [DK074468]; National

Institutes of Health [DK074468]

FX Foundation for the National Institutes of Health, Grant/Award Number:

DK074468; National Institutes of Health, Grant/Award Number: DK074468

CR Ahrens KR, 2012, J PSYCHOSOM RES, V72, P364, DOI 10.1016/j.jpsychores.2012.02.002

Baek RN, 2014, ANN BEHAV MED, V48, P145, DOI 10.1007/s12160-013-9585-4

Barry J., 2015, AUSTR I MALE HLTH ST, V4, P63

BRETHERTON I, 1992, DEV PSYCHOL, V28, P759, DOI 10.1037/0012-1649.28.5.759

Ciechanowski P, 2004, PSYCHOSOM MED, V66, P720, DOI 10.1097/01.psy.0000138125.59122.23

Ciechanowski P, 2010, DIABETES CARE, V33, P539, DOI 10.2337/dc09-1298

Ciechanowski PS, 2006, MED CARE, V44, P283, DOI 10.1097/01.mlr.0000199695.03840.0d

Ciechanowski PS, 2005, J PSYCHOSOM RES, V58, P139, DOI 10.1016/j.jpsychores.2004.07.009

Ciechanowski PS, 2001, AM J PSYCHIAT, V158, P29, DOI 10.1176/appi.ajp.158.1.29

Fisher L, 2008, DIABETIC MED, V25, P1096, DOI 10.1111/j.1464-5491.2008.02533.x

Fisher L, 2014, DIABETIC MED, V31, P764, DOI 10.1111/dme.12428

Fisher L, 2012, DIABETES CARE, V35, P259, DOI 10.2337/dc11-1572

Fisher L, 2010, DIABETES CARE, V33, P23, DOI 10.2337/dc09-1238

Griffin D, 1994, ADV PERSONAL RELATIO, P17

Gross T M, 2000, Diabetes Technol Ther, V2, P49, DOI 10.1089/152091500316737

Liu LJ, 2011, ANN EPIDEMIOL, V21, P26, DOI 10.1016/j.annepidem.2010.10.012

Lustman PJ, 2000, DIABETES CARE, V23, P934, DOI 10.2337/diacare.23.7.934

Melloni C, 2010, CIRC-CARDIOVASC QUAL, V3, P135, DOI 10.1161/CIRCOUTCOMES.110.868307

Mondesir FL, 2016, J GERONTOL B-PSYCHOL, V71, P1081, DOI 10.1093/geronb/gbv061

Muthen L. K., 1998, MPLUS USERS GUIDE

Olson MM, 2010, INT J PSYCHIAT MED, V40, P289, DOI 10.2190/PM.40.3.e

POLONSKY WH, 1995, DIABETES CARE, V18, P754, DOI 10.2337/diacare.18.6.754

Radin MS, 2014, J GEN INTERN MED, V29, P388, DOI 10.1007/s11606-013-2595-x

RADLOFF L S, 1977, Applied Psychological Measurement, V1, P385, DOI 10.1177/014662167700100306

Rivellese AA, 2010, NUTR METAB CARDIOVAS, V20, P474, DOI 10.1016/j.numecd.2010.01.008

Saunders R, 2011, ATTACH HUM DEV, V13, P403, DOI 10.1080/14616734.2011.584405

SCHARFE E, 1994, PERS RELATIONSHIP, V1, P23, DOI DOI 10.1111/J.1475-6811.1994.TB00053.X

Snoek FJ, 2000, DIABETES CARE, V23, P1305, DOI 10.2337/diacare.23.9.1305

Spencer-Bonilla G, 2017, BMJ OPEN, V7

NR 29

TC 0

Z9 0

U1 2

U2 2

PU WILEY

PI HOBOKEN

PA 111 RIVER ST, HOBOKEN 07030-5774, NJ USA

SN 1532-3005

EI 1532-2998

J9 STRESS HEALTH

JI Stress Health

PD AUG

PY 2018

VL 34

IS 3

BP 462

EP 467

DI 10.1002/smi.2795

PG 6

WC Psychology, Applied; Psychiatry; Psychology

SC Psychology; Psychiatry

GA GP9QK

UT WOS:000441251300014

PM 29327498

DA 2019-01-08

ER

PT J

AU Chew, BH

Vos, RC

Pouwer, F

Rutten, GEHM

AF Chew, Boon-How

Vos, Rimke C.

Pouwer, Frans

Rutten, Guy E. H. M.

TI The associations between diabetes distress and self-efficacy, medication

adherence, self-care activities and disease control depend on the way

diabetes distress is measured: Comparing the DDS-17, DDS-2 and the

PAID-5

SO DIABETES RESEARCH AND CLINICAL PRACTICE

LA English

DT Article

DE Diabetes distress; Epidemiologic measurements; Type 2 diabetes mellitus;

Self-efficacy; Self-care activities; Medication adherence

ID QUALITY-OF-LIFE; EMOTIONAL DISTRESS; PROBLEM AREAS; TYPE-2; HEALTH;

SCALE; MELLITUS; DEPRESSION; DETERMINANTS; MANAGEMENT

AB Aims: To examine whether diabetes distress (DD), when measured by three different instruments, was associated differently with self-efficacy, self-care activity, medication adherence and disease control in people with Type 2 diabetes mellitus.

Methods: A cross-sectional study in three health clinics. DD was assessed with the 17-item Diabetes Distress Scale, the 2-item DDS-2 (DDS-2) and the 5-item Problem Areas in Diabetes Scale (PAID-5). Dependent variables included self-efficacy, self-care activities, medication adherence, HbA1c, systolic and diastolic blood pressure (SBP, DBP). Multiple linear and logistic regression were used in analyses.

Results: In total 338 participants (56% women), with a mean age of 61 years and diabetes duration of 9.8 years, were included. DDS-2 was an independent determinant of SBP (beta = 1.89, 95% CI 0.14, 3.64), DBP (beta = 1.19, 95% CI 0.16, 2.21) and blood pressure target (OR = 2.09, 95% CI 1.12, 3.83). PAID-5 was an independent determinant of medication adherence (adjusted beta = -0.05, 95% CI -0.08, -0.01) and self-care activities (OR = 0.50, 95% CI 0.26, 0.99).

Conclusions: Associations of DD with important aspects of diabetes care are substantially influenced by confounders and depend on the way DD is measured. Our findings call for a judicious use of different DD measures in clinical practice and research.

The study is registered on ClinicalTrials.gov (NCT02730754). (C) 2018 Elsevier B.V. All rights reserved.

C1 [Chew, Boon-How] Univ Putra Malaysia, Fac Med & Hlth Sci, Dept Family Med, Serdang 43400, Selangor, Malaysia.

[Chew, Boon-How; Vos, Rimke C.; Rutten, Guy E. H. M.] Univ Utrecht, Univ Med Ctr Utrecht, Julius Ctr Hlth Sci & Primary Care, Huispost Str 6-131,POB 85500, NL-3508 GA Utrecht, Netherlands.

[Pouwer, Frans] Univ Southern Denmark, Dept Psychol, Odense, Denmark.

RP Chew, BH (reprint author), Univ Putra Malaysia, Fac Med & Hlth Sci, Dept Family Med, Serdang 43400, Selangor, Malaysia.

EM chewboonhow@upm.edu.my

OI Chew, Boon How/0000-0002-8627-6248

FU Ministry of Higher Education Malaysia [KPT(BS)730928075371]; Universiti

Putra Malaysia [UPM/TNCPI/RMC/5.1.17/F5(16)]

FX This work was partly supported by the Ministry of Higher Education

Malaysia (grant numbers KPT(BS)730928075371, 2013) and Universiti Putra

Malaysia's journal publication awards (award numbers

UPM/TNCPI/RMC/5.1.17/F5(16)). The funding bodies had no role in the

design of the study and collection, analysis, and interpretation of data

and in writing the manuscript.

CR Aghili R, 2016, CAN J DIABETES, V40, P424, DOI 10.1016/j.jcjd.2016.02.014

Al-Qazaz HK, 2010, DIABETES RES CLIN PR, V90, P216, DOI 10.1016/j.diabres.2010.08.012

[Anonymous], 2017, DIABETES CARE, V40, pS1, DOI 10.2337/dc17-S001

Chew B H, 2015, Malays Fam Physician, V10, P22

Chew BH, 2016, PLOS ONE, V11, DOI 10.1371/journal.pone.0152095

Chew BH, 2015, HEALTH QUAL LIFE OUT, V13, DOI 10.1186/s12955-015-0384-4

Chew BH, 2015, PATIENT PREFER ADHER, V9, P639, DOI 10.2147/PPA.S81612

Chew BH, 2015, THER CLIN RISK MANAG, V11, P669, DOI 10.2147/TCRM.S81623

Chin YW, 2017, BMC FAM PRACT, V18, DOI 10.1186/s12875-017-0601-9

Dennick K, 2017, J DIABETES COMPLICAT, V31, P898, DOI 10.1016/j.jdiacomp.2016.12.018

Devarajooh C, 2017, PLOS ONE, V12, DOI 10.1371/journal.pone.0175096

Fenwick EK, 2018, J HEALTH PSYCHOL, V23, P667, DOI 10.1177/1359105316642006

Fisher L, 2014, DIABETIC MED, V31, P764, DOI 10.1111/dme.12428

Fisher L, 2008, ANN FAM MED, V6, P246, DOI 10.1370/afm.842

Fisher L, 2012, DIABETES CARE, V35, P259, DOI 10.2337/dc11-1572

Gonzalez JS, 2016, DIABETES CARE, V39, P2182, DOI 10.2337/dc16-1657

How Chew Boon, 2011, Ment Health Fam Med, V8, P21

Jannoo Z, 2017, J CLIN TRANSL ENDOCR, V9, P48, DOI 10.1016/j.jcte.2017.07.003

Jeppesen KM, 2009, ANN FAM MED, V7, P24, DOI 10.1370/afm.919

Kasteleyn MJ, 2015, DIABETIC MED, V32, P1617, DOI 10.1111/dme.12743

Kaur G, 2013, BMC FAM PRACT, V14, DOI 10.1186/1471-2296-14-69

Kroenke K, 2001, J GEN INTERN MED, V16, P606, DOI 10.1046/j.1525-1497.2001.016009606.x

Lee EH, 2014, HEALTH QUAL LIFE OUT, V12, DOI 10.1186/s12955-014-0142-z

Lee J, 2015, INT J NURS STUD, V52, P1868, DOI 10.1016/j.ijnurstu.2015.07.004

Lee YK, 2013, PLOS ONE, V8, P80051

Luo XP, 2015, INT J ENV RES PUB HE, V12, P11304, DOI 10.3390/ijerph120911304

McGuire BE, 2010, DIABETOLOGIA, V53, P66, DOI 10.1007/s00125-009-1559-5

Mohd Razali N, 2014, MATH COMPUT METHODS, P200

MORISKY DE, 1986, MED CARE, V24, P67, DOI 10.1097/00005650-198601000-00007

Morris Nancy S, 2006, BMC Fam Pract, V7, P21, DOI 10.1186/1471-2296-7-21

Ogbera A, 2011, J DIABETES, V3, P348, DOI 10.1111/j.1753-0407.2011.00156.x

Pintaudi B, 2015, J PSYCHOSOM RES, V79, P348, DOI 10.1016/j.jpsychores.2015.08.010

POLONSKY WH, 1995, DIABETES CARE, V18, P754, DOI 10.2337/diacare.18.6.754

Schmitt A, 2016, DIABETIC MED, V33, P835, DOI 10.1111/dme.12887

Sharoni SKA, 2012, NURS HEALTH SCI, V14, P38, DOI 10.1111/j.1442-2018.2011.00658.x

Ting RZW, 2011, DIABETES CARE, V34, P1094, DOI 10.2337/dc10-1612

Toobert DJ, 2000, DIABETES CARE, V23, P943, DOI 10.2337/diacare.23.7.943

van der Bijl J, 1999, J ADV NURS, V30, P352, DOI 10.1046/j.1365-2648.1999.01077.x

Venkataraman K, 2015, PLOS ONE, V10, DOI 10.1371/journal.pone.0136759

Walker RJ, 2014, GEN HOSP PSYCHIAT, V36, P662, DOI 10.1016/j.genhosppsych.2014.06.011

Wang RH, 2017, J ADV NURS, V73, P1137, DOI 10.1111/jan.13201

Winchester RJ, 2016, J DIABETES COMPLICAT, V30, P312, DOI 10.1016/j.jdiacomp.2015.11.010

Yan J, 2014, J EVAL CLIN PRACT, V20, P311, DOI 10.1111/jep.12125

NR 43

TC 0

Z9 0

U1 3

U2 3

PU ELSEVIER IRELAND LTD

PI CLARE

PA ELSEVIER HOUSE, BROOKVALE PLAZA, EAST PARK SHANNON, CO, CLARE, 00000,

IRELAND

SN 0168-8227

EI 1872-8227

J9 DIABETES RES CLIN PR

JI Diabetes Res. Clin. Pract.

PD AUG

PY 2018

VL 142

BP 74

EP 84

DI 10.1016/j.diabres.2018.05.021

PG 11

WC Endocrinology & Metabolism

SC Endocrinology & Metabolism

GA GO7YL

UT WOS:000440297500009

PM 29802952

DA 2019-01-08

ER

PT J

AU Nichols, J

Vallis, M

Boutette, S

Casey, CG

Yu, CH

AF Nichols, Jennica

Vallis, Michael

Boutette, Stephanie

Casey, Carolyn Gall

Yu, Catherine H.

TI A Canadian Cross-Sectional Survey on Psychosocial Supports for Adults

Living With Type 1 or 2 Diabetes: Health-Care Providers' Awareness,

Capacity and Motivation

SO CANADIAN JOURNAL OF DIABETES

LA English

DT Article

DE Capacity-Opportunity-Motivation Behaviour (COM-B); diabetes; mental

health psychosocial issues; self-management

ID PSYCHOLOGICAL INSULIN-RESISTANCE; NEEDS 2ND; NATIONAL BENCHMARKING;

DISTRESS; ATTITUDES; PEOPLE; WISHES; HYPOGLYCEMIA; MANAGEMENT; BARRIERS

AB Objectives: Addressing psychosocial issues is critical for diabetes self-management. This work explores health-care professionals' (HCPs') 1) perceived relevance of various psychosocial issues in diabetes management and 2) confidence in working on these issues within then services.

Methods: An online cross-sectional survey was developed based on the Capacity-Opportunity-Motivation Behaviour Model. It assessed self-rated confidence in supporting patients with psychosocial issues (capability), perceived relevance of these issues (motivation) and facilitators of skill development (oppor tunity). An e-mail invitation was sent to all Diabetes Canada's professional members, conference delegates and committee members. Qualitative responses were analyzed using thematic analysis.

Results: Of the 260 responses received (25% response rate), many were Diabetes Canada professional members (83%) and/or certified diabetes educators (66%). The largest professional groups in the sample were registered nurses (44%) and registered dietitians (33%). All psychosocial issues were perceived as somewhat or extremely important by at least 80% of respondents (range, 80% to 97%). However, HCPs were less confident in supporting their patients with these psychosocial issues; significantly fewer respondents reported that they felt somewhat or extremely confident (range, 26% to 62%). Depression (80%) and anxiety (80%) were the issues in which guidance was most desired. Most respondents wanted some form of formal self-management support training (83%). Preferred training methods included in-person workshops (56%), webinars (56%) and conference sessions (51%).

Conclusions: Motivation to address psychosocial issues in diabetes was high, but capacity to do so and opportunity to learn how were both low. These findings can be used to develop a targeted strategy to help address this gap. (C) 2017 Canadian Diabetes Association.

C1 [Nichols, Jennica; Boutette, Stephanie; Casey, Carolyn Gall] Diabet Canada, Toronto, ON, Canada.

[Vallis, Michael] Dalhousie Univ, Halifax, NS, Canada.

[Vallis, Michael] Capital Hlth, Halifax, NS, Canada.

[Yu, Catherine H.] St Michaels Hosp, Toronto, ON, Canada.

RP Yu, CH (reprint author), St Michaels Hosp, Ctr Diabet & Metab, 30 Bond St, Toronto, ON M5B 1W8, Canada.

EM YuCa@smh.ca

OI Vallis, Michael/0000-0002-0165-5936

CR Battersby MW, 2008, AUST J PRIM HEALTH, V14, P66, DOI 10.1071/PY08009

Booth G, 2013, CAN J DIABETES, V37, pS4, DOI 10.1016/j.jcjd.2013.01.010

Braun V., 2008, QUALITATIVE RES PSYC, V3, P77, DOI DOI 10.1191/1478088706QP0630A

Burns KK, 2013, DIABETIC MED, V30, P778, DOI 10.1111/dme.12239

Canadian Diabetes Association Clinical Practice Guidelines Expert Committee, 2008, CAN J DIABETES S1, V32, pS1, DOI DOI 10.1503/CMAJ.080554

Canadian Institute for Health Information, 2011, CAN HLTH CAR PROV 19

Fisher L, 2008, ANN FAM MED, V6, P246, DOI 10.1370/afm.842

Fisher L, 2016, J DIABETES COMPLICAT, V30, P1123, DOI 10.1016/j.jdiacomp.2016.03.032

Fisher L, 2012, DIABETES CARE, V35, P259, DOI 10.2337/dc11-1572

Fisher L, 2010, DIABETES CARE, V33, P23, DOI 10.2337/dc09-1238

Frost MH, 1997, CANCER NURS, V20, P350, DOI 10.1097/00002820-199710000-00006

Funnell MM, 2008, DIABETES CARE, V31, pS97, DOI 10.2337/dc08-S097

Glasgow RE, 2006, HEALTH PROMOT INT, V21, P245, DOI 10.1093/heapro/dal017

Gonder-Frederick LA, 2013, DIABETIC MED, V30, P603, DOI 10.1111/dme.12129

Greenberg R, 2008, NEW TOOL MOTIVATE PA

Hagger V, 2016, CURR DIABETES REP, V16, DOI 10.1007/s11892-015-0694-2

Hendrieckx C, 2014, DIABETES RES CLIN PR, V103, P430, DOI 10.1016/j.diabres.2013.12.005

Holmes-Truscott E, 2016, PRIM CARE DIABETES, V10, P75, DOI 10.1016/j.pcd.2015.06.006

Houlden R, 2003, CAN J DIABETES S2, V27, pS50

Institute for Healthcare Communication, CHOIC CHANG MOT HEAL

Kovacs Burns K, 2015, DIABETES RES TREAT, V2, P125

Kreider KE, 2017, DIABETES THER, V8, P1, DOI 10.1007/s13300-017-0231-1

Larkin ME, 2008, DIABETES EDUCATOR, V34, P511, DOI 10.1177/0145721708317869

Michie S, 2011, IMPLEMENT SCI, V6, DOI 10.1186/1748-5908-6-42

Miller W.R., 1995, MOTIVATIONAL ENHANCE

Miller WR, 2012, MOTIVATIONAL INTERVI

Nicolucci A, 2013, DIABETIC MED, V30, P767, DOI 10.1111/dme.12245

Nouwen A, 2015, DIABETIC MED, V32, P1261, DOI 10.1111/dme.12863

Peyrot M, 2005, DIABETIC MED, V22, P1379, DOI 10.1111/j.1464-5491.2005.01644.x

Pfizer Canada, 2012, 3 MIN EMP

Polonsky WH, 2015, J DIABETES COMPLICAT, V29, P1171, DOI 10.1016/j.jdiacomp.2015.08.002

Polonsky WH, 2005, DIABETES CARE, V28, P2543, DOI 10.2337/diacare.28.10.2543

Polonsky WH, 2004, CLIN DIABETES, V22, P147

Polonsky WH, 2017, DIABETES TECHNOL THE, V19, P131, DOI 10.1089/dia.2016.0366

Polonsky WH, 2011, CURR MED RES OPIN, V27, P1169, DOI 10.1185/03007995.2011.573623

Powers MA, 2017, DIABETES EDUCATOR, V43, P105, DOI 10.1177/0145721716680888

R Core Team, 2016, R LANG ENV STAT COMP

Robinson DJ, 2013, CAN J DIABETES, V37, pS87, DOI 10.1016/j.jcjd.2013.01.026

RUBIN RR, 1992, DIABETES CARE, V15, P1640, DOI 10.2337/diacare.15.11.1640

Rubin RR, 2001, J CLIN PSYCHOL, V57, P457, DOI 10.1002/jclp.1041

Stuckey HL, 2014, DIABETES CARE, V37, P2466, DOI 10.2337/dc13-2536

Vallis M, 2016, CAN J DIABETES, V40, P234, DOI 10.1016/j.jcjd.2015.11.002

Vallis M, 2014, CURR DIABETES REV, V10, P364, DOI 10.2174/1573399810666141113115026

von Elm E, 2008, GAC SANIT, V22, P144, DOI 10.1157/13119325

NR 44

TC 0

Z9 0

U1 3

U2 3

PU ELSEVIER SCIENCE BV

PI AMSTERDAM

PA PO BOX 211, 1000 AE AMSTERDAM, NETHERLANDS

SN 1499-2671

J9 CAN J DIABETES

JI Can. J. Diabetes

PD AUG

PY 2018

VL 42

IS 4

BP 389

EP +

DI 10.1016/j.jcjd.2017.09.004

PG 8

WC Endocrinology & Metabolism

SC Endocrinology & Metabolism

GA GO6XI

UT WOS:000440196700009

PM 29129456

DA 2019-01-08

ER

PT J

AU Hood, KK

Iturralde, E

Rausch, J

Weissberg-Benchell, J

AF Hood, Korey K.

Iturralde, Esti

Rausch, Joseph

Weissberg-Benchell, Jill

TI Preventing Diabetes Distress in Adolescents With Type 1 Diabetes:

Results 1 Year After Participation in the STePS Program

SO DIABETES CARE

LA English

DT Article

ID AUTOMATIC THOUGHTS QUESTIONNAIRE; GLYCEMIC CONTROL; DEPRESSIVE SYMPTOMS;

CLINICAL-TRIALS; CHILDREN; GROWTH; INVENTORY; ADULTS; MODEL; YOUTH

AB OBJECTIVE

The aim is to report 1-year outcomes of the Supporting Teens Problem Solving (STePS) study, a randomized controlled trial comparing a distress and depression prevention program with a diabetes education program for adolescents with type 1 diabetes.

RESEARCH DESIGN AND METHODS

With 264 adolescents in two locations (Chicago and San Francisco Bay Area), a randomized controlled trial was conducted comparing the Penn Resilience Program for type 1 diabetes (PRP T1D) to Advanced Diabetes Education. Interventions lasted 4.5 months, and assessments were conducted at baseline, and 4.5, 8, 12, and 16 months. Outcomes of interest were diabetes distress (DD), depressive symptoms, resilience, diabetes self-management, and glycemic control. Latent growth curve modeling was used to test between-group differences over time.

RESULTS

Results indicate that there was acceptable randomization and exposure to interventions, and that exposure to PRP T1D was associated with substantial reductions in DD. In addition, stable glycemic control, resilience characteristics, and depressive symptoms were observed 1 year post-treatment. Diabetes management deteriorated in both groups.

CONCLUSIONS

Intervening before symptoms of psychological distress start can prevent the development of the DD commonly seen in adolescents with type 1 diabetes. The STePS program represents a promising prevention program, and future reports on 2- and 3-year outcomes will explore benefits over longer periods of time.

C1 [Hood, Korey K.; Iturralde, Esti] Stanford Univ, Dept Pediat, Sch Med, Stanford, CA 94305 USA.

[Rausch, Joseph] Ohio State Univ, Dept Pediat, Res Inst, Nationwide Childrens Hosp, Columbus, OH 43210 USA.

[Weissberg-Benchell, Jill] Northwestern Univ, Dept Psychiat & Behav Sci, Ann & Robert H Lurie Childrens Hosp Chicago, Feinberg Sch Med, Chicago, IL 60611 USA.

RP Hood, KK (reprint author), Stanford Univ, Dept Pediat, Sch Med, Stanford, CA 94305 USA.

EM kkhood@stanford.edu

FU National Institute of Diabetes and Digestive and Kidney Diseases

[R01-090030]

FX This study was supported by funding from the National Institute of

Diabetes and Digestive and Kidney Diseases (grant R01-090030).

CR Feingold A, 2009, PSYCHOL METHODS, V14, P43, DOI 10.1037/a0014699

Fisher L, 2010, DIABETES CARE, V33, P1034, DOI 10.2337/dc09-2175

Garrison MM, 2005, DIABETES CARE, V28, P2150, DOI 10.2337/diacare.28.9.2150

Gillham JE, 2006, J ABNORM CHILD PSYCH, V34, P203, DOI 10.1007/s10802-005-9014-7

Grey M, 2002, J PSYCHOSOM RES, V53, P907, DOI 10.1016/S0022-3999(02)00312-4

Hagger V, 2016, CURR DIABETES REP, V16, DOI 10.1007/s11892-015-0694-2

HARRELL TH, 1983, J CONSULT CLIN PSYCH, V51, P721, DOI 10.1037//0022-006X.51.5.721

Hessler DM, 2017, DIABETIC MED, V34, P1228, DOI 10.1111/dme.13381

Hilliard ME, 2012, CURR DIABETES REP, V12, P739, DOI 10.1007/s11892-012-0314-3

HOLLON SD, 1980, COGNITIVE THER RES, V4, P383, DOI 10.1007/BF01178214

Hood KK, 2006, DIABETES CARE, V29, P1389, DOI 10.2337/dc06-0087

Hood KK, 2014, J ADOLESCENT HEALTH, V55, P498, DOI 10.1016/j.jadohealth.2014.03.011

Hu LT, 1999, STRUCT EQU MODELING, V6, P1, DOI 10.1080/10705519909540118

Jaser SS, 2017, ANN BEHAV MED, V51, P30, DOI 10.1007/s12160-016-9825-5

Kazdin A. E., 1990, PSYCHOL ASSESSMENT J, V2, P73, DOI DOI 10.1037/1040-3590.2.1.73

Kovacs M, 2003, CHILDRENS DEPRESSION

MaydeuOlivares A, 1996, COGNITIVE THER RES, V20, P115, DOI 10.1007/BF02228030

McArdle JJ, 2009, ANNU REV PSYCHOL, V60, P577, DOI 10.1146/annurev.psych.60.110707.163612

Miller KM, 2015, DIABETES CARE, V38, P971, DOI 10.2337/dc15-0078

Nicolucci A, 2013, DIABETIC MED, V30, P767, DOI 10.1111/dme.12245

Reivich K., 2005, HDB RESILIENCE CHILD, P223

Riley AR, 2015, DIABETES CARE, V38, P1435, DOI 10.2337/dc14-2519

Sandler IN, 2000, CHILD DEV, V71, P1099, DOI 10.1111/1467-8624.00212

Stull DE, 2008, QUAL LIFE RES, V17, P47, DOI 10.1007/s11136-007-9290-5

Weinger K, 2005, DIABETES CARE, V28, P1346, DOI 10.2337/diacare.28.6.1346

Weissberg-Benchell J, 2014, 74 ANN SCI SESS AM D

Weissberg-Benchell J, 2016, CONTEMP CLIN TRIALS, V49, P78, DOI 10.1016/j.cct.2016.05.006

Weissberg-Benchell J, 2011, PEDIATR DIABETES, V12, P341, DOI 10.1111/j.1399-5448.2010.00720.x

Wu AD, 2010, SOC INDIC RES, V97, P123, DOI 10.1007/s11205-009-9496-8

NR 29

TC 1

Z9 1

U1 2

U2 2

PU AMER DIABETES ASSOC

PI ALEXANDRIA

PA 1701 N BEAUREGARD ST, ALEXANDRIA, VA 22311-1717 USA

SN 0149-5992

EI 1935-5548

J9 DIABETES CARE

JI Diabetes Care

PD AUG 1

PY 2018

VL 41

IS 8

BP 1623

EP 1630

DI 10.2337/dc17-2556

PG 8

WC Endocrinology & Metabolism

SC Endocrinology & Metabolism

GA GN7EC

UT WOS:000439288600012

PM 29921624

DA 2019-01-08

ER

PT J

AU Hartzler, ML

Shenk, M

Williams, J

Schoen, J

Dunn, T

Anderson, D

AF Hartzler, Melody L.

Shenk, McKenzie

Williams, Julie

Schoen, James

Dunn, Thomas

Anderson, Douglas

TI Impact of Collaborative Shared Medical Appointments on Diabetes Outcomes

in a Family Medicine Clinic

SO DIABETES EDUCATOR

LA English

DT Article

ID RANDOMIZED-CONTROLLED-TRIAL; SELF-MANAGEMENT EDUCATION; QUALITY-OF-LIFE;

GROUP VISITS; PRIMARY-CARE; DEPRESSIVE SYMPTOMS; COMORBID DEPRESSION;

VULNERABLE PATIENTS; FOLLOW-UP; MELLITUS

AB Purpose The purpose of this study is to evaluate the impact of a collaborative diabetes shared medical appointment on patient outcomes in an urban family medicine practice.

Methods Fifty-nine patients were enrolled to participate in multiple shared medical appointments (SMAs) over 12 months. Baseline data included hemoglobin (A1C), lipids, systolic blood pressure (SBP), weight, adherence to American Diabetes Association (ADA) guidelines, and surveys, including the Problem Areas in Diabetes (PAID-2) scale and the Spoken Knowledge in Low Literacy in Diabetes Scale (SKILLD). A1C and SBP were evaluated at each visit. Lipid control was assessed at baseline and at 6 and 12 months. Adherence to ADA guidelines, SKILLD and PAID-2 survey scores, and number of antihyperglycemic and antihypertensive medications were also evaluated at 12 months.

Results Thirty-eight patients completed the study. Compared with baseline, A1C and low-density lipoprotein cholesterol (LDL-C) levels decreased significantly over 12 months (P < .001 and P = .004, respectively). More patients became compliant with the ADA guidelines throughout the course of the study. Specifically, more patients achieved the LDL-C goal of 100 mg/dL (2.59 mmol/L; P < .001), were prescribed appropriate antihypertensive medications (P < .001) and aspirin (P < .001), and received the pneumonia vaccine (P < .001). PAID-2 and SKILLD survey scores also significantly improved over the course of the study (P .001 and P = .003, respectively).

Conclusion Short-term interdisciplinary SMAs decreased A1C and LDL-C, improved patient adherence to ADA guidelines, improved emotional distress related to diabetes, and increased knowledge of diabetes.

C1 [Hartzler, Melody L.; Shenk, McKenzie; Anderson, Douglas] Cedarville Univ, Sch Pharm, 251 N Main St, Cedarville, OH 45314 USA.

[Williams, Julie] Wright State Univ, Sch Profess Psychol, Dayton, OH 45435 USA.

[Schoen, James] Grandview Med Ctr, Family Med, Dayton, OH USA.

[Dunn, Thomas] Kettering Phys Network, Family Med, Dayton, OH USA.

RP Hartzler, ML (reprint author), Cedarville Univ, Sch Pharm, 251 N Main St, Cedarville, OH 45314 USA.

EM mhartzler@cedarville.edu

FU Internal Grant by Cedarville University School of Pharmacy

FX Internal Grant by Cedarville University School of Pharmacy.

CR American Diabetes Association, 2013, EC COSTS DIAB US 201

American Diabetes Association, 2017, STAT DIAB

Anderson RJ, 2001, DIABETES CARE, V24, P1069, DOI 10.2337/diacare.24.6.1069

Bassett J, 2012, J DIABETES METAB, V3, DOI 10.4172/2155-6156.1000210

Black SA, 2003, DIABETES CARE, V26, P2822, DOI 10.2337/diacare.26.10.2822

Brennan J, 2011, AM J LIFESTYLE MED, V5, P69, DOI 10.1177/1559827610378331

Christie D, 2014, HEALTH TECHNOL ASSES, V18, P1, DOI 10.3310/hta18200

Clancy DE, 2008, AM J MANAG CARE, V14, P39

Clancy DE, 2007, J GEN INTERN MED, V22, P620, DOI 10.1007/s11606-007-0150-3

Cohen LB, 2011, DIABETES EDUCATOR, V37, P801, DOI 10.1177/0145721711423980

Culhane-Pera KA, 2005, J HEALTH CARE POOR U, V16, P315, DOI 10.1353/hpu.2005.0030

Dickman K, 2012, J AM ACAD NURSE PRAC, V24, P43, DOI 10.1111/j.1745-7599.2011.00660.x

Edelman D, 2012, SHARED MED APPOINTME

Edelman D, 2015, J GEN INTERN MED, V30, P99, DOI 10.1007/s11606-014-2978-7

Edelman D, 2010, ANN INTERN MED, V152, P689, DOI 10.7326/0003-4819-152-11-201006010-00001

Eisenstat SA, 2013, CURR DIABETES REP, V13, P177, DOI 10.1007/s11892-012-0349-5

Fisher L, 2013, DIABETES CARE, V36, P2551, DOI 10.2337/dc12-2493

Fisher L, 2010, DIABETES CARE, V33, P23, DOI 10.2337/dc09-1238

Glasgow RE, 2004, J GEN INTERN MED, V19, P1167, DOI 10.1111/j.1525-1497.2004.30425.x

Golden SH, 2008, JAMA-J AM MED ASSOC, V299, P2751, DOI 10.1001/jama.299.23.2751

Goldney RD, 2004, DIABETES CARE, V27, P1066, DOI 10.2337/diacare.27.5.1066

Golstein MK, 2007, VET AFF HLTH SERV RE

Gonzalez JS, 2008, DIABETES CARE, V31, P2398, DOI 10.2337/dc08-1341

Gregg JA, 2007, J CONSULT CLIN PSYCH, V75, P336, DOI 10.1037/0022-006X.75.2.336

Hermanns N, 2006, DIABETOLOGIA, V49, P469, DOI 10.1007/s00125-005-0094-2

Hermanns N, 2015, DIABETES CARE, V38, P551, DOI 10.2337/dc14-1416

Heyworth L, 2014, ANN FAM MED, V12, P324, DOI 10.1370/afm.1660

Johnson JA, 2014, DIABETES CARE, V37, P3220, DOI 10.2337/dc14-1308

Katon W, 2008, J GEN INTERN MED, V23, P1571, DOI 10.1007/s11606-008-0731-9

Katon W, 2012, ARCH GEN PSYCHIAT, V69, P506, DOI 10.1001/archgenpsychiatry.2011.1548

Katon WJ, 2010, NEW ENGL J MED, V363, P2611, DOI 10.1056/NEJMoa1003955

McGuire BE, 2010, DIABETOLOGIA, V53, P66, DOI 10.1007/s00125-009-1559-5

Naik AD, 2011, ARCH INTERN MED, V171, P453, DOI 10.1001/archinternmed.2011.70

Noffsinger E. B., 1999, PERMANENTE J, V3, P58

Peikes D, 2009, JAMA-J AM MED ASSOC, V301, P603, DOI 10.1001/jama.2009.126

Pena-Purcell NC, 2011, DIABETES EDUCATOR, V37, P770, DOI 10.1177/0145721711423319

Polonsky WH, 2005, DIABETES CARE, V28, P626, DOI 10.2337/diacare.28.3.626

Ramser KL, 2008, DIABETES SPECTR, V21, P209, DOI DOI 10.2377/DIASPECT.21.3.209

Ridgeway NA, 1999, SOUTHERN MED J, V92, P667, DOI 10.1097/00007611-199907000-00004

Rothman RL, 2005, DIABETES EDUCATOR, V31, P215, DOI 10.1177/0145721705275002

Rygg LO, 2012, PATIENT EDUC COUNS, V86, P98, DOI 10.1016/j.pec.2011.04.008

Schillinger D, 2009, DIABETES CARE, V32, P559, DOI 10.2337/dc08-0787

Sikon A, 2010, ANN INTERN MED, V152, P745, DOI 10.7326/0003-4819-152-11-201006010-00012

Sullivan MD, 2012, DIABETES CARE, V35, P1708, DOI 10.2337/dc11-1791

Sumego M, 2014, ACP J CLUB, V160, P1

Taveira TH, 2011, ANN PHARMACOTHER, V45, P1346, DOI 10.1345/aph.1Q212

Taveira TH, 2010, DIABETES EDUCATOR, V36, P109, DOI 10.1177/0145721709352383

Trento M, 2005, NUTR METAB CARDIOVAS, V15, P293, DOI 10.1016/j.numecd.2004.12.005

Trento M, 2004, DIABETES CARE, V27, P670, DOI 10.2337/diacare.27.3.670

Trento M, 2002, DIABETOLOGIA, V45, P1231, DOI 10.1007/s00125-002-0904-8

Trento M, 2001, DIABETES CARE, V24, P995, DOI 10.2337/diacare.24.6.995

Trott HW, 2012, THESIS

Vachon GC, 2007, J NATL MED ASSOC, V99, P1327

Wagner EH, 2001, DIABETES CARE, V24, P695, DOI 10.2337/diacare.24.4.695

Welch GW, 1997, DIABETES CARE, V20, P760, DOI 10.2337/diacare.20.5.760

Wheelock C, 2009, FAM MED, V41, P116

NR 56

TC 0

Z9 0

U1 11

U2 11

PU SAGE PUBLICATIONS INC

PI THOUSAND OAKS

PA 2455 TELLER RD, THOUSAND OAKS, CA 91320 USA

SN 0145-7217

EI 1554-6063

J9 DIABETES EDUCATOR

JI Diabetes Educ.

PD AUG

PY 2018

VL 44

IS 4

BP 361

EP 372

DI 10.1177/0145721718776597

PG 12

WC Endocrinology & Metabolism; Public, Environmental & Occupational Health

SC Endocrinology & Metabolism; Public, Environmental & Occupational Health

GA GO0KP

UT WOS:000439617600004

PM 29792133

DA 2019-01-08

ER

PT J

AU Piatt, GA

Rodgers, EA

Xue, LS

Zgibor, JC

AF Piatt, Gretchen A.

Rodgers, Elizabeth A.

Xue, Lingshu

Zgibor, Janice C.

TI Integration and Utilization of Peer Leaders for Diabetes Self-Management

Support: Results From Project SEED (Support, Education, and Evaluation

in Diabetes)

SO DIABETES EDUCATOR

LA English

DT Article

ID COMMUNITY-HEALTH WORKERS; RANDOMIZED CONTROLLED-TRIAL; TRAINING-PROGRAM;

CHRONIC DISEASE; CARE; PREVENTION; MELLITUS; INTERVENTION; OUTCOMES;

ADULTS

AB Purpose The purpose of the study was to evaluate the effectiveness of a peer leader-led (PL) diabetes self-management support (DSMS) group in achieving and maintaining improvements in A1C, self-monitoring of blood glucose (SMBG), and diabetes distress in individuals with diabetes. Diabetes self-management support is critical; however, effective, sustainable support models are scarce.

Methods The study was a cluster randomized controlled trial of 221 people with diabetes from 6 primary care practices. Practices and eligible participants (mean age: 63.0 years, 63.8% female, 96.8% white, 28.5% at or below poverty level, 32.5% using insulin, A1C 7%: 54.2%) were randomized to diabetes self-management education (DSME) + PL DSMS (n = 119) or to enhanced usual care (EUC) (DSME + traditional DSMS with no PL; n = 102). Data were collected at baseline, after DSME (6 weeks), after DSMS (6 months), and after telephonic DSMS (12 months).

Results Decreases in A1C occurred between baseline and post-DSME in both groups. Both groups sustained improvements during DSMS, but A1C levels increased during telephonic DSMS. Improvements in self-monitoring of blood glucose were observed in both groups following DSME and were sustained throughout. At study end, the intervention group was 4.3 times less likely to have diabetes regimen-related distress compared to EUC.

Conclusions PL DSMS is as effective as traditional DSMS in helping participants to maintain glycemic control and self-monitoring of blood glucose (SMBG) and more effective at improving distress. With increasing diabetes prevalence and shortage of diabetes educators, it is important to integrate and use low-cost interventions in high-risk communities that build on available resources.

C1 [Piatt, Gretchen A.] Univ Michigan, Sch Med, Dept Learning Hlth Sci, Ann Arbor, MI USA.

[Rodgers, Elizabeth A.] Gateway Hlth Plan, Pittsburgh, PA USA.

[Rodgers, Elizabeth A.; Xue, Lingshu] Univ Pittsburgh, Grad Sch Publ Hlth, Dept Epidemiol, Pittsburgh, PA 15260 USA.

[Zgibor, Janice C.] Univ S Florida, Coll Publ Hlth, Tampa, FL USA.

RP Piatt, GA (reprint author), Univ Michigan, Dept Learning Hlth Sci, 1111 E Catherine St,Room 222, Ann Arbor, MI 48109 USA.

EM piattg@umich.edu

FU educational grant from Eli Lilly and Company

FX Bringing Research in Diabetes to Global Environments and Systems

(BRiDGES), an International Diabetes Federation project supported by an

educational grant from Eli Lilly and Company.

CR American Association of Diabetes Educators, 2010, SUST MOD DIAB SELF M

Anderson G, 2004, PUBLIC HEALTH REP, V119, P263, DOI 10.1016/j.phr.2004.04.005

Balagopal P, 2012, DIABETES EDUCATOR, V38, P822, DOI 10.1177/0145721712459890

Beck J, 2017, DIABETES EDUCATOR, V43, P449, DOI 10.1177/0145721717722968

Cherrington A, 2008, J HEALTH CARE POOR U, V19, P1044, DOI 10.1353/hpu.0.0077

Dale JR, 2012, DIABETIC MED, V29, P1361, DOI 10.1111/j.1464-5491.2012.03749.x

Dennis CL, 2003, INT J NURS STUD, V40, P321, DOI 10.1016/S0020-7489(02)00092-5

Eng Eugenia, 1992, FAM COMMUNITY HEALTH, V15, P24, DOI DOI 10.1097/00003727-199204000-00005

Fischman Josh, 2005, US News World Rep, V138, P44

Fitzgerald JT, 1996, EVAL HEALTH PROF, V19, P208, DOI 10.1177/016327879601900205

Funnell MM, 2010, FAM PRACT, V27, pI17, DOI 10.1093/fampra/cmp027

Funnell MM, 2005, DIABETES EDUCATOR, V31, P53, DOI 10.1177/0145721704273166

Funnell MM, 2005, DIABETES ED, V31, P61

Funnell MM, 2007, DIABETES SPECTR, V20, P221, DOI DOI 10.2337/DIASPECT.20.4.221

Harris M. I., 1995, DIABETES AM, P1

Institute of Medicine, 2001, CROSS QUAL CHASM NEW

Lasater TM, 1997, ANN EPIDEMIOL, V7, pS46, DOI 10.1016/S1047-2797(97)80007-5

Lorig K, 2009, DIABETES EDUCATOR, V35, P641, DOI 10.1177/0145721709335006

Millay JM, ACCURATELY MEASURING

Moskowitz D, 2013, J GEN INTERN MED, V28, P938, DOI 10.1007/s11606-013-2367-7

National Certification Board for Diabetes Educators, 2017, SUMM 2016 CERT EX RE

Norris SL, 2006, DIABETIC MED, V23, P544, DOI 10.1111/j.1464-5491.2006.01845.x

Norris SL, 2002, DIABETES CARE, V25, P1159, DOI 10.2337/diacare.25.7.1159

Norris SL, 2002, AM J PREV MED, V22, P39, DOI 10.1016/S0749-3797(02)00424-5

Norris SL, 2001, DIABETES CARE, V24, P561, DOI 10.2337/diacare.24.3.561

O'Connor A, 2012, PUBLIC HEALTH, V126, P813, DOI 10.1016/j.puhe.2012.05.029

PADGETT D, 1988, J CLIN EPIDEMIOL, V41, P1007, DOI 10.1016/0895-4356(88)90040-6

Polonsky WH, 2005, DIABETES CARE, V28, P626, DOI 10.2337/diacare.28.3.626

SHAMOON H, 1993, NEW ENGL J MED, V329, P977

Siminerio L, 2013, DIABETES EDUCATOR, V39, P705, DOI 10.1177/0145721713492570

Stewart MJ, 1995, INT J NURS STUD, V32, P535, DOI 10.1016/0020-7489(95)00014-9

Swider SM, 2002, PUBLIC HEALTH NURS, V19, P11, DOI 10.1046/j.1525-1446.2002.19003.x

Tang TS, 2013, DIABETIC MED, V30, P746, DOI 10.1111/dme.12179

Tang TS, 2014, DIABETES CARE, V37, P1525, DOI 10.2337/dc13-2161

Tang TS, 2012, DIABETES EDUCATOR, V38, P519, DOI 10.1177/0145721712447982

Tang TS, 2011, PATIENT EDUC COUNS, V85, P160, DOI 10.1016/j.pec.2010.12.013

Tang TS, 2011, DIABETES EDUCATOR, V37, P67, DOI 10.1177/0145721710387308

Thompson JR, 2007, DIABETES EDUCATOR, V33, p159S, DOI 10.1177/01457207304077

Toobert DJ, 2000, DIABETES CARE, V23, P943, DOI 10.2337/diacare.23.7.943

UK Prospective Diabetes Study (UKPDS) Group, 1998, LANCET, V352, P837, DOI DOI 10.1016/S0140-6736(98)07019-6

US Census Bureau, 2015, QUICKFACTS FAYETT CO

Wing RR, 2010, ARCH INTERN MED, V170, P1566, DOI 10.1001/archinternmed.2010.334

2018, DIABETES CARE S1, V41, pS38

1979, JAMA, V242, P2562

NR 44

TC 1

Z9 1

U1 5

U2 5

PU SAGE PUBLICATIONS INC

PI THOUSAND OAKS

PA 2455 TELLER RD, THOUSAND OAKS, CA 91320 USA

SN 0145-7217

EI 1554-6063

J9 DIABETES EDUCATOR

JI Diabetes Educ.

PD AUG

PY 2018

VL 44

IS 4

BP 373

EP 382

DI 10.1177/0145721718777855

PG 10

WC Endocrinology & Metabolism; Public, Environmental & Occupational Health

SC Endocrinology & Metabolism; Public, Environmental & Occupational Health

GA GO0KP

UT WOS:000439617600005

PM 29806788

DA 2019-01-08

ER

PT J

AU Sheikh, MA

AF Sheikh, Mashhood Ahmed

TI Coloring of the past via respondent's current psychological state,

mediation, and the association between childhood disadvantage and

morbidity in adulthood

SO JOURNAL OF PSYCHIATRIC RESEARCH

LA English

DT Article

DE Childhood disadvantage; Social epidemiology; Social causation; Life

course; Anchoring; Recall bias; Measurement error; Differential error;

Non-differential error; Reliability; Validity

ID RETROSPECTIVE REPORTS; AUTOBIOGRAPHICAL MEMORY; MALTREATMENT RESEARCH;

REPORTING BEHAVIOR; RECALL BIAS; EXPERIENCES; HEALTH; DISORDERS; ABUSE;

DISTRESS

AB Objective: Many researchers view retrospective reports with skepticism. Indeed, the observed association between retrospectively-reported childhood disadvantage (CD) and morbidity in adulthood has been criticized as an artefactual correlation driven by the psychological state of the respondent at the time of reporting (current psychological state). The aim of this study was to assess the role of current psychological state in the association between childhood disadvantage and morbidity in adulthood.

Methods: The present analysis used cross-sectional data collected in 2007-2008 within the framework of the Tromso Study (N = 10,765), a representative study of adult men and women in Norway. The association between CD and the physical health outcomes heart attack, angina pectoris, chronic bronchitis/emphysema/COPD, diabetes mellitus, hypothyroid/low metabolism, migraine, hypertension, and comorbidity (i.e., the sum of these physical health outcomes) was assessed with Poisson regression models. Relative risks (RR) and 95% confidence intervals (CI) were estimated. A wide range of indicators of respondents' current psychological state were included in the models to assess the % attenuation in estimates.

Results: CD was associated with an increased risk of heart attack, angina pectoris, chronic bronchitis/emphysema/COPD, diabetes mellitus, hypothyroid/low metabolism, migraine, hypertension, and comorbidity (p < 0.05), independent of respondents' current psychological state. A sizeable proportion (23-42%) of the association between CD and physical health outcomes was driven by recall bias or mediation via respondents' current psychological state. Controlling for indicators of current psychological state reduced the strength of associations between CD and physical health outcomes; however, the independent associations remained in the same direction.

Conclusion: The association between retrospectively-reported CD and physical health outcomes in adulthood is not driven entirely by respondent's current psychological state.

C1 [Sheikh, Mashhood Ahmed] Univ Tromso, Dept Community Med, Tromso, Norway.

RP Sheikh, MA (reprint author), Univ Tromso, Dept Community Med, Tromso, Norway.

EM mashhood.a.sheikh@uit.no

OI Sheikh, Mashhood Ahmed/0000-0002-3212-592X

CR ANESHENSEL CS, 1987, J HEALTH SOC BEHAV, V28, P232, DOI 10.2307/2136843

Beckett M, 2001, J HUM RESOUR, V36, P593, DOI 10.2307/3069631

Beckett M, 2000, AM J EPIDEMIOL, V151, P315

BOWER GH, 1981, AM PSYCHOL, V36, P129, DOI 10.1037//0003-066X.36.2.129

BRADBURN NM, 1987, SCIENCE, V236, P157, DOI 10.1126/science.3563494

Bremner JD, 1999, BIOL PSYCHIAT, V45, P797, DOI 10.1016/S0006-3223(99)00009-8

BREWIN CR, 1988, COGNITIVE FDN CLIN P

Brown M, 2014, LONGITUD LIFE COURSE, V5, P64, DOI 10.14301/llcs.v5i1.232

Campbell JA, 2016, AM J PREV MED, V50, P344, DOI 10.1016/j.amepre.2015.07.022

Deary IJ, 2009, BRIT MED BULL, V92, P135, DOI 10.1093/bmb/ldp033

Dube SR, 2004, CHILD ABUSE NEGLECT, V28, P729, DOI 10.1016/j.chiabu.2003.08.009

Engstad T, 2000, STROKE, V31, P1602, DOI 10.1161/01.STR.31.7.1602

Falkegard M, 2015, ACTA OBSTET GYN SCAN, V94, P28, DOI 10.1111/aogs.12514

Femina D, 1990, CHILD ABUSE NEGLECT, V14, P227, DOI DOI 10.1016/0145-2134(90)90033-P

Fergusson DM, 2011, INT J METH PSYCH RES, V20, P93, DOI 10.1002/mpr.337

Fergusson DM, 2000, PSYCHOL MED, V30, P529, DOI 10.1017/S0033291799002111

FIELD D, 1981, INT J BEHAV DEV, V4, P77, DOI 10.1177/016502548100400106

Gerlsma C, 1997, PERS INDIV DIFFER, V23, P271, DOI 10.1016/S0191-8869(97)00033-0

Gilbert D. T., 2006, STUMBLING HAPPINESS

Goodman G. S., 2016, DEV PSYCHOPATHOLOGY

Haas SA, 2007, DEMOGRAPHY, V44, P113, DOI 10.1353/dem.2007.0003

HALVERSON CF, 1988, J PERS, V56, P435, DOI 10.1111/j.1467-6494.1988.tb00895.x

Hardt J, 2004, J CHILD PSYCHOL PSYC, V45, P260, DOI 10.1111/j.1469-7610.2004.00218.x

Hardt J, 2010, PSYCHOL REP, V107, P425, DOI 10.2466/02.04.09.10.16.21.PR0.107.5.425-440

Havari E, 2015, EUR J POPUL, V31, P233, DOI 10.1007/s10680-014-9332-y

HERZOG AR, 1989, PSYCHOL AGING, V4, P173, DOI 10.1037/0882-7974.4.2.173

Jacobsen BK, 2012, INT J EPIDEMIOL, V41, P961, DOI 10.1093/ije/dyr049

Jorgensen RS, 1996, PSYCHOL BULL, V120, P293, DOI 10.1037/0033-2909.120.2.293

Kendall-Tackett K, 2004, CHILD ABUSE NEGLECT, V28, P723, DOI 10.1016/j.chiabu.2004.02.002

Krieger N, 1998, AM J EPIDEMIOL, V147, P704

Kuh D., 2004, LIFE COURSE APPROACH, P371

LEWINSOHN PM, 1987, J PERS SOC PSYCHOL, V52, P611, DOI 10.1037//0022-3514.52.3.611

LEWIS M, 1995, J AM ACAD CHILD PSY, V34, P405, DOI 10.1097/00004583-199504000-00007

Lindsay DS, 2004, MEMORY, V12, P27, DOI 10.1080/09658210244000243

MATT GE, 1992, CLIN PSYCHOL REV, V12, P227, DOI 10.1016/0272-7358(92)90116-P

McFarland C, 1998, J PERS SOC PSYCHOL, V75, P1424, DOI 10.1037/0022-3514.75.6.1424

Memon A., 1997, LEGAL CRIMINOL PSYCH, V2, P131

Mock S. E., 2011, FRONT PSYCHOL, V1

Offer D, 2000, J AM ACAD CHILD PSY, V39, P735, DOI 10.1097/00004583-200006000-00012

Pan A, 2010, ARCH INTERN MED, V170, P1884, DOI 10.1001/archinternmed.2010.356

PARKS ED, 1995, PSYCHIATRY, V58, P199, DOI 10.1080/00332747.1995.11024726

Pinto R, 2014, J FAM VIOLENCE, V29, P431, DOI 10.1007/s10896-014-9602-9

Pinto RJ, 2013, CHILD ABUSE REV, V22, P354, DOI 10.1002/car.2232

Raphael Karen G, 2004, Curr Pain Headache Rep, V8, P99, DOI 10.1007/s11916-004-0023-y

Raphael KG, 2005, CLIN J PAIN, V21, P371, DOI 10.1097/01.ajp.0000149794.70627.18

RAPHAEL KG, 1994, J CLIN EPIDEMIOL, V47, P555, DOI 10.1016/0895-4356(94)90302-6

Reuben A, 2016, J CHILD PSYCHOL PSYC, V57, P1103, DOI 10.1111/jcpp.12621

ROBINS LN, 1985, AM J ORTHOPSYCHIAT, V55, P27, DOI 10.1111/j.1939-0025.1985.tb03419.x

ROSS M, 1989, PSYCHOL REV, V96, P341, DOI 10.1037/0033-295X.96.2.341

Scott KM, 2011, ARCH GEN PSYCHIAT, V68, P838, DOI 10.1001/archgenpsychiatry.2011.77

Scott KM, 2008, PSYCHOSOM MED, V70, P1035, DOI 10.1097/PSY.0b013e318187a2fb

Sheikh MA, 2018, EUR CHILD ADOLES PSY, V27, P481, DOI 10.1007/s00787-017-1090-z

Sheikh MA, 2018, J PSYCHOSOM RES, V108, P78, DOI 10.1016/j.jpsychores.2018.03.001

Sheikh MA, 2018, J PSYCHIATR RES, V101, P50, DOI 10.1016/j.jpsychires.2018.02.025

Sheikh MA, 2018, INT J PUBLIC HEALTH, V63, P181, DOI 10.1007/s00038-017-1021-2

Sheikh MA, 2018, J AFFECT DISORDERS, V229, P206, DOI 10.1016/j.jad.2017.12.051

Sheikh MA, 2018, J AFFECT DISORDERS, V226, P21, DOI 10.1016/j.jad.2017.09.015

Sheikh MA, 2016, SAGE OPEN MED, V4, DOI 10.1177/2050312115622857

Sheikh MA, 2014, LIPIDS HEALTH DIS, V13, DOI 10.1186/1476-511X-13-164

SINGER JA, 1988, CLIN PSYCHOL REV, V8, P211, DOI 10.1016/0272-7358(88)90060-8

Smith JP, 2009, DEMOGRAPHY, V46, P387, DOI 10.1353/dem.0.0058

STEELE GP, 1980, PSYCHOL MED, V10, P301, DOI 10.1017/S0033291700044056

STROMGREN LS, 1977, ACTA PSYCHIAT SCAND, V56, P109, DOI 10.1111/j.1600-0447.1977.tb06670.x

Sullivan H. S., 1973, CLIN STUDIES PSYCHIA

Susser E, 2012, SCHIZOPHRENIA BULL, V38, P672, DOI 10.1093/schbul/sbs074

TAYLOR SE, 1988, PSYCHOL BULL, V103, P193, DOI 10.1037/0033-2909.103.2.193

Tietjen GE, 2010, EXPERT REV NEUROTHER, V10, P1625, DOI [10.1586/ern.10.152, 10.1586/ERN.10.152]

von Fintel D, 2016, SOC INDIC RES, V126, P119, DOI 10.1007/s11205-015-0896-7

Vuolo M, 2014, DEMOGRAPHY, V51, P1999, DOI 10.1007/s13524-014-0344-3

Ward MM, 2011, BMC MED RES METHODOL, V11, DOI 10.1186/1471-2288-11-147

White HR, 2007, PSYCHOL REP, V101, P1079, DOI 10.2466/PR0.101.4.1079-1094

Widom CS, 2007, ARCH GEN PSYCHIAT, V64, P49

Widom CS, 2004, CHILD ABUSE NEGLECT, V28, P715, DOI 10.1016/j.chiabu.2004.03.009

WILLIAMS LM, 1994, J CONSULT CLIN PSYCH, V62, P1167, DOI 10.1037/0022-006X.62.6.1167

Yarrow M R, 1970, Monogr Soc Res Child Dev, V35, P1

NR 75

TC 3

Z9 3

U1 3

U2 3

PU PERGAMON-ELSEVIER SCIENCE LTD

PI OXFORD

PA THE BOULEVARD, LANGFORD LANE, KIDLINGTON, OXFORD OX5 1GB, ENGLAND

SN 0022-3956

EI 1879-1379

J9 J PSYCHIATR RES

JI J. Psychiatr. Res.

PD AUG

PY 2018

VL 103

BP 173

EP 181

DI 10.1016/j.jpsychires.2018.05.027

PG 9

WC Psychiatry

SC Psychiatry

GA GM3LA

UT WOS:000438004900025

PM 29883925

DA 2019-01-08

ER

PT J

AU Wardian, JL

Tate, J

Folaron, I

Graybill, S

True, M

Sauerwein, T

AF Wardian, Jana L.

Tate, Joshua

Folaron, Irene

Graybill, Sky

True, Mark

Sauerwein, Tom

TI Who's distressed? A comparison of diabetes-related distress by type of

diabetes and medication

SO PATIENT EDUCATION AND COUNSELING

LA English

DT Article

DE Diabetes-related distress; Regimen distress; Emotional burden; Type 1

diabetes; Type 2 diabetes

ID PSYCHOLOGICAL INSULIN-RESISTANCE; EMOTIONAL BURDEN; GLYCEMIC CONTROL;

SELF-MANAGEMENT; SOCIAL SUPPORT; ADULTS; SCALE; AUSTRALIA; THERAPY;

IMPACT

AB Objective: We hypothesized that diabetes-related distress would vary by type of diabetes and medication regimen [Type 1 diabetes (T1DM), Type 2 diabetes with insulin use (T2DM-i), Type 2 diabetes without insulin use (T2DM)]. Thus, the aim of this study was to identify groups with elevated diabetes-related distress.

Methods: We administered the 17-item Diabetes-related Distress Scale (DDS-17) to 585 patients. We collected demographics, medications, and lab results from patient records.

Results: Patients were categorized by type of diabetes and medication: T1DM (n = 149); T2DM-i (n = 333); and T2DM (n = 103). ANOVA revealed significant differences in sample characteristics. ANCOVA were conducted on all four DDS-17 domains [Emotional Burden (EB); Physician-related Distress (PD); Regimen-related Distress (RD); and Interpersonal Distress (ID)]; covariates included in the models were sex, age, duration of diabetes, BMI, and HbA1c. EB was significantly lower in T1DM than T2DM-i, p < 0.05. In addition, RD was significantly lower in T1DM than either T2DM-i, p < 0.05 and T2DM, p < 0.05.

Conclusions: EB and RD are higher for those with type 2 diabetes. Thus, interventions to reduce EB and RD need to be considered for patients with type 2 diabetes.

Implications: DDS-17 is useful in identifying diabetes-related distress in patients with diabetes. Efforts need to be made to reduce EB and RD. Published by Elsevier B.V.

C1 [Wardian, Jana L.; Sauerwein, Tom] Wilford Hall Ambulatory Surg Ctr, Diabet Ctr Excellence, Lackland AFB, TX USA.

[Tate, Joshua; Folaron, Irene; Graybill, Sky; True, Mark] San Antonio Mil Med Ctr, Serv Endocrinol, Ft Sam Houston, TX 78234 USA.

RP Wardian, JL (reprint author), Wilford Hall Ambulatory Surg Ctr, Diabet Ctr Excellence, 1100 Wilford Hall Loop, Jbsa Lackland AFB, TX 78236 USA.

EM Jana.L.Wardian.ctr@mail.mil

OI Wardian, Jana/0000-0003-3025-686X

CR American Diabetes Association (ADA), 2017, DIAB CAR S1, V40

Baek RN, 2014, ANN BEHAV MED, V48, P145, DOI 10.1007/s12160-013-9585-4

Benroubi M, 2011, DIABETES RES CLIN PR, V93, pS97, DOI 10.1016/S0168-8227(11)70021-3

Bushatz A., 2015, MILITARY DIVORCE RAT

Fenwick E. K., 2016, J HEALTH PSYCHOL

Fisher L, 2008, DIABETIC MED, V25, P1096, DOI 10.1111/j.1464-5491.2008.02533.x

Fisher L, 2009, DIABETIC MED, V26, P622, DOI 10.1111/j.1464-5491.2009.02730.x

Fisher L, 2008, ANN FAM MED, V6, P246, DOI 10.1370/afm.842

Fisher L, 2007, DIABETES CARE, V30, P542, DOI 10.2337/dc06-1614

Fisher L, 2012, DIABETES CARE, V35, P259, DOI 10.2337/dc11-1572

Fisher L, 2010, DIABETES CARE, V33, P23, DOI 10.2337/dc09-1238

Gray M, 2017, METHODS MOL BIOL, V1519, P1, DOI 10.1007/978-1-4939-6581-6_1

Hessler D, 2016, J PEDIATR PSYCHOL, V41, P750, DOI 10.1093/jpepsy/jsw002

Holmes-Truscott E, 2016, PRIM CARE DIABETES, V10, P75, DOI 10.1016/j.pcd.2015.06.006

Holmes-Truscott E, 2015, DIABETIC MED, V32, P1297, DOI 10.1111/dme.12729

Joensen LE, 2016, PRIM CARE DIABETES, V10, P41, DOI 10.1016/j.pcd.2015.06.007

Joensen LE, 2013, DIABETES RES CLIN PR, V101, P278, DOI 10.1016/j.diabres.2013.07.001

Jones A, 2016, PRIM CARE DIABETES, V10, P83, DOI 10.1016/j.pcd.2015.03.008

Karney BR, 2012, J FAM ISSUES, V33, P1572, DOI 10.1177/0192513X12439690

Phillips Patrick, 2005, Rev Diabet Stud, V2, P35, DOI 10.1900/RDS.2005.2.35

Polonsky WH, 2005, DIABETES CARE, V28, P626, DOI 10.2337/diacare.28.3.626

Polonsky WH, 2011, CURR MED RES OPIN, V27, P1169, DOI 10.1185/03007995.2011.573623

Ramkisson S, 2016, JEMDSA, V21, P35, DOI 10.1080/16089677.2016.1205822

Sauerwein TJ, 2016, MIL MED, V181, P407, DOI 10.7205/MILMED-D-15-00007

Schmitt A, 2016, DIABETIC MED, V33, P835, DOI 10.1111/dme.12887

Slean GR, 2012, MED CARE, V50, pS69, DOI 10.1097/MLR.0b013e3182641127

Strom JL, 2012, CURR DIABETES REP, V12, P769, DOI 10.1007/s11892-012-0317-0

Wardian J, 2014, SOC WORK HEALTH CARE, V53, P364, DOI 10.1080/00981389.2014.884038

Wardian Jana L, 2017, Clin Diabetes, V35, P329, DOI 10.2337/cd16-0061

Young-Hyman D, 2016, DIABETES CARE, V39, P2126, DOI 10.2337/dc16-2053

NR 30

TC 1

Z9 1

U1 1

U2 1

PU ELSEVIER IRELAND LTD

PI CLARE

PA ELSEVIER HOUSE, BROOKVALE PLAZA, EAST PARK SHANNON, CO, CLARE, 00000,

IRELAND

SN 0738-3991

J9 PATIENT EDUC COUNS

JI Patient Educ. Couns.

PD AUG

PY 2018

VL 101

IS 8

BP 1490

EP 1495

DI 10.1016/j.pec.2018.03.001

PG 6

WC Public, Environmental & Occupational Health; Social Sciences,

Interdisciplinary

SC Public, Environmental & Occupational Health; Social Sciences - Other

Topics

GA GL9MH

UT WOS:000437636700021

PM 29525142

DA 2019-01-08

ER

PT J

AU Hessler, DM

Fisher, L

Polonsky, WH

Bowyer, V

Potter, M

AF Hessler, Danielle M.

Fisher, L.

Polonsky, W. H.

Bowyer, V

Potter, M.

TI Motivation and attitudes toward changing health (MATCH): A new

patient-reported measure to inform clinical conversations

SO JOURNAL OF DIABETES AND ITS COMPLICATIONS

LA English

DT Article

DE Motivation; Behavior change; Patient reported outcome; Type 2 diabetes;

Pragmatic measure

ID DIABETES DISTRESS SCALE; SELF-MANAGEMENT; RECOMMENDATIONS; EFFICACY;

PHQ-9; NEED

AB Objective: To identify and assess patient motivation to initiate or maintain behavior changes.

Methods: Attitudinal statements were developed from structured patient interviews and translated into 18 survey items. Items were analyzed with exploratory factor analysis (EFA).

Results: An EFA with 340 type 2 diabetes patients identified three areas of patient attitudes toward changing health behaviors: (1) willingness to make changes (3 items; alpha = 0.69), (2) perceived ability to make or maintain changes (3 items; alpha = 0.74), and (3) and feeling changes are worthwhile (3 items; alpha = 0.61). Greater perceived ability and feelings of worthwhileness were associated with positive psychosocial and behavioral management indicators. All three areas were associated with confidence and attitudes toward making a specific behavioral change (e.g., improve diet).

Conclusions: MATCH is an internally consistent and valid 9-item scale that provides a profile of factors influencing motivation that can be used in clinical and research settings. (C) 2018 Elsevier Inc. All rights reserved.

C1 [Hessler, Danielle M.; Fisher, L.; Bowyer, V; Potter, M.] Univ Calif San Francisco, Dept Family & Community Med, Box 0900, San Francisco, CA 94143 USA.

[Polonsky, W. H.] Behav Diabet Inst, San Diego, CA USA.

RP Hessler, DM (reprint author), Univ Calif San Francisco, Dept Family & Community Med, Box 0900, San Francisco, CA 94143 USA.

EM danielle.hessler@ucsf.edu

FU NIH [DK108039]

FX This research was funded by NIH Grant DK108039.

CR Ajzen I., 1985, ACTION CONTROL COGNI, P11, DOI DOI 10.1007/978-3-642-69746-3_2

Anderson RM, 2000, DIABETES CARE, V23, P739, DOI 10.2337/diacare.23.6.739

BAGOZZI RP, 1989, SOC PSYCHOL QUART, V52, P266, DOI 10.2307/2786991

BANDURA A, 1977, PSYCHOL REV, V84, P191, DOI 10.1037/0033-295X.84.2.191

Bandura A., 1997, COGNITIVE THERAPY RE, V1, P287, DOI DOI 10.1007/BF01663995

Coulter A, 2007, BMJ-BRIT MED J, V335, P24, DOI 10.1136/bmj.39246.581169.80

DiMatteo MR, 2004, MED CARE, V42, P200, DOI 10.1097/01.mlr.0000114908.90348.f9

Fisher L, 2012, DIABETES CARE, V35, P259, DOI 10.2337/dc11-1572

Funnell MM, 2004, CLIN DIABETES, V22, P123, DOI DOI 10.2337/DIACLIN.22.3.123

Glasgow RE, 2013, AM J PREV MED, V45, P237, DOI 10.1016/j.amepre.2013.03.010

Hessler DM, 2011, PATIENT EDUC COUNS, V85, P154, DOI 10.1016/j.pec.2010.10.030

Hessler DM, 2012, J HEALTH PSYCHOL, V17, P535, DOI 10.1177/1359105311422120

Hibbard JH, 2004, HEALTH SERV RES, V39, P1005, DOI 10.1111/j.1475-6773.2004.00269.x

Jordan JE, 2008, MED J AUSTRALIA, V189, pS9

Kroenke K, 2001, J GEN INTERN MED, V16, P606, DOI 10.1046/j.1525-1497.2001.016009606.x

Lorig K, 1996, OUTCOME MEASURES HLT

Michie S, 2014, BEHAV CHANGE WHEEL G

Moss-Morris R, 2002, PSYCHOL HEALTH, V17, P1, DOI 10.1080/08870440290001494

Polonsky WH, 2005, DIABETES CARE, V28, P626, DOI 10.2337/diacare.28.3.626

Rollnick S., 1992, J MENTAL HLTH, V1, P25, DOI DOI 10.3109/09638239209034509

Rollnick S, 2007, MOTIVATIONAL INTERVI

Ruggiero L, 1997, DIABETES CARE, V20, P568, DOI 10.2337/diacare.20.4.568

Ryan RM, 2000, AM PSYCHOL, V55, P68, DOI 10.1037/0003-066X.55.1.68

Schmitt A, 2013, HEALTH QUAL LIFE OUT, V11, DOI 10.1186/1477-7525-11-138

Toobert DJ, 2000, DIABETES CARE, V23, P943, DOI 10.2337/diacare.23.7.943

Wells TS, 2013, J AFFECT DISORDERS, V148, P77, DOI 10.1016/j.jad.2012.11.052

NR 26

TC 0

Z9 0

U1 4

U2 4

PU ELSEVIER SCIENCE INC

PI NEW YORK

PA 360 PARK AVE SOUTH, NEW YORK, NY 10010-1710 USA

SN 1056-8727

EI 1873-460X

J9 J DIABETES COMPLICAT

JI J. Diabetes Complications

PD JUL

PY 2018

VL 32

IS 7

BP 665

EP 669

DI 10.1016/j.jdiacomp.2018.04.009

PG 5

WC Endocrinology & Metabolism

SC Endocrinology & Metabolism

GA GM1GP

UT WOS:000437814400008

PM 29887299

DA 2019-01-08

ER

PT J

AU Rovner, BW

Casten, RJ

AF Rovner, Barry W.

Casten, Robin J.

TI Health Beliefs and Medication Adherence in Black Patients with Diabetes

and Mild Cognitive Impairment

SO AMERICAN JOURNAL OF GERIATRIC PSYCHIATRY

LA English

DT Article

DE Mild cognitive impairment; diabetes; medication adherence; health

beliefs; blacks

ID DEMENTIA; VALIDITY

AB Objectives: To evaluate determinants of medication adherence and glycemic control in black patients with diabetes and mild cognitive impairment (MCI). Methods: Cross-sectional study of 143 participants with mean age of 68.8 (SD: 6.7) years; 66.4% were women. Results: Eighty-seven participants (60.8%) self-reported medication nonadherence; they had more negative beliefs about medicines, greater diabetes-related distress, and more difficulty with daily living activities and affording medications than adherent participants. There were no group differences in cognition, depressive symptoms, or glycemic control. Glycemic control negatively correlated with regimen distress, emotional burden, interpersonal distress, beliefs that physicians overprescribe medications, and beliefs that medications are harmful. Conclusions: Beliefs about medications, diabetes-related distress, functional disability, and medication affordability are associated with medication nonadherence in black individuals with diabetes and MCI. Interventions that respect personal health beliefs and compensate for impaired cognition may improve medication adherence and glycemic control in this population.

C1 [Rovner, Barry W.] Thomas Jefferson Univ, Dept Neurol, Sidney Kimmel Med Coll, Philadelphia, PA 19107 USA.

[Rovner, Barry W.] Thomas Jefferson Univ, Dept Psychiat, Sidney Kimmel Med Coll, Philadelphia, PA 19107 USA.

[Rovner, Barry W.] Thomas Jefferson Univ, Dept Ophthalmol, Sidney Kimmel Med Coll, Philadelphia, PA 19107 USA.

[Casten, Robin J.] Thomas Jefferson Univ, Dept Psychiat & Human Behav, Sidney Kimmel Med Coll, Philadelphia, PA 19107 USA.

RP Rovner, BW (reprint author), Jefferson Hosp Neurosci, 900 Walnut St,Suite 200, Philadelphia, PA 19107 USA.

EM barry.rovner@jefferson.edu

FU National Institute of Diabetes and Digestive and Kidney Diseases (NIDDK)

[R01 DK102609-01]

FX This study was supported by the National Institute of Diabetes and

Digestive and Kidney Diseases (NIDDK; grant R01 DK102609-01).

CR Ali MK, 2013, NEW ENGL J MED, V368, P1613, DOI 10.1056/NEJMsa1213829

Crane PK, 2013, NEW ENGL J MED, V369, P540, DOI 10.1056/NEJMoa1215740

Galasko D, 2006, ALZ DIS ASSOC DIS, V20, pS152, DOI 10.1097/01.wad.0000213873.25053.2b

Horne R, 1999, PSYCHOL HEALTH, V14, P1, DOI 10.1080/08870449908407311

Kroenke K, 2001, J GEN INTERN MED, V16, P606, DOI 10.1046/j.1525-1497.2001.016009606.x

MORISKY DE, 1986, MED CARE, V24, P67, DOI 10.1097/00005650-198601000-00007

Morris JC, 2006, ALZ DIS ASSOC DIS, V20, P210, DOI 10.1097/01.wad.0000213865.09806.92

National Institute of Diabetes and Digestive and Kidney Diseases, 2005, NIH PUBLICATION, V02-3266

Polonsky WH, 2005, DIABETES CARE, V28, P626, DOI 10.2337/diacare.28.3.626

Weinger K, 2005, DIABETES CARE, V28, P1346, DOI 10.2337/diacare.28.6.1346

NR 10

TC 0

Z9 0

U1 6

U2 6

PU ELSEVIER SCIENCE INC

PI NEW YORK

PA 360 PARK AVE SOUTH, NEW YORK, NY 10010-1710 USA

SN 1064-7481

EI 1545-7214

J9 AM J GERIAT PSYCHIAT

JI Am. J. Geriatr. Psychiatr.

PD JUL

PY 2018

VL 26

IS 7

BP 812

EP 816

DI 10.1016/j.jagp.2018.03.012

PG 5

WC Geriatrics & Gerontology; Gerontology; Psychiatry

SC Geriatrics & Gerontology; Psychiatry

GA GL0BZ

UT WOS:000436629200011

PM 29673896

DA 2019-01-08

ER

PT J

AU Spencer, MS

Kieffer, EC

Sinco, B

Piatt, G

Palmisano, G

Hawkins, J

Lebron, A

Espitia, N

Tang, T

Funnell, M

Heisler, M

AF Spencer, Michael S.

Kieffer, Edith C.

Sinco, Brandy

Piatt, Gretchen

Palmisano, Gloria

Hawkins, Jaclynn

Lebron, Alana

Espitia, Nicolaus

Tang, Tricia

Funnell, Martha

Heisler, Michele

TI Outcomes at 18 Months From a Community Health Worker and Peer Leader

Diabetes Self-Management Program for Latino Adults

SO DIABETES CARE

LA English

DT Article

ID RANDOMIZED CONTROLLED-TRIAL; CHRONIC DISEASE MANAGEMENT;

AFRICAN-AMERICAN; GLYCEMIC CONTROL; CARE PROFILE; INTERVENTION; SUPPORT;

DETROIT; RELIABILITY; EDUCATION

AB OBJECTIVEThis study evaluated the effectiveness of a community health worker (CHW) diabetes self-management education (DSME) program, followed by two different approaches to maintain improvements in HbA(1c) and other clinical and patient-centered outcomes over 18 months.RESEARCH DESIGN AND METHODSThe study randomized 222 Latino adults with type 2 diabetes and poor glycemic control from a federally qualified health center to 1) a CHW-led, 6-month DSME program or 2) enhanced usual care (EUC). After the 6-month program, participants randomized to the CHW-led DSME were further randomized to 1) 12 months of CHW-delivered monthly telephone outreach (CHW-only) or 2) 12 months of weekly group sessions delivered by peer leaders (PLs) with telephone outreach to those unable to attend (CHW+PL). The primary outcome was HbA(1c). Secondary outcomes were blood pressure, lipid levels, diabetes distress, depressive symptoms, understanding of diabetes self-management, and diabetes social support. Assessments were conducted at baseline and at 6, 12, and 18 months.RESULTSParticipants in the CHW intervention at the 6-month follow-up had greater decreases in HbA(1c) (-0.45% [95% CI -0.87, -0.03]; P < 0.05) and in diabetes distress (-0.3 points [95% CI -0.6, -0.03]; P < 0.05) compared with EUC. CHW+PL participants maintained HbA(1c) improvements at 12 and 18 months, and CHW-only participants maintained improvements in diabetes distress at 12 and 18 months. CHW+PL participants also had significantly fewer depressive symptoms at 18 months compared with EUC (-2.2 points [95% CI -4.1, -0.3]; P < 0.05). Participants in CHW-led DSME had significant improvements in diabetes social support and in understanding of diabetes self-management at 6 months relative to EUC, but these intervention effects were not sustained at 18 months.CONCLUSIONSThis study demonstrates the effectiveness of a 6-month CHW intervention on key diabetes outcomes and of a volunteer PL program in sustaining key achieved gains. These are scalable models for health care centers in low-resource settings for achieving and maintaining improvements in key diabetes outcomes.

C1 [Spencer, Michael S.; Kieffer, Edith C.; Sinco, Brandy; Piatt, Gretchen; Espitia, Nicolaus; Funnell, Martha; Heisler, Michele] Univ Michigan, Ann Arbor, MI 48109 USA.

[Palmisano, Gloria] Community Hlth & Social Serv CHASS, Detroit, MI USA.

[Hawkins, Jaclynn] Michigan State Univ, E Lansing, MI 48824 USA.

[Lebron, Alana] Univ Calif Irvine, Irvine, CA USA.

[Tang, Tricia] Univ British Columbia, Vancouver, BC, Canada.

RP Spencer, MS (reprint author), Univ Michigan, Ann Arbor, MI 48109 USA.

EM spencerm1014@gmail.com

FU Peers for Progress grant from the American Association of Family

Physicians Foundation; National Institute of Diabetes and Digestive and

Kidney Diseases [P30-DK092926, R18-DK-0785501A1]; Centers for Disease

Control and Prevention [U50/CCU417409]

FX This research was supported by a Peers for Progress grant from the

American Association of Family Physicians Foundation, by the National

Institute of Diabetes and Digestive and Kidney Diseases (grant

P30-DK092926 to the Michigan Center for Diabetes Translational Research

and grant R18-DK-0785501A1), and by the Centers for Disease Control and

Prevention (cooperative agreement no. U50/CCU417409). The funding

sources had no role in the study design; data collection; administration

of the interventions; analysis, interpretation, or reporting of data; or

decision to submit the findings for publication.

CR American Public Health Association, COMM HLTH WORK

Anderson R., 2003, P 22 ANN S PRINC DIS, P3, DOI 10.1145/872035.872036

Arsie MP, 2000, ACTA DIABETOL, V37, P1, DOI 10.1007/s005920070028

Baig AA, 2015, J GEN INTERN MED, V30, P1481, DOI 10.1007/s11606-015-3339-x

Barrera M, 2002, AM J COMMUN PSYCHOL, V30, P637, DOI 10.1023/A:1016369114780

de Vries L, 2014, BMC ENDOCR DISORD, V14, DOI 10.1186/1472-6823-14-21

EDWARDS D, 1987, BIOMETRICS, V43, P913, DOI 10.2307/2531545

Emmons KM, 2001, AM J PREV MED, V20, P68, DOI 10.1016/S0749-3797(00)00254-3

Feathers JT, 2007, DIABETES EDUCATOR, V33, P509, DOI 10.1177/0145721707301371

Feathers JT, 2005, AM J PUBLIC HEALTH, V95, P1552, DOI 10.2105/AJPH.2005.066134

FISHER RA, 1942, DESIGN EXPT

Fitzgerald JT, 1996, EVAL HEALTH PROF, V19, P208, DOI 10.1177/016327879601900205

Fitzgerald JT, 1998, EVAL HEALTH PROF, V21, P52, DOI 10.1177/016327879802100103

Funnel MM, 2004, MED MANAGEMENT TYPE, P124

Funnell MM, 2004, CLIN DIABETES, V22, P123, DOI DOI 10.2337/DIACLIN.22.3.123

Haas L, 2012, DIABETES CARE, V35, P2393, DOI 10.2337/dc12-1707

Kalbfleisch J. D., 2011, STAT ANAL FAILURE TI

Kieffer EC, 2004, ETHNIC DIS, V14, P27

Kim K, 2016, AM J PUBLIC HEALTH, V106, pE3, DOI 10.2105/AJPH.2015.302987

Kroenke K, 2001, J GEN INTERN MED, V16, P606, DOI 10.1046/j.1525-1497.2001.016009606.x

Norris SL, 2006, DIABETIC MED, V23, P544, DOI 10.1111/j.1464-5491.2006.01845.x

Norris SL, 2002, DIABETES CARE, V25, P1159, DOI 10.2337/diacare.25.7.1159

Norris SL, 2001, DIABETES CARE, V24, P561, DOI 10.2337/diacare.24.3.561

Palmas W, 2014, DIABETES CARE, V37, P963, DOI 10.2337/dc13-2142

Piatt GA, 2006, DIABETES CARE, V29, P811, DOI 10.2337/diacare.29.04.06.dc05-1785

Piatt GA, 2010, DIABETES EDUCATOR, V36, P301, DOI 10.1177/0145721710361388

Piette JD, 2001, EVIDENCE BASED DIABE, P207

Polonsky WH, 2005, DIABETES CARE, V28, P626, DOI 10.2337/diacare.28.3.626

ROGERS EJ, 1993, CLIN CHEM, V39, P860

Rubin D., 2004, MULTIPLE IMPUTATION

Shah M, 2013, CURR DIABETES REP, V13, P163, DOI 10.1007/s11892-012-0359-3

Spencer MS, 2011, AM J PUBLIC HEALTH, V101, P2253, DOI 10.2105/AJPH.2010.300106

Tang TS, 2014, DIABETES CARE, V37, P1525, DOI 10.2337/dc13-2161

Tang TS, 2014, DIABETES EDUCATOR, V40, P223, DOI 10.1177/0145721713520569

Tang TS, 2012, DIABETES RES CLIN PR, V95, P85, DOI 10.1016/j.diabres.2011.04.003

Thom DH, 2013, ANN FAM MED, V11, P137, DOI 10.1370/afm.1443

van Buuren S, 2007, STAT METHODS MED RES, V16, P219, DOI 10.1177/0962280206074463

Wagner EH, 2000, BMJ-BRIT MED J, V320, P569, DOI 10.1136/bmj.320.7234.569

Xue L, 2014, EFFECTIVENESS PEER L

NR 39

TC 1

Z9 1

U1 5

U2 5

PU AMER DIABETES ASSOC

PI ALEXANDRIA

PA 1701 N BEAUREGARD ST, ALEXANDRIA, VA 22311-1717 USA

SN 0149-5992

EI 1935-5548

J9 DIABETES CARE

JI Diabetes Care

PD JUL 1

PY 2018

VL 41

IS 7

BP 1414

EP 1422

DI 10.2337/dc17-0978

PG 9

WC Endocrinology & Metabolism

SC Endocrinology & Metabolism

GA GK2AH

UT WOS:000435926000025

PM 29703724

DA 2019-01-08

ER

PT J

AU Kampling, H

Mittag, O

Herpertz, S

Baumeister, H

Kulzer, B

Petrak, F

AF Kampling, Hanna

Mittag, Oskar

Herpertz, Stephan

Baumeister, Harald

Kulzer, Bernd

Petrak, Frank

TI Can trajectories of glycemic control be predicted by depression,

anxiety, or diabetes-related distress in a prospective cohort of adults

with newly diagnosed type 1 diabetes? Results of a five-year follow-up

from the German multicenter diabetes cohort study (GMDC-Study)

SO DIABETES RESEARCH AND CLINICAL PRACTICE

LA English

DT Article

DE Adults; Depression; Longitudinal data; Onset cohort; Glycemic control;

Type 1 diabetes

ID QUALITY-OF-LIFE; COGNITIVE-BEHAVIORAL THERAPY; EMOTIONAL DISTRESS;

COMPLICATIONS; PREVALENCE; SYMPTOMS; HBA(1C); EPIDEMIOLOGY; ASSOCIATION;

DISORDERS

AB Aims: The longitudinal association between glycemic control with depression, anxiety or diabetes-related distress in type 1 diabetes is poorly understood. Therefore, we examined long-term trajectories of HbA1c in a new-onset cohort of adults with type 1 diabetes, and analyzed associations with depression, anxiety, and diabetes-related distress.

Methods: We included 313 newly diagnosed adults with type 1 diabetes in a prospective multicenter cohort study. Depression, anxiety, and diabetes-related distress were assessed starting with the diabetes diagnosis and at five annual surveys. HbA1c-measurements started with the one-year follow-up. HbA1c trajectories were analyzed applying Growth mixture modeling, while prediction of membership in the trajectories classes was analyzed Onset cohort using multiple regression, and one-way ANOVA/Chi2 to identify differences between Glycemic control classes.

Results: Average HbA1c increased constantly: follow-up at 1-year 6.5% (48 mmol/mol), 2- years 6.9% (52 mmol/mol), 3-years 7.1% (54 mmol/mol), 4-years 7.1% (54 mmol/mol), and 5-years 7.4% (57 mmol/mol). HbA1c trajectories included one 'good control' and three 'poor control' (52% of patients) classes. At the five-year follow-up, mean HbA1c was 6.3% (45 mmol/mol) in the 'good control' class, and ranging from 7.9% (63 mmol/mol) to 9.0% (75 mmol/mol) in the three 'poor control' classes. Classes were neither predicable, nor differentiated by depression, anxiety, or diabetes-related distress.

Conclusions: We identified distinct trajectories of glycemic control. Depression and anxiety were highly prevalent but they neither predicted 'poor'/'good' glycemic control trajectories nor were they associated with glycemic control at any assessment point. (C) 2018 Elsevier B.V. All rights reserved.

C1 [Kampling, Hanna; Mittag, Oskar] Univ Freiburg, Fac Med, Med Ctr, Sect Hlth Care Res & Rehabil Res, Hugstetter Str 49, D-79115 Freiburg, Germany.

[Herpertz, Stephan; Petrak, Frank] Ruhr Univ Bochum, Dept Psychosomat Med & Psychotherapy, LWL Univ Clin Bochum, Alexandrinenstr 1-3, D-44791 Bochum, Germany.

[Baumeister, Harald] Univ Ulm, Inst Psychol & Educ, Dept Clin Psychol & Psychotherapy, Albert Einstein Allee 47, D-89069 Ulm, Germany.

[Kulzer, Bernd] Diabet Ctr Mergentheim, Klotzbucher Str 12, D-97980 Bad Mergentheim, Germany.

[Kulzer, Bernd] Diabet Acad Mergentheim, Res Inst, Klotzbucher Str 12, D-97980 Bad Mergentheim, Germany.

[Kulzer, Bernd] Otto Friedrich Univ Bamberg, Dept Clin Psychol & Psychotherapy, Markuspl 3, D-96047 Bamberg, Germany.

[Petrak, Frank] Ctr Psychotherapy Wiesbaden, Sonnenberger Str 20, D-65193 Wiesbaden, Germany.

RP Kampling, H (reprint author), Univ Freiburg, Fac Med, Med Ctr, Sect Hlth Care Res & Rehabil Res, Hugstetter Str 49, D-79106 Freiburg, Germany.

EM hanna.kampling@uniklinik-freiburg.de;

oskar.mittag@uniklinik-freiburg.de; stephan.herpertz@ruhr-uni-bochum.de;

harald.baumeister@uni-ulm.de; kulzer@diabetes-zentrum.de;

mail@dr-frank-petrak.de

OI Petrak, Frank/0000-0001-5985-5845; Kampling, Hanna/0000-0002-5213-0179

FU German Diabetes Association (DDG; Deutsche Diabetes Gesellschaft) in

Germany; University Medical Centre - Johannes Gutenberg-University Mainz

in Germany; Novo Nordisk in Germany; Berlin-Chemie AG in Germany

FX The study was supported by the German Diabetes Association (DDG;

Deutsche Diabetes Gesellschaft), University Medical Centre - Johannes

Gutenberg-University Mainz, Novo Nordisk, Berlin-Chemie AG, all

institutions are in Germany.

CR American Diabetes Association, 2017, Diabetes Care, V40, pS11

American Psychiatric Association APA, 1994, DIAGN STAT MAN MENT

Asparouhov T, 2014, VARIABLE SPECFIC ENT

Baumeister H, 2012, COCHRANE DB SYST REV, DOI 10.1002/14651858.CD008381.pub2

Berge Line Iden, 2013, BMC Res Notes, V6, P543, DOI 10.1186/1756-0500-6-543

DCCT Res Grp, 1988, DIABETES CARE, V11, P725

Maia ACCD, 2014, REV ASSOC MED BRAS, V60, P59, DOI 10.1590/1806-9282.60.01.013

Derogatis L. R., 1995, SYMPTOM CHECKLISTE D

DeVries JH, 2004, DIABETIC MED, V21, P1263, DOI 10.1111/j.1464-5491.2004.01386.x

Fisher L, 2015, J DIABETES COMPLICAT, V29, P572, DOI 10.1016/j.jdiacomp.2015.01.012

Fisher L, 2010, DIABETES CARE, V33, P23, DOI 10.2337/dc09-1238

Franciosi M, 2013, NUTR METAB CARDIOVAS, V23, P606, DOI 10.1016/j.numecd.2012.03.002

Golden SH, 2008, JAMA-J AM MED ASSOC, V299, P2751, DOI 10.1001/jama.299.23.2751

Gonzalez JS, 2011, DIABETES CARE, V34, P236, DOI 10.2337/dc10-1970

Hirsch A, 2000, QUAL LIFE RES, V9, P207, DOI 10.1023/A:1008959810698

Ismail K, 2017, DIABETOLOGIA, V60, P2092, DOI 10.1007/s00125-017-4367-3

Jung T, 2008, SOC PERSONAL PSYCHOL, V2, P302, DOI 10.1111/j.1751-9004.2007.00054.x

Kampling H, 2017, DIABETOLOGIA, V60, P60, DOI 10.1007/s00125-016-4123-0

Kong Lulu, 2013, J Biomed Res, V27, P380, DOI 10.7555/JBR.27.20120112

Korczak DJ, 2011, DIABETOLOGIA, V54, P2483, DOI 10.1007/s00125-011-2240-3

Lloyd CE, 2010, DIABETIC MED, V27, P234, DOI 10.1111/j.1464-5491.2009.02896.x

Luyckx JRK, 2015, HEALTH PSYCHOL, V34, P1058, DOI 10.1037/hea0000212

Margraf J., 1995, DIAGNOSTIC INTERVIEW

McKnight JA, 2015, DIABETIC MED, V32, P1036, DOI 10.1111/dme.12676

Melin EO, 2013, EUR J ENDOCRINOL, V168, P861, DOI 10.1530/EJE-13-0137

Miller RG, 2016, DIABETES CARE, V39, P2296, DOI 10.2337/dc16-1162

Moulton C. D., 2015, ENDOCRINOLOGY, V3, P461, DOI [10.1016/S2213-8587(15)00134-5, DOI 10.1016/S2213-8587(15)00134-5]

Nefs G, 2012, DIABETOLOGIA, V55, P608, DOI 10.1007/s00125-011-2411-2

Petrak F, 2003, DIABETES-METAB RES, V19, P216, DOI 10.1002/dmrr.374

Petrak F, 2015, LANCET DIABETES ENDO, V3, P472, DOI 10.1016/S2213-8587(15)00045-5

Petrak F, 2015, DIABETES CARE, V38, P767, DOI 10.2337/dc14-1599

Pouwer F, 2010, DIABETIC MED, V27, P217, DOI 10.1111/j.1464-5491.2009.02903.x

Rose M, 2002, DIABETES CARE, V25, P35, DOI 10.2337/diacare.25.1.35

Roy T, 2012, J AFFECT DISORDERS, V142, pS8, DOI 10.1016/S0165-0327(12)70004-6

Smith-Palmer J, 2016, CLINICOECONOMIC OUTC, V8, P559, DOI 10.2147/CEOR.S114699

Snoek FJ, 2008, DIABETIC MED, V25, P1337, DOI 10.1111/j.1464-5491.2008.02595.x

Snoek FJ, 2015, LANCET DIABETES ENDO, V3, P450, DOI 10.1016/S2213-8587(15)00135-7

Strandberg RB, 2015, DIABETIC MED, V32, P1304, DOI 10.1111/dme.12781

Strandberg RB, 2014, J PSYCHOSOM RES, V77, P174, DOI 10.1016/j.jpsychores.2014.06.015

Sultan S, 2008, PSYCHOL HEALTH, V23, P73, DOI 10.1080/14768320701205218

Tanenbaum ML, 2012, DIABETES EDUCATOR, V38, P695, DOI 10.1177/0145721712452795

Uher R, 2010, PSYCHOL MED, V40, P1367, DOI 10.1017/S0033291709991528

Waadt S, 1995, DIABETES PSYCHOL DIA, P17

World Health Organisation, 2011, US GLYC HAEM HBA1C D

Zoffmann V, 2014, DIABETIC MED, V31, P493, DOI 10.1111/dme.12363

NR 45

TC 1

Z9 1

U1 0

U2 0

PU ELSEVIER IRELAND LTD

PI CLARE

PA ELSEVIER HOUSE, BROOKVALE PLAZA, EAST PARK SHANNON, CO, CLARE, 00000,

IRELAND

SN 0168-8227

EI 1872-8227

J9 DIABETES RES CLIN PR

JI Diabetes Res. Clin. Pract.

PD JUL

PY 2018

VL 141

BP 106

EP 117

DI 10.1016/j.diabres.2018.04.017

PG 12

WC Endocrinology & Metabolism

SC Endocrinology & Metabolism

GA GK8IC

UT WOS:000436462900013

PM 29698712

DA 2019-01-08

ER

PT J

AU Sina, M

Graffy, J

Simmons, D

AF Sina, Maryam

Graffy, Jonathan

Simmons, David

TI Associations between barriers to self-care and diabetes complications

among patients with type 2 diabetes

SO DIABETES RESEARCH AND CLINICAL PRACTICE

LA English

DT Article

DE Type 2 diabetes; Complications; Psychological; Health services

ID GLYCEMIC CONTROL; MULTIETHNIC COMMUNITY; INSULIN INITIATION; MANAGEMENT;

POPULATION; DEPRESSION; IMPACT; PREVALENCE; DISTRESS; EFFICACY

AB Aims: To determine which barriers to care are associated with type 2 diabetes complications in an area in rural East England.

Methods: 3649 individuals with type 2 diabetes from 62 general practices were contacted via postal invitation which included a 33 item Barriers-to-Diabetes-Care Survey. Barriers were grouped into five priori major categories: educational, physical, psychological, psychosocial, and systems. The associations of reported barriers, both individually and as a group, with self-reported complications were assessed using logistic regression.

Results: 39.5% of participants had self-reported diabetes complications. Physical health barriers (OR = 3.3; 95%CI: 2.7, 4.0), systems barriers (OR = 1.6; 95%CI: 1.3, 2.0) and psychological barriers (OR = 1.3 (95%CI: 1.1, 1.5) were associated with diabetes complications. In subcategories, presence of comorbidities (OR = 4.8; 95%CI: 3.9, 5.9), financial difficulties (OR = 1.7; 95%CI: 1.3, 2.1), absence of services (OR = 2.0; 95%CI: 1.4, 3.0), feeling others should bear more financial responsibility for their care (OR = 1.6 (95%CI: 1.1, 2.1), no access to diabetes service (OR = 1.3; 95%CI: 1.1, 1.5), feeling worried about their diabetes (OR = 1.5; 95%CI: 1.2, 2.0) and lack of readiness to exercise (OR = 1.4; 95%CI: 1.2, 1.7) were associated with diabetes complications.

Conclusions: Barriers to self-care are significantly more common among those with, than those without, diabetes complications. Systematic identification and management of different barriers to self-care could help personalise care for those with diabetes related complications. (C) 2018 Elsevier B.V. All rights reserved.

C1 [Sina, Maryam; Simmons, David] Western Sydney Univ, Sch Med, Campbelltown, NSW, Australia.

[Graffy, Jonathan] Univ Cambridge, Dept Publ Hlth & Primary Care, Primary Care Unit, Cambridge CB2 0SR, England.

RP Simmons, D (reprint author), Western Sydney Univ, Sch Med, Macarthur Clin Sch, Locked Bag 1797, Campbelltown, NSW 2751, Australia.

EM da.simmons@westernsydney.edu.au

FU Peers for Progress

FX We thank Peers for Progress for funding this work.

CR Aljasem LI, 2001, DIABETES EDUCATOR, V27, P393, DOI 10.1177/014572170102700309

Ascher-Svanum H, 2015, DIABETES THER, V6, P303, DOI 10.1007/s13300-015-0118-y

BARR M, 2006, ADV MED HOME PATIENT

Bayliss Elizabeth A, 2003, Ann Fam Med, V1, P15, DOI 10.1370/afm.4

Benroubi M, 2011, DIABETES RES CLIN PR, V93, pS97, DOI 10.1016/S0168-8227(11)70021-3

Boyd CM, 2005, JAMA-J AM MED ASSOC, V294, P716, DOI 10.1001/jama.294.6.716

Casagrande SS, 2013, DIABETES CARE, V36, P2271, DOI 10.2337/dc12-2258

Cheung BMY, 2009, AM J MED, V122, P443, DOI 10.1016/j.amjmed.2008.09.047

Comino EJ, 2015, BMC HEALTH SERV RES, V15, DOI 10.1186/s12913-014-0666-2

de Groot M, 2001, PSYCHOSOM MED, V63, P619, DOI 10.1097/00006842-200107000-00015

Dillman D., 2007, MAIL INTERNET SURVEY

Gao JL, 2013, BMC FAM PRACT, V14, DOI 10.1186/1471-2296-14-66

Harvey JN, 2015, DIABET METAB SYND OB, V8, P29, DOI 10.2147/DMSO.S44352

Herbert MS, 2013, DIABETIC MED, V30, P1466, DOI 10.1111/dme.12264

Karter AJ, 2010, DIABETES CARE, V33, P733, DOI 10.2337/dc09-1184

Kerr EA, 2007, J GEN INTERN MED, V22, P1635, DOI 10.1007/s11606-007-0313-2

Krichbaum K, 2003, DIABETES EDUCATOR, V29, P653, DOI 10.1177/014572170302900411

Lago RM, 2007, NAT CLIN PRACT ENDOC, V3, P667, DOI 10.1038/ncpendmet0638

Larkin ME, 2008, DIABETES EDUCATOR, V34, P511, DOI 10.1177/0145721708317869

Leyva B, 2011, ETHNIC DIS, V21, P322

Martin Leslie R, 2005, Ther Clin Risk Manag, V1, P189

Munshi MN, 2013, DIABETES CARE, V36, P543, DOI 10.2337/dc12-1303

Murray Elizabeth, 2012, Med 2 0, V1, pe3, DOI 10.2196/med20.1741

Nam S, 2011, DIABETES RES CLIN PR, V93, P1, DOI 10.1016/j.diabres.2011.02.002

Norris SL, 2001, DIABETES CARE, V24, P561, DOI 10.2337/diacare.24.3.561

Peyrot M, 2005, DIABETIC MED, V22, P1379, DOI 10.1111/j.1464-5491.2005.01644.x

Piatt GA, 2011, PATIENT EDUC COUNS, V82, P266, DOI 10.1016/j.pec.2010.04.005

Robbins I, 2013, BMJ OPEN, V3, DOI 10.1136/bmjopen-2013-003178

SAS Institute, 2013, SAS SYST WIND

Simmons D, 1998, DIABETIC MED, V15, P958, DOI 10.1002/(SICI)1096-9136(1998110)15:11<958::AID-DIA687>3.0.CO;2-9

Simmons D, 2001, DIABETES SPECTRUM, V14, P10

Simmons D, 2007, AUST J RURAL HEALTH, V15, P296, DOI 10.1111/j.1440-1584.2007.00903.x

Simmons D, 2007, DIABETES CARE, V30, P490, DOI 10.2337/dc06-2338

Simmons D, 2015, PLOS ONE, V10, DOI 10.1371/journal.pone.0120277

Sparring V, 2013, BMC PUBLIC HEALTH, V13, DOI 10.1186/1471-2458-13-377

Strauss K, 2006, J GEN INTERN MED, V21, P378, DOI 10.1111/j.1525-1497.2006.00386.x

Vanasse A, 2010, RURAL REMOTE HEALTH, V10

Zgibor JC, 2002, DIABETES CARE, V25, P1772, DOI 10.2337/diacare.25.10.1772

Zgibor JC, 2001, DIABETES SPECTRUM, V14, P23, DOI DOI 10.2337/DIASPECT.14.1.23

NR 39

TC 0

Z9 0

U1 11

U2 11

PU ELSEVIER IRELAND LTD

PI CLARE

PA ELSEVIER HOUSE, BROOKVALE PLAZA, EAST PARK SHANNON, CO, CLARE, 00000,

IRELAND

SN 0168-8227

EI 1872-8227

J9 DIABETES RES CLIN PR

JI Diabetes Res. Clin. Pract.

PD JUL

PY 2018

VL 141

BP 126

EP 131

DI 10.1016/j.diabres.2018.04.031

PG 6

WC Endocrinology & Metabolism

SC Endocrinology & Metabolism

GA GK8IC

UT WOS:000436462900015

PM 29689320

DA 2019-01-08

ER

PT J

AU Berry, E

Davies, M

Dempster, M

AF Berry, Emma

Davies, Mark

Dempster, Martin

TI Managing Type 2 diabetes as a couple: The influence of partners' beliefs

on diabetes distress over time

SO DIABETES RESEARCH AND CLINICAL PRACTICE

LA English

DT Article

DE Type 2 diabetes mellitus; Psychological adjustment; Social support;

Interpersonal relations

ID RANDOMIZED CLINICAL-TRIAL; PROSTATE-CANCER PATIENTS; ILLNESS

REPRESENTATIONS; PSYCHOLOGICAL DISTRESS; RELATIONSHIP QUALITY; EMOTIONAL

DISTRESS; INTERVENTION; DEPRESSION; BEHAVIORS; ADHERENCE

AB Aims: Partners and spouses have an important role in supporting healthy self-care in adults with Type 2 diabetes. While evidence has shown that the beliefs held by people with diabetes influence emotional wellbeing, little is known about the long-term impact of partners' illness beliefs on diabetes distress.

Methods: Persons with Type 2 diabetes (pwt2d) and their partners completed a questionnaire at baseline (N = 75 couples) and 12 months later (N = 45 couples). Measures included demographic/clinical parameters, the Revised Illness Perception questionnaire, and the Diabetes Distress Scale. A repeated measures ANOVA was used to examine change in measures over time. Multiple regression and moderation analysis were used to explore the indirect influence of partners beliefs on diabetes distress at baseline and follow-up.

Results: Illness perceptions and diabetes distress in pwt2d and partners did not change overtime. Partners' beliefs about the controllability, chronicity, and predictability of symptoms of diabetes moderated the relationship between the corresponding pwt2d beliefs and diabetes distress. These indirect effects were observed across both time points.

Conclusions: Conflicting illness perceptions about the controllability and chronicity of diabetes, and congruous negative perceptions about diabetes symptoms among couples sustain distress overtime. Targeting the beliefs of couples to improve communication and understanding may reduce diabetes distress. (C) 2018 Elsevier B.V. All rights reserved.

C1 [Berry, Emma; Dempster, Martin] Queens Univ Belfast, Sch Psychol, David Keir Bldg,18-30 Malone Rd, Belfast BT9 5BN, Antrim, North Ireland.

[Davies, Mark] Belfast City Hosp, Dept Clin Psychol, Belfast, Antrim, North Ireland.

RP Berry, E (reprint author), Queens Univ Belfast, Sch Psychol, David Keir Bldg,18-30 Malone Rd, Belfast BT9 5BN, Antrim, North Ireland.

EM eberry05@qub.ac.uk; mark.davies@belfasttrust.hscni.net;

m.dempster@qub.ac.uk

OI , Martin/0000-0001-9499-3186

FU Department of Education and Learning at Queen's University Belfast

FX This study was funded by the Department of Education and Learning at

Queen's University Belfast.

CR Agren S, 2012, J CARD FAIL, V18, P359, DOI 10.1016/j.cardfail.2012.01.014

Allison P. D., 1990, SOCIOL METHODOL, P93, DOI DOI 10.2307/271083

[Anonymous], 2018, TYP 2 DIAB AD MAN, P1

BECK AT, 1988, CLIN PSYCHOL REV, V8, P77, DOI 10.1016/0272-7358(88)90050-5

Berry E, 2017, PSYCHOL HEALTH MED, V22, P1118, DOI 10.1080/13548506.2017.1281976

Berry E, 2017, PATIENT EDUC COUNS, V100, P1287, DOI 10.1016/j.pec.2017.02.015

Cleal B, 2017, DIABETIC MED, V34, P108, DOI 10.1111/dme.13187

Cohen J, 1988, STAT POWER ANAL BEHA

CRONBACH LJ, 1951, PSYCHOMETRIKA, V16, P297, DOI DOI 10.1007/BF02310555

Dempster M, 2011, DIABETIC MED, V28, P487, DOI 10.1111/j.1464-5491.2010.03214.x

Dempster M, 2011, J PSYCHOSOM RES, V70, P432, DOI 10.1016/j.jpsychores.2010.07.007

Dimitraki G, 2014, INT J BEHAV MED, V21, P230, DOI 10.1007/s12529-013-9296-z

Ervasti J, 2016, DIABETIC MED, V33, P208, DOI 10.1111/dme.12821

Fisher L, 2008, DIABETIC MED, V25, P1096, DOI 10.1111/j.1464-5491.2008.02533.x

Fisher L, 2012, DIABETES CARE, V35, P259, DOI 10.2337/dc11-1572

Fisher L, 2010, DIABETES CARE, V33, P23, DOI 10.2337/dc09-1238

Gonzalez JS, 2015, HEALTH PSYCHOL, V34, P505, DOI 10.1037/hea0000131

Pereira MG, 2016, PSYCHOL HEALTH MED, V21, P707, DOI 10.1080/13548506.2015.1118517

Heinrichs N, 2012, ANN BEHAV MED, V43, P239, DOI 10.1007/s12160-011-9314-9

Hessler DM, 2011, PATIENT EDUC COUNS, V85, P154, DOI 10.1016/j.pec.2010.10.030

Kuijer RG, 2004, PSYCHO-ONCOLOGY, V13, P321, DOI 10.1002/pon.749

Meltzer D, 2000, Eff Clin Pract, V3, P7

Miller-Matero LR, 2017, PSYCHOL HEALTH MED, V22, P19, DOI 10.1080/13548506.2016.1191659

Mishel MH, 2002, CANCER-AM CANCER SOC, V94, P1854, DOI 10.1002/cncr.10390

Moss-Morris R, 2002, PSYCHOL HEALTH, V17, P1, DOI 10.1080/08870440290001494

Northouse LL, 2007, CANCER, V110, P2809, DOI 10.1002/cncr.23114

Polonsky WH, 2005, DIABETES CARE, V28, P626, DOI 10.2337/diacare.28.3.626

Polonsky WH, 2016, PATIENT PREFER ADHER, V10, P1299, DOI 10.2147/PPA.S106821

Polonsky WH, 2016, DIABETES TECHNOL THE, V18, P292, DOI 10.1089/dia.2015.0451

Searle A, 2007, J PSYCHOSOM RES, V63, P175, DOI 10.1016/j.jpyschores.2007.02.006

Sekhar TVDS, 2013, J CLIN DIAGN RES, V7, P857, DOI 10.7860/JCDR/2013/5726.2958

Skinner TC, 2011, PSYCHOL HEALTH, V26, P321, DOI 10.1080/08870440903411039

Stoop CH, 2014, DIABETIC MED, V31, P1252, DOI 10.1111/dme.12472

Trief PM, 2016, DIABETES CARE, V39, P2165, DOI 10.2337/dc16-0035

Weinman J, 2000, BR J HLTH PSYCHOL, P263

White P, 2009, DIABETES EDUCATOR, V35, P810, DOI 10.1177/0145721709340930

Wu LM, 2013, PSYCHOL HEALTH, V28, P355, DOI 10.1080/08870446.2012.722219

NR 37

TC 0

Z9 0

U1 0

U2 0

PU ELSEVIER IRELAND LTD

PI CLARE

PA ELSEVIER HOUSE, BROOKVALE PLAZA, EAST PARK SHANNON, CO, CLARE, 00000,

IRELAND

SN 0168-8227

EI 1872-8227

J9 DIABETES RES CLIN PR

JI Diabetes Res. Clin. Pract.

PD JUL

PY 2018

VL 141

BP 244

EP 255

DI 10.1016/j.diabres.2018.05.020

PG 12

WC Endocrinology & Metabolism

SC Endocrinology & Metabolism

GA GK8IC

UT WOS:000436462900029

PM 29775677

DA 2019-01-08

ER

PT J

AU Ruiz-Aranda, D

Zysberg, L

Garcia-Linares, E

Castellano-Guerrero, AM

Martinez-Brocca, MA

Gutierrez-Colosia, MR

AF Ruiz-Aranda, Desiree

Zysberg, Leehu

Garcia-Linares, Ernesto

Maria Castellano-Guerrero, Ana

Asuncion Martinez-Brocca, Maria

Gutierrez-Colosia, Mencia R.

TI Emotional abilities and HbA1c levels in patients with type 1 diabetes

SO PSYCHONEUROENDOCRINOLOGY

LA English

DT Article

DE Type 1 diabetes mellitus; HbA1c; Emotional abilities; Distress;

Emotional intelligence

ID SELF-EFFICACY; INTELLIGENCE; HEALTH; ADOLESCENTS; MANAGEMENT; DISTRESS;

METAANALYSIS; COMPETENCE; DEPRESSION; BEHAVIORS

AB In recent years a growing body of research is focused on the relationships between emotions and health. When it comes to diabetes, findings suggest that distress might play a key role in the acquisition and maintenance of health habits associated with diabetic management. This report describes two studies examining the roles of emotional abilities in diabetic management from two different conceptual points of view using two culturally different samples. In study 1, we examined the relationship between emotional intelligence and HbA1c levels in a sample of eighty-five patients with type 1 diabetes mellitus (DM1) in Israel. In study 2, we examined the relationship between specific emotional regulation strategies and HbA1c in sixty-seven adolescents with DM1, while examining the mediating role of distress in this association. The results showed a negative association between emotional intelligence and HbA1c levels, even after controlling for potential intervening factors. We found that the relationship between difficulties in emotion regulation and HbA1c seemed to be mediated by diabetes-related distress. These findings may aid in the design of psychological models for future research as well as interventions aimed at improving emotional abilities in people with DM1.

C1 [Ruiz-Aranda, Desiree; Gutierrez-Colosia, Mencia R.] Univ Loyola Andalucia, Seville, Spain.

[Zysberg, Leehu] Gordon Coll Educ, Haifa, Israel.

[Garcia-Linares, Ernesto] Univ Nacl Educ Distancia, Madrid, Spain.

[Maria Castellano-Guerrero, Ana; Asuncion Martinez-Brocca, Maria] Hosp Univ Virgen Macarena, UGC Endocrinol & Nutr, Seville, Spain.

[Asuncion Martinez-Brocca, Maria] Univ Seville, CSIC, Hosp Univ Virgen Rocio, Inst Biomed Sevilla IBiS, Seville, Spain.

RP Gutierrez-Colosia, MR (reprint author), Univ Loyola Andalucia, Seville, Spain.

EM menciaruiz@uloyola.es

RI Ruiz Gutierrez-Colosia, Mencia/N-4515-2014

OI Ruiz Gutierrez-Colosia, Mencia/0000-0002-2631-0205

CR American Diabetes Association [ADA], 2015, FAST FACTS DAT STAT

Ashraff Suhel, 2013, Oman Med J, V28, P159, DOI 10.5001/omj.2013.46

Baek RN, 2014, ANN BEHAV MED, V48, P145, DOI 10.1007/s12160-013-9585-4

BARON RM, 1986, J PERS SOC PSYCHOL, V51, P1173, DOI 10.1037//0022-3514.51.6.1173

Belendez M., 2014, CLIN PRACT, V106, P93

Berg CA, 2011, J PEDIATR PSYCHOL, V36, P329, DOI 10.1093/jpepsy/jsq088

Beveridge RM, 2006, J PEDIATR PSYCHOL, V31, P818, DOI 10.1093/jpepsy/jsj094

Brackett MA, 2006, J PERS SOC PSYCHOL, V91, P780, DOI 10.1037/0022-3514.91.4.780

Buchberger B, 2016, PSYCHONEUROENDOCRINO, V70, P70, DOI 10.1016/j.psyneuen.2016.04.019

Coccaro E., 2016, PRIM CARE DIABETES, P334

Fernandez-Abascal EG, 2015, FRONT PSYCHOL, V6, DOI 10.3389/fpsyg.2015.00317

Fisher L, 2009, DIABETIC MED, V26, P622, DOI 10.1111/j.1464-5491.2009.02730.x

Fisher L, 2012, DIABETES CARE, V35, P259, DOI 10.2337/dc11-1572

Fortenberry KT, 2009, ANN BEHAV MED, V37, P1, DOI 10.1007/s12160-009-9086-7

Gemez-Rico I., 2015, AN PEDIAT, V82, P43

Gonzalez JS, 2016, AM PSYCHOL, V71, P539, DOI 10.1037/a0040388

Gonzalez JS, 2011, DIABETES CARE, V34, P236, DOI 10.2337/dc10-1970

Gratz KL, 2004, J PSYCHOPATHOL BEHAV, V26, P41, DOI 10.1023/B:JOBA.0000007455.08539.94

Haves G., 2008, CLIN SALUD, V19, P139

Hughes AE, 2012, J PEDIATR PSYCHOL, V37, P925, DOI 10.1093/jpepsy/jss062

Hunter CM, 2016, AM PSYCHOL, V71, P515, DOI 10.1037/a0040344

Janzen-Claude J. A., 2014, J HEALTH PSYCHOL, V9, P312

Kroger J., 2007, IDENTITY DEV ADOLESC

Kyngas H, 2000, J CLIN NURS, V9, P549, DOI 10.1046/j.1365-2702.2000.00368.x

MacKinnon DP, 2004, MULTIVAR BEHAV RES, V39, P99, DOI 10.1207/s15327906mbr3901_4

Martins A, 2010, PERS INDIV DIFFER, V49, P554, DOI 10.1016/j.paid.2010.05.029

Mayer J. D, 1997, EMOTIONAL DEV EMOTIO, P3

Mikolajczak M, 2015, EMOTION, V15, P653, DOI 10.1037/emo0000034

Naranjo D, 2012, CURR DIABETES REP, V12, P729, DOI 10.1007/s11892-012-0319-y

Polonsky WH, 2005, DIABETES CARE, V28, P626, DOI 10.2337/diacare.28.3.626

Preacher KJ, 2004, BEHAV RES METH INS C, V36, P717, DOI 10.3758/BF03206553

Ryden A, 2016, PLOS ONE, V11, DOI 10.1371/journal.pone.0164977

Sato Asako, 2014, Rinsho Byori, V62, P45

Schutte NS, 2007, PERS INDIV DIFFER, V42, P921, DOI 10.1016/j.paid.2006.09.003

Stupiansky NW, 2013, J PEDIATR PSYCHOL, V38, P247, DOI 10.1093/jpepsy/jss110

Unnikrishnan R., 2016, CLIN DIABETES ENDOCR, V2, P1

Whittemore R, 2010, NURS OUTLOOK, V58, P242, DOI 10.1016/j.outlook.2010.05.001

Yalcin BM, 2008, DIABETES EDUCATOR, V34, P1013, DOI 10.1177/0145721708327303

Zysberg L., 2016, J ENDOCRINOL DIABETE, V3, P1

Zysberg L, 2017, J HEALTH PSYCHOL, V22, P158, DOI 10.1177/1359105315596373

Zysberg L, 2011, J PSYCHOEDUC ASSESS, V29, P27, DOI 10.1177/0734282910365059

NR 41

TC 0

Z9 0

U1 1

U2 2

PU PERGAMON-ELSEVIER SCIENCE LTD

PI OXFORD

PA THE BOULEVARD, LANGFORD LANE, KIDLINGTON, OXFORD OX5 1GB, ENGLAND

SN 0306-4530

J9 PSYCHONEUROENDOCRINO

JI Psychoneuroendocrinology

PD JUL

PY 2018

VL 93

BP 118

EP 123

DI 10.1016/j.psyneuen.2018.04.015

PG 6

WC Endocrinology & Metabolism; Neurosciences; Psychiatry

SC Endocrinology & Metabolism; Neurosciences & Neurology; Psychiatry

GA GI8BU

UT WOS:000434747600015

PM 29723781

DA 2019-01-08

ER

PT J

AU Gomez-Peralta, TG

Gonzalez-Castro, TB

Fresan, A

Tovilla-Zarate, CA

Juarez-Rojop, IE

Villar-Soto, M

Hernandez-Diaz, Y

Lopez-Narvaez, ML

Ble-Castillo, JL

Perez-Hernandez, N

Rodriguez-Perez, JM

AF Guadalupe Gomez-Peralta, Tania

Beatriz Gonzalez-Castro, Thelma

Fresan, Ana

Alfonso Tovilla-Zarate, Carlos

Esther Juarez-Rojop, Isela

Villar-Soto, Mario

Hernandez-Diaz, Yazmin

Lilia Lopez-Narvaez, Maria

Ble-Castillo, Jorge L.

Perez-Hernandez, Nonanzit

Manuel Rodriguez-Perez, Jose

TI Risk Factors and Prevalence of Suicide Attempt in Patients with Type 2

Diabetes in the Mexican Population

SO INTERNATIONAL JOURNAL OF ENVIRONMENTAL RESEARCH AND PUBLIC HEALTH

LA English

DT Article

DE suicide; depression; diabetes; Mexican population

ID BODY-MASS INDEX; QUALITY-OF-LIFE; EMOTIONAL DISTRESS; PROBLEM AREAS;

CHOLESTEROL; ADULTS; DEPRESSION; IDEATION; MELLITUS; INSULIN

AB Background: It has been proposed that the risk of death by suicide is higher in patients with diabetes than in the general population. Therefore, it is necessary to investigate the risk factors of suicidal behavior in patients with type 2 diabetes. The aim of the present study was to analyze the prevalence of suicide attempt and determine the risk factors of suicide attempt, in patients with type 2 diabetes in a Mexican population. Methods: Clinic characteristics, anthropometric measurements, biochemical levels, depression, and suicidal behavior were evaluated in 185 Mexican patients with type 2 diabetes. A multivariate logistic regression analysis was performed to find predictive factors of suicide attempt. Results: 11.4% of patients reported previous suicide attempts n = 21). Younger patients (OR: 3.63, 95% CI: 1.29-10.19), having depression (OR: 3.33, 95% CI: 1.13-9.76) and normal BMI (OR: 3.14, 95% CI: 1.11-8.83), were predictive factors of suicide attempt. No other variables in the study showed statistical significance. Conclusions: Our results showed a high prevalence of suicidal behavior in patients with type 2 diabetes. We found that younger age, depression and normal BMI could be risk factors of suicide attempt in these patients. Therefore, psychiatric interventions to prevent depression and suicidal behavior in this population are necessary. New studies using larger samples are necessary to replicate and confirm these results.

C1 [Guadalupe Gomez-Peralta, Tania; Alfonso Tovilla-Zarate, Carlos] Univ Juarez Autonoma Tabasco, Div Acad Multidisciplinaria Comalcalco, Comalcalco 86025, Tabasco, Mexico.

[Beatriz Gonzalez-Castro, Thelma; Hernandez-Diaz, Yazmin] Univ Juarez Autonoma Tabasco, Div Acad Multidisciplinaria Jalpa de Mendez, Jalpa De Mendez 86200, Tabasco, Mexico.

[Fresan, Ana] Inst Nacl Psiquiatria Ramon de la Fuente Muniz, Subdirecc Invest Clin, Ciudad De Mexico 14370, Mexico.

[Esther Juarez-Rojop, Isela; Ble-Castillo, Jorge L.] Univ Juarez Autonoma Tabasco, Div Acad Ciencias Salud, Villahermosa 86140, Tabasco, Mexico.

[Villar-Soto, Mario] Hosp Alta Especialidad Gustavo A Rovirosa Perez, Secretaria Salud, Villahermosa 86140, Tabasco, Mexico.

[Lilia Lopez-Narvaez, Maria] Hosp Gen Yajalon Dr Manuel Velasco Suarez, Secretaria Salud, Yajalon 29930, Chiapas, Mexico.

[Perez-Hernandez, Nonanzit; Manuel Rodriguez-Perez, Jose] Inst Nacl Cardiol Ignacio Chavez, Dept Biol Mol, Ciudad De Mexico 14080, Mexico.

RP Tovilla-Zarate, CA (reprint author), Univ Juarez Autonoma Tabasco, Div Acad Multidisciplinaria Comalcalco, Comalcalco 86025, Tabasco, Mexico.; Juarez-Rojop, IE (reprint author), Univ Juarez Autonoma Tabasco, Div Acad Ciencias Salud, Villahermosa 86140, Tabasco, Mexico.

EM tanyperalta93@gmail.com; thelma.glez.castro@gmail.com;

fresan@imp.edu.mx; alfonso_tovillaz@yahoo.com.mx;

iselajuarezrojop@hotmail.com; mariovillarsoto@hotmail.com;

yazmin.hdez.diaz@gmail.com; dralilialonar@yahoo.com.mx;

jblecastillo@hotmail.com; unicanona@yahoo.com.mx;

josemanuel_rodriguezperez@yahoo.com.mx

OI Perez-Hernandez, Nonanzit/0000-0003-4786-508X; Hernandez-Diaz,

Yazmin/0000-0003-1200-2292; Rodriguez-Perez, Jose

Manuel/0000-0002-3802-0897; BLE-CASTILLO, JORGE L./0000-0002-1376-1461;

Gonzalez Castro, Thelma Beatriz/0000-0001-8046-6908; Tovilla-Zarate,

Carlos Alfonso/0000-0001-8170-8171

FU Universidad Juarez Autonoma de Tabasco [UJAT-IB-2105-05]

FX This research was funded by Universidad Juarez Autonoma de Tabasco grant

number UJAT-IB-2105-05.

CR Apiquian R., 2000, EVALUATION PSYCHOPAT, P49

Ayyappa KA, 2017, NUTR METAB, V14, DOI 10.1186/s12986-016-0155-1

Bartoli F, 2017, PROG NEURO-PSYCHOPH, V79, P90, DOI 10.1016/j.pnpbp.2017.06.008

Bartoli F, 2017, CLIN BIOCHEM, V50, P274, DOI 10.1016/j.clinbiochem.2016.11.035

Batty GD, 2012, J EPIDEMIOL COMMUN H, V66, P1196, DOI 10.1136/jech-2011-200464corr1

Beck A.T., 1974, PREDICTION SUICIDE

Belendez M, 2014, DIABETES RES CLIN PR, V106, pE93, DOI 10.1016/j.diabres.2014.09.012

Vazquez MB, 2015, GAC SANIT, V29, P300, DOI 10.1016/j.gaceta.2015.02.005

Bendix M, 2017, PSYCHONEUROENDOCRINO, V81, P1, DOI 10.1016/j.psyneuen.2017.03.019

Branco JC, 2017, PSYCHOL HEALTH MED, V22, P275, DOI 10.1080/13548506.2016.1164870

Butwicka A, 2016, DIABETES CARE, V39, P495, DOI 10.2337/dc16-er03

Cantarelli MD, 2014, PSYCHIAT RES, V220, P745, DOI 10.1016/j.psychres.2014.10.017

Cheah YK, 2018, PSYCHIAT RES, V261, P319, DOI 10.1016/j.psychres.2017.12.086

Chung JH, 2014, J PSYCHOSOM RES, V77, P457, DOI 10.1016/j.jpsychores.2014.08.008

Cohen-Manheim I, 2018, EUR J PUBLIC HEALTH, V28, P496, DOI 10.1093/eurpub/ckx194

Conti C, 2017, FRONT PSYCHIATRY, V8, DOI 10.3389/fpsyt.2017.00040

Davila Cervantes CA, 2015, SALUD COLECT, V11, P471, DOI 10.18294/sc.2015.784

Davis WA, 2015, INTERN MED J, V45, P976, DOI 10.1111/imj.12853

Deschenes SS, 2018, J AFFECT DISORDERS, V238, P24, DOI 10.1016/j.jad.2018.05.029

Dreber H, 2015, PLOS ONE, V10, DOI 10.1371/journal.pone.0145273

Dutton GR, 2013, INT J OBESITY, V37, P1282, DOI 10.1038/ijo.2012.224

FOSTER HD, 1988, AM J PSYCHIAT, V145, P272

Gamboa CM, 2017, J AM HEART ASSOC, V6, DOI 10.1161/JAHA.116.004264

GOLDSTON DB, 1994, J AM ACAD CHILD PSY, V33, P240, DOI 10.1097/00004583-199402000-00013

Golomb BA, 2002, J CLIN EPIDEMIOL, V55, P767, DOI 10.1016/S0895-4356(02)00407-9

Gylfadottir Sandra Sif, 2017, Scand J Pain, V16, P173, DOI 10.1016/j.sjpain.2017.04.026

HAMILTON M, 1960, J NEUROL NEUROSUR PS, V23, P56, DOI 10.1136/jnnp.23.1.56

Handley TE, 2016, DIABETIC MED, V33, P1582, DOI 10.1111/dme.13022

Igwe MN, 2013, AFR HEALTH SCI, V13, P68, DOI 10.4314/ahs.v13i1.10

INEGI, EST MORT

Jin H, 2015, METHOD INFORM MED, V54, P553, DOI 10.3414/ME14-02-0009

Lee EH, 2014, HEALTH QUAL LIFE OUT, V12, DOI 10.1186/s12955-014-0142-z

Lofman S, 2012, J PSYCHOSOM RES, V73, P268, DOI 10.1016/j.jpsychores.2012.08.002

Macip SG, 2000, SALUD MENT, V23, P21

Mantantzis K, 2017, PSYCHOL AGING, V32, P203, DOI 10.1037/pag0000169

Marklund M, 2017, J NUTR, V147, P2118, DOI 10.3945/jn.117.254250

McGuire BE, 2010, DIABETOLOGIA, V53, P66, DOI 10.1007/s00125-009-1559-5

Mukamal KJ, 2007, ARCH INTERN MED, V167, P468, DOI 10.1001/archinte.167.5.468

Mukamal KJ, 2010, EPIDEMIOLOGY, V21, P82, DOI 10.1097/EDE.0b013e3181c1fa2d

Mukamal KJ, 2009, OBESITY, V17, P532, DOI 10.1038/oby.2008.538

MULDOON MF, 1990, BRIT MED J, V301, P309, DOI 10.1136/bmj.301.6747.309

Myers AK, 2017, CURR DIABETES REV, V13, P251, DOI 10.2174/1573399812666161005163618

Onat A, 2017, TURK KARDIYOL DERN A, V45, P590, DOI 10.5543/tkda.2017.38085

Ozdemir O, 2017, PSYCHIAT RES, V258, P171, DOI 10.1016/j.psychres.2016.11.043

Park S, 2018, PSYCHIAT RES, V261, P143, DOI 10.1016/j.psychres.2017.12.055

Petrak F, 2015, LANCET DIABETES ENDO, V3, P472, DOI 10.1016/S2213-8587(15)00045-5

Pompili M, 2014, J PSYCHOSOM RES, V76, P352, DOI 10.1016/j.jpsychores.2014.02.009

Pompili M, 2009, PSYCHOSOMATICS, V50, P16, DOI 10.1176/appi.psy.50.1.16

Radobuljac MD, 2009, PEDIATR DIABETES, V10, P424, DOI 10.1111/j.1399-5448.2009.00501.x

Rojas-Martinez R, 2018, SALUD PUBLICA MEXICO, V60, P224, DOI 10.21149/8566

Sarkar Siddharth, 2014, Indian J Endocrinol Metab, V18, P468, DOI 10.4103/2230-8210.137487

Song HB, 2016, J AFFECT DISORDERS, V197, P21, DOI 10.1016/j.jad.2016.02.035

Tabb KM, 2017, J PSYCHOSOM RES, V103, P113, DOI 10.1016/j.jpsychores.2017.10.009

Tovilla-Zarate C, 2012, PLOS ONE, V7, DOI 10.1371/journal.pone.0036887

Wagner B, 2013, DEPRESS ANXIETY, V30, P975, DOI 10.1002/da.22105

Wang B, 2017, EUR J ENDOCRINOL, V177, pR169, DOI 10.1530/EJE-16-0952

Wang Y, 2016, COMPR PSYCHIAT, V71, P11, DOI 10.1016/j.comppsych.2016.08.006

Webb RT, 2014, DIABETES CARE, V37, P2276, DOI 10.2337/dc14-0005

Wu SQ, 2016, J PSYCHIATR NEUROSCI, V41, P56, DOI 10.1503/jpn.150079

NR 59

TC 1

Z9 1

U1 6

U2 6

PU MDPI

PI BASEL

PA ST ALBAN-ANLAGE 66, CH-4052 BASEL, SWITZERLAND

SN 1660-4601

J9 INT J ENV RES PUB HE

JI Int. J. Environ. Res. Public Health

PD JUN

PY 2018

VL 15

IS 6

AR 1198

DI 10.3390/ijerph15061198

PG 10

WC Environmental Sciences; Public, Environmental & Occupational Health

SC Environmental Sciences & Ecology; Public, Environmental & Occupational

Health

GA GK8SE

UT WOS:000436496900149

PM 29880751

OA DOAJ Gold

DA 2019-01-08

ER

PT J

AU Mulligan, K

McBain, H

Lamontagne-Godwin, F

Chapman, J

Flood, C

Haddad, M

Jones, J

Simpson, A

AF Mulligan, Kathleen

McBain, Hayley

Lamontagne-Godwin, Frederique

Chapman, Jacqui

Flood, Chris

Haddad, Mark

Jones, Julia

Simpson, Alan

TI Barriers to effective diabetes management - a survey of people with

severe mental illness

SO BMC PSYCHIATRY

LA English

DT Article

DE Diabetes; Severe mental illness; Service users; Self-management;

Theoretical domains framework

ID RANDOMIZED CONTROLLED-TRIAL; OLDER SCHIZOPHRENIA-PATIENTS; LIFE-STYLE

INTERVENTION; SELF-MANAGEMENT; MELLITUS; CARE; COMPLICATIONS;

METAANALYSIS; INDIVIDUALS; MEDICATIONS

AB Background: People with severe mental illnesses (SMI) such as schizophrenia and bipolar disorder have an increased risk of developing type 2 diabetes and have poorer health outcomes than those with diabetes alone. To maintain good diabetes control, people with diabetes are advised to engage in several self-management behaviours. The aim of this study was to identify barriers or enablers of diabetes self-management experienced by people with SMI.

Methods: Adults with type 2 diabetes and SMI were recruited through UK National Health Service organisations and mental health and diabetes charities. Participants completed an anonymous survey consisting of. Summary of Diabetes Self-Care Activities (SDSCA); CORE-10 measure of psychological distress; a measure of barriers and enablers of diabetes self-management based on the Theoretical Domains Framework; Diabetes UK care survey on receipt of 14 essential aspects of diabetes healthcare. To identify the strongest explanatory variables of SDSCA outcomes, significant variables (p < .05) identified from univariate analyses were entered into multiple regressions.

Results: Most of the 77 participants had bipolar disorder (42%) or schizophrenia (36%). They received a mean of 7.6 (SD 3.0) diabetes healthcare essentials. Only 28.6% had developed a diabetes care plan with their health professional and only 40% reported receiving specialist psychological support. Engagement in self-management activities was variable. Participants reported taking medication on 6.1 (SD 2.0) days in the previous week but other behaviours were less frequent general diet 4.1 (2.3) days; specific diet 3.6 (1.8) days, taking exercise 2.4 (2.1) days and checking feet on 1.7 (1.8) days. Smoking prevalence was 44%. Participants reported finding regular exercise and following a healthy diet particularly difficult. Factors associated with diabetes self-management included: the level of diabetes healthcare and support received; emotional wellbeing; priority given to diabetes; perceived ability to manage diabetes or establish a routine to do so; and perceived consequences of diabetes self-management.

Conclusions: Several aspects of diabetes healthcare and self-management are suboptimal in people with SMI. There is a need to improve diabetes self-management support for this population by integrating diabetes action plans into care planning and providing adequate psychological support to help people with SMI manage their diabetes.

C1 [Mulligan, Kathleen; McBain, Hayley; Flood, Chris; Haddad, Mark; Simpson, Alan] City Univ London, Sch Hlth Sci, Myddelton St, London EC1V 0HB, England.

[Mulligan, Kathleen; Simpson, Alan] East London NHS Fdn Trust, London, England.

[Lamontagne-Godwin, Frederique] Univ West London, Sch Human & Social Sci, London, England.

[Chapman, Jacqui] East London NHS Fdn Trust, Diabet Specialist Nursing Serv, London, England.

[Jones, Julia] Univ Hertfordshire, CRIPACC, Hatfield, Herts, England.

RP Mulligan, K (reprint author), City Univ London, Sch Hlth Sci, Myddelton St, London EC1V 0HB, England.; Mulligan, K (reprint author), East London NHS Fdn Trust, London, England.

EM Kathleen.Mulligan.1@city.ac.uk

FU Barts Charity [477/2314]

FX This study was funded by a grant from Barts Charity, reference number:

477/2314. The funding body did not have any role in the design of the

study or collection, analysis, or interpretation of data or in writing

the manuscript.

CR Babyak MA, 2004, PSYCHOSOM MED, V66, P411, DOI 10.1097/01.psy.0000127692.23278.a9

Barkham M, 2012, COUNSELLING PSYCHOTH, V13, P3, DOI DOI 10.1080/14733145.2012.729069

Becker T, 2011, DIABETES CARE, V34, P398, DOI 10.2337/dc10-1139

Blixen CE, 2016, AM J HEALTH BEHAV, V40, P194, DOI 10.5993/AJHB.40.2.4

Cane J, 2012, IMPLEMENT SCI, V7, DOI 10.1186/1748-5908-7-37

Care Quality Commission, 2016, 2016 COMM MENT HLTH

Chen SR, 2014, J PSYCHIATR MENT HLT, V21, P414, DOI 10.1111/jpm.12101

Chrvala CA, 2016, PATIENT EDUC COUNS, V99, P926, DOI 10.1016/j.pec.2015.11.003

Clark LA, 1995, PSYCHOL ASSESSMENT, V7, P309, DOI 10.1037/1040-3590.7.3.309

Cook JA, 2013, PSYCHIATR REHABIL J, V36, P250, DOI 10.1037/prj0000028

Cook JA, 2012, SCHIZOPHRENIA BULL, V38, P881, DOI 10.1093/schbul/sbr012

Corker E, 2016, ACTA PSYCHIAT SCAND, V134, P6, DOI 10.1111/acps.12610

Davies MJ, 2008, BRIT MED J, V336, P491, DOI 10.1136/bmj.39474.922025.BE

De Hert M, 2011, WORLD PSYCHIATRY, V10, P52

Diabetes UK, 2014, DIAB 15 HEALTHC ESS

Diabetes UK, 2014, CARE SURVEY RESULTS

Edwards PJ, 2009, COCHRANE DB SYST REV, DOI 10.1002/14651858.MR000008.pub4

Fagard Robert H, 2009, Prim Care Diabetes, V3, P205, DOI 10.1016/j.pcd.2009.09.003

Garson GD, 2015, MISSING VALUES ANAL

Graham JW, 2009, ANNU REV PSYCHOL, V60, P549, DOI 10.1146/annurev.psych.58.110405.085530

Health and Social Care Information Centre, 2016, SMOKING RATES IN PEO

Krakow D, 2007, EUR DIABETES NURSING, V4, P106

Kreyenbuhl J, 2010, SCHIZOPHRENIA BULL, V36, P428, DOI 10.1093/schbul/sbn106

Look AHEAD Research Group, 2010, ARCH INTERN MED, V170, P1566

Mathur R, 2012, BRIT J GEN PRACT, V62, DOI 10.3399/bjgp12X653642

McBain H., 2016, COCHRANE DB SYST REV, V4

McKibbin CL, 2006, SCHIZOPHR RES, V86, P36, DOI 10.1016/j.schres.2006.05.010

McKibbin CL, 2010, SCHIZOPHR RES, V121, P203, DOI 10.1016/j.schres.2009.09.039

Michie S, 2008, APPL PSYCHOL-INT REV, V57, P660, DOI 10.1111/j.1464-0597.2008.00341.x

Mulligan K, 2017, HEALTH EXPECT, V20, P1020, DOI 10.1111/hex.12543

Nicolucci A, 2013, DIABETIC MED, V30, P767, DOI 10.1111/dme.12245

Office for National Statistics, 2016, AD SMOK HAB UK 2016, P2017

Office for National Statistics, 2013, GRAD UK LAB MARK 201

Osborn DPJ, 2008, BMC PSYCHIATRY, V8, DOI 10.1186/1471-244X-8-84

Peckham E, 2017, BMC PSYCHIATRY, V17, DOI 10.1186/s12888-017-1419-7

Queen Mary Univeristy of London. Diabetes Research Lay Panel Group, 2017, ENGAGEMENT AND ENTER

Royal College of Psychiatrists, 2016, IMPR PHYS HLTH AD SE

Rubin DB, 2009, MULTIPLE IMPUTATION

Simpson A, 2016, BMC PSYCHIATRY, V16, DOI 10.1186/s12888-016-0858-x

Simpson A, 2014, J PSYCHOSOC NURS MEN, V52, P22, DOI 10.3928/02793695-20131126-04

Steinsbekk A, 2012, BMC HEALTH SERV RES, V12, DOI 10.1186/1472-6963-12-213

Stubbs B, 2015, ACTA PSYCHIAT SCAND, V132, P144, DOI 10.1111/acps.12439

Taylor S, 2014, HLTH SERV DELIV RES, V2, P53, DOI DOI 10.3310/HSDR02530

Tempelaar WM, 2017, PSYCHOL MED, V47, P776, DOI 10.1017/S0033291716002877

Toobert DJ, 2000, DIABETES CARE, V23, P943, DOI 10.2337/diacare.23.7.943

Vinogradova Y, 2010, BRIT J PSYCHIAT, V197, P272, DOI 10.1192/bjp.bp.109.074674

Walker ER, 2015, JAMA PSYCHIAT, V72, P334, DOI 10.1001/jamapsychiatry.2014.2502

Ward M, 2015, LANCET PSYCHIAT, V2, P431, DOI 10.1016/S2215-0366(15)00007-3

Whyte S, 2007, DIABETIC MED, V24, P1442, DOI 10.1111/j.1464-5491.2007.02324.x

Wu CS, 2015, BRIT J PSYCHIAT, V207, P450, DOI 10.1192/bjp.bp.113.143925

NR 50

TC 1

Z9 1

U1 9

U2 9

PU BMC

PI LONDON

PA CAMPUS, 4 CRINAN ST, LONDON N1 9XW, ENGLAND

SN 1471-244X

J9 BMC PSYCHIATRY

JI BMC Psychiatry

PD JUN 1

PY 2018

VL 18

AR 165

DI 10.1186/s12888-018-1744-5

PG 15

WC Psychiatry

SC Psychiatry

GA GI4AC

UT WOS:000434312300003

PM 29859061

OA DOAJ Gold, Green Published

DA 2019-01-08

ER

PT J

AU Hansen, UM

Olesen, K

Browne, JL

Skinner, TC

Willaing, I

AF Hansen, Ulla M.

Olesen, Kasper

Browne, Jessica L.

Skinner, Timothy C.

Willaing, Ingrid

TI A call for inclusion of work-related diabetes distress in the spectrum

of diabetes management: Results from a cross-sectional survey among

working people with type 1 diabetes

SO DIABETES RESEARCH AND CLINICAL PRACTICE

LA English

DT Article

DE Type 1 diabetes mellitus; Work-related diabetes distress; Well-being;

Diabetes distress

ID DEPRESSION; ADULTS

AB Aim: Diabetes distress captures a range of emotional responses and reactions to life with diabetes and is considered a part of the experience of managing diabetes and its treatment Given the importance of the social context of work life for people of working age we set out to explore whether work-related diabetes distress is a distinct and important dimension of diabetes-related emotional distress in working people with type 1 diabetes.

Methods: A questionnaire with self-reported measures of psychosocial health and well-being at work was completed by 1126 working people with type 1 diabetes from a specialist diabetes clinic in Denmark. Work-related diabetes distress was assessed with two questions about worry and exhaustion related to reconciling work life and diabetes. Diabetes-related emotional distress was assessed with the Problem Areas in Diabetes scale (PAID-5), a short form version of the full PAID scale. We performed inter-item correlation analyses, exploratory factor analysis, and hierarchical multiple regression analyses.

Results: Inter-item correlations and exploratory factor analysis indicated that work-related diabetes distress was distinct from diabetes-related emotional distress. Further, work-related diabetes distress was found to be a unique contributor to work ability, quality of life, intentional hyperglycaemia at work, and absenteeism, after adjusting for covanates and diabetes-related emotional distress.

Conclusions: The findings suggest that work-related diabetes distress captures an aspect of distress so far unaccounted for in workers with type 1 diabetes Further studies are needed to strengthen the conceptual basis of work-related diabetes distress, explore its clinical usefulness and clarify its risk factors. (c) 2018 Elsevier B.V All rights reserved.

C1 [Hansen, Ulla M.; Olesen, Kasper; Willaing, Ingrid] Steno Diabet Ctr Copenhagen, Gentofte, Denmark.

[Hansen, Ulla M.] Univ Southern Denmark, Natl Inst Publ Hlth, Copenhagen, Denmark.

[Browne, Jessica L.] Deakin Univ, Sch Psychol, Geelong, Vic, Australia.

[Browne, Jessica L.] Diabet Victoria, Australian Ctr Behav Res Diabet, Melbourne, Vic, Australia.

[Skinner, Timothy C.] Univ Copenhagen, Dept Psychol, Copenhagen, Denmark.

RP Hansen, UM (reprint author), Steno Diabet Ctr Copenhagen, Diabet Management Res, Niels Steensens Vej 6, DK-2820 Gentofte, Denmark.

EM Ulla.moeller.hansen@regionh.dk

OI Willaing, Ingrid/0000-0002-3082-8816; Skinner,

Timothy/0000-0002-0018-6963; Hansen, Ulla Moller/0000-0001-8416-9783

FU Innovation Fund Denmark

FX This work was supported by an unrestricted grant from Innovation Fund

Denmark.

CR Amer Diabet Assoc, 2018, DIABETES CARE, V41, pS38, DOI 10.2337/dc18-S004

Balfe MBR, 2014, HEALTH PLACE, V26, P7

Brown TA, 2015, CONFIRMATORY FACTOR

Centers for Disease Control and Prevention, 2000, MEAS HLTH DAYS

El Fassi M, 2013, BMC PUBLIC HEALTH, V13, DOI 10.1186/1471-2458-13-305

Fisher L, 2014, DIABETIC MED, V31, P764, DOI 10.1111/dme.12428

Fisher L, 2015, J DIABETES COMPLICAT, V29, P572, DOI 10.1016/j.jdiacomp.2015.01.012

Fisher L, 2013, DIABETES CARE, V36, P2551, DOI 10.2337/dc12-2493

Golenbock S, 2017, QUAL LIFE RES, V26, P1839, DOI 10.1007/s11136-017-1523-7

Gonzalez JS, 2011, DIABETES CARE, V34, P236, DOI 10.2337/dc10-1970

Hakkarainen P, 2016, J OCCUP MED TOXICOL, V11, DOI 10.1186/s12995-016-0099-4

Hansen UM, 2016, P 48 ANN C NORD ERG, P222

Hessler DM, 2017, DIABETIC MED, V34, P1228, DOI 10.1111/dme.13381

Hinder S, 2012, BMC HEALTH SERV RES, V12, DOI 10.1186/1472-6963-12-83

McGuire BE, 2010, DIABETOLOGIA, V53, P66, DOI 10.1007/s00125-009-1559-5

Nielsen HB, 2016, DIABETES RES CLIN PR, V121, P62, DOI 10.1016/j.diabres.2016.08.021

Persson S, 2018, DIABETOLOGIA, V61, P342, DOI 10.1007/s00125-017-4472-3

Persson S, 2016, ECON HUM BIOL, V23, P180, DOI 10.1016/j.ehb.2016.09.003

POLONSKY WH, 1995, DIABETES CARE, V18, P754, DOI 10.2337/diacare.18.6.754

Polonsky WH, 2005, DIABETES CARE, V28, P626, DOI 10.2337/diacare.28.3.626

Reddy M, 2016, BMJ-BRIT MED J, V352, DOI 10.1136/bmj.i998

Schulte P, 2010, SCAND J WORK ENV HEA, V36, P422, DOI 10.5271/sjweh.3076

Sirgy MJ., 2012, WORK QUALITY LIFE ET, P35

Thurstone LL, 1947, MULTIPLE FACTOR ANAL

Tuomi K, 1994, WORK ABILITY INDEX

World Health Organization Quality of Life Group, 1998, PSYCHOL MED, V28, P551, DOI DOI 10.1017/S0033291798006667.

Young-Hyman D, 2016, DIABETES CARE, V39, P2126, DOI 10.2337/dc16-2053

NR 27

TC 0

Z9 0

U1 1

U2 1

PU ELSEVIER IRELAND LTD

PI CLARE

PA ELSEVIER HOUSE, BROOKVALE PLAZA, EAST PARK SHANNON, CO, CLARE, 00000,

IRELAND

SN 0168-8227

EI 1872-8227

J9 DIABETES RES CLIN PR

JI Diabetes Res. Clin. Pract.

PD JUN

PY 2018

VL 140

BP 139

EP 147

DI 10.1016/j.diabres.2018.03.040

PG 9

WC Endocrinology & Metabolism

SC Endocrinology & Metabolism

GA GI1CM

UT WOS:000434107600016

PM 29604390

DA 2019-01-08

ER

PT J

AU Hagiwara, Y

Kasai, J

Nakanishi, S

Saigusa, Y

Miyagi, E

Aoki, S

AF Hagiwara, Yuko

Kasai, Junko

Nakanishi, Sayuri

Saigusa, Yusuke

Miyagi, Etsuko

Aoki, Shigeru

TI Should the IADPSG criteria be applied when diagnosing early-onset

gestational diabetes?

SO DIABETES RESEARCH AND CLINICAL PRACTICE

LA English

DT Article

DE Gestational diabetes mellitus (GDM); 75g oral glucose tolerance test

(OGTT); Early-onset GDM; The International Association of Diabetes and

Pregnancy Study Groups (IADPSG) criteria; Pregnancy outcomes

ID GLUCOSE-TOLERANCE TEST; CARDIOVASCULAR-DISEASE; RETROSPECTIVE COHORT;

PREGNANCY OUTCOMES; MATERNAL OBESITY; MELLITUS; WOMEN; HYPERGLYCEMIA;

OBSTETRICS; RISK

AB Aims: To investigate whether the broad interpretation of the International Association of Diabetes and Pregnancy Study Groups (IADPSG) criteria with application to the early pregnancy, which is adopted as the standard in Japan, is appropriate.

Methods: We conducted this investigation by comparing diabetes-related adverse pregnancy outcomes among women treated for gestational diabetes mellitus (GDM) following an early-pregnancy diagnosis (early-onset GDM, n = 528) and those treated for GDM following a mid-pregnancy diagnosis, which is the international standard (Mid-term-onset GDM, n = 147).

Results: Gestational weight gain was significantly lower in the early-onset GDM group (7.5 kg) than in the mid-term-onset GDM group (8.4 kg). The frequency of hypertensive disorders of pregnancy tended to be lower in the early-onset GDM group (5.6% vs 8.8%, p = 0.085), but infant birth weight did not differ significantly between the groups. No between-group difference was observed in macrosomia, large-for-gestational-age (LGA), small-for-gestational age (SGA), low Apgar score, shoulder dystocia, cesarean delivery, NICU admission, hyperbilirubinemia, neonatal hypoglycemia, or respiratory distress syndrome The frequency of LGA showed a significant association with pre-pregnancy body mass index, but did not differ according to the timing of therapy initiation.

Conclusions: We could not find the effectiveness of therapeutic interventions initiated after GDM diagnosis in the early pregnancy based on the IADPSG criteria, compared with therapeutic interventions after a mid-pregnancy GDM diagnosis. It was suggested that the IADPSG criteria for diagnosing GDM at 24-28 weeks' gestation should not be applied to Japanese women in the early pregnancy by a broader interpretation. (c) 2018 Elsevier B.V All rights reserved.

C1 [Hagiwara, Yuko; Kasai, Junko; Nakanishi, Sayuri; Aoki, Shigeru] Yokohama City Univ, Med Ctr, Perinatal Ctr Matern & Neonates, Yokohama, Kanagawa, Japan.

[Saigusa, Yusuke] Yokohama City Univ, Grad Sch Med, Dept Biostat, Yokohama, Kanagawa, Japan.

[Miyagi, Etsuko] Yokohama City Univ, Sch Med, Dept Obstet & Gynecol, Yokohama, Kanagawa, Japan.

RP Aoki, S (reprint author), Yokohama City Univ, Med Ctr, Perinatal Ctr Matern & Neonate, Minami Ku, 4-57 Urafunecho, Yokohama, Kanagawa 2320024, Japan.

EM smyyaoki@yahoo.co.jp

OI Miyagi, Etsuko/0000-0002-1943-594X

CR American Diabetes Association, 2017, Diabetes Care, V40, pS11

Bellamy L, 2009, LANCET, V373, P1773, DOI 10.1016/S0140-6736(09)60731-5

Benhalima K, 2015, DIABETOLOGIA, V58, P1422, DOI 10.1007/s00125-015-3615-7

Catalano PM, 2006, BJOG-INT J OBSTET GY, V113, P1126, DOI 10.1111/j.1471-0528.2006.00989.x

CATALANO PM, 1991, AM J OBSTET GYNECOL, V165, P1667, DOI 10.1016/0002-9378(91)90012-G

Clausen TD, 2009, J CLIN ENDOCR METAB, V94, P2464, DOI 10.1210/jc.2009-0305

Committee on Practice Bulletins--Obstetrics, 2013, Obstet Gynecol, V122, P406, DOI 10.1097/01.AOG.0000433006.09219.f1

Corrado F, 2012, DIABETES METAB, V38, P458, DOI 10.1016/j.diabet.2012.03.006

Crowther CA, 2005, NEW ENGL J MED, V352, P2477, DOI 10.1056/NEJMoa042973

Dabelea D, 2000, J Matern Fetal Med, V9, P83, DOI 10.1002/(SICI)1520-6661(200001/02)9:1<83::AID-MFM17>3.0.CO;2-O

Enomoto K, 2016, PLOS ONE, V11, DOI 10.1371/journal.pone.0157081

HAPO study cooperative research group, 2008, NEW ENGL J MED, V122, P406

Hillier TA, 2007, DIABETES CARE, V30, P2287, DOI 10.2337/dc06-2361

Horie I, 2015, DIABETES RES CLIN PR, V107, P400, DOI 10.1016/j.diabres.2014.12.011

Kim C, 2002, DIABETES CARE, V25, P1862, DOI 10.2337/diacare.25.10.1862

KUHL C, 1975, ACTA ENDOCRINOL-COP, V79, P709, DOI 10.1530/acta.0.0790709

Landon MB, 2009, NEW ENGL J MED, V361, P1339, DOI 10.1056/NEJMoa0902430

Lee AJ, 2007, DIABETES CARE, V30, P878, DOI 10.2337/dc06-1816

LIND T, 1973, J OBSTET GYN BR COMM, V80, P1033

McIntyre HD, 2012, DIABETES CARE, V38, P458

Metzger BE, 2010, DIABETES CARE, V33, P676, DOI 10.2337/dc09-1848

Meyer WJ, 1996, J REPROD MED, V41, P675

Minakami H, 2014, J OBSTET GYNAECOL RE, V40, P1469, DOI 10.1111/jog.12419

Morikawa M, 2010, DIABETES RES CLIN PR, V90, P339, DOI 10.1016/j.diabres.2010.08.023

Moyer VA, 2014, ANN INTERN MED, V160, P414, DOI 10.7326/M13-2905

Pace R, 2017, AM J EPIDEMIOL, V186, P1115, DOI 10.1093/aje/kwx263

Page KA, 2014, J PEDIATR-US, V164, P807, DOI 10.1016/j.jpeds.2013.11.063

PETTITT DJ, 1988, DIABETES, V37, P622, DOI 10.2337/diabetes.37.5.622

Reece EA, 2004, DIABETES IN WOMEN, P159

Riskin-Mashiah S, 2009, DIABETES CARE, V32, P1639, DOI 10.2337/dc09-0688

Sacks DA, 2012, DIABETES CARE, V35, P526, DOI 10.2337/dc11-1641

Seino Y, 2010, J DIABETES INVEST, V1, P1, DOI 10.1111/j.2040-1124.2010.00005.x

Seshiah V, 2008, DIABETES RES CLIN PR, V80, P199, DOI 10.1016/j.diabres.2007.12.008

Shah BR, 2008, DIABETES CARE, V31, P1668, DOI 10.2337/dc08-0706

SILVERMAN BL, 1995, DIABETES CARE, V18, P611, DOI 10.2337/diacare.18.5.611

SUPER DM, 1991, DIABETES CARE, V14, P288, DOI 10.2337/diacare.14.4.288

Zhu WW, 2013, DIABETES CARE, V36, P586, DOI 10.2337/dc12-1157

NR 37

TC 0

Z9 0

U1 0

U2 0

PU ELSEVIER IRELAND LTD

PI CLARE

PA ELSEVIER HOUSE, BROOKVALE PLAZA, EAST PARK SHANNON, CO, CLARE, 00000,

IRELAND

SN 0168-8227

EI 1872-8227

J9 DIABETES RES CLIN PR

JI Diabetes Res. Clin. Pract.

PD JUN

PY 2018

VL 140

BP 154

EP 161

DI 10.1016/j.diabres.2018.03.048

PG 8

WC Endocrinology & Metabolism

SC Endocrinology & Metabolism

GA GI1CM

UT WOS:000434107600018

PM 29621563

DA 2019-01-08

ER

PT J

AU Ishii, H

Shin, H

Tosaki, T

Haga, T

Nakajima, Y

Shiraiwa, T

Watanabe, N

Koizumi, M

Nakajima, H

Okada, S

Mashitani, T

Mohri, T

Akai, Y

AF Ishii, Hitoshi

Shin, Hiroki

Tosaki, Takahiro

Haga, Tatsuya

Nakajima, Yoshiki

Shiraiwa, Toshihiko

Watanabe, Nobuaki

Koizumi, Miyuki

Nakajima, Hiroki

Okada, Sadanori

Mashitani, Tsuyoshi

Mohri, Takako

Akai, Yasuhiro

TI Reproducibility and Validity of a Questionnaire Measuring Treatment

Burden on Patients with Type 2 Diabetes: Diabetic Treatment Burden

Questionnaire (DTBQ)

SO DIABETES THERAPY

LA English

DT Article

DE Pharmacotherapy; Questionnaire; Reproducibility; Treatment burden; Type

2 diabetes mellitus; Validity

ID QUALITY-OF-LIFE; TREATMENT ATTRIBUTES IMPORTANT; TREATMENT SATISFACTION;

MEDICATION ADHERENCE; GLYCEMIC CONTROL; MELLITUS; THERAPY; PREFERENCES;

INJECTION; DISTRESS

AB Introduction: To measure the burden of pharmacotherapy on patients with type 2 diabetes mellitus (T2DM), we developed the Diabetes Treatment Burden Questionnaire (DTBQ), a patient-administered questionnaire composed of 18 questions, and evaluated its reproducibility and validity.

Methods: We enrolled 240 patients with T2DM under pharmacotherapy over 20 years of age at seven institutes in Japan. Their physicians filled out report forms on patient backgrounds, and the patients answered both the DTBQ and the Diabetes Treatment Satisfaction Questionnaire (DTSQ). For evaluation of reproducibility, 48 of the enrolled subjects completed a 2nd DTBQ at home after leaving the medical institutes.

Results: Statistical analyses were performed for two sets of subjects, the validity analysis set (N = 236) and the reproducibility analysis set (N = 47). Factor analysis found a simple structure in the DTBQ item scores using a three-factor model with varimax rotation; the three subscales were designated as "implementation burden", "flexibility burden", and "blood glucose control burden". All intraclass correlation coefficients for the subscale scores were 0.8 or higher, indicating high reproducibility. Negative correlations were observed between the DTSQ satisfaction score and the DTBQ subscale scores. Moreover, as the dosing frequency of diabetic medicines increased, the DTBQ total score (total burden score) also became higher. Likewise, expected associations were observed between patient backgrounds and DTSQ scores.

Conclusion: The DTBQ has adequate reproducibility and validity as a measurement scale for treatment burden on T2DM patients.

C1 [Ishii, Hitoshi; Koizumi, Miyuki; Nakajima, Hiroki; Okada, Sadanori; Mashitani, Tsuyoshi; Mohri, Takako; Akai, Yasuhiro] Nara Med Univ, Dept Diabetol, Nara, Japan.

[Shin, Hiroki] Shin Clin, Tokyo, Japan.

[Tosaki, Takahiro] TDE Healthcare Corp, TOSAKI Clin Diabet & Endocrinol, Nagoya, Aichi, Japan.

[Haga, Tatsuya] Haga Diabet Clin, Nagoya, Aichi, Japan.

[Nakajima, Yoshiki] Nakajima Diabet Clin, Osaka, Japan.

[Shiraiwa, Toshihiko] Shiraiwa Med Clin, Osaka, Japan.

[Watanabe, Nobuaki] Watanabe Med Clin, Nishinomiya, Hyogo, Japan.

RP Ishii, H (reprint author), Nara Med Univ, Dept Diabetol, Nara, Japan.

EM hits1@naramed-u.ac.jp

FU Eli Lilly Japan (Kobe, Japan)

FX This study was funded by Eli Lilly Japan (Kobe, Japan). No funding was

received for the article processing charges and medical

writing/editorial assistance.

CR Ali MK, 2013, NEW ENGL J MED, V368, P1613, DOI 10.1056/NEJMsa1213829

American Diabetes Association, 2017, Diabetes Care, V40, pS6

Bailey CJ, 2011, INT J CLIN PRACT, V65, P314, DOI 10.1111/j.1742-1241.2010.02544.x

Best JH, 2009, DIABETIC MED, V26, P722, DOI 10.1111/j.1464-5491.2009.02752.x

Blonde L, 2012, ADV THER, V29, P1, DOI 10.1007/s12325-011-0094-1

Brunton S, 2016, CLIN THER, V38, P582, DOI 10.1016/j.clinthera.2016.01.014

Burness CB, 2015, BIODRUGS, V29, P407, DOI 10.1007/s40259-015-0143-4

Confavreux CB, 2012, EUR J ENDOCRINOL, V166, P735, DOI 10.1530/EJE-11-0959

Cramer JA, 2004, DIABETES CARE, V27, P1218, DOI 10.2337/diacare.27.5.1218

de Climens AR, 2015, CURR MED RES OPIN, V31, P643, DOI 10.1185/03007995.2015.1020364

Dennick K, 2017, J DIABETES COMPLICAT, V31, P898, DOI 10.1016/j.jdiacomp.2016.12.018

Donnelly LA, 2007, QJM-INT J MED, V100, P345, DOI 10.1093/qjmed/hcm031

Evans PMS, 2016, EXPERT OPIN PHARMACO, V17, P1947, DOI 10.1080/14656566.2016.1218472

Farmer AJ, 2016, DIABETES CARE, V39, P258, DOI 10.2337/dc15-1194

Garcia-Perez LE, 2013, DIABETES THER, V4, P175, DOI 10.1007/s13300-013-0034-y

Gelhorn HL, 2016, PATIENT PREFER ADHER, V10, P1337, DOI 10.2147/PPA.S109289

Gelhorn HL, 2015, PATIENT PREFER ADHER, V9, P1611, DOI 10.2147/PPA.S90842

Hauber AB, 2016, CURR MED RES OPIN, V32, P251, DOI 10.1185/03007995.2015.1117433

Hauber AB, 2015, DIABETES THER, V6, P75, DOI 10.1007/s13300-015-0094-2

Hauber AB, 2013, PATIENT PREFER ADHER, V7, P937, DOI 10.2147/PPA.S43465

Ishii H, 2008, DIABETES RES CLIN PR, V81, P169, DOI 10.1016/j.diabres.2008.03.020

Ishii H, 2012, DIABETOL INT, V3, P152, DOI 10.1007/s13340-012-0074-y

Kaku K, 2015, EXPERT OPIN PHARMACO, V16, P2539, DOI 10.1517/14656566.2015.1099630

Kirkman MS, 2015, DIABETES CARE, V38, P604, DOI 10.2337/dc14-2098

Kishimoto H, 2015, ARCH OSTEOPOROS, V10, DOI 10.1007/s11657-015-0231-6

Lglay K, 2015, CLIN THER, V37, P1813, DOI 10.1016/j.clinthera.2015.05.505

Nguyen H, 2017, ADV THER, V34, P658, DOI 10.1007/s12325-016-0470-y

Peron EP, 2015, CLIN GERIATR MED, V31, P17, DOI 10.1016/j.cger.2014.08.017

Polonsky WH, 2011, DIABETES OBES METAB, V13, P144, DOI 10.1111/j.1463-1326.2010.01327.x

POLONSKY WH, 1995, DIABETES CARE, V18, P754, DOI 10.2337/diacare.18.6.754

Polonsky WH, 2005, DIABETES CARE, V28, P626, DOI 10.2337/diacare.28.3.626

Polonsky WH, 2016, PATIENT PREFER ADHER, V10, P1299, DOI 10.2147/PPA.S106821

Pouwer F, 1998, QUAL LIFE RES, V7, P33

Qiao Q, 2016, DIABET METAB SYND OB, V9, P201, DOI 10.2147/DMSO.S99732

Qin L, 2017, DIABETES THER, V8, P335, DOI 10.1007/s13300-017-0237-8

Qin L, 2017, DIABETES THER, V8, P321, DOI 10.1007/s13300-017-0230-2

Reaney M, 2015, DIABETES OBES METAB, V17, P896, DOI 10.1111/dom.12527

Schernthaner G, 2010, DIABETIC MED, V27, P739, DOI 10.1111/j.1464-5491.2010.03000.x

Thrasher J, 2017, AM J CARDIOL, V120, pS4, DOI 10.1016/j.amjcard.2017.05.009

NR 39

TC 1

Z9 1

U1 1

U2 1

PU SPRINGER HEIDELBERG

PI HEIDELBERG

PA TIERGARTENSTRASSE 17, D-69121 HEIDELBERG, GERMANY

SN 1869-6953

EI 1869-6961

J9 DIABETES THER

JI Diabetes Ther.

PD JUN

PY 2018

VL 9

IS 3

BP 1001

EP 1019

DI 10.1007/s13300-018-0414-4

PG 19

WC Endocrinology & Metabolism

SC Endocrinology & Metabolism

GA GI1ZN

UT WOS:000434169400010

PM 29600503

OA DOAJ Gold

DA 2019-01-08

ER

PT J

AU Clarke, J

Proudfoot, J

Vatiliotis, V

Verge, C

Holmes-Walker, DJ

Campbell, L

Wilhelm, K

Moravac, C

Indu, PS

Bridgett, M

AF Clarke, Janine

Proudfoot, Judy

Vatiliotis, Veronica

Verge, Charles

Holmes-Walker, Deborah J.

Campbell, Lesley

Wilhelm, Kay

Moravac, Catherine

Indu, Pillaveetil S.

Bridgett, Madeleine

TI Attitudes towards mental health, mental health research and digital

interventions by young adults with type 1 diabetes: A qualitative

analysis

SO HEALTH EXPECTATIONS

LA English

DT Article

DE digital interventions; psychosocial support; type 1 diabetes

ID ADOLESCENTS; DEPRESSION; DISTRESS; SERVICES; PEOPLE

AB BackgroundYoung people with type 1 diabetes are at increased risk of mental disorders. Whereas treatment need is high, difficulty recruiting young people with type 1 diabetes into psychosocial studies complicates development, testing and dissemination of these interventions.

ObjectiveInterviews with young adults with type 1 diabetes were conducted to examine attitudes towards mental health and mental health research, including barriers and motivators to participation in mental health studies and preferred sources of mental health support. The interviews were audio-taped, transcribed and evaluated via thematic analysis.

Setting and participantsYoung adults with type 1 diabetes were recruited via social media channels of 3 advocacy organizations. A total of 31 young adults (26 females and 5 males) with an average age of 22years were interviewed between October 2015 and January 2016.

ResultsParticipants were largely unaware of their increased vulnerability to common mental health problems and knew little about mental health research. Major barriers to participation included perceived stigma and lifestyle issues and low levels of trust in researchers. Opportunities to connect with peers and help others were described as key motivators. Psychological distress was considered normal within the context of diabetes. A need for some level of human contact in receiving psychosocial support was expressed.

Discussion and conclusionFindings provide valuable insights into the complex dynamics of engaging young adults with type 1 diabetes in mental health studies. Interviewees provided practical suggestions to assist investigation and delivery of psychosocial interventions for this vulnerable group.

C1 [Clarke, Janine; Vatiliotis, Veronica] Black Dog Inst, Sydney, NSW, Australia.

[Proudfoot, Judy] Black Dog Inst, eHlth Implementat & Policy, Sydney, NSW, Australia.

[Clarke, Janine] UNSW Australia, Sydney, NSW, Australia.

[Proudfoot, Judy] UNSW Australia, eHlth Implementat & Policy, Sydney, NSW, Australia.

[Verge, Charles] Sydney Childrens Hosp, Endocrinol, Sydney, NSW, Australia.

[Verge, Charles] Sydney Childrens Hosp, Sydney, NSW, Australia.

[Verge, Charles] UNSW Australia, Sch Womens & Childrens Hlth, Endocrinol, Sydney, NSW, Australia.

[Verge, Charles] UNSW Australia, Sch Womens & Childrens Hlth, Sydney, NSW, Australia.

[Holmes-Walker, Deborah J.] Westmead Hosp, Dept Diabet & Endocrinol, Westmead, NSW, Australia.

[Campbell, Lesley] St Vincents Hosp, Dept Endocrinol, Med, Sydney, NSW, Australia.

[Wilhelm, Kay] St Vincents Hosp, Urban Mental Res Inst, Sydney, NSW, Australia.

[Moravac, Catherine] Univ Toronto, Toronto, ON, Canada.

[Indu, Pillaveetil S.] Govt Med Coll, Trivandrum, Kerala, India.

[Indu, Pillaveetil S.] Govt Med Coll, Community Med, Trivandrum, Kerala, India.

[Bridgett, Madeleine] 6 St James Hall Chambers, Sydney, NSW, Australia.

RP Clarke, J (reprint author), Black Dog Inst, Sydney, NSW, Australia.

EM janine.clarke@blackdog.org.au

OI Indu, Pillaveetil Sathyadas/0000-0002-2282-7661

FU Australian Rotary Health Research Fund

FX Australian Rotary Health Research Fund

CR Australia D, 2006, YOUNG ADULTS DIABETE

Australian Bureau of Statistics, 2007, NAT SURV MENT HLTH W

Balfe M, 2012, CIN-COMPUT INFORM NU, V30, P511, DOI 10.1097/NXN.0b013e31826e4fca

Bernstein CM, 2013, CLIN PEDIATR, V52, P10, DOI 10.1177/0009922812459950

Bradford S, 2014, CHILD ADOL MENT H-UK, V19, P39, DOI 10.1111/camh.12002

Bryden KS, 2003, DIABETES CARE, V26, P1052, DOI 10.2337/diacare.26.4.1052

Burns J, 2014, PSYCHOL RES BEHAV MA, V7, P303, DOI 10.2147/PRBM.S49151

Burns JM, 2010, MED J AUSTRALIA, V192, pS22

Clarke J, 2016, JMIR MENT HEALTH, V3, DOI 10.2196/mental.4656

Clarke V., 2014, ENCY CRITICAL PSYCHO, P1947, DOI DOI 10.1007/978-1-4614-5583-7

Corrigan PW, 2000, CLIN PSYCHOL-SCI PR, V7, P48, DOI 10.1093/clipsy/7.1.48

Cunradi CB, 2005, SUBST USE MISUSE, V40, P171, DOI 10.1081/JA-200048447

de Wit M, 2011, PEDIATR DIABETES, V12, P172, DOI 10.1111/j.1399-5448.2010.00673.x

Ellis L. A., 2014, Clinical Investigation, V4, P881, DOI 10.4155/cli.14.61

Ellis LA, 2013, BMC PSYCHIATRY, V13, DOI 10.1186/1471-244X-13-119

Ellis LA, 2012, J MED INTERNET RES, V14, P338, DOI 10.2196/jmir.2291

Fenner Y, 2012, J MED INTERNET RES, V14, DOI 10.2196/jmir.1978

Fisher L, 2014, DIABETIC MED, V31, P764, DOI 10.1111/dme.12428

Fisher L, 2008, ANN FAM MED, V6, P246, DOI 10.1370/afm.842

Fogarty AS, 2017, JMIR MENT HEALTH, V4, DOI 10.2196/mental.7769

Frew PM, 2010, J CLIN EPIDEMIOL, V63, P1110, DOI 10.1016/j.jclinepi.2009.12.002

Galea S, 2007, ANN EPIDEMIOL, V17, P643, DOI 10.1016/j.annepidem.2007.03.013

Gorman JR, 2014, J ADOLESC YOUNG ADUL, V3, P59, DOI 10.1089/jayao.2013.0031

Harris MF, 2011, HEALTH POLICY, V101, P140, DOI 10.1016/j.healthpol.2010.10.019

Krischer Jeffrey, 2017, J Med Internet Res, V19, pe50, DOI 10.2196/jmir.6798

Kroenke K, 2002, PSYCHIAT ANN, V32, P509, DOI 10.3928/0048-5713-20020901-06

Miller SM, 2013, PSYCHO-ONCOLOGY, V22, P481, DOI 10.1002/pon.3043

Newington L, 2014, BMC MED RES METHODOL, V14, DOI 10.1186/1471-2288-14-10

Ramo Danielle E, 2014, Internet Interv, V1, P58

Shaghaghi A, 2011, HEALTH PROMOT PERSPE, V1, P86, DOI 10.5681/hpp.2011.009

Treweek S, 2013, BMJ OPEN, V3, DOI 10.1136/bmjopen-2012-002360

Weiner B, 1995, JUDGMENTS RESPONSIBI

White M, 2017, LANCET CHILD ADOLESC, V1, P274, DOI 10.1016/S2352-4642(17)30089-5

NR 33

TC 0

Z9 0

U1 8

U2 8

PU WILEY

PI HOBOKEN

PA 111 RIVER ST, HOBOKEN 07030-5774, NJ USA

SN 1369-6513

EI 1369-7625

J9 HEALTH EXPECT

JI Health Expect.

PD JUN

PY 2018

VL 21

IS 3

BP 668

EP 677

DI 10.1111/hex.12662

PG 10

WC Health Care Sciences & Services; Health Policy & Services; Public,

Environmental & Occupational Health

SC Health Care Sciences & Services; Public, Environmental & Occupational

Health

GA GI1DY

UT WOS:000434112100013

PM 29319923

OA DOAJ Gold

DA 2019-01-08

ER

PT J

AU McLachlan, KJJ

Gale, CR

AF McLachlan, Kyle J. J.

Gale, Catharine R.

TI The effects of psychological distress and its interaction with

socioeconomic position on risk of developing four chronic diseases

SO JOURNAL OF PSYCHOSOMATIC RESEARCH

LA English

DT Article

DE Psychological distress; Depression; Arthritis; Cardiovascular disease;

Chronic obstructive pulmonary disease; Diabetes

ID CORONARY-HEART-DISEASE; OBSTRUCTIVE PULMONARY-DISEASE; GENERAL-PRACTICE;

NEIGHBORHOOD COHESION; CARDIOVASCULAR EVENTS; RHEUMATOID-ARTHRITIS;

INFLAMMATORY MARKERS; PSYCHOSOCIAL FACTORS; ARTERY-DISEASE; DEPRESSION

AB Objective: To examine the relationship between psychological distress and risk of developing arthritis, cardiovascular disease, chronic obstructive pulmonary disease and diabetes across the range of distress severity, investigate the mediating roles of health behaviours and explore whether the associations vary with socioeconomic position.

Methods: Participants were 16,485 adults from the UK Household Longitudinal Study We examined prospective relationships between psychological distress at baseline (measured using the 12-item General Health Questionnaire) and incidence of arthritis, cardiovascular disease, chronic obstructive pulmonary disease and diabetes (measured using self-report) over 3 years using logistic regression. We then examined the mediating effects of health behaviours and investigated whether the associations varied with socioeconomic position.

Results: Distress significantly increased risk of incident arthritis, cardiovascular disease and chronic obstructive pulmonary disease in a dose-response pattern after controlling for age, sex, socioeconomic position, neighbourhood cohesion, marital status, BMI and baseline disease. High levels of distress (GHQ 7) increased risk of arthritis (OR 2.22; 1.58-2.13), cardiovascular disease (OR 3.06; 1.89-4.98) and chronic obstructive pulmonary disease (OR 3.25; 1.47-7.18). These associations were partially mediated by smoking status but remained significant after controlling for smoking status, diet and exercise. Distress significantly predicted incident diabetes in manual socioeconomic groups only. Effect sizes did not vary with socioeconomic position for arthritis, cardiovascular disease and chronic obstructive pulmonary disease.

Conclusion: Psychological distress increases risk of incident arthritis, cardiovascular disease and chronic obstructive pulmonary disease in a dose-response pattern, even at low and moderate distress levels. Future research should investigate the mediating role of inflammatory biomarkers.

C1 [McLachlan, Kyle J. J.] Univ Edinburgh, Edinburgh Med Sch, Coll Med & Vet Med, Edinburgh, Midlothian, Scotland.

[McLachlan, Kyle J. J.] Univ Edinburgh, Dept Psychol, Edinburgh, Midlothian, Scotland.

[Gale, Catharine R.] Univ Edinburgh, Dept Psychol, Ctr Cognit Ageing & Cognit Epidemiol, Edinburgh, Midlothian, Scotland.

[Gale, Catharine R.] Univ Southampton, MRC Lifecourse Epidemiol Unit, Southampton, Hants, England.

RP Gale, CR (reprint author), Southampton Gen Hosp, MRC Lifecourse Epidemiol Unit, Southampton SO16 6YD, Hants, England.

EM crg@mrc.soton.ac.uk

FU MRC; BBSRC part of the cross council Lifelong Health and Wellbeing

Initiative [MR/K026992/1]

FX CRG is a member of the University of Edinburgh Centre for Cognitive

Ageing and Cognitive Epidemiology, funded by the MRC and BBSRC part of

the cross council Lifelong Health and Wellbeing Initiative

(MR/K026992/1).

CR Aalto AM, 2012, PSYCHIAT RES, V197, P163, DOI 10.1016/j.psychres.2011.09.008

Atlantis E, 2013, CHEST, V144, P766, DOI 10.1378/chest.12-1911

Batty G. D., 2017, BMJ, V356

Batty GD, 2014, ATHEROSCLEROSIS, V236, P385, DOI 10.1016/j.atherosclerosis.2014.06.025

Bjertness E, 2010, BMC PUBLIC HEALTH, V10, DOI 10.1186/1471-2458-10-602

Brotman DJ, 2007, LANCET, V370, P1089, DOI 10.1016/S0140-6736(07)61305-1

Buck N, 2012, LONGITUD LIFE COURSE, V3, P5

BUCKNER JC, 1988, AM J COMMUN PSYCHOL, V16, P771, DOI 10.1007/BF00930892

Celiker R, 2001, J MUSCULOSKELET PAIN, V9, P35, DOI 10.1300/J094v09n01_05

Cesari M, 2003, CIRCULATION, V108, P2317, DOI 10.1161/01.CIR.0000097109.90783.FC

CHAN KWA, 1994, ARTHRITIS RHEUM-US, V37, P814, DOI 10.1002/art.1780370606

Chida Y, 2008, NAT CLIN PRACT ONCOL, V5, P466, DOI 10.1038/ncponc1134

Dahl M, 2001, AM J RESP CRIT CARE, V164, P1008, DOI 10.1164/ajrccm.164.6.2010067

Dixon KE, 2007, HEALTH PSYCHOL, V26, P241, DOI 10.1037/0278-6133.26.3.241

Do DP, 2010, PSYCHOSOM MED, V72, P613, DOI 10.1097/PSY.0b013e3181e2cca5

Elliott J, 2014, SOC SCI MED, V107, P44, DOI 10.1016/j.socscimed.2014.02.027

Engum A, 2007, J PSYCHOSOM RES, V62, P31, DOI 10.1016/j.jpsychores.2006.07.009

Eriksson AK, 2008, DIABETIC MED, V25, P834, DOI 10.1111/j.1464-5491.2008.02463.x

Ferrucci L, 1999, J AM GERIATR SOC, V47, P639, DOI 10.1111/j.1532-5415.1999.tb01583.x

Goldberg D., 1988, USERS GUIDE GEN HLTH

Goldberg D., 1978, MANUAL OF THE GHQ

GOLDBERG DP, 1970, BMJ-BRIT MED J, V2, P439, DOI 10.1136/bmj.2.5707.439

Goldberg DP, 1997, PSYCHOL MED, V27, P191, DOI 10.1017/S0033291796004242

Golden SH, 2008, JAMA-J AM MED ASSOC, V299, P2751, DOI 10.1001/jama.299.23.2751

Hamer M, 2008, J AM COLL CARDIOL, V52, P2156, DOI 10.1016/j.jacc.2008.08.057

Hankins Matthew, 2008, Clin Pract Epidemiol Ment Health, V4, P10, DOI 10.1186/1745-0179-4-10

Hemingway H, 1999, BMJ-BRIT MED J, V318, P1460, DOI 10.1136/bmj.318.7196.1460

KESSLER RC, 1995, EPIDEMIOL REV, V17, P192, DOI 10.1093/oxfordjournals.epirev.a036176

Klebanoff MA, 2008, AM J EPIDEMIOL, V168, P355, DOI 10.1093/aje/kwn071

Knies G., 2014, UNDERSTANDING SOC UK

Knol MJ, 2006, DIABETOLOGIA, V49, P837, DOI 10.1007/s00125-006-0159-x

Kriegsman DMW, 1996, J CLIN EPIDEMIOL, V49, P1407, DOI 10.1016/S0895-4356(96)00274-0

Lacasse Y, 1996, LANCET, V348, P1115, DOI 10.1016/S0140-6736(96)04201-8

Lazzarino AI, 2013, PSYCHOSOM MED, V75, P311, DOI 10.1097/PSY.0b013e3182898e6d

Lazzarino AI, 2013, JAMA INTERN MED, V173, P22, DOI 10.1001/2013.jamainternmed.951

Lett HS, 2004, PSYCHOSOM MED, V66, P305, DOI 10.1097/01.psy.0000126207.43307.c0

Linden W, 1996, ARCH INTERN MED, V156, P745, DOI 10.1001/archinte.156.7.745

Lochner K, 1999, HEALTH PLACE, V5, P259, DOI 10.1016/S1353-8292(99)00016-7

March LM, 1998, OSTEOARTHR CARTILAGE, V6, P87, DOI 10.1053/joca.1997.0098

Matthews KA, 2011, ANNU REV PSYCHOL, V62, P501, DOI 10.1146/annurev.psych.031809.130711

Mezuk B, 2008, DIABETES CARE, V31, P2383, DOI 10.2337/dc08-0985

Mikkelsen RL, 2004, NORD J PSYCHIAT, V58, P65, DOI 10.1080/08039480310000824

Mitchell AJ, 2009, LANCET, V374, P609, DOI 10.1016/S0140-6736(09)60879-5

Mulder M, 1998, INT J EPIDEMIOL, V27, P199, DOI 10.1093/ije/27.2.199

Murray CJL, 2013, LANCET, V381, P997, DOI 10.1016/S0140-6736(13)60355-4

NICE, 2009, DEPR AD REC MAN

Nicholson A, 2006, EUR HEART J, V27, P2763, DOI 10.1093/eurheartj/ehl338

Pai JK, 2004, NEW ENGL J MED, V351, P2599, DOI 10.1056/NEJMoa040967

Pan A, 2011, JAMA-J AM MED ASSOC, V306, P1241, DOI 10.1001/jama.2011.1282

Patten SB, 2008, GEN HOSP PSYCHIAT, V30, P407, DOI [10.1016/j.genhosppsych.2008.05.001, 10.1016/j.genhosppsych.2008.05.00l]

Peckham Stephen, 2011, Qual Prim Care, V19, P317

Peduzzi P, 1996, J CLIN EPIDEMIOL, V49, P1373, DOI 10.1016/S0895-4356(96)00236-3

Pembroke TPI, 2006, J EPIDEMIOL COMMUN H, V60, P789, DOI 10.1136/jech.2005.042150

Pitsavos C, 2006, ATHEROSCLEROSIS, V185, P320, DOI 10.1016/j.atherosclerosis.2005.06.001

Rasul F, 2005, J EPIDEMIOL COMMUN H, V59, P140, DOI 10.1136/jech.2004.019786

ROBERTS RE, 1990, SOC SCI MED, V31, P527, DOI 10.1016/0277-9536(90)90087-9

Roest AM, 2010, J AM COLL CARDIOL, V56, P38, DOI 10.1016/j.jacc.2010.03.034

RUSS TC, 2012, BRIT MED J, V345, DOI [10.1136/bmj.e4933, DOI 10.1136/BMJ.E4933]

Russ TC, 2015, GASTROENTEROLOGY, V148, P958, DOI 10.1053/j.gastro.2015.02.004

Sargent-Cox K, 2014, PREV MED, V58, P75, DOI 10.1016/j.ypmed.2013.10.017

Scott KM, 2011, ARCH GEN PSYCHIAT, V68, P838, DOI 10.1001/archgenpsychiatry.2011.77

Stansfeld SA, 2002, INT J EPIDEMIOL, V31, P248, DOI 10.1093/ije/31.1.248

Steptoe A, 2012, NAT REV CARDIOL, V9, P360, DOI 10.1038/nrcardio.2012.45

STERNE JAC, 2009, BRIT MED J, V339, DOI DOI 10.1136/BMJ.B2393

Takahashi TU, 2003, RESPIROLOGY, V8, P504, DOI 10.1046/j.1440-1843.2003.00501.x

UNGER DG, 1985, AM J COMMUN PSYCHOL, V13, P139, DOI 10.1007/BF00905726

Van der Kooy K, 2007, INT J GERIATR PSYCH, V22, P613, DOI 10.1002/gps.1723

van Schayck CP, 2002, BRIT MED J, V324, P1370, DOI 10.1136/bmj.324.7350.1370

Vedsted P, 2005, PUBLIC HEALTH, V119, P118, DOI 10.1016/j.puhe.2004.03.007

NR 69

TC 0

Z9 0

U1 3

U2 3

PU PERGAMON-ELSEVIER SCIENCE LTD

PI OXFORD

PA THE BOULEVARD, LANGFORD LANE, KIDLINGTON, OXFORD OX5 1GB, ENGLAND

SN 0022-3999

EI 1879-1360

J9 J PSYCHOSOM RES

JI J. Psychosomat. Res.

PD JUN

PY 2018

VL 109

BP 79

EP 85

DI 10.1016/j.jpsychores.2018.04.004

PG 7

WC Psychiatry

SC Psychiatry

GA GH2ZS

UT WOS:000433271100013

PM 29680578

OA Other Gold, Green Published

DA 2019-01-08

ER

PT J

AU Lee, AA

Piette, JD

Heisler, M

Rosland, AM

AF Lee, Aaron A.

Piette, John D.

Heisler, Michele

Rosland, Ann-Marie

TI Diabetes Distress and Glycemic Control: The Buffering Effect of Autonomy

Support From Important Family Members and Friends

SO DIABETES CARE

LA English

DT Article

ID SOCIAL SUPPORT; MEDICATION ADHERENCE; EMOTIONAL DISTRESS; HEALTH;

ADULTS; LIFE; INTERVENTION; ASSOCIATION; MANAGEMENT; EDUCATION

AB OBJECTIVE

To examine whether autonomy support (defined as social support for an individual's personal agency) for diabetes management from in formal health supporters (family/friends) reduces the detrimental effects of diabetes distress on glycemic control.

RESEARCH DESIGN AND METHODS

Three hundred eight veterans with type 2 diabetes and one or more risk factors for diabetes complications completed a survey that included measures of diabetes distress and perceived autonomy support from their main informal health supporter. Hemoglobin A(1c) (HbA(1c)) data from 12 months before and after the survey were extracted from electronic medical records. Linear mixed modeling examined the main effects and interaction of autonomy support and diabetes distress on repeated measures of HbA(1c) over the 12 months after the survey, controlling for mean prior 12-month HbA(1c), time, insulin use, age, and race/ethnicity.

RESULTS

Diabetes distress (B = 0.12 [SE 0.05]; P = 0.023) was associated with higher and autonomy support (B = -0.16 [SE 0.07]; P = 0.032) with lower subsequent HbA(1c) levels. Autonomy support moderated the relationship between diabetes distress and HbA1c (B = -0.13 [SE 0.06]; P = 0.027). Greater diabetes distress was associated with higher HbA(1c) at low (B = 0.21 [SE 07]; P = 0.002) but not high (B = 0.01 [SE 0.07]; P = 0.890) levels of autonomy support.

CONCLUSIONS

Autonomy support from main health supporters may contribute to better glycemic control by ameliorating the effects of diabetes distress. Interventions that reduce diabetes distress and enhance the autonomy supportiveness of informal supporters may be effective approaches to improving glycemic control.

C1 [Lee, Aaron A.; Piette, John D.; Heisler, Michele] VA Ctr Clin Management Res, Ann Arbor, MI 48105 USA.

[Piette, John D.; Heisler, Michele] Univ Michigan, Sch Publ Hlth, Dept Hlth Behav & Hlth Educ, Ann Arbor, MI 48109 USA.

[Piette, John D.; Heisler, Michele] Univ Michigan, Inst Healthcare Policy & Innovat, Ann Arbor, MI 48109 USA.

[Heisler, Michele] Univ Michigan, Sch Med, Dept Internal Med, Ann Arbor, MI USA.

[Rosland, Ann-Marie] VA Ctr Hlth Equ Res & Promot, Pittsburgh, PA USA.

[Rosland, Ann-Marie] Univ Pittsburgh, Sch Med, Div Gen Internal Med, Pittsburgh, PA USA.

RP Lee, AA (reprint author), VA Ctr Clin Management Res, Ann Arbor, MI 48105 USA.

EM aaronlee@med.umich.edu

FU VA Center for Clinical Management Research; VA Veterans Integrated

Service Network 11 Patient Aligned Care Team Demonstration Laboratory;

Michigan Center for Diabetes Translational Research (National Institute

of Diabetes and Digestive and Kidney Diseases) [P30-DK-092926]

FX This study was supported by the VA Center for Clinical Management

Research, the VA Veterans Integrated Service Network 11 Patient Aligned

Care Team Demonstration Laboratory, and the Michigan Center for Diabetes

Translational Research (National Institute of Diabetes and Digestive and

Kidney Diseases grant P30-DK-092926). J.D.P. is a VA senior Research

Career Scientist.

CR Aikens JE, 2012, DIABETES CARE, V35, P2472, DOI 10.2337/dc12-0181

American Diabetes Association, 2017, Diabetes Care, V40, pS48

Baek RN, 2014, ANN BEHAV MED, V48, P145, DOI 10.1007/s12160-013-9585-4

COHEN S, 1985, PSYCHOL BULL, V98, P310, DOI 10.1037//0033-2909.98.2.310

COHEN S, 2004, SOCIAL SUPPORT MEASU, V59, P676, DOI DOI 10.1093/MED:PSYCH/9780195126709.003.0001

Cramer JA, 2005, DIABETES CARE, V28, P78, DOI 10.2337/diacare.28.1.78

Du YF, 2014, CLIN INTERV AGING, V9, P1963, DOI 10.2147/CIA.S53482

Fisher L, 2008, DIABETIC MED, V25, P1096, DOI 10.1111/j.1464-5491.2008.02533.x

Fisher L, 2014, DIABETIC MED, V31, P764, DOI 10.1111/dme.12428

Fisher L, 2009, DIABETIC MED, V26, P622, DOI 10.1111/j.1464-5491.2009.02730.x

Fisher L, 2008, ANN FAM MED, V6, P246, DOI 10.1370/afm.842

Fisher L, 2013, DIABETES CARE, V36, P2551, DOI 10.2337/dc12-2493

Fisher L, 2012, DIABETES CARE, V35, P259, DOI 10.2337/dc11-1572

Fisher L, 2010, DIABETES CARE, V33, P23, DOI 10.2337/dc09-1238

Funnell MM, 2007, DIABETES SPECTR, V20, P221, DOI DOI 10.2337/DIASPECT.20.4.221

GRIFFITH LS, 1990, INT J PSYCHIAT MED, V20, P365, DOI 10.2190/APH4-YMBG-NVRL-VLWD

Guideline Oversight Group, 2017, TYP 2 DIAB SCREEN TR

Hessler D, 2014, DIABETES CARE, V37, P617, DOI 10.2337/dc13-0762

Hilliard ME, 2016, CURR DIABETES REP, V16, DOI 10.1007/s11892-016-0761-3

Langa KM, 2002, J GERONTOL B-PSYCHOL, V57, pS177, DOI 10.1093/geronb/57.3.S177

Martire LM, 2007, CURR DIR PSYCHOL SCI, V16, P90, DOI 10.1111/j.1467-8721.2007.00482.x

Miller TA, 2013, DIABETES METAB SYNDR, V6, P421, DOI 10.2147/DMSO.S36368

POLONSKY WH, 1995, DIABETES CARE, V18, P754, DOI 10.2337/diacare.18.6.754

Polonsky WH, 2005, DIABETES CARE, V28, P626, DOI 10.2337/diacare.28.3.626

Powers MA, 2015, DIABETES EDUCATOR, V41, P417, DOI 10.1177/0145721715588904

Preacher KJ, 2006, J EDUC BEHAV STAT, V31, P437, DOI 10.3102/10769986031004437

Rosland AM, 2010, CHRONIC ILLN, V6, P22, DOI 10.1177/1742395309354608

Ryan R. M., 1996, HDB SOCIAL SUPPORT F, P249, DOI DOI 10.1007/978-1-4899-1388-3_11

Strandberg RB, 2015, DIABETIC MED, V32, P1304, DOI 10.1111/dme.12781

Strom JL, 2012, CURR DIABETES REP, V12, P769, DOI 10.1007/s11892-012-0317-0

Sullivan M. D., 2012, CLIN DIABETES, V30, P101

Thoits PA, 2011, J HEALTH SOC BEHAV, V52, P145, DOI 10.1177/0022146510395592

Uchino BN, 2009, PERSPECT PSYCHOL SCI, V4, P236, DOI 10.1111/j.1745-6924.2009.01122.x

Wagner JA, 2015, J IMMIGR MINOR HEALT, V17, P566, DOI 10.1007/s10903-013-9948-8

Williams GC, 2005, PATIENT EDUC COUNS, V57, P39, DOI 10.1016/j.pec.2004.04.001

Williams GC, 1998, HEALTH PSYCHOL, V17, P269, DOI 10.1037//0278-6133.17.3.269

WILLIAMS GC, 2006, FAMILIES SYSTEMS HLT, V24, P179, DOI DOI 10.1037/1091-7527.24.2.179

Williams GC, 2007, HEALTH PSYCHOL, V26, P728, DOI 10.1037/0278-6133.26.6.728

Williams GC, 2009, DIABETES EDUCATOR, V35, P484, DOI 10.1177/0145721709333856

Zagarins SE, 2012, J BEHAV MED, V35, P299, DOI 10.1007/s10865-011-9359-z

NR 40

TC 1

Z9 1

U1 6

U2 6

PU AMER DIABETES ASSOC

PI ALEXANDRIA

PA 1701 N BEAUREGARD ST, ALEXANDRIA, VA 22311-1717 USA

SN 0149-5992

EI 1935-5548

J9 DIABETES CARE

JI Diabetes Care

PD JUN 1

PY 2018

VL 41

IS 6

BP 1157

EP 1163

DI 10.2337/dc17-2396

PG 7

WC Endocrinology & Metabolism

SC Endocrinology & Metabolism

GA GG4NI

UT WOS:000432673000020

PM 29599295

DA 2019-01-08

ER

PT J

AU Kumar, S

Moseson, H

Uppal, J

Juusola, JL

AF Kumar, Shefali

Moseson, Heidi

Uppal, Jaspreet

Juusola, Jessie L.

TI A Diabetes Mobile App With In-App Coaching From a Certified Diabetes

Educator Reduces A1C for Individuals With Type 2 Diabetes

SO DIABETES EDUCATOR

LA English

DT Article

ID SELF-MANAGEMENT EDUCATION; GLYCEMIC CONTROL; POSITION STATEMENT;

DISTRESS SCALE; DEPRESSION; ASSOCIATION; MELLITUS; TRIALS; ADULTS; CARE

AB Purpose There are currently many diabetes apps available, but there is limited evidence demonstrating clinical impact. The purpose of this study is to evaluate the impact of a diabetes mobile app with in-app coaching by a certified diabetes educator on glycemic control for individuals with type 2 diabetes.

Methods A 12 week-long single-arm intent-to-treat trial evaluated the impact of a diabetes mobile app and coaching program (One Drop | Mobile With One Drop | Experts), which facilitated tracking of self-care and included an in-app diabetes education program, on A1C for individuals with type 2 diabetes and an A1C 7.5% (58 mmol/mol). An online study platform (Achievement Studies, Evidation Health Inc, San Mateo, CA) was used to screen, consent, and enroll participants; collect study data; and track participants' progress throughout the study. Baseline and study end A1C measurements as well as questionnaire data from participants were collected.

Results Participants (n = 146) were 52 9 years old, 71% female, 25% black or Hispanic, diagnosed with diabetes for 11 +/- 7 years, and with a mean baseline A1C of 9.87% +/- 2.0 (84 mmol/mol). In adjusted repeated measures models, mean A1C improved by -0.86% among study completers (n = 127), -0.96% among active users of the app and coaching program (n = 93), and -1.32% among active users with a baseline A1C 9.0% (75 mmol/mol) (n = 53).

Conclusions This program was associated with a clinically meaningful and significant reduction in A1C and can potentially increase access to effective diabetes self-management education and support for individuals with diabetes.

C1 [Kumar, Shefali; Moseson, Heidi; Uppal, Jaspreet; Juusola, Jessie L.] Evidat Hlth, 167 2nd Ave, San Mateo, CA 94401 USA.

RP Kumar, S (reprint author), Evidat Hlth, 167 2nd Ave, San Mateo, CA 94401 USA.

EM skumar@evidation.com

FU Informed Data Systems, Inc.

FX Informed Data Systems, Inc.

CR Anderson RM, 2003, DIABETES CARE, V26, P1641, DOI 10.2337/diacare.26.5.1641-a

Cahn A, 2018, J DIABETES, V10, P10, DOI 10.1111/1753-0407.12606

Centers for Disease Control and Prevention, 2016, DIABETES

Chrvala CA, 2016, PATIENT EDUC COUNS, V99, P926, DOI 10.1016/j.pec.2015.11.003

de Groot M, 2012, CURR DIABETES REP, V12, P157, DOI 10.1007/s11892-012-0261-z

Eng DS, 2013, PEDIATR DIABETES, V14, P231, DOI 10.1111/pedi.12034

Fisher L, 2008, ANN FAM MED, V6, P246, DOI 10.1370/afm.842

Fisher L, 2012, DIABETES CARE, V35, P259, DOI 10.2337/dc11-1572

Fisher L, 2010, DIABETES CARE, V33, P23, DOI 10.2337/dc09-1238

Hood M, 2016, J BEHAV MED, V39, P981, DOI 10.1007/s10865-016-9765-3

Hou C, 2016, DIABETES CARE, V39, P2089, DOI 10.2337/dc16-0346

Huang ES, 2014, JAMA INTERN MED, V174, P251, DOI 10.1001/jamainternmed.2013.12956

Hunt CW, 2015, WORLD J DIABETES, V6, P225, DOI 10.4239/wjd.v6.i2.225

Kreider KE, 2017, DIABETES THER, V8, P1, DOI 10.1007/s13300-017-0231-1

Lin EHB, 2004, DIABETES CARE, V27, P2154, DOI 10.2337/diacare.27.9.2154

Lustman PJ, 2000, DIABETES CARE, V23, P934, DOI 10.2337/diacare.23.7.934

Polonsky WH, 2005, DIABETES CARE, V28, P626, DOI 10.2337/diacare.28.3.626

Powers MA, 2015, DIABETES EDUCATOR, V41, P417, DOI 10.1177/0145721715588904

Research2Guidance, 2017, MHEALTH APP DEV EC

Rosa C, 2015, CONTEMP CLIN TRIALS, V45, P41, DOI 10.1016/j.cct.2015.07.007

UK Prospective Diabetes Study (UKPDS) Group, 1998, LANCET, V352, P854, DOI DOI 10.1016/S0140-6736(98)07037-8

White IR, 2011, STAT MED, V30, P377, DOI 10.1002/sim.4067

Young-Hyman D, 2016, DIABETES CARE, V39, P2126, DOI 10.2337/dc16-2053

NR 23

TC 2

Z9 2

U1 5

U2 10

PU SAGE PUBLICATIONS INC

PI THOUSAND OAKS

PA 2455 TELLER RD, THOUSAND OAKS, CA 91320 USA

SN 0145-7217

EI 1554-6063

J9 DIABETES EDUCATOR

JI Diabetes Educ.

PD JUN

PY 2018

VL 44

IS 3

BP 225

EP 236

DI 10.1177/0145721718765650

PG 12

WC Endocrinology & Metabolism; Public, Environmental & Occupational Health

SC Endocrinology & Metabolism; Public, Environmental & Occupational Health

GA GG7RB

UT WOS:000432894500002

PM 29575982

DA 2019-01-08

ER

PT J

AU Hagger, V

Hendrieckx, C

Cameron, F

Pouwer, F

Skinner, TC

Speight, J

AF Hagger, Virginia

Hendrieckx, Christel

Cameron, Fergus

Pouwer, Frans

Skinner, Timothy C.

Speight, Jane

TI Diabetes distress is more strongly associated with HbA1c than depressive

symptoms in adolescents with type 1 diabetes: Results from Diabetes

MILES Youth-Australia

SO PEDIATRIC DIABETES

LA English

DT Article

DE adolescent; depression; emotions; hemoglobin A1c; type 1 diabetes

ID GLYCEMIC CONTROL; METABOLIC-CONTROL; CONTROLLED-TRIAL; ADULTS; TIME;

PREDICTORS; DISORDERS; CHILDREN; OUTCOMES; CARE

AB Background: Glycated hemoglobin (HbA1c) is higher during adolescence than at any other life stage. Some research among adolescents indicates that depressive symptoms are associated with suboptimal HbA1c. However, research among adults suggests diabetes distress is a stronger predictor of HbA1c than depressive symptoms.

Objective: To determine the relative contributions of depressive symptoms and diabetes distress to explain the variance in HbA1c among adolescents with type 1 diabetes.

Participants and methods: Diabetes MILES Youth Study respondents aged 13 to 19 years completed questionnaires assessing depressive symptoms (Patient Health Questionnaire for Adolescents: PHQA-8), diabetes distress (Problem Areas in Diabetes-Teen version: PAID-T), and self-reported socio-demographic and clinical variables, including their most recent HbA1c. Stepwise hierarchical multiple regression was conducted to examine the contributions of depressive symptoms and diabetes distress to HbA1c.

Results: Participants (N=450) had a (mean +/- SD) age of 15.7 +/- 1.9 years; diabetes duration of 6.9 +/- 4.3 years; and 38% (n=169) were male. Twenty-one percent (n=96) experienced moderate-to-severe depressive symptoms (PHQA-8 >= 11) and 36% (n=162) experienced high diabetes distress (PAID-T>90). In the final regression model, HbA1c was explained by: diabetes duration (beta =.14, P=.001), self-monitoring of blood glucose (beta=-.20, P<.001), and diabetes distress (beta=.30, P<.001). Following the addition of diabetes distress, depressive symptoms were no longer significantly associated with HbA1c (P=.551). The final model explained 18% of the variance in HbA1c.

Conclusions: Consistent with evidence from studies among adults, diabetes distress mediated the relationship between depressive symptoms and HbA1c among adolescents with type 1 diabetes. These findings suggest that clinicians need to be aware of diabetes distress.

C1 [Hagger, Virginia; Hendrieckx, Christel; Speight, Jane] Deakin Univ, Sch Psychol, Ctr Social & Early Emot Dev, Geelong, Vic, Australia.

[Hagger, Virginia; Hendrieckx, Christel; Speight, Jane] Australian Ctr Behav Res Diabet, Diabet Victoria, Melbourne, Vic, Australia.

[Cameron, Fergus] Royal Childrens Hosp, Murdoch Childrens Res Inst, Melbourne, Vic, Australia.

[Pouwer, Frans] Univ Southern Denmark, Dept Psychol, Odense, Denmark.

[Skinner, Timothy C.] Charles Darwin Univ, Sch Psychol & Clin Sci, Darwin, NT, Australia.

[Skinner, Timothy C.] Baker Heart & Diabet Inst, Melbourne, Vic, Australia.

[Speight, Jane] AHP Res, Hornchurch, England.

RP Hagger, V (reprint author), Australian Ctr Behav Res Diabet, 570 Elizabeth St, Melbourne, Vic 3000, Australia.

EM vhagger@acbrd.org.au

OI Speight, Jane/0000-0002-1204-6896; Hagger, Virginia/0000-0003-3845-2814;

Skinner, Timothy/0000-0002-0018-6963

FU National Diabetes Services Scheme; Deakin University; Diabetes Victoria

FX National Diabetes Services Scheme; Deakin University; Diabetes Victoria

CR AMIEL SA, 1986, NEW ENGL J MED, V315, P215, DOI 10.1056/NEJM198607243150402

Anderson BJ, 2009, FAM SYST HEALTH, V27, P141, DOI 10.1037/a0015759

BLANZ BJ, 1993, DIABETES CARE, V16, P1579, DOI 10.2337/diacare.16.12.1579

Bryden KS, 2003, DIABETES CARE, V26, P1052, DOI 10.2337/diacare.26.4.1052

Butwicka A, 2012, DIABETES CARE, V35, P2133, DOI 10.2337/dc12-0160

Christie D, 2016, BMJ OPEN DIAB RES CA, V4, DOI 10.1136/bmjdrc-2015-000165

Corathers SD, 2013, PEDIATRICS, V132, pE1395, DOI 10.1542/peds.2013-0681

Cuijpers P, 2008, EUR CHILD ADOLES PSY, V17, P32, DOI 10.1007/s00787-007-0631-2

De Wit M, 2008, DIABETES CARE, V31, P1521, DOI 10.2337/dc08-0394

Ellis DA, 2005, PEDIATRICS, V116, pE826, DOI 10.1542/peds.2005-0638

Fisher L, 2016, DIABETIC MED, V33, P1590, DOI 10.1111/dme.12973

Fisher L, 2016, J DIABETES COMPLICAT, V30, P1123, DOI 10.1016/j.jdiacomp.2016.03.032

Hackorth NJ, 2013, AUST PSYCHOL, V48, P360, DOI [DOI 10.1111/AP.12007, 10.1111/ap.12007]

Hagger V, 2017, DIABETES CARE, V40, P1462, DOI 10.2337/dc17-0441

Hagger Virginia, 2016, BMC Psychol, V4, P42, DOI 10.1186/s40359-016-0149-9

Hagger V, 2016, CURR DIABETES REP, V16, DOI 10.1007/s11892-015-0694-2

Handley TE, 2016, DIABETIC MED, V33, P1582, DOI 10.1111/dme.13022

Heiervang E, 2011, SOC PSYCH PSYCH EPID, V46, P69, DOI 10.1007/s00127-009-0171-9

Helgeson VS, 2010, J PEDIATR PSYCHOL, V35, P1161, DOI 10.1093/jpepsy/jsq011

Helgeson VS, 2009, J PEDIATR PSYCHOL, V34, P254, DOI 10.1093/jpepsy/jsn079

Hermanns N, 2013, DIABETES RES CLIN PR, V102, P149, DOI 10.1016/j.diabres.2013.10.009

Herzer M, 2010, J PEDIATR PSYCHOL, V35, P415, DOI 10.1093/jpepsy/jsp063

Hessler DM, 2017, DIABETIC MED, V34, P1228, DOI 10.1111/dme.13381

Hood KK, 2006, DIABETES CARE, V29, P1389, DOI 10.2337/dc06-0087

IDF ISP AD, 2011, GLOB IDF ISPAD GUID

Iturralde E, 2017, HEALTH PSYCHOL, V36, P236, DOI 10.1037/hea0000445

Kokkonen J, 1997, NORD J PSYCHIAT, V51, P165, DOI 10.3109/08039489709109091

Kroenke K, 2009, J AFFECT DISORDERS, V114, P163, DOI 10.1016/j.jad.2008.06.026

Lasaite L, 2016, J DIABETES COMPLICAT, V30, P1500, DOI 10.1016/j.jdiacomp.2016.08.013

Levine BS, 2001, J PEDIATR-US, V139, P197, DOI 10.1067/mpd.2001.116283

Lewin AB, 2009, J PEDIATR PSYCHOL, V34, P999, DOI 10.1093/jpepsy/jsp032

McGrady Meghan E, 2010, Diabetes Res Clin Pract, V88, pe35, DOI 10.1016/j.diabres.2010.03.025

Miller KM, 2015, DIABETES CARE, V38, P971, DOI 10.2337/dc15-0078

Plener PL, 2015, PEDIATR DIABETES, V16, P58, DOI 10.1111/pedi.12130

POLONSKY WH, 1995, DIABETES CARE, V18, P754, DOI 10.2337/diacare.18.6.754

Richardson LP, 2014, JAMA-J AM MED ASSOC, V312, P809, DOI 10.1001/jama.2014.9259

Richardson LP, 2010, PEDIATRICS, V126, P1117, DOI 10.1542/peds.2010-0852

Serlachius A, 2014, J HEALTH PSYCHOL, V21, P1157

Snoek FJ, 2015, LANCET DIABETES ENDO, V3, P450, DOI 10.1016/S2213-8587(15)00135-7

van Bastelaar KMP, 2010, DIABETIC MED, V27, P798, DOI 10.1111/j.1464-5491.2010.03025.x

Weissberg-Benchell J, 2011, PEDIATR DIABETES, V12, P341, DOI 10.1111/j.1399-5448.2010.00720.x

Wisting L, 2016, BMJ OPEN DIAB RES CA, V4, DOI 10.1136/bmjdrc-2016-000203

NR 42

TC 1

Z9 1

U1 4

U2 6

PU WILEY

PI HOBOKEN

PA 111 RIVER ST, HOBOKEN 07030-5774, NJ USA

SN 1399-543X

EI 1399-5448

J9 PEDIATR DIABETES

JI Pediatr. Diabetes

PD JUN

PY 2018

VL 19

IS 4

BP 840

EP 847

DI 10.1111/pedi.12641

PG 8

WC Endocrinology & Metabolism; Pediatrics

SC Endocrinology & Metabolism; Pediatrics

GA GF5SD

UT WOS:000432027100036

PM 29383803

DA 2019-01-08

ER

PT J

AU Chew, BH

Vos, RC

Stellato, RK

Ismail, M

Rutten, GEHM

AF Chew, B. H.

Vos, R. C.

Stellato, R. K.

Ismail, M.

Rutten, G. E. H. M.

TI The effectiveness of an emotion-focused educational programme in

reducing diabetes distress in adults with Type 2 diabetes mellitus

(VEMOFIT): a cluster randomized controlled trial

SO DIABETIC MEDICINE

LA English

DT Article

ID QUALITY-OF-LIFE; SELF-CARE; PSYCHOMETRIC PROPERTIES; GLYCEMIC CONTROL;

HEALTH; SCALE; DEPRESSION; MANAGEMENT; INTERVENTIONS; METAANALYSIS

AB AimsTo evaluate the effectiveness of a brief, value-based emotion-focused educational programme (VEMOFIT) in Malay adults with Type 2 diabetes mellitus compared with a programme of active listening to participants' emotional experiences, social support and their opinion on the health clinic diabetes care services (attention control).

MethodsMalay adults with severe diabetes distress [Diabetes Distress Scale (DDS-17) mean score 3] were included. VEMOFIT consisted of four biweekly group sessions, a booster session after 3 months and a follow-up 6 months post intervention. The attention control programme consisted of three sessions over the same period. Outcomes included diabetes distress, depressive symptoms, self-efficacy and disease control. Required total sample size was 165.

ResultsParticipants (n = 124) were randomized to either VEMOFIT (n = 53) or the attention control programme (n = 71). Participants had a mean (sd) age of 55.7 (9.7) years, median diabetes duration of 7.0 (8.0) years and mean HbA(1c) level of 82 mmol/mol (9.7%). The mean DDS-17 level decreased significantly in both the VEMOFIT and the attention control programmes (3.4 to 2.9 vs. 3.1 to 2.7, respectively). The adjusted between-group DDS-17 difference was not significant [-0.01, 95% confidence interval (CI) -0.38, 0.35]. The proportion of individuals with severe diabetes distress decreased in both groups, from 89% to 47% vs. 69% to 39% (odds ratio 0.88; 95% CI 0.26, 2.90). Other outcomes did not differ between groups.

ConclusionsBoth interventions decreased diabetes distress significantly. The theory-based VEMOFIT programme was not superior to the attention control programme. The latter approach is a simpler way to decrease severe diabetes distress (Trial registration: NCT02730078; NMRR-15-1144-24803).

C1 [Chew, B. H.] Univ Putra Malaysia, Dept Family Med, Serdang, Selangor, Malaysia.

[Chew, B. H.; Vos, R. C.; Stellato, R. K.; Rutten, G. E. H. M.] Univ Utrecht, Univ Med Ctr Utrecht, Julius Ctr Hlth Sci & Primary Care, Utrecht, Netherlands.

[Ismail, M.] Hlth Clin Seremban 2, Seremban, Negeri Sembilan, Malaysia.

RP Chew, BH (reprint author), Univ Putra Malaysia, Dept Family Med, Serdang, Selangor, Malaysia.; Chew, BH (reprint author), Univ Utrecht, Univ Med Ctr Utrecht, Julius Ctr Hlth Sci & Primary Care, Utrecht, Netherlands.

EM chewboonhow@upm.edu.my

OI Chew, Boon How/0000-0002-8627-6248

FU Malaysian MOH-NIH Research Grant (MRG); Ministry of Higher Education

Malaysia

FX The trial was supported by the Malaysian MOH-NIH Research Grant (MRG)

and Ministry of Higher Education Malaysia. These funding sources had no

role in the design or execution of this study.

CR Aikens JE, 2012, DIABETES CARE, V35, P2472, DOI 10.2337/dc12-0181

Berry E, 2017, PSYCHOL HEALTH MED, V22, P1118, DOI 10.1080/13548506.2017.1281976

Broadbent E, 2015, PSYCHOL HEALTH, V30, P1361, DOI 10.1080/08870446.2015.1070851

Chew B H, 2015, Malays Fam Physician, V10, P22

Chew BH, 2017, COCHRANE DB SYST REV, V9

Chew BH, 2017, BMC MED RES METHODOL, V17, DOI 10.1186/s12874-017-0394-5

Chew BH, 2017, BMC ENDOCR DISORD, V17, DOI 10.1186/s12902-017-0172-8

Chew BH, 2016, PLOS ONE, V11, DOI 10.1371/journal.pone.0152095

Chew BH, 2015, HEALTH QUAL LIFE OUT, V13, DOI 10.1186/s12955-015-0384-4

Davies MJ, 2008, BRIT MED J, V336, P491, DOI 10.1136/bmj.39474.922025.BE

Fisher L, 2014, DIABETIC MED, V31, P764, DOI 10.1111/dme.12428

Fisher L, 2012, DIABETES CARE, V35, P259, DOI 10.2337/dc11-1572

Fisher L, 2010, DIABETES CARE, V33, P1034, DOI 10.2337/dc09-2175

Harper A, 1998, PSYCHOL MED, V28, P551

Hasanah C I, 2003, Med J Malaysia, V58, P79

Kroenke K, 2001, J GEN INTERN MED, V16, P606, DOI 10.1046/j.1525-1497.2001.016009606.x

Martinez K, 2018, PSYCHOL HEALTH MED, V23, P171, DOI 10.1080/13548506.2017.1339892

NICE guideline NG 28, 2015, TYP 2 DIAB AD MAN

Ogbera A, 2011, J DIABETES, V3, P348, DOI 10.1111/j.1753-0407.2011.00156.x

Polonsky WH, 2005, DIABETES CARE, V28, P626, DOI 10.2337/diacare.28.3.626

RADLOFF L S, 1977, Applied Psychological Measurement, V1, P385, DOI 10.1177/014662167700100306

Sharoni SKA, 2012, NURS HEALTH SCI, V14, P38, DOI 10.1111/j.1442-2018.2011.00658.x

Sherina M S, 2012, Med J Malaysia, V67, P309

Skevington SM, 2004, QUAL LIFE RES, V13, P299, DOI 10.1023/B:QURE.0000018486.91360.00

Sturt J, 2015, INT DIAB NURS, V12, P40, DOI DOI 10.1179/2057332415Y.0000000004

Toobert DJ, 2000, DIABETES CARE, V23, P943, DOI 10.2337/diacare.23.7.943

van der Bijl J, 1999, J ADV NURS, V30, P352, DOI 10.1046/j.1365-2648.1999.01077.x

Yi JP, 2008, BRIT J HEALTH PSYCH, V13, P311, DOI 10.1348/135910707X186994

Zeh P, 2012, DIABETIC MED, V29, P1237, DOI 10.1111/j.1464-5491.2012.03701.x

Zhao FF, 2017, J ADV NURS, V73, P812, DOI 10.1111/jan.13163

NR 30

TC 1

Z9 1

U1 2

U2 7

PU WILEY

PI HOBOKEN

PA 111 RIVER ST, HOBOKEN 07030-5774, NJ USA

SN 0742-3071

EI 1464-5491

J9 DIABETIC MED

JI Diabetic Med.

PD JUN

PY 2018

VL 35

IS 6

BP 750

EP 759

DI 10.1111/dme.13615

PG 10

WC Endocrinology & Metabolism

SC Endocrinology & Metabolism

GA GF5DD

UT WOS:000431985000010

PM 29505098

DA 2019-01-08

ER

PT J

AU Lloyd, CE

Nouwen, A

Sartorius, N

Ahmed, HU

Alvarez, A

Bahendeka, S

Basangwa, D

Bobrov, AE

Boden, S

Bulgari, V

Burti, L

Chaturvedi, SK

Cimino, LC

Gaebel, W

de Girolamo, G

Gondek, TM

de Braude, MG

Guntupalli, A

Heinze, MG

Ji, L

Hong, X

Khan, A

Kiejna, A

Kokoszka, A

Kamala, T

Lalic, NM

Tosevski, DL

Mankovsky, B

Li, M

Musau, A

Mussig, K

Ndetei, D

Rabbani, G

Srikanta, SS

Starostina, EG

Shevchuk, M

Taj, R

Vukovic, O

Wolwer, W

Xin, Y

AF Lloyd, C. E.

Nouwen, A.

Sartorius, N.

Ahmed, H. U.

Alvarez, A.

Bahendeka, S.

Basangwa, D.

Bobrov, A. E.

Boden, S.

Bulgari, V.

Burti, L.

Chaturvedi, S. K.

Cimino, L. C.

Gaebel, W.

de Girolamo, G.

Gondek, T. M.

Guinzbourg de Braude, M.

Guntupalli, A.

Heinze, M. G.

Ji, L.

Hong, X.

Khan, A.

Kiejna, A.

Kokoszka, A.

Kamala, T.

Lalic, N. M.

Tosevski, D. Lecic

Mankovsky, B.

Li, M.

Musau, A.

Muessig, K.

Ndetei, D.

Rabbani, G.

Srikanta, S. S.

Starostina, E. G.

Shevchuk, M.

Taj, R.

Vukovic, O.

Woelwer, W.

Xin, Y.

TI Prevalence and correlates of depressive disorders in people with Type 2

diabetes: results from the International Prevalence and Treatment of

Diabetes and Depression (INTERPRET-DD) study, a collaborative study

carried out in 14 countries

SO DIABETIC MEDICINE

LA English

DT Article

ID NEUROPSYCHIATRIC INTERVIEW MINI; EMOTIONAL-PROBLEMS; DISTRESS;

EPIDEMIOLOGY; METAANALYSIS; ASSOCIATION; SYMPTOMS; MELLITUS; VALIDITY;

CARE

AB AimsTo assess the prevalence and management of depressive disorders in people with Type 2 diabetes in different countries.

MethodsPeople with diabetes aged 18-65 years and treated in outpatient settings were recruited in 14 countries and underwent a psychiatric interview. Participants completed the Patient Health Questionnaire and the Problem Areas in Diabetes scale. Demographic and medical record data were collected.

ResultsA total of 2783 people with Type 2 diabetes (45.3% men, mean duration of diabetes 8.8 years) participated. Overall, 10.6% were diagnosed with current major depressive disorder and 17.0% reported moderate to severe levels of depressive symptomatology (Patient Health Questionnaire scores >9). Multivariable analyses showed that, after controlling for country, current major depressive disorder was significantly associated with gender (women) (P<0.0001), a lower level of education (P<0.05), doing less exercise (P<0.01), higher levels of diabetes distress (P<0.0001) and a previous diagnosis of major depressive disorder (P<0.0001). The proportion of those with either current major depressive disorder or moderate to severe levels of depressive symptomatology who had a diagnosis or any treatment for their depression recorded in their medical records was extremely low and non-existent in many countries (0-29.6%).

ConclusionsOur international study, the largest of this type ever undertaken, shows that people with diabetes frequently have depressive disorders and also significant levels of depressive symptoms. Our findings indicate that the identification and appropriate care for psychological and psychiatric problems is not the norm and suggest a lack of the comprehensive approach to diabetes management that is needed to improve clinical outcomes.

C1 [Lloyd, C. E.; Boden, S.; Guntupalli, A.] Open Univ, Sch Hlth Wellbeing & Social Care, Milton Keynes, Bucks, England.

[Nouwen, A.] Middlesex Univ, Dept Psychol, London, England.

[Sartorius, N.] Assoc Improvement Mental Hlth Programmes, Geneva, Switzerland.

[Ahmed, H. U.] Natl Inst Mental Hlth NIMH, Child Adolescent & Family Psychiat, Dhaka, Bangladesh.

[Alvarez, A.; Guinzbourg de Braude, M.] Hosp Italiano Buenos Aires, Buenos Aires, DF, Argentina.

[Bahendeka, S.; Basangwa, D.] Uganda Martyrs Univ, Mother Kevin Post Grad Med Sch, Kampala, Uganda.

[Bobrov, A. E.] Fed Med Res Ctr Psychiat & Narcol, Moscow, Russia.

[Bulgari, V.; de Girolamo, G.] St John God Clin Res Ctr, Psychiat Epidemiol & Evaluat Unit, Brescia, Italy.

[Bulgari, V.] Univ Cattolica Sacro Cuore, PhD Sch Psychol, Milan, Italy.

[Burti, L.] Univ Verona, Dept Neurosci Biomed & Movement Sci, Verona, Italy.

[Chaturvedi, S. K.] Natl Inst Mental Hlth & Neurosci, Bangalore, Karnataka, India.

[Cimino, L. C.] Indiana Univ, Indiana, PA USA.

[Gaebel, W.; Woelwer, W.] Heinrich Heine Univ, Fac Med, Dept Psychiat & Psychotherapy, Dusseldorf, Germany.

[Gondek, T. M.] Wroclaw Med Univ, Dept Psychiat, Wroclaw, Poland.

[Heinze, M. G.] Univ Nacl Autonoma Mexico, Mexico City, DF, Mexico.

[Ji, L.] Peking Univ, Peoples Hosp, Beijing, Peoples R China.

[Hong, X.] Chinese Acad Med Sci, Dept Psychol Med, Beijing, Peoples R China.

[Hong, X.] Peking Union Med Coll, Beijing, Peoples R China.

[Khan, A.; Taj, R.] Pakistan Inst Med Sci, Islamabad, Pakistan.

[Kiejna, A.] Univ Lower Silesia, Wroclaw, Poland.

[Kiejna, A.] Med Univ, Dept Psychiat, Wroclaw, Poland.

[Kokoszka, A.] Med Univ Warsaw, Dept Psychiat 2, Warsaw, Poland.

[Kamala, T.] Diabet Ctr, Bangalore, Karnataka, India.

[Kamala, T.; Srikanta, S. S.] Jnana Sanjeevini Med Ctr, Bangalore, Karnataka, India.

[Lalic, N. M.] Univ Belgrade, Serbian Acad Sci & Arts, Clin Endocrinol, Sch Med, Belgrade, Serbia.

[Tosevski, D. Lecic; Vukovic, O.] Univ Belgrade, Serbian Acad Sci & Arts, Inst Mental Hlth, Sch Med, Belgrade, Serbia.

[Mankovsky, B.] Natl Med Acad Postgrad Educ, Dept Diabetol, Kiev, Ukraine.

[Li, M.] Peking Univ, Sch Nursing, Beijing, Peoples R China.

[Musau, A.] Africa Mental Hlth Fdn, Nairobi, Kenya.

[Muessig, K.] German Diabet Ctr, Inst Clin Diabetol, Dusseldorf, Germany.

[Muessig, K.] Heinrich Heine Univ Dusseldorf, Leibniz Ctr Diabet Res, Dusseldorf, Germany.

[Muessig, K.] Heinrich Heine Univ, Fac Med, Dept Endocrinol & Diabetol, Dusseldorf, Germany.

[Muessig, K.] German Ctr Diabet Res DZD, Munich, Germany.

[Ndetei, D.] Univ Nairobi, Africa Mental Hlth Fdn, Nairobi, Kenya.

[Rabbani, G.] Popular Med Coll, Dhaka, Bangladesh.

[Srikanta, S. S.] Samatvam Endocrinol Diabet Ctr, Bangalore, Karnataka, India.

[Starostina, E. G.] Moscow Reg Clin & Res Inst, Dept Endocrinol, Moscow, Russia.

[Shevchuk, M.] Natl Med Acad Postgrad Educ, Dept Diabetol, Kiev, Ukraine.

[Xin, Y.] Peking Univ, Clin Res Ctr, Natl Clin Res Ctr Mental Disorders,Hosp 6, Inst Mental Hlth,Key Lab Mental Hlth,Minist Hlth, Beijing, Peoples R China.

RP Lloyd, CE (reprint author), Open Univ, Sch Hlth Wellbeing & Social Care, Milton Keynes, Bucks, England.

EM Cathy.Lloyd@open.ac.uk

RI Kokoszka, Andrzej/O-7454-2018

OI Kokoszka, Andrzej/0000-0003-4518-5494; Mankovsky,

Boris/0000-0001-8289-3604; de Girolamo, Giovanni/0000-0002-1611-8324;

Lloyd, Cathy/0000-0002-8863-3069

FU AIMH; Eli Lilly company; Sanofi company

FX This study was carried out under the aegis of the Association for the

Improvement of Mental Health Programmes (AIMH) (and supported by AIMH

and the collaborating institutions). The meetings of the investigators

and data entry were in part supported by unrestricted grants by Eli

Lilly and Sanofi companies.

CR Ali S, 2006, DIABETIC MED, V23, P1165, DOI 10.1111/j.1464-5491.2006.01943.x

[Anonymous], 2017, Diabetes Care, V40, pS4

Ballinger GA, 2004, ORGAN RES METHODS, V7, P127, DOI 10.1177/1094428104263672

Baumeister H, 2012, COCHRANE DB SYST REV, V12, DOI DOI 10.1002/14651858

Chan J, 2010, DEPRESSION DIABETES, P143

de Groot M, 2016, DIABETES CARE, V39, P2174, DOI 10.2337/dc16-1145

Diabetes UK, 15 HEALTHC ESS CAR Y

Fisher L, 2014, DIABETIC MED, V31, P764, DOI 10.1111/dme.12428

Gonzalez JS, 2008, DIABETIC MED, V25, P1102, DOI 10.1111/j.1464-5491.2008.02535.x

Hermanns N, 2006, DIABETOLOGIA, V49, P469, DOI 10.1007/s00125-005-0094-2

Hosmer D.W., 2000, APPL LOGISTIC REGRES

Hossain MD, 2014, BMC PSYCHIATRY, V14, DOI 10.1186/s12888-014-0216-9

Ishizawa K, 2016, J DIABETES COMPLICAT, V30, P597, DOI 10.1016/j.jdiacomp.2016.02.004

Kirmayer LJ, 2017, SOC SCI MED, V183, P163, DOI 10.1016/j.socscimed.2017.04.034

Kroenke K, 2001, J GEN INTERN MED, V16, P606, DOI 10.1046/j.1525-1497.2001.016009606.x

Lloyd CE, 2015, DIABETIC MED, V32, P925, DOI 10.1111/dme.12719

Lloyd CE, 2012, J AFFECT DISORDERS, V142, pS22, DOI 10.1016/S0165-0327(12)70005-8

Lysy Z, 2008, DIABETIC MED, V25, P1133, DOI 10.1111/j.1464-5491.2008.02545.x

Mander A, 2007, HOTDECK STATA MODULE

Mendenhall E, 2015, SOC SCI MED, V146, P11, DOI 10.1016/j.socscimed.2015.10.015

Mommersteeg PMC, 2013, DIABETIC MED, V30, pE208, DOI 10.1111/dme.12193

Nefs G, 2012, DIABETOLOGIA, V55, P608, DOI 10.1007/s00125-011-2411-2

Nicolucci A, 2013, DIABETIC MED, V30, P767, DOI 10.1111/dme.12245

Nouwen A, 2015, DIABETIC MED, V32, P1261, DOI 10.1111/dme.12863

Nouwen A, 2010, DIABETOLOGIA, V53, P2480, DOI 10.1007/s00125-010-1874-x

Petrak F, 2015, LANCET DIABETES ENDO, V3, P472, DOI 10.1016/S2213-8587(15)00045-5

POLONSKY WH, 1995, DIABETES CARE, V18, P754, DOI 10.2337/diacare.18.6.754

Pouwer F, 2006, PATIENT EDUC COUNS, V60, P235, DOI 10.1016/j.pec.2005.01.009

Roy T, 2012, J AFFECT DISORDERS, V142, pS8, DOI 10.1016/S0165-0327(12)70004-6

RUBIN DB, 1986, J AM STAT ASSOC, V81, P366, DOI 10.2307/2289225

Sheehan DV, 1997, EUR PSYCHIAT, V12, P232, DOI 10.1016/S0924-9338(97)83297-X

Sheehan DV, 1998, J CLIN PSYCHIAT, V59, P34, DOI 10.4088/JCP.09m05305whi

Snoek FJ, 2015, LANCET DIABETES ENDO, V3, P450, DOI 10.1016/S2213-8587(15)00135-7

Van der Feltz-Cornelis CM, 2010, GEN HOSP PSYCHIAT, V32, P380, DOI 10.1016/j.genhosppsych.2010.03.011

Van Dornan FEP, 2013, PLOS ONE, V8, pe57

World Health Organization, 2006, DEF DIAGN DIAB MELL

NR 36

TC 3

Z9 3

U1 16

U2 21

PU WILEY

PI HOBOKEN

PA 111 RIVER ST, HOBOKEN 07030-5774, NJ USA

SN 0742-3071

EI 1464-5491

J9 DIABETIC MED

JI Diabetic Med.

PD JUN

PY 2018

VL 35

IS 6

BP 760

EP 769

DI 10.1111/dme.13611

PG 10

WC Endocrinology & Metabolism

SC Endocrinology & Metabolism

GA GF5DD

UT WOS:000431985000011

PM 29478265

DA 2019-01-08

ER

PT J

AU Ju, C

Shi, R

Yao, L

Ye, X

Jia, M

Han, J

Yang, T

Lu, Q

Jin, H

Cai, X

Yuan, S

Xie, B

Yu, X

Coufal, MM

Fisher, EB

Sun, Z

AF Ju, C.

Shi, R.

Yao, L.

Ye, X.

Jia, M.

Han, J.

Yang, T.

Lu, Q.

Jin, H.

Cai, X.

Yuan, S.

Xie, B.

Yu, X.

Coufal, M. M.

Fisher, E. B.

Sun, Z.

TI Effect of peer support on diabetes distress: a cluster randomized

controlled trial

SO DIABETIC MEDICINE

LA English

DT Article

ID SELF-MANAGEMENT EDUCATION; GLYCEMIC CONTROL; ADULTS; CARE; DEPRESSION;

COMMUNITY; OUTCOMES

AB AimTo investigate whether peer support would reduce diabetes distress and improve glycaemic control when added to usual diabetes education among adults with Type 2 diabetes in China.

MethodsWe conducted a cluster randomized trial involving 400 adults with Type 2 diabetes from eight communities in Nanjing. All participants received usual education for an average of 2 h each month from physicians, certified diabetes educators, dieticians, psychologists and podiatric nurses. Peer support was led by trained peer leaders and included diabetes knowledge- and skills-sharing at least once a month, as well as peer-to-peer communication. The primary outcome was diabetes distress measured using the Diabetes Distress Scale at 12 months. Secondary outcomes included fasting plasma glucose, 2-h postprandial glucose and HbA(1c) concentration. Outcome data were collected from all participants at baseline, 6 months and 12 months.

ResultsFrom 2012 to 2013, there were 200 participants in each study arm at baseline. Compared with the usual education arm, the peer support with usual education arm had greater reductions in regimen-related distress (1.4 0.6 vs 1.2 0.4; P=0.004) and total distress (1.3 0.4 vs 1.2 0.3; P=0.038) at 6 months. At 12 months, the scores for emotional burden (1.2 +/- 0.3 vs 1.4 +/- 0.6; P=0.002), physician-related distress (1.1 +/- 0.3 vs 1.3 +/- 0.4; P=0.001) and total scores (1.2 +/- 0.3 vs 1.3 +/- 0.4; P=0.002) were significantly lower in the peer support with usual education arm than in the usual education arm. Fasting plasma glucose levels were lower in the peer support with usual education arm than in the usual education arm at 6 months (7.5 +/- 1.95 vs 8.0 +/- 2.2; P=0.044) and 12 months (7.0 +/- 2.3 vs 7.6 +/- 1.5; P=0.008).

ConclusionsBeyond the benefits of usual education, peer support was effective in reducing diabetes distress for Type 2 diabetes mellitus. (Clinical Trials Registry no: NCT02119572)

C1 [Ju, C.; Shi, R.; Yao, L.; Ye, X.; Jia, M.; Han, J.; Yang, T.; Lu, Q.; Jin, H.; Cai, X.; Yuan, S.; Xie, B.; Sun, Z.] Zhongda Hosp, Dept Endocrinol, Sch Med, Inst Diabet, Nanjing, Jiangsu, Peoples R China.

[Yu, X.] Southeast Univ, Sch Publ Hlth, Dept Epidemiol & Biostat, Nanjing, Jiangsu, Peoples R China.

[Coufal, M. M.] Asian Ctr Hlth Educ, Plano, AS USA.

[Fisher, E. B.] Univ N Carolina, Gillings Sch Global Publ Hlth, Dept Hlth Behav, Chapel Hill, NC USA.

RP Sun, Z (reprint author), Zhongda Hosp, Dept Endocrinol, Sch Med, Inst Diabet, Nanjing, Jiangsu, Peoples R China.

EM sunzilin1963@126.com

FU Jiangsu Provincial Special Programme of Medical Science [BK2014010]

FX This study was supported by the Jiangsu Provincial Special Programme of

Medical Science (BK2014010).

CR Aghili R, 2016, CAN J DIABETES, V40, P424, DOI 10.1016/j.jcjd.2016.02.014

Brownson CA, 2009, PATIENT, V2, P5, DOI 10.2165/01312067-200902010-00002

Campbell MK, 2012, BMJ-BRIT MED J, V345, DOI 10.1136/bmj.e5661

Dale JR, 2012, DIABETIC MED, V29, P1361, DOI 10.1111/j.1464-5491.2012.03749.x

Dennis CL, 2003, INT J NURS STUD, V40, P321, DOI 10.1016/S0020-7489(02)00092-5

Due-Christensen M, 2016, PATIENT EDUC COUNS, V99, P1978, DOI 10.1016/j.pec.2016.07.023

Fisher Edwin B, 2017, Clin Diabetes Endocrinol, V3, P4, DOI 10.1186/s40842-017-0042-3

Fisher L, 2008, ANN FAM MED, V6, P246, DOI 10.1370/afm.842

Fisher L, 2007, DIABETES CARE, V30, P542, DOI 10.2337/dc06-1614

Heisler M, 2010, ANN INTERN MED, V153, P507, DOI 10.7326/0003-4819-153-8-201010190-00007

Heisler M, 2010, FAM PRACT, V27, pI23, DOI 10.1093/fampra/cmp003

Kong Lulu, 2013, J Biomed Res, V27, P380, DOI 10.7555/JBR.27.20120112

Lustman PJ, 2000, DIABETES CARE, V23, P934, DOI 10.2337/diacare.23.7.934

Norris SL, 2001, DIABETES CARE, V24, P561, DOI 10.2337/diacare.24.3.561

Peyrot M, 2008, DIABETES EDUCATOR, V34, P90, DOI 10.1177/0145721707312399

Polonsky WH, 2005, DIABETES CARE, V28, P626, DOI 10.2337/diacare.28.3.626

Powers MA, 2017, DIABETES EDUCATOR, V43, P40, DOI 10.1177/0145721716689694

Schulz KF, 2010, PLOS MED, V7, DOI 10.1371/journal.pmed.1000251

Tang TS, 2015, DIABETIC MED, V32, P1077, DOI 10.1111/dme.12655

Tang TS, 2014, DIABETES CARE, V37, P1525, DOI 10.2337/dc13-2161

Tang TS, 2005, DIABETES EDUCATOR, V31, P91, DOI 10.1177/0145721704273231

Whittle J, 2014, JAMA INTERN MED, V174, P982, DOI 10.1001/jamainternmed.2014.654

Xu Y, 2013, JAMA-J AM MED ASSOC, V310, P948, DOI 10.1001/jama.2013.168118

Zhang XX, 2016, PATIENT EDUC COUNS, V99, P186, DOI 10.1016/j.pec.2015.09.006

NR 24

TC 1

Z9 1

U1 5

U2 9

PU WILEY

PI HOBOKEN

PA 111 RIVER ST, HOBOKEN 07030-5774, NJ USA

SN 0742-3071

EI 1464-5491

J9 DIABETIC MED

JI Diabetic Med.

PD JUN

PY 2018

VL 35

IS 6

BP 770

EP 775

DI 10.1111/dme.13625

PG 6

WC Endocrinology & Metabolism

SC Endocrinology & Metabolism

GA GF5DD

UT WOS:000431985000012

PM 29574995

DA 2019-01-08

ER

PT J

AU Lum, ZK

Tsou, KYK

Lee, JYC

AF Lum, Z. K.

Tsou, K. Y. K.

Lee, J. Y-C

TI Mediators of medication adherence and glycaemic control and their

implications for direct outpatient medical costs: a cross-sectional

study

SO DIABETIC MEDICINE

LA English

DT Article

ID TYPE-2 DIABETES-MELLITUS; SELF-MANAGEMENT; DISTRESS; OUTCOMES; MODEL;

SINGAPORE; BARRIERS; ILLNESS; ADULTS

AB AimsTo investigate the effects of diabetes-related distress and perception of hyperglycaemia on self-reported medication adherence and glycaemic control, as measured by HbA(1c), and to compare the cost outcomes in patients with sub-optimally vs uncontrolled Type 2 diabetes mellitus.

MethodsWe conducted a retrospective cross-sectional study that involved the review of a chronic disease database in Singapore. Data on clinical characteristics, diabetes-related distress, perception of hyperglycaemia, self-reported medication adherence and costs were obtained from the database. Mediation analyses were conducted using a linear regression-based approach. A final path model was built to illustrate the sequential mediating effects of diabetes-related distress and perception on the association of medication adherence and HbA(1c) concentration.

ResultsDiabetes-related distress and perception of hyperglycaemia were significantly associated with medication adherence and HbA(1c) concentration. Mediation analyses showed a significant indirect effect of diabetes-related distress and perception of hyperglycaemia on medication adherence and HbA(1c) concentration. People with uncontrolled diabetes were found to incur significantly higher total direct medical costs than those with sub-optimally controlled diabetes (P = 0.034), with medication cost as the main cost driver (66.6%).

ConclusionsIdentifying the influence of the sequential mediating effects of distress and perception was important in understanding the pathway between medication adherence and glycaemic control. This suggests the importance of a team-based approach to address these mediators and thus improve glycaemic control. Poor glycaemic control was also found to be associated with higher direct medical costs.

C1 [Lum, Z. K.; Lee, J. Y-C] Natl Univ Singapore, Dept Pharm, Fac Sci, Singapore, Singapore.

[Tsou, K. Y. K.] Natl Univ Polyclin, Singapore, Singapore.

RP Lee, JYC (reprint author), Natl Univ Singapore, Dept Pharm, Fac Sci, Singapore, Singapore.

EM phalycj@nus.edu.sg

FU Health Services Research Competitive Research Grant from the Ministry of

Health, Singapore [HSRG/11MAY/016]

FX This study was supported by the Health Services Research Competitive

Research Grant from the Ministry of Health, Singapore (HSRG/11MAY/016).

The funder was not involved in the design and conduct of the study,

collection, management, analysis and interpretation of the data,

preparation, review and approval of the manuscript, or decision to

submit the manuscript for publication.

CR Ahola AJ, 2013, DIABETIC MED, V30, P413, DOI 10.1111/dme.12105

Aikens JE, 2012, DIABETES CARE, V35, P2472, DOI 10.2337/dc12-0181

American Diabetes Association, DIABETES CARE, V40, pS33

BARON RM, 1986, J PERS SOC PSYCHOL, V51, P1173, DOI 10.1037//0022-3514.51.6.1173

BRADLEY C, 1990, DIABETIC MED, V7, P445, DOI 10.1111/j.1464-5491.1990.tb01421.x

Chew BH, 2015, PATIENT PREFER ADHER, V9, P639, DOI 10.2147/PPA.S81612

Cummings DM, 2014, ANN PHARMACOTHER, V48, P970, DOI 10.1177/1060028014536532

Fisher L, 2014, DIABETIC MED, V31, P764, DOI 10.1111/dme.12428

Gellad WF, 2011, AM J GERIATR PHARMAC, V9, P11, DOI 10.1016/j.amjopharm.2011.02.004

Gonzalez JS, 2015, HEALTH PSYCHOL, V34, P505, DOI 10.1037/hea0000131

Hayes A. F., 2013, INTRO MEDIATION MODE

Krass I, 2015, DIABETIC MED, V32, P725, DOI 10.1111/dme.12651

Leventhal H, 2016, J BEHAV MED, V39, P935, DOI 10.1007/s10865-016-9782-2

Lin L-K, 2017, BMJ OPEN DIABETES RE, V5

Miller-Matero LR, 2017, PSYCHOL HEALTH MED, V22, P19, DOI 10.1080/13548506.2016.1191659

Ministry of Health Singapore, 2006, DIAB MELL MOH CLIN P

Ng CS, 2014, DIABETES RES CLIN PR, V105, P151, DOI 10.1016/j.diabres.2014.03.020

Ogurtsova K, 2017, DIABETES RES CLIN PR, V128, P40, DOI 10.1016/j.diabres.2017.03.024

Okumura Y, 2013, GEN HOSP PSYCHIAT, V35, P233, DOI 10.1016/j.genhosppsych.2012.12.013

Pintaudi B, 2015, J PSYCHOSOM RES, V79, P348, DOI 10.1016/j.jpsychores.2015.08.010

Png ME, 2016, BMC PUBLIC HEALTH, V16, DOI 10.1186/s12889-016-2827-1

Sapkota S, 2015, PLOS ONE, V10, DOI 10.1371/journal.pone.0128581

Siaw MYL, 2017, J CLIN PHARM THER, V42, P475, DOI 10.1111/jcpt.12536

Sobel M. E., 1982, SOCIOL METHODOL, P290, DOI DOI 10.2307/270723

Venkataraman K, 2015, PLOS ONE, V10, DOI 10.1371/journal.pone.0136759

Wang Y, 2012, DIABETIC MED, V29, pE338, DOI 10.1111/j.1464-5491.2012.03733.x

Wang Y, 2012, THROMB HAEMOSTASIS, V108, P789, DOI 10.1160/TH12-05-0368

Wilmot EG, 2012, DIABETOLOGIA, V55, P2895, DOI 10.1007/s00125-012-2677-z

Wong LY, 2017, J DIABETES, V9, P65, DOI 10.1111/1753-0407.12384

Zhao Y, 2015, PATIENT EDUC COUNS, V98, P669, DOI 10.1016/j.pec.2015.02.007

NR 30

TC 2

Z9 2

U1 2

U2 5

PU WILEY

PI HOBOKEN

PA 111 RIVER ST, HOBOKEN 07030-5774, NJ USA

SN 0742-3071

EI 1464-5491

J9 DIABETIC MED

JI Diabetic Med.

PD JUN

PY 2018

VL 35

IS 6

BP 807

EP 815

DI 10.1111/dme.13619

PG 9

WC Endocrinology & Metabolism

SC Endocrinology & Metabolism

GA GF5DD

UT WOS:000431985000016

PM 29532548

DA 2019-01-08

ER

PT J

AU McGill, DE

Volkening, LK

Butler, DA

Harrington, KR

Katz, ML

Laffel, LM

AF McGill, Dayna E.

Volkening, Lisa K.

Butler, Deborah A.

Harrington, Kara R.

Katz, Michelle L.

Laffel, Lori M.

TI Baseline Psychosocial Characteristics Predict Frequency of Continuous

Glucose Monitoring in Youth with Type 1 Diabetes

SO DIABETES TECHNOLOGY & THERAPEUTICS

LA English

DT Article

DE Continuous glucose monitoring; Depressive symptoms; Diabetes burden;

Quality of life; Youth; Technology

ID RANDOMIZED CLINICAL-TRIAL; QUALITY-OF-LIFE; GLYCEMIC CONTROL; FOLLOW-UP;

TIME; ADULTS; CHILDREN; ADOLESCENTS; DEPRESSION; DISTRESS

AB Background: Consistent use of continuous glucose monitoring (CGM) has been associated with improved glycemic control in youth with type 1 diabetes (T1D). There are many barriers to device uptake and continued use. There is a need to understand patient-specific characteristics when considering CGM. We evaluated patterns of CGM use and associations between baseline psychosocial measures and frequency of CGM use over 1 year. Methods: Youth with T1D (n=120), ages 8-18 years, completed questionnaires at CGM initiation and after 6 and 12 months assessing depressive symptoms, diabetes burden, and diabetes-specific and generic quality of life (QOL). Results: Youth (51% male and 95% white) had mean age 12.72.7 years, diabetes duration 6.1 +/- 3.6 years, and glycated hemoglobin (HbA(1c)) 8.0 +/- 0.8%. Over 1 year, 35% of youth used CGM 6 to 7 days per week, 45% used CGM 3-5 days per week, and 20% used CGM only 0-2 days per week. Youth who used CGM 3-7 days per week over 12 months had lower HbA(1c) at months 9 and 12 than youth who used CGM 0-2 days per week (9 months: 7.9 +/- 0.9% vs. 8.5 +/- 1.1%, P=0.006 and 12 months: 8.0 +/- 0.9% vs. 8.5 +/- 1.1%, P=0.02). Those using CGM 0-2 days per week had greater endorsement of depressive symptoms and diabetes burden and reported lower QOL at baseline compared with those using CGM 3-7 days per week. Conclusions: CGM use for 3 or more days per week over 12 months had a protective effect on HbA(1c). Providers should consider addressing psychosocial parameters when initiating CGM to maximize uptake and promote continued use in youth with T1D.

C1 [McGill, Dayna E.; Volkening, Lisa K.; Butler, Deborah A.; Harrington, Kara R.; Katz, Michelle L.; Laffel, Lori M.] Harvard Med Sch, Sect Clin Behav & Outcomes Res, Pediat Adolescent & Young Adult Sect, Joslin Diabet Ctr, One Joslin Pl, Boston, MA 02215 USA.

RP Laffel, LM (reprint author), Harvard Med Sch, Sect Clin Behav & Outcomes Res, Pediat Adolescent & Young Adult Sect, Joslin Diabet Ctr, One Joslin Pl, Boston, MA 02215 USA.

EM lori.laffel@joslin.harvard.edu

FU National Institutes of Health [K12DK094721, T32DK007260, R01DK089349,

P30DK036836, K23HL125976]; Katherine Adler Astrove Youth Education Fund;

Maria Griffin Drury Pediatric Fund; Eleanor Chesterman Beatson Fund

FX This research was supported by the National Institutes of Health under

grants K12DK094721, T32DK007260, R01DK089349, P30DK036836, and

K23HL125976; the Katherine Adler Astrove Youth Education Fund; the Maria

Griffin Drury Pediatric Fund; and the Eleanor Chesterman Beatson Fund.

The content is solely the responsibility of the authors and does not

necessarily represent the official views of these organizations. The

study sponsors were not involved in designing the study; collecting,

analyzing, or interpreting the data; writing the article; or deciding to

submit the article for publication. Portions of this article were

presented as an abstract at the 77th Scientific Sessions of the American

Diabetes Association, June 9-13, 2017, San Diego, CA.

CR Beck RW, 2017, JAMA-J AM MED ASSOC, V317, P371, DOI 10.1001/jama.2016.19975

Bjornstad P, 2016, DIABETES, V65, pA138

Chase HP, 2010, DIABETES TECHNOL THE, V12, P507, DOI 10.1089/dia.2010.0021

Deis D, 2006, DIABETES CARE, V29, P2730, DOI 10.2337/dc06-1134

FENDRICH M, 1990, AM J EPIDEMIOL, V131, P538, DOI 10.1093/oxfordjournals.aje.a115529

Fisher L, 2016, DIABETIC MED, V33, P1590, DOI 10.1111/dme.12973

Fisher L, 2014, DIABETIC MED, V31, P764, DOI 10.1111/dme.12428

Giani Elisa, 2017, J Diabetes Sci Technol, V11, P476, DOI 10.1177/1932296816676280

Hagger V, 2018, PEDIATR DIABETES, V19, P840, DOI 10.1111/pedi.12641

Hessler DM, 2017, DIABETIC MED, V34, P1228, DOI 10.1111/dme.13381

Markowitz JT, 2015, DIABETES SCI TECHNOL, V9, P1080

Miller KM, 2015, DIABETES CARE, V38, P971, DOI 10.2337/dc15-0078

Phillip M, 2012, PEDIATR DIABETES, V13, P215, DOI 10.1111/j.1399-5448.2011.00849.x

Pickup JC, 2011, BRIT MED J, V343, DOI 10.1136/bmj.d3805

Polonsky WH, 2017, DIABETES CARE, V40, P736, DOI 10.2337/dc17-0133

Polonsky WH, 2013, DIABETES TECHNOL THE, V15, P295, DOI 10.1089/dia.2012.0298

Scaramuzza AE, 2011, DIABETES TECHNOL THE, V13, P99, DOI 10.1089/dia.2010.0119

Tamborlane WV, 2008, NEW ENGL J MED, V359, P1464, DOI 10.1056/NEJMoa0805017

Tanenbaum ML, 2017, DIABETES CARE, V40, P181, DOI 10.2337/dc16-1536

Tansey M, 2011, DIABETIC MED, V28, P1118, DOI 10.1111/j.1464-5491.2011.03368.x

Van Name MA, 2018, PEDIATR DIABETES, V19, P114, DOI 10.1111/pedi.12525

Varni JW, 2001, MED CARE, V39, P800, DOI 10.1097/00005650-200108000-00006

Varni JW, 2003, DIABETES CARE, V26, P631, DOI 10.2337/diacare.26.3.631

Wong JC, 2014, DIABETES CARE, V37, P2702, DOI 10.2337/dc14-0303

NR 24

TC 0

Z9 0

U1 2

U2 2

PU MARY ANN LIEBERT, INC

PI NEW ROCHELLE

PA 140 HUGUENOT STREET, 3RD FL, NEW ROCHELLE, NY 10801 USA

SN 1520-9156

EI 1557-8593

J9 DIABETES TECHNOL THE

JI Diabetes Technol. Ther.

PD JUN

PY 2018

VL 20

IS 6

BP 434

EP 439

DI 10.1089/dia.2018.0037

EA MAY

EY 2018

PG 6

WC Endocrinology & Metabolism

SC Endocrinology & Metabolism

GA GJ8QT

UT WOS:000431467600001

PM 29727245

DA 2019-01-08

ER

PT J

AU Makrilakis, K

Liatis, S

Tsiakou, A

Stathi, C

Papachristoforou, E

Perrea, D

Katsilambros, N

Kontodimopoulos, N

Niakas, D

AF Makrilakis, Konstantinos

Liatis, Stavros

Tsiakou, Afroditi

Stathi, Chryssoula

Papachristoforou, Eleftheria

Perrea, Despoina

Katsilambros, Nicholas

Kontodimopoulos, Nikolaos

Niakas, Dimitrios

TI Comparison of health-related quality of Life (HRQOL) among patients with

pre-diabetes, diabetes and normal glucose tolerance, using the 15D-HRQOL

questionnaire in Greece: the DEPLAN study

SO BMC ENDOCRINE DISORDERS

LA English

DT Article

DE Diabetes mellitus; Pre-diabetes; Quality of life; HRQOL-15D

questionnaire

ID DE-PLAN PROJECT; PSYCHOLOGICAL DISTRESS; NEUROTROPHIC FACTOR;

PHYSICAL-ACTIVITY; HIGH-RISK; METABOLISM; DEPRESSION; STYLE; POPULATION;

COHORT

AB Background: Diabetes mellitus is usually preceded by a pre-diabetic stage before the clinical presentation of the disease, the influence of which on persons' quality of life is not adequately elucidated. The purpose of this study was to compare the Health Related Quality of Life (HRQOL) of persons with pre diabetes with that of diabetes or normal glucose tolerance (NGT), using the validated HRQOL-15D questionnaire.

Methods: The HRQOL-1 5D scores of 172 people with pre-diabetes (108 with Impaired fasting Glucose [ITG], 64 with Impaired Glucose Tolerance [IGT], aged 58.3 +/- 10.3 years) and 198 with NGT (aged 54.4 +/- 10.1 years) from the Greek part of the DEPLAN study (Diabetes in Europe - Prevention using Lifestyle, Physical Activity and Nutritional Intervention), were compared to 100 diabetes patients' scores (aged 60.9 +/- 12.5 years, diabetes duration 17.0 +/- 10.0 years, HbAlc 7.2 +/- 1.2%), derived from the outpatient Diabetes Clinic of a University Hospital.

Results: The diabetes patients' HRQOL-15D score (0.8605) was significantly lower than the pre-diabetes' (0.9008) and the controls' (0.9092) (p < 0.001). There were no differences in the total score between the controls and the group with pre-diabetes. However, examination of individual parameters of the score showed that people with IGT had lower scores compared to the control group, as related to the parameters of "mobility" and "psychological distress". No differences were found in any component of the HRQOL-15D score between the control group and the IPG group, nor between the two groups with pre-diabetes (IPG vs. IGT).

Conclusions: Persons with pre diabetes had a similar HRQOL score with healthy individuals, and a higher score than persons with diabetes. Specific components of the score, however, were lower in the IGT group compared to the controls. These findings help clarify the issue of HRQOL of persons with pre-diabetes and its possible impact on prevention.

C1 [Makrilakis, Konstantinos; Liatis, Stavros; Tsiakou, Afroditi; Stathi, Chryssoula; Papachristoforou, Eleftheria; Katsilambros, Nicholas; Niakas, Dimitrios] Univ Athens, Dept Propaedeut Med 1, Med Sch, Laiko Gen Hosp, 17 Ag Thoma St, Athens 11527, Greece.

[Perrea, Despoina; Katsilambros, Nicholas] Univ Athens, Med Sch, Lab Expt Surg & Surg Res Christeas Hall, Athens, Greece.

[Kontodimopoulos, Nikolaos; Niakas, Dimitrios] Hellen Open Univ, Patras, Greece.

RP Makrilakis, K (reprint author), Univ Athens, Dept Propaedeut Med 1, Med Sch, Laiko Gen Hosp, 17 Ag Thoma St, Athens 11527, Greece.

EM kmakrila@med.uoa.gr

FU Commission of the European Communities, Directorate C - Public Health

[2004310]; Bristol-Myers-Squibb, Greece

FX This project was partly funded by the Commission of the European

Communities, Directorate C - Public Health, grant agreement No. 2004310.

Under the rules of the agreement, it was also partly co-funded by the

private sector and in this case it was supported by an unrestricted

educational grant from Bristol-Myers-Squibb, Greece.

CR Adriaanse MC, 2008, DIABETIC MED, V25, P843, DOI 10.1111/j.1464-5491.2008.02464.x

Amer Diabet Assoc, 2016, DIABETES CARE, V39, pS13, DOI 10.2337/dc16-S005

Anagnostopoulos F, 2013, QUAL LIFE RES, V22, P1973, DOI 10.1007/s11136-013-0348-2

Burroughs TE, 2004, DIABETES SPECTRUM, V17, P41, DOI DOI 10.2337/DIASPECT.17.1.41

Castren E, 2010, CNS DRUGS, V24, P1, DOI 10.2165/11530010-000000000-00000

Cederlund RI, 2009, J DIABETES COMPLICAT, V23, P32, DOI 10.1016/j.jdiacomp.2007.09.002

Chittleborough CR, 2006, QUAL LIFE RES, V15, P687, DOI 10.1007/s11136-005-3570-8

El Achhab Y, 2008, DIABETES RES CLIN PR, V80, P171, DOI 10.1016/j.diabres.2007.12.020

Eriksson AK, 2008, DIABETIC MED, V25, P834, DOI 10.1111/j.1464-5491.2008.02463.x

FRIEDEWALD WT, 1972, CLIN CHEM, V18, P499

Golden SH, 2008, JAMA-J AM MED ASSOC, V299, P2751, DOI 10.1001/jama.299.23.2751

GREENFIELD S, 1992, MED CARE, V30, pMS23, DOI 10.1097/00005650-199205001-00003

Hiltunen L, 1999, DIABETES RES CLIN PR, V46, P161, DOI 10.1016/S0168-8227(99)00082-0

Holt RIG, 2014, CURR DIABETES REP, V14, DOI 10.1007/s11892-014-0491-3

Holt RIG, 2012, J AFFECT DISORDERS, V142, pS1, DOI 10.1016/S0165-0327(12)00632-5

Hunger M, 2014, QUAL LIFE RES, V23, P2515, DOI 10.1007/s11136-014-0689-5

Kontodimopoulos N, 2012, EUR J HEALTH ECON, V13, P111, DOI 10.1007/s10198-010-0290-y

Krabbe KS, 2007, DIABETOLOGIA, V50, P431, DOI 10.1007/s00125-006-0537-4

Li J, 2013, INT J BEHAV MED, V20, P495, DOI 10.1007/s12529-012-9255-0

Liatis S, 2016, DIABETES RES CLIN PR, V118, P162, DOI 10.1016/j.diabres.2016.06.018

Lindstrom J, 2003, DIABETES CARE, V26, P725, DOI 10.2337/diacare.26.3.725

Makrilakis K, 2011, DIABETES METAB, V37, P144, DOI 10.1016/j.diabet.2010.09.006

Makrilakis K, 2010, DIABETIC MED, V27, P459, DOI 10.1111/j.1464-5491.2010.02918.x

Marcuello C, 2012, INT J ENDOCRINOL, DOI 10.1155/2012/872305

Milman S, 2011, MED CLIN N AM, V95, P309, DOI 10.1016/j.mcna.2010.11.004

Neumann A, 2014, HEALTH QUAL LIFE OUT, V12, DOI 10.1186/s12955-014-0150-z

Nouwen A, 2011, DIABETES CARE, V34, P752, DOI 10.2337/dc10-1414

Schwarz PEH, 2008, EXP CLIN ENDOCR DIAB, V116, P167, DOI 10.1055/s-2007-992115

Seppala T, 2013, PRIM CARE DIABETES, V7, P223, DOI 10.1016/j.pcd.2013.03.001

Sikdar KC, 2010, QUAL LIFE RES, V19, P781, DOI 10.1007/s11136-010-9641-5

Sintonen H, 2001, ANN MED, V33, P328, DOI 10.3109/07853890109002086

Solli O, 2010, HEALTH QUAL LIFE OUT, V8, DOI 10.1186/1477-7525-8-18

Tabak AG, 2012, LANCET, V379, P2279, DOI 10.1016/S0140-6736(12)60283-9

Tapp R, 2010, DIABETES CARE, V33, P757, DOI 10.2337/dc09-1107

Tapp RJ, 2006, DIABETES RES CLIN PR, V74, P154, DOI 10.1016/j.diabres.2006.03.012

Taylor LM, 2010, DIABETES RES CLIN PR, V90, P15, DOI 10.1016/j.diabres.2010.04.011

Vaatainen S, 2014, QUAL LIFE RES, V23, P1935, DOI 10.1007/s11136-014-0638-3

Virtanen M, 2014, DIABETES CARE, V37, P2091, DOI 10.2337/dc13-2725

Wexler DJ, 2006, DIABETOLOGIA, V49, P1489, DOI 10.1007/s00125-006-0249-9

Williams JR, 2008, B WORLD HEALTH ORGAN, V86, P650, DOI 10.2471/BLT.08.050955

Wong E, 2013, LANCET DIABETES ENDO, V1, P106, DOI 10.1016/S2213-8587(13)70046-9

Cos FX, 2015, DIABETES RES CLIN PR, V109, P149, DOI 10.1016/j.diabres.2015.04.016

NR 42

TC 0

Z9 0

U1 0

U2 0

PU BIOMED CENTRAL LTD

PI LONDON

PA 236 GRAYS INN RD, FLOOR 6, LONDON WC1X 8HL, ENGLAND

SN 1472-6823

J9 BMC ENDOCR DISORD

JI BMC Endocr. Disord.

PD MAY 29

PY 2018

VL 18

AR 32

DI 10.1186/s12902-018-0261-3

PG 10

WC Endocrinology & Metabolism

SC Endocrinology & Metabolism

GA GI0SA

UT WOS:000434078500002

PM 29843700

OA DOAJ Gold

DA 2019-01-08

ER

PT J

AU Herder, C

Schmitt, A

Budden, F

Reimer, A

Kulzer, B

Roden, M

Haak, T

Hermanns, N

AF Herder, Christian

Schmitt, Andreas

Budden, Florian

Reimer, Andre

Kulzer, Bernhard

Roden, Michael

Haak, Thomas

Hermanns, Norbert

TI Longitudinal associations between biomarkers of inflammation and changes

in depressive symptoms in patients with type 1 and type 2 diabetes

SO PSYCHONEUROENDOCRINOLOGY

LA English

DT Article

DE Inflammation; Biomarkers; Type 1 diabetes; Type 2 diabetes; Depression

ID RECENTLY DIAGNOSED TYPE-1; C-REACTIVE PROTEIN; ANTIINFLAMMATORY

TREATMENT; GLYCEMIC CONTROL; HS-CRP; METAANALYSIS; ADIPONECTIN; PEOPLE;

RISK; IL-6

AB Background: Depressive disorders represent a frequent comorbidity of type 1 diabetes (T1D) and type 2 diabetes (T2D). Subclinical inflammation increases the risk of depressive symptoms in the general population, but the relationship appears complex and bidirectional, and longitudinal data from patients with diabetes are lacking. Therefore, this study aimed to analyse associations between changes in depressive symptoms and changes in biomarkers of inflammation in patients with T1D and T2D and to investigate the hypothesis that higher baseline levels of biomarkers of inflammation are related to a less pronounced reduction of depressive symptoms over time.

Methods: Depressive symptoms and systemic levels of six biomarkers of inflammation were assessed in 168 individuals with T1D and 103 individuals with T2D who participated in baseline and 1-year follow-up examinations. Data were obtained from two matching randomised controlled trials addressing diabetes distress and depressive symptoms. Longitudinal associations between biomarkers and depressive symptoms were estimated using linear regression models adjusting for multiple confounders.

Results: In patients with T2D, reductions in depressive symptoms were associated with reductions in high-sensitivity C-reactive protein (hsCRP), interleukin (IL)-18 and IL-1 receptor antagonist (IL-1RA) (P <= 0.016), whereas no associations were seen for IL-6, CCL2 and adiponectin. Higher CCL2 levels at baseline were associated with lower subsequent reduction in depressive symptoms (P = 0.018). Neither baseline levels nor changes in biomarkers in subclinical inflammation were associated with changes in depressive symptoms in patients with T1D.

Conclusions: Reductions of depressive symptoms were longitudinally associated with reductions in biomarkers of inflammation in patients with T2D. Higher baseline CCL2 levels were related with lower reduction of depressive symptoms in this group. No such associations were observed in patients with T1D, suggesting that risk factors and pathomechanisms linking inflammation and depression may differ between diabetes types.

C1 [Herder, Christian; Budden, Florian; Roden, Michael] Heinrich Heine Univ Dusseldorf, Inst Clin Diabetol, German Diabet Ctr, Leibniz Ctr Diabet Res, Aufm Hennekamp 65, D-40225 Dusseldorf, Germany.

[Herder, Christian; Schmitt, Andreas; Budden, Florian; Reimer, Andre; Kulzer, Bernhard; Roden, Michael; Hermanns, Norbert] German Ctr Diabet Res DZD, Munich, Germany.

[Schmitt, Andreas; Reimer, Andre; Kulzer, Bernhard; Haak, Thomas; Hermanns, Norbert] Res Inst Diabet Acad Mergentheim FIDAM, Bad Mergentheim, Germany.

[Kulzer, Bernhard; Hermanns, Norbert] Otto Friedrich Univ Bamberg, Dept Clin Psychol & Psychotherapy, Bamberg, Germany.

[Roden, Michael] Heinrich Heine Univ Dusseldorf, Med Fac, Div Endocrinol & Diabetol, Dusseldorf, Germany.

RP Herder, C (reprint author), Heinrich Heine Univ Dusseldorf, Inst Clin Diabetol, German Diabet Ctr, Leibniz Ctr Diabet Res, Aufm Hennekamp 65, D-40225 Dusseldorf, Germany.

EM christian.herder@ddz.uni-duesseldorf.de; schmitt@diabetes-zentrum.de;

florian.budden@gmx.de; reimer@diabetes-zentrum.de;

kulzer@diabetes-zentrum.de; michael.roden@ddz.uni-duesseldorf.de;

haak@diabetes-zentrum.de; hermanns@diabetes-zentrum.de

OI Schmitt, Andreas/0000-0002-5913-1457

FU German Federal Ministry of Education and Research (BMBF) [FKZ 01G10809,

FKZ 01GI1107]; German Federal Ministry of Health (BMG); Ministry of

Culture and Science of the State North Rhine-Westphalia; German Federal

Ministry of Education and Research (BMBF)

FX This study was supported by the German Federal Ministry of Education and

Research (BMBF) (FKZ 01G10809 and FKZ 01GI1107). The German Diabetes

Center is funded by the German Federal Ministry of Health (BMG) and the

Ministry of Culture and Science of the State North Rhine-Westphalia.

This study was also supported in part by a grant from the German Federal

Ministry of Education and Research (BMBF) to the German Center for

Diabetes Research (DZD e.V.). The funders had no role in study design,

data collection, data analysis, data interpretation, writing of the

report and decision to publish the manuscript.

CR Andrews G, 1998, SOC PSYCH PSYCH EPID, V33, P80, DOI 10.1007/s001270050026

Baumeister D, 2016, PSYCHOPHARMACOLOGY, V233, P1575, DOI 10.1007/s00213-015-4044-5

Berk M, 2013, BMC MED, V11, DOI 10.1186/1741-7015-11-74

Buchberger B, 2016, PSYCHONEUROENDOCRINO, V70, P70, DOI 10.1016/j.psyneuen.2016.04.019

Doyle TA, 2013, J PSYCHOSOM RES, V75, P419, DOI 10.1016/j.jpsychores.2013.08.006

Eyre HA, 2016, PROG NEURO-PSYCHOPH, V68, P1, DOI 10.1016/j.pnpbp.2016.02.006

Gimeno D, 2009, PSYCHOL MED, V39, P413, DOI 10.1017/S0033291708003723

Hannestad J, 2011, NEUROPSYCHOPHARMACOL, V36, P2452, DOI 10.1038/npp.2011.132

Hautzinger M, 2012, ADS ALLGEMEINE DEPRE

Hautzinger M, 1988, DIAGNOSTICA, V34, P167

Hayashino Y, 2014, DIABETES CARE, V37, P2459, DOI 10.2337/dc13-2312

Herder C., 2018, TRANSL PSYCHIAT, DOI [10.1038/s41398-017-009-2, DOI 10.1038/S41398-017-009-2]

Herder C, 2017, ARTERIOSCL THROM VAS, V37, P1222, DOI 10.1161/ATVBAHA.117.309307

Herder C, 2017, BRAIN BEHAV IMMUN, V61, P137, DOI 10.1016/j.bbi.2016.12.025

Herder C, 2017, HEART, V103, P63, DOI 10.1136/heartjnl-2015-309181

Hermanns N, 2015, DIABETES CARE, V38, P551, DOI 10.2337/dc14-1416

Hiles SA, 2012, PSYCHOL MED, V42, P2015, DOI 10.1017/S0033291712000128

Hiles SA, 2012, BRAIN BEHAV IMMUN, V26, P1180, DOI 10.1016/j.bbi.2012.06.001

Holt RIG, 2014, DIABETES CARE, V37, P2067, DOI 10.2337/dc13-2134

Hood KK, 2012, DIABETES CARE, V35, P2443, DOI 10.2337/dc11-2329

Hotta K, 2000, ARTERIOSCL THROM VAS, V20, P1595, DOI 10.1161/01.ATV.20.6.1595

Howren MB, 2009, PSYCHOSOM MED, V71, P171, DOI 10.1097/PSY.0b013e3181907c1b

Imagawa A, 2002, DIABETES CARE, V25, P1665, DOI 10.2337/diacare.25.9.1665

Ismail K, 2017, DIABETOLOGIA, V60, P2092, DOI 10.1007/s00125-017-4367-3

Kaptoge S, 2014, EUR HEART J, V35, P578, DOI 10.1093/eurheartj/eht367

Kohler O, 2016, CURR NEUROPHARMACOL, V14, P732, DOI 10.2174/1570159X14666151208113700

Kohler O, 2014, JAMA PSYCHIAT, V71, P1381, DOI 10.1001/jamapsychiatry.2014.1611

Korczak DJ, 2011, DIABETOLOGIA, V54, P2483, DOI 10.1007/s00125-011-2240-3

Laake JPS, 2014, DIABETES CARE, V37, P2186, DOI 10.2337/dc13-2522

Leslie RDG, 2008, DIABETES-METAB RES, V24, P511, DOI 10.1002/dmrr.877

Meurs M, 2016, PSYCHOSOM MED, V78, P233, DOI 10.1097/PSY.0000000000000255

Milaneschi Y, 2009, BIOL PSYCHIAT, V65, P973, DOI [10.1016/j.biopsych.2008.11.01, 10.1016/j.biopsych.2008.11.011]

Miller AH, 2016, NAT REV IMMUNOL, V16, P22, DOI 10.1038/nri.2015.5

Moulton C. D., 2015, ENDOCRINOLOGY, V3, P461, DOI [10.1016/S2213-8587(15)00134-5, DOI 10.1016/S2213-8587(15)00134-5]

Musselman DL, 2003, BIOL PSYCHIAT, V54, P317, DOI 10.1016/S0006-3223(03)00569-9

Nouwen A, 2010, DIABETOLOGIA, V53, P2480, DOI 10.1007/s00125-010-1874-x

Pae CU, 2014, PSYCHIAT INVEST, V11, P217, DOI 10.4306/pi.2014.11.3.217

Pham MN, 2011, DIABETOLOGIA, V54, P1630, DOI 10.1007/s00125-011-2088-6

Pouwer F, 2017, DIABETOLOGIA, V60, P30, DOI 10.1007/s00125-016-4154-6

Prossin AR, 2016, MOL PSYCHIATR, V21, P243, DOI 10.1038/mp.2015.110

RADLOFF L S, 1977, Applied Psychological Measurement, V1, P385, DOI 10.1177/014662167700100306

Schamarek I, 2016, EUR J ENDOCRINOL, V174, P433, DOI 10.1530/EJE-15-1010

Schloot NC, 2016, DIABETES CARE, V39, P1932, DOI 10.2337/dc16-0293

Schmitt A, 2017, J DIABETES COMPLICAT, V31, P1608, DOI 10.1016/j.jdiacomp.2017.08.004

Strawbridge R, 2015, EUR NEUROPSYCHOPHARM, V25, P1532, DOI 10.1016/j.euroneuro.2015.06.007

Stuart MJ, 2014, NEUROSCI BIOBEHAV R, V42, P93, DOI 10.1016/j.neubiorev.2014.02.001

Stuart MJ, 2012, NEUROSCI BIOBEHAV R, V36, P658, DOI 10.1016/j.neubiorev.2011.10.001

Tabak AG, 2014, LANCET DIABETES ENDO, V2, P236, DOI 10.1016/S2213-8587(13)70139-6

Valkanova V, 2013, J AFFECT DISORDERS, V150, P736, DOI 10.1016/j.jad.2013.06.004

Vilagut G, 2016, PLOS ONE, V11, DOI 10.1371/journal.pone.0155431

Zahn D, 2016, DIABETES CARE, V39, pE171, DOI 10.2337/dc16-0710

NR 51

TC 1

Z9 1

U1 0

U2 0

PU PERGAMON-ELSEVIER SCIENCE LTD

PI OXFORD

PA THE BOULEVARD, LANGFORD LANE, KIDLINGTON, OXFORD OX5 1GB, ENGLAND

SN 0306-4530

J9 PSYCHONEUROENDOCRINO

JI Psychoneuroendocrinology

PD MAY

PY 2018

VL 91

BP 216

EP 225

DI 10.1016/j.psyneuen.2018.02.032

PG 10

WC Endocrinology & Metabolism; Neurosciences; Psychiatry

SC Endocrinology & Metabolism; Neurosciences & Neurology; Psychiatry

GA GK5KT

UT WOS:000436214700026

PM 29525039

DA 2019-01-08

ER

PT J

AU Bak, E

Marcisz, C

Krzeminska, S

Dobrzyn-Matusiak, D

Foltyn, A

Drosdzol-Cop, A

AF Bak, Ewelina

Marcisz, Czeslaw

Krzeminska, Sylwia

Dobrzyn-Matusiak, Dorota

Foltyn, Agnieszka

Drosdzol-Cop, Agnieszka

TI Does Type 1 Diabetes Modify Sexuality and Mood of Women and Men?

SO INTERNATIONAL JOURNAL OF ENVIRONMENTAL RESEARCH AND PUBLIC HEALTH

LA English

DT Article

DE sexual disorders; type 1 diabetes; depression; illness acceptance

ID ERECTILE DYSFUNCTION; RISK-FACTORS; PREVALENCE; MELLITUS; DISTRESS;

COHORT; INDEX

AB Background: Sexual disorders occurring in women and men with type 1 diabetes have not been sufficiently investigated and described until now. This study attempts to evaluate sexuality in women and men. Methods: Altogether, the study comprised 115 patients with type 1 diabetes and 105 healthy people constituting the control group. All the studied persons underwent survey studies determining sexuality using the Female Sexual Function Index (FSFI-19) in women and the International Index of Erectile Function (IIEF-15) in men, and the occurrence of depression using the Beck Depression Inventory. The acceptance of illness among patients with diabetes was examined using the Acceptance of Illness Scale questionnaire. Results: In 35% of the examined women with diabetes, the study demonstrated sexual dysfunction as determined by total FSFI. The point values of all the investigated FSFI domains were significantly lower in women with diabetes than in healthy ones (p < 0.001). Erectile dysfunction occurred in 50% of the studied men with diabetes and in 23% of the control group of men (p = 0.0017). Conclusions: Type 1 diabetes leads to sexual disorders which occur in 1/3 of women and in 1/2 of men. Sexual disorders in patients with diabetes more frequently occur in men, persons with coexisting complications of diabetes, and in those with a concentration of glycated hemoglobin higher than 6.5%.

C1 [Bak, Ewelina] Univ Bielsko Biala, Dept Nursing, Fac Hlth Sci, PL-43309 Bielsko Biala, Poland.

[Marcisz, Czeslaw] Med Univ Silesia, Sch Hlth Sci, Dept Gerontol & Geriatr Nursing, PL-40752 Katowice, Poland.

[Krzeminska, Sylwia] Med Univ, Dept Clin Nursing, Fac Hlth Sci, PL-50367 Wroclaw, Poland.

[Dobrzyn-Matusiak, Dorota] Med Univ Silesia, Sch Hlth Sci, Dept Nursing Propaedeut, PL-40752 Katowice, Poland.

[Foltyn, Agnieszka] Med Univ Silesia, Sch Hlth Sci, Dept Social & Humanist Studies, Chair Philosophy & Bioeth, PL-40752 Katowice, Poland.

[Drosdzol-Cop, Agnieszka] Med Univ Silesia, Sch Hlth Sci Katowice, Chair Womans Hlth, PL-40752 Katowice, Poland.

RP Bak, E (reprint author), Univ Bielsko Biala, Dept Nursing, Fac Hlth Sci, PL-43309 Bielsko Biala, Poland.

EM ewelina.bak76@wp.pl; klinwewtychy@poczta.onet.pl; s.krzeminska@wp.pl;

dorotadobrzyn@op.pl; agnieszka_foltyn@wp.pl; cor111@poczta.onet.pl

OI Dobrzyn-Matusiak, Dorota/0000-0001-6042-8859; Drosdzol-Cop,

Agnieszka/0000-0003-1249-5840; Bak, Ewelina/0000-0002-2014-3717

CR Abu Ali RM, 2008, DIABETES CARE, V31, P1580, DOI 10.2337/dc08-0081

Addis IB, 2006, OBSTET GYNECOL, V107, P755, DOI 10.1097/01.AOG.0000202398.27428.e2

Bak E, 2017, INT J ENV RES PUB HE, V14, DOI 10.3390/ijerph14091073

Basson RJ, 2001, J SEX REPROD MED, V1, P11

Basu A, 2004, DRUGS, V64, P2667, DOI 10.2165/00003495-200464230-00004

Beck AT, 1996, BECK DEPRESSION INVE

Bonierbale M, 2003, CURR MED RES OPIN, V19, P114, DOI 10.1185/030079902125001461

Brooks M., 1977, MED ASPECTS HUM SEX, V8, P63

Brown JS, 2005, DIABETES CARE, V28, P177, DOI 10.2337/diacare.28.1.177

Clayton AH, 2007, J SEX MED, V4, P260, DOI 10.1111/j.1743-6109.2007.00609.x

Costabile RA, 2003, J UROLOGY, V170, pS35, DOI 10.1097/01.ju.0000077448.31995.99

Dembe K., 2002, DIABETOL POL, V9, P82

Dembe K., 2004, SEKSUOLOGIA POLSKA, V2, P51

Dimitropoulos K, 2012, J SEX MED, V9, P1374, DOI 10.1111/j.1743-6109.2012.02664.x

Doruk H, 2005, ARCH ANDROLOGY, V51, P1, DOI 10.1080/014850190512798

Enzlin P, 2003, DIABETES CARE, V26, P409, DOI 10.2337/diacare.26.2.409

Enzlin P, 1998, DIABETIC MED, V15, P809, DOI 10.1002/(SICI)1096-9136(199810)15:10<809::AID-DIA689>3.0.CO;2-Z

Enzlin P, 2002, DIABETES CARE, V25, P672, DOI 10.2337/diacare.25.4.672

Enzlin P, 2009, DIABETES CARE, V32, P780, DOI 10.2337/dc08-1164

Ferenidou F, 2008, J SEX MED, V5, P631, DOI 10.1111/j.1743-6109.2007.00644.x

Hatzichristou D, 2004, J SEX MED, V1, P49, DOI 10.1111/j.1743-6109.2004.10108.x

Hayes RD, 2008, J SEX MED, V5, P1681, DOI 10.1111/j.1743-6109.2008.00838.x

JENSEN SB, 1986, ARCH SEX BEHAV, V15, P271, DOI 10.1007/BF01550362

Juczynski Z, 2001, MEASUREMENT TOOLS PR

KOLODNY RC, 1971, DIABETES, V20, P557, DOI 10.2337/diab.20.8.557

Kouidrat Y, 2017, DIABETIC MED, V34, P1185, DOI 10.1111/dme.13403

Laumann EO, 2005, INT J IMPOT RES, V17, P39, DOI 10.1038/sj.ijir.3901250

Mazzilli R, 2015, DIABET METAB SYND OB, V8, P97, DOI 10.2147/DMSO.S71376

Montenero P., 1973, ANNUELLES DIABETOLOG, V11-13, P91

Muniyappa Ranganath, 2005, Curr Diab Rep, V5, P230, DOI 10.1007/s11892-005-0014-3

Nowosielski K, 2010, J SEX MED, V7, P723, DOI 10.1111/j.1743-6109.2009.01561.x

Penson DF, 2009, J SEX MED, V6, P1969, DOI 10.1111/j.1743-6109.2009.01292.x

Rawa B, 2010, INT MED J, V17, P179

Rosen R, 2000, J SEX MARITAL THER, V26, P191, DOI 10.1080/009262300278597

Rosen RC, 1997, UROLOGY, V49, P822, DOI 10.1016/S0090-4295(97)00238-0

Salonia A, 2006, DIABETES CARE, V29, P312, DOI 10.2337/diacare.29.02.06.dc05-1067

SCHIAVI RC, 1995, PSYCHIAT CLIN N AM, V18, P7, DOI 10.1016/S0193-953X(18)30067-4

Shiften JL, 2008, OBSTET GYNECOL, V112, P970, DOI 10.1097/AOG.0b013e3181898cdb

Tagliabue M, 2011, J SEX MED, V8, P1694, DOI 10.1111/j.1743-6109.2011.02262.x

West SL, 2008, ARCH INTERN MED, V168, P1441, DOI 10.1001/archinte.168.13.1441

NR 40

TC 0

Z9 0

U1 1

U2 1

PU MDPI

PI BASEL

PA ST ALBAN-ANLAGE 66, CH-4052 BASEL, SWITZERLAND

SN 1660-4601

J9 INT J ENV RES PUB HE

JI Int. J. Environ. Res. Public Health

PD MAY

PY 2018

VL 15

IS 5

AR 958

DI 10.3390/ijerph15050958

PG 14

WC Environmental Sciences; Public, Environmental & Occupational Health

SC Environmental Sciences & Ecology; Public, Environmental & Occupational

Health

GA GJ3LS

UT WOS:000435197300126

PM 29751592

OA DOAJ Gold

DA 2019-01-08

ER

PT J

AU Adams, C

Stears, A

Savage, D

Deaton, C

AF Adams, Claire

Stears, Anna

Savage, David

Deaton, Christi

TI "We're stuck with what we've got": The impact of lipodystrophy on body

image

SO JOURNAL OF CLINICAL NURSING

LA English

DT Article

DE anxiety; body image and sexuality; depression; diabetes; emotional

distress; endocrine disorders; femininity and daily life; health

psychology; metabolic syndrome

ID QUALITY-OF-LIFE; HIV; MANAGEMENT; DISFIGUREMENT; PERCEPTIONS; ADHERENCE;

DIAGNOSIS; DISORDER; RARE

AB Aims and objectivesTo evaluate the impact of lipodystrophy on body image and how this affects patients' daily lives.

BackgroundLipodystrophy refers to a group of rare conditions characterised by generalised or partial lack of body fat and is associated with severe metabolic problems, for example, severe insulin resistance, diabetes and pancreatitis. In addition to its metabolic effect, lack of adipose tissue may have a major impact on appearance and cause distressing physical changes. While global research has focused on diagnosis and management, there is no published work investigating the psychological effects of lipodystrophy on body image.

MethodsFollowing ethical approval, participants with lipodystrophy were purposively sampled from the National Severe Insulin Resistance Service in Cambridge, UK, and invited to take part in a semi-structured interview. Eleven (10 female, one male) interviews were conducted and digitally recorded. Data were analysed using an inductive thematic approach.

ResultsFour main themes were identified in the data set; Always feeling appearance was different, a better understanding of lipodystrophy is needed, feeling accepted and there's more to lipodystrophy than managing symptoms. Participants spoke of distressing cosmetic effects related to lack of fat tissue and other changes related to lipodystrophy, contributing to negative body image. For some, negative body image led to feelings of worthlessness impacting daily life and adherence to treatment. Psychological support was lacking but desired by participants.

ConclusionLipodystrophy contributes to negative body image affecting patients' daily lives. Patients wanted psychological support alongside medical management. Further research is needed to determine how best to deliver psychological support and to evaluate its impact on well-being and metabolic management.

Relevance to clinical practiceThe effects of rare diseases such as lipodystrophy on appearance can be distressing for patients. Support beyond medical management is needed to improve patients' daily lives and help them to live well with appearance-altering conditions.

C1 [Adams, Claire; Savage, David] Univ Cambridge, Metab Res Labs, Addenbrookes Treatment Ctr, Wellcome Trust MRC Inst Metab Sci,Addenbrookes Ho, Cambridge, England.

[Adams, Claire; Stears, Anna] Cambridge Univ Hosp NHS Fdn Trust, Natl Severe Insulin Resistance Serv, Addenbrookes Treatment Ctr, Cambridge, England.

[Deaton, Christi] Univ Cambridge, Sch Clin Med, Natl Severe Insulin Resistance Serv, Cambridge Inst Publ Hlth, Cambridge, England.

RP Adams, C (reprint author), Univ Cambridge, Metab Res Labs, Addenbrookes Treatment Ctr, Wellcome Trust MRC Inst Metab Sci,Addenbrookes Ho, Cambridge, England.

EM ca337@medschl.cam.ac.uk

FU Addenbrooke's Charitable Trust

FX This research was funded by the Addenbrooke's Charitable Trust and was

conducted as part of a National Institute of Health Research Masters in

Clinical Research studentship.

CR Bessell Alyson, 2012, Br J Nurs, V21, P1138

Bowling A., 2009, RES METHODS HLTH INV

Braun V., 2008, QUALITATIVE RES PSYC, V3, P77, DOI DOI 10.1191/1478088706QP0630A

Brown RJ, 2016, J CLIN ENDOCR METAB, V101, P4500, DOI 10.1210/jc.2016-2466

Burgoyne R, 2005, QUAL LIFE RES, V14, P981, DOI 10.1007/s11136-004-2580-2

Cadogan J., 2012, OXFORD HDB PSYCHOL A, P486

Calderoni DR, 2011, J PLAST RECONSTR AES, V64, P1248, DOI 10.1016/j.bjps.2011.02.009

Cash TF, 2005, J PSYCHOSOM RES, V58, P191, DOI 10.1016/j.jpsychores.2004.07.008

Cash Thomas F, 2004, Body Image, V1, P279, DOI 10.1016/S1740-1445(03)00023-8

Changing Faces, 2017, DISF UK DISF

Chiquette Elaine, 2017, Diabetes Metab Syndr Obes, V10, P375, DOI 10.2147/DMSO.S130810

Clarke A, 2001, PATIENT EDUC COUNS, V43, P305

Collins E, 2000, AIDS Read, V10, P546

Green J, 2009, QUALITATIVE METHODS

Grenard JL, 2011, J GEN INTERN MED, V26, P1175, DOI 10.1007/s11606-011-1704-y

Grogan S., 2012, OXFORD HDB PSYCHOL A, P581

Grogan Sara, 2008, BODY IMAGE UNDERSTAN

Gunter B., 2012, OXFORD HDB PSYCHOL A, P455

Hale ED, 2015, RHEUMATOLOGY, V54, P1220, DOI 10.1093/rheumatology/keu448

Halliwell E., 2012, OXFORD HDB PSYCHOL A, P217

Huang-Doran I, 2010, J ENDOCRINOL, V207, P245, DOI 10.1677/JOE-10-0272

Hughes JMF, 2011, J PLAST RECONSTR AES, V64, pE121, DOI 10.1016/j.bjps.2011.01.001

Huyard C, 2009, HEALTH EXPECT, V12, P361, DOI 10.1111/j.1369-7625.2009.00552.x

Jackson SNJ, 1997, QJM-INT J MED, V90, P27

Kelly JS, 2009, AIDS CARE, V21, P1560, DOI 10.1080/09540120902923014

Lenert LA, 2002, AM J MED, V113, P229, DOI 10.1016/S0002-9343(02)01156-7

Limb L, 2010, EXPERIENCES RARE DIS

Malterud K, 2016, QUAL HEALTH RES, V26, P1753, DOI 10.1177/1049732315617444

Oette M, 2002, AIDS PATIENT CARE ST, V16, P413, DOI 10.1089/108729102760330254

Patton M, 2002, QUALITATIVE RES EVAL

Plankey M, 2009, AIDS BEHAV, V13, P53, DOI 10.1007/s10461-008-9444-7

Power R, 2003, SEX TRANSM INFECT, V79, P137, DOI 10.1136/sti.79.2.137

Public Health England, 2015, HIV UK STAT 2015

QSR International Pty Ltd, 2012, NVIVO QUAL DAT AN SO

Reynolds NR, 2006, AIDS CARE, V18, P663, DOI 10.1080/09540120500287051

Robbins AL, 2015, TRENDS MOL MED, V21, P433, DOI 10.1016/j.molmed.2015.04.004

RUDD NA, 2001, CLOTH TEXT RES J, V19, P120

Rumsey Nichola, 2004, Body Image, V1, P83, DOI 10.1016/S1740-1445(03)00005-6

Semple RK, 2010, GENETIC DIAGNOSIS OF ENDOCRINE DISORDERS, P39, DOI 10.1016/B978-0-12-374430-2.00004-3

Stears A, 2014, CLIN LIPIDOL, V9, P235, DOI 10.2217/CLP.14.13

Thompson A, 2001, CLIN PSYCHOL REV, V21, P663, DOI 10.1016/S0272-7358(00)00056-8

Tiggemann M., 2007, PSYCHOL MEN MASCULIN, V8, P15, DOI DOI 10.1037/1524-9220.8.1.15

Veale D., 2009, OVERCOMING BODY IMAG

Voelker DK, 2015, ADOLESC HEALTH MED T, V6, P149, DOI 10.2147/AHMT.S68344

Want SC, 2009, BODY IMAGE, V6, P257, DOI 10.1016/j.bodyim.2009.07.008

NR 45

TC 0

Z9 0

U1 2

U2 2

PU WILEY

PI HOBOKEN

PA 111 RIVER ST, HOBOKEN 07030-5774, NJ USA

SN 0962-1067

EI 1365-2702

J9 J CLIN NURS

JI J. Clin. Nurs.

PD MAY

PY 2018

VL 27

IS 9-10

BP 1958

EP 1968

DI 10.1111/jocn.14342

PG 11

WC Nursing

SC Nursing

GA GI1KB

UT WOS:000434128200021

PM 29516553

DA 2019-01-08

ER

PT J

AU McGavock, J

Durksen, A

Wicklow, B

Malik, S

Sellers, EAC

Blydt-Hansen, T

Chateau, D

Dart, A

AF McGavock, Jonathan

Durksen, Anita

Wicklow, Brandy

Malik, Sayma

Sellers, Elizabeth A. C.

Blydt-Hansen, Tom

Chateau, Dan

Dart, Allison

TI Determinants of Readiness for Adopting Healthy Lifestyle Behaviors Among

Indigenous Adolescents with Type 2 Diabetes in Manitoba, Canada: A

Cross-Sectional Study

SO OBESITY

LA English

DT Article

ID PHYSICAL-ACTIVITY; SCREENING MEASURE; GLYCEMIC CONTROL; MENTAL-HEALTH;

DEPRESSIVE SYMPTOMS; PRIMARY-CARE; YOUTH; CHILDREN; INTERVENTION;

MANAGEMENT

AB ObjectiveThe aim of this study was to determine whether the readiness for adopting healthy lifestyle behaviors was associated with mental health and comorbid conditions in youth with T2D.

MethodsA cross-sectional comparison of various measures of mental health (distress, stress, resilience) and comorbid conditions (glycated hemoglobin, adiposity, hypertension) was conducted within a cohort of indigenous youth with T2D living in Canada, stratified according to their readiness to adopt healthy lifestyle behaviors based on Prochaska's transtheoretical model.

ResultsWithin the entire cohort (n=162), only 14% were considered ready to adopt all healthy lifestyle behaviors. The readiness to adopt all lifestyle behaviors was associated with higher positive mental health (47 vs. 39 units; P<0.05) and sense of mastery (40 vs. 37 units; P<0.05), lower perceived stress (27 vs. 29 units; P<0.05) and distress (8 vs. 10 units; P<0.05), and better glycemic control (HbA1c: 8.42.6% vs. 9.7 +/- 2.8%; P<0.05) compared with youth who were not ready to adopt all lifestyle behaviors.

ConclusionsThe readiness for adopting healthy lifestyle behaviors is low among adolescents with T2D. Being ready to adopt healthy lifestyle behaviors is associated with better mental health and glycemic control.

C1 [McGavock, Jonathan; Wicklow, Brandy; Sellers, Elizabeth A. C.; Dart, Allison] Univ Manitoba, Rady Fac Hlth Sci, Dept Pediat & Child Hlth, Winnipeg, MB, Canada.

[McGavock, Jonathan; Durksen, Anita; Wicklow, Brandy; Sellers, Elizabeth A. C.; Dart, Allison] Univ Manitoba, Childrens Hosp Res Inst Manitoba, Winnipeg, MB, Canada.

[McGavock, Jonathan; Wicklow, Brandy; Sellers, Elizabeth A. C.; Dart, Allison] Univ Manitoba, Diabet Res Envisioned & Accomplished Manitoba DRE, Winnipeg, MB, Canada.

[Malik, Sayma] Univ Manitoba, Dept Clin Psychol, Winnipeg, MB, Canada.

[Blydt-Hansen, Tom] BC Childrens Hosp, Nephrol Sect, Dept Pediat, Vancouver, BC, Canada.

[Chateau, Dan] Univ Manitoba, Manitoba Ctr Hlth Policy, Fac Hlth Sci, Winnipeg, MB, Canada.

RP McGavock, J (reprint author), Univ Manitoba, Rady Fac Hlth Sci, Dept Pediat & Child Hlth, Winnipeg, MB, Canada.; McGavock, J (reprint author), Univ Manitoba, Childrens Hosp Res Inst Manitoba, Winnipeg, MB, Canada.; McGavock, J (reprint author), Univ Manitoba, Diabet Res Envisioned & Accomplished Manitoba DRE, Winnipeg, MB, Canada.

EM jmcgavock@chrim.ca

OI Blydt-Hansen, Tom/0000-0002-8864-0197

FU Canadian Diabetes Association; Research Manitoba; Canadian Institutes of

Child Health; Children's Hospital Research Institute through the

Diabetes Research Envisioned and Accomplished in Manitoba (DREAM)

research theme

FX This study was funded by grants from the Canadian Diabetes Association,

Research Manitoba, the Canadian Institutes of Child Health, and the

Children's Hospital Research Institute through the Diabetes Research

Envisioned and Accomplished in Manitoba (DREAM) research theme. JM holds

a Canadian Institutes of Health Research Applied Public Health Chair in

Resilience and Obesity in Youth.

CR Anderson BJ, 2011, DIABETES CARE, V34, P2205, DOI 10.2337/dc11-0431

Berkowitz RI, 2018, PEDIATR DIABETES, V19, P191, DOI 10.1111/pedi.12555

Copeland KC, 2013, PEDIATRICS, V131, P364, DOI 10.1542/peds.2012-3494

Dart AB, 2014, CAN J DIABETES, V38, P349, DOI 10.1016/j.jcjd.2014.07.224

Dray J, 2017, J AM ACAD CHILD PSY, V56, P813, DOI 10.1016/j.jaac.2017.07.780

Goldenberg R, 2013, CAN J DIABETES, V37, pS8, DOI 10.1016/j.jcjd.2013.01.011

Hagler AS, 2006, ANN BEHAV MED, V31, P186, DOI 10.1207/s15324796abm3102_11

Holmen H, 2016, BMJ OPEN DIAB RES CA, V4, DOI 10.1136/bmjdrc-2016-000193

Hood KK, 2014, J ADOLESCENT HEALTH, V55, P498, DOI 10.1016/j.jadohealth.2014.03.011

Hood KK, 2012, DIABETES CARE, V35, P2443, DOI 10.2337/dc11-2329

Huynh E, 2015, CAN J DIABETES, V39, P484, DOI 10.1016/j.jcjd.2015.09.093

Jones H, 2003, DIABETES CARE, V26, P732, DOI 10.2337/diacare.26.3.732

Kann L., 2016, MMWR SURVEILL SUMM, V65, P1, DOI DOI 10.15585/MMWR.SS6506A1

Kramer JR, 2000, ANN BEHAV MED, V22, P17, DOI 10.1007/BF02895164

Laforge RG, 1999, PREV MED, V28, P61, DOI 10.1006/pmed.1998.0384

Lamers SMA, 2011, J CLIN PSYCHOL, V67, P99, DOI 10.1002/jclp.20741

Lawrence JM, 2006, PEDIATRICS, V117, P1348, DOI 10.1542/peds.2005-1398

Mastellos N, 2014, COCHRANE DB SYST REV, DOI 10.1002/14651858.CD008066.pub3

McFarlane P, 2013, CAN J DIABETES, V37, pS129, DOI 10.1016/j.jcjd.2013.01.037

McGavock J, 2015, CURR DIABETES REP, V15, DOI 10.1007/s11892-014-0568-z

Meehan C, 2016, J PEDIATR-US, V170, P20, DOI 10.1016/j.jpeds.2015.11.015

Mitchell CM, 2011, PSYCHOL ASSESSMENT, V23, P752, DOI 10.1037/a0023288

Mulvaney SA, 2006, DIABETES CARE, V29, P993, DOI 10.2337/dc05-2099

Mulvaney SA, 2008, DIABETES EDUCATOR, V34, P674, DOI 10.1177/0145721708320902

Nadeau KJ, 2016, DIABETES CARE, V39, P1635, DOI 10.2337/dc16-1066

Norcross JC, 2011, J CLIN PSYCHOL, V67, P143, DOI 10.1002/jclp.20758

Panagiotopoulos C, 2013, CAN J DIABETES, V37, pS163, DOI 10.1016/j.jcjd.2013.01.043

Patrick K, 2006, ARCH PEDIAT ADOL MED, V160, P128, DOI 10.1001/archpedi.160.2.128

Plotnikoff RC, 2010, ANN BEHAV MED, V40, P138, DOI 10.1007/s12160-010-9193-5

Prince-Embury S, 2008, CAN J SCH PSYCHOL, V23, P41, DOI 10.1177/0829573508316592

Prochaska JJ, 2004, J ADOLESCENT HEALTH, V34, P163, DOI 10.1016/j.jadohealth.2003.07.001

Prochaska JJ, 2001, ARCH PEDIAT ADOL MED, V155, P554, DOI 10.1001/archpedi.155.5.554

Prochaska JJ, 2001, PREV MED, V33, P699, DOI 10.1006/pmed.2001.0951

PROCHASKA JO, 1992, AM PSYCHOL, V47, P1102, DOI 10.1037//0003-066X.47.9.1102

Protudjer JLP, 2014, CAN J DIABETES, V38, P229, DOI 10.1016/j.jcjd.2014.05.008

Reinehr T, 2008, HORM RES, V69, P107, DOI 10.1159/000111814

Robinson DJ, 2013, CAN J DIABETES, V37, pS87, DOI 10.1016/j.jcjd.2013.01.026

Sellers E, 2000, LANCET, V355, P1156, DOI 10.1016/S0140-6736(00)02067-5

Silverstein J, 2015, DIABETES CARE, V38, P2341, DOI 10.2337/dc15-0982

Springer SC, 2013, PEDIATRICS, V131, pE648, DOI 10.1542/peds.2012-3496

Wilfley DE, 2010, INT J OBESITY, V34, P217, DOI 10.1038/ijo.2009.195

Wittmeier KDM, 2012, PAED CHILD HEALT-CAN, V17, P129, DOI 10.1093/pch/17.3.129

Zeitler P, 2012, NEW ENGL J MED, V366, P2247, DOI 10.1056/NEJMoa1109333

NR 43

TC 1

Z9 1

U1 0

U2 0

PU WILEY

PI HOBOKEN

PA 111 RIVER ST, HOBOKEN 07030-5774, NJ USA

SN 1930-7381

EI 1930-739X

J9 OBESITY

JI Obesity

PD MAY

PY 2018

VL 26

IS 5

BP 910

EP 915

DI 10.1002/oby.22148

PG 6

WC Endocrinology & Metabolism; Nutrition & Dietetics

SC Endocrinology & Metabolism; Nutrition & Dietetics

GA GD7GG

UT WOS:000430678000018

PM 29533530

DA 2019-01-08

ER

PT J

AU Hilliard, ME

De Wit, M

Wasserman, RM

Butler, AM

Evans, M

Weissberg-Benchell, J

Anderson, BJ

AF Hilliard, Marisa E.

De Wit, Maartje

Wasserman, Rachel M.

Butler, Ashley M.

Evans, Meredyth

Weissberg-Benchell, Jill

Anderson, Barbara J.

TI Screening and support for emotional burdens of youth with type 1

diabetes: Strategies for diabetes care providers

SO PEDIATRIC DIABETES

LA English

DT Article

DE ambulatory care; behavioral medicine; diabetes mellitus;

insulin-dependent; emotional adjustment

ID QUALITY-OF-LIFE; RANDOMIZED CONTROLLED-TRIAL; SHARED DECISION-MAKING;

MENTAL-HEALTH PROBLEMS; GLYCEMIC CONTROL; DEPRESSIVE SYMPTOMS;

DIFFICULTIES QUESTIONNAIRE; PSYCHOMETRIC PROPERTIES; DELIVERED

INTERVENTION; MEDICAL APPOINTMENTS

AB Multiple sources of burden for youth with type 1 diabetes (T1D) impact key outcomes including quality of life, self-management, and glycemic control. Professional diabetes organizations recommend diabetes care providers screen for psychosocial and behavioral challenges and implement strategies to support youth with T1D. The purpose of this article is to review the literature and recommend practical strategies medical providers can use for screening and behavioral support for youth with diabetes and their families. As part of their routine medical care, diabetes care providers are well-positioned to identify and intervene to address emotional distress related to the burdens of living with diabetes. In collaboration with multidisciplinary team members, including psychologists and mental health professionals, medical providers may be able to successfully implement brief behavioral strategies for screening and providing emotional support.

C1 [Hilliard, Marisa E.; Wasserman, Rachel M.; Butler, Ashley M.; Anderson, Barbara J.] Baylor Coll Med, Dept Pediat, Psychol Sect, 1102 Bates Ave,Suite 940, Houston, TX 77030 USA.

[Hilliard, Marisa E.; Wasserman, Rachel M.; Butler, Ashley M.; Anderson, Barbara J.] Texas Childrens Hosp, 1102 Bates Ave,Suite 940, Houston, TX 77030 USA.

[De Wit, Maartje] Vrije Univ Amsterdam Med Ctr, Dept Med Psychol, Amsterdam, Netherlands.

[Evans, Meredyth; Weissberg-Benchell, Jill] Northwestern Univ, Ann & Robert H Lurie Childrens Hosp Chicago, Feinberg Sch Med, Chicago, IL 60611 USA.

RP Hilliard, ME (reprint author), Baylor Coll Med, Dept Pediat, Psychol Sect, 1102 Bates Ave,Suite 940, Houston, TX 77030 USA.; Hilliard, ME (reprint author), Texas Childrens Hosp, 1102 Bates Ave,Suite 940, Houston, TX 77030 USA.

EM marisa.hilliard@bcm.edu

OI Hilliard, Marisa/0000-0002-8813-629X

FU NIH [K12 DK097696]

FX NIH, Grant/Award number: K12 DK097696

CR American Diabetes Association, 2017, Diabetes Care, V40, pS105

Anderson BJ, 1999, DIABETES CARE, V22, P713, DOI 10.2337/diacare.22.5.713

Angold A, 1995, INT J METHOD PSYCH, V5, P237

Balfe M, 2013, BMC ENDOCR DISORD, V13, DOI 10.1186/1472-6823-13-25

Boogerd EA, 2015, J CLIN NURS, V24, P2143, DOI 10.1111/jocn.12789

Boogerd EA, 2014, PEDIATR DIABETES, V15, P394, DOI 10.1111/pedi.12103

Caccavale LJ, 2015, J PEDIATR PSYCHOL, V40, P500, DOI 10.1093/jpepsy/jsu113

Cella D, 2010, J CLIN EPIDEMIOL, V63, P1179, DOI 10.1016/j.jclinepi.2010.04.011

Channon SJ, 2007, DIABETES CARE, V30, P1390, DOI 10.2337/dc06-2260

Chorpita BF, 1997, BEHAV RES THER, V35, P569, DOI 10.1016/S0005-7967(96)00116-7

Commissariat PV, 2018, PEDIATR DIABETES, V19, P85, DOI 10.1111/pedi.12523

Corathers SD, 2015, CURR DIABETES REP, V15, DOI 10.1007/s11892-015-0664-8

Corathers SD, 2013, PEDIATRICS, V132, pE1395, DOI 10.1542/peds.2013-0681

De Brock AJLL, 2006, NOSI R NIJMEEGSE OUD

de Wit M, 2012, PEDIATR DIABETES, V13, P638, DOI 10.1111/j.1399-5448.2012.00872.x

de Wit M, 2011, PEDIATR DIABETES, V12, P172, DOI 10.1111/j.1399-5448.2010.00673.x

de Wit M, 2010, PEDIATR DIABETES, V11, P175, DOI 10.1111/j.1399-5448.2009.00543.x

De Wit M, 2008, DIABETES CARE, V31, P1521, DOI 10.2337/dc08-0394

Delamater AM, 2014, PEDIATR DIABETES, V15, P232, DOI 10.1111/pedi.12191

Driscoll KA, 2016, CURR DIABETES REP, V16, P1

Drotar D, 2012, J PEDIATR PSYCHOL, V37, P479, DOI 10.1093/jpepsy/jss065

Eilander M, 2016, PEDIATR DIABETES, V17, P112, DOI 10.1111/pedi.12237

Ellis DA, 2005, PEDIATRICS, V116, pE826, DOI 10.1542/peds.2005-0638

Evans M, 2016, DIABETES S1, V76, P785

Fisher L, 2016, J DIABETES COMPLICAT, V30, P1123, DOI 10.1016/j.jdiacomp.2016.03.032

Gallo LC, 2003, PSYCHOL BULL, V129, P10, DOI 10.1037//0033-2909.129.1.10

Gardner W, 2002, MED CARE, V40, P812, DOI 10.1097/01.MLR.0000025436.30093.77

Garvey KC, 2016, DIABETES CARE, V39, P190, DOI 10.2337/dc15-1775

Gayes LA, 2014, J CONSULT CLIN PSYCH, V82, P521, DOI 10.1037/a0035917

Goodman R, 2001, J AM ACAD CHILD PSY, V40, P1337, DOI 10.1097/00004583-200111000-00015

Grey M, 2000, J PEDIATR-US, V137, P107, DOI 10.1067/mpd.2000.106568

Hapunda G, 2015, BMC ENDOCR DISORD, V15, DOI 10.1186/s12902-015-0013-6

Harris MA, 2015, DIABETES CARE, V38, P1427, DOI 10.2337/dc14-2469

Harris MA, 2014, DIABETES CARE, V37, pE125, DOI 10.2337/dc13-2232

Haverman L, 2013, PEDIATRICS, V131, pE533, DOI 10.1542/peds.2012-0958

Hex N, 2012, DIABETIC MED, V29, P855, DOI 10.1111/j.1464-5491.2012.03698.x

Hilliard ME, 2017, J PEDIATR PSYCHOL, V42, P995, DOI 10.1093/jpepsy/jsx086

Hilliard ME, 2017, DIABETES, V66, pA225

Hilliard ME, 2017, DIABETES CARE, V40, P849, DOI 10.2337/dc16-2688

Hilliard ME, 2016, AM PSYCHOL, V71, P590, DOI 10.1037/a0040359

Hilliard ME, 2016, CURR DIABETES REP, V16, DOI 10.1007/s11892-016-0761-3

Hilliard ME, 2015, DIABETES, V64, pA213

Hilliard ME, 2013, HEALTH PSYCHOL, V32, P388, DOI 10.1037/a0027811

Hilliard ME, 2013, J ADOLESCENT HEALTH, V52, P28, DOI 10.1016/j.jadohealth.2012.05.009

Hilliard ME, 2012, CURR DIABETES REP, V12, P739, DOI 10.1007/s11892-012-0314-3

Hood KK, 2007, DIABETES CARE, V30, P1764, DOI 10.2337/dc06-2358

Hood KK, 2014, J ADOLESCENT HEALTH, V55, P498, DOI 10.1016/j.jadohealth.2014.03.011

Hood KK, 2010, DIABETES CARE, V33, P1658, DOI 10.2337/dc09-2268

Huston SA, 2016, PEDIATR DIABETES, V17, P623, DOI 10.1111/pedi.12347

Jaser SS, 2011, CURR DIABETES REP, V11, P480, DOI 10.1007/s11892-011-0222-y

Jensen SA, 2006, PSYCHOL ASSESSMENT, V18, P346, DOI 10.1037/1040-3590.18.3.346

Johnson B, 2013, DIABETIC MED, V30, P199, DOI 10.1111/j.1464-5491.2012.03721.x

Johnson JG, 2002, J ADOLESCENT HEALTH, V30, P196, DOI 10.1016/S1054-139X(01)00333-0

Kazak AE, 2017, J PEDIATR PSYCHOL, V42, P700, DOI 10.1093/jpepsy/jsw110

Kolko DJ, 2010, J DEV BEHAV PEDIATR, V31, P393, DOI 10.1097/DBP.0b013e3181dff307

KOVACS M, 1985, PSYCHOPHARMACOL BULL, V21, P995

Kroenke K, 2001, J GEN INTERN MED, V16, P606, DOI 10.1046/j.1525-1497.2001.016009606.x

Lavigne JV, 2016, J PEDIATR PSYCHOL, V41, P1081, DOI 10.1093/jpepsy/jsw048

Loeb DF, 2012, J GEN INTERN MED, V27, P945, DOI 10.1007/s11606-012-2005-9

Lord JH, 2015, J PEDIATR PSYCHOL, V40, P132, DOI 10.1093/jpepsy/jsu078

Main A, 2014, J PEDIATR PSYCHOL, V39, P1104, DOI 10.1093/jpepsy/jsu062

Matthews KA, 2011, ANNU REV PSYCHOL, V62, P501, DOI 10.1146/annurev.psych.031809.130711

Mayor S, 2016, BMJ-BRIT MED J, V352, DOI 10.1136/bmj.i394

Monaghan M, 2015, CLIN PEDIATR, V54, P1346, DOI 10.1177/0009922815581833

Mullins LL, 2011, J PEDIATR PSYCHOL, V36, P249, DOI 10.1093/jpepsy/jsq080

National Institutes of Health, 2016, DOM FRAM PROMIS PED

Northam EA, 2010, DIABETES CARE, V33, P1430, DOI 10.2337/dc09-2232

Noser AE, 2017, PEDIATR DIABETES, V18, P614, DOI 10.1111/pedi.12471

Petrovski G, 2015, DIABETES CARE, V38, pe51, DOI 10.2337/dc14-2487

Powell PW, 2014, CURR DIABETES REP, V14, DOI 10.1007/s11892-014-0531-z

Quittner AL, 2016, THORAX, V71, P26, DOI 10.1136/thoraxjnl-2015-207488

RADLOFF L S, 1977, Applied Psychological Measurement, V1, P385, DOI 10.1177/014662167700100306

Raymond Jennifer K, 2015, Diabetes Spectr, V28, P68, DOI 10.2337/diaspect.28.1.68

Redondo MJ, 2017, DIABETES RES CLIN PR, V129, P203, DOI 10.1016/j.diabres.2017.05.006

Reynolds KA, 2011, ANN BEHAV MED, V42, P29, DOI 10.1007/s12160-011-9262-4

Rijswijk C, 2010, PATIENT EDUC COUNS, V79, P351, DOI 10.1016/j.pec.2010.04.016

Robling M, 2012, BRIT MED J, V344, DOI 10.1136/bmj.e2359

Rumburg TM, 2017, PEDIATR DIABETES, V18, P67, DOI 10.1111/pedi.12350

Sanchez I, 2011, DIABETES EDUCATOR, V37, P381, DOI 10.1177/0145721711401667

Schabert J, 2013, PATIENT, V6, P1, DOI 10.1007/s40271-012-0001-0

Schwartz DD, 2011, DIABETES CARE, V34, P326, DOI 10.2337/dc10-1553

Silverstein J, 2015, DIABETES CARE, V38, P2341, DOI 10.2337/dc15-0982

Siu AL, 2016, ANN INTERN MED, V164, DOI 10.7326/P16-9006

Spitzer RL, 2006, ARCH INTERN MED, V166, P1092, DOI 10.1001/archinte.166.10.1092

Tao B, 2010, PLOS ONE, V5, DOI 10.1371/journal.pone.0011501

Thompson D, 2016, JMIR RES PROTOC, V5, P118, DOI 10.2196/resprot.5817

van Widenfelt BM, 2003, EUR CHILD ADOLES PSY, V12, P281, DOI 10.1007/s00787-003-0341-3

Varni JW, 2001, MED CARE, V39, P800, DOI 10.1097/00005650-200108000-00006

Walker Ashby F, 2015, Clin Diabetes, V33, P62, DOI 10.2337/diaclin.33.2.62

Wei C, 2018, PEDIATR DIABETES, V19, P106, DOI 10.1111/pedi.12519

Weinger K., 2003, DIABETES SPECTRUM, V16, P104, DOI DOI 10.2337/

Weissberg-Benchell J, 2011, PEDIATR DIABETES, V12, P341, DOI 10.1111/j.1399-5448.2010.00720.x

Weitzman C, 2015, PEDIATRICS, V135, P384, DOI 10.1542/peds.2014-3716

Whittemore R, 2012, NURS RES, V61, P395, DOI 10.1097/NNR.0b013e3182690a29

Whittemore R, 2012, DIABETES EDUCATOR, V38, P562, DOI 10.1177/0145721712445216

Wiley J, 2014, PATIENT PREFER ADHER, V8, P423, DOI 10.2147/PPA.S57707

Wissow L, 2011, PATIENT EDUC COUNS, V82, P226, DOI 10.1016/j.pec.2010.03.019

Wolpert HA, 2001, BRIT MED J, V323, P994, DOI 10.1136/bmj.323.7319.994

Wright DR, 2016, JAMA PEDIATR, V170, P1048, DOI 10.1001/jamapediatrics.2016.1721

Wyatt KD, 2015, ACAD PEDIATR, V15, P573, DOI 10.1016/j.acap.2015.03.011

Wysocki T, 2006, J PEDIATR PSYCHOL, V31, P928, DOI 10.1093/jpepsy/jsj098

Wysocki T, 2016, DIABETES EDUCATOR, V42, P395, DOI 10.1177/0145721716647492

Wysocki T, 2012, PEDIATR DIABETES, V13, P432, DOI 10.1111/j.1399-5448.2011.00823.x

Wysocki T, 2009, J PEDIATR PSYCHOL, V34, P869, DOI 10.1093/jpepsy/jsn136

Young-Hyman D, 2016, DIABETES CARE, V39, P2126, DOI 10.2337/dc16-2053

NR 105

TC 1

Z9 1

U1 4

U2 5

PU WILEY

PI HOBOKEN

PA 111 RIVER ST, HOBOKEN 07030-5774, NJ USA

SN 1399-543X

EI 1399-5448

J9 PEDIATR DIABETES

JI Pediatr. Diabetes

PD MAY

PY 2018

VL 19

IS 3

BP 534

EP 543

DI 10.1111/pedi.12575

PG 10

WC Endocrinology & Metabolism; Pediatrics

SC Endocrinology & Metabolism; Pediatrics

GA GE0QW

UT WOS:000430921600029

PM 28940936

DA 2019-01-08

ER

PT J

AU Foster, ED

Bridges, ND

Feurer, ID

Eggerman, TL

Hunsicker, LG

Alejandro, R

AF Foster, Eric D.

Bridges, Nancy D.

Feurer, Irene D.

Eggerman, Thomas L.

Hunsicker, Lawrence G.

Alejandro, Rodolfo

CA Clinical Islet Transplantation Con

TI Improved Health-Related Quality of Life in a Phase 3 Islet

Transplantation Trial in Type 1 Diabetes Complicated by Severe

Hypoglycemia

SO DIABETES CARE

LA English

DT Article

ID FALSE DISCOVERY RATE; PANCREAS TRANSPLANTATION; TOTAL PANCREATECTOMY;

DISTRESS SCALE; FOLLOW-UP; FEAR; MELLITUS; IMPACT; ADULTS; OUTCOMES

AB OBJECTIVE

Attaining glycemic targets without severe hypoglycemic events (SHEs) is a challenging treatment goal for patients with type 1 diabetes complicated by impaired awareness of hypoglycemia (IAH). The CIT Consortium Protocol 07 (CIT-07) trial showed islet transplantation to be an effective treatment for subjects with IAH and intractable SHEs. We evaluated health-related quality of life (HRQOL), functional health status, and health utility before and after pancreatic islet transplantation in CIT-07 trial participants.

RESEARCH DESIGN AND METHODS

Four surveys, the Diabetes Distress Scale (DDS), the Hypoglycemic Fear Survey (HFS), the Short Form 36 Health Survey (SF-36), and the EuroQoL 5 Dimensions (EQ-5D), were administered repeatedly before and after islet transplantation. Summary statistics and longitudinal modeling were used to describe changes in survey scores from baseline and to characterize change in relation to a minimally important difference (MID) threshold of half an SD.

RESULTS

Improvements in condition-specific HRQOL met the MID threshold. Reductions from baseline in the DDS total score and its four DDS subscales (all P <= 0.0013) and in the HFS total score and its two subscales (all P < 0.0001) were observed across all time points. Improvements were observed after both 1 and 2 years for the EQ-5D visual analog scale (both P < 0.0001).

CONCLUSIONS

In CIT-07, 87.5% of the subjects achieved the primary end point of freedom from SHE along with glycemic control (HbA(1c) < 7% [< 53 mmol/mol]) at 1 year post-initial islet transplantation. The same subjects reported consistent, statistically significant, and clinically meaningful improvements in condition-specific HRQOL as well as self-assessments of overall health.

C1 [Foster, Eric D.; Hunsicker, Lawrence G.] Univ Iowa, Dept Biostat, Clin Trials Stat & Data Management Ctr, Iowa City, IA 52242 USA.

[Bridges, Nancy D.] NIAID, NIH, 9000 Rockville Pike, Bethesda, MD 20892 USA.

[Feurer, Irene D.] Vanderbilt Univ, Dept Surg, Med Ctr, Nashville, TN 37240 USA.

[Feurer, Irene D.] Vanderbilt Univ, Med Ctr, Dept Biostat, Nashville, TN USA.

[Feurer, Irene D.] Vanderbilt Transplant Ctr, Nashville, TN USA.

[Eggerman, Thomas L.] NIDDK, NIH, Bethesda, MD USA.

[Alejandro, Rodolfo] Univ Miami, Miller Sch Med, Diabet Res Inst, Miami, FL 33136 USA.

RP Foster, ED (reprint author), Univ Iowa, Dept Biostat, Clin Trials Stat & Data Management Ctr, Iowa City, IA 52242 USA.

EM eric-foster@uiowa.edu

FU National Institute of Allergy and Infectious Diseases (NIAID); National

Institute of Diabetes and Digestive and Kidney Diseases (NIDDK); NIDDK

at Emory University [NIAID U01-AI-089317]; NIDDK at Northwestern

University [NIAID U01-AI-089316]; NIDDK at the University of Alberta,

Edmonton [NIAID U01-AI-065191]; NIDDK at the University of California,

San Francisco [NIDDK U01-DK-085531]; NIDDK at the University of

Illinois, Chicago [NIDDK 5U01-DK-070431-10]; NIDDK at the University of

Iowa [NIDDK U01-DK-070431]; NIDDK at the University of Miami [NIDDK

U01-DK-070460]; NIDDK at the University of Minnesota [NIAID

U01-AI-065193]; NIDDK at the University of Pennsylvania [NIDDK

U01-DK-070430]; NIDDK at Uppsala University [NIAID U01-AI-065192]; Emory

University [UL1-TR-000454]; Northwestern University [5UL1-RR-025741,

8UL1-TR-000150]; University of California, San Francisco

[UL1-TR-000004]; University of Illinois, Chicago [UL1-TR-000050];

University of Miami [1UL1-TR-000460]; University of Minnesota

[5M01-RR-000400, UL1-TR-000114]; University of Pennsylvania

[M01-RR00040, UL1-TR-000003]

FX This research was funded by the National Institute of Allergy and

Infectious Diseases (NIAID) and the National Institute of Diabetes and

Digestive and Kidney Diseases (NIDDK), ClinicalTrials.gov no.

NCT00434811. This research was supported by the following grants from

the NIAID and the NIDDK: at Emory University, NIAID U01-AI-089317; at

Northwestern University, NIAID U01-AI-089316; at the University of

Alberta, Edmonton, NIAID U01-AI-065191; at the University of California,

San Francisco, NIDDK U01-DK-085531; at the University of Illinois,

Chicago, NIDDK 5U01-DK-070431-10; at the University of Iowa, NIDDK

U01-DK-070431; at the University of Miami, NIDDK U01-DK-070460; at the

University of Minnesota, NIAID U01-AI-065193; at the University of

Pennsylvania, NIDDK U01-DK-070430; and at Uppsala University, NIAID

U01-AI-065192. In addition, the study was supported by the following

General Clinical Research Center grants and Clinical and Translational

Science Awards: at Emory University, UL1-TR-000454; at Northwestern

University, 5UL1-RR-025741 and 8UL1-TR-000150; at the University of

California, San Francisco, UL1-TR-000004; at the University of Illinois,

Chicago, UL1-TR-000050; at the University of Miami, 1UL1-TR-000460; at

the University of Minnesota, 5M01-RR-000400 and UL1-TR-000114; and at

the University of Pennsylvania, M01-RR00040 and UL1-TR-000003.

CR Ahroni JH, 2000, J DIABETES COMPLICAT, V14, P31, DOI 10.1016/S1056-8727(00)00066-0

[Anonymous], 1997, DIABETES, V46, P271

Barshes NR, 2005, TRANSPLANTATION, V79, P1727, DOI 10.1097/01.TP.0000160816.21799.F5

Bellin MD, 2011, CLIN GASTROENTEROL H, V9, P793, DOI 10.1016/j.cgh.2011.04.024

Benhamou PY, 2009, DIABETIC MED, V26, P617, DOI 10.1111/j.1464-5491.2009.02731.x

BENJAMINI Y, 1995, J R STAT SOC B, V57, P289

Bjorner JB, 2013, VALUE HEALTH, V16, P993, DOI 10.1016/j.jval.2013.06.022

Bohme P, 2013, DIABETES METAB, V39, P63, DOI 10.1016/j.diabet.2012.10.006

Chinnakotla S, 2014, J AM COLL SURGEONS, V218, P530, DOI 10.1016/j.jamcollsurg.2013.12.037

COX DJ, 1987, DIABETES CARE, V10, P617, DOI 10.2337/diacare.10.5.617

Cryer PE, 2014, DIABETES, V63, P2188, DOI 10.2337/db14-0059

Cure P, 2008, TRANSPLANTATION, V85, P801, DOI [10.1097/TP.0b013c318166a27b, 10.1097/TP.0b013e318166a27b]

DCCT Res Grp, 1988, DIABETES CARE, V11, P725

Fisher L, 2012, DIABETES CARE, V35, P259, DOI 10.2337/dc11-1572

Glickman ME, 2014, J CLIN EPIDEMIOL, V67, P850, DOI 10.1016/j.jclinepi.2014.03.012

Hart HE, 2003, QUAL LIFE RES, V12, P1089, DOI 10.1023/A:1026197119569

Hering BJ, 2016, DIABETES CARE, V39, P1230, DOI 10.2337/dc15-1988

IRVINE AA, 1992, HEALTH PSYCHOL, V11, P135, DOI 10.1037/0278-6133.11.2.135

JACOBSON AM, 1994, DIABETES CARE, V17, P267, DOI 10.2337/diacare.17.4.267

Janssen MF, 2011, DIABETIC MED, V28, P395, DOI 10.1111/j.1464-5491.2010.03136.x

Johnson JA, 2004, DIABETES CARE, V27, P624, DOI 10.2337/diacare.27.2.624

Martyn-Nemeth P, 2016, J DIABETES COMPLICAT, V30, P167, DOI 10.1016/j.jdiacomp.2015.09.003

Mehrabi A, 2014, PANCREAS, V43, P1190, DOI 10.1097/MPA.0000000000000181

Norman GR, 2003, MED CARE, V41, P582, DOI 10.1097/00005650-200305000-00004

Poggioli R, 2006, AM J TRANSPLANT, V6, P371, DOI 10.1111/j.1600-6143.2005.01174.x

Polonsky WH, 2005, DIABETES CARE, V28, P626, DOI 10.2337/diacare.28.3.626

Polonsky WH, 2017, DIABETES CARE, V40, P736, DOI 10.2337/dc17-0133

Radosevich DM, 2013, CLIN TRANSPLANT, V27, pE715, DOI 10.1111/ctr.12265

Reenen MV, 2015, EQ 5D 3L USER GUIDE

Ricordi C, 2016, DIABETES, V65, P3418, DOI 10.2337/db16-0234

Rubin RR, 2012, DIABETES TECHNOL THE, V14, P143, DOI 10.1089/dia.2011.0162

Ryan EA, 2005, DIABETES, V54, P2060, DOI 10.2337/diabetes.54.7.2060

Scalea JR, 2018, CLIN TRANSPLANT, V32, DOI 10.1111/ctr.13177

SHAMOON H, 1993, NEW ENGL J MED, V329, P977

Shaw JW, 2005, MED CARE, V43, P203, DOI 10.1097/00005650-200503000-00003

Speight J, 2010, DIABETIC MED, V27, P812, DOI 10.1111/j.1464-5491.2010.03029.x

Tharavanij T, 2008, TRANSPLANTATION, V86, P1161, DOI 10.1097/TP.0b013e31818a7f45

Toso C, 2007, TRANSPLANTATION, V84, P664, DOI 10.1097/01.tp.0000280550.01028.89

Vanstone Meredith, 2015, Ont Health Technol Assess Ser, V15, P1

Ware Jr JE, 2000, SF 36 HLTH SURVEY MA, V10, P24

WILLIAMS A, 1990, HEALTH POLICY, V16, P199

NR 41

TC 4

Z9 4

U1 3

U2 7

PU AMER DIABETES ASSOC

PI ALEXANDRIA

PA 1701 N BEAUREGARD ST, ALEXANDRIA, VA 22311-1717 USA

SN 0149-5992

EI 1935-5548

J9 DIABETES CARE

JI Diabetes Care

PD MAY

PY 2018

VL 41

IS 5

BP 1001

EP 1008

DI 10.2337/dc17-1779

PG 8

WC Endocrinology & Metabolism

SC Endocrinology & Metabolism

GA GD4EJ

UT WOS:000430456100020

PM 29563196

DA 2019-01-08

ER

PT J

AU Holmes-Truscott, E

Browne, JL

Ventura, AD

Pouwer, F

Speight, J

AF Holmes-Truscott, E.

Browne, J. L.

Ventura, A. D.

Pouwer, F.

Speight, J.

TI Diabetes stigma is associated with negative treatment appraisals among

adults with insulin-treated Type 2 diabetes: results from the second

Diabetes MILES - Australia (MILES-2) survey

SO DIABETIC MEDICINE

LA English

DT Article

ID SOCIAL STIGMA; SELF-MANAGEMENT; THERAPY; EXPERIENCES; ADHERENCE;

ATTITUDES; IMPACT; SCALE; CARE

AB AimTo explore factors associated with negative insulin appraisals among adults with Type 2 diabetes, including perceived and experienced diabetes stigma.

MethodsThe second Diabetes MILES - Australia study (MILES-2) is a national survey of adults with diabetes, focused on behavioural and psychosocial issues. Subgroup analyses were conducted on the responses of 456 adults with insulin-treated Type 2 diabetes (38% women; mean sd age: 61.2 8.8 years; diabetes duration: 14.5 7.5 years; years using insulin: 6.4 5.5). Participants completed validated measures of perceived and experienced diabetes stigma (Type 2 Diabetes Stigma Assessment Scale), insulin appraisals [Insulin Treatment Appraisal Scale (ITAS)] and known correlates of insulin appraisals: diabetes-specific distress (Problem Areas In Diabetes scale) and diabetes-specific self-efficacy (Confidence in Diabetes Self-care scale). A multiple linear regression was conducted (N = 279) to determine the contribution of those variables found to be associated with ITAS Negative scores.

ResultsUnivariable analyses revealed negative insulin appraisals were associated with demographic and self-care characteristics (age, employment status, BMI, years using insulin, injections per day), self-efficacy, diabetes-specific distress and diabetes stigma (all P < 0.01). Number of injections per day [regression coefficient [95% confidence interval]: 0.74 [0.08, 1.40]; P = 0.028], self-efficacy [-0.12 [-0.19, -0.06]; P < 0.001] and diabetes stigma [0.39 (0.31, 0.46); P < 0.001) significantly and independently contributed to the final multivariable model, explaining 58% of the variance in ITAS Negative scores. The independent contribution of diabetes-specific distress was suppressed following the inclusion of diabetes stigma.

ConclusionsThis study represents the first step in understanding the relationship between perceived and experienced diabetes stigma and negative insulin appraisals, and provides quantitative evidence for the strong, independent relationship between these two important constructs.

C1 [Holmes-Truscott, E.; Browne, J. L.; Ventura, A. D.; Speight, J.] Deakin Univ, Sch Psychol, Geelong, Vic, Australia.

[Holmes-Truscott, E.; Browne, J. L.; Ventura, A. D.; Speight, J.] Diabet Victoria, Australian Ctr Behav Res Diabet, Melbourne, Vic, Australia.

[Pouwer, F.] Univ Southern Denmark, Dept Psychol, Odense, Denmark.

[Speight, J.] AHP Res, Hornchurch, England.

RP Holmes-Truscott, E (reprint author), Deakin Univ, Sch Psychol, Geelong, Vic, Australia.; Holmes-Truscott, E (reprint author), Diabet Victoria, Australian Ctr Behav Res Diabet, Melbourne, Vic, Australia.

EM etruscott@acbrd.org.au

OI Holmes-Truscott, Elizabeth/0000-0001-9139-4663; Speight,

Jane/0000-0002-1204-6896

FU Diabetes Victoria; Deakin University; Sanofi ANZ

FX The key research personnel on the second Diabetes MILES - Australia

study were supported by core funding provided to The Australian Centre

for Behavioural Research in Diabetes (ACBRD) by Diabetes Victoria and

Deakin University. Recruitment activities and the development of the

study website were funded through an unrestricted educational grant (to

the ACBRD) from Sanofi ANZ. Sanofi ANZ was not involved in the study

design, data collection, or data analysis and had no input on the

preparation of this manuscript or the decision to submit for

publication.

CR Browne JL, 2017, BMJ OPEN, V7, DOI 10.1136/bmjopen-2016-012926

Browne JL, 2016, DIABETES CARE, V39, P2141, DOI 10.2337/dc16-0117

Browne JL, 2014, BMJ OPEN, V4, DOI 10.1136/bmjopen-2014-005625

Browne JL, 2013, BMJ OPEN, V3, DOI 10.1136/bmjopen-2013-003384

Davies MJ, 2013, DIABETIC MED, V30, P512, DOI 10.1111/dme.12128

Holmes-Truscott E, 2015, DIABETIC MED, V32, P1297, DOI 10.1111/dme.12729

Holmes-Truscott E, 2016, J DIABETES COMPLICAT, V30, P1151, DOI 10.1016/j.jdiacomp.2016.03.027

Holmes-Truscott E, 2014, HEALTH QUAL LIFE OUT, V12, DOI 10.1186/1477-7525-12-87

Jenkins N, 2011, DIABETIC MED, V28, P543, DOI 10.1111/j.1464-5491.2010.03200.x

Lowe J, 2008, DIABETES RES CLIN PR, V80, P439, DOI 10.1016/j.diabres.2008.02.003

Phelan SM, 2015, OBES REV, V16, P319, DOI 10.1111/obr.12266

Polinski JM, 2013, DIABETES EDUCATOR, V39, P53, DOI 10.1177/0145721712467696

POLONSKY WH, 1995, DIABETES CARE, V18, P754, DOI 10.2337/diacare.18.6.754

Rintamaki LS, 2006, AIDS PATIENT CARE ST, V20, P359, DOI 10.1089/apc.2006.20.359

Schabert J, 2013, PATIENT, V6, P1, DOI 10.1007/s40271-012-0001-0

Shah RB, 2016, INT J PHARM INVESTIG, V6, P1, DOI 10.4103/2230-973X.176456

Shiu ATY, 2003, J CLIN NURS, V12, P149, DOI 10.1046/j.1365-2702.2003.00735.x

Snoek FJ, 2007, HEALTH QUAL LIFE OUT, V5, DOI 10.1186/1477-7525-5-69

Stuckey HL, 2014, DIABETES CARE, V37, P2466, DOI 10.2337/dc13-2536

van der Ven NCW, 2003, DIABETES CARE, V26, P713, DOI 10.2337/diacare.26.3.713

NR 20

TC 1

Z9 1

U1 4

U2 5

PU WILEY

PI HOBOKEN

PA 111 RIVER ST, HOBOKEN 07030-5774, NJ USA

SN 0742-3071

EI 1464-5491

J9 DIABETIC MED

JI Diabetic Med.

PD MAY

PY 2018

VL 35

IS 5

BP 658

EP 662

DI 10.1111/dme.13598

PG 5

WC Endocrinology & Metabolism

SC Endocrinology & Metabolism

GA GC9LN

UT WOS:000430118900016

PM 29417612

DA 2019-01-08

ER

PT J

AU Castonguay, A

Miquelon, P

AF Castonguay, Alexandre

Miquelon, Paule

TI Motivational profiles, accelerometer-derived physical activity, and

acute diabetes-related symptoms in adults with type 2 diabetes

SO BMC PUBLIC HEALTH

LA English

DT Article

DE Type 2 diabetes; Physical activity behavior; Accelerometer;

Self-determination theory; Motivational profile; Diabetes-related

symptoms

ID SELF-DETERMINATION THEORY; DETERMINATION THEORY PERSPECTIVE; POWER

ANALYSIS PROGRAM; PSYCHOMETRIC EVALUATION; AUTONOMOUS MOTIVATION;

GLYCEMIC CONTROL; CLUSTER-ANALYSIS; GLUCOSE CONTROL; RISK-FACTORS;

EXERCISE

AB Background: Using self-determination theory, the objective of this study was to examine, over a one-month period, how physical activity (PA) motivation would influence accelerometer-derived PA behavior, and ultimately, acute diabetes-related symptoms burden among adults with type 2 diabetes (T2D adults). Using both a person and variable-centered approach, this objective was attained by means of: 1) investigating the indirect effect of PA participation on the relationship between PA motivation and acute diabetes-related symptom burden and 2) examining whether participants who met PA recommendations (i.e., 150 min of moderate-to-vigorous PA per week) would experience less acute diabetes-related symptom burden over a one-month period.

Methods: A two-wave prospective longitudinal design was used. At time 1, participants completed a questionnaire assessing their PA motivation and were asked to wear an ActiGraph GT3x accelerometer for four consecutive weeks. At time 2, they completed a short questionnaire assessing their acute diabetes-related symptoms (i.e., symptoms related to fatigue, cognitive distress, hyperglycemia, and hypoglycemia). The final sample includes 165 adults (89 or 53.61% women) aged from 26 to 75 years (M = 62.05, SD = 8.75) with T2D, which provided at least 21 valid days of accelerometer-derived data.

Results: First, results of a path analysis demonstrated that over a one-month period, the average number of minutes spent practicing moderate to vigorous PA per week mediated the relationship between intrinsic and external PA motivation and the level of burden associated with the following diabetes-related symptoms: fatigue, cognitive distress, and hyperglycemia. In addition, results of covariance analyses showed that participants meeting PA recommendations also reported significantly less burden associated with these three symptoms over a month period. Then, the existence of four motivational profiles (Self-Determined, High Introjected, Low Motivation, and Non-Self-Determined) was confirmed using a k-means analysis. Results of covariance and chi-square analyses further showed, respectively, that compared to other motivational profiles, the Self-Determined profile was associated with a higher score on weekly PA participation and meeting PA recommendations.

Conclusions: The results highlight the importance of promoting autonomous motives for PA participation among T2D adults. They also suggest that T2D adults meeting PA recommendations experience less acute diabetes-related symptoms burden, which further support the importance of their PA motivation.

C1 [Castonguay, Alexandre; Miquelon, Paule] Univ Quebec Trois Rivieres, Dept Psychol, 3351 Boul Borges,CP 500, Trois Rivieres, PQ G9A 5H7, Canada.

RP Castonguay, A (reprint author), Univ Quebec Trois Rivieres, Dept Psychol, 3351 Boul Borges,CP 500, Trois Rivieres, PQ G9A 5H7, Canada.

EM Alexandre.Castonguay@uqtr.ca

FU Fonds de recherche du Quebec - Societe et culture; career research grant

from the Fonds de recherche du Quebec - Sante

FX This study was supported by a doctoral award from the Fonds de recherche

du Quebec - Societe et culture to the first author (years 2016-2018) and

a career research grant from the Fonds de recherche du Quebec - Sante to

the second author (2012-2016).

CR Arbuckle RA, 2009, VALUE HEALTH, V12, P1168, DOI 10.1111/j.1524-4733.2009.00571.x

Barrett JE, 2007, DIABETES EDUCATOR, V33, P128, DOI 10.1177/0145721706297453

Bowen N. K., 2011, STRUCTURAL EQUATION

Brunet J, 2015, PERS INDIV DIFFER, V84, P2, DOI 10.1016/j.paid.2015.02.034

Brunet J, 2013, PSYCHO-ONCOLOGY, V22, P2245, DOI 10.1002/pon.3287

Bryant S., 2016, EMOTIONAL RESPONSE E

Castonguay A, 2017, HEALTH PSYCHOL BEHAV, V5, P110, DOI 10.1080/21642850.2016.1272416

Centers for Disease Control and Prevention, 2017, NAT DIAB STAT REP 20

Chernick M., 2007, BOOTSTRAP METHODS GU

Chudyk A, 2011, DIABETES CARE, V34, P1228, DOI 10.2337/dc10-1881

Cohen J, 1988, STAT POWER ANAL BEHA

Colberg SR, 2016, DIABETES CARE, V39, P2065, DOI 10.2337/dc16-1728

Colberg Sheri R, 2010, Diabetes Care, V33, pe147, DOI 10.2337/dc10-9990

Davis CM., 2017, ASS PHYS SELF DISREP

Deci E. L, 2002, HDB SELF DETERMINATI

Deci EL, 2000, PSYCHOL INQ, V11, P227, DOI 10.1207/S15327965PLI1104_01

Dyrstad SM, 2014, MED SCI SPORT EXER, V46, P99, DOI 10.1249/MSS.0b013e3182a0595f

Efron B, 1993, INTRO BOOTSTRAP

Erdfelder E, 1996, BEHAV RES METH INS C, V28, P1, DOI 10.3758/BF03203630

Faul F, 2007, BEHAV RES METHODS, V39, P175, DOI 10.3758/BF03193146

Fennell CG., 2016, EFFECTS 16 WEEK EXER

Fortier MS, 2012, J HEALTH PSYCHOL, V17, P87, DOI 10.1177/1359105311408948

Freedson PS, 1998, MED SCI SPORT EXER, V30, P777, DOI 10.1097/00005768-199805000-00021

Friederichs Stijn Ah, 2015, BMC Psychol, V3, P1, DOI 10.1186/s40359-015-0059-2

Gagne M, 2005, J ORGAN BEHAV, V26, P331, DOI 10.1002/job.322

Gourlan M, 2016, BEHAV MED, V42, P227, DOI 10.1080/08964289.2014.1001810

Grace A, 2017, CARDIOVASC DIABETOL, V16, DOI 10.1186/s12933-017-0518-6

GROOTENHUIS PA, 1994, DIABETIC MED, V11, P253, DOI 10.1111/j.1464-5491.1994.tb00268.x

Guerin E., 2012, REV PHENEPS PHENEX J, V4, P1

Hair J, 2009, MULTIVARIATE DATA AN

Healey J., 2013, THESIS

Hendelman D, 2000, MED SCI SPORT EXER, V32, pS442, DOI 10.1097/00005768-200009001-00002

Hu LT, 1999, STRUCT EQU MODELING, V6, P1, DOI 10.1080/10705519909540118

Koponen AM, 2017, PSYCHOL HEALTH MED, V22, P332, DOI 10.1080/13548506.2016.1154179

Krishnan S, 2015, AM J HEALTH BEHAV, V39, P109, DOI 10.5993/AJHB.39.1.12

Leiter LA, 2013, CAN J DIABETES, V37, P82, DOI 10.1016/j.jcjd.2013.02.055

Ly TT, 2014, DIABETES CARE, V37, P2310, DOI 10.2337/dc14-0147

MacKinnon D., 2008, INTRO STAT MEDIATION

Markland D, 2004, J SPORT EXERCISE PSY, V26, P191, DOI 10.1123/jsep.26.2.191

Matsumoto H., 2004, INT J SPORT HLTH SCI, V2, P89, DOI DOI 10.5432/IJSHS.2.89

Migueles JH, 2017, SPORTS MED, V47, P1821, DOI 10.1007/s40279-017-0716-0

Miquelon P, 2016, INT J SPORT EXERCISE, V15, P1, DOI DOI 10.1080/1612197X.2016.1155637

Miquelon P, 2016, CAN J DIABETES, V40, P399, DOI 10.1016/j.jcjd.2016.02.009

Morrato EH, 2007, DIABETES CARE, V30, P203, DOI 10.2337/dc06-1128

Naegeli AN, 2010, DIABETES METAB SYNDR, V3, P27, DOI 10.2147/DMSO.S9465

Patrick H, 2014, PSYCHOL INQ, V25, P101, DOI 10.1080/1047840X.2014.878682

Phillips LA, 2018, PERS SOC PSYCHOL B, V44, P49, DOI 10.1177/0146167217733068

Pintrich PR, 2003, J EDUC PSYCHOL, V95, P667, DOI 10.1037/0022-0663.95.4.667

Plotnikoff RC, 2011, J OBES, DOI 10.1155/2011/561432

Public Health Agency of Canada, 2002, DIAB CAN

Public Health Agency of Canada, 2011, DIAB CAN FACTS FIG P

Rea LM, 2014, DESIGNING CONDUCTING

Scarapicchia TMF, 2014, PREV MED, V66, P45, DOI 10.1016/j.ypmed.2014.05.017

Sebire SJ, 2011, J SPORT EXERCISE PSY, V33, P175, DOI 10.1123/jsep.33.2.175

Senecal C, 2000, HEALTH PSYCHOL, V19, P452, DOI 10.1037//0278-6133.19.5.452

Sigal RJ, 2013, CAN J DIABETES, V37, pS40, DOI 10.1016/j.jcjd.2013.01.018

Snowling NJ, 2006, DIABETES CARE, V29, P2518, DOI 10.2337/dc06-1317

Standage M, 2008, J SPORT EXERCISE PSY, V30, P337, DOI 10.1123/jsep.30.4.337

Stephan Y, 2010, PSYCHOL WOMEN QUART, V34, P339, DOI 10.1111/j.1471-6402.2010.01579.x

Strath SJ, 2013, CIRCULATION, V128, P2259, DOI 10.1161/01.cir.0000435708.67487.da

Swartz AM, 2000, MED SCI SPORT EXER, V32, pS450, DOI 10.1097/00005768-200009001-00003

Sweet SN, 2009, PSYCHOL HEALTH MED, V14, P419, DOI 10.1080/13548500903111806

Tabachnick B. G., 2013, USING MULTIVARIATE S

Teixeira PJ, 2012, INT J BEHAV NUTR PHY, V9, DOI 10.1186/1479-5868-9-78

Troiano RP, 2008, MED SCI SPORT EXER, V40, P181, DOI 10.1249/mss.0b013e31815a51b3

Umpierre D, 2013, DIABETOLOGIA, V56, P242, DOI 10.1007/s00125-012-2774-z

Umpierre D, 2011, JAMA-J AM MED ASSOC, V305, P1790, DOI 10.1001/jama.2011.576

Urich-French S, 2009, J SPORT EXERCISE PSY, V31, P358

Vallerand RJ, 1997, ADV EXP SOC PSYCHOL, V29, P271, DOI 10.1016/S0065-2601(08)60019-2

Vansteenkiste M, 2009, J EDUC PSYCHOL, V101, P671, DOI 10.1037/a0015083

Wang CKJ, 2001, J SPORT EXERCISE PSY, V23, P1

Williams GC, 2004, HEALTH PSYCHOL, V23, P58, DOI 10.1037/0278-6133.23.1.58

Yu C-A, 2015, MOTIVATION PHYS ACTI

NR 73

TC 0

Z9 0

U1 2

U2 5

PU BMC

PI LONDON

PA CAMPUS, 4 CRINAN ST, LONDON N1 9XW, ENGLAND

SN 1471-2458

J9 BMC PUBLIC HEALTH

JI BMC Public Health

PD APR 10

PY 2018

VL 18

AR 469

DI 10.1186/s12889-018-5376-y

PG 15

WC Public, Environmental & Occupational Health

SC Public, Environmental & Occupational Health

GA GC5TN

UT WOS:000429851700008

PM 29636035

OA DOAJ Gold, Green Published

DA 2019-01-08

ER

PT J

AU Bani-issa, W

Al-Shujairi, AM

Patrick, L

AF Bani-issa, Wegdan

Al-Shujairi, Arwa M.

Patrick, Linda

TI Association between quality of sleep and health-related quality of life

in persons with diabetes mellitus type 2

SO JOURNAL OF CLINICAL NURSING

LA English

DT Article

DE daytime dysfunction; diabetes mellitus type 2; health-related quality of

life; sleep quality; subjective sleep quality; use of sleep medication

ID RESTLESS LEGS SYNDROME; GLYCEMIC CONTROL; PEOPLE; ADULTS; APNEA; RISK;

CARE; DISTURBANCES; DEPRESSION; IMPACT

AB Aim and objectives: To estimate the relationship of sleep quality with health-related quality of life (HRQOL) in persons with diabetes mellitus type 2 (DMT2) living in the United Arab Emirates (UAE).

Background: DMT2 is an epidemic health condition in the UAE that has enormous impacts on heath, and consequent effects on HRQOL. However, because of an absence of screening for quality of sleep, people with DMT2 who experience poor sleep are likely to go untreated, which may compound the distressing impacts of DMT2 on their HRQOL.

Design: This is a cross-sectional quantitative research design.

Methods: A sample of 268 participants with DMT2 were recruited from community healthcare settings in the UAE using cluster sampling. Participants completed questionnaires, including the Pittsburgh Sleep Quality Index (PSQI) and the World Health Organization HRQOL. Data analysis used descriptive and correlational statistics.

Results: Of the 268 participants, 34% identified as poor sleepers and 55% had poor HRQOL. Poor sleepers showed significantly lower scores for HRQOL than good sleepers. The global PSQI scores were found to be independently predictive of global HRQOL. Subjective perceptions of sleep quality, the use of sleep medications and impaired daytime functioning were the variables found to have the highest correlations with global HRQOL and its four domains.

Conclusions: This study found that people with DMT2 who indicate experiencing poor quality sleep are more likely to show a negative correlation with HRQOL. Additional research is needed to investigate how poor sleep may impact the health of people with DMT2.

C1 [Bani-issa, Wegdan] Univ Sharjah, Dept Nursing, Coll Hlth Sci, Sharjah, U Arab Emirates.

[Al-Shujairi, Arwa M.] Univ Sharjah, Res Inst Med & Hlth Sci, Sharjah, U Arab Emirates.

[Patrick, Linda] Univ Windsor, Fac Nursing, Windsor, CT USA.

RP Bani-issa, W (reprint author), Univ Sharjah, Dept Nursing, Coll Hlth Sci, Sharjah, U Arab Emirates.

EM wbaniissa@sharjah.ac.ae

FU Health Promotion Research Group/Research Institute for Medical and

Health Sciences Research/University of Sharjah, United Arab Emirates

[150310]

FX This work was supported by the Health Promotion Research Group/Research

Institute for Medical and Health Sciences Research/University of

Sharjah, United Arab Emirates. Grant number: 150310.

CR Anderson RM, 2000, DIABETES EDUCATOR, V26, P597, DOI 10.1177/014572170002600405

[Anonymous], 2017, Diabetes Care, V40, pS4

[Anonymous], 1996, PROGR MENT HLTH

Anothaisintawee T, 2016, SLEEP MED REV, V30, P11, DOI 10.1016/j.smrv.2015.10.002

BaHammam AS, 2011, ANN THORAC MED, V6, P187, DOI 10.4103/1817-1737.84771

Bani-Issa W., 2011, Eastern Mediterranean Health Journal, V17, P825

Silva PAB, 2014, REV SAUDE PUBL, V48, P390, DOI 10.1590/S0034-8910.2014048004912

Barone MTU, 2011, DIABETES RES CLIN PR, V91, P129, DOI 10.1016/j.diabres.2010.07.011

BUYSSE DJ, 1989, PSYCHIAT RES, V28, P193, DOI 10.1016/0165-1781(89)90047-4

Cass AR, 2013, FAM MED, V45, P492

Chasens Eileen R, 2016, Diabetes Spectr, V29, P20, DOI 10.2337/diaspect.29.1.20

Chasens ER, 2014, APPL NURS RES, V27, P237, DOI 10.1016/j.apnr.2014.02.006

Dean A. G., 2016, OPENEPI OPEN SOURCE

Eljedi A, 2006, BMC PUBLIC HEALTH, V6, DOI 10.1186/1471-2458-6-268

Grimaldi D, 2014, DIABETES CARE, V37, P355, DOI 10.2337/dc13-0933

Herman WH, 2015, DIABETES CARE, V38, P1449, DOI 10.2337/dc14-2459

Institute of Medicine, 2006, SLEEP DISORDERS SLEE, DOI [10. 17226/11617, DOI 10.17226/11617]

Kim TH, 2016, SLEEP MED, V27-28, P72, DOI 10.1016/j.sleep.2016.07.023

Kuznetsov L, 2015, DIABETES-METAB RES, V31, P69, DOI 10.1002/dmrr.2559

Lecube A, 2016, PLOS ONE, V11, DOI 10.1371/journal.pone.0157579

Lou PA, 2015, DIABETES RES CLIN PR, V107, P69, DOI 10.1016/j.diabres.2014.09.060

Lou PA, 2012, BMJ OPEN, V2, DOI 10.1136/bmjopen-2012-000956

Luyster FS, 2011, DIABETES EDUCATOR, V37, P347, DOI 10.1177/0145721711400663

Maglione JE, 2014, SLEEP, V37, P1179, DOI 10.5665/sleep.3834

Merlino G, 2010, DIABETES EDUCATOR, V36, P79, DOI 10.1177/0145721709351252

Nedeltcheva AV, 2014, CURR OPIN ENDOCRINOL, V21, P293, DOI 10.1097/MED.0000000000000082

Plantinga L, 2012, PREV CHRONIC DIS, V9, DOI 10.5888/pcd9.110244

Rubin RR, 1999, DIABETES-METAB RES, V15, P205, DOI 10.1002/(SICI)1520-7560(199905/06)15:3<205::AID-DMRR29>3.0.CO;2-O

Shackman G., 2001, ALB AM STAT ASS ALB

Shaw JE, 2008, DIABETES RES CLIN PR, V81, P2, DOI 10.1016/j.diabres.2008.04.025

Sicree R., 2015, GLOBAL BURDEN DIABET

Siersma V, 2014, DIABETES CARE, V37, P694, DOI 10.2337/dc13-1212

Skomro RP, 2001, SLEEP MED, V2, P417, DOI 10.1016/S1389-9457(01)00110-1

Skomro R. P., 2008, SLEEP QUALITY LIFE C, P461, DOI 10. 1007/978-1-60327-343-5

Sparring V, 2013, BMC PUBLIC HEALTH, V13, DOI 10.1186/1471-2458-13-377

SRIDHAR GR, 1994, DIABETES RES CLIN PR, V23, P183, DOI 10.1016/0168-8227(94)90103-1

Storgaard H, 2014, DIABETIC MED, V31, P1460, DOI 10.1111/dme.12477

Suleiman KH, 2010, WESTERN J NURS RES, V32, P250, DOI 10.1177/0193945909348230

Sullivan P., 2016, CIRCULATION CARDI S2, V9, pA160

Tang YZ, 2014, CHINESE MED J-PEKING, V127, P3543, DOI 10.3760/cma.j.issn.0366-6999.20141512

Tsai YW, 2012, FAM PRACT, V29, P30, DOI 10.1093/fampra/cmr041

Wadden TA, 2014, DIABETES CARE, V37, P1544, DOI 10.2337/dc13-1928

Wandell PE, 2005, SCAND J PRIM HEALTH, V23, P68, DOI 10.1080/02813430510015296

Zhou B, 2016, LANCET, V387, P1513, DOI 10.1016/S0140-6736(16)00618-8

NR 44

TC 0

Z9 0

U1 2

U2 2

PU WILEY

PI HOBOKEN

PA 111 RIVER ST, HOBOKEN 07030-5774, NJ USA

SN 0962-1067

EI 1365-2702

J9 J CLIN NURS

JI J. Clin. Nurs.

PD APR

PY 2018

VL 27

IS 7-8

BP 1653

EP 1661

DI 10.1111/jocn.14221

PG 9

WC Nursing

SC Nursing

GA GD9HZ

UT WOS:000430825100072

PM 29266588

DA 2019-01-08

ER

PT J

AU Kuniss, N

Muller, UA

Kloos, C

Wolf, G

Kramer, G

AF Kuniss, Nadine

Mueller, Ulrich Alfons

Kloos, Christof

Wolf, Gunter

Kramer, Guido

TI Reduction of HbA1c and diabetes-related distress after intervention in a

diabetes day care clinic in people with type 2 diabetes but not with

type 1 diabetes

SO EXPERIMENTAL AND CLINICAL ENDOCRINOLOGY & DIABETES

LA English

DT Article

DE diabetes day care clinic; diabetes-related distress; PAID questionnaire

ID RANDOMIZED CONTROLLED-TRIAL; QUALITY-OF-LIFE; INSULIN-TREATMENT;

TEACHING PROGRAM; EMOTIONAL DISTRESS; PROBLEM AREAS; ROUTINE CARE;

DEPRESSION; EDUCATION; MANAGEMENT

AB Objective The aim of this prospective, longitudinal study was to assess diabetes-related distress in people with diabetes mellitus type 1 (DM1) and type 2 (DM2) treated for diabetes-related problems and to evaluate, whether distress by diabetes are reduced after intervention in a diabetes day care clinic (DDC).

Methods Diabetes-related burden was assessed with the "Problem Area In Diabetes (PAID) questionnaire before (T0), directly after (T1) and 6 months after intervention (T2) in the DDC in 72 people (18 DM1, 54 DM2) admitted with a diabetes-specific problem to a DDC in a University department for metabolic diseases. A PAID score of >= 40 was considered as high diabetes-related distress.

Results The PAID score in people with DM1 was not significantly different between T0, T1 and T2. Furthermore, neither HbA1c nor insulin dose did change at T2. In participants with DM2, the PAID score decreased significantly from T0 to T1 (18.4 +/- 15.9 vs. 15.4 +/- 13.0, p=0.042) and preserved the benefit at T2 (15.0 +/- 13.3). In addition, there was a significant reduction in HbA1c (-1.2%, p<0.001) and body weight (-1.3 kg, p=0.038) between T0 and T2. Insulin dose per day was 15.0 IU/day lower at T1 (p=0.006) and 9.0 IU/day lower at T2 (p=0.212) in comparison to T0.

Conclusions Diabetes-related distress was reduced after a problem-oriented intervention in a DDC in people with DM2 but not with DM1. Although, all participants at tertiary care level had serious diabetes-specific problems before intervention, mean PAID score was far under the threshold of 40 points thus distress was low.

C1 [Kuniss, Nadine; Mueller, Ulrich Alfons; Kloos, Christof; Wolf, Gunter; Kramer, Guido] Jena Univ Hosp, Dept Internal Med 3, Jena, Germany.

RP Kuniss, N (reprint author), Jena Univ Hosp, Endocrinol & Metab Disorders, Dept Internal Med 3, Klinikum 1, D-07740 Jena, Germany.

EM nadine.kuniss@med.uni-jena.de

CR Amiel S, 2002, BRIT MED J, V325, P746

Bradley C, 1999, QUAL LIFE RES, V8, P79, DOI 10.1023/A:1026485130100

Bradley C, 1994, DIABETES TREATMENT S

Cooke D, 2013, DIABETES CARE, V36, P270, DOI 10.2337/dc12-0080

Dulon M, 2003, GESUNDHEITSWESEN, V65, P629

German Diabetes Association (DDG), 2015, GUID REC TREAT I CER

Gru<ss>er M, 2012, STRUKTURIERTES BEHAN

Gru<ss>er M, 2009, STRUKTURIERTES BEHAN

Gruesser M, 1996, PATIENT EDUC COUNS, V29, P123, DOI 10.1016/0738-3991(96)00941-X

Hagen B, 2015, QUALITY ASSURANCE RE

Hermanns N, 2006, DIABETOLOGIA, V49, P469, DOI 10.1007/s00125-005-0094-2

Hermanns N, 2015, DIABETES CARE, V38, P551, DOI 10.2337/dc14-1416

Jorgens V, 2011, STRUKTURIERTES BEHAN

Jorgens V, 2013, STRUKTURIERTES BEHAN

Kalfhaus J, 2000, DIABETES METAB, V26, P197

Kulzer BAC, 2013, S2 GUIDELINE PSYCHOS

Kuniss N, 2016, EXP CLIN ENDOCR DIAB, V124, P307, DOI 10.1055/s-0042-103685

Kuniss N, 2016, ACTA DIABETOL, V53, P833, DOI 10.1007/s00592-016-0881-x

MUHLHAUSER I, 1987, DIABETOLOGIA, V30, P681, DOI 10.1007/BF00296989

Muller UA, 1998, DIABETES METAB, V24, P251

Petrak F, 2011, DIABETES STOFFWECH H, V20, P7

PIEBER TR, 1995, DIABETES CARE, V18, P625, DOI 10.2337/diacare.18.5.625

Pintaudi B, 2015, J PSYCHOSOM RES, V79, P348, DOI 10.1016/j.jpsychores.2015.08.010

POLONSKY WH, 1995, DIABETES CARE, V18, P754, DOI 10.2337/diacare.18.6.754

Pouwer F, 2005, DIABETES RES CLIN PR, V70, P166, DOI 10.1016/j.diabres.2005.03.031

Schmitt A, 2015, J BEHAV MED, V38, P556, DOI 10.1007/s10865-014-9604-3

Schumann M., 2013, ELECT MED INFORM SYS

Seaquist ER, 2013, DIABETES CARE, V36, P1384, DOI 10.2337/dc12-2480

SHAMOON H, 1993, NEW ENGL J MED, V329, P977

Snoek FJ, 2000, DIABETES CARE, V23, P1305, DOI 10.2337/diacare.23.9.1305

Speight J, 2016, DIABETES RES CLIN PR, V112, P65, DOI 10.1016/j.diabres.2015.11.002

STAROSTINA EG, 1994, DIABETOLOGIA, V37, P170, DOI 10.1007/s001250050089

Stoop CH, 2014, DIABETIC MED, V31, P1252, DOI 10.1111/dme.12472

Trento M, 2017, ACTA DIABETOL, V54, P415, DOI 10.1007/s00592-016-0935-0

Welch GW, 1997, DIABETES CARE, V20, P760, DOI 10.2337/diacare.20.5.760

WHO, 1998, INF PACK MAST DEPR P

NR 36

TC 0

Z9 0

U1 1

U2 1

PU JOHANN AMBROSIUS BARTH VERLAG MEDIZINVERLAGE HEIDELBERG GMBH

PI STUTTGART

PA RUEDIGERSTR 14, D-70469 STUTTGART, GERMANY

SN 0947-7349

EI 1439-3646

J9 EXP CLIN ENDOCR DIAB

JI Exp. Clin. Endocrinol. Diabet.

PD APR

PY 2018

VL 126

IS 4

BP 242

EP 248

DI 10.1055/s-0043-112862

PG 7

WC Endocrinology & Metabolism

SC Endocrinology & Metabolism

GA GD3YR

UT WOS:000430440200006

PM 28704855

DA 2019-01-08

ER

PT J

AU Hayashino, Y

Okamura, S

Mashitani, T

Tsujii, S

Ishii, H

AF Hayashino, Yasuaki

Okamura, Shintaro

Mashitani, Tsuyoshi

Tsujii, Satoru

Ishii, Hitoshi

CA Diabet Distress Care Registry

TI Association of proton pump inhibitor use with the risk of the

development or progression of albuminuria among Japanese patients with

diabetes: A prospective cohort study [Diabetes Distress and Care

Registry at Tenri (DDCRT 16)]

SO DIABETES RESEARCH AND CLINICAL PRACTICE

LA English

DT Article

DE Diabetes; Proton pump inhibitor; Diabetic nephropathy; Kidney; Renal

disease; Cohort study; Epidemiology; Adults

ID ACUTE INTERSTITIAL NEPHRITIS; TYPE-2; NEPHROTOXICITY; NEPHROPATHY;

OMEPRAZOLE; MELLITUS; DISEASE; KIDNEY

AB Aims: We aimed to determine the prospective association between proton pump inhibitor (PPI) use and the subsequent risk of the development or progression of albuminuria or eGFR.

Methods: Longitudinal data of patients with diabetes were obtained from a large Japanese diabetes registry. To assess the independent correlation between PPI use and the development or progression of urine microalbuminuria, the time-varying Cox proportional hazards model was used with adjustment for potential confounders.

Results: The mean patient age, body-mass index (BMI), and hemoglobin A1c (HbA1c) levels were 65.7 y, 24.5 kg/m(2), and 7.5% (57.9 mmol/mol), respectively. In 1711 patients without albuminuria, we observed 599 cases with development of albuminuria over median follow-up of 4.0 years, and in 1279 patients with microalbuminuria, 290 cases with urinary albuminuria progression over 4.0 years, and 257 eGFR decline cases over 3.8 years. PPI use was not associated with the development of albuminuria (HR = 0.88; 95% CI, 0.77-1.01; p=.058), progression of albuminuria (HR = 1.24; 95% CI, 0.87-1.79; p=.236), nor eGFR decline (HR = 1.05; 95% CI, 0.81-1.34; p=.973) even in a propensity score-adjusted model with time-varyingly updating PPI use information.

Conclusions: In conclusion, PPI use was not associated with the subsequent risk of development or progression of albuminuria, or eGFR decline in patients with diabetes. (C) 2018 Elsevier B.V. All rights reserved.

C1 [Hayashino, Yasuaki; Okamura, Shintaro; Tsujii, Satoru] Tenri Hosp, Dept Endocrinol, 200 Mishima Cho, Tenri, Nara 6328552, Japan.

[Mashitani, Tsuyoshi; Ishii, Hitoshi] Nara Med Univ, Dept Diabetol, Kashihara, Nara 6348521, Japan.

RP Hayashino, Y (reprint author), Tenri Hosp, Dept Endocrinol, 200 Mishima Cho, Tenri, Nara 6328552, Japan.

EM hayasino-y@umin.net

FU Manpei Suzuki Diabetes Foundation; Japan Society for the Promotion of

Science (JSPS) KAKENHI [25460641]

FX This study was partially supported by the Manpei Suzuki Diabetes

Foundation and Japan Society for the Promotion of Science (JSPS) KAKENHI

(Grant No. 25460641); however, they played no role in the study design

or conduct, data collection, analysis, or interpretation, and the

preparation, review, or approval of the manuscript.

CR Afghahi H, 2011, NEPHROL DIAL TRANSPL, V26, P1236, DOI 10.1093/ndt/gfq535

Antoniou Tony, 2015, CMAJ Open, V3, pE166, DOI 10.9778/cmajo.20140074

Berney-Meyer L, 2014, NEPHROLOGY, V19, P359, DOI 10.1111/nep.12226

Blank ML, 2014, KIDNEY INT, V86, P837, DOI 10.1038/ki.2014.74

Brewster UC, 2007, CLIN NEPHROL, V68, P65

Danziger J, 2013, KIDNEY INT, V83, P692, DOI 10.1038/ki.2012.452

Dial S, 2004, CAN MED ASSOC J, V171, P33, DOI 10.1503/cmaj.1040876

Forgacs I, 2008, BMJ-BRIT MED J, V336, P2, DOI 10.1136/bmj.39406.449456.BE

Gai ZB, 2016, ANTIMICROB AGENTS CH, V60, P5573, DOI 10.1128/AAC.00907-16

Gerstein HC, 2001, JAMA-J AM MED ASSOC, V286, P421, DOI 10.1001/jama.286.4.421

Grant K, 2006, PHARM WORLD SCI, V28, P189, DOI 10.1007/s11096-006-9028-4

Hacker K, 2015, PLOS ONE, V10, DOI 10.1371/journal.pone.0136451

Hayashino Y, 2014, DIABETES CARE, V37, P2459, DOI 10.2337/dc13-2312

Ikemura K, 2017, CANCER CHEMOTH PHARM, V79, P943, DOI 10.1007/s00280-017-3296-7

Lambert AA, 2015, PLOS ONE, V10, DOI 10.1371/journal.pone.0128004

Lazarus B, 2016, JAMA INTERN MED, V176, P238, DOI 10.1001/jamainternmed.2015.7193

Mashitani T, 2015, ACTA DIABETOL, V52, P639, DOI 10.1007/s00592-015-0725-0

Mashitani T, 2014, DIABETES CARE, V37, P252, DOI 10.2337/dc13-0407

Matsuo S, 2009, AM J KIDNEY DIS, V53, P982, DOI 10.1053/j.ajkd.2008.12.034

Molitch Mark E, 2004, Diabetes Care, V27 Suppl 1, pS79

Nies AT, 2011, PLOS ONE, V6, DOI 10.1371/journal.pone.0022163

Perazella MA, 2015, EXPERT REV CLIN PHAR, V8, P367, DOI 10.1586/17512433.2015.1045489

Perazella MA, 2010, NAT REV NEPHROL, V6, P461, DOI 10.1038/nrneph.2010.71

Pham PCT, 2005, CLIN NEPHROL, V63, P429

Praga M, 2010, KIDNEY INT, V77, P956, DOI 10.1038/ki.2010.89

Ritz E, 1999, NEW ENGL J MED, V341, P1127, DOI 10.1056/NEJM199910073411506

RUFFENACH SJ, 1992, AM J MED, V93, P472, DOI 10.1016/0002-9343(92)90181-A

Sakaguchi Y, 2012, DIABETES CARE, V35, P1591, DOI 10.2337/dc12-0226

Seino Y, 2010, J DIABETES INVEST, V1, P1, DOI 10.1111/j.2040-1124.2010.00005.x

Xie Y, 2016, J AM SOC NEPHROL, V27, P3153, DOI 10.1681/ASN.2015121377

Yang YX, 2006, JAMA-J AM MED ASSOC, V296, P2947, DOI 10.1001/jama.296.24.2947

NR 31

TC 0

Z9 0

U1 0

U2 0

PU ELSEVIER IRELAND LTD

PI CLARE

PA ELSEVIER HOUSE, BROOKVALE PLAZA, EAST PARK SHANNON, CO, CLARE, 00000,

IRELAND

SN 0168-8227

EI 1872-8227

J9 DIABETES RES CLIN PR

JI Diabetes Res. Clin. Pract.

PD APR

PY 2018

VL 138

BP 1

EP 7

DI 10.1016/j.diabres.2018.01.022

PG 7

WC Endocrinology & Metabolism

SC Endocrinology & Metabolism

GA GD4AS

UT WOS:000430445500001

PM 29382588

DA 2019-01-08

ER

PT J

AU Boden, MT

Gala, S

AF Boden, Matthew Tyler

Gala, Sasha

TI Exploring correlates of diabetes-related stress among adults with Type 1

diabetes in the T1D exchange clinic registry

SO DIABETES RESEARCH AND CLINICAL PRACTICE

LA English

DT Article

DE Type 1 diabetes; Stress; Distress; HbA1c

ID FACTOR SURVEILLANCE SYSTEM; HEALTH INTERVIEW SURVEY; QUALITY-OF-LIFE;

METABOLIC-CONTROL; GLYCEMIC CONTROL; EMOTIONAL DISTRESS; REGIMEN

ADHERENCE; NATIONAL-HEALTH; MELLITUS; OUTCOMES

AB Aims: To explore relations between diabetes-related stress and multiple sociodemographic, diabetes health, other health, and treatment-related variables among a large sample of adults with Type 1 Diabetes (T1D).

Methods: The sample consisted of 10,821 adults (over 18 years old) enrolled in the T1D Exchange Clinic Registry. The T1D Exchange clinic network consists of 67 diabetes clinical centers throughout the United States selected to broadly represent pediatric and adult patients with T1D. Variables were assessed through participant self-report and extraction of clinic chart data. Univariate and multiple linear regression (with simultaneous entry of all predictors) analyses were conducted.

Results: Robustly associated with increased diabetes-related stress across analyses were multiple sociodemographic (female [vs. male], native Hawaiian/other Pacific islander [vs. white/Caucasian], decreased age and diabetes duration), diabetes health (higher HbA1c), other health (lower general health, presence of major life stress and depression, less physical activity), and treatment related variables (use of injections/pen or combination injection/pen/pump [vs. pump], use of CGM, increased frequency of missing insulin doses and BG checking, decreased frequency of BG checking prior to bolus, receipt of mental health treatment).

Conclusions: We replicated and extended research demonstrating that diabetes-related stress among people with T1D occurs at higher levels among those with particular sociodemographic characteristics and is associated with a range poorer diabetes health and other health variables, and multiple treatment-related variables. The strong incremental prediction of diabetes-related stress by multiple variables in our study suggests that a multivariable, personalized approach may increase the effectiveness of treatments for diabetes-related stress. Published by Elsevier Ireland Ltd.

C1 [Boden, Matthew Tyler] VA Palo Alto Hlth Care Syst, Ctr Innovat Implementat, Menlo Pk, CA USA.

[Gala, Sasha] VA Palo Alto Hlth Care Syst, Natl Ctr PTSD, Menlo Pk, CA USA.

RP Boden, MT (reprint author), 795 Willow Rd,152-MPD, Menlo Pk, CA 94025 USA.

EM Matthew.Boden@va.gov

CR Aikens JE, 2012, DIABETES CARE, V35, P2472, DOI 10.2337/dc12-0181

AIKENS JE, 1992, J CONSULT CLIN PSYCH, V60, P113, DOI 10.1037/0022-006X.60.1.113

Cohen J., 2013, APPL MULTIPLE REGRES

Cox DJ, 1994, HEALTH PSYCHOL, V3, P63

Dollinger SJ, 2009, J GEN PSYCHOL, V136, P231, DOI 10.3200/GENP.136.3.231-242

DURBIN J, 1971, BIOMETRIKA, V58, P1

Eicker F., 1967, P 5 BERK S MATH STAT, V1, P59

Farrell SP, 2004, J ADOLESCENT HEALTH, V34, P461, DOI 10.1016/S1054-139X(03)00215-5

Fisher L, 2007, DIABETES CARE, V30, P542, DOI 10.2337/dc06-1614

Fisher L, 2016, J DIABETES COMPLICAT, V30, P1123, DOI 10.1016/j.jdiacomp.2016.03.032

Fisher L, 2010, DIABETES CARE, V33, P23, DOI 10.2337/dc09-1238

Hermanns N, 2009, DIABETES TECHNOL THE, V11, P275, DOI 10.1089/dia.2008.0078

Huber P. J., 1967, P 5 BERK S MATH STAT, V1, P221

Iturralde E, 2017, HEALTH PSYCHOL, V36, P236, DOI 10.1037/hea0000445

Jamieson S, 2004, MED EDUC, V38, P1217, DOI 10.1111/j.1365-2929.2004.02012.x

KEMMER FW, 1986, NEW ENGL J MED, V314, P1078, DOI 10.1056/NEJM198604243141704

Li CY, 2012, PREV MED, V54, P381, DOI 10.1016/j.ypmed.2012.04.003

Lloyd CE, 2010, DIABETIC MED, V27, P234, DOI 10.1111/j.1464-5491.2009.02896.x

Lynam DR, 2006, ASSESSMENT, V13, P328, DOI 10.1177/1073191106290562

Mauss IB, 2005, EMOTION, V5, P175, DOI 10.1037/1528-3542.5.2.175

McGuire BE, 2010, DIABETOLOGIA, V53, P66, DOI 10.1007/s00125-009-1559-5

Nelson DE, 2003, AM J PUBLIC HEALTH, V93, P1335, DOI 10.2105/AJPH.93.8.1335

Nicolucci A, 2013, DIABETIC MED, V30, P767, DOI 10.1111/dme.12245

Norman G, 2010, ADV HEALTH SCI EDUC, V15, P625, DOI 10.1007/s10459-010-9222-y

Pena EA, 2006, J AM STAT ASSOC, V101, P341, DOI 10.1198/016214505000000637

Peyrot M, 1999, J HEALTH SOC BEHAV, V40, P141, DOI 10.2307/2676370

Polonsky WH, 2005, DIABETES CARE, V28, P626, DOI 10.2337/diacare.28.3.626

R Core Team, 2013, R LANG ENV STAT COMP

REES WL, 1976, BRIT J PSYCHIAT, V128, P3, DOI 10.1192/bjp.128.1.3

Riazi A, 2004, DIABETES RES CLIN PR, V66, P237, DOI 10.1016/j.diabres.2004.04.001

Strandberg RB, 2015, DIABETIC MED, V32, P1304, DOI 10.1111/dme.12781

Sturt J, 2015, INT DIAB NURS, V12, P40, DOI DOI 10.1179/2057332415Y.0000000004

Sturt J, 2015, CURR DIABETES REP, V15, P1, DOI DOI 10.1007/S11892-015-0660-Z

WATSON D, 1989, PSYCHOL REV, V96, P234, DOI 10.1037//0033-295X.96.2.234

Weinger K, 2001, PATIENT EDUC COUNS, V42, P123, DOI 10.1016/S0738-3991(00)00098-7

Welch GW, 1997, DIABETES CARE, V20, P760, DOI 10.2337/diacare.20.5.760

WHITE H, 1980, ECONOMETRICA, V48, P817, DOI 10.2307/1912934

Young-Hyman D, 2016, DIABETES CARE, V39, P2126, DOI 10.2337/dc16-2053

Zimmerman M, 2006, J CLIN PSYCHIAT, V67, P1536, DOI 10.4088/JCP.v67n1007

Zoffmann V, 2014, DIABETIC MED, V31, P493, DOI 10.1111/dme.12363

NR 40

TC 0

Z9 0

U1 0

U2 0

PU ELSEVIER IRELAND LTD

PI CLARE

PA ELSEVIER HOUSE, BROOKVALE PLAZA, EAST PARK SHANNON, CO, CLARE, 00000,

IRELAND

SN 0168-8227

EI 1872-8227

J9 DIABETES RES CLIN PR

JI Diabetes Res. Clin. Pract.

PD APR

PY 2018

VL 138

BP 211

EP 219

DI 10.1016/j.diabres.2017.10.012

PG 9

WC Endocrinology & Metabolism

SC Endocrinology & Metabolism

GA GD4AS

UT WOS:000430445500026

PM 29107758

DA 2019-01-08

ER

PT J

AU Poon, AWC

Curtis, J

Ward, P

Loneragan, C

Lappin, J

AF Poon, Abner Weng Cheong

Curtis, Jackie

Ward, Philip

Loneragan, Celia

Lappin, Julia

TI Physical and psychological health of carers of young people with first

episode psychosis

SO AUSTRALASIAN PSYCHIATRY

LA English

DT Article

DE carer; first episode psychosis; physical health; mental health; youth

ID SCHIZOPHRENIA; CAREGIVERS

AB Objective: Carers of people with psychosis may experience psychological distress and caregiving burden. However, few studies have examined both psychological and physical health of carers of young people with first episode psychosis (FEP).

Method: A total of 32 young people with FEP and 42 of their carers were recruited from a mental health service. Standardised scales were administered to assess carers' psychological distress and risk for development of Type 2 diabetes. Their body mass index, waist circumference and blood pressure were measured.

Results: A total of 24% (n = 10) of carers experienced high/very high psychological distress and 39.0% (n = 16) had high risk for Type 2 diabetes. It was common for carers to be overweight (n = 33, 78.6%) and to have hypertension (n = 14, 33.3%). Carers' higher levels of psychological distress were associated with shorter duration of illness in the young person.

Conclusions: Caring for a young person with FEP is associated with poor physical and psychological health. Findings show the importance of supporting carers' physical and psychological health early in treatment of young people with FEP.

C1 [Poon, Abner Weng Cheong] Univ New South Wales, Sch Social Sci, Morven Brown Bldg, Sydney, NSW 2052, Australia.

[Curtis, Jackie] South Eastern Sydney Local Hlth Dist, Youth Mental Hlth, Sydney, NSW, Australia.

[Curtis, Jackie; Loneragan, Celia] Univ New South Wales, Sydney, NSW, Australia.

[Ward, Philip; Lappin, Julia] Univ New South Wales, Sch Psychiat, Sydney, NSW, Australia.

[Ward, Philip] Univ New South Wales, South Western Sydney Local Hlth Dist, Schizophrenia Res Unit, Sydney, NSW, Australia.

RP Poon, AWC (reprint author), Univ New South Wales, Sch Social Sci, Morven Brown Bldg, Sydney, NSW 2052, Australia.

EM w.poon@unsw.edu.au

OI Poon, Weng Cheong/0000-0003-2863-5916

FU School of Social Sciences, University of New South Wales Australia [SPG

2015]

FX This research project was supported by the School of Social Sciences,

University of New South Wales Australia (grant number: SPG 2015 - 13

July 2015).

CR Addington J, 2003, ACTA PSYCHIAT SCAND, V108, P285, DOI 10.1034/j.1600-0447.2003.00153.x

American Psychiatric Association, 2000, DIAGN STAT MAN MENT

Australian Bureau of Statistics, 2012, AUSTR HLTH SURV 1 RE

Australian Bureau of Statistics, 2015, NAT HLTH SURV 1 RES

Awad AG, 2008, PHARMACOECONOMICS, V26, P149, DOI 10.2165/00019053-200826020-00005

Bradford S, 2015, BMC PSYCHIATRY, V15, DOI 10.1186/s12888-015-0694-4

Cotton SM, 2013, SCHIZOPHR RES, V146, P118, DOI 10.1016/j.schres.2013.02.008

Curtis J, 2016, EARLY INTERV PSYCHIA, V10, P267, DOI 10.1111/eip.12230

Department of Health and Ageing, 2010, AUSTR TYP 2 DIAB RIS

Gupta S, 2015, BMC PSYCHIATRY, V15, DOI 10.1186/s12888-015-0547-1

IBM Corp, 2012, IBM SPSS STAT WINDOW

Kessler RC, 2002, PSYCHOL MED, V32, P959, DOI 10.1017/S0033291702006074

Poon AWC, 2017, EPIDEMIOL PSYCH SCI, V26, P265, DOI 10.1017/S2045796015001195

Sin J, 2016, SOC PSYCH PSYCH EPID, V51, P1247, DOI 10.1007/s00127-016-1222-7

Tanamas SK, 2013, AUSDIAB 2012 AUSTR D

Thunyadee C, 2015, NURS HEALTH SCI, V17, P412, DOI 10.1111/nhs.12205

World Health Organization, 1992, INT CLASS DIS

NR 17

TC 1

Z9 1

U1 0

U2 5

PU SAGE PUBLICATIONS LTD

PI LONDON

PA 1 OLIVERS YARD, 55 CITY ROAD, LONDON EC1Y 1SP, ENGLAND

SN 1039-8562

EI 1440-1665

J9 AUSTRALAS PSYCHIATRY

JI Australas. Psychiatry

PD APR

PY 2018

VL 26

IS 2

BP 184

EP 188

DI 10.1177/1039856217748250

PG 5

WC Psychiatry

SC Psychiatry

GA GC4LL

UT WOS:000429755900014

PM 29334229

DA 2019-01-08

ER

PT J

AU Hood, S

Irby-Shasanmi, A

de Groot, M

Martin, E

LaJoie, AS

AF Hood, Sula

Irby-Shasanmi, Amy

de Groot, Mary

Martin, Elissabeth

LaJoie, Andrew S.

TI Understanding Diabetes-Related Distress Characteristics and Psychosocial

Support Preferences of Urban African American Adults Living With Type 2

Diabetes: A Mixed-Methods Study

SO DIABETES EDUCATOR

LA English

DT Article

ID SELF-MANAGEMENT; DEPRESSION; WOMEN; CARE; SCALE; CHALLENGE; DISEASE;

PROFILE; IMPACT; STIGMA

AB Purpose The purpose of this study is to understand diabetes-related distress (DRD) characteristics and identify psychosocial support preferences of urban African American adults living with type 2 diabetes (T2DM).

Methods A 2-phase, mixed-methods sequential explanatory study design was used to gather data. In phase 1, a purposive sample of participants (N = 155) was recruited and asked to complete a written survey. The Diabetes Distress Scale (DDS17) was used to assess DRD, including subscalesemotional burden (EB), regimen distress (RD), interpersonal distress (ID), and physician distress (PD). In phase 2, a subset of phase 1 participants (N = 23) volunteered to attend 1 of 4 gender-stratified follow-up focus groups to contextualize the quantitative survey results.

Results Survey findings indicate that on average, participants had moderate levels of DRD (aggregate), RD, and EB but had low ID and PD. During follow-up focus groups, participants described RD and EB as their primary distress types and emphasized that clinicians should prioritize the mental health aspects of T2DM similarly to its physical aspects. Participants expressed a desire for culturally appropriate peer support groups as a psychosocial support resource for distress coping and specifically requested the development of gender-stratified groups and groups for young adults.

Conclusions Results support the need to screen for and address diabetes-related distress among African American patients with T2DM. Findings also inform the development of culturally appropriate psychosocial support resources to facilitate diabetes-related distress coping.

C1 [Hood, Sula] Indiana Univ, Richard M Fairbanks Sch Publ Hlth, Dept Social & Behav Sci, Off 6051,1050 Wishard Blvd, Indianapolis, IN 46202 USA.

[Irby-Shasanmi, Amy] Univ West Georgia, Dept Sociol, Carrollton, GA USA.

[de Groot, Mary] Indiana Univ Sch Med, Div Endocrinol, Indianapolis, IN 46202 USA.

[Martin, Elissabeth] Univ Arkansas Med Sci, Coll Med, Little Rock, AR 72205 USA.

[LaJoie, Andrew S.] Univ Louisville, Dept Hlth Promot & Behav Sci, Sch Publ Hlth & Informat Sci, Louisville, KY 40292 USA.

RP Hood, S (reprint author), Indiana Univ, Richard M Fairbanks Sch Publ Hlth, Dept Social & Behav Sci, Off 6051,1050 Wishard Blvd, Indianapolis, IN 46202 USA.

EM sulahood@iu.edu

FU University of Louisville Commission on Diversity and Racial Equality;

Southern Regional Education Board

FX This research was supported by a Signature Partnership Grant from the

University of Louisville Commission on Diversity and Racial Equality and

a Doctoral Scholar Dissertation Award from the Southern Regional

Education Board. A special thank you to Dr A. O'tayo Lalude of the

Westside Family Medical Center and Dr Gina Pariser of the Active Steps

for Diabetes program for assistance with participant recruitment.

CR 100 Black Men of America Inc, WHAT THEY SEE IS WHA

Aghili R, 2017, PRIM CARE DIABETES, V11, P467, DOI 10.1016/j.pcd.2017.05.001

Akindana Adeola, 2016, AADE Pract, V4, P44

Anderson RM, 1996, DIABETES EDUCATOR, V22, P28, DOI 10.1177/014572179602200104

[Anonymous], 2017, DIABETES CARE, V40, pS1, DOI 10.2337/dc17-S001

Bogner HR, 2010, DIABETES EDUCATOR, V36, P284, DOI 10.1177/0145721709356115

Byrd GS, 2011, J NATL MED ASSOC, V103, P480, DOI 10.1016/S0027-9684(15)30361-8

Centers for Disease Control and Prevention, 2017, NAT DIAB STAT REP 20

Chlebowy DO, 2010, DIABETES EDUCATOR, V36, P897, DOI 10.1177/0145721710385579

Conner KO, 2010, AM J GERIAT PSYCHIAT, V18, P531, DOI 10.1097/JGP.0b013e3181cc0366

Crabtree K, 2015, DIABETES EDUCATOR, V41, P118, DOI 10.1177/0145721714557043

Creswell J. W., 2011, DESIGNING CONDUCTING

DeCoster VA, 2003, SOC WORK HEALTH CARE, V36, P79, DOI 10.1300/J010v36n04_05

Dismuke CE, 2014, INT J PSYCHIAT MED, V48, P135, DOI 10.2190/PM.48.2.f

Egede LE, 2002, DIABETES EDUCATOR, V28, P258, DOI 10.1177/014572170202800211

Egede LE, 2006, MED CLIN N AM, V90, P627, DOI 10.1016/j.mcna.2006.04.001

Elo S, 2008, J ADV NURS, V62, P107, DOI 10.1111/j.1365-2648.2007.04569.x

Feifer C, 1999, PROF PSYCHOL-RES PR, V30, P14, DOI 10.1037//0735-7028.30.1.14

Fisher L, 2008, DIABETIC MED, V25, P1096, DOI 10.1111/j.1464-5491.2008.02533.x

Fisher L, 2012, DIABETES CARE, V35, P259, DOI 10.2337/dc11-1572

Fitzgerald JT, 1996, EVAL HEALTH PROF, V19, P208, DOI 10.1177/016327879601900205

Gonzalez JS, 2011, DIABETES CARE, V34, P236, DOI 10.2337/dc10-1970

Hausmann LRM, 2010, PATIENT PREFER ADHER, V4, P291, DOI 10.2147/PPA.S12353

Hawkins JL, 2015, J AGING PHYS ACTIV, V23, P161, DOI 10.1123/japa.2013-0171

Hessler DM, 2012, J HEALTH PSYCHOL, V17, P535, DOI 10.1177/1359105311422120

Jones RA, 2008, DIABETES EDUCATOR, V34, P318, DOI 10.1177/0145721708314485

Kasteleyn MJ, 2015, DIABETIC MED, V32, P1617, DOI 10.1111/dme.12743

Lehrer HM, 2017, DIABETES EDUCATOR, V43, P367, DOI 10.1177/0145721717714894

Lennon-Dearing R, 2008, J HIV-AIDS SOC SERV, V7, P27, DOI 10.1080/15381500802093142

Li CY, 2009, INT J PUBLIC HEALTH, V54, P43, DOI 10.1007/s00038-009-0006-1

Miller ST, 2011, J HEALTH CARE POOR U, V22, P1239, DOI 10.1353/hpu.2011.0110

Pariser G, 2014, DIABETES EDUCATOR, V40, P60, DOI 10.1177/0145721713514281

Penckofer S, 2007, DIABETES EDUCATOR, V33, P680, DOI 10.1177/0145721707304079

Polonsky WH, 2005, DIABETES CARE, V28, P626, DOI 10.2337/diacare.28.3.626

Pouwer F, 2009, NAT REV ENDOCRINOL, V5, P665, DOI 10.1038/nrendo.2009.214

Saldana J, 2013, CODING MANUAL QUALIT

Samuel-Hodge CD, 2000, DIABETES CARE, V23, P928, DOI 10.2337/diacare.23.7.928

Schiotz ML, 2012, DIABETIC MED, V29, P654, DOI 10.1111/j.1464-5491.2011.03485.x

Spencer MS, 2006, J HEALTH CARE POOR U, V17, P88, DOI 10.1353/hpu.2006.0095

Stang I., 2008, INT J MENT HEALTH PR, V10, P15, DOI DOI 10.1080/14623730.2008.9721759

Tang TS, 2011, PATIENT EDUC COUNS, V85, P160, DOI 10.1016/j.pec.2010.12.013

Toobert DJ, 2000, DIABETES CARE, V23, P943, DOI 10.2337/diacare.23.7.943

US Department of Health and Human Services Office of Minority Health, 2016, DIAB AFR AM

Verhaak PFM, 2005, SOC SCI MED, V60, P789, DOI 10.1016/j.socscimed.2004.06.012

Wagner JA, 2009, DIABETES RES CLIN PR, V86, P111, DOI 10.1016/j.diabres.2009.08.004

Ward EC, 2013, NURS RES, V62, P185, DOI 10.1097/NNR.0b013e31827bf533

Young-Hyman D, 2016, DIABETES CARE, V39, P2126, DOI 10.2337/dc16-2053

NR 47

TC 0

Z9 0

U1 2

U2 3

PU SAGE PUBLICATIONS INC

PI THOUSAND OAKS

PA 2455 TELLER RD, THOUSAND OAKS, CA 91320 USA

SN 0145-7217

EI 1554-6063

J9 DIABETES EDUCATOR

JI Diabetes Educ.

PD APR

PY 2018

VL 44

IS 2

BP 144

EP 157

DI 10.1177/0145721718754325

PG 14

WC Endocrinology & Metabolism; Public, Environmental & Occupational Health

SC Endocrinology & Metabolism; Public, Environmental & Occupational Health

GA GC7EH

UT WOS:000429955100004

PM 29375023

DA 2019-01-08

ER

PT J

AU Halepian, L

Saleh, MB

Hallit, S

Khabbaz, LR

AF Halepian, Lucine

Saleh, Mary Bou

Hallit, Souheil

Khabbaz, Lydia Rabbaa

TI Adherence to Insulin, Emotional Distress, and Trust in Physician Among

Patients with Diabetes: A Cross-Sectional Study

SO DIABETES THERAPY

LA English

DT Article

DE Adherence; Diabetes; Emotional distress; Insulin; Trust in physician

ID MEDICATION ADHERENCE; GLYCEMIC CONTROL; SELF-MANAGEMENT; SOCIAL SUPPORT;

DEPRESSION; CARE; ASSOCIATION; MELLITUS; HEALTH; SCALE

AB Introduction: Type 2 diabetes represents a significant public health issue, with increasing prevalence in developing countries while adherence to insulin treatment remains a challenge. No studies have evaluated the relationship between adherence to insulin, diabetes-related distress, and trust in physician among persons with diabetes. Our objectives were to evaluate treatment adherence to insulin, emotional distress (using the Problem Areas in Diabetes Questionnaire, PAID), trust in physician, and to examine associations between them among Lebanese patients with diabetes.

Methods: This cross-sectional study, conducted in all districts of Lebanon between August 2016 and April 2017, enrolled 135 adult patients.

Results: The mean percentage score of adherence to insulin was 79.7 +/- 19.94. A significantly higher mean adherence score was found in non-sedentary (81.96) compared to sedentary patients (67.41) (p = 0.017), with no difference between gender, employment, rural vs non-rural residence, or familial history of diabetes. In addition, no significant relationship was seen between adherence score and education level, smoking, or alcohol intake. A significant positive association was found between trust in physician and adherence scores, whereas a significant but negative one was found between PAID and adherence scores. The results of linear regressions showed that a secondary level of education (beta = -13.48) significantly decreased the trust in physician score, whereas the total number of oral antidiabetics (beta = 0.93) increased it. Having a sedentary lifestyle (beta = -12.73) and smoking < 3 waterpipes/week compared to no smoking (beta = -16.82) significantly decreased the adherence score. Female gender (beta = 10.46), smoking < 3 waterpipes (beta = 27.42) and 3+ waterpipes/week (beta = 17.95) significantly increased the PAID score.

Conclusion: Trust in physician is associated with an increased adherence and with decreased diabetes-related distress. This distress was also associated with poor adherence in our study.

C1 [Halepian, Lucine; Saleh, Mary Bou; Khabbaz, Lydia Rabbaa] St Joseph Univ Beirut, Pharmacol Clin Pharm & Drug Qual Control Lab, Beirut, Lebanon.

[Halepian, Lucine; Saleh, Mary Bou; Hallit, Souheil; Khabbaz, Lydia Rabbaa] St Joseph Univ Beirut, Fac Pharm, Beirut, Lebanon.

RP Khabbaz, LR (reprint author), St Joseph Univ Beirut, Pharmacol Clin Pharm & Drug Qual Control Lab, Beirut, Lebanon.; Hallit, S; Khabbaz, LR (reprint author), St Joseph Univ Beirut, Fac Pharm, Beirut, Lebanon.

EM souheilhallit@hotmail.com; Lydia.khabbaz@usj.edu.lb

OI Hallit, Souheil/0000-0001-6918-5689

CR Aikens JE, 2013, DIABETIC MED, V30, P338, DOI 10.1111/dme.12046

Aikens JE, 2012, DIABETES CARE, V35, P2472, DOI 10.2337/dc12-0181

Al-Qazaz HK, 2010, DIABETES RES CLIN PR, V90, P216, DOI 10.1016/j.diabres.2010.08.012

Alazri MH, 2003, DIABETIC MED, V20, P486, DOI 10.1046/j.1464-5491.2003.00957.x

Alimena S, 2016, MED PROBL PERFORM AR, V31, P166, DOI 10.21091/mppa.2016.3030

ANDERSON LA, 1990, PSYCHOL REP, V67, P1091, DOI 10.2466/pr0.1990.67.3f.1091

Beck Rainer S, 2002, J Am Board Fam Pract, V15, P25

Bell RA, 2013, AM J HEALTH BEHAV, V37, P660, DOI 10.5993/AJHB.37.5.10

Ciechanowski PS, 2000, ARCH INTERN MED, V160, P3278, DOI 10.1001/archinte.160.21.3278

DiMatteo MR, 2004, HEALTH PSYCHOL, V23, P207, DOI 10.1037/0278-6133.23.2.207

Dong XQ, 2017, J GERONTOL A-BIOL, V72, pS125, DOI 10.1093/gerona/glx036

Egede LE, 2004, DIABETES CARE, V27, P1751, DOI 10.2337/diacare.27.7.1751

Farsaei S, 2014, PRIM CARE DIABETES, V8, P338, DOI 10.1016/j.pcd.2014.03.001

Fisher L, 2007, DIABETES CARE, V30, P542, DOI 10.2337/dc06-1614

Fisher L, 2015, J DIABETES COMPLICAT, V29, P572, DOI 10.1016/j.jdiacomp.2015.01.012

Forum for Injection Technique, 2015, UK INJ TECHN REC

Garfield S, 2012, BMC HEALTH SERV RES, V12, DOI 10.1186/1472-6963-12-350

Golin CE, 2001, DIABETES EDUCATOR, V27, P875, DOI 10.1177/014572170102700613

He XN, 2017, PATIENT PREFER ADHER, V11, P237, DOI 10.2147/PPA.S123389

Heisler M, 2007, J GERONTOL A-BIOL, V62, P1435, DOI 10.1093/gerona/62.12.1435

Hermanns N, 2006, DIABETOLOGIA, V49, P469, DOI 10.1007/s00125-005-0094-2

Kalsingh Maria Jusler, 2017, J Family Med Prim Care, V6, P34, DOI 10.4103/2249-4863.214966

Kanbara S, 2008, DIABETES RES CLIN PR, V80, P56, DOI 10.1016/j.diabres.2007.12.01S

Karter AJ, 2010, DIABETES CARE, V33, P733, DOI 10.2337/dc09-1184

Kumar Nithin, 2017, J Nat Sci Biol Med, V8, P216, DOI 10.4103/0976-9668.210008

Lobo CP, 2014, PRIM CARE COMPANION, V16

Lu M, 2008, AIDS BEHAV, V12, P86, DOI 10.1007/s10461-007-9261-4

Maeda U, 2013, INT J BEHAV MED, V20, P88, DOI 10.1007/s12529-011-9215-0

Manan MM, 2014, J CLIN DIAGN RES, V8, pJC5, DOI 10.7860/JCDR/2014/10481.5309

Mayer-Davis EJ, 2006, J AM DIET ASSOC, V106, P689, DOI 10.1016/j.jada.2006.02.002

National Collaborating Centre for Chronic Conditions (UK), 2008, TYP 2 DIAB NAT CLIN

NICE, 2004, TYP 1 DIAB DIAGN MAN

Ogurtsova K, 2017, DIABETES RES CLIN PR, V128, P40, DOI 10.1016/j.diabres.2017.03.024

Polonsky W, 1996, UNDERSTANDING TREATI, P183

Preston CC, 2000, ACTA PSYCHOL, V104, P1, DOI 10.1016/S0001-6918(99)00050-5

Ramkisson Samantha, 2017, Afr J Prim Health Care Fam Med, V9, pe1, DOI 10.4102/phcfm.v9i1.1405

Riaz M, 2014, PAK J MED SCI, V30, P233

Rodriguez KM, 2013, CLIN THER, V35, P170, DOI 10.1016/j.clinthera.2013.01.002

Rolfe A, 2014, COCHRANE DB SYST REV, DOI 10.1002/14651858.CD004134.pub3

Rubin DJ, 2010, ENDOCR PRACT, V16, P408, DOI 10.4158/EP09275.OR

*/* S 11, 2012, DIABETES CARE S1, V35, pS1, DOI DOI 10.2337/DC35-S011

Sapkota S, 2015, PLOS ONE, V10, DOI 10.1371/journal.pone.0128581

Schiotz ML, 2012, DIABETIC MED, V29, P654, DOI 10.1111/j.1464-5491.2011.03485.x

Schoenthaler AM, 2012, DIABETES EDUCATOR, V38, P397, DOI 10.1177/0145721712440333

Shao YC, 2017, J DIABETES RES, DOI 10.1155/2017/2804178

Slean GR, 2012, MED CARE, V50, pS69, DOI 10.1097/MLR.0b013e3182641127

Smith SK, 2009, SOC SCI MED, V69, P1805, DOI 10.1016/j.socscimed.2009.09.056

Solberg LI, 2004, ANN FAM MED, V2, P26, DOI 10.1370/afm.36

Ulvi Osman Saleem, 2009, JPMA Journal of the Pakistan Medical Association, V59, P798

Welch GW, 1997, DIABETES CARE, V20, P760, DOI 10.2337/diacare.20.5.760

White RO, 2013, J HEALTH CARE POOR U, V24, P1756, DOI 10.1353/hpu.2013.0177

Wong MCS, 2015, J CLIN PHARMACOL, V55, P279, DOI 10.1002/jcph.408

World Health Organization, DIABETES

NR 53

TC 0

Z9 0

U1 1

U2 2

PU SPRINGER HEIDELBERG

PI HEIDELBERG

PA TIERGARTENSTRASSE 17, D-69121 HEIDELBERG, GERMANY

SN 1869-6953

EI 1869-6961

J9 DIABETES THER

JI Diabetes Ther.

PD APR

PY 2018

VL 9

IS 2

BP 713

EP 726

DI 10.1007/s13300-018-0389-1

PG 14

WC Endocrinology & Metabolism

SC Endocrinology & Metabolism

GA GA6UO

UT WOS:000428470000019

PM 29520741

OA DOAJ Gold

DA 2019-01-08

ER

PT J

AU Hsu, HC

Lee, YJ

Wang, RH

AF Hsu, Hui-Chun

Lee, Yau-Jiunn

Wang, Ruey-Hsia

TI Influencing Pathways to Quality of Life and HbA1c in Patients With

Diabetes: A Longitudinal Study That Inform Evidence-Based Practice

SO WORLDVIEWS ON EVIDENCE-BASED NURSING

LA English

DT Article

DE type 2 diabetes mellitus; model testing; hemoglobin A1C; quality of

life; diabetes distress; social support; self-care behaviors

ID SELF-CARE ACTIVITIES; GLYCEMIC CONTROL; SOCIAL SUPPORT;

CLINICAL-OUTCOMES; HEALTH LITERACY; PROBLEM AREAS; OLDER-ADULTS;

DISTRESS; MELLITUS; ASSOCIATIONS

AB BackgroundDetermining possible associated factors and the influencing pathways to hemoglobin A1C (HbA1C) levels and quality of life (QoL) will facilitate the development of effective interventions to improve the physical and psychosocial health of patients with type 2 diabetes mellitus (T2DM).

ObjectivesTo test a hypothesized model that addressed the pathways among personal characteristics, social support, diabetes distress, and self-care behaviors to HbA1C and QoL.

MethodsA total of 382 adults with T2DM were recruited. Self-reported questionnaires and medical records were used to collect data regarding personal characteristics, diabetes distress, and social support at baseline. The self-care behaviors characters were collected 6 months later, as well as QoL and HbA1C levels 1 year later.

ResultsThe 12-month QoL directly affected 12-month HbA1C levels. The 6-month self-care behaviors directly affected 12-month QoL, and indirectly affected 12-month HbA1C levels through 12-month QoL. Baseline diabetes distress directly affected 12-month QoL. Moreover, baseline diabetes distress indirectly affected 12-month HbA1C levels through 12-month QoL. Baseline social support directly affected baseline diabetes distress and 6-month self-care behaviors. In addition, baseline social support indirectly affected 12-month QoL through baseline diabetes distress. Baseline social support also indirectly affected 12-month QoL through 6-month self-care behaviors.

Linking Evidence to ActionEnhancing QoL is important to improve HbA1C levels. Enhancing self-care behaviors is essential to improve subsequent HbA1C control and QoL. Reducing diabetes distress is crucial to improve subsequent QoL. Improving social support is suggested a favorable strategy to reduce diabetes distress and enhance subsequent self-care behaviors in patients with T2DM.

C1 [Hsu, Hui-Chun] Lees Endocrinol Clin, Dept Diabet Management, Pingtung, Taiwan.

[Lee, Yau-Jiunn] Lees Endocrinol Clin, Pingtung, Taiwan.

[Wang, Ruey-Hsia] Kaohsiung Med Univ, Coll Nursing, 100 Shih Chuan,1st Rd, Kaohsiung 807, Taiwan.

[Wang, Ruey-Hsia] Kaohsiung Med Univ Hosp, Dept Med Res, 100 Shih Chuan,1st Rd, Kaohsiung 807, Taiwan.

RP Wang, RH (reprint author), Kaohsiung Med Univ, Coll Nursing, 100 Shih Chuan,1st Rd, Kaohsiung 807, Taiwan.; Wang, RH (reprint author), Kaohsiung Med Univ Hosp, Dept Med Res, 100 Shih Chuan,1st Rd, Kaohsiung 807, Taiwan.

EM wrhsia@kmu.edu.tw

FU National Science Council, Taiwan [NSC-102-2628-B-037-012-MY3]

FX Study funding was supported by the National Science Council, Taiwan

(NSC-102-2628-B-037-012-MY3).

CR Aikens JE, 2012, DIABETES CARE, V35, P2472, DOI 10.2337/dc12-0181

Al Hayek AA, 2014, DIABETES METAB J, V38, P220, DOI 10.4093/dmj.2014.38.3.220

[Anonymous], 2017, Diabetes Care, V40, pS4

Baek RN, 2014, ANN BEHAV MED, V48, P145, DOI 10.1007/s12160-013-9585-4

Bot M, 2013, DIABETIC MED, V30, pE115, DOI 10.1111/dme.12082

Bowen PG, 2015, J GERONTOL NURS, V41, P21, DOI 10.3928/00989134-20151008-44

Chao CY, 2014, OBES RES CLIN PRACT, V8, pE212, DOI 10.1016/j.orcp.2012.07.002

Cheng JS, 2015, MED CARE, V53, P116, DOI 10.1097/MLR.0000000000000288

Chew BH, 2015, HEALTH QUAL LIFE OUT, V13, DOI 10.1186/s12955-015-0384-4

Chew BH, 2014, WORLD J DIABETES, V5, P796, DOI 10.4239/wjd.v5.i6.796

Cohen S, 2007, JAMA-J AM MED ASSOC, V298, P1685, DOI 10.1001/jama.298.14.1685

Cummings DM, 2014, ANN PHARMACOTHER, V48, P970, DOI 10.1177/1060028014536532

Dismuke CE, 2014, INT J PSYCHIAT MED, V48, P135, DOI 10.2190/PM.48.2.f

DSouza MS, 2015, GSTF J NURSING HLTH, V2, P29

Egede LE, 2014, ANN PHARMACOTHER, V48, P562, DOI 10.1177/1060028014526362

Fisher L, 2010, DIABETES CARE, V33, P23, DOI 10.2337/dc09-1238

Gonzalez JS, 2015, HEALTH PSYCHOL, V34, P505, DOI 10.1037/hea0000131

Hsu HC, 2013, J NURS RES, V21, P212, DOI 10.1097/01.jnr.0000432048.31921.e2

Hu LT, 1999, STRUCT EQU MODELING, V6, P1, DOI 10.1080/10705519909540118

Ikeda K, 2014, PLOS ONE, V9, DOI 10.1371/journal.pone.0109323

International Diabetes Federation, 2015, IDF DIAB ATL 7, V7

Kamuhabwa AR, 2014, DRUG HEALTHC PATIENT, V6, P155, DOI 10.2147/DHPS.S68786

Karlsen B, 2014, INT J NURS STUD, V51, P438, DOI 10.1016/j.ijnurstu.2013.06.016

Kiadaliri AA, 2013, J DIABETES METAB DIS, V12, DOI 10.1186/2251-6581-12-54

Kuznetsov L, 2014, DIABETES RES CLIN PR, V104, P281, DOI 10.1016/j.diabres.2013.12.029

Laxy M, 2014, DIABETES CARE, V37, P1604, DOI 10.2337/dc13-2533

Lee EH, 2016, ASIAN NURS RES, V10, P82, DOI 10.1016/j.anr.2016.01.005

Lee EH, 2012, DIABETES RES CLIN PR, V95, P76, DOI 10.1016/j.diabres.2011.08.022

Lee YJ, 2016, PATIENT EDUC COUNS, V99, P287, DOI 10.1016/j.pec.2015.08.021

Ma WY, 2012, J DIABETES COMPLICAT, V26, P296, DOI 10.1016/j.jdiacomp.2012.03.028

Ministry of Health and Welfare, 2017, 2016 STAT CAUS DEATH

Ministry of Health and Welfare, 2013, 2013 HLTH PROM ADM A

Murphy ME, 2017, BMJ OPEN, V7, DOI 10.1136/bmjopen-2016-015135

Nichols GA, 2014, DIABETES CARE, V37, P167, DOI 10.2337/dc13-1300

Nicklett EJ, 2013, J GERONTOL B-PSYCHOL, V68, P933, DOI 10.1093/geronb/gbt100

Osborn CY, 2010, PATIENT EDUC COUNS, V79, P49, DOI 10.1016/j.pec.2009.07.016

Ouyang CM, 2015, ASIA PAC J CLIN NUTR, V24, P438, DOI 10.6133/apjcn.2015.24.3.03

Pandit AU, 2014, J EPIDEMIOL COMMUN H, V68, P557, DOI 10.1136/jech-2013-203063

Polit D. F., 2012, NURSING RES GENERATI

Polit DF, 2007, RES NURS HEALTH, V30, P459, DOI 10.1002/nur.20199

Preacher K. J., 2006, COMPUTING POWER MINI

Rad Ghalmreza Sharfi, 2013, J Educ Health Promot, V2, P62, DOI 10.4103/2277-9531.120864

Rajasekharan D, 2015, ANN MED HEALTH SCI R, V5, P59, DOI 10.4103/2141-9248.149791

Reddy J, 2013, PSYCHOSOMATICS, V54, P44, DOI 10.1016/j.psym.2012.08.004

Saleh F, 2014, BMC PUBLIC HEALTH, V14, DOI [10.1186/1471-2458-14-431, 10.1186/1471-2458-14-433]

Schumacker RE, 2010, BEGINNERS GUIDE STRU

Sidhu R., 2016, CANADA J DIABETES, V40, pS70

Ullman JB, 2006, J PERS ASSESS, V87, P35, DOI 10.1207/s15327752jpa8701_03

Wang RH, 2017, J ADV NURS, V73, P1137, DOI 10.1111/jan.13201

Wang RH, 2016, PATIENT EDUC COUNS, V99, P1626, DOI 10.1016/j.pec.2016.04.001

Wang RH, 2011, J ADV NURS, V67, P2658, DOI 10.1111/j.1365-2648.2011.05701.x

Weir JP, 2005, J STRENGTH COND RES, V19, P231, DOI 10.1519/15184.1

Yu DSF, 2004, RES NURS HEALTH, V27, P135, DOI 10.1002/nur.20008

Zhang P, 2010, DIABETES RES CLIN PR, V87, P293, DOI 10.1016/j.diabres.2010.01.026

NR 54

TC 0

Z9 0

U1 2

U2 2

PU WILEY

PI HOBOKEN

PA 111 RIVER ST, HOBOKEN 07030-5774, NJ USA

SN 1545-102X

EI 1741-6787

J9 WORLDV EVID-BASED NU

JI Worldviews Evid.-Based Nurs.

PD APR

PY 2018

VL 15

IS 2

BP 104

EP 112

DI 10.1111/wvn.12275

PG 9

WC Nursing

SC Nursing

GA GB7YN

UT WOS:000429291100004

PM 29443437

DA 2019-01-08

ER

PT J

AU Dogba, MJ

Dipankui, MT

Dansokho, SC

Legare, F

Witteman, HO

AF Dogba, Maman Joyce

Dipankui, Mylene Tantchou

Dansokho, Selma Chipenda

Legare, France

Witteman, Holly O.

TI Diabetes-related complications: Which research topics matter to diverse

patients and caregivers?

SO HEALTH EXPECTATIONS

LA English

DT Article

DE diabetes; diabetes-related complications; participatory research;

patient engagement; patient involvement; patient-oriented research;

stakeholder voice; under-represented populations

ID QUALITY-OF-LIFE; VULNERABLE POPULATIONS; MIXED METHODS; RESEARCH

PRIORITIES; FEAR; TYPE-1; HYPOGLYCEMIA; DISTRESS; INVOLVEMENT;

PREVALENCE

AB BackgroundDiabetes is a chronic disease with increasing prevalence worldwide. Although research has improved its treatment and management, little is known about which research topics matter to people living with diabetes, particularly among under-represented groups.

ObjectivesTo explore the importance of research topics among a diverse range of people living with any type of diabetes or caring for someone living with any type of diabetes.

MethodsWe used a convergent mixed-method design with quantitative and qualitative aspects. We surveyed a national sample of people living with diabetes and caregivers of people with diabetes, asking them to rate the importance of 10 predetermined important research topics. We also held three focus groups in two major cities to explore research concerns of people who are under-represented in research.

Results469 adults (57% men, 42% women) in Canada completed the online survey, indicating that all 10 areas of research mattered to them, with the highest ratings accorded to preventing and treating kidney, eye and nerve complications. Fourteen individuals participated in three focus groups and similarly noted the importance of research on those three complications. Additionally, focus group participants also noted the importance of research around daily management. No new topics were identified.

ConclusionsThis study confirmed the importance of research topics among a population of people living with or caring for someone with diabetes. Findings from this study were used to inform the vision for Diabetes Action Canadaa pan-Canadian Strategy for Patient-Oriented Research (SPOR) Network on diabetes and its complications.

C1 [Dogba, Maman Joyce; Dipankui, Mylene Tantchou; Dansokho, Selma Chipenda; Legare, France; Witteman, Holly O.] Univ Laval, Dept Family & Emergency Med, Fac Med, Quebec City, PQ, Canada.

[Dogba, Maman Joyce; Legare, France; Witteman, Holly O.] Univ Laval, Off Educ & Profess Dev, Fac Med, Quebec City, PQ, Canada.

[Witteman, Holly O.] CHU Quebec, Res Ctr, Hlth Populat & Best Hlth Practices Axis, Quebec City, PQ, Canada.

RP Dogba, MJ (reprint author), Univ Laval, Dept Family & Emergency Med, Fac Med, Quebec City, PQ, Canada.

EM Joyce-maman.dogba@fmed.ulaval.ca

RI Legare, France/O-6971-2017

FU Canadian Institutes of Health Research [CIHR SCD 139932]

FX This study was funded through a subgrant of the Canadian Institutes of

Health Research (CIHR SCD 139932)

CR [Anonymous], 2006, AM J MANAG CARE, V12, pS348

Association Canadienne du Diabete, 2009, UNTS EC COUT DIAB CA

Barnard KD, 2012, PSYCHOL DIABETES CAR, P1

Boote J, 2002, HEALTH POLICY, V61, P213, DOI 10.1016/S0168-8510(01)00214-7

Braun V., 2006, QUALITATIVE RES PSYC, V3, P77, DOI DOI 10.1191/1478088706QP0630A

Canadian Diabetes Association, 2011, DIAB CAN TIPP POINT

Candib LM, 2007, ANN FAM MED, V5, P547, DOI 10.1370/afm.754

Chalmers I, 2014, LANCET, V383, P156, DOI 10.1016/S0140-6736(13)62229-1

Chalmers I, 2009, LANCET, V374, P86, DOI 10.1016/S0140-6736(09)60329-9

COX DJ, 1987, DIABETES CARE, V10, P617, DOI 10.2337/diacare.10.5.617

Creswell JW, 2004, ANN FAM MED, V2, P7, DOI 10.1370/afm.104

Creswell JW, 2007, DESIGNING CONDUCTING, P58

Dugas M, 2017, BMC MED INFORM DECIS, V17, DOI 10.1186/s12911-016-0399-8

Fidler Carrie, 2011, J Med Econ, V14, P646, DOI 10.3111/13696998.2011.610852

Fisher L, 2008, DIABETIC MED, V25, P1096, DOI 10.1111/j.1464-5491.2008.02533.x

Fisher L, 2016, J DIABETES COMPLICAT, V30, P1123, DOI 10.1016/j.jdiacomp.2016.03.032

Fisher L, 2012, DIABETES CARE, V35, P259, DOI 10.2337/dc11-1572

Gadsby R, 2012, DIABETIC MED, V29, P1321, DOI 10.1111/j.1464-5491.2012.03755.x

Green ML, 1989, J PEDIATR PSYCHOL, V15, P9

Hajos TRS, 2014, DIABETES CARE, V37, P102, DOI 10.2337/dc13-0971

Hendricks LE, 1998, DIABETES EDUCATOR, V24, P168, DOI 10.1177/014572179802400206

Irvine A, 1992, HEALTH PSYCHOL, V11, P4

Jodar-Gimeno E, 2015, REV CLIN ESP, V215, P91, DOI 10.1016/j.rce.2014.07.009

KITZINGER J, 1995, BRIT MED J, V311, P299, DOI 10.1136/bmj.311.7000.299

Knoll Megan, 2012, BMC Res Notes, V5, P572, DOI 10.1186/1756-0500-5-572

Laurance J, 2014, HEALTH AFFAIR, V33, P1627, DOI 10.1377/hlthaff.2014.0375

Mulhall A, 2003, J ADV NURS, V41, P306, DOI 10.1046/j.1365-2648.2003.02514.x

Ocloo J, 2016, BMJ QUAL SAF, V25, P626, DOI 10.1136/bmjqs-2015-004839

Patton M. Q., 2001, QUALITATIVE RES EVAL

Peyrot M, 2014, CURR MED RES OPIN, V30, P2241, DOI 10.1185/03007995.2014.947023

Pluye P, 2014, ANNU REV PUBL HEALTH, V35, P29, DOI 10.1146/annurev-publhealth-032013-182440

POLONSKY WH, 1992, HEALTH PSYCHOL, V11, P199, DOI 10.1037/0278-6133.11.3.199

Polonsky WH, 2005, DIABETES CARE, V28, P626, DOI 10.2337/diacare.28.3.626

Qualtrics, 2015, WORLDS LEAD ENT SURV

Rabiee F, 2004, P NUTR SOC, V63, P655, DOI 10.1079/PNS2004399

Sakane N, 2015, J DIABETES INVEST, V6, P567, DOI 10.1111/jdi.12340

Shaghaghi A, 2011, HEALTH PROMOT PERSPE, V1, P86, DOI 10.5681/hpp.2011.009

Shaw JE, 2010, DIABETES RES CLIN PR, V87, P4, DOI 10.1016/j.diabres.2009.10.007

Stewart RJ, 2011, HEALTH EXPECT, V14, P439, DOI 10.1111/j.1369-7625.2010.00648.x

Taylor EP, 2005, DIABETES-METAB RES, V21, P264, DOI 10.1002/dmrr.524

UyBico SJ, 2007, J GEN INTERN MED, V22, P852, DOI 10.1007/s11606-007-0126-3

Vaismoradi M, 2013, NURS HEALTH SCI, V15, P398, DOI 10.1111/nhs.12048

Vanasse A, 2015, BMC HEALTH SERV RES, V15, DOI 10.1186/s12913-015-0824-1

Whiting DR, 2011, DIABETES RES CLIN PR, V94, P311, DOI 10.1016/j.diabres.2011.10.029

Wilson J, 1999, BMJ-BRIT MED J, V319, P771, DOI 10.1136/bmj.319.7212.771

World Health Organization, 2016, GLOB REP DIAB

Zhou B, 2016, LANCET, V387, P1513, DOI 10.1016/S0140-6736(16)00618-8

NR 47

TC 0

Z9 0

U1 4

U2 6

PU WILEY

PI HOBOKEN

PA 111 RIVER ST, HOBOKEN 07030-5774, NJ USA

SN 1369-6513

EI 1369-7625

J9 HEALTH EXPECT

JI Health Expect.

PD APR

PY 2018

VL 21

IS 2

BP 549

EP 559

DI 10.1111/hex.12649

PG 11

WC Health Care Sciences & Services; Health Policy & Services; Public,

Environmental & Occupational Health

SC Health Care Sciences & Services; Public, Environmental & Occupational

Health

GA GA5VT

UT WOS:000428402700016

PM 29165920

OA DOAJ Gold, Green Published

DA 2019-01-08

ER

PT J

AU Polonsky, WH

Fisher, L

Hessler, D

AF Polonsky, W. H.

Fisher, L.

Hessler, D.

TI The impact of non-severe hypoglycemia on quality of life in patients

with type 2 diabetes

SO JOURNAL OF DIABETES AND ITS COMPLICATIONS

LA English

DT Article

DE Hypoglycemia; Perceived burden, type 2 diabetes; Quality of life;

Hypoglycemic fear; Diabetes distress

ID EVENTS; BURDEN; DEPRESSION; MANAGEMENT; DISTRESS; MELLITUS; ADULTS;

PRODUCTIVITY; WORRY; PHQ-9

AB Aims: To determine the impact of frequency of non-severe hypoglycemic events (NSHE) and the perceived burden of NSHE on quality of life (QOL) over time.

Methods: T2D adults (n = 424) were re-contacted two years after initial QOL assessment. Responding subjects (n = 290) reported the frequency and burden of NSHE over time and completed six genetic and diabetes-specific QOL measures.

Results: Most subjects (86%) reported >= one NSHE overtime. Higher frequency of NSHE was significantly associated with decrements in QOL Greater perceived burden of NSHE was significantly linked to decreases in QOL over time for all six QOL measures. Interaction terms indicated that participants with a higher frequency of NSHE and higher perceived burden reported the greatest decrease in QOL; participants who experienced frequent NSHE but did not perceive these events as burdensome evidenced little worsening in QOL over time.

Conclusions: NSHE have a negative impact on QOL over time in T2D adults. However, it is not just the occurrence of NSHE that affects QOL; it is the individual's felt burden of these events that is critical. The greatest reductions in QOL are seen among those subjects reporting a higher frequency of NSHE and indicating that such events are burdensome. (C) 2018 Elsevier Inc. All rights reserved.

C1 [Polonsky, W. H.] Univ Calif San Diego, Dept Psychiat, San Diego, CA 92103 USA.

[Polonsky, W. H.] Behav Diabet Inst, San Diego, CA USA.

[Fisher, L.; Hessler, D.] Univ Calif San Francisco, Dept Family & Community Med, San Francisco, CA 94143 USA.

RP Polonsky, WH (reprint author), POB 2148, Del Mar, CA 92014 USA.

EM whp@behavioraldiabetes.org

FU Merck

FX This investigator-initiated study was supported by Merck.

CR Barendse S, 2012, DIABETIC MED, V29, P293, DOI 10.1111/j.1464-5491.2011.03416.x

BENJAMINI Y, 1995, J R STAT SOC B, V57, P289

Brod M, 2013, DIABETES OBES METAB, V15, P546, DOI 10.1111/dom.12070

Brod M, 2013, VALUE HEALTH, V16, P1140, DOI 10.1016/j.jval.2013.09.002

Brod M, 2013, QUAL LIFE RES, V22, P997, DOI 10.1007/s11136-012-0234-3

Davis RE, 2005, CURR MED RES OPIN, V21, P1477, DOI 10.1185/030079905X61929

Fisher I., 2016, DIABET MED, V33

Fisher L, 2014, DIABETIC MED, V31, P764, DOI 10.1111/dme.12428

Fulcher Gregory, 2014, J Med Econ, V17, P751, DOI 10.3111/13696998.2014.946992

Gilet H, 2012, VALUE HEALTH, V15, P1036, DOI 10.1016/j.jval.2012.06.002

Green AJ, 2012, DIABETES RES CLIN PR, V96, P313, DOI 10.1016/j.diabres.2012.01.002

Hajos TRS, 2013, DIABETIC MED, V30, pE63, DOI 10.1111/dme.12040

Hajos TRS, 2014, DIABETES CARE, V37, P102, DOI 10.2337/dc13-0971

Hessler DM, 2017, DIABETIC MED, V34, P1228, DOI 10.1111/dme.13381

Irvine A, 1994, HDB PSYCHOL DIABETES

Kroenke K, 2001, J GEN INTERN MED, V16, P606, DOI 10.1046/j.1525-1497.2001.016009606.x

Marrett Elizabeth, 2011, BMC Res Notes, V4, P251, DOI 10.1186/1756-0500-4-251

Pedersen-Bjergaard U, 2003, DIABETES-METAB RES, V19, P232, DOI 10.1002/dmrr.377

Polonsky WH, 2015, J DIABETES COMPLICAT, V29, P1171, DOI 10.1016/j.jdiacomp.2015.08.002

Polonsky WH, 2014, DIABETIC MED, V31, P40, DOI 10.1111/dme.12275

Polonsky WH, 2005, DIABETES CARE, V28, P626, DOI 10.2337/diacare.28.3.626

Sakane N, 2015, J DIABETES INVEST, V6, P567, DOI 10.1111/jdi.12340

Sheu WHH, 2012, DIABETES RES CLIN PR, V96, P141, DOI 10.1016/j.diabres.2011.12.027

Shi LZ, 2014, HEALTH QUAL LIFE OUT, V12, DOI 10.1186/s12955-014-0167-3

Spitzer RL, 2006, ARCH INTERN MED, V166, P1092, DOI 10.1001/archinte.166.10.1092

Wells TS, 2013, J AFFECT DISORDERS, V148, P77, DOI 10.1016/j.jad.2012.11.052

Wieringa TH, 2017, J ENDOCRINOL INVESTI

Williams SA, 2012, J DIABETES COMPLICAT, V26, P399, DOI 10.1016/j.jdiacomp.2012.05.002

Williams SA, 2011, DIABETES RES CLIN PR, V91, P363, DOI 10.1016/j.diabres.2010.12.027

NR 29

TC 0

Z9 0

U1 3

U2 3

PU ELSEVIER SCIENCE INC

PI NEW YORK

PA 360 PARK AVE SOUTH, NEW YORK, NY 10010-1710 USA

SN 1056-8727

EI 1873-460X

J9 J DIABETES COMPLICAT

JI J. Diabetes Complications

PD APR

PY 2018

VL 32

IS 4

BP 373

EP 378

DI 10.1016/j.jdiacomp.2018.01.014

PG 6

WC Endocrinology & Metabolism

SC Endocrinology & Metabolism

GA GA5GP

UT WOS:000428361500005

PM 29496364

DA 2019-01-08

ER

PT J

AU Schmitt, A

Reimer, A

Kulzer, B

Icks, A

Paust, R

Roelver, KM

Kaltheuner, M

Ehrmann, D

Krichbaum, M

Haak, T

Hermanns, N

AF Schmitt, Andreas

Reimer, Andre

Kulzer, Bernhard

Icks, Andrea

Paust, Rainer

Roelver, Klaus-Martin

Kaltheuner, Matthias

Ehrmann, Dominic

Krichbaum, Michael

Haak, Thomas

Hermanns, Norbert

TI Measurement of psychological adjustment to diabetes with the diabetes

acceptance scale

SO JOURNAL OF DIABETES AND ITS COMPLICATIONS

LA English

DT Article

DE Diabetes integration; Illness acceptance; Psychological adjustment; Self

and identity; Diabetes-related distress

ID QUALITY-OF-LIFE; PERCEPTION QUESTIONNAIRE; SELF-MANAGEMENT; DEPRESSION;

METAANALYSIS; DISTRESS; MELLITUS; ILLNESS; IDENTITY; PATIENT

AB Aims: To develop a psychometric measure of diabetes acceptance.,

Methods: An item pool was developed and pilot-tested using a sample of 220 people with diabetes; item selection resulted in the 20-item 'Diabetes Acceptance Scale (DAS)'. 606 people with diabetes were then cross-sectionally assessed with the DAS to evaluate its reliability, validity and clinical utility; concurrent measurements included diabetes-related coping (FQCI), diabetes distress (PAID-5), depressive symptoms (PHQ-9), quality of life (EQ-5D), self-management (DSMQ), glycaemic control (HbA(1c)) and complications.

Results: Internal reliability was high (Cronbach's alpha = 0.96). Factorial and criterion-related results supported validity. Higher diabetes acceptance scores correlated with more functional coping styles, lower distress and depression levels, higher treatment adherence, better glycaemic control and better quality of life (all P < .001). Persons with low diabetes acceptance (22% of the sample) were four times more likely to have HbA(1c) values over 9.0% (75 mmol/mol), two times more likely to be diagnosed with long-term complications and each over two times more likely to have had episodes of severe hypoglycaemia and ketoacidosis in the past year; the prevalence of major depression in this group was fivefold increased (all P < .05).

Conclusions: The DAS is a reliable and valid tool to measure diabetes acceptance. It may help identify patients with significant problems of accepting diabetes, a putative high-risk group in need of tailored care and support. (C) 2018 Elsevier Inc. All rights reserved.

C1 [Schmitt, Andreas; Reimer, Andre; Kulzer, Bernhard; Ehrmann, Dominic; Krichbaum, Michael; Haak, Thomas; Hermanns, Norbert] Diabet Acad Mergentheim FIDAM, Res Inst, Johann Hammer Str 24, D-97980 Bad Mergentheim, Germany.

[Schmitt, Andreas; Reimer, Andre; Kulzer, Bernhard; Ehrmann, Dominic; Krichbaum, Michael; Haak, Thomas; Hermanns, Norbert] Diabet Ctr Mergentheim DZM, Theodor Klotzbuecher Str 12, D-97980 Bad Mergentheim, Germany.

[Schmitt, Andreas; Reimer, Andre; Kulzer, Bernhard; Icks, Andrea; Hermanns, Norbert] German Ctr Diabet Res DZD, Ingolstaedter Landstr 1, D-85764 Neuherberg, Germany.

[Kulzer, Bernhard; Hermanns, Norbert] Otto Friedrich Univ Bamberg, Dept Psychol, Markuspl 3, D-96047 Bamberg, Germany.

[Icks, Andrea] German Diabet Ctr DDZ, Inst Hlth Serv Res & Hlth Econ, Aufm Hennekamp 65, D-40225 Dusseldorf, Germany.

[Icks, Andrea] Heinrich Heine Univ, Inst Hlth Serv Res & Hlth Econ, Fac Med, Moorenstr 5, D-40225 Dusseldorf, Germany.

[Paust, Rainer] Elisabeth Hosp, Inst Psychosocial Med, Klara Kopp Weg 1, D-45138 Essen, Germany.

[Roelver, Klaus-Martin] Christian Hosp Quakenbrueck, Diabet Ctr Quakenbrueck, Danziger Str 2, D-49610 Quakenbrueck, Germany.

[Kaltheuner, Matthias] Specialised Diabet Practice Leverkusen, Kalkstr 117, D-51377 Leverkusen, Germany.

RP Schmitt, A (reprint author), Diabet Ctr Mergentheim, Theodor Klotzbuecher Str 12, D-97980 Bad Mergentheim, Germany.

EM schmitt@diabetes-zentrum.de

OI Krichbaum, Michael/0000-0002-5937-1266; Schmitt,

Andreas/0000-0002-5913-1457; Ehrmann, Dominic/0000-0002-5794-5596

FU German Center for Diabetes Research (DZD) [82DZD01101]; German Diabetes

Foundation (DDS) [375.10.15]

FX This work was supported by the German Center for Diabetes Research (DZD)

[grant number 82DZD01101]; and the German Diabetes Foundation (DDS)

[grant number 375.10.15]. The funders were not involved in decisions

regarding study design; collection, analysis and interpretation of data;

writing of the report; and in the decision to submit the article for

publication.

CR ANDERSON RM, 1986, DIABETIC MED, V3, P85, DOI 10.1111/j.1464-5491.1986.tb00715.x

Broadbent E, 2015, PSYCHOL HEALTH, V30, P1361, DOI 10.1080/08870446.2015.1070851

Cohen GL, 2014, ANNU REV PSYCHOL, V65, P333, DOI 10.1146/annurev-psych-010213-115137

Dempster M, 2011, DIABETIC MED, V28, P487, DOI 10.1111/j.1464-5491.2010.03214.x

Dion GA, 1990, THESIS

Dlugosch GE, 2002, PSYCHODIABETOLOGIE, P150

Garay-Sevilla ME, 1999, DIABETIC MED, V16, P238, DOI 10.1046/j.1464-5491.1999.00033.x

Gfeller R., 1987, VERHALTENSMEDIZIN DI, P63

Gois CJ, 2012, ACTA DIABETOL, V49, pS33, DOI 10.1007/s00592-010-0191-7

Gonzalez JS, 2008, DIABETES CARE, V31, P2398, DOI 10.2337/dc08-1341

Gregg JA, 2007, J CONSULT CLIN PSYCH, V75, P336, DOI 10.1037/0022-006X.75.2.336

Hardt J, 2003, Z KL PSYCH PSYCHOTH, V32, P41, DOI 10.1026//1616-3443.32.1.41

Henkel V, 2003, BRIT MED J, V326, P200, DOI 10.1136/bmj.326.7382.200

Hinz A, 2014, QUAL LIFE RES, V23, P443, DOI 10.1007/s11136-013-0498-2

Hu LT, 1998, PSYCHOL METHODS, V3, P424, DOI 10.1037//1082-989X.3.4.424

Isla Pera Pilar, 2008, Endocrinol Nutr, V55, P78

Kamody RC, 2017, BEHAV MED

Kiibler-Ross E, 1969, DEATH DYING

Lewko J, 2007, Adv Med Sci, V52 Suppl 1, P144

Lowe B, 2004, J AFFECT DISORDERS, V78, P131, DOI 10.1016/S0165-0327(02)00237-9

McDonald Patricia Elaine, 2011, Issues Ment Health Nurs, V32, P416, DOI 10.3109/01612840.2010.551449

McGuire BE, 2010, DIABETOLOGIA, V53, P66, DOI 10.1007/s00125-009-1559-5

Mezuk B, 2008, DIABETES CARE, V31, P2383, DOI 10.2337/dc08-0985

Misra R, 2008, J DIABETES COMPLICAT, V22, P217, DOI 10.1016/j.jdiacomp.2006.09.002

Oris L, 2016, DIABETES CARE, V39, P757, DOI 10.2337/dc15-2559

Richardson A, 2001, J ADV NURS, V33, P758, DOI 10.1046/j.1365-2648.2001.01717.x

Sanden-Eriksson B, 2000, J ADV NURS, V31, P1393, DOI 10.1046/j.1365-2648.2000.01410.x

Schmitt A, 2016, DIABETIC MED, V33, P835, DOI 10.1111/dme.12887

Schmitt A, 2013, DIABETES STOFFWECH H, V22, P9

Schmitt A, 2014, DIABETIC MED, V31, P1446, DOI 10.1111/dme.12553

Schmitt A, 2017, J DIABETES COMPLICAT, V31, P1608, DOI 10.1016/j.jdiacomp.2017.08.004

Schmitt A, 2017, J PSYCHOSOM RES, V94, P17, DOI [10.1016/jjpsychores.2016.12.015, 10.1016/j.jpsychores.2016.12.015]

Schmitt A, 2016, PLOS ONE, V11, DOI 10.1371/journal.pone.0150774

Schmitt A, 2013, HEALTH QUAL LIFE OUT, V11, DOI 10.1186/1477-7525-11-138

SCHULZ R, 1974, OMEGA-J DEATH DYING, V5, P137, DOI 10.2190/HYRB-7VQK-VU9Y-7L5D

Snoek FJ, 2015, LANCET DIABETES ENDO, V3, P450, DOI 10.1016/S2213-8587(15)00135-7

Strandberg RB, 2014, J PSYCHOSOM RES, V77, P174, DOI 10.1016/j.jpsychores.2014.06.015

Sturgeon JA, 2010, CURR PAIN HEADACHE R, V14, P105, DOI 10.1007/s11916-010-0095-9

Templeton GF, 2011, COMMUN ASSOC INF SYS, V28, P41

Tilden B, 2005, QUAL HEALTH RES, V15, P312, DOI 10.1177/1049732304272965

Topp CW, 2015, PSYCHOTHER PSYCHOSOM, V84, DOI 10.1159/000376585

Weinman J, 1996, PSYCHOL HEALTH, V11, P431, DOI 10.1080/08870449608400270

WELCH G, 1996, MULTIVAR EXP CLIN R, V11, P75

Wilson AL, 2017, ARCH PUBLIC HEALTH, V75, DOI 10.1186/s13690-017-0222-8

NR 44

TC 1

Z9 1

U1 9

U2 11

PU ELSEVIER SCIENCE INC

PI NEW YORK

PA 360 PARK AVE SOUTH, NEW YORK, NY 10010-1710 USA

SN 1056-8727

EI 1873-460X

J9 J DIABETES COMPLICAT

JI J. Diabetes Complications

PD APR

PY 2018

VL 32

IS 4

BP 384

EP 392

DI 10.1016/j.jdiacomp.2018.01.005

PG 9

WC Endocrinology & Metabolism

SC Endocrinology & Metabolism

GA GA5GP

UT WOS:000428361500007

PM 29439862

DA 2019-01-08

ER

PT J

AU Rhee, TG

Westberg, SM

Harris, IM

AF Rhee, Taeho G.

Westberg, Sarah M.

Harris, Ila M.

TI Complementary and alternative medicine in US adults with diabetes:

Reasons for use and perceived benefits

SO JOURNAL OF DIABETES

LA English

DT Article

DE Complementary and alternative medicine (CAM); Diabetes; Health

promotion; Wellness; Well-being

ID MODERATE MENTAL DISTRESS; MELLITUS; HEALTH; ASSOCIATION; THERAPIES;

PEOPLE; CARE; CAM

AB BackgroundAlthough complementary and alternative medicine (CAM) is increasingly used, little is known about the reasons for CAM use (treatment, wellness, or both), or the self-reported perceived benefits among US adults with diabetes. In this study we estimated prevalence rates of overall and specific types of CAM, as well as the perceived benefits of CAM, by reason for use among US diabetic adults.

MethodsCross-sectional data from the 2012 National Health Interview Survey, which represents non-institutionalized adults with diabetes (n=3386 unweighted), were used to estimate prevalence rates of CAM use by reason. Multivariate logistic regression analyses were used to investigate the odds of perceived benefits of CAM by reason for use after controlling for covariates.

ResultsOf US diabetic adults, 26.2% reported using some form of CAM in the past year. Of these, 56.7% used CAM for both treatment and wellness, 28.3% used CAM for wellness only, and 15.0% used CAM for treatment only. Regardless of reasons for use, most commonly used CAM were herbal therapies (56.9%), followed by chiropractic (25.3%) and massage (20.2%). Those using CAM for a combination of both treatment and wellness had a higher likelihood of self-reporting a better sense of control over their health (P=0.011) and improved overall health and feeling better (P=0.014) than those using CAM for treatment only.

ConclusionAlthough CAM may be a promising approach to improving health-related quality of life, future research should address efficacy and patient safety.

C1 [Rhee, Taeho G.] Yale Univ, Sch Med, Dept Internal Med, 333 Cedar St,POB 208025, New Haven, CT 06520 USA.

[Rhee, Taeho G.; Westberg, Sarah M.; Harris, Ila M.] Univ Minnesota, Coll Pharm, Dept Pharmaceut Care & Hlth Syst, Minneapolis, MN 55455 USA.

[Harris, Ila M.] Univ Minnesota, Sch Med, Dept Family Med & Community Hlth, Minneapolis, MN 55455 USA.

RP Rhee, TG (reprint author), Yale Univ, Sch Med, Dept Internal Med, 333 Cedar St,POB 208025, New Haven, CT 06520 USA.

EM tgrhee.research@gmail.com

CR American Diabetes Association, 2017, Diabetes Care, V40, pS11

[Anonymous], 2017, Diabetes Care, V40, pS4

Barnes P. M., 2008, COMPLEMENTARY ALTERN

Centers for Disease Control and Prevention, 2017, NAT DIAB STAT REP 20

Centers for Disease Control and Prevention, 2015, NAT HLTH INT SURV

Centers for Disease Control and Prevention, 2012, NAT HLTH INT SURV NH

Chang HY, 2007, J ADV NURS, V58, P307, DOI 10.1111/j.1365-2648.2007.04291.x

Clarke Tainya C, 2015, Natl Health Stat Report, P1

Cowan RP, 2014, HEADACHE, V54, P1097, DOI 10.1111/head.12364

Davis MA, 2011, HEALTH SERV RES, V46, P1402, DOI 10.1111/j.1475-6773.2011.01270.x

Eisenberg DM, 2001, ANN INTERN MED, V135, P344, DOI 10.7326/0003-4819-135-5-200109040-00011

Fabian E, 2011, ANN NUTR METAB, V58, P101, DOI 10.1159/000326765

Garber AJ, 2016, ENDOCR PRACT, V22, P84, DOI 10.4158/EP151126.CS

Johnson PJ, 2016, MATURITAS, V89, P36, DOI 10.1016/j.maturitas.2016.04.012

Leach MJ, 2012, COCHRANE DB SYST REV, DOI 10.1002/14651858.CD007170.pub2

LIU P., 2004, COCHRANE DB SYST REV, V3

Nahin RL, 2009, COSTS COMPLEMENTARY

National Center for Complementary and Integrative Health, 2015, STAT NAT HLTH INT SU

Odegard PS, 2011, DIABETES EDUCATOR, V37, P419, DOI 10.1177/0145721711401668

Ooi C.P., 2012, COCHRANE DB SYST REV, V8

Ooi CP, 2013, COCHRANE DB SYST REV, V9

Popli U, 2014, ALTERN THER HEALTH M, V20, P24

Prochaska JJ, 2012, INT J METH PSYCH RES, V21, P88, DOI 10.1002/mpr.1349

Rhee TG, 2017, COMPLEMENT THER CLIN, V29, P9, DOI 10.1016/j.ctcp.2017.07.003

Rhee TG, 2017, J PRIM CARE COMMUNIT, V8, P43, DOI 10.1177/2150131916671229

Rhee TG, 2017, HEADACHE, V57, P97, DOI 10.1111/head.12986

Roglic G, 2016, GLOB REP DIAB

Shane-McWhorter L, 2013, DIAB SPECT, V26, P259

Spinks J, 2013, EUR J INTEGR MED, V5, P44, DOI 10.1016/j.eujim.2012.08.003

Sridharan K, 2011, COCHRANE DB SYST REV, V12

Stata Corp, 2013, SURV DAT REF MAN

Upchurch DM, 2015, BMC COMPLEM ALTERN M, V15, DOI 10.1186/s12906-015-0886-y

Ward BW, 2014, PREV CHRONIC DIS, V11, DOI 10.5888/pcd11.130389

NR 33

TC 3

Z9 3

U1 2

U2 7

PU WILEY

PI HOBOKEN

PA 111 RIVER ST, HOBOKEN 07030-5774, NJ USA

SN 1753-0393

EI 1753-0407

J9 J DIABETES

JI J. Diabetes

PD APR

PY 2018

VL 10

IS 4

BP 310

EP 319

DI 10.1111/1753-0407.12607

PG 10

WC Endocrinology & Metabolism

SC Endocrinology & Metabolism

GA GA6JF

UT WOS:000428438500007

PM 28898555

DA 2019-01-08

ER

PT J

AU Whittemore, R

Delvy, R

McCarthy, MM

AF Whittemore, Robin

Delvy, Roberta

McCarthy, Margaret M.

TI The Experience of Partners of Adults with Type 1 Diabetes: an

Integrative Review

SO CURRENT DIABETES REPORTS

LA English

DT Review

DE Type 1 diabetes; Adults; Partners; Hypoglycemia; Significant others;

Marriage

ID QUALITY-OF-LIFE; EXCHANGE CLINIC REGISTRY; SELF-MANAGEMENT BEHAVIOR;

GLYCEMIC CONTROL; MARITAL RELATIONSHIP; SEVERE HYPOGLYCEMIA;

YOUNG-ADULTS; ASSOCIATION; IMPACT; SPOUSES

AB Purpose of Review The purpose of this review was to synthesize the research on the experience of partners living with adults with type 1 diabetes (T1D).

Recent Findings Eleven studies were included in the review. Three themes on the experience of living with a person with T1D were identified: the undercurrent of hypoglycemia, partners' involvement in diabetes care, and the impact on partners' lives. Due to considerable fear of hypoglycemia, partners had pervasive and deliberate ways in which they made attempts to minimize hypoglycemia in the person with diabetes and its cascade to a health emergency. As a result, partners of adults with T1D experienced considerable distress and disrupted lives. Partners also expressed a need for more support from family, friends, and health professionals.

Summary Research is needed on the partner experience across the lifespan and the specific supportive services they need in order to optimize their health outcomes.

C1 [Whittemore, Robin; Delvy, Roberta] Yale Sch Nursing, West Haven, CT 06516 USA.

[McCarthy, Margaret M.] NYU, Rory Meyers Coll Nursing, New York, NY USA.

RP Whittemore, R (reprint author), Yale Sch Nursing, West Haven, CT 06516 USA.

EM Robin.whittemore@yale.edu

CR American Diabetes A. 6, 2017, DIABETES CARE S1, V40, pS48, DOI DOI 10.2337/DC17-S009

American Diabetes Association, 2017, Diabetes Care, V40, pS114

Anderson Barbara J, 2003, Curr Diab Rep, V3, P134, DOI 10.1007/s11892-003-0037-6

[Anonymous], TYP 1 DIAB FACTS

Bailey TS, 2016, ENDOCR PRACT, V22, P231, DOI 10.4158/EP151124.CS

Balfe M, 2014, HEALTH PLACE, V26, P180, DOI 10.1016/j.healthplace.2013.12.016

Balfe M, 2013, BMC ENDOCR DISORD, V13, DOI 10.1186/1472-6823-13-25

Barnard Katharine, 2016, J Diabetes Sci Technol, V10, P824, DOI 10.1177/1932296816645365

Beck RW, 2009, DIABETES CARE, V32, P2047, DOI 10.2337/dc09-0846

Benjamin EJ, 2017, CIRCULATION, V135, pE146, DOI 10.1161/CIR.0000000000000485

Chaytor Naomi S, 2016, Diabetes Spectr, V29, P219

Critical Appraisal Skills Programme, CASP QUAL CHECKL

Dhaliwal Ruban, 2014, Diabetes Spectr, V27, P9, DOI 10.2337/diaspect.27.1.9

DuBose SN, 2016, DIABETES TECHNOL THE, V18, P765, DOI 10.1089/dia.2016.0268

Fisher L, 2016, J DIABETES COMPLICAT, V30, P1123, DOI 10.1016/j.jdiacomp.2016.03.032

GonderFrederick L, 1997, DIABETES CARE, V20, P1543, DOI 10.2337/diacare.20.10.1543

Hagedoorn M, 2006, DIABETIC MED, V23, P271, DOI 10.1111/j.1464-5491.2006.01794.x

Joensen LE, 2016, PRIM CARE DIABETES, V10, P41, DOI 10.1016/j.pcd.2015.06.007

Joensen LE, 2013, DIABETES RES CLIN PR, V101, P278, DOI 10.1016/j.diabres.2013.07.001

Jorgensen HV, 2003, DIABETES CARE, V26, P1106, DOI 10.2337/diacare.26.4.1106

Joseph NT, 2014, PSYCHOSOM MED, V76, P347, DOI 10.1097/PSY.0000000000000071

Jull J, 2016, CAN J DIABETES, V40, P164, DOI 10.1016/j.jcjd.2015.09.080

King J, 2015, DIABETES EDUCATOR, V41, P698, DOI 10.1177/0145721715606223

King PS, 2014, HEALTH PSYCHOL, V33, P424, DOI 10.1037/a0032804

Lawton J, 2014, DIABETES CARE, V37, P109, DOI 10.2337/dc13-1154

Livingstone SJ, 2015, JAMA-J AM MED ASSOC, V313, P37, DOI 10.1001/jama.2014.16425

Marathe PH, 2017, J DIABETES, V9, P320, DOI 10.1111/1753-0407.12524

Martyn-Nemeth P, 2017, J DIABETES COMPLICAT, V31, P735, DOI 10.1016/j.jdiacomp.2016.12.015

McCarthy MM, 2016, DIABETES EDUCATOR, V42, P108, DOI 10.1177/0145721715620021

McCrimmon RJ, 2010, DIABETES, V59, P2333, DOI 10.2337/db10-0103

Miller KM, 2015, DIABETES CARE, V38, P971, DOI 10.2337/dc15-0078

Morris M, 2006, J DIABETES NURSING, V10, P194

National Heart Lung and Blood Institute, QUAL ASS TOOL OBS CO

Nefs G, 2015, DIABETIC MED, V32, P1289, DOI 10.1111/dme.12739

Nielsen HB, 2016, DIABETES RES CLIN PR, V121, P62, DOI 10.1016/j.diabres.2016.08.021

Peinemann F, 2013, COCHRANE DB SYST REV, DOI [10.1002/14651858.CD008216.pub4, 10.1371/journal.pmed.1000097, 10.1136/bmj.c869, 10.1136/bmj.b2535, 10.1016/j.jclinepi.2010.02.006, 10.1002/14651858.CD008242.pub3, 10.1016/j.jclinepi.2010.03.004]

Polonsky WH, 2016, DIABETES TECHNOL THE, V18, P292, DOI 10.1089/dia.2015.0451

Rajaram SS, 1997, SOCIOL HEALTH ILL, V19, P281, DOI 10.1111/1467-9566.ep11057161

Rickels MR, 2017, J CLIN ENDOCR METAB

Rintala TM, 2013, J FAM NURS, V19, P3, DOI 10.1177/1074840712471899

Ritholz MD, 2014, DIABETIC MED, V31, P47, DOI 10.1111/dme.12276

Schokker MC, 2010, J FAM PSYCHOL, V24, P578, DOI 10.1037/a0021009

Secrest AM, 2010, DIABETES, V59, P3216, DOI 10.2337/db10-0862

Snell-Bergeon JK, 2012, J CARDIOVASC TRANSL, V5, P446, DOI 10.1007/s12265-012-9363-x

Stahl M, 1998, DIABETIC MED, V15, P619, DOI 10.1002/(SICI)1096-9136(199807)15:7<619::AID-DIA619>3.0.CO;2-T

Sullivan-Bolyai S, 2003, J Pediatr Nurs, V18, P21, DOI 10.1053/jpdn.2003.4

Trief PM, 2017, J HEALTH PSYCHOL, V22, P446, DOI 10.1177/1359105315605654

Trief PM, 2014, DIABETES CARE, V37, P1563, DOI 10.2337/dc13-1867

Trief PM, 2013, DIABETES CARE, V36, P2483, DOI 10.2337/dc12-1718

Trief PM, 2002, DIABETES CARE, V25, P1154, DOI 10.2337/diacare.25.7.1154

Trief PM, 2001, DIABETES CARE, V24, P1384, DOI 10.2337/diacare.24.8.1384

Vaismoradi M, 2013, NURS HEALTH SCI, V15, P398, DOI 10.1111/nhs.12048

Weinstock RS, 2016, DIABETES CARE, V39, P603, DOI 10.2337/dc15-1426

Whittemore R, 2005, J ADV NURS, V52, P546, DOI 10.1111/j.1365-2648.2005.03621.x

Whittemore R, 2012, DIABETES EDUCATOR, V38, P562, DOI 10.1177/0145721712445216

Wong ND, 2016, DIABETES CARE, V39, P668, DOI 10.2337/dc15-2439

Young-Hyman D, 2016, DIABETES CARE, V39, P2126, DOI 10.2337/dc16-2053

NR 57

TC 0

Z9 0

U1 3

U2 8

PU CURRENT MEDICINE GROUP

PI PHILADELPHIA

PA 400 MARKET STREET, STE 700, PHILADELPHIA, PA 19106 USA

SN 1534-4827

EI 1539-0829

J9 CURR DIABETES REP

JI Curr. Diabetes Rep.

PD APR

PY 2018

VL 18

IS 4

AR 19

DI 10.1007/s11892-018-0986-4

PG 10

WC Endocrinology & Metabolism

SC Endocrinology & Metabolism

GA FZ9TG

UT WOS:000427953900003

PM 29500739

DA 2019-01-08

ER

PT J

AU Pyatak, EA

Carandang, K

Vigen, CLP

Blanchard, J

Diaz, J

Concha-Chavez, A

Sequeira, PA

Wood, JR

Whittemore, R

Spruijt-Metz, D

Peters, AL

AF Pyatak, Elizabeth A.

Carandang, Kristine

Vigen, Cheryl L. P.

Blanchard, Jeanine

Diaz, Jesus

Concha-Chavez, Alyssa

Sequeira, Paola A.

Wood, Jamie R.

Whittemore, Robin

Spruijt-Metz, Donna

Peters, Anne L.

TI Occupational Therapy Intervention Improves Glycemic Control and Quality

of Life Among Young Adults With Diabetes: the Resilient, Empowered,

Active Living with Diabetes (REAL Diabetes) Randomized Controlled Trial

SO DIABETES CARE

LA English

DT Article

ID SELF-CARE BEHAVIORS; EMERGING ADULTS; MANAGEMENT INTERVENTION;

TRANSITION PROGRAM; TYPE-1; ADHERENCE; OUTCOMES; SUPPORT; SCALE;

QUESTIONNAIRE

AB OBJECTIVE

To assess the efficacy of a manualized occupational therapy (OT) intervention (Resilient, Empowered, Active Living with Diabetes [REAL Diabetes]) to improve glycemic control and psychosocial well-being among ethnically diverse young adults with low socioeconomic status (SES) who have type 1 or type 2 diabetes.

RESEARCH DESIGN AND METHODS

Eighty-one young adults (age 22.6 +/- 3.5 years; hemoglobin A(1c) [HbA(1c)] = 10.8%/95 mmol/mol +/- 1.9%/20.8 mmol/mol) were randomly assigned to the REAL Diabetes intervention group (IG) or an attention control group (CG) over 6 months. IG participants received biweekly sessions guided by a manual composed of seven content modules; CG participants received standardized educational materials and biweekly phone calls. Blinded assessors collected data at baseline and 6 months. The primary outcome was HbA(1c); secondary outcomes included diabetes self-care, diabetes-related quality of life (QOL), diabetes distress, depressive symptoms, and life satisfaction. Change scores were analyzed using Wilcoxon rank sum tests.

RESULTS

Intent-to-treat analyses showed that IG participants showed significant improvement in HbA(1c) (-0.57%/6.2 mmol/mol vs. +0.36%/3.9 mmol/mol, P = 0.01), diabetes-related QOL (+0.7 vs. +0.15, P = 0.04), and habit strength for checking blood glucose (+3.9 vs. +1.7, P = 0.05) as compared with CG participants. There was no statistically significant effect modification by sex, ethnicity, diabetes type, recruitment site, or SES. No study-related serious adverse events were reported.

CONCLUSIONS

The REAL Diabetes intervention improved blood glucose control and diabetes-related QOL among a typically hard-to-reach population, thus providing evidence that a structured OT intervention may be beneficial in improving both clinical and psychosocial outcomes among individuals with diabetes.

C1 [Pyatak, Elizabeth A.; Carandang, Kristine; Vigen, Cheryl L. P.; Blanchard, Jeanine; Diaz, Jesus; Concha-Chavez, Alyssa] Univ Southern Calif, Chan Div Occupat Sci & Occupat Therapy, Los Angeles, CA 90007 USA.

[Sequeira, Paola A.] Univ Southern Calif, Dept Pediat, Los Angeles, CA USA.

[Wood, Jamie R.] Childrens Hosp Los Angeles, Los Angeles, CA USA.

[Whittemore, Robin] Yale Sch Nursing, New Haven, CT USA.

[Spruijt-Metz, Donna] Univ Southern Calif, Ctr Econ & Social Res, Los Angeles, CA USA.

[Peters, Anne L.] Univ Southern Calif, Div Endocrinol, Beverly Hills, CA USA.

RP Pyatak, EA (reprint author), Univ Southern Calif, Chan Div Occupat Sci & Occupat Therapy, Los Angeles, CA 90007 USA.

EM beth.pyatak@usc.edu

OI Pyatak, Elizabeth/0000-0003-3280-8912

FU National Institute of Diabetes and Digestive and Kidney Diseases at the

National Institutes of Health [1K01 DK099202-01A1]

FX This work was supported by the National Institute of Diabetes and

Digestive and Kidney Diseases at the National Institutes of Health (1K01

DK099202-01A1).; The content is solely the responsibility of the authors

and does not represent the official view of the National Institutes of

Health.

CR Agarwal S, 2017, DIABETES EDUCATOR, V43, P87, DOI 10.1177/0145721716677098

American Occupational Therapy Association, 2015, AOTA FACT SHEET THE

Anderson RM, 2003, DIABETES CARE, V26, P1641, DOI 10.2337/diacare.26.5.1641-a

Berkowitz RI, 2018, PEDIATR DIABETES, V19, P191, DOI 10.1111/pedi.12555

Bradley C, 1999, QUAL LIFE RES, V8, P79, DOI 10.1023/A:1026485130100

Brown M, 2004, J HEAD TRAUMA REHAB, V19, P459, DOI 10.1097/00001199-200411000-00004

Dabelea D, 2017, JAMA-J AM MED ASSOC, V317, P825, DOI 10.1001/jama.2017.0686

DIENER E, 1985, J PERS ASSESS, V49, P71, DOI 10.1207/s15327752jpa4901_13

Garcia AA, 2001, DIABETES CARE, V24, P16, DOI 10.2337/diacare.24.1.16

Gardner B, 2012, INT J BEHAV NUTR PHY, V9, DOI 10.1186/1479-5868-9-102

Garvey J, 2015, BMC FAM PRACT, V16, DOI 10.1186/s12875-015-0267-0

Glasgow RE, 2004, J BEHAV MED, V27, P477, DOI 10.1023/B:JOBM.0000047611.81027.71

Gonzalez JS, 2016, AM PSYCHOL, V71, P539, DOI 10.1037/a0040388

Haltiwanger EP, 2012, AM J OCCUP THER, V66, P447, DOI 10.5014/ajot.2012.004457

Harris PA, 2009, J BIOMED INFORM, V42, P377, DOI 10.1016/j.jbi.2008.08.010

Hendricks M, 2013, DIABETES EDUCATOR, V39, P195, DOI 10.1177/0145721713475840

Hollingshead AB, 1975, 4 FACTOR INDEX SOCIA

Holmes-Walker DJ, 2007, DIABETIC MED, V24, P764, DOI 10.1111/j.1464-5491.2007.02152.x

Jackson J, 1998, AM J OCCUP THER, V52, P326, DOI 10.5014/ajot.52.5.326

Johnson B, 2014, DIABETIC MED, V31, P227, DOI 10.1111/dme.12305

Kibbey KJ, 2013, DIABETIC MED, V30, P878, DOI 10.1111/dme.12227

Kroenke K, 2009, J AFFECT DISORDERS, V114, P163, DOI 10.1016/j.jad.2008.06.026

Lado JJ, 2016, ENDOCRIN METAB CLIN, V45, P453, DOI 10.1016/j.ecl.2016.01.002

Markowitz JT, 2012, DIABETIC MED, V29, P522, DOI 10.1111/j.1464-5491.2011.03537.x

Markowitz Jessica T, 2014, J Diabetes Sci Technol, V8, P1029, DOI 10.1177/1932296814540130

McGuire BE, 2010, DIABETOLOGIA, V53, P66, DOI 10.1007/s00125-009-1559-5

O'Toole L, 2013, AUST OCCUP THER J, V60, P30, DOI 10.1111/1440-1630.12008

Peters A, 2011, DIABETES CARE, V34, P2477, DOI 10.2337/dc11-1723

Peyrot M, 1999, J HEALTH SOC BEHAV, V40, P141, DOI 10.2307/2676370

Pillay J, 2015, ANN INTERN MED, V163, P836, DOI 10.7326/M15-1399

Pillay J, 2015, ANN INTERN MED, V163, P848, DOI 10.7326/M15-1400

Pyatak EA, 2017, CONTEMP CLIN TRIALS, V54, P8, DOI 10.1016/j.cct.2016.12.025

Pyatak EA, 2015, OTJR-OCCUP PART HEAL, V35, P187, DOI 10.1177/1539449215584310

Raymond JK, 2016, DIABETES TECHNOL THE, V18, P385, DOI 10.1089/dia.2015.0425

Rhodes ET, 2012, DIABETIC MED, V29, P453, DOI 10.1111/j.1464-5491.2011.03542.x

Schwartz JK, 2016, AM J OCCUP THER, V70, DOI 10.5014/ajot.2016.021006

Sequeira PA, 2015, DIABETES CARE, V38, P1412, DOI 10.2337/dc14-2577

Simon AU, 2017, AM J OCCUP THER, V71, DOI 10.5014/ajot.2017.025502

Smalls BL, 2015, BMC HEALTH SERV RES, V15, DOI 10.1186/s12913-015-1086-7

Steinbeck KS, 2015, PEDIATR DIABETES, V16, P634, DOI 10.1111/pedi.12229

Toobert DJ, 2000, DIABETES CARE, V23, P943, DOI 10.2337/diacare.23.7.943

Wood Jamie R, 2012, J Diabetes Sci Technol, V6, P380

Wood W., 2016, BEHAV SCI POLICY, V2, P71, DOI DOI 10.1353/BSP.2016.0008

NR 43

TC 2

Z9 2

U1 6

U2 11

PU AMER DIABETES ASSOC

PI ALEXANDRIA

PA 1701 N BEAUREGARD ST, ALEXANDRIA, VA 22311-1717 USA

SN 0149-5992

EI 1935-5548

J9 DIABETES CARE

JI Diabetes Care

PD APR 1

PY 2018

VL 41

IS 4

BP 696

EP 704

DI 10.2337/dc17-1634

PG 9

WC Endocrinology & Metabolism

SC Endocrinology & Metabolism

GA GA0JT

UT WOS:000428000000027

PM 29351961

DA 2019-01-08

ER

PT J

AU Vanlalhruaii

Dasgupta, R

Ramachandran, R

Mathews, JE

Regi, A

Thomas, N

Gupta, V

Visalakshi, P

Asha, HS

Paul, T

Thomas, N

AF Vanlalhruaii

Dasgupta, Riddhi

Ramachandran, Roshna

Mathews, Jiji E.

Regi, Annie

Thomas, Niranjan

Gupta, Vijay

Visalakshi, P.

Asha, H. S.

Paul, Thomas

Thomas, Nihal

TI How safe is metformin when initiated in early pregnancy? A retrospective

5-year study of pregnant women with gestational diabetes mellitus from

India

SO DIABETES RESEARCH AND CLINICAL PRACTICE

LA English

DT Article

ID POLYCYSTIC-OVARY-SYNDROME; 1ST TRIMESTER; OUTCOMES; INSULIN; BIRTH;

HYPERGLYCEMIA; INFANTS; RISK

AB Background: The initiation of metformin in early pregnancy in Gestational Diabetes mellitus (GDM) remains controversial. The aim of our study was to assess the influence of Metformin on maternal and fetal outcomes when initiated within the first trimester of pregnancy in GDM.

Methods and materials: A retrospective analysis of 540 women with diabetes complicating pregnancy (IADPSG criteria) over five years (January 2011 to May 2016) was done. The study population comprised of patients initiated on (a) metformin within the first trimester (Group A:n = 186), (b) metformin after the first trimester (Group B:n = 203) and (c) insulin at any time during their pregnancy (Group C:n = 151). The primary outcomes compared were prematurity, respiratory distress, birth trauma, 5-min APGAR score, neonatal hypoglycaemia and need for phototherapy, while secondary outcomes compared were neonatal anthropometric measurements, maternal glycemic control, maternal hypertensive complications, postpartum glucose tolerance.

Results: Individual and composite primary or secondary outcomes in group A were similar to Groups B and C, though numerically higher premature births were seen in Group A. There was a 1.3% overall incidence of stillbirths/IUD, while 1.11% congenital anomalies were noted of which 2.15% were in group A and 1.32% were in Group C (p = .16).

Conclusions: The initiation of metformin within the first trimester of pregnancy has no significant adverse maternal or fetal outcomes. However, vigilance for premature births is recommended in women exposed to metformin in early pregnancy. (C) 2018 Elsevier B.V. All rights reserved.

C1 [Vanlalhruaii; Dasgupta, Riddhi; Ramachandran, Roshna; Asha, H. S.; Paul, Thomas; Thomas, Nihal] CMC, Dept Endocrinol Diabet & Metab, Vellore 632004, Tamil Nadu, India.

[Mathews, Jiji E.; Regi, Annie] CMC, Dept Obstet & Gynecol, Vellore, Tamil Nadu, India.

[Thomas, Niranjan; Gupta, Vijay] CMC, Dept Neonatol, Vellore, Tamil Nadu, India.

[Visalakshi, P.] CMC, Dept Stat, Vellore, Tamil Nadu, India.

RP Dasgupta, R (reprint author), CMC, Dept Endocrinol Diabet & Metab, Vellore 632004, Tamil Nadu, India.

EM riddhi_dg@rediffmail.com

CR Balani J, 2009, DIABETIC MED, V26, P798, DOI 10.1111/j.1464-5491.2009.02780.x

Ben-Haroush A, 2004, EUR J OBSTET GYN R B, V115, P125, DOI 10.1016/j.ejogrb.2003.11.027

Blumer I, 2013, J CLIN ENDOCR METAB, V98, P4227, DOI 10.1210/jc.2013-2465

Casey BM, 2001, NEW ENGL J MED, V344, P467, DOI 10.1056/NEJM200102153440701

Cassina M, 2014, HUM REPROD UPDATE, V20, P656, DOI 10.1093/humupd/dmu022

Coustan DR, 2010, AM J OBSTET GYNECOL, V202, DOI 10.1016/j.ajog.2010.04.006

George A, 2015, AUST NZ J OBSTET GYN, V55, P47, DOI 10.1111/ajo.12276

Gilbert CJ, 2005, CAN FAM PHYSICIAN, V51, P1070

Gui J, 2013, PLOS ONE, V8, DOI 10.1371/journal.pone.0064585

Ivan Ngai, 2014, OBSTET GYNECOLOGY, DOI [10.1097/01.AOG.0000447165.22404.99, DOI 10.1097/01.AOG.0000447165.22404.99]

Kamana KC, 2015, ANN NUTR METAB, V66, P14, DOI 10.1159/000371628

Kitzmiller JL, 2007, DIABETES CARE, V30, pS225, DOI 10.2337/dc07-s221

Kock K, 2010, J MATERN-FETAL NEO M, V23, P1004, DOI 10.3109/14767050903551392

Koren G, 2006, CAN FAM PHYSICIAN, V52, P171

Lautatzis ME, 2013, METABOLISM, V62, P1522, DOI 10.1016/j.metabol.2013.06.006

Metzger BE, 1998, DIABETES CARE, V21, pB161

Metzger BE, 2008, NEW ENGL J MED, V358, P1991, DOI 10.1056/NEJMoa0707943

Poolsup N, 2014, PLOS ONE, V9, DOI 10.1371/journal.pone.0109985

Roberts JM, 2013, OBSTET GYNECOL, V122, P1122, DOI 10.1097/01.AOG.0000437382.03963.88

Rowan JA, 2008, NEW ENGL J MED, V358, P2003, DOI 10.1056/NEJMoa0707193

Seaton SE, 2011, NEONATOLOGY, V100, P398, DOI 10.1159/000325916

Singh A, 2016, J OBSTET GYN INDIA, V66, P77, DOI 10.1007/s13224-015-0785-7

Sivalingam VN, 2014, HUM REPROD UPDATE, V20, P853, DOI 10.1093/humupd/dmu037

Zeng XL, 2016, MEDICINE, V95, DOI 10.1097/MD.0000000000004526

NR 24

TC 2

Z9 2

U1 0

U2 0

PU ELSEVIER IRELAND LTD

PI CLARE

PA ELSEVIER HOUSE, BROOKVALE PLAZA, EAST PARK SHANNON, CO, CLARE, 00000,

IRELAND

SN 0168-8227

EI 1872-8227

J9 DIABETES RES CLIN PR

JI Diabetes Res. Clin. Pract.

PD MAR

PY 2018

VL 137

BP 47

EP 55

DI 10.1016/j.diabres.2018.01.002

PG 9

WC Endocrinology & Metabolism

SC Endocrinology & Metabolism

GA GC6KZ

UT WOS:000429901700006

PM 29325773

DA 2019-01-08

ER

PT J

AU Trump, LJ

Novak, JR

Anderson, JR

Mendenhall, TJ

Johnson, MD

Scheufler, AC

Wilcox, A

Lewis, VL

Robbins, DC

AF Trump, Lisa J.

Novak, Joshua R.

Anderson, Jared R.

Mendenhall, Tai J.

Johnson, Matthew D.

Scheufler, Ann C.

Wilcox, Allison

Lewis, Virginia L.

Robbins, David C.

TI Evaluative Coping, Emotional Distress, and Adherence in Couples With

Type 2 Diabetes

SO FAMILIES SYSTEMS & HEALTH

LA English

DT Article

DE couples; diabetes; evaluative coping; emotional distress; spousal

support

ID QUALITY-OF-LIFE; SELF-CARE; EXERCISE ADHERENCE; SPOUSAL SUPPORT; CHRONIC

ILLNESS; MEDICATION ADHERENCE; GENDER-DIFFERENCES; GLUCOSE CONTROL;

SOCIAL SUPPORT; OLDER-ADULTS

AB Introduction: Spousal support is one of the strongest and most consistent predictors of Type 2 diabetes treatment adherence. However, the effects of both spouses' evaluations of dyadic coping on emotional distress and patients' physical health remain largely unknown. Method: Dyadic data from 117 married couples in which one member is diagnosed with Type 2 diabetes were evaluated in two separate models to explore the associations between (a) patients' and spouses' depression symptoms and patients' adherence to dietary and exercise regimens, and (b) patients' and spouses' acute stress levels and patients' adherence to dietary and exercise regimens. Finally, evaluative dyadic coping was included as a possible moderator between these associations. Results: Results from an actor-partner interdependence model revealed significant actor effects of patients' depression symptoms on patients' adherence to dietary and exercise regimens. Spouses' evaluation of dyadic coping attenuated the direct paths between spouses' depression symptoms and patients' adherence to dietary regimens. No direct pathways were found from patients' or spouses' acute stress to patients' adherence to dietary and exercise regimens. However, spouses' evaluation of dyadic coping attenuated the direct paths between spouses' acute stress and patients' adherence to dietary regimens. Discussion: Tapping into spouses' evaluations of dyadic coping has significant implications for patients' diabetes health outcomes (e.g., adherence to dietary and exercise treatment regimens). Findings from this study highlight the need for systemic interventions targeting both partners.

C1 [Trump, Lisa J.; Mendenhall, Tai J.] Univ Minnesota, Dept Family Social Sci, 290 McNeal Hall,1985 Buford Ave, St Paul, MN 55108 USA.

[Novak, Joshua R.] Utah State Univ, Dept Family Consumer & Human Dev, Logan, UT 84322 USA.

[Anderson, Jared R.] Kansas State Univ, Dept Family Studies & Human Serv, Manhattan, KS 66506 USA.

[Johnson, Matthew D.] Univ Alberta, Dept Human Ecol, Edmonton, AB, Canada.

[Scheufler, Ann C.; Wilcox, Allison; Lewis, Virginia L.; Robbins, David C.] Univ Kansas, Sch Med, Lawrence, KS 66045 USA.

RP Trump, LJ (reprint author), Univ Minnesota, Dept Family Social Sci, 290 McNeal Hall,1985 Buford Ave, St Paul, MN 55108 USA.

EM buch0245@umn.edu

FU CTSA grant from NCRR [UL1TR000001]; NCATS

FX REDCap at University of Kansas Medical Center is supported by CTSA grant

(Award UL1TR000001) from NCRR and NCATS awarded to the University of

Kansas Medical Center for Frontiers: The Heartland Institute for

Clinical and Translational Research.

CR American Diabetes Association, 2014, DEPRESSION

Anderson JR, 2016, J BEHAV MED, V39, P1020, DOI 10.1007/s10865-016-9796-9

ANTONUCCI TC, 1987, SEX ROLES, V17, P737, DOI 10.1007/BF00287685

August KJ, 2013, J FAM PSYCHOL, V27, P712, DOI 10.1037/a0034181

August KJ, 2010, SOC SCI MED, V71, P1831, DOI 10.1016/j.socscimed.2010.08.022

Awadalla AW, 2006, J NATL MED ASSOC, V98, P727

Berg CA, 2007, PSYCHOL BULL, V133, P920, DOI 10.1037/0033-2909.133.6.920

Berg CA, 2009, J FAM PSYCHOL, V23, P521, DOI 10.1037/a0015556

Beverly EA, 2008, HEALTH EDUC BEHAV, V35, P707, DOI 10.1177/1090198107299787

Beverly EA, 2010, HEALTH EDUC RES, V25, P211, DOI 10.1093/her/cyn032

Bodenmann G., 2008, MULTIDIMENSION UNPUB

Bodenmann G., 2008, DYADIC COPING INVENT

Bodenmann G, 2011, EUR PSYCHOL, V16, P255, DOI 10.1027/1016-9040/a000068

Choi SE, 2009, DIABETES EDUCATOR, V35, P978, DOI 10.1177/0145721709349220

Ciechanowski PS, 2003, GEN HOSP PSYCHIAT, V25, P246, DOI 10.1016/S0163-8343(03)00055-0

Conger RD, 1999, J PERS SOC PSYCHOL, V76, P54, DOI 10.1037//0022-3514.76.1.54

Corrigan FM, 2011, J PSYCHOPHARMACOL, V25, P17, DOI 10.1177/0269881109354930

Denham SA, 2007, FAMILIES SYSTEMS HLT, V25, P36, DOI DOI 10.1037/1091-7527.25.1.36

Denton M, 2004, SOC SCI MED, V58, P2585, DOI 10.1016/j.socscimed.2003.09.008

Dimitraki G, 2014, INT J BEHAV MED, V21, P230, DOI 10.1007/s12529-013-9296-z

Fisher L, 2002, DIABETES CARE, V25, P1564, DOI 10.2337/diacare.25.9.1564

Fisher L, 2009, DIABETIC MED, V26, P622, DOI 10.1111/j.1464-5491.2009.02730.x

Franks MM, 2012, AGING MENT HEALTH, V16, P902, DOI 10.1080/13607863.2012.674486

Gonzalez JS, 2007, DIABETES CARE, V30, P2222, DOI 10.2337/dc07-0158

Gonzalez JS, 2008, DIABETES CARE, V31, P2398, DOI 10.2337/dc08-1341

Gorchoff SM, 2008, PSYCHOL SCI, V19, P1194, DOI 10.1111/j.1467-9280.2008.02222.x

Heffner KL, 2004, J BEHAV MED, V27, P233, DOI 10.1023/B:JOBM.0000028497.79129.ad

Hu LT, 1999, STRUCT EQU MODELING, V6, P1, DOI 10.1080/10705519909540118

International Diabetes Federation, 2015, DIABETES ATLAS

Johnson MD, 2016, J FAM PSYCHOL, V30, P203, DOI 10.1037/fam0000161

Johnson MD, 2014, HEALTH PSYCHOL, V33, P841, DOI 10.1037/hea0000054

Johnson MD, 2013, J FAM PSYCHOL, V27, P722, DOI 10.1037/a0034006

Jorwal P., 2015, J SOCIAL HLTH DIABET, V3, P95, DOI DOI 10.4103/2321-0656.152806

Kroenke K, 2001, J GEN INTERN MED, V16, P606, DOI 10.1046/j.1525-1497.2001.016009606.x

Martire LM, 2002, HEALTH PSYCHOL, V21, P167, DOI 10.1037//0278-6133.21.2.167

Martire LM, 2010, ANN BEHAV MED, V40, P325, DOI 10.1007/s12160-010-9216-2

Muthen L. K., 1998, MPLUS USERS GUIDE

National Institute of Mental Health, 2011, DEPRESSION DIABETES

Novak M, 2013, DIABETIC MED, V30, pE8, DOI 10.1111/dme.12037

Osborn CY, 2014, WESTERN J NURS RES, V36, P1091, DOI 10.1177/0193945914524639

Polonsky William H, 2002, Curr Diab Rep, V2, P153, DOI 10.1007/s11892-002-0075-5

Rook KS, 2016, J HEALTH PSYCHOL, V21, P2538, DOI 10.1177/1359105315581064

Roper SO, 2009, FAM RELAT, V58, P460, DOI 10.1111/j.1741-3729.2009.00566.x

Tang TS, 2008, DIABETES EDUCATOR, V34, P266, DOI 10.1177/0145721708315680

Toobert DJ, 2000, DIABETES CARE, V23, P943, DOI 10.2337/diacare.23.7.943

Trief P., 2003, FAMILIES SYSTEMS HLT, V21, P57, DOI [DOI 10.1037/H0089502, 10.1037/h0089502]

Vassilev I, 2011, CHRONIC ILLN, V7, P60, DOI 10.1177/1742395310383338

Whittemore R, 2005, J CLIN NURS, V14, P195, DOI 10.1111/j.1365-2702.2004.00937.x

NR 48

TC 0

Z9 0

U1 1

U2 3

PU EDUCATIONAL PUBLISHING FOUNDATION-AMERICAN PSYCHOLOGICAL ASSOC

PI WASHINGTON

PA 750 FIRST ST, NE, WASHINGTON, DC 20002-4242 USA

SN 1091-7527

EI 1939-0602

J9 FAM SYST HEALTH

JI Fam. Syst. Health

PD MAR

PY 2018

VL 36

IS 1

BP 87

EP 96

DI 10.1037/fsh0000302

PG 10

WC Health Care Sciences & Services; Family Studies; Public, Environmental &

Occupational Health

SC Health Care Sciences & Services; Family Studies; Public, Environmental &

Occupational Health

GA GB1YQ

UT WOS:000428848000010

PM 29172625

DA 2019-01-08

ER

PT J

AU Lobo, JM

Anderson, R

Stukenborg, GJ

McCall, A

Kang, H

Camacho, F

Sohn, MW

AF Lobo, Jennifer M.

Anderson, Roger

Stukenborg, George J.

McCall, Anthony

Kang, Hyojung

Camacho, Fabian

Sohn, Min-Woong

TI Disparities in the Use of Diabetes Screening in Appalachia

SO JOURNAL OF RURAL HEALTH

LA English

DT Article

DE Appalachia; diabetes; health disparities; rural; screening

ID CANCER

AB PurposeThe Appalachian region presents disproportionately high rates of chronic disease compared to the rest of the United States. Early diagnosis of diabetes through screening is an important step in reducing diabetes complications. This study examines disparities in the use of diabetes screening in Appalachia.

MethodsWe analyzed 2009 and 2010 Behavioral Risk Factor Surveillance System data for 96,111 adults aged 45 years from 11 Appalachian states. Based on economic status, Appalachian counties were grouped into distressed (least affluent), at-risk, transitional, and competitive (most affluent). Logistic regression analyses were used to estimate the statistical significance and effect size of factors associated with diabetes screening.

ResultsCompetitive counties had the highest rate of diabetes screening (65.4%). At-risk counties had the lowest rate (60.3%), about 7.8% lower compared to competitive counties (P < .001). After adjusting for socioeconomic factors, differences in screening rates between county economic levels in Appalachia were not statistically significant. Among respondents 65 years, at-risk counties had an 8.1% lower screening rate compared to competitive counties; this difference was not adequately explained by differences in socioeconomic factors. Screening rates in distressed and transitional counties were not significantly different from competitive counties in unadjusted or adjusted models.

ConclusionsAt-risk counties had significantly lower screening rates than competitive counties. They should receive more policy attention similar to that received by distressed counties. Social policies that improve socioeconomic status and educational attainment, and health policies that reduce barriers to access to care may reduce disparities in diabetes screening rates in the less affluent Appalachian counties.

C1 [Lobo, Jennifer M.; Anderson, Roger; Stukenborg, George J.; Camacho, Fabian; Sohn, Min-Woong] Univ Virginia, Sch Med, Dept Publ Hlth Sci, 3rd Floor,Rm 3181, Charlottesville, VA 22908 USA.

[McCall, Anthony] Univ Virginia, Sch Med, Internal Med, Charlottesville, VA 22908 USA.

[Kang, Hyojung] Univ Virginia, Dept Syst & Informat Engn, Charlottesville, VA USA.

RP Sohn, MW (reprint author), Univ Virginia, Sch Med, Dept Publ Hlth Sci, 3rd Floor,Rm 3181, Charlottesville, VA 22908 USA.

EM msohn@virginia.edu

FU Agency for Healthcare Research and Quality [R01HS018542]

FX The authors gratefully acknowledge the financial support from the Agency

for Healthcare Research and Quality (R01HS018542). The paper presents

the findings and conclusions of the authors; it does not necessarily

represent the Agency for Healthcare Research and Quality.

CR American Diabetes Association, 2002, DIABETES CARE, V25, pS21

American Diabetes Association, 2015, AM DIAB ASS DIS SCOP

Anderson RT, 2014, HEALTH SERV RES, V49, P546, DOI 10.1111/1475-6773.12108

[Anonymous], 2016, HLTH PEOPL 2020 DIAB

Appalachian Regional Commission, 2013, COUNTY EC STAT APP

Appalachian Regional Commission, 2010, MOV APP FORW APP REG

Ashkenazy R, 2006, J GEN INTERN MED, V21, P386, DOI 10.1111/j.1525-1497.2006.00403.x

Barker L, 2011, POPUL HEALTH METR, V9, DOI 10.1186/1478-7954-9-54

Barker L, 2010, PREV CHRONIC DIS, V7

Berg AO, 2003, ANN INTERN MED, V138, P212, DOI 10.7326/0003-4819-138-3-200302040-00014

Crespo R, 2011, HEALTH PROMOT PRACT, V12, P544, DOI 10.1177/1524839910377965

Denham SA, 2012, APPALACHIAN HEALTH AND WELL-BEING, P131

Greever C, 2014, PUBLIC HLTH APPALACH, V35, P46

Health Resources and Services Administration, 2013, AR HLTH RES FIL AHRF

Hopenhayn C, 2008, CANCER, V113, P2974, DOI 10.1002/cncr.23749

Johnson CL, 2013, VITAL HLTH STAT, V2, P2013

Johnson LK, 2015, RURAL REMOTE HEALTH, V15

Keppel Kenneth, 2005, Vital Health Stat 2, P1

Mashaw AJ, 2014, AM J CLIN MED, V10, P64

Misra R, 2016, J DIABETES RES, DOI 10.1155/2016/2456518

Pedhazur E. J., 1982, MULTIPLE REGRESSION

Petrocelli JV, 2003, MEAS EVAL COUNS DEV, V36, P9

Rao J.N.K., 2003, SMALL AREA ESTIMATIO

Shrestha SS, 2012, OPEN DIABETES J, V5, P29

Siu AL, 2015, ANN INTERN MED, V163, P861, DOI 10.7326/M15-2345

Tan X, 2016, RES SOC ADM PHARM

Yao N, 2016, J RURAL HEALTH

Zhuo XH, 2012, HEALTH AFFAIR, V31, P50, DOI 10.1377/hlthaff.2011.1115

NR 28

TC 0

Z9 0

U1 0

U2 0

PU WILEY

PI HOBOKEN

PA 111 RIVER ST, HOBOKEN 07030-5774, NJ USA

SN 0890-765X

EI 1748-0361

J9 J RURAL HEALTH

JI J. Rural Health

PD SPR

PY 2018

VL 34

IS 2

BP 173

EP 181

DI 10.1111/jrh.12247

PG 9

WC Health Care Sciences & Services; Health Policy & Services; Public,

Environmental & Occupational Health

SC Health Care Sciences & Services; Public, Environmental & Occupational

Health

GA GB8KW

UT WOS:000429325800008

PM 28686786

DA 2019-01-08

ER

PT J

AU Pacanowski, CR

Linde, JA

Faulconbridge, LF

Coday, M

Safford, MM

Chen, HY

Yanovski, SZ

Ewing, LJ

Wing, R

Jeffery, RW

AF Pacanowski, Carly R.

Linde, Jennifer A.

Faulconbridge, Lucy F.

Coday, Mace

Safford, Monika M.

Chen, Haiying

Yanovski, Susan Z.

Ewing, Linda J.

Wing, Rena

Jeffery, Robert W.

TI Psychological Status and Weight Variability Over Eight Years: Results

From Look AHEAD

SO HEALTH PSYCHOLOGY

LA English

DT Article

DE body weight; mental health; depressive symptoms; binge eating; body

weight change

ID BINGE-EATING DISORDER; LIFE-STYLE INTERVENTION; HEALTH SURVEY SF-36;

OBESE PERSONS; LOSS MAINTENANCE; WOMEN; TRIAL; DEPRESSION; INVENTORY;

CONSEQUENCES

AB Objective: Cross-sectional studies suggest an association between weight cycling and psychological status. Although this is often interpreted as suggesting that weight cycles "cause" psychological distress, the relationship could be bidirectional. This study provides a prospective analysis of the bidirectional association between weight variability and psychological status over an 8-year period in overweight/obese adults with Type 2 diabetes. Method: Data were from the first 8 years of Look AHEAD, a randomized controlled trial comparing health outcomes in individuals with Type 2 diabetes assigned to an intensive lifestyle intervention designed to produce weight loss or a diabetes education and support control group. Psychological status (mental health, depressive symptoms, binge eating) was assessed via surveys and were examined in relation to weight variability at both baseline and year 8. Weight variability was derived from 8 possible annual measurements from participants who had a minimum of 3 consecutive body weight measurements (N = 4,774) and operationalized as the number of year-to-year cycles and the coefficient of variation across all available weight measurements. Results: Controlling for study group, higher baseline scores on mental health (Short Form-36 Mental Component Summary) and lower levels of depressive symptomatology (Beck Depression Inventory) and binge eating (Questionnaire on Eating and Weight Patterns) were associated with significantly less subsequent weight variability. The prospective association between weight variability and psychological status at year 8 was less robust. Conclusions: These results suggest that the cross-sectional relationship between weight variability and psychological status is due primarily to poorer psychological function preceding greater weight instability.

C1 [Pacanowski, Carly R.] Univ Delaware, Dept Behav Hlth & Nutr, 026 North Coll Ave,015 Carpenter Sports Bldg, Newark, DE 19716 USA.

[Linde, Jennifer A.] Univ Minnesota, Div Epidemiol & Community Hlth, Minneapolis, MN 55455 USA.

[Faulconbridge, Lucy F.] Univ Penn, Pearlman Sch Med, Philadelphia, PA 19104 USA.

[Coday, Mace] Univ Tennessee, Hlth Sci Ctr, Knoxville, TN 37996 USA.

[Safford, Monika M.] Cornell Univ, Weill Cornell Med Coll, Dept Med, Ithaca, NY 14853 USA.

[Chen, Haiying] Wake Forest Univ, Bowman Gray Sch Med, Dept Biostat, Winston Salem, NC 27109 USA.

[Yanovski, Susan Z.] NIDDK, NIH, Bethesda, MD 20892 USA.

[Ewing, Linda J.] Univ Pittsburgh, Dept Psychiat, Pittsburgh, PA 15260 USA.

[Wing, Rena] Brown Univ, Dept Psychiat & Human Behav, Providence, RI 02912 USA.

[Jeffery, Robert W.] Univ Minnesota, Div Epidemiol & Community Hlth, Sch Publ Hlth, Minneapolis, MN 55455 USA.

RP Pacanowski, CR (reprint author), Univ Delaware, Dept Behav Hlth & Nutr, 026 North Coll Ave,015 Carpenter Sports Bldg, Newark, DE 19716 USA.

EM cpacanow@udel.edu

OI Pacanowski, Carly/0000-0001-6404-0886

FU [T32 DK 083250]; [T32 MH 082761]

FX The first author was supported by Grants T32 DK 083250 and T32 MH

082761.

CR Anderson JW, 2001, AM J CLIN NUTR, V74, P579

Bartlett SJ, 1996, J CONSULT CLIN PSYCH, V64, P587, DOI 10.1037//0022-006X.64.3.587

BECK AT, 1961, ARCH GEN PSYCHIAT, V4, P561

BECK AT, 1988, CLIN PSYCHOL REV, V8, P77, DOI 10.1016/0272-7358(88)90050-5

Brancati F, 2003, CONTROL CLIN TRIALS, V24, P610, DOI 10.1016/S0197-2456(03)00064-3

BROWNELL KD, 1994, ARCH INTERN MED, V154, P1325, DOI 10.1001/archinte.154.12.1325

Casazza K, 2013, NEW ENGL J MED, V368, P446, DOI 10.1056/NEJMsa1208051

Chen HN, 2010, COMMUN STAT-SIMUL C, V39, P860, DOI 10.1080/03610911003650383

Chua JL, 2004, INT J OBESITY, V28, P606, DOI 10.1038/sj.ijo.0802595

Clark M M, 2000, Eat Behav, V1, P47, DOI 10.1016/S1471-0153(00)00009-X

Cutter G, 1996, ANN BEHAV MED, V18, P280, DOI 10.1007/BF02895290

Dalle Grave R, 1996, EAT DISORD, V4, P67

de Zwaan M, 2015, APPETITE, V91, P336, DOI 10.1016/j.appet.2015.04.064

Diabetes Prevention Program ( DPP) Research Group, 2002, DIABETES CARE, V25, P2165, DOI DOI 10.2337/DIACARE.25.12.2165

Elfhag K, 2005, OBES REV, V6, P67, DOI 10.1111/j.1467-789X.2005.00170.x

Fairburn CG, 2008, COGNITIVE BEHAV THER

FOREYT JP, 1995, INT J EAT DISORDER, V17, P263, DOI 10.1002/1098-108X(199504)17:3<263::AID-EAT2260170307>3.0.CO;2-N

Forman EM, 2016, OBESITY, V24, P2050, DOI 10.1002/oby.21601

Foster GD, 1996, J CONSULT CLIN PSYCH, V64, P752

Foster GD, 1997, OBES RES, V5, P474, DOI 10.1002/j.1550-8528.1997.tb00674.x

French SA, 1994, AM PSYCHOL ASS, V13, P195, DOI DOI 10.1037/0278-6133.13.3.195

Friedman MA, 1998, J CONSULT CLIN PSYCH, V66, P646, DOI 10.1037/0022-006X.66.4.646

Gallup, 2014, AM EFF LOS WEIGHT ST

Geliebter Allan, 2003, Eat Behav, V3, P341, DOI 10.1016/S1471-0153(02)00100-9

GORMALLY J, 1982, ADDICT BEHAV, V7, P47, DOI 10.1016/0306-4603(82)90024-7

Hagger MS, 2010, PSYCHOL BULL, V136, P495, DOI 10.1037/a0019486

Hays RD, 2000, PHARMACOECONOMICS, V18, P419, DOI 10.2165/00019053-200018050-00001

JAESCHKE R, 1989, CONTROL CLIN TRIALS, V10, P407, DOI 10.1016/0197-2456(89)90005-6

Kensinger GJ, 1998, J AM DIET ASSOC, V98, P863, DOI 10.1016/S0002-8223(98)00199-0

Knowler WC, 2002, NEW ENGL J MED, V346, P393

Kroenke K, 2001, J GEN INTERN MED, V16, P606, DOI 10.1046/j.1525-1497.2001.016009606.x

KUEHNEL RH, 1994, INT J EAT DISORDER, V15, P321

Manikandan S, 2010, J PHARMACOL PHARMACO, V1, P126, DOI 10.4103/0976-500X.72373

Marchesini G, 2004, INT J OBESITY, V28, P1456, DOI 10.1038/sj.ijo.0802741

MCHORNEY CA, 1993, MED CARE, V31, P247, DOI 10.1097/00005650-199303000-00006

McNair D. M, 1981, PROFILE MOOD STATES

Messier L, 2014, CAN J DIABETES, V38, P456, DOI 10.1016/j.jcjd.2014.01.005

Montani JP, 2015, OBES REV, V16, P7, DOI 10.1111/obr.12251

Osborn RL, 2011, ETHNIC DIS, V21, P274

Sherwood NE, 1999, INT J OBESITY, V23, P485, DOI 10.1038/sj.ijo.0800846

Simkin-Silverman LR, 1998, INT J EAT DISORDER, V24, P175, DOI 10.1002/(SICI)1098-108X(199809)24:2<175::AID-EAT7>3.0.CO;2-B

SPITZER RL, 1993, INT J EAT DISORDER, V13, P137

SPITZER RL, 1992, INT J EAT DISORDER, V11, P191, DOI 10.1002/1098-108X(199204)11:3<191::AID-EAT2260110302>3.0.CO;2-S

Stein RI, 2007, INT J EAT DISORDER, V40, P195, DOI 10.1002/eat.20352

Stevens J, 2006, INT J OBESITY, V30, P391, DOI 10.1038/sj.ijo.0803175

Stojek MMK, 2017, INT J EAT DISORDER, V50, P551, DOI 10.1002/eat.22636

Strychar I, 2009, J AM DIET ASSOC, V109, P718, DOI 10.1016/j.jada.2008.12.026

Venditti EM, 1996, J CONSULT CLIN PSYCH, V64, P400

Vergnaud AC, 2008, INT J OBESITY, V32, P315, DOI 10.1038/sj.ijo.0803739

Wadden TA, 2006, OBESITY, V14, p99S, DOI 10.1038/oby.2006.289

Wadden TA, 2006, OBESITY, V14, P737

WARE JE, 1992, MED CARE, V30, P473, DOI 10.1097/00005650-199206000-00002

Wesche-Thobaben J, 2011, CLIN TRIALS, V8, P320, DOI 10.1177/1740774511405858

NR 53

TC 0

Z9 0

U1 0

U2 3

PU AMER PSYCHOLOGICAL ASSOC

PI WASHINGTON

PA 750 FIRST ST NE, WASHINGTON, DC 20002-4242 USA

SN 0278-6133

EI 1930-7810

J9 HEALTH PSYCHOL

JI Health Psychol.

PD MAR

PY 2018

VL 37

IS 3

BP 238

EP 246

DI 10.1037/hea0000547

PG 9

WC Psychology, Clinical; Psychology

SC Psychology

GA FY6XL

UT WOS:000427006100004

PM 29504788

DA 2019-01-08

ER

PT J

AU Fujita, N

Yamamoto, Y

Hayashino, Y

Kuwata, H

Okamura, S

Iburi, T

Furuya, M

Kitatani, M

Yamazaki, S

Ishii, H

Tsujii, S

Inagaki, N

Fukuhara, S

AF Fujita, Naotaka

Yamamoto, Yosuke

Hayashino, Yasuaki

Kuwata, Hirohito

Okamura, Shintaro

Iburi, Tadao

Furuya, Miyuki

Kitatani, Masako

Yamazaki, Shin

Ishii, Hitoshi

Tsujii, Satoru

Inagaki, Nobuya

Fukuhara, Shunichi

CA Tenri Study Grp

TI Real-life glycemic control in patients with type 2 diabetes treated with

insulin therapy: A prospective, longitudinal cohort study (Diabetes

Distress and Care Registry at Tenri [DDCRT 9])

SO JOURNAL OF DIABETES INVESTIGATION

LA English

DT Article

DE Cohort study; Insulin therapy; Type 2 diabetes

ID QUALITY-OF-LIFE; JAPANESE PATIENTS; CARDIOVASCULAR-DISEASE; WEIGHT-GAIN;

MELLITUS; HYPOGLYCEMIA; ASSOCIATION; BASAL; COMPLICATIONS; QUESTIONNAIRE

AB Aims/IntroductionWe investigated the association between four insulin regimens, and increase in glycated hemoglobin (HbA1c) and insulin dose in a real-life clinical setting because there are no data about them among insulin regimens.

Materials and MethodsParticipants included 757 patients with type 2 diabetes having been treated with insulin therapy for more than 1 year. The four insulin regimens were regimen 1 (long-acting insulin, once daily), regimen 2 (biphasic insulin, twice daily), regimen 3 (biphasic insulin, three times daily) and regimen 4 (basal-bolus therapy). Main outcomes were increases in HbA1c levels >0.5% and increases in daily insulin units after 1 year. We carried out multivariable analyses to examine differences in glycemic control and insulin dose with adjustment for possible confounders.

ResultsMean HbA1c level and duration of insulin therapy were 7.8% and 11.3 years, respectively. HbA1c levels increased by >0.5% at follow up in 22.8, 24.9, 20.7, and 29.3% of participants using regimen 1, 2, 3 and 4, respectively, with no significant differences between groups. Daily insulin doses increased in 62.3, 68.8, 65.3 and 38.6% of patients, respectively (P < 0.001). Multivariable regression analysis showed that patients who received regimen 4 had significantly lower odds of requiring future insulin dose increases than those who had received regimen 2 (adjusted odds ratio 0.24, 95% confidence interval 0.14-0.41; P < 0.001).

ConclusionsMany patients receiving insulin therapy showed increases in HbA1c levels and insulin doses 1 year later. The smallest increase in insulin dose was observed in the basal-bolus therapy group compared with other regimens.

C1 [Fujita, Naotaka; Yamamoto, Yosuke; Hayashino, Yasuaki; Yamazaki, Shin; Fukuhara, Shunichi] Kyoto Univ, Dept Healthcare Epidemiol, Sch Publ Hlth, Grad Sch Med, Kyoto, Japan.

[Fujita, Naotaka; Inagaki, Nobuya] Kyoto Univ, Dept Diabet Endocrinol & Nutr, Grad Sch Med, Kyoto, Japan.

[Hayashino, Yasuaki; Kuwata, Hirohito; Okamura, Shintaro; Iburi, Tadao; Furuya, Miyuki; Kitatani, Masako; Tsujii, Satoru] Tenri Hosp, Dept Endocrinol, Tenri, Nara, Japan.

[Ishii, Hitoshi] Nara Med Univ, Dept Diabetol, Kashihara, Nara, Japan.

RP Tsujii, S (reprint author), Tenri Hosp, Dept Endocrinol, Tenri, Nara, Japan.

EM stsujii@tenriyorozu.jp

OI Tsujii, Satoru/0000-0002-1834-6515

FU Manpei Suzuki Diabetes Foundation; JSPS KAKENHI [25460641]

FX We would like to especially thank Yukari Moritsuji, Yuki Fujita, Noriko

Nakamura and Yoko Sakamoto for their clerical support. This study was

partially supported by the Manpei Suzuki Diabetes Foundation and JSPS

KAKENHI (grant number: 25460641); however, they played no role in the

study design or conduct; data collection, analysis or interpretation; or

the preparation, review or approval of the manuscript.

CR Ali MK, 2013, NEW ENGL J MED, V368, P1613, DOI 10.1056/NEJMsa1213829

Buse JB, 2011, DIABETES CARE, V34, P249, DOI 10.2337/dc10-1701

DANDONA P, 1979, BMJ-BRIT MED J, V1, P1183, DOI 10.1136/bmj.1.6172.1183

Desouza C, 2003, DIABETES CARE, V26, P1485, DOI 10.2337/diacare.26.5.1485

Gaede P, 2003, NEW ENGL J MED, V348, P383, DOI 10.1056/NEJMoa021778

Gerstein HC, 2008, NEW ENGL J MED, V358, P2545, DOI 10.1056/NEJMoa0802743

Green AJ, 2012, DIABETES RES CLIN PR, V96, P313, DOI 10.1016/j.diabres.2012.01.002

Hayashino Y, 2013, EXP CLIN ENDOCR DIAB, V121, P628, DOI 10.1055/s-0033-1355424

Hayashino Y, 2014, DIABETES CARE, V37, P2947, DOI 10.2337/dc14-1357

Hayashino Y, 2014, DIABETES CARE, V37, P2459, DOI 10.2337/dc13-2312

Hayashino Y, 2012, DIABETES RES CLIN PR, V97, P405, DOI 10.1016/j.diabres.2012.04.005

Holman RR, 2007, NEW ENGL J MED, V357, P1716, DOI 10.1056/NEJMoa075392

Hotta N, 2010, J DIABETES INVEST, V1, P66, DOI 10.1111/j.2040-1124.2010.00019.x

International Diabetes Federation, 2015, IDF DIABETES ATLAS

Inzucchi SE, 2015, DIABETOLOGIA, V58, P429, DOI 10.1007/s00125-014-3460-0

Ishii H, 2008, DIABETES RES CLIN PR, V81, P169, DOI 10.1016/j.diabres.2008.03.020

Ishii H, 2012, PLOS ONE, V7, DOI 10.1371/journal.pone.0036361

Ishii Hitoshi, 2012, J Med Econ, V15, P556, DOI 10.3111/13696998.2012.665111

Jansen HJ, 2014, DIABETES CARE, V37, P2710, DOI 10.2337/dc13-1205

Japan Diabetes Society, 2014, TREATM GUID DIAB 201, P13

Kobayashi M, 2006, DIABETES RES CLIN PR, V73, P198, DOI 10.1016/j.diabres.2006.01.013

Mashitani T, 2015, ACTA DIABETOL, V52, P639, DOI 10.1007/s00592-015-0725-0

Mashitani T, 2014, DIABETES CARE, V37, P252, DOI 10.2337/dc13-0407

Mashitani T, 2013, DIABETES RES CLIN PR, V100, P189, DOI 10.1016/j.diabres.2013.03.006

Norhammar A, 2013, DIABETOLOGIA, V56, P1, DOI 10.1007/s00125-012-2694-y

OHKUBO Y, 1995, DIABETES RES CLIN PR, V28, P103, DOI 10.1016/0168-8227(95)01064-K

POLONSKY WH, 1995, DIABETES CARE, V18, P754, DOI 10.2337/diacare.18.6.754

Purnell JQ, 1998, JAMA-J AM MED ASSOC, V280, P140, DOI 10.1001/jama.280.2.140

Seino Y, 2010, J DIABETES INVEST, V1, P1, DOI 10.1111/j.2040-1124.2010.00005.x

Siegmund T, 2007, EXP CLIN ENDOCR DIAB, V115, P349, DOI 10.1055/s-2007-973082

Tsujii S, 2012, DIABETIC MED, V29, P1451, DOI 10.1111/j.1464-5491.2012.03647.x

Turner RC, 1998, LANCET, V352, P837

UK Prospective Diabetes Study (UKPDS) Group, 1998, LANCET, V352, P854, DOI DOI 10.1016/S0140-6736(98)07037-8

Wu T, 2015, DIABETES THER, V6, P273, DOI 10.1007/s13300-015-0116-0

NR 34

TC 0

Z9 0

U1 0

U2 0

PU WILEY

PI HOBOKEN

PA 111 RIVER ST, HOBOKEN 07030-5774, NJ USA

SN 2040-1124

J9 J DIABETES INVEST

JI J. Diabetes Investig.

PD MAR

PY 2018

VL 9

IS 2

BP 294

EP 302

DI 10.1111/jdi.12693

PG 9

WC Endocrinology & Metabolism

SC Endocrinology & Metabolism

GA FY2DZ

UT WOS:000426626900011

PM 28494126

OA DOAJ Gold, Green Published

DA 2019-01-08

ER

PT J

AU Rathmann, W

Kuss, O

Anderson, D

Busch, S

Hahn, M

Engelhard, J

Zou, M

Kostev, K

AF Rathmann, Wolfgang

Kuss, Oliver

Anderson, Doerte

Busch, Stefan

Hahn, Michael

Engelhard, Johanna

Zou, Ming

Kostev, Karel

TI Increased depression symptom score in newly diagnosed type 2 diabetes

patients

SO PSYCHIATRY RESEARCH

LA English

DT Article

DE Type 2 diabetes; Depression; Primary care; Diabetes distress

ID QUALITY-OF-LIFE; TREATMENT SATISFACTION; ANXIETY DISORDERS; GLYCEMIC

CONTROL; PRIMARY-CARE; HEALTH; DATABASE; ASSOCIATION; PREVALENCE;

DISTRESS

AB The aim was to investigate the association between diabetes duration and depressive symptoms in type 2 diabetes. The DIAREG registry used data of a nationwide general medicine practice database (Disease Analyzer, Germany) augmented by prospective data from patient reported outcomes (PRO) including Center of Epidemiological Studies Depression Scale (CES-D: < 16 vs. >= 16) and the SF-36. Multiple logistic regression models were used to investigate the association of diabetes duration with PROs. Overall, 1807 type 2 diabetes patients were registered in 108 practices. From 270 (15%) patients complete PRO could be collected, which were similar with respect to age, sex, body mass index, HbA1c, diabetes duration and treatment to patients with incomplete data (p >= 0.05). Patients with a longer diabetes duration (reference: < 2 years) displayed a significantly increased odds of having no indication of depression (CES-D < 16: 66%) (Odds Ratio, 95%Cl: 2- < 5 years: 5.9, 1.2-29.6; 5- < 10 years: 6.2, 1.3-28.7; >= 10 years: 5.6, 1.2-23.1), after adjusting for age, sex, BMI, diabetes complications, antidepressants and glucose-lowering treatment. Patients with < 2 years diabetes duration also exhibited a significantly decreased mean mental component score (SF-36: < 2 years: 50, 2- < 5 years: 69). In type 2 diabetes with a short duration an increased depressive symptom score was observed.

C1 [Rathmann, Wolfgang; Kuss, Oliver] Heinrich Heine Univ, Inst Biometr & Epidemiol, Leibniz Inst Diabet Res, German Diabet Ctr, Dusseldorf, Germany.

[Anderson, Doerte; Busch, Stefan] AstraZeneca, Med Dept, Wedel, Germany.

[Hahn, Michael] Univ Clin, Hamburg, Germany.

[Engelhard, Johanna; Kostev, Karel] IQVIA, Frankfurt, Germany.

[Zou, Ming] IQVIA, Basel, Switzerland.

RP Rathmann, W (reprint author), Heinrich Heine Univ, Inst Biometr & Epidemiol, Leibniz Inst Diabet Res, German Diabet Ctr, Dusseldorf, Germany.

EM rathmann@ddz.uni-duesseldorf.de

FU AstraZeneca, Germany

FX The establishment of the DIAREG registry and the present analysis was

supported by an unlimited grant by AstraZeneca, Germany. AstraZeneca had

no influence on preparation of the article and in the current study

design, in the analysis and interpretation of data and in the decision

to submit the article for publication.

CR Almeida OP, 2016, MATURITAS, V86, P3, DOI 10.1016/j.maturitas.2016.01.003

Altinok Ali, 2016, J Family Med Prim Care, V5, P302

Anderson RJ, 2001, DIABETES CARE, V24, P1069, DOI 10.2337/diacare.24.6.1069

Arshad AR, 2016, J PAK MED ASSOC, V66, P425

Bassett J, 2012, J DIABETES METAB, V3, DOI 10.4172/2155-6156.1000210

Becher H, 2009, INT J CLIN PHARM TH, V47, P617

Biderman A, 2009, FAM PRACT, V26, P102, DOI 10.1093/fampra/cmp007

BRADLEY C, 1994, DIABETIC MED, V11, P510, DOI 10.1111/j.1464-5491.1994.tb00316.x

Bradley C, 1999, QUAL LIFE RES, V8, P79, DOI 10.1023/A:1026485130100

Carter J, 2016, J PSYCHOSOM RES, V90, P91, DOI 10.1016/j.jpsychores.2016.09.013

Carter J, 2016, PSYCHONEUROENDOCRINO, V69, P123, DOI 10.1016/j.psyneuen.2016.04.002

Chiu CJ, 2017, INT PSYCHOGERIATR, V29, P281, DOI 10.1017/S1041610216001630

Ciechanowski PS, 2000, ARCH INTERN MED, V160, P3278, DOI 10.1001/archinte.160.21.3278

Donald M, 2013, HEALTH QUAL LIFE OUT, V11, DOI 10.1186/1477-7525-11-170

Ellert U, 2004, Bundesgesundheitsblatt Gesundheitsforschung Gesundheitsschutz, V47, P1027, DOI 10.1007/s00103-004-0933-1

Fisher L, 2008, DIABETIC MED, V25, P1096, DOI 10.1111/j.1464-5491.2008.02533.x

Fisher L, 2008, ANN FAM MED, V6, P246, DOI 10.1370/afm.842

Golden SH, 2008, JAMA-J AM MED ASSOC, V299, P2751, DOI 10.1001/jama.299.23.2751

Hermanns N, 2005, DIABETIC MED, V22, P293, DOI 10.1111/j.1464-5491.2005.01414.x

Hermanns N, 2013, PRIM CARE DIABETES, V7, P1, DOI 10.1016/j.pcd.2012.11.002

Holt RIG, 2014, CURR DIABETES REP, V14, DOI 10.1007/s11892-014-0491-3

Hood KK, 2014, J ADOLESCENT HEALTH, V55, P498, DOI 10.1016/j.jadohealth.2014.03.011

Ismail K., 2017, DIABETOL	0803

Kowall B, 2015, DIABETES CARE, V38, P59, DOI 10.2337/dc14-0977

Lin EHB, 2010, DIABETES CARE, V33, P264, DOI 10.2337/dc09-1068

Lunghi C, 2016, MEDICINE, V95, DOI 10.1097/MD.0000000000003514

Lustman PJ, 2000, DIABETES CARE, V23, P934, DOI 10.2337/diacare.23.7.934

Nicolucci A, 2009, NUTR METAB CARDIOVAS, V19, P45, DOI 10.1016/j.numecd.2007.12.005

RADLOFF L S, 1977, Applied Psychological Measurement, V1, P385, DOI 10.1177/014662167700100306

Rathmann W, 2016, DIABETES OBES METAB, V18, P840, DOI 10.1111/dom.12673

Redekop WK, 2002, DIABETES CARE, V25, P458, DOI 10.2337/diacare.25.3.458

Siegel E. G., 2016, DTSCH GESUNDHEITSBER

Thoolen BJ, 2006, DIABETES CARE, V29, P2257, DOI 10.2337/dc06-0617

Wexler DJ, 2006, DIABETOLOGIA, V49, P1489, DOI 10.1007/s00125-006-0249-9

NR 34

TC 1

Z9 1

U1 1

U2 9

PU ELSEVIER IRELAND LTD

PI CLARE

PA ELSEVIER HOUSE, BROOKVALE PLAZA, EAST PARK SHANNON, CO, CLARE, 00000,

IRELAND

SN 0165-1781

J9 PSYCHIAT RES

JI Psychiatry Res.

PD MAR

PY 2018

VL 261

BP 259

EP 263

DI 10.1016/j.psychres.2017.12.091

PG 5

WC Psychiatry

SC Psychiatry

GA FX6UK

UT WOS:000426222600042

PM 29329045

DA 2019-01-08

ER

PT J

AU Deary, V

Hagenaars, SP

Harris, SE

Hill, WD

Davies, G

Liewald, DCM

McIntosh, AM

Gale, CR

Deary, IJ

AF Deary, V.

Hagenaars, S. P.

Harris, S. E.

Hill, W. D.

Davies, G.

Liewald, D. C. M.

McIntosh, A. M.

Gale, C. R.

Deary, I. J.

CA Int Consortium Blood Pressure GWAS

CHARGE Consortium Aging Lon

CHARGE Consortium Inflammation Grp

TI Genetic contributions to self-reported tiredness

SO MOLECULAR PSYCHIATRY

LA English

DT Article

ID GENOME-WIDE ASSOCIATION; MULTISYSTEM BIOLOGICAL RISK;

CHRONIC-FATIGUE-SYNDROME; CANCER-RELATED FATIGUE; UK BIOBANK N=112151;

MULTIPLE-SCLEROSIS; OLDER-ADULTS; PSYCHOLOGICAL DISTRESS; COGNITIVE

FUNCTIONS; MENTAL-HEALTH

AB Self-reported tiredness and low energy, often called fatigue, are associated with poorer physical and mental health. Twin studies have indicated that this has a heritability between 6 and 50%. In the UK Biobank sample (N = 108 976), we carried out a genome-wide association study (GWAS) of responses to the question, 'Over the last two weeks, how often have you felt tired or had little energy?' Univariate GCTA-GREML found that the proportion of variance explained by all common single-nucleotide polymorphisms for this tiredness question was 8.4% (s.e. = 0.6%). GWAS identified one genome-wide significant hit (Affymetrix id 1: 64178756_C_T; P = 1.36 x 10(-11)). Linkage disequilibrium score regression and polygenic profile score analyses were used to test for shared genetic aetiology between tiredness and up to 29 physical and mental health traits from GWAS consortia. Significant genetic correlations were identified between tiredness and body mass index (BMI), C-reactive protein, high-density lipoprotein (HDL) cholesterol, forced expiratory volume, grip strength, HbA1c, longevity, obesity, self-rated health, smoking status, triglycerides, type 2 diabetes, waist-hip ratio, attention deficit hyperactivity disorder, bipolar disorder, major depressive disorder, neuroticism, schizophrenia and verbal-numerical reasoning (absolute rg effect sizes between 0.02 and 0.78). Significant associations were identified between tiredness phenotypic scores and polygenic profile scores for BMI, HDL cholesterol, low-density lipoprotein cholesterol, coronary artery disease, C-reactive protein, HbA1c, height, obesity, smoking status, triglycerides, type 2 diabetes, waist-hip ratio, childhood cognitive ability, neuroticism, bipolar disorder, major depressive disorder and schizophrenia (standardised beta's had absolute values < 0.03). These results suggest that tiredness is a partly heritable, heterogeneous and complex phenomenon that is phenotypically and genetically associated with affective, cognitive, personality and physiological processes.

C1 [Deary, V.; Liewald, D. C. M.] Northumbria Univ, Dept Psychol, Newcastle NE1 8ST, England.

[Hagenaars, S. P.; Harris, S. E.; Hill, W. D.; Davies, G.; Liewald, D. C. M.; Gale, C. R.; Deary, I. J.] Univ Edinburgh, Ctr Cognit Ageing & Cognit Epidemiol, Edinburgh, Midlothian, Scotland.

[Hagenaars, S. P.; Hill, W. D.; Davies, G.; Gale, C. R.; Deary, I. J.] Univ Edinburgh, Dept Psychol, 7 George Sq, Edinburgh EH8 9JZ, Midlothian, Scotland.

[Hagenaars, S. P.; McIntosh, A. M.] Univ Edinburgh, Div Psychiat, Edinburgh, Midlothian, Scotland.

[Harris, S. E.] Univ Edinburgh, Med Genet Sect, Ctr Genom & Expt Med, Edinburgh, Midlothian, Scotland.

[Harris, S. E.] Western Gen Hosp, MRC Inst Genet & Mol Med, Edinburgh, Midlothian, Scotland.

[Gale, C. R.] Univ Southampton, MRC Lifecourse Epidemiol Unit, Southampton, Hants, England.

RP Deary, V (reprint author), Northumbria Univ, Dept Psychol, Newcastle NE1 8ST, England.; Deary, IJ (reprint author), Univ Edinburgh, Dept Psychol, 7 George Sq, Edinburgh EH8 9JZ, Midlothian, Scotland.; Deary, V (reprint author), Northumbria Univ, Dept Psychol, Newcastle NE1 8ST, England.

EM vincent.deary@northumbria.ac.uk; i.deary@ed.ac.uk

RI Deary, Ian/C-6297-2009; McIntosh, Andrew/B-9379-2008

OI Deary, Ian/0000-0002-1733-263X; McIntosh, Andrew/0000-0002-0198-4588;

Hagenaars, Saskia/0000-0001-9697-8596

FU University of Edinburgh Centre for Cognitive Ageing and Cognitive

Epidemiology, part of the cross council Lifelong Health and Wellbeing

Initiative [MR/K026992/1]; BBSRC; Age UK (Disconnected Mind Project);

Medical Research Council (MRC)

FX This research has been conducted using the UK Biobank Resource. The work

was undertaken in The University of Edinburgh Centre for Cognitive

Ageing and Cognitive Epidemiology, part of the cross council Lifelong

Health and Wellbeing Initiative (MR/K026992/1). Funding from the BBSRC,

Age UK (Disconnected Mind Project) and Medical Research Council (MRC) is

gratefully acknowledged.

CR Alberti KGMM, 2005, LANCET, V366, P1059, DOI 10.1016/S0140-6736(05)67402-8

Alves G, 2004, NEUROLOGY, V63, P1908, DOI 10.1212/01.WNL.0000144277.06917.CC

Aristotle, 1999, PHYSICS

Bandeen-Roche K, 2006, J GERONTOL A-BIOL, V61, P262, DOI 10.1093/gerona/61.3.262

Barsevick A, 2010, QUAL LIFE RES, V19, P1419, DOI 10.1007/s11136-010-9757-7

BENJAMINI Y, 1995, J R STAT SOC B, V57, P289

Booth T, 2013, AM J HUM BIOL, V25, P538, DOI 10.1002/ajhb.22406

Bower JE, 2014, NAT REV CLIN ONCOL, V11, P597, DOI 10.1038/nrclinonc.2014.127

Brown PJ, 2014, AM J GERIAT PSYCHIAT, V22, P1083, DOI 10.1016/j.jagp.2013.04.010

Bulik-Sullivan B, 2015, NAT GENET, V47, P1236, DOI 10.1038/ng.3406

Bulik-Sullivan BK, 2015, NAT GENET, V47, P291, DOI 10.1038/ng.3211

Bultmann U, 2002, INT ARCH OCC ENV HEA, V75, P259, DOI 10.1007/s00420-001-0294-0

Clark Gary D, 2015, Enzymes, V38, P37, DOI 10.1016/bs.enz.2015.09.009

Cullen W, 2002, IRISH J MED SCI, V171, P10, DOI 10.1007/BF03168931

Davies G, 2016, MOL PSYCHIATR, V21, P758, DOI 10.1038/mp.2016.45

de Leeuw CA, 2015, PLOS COMPUT BIOL, V11, DOI 10.1371/journal.pcbi.1004219

de Miguel FJ, 2014, CANCER RES, V74, P1105, DOI 10.1158/0008-5472.CAN-13-1481

Deary IJ, 2012, GERONTOLOGY, V58, P545, DOI 10.1159/000341157

Demyttenaere K, 2005, INT J NEUROPSYCHOPH, V8, P93, DOI 10.1017/S1461145704004729

Dixon JB, 2010, MOL CELL ENDOCRINOL, V316, P104, DOI 10.1016/j.mce.2009.07.008

Dolan P., 2013, J GERONTOL B-PSYCHOL, V70, P576

Euesden J, 2015, BIOINFORMATICS, V31, P1466, DOI 10.1093/bioinformatics/btu848

Felger JC, 2012, PSYCHOL MED, V42, P1591, DOI 10.1017/S0033291711002868

Fernandez-Munoz JJ, 2015, ACTA NEUROL SCAND, V132, P118, DOI 10.1111/ane.12370

Finucane HK, 2015, NAT GENET, V47, P1228, DOI 10.1038/ng.3404

Franssen PML, 2003, J PSYCHOSOM RES, V54, P339, DOI 10.1016/S0022-3999(02)00395-1

Fried LP, 2001, J GERONTOL A-BIOL, V56, pM146, DOI 10.1093/gerona/56.3.M146

Fritschi C, 2010, J PSYCHOSOM RES, V69, P33, DOI 10.1016/j.jpsychores.2010.01.021

Gale CR, 2016, TRANSL PSYCHIAT, V6, DOI 10.1038/tp.2016.56

Griffiths L, 2014, DIGEST DIS, V32, P615, DOI 10.1159/000360515

Hagenaars SP, 2016, MOL PSYCHIATR, V21, P1624, DOI 10.1038/mp.2015.225

Harris SE, 2016, INT J EPIDEMIOL

Hewlett S, 2005, ARTHRIT RHEUM-ARTHR, V53, P697, DOI 10.1002/art.21450

Hickie I, 1999, PSYCHOL MED, V29, P259, DOI 10.1017/S0033291798007934

Hill WD, 2016, TRANSL PSYCHIAT, V6, DOI 10.1038/tp.2016.246

Hoenicka J, 2007, NEUROTOX RES, V11, P61, DOI 10.1007/BF03033483

Hur YM, 2012, TWIN RES HUM GENET, V15, P720, DOI 10.1017/thg.2012.60

Jarosz PA, 2014, WOMEN HEALTH ISS, V24, pE447, DOI 10.1016/j.whi.2014.04.005

Juster RP, 2010, NEUROSCI BIOBEHAV R, V35, P2, DOI 10.1016/j.neubiorev.2009.10.002

Kitamura H, 2013, J OCCUP HEALTH, V55, P1

Klimas NG, 2012, BRAIN BEHAV IMMUN, V26, P1202, DOI 10.1016/j.bbi.2012.06.006

KRUPP LB, 1988, ARCH NEUROL-CHICAGO, V45, P435, DOI 10.1001/archneur.1988.00520280085020

Landmark-Hoyvik H, 2009, PHARMACOGENOMICS J, V9, P333, DOI 10.1038/tpj.2009.27

Landmark-Hoyvik H, 2010, PM&R, V2, P456, DOI 10.1016/j.pmrj.2010.04.003

Lasselin J, 2012, BRAIN BEHAV IMMUN, V26, P1211, DOI 10.1016/j.bbi.2012.03.003

LEWIS G, 1992, J EPIDEMIOL COMMUN H, V46, P92, DOI 10.1136/jech.46.2.92

Marchini J, 2007, NAT GENET, V39, P906, DOI 10.1038/ng2088

Meng HD, 2010, J AM GERIATR SOC, V58, P2033, DOI 10.1111/j.1532-5415.2010.03088.x

Ng WF, 2010, RHEUMATOLOGY, V49, P844, DOI 10.1093/rheumatology/keq009

PAWLIKOWSKA T, 1994, BRIT MED J, V308, P763, DOI 10.1136/bmj.308.6931.763

Purcell SM, 2009, NATURE, V460, P748, DOI 10.1038/nature08185

Qi QB, 2012, HUM MOL GENET, V21, P4774, DOI 10.1093/hmg/dds300

Ricci JA, 2007, J OCCUP ENVIRON MED, V49, P1, DOI 10.1097/01.jom.0000249782.60321.2a

Russell B, 2013, GENOME MED, V5, DOI 10.1186/gm420

Sailer A, 2012, CURR NEUROL NEUROSCI, V12, P227, DOI 10.1007/s11910-012-0267-6

Schlauch KA, 2016, TRANSL PSYCHIAT, V6, DOI 10.1038/tp.2015.208

Schur E, 2007, TWIN RES HUM GENET, V10, P729, DOI 10.1375/twin.10.5.729

Seeman T, 2010, AM J HUM BIOL, V22, P463, DOI 10.1002/ajhb.21018

Skerrett TN, 2006, J PSYCHOSOM RES, V61, P587, DOI 10.1016/j.jpsychores.2006.04.018

Smith AK, 2011, NEUROPSYCHOBIOLOGY, V64, P183, DOI 10.1159/000326692

Smith DJ, 2013, PLOS ONE, V8, DOI 10.1371/journal.pone.0075362

Spitzer RL, 1999, JAMA-J AM MED ASSOC, V282, P1737, DOI 10.1001/jama.282.18.1737

Sudlow C, 2015, PLOS MED, V12, DOI 10.1371/journal.pmed.1001779

Sugimoto M, 2012, PLOS ONE, V7, DOI 10.1371/journal.pone.0041267

Sullivan PF, 2005, PSYCHOL MED, V35, P1327, DOI 10.1017/S0033291705005222

The National Institute of Mental Health, 2016, REQ INF RFI INP PLAN

Theorell-Haglow J, 2006, SLEEP, V29, P751, DOI 10.1093/sleep/29.6.751

Tomas C., 2013, ISRN NEUROSCI, V2013, DOI DOI 10.1155/2013/784520

van Kessel K, 2006, J PSYCHOSOM RES, V61, P583, DOI 10.1016/j.jpsychores.2006.03.006

van't Leven M, 2010, EUR J PUBLIC HEALTH, V20, P251, DOI 10.1093/eurpub/ckp113

Wain LV, 2015, LANCET RESP MED, V3, P769, DOI 10.1016/S2213-2600(15)00283-0

WATSON D, 1989, PSYCHOL REV, V96, P234, DOI 10.1037//0033-295X.96.2.234

Wessely S, 2001, ANN INTERN MED, V134, P838, DOI 10.7326/0003-4819-134-9_Part_2-200105011-00007

Yang JA, 2010, NAT GENET, V42, P565, DOI 10.1038/ng.608

NR 74

TC 5

Z9 5

U1 7

U2 10

PU NATURE PUBLISHING GROUP

PI LONDON

PA MACMILLAN BUILDING, 4 CRINAN ST, LONDON N1 9XW, ENGLAND

SN 1359-4184

EI 1476-5578

J9 MOL PSYCHIATR

JI Mol. Psychiatr.

PD MAR

PY 2018

VL 23

IS 3

BP 609

EP 620

DI 10.1038/mp.2017.5

PG 12

WC Biochemistry & Molecular Biology; Neurosciences; Psychiatry

SC Biochemistry & Molecular Biology; Neurosciences & Neurology; Psychiatry

GA FW8MH

UT WOS:000425588100016

PM 28194004

OA Other Gold, Green Published

HC Y

HP N

DA 2019-01-08

ER

PT J

AU Zhu, BQ

Quinn, L

Fritschi, C

AF Zhu, Bingqian

Quinn, Laurie

Fritschi, Cynthia

TI Relationship and variation of diabetes related symptoms, sleep

disturbance and sleep-related impairment in adults with type 2 diabetes

SO JOURNAL OF ADVANCED NURSING

LA English

DT Article

DE fatigue; neuropathy; nursing; sleep disturbance; sleep-related

impairment; symptom; type 2 diabetes

ID PHYSICAL-ACTIVITY; GLYCEMIC CONTROL; GLUCOSE-METABOLISM; BREAST-CANCER;

OLDER-ADULTS; RISK-FACTORS; MELLITUS; FATIGUE; QUALITY; METAANALYSIS

AB AimThe objective of this study was to examine whether diabetes-related symptoms (e.g. fatigue, neuropathic pain, diabetes distress and depressive symptoms) were related to sleep disturbance and sleep-related impairment in adults with type 2 diabetes while controlling for potential covariates.

BackgroundIn people with type 2 diabetes, sleep disturbance and sleep-related impairment are common and likely associated with diabetes-related symptoms. However, limited research has investigated the predictive ability of diabetes-related symptoms on sleep.

DesignA correlational, cross-sectional design was used.

MethodsData were collected at a large university in the Midwestern United States from September 2013-March 2014. Multiple linear regression analyses were used to examine the relationship of diabetes-related symptoms (fatigue, neuropathic pain, distress and depressive symptoms) to sleep disturbance and sleep-related impairment. The instruments included Patient-Reported Outcomes Measurement Information System instruments, Diabetes Symptom Checklist and Diabetes Distress Scale.

FindingsIn this study of adults with type 2 diabetes (N=90; 52.2% female, mean age 57.4years), gender, A1C, neuropathic pain and fatigue were significantly related to sleep disturbance when age, diabetes duration, depressive symptoms and distress were controlled. Those variables collectively explained 52% of the variation in sleep disturbance. Fatigue was significantly associated with sleep-related impairment when the same covariates were controlled.

ConclusionFindings suggested that diabetes-related symptoms, including neuropathic pain and fatigue, are strongly related to sleep disturbance and sleep-related impairment in adults with type 2 diabetes, underscoring the need to include detailed assessments of neuropathic pain and fatigue when evaluating sleep.

C1 [Zhu, Bingqian; Quinn, Laurie; Fritschi, Cynthia] Univ Illinois, Coll Nursing, Chicago, IL USA.

RP Fritschi, C (reprint author), Univ Illinois, Coll Nursing, Dept Biobehav Hlth Sci, Chicago, IL 60607 USA.

EM fritschi@uic.edu

OI Fritschi, Cynthia/0000-0001-5447-8315

FU National Institutes of Health/National Institute for Nursing Research

[K99 R00 NR012219]

FX This work was supported in part by the National Institutes of

Health/National Institute for Nursing Research K99 R00 NR012219 (CF).

The funders had no role in research design, data collection, and

analysis, or manuscript preparation.

CR American Diabetes Association, 2017, DIABETES CARE S1, V40, pS25, DOI DOI 10.2337/DC17-S006

Anothaisintawee T, 2016, SLEEP MED REV, V30, P11, DOI 10.1016/j.smrv.2015.10.002

Arber S, 2009, SOC SCI MED, V68, P281, DOI 10.1016/j.socscimed.2008.10.016

Arbuckle RA, 2009, VALUE HEALTH, V12, P1168, DOI 10.1111/j.1524-4733.2009.00571.x

Barone MTU, 2011, DIABETES RES CLIN PR, V91, P129, DOI 10.1016/j.diabres.2010.07.011

Bode Bruce W, 2007, J Diabetes Sci Technol, V1, P405

Bromley LE, 2012, SLEEP, V35, P977, DOI 10.5665/sleep.1964

Carpenter JS, 2004, ONCOL NURS FORUM, V31, P591, DOI 10.1188/04.ONF.591-598

Cella D, 2007, MED CARE, V45, pS3, DOI 10.1097/01.mlr.0000258615.42478.55

Cella D, 2010, J CLIN EPIDEMIOL, V63, P1179, DOI 10.1016/j.jclinepi.2010.04.011

Centers for Disease Control and Prevention, 2017, NAT DIAB STAT REP

Cuellar NG, 2008, J CLIN SLEEP MED, V4, P50

Dillon HR, 2015, J GERONTOL B-PSYCHOL, V70, P46, DOI 10.1093/geronb/gbu035

GROOTENHUIS PA, 1994, DIABETIC MED, V11, P253, DOI 10.1111/j.1464-5491.1994.tb00268.x

Gupta S, 2016, DIABETES METAB SYND, V10, P213, DOI 10.1016/j.dsx.2016.06.009

Ho SY, 2015, J PAIN SYMPTOM MANAG, V49, P707, DOI 10.1016/j.jpainsymman.2014.09.009

Khanna D, 2012, VALUE HEALTH, V15, P128, DOI 10.1016/j.jval.2011.08.006

Knutson KL, 2007, SLEEP MED REV, V11, P163, DOI 10.1016/j.smrv.2007.01.002

Knutson KL, 2006, ARCH INTERN MED, V166, P1768, DOI 10.1001/archinte.166.16.1768

Knutson KL, 2011, DIABETES CARE, V34, P1171, DOI 10.2337/dc10-1962

Kredlow MA, 2015, J BEHAV MED, V38, P427, DOI 10.1007/s10865-015-9617-6

Lambiase MJ, 2013, MED SCI SPORT EXER, V45, P2362, DOI 10.1249/MSS.0b013e31829e4cea

Lamond N, 2000, SLEEP, V23, P415

Lee SWH, 2017, SLEEP MED REV, V31, P91, DOI 10.1016/j.smrv.2016.02.001

Mahmood WAW, 2013, INT J ENDOCRINOL, DOI 10.1155/2013/341506

McArdle W. D., 2001, EXERCISE PHYSL, P752

Nasseri R, 2015, INT J DIABETES DEV C, V35, P454, DOI 10.1007/s13410-015-0314-3

Nefs G, 2015, DIABETES RES CLIN PR, V109, P466, DOI 10.1016/j.diabres.2015.07.008

Nerenz DR, 2011, VALUE HEALTH, V14, pA90, DOI 10.1016/j.jval.2011.02.506

Ohayon MM, 2004, SLEEP, V27, P1255, DOI 10.1093/sleep/27.7.1255

Osonoi Y, 2015, BMC ENDOCR DISORD, V15, DOI 10.1186/s12902-015-0026-1

Ozturk ZA, 2015, AGING CLIN EXP RES, V27, P533, DOI 10.1007/s40520-014-0293-0

Parikh RM, 2007, MED HYPOTHESES, V68, P1272, DOI 10.1016/j.mehy.2006.10.038

Patrick D. L., 2008, COCHRANE HDB SYSTEMA, P531

Perrin NE, 2017, DIABETIC MED, V34, P1508, DOI 10.1111/dme.13448

Polonsky WH, 2005, DIABETES CARE, V28, P626, DOI 10.2337/diacare.28.3.626

PROMIS, 2017, PAT REP OUTC MEAS IN

PROMIS, 2017, INTR PROMIS LIST AUD

Ramtahal R, 2015, J EPIDEMIOL GLOB HEA, V5, pS35, DOI 10.1016/j.jegh.2015.05.003

Reutrakul S, 2014, ANN NY ACAD SCI, V1311, P151, DOI 10.1111/nyas.12355

Shan ZL, 2015, DIABETES CARE, V38, P529, DOI 10.2337/dc14-2073

Shaw JE, 2010, DIABETES RES CLIN PR, V87, P4, DOI 10.1016/j.diabres.2009.10.007

Sherrill DL, 1998, ARCH INTERN MED, V158, P1894, DOI 10.1001/archinte.158.17.1894

Smagula SF, 2016, SLEEP MED REV, V25, P21, DOI 10.1016/j.smrv.2015.01.003

Sun NQ, 2016, BMJ OPEN, V6, DOI 10.1136/bmjopen-2016-012540

Taylor-Piliae RE, 2006, AM J EPIDEMIOL, V164, P598, DOI 10.1093/aje/kwj248

Taylor-Piliae RE, 2010, J PHYS ACT HEALTH, V7, P87, DOI 10.1123/jpah.7.1.87

Tomas-Carus P, 2016, J SPORT MED PHYS FIT, V56, P572

Trento M, 2008, ACTA DIABETOL, V45, P225, DOI 10.1007/s00592-008-0047-6

Tsai YW, 2012, FAM PRACT, V29, P30, DOI 10.1093/fampra/cmr041

Yu L, 2012, BEHAV SLEEP MED, V10, P6, DOI 10.1080/15402002.2012.636266

Zhu BQ, 2017, J CLIN NURS, V26, P4053, DOI 10.1111/jocn.13899

NR 52

TC 0

Z9 0

U1 5

U2 9

PU WILEY

PI HOBOKEN

PA 111 RIVER ST, HOBOKEN 07030-5774, NJ USA

SN 0309-2402

EI 1365-2648

J9 J ADV NURS

JI J. Adv. Nurs.

PD MAR

PY 2018

VL 74

IS 3

BP 689

EP 697

DI 10.1111/jan.13482

PG 9

WC Nursing

SC Nursing

GA FV5RK

UT WOS:000424639900019

PM 29114911

DA 2019-01-08

ER

PT J

AU Beach, LB

Elasy, TA

Gonzales, G

AF Beach, Lauren B.

Elasy, Tom A.

Gonzales, Gilbert

TI Prevalence of Self-Reported Diabetes by Sexual Orientation: Results from

the 2014 Behavioral Risk Factor Surveillance System

SO LGBT HEALTH

LA English

DT Article

DE Behavioral Risk Factor Surveillance System; bisexual; diabetes; gay;

health disparities; lesbian

ID PHYSICAL HEALTH; UNITED-STATES; BISEXUAL ADULTS; MENTAL-HEALTH;

PSYCHOLOGICAL DISTRESS; MINORITY ADOLESCENTS; STRUCTURAL STIGMA;

NATIONAL-HEALTH; YOUNG-ADULTS; GAY

AB Purpose: This study aimed to compare the prevalence of self-reported diabetes and diabetes risk factors among adult sexual minority and heterosexual populations in the United States. Methods: Data from the 2014 Behavioral Risk Factor Surveillance System for 3776 lesbian, gay, and bisexual (LGB) adults and 142,852 heterosexual adults aged 18 years and older were used to estimate the prevalence of diabetes. Binomial logistic regression models were used to compare the odds of diabetes by sexual orientation. Results: Sexual minorities were younger and more racially diverse than heterosexuals. Gay men less often and lesbian and bisexual women more often reported a body mass index of 30kg/m(2) or higher than heterosexuals. Overall, 14.2% of bisexual men, 11.4% of gay men, and 10.8% of heterosexual men reported a lifetime diabetes diagnosis, as did 8.5% of lesbian women, 5.7% of bisexual women, and 10.2% of heterosexual women. After controlling for multiple factors, gay (odds ratio [OR]=1.50; confidence interval [95% CI]=1.09-2.07) and bisexual men [OR=1.55; 95% CI=1.00-2.07] were more likely to report a lifetime diabetes diagnosis than heterosexual men. Similar differences were not found for lesbian [OR=1.22; 95% CI=0.76-1.95] or bisexual women [OR=0.88; 95% CI=0.62-1.26]. Conclusion: Sexual minorities may be at increased risk for diabetes than their heterosexual peers. This may be due partly to the chronic stressors associated with being a member of a marginalized population. Future research should explore the underlying causes and consequences of LGB diabetes disparities and elucidate best practices to improve diabetes screening and care for these vulnerable patient populations.

C1 [Beach, Lauren B.; Elasy, Tom A.] Vanderbilt Univ, Med Ctr, Dept Med, Div Nephrol, Nashville, TN USA.

[Gonzales, Gilbert] Vanderbilt Univ, Sch Med, Dept Hlth Policy, Nashville, TN 37212 USA.

RP Beach, LB (reprint author), Vanderbilt Univ, Med Ctr, Dept Med, Div Nephrol,Med Ctr East, 1161 21st Ave South,Suite 6100, Nashville, TN 37212 USA.

EM lauren.b.beach@vanderbilt.edu

OI Beach, Lauren/0000-0003-3302-5047

FU Vanderbilt University Medical Center Renal Biology Disease Training

Program; National Institutes of Health/National Institute of Diabetes

and Digestive and Kidney Diseases (NIH/NIDDK) [T32 5T32DK007569];

NIH/National Institute on Minority Health and Health Disparities;

Vanderbilt Center for Diabetes Translation Research [NIH/NIDDK

5P30DK092986]; Vanderbilt Diabetes Research and Training Center

[NIH/NIDDK P30DK020593]

FX L.B. and G.G. contributed to the study design, conducted the analyses,

and wrote the article. T.E. reviewed/edited the article and contributed

to the study design. L.B. is supported by the Vanderbilt University

Medical Center Renal Biology Disease Training Program, the National

Institutes of Health/National Institute of Diabetes and Digestive and

Kidney Diseases (NIH/NIDDK) T32 5T32DK007569 and a Loan Repayment Award

from NIH/National Institute on Minority Health and Health Disparities.

T.E. is supported by the Vanderbilt Center for Diabetes Translation

Research, NIH/NIDDK 5P30DK092986, and the Vanderbilt Diabetes Research

and Training Center, NIH/NIDDK P30DK020593. We thank Drs. Kim Unertl and

Amelia Maiga of Vanderbilt University Medical Center and Dr. Faheemah

Mustafaa of the University of Michigan for providing helpful comments on

the article draft. Dr. Lauren Beach takes responsibility for the

contents of this article.

CR Anyanwagu U, 2016, CLIN PHARMACOL THER, V99, P390, DOI 10.1002/cpt.274

Blosnich JR, 2014, AM J PREV MED, V46, P337, DOI 10.1016/j.amepre.2013.11.010

Blosnich JR, 2013, ANN EPIDEMIOL, V23, P448, DOI 10.1016/j.annepidem.2013.04.009

Bolton SL, 2011, CAN J PSYCHIAT, V56, P35, DOI 10.1177/070674371105600107

Bostwick WB, 2010, AM J PUBLIC HEALTH, V100, P468, DOI 10.2105/AJPH.2008.152942

Brown TT, 2005, ARCH INTERN MED, V165, P1179, DOI 10.1001/archinte.165.10.1179

Centers for Disease Control and Prevention, 2013, BRFSS DAT US GUID

Centers for Disease Control and Prevention, 2015, BEH RISK FACT SURV S

Cochran SD, 2003, J CONSULT CLIN PSYCH, V71, P53, DOI 10.1037/0022-006X.71.1.53

Cochran SD, 2007, AM J PUBLIC HEALTH, V97, P2048, DOI 10.2105/AJPH.2006.087254

Conron KJ, 2010, AM J PUBLIC HEALTH, V100, P1953, DOI 10.2105/AJPH.2009.174169

Corliss HL, 2011, AM J PUBLIC HEALTH, V101, P1683, DOI 10.2105/AJPH.2011.300155

Dilley JA, 2010, AM J PUBLIC HEALTH, V100, P460, DOI 10.2105/AJPH.2007.130336

Djuric Zora, 2008, Open Biomark J, V1, P7, DOI 10.2174/1875318300801010007

Eliason MJ, 2014, LGBT HEALTH, V1, P259, DOI 10.1089/lgbt.2014.0026

Everett B, 2013, J COMMUN HEALTH, V38, P588, DOI 10.1007/s10900-013-9655-3

Fallin A, 2015, PREV MED, V74, P123, DOI 10.1016/j.ypmed.2014.11.026

Farmer GW, 2013, AM J PUBLIC HEALTH, V103, P1845, DOI 10.2105/AJPH.2013.301258

Farmer GW, 2013, J EPIDEMIOL COMMUN H, V67, P772, DOI 10.1136/jech-2013-202658

Fredriksen-Goldsen KI, 2017, AM J PUBLIC HEALTH, V107, P1332, DOI 10.2105/AJPH.2017.303922

Fredriksen-Goldsen KI, 2013, AM J PUBLIC HEALTH, V103, P1802, DOI 10.2105/AJPH.2012.301110

Friedman MR, 2014, PLOS ONE, V9, DOI 10.1371/journal.pone.0087139

Garland-Forshee RY, 2014, PREV CHRONIC DIS, V11, DOI 10.5888/pcd11.140126

Goldbach JT, 2014, PREV SCI, V15, P350, DOI 10.1007/s11121-013-0393-7

Gonzales G, 2016, JAMA INTERN MED, V176, P1344, DOI 10.1001/jamainternmed.2016.3432

Gruskin EP, 2006, BMC PUBLIC HEALTH, V6, DOI 10.1186/1471-2458-6-241

Hammerman A, 2008, ANN PHARMACOTHER, V42, P1316, DOI 10.1345/aph.1L015

Hatzenbuehler ML, 2016, AM PSYCHOL, V71, P742, DOI 10.1037/amp0000068

Hatzenbuehler ML, 2014, ANN BEHAV MED, V47, P39, DOI 10.1007/s12160-013-9556-9

Heidemann DL, 2016, ETHNIC DIS, V26, P85, DOI 10.18865/ed.26.1.85

Hirshfield S, 2016, AM J MENS HLTH

Institute of Medicine (US) Committee on Lesbian Gay Bisexual and Transgender Health Issues and Research Gaps and Opportunities, 2011, HLTH LESB GAY BISEX

Joseph JJ, 2017, ANN NY ACAD SCI, V1391, P20, DOI 10.1111/nyas.13217

Kelly SJ, 2015, ANNU REV PUBL HEALTH, V36, P441, DOI 10.1146/annurev-publhealth-031914-122921

Lick DJ, 2013, PERSPECT PSYCHOL SCI, V8, P521, DOI 10.1177/1745691613497965

Lindley LL, 2012, AM J PUBLIC HEALTH, V102, P1177, DOI 10.2105/AJPH.2011.300262

Lumley T., 2004, J STAT SOFTW, V9, P1, DOI DOI 10.18637/JSS.V009.I08

Marshal MP, 2013, J YOUTH ADOLESCENCE, V42, P1243, DOI 10.1007/s10964-013-9970-0

Meyer IH, 2003, PSYCHOL BULL, V129, P674, DOI 10.1037/0033-2909.129.5.674

Mustanski BS, 2010, AM J PUBLIC HEALTH, V100, P2426, DOI 10.2105/AJPH.2009.178319

Pierannunzi Carol, 2012, Morbidity and Mortality Weekly Report, V61, P410

Puckrein GA, 2015, ETHNIC DIS, V25, P521, DOI 10.18865/ed.25.4.521

R Core Development Team, 2016, R LANG ENV STAT COMP

Sell RL, 2014, AM J PUBLIC HEALTH, V104, P967, DOI 10.2105/AJPH.2013.301814

Simoni JM, 2017, J HOMOSEXUAL, V64, P32, DOI 10.1080/00918369.2016.1174021

Slavich GM, 2014, PSYCHOL BULL, V140, P774, DOI 10.1037/a0035302

Tomedi LE, 2013, HLTH INEQUITIES SEXU

Turi KN, 2017, APPL GEOGR, V85, P62, DOI 10.1016/j.apgeog.2017.05.005

VanKim NA, 2010, AM J PUBLIC HEALTH, V100, P2392, DOI 10.2105/AJPH.2009.186270

Ward BW, 2015, PREV CHRONIC DIS, V12, DOI 10.5888/pcd12.150292

NR 50

TC 2

Z9 2

U1 1

U2 2

PU MARY ANN LIEBERT, INC

PI NEW ROCHELLE

PA 140 HUGUENOT STREET, 3RD FL, NEW ROCHELLE, NY 10801 USA

SN 2325-8292

EI 2325-8306

J9 LGBT HEALTH

JI LGBT Health

PD MAR

PY 2018

VL 5

IS 2

BP 121

EP 130

DI 10.1089/lgbt.2017.0091

EA JAN

EY 2018

PG 10

WC Public, Environmental & Occupational Health

SC Public, Environmental & Occupational Health

GA FY1GM

UT WOS:000423533400001

PM 29377760

DA 2019-01-08

ER

PT J

AU Schinckus, L

Dangoisse, F

Van den Broucke, S

Mikolajczak, M

AF Schinckus, Louise

Dangoisse, Florence

Van den Broucke, Stephan

Mikolajczak, Moira

TI When knowing is not enough: Emotional distress and depression reduce the

positive effects of health literacy on diabetes self-management

SO PATIENT EDUCATION AND COUNSELING

LA English

DT Article

DE Diabetes; Health literacy; Self-efficacy; Depression; Distress

ID GLYCEMIC CONTROL; TYPE-2; OUTCOMES; CARE; ADHERENCE; MELLITUS; ANXIETY;

WELL; METAANALYSIS; ASSOCIATION

AB Objectives: Adequate self-management activities are important predictors of diabetes outcomes. As diabetes literacy and self-efficacy are strong predictors of diabetes self-care, self-management education programs focus essentially on these factors. This study investigated whether emotional distress or depression moderates the relation between health literacy, self-efficacy and diabetes self-care behaviors.

Methods: 128 people with type 2 diabetes were recruited in hospitals, through general practitioners and via a diabetes website, and completed a questionnaire assessing health literacy, self-efficacy, diabetes-related distress, depression and self-care behaviors.

Results: Multiple regression analysis confirms that health literacy and self-efficacy significantly predict reported self-care behaviors. Additional regression analyses reveal that distress or depression do not predict self-care behaviors directly, but moderate the effect of health literacy, which has a weaker impact in patients experiencing distress or depression. In contrast, distress and depression do not moderate the effect of self-efficacy on diabetes self-care behaviors.

Conclusion: Emotional distress, whether related to diabetes or not, prevents patients from acting on their competence to perform adequate self-management behaviors.

Practice implications: Diabetes Professionals should pay more attention to the patients' affective state and its influence on self-care. Psychological support should be integrated in the care for people suffering from type 2 diabetes. (C) 2017 Published by Elsevier Ireland Ltd.

C1 [Schinckus, Louise; Dangoisse, Florence; Van den Broucke, Stephan; Mikolajczak, Moira] Catholic Univ Louvain, Psychol Sci Res Inst, Louvain La Neuve, Belgium.

RP Schinckus, L (reprint author), Catholic Univ Louvain, Psychol Sci Res Inst, 10 Pl Cardinal Mercier,Bte L3-05-01, B-1348 Louvain La Neuve, Belgium.

EM schinckuslouise@gmail.com

OI Mikolajczak, Moira/0000-0002-7333-1578

FU Psychological Sciences Research Institute, Catholic University of

Louvain, Louvain-la-Neuve, Belgium

FX This work was supported by the Psychological Sciences Research

Institute, Catholic University of Louvain, Louvain-la-Neuve, Belgium.

CR AJZEN I, 1991, ORGAN BEHAV HUM DEC, V50, P179, DOI 10.1016/0749-5978(91)90020-T

Anderson RJ, 2001, DIABETES CARE, V24, P1069, DOI 10.2337/diacare.24.6.1069

[Anonymous], 2011, AADE 7 SELF CAR BEH

Association Belge du Diabete, 2014, VIV VOTR DIAB TOUT S

BANDURA A, 1991, ORGAN BEHAV HUM DEC, V50, P248, DOI 10.1016/0749-5978(91)90022-L

BANDURA A, 1982, AM PSYCHOL, V37, P122, DOI 10.1037//0003-066X.37.2.122

Barnard K. D., 2012, PSYCHOL DIABETES CAR

BARTLETT EE, 1986, PATIENT EDUC COUNS, V8, P135, DOI 10.1016/0738-3991(86)90085-6

Baudrant-Boga M., 2009, THESIS

Berkman ND, 2011, ANN INTERN MED, V155, P97, DOI 10.7326/0003-4819-155-2-201107190-00005

Black SA, 2003, DIABETES CARE, V26, P2822, DOI 10.2337/diacare.26.10.2822

Brasseur S, 2013, PLOS ONE, V8, DOI 10.1371/journal.pone.0062635

Cherrington A, 2010, J BEHAV MED, V33, P81, DOI 10.1007/s10865-009-9233-4

Delahanty LM, 2007, DIABETIC MED, V24, P48, DOI 10.1111/j.1464-5491.2007.02028.x

DeWalt DA, 2004, J GEN INTERN MED, V19, P1228, DOI 10.1111/j.1525-1497.2004.40153.x

Engum A, 2007, J PSYCHOSOM RES, V62, P31, DOI 10.1016/j.jpsychores.2006.07.009

Fisher L, 2008, DIABETIC MED, V25, P1096, DOI 10.1111/j.1464-5491.2008.02533.x

Gonzalez JS, 2008, DIABETOLOGIA, V51, P1822, DOI 10.1007/s00125-008-1113-x

Gonzalez JS, 2015, HEALTH PSYCHOL, V34, P505, DOI 10.1037/hea0000131

Hajos TRS, 2011, PATIENT EDUC COUNS, V85, P258, DOI 10.1016/j.pec.2010.08.019

Harkness E, 2010, DIABETES CARE, V33, P926, DOI 10.2337/dc09-1519

Heisler M, 2003, DIABETES CARE, V26, P738, DOI 10.2337/diacare.26.3.738

Ishikawa H, 2008, DIABETES CARE, V31, P874, DOI 10.2337/dc07-1932

Karahan TF, 2009, TURK KLIN TIP BILIM, V29, P16

Kotsou I, 2011, J APPL PSYCHOL, V96, P827, DOI 10.1037/a0023047

Lloyd C. E., 2013, SCREEN DEPRESS PSYCH, P87

Mann DM, 2009, J BEHAV MED, V32, P278, DOI 10.1007/s10865-009-9202-y

Millan-Ferro A, 2009, CONTEMP DIABETES, P115, DOI 10.1007/978-1-60327-208-7_8

Nelis D, 2011, EMOTION, V11, P354, DOI 10.1037/a0021554

Ngcobo M., 2008, EUR DIABETES NURSING, V11, P133, DOI [10.4314/ajpsy.v11i2.30266, DOI 10.1002/EDN.72]

Pelikan JM, 2014, MEASURING COMPREHENS

Polonsky WH, 2005, DIABETES CARE, V28, P626, DOI 10.2337/diacare.28.3.626

Powell CK, 2007, DIABETES EDUCATOR, V33, P144, DOI 10.1177/0145721706297452

Rhee MK, 2005, DIABETES EDUCATOR, V31, P240, DOI 10.1177/0145721705274927

ROGERS RW, 1975, J PSYCHOL, V91, P93, DOI 10.1080/00223980.1975.9915803

Schillinger D, 2002, JAMA-J AM MED ASSOC, V288, P475, DOI 10.1001/jama.288.4.475

Schmitt A, 2013, HEALTH QUAL LIFE OUT, V11, DOI 10.1186/1477-7525-11-138

Sorensen K, 2015, EUR J PUBLIC HEALTH, V25, P1053, DOI 10.1093/eurpub/ckv043

Thompson C, 2001, BRIT J PSYCHIAT, V179, P450, DOI 10.1192/bjp.179.5.450

van der Bijl J, 1999, J ADV NURS, V30, P352, DOI 10.1046/j.1365-2648.1999.01077.x

Yalcin BM, 2008, DIABETES EDUCATOR, V34, P1013, DOI 10.1177/0145721708327303

NR 41

TC 1

Z9 1

U1 6

U2 8

PU ELSEVIER IRELAND LTD

PI CLARE

PA ELSEVIER HOUSE, BROOKVALE PLAZA, EAST PARK SHANNON, CO, CLARE, 00000,

IRELAND

SN 0738-3991

J9 PATIENT EDUC COUNS

JI Patient Educ. Couns.

PD FEB

PY 2018

VL 101

IS 2

BP 324

EP 330

DI 10.1016/j.pec.2017.08.006

PG 7

WC Public, Environmental & Occupational Health; Social Sciences,

Interdisciplinary

SC Public, Environmental & Occupational Health; Social Sciences - Other

Topics

GA FX9SQ

UT WOS:000426445700020

PM 28855062

DA 2019-01-08

ER

PT J

AU Masters, RK

Tilstra, AM

Simon, DH

AF Masters, Ryan K.

Tilstra, Andrea M.

Simon, Daniel H.

TI Explaining recent mortality trends among younger and middle-aged White

Americans

SO INTERNATIONAL JOURNAL OF EPIDEMIOLOGY

LA English

DT Article

DE USA; midlife mortality; obesogenic; opiates; despair deaths; mortality

trends

ID UNITED-STATES; LIFE EXPECTANCY; ANTIRETROVIRAL THERAPY; DISEASE; COHORT;

INCREASES; PERIOD; WOMEN; DRUG

AB Background: Recent research has suggested that increases in mortality among middle-aged US Whites are being driven by suicides and poisonings from alcohol and drug use. Increases in these 'despair' deaths have been argued to reflect a cohort-based epidemic of pain and distress among middle-aged US Whites.

Methods: We examine trends in all-cause and cause-specific mortality rates among younger and middle-aged US White men and women between 1980 and 2014, using official US mortality data. We estimate trends in cause-specific mortality from suicides, alcohol-related deaths, drug-related deaths, 'metabolic diseases' (i.e. deaths from heart diseases, diabetes, obesity and/or hypertension), and residual deaths from extrinsic causes (i.e. causes external to the body). We examine variation in mortality trends by gender, age and cause of death, and decompose trends into period-and cohort-based variation.

Results: Trends in middle-aged US White mortality vary considerably by cause and gender. The relative contribution to overall mortality rates from drug-related deaths has increased dramatically since the early 1990s, but the contributions from suicide and alcohol-related deaths have remained stable. Rising mortality from drug-related deaths exhibit strong period-based patterns. Declines in deaths from metabolic diseases have slowed for middle-aged White men and have stalled for middle-aged White women, and exhibit strong cohort-based patterns.

Conclusions: We find little empirical support for the pain-and distress-based explanations for rising mortality in the US White population. Instead, recent mortality increases among younger and middle-aged US White men and women have likely been shaped by the US opiate epidemic and an expanding obesogenic environment.

C1 [Masters, Ryan K.; Tilstra, Andrea M.; Simon, Daniel H.] Univ Colorado, Dept Sociol, Boulder, CO 80309 USA.

[Masters, Ryan K.; Tilstra, Andrea M.; Simon, Daniel H.] Univ Colorado, Inst Behav Sci, Populat Program, Boulder, CO 80309 USA.

RP Masters, RK (reprint author), Univ Colorado, Dept Sociol, Boulder, CO 80309 USA.; Masters, RK (reprint author), Univ Colorado, Inst Behav Sci, Populat Program, Boulder, CO 80309 USA.

EM Ryan.Masters@Colorado.EDU

CR [Anonymous], 1990, Q POP EST 1980 1990

[Anonymous], 2014, BRIDG RAC POP EST 19

Boyd MA, 2009, CURR OPIN HIV AIDS, V4, P194, DOI 10.1097/COH.0b013e328329fc8d

Calcaterra S, 2013, DRUG ALCOHOL DEPEN, V131, P263, DOI 10.1016/j.drugalcdep.2012.11.018

Carnes BA, 2006, BIOGERONTOLOGY, V7, P183, DOI 10.1007/s10522-006-9020-3

Case A, 2015, P NATL ACAD SCI USA, V112, P15078, DOI 10.1073/pnas.1518393112

Cassidy J, 2015, NEW YORKER

Cicero TJ, 2014, JAMA PSYCHIAT, V71, P821, DOI 10.1001/jamapsychiatry.2014.366

Douthat R, 2015, NY TIMES

Dowell D, 2016, JAMA-J AM MED ASSOC, V315, P1624, DOI 10.1001/jama.2016.1464

Gelman A, 2016, P NATL ACAD SCI USA, V113, pE816, DOI 10.1073/pnas.1523465113

Hedegaard H., 2015, DRUG POISONING DEATH

Hogg R, 2008, LANCET, V372, P293, DOI 10.1016/S0140-6736(08)61113-7

Kochanek Kenneth D., 2016, EFFECT CHANGES SELEC

Krugman P, 2015, NY TIMES

Manchikanti L, 2012, PAIN PHYSICIAN, V15, pES9

Masters RK, 2017, BIODEMOGR SOC BIOL, V63, P31, DOI 10.1080/19485565.2016.1248892

Masters RK, 2014, DEMOGRAPHY, V51, P2047, DOI 10.1007/s13524-014-0343-4

Meara E, 2015, P NATL ACAD SCI USA, V112, P15006, DOI 10.1073/pnas.1519763112

Montez JK, 2014, AM J PUBLIC HEALTH, V104, pE5, DOI 10.2105/AJPH.2014.302146

National Center for Health Statistics, 2016, Q PROV EST SEL CAUS

NCHS, 1968, MORT MULT CAUS DEATH

Olshansky SJ, 2012, HEALTH AFFAIR, V31, P1803, DOI 10.1377/hlthaff.2011.0746

Olshansky SJ, 2005, NEW ENGL J MED, V352, P1138, DOI 10.1056/NEJMsr043743

Quinones S., 2015, DREAMLAND TRUE TALE

Reither EN, 2011, HEALTH AFFAIR, V30, P1562, DOI 10.1377/hlthaff.2011.0092

Rudd RA, 2016, AM J TRANSPLANT, V16, P1323, DOI 10.1111/ajt.13776

Rudd RA, 2014, MMWR-MORBID MORTAL W, V63, P849

Saslow E, 2016, WASHINGTON POST

Tavernise S., 2016, NY TIMES

Woolf S. H., 2013, US HLTH INT PERSPECT

Yang Y, 2008, DEMOGRAPHY, V45, P387, DOI 10.1353/dem.0.0000

Zee AV, 2009, AM J PUBLIC HEALTH, V99, P221, DOI 10.2105/AJPH.2007.131714

NR 33

TC 6

Z9 6

U1 2

U2 6

PU OXFORD UNIV PRESS

PI OXFORD

PA GREAT CLARENDON ST, OXFORD OX2 6DP, ENGLAND

SN 0300-5771

EI 1464-3685

J9 INT J EPIDEMIOL

JI Int. J. Epidemiol.

PD FEB

PY 2018

VL 47

IS 1

BP 81

EP 88

DI 10.1093/ije/dyx127

PG 8

WC Public, Environmental & Occupational Health

SC Public, Environmental & Occupational Health

GA FX5UZ

UT WOS:000426148300020

PM 29040539

DA 2019-01-08

ER

PT J

AU Whittemore, R

Zincavage, RM

Jaser, SS

Grey, M

Coleman, JL

Collett, D

Delvy, R

Ibrahim, BB

Marceau, LD

AF Whittemore, Robin

Zincavage, Rebekah M.

Jaser, Sarah S.

Grey, Margaret

Coleman, Julia L.

Collett, David

Delvy, Roberta

Ibrahim, Bridget Basile

Marceau, Lisa D.

TI Development of an eHealth Program for Parents of Adolescents With Type 1

Diabetes

SO DIABETES EDUCATOR

LA English

DT Article

ID PSYCHOSOCIAL ADJUSTMENT; DEPRESSIVE SYMPTOMS; CONTROLLED-TRIAL; GLYCEMIC

CONTROL; YOUNG-CHILDREN; MOTHERS; FAMILY; YOUTH; INTERVENTION; MELLITUS

AB Purpose The purpose of this study was to understand the experience of parenting an adolescent with type 1 diabetes (T1DM), to develop a prototype of an eHealth program for parents of adolescents with T1DM, and to evaluate the prototype content and acceptability from the perspective of parents and health care providers.

Methods A multiphase method was used generating both qualitative and quantitative data at multiple time points. There were 27 parents of adolescents aged 12 to 18 years with T1DM and 16 health care providers who participated in semistructured interviews to identify parental challenges; 53 parents and 27 providers evaluated the prototype. Thematic content analysis was used to analyze interview transcripts, and descriptive statistics were used to summarize survey data.

Results Challenges experienced by parents of adolescents with T1DM included understanding the developmental and hormonal changes of adolescence that affect diabetes care, feeling tension between adolescent independence and parent control, communicating without nagging or conflict, transferring diabetes care responsibility safely, dealing with feelings of stress and distress, and perceiving a lack of resources for T1DM care and insufficient personal time for self-care. In the prototype evaluation, both parents and providers found content to be relevant and provided feedback to guide the development of the full program.

Conclusions Parents of adolescents with T1DM and providers expressed a need for parents to have more support in transitioning diabetes care from parent to adolescent. eHealth programs offer an ideal way to address these needs and ultimately can be linked to electronic medical records improving quality and efficiency of health care in this population.

C1 [Whittemore, Robin; Grey, Margaret; Collett, David; Delvy, Roberta; Ibrahim, Bridget Basile] Yale Sch Nursing, POB 27399, West Haven, CT 06516 USA.

[Zincavage, Rebekah M.; Coleman, Julia L.; Marceau, Lisa D.] New England Res Inst, 9 Galen St, Watertown, MA 02172 USA.

[Jaser, Sarah S.] Vanderbilt Univ, Med Ctr, Nashville, TN USA.

RP Whittemore, R (reprint author), Yale Sch Nursing, POB 27399, West Haven, CT 06516 USA.

EM Robin.whittemore@yale.edu

FU National Institute of Diabetes and Digestive and Kidney Diseases

[1R43DK098857]

FX This project was funded by a grant (1R43DK098857) from the National

Institute of Diabetes and Digestive and Kidney Diseases.

CR Anderson BJ, 1999, DIABETES CARE, V22, P713, DOI 10.2337/diacare.22.5.713

Borus JS, 2010, CURR OPIN PEDIATR, V22, P405, DOI 10.1097/MOP.0b013e32833a46a7

Bowes S, 2009, J ADV NURS, V65, P992, DOI 10.1111/j.1365-2648.2009.04963.x

Buckloh LM, 2008, DIABETES CARE, V31, P1516, DOI 10.2337/dc07-2349

CADMAN D, 1991, PEDIATRICS, V87, P884

Cameron LD, 2007, J PEDIATR PSYCHOL, V32, P733, DOI 10.1093/jpepsy/jslo53

Colorafi KJ, 2016, HERD-HEALTH ENV RES, V9, P16, DOI 10.1177/1937586715614171

Eckshtain D, 2010, J PEDIATR PSYCHOL, V35, P426, DOI 10.1093/jpepsy/jsp068

Ellis DA, 2007, J CONSULT CLIN PSYCH, V75, P168, DOI 10.1037/0022-006X.75.1.168

Gale EAM, 2002, DIABETES, V51, P3353, DOI 10.2337/diabetes.51.12.3353

Green J, 2009, QUALITATIVE METHODS

Grey M, 2009, RES NURS HEALTH, V32, P405, DOI 10.1002/nur.20336

Howe CJ, 2012, J PEDIATR NURS, V27, P119, DOI 10.1016/j.pedn.2010.10.006

Jaser SS, 2008, J PEDIATR PSYCHOL, V33, P509, DOI 10.1093/jpepsy/jsm104

Jaser SS, 2010, J PEDIATR PSYCHOL, V35, P738, DOI 10.1093/jpepsy/jsp098

Jaser SS, 2009, CHILD HEALTH CARE, V38, P91, DOI 10.1080/02739610902813229

Kaugars AS, 2011, PEDIATR DIABETES, V12, P547, DOI 10.1111/j.1399-5448.2010.00737.x

Kim H, 2017, RES NURS HEALTH, V40, P23, DOI 10.1002/nur.21768

KOVACS M, 1990, J CONSULT CLIN PSYCH, V58, P189, DOI 10.1037//0022-006X.58.2.189

Laffel LMB, 2003, J PEDIATR-US, V142, P409, DOI 10.1067/mpd.2003.138

Landolt MA, 2002, J PEDIATR PSYCHOL, V27, P647, DOI 10.1093/jpepsy/27.7.647

Leonard Barbara J, 2005, J Pediatr Nurs, V20, P405, DOI 10.1016/j.pedn.2005.03.010

Liese AD, 2006, PEDIATRICS, V118, P1510, DOI 10.1542/peds.2006-0690

Livingston G., 2009, LATINOS ONLINE 2006

Lofland John, 1995, ANAL SOCIAL SETTINGS

Mitchell SJ, 2009, FAM SYST HEALTH, V27, P314, DOI 10.1037/a0018191

Pew Research Center, 2009, DEM TEEN INT US

Spencer J, 2010, PEDIATR DIABETES, V11, P364, DOI 10.1111/j.1399-5448.2009.00603.x

Streisand R, 2005, J PEDIATR PSYCHOL, V30, P513, DOI 10.1093/jpepsy/jsi076

Sullivan-Bolyai S, 2003, J Pediatr Nurs, V18, P21, DOI 10.1053/jpdn.2003.4

Weinger K, 2001, J ADOLESCENT HEALTH, V29, P330, DOI 10.1016/S1054-139X(01)00270-1

Whittemore R, 2012, DIABETES EDUCATOR, V38, P562, DOI 10.1177/0145721712445216

Wysocki T, 2000, J PEDIATR PSYCHOL, V25, P23, DOI 10.1093/jpepsy/25.1.23

Young MT, 2014, CURR DIABETES REP, V14, DOI 10.1007/s11892-014-0546-5

Young-Hyman D, 2016, DIABETES CARE, V39, P2126, DOI 10.2337/dc16-2053

NR 35

TC 0

Z9 0

U1 1

U2 5

PU SAGE PUBLICATIONS INC

PI THOUSAND OAKS

PA 2455 TELLER RD, THOUSAND OAKS, CA 91320 USA

SN 0145-7217

EI 1554-6063

J9 DIABETES EDUCATOR

JI Diabetes Educ.

PD FEB

PY 2018

VL 44

IS 1

BP 72

EP 82

DI 10.1177/0145721717748606

PG 11

WC Endocrinology & Metabolism; Public, Environmental & Occupational Health

SC Endocrinology & Metabolism; Public, Environmental & Occupational Health

GA FX3SY

UT WOS:000425993800008

PM 29262747

DA 2019-01-08

ER

PT J

AU Purewal, R

Fisher, PL

AF Purewal, Rebecca

Fisher, Peter L.

TI The contribution of illness perceptions and metacognitive beliefs to

anxiety and depression in adults with diabetes

SO DIABETES RESEARCH AND CLINICAL PRACTICE

LA English

DT Article

DE Diabetes; Anxiety; Depression; Metacognitive beliefs; Illness

perceptions

ID QUALITY-OF-LIFE; EMOTIONAL DISTRESS; GLYCEMIC CONTROL;

PARKINSONS-DISEASE; GENERAL-POPULATION; SUBSTANCE USE; HEALTH;

REPRESENTATIONS; ASSOCIATION; DISORDER

AB Aims: Anxiety and depression are highly prevalent in people with diabetes (PwD). The most widely used psychological model to explain anxiety and depression in PwD is the Common-Sense Model, which gives a central role to illness perceptions. The Self-Regulatory Executive Function (S-REF) model proposes metacognitive beliefs are key to understanding the development and maintenance of emotional disorders. To test the potential utility of the S-REF model in PwD, the study explored if metacognitive beliefs explained additional variance in anxiety and depression after controlling for demographic and illness perceptions.

Methods: 614 adults with either Type 1 (n = 335) or Type 2 (n = 279) diabetes participated in a cross sectional online survey. All participants completed questionnaires on anxiety, depression, illness perceptions and metacognitive beliefs.

Results: Regression analyses showed that metacognitive beliefs were associated with anxiety and depression in PwD and explained additional variance in both anxiety and depression after controlling for demographics and illness perceptions.

Conclusions: This is the first study to demonstrate that metacognitive beliefs are associated with anxiety and depression in PwD. The clinical implications of the study are illustrated. Crown Copyright (C) 2017 Published by Elsevier Ireland Ltd. All rights reserved.

C1 [Purewal, Rebecca; Fisher, Peter L.] Univ Liverpool, Psychol Sci, Liverpool, Merseyside, England.

[Fisher, Peter L.] St Olavs Univ Hosp, Nidaros DPS, Div Psychiat, Trondheim, Norway.

RP Fisher, PL (reprint author), Univ Liverpool, Peter Fisher Inst Psychol Hlth & Soc, Whelan Bldg,Brownlow Hill, Liverpool, Merseyside, England.

EM peter.fisher@liverpool.ac.uk

OI Fisher, Peter/0000-0002-7388-720X

CR Allott R, 2005, BRIT J PSYCHIAT, V187, P182, DOI 10.1192/bjp.187.2.182

Baumeister H, 2012, COCHRANE DB SYST REV, P12

Brown RG, 2015, J PSYCHOSOM RES, V78, P143, DOI 10.1016/j.jpsychores.2014.09.021

Ciechanowski PS, 2000, ARCH INTERN MED, V160, P3278, DOI 10.1001/archinte.160.21.3278

Cook SA, 2015, COGNITIVE THER RES, V39, P51, DOI 10.1007/s10608-014-9640-x

Cook SA, 2015, HEALTH PSYCHOL, V34, P207, DOI 10.1037/hea0000096

Cook SA, 2014, PLOS ONE, V9, DOI 10.1371/journal.pone.0107302

Cukierman-Yaffe T, 2014, DIABETES-METAB RES, V30, P341, DOI 10.1002/dmrr.2507

Ducat L, 2015, DIABETES CARE, V38, P333, DOI 10.2337/dc14-1383

Gonzalez JS, 2008, DIABETES CARE, V31, P2398, DOI 10.2337/dc08-1341

Hampson SE, 2000, BRIT J HEALTH PSYCH, V5, P27, DOI 10.1348/135910700168748

Hudson JL, 2014, J PSYCHOSOM RES, V76, P265, DOI 10.1016/j.jpsychores.2014.02.004

Jeeva F, 2013, INT J TECHNOL ASSESS, V29, P384, DOI 10.1017/S0266462313000445

Kessler RC, 2003, JAMA-J AM MED ASSOC, V289, P3095, DOI 10.1001/jama.289.23.3095

Kroenke K, 2001, J GEN INTERN MED, V16, P606, DOI 10.1046/j.1525-1497.2001.016009606.x

Leventhal H, 1980, CONTRIBUTIONS MED PS, V2, P7

Li C, 2008, DIABETIC MED, V25, P878, DOI 10.1111/j.1464-5491.2008.02477.x

Lim AKH, 2014, INT J NEPHROL RENOV, V7, P361, DOI 10.2147/IJNRD.S40172

Lowe B, 2008, MED CARE, V46, P266, DOI 10.1097/MLR.0b013e318160d093

Lustman PJ, 2005, J DIABETES COMPLICAT, V19, P113, DOI 10.1016/j.jdiacomp.2004.01.002

Lustman PJ, 2000, DIABETES CARE, V23, P934, DOI 10.2337/diacare.23.7.934

Maher-Edwards L, 2012, CLIN PSYCHOL PSYCHOT, V19, P552, DOI 10.1002/cpp.757

Mollema ED, 2001, J PSYCHOSOM RES, V51, P665, DOI 10.1016/S0022-3999(01)00229-X

Molosankwe I, 2012, J AFFECT DISORDERS, V142, pS42, DOI 10.1016/S0165-0327(12)70008-3

Moussavi S, 2007, LANCET, V370, P851, DOI 10.1016/S0140-6736(07)61415-9

Paschalides C, 2004, J PSYCHOSOM RES, V57, P557, DOI 10.1016/j.jpsychores.2004.03.006

Renn BN, 2011, CLIN PSYCHOL REV, V31, P1239, DOI 10.1016/j.cpr.2011.08.001

Roy T, 2012, J AFFECT DISORDERS, V142, pS8, DOI 10.1016/S0165-0327(12)70004-6

Schram MT, 2009, CURR DIABETES REV, V5, P112, DOI 10.2174/157339909788166828

Skinner TC, 2003, DIABETIC MED, V20, P283, DOI 10.1046/j.1464-5491.2003.00923.x

Sommerfield AJ, 2003, DIABETES CARE, V26, P390, DOI 10.2337/diacare.26.2.390

Spada MM, 2008, PERS INDIV DIFFER, V45, P238, DOI 10.1016/j.paid.2008.04.005

Spitzer RL, 2006, ARCH INTERN MED, V166, P1092, DOI 10.1001/archinte.166.10.1092

Steed L, 2003, PATIENT EDUC COUNS, V51, P5, DOI 10.1016/S0738-3991(02)00213-6

Stoop CH, 2015, J AFFECT DISORDERS, V184, P269, DOI 10.1016/j.jad.2015.05.063

Toobert DJ, 2000, DIABETES CARE, V23, P943, DOI 10.2337/diacare.23.7.943

Van der Feltz-Cornelis CM, 2010, GEN HOSP PSYCHIAT, V32, P380, DOI 10.1016/j.genhosppsych.2010.03.011

Van Steenbergen-Weijenburg KM, 2010, BMC HLTH SERV RES, V10

Wells A, 2004, BEHAV RES THER, V42, P385, DOI 10.1016/S0005-7967(03)00147-5

Wells A., 1994, ATTENTION EMOTION CL

Wells A., 2009, METACOGNITIVE THERAP

WELLS KB, 1989, AM J PSYCHIAT, V146, P1440

WELLS KB, 1989, GEN HOSP PSYCHIAT, V11, P320, DOI 10.1016/0163-8343(89)90119-9

NR 43

TC 2

Z9 2

U1 1

U2 3

PU ELSEVIER IRELAND LTD

PI CLARE

PA ELSEVIER HOUSE, BROOKVALE PLAZA, EAST PARK SHANNON, CO, CLARE, 00000,

IRELAND

SN 0168-8227

EI 1872-8227

J9 DIABETES RES CLIN PR

JI Diabetes Res. Clin. Pract.

PD FEB

PY 2018

VL 136

BP 16

EP 22

DI 10.1016/j.diabres.2017.11.029

PG 7

WC Endocrinology & Metabolism

SC Endocrinology & Metabolism

GA FX2GP

UT WOS:000425877900003

PM 29203257

DA 2019-01-08

ER

PT J

AU Hayashino, Y

Tsujii, S

Ishii, H

AF Hayashino, Yasuaki

Tsujii, Satoru

Ishii, Hitoshi

CA Tenri Study Grp

TI Association of diabetes therapy-related quality of life and physical

activity levels in patients with type 2 diabetes receiving medication

therapy: the Diabetes Distress and Care Registry at Tenri (DDCRT 17)

SO ACTA DIABETOLOGICA

LA English

DT Article

DE Diabetes; Physical activity; Diabetes therapy; Quality of life

ID RANDOMIZED CONTROLLED-TRIAL; GLYCEMIC CONTROL; JAPANESE PATIENTS;

HEALTH; HYPOGLYCEMIA; EXERCISE; MELLITUS; PROGRAM; PEOPLE; ADULTS

AB To examine the association between diabetes therapy-related quality of life (DTR-QOL) and physical activity levels, and identify factors associated with high diabetes therapy-related quality of life.

Cross-sectional data from 2970 patients with type 2 diabetes in a Japanese diabetes registry were assessed for independent correlations between DTR-QOL (domains 1-4) and high physical activity levels. Data collected by the International Physical Activity Questionnaire were analyzed by logistic regression and adjusted for potential confounders.

The mean patient age, BMI and HbA1c level were 65.8 years, 24.7 kg/m(2) and 7.6% (58.7 mmol/mol), respectively. Univariate analysis showed that DTR-QOL domain 1, 2 and 4 scores were significantly associated with physical activity levels (p = 0.0046, p = 0.0004 and p < 0.001, respectively, but domain 3 score was not (p = 0.5073). In a multivariable-adjusted logistic regression model, odds ratios (ORs) of DTR-QOL domains 1, 3 and 4 were independently associated with high physical activity (ORs for 2nd to 4th quartile and p for trend; [domain 1] 1.16, 1.56, 1.22, p = 0.032; [domain 3] 1.45, 1.55, 1.38, p = 0.049; [domain 4] 1.09, 1.30, 1.51, p = 0.001, respectively), but domain 2 was not (ORs for 2nd to 4th quartile and p for trend; 1.19, 1.26, 1.23, p = 0.096).

High diabetes therapy-related QOL scores were associated with high levels of physical activity in patients with type 2 diabetes. Because this is a cross-sectional study, further study is needed to evaluate the causal association between therapy-related QOL and physical activity.

C1 [Hayashino, Yasuaki; Tsujii, Satoru] Tenri Hosp, Dept Endocrinol, 200 Mishima Cho, Tenri, Nara 6328552, Japan.

[Ishii, Hitoshi] Nara Med Univ, Dept Diabetol, Kashihara, Nara 6348521, Japan.

RP Hayashino, Y (reprint author), Tenri Hosp, Dept Endocrinol, 200 Mishima Cho, Tenri, Nara 6328552, Japan.

EM hayasino-y@umin.net

OI Hayashino, Yasuaki/0000-0002-2932-8478

FU Manpei Suzuki Diabetes Foundation; JSPS KAKENHI [25460641]

FX This study was partially supported by the Manpei Suzuki Diabetes

Foundation and JSPS KAKENHI (Grant Number 25460641). These agencies did

not participate in the study design or conduct, data collection,

analysis or interpretation, preparation, review or approval of the

manuscript. We would like to especially thank Yukari Moritsuji, Yuki

Fujita, Noriko Nakamura and Yoko Sakamoto (Department of Endocrinology,

Tenri Hospital) for their clerical support. The authors would like to

thank Enago (www.enago.jp) for the English language review.

CR Balducci S, 2004, DIABETES CARE, V27, P841, DOI 10.2337/diacare.27.3.841

Bjorner JB, 2013, VALUE HEALTH, V16, P993, DOI 10.1016/j.jval.2013.06.022

Chatterjee S, 2016, DIABETES CARE, V39, P300, DOI 10.2337/dc15-1588

Craig CL, 2003, MED SCI SPORT EXER, V35, P1381, DOI 10.1249/01.MSS.0000078924.61453.FB

Domgaard M, 2015, POSTGRAD MED, V127, P438, DOI 10.1080/00325481.2015.1045815

Dunstan DW, 2006, DIABETES CARE, V29, P2586, DOI 10.2337/dc06-1310

Hakkinen A, 2009, DISABIL REHABIL, V31, P799, DOI 10.1080/08916930802354930

Hayashino Y, 2012, DIABETES RES CLIN PR, V97, P405, DOI 10.1016/j.diabres.2012.04.005

Hopkins D, 2012, DIABETES CARE, V35, P1638, DOI 10.2337/dc11-1579

Hu J, 2010, J COMMUN HEALTH NURS, V27, P70, DOI 10.1080/07370011003704933

Inzucchi SE, 2012, DIABETOLOGIA, V55, P1577, DOI 10.1007/s00125-012-2534-0

Ishii Hitoshi, 2012, J Med Econ, V15, P556, DOI 10.3111/13696998.2012.665111

Jacobson Alan M, 2004, Endocr Pract, V10, P502

Kirk A, 2004, DIABETOLOGIA, V47, P821, DOI 10.1007/s00125-004-1396-5

Kueh YC, 2015, HEALTH QUAL LIFE OUT, V13, DOI 10.1186/s12955-015-0303-8

Maddigan SL, 2005, QUAL LIFE RES, V14, P1489, DOI 10.1007/s11136-005-0586-z

Mashitani T, 2015, ACTA DIABETOL, V52, P639, DOI 10.1007/s00592-015-0725-0

Mashitani T, 2013, DIABETES RES CLIN PR, V100, P189, DOI 10.1016/j.diabres.2013.03.006

Murata GH, 2004, BIOMED PHARMACOTHER, V58, P551, DOI 10.1016/j.biopha.2004.09.001

Nakamura A, 2010, J DIABETES INVEST, V1, P208, DOI 10.1111/j.2040-1124.2010.00046.x

Nicolucci A, 2012, DIABETOLOGIA, V55, P579, DOI 10.1007/s00125-011-2425-9

Nicolucci A, 2011, ARCH INTERN MED, V171, P1951, DOI 10.1001/archinternmed.2011.561

Praet SFE, 2008, DIABETOLOGIA, V51, P736, DOI 10.1007/s00125-008-0950-y

Praet SFE, 2009, ACTA DIABETOL, V46, P263, DOI 10.1007/s00592-009-0129-0

SCHNEIDER SH, 1992, DIABETES CARE, V15, P1800, DOI 10.2337/diacare.15.11.1800

Schram MT, 2009, CURR DIABETES REV, V5, P112, DOI 10.2174/157339909788166828

Schwartz SS, 2013, DRUGS CONTEXT, V2013

Seaquist ER, 2013, J CLIN ENDOCR METAB, V98, P1845, DOI 10.1210/jc.2012-4127

Shi LZ, 2014, HEALTH QUAL LIFE OUT, V12, DOI 10.1186/s12955-014-0167-3

Shultz JA, 2001, J HEALTH COMMUN, V6, P99, DOI 10.1080/108107301750254457

Tancredi M, 2015, NEW ENGL J MED, V373, P1720, DOI 10.1056/NEJMoa1504347

Tsujii S, 2012, DIABETIC MED, V29, P1451, DOI 10.1111/j.1464-5491.2012.03647.x

Watkins KW, 2000, DIABETES CARE, V23, P1511, DOI 10.2337/diacare.23.10.1511

Yong YM, 2015, DIABETES METAB J, V39, P154, DOI 10.4093/dmj.2015.39.2.154

Zhang P, 2012, DIABETES CARE, V35, P2250, DOI 10.2337/dc11-2478

NR 35

TC 0

Z9 0

U1 1

U2 2

PU SPRINGER-VERLAG ITALIA SRL

PI MILAN

PA VIA DECEMBRIO, 28, MILAN, 20137, ITALY

SN 0940-5429

EI 1432-5233

J9 ACTA DIABETOL

JI Acta Diabetol.

PD FEB

PY 2018

VL 55

IS 2

BP 165

EP 173

DI 10.1007/s00592-017-1080-0

PG 9

WC Endocrinology & Metabolism

SC Endocrinology & Metabolism

GA FW4JM

UT WOS:000425279400007

PM 29188385

DA 2019-01-08

ER

PT J

AU Choi, WH

Seo, YM

Ha, Y

AF Choi, Won-Hee

Seo, Yeong-Mi

Ha, Yeongmi

TI Evaluation of factors related to glycaemic control among South Korean

patients with type 2 diabetes

SO INTERNATIONAL JOURNAL OF NURSING PRACTICE

LA English

DT Article

DE diabetes mellitus; glycaemic index; medication adherence; nurses;

self-care

ID EMOTIONAL DISTRESS; MELLITUS; DEPRESSION; COMPLICATIONS; ASSOCIATIONS;

ANXIETY; SCALE; LIFE

AB AimsTo examine specific self-care behaviours, depression, and diabetes-related stress among South Korean patients with type 2 diabetes and to evaluate whether these factors are related to glycaemic control.

MethodsThis cross-sectional study included 171 patients with type 2 diabetes who visited an endocrinology clinic. A structured questionnaire and electronic medical records were used to collect data regarding self-care behaviours, depression, diabetes-related distress, and glycaemic control between May 2015 and July 2015.

ResultsCompared with the group with good glycaemic control, the group with poor glycaemic control had significantly lower values for medication adherence and significantly greater values for regimen-related distress. Depression was not significantly associated with glycaemic control. In logistic regression analysis, only medication adherence was independently associated with glycaemic control.

ConclusionsMedication adherence should be continuously emphasized and monitored in clinical practice to effectively manage glycaemic control among patients with type 2 diabetes. Furthermore, consideration of diabetes-related distress may help improve glycaemic control among patients with type 2 diabetes.

C1 [Choi, Won-Hee] Kyungsung Univ, Dept Nursing, Busan, South Korea.

[Seo, Yeong-Mi] Gyeongnam Natl Univ Sci & Technol, Dept Nursing, 33 Dongjin Ro, Jinju 52725, Gyeongnam, South Korea.

[Ha, Yeongmi] Gyeongsang Natl Univ, Inst Hlth Sci, Coll Nursing, Jinju, South Korea.

RP Seo, YM (reprint author), Gyeongnam Natl Univ Sci & Technol, Dept Nursing, 33 Dongjin Ro, Jinju 52725, Gyeongnam, South Korea.

EM asfirst@gntech.ac.kr

CR Aikens JE, 2012, DIABETES CARE, V35, P2472, DOI 10.2337/dc12-0181

[Anonymous], 2015, DIABETES CARE, V38, pS33, DOI 10.2337/dc15-S009

[Anonymous], 1995, DIABETES, V44, P968

Calhoun D, 2010, J DIABETES COMPLICAT, V24, P217, DOI 10.1016/j.jdiacomp.2009.03.005

Choi E, 2007, THESIS

Chon K.K., 2001, KOREAN J HLTH PSYCHO, V6, P59

Danaei G, 2011, LANCET, V378, P31, DOI 10.1016/S0140-6736(11)60679-X

Faul F, 2009, BEHAV RES METHODS, V41, P1149, DOI 10.3758/BRM.41.4.1149

Feldman BS, 2014, PLOS ONE, V9, DOI 10.1371/journal.pone.0108145

Fisher L, 2012, DIABETES CARE, V35, P259, DOI 10.2337/dc11-1572

Fisher L, 2010, DIABETES CARE, V33, P1034, DOI 10.2337/dc09-2175

Fisher L, 2010, DIABETES CARE, V33, P23, DOI 10.2337/dc09-1238

Forrest KYZ, 2000, ATHEROSCLEROSIS, V148, P159, DOI 10.1016/S0021-9150(99)00217-8

GOLDSTON DB, 1995, HEALTH PSYCHOL, V14, P409, DOI 10.1037/0278-6133.14.5.409

강혜연, 2012, [The Journal of Fundamentals of Nursing, 기본간호학회지], V19, P168

Hessler D, 2014, DIABETES CARE, V37, P617, DOI 10.2337/dc13-0762

Ji E. J., 2015, J KOREAN ACAD FUNDAM, V22, P433

Korean Diabetes Association, 2015, KOR DIAB MELL FACT S

Ministry of Health and Welfare, 2014, KOR HLTH STAT 2013 K

Osborn CY, 2016, J CLIN PHARM THER, V41, P256, DOI 10.1111/jcpt.12360

Parildar H, 2015, PAK J MED SCI, V31, P19, DOI 10.12669/pjms.311.6011

Park Ju-Young, 2010, [The Korean Academic Society of Adult Nursing, 성인간호학회지], V22, P271

Park YS, 2005, KOREAN J FAM MED, V26, P744

Paschalides C, 2004, J PSYCHOSOM RES, V57, P557, DOI 10.1016/j.jpsychores.2004.03.006

Polonsky WH, 2005, DIABETES CARE, V28, P626, DOI 10.2337/diacare.28.3.626

RADLOFF L S, 1977, Applied Psychological Measurement, V1, P385, DOI 10.1177/014662167700100306

Sekhar TVDS, 2013, J CLIN DIAGN RES, V7, P857, DOI 10.7860/JCDR/2013/5726.2958

Seo Y. M., 2008, THESIS

SHAMOON H, 1993, NEW ENGL J MED, V329, P977

Strandberg RB, 2014, J PSYCHOSOM RES, V77, P174, DOI 10.1016/j.jpsychores.2014.06.015

Stratton IM, 2000, BRIT MED J, V321, P405, DOI 10.1136/bmj.321.7258.405

SURWIT RS, 1992, DIABETES CARE, V15, P1413, DOI 10.2337/diacare.15.10.1413

Turner RC, 1998, LANCET, V352, P854

Turner RC, 1998, LANCET, V352, P837

이주안, 2014, [The Journal of Fundamentals of Nursing, 기본간호학회지], V21, P243, DOI 10.7739/jkafn.2014.21.3.243

부선주, 2012, [The Korean Academic Society of Adult Nursing, 성인간호학회지], V24, P406

NR 36

TC 0

Z9 0

U1 3

U2 5

PU WILEY

PI HOBOKEN

PA 111 RIVER ST, HOBOKEN 07030-5774, NJ USA

SN 1322-7114

EI 1440-172X

J9 INT J NURS PRACT

JI Int. J. Nurs. Pract.

PD FEB

PY 2018

VL 24

IS 1

AR e12616

DI 10.1111/ijn.12616

PG 8

WC Nursing

SC Nursing

GA FU8YL

UT WOS:000424142400013

PM 29205693

OA Green Published, Other Gold

DA 2019-01-08

ER

PT J

AU Grammes, J

Schafer, M

Benecke, A

Low, U

Klostermann, AL

Kubiak, T

Witthoft, M

AF Grammes, Jennifer

Schaefer, Manuela

Benecke, Andrea

Loew, Ulrike

Klostermann, Alena-Lynn

Kubiak, Thomas

Witthoeft, Michael

TI Fear of hypoglycemia in patients with type 2 diabetes: The role of

interoceptive accuracy and prior episodes of hypoglycemia

SO JOURNAL OF PSYCHOSOMATIC RESEARCH

LA English

DT Article

DE Fear of hypoglycemia; Heartbeat perception; Hypoglycemia Fear Survey;

Interoceptive accuracy; Type 2 diabetes mellitus

ID MEDICALLY UNEXPLAINED SYMPTOMS; QUALITY-OF-LIFE; TACTILE PERCEPTUAL

PROCESSES; POOR GLYCEMIC CONTROL; HEARTBEAT PERCEPTION; PANIC DISORDER;

SOMATOFORM DISORDERS; HEALTH ANXIETY; SOMATOSENSORY AMPLIFICATION;

IMPAIRED AWARENESS

AB Objective: Fear of hypoglycemia (FoH) is a limiting factor for diabetes self-management and can have detrimental effects on quality of life. However, relatively little is known about its underlying mechanisms. In line with findings on patients with anxiety disorders, we hypothesized that interoceptive accuracy (IA) might be positively linked to FoH in patients with type 2 diabetes (T2DM).

Methods: 133 patients with T2DM were screened according to the extreme quartiles of the Hypoglycemia Fear Survey worry subscale (HFS-W). Overall, 66 participants (HFS-W < 4; HFS-W > 17) were included in the present study. Participants completed questionnaires on sociodemographic and diabetes-related measures. Accuracy of heartbeat perception was assessed using the mental tracking task.

Results: Contrary to expectations, IA did not differ significantly between patients with low and high FoH. A linear regression analysis demonstrated that the experience of mild hypoglycemia (beta = 0.32, p <= 0.01) and its interaction with IA (beta = 0.26, p = 0.040) were significant predictors of FoH, indicating that low IA and a history of experiencing mild hypoglycemia are positively associated with FoH.

Conclusion: Our findings suggest a positive association of low IA in combination with prior episodes of hypoglycemia and FoH in patients with T2DM. The results are in line with recent findings on IA in patients with chronic somatic symptom distress more generally and contribute to our understanding of the relations between interoception, body related fears, and physical symptom perception.

C1 [Grammes, Jennifer; Kubiak, Thomas] Johannes Gutenberg Univ Mainz, Hlth Psychol, Binger Str 14-16, D-55122 Mainz, Germany.

[Schaefer, Manuela; Benecke, Andrea; Loew, Ulrike; Klostermann, Alena-Lynn] Johannes Gutenberg Univ Mainz, Outpatient Clin Psychotherapy, Mainz, Germany.

[Witthoeft, Michael] Johannes Gutenberg Univ Mainz, Clin Psychol Psychotherapy & Expt Psychopathol, Mainz, Germany.

RP Grammes, J (reprint author), Johannes Gutenberg Univ Mainz, Hlth Psychol, Binger Str 14-16, D-55122 Mainz, Germany.

EM jegramme@uni-mainz.de

RI Kubiak, Thomas/D-4655-2009

OI Kubiak, Thomas/0000-0003-0095-9265

CR Al Hayek AA, 2015, MED PRIN PRACT, V24, P222, DOI 10.1159/000375306

Amiel SA, 2008, DIABETIC MED, V25, P245, DOI 10.1111/j.1464-5491.2007.02341.x

Anderbro T, 2010, DIABETIC MED, V27, P1151, DOI 10.1111/j.1464-5491.2010.03078.x

Anderbro T, 2015, ACTA DIABETOL, V52, P581, DOI 10.1007/s00592-014-0694-8

Anderson RJ, 2002, INT J PSYCHIAT MED, V32, P235, DOI 10.2190/KLGD-4H8D-4RYL-TWQ8

ANTONY MM, 1995, J ANXIETY DISORD, V9, P355, DOI 10.1016/0887-6185(95)00017-I

Barendse S, 2012, DIABETIC MED, V29, P293, DOI 10.1111/j.1464-5491.2011.03416.x

Barlow D., 1988, INFORM PROCESSING DE

BARSKY AJ, 1990, BRIT J PSYCHIAT, V157, P404, DOI 10.1192/bjp.157.3.404

Beck A. T., 1985, ANXIETY DISORDERS PH

Bornemann B, 2017, PSYCHOPHYSIOLOGY, V54, P469, DOI 10.1111/psyp.12790

Bouton ME, 2001, PSYCHOL REV, V108, P4, DOI 10.1037//0033-295X.108.1.4

Brown RJ, 2004, PSYCHOL BULL, V130, P793, DOI 10.1037/0033-2909.130.5.793

Ceunen E, 2013, BIOL PSYCHOL, V92, P426, DOI 10.1016/j.biopsycho.2012.09.012

CLARK DM, 1986, BEHAV RES THER, V24, P461, DOI 10.1016/0005-7967(86)90011-2

COHEN J, 1992, PSYCHOL BULL, V112, P155, DOI 10.1037//0033-2909.112.1.155

Cohen J, 1988, STAT POWER ANAL BEHA

Cortina JM, 1998, J APPL PSYCHOL, V83, P798, DOI 10.1037/0021-9010.83.5.798

COX DJ, 1987, DIABETES CARE, V10, P617, DOI 10.2337/diacare.10.5.617

Crucianelli L., 2017, CORTEX IN PRESS

De Berardis D, 2007, PSYCHOSOMATICS, V48, P239, DOI 10.1176/appi.psy.48.3.239

de Groot M, 2001, PSYCHOSOM MED, V63, P619, DOI 10.1097/00006842-200107000-00015

Domschke K, 2010, CLIN PSYCHOL REV, V30, P1, DOI 10.1016/j.cpr.2009.08.008

Garfinkel SN, 2015, BIOL PSYCHOL, V104, P65, DOI 10.1016/j.biopsycho.2014.11.004

GOLD AE, 1994, DIABETES CARE, V17, P697, DOI 10.2337/diacare.17.7.697

Gonder-Frederick LA, 2006, PEDIATR DIABETES, V7, P215, DOI 10.1111/j.1399-5448.2006.00182.x

Grammes J, 2017, DIABETIC MED, V34, P1765, DOI 10.1111/dme.13506

Green AJ, 2012, DIABETES RES CLIN PR, V96, P313, DOI 10.1016/j.diabres.2012.01.002

Hajos TRS, 2014, DIABETES CARE, V37, P102, DOI 10.2337/dc13-0971

Hay L C, 2003, Diabetes Technol Ther, V5, P19, DOI 10.1089/152091503763816427

Henderson JN, 2003, DIABETIC MED, V20, P1016, DOI 10.1046/j.1464-5491.2003.01072.x

HEPBURN DA, 1993, DIABETIC MED, V10, P231, DOI 10.1111/j.1464-5491.1993.tb00050.x

HEPBURN DA, 1994, DIABETES CARE, V17, P1273, DOI 10.2337/diacare.17.11.1273

Herbert BM, 2010, PSYCHOPHYSIOLOGY, V47, P342, DOI 10.1111/j.1469-8986.2009.00931.x

Hermanns N, 2007, DIABETES-METAB RES, V23, P528, DOI 10.1002/dmrr.710

Irvin A, 1994, HDB PSYCHOL DIABETES, P133

Jodar-Gimeno E, 2015, REV CLIN ESP, V215, P91, DOI 10.1016/j.rce.2014.07.009

Katzer A, 2012, J ABNORM PSYCHOL, V121, P530, DOI 10.1037/a0026536

Katzer A, 2011, J PSYCHOSOM RES, V71, P335, DOI 10.1016/j.jpsychores.2011.03.009

King P, 1998, DIABETES CARE, V21, P341, DOI 10.2337/diacare.21.3.341

Krautwurst S, 2016, J ABNORM PSYCHOL, V125, P1179, DOI 10.1037/abn0000210

Krautwurst S, 2014, J BEHAV THER EXP PSY, V45, P303, DOI 10.1016/j.jbtep.2014.02.001

Ludewig S, 2005, J PSYCHIATR NEUROSCI, V30, P37

Lustman PJ, 2000, DIABETES CARE, V23, P934, DOI 10.2337/diacare.23.7.934

Martin-Timon I, 2015, WORLD J DIABETES, V6, P912, DOI 10.4239/wjd.v6.i7.912

Matza L. S., 2007, HLTH QUAL LIFE OUTCO, V5

NARING GWB, 1995, PSYCHOTHER PSYCHOSOM, V63, P193

Olatunji BO, 2007, BEHAV THER, V38, P392, DOI 10.1016/j.beth.2006.09.002

Pennebaker J. W., 1982, PHYSL PHYS SYMPTOMS

Petersen S, 2015, FRONT PSYCHOL, V6, DOI 10.3389/fpsyg.2015.00732

Pollatos O, 2007, J ANXIETY DISORD, V21, P931, DOI 10.1016/j.janxdis.2006.12.004

Pollatos O, 2011, J PSYCHOSOM RES, V71, P232, DOI 10.1016/j.jpsychores.2011.03.012

Polonsky WH, 2015, J DIABETES COMPLICAT, V29, P1171, DOI 10.1016/j.jdiacomp.2015.08.002

POLONSKY WH, 1992, HEALTH PSYCHOL, V11, P199, DOI 10.1037/0278-6133.11.3.199

Preacher KJ, 2015, ENCY CLIN PSYCHOL, P1189

Richards JC, 2003, COGNITIVE THER RES, V27, P447, DOI 10.1023/A:1025476514714

Richards JC, 2000, J ANXIETY DISORD, V14, P413, DOI 10.1016/S0887-6185(00)00031-1

Rosendal M, 2017, BMC FAM PRACT, V18, DOI 10.1186/s12875-017-0592-6

Sakane N, 2015, J DIABETES INVEST, V6, P567, DOI 10.1111/jdi.12340

Schachinger H, 2005, J BEHAV MED, V28, P587, DOI 10.1007/s10865-005-9026-3

SCHACHTER S, 1964, ADV EXP SOC PSYCHOL, V1, P49, DOI 10.1016/S0065-2601(08)60048-9

Schaefer M, 2014, BIOL PSYCHOL, V101, P69, DOI [10.1016/j.biopsycho.2014.06.012, 10.1016/j.biopsycho.2014.05.012]

Schaefer M, 2012, J ABNORM PSYCHOL, V121, P719, DOI 10.1037/a0028509

SCHANDRY R, 1981, PSYCHOPHYSIOLOGY, V18, P154

Shi L. Z., 2014, HLTH QUAL LIFE OUTCO, V12

SMITH RC, 1991, J GEN INTERN MED, V6, P168, DOI 10.1007/BF02598318

Stargardt T., 2009, HLTH QUAL LIFE OUTCO, V7

Sturges LV, 1996, J ANXIETY DISORD, V10, P283, DOI 10.1016/0887-6185(96)00012-6

ter Braak EWMT, 2000, DIABETES CARE, V23, P1467, DOI 10.2337/diacare.23.10.1467

Van den Bergh O, 2017, NEUROSCI BIOBEHAV R, V74, P185, DOI 10.1016/j.neubiorev.2017.01.015

Vexiau P, 2008, DIABETES OBES METAB, V10, P16, DOI 10.1111/j.1463-1326.2008.00883.x

Wiens S, 2000, COGNITION EMOTION, V14, P417, DOI 10.1080/026999300378905

Wild D, 2007, PATIENT EDUC COUNS, V68, P10, DOI 10.1016/j.pec.2007.05.003

Williams SA, 2011, DIABETES RES CLIN PR, V91, P363, DOI 10.1016/j.diabres.2010.12.027

Zammitt NN, 2005, DIABETES CARE, V28, P2948, DOI 10.2337/diacare.28.12.2948

Zoellner LA, 1999, BEHAV RES THER, V37, P1141, DOI 10.1016/S0005-7967(98)00202-2

NR 76

TC 0

Z9 0

U1 0

U2 6

PU PERGAMON-ELSEVIER SCIENCE LTD

PI OXFORD

PA THE BOULEVARD, LANGFORD LANE, KIDLINGTON, OXFORD OX5 1GB, ENGLAND

SN 0022-3999

EI 1879-1360

J9 J PSYCHOSOM RES

JI J. Psychosomat. Res.

PD FEB

PY 2018

VL 105

BP 58

EP 63

DI 10.1016/j.jpsychores.2017.12.010

PG 6

WC Psychiatry

SC Psychiatry

GA FT6HV

UT WOS:000423256000008

PM 29332635

DA 2019-01-08

ER

PT J

AU Inman, M

Nour, MA

AF Inman, Mark

Nour, Munier A.

TI Insulin-mediated pseudoacromegaly: a report of two pediatric patients

SO JOURNAL OF PEDIATRIC ENDOCRINOLOGY & METABOLISM

LA English

DT Article

DE acromegaly; diabetes mellitus; insulin resistance; type 2

ID RESISTANCE; HYPERINSULINISM; FIBROBLASTS; RECEPTOR; 3-KINASE; OBESITY

AB Background: Insulin-mediated pseudoacromegaly is a rarely described pediatric phenotype. We present two patients displaying excessive growth associated with marked acanthosis nigricans, hyperinsulinemia and metabolic dysregulation.

Case presentation: Both patients, of First Nations descent, presented with excessive growth - patient one at 3.92 years (height z-score +3.75) and patient two at 9.0 years (height z-score 5.15). Insulin-like growth factor-1 (IGF-1) levels were normal with appropriate growth hormone suppression, yet marked hyperinsulinemia. Prepubescent growth velocities exceeded 9 cm/year, resulting in final adult height predictions exceeding 3 standard deviations (SDs) of predicted. Clinical courses were complicated by type 2 diabetes, marked acanthosis nigricans and long-standing psychosocial distress.

Conclusions: Pediatric patients with insulin-mediated pseudoacromegaly are at risk of significant physical, metabolic and psychosocial comorbidities. Unlike adults, the implications in childhood prompt consideration for therapies to decelerate linear growth and avert progression to metabolic dysregulation. Increased recognition of this condition may improve pathophysiological understanding, diagnostic criteria and therapeutic options.

C1 [Inman, Mark; Nour, Munier A.] Univ Saskatchewan, Dept Pediat, 103 Hosp Dr, Saskatoon, SK S7N 0W8, Canada.

RP Inman, M (reprint author), Univ Saskatchewan, Dept Pediat, 103 Hosp Dr, Saskatoon, SK S7N 0W8, Canada.

EM mark.inman@usask.ca

CR Dib K, 1998, J CLIN INVEST, V101, P1111, DOI 10.1172/JCI119884

FLIER JS, 1993, J CLIN ENDOCR METAB, V76, P1533, DOI 10.1210/jc.76.6.1533

FRADKIN JE, 1989, NEW ENGL J MED, V320, P640

Kausch C, 1999, EXP CLIN ENDOCR DIAB, V107, P148, DOI 10.1055/s-0029-1212090

Kumar S, 1996, J CLIN ENDOCR METAB, V81, P3465, DOI 10.1210/jc.81.10.3465

Littlejohn EE, 2007, J PEDIATR ENDOCR MET, V20, P41

Sam AH, 2011, HORM-INT J ENDOCRINO, V10, P156, DOI 10.14310/horm.2002.1306

Slaaby R, 2006, J BIOL CHEM, V281, P25869, DOI 10.1074/jbc.M605189200

Srinivasan S, 2003, ARCH DIS CHILD, V88, P332, DOI 10.1136/adc.88.4.332

Yaqub Abid, 2008, W V Med J, V104, P12

NR 10

TC 0

Z9 0

U1 1

U2 1

PU WALTER DE GRUYTER GMBH

PI BERLIN

PA GENTHINER STRASSE 13, D-10785 BERLIN, GERMANY

SN 0334-018X

EI 2191-0251

J9 J PEDIATR ENDOCR MET

JI J. Pediatr. Endocrinol. Metab.

PD FEB

PY 2018

VL 31

IS 2

BP 235

EP 238

DI 10.1515/jpem-2017-0398

PG 4

WC Endocrinology & Metabolism; Pediatrics

SC Endocrinology & Metabolism; Pediatrics

GA FU2KD

UT WOS:000423677900018

PM 29252200

DA 2019-01-08

ER

PT J

AU Tanenbaum, ML

Adams, RN

Gonzalez, JS

Hanes, SJ

Hood, KK

AF Tanenbaum, M. L.

Adams, R. N.

Gonzalez, J. S.

Hanes, S. J.

Hood, K. K.

TI Adapting and validating a measure of diabetes-specific self-compassion

SO JOURNAL OF DIABETES AND ITS COMPLICATIONS

LA English

DT Article

DE Self-compassion; Type 1 diabetes; Mindfulness; Scale validation;

Confirmatory factor analysis

ID RANDOMIZED CONTROLLED-TRIAL; QUALITY-OF-LIFE; STRESS-REDUCTION; GLYCEMIC

CONTROL; MINDFULNESS; MANAGEMENT; ADOLESCENTS; ACCEPTANCE; THERAPY;

HEALTH

AB Aims: Self-compassion (SC), or treating oneself with kindness when dealing with personal challenges, has not been rigorously examined in people with T1D. SC has been shown to buffer against negative emotions and to be linked to improved health outcomes, but diabetes-specific SC has not been studied. This study aimed to adapt the Self-Compassion Scale and validate it for a diabetes-specific population.

Methods: We developed and validated a diabetes-specific version of the Self-Compassion Scale (Neff, 2003) in a sample of adults with T1D (N = 542; 65% female; 97% non-Hispanic White; M age 41, SD = 15.7; M Alc = 73, SD = 1; 72% insulin pump users; 50% continuous glucose monitoring [CCM] users). Confirmatory factor analyses (CFA), and reliability and construct validity analyses were conducted. Validity measures included diabetes distress, diabetes empowerment, diabetes numeracy, and Al c.

Results: A two-factor bi-factor structure showed best fit, providing support for use of the adapted scale (SCS-D) as a unitary construct The 19-item unidimensional SCS-D demonstrated excellent internal consistency (a = 0.94; range of item-total correlations: 0.52-0.71) and construct validity. As hypothesized, higher SCS-D was associated with less distress, greater empowerment, and lower Aic, and was not associated with numeracy.

Conclusions: The SCS-D is a reliable and valid measure of diabetes-specific self-compassion in adults with T1D. (C) 2017 Elsevier Inc. All rights reserved.

C1 [Tanenbaum, M. L.; Adams, R. N.; Hanes, S. J.; Hood, K. K.] Stanford Univ, Sch Med, Dept Pediat, 780 Welch Rd, Palo Alto, CA 94304 USA.

[Gonzalez, J. S.] Yeshiva Univ, Ferkauf Grad Sch Psychol, 1300 Morris Pk Ave,Rousso Bldg, Bronx, NY 10461 USA.

[Gonzalez, J. S.] Albert Einstein Coll Med, Diabet Res Ctr, 1300 Morris Pk Ave, Bronx, NY 10461 USA.

RP Tanenbaum, ML (reprint author), Stanford Univ, Sch Med, Dept Pediat, 780 Welch Rd, Palo Alto, CA 94304 USA.

EM mollyt@stanford.edu; rnadams@stanford.edu;

jeffrey.gonzalez@einstein.yu.edu; sjhanes@stanford.edu;

kkhood@stanford.edu

CR Abernathy P, 2015, WHY I TOLD MY DIABET

Anderson RM, 2003, DIABETES CARE, V26, P1641, DOI 10.2337/diacare.26.5.1641-a

Archer A, 2014, PRACT DIABETES, V31, P102, DOI 10.1002/pdi.1842

Balfe M, 2013, BMC ENDOCR DISORD, V13, DOI 10.1186/1472-6823-13-25

Basco M., 1998, DIABETES SPECTRUM, V11, P43

Brion JM, 2014, J HEALTH PSYCHOL, V19, P218, DOI 10.1177/1359105312467391

Broom D, 2004, SOC SCI MED, V58, P2371, DOI 10.1016/j.socscimed.2003.09.002

Costa J, 2016, CLIN PSYCHOL PSYCHOT, V23, P460, DOI 10.1002/cpp.1974

Davies NJ, 2012, LEARNING SELF COMPAS

Esbitt SA, 2015, COGN BEHAV PRACT, V22, P393, DOI 10.1016/j.cbpra.2014.02.006

Fisher L, 2015, J DIABETES COMPLICAT, V29, P572, DOI 10.1016/j.jdiacomp.2015.01.012

Folias A, 2014, INVESTIGATION PRESEN

Friis AM, 2015, DIABETIC MED, V32, P1634, DOI 10.1111/dme.12774

Friis A. M., 2016, DIABETES CARE

Friis Anna M, 2015, Diabetes Spectr, V28, P252, DOI 10.2337/diaspect.28.4.252

Graham CD, 2016, CLIN PSYCHOL REV, V46, P46, DOI 10.1016/j.cpr.2016.04.009

Gregg JA, 2007, J CONSULT CLIN PSYCH, V75, P336, DOI 10.1037/0022-006X.75.2.336

Grylli V, 2005, J PSYCHOSOM RES, V59, P65, DOI 10.1016/j.jpsychores.2005.02.010

Hartmann M, 2012, DIABETES CARE, V35, P945, DOI 10.2337/dc11-1343

Hu LT, 1999, STRUCT EQU MODELING, V6, P1, DOI 10.1080/10705519909540118

Huizinga MM, 2008, BMC HEALTH SERV RES, V8, DOI 10.1186/1472-6963-8-96

Iturralde E, 2017, HEALTH PSYCHOL, V36, P236, DOI 10.1037/hea0000445

Jaser SS, 2011, CHILD CARE HLTH DEV, V37, P335, DOI 10.1111/j.1365-2214.2010.01184.x

Joensen LE, 2016, PRIM CARE DIABETES, V10, P41, DOI 10.1016/j.pcd.2015.06.007

Kline R. B., 2011, PRINCIPLES PRACTICE

Lopez A, 2015, PLOS ONE, V10, DOI 10.1371/journal.pone.0132940

Loucks EB, 2016, AM J HEALTH BEHAV, V40, P258, DOI 10.5993/AJHB.40.2.11

Loucks EB, 2015, CURR CARDIOL REP, V17, DOI 10.1007/s11886-015-0668-7

Miller KM, 2015, DIABETES CARE, V38, P971, DOI 10.2337/dc15-0078

Muthen L. K., 1998, MPLUS USERS GUIDE

Neff KD, 2017, J PERS ASSESS, P1

Neff K, 2003, SELF IDENTITY, V2, P85, DOI 10.1080/15298860390129863

Neff KD, 2016, MINDFULNESS, V7, P264, DOI 10.1007/s12671-015-0479-3

Neff KD, 2003, SELF IDENTITY, V2, P223, DOI 10.1080/15298860390209035

Pinto-Gouveia J, 2014, CLIN PSYCHOL PSYCHOT, V21, P311, DOI 10.1002/cpp.1838

Raes F, 2011, CLIN PSYCHOL PSYCHOT, V18, P250, DOI 10.1002/cpp.702

Rosenzweig S, 2007, ALTERN THER HEALTH M, V13, P36

Schmitt A, 2014, DIABETIC MED, V31, P1446, DOI 10.1111/dme.12553

Schroevers MJ, 2016, MIND, P1

Shayeghian Z, 2016, PLOS ONE, V11, DOI 10.1371/journal.pone.0166599

Sirois Fuschia M, 2015, Prev Med Rep, V2, P218, DOI 10.1016/j.pmedr.2015.03.006

Sirois FM, 2015, HEALTH PSYCHOL, V34, P661, DOI 10.1037/hea0000158

Sirois FM, 2015, SELF IDENTITY, V14, P334, DOI 10.1080/15298868.2014.996249

Tanenbaum ML, 2016, DIABETES CARE

Tanenbaum ML, 2017, DIABETES CARE, V40, P181, DOI 10.2337/dc16-1536

Terry ML, 2013, PERS SOC PSYCHOL B, V39, P911, DOI 10.1177/0146167213488213

Terry ML, 2011, SELF IDENTITY, V10, P352, DOI 10.1080/15298868.2011.558404

van Son J, 2014, J PSYCHOSOM RES, V77, P81, DOI 10.1016/j.jpsychores.2014.03.013

Vanstone Meredith, 2015, Ont Health Technol Assess Ser, V15, P1

Yi-Frazier JP, 2015, J HEALTH PSYCHOL, V20, P1196, DOI 10.1177/1359105313509846

Young Laura A, 2009, Pract Diabetes Int, V26, P222, DOI 10.1002/pdi.1380

Young-Hyman D, 2016, DIABETES CARE, V39, P2126, DOI 10.2337/dc16-2053

NR 52

TC 1

Z9 1

U1 4

U2 11

PU ELSEVIER SCIENCE INC

PI NEW YORK

PA 360 PARK AVE SOUTH, NEW YORK, NY 10010-1710 USA

SN 1056-8727

EI 1873-460X

J9 J DIABETES COMPLICAT

JI J. Diabetes Complications

PD FEB

PY 2018

VL 32

IS 2

BP 196

EP 202

DI 10.1016/j.jdiacomp.2017.10.009

PG 7

WC Endocrinology & Metabolism

SC Endocrinology & Metabolism

GA FT8QT

UT WOS:000423419000013

PM 29157869

DA 2019-01-08

ER

PT J

AU Thong, MSY

Mols, F

van de Poll-Franse, LV

Sprangers, MAG

van der Rijt, CCD

Barsevick, AM

Knoop, H

Husson, O

AF Thong, Melissa S. Y.

Mols, Floortje

van de Poll-Franse, Lonneke V.

Sprangers, Mirjam A. G.

van der Rijt, Carin C. D.

Barsevick, Andrea M.

Knoop, Hans

Husson, Olga

TI Identifying the subtypes of cancer-related fatigue: results from the

population-based PROFILES registry

SO JOURNAL OF CANCER SURVIVORSHIP

LA English

DT Article

DE Cancer; Latent class analysis; Fatigue subtypes; Population-based;

Survivors

ID QUALITY-OF-LIFE; RANDOMIZED CONTROLLED-TRIAL; POSITIVE AFFECT ANHEDONIA;

PHYSICAL-ACTIVITIES; EVENING FATIGUE; CLINICAL-TRIALS; SURVIVORS;

INSTRUMENT; BEHAVIOR; QUESTIONNAIRE

AB Little research has been done to identify possible cancer-related fatigue (CRF) subtypes and to classify cancer survivors accordingly. We aimed to identify CRF subtypes in a large population-based sample of (long term) stage I-III colorectal cancer survivors. We also identified factors associated with the CRF subtypes.

Respondents completed the Multidimensional Fatigue Inventory and other validated questionnaires on anxiety and reduced positive affect (anhedonia), sleep quality, and lifestyle factors (body mass index and physical activity). Latent class analysis was used to derive the CRF subtypes. Factors associated with the derived CRF subtypes were determined with multinomial logistic regression.

Three CRF classes were identified: class 1 (no fatigue and distress, n = 644, 56%), class 2 (low fatigue, moderate distress, n = 256, 22%), and class 3 (high fatigue, moderate distress, n = 256, 22%). Multinomial logistic regression results show that survivors in class 3 were more likely to be female, were treated with radiotherapy, have comorbid diabetes mellitus, and be overweight/obese than survivors in class 1 (reference). Survivors in classes 2 and 3 were also more likely to have comorbid heart condition, report poorer sleep quality, experience anhedonia, and report more anxiety symptoms when compared with survivors in class 1.

Three distinct classes of CRF were identified which could be differentiated with sleep quality, anxiety, anhedonia, and lifestyle factors.

The identification of CRF subtypes with distinct characteristics suggests that interventions should be targeted to the CRF subtype.

C1 [Thong, Melissa S. Y.; Sprangers, Mirjam A. G.; Knoop, Hans] Univ Amsterdam, Amsterdam Publ Hlth Res Inst, Dept Med Psychol, Acad Med Ctr, POB 22660, NL-1100 DD Amsterdam, Netherlands.

[Mols, Floortje; van de Poll-Franse, Lonneke V.] Netherlands Comprehens Canc Org, Utrecht, Netherlands.

[Mols, Floortje; van de Poll-Franse, Lonneke V.] Tilburg Univ, CoRPS Ctr Res Psychol Somat Dis, Dept Med & Clin Psychol, Tilburg, Netherlands.

[van de Poll-Franse, Lonneke V.] Netherlands Canc Inst, Div Psychosocial Res & Epidemiol, Amsterdam, Netherlands.

[van der Rijt, Carin C. D.] Erasmus MC Canc Inst, Dept Med Oncol, Rotterdam, Netherlands.

[Barsevick, Andrea M.] Thomas Jefferson Univ, Dept Med Oncol, Philadelphia, PA 19107 USA.

[Knoop, Hans] Vrije Univ Amsterdam, Amsterdam Publ Hlth Res Inst, Dept Med Psychol, Expert Ctr Chron Fatigue,Med Ctr, Amsterdam, Netherlands.

[Husson, Olga] Radboud Univ Nijmegen, Dept Med Psychol, Med Ctr, Nijmegen, Netherlands.

RP Thong, MSY (reprint author), Univ Amsterdam, Amsterdam Publ Hlth Res Inst, Dept Med Psychol, Acad Med Ctr, POB 22660, NL-1100 DD Amsterdam, Netherlands.

EM s.y.thong@amc.uva.nl

RI Husson, Olga/L-1339-2015; Knoop, Hans/D-3264-2009

OI Husson, Olga/0000-0002-1387-8686; Knoop, Hans/0000-0001-7763-3517

FU VENI grant from the Netherlands Organization for Scientific Research

[451-10-041]; Medium Investment Grant from the Netherlands Organisation

for Scientific Research (NWO), The Hague [480-08-009]; Medium Investment

Grant from the Netherlands Organisation for Scientific Research (NWO),

The Netherlands; Dutch Cancer Society [KUN2015-7527]; the Netherlands

Organization for Scientific Research [451-10-041]

FX The data collection of this study was funded in part by a VENI grant

from the Netherlands Organization for Scientific Research (#451-10-041)

awarded to Dr. Floortje Mols, and a Medium Investment Grant from the

Netherlands Organisation for Scientific Research (NWO#480-08-009), The

Hague, and The Netherlands. Dr. Olga Husson (KUN2015-7527) is supported

by a Social Psychology Fellowship from the Dutch Cancer Society, and Dr.

Floortje Mols is supported by a VENI grant (#451-10-041) from the

Netherlands Organization for Scientific Research. These funding agencies

had no further role in study design, in the collection, analysis, and

interpretation of data, in the writing of the report, and in the

decision to submit the paper for publication.

CR AARONSON NK, 1993, J NATL CANCER I, V85, P365, DOI 10.1093/jnci/85.5.365

AINSWORTH BE, 1993, MED SCI SPORT EXER, V25, P71, DOI 10.1249/00005768-199301000-00011

Ainsworth BE, 2000, MED SCI SPORT EXER, V32, pS498, DOI 10.1097/00005768-200009001-00009

Altman DG, 2006, BRIT MED J, V332, P1080, DOI 10.1136/bmj.332.7549.1080

Barsevick AM, 2013, JNCI-J NATL CANCER I, V105, P1432, DOI 10.1093/jnci/djt242

Bennett BK, 2014, PSYCHOSOM MED, V76, P379, DOI 10.1097/PSY.0000000000000061

Berger AM, 2010, J NATL COMPR CANC NE, V8, P904, DOI 10.6004/jnccn.2010.0067

Bower JE, 2006, CANCER, V106, P751, DOI 10.1002/cncr.21671

Bower JE, 2011, J CLIN ONCOL, V29, P3517, DOI 10.1200/JCO.2011.36.1154

BUYSSE DJ, 1989, PSYCHIAT RES, V28, P193, DOI 10.1016/0165-1781(89)90047-4

Carpenter JS, 1998, J PSYCHOSOM RES, V45, P5, DOI 10.1016/S0022-3999(97)00298-5

Cheng KKF, 2011, CRIT REV ONCOL HEMAT, V78, P127, DOI 10.1016/j.critrevonc.2010.03.002

Cramp F, 2012, COCHRANE DB SYST REV, DOI DOI 10.1002/14651858.CD006145

Damen NL, 2013, EUR J PREV CARDIOL, V20, P127, DOI 10.1177/2047487312436452

de Raaf PJ, 2013, PSYCHO-ONCOLOGY, V22, P1919, DOI 10.1002/pon.3225

de Raaf PJ, 2013, CANC RELATED FATIGUE

de Raaf PJ, 2012, J PAIN SYMPTOM MANAG, V44, P823, DOI 10.1016/j.jpainsymman.2011.12.279

Dennett AM, 2016, J PHYSIOTHER, V62, P68, DOI 10.1016/j.jphys.2016.02.012

Denollet J, 2008, J INTERN MED, V263, P203, DOI 10.1111/j.1365-2796.2007.01870.x

Fayers PM, 1995, BRUSSELS BELG, V11, P1

Garland SN, 2014, NEUROPSYCH DIS TREAT, V10, P1113, DOI 10.2147/NDT.S47790

Gielissen MFM, 2006, J CLIN ONCOL, V24, P4882, DOI 10.1200/JCO.2006.06.8270

Goedendorp MM, 2012, CANCER-AM CANCER SOC, V118, P3833, DOI 10.1002/cncr.26226

Goedendorp MM, 2009, COCHRANE DB SYST REV, V21, DOI DOI 10.1002/14651858.CD006953.PUB2

Grimmett C, 2011, QUAL LIFE RES, V20, P1237, DOI 10.1007/s11136-011-9855-1

Hofman M, 2007, ONCOLOGIST, V12, P4, DOI 10.1634/theoncologist.12-S1-4

Holzner B, 2002, ANN ONCOL, V13, P965, DOI 10.1093/annonc/mdf122

Janssen-Heijnen MLG LW, 2005, RESULTS 50 YEARS CAN

Kim HJ, 2012, CANCER NURS, V35, pE1, DOI 10.1097/NCC.0b013e318233a811

Koutoukidis DA, 2015, NUTR REV, V73, P399, DOI 10.1093/nutrit/nuu063

Lengacher CA, 2016, J CLIN ONCOL, V34, P2827, DOI 10.1200/JCO.2015.65.7874

Magidson Jay, 2004, SAGE HDB QUANTITATIV, P175, DOI DOI 10.4135/9781412986311.N10

Miaskowski C, 2010, J PAIN SYMPTOM MANAG, V40, P531, DOI 10.1016/j.jpainsymman.2009.12.006

Mustian K.M., 2017, JAMA ONCOL

Olsson I, 2005, BMC PSYCHIATRY, V5, DOI 10.1186/1471-244X-5-46

Pols MA, 1997, INT J EPIDEMIOL, V26, pS181, DOI 10.1093/ije/26.suppl_1.S181

Prue G, 2006, EUR J CANCER, V42, P846, DOI 10.1016/j.ejca.2005.11.026

Reeves WC, 2005, BMC MED, V3, DOI 10.1186/1741-7015-3-19

Rich T, 2005, CLIN CANCER RES, V11, P1757, DOI 10.1158/1078-0432.CCR-04-2000

Sangha O, 2003, ARTHRIT RHEUM-ARTHR, V49, P156, DOI 10.1002/art.10993

Scott JA, 2011, ONCOL NURS FORUM, V38, P368, DOI 10.1188/11.ONF.E191-E203

SMETS EMA, 1995, J PSYCHOSOM RES, V39, P315, DOI 10.1016/0022-3999(94)00125-O

Smets EMA, 1996, BRIT J CANCER, V73, P241, DOI 10.1038/bjc.1996.42

UICC, TNM ATL ILL GUID TNM

van de Poll-Franse LV, 2011, EUR J CANCER, V47, P2188, DOI 10.1016/j.ejca.2011.04.034

Vermunt J. K, 2013, LATENT GOLD 5 0 UPGR

Whitehead L, 2009, J PAIN SYMPTOM MANAG, V37, P107, DOI 10.1016/j.jpainsymman.2007.08.019

Wright F, 2015, J PAIN SYMPTOM MANAG, V50, P163, DOI 10.1016/j.jpainsymman.2015.02.015

Wright F, 2015, J PAIN SYMPTOM MANAG, V50, P176, DOI 10.1016/j.jpainsymman.2015.02.016

Yennurajalingam S, 2014, CANCER J, V20, P319, DOI 10.1097/PPO.0000000000000069

ZIGMOND AS, 1983, ACTA PSYCHIAT SCAND, V67, P361, DOI 10.1111/j.1600-0447.1983.tb09716.x

NR 51

TC 3

Z9 3

U1 3

U2 4

PU SPRINGER

PI NEW YORK

PA 233 SPRING ST, NEW YORK, NY 10013 USA

SN 1932-2259

EI 1932-2267

J9 J CANCER SURVIV

JI J. Cancer Surviv.-Res. Pract.

PD FEB

PY 2018

VL 12

IS 1

BP 38

EP 46

DI 10.1007/s11764-017-0641-0

PG 9

WC Oncology; Social Sciences, Biomedical

SC Oncology; Biomedical Social Sciences

GA FU1CJ

UT WOS:000423586600005

PM 28889367

OA Green Published, Other Gold

DA 2019-01-08

ER

PT J

AU Wieringa, TH

de Wit, M

Twisk, JWR

Snoek, FJ

AF Wieringa, T. H.

de Wit, M.

Twisk, J. W. R.

Snoek, F. J.

TI Does hypoglycaemia affect the improvement in QoL after the transition to

insulin in people with type 2 diabetes?

SO JOURNAL OF ENDOCRINOLOGICAL INVESTIGATION

LA English

DT Article

DE Diabetes symptom burden; Emotional well-being; Hypoglycaemia;

Hypoglycaemia fear; Insulin initiation; Quality of life

ID QUALITY-OF-LIFE; HEALTH-RELATED QUALITY; TREATMENT MODALITIES; GLYCEMIC

CONTROL; MELLITUS; IMPACT; FREQUENCY; GLARGINE; OUTCOMES; BURDEN

AB Purpose Quality of Life (QoL) of insulin-naive people with type 2 diabetes mellitus (T2DM) improves after transition to insulin. Little is known about the role of hypoglycaemia in this context. Secondary analyses of the Study of the Psychological Impact in Real care of Initiating insulin glargine Treatment (SPIRIT) aimed to investigate the relationship between hypoglycaemia and QoL when transitioning to insulin.

Methods Insulin-naive Dutch people with T2DM in suboptimal glycaemic control (HbA1c >53 mmol/mol; 7.0%) on maximum dose of oral glucose-lowering medications were included from 363 primary care practices (n = 911). Participants started insulin glargine and completed QoL-questionnaires (WHO-5 Well-being Index (WHO-5; emotional well-being), Hypoglycaemia Fear Survey-worry scale (HFS-w; hypoglycaemia fear) and Diabetes Symptom Checklist-revised (DSC-r; diabetes symptom distress) at baseline, 3 and 6 months follow-up. Linear GEE analyses were used to investigate the association between symptomatic, nocturnal, severe hypoglycaemia (number of episodes during 3 months prior to visit) and QoL over time.

Results 52.5% men participated, mean age 62.2 years (SD +/- 10.92), and median HbA1c 67 mmol/mol (range 61-77) (8.3%). More symptomatic hypoglycaemic episodes were associated with higher HFS-w and DSC-r scores (P < 0.01). Experiencing multiple nocturnal or severe episodes was related to higher symptom distress as well, when compared to no episodes. These associations did not change significantly over time.

Conclusions Hypoglycaemia is associated with lower QoL in terms of hypoglycaemia fear and diabetes symptom distress. The transition to insulin does not affect this relationship, suggesting hypoglycaemia in itself has a detrimental effect on diabetes-related QoL independent of treatment regimen.

C1 [Wieringa, T. H.; de Wit, M.; Snoek, F. J.] Vrije Univ Amsterdam Med Ctr, Dept Med Psychol, De Boelelaan 1117, NL-1081 HV Amsterdam, Netherlands.

[Wieringa, T. H.; de Wit, M.; Twisk, J. W. R.; Snoek, F. J.] Amsterdam Publ Hlth Res Inst, Amsterdam, Netherlands.

[Twisk, J. W. R.] Vrije Univ Amsterdam Med Ctr, Dept Clin Epidemiol & Biostat, De Boelelaan 1089a, NL-1081 HV Amsterdam, Netherlands.

[Snoek, F. J.] AMC, Dept Med Psychol, Meibergdreef 9, NL-1100 DD Amsterdam, Netherlands.

RP Wieringa, TH (reprint author), Vrije Univ Amsterdam Med Ctr, Dept Med Psychol, De Boelelaan 1117, NL-1081 HV Amsterdam, Netherlands.; Wieringa, TH (reprint author), Amsterdam Publ Hlth Res Inst, Amsterdam, Netherlands.

EM t.wieringa@vumc.nl; m.dewit@vumc.nl; jwr.twisk@vumc.nl; fj.snoek@vumc.nl

FU Sanofi

FX This study was supported by an unrestricted grant from Sanofi.

CR Arbuckle RA, 2009, VALUE HEALTH, V12, P1168, DOI 10.1111/j.1524-4733.2009.00571.x

Barendse S, 2012, DIABETIC MED, V29, P293, DOI 10.1111/j.1464-5491.2011.03416.x

Boyle PJ, 2007, SOUTH MED J, V100, P175, DOI 10.1097/01.smj.0000242866.81791.70

Chelliah A, 2004, DRUG AGING, V21, P511, DOI 10.2165/00002512-200421080-00003

COX DJ, 1987, DIABETES CARE, V10, P617, DOI 10.2337/diacare.10.5.617

Donnelly LA, 2005, DIABETIC MED, V22, P749, DOI 10.1111/j.1464-5491.2005.01501.x

Gilet H, 2012, VALUE HEALTH, V15, P1036, DOI 10.1016/j.jval.2012.06.002

Green AJ, 2012, DIABETES RES CLIN PR, V96, P313, DOI 10.1016/j.diabres.2012.01.002

Hajos TRS, 2011, DIABETIC MED, V28, P1096, DOI 10.1111/j.1464-5491.2011.03329.x

Harris S, 2014, CAN J DIABETES, V38, P45, DOI 10.1016/j.jcjd.2013.09.001

Hartman I, 2008, CLIN MED RES, V6, P54, DOI 10.3121/cmr.2008.793

Heinemann L, 2000, DIABETES CARE, V23, P644, DOI 10.2337/diacare.23.5.644

Heller SR, 2007, DIABETOLOGIA, V50, P1140, DOI 10.1007/s00125-007-0599-y

Horvath K, 2007, COCHRANE DB SYST REV

Inzucchi SE, 2015, DIABETES CARE, V38, P140, DOI 10.2337/dc14-2441

Marrett Elizabeth, 2011, BMC Res Notes, V4, P251, DOI 10.1186/1756-0500-4-251

Murata GH, 2004, DIABETES RES CLIN PR, V65, P61, DOI 10.1016/j.diabres.2003.11.013

Schafer JL, 2002, PSYCHOL METHODS, V7, P147, DOI 10.1037//1082-989X.7.2.147

WHO, 1998, WHO M STOCKH SWED 12

Williams SA, 2012, J DIABETES COMPLICAT, V26, P399, DOI 10.1016/j.jdiacomp.2012.05.002

Williams SA, 2011, DIABETES RES CLIN PR, V91, P363, DOI 10.1016/j.diabres.2010.12.027

Zammitt NN, 2005, DIABETES CARE, V28, P2948, DOI 10.2337/diacare.28.12.2948

NR 22

TC 0

Z9 0

U1 1

U2 1

PU SPRINGER

PI NEW YORK

PA 233 SPRING ST, NEW YORK, NY 10013 USA

SN 1720-8386

J9 J ENDOCRINOL INVEST

JI J. Endocrinol. Invest.

PD FEB

PY 2018

VL 41

IS 2

BP 249

EP 258

DI 10.1007/s40618-017-0744-5

PG 10

WC Endocrinology & Metabolism

SC Endocrinology & Metabolism

GA FT8FB

UT WOS:000423387100012

PM 28803366

OA Green Published, Other Gold

DA 2019-01-08

ER

PT J

AU Wu, FL

Wu, EC

Chang, YC

Hu, WY

Juang, JH

Yeh, MC

AF Wu, Fei-Ling

Wu, En-Chang

Chang, Yue-Cune

Hu, Wen-Yu

Juang, Jyuhn-Huarng

Yeh, Mei Chang

TI Factors Affecting the Ability of People With Diabetes to Avoid

Hypoglycemia

SO JOURNAL OF NURSING RESEARCH

LA English

DT Article

DE problem solving; negative mood; diabetes; hypoglycemia

ID MANAGEMENT

AB Background: Hypoglycemia is recognized as a limiting factor in diabetes management. Fear of experiencing hypoglycemia may lead to lower quality of life, impaired glycemic control, and emotional distress, all of which impair the ability of patients to self-manage their diabetes effectively. Problem solving is central to diabetes self-management and may help patients achieve effective self-care of their disease.

Purpose: The aim of this study was to investigate the ability of people with diabetes to avoid hypoglycemia and to explore associated factors.

Methods: A cross-sectional, descriptive design was used for the study. Data were collected using a demographic and disease characteristics datasheet, the Hypoglycemic Problem Solving Scale, and the Disease-Associated Negative Mood Scale.

Results: Three hundred thirteen participants were recruited, with a mean age of 55.49 years. The average item score for the questions on hypoglycemic problem-solving ability was 2.43 (SD = 0.75). In comparing Hypoglycemic Problem Solving Scale subscales, participants scored highest on the problem orientation subscales and lowest on the problem-solving skills subscales. Multiple regression analysis revealed that being younger and unmarried and having a higher level of education, a diagnosis of Type 1 diabetes, and a lower negative mood score were each significantly associated with greater problem-solving ability as regards hypoglycemic events.

Conclusions/Implications for Practice: We suggest that patients with diabetes, especially those who are older or with lower levels of education, receive disease-related psychological interventions and that healthcare professionals teach problem-solving abilities in conjunction with hypoglycemia management.

C1 [Wu, Fei-Ling] Chang Gung Univ Sci & Technol, Dept Nursing, Taoyuan, Taiwan.

[Wu, En-Chang] Natl Taiwan Univ, Dept Psychol, Taipei, Taiwan.

[Chang, Yue-Cune] Tamkang Univ, Dept Math, New Taipei, Taiwan.

[Hu, Wen-Yu; Yeh, Mei Chang] Natl Taiwan Univ, Sch Nursing, Coll Med, Taipei, Taiwan.

[Juang, Jyuhn-Huarng] Chang Gung Univ, Div Endocrinol & Metab, Dept Internal Med, Chang Gung Mem Hosp, Taoyuan, Taiwan.

[Juang, Jyuhn-Huarng] Chang Gung Univ, Sch Med, Coll Med, Taoyuan, Taiwan.

RP Yeh, MC (reprint author), 1,Sect 1,Jen Ai Rd, Taipei 10051, Taiwan.

EM mchang@ntu.edu.tw

FU Ministry of Science and Technology [NSC 102-2314-B-255-002-MY3]

FX This research was supported by Grant NSC 102-2314-B-255-002-MY3 from the

Ministry of Science and Technology.

CR American Association of Diabetes Educators, 2009, AADE7 SELF CAR BEH

Anderson B. J., 2002, PRACTICAL PSYCHOL DI, P113

Brodovicz KG, 2013, CURR MED RES OPIN, V29, P101, DOI 10.1185/03007995.2012.754744

Chang F.M., 2014, FORMOSA JOURNAL OF C, V22, P67, DOI [10. 6168/FJCP. 2014. 2202. 01, DOI 10.6168/FJCP.2014.2202.01]

Cook S, 2001, DIABETES EDUCATOR, V27, P865, DOI 10.1177/014572170102700612

D'Zurilla T. J., 2007, PROBLEM SOLVING THER

D'Zurilla TJ, 1998, PERS INDIV DIFFER, V25, P241, DOI 10.1016/S0191-8869(98)00029-4

DAVIS TM, 2010, THE JOURNAL OF CLINI, V95, P2240

DZURILLA TJ, 1971, J ABNORM PSYCHOL, V78, P107, DOI 10.1037/h0031360

Elliott TR, 2001, J CLIN PSYCHOL MED S, V8, P283, DOI 10.1023/A:1011920914079

Faul F, 2007, BEHAV RES METHODS, V39, P175, DOI 10.3758/BF03193146

Fisher EB, 2007, DIABETES EDUCATOR, V33, P1080, DOI 10.1177/0145721707309808

Fitzpatrick SL, 2013, DIABETES RES CLIN PR, V100, P145, DOI 10.1016/j.diabres.2012.12.016

Health Promotion Administration Taiwan ROC, 2015, 2014 HEALTH PROMOTIO

Hill-Briggs F, 2007, DIABETES EDUCATOR, V33, P1032, DOI 10.1177/0145721707308412

Hill-Briggs F, 2011, J GEN INTERN MED, V26, P972, DOI 10.1007/s11606-011-1689-6

Kerns RD, 2002, ANN BEHAV MED, V24, P100, DOI 10.1207/S15324796ABM2402_06

Leese GP, 2003, DIABETES CARE, V26, P1176, DOI 10.2337/diacare.26.4.1176

Leiter LA, 2005, CAN J DIABETES, V29, P186

Lilly CL, 2014, PREV CHRONIC DIS, V11, DOI 10.5888/pcd11.130249

Murata GH, 2004, DIABETES RES CLIN PR, V65, P61, DOI 10.1016/j.diabres.2003.11.013

Nezu AM, 2004, BEHAV THER, V35, P1, DOI 10.1016/S0005-7894(04)80002-9

Walls ML, 2014, DIABETES EDUCATOR, V40, P319, DOI 10.1177/0145721714524282

Wang J, 2012, DIABETES EDUCATOR, V38, P207, DOI 10.1177/0145721712440331

Wild D, 2007, PATIENT EDUC COUNS, V68, P10, DOI 10.1016/j.pec.2007.05.003

Wu FL, 2011, J CLIN NURS, V20, P2277, DOI 10.1111/j.1365-2702.2011.03725.x

WU FL, 2016, THE JOURNAL OF INTER, V44, P592

Wu Y.C., 2008, RESEARCH GRANT DOH97

Yeung S, 2007, J CLIN NURS, V16, P344, DOI 10.1111/j.1365-2702.2005.01537.x

NR 29

TC 1

Z9 1

U1 3

U2 8

PU LIPPINCOTT WILLIAMS & WILKINS

PI PHILADELPHIA

PA TWO COMMERCE SQ, 2001 MARKET ST, PHILADELPHIA, PA 19103 USA

SN 1682-3141

J9 J NURS RES

JI J. Nurs. Res.

PD FEB

PY 2018

VL 26

IS 1

BP 44

EP 51

DI 10.1097/JNR.0000000000000198

PG 8

WC Nursing

SC Nursing

GA FT8ZQ

UT WOS:000423442100007

PM 28079649

DA 2019-01-08

ER

PT J

AU McGill, DE

Volkening, LK

Pober, DM

Muir, AB

Young-Hyman, DL

Laffel, LM

AF McGill, Dayna E.

Volkening, Lisa K.

Pober, David M.

Muir, Andrew B.

Young-Hyman, Deborah L.

Laffel, Lori M.

TI Depressive Symptoms at Critical Times in Youth With Type 1 Diabetes:

Following Type 1 Diabetes Diagnosis and Insulin Pump Initiation

SO JOURNAL OF ADOLESCENT HEALTH

LA English

DT Article

DE Type 1 diabetes; Adolescent; Depression; New-onset; Insulin pump;

Honeymoon; HbA1c

ID GLYCEMIC CONTROL; ADOLESCENTS; CHILDREN; MELLITUS; ASSOCIATION;

PREVALENCE; MANAGEMENT; DISTRESS; INFUSION; CARE

AB Purpose: Depressive symptoms occur at various times during the life cycle in persons with type 1 diabetes. We investigated depressive symptoms prospectively in youth with new-onset type 1 diabetes and in those beginning pump therapy.

Methods: Youth with type 1 diabetes (N = 96), ages 10-17 years, completed the Children's Depression Inventory (CDI) at baseline and at 1, 6, and 12 months after diabetes onset or pump start; scores >= 13 indicated clinical elevation. The change in depressive symptoms and the association between CDI score and hemoglobin A1c (HbA1c) level were assessed over 1 year.

Results: The new-onset group (n = 54) had an HbA1c level of 11.4% +/- 2.5%. The pump group (n = 42) had a diabetes duration of 4.1 +/- 3.4 years and an HbA1c level of 8.3% +/- 1.3%. The baseline median CDI was 5.0 in both groups and remained low over time (ranging from 2.0 to 3.5). Most youth (new onset 72%, pump 81%) scored < 13 at all times. Those with a CDI score of >= 13 in month 1 had 9-fold (95% confidence interval: 3-28) and 11-fold (95% confidence interval: 3-38) higher risks of CDI score of >= 13 at 6 and 12 months, respectively, than those with a CDI score of < 13. New-onset youth with a CDI score of >= 13 in month 1 had a higher HbA1c level at 6 months (8.3% +/- 1.7%) than new-onset youth with a CDI score of < 13 (7.2% +/- 1.6%, p = .04).

Conclusions: CDI scores over 1 year were similar in the new-onset and pump groups. Youth with elevated CDI in the first month after diagnosis or pump start were significantly more likely to have a CDI score of >= 13 at 6 or 12 months, supporting recommendations to screen for depressive symptoms because of persistence over time. Those with new-onset diabetes and depressive symptoms in the first month had higher HbA1c at 6 months; confirmatory research is needed. (C) 2017 Society for Adolescent Health and Medicine. All rights reserved.

C1 [McGill, Dayna E.; Volkening, Lisa K.; Pober, David M.; Laffel, Lori M.] Harvard Med Sch, Joslin Diabet Ctr, Sect Clin Behav & Outcomes Res, Pediat Adolescent & Young Adult Sect, Boston, MA USA.

[Muir, Andrew B.] Emory Univ, Sch Med, Dept Pediat, Atlanta, GA 30322 USA.

[Young-Hyman, Deborah L.] Georgia Hlth Sci Univ, Georgia Prevent Inst, Augusta, GA USA.

[Young-Hyman, Deborah L.] NIH, Off Behav & Social Sci Res, Off Director, Bethesda, MD 20892 USA.

RP Laffel, LM (reprint author), Joslin Diabet Ctr, Sect Clin Behav & Outcomes Res, One Joslin Pl, Boston, MA 02215 USA.

EM lori.laffel@joslin.harvard.edu

FU National Institutes of Health [T32DK007260, P30DK036836]; American

Diabetes Association [07-08-CR66]; Katherine Adler Astrove Youth

Education Fund; Maria Griffin Drury Pediatric Fund; Eleanor Chesterman

Beatson Fund

FX This research was supported by the National Institutes of Health under

grants T32DK007260 and P30DK036836, the American Diabetes Association

under grant 07-08-CR66, the Katherine Adler Astrove Youth Education

Fund, the Maria Griffin Drury Pediatric Fund, and the Eleanor Chesterman

Beatson Fund.

CR American Diabetes Association, 2017, Diabetes Care, V40, pS6

Boland EA, 1999, DIABETES CARE, V22, P1779, DOI 10.2337/diacare.22.11.1779

Borus JS, 2010, CURR OPIN PEDIATR, V22, P405, DOI 10.1097/MOP.0b013e32833a46a7

Center for Behavioral Health Statistics and Quality, 2016, HHS PUBL SMA, VH-51

Corathers SD, 2013, PEDIATRICS, V132, pE1395, DOI 10.1542/peds.2013-0681

Delamater AM, 2014, PEDIATR DIABETES, V15, P232, DOI 10.1111/pedi.12191

Fisher L, 2016, DIABETIC MED, V33, P1590, DOI 10.1111/dme.12973

Fisher L, 2015, J DIABETES COMPLICAT, V29, P572, DOI 10.1016/j.jdiacomp.2015.01.012

GREY M, 1995, DIABETES CARE, V18, P1330, DOI 10.2337/diacare.18.10.1330

Hagger V, 2016, CURR DIABETES REP, V16, DOI 10.1007/s11892-015-0694-2

Hirose M, 2012, CURR DIABETES REP, V12, P711, DOI 10.1007/s11892-012-0313-4

Hood KK, 2006, DIABETES CARE, V29, P1389, DOI 10.2337/dc06-0087

Hood KK, 2014, J ADOLESCENT HEALTH, V55, P498, DOI 10.1016/j.jadohealth.2014.03.011

Hood KK, 2012, DIABETES CARE, V35, P2443, DOI 10.2337/dc11-2329

Hood KK, 2011, PEDIATR DIABETES, V12, P718, DOI 10.1111/j.1399-5448.2011.00771.x

Hosoya T, 2012, INTERNAL MED, V51, P263, DOI 10.2169/internalmedicine.51.5768

Kessler RC, 2012, ARCH GEN PSYCHIAT, V69, P372, DOI 10.1001/archgenpsychiatry.2011.160

Kokoszka A, 2009, EUR PSYCHIAT, V24, P425, DOI 10.1016/j.eurpsy.2009.04.002

KOVACS M, 1985, PSYCHOPHARMACOL BULL, V21, P995

KOVACS M, 1990, J PEDIATR PSYCHOL, V15, P619, DOI 10.1093/jpepsy/15.5.619

KOVACS M, 1985, J PEDIATR-US, V106, P827, DOI 10.1016/S0022-3476(85)80368-1

Kuczmarski R. J., 2000, VITAL HLTH STAT, V11, P1, DOI DOI 10.1016/J.BBRC.2015.06.114

Lichtenberger-Geslin L, 2013, ARCH PEDIATRIE, V20, P248, DOI 10.1016/j.arcped.2012.12.011

Matlock KA, 2017, J ADOLESCENT HEALTH, V61, P471, DOI 10.1016/j.jadohealth.2017.05.004

McGrady Meghan E, 2010, Diabetes Res Clin Pract, V88, pe35, DOI 10.1016/j.diabres.2010.03.025

Mortensen HB, 2009, DIABETES CARE, V32, P1384, DOI 10.2337/dc08-1987

Moulton C. D., 2015, ENDOCRINOLOGY, V3, P461, DOI [10.1016/S2213-8587(15)00134-5, DOI 10.1016/S2213-8587(15)00134-5]

Mueller-Godeffroy E, 2009, DIABETIC MED, V26, P493, DOI 10.1111/j.1464-5491.2009.02707.x

Prager LM, 2009, PEDIATR REV, V30, P199, DOI 10.1542/pir.30-6-199

Rushton JL, 2002, J AM ACAD CHILD PSY, V41, P199, DOI 10.1097/00004583-200202000-00014

Silverstein J, 2015, DIABETES CARE, V38, P2341, DOI 10.2337/dc15-0982

Stahl-Pehe A, 2014, PEDIATR DIABETES, V15, P436, DOI 10.1111/pedi.12096

Stewart SM, 2005, PEDIATRICS, V115, P1315, DOI 10.1542/peds.2004-1717

Whittemore Robin, 2002, Pediatr Diabetes, V3, P135, DOI 10.1034/j.1399-5448.2002.30303.x

Willi SM, 2015, PEDIATRICS, V135, P424, DOI 10.1542/peds.2014-1774

Young-Hyman D, 2016, DIABETES CARE, V39, P2126, DOI 10.2337/dc16-2053

Young-Hyman Deborah L, 2016, J Diabetes Sci Technol, V10, P845, DOI 10.1177/1932296816645118

Zdunczyk B, 2014, DIABETES-METAB RES, V30, P333, DOI 10.1002/dmrr.2499

NR 38

TC 0

Z9 0

U1 2

U2 5

PU ELSEVIER SCIENCE INC

PI NEW YORK

PA 360 PARK AVE SOUTH, NEW YORK, NY 10010-1710 USA

SN 1054-139X

EI 1879-1972

J9 J ADOLESCENT HEALTH

JI J. Adolesc. Health

PD FEB

PY 2018

VL 62

IS 2

BP 219

EP 225

DI 10.1016/j.jadohealth.2017.09.017

PG 7

WC Psychology, Developmental; Public, Environmental & Occupational Health;

Pediatrics

SC Psychology; Public, Environmental & Occupational Health; Pediatrics

GA FS8QV

UT WOS:000422678400016

PM 29212599

DA 2019-01-08

ER

PT J

AU Vallis, M

Willaing, I

Holt, RIG

AF Vallis, M.

Willaing, I.

Holt, R. I. G.

TI Emerging adulthood and Type 1 diabetes: insights from the DAWN2 Study

SO DIABETIC MEDICINE

LA English

DT Article

ID QUALITY-OF-LIFE; PATIENT ASSESSMENT; PSYCHOMETRIC PROPERTIES;

EMPOWERMENT SCALE; SELF-MANAGEMENT; SHORT-FORM; CARE; PEOPLE;

HYPOGLYCEMIA; VALIDATION

AB Aims To compare clinical, psychological, education and social variables in emerging adults (aged 18-30years) with Type 1 diabetes with their adult counterparts aged >30years.

Methods A single assessment multinational sample was surveyed as part of the larger second Diabetes Attitudes, Wishes and Needs (DAWN2) study. Participants completed a series of surveys incorporating demographic as well as clinical questions (comorbidities, hypoglycaemia) and validated self-report scales concerning psychosocial (health impact, quality of life, beliefs and attitudes, self-management behaviours, healthcare experience and family support) and diabetes education factors.

Results Emerging adults differed from adults aged >30years with regard to a number of psychosocial variables. Emerging adults reported better overall quality of life, social support and support from their healthcare team compared with adults aged >30years of age; however, emerging adults experienced greater diabetes-specific distress and were less engaged in self-management. Diabetes education was related to a number of indicators, while experience of discrimination was harmful, but these impacts did not differ between emerging adults and adults aged >30years. An analysis of geographical regions suggested that emerging adults in North America and Europe had better well-being than older adults, while the opposite was observed in Asia.

Conclusions Emerging adults, particularly those in the later phase (ages 25-30years) are especially at risk in terms of diabetes-specific distress. There is a need for novel interventions to meet the needs of these vulnerable emerging adults more effectively.

C1 [Vallis, M.] Nova Scotia Hlth Author, Behav Change Inst, Halifax, NS, Canada.

[Vallis, M.] Dalhousie Univ, Halifax, NS, Canada.

[Willaing, I.] Steno Diabet Ctr, Hlth Promot Res, Diabet Management Res, Copenhagen, Denmark.

[Holt, R. I. G.] Univ Southampton, Human Dev & Hlth Acad Unit, Fac Med, Southampton, Hants, England.

RP Vallis, M (reprint author), Nova Scotia Hlth Author, Behav Change Inst, Halifax, NS, Canada.; Vallis, M (reprint author), Dalhousie Univ, Halifax, NS, Canada.

EM tvallis@dal.ca

OI Holt, Richard/0000-0001-8911-6744; Vallis, Michael/0000-0002-0165-5936;

Willaing, Ingrid/0000-0002-3082-8816

FU Novo Nordisk

FX Novo Nordisk funded the DAWN2 study, which was overseen by the DAWN2

Study Group (see www.dawnstudy.com).

CR Abidi S, 2014, CAN J DIABETES, V38, P205, DOI 10.1016/j.jcjd.2014.03.006

Anderson RM, 2003, DIABETES CARE, V26, P1641, DOI 10.2337/diacare.26.5.1641-a

Anderson RM, 2000, DIABETES CARE, V23, P739, DOI 10.2337/diacare.23.6.739

Arnett JJ, 2000, AM PSYCHOL, V55, P469, DOI 10.1037/0003-066X.55.5.469

Barnard KD, 2012, DIABETIC MED, V29, P1358, DOI 10.1111/j.1464-5491.2012.03727.x

Bech P, 1996, PSYCHOTHER PSYCHOSOM, V65, P183, DOI 10.1159/000289073

Crowley R, 2011, ARCH DIS CHILD, V96, P548, DOI 10.1136/adc.2010.202473

Fisher L, 2010, DIABETES CARE, V33, P23, DOI 10.2337/dc09-1238

Glasgow RE, 2005, DIABETES CARE, V28, P2655, DOI 10.2337/diacare.28.11.2655

Glasgow RE, 2005, MED CARE, V43, P436, DOI 10.1097/01.mlr.0000160375.47920.8c

Goetz Katja, 2012, Am J Manag Care, V18, pe55

Hajos TRS, 2013, DIABETIC MED, V30, pE63, DOI 10.1111/dme.12040

Harper A, 1998, PSYCHOL MED, V28, P551

Helgeson VS, 2013, DIABETIC MED, V30, P610, DOI 10.1111/dme.12067

Hendrieckx C, 2014, DIABETES RES CLIN PR, V103, P430, DOI 10.1016/j.diabres.2013.12.005

Janssen MF, 2013, QUAL LIFE RES, V22, P1717, DOI 10.1007/s11136-012-0322-4

Lee EH, 2014, HEALTH QUAL LIFE OUT, V12, DOI 10.1186/s12955-014-0142-z

McGuire BE, 2010, DIABETOLOGIA, V53, P66, DOI 10.1007/s00125-009-1559-5

Nicolucci A, 2013, DIABETIC MED, V30, P767, DOI 10.1111/dme.12245

Parkin T, 2003, DIABETIC MED, V20, P909, DOI 10.1046/j.1464-5491.2003.01056.x

Peyrot M, 2013, DIABETES RES CLIN PR, V99, P174, DOI 10.1016/j.diabres.2012.11.016

POLONSKY WH, 1995, DIABETES CARE, V18, P754, DOI 10.2337/diacare.18.6.754

Rick J, 2012, BMC HEALTH SERV RES, V12, DOI 10.1186/1472-6963-12-293

Schmitt A, 2013, HEALTH QUAL LIFE OUT, V11, DOI 10.1186/1477-7525-11-138

Schram MT, 2009, CURR DIABETES REV, V5, P112, DOI 10.2174/157339909788166828

Sheehan AM, 2015, DIABETIC MED, V32, P440, DOI 10.1111/dme.12639

Skevington SM, 2004, QUAL LIFE RES, V13, P299, DOI 10.1023/B:QURE.0000018486.91360.00

Speight J, 2009, DIABETIC MED, V26, P315, DOI 10.1111/j.1464-5491.2009.02682.x

Szende A, 2007, EQ D VALUE SETS INVE

Toobert DJ, 2000, DIABETES CARE, V23, P943, DOI 10.2337/diacare.23.7.943

Vallis M, 2014, CURR DIABETES REV, V10, P364, DOI 10.2174/1573399810666141113115026

Yu CH, 2016, PATIENT PREFER ADHER, V10, P371, DOI 10.2147/PPA.S102574

NR 32

TC 2

Z9 2

U1 0

U2 10

PU WILEY

PI HOBOKEN

PA 111 RIVER ST, HOBOKEN 07030-5774, NJ USA

SN 0742-3071

EI 1464-5491

J9 DIABETIC MED

JI Diabetic Med.

PD FEB

PY 2018

VL 35

IS 2

BP 203

EP 213

DI 10.1111/dme.13554

PG 11

WC Endocrinology & Metabolism

SC Endocrinology & Metabolism

GA FS6IX

UT WOS:000419902200006

PM 29171084

DA 2019-01-08

ER

PT J

AU Elamoshy, R

Bird, Y

Thorpe, LU

Moraros, J

AF Elamoshy, Rasha

Bird, Yelena

Thorpe, Lilian Ulrica

Moraros, John

TI Examining the association between diabetes, depressive symptoms, and

suicidal ideation among Aboriginal Canadian peoples living off-reserve:

a cross-sectional, population-based study

SO DIABETES METABOLIC SYNDROME AND OBESITY-TARGETS AND THERAPY

LA English

DT Article

DE aboriginal; Canada; diabetes; depression; depressive symptoms; suicidal

ideation

ID PSYCHOLOGICAL DISTRESS; ALCOHOL-CONSUMPTION; RISK-FACTOR; TYPE-2;

ANXIETY; HEALTH; MELLITUS; INEQUALITIES; METAANALYSIS; COMORBIDITY

AB Background: Diabetes is a prevalent chronic condition that has been linked to depression and suicidal behavior. The Aboriginal peoples of Canada are known to suffer from significant health disparities and higher burden of physical and mental illnesses. The purpose of this study was to assess whether diabetes is associated with higher depressive symptoms and lifetime suicidal ideation among Aboriginal Canadian peoples living off-reserve.

Methods: Data were obtained from the Aboriginal Peoples Survey, 2012. Depressive symptoms were evaluated by a modified version of the previously validated K-10 scale, while diabetes and suicidal ideation were self-reported. A secondary analysis was conducted on a weighted sample of 689,860 participants for depressive symptoms (9.25% diabetics) and 694,960 for suicidal ideation (9.39% diabetics). Descriptive statistics and multiple logistic regression analysis were conducted.

Results : Our study found that the prevalence of depressive symptoms was higher among diabetics (17.53%) compared with nondiabetics (11.12%; OR = 1.70, 95% CI: 1.22-1.61). After adjusting for sociodemographic variables, smoking/alcohol use/drug use, anxiety disorders, and other chronic illnesses, diabetes was still significantly associated with depressive symptoms (aOR = 1.46, 95% CI: 1.03-2.07). Additionally, diabetics (23.86%) were more likely to report suicidal ideation compared with nondiabetics (18.71%; OR = 1.36, 95% CI: 1.05-1.77). Controlling for the effect of sociodemographics and health-related behaviors, diabetes was still associated with higher risk of reporting suicidal ideation (aOR = 1.40, 95% CI: 1.05-1.88).

Conclusion: Our results suggest that the Aboriginal Canadian diabetic patients living off-reserve are at higher risk of depressive symptoms and suicidal ideation. Culturally appropriate co-screening strategies need to be implemented in primary health care settings to provide the supports necessary for this vulnerable population. Further research is needed to fully elucidate the nature of these associations in order to develop effective intervention and treatment approaches.

C1 [Elamoshy, Rasha; Bird, Yelena; Moraros, John] Univ Saskatchewan, Sch Publ Hlth, 104 Clin Pl, Saskatoon, SK S7N 2Z4, Canada.

[Thorpe, Lilian Ulrica] Univ Saskatchewan, Community Hlth & Epidemiol Dept, Saskatoon, SK, Canada.

RP Moraros, J (reprint author), Univ Saskatchewan, Sch Publ Hlth, 104 Clin Pl, Saskatoon, SK S7N 2Z4, Canada.

EM john.moraros@usask.ca

OI Thorpe, Lilian/0000-0003-2547-4205

CR Agardh E, 2011, INT J EPIDEMIOL, V40, P804, DOI 10.1093/ije/dyr029

American Psychiatric Association, 2013, DIAGN STAT MAN MENT

Anderson RJ, 2001, DIABETES CARE, V24, P1069, DOI 10.2337/diacare.24.6.1069

Armitage A., 1995, COMP POLICY ABORIGIN

Australian Government, 2001, US KESSL PSYCH DISTR

Australian Institute of Health and Welfare, 2009, MEAS SOC EM WELLB AB

Bird Y, 2015, INT J EQUITY HEALTH, V14, DOI 10.1186/s12939-015-0237-0

Borges G, 2006, PSYCHOL MED, V36, P1747, DOI 10.1017/S0033291706008786

BRIEN SE, 2011, BMJ-BRIT MED J, V342, DOI DOI 10.1136/BMJ.D636

Bursac Z, 2008, SOURCE CODE BIOL MED, V3, DOI 10.1186/1751-0473-3-17

Cairney J, 2007, CAN J PSYCHIAT, V52, P111, DOI 10.1177/070674370705200209

Caldwell TM, 2002, ADDICTION, V97, P583, DOI 10.1046/j.1360-0443.2002.00092.x

Cameron BL, 2014, ADV NURS SCI, V37, pE1, DOI 10.1097/ANS.0000000000000039

Canadian Diabetes Association, 2001, DIAB CAN TIPP POINT

Canadian Diabetes Association, 2018, TYP 2 DIAB AB PEOPL

Centre for Chronic Disease Prevention and Control Diabetes in Canada: Facts and figures from a public health perspective, 2011, CHRON DIS SURV MON D

Centre for Suicide Prevention, IND SUIC PREV

Chaufan C., 2009, HUMANITY SOC, V33, P74, DOI DOI 10.1177/016059760903300106

Daniel M, 2001, PATIENT EDUC COUNS, V43, P85, DOI 10.1016/S0738-3991(00)00153-1

Davis TME, 2015, DIABETES RES CLIN PR, V107, P384, DOI 10.1016/j.diabres.2014.12.014

Elias B, 2012, SOC SCI MED, V74, P1560, DOI 10.1016/j.socscimed.2012.01.026

Freeman A, 2016, BMC PUBLIC HEALTH, V16, DOI 10.1186/s12889-016-3638-0

Furukawa TA, 2003, PSYCHOL MED, V33, P357, DOI 10.1017/S0033291702006700

Gorrell P., SURVEY ANAL OPTIONS

Gracey M, 2009, LANCET, V374, P65, DOI 10.1016/S0140-6736(09)60914-4

Graham K, 2007, ALCOHOL CLIN EXP RES, V31, P78, DOI 10.1111/j.1530-0277.2006.00274.x

GRANT BF, 1995, DRUG ALCOHOL DEPEN, V39, P197, DOI 10.1016/0376-8716(95)01160-4

Hammen C, 2005, ANNU REV CLIN PSYCHO, V1, P293, DOI 10.1146/annurev.clinpsy.1.102803.143938

Han SJ, 2013, DIABETES RES CLIN PR, V101, pE14, DOI 10.1016/j.diabres.2013.06.012

Harris SB, 2013, CAN J DIABETES, V37, pS191, DOI 10.1016/j.jcjd.2013.01.046

Howard HA, 2014, MED ANTHROPOL, V33, P529, DOI 10.1080/01459740.2013.828722

Joseph B, 2018, INDIAN ACT PASS SYST

Kessler RC, 2003, ARCH GEN PSYCHIAT, V60, P184, DOI 10.1001/archpsyc.60.2.184

Kirmayer LJ, 2009, CURRENT APPROACHES A, P14

Klonsky ED, 2016, ANNU REV CLIN PSYCHO, V12, P307, DOI 10.1146/annurev-clinpsy-021815-093204

Loppie Reading C., 2009, HLTH INEQUALITIES SO

Lorant V, 2003, AM J EPIDEMIOL, V157, P98, DOI 10.1093/aje/kwf182

Mezuk B, 2008, DIABETES CARE, V31, P2383, DOI 10.2337/dc08-0985

Midi H, 2010, J INTERDISCIP MATH, V13, P253, DOI 10.1080/09720502.2010.10700699

Nouwen A, 2010, DIABETOLOGIA, V53, P2480, DOI 10.1007/s00125-010-1874-x

Pan A, 2010, ARCH INTERN MED, V170, P1884, DOI 10.1001/archinternmed.2010.356

Pelletier C, 2012, CHRON DIS INJ CAN, V33, P53

Pouwer F, 2010, DISCOV MED, V9, P112

Public Health Agency of Canada, 2011, ARCH HUM FAC MENT HL

Public Health Agency of Canada, 2011, DIAB CAN FACTS FIG P

Rodgers B, 2000, ADDICTION, V95, P1833, DOI 10.1046/j.1360-0443.2000.9512183312.x

Rotella F, 2013, DIABETES RES CLIN PR, V99, P98, DOI 10.1016/j.diabres.2012.11.022

Roy T, 2012, J AFFECT DISORDERS, V142, pS8, DOI 10.1016/S0165-0327(12)70004-6

Schierhout G, 2013, INT J MENT HEALTH SY, V7, DOI 10.1186/1752-4458-7-16

Schilling EA, 2009, J ADOLESCENT HEALTH, V44, P335, DOI 10.1016/j.jadohealth.2008.08.006

Si DM, 2011, AUST FAM PHYSICIAN, V40, P331

Singer J, 2014, CLIN OBES, V4, P316, DOI 10.1111/cob.12074

Skogen JC, 2009, ADDICTION, V104, P1519, DOI 10.1111/j.1360-0443.2009.02659.x

Statistics Canada. Life expectancy, LIF EXP

Tabb KM, 2017, J PSYCHOSOM RES, V103, P113, DOI 10.1016/j.jpsychores.2017.10.009

Thompson EA, 2005, SUICIDE LIFE-THREAT, V35, P14, DOI 10.1521/suli.35.1.14.59266

Tjepkema M, 2002, HLTH REP S, V13, P73

Wang B, 2017, EUR J ENDOCRINOL, V177, pR169, DOI 10.1530/EJE-16-0952

Wang Y, 2016, COMPR PSYCHIAT, V71, P11, DOI 10.1016/j.comppsych.2016.08.006

World Health Organization, 2018, SOCIAL DETERMINANTS

Yu CHY, 2007, DIABETES RES CLIN PR, V78, P159, DOI 10.1016/j.diabres.2007.03.022

NR 61

TC 0

Z9 0

U1 1

U2 1

PU DOVE MEDICAL PRESS LTD

PI ALBANY

PA PO BOX 300-008, ALBANY, AUCKLAND 0752, NEW ZEALAND

SN 1178-7007

J9 DIABET METAB SYND OB

JI Diabetes Metab. Syndr. Obes.

PY 2018

VL 11

BP 767

EP 780

DI 10.2147/DMSO.S184058

PG 14

WC Endocrinology & Metabolism

SC Endocrinology & Metabolism

GA HB8VH

UT WOS:000451368600001

PM 30538514

OA DOAJ Gold

DA 2019-01-08

ER

PT J

AU Bao, HL

Liu, JR

Ye, J

AF Bao, Huilan

Liu, Junru

Ye, Jun

TI INFLUENCING FACTORS OF THE DIABETES DISTRESS AMONG CHINESE PATIENTS WITH

TYPE 2 DIABETES MELLITUS

SO PSYCHIATRIA DANUBINA

LA English

DT Article

DE T2DM; Diabetes Distress; influencing factors

ID EMOTIONAL DISTRESS; PROBLEM AREAS; ADULTS; DEPRESSION; MANAGEMENT;

SUPPORT; SCALE; SLEEP; PAID

AB Background: Patients with Diabetes Mellitus (DM) are required to have long-term treatment regimen and strict self-management, which thus might lead to the Diabetes Distress (DD). Patients' DD varies in different regions with different levels of medical conditions. For improving the treatment effect of the patients with the Type-2-Diabetes-Mellitus (T2DM), this study explores the influencing factors of the patients' DD in the regions where the medical treatment are at low level.

Subjects and methods: In this study, 167 adult patients with T2DM were selected from the People's Hospital of Jinhua city, an A-grade hospital of a small-mid-sized city in Eastern China. Based on these samples, the Diabetes Distress Scale with 17 items (DDS17) was adopted to measure the degree of patients' DD, and then regression analyses were carried out to investigate the influencing factors for their DD.

Results: The T2DM patients with moderate and high levels of DD take up 54.5% of the samples investigated in this study. According to the Spearman correlation analysis, sleep time, physical exercise, diet control, treatment regimen, TG and HbA1c significantly affect the patients' DD. Through the multivariate linear regression analysis, this study shows that (1) sleep time significantly influences the T2DM patients' DD (beta=-0.213, P=0.034); (2) sleep time also impacts emotional burden (beta=-0.379, P=0.027); (3) physical exercise (beta=-0.195, P=0.002), treatment regimen (beta=0.158, P=0.026) and diet control (beta=0.158, P=0.026) are the three major influencing factors for regimen-related distress.

Conclusion: In the small-mid-sized city where the treatment regimen is not highly advanced, to alleviate the T2DM patients' DD, the medical staff should suggest the patients to improve sleep quality and exercise more, help them positively understand the insulin infusion therapy and take proper diet control.

C1 [Bao, Huilan; Liu, Junru; Ye, Jun] Peoples Hosp Jinhua, Endocrinol Dept, Jinhua 321000, Peoples R China.

RP Bao, HL (reprint author), Peoples Hosp Jinhua, Endocrinol Dept, Jinhua 321000, Peoples R China.

EM huilanbao@outlook.com

CR Baek RN, 2014, ANN BEHAV MED, V48, P145, DOI 10.1007/s12160-013-9585-4

Cappuccio FP, 2010, DIABETES CARE, V33, P414, DOI 10.2337/dc09-1124

de Vries L, 2014, BMC ENDOCR DISORD, V14, DOI 10.1186/1472-6823-14-21

Delahanty LM, 2007, DIABETIC MED, V24, P48, DOI 10.1111/j.1464-5491.2007.02028.x

Fisher L, 2012, DIABETES CARE, V35, P259, DOI 10.2337/dc11-1572

Graue M, 2012, INT J NURS STUD, V49, P174, DOI 10.1016/j.ijnurstu.2011.08.007

Hessler D, 2014, DIABETES CARE, V37, P617, DOI 10.2337/dc13-0762

Ikeda K, 2014, PLOS ONE, P9

Ip M, 2007, SLEEP MED CLIN, V2, P19, DOI 10.1016/j.jsmc.2006.12.002

Jones A, 2016, PRIM CARE DIABETES, V10, P83, DOI 10.1016/j.pcd.2015.03.008

Karlsen B, 2012, J ADV NURS, V68, P391, DOI 10.1111/j.1365-2648.2011.05751.x

Kasteleyn MJ, 2015, DIABETIC MED, V32, P1617, DOI 10.1111/dme.12743

Li C, 2008, DIABETIC MED, V25, P878, DOI 10.1111/j.1464-5491.2008.02477.x

Makine C, 2009, DIABETIC MED, V26, P28, DOI 10.1111/j.1464-5491.2008.02606.x

Nouwen A, 2015, DIABETIC MED, V32, P1261, DOI 10.1111/dme.12863

POLONSKY WH, 1995, DIABETES CARE, V18, P754, DOI 10.2337/diacare.18.6.754

Polonsky WH, 2005, DIABETES CARE, V28, P626, DOI 10.2337/diacare.28.3.626

Reddy J, 2013, PSYCHOSOMATICS, V54, P44, DOI 10.1016/j.psym.2012.08.004

Snoek FJ, 2011, DIABETES CARE, V34, P601, DOI 10.2337/dc10-1552

Strandberg RB, 2015, DIABETIC MED, V32, P1304, DOI 10.1111/dme.12781

Thorne SE, 2001, PATIENT EDUC COUNS, V42, P81, DOI 10.1016/S0738-3991(00)00095-1

Wagner JA, 2007, DIABETIC MED, V24, P211, DOI 10.1111/j.1464-5491.2007.02044.x

Wardian J, 2014, SOC WORK HEALTH CARE, V53, P364, DOI 10.1080/00981389.2014.884038

NR 23

TC 0

Z9 0

U1 3

U2 3

PU MEDICINSKA NAKLADA

PI ZAGREB

PA VLASKA 69, HR-10000 ZAGREB, CROATIA

SN 0353-5053

J9 PSYCHIAT DANUB

JI Psychiatr. Danub.

PY 2018

VL 30

IS 4

BP 459

EP 465

DI 10.24869/psyd.2018.459

PG 7

WC Psychiatry

SC Psychiatry

GA HB3ZN

UT WOS:000450991500011

PM 30439806

DA 2019-01-08

ER

PT J

AU Lacomba-Trejo, L

Valero-Moreno, S

Casana-Granell, S

Prado-Gasco, VJ

Perez-Marin, M

Montoya-Castilla, I

AF Lacomba-Trejo, Laura

Valero-Moreno, Selene

Casana-Granell, Sara

Javier Prado-Gasco, Vicente

Perez-Marin, Marian

Montoya-Castilla, Inmaculada

TI Questionnaire on adaptation to type 1 diabetes among children and its

relationship to psychological disorders

SO REVISTA LATINO-AMERICANA DE ENFERMAGEM

LA English

DT Article

DE Adaptive Response; Diabetes Mellitus Type I; Pediatrics; Anxiety;

Depression; Psychometric

ID HOSPITAL ANXIETY; DEPRESSION SCALE; ADOLESCENTS; PREVALENCE; YOUTH

AB Objective: to study the psychometric properties of an adaptive disease response questionnaire for use with Spanish children with type 1 diabetes; to analyse this response in this sample and to observe the relationship between adaptive response and levels of anxiety-depression. Method: a total of 100 patients with type 1 diabetes aged between nine and 16 years (M=12.28, SD=1.78) participated in the study, of which 59% were children. Data was collected in public hospitals via interviews using the Adaptive Disease Response Questionnaire and Anxiety and Depression Scale. The data was analysed using Pearson correlations, multiple hierarchical linear regressions, Student's t Test for independent samples, and Cohen's d effect size to determine reliability and validity. Result: the instrument was shown to have adequate psychometric properties. Adaptive response was generally high. Adaptive response is negatively related to emotional distress, being a better predictor of depression than of anxiety. There was no association betwee adaptation and sex and age. Conclusion: promoting a better adaptive response appears to reduce emotional distress, especially in the case of depression, regardless of the age or gender of the patients.

C1 [Lacomba-Trejo, Laura; Valero-Moreno, Selene; Casana-Granell, Sara; Javier Prado-Gasco, Vicente; Perez-Marin, Marian; Montoya-Castilla, Inmaculada] Univ Valencia, Fac Psicol, Valencia, Comunidad Valen, Spain.

RP Perez-Marin, M (reprint author), Univ Valencia, Fac Psicol, Valencia, Comunidad Valen, Spain.

EM marian.perez@uv.es

RI Perez Marin, Maria Antonia/G-9394-2011

OI Perez Marin, Maria Antonia/0000-0003-3532-8818

FU Asociacion Espanola de Psicologia Clinica y Psicopatologia; Federacion

Espanola de Asociaciones de Terapia Familiar; Generalitat Valenciana

[ACIF17]; Fondo Social Europeo; Instituto de Investigacion La Fe;

Hospital Universitario y Politecnico La Fe; Universitat de Valencia

(VLC-BIOMED); Universitat de Valencia

FX Supported by Asociacion Espanola de Psicologia Clinica y Psicopatologia,

Federacion Espanola de Asociaciones de Terapia Familiar, Instituto de

Investigacion La Fe, Hospital Universitario y Politecnico La Fe and

Universitat de Valencia (VLC-BIOMED), initiation grant to research from

Universitat de Valencia and predoctoral research contract of the

Generalitat Valenciana (ACIF17) and Fondo Social Europeo.

CR Bilbao-Cercós Ana, 2014, Clínica y Salud, V25, P57, DOI 10.5093/cl2014a5

Carballal Marino Marta, 2018, An Pediatr (Barc), V89, P153, DOI 10.1016/j.anpedi.2017.10.007

Cardila F, 2015, EUR J INVEST HEALTH, V5, P267, DOI 10.1989/ejihpe.v5i2.118

Caro I., 1992, B PSICOL, V36, P43

Castro-Espinoza José Martín, 2015, Aquichán, V15, P52, DOI 10.5294/aqui.2015.15.1.6

Chan YF, 2010, QUAL LIFE RES, V19, P865, DOI 10.1007/s11136-010-9645-1

Conde Barreiro S, 2014, AN PEDIAT, V81, P139, DOI [10.1016/j.anpedi.2013.12.010, DOI 10.1016/J.ANPEDI.2013.12.010]

Gimenez-Espert MD, 2017, REV LAT-AM ENFERM, V25, DOI 10.1590/1518-8345.2018.2969

Flores-Carvajal D, 2016, REV CHIL PEDIATR-CHI, V87, P169, DOI 10.1016/j.rchipe.2015.08.007

Garcia A, 2014, CALIDAD VIDA SALUD, V7, P42

Jacob AA, 2018, J RES MED SCI, V6, P177, DOI [10.18203/2320-6012.ijrms20175716, DOI 10.18203/2320-6012.IJRMS20175716]

Johnson B, 2013, DIABETIC MED, V30, P199, DOI 10.1111/j.1464-5491.2012.03721.x

Korczak DJ, 2016, J DEPRESS ANXIETY, V217, DOI [10.4200/2167-1044.1000, DOI 10.4200/2167-1044.1000]

Kristensen LJ, 2014, PLOS ONE, V9, DOI 10.1371/journal.pone.0097543

Lacomba-Trejo L, 2017, CALID VIDA SALUD, V10, P11

Markowitz JT, 2015, CURR DIABETES REV, V11, P231, DOI 10.2174/1573399811666150421114146

Mihalca AM, 2015, PSYCHIATR POL, V49, P1071, DOI 10.12740/PP/38139

Santos FRM, 2013, DIABETOL METAB SYNDR, V5, DOI 10.1186/1758-5996-5-55

Ortuno-Sierra J, 2014, REV PSIQUIATR SALUD, V7, P121, DOI 10.1016/j.rpsm.2013.12.003

Perez-Marin M, 2015, ARCH ARGENT PEDIATR, V113, P158, DOI [10.5546/aap.2015.158, 10.5546/aap.2015.eng.158]

PERRIN JM, 1993, PEDIATRICS, V92, P876

Portilla L, 1995, REV CUBANA PSICOL, V12, P193

Quesada AB, 2014, INT J DEV ED PSYCHOL, V4, P569, DOI 10.17060/ijodaep.2014.n1.v4.832

Quesada-Conde AB, 2016, INT J DEV ED PSYCHOL, V2, P251, DOI [10.17060/ijodaep.2016.n1.v2.215, DOI 10.17060/IJODAEP.2016.N1.V2.215]

Rasbach L, 2015, DIABETES EDUCATOR, V41, P43, DOI 10.1177/0145721714550254

Riaz M, 2017, PAK J MED SCI, V33, P1318, DOI 10.12669/pjms.336.13911

Silverstein J, 2015, DIABETES CARE, V38, P2341, DOI 10.2337/dc15-0982

Valanzuela C, 2016, QUADERNS DIGITAL, V82, P184

ZIGMOND AS, 1983, ACTA PSYCHIAT SCAND, V67, P361, DOI 10.1111/j.1600-0447.1983.tb09716.x

NR 29

TC 0

Z9 0

U1 0

U2 0

PU UNIV SAO PAULO, ESCOLA DE ENFERMAGEM DE RIBEIRAO PRETO

PI RIBEIRAO PRETO

PA AV BANDEIRANTES, 3900, RIBEIRAO PRETO, SP 14040-902 A, BRAZIL

SN 1518-8345

J9 REV LAT-AM ENFERM

JI Rev. Latino-Am. Enfermagem

PY 2018

VL 26

AR e3088

DI 10.1590/1518-8345.2759.3088

PG 9

WC Nursing

SC Nursing

GA HB2KV

UT WOS:000450870700001

PM 30462792

OA DOAJ Gold

DA 2019-01-08

ER

PT J

AU Darwish, L

Beroncal, E

Sison, MV

Swardfager, W

AF Darwish, Lina

Beroncal, Erika

Sison, Ma Veronica

Swardfager, Walter

TI Depression in people with type 2 diabetes: current perspectives

SO DIABETES METABOLIC SYNDROME AND OBESITY-TARGETS AND THERAPY

LA English

DT Review

DE type 2 diabetes; depression; mood; complications

ID PLACEBO-CONTROLLED TRIAL; RANDOMIZED CONTROLLED-TRIAL; IMPAIRED GLUCOSE

REGULATION; EDID RESEARCH CONSORTIUM; SERIOUS MENTAL-ILLNESS; ALL-CAUSE

MORTALITY; WELL-BEING INDEX; GLYCEMIC CONTROL; MENDELIAN RANDOMIZATION;

COMORBID DEPRESSION

AB Type 2 diabetes mellitus (T2DM) is associated with depressive symptoms, and comorbid depression in those with T2DM has been associated with adverse clinical profiles. Recognizing and addressing psychological symptoms remain significant clinical challenges in T2DM. Possible mediators of the reciprocal relationship between T2DM and depression may include physical activity levels, effectiveness of self-management, distress associated with a new T2DM diagnosis, and frailty associated with advanced diabetes duration. The latter considerations contribute to a "J-shaped" trajectory from the time of diagnosis. There remain significant challenges to screening for clinical risks associated with psychological symptoms in T2DM; poorer outcomes may be associated with major depressive episodes, isolated (eg, anhedonic), or subsyndromal depressive symptoms, depressive-like symptoms more specific to T2DM (eg, diabetes-related distress), apathy or fatigue. In this review, we discuss current perspectives on depression in the context of T2DM with implications for screening and management of these highly comorbid conditions.

C1 [Darwish, Lina; Beroncal, Erika; Sison, Ma Veronica; Swardfager, Walter] Sunnybrook Res Inst, Hurvitz Brain Sci Program, 2075 Bayview Ave, Toronto, ON M4N 3M5, Canada.

[Darwish, Lina; Beroncal, Erika; Sison, Ma Veronica; Swardfager, Walter] Univ Toronto, Dept Pharmacol & Toxicol, Toronto, ON, Canada.

[Darwish, Lina; Beroncal, Erika; Sison, Ma Veronica; Swardfager, Walter] Univ Hlth Network, Cardiac Rehabil Program, Toronto Rehabil Inst, Toronto, ON, Canada.

[Darwish, Lina; Swardfager, Walter] Canadian Partnership Stroke Recovery, Toronto, ON, Canada.

RP Swardfager, W (reprint author), Sunnybrook Res Inst, Hurvitz Brain Sci Program, 2075 Bayview Ave, Toronto, ON M4N 3M5, Canada.

EM w.swardfager@utoronto.ca

CR Almeida OP, 2012, TRANSL PSYCHIAT, V2, DOI 10.1038/tp.2012.79

Almeida OP, 2008, INT PSYCHOGERIATR, V20, P645, DOI 10.1017/S1041610207006473

Almeida OP, 2016, MATURITAS, V86, P3, DOI 10.1016/j.maturitas.2016.01.003

Almeida OP, 2015, J AM MED DIR ASSOC, V16, P296, DOI 10.1016/j.jamda.2014.10.023

American Psychiatric Association, 2013, DIAGN STAT MAN MENT

American Psychiatric Association, 2013, DSM 5

Anderberg RH, 2016, PSYCHONEUROENDOCRINO, V65, P54, DOI 10.1016/j.psyneuen.2015.11.021

Anderson RJ, 2001, DIABETES CARE, V24, P1069, DOI 10.2337/diacare.24.6.1069

Arshad AR, 2016, J PAK MED ASSOC, V66, P425

Aujla N, 2010, DIABETIC MED, V27, P896, DOI 10.1111/j.1464-5491.2010.03042.x

Bauer AM, 2014, J GEN INTERN MED, V29, P1139, DOI 10.1007/s11606-014-2845-6

Bech P, 1996, PSYCHOTHER PSYCHOSOM, V65, P183, DOI 10.1159/000289073

BECK AT, 1988, CLIN PSYCHOL REV, V8, P77, DOI 10.1016/0272-7358(88)90050-5

Bogner HR, 2012, ANN FAM MED, V10, P15, DOI 10.1370/afm.1344

Brewster P, 2015, GERONTOLOGIST, V55, P525

Brouwers EPM, 2001, J PSYCHOSOM RES, V51, P659, DOI 10.1016/S0022-3999(01)00245-8

Bruce DG, 2015, AM J GERIAT PSYCHIAT, V23, P615, DOI 10.1016/j.jagp.2014.09.010

Carleton RN, 2013, PLOS ONE, V8, DOI 10.1371/journal.pone.0058067

Carter J, 2016, J PSYCHOSOM RES, V90, P91, DOI 10.1016/j.jpsychores.2016.09.013

Carter J, 2016, PSYCHONEUROENDOCRINO, V69, P123, DOI 10.1016/j.psyneuen.2016.04.002

Cebul RD, 2011, NEW ENGL J MED, V365, P825, DOI 10.1056/NEJMsa1102519

Chiu CJ, 2017, INT PSYCHOGERIATR, V29, P281, DOI 10.1017/S1041610216001630

Cummings DM, 2016, DIABETES CARE, V39, P101, DOI 10.2337/dc15-1174

Davis LM, 2009, NEUROENDOCRINOLOGY, V89, P152, DOI 10.1159/000170586

de Cock ESA, 2011, BMC PSYCHIATRY, V11, DOI 10.1186/1471-244X-11-141

de Groot M, 1999, J PSYCHOSOM RES, V46, P425, DOI 10.1016/S0022-3999(99)00014-8

Deschenes SS, 2017, PSYCHOSOM MED, V79, P603, DOI 10.1097/PSY.0000000000000447

Drivsholm T, 2005, DIABETOLOGIA, V48, P210, DOI 10.1007/s00125-004-1625-y

Eaton WW, 1996, DIABETES CARE, V19, P1097, DOI 10.2337/diacare.19.10.1097

Fisher L, 2008, DIABETIC MED, V25, P1096, DOI 10.1111/j.1464-5491.2008.02533.x

Fisher L, 2014, DIABETIC MED, V31, P764, DOI 10.1111/dme.12428

Fisher L, 2007, DIABETES CARE, V30, P542, DOI 10.2337/dc06-1614

Fisher L, 2012, DIABETES CARE, V35, P259, DOI 10.2337/dc11-1572

Fisher L, 2010, DIABETES CARE, V33, P23, DOI 10.2337/dc09-1238

Fritschi C, 2010, J PSYCHOSOM RES, V69, P33, DOI 10.1016/j.jpsychores.2010.01.021

Georgiades A, 2007, PSYCHOSOM MED, V69, P235, DOI 10.1097/PSY.0b013e318042588d

Gibson EL, 2006, PHYSIOL BEHAV, V89, P53, DOI 10.1016/j.physbeh.2006.01.024

Goldberg D, 2011, WORLD PSYCHIATRY, V10, P226

Gonzalez JS, 2008, DIABETES CARE, V31, P2398, DOI 10.2337/dc08-1341

Gunzler DD, 2015, STAT MED, V34, P3246, DOI 10.1002/sim.6541

Hajos TRS, 2013, DIABETIC MED, V30, pE63, DOI 10.1111/dme.12040

Hartwig FP, 2016, SCI REP-UK, V6, DOI 10.1038/srep32730

Hermanns N, 2013, PRIM CARE DIABETES, V7, P1, DOI 10.1016/j.pcd.2012.11.002

Herrera JJ, 2016, EUR J NEUROSCI, V43, P1190, DOI 10.1111/ejn.13193

Holmes-Truscott E, 2016, PRIM CARE DIABETES, V10, P75, DOI 10.1016/j.pcd.2015.06.006

Hood KK, 2014, J ADOLESCENT HEALTH, V55, P498, DOI 10.1016/j.jadohealth.2014.03.011

Hung CF, 2014, BRIT J PSYCHIAT, V205, P24, DOI 10.1192/bjp.bp.113.130419

Ismail K, 2017, DIABETOLOGIA, V60, P2092, DOI 10.1007/s00125-017-4367-3

Jahoda M., 1958, CURRENT CONCEPTS POS, V61

Johnson JA, 2012, BMC HEALTH SERV RES, V12, DOI 10.1186/1472-6963-12-258

Kappelmann N, 2018, MOL PSYCHIATR, V23, P335, DOI 10.1038/mp.2016.167

Kasteleyn MJ, 2015, DIABETIC MED, V32, P1617, DOI 10.1111/dme.12743

Katon W, 2004, DIABETES CARE, V27, P914, DOI 10.2337/diacare.27.4.914

Katon WJ, 2010, INT J GERIATR PSYCH, V25, P466, DOI 10.1002/gps.2363

Katon WJ, 2010, J GEN INTERN MED, V25, P423, DOI 10.1007/s11606-009-1248-6

Katon WJ, 2005, DIABETES CARE, V28, P2668, DOI 10.2337/diacare.28.11.2668

Khamseh ME, 2011, BMC PSYCHIATRY, V22, P1619

Kivimaki M, 2011, BIOL PSYCHIAT, V70, P978, DOI 10.1016/j.biopsych.2011.07.008

Laake J-PS, 2014, DIABETES CARE, V1, P1

Lavretsky H, 2008, INT J GERIATR PSYCH, V23, P1040, DOI 10.1002/gps.2030

Lawlor DA, 2011, J INTERN MED, V269, P525, DOI 10.1111/j.1365-2796.2011.02343.x

Leventhal AM, 2012, AM J HEALTH BEHAV, V36, P860, DOI 10.5993/AJHB.36.6.12

Lin EHB, 2004, DIABETES CARE, V27, P2154, DOI 10.2337/diacare.27.9.2154

Lin EHB, 2009, ANN FAM MED, V7, P414, DOI 10.1370/afm.998

Lobbestael J, 2011, CLIN PSYCHOL PSYCHOT, V18, P75, DOI 10.1002/cpp.693

Molina MAL, 2014, PSYCHOL HEALTH MED, V19, P136, DOI 10.1080/13548506.2013.793369

Lunghi C, 2016, MEDICINE, V95, DOI 10.1097/MD.0000000000003514

Lustman PJ, 2007, DIABETES CARE, V30, P459, DOI 10.2337/dc06-1769

Lustman PJ, 1997, PSYCHOSOM MED, V59, P24, DOI 10.1097/00006842-199701000-00004

Lustman PJ, 2006, ARCH GEN PSYCHIAT, V63, P521, DOI 10.1001/archpsyc.63.5.521

Lustman PJ, 2005, J DIABETES COMPLICAT, V19, P113, DOI 10.1016/j.jdiacomp.2004.01.002

Lustman PJ, 1998, ANN INTERN MED, V129, P613, DOI 10.7326/0003-4819-129-8-199810150-00005

Madden KM, 2013, DIABETES METAB SYNDR, V6, P233, DOI 10.2147/DMSO.S32951

Manea L, 2016, J AFFECT DISORDERS, V203, P382, DOI 10.1016/j.jad.2016.06.003

MARIN RS, 1991, PSYCHIAT RES, V38, P143, DOI 10.1016/0165-1781(91)90040-V

Markowitz SM, 2011, PSYCHOSOMATICS, V52, P1, DOI 10.1016/j.psym.2010.11.007

Mendlewicz J, 2006, INT CLIN PSYCHOPHARM, V21, P227, DOI 10.1097/00004850-200607000-00005

Mommersteeg PMC, 2013, DIABETIC MED, V30, pE208, DOI 10.1111/dme.12193

Moran C, 2013, DIABETES CARE, V36, P4036, DOI 10.2337/dc13-0143

Morley JE, 2008, CLIN GERIATR MED, V24, P455, DOI 10.1016/j.cger.2008.03.004

Moskowitz JT, 2008, HEALTH PSYCHOL, V27, pS73, DOI 10.1037/0278-6133.27.1.S73

Mota-Pereira J, 2011, J PSYCHIATR RES, V45, P1005, DOI 10.1016/j.jpsychires.2011.02.005

Moulton C. D., 2015, ENDOCRINOLOGY, V3, P461, DOI [10.1016/S2213-8587(15)00134-5, DOI 10.1016/S2213-8587(15)00134-5]

Naicker K, 2017, PSYCHONEUROENDOCRINO, V84, P116, DOI 10.1016/j.psyneuen.2017.07.002

Nefs G, 2012, J PSYCHIATR RES, V46, P549, DOI 10.1016/j.jpsychires.2012.01.013

Nefs G, 2016, BRIT J PSYCHIAT, V209, P142, DOI 10.1192/bjp.bp.114.154781

Nouwen A, 2010, DIABETOLOGIA, V53, P2480, DOI 10.1007/s00125-010-1874-x

Nouwen A, 2011, DIABETES CARE, V34, P752, DOI 10.2337/dc10-1414

Novak V, 2006, DIABETES CARE, V29, P1529, DOI 10.2337/dc06-0261

Padala PR, 2018, AM J PSYCHIAT, V175, P159, DOI 10.1176/appi.ajp.2017.17030316

Park H, 2015, J ADV NURS, V71, P1650, DOI 10.1111/jan.12632

Park M, 2013, GEN HOSP PSYCHIAT, V35, P217, DOI 10.1016/j.genhosppsych.2013.01.006

Petrak F, 2015, DIABETES CARE, V38, P767, DOI 10.2337/dc14-1599

Pibernik-Okanovic M, 2005, DIABETIC MED, V22, P942, DOI 10.1111/j.1464-5491.2005.01528.x

Polonsky WH, 2005, DIABETES CARE, V28, P626, DOI 10.2337/diacare.28.3.626

Pruzin JJ, 2017, ALZ DIS ASSOC DIS, V31, P41, DOI 10.1097/WAD.0000000000000172

Raison CL, 2013, JAMA PSYCHIAT, V70, P31, DOI 10.1001/2013.jamapsychiatry.4

Raison CL, 2003, AM J PSYCHIAT, V160, P1554, DOI 10.1176/appi.ajp.160.9.1554

Renn BN, 2011, CLIN PSYCHOL REV, V31, P1239, DOI 10.1016/j.cpr.2011.08.001

Rethorst CD, 2009, SPORTS MED, V39, P491, DOI 10.2165/00007256-200939060-00004

Rosenberg PB, 2013, J CLIN PSYCHIAT, V74, P810, DOI 10.4088/JCP.12m08099

Rotella F, 2013, DIABETES RES CLIN PR, V99, P98, DOI 10.1016/j.diabres.2012.11.022

Rotella F, 2013, J CLIN PSYCHIAT, V74, P32, DOI 10.4088/JCP.12r07922

Sajatovic M, 2016, ARCH PSYCHIAT NURS, V30, P226, DOI 10.1016/j.apnu.2015.11.004

Sajatovic M, 2015, PSYCHIAT SERV, V66, P197, DOI 10.1176/appi.ps.201300538

Schmitz N, 2018, BRIT J PSYCHIAT, V212, P96, DOI 10.1192/bjp.2017.26

Scranton R, 2010, EXPERT OPIN PHARMACO, V11, P269, DOI 10.1517/14656560903501544

Sharma S, 2013, INT J OBESITY, V37, P382, DOI 10.1038/ijo.2012.48

Snoek FJ, 2015, LANCET DIABETES ENDO, V3, P450, DOI 10.1016/S2213-8587(15)00135-7

Snowling NJ, 2006, DIABETES CARE, V29, P2518, DOI 10.2337/dc06-1317

Stuart MJ, 2012, NEUROSCI BIOBEHAV R, V36, P658, DOI 10.1016/j.neubiorev.2011.10.001

Sun JC, 2015, DIABETIC MED, V32, P935, DOI 10.1111/dme.12649

Swardfager W, 2016, DIABETIC MED, V33, P529, DOI 10.1111/dme.12872

Swardfager W, 2017, NEUROREHAB NEURAL RE, V31, P48, DOI 10.1177/1545968316656054

Swardfager W, 2016, NEUROSCI BIOBEHAV R, V64, P148, DOI 10.1016/j.neubiorev.2016.02.017

Swardfager W, 2011, J CLIN PSYCHIAT, V72, P1181, DOI 10.4088/JCP.09m05810blu

Tabak AG, 2014, LANCET DIABETES ENDO, V2, P236, DOI 10.1016/S2213-8587(13)70139-6

Tanenbaum ML, 2013, J AFFECT DISORDERS, V150, P533, DOI 10.1016/j.jad.2013.01.029

Teresi Jeanne A, 2008, Psychol Sci Q, V50, P538

Thombs BD, 2016, INT J METH PSYCH RES, V25, P145, DOI 10.1002/mpr.1504

Tiemeier H, 2002, J NEUROL NEUROSUR PS, V73, P34, DOI 10.1136/jnnp.73.1.34

Treadway MT, 2011, NEUROSCI BIOBEHAV R, V35, P537, DOI 10.1016/j.neubiorev.2010.06.006

Tsenkova VK, 2008, HEALTH PSYCHOL, V27, pS163, DOI 10.1037/0278-6133.27.2(Suppl.).S163

Tuohy A, 2008, BRIT J CLIN PSYCHOL, V47, P153, DOI 10.1348/014466507X238608

Van der Feltz-Cornelis CM, 2010, GEN HOSP PSYCHIAT, V32, P380, DOI 10.1016/j.genhosppsych.2010.03.011

van der Heijden MMP, 2013, DIABETOLOGIA, V56, P1210, DOI 10.1007/s00125-013-2871-7

van der Ven NCW, 2005, DIABETIC MED, V22, P1619

van Dijk SEM, 2018, QUAL LIFE RES, V27, P1415, DOI 10.1007/s11136-018-1782-y

VANSTEENBERGENWEIJ, 2010, BMC HEALTH SERV RES, V10, DOI DOI 10.1186/1472-6963-10-235

Wahlqvist ML, 2012, BMC MED, V10, DOI 10.1186/1741-7015-10-150

Wang YP, 2013, REV BRAS PSIQUIATR, V35, P416, DOI 10.1590/1516-4446-2012-1048

Wang YP, 2013, CLINICS, V68, P1274, DOI 10.6061/clinics/2013(09)15

NR 132

TC 1

Z9 1

U1 3

U2 3

PU DOVE MEDICAL PRESS LTD

PI ALBANY

PA PO BOX 300-008, ALBANY, AUCKLAND 0752, NEW ZEALAND

SN 1178-7007

J9 DIABET METAB SYND OB

JI Diabetes Metab. Syndr. Obes.

PY 2018

VL 11

BP 333

EP 343

DI 10.2147/DMSO.S106797

PG 11

WC Endocrinology & Metabolism

SC Endocrinology & Metabolism

GA GM9RS

UT WOS:000438590800001

PM 30022843

OA DOAJ Gold

DA 2019-01-08

ER

PT J

AU Sympa, P

Vlachou, E

Kazakos, K

Govina, O

Stamatiou, G

Lavdaniti, M

AF Sympa, Paraskevi

Vlachou, Eugenia

Kazakos, Kyriakos

Govina, Ourania

Stamatiou, George

Lavdaniti, Maria

TI Depression and Self-Efficacy in Patients with Type 2 Diabetes in

Northern Greece

SO ENDOCRINE METABOLIC & IMMUNE DISORDERS-DRUG TARGETS

LA English

DT Article

DE Depression; anxiety; distress; self-efficacy; diabetes mellitus; Greece

ID BODY-MASS INDEX; GLYCEMIC CONTROL; MELLITUS; ANXIETY; ADULTS; DISORDERS;

ADHERENCE; DISTRESS

AB Background: Depression represents one of the most common disorders in diabetic patients and frequently complicates the health care of this population. Poor self-efficacy has been associated with increased depressive symptoms as well as poor glycemic control.

Objective: To assess depression and self-efficacy in adults with type 2 diabetes in Northern Greece and to explore the factors which may affect them in this group of population.

Method: A descriptive study was conducted in a tertiary hospital in the largest city of Northern Greece. The study group included a convenience sample of 170 adults with type 2 diabetes mellitus who completed the General Health Questionnaire-28 (GHQ-28) and the Diabetes Empowerment Scale- short form (DES) questionnaire.

Results: The overall rate of diabetic patients showing psychological distress in the present study was 50.6%. Adults with low and moderate income experienced higher levels of depression and anxiety, compared to those with high economical status (p<0,001). Also, adults who graduated elementary education experienced higher levels of depressive and anxiety symptoms than those with a higher educational level (p =0,038). There was a statistically significant difference between age (p<0.001), type of residence (p=0.019), family status (p=0,002), financial status (p<0.001) and self-efficacy. Also, there was a negative correlation between BMI and self-efficacy (r=-0.206, p<0.001) and a negative correlation between depression and self-efficacy scale (r=-0.439, p<0.001)

Conclusion: The results of the present study highlight the importance of well-planned interventions in order to reduce depression and increase self-efficacy in diabetic adults and to help them further improve their quality of life.

C1 [Sympa, Paraskevi; Kazakos, Kyriakos; Stamatiou, George; Lavdaniti, Maria] Alexander Technol Educ Inst, Nursing Dept, Postgrad Program Diabet Care, Thessaloniki, Greece.

[Vlachou, Eugenia; Govina, Ourania] Technol Educ Inst Athens, Nursing Dept, Athens, Greece.

RP Lavdaniti, M (reprint author), Alexander Technol Educ Inst Thessaloniki, Thessaloniki, Greece.

EM maria_lavdaniti@yahoo.gr

CR Abazarian Elaheh, 2015, Adv Biomed Res, V4, P112, DOI 10.4103/2277-9175.157830

Al-Amer R, 2016, ISSUES MENT HEALTH N, V37, P744, DOI 10.1080/01612840.2016.1208692

Al-Khawaldeh OA, 2012, J DIABETES COMPLICAT, V26, P10, DOI 10.1016/j.jdiacomp.2011.11.002

Anderson RM, 2003, DIABETES CARE, V26, P1641, DOI 10.2337/diacare.26.5.1641-a

Anderson RM, 2000, DIABETES CARE, V23, P739, DOI 10.2337/diacare.23.6.739

Asuzu CC, 2017, J DIABETES COMPLICAT, V31, P169, DOI 10.1016/j.jdiacomp.2016.09.013

Cherrington A, 2010, J BEHAV MED, V33, P81, DOI 10.1007/s10865-009-9233-4

Chew B.H., 2016, PLOS ONE, V22

Collins MM, 2009, DIABETIC MED, V26, P153, DOI 10.1111/j.1464-5491.2008.02648.x

Das-Munshi J, 2007, PSYCHOSOM MED, V69, P543, DOI 10.1097/PSY.0b013e3180cc3062

Dehghan H, 2017, J DIABETES METAB DIS, V16, DOI 10.1186/s40200-016-0285-z

Demmer RT, 2015, PSYCHOSOM MED, V77, P467, DOI 10.1097/PSY.0000000000000169

Devarajooh C, 2017, PLOS ONE, V12, DOI 10.1371/journal.pone.0175096

DeVellis BM, 2001, HANDBOOK OF HEALTH PSYCHOLOGY, P235

Giaglis G.D., 2003, GREEK LANGUAGE 8O PA

Goldberg D., 1978, MANUAL GEN HLTH QUES

Hermanns N, 2005, DIABETIC MED, V22, P293, DOI 10.1111/j.1464-5491.2005.01414.x

Ikeda K, 2003, PSYCHOL REP, V92, P540, DOI 10.2466/PR0.92.2.540-544

Ilioudi Stamatia, 2010, Int J Electron Healthc, V5, P327, DOI 10.1504/IJEH.2010.036205

Indelicato L, 2017, NUTR METAB CARDIOVAS, V27, P300, DOI 10.1016/j.numecd.2017.01.006

Karaiskos D, 2013, INT J CLIN PRACT, V67, P257, DOI 10.1111/ijcp.12112

Kessler RC, 2013, ANNU REV PUBL HEALTH, V34, P119, DOI 10.1146/annurev-publhealth-031912-114409

Mousleh J., 2000, GERMAN J PSYCHIAT, V3, P1

Moutzoukis X., 1990, PSYCHIAT HOSP THESSA

Papathanasiou Athanasia, 2008, Ment Health Fam Med, V5, P85

Rajput Rajesh, 2016, Indian J Endocrinol Metab, V20, P746

Sacco WP, 2007, HEALTH PSYCHOL, V26, P693, DOI 10.1037/0278-6133.26.6.693

Sacco WP, 2005, HEALTH PSYCHOL, V24, P630, DOI 10.1037/0278-6133.6.630

Shao YC, 2017, NEUROPSYCH DIS TREAT, V13, P855, DOI 10.2147/NDT.S128432

Trento M, 2015, ACTA DIABETOL, V52, P1157, DOI 10.1007/s00592-015-0806-0

Willmott SA, 2004, SOC PSYCH PSYCH EPID, V39, P613, DOI 10.1007/s00127-004-0801-1

World Health Organization, 2016, DIABETES

Wu SFV, 2013, NURS HEALTH SCI, V15, P213, DOI 10.1111/nhs.12022

NR 33

TC 1

Z9 1

U1 3

U2 3

PU BENTHAM SCIENCE PUBL LTD

PI SHARJAH

PA EXECUTIVE STE Y-2, PO BOX 7917, SAIF ZONE, 1200 BR SHARJAH, U ARAB

EMIRATES

SN 1871-5303

EI 2212-3873

J9 ENDOCR METAB IMMUNE

JI Endocr. Metab. Immune Disord.-Drug Targets

PY 2018

VL 18

IS 4

BP 371

EP 378

DI 10.2174/1871530317666171120154002

PG 8

WC Endocrinology & Metabolism; Immunology; Pharmacology & Pharmacy

SC Endocrinology & Metabolism; Immunology; Pharmacology & Pharmacy

GA GJ5PP

UT WOS:000435434400012

PM 29165096

DA 2019-01-08

ER

PT J

AU Pernomian, L

Moreira, JD

Gomes, MS

AF Pernomian, Larissa

Moreira, Josimar Dornelas

Gomes, Mayara Santos

TI In the View of Endothelial Microparticles: Novel Perspectives for

Diagnostic and Pharmacological Management of Cardiovascular Risk during

Diabetes Distress

SO JOURNAL OF DIABETES RESEARCH

LA English

DT Review

ID CORONARY-ARTERY-DISEASE; CHRONIC MILD STRESS; ANGIOTENSIN-II;

PSYCHOLOGICAL DISTRESS; MYOCARDIAL-INFARCTION; HYPERTENSIVE PATIENTS;

VASCULAR DYSFUNCTION; DEPRESSIVE SYMPTOMS; CLINICAL DEPRESSION; GLYCEMIC

CONTROL

AB Acute or chronic exposure to diabetes-related stressors triggers a specific psychological and behavior stress syndrome called diabetes distress, which underlies depressive symptoms in most diabetic patients. Distressed and/or depressive diabetic adults exhibit higher rates of cardiovascular mortality and morbidity, which have been correlated to macrovascular complications evoked by diabetic behavior stress. Recent experimental findings clearly point out that oxidative stress accounts for the vascular dysfunction initiated by the exposure to life stressors in diabetic conditions. Moreover, oxidative stress has been described as the main autocrine and paracrine mechanism of cardiovascular damage induced by endothelial microparticles (anuclear ectosomal microvesicles released from injured endothelial cells) in diabetic subjects. Such robust relationship between oxidative stress and cardiovascular diseases strongly suggests a critical role for endothelial microparticles as the primer messengers of the redox-dependent vascular dysfunction underlying diabetes distress. Here, we provide novel perspectives opened in the view of endothelial microparticles as promising diagnostic and pharmacotherapeutic biomarkers of cardiovascular risk in distressed diabetic patients.

C1 [Pernomian, Larissa] Univ Sao Paulo, Fac Pharmaceut Sci Ribeirao Preto, Dept Biosci Appl Pharm, Ribeirao Preto, SP, Brazil.

[Moreira, Josimar Dornelas] Univ Fed Minas Gerais, Fac Pharm, Dept Clin & Toxicol Anal, Belo Horizonte, MG, Brazil.

[Gomes, Mayara Santos] Univ Sao Paulo, Fac Pharmaceut Sci Ribeirao Preto, Dept Phys & Chem, Ribeirao Preto, SP, Brazil.

RP Pernomian, L (reprint author), Univ Sao Paulo, Fac Pharmaceut Sci Ribeirao Preto, Dept Biosci Appl Pharm, Ribeirao Preto, SP, Brazil.

EM l.pernomian@usp.br

FU Coordination for the Improvement of Higher Education Personnel (CAPES);

CAPES (PNPD/CAPES); Sao Paulo Research Foundation (FAPESP, Brazil)

[2014/17740-0, 2013/08216-2, 2012/00640-7, 2012/09019-3, 2009/01005-0]

FX The authors thank Professor Cristina Ribeiro de Barros Cardoso for her

scientific support. This work was supported by the Coordination for the

Improvement of Higher Education Personnel (CAPES), by the National

Postdoctorate Program of CAPES (PNPD/CAPES), and by the Sao Paulo

Research Foundation (FAPESP, Brazil, Grant nos. 2014/17740-0,

2013/08216-2, 2012/00640-7, 2012/09019-3, and 2009/01005-0).

CR Adriaanse MC, 2008, DIABETES CARE, V31, P2268, DOI 10.2337/dc08-1074

Amabile N, 2005, J AM SOC NEPHROL, V16, P3381, DOI 10.1681/ASN.2005050535

Anderson RJ, 2002, INT J PSYCHIAT MED, V32, P235, DOI 10.2190/KLGD-4H8D-4RYL-TWQ8

Burger D, 2016, OXID MED CELL LONGEV, V2016, DOI DOI 10.1155/2016/5047954

Chan S, 2018, TOXICOL APPL PHARM, V338, P148, DOI 10.1016/j.taap.2017.11.005

Chung IM, 2010, ATHEROSCLEROSIS, V213, P109, DOI 10.1016/j.atherosclerosis.2010.08.052

Coco H, 2017, J PHARM PHARMACOL, V69, P52, DOI 10.1111/jphp.12659

Cohen S, 2016, PERSPECT PSYCHOL SCI, V11, P456, DOI 10.1177/1745691616646305

Costa-Ferreira W, 2016, FRONT PHARMACOL, V7, DOI 10.3389/fphar.2016.00262

Dalsgaard EM, 2014, DIABETOLOGIA, V57, P710, DOI 10.1007/s00125-014-3165-4

Deng F., 2016, BIOMED RES INT, V2016, DOI DOI 10.1155/2016/980202627803933

Egede LE, 2005, DIABETES CARE, V28, P1339, DOI 10.2337/diacare.28.6.1339

Esbitt S. A., 2013, SCREENING DEPRESSION

Esposito K, 2007, INT J IMPOT RES, V19, P161, DOI 10.1038/sj.ijir.3901500

Feng B, 2010, ATHEROSCLEROSIS, V208, P264, DOI 10.1016/j.atherosclerosis.2009.06.037

Fisher EB, 2012, J AFFECT DISORDERS, V142, pS56, DOI 10.1016/S0165-0327(12)70009-5

Fisher L, 2014, DIABETIC MED, V31, P764, DOI 10.1111/dme.12428

Fisher L, 2007, DIABETES CARE, V30, P542, DOI 10.2337/dc06-1614

Fisher L, 2010, DIABETES CARE, V33, P1034, DOI 10.2337/dc09-2175

Fisher L, 2010, DIABETES CARE, V33, P23, DOI 10.2337/dc09-1238

Franca CN, 2015, ARQ BRAS CARDIOL, V104, P169, DOI 10.5935/abc.20140210

Gask L, 2011, CHRONIC ILLN, V7, P239, DOI 10.1177/1742395311403636

Gero D, 2013, DIABETES, V62, P953, DOI [10.2337/db12-0789, 10.2337/db12-0789/-/DC1]

Gonzalez JS, 2011, DIABETES CARE, V34, P236, DOI 10.2337/dc10-1970

Groeschel M, 2011, AM J PHYSIOL-RENAL, V300, pF1, DOI 10.1152/ajprenal.00208.2010

Hackett RA, 2016, CURR CARDIOL REP, V18, DOI 10.1007/s11886-016-0771-4

Jansen F, 2013, CARDIOVASC RES, V98, P94, DOI 10.1093/cvr/cvt013

Khuwaja AK, 2010, DIABETOL METAB SYNDR, V2, DOI 10.1186/1758-5996-2-72

Koga H, 2005, J AM COLL CARDIOL, V45, P1622, DOI 10.1016/j.jacc.2005.02.047

Kreider KE, 2017, DIABETES THER, V8, P1, DOI 10.1007/s13300-017-0231-1

Kyritsi EM, 2017, HORM-INT J ENDOCRINO, V16, P42, DOI 10.14310/horm.2002.1718

Lanuti P, 2012, J IMMUNOL METHODS, V380, P16, DOI 10.1016/j.jim.2012.03.007

Lee SK, 2014, THROMB HAEMOSTASIS, V112, P580, DOI 10.1160/TH13-11-0975

Leroyer AS, 2008, DIABETES METAB, V34, pS27, DOI 10.1016/S1262-3636(08)70100-9

Leroyer AS, 2007, J AM COLL CARDIOL, V49, P772, DOI 10.1016/j.jacc.2006.10.053

Lichtenauer M, 2017, CLIN HEMORHEOL MICRO, V67, P101, DOI 10.3233/CH-170273

Lin EHB, 2010, DIABETES CARE, V33, P264, DOI 10.2337/dc09-1068

Lloyd CE, 2010, DIABETIC MED, V27, P234, DOI 10.1111/j.1464-5491.2009.02896.x

Matchkov VV, 2015, AM J PHYSIOL-REG I, V309, pR814, DOI 10.1152/ajpregu.00337.2014

Matsuura N, 2015, AM J PHYSIOL-HEART C, V308, pH1275, DOI 10.1152/ajpheart.00906.2014

Mobarrez F, 2012, THROMB RES, V129, P95, DOI 10.1016/j.thromres.2011.09.027

Moreira JD, 2016, EUR J PHARMACOL, V783, P11, DOI 10.1016/j.ejphar.2016.04.050

Moreira JD, 2015, EUR J PHARMACOL, V765, P503, DOI 10.1016/j.ejphar.2015.09.020

Morel O, 2003, J THROMB HAEMOST, V1, P171, DOI 10.1046/j.1538-7836.2003.00010.x

Morel O, 2006, ARTERIOSCL THROM VAS, V26, P2594, DOI 10.1161/01.ATV.0000246775.14471.26

Nomura S, 2005, J HUM HYPERTENS, V19, P551, DOI 10.1038/sj.jhh.1001863

Nomura S, 2007, J HUM HYPERTENS, V21, P38, DOI 10.1038/sj.jhh.1002100

Nomura S, 2005, J THROMB THROMBOLYS, V18, P177, DOI 10.1007/s11239-005-0343-8

Nomura S, 2016, J ATHEROSCLER THROMB, V23, P1, DOI 10.5551/jat.32326

Nomura S, 2009, J ATHEROSCLER THROMB, V16, P83, DOI 10.5551/jat.E091

Pernomian L, 2012, EUR J PHARMACOL, V678, P15, DOI 10.1016/j.ejphar.2011.12.036

Puddu Paolo, 2010, Can J Cardiol, V26, P140

Roohafza H, 2016, ARYA ATHEROSCLER, V12, P166

Roopan S, 2017, ACTA NEUROPSYCHIATR, V29, P127, DOI 10.1017/neu.2016.54

Roy T, 2012, J AFFECT DISORDERS, V142, pS8, DOI 10.1016/S0165-0327(12)70004-6

Rustad JK, 2011, PSYCHONEUROENDOCRINO, V36, P1276, DOI 10.1016/j.psyneuen.2011.03.005

Sabatier F, 2002, DIABETES, V51, P2840, DOI 10.2337/diabetes.51.9.2840

Sabatier F, 2009, J CELL MOL MED, V13, P454, DOI 10.1111/j.1582-4934.2008.00639.x

Safren SA, 2014, DIABETES CARE, V37, P625, DOI [10.2337/dc13-0816, 10.2337/dc16-er06]

Santilli F, 2016, THROMB HAEMOSTASIS, V116, P220, DOI 10.1160/TH16-03-0176

Scherrer JF, 2011, DIABETES CARE, V34, P1729, DOI 10.2337/dc11-0031

Semenkovich K, 2015, DRUGS, V75, P577, DOI 10.1007/s40265-015-0347-4

Shen JD, 2017, METAB BRAIN DIS, V32, P1163, DOI 10.1007/s11011-017-0017-1

Suades R, 2013, THROMB HAEMOSTASIS, V110, P366, DOI 10.1160/TH13-03-0238

Tanenbaum ML, 2012, DIABETES EDUCATOR, V38, P695, DOI 10.1177/0145721712452795

Tramontano AF, 2004, BIOCHEM BIOPH RES CO, V320, P34, DOI 10.1016/j.bbrc.2004.05.127

Tushuizen ME, 2007, DIABETES CARE, V30, P728, DOI 10.2337/dc06-1473

van Bodegom M, 2017, FRONT CELL NEUROSCI, V11, DOI 10.3389/fncel.2017.00087

van Dooren FEP, 2013, PLOS ONE, V8, DOI 10.1371/journal.pone.0057058

Werner N, 2006, ARTERIOSCL THROM VAS, V26, P112, DOI 10.1161/01.ATV.0000191634.13057.15

Wheatcroft SB, 2013, DIABETES, V62, P698, DOI 10.2337/db12-1530

Winchester RJ, 2016, J DIABETES COMPLICAT, V30, P312, DOI 10.1016/j.jdiacomp.2015.11.010

Yang SJ, 2014, J HYPERTENS, V32, P762, DOI 10.1097/HJH.0000000000000083

Zannas AS, 2017, MOL PSYCHIATR, V22, P640, DOI 10.1038/mp.2017.35

NR 74

TC 0

Z9 0

U1 0

U2 0

PU HINDAWI LTD

PI LONDON

PA ADAM HOUSE, 3RD FLR, 1 FITZROY SQ, LONDON, W1T 5HF, ENGLAND

SN 2314-6745

EI 2314-6753

J9 J DIABETES RES

JI J. Diabetes Res.

PY 2018

AR 9685205

DI 10.1155/2018/9685205

PG 7

WC Endocrinology & Metabolism; Medicine, Research & Experimental

SC Endocrinology & Metabolism; Research & Experimental Medicine

GA GH4WU

UT WOS:000433413000001

PM 29862304

OA DOAJ Gold

DA 2019-01-08

ER

PT J

AU Gillani, SW

Ansari, IA

Zaghloul, HA

Abdul, MIM

Sulaiman, SAS

Baig, MR

Rathore, HA

AF Gillani, Syed Wasif

Ansari, Irfan Altaf

Zaghloul, Hisham A.

Abdul, Mohi Iqbal Mohammad

Sulaiman, Syed Azhar Syed

Baig, Mirza R.

Rathore, Hassaan Anwar

TI Women with Type 1 Diabetes Mellitus: Effect of Disease and

Psychosocial-Related Correlates on Health-Related Quality of Life

SO JOURNAL OF DIABETES RESEARCH

LA English

DT Article

ID CARE; MANAGEMENT; SUPPORT; INSULIN; SCALE

AB Background. This study is aimed at investigating the various disease-specific and health-related psychosocial concepts of HRQOL among insulin-dependent diabetes mellitus (IDDM) and understanding the gender differences in HRQOL among IDDM patients. Methods. A cross-sectional observational study was conducted to assess the effect of health-related and psychosocial correlates on HRQOL of IDDM patients in Penang, Malaysia. The participants were recruited from five governmental diabetic clinics. Patients with insulin use only, IDDM diagnosed at least 1 year earlier, were identified from clinical registers. The sample was then age stratified for 20-64 years, and severe complications (e. g., end-stage renal failure, hemodialysis, and liver cirrhosis) were excluded; a total of 1003 participants were enrolled in the study. Multivariate regression analysis was used to predict the response. Results. A total of 853 (100%) participants were enrolled and completed the study. Women exhibited significantly higher/ better mental health (p < 0 013) and health perception scores (p < 0 001) despite high prevalence of impaired role (49.2%), social (24.2%), and physical (40.5%) functionings as compared to men. Women with longer diabetes exposure and uncontrolled glycemic levels (HbA1c) have poorer HRQOL. Availability of social support showed no significant association with either HRQOL or diabetes distress levels. Diabetes distress levels remained not associated with social support. Women also showed significantly higher association with health perception (15% versus 13% men, p < 0 001) and mental health (13% versus 11% men, p < 0 001) in diabetes-specific psychosocial factors. Thus, among women alone, diabetes-related specific and psychosocial factors explained 15% and 13% of variations in HRQOL extents, respectively. Conclusion. Women exhibit extensive and significant patterns with health-related factors and diabetes-specific psychosocial factors (self-efficacy, social support, and DLC) to improve HRQOL. Also, women have significantly high reported distress levels and low social functioning compared to men.

C1 [Gillani, Syed Wasif; Zaghloul, Hisham A.; Abdul, Mohi Iqbal Mohammad] Taibah Univ, Coll Pharm, Al Madinah Al Munawarah, Saudi Arabia.

[Ansari, Irfan Altaf] Taibah Univ, Coll Med, Dept Pathol, Al Madinah Al Munawarah, Saudi Arabia.

[Sulaiman, Syed Azhar Syed; Rathore, Hassaan Anwar] USM, Sch Pharmaceut Sci, George Town, Malaysia.

[Baig, Mirza R.] Dubai Pharm Coll, Dubai, U Arab Emirates.

RP Gillani, SW (reprint author), Taibah Univ, Coll Pharm, Al Madinah Al Munawarah, Saudi Arabia.

EM wasifgillani@gmail.com

CR Adejoh SO, 2014, SAGE OPEN, V4, DOI 10.1177/2158244014539966

[Anonymous], 2017, MED OUTCOMES STUDY H

Bains SS, 2011, DIABETES TECHNOL THE, V13, P335, DOI 10.1089/dia.2010.0160

Chao Jingdong, 2005, Res Social Adm Pharm, V1, P508, DOI 10.1016/j.sapharm.2005.09.002

Domgaard M, 2015, POSTGRAD MED, V127, P438, DOI 10.1080/00325481.2015.1045815

FERRARO LA, 1987, PSYCHOL REP, V61, P763, DOI 10.2466/pr0.1987.61.3.763

Garfield S, 2011, BMC MED RES METHODOL, V11, DOI 10.1186/1471-2288-11-149

Gillani SW, 2018, DIABETOL METAB SYNDR, V10, DOI 10.1186/s13098-018-0325-6

Gillani SW, 2017, CARDIOVASC DIABETOL, V16, DOI 10.1186/s12933-017-0584-9

Gillani SW, 2017, DIABETOL METAB SYNDR, V9, DOI 10.1186/s13098-017-0257-6

Gillani SW, 2017, ACTA PHARMACEUT, V67, P71, DOI 10.1515/acph-2017-0008

Gillani SW, 2016, CURR PHARM DESIGN, V22, P6469, DOI 10.2174/1381612822666160813235704

Gillani SW, 2016, CURR DIABETES REV, V12, P279, DOI 10.2174/1573399811666150520094631

HARRIS R, 1985, SOUTHERN MED J, V78, P162, DOI 10.1097/00007611-198502000-00011

Hays R. D., RAND CORPORATION MON

Hemkens LG, 2009, DIABETOLOGIA, V52, P1732, DOI 10.1007/s00125-009-1418-4

International Diabetes Federation (IDF), 2013, AB DIAB 2013

Inzucchi SE, 2015, DIABETES CARE, V38, P140, DOI 10.2337/dc14-2441

La Greca AM, 2002, J PEDIATR PSYCHOL, V27, P665, DOI 10.1093/jpepsy/27.8.665

Osborn CY, 2010, PATIENT EDUC COUNS, V79, P49, DOI 10.1016/j.pec.2009.07.016

Persell SD, 2004, PREV MED, V39, P746, DOI 10.1016/j.ypmed.2004.02.045

Santos LM, 2015, SOC SCI RES, V50, P139, DOI 10.1016/j.ssresearch.2014.11.009

SCHAFER LC, 1986, DIABETES CARE, V9, P179, DOI 10.2337/diacare.9.2.179

Tabak AG, 2009, LANCET, V373, P2215, DOI 10.1016/S0140-6736(09)60619-X

Vartanian LR, 2007, AM J PUBLIC HEALTH, V97, P667, DOI 10.2105/AJPH.2005.083782

von Arx LB, 2014, PATIENT, V7, P283, DOI 10.1007/s40271-014-0057-0

Welch GW, 1997, DIABETES CARE, V20, P760, DOI 10.2337/diacare.20.5.760

NR 27

TC 0

Z9 0

U1 2

U2 2

PU HINDAWI LTD

PI LONDON

PA ADAM HOUSE, 3RD FLR, 1 FITZROY SQ, LONDON, W1T 5HF, ENGLAND

SN 2314-6745

EI 2314-6753

J9 J DIABETES RES

JI J. Diabetes Res.

PY 2018

AR 4079087

DI 10.1155/2018/4079087

PG 10

WC Endocrinology & Metabolism; Medicine, Research & Experimental

SC Endocrinology & Metabolism; Research & Experimental Medicine

GA GF5PX

UT WOS:000432021100001

PM 29854822

OA DOAJ Gold

DA 2019-01-08

ER

PT J

AU Aljuaid, MO

Almutairi, AM

Assiri, MA

Almalki, DM

Alswat, K

AF Aljuaid, Majed O.

Almutairi, Abdulmajeed M.

Assiri, Mohammed A.

Almalki, Dhifallah M.

Alswat, Khaled

TI Diabetes-Related Distress Assessment among Type 2 Diabetes Patients

SO JOURNAL OF DIABETES RESEARCH

LA English

DT Article

ID GLYCEMIC CONTROL; CARE LEVEL; DEPRESSION; MELLITUS; PEOPLE; BURDEN

AB Background and Objectives. Diabetes is one of the most common chronic diseases; it is a debilitating and hard to live with. Diabetes-related distress (DRD) refers to the emotional and behavioral changes caused by diabetes. Our study aims to assess the prevalence of DRD among type 2 diabetes (T2D) patients using Diabetes Distress Scale-17 items (DDS-17) and its relation to complications and treatment modalities. Methods. A cross-sectional study of adult T2D patients with follow-up visits at the Diabetes and Endocrinology Center in Taif, Saudi Arabia, between January and July 2017. We excluded patients with other forms of diabetes, untreated hypothyroidism, and psychiatric illness. The total score of DDS-17 was calculated by summing the 17 items' results and then dividing the total by 17. If the total score was >2, then it was considered as clinically significant results (moderate distress), but if it is >= 3, then it is classified as a high distress. Results. A total of 509 T2D patients with a mean age of 58 +/- 14 years were included. The majority of participants were male, married, not college educated, and reported a sedentary lifestyle. We found 25% of the screened T2D patients have moderate to high DRD. Regarding the DRD components, emotional distress was the most prevalent followed by physician-related distress. HabA1c was significantly higher in those with high combined distress and high emotional distress compared to those with mild/moderate distress (p = 0 015 and 0.030, resp.). Conclusion. Our study shows that DRD is a medically relevant issue that clinicians need to address. Despite observing a low prevalence of DRD compared to other studies, we found significant correlations between DRD scores and HabA1c, triglyceride levels, BMI, T2D duration, and interval between visits.

C1 [Aljuaid, Majed O.; Almutairi, Abdulmajeed M.; Assiri, Mohammed A.; Alswat, Khaled] Taif Univ, Sch Med, At Taif, Saudi Arabia.

[Almalki, Dhifallah M.] Prince Mansoure Hosp, Diabet & Endocrinol Ctr, At Taif, Saudi Arabia.

RP Alswat, K (reprint author), Taif Univ, Sch Med, At Taif, Saudi Arabia.

EM dr_kswat@hotmail.com

OI Aljuaid, Majed/0000-0001-9529-4803

CR Alvani S. Reza, 2016, PRACTICE CLIN PSYCHO, V3, P167

Berry E, 2015, POSTGRAD MED J, V91, P278, DOI 10.1136/postgradmedj-2014-133017

Chew BH, 2016, PLOS ONE, V11, DOI 10.1371/journal.pone.0152095

Chew BH, 2015, HEALTH QUAL LIFE OUT, V13, DOI 10.1186/s12955-015-0384-4

Egede LE, 2004, DIABETES CARE, V27, P1751, DOI 10.2337/diacare.27.7.1751

Fisher L, 2008, ANN FAM MED, V6, P246, DOI 10.1370/afm.842

Fisher L, 2015, J DIABETES COMPLICAT, V29, P572, DOI 10.1016/j.jdiacomp.2015.01.012

Fisher L, 2010, DIABETES CARE, V33, P1034, DOI 10.2337/dc09-2175

Fritschi C, 2010, J PSYCHOSOM RES, V69, P33, DOI 10.1016/j.jpsychores.2010.01.021

Funnell MM, 2006, CLIN DIABETES, V24, P154

Gideon E., 2014, P NAT C UND RES NCUR, P204

Islam MR, 2013, INT J MED BIOMED RES, V2, P113, DOI DOI 10.14194/IJMBR.224

Kalra S, 2017, J PAK MED ASSOC, V67, P1116

Kuniss N, 2017, ACTA DIABETOL, V54, P471, DOI 10.1007/s00592-017-0972-3

Kuniss N, 2016, EXP CLIN ENDOCR DIAB, V124, P307, DOI 10.1055/s-0042-103685

Kuniss N., 2017, EXPT CLIN ENDOCRINOL

Mathers CD, 2006, PLOS MED, V3, DOI 10.1371/journal.pmed.0030442

Nicolucci A, 2013, DIABETIC MED, V30, P767, DOI 10.1111/dme.12245

Ogurtsova K, 2017, DIABETES RES CLIN PR, V128, P40, DOI 10.1016/j.diabres.2017.03.024

Polonsky WH, 2005, DIABETES CARE, V28, P626, DOI 10.2337/diacare.28.3.626

Reimer A, 2017, PLOS ONE, V12, DOI 10.1371/journal.pone.0181218

Tol A, 2012, J DIABETES METAB DIS, V11, DOI 10.1186/2251-6581-11-20

Wong EM, 2017, CAN J DIABETES, V41, P362, DOI 10.1016/j.jcjd.2016.11.006

Zhou HH, 2017, PSYCHIAT RES, V252, P45, DOI 10.1016/j.psychres.2017.02.049

NR 24

TC 0

Z9 0

U1 1

U2 1

PU HINDAWI LTD

PI LONDON

PA ADAM HOUSE, 3RD FLR, 1 FITZROY SQ, LONDON, W1T 5HF, ENGLAND

SN 2314-6745

EI 2314-6753

J9 J DIABETES RES

JI J. Diabetes Res.

PY 2018

AR 7328128

DI 10.1155/2018/7328128

PG 10

WC Endocrinology & Metabolism; Medicine, Research & Experimental

SC Endocrinology & Metabolism; Research & Experimental Medicine

GA GC2EO

UT WOS:000429595200001

PM 29770340

OA DOAJ Gold, Green Published

DA 2019-01-08

ER

PT J

AU Madva, EN

Gomez-Bernal, F

Millstein, RA

Celano, CM

Park, ER

Mastromauro, CA

Albanese, AM

Beale, EE

Huffman, JC

AF Madva, Elizabeth N.

Gomez-Bernal, Federico

Millstein, Rachel A.

Celano, Christopher M.

Park, Elyse R.

Mastromauro, Carol A.

Albanese, Ariana M.

Beale, Eleanor E.

Huffman, Jeff C.

TI Magnitude and sources of distress in mid-life adults with chronic

medical illness: an exploratory mixed-methods analysis

SO PSYCHOLOGY HEALTH & MEDICINE

LA English

DT Article

DE Acute coronary syndrome; chronic medical conditions; diabetes; heart

failure; mid-life

ID CORONARY-HEART-DISEASE; POSITIVE-AFFECT; PHYSICAL-ACTIVITY; HEALTH

BEHAVIORS; UNITED-STATES; OLDER-ADULTS; MORTALITY; DEPRESSION; OPTIMISM;

ANXIETY

AB Most mid-life adults have at least one chronic medical condition (CMC) and are at risk for developing additional CMCs. Stressors specific to this life stage may contribute to CMC development by hindering healthy behaviors. The goal of this study was to compare sources and intensity of distress, as they relate to health behaviors, between mid-life and non-mid-life adults with CMCs. We utilized a mixed-methods approach by analyzing quantitative self-report measures of psychiatric symptoms and psychological well-being, with in-depth, semi-structured qualitative interviews to identify sources of stress in three cohorts of patients with CMCs (heart failure, type 2 diabetes, and coronary artery disease). Between-group differences on self-report measures were compared via independent samples t-tests and relevant themes from interview transcripts were compared via chi-square analysis. We found that mid-life participants (n = 30) reported greater psychological distress (depression/anxiety) than non-mid-life (n = 62) participants (Hospital Anxiety and Depression Scale scores 13.8 [SD 7.3] vs. 10.6 [SD 6.6]; t(90)=2.13; p = .035), and qualitative analysis revealed several specific sources of stress significantly more common (p < .001) in mid-life adults. Interventions targeting the needs of this population could reduce distress, improve health behaviors, and have a major impact on public health.

C1 [Madva, Elizabeth N.; Millstein, Rachel A.; Celano, Christopher M.; Park, Elyse R.; Huffman, Jeff C.] Harvard Med Sch, Dept Psychiat, Boston, MA 02115 USA.

[Madva, Elizabeth N.; Gomez-Bernal, Federico; Millstein, Rachel A.; Celano, Christopher M.; Park, Elyse R.; Mastromauro, Carol A.; Albanese, Ariana M.; Beale, Eleanor E.; Huffman, Jeff C.] Massachusetts Gen Hosp, Dept Psychiat, Boston, MA 02114 USA.

RP Huffman, JC (reprint author), Harvard Med Sch, Dept Psychiat, Boston, MA 02115 USA.; Huffman, JC (reprint author), Massachusetts Gen Hosp, Dept Psychiat, Boston, MA 02114 USA.

EM jhuffman@partners.org

FU NHLBI NIH HHS [R01 HL113272, K23 HL135277, K23 HL123607]; NIDDK NIH HHS

[R21 DK109313]

CR Almeida DM, 2004, MAC FDN MEN, P425

Badley EM, 2010, ARTHRIT CARE RES, V62, P308, DOI 10.1002/acr.20100

Blackwell DL, 2012, VITAL HLTH STAT, V10, P1

Boehm JK, 2013, AM J CARDIOL, V111, P1425, DOI 10.1016/j.amjcard.2013.01.292

Bolier L, 2013, BMC PUBLIC HEALTH, V13, DOI 10.1186/1471-2458-13-119

Bouchard C, 1993, PHYS ACTIVITY FITNES

Boylan JM, 2015, PSYCHOSOM MED, V77, P548, DOI 10.1097/PSY.0000000000000192

Brim O. G., 2004, HLTH ARE WE NATL STU

CDC, 2011, DIAB SUCC OPP POP BA

CDC, 2009, CTR DIS CONTR PREV C

CDC, 2012, NAT CTR HLTH STAT HL

Celano CM, 2013, CURR DIABETES REP, V13, P917, DOI 10.1007/s11892-013-0430-8

Celano CM, 2013, CIRC-CARDIOVASC QUAL, V6, P498, DOI 10.1161/CIRCOUTCOMES.111.000049

Charlson FJ, 2016, MENTAL NEUROLOGICAL, V4, P41, DOI [10.1596/978-1-4648-0426-7, DOI 10.1596/978-1-4648-0426-7]

Chida Y, 2008, PSYCHOSOM MED, V70, P741, DOI 10.1097/PSY.0b013e31818105ba

CORYELL W, 1988, PSYCHIAT CLIN N AM, V11, P433

Dainese SM, 2011, GEROPSYCH, V24, P19, DOI 10.1024/1662-9647/a000032

Davis JC, 2015, BMC GERIATR, V15, DOI 10.1186/s12877-015-0076-2

Gathuru I. M., 2016, SOCIAL PHARM J SPJ, V1

Gillette DB, 2015, J GERIATR PHYS THER, V38, P194, DOI 10.1519/JPT.0000000000000041

Giltay EJ, 2004, ARCH GEN PSYCHIAT, V61, P1126, DOI 10.1001/archpsyc.61.11.1126

GRZYWACZ J, 2000, EC NEUROSCI, V2, P41

Guest G, 2006, FIELD METHOD, V18, P59, DOI 10.1177/1525822X05279903

Hevey D, 2014, J BEHAV MED, V37, P300, DOI 10.1007/s10865-012-9487-0

Huffman J. C., 2015, J DIABETES RES, V2015

Huffman JC, 2016, J HAPPINESS STUD, V17, P1985, DOI 10.1007/s10902-015-9681-1

Lachman ME, 2015, INT J BEHAV DEV, V39, P20, DOI 10.1177/0165025414533223

LINN MW, 1985, AM J PUBLIC HEALTH, V75, P502, DOI 10.2105/AJPH.75.5.502

McPhail SM, 2014, CLIN INTERV AGING, V9, P2113, DOI 10.2147/CIA.S72731

Pederson JL, 2016, J HOSP MED, V11, P373, DOI 10.1002/jhm.2547

Peterson JC, 2012, ARCH INTERN MED, V172, P329, DOI 10.1001/archinternmed.2011.1311

Piazza JR, 2007, J GERONTOL B-PSYCHOL, V62, pP313, DOI 10.1093/geronb/62.6.P313

Pratt Laura A, 2014, NCHS Data Brief, P1

Saint Onge JM, 2014, J GERONTOL B-PSYCHOL, V69, P622, DOI 10.1093/geronb/gbu009

SCHEIER MF, 1994, J PERS SOC PSYCHOL, V67, P1063, DOI 10.1037/0022-3514.67.6.1063

Sin NL, 2015, PSYCHOSOM MED, V77, P1058, DOI 10.1097/PSY.0000000000000238

Steptoe A, 2015, LANCET, V385, P640, DOI 10.1016/S0140-6736(13)61489-0

Steptoe A, 2009, J PERS, V77, P1747, DOI 10.1111/j.1467-6494.2009.00599.x

Sun Q, 2010, ARCH INTERN MED, V170, P194, DOI 10.1001/archinternmed.2009.503

Tsenkova VK, 2016, ANN BEHAV MED, V50, P836, DOI 10.1007/s12160-016-9810-z

Valenstein M, 2001, ANN INTERN MED, V134, P345, DOI 10.7326/0003-4819-134-5-200103060-00007

Walker ER, 2015, JAMA PSYCHIAT, V72, P334, DOI 10.1001/jamapsychiatry.2014.2502

WATSON D, 1988, J PERS SOC PSYCHOL, V54, P1063, DOI 10.1037/0022-3514.54.6.1063

White DK, 2012, ARTHRIT CARE RES, V64, P1312, DOI 10.1002/acr.21694

ZIGMOND AS, 1983, ACTA PSYCHIAT SCAND, V67, P361, DOI 10.1111/j.1600-0447.1983.tb09716.x

NR 45

TC 0

Z9 0

U1 1

U2 3

PU ROUTLEDGE JOURNALS, TAYLOR & FRANCIS LTD

PI ABINGDON

PA 2-4 PARK SQUARE, MILTON PARK, ABINGDON OX14 4RN, OXON, ENGLAND

SN 1354-8506

EI 1465-3966

J9 PSYCHOL HEALTH MED

JI Psychol. Health Med.

PY 2018

VL 23

IS 5

BP 555

EP 566

DI 10.1080/13548506.2017.1384554

PG 12

WC Public, Environmental & Occupational Health

SC Public, Environmental & Occupational Health

GA GB4NB

UT WOS:000429035800009

PM 28984158

DA 2019-01-08

ER

PT J

AU Pinidiyapathirage, J

O'Shannessy, M

Harte, J

Brumby, S

Kitchener, S

AF Pinidiyapathirage, Janani

O'Shannessy, Megan

Harte, Jane

Brumby, Susan

Kitchener, Scott

TI Chronic Disease and Health Risk Behaviors Among Rural Agricultural

Workforce in Queensland

SO JOURNAL OF AGROMEDICINE

LA English

DT Article

DE Agricultural workers; behavioral risk factors; chronic disease; rural

Queensland

ID MALE FARMERS; AUSTRALIA

AB Introduction: Little is known of the lifestyle behaviors and prevalence of chronic disease in the Australian agricultural workforce. This study aimed to assess behavioral risk factors and the prevalence of chronic disease among attendees of agricultural events in rural Queensland. Methods: Data on lifestyle risk factors and prevalence of diabetes and cardiovascular diseases were collected from participants in four separate cross-sectional studies in rural southern Queensland. Anthropometric measures, blood pressure, serum cholesterol, and glucose levels of consenting participants were assessed by trained medical students under the supervision of rural clinicians. Data were analyzed using SPSS 22 statistical software package and t-tests and chisquare tests were used to compare differences between groups. Results: A total of 702 attendees participated; the majority were agricultural workers (n = 393). Greater psychological distress was reported among participants from these rural communities (42%) than in the Australian population (31%); however, levels of psychological distress was similar between agricultural workers and others in the sample. Fewer people in these agricultural communities reported smoking (10%), and they reported being more active (86%) than the average Australian, but a greater proportion reported high-risk alcohol consumption (53%) and were found to be hypertensive (31%). These findings were accentuated among agricultural workers. Conclusion: This method of investigation both raises awareness in the community and identifies health risks for further management in a group that has otherwise been poorly defined. Resident agricultural workers have different health risks and behaviors, though psychological distress appears to be borne across these communities.

C1 [Pinidiyapathirage, Janani; Harte, Jane; Kitchener, Scott] Univ Southern Queensland, Agr Hlth & Med Res Grp, Toowoomba, Qld, Australia.

[O'Shannessy, Megan; Harte, Jane; Kitchener, Scott] Griffith Univ, Rural Hlth Stream, Toowoomba, Qld, Australia.

[Brumby, Susan] Deakin Univ, Sch Med, Waurn Ponds, Australia.

[Brumby, Susan] Western Dist Hlth Serv, Natl Ctr Farmer Hlth, Hamilton, Vic, Australia.

RP Kitchener, S (reprint author), Griffith Univ, Rural Stream Campus,POB 2076, Toowoomba, Qld 4350, Australia.

EM s.kitchener@qrme.org.au

FU Queensland Rural Medical Education; Griffith Rural Health Stream

FX This work was supported by the Queensland Rural Medical Education;

Griffith Rural Health Stream.

CR Australian Bureau of Statistics, 2014, AUSTR HLTH SURV BIOM

Australian Bureau of Statistics, 2015, NAT HLTH SURV 1 RES

Australian Institute of Health and Welfare (AIHW), 2008, RURAL HLTH SERIES, V9

Brumby SA, 2009, RURAL REMOTE HEALTH, V9

Brumby S, 2013, J RURAL HEALTH, V29, P311, DOI 10.1111/jrh.12001

Chen L, 2010, MED J AUSTRALIA, V192, P197

Fragar L, 2011, AUST J RURAL HEALTH, V19, P179, DOI 10.1111/j.1440-1584.2011.01209.x

HEGNEY D, 1993, AUST J RURAL HEALTH, V1, P27, DOI DOI 10.1111/J.1440-1584.1993.TB00077.X

Kennedy A, 2014, RURAL REMOTE HEALTH, V14

Kessler RC, 2003, ARCH GEN PSYCHIAT, V60, P184, DOI 10.1001/archpsyc.60.2.184

Langsted A, 2008, CIRCULATION, V118, P2047, DOI 10.1161/CIRCULATIONAHA.108.804146

Lower T, 2011, J AGROMEDICINE, V16, P292, DOI 10.1080/1059924X.2011.605726

Miller PG, 2010, AUST J RURAL HEALTH, V18, P110, DOI 10.1111/j.1440-1584.2010.01133.x

Monaghan N, 2017, J AGROMEDICINE, V22, P100, DOI 10.1080/1059924X.2017.1282907

Morrissey SA, 2007, AUST J RURAL HEALTH, V15, P120, DOI 10.1111/j.1440-1584.2007.00865.x

MUELLEMAN RL, 1993, J TRAUMA, V35, P717, DOI 10.1097/00005373-199311000-00011

National Health and Medical Research Council, 2009, AUSTR GUID RED HLTH

Roy P, 2013, AUST J RURAL HEALTH, V21, P3, DOI 10.1111/ajr.12008

Smith KB, 2008, AUST J RURAL HEALTH, V16, P56, DOI 10.1111/j.1440-1584.2008.00953.x

Sorensen Tony, 2011, International Journal of Foresight and Innovation Policy, V7, P192, DOI 10.1504/IJFIP.2011.040074

Thomas SL, 2014, BMC FAM PRACT, V15, DOI 10.1186/1471-2296-15-143

World Health Organisation, 2006, BMI CLASS

NR 22

TC 1

Z9 1

U1 2

U2 3

PU TAYLOR & FRANCIS INC

PI PHILADELPHIA

PA 530 WALNUT STREET, STE 850, PHILADELPHIA, PA 19106 USA

SN 1059-924X

EI 1545-0813

J9 J AGROMEDICINE

JI J. Agromedicine

PY 2018

VL 23

IS 1

BP 32

EP 39

DI 10.1080/1059924X.2017.1387634

PG 8

WC Public, Environmental & Occupational Health

SC Public, Environmental & Occupational Health

GA FZ8FJ

UT WOS:000427842100008

PM 28976267

DA 2019-01-08

ER

PT J

AU Dirik, G

Gocek-Yorulmaz, E

AF Dirik, Gulay

Gocek-Yorulmaz, Ezgi

TI Positive Sides of the Disease: Posttraumatic Growth in Adults with Type

2 Diabetes

SO BEHAVIORAL MEDICINE

LA English

DT Article

DE posttraumatic growth; perceived social support; self-efficacy; coping

and perceived expressed emotions

ID STRESS-RELATED GROWTH; PERCEIVED SOCIAL SUPPORT; EXPRESSED EMOTION;

BREAST-CANCER; COPING STRATEGIES; SELF-EFFICACY; PREVALENCE;

DETERMINANTS; METAANALYSIS; SURVIVORS

AB Type 2 diabetes is a chronic disease that can causes positive changes, as well as psychological distress. The aim of the this study is to investigate the posttraumatic growth level of adults with type 2 diabetes and to identify the relationship among perceived social support, coping, diabetes management self-efficacy, perceived expressed emotions, and PTG in diabetes patients from non-western, developing country. The findings revealed that patients experience a moderate level of posttraumatic growth. Regression analyses showed that the severity of the disease, educational level, perceived social support, problem-focused coping, optimistic coping and the criticism/hostility dimension of expressed emotions were associated significantly with posttraumatic growth. In the conclusion part, these findings were consistent with the posttraumatic growth theory. The study reveals that professionals aiming to increase the posttraumatic growth of diabetes patients may need to focus on and facilitate perceived social support, adaptive coping strategies, and perceived expressed emotions to facilitate growth experiences.

C1 [Dirik, Gulay] Dokuz Eylul Univ, Dept Psychol, Izmir, Turkey.

[Gocek-Yorulmaz, Ezgi] Izmir Univ, Dept Psychol, Izmir, Turkey.

RP Dirik, G (reprint author), Dokuz Eylul Univ, Dept Psychol, Izmir, Turkey.

EM gdirik2@gmail.com

CR American Diabetes Association, 2015, DIAB BAS

Anderson RJ, 2001, DIABETES CARE, V24, P1069, DOI 10.2337/diacare.24.6.1069

Barrowclough C, 2003, CLIN PSYCHOL REV, V23, P849, DOI 10.1016/S0272-7358(03)00075-8

Batu G, 2008, THESIS

Bellizzi KM, 2006, HEALTH PSYCHOL, V25, P47, DOI 10.1037/0278-6133.25.1.47

Benight CC, 2004, BEHAV RES THER, V42, P1129, DOI 10.1016/j.brat.2003.08.008

Berksun O., 1992, THESIS

Carver CS, 1998, J SOC ISSUES, V54, P245, DOI 10.1111/0022-4537.641998064

Collins MM, 2009, DIABETIC MED, V26, P153, DOI 10.1111/j.1464-5491.2008.02648.x

Cordova MJ, 2001, HEALTH PSYCHOL, V20, P176, DOI 10.1037//0278-6133.20.3.176

Dirik G, 2008, J CLIN PSYCHOL MED S, V15, P193, DOI 10.1007/s10880-008-9115-x

EKER D, 1995, SOC PSYCH PSYCH EPID, V30, P121, DOI 10.1007/BF00802040

FOLKMAN S, 1985, J PERS SOC PSYCHOL, V48, P150, DOI 10.1037/0022-3514.48.1.150

Helgeson VS, 2008, J PEDIATR PSYCHOL, V33, P497, DOI 10.1093/jpepsy/jsm081

Jansen L, 2011, BRIT J CANCER, V105, P1158, DOI 10.1038/bjc.2011.335

Karanci AN, 2007, STRESS HEALTH, V23, P315, DOI 10.1002/smi.1154

Karanci NA, 1999, N AM J PSYCHOL, V1, P189

Karlsen B., 2004, PSYCHOL HEALTH MED, V9, P53

Kesimci A, 2005, CURR PSYCHOL, V24, P68, DOI 10.1007/s12144-005-1005-x

King S, 2000, SCHIZOPHR RES, V45, P65, DOI 10.1016/S0920-9964(99)00174-7

Kirk JK, 2013, EXPERT REV ENDOCRINO, V8, P379, DOI 10.1586/17446651.2013.811895

Koch T, 2000, J CLIN NURS, V9, P247, DOI 10.1046/j.1365-2702.2000.00341.x

Kroo A, 2011, J LOSS TRAUMA, V16, P440, DOI 10.1080/15325024.2011.575705

Lotfi-Kashani F, 2014, IRAN J CANCER PREV, V7, P115

o Usta-Yeilbalkan, 2001, THESIS

o Yalcnkaya Alkar, 2014, J COGN BEHAV PSYCHOT, V3, P23

Polatinsky S, 2000, J TRAUMA STRESS, V13, P709, DOI 10.1023/A:1007870419116

Prati G, 2009, J LOSS TRAUMA, V14, P364, DOI 10.1080/15325020902724271

Samuel-Hodge CD, 2008, DIABETES EDUCATOR, V34, P501, DOI 10.1177/0145721708316946

Satman I, 2002, DIABETES CARE, V25, P1551, DOI 10.2337/diacare.25.9.1551

Senol-Durak E, 2014, SOC INDIC RES, V118, P729, DOI 10.1007/s11205-013-0435-3

Sheikh AI, 2004, J CLIN PSYCHOL MED S, V11, P265, DOI 10.1023/B:JOCS.0000045346.76242.73

STEVENSON K, 1991, PSYCHOTHER PSYCHOSOM, V55, P170, DOI 10.1159/000288426

Tedeschi R. G., 1995, TRAUMA TRANSFORMATIO

Tedeschi RG, 1996, J TRAUMA STRESS, V9, P455, DOI 10.1007/BF02103658

THOITS PA, 1986, J CONSULT CLIN PSYCH, V54, P416, DOI 10.1037//0022-006X.54.4.416

Updegraff JA, 2002, PERS SOC PSYCHOL B, V28, P382, DOI 10.1177/0146167202286009

van der Bijl J, 1999, J ADV NURS, V30, P352, DOI 10.1046/j.1365-2648.1999.01077.x

Wearden AJ, 2000, J PSYCHOSOM RES, V49, P125, DOI 10.1016/S0022-3999(00)00141-0

WILD ND, 2003, J AGGRESS MALTREAT T, V8, P97, DOI DOI 10.1300/J146V08N04_05

Wild S, 2004, DIABETES CARE, V27, P1047, DOI 10.2337/diacare.27.5.1047

Yamakawa M, 2008, INT J NURS STUD, V45, P1032, DOI 10.1016/j.ijnurstu.2007.05.003

Yorulmaz H, 2010, PROCD SOC BEHV, V5, P2313, DOI 10.1016/j.sbspro.2010.07.456

ZIMET GD, 1988, J PERS ASSESS, V52, P30, DOI 10.1207/s15327752jpa5201_2

NR 44

TC 1

Z9 1

U1 3

U2 7

PU ROUTLEDGE JOURNALS, TAYLOR & FRANCIS LTD

PI ABINGDON

PA 2-4 PARK SQUARE, MILTON PARK, ABINGDON OX14 4RN, OXON, ENGLAND

SN 0896-4289

EI 1940-4026

J9 BEHAV MED

JI Behav. Med.

PY 2018

VL 44

IS 1

BP 1

EP 10

DI 10.1080/08964289.2016.1173635

PG 10

WC Behavioral Sciences; Psychiatry

SC Behavioral Sciences; Psychiatry

GA FX0VU

UT WOS:000425766200001

PM 27115317

DA 2019-01-08

ER

PT J

AU Van Buren, DJ

Wilfley, DE

Marcus, MD

Anderson, B

Abramson, NW

Berkowitz, R

Ievers-Landis, C

Trief, P

Yasuda, P

Hirst, K

AF Van Buren, Dorothy J.

Wilfley, Denise E.

Marcus, Marsha D.

Anderson, Barbara

Abramson, Natalie Walders

Berkowitz, Robert

Ievers-Landis, Carolyn

Trief, Paula

Yasuda, Patrice

Hirst, Kathryn

CA TODAY Study Grp

TI Depressive symptoms and glycemic control in youth with type 2 diabetes

participating in the TODAY clinical trial

SO DIABETES RESEARCH AND CLINICAL PRACTICE

LA English

DT Article

DE Adolescent; Depressive symptoms; Type 2 diabetes

ID ADOLESCENTS; DISTRESS; CHILDREN

AB The relationship between depressive symptoms and glycemic control in youth with type 2 diabetes was assessed at baseline (n = 682), 6, and/or 24 months (n = 576). Neither baseline nor persistence of depressive symptoms was significantly associated with maintenance of glycemic control. Nevertheless, depressive symptoms were common, suggesting the importance of repeated screening. (C) 2017 Elsevier B.V. All rights reserved.

C1 [Van Buren, Dorothy J.; Wilfley, Denise E.] Washington Univ, Sch Med, St Louis, MO 63130 USA.

[Marcus, Marsha D.] Univ Pittsburgh, Sch Med, Pittsburgh, PA 15260 USA.

[Anderson, Barbara] Baylor Coll Med, Houston, TX 77030 USA.

[Abramson, Natalie Walders] Univ Colorado, Sch Med, Dept Pediat, Boulder, CO 80309 USA.

[Berkowitz, Robert] Univ Penn, Philadelphia, PA 19104 USA.

[Ievers-Landis, Carolyn] Case Western Reserve Univ, Sch Med, Cleveland, OH 44106 USA.

[Trief, Paula] SUNY Upstate Med Univ, Syracuse, NY 13210 USA.

[Yasuda, Patrice] Childrens Hosp Los Angeles, Los Angeles, CA 90027 USA.

[Hirst, Kathryn] George Washington Univ, Biostat Ctr, 6110 Execut Blvd,Suite 750, Rockville, MD 20852 USA.

RP Hirst, K (reprint author), George Washington Univ, Biostat Ctr, 6110 Execut Blvd,Suite 750, Rockville, MD 20852 USA.

EM today@bsc.gwu.edu

OI Trief, Paula/0000-0002-2827-5722

FU NIDDK; NIH Office of the Director [U01-DK61212, U01-DK61230,

U01-DK61239, U01-DK61242, U01-DK61254]; National Center for Research

Resources (NCRR) General Clinical Research Centers Program

[M01-RR00036]; NCRR Clinical and Translational Science Awards

[UL1-RR024992]; Becton; Dickinson and Company; Bristol-Myers Squibb; Eli

Lilly and Company; GlaxoSmithKline; LifeScan, Inc.; Pfizer; Sanofi

Aventis

FX This work was completed with funding from NIDDK and the NIH Office of

the Director through grants U01-DK61212, U01-DK61230, U01-DK61239,

U01-DK61242, and U01-DK61254; the National Center for Research Resources

(NCRR) General Clinical Research Centers Program through grants

M01-RR00036 (Washington University School of Medicine); and from the

NCRR Clinical and Translational Science Awards through grants

UL1-RR024992 (Washington University in St. Louis). The content is solely

the responsibility of the authors and does not necessarily represent the

official views of the National Institutes of Health. The NIDDK project

office was involved in all aspects of the study, including: design and

conduct; collection, management, analysis, and interpretation of the

data; review and approval of the manuscript; and decision to submit the

manuscript for publication.; The TODAY Study Group thanks the following

companies for donations in support of the study's efforts: Becton,

Dickinson and Company; Bristol-Myers Squibb; Eli Lilly and Company;

GlaxoSmithKline; LifeScan, Inc.; Pfizer; Sanofi Aventis. We also

gratefully acknowledge the participation and guidance of the American

Indian partners associated with the clinical center located at the

University of Oklahoma Health Sciences Center, including members of the

Absentee Shawnee Tribe, Cherokee Nation, Chickasaw Nation, Choctaw

Nation of Oklahoma, and Oklahoma City Area Indian Health Service; the

opinions expressed in this paper are those of the authors and do not

necessarily reflect the views of the respective Tribal and Indian Health

Service Institution Review Boards or their members.

CR [Anonymous], 2016, DIABETES CARE, V39, pS1, DOI 10.2337/dc16-S001

Beck AT, 1996, BECK DEPRESSION INVE

Dever M, 2016, J PEDIATR NURS, V31, P117, DOI 10.1016/j.pedn.2015.11.004

Fisher L, 2012, DIABETES CARE, V35, P259, DOI 10.2337/dc11-1572

Fisher L, 2010, DIABETES CARE, V33, P1034, DOI 10.2337/dc09-2175

Hood KK, 2006, DIABETES CARE, V29, P1389, DOI 10.2337/dc06-0087

Kovacs M., 1992, CHILDRENS DEPRESSION

Lustman PJ, 2005, J DIABETES COMPLICAT, V19, P113, DOI 10.1016/j.jdiacomp.2004.01.002

Merikangas KR, 2010, J AM ACAD CHILD PSY, V49, P980, DOI 10.1016/j.jaac.2010.05.017

Pinhas-Hamiel O, 2007, PEDIATR DIABETES, V8, P16, DOI 10.1111/j.1399-5448.2007.00330.x

Zeitler P, 2012, NEW ENGL J MED, V366, P2247, DOI 10.1056/NEJMoa1109333

NR 11

TC 0

Z9 0

U1 0

U2 0

PU ELSEVIER IRELAND LTD

PI CLARE

PA ELSEVIER HOUSE, BROOKVALE PLAZA, EAST PARK SHANNON, CO, CLARE, 00000,

IRELAND

SN 0168-8227

EI 1872-8227

J9 DIABETES RES CLIN PR

JI Diabetes Res. Clin. Pract.

PD JAN

PY 2018

VL 135

BP 85

EP 87

DI 10.1016/j.diabres.2017.11.008

PG 3

WC Endocrinology & Metabolism

SC Endocrinology & Metabolism

GA FU4ON

UT WOS:000423832500014

PM 29146120

DA 2019-01-08

ER

PT J

AU Lin, CE

Chen, LF

Chou, PH

Chung, CH

AF Lin, Ching-En

Chen, Li-Fen

Chou, Po-Han

Chung, Chi-Hsiang

TI Increased prevalence and risk of anxiety disorders in adults with

tinnitus: A population-based study in Taiwan

SO GENERAL HOSPITAL PSYCHIATRY

LA English

DT Article

DE Anxiety disorder; Tinnitus; Prevalence; National Health Insurance

Research Database; NHIRD

ID MAJOR DEPRESSIVE DISORDER; DORSAL COCHLEAR NUCLEUS; PSYCHIATRIC

COMORBIDITIES; MENIERES-DISEASE; MENTAL-DISORDERS; PANIC DISORDER;

HEARING-LOSS; FOLLOW-UP; ASSOCIATION; STROKE

AB Objective: Tinnitus is a common disorder that may cause psychological distress and anxiety. The aim of this study was to investigate the association between anxiety disorders (ADs) and tinnitus in a large population.

Method: We conducted a cross-sectional study using the National Health Insurance Research Database in Taiwan. Study subjects included 14,772 patients with tinnitus and 709,963 people in the general population who sought treatment in 2005. Distributions in ADs, age, sex, and medical comorbidities were compared between groups using chi-squared tests. Multivariate logistic regression models adjusted for age, sex, and medical comorbidities were used to analyze the association between tinnitus and ADs.

Results: Prevalence of ADs in tinnitus and general population groups was 3.9% and 1.5%, respectively, and this difference was significant (P < 0.001). Diabetes mellitus, hypertension, hyperlipidemia, concussion or head injury, Meniere's disease, sensorineural hearing impairment, renal disease, coronary artery disease, and cerebrovascular disease were significantly more prevalent in the tinnitus group (all P-values < 0.001). Multivariate logistic regression model demonstrated that patients with tinnitus were significantly associated with increased risk of ADs (adjusted OR = 1.99; 95% CI = 1.81-2.19; P < 0.001).

Conclusion: Because the risk of ADs was significantly higher in patients with tinnitus than in the general population, physicians should be aware of the importance of psychological factors in tinnitus management.

C1 [Lin, Ching-En] Taipei Tzu Chi Hosp, Buddhist Tzu Chi Med Fdn, 289 Jianguo Rd, New Taipei, Taiwan.

[Lin, Ching-En] Tzu Chi Univ, Sch Med, Hualien, Taiwan.

[Chen, Li-Fen] Triserv Gen Hosp, Penghu Branch, Taipei, Taiwan.

[Chen, Li-Fen] Natl Def Med Ctr, Sch Med, Taipei, Taiwan.

[Chou, Po-Han] Harvard TH Chan Sch Publ Hlth, Dept Epidemiol, 677 Huntington Ave, Boston, MA 02115 USA.

[Chou, Po-Han] Natl Yang Ming Univ, Fac Med, Taipei, Taiwan.

[Chou, Po-Han] Natl Chiao Tung Univ, Dept Biol Sci & Technol, Hsinchu, Taiwan.

[Chung, Chi-Hsiang] Triserv Gen Hosp, Dept Med Res, Taipei, Taiwan.

[Chung, Chi-Hsiang] Natl Def Med Ctr, Sch Publ Hlth, Taipei, Taiwan.

RP Lin, CE (reprint author), Taipei Tzu Chi Hosp, Buddhist Tzu Chi Med Fdn, 289 Jianguo Rd, New Taipei, Taiwan.; Chou, PH (reprint author), Harvard TH Chan Sch Publ Hlth, Dept Epidemiol, 677 Huntington Ave, Boston, MA 02115 USA.

EM chingen_lin@tzuchi.com.tw; pchou@hsph.havard.edu

CR Alonso J, 2007, J CLIN PSYCHIAT, V68, P3

Asnaani A, 2010, J NERV MENT DIS, V198, P551, DOI 10.1097/NMD.0b013e3181ea169f

Ayerbe L, 2013, AGE AGEING

Baigi A, 2011, EAR HEARING, V32, P787, DOI 10.1097/AUD.0b013e31822229bd

Belli S, 2008, EUR ARCH OTO-RHINO-L, V265, P279, DOI 10.1007/s00405-007-0440-8

Bhatt JM, 2017, LARYNGOSCOPE, V127, P466, DOI 10.1002/lary.26107

Borghi C, 2011, BMC MED, V9, DOI 10.1186/1741-7015-9-80

Chen YH, 2010, CAN J PSYCHIAT, V55, P43, DOI 10.1177/070674371005500107

Cheng T- M., 2010, SIX COUNTRIES SIX RE, P171

Chien IC, 2013, J PSYCHOSOM RES, V75, P270, DOI 10.1016/j.jpsychores.2013.06.003

Chou PH, 2014, PSYCHOSOMATICS, V55, P155, DOI 10.1016/j.psym.2013.06.003

Chou PH, 2013, EUR CHILD ADOLES PSY, V22, P301, DOI 10.1007/s00787-012-0369-3

Chou PH, 2012, PSYCHOSOMATICS, V53, P463, DOI 10.1016/j.psym.2012.03.007

Chung SD, 2015, EUR ARCH OTO-RHINO-L, V272, P2673, DOI 10.1007/s00405-014-3235-8

Cukor D, 2008, CLIN J AM SOC NEPHRO, V3, P1752, DOI 10.2215/CJN.01120308

Dortland AKBV, 2010, ACTA PSYCHIAT SCAND, V122, P30, DOI 10.1111/j.1600-0447.2010.01565.x

Figueiredo RR, 2017, MENIERES DIS TINNITU

Fure B, 2006, INT J GERIATR PSYCH, V21, P382, DOI 10.1002/gps.1482

Furukawa M, 2013, ACTA OTO-LARYNGOL, V133, P584, DOI 10.3109/00016489.2012.759274

Granjeiro RC, 2013, OTOLARYNG HEAD NECK, V148, P658, DOI 10.1177/0194599812473554

Gul AI, 2015, NEUROPSYCH DIS TREAT, V11, P413, DOI 10.2147/NDT.S77786

Hafeez F, 1999, J Stroke Cerebrovasc Dis, V8, P217, DOI 10.1016/S1052-3057(99)80070-6

Ho CH, 2014, BMJ OPEN, V4, DOI 10.1136/bmjopen-2014-005269

Hoffman HJ, 2004, TINNITUS THEORY MANA, P16

Holmes S, 2008, REV CLIN GERONTOL, V18, P269, DOI 10.1017/S0959259809002883

Huang CJ, 2011, GEN HOSP PSYCHIAT, V33, P8, DOI 10.1016/j.genhosppsych.2010.10.008

Jacques D, 2013, PSYCHIAT DANUB, V25, pS102

Jalessi Maryam, 2013, Acta Med Iran, V51, P886

Kaltenbach JA, 2006, HEARING RES, V216, P224, DOI 10.1016/j.heares.2006.01.002

Kaltenbach JA, 2008, AM J AUDIOL, V17, pS148, DOI 10.1044/1059-0889(2008/08-0004)

Kanzaki J, 2015, ACTA OTO-LARYNGOL, V135, P447, DOI 10.3109/00016489.2014.990581

Kazmierczak H, 2001, Int Tinnitus J, V7, P54

Kim HJ, 2015, PLOS ONE, V10, DOI 10.1371/journal.pone.0127578

Liao SC, 2012, PSYCHOL MED, V42, P1227, DOI 10.1017/S0033291711002364

Minen MT, 2014, J NEUROL NEUROSUR PS, V85, P1138, DOI 10.1136/jnnp-2013-307339

Olafiranye O, 2011, Mind Brain, V2, P32

Pattyn T, 2015, HEARING RES, V30, pe11

Preljevic VT, 2013, GEN HOSP PSYCHIAT, V35, P619, DOI 10.1016/j.genhosppsych.2013.05.006

Schmieder RE, 2013, J HYPERTENS, V31, P610, DOI 10.1097/HJH.0b013e32835d6e53

Shargorodsky J, 2010, JAMA-J AM MED ASSOC, V304, P772, DOI 10.1001/jama.2010.1124

Shargorodsky J, 2010, AM J MED, V123, P711, DOI 10.1016/j.amjmed.2010.02.015

Shargorodsky M, 2003, AM J HYPERTENS, V16, P617, DOI 10.1016/S0895-7061(03)00911-7

Smith KJ, 2013, J PSYCHOSOM RES, V74, P89, DOI 10.1016/j.jpsychores.2012.11.013

SPSS I, 2013, IBM SPSS STAT 22

Steel Z, 2014, INT J EPIDEMIOL, V43, P476, DOI 10.1093/ije/dyu038

Strumila R, 2017, PSYCHIAT QUART, P1

Tully PJ, 2013, PSYCHOL HEALTH MED, V18, P627, DOI 10.1080/13548506.2012.749355

Tyler R. S., 1983, SPEECH HEAR DISORD, V48, P150, DOI [10.1044/jshd.4802.150, DOI 10.1044/JSHD.4802.150]

VANREEDT DAK, 2013, PSYCHONEUROENDOCRINO, V38, P209, DOI DOI 10.1016/J.PSYNEUEN.2012.05.017

Wallhausser-Franke E, 2012, PLOS ONE, V7, DOI 10.1371/journal.pone.0034583

NR 50

TC 2

Z9 2

U1 0

U2 0

PU ELSEVIER SCIENCE INC

PI NEW YORK

PA 360 PARK AVE SOUTH, NEW YORK, NY 10010-1710 USA

SN 0163-8343

EI 1873-7714

J9 GEN HOSP PSYCHIAT

JI Gen. Hosp. Psych.

PD JAN-FEB

PY 2018

VL 50

BP 131

EP 136

DI 10.1016/j.genhosppsych.2017.11.006

PG 6

WC Psychiatry

SC Psychiatry

GA FV1RU

UT WOS:000424343000020

PM 29190572

DA 2019-01-08

ER

PT J

AU Fisher, L

Hessler, D

Polonsky, W

Strycker, L

Guzman, S

Bowyer, V

Blumer, I

Masharani, U

AF Fisher, Lawrence

Hessler, Danielle

Polonsky, William

Strycker, Lisa

Guzman, Susan

Bowyer, Vicky

Blumer, Ian

Masharani, Umesh

TI Emotion regulation contributes to the development of diabetes distress

among adults with type 1 diabetes

SO PATIENT EDUCATION AND COUNSELING

LA English

DT Article

DE Diabetes; Emotion management; Diabetes distress

ID SELF-REGULATION; DEPRESSION; INTERVENTION; ILLNESS; QUESTIONNAIRE;

MINDFULNESS; VALIDATION; MANAGEMENT; SYMPTOMS; VALIDITY

AB Objective: To demonstrate how maladaptive emotion regulation (ER) can lead to diabetes distress (DD), with subsequent effects on management and metabolic outcomes among adults with type 1 diabetes.

Methods: Data are based on pre-intervention assessment for a random controlled trial to reduce DD. Patients were recruited in California, Oregon, Arizona and Ontario, Canada. After screening and consent, patients completed an online assessment and released their most recent laboratory HbA1C. Structural equation modeling was used to define an ER measurement model and test for significant pathways.

Results: Three ER mechanisms combined into a single construct: emotion processing, non-judgment of emotions, non-reactivity to emotions. Models indicated a significant pathway from ER and cognitions to DD to disease management to metabolic control.

Conclusions: As hypothesized, the three ER mechanisms formed a single, coherent ER construct. Patients with poor ER reported high DD; and high DD was linked to poor diabetes management and poor metabolic control.

Practice implications: Identifying both the level of DD and the ER mechanisms that lead to high DD should be explored in clinical settings. Helping T1Ds to become more aware, less judgmental and less reactive behaviorally to what they feel about diabetes and its management may reduce DD. Crown Copyright (c) 2017 Published by Elsevier Ireland Ltd. All rights reserved.

C1 [Fisher, Lawrence; Hessler, Danielle; Bowyer, Vicky] UCSF, Dept Family & Community Med, San Francisco, CA USA.

[Polonsky, William] Univ Calif San Diego, Dept Psychiat, Behav Diabet Inst, San Diego, CA USA.

[Strycker, Lisa] Oregon Res Inst, Eugene, OR 97403 USA.

[Guzman, Susan] Behav Diabet Inst, San Diego, CA USA.

[Blumer, Ian] Charles H Best Diabet Ctr, Whitby, ON, Canada.

[Masharani, Umesh] UCSF, Dept Med, San Francisco, CA USA.

RP Fisher, L (reprint author), UCSF, Box 0900, San Francisco, CA 94143 USA.

EM larry.fisher@ucsf.edu; Danielle.hessler@ucsf.edu;

whp@behavioraldiabetes.org; lisas@ori.org; sguzman1@san.rr.com;

vicky.bowyer@ucsf.edu; ian@ianblumer.com; umesh.masharani@ucsf.edu

FU National Institutes of Health [RO1DK094863, R18DK108039]

FX This work was supported by the National Institutes of Health

RO1DK094863, R18DK108039.

CR Baer RA, 2006, ASSESSMENT, V13, P27, DOI 10.1177/1073191105283504

Baer RA, 2008, ASSESSMENT, V15, P329, DOI 10.1177/1073191107313003

Barlow J, 2002, PATIENT EDUC COUNS, V48, P177, DOI 10.1016/S0738-3991(02)00032-0

Berking M, 2012, J BEHAV THER EXP PSY, V43, P931, DOI 10.1016/j.jbtep.2011.09.009

Blair C, 2008, DEV PSYCHOPATHOL, V20, P899, DOI 10.1017/S0954579408000436

Diedrich A., 2016, PSYCHOL PSYCHOTHER-T, V89, P1

Fisher L, 2008, DIABETIC MED, V25, P1096, DOI 10.1111/j.1464-5491.2008.02533.x

Fisher L, 2014, DIABETIC MED, V31, P764, DOI 10.1111/dme.12428

Fisher L, 2015, DIABETIC MED

Fisher L, 2007, DIABETES CARE, V30, P542, DOI 10.2337/dc06-1614

Fisher L, 2015, J DIABETES COMPLICAT, V29, P572, DOI 10.1016/j.jdiacomp.2015.01.012

Fisher L, 2013, DIABETES CARE, V36, P2551, DOI 10.2337/dc12-2493

Fisher L, 2012, PATIENT EDUC COUNS, V86, P372, DOI 10.1016/j.pec.2011.07.002

Fisher L, 2010, DIABETES CARE, V33, P23, DOI 10.2337/dc09-1238

Gonzalez JS, 2011, DIABETES CARE, V34, P236, DOI 10.2337/dc10-1970

Gratz KL, 2004, J PSYCHOPATHOL BEHAV, V26, P41, DOI 10.1023/B:JOBA.0000007455.08539.94

Gross J. J, 2007, HDB EMOTION REGULATI, P3, DOI DOI 10.'1080/00140130600971135

Gu J, 2015, CLIN PSYCHOL REV, V37, P1, DOI 10.1016/j.cpr.2015.01.006

Guendelman S, 2017, FRONT PSYCHOL, V8, P1, DOI 10.3389/fpsyg.2017.00220

Jazaieri H, 2015, CURR PSYCHIAT REP, V17, DOI 10.1007/s11920-014-0531-3

Joormann J, 2016, BEHAV RES THER, V86, P35, DOI 10.1016/j.brat.2016.07.007

Kroenke K, 2002, PSYCHOSOM MED, V64, P258, DOI 10.1097/00006842-200203000-00008

Lansing AH, 2014, J PEDIATR PSYCHOL, V39, P1091, DOI 10.1093/jpepsy/jsu067

Maes S, 2005, APPL PSYCHOL-INT REV, V54, P267, DOI 10.1111/j.1464-0597.2005.00210.x

Miller W. R., 2013, MOTIVATIONAL INTERVI

Moss-Morris R, 2002, PSYCHOL HEALTH, V17, P1, DOI 10.1080/08870440290001494

Neff KD, 2007, J RES PERS, V41, P139, DOI 10.1016/j.jrp.2006.03.004

Nigg JT, 2017, J CHILD PSYCHOL PSYC, V58, P361, DOI 10.1111/jcpp.12675

Quoidbach J, 2015, PSYCHOL BULL, V141, P655, DOI 10.1037/a0038648

Stanton AL, 2000, J PERS SOC PSYCHOL, V78, P1150, DOI 10.1037//0022-3514.78.6.1150

Stanton K, 2016, J ABNORM PSYCHOL, V125, P960, DOI 10.1037/abn0000208

Sturt J., 2016, INT DIABETIC NURS, V12, P40

Tang T. S., 2011, PEER LEADER TRAINING

Tsjii S., 2012, DIABETIC MED, V29, P1451

Zagarins SE, 2012, J BEHAV MED, V35, P299, DOI 10.1007/s10865-011-9359-z

NR 35

TC 2

Z9 2

U1 2

U2 4

PU ELSEVIER IRELAND LTD

PI CLARE

PA ELSEVIER HOUSE, BROOKVALE PLAZA, EAST PARK SHANNON, CO, CLARE, 00000,

IRELAND

SN 0738-3991

J9 PATIENT EDUC COUNS

JI Patient Educ. Couns.

PD JAN

PY 2018

VL 101

IS 1

BP 124

EP 131

DI 10.1016/j.pec.2017.06.036

PG 8

WC Public, Environmental & Occupational Health; Social Sciences,

Interdisciplinary

SC Public, Environmental & Occupational Health; Social Sciences - Other

Topics

GA FT9LT

UT WOS:000423474300018

PM 28739179

DA 2019-01-08

ER

PT J

AU Goldberg, A

Ebraheem, Z

Freiberg, C

Ferarro, R

Chai, S

Gottfried, OD

AF Goldberg, Alon

Ebraheem, Zaheera

Freiberg, Cynthia

Ferarro, Rachel

Chai, Sharon

Gottfried, Orna Dally

TI Sweet and Sensitive: Sensory Processing Sensitivity and Type 1 Diabetes

SO JOURNAL OF PEDIATRIC NURSING-NURSING CARE OF CHILDREN & FAMILIES

LA English

DT Article

DE Sensory processing sensitivity; Autoimmune disease; Type 1 diabetes

ID PSYCHOLOGICAL DISTRESS; BEHAVIORAL-INHIBITION; AUTOIMMUNE-DISEASES;

GLYCEMIC CONTROL; STRESS; DEPRESSION; CHILDHOOD; DISORDER; CHILDREN;

ANXIETY

AB Objective: Sensory processing sensitivity (SPS) is a recently proposed construct that refers to a genetically influenced tendency to more strongly and deeply process a variety of information. The aim of the study was to examine whether SPS is associated with an autoimmune disease such as type 1 diabetes (T1D).

Research design and methods: Participants were 128 adolescents (62 with T1D and 66 comparisons [without autoimmune disease]) and their parents who completed the Highly Sensitive Person Scale (HSPS) questionnaire, assessing SPS level.

Results: Higher levels of SPS were found in the T1D group than in the comparison group. Furthermore, the frequency of SPS trait was significantly higher in the T1D group than in the comparison group.

Conclusions: T1D is associated with higher levels of SPS. Hence, there is a need to develop interventions, treatments, and care focused on the needs of T1D patients with SPS temperament, aimed at better treatment adherence. Furthermore, longitudinal research is needed to evaluate whether SPS is a risk factor in the development of T1D. (c) 2017 Elsevier Inc. All rights reserved.

C1 [Goldberg, Alon] Tel Hai Coll, Dept Educ, Upper Galilee, Israel.

[Ebraheem, Zaheera; Ferarro, Rachel] Ziv Hosp, Ctr Juvenile Diabet & Pediat Endocrinol, Safed, Israel.

[Ebraheem, Zaheera; Ferarro, Rachel] Ziv Hosp, Pediat Outpatient Clin, Safed, Israel.

[Freiberg, Cynthia] Bar Ilan Univ, Ziv Hosp, Sch Med, Safed, Israel.

[Chai, Sharon] Tel Hai Coll, Dept Field Practice, Upper Galilee, Israel.

[Gottfried, Orna Dally] Bar Ilan Univ, Ziv Hosp, Sch Med, Ctr Juvenile Diabet & Pediat Endocrinol, Safed, Israel.

[Gottfried, Orna Dally] Bar Ilan Univ, Ziv Hosp, Sch Med, Pediat Outpatient Clin, Safed, Israel.

RP Goldberg, A (reprint author), Tel Hai Coll, Dept Educ, Upper Galilee, Israel.

EM goldbergim@gmail.com

CR Acevedo BP, 2014, BRAIN BEHAV, V4, P580, DOI 10.1002/brb3.242

Aron A, 2010, SOC COGN AFFECT NEUR, V5, P219, DOI 10.1093/scan/nsq028

Aron EN, 2012, PERS SOC PSYCHOL REV, V16, P262, DOI 10.1177/1088868311434213

Aron EN, 1997, J PERS SOC PSYCHOL, V73, P345, DOI 10.1037/0022-3514.73.2.345

Aron EN, 2005, PERS SOC PSYCHOL B, V31, P181, DOI 10.1177/0146167204271419

Benham G, 2006, PERS INDIV DIFFER, V40, P1433, DOI 10.1016/j.paid.2005.11.021

CARTER WR, 1987, DIABETOLOGIA, V30, P674

CARVER CS, 1994, J PERS SOC PSYCHOL, V67, P319, DOI 10.1037/0022-3514.67.2.319

Compas BE, 2012, ANNU REV CLIN PSYCHO, V8, P455, DOI 10.1146/annurev-clinpsy-032511-143108

Cunningham NR, 2011, J PEDIATR PSYCHOL, V36, P196, DOI 10.1093/jpepsy/jsq071

Dunn W, 2001, AM J OCCUP THER, V55, P608, DOI 10.5014/ajot.55.6.608

Eysenck H. J, 1991, HDB PERSONALITY, P244

Gray J. A., 1991, EXPLORATIONS TEMPERA, P105, DOI DOI 10.1007/978-1-4899-0643-4_8

Hartman S, 2016, FAM PROCESS, V55, P700, DOI 10.1111/famp.12161

HAWKINS NG, 1957, AM REV TUBERC PULM, V75, P768

Herrmann M, 2000, RHEUM DIS CLIN N AM, V26, P737, DOI 10.1016/S0889-857X(05)70167-8

Hofherr SE, 2011, PLOS ONE, V6, DOI 10.1371/journal.pone.0017076

Homberg JR, 2016, NEUROSCI BIOBEHAV R, V71, P472, DOI 10.1016/j.neubiorev.2016.09.029

Jerome EM, 2005, PERS INDIV DIFFER, V38, P1341, DOI 10.1016/j.paid.2004.08.016

Johnson SL, 2003, J PSYCHOPATHOL BEHAV, V25, P25, DOI 10.1023/A:1022247919288

Juvenile Diabetes Research Foundation, 2016, TYP 1 DIAB FACTS

Kagan J, 1997, CHILD DEV, V68, P139, DOI 10.2307/1131931

Kinnealey M, 1999, OCCUPATIONAL THERAPY, V6, P195, DOI DOI 10.1002/OTI.97

Landolt MA, 2005, J AM ACAD CHILD PSY, V44, P682, DOI 10.1097/01-chi.0000161645.98022.35

Liss M, 2005, PERS INDIV DIFFER, V39, P1429, DOI 10.1016/j.paid.2005.05.007

Liss M, 2008, PERS INDIV DIFFER, V45, P255, DOI 10.1016/j.paid.2008.04.009

Luyckx K, 2010, DIABETES CARE, V33, P1424, DOI 10.2337/dc09-2017

Meyer B, 2000, J PERS DISORD, V14, P233, DOI 10.1521/pedi.2000.14.3.233

Neal JA, 2002, BRIT J CLIN PSYCHOL, V41, P361, DOI 10.1348/014466502760387489

Persson LO, 1999, SCAND J RHEUMATOL, V28, P137

Pluess M, 2015, CHILD DEV PERSPECT, V9, P138, DOI 10.1111/cdep.12120

Pluess M, 2013, PSYCHOL BULL, V139, P901, DOI 10.1037/a0030196

Renzaho AMN, 2014, EUR J PUBLIC HEALTH, V24, P57, DOI 10.1093/eurpub/ckt007

Shepshelovich D, 2006, LUPUS, V15, P183, DOI 10.1191/0961203306lu2274rr

SHOENFELD Y, 1989, IMMUNOL TODAY, V10, P123, DOI 10.1016/0167-5699(89)90245-4

Shoenfeld Y, 2008, ISR MED ASSOC J, V10, P8

Silverstein J, 2005, DIABETES CARE, V28, P186, DOI 10.2337/diacare.28.1.186

Stojanovich L, 2008, AUTOIMMUN REV, V7, P209, DOI 10.1016/j.autrev.2007.11.007

Stojanovich L, 2010, AUTOIMMUN REV, V9, pA271, DOI 10.1016/j.autrev.2009.11.014

Storch EA, 2004, CLIN PEDIATR, V43, P467, DOI 10.1177/000992280404300508

Wolfe F, 1999, SCAND J RHEUMATOL, V28, P131, DOI 10.1080/03009749950154185

NR 41

TC 1

Z9 1

U1 4

U2 8

PU ELSEVIER SCIENCE INC

PI NEW YORK

PA 360 PARK AVE SOUTH, NEW YORK, NY 10010-1710 USA

SN 0882-5963

J9 J PEDIATR NURS

JI J. Pediatr. Nurs.

PD JAN-FEB

PY 2018

VL 38

BP E35

EP E38

DI 10.1016/j.pedn.2017.10.015

PG 4

WC Nursing; Pediatrics

SC Nursing; Pediatrics

GA FT3GJ

UT WOS:000423035000008

PM 29100650

DA 2019-01-08

ER

PT J

AU Deschenes, SS

Burns, RJ

Schmitz, N

AF Deschenes, Sonya S.

Burns, Rachel J.

Schmitz, Norbert

TI Trajectories of anxiety symptoms and associations with incident

cardiovascular disease in adults with type 2 diabetes

SO JOURNAL OF PSYCHOSOMATIC RESEARCH

LA English

DT Article

DE Anxiety; Cardiovascular disease; Latent trajectories; Type 2 diabetes

ID COMORBID ANXIETY; RISK; DEPRESSION; DISORDERS; INDIVIDUALS; MORTALITY;

DISTRESS; MELLITUS; OUTCOMES; HEALTH

AB Background: Trajectories of anxiety symptoms in people with type 2 diabetes (T2D) and their associations with diabetes health outcomes have not been investigated. This study examined latent longitudinal trajectories of anxiety symptoms in adults with T2D and their associations with incident cardiovascular disease (CVD).

Methods: Data were from the Evaluation of Diabetes Treatment Study, a community-based cohort study of adults aged 40-76 years with T2D. Anxiety and CVD were assessed by self-report at baseline and at four annual follow-up assessments. N = 832 participants without cardiovascular disease at baseline and 12-month follow-up were included in the present study. Group-based trajectories of anxiety at baseline, 12-month follow-up, and 24 month follow-up were modelled using latent class growth modeling. Associations between anxiety trajectories and CVD reported at 24-, 36-, or 48-month follow-ups were examined with logistic regression analysis adjusted for sociodemographic and lifestyle characteristics.

Results: Four distinct anxiety trajectories were identified, reflecting chronically low (39.4%), chronically moderate-low (47.4%), chronically moderate-high (11.1%), and chronically high (2.2%) anxiety. The likelihood of CVD was greater for the chronically moderate-low (OR = 2.23, 95% CI = 1.36-3.66), chronically moderate high (OR = 3.05, 95% CI = 1.54-6.02), and chronically high (OR = 3.61, 95% CI = 1.09-12.00) anxiety trajectory groups compared to the chronically low anxiety group.

Conclusion: The identified latent trajectories reflected three groups with chronic courses of anxiety symptoms at different levels of severity and one group with chronically low levels of anxiety. Chronic anxiety, even at subthreshold levels, was associated with an increased risk of CVD among people with T2D.

C1 [Deschenes, Sonya S.; Schmitz, Norbert] McGill Univ, Dept Psychiat, Montreal, PQ, Canada.

[Deschenes, Sonya S.; Schmitz, Norbert] Douglas Mental Hlth Univ Inst, 6875 Boul Lasalle,Frank B Common Pavil,F2117-2, Montreal, PQ H4H 1R3, Canada.

[Burns, Rachel J.] Carleton Univ, Dept Psychol, Ottawa, ON, Canada.

[Schmitz, Norbert] McGill Univ, Dept Epidemiol Biostat & Occupat Hlth, Montreal, PQ, Canada.

RP Deschenes, SS (reprint author), Douglas Mental Hlth Univ Inst, 6875 Boul Lasalle,Frank B Common Pavil,F2117-2, Montreal, PQ H4H 1R3, Canada.

EM sonya.deschenes@mail.mcgill.ca

RI Schmitz, Norbert/A-5177-2010

OI Schmitz, Norbert/0000-0001-7777-6323

FU Canadian Institutes of Health Research [MOP-106514]; Canadian Institutes

of Health Research

FX This work was supported by an Operating Grant from the Canadian

Institutes of Health Research (MOP-106514). Sonya Deschenes is supported

by a fellowship from the Canadian Institutes of Health Research.

CR Anderson RJ, 2002, INT J PSYCHIAT MED, V32, P235, DOI 10.2190/KLGD-4H8D-4RYL-TWQ8

Andruff H, 2009, TUTOR QUANT METHODS, V5, P11, DOI 10.20982/tqmp.05.1.p011

Bruce DG, 2016, DEPRESS ANXIETY, V33, P960, DOI 10.1002/da.22523

Chandola T, 2006, BRIT MED J, V332, P521, DOI 10.1136/bmj.38693.435301.80

Deschenes SS, 2016, J DIABETES, V8, P854, DOI 10.1111/1753-0407.12368

Edwards LE, 2012, J PSYCHOSOM RES, V73, P418, DOI 10.1016/j.jpsychores.2012.09.018

Enders CK, 2011, REHABIL PSYCHOL, V56, P267, DOI 10.1037/a0025579

Fincke BG, 2005, J AMBUL CARE MANAG, V28, P262

Fisher L, 2008, DIABETIC MED, V25, P1096, DOI 10.1111/j.1464-5491.2008.02533.x

Hasan SS, 2016, PUBLIC HEALTH, V130, P21, DOI 10.1016/j.puhe.2015.07.022

Holmes S.E., 2017, INT J GERIATR PSYCHI

Jones BL, 2001, SOCIOL METHOD RES, V29, P374, DOI 10.1177/0049124101029003005

Jones BL, 2013, SOCIOL METHOD RES, V42, P608, DOI 10.1177/0049124113503141

Kroenke K, 2001, J GEN INTERN MED, V16, P606, DOI 10.1046/j.1525-1497.2001.016009606.x

Licht CMM, 2009, PSYCHOSOM MED, V71, P508, DOI 10.1097/PSY.0b013e3181a292a6

Lipscombe C, 2015, J AFFECT DISORDERS, V188, P160, DOI 10.1016/j.jad.2015.08.003

Baena-Diez JM, 2016, DIABETES CARE, V39, P1987, DOI 10.2337/dc16-0614

Nagin D, 2005, GROUP BASED MODELING

Naicker K, 2017, DIABETES CARE, V40, P352, DOI 10.2337/dc16-2018

Park J, 2014, HEALTH REP, V25, P12

Peyrot M, 2005, DIABETIC MED, V22, P1379, DOI 10.1111/j.1464-5491.2005.01644.x

Schmitz N, 2014, DIABETES CARE, V37, P970, DOI 10.2337/dc13-1832

Smith KJ, 2013, J PSYCHOSOM RES, V74, P89, DOI 10.1016/j.jpsychores.2012.11.013

Smith KJ, 2013, PSYCHOSOMATICS, V54, P35, DOI 10.1016/j.psym.2012.08.002

Spitzer RL, 2006, ARCH INTERN MED, V166, P1092, DOI 10.1001/archinte.166.10.1092

Teesson M, 2011, AUST NZ J PSYCHIAT, V45, P939, DOI 10.3109/00048674.2011.614590

Tsai MT, 2016, J AFFECT DISORDERS, V202, P178, DOI 10.1016/j.jad.2016.03.060

Whitworth SR, 2017, DIABETIC MED, V34, P1108, DOI 10.1111/dme.13372

World Health Organization, 2000, WHO TECHN REP SER

Young-Hyman D, 2016, DIABETES CARE, V39, P2126, DOI 10.2337/dc16-2053

NR 30

TC 0

Z9 0

U1 0

U2 2

PU PERGAMON-ELSEVIER SCIENCE LTD

PI OXFORD

PA THE BOULEVARD, LANGFORD LANE, KIDLINGTON, OXFORD OX5 1GB, ENGLAND

SN 0022-3999

EI 1879-1360

J9 J PSYCHOSOM RES

JI J. Psychosomat. Res.

PD JAN

PY 2018

VL 104

BP 95

EP 100

DI 10.1016/j.jpsychores.2017.11.015

PG 6

WC Psychiatry

SC Psychiatry

GA FT2XW

UT WOS:000423010300014

PM 29275792

DA 2019-01-08

ER

PT J

AU Cohen, A

Dias, A

Azariah, F

Krishna, RN

Sequeira, M

Abraham, S

Cuijpers, P

Morse, JQ

Reynolds, CF

Patel, V

AF Cohen, Alex

Dias, Amit

Azariah, Fredric

Krishna, Revathi N.

Sequeira, Miriam

Abraham, Sherin

Cuijpers, Pim

Morse, Jennifer Q.

Reynolds, Charles F., III

Patel, Vikram

TI Aging and well-being in Goa, India: a qualitative study

SO AGING & MENTAL HEALTH

LA English

DT Article

DE Late-life depression; prevention; Goa; India; qualitative research

ID LATE-LIFE DEPRESSION; PRIMARY-CARE; OLDER-ADULTS; EARLY INTERVENTION;

SUICIDAL IDEATION; MAJOR DEPRESSION; MENTAL-HEALTH; PREVENTION; TRIAL;

INEQUALITIES

AB Objectives: The population of India is aging rapidly. This demographic shift brings with it a host of challenges to the health and well-being of older adults, including the increased prevalence of non-communicable diseases, among them depressive disorders. In this paper, we report on qualitative research intended to inform the development of a locally acceptable and appropriate intervention to improve the well-being of older adults in Goa, India and, specifically, to prevent late-life depression.Method: Semi-structured interviews with 20 individuals, aged 60years and older, attending two primary care clinics in Goa, India. Transcripts were reviewed to identify emerging themes, a coding scheme was developed and thematic analyses were conducted.Results: Analyses of the interview transcripts revealed the following key themes: (1) notions of old age tended to be negative and there were widespread fears of becoming widowed or incapacitated; (2) the most frequently reported health conditions were joint pain, diabetes and heart disease; (3) emotional distress was described using the terms tension', stress', worry' and thinking'; (4) family issues often involved financial matters, difficult relationships with daughters-in-law and conflicted feelings about living with the family or independently; (5) other than a pension scheme, participants did not know of community resources available to older adults.Conclusions: Our findings are in general agreement with those of previous research, and with our experiences of working with older adults in Pittsburgh and the Netherlands. This research will inform the development of an intervention to prevent depression in older adults in Goa.

C1 [Cohen, Alex; Patel, Vikram] London Sch Hyg & Trop Med, Fac Epidemiol & Populat Hlth, Dept Populat Hlth, London, England.

[Dias, Amit] Goa Med Coll, Dept Prevent & Social Med, Bambolim, Goa, India.

[Dias, Amit; Azariah, Fredric; Krishna, Revathi N.; Sequeira, Miriam; Abraham, Sherin; Patel, Vikram] Sangath, Socorro, Goa, India.

[Cuijpers, Pim] Vrije Univ Amsterdam, Dept Clin Psychol, Amsterdam, Netherlands.

[Morse, Jennifer Q.] Chatham Univ, Sch Hlth Sci, Grad Psychol Programs, Pittsburgh, PA USA.

[Reynolds, Charles F., III] Univ Pittsburgh, Sch Med, Dept Psychiat, Pittsburgh, PA USA.

[Reynolds, Charles F., III] Univ Pittsburgh, Grad Sch Publ Hlth, Dept Behav & Community Hlth Sci, Pittsburgh, PA USA.

RP Cohen, A (reprint author), London Sch Hyg & Trop Med, Fac Epidemiol & Populat Hlth, Dept Populat Hlth, London, England.

EM alex.cohen@lshtm.ac.uk

FU US National Institute of Mental Health [R34MH096997, P30MH90333]

FX This research was supported by funding from the US National Institute of

Mental Health [R34MH096997 and P30MH90333].

CR Census of India, 2011, POP REL COMM

Charlson FJ, 2016, LANCET, V388, P376, DOI 10.1016/S0140-6736(16)30590-6

Cuijpers P, 2014, AM J PSYCHIAT, V171, P453, DOI 10.1176/appi.ajp.2013.13030325

Diniz BS, 2014, DEPRESS ANXIETY, V31, P787, DOI 10.1002/da.22226

Diniz BS, 2013, BRIT J PSYCHIAT, V202, P329, DOI 10.1192/bjp.bp.112.118307

Gilman SE, 2013, SOC PSYCH PSYCH EPID, V48, P59, DOI 10.1007/s00127-012-0575-9

Gilman SE, 2013, EPIDEMIOLOGY, V24, P14, DOI 10.1097/EDE.0b013e3182762403

Grover S., 2015, J GERIATRIC MENTAL H, V2, P4, DOI DOI 10.4103/2348-9995.161376

Haringsma R, 2006, INT PSYCHOGERIATR, V18, P307, DOI 10.1017/S104461020500253X

He W, 2012, SHADES GRAY CROSS CO

Institute of Applied Manpower Research Planning Commission Government of India, 2011, IND HUM DEV REP 2011

Jimenez DE, 2015, AM J GERIAT PSYCHIAT, V23, P548, DOI 10.1016/j.jagp.2014.08.001

Lakshmi Vijayakumar, 2010, Indian Journal of Psychiatry, V52, P291, DOI 10.4103/0019-5545.69255

Moussavi S, 2007, LANCET, V370, P851, DOI 10.1016/S0140-6736(07)61415-9

Okereke Olivia I, 2013, Focus (Am Psychiatr Publ), V11, P22

Onrust S, 2010, DEATH STUD, V34, P777, DOI 10.1080/07481181003761252

Patel V, 2001, PSYCHOL MED, V31, P29, DOI 10.1017/S0033291799003098

Patel V, 2011, LANCET, V377, P413, DOI 10.1016/S0140-6736(10)61188-9

Patel V, 2010, LANCET, V376, P2086, DOI 10.1016/S0140-6736(10)61508-5

Reynolds CF, 2014, PSYCHIAT SERV, V65, P765, DOI 10.1176/appi.ps.201300216

Reynolds CF, 2012, ANNU REV PUBL HEALTH, V33, P123, DOI 10.1146/annurev-publhealth-031811-124544

SARTORIUS N, 1993, ARCH GEN PSYCHIAT, V50, P819

Schoevers RA, 2006, AM J PSYCHIAT, V163, P1611, DOI 10.1176/appi.ajp.163.9.1611

Smits F, 2008, AM J GERIAT PSYCHIAT, V16, P444, DOI 10.1097/JGP.0b013e3181662ab6

Stahl ST, 2014, AM J PSYCHIAT, V171, P499, DOI 10.1176/appi.ajp.2013.13101373

United States Census Bureau, 2016, INT DAT BAS

van Zoonen K, 2014, INT J EPIDEMIOL, V43, P318, DOI 10.1093/ije/dyt175

van't Veer-Tazelaar PJ, 2011, AM J GERIAT PSYCHIAT, V19, P230, DOI 10.1097/JGP.0b013e3181faee4d

van't Veer-Tazelaar PJ, 2009, ARCH GEN PSYCHIAT, V66, P297, DOI 10.1001/archgenpsychiatry.2008.555

Vaz NC, 2011, NATL MED J INDIA, V24, P16

Zegwaard MI, 2013, BMC PSYCHIATRY, V13, DOI 10.1186/1471-244X-13-103

NR 31

TC 1

Z9 1

U1 1

U2 4

PU ROUTLEDGE JOURNALS, TAYLOR & FRANCIS LTD

PI ABINGDON

PA 2-4 PARK SQUARE, MILTON PARK, ABINGDON OX14 4RN, OXON, ENGLAND

SN 1360-7863

EI 1364-6915

J9 AGING MENT HEALTH

JI Aging Ment. Health

PY 2018

VL 22

IS 2

BP 168

EP 174

DI 10.1080/13607863.2016.1236239

PG 7

WC Geriatrics & Gerontology; Gerontology; Psychiatry

SC Geriatrics & Gerontology; Psychiatry

GA FS5ZD

UT WOS:000419876800002

PM 27689842

OA Green Published

DA 2019-01-08

ER

PT J

AU Gonzalez-Cantu, A

Mireles-Zavala, L

Rodriguez-Romo, A

Olavide-Aguilar, E

De la Garza-Hernandez, NE

Romero-Ibarguengoitia, ME

AF Gonzalez-Cantu, Arnulfo

Mireles-Zavala, Leonor

Rodriguez-Romo, Anabel

Olavide-Aguilar, Elizabeth

Eloisa De la Garza-Hernandez, Natalia

Elena Romero-Ibarguengoitia, Maria

TI Eating behaviors and emotional distress are predicted by treatment and

adverse outcome in patients with type 2 diabetes

SO PSYCHOLOGY HEALTH & MEDICINE

LA English

DT Article

DE Depression; anxiety; type 2 diabetes; eating behaviors

ID PSYCHOMETRIC PROPERTIES; CARDIOVASCULAR-DISEASE; GLYCEMIC CONTROL;

DEPRESSION; PREVALENCE; POPULATION; ADULTS; ASSOCIATION; ANXIETY;

MORTALITY

AB Depression and anxiety are hightly prevalent among patients with Type 2 Diabetes (T2D), however not commonly related to outcomes, treatment and comorbidities. Eating behaviors could also have an implication. To evaluate the relation between mood and eating behaviors with demographic, physical, treatment, biochemical profiles and chronic comorbidities in T2D we conducted this exploratory cross sectional study in a population from Mexico. Hospital Anxiety and Depression Scale and the Three Factor Eating Questionnaire Revised 21 (TFEQ-R21) were correlated with age, gender, blood pressure, treatment and comorbidities (nephropathy, neuropathy, retinopathy and cardiopathy). Multi-linear regression models and 2(k) factorial analyses were conducted. Sixty-one patients (31 male) 55years old (SD 13) with at least 5years of T2D were included. Anxiety correlated with depression (r=0.25, p<0.05). Gender (women) (std B 0.026, p<0.001), insulin therapy (std B 0.3, p=0.11), systolic blood pressure (std B 0.263, p=0.02) and cardiovascular disease (std B 0.232, p=0.035) predicted depression. Insulin therapy had a positive effect in cognitive restraint. (std B 0.32, p=0.001). Age (std B 0.37, p=0.003) and systolic blood pressure (std B 0.237, p=0.048) predicted positively emotional eating (not previously published). 2(k) factorial analyses proved additive interaction between complications and insulin therapy with depression. This study supports the relation between mood disorders and eating behaviors with demographic, physical, treatment, biochemical profiles and chronic comorbidities in T2D and the additive interaction between factors and mood disorders.

C1 [Gonzalez-Cantu, Arnulfo] Ctr Invest Matemat CIMAT, Internal Med, Nuevo Leon, Mexico.

[Mireles-Zavala, Leonor; Rodriguez-Romo, Anabel; Olavide-Aguilar, Elizabeth] Inst Seguridad & Serv Sociales Trabajadores Estad, Internal Med, Nuevo Leon, Mexico.

[Elena Romero-Ibarguengoitia, Maria] Univ Nacl Autonoma Mexico, Hosp Gen Mexico, Res Dept, Mexico City, DF, Mexico.

RP Romero-Ibarguengoitia, ME (reprint author), Univ Nacl Autonoma Mexico, Hosp Gen Mexico, Res Dept, Mexico City, DF, Mexico.

EM elenaromero83@gmail.com

CR Alegre-Diaz J, 2016, NEW ENGL J MED, V375, P1961, DOI 10.1056/NEJMoa1605368

Alonso-Moran E, 2014, BMC PUBLIC HEALTH, V14, DOI 10.1186/1471-2458-14-769

Anderson RJ, 2001, DIABETES CARE, V24, P1069, DOI 10.2337/diacare.24.6.1069

[Anonymous], 2017, DIABETES CARE, V40, pS1, DOI 10.2337/dc17-S001

Berge LI, 2015, PLOS ONE, V10, DOI 10.1371/journal.pone.0127161

Bhat SK, 2017, J HYPERTENS, V35, P1983, DOI [10.1097/HJH.0000000000001410, 10.1097/hjh.0000000000001410]

Bruce DG, 2016, DEPRESS ANXIETY, V33, P960, DOI 10.1002/da.22523

Cappelleri JC, 2009, INT J OBESITY, V33, P611, DOI 10.1038/ijo.2009.74

Cerrelli F, 2005, Eat Weight Disord, V10, P251

Chaudhary Rupesh, 2017, Indian J Psychol Med, V39, P342, DOI 10.4103/IJPSYM.IJPSYM_35_17

COLIZZI V, 1979, ANNALES DIMMUNOLOGIE, V100, P659

Collins MM, 2009, DIABETIC MED, V26, P153, DOI 10.1111/j.1464-5491.2008.02648.x

Dziemidok P, 2016, PSYCHIATR POL, V50, P407, DOI 10.12740/PP/OnlineFirst/42352

Golden SH, 2007, PSYCHOSOM MED, V69, P529, DOI 10.1097/PSY.0b013e3180f61c5c

Heckbert SR, 2010, J GEN INTERN MED, V25, P524, DOI 10.1007/s11606-010-1272-6

Herrero MJ, 2003, GEN HOSP PSYCHIAT, V25, P277, DOI 10.1016/S0163-8343(03)00043-4

Hessler DM, 2011, PATIENT EDUC COUNS, V85, P154, DOI 10.1016/j.pec.2010.10.030

Jauregui-Lobera I, 2014, NUTRIENTS, V6, P5619, DOI 10.3390/nu6125619

Jimenez-Cruz A., 2015, NUTR HOSP, V31, P2775, DOI [10.3305/nh.2015.31.6.8845, DOI 10.3305/NH.2015.31.6.8845]

Karlsson J, 2000, INT J OBESITY, V24, P1715, DOI 10.1038/sj.ijo.0801442

KATON WTHE, 2008, THE AMERICAN JOURNAL, V121, pS8, DOI DOI 10.1016/J.AMJMED.2008.09.008

Khullar S, 2016, COMMUNITY MENT HLT J, V52, P479, DOI 10.1007/s10597-015-9985-y

Kimbro LB, 2012, INT J ENDOCRINOL, DOI 10.1155/2012/747460

Lerman I, 2009, ENDOCR PRACT, V15, P41, DOI 10.4158/EP.15.1.41

Loffler A, 2015, PLOS ONE, V10, DOI 10.1371/journal.pone.0133977

Lustman PJ, 2000, DIABETES CARE, V23, P934, DOI 10.2337/diacare.23.7.934

Nichols GA, 2003, DIABETES CARE, V26, P744, DOI 10.2337/diacare.26.3.744

Pan A, 2010, ARCH INTERN MED, V170, P1884, DOI 10.1001/archinternmed.2010.356

Rawshani A, 2017, NEW ENGL J MED, V376, P1407, DOI 10.1056/NEJMoa1608664

Sacco WP, 2007, HEALTH PSYCHOL, V26, P693, DOI 10.1037/0278-6133.26.6.693

Shah BM, 2015, J DIABETES COMPLICAT, V29, P794, DOI 10.1016/j.jdiacomp.2015.04.009

Takasaki Keiko, 2016, BMJ Open Diabetes Res Care, V4, pe000310, DOI 10.1136/bmjdrc-2016-000310

Trento M, 2014, J ENDOCRINOL INVEST, V37, P79, DOI 10.1007/s40618-013-0028-7

Trento M, 2012, ACTA DIABETOL, V49, P199, DOI 10.1007/s00592-011-0275-z

Wahlqvist ML, 2012, BMC MED, V10, DOI 10.1186/1741-7015-10-150

Wang LC, 2015, BMC PUBLIC HEALTH, V15, DOI 10.1186/s12889-015-1567-y

Wild S, 2004, DIABETES CARE, V27, P1047, DOI 10.2337/diacare.27.5.1047

Yu MK, 2013, NEPHRON CLIN PRACT, V124, P106, DOI 10.1159/000355551

NR 38

TC 0

Z9 0

U1 3

U2 8

PU ROUTLEDGE JOURNALS, TAYLOR & FRANCIS LTD

PI ABINGDON

PA 2-4 PARK SQUARE, MILTON PARK, ABINGDON OX14 4RN, OXON, ENGLAND

SN 1354-8506

EI 1465-3966

J9 PSYCHOL HEALTH MED

JI Psychol. Health Med.

PY 2018

VL 23

IS 3

BP 325

EP 336

DI 10.1080/13548506.2017.1363897

PG 12

WC Public, Environmental & Occupational Health

SC Public, Environmental & Occupational Health

GA FS6KK

UT WOS:000419906100009

PM 28871828

DA 2019-01-08

ER

PT J

AU Van Gampelaere, C

Vervoort, T

Luyckx, K

De Paepe, A

Van Aken, S

Goubert, L

AF Van Gampelaere, Cynthia

Vervoort, Tine

Luyckx, Koen

De Paepe, Annick

Van Aken, Sara

Goubert, Liesbet

TI Maternal distress in the context of their child's type 1 diabetes:

exploring the role of adaptive maternal emotion regulation on child

outcomes

SO PSYCHOLOGY HEALTH & MEDICINE

LA English

DT Article

DE Pediatric psychology; diabetes mellitus type 1; parental distress;

cognitive emotion regulation

ID PEDIATRIC PARENTING STRESS; DEPRESSIVE SYMPTOMS; YOUNG-CHILDREN;

PSYCHOLOGICAL DISTRESS; PSYCHIATRIC-DISORDERS; INTERVENTION TARGETS;

HOSPITAL ANXIETY; DUTCH VERSION; DOWN-SYNDROME; CHRONIC PAIN

AB Parents of children with Type 1 diabetes (T1D) experience high levels of distress, which may negatively impact child functioning. However, little is known about mechanisms that may buffer the adverse impact of parental distress. The current study explored the possible buffering role of maternal adaptive cognitive emotion regulation (CER) for the relationship between maternal distress and child psychological functioning. Forty-three children with T1D (8-15years) completed measures assessing trait anxiety and depressive symptoms. Their mothers reported on general distress, illness-related parenting stress, and adaptive CER. Maternal illness-related parenting stress (but not general distress) was significantly associated with child psychological functioning. No buffering role for maternal adaptive CER was observed. As the current study is rather preliminary, future research using other methods to examine maternal adaptive CER, and examining other parental variables that may buffer against the negative impact of parental distress is warranted.

C1 [Van Gampelaere, Cynthia; Vervoort, Tine; De Paepe, Annick; Goubert, Liesbet] Univ Ghent, Dept Expt Clin & Hlth Psychol, Ghent, Belgium.

[Luyckx, Koen] Univ Leuven, Dept Sch Psychol & Dev Context, Leuven, Belgium.

[Van Aken, Sara] Univ Hosp Ghent, Dept Pediat, Ghent, Belgium.

RP Van Gampelaere, C (reprint author), Univ Ghent, Dept Expt Clin & Hlth Psychol, Ghent, Belgium.

EM Cynthia.VanGampelaere@UGent.be

OI Goubert, Liesbet/0000-0002-7657-5148; De Paepe,

Annick/0000-0002-9366-3692

FU Fund for Scientific Research - Flanders (FWO, Belgium) [11V9518N]

FX This work was supported by Fund for Scientific Research - Flanders (FWO,

Belgium), grant number 11V9518N.

CR Aktar E, 2014, J CHILD PSYCHOL PSYC, V55, P77, DOI 10.1111/jcpp.12121

Bakker F. C., 1989, MANUAL DUTCH VERSION

Butwicka A, 2016, PSYCHOSOMATICS, V57, P185, DOI 10.1016/j.psym.2015.11.001

Cameron LD, 2007, J PEDIATR PSYCHOL, V32, P733, DOI 10.1093/jpepsy/jslo53

Chow ET, 2016, J PAIN, V17, P729, DOI 10.1016/j.jpain.2016.02.014

Cline GD, 2011, J CLIN PSYCHOL MED S, V18, P416, DOI 10.1007/s10880-011-9266-z

Durlak JA, 2009, J PEDIATR PSYCHOL, V34, P917, DOI 10.1093/jpepsy/jsp004

Garnefski N, 2001, PERS INDIV DIFFER, V30, P1311, DOI 10.1016/S0191-8869(00)00113-6

Geisbusch CM, 2015, MONATSSCHR KINDERH, V163, P696, DOI 10.1007/s00112-015-3357-0

Glidden LM, 2006, J INTELL DISABIL RES, V50, P949, DOI 10.1111/j.1365-2788.2006.00929.x

Goubert L., 2013, OXFORD TXB PEDIAT PA, P95

Haugstvedt A, 2010, DIABETIC MED, V27, P72, DOI 10.1111/j.1464-5491.2009.02867.x

Helgeson VS, 2012, J PEDIATR PSYCHOL, V37, P467, DOI 10.1093/jpepsy/jsr110

Hilliard ME, 2011, CHILD CARE HLTH DEV, V37, P224, DOI 10.1111/j.1365-2214.2010.01162.x

Hilliard ME, 2015, J PEDIATR PSYCHOL, V40, P835, DOI 10.1093/jpepsy/jsv072

Hoorelbeke K, 2016, J AFFECT DISORDERS, V195, P96, DOI 10.1016/j.jad.2016.02.001

Jaser SS, 2008, J PEDIATR PSYCHOL, V33, P509, DOI 10.1093/jpepsy/jsm104

Jaser SS, 2014, MATERN CHILD HLTH J, V18, P101, DOI 10.1007/s10995-013-1239-4

KOVACS M, 1981, ACTA PAEDOPSYCHIATR, V46, P305

Kovacs M, 1997, DIABETES CARE, V20, P36, DOI 10.2337/diacare.20.1.36

Kraaij V, 2015, J CLIN PSYCHOL MED S, V22, P45, DOI 10.1007/s10880-015-9417-8

Kraaij V, 2012, J ADOLESCENCE, V35, P1593, DOI 10.1016/j.adolescence.2012.06.007

Lowes L, 2005, J ADV NURS, V50, P253, DOI 10.1111/j.1365-2648.2005.03388.x

Mullins LL, 2004, CHILD HEALTH CARE, V33, P21, DOI 10.1207/s15326888chc3301_2

Nieuwesteeg AM, 2016, EUR J PEDIATR, V175, P329, DOI 10.1007/s00431-015-2631-4

Norizan A, 2010, J INTELL DISABIL RES, V54, P992, DOI 10.1111/j.1365-2788.2010.01324.x

Patterson CC, 2012, DIABETOLOGIA, V55, P2142, DOI 10.1007/s00125-012-2571-8

Patton SR, 2008, J CLIN PSYCHOL MED S, V15, P252, DOI 10.1007/s10880-008-9123-x

Patton SR, 2011, J CLIN PSYCHOL MED S, V18, P345, DOI 10.1007/s10880-011-9256-1

Roelofs J, 2010, PSYCHOL ASSESSMENT, V22, P866, DOI 10.1037/a0020593

Sieberg CB, 2011, J PEDIATR PSYCHOL, V36, P1043, DOI 10.1093/jpepsy/jsr043

Spielberger C. D., 1973, STATE TRAIT ANXIETY

Spinhoven P, 1997, PSYCHOL MED, V27, P363, DOI 10.1017/S0033291796004382

Streisand R, 2005, J PEDIATR PSYCHOL, V30, P513, DOI 10.1093/jpepsy/jsi076

Streisand R, 2008, PATIENT EDUC COUNS, V73, P333, DOI 10.1016/j.pec.2008.06.014

van der Veek SMC, 2009, INTELLECT DEV DISAB, V47, P295, DOI 10.1352/1934-9556-47.4.295

Vanderhasselt MA, 2014, J BEHAV THER EXP PSY, V45, P291, DOI 10.1016/j.jbtep.2014.01.003

Vervoort T, 2011, PSYCHOL REP, V109, P879, DOI 10.2466/07.15.16.21.PR0.109.6.879-895

Vrijmoet-Wiersma CMJ, 2010, PSYCHO-ONCOLOGY, V19, P368, DOI 10.1002/pon.1571

Whittemore R, 2012, DIABETES EDUCATOR, V38, P562, DOI 10.1177/0145721712445216

Williams LB, 2009, DIABETIC MED, V26, P908, DOI 10.1111/j.1464-5491.2009.02794.x

ZIGMOND AS, 1983, ACTA PSYCHIAT SCAND, V67, P361, DOI 10.1111/j.1600-0447.1983.tb09716.x

NR 42

TC 0

Z9 0

U1 2

U2 9

PU ROUTLEDGE JOURNALS, TAYLOR & FRANCIS LTD

PI ABINGDON

PA 2-4 PARK SQUARE, MILTON PARK, ABINGDON OX14 4RN, OXON, ENGLAND

SN 1354-8506

EI 1465-3966

J9 PSYCHOL HEALTH MED

JI Psychol. Health Med.

PY 2018

VL 23

IS 3

BP 337

EP 346

DI 10.1080/13548506.2017.1394475

PG 10

WC Public, Environmental & Occupational Health

SC Public, Environmental & Occupational Health

GA FS6KK

UT WOS:000419906100010

PM 29057673

DA 2019-01-08

ER

PT J

AU Dieter, T

Lauerer, J

AF Dieter, Taylor

Lauerer, Joy

TI Depression or Diabetes Distress?

SO PERSPECTIVES IN PSYCHIATRIC CARE

LA English

DT Article

AB PURPOSEThe purpose of this paper is to summarize the need for improved recognition of depression versus diabetes distress and discuss recommendations for appropriate screening tools.

PRACTICE IMPLICATIONSIn order to assess for both depression and diabetes distress, a combined approach of using both the WHO-5 and the PAID-20 is a simple and reliable method for detecting depression and assisting in determining the focus of specific aspects of diabetes management based on the patient's reported depressive symptoms.

CONCLUSIONSRoutine screening for depression and diabetes distress is essential for improving quality of life and reducing healthcare cost in patients with diabetes.

C1 [Dieter, Taylor] Med Univ South Carolina, Charleston, SC 29425 USA.

[Lauerer, Joy] Med Univ South Carolina, Sch Nursing, Charleston, SC USA.

RP Dieter, T (reprint author), Med Univ South Carolina, Charleston, SC 29425 USA.

EM coatest@musc.edu

CR American Diabetes Association, 2016, DIABETES, V39, pS13, DOI DOI 10.2337/DC16-S005

American Psychiatric Association, 2013, DIAGN STAT MAN MENT

Balhara Yatan Pal Singh, 2011, Indian J Endocrinol Metab, V15, P274, DOI 10.4103/2230-8210.85579

Chew BH, 2016, PLOS ONE, V11, DOI 10.1371/journal.pone.0152095

Esbitt S., 2013, SCREENING DEPRESSION, V2, P22, DOI 10. 1007/978-0-85729-751-8_2

Hermanns N, 2013, PRIM CARE DIABETES, V7, P1, DOI 10.1016/j.pcd.2012.11.002

Holt RIG, 2014, CURR DIABETES REP, V14, DOI 10.1007/s11892-014-0491-3

Markowitz SM, 2011, PSYCHOSOMATICS, V52, P1, DOI 10.1016/j.psym.2010.11.007

Novo Nordisk, 2006, PROBL AR DIAB QUEST

Polonsky WH, 2005, DIABETES CARE, V28, P626, DOI 10.2337/diacare.28.3.626

Sarkar Siddharth, 2014, Indian J Endocrinol Metab, V18, P468, DOI 10.4103/2230-8210.137487

Topp CW, 2015, PSYCHOTHER PSYCHOSOM, V84, DOI 10.1159/000376585

Twist K, 2013, PSYCHOSOM MED, V75, P791, DOI 10.1097/PSY.0b013e3182a2b108

World Health Organization, 2016, DEPRESSION

World Health Organization, 2016, 10 FACTS DIAB

Wu Shu-Fang Vivienne, 2014, Depress Res Treat, V2014, P239490, DOI 10.1155/2014/239490

NR 16

TC 2

Z9 2

U1 0

U2 1

PU WILEY

PI HOBOKEN

PA 111 RIVER ST, HOBOKEN 07030-5774, NJ USA

SN 0031-5990

EI 1744-6163

J9 PERSPECT PSYCHIATR C

JI Perspect. Psychiatr. Care

PD JAN

PY 2018

VL 54

IS 1

BP 84

EP 87

DI 10.1111/ppc.12203

PG 4

WC Nursing; Psychiatry

SC Nursing; Psychiatry

GA FS4DW

UT WOS:000419738700012

PM 28090642

DA 2019-01-08

ER

PT J

AU Milaniak, I

Wilczek-Ruzyczka, E

Wierzbicki, K

Piatek, J

Kedziora, A

Przybylowski, P

AF Milaniak, Irena

Wilczek-Ruzyczka, Ewa

Wierzbicki, Karol

Piatek, Jacek

Kedziora, Anna

Przybylowski, Piotr

TI The effect of clinical variables on distress and depressive symptoms

among heart transplant recipients

SO HEART & LUNG

LA English

DT Article

DE Comorbidities; Stress; Depression; Heart transplantation

ID QUALITY-OF-LIFE; INTERNATIONAL SOCIETY; ORGAN-TRANSPLANTATION;

PSYCHIATRIC ASPECTS; SHORT-FORM; PREVALENCE; METAANALYSIS; INVENTORY;

VALIDITY; RISK

AB Background: Heart transplantation (HTx) is the standard treatment for end-stage cardiomyopathy and coronary artery disease. Although major improvements have been made in the prevention and treatment of acute graft rejection, comorbidities still limit the long-term survival of heart transplant recipients. The risk of poor outcome, such us major health status aggravation and death, can stimulate the occurrence of depression and stress in this population. The aim of this study was to determine the impact of comorbidities on depressive symptoms and distress among heart transplant recipients.

Material/methods: The sample included 131 HTx recipients from one site. Data were collected during a follow-up in-hospital appointment, using the questionnaires assessing depression (Beck Depression Inventory Short Form) and stress (Perceived Stress Scale-10). Statistical analyses included descriptive statistics, Pearson correlations, t-tests, and generalized linear models.

Results: Study patients were 75.6% (n = 97) male, 100% (n = 131) Caucasian, 74% (n = 89) married, with the mean age of 54 years at time of heart transplantation. Nearly half of the participants (40.5%) presented depression symptoms at the time of evaluation. Severe stress was observed in 30% of individuals. Depression symptoms and severe distress were more commonly observed in patients with many comorbidities, requiring multiple drug therapy, and high NYHA score. Moreover, in a multivariate logistic regression, depression and high distress level occurrence were predicted by the following independent factors: cardiac allograft vasculopathy, cancer, diabetes, higher NYHA score, and comorbidities.

Conclusions: The prevalence of depression and severe distress is common among heart transplant recipients. Patients with many comorbidities are at higher risk of psychological indisposition. (C) 2017 Elsevier Inc. All rights reserved.

C1 [Milaniak, Irena; Wierzbicki, Karol; Kedziora, Anna] John Paul 2 Hosp, Cardiovasc Surg & Transplantol Dept, Ul Pradnicka 80, PL-31202 Krakow, Poland.

[Milaniak, Irena] State Higher Vocat Sch, Inst Hlth, Nowysacz, Poland.

[Milaniak, Irena] Andrzej FryczModrzewski Krakow Univ, Fac Hlth & Med Sci, Krakow, Poland.

[Wilczek-Ruzyczka, Ewa] Andrzej FryczModrzewski Krakow Univ, Fac Psychol & Humanities, Krakow, Poland.

[Wierzbicki, Karol] Jagiellonian Univ, Med Coll, Krakow, Poland.

[Przybylowski, Piotr] Jagiellonian Univ, Med Coll, Chair Gen Surg 1, Krakow, Poland.

[Przybylowski, Piotr] Silesian Ctr Heart Dis, Zabrze, Poland.

[Piatek, Jacek] Jagiellonian Univ, John Paul Hosp 2, Coll Med, Dept Cardiovasc Surg & Transplantol, Krakow, Poland.

RP Milaniak, I (reprint author), John Paul 2 Hosp, Cardiovasc Surg & Transplantol Dept, Ul Pradnicka 80, PL-31202 Krakow, Poland.

EM irenem@poczta.onet.pl

OI , IRENA/0000-0002-6649-7545

CR Anderson RJ, 2001, DIABETES CARE, V24, P1069, DOI 10.2337/diacare.24.6.1069

CHIBNALL JT, 1994, CLIN J PAIN, V10, P261, DOI 10.1097/00002508-199412000-00004

COHEN S, 1983, J HEALTH SOC BEHAV, V24, P385, DOI 10.2307/2136404

Cristiano YH, 2012, TRANSPLANT J AUSTRAL, V21, P16

Dew MA, 2001, PSYCHOSOMATICS, V42, P300, DOI 10.1176/appi.psy.42.4.300

DiMartini A, 2008, CRIT CARE CLIN, V24, P949, DOI 10.1016/j.ccc.2008.05.001

Dobbels F, 2004, TRANSPLANT INT, V17, P424, DOI 10.1007/s00147-004-0732-5

Furlanetto LM, 2005, J AFFECT DISORDERS, V86, P87, DOI 10.1016/j.jad.2004.12.011

HOUSE RM, 1988, JAMA-J AM MED ASSOC, V260, P535, DOI 10.1001/jama.260.4.535

Hsu RB, 2005, J FORMOS MED ASSOC, V104, P482

Jalowiec A, 2007, J CARDIOPULM REHABIL, V27, P24

Kugler C, 2014, J PSYCHOSOM RES, V77, P128, DOI 10.1016/j.jpsychores.2014.06.007

Lund LH, 2016, J HEART LUNG TRANSPL, V35, P1158, DOI 10.1016/j.healun.2016.08.017

Mehra MR, 2010, J HEART LUNG TRANSPL, V29, P717, DOI 10.1016/j.healun.2010.05.017

Moskalewicz J, 2012, EPIDEMIOLOGIA ZABURZ

Palmer S, 2013, KIDNEY INT, V84, P179, DOI 10.1038/ki.2013.77

Ravven S, 2013, HARVARD REV PSYCHIAT, V21, P59, DOI 10.1097/HRP.0b013e31828a3612

Reyes CJ, 2004, J HEART LUNG TRANSPL, V23, P614, DOI 10.1016/S1053-2498(03)00310-3

Riley A. A., 2009, JNP-J NURSE PRACT, V5, P523, DOI DOI 10.1016/J.NURPRA.2009.03.020

Ruzyczka EW, 2011, TRANSPL P, V43, P3076, DOI 10.1016/j.transproceed.2011.07.012

Rybarczyk B, 2007, REHABIL PSYCHOL, V52, P206, DOI 10.1037/0090-5550.52.2.206

Stiefel P, 2013, J HEALTH PSYCHOL, V18, P680, DOI 10.1177/1359105312454909

NR 22

TC 0

Z9 0

U1 1

U2 1

PU MOSBY-ELSEVIER

PI NEW YORK

PA 360 PARK AVENUE SOUTH, NEW YORK, NY 10010-1710 USA

SN 0147-9563

EI 1527-3288

J9 HEART LUNG

JI Heart Lung

PD JAN-FEB

PY 2018

VL 47

IS 1

BP 68

EP 72

DI 10.1016/j.hrtlng.2017.09.008

PG 5

WC Cardiac & Cardiovascular Systems; Nursing; Respiratory System

SC Cardiovascular System & Cardiology; Nursing; Respiratory System

GA FQ2TD

UT WOS:000418210000010

PM 29103660

DA 2019-01-08

ER

PT J

AU Martinez, K

Lockhart, S

Davies, M

Lindsay, JR

Dempster, M

AF Martinez, Kelly

Lockhart, Sam

Davies, Mark

Lindsay, John R.

Dempster, Martin

TI Diabetes distress, illness perceptions and glycaemic control in adults

with type 2 diabetes

SO PSYCHOLOGY HEALTH & MEDICINE

LA English

DT Article

DE Type 2 diabetes; distress; glycaemic control; illness perceptions

ID INTERVENTION; SCALE

AB The emotional distress associated with adjusting to and living with diabetes has been termed diabetes distress. Diabetes distress is associated with glycaemic control but interventions to reduce diabetes distress have failed to consistently improve diabetes control. Various illness perceptions have previously been linked with both diabetes distress and glycaemic control but interrelationships between these features have not been previously investigated. We hypothesised that illness perceptions mediate the relationship between diabetes distress and glycaemia. Participants with type 2 diabetes attending diabetes outpatient clinics (n=84) provided demographic and clinical information and completed the Diabetes Distress Scale-17 and the Brief Illness Perceptions Questionnaire. Using regression analysis we demonstrated that the illness perceptions of personal control, regimen-related distress, socioeconomic status and insulin use were significant contributors in the final model predicting HbA(1c). Higher levels of personal control were associated with better glycaemic control. Conversely, regimen-related distress was associated with hyperglycaemia. Mediation analyses showed that the relationship between regimen-related distress and HbA(1c) was mediated by personal control. Our work suggests that psychological interventions designed to reduce diabetes distress may be more efficacious in improving glycaemic control if they address an individual's perception of personal control.

C1 [Martinez, Kelly; Dempster, Martin] Queens Univ Belfast, Sch Psychol, Belfast, Antrim, North Ireland.

[Lockhart, Sam] Queens Univ Belfast, Sch Med Dent & Biol Sci, Belfast, Antrim, North Ireland.

[Davies, Mark] Belfast City Hosp, Dept Psychol, Belfast, Antrim, North Ireland.

[Lindsay, John R.] Mater Hosp, Belfast, Antrim, North Ireland.

[Lockhart, Sam] Altnagelvin Hosp, Derry, North Ireland.

RP Lockhart, S (reprint author), Queens Univ Belfast, Sch Med Dent & Biol Sci, Belfast, Antrim, North Ireland.; Lockhart, S (reprint author), Altnagelvin Hosp, Derry, North Ireland.

EM slockhart05@doctors.org.uk

OI Lockhart, Sam/0000-0003-2092-4350; , Martin/0000-0001-9499-3186

CR BARON RM, 1986, J PERS SOC PSYCHOL, V51, P1173, DOI 10.1037//0022-3514.51.6.1173

Berry E, 2015, POSTGRAD MED J, V91, P278, DOI 10.1136/postgradmedj-2014-133017

Bridges HA, 2016, J HEALTH PSYCHOL, V21, P1956, DOI 10.1177/1359105315569094

Broadbent E, 2006, J PSYCHOSOM RES, V60, P631, DOI 10.1016/j.jpsychores.2005.10.020

Cummings DM, 2014, ANN PHARMACOTHER, V48, P970, DOI 10.1177/1060028014536532

Fisher L, 2007, DIABETES CARE, V30, P542, DOI 10.2337/dc06-1614

Fisher L, 2013, DIABETES CARE, V36, P2551, DOI 10.2337/dc12-2493

Fisher L, 2010, DIABETES CARE, V33, P23, DOI 10.2337/dc09-1238

Gonzalez JS, 2015, HEALTH PSYCHOL, V34, P505, DOI 10.1037/hea0000131

Hessler D, 2014, DIABETES CARE, V37, P617, DOI 10.2337/dc13-0762

Keogh KM, 2011, AM J MANAG CARE, V17, P105

Leventhal H., 2003, SELF REGULATION HLTH, V1, P42

Mc Sharry J, 2011, DIABETIC MED, V28, P1300, DOI 10.1111/j.1464-5491.2011.03298.x

Northern Ireland Statistics and Research Agency, 2010, NO IR MULT DEPR MEAS

Pandit AU, 2014, J EPIDEMIOL COMMUN H, V68, P557, DOI 10.1136/jech-2013-203063

Polonsky WH, 2005, DIABETES CARE, V28, P626, DOI 10.2337/diacare.28.3.626

Strandberg RB, 2015, DIABETIC MED, V32, P1304, DOI 10.1111/dme.12781

Welch GW, 1997, DIABETES CARE, V20, P760, DOI 10.2337/diacare.20.5.760

World Health Organization, 2016, GLOB REP DIAB

NR 19

TC 3

Z9 3

U1 3

U2 6

PU ROUTLEDGE JOURNALS, TAYLOR & FRANCIS LTD

PI ABINGDON

PA 2-4 PARK SQUARE, MILTON PARK, ABINGDON OX14 4RN, OXON, ENGLAND

SN 1354-8506

EI 1465-3966

J9 PSYCHOL HEALTH MED

JI Psychol. Health Med.

PY 2018

VL 23

IS 2

BP 171

EP 177

DI 10.1080/13548506.2017.1339892

PG 7

WC Public, Environmental & Occupational Health

SC Public, Environmental & Occupational Health

GA FP4RU

UT WOS:000417604900005

PM 28610449

OA Green Published

DA 2019-01-08

ER

PT J

AU Al-Yateem, N

Rossiter, R

Robb, W

Ahmad, A

Elhalik, MS

Albloshi, S

Slewa-Younan, S

AF Al-Yateem, Nabeel

Rossiter, Rachel

Robb, Walter

Ahmad, Alaa

Elhalik, Mahmoud Saleh

Albloshi, Sumaya

Slewa-Younan, Shameran

TI Mental health literacy among pediatric hospital staff in the United Arab

Emirates

SO BMC PSYCHIATRY

LA English

DT Article

DE Cross-cultural care; Early intervention; Health literacy; Health

promotion; Mental health; Professional practice gaps; Pediatric care

ID ADULT SERVICES; BELIEFS; PROFESSIONALS; PSYCHIATRISTS; TRANSITION;

DISORDERS; KNOWLEDGE; ILLNESS

AB Background: In the United Arab Emirates (UAE) 35% of the population are aged 0-24 years. A significant proportion of these young people are living with chronic conditions (e.g., asthma, type 1 diabetes, cardiac conditions, and geneticallytransmitted conditions such as thalassemia and cystic fibrosis). This group has increased vulnerability to developmental delays and mental health problems, and is increasingly coming to the attention of service providers in mainstream schools, primary healthcare centers, and pediatric hospitals. Despite the government directing attention to improving the mental health of the UAE population, there is concern that mental health services are not growing at the rate needed to meet the mental health needs of children and young people with chronic conditions.

Method: A cross sectional survey design was used to determine the mental health literacy of nurses and other healthcare professionals working with children with chronic illnesses. Participants completed a culturally-adapted mental health literacy questionnaire comprising three vignettes of fictional characters meeting diagnostic criteria for posttraumatic stress disorder, psychosis, and depression with suicidal thoughts. Participants also completed the Kessler Psychological Distress Scale (K10).

Results: Participants were 317 healthcare professionals from across the UAE. The majority were nurses. Correct identification of the diagnosis for each vignette was limited, with the highest level of accuracy achieved for the psychosis vignette (n = 113, 54.3%). Accurate identification of appropriate evidence-based interventions was also limited. K10 scores indicated 40% of participants had moderate to high levels of psychological distress.

Conclusions: These findings are concerning and provide important data to inform the development of undergraduate and continuing education programs for nurses. The K10 scores suggest healthcare professionals are under considerable stress, highlighting the need to support healthcare professionals who experience multiple psychosocial stressors.

C1 [Al-Yateem, Nabeel] Univ Sharjah, Dept Nursing, Coll Hlth Sci, POB 27272, Sharjah, U Arab Emirates.

[Al-Yateem, Nabeel] Charles Sturt Univ, Sch Nursing Midwifery & Indigenous Hlth, Fac Sci, Orange, NSW, Australia.

[Al-Yateem, Nabeel] Univ Sharjah, RIMHS, Sharjah, U Arab Emirates.

[Rossiter, Rachel] Charles Sturt Univ, Sch Nursing Midwifery & Indigenous Hlth, Orange, NSW, Australia.

[Robb, Walter] Griffith Univ, Menzies Hlth Inst, Brisbane, Qld, Australia.

[Ahmad, Alaa] SKMC, Abu Dhabi, U Arab Emirates.

[Elhalik, Mahmoud Saleh] Latifa Hosp, Dept & Neonatol Unit, Dubai, U Arab Emirates.

[Albloshi, Sumaya] Minist Hlth & Prevent, Dubai, U Arab Emirates.

[Slewa-Younan, Shameran] Western Sydney Univ, Ctr Hlth Res, Sch Med, Mental Hlth, Sydney, NSW, Australia.

[Slewa-Younan, Shameran] Univ Melbourne, Ctr Mental Hlth, Melbourne Sch Populat & Global Hlth, Melbourne, Vic, Australia.

RP Al-Yateem, N (reprint author), Univ Sharjah, Dept Nursing, Coll Hlth Sci, POB 27272, Sharjah, U Arab Emirates.; Al-Yateem, N (reprint author), Charles Sturt Univ, Sch Nursing Midwifery & Indigenous Hlth, Fac Sci, Orange, NSW, Australia.; Al-Yateem, N (reprint author), Univ Sharjah, RIMHS, Sharjah, U Arab Emirates.

EM Nalyateem@sharjah.ac.ae

OI Rossiter, Rachel/0000-0002-6147-861X

FU AL Jalila Foundation, United Arab Emirates

FX This research was funded by AL Jalila Foundation, United Arab Emirates.

The funding body had no role in the design of the study, the collection,

analysis, and interpretation of data, and in writing the manuscript.

CR Al-Darmaki FR, 2015, PSYCHOL INT NEWSL, V2015, P1

Al-Yateem N, 2016, J SPEC PEDIATR NURS, P1

Al-Yateem Nabeel, 2013, Nurs Child Young People, V25, P29, DOI 10.7748/ncyp2013.06.25.5.29.e175

Al-Yateem Nabeel, 2012, Br J Nurs, V21, P850

Annunziato RA, 2007, PEDIATR TRANSPLANT, V11, P608, DOI 10.1111/j.1399-3046.2007.00689.x

Annunziato RA, 2013, J PEDIATR-US, V163, P1628, DOI 10.1016/j.jpeds.2013.07.031

Chikaodiri AN, 2010, MENT ILLN, V2, DOI 10.4081/mi.2010.e1

CIA, 2016, WORLD FACTBOOK

Coyne I., 2015, AN INT J PUBLIC PART, V18, P796

Ekra EMR, 2012, INT J QUAL STUD HEAL, V7, DOI 10.3402/qhw.v7i0.18694

Ghaffar A, 2010, SUMMARY NATL PRIORIT

Jones WR, 2013, EUR EAT DISORD REV, V21, P84, DOI 10.1002/erv.2155

Jorm AF, 1997, MED J AUSTRALIA, V166, P182

Jorm AF, 2012, AM PSYCHOL, V67, P231, DOI 10.1037/a0025957

Liu W, 2011, INT NURS REV, V58, P361, DOI 10.1111/j.1466-7657.2011.00883.x

MacCarthy Dan, 2013, Perm J, V17, P14, DOI 10.7812/TPP/13-033

Maindonald J., 2006, DATA ANAL GRAPHICS U

Mayer CH, 2014, INT REV PSYCHIATR, V26, P265, DOI 10.3109/09540261.2013.866076

Mohamed-Kaloo Z, 2014, SAMJ S AFR MED J, V104, P350, DOI [10.7196/SAMJ.7863, 10.7196/samj.7863]

Morgan AJ, 2014, AUST NZ J PSYCHIAT, V48, P442, DOI 10.1177/0004867413512686

Reavley NJ, 2012, EARLY INTERV PSYCHIA, V6, P45, DOI 10.1111/j.1751-7893.2011.00314.x

Rutishauser C, 2011, EUR J PEDIATR, V170, P865, DOI 10.1007/s00431-010-1364-7

Sayed MA, 2015, INT J EMERG MENT HLT, V17, P661

Schumacher K, 2010, TRANSITIONS THEORY M

Slewa-Younan S, 2014, BMC PSYCHIATRY, V14, DOI 10.1186/s12888-014-0320-x

Sulaiman-Hill Cheryl Mr, 2010, BMC Res Notes, V3, P237, DOI 10.1186/1756-0500-3-237

The American Psychiatric Association, 1995, DIAGN STAT MAN MENT

US-UAE Business Council, 2014, UAE HEALTHC SECT

NR 28

TC 0

Z9 0

U1 2

U2 10

PU BIOMED CENTRAL LTD

PI LONDON

PA 236 GRAYS INN RD, FLOOR 6, LONDON WC1X 8HL, ENGLAND

SN 1471-244X

J9 BMC PSYCHIATRY

JI BMC Psychiatry

PD DEC 8

PY 2017

VL 17

AR 390

DI 10.1186/s12888-017-1556-z

PG 12

WC Psychiatry

SC Psychiatry

GA FP3SK

UT WOS:000417537900001

PM 29216871

OA DOAJ Gold, Green Published

DA 2019-01-08

ER

PT J

AU Tanaka, R

Trief, PM

Scales, K

Weinstock, RS

AF Tanaka, Rika

Trief, Paula M.

Scales, Kasandra

Weinstock, Ruth S.

TI "Miscarried Helping" in Adults With Type 2 Diabetes: Helping for Health

Inventory-Couples

SO FAMILIES SYSTEMS & HEALTH

LA English

DT Article

DE miscarried helping; Type 2 diabetes; marital satisfaction

ID RELATIONSHIP SATISFACTION; MARITAL RELATIONSHIP; SUPPORT;

OVERPROTECTION; RELIABILITY; ADJUSTMENT; ADHERENCE; DISTRESS; PARTNERS;

VALIDITY

AB Objective: Family-partner support has been associated with better blood glucose control, self-care adherence, and quality of life in adult patients with Type 2 diabetes (T2D). "Miscarried helping" has described interactions between youth with chronic diseases and their family members, in which a family member is helpful, but efforts are perceived as negative by the patient. Miscarried helping has not, however, been measured in adults with diabetes, which was the purpose of our study. Method: Data from a randomized clinical trial (n = 268) were analyzed to establish the psychometric properties and correlates of an adaptation of a measure of miscarried helping developed in pediatric populations, for use with adults with T2D. Results: The Helping for Health Inventory-Couples Version (HHI-C) was found to have three underlying factors and demonstrated adequate internal consistency across time (alpha at baseline = .86, 4 months = .87, 8 months = .86, and 12 months = .83) and showed high test-retest reliability (p <.01) over a 12-month period. Convergent validity was partially supported, because baseline HHI-C was positively associated with maladaptive conflict resolution strategies (p = .03) and negatively associated with adaptive conflict resolution strategies (p = .04) and diabetes knowledge (p < .01). The HHI-C did not correlate with body mass index or hemoglobin A1c, a measure of glycemic control. The HHI-C was positively associated with diabetes distress (p < .01) and depressive symptoms (p = .01). Discussion: This study is the 1st known reporting of the psychometric properties of a measure of miscarried helping for adults with T2D. This valid measure of miscarried helping could be useful in future studies evaluating novel, relationship-based approaches to assist adults with T2D in disease management.

C1 [Tanaka, Rika] Toronto Gen Hosp, Behav Cardiol Res Unit, 585 Univ Ave,6N-618, Toronto, ON M5G 2C2, Canada.

[Tanaka, Rika] Univ Toronto, Dept Psychiat, Toronto, ON, Canada.

[Trief, Paula M.; Scales, Kasandra] SUNY Upstate Med Univ, Dept Psychiat & Behav Sci, Syracuse, NY 13210 USA.

[Weinstock, Ruth S.] SUNY Upstate Med Univ, Dept Med, Syracuse, NY 13210 USA.

RP Tanaka, R (reprint author), Toronto Gen Hosp, Behav Cardiol Res Unit, 585 Univ Ave,6N-618, Toronto, ON M5G 2C2, Canada.

EM rika.tanaka@uhnresearch.ca

FU National Institutes of Health (NIH) [1R18DK080867-01A2]

FX This research was supported in part by National Institutes of Health

(NIH) Grant 1R18DK080867-01A2. We would like to acknowledge our

colleagues whose work on the Diabetes Support Project provided the data

for this article, including Lawrence Fisher and Danielle Hessler of

University of California, San Francisco; Jonathan Sandberg of Brigham

Young University; and Jacqueline Dimmock and Patricia Forken of SUNY

Upstate Medical University. We would also like to thank Michael Harris

of Oregon Health Sciences University for graciously granting permission

for us to adapt the Helping for Health Inventory to use with couples.

Finally, we thank the patients and partners who generously shared their

time, thoughts, and feelings with us and the diabetes educators who

provided skilled interventions. The content is solely the responsibility

of the authors and does not necessarily reflect the official views of

the National Institutes of Health or its affiliates.

CR ANDERSON BJ, 1991, ADVANCES IN CHILD HEALTH PSYCHOLOGY, P167

BUSBY DM, 1995, J MARITAL FAM THER, V21, P289, DOI 10.1111/j.1752-0606.1995.tb00163.x

COYNE JC, 1991, J PERS SOC PSYCHOL, V61, P404, DOI 10.1037/0022-3514.61.3.404

Davidson N. K., 2012, DEALING DIABETES POL

DEURENBERG P, 1991, BRIT J NUTR, V65, P105, DOI 10.1079/BJN19910073

Fales JL, 2014, J PEDIATR PSYCHOL, V39, P427, DOI 10.1093/jpepsy/jsu003

Fisher L, 2000, J FAM PRACTICE, V49, P561

Fisher L., 2006, FAMILIES SYSTEMS HLT, V24, P373, DOI DOI 10.1037/1091-7527.24.4.373

Fitzgerald JT, 1998, DIABETES CARE, V21, P706, DOI 10.2337/diacare.21.5.706

GARAYSEVILA ME, 1995, J DIABETES COMPLICAT, V9, P81, DOI 10.1016/1056-8727(94)00021-F

Gonder-Frederick LA, 2002, J CONSULT CLIN PSYCH, V70, P611, DOI 10.1037//0022-006X.70.3.611

Hagedoorn M, 2006, DIABETIC MED, V23, P271, DOI 10.1111/j.1464-5491.2006.01794.x

Harris M. A., 2008, FAMILIES SYSTEMS HLT, V26, P393, DOI [10.1037/a0014232, DOI 10.1037/A0014232]

Hinnen C, 2008, BRIT J HEALTH PSYCH, V13, P737, DOI 10.1348/135910707X252431

International Diabetes Federation, 2017, IDF DIAB ATL

Kroenke K, 2009, J AFFECT DISORDERS, V114, P163, DOI 10.1016/j.jad.2008.06.026

Kuijer RG, 2000, J SOC CLIN PSYCHOL, V19, P256, DOI 10.1521/jscp.2000.19.2.256

KURDEK LA, 1994, J MARRIAGE FAM, V56, P705, DOI 10.2307/352880

LOCKE HJ, 1959, MARRIAGE FAM LIVING, V21, P251, DOI 10.2307/348022

NATHAN DM, 1984, NEW ENGL J MED, V310, P341, DOI 10.1056/NEJM198402093100602

Nunnally J. C., 1978, PSYCHOMETRIC THEORY

Polonsky W.H., 1995, DIABETES FORECAST, V12, P21

Polonsky WH, 2005, DIABETES CARE, V28, P626, DOI 10.2337/diacare.28.3.626

Rook KS, 2011, J SOC PERS RELAT, V28, P772, DOI 10.1177/0265407510391335

Roszler J., 2016, COPING DIABETES POLI

SCHMALING K, 2000, PSYCHOL COUPLES ILLN

Schokker MC, 2010, J FAM PSYCHOL, V24, P578, DOI 10.1037/a0021009

Trief PM, 2017, J HEALTH PSYCHOL, V22, P446, DOI 10.1177/1359105315605654

Trief PM, 2016, DIABETES CARE, V39, P2165, DOI 10.2337/dc16-0035

Trief PM, 2011, TRANSL BEHAV MED, V1, P461, DOI 10.1007/s13142-011-0057-8

Trief PM, 2004, ANN BEHAV MED, V27, P148, DOI 10.1207/s15324796abm2703_2

Trief PM, 2002, DIABETES CARE, V25, P1154, DOI 10.2337/diacare.25.7.1154

Trief PM, 2001, DIABETES CARE, V24, P1384, DOI 10.2337/diacare.24.8.1384

Turner RC, 1998, LANCET, V352, P837

Yang W, 2013, DIABETES CARE, V36, P1033, DOI DOI 10.2337/DC12-2625

Yang Y, 2013, DIABETES CARE, V36, P978, DOI 10.2337/dc12-0227

NR 36

TC 0

Z9 0

U1 1

U2 2

PU EDUCATIONAL PUBLISHING FOUNDATION-AMERICAN PSYCHOLOGICAL ASSOC

PI WASHINGTON

PA 750 FIRST ST, NE, WASHINGTON, DC 20002-4242 USA

SN 1091-7527

EI 1939-0602

J9 FAM SYST HEALTH

JI Fam. Syst. Health

PD DEC

PY 2017

VL 35

IS 4

BP 409

EP 419

DI 10.1037/fsh0000289

PG 11

WC Health Care Sciences & Services; Family Studies; Public, Environmental &

Occupational Health

SC Health Care Sciences & Services; Family Studies; Public, Environmental &

Occupational Health

GA FR0OR

UT WOS:000418763000003

PM 29283609

DA 2019-01-08

ER

PT J

AU Mora, P

Buskirk, A

Lyden, M

Parkin, CG

Borsa, L

Petersen, B

AF Mora, Pablo

Buskirk, Ann

Lyden, Maureen

Parkin, Christopher G.

Borsa, Lena

Petersen, Bettina

TI Use of a Novel, Remotely Connected Diabetes Management System Is

Associated with Increased Treatment Satisfaction, Reduced Diabetes

Distress, and Improved Glycemic Control in Individuals with

Insulin-Treated Diabetes: First Results from the Personal Diabetes

Management Study

SO DIABETES TECHNOLOGY & THERAPEUTICS

LA English

DT Article

DE mHealth; Type 1 diabetes; Type 2 diabetes; Insulin; Multiple daily

injections; Self-monitoring of blood glucose

ID BLOOD-GLUCOSE; CLINICAL INERTIA; MEDICATION NONADHERENCE; PEOPLE;

IMPACT; CARE; HOSPITALIZATION; MORTALITY; MELLITUS; THERAPY

AB Background: The ability to automatically transfer data to clinicians and receive timely guidance in therapy adjustments through remote and in-office consults can positively impact patients' perceptions about quality of care, which is positively associated with clinical outcomes. We assessed the impact of using the Accu-Chek Connect diabetes management system on treatment satisfaction, diabetes distress, and glycemic control in adults with type 1 diabetes and insulin-treated type 2 diabetes. Subjects and Methods: This 6-month, prospective, multicenter, single-arm study assessed the impact of using the system on treatment satisfaction and glycemic control among 87 adults with insulin-treated diabetes (multiple daily insulin injections and basal only), with 8.8%1.6% glycated hemoglobin (HbA1c) at baseline. The Diabetes Treatment Satisfaction Questionnaire-status (DTSQs) and Diabetes Distress Scale (DDS) were administered at baseline, and the Diabetes Treatment Satisfaction Questionnaire-change (DTSQc) and DDS at 6 months. Changes in HbA1c, average blood glucose (BG), and other metrics were also assessed. Results: Improvements in DTSQc scores were observed at 6 months with a total mean (standard deviation) score of 14.3 +/- 5.1. Significant reductions in total mean DDS scores from baseline to 6 months were also observed, from 2.0 +/- 0.8 to 1.7 +/- 0.7, P<0.0001. A significant reduction in regimen-related distress was notable, from moderate distress (2.4 +/- 1.0) to not distressed (1.9 +/- 0.9), P<0.0001). Significant reductions in mean HbA1c (-0.9 +/- 1.6, P<0.0001) and mean BG (-24.8 +/- 50.8, P<0.0001) were observed. Conclusions: Use of the Accu-Chek Connect diabetes management system is associated with increased treatment satisfaction and improved glycemic control among individuals with insulin-treated diabetes. NCT02600845 (www.clinicaltrials.gov).

C1 [Mora, Pablo] Dallas Diabet Res Ctr, Dallas, TX USA.

[Buskirk, Ann; Borsa, Lena] Roche Diabet Care, Indianapolis, IN USA.

[Lyden, Maureen] Biostat Int, Tampa, FL USA.

[Parkin, Christopher G.] CGParkin Commun Inc, 932 Vista Lago Way, Boulder City, NV 89005 USA.

[Petersen, Bettina] Roche Diabet Care GmbH, Mannheim, Germany.

RP Parkin, CG (reprint author), CGParkin Commun Inc, 932 Vista Lago Way, Boulder City, NV 89005 USA.

EM chris@cgparkin.org

FU Roche Diabetes Care

FX The authors wish to thank our investigators and their patients for

participating in this study. The Bluetooth word mark and logos are

registered trademarks owned by the Bluetooth SIG, Inc., and any use of

such marks by Roche Diabetes Care is under license. Roche Diabetes Care

provided funding for the study.

CR Al Shahrani Abeer, 2014, J Family Med Prim Care, V3, P5, DOI 10.4103/2249-4863.130254

Ali MK, 2013, NEW ENGL J MED, V369, P287, DOI 10.1056/NEJMc1306652

Auerbach SM, 2002, J BEHAV MED, V25, P17, DOI 10.1023/A:1013585617303

Bosi E, 2013, DIABETES CARE, V36, P2887, DOI 10.2337/dc13-0092

Bradley C, 2007, HEALTH QUAL LIFE OUT, V5, DOI 10.1186/1477-7525-5-57

BRODY DS, 1980, J CHRON DIS, V33, P57, DOI 10.1016/0021-9681(80)90086-7

Brown JB, 2003, AM J MANAG CARE, V9, P213

Carls GS, 2016, AM DIAB ASS 76 SCI S

Currie CJ, 2013, J DIABETES COMPLICAT, V27, P219, DOI 10.1016/j.jdiacomp.2012.10.006

Doyle C, 2013, BMJ OPEN, V3, DOI 10.1136/bmjopen-2012-001570

Egede LE, 2014, ANN PHARMACOTHER, V48, P562, DOI 10.1177/1060028014526362

Faridi Z, 2008, J EVAL CLIN PRACT, V14, P465, DOI 10.1111/j.1365-2753.2007.00881.x

Fisher L, 2011, CURR MED RES OPIN, V27, P39, DOI 10.1185/03007995.2011.619176

Franciosi M, 2011, DIABETIC MED, V28, P789, DOI 10.1111/j.1464-5491.2011.03268.x

Given JE, 2013, DIABETIC MED, V30, P901, DOI 10.1111/dme.12130

Haynes RB, 1979, COMPLIANCE HLTH CARE

Hirsch Irl B, 2004, Endocr Pract, V10, P67

Ho PM, 2006, ARCH INTERN MED, V166, P1836, DOI 10.1001/archinte.166.17.1836

Holmen H, 2014, JMIR MHEALTH UHEALTH, V2, DOI 10.2196/mhealth.3882

Kato N, 2013, J DIABETES INVEST, V4, P450, DOI 10.1111/jdi.12072

Kazlauskaite R, 2009, DIABETES TECHNOL THE, V11, P385, DOI 10.1089/dia.2008.0111

Khunti K, 2016, DIABETES OBES METAB, V18, P401, DOI 10.1111/dom.12626

Khunti K, 2013, DIABETES CARE, V36, P3411, DOI 10.2337/dc13-0331

Klonoff David C, 2013, J Diabetes Sci Technol, V7, P749

Lalic Nebojsa M, 2017, J Diabetes Sci Technol, V11, P746, DOI 10.1177/1932296816681323

LEY P, 1982, BRIT J CLIN PSYCHOL, V21, P241, DOI 10.1111/j.2044-8260.1982.tb00562.x

Ley P, 1979, RES PSYCHOL MED

Liang X, 2011, DIABETIC MED, V28, P455, DOI 10.1111/j.1464-5491.2010.03180.x

Nichols GA, 2007, J GEN INTERN MED, V22, P453, DOI 10.1007/s11606-007-0139-y

Polonsky WH, 2005, DIABETES CARE, V28, P626, DOI 10.2337/diacare.28.3.626

Polonsky WH, 2011, DIABETES TECHNOL THE, V13, P797, DOI 10.1089/dia.2011.0073

Sokol MC, 2005, MED CARE, V43, P521, DOI 10.1097/01.mlr.0000163641.86870.af

World Medical Association, DECLARATION OF HELSI

Ziemer DC, 2005, DIABETES EDUCATOR, V31, P564, DOI 10.1177/0145721705279050

NR 34

TC 1

Z9 1

U1 0

U2 2

PU MARY ANN LIEBERT, INC

PI NEW ROCHELLE

PA 140 HUGUENOT STREET, 3RD FL, NEW ROCHELLE, NY 10801 USA

SN 1520-9156

EI 1557-8593

J9 DIABETES TECHNOL THE

JI Diabetes Technol. Ther.

PD DEC

PY 2017

VL 19

IS 12

BP 715

EP 722

DI 10.1089/dia.2017.0206

PG 8

WC Endocrinology & Metabolism

SC Endocrinology & Metabolism

GA FQ1XU

UT WOS:000418152000004

PM 29027812

OA Other Gold, Green Published

DA 2019-01-08

ER

PT J

AU Capistrant, BD

Friedemann-Sanchez, G

Novak, LK

Zuijdwijk, C

Ogle, GD

Pendsey, S

AF Capistrant, Benjamin D.

Friedemann-Sanchez, Greta

Novak, Lindsey K.

Zuijdwijk, Caroline

Ogle, Graham D.

Pendsey, Sharad

TI Mental health and well-being among type 1 diabetes caregivers in India:

Evidence from the IDREAM study

SO DIABETES RESEARCH AND CLINICAL PRACTICE

LA English

DT Article

DE Type 1 diabetes; Depression; Well-being; Caregiver

ID GLYCEMIC CONTROL; DIAGNOSTIC METAANALYSIS; PRIMARY-CARE; CHILDREN;

DEPRESSION; PARENTS; BURDEN; DISTRESS; STRESS; PHQ-9

AB Aims: Although more than half of the world's children with T1D live in developing countries, still little is known about how caregiving for children with T1D affects the parent/caregivers' health in low-and middle-income country settings.

Methods: Caregivers of 178 children with T1D from a specialized diabetes clinic in Maharashtra, India were surveyed. Ordered and standard logistic regression models adjusted for caregiver, household and child characteristics, were fit to estimate the association of caregiving burden (objective caregiving burden and subjective caregiving burden (Zarit Burden Inventory - tertiles)) with caregiver depression (Patient Health Questionnaire [PHQ-9]) and well-being (CDC Unhealthy Days) outcomes.

Results: Caregivers with high subjective caregiving burden had a 41% probability of most severe depression category (probability: 0.41, 95% CI: 0.25, 0.57) and an 39% probability of low well-being (probability: 0.39, 95% CI: 0.27, 0.51), compared to caregivers with low subjective burden. Caregivers with high subjective caregiving burden and high objective direct caregiving burden had an adjusted 30% probability of elevated depressive symptoms (PHQ >= 10).

Conclusions: Among Indian T1D caregivers, high subjective caregiving burden and objective direct caregiving burden were associated with a high risk for caregiver depression and poorer well-being. (C) 2017 Elsevier B.V. All rights reserved.

C1 [Capistrant, Benjamin D.] Smith Coll, Stat & Data Sci, Burton Hall 115,44 Coll Lane, Northampton, MA 01063 USA.

[Capistrant, Benjamin D.] Univ Minnesota, Sch Publ Hlth, West Bank Off Bldg,1300 S 2nd St,Suite 300, Minneapolis, MN 55455 USA.

[Friedemann-Sanchez, Greta] Univ Minnesota, Humphrey Sch Publ Affairs, Humphrey Sch 267, 301 19th Ave South, Minneapolis, MN 55455 USA.

Colby Coll, Dept Econ, 5237 Mayflower Hill Dr, Waterville, ME USA.

[Zuijdwijk, Caroline] Childrens Hosp Eastern Ontario, CHEO Res Inst, Div Endocrinol & Metab, 401 Smyth Rd, Ottawa, ON K1H 8L1, Canada.

[Ogle, Graham D.] Int Diabet Federat, Life Child, GPO Box 9824, Sydney, NSW 2001, Australia.

[Pendsey, Sharad] Diabet Res Educ & Management Trust, Shreeniwas Opposite Dhantoli Pk, Nagpur 440012, Maharashtra, India.

RP Capistrant, BD (reprint author), Smith Coll, Stat & Data Sci, Burton Hall 115,44 Coll Lane, Northampton, MA 01063 USA.; Capistrant, BD (reprint author), Univ Minnesota, Sch Publ Hlth, West Bank Off Bldg,1300 S 2nd St,Suite 300, Minneapolis, MN 55455 USA.

EM bcapistrant@smith.edu; gfs@umn.edu; lindsey.novak@colby.edu;

czuijdwijk@cheo.on.ca; grahamo@diabetesnsw.com.au;

sharadpendsey2015@g-mail.com

OI Ogle, Graham/0000-0002-2022-0866; Friedemann-Sanchez,

Greta/0000-0002-0493-7218

FU University of Minnesota [22782]; C-Voter News Services PvT. Ltd.

FX This work was possible by the generous support of a University of

Minnesota Grant-in-Aid-of-Research, Artistry and Scholarship (GIA)

#22782 and C-Voter News Services PvT. Ltd.

CR ANDERSON BJ, 1990, J PEDIATR PSYCHOL, V15, P477, DOI 10.1093/jpepsy/15.4.477

Balarajan Y, 2011, LANCET, V377, P505, DOI 10.1016/S0140-6736(10)61894-6

Barik D, 2016, FRONT PUBLIC HEALTH, V4, DOI 10.3389/fpubh.2016.00268

Barile JP, 2013, QUAL LIFE RES, V22, P1201, DOI 10.1007/s11136-012-0246-z

Barola A, 2016, VALUE HEALTH, V19, pA201, DOI 10.1016/j.jval.2016.03.1291

Bedard M, 2001, GERONTOLOGIST, V41, P652, DOI 10.1093/geront/41.5.652

Berman P., 2010, Economic and Political Weekly, V45, P65

Bhadada SK, 2011, J GASTROEN HEPATOL, V26, P378, DOI 10.1111/j.1440-1746.2010.06508.x

Binnendijk E, 2012, TROP MED INT HEALTH, V17, P1376, DOI 10.1111/j.1365-3156.2012.03070.x

Burns KK, 2013, DIABETIC MED, V30, P778, DOI 10.1111/dme.12239

Butler DA, 2008, PEDIATR DIABETES, V9, P373, DOI 10.1111/j.1399-5448.2008.00370.x

Control CfD Prevention, 2000, MEAS HLTH DAYS POP A, P40

Cunningham NR, 2011, J PEDIATR PSYCHOL, V36, P196, DOI 10.1093/jpepsy/jsq071

DATTA SS, 2002, J LEARN DISABIL, V6, P337, DOI DOI 10.1177/146900470200600401

Dilworth-Anderson P, 2005, J GERONTOL B-PSYCHOL, V60

Divan G, 2012, AUTISM RES, V5, P190, DOI 10.1002/aur.1225

Friedemann-Sanchez G, 2017, PEDIAT DIABETES

FRIEDEMANNSANCHEZ, 2012, FEMINIST EC, V18, P55, DOI DOI 10.1080/13545701.2012.714471

Gupta R, 2009, AFFILIA J WOM SOC WO, V24, P69, DOI 10.1177/0886109908326998

HAUENSTEIN EJ, 1989, DIABETES CARE, V12, P18, DOI 10.2337/diacare.12.1.18

Haugstvedt A, 2011, PEDIATR DIABETES, V12, P107, DOI 10.1111/j.1399-5448.2010.00661.x

Helgeson VS, 2007, PEDIAT PSYCHOL, V34

Kahana S, 2008, J PEDIATR PSYCHOL, V33, P590, DOI 10.1093/jpepsy/jsm128

Kalra S., 2015, J SOCIAL HLTH DIABET, V3, P56, DOI [10.4103/2321-0656.140896, DOI 10.4103/2321-0656.140896]

Kalra S, 2014, J SOC HLTH DIABETES, V2, P3

Kroenke K, 2002, PSYCHIAT ANN, V32, P509, DOI 10.3928/0048-5713-20020901-06

Kroenke K, 2001, J GEN INTERN MED, V16, P606, DOI 10.1046/j.1525-1497.2001.016009606.x

Landolt MA, 2005, J AM ACAD CHILD PSY, V44, P682, DOI 10.1097/01-chi.0000161645.98022.35

Li CY, 2007, DIABETES CARE, V30, P1770, DOI 10.2337/dc06-2571

Manea L, 2015, GEN HOSP PSYCHIAT, V37, P67, DOI 10.1016/j.genhosppsych.2014.09.009

Minhas A, 2015, INT REV PSYCHIATR, V27, P247, DOI 10.3109/09540261.2015.1049128

Mitchell AJ, 2016, BJPSYCH OPEN, V2, P127, DOI 10.1192/bjpo.bp.115.001685

Monaghan M, 2011, PATIENT EDUC COUNS, V82, P271, DOI 10.1016/j.pec.2010.04.007

Mukama LJ, 2013, PEDIATR DIABETES, V14, P211, DOI 10.1111/pedi.12005

Patel V, 2008, PSYCHOL MED, V38, P221, DOI 10.1017/S0033291707002334

Pearlin L. I., 1999, HDB SOCIOLOGY MENTAL, P395, DOI DOI 10.1007/0-387-36223-1_19

PEARLIN LI, 1990, GERONTOLOGIST, V30, P583, DOI 10.1093/geront/30.5.583

Peyrot M, 2013, DIABETES RES CLIN PR, V99, P174, DOI 10.1016/j.diabres.2012.11.016

POLONSKY WH, 1995, DIABETES CARE, V18, P754, DOI 10.2337/diacare.18.6.754

Poongothai S, 2009, PLOS ONE, V4, DOI 10.1371/journal.pone.0007185

Rastogi D, 2009, J ASTHMA, V46, P911, DOI 10.3109/02770900903191323

Snoek FJ, 2015, LANCET DIABETES ENDO, V3, P450, DOI 10.1016/S2213-8587(15)00135-7

Streisand R, 2008, PATIENT EDUC COUNS, V73, P333, DOI 10.1016/j.pec.2008.06.014

Tsiouli E, 2014, EXPLORE-NY, V10, P88, DOI 10.1016/j.explore.2013.12.004

Whittemore R, 2012, DIABETES EDUCATOR, V38, P562, DOI 10.1177/0145721712445216

Zahran Hatice S., 2005, Morbidity and Mortality Weekly Report, V54, P1

NR 46

TC 0

Z9 0

U1 2

U2 4

PU ELSEVIER IRELAND LTD

PI CLARE

PA ELSEVIER HOUSE, BROOKVALE PLAZA, EAST PARK SHANNON, CO, CLARE, 00000,

IRELAND

SN 0168-8227

EI 1872-8227

J9 DIABETES RES CLIN PR

JI Diabetes Res. Clin. Pract.

PD DEC

PY 2017

VL 134

BP 168

EP 177

DI 10.1016/j.diabres.2017.10.009

PG 10

WC Endocrinology & Metabolism

SC Endocrinology & Metabolism

GA FP0TC

UT WOS:000417318700020

PM 29054484

DA 2019-01-08

ER

PT J

AU Hernar, I

Haltbakk, J

Brostrom, A

AF Hernar, Ingvild

Haltbakk, Johannes

Brostrom, Anders

TI Differences in depression, treatment satisfaction and injection

behaviour in adults with type 1 diabetes and different degrees of

lipohypertrophy

SO JOURNAL OF CLINICAL NURSING

LA English

DT Article

DE depressive symptoms; diabetes specialist nurses; health behaviour;

insulin injection therapy; lipohypertrophy; self-assessment tools;

self-management of injection technique; treatment satisfaction; type 1

diabetes

ID PATIENT ACTIVATION; GLYCEMIC CONTROL; EMOTIONAL DISTRESS;

SELF-MANAGEMENT; D PERSONALITY; PSYCHOMETRIC PROPERTIES; INSULIN;

HEALTH; PEOPLE; SCALE

AB Aims and objectivesTo assess the prevalence of lipohypertrophy, and to compare differences in external, personal and regimen factors in adults with type 1 diabetes and different degrees of lipohypertrophy.

BackgroundSuboptimal insulin injection behaviour is associated with lipohypertrophy, which may affect insulin absorption and lead to blood glucose fluctuations. Few, if any studies have investigated how external, personal and regimen factors differ in people with type 1 diabetes and different degrees of lipohypertrophy.

DesignA cross-sectional study including adults with type 1 diabetes at a diabetes outpatient clinic in a Norwegian university hospital.

MethodsParticipants (n=215) were included consecutively at scheduled appointments. Sociodemographic, diabetes and insulin treatment data, and self-report questionnaires concerning patient activation (Patient Activation Measure), depression (Patient Health Questionnaire-2), diabetes distress (Diabetes Distress Scale), type D personality (14-item Type D scale), treatment satisfaction (Insulin Treatment Satisfaction Questionnaire) and motivation (Treatment Self-Regulation Questionnaire), were collected. Lipohypertrophic injection sites were identified by palpation by diabetes specialist nurses.

ResultsLipohypertrophy was present in 53% and was more frequent in insulin pen users (63%) compared to insulin pump users (34%). Participants with two or more lipohypertrophic areas had higher depression scores, lower treatment satisfaction with glycaemic control, higher bolus doses and reported suboptimal injection behaviour compared to those with no lipohypertrophic areas. There were no differences in patient activation, diabetes distress, type D personality or motivation between the groups.

Discussion and conclusionCompared to pump treatment, pen treatment requires greater awareness of injection technique. Symptoms of depression and lower treatment satisfaction might affect diabetes self-management and glycaemic control, but the association with lipohypertrophy needs further exploration.

Relevance to clinical practiceLipohypertrophy is more frequent in insulin pen users compared to pump users. Nurses should focus on injection technique education, and should also consider screening for depressive symptoms and treatment satisfaction as these factors could be associated with development of lipohypertrophy.

C1 [Hernar, Ingvild] Haukeland Hosp, Dept Med, Bergen, Norway.

[Hernar, Ingvild; Haltbakk, Johannes; Brostrom, Anders] Western Norway Univ Appl Sci, Fac Hlth & Social Sci, Dept Nursing, Bergen, Norway.

[Brostrom, Anders] Jonkoping Univ, Dept Nursing Sci, Jonkoping, Sweden.

[Brostrom, Anders] Univ Hosp Linkoping, Dept Clin Neurophysiol, Linkoping, Sweden.

RP Hernar, I (reprint author), Western Norway Univ Appl Sci, Bergen, Norway.

EM ingvild.hernar@hvl.no

FU Norwegian Nurses Organisation

FX Ingvild Hernar received a 10,000 NOK scholarship from the Norwegian

Nurses Organisation for her Master's Programme.

CR Anderson RT, 2004, CLIN THER, V26, P565, DOI 10.1016/S0149-2918(04)90059-8

Atkinson MA, 2014, LANCET, V383, P69, DOI 10.1016/S0140-6736(13)60591-7

Barnard KD, 2014, DIABETIC MED, V31, P522, DOI 10.1111/dme.12400

Bergvik S, 2010, SCAND J PSYCHOL, V51, P334, DOI 10.1111/j.1467-9450.2009.00793.x

Blanco M, 2013, Diabetes Metab, V39, P445, DOI 10.1016/j.diabet.2013.05.006

Brod M, 2007, QUAL LIFE RES, V16, P1053, DOI 10.1007/s11136-007-9209-1

Brod M, 2009, QUAL LIFE RES, V18, P23, DOI 10.1007/s11136-008-9419-1

De Coninck C, 2010, J DIABETES, V2, P168, DOI 10.1111/j.1753-0407.2010.00077.x

Deci E. L, 2002, HDB SELF DETERMINATI

Denollet J, 2005, PSYCHOSOM MED, V67, P89, DOI 10.1097/01.psy.0000149256.81953.49

Fisher L, 2012, DIABETES CARE, V35, P259, DOI 10.2337/dc11-1572

Frid A, 2010, DIABETES METAB, V36, pS3, DOI 10.1016/S1262-3636(10)70002-1

Garmo A, 2013, DIABETIC MED, V30, P717, DOI 10.1111/dme.12155

Gentile S, 2011, ACTA DIABETOL, V48, P121, DOI 10.1007/s00592-009-0172-x

Gin H, 2005, DIABETES METAB, V31, P7, DOI 10.1016/S1262-3636(07)70160-X

Grassi G, 2014, J CLIN TRANSL ENDOCR, V1, P145, DOI 10.1016/j.jcte.2014.07.006

Graue M, 2012, INT J NURS STUD, V49, P174, DOI 10.1016/j.ijnurstu.2011.08.007

Hauner H, 1996, EXP CLIN ENDOCR DIAB, V104, P106, DOI 10.1055/s-0029-1211431

Hendriks M, 2014, BMC HEALTH SERV RES, V14, DOI 10.1186/1472-6963-14-393

Hibbard JH, 2005, HEALTH SERV RES, V40, P1918, DOI 10.1111/j.1475-6773.2005.00438.x

International Diabetes Federation, 2015, IDF DIABETES ATLAS

Joensen LE, 2013, DIABETES RES CLIN PR, V101, P278, DOI 10.1016/j.diabres.2013.07.001

Johansson UB, 2005, DIABETES CARE, V28, P2025, DOI 10.2337/diacare.28.8.2025

Jones A, 2015, DIABETIC MED, V32, P155, DOI 10.1111/dme.12620

Jones A., 2016, J DIABETES RES, V2016

KADIYALA P, 2014, THE BRITISH JOURNAL, V14, P131

Kordonouri O, 2002, DIABETES CARE, V25, P634, DOI 10.2337/diacare.25.3.634

Kroenke K, 2003, MED CARE, V41, P1284, DOI 10.1097/01.MLR.0000093487.78664.3C

LAMAR CP, 1950, JAMA-J AM MED ASSOC, V142, P1350, DOI 10.1001/jama.1950.02910350020007

Lowe B, 2005, J PSYCHOSOM RES, V58, P163, DOI 10.1016/j.jpsychores.2004.09.006

Mapi Research Trust, 2014, PERS COMM REG TRANSL

Mohn J., 2011, THESIS

Moljord IEO, 2015, PATIENT EDUC COUNS, V98, P1410, DOI 10.1016/j.pec.2015.06.009

Mols F, 2010, GEN HOSP PSYCHIAT, V32, P66, DOI 10.1016/j.genhosppsych.2009.09.010

Nathan DM, 2014, DIABETES CARE, V37, P9, DOI 10.2337/dc13-2112

Nefs G, 2015, DIABETES RES CLIN PR, V108, P94, DOI 10.1016/j.diabres.2015.01.015

Nicolucci A, 2013, DIABETIC MED, V30, P767, DOI 10.1111/dme.12245

Oxford Outcomes, 2014, ICON LANG SERV

Ozcan S, 2014, DIABETES RES CLIN PR, V106, P35, DOI 10.1016/j.diabres.2014.07.023

Peyrot M, 2005, DIABETIC MED, V22, P1379, DOI 10.1111/j.1464-5491.2005.01644.x

PHQ Screeners, 2013, PAT HLTH QUEST PHQ S

Polonsky WH, 2005, DIABETES CARE, V28, P626, DOI 10.2337/diacare.28.3.626

Richardson T, 2003, AM J CLIN DERMATOL, V4, P661, DOI 10.2165/00128071-200304100-00001

Rijken M, 2014, PATIENT EDUC COUNS, V97, P383, DOI 10.1016/j.pec.2014.09.006

Rogvi S, 2012, DIABETIC MED, V29, pe382, DOI 10.1111/j.1464-5491.2012.03703.x

Saez-de Ibarra L, 1998, PRACT DIABETES INT, V15, P9

Saltiel-Berzin R, 2012, DIABETES EDUCATOR, V38, P635, DOI 10.1177/0145721712455107

Sawatkar GU, 2013, J EUR ACAD DERMATOL, V27, P1321, DOI 10.1111/jdv.12068

Siemens Healthcare, 2016, DCA VANTAGE ANALYZER

Skrivarhaug T., 2015, NORWEGIAN CHILDHOOD

Snoek FJ, 2015, LANCET DIABETES ENDO, V3, P450, DOI 10.1016/S2213-8587(15)00135-7

Speight J, 2009, DIABETIC MED, V26, P315, DOI 10.1111/j.1464-5491.2009.02682.x

Strandberg RB, 2014, J PSYCHOSOM RES, V77, P174, DOI 10.1016/j.jpsychores.2014.06.015

van Bastelaar KMP, 2010, DIABETIC MED, V27, P798, DOI 10.1111/j.1464-5491.2010.03025.x

vansMunster H. E., 2014, EUR DIABETES NURS, V11, P79

Vardar B, 2007, DIABETES RES CLIN PR, V77, P231, DOI 10.1016/j.diabres.2006.12.023

Williams GC, 2004, HEALTH PSYCHOL, V23, P58, DOI 10.1037/0278-6133.23.1.58

Yeh HC, 2012, ANN INTERN MED, V157, P336, DOI 10.7326/0003-4819-157-5-201209040-00508

NR 58

TC 1

Z9 1

U1 3

U2 5

PU WILEY

PI HOBOKEN

PA 111 RIVER ST, HOBOKEN 07030-5774, NJ USA

SN 0962-1067

EI 1365-2702

J9 J CLIN NURS

JI J. Clin. Nurs.

PD DEC

PY 2017

VL 26

IS 23-24

BP 4583

EP 4596

DI 10.1111/jocn.13801

PG 14

WC Nursing

SC Nursing

GA FN9AK

UT WOS:000416319600071

PM 28295770

DA 2019-01-08

ER

PT J

AU Levesque, C

AF Levesque, Celia

TI Therapeutic Lifestyle Changes for Diabetes Mellitus

SO NURSING CLINICS OF NORTH AMERICA

LA English

DT Article

DE Diabetes mellitus; Medical nutrition therapy; Physical activity; Smoking

cessation; Diabetes distress; Diabetes self-management education;

Diabetes self-management support

ID AMERICAN ASSOCIATION; GLYCEMIC CONTROL; TYPE-2; STATEMENT; EDUCATION;

RAMADAN

AB Diabetes mellitus is a common chronic disease affecting approximately 9% of the US population. Successful management of diabetes demands constant self-management on the part of the patient. The patient must balance diabetes medications, blood glucose monitoring, food intake, physical activity, and management of diabetes-related acute and chronic complications. The patient is often bombarded with misinformation from friends, relatives, and such sources as the Internet and social media. This article discusses the current recommendations for diabetes self-management education and skills including medical nutrition therapy, physical activity, smoking cessation, and assessment for diabetes distress.

C1 [Levesque, Celia] MD Anderson Canc Ctr, Dept Endocrine Neoplasia & Hormonal Disorders, 1515 Holcomb Blvd, Houston, TX 77030 USA.

RP Levesque, C (reprint author), MD Anderson Canc Ctr, Dept Endocrine Neoplasia & Hormonal Disorders, 1515 Holcomb Blvd, Houston, TX 77030 USA.

EM clevesqu@mdanderson.org

CR Ahmedani MY, 2016, PAK J MED SCI, V32, P846, DOI 10.12669/pjms.324.9904

American Diabetes Association, 2017, J DIABETES S1, V40, pS1

Boucher Jackie L, 2017, Diabetes Spectr, V30, P72, DOI 10.2337/ds16-0074

Campbell Amy P, 2017, Diabetes Spectr, V30, P76, DOI 10.2337/ds16-0084

Centers for Disease Control and Prevention, 2014, NAT DIAB STAT REP ES

Chong Shanley, 2017, Diabetes Spectr, V30, P43, DOI 10.2337/ds15-0044

Eid Yara M, 2017, Diabetes Spectr, V30, P36, DOI 10.2337/ds15-0058

Foster GD, 2013, NUTR DIABETES, V3, DOI 10.1038/nutd.2013.3

Garber AJ, 2016, ENDOCR PRACT, V22, P84, DOI 10.4158/EP151126.CS

Healy SJ, 2013, DIABETES CARE, V36, P2960, DOI 10.2337/dc13-0108

Ibrahim M, 2015, BMJ OPEN DIAB RES CA, V3, DOI 10.1136/bmjdrc-2015-000108

Lessan N, 2015, DIABETES METAB, V41, P28, DOI 10.1016/j.diabet.2014.11.004

Pan A, 2015, LANCET DIABETES ENDO, V3, P958, DOI 10.1016/S2213-8587(15)00316-2

Pawlak Roman, 2017, Diabetes Spectr, V30, P82, DOI 10.2337/ds16-0057

Powers Margaret A, 2017, Diabetes Spectr, V30, P51, DOI 10.2337/ds16-0078

Powers MA, 2017, DIABETES EDUCATOR, V43, P40, DOI 10.1177/0145721716689694

Salas-Salvado J, 2016, J NUTR, V146, p920S, DOI 10.3945/jn.115.218487

NR 17

TC 0

Z9 0

U1 3

U2 8

PU W B SAUNDERS CO-ELSEVIER INC

PI PHILADELPHIA

PA 1600 JOHN F KENNEDY BOULEVARD, STE 1800, PHILADELPHIA, PA 19103-2899 USA

SN 0029-6465

EI 1558-1357

J9 NURS CLIN N AM

JI Nurs. Clin. North Am.

PD DEC

PY 2017

VL 52

IS 4

BP 679

EP +

DI 10.1016/j.cnur.2017.07.012

PG 15

WC Nursing

SC Nursing

GA FO1FH

UT WOS:000416501600014

PM 29080584

DA 2019-01-08

ER

PT J

AU Raymaekers, K

Oris, L

Prikken, S

Moons, P

Goossens, E

Weets, I

Luyckx, K

AF Raymaekers, Koen

Oris, Leen

Prikken, Sofie

Moons, Philip

Goossens, Eva

Weets, Ilse

Luyckx, Koen

TI The Role of Peers for Diabetes Management in Adolescents and Emerging

Adults With Type 1 Diabetes: A Longitudinal Study

SO DIABETES CARE

LA English

DT Article

ID GLYCEMIC CONTROL; METABOLIC-CONTROL; SELF-MANAGEMENT; CARE; SUPPORT;

INVENTORY; FRIENDS; PARENT; ORIENTATION; DISTRESS

AB OBJECTIVE

The increasing importance of peers in adolescence and emerging adulthood has been widely acknowledged. However, longitudinal research linking the peer context to diabetes management and outcomes is scarce. The present longitudinal study in a large sample of youths with type 1 diabetes related both positive and negative peer variables to diabetes outcomes over a time interval of 1 year.

RESEARCH DESIGN AND METHODS

Our sample consisted of 467 adolescents (14-17 years of age) and emerging adults (18-25 years of age) with type 1 diabetes who participated in a two-wave longitudinal study. Questionnaires tapped into peer support, extreme peer orientation, parental responsiveness, diabetes-related distress, and treatment adherence. HbA(1c) values were obtained from the treating physicians of patients. Cross-lagged analysis from a structural equation modeling approach was performed to assess the directionality of effects.

RESULTS

Peer support negatively predicted diabetes-related distress over time. Extreme peer orientation positively predicted treatment distress over time. Parental responsiveness negatively predicted food distress over time. Treatment adherence negatively predicted extreme peer orientation, treatment distress, and HbA(1c) values over time. For emerging adults specifically, there was a reciprocal relationship between HbA(1c) values and extreme peer orientation because they positively predicted each other.

CONCLUSIONS

This study highlights the importance of peers in predicting the functioning of youths with type 1 diabetes. Additionally, treatment adherence at baseline was found to negatively predict extreme peer orientation, treatment distress, and worse glycemic control over time. In sum, the current study underscores the importance of the peer context for adolescents and emerging adults with type 1 diabetes.

C1 [Raymaekers, Koen; Oris, Leen; Prikken, Sofie; Moons, Philip; Goossens, Eva; Luyckx, Koen] Univ Leuven, Leuven, Belgium.

[Oris, Leen; Goossens, Eva] Res Fdn Flanders, Brussels, Belgium.

[Moons, Philip] Univ Gothenburg, Gothenburg, Sweden.

[Weets, Ilse] Free Univ Brussels, Brussels, Belgium.

RP Raymaekers, K (reprint author), Univ Leuven, Leuven, Belgium.

EM koen.raymaekers@kuleuven.be

OI Raymaekers, Koen/0000-0002-5571-4081

FU Research Foundation Flanders (FWO) [G.0B35.14N]

FX Funding was provided by Research Foundation Flanders (FWO) through

Research Project G.0B35.14N to K.L.

CR [Anonymous], 2016, DIABETES CARE S1, V39, pS86

ARMSDEN GC, 1987, J YOUTH ADOLESCENCE, V16, P427, DOI 10.1007/BF02202939

Arnett JJ, 2000, AM PSYCHOL, V55, P469, DOI 10.1037/0003-066X.55.5.469

Berg CA, 2008, DIABETES CARE, V31, P678, DOI 10.2337/dc07-1678

Berlin K. S., 2006, FAMILIES SYSTEMS HLT, V24, P33, DOI DOI 10.1037/1091-7527.24.1.33

Beyers W, 2003, J YOUTH ADOLESCENCE, V32, P351, DOI 10.1023/A:1024922031510

BRONFENBRENNER U, 1986, DEV PSYCHOL, V22, P723, DOI 10.1037//0012-1649.22.6.723

Brown B. B., 2004, HDB ADOLESCENT PSYCH, V2, P363, DOI DOI 10.1002/9780471726746.CH12

Carcone AI, 2011, J DEV BEHAV PEDIATR, V32, P581, DOI 10.1097/DBP.0b013e31822c1a27

Cross CP, 2011, PSYCHOL BULL, V137, P97, DOI 10.1037/a0021591

Doe E., 2016, J HLTH PSYCHOL

Drew LM, 2010, J FAM PSYCHOL, V24, P299, DOI 10.1037/a0019352

Enders CK, 2010, APPL MISSING DATA AN

Enzlin P, 2002, PATIENT EDUC COUNS, V48, P139, DOI 10.1016/S0738-3991(02)00009-5

Fisher EB, 2012, HEALTH AFFAIR, V31, P130, DOI 10.1377/hlthaff.2011.0914

FULIGNI AJ, 1993, DEV PSYCHOL, V29, P622, DOI 10.1037/0012-1649.29.4.622

Hains AA, 2007, J PEDIATR PSYCHOL, V32, P561, DOI 10.1093/jpepsy/jsl040

Hausmann LRM, 2010, PATIENT PREFER ADHER, V4, P291, DOI 10.2147/PPA.S12353

Helgeson VS, 2015, J PEDIATR PSYCHOL, V40, P359, DOI 10.1093/jpepsy/jsu069

Helgeson VS, 2014, HEALTH PSYCHOL, V33, P1125, DOI 10.1037/a0033511

Helgeson VS, 2009, J PEDIATR PSYCHOL, V34, P254, DOI 10.1093/jpepsy/jsn079

Karlsson A, 2008, INT J NURS STUD, V45, P562, DOI 10.1016/j.ijnurstu.2006.08.022

Kline R. B., 2015, PRINCIPLES PRACTICE

LAGRECA AM, 1995, J PEDIATR PSYCHOL, V20, P449, DOI 10.1093/jpepsy/20.4.449

LITTLE RJA, 1988, J AM STAT ASSOC, V83, P1198, DOI 10.2307/2290157

Luyckx K, 2016, J HEALTH PSYCHOL, V21, P540, DOI 10.1177/1359105314531467

Luyckx K, 2010, DIABETES CARE, V33, P1424, DOI 10.2337/dc09-2017

Oris L, 2016, DIABETES CARE, V39, P757, DOI 10.2337/dc15-2559

Palladino DK, 2012, J PEDIATR PSYCHOL, V37, P591, DOI 10.1093/jpepsy/jss009

POLONSKY WH, 1995, DIABETES CARE, V18, P754, DOI 10.2337/diacare.18.6.754

Ramchandani N, 2000, DIABETES EDUCATOR, V26, P656, DOI 10.1177/014572170002600413

SCHAEFER ES, 1965, CHILD DEV, V36, P413, DOI 10.2307/1126465

Schumacker RE, 2004, BEGINNERS GUIDE STRU

Silverstein J, 2005, DIABETES CARE, V28, P186, DOI 10.2337/diacare.28.1.186

Snoek FJ, 2000, DIABETES CARE, V23, P1305, DOI 10.2337/diacare.23.9.1305

Soenens B, 2006, DEV PSYCHOL, V42, P305, DOI 10.1073/0012-1649.42.2.305

Walker Ashby F, 2015, Clin Diabetes, V33, P62, DOI 10.2337/diaclin.33.2.62

Weinger K, 2005, DIABETES CARE, V28, P1346, DOI 10.2337/diacare.28.6.1346

Weissberg-Benchell J, 2007, DIABETES CARE, V30, P2441, DOI 10.2337/dc07-1249

Wiebe DJ, 2016, AM PSYCHOL, V71, P526, DOI 10.1037/a0040355

Wysocki Tim, 2006, Curr Diab Rep, V6, P117, DOI 10.1007/s11892-006-0022-y

NR 41

TC 3

Z9 3

U1 2

U2 8

PU AMER DIABETES ASSOC

PI ALEXANDRIA

PA 1701 N BEAUREGARD ST, ALEXANDRIA, VA 22311-1717 USA

SN 0149-5992

EI 1935-5548

J9 DIABETES CARE

JI Diabetes Care

PD DEC 1

PY 2017

VL 40

IS 12

BP 1678

EP 1684

DI 10.2337/dc17-0643

PG 7

WC Endocrinology & Metabolism

SC Endocrinology & Metabolism

GA FN3AO

UT WOS:000415868000015

PM 29138272

DA 2019-01-08

ER

PT J

AU Dickinson, JK

Guzman, SJ

Maryniuk, MD

O'Brian, CA

Kadohiro, JK

Jackson, RA

D'Hondt, N

Montgomery, B

Close, KL

Funnell, MM

AF Dickinson, Jane K.

Guzman, Susan J.

Maryniuk, Melinda D.

O'Brian, Catherine A.

Kadohiro, Jane K.

Jackson, Richard A.

D'Hondt, Nancy

Montgomery, Brenda

Close, Kelly L.

Funnell, Martha M.

TI The Use of Language in Diabetes Care and Education

SO DIABETES CARE

LA English

DT Article

ID PSYCHOLOGICAL INSULIN-RESISTANCE; WEIGHT BIAS INTERNALIZATION; PATIENTS

PREFERRED TERMS; PERSON-1ST LANGUAGE; PATIENT EMPOWERMENT; EMOTIONAL

DISTRESS; DESCRIBING OBESITY; SELF-MANAGEMENT; NEGATIVE-WORDS; CENTERED

CARE

AB Language is powerful and can have a strong impact on perceptions as well as behavior. A task force, consisting of representatives from the American Association of Diabetes Educators (AADE) and the American Diabetes Association (ADA), convened to discuss language in diabetes care and education. This document represents the expert opinion of the task force. The literature supports the need for a language movement in diabetes care and education. There are effective ways of communicating about diabetes. This article provides recommendations for language used by health care professionals and others when discussing diabetes through spoken or written words-whether directed to people with diabetes, colleagues, or the general public, as well as research questions related to language and diabetes.

C1 [Dickinson, Jane K.] Columbia Univ, Teachers Coll, Dept Hlth & Behav Studies, New York, NY 10027 USA.

[Guzman, Susan J.] Behav Diabet Inst, Clin Educ Serv, San Diego, CA USA.

[Maryniuk, Melinda D.] Joslin Diabet Ctr, Clin Educ Programs, Boston, MA 02215 USA.

[O'Brian, Catherine A.] Amer Assoc Diabet Educ, Dept Sci & Practice, Chicago, IL USA.

[Kadohiro, Jane K.] Diabet Educ & Support Consulting Serv, Reno, NV USA.

[Jackson, Richard A.] Grassroots Diabet, Boston, MA USA.

[D'Hondt, Nancy] Ascens St John Hosp, Detroit, MI USA.

[Montgomery, Brenda] AstraZeneca, Bothell, WA USA.

[Close, Kelly L.] Close Concerns & diaTribe Fdn, San Francisco, CA USA.

[Funnell, Martha M.] Univ Michigan, Med Sch, Ann Arbor, MI USA.

RP Dickinson, JK (reprint author), Columbia Univ, Teachers Coll, Dept Hlth & Behav Studies, New York, NY 10027 USA.

EM dickinson@tc.columbia.edu

CR Aikens JE, 2012, DIABETES CARE, V35, P2472, DOI 10.2337/dc12-0181

Aikens JE, 2005, DIABETES EDUCATOR, V31, P681, DOI 10.1177/0145721705280829

Anderson RM, 2000, DIABETES EDUCATOR, V26, P597, DOI 10.1177/014572170002600405

Anderson RM, 2010, PATIENT EDUC COUNS, V79, P277, DOI 10.1016/j.pec.2009.07.025

[Anonymous], 2017, DIABETES CARE, V40, pS1, DOI 10.2337/dc17-S001

[Anonymous], 2001, CROSSING QUALITY CHA

[Anonymous], 2013, PEOPL 1 LANG OB

Ayala J, 2014, WESTERN J NURS RES, V36, P1238, DOI 10.1177/0193945913518848

Barnish M, 2014, OPEN J MOD LINGUIST, V4, P5505

Benedetti F, 1997, PROG NEUROBIOL, V52, P109, DOI 10.1016/S0301-0082(97)00006-3

Benedetti F, 2002, EVAL HEALTH PROF, V25, P369, DOI 10.1177/0163278702238051

Berg FM, 1998, HLTH WEIGHT J, V12, P12

Beverly EA, 2012, J GEN INTERN MED, V27, P1180, DOI 10.1007/s11606-012-2070-0

Bissell P, 2004, SOC SCI MED, V58, P851, DOI 10.1016/S0277-9536(03)00259-4

Bootle S, 2015, DIABETES RES CLIN PR, V109, P6, DOI 10.1016/j.diabres.2015.04.004

Broom D, 2004, SOC SCI MED, V58, P2371, DOI 10.1016/j.socscimed.2003.09.002

Brown A, 2016, DO WE REDUCE DIABETE

Browne JL, 2014, BMJ OPEN, V4, DOI 10.1136/bmjopen-2014-005625

Browne JL, 2013, BMJ OPEN, V3, DOI 10.1136/bmjopen-2013-003384

Brummel-Smith K, 2016, J AM GERIATR SOC, V64, P15, DOI 10.1111/jgs.13866

CHROUSOS GP, 1995, NEW ENGL J MED, V332, P1351, DOI 10.1056/NEJM199505183322008

Chun H, 2007, ADV CONSUM RES, V34, P715

Collier R, 2012, CAN MED ASSOC J, V184, P1977, DOI 10.1503/cmaj.109-4319

Dellasega C, 2012, DIABETES RES CLIN PR, V95, P37, DOI 10.1016/j.diabres.2011.08.011

Diab P, 2012, JEMDSA, V17, P52, DOI 10.1080/22201009.2012.10872274

Dickinson Jane K, 2017, Diabetes Spectr, V30, P11, DOI 10.2337/ds15-0054

Dickinson Jane K, 2017, Clin Diabetes, V35, P51, DOI 10.2337/cd16-0014

Dickinson JK, 2015, DIABETES EDUCATOR, V41, P665, DOI 10.1177/0145721715608952

Dickinson JK, 2017, DIABETES SPECTR

Dunn DS, 2015, AM PSYCHOL, V70, P255, DOI 10.1037/a0038636

Dunning T, 2017, DIABETES EDUCATOR, V43, P18, DOI 10.1177/0145721716683449

Durso LE, 2008, OBESITY, V16, pS80, DOI 10.1038/oby.2008.448

Ellis S, 2014, J NUTR EDUC BEHAV, V46, P299, DOI 10.1016/j.jneb.2013.08.006

Fisher L, 2012, DIABETES CARE, V35, P259, DOI 10.2337/dc11-1572

Fleischman S, 1999, J MED HUMANITIES, V20, P3

Fleitas J., 2003, MCN-AM J MATERN-CHIL, V28, P389

Fleitas Joan, 2003, MCN Am J Matern Child Nurs, V28, P384

Frosch DL, 2012, HEALTH AFFAIR, V31, P1030, DOI 10.1377/hlthaff.2011.0576

Funnell MM, 2016, PATIENT EDUC COUNS, V99, P1921, DOI 10.1016/j.pec.2016.10.010

Glasgow RE, 1999, DIABETES CARE, V22, P2090, DOI 10.2337/diacare.22.12.2090

Gonzalez JS, 2011, DIABETES CARE, V34, P236, DOI 10.2337/dc10-1970

Gottlieb LN, 2014, AM J NURS, V114, P24, DOI 10.1097/01.NAJ.0000453039.70629.e2

Gredig D, 2017, HEALTH SOC CARE COMM, V25, P1620, DOI 10.1111/hsc.12376

Greenfield C, 2011, AUST J RURAL HEALTH, V19, P15, DOI 10.1111/j.1440-1584.2010.01171.x

Haque M, 2005, SAMJ S AFR MED J, V95, P798

HARDIN C, 1993, SOC COGNITION, V11, P277, DOI 10.1521/soco.1993.11.3.277

HOLM S, 1993, J MED ETHICS, V19, P108, DOI 10.1136/jme.19.2.108

Hoover J, 1980, Diabetes Educ, V6, P9, DOI 10.1177/014572178000600101

International Diabetes Federation, 2014, INT DIAB FED LANG PH

Iverson C, 2007, AMA MANUAL STYLE, P416

Jensen ME, 2013, J AM PSYCHIAT NURSES, V19, P146, DOI 10.1177/1078390313489729

Jernigan K, 2009, BRAILLE MONITOR

Kalra S., 2015, J SOCIAL HLTH DIABET, V3, P56, DOI [10.4103/2321-0656.140896, DOI 10.4103/2321-0656.140896]

Karter AJ, 2010, DIABETES CARE, V33, P733, DOI 10.2337/dc09-1184

Kendrick JM, 2005, JOGNN-J OBST GYN NEO, V34, P329, DOI 10.1177/0884217505276306

Link BG, 2006, LANCET, V367, P528, DOI 10.1016/S0140-6736(06)68184-1

Liu Nancy F, 2017, Clin Diabetes, V35, P27, DOI 10.2337/cd16-0020

Lloyd C, 2005, DIABETES SPECTR, V18, P121, DOI [DOI 10.2337/DIASPECT.18.2.121, 10.2337/diaspect.18.2.121]

Lydecker JA, 2016, INT J CLIN PRACT, V70, P682, DOI 10.1111/ijcp.12835

Marrero DG, 2013, DIABETES CARE, V36, P463, DOI 10.2337/dc12-2305

Matthews SM, 2009, PATIENT EDUC COUNS, V76, P31, DOI 10.1016/j.pec.2008.11.022

Meadows A, 2016, FRONT PSYCHOL, V7, DOI 10.3389/fpsyg.2016.01527

Nakar S, 2007, J DIABETES COMPLICAT, V21, P220, DOI 10.1016/j.jdiacomp.2006.02.004

National Center for Health Statistics, INT CLASS DIS 9 REV

National Center for Health Statistics Centers for Disease Control and Prevention, INT CLASS DIS 10 REV

Nicolucci A, 2013, DIABETIC MED, V30, P767, DOI 10.1111/dme.12245

Norman Debbie, 2003, Br J Nurs, V12, P1256

Ott J, 2012, CLIN J PAIN, V28, P324, DOI 10.1097/AJP.0b013e3182321cc3

Paget L, 2012, PATIENT CLIN COMMUNI

Pearl RL, 2017, OBESITY, V25, P317, DOI 10.1002/oby.21716

Peyrot M, 2005, DIABETES CARE, V28, P2673, DOI 10.2337/diacare.28.11.2673

Peyrot M, 2013, DIABETES RES CLIN PR, V99, P174, DOI 10.1016/j.diabres.2012.11.016

Polonsky WH, 2005, DIABETES CARE, V28, P2543, DOI 10.2337/diacare.28.10.2543

Polonsky WH, 2017, DIABETES RES CLIN PR, V127, P265, DOI 10.1016/j.diabres.2017.03.016

Polonsky WH, 2011, CURR MED RES OPIN, V27, P1169, DOI 10.1185/03007995.2011.573623

Polonsky WH, 2010, DIABETES EDUCATOR, V36, P828, DOI 10.1177/0145721710378539

Puhl R, 2013, INT J OBESITY, V37, P612, DOI 10.1038/ijo.2012.110

Rathert C, 2013, MED CARE RES REV, V70, P351, DOI 10.1177/1077558712465774

Ritholz MD, 2014, CHRONIC ILLN, V10, P303, DOI 10.1177/1742395314525647

Ritter A, 2016, HEALTHCARE, V4, P54

ROSENTHAL R, 1994, CURR DIR PSYCHOL SCI, V3, P176, DOI 10.1111/1467-8721.ep10770698

Rosenthal Robert, 1968, PYGMALION CLASSROOM

Saleebey D, 1996, SOC WORK, V41, P296

Schabert J, 2013, PATIENT, V6, P1, DOI 10.1007/s40271-012-0001-0

Schaffer R, 2016, ENDOCRINE TODAY

SHAMOON H, 1993, NEW ENGL J MED, V329, P977

Shiu ATY, 2003, J CLIN NURS, V12, P149, DOI 10.1046/j.1365-2702.2003.00735.x

Speight J, 2012, DIABETES RES CLIN PR, V97, P425, DOI 10.1016/j.diabres.2012.03.015

Strandberg RB, 2014, J PSYCHOSOM RES, V77, P174, DOI 10.1016/j.jpsychores.2014.06.015

Swift JA, 2013, PATIENT EDUC COUNS, V91, P186, DOI 10.1016/j.pec.2012.12.008

Texas Department of Aging and Disability Services, 2013, PERS 1 RESP LANG

Vartanian LR, 2008, J HEALTH PSYCHOL, V13, P131, DOI 10.1177/1359105307084318

Vishwanath A, 2014, HEALTH COMMUN, V29, P516, DOI 10.1080/10410236.2013.777685

Volger S, 2012, OBESITY, V20, P147, DOI 10.1038/oby.2011.217

Wadden TA, 2003, OBES RES, V11, P1140, DOI 10.1038/oby.2003.155

Wang F, 2008, MINERVA ANESTESIOL, V74, P353

Ward SH, 2009, J GEN INTERN MED, V24, P579, DOI 10.1007/s11606-009-0922-z

Weaver J, 2016, J SOC PSYCHOL, V156, P179, DOI 10.1080/00224545.2015.1076761

Weinger Katie, 2015, Diabetes Spectr, V28, P146, DOI 10.2337/diaspect.28.2.146

Weinger K, 2011, ARCH INTERN MED, V171, P1990, DOI 10.1001/archinternmed.2011.502

Winkley K, 2015, DIABETIC MED, V32, P120, DOI 10.1111/dme.12556

Yelovich MC, 2016, J EVAL CLIN PRACT, V22, P558, DOI 10.1111/jep.12561

Young-Hyman D, 2016, DIABETES CARE, V39, P2126, DOI 10.2337/dc16-2053

Zolnierek KBH, 2009, MED CARE, V47, P826, DOI 10.1097/MLR.0b013e31819a5acc

NR 104

TC 3

Z9 3

U1 1

U2 3

PU AMER DIABETES ASSOC

PI ALEXANDRIA

PA 1701 N BEAUREGARD ST, ALEXANDRIA, VA 22311-1717 USA

SN 0149-5992

EI 1935-5548

J9 DIABETES CARE

JI Diabetes Care

PD DEC 1

PY 2017

VL 40

IS 12

BP 1790

EP 1799

DI 10.2337/dci17-0041

PG 10

WC Endocrinology & Metabolism

SC Endocrinology & Metabolism

GA FN3AO

UT WOS:000415868000030

PM 29042412

OA Bronze

DA 2019-01-08

ER

PT J

AU Nilsson, S

Hanberger, L

Olinder, AL

Forsner, M

AF Nilsson, Stefan

Hanberger, Lena

Olinder, Anna Lindholm

Forsner, Maria

TI The Faces Emotional Coping Scale as a self-reporting instrument for

coping with needle-related procedures: An initial validation study with

children treated for type 1 diabetes

SO JOURNAL OF CHILD HEALTH CARE

LA English

DT Article

DE Child; coping; needle-related procedures; pain; pain unpleasantness

ID PAIN SCALES; VENIPUNCTURE; INTENSITY; PARENTS; FEAR; UNPLEASANTNESS;

ASSOCIATION; PERSPECTIVE; DISTRESS; RATINGS

AB The aim of this study was to determine the concurrent and content validity, sensitivity and inter-rater reliability of the Faces Emotional Coping Scale (FECS) to evaluate the children's anticipation of the level of emotional coping in conjunction with a venepuncture. A total of 153 children with type 1 diabetes and 86 of their parents participated in the study. The age of the children, 76 of whom were boys, ranged from 7 to 18 years. The child and his or her parent reported the child's coping ability, and the child reported the pain intensity and unpleasantness of a venepuncture. The child also wrote a short narrative about his or her experience of the needle procedure. The FECS correlated negatively with the Coloured Analogue Scale and the Facial Affective Scale and positively with the FECS by proxy. The narratives of 90 children correlated negatively with the FECS. Younger children reported significantly lower scores than older children did regarding their ability to cope with a venepuncture. The children's scores on the FECS showed good agreement with the parents' scores. In this study, the FECS was deemed valid for measuring children's ability to cope with their emotions when undergoing needle-related procedures like venepuncture.

C1 [Nilsson, Stefan] Univ Gothenburg, Inst Hlth & Care Sci, Box 457, S-40530 Gothenburg, Sweden.

[Hanberger, Lena] Linkoping Univ, Div Nursing Sci, Dept Med & Hlth Sci, Linkoping, Sweden.

[Olinder, Anna Lindholm] Karolinska Inst, Sachs Children & Youths Hosp, Dept Clin Sci & Educ, Sodersjukhuset, Stockholm, Sweden.

[Forsner, Maria] Dalarna Univ, Dept Hlth & Social Sci, Caring Sci, Falun, Sweden.

[Forsner, Maria] Umea Univ, Dept Nursing, Umea, Sweden.

RP Nilsson, S (reprint author), Univ Gothenburg, Inst Hlth & Care Sci, Box 457, S-40530 Gothenburg, Sweden.

EM stefan.nilsson.4@gu.se

FU Diabetes stiftelsen; Novo Nordisk; Ypsomed; Infocare

FX The author(s) disclosed receipt of the following financial support for

the research, authorship, and/or publication of this article: This work

was supported by grants from the Diabetes stiftelsen, Novo Nordisk,

Ypsomed and finally Infocare.

CR Aydin D, 2016, J CLIN NURS, V25, P2328, DOI 10.1111/jocn.13321

Bulloch B, 2002, PEDIATRICS, V110, DOI 10.1542/peds.110.3.e33

Cemeroglu AP, 2015, ENDOCR PRACT, V21, P46, DOI 10.4158/EP14252.OR

Duff AJA, 2003, ARCH DIS CHILD, V88, P931, DOI 10.1136/adc.88.10.931

Fang H, 2016, SPRINGERPLUS, V5, DOI 10.1186/s40064-016-3613-3

Ferrari M, 2016, J HLTH PSYCHOL

Forsner M, 2016, J CHILD HEALTH CARE, V20, P365, DOI 10.1177/1367493515598646

Goodenough B, 1999, EUR J PAIN-LONDON, V3, P301, DOI 10.1016/S1090-3801(99)90012-5

Heinrich KH, 2016, VALUE HEALTH, V19, pA199, DOI 10.1016/j.jval.2016.03.1325

Hodgins MJ, 1997, J PAIN SYMPTOM MANAG, V13, P274, DOI 10.1016/S0885-3924(96)00328-4

Hofman PL, 2010, DIABETES CARE, V33, P1940, DOI 10.2337/dc10-0871

Howe CJ, 2011, MCN-AM J MATERN-CHIL, V36, P25, DOI 10.1097/NMC.0b013e3181fc6093

Hsieh HF, 2005, QUAL HEALTH RES, V15, P1277, DOI 10.1177/1049732305276687

Jaaniste T, 2016, PAIN, V157, P2399, DOI 10.1097/j.pain.0000000000000666

Kamper SJ, 2016, CHIROPR MAN THER, V24, DOI 10.1186/s12998-016-0104-0

Kamsvag-Magnusson T, 2014, ACTA PAEDIATR, V103, P630, DOI 10.1111/apa.12627

Karlsson K, 2014, INT J QUAL STUD HEAL, V9, DOI 10.3402/qhw.v9.23063

Kennedy RM, 2008, PEDIATRICS, V122, pS130, DOI DOI 10.1542/PEDS.2008-1055E

King N., 1994, QUALITATIVE METHODS, P14

Laerd Statistics, 2012, CONC PRED VAL

Ljusegren G, 2012, CHILD CARE HLTH DEV, V38, P464, DOI 10.1111/j.1365-2214.2011.01262.x

McCarthy AM, 2010, NURS RES, V59, P407, DOI 10.1097/NNR.0b013e3181f80ed5

McGrath P, 1996, PAIN, V64, P435, DOI 10.1016/0304-3959(95)00171-9

McMurtry C Meghan, 2015, Clin J Pain, V31, pS3, DOI 10.1097/AJP.0000000000000272

Melzack R, 1999, PAIN, pS121

Nilsson S, 2014, NURS RES PRACT, V2014

Nilsson Stefan, 2016, Nurs Child Young People, V28, P22, DOI 10.7748/ncyp.28.3.22.s21

Nilsson S, 2012, NURS RES PRACT, DOI 10.1155/2012/932570

Nilsson S, 2015, DEV NEUROREHABIL, V18, P162, DOI 10.3109/17518423.2013.801529

Perrott DA, 2004, EUR J PAIN, V8, P119, DOI 10.1016/S1090-3801(03)00087-9

Place MA, 2016, J CHILD HEALTH CARE, V20, P55, DOI 10.1177/1367493514551307

Randall D, 2012, J CHILD HEALTH CARE, V16, P305, DOI 10.1177/1367493512443907

Reid GJ, 1998, PAIN, V76, P83, DOI 10.1016/S0304-3959(98)00029-3

Richter M, 2010, PAIN, V148, P198, DOI 10.1016/j.pain.2009.08.009

Rindstedt Camilla, 2013, Commun Med, V10, P51

Rzeszut JR, 2011, J PEDIATR NURS, V26, P589, DOI 10.1016/j.pedn.2011.09.003

Sparapani Valeria de Cassia, 2015, Pediatr Nurs, V41, P17

Stanford EA, 2006, PAIN, V120, P16, DOI 10.1016/j.pain.2005.10.004

Streiner DL, 2015, HLTH MEASUREMENT SCA

Thompson S, 2016, J CHILD HEALTH CARE, V20, P267, DOI 10.1177/1367493515598643

Tsze DS, 2013, PEDIATRICS, V132, pE971, DOI 10.1542/peds.2013-1509

Twycross A, 2015, CLIN J PAIN, V31, P707, DOI 10.1097/AJP.0000000000000165

Uman LS, 2006, COCHRANE DB SYST REV, DOI 10.1002/14651858.CD005179

Vantaa Benjaminsson M, 2017, CLIN NURSING STUDIES, V5, P13

Varni JW, 1996, PAIN, V67, P141, DOI 10.1016/0304-3959(96)03077-1

von Baeyer CL, 2004, J PAIN, V5, P241, DOI 10.1016/j.jpain.2004.05.001

Walco Gary A, 2008, Pediatrics, V122 Suppl 3, pS125, DOI 10.1542/peds.2008-1055d

Zhou HQ, 2008, J ADV NURS, V63, P334, DOI 10.1111/j.1365-2648.2008.04694.x

NR 48

TC 0

Z9 0

U1 2

U2 7

PU SAGE PUBLICATIONS LTD

PI LONDON

PA 1 OLIVERS YARD, 55 CITY ROAD, LONDON EC1Y 1SP, ENGLAND

SN 1367-4935

EI 1741-2889

J9 J CHILD HEALTH CARE

JI J. Child Health Care

PD DEC

PY 2017

VL 21

IS 4

BP 392

EP 403

DI 10.1177/1367493517729041

PG 12

WC Nursing; Pediatrics

SC Nursing; Pediatrics

GA FN4CL

UT WOS:000415950500004

PM 29110524

OA Green Published

DA 2019-01-08

ER

PT J

AU Dickinson, JK

Guzman, SJ

Maryniuk, MD

O'Brian, CA

Kadohiro, JK

Jackson, RA

D'Hondt, N

Montgomery, B

Close, KL

Funnell, MM

AF Dickinson, Jane K.

Guzman, Susan J.

Maryniuk, Melinda D.

O'Brian, Catherine A.

Kadohiro, Jane K.

Jackson, Richard A.

D'Hondt, Nancy

Montgomery, Brenda

Close, Kelly L.

Funnell, Martha M.

TI The Use of Language in Diabetes Care and Education

SO DIABETES EDUCATOR

LA English

DT Article

ID PSYCHOLOGICAL INSULIN-RESISTANCE; WEIGHT BIAS INTERNALIZATION; PATIENTS

PREFERRED TERMS; PERSON-1ST LANGUAGE; PATIENT EMPOWERMENT; EMOTIONAL

DISTRESS; DESCRIBING OBESITY; SELF-MANAGEMENT; NEGATIVE-WORDS; SOCIAL

STIGMA

AB Language is powerful and can have a strong impact on perceptions as well as behavior. A task force, consisting of representatives from the American Association of Diabetes Educators and the American Diabetes Association, convened to discuss language in diabetes care and education. The literature supports the need for a language movement in diabetes care and education. There are effective ways of communicating about diabetes. This article provides recommendations for language used by health care professionals and others when discussing diabetes through spoken or written words, whether directed to people with diabetes, colleagues, or the general public, as well as research questions related to language and diabetes.

C1 [Dickinson, Jane K.] Columbia Univ, Teachers Coll, Box 35,525 West 120th St, New York, NY 10027 USA.

[Guzman, Susan J.] Behav Diabet Inst, San Diego, CA USA.

[Maryniuk, Melinda D.] Joslin Diabet Ctr, Boston, MA 02215 USA.

[O'Brian, Catherine A.] Amer Assoc Diabet Educ, Chicago, IL USA.

[Kadohiro, Jane K.] Univ Hawaii Manoa, Honolulu, HI 96822 USA.

[Jackson, Richard A.] Grassroots Diabet, Boston, MA USA.

[D'Hondt, Nancy] Ascens St John Providence Hosp & Med Ctr, Detroit, MI USA.

[Montgomery, Brenda] Astra Zeneca, Seattle, WA USA.

[Close, Kelly L.] Close Concerns, San Francisco, CA USA.

[Funnell, Martha M.] Michigan Ctr Diabet Translat Res, Ann Arbor, MI USA.

RP Dickinson, JK (reprint author), Columbia Univ, Teachers Coll, Box 35,525 West 120th St, New York, NY 10027 USA.

EM Dickinson@tc.columbia.edu

CR Aikens JE, 2012, DIABETES CARE, V35, P2472, DOI 10.2337/dc12-0181

Aikens JE, 2005, DIABETES EDUCATOR, V31, P681, DOI 10.1177/0145721705280829

Anderson RM, 2000, DIABETES EDUCATOR, V26, P597, DOI 10.1177/014572170002600405

Anderson RM, 2010, PATIENT EDUC COUNS, V79, P277, DOI 10.1016/j.pec.2009.07.025

[Anonymous], 2017, DIABETES CARE, V40, pS1, DOI 10.2337/dc17-S001

[Anonymous], 2001, CROSSING QUALITY CHA

[Anonymous], 2013, PEOPL 1 LANG OB

[Anonymous], INT CLASS DIS

Ayala J, 2014, WESTERN J NURS RES, V36, P1238, DOI 10.1177/0193945913518848

Barnish M, 2014, OPEN J MOD LINGUIST, V4, P5505

Benedetti F, 1997, PROG NEUROBIOL, V52, P109, DOI 10.1016/S0301-0082(97)00006-3

Benedetti F, 2002, EVAL HEALTH PROF, V25, P369, DOI 10.1177/0163278702238051

Berg FM, 1998, HLTH WEIGHT J, V12, P12

Beverly EA, 2012, J GEN INTERN MED, V27, P1180, DOI 10.1007/s11606-012-2070-0

Bissell P, 2004, SOC SCI MED, V58, P851, DOI 10.1016/S0277-9536(03)00259-4

Bootle S, 2015, DIABETES RES CLIN PR, V109, P6, DOI 10.1016/j.diabres.2015.04.004

Broom D, 2004, SOC SCI MED, V58, P2371, DOI 10.1016/j.socscimed.2003.09.002

Brown A, 2016, DO WE REDUCE DIABETE

Browne JL, 2014, BMJ OPEN, V4, DOI 10.1136/bmjopen-2014-005625

Browne JL, 2013, BMJ OPEN, V3, DOI 10.1136/bmjopen-2013-003384

Brummel-Smith K, 2016, J AM GERIATR SOC, V64, P15, DOI 10.1111/jgs.13866

CHROUSOS GP, 1995, NEW ENGL J MED, V332, P1351, DOI 10.1056/NEJM199505183322008

Chun H, 2007, ADV CONSUM RES, V34, P715

Collier R, 2012, CAN MED ASSOC J, V184, P1977, DOI 10.1503/cmaj.109-4319

Dellasega C, 2012, DIABETES RES CLIN PR, V95, P37, DOI 10.1016/j.diabres.2011.08.011

Diab P, 2012, JEMDSA, V17, P52, DOI 10.1080/22201009.2012.10872274

Dickinson Jane K, 2017, Diabetes Spectr, V30, P11, DOI 10.2337/ds15-0054

Dickinson Jane K, 2017, Clin Diabetes, V35, P51, DOI 10.2337/cd16-0014

Dickinson JK, 2015, DIABETES EDUCATOR, V41, P665, DOI 10.1177/0145721715608952

Dickinson JK, 2017, DIABETES SPECTR

Dunn DS, 2015, AM PSYCHOL, V70, P255, DOI 10.1037/a0038636

Dunning T, 2017, DIABETES EDUCATOR, V43, P18, DOI 10.1177/0145721716683449

Durso LE, 2008, OBESITY, V16, pS80, DOI 10.1038/oby.2008.448

Ellis S, 2014, J NUTR EDUC BEHAV, V46, P299, DOI 10.1016/j.jneb.2013.08.006

Fisher L, 2012, DIABETES CARE, V35, P259, DOI 10.2337/dc11-1572

Fleischman S, 1999, J MED HUMANITIES, V20, P3

Fleitas Joan, 2003, MCN Am J Matern Child Nurs, V28, P384

Frosch DL, 2012, HEALTH AFFAIR, V31, P1030, DOI 10.1377/hlthaff.2011.0576

Funnell MM, 2016, PATIENT EDUC COUNS, V99, P1921, DOI 10.1016/j.pec.2016.10.010

Glasgow RE, 1999, DIABETES CARE, V22, P2090, DOI 10.2337/diacare.22.12.2090

Gonzalez JS, 2011, DIABETES CARE, V34, P236, DOI 10.2337/dc10-1970

Gottlieb LN, 2014, AM J NURS, V114, P24, DOI 10.1097/01.NAJ.0000453039.70629.e2

Gredig D, 2017, HEALTH SOC CARE COMM, V25, P1620, DOI 10.1111/hsc.12376

Greenfield C, 2011, AUST J RURAL HEALTH, V19, P15, DOI 10.1111/j.1440-1584.2010.01171.x

Haque M, 2005, SAMJ S AFR MED J, V95, P798

HARDIN C, 1993, SOC COGNITION, V11, P277, DOI 10.1521/soco.1993.11.3.277

HOLM S, 1993, J MED ETHICS, V19, P108, DOI 10.1136/jme.19.2.108

Hoover J, 1980, Diabetes Educ, V6, P9, DOI 10.1177/014572178000600101

International Diabetes Federation, 2014, INT DIAB FED LANG PH

Iverson Ch., 2007, AMA MANUAL OF STYLE

Jensen ME, 2013, J AM PSYCHIAT NURSES, V19, P146, DOI 10.1177/1078390313489729

Jernigan K, 2009, BRAILLE MONITOR

Kalra S, 2015, J SOC HLTH DIABETES, V3, P3

Karter AJ, 2010, DIABETES CARE, V33, P733, DOI 10.2337/dc09-1184

Kendrick JM, 2005, JOGNN-J OBST GYN NEO, V34, P329, DOI 10.1177/0884217505276306

Link BG, 2006, LANCET, V367, P528, DOI 10.1016/S0140-6736(06)68184-1

Liu Nancy F, 2017, Clin Diabetes, V35, P27, DOI 10.2337/cd16-0020

Lloyd C, 2005, DIABETES SPECTR, V18, P121, DOI [DOI 10.2337/DIASPECT.18.2.121, 10.2337/diaspect.18.2.121]

Lydecker JA, 2016, INT J CLIN PRACT, V70, P682, DOI 10.1111/ijcp.12835

Marrero DG, 2013, DIABETES CARE, V36, P463, DOI 10.2337/dc12-2305

Matthews SM, 2009, PATIENT EDUC COUNS, V76, P31, DOI 10.1016/j.pec.2008.11.022

Meadows A, 2016, FRONT PSYCHOL, V7, DOI 10.3389/fpsyg.2016.01527

Nakar S, 2007, J DIABETES COMPLICAT, V21, P220, DOI 10.1016/j.jdiacomp.2006.02.004

Nicolucci A, 2013, DIABETIC MED, V30, P767, DOI 10.1111/dme.12245

Norman Debbie, 2003, Br J Nurs, V12, P1256

Ott J, 2012, CLIN J PAIN, V28, P324, DOI 10.1097/AJP.0b013e3182321cc3

Paget L, 2012, PATIENT CLIN COMMUNI

Pearl RL, 2017, OBESITY, V25, P317, DOI 10.1002/oby.21716

Peyrot M, 2005, DIABETES CARE, V28, P2673, DOI 10.2337/diacare.28.11.2673

Peyrot M, 2013, DIABETES RES CLIN PR, V99, P174, DOI 10.1016/j.diabres.2012.11.016

Polonsky WH, 2005, DIABETES CARE, V28, P2543, DOI 10.2337/diacare.28.10.2543

Polonsky WH, 2017, DIABETES RES CLIN PR, V127, P265, DOI 10.1016/j.diabres.2017.03.016

Polonsky WH, 2011, CURR MED RES OPIN, V27, P1169, DOI 10.1185/03007995.2011.573623

Polonsky WH, 2010, DIABETES EDUCATOR, V36, P828, DOI 10.1177/0145721710378539

Puhl R, 2013, INT J OBESITY, V37, P612, DOI 10.1038/ijo.2012.110

Rathert C, 2013, MED CARE RES REV, V70, P351, DOI 10.1177/1077558712465774

Ritholz MD, 2014, CHRONIC ILLN, V10, P303, DOI 10.1177/1742395314525647

Ritter A, 2016, HEALTHCARE, V4, P54

ROSENTHAL R, 1994, CURR DIR PSYCHOL SCI, V3, P176, DOI 10.1111/1467-8721.ep10770698

Rosenthal Robert, 1968, PYGMALION CLASSROOM

Saleebey D, 1996, SOC WORK, V41, P296

Schabert J, 2013, PATIENT, V6, P1, DOI 10.1007/s40271-012-0001-0

Schaffer R, 2016, ENDOCR TODAY

SHAMOON H, 1993, NEW ENGL J MED, V329, P977

Shiu ATY, 2003, J CLIN NURS, V12, P149, DOI 10.1046/j.1365-2702.2003.00735.x

Speight J, 2012, DIABETES RES CLIN PR, V97, P425, DOI 10.1016/j.diabres.2012.03.015

Strandberg RB, 2014, J PSYCHOSOM RES, V77, P174, DOI 10.1016/j.jpsychores.2014.06.015

Swift JA, 2013, PATIENT EDUC COUNS, V91, P186, DOI 10.1016/j.pec.2012.12.008

Texas Department of Aging and Disability Services, 2013, PERS 1 RESP LANG

Vartanian LR, 2008, J HEALTH PSYCHOL, V13, P131, DOI 10.1177/1359105307084318

Vishwanath A, 2014, HEALTH COMMUN, V29, P516, DOI 10.1080/10410236.2013.777685

Volger S, 2012, OBESITY, V20, P147, DOI 10.1038/oby.2011.217

Wadden TA, 2003, OBES RES, V11, P1140, DOI 10.1038/oby.2003.155

Wang F, 2008, MINERVA ANESTESIOL, V74, P353

Ward SH, 2009, J GEN INTERN MED, V24, P579, DOI 10.1007/s11606-009-0922-z

Weaver J, 2016, J SOC PSYCHOL, V156, P179, DOI 10.1080/00224545.2015.1076761

Weinger Katie, 2015, Diabetes Spectr, V28, P146, DOI 10.2337/diaspect.28.2.146

Weinger K, 2011, ARCH INTERN MED, V171, P1990, DOI 10.1001/archinternmed.2011.502

Winkley K, 2015, DIABETIC MED, V32, P120, DOI 10.1111/dme.12556

Yelovich MC, 2016, J EVAL CLIN PRACT, V22, P558, DOI 10.1111/jep.12561

Young-Hyman D, 2016, DIABETES CARE, V39, P2126, DOI 10.2337/dc16-2053

Zolnierek KBH, 2009, MED CARE, V47, P826, DOI 10.1097/MLR.0b013e31819a5acc

NR 102

TC 3

Z9 3

U1 4

U2 8

PU SAGE PUBLICATIONS INC

PI THOUSAND OAKS

PA 2455 TELLER RD, THOUSAND OAKS, CA 91320 USA

SN 0145-7217

EI 1554-6063

J9 DIABETES EDUCATOR

JI Diabetes Educ.

PD DEC

PY 2017

VL 43

IS 6

BP 551

EP 564

DI 10.1177/0145721717735535

PG 14

WC Endocrinology & Metabolism; Public, Environmental & Occupational Health

SC Endocrinology & Metabolism; Public, Environmental & Occupational Health

GA FM7DJ

UT WOS:000415231200001

PM 29040034

DA 2019-01-08

ER

PT J

AU Heitkemper, EM

Mamykina, L

Tobin, JN

Cassells, A

Smaldone, A

AF Heitkemper, Elizabeth M.

Mamykina, Lena

Tobin, Jonathan N.

Cassells, Andrea

Smaldone, Arlene

TI Baseline Characteristics and Technology Training of Underserved Adults

With Type 2 Diabetes in the Mobile Diabetes Detective (MoDD) Randomized

Controlled Trial

SO DIABETES EDUCATOR

LA English

DT Article

ID SELF-MANAGEMENT EDUCATION; IMPROVE GLYCEMIC CONTROL; CARE;

QUESTIONNAIRE; METAANALYSIS; DISPARITIES; NUTRITION; DISTRESS; MELLITUS;

SUPPORT

AB Purpose The purpose of this study is to describe the characteristics and technology training needs of underserved adults with type 2 diabetes mellitus (T2DM) who participated in a health information technology (HIT) diabetes self-management education (DSME) intervention.

Methods The baseline physiological, psychosocial, and technology use characteristics for 220 adults with poorly controlled T2DM were evaluated. Intervention participants received a 1-time intervention training, which included basic technology help, introduction to the Mobile Diabetes Detective (MoDD) website and text message features, and account activation that included subject-specific tailoring. Four additional on-site sessions for participants needing computer or Internet access or technology support were made available based on need. Data regarding on-site visits for usual care were collected. Data were analyzed using descriptive statistics and bivariate analysis.

Results The participants were predominately Hispanic and female with a baseline mean A1C of 10% (86 mmol/mol). Only half of the participants regularly used computers or text messages in daily life. The average introductory MoDD training session lasted 73.6 minutes. Following training, approximately one-third (35%) of intervention participants returned for basic and MoDD-specific technology assistance at their federally qualified health center. The most frequently reported duration for the extra training sessions was 30 to 45 minutes.

Conclusions Training and support needs were greater than anticipated. Diabetes educators should assess technology abilities prior to implementing health information technology (HIT) diabetes self-management education (DSME) in underserved adults. Future research must invest resources in technology access, anticipate subject training, and develop new training approaches to ensure HIT DSME use and engagement.

C1 [Heitkemper, Elizabeth M.; Smaldone, Arlene] Columbia Univ, Med Ctr, Sch Nursing, New York, NY 10032 USA.

[Mamykina, Lena] Columbia Univ, Med Ctr, Dept Biomed Informat, New York, NY 10032 USA.

[Tobin, Jonathan N.; Cassells, Andrea] Clin Directors Network CDN Inc, New York, NY USA.

[Tobin, Jonathan N.] Rockefeller Univ, Ctr Clin & Translat Sci, 1230 York Ave, New York, NY 10021 USA.

[Smaldone, Arlene] Columbia Univ, Coll Dent Med, Med Ctr, Dept Dent Behav Sci, New York, NY 10032 USA.

RP Heitkemper, EM (reprint author), Columbia Univ, Sch Nursing, 617 West 168th St, New York, NY 10032 USA.

EM emh2215@cumc.columbia.edu

FU National Institute of Nursing Research Training [NINR T32NR007969];

National Institute of Diabetes and Digestive and Kidney Diseases

[R01DK090372]

FX This work was supported by the National Institute of Nursing Research

Training grant NINR T32NR007969 and the National Institute of Diabetes

and Digestive and Kidney Diseases grant R01DK090372

CR American Diabetes Association, 2017, J DIABETES S1, V40, pS1

[Anonymous], 2012, DIGITAL DIFFERENCES

Attridge M, 2014, COCHRANE DB SYST REV, DOI 10.1002/14651858.CD006424.pub3

Chatterjee S, 2015, POSTGRAD MED J, V91, P612, DOI 10.1136/postgradmedj-2014-133200

Cole-Lewis HJ, 2016, INT J MED INFORM, V85, P96, DOI 10.1016/j.ijmedinf.2015.08.003

Czaja SJ, 2015, HUM FACTORS, V57, P491, DOI 10.1177/0018720814549238

Ferdinand KC, 2015, CURR MED RES OPIN, V31, P913, DOI 10.1185/03007995.2015.1029894

Ferguson S, 2015, DIABETES EDUCATOR, V41, P472, DOI 10.1177/0145721715584404

Glasgow RE, 2004, J BEHAV MED, V27, P477, DOI 10.1023/B:JOBM.0000047611.81027.71

Glasgow RE, 2007, DIABETES CARE, V30, P33, DOI 10.2337/dc06-1390

Harris PA, 2009, J BIOMED INFORM, V42, P377, DOI 10.1016/j.jbi.2008.08.010

Hawthorne K, 2010, DIABETIC MED, V27, P613, DOI 10.1111/j.1464-5491.2010.02954.x

Heitkemper EM, 2017, J AM MED INFORM ASSN, V24, P1024, DOI 10.1093/jamia/ocx025

Horigan G, 2017, DIABETIC MED, V34, P14, DOI 10.1111/dme.13120

Lai Albert M, 2006, AMIA Annu Symp Proc, P459

Lanting LC, 2005, DIABETES CARE, V28, P2280, DOI 10.2337/diacare.28.9.2280

Li R, 2014, MMWR-MORBID MORTAL W, V63, P1045

Liang X, 2011, DIABETIC MED, V28, P455, DOI 10.1111/j.1464-5491.2010.03180.x

Lowe B, 2005, J PSYCHOSOM RES, V58, P163, DOI 10.1016/j.jpsychores.2004.09.006

Mamykina L, 2016, J AM MED INFORM ASSN, V23, P129, DOI 10.1093/jamia/ocv169

Mamykina L, 2008, CHI 2008: 26TH ANNUAL CHI CONFERENCE ON HUMAN FACTORS IN COMPUTING SYSTEMS VOLS 1 AND 2, CONFERENCE PROCEEDINGS, P477

Office of Disease Prevention and Health Promotion Department of Health and Human Services, DIAB OBJ HLTH PEOPL

Oster Natalia Vukshich, 2006, Dis Manag, V9, P167, DOI 10.1089/dis.2006.9.167

Pal K, 2013, COCHRANE DB SYST REV, DOI 10.1002/14651858.CD008776.pub2

POLONSKY WH, 1995, DIABETES CARE, V18, P754, DOI 10.2337/diacare.18.6.754

Powers MA, 2017, DIABETES EDUCATOR, V43, P40, DOI 10.1177/0145721716689694

Prochaska JO, 1997, AM J HEALTH PROMOT, V12, P38, DOI 10.4278/0890-1171-12.1.38

PROCHASKA JO, 1982, PSYCHOTHER-THEOR RES, V19, P276, DOI 10.1037/h0088437

Ricci-Cabello I, 2014, BMC ENDOCR DISORD, V14, DOI 10.1186/1472-6823-14-60

Ritter PL, 2016, DIABETES EDUCATOR, V42, P167, DOI 10.1177/0145721716628648

Robinson KS, 2010, CIN-COMPUT INFORM NU, V28, P172, DOI 10.1097/NCN.0b013e3181d785d5

Rovner AJ, 2012, DIABETES CARE, V35, P1643, DOI 10.2337/dc11-2371

Shah Viral N, 2015, Clin Diabetes Endocrinol, V1, P16, DOI 10.1186/s40842-015-0016-2

Shaw K, 2011, DIABETES EDUCATOR, V37, P813, DOI 10.1177/0145721711424618

Starren J, 2005, CIN-COMPUT INFORM NU, V23, P181, DOI 10.1097/00024665-200507000-00004

Toobert DJ, 2000, DIABETES CARE, V23, P943, DOI 10.2337/diacare.23.7.943

Vincent D, 2008, NURS RES, V57, P101, DOI 10.1097/01.NNR.0000313484.18670.ab

Welch G, 2003, DIABETIC MED, V20, P69, DOI 10.1046/j.1464-5491.2003.00832.x

Welch G, 2007, ETHNIC DIS, V17, P541

NR 39

TC 0

Z9 0

U1 2

U2 9

PU SAGE PUBLICATIONS INC

PI THOUSAND OAKS

PA 2455 TELLER RD, THOUSAND OAKS, CA 91320 USA

SN 0145-7217

EI 1554-6063

J9 DIABETES EDUCATOR

JI Diabetes Educ.

PD DEC

PY 2017

VL 43

IS 6

BP 576

EP 588

DI 10.1177/0145721717737367

PG 13

WC Endocrinology & Metabolism; Public, Environmental & Occupational Health

SC Endocrinology & Metabolism; Public, Environmental & Occupational Health

GA FM7DJ

UT WOS:000415231200003

PM 29059017

OA Green Accepted

DA 2019-01-08

ER

PT J

AU DiNardo, M

Saba, S

Greco, CM

Broyles, L

Terhorst, L

Chasens, ER

Bair, B

Charron-Prochownik, D

Cohen, S

AF DiNardo, Monica

Saba, Shaddy

Greco, Carol M.

Broyles, Lauren

Terhorst, Lauren

Chasens, Eileen R.

Bair, Brandi

Charron-Prochownik, Denise

Cohen, Susan

TI A Mindful Approach to Diabetes Self-Management Education and Support for

Veterans

SO DIABETES EDUCATOR

LA English

DT Article

ID RANDOMIZED CONTROLLED-TRIAL; QUALITY-OF-LIFE; STRESS-REDUCTION

INTERVENTION; GLYCEMIC CONTROL; EMPOWERMENT SCALE; MEDITATION;

DEPRESSION; DISTRESS; MEDICINE; ADULTS

AB Purpose The purpose of this study is to determine feasibility, satisfaction, and preliminary effects of Mindful Stress Reduction in Diabetes Education (Mind-STRIDE), a mindfulness-based intervention for veterans.

Methods The study used a single-group pretest-posttest repeated-measures design. The 90-minute Mind-STRIDE training, adapted from Mindfulness Based Stress Reduction (MBSR), was provided as the final component of a half-day diabetes self-management education class at a Veterans Affairs (VA) outpatient diabetes clinic. Following initial training, participants were asked to practice mindfulness at home for 10 minutes each day during the 3-month study. Study recruitment and retention were calculated as rates. Veteran and diabetes educator satisfaction were assessed by rating scales and open-ended comments. Psychosocial-behavioral and metabolic outcomes were assessed at baseline and 3 months after initial training. Bivariate correlations were performed to describe relationships between mindfulness and other outcome variables. Gain scores and Wilcoxon matched-pair signed rank tests were used to assess pre to post changes; Cohen's d was applied to estimate the magnitude of effects.

Results Twenty-eight of 49 eligible veterans (57%) enrolled in the study. Of those, 11 veterans (39%) demonstrated participation in home practice, and 20 veterans (71%) completed the study. Overall, participants and diabetes educators were highly satisfied with the Mind-STRIDE intervention. Significant improvements were found in diabetes distress, diabetes self-efficacy, diabetes self-management behaviors, mindful-describing, and A1C.

Conclusion Results suggest feasibility, satisfaction, and positive preliminary effects. Efficacy testing by randomized controlled trial with analysis of covariance structures is warranted.

C1 [DiNardo, Monica; Saba, Shaddy; Broyles, Lauren; Bair, Brandi] Vet Affairs Pittsburgh Healthcare Syst, Ctr Hlth Equ Res & Promot, Pittsburgh, PA USA.

[Saba, Shaddy] Univ Pittsburgh, Med Ctr, Dept Anesthesiol, Pittsburgh, PA USA.

[Greco, Carol M.] Univ Pittsburgh, Sch Med, Pittsburgh, PA USA.

[Terhorst, Lauren] Univ Pittsburgh, Sch Hlth & Rehabil Sci, Pittsburgh, PA USA.

[Chasens, Eileen R.; Bair, Brandi; Charron-Prochownik, Denise; Cohen, Susan] Univ Pittsburgh, Sch Nursing, Pittsburgh, PA 15261 USA.

RP DiNardo, M (reprint author), VA Pittsburgh Healthcare Syst, Univ Dr,151C, Pittsburgh, PA 15240 USA.

EM Monica.Dinardo@va.gov

RI Terhorst, Lauren/H-7942-2018

OI Cohen, Susan/0000-0002-3860-4681

FU American Association of Diabetes Educators Education AADE Research

Foundation; AADE Advanced Practice Community of Interest Scholarship and

Research Grant [AADE-AP 2012-001]

FX This study was supported by a 2012 American Association of Diabetes

Educators Education AADE Research Foundation and AADE Advanced Practice

Community of Interest Scholarship and Research Grant (AADE-AP 2012-001)

awarded to the principal investigator.

CR Anderson RM, 2003, DIABETES CARE, V26, P1641, DOI 10.2337/diacare.26.5.1641-a

Anderson RM, 2000, DIABETES CARE, V23, P739, DOI 10.2337/diacare.23.6.739

Baer RA, 2006, ASSESSMENT, V13, P27, DOI 10.1177/1073191105283504

Bergomi C, 2013, MINDFULNESS, V4, P191, DOI 10.1007/s12671-012-0110-9

Bishop SR, 2004, CLIN PSYCHOL-SCI PR, V11, P230, DOI 10.1093/clipsy/bph077

Bormann JE, 2014, MED CARE, V52, pS13, DOI 10.1097/MLR.0000000000000200

Carlson LE, 2005, INT J BEHAV MED, V12, P278, DOI 10.1207/s15327558ijbm1204_9

Charron-Prochownik D, 2006, DIABETES SPECTR, V19, P220

Cohen J, 1988, STAT POWER ANAL BEHA

COHEN S, 1983, J HEALTH SOC BEHAV, V24, P385, DOI 10.2307/2136404

Diabetes-QUERI Executive Committee, 2013, QUAL ENH RES IN FACT

Fabian E, 2011, ANN NUTR METAB, V58, P101, DOI 10.1159/000326765

Fisher EB, 2007, DIABETES EDUCATOR, V33, P1080, DOI 10.1177/0145721707309808

Fisher L, 2008, ANN FAM MED, V6, P246, DOI 10.1370/afm.842

Fisher L, 2012, DIABETES CARE, V35, P259, DOI 10.2337/dc11-1572

Fitzgerald JT, 1996, EVAL HEALTH PROF, V19, P208, DOI 10.1177/016327879601900205

Friis AM, 2016, DIABETES CARE, V39, P1963, DOI 10.2337/dc16-0416

Gould CE, 2015, INT J GERIATR PSYCH, V30, P623, DOI 10.1002/gps.4193

Gregg JA, ACCEPTANCE COMMITMEN

Gregg JA, 2007, J CONSULT CLIN PSYCH, V75, P336, DOI 10.1037/0022-006X.75.2.336

Gross CR, 2014, WILEY BLACKWELL HANDBOOK OF MINDFULNESS, VOLS I AND II, P898

Grossman P, 2007, PSYCHOTHER PSYCHOSOM, V76, P226, DOI 10.1159/000101501

Hartmann M, 2012, DIABETES CARE, V35, P945, DOI 10.2337/dc11-1343

Kabat-Zinn J, 1998, PSYCHOSOM MED, V60, P625, DOI 10.1097/00006842-199809000-00020

Kabat-Zinn J., 1990, FULL CATASTROPHE LIV

KABATZINN J, 1982, GEN HOSP PSYCHIAT, V4, P33, DOI 10.1016/0163-8343(82)90026-3

Kearney DJ, 2012, J CLIN PSYCHOL, V68, P101, DOI 10.1002/jclp.20853

Khunti K, 2012, BRIT MED J, V344, DOI 10.1136/bmj.e2333

King AP, 2016, FRONT PSYCHIATRY, V7, DOI 10.3389/fpsyt.2016.00154

Kligler Benjamin, 2014, Journal of the National Cancer Institute Monographs, P292, DOI 10.1093/jncimonographs/lgu039

KOENIG RJ, 1976, DIABETES, V25, P230, DOI 10.2337/diabetes.25.3.230

Lang AJ, 2012, BEHAV MODIF, V36, P759, DOI 10.1177/0145445512441200

Ludwig DS, 2008, JAMA-J AM MED ASSOC, V300, P1350, DOI 10.1001/jama.300.11.1350

Merkes M, 2010, AUST J PRIM HEALTH, V16, P200, DOI 10.1071/PY09063

Miller CK, 2012, J ACAD NUTR DIET, V112, P1835, DOI 10.1016/j.jand.2012.07.036

Morone NE, 2008, PAIN, V134, P310, DOI 10.1016/j.pain.2007.04.038

Mulcahy K, 2003, DIABETES EDUCATOR, V29, P768, DOI 10.1177/014572170302900509

Norris SL, 2002, DIABETES CARE, V25, P1159, DOI 10.2337/diacare.25.7.1159

Peyrot M, 2005, DIABETIC MED, V22, P1379, DOI 10.1111/j.1464-5491.2005.01644.x

Powers MA, 2017, DIABETES EDUCATOR, V43, P40, DOI 10.1177/0145721716689694

Pradhan EK, 2007, ARTHRIT RHEUM-ARTHR, V57, P1134, DOI 10.1002/art.23010

Rosenzweig S, 2010, J PSYCHOSOM RES, V68, P29, DOI 10.1016/j.jpsychores.2009.03.010

Rosenzweig S, 2007, ALTERN THER HEALTH M, V13, P36

Shapiro D, 1980, SCI MEDITATION THEOR

Tacon AM, 2003, FAM COMMUNITY HEALTH, V26, P25, DOI 10.1097/00003727-200301000-00004

Teasdale JD, 2000, J CONSULT CLIN PSYCH, V68, P615, DOI 10.1037/0022-006X.68.4.615

Teixeira E, 2010, HOLIST NURS PRACT, V24, P277, DOI 10.1097/HNP.0b013e3181f1add2

Tilburt JC, 2009, ARCH INTERN MED, V169, P670, DOI 10.1001/archinternmed.2009.49

van Son J, 2013, DIABETES CARE, V36, P823, DOI 10.2337/dc12-1477

Wahbeh Helane, 2011, Mindfulness (N Y), V2, P219

Walsh R, 2006, AM PSYCHOL, V61, P227, DOI 10.1037/0003-066X.61.3.227

Witek-Janusek L, 2008, BRAIN BEHAV IMMUN, V22, P969, DOI 10.1016/j.bbi.2008.01.012

Young-Hyman D, 2017, DIABETES CARE, V40, P726, DOI 10.2337/dc17-er05

Youssef NA, 2013, ARCH SUICIDE RES, V17, P106, DOI 10.1080/13811118.2013.776445

Zagarins SE, 2012, J BEHAV MED, V35, P299, DOI 10.1007/s10865-011-9359-z

Zeidan F, 2010, CONSCIOUS COGN, V19, P597, DOI 10.1016/j.concog.2010.03.014

NR 56

TC 0

Z9 0

U1 7

U2 13

PU SAGE PUBLICATIONS INC

PI THOUSAND OAKS

PA 2455 TELLER RD, THOUSAND OAKS, CA 91320 USA

SN 0145-7217

EI 1554-6063

J9 DIABETES EDUCATOR

JI Diabetes Educ.

PD DEC

PY 2017

VL 43

IS 6

BP 608

EP 620

DI 10.1177/0145721717738019

PG 13

WC Endocrinology & Metabolism; Public, Environmental & Occupational Health

SC Endocrinology & Metabolism; Public, Environmental & Occupational Health

GA FM7DJ

UT WOS:000415231200006

PM 29078735

DA 2019-01-08

ER

PT J

AU Browne, JL

Ventura, AD

Mosely, K

Speight, J

AF Browne, J. L.

Ventura, A. D.

Mosely, K.

Speight, J.

TI Measuring Type 1 diabetes stigma: development and validation of the Type

1 Diabetes Stigma Assessment Scale (DSAS-1)

SO DIABETIC MEDICINE

LA English

DT Article

ID NEUROLOGICAL CONDITIONS; PUBLIC-HEALTH; DISTRESS; EPILEPSY; ILLNESS;

OBESITY; PEOPLE; BIAS; METAANALYSIS; DISORDER

AB Aims To develop and validate a self-report measure designed to assess perceived and experienced stigma for adults with Type 1 diabetes: the Type 1 Diabetes Stigma Assessment Scale (DSAS-1).

Methods A large item-pool (64 items) was drafted based on qualitative data from interviews with 27 adults with Type 1 diabetes. Eleven adults with Type 1 diabetes completed the draft questionnaire (responding to items using a five-point Likert scale), and participated in cognitive debriefing interviews. Based on their feedback, the item-pool was reduced and refined. Adults with Type 1 diabetes (N=898) completed an online survey including the draft stigma questionnaire (41 items) and other validated measures. Psychometric validation included principal components analysis and confirmatory factor analysis (split samples), internal consistency reliability assessment and Spearman's rho correlations.

Results Scale reduction techniques resulted in 19 items (alpha=0.93). An unforced three-factor solution suggested three subscales: Treated Differently (six items, alpha=0.89); Blame and Judgement (six items, alpha=0.88); and Identity Concerns (seven items, alpha =0.89). This was corroborated with a confirmatory factor analysis, which demonstrated reasonable model fit with the three factors; less so for a single-factor model. Satisfactory concurrent, convergent and discriminant validity were demonstrated.

ConclusionsThe 19-item DSAS-1 is a valid and reliable measure of the perceptions and experiences of Type 1 diabetes stigma. This novel, relatively brief measure has satisfactory psychometric properties. The DSAS-1 is now available for investigations into the nature and magnitude of the relationships between diabetes stigma and diabetes self-care behaviours and outcomes.

C1 [Browne, J. L.; Ventura, A. D.; Speight, J.] Deakin Univ, Sch Psychol, Geelong, Vic, Australia.

[Browne, J. L.; Ventura, A. D.; Speight, J.] Australian Ctr Behav Res Diabet, Diabet Victoria, Melbourne, Vic, Australia.

[Mosely, K.] BodyMatters Australasia, Sydney, NSW, Australia.

[Speight, J.] AHP Res, Hornchurch, England.

RP Browne, JL (reprint author), Deakin Univ, Sch Psychol, Geelong, Vic, Australia.; Browne, JL (reprint author), Australian Ctr Behav Res Diabet, Diabet Victoria, Melbourne, Vic, Australia.

EM jbrowne@acbrd.org.au

OI Speight, Jane/0000-0002-1204-6896

FU Diabetes Australia Research Trust grant; Sanofi; Diabetes Victoria;

Deakin University

FX This design and development of the questionnaire was funded by a

Diabetes Australia Research Trust grant, while an unrestricted

educational grant from Sanofi enabled us to enhance the sample size for

the validation study. Neither of the funding bodies played a role in the

study design, data collection or analysis, or interpretation or write-up

of the results. J.L.B. and J.S. were supported by core funding to The

Australian Centre for Behavioural Research in Diabetes derived from the

collaboration between Diabetes Victoria and Deakin University.

CR Aikins ADG, 2006, J COMMUNITY APPL SOC, V16, P426, DOI 10.1002/casp.892

Barney LJ, 2006, AUST NZ J PSYCHIAT, V40, P51, DOI 10.1080/j.1440-1614.2006.01741.x

Bethlehem J, 2010, INT STAT REV, V78, P161, DOI 10.1111/j.1751-5823.2010.00112.x

Browne JL, 2016, DIABETES CARE, V39, P2141, DOI 10.2337/dc16-0117

Browne JL, 2014, BMJ OPEN, V4, DOI 10.1136/bmjopen-2014-005625

Browne JL, 2013, BMJ OPEN, V3, DOI 10.1136/bmjopen-2013-003384

Cataldo Janine K, 2011, Oncol Nurs Forum, V38, pE46, DOI 10.1188/11.ONF.E46-E54

Costello AB, 2005, PRACTICAL ASSESSMENT, V10, P1, DOI DOI 10.1316/S0782-051X03024046-9

Crawford JR, 2003, MEMORY, V11, P261, DOI 10.1080/09658210244000027

Della LJ, 2011, J RURAL HEALTH, V27, P3, DOI 10.1111/j.1748-0361.2010.00311.x

Earnshaw VA, 2013, J BEHAV MED, V36, P270, DOI 10.1007/s10865-012-9422-4

Eysenbach G, 2002, J MED INTERNET RES, V4, DOI 10.2196/jmir.4.2.e13

Fisher L, 2015, J DIABETES COMPLICAT, V29, P572, DOI 10.1016/j.jdiacomp.2015.01.012

Hatzenbuehler ML, 2013, AM J PUBLIC HEALTH, V103, P813, DOI 10.2105/AJPH.2012.301069

Hayward P, 1997, J MENT HEALTH, V6, P345, DOI DOI 10.1080/09638239718671

Hu LT, 1999, STRUCT EQU MODELING, V6, P1, DOI 10.1080/10705519909540118

Hutcheson G. D, 1999, MULTIVARIATE SOCIAL

Jacoby A, 2005, LANCET NEUROL, V4, P171, DOI 10.1016/S1474-4422(05)01014-8

JACOBY A, 1994, SOC SCI MED, V38, P269, DOI 10.1016/0277-9536(94)90396-4

Kato A, 2014, HEALTH QUAL LIFE OUT, V12, DOI 10.1186/s12955-014-0179-z

Kroenke K, 2009, J AFFECT DISORDERS, V114, P163, DOI 10.1016/j.jad.2008.06.026

Link BG, 2001, ANNU REV SOCIOL, V27, P363, DOI 10.1146/annurev.soc.27.1.363

Livingston JD, 2010, SOC SCI MED, V71, P2150, DOI 10.1016/j.socscimed.2010.09.030

MacLeod JS, 2003, EPILEPSY BEHAV, V4, P112, DOI 10.1016/S1525-5050(03)00007-6

Major B, 2005, ANNU REV PSYCHOL, V56, P393, DOI 10.1146/annurev.psych.56.091103.070137

Mak WWS, 2007, SOC SCI MED, V65, P245, DOI 10.1016/j.socscimed.2007.03.015

Molina Y, 2013, INT J BEHAV MED, V20, P450, DOI 10.1007/s12529-012-9243-4

Nicolucci A, 2013, DIABETIC MED, V30, P767, DOI 10.1111/dme.12245

POLONSKY WH, 1995, DIABETES CARE, V18, P754, DOI 10.2337/diacare.18.6.754

Polonsky WH, 2005, DIABETES CARE, V28, P626, DOI 10.2337/diacare.28.3.626

Polonsky WH, 2009, AM DIAB ASS 69 SCI S

Puhl R, 2001, OBES RES, V9, P788, DOI 10.1038/oby.2001.108

Puhl RM, 2008, HEALTH EDUC RES, V23, P347, DOI 10.1093/her/cym052

Puhl RM, 2010, AM J PUBLIC HEALTH, V100, P1019, DOI 10.2105/AJPH.2009.159491

Puhl RM, 2009, OBESITY, V17, P941, DOI 10.1038/oby.2008.636

Rao D, 2009, QUAL LIFE RES, V18, P585, DOI 10.1007/s11136-009-9475-1

Rosenberg M., 1965, SOC ADOLESCENT CHILD

Schabert J, 2013, PATIENT, V6, P1, DOI 10.1007/s40271-012-0001-0

Shrive Fiona M, 2006, BMC Med Res Methodol, V6, P57, DOI 10.1186/1471-2288-6-57

Spitzer RL, 2006, ARCH INTERN MED, V166, P1092, DOI 10.1001/archinte.166.10.1092

Steiger JH, 2007, PERS INDIV DIFFER, V42, P893, DOI 10.1016/j.paid.2006.09.017

Valdiserri RO, 2002, AM J PUBLIC HEALTH, V92, P341, DOI 10.2105/AJPH.92.3.341

van der Ven NCW, 2003, DIABETES CARE, V26, P713, DOI 10.2337/diacare.26.3.713

Wheaton B, 1977, SOCIOL METHODOL, V8, P84, DOI [10.2307/270754, DOI 10.2307/270754, 10. 2307/270754]

NR 44

TC 2

Z9 2

U1 2

U2 9

PU WILEY

PI HOBOKEN

PA 111 RIVER ST, HOBOKEN 07030-5774, NJ USA

SN 0742-3071

EI 1464-5491

J9 DIABETIC MED

JI Diabetic Med.

PD DEC

PY 2017

VL 34

IS 12

BP 1773

EP 1782

DI 10.1111/dme.13507

PG 10

WC Endocrinology & Metabolism

SC Endocrinology & Metabolism

GA FM6TJ

UT WOS:000415191800017

PM 28891210

DA 2019-01-08

ER

PT J

AU Weissberg-Benchell, J

Hessler, D

Fisher, L

Russell, SJ

Polonsky, WH

AF Weissberg-Benchell, Jill

Hessler, Danielle

Fisher, Lawrence

Russell, Steven J.

Polonsky, William H.

TI Impact of an Automated Bihormonal Delivery System on Psychosocial

Outcomes in Adults with Type 1 Diabetes

SO DIABETES TECHNOLOGY & THERAPEUTICS

LA English

DT Article

DE Bionic pancreas; Psychosocial outcomes; Artificial pancreas

ID EXCHANGE CLINIC REGISTRY; ARTIFICIAL PANCREAS; CLOSED-LOOP;

PEDIATRIC-PATIENTS; INSULIN DELIVERY; BIONIC PANCREAS; GLUCOSE CONTROL;

CHILDREN; DISCONTINUATION; ADOLESCENTS

AB Objectives: The study assessed the psychosocial impact of the bihormonal bionic pancreas (BP) on adults in a real-world outpatient setting.

Research Design and Methods: Thirty-nine adults with type 1 diabetes at four study centers across the U.S. participated in a two-arm, random-order, crossover design study: 11 days with the BP and 11 days with their usual care (UC). Psychosocial questionnaires were administered before the first study arm, at the end of the first study arm, and at the end of the second study arm.

Results: The mean age of participants was 33 years; mean diabetes duration was 17 years; and 84% were non-Hispanic Caucasian. Significantly greater improvements in psychosocial outcomes were found following the use of BP versus UC; these included reductions in diabetes-related distress (P<0.001) with the greatest drops in distress related to hypoglycemia and to eating constraints, and greater treatment satisfaction (P<0.05). The majority of BP users described their experience as positive with a reduction in worrying about low (72%) and high (69%) blood sugars. The majority reported trusting the device (69%) and feeling less burdened by the BP than their usual method of diabetes care (64%). Concerns about the burden of the BP were also expressed, with >75% of users highlighting the burden of carrying around the equipment and the need to change glucagon daily, and more than half of the participants reporting concerns about wearability, discomfort, the time it took to correct out-of-range numbers, and glitches in the system.

Conclusions: Overall, participants report substantial psychosocial benefits accruing from the BP relative to their usual method of diabetes care. However, participants also reported a number of burdens associated with the system. Future versions of the BP device should be designed with the goal of addressing these concerns, and studies with larger, more diverse samples, and with more technology-naive participants are needed.

C1 [Weissberg-Benchell, Jill] Ann & Robert H Lurie Childrens Hosp Chicago, 225 East Chicago Ave,Box 10, Chicago, IL 60611 USA.

[Hessler, Danielle; Fisher, Lawrence; Polonsky, William H.] Univ Calif San Francisco, Dept Family & Community Med, San Francisco, CA 94143 USA.

[Russell, Steven J.] Massachusetts Gen Hosp, Diabet Res Ctr, Boston, MA 02114 USA.

[Polonsky, William H.] Behav Diabet Inst, San Diego, CA USA.

RP Weissberg-Benchell, J (reprint author), Ann & Robert H Lurie Childrens Hosp Chicago, 225 East Chicago Ave,Box 10, Chicago, IL 60611 USA.

EM jwbenchell@luriechildrens.org

CR Atlas E, 2010, DIABETES CARE, V33, P1072, DOI 10.2337/dc09-1830

Babar GS, 2009, DIABETES TECHNOL THE, V11, P131, DOI 10.1089/dia.2008.0042

Bech P, 1999, ACTA ANAESTH SCAND, V43, P893, DOI 10.1034/j.1399-6576.1999.430906.x

Beck RW, 2012, J CLIN ENDOCR METAB, V97, P4383, DOI 10.1210/jc.2012-1561

Blauw H, 2013, DIABETES TECHNOL THE, V15, P619, DOI 10.1089/dia.2013.0196

BRADLEY C, 1990, DIABETIC MED, V7, P445, DOI 10.1111/j.1464-5491.1990.tb01421.x

Bradley C., 1994, HDB PSYCHOL DIABETES, P111

Breton M, 2012, DIABETES, V61, P2230, DOI 10.2337/db11-1445

Buckingham Bruce, 2005, Diabetes Technol Ther, V7, P440, DOI 10.1089/dia.2005.7.440

Castle JR, 2010, DIABETES CARE, V33, P1282, DOI 10.2337/dc09-2254

Cobelli C, 2011, DIABETES, V60, P2672, DOI 10.2337/db11-0654

de Vries L, 2011, PEDIATR DIABETES, V12, P506, DOI 10.1111/j.1399-5448.2010.00701.x

El-Khatib F, 2010, SCI TRANS MED, V4, P1374

El-Khatib FH, 2017, LANCET, V389, P369, DOI 10.1016/S0140-6736(16)32567-3

El-Khatib FH, 2014, J CLIN ENDOCR METAB, V99, P1701, DOI 10.1210/jc.2013-4151

Fisher L, 2015, J DIABETES COMPLICAT, V29, P572, DOI 10.1016/j.jdiacomp.2015.01.012

Fisher L, 2012, DIABETES CARE, V35, P259, DOI 10.2337/dc11-1572

Hofer SE, 2010, PEDIATR DIABETES, V11, P116, DOI 10.1111/j.1399-5448.2009.00546.x

Hovorka R, 2010, LANCET, V375, P743, DOI 10.1016/S0140-6736(09)61998-X

Lombardo F, 2012, ACTA DIABETOL, V49, pS99, DOI 10.1007/s00592-011-0344-3

Mauras N, 2012, DIABETES CARE, V35, P204, DOI 10.2337/dc11-1746

Miller KM, 2015, DIABETES CARE, V38, P971, DOI 10.2337/dc15-0078

Nathan DM, 2014, DIABETES CARE, V37, P9, DOI 10.2337/dc13-2112

O'Connell Michele A, 2008, J Diabetes Sci Technol, V2, P91

Russell SJ, 2016, LANCET DIABETES ENDO, V4, P233, DOI 10.1016/S2213-8587(15)00489-1

Russell SJ, 2014, NEW ENGL J MED, V371, P313, DOI 10.1056/NEJMoa1314474

Russell SJ, 2012, DIABETES CARE, V35, P2148, DOI [10.2337/dc12-0071, 10.2337/dc12-0071/-/DC1]

Staehr Johansen K, 1989, MULTICENTRE CONTINUO

Steil GM, 2006, DIABETES, V55, P3344, DOI 10.2337/db06-0419

Weinzimer SA, 2008, DIABETES CARE, V31, P934, DOI 10.2337/dc07-1967

NR 30

TC 2

Z9 2

U1 0

U2 6

PU MARY ANN LIEBERT, INC

PI NEW ROCHELLE

PA 140 HUGUENOT STREET, 3RD FL, NEW ROCHELLE, NY 10801 USA

SN 1520-9156

EI 1557-8593

J9 DIABETES TECHNOL THE

JI Diabetes Technol. Ther.

PD DEC

PY 2017

VL 19

IS 12

BP 723

EP 729

DI 10.1089/dia.2017.0174

EA NOV

EY 2017

PG 7

WC Endocrinology & Metabolism

SC Endocrinology & Metabolism

GA FQ1XU

UT WOS:000414637900001

PM 29106311

DA 2019-01-08

ER

PT J

AU Weissberg-Benchell, J

Rychlik, K

AF Weissberg-Benchell, Jill

Rychlik, Karen

TI Diabetes camp matters: Assessing families' views of their diabetes camp

experience

SO PEDIATRIC DIABETES

LA English

DT Article

DE adaptation; camping; pediatric psychology; psychological; type 1

diabetes

ID ADOLESCENTS; CHILDREN; CARE

AB BACKGROUNDCamp offers a safe and fun environment for learning new information about diabetes care and for trying new self-care skills. It is a place where children and teens are able to meet with others who also have diabetes and who share their experiences.

MATERIALS AND METHODSThis study assessed self-reports before and after attending diabetes camp on diabetes-specific emotional distress, diabetes-specific quality of life, and self-care behaviors by surveying campers and their parents from over 42 diabetes-specific summer camps across the United States. Parents (N=413), Teenagers (N=154), and Children (N=116) completed both pre- and post-camp surveys.

RESULTSParents reported higher levels of diabetes-specific emotional distress than their campers did. Both parents and children reported significant improvements in their own distress after camp. Teens reported improvements in distress but they were not statistically significant. Youth report higher levels of self-care skills than parents believe their children possess. Parents, Teens, and Children all reported significant improvements in the camper's self-care skills after camp. First time campers' and their parents' perspectives regarding self-care skills are consistent with veteran campers after attending camp. Self-reports post-camp reveals that campers and their parents see camp as a place where youth feel they are with others who really understand what it is like to live with diabetes. Respondents also report that camp is a place where youth are exposed to new technologies and where campers can try new self-care tasks.

C1 [Weissberg-Benchell, Jill] Ann & Robert H Lurie Childrens Hosp Chicago, Child & Adolescent Psychiat, 225 East Chicago Ave,Box 10, Chicago, IL 60611 USA.

[Rychlik, Karen] Ann & Robert H Lurie Childrens Hosp Chicago, Stanley Manne Childrens Res Inst, Stat Core, Chicago, IL 60611 USA.

RP Weissberg-Benchell, J (reprint author), Ann & Robert H Lurie Childrens Hosp Chicago, Child & Adolescent Psychiat, 225 East Chicago Ave,Box 10, Chicago, IL 60611 USA.

EM jwbenchell@luriechildrens.org

CR Briery BG, 1999, J PEDIATR PSYCHOL, V24, P183, DOI 10.1093/jpepsy/24.2.183

de Wit M, 2012, PEDIATR DIABETES, V13, P638, DOI 10.1111/j.1399-5448.2012.00872.x

Evans M., 2016, 76 ANN SCI SESS AM D

Evans M, 2016, SOC PED PSYCH ANN C

Fegan-Bohn K, CURR DIAB R IN PRESS

Nabors Laura A, 2014, Diabetes Spectr, V27, P257, DOI 10.2337/diaspect.27.4.257

Odar C, 2013, J PEDIATR PSYCHOL, V38, P398, DOI 10.1093/jpepsy/jss176

Pihoker C, 2014, PEDIATR DIABETES, V15, P86, DOI 10.1111/pedi.12181

Redon I, 2014, PEDIATR DIABETES, V15, P329, DOI 10.1111/pedi.12062

Santiprabhob J, 2008, PATIENT EDUC COUNS, V73, P60, DOI 10.1016/j.pec.2008.05.023

Wang YCA, 2008, PEDIATR DIABETES, V9, P29, DOI 10.1111/j.1399-5448.2007.00285.x

Weissberg-Benchell J, 2014, 74 ANN SCI SESS AM D

Weissberg-Benchell J, 2011, PEDIATR DIABETES, V12, P341, DOI 10.1111/j.1399-5448.2010.00720.x

Weissberg-Benchell J, 2009, J PEDIATR PSYCHOL, V34, P977, DOI 10.1093/jpepsy/jsp003

NR 14

TC 3

Z9 3

U1 0

U2 3

PU WILEY

PI HOBOKEN

PA 111 RIVER ST, HOBOKEN 07030-5774, NJ USA

SN 1399-543X

EI 1399-5448

J9 PEDIATR DIABETES

JI Pediatr. Diabetes

PD DEC

PY 2017

VL 18

IS 8

BP 853

EP 860

DI 10.1111/pedi.12499

PG 8

WC Endocrinology & Metabolism; Pediatrics

SC Endocrinology & Metabolism; Pediatrics

GA FM4SP

UT WOS:000415012600027

PM 28156056

DA 2019-01-08

ER

PT J

AU Holmes-Truscott, E

Furler, J

Blackberry, I

O'Neal, DN

Speight, J

AF Holmes-Truscott, Elizabeth

Furler, John

Blackberry, Irene

O'Neal, David N.

Speight, Jane

TI Predictors of insulin uptake among adults with type 2 diabetes in the

Stepping Up Study

SO DIABETES RESEARCH AND CLINICAL PRACTICE

LA English

DT Article

DE Insulin therapy; Psychological insulin resistance; Attitudes; Primary

care

ID EUROPEAN ASSOCIATION; STARTING INSULIN; THERAPY; RESISTANCE;

HYPERGLYCEMIA; MANAGEMENT; INITIATION; STATEMENT; ATTITUDES; PEOPLE

AB Aims: We aimed to investigate predictors of insulin uptake, and change in insulin appraisals, among adults with type 2 diabetes mellitus (T2DM) who participated in the Stepping Up trial.

Methods: The Stepping Up model of care, supporting timely insulin initiation in primary care, was evaluated in a two-armed cluster-randomised controlled trial. Participants were 266 adults (mean +/- SD age 62 +/- 10 years; 39% women) with T2DM (median (IQR) duration 8.5 (5, 13) years) from 74 primary care practices (Stepping Up intervention: 57%, control 43%). At 12 months, 47% (n = 126) had commenced insulin. Controlling for randomisation, logistic regression was used to explore baseline predictors of insulin uptake, including: demographic and clinical characteristics, emotional wellbeing (depressive symptoms and diabetes-related distress), insulin treatment appraisals, and, 'willingness' to initiate insulin. Two-way analysis of variance examined effects of, and interaction between, randomisation and insulin uptake on 12-month change in insulin appraisals.

Results: Participants using insulin at 12 months were more likely (all p < 0.05) than those with non-insulin-treated T2DM to report: lower socioeconomic status, higher baseline HbA1c (median difference: 0.3%; 3 mmol/mol), greater willingness to commence insulin (very willing: 27% vs 12%), and less negative and more positive insulin appraisals. All contributed significantly to the final model (chi(2)(8) = 92.1, p < 0.001) except insulin appraisals. Regardless of trial allocation, those initiating insulin reported significantly greater reductions in negative insulin appraisals.

Conclusions: Controlling for randomisation, 12-month insulin use was predicted by higher baseline HbA1c and 'willingness' to use insulin if recommended. Negative insulin appraisals reduced following insulin initiation. (C) 2017 Elsevier B.V. All rights reserved.

C1 [Holmes-Truscott, Elizabeth; Speight, Jane] Deakin Univ, Sch Psychol, 1 Gheringhap St, Geelong, Vic 3220, Australia.

[Holmes-Truscott, Elizabeth; Speight, Jane] Australian Ctr Behav Res Diabet, Diabet Victoria, 570 Elizabeth St, Melbourne, Vic 3000, Australia.

[Furler, John; Blackberry, Irene] Univ Melbourne, Dept Gen Practice, 200 Berkeley St, Carlton, Vic 3052, Australia.

[Blackberry, Irene] La Trobe Univ, Australian Inst Primary Care & Ageing, John Richards Initiat, POB 821, Wodonga, Vic 3689, Australia.

[O'Neal, David N.] Univ Melbourne, St Vincents Hosp Melbourne, Dept Med, Fitzroy, Vic 3065, Australia.

[Speight, Jane] AHP Res, 16 Walden Way, Hornchurch RM11 2LB, England.

RP Holmes-Truscott, E (reprint author), Australian Ctr Behav Res Diabet, 570 Elizabeth St, Melbourne, Vic 3000, Australia.

EM etruscott@acbrd.org.au

OI Holmes-Truscott, Elizabeth/0000-0001-9139-4663; Speight,

Jane/0000-0002-1204-6896

FU Australian National Health and Medical Research Council [APP1023738];

Roche Diabetes Care Pty Ltd; RACGP Foundation RACGP/Independent

Practitioner Network Pty Ltd (IPN); Australian Postgraduate Award/Deakin

University PhD scholarship; NHMRC/PHCRED Career Development Fellowship;

Australian Centre for Behavioural Research in Diabetes by Diabetes

Victoria and Deakin University

FX The Stepping Up trial was funded substantively by the Australian

National Health and Medical Research Council (Project Grant:

APP1023738). It was also supported by an unrestricted

educational/research grant by Roche Diabetes Care Pty Ltd, an RACGP

Foundation RACGP/Independent Practitioner Network Pty Ltd (IPN) Grant

and in-kind support from Sanofi. The funders of Stepping Up were not

involved in trial study design, data collection or analysis,

interpretation of results or manuscript preparation and submission.; EHT

is supported by an Australian Postgraduate Award/Deakin University PhD

scholarship. JF is supported by an NHMRC/PHCRED Career Development

Fellowship. JS is supported by the core funding provided to The

Australian Centre for Behavioural Research in Diabetes by Diabetes

Victoria and Deakin University.

CR [Anonymous], 1995, Diabetes, V44, P1249

Australian Bureau of Statistics, 2011, SOC EC IND AR 2011

Barnett K, 2012, LANCET, V380, P37, DOI 10.1016/S0140-6736(12)60240-2

Bogatean MP, 2004, PRACT DIABETES INT, V21, P247, DOI DOI 10.1002/PDI.670

Danne T, 2015, BMC ENDOCR DISORD, V15, DOI 10.1186/s12902-015-0051-0

Furler J, 2015, ADS ADEA ANN SCI M A

Furler JS, 2014, IMPLEMENT SCI, V9, DOI 10.1186/1748-5908-9-20

Hermanns N, 2010, HEALTH QUAL LIFE OUT, V8, DOI 10.1186/1477-7525-8-113

Holmes-Truscott E, 2016, DIABETES RES CLIN PR, V114, P126, DOI 10.1016/j.diabres.2015.12.011

Inzucchi SE, 2015, DIABETES CARE, V38, P140, DOI 10.2337/dc14-2441

Keij SM, 2016, DIABETIC MED, V33, P170

Khunti K, 2013, DIABETES CARE, V36, P3411, DOI 10.2337/dc13-0331

Khunti S, 2015, BRIT J DIABETES VASC, V15, P65, DOI 10.15277/bjdvd.2015.019

Kroenke K, 2001, J GEN INTERN MED, V16, P606, DOI 10.1046/j.1525-1497.2001.016009606.x

Nam S, 2011, DIABETES RES CLIN PR, V93, P1, DOI 10.1016/j.diabres.2011.02.002

Nathan D, 2009, DIABETOLOGIA, V52, P17, DOI 10.1007/s00125-008-1157-y

Nefs G, 2013, PLOS ONE, V8, DOI 10.1371/journal.pone.0078865

Ng CJ, 2015, INT J CLIN PRACT, V69, P1050, DOI 10.1111/ijcp.12691

Odawara M, 2016, CURR MED RES OPIN, V32, P681, DOI 10.1185/03007995.2015.1136605

Peyrot M, 2005, DIABETES CARE, V28, P2673, DOI 10.2337/diacare.28.11.2673

POLONSKY WH, 1995, DIABETES CARE, V18, P754, DOI 10.2337/diacare.18.6.754

Polonsky WH, 2005, DIABETES CARE, V28, P2543, DOI 10.2337/diacare.28.10.2543

Polonsky WH, 2011, CURR MED RES OPIN, V27, P1169, DOI 10.1185/03007995.2011.573623

Snoek FJ, 2011, DIABETES CARE, V34, P601, DOI 10.2337/dc10-1552

Snoek FJ, 2007, HEALTH QUAL LIFE OUT, V5, DOI 10.1186/1477-7525-5-69

Speight J, 2011, DIABETES MILES AUSTR

The Royal Australian College of General Practitioners and Diabetes Australia, 2014, GEN PRACT MAN TYP 2

Turner RC, 1998, LANCET, V352, P837

NR 28

TC 2

Z9 2

U1 0

U2 1

PU ELSEVIER IRELAND LTD

PI CLARE

PA ELSEVIER HOUSE, BROOKVALE PLAZA, EAST PARK SHANNON, CO, CLARE, 00000,

IRELAND

SN 0168-8227

EI 1872-8227

J9 DIABETES RES CLIN PR

JI Diabetes Res. Clin. Pract.

PD NOV

PY 2017

VL 133

BP 204

EP 210

DI 10.1016/j.diabres.2017.01.002

PG 7

WC Endocrinology & Metabolism

SC Endocrinology & Metabolism

GA FM9WU

UT WOS:000415623700025

PM 29122108

DA 2019-01-08

ER

PT J

AU Vileikyte, L

Crews, RT

Reeves, ND

AF Vileikyte, Loretta

Crews, Ryan T.

Reeves, Neil D.

TI Psychological and Biomechanical Aspects of Patient Adaptation to

Diabetic Neuropathy and Foot Ulceration

SO CURRENT DIABETES REPORTS

LA English

DT Review

DE Diabetic symmetric polyneuropathy; Pain; Unsteadiness; Foot ulcers;

Depression; Illness cognition and emotion; Offloading adherence;

Physical activity

ID RANDOMIZED CONTROLLED-TRIAL; QUALITY-OF-LIFE; NORD-TRONDELAG HEALTH;

CUSTOM-MADE FOOTWEAR; PERIPHERAL NEUROPATHY; OLDER-ADULTS; AEROBIC

EXERCISE; SINGLE-BLIND; HIGH-RISK; DEPRESSIVE SYMPTOMS

AB Purpose of Review The purpose of this review was to elucidate how psychological and biomechanical factors interrelate in shaping patients' experience with diabetic symmetric polyneuropathy (DSPN) and its sequela-diabetic foot ulceration (DFU).

Recent Findings Recent findings emphasize the importance not only of neuropathic pain but also of other DSPN symptoms, such as unsteadiness. We highlight the negative spiral between unsteadiness, falls, and psychological distress. Moreover, unsteadiness is a key determinant of non-adherence to offloading resulting in the delayed DFU healing. While depression is an established predictor of incident DFU, findings linking depression and DFU healing remain inconclusive. Examination of physical activity in DFU development and healing represents the most recent application of research to this field.

Summary Research evidence indicates that DSPN markedly impairs physical and emotional functioning and suggests that there is an unmet need for the development of multifaceted interventions that address both psychological distress and biomechanical challenges experienced by patients with this debilitating complication of diabetes.

C1 [Vileikyte, Loretta] Univ Manchester, Dept Med, Manchester M13 9WL, Lancs, England.

[Crews, Ryan T.] Rosalind Franklin Univ Med & Sci, William M Scholl Coll Podiatr Med, N Chicago, IL 60064 USA.

[Reeves, Neil D.] Manchester Metropolitan Univ, Fac Sci & Engn, Sch Healthcare Sci, Manchester M1 5GD, Lancs, England.

RP Vileikyte, L (reprint author), Univ Manchester, Dept Med, Manchester M13 9WL, Lancs, England.

EM lvileikyte@med.miami.edu

OI Reeves, Neil/0000-0001-9213-4580

FU NIH (NIDDK) [R01-DK-071066]

FX We thank the NIH (NIDDK R01-DK-071066) for their generous support of

some of the studies reported in this review.

CR Ahroni JH, 2000, J DIABETES COMPLICAT, V14, P31, DOI 10.1016/S1056-8727(00)00066-0

Arts MLJ, 2014, J REHABIL MED, V46, P357, DOI 10.2340/16501977-1272

Asmundson GJG, 2009, DEPRESS ANXIETY, V26, P888, DOI 10.1002/da.20600

Brown SJ, 2015, DIABETES CARE, V38, P1116, DOI 10.2337/dc14-1982

Brownlee S., 2000, HDB SELF REGULATION, P369, DOI DOI 10.1016/B978-012109890-2/50041-X

Bus SA, 2016, DIABETES-METAB RES, V32, P195, DOI 10.1002/dmrr.2738

Clemson L, 2004, J AM GERIATR SOC, V52, P1487, DOI 10.1111/j.1532-5415.2004.52411.x

Colberg Sheri R, 2010, Diabetes Care, V33, pe147, DOI 10.2337/dc10-9990

Crews RT, 2016, DIABETES-METAB RES, V32, P791, DOI 10.1002/dmrr.2817

Crews RT, 2016, DIABETES CARE, V39, P1371, DOI 10.2337/dc15-2373

Crews Ryan T, 2013, J Aging Res, V2013, P342650, DOI 10.1155/2013/342650

Crews RT, 2012, CLIN BIOMECH, V27, P725, DOI 10.1016/j.clinbiomech.2012.03.001

de Groot M, 2001, PSYCHOSOM MED, V63, P619, DOI 10.1097/00006842-200107000-00015

DeMott TK, 2007, AM J PHYS MED REHAB, V86, P125, DOI 10.1097/PHM.0b013e31802ee1d1

Dixit S, 2016, AM J PHYS MED REHAB, V95, P516, DOI 10.1097/PHM.0000000000000426

Dixit S, 2014, QUAL LIFE RES, V23, P1629, DOI 10.1007/s11136-013-0602-7

Dixit S, 2014, J DIABETES COMPLICAT, V28, P332, DOI 10.1016/j.jdiacomp.2013.12.006

DUNN JE, 1992, AM J PUBLIC HEALTH, V82, P395, DOI 10.2105/AJPH.82.3.395

Emery CF, 2005, J GERONTOL A-BIOL, V60, P1432, DOI 10.1093/gerona/60.11.1432

French DP, 2013, J PSYCHOSOM RES, V74, P327, DOI 10.1016/j.jpsychores.2012.12.008

Glaser R, 2005, NAT REV IMMUNOL, V5, P243, DOI 10.1038/nri1571

Gleeson M, 2011, NAT REV IMMUNOL, V11, P607, DOI 10.1038/nri3041

Gonzalez JS, 2011, DIABETOLOGIA, V54, P205, DOI 10.1007/s00125-010-1926-2

Gonzalez JS, 2010, DIABETOLOGIA, V53, P2241, DOI 10.1007/s00125-010-1821-x

Gonzalez JS, 2008, DIABETES CARE, V31, P2398, DOI 10.2337/dc08-1341

Goodworth AD, 2014, J PROSTHET ORTHOT, V26, P6

Gore M, 2005, J PAIN SYMPTOM MANAG, V30, P374, DOI 10.1016/j.jpainsymman.2005.04.009

Gore M, 2006, J PAIN, V7, P892, DOI 10.1016/j.jpain.2006.04.013

Gouin JP, 2011, IMMUNOL ALLERGY CLIN, V31, P81, DOI 10.1016/j.iac.2010.09.010

Grewal GS, 2015, GERONTOLOGY, V61, P567, DOI 10.1159/000371846

Handsaker JC, 2016, DIABETIC MED, V33, P97, DOI 10.1111/dme.12841

Hazari A, 2016, SPRINGERPLUS, V5, DOI 10.1186/s40064-016-3405-9

Ismail K, 2007, DIABETES CARE, V30, P1473, DOI 10.2337/dc06-2313

Iversen MM, 2015, J DIABETES COMPLICAT, V29, P20, DOI 10.1016/j.jdiacomp.2014.09.006

Iversen MM, 2009, BMC ENDOCR DISORD, V9, DOI 10.1186/1472-6823-9-18

Jensen MP, 2007, NEUROLOGY, V68, P1178, DOI 10.1212/01.wnl.0000259085.61898.9e

Kanchanasamut W, 2017, DIABET FOOT ANKLE, V8, DOI 10.1080/2000625X.2017.1287239

Katon WJ, 2010, INT J GERIATR PSYCH, V25, P466, DOI 10.1002/gps.2363

Kelly C, 2013, J AM PODIAT MED ASSN, V103, P480, DOI 10.7547/1030480

Keylock KT, 2008, AM J PHYSIOL-REG I, V294, pR179, DOI 10.1152/ajpregu.00177.2007

Kluding PM, 2015, PHYS THER, V95, P223, DOI 10.2522/ptj.20140108

Kluding PM, 2012, J DIABETES COMPLICAT, V26, P424, DOI 10.1016/j.jdiacomp.2012.05.007

Lavery L A, 1998, Ostomy Wound Manage, V44, P26

Marchettini P, 2016, EUR J PAIN, V20, P472, DOI 10.1002/ejp.763

McKee KJ, 2002, DISABIL REHABIL, V24, P327, DOI 10.1080/09638280110093686

Mendes R, 2013, POSTGRAD MED J, V89, P715, DOI 10.1136/postgradmedj-2013-132222

Monami M, 2008, J AM PODIAT MED ASSN, V98, P130, DOI 10.7547/0980130

Morrison S, 2014, J DIABETES COMPLICAT, V28, P715, DOI 10.1016/j.jdiacomp.2014.04.007

Mueller MJ, 2013, ARCH PHYS MED REHAB, V94, P829, DOI 10.1016/j.apmr.2012.12.015

Najafi B, 2016, J DIABETES SCI TECHN, V11, P657

Otto M, 2007, EUR J PAIN, V11, P572, DOI 10.1016/j.ejpain.2006.08.003

Padua L, 2001, EUR NEUROL, V45, P214, DOI 10.1159/000052132

Pence BD, 2014, ADV WOUND CARE, V3, P71, DOI 10.1089/wound.2012.0377

Pence BD, 2012, MED SCI SPORT EXER, V44, P1846, DOI 10.1249/MSS.0b013e31825a5971

Perrin BM, 2014, DIABETIC MED, V31, P102, DOI 10.1111/dme.12287

Pop-Busui R, 2017, DIABETES CARE, V40, P136, DOI 10.2337/dc16-2042

Razjouyan Javad, 2017, J Diabetes Sci Technol, V11, P685, DOI 10.1177/1932296817705397

Reeves ND, 2017, PRELIMINARY OBS DIAB, V40, pe51

Schwartz AV, 2008, DIABETES CARE, V31, P391, DOI 10.2337/dc07-1152

Schwenk M, 2013, J AM PODIAT MED ASSN, V103, P534, DOI 10.7547/1030534

Selvarajah D, 2014, DIABETES VASC DIS RE, V11, P218, DOI 10.1177/1479164114522135

Singleton JR, 2015, ANN NEUROL, V77, P146, DOI 10.1002/ana.24310

Tan MP, 2016, GERIATR GERONTOL INT, V16, P111, DOI 10.1111/ggi.12446

Tesfaye S, 2011, DIABETES-METAB RES, V27, P629, DOI 10.1002/dmrr.1225

van Deursen R, 2008, DIABETES-METAB RES, V24, pS96, DOI 10.1002/dmrr.827

VanRoss ER, 2009, ARCH PHYS MED REHAB, V90, P610, DOI 10.1016/j.apmr.2008.10.026

Vedhara K, 2010, DIABETOLOGIA, V53, P1590, DOI 10.1007/s00125-010-1743-7

Vickrey BG, 2000, NEUROREHAB NEURAL RE, V14, P93, DOI 10.1177/154596830001400202

Vileikyte L, 2005, DIABETES CARE, V28, P2378, DOI 10.2337/diacare.28.10.2378

Vileikyte L, 2003, DIABETES CARE, V26, P2549, DOI 10.2337/diacare.26.9.2549

Vileikyte L, 2009, DIABETOLOGIA, V52, P1265, DOI 10.1007/s00125-009-1363-2

Vileikyte L, 2006, DIABETES CARE, V29, P2617, DOI 10.2337/dc06-1550

Vileikyte L, 2017, DIABETES, V66, pA168

Vinik Etta J, 2005, Diabetes Technol Ther, V7, P497, DOI 10.1089/dia.2005.7.497

Waaijman R, 2013, DIABETES CARE, V36, P1613, DOI 10.2337/dc12-1330

Williams LH, 2010, AM J MED, V123, P748, DOI 10.1016/j.amjmed.2010.01.023

Winkley K, 2012, DIABETOLOGIA, V55, P303, DOI 10.1007/s00125-011-2359-2

Yoo M, 2015, PAIN MED, V16, P1482, DOI 10.1111/pme.12743

Young-Hyman D, 2016, DIABETES CARE, V39, P2126, DOI 10.2337/dc16-2053

NR 79

TC 0

Z9 1

U1 2

U2 9

PU CURRENT MEDICINE GROUP

PI PHILADELPHIA

PA 400 MARKET STREET, STE 700, PHILADELPHIA, PA 19106 USA

SN 1534-4827

EI 1539-0829

J9 CURR DIABETES REP

JI Curr. Diabetes Rep.

PD NOV

PY 2017

VL 17

IS 11

AR 109

DI 10.1007/s11892-017-0945-5

PG 11

WC Endocrinology & Metabolism

SC Endocrinology & Metabolism

GA FJ9BZ

UT WOS:000413064200009

PM 28942488

DA 2019-01-08

ER

PT J

AU Hagger, V

Hendrieckx, C

Cameron, F

Pouwer, F

Skinner, TC

Speight, J

AF Hagger, Virginia

Hendrieckx, Christel

Cameron, Fergus

Pouwer, Frans

Skinner, Timothy C.

Speight, Jane

TI Cut Points for Identifying Clinically Significant Diabetes Distress in

Adolescents With Type 1 Diabetes Using the PAID-T: Results From Diabetes

MILES Youth-Australia

SO DIABETES CARE

LA English

DT Article

ID PROBLEM-AREAS; SCALE; ADULTS; RELIABILITY; DISORDERS; VALIDITY

AB OBJECTIVE

To establish cut point(s) for the Problem Areas in Diabetes-teen version (PAID-T) scale to identify adolescents with clinically meaningful, elevated diabetes distress.

RESEARCH DESIGN AND METHODS

Data were available from the Diabetes Management and Impact for Long-term Empowerment and Success (MILES) Youth-Australia Study, a national survey assessing various psychosocial indicators among self-selected National Diabetes Services Scheme registrants. Participants in the current study (n = 537) were (mean SD) 16 2 years old, had type 1 diabetes for 6 +/- 4 years, and 62% (n = 334) were girls. They completed measures of diabetes distress (PAID-T) and depressive symptoms (Patient Health Questionnaire for Adolescents) and self-reported their most recent HbA(1c) and frequency of self-monitoring of blood glucose (SMBG). Relationships between the PAID-T and the psychological and clinical variables were examined to identify a clinically meaningful threshold for elevated diabetes distress. ANOVA was used to test whether these variables differed by levels of distress.

RESULTST

wo cut points distinguished none-to-mild (<70), moderate (70-90), and high (>90) diabetes distress. Moderate distress was experienced by 18% of adolescents and high distress by 36%. Mean depressive symptoms, self-reported HbA(1c), and SMBG differed significantly across the three levels of diabetes distress (all P < 0.001), with moderate-to-large effect sizes.

CONCLUSIONS

Using the PAID-T, this study defined two clinically meaningful cut points to distinguish none-to-mild, moderate, and high diabetes distress in adolescents (aged 13-19). Based on these cut points, most respondents experienced at least moderate diabetes distress, which was clinically significant. Establishing thresholds for elevated diabetes distress will aid clinicians and researchers to interpret PAID-T scores, prompt discussion and intervention for those with unmet needs, and enable the effectiveness of interventions to be evaluated.

C1 [Hagger, Virginia; Hendrieckx, Christel; Speight, Jane] Deakin Univ, Sch Psychol, Ctr Social & Early Emot Dev, Geelong, Vic, Australia.

[Hagger, Virginia; Hendrieckx, Christel; Speight, Jane] Diabet Victoria, Australian Ctr Behav Res Diabet, Melbourne, Vic, Australia.

[Cameron, Fergus] Royal Childrens Hosp, Melbourne, Vic, Australia.

[Cameron, Fergus] Murdoch Childrens Res Inst, Melbourne, Vic, Australia.

[Pouwer, Frans] Univ Southern Denmark, Dept Psychol, Odense, Denmark.

[Skinner, Timothy C.] Charles Darwin Univ, Sch Psychol & Clin Sci, Darwin, NT, Australia.

[Skinner, Timothy C.] Baker Heart & Diabet Inst, Melbourne, Vic, Australia.

[Speight, Jane] Appl Hlth Psychol Res, Hornchurch, Essex, England.

RP Hagger, V (reprint author), Deakin Univ, Sch Psychol, Ctr Social & Early Emot Dev, Geelong, Vic, Australia.

EM vhagger@acbrd.org.au

OI Skinner, Timothy/0000-0002-0018-6963; Hagger,

Virginia/0000-0003-3845-2814; Speight, Jane/0000-0002-1204-6896

FU NDSS, an initiative of the Australian Government; Diabetes Victoria;

Deakin University; Diabetes Australia

FX The Diabetes MILES Youth-Australia 2014 Survey was funded by the NDSS,

an initiative of the Australian Government administered with assistance

from Diabetes Australia. C.H. and J.S. are supported by core funding to

The Australian Centre for Behavioural Research in Diabetes derived from

the collaboration between Diabetes Victoria and Deakin University.

CR Amsberg S, 2008, INT J NURS STUD, V45, P1319, DOI 10.1016/j.ijnurstu.2007.09.010

Australian Bureau of Statistics, 2011, CENS POP HOUS SOC IN

Butwicka A, 2012, DIABETES CARE, V35, P2133, DOI 10.2337/dc12-0160

Chae M, 2014, J DIABETES METAB DIS, V13, DOI 10.1186/2251-6581-13-14

Cohen J., 1992, CURRENT DIRECTIONS P, V1, P98, DOI [10.1111/1467-8721.ep10768783, DOI 10.1111/1467-8721.EP10768783]

Fisher L, 2016, DIABETIC MED, V33, P1590, DOI 10.1111/dme.12973

Fisher L, 2016, J DIABETES COMPLICAT, V30, P1123, DOI 10.1016/j.jdiacomp.2016.03.032

Fisher L, 2015, J DIABETES COMPLICAT, V29, P572, DOI 10.1016/j.jdiacomp.2015.01.012

Fisher L, 2013, DIABETES CARE, V36, P2551, DOI 10.2337/dc12-2493

Fisher L, 2012, DIABETES CARE, V35, P259, DOI 10.2337/dc11-1572

Graue M, 2012, INT J NURS STUD, V49, P174, DOI 10.1016/j.ijnurstu.2011.08.007

Hackorth NJ, 2013, AUST PSYCHOL, V48, P360, DOI [DOI 10.1111/AP.12007, 10.1111/ap.12007]

Hagger Virginia, 2016, BMC Psychol, V4, P42, DOI 10.1186/s40359-016-0149-9

Hagger V, 2016, CURR DIABETES REP, V16, DOI 10.1007/s11892-015-0694-2

Handley TE, 2016, DIABETIC MED, V33, P1582, DOI 10.1111/dme.13022

Hermanns N, 2006, DIABETOLOGIA, V49, P469, DOI 10.1007/s00125-005-0094-2

Hermanns Norbert, 2007, Diabetes Care, V30, pe101, DOI 10.2337/dc07-1019

Iturralde E, 2017, HEALTH PSYCHOL, V36, P236, DOI 10.1037/hea0000445

Johnson JG, 2002, J ADOLESCENT HEALTH, V30, P196, DOI 10.1016/S1054-139X(01)00333-0

Kibbey KJ, 2013, DIABETIC MED, V30, P878, DOI 10.1111/dme.12227

Kroenke K, 2009, J AFFECT DISORDERS, V114, P163, DOI 10.1016/j.jad.2008.06.026

Lasaite L, 2016, J DIABETES COMPLICAT, V30, P1500, DOI 10.1016/j.jdiacomp.2016.08.013

Lewin AB, 2009, J PEDIATR PSYCHOL, V34, P999, DOI 10.1093/jpepsy/jsp032

POLONSKY WH, 1995, DIABETES CARE, V18, P754, DOI 10.2337/diacare.18.6.754

ROSE G, 1978, BRIT MED J, V2, P873, DOI 10.1136/bmj.2.6141.873

Snoek FJ, 2011, DIABETES CARE, V34, P601, DOI 10.2337/dc10-1552

Sturt J, 2015, INT DIAB NURS, V12, P40, DOI DOI 10.1179/2057332415Y.0000000004

Weissberg-Benchell J, 2011, PEDIATR DIABETES, V12, P341, DOI 10.1111/j.1399-5448.2010.00720.x

Welch GW, 1997, DIABETES CARE, V20, P760, DOI 10.2337/diacare.20.5.760

NR 29

TC 2

Z9 2

U1 1

U2 2

PU AMER DIABETES ASSOC

PI ALEXANDRIA

PA 1701 N BEAUREGARD ST, ALEXANDRIA, VA 22311-1717 USA

SN 0149-5992

EI 1935-5548

J9 DIABETES CARE

JI Diabetes Care

PD NOV 1

PY 2017

VL 40

IS 11

BP 1462

EP 1468

DI 10.2337/dc17-0441

PG 7

WC Endocrinology & Metabolism

SC Endocrinology & Metabolism

GA FK5RE

UT WOS:000413557200014

PM 28882887

DA 2019-01-08

ER

PT J

AU Perrin, NE

Davies, MJ

Robertson, N

Snoek, FJ

Khunti, K

AF Perrin, N. E.

Davies, M. J.

Robertson, N.

Snoek, F. J.

Khunti, K.

TI The prevalence of diabetes-specific emotional distress in people with

Type 2 diabetes: a systematic review and meta-analysis

SO DIABETIC MEDICINE

LA English

DT Review

ID PROBLEM AREAS; GLYCEMIC CONTROL; DEPRESSION; ADULTS; PAID; RECOGNITION;

ASSOCIATION; ADHERENCE; SYMPTOMS; VALIDITY

AB AimsPsychological comorbidity, such as depression and/or diabetes-specific emotional distress (diabetes distress), is widespread in people with Type 2 diabetes and is associated with poorer treatment outcomes. Although extensive research into the prevalence of depression has been conducted, the same attention has not been given to diabetes distress. The aim of this systematic review was to determine the overall prevalence of diabetes distress in people with Type 2 diabetes. MethodsSeven databases were searched to identify potentially relevant studies; eligible studies (adult population aged > 18 years with Type 2 diabetes and an outcome measure of diabetes distress) were selected and appraised independently by two reviewers. Multiple fixed- and random-effects meta-analyses were performed to synthesize the data; with primary analyses to determine the overall prevalence of diabetes distress in people with Type 2 diabetes, and secondary meta-analyses and meta-regression to explore the prevalence across different variables.

ResultsFifty-five studies (n = 36 998) were included in the meta-analysis and demonstrated an overall prevalence of 36% for diabetes distress in people with Type 2 diabetes. Prevalence of diabetes distress was significantly higher in samples with a higher prevalence of comorbid depressive symptoms and a female sample majority.

ConclusionsDiabetes distress is a prominent issue in people with Type 2 diabetes that is associated with female gender and comorbid depressive symptoms. It is important to consider the relationship between diabetes distress and depression, and the significant overlap between conditions. Further work is needed to explore psychological comorbidity in Type 2 diabetes to better understand how best to identify and appropriately treat individuals.

C1 [Perrin, N. E.; Davies, M. J.; Khunti, K.] Univ Leicester, Coll Med Biol Sci & Psychol, Diabet Res Ctr, Leicester, Leics, England.

[Robertson, N.] Univ Leicester, Coll Med Biol Sci & Psychol, Sch Psychol, Leicester, Leics, England.

[Snoek, F. J.] Vrije Univ Amsterdam, Med Ctr, Dept Med Psychol, Acad Med Ctr Amsterdam, Amsterdam, Netherlands.

RP Perrin, NE (reprint author), Univ Leicester, Coll Med Biol Sci & Psychol, Diabet Res Ctr, Leicester, Leics, England.

EM n.e.perrin@gmail.com

OI Davies, Melanie/0000-0002-9987-9371

FU National Institute for Health Research Collaboration for Leadership in

Applied Health Research and Care - Leicestershire, Northamptonshire and

Rutland (NIHR CLAHRC - LNR); Leicester Clinical Trials Unit; NIHR

Leicester-Loughborough Diet, Lifestyle and Physical Activity Biomedical

Research Unit; University Hospitals of Leicester NHS Trust; Loughborough

University; University of Leicester

FX We acknowledge support from the National Institute for Health Research

Collaboration for Leadership in Applied Health Research and Care -

Leicestershire, Northamptonshire and Rutland (NIHR CLAHRC - LNR), the

Leicester Clinical Trials Unit and the NIHR Leicester-Loughborough Diet,

Lifestyle and Physical Activity Biomedical Research Unit which is a

partnership between University Hospitals of Leicester NHS Trust,

Loughborough University and the University of Leicester.

CR Adriaanse MC, 2008, DIABETES CARE, V31, P2268, DOI 10.2337/dc08-1074

Adriaanse MC, 2005, QUAL LIFE RES, V14, P1501, DOI 10.1007/s11136-004-0028-3

Ali S, 2006, DIABETIC MED, V23, P1165, DOI 10.1111/j.1464-5491.2006.01943.x

Anderson RJ, 2001, DIABETES CARE, V24, P1069, DOI 10.2337/diacare.24.6.1069

Asghar S, 2007, DIABETIC MED, V24, P872, DOI 10.1111/j.1464-5491.2007.02136.x

Center for Reviews and Dissemination, 2009, SYSTEMATIC REV CRDS

Charmaz K., 1995, MENS HLTH ILLNESS GE, P266

Dennick K, 2015, INT DIABETES NURS, V12, P93

Doyal L, 2000, SOC SCI MED, V51, P931, DOI 10.1016/S0277-9536(00)00072-1

Egede LE, 2010, DIABETES RES CLIN PR, V87, P302, DOI 10.1016/j.diabres.2010.01.024

Egger M, 1997, BMJ-BRIT MED J, V315, P629, DOI 10.1136/bmj.315.7109.629

Fisher L, 2008, DIABETIC MED, V25, P1096, DOI 10.1111/j.1464-5491.2008.02533.x

Fisher L, 2014, DIABETIC MED, V31, P764, DOI 10.1111/dme.12428

Fisher L, 2009, DIABETIC MED, V26, P622, DOI 10.1111/j.1464-5491.2009.02730.x

Fisher L, 2001, DIABETES CARE, V24, P1751, DOI 10.2337/diacare.24.10.1751

Fisher L, 2007, DIABETES CARE, V30, P542, DOI 10.2337/dc06-1614

Fisher L, 2015, J DIABETES COMPLICAT, V29, P572, DOI 10.1016/j.jdiacomp.2015.01.012

Fisher L, 2012, DIABETES CARE, V35, P259, DOI 10.2337/dc11-1572

Fisher L, 2010, DIABETES CARE, V33, P23, DOI 10.2337/dc09-1238

FREEMAN MF, 1950, ANN MATH STAT, V21, P607, DOI 10.1214/aoms/1177729756

Gonzalez JS, 2007, DIABETES CARE, V30, P2222, DOI 10.2337/dc07-0158

Gonzalez JS, 2015, HEALTH PSYCHOL, V34, P505, DOI 10.1037/hea0000131

Gonzalez JS, 2011, DIABETES CARE, V34, P236, DOI 10.2337/dc10-1970

Gonzalez JS, 2008, DIABETES CARE, V31, P2398, DOI 10.2337/dc08-1341

Hermanns N, 2006, DIABETOLOGIA, V49, P469, DOI 10.1007/s00125-005-0094-2

Higgins JPT, 2003, BRIT MED J, V327, P557, DOI 10.1136/bmj.327.7414.557

Holt RIG, 2014, CURR DIABETES REP, V14, DOI 10.1007/s11892-014-0491-3

Hudson DL, 2013, AM J MANAG CARE, V19, P344

Katon WJ, 2004, MED CARE, V42, P1222, DOI 10.1097/00005650-200412000-00009

Loney P L, 1998, Chronic Dis Can, V19, P170

Lustman PJ, 2005, J DIABETES COMPLICAT, V19, P113, DOI 10.1016/j.jdiacomp.2004.01.002

Lustman PJ, 2000, DIABETES CARE, V23, P934, DOI 10.2337/diacare.23.7.934

McGuire BE, 2010, DIABETOLOGIA, V53, P66, DOI 10.1007/s00125-009-1559-5

Moulton CD, 2015, LANCET DIABETES ENDO, V3, P461, DOI 10.1016/S2213-8587(15)00134-5

Nyaga VN, 2014, ARCH PUBLIC HEALTH, V72, DOI 10.1186/2049-3258-72-39

POLONSKY WH, 1995, DIABETES CARE, V18, P754, DOI 10.2337/diacare.18.6.754

Polonsky WH, 2005, DIABETES CARE, V28, P626, DOI 10.2337/diacare.28.3.626

Pouwer F, 2006, PATIENT EDUC COUNS, V60, P235, DOI 10.1016/j.pec.2005.01.009

Pouwer F, 2005, DIABETES RES CLIN PR, V70, P166, DOI 10.1016/j.diabres.2005.03.031

Pouwer F, 2009, NAT REV ENDOCRINOL, V5, P665, DOI 10.1038/nrendo.2009.214

Schmitt A, 2016, DIABETIC MED, V33, P835, DOI 10.1111/dme.12887

Sigurdardottir AK, 2008, INT J NURS STUD, V45, P526, DOI 10.1016/j.ijnurstu.2006.10.008

Snoek FJ, 2000, DIABETES CARE, V23, P1305, DOI 10.2337/diacare.23.9.1305

Snoek FJ, 2015, LANCET DIABETES ENDO, V3, P450, DOI 10.1016/S2213-8587(15)00135-7

Snoek FJ, 2012, DIABETES CARE, V35, P2128, DOI 10.2337/dc11-1326

van Bastelaar KMP, 2010, DIABETIC MED, V27, P798, DOI 10.1111/j.1464-5491.2010.03025.x

Van der Feltz-Cornelis CM, 2010, GEN HOSP PSYCHIAT, V32, P380, DOI 10.1016/j.genhosppsych.2010.03.011

Welch G, 2003, DIABETIC MED, V20, P69, DOI 10.1046/j.1464-5491.2003.00832.x

Welch GW, 1997, DIABETES CARE, V20, P760, DOI 10.2337/diacare.20.5.760

World Health Organization, 2017, WHO MEMB STAT 6 REG

NR 50

TC 8

Z9 9

U1 3

U2 12

PU WILEY

PI HOBOKEN

PA 111 RIVER ST, HOBOKEN 07030-5774, NJ USA

SN 0742-3071

EI 1464-5491

J9 DIABETIC MED

JI Diabetic Med.

PD NOV

PY 2017

VL 34

IS 11

BP 1508

EP 1520

DI 10.1111/dme.13448

PG 13

WC Endocrinology & Metabolism

SC Endocrinology & Metabolism

GA FK0JC

UT WOS:000413165100004

PM 28799294

OA Bronze

DA 2019-01-08

ER

PT J

AU Ferrari, M

Dal Cin, M

Steele, M

AF Ferrari, M.

Dal Cin, M.

Steele, M.

TI Self-compassion is associated with optimum self-care behaviour, medical

outcomes and psychological well-being in a cross-sectional sample of

adults with diabetes

SO DIABETIC MEDICINE

LA English

DT Article

ID RANDOMIZED CONTROLLED-TRIAL; GLYCEMIC CONTROL; METABOLIC-CONTROL;

DEPRESSION; LOCUS; ADHERENCE; INTERVENTION; METAANALYSIS; DISTRESS;

SUPPORT

AB AimTo investigate the role of self-compassion in diabetes outcomes. Self-compassion is a construct which may be relevant to chronic conditions, given its focus on compassion toward oneself, especially in times of difficulty.

MethodsIn this cross-sectional study we collected data online from 310 adults diagnosed with diabetes. The questionnaire measured three primary outcomes: self-management behaviours; HbA(1c) levels and psychological well-being. Potential predictors were also assessed, including self-compassion, locus of control, social support and demographics.

ResultsMultiple regression analyses showed that self-compassion had the most consistent association with better outcomes, including all forms of self-management behaviour, HbA(1c) levels and psychological well-being. Self-compassion was independently associated with 55.1% of the variance in well-being. Internal locus of control was also significantly associated with better well-being and HbA(1c) outcomes. Chance and external locus of control and social support were generally associated with poorer outcomes.

ConclusionsHigher levels of self-compassion are typically associated with improved self-management behaviour, medical outcomes and psychological well-being in adults with diabetes mellitus. The present findings suggest that self-compassion may be a parsimonious and suitable intervention target. Future interventions and consultations with medical professionals may benefit from fostering self-compassion in adults with diabetes mellitus.

What's new?

In a cross-sectional study in adults diagnosed with diabetes mellitus (N = 310) we found that self-compassion was consistently associated with better behavioural (self-management behaviours, exercise, blood glucose checking and healthcare use), metabolic (HbA(1c)) and psychological (well-being) outcomes. Independently, self-compassion accounted for a large proportion of variance in the outcomes compared with other known predictors, such as locus of control, social support and demographic variables (age, gender, education and relationship status). Our findings demonstrate the relevance of self-compassion to health behaviour in adults with diabetes mellitus, and empirically support the development of holistic psychological interventions targeting self-compassion for adults with diabetes mellitus.

C1 [Ferrari, M.; Dal Cin, M.] Australian Catholic Univ, Sch Psychol, Strathfield, NSW, Australia.

[Ferrari, M.] Univ Sydney, Sch Psychol, Sydney, NSW, Australia.

[Steele, M.] Australian Catholic Univ, Sch Allied Hlth, Brisbane, Qld, Australia.

RP Ferrari, M (reprint author), Australian Catholic Univ, Sch Psychol, Strathfield, NSW, Australia.; Ferrari, M (reprint author), Univ Sydney, Sch Psychol, Sydney, NSW, Australia.

EM m.ferrari037@gmail.com

OI Steele, Michael/0000-0001-9628-3057

CR BRADLEY C, 1994, HDB PSYCHOL DIABETES, P89

DeCoster VA, 2003, SOC WORK HEALTH CARE, V36, P79, DOI 10.1300/J010v36n04_05

DiMatteo MR, 2004, HEALTH PSYCHOL, V23, P207, DOI 10.1037/0278-6133.23.2.207

FERRARO LA, 1987, PSYCHOL REP, V61, P763, DOI 10.2466/pr0.1987.61.3.763

Friis AM, 2015, DIABETIC MED, V32, P1634, DOI 10.1111/dme.12774

Friis AM, 2016, DIABETES CARE, V39, P1963, DOI 10.2337/dc16-0416

Gonzalez JS, 2007, DIABETES CARE, V30, P2222, DOI 10.2337/dc07-0158

Houle J, 2015, DIABETIC MED, V32, P1247, DOI 10.1111/dme.12686

Hunter CM, 2016, AM PSYCHOL, V71, P515, DOI 10.1037/a0040344

Indelicato L, 2017, DIABETIC MED, V34, P691, DOI 10.1111/dme.13321

Jaacks LM, 2016, BEST PRACT RES CL EN, V30, P331, DOI 10.1016/j.beem.2016.05.003

Janowski K, 2013, PLOS ONE, V8, DOI 10.1371/journal.pone.0063920

Knittle K, 2016, ARTHRIT CARE RES, V68, P203, DOI 10.1002/acr.22647

Lloyd CE, 2000, DIABETIC MED, V17, P198, DOI 10.1046/j.1464-5491.2000.00260.x

Lustman PJ, 2005, J DIABETES COMPLICAT, V19, P113, DOI 10.1016/j.jdiacomp.2004.01.002

MacBeth A, 2012, CLIN PSYCHOL REV, V32, P545, DOI 10.1016/j.cpr.2012.06.003

Neff KD, 2007, J RES PERS, V41, P908, DOI 10.1016/j.jrp.2006.08.002

Neff KD, 2003, SELF IDENTITY, V2, P223, DOI 10.1080/15298860390209035

Neff KD, 2009, J PERS, V77, P23, DOI 10.1111/j.1467-6494.2008.00537.x

Pfattheicher S, 2017, EUR J PERSONALITY, V31, P160, DOI 10.1002/per.2097

Powers MA, 2015, DIABETES EDUCATOR, V41, P417, DOI 10.1177/0145721715588904

Przezdziecki A, 2013, PSYCHO-ONCOLOGY, V22, P1872, DOI 10.1002/pon.3230

Raes F, 2011, CLIN PSYCHOL PSYCHOT, V18, P250, DOI 10.1002/cpp.702

Russo GT, 2016, DIABETES TECHNOL THE, V18, P421, DOI 10.1089/dia.2015.0358

SCHAFER LC, 1986, DIABETES CARE, V9, P179, DOI 10.2337/diacare.9.2.179

Schmitt A, 2013, HEALTH QUAL LIFE OUT, V11, DOI 10.1186/1477-7525-11-138

Sirois FM, 2015, HEALTH PSYCHOL, V34, P661, DOI 10.1037/hea0000158

Stenstrom U, 1998, BRIT J HEALTH PSYCH, V3, P15, DOI 10.1111/j.2044-8287.1998.tb00552.x

Tabachnick B. G., 2007, USING MULTIVARIATE S

Wren AA, 2012, J PAIN SYMPTOM MANAG, V43, P759, DOI 10.1016/j.jpainsymman.2011.04.014

NR 30

TC 1

Z9 1

U1 4

U2 14

PU WILEY

PI HOBOKEN

PA 111 RIVER ST, HOBOKEN 07030-5774, NJ USA

SN 0742-3071

EI 1464-5491

J9 DIABETIC MED

JI Diabetic Med.

PD NOV

PY 2017

VL 34

IS 11

BP 1546

EP 1553

DI 10.1111/dme.13451

PG 8

WC Endocrinology & Metabolism

SC Endocrinology & Metabolism

GA FK0JC

UT WOS:000413165100008

PM 28799282

OA Bronze

DA 2019-01-08

ER

PT J

AU Khan, S

Yao, Z

Shah, BR

AF Khan, S.

Yao, Z.

Shah, B. R.

TI Gestational diabetes care and outcomes for refugee women: a

population-based cohort study

SO DIABETIC MEDICINE

LA English

DT Article

ID PREGNANCY OUTCOMES; BIRTH OUTCOMES; UNITED-STATES; HEALTH-STATUS;

RISK-FACTORS; PREVALENCE; IMMIGRANTS; CANADA; MELLITUS; DISEASE

AB AimTo determine the prevalence of adverse clinical outcomes, the rates of healthcare utilization, and the incidence of post-partum Type 2 diabetes in refugees with gestational diabetes (GDM), compared with other immigrants and non-immigrants.

MethodsA population-based cohort study was conducted using healthcare databases in Ontario, Canada. Over 40000 women with GDM having singleton live births between 2002 and 2014 were identified. We identified GDM adverse outcomes such as macrosomia, pre-eclampsia and respiratory distress syndrome. Antenatal and newborn healthcare utilization were ascertained. Women were then followed for diagnosis of diabetes post-partum.

ResultsBoth refugees and other immigrants had a lower rate than non-immigrants of many adverse GDM outcomes, including pre-eclampsia [relative risk (RR) 0.65, 95% confidence interval (95% CI) 0.44-0.95 and 0.61, 95% CI 0.52-0.72, respectively], preterm birth (RR 0.87, 95% CI 0.75-0.995 and 0.85, 95% CI 0.80-0.91, respectively), and respiratory distress syndrome (RR 0.83, 95% CI 0.70-0.97 and 0.78, 95% CI 0.72-0.84, respectively). However, refugees were less likely to attend well-baby care in time for the first routine vaccination (RR 0.92, 95% CI 0.88-0.95). Incidence of post-partum diabetes was high in all groups, but refugee women were at increased risk (hazard ratio 1.23, 95% CI 1.11-1.37).

ConclusionsDespite different circumstances leading to migration, refugees have a similar healthy immigrant effect' to other immigrants, with respect to adverse GDM outcomes. However, newborns of refugees were less likely to have well-baby care, and refugee women were also at especially high risk of developing diabetes post-partum. These are both important public health issues.

C1 [Khan, S.; Shah, B. R.] Univ Toronto, Dept Med, Toronto, ON, Canada.

[Yao, Z.; Shah, B. R.] Inst Clin Evaluat Sci, Toronto, ON, Canada.

[Shah, B. R.] Sunnybrook Hlth Sci Ctr, Toronto, ON, Canada.

RP Shah, BR (reprint author), Univ Toronto, Dept Med, Toronto, ON, Canada.; Shah, BR (reprint author), Inst Clin Evaluat Sci, Toronto, ON, Canada.; Shah, BR (reprint author), Sunnybrook Hlth Sci Ctr, Toronto, ON, Canada.

EM baiju.shah@ices.on.ca

FU Ontario Ministry of Health and Long-Term Care (MOHLTC)

FX The study relied on data and information compiled and provided by the

Canadian Institute of Health Information (CIHI). The Institute for

Clinical Evaluative Sciences (ICES) is a non-profit research institute

funded by the Ontario Ministry of Health and Long-Term Care (MOHLTC).

The opinions, results and conclusions reported in this paper are those

of the authors. No endorsement by CIHI, ICES or MOHLTC is intended or

should be inferred.

CR Anand SS, 2000, LANCET, V356, P279, DOI 10.1016/S0140-6736(00)02502-2

Bellamy L, 2009, LANCET, V373, P1773, DOI 10.1016/S0140-6736(09)60731-5

Buchanan TA, 2001, J CLIN ENDOCR METAB, V86, P989, DOI 10.1210/jc.86.3.989

Creatore MI, 2010, CAN MED ASSOC J, V182, P781, DOI 10.1503/cmaj.091551

Crowther CA, 2005, NEW ENGL J MED, V352, P2477, DOI 10.1056/NEJMoa042973

Glazier R. H., 2007, NEIGHBOURHOOD ENV RE

Hux JE, 2002, DIABETES CARE, V25, P512, DOI 10.2337/diacare.25.3.512

Janevic T, 2011, SOC SCI MED, V73, P429, DOI 10.1016/j.socscimed.2011.05.041

Kandasamy T, 2014, J OBSTET GYNAECOL CA, V36, P296, DOI 10.1016/S1701-2163(15)30604-6

McDonald JT, 2004, SOC SCI MED, V59, P1613, DOI 10.1016/j.socscimed.2004.02.004

Metzger BE, 2008, NEW ENGL J MED, V358, P1991, DOI 10.1056/NEJMoa0707943

Miller LS, 2016, MATERN CHILD HLTH J, V20, P484, DOI 10.1007/s10995-015-1846-3

Mukerji G, 2012, DIABETOLOGIA, V55, P2148, DOI 10.1007/s00125-012-2549-6

Newbold B, 2009, ETHNIC HEALTH, V14, P315, DOI 10.1080/13557850802609956

Ng Edward, 2011, Health Rep, V22, P25

Okrainec K, 2015, AM HEART J, V170, P123, DOI 10.1016/j.ahj.2015.04.009

Pottie K, 2011, CAN MED ASSOC J, V183, pE824, DOI 10.1503/cmaj.090313

Redditt VJ, 2015, CAN FAM PHYSICIAN, V61, pE310

Shah BR, 2010, BMC MED RES METHODOL, V10, DOI 10.1186/1471-2288-10-42

Statistics Canada, 2013, IMM ETHN DIV 2011 NA

UNHCR, UNHCR VIEWP REF MIGR

Vang Z, 2015, POPULATION CHANGE LI, V3, P6

Wanigaratne S, 2016, MATERN CHILD HLTH J, V20, P2189, DOI 10.1007/s10995-016-2047-4

Wanigaratne S, 2016, J EPIDEMIOL COMMUN H, V70, P622, DOI 10.1136/jech-2015-206529

WASSE H, 1994, AM J PUBLIC HEALTH, V84, P1505, DOI 10.2105/AJPH.84.9.1505

Yeung RO, 2017, J DIABETES COMPLICAT, V31, P529, DOI 10.1016/j.jdiacomp.2016.10.016

Yun K, 2012, J IMMIGR MINOR HEALT, V14, P933, DOI 10.1007/s10903-012-9618-2

Zou GY, 2004, AM J EPIDEMIOL, V159, P702, DOI 10.1093/aje/kwh090

NR 28

TC 2

Z9 2

U1 0

U2 6

PU WILEY

PI HOBOKEN

PA 111 RIVER ST, HOBOKEN 07030-5774, NJ USA

SN 0742-3071

EI 1464-5491

J9 DIABETIC MED

JI Diabetic Med.

PD NOV

PY 2017

VL 34

IS 11

BP 1608

EP 1614

DI 10.1111/dme.13440

PG 7

WC Endocrinology & Metabolism

SC Endocrinology & Metabolism

GA FK0JC

UT WOS:000413165100016

PM 28779484

DA 2019-01-08

ER

PT J

AU Quiles, NN

Ciccolo, JT

Garber, CE

AF Quiles, Norberto N.

Ciccolo, Joseph T.

Garber, Carol Ewing

TI Association Between Physical Activity, Depression, and Diabetes in

Urban-Dwelling People Living with HIV

SO JANAC-JOURNAL OF THE ASSOCIATION OF NURSES IN AIDS CARE

LA English

DT Article

DE cardiovascular disease; exercise; mental health; metabolic disease

ID CARDIOVASCULAR-DISEASE; DRUG-USERS; EXERCISE; ADULTS; RISK; VALIDITY;

COHORT; TIME

AB Physical activity (PA) may improve physical and mental health in people living with HIV (PLWH). However, the associations between PA participation and physical and mental health problems of PLWH in urban settings remain largely unknown. Our objective was to determine the relationships between PA and physical and mental health in urban-dwelling PLWH. There were 289 adult PLWH who responded to an electronic survey including questions on PA and current physical and mental health. The associations between physical and mental health and PA were investigated using linear and logistic regression. A large proportion of participants met recommended volumes of PA. Eighty-three percent of respondents reported symptoms of severe mental distress. Diabetes mellitus was associated with a lower total volume of PA (p = .035). Similarly, depression was negatively associated with muscle-strengthening exercise participation (p = .030). Sufficient amounts of aerobic activity and/or muscle strengthening exercise are associated with better physical and mental health. Copyright (C) 2017 Association of Nurses in AIDS Care

C1 [Quiles, Norberto N.] CUNY Queens Coll, Dept Family Nutr & Exercise Sci, Flushing, NY 11367 USA.

[Ciccolo, Joseph T.; Garber, Carol Ewing] Columbia Univ, Teachers Coll, Dept Biobehav Sci, New York, NY 10027 USA.

RP Quiles, NN (reprint author), CUNY Queens Coll, Dept Family Nutr & Exercise Sci, Flushing, NY 11367 USA.

EM norberto.quilesgonzalez@qc.cuny.edu

FU Teachers College Deans Grant for Student Research

FX This project was supported in part by the Teachers College Deans Grant

for Student Research. All authors declare no financial interests or

potential conflicts of interest. We thank Abdulaziz Alnafesah for his

assistance conducting this study.

CR Arseniou S, 2014, PSYCHIAT CLIN NEUROS, V68, P96, DOI 10.1111/pcn.12097

Bauman A, 2011, AM J PREV MED, V41, P228, DOI 10.1016/j.amepre.2011.05.003

Blashill AJ, 2013, ANN BEHAV MED, V46, P149, DOI 10.1007/s12160-013-9489-3

Centers for Disease Control and Prevention, 2014, STAT IND REP PHYS AC

Centers for Disease Control and Prevention, 2013, BEH RISK FACT SURV S

Centers for Disease Control and Prevention, 2013, HIV SURV REP

Clingerman Evelyn M, 2003, J Assoc Nurses AIDS Care, V14, P59, DOI 10.1177/1055329003255284

Codella R., 2005, SPORT SCI HLTH, V1, P81

Colberg SR, 2010, MED SCI SPORT EXER, V42, P2282, DOI 10.1249/MSS.0b013e3181eeb61c

Cossman Ronald E, 2008, J Health Hum Serv Adm, V30, P503

De Socio GV, 2014, AM J HYPERTENS, V27, P222, DOI 10.1093/ajh/hpt182

Della Justina LB, 2014, REV SOC BRAS MED TRO, V47, P30, DOI 10.1590/0037-8682-0240-2013

Fahimi Mansour, 2008, Prev Chronic Dis, V5, pA80

Fillipas S., 2013, J AIDS CLIN RES, V9, P2

Fillipas S, 2010, J ASSOC NURSE AIDS C, V21, P173, DOI 10.1016/j.jana.2009.11.003

Gavrila A, 2003, CLIN INFECT DIS, V36, P1593, DOI 10.1086/375225

Hare DL, 2014, EUR HEART J, V35, P1365, DOI 10.1093/eurheartj/eht462

Healy GN, 2008, MED SCI SPORT EXER, V40, P639, DOI 10.1249/MSS.0b013e3181607421

International Physical Activity Questionnaire, 2005, GUID DAT PROC AN INT

Islam FM, 2012, HIV MED, V13, P453, DOI 10.1111/j.1468-1293.2012.00996.x

Katzmarzyk PT, 2009, MED SCI SPORT EXER, V41, P998, DOI 10.1249/MSS.0b013e3181930355

Kessler RC, 2002, PSYCHOL MED, V32, P959, DOI 10.1017/S0033291702006074

Korkiakangas EE, 2009, HEALTH PROMOT INT, V24, P416, DOI 10.1093/heapro/dap031

Segatto AFM, 2011, REV SOC BRAS MED TRO, V44, P420, DOI 10.1590/S0037-86822011000400004

Ong AD, 2000, J APPL SOC PSYCHOL, V30, P1691, DOI 10.1111/j.1559-1816.2000.tb02462.x

Pellowski JA, 2013, AM PSYCHOL, V68, P197, DOI 10.1037/a0032694

Physical Activity Guidelines Advisory Committee, 2008, PHYS ACT GUID ADV CO

Pierannunzi C, 2013, BMC MED RES METHODOL, V13, DOI 10.1186/1471-2288-13-49

Pilu Alessandra, 2007, Clin Pract Epidemiol Ment Health, V3, P8, DOI 10.1186/1745-0179-3-8

Ramirez-Marrero FA, 2004, J ASSOC NURSE AIDS C, V15, P68, DOI 10.1177/1055329003261966

Ramirez-Marrero FA, 2008, J ASSOC NURSE AIDS C, V19, P283, DOI 10.1016/j.jana.2008.04.003

Roshanaei-Moghaddam B, 2009, GEN HOSP PSYCHIAT, V31, P306, DOI 10.1016/j.genhosppsych.2009.04.002

Schuelter-Trevisol F, 2012, CURR HIV RES, V10, P487, DOI 10.2174/157016212802429794

Smit E, 2006, AIDS CARE, V18, P1040, DOI 10.1080/09540120600580926

Spies G, 2009, AIDS CARE, V21, P1163, DOI 10.1080/09540120902729965

Tang AM, 2010, AM J DRUG ALCOHOL AB, V36, P78, DOI 10.3109/00952990903544851

U.S. Census Bureau, 2015, SEL RAC SEX ED INC C

U.S. Department of Health and Human Services, 2016, ANN UPD HLTH HUM SER

Willig AL, 2014, CURR HIV-AIDS REP, V11, P35, DOI 10.1007/s11904-013-0191-7

Wyker B., 2013, SELF REPORTED ACCELE

Yore MM, 2007, MED SCI SPORT EXER, V39, P1267, DOI 10.1249/mss.0b013e3180618bbe

NR 41

TC 0

Z9 0

U1 2

U2 9

PU ELSEVIER SCIENCE INC

PI NEW YORK

PA 360 PARK AVE SOUTH, NEW YORK, NY 10010-1710 USA

SN 1055-3290

EI 1552-6917

J9 J ASSOC NURSE AIDS C

JI J. Assoc. Nurses Aids Care

PD NOV-DEC

PY 2017

VL 28

IS 6

BP 838

EP 848

DI 10.1016/j.jana.2017.06.015

PG 11

WC Nursing

SC Nursing

GA FL4RR

UT WOS:000414218900002

PM 28743495

DA 2019-01-08

ER

PT J

AU Clapin, H

Hop, L

Ritchie, E

Jayabalan, R

Evans, M

Browne-Cooper, K

Peter, S

Vine, J

Jones, TW

Davis, EA

AF Clapin, H.

Hop, L.

Ritchie, E.

Jayabalan, R.

Evans, M.

Browne-Cooper, K.

Peter, S.

Vine, J.

Jones, T. W.

Davis, E. A.

TI Home-based vs inpatient education for children newly diagnosed with type

1 diabetes

SO PEDIATRIC DIABETES

LA English

DT Article

DE ambulatory; education; pediatric; randomized-controlled trial; type 1

diabetes mellitus

ID YOUNG-PEOPLE; MANAGEMENT; CHILDHOOD; OUTPATIENT; EXPERIENCE; DISTRESS;

FAMILIES; OUTCOMES; PARENTS; CARE

AB Background: Initial management of children diagnosed with type 1 diabetes (T1D) varies worldwide with sparse high quality evidence regarding the impact of different models of care.

Aim: To compare the inpatient model of care with a hybrid home-based alternative, examining metabolic and psychosocial outcomes, diabetes knowledge, length of stay, and patient satisfaction.

Subjects and Methods: The study design was a randomized-controlled trial. Inclusion criteria were: newly diagnosed T1D, aged 3 to 16 years, living within approximately 1 hour of the hospital, English-speaking, access to transport, absence of significant medical or psychosocial comorbidity. Patients were randomized to standard care with a 5 to 6 day initial inpatient stay or discharge after 2 days for home-based management. All patients received practical skills training in the first 48 hours. The intervention group was visited twice/day by a nurse for 2 days to assist with injections, then a multi-disciplinary team made 3 home visits over 2 weeks to complete education. Patients were followed up for 12 months. Clinical outcomes included HbA1c, hypoglycemia, and diabetes-related readmissions. Surveys measured patient satisfaction, diabetes knowledge, family impact, and quality of life.

Results: Fifty patients were recruited, 25 to each group. There were no differences in medical or psychosocial outcomes or diabetes knowledge. Average length of admission was 1.9 days shorter for the intervention group. Families indicated that with hindsight, most would choose home-over hospital-based management.

Conclusions: With adequate support, children newly diagnosed with T1D can be safely managed at home following practical skills training.

C1 [Clapin, H.; Hop, L.; Ritchie, E.; Jayabalan, R.; Evans, M.; Browne-Cooper, K.; Jones, T. W.; Davis, E. A.] Princess Margaret Hosp Children, Dept Endocrinol & Diabet, GPO Box D184, Perth, WA 6840, Australia.

[Clapin, H.; Evans, M.; Jones, T. W.; Davis, E. A.] Univ Western Australia, Telethon Kids Inst, Perth, WA, Australia.

[Peter, S.; Vine, J.] Princess Margaret Hosp Children, Hosp Home, Perth, WA, Australia.

RP Clapin, H (reprint author), Princess Margaret Hosp Children, Dept Endocrinol & Diabet, GPO Box D184, Perth, WA 6840, Australia.

EM helen.clapin@health.wa.gov.au

FU PMH Foundation; WA Department of Health; Paediatric Medicine Clinical

Care Unit of PMH

FX PMH Foundation; WA Department of Health; Paediatric Medicine Clinical

Care Unit of PMH

CR AIHW, 2010, DIAB SER

CHASE HP, 1992, CLIN PEDIATR, V31, P450, DOI 10.1177/000992289203100801

Clar C, 2007, COCHRANE DB SYST REV, V2

DCCT Res Grp, 1988, DIABETES CARE, V11, P725

Dougherty G, 1999, PEDIATRICS, V103, P122, DOI 10.1542/peds.103.1.122

Eigenmann CA, 2011, PRACT DIABETES, V28, P166, DOI 10.1002/pdi.1586

Funnel MM, 2009, DIABETES CARE, V32, pS87, DOI 10.2337/dc09-S087

Grey M, 2011, MCN-AM J MATERN-CHIL, V36, P214, DOI 10.1097/NMC.0b013e3182184c8f

Haynes A, 2004, DIABETOLOGIA, V47, P866, DOI 10.1007/s00125-004-1385-8

Ingersoll G M, 1991, Diabetes Educ, V17, P114, DOI 10.1177/014572179101700219

Jasinski CF, 2013, BMC PEDIATR, V13, DOI 10.1186/1471-2431-13-55

KAZAK A, 2001, FAMILIES SYSTEMS HLT, V19, P303, DOI DOI 10.1037/H0089454

Koizumi S, 1992, J Pediatr Nurs, V7, P154

Konrad K, 2016, PEDIATR DIABETES, V17, P483, DOI 10.1111/pedi.12330

Lee P D, 1992, Tex Med, V88, P64

Lowes L, 2004, ARCH DIS CHILD, V89, P934, DOI 10.1136/adc.2003.035949

Lowes L, 2004, DIABETIC MED, V21, P531, DOI 10.1111/j.1464-5491.2004.01193.x

Markowitz JT, 2012, DIABETIC MED, V29, P526, DOI 10.1111/j.1464-5491.2011.03434.x

Morgan-Trimmer S, 2016, DIABETIC MED, V33, P119, DOI 10.1111/dme.12891

Pai ALH, 2008, J PEDIATR PSYCHOL, V33, P50, DOI 10.1093/jpepsy/jsm053

Patterson CC, 2009, LANCET, V373, P2027, DOI 10.1016/S0140-6736(09)60568-7

POLONSKY WH, 1995, DIABETES CARE, V18, P754, DOI 10.2337/diacare.18.6.754

Samuelsson U, 2014, PEDIATR DIABETES, V15, P229, DOI 10.1111/pedi.12085

Scott A, 2001, DIABETIC MED, V18, P861, DOI 10.1046/j.1464-5491.2001.00627.x

SIMELL T, 1993, DIABETIC MED, V10, P855, DOI 10.1111/j.1464-5491.1993.tb00180.x

Siminerio LM, 1999, DIABETES EDUCATOR, V25, P895, DOI 10.1177/014572179902500607

Srinivasan S, 2004, MED J AUSTRALIA, V180, P277

SWIFT PGF, 1993, BRIT MED J, V307, P96, DOI 10.1136/bmj.307.6896.96

Tiberg I, 2014, EUR DIABETES NURSING, V11, P70

Tonyushkina KN, 2014, PEDIATR DIABETES, V15, P135, DOI 10.1111/pedi.12069

Townson JK, 2011, BMC PEDIATR, V11, DOI 10.1186/1471-2431-11-7

Wennick A, 2006, J FAM NURS, V12, P368, DOI 10.1177/1074840706296724

NR 32

TC 1

Z9 1

U1 5

U2 11

PU WILEY

PI HOBOKEN

PA 111 RIVER ST, HOBOKEN 07030-5774, NJ USA

SN 1399-543X

EI 1399-5448

J9 PEDIATR DIABETES

JI Pediatr. Diabetes

PD NOV

PY 2017

VL 18

IS 7

BP 579

EP 587

DI 10.1111/pedi.12466

PG 9

WC Endocrinology & Metabolism; Pediatrics

SC Endocrinology & Metabolism; Pediatrics

GA FK2XY

UT WOS:000413348000011

PM 27807908

DA 2019-01-08

ER

PT J

AU Forsander, G

Bogelund, M

Haas, J

Samuelsson, U

AF Forsander, Gun

Bogelund, Mette

Haas, Josephine

Samuelsson, Ulf

TI Adolescent life with diabetes-Gender matters for level of distress.

Experiences from the national TODS study

SO PEDIATRIC DIABETES

LA English

DT Article

DE adolescent; diabetes; distress; female; gender

ID METABOLIC-CONTROL; GLYCEMIC CONTROL; CHILDREN; RISK; COMPLICATIONS;

MORTALITY; BRAIN; SEX; AGE; DIAGNOSIS

AB Objective: To examine the relationship between diabetes distress and gender, and the association with glycemic control, social support, health behaviors, and socio-economic status.

Methods: All adolescents, aged 15 to 18 years, in the national, pediatric diabetes registry SWE-DIABKIDS with type 1 diabetes were invited to complete an online questionnaire. A total of 2112 teenagers were identified.

Results: 453 complete responses were valid for analyses. Young women scored significantly higher on the distress-screening instrument DDS-2. Almost half of the female respondents exhibited moderate to severe diabetes distress-more than twice the proportion than among male respondents (44% vs 19%). Females reported twice as high scores on the fear of hypoglycemia scale (P < 0.0001) and had a higher HbA1c value than males (P < 0.0001). Gender was highly correlated with distress level even when controlling for multiple factors that may affect distress (parameter(female) = 0.4, P = 0.0003). Particular social problems were highly significant, that is, those who trust that their parents can handle their diabetes when necessary were significantly less distressed than others (P = 0.018). Higher HbA1c levels were associated with higher distress scores (P = 0.0005 [female], P = 0.0487 [male]).

Conclusions: Diabetes-related distress is a great burden for adolescents living with diabetes. Actively involved family and friends may reduce diabetes distress, but female adolescents appear to be particularly vulnerable and may need extra focus and support. Our findings indicate that pediatric diabetes teams working with teenagers must intensify the care during this vulnerable period of life in order to reduce the risk of both psychological and vascular complications in young adults.

C1 [Forsander, Gun] Univ Gothenburg, Sahlgrenska Acad, Inst Clin Sci, Dept Pediat, S-41685 Gothenburg, Sweden.

[Forsander, Gun] Sahlgrens Univ Hosp, Queen Silvia Childrens Hosp, S-41685 Gothenburg, Sweden.

[Bogelund, Mette] Incentive, Holte Stationsvej 14,1, DK-2840 Holte, Denmark.

[Haas, Josephine] Karolinska Inst, Sodersjukhuset, Dept Clin Sci & Educ, Stockholm, Sweden.

[Samuelsson, Ulf] Linkoping Univ, Div Pediat, Dept Clin & Expt Med, Linkoping, Sweden.

RP Bogelund, M (reprint author), Incentive, Holte Stationsvej 14,1, DK-2840 Holte, Denmark.

EM mb@incentive.dk

FU NovoNordisk

FX NovoNordisk

CR Akesson K, 2015, PEDIATR DIABETES, V16, P581, DOI 10.1111/pedi.12219

Arbelaez AM, 2013, PEDIATR DIABETES, V14, P541, DOI 10.1111/pedi.12088

Benitez-Aguirre P, 2015, INVEST OPHTH VIS SCI, V56, P571, DOI 10.1167/iovs.14-15147

Casey BJ, 2008, ANN NY ACAD SCI, V1124, P111, DOI 10.1196/annals.1440.010

Chaplin JE, 2009, ACTA PAEDIATR, V98, P361, DOI 10.1111/j.1651-2227.2008.01066.x

Cho YH, 2014, PEDIATR DIABETES, V15, P18, DOI 10.1111/pedi.12112

Colver A, 2013, ARCH DIS CHILD, V98, P902, DOI 10.1136/archdischild-2013-303945

Delamater AM, 2014, PEDIATR DIABETES, V15, P232, DOI 10.1111/pedi.12191

Fisher L, 2008, ANN FAM MED, V6, P246, DOI 10.1370/afm.842

Gallego PH, 2008, BRIT MED J, V337, DOI 10.1136/bmi.a918

Graue M, 2004, DIABETES CARE, V27, P1313, DOI 10.2337/diacare.27.6.1313

Hanberger L, 2008, DIABETES CARE, V31, P927, DOI 10.2337/dc07-1863

Hochhauser CJ, 2008, PEDIATR DIABETES, V9, P303, DOI 10.1111/j.1399-5448.2008.00395.x

Holl RW, 1999, DIABETES CARE, V22, P1555, DOI 10.2337/diacare.22.9.1555

Holl RW, 2003, EUR J PEDIATR, V162, P22, DOI 10.1007/s00431-002-1037-2

HOLMES CS, 1992, J CONSULT CLIN PSYCH, V60, P698, DOI 10.1037/0022-006X.60.5.698

Huston SA, 2016, PEDIATR DIABETES, V17, P623, DOI 10.1111/pedi.12347

Huxley RR, 2015, LANCET DIABETES ENDO, V3, P198, DOI 10.1016/S2213-8587(14)70248-7

Jeffery AN, 2014, PEDIATR DIABETES, V15, P599, DOI 10.1111/pedi.12125

Joensen LE, 2013, DIABETIC MED, V30, P1132, DOI 10.1111/dme.12241

Kyngas H, 2001, J CLIN NURS, V10, P767, DOI 10.1046/j.1365-2702.2001.00538.x

LAGRECA AM, 1995, CHILD HEALTH CARE, V24, P61, DOI 10.1207/s15326888chc2401_6

Laing SP, 2003, DIABETOLOGIA, V46, P760, DOI 10.1007/s00125-003-1116-6

Lind M, 2014, NEW ENGL J MED, V371, P1972, DOI 10.1056/NEJMoa1408214

Nationella Diabetesregistret (NDR), SWED

Nylander C, 2014, ACTA PAEDIATR, V103, P194, DOI 10.1111/apa.12461

Polonsky WH, 2005, DIABETES CARE, V28, P626, DOI 10.2337/diacare.28.3.626

Samuelsson U, 2016, J DIABETES COMPLICAT, V30, P917, DOI 10.1016/j.jdiacomp.2016.02.007

Schoenaker DAJM, 2014, J CLIN ENDOCR METAB, V99, P800, DOI 10.1210/jc.2013-2824

Schoenle EJ, 2002, DIABETOLOGIA, V45, P108, DOI 10.1007/s125-002-8250-6

Schwab KO, 2010, PEDIATR DIABETES, V11, P357, DOI 10.1111/j.1399-5448.2010.00665.x

Setoodeh A, 2012, INDIAN J PEDIATR, V79, P896, DOI 10.1007/s12098-011-0613-8

Statistics Sweden, 2012, WOM MEN SWED FACTS F

Sundberg F, 2012, ACTA PAEDIATR, V101, P1164, DOI 10.1111/j.1651-2227.2012.02803.x

NR 34

TC 2

Z9 2

U1 2

U2 7

PU WILEY

PI HOBOKEN

PA 111 RIVER ST, HOBOKEN 07030-5774, NJ USA

SN 1399-543X

EI 1399-5448

J9 PEDIATR DIABETES

JI Pediatr. Diabetes

PD NOV

PY 2017

VL 18

IS 7

BP 651

EP 659

DI 10.1111/pedi.12478

PG 9

WC Endocrinology & Metabolism; Pediatrics

SC Endocrinology & Metabolism; Pediatrics

GA FK2XY

UT WOS:000413348000021

PM 28004484

DA 2019-01-08

ER

PT J

AU Vancampfort, D

Koyanagi, A

Ward, PB

Veronese, N

Carvalho, AF

Solmi, M

Mugisha, J

Rosenbaum, S

De Hert, M

Stubbs, B

AF Vancampfort, Davy

Koyanagi, Ai

Ward, Philip B.

Veronese, Nicola

Carvalho, Andre F.

Solmi, Marco

Mugisha, James

Rosenbaum, Simon

De Hert, Marc

Stubbs, Brendon

TI Perceived Stress and Its Relationship With Chronic Medical Conditions

and Multimorbidity Among 229,293 Community-Dwelling Adults in 44 Low-

and Middle-Income Countries

SO AMERICAN JOURNAL OF EPIDEMIOLOGY

LA English

DT Article

DE chronic conditions; low- and middle-income countries; multimorbidity;

perceived stress; poverty

ID RANDOMIZED CONTROLLED-TRIAL; MAJOR DEPRESSIVE EPISODES; HEALTH-CARE

UTILIZATION; CULTURALLY ADAPTED CBT; CHRONIC DISEASES; ASSOCIATION;

DISTRESS; EXPENDITURES; POPULATION; TRANSITION

AB In this study, we assessed the association of chronic medical conditions and multimorbidity with perceived stress among community-dwelling adults in 44 low- and middle-income countries. Data from the World Health Survey (2002-2004), including 229,293 adults, were analyzed. A perceived stress score (range, 0 (lowest stress)-100 (highest stress)) was computed on the basis of 2 questions from the Perceived Stress Scale. Eleven chronic conditions were assessed. Multivariable linear regression analyses were conducted to explore the associations. All chronic conditions were associated with significantly higher mean perceived stress scores, with the exception of edentulism. The associations were particularly strong for depression (beta = 14.71, 95% confidence interval (CI): 13.68, 15.74), visual impairment (beta = 10.66, 95% CI: 8.09, 13.23), and schizophrenia (beta = 9.98, 95% CI: 7.71, 12.24). Compared with no chronic conditions, the beta coefficients for perceived stress with the presence of 1, 2, 3, and >= 4 chronic conditions were 5.58 (95% CI: 4.94, 6.23), 9.58 (95% CI: 8.67, 10.49), 14.15 (95% CI: 12.63, 15.67), and 20.17 (95% CI: 18.29, 22.05), respectively. The associations with perceived stresswere significantly stronger among the poorest individuals for arthritis, asthma, diabetes, edentulism, and >= 4 chronic conditions. Our data suggest that a range of chronic conditions and multimorbidity are associated with greatly increased perceived stress among people in low- and middle-income countries, and that the poorest persons may be a particularly vulnerable group.

C1 [Vancampfort, Davy] Katholieke Univ Leuven, Dept Rehabil Sci, Biomed Sci Grp, Leuven, Belgium.

[Vancampfort, Davy; De Hert, Marc] Katholieke Univ Leuven, Univ Psychiat Ctr, Leuven Kortenberg, Belgium.

[Koyanagi, Ai] Univ Barcelona, Res & Dev Unit, Fundacio St Joan de Deu, Parc Sanitari St Joan de Deu, Barcelona, Spain.

[Koyanagi, Ai] Inst Salud Carlos III, Ctr Invest Biomed Red Salud Mental, Monforte De Lemos, Spain.

[Koyanagi, Ai] Inst Salud Carlos III, Ctr Invest Biomed Red Salud Mental, Madrid, Spain.

[Ward, Philip B.] Univ New South Wales, Sch Psychiat, Fac Med, Sydney, NSW, Australia.

[Ward, Philip B.] Ingham Inst Appl Med Res, Schizophrenia Res Unit, Liverpool, Merseyside, England.

[Veronese, Nicola] Univ Padua, Inst Clin Res & Educ Med, Geriatr Div, Dept Med, Padua, Italy.

[Veronese, Nicola] Inst Clin Res & Educ Med, Padua, Italy.

[Carvalho, Andre F.] Univ Fed Ceara, Translat Psychiat Res Grp, Fac Med, Fortaleza, Ceara, Brazil.

[Carvalho, Andre F.] Univ Fed Ceara, Dept Clin Med, Fac Med, Fortaleza, Ceara, Brazil.

[Solmi, Marco] Univ Padua, Dept Neurosci, Padua, Italy.

[Mugisha, James] Kyambogo Univ, Dept Sociol & Social Sci, Kampala, Uganda.

[Mugisha, James] Butabika Natl Referral & Mental Hlth Hosp, Kampala, Uganda.

[Rosenbaum, Simon] Univ New South Wales, Sch Med Sci, Exercise Physiol Dept, Sydney, NSW, Australia.

[Stubbs, Brendon] South London & Maudsley NHS Fdn Trust, Physiotherapy Dept, London, England.

[Stubbs, Brendon] Kings Coll London, Inst Psychiat Psychol & Neurosci, Hlth Serv & Populat Res Dept, London, England.

RP Vancampfort, D (reprint author), Katholieke Univ Leuven, Univ Psychiat Ctr, Leuvensesteenweg 517, B-3070 Kortenberg, Belgium.

EM Davy.Vancampfort@kuleuven.be

RI Stubbs, Brendon/X-1904-2018; Koyanagi, Ai/D-3833-2018; Ward,

Philip/E-3959-2010

OI Stubbs, Brendon/0000-0001-7387-3791; Koyanagi, Ai/0000-0002-9565-5004;

Ward, Philip/0000-0002-5779-7722

FU National Institute for Health Research Collaboration for Leadership in

Applied Health Research and Care (United Kingdom).; Miguel Servet

contract [CP13/00150, PI15/00862]; Instituto de Salud Carlos III

(General Branch Evaluation and Promotion of Health Research); European

Regional Development Fund; Society for Mental Health Research

(Australia); Research Foundation-Flanders

FX There was no funding for the current work. B.S. receives financial

support from the National Institute for Health Research Collaboration

for Leadership in Applied Health Research and Care (United Kingdom).

A.K.'s work was supported by a Miguel Servet contract (projects

CP13/00150 and PI15/00862), integrated into the National Research,

Development, and Innovation program and funded by the Instituto de Salud

Carlos III (General Branch Evaluation and Promotion of Health Research)

and the European Regional Development Fund. S.R. was supported by a

Society for Mental Health Research (Australia) Early Career Fellowship.

D.V. was supported by the Research Foundation-Flanders.

CR Aikens JE, 2012, DIABETES CARE, V35, P2472, DOI 10.2337/dc12-0181

Arokiasamy P, 2015, BMC MED, V13, DOI 10.1186/s12916-015-0402-8

Barnett K, 2012, LANCET, V380, P37, DOI 10.1016/S0140-6736(12)60240-2

Cifuentes M, 2008, SOC SCI MED, V67, P529, DOI 10.1016/j.socscimed.2008.04.003

COHEN S, 1983, J HEALTH SOC BEHAV, V24, P385, DOI 10.2307/2136404

DeVylder JE, 2016, SCHIZOPHRENIA BULL, V42, P1353, DOI 10.1093/schbul/sbw044

Diederichs C, 2011, J GERONTOL A-BIOL, V66, P301, DOI 10.1093/gerona/glq208

Errisuriz VL, 2016, EAT BEHAV, V22, P211, DOI 10.1016/j.eatbeh.2016.06.008

Fortin Martin, 2004, Health Qual Life Outcomes, V2, P51, DOI 10.1186/1477-7525-2-51

Fortin M, 2012, ANN FAM MED, V10, P142, DOI 10.1370/afm.1337

Freeman EE, 2013, PLOS ONE, V8, DOI 10.1371/journal.pone.0063315

Gallo JJ, 2016, J GEN INTERN MED, V31, P380, DOI 10.1007/s11606-015-3524-y

Goldhaber-Fiebert JD, 2011, INT J EPIDEMIOL, V40, P417, DOI 10.1093/ije/dyq238

Goldstein DS, 2002, STRESS, V5, P54, DOI 10.1080/10253890290012345

Huffman MD, 2011, PLOS ONE, V6, DOI 10.1371/journal.pone.0020821

Lavoie KL, 2010, CHEST, V137, P1324, DOI 10.1378/chest.09-1979

Lee EH, 2012, ASIAN NURS RES, V6, P121, DOI 10.1016/j.anr.2012.08.004

Lehnert T, 2011, MED CARE RES REV, V68, P387, DOI 10.1177/1077558711399580

Leive A, 2008, B WORLD HEALTH ORGAN, V86, P849, DOI 10.2471/BLT.07.049403

Loerbroks A, 2012, INT J EPIDEMIOL, V41, P1436, DOI 10.1093/ije/dys123

Makinen M, 2000, B WORLD HEALTH ORGAN, V78, P55

McEwen BS, 2008, EUR J PHARMACOL, V583, P174, DOI 10.1016/j.ejphar.2007.11.07t

McEwen BS, 2015, METABOLISM, V64, pS2, DOI 10.1016/j.metabol.2014.10.029

Moussavi S, 2007, LANCET, V370, P851, DOI 10.1016/S0140-6736(07)61415-9

Murray CJL, 2015, LANCET, V386, P2145, DOI 10.1016/S0140-6736(15)61340-X

Naeem F, 2014, J AFFECT DISORDERS, V156, P224, DOI 10.1016/j.jad.2013.10.051

Naeem F, 2015, SCHIZOPHR RES, V164, P143, DOI 10.1016/j.schres.2015.02.015

Nuevo R, 2012, SCHIZOPHRENIA BULL, V38, P475, DOI 10.1093/schbul/sbq099

Oni T, 2014, BMC PUBLIC HEALTH, V14, DOI 10.1186/1471-2458-14-575

Pandey KR, 2016, PLOS ONE, V11, DOI 10.1371/journal.pone.0155293

Pati S, 2014, BMC HEALTH SERV RES, V14, DOI 10.1186/1472-6963-14-451

Phillips A. C., 2013, ENCY BEHAV MED, P1453

Prior A, 2016, AM J EPIDEMIOL, V184, P199, DOI 10.1093/aje/kwv324

ROSE GA, 1962, B WORLD HEALTH ORGAN, V27, P645

RUSS TC, 2012, BRIT MED J, V345, DOI [10.1136/bmj.e4933, DOI 10.1136/BMJ.E4933]

Salisbury C, 2012, LANCET, V380, P7, DOI 10.1016/S0140-6736(12)60482-6

Santiago T, 2015, CURR PHARM DESIGN, V21, P257

Sladek MR, 2016, BIOL PSYCHOL, V117, P8, DOI 10.1016/j.biopsycho.2016.02.003

Stults-Kolehmainen MA, 2014, SPORTS MED, V44

Swartz JA, 2014, AM J PUBLIC HEALTH, V104, P2350, DOI 10.2105/AJPH.2014.302165

Tola HH, 2015, GLOBAL HEALTH ACTION, V8, DOI 10.3402/gha.v8.29019

Tomasdottir MO, 2015, PLOS ONE, V10, DOI 10.1371/journal.pone.0130591

Uijen Annemarie A, 2008, Eur J Gen Pract, V14 Suppl 1, P28, DOI 10.1080/13814780802436093

Whiteford HA, 2013, LANCET, V382, P1575, DOI 10.1016/S0140-6736(13)61611-6

World Health Organization, HLTH STAT INF SYST

Xu K, 2007, HEALTH AFFAIR, V26, P972, DOI 10.1377/hlthaff.26.4.972

Yoon SJ, 2016, ASIA PAC J CLIN NUTR, V25, P316, DOI 10.6133/apjcn.2016.25.2.23

NR 47

TC 6

Z9 6

U1 1

U2 5

PU OXFORD UNIV PRESS INC

PI CARY

PA JOURNALS DEPT, 2001 EVANS RD, CARY, NC 27513 USA

SN 0002-9262

EI 1476-6256

J9 AM J EPIDEMIOL

JI Am. J. Epidemiol.

PD OCT 15

PY 2017

VL 186

IS 8

BP 979

EP 989

DI 10.1093/aje/kwx159

PG 11

WC Public, Environmental & Occupational Health

SC Public, Environmental & Occupational Health

GA FJ5NK

UT WOS:000412798300011

PM 28637230

DA 2019-01-08

ER

PT J

AU Ognean, L

Boanta, O

Visa, G

Grosu, F

Sofariu, C

Gafencu, M

Matei, C

Iurian, S

AF Ognean, L.

Boanta, O.

Visa, G.

Grosu, F.

Sofariu, C.

Gafencu, M.

Matei, C.

Iurian, S.

TI HYDROCEPHALY, SCHIZENCEPHALY, SPONDYLOCOSTAL DYSPLASIA, AND

HYPOPARATHYROIDISM IN AN INFANT OF A DIABETIC MOTHER

SO ACTA ENDOCRINOLOGICA-BUCHAREST

LA English

DT Article

DE diabetic mother; infant; hydrocephaly; hypoparathyroidism;

schizencephaly; spondylocostal dysplasia

ID NEWBORN-INFANTS; BIRTH-DEFECTS; PREGNANCY; MELLITUS; POPULATION; WOMEN;

OUTCOMES; TYPE-1; PREVALENCE; ANOMALIES

AB Context. Diabetes mellitus is the most frequent chronic complication in pregnancy and continues to contribute to increased perinatal morbidity and mortality in newborns. Macrosomia, respiratory distress syndrome, metabolic and electrolytic disturbances, and increased rates of congenital structural defects are well-known neonatal complications associated with maternal diabetes, even if well-controlled.

Case report. A macrosomic infant born from an insulin-dependent mother, with uncontrolled diabetes and lack of adequate prenatal care, prenatally diagnosed with hydrocephaly showed a complicated postnatal course. Initial respiratory distress syndrome and transient hypoglycemia, rapidly corrected under treatment, were followed by persistent hypocalcemia and hyperphosphatemia due to hypoparathyroidism and evolving hydrocephaly. Ventriculoperitoneal shunting was followed by resolution of hypocalcemia, but seizures associated with schizencephaly and recurrent respiratory tract infections, aggravated by spondylocostal dysplasia, concurred to infant's demise at the age of 5 months.

Conclusions. The reported case is rare due to multiple aspects: persistent hypoparathyroidism, uncommon association of schizencephaly, and even rarely association with spondylocostal dysplasia, all these conditions requiring a multidisciplinary therapeutic approach. Also, the reported case is evocative for challenges associated with infants born from diabetic mothers.

C1 [Ognean, L.; Boanta, O.] Lucian Blaga Univ, Fac Med, Dept Neonatol, Sibiu, Romania.

[Grosu, F.] Lucian Blaga Univ, Fac Med, Dept Radiol, Sibiu, Romania.

[Matei, C.] Lucian Blaga Univ, Fac Med, Dept Neurosurg, Sibiu, Romania.

[Iurian, S.] Lucian Blaga Univ, Fac Med, Pediat Hosp, Sibiu, Romania.

[Visa, G.] Clin Hosp Pediat, Dept Neurol, Sibiu, Romania.

[Sofariu, C.] Clin Hosp Pediat, Childrens Neurol Dis Res Ctr & Telemed CEFORATEN, Sibiu, Romania.

[Gafencu, M.] Victor Babes Univ Med & Pharm, Dept Pediat, Timisoara, Romania.

[Gafencu, M.] Louis Turcanu Emergency Children Peritoneal Dialy, Timisoara, Romania.

RP Gafencu, M (reprint author), Victor Babes Univ Med & Pharm, Dept Nephrol, 2nd E Murgu Square, Timisoara 300350, Timis, Romania.

EM mgafencu@umft.ro

CR Allen VM, 2007, J OBSTET GYNAECOL CA, V29, P927, DOI 10.1016/S1701-2163(16)32653-6

BECERRA JE, 1990, PEDIATRICS, V85, P1

Bentham J, 2016, ELIFE, V5, DOI 10.7554/eLife.13410

Bodmer-Roy S, 2012, OBSTET GYNECOL, V120, P746, DOI 10.1097/AOG.0b013e31826994ec

BUCHANAN TA, 1994, ANNU REV MED, V45, P245

Casson IF, 1997, BRIT MED J, V315, P275, DOI 10.1136/bmj.315.7103.275

Ceausu I, 2016, ACTA ENDOCRINOL-BUCH, V12, P500, DOI 10.4183/aeb.2016.500

Celik O, 2011, ACTA ENDOCRINOL-BUCH, V7, P545, DOI 10.4183/aeb.2011.545

Chen YZ, 2009, POPUL HEALTH MANAG, V12, P165, DOI 10.1089/pop.2009.12303

Cohen RS, 2012, NEOREVIEWS, V13, pe486

Das S, 2009, ARCH DIS CHILD-FETAL, V94, pF419, DOI 10.1136/adc.2008.156026

Das SU, 2012, J NEONATOLOGY RES, V2, P130

DEMARINI S, 1994, OBSTET GYNECOL, V83, P918, DOI 10.1097/00006250-199406000-00003

Gheorman L, 2011, ROM J MORPHOL EMBRYO, V52, P1127

Gilbert-Barness E, 2010, ANN CLIN LAB SCI, V40, P99

HILLMAN LS, 1977, PEDIATR RES, V11, P739, DOI 10.1203/00006450-197706000-00009

Jain A, 2010, INDIAN J PEDIATR, V77, P1123, DOI 10.1007/s12098-010-0176-0

Lawrence JM, 2008, DIABETES CARE, V31, P899, DOI 10.2337/dc07-2345

LOUGHEAD JL, 1991, J AM COLL NUTR, V10, P123

Macintosh MCM, 2006, BRIT MED J, V333, P177, DOI 10.1136/bmj.38856.692986.AE

McFarland M B, 1998, J Matern Fetal Med, V7, P292

Nold JL, 2004, PEDIATR CLIN N AM, V51, P619, DOI 10.1016/j.pcl.2004.01.003

Oakley GP, 2012, AM J OBSTET GYNECOL, V206, P179, DOI 10.1016/j.ajog.2011.12.019

Ozkinay F, 1996, TURKISH J PEDIATR, V38, P381

Panaitescu AM, 2016, ACTA ENDOCRINOL-BUCH, V12, P331, DOI 10.4183/aeb.2016.331

Patil MB, 2012, INDIAN PEDIATR, V49, P670

PEDERSEN J, 1954, ACTA ENDOCRINOL-COP, V16, P330, DOI 10.1530/acta.0.0160330

Persson B, 1998, DIABETES CARE, V21, pB79

Persson M, 2011, DIABETES CARE, V34, P1145, DOI 10.2337/dc10-2406

Prabha FCS, 2010, CURR PEDIAT RES, V14, P83

Rafiq W., 2015, INT J CONT PEDIAT, V2, P114, DOI [10.5455/2349-3291.ijcp20150510, DOI 10.5455/2349-3291.IJCP20150510]

Rosenn B, 1996, PEDIATR ANN, V25, P215, DOI 10.3928/0090-4481-19960401-09

ROSENN B, 1991, ANN CLIN LAB SCI, V21, P153

Rozance PJ, 2006, BIOL NEONATE, V90, P74, DOI 10.1159/000091948

Schwartz R, 2000, SEMIN PERINATOL, V24, P120, DOI 10.1053/sp.2000.6363

Stoll BJ, 2003, NELSON TXB PAEDIAT, P614

Tyrala EE, 1996, OBSTET GYN CLIN N AM, V23, P221, DOI 10.1016/S0889-8545(05)70253-9

Ullmo S, 2007, EUR HEART J, V28, P1319, DOI 10.1093/eurheartj/ehl416

WIDNESS JA, 1990, DIABETOLOGIA, V33, P378, DOI 10.1007/BF00404643

Wild S, 2004, DIABETES CARE, V27, P1047, DOI 10.2337/diacare.27.5.1047

Yang J, 2006, OBSTET GYNECOL, V108, P644, DOI 10.1097/01.AOG.0000231688.08263.47

NR 41

TC 0

Z9 0

U1 2

U2 4

PU EDITURA ACAD ROMANE

PI BUCURESTI

PA CALEA 13 SEPTEMBRIE NR 13, SECTOR 5, BUCURESTI 050711, ROMANIA

SN 1841-0987

EI 1843-066X

J9 ACTA ENDOCRINOL-BUCH

JI Acta Endocrinol.

PD OCT-DEC

PY 2017

VL 13

IS 4

BP 494

EP 501

DI 10.4183/aeb.2017.494

PG 8

WC Endocrinology & Metabolism

SC Endocrinology & Metabolism

GA FT4QQ

UT WOS:000423140200015

DA 2019-01-08

ER

PT J

AU Halliday, JA

Hendrieckx, C

Busija, L

Browne, JL

Nefs, G

Pouwer, F

Speight, J

AF Halliday, Jennifer A.

Hendrieckx, Christel

Busija, Lucy

Browne, Jessica L.

Nefs, Giesje

Pouwer, Francois

Speight, Jane

TI Validation of the WHO-5 as a first-step screening instrument for

depression in adults with diabetes: Results from Diabetes MILES -

Australia

SO DIABETES RESEARCH AND CLINICAL PRACTICE

LA English

DT Article

DE Diabetes mellitus; Depression; Surveys and questionnaires;

Psychometrics; Factor analysis; statistical; ROC curve

ID WELL-BEING-INDEX; PRIMARY-CARE; OUTPATIENTS; PEOPLE; ASSOCIATION;

DISTRESS; VALIDITY; QUALITY; TOOLS; PHQ-9

AB Aims: Screening for depression is recommended internationally. The World Health Organization's 5-item Well-being Index (WHO-5) is used clinically to screen for depression but its empirical suitability for this purpose is not well documented. We investigated the psychometric properties of the WHO-5 and its suitability for identifying likely depression in Australian adults with diabetes.

Methods: The Diabetes MILES -Australia study dataset provided a sample of N = 3249 who completed the WHO-5 (positively-worded 5-item measure of emotional well-being) and the PHQ-9 (9-item measure of depressive symptoms). Analyses were conducted for the full sample, and separately by diabetes type and treatment (type 1, non-insulin-treated type 2, and insulin-treated type 2 diabetes). Construct (convergent and factorial) validity and reliability of the WHO-5 were examined. ROC analyses were used to examine the sensitivity and specificity of the WHO-5 as a depression screening instrument, comparing two commonly used WHO-5 cut-off values (<= 7 and < 13) with the PHQ-9.

Results: For the whole sample, the WHO-5 demonstrated satisfactory internal consistency reliability (a = 0.90) and convergent validity with the PHQ-9 (r = -0.73, p < 0.001). Confirmatory factor analysis partially supported factorial validity:.2(5) = 834.94, p < 0.001; RMSEA = 0.23, 90% CI 0.21-0.24; CFI = 0.98, TLI = 0.96; factor loadings = 0.78-0.92. The AUC was 0.87 (95% CI: 0.86-0.89, p < 0.001). The sensitivity/specificity of the WHO-5 for detecting likely depression was 0.44/ 0.96 for the <= 7 cut-off, and 0.79/ 0.79 for the < 13 cut-off, with similar findings by diabetes type and treatment.

Conclusions: These findings support use of a WHO-5 cut-point of <13 to identify likely depression in Australian adults with diabetes, regardless of type/treatment. (C) 2017 Elsevier B.V. All rights reserved.

C1 [Halliday, Jennifer A.; Hendrieckx, Christel; Browne, Jessica L.; Speight, Jane] Deakin Univ, Sch Psychol, 1 Geringhap St, Geelong, Vic 3220, Australia.

[Halliday, Jennifer A.; Hendrieckx, Christel; Browne, Jessica L.; Speight, Jane] Australian Ctr Behav Res Diabet, Diabet Victoria, 570 Elizabeth St, Melbourne, Vic 3000, Australia.

[Busija, Lucy] Australian Catholic Univ, Inst Hlth & Ageing, Level 6,215 Spring St, Melbourne, Vic 3000, Australia.

[Nefs, Giesje] Tilburg Univ, Ctr Res Psychol & Somat Disorders CoRPS, Dept Med & Clin Psychol, POB 90153, NL-5000 LE Tilburg, Netherlands.

[Nefs, Giesje] Ctr Pediat & Adolescent Diabet Care & Res, Blaak 6, NL-3011 TA Rotterdam, Netherlands.

[Pouwer, Francois] Univ Southern Denmark, Dept Psychol, Campusvej 55, DK-5230 Odense M, Denmark.

[Speight, Jane] AHP Res, Hornchurch, Essex, England.

RP Halliday, JA (reprint author), Deakin Univ, Sch Psychol, 1 Geringhap St, Geelong, Vic 3220, Australia.

EM j.halliday@deakin.edu.au

RI Nefs, Giesje/C-9992-2018

OI Speight, Jane/0000-0002-1204-6896

FU National Diabetes Services Scheme (NDSS) Strategic Development Grant;

Sanofi-Aventis; NDSS Mental Health and Diabetes Priority Area; Diabetes

Victoria; Deakin University

FX The Diabetes MILES - Australia 2011 Survey was funded by a National

Diabetes Services Scheme (NDSS) Strategic Development Grant. The NDSS is

an initiative of the Australia Government administered with the

assistance of Diabetes Australia. The Diabetes MILES Study was also

supported by an unrestricted educational grant from Sanofi-Aventis. The

funding sources had no role in the design, data collection, analysis or

interpretation of the study, or in the decision to submit the manuscript

for publication. JH was funded by the NDSS Mental Health and Diabetes

Priority Area. The contributions of JS, CH and JB are supported by core

funding provided by Diabetes Victoria and Deakin University to The

Australian Centre for Behavioural Research in Diabetes.

CR Awata S, 2007, PSYCHIAT CLIN NEUROS, V61, P112, DOI 10.1111/j.1440-1819.2007.01619.x

Bech P., 2004, QUALITY LIFE NEWSLET, V32, P15

Browne MW, 2002, PSYCHOL METHODS, V7, P403, DOI 10.1037//1082-989X.7.4.403

Byrne JL, 2017, DIABETIC MED, V34, P1074, DOI 10.1111/dme.13334

Cohen J, 1988, STAT POWER ANAL BEHA

Davies MJ, 2007, PRACT DIAB INT, V24, P483

de Wit M, 2007, DIABETES CARE, V30, P2003, DOI 10.2337/dc07-0447

Fischer JE, 2003, INTENS CARE MED, V29, P1043, DOI 10.1007/s00134-003-1761-8

Fisher L, 2014, DIABETIC MED, V31, P764, DOI 10.1111/dme.12428

Furuya M, 2013, ACTA DIABETOL, V50, P117, DOI 10.1007/s00592-010-0219-z

Hajos TRS, 2012, DIABETIC MED, V30, pe63

Halliday JA, 2015, DIABETIC MED, V32, P1393, DOI 10.1111/dme.12768

Hendrieckx C, 2014, DIABETES RES CLIN PR, V103, P430, DOI 10.1016/j.diabres.2013.12.005

Hendrieckx C, 2012, AUSTR DIAB SOC AUSTR

Hendrieckx C, 2016, HDB HLTH PROFESSIONA

Henkel V, 2004, GEN HOSP PSYCHIAT, V26, P190, DOI 10.1016/j.genhosppsych.2004.02.003

Hochberg G, 2012, DIABETES METAB, V38, P515, DOI 10.1016/j.diabet.2012.06.002

Kroenke K, 2001, J GEN INTERN MED, V16, P606, DOI 10.1046/j.1525-1497.2001.016009606.x

Lamers F, 2008, J CLIN EPIDEMIOL, V61, P679, DOI 10.1016/j.jclinepi.2007.07.018

Lloyd CE, 2012, DIABETIC MED, V29, P140, DOI 10.1111/j.1464-5491.2011.03481.x

Lloyd CE, 2013, SCREENING DEPRESSION

Lombardo P, 2011, BMC MED, V9, DOI 10.1186/1741-7015-9-114

Mosely K, 2010, DIABETES RES CLIN PR, V87, P11

Muthen B, 1997, PSYCHOMETRIKA, V1, P1

Novo Nordisk, 2015, THE DAWN PROGR

Nunnally JC, 1994, PSYCHOMETRIC THEORY

Pignone MP, 2002, ANN INTERN MED, V136, P765, DOI 10.7326/0003-4819-136-10-200205210-00013

Pouwer F, 2006, PATIENT EDUC COUNS, V60, P235, DOI 10.1016/j.pec.2005.01.009

Pouwer F, 2001, DIABETES CARE, V24, P1929, DOI 10.2337/diacare.24.11.1929

Pouwer F, 2013, SCREENING DEPRESSION, P199

Pouwer F, 2013, ENDOCRIN METAB CLIN, V42, P529, DOI 10.1016/j.ecl.2013.05.002

Pouwer F, 2009, NAT REV ENDOCRINOL, V5, P665, DOI 10.1038/nrendo.2009.214

Psychiatric Research Unit at the Mental Health Centre, 2014, WHO 5 WELL BEING IND

RADLOFF L S, 1977, Applied Psychological Measurement, V1, P385, DOI 10.1177/014662167700100306

Shea S, 2003, DIABETOLOGIA, V46, pA85

Snoek FJ, 2015, LANCET DIABETES ENDO, V3, P450, DOI 10.1016/S2213-8587(15)00135-7

Snoek FJ, 2011, DIABETES CARE, V34, P601, DOI 10.2337/dc10-1552

Speight J, 2012, BEHALF DIABETES MILE

Speight J, 2012, BMC PUBLIC HEALTH, V12, DOI 10.1186/1471-2458-12-120

Spitzer RL, INSTRUCTION MANUAL I

Tarolla E, 2012, RIV PSICHIATR, V47, P304, DOI 10.1708/1139.12557

Terwee CB, 2007, J CLIN EPIDEMIOL, V60, P34, DOI 10.1016/j.jclinepi.2006.03.012

The Royal Australian College of General Practitioners, 2016, GEN PRACT MAN TYP 2

Topp CW, 2015, PSYCHOTHER PSYCHOSOM, V84, DOI 10.1159/000376585

VANSTEENBERGENWEIJ, 2010, BMC HEALTH SERV RES, V10, DOI DOI 10.1186/1472-6963-10-235

West SG, 1995, STRUCTURAL EQUATION, P56, DOI DOI 10.1016/J.STUEDUC.2012.09.001

YOUDEN WJ, 1950, CANCER, V3, P32, DOI 10.1002/1097-0142(1950)3:1<32::AID-CNCR2820030106>3.0.CO;2-3

Young-Hyman D, 2016, DIABETES CARE, V39, P2126, DOI 10.2337/dc16-2053

Yu C. Y., 2002, EVALUATING CUTOFF CR

NR 49

TC 2

Z9 2

U1 4

U2 4

PU ELSEVIER IRELAND LTD

PI CLARE

PA ELSEVIER HOUSE, BROOKVALE PLAZA, EAST PARK SHANNON, CO, CLARE, 00000,

IRELAND

SN 0168-8227

EI 1872-8227

J9 DIABETES RES CLIN PR

JI Diabetes Res. Clin. Pract.

PD OCT

PY 2017

VL 132

BP 27

EP 35

DI 10.1016/j.diabres.2017.07.005

PG 9

WC Endocrinology & Metabolism

SC Endocrinology & Metabolism

GA FM6WQ

UT WOS:000415202800004

PM 28783530

DA 2019-01-08

ER

PT J

AU Raymond, KL

Kannis-Dymand, L

Lovell, GP

AF Raymond, Karren-Lee

Kannis-Dymand, Lee

Lovell, Geoff P.

TI A graduated food addiction classifications approach significantly

differentiates depression, anxiety and stress among people with type 2

diabetes

SO DIABETES RESEARCH AND CLINICAL PRACTICE

LA English

DT Article

DE Type 2 diabetes mellitus; Depression; Anxiety; Stress; Processed food

addiction; Severity levels; Processed food use disorder

ID BODY-MASS INDEX; SUBSTANCE USE; MULTIMORBIDITY; DISORDERS; HEALTH;

ADULTS; RISK; PREVALENCE; VALIDATION; DISTRESS

AB Aims: To examine differences in depression, anxiety, and stress across people with type 2 diabetes mellitus (t2d) classified according to a four level processed food addiction (PFA) severity indicator dichotomy.

Methods: Four hundred and eight participants with a t2d diagnoses completed an online survey including the Yale Food Addiction Scale (YFAS) and the DASS-21. Based on YFAS symptom counts participants were classified as either: non-PFA; mild-PFA; moderatePFA; or severe-PFA.

Results: Multivariate, lambda = 0.422, F(9,978.51) = 46.286, p < 0.001, n(p)(2) = 0.250, and univariate analyses of variance demonstrated that depression F(3,408) = 159.891, p < 0.001, n(p)(2) = 0.543, anxiety F(3,408) = 127.419, p < 0.001, np 2 = 0.486, and stress scores F(3,408) = 129.714, p < 0.001, n(p)(2) = 0.491, significantly and meaningfully increased from one PFA classification level to the next. Furthermore, the proportion of participants with more severe classifications of depression chi(2) (12) = 297.820, p < 0.001, anxiety chi(2) (12) = 271.805, p < 0.001, and stress chi(2) (12) = 240.875, p < 0.001, were significantly higher in the more severe PFA groupings.

Conclusion: For people with t2d, PFA is an important and meaningful associate of depression, anxiety, and stress, and that the adopted four level PFA severity indicator dichotomy is valid and useful. (C) 2017 Elsevier B. V. All rights reserved.

C1 [Raymond, Karren-Lee; Kannis-Dymand, Lee; Lovell, Geoff P.] Univ Sunshine Coast, Sch Social Sci, Locked Bag 4, Maroochydore, Qld 4558, Australia.

RP Raymond, KL (reprint author), Univ Sunshine Coast, Sch Social Sci, Locked Bag 4, Maroochydore, Qld 4558, Australia.

EM karren-lee.raymond@research.usc.edu.au; lkannisd@usc.edu.au;

glovell@usc.edu.au

CR American Psychiatric Association, 2013, DIAGN STAT MAN MENT

American Psychiatric Association, 2000, DIAGN STAT MAN MENT

Barnett K, 2012, LANCET, V380, P37, DOI 10.1016/S0140-6736(12)60240-2

Begin C., 2012, HEALTH, V4, P1492, DOI DOI 10.4236/HEALTH.2012.412A214

Bener A, 2011, ONCOL REV, V5, P5, DOI 10.1007/s12156-010-0061-0

Bruinsma K, 1999, J AM DIET ASSOC, V99, P1249, DOI 10.1016/S0002-8223(99)00307-7

Burmeister JM, 2013, APPETITE, V60, P103, DOI 10.1016/j.appet.2012.09.013

Chien IC, 2016, J PSYCHOSOM RES, V86, P47, DOI 10.1016/j.jpsychores.2016.05.003

Collins MM, 2009, DIABETIC MED, V26, P153, DOI 10.1111/j.1464-5491.2008.02648.x

Diabetes Australia, 2016, DIAB GLOB

Fisher L, 2008, DIABETIC MED, V25, P1096, DOI 10.1111/j.1464-5491.2008.02533.x

Fortin M, 2007, BMJ-BRIT MED J, V334

Gearhardt AN, 2013, EAT BEHAV, V14, P508, DOI 10.1016/j.eatbeh.2013.07.002

Gearhardt AN, 2009, APPETITE, V52, P430, DOI 10.1016/j.appet.2008.12.003

Global Burden of Disease, 2015, LANCET

Gorber SC, 2007, OBES REV, V8, P307, DOI 10.1111/j.1467-789X.2007.00347.x

Guariguata L, 2014, DIABETES RES CLIN PR, V103, P137, DOI 10.1016/j.diabres.2013.11.002

Hebebrand J, 2014, NEUROSCI BIOBEHAV R, V47, P295, DOI 10.1016/j.neubiorev.2014.08.016

Henry JD, 2005, BRIT J CLIN PSYCHOL, V44, P227, DOI 10.1348/014466505X29657

Hu FB, 2011, DIABETES CARE, V34, P1249, DOI 10.2337/dc11-0442

Ifland JR, 2009, MED HYPOTHESES, V72, P518, DOI 10.1016/j.mehy.2008.11.035

Kelly TM, 2012, ADDICT BEHAV, V37, P11, DOI 10.1016/j.addbeh.2011.09.010

Lai HMX, 2015, DRUG ALCOHOL DEPEN, V154, P1, DOI 10.1016/j.drugalcdep.2015.05.031

Lian J, 2014, CURR MED RES OPIN, V30, P2233, DOI 10.1185/03007995.2014.952716

Lovibond SH, 1995, MANUAL DEPRESSION AN

Lerma-Cabrera JM, 2016, NUTR J, V15, DOI 10.1186/s12937-016-0124-6

Mercer SW, 2009, FAM PRACT, V26, P79, DOI 10.1093/fampra/cmp020

Meule A, 2012, DIAGNOSTICA, V58, P115, DOI 10.1026/0012-1924/a000047

Mezuk B, 2008, DIABETES CARE, V31, P2383, DOI 10.2337/dc08-0985

Moodie R, 2013, LANCET, V381, P670, DOI 10.1016/S0140-6736(12)62089-3

Moubarac JC, 2014, CURR OBES REP, V3, P256, DOI 10.1007/s13679-014-0092-0

Murphy CM, 2014, APPETITE, V73, P45, DOI 10.1016/j.appet.2013.10.008

Pelchat ML, 2009, J NUTR, V139, P620, DOI 10.3945/jn.108.097816

Pivarunas B, 2015, EAT BEHAV, V19, P9, DOI 10.1016/j.eatbeh.2015.06.007

Quello Susan B, 2005, Sci Pract Perspect, V3, P13

Raymond K-L, 2016, J HEALTH PSYCHOL, P1

Raymond KL, 2016, J DIABETES COMPLICAT, V30, P651, DOI 10.1016/j.jdiacomp.2016.01.020

Schult EM, 2016, APPETITE

Schulte EM, 2015, PLOS ONE

Sinha R, 2008, ANN NY ACAD SCI, V1141, P105, DOI 10.1196/annals.1441.030

Sinha R, 2013, BIOL PSYCHIAT, V73, P827, DOI 10.1016/j.biopsych.2013.01.032

Tann SS, 2007, AM INDIAN ALASKA NAT, V14, P1

Tolliver BK, 2015, DIALOGUES CLIN NEURO, V17, P181

Tovilla-Zarate C, 2011, PLOS ONE, V7, P5

Uijen Annemarie A, 2008, Eur J Gen Pract, V14 Suppl 1, P28, DOI 10.1080/13814780802436093

van Oostrom SH, 2012, BMC PUBLIC HEALTH, V12, DOI 10.1186/1471-2458-12-715

Whiteford HA, GLOBAL BURDEN DIS AT

Whitworth SR, 2016, DIABETES RES CLIN PR, V122, P190, DOI 10.1016/j.diabres.2016.10.023

Wu LT, 2015, DRUG ALCOHOL DEPEN, V156, P162, DOI 10.1016/j.drugalcdep.2015.09.003

NR 49

TC 0

Z9 0

U1 0

U2 1

PU ELSEVIER IRELAND LTD

PI CLARE

PA ELSEVIER HOUSE, BROOKVALE PLAZA, EAST PARK SHANNON, CO, CLARE, 00000,

IRELAND

SN 0168-8227

EI 1872-8227

J9 DIABETES RES CLIN PR

JI Diabetes Res. Clin. Pract.

PD OCT

PY 2017

VL 132

BP 95

EP 101

DI 10.1016/j.diabres.2017.07.028

PG 7

WC Endocrinology & Metabolism

SC Endocrinology & Metabolism

GA FM6WQ

UT WOS:000415202800011

PM 28826026

DA 2019-01-08

ER

PT J

AU Hoogendoorn, CJ

Roy, JF

Gonzalez, JS

AF Hoogendoorn, Claire J.

Roy, Juan F.

Gonzalez, Jeffrey S.

TI Shared Dysregulation of Homeostatic Brain-Body Pathways in Depression

and Type 2 Diabetes

SO CURRENT DIABETES REPORTS

LA English

DT Review

DE Type 2 diabetes; Depression; Biological pathways;

Hypothalamic-pituitary-adrenal axis; Brain-gut-microbiome axis

ID RANDOMIZED CONTROLLED-TRIALS; MIDDLE-AGED MEN; GUT MICROBIOTA; HPA AXIS;

GLUCOSE-METABOLISM; INSULIN-RESISTANCE; MAJOR DEPRESSION; IMMUNE-SYSTEM;

SECRETORY PATTERN; CIRCADIAN-RHYTHM

AB Purpose of Review The purpose of this review is to provide an overview of shared dysregulation of the hypothalamic-pituitary-adrenal (HPA) and brain-gut-microbiome (BGM) axes associated with depression and type 2 diabetes (T2D). Clinical implications and future research are also discussed.

Recent Findings Both depression and T2D are associated with dysregulation of the HPA and BGM axes. These pathways regulate immune function, glucose metabolism, and sleep, which are altered in both illnesses. Dysregulation of homeostatic brain-body pathways may be positively influenced through different therapeutic actions, including psychotherapy, healthy eating, physical activity, sleep promotion, and certain anti-inflammatory or antidepressant medications.

Summary While the causal nature of the relationship between depression and T2D remains unclear, these conditions share dysregulation of homeostatic brain-body pathways that are central to mental and physical health. Better understanding of this dysregulation may provide opportunities for interventions that could benefit both conditions. Future research should examine the additive burden of depression and T2D on HPA and BGM dysregulation and better differentiate depression from emotional distress.

C1 [Hoogendoorn, Claire J.; Roy, Juan F.; Gonzalez, Jeffrey S.] Yeshiva Univ, Ferkauf Grad Sch Psychol, 1165 Morris Pk Ave,Rousso Bldg, Bronx, NY 10461 USA.

[Gonzalez, Jeffrey S.] Albert Einstein Coll Med, Dept Med Endocrinol, Bronx, NY 10467 USA.

RP Hoogendoorn, CJ (reprint author), Yeshiva Univ, Ferkauf Grad Sch Psychol, 1165 Morris Pk Ave,Rousso Bldg, Bronx, NY 10461 USA.

EM Claire.hoogendoorn@yu.edu; juan.roy-delgado@yu.edu;

jeffrey.gonzalez@einstein.yu.edu

FU National Institutes of Health [R18 DK098742, R01 DK104845, P30

DK111022]; Drs. David and Jane Willner Bloomgarden Family Fellowship

Fund

FX Dr. Gonzalez's effort is partialy supported by grants from the National

Institutes of Health: R18 DK098742, R01 DK104845 and P30 DK111022.

Support was also provided by The Drs. David and Jane Willner Bloomgarden

Family Fellowship Fund.

CR Ali S, 2006, DIABETIC MED, V23, P1165, DOI 10.1111/j.1464-5491.2006.01943.x

American Psychiatric Association, 2013, DIAGN STAT MAN MENT

Anderson RJ, 2001, DIABETES CARE, V24, P1069, DOI 10.2337/diacare.24.6.1069

Aronsohn RS, 2010, AM J RESP CRIT CARE, V181, P507, DOI 10.1164/rccm.200909-1423OC

Baganz NL, 2013, ACS CHEM NEUROSCI, V4, P48, DOI 10.1021/cn300186b

Bailey MT, 2010, INFECT IMMUN, V78, P1509, DOI 10.1128/IAI.00862-09

Beishuizen A, 2003, J ENDOTOXIN RES, V9, P3, DOI 10.1179/096805103125001298

Bhaskar Swapna, 2016, J Family Med Prim Care, V5, P780, DOI 10.4103/2249-4863.201153

Blalock JE, 2005, J INTERN MED, V257, P126, DOI 10.1111/j.1365-2796.2004.01441.x

Blumenthal JA, 2007, PSYCHOSOM MED, V69, P587, DOI 10.1097/PSY.0b013e318148c19a

Boulange CL, 2016, GENOME MED, V8, DOI 10.1186/s13073-016-0303-2

Boyle JP, 2010, POPUL HEALTH METR, V8, DOI 10.1186/1478-7954-8-29

Campayo A, 2010, AM J PSYCHIAT, V167, P580, DOI 10.1176/appi.ajp.2009.09010038

Chakravarthy MV, 2004, J APPL PHYSIOL, V96, P3, DOI 10.1152/japplphysiol.00757.2003

Chan O, 2003, Minerva Endocrinol, V28, P87

Charmandari E, 2005, ANNU REV PHYSIOL, V67, P259, DOI 10.1146/annurev.physiol.67.040403.120816

Cohen S, 2012, P NATL ACAD SCI USA, V109, P5995, DOI 10.1073/pnas.1118355109

Cryan JF, 2012, NAT REV NEUROSCI, V13, P701, DOI 10.1038/nrn3346

DALLMAN MF, 1993, FRONT NEUROENDOCRIN, V14, P303, DOI 10.1006/frne.1993.1010

Dantzer R, 2008, NAT REV NEUROSCI, V9, P46, DOI 10.1038/nrn2297

de Groot M, 2012, CURR DIABETES REP, V12, P157, DOI 10.1007/s11892-012-0261-z

de Jonge P, 2006, DIABETOLOGIA, V49, P2627, DOI 10.1007/s00125-006-0442-x

de Weerth C, 2003, EARLY HUM DEV, V73, P39, DOI 10.1016/S0378-3782(03)00074-4

Desan PH, 2000, AM J MED GENET, V96, P418, DOI 10.1002/1096-8628(20000612)96:3<418::AID-AJMG34>3.0.CO;2-S

Deuschle M, 2013, CURR OPIN PSYCHIATR, V26, P60, DOI 10.1097/YCO.0b013e32835a4206

Dhabhar FS, 2009, NEUROIMMUNOMODULAT, V16, P300, DOI 10.1159/000216188

Dinan TG, 2013, NEUROGASTROENT MOTIL, V25, P713, DOI 10.1111/nmo.12198

Dinan TG, 1996, LIFE SCI, V58, P1683, DOI 10.1016/0024-3205(96)00066-5

Donath MY, 2011, NAT REV IMMUNOL, V11, P98, DOI 10.1038/nri2925

Dowlati Y, 2010, BIOL PSYCHIAT, V67, P446, DOI 10.1016/j.biopsych.2009.09.033

Dupuis J, 2010, NAT GENET, V42, P105, DOI 10.1038/ng.520

Eskandari F, 2002, ANN NY ACAD SCI, V966, P20, DOI 10.1111/j.1749-6632.2002.tb04198.x

Ferrari E, 2001, BRAIN RES REV, V37, P294, DOI 10.1016/S0165-0173(01)00133-3

Fisher L, 2008, DIABETIC MED, V25, P1096, DOI 10.1111/j.1464-5491.2008.02533.x

Forslund K, 2015, NATURE, V528, P262, DOI 10.1038/nature15766

Franchimont DP, 2008, NEUROIMMUNE, V7, P349

Germain A, 2008, HUM PSYCHOPHARM CLIN, V23, P571, DOI 10.1002/hup.964

Gonzalez JS, 2007, DIABETES CARE, V30, P2222, DOI 10.2337/dc07-0158

Gonzalez JS, 2008, DIABETES CARE, V31, P2398, DOI 10.2337/dc08-1341

Grenham S, 2011, FRONT PHYSIOL, V2, DOI 10.3389/fphys.2011.00094

Guseva D, 2014, INFLAMM BOWEL DIS, V20, P1516, DOI 10.1097/MIB.0000000000000150

Hawrelak Jason A, 2004, Altern Med Rev, V9, P180

Hiller-Sturmhofel S, 1998, ALCOHOL HEALTH RES W, V22, P153

Himmerich H, 2006, BIOL PSYCHIAT, V60, P882, DOI 10.1016/j.biopsych.2006.03.075

HOLDEMAN LV, 1976, APPL ENVIRON MICROB, V31, P359

Hotamisligil GS, 2006, NATURE, V444, P860, DOI 10.1038/nature05485

Ilan Y, 2010, P NATL ACAD SCI USA, V107, P9765, DOI 10.1073/pnas.0908771107

Ismail K, 2004, LANCET, V363, P1589, DOI 10.1016/S0140-6736(04)16202-8

Kahn SE, 2014, LANCET, V383, P1068, DOI 10.1016/S0140-6736(13)62154-6

Kan C, 2013, DIABETES CARE, V36, P480, DOI 10.2337/dc12-1442

Kasinska MA, 2015, POL ARCH MED WEWN, V125, P803, DOI 10.20452/pamw.3156

Kaye J, 2004, J NEUROENDOCRINOL, V16, P256, DOI 10.1111/j.0953-8194.2004.01158.x

Kivimaki M, 2010, DIABETES CARE, V33, P2611, DOI 10.2337/dc10.1187

Knowles SR, 2008, BIOL PSYCHOL, V77, P132, DOI 10.1016/j.biopsycho.2007.09.010

Knutson KL, 2007, SLEEP MED REV, V11, P163, DOI 10.1016/j.smrv.2007.01.002

Kudielka BM, 2004, PSYCHONEUROENDOCRINO, V29, P983, DOI 10.1016/j.psyneuen.2003.08.009

Laake JPS, 2014, DIABETES CARE, V37, P2186, DOI 10.2337/dc13-2522

Lambillotte C, 1997, J CLIN INVEST, V99, P414, DOI 10.1172/JCI119175

Ley RE, 2010, CURR OPIN GASTROEN, V26, P5, DOI 10.1097/MOG.0b013e328333d751

Lustman PJ, 2000, DIABETES CARE, V23, P934, DOI 10.2337/diacare.23.7.934

Macia L, 2012, IMMUNOL REV, V245, P164, DOI 10.1111/j.1600-065X.2011.01080.x

Magouliotis DE, 2017, OBES SURG, P1

Manber R, 2011, J CLIN SLEEP MED, V7, P645, DOI 10.5664/jcsm.1472

McEwen BS, 2000, NEUROPSYCHOPHARMACOL, V22, P108, DOI 10.1016/S0893-133X(99)00129-3

McEwen BS, 1998, ANN NY ACAD SCI, V840, P33, DOI 10.1111/j.1749-6632.1998.tb09546.x

Miller DB, 2002, METABOLISM, V51, P5, DOI 10.1053/meta.2002.33184

Miller GE, 2007, PSYCHOL BULL, V133, P25, DOI 10.1037/0033-2909.133.1.25

Moreno-Indias I, 2015, EUR RESPIR J, V45, P1055, DOI 10.1183/09031936.00184314

Moulton CD, 2015, LANCET DIABETES ENDO, V3, P461, DOI 10.1016/S2213-8587(15)00134-5

Murri MB, 2014, PSYCHONEUROENDOCRINO, V41, P46, DOI 10.1016/j.psyneuen.2013.12.004

Naseribafrouei A, 2014, NEUROGASTROENT MOTIL, V26, P1155, DOI 10.1111/nmo.12378

National Institutes of Mental Health, MENTAL HLTH

Naughton M, 2000, HUM PSYCHOPHARM CLIN, V15, P397, DOI 10.1002/1099-1077(200008)15:6<397::AID-HUP212>3.3.CO;2-C

Nemeroff CB, 2004, CNS SPECTRUMS, V9, P23, DOI 10.1017/S1092852900025475

Nouwen A, 2011, DIABETES CARE, V34, P752, DOI 10.2337/dc10-1414

O'Mahony SM, 2015, BEHAV BRAIN RES, V277, P32, DOI 10.1016/j.bbr.2014.07.027

Pariante CM, 2001, BIOL PSYCHIAT, V49, P391, DOI 10.1016/S0006-3223(00)01088-X

Pirbaglou M, 2016, NUTR RES, V36, P889, DOI 10.1016/j.nutres.2016.06.009

Qin JJ, 2012, NATURE, V490, P55, DOI 10.1038/nature11450

Raison CL, 2006, TRENDS IMMUNOL, V27, P24, DOI 10.1016/j.it.2005.11.006

Reutrakul S, 2014, ANN NY ACAD SCI, V1311, P151, DOI 10.1111/nyas.12355

Rhee SH, 2009, NAT REV GASTRO HEPAT, V6, P306, DOI 10.1038/nrgastro.2009.35

Rosmond R, 2000, OBES RES, V8, P445, DOI 10.1038/oby.2000.55

Rosmond R, 2003, MED SCI MONITOR, V9, P35

Roy Monique, 1991, Journal of Diabetic Complications, V5, P218, DOI 10.1016/0891-6632(91)90079-5

Rubin RR, 2008, DIABETES CARE, V31, P420, DOI 10.2337/dc07-182T

Sapolsky RM, 2000, ENDOCR REV, V21, P55, DOI 10.1210/er.21.1.55

Serretti A, 2010, J CLIN PSYCHIAT, V71, P1259, DOI 10.4088/JCP.09r05346blu

Snoek FJ, 2015, LANCET DIABETES ENDO, V3, P450, DOI 10.1016/S2213-8587(15)00135-7

Steptoe A, 2003, PSYCHOL MED, V33, P667, DOI 10.1017/S0033291702007250

Stetler C, 2011, PSYCHOSOM MED, V73, P114, DOI 10.1097/PSY.0b013e31820ad12b

Swain MG, 2000, CLIN SCI, V99, P1, DOI 10.1042/CS19990372

Trinder M, 2015, UNIV TOR MED J, V92, P52

Tsatsoulis A, 2006, ANN NY ACAD SCI, V1083, P196, DOI 10.1196/annals.1367.020

Turnbaugh PJ, 2009, NATURE, V457, P480, DOI 10.1038/nature07540

Vedder H, 2008, NEUROIMMUNE, V7, P17

Vrieze A, 2012, GASTROENTEROLOGY, V143, P913, DOI 10.1053/j.gastro.2012.06.031

Walker BR, 2007, EUR J ENDOCRINOL, V157, P545, DOI 10.1530/EJE-07-0455

Yano JM, 2015, CELL, V161, P264, DOI 10.1016/j.cell.2015.02.047

Zhang JP, 2006, PSYCHOSOM MED, V68, P718, DOI 10.1097/01.psy.0000228343.89466.11

NR 100

TC 2

Z9 2

U1 2

U2 12

PU CURRENT MEDICINE GROUP

PI PHILADELPHIA

PA 400 MARKET STREET, STE 700, PHILADELPHIA, PA 19106 USA

SN 1534-4827

EI 1539-0829

J9 CURR DIABETES REP

JI Curr. Diabetes Rep.

PD OCT

PY 2017

VL 17

IS 10

AR 90

DI 10.1007/s11892-017-0923-y

PG 10

WC Endocrinology & Metabolism

SC Endocrinology & Metabolism

GA FJ9BY

UT WOS:000413063800009

PM 28815394

DA 2019-01-08

ER

PT J

AU Kuwata, H

Okamura, S

Hayashino, Y

Tsujii, S

Ishii, H

AF Kuwata, Hirohito

Okamura, Shintaro

Hayashino, Yasuaki

Tsujii, Satoru

Ishii, Hitoshi

CA Tenri Study Grp

TI Serum uric acid levels are associated with increased risk of newly

developed diabetic retinopathy among Japanese male patients with type 2

diabetes: A prospective cohort study (diabetes distress and care

registry at Tenri [DDCRT 13])

SO DIABETES-METABOLISM RESEARCH AND REVIEWS

LA English

DT Article

DE cohort; diabetic retinopathy; epidemiology; human; type 2 diabetes; uric

acid

ID KIDNEY-DISEASE PROGRESSION; CARDIOVASCULAR MORTALITY; ATHEROSCLEROSIS;

COMPLICATIONS; HYPERURICEMIA; ALBUMINURIA; PREVALENCE; ADULTS

AB Objective: We assessed the prospective association between baseline serum uric acid levels and consequent risk of developing diabetic retinopathy.

Research Design and Methods: Data for 1839 type 2 diabetes patients without diabetic retinopathy were obtained from a Japanese diabetes registry. A Cox proportional hazards model with time-varying exposure information by sex was used and adjusted for potential confounders to assess the independent correlations between baseline serum uric acid levels and incidence rate of diabetic retinopathy.

Results: Newly developed diabetic retinopathy was recognized in 188 patients (10.2%) during the observation period of 2years. Compared to the first serum uric acid quartile level, the multivariate adjusted hazards ratio for diabetic retinopathy development in male patients was 1.97 (95% CI, 1.14-3.41; P=.015), 1.92 (95% CI, 1.18-3.13; P=.008), and 2.17 (95% CI, 1.40-3.37; P=.001) for the second, third, and fourth serum uric acid quartile levels, respectively. But this was not the case with female patients.

Conclusion: Higher serum uric acid levels were associated with increased risk of developing diabetic retinopathy in male patients with type 2 diabetes, but not in female patients. Serum uric acid may be a useful biomarker for predicting the future risk of developing diabetic retinopathy in male patients with type 2 diabetes.

C1 [Kuwata, Hirohito; Ishii, Hitoshi] Nara Med Univ, Dept Diabetol, Kashihara, Nara, Japan.

[Okamura, Shintaro; Hayashino, Yasuaki; Tsujii, Satoru] Tenri Hosp, Dept Endocrinol, 200 Mishima Cho, Tenri, Nara 6328552, Japan.

RP Tsujii, S (reprint author), Tenri Hosp, Dept Endocrinol, 200 Mishima Cho, Tenri, Nara 6328552, Japan.

EM stsujii@tenriyorozu.jp

OI Tsujii, Satoru/0000-0002-1834-6515

FU Manpei Suzuki Diabetes Foundation; JSPS KAKENHI [25460641]

FX Manpei Suzuki Diabetes Foundation and JSPS KAKENHI, Grant/Award Number:

25460641

CR AKIZUKI S, 1982, ANN RHEUM DIS, V41, P272, DOI 10.1136/ard.41.3.272

Bjornstad P, 2014, ACTA DIABETOL, V51, P783, DOI 10.1007/s00592-014-0611-1

Buch H, 2004, OPHTHALMOLOGY, V111, P53, DOI 10.1016/j.ophtha.2003.05.010

Cai XL, 2006, CHINESE MED J-PEKING, V119, P822

De Cosmo S, 2015, CLIN J AM SOC NEPHRO, V10, P1921, DOI 10.2215/CJN.03140315

Fang J, 2000, JAMA-J AM MED ASSOC, V283, P2404, DOI 10.1001/jama.283.18.2404

Feig DI, 2008, NEW ENGL J MED, V359, P1811, DOI 10.1056/NEJMra0800885

Fenwick E, 2012, CLIN EXP OPHTHALMOL, V40, P27, DOI 10.1111/j.1442-9071.2011.02599.x

Filiopoulos V, 2012, RENAL FAILURE, V34, P510, DOI 10.3109/0886022X.2011.653753

Fong DS, 1999, AM J OPHTHALMOL, V127, P137, DOI 10.1016/S0002-9394(98)00309-2

FREEDMAN DS, 1995, AM J EPIDEMIOL, V141, P637, DOI 10.1093/oxfordjournals.aje.a117479

Fu CC, 2009, ACTA DIABETOL, V46, P127, DOI 10.1007/s00592-008-0069-0

FUKUDA M, 1983, Tohoku Journal of Experimental Medicine, V141, P331

Fukui M, 2008, METABOLISM, V57, P625, DOI 10.1016/j.metabol.2007.12.005

Goicoechea M, 2010, CLIN J AM SOC NEPHRO, V5, P1388, DOI 10.2215/CJN.01580210

Hakoda M, 2005, J RHEUMATOL, V32, P906

Hayashino Y, 2016, ACTA DIABETOL, V53, P599, DOI 10.1007/s00592-015-0825-x

Hoieggen A, 2004, KIDNEY INT, V65, P1041, DOI 10.1111/j.1523-1755.2004.00484.x

KABASAKALIAN P, 1973, Clinical Chemistry, V19, P522

Kawasaki R, 2011, DIABETOLOGIA, V54, P2288, DOI 10.1007/s00125-011-2199-0

KLEIN R, 1984, ARCH OPHTHALMOL-CHIC, V102, P520, DOI 10.1001/archopht.1984.01040030398010

Krizova L, 2011, OPHTHALMIC RES, V46, P73, DOI 10.1159/000322994

Kushiyama A, 2012, ARTERIOSCL THROM VAS, V32, P291, DOI 10.1161/ATVBAHA.111.234559

Kuwata H, 2016, DIABETOL INT, V7, P352, DOI 10.1007/s13340-016-0254-2

Lee JJ, 2014, DIABETES RES CLIN PR, V106, P366, DOI 10.1016/j.diabres.2014.07.027

LEVINE W, 1989, J CLIN EPIDEMIOL, V42, P257, DOI 10.1016/0895-4356(89)90061-9

Maahs DM, 2013, CURR DIABETES REP, V13, P550, DOI 10.1007/s11892-013-0381-0

Matsuo S, 2009, AM J KIDNEY DIS, V53, P982, DOI 10.1053/j.ajkd.2008.12.034

Mercuro G, 2004, AM J CARDIOL, V94, P932, DOI 10.1016/j.amjcard.2004.06.032

OLUKOGA AO, 1991, DIABETES RES CLIN PR, V14, P99, DOI 10.1016/0168-8227(91)90115-T

REUNANEN A, 1982, ACTA MED SCAND, P49

ROUBENOFF R, 1990, RHEUM DIS CLIN N AM, V16, P539

Sakata K, 2001, EUR J EPIDEMIOL, V17, P461, DOI 10.1023/A:1013735717961

Seino Y, 2010, J DIABETES INVEST, V1, P1, DOI 10.1111/j.2040-1124.2010.00005.x

Tomita M, 2000, J Epidemiol, V10, P403

Tsujii S, 2012, DIABETIC MED, V29, P1451, DOI 10.1111/j.1464-5491.2012.03647.x

YANKO L, 1983, BRIT J OPHTHALMOL, V67, P759, DOI 10.1136/bjo.67.11.759

NR 37

TC 2

Z9 2

U1 1

U2 2

PU WILEY

PI HOBOKEN

PA 111 RIVER ST, HOBOKEN 07030-5774, NJ USA

SN 1520-7560

J9 DIABETES-METAB RES

JI Diabetes-Metab. Res. Rev.

PD OCT

PY 2017

VL 33

IS 7

AR e2905

DI 10.1002/dmrr.2905

PG 9

WC Endocrinology & Metabolism

SC Endocrinology & Metabolism

GA FI7GU

UT WOS:000412165800002

PM 28444955

DA 2019-01-08

ER

PT J

AU van Beers, CAJ

de Wit, M

Kleijer, SJ

Geelhoed-Duijvestijn, PH

DeVries, JH

Kramer, MHH

Diamant, M

Serne, EH

Snoek, FJ

AF van Beers, Cornelis A. J.

de Wit, Maartje

Kleijer, Susanne J.

Geelhoed-Duijvestijn, Petronella H.

DeVries, J. Hans

Kramer, Mark H. H.

Diamant, Michaela

Serne, Erik H.

Snoek, Frank J.

TI Continuous Glucose Monitoring in Patients with Type 1 Diabetes and

Impaired Awareness of Hypoglycemia: Also Effective in Patients with

Psychological Distress?

SO DIABETES TECHNOLOGY & THERAPEUTICS

LA English

DT Article

DE Type 1 diabetes; Continuous glucose monitoring; Impaired awareness of

hypoglycemia; Psychological distress

ID QUALITY-OF-LIFE; RANDOMIZED CLINICAL-TRIAL; PUMP THERAPY; ADULTS; TIME;

FEAR; INTERVENTION; PREVALENCE; MELLITUS; OUTCOMES

AB The aim of this study was to evaluate whether psychological distress modifies the effect of continuous glucose monitoring (CGM) in patients with type 1 diabetes (T1D) and impaired awareness of hypoglycemia. Fifty-two patients with T1D and impaired awareness of hypoglycemia participated in an earlier reported randomized crossover trial with two 16-week intervention periods comparing CGM with self-monitoring of blood glucose (SMBG). During the CGM phase, time spent in euglycemia (4-10mmol/L), the primary outcome, was 9.6% higher compared with the SMBG phase (P<0.0001). Psychological distress was operationalized as low emotional well-being (World Health Organization Well-being Index 5 [WHO-5] < 50), high diabetes-related distress (Problem Areas in Diabetes 5 [PAID-5] 8), and/or high fear of hypoglycemia (Hypoglycemia Fear Survey [HFS] Worry > mean HFS Worry score +1 standard deviation). Modifying effects were assessed by analyzing psychological distress scorexinterventioninteraction effects. Results showed that both the low emotional well-being group and normal emotional well-being group had equal glycemic outcomes during the CGM phase. High diabetes distress and elevated fear of hypoglycemia did not result in significant interaction effects for glycemic outcomes. This study demonstrated that CGM is equally effective in terms of glycemic improvements in high versus low distressed patients with T1D and impaired awareness of hypoglycemia.

C1 [van Beers, Cornelis A. J.; Kleijer, Susanne J.; Kramer, Mark H. H.; Diamant, Michaela; Serne, Erik H.] Vrije Univ Amsterdam, Med Ctr, Dept Internal Med, Amsterdam, Netherlands.

[de Wit, Maartje; Snoek, Frank J.] Vrije Univ Amsterdam, Med Ctr, Dept Med Psychol, Amsterdam, Netherlands.

[Geelhoed-Duijvestijn, Petronella H.] Med Ctr Haaglanden, Dept Internal Med, The Hague, Netherlands.

[DeVries, J. Hans] Univ Amsterdam, Acad Med Ctr, Dept Endocrinol, Amsterdam, Netherlands.

[Snoek, Frank J.] Univ Amsterdam, Acad Med Ctr, Dept Med Psychol, Amsterdam, Netherlands.

RP van Beers, CAJ (reprint author), Vrije Univ Amsterdam, Med Ctr, Diabet Ctr, Dept Internal Med, Room ZH-4A63,Boelelaan 1117, NL-1081 HV Amsterdam, Netherlands.

EM c.vanbeers@vumc.nl

FU Eli Lilly; Sanofi; AstraZeneca; Boehringer Ingelheim; Novo Nordisk;

Abbott; Dexcom; Medtronic; Roche Diabetes Care; Senseonics

FX This research received funding from Eli Lilly and Sanofi. Medtronic

provided continuous glucose monitoring devices. Through M.H.H.K. and

M.D., the VU University Medical Center received research grants from

AstraZeneca, Boehringer Ingelheim, Novo Nordisk, and Sanofi. J.H.D. or

his institution received research support and speaker's fees from

Abbott, Dexcom, Medtronic, Roche Diabetes Care, and Senseonics.

P.H.G.-D. received speaker's fees from Abbott and Medtronic and is a

member of the advisory board of Medtronic.

CR Barendse S, 2012, DIABETIC MED, V29, P293, DOI 10.1111/j.1464-5491.2011.03416.x

Beck RW, 2010, DIABETES CARE, V33, P2175, DOI 10.2337/dc10-0331

Choudhary P, 2013, DIABETES CARE, V36, P4160, DOI 10.2337/dc13-0939

COX DJ, 1987, DIABETES CARE, V10, P617, DOI 10.2337/diacare.10.5.617

Fisher L, 2016, J DIABETES COMPLICAT, V30, P1123, DOI 10.1016/j.jdiacomp.2016.03.032

Geddes J, 2008, DIABETIC MED, V25, P501, DOI 10.1111/j.1464-5491.2008.02413.x

GOLD AE, 1994, DIABETES CARE, V17, P697, DOI 10.2337/diacare.17.7.697

Hajos TRS, 2013, DIABETIC MED, V30, pE63, DOI 10.1111/dme.12040

Hajos TRS, 2014, DIABETES CARE, V37, P102, DOI 10.2337/dc13-0971

Hermanides J, 2011, DIABETIC MED, V28, P1158, DOI 10.1111/j.1464-5491.2011.03256.x

Lustman PJ, 2000, DIABETES CARE, V23, P934, DOI 10.2337/diacare.23.7.934

Ly TT, 2013, JAMA-J AM MED ASSOC, V310, P1240, DOI 10.1001/jama.2013.277818

McGuire BE, 2010, DIABETOLOGIA, V53, P66, DOI 10.1007/s00125-009-1559-5

Nicolucci A, 2013, DIABETIC MED, V30, P767, DOI 10.1111/dme.12245

Peters AL, 2016, J CLIN ENDOCR METAB, V101, P3922, DOI 10.1210/jc.2016-2534

Pickup JC, 2015, DIABETES CARE, V38, P544, DOI 10.2337/dc14-1855

Pickup JC, 2011, BRIT MED J, V343, DOI 10.1136/bmj.d3805

Polonsky WH, 2017, DIABETES CARE, V40, P736, DOI 10.2337/dc17-0133

Ritholz MD, 2010, DIABETIC MED, V27, P1060, DOI 10.1111/j.1464-5491.2010.03061.x

Rondags SMPA, 2016, DIABETES CARE, V39, P2190, DOI 10.2337/dc16-1614

Rubin RR, 2012, DIABETES TECHNOL THE, V14, P143, DOI 10.1089/dia.2011.0162

Rubin RR, 2001, J CLIN PSYCHOL, V57, P457, DOI 10.1002/jclp.1041

Snoek FJ, 2011, DIABETES CARE, V34, P601, DOI 10.2337/dc10-1552

Tanenbaum ML, 2017, DIABETES CARE, V40, P181, DOI 10.2337/dc16-1536

van Beers CAJ, 2016, LANCET DIABETES ENDO, V4, P893, DOI 10.1016/S2213-8587(16)30193-0

van Beers CAJ, 2015, BMC ENDOCR DISORD, V15, DOI 10.1186/s12902-015-0040-3

Wild D, 2007, PATIENT EDUC COUNS, V68, P10, DOI 10.1016/j.pec.2007.05.003

NR 27

TC 0

Z9 0

U1 0

U2 0

PU MARY ANN LIEBERT, INC

PI NEW ROCHELLE

PA 140 HUGUENOT STREET, 3RD FL, NEW ROCHELLE, NY 10801 USA

SN 1520-9156

EI 1557-8593

J9 DIABETES TECHNOL THE

JI Diabetes Technol. Ther.

PD OCT

PY 2017

VL 19

IS 10

BP 595

EP 599

DI 10.1089/dia.2017.0141

PG 5

WC Endocrinology & Metabolism

SC Endocrinology & Metabolism

GA FK1XT

UT WOS:000413277600009

PM 28836833

DA 2019-01-08

ER

PT J

AU Sominsky, L

Hodgson, DM

McLaughlin, EA

Smith, R

Wall, HM

Spencer, SJ

AF Sominsky, Luba

Hodgson, Deborah M.

McLaughlin, Eileen A.

Smith, Roger

Wall, Hannah M.

Spencer, Sarah J.

TI Linking Stress and Infertility: A Novel Role for Ghrelin

SO ENDOCRINE REVIEWS

LA English

DT Review

ID POLYCYSTIC-OVARY-SYNDROME; GONADOTROPIN-RELEASING-HORMONE; IN-VITRO

FERTILIZATION; PITUITARY-ADRENAL AXIS; GESTATIONAL DIABETES-MELLITUS;

DES-ACYL GHRELIN; MAJOR DEPRESSIVE DISORDER; MESSENGER-RNA EXPRESSION;

FEMALE RHESUS-MONKEYS; QUALITY-OF-LIFE

AB Infertility affects a remarkable one in four couples in developing countries. Psychological stress is a ubiquitous facet of life, and although stress affects us all at some point, prolonged or unmanageable stress may become harmful for some individuals, negatively impacting on their health, including fertility. For instance, women who struggle to conceive are twice as likely to suffer from emotional distress than fertile women. Assisted reproductive technology treatments place an additional physical, emotional, and financial burden of stress, particularly on women, who are often exposed to invasive techniques associated with treatment. Stress-reduction interventions can reduce negative affect and in some cases to improve in vitro fertilization outcomes. Although it has been well-established that stress negatively affects fertility in animal models, human research remains inconsistent due to individual differences and methodological flaws. Attempts to isolate single causal links between stress and infertility have not yet been successful due to their multifaceted etiologies. In this review, we will discuss the current literature in the field of stress-induced reproductive dysfunction based on animal and human models, and introduce a recently unexplored link between stress and infertility, the gut-derived hormone, ghrelin. We also present evidence from recent seminal studies demonstrating that ghrelin has a principal role in the stress response and reward processing, as well as in regulating reproductive function, and that these roles are tightly interlinked. Collectively, these data support the hypothesis that stress may negatively impact upon fertility at least in part by stimulating a dysregulation in ghrelin signaling.

C1 [Sominsky, Luba; Wall, Hannah M.; Spencer, Sarah J.] RMIT Univ, Sch Hlth & Biomed Sci, Melbourne, Vic 3083, Australia.

[Hodgson, Deborah M.] Univ Newcastle, Sch Psychol, Fac Sci & IT, Callaghan, NSW 2308, Australia.

[McLaughlin, Eileen A.] Univ Auckland, Sch Biol Sci, Fac Sci, Auckland 1010, New Zealand.

[McLaughlin, Eileen A.] Univ Newcastle, Sch Environm & Life Sci, Fac Sci & IT, Callaghan, NSW 2308, Australia.

[Smith, Roger] Hunter Med Res Inst, Mothers & Babies Res Ctr, Lookout Rd, New Lambton Hts, NSW 2305, Australia.

[Smith, Roger] Univ Newcastle, Prior Res Ctr Reprod Sci, Callaghan, NSW 2308, Australia.

RP Sominsky, L (reprint author), RMIT Univ, Sch Hlth & Biomed Sci, Melbourne, Vic 3083, Australia.

EM luba.sominsky@rmit.edu.au

RI Spencer, Sarah/D-5777-2018

OI McLaughlin, Eileen/0000-0002-5837-3876; Sominsky,

Luba/0000-0002-0674-8824

FU Australian Research Council (ARC) [DP130100508]

FX This work is supported by a Discovery Project Grant from the Australian

Research Council (ARC) to S.J.S. (DP130100508). S.J.S. is an ARC Future

Fellow (FT110100084) and an RMIT University VC Senior Research Fellow.

CR Abizaid A, 2011, NEUROSCIENCE, V192, P500, DOI 10.1016/j.neuroscience.2011.06.001

Abizaid A, 2006, J CLIN INVEST, V116, P3229, DOI 10.1172/JCI29867

Abu El-Magd M, 2016, GEN COMP ENDOCR, V237, P131, DOI 10.1016/j.ygcen.2016.08.016

Abu-Musa AA, 2007, FERTIL STERIL, V88, P1579, DOI 10.1016/j.fertnstert.2007.01.067

Achache H, 2006, HUM REPROD UPDATE, V12, P731, DOI 10.1093/humupd/dml004

Acromite Michael, 2004, Hormones (Athens), V3, P46

Aghajanova L, 2010, REPROD SCI, V17, P823, DOI 10.1177/1933719110371683

AGUADO LI, 1984, ENDOCRINOLOGY, V114, P1845, DOI 10.1210/endo-114-5-1845

AGUADO LI, 1982, ENDOCRINOLOGY, V110, P1124, DOI 10.1210/endo-110-4-1124

Aiken C. E., 2015, NATURE SCI REPORTS, V5, P16175, DOI DOI 10.1038/SREP16175)

Akema T, 2005, J NEUROENDOCRINOL, V17, P672, DOI 10.1111/j.1365-2826.2005.01358.x

Allas S, 2016, DIABETES OBES METAB, V18, P868, DOI 10.1111/dom.12675

ALTEMUS M, 1995, J CLIN ENDOCR METAB, V80, P2954, DOI 10.1210/jc.80.10.2954

Alvarez-Crespo M, 2012, PLOS ONE, V7, DOI 10.1371/journal.pone.0046321

Ananth CV, 2013, BMJ-BRIT MED J, V347, DOI 10.1136/bmj.f6564

Ananth CV, 2010, EPIDEMIOLOGY, V21, P118, DOI 10.1097/EDE.0b013e3181c297af

Anderheim L, 2005, HUM REPROD, V20, P2969, DOI 10.1093/humrep/dei219

Anderson K, 2010, AUST NZ J OBSTET GYN, V50, P8, DOI 10.1111/j.1479-828X.2009.01119.x

Andrews ZB, 2009, J NEUROSCI, V29, P14057, DOI 10.1523/JNEUROSCI.3890-09.2009

Angelidis G, 2012, REPROD SCI, V19, P903, DOI 10.1177/1933719112443880

Arck PC, 2008, REPROD BIOMED ONLINE, V17, P101, DOI 10.1016/S1472-6483(10)60300-8

Arusoglu G, 2013, J CLIN ENDOCR METAB, V98, P4475, DOI 10.1210/jc.2013-1526

Arvat E, 2001, J CLIN ENDOCR METAB, V86, P1169, DOI 10.1210/jc.86.3.1169

Asakawa A, 2001, NEUROENDOCRINOLOGY, V74, P143, DOI 10.1159/000054680

Aydin S, 2008, J PHYSIOL BIOCHEM, V64, P51, DOI 10.1007/BF03168234

Aydin S, 2006, PEPTIDES, V27, P878, DOI 10.1016/j/peptides.2005.08.006

Bale TL, 2010, BIOL PSYCHIAT, V68, P314, DOI 10.1016/j.biopsych.2010.05.028

Banks WA, 2002, J PHARMACOL EXP THER, V302, P822, DOI 10.1124/jpet.102.034827

BARBARINO A, 1989, METABOLISM, V38, P504, DOI 10.1016/0026-0495(89)90208-4

Barbazanges A, 1996, J NEUROSCI, V16, P3943

Barber TM, 2008, EUR J ENDOCRINOL, V158, P511, DOI 10.1530/EJE-07-0683

Barim AO, 2009, CLIN BIOCHEM, V42, P1076, DOI 10.1016/j.clinbiochem.2009.02.020

Barreiro ML, 2004, ENDOCRINOLOGY, V145, P4825, DOI 10.1210/en.2004-0732

Barreiro ML, 2003, BIOL REPROD, V68, P1631, DOI 10.1095/biolreprod.102.008862

Barreiro ML, 2002, BIOL REPROD, V67, P1768, DOI 10.1095/biolreprod.102.006965

Bayliss JA, 2016, J NEUROCHEM, V137, P460, DOI 10.1111/jnc.13576

BENJONATHAN N, 1985, ENDOCR REV, V6, P564, DOI 10.1210/edrv-6-4-564

Bernuci MP, 2008, ENDOCRINOLOGY, V149, P2907, DOI 10.1210/en.2007-1254

Bertoldi ML, 2011, HORM METAB RES, V43, P494, DOI 10.1055/s-0031-1277228

Berton O, 2006, SCIENCE, V311, P864, DOI 10.1126/science.1120972

Bhongade MB, 2015, ANDROLOGIA, V47, P336, DOI 10.1111/and.12268

Bideci A, 2008, J OBSTET GYNAECOL RE, V34, P578, DOI 10.1111/j.1447-0756.2008.00819.x

Bigelow PL, 1998, FERTIL STERIL, V69, P11, DOI 10.1016/S0015-0282(97)00437-8

Bilger M, 2001, ENDOCRINOLOGY, V142, P2102, DOI 10.1210/en.142.5.2102

BLACKBURN JR, 1992, PROG NEUROBIOL, V39, P247, DOI 10.1016/0301-0082(92)90018-A

Bock J, 2015, BIOL PSYCHIAT, V78, P315, DOI 10.1016/j.biopsych.2015.02.036

Bodnar LM, 2005, ANN EPIDEMIOL, V15, P475, DOI 10.1016/j.annepidem.2004.12.008

BOIVIN J, 1995, FERTIL STERIL, V64, P802, DOI 10.1016/S0015-0282(16)57858-3

Boivin J, 2005, FERTIL STERIL, V83, P1745, DOI 10.1016/j.fertnstert.2004.12.039

Boivin J, 2011, BRIT MED J, V342, DOI 10.1136/bmj.d223

Boivin J, 2003, SOC SCI MED, V57, P2325, DOI 10.1016/S0277-9536(03)00138-2

Bowman RE, 2002, NEUROSCIENCE, V113, P401, DOI 10.1016/S0306-4522(02)00156-2

Breen KM, 2007, ENDOCRINOLOGY, V148, P1882, DOI 10.1210/en.2006-0973

Breen KM, 2012, MOL ENDOCRINOL, V26, P1716, DOI 10.1210/me.2011-1327

Breen KM, 2004, ENDOCRINOLOGY, V145, P2739, DOI 10.1210/en.2004-0123

Breen KM, 2004, ENDOCRINOLOGY, V145, P692, DOI 10.1210/en.2003-1114

Briggs DI, 2014, ENDOCRINOLOGY, V155, P2411, DOI 10.1210/en.2013-1861

Briggs DI, 2013, ENDOCRINOLOGY, V154, P709, DOI 10.1210/en.2012-1421

Briggs DI, 2010, ENDOCRINOLOGY, V151, P4745, DOI 10.1210/en.2010-0556

Broglio F, 2004, J CLIN ENDOCR METAB, V89, P3062, DOI 10.1210/jc.2003-031964

Brugh VM, 2004, MED CLIN N AM, V88, P367, DOI 10.1016/S0025-7125(03)00150-0

Brugo-Olmedo Santiago, 2001, Reprod Biomed Online, V2, P41

Brunner RL, 2005, ARCH INTERN MED, V165, P1976, DOI 10.1001/archinte.165.17.1976

Buller K, 2001, NEUROENDOCRINOLOGY, V73, P129, DOI 10.1159/000054629

Buller KM, 2003, STRESS, V6, P11, DOI 10.1080/1025389031000092313

Burkett JP, 2012, PSYCHOPHARMACOLOGY, V224, P1, DOI 10.1007/s00213-012-2794-x

Cabral A, 2014, J NEUROENDOCRINOL, V26, P542, DOI 10.1111/jne.12168

Cabral A, 2016, PSYCHONEUROENDOCRINO, V67, P27, DOI 10.1016/j.psyneuen.2016.01.027

Cabral A, 2012, PLOS ONE, V7, DOI 10.1371/journal.pone.0031462

Cai G, 2016, SCI REP-UK, V6, DOI 10.1038/srep21097

Camina JP, 2006, J NEUROENDOCRINOL, V18, P65, DOI 10.1111/j.1365-2826.2005.01379.x

Caminos JE, 2003, ENDOCRINOLOGY, V144, P5089, DOI 10.1210/en.2003-0529

Caminos JE, 2003, ENDOCRINOLOGY, V144, P1594, DOI 10.1210/en.2002-221058

Campagne DM, 2006, HUM REPROD, V21, P1651, DOI 10.1093/humrep/del078

Cannon W. B., 1929, BODILY CHANGES PAIN

Cannon WB, 1935, AM J MED SCI, V189, P1

Castellano JM, 2005, ENDOCRINOLOGY, V146, P3917, DOI 10.1210/en.2005-0337

Cates PS, 2004, STRESS, V7, P113, DOI 10.1080/102589042000218988

Catherino WH, 2011, FERTIL STERIL, V95, P2462, DOI 10.1016/j.fertnstert.2011.05.067

Cesur G, 2012, J PHYSIOL SCI, V62, P185, DOI 10.1007/s12576-012-0193-z

Chan CB, 2004, FEBS LETT, V577, P147, DOI 10.1016/j.febslet.2004.08.088

Chan KA, 2015, BIOL REPROD, V92, DOI 10.1095/biolreprod.114.124149

Chen HY, 2004, ENDOCRINOLOGY, V145, P2607, DOI 10.1210/en.2003-1596

Chen T, 2008, J ENDOCRINOL, V199, P253, DOI 10.1677/JOE-08-0325

Christo K, 2008, CLIN ENDOCRINOL, V69, P628, DOI 10.1111/j.1365-2265.2008.03237.x

Chu SY, 2007, DIABETES CARE, V30, P2070, DOI 10.2337/dc06-2559a

Chuang JC, 2011, J CLIN INVEST, V121, P2684, DOI 10.1172/JCI57660

Chuang Jen-Chieh, 2010, Int J Pept, V2010, DOI 10.1155/2010/460549

Clarkson J, 2009, J NEUROENDOCRINOL, V21, P673, DOI 10.1111/j.1365-2826.2009.01892.x

Clifford PS, 2012, ADDICT BIOL, V17, P956, DOI 10.1111/j.1369-1600.2011.00339.x

COHENBECKER IR, 1986, NEUROENDOCRINOLOGY, V42, P328, DOI 10.1159/000124459

Collden G, 2015, MOL METAB, V4, P15, DOI 10.1016/j.molmet.2014.10.003

Cordoba-Chacon J, 2011, ENDOCRINOLOGY, V152, P4800, DOI 10.1210/en.2011-1542

Cortelazzi D, 2003, EUR J ENDOCRINOL, V149, P111, DOI 10.1530/eje.0.1490111

Cottrell EC, 2009, FRONT BEHAV NEUROSCI, V3, DOI 10.3389/neuro.08.019.2009

Coussons-Read ME, 2007, BRAIN BEHAV IMMUN, V21, P343, DOI 10.1016/j.bbi.2006.08.006

CUMMING DC, 1983, J CLIN ENDOCR METAB, V57, P671, DOI 10.1210/jcem-57-3-671

Currie PJ, 2012, BEHAV BRAIN RES, V226, P96, DOI 10.1016/j.bbr.2011.08.037

Currie PJ, 2010, PHARMACOL BIOCHEM BE, V97, P152, DOI 10.1016/j.pbb.2010.05.027

Currie PJ, 2005, AM J PHYSIOL-REG I, V289, pR353, DOI 10.1152/ajpregu.00756.2004

Daniells S, 2003, DIABETES CARE, V26, P385, DOI 10.2337/diacare.26.2.385

Day TA, 2005, PROG NEURO-PSYCHOPH, V29, P1195, DOI 10.1016/j.pnpbp.2005.08.005

Dayas CV, 1999, EUR J NEUROSCI, V11, P2312, DOI 10.1046/j.1460-9568.1999.00645.x

Dayas CV, 2001, EUR J NEUROSCI, V14, P1143, DOI 10.1046/j.0953-816x.2001.01733.x

de Jonge J, 2000, SOC SCI MED, V50, P1317, DOI 10.1016/S0277-9536(99)00388-3

de Klerk C, 2008, HUM REPROD, V23, P112, DOI 10.1093/humrep/dem357

de Liz TM, 2005, HUM REPROD, V20, P1324, DOI 10.1093/humrep/deh743

De Souza MJ, 2004, J CLIN ENDOCR METAB, V89, P3536, DOI 10.1210/jc.2003-032007

De Vriendt T, 2009, NUTR METAB CARDIOVAS, V19, P511, DOI 10.1016/j.numecd.2009.02.009

De Vriese C, 2004, ENDOCRINOLOGY, V145, P4997, DOI 10.1210/en.2004-0569

Delhanty PJD, 2014, FRONT HORM RES, V42, P163, DOI 10.1159/000358345

Delhanty PJD, 2013, FASEB J, V27, P1690, DOI 10.1096/fj.12-221143

Delhanty PJD, 2012, EUR J ENDOCRINOL, V167, P601, DOI 10.1530/EJE-12-0456

Delhanty PJD, 2010, PLOS ONE, V5, DOI 10.1371/journal.pone.0011749

DEMYTTENAERE K, 1992, PSYCHONEUROENDOCRINO, V17, P655, DOI 10.1016/0306-4530(92)90024-2

Diano S, 2006, NAT NEUROSCI, V9, P381, DOI 10.1038/nn1656

Dickson SL, 2010, NEUROSCIENCE, V171, P1180, DOI 10.1016/j.neuroscience.2010.10.005

Diedrich K, 2007, HUM REPROD UPDATE, V13, P365, DOI 10.1093/humupd/dmm011

Disse E, 2010, PHYSIOL BEHAV, V101, P277, DOI 10.1016/j.physbeh.2010.05.017

DiVall SA, 2010, J CLIN INVEST, V120, P2900, DOI 10.1172/JCI41069

Domar AD, 2000, FERTIL STERIL, V73, P805, DOI 10.1016/S0015-0282(99)00493-8

Domar AD, 2015, FERTIL STERIL, V104, P440, DOI 10.1016/j.fertnstert.2015.05.009

Donarelli Zaira, 2016, Reprod Biomed Soc Online, V3, P16, DOI 10.1016/j.rbms.2016.10.001

Dorfman A, 2003, BIOL REPROD, V68, P2038, DOI 10.1095/biolreprod.102.008318

Douglas AJ, 2003, ENDOCRINOLOGY, V144, P5268, DOI 10.1210/en.2003-0461

Dovolou E, 2014, THERIOGENOLOGY, V81, P565, DOI 10.1016/j.theriogenology.2013.11.009

Du C, 2010, REPROD DOMEST ANIM, V45, P92, DOI 10.1111/j.1439-0531.2008.01259.x

Dunaif A, 1997, ENDOCR REV, V18, P774, DOI 10.1210/er.18.6.774

Dundar NO, 2010, PEDIATR INT, V52, P622, DOI 10.1111/j.1442-200X.2010.03100.x

Dunn AJ, 2004, ANN NY ACAD SCI, V1018, P25, DOI 10.1196/annals.1296.003

Ebbesen SMS, 2009, HUM REPROD, V24, P2173, DOI 10.1093/humrep/dep185

Egecioglu E, 2016, ADDICT BIOL, V21, P348, DOI 10.1111/adb.12202

Egecioglu E, 2010, ADDICT BIOL, V15, P304, DOI 10.1111/j.1369-1600.2010.00216.x

Ehrmann DA, 2005, NEW ENGL J MED, V352, P1223, DOI 10.1056/NEJMra041536

El-Eshmawy MM, 2010, REPROD BIOL ENDOCRIN, V8, DOI 10.1186/1477-7827-8-153

Elsenbruch S, 2007, HUM REPROD, V22, P869, DOI 10.1093/humrep/del432

Entringer S, 2013, NESTLE NUTR WORKS SE, V74, P107, DOI 10.1159/000348454

Entringer S, 2008, AM J OBSTET GYNECOL, V199, DOI 10.1016/j.ajog.2008.03.006

Eugster A, 2004, J PSYCHOSOM OBST GYN, V25, P57, DOI 10.1080/01674820410001737441

Evans JJ, 1999, ENDOCR REV, V20, P46, DOI 10.1210/er.20.1.46

Faron-Gorecka A, 2014, BRAIN RES, V1555, P48, DOI 10.1016/j.brainres.2014.01.031

Fassnacht M, 2003, J CLIN ENDOCR METAB, V88, P2760, DOI 10.1210/jc.2002-021875

Fenster L, 1997, J ANDROL, V18, P194

Ferguson AV, 2008, EXPERT OPIN THER TAR, V12, P717, DOI [10.1517/14728220802138252, 10.1517/14728222.12.6.717 ]

Fernandez-Fernandez R, 2005, NEUROENDOCRINOLOGY, V82, P245, DOI 10.1159/000092753

Fernandez-Fernandez R, 2005, ENDOCRINOLOGY, V146, P3018, DOI 10.1210/en.2004-1622

Fernandez-Fernandez R, 2004, NEUROSCI LETT, V362, P103, DOI 10.1016/j.neulet.2004.03.003

Fernandez-Fernandez R, 2007, REPRODUCTION, V133, P1223, DOI 10.1530/REP-06-0227

Ferrara A, 2007, DIABETES CARE, V30, pS141, DOI 10.2337/dc07-s206

Feuer S, 2012, BIRTH DEFECTS RES C, V96, P299, DOI 10.1002/bdrc.21022

Forbes S, 2009, NEUROSCI LETT, V460, P143, DOI 10.1016/j.neulet.2009.05.060

Frazao R, 2014, AM J PHYSIOL-ENDOC M, V306, pE606, DOI 10.1152/ajpendo.00211.2013

Frederiksen Y, 2015, BMJ OPEN, V5, DOI 10.1136/bmjopen-2014-006592

Fuglsang J, 2005, CLIN ENDOCRINOL, V62, P554, DOI 10.1111/j.1365-2265.2005.02257.x

Furness JB, 2011, NEUROSCIENCE, V193, P1, DOI 10.1016/j.neuroscience.2011.07.063

Furuta M, 2001, BIOCHEM BIOPH RES CO, V288, P780, DOI 10.1006/bbrc.2001.5854

Gambineri A, 2003, J ENDOCRINOL INVEST, V26, P629, DOI 10.1007/BF03347020

Gameiro S, 2015, HUM REPROD, V30, P2476, DOI 10.1093/humrep/dev177

Gao Y, 2016, REPROD SCI, V23, P1148, DOI 10.1177/1933719116630416

Garcia JM, 2015, BIOL REPROD, V93, DOI 10.1095/biolreprod.115.129759

Garcia MC, 2007, REPRODUCTION, V133, P531, DOI 10.1530/REP-06-0249

Garcia-Rudaz C, 1998, CLIN ENDOCRINOL, V49, P221, DOI 10.1046/j.1365-2265.1998.00511.x

Gaytan F, 2005, J CLIN ENDOCR METAB, V90, P1798, DOI 10.1210/jc.2004-1532

Gaytan F, 2004, J CLIN ENDOCR METAB, V89, P400, DOI 10.1210/jc.2003-031375

Gaytan F, 2003, J CLIN ENDOCR METAB, V88, P879, DOI 10.1210/jc.2002-021196

Gelbaya TA, 2014, OBSTET GYNECOL SURV, V69, P109, DOI 10.1097/OGX.0000000000000043

Geraghty AC, 2015, ELIFE, V4, DOI 10.7554/eLife.04316

Gibson W, 2010, METABOLISM, V59, P841, DOI 10.1016/j.metabol.2009.09.033

Glintborg D, 2006, EUR J ENDOCRINOL, V155, P337, DOI 10.1530/eje.1.02207

Glynn LM, 2007, EARLY HUM DEV, V83, P675, DOI 10.1016/j.earlhumdev.2007.01.003

Gnanapavan S, 2002, J CLIN ENDOCR METAB, V87, P2988, DOI 10.1210/jc.87.6.2988

Goldstone AP, 2009, EUR J NEUROSCI, V30, P1625, DOI 10.1111/j.1460-9568.2009.06949.x

Gollenberg AL, 2010, FERTIL STERIL, V93, P1104, DOI 10.1016/j.fertnstert.2008.12.018

Gonzalez R, 2010, REPROD TOXICOL, V29, P198, DOI 10.1016/j.reprotox.2009.10.009

GORDON M, 1982, J PEDIATR-US, V101, P477, DOI 10.1016/S0022-3476(82)80093-0

Gottsch ML, 2011, ENDOCRINOLOGY, V152, P4298, DOI 10.1210/en.2011-1521

Gourounti K, 2011, WOMEN HEALTH, V51, P321, DOI 10.1080/03630242.2011.574791

Govic A, 2008, BEHAV BRAIN RES, V190, P140, DOI 10.1016/j.bbr.2008.02.013

Grattan DR, 2008, J NEUROENDOCRINOL, V20, P752, DOI 10.1111/j.1365-2826.2008.01736.x

Greil AL, 2010, SOCIOL HEALTH ILL, V32, P140, DOI 10.1111/j.1467-9566.2009.01213.x

Grey KR, 2013, PSYCHONEUROENDOCRINO, V38, P1178, DOI 10.1016/j.psyneuen.2012.11.002

GROSSMAN A, 1981, CLIN ENDOCRINOL, V14, P41, DOI 10.1111/j.1365-2265.1981.tb00363.x

Gualillo O, 2001, ENDOCRINOLOGY, V142, P788, DOI 10.1210/en.142.2.788

Gutierrez JA, 2008, P NATL ACAD SCI USA, V105, P6320, DOI 10.1073/pnas.0800708105

Hammerli K, 2009, HUM REPROD UPDATE, V15, P279, DOI 10.1093/humupd/dmp002

Hahn JD, 2003, J NEUROENDOCRINOL, V15, P732, DOI 10.1046/j.1365-2826.2003.01056.x

Han SK, 2002, ENDOCRINOLOGY, V143, P1459, DOI 10.1210/en.143.4.1459

Handa RJ, 2012, NEUROENDOCRINOLOGY, V96, P111, DOI 10.1159/000338397

Hansson C, 2011, NEUROSCIENCE, V180, P201, DOI 10.1016/j.neuroscience.2011.02.002

Hansson C, 2013, PSYCHIAT RES, V206, P22, DOI 10.1016/j.psychres.2012.09.051

Harrison JL, 2007, REPROD BIOL ENDOCRIN, V5, DOI 10.1186/1477-7827-5-25

Hays J, 2003, NEW ENGL J MED, V348, P1839, DOI 10.1056/NEJMoa030311

HECKERT L, 1993, RECENT PROG HORM RES, V48, P61

Hehir MP, 2008, AM J OBSTET GYNECOL, V198, DOI 10.1016/j.ajog.2007.09.040

Heider U, 2001, FERTIL STERIL, V75, P1141, DOI 10.1016/S0015-0282(01)01805-2

Heine PA, 2000, P NATL ACAD SCI USA, V97, P12729, DOI 10.1073/pnas.97.23.12729

HERBISON AE, 1991, EXP BRAIN RES, V87, P345

Herman JP, 2005, PROG NEURO-PSYCHOPH, V29, P1201, DOI 10.1016/j.pnpbp.2005.08.006

Herman JP, 2003, FRONT NEUROENDOCRIN, V24, P151, DOI 10.1016/j.yfrne.2003.07.001

HERNANDEZ ER, 1988, ENDOCRINOLOGY, V122, P1592, DOI 10.1210/endo-122-4-1592

Hernandez-Diaz S, 2009, BRIT MED J, V338, DOI 10.1136/bmj.b2255

Herod SM, 2011, AM J PHYSIOL-ENDOC M, V300, pE19, DOI 10.1152/ajpendo.00224.2010

Hjollund NHI, 2004, EPIDEMIOLOGY, V15, P114, DOI 10.1097/01.ede.0000100290.908884a

Hjollund NHI, 2004, EPIDEMIOLOGY, V15, P21, DOI 10.1097/01.edu.0000100289.82156.8b

Hlatky MA, 2002, JAMA-J AM MED ASSOC, V287, P591, DOI 10.1001/jama.287.5.591

Hoffman Elizabeth R, 2011, Expert Rev Obstet Gynecol, V6, P403

Hoffman S, 2000, HEALTH PSYCHOL, V19, P535, DOI 10.1037/0278-6133.19.6.535

Homan P, 2013, PSYCHONEUROENDOCRINO, V38, P1545, DOI 10.1016/j.psyneuen.2012.12.024

Hou GL, 2014, J SEX MED, V11, P653, DOI 10.1111/jsm.12416

Houjeghani S, 2012, INT J FERTIL STERIL, V6, P117

Howard AD, 1996, SCIENCE, V273, P974, DOI 10.1126/science.273.5277.974

Hu GX, 2008, STEROIDS, V73, P1018, DOI 10.1016/j.steroids.2007.12.020

Hu L, 2008, MOL ENDOCRINOL, V22, P2250, DOI 10.1210/me.2008-0192

Hull EM, 2004, PHYSIOL BEHAV, V83, P291, DOI 10.1016/j.physbeh.2004.08.018

Hunt KJ, 2007, OBSTET GYN CLIN N AM, V34, P173, DOI 10.1016/j.ogc.2007.03.002

Ikezaki A, 2002, DIABETES, V51, P3408, DOI 10.2337/diabetes.51.12.3408

Ilcol YO, 2007, ACTA PAEDIATR, V96, P1632, DOI 10.1111/j.1651-2227.2007.00493.x

Inal HA, 2016, J ENDOCRINOL INVEST, V39, P431, DOI 10.1007/s40618-015-0392-6

Inhoff T, 2008, PEPTIDES, V29, P2159, DOI 10.1016/j.peptides.2008.09.014

Inhorn MC, 2015, HUM REPROD UPDATE, V21, P411, DOI 10.1093/humupd/dmv016

Inoue Y, 2010, BIOCHEM BIOPH RES CO, V393, P455, DOI 10.1016/j.bbrc.2010.02.022

Iqbal J, 2006, ENDOCRINOLOGY, V147, P510, DOI 10.1210/en.2005-1048

Irwig MS, 2004, NEUROENDOCRINOLOGY, V80, P264, DOI 10.1159/000083140

Ishikawa T, 2007, J ANDROL, V28, P320, DOI 10.2164/jandrol.106.000810

Ishitobi Y, 2012, NEUROPSYCHOBIOLOGY, V66, P185, DOI 10.1159/000339948

Iwasa T, 2014, HORM BEHAV, V66, P309, DOI 10.1016/j.yhbeh.2014.06.007

Janevic T, 2014, FERTIL STERIL, V102, P530, DOI 10.1016/j.fertnstert.2014.04.021

Jaremka LM, 2014, PSYCHONEUROENDOCRINO, V48, P178, DOI 10.1016/j.psyneuen.2014.06.018

Jarrell H, 2008, PHYSIOL BEHAV, V93, P807, DOI 10.1016/j.physbeh.2007.11.042

JARRY H, 1988, ACTA ENDOCRINOL-COP, V118, P573, DOI 10.1530/acta.0.1180573

Jasoni CL, 2005, NEUROENDOCRINOLOGY, V82, P320, DOI 10.1159/000093155

Jerlhag E, 2007, ADDICT BIOL, V12, P6, DOI 10.1111/j.1369-1600.2006.00041.x

Jerlhag E, 2011, DRUG ALCOHOL DEPEN, V117, P126, DOI 10.1016/j.drugalcdep.2011.01.010

Jerlhag E, 2010, PSYCHOPHARMACOLOGY, V211, P415, DOI 10.1007/s00213-010-1907-7

Jerlhag E, 2009, P NATL ACAD SCI USA, V106, P11318, DOI 10.1073/pnas.0812809106

JIMENA P, 1992, ACTA ENDOCRINOL-COP, V127, P403, DOI 10.1530/acta.0.1270403

Joachim R, 2003, STEROIDS, V68, P931, DOI 10.1016/j.steroids.2003.08.010

Johnstone HA, 2000, J NEUROENDOCRINOL, V12, P811, DOI 10.1046/j.1365-2826.2000.00525.x

Johnstone LE, 2005, STRESS, V8, P59, DOI 10.1080/10253890500095283

Julien M, 2012, EUR J PHARM SCI, V47, P625, DOI 10.1016/j.ejps.2012.07.014

Kageyama K, 2012, PEPTIDES, V33, P149, DOI 10.1016/j.peptides.2011.11.020

Kammerer M., 2002, BMC PREGNANCY CHILDB, V2, P8, DOI DOI 10.1186/1471-2393-2-8

Kan C, 2013, DIABETES CARE, V36, P480, DOI 10.2337/dc12-1442

Kanayama N, 1997, J HYPERTENS, V15, P383, DOI 10.1097/00004872-199715040-00009

Kaur S, 2010, ALCOHOL CLIN EXP RES, V34, P1525, DOI 10.1111/j.1530-0277.2010.01237.x

Kawakami A, 2008, STRESS, V11, P363, DOI 10.1080/10253890701820257

Kawamura K, 2003, ENDOCRINOLOGY, V144, P2623, DOI 10.1210/en.2003-0033

Keen KL, 1999, ENDOCRINOLOGY, V140, P5257, DOI 10.1210/en.140.11.5257

Keen-Rhinehart E, 2009, MOL PSYCHIATR, V14, P37, DOI 10.1038/mp.2008.91

Kenny R, 2013, AM J PHYSIOL-REG I, V304, pR980, DOI 10.1152/ajpregu.00594.2012

Kern A, 2012, NEURON, V73, P317, DOI 10.1016/j.neuron.2011.10.038

Khatun S, 1999, EUR J OBSTET GYN R B, V86, P89, DOI 10.1016/S0301-2115(99)00050-0

Kheradmand A, 2009, ANDROLOGIA, V41, P335, DOI 10.1111/j.1439-0272.2009.00932.x

Kheradmand A, 2009, TISSUE CELL, V41, P311, DOI 10.1016/j.tice.2009.01.002

Kheradmand A, 2009, TISSUE CELL, V41, P105, DOI 10.1016/j.tice.2008.07.006

Kheradmand A, 2011, REGUL PEPTIDES, V167, P97, DOI 10.1016/j.regpep.2010.12.002

Kierson JA, 2006, ACTA PAEDIATR, V95, P991, DOI 10.1080/08035250600669769

Kim C, 2002, DIABETES CARE, V25, P1862, DOI 10.2337/diacare.25.10.1862

Kinsey-Jones JS, 2009, J NEUROENDOCRINOL, V21, P20, DOI 10.1111/j.1365-2826.2008.01807.x

Kirby ED, 2009, P NATL ACAD SCI USA, V106, P11324, DOI 10.1073/pnas.0901176106

Kishimoto I, 2012, J CARDIOL, V59, P8, DOI 10.1016/j.jjcc.2011.11.002

Klonoff-Cohen H, 2001, FERTIL STERIL, V76, P675, DOI 10.1016/S0015-0282(01)02008-8

Kluge M, 2007, J CLIN ENDOCR METAB, V92, P3202, DOI 10.1210/jc.2007-0593

Kluge M, 2013, J PSYCHIATR RES, V47, P1236, DOI 10.1016/j.jpsychires.2013.05.010

Kluge M, 2012, REPROD SCI, V19, pNP3, DOI 10.1177/1933719112459242

Kluge M, 2012, J CLIN ENDOCR METAB, V97, pE448, DOI 10.1210/jc.2011-2607

Kluge M, 2011, J PSYCHIATR RES, V45, P421, DOI 10.1016/j.jpsychires.2010.09.002

Kluge M, 2009, CYTOKINE GROWTH F R, V20, P339, DOI 10.1016/j.cytogfr.2009.07.003

Kluge M, 2009, CLIN ENDOCRINOL, V70, P920, DOI 10.1111/j.1365-2265.2008.03440.x

Kluge M, 2009, NEUROPSYCHOBIOLOGY, V59, P199, DOI 10.1159/000223731

KNOBIL E, 1988, HUM REPROD, V3, P29, DOI 10.1093/oxfordjournals.humrep.a136647

Kodomari I, 2009, NEUROCHEM INT, V54, P222, DOI 10.1016/j.neuint.2008.11.006

KOIKE K, 1991, HORM RES, V35, P5, DOI 10.1159/000181921

Kojima M, 1999, NATURE, V402, P656, DOI 10.1038/45230

Komarowska H, 2013, NEUROENDOCRINOL LETT, V34, P669

Komesaroff PA, 1999, J CLIN ENDOCR METAB, V84, P606, DOI 10.1210/jc.84.2.606

KONONEN J, 1993, MOL CELL ENDOCRINOL, V93, P97, DOI 10.1016/0303-7207(93)90144-9

Korek E, 2013, PRZ GASTROENTEROL, V8, P383, DOI 10.5114/pg.2013.39922

Kristenssson E, 2006, REGUL PEPTIDES, V134, P114, DOI 10.1016/j.regpep.2006.02.003

Krsmanovic LZ, 2009, TRENDS ENDOCRIN MET, V20, P402, DOI 10.1016/j.tem.2009.05.002

Krsmanovic LZ, 2003, P NATL ACAD SCI USA, V100, P2969, DOI 10.1073/pnas.05305708100

Krsmanovic LZ, 1996, TRENDS ENDOCRIN MET, V7, P56, DOI 10.1016/1043-2760(96)00007-0

KRSMANOVIC LZ, 1993, P NATL ACAD SCI USA, V90, P3908, DOI 10.1073/pnas.90.9.3908

Ku JM, 2016, CLIN SCI, V130, P1545, DOI 10.1042/CS20160077

Ku JM, 2015, ENDOCRINOLOGY, V156, P280, DOI 10.1210/en.2014-1415

Kurki T, 2000, OBSTET GYNECOL, V95, P487, DOI 10.1016/S0029-7844(99)00602-X

Kurt E, 2007, NEUROSCI LETT, V426, P49, DOI 10.1016/j.neulet.2007.08.018

Lanfranco F, 2008, J CLIN ENDOCR METAB, V93, P3633, DOI 10.1210/jc.2008-0049

Lappas M, 2015, J DIABETES, V7, P506, DOI 10.1111/1753-0407.12209

LARA HE, 1990, ENDOCRINOLOGY, V126, P364, DOI 10.1210/endo-126-1-364

Larson-Meyer DE, 2010, AM J CLIN NUTR, V91, P366, DOI 10.3945/ajcn.2009.28616

Laszlo KD, 2013, HYPERTENSION, V62, P183, DOI 10.1161/HYPERTENSIONAHA.111.00550

Latendresse G, 2009, J MIDWIFERY WOM HEAL, V54, P8, DOI 10.1016/j.jmwh.2008.08.001

Lewicka S, 2003, HUM REPROD, V18, P1613, DOI 10.1093/humrep/deg352

Li LY, 2011, FERTIL STERIL, V96, P1116, DOI 10.1016/j.fertnstert.2011.08.017

LI PS, 1983, BIOL REPROD, V29, P25, DOI 10.1095/biolreprod29.1.25

Li W, 2014, CELL DEATH DIS, V5, DOI 10.1038/cddis.2014.223

Li XF, 2006, J NEUROENDOCRINOL, V18, P602, DOI 10.1111/j.1365-2826.2006.01450.x

Li XF, 2011, J NEUROENDOCRINOL, V23, P3, DOI 10.1111/j.1365-2826.2010.02071.x

Li XF, 2009, STRESS, V12, P259, DOI 10.1080/10253890802379922

Li XF, 2005, ENDOCRINOLOGY, V146, P318, DOI 10.1210/en.2004-0950

Li XF, 2004, ENDOCRINOLOGY, V145, P1556, DOI 10.1210/en.2003-1609

Li XF, 2009, PLOS ONE, V4, DOI 10.1371/journal.pone.0008334

Li XF, 2015, J MOL ENDOCRINOL, V55, P9, DOI 10.1530/JME-15-0084

Lin YS, 2012, J NEUROENDOCRINOL, V24, P477, DOI 10.1111/j.1365-2826.2011.02270.x

Lin YS, 2011, ENDOCRINOLOGY, V152, P545, DOI 10.1210/en.2010-1003

Lindsay JR, 2005, ENDOCR REV, V26, P775, DOI 10.1210/er.2004-0025

Lintsen AME, 2009, HUM REPROD, V24, P1092, DOI 10.1093/humrep/den491

LIU JH, 1990, AM J OBSTET GYNECOL, V163, P1732, DOI 10.1016/0002-9378(90)91437-H

Lockie SH, 2015, PSYCHONEUROENDOCRINO, V62, P114, DOI 10.1016/j.psyneuen.2015.08.004

Lorenzi T, 2009, CYTOKINE GROWTH F R, V20, P137, DOI 10.1016/j.cytogfr.2009.02.003

Louis GMB, 2011, FERTIL STERIL, V95, P2184, DOI 10.1016/j.fertnstert.2010.06.078

Lutter M, 2008, NAT NEUROSCI, V11, P752, DOI 10.1038/nn.2139

Lydon K, 2012, Ir Med J, V105, P26

Lynch CD, 2014, HUM REPROD, V29, P1067, DOI 10.1093/humrep/deu032

Macfarlane MS, 2000, ANIM REPROD SCI, V63, P167, DOI 10.1016/S0378-4320(00)00179-2

MACLUSKY NJ, 1988, BRAIN RES, V439, P391, DOI 10.1016/0006-8993(88)91501-6

Magiakou MA, 1996, CLIN ENDOCRINOL, V44, P419, DOI 10.1046/j.1365-2265.1996.683505.x

Magiakou MA, 1996, J CLIN ENDOCR METAB, V81, P1912, DOI 10.1210/jc.81.5.1912

Maheshwari A, 2008, HUM REPROD, V23, P538, DOI 10.1093/humrep/dem431

Makino Y, 2002, HYPERTENSION, V39, P781, DOI 10.1161/hy0302.105221

Malik S, 2008, CELL METAB, V7, P400, DOI 10.1016/j.cmet.2008.03.007

Mao YJ, 2016, CURR HYPERTENS REP, V18, DOI 10.1007/s11906-015-0622-5

Martin JR, 2011, ENDOCRINOLOGY, V152, P2060, DOI 10.1210/en.2010-1485

Martin SA, 2014, J SEX MED, V11, P1136, DOI 10.1111/jsm.12483

Martini AC, 2006, ENDOCRINOLOGY, V147, P2374, DOI 10.1210/en.2005-1422

Mascarenhas MN, 2012, PLOS MED, V9, DOI 10.1371/journal.pmed.1001356

Matsuo K, 2012, BRAIN RES, V1473, P185, DOI 10.1016/j.brainres.2012.07.027

MATSUZAKI T, 1994, FERTIL STERIL, V62, P1143

Matthiesen SMS, 2011, HUM REPROD, V26, P2763, DOI 10.1093/humrep/der246

Mauvais-Jarvis F, 2013, ENDOCR REV, V34, P309, DOI 10.1210/er.2012-1055

McGovern-Gooch KR, 2016, ENDOCRINOLOGY, V157, P4330, DOI 10.1210/en.2016-1657

McKee KK, 1997, MOL ENDOCRINOL, V11, P415, DOI 10.1210/me.11.4.415

McLaughlin T, 2004, J CLIN ENDOCR METAB, V89, P1630, DOI 10.1210/jc.2003-031572

MELIS GB, 1987, J CLIN ENDOCR METAB, V65, P237, DOI 10.1210/jcem-65-2-237

MERARI D, 1992, J ASSIST REPROD GEN, V9, P161, DOI 10.1007/BF01203757

Luque EM, 2014, REPRODUCTION, V148, P159, DOI 10.1530/REP-14-0129

Messini CI, 2011, J ENDOCRINOL INVEST, V34, P276, DOI 10.3275/7102

Meyer RM, 2014, MOL PSYCHIATR, V19, P1284, DOI 10.1038/mp.2013.135

MICHAEL AE, 1993, CLIN ENDOCRINOL, V38, P641, DOI 10.1111/j.1365-2265.1993.tb02147.x

Michael AE, 2008, HUM REPROD UPDATE, V14, P497, DOI 10.1093/humupd/dmn021

Michopoulos V, 2013, NEUROSCIENCE, V250, P300, DOI 10.1016/j.neuroscience.2013.07.002

Michopoulos V, 2011, PHYSIOL BEHAV, V102, P382, DOI 10.1016/j.physbeh.2010.11.031

Michopoulos V, 2010, ENDOCRINE, V38, P227, DOI 10.1007/s12020-010-9378-5

Michopoulos V, 2009, BIOL REPROD, V81, P1154, DOI 10.1095/biolreprod.109.079038

Micic D, 2007, J ENDOCRINOL INVEST, V30, P820, DOI 10.1007/BF03349222

Millar RP, 2005, ANIM REPROD SCI, V88, P5, DOI 10.1016/j.anireprosci.2005.05.032

Miller DW, 2005, REPROD BIOL ENDOCRIN, V3, DOI 10.1186/1477-7827-3-60

Mircea CN, 2007, J OBSTET GYNAECOL CA, V29, P887, DOI 10.1016/S1701-2163(16)32661-5

Mitkov M, 2008, GYNECOL ENDOCRINOL, V24, P625, DOI 10.1080/09513590802302233

Monteleone P, 2012, NEUROPSYCHOBIOLOGY, V66, P230, DOI 10.1159/000341877

Moran LJ, 2004, J CLIN ENDOCR METAB, V89, P3337, DOI 10.1210/jc.2003-031583

Moretti E, 2014, ANDROLOGIA, V46, P979, DOI 10.1111/and.12183

Muccioli G, 2011, PEPTIDES, V32, P2514, DOI 10.1016/j.peptides.2011.10.020

Nakahara K, 2006, ENDOCRINOLOGY, V147, P1333, DOI 10.1210/en.2005-0708

Nakashima K, 2008, PSYCHIAT GENET, V18, P257, DOI 10.1097/YPG.0b013e328306c979

Nakazato M, 2001, NATURE, V409, P194, DOI 10.1038/35051587

Naleid AM, 2005, PEPTIDES, V26, P2274, DOI 10.1016/j.peptides.2005.04.025

Nepomnaschy PA, 2006, P NATL ACAD SCI USA, V103, P3938, DOI 10.1073/pnas.0511183103

Nepomnaschy PA, 2004, AM J HUM BIOL, V16, P523, DOI 10.1002/ajhb.20057

Nepomnaschy PA, 2007, ANN NY ACAD SCI, V1113, P350, DOI 10.1196/annals.1391.028

Ness RB, 2006, AM J OBSTET GYNECOL, V195, P40, DOI 10.1016/j.ajog.2005.07.049

Nestler EJ, 2010, NAT NEUROSCI, V13, P1161, DOI 10.1038/nn.2647

Ng PC, 2005, CLIN ENDOCRINOL, V63, P217, DOI 10.1111/j.1365-2265.2005.02328.x

Nonoshita A, 2010, PLACENTA, V31, P358, DOI 10.1016/j.placenta.2010.02.013

NORMAN RL, 1992, NEUROENDOCRINOLOGY, V55, P405, DOI 10.1159/000126151

Nouri K, 2011, FERTIL STERIL, V96, P69, DOI 10.1016/j.fertnstert.2011.04.063

O'Brien M, 2010, REPROD BIOL ENDOCRIN, V8, DOI 10.1186/1477-7827-8-55

Ochi M, 2008, LIFE SCI, V82, P862, DOI 10.1016/j.lfs.2008.01.020

Ohkawa N, 2010, ACTA PAEDIATR, V99, P37, DOI 10.1111/j.1651-2227.2009.01516.x

Ohkura S, 2009, PEPTIDES, V30, P49, DOI 10.1016/j.peptides.2008.08.004

Olivius C, 2004, FERTIL STERIL, V81, P258, DOI 10.1016/j.fertnster.2003.06.029

Orio F, 2003, J CLIN ENDOCR METAB, V88, P942, DOI 10.1210/jc.2002-021451

Orr ST, 2002, AM J EPIDEMIOL, V156, P797, DOI 10.1093/aje/kwf131

Orr ST, 2007, PSYCHOSOM MED, V69, P566, DOI 10.1097/PSY.0b013e3180cac25d

Otto B, 2001, EUR J ENDOCRINOL, V145, pR5

Ozcan B, 2014, EUR J ENDOCRINOL, V170, P799, DOI 10.1530/EJE-13-0347

Ozsoy S, 2014, PSYCHIAT INVEST, V11, P167, DOI 10.4306/pi.2014.11.2.167

Pacifico L, 2009, BONE, V45, P274, DOI 10.1016/j.bone.2009.04.204

Pagotto U, 2002, J CLIN ENDOCR METAB, V87, P5625, DOI 10.1210/jc.2002-020776

Palik E, 2007, DIABETES RES CLIN PR, V76, P351, DOI 10.1016/j.diabres.2006.09.005

Panidis D, 2005, HUM REPROD, V20, P2127, DOI 10.1093/humrep/dei055

Pantham P, 2015, PLACENTA, V36, P709, DOI 10.1016/j.placenta.2015.04.006

Papadimitriou A, 2009, NEUROIMMUNOMODULAT, V16, P265, DOI 10.1159/000216184

Papotti M, 2000, J CLIN ENDOCR METAB, V85, P3803, DOI 10.1210/jc.85.10.3803

Paredes A, 1998, ENDOCRINE, V8, P309, DOI 10.1385/ENDO:8:3:309

Parker VJ, 2010, J REPROD IMMUNOL, V85, P86, DOI 10.1016/j.jri.2009.10.011

Patel SS, 2007, J OBSTET GYNAECOL, V27, P455, DOI 10.1080/01443610701406125

Patterson ZR, 2010, EUR J NEUROSCI, V32, P632, DOI 10.1111/j.1460-9568.2010.07310.x

PEARCE GP, 1988, J REPROD FERTIL, V83, P413

Pearson S, 2010, DIABETES CARE, V33, P1128, DOI 10.2337/dc09-1940

PERRIN M, 1995, P NATL ACAD SCI USA, V92, P2969, DOI 10.1073/pnas.92.7.2969

Petersen PS, 2009, ENDOCRINOLOGY, V150, P4920, DOI 10.1210/en.2008-1638

Pfaus JG, 2009, J SEX MED, V6, P1506, DOI 10.1111/j.1743-6109.2009.01309.x

Pinar M, 2008, PROG NEURO-PSYCHOPH, V32, P135, DOI 10.1016/j.pnpbp.2007.07.028

Pinheiro AP, 2007, INT J EAT DISORDER, V40, P424, DOI 10.1002/eat.20388

Pitchers KK, 2010, GENES BRAIN BEHAV, V9, P831, DOI 10.1111/j.1601-183X.2010.00621.x

Pitchers KK, 2012, PLOS ONE, V7, DOI 10.1371/journal.pone.0034700

Pitchers KK, 2010, BIOL PSYCHIAT, V67, P872, DOI 10.1016/j.biopsych.2009.09.036

PRADER A, 1975, CLIN ENDOCRINOL META, V4, P143, DOI 10.1016/S0300-595X(75)80037-5

Prieto-Garcia L, 2015, PSYCHONEUROENDOCRINO, V62, P392, DOI 10.1016/j.psyneuen.2015.09.009

Qiu CF, 2009, AM J HYPERTENS, V22, P397, DOI 10.1038/ajh.2008.366

Quant HS, 2013, J ASSIST REPROD GEN, V30, P1451, DOI 10.1007/s10815-013-0098-7

Rajkhowa M, 2006, HUM REPROD, V21, P358, DOI 10.1093/humrep/dei355

Rak A, 2009, GROWTH HORM IGF RES, V19, P68, DOI 10.1016/j.ghir.2008.08.006

Rak-Mardyla A, 2012, THERIOGENOLOGY, V77, P1505, DOI 10.1016/j.theriogenology.2011.11.017

Rak-Mardyla A, 2015, REPROD SCI, V22, P469, DOI 10.1177/1933719114549854

Rak-Mardyla A, 2010, REPROD BIOL, V10, P159, DOI 10.1016/S1642-431X(12)60058-8

Rannikko A, 1996, J ENDOCRINOL, V151, P29, DOI 10.1677/joe.0.1510029

Raso GM, 2007, J ENDOCRINOL, V194, P611, DOI 10.1677/JOE-07-0159

Raspopow K, 2014, APPETITE, V74, P35, DOI 10.1016/j.appet.2013.11.018

Raspopow K, 2010, HORM BEHAV, V58, P677, DOI 10.1016/j.yhbeh.2010.06.003

Rediger A, 2011, J BIOL CHEM, V286, P39623, DOI 10.1074/jbc.M111.287607

Reding K, 2012, HORM BEHAV, V62, P612, DOI 10.1016/j.yhbeh.2012.09.010

Reichenbach A, 2012, ENDOCRINOLOGY, V153, P5452, DOI 10.1210/en.2012-1622

Richardson AC, 2007, OBSTET GYN CLIN N AM, V34, P213, DOI 10.1016/j.ogc.2007.04.001

Riedl M, 2007, J INTERN MED, V262, P458, DOI 10.1111/j.1365-2796.2007.01832.x

RIVEST S, 1991, ENDOCRINOLOGY, V129, P2049, DOI 10.1210/endo-129-4-2049

RIVIER C, 1984, ENDOCRINOLOGY, V114, P914, DOI 10.1210/endo-114-3-914

Rivier C, 2002, NEUROIMMUNOMODULAT, V10, P17, DOI 10.1159/000064411

RIVIER C, 1991, BIOL REPROD, V45, P523, DOI 10.1095/biolreprod45.4.523

Rizwan MZ, 2012, ENDOCRINOLOGY, V153, P3770, DOI 10.1210/en.2012-1133

Roa J, 2010, MOL CELL ENDOCRINOL, V324, P87, DOI 10.1016/j.mce.2009.12.018

Roney JR, 2015, ADAPT HUM BEHAV PHYS, V1, P30, DOI 10.1007/s40750-014-0004-2

Rouach V, 2007, PSYCHONEUROENDOCRINO, V32, P693, DOI 10.1016/j.psyneuen.2007.04.010

Rubinfeld H, 2004, EUR J ENDOCRINOL, V151, P787, DOI 10.1530/eje.0.1510787

Sacher J, 2012, J AFFECT DISORDERS, V140, P142, DOI 10.1016/j.jad.2011.08.001

Sagsoz N, 2009, FERTIL STERIL, V92, P660, DOI 10.1016/j.fertnstert.2008.07.008

Sakata I, 2009, REGUL PEPTIDES, V155, P91, DOI 10.1016/j.regpep.2009.04.001

Sandberg DE, 2002, BEST PRACT RES CL EN, V16, P449, DOI 10.1053/beem.2002.0211

Sapolsky RM, 2000, ENDOCR REV, V21, P55, DOI 10.1210/er.21.1.55

Sapolsky RM, 2000, ARCH GEN PSYCHIAT, V57, P925, DOI 10.1001/archpsyc.57.10.925

Schanze A, 2008, NEUROPSYCHOBIOLOGY, V57, P126, DOI 10.1159/000138915

Schellekens H, 2013, VITAM HORM, V91, P285, DOI 10.1016/B978-0-12-407766-9.00013-4

Schellekens H, 2013, J BIOL CHEM, V288, P181, DOI 10.1074/jbc.M112.382473

Schellekens H, 2012, PHARMACOL THERAPEUT, V135, P316, DOI 10.1016/j.pharmthera.2012.06.004

Schliep KC, 2015, EPIDEMIOLOGY, V26, P177, DOI 10.1097/EDE.0000000000000238

Schmid DA, 2006, NEUROPSYCHOPHARMACOL, V31, P832, DOI 10.1038/sj.npp.1300923

Schmid DA, 2005, NEUROPSYCHOPHARMACOL, V30, P1187, DOI 10.1038/sj.npp.1300670

Schneider LF, 2008, FERTIL STERIL, V90, P121, DOI 10.1016/j.fertnstert.2007.06.002

Schofl C, 2002, J CLIN ENDOCR METAB, V87, P4607, DOI 10.1210/jc.2002-020505

SCHREIBER JR, 1982, STEROIDS, V39, P569, DOI 10.1016/0039-128X(82)90057-5

SCHULTE HM, 1990, CLIN ENDOCRINOL, V33, P99, DOI 10.1111/j.1365-2265.1990.tb00470.x

SCHWARTZ R, 1994, DIABETES CARE, V17, P640, DOI 10.2337/diacare.17.7.640

SCOTT CJ, 1993, ENDOCRINOLOGY, V133, P2904, DOI 10.1210/en.133.6.2904

Selye H, 1998, J NEUROPSYCH CLIN N, V10, P230

SELYE H, 1946, J CLIN ENDOCR METAB, V6, P117, DOI 10.1210/jcem-6-2-117

Seminara SB, 2003, NEW ENGL J MED, V349, P1614, DOI 10.1056/NEJMoa035322

Sen TA, 2011, J PEDIATR ADOL GYNEC, V24, P211, DOI 10.1016/j.jpag.2011.02.007

Sen Tolta Altug, 2010, J Clin Res Pediatr Endocrinol, V2, P117, DOI 10.4274/jcrpe.v2i3.117

Sepa A, 2005, DIABETES CARE, V28, P290, DOI 10.2337/diacare.28.2.290

Shah SN, 2010, HORM BEHAV, V57, P291, DOI 10.1016/j.yhbeh.2009.12.010

Shalitin S, 2003, INT J OBESITY, V27, P869, DOI 10.1038/sj.ijo.0802328

Shansky RM, 2010, CEREB CORTEX, V20, P2560, DOI 10.1093/cercor/bhq003

Shansky RM, 2004, MOL PSYCHIATR, V9, P531, DOI 10.1038/sj.mp.4001435

Sheiner EK, 2002, J OCCUP ENVIRON MED, V44, P1093, DOI 10.1097/01.jom.0000044116.59147.64

Shepard JD, 2000, BRAIN RES, V861, P288, DOI 10.1016/S0006-8993(00)02019-9

Shibata K, 2004, PEPTIDES, V25, P279, DOI 10.1016/j.peptides.2004.01.011

Shimbara T, 2004, NEUROSCI LETT, V369, P75, DOI 10.1016/j.neulet.2004.07.060

Sibai BM, 2006, SEMIN PERINATOL, V30, P16, DOI 10.1053/j.semperi.2006.01.008

SILVERMAN BL, 1995, DIABETES CARE, V18, P611, DOI 10.2337/diacare.18.5.611

Simon C, 2000, BEST PRACT RES CL OB, V14, P815, DOI 10.1053/beog.2000.0121

Simon C, 1998, J REPROD IMMUNOL, V39, P117, DOI 10.1016/S0165-0378(98)00017-5

Skibicka KP, 2011, NEUROSCIENCE, V180, P129, DOI 10.1016/j.neuroscience.2011.02.016

Skibicka KP, 2012, ADDICT BIOL, V17, P95, DOI 10.1111/j.1369-1600.2010.00294.x

Slattery DA, 2008, J PHYSIOL-LONDON, V586, P377, DOI 10.1113/jphysiol.2007.145896

Smeenk JMJ, 2004, FERTIL STERIL, V81, P262, DOI 10.1016/j.fertnstert.2003.09.027

Smeenk JMJ, 2001, HUM REPROD, V16, P1420, DOI 10.1093/humrep/16.7.1420

Smith JT, 2007, ENDOCRINOLOGY, V148, P1150, DOI 10.1210/en.2006-1435

Smith JT, 2013, PEPTIDES, V47, P45, DOI 10.1016/j.peptides.2013.06.012

Smith JT, 2013, ADV EXP MED BIOL, V784, P275, DOI 10.1007/978-1-4614-6199-9_13

Smith JT, 2005, ENDOCRINOLOGY, V146, P3686, DOI 10.1210/en.2005-0488

SMITH R, 1981, CLIN ENDOCRINOL, V15, P291, DOI 10.1111/j.1365-2265.1981.tb00668.x

Smith RG, 1997, ENDOCR REV, V18, P621, DOI 10.1210/er.18.5.621

Sominsky L, 2017, PLOS ONE, V12, DOI 10.1371/journal.pone.0173498

Sominsky L, 2017, NEUROPHARMACOLOGY, V113, P21, DOI 10.1016/j.neuropharm.2016.09.023

Sominsky L, 2014, FRONT PSYCHOL, V5, DOI 10.3389/fpsyg.2014.00434

Sominsky L, 2012, HORM BEHAV, V62, P345, DOI 10.1016/j.yhbeh.2012.02.005

Soriano-Guillen L, 2004, J PEDIATR-US, V144, P30, DOI 10.1016/j.jpeds.2003.08.050

Soriano-Guillen L, 2004, J PEDIATR-US, V144, P36, DOI 10.1016/j.jpeds.2003.10.036

Speakman JR, 2008, PHILOS T R SOC B, V363, P375, DOI 10.1098/rstb.2007.2145

Spencer SJ, 2015, BIOL PSYCHIAT, V78, P19, DOI 10.1016/j.biopsych.2014.10.021

Spencer SJ, 2012, BIOL PSYCHIAT, V72, P457, DOI 10.1016/j.biopsych.2012.03.010

Spencer SJ, 2011, STRESS, V14, P233, DOI 10.3109/10253890.2010.534831

STALKER A, 1989, AM J ANAT, V186, P369, DOI 10.1002/aja.1001860406

Stark R, 2016, ENDOCRINOLOGY, V157, P3946, DOI 10.1210/en.2016-1306

Steculorum SM, 2015, J CLIN INVEST, V125, P846, DOI 10.1172/JCI73688

Stengel A, 2011, PEPTIDES, V32, P2208, DOI 10.1016/j.peptides.2011.07.002

Sullivan EC, 2011, DEV PSYCHOBIOL, V53, P96, DOI 10.1002/dev.20483

SUTER DE, 1989, BIOL REPROD, V41, P1091, DOI 10.1095/biolreprod41.6.1091

SUTER DE, 1985, ENDOCRINOLOGY, V117, P849, DOI 10.1210/endo-117-3-849

SUTER DE, 1988, AM J PHYSIOL, V254, pE595

Suzuki H, 2010, REPROD DOMEST ANIM, V45, P927, DOI 10.1111/j.1439-0531.2009.01352.x

Sverrisdottir YB, 2008, AM J PHYSIOL-ENDOC M, V294, pE576, DOI 10.1152/ajpendo.00725.2007

Szczepankiewicz D, 2010, J PHYSIOL PHARMACOL, V61, P443

Taati M, 2012, J PHYSIOL BIOCHEM, V68, P91, DOI 10.1007/s13105-011-0122-2

Takaya K, 2000, J CLIN ENDOCR METAB, V85, P4908, DOI 10.1210/jc.85.12.4908

Takiuti NH, 2002, AM J OBSTET GYNECOL, V186, P544, DOI 10.1067/mob.2002.121102

Tanaka K, 2003, J CLIN ENDOCR METAB, V88, P2335, DOI 10.1210/jc.2002-021024

Vulliemoz NR, 2004, J CLIN ENDOCR METAB, V89, P5718, DOI 10.1210/jc.2004-1244

Traslavina GAA, 2012, ENDOCRINOLOGY, V153, P4838, DOI 10.1210/en.2012-1333

Tanaka M, 2003, PSYCHONEUROENDOCRINO, V28, P829, DOI 10.1016/S0306-4530(02)00066-5

Tawadros N, 2007, MOL HUM REPROD, V13, P483, DOI 10.1093/molehr/gam029

Taylor BD, 2015, HYPERTENSION, V65, P594, DOI 10.1161/HYPERTENSIONAHA.114.03979

Teede H, 2010, BMC MED, V8, DOI 10.1186/1741-7015-8-41

Telejko B, 2010, EXP CLIN ENDOCR DIAB, V118, P87, DOI 10.1055/s-0029-1238313

Tena-Sempere M, 2004, NEUROENDOCRINOLOGY, V79, P133, DOI 10.1159/000077271

Tena-Sempere M, 2002, ENDOCRINOLOGY, V143, P717, DOI 10.1210/en.143.2.717

Tetsuka M, 1999, BIOL REPROD, V60, P330, DOI 10.1095/biolreprod60.2.330

Tham E, 2009, AM J PHYSIOL-ENDOC M, V296, pE1093, DOI 10.1152/ajpendo.90866.2008

THEORELL T, 1990, J INTERN MED, V227, P31, DOI 10.1111/j.1365-2796.1990.tb00115.x

THIERING P, 1993, J PSYCHOSOM RES, V37, P481, DOI 10.1016/0022-3999(93)90004-Y

Tilbrook AJ, 2002, STRESS, V5, P83, DOI 10.1080/10253890290027912

Tilbrook AJ, 1999, J ENDOCRINOL, V160, P469, DOI 10.1677/joe.0.1600469

Tilbrook AJ, 2000, REV REPROD, V5, P105, DOI 10.1530/revreprod/5.2.105

Tolle V, 2003, J CLIN ENDOCR METAB, V88, P109, DOI 10.1210/jc.2002-020645

Torner L, 2002, EUR J NEUROSCI, V15, P1381, DOI 10.1046/j.1460-9568.2002.01965.x

Torner L, 2016, FRONT ENDOCRINOL, V7, DOI 10.3389/fendo.2016.00025

Torner L, 2002, STRESS, V5, P249, DOI 10.1080/1025389021000048638

Tschop M, 2001, DIABETES, V50, P707, DOI 10.2337/diabetes.50.4.707

Tschop M, 2000, NATURE, V407, P908, DOI 10.1038/35038090

Tsilchorozidou T, 2003, J CLIN ENDOCR METAB, V88, P5907, DOI 10.1210/jc.2003-030240

Tuncel OK, 2016, J CHILD ADOL PSYCHOP, V26, P733, DOI 10.1089/cap.2015.0149

Turgut A, 2015, CLIN EXP OBSTET GYN, V42, P473

Turner K, 2013, PLOS ONE, V8, DOI 10.1371/journal.pone.0063743

Ubuka T, 2009, PLOS ONE, V4, DOI 10.1371/journal.pone.0008400

VADAKKADATH MS, 2005, CELL MOL LIFE SCI, V62, P257, DOI DOI 10.1007/S00018-004-4381-3

VALE W, 1981, SCIENCE, V213, P1394, DOI 10.1126/science.6267699

Van Merris V, 2007, REPROD TOXICOL, V23, P32, DOI 10.1016/j.reprotox.2006.08.008

Van Pett K, 2000, J COMP NEUROL, V428, P191, DOI 10.1002/1096-9861(20001211)428:2<191::AID-CNE1>3.0.CO;2-U

Varnagy A, 2013, J REPROD MED, V58, P305

Vassiliadi DA, 2009, J CLIN ENDOCR METAB, V94, P3558, DOI 10.1210/jc.2009-0837

Verhaak CM, 2001, FERTIL STERIL, V76, P525, DOI 10.1016/S0015-0282(01)01931-8

Viani I, 2008, J CLIN ENDOCR METAB, V93, P1476, DOI 10.1210/jc.2007-2063

Vianna P, 2011, MED HYPOTHESES, V77, P188, DOI 10.1016/j.mehy.2011.04.007

Vidal C, 2008, HUM REPROD, V23, P958, DOI 10.1093/humrep/den020

Visser-van Balen H, 2006, ARCH DIS CHILD, V91, P433, DOI 10.1136/adc.2005.086942

Vitalis EA, 2000, P NATL ACAD SCI USA, V97, P1861, DOI 10.1073/pnas.040545197

von Borell E, 2007, HORM BEHAV, V52, P130, DOI 10.1016/j.yhbeh.2007.03.014

Vulliemoz NR, 2008, ENDOCRINOLOGY, V149, P869, DOI 10.1210/en.2007-1350

Wahab F, 2015, J ENDOCRINOL, V225, pR49, DOI 10.1530/JOE-14-0688

Wahab F, 2008, LIFE SCI, V83, P633, DOI 10.1016/j.lfs.2008.09.001

Walf AA, 2006, NEUROPSYCHOPHARMACOL, V31, P1097, DOI 10.1038/sj.npp

Walker AK, 2011, BRAIN BEHAV IMMUN, V25, P674, DOI 10.1016/j.bbi.2011.01.004

Walker JJ, 2010, P ROY SOC B-BIOL SCI, V277, P1627, DOI 10.1098/rspb.2009.2148

Wang JW, 2015, EXP THER MED, V9, P1909, DOI 10.3892/etm.2015.2341

Wang ZG, 2013, THERIOGENOLOGY, V79, P695, DOI 10.1016/j.theriogenology.2012.11.026

Wasko R, 2004, HORM METAB RES, V36, P170, DOI 10.1055/s-2004-814341

Wellman PJ, 2008, REGUL PEPTIDES, V146, P33, DOI 10.1016/j.regpep.2007.07.007

Wellman PJ, 2011, REGUL PEPTIDES, V172, P77, DOI 10.1016/j.regpep.2011.08.014

Whatmore AJ, 2003, CLIN ENDOCRINOL, V59, P649, DOI 10.1046/j.1365-2265.2003.01903.x

Wigger A, 1999, ENDOCRINOLOGY, V140, P2843, DOI 10.1210/en.140.6.2843

WILLIAMS CL, 1990, NEUROENDOCRINOLOGY, V52, P133, DOI 10.1159/000125563

Williams DL, 2005, J NUTR, V135, P1320

Windle RJ, 1998, ENDOCRINOLOGY, V139, P443, DOI 10.1210/en.139.2.443

Wischmann TH, 2003, J ASSIST REPROD GEN, V20, P485, DOI 10.1023/B:JARG.0000013648.74404.9d

Wood GE, 1998, P NATL ACAD SCI USA, V95, P4066, DOI 10.1073/pnas.95.7.4066

Wu LM, 2012, PLOS ONE, V7, DOI 10.1371/journal.pone.0052331

Wu LM, 2012, BIOL REPROD, V86, DOI 10.1095/biolreprod.111.093468

Wu M, 2009, J PHYSIOL-LONDON, V587, P1401, DOI 10.1113/jphysiol.2008.166447

Wu WG, 2015, PREGNANCY HYPERTENS, V5, P263, DOI 10.1016/j.preghy.2015.09.002

Xiao EN, 2007, ENDOCRINOLOGY, V148, P841, DOI 10.1210/en.2006-1074

Yang H, 2008, BRAIN BEHAV IMMUN, V22, P1138, DOI 10.1016/j.bbi.2008.06.003

Yang JG, 1999, BIOL REPROD, V60, P929, DOI 10.1095/biolreprod60.4.929

Yang J, 2008, CELL, V132, P387, DOI 10.1016/j.cell.2008.01.017

Yap BK, 1996, J CHROMATOGR B, V687, P117, DOI 10.1016/S0378-4347(96)00017-5

Yazawa H, 2000, HUM REPROD, V15, P1917, DOI 10.1093/humrep/15.9.1917

Yazawa H, 1999, HUM REPROD, V14, P1806, DOI 10.1093/humrep/14.7.1806

YEN SSC, 1970, J CLIN ENDOCR METAB, V30, P435, DOI 10.1210/jcem-30-4-435

Young AJ, 2006, P NATL ACAD SCI USA, V103, P12005, DOI 10.1073/pnas.0510038103

Yu Y., 2013, AM J OBSTET GYNECOL, V209, p438 e431

Zhang K, 2007, J REPROD DEVELOP, V53, P647, DOI 10.1262/jrd.18140

Zhang SC, 2013, OBSTET GYNECOL SURV, V68, P825, DOI 10.1097/OGX.0000000000000001

ZHANG SM, 1995, BRIT MED J, V311, P1139, DOI 10.1136/bmj.311.7013.1139

Zhu CC, 2013, FERTIL STERIL, V99, P918, DOI 10.1016/j.fertnstert.2012.11.022

Zhu XR, 2006, J BIOL CHEM, V281, P38867, DOI 10.1074/jbc.M607955200

Zigman JM, 2006, J COMP NEUROL, V494, P528, DOI 10.1002/cne.20823

Zorn B, 2002, HUM REPROD, V17, P3173, DOI 10.1093/humrep/17.12.3173

Zorn B, 2008, INT J ANDROL, V31, P557, DOI 10.1111/j.1365-2605.2007.00806.x

NR 579

TC 1

Z9 1

U1 4

U2 21

PU OXFORD UNIV PRESS INC

PI CARY

PA JOURNALS DEPT, 2001 EVANS RD, CARY, NC 27513 USA

SN 0163-769X

EI 1945-7189

J9 ENDOCR REV

JI Endocr. Rev.

PD OCT 1

PY 2017

VL 38

IS 5

BP 432

EP 467

DI 10.1210/er.2016-1133

PG 36

WC Endocrinology & Metabolism

SC Endocrinology & Metabolism

GA FJ5EJ

UT WOS:000412769300002

PM 28938425

DA 2019-01-08

ER

PT J

AU van de Pavert, I

Sunderland, M

Luijten, M

Slade, T

Teesson, M

AF van de Pavert, Iris

Sunderland, Matthew

Luijten, Maartje

Slade, Tim

Teesson, Maree

TI The general relationship between internalizing psychopathology and

chronic physical health conditions: a population-based study

SO SOCIAL PSYCHIATRY AND PSYCHIATRIC EPIDEMIOLOGY

LA English

DT Article

DE Internalizing; Comorbidity; Physical health; Obesity; Tobacco use;

Latent variable models

ID SUBSTANCE USE DISORDERS; MENTAL-DISORDERS; EMOTIONAL DISORDERS;

DIABETES-MELLITUS; META-STRUCTURE; DSM-IV; DEPRESSION; COMORBIDITY;

OBESITY; ADULTS

AB Studies have consistently demonstrated a reciprocal relationship between internalizing disorders and several chronic physical health conditions. Yet, much of the extant literature fails to take into account the role of comorbidity among internalizing disorders when examining the relationship with poor physical health. The current study applied latent variable modelling to investigate the shared and specific relationships between internalizing (fear and distress factors) and a range of physical health conditions. Data comprised 8841 respondents aged 16-85 years who took part in the 2007 Australian National Survey of Mental Health and Wellbeing. Multiple indicator, multiple causes models were used to parse the shared and specific relationships between internalizing disorders and variables associated with poor physical health. The study found that several physical conditions were significantly related to mean levels of fear and distress. The results were broadly similar but minor differences emerged depending on whether lifetime or past 12 months indicators of mental disorders and physical conditions were utilized in the model. Finally, the results demonstrated that the association between individual mental disorders and physical health conditions are better accounted for by indirect relationships with broad transdiagnostic dimensions rather than including additional disorder-specific relationships. The results indicate that researchers should focus on common mechanisms across multiple internalizing disorders and poor physical health when developing prevention and treatment initiatives.

C1 [van de Pavert, Iris; Luijten, Maartje] Radboud Univ Nijmegen, Inst Behav Sci, Nijmegen, Netherlands.

[Sunderland, Matthew; Slade, Tim; Teesson, Maree] UNSW Sydney, Ctr Res Excellence Mental Hlth & Subst Use, Natl Drug & Alcohol Res Ctr, Sydney, NSW 2052, Australia.

RP Sunderland, M (reprint author), UNSW Sydney, Ctr Res Excellence Mental Hlth & Subst Use, Natl Drug & Alcohol Res Ctr, Sydney, NSW 2052, Australia.

EM Matthews@unsw.edu.au

OI Sunderland, Matthew/0000-0001-8452-364X

CR Andrews G, 2009, PSYCHOL MED, V39, P1993, DOI 10.1017/S0033291709990250

[Anonymous], 2009, NAT SURV MENT HLTH W

Baumeister D, 2014, SOC PSYCH PSYCH EPID, V49, P841, DOI 10.1007/s00127-014-0887-z

Campayo A, 2010, AM J PSYCHIAT, V167, P580, DOI 10.1176/appi.ajp.2009.09010038

Clarke DM, 2009, MED J AUSTRALIA, V190, pS52

Conway CC, 2012, J ABNORM PSYCHOL, V121, P754, DOI 10.1037/a0027457

Courtney KE, 2009, PSYCHOL BULL, V135, P142, DOI 10.1037/a0014414

Crane PK, 2009, GLOBAL PERSPECTIVES, P239

Degenhardt L, 2001, Nicotine Tob Res, V3, P225

Eaton NR, 2013, J ABNORM PSYCHOL, V122, P86, DOI 10.1037/a0029598

Farchione TJ, 2012, BEHAV THER, V43, P666, DOI 10.1016/j.beth.2012.01.001

Goldberg DP, 2009, PSYCHOL MED, V39, P2043, DOI 10.1017/S0033291709990298

Hettema JM, 2006, AM J PSYCHIAT, V163, P857, DOI 10.1176/appi.ajp.163.5.857

Hu LT, 1999, STRUCT EQU MODELING, V6, P1, DOI 10.1080/10705519909540118

Kendler KS, 2003, ARCH GEN PSYCHIAT, V60, P929, DOI 10.1001/archpsyc.60.9.929

Kessler RC, 2004, INT J METH PSYCH RES, V13, P93, DOI 10.1002/mpr.168

Kessler RC, 2005, ARCH GEN PSYCHIAT, V62, P617, DOI 10.1001/archpsyc.62.6.617

Kotov R, 2017, J ABNORM PSYCHOL, V126, P454, DOI 10.1037/abn0000258

Krueger RF, 2001, PERS INDIV DIFFER, V30, P1245, DOI 10.1016/S0191-8869(00)00106-9

Krueger RF, 1999, ARCH GEN PSYCHIAT, V56, P921, DOI 10.1001/archpsyc.56.10.921

Krueger RE, 2006, ANNU REV CLIN PSYCHO, V2, P111, DOI 10.1146/annurev.clinpsy.2.022305.095213

Kruse J, 2003, DIABETES CARE, V26, P1841, DOI 10.2337/diacare.26.6.1841

Lahey BB, 2014, JAMA PSYCHIAT, V71, P989, DOI 10.1001/jamapsychiatry.2014.359

Lasser K, 2000, JAMA-J AM MED ASSOC, V284, P2606, DOI 10.1001/jama.284.20.2606

Markon KE, 2004, BEHAV GENET, V34, P593, DOI 10.1007/s10519-004-5587-0

McLaughlin KA, 2011, BEHAV RES THER, V49, P186, DOI 10.1016/j.brat.2010.12.006

Muthen LK, 2015, MPLUS USERS GUIDE

O'Connor KA, 2003, BRAIN RES, V991, P123, DOI 10.1016/j.brainres.2003.08.006

Raftery AE, 1995, SOCIOL METHODOL, V25, P111, DOI 10.2307/271063

Raison CL, 2006, TRENDS IMMUNOL, V27, P24, DOI 10.1016/j.it.2005.11.006

Saydah SH, 2003, DIABETES-METAB RES, V19, P202, DOI 10.1002/dmrr.353

Scott KM, 2008, INT J OBESITY, V32, P192, DOI 10.1038/sj.ijo.0803701

Slade T, 2007, SOC PSYCH PSYCH EPID, V42, P554, DOI 10.1007/s00127-007-0200-5

Slade T, 2006, PSYCHOL MED, V36, P1593, DOI 10.1017/S0033291706008452

Steptoe A, 2007, BRAIN BEHAV IMMUN, V21, P901, DOI 10.1016/j.bbi.2007.03.011

Strine TW, 2008, GEN HOSP PSYCHIAT, V30, P127, DOI 10.1016/j.genhosppsych.2007.12.008

Sturm R, 2002, HEALTH AFFAIR, V21, P245, DOI 10.1377/hlthaff.21.2.245

Teesson M, 2011, AUST NZ J PSYCHIAT, V45, P939, DOI 10.3109/00048674.2011.614590

Whiteford HA, 2013, LANCET, V382, P1575, DOI 10.1016/S0140-6736(13)61611-6

Wright AGC, 2013, J ABNORM PSYCHOL, V122, P281, DOI 10.1037/a0030133

NR 40

TC 1

Z9 1

U1 0

U2 1

PU SPRINGER HEIDELBERG

PI HEIDELBERG

PA TIERGARTENSTRASSE 17, D-69121 HEIDELBERG, GERMANY

SN 0933-7954

EI 1433-9285

J9 SOC PSYCH PSYCH EPID

JI Soc. Psychiatry Psychiatr. Epidemiol.

PD OCT

PY 2017

VL 52

IS 10

BP 1257

EP 1265

DI 10.1007/s00127-017-1422-9

PG 9

WC Psychiatry

SC Psychiatry

GA FJ2IH

UT WOS:000412548200006

PM 28744565

DA 2019-01-08

ER

PT J

AU Aghili, R

Ridderstrale, M

Kia, M

Valojerdi, AE

Malek, M

Farshchi, A

Khamseh, ME

AF Aghili, Rokhsareh

Ridderstrale, Martin

Kia, Maryam

Valojerdi, Ameneh Ebrahim

Malek, Mojtaba

Farshchi, Amir

Khamseh, Mohammad Ebrahim

TI The challenge of living with diabetes in women and younger adults: A

structural equation model

SO PRIMARY CARE DIABETES

LA English

DT Article

DE Diabetes; Type 2; Age; Gender; Psychosocial factors

ID PHYSICAL-DISABILITY; GENDER; EDUCATION; MEN; AGE

AB Background: Attitudes toward diabetes care are different between genders and age-groups. Furthermore, diabetes related challenges may cause psychosocial problems. Therefore, we were to compare the psychosocial status and glycemic control in women and men with type 2 diabetes (T2D) in different age-groups.

Methods: 441 adults with T2D were recruited. Demographic, self-care behavior, resources and affective variables as well as the health related quality of life (HRQoL) were measured. The median age of 55 was used as the cut-off for the age comparison. Structured equation modeling (SEM) investigated the relationship between age, gender, psychosocial factors and glycemic control.

Results: Finally, 203 women and 177 men completed the study (86.1%). There was no significant difference in mean duration of T2D, or glycemic control between genders or age-groups. Women, especially those below the median age of 55, had significantly higher level of diabetes-related distress (2.16 +/- 1 0.94 vs. 1.92 +/- 0.81), depression (9.67 +/- 5.37 vs. 7.54 +/- 5.06), and anxiety (19.81 +/- 12.04 vs. 12.81 +/- 9.04, P < 0.05 for all comparisons), while people above the age of 55 reported better self-management and patient-physician relationship. HRQoL was lower in women compared to men (0.77 +/- 0.23 vs. 0.81 +/- 0.18, P=0.02). The final SEM suggested that the effect (standardized beta coefficient) of gender and age on affective variables was 0.25 and -0.19 (P < 0.05), respectively, though psychosocial factors did not directly influence HbA1c.

Conclusions: This study shows that psychosocial factors are associated with age and gender in patients with T2D; with younger women demonstrating higher level of depressive symptoms, anxiety, and diabetes-related distress independent of status of glycemic control. (C) 2017 Primary Care Diabetes Europe. Published by Elsevier Ltd. All rights reserved.

C1 [Aghili, Rokhsareh; Valojerdi, Ameneh Ebrahim; Farshchi, Amir; Khamseh, Mohammad Ebrahim] Iran Univ Med Sci, Inst Endocrinol & Metab, Endocrine Res Ctr, Tehran, Iran.

[Ridderstrale, Martin] Lund Univ, Skane Univ Hosp Malmo, Clin Obes, Dept Clin Sci, S-20502 Malmo, Sweden.

[Kia, Maryam] Univ Tehran Med Sci, Ziaeian Hosp, Dept Internal Med, Tehran, Iran.

[Malek, Mojtaba] Iran Univ Med Sci, Inst Endocrinol & Metab, Res Ctr Prevent Cardiovasc Dis, Tehran, Iran.

[Farshchi, Amir] Univ Tehran Med Sci, Sch Pharm, Dept Pharmacoecon & Pharmaceut Adm, Tehran, Iran.

RP Khamseh, ME (reprint author), Valadi St,Behafarin St,Karimkhan Ave,Vali Asr Sq, Tehran, Iran.

EM aghili.r@tak.iums.ac.ir; martin.ridderstrale@med.lu.se;

mkia220@gmail.com; ebrahimv86@gmail.com; malek.m@iums.acir;

farshchi_a@razi.tums.ac.ir; khamseh.m@iums.ac.ir

RI Khamseh, Mohammad Ebrahim/L-9391-2018

FU Iran University of Medical Sciences [93-02-122-24800]

FX This study was funded and supported by Iran University of Medical

Sciences; grant number: 93-02-122-24800. The authors wish to thank all

people who participated in this study.

CR Aamir Sayed, 2012, MIDDLE E J PSYCHIAT, V3, P16

Aghili R, 2013, INT NURS REV, V60, P267, DOI 10.1111/inr.12007

Aghili R, 2016, CAN J DIABETES, V40, P424, DOI 10.1016/j.jcjd.2016.02.014

Amer Diabet Assoc, 2016, DIABETES CARE, V39, pS23, DOI 10.2337/dc16-S006

Bruce DG, 2005, DIABETES CARE, V28, P2441, DOI 10.2337/diacare.28.10.2441

Chiu CJ, 2011, ANN BEHAV MED, V41, P71, DOI 10.1007/s12160-010-9226-0

Chiu CJ, 2011, GERONTOLOGIST, V51, P51, DOI 10.1093/geront/gnq069

Fisher L, 2014, DIABETIC MED, V31, P764, DOI 10.1111/dme.12428

Gao J, 2013, J CHEM-NY, DOI 10.1155/2013/719179

Gucciardi E, 2008, CAN FAM PHYSICIAN, V54, P219

HIBBARD JH, 1983, SOC SCI MED, V17, P129, DOI 10.1016/0277-9536(83)90246-0

Maggi S, 2004, DIABETOLOGIA, V47, P1957, DOI 10.1007/s00125-004-1555-8

Peyrot M, 1999, J HEALTH SOC BEHAV, V40, P141, DOI 10.2307/2676370

Ponzo MG, 2006, BEHAV MED, V31, P153, DOI 10.3200/BMED.31.4.153-160

Pound N, 1996, DIABETIC MED, V13, P510, DOI 10.1002/(SICI)1096-9136(199606)13:6<510::AID-DIA122>3.0.CO;2-W

Rintala TM, 2013, J FAM NURS, V19, P3, DOI 10.1177/1074840712471899

SASISEKHAR TVD, 2013, IOSR JDMS, V4, P48

Schieman S, 2002, J AGING HEALTH, V14, P260, DOI 10.1177/089826430201400205

Shalev V, 2005, PUBLIC HEALTH, V119, P45, DOI 10.1016/j.puhe.2004.03.004

Siddiqui Muhammad A, 2013, Mater Sociomed, V25, P140, DOI 10.5455/msm.2013.25.140-142

Sriram V., 2001, INT J DIABETES DEV C, V21

Stofan JR, 1998, AM J PUBLIC HEALTH, V88, P1807, DOI 10.2105/AJPH.88.12.1807

Trief PM, 2003, AGE AGEING, V32, P613, DOI 10.1093/ageing/afg105

Wray LA, 2005, J GERONTOL A-BIOL, V60, P1206, DOI 10.1093/gerona/60.9.1206

NR 24

TC 2

Z9 2

U1 1

U2 10

PU ELSEVIER SCI LTD

PI OXFORD

PA THE BOULEVARD, LANGFORD LANE, KIDLINGTON, OXFORD OX5 1GB, OXON, ENGLAND

SN 1751-9918

EI 1878-0210

J9 PRIM CARE DIABETES

JI Prim. Care Diabetes

PD OCT

PY 2017

VL 11

IS 5

BP 467

EP 473

DI 10.1016/j.pcd.2017.05.001

PG 7

WC Endocrinology & Metabolism; Primary Health Care

SC Endocrinology & Metabolism; General & Internal Medicine

GA FI3HO

UT WOS:000411848800009

PM 28579058

DA 2019-01-08

ER

PT J

AU Vloemans, AF

van Beers, CAJ

de Wit, M

Cleijne, W

Rondags, SM

Geelhoed-Duijvestijn, PH

Kramer, MHH

Serne, EH

Snoek, FJ

AF Vloemans, A. F.

van Beers, C. A. J.

de Wit, M.

Cleijne, W.

Rondags, S. M.

Geelhoed-Duijvestijn, P. H.

Kramer, M. H. H.

Serne, E. H.

Snoek, F. J.

TI Keeping safe. Continuous glucose monitoring (CGM) in persons with Type 1

diabetes and impaired awareness of hypoglycaemia: a qualitative study

SO DIABETIC MEDICINE

LA English

DT Article

ID RANDOMIZED CONTROLLED-TRIAL; ADULTS; MANAGEMENT; BENEFITS

AB AimTo further our understanding of individual use and experience of continuous glucose monitoring (CGM) in adults with Type 1 diabetes and impaired awareness of hypoglycaemia, we conducted a qualitative study supplementary to a randomized controlled trial, using semi-structured interviews.

MethodsTwenty-three participants of the IN CONTROL trial were interviewed within 4weeks after the last study visit. The interview centred around experiences of CGM, taking into account the person's expectations prior to the trial. The interview was semi-structured, using open-ended questions and, if needed, prompts were offered to elicit further responses. Using thematic analysis, the interview transcripts were coded independently by three members of the research team. The consolidated criteria for reporting qualitative research (COREQ) were followed.

ResultsOverall, CGM was experienced as helpful in gaining more insight into glucose variability, and temporarily improved sense of control, reduced distress and made participants less dependent on others. However, some participants experienced confrontation with CGM output as intrusive, while some reported frustration due to failing technique and difficulty trusting the device. Participants reported active and passive self-management behaviours mirroring individual differences in attitudes and coping styles.

ConclusionsIn adults with Type 1 diabetes at risk of recurrent hypoglycaemia due to impaired awareness of hypoglycaemia, CGM use enhances a sense of control and safety for most, but not all. Future studies should further explore differential use of CGM in this population in the context of active and passive self-management styles.

C1 [Vloemans, A. F.; de Wit, M.; Cleijne, W.; Rondags, S. M.; Snoek, F. J.] Vrije Univ Amsterdam, Med Ctr, Dept Med Psychol, Amsterdam, Netherlands.

[van Beers, C. A. J.; Kramer, M. H. H.; Serne, E. H.] Vrije Univ Amsterdam, Med Ctr, Dept Internal Med, Amsterdam, Netherlands.

[Geelhoed-Duijvestijn, P. H.] Med Ctr Haaglanden, Dept Internal Med, The Hague, Netherlands.

[Snoek, F. J.] Univ Amsterdam, Dept Med Psychol, Acad Med Ctr, Amsterdam, Netherlands.

RP van Beers, CAJ (reprint author), Vrije Univ Amsterdam, Med Ctr, Dept Internal Med, Amsterdam, Netherlands.

EM c.vanbeers@vumc.nl

FU Eli Lilly; Sanofi

FX The IN CONTROL trial received funding from Eli Lilly and Sanofi.

Medtronic supplied CGM devices.

CR Beck RW, 2010, DIABETES CARE, V33, P2175, DOI 10.2337/dc10-0331

Bergenstal RM, 2016, JAMA-J AM MED ASSOC, V316, P1407, DOI 10.1001/jama.2016.11708

Bolinder J, 2016, LANCET, V388, P2254, DOI 10.1016/S0140-6736(16)31535-5

Borges Uirassu Jr, 2016, J Diabetes Sci Technol, V10, P633, DOI 10.1177/1932296816634736

Braun V., 2006, QUALITATIVE RES PSYC, V3, P77, DOI DOI 10.1191/1478088706QP0630A

Choudhary P, 2013, DIABETES CARE, V36, P4160, DOI 10.2337/dc13-0939

Cox DJ, 2001, DIABETES CARE, V24, P637, DOI 10.2337/diacare.24.4.637

Cryer PE, 2002, DIABETOLOGIA, V45, P937, DOI 10.1007/s00125-002-0822-9

Hermanides J, 2011, DIABETIC MED, V28, P1158, DOI 10.1111/j.1464-5491.2011.03256.x

Kubiak Thomas, 2016, J Diabetes Sci Technol, V10, P859, DOI 10.1177/1932296816651450

Litchman ML, 2017, J DIABETES SCI TECHN

Ly TT, 2013, JAMA-J AM MED ASSOC, V310, P1240, DOI 10.1001/jama.2013.277818

Miller KM, 2015, DIABETES CARE, V38, P971, DOI 10.2337/dc15-0078

Pickup JC, 2015, DIABETES CARE, V38, P544, DOI 10.2337/dc14-1855

Pickup JC, 2011, BRIT MED J, V343, DOI 10.1136/bmj.d3805

Polonsky WH, 2013, DIABETES TECHNOL THE, V15, P295, DOI 10.1089/dia.2012.0298

Ritholz MD, 2014, DIABETIC MED, V31, P47, DOI 10.1111/dme.12276

Ritholz MD, 2010, DIABETIC MED, V27, P1060, DOI 10.1111/j.1464-5491.2010.03061.x

Rondags SMPA, 2016, DIABETES CARE, V39, P2190, DOI 10.2337/dc16-1614

Russell Cynthia K, 2003, Evid Based Nurs, V6, P36

Schmidt Signe, 2012, J Diabetes Sci Technol, V6, P986

Tong A, 2007, INT J QUAL HEALTH C, V19, P349, DOI 10.1093/intqhc/mzm042

van Beers CAJ, 2016, LANCET DIABETES ENDO, V4, P893, DOI 10.1016/S2213-8587(16)30193-0

Yeoh E, 2015, DIABETES CARE, V38, P1592, DOI 10.2337/dc15-0102

NR 24

TC 3

Z9 3

U1 2

U2 8

PU WILEY

PI HOBOKEN

PA 111 RIVER ST, HOBOKEN 07030-5774, NJ USA

SN 0742-3071

EI 1464-5491

J9 DIABETIC MED

JI Diabetic Med.

PD OCT

PY 2017

VL 34

IS 10

BP 1470

EP 1476

DI 10.1111/dme.13429

PG 7

WC Endocrinology & Metabolism

SC Endocrinology & Metabolism

GA FH3DP

UT WOS:000411025300021

PM 28731509

DA 2019-01-08

ER

PT J

AU Castensoe-Seidenfaden, P

Teilmann, G

Kensing, F

Hommel, E

Olsen, BS

Husted, GR

AF Castensoe-Seidenfaden, Pernille

Teilmann, Grete

Kensing, Finn

Hommel, Eva

Olsen, Birthe Susanne

Husted, Gitte Reventlov

TI Isolated thoughts and feelings and unsolved concerns: adolescents' and

parents' perspectives on living with type 1 diabetes - a qualitative

study using visual storytelling

SO JOURNAL OF CLINICAL NURSING

LA English

DT Article

DE adolescence; qualitative methods; self-management; transition; type 1

diabetes; visual storytelling

ID DETERMINATION-YOUTH INTERVENTION; GLYCEMIC CONTROL; EMOTIONAL DISTRESS;

SELF-MANAGEMENT; EMERGING ADULTS; LIFE SKILLS; CHILDREN; CARE;

HYPOGLYCEMIA; FEAR

AB Aims and objectivesTo explore and describe the experiences of adolescents and their parents living with type 1 diabetes, to identify their needs for support to improve adolescents' self-management skills in the transition from child- to adulthood.

BackgroundAdolescents with type 1 diabetes often experience deteriorating glycaemic control and distress. Parents are important in adolescents' ability to self-manage type 1 diabetes, but they report anxiety and frustrations. A better understanding of the challenges adolescents and parents face, in relation to the daily self-management of type 1 diabetes, is important to improve clinical practice.

DesignA qualitative explorative study using visual storytelling as part of individual interviews.

MethodsA purposive sample of nine adolescents and their parents (seven mothers, six fathers) took photographs illustrating their experiences living with type 1 diabetes. Subsequently, participants were interviewed individually guided by participants' photographs and a semistructured interview guide. Interviews were analysed using thematic analysis.

ResultsFour major themes were consistent across adolescents and their parents: (1) striving for safety, (2) striving for normality, (3) striving for independence and (4) worrying about future. Although adolescents and parents had same concerns and challenges living with type 1 diabetes, they were experienced differently. Their thoughts and feelings mostly remained isolated and their concerns and challenges unsolved.

ConclusionsThe concerns and challenges adolescents and their parents face in the transition from child- to adulthood are still present despite new treatment modalities. Parents are fundamental in supporting the adolescents' self-management-work; however, the parties have unspoken concerns and challenges.

Relevance to clinical practiceHealthcare providers should address the parties' challenges and concerns living with type 1 diabetes to diminish worries about future including fear of hypoglycaemia, the burden of type 1 diabetes and the feeling of being incompetent in diabetes self-management. It is important to focus on supporting both adolescents and their parents, and to provide a shared platform for communication.

C1 [Castensoe-Seidenfaden, Pernille; Teilmann, Grete] Nordsjaellands Hosp, Pediat & Adolescent Dept, Dyrehavevej 29, DK-3400 Hillerod, Denmark.

[Kensing, Finn] Univ Copenhagen, Dept Comp Sci, Copenhagen, Denmark.

[Hommel, Eva] Steno Diabet Ctr, Gentofte, Denmark.

[Olsen, Birthe Susanne] Herlev Hosp, Dept Pediat, Herlev, Denmark.

[Husted, Gitte Reventlov] Steno Diabet Ctr, Hlth Promot Res, Gentofte, Denmark.

RP Castensoe-Seidenfaden, P (reprint author), Nordsjaellands Hosp, Pediat & Adolescent Dept, Dyrehavevej 29, DK-3400 Hillerod, Denmark.

EM pernille.castensoee-seidenfaden@regionh.dk

FU Nordsjaellands Hospital, University of Copenhagen; Regionradets

Foundation for Particularly Vulnerable Citizens, the Capital Region,

Denmark

FX This study was funded by a Research Grant from Nordsjaellands Hospital,

University of Copenhagen and Regionradets Foundation for Particularly

Vulnerable Citizens, the Capital Region, Denmark.

CR Babler E, 2015, DIABETES EDUCATOR, V41, P351, DOI 10.1177/0145721715579108

Braun V., 2006, QUALITATIVE RES PSYC, V3, P77, DOI DOI 10.1191/1478088706QP0630A

Burke K, 2012, J ADOLESCENCE, V35, P1315, DOI 10.1016/j.adolescence.2012.05.002

Cardwell CR, 2005, ARCH DIS CHILD, V90, P468, DOI 10.1136/adc.2004.061150

Carroll AE, 2006, DIABETIC MED, V23, P1222, DOI 10.1111/j.1464-5491.2006.01961.x

Cheung Ruth, 2006, J Sch Nurs, V22, P53

Collier J., 1986, VISUAL ANTHR PHOTOGR

Court JM, 2009, PEDIATR DIABETES, V10, P185, DOI 10.1111/j.1399-5448.2009.00586.x

Crowley R, 2011, ARCH DIS CHILD, V96, P548, DOI 10.1136/adc.2010.202473

Denzin NK, 2000, HDB QUALITATIVE RES

Drew SE, 2010, QUAL HEALTH RES, V20, P1677, DOI 10.1177/1049732310377455

Freckleton E, 2014, INT J BEHAV MED, V21, P804, DOI 10.1007/s12529-013-9360-8

Freeborn D, 2013, J CLIN NURS, V22, P1890, DOI 10.1111/jocn.12046

Gallagher J. M., 1981, LEARNING THEORY PIAG

Garvey K, 2015, PEDIATR CLIN N AM, V62, P873, DOI 10.1016/j.pcl.2015.04.005

Glindorf M, 2014, PAID 1 IS VALUABLE T

Grey M, 2000, J PEDIATR-US, V137, P107, DOI 10.1067/mpd.2000.106568

Hampson SE, 2000, DIABETES CARE, V23, P1416, DOI 10.2337/diacare.23.9.1416

Hanna K M, 2001, Issues Compr Pediatr Nurs, V24, P209

Haugstvedt A, 2010, DIABETIC MED, V27, P72, DOI 10.1111/j.1464-5491.2009.02867.x

Haugstvedt A, 2011, PEDIATR DIABETES, V12, P107, DOI 10.1111/j.1399-5448.2010.00661.x

Husted GR, 2014, J ADV NURS, V70, P2634, DOI 10.1111/jan.12413

Husted GR, 2014, TRIALS, V15, DOI 10.1186/1745-6215-15-321

Huus Karina, 2007, Paediatr Nurs, V19, P29

Inzucchi SE, 2009, CECIL MED, P1727

Joensen LE, 2016, PATIENT PREFER ADHER, V10, P1443, DOI 10.2147/PPA.S111696

Kovacs M, 1997, DIABETES CARE, V20, P36, DOI 10.2337/diacare.20.1.36

Luyckx K, 2008, J ADOLESCENT HEALTH, V43, P451, DOI 10.1016/j.jadohealth.2008.04.005

Luyckx K, 2009, DIABETES CARE, V32, P797, DOI 10.2337/dc08-1990

Marshall M, 2009, J CLIN NURS, V18, P1703, DOI 10.1111/j.1365-2702.2008.02737.x

Mattelmaki T, 2005, APPL PROBES FROM INS

NICE, 2016, TRANS CHILDR AD SERV

Olsson E, 2015, ACTION RES-LONDON, V13, P9, DOI 10.1177/1476750314566216

Palmer DL, 2011, J PEDIATR PSYCHOL, V36, P596, DOI 10.1093/jpepsy/jsq019

Patton M., 1990, QUALITATIVE EVALUATI

Peters A, 2011, DIABETES CARE, V34, P2477, DOI 10.2337/dc11-1723

Pillay J, 2015, ANN INTERN MED, V163, P836, DOI 10.7326/M15-1399

Rasmussen B, 2011, J CLIN NURS, V20, P1981, DOI 10.1111/j.1365-2702.2010.03657.x

Sakane N, 2015, J DIABETES INVEST, V6, P567, DOI 10.1111/jdi.12340

Schilling LS, 2002, J ADV NURS, V37, P87, DOI 10.1046/j.1365-2648.2002.02061.x

Schilling LS, 2009, NURS RES, V58, P228, DOI 10.1097/NNR.0b013e3181ac142a

Stuckey HL, 2015, CLIN THER, V37, P1986, DOI 10.1016/j.clinthera.2015.06.010

Tsiouli E, 2013, CAN FAM PHYSICIAN, V59, P143

Vallis M, 2014, CURR DIABETES REV, V10, P364, DOI 10.2174/1573399810666141113115026

Van Ryzin MJ, 2012, DEV PSYCHOPATHOL, V24, P1139, DOI 10.1017/S0954579412000582

Viklund G, 2009, J CLIN NURS, V18, P3262, DOI 10.1111/j.1365-2702.2009.02963.x

Young MT, 2014, CURR DIABETES REP, V14, DOI 10.1007/s11892-014-0546-5

NR 47

TC 4

Z9 4

U1 2

U2 12

PU WILEY

PI HOBOKEN

PA 111 RIVER ST, HOBOKEN 07030-5774, NJ USA

SN 0962-1067

EI 1365-2702

J9 J CLIN NURS

JI J. Clin. Nurs.

PD OCT

PY 2017

VL 26

IS 19-20

BP 3018

EP 3030

DI 10.1111/jocn.13649

PG 13

WC Nursing

SC Nursing

GA FG6YI

UT WOS:000410546400019

PM 27865017

DA 2019-01-08

ER

PT J

AU Ismail, K

Moulton, CD

Winkley, K

Pickup, JC

Thomas, SM

Sherwood, RA

Stahl, D

Amiel, SA

AF Ismail, Khalida

Moulton, Calum D.

Winkley, Kirsty

Pickup, John C.

Thomas, Stephen M.

Sherwood, Roy A.

Stahl, Daniel

Amiel, Stephanie A.

TI The association of depressive symptoms and diabetes distress with

glycaemic control and diabetes complications over 2 years in newly

diagnosed type 2 diabetes: a prospective cohort study

SO DIABETOLOGIA

LA English

DT Article

DE Depressive symptoms; Diabetes distress; Glycaemic control; Inflammation;

Macrovascular complications; Microvascular complications; Type 2

diabetes

ID EMOTIONAL DISTRESS; CONTROLLED-TRIAL; CARDIOVASCULAR COMPLICATIONS;

PROBLEM AREAS; MISSING DATA; FOLLOW-UP; MELLITUS; CARE; METAANALYSIS;

MORTALITY

AB Aims/hypothesis We examined the associations between depressive symptoms and diabetes distress with glycaemic control and diabetes complications over 2 years, after diagnosis of type 2 diabetes.

Methods In a multi-ethnic, primary care cohort (n = 1735) of adults, all with recent (< 6 months) diagnosis of type 2 diabetes, we measured the associations between depressive symptoms (Patient Health Questionnaire-9 [PHQ-9] score = 10) and diabetes distress (Problem Areas in Diabetes [PAID] score = 40), with change in 2 year HbA(1c) as the primary outcome and with incident rates of diabetes complications as secondary outcomes. Multivariate models were used to account for potential confounders.

Results Of the 1651 participants (95.2%) of the total primary care cohort with available baseline PHQ-9 and PAID scores, mean +/- SD age was 56.2 +/- 11.1 years, 55.1% were men and 49.1% were of non-white ethnicity; 232 (14.1%) and 111 (6.7%) had depressive symptoms and diabetes distress, respectively. After adjustment for confounders, depressive symptoms were not associated with worsening HbA(1c). After adjustment for age, sex, ethnicity, vascular risk factors and diabetes treatments, depressive symptoms were associated with increased risk of incident macrovascular complications (OR 2.78 [95% CI 1.19, 6.49], p = 0.018) but notmicrovascular complications. This was attenuated (p = 0.09) after adjustment for IL-1 receptor antagonist concentration. Diabetes distress was not associated with worsening HbA1c or incident complications.

Conclusions/interpretation In the first 2 years of type 2 diabetes, the effect of depressive symptoms and diabetes distress on glycaemic control is minimal. There was, however, an association between depressive symptoms and incidence of macrovascular complications. Elevated innate inflammation may be common to both depression and macrovascular diabetes complications, but these findings require replication.

C1 [Ismail, Khalida; Moulton, Calum D.; Winkley, Kirsty] Kings Coll London, Dept Psychol Med, Inst Psychiat Psychol & Neurosci, London SE5 9RJ, England.

[Pickup, John C.; Amiel, Stephanie A.] Kings Coll London, Div Diabet & Nutr Sci, Fac Life Sci & Med, London, England.

[Thomas, Stephen M.] Guys & St Thomas NHS Fdn Trust, Dept Endocrinol & Diabet, London, England.

[Sherwood, Roy A.] Kings Coll Hosp NHS Fdn Trust, Dept Clin Biochem, London, England.

[Stahl, Daniel] Kings Coll London, Dept Biostat, Inst Psychiat Psychol & Neurosci, London, England.

RP Ismail, K (reprint author), Kings Coll London, Dept Psychol Med, Inst Psychiat Psychol & Neurosci, London SE5 9RJ, England.

EM khalida.2.ismail@kcl.ac.uk

RI Stahl, Daniel/B-9713-2011

OI Stahl, Daniel/0000-0001-7987-6619; Winkley, Kirsty/0000-0002-1725-6040;

ismail, khalida/0000-0001-6084-449X

FU National Institute for Health Research (NIHR) under its Programme Grants

for Applied Research scheme [RP-PG-0606-1142]; NIHR Biomedical Research

Centre at South London and South London and Maudsley NHS Foundation

Trust; EFSD/Lilly Mental Health in Diabetes Programme; NIHR Biomedical

Research Centre for Mental Health at the South London and Maudsley NHS

Foundation Trust; Institute of Psychiatry, Psychology and Neuroscience,

King's College London; Royal College of Physicians of Edinburgh

FX This report presents independent research funded by the National

Institute for Health Research (NIHR) under its Programme Grants for

Applied Research scheme (RP-PG-0606-1142) and part-funded by the NIHR

Biomedical Research Centre at South London and South London and Maudsley

NHS Foundation Trust. Additional funding was received from the

EFSD/Lilly Mental Health in Diabetes Programme (2010) and the NIHR

Biomedical Research Centre for Mental Health at the South London and

Maudsley NHS Foundation Trust and Institute of Psychiatry, Psychology

and Neuroscience, King's College London. The views expressed in this

publication are those of the authors and not necessarily those of the

NHS, the NIHR or the Department of Health. CM receives salary support

from a fellowship funded by the Royal College of Physicians of

Edinburgh. The study sponsor was not involved in the design of the

study; the collection, analysis, and interpretation of data; writing the

report; or the decision to submit the report for publication.

CR Aikens JE, 2009, DIABETES CARE, V32, P1177, DOI 10.2337/dc09-0071

Alberti KGMM, 1998, DIABETIC MED, V15, P539, DOI 10.1002/(SICI)1096-9136(199807)15:7<539::AID-DIA668>3.0.CO;2-S

Anderson RJ, 2001, DIABETES CARE, V24, P1069, DOI 10.2337/diacare.24.6.1069

Bruce DG, 2005, DIABETOLOGIA, V48, P2532, DOI 10.1007/s00125-005-0024-3

Chiu CJ, 2010, SOC PSYCH PSYCH EPID, V45, P67, DOI 10.1007/s00127-009-0043-3

Cummings DM, 2016, DIABETES CARE, V39, P101, DOI 10.2337/dc15-1174

Dalsgaard EM, 2014, DIABETOLOGIA, V57, P710, DOI 10.1007/s00125-014-3165-4

de Groot M, 2001, PSYCHOSOM MED, V63, P619, DOI 10.1097/00006842-200107000-00015

Department for Communities and Local Government DCLG, 2010, ENGL IND DEPR 2010

Dirmaier J, 2010, PSYCHOTHER PSYCHOSOM, V79, P172, DOI 10.1159/000296135

Fenwick EK, 2018, J HEALTH PSYCHOL, V23, P667, DOI 10.1177/1359105316642006

Fisher L, 2010, DIABETES CARE, V33, P23, DOI 10.2337/dc09-1238

Herder C, 2015, TRENDS ENDOCRIN MET, V26, P551, DOI 10.1016/j.tem.2015.08.001

Hermanns N, 2015, DIABETES CARE, V38, P551, DOI 10.2337/dc14-1416

Kasteleyn MJ, 2015, DIABETIC MED, V32, P1617, DOI 10.1111/dme.12743

Katon WJ, 2010, NEW ENGL J MED, V363, P2611, DOI 10.1056/NEJMoa1003955

Katon WJ, 2010, INT J GERIATR PSYCH, V25, P466, DOI 10.1002/gps.2363

Katon WJ, 2004, J GEN INTERN MED, V19, P1192, DOI 10.1111/j.1525-1497.2004.30405.x

Kroenke K, 2001, J GEN INTERN MED, V16, P606, DOI 10.1046/j.1525-1497.2001.016009606.x

Laake JPS, 2014, DIABETES CARE, V37, P2186, DOI 10.2337/dc13-2522

Lustman PJ, 2006, ARCH GEN PSYCHIAT, V63, P521, DOI 10.1001/archpsyc.63.5.521

Lustman PJ, 2000, DIABETES CARE, V23, P934, DOI 10.2337/diacare.23.7.934

Lustman PJ, 1998, ANN INTERN MED, V129, P613, DOI 10.7326/0003-4819-129-8-199810150-00005

Mezuk B, 2008, DIABETES CARE, V31, P2383, DOI 10.2337/dc08-0985

Nouwen A, 2010, DIABETOLOGIA, V53, P2480, DOI 10.1007/s00125-010-1874-x

Palta P, 2014, J DIABETES COMPLICAT, V28, P798, DOI 10.1016/j.jdiacomp.2014.06.014

Pickup JC, 2003, DIABETIC MED, V20, P723, DOI 10.1046/j.1464-5491.2003.00990.x

POLONSKY WH, 1995, DIABETES CARE, V18, P754, DOI 10.2337/diacare.18.6.754

RAYMOND MR, 1986, EVAL HEALTH PROF, V9, P395, DOI 10.1177/016327878600900401

Roth PL, 1999, ORGAN RES METHODS, V2, P211, DOI 10.1177/109442819923001

Schmitt A, 2014, DIABETIC MED, V31, P1446, DOI 10.1111/dme.12553

Schmitt A, 2015, J BEHAV MED, V38, P556, DOI 10.1007/s10865-014-9604-3

Snoek FJ, 2000, DIABETES CARE, V23, P1305, DOI 10.2337/diacare.23.9.1305

Snoek FJ, 2015, LANCET DIABETES ENDO, V3, P450, DOI 10.1016/S2213-8587(15)00135-7

Snoek FJ, 2012, DIABETES CARE, V35, P2128, DOI 10.2337/dc11-1326

Stoop CH, 2014, DIABETIC MED, V31, P1252, DOI 10.1111/dme.12472

Ting RZ, J AFFECT DISORD, V149, P129

Tsujii S, 2012, DIABETIC MED, V29, P1451, DOI 10.1111/j.1464-5491.2012.03647.x

Twist K, 2013, PSYCHOSOM MED, V75, P791, DOI 10.1097/PSY.0b013e3182a2b108

van Bastelaar KMP, 2010, DIABETIC MED, V27, P798, DOI 10.1111/j.1464-5491.2010.03025.x

Welch GW, 1997, DIABETES CARE, V20, P760, DOI 10.2337/diacare.20.5.760

Winkley K, 2013, DIABETOLOGIA, V56, P1272, DOI 10.1007/s00125-013-2873-5

Winkley K, 2012, DIABETOLOGIA, V55, P303, DOI 10.1007/s00125-011-2359-2

NR 43

TC 4

Z9 5

U1 2

U2 6

PU SPRINGER

PI NEW YORK

PA 233 SPRING ST, NEW YORK, NY 10013 USA

SN 0012-186X

EI 1432-0428

J9 DIABETOLOGIA

JI Diabetologia

PD OCT

PY 2017

VL 60

IS 10

BP 2092

EP 2102

DI 10.1007/s00125-017-4367-3

PG 11

WC Endocrinology & Metabolism

SC Endocrinology & Metabolism

GA FF3BK

UT WOS:000408770300026

PM 28776084

OA Other Gold, Green Published

DA 2019-01-08

ER

PT J

AU Gamble, S

Mawokomatanda, T

Xu, F

Chowdhury, PP

Pierannunzi, C

Flegel, D

Garvin, W

Town, M

AF Gamble, Sonya

Mawokomatanda, Tebitha

Xu, Fang

Chowdhury, Pranesh P.

Pierannunzi, Carol

Flegel, David

Garvin, William

Town, Machell

TI Surveillance for Certain Health Behaviors and Conditions Among States

and Selected Local Areas - Behavioral Risk Factor Surveillance System,

United States, 2013 and 2014

SO MMWR SURVEILLANCE SUMMARIES

LA English

DT Article

ID AMERICAN-HEART-ASSOCIATION; JOINT CONSENSUS STATEMENT; SLEEP RESEARCH

SOCIETY; MENTAL DISTRESS STATUS; SERVICES TASK-FORCE; RECOMMENDED

AMOUNT; ADULTS; DISEASE; US; CARE

AB Problem: Chronic diseases and conditions (e.g., heart diseases, stroke, arthritis, and diabetes) are the leading causes of morbidity and mortality in the United States. These conditions are costly to the U.S. economy, yet they are often preventable or controllable. Behavioral risk factors (e.g., excessive alcohol consumption, tobacco use, poor diet, frequent mental distress, and insufficient sleep) are linked to the leading causes of morbidity and mortality. Adopting positive health behaviors (e.g., staying physically active, quitting tobacco use, obtaining routine physical checkups, and checking blood pressure and cholesterol levels) can reduce morbidity and mortality from chronic diseases and conditions. Monitoring the health risk behaviors, chronic diseases and conditions, access to health care, and use of preventive health services at multilevel public health points (states, territories, and metropolitan and micropolitan statistical areas [MMSA]) can provide important information for development and evaluation of health intervention programs.

Reporting Period: 2013 and 2014.

Description of the System: The Behavioral Risk Factor Surveillance System (BRFSS) is an ongoing, state-based, random-digitdialed telephone survey of noninstitutionalized adults aged >= 18 years residing in the United States. BRFSS collects data on health risk behaviors, chronic diseases and conditions, access to health care, and use of preventive health services and practices related to the leading causes of death and disability in the United States and participating territories. This is the first BRFSS report to include age-adjusted prevalence estimates. For 2013 and 2014, these age-adjusted prevalence estimates are presented for all 50 states, the District of Columbia, the Commonwealth of Puerto Rico, Guam, and selected MMSA.

Results: Age-adjusted prevalence estimates of health status indicators, health care access and preventive practices, health risk behaviors, chronic diseases and conditions, and cardiovascular conditions vary by state, territory, and MMSA. Each set of proportions presented refers to the range of age-adjusted prevalence estimates of selected BRFSS measures as reported by survey respondents. The following are estimates for 2013. Adults reporting frequent mental distress: 7.7%-15.2% in states and territories and 6.3%-19.4% in MMSA. Adults with inadequate sleep: 27.6%-49.2% in states and territories and 26.5%-44.4% in MMSA. Adults aged 18-64 years having health care coverage: 66.9%-92.4% in states and territories and 60.5%-97.6% in MMSA. Adults identifying as current cigarette smokers: 10.1%-28.8% in states and territories and 6.1%-33.6% in MMSA. Adults reporting binge drinking during the past month: 10.5%-25.2% in states and territories and 7.2%-25.3% in MMSA. Adults with obesity: 21.0%-35.2% in states and territories and 12.1%-37.1% in MMSA. Adults aged >= 45 years with some form of arthritis: 30.6%-51.0% in states and territories and 27.6%-52.4% in MMSA. Adults aged >= 45 years who have had coronary heart disease: 7.4%-17.5% in states and territories and 6.2%-20.9% in MMSA. Adults aged >= 45 years who have had a stroke: 3.1%-7.5% in states and territories and 2.3%-9.4% in MMSA. Adults with high blood pressure: 25.2%-40.1% in states and territories and 22.2%-42.2% in MMSA. Adults with high blood cholesterol: 28.8%-38.4% in states and territories and 26.3%-39.6% in MMSA.

The following are estimates for 2014. Adults reporting frequent physical distress: 7.8%-16.0% in states and territories and 6.2%-18.5% in MMSA. Women aged 21-65 years who had a Papanicolaou test during the past 3 years: 67.7%-87.8% in states and territories and 68.0%-94.3% in MMSA. Adults aged 50-75 years who received colorectal cancer screening on the basis of the 2008 U.S. Preventive Services Task Force recommendation: 42.8%-76.7% in states and territories and 49.1%-79.6% in MMSA. Adults with inadequate sleep: 28.4%-48.6% in states and territories and 25.4%-45.3% in MMSA. Adults reporting binge drinking during the past month: 10.7%-25.1% in states and territories and 6.7%-26.3% in MMSA. Adults aged >= 45 years who have had coronary heart disease: 8.0%-17.1% in states and territories and 7.6%-19.2% in MMSA. Adults aged >= 45 years with some form of arthritis: 31.2%-54.7% in states and territories and 28.4%-54.7% in MMSA. Adults with obesity: 21.0%-35.9% in states and territories and 19.7%-42.5% in MMSA.

Interpretation: Prevalence of certain chronic diseases and conditions, health risk behaviors, and use of preventive health services varies among states, territories, and MMSA. The findings of this report highlight the need for continued monitoring of health status, health care access, health behaviors, and chronic diseases and conditions at state and local levels.

Public Health Action: State and local health departments and agencies can continue to use BRFSS data to identify populations at risk for certain unhealthy behaviors and chronic diseases and conditions. Data also can be used to design, monitor, and evaluate public health programs at state and local levels.

C1 [Gamble, Sonya; Mawokomatanda, Tebitha; Xu, Fang; Chowdhury, Pranesh P.; Pierannunzi, Carol; Garvin, William; Town, Machell] CDC, Natl Ctr Chron Dis Prevent & Hlth Promot, Div Populat Hlth, Atlanta, GA 30333 USA.

[Flegel, David] Northrop Grumman Corp, Atlanta, GA USA.

RP Mawokomatanda, T (reprint author), CDC, Natl Ctr Chron Dis Prevent & Hlth Promot, Div Populat Hlth, Atlanta, GA 30333 USA.

EM chn4@cdc.gov

CR American Association for Public Opinion Research, 2016, STAND DEF FIN DISP C

Barbour KE, 2017, MMWR-MORBID MORTAL W, V66, P246, DOI 10.15585/mmwr.mm6609e1

Benard VB, 2014, MMWR-MORBID MORTAL W, V63, P1004

Bouchery EE, 2011, AM J PREV MED, V41, P516, DOI 10.1016/j.amepre.2011.06.045

CDC, 2012, CHRON DIS OV

CDC, 2016, TABL SUMM HLTH STAT

CDC, 2017, ARTHR AW MONTH

CDC, 2011, STRAT PREV OB OTH CH

*CDC, 2000, MEAS HLTH DAYS POP A

CDC, 2017, FACT SHEETS BING DRI

CDC, 2016, BEH RISK FACT SURV S

CDC, 2015, BEH RISK FACT SURV S

CDC Centers for Disease Control and Prevention, 2016, PHYS ACTIVITY HLTH

CDC National Center for Chronic Disease Prevention and Health Promotion, 2014, HLTH CONSMOK 50 YE

Centers for Disease Control, 2016, AD OB FACTS

Centers for Disease Control and Prevention (CDC), 2014, BEH RISK FACT SURV S

Chowdhury PP, 2016, MMWR SURVEILL SUMM, V65, P1, DOI 10.15585/mmwr.ss6504a1

Dwyer-Lindgren L, 2015, AM J PUBLIC HEALTH, V105, P1120, DOI 10.2105/AJPH.2014.302313

Ferrari AJ, 2013, PLOS MED, V10, DOI 10.1371/journal.pmed.1001547

Fox J. B., 2010, Morbidity and Mortality Weekly Report, V59, P1448

Heron Melonie, 2016, Natl Vital Stat Rep, V65, P1

Hoffman C, 2008, ANN NY ACAD SCI, V1136, P149, DOI 10.1196/annals.1425.007

Kanny Dafna, 2012, Morbidity and Mortality Weekly Report, V61, P14

King Brian, 2011, Morbidity and Mortality Weekly Report, V60, P1207

Klabunde CN, 2013, MMWR-MORBID MORTAL W, V62, P881

Kochanek KD, 2014, NATL VITAL STAT REPO, V65, P1

Lee IM, 2012, LANCET, V380, P219, DOI 10.1016/S0140-6736(12)61031-9

Li C, 2012, PREV MED, V54, P381

LIU Y, 2009, ELECTRON J DIFF 0112

May AL, 2013, MMWR SUPPL S3, V62

Moyer VA, 2012, ANN INTERN MED, V156, P880, DOI 10.7326/0003-4819-156-12-201206190-00424

Mozaffarian D, 2016, CIRCULATION, V133, P447, DOI 10.1161/CIR.0000000000000366

Mozaffarian D, 2015, CIRCULATION, V131, pE29, DOI 10.1161/CIR.0000000000000152

Pew Research Center, 2016, MOB FACT SHEET

Pierannunzi C, 2004, BMC MED RES METHODOL, V2013, P13, DOI DOI 10.1186/1471-2288-13-49

Pierannunzi C, 2015, AM ASS PUBL OP RES A

Sherry B., 2010, Morbidity and Mortality Weekly Report, V59, P951

Simon JG, 2005, EUR J PUBLIC HEALTH, V15, P200, DOI 10.1093/eurpub/cki102

Stevens JA, 2006, INJURY PREV, V12, P290, DOI 10.1136/ip.2005.011015

Strine TW, 2004, PREV MED, V39, P191, DOI 10.1016/j.ypmed.2004.01.022

Strine TW, 2004, AM J PREV MED, V26, P213, DOI 10.1016/j.amepre.2003.11.002

US Centers for Disease Control and Prevention (CDC), 2010, TOB SMOK CAUS DIS BI

US Department of Health and Human Services, 2001, HLTH PEOPL 2010 AG A

Watson NF, 2015, SLEEP, V38, P1161, DOI 10.5665/sleep.4886

Watson NF, 2015, SLEEP, V38, P843, DOI 10.5665/sleep.4716

Whiteford HA, 2013, LANCET, V382, P1575, DOI 10.1016/S0140-6736(13)61611-6

World Health Organization [WHO], 2013, WHO REP GLOB TOB EP

Xu J, 2013, NATL VITAL STAT REP, V64, P1

Xuan ZM, 2015, AM J PUBLIC HEALTH, V105, P816, DOI 10.2105/AJPH.2014.302112

Zack MM, 2004, PUBLIC HEALTH REP, V119, P493, DOI 10.1016/j.phr.2004.07.007

Zack MM, 2013, MMWR SUPPL S3, V62

Zauber AG, 2008, ANN INTERN MED, V149, P659, DOI 10.7326/0003-4819-149-9-200811040-00244

NR 52

TC 5

Z9 5

U1 1

U2 3

PU CENTERS DISEASE CONTROL

PI ATLANTA

PA 1600 CLIFTON RD, ATLANTA, GA 30333 USA

SN 1545-8636

J9 MMWR SURVEILL SUMM

JI MMWR Surv. Summ.

PD SEP 15

PY 2017

VL 66

IS 16

BP 1

EP 139

PG 139

WC Public, Environmental & Occupational Health

SC Public, Environmental & Occupational Health

GA FM4IG

UT WOS:000414978200001

PM 28910267

OA Bronze, Green Published

DA 2019-01-08

ER

PT J

AU Assari, S

Lee, DB

Nicklett, EJ

Lankarani, MM

Piette, JD

Aikens, JE

AF Assari, Shervin

Lee, Daniel B.

Nicklett, Emily Joy

Lankarani, Maryam Moghani

Piette, John D.

Aikens, James E.

TI Racial Discrimination in Health Care Is Associated with Worse Glycemic

Control among Black Men but Not Black Women with Type 2 Diabetes

SO FRONTIERS IN PUBLIC HEALTH

LA English

DT Article

DE Blacks; African-Americans; gender; racial discrimination; diabetes

ID AFRICAN-AMERICAN MEN; SELF-RATED HEALTH; STRESSFUL LIFE EVENTS;

SICKLE-CELL-DISEASE; PERCEIVED DISCRIMINATION; DEPRESSIVE SYMPTOMS;

UNITED-STATES; PSYCHOLOGICAL DISTRESS; CULTURAL COMPETENCE;

GENDER-DIFFERENCES

AB Background: A growing body of research suggests that racial discrimination may affect the health of Black men and Black women differently.

Aims: This study examined Black patients with diabetes mellitus (DM) in order to test gender differences in (1) levels of perceived racial discrimination in health care and (2) how perceived discrimination relates to glycemic control.

Methods: A total of 163 Black patients with type 2 DM (78 women and 85 men) provided data on demographics (age and gender), socioeconomic status, perceived racial discrimination in health care, self-rated health, and hemoglobin A1c (HbA1c). Data were analyzed using linear regression.

Results: Black men reported more racial discrimination in health care than Black women. Although racial discrimination in health care was not significantly associated with HbA1c in the pooled sample (b = 0.20, 95% CI = -0.41 -0.80), gender-stratified analysis indicated an association between perceived discrimination and higher HbA1c levels for Black men (b = 0.86, 95% confidence intervals (CI) = 0.01-1.73) but not Black women (b = -0.31, 95% CI = -1.17 to -0.54).

Conclusion: Perceived racial discrimination in diabetes care may be more salient for glycemic control of Black men than Black women. Scholars and clinicians should take gender into account when considering the impacts of race-related discrimination experiences on health outcomes. Policies should reduce racial discrimination in the health care.

C1 [Assari, Shervin; Lankarani, Maryam Moghani] Univ Michigan, Dept Psychiat, Ann Arbor, MI 48109 USA.

[Assari, Shervin] Univ Michigan, Sch Publ Hlth, Ctr Res Ethn Culture & Hlth, Ann Arbor, MI 48109 USA.

[Lee, Daniel B.; Piette, John D.] Univ Michigan, Sch Publ Hlth, Dept Hlth Behav & Hlth Educ, Ann Arbor, MI 48109 USA.

[Lee, Daniel B.] Univ Michigan, Sch Publ Hlth, Prevent Res Ctr, Ann Arbor, MI 48109 USA.

[Nicklett, Emily Joy] Univ Michigan, Sch Social Work, Ann Arbor, MI 48109 USA.

[Piette, John D.] Vet Affairs Ctr Clin Management Res, Ann Arbor, MI USA.

[Piette, John D.] Michigan Med, Dept Internal Med, Ann Arbor, MI USA.

[Aikens, James E.] Michigan Med, Dept Family Med, Ann Arbor, MI USA.

RP Assari, S (reprint author), Univ Michigan, Dept Psychiat, Ann Arbor, MI 48109 USA.; Assari, S (reprint author), Univ Michigan, Sch Publ Hlth, Ctr Res Ethn Culture & Hlth, Ann Arbor, MI 48109 USA.

EM assari@umich.edu

FU National Institutes of Health [R01DK066016]; Heinz C. Prechter Bipolar

Research Fund; Richard Tam Foundation at the University of Michigan

Depression Center; Eunice Kennedy Shriver National Institute of Child

Health and Development (NICHD) [T32 HD 79350-2]

FX This study was funded by the National Institutes of Health

(R01DK066016). SA is supported by the Heinz C. Prechter Bipolar Research

Fund and the Richard Tam Foundation at the University of Michigan

Depression Center. Second author is supported by a grant from the Eunice

Kennedy Shriver National Institute of Child Health and Development

(NICHD) (T32 HD 79350-2).

CR Aikens JE, 2012, DIABETES CARE, V35, P2472, DOI 10.2337/dc12-0181

Aikens JE, 2009, DIABETES CARE, V32, P1177, DOI 10.2337/dc09-0071

Anderson LM, 2003, AM J PREV MED, V24, P68, DOI 10.1016/S0749-3797(02)00657-8

Assari S, 2018, J RACIAL ETHN HEALTH, V5, P243, DOI 10.1007/s40615-017-0364-y

Assari S, 2016, SAGE OPEN MED, V4, DOI 10.1177/2050312116666975

Assari S, 2017, CHILDREN-BASEL, V4, DOI 10.3390/children4020014

Assari S, 2017, FRONT PSYCHIATRY, V8, DOI 10.3389/fpsyt.2017.00023

Assari S, 2016, IRAN J PSYCHIATRY BE, V10, DOI 10.17795/ijpbs-3458

Assari S, 2016, ARCH TRAUMA RES, V5, DOI 10.5812/atr.31475

Assari S, 2016, J RACIAL ETHN HEALTH, V3, P349, DOI 10.1007/s40615-015-0160-5

Assari S, 2016, ANN EPIDEMIOL, V26, P106, DOI 10.1016/j.annepidem.2015.11.006

Assari S, 2015, J RACIAL ETHN HEALTH, V2, P200, DOI 10.1007/s40615-014-0064-9

Assari S, 2015, INT J PREVENTIVE MED, V6, DOI [10.4103/2008-7802.17043, 10.4103/2008-7802.170431]

Assari S, 2015, SOCIETIES, V5, P151, DOI 10.3390/soc5010151

Assari S, 2014, J DIABETES METAB DIS, V13, DOI 10.1186/2251-6581-13-36

Banks KH, 2006, COMMUNITY MENT HLT J, V42, P555, DOI 10.1007/s10597-006-9052-9

Bertakis KD, 2000, J FAM PRACTICE, V49, P147

BOWMAN PJ, 1985, J AM ACAD CHILD PSY, V24, P134, DOI 10.1016/S0002-7138(09)60438-6

Brodish Amanda B, 2011, Race Soc Probl, V3, P160

Broman C.L., 2000, J BLACK PSYCHOL, V26, P165, DOI DOI 10.1177/0095798400026002003

Brondolo E, 2008, J SOC CLIN PSYCHOL, V27, P150, DOI 10.1521/jscp.2008.27.2.150

Brondolo E, 2009, J BEHAV MED, V32, P64, DOI 10.1007/s10865-008-9193-0

BROWN TN, 2000, RACE SOC, V0002

Browne I, 2003, ANNU REV SOCIOL, V29, P487, DOI 10.1146/annurev.soc.29.010202.100016

Caldwell CH, 2013, PSYCHOL MEN MASCULIN, V14, P47, DOI 10.1037/a0029105

Canady RB, 2008, WOMEN HEALTH ISS, V18, P292, DOI 10.1016/j.whi.2008.04.003

Carter JS, 1996, ANN INTERN MED, V125, P221, DOI 10.7326/0003-4819-125-3-199608010-00011

Caughy MO, 2004, AM J PUBLIC HEALTH, V94, P2118, DOI 10.2105/AJPH.94.12.2118

Chen FM, 2005, ANN FAM MED, V3, P138, DOI 10.1370/afm.282

Clark R, 1999, AM PSYCHOL, V54, P805, DOI 10.1037//0003-066X.54.10.805

Clifford A, 2015, INT J QUAL HEALTH C, V27, P89, DOI 10.1093/intqhc/mzv010

Coard S. J., 2004, J CHILD FAM STUD, V13, P277, DOI DOI 10.1023/B:JCFS.0000022035.07171.F8

Cook CB, 2000, DIABETES CARE, V23, P319, DOI 10.2337/diacare.23.3.319

Davis JE, 2003, URBAN EDUC, V38, P515, DOI 10.1177/0042085903256220

Forsyth J, 2014, PSYCHOSOM MED, V76, P229, DOI 10.1097/PSY.0000000000000043

Foynes MM, 2013, CULT DIVERS ETHN MIN, V19, P111, DOI 10.1037/a0030567

Gallagher RW, 2015, NURS EDUC TODAY, V35, P333, DOI 10.1016/j.nedt.2014.10.021

Hammond WP, 2012, AM J PUBLIC HEALTH, V102, pS232, DOI 10.2105/AJPH.2011.300485

Hammond WP, 2010, J GEN INTERN MED, V25, P1300, DOI 10.1007/s11606-010-1481-z

Hammond WP, 2010, AM J COMMUN PSYCHOL, V45, P87, DOI 10.1007/s10464-009-9280-6

Harnois CE, 2011, ETHNIC RACIAL STUD, V34, P1006, DOI 10.1080/01419870.2010.516836

Hausmann LRM, 2010, ETHNIC DIS, V20, P40

Hawkins J, 2017, AM J MENS HEALTH, V11, P344, DOI 10.1177/1557988316681125

Haywood C, 2014, J PAIN SYMPTOM MANAG, V48, P934, DOI 10.1016/j.jpainsymman.2014.02.002

Haywood C, 2014, J GEN INTERN MED, V29, P1657, DOI 10.1007/s11606-014-2986-7

Himmelstein MS, 2015, PSYCHOL HEALTH, V30, P253, DOI 10.1080/08870446.2014.966104

Honora DT, 2002, ADOLESCENCE, V37, P301

Horvat L, 2014, COCHRANE DB SYST REV, DOI 10.1002/14651858.CD009405.pub2

Hudson DL, 2012, J MENS HEALTH, V9, P127, DOI 10.1016/j.jomh.2012.03.006

Infatunji M., 2016, SOCIOL RACE ETHN, V2, P263, DOI [10.1177/2332649215613532, DOI 10.1177/2332649215613532]

Jackson J. S, 1995, ETHNIC DIS, V6, P132

Jackson JS, 2010, AM J PUBLIC HEALTH, V100, P933, DOI 10.2105/AJPH.2008.143446

Kelly MM, 2008, DEPRESS ANXIETY, V25, P839, DOI 10.1002/da.20341

Kessler RC, 1999, J HEALTH SOC BEHAV, V40, P208, DOI 10.2307/2676349

Krieger N, 1996, AM J PUBLIC HEALTH, V86, P1370, DOI 10.2105/AJPH.86.10.1370

KRIEGER N, 1990, SOC SCI MED, V30, P1273, DOI 10.1016/0277-9536(90)90307-E

Krieger N, 2012, AM J PUBLIC HEALTH, V102, P936, DOI 10.2105/AJPH.2011.300544

Landrine H., 1996, J BLACK PSYCHOL, V22, P144, DOI DOI 10.1177/00957984960222002

Lie DA, 2011, J GEN INTERN MED, V26, P317, DOI 10.1007/s11606-010-1529-0

Mayor E, 2015, FRONT PSYCHOL, V6, DOI 10.3389/fpsyg.2015.00779

McLaughlin KA, 2010, AM J PUBLIC HEALTH, V100, P1477, DOI 10.2105/AJPH.2009.181586

Mezuk B, 2013, SOC MENT HEALTH, V3, P79, DOI 10.1177/2156869313480892

Nam C., 1983, SOCIOECONOMIC APPROA

Neblett EW, 2016, COGN BEHAV PRACT, V23, P385, DOI 10.1016/j.cbpra.2016.05.002

Noguera PA, 2003, URBAN EDUC, V38, P431, DOI 10.1177/0042085903254969

Odom EC, 2010, J MARRIAGE FAM, V72, P346, DOI 10.1111/j.1741-3737.2010.00704.x

Ong AD, 2009, J PERS SOC PSYCHOL, V96, P1259, DOI 10.1037/a0015335

Pascoe EA, 2009, PSYCHOL BULL, V135, P531, DOI 10.1037/a0016059

Peek ME, 2011, MED CARE, V49, P618, DOI 10.1097/MLR.0b013e318215d925

Piette JD, 2006, PATIENT EDUC COUNS, V60, P41, DOI 10.1016/j.pec.2004.12.001

Roderick M, 2003, URBAN EDUC, V38, P538, DOI 10.1177/0042085903256221

Ryan AM, 2008, J HEALTH CARE POOR U, V19, P149, DOI 10.1353/hpu.2008.0005

Schnittker J, 2014, PLOS ONE, V9, DOI 10.1371/journal.pone.0084933

Schulz AJ, 2006, AM J PUBLIC HEALTH, V96, P1265, DOI 10.2105/AJPH.2005.064543

Seaton EK, 2011, CHILD DEV, V82, P1850, DOI 10.1111/j.1467-8624.2011.01651.x

Sellers RM, 2006, J RES ADOLESCENCE, V16, P187, DOI 10.1111/j.1532-7795.2006.00128.x

Sellers RM, 2003, J HEALTH SOC BEHAV, V44, P302, DOI 10.2307/1519781

Sellers RM, 2003, J PERS SOC PSYCHOL, V84, P1079, DOI 10.1037/0022-3514.84.5.1079

SIMPSON AW, 1983, AM EDUC RES J, V20, P183, DOI 10.2307/1162593

Singh-Manoux A, 2006, J EPIDEMIOL COMMUN H, V60, P364, DOI 10.1136/jech.2005.039883

Slaughter-Acey JC, 2013, WOMEN HEALTH ISS, V23, pE381, DOI 10.1016/j.whi.2013.08.001

Smalls C, 2007, J BLACK PSYCHOL, V33, P299, DOI 10.1177/0095798407302541

Swanson DP, 2003, URBAN EDUC, V38, P608, DOI 10.1177/0042085903256218

Thomas A., 2016, J MEN STUD, V24, P185, DOI DOI 10.1177/1060826516641104

Torres L, 2010, CULT DIVERS ETHN MIN, V16, P561, DOI 10.1037/a0020652

Trivedi AN, 2006, J GEN INTERN MED, V21, P553, DOI 10.1111/j.1525-1497.2006.00413.x

Utsey SO, 2008, J COUNS PSYCHOL, V55, P49, DOI 10.1037/0022-0167.55.1.49

Varner F, 2013, CHILD DEV, V84, P875, DOI 10.1111/cdev.12021

Wagner J, 2007, DIABETES CARE, V30, P364, DOI 10.2337/dc06-1756

Walker RL, 2014, SUICIDE LIFE-THREAT, V44, P548, DOI 10.1111/sltb.12089

Wang JJ, 2013, INT J PALLIAT NURS, V19, P347, DOI 10.12968/ijpn.2013.19.7.347

Williams DR, 2009, J BEHAV MED, V32, P20, DOI 10.1007/s10865-008-9185-0

Williams DR, 2003, AM J PUBLIC HEALTH, V93, P200, DOI 10.2105/AJPH.93.2.200

NR 96

TC 4

Z9 4

U1 0

U2 2

PU FRONTIERS MEDIA SA

PI LAUSANNE

PA AVENUE DU TRIBUNAL FEDERAL 34, LAUSANNE, CH-1015, SWITZERLAND

SN 2296-2565

J9 FRONT PUBLIC HEALTH

JI Front. Public Health

PD SEP 12

PY 2017

VL 5

AR UNSP 235

DI 10.3389/fpubh.2017.00235

PG 9

WC Public, Environmental & Occupational Health

SC Public, Environmental & Occupational Health

GA FH2ME

UT WOS:000410972700001

PM 28955703

OA DOAJ Gold, Green Published

DA 2019-01-08

ER

PT J

AU Lin, KK

Park, C

Li, MZ

Wang, XD

Li, XS

Li, W

Quinn, L

AF Lin, Keke

Park, Chang

Li, Mingzi

Wang, Xiudong

Li, Xiushu

Li, Wei

Quinn, Laurie

TI Effects of depression, diabetes distress, diabetes self-efficacy, and

diabetes self-management on glycemic control among Chinese population

with type 2 diabetes mellitus

SO DIABETES RESEARCH AND CLINICAL PRACTICE

LA English

DT Article

DE Diabetes; Glycemic control; Diabetes self-management; Diabetes

self-efficacy; Depression; Diabetes distress

ID SOCIAL DETERMINANTS; HEALTH; ADULTS; CARE; VALIDATION; ADHERENCE;

OUTCOMES; WOMEN

AB Aim: To examine the direct and indirect effects of depression, diabetes distress, diabetes self-efficacy and diabetes self-management on glycemic control among a group of T2DM patients in China.

Method: A convenience sample of 254 participants were selected from three outpatient departments in Beijing, China. They were surveyed using a self-administered questionnaire. Diabetes-related information was retrieved from their medical records. Descriptive statistics, independent student t tests, Chi-square tests, correlation analyses and Generalized Structural Equation Modeling were used.

Results: Only 91 (35.82%) participants achieved optimal glycemic control of HbA1c < 7.0% (53 mmol/mol). Only diabetes self-management had a direct effect on glycemic control (OR = 0.95, P < 0.001). Depression and diabetes distress had only indirect effects on glycemic control through both diabetes self-efficacy and diabetes self-management. Diabetes selfefficacy only had an indirect effect on glycemic control through diabetes self-management.

Conclusions: Glycemic control among Chinese population with T2DM was suboptimal. Future interventions should focus on decreasing depressive symptoms and diabetes distress levels, and, therefore, improve diabetes self-efficacy and self-management practices and, ultimately, reach the optimal goal of glycemic control. (C) 2017 Elsevier B.V. All rights reserved.

C1 [Lin, Keke; Park, Chang; Quinn, Laurie] Univ Illinois, Coll Nursing, Chicago, IL USA.

[Lin, Keke; Li, Mingzi] Peking Univ, Hlth Sci Ctr, Sch Nursing, 38 Xueyuan Rd, Beijing 100191, Peoples R China.

[Wang, Xiudong] Beijing Jingmei Grp Gen Hosp, Dept Endocrinol, Beijing, Peoples R China.

[Li, Xiushu] Peking Univ, Peoples Hosp, Emergency Room, Beijing, Peoples R China.

[Li, Wei] Peking Univ, Dept Endocrinol, Hosp 1, Beijing, Peoples R China.

RP Li, MZ (reprint author), Peking Univ, Hlth Sci Ctr, Sch Nursing, 38 Xueyuan Rd, Beijing 100191, Peoples R China.

EM limingzi@bjmu.edu.cn

CR Ahmad NS, 2014, J DIABETES INVEST, V5, P563, DOI 10.1111/jdi.12175

Al-Khawaldeh OA, 2012, J DIABETES COMPLICAT, V26, P10, DOI 10.1016/j.jdiacomp.2011.11.002

American Diabetes Association, STAND MED CAR DIAB 2

Asuzu CC, 2016, J DIABETES COMPLICAT

Chen R, 2015, MED SCI MONITOR, V21, P1440, DOI 10.12659/MSM.892246

Ciccone MM, 2010, VASC HEALTH RISK MAN, V6, P297

COHEN J, 1992, PSYCHOL BULL, V112, P155, DOI 10.1037//0033-2909.112.1.155

Cosansu G, 2014, J TRANSCULT NURS, V25, P51, DOI 10.1177/1043659613504112

Cummings DM, 2014, ANN PHARMACOTHER, V48, P970, DOI 10.1177/1060028014536532

Faul F, 2007, BEHAV RES METHODS, V39, P175, DOI 10.3758/BF03193146

Fisher L, 2010, DIABETES CARE, V33, P1034, DOI 10.2337/dc09-2175

Fisher L, 2010, DIABETES CARE, V33, P23, DOI 10.2337/dc09-1238

Gao JL, 2013, BMC FAM PRACT, V14, DOI 10.1186/1471-2296-14-66

Gonzalez JS, 2015, HEALTH PSYCHOL, V34, P505, DOI 10.1037/hea0000131

Guo XH, 2012, CHINESE MED J-PEKING, V125, P4175, DOI 10.3760/cma.j.issn.0366-6999.2012.23.003

International Diabetes Federation, IDF DIABETES ATLAS

[嵇加佳 Ji Jiajia], 2014, [中华护理杂志, Chinese Journal of Nursing], V49, P617

Ji LN, 2013, BMC PUBLIC HEALTH, V13, DOI 10.1186/1471-2458-13-602

Li MZ., 2012, STUDY APPL DEPRESSIO

Liu Ming, 2014, Beijing Da Xue Xue Bao Yi Xue Ban, V46, P782

Lu JM, 2014, PATIENT PREFER ADHER, V8, P1161, DOI 10.2147/PPA.S66915

Peyrot M, 2005, DIABETIC MED, V22, P1379, DOI 10.1111/j.1464-5491.2005.01644.x

Qteishat RR, 2015, DIABETES METABOL SYN

SHAMOON H, 1993, NEW ENGL J MED, V329, P977

Shiu ATY, 2003, DIABETES CARE, V26, P2817, DOI 10.2337/diacare.26.10.2817

[孙胜男 Sun Shengnan], 2011, [中华护理杂志, Chinese Journal of Nursing], V46, P229

Toobert DJ, 2000, DIABETES CARE, V23, P943, DOI 10.2337/diacare.23.7.943

Tsujii S, 2012, DIABETIC MED, V29, P1451, DOI 10.1111/j.1464-5491.2012.03647.x

Turner RC, 1998, LANCET, V352, P837

Walker RJ, 2015, DIABETES RES CLIN PR, V110, P193, DOI 10.1016/j.diabres.2015.09.007

Walker RJ, 2015, DIABETES TECHNOL THE, V17, P80, DOI 10.1089/dia.2014.0166

Walker RJ, 2014, GEN HOSP PSYCHIAT, V36, P662, DOI 10.1016/j.genhosppsych.2014.06.011

Walker RJ, 2014, BMC ENDOCR DISORD, V14, DOI 10.1186/1472-6823-14-82

Xu Y, 2008, RES NURS HEALTH, V31, P613, DOI 10.1002/nur.20293

Xu Y, 2013, JAMA-J AM MED ASSOC, V310, P948, DOI 10.1001/jama.2013.168118

Yeung A, 2008, COMPR PSYCHIAT, V49, P211, DOI 10.1016/j.comppsych.2006.06.002

NR 36

TC 2

Z9 3

U1 2

U2 11

PU ELSEVIER IRELAND LTD

PI CLARE

PA ELSEVIER HOUSE, BROOKVALE PLAZA, EAST PARK SHANNON, CO, CLARE, 00000,

IRELAND

SN 0168-8227

EI 1872-8227

J9 DIABETES RES CLIN PR

JI Diabetes Res. Clin. Pract.

PD SEP

PY 2017

VL 131

BP 179

EP 186

DI 10.1016/j.diabres.2017.03.013

PG 8

WC Endocrinology & Metabolism

SC Endocrinology & Metabolism

GA FM6VC

UT WOS:000415197900020

PM 28756132

DA 2019-01-08

ER

PT J

AU Massetti, GM

Thomas, CC

King, J

Ragan, K

Lunsford, NB

AF Massetti, Greta M.

Thomas, Cheryll C.

King, Jessica

Ragan, Kathleen

Lunsford, Natasha Buchanan

TI Mental Health Problems and Cancer Risk Factors Among Young Adults

SO AMERICAN JOURNAL OF PREVENTIVE MEDICINE

LA English

DT Article

ID SERVICES TASK-FORCE; PHYSICAL HEALTH; CERVICAL-CANCER; UNITED-STATES;

ILLNESS; METAANALYSIS; DEPRESSION; DISORDERS; MORTALITY; SYMPTOMS

AB Introduction: Chronic mental health problems often emerge in young adulthood, when adults begin to develop lifelong health behaviors and access preventive health services. The associations between mental health problems and modifiable cancer risk factors in young adulthood are not well understood.

Methods: In 2016, the authors analyzed 2014 Behavioral Risk Factor Surveillance System data on demographic characteristics, health service access and use, health status, and cancer risk factors (tobacco use, alcohol use, overweight or obesity, physical activity, and sleep) for 90,821 young adults aged 18-39 years with mental health problems (depressive disorder or frequent mental distress) compared to other young adults.

Results: Mental health problems were associated with white race; less than a high school education; lower income; being out of work or unable to work; being uninsured (for men only); poor health; previous diagnosis of asthma, skin cancer, or diabetes; and not having a recent checkup. After controlling for demographic characteristics, health service use, and health status, mental health problems among young adults were associated with smoking, binge drinking, inadequate sleep, having no leisure time physical activity, and being overweight or obese (among women only). Cervical cancer screening was not associated with mental health problems after controlling for demographic characteristics, health service use, and health status.

Conclusions: Mental health problems in young adulthood were associated with potentially modifiable factors and behaviors that increase risk for cancer. Efforts to prevent cancer and promote health must attend to mental health disparities to meet the needs of young adults. Published by Elsevier Inc. on behalf of American Journal of Preventive Medicine.

C1 [Massetti, Greta M.; Thomas, Cheryll C.; King, Jessica; Ragan, Kathleen; Lunsford, Natasha Buchanan] Ctr Dis Control & Prevent, Div Canc Prevent & Control, Natl Ctr Chron Dis Prevent & Hlth Promot, 4770 Buford Highway,NE MS F-76, Atlanta, GA 30341 USA.

RP Massetti, GM (reprint author), Ctr Dis Control & Prevent, Div Canc Prevent & Control, Natl Ctr Chron Dis Prevent & Hlth Promot, 4770 Buford Highway,NE MS F-76, Atlanta, GA 30341 USA.

EM gmassetti@cdc.gov

FU U.S. Centers for Disease Control and Prevention (CDC), an Agency of the

U.S. Department of Health and Human Services [200-2017-M-94637]

FX Publication of this article was supported by the U.S. Centers for

Disease Control and Prevention (CDC), an Agency of the U.S. Department

of Health and Human Services, under contract number: 200-2017-M-94637.

The findings and conclusions in this article are those of the authors

and do not necessarily represent the official position of CDC. n.

CR BEEKMAN ATF, 1995, SOC PSYCH PSYCH EPID, V30, P32, DOI 10.1007/BF00784432

Brown DW, 2003, J OCCUP ENVIRON MED, V45, P1159, DOI 10.1097/01.jom.0000094994.09.655.0f

Center for Behavioral Health Statistics and Quality, HHS PUBL

Charara R, 2016, PREV MED, V89, P292, DOI 10.1016/j.ypmed.2016.06.011

Chesher NJ, 2012, AM J ADDICTION, V21, P1, DOI 10.1111/j.1521-0391.2011.00196.x

Clarke TC, 2015, EARLY RELEASE SELECT

Eaton WW, 1997, ARCH GEN PSYCHIAT, V54, P993

Gallicchio L, 2009, J SLEEP RES, V18, P148, DOI 10.1111/j.1365-2869.2008.00732.x

Gardner MJ, BR MED J CLIN RES ED, P746

Gfroerer J, 2013, MMWR-MORBID MORTAL W, V62, P81

Lando James, 2006, Prev Chronic Dis, V3, pA61

Moriarty David G, 2003, Health Qual Life Outcomes, V1, P37, DOI 10.1186/1477-7525-1-37

Moyer VA, 2012, ANN INTERN MED, V156, P880, DOI 10.7326/0003-4819-156-12-201206190-00424

National Center for Health Statistics, 2010, HLTH US 2009 SPEC FE, P458

O'Connor E, 2016, JAMA-J AM MED ASSOC, V315, P388, DOI 10.1001/jama.2015.18948

Office of Disease Prevention and Health Promotion, HLTH PEOPL 2020

Robson D, 2007, INT J NURS STUD, V44, P457, DOI 10.1016/j.ijnurstu.2006.07.013

Schottenfeld D., 2006, CANC EPIDEMIOLOGY PR

Siu AL, 2016, JAMA-J AM MED ASSOC, V315, P380, DOI 10.1001/jama.2015.18392

Stanley SL, 2014, J COMMUN HEALTH, V39, P400, DOI 10.1007/s10900-013-9778-6

Vanhoof JMM, 2014, J HEART LUNG TRANSPL, V33, P800, DOI 10.1016/j.healun.2014.04.003

Walker ER, 2015, JAMA PSYCHIAT, V72, P334, DOI 10.1001/jamapsychiatry.2014.2502

NR 22

TC 2

Z9 2

U1 6

U2 6

PU ELSEVIER SCIENCE INC

PI NEW YORK

PA 360 PARK AVE SOUTH, NEW YORK, NY 10010-1710 USA

SN 0749-3797

EI 1873-2607

J9 AM J PREV MED

JI Am. J. Prev. Med.

PD SEP

PY 2017

VL 53

IS 3

SU 1

SI SI

BP S30

EP S39

DI 10.1016/j.amepre.2017.04.023

PG 10

WC Public, Environmental & Occupational Health; Medicine, General &

Internal

SC Public, Environmental & Occupational Health; General & Internal Medicine

GA FM6TN

UT WOS:000415192400005

PM 28818243

OA Other Gold

DA 2019-01-08

ER

PT J

AU Trump, LJ

Mendenhall, TJ

AF Trump, Lisa J.

Mendenhall, Tai J.

TI Community Health Workers in Diabetes Care: A Systematic Review of

Randomized Controlled Trials

SO FAMILIES SYSTEMS & HEALTH

LA English

DT Review

DE chronic illness; community health workers; diabetes; type II diabetes

ID AFRICAN-AMERICAN ADULTS; SELF-MANAGEMENT; MEXICAN-AMERICANS; GLYCEMIC

CONTROL; SOCIAL SUPPORT; INTERVENTION; LATINOS; EDUCATION; OUTCOMES;

PROGRAM

AB Introduction: Maintaining optimal self-care in managing Type 2 diabetes is a common struggle for patients due to several barriers, including access to quality services, financial insecurity and/or lack of insurance, and emotional distress. Consequently, morbidity and mortality rates are high, alongside rising health care costs. Alternative approaches that address common barriers require further investigation. This systematic review of randomized controlled trials examines the effectiveness of using community health workers (CHWs) in Type 2 diabetes care. This effort is warranted to orient practitioners and researchers to the state of existing knowledge, and to direct clinical practice and future research. Method: Data were extracted from 17 peer-reviewed articles; they were examined with respect to theory integration, CHW intervention design, outcome variables, and findings. Results: Approximately one-third of articles explicitly integrated theory into their research conceptualization and design. There was great variation across intervention dosages, attrition rates, and methods of CHW training. Main foci across studies' findings suggest that a CHW intervention has significant impacts on physical health outcomes, diabetes knowledge, self-care behaviors, and emotional distress and well-being. Discussion: Principal implications relate to the need for more research regarding CHW intervention types and methods, and further investigation about the mechanisms of change within a CHW-delivered intervention. Findings support the case for more CHWs in treatment teams to bridge patients with the medical system. This research will serve to better equip providers in the support of patients managing Type 2 diabetes and advance the Triple Aim of health care.

C1 [Trump, Lisa J.; Mendenhall, Tai J.] Univ Minnesota, Dept Family Social Sci, 1985 Buford Ave,290 McNeal Hall, St Paul, MN 55108 USA.

RP Trump, LJ (reprint author), Univ Minnesota, Dept Family Social Sci, 1985 Buford Ave,290 McNeal Hall, St Paul, MN 55108 USA.

EM buch0245@umn.edu; mend0009@umn.edu

CR Ali MK, 2013, NEW ENGL J MED, V368, P1613, DOI 10.1056/NEJMsa1213829

Anderson R. M., 2005, ART EMPOWERMENT STOR

Babamoto KS, 2009, HEALTH EDUC BEHAV, V36, P113, DOI 10.1177/1090198108325911

Bandura A, 2001, MEDIA PSYCHOL, V3, P265, DOI 10.1207/S1532785XMEP0303_03

Batts ML, 2001, DIABETES EDUCATOR, V27, P405, DOI 10.1177/014572170102700310

Bureau of Labor Statistics, 2015, OCC EMPL STAT

Carolan-Olah MC, 2013, BMC HEALTH SERV RES, V13, DOI 10.1186/1472-6963-13-447

Corkery E, 1997, DIABETES CARE, V20, P254, DOI 10.2337/diacare.20.3.254

Dumville JC, 2006, BMJ-BRIT MED J, V332, P969, DOI 10.1136/bmj.332.7547.969

Fewtrell MS, 2008, ARCH DIS CHILD, V93, P458, DOI 10.1136/adc.2007.127316

Gary TL, 2003, PREV MED, V37, P23, DOI 10.1016/S0091-7435(03)00040-9

GREEN LW, 1991, HLTH PROMOTION PLANN, P22

Heisler M, 2014, ANN INTERN MED, V161, pS13, DOI 10.7326/M13-3012

Katon WJ, 2013, J PSYCHOSOM RES, V74, P533, DOI 10.1016/j.jpsychores.2013.04.005

Kelly M, 2010, FAM PRACT, V27, P285, DOI 10.1093/fampra/cmp077

Kenya S, 2014, CLIN THER, V36, P485, DOI 10.1016/j.clinthera.2013.12.009

Kollannoor-Samuel G, 2016, AM J PUBLIC HEALTH, V106, P1059, DOI 10.2105/AJPH.2016.303091

Little TV, 2012, CURR DIABETES REP, V14, P558, DOI 10.1007/s11892-014-0558-1

Lyles CM, 2007, AM J PUBLIC HEALTH, V97, P133, DOI 10.2105/AJPH.2005.076182

Mayo Foundation for Medical Education and Research, 2016, TEST ID HBA1C HEM AL

McDermott RA, 2015, BMC HEALTH SERV RES, V15, DOI 10.1186/s12913-015-0695-5

McEwen MM, 2010, PUBLIC HEALTH NURS, V27, P310, DOI 10.1111/j.1525-1446.2010.00860.x

Miller W. R., 1991, MOTIVATIONAL INTERVI

National Institute of Mental Health, 2011, DEPRESSION DIABETES

Navaneethan SD, 2010, NEPHROLOGY, V15, P732, DOI 10.1111/j.1440-1797.2010.01428.x

Norris SL, 2006, DIABETIC MED, V23, P544, DOI 10.1111/j.1464-5491.2006.01845.x

Palmas W, 2014, DIABETES CARE, V37, P963, DOI 10.2337/dc13-2142

Perez-Escamilla R, 2015, DIABETES CARE, V38, P197, DOI 10.2337/dc14-0327

Perry HB, 2014, ANNU REV PUBL HEALTH, V35, P399, DOI 10.1146/annurev-publhealth-032013-182354

Prezio EA, 2013, DIABETES RES CLIN PR, V100, P19, DOI 10.1016/j.diabres.2013.01.027

Prochaska JO, 1997, AM J HEALTH PROMOT, V12, P38, DOI 10.4278/0890-1171-12.1.38

PROCHASKA JO, 2002, HLTH BEHAV HLTH ED T

Rosen L, 2006, AM J PUBLIC HEALTH, V96, P1181, DOI 10.2105/AJPH.2004.061713

Rothschild SK, 2014, AM J PUBLIC HEALTH, V104, P1540, DOI 10.2105/AJPH.2013.301439

Santos-Longhurst A., 2014, TYPE 2 DIABETES STAT

Spencer Michael S, 2013, Race Soc Probl, V5, P137

Spencer MS, 2011, AM J PUBLIC HEALTH, V101, P2253, DOI 10.2105/AJPH.2010.300106

Tang TS, 2008, DIABETES EDUCATOR, V34, P266, DOI 10.1177/0145721708315680

Tang TS, 2014, DIABETES CARE, V37, P1525, DOI 10.2337/dc13-2161

Wagner J, 2015, TRANSL BEHAV MED, V5, P415, DOI 10.1007/s13142-015-0332-1

Wagner JA, 2016, DIABETES RES CLIN PR, V120, P162, DOI 10.1016/j.diabres.2016.07.022

Whittemore R, 2005, J CLIN NURS, V14, P195, DOI 10.1111/j.1365-2702.2004.00937.x

Zgibor JC, 2001, DIABETES SPECTRUM, V14, P23, DOI DOI 10.2337/DIASPECT.14.1.23

NR 43

TC 3

Z9 3

U1 1

U2 1

PU EDUCATIONAL PUBLISHING FOUNDATION-AMERICAN PSYCHOLOGICAL ASSOC

PI WASHINGTON

PA 750 FIRST ST, NE, WASHINGTON, DC 20002-4242 USA

SN 1091-7527

EI 1939-0602

J9 FAM SYST HEALTH

JI Fam. Syst. Health

PD SEP

PY 2017

VL 35

IS 3

BP 320

EP 340

DI 10.1037/fsh0000283

PG 21

WC Health Care Sciences & Services; Family Studies; Public, Environmental &

Occupational Health

SC Health Care Sciences & Services; Family Studies; Public, Environmental &

Occupational Health

GA FK9LW

UT WOS:000413833700007

PM 28639794

DA 2019-01-08

ER

PT J

AU Yi-Frazier, JP

Fladeboe, K

Klein, V

Eaton, L

Wharton, C

McCauley, E

Rosenberg, AR

AF Yi-Frazier, Joyce P.

Fladeboe, Kaitlyn

Klein, Victoria

Eaton, Lauren

Wharton, Claire

McCauley, Elizabeth

Rosenberg, Abby R.

TI Promoting Resilience in Stress Management for Parents (PRISM-P): An

Intervention for Caregivers of Youth With Serious Illness

SO FAMILIES SYSTEMS & HEALTH

LA English

DT Article

DE caregiver stress; resilience; Type 1 diabetes; cancer

ID RANDOMIZED CONTROLLED-TRIALS; FAMILY RESILIENCE; MENTAL-HEALTH;

GENERAL-POPULATION; PSYCHOLOGICAL DISTRESS; PSYCHOSOCIAL CARE; PEDIATRIC

CANCER; YOUNG-ADULTS; CHILDREN; SCALE

AB Introduction: It is well-known that parental stress and coping impacts the well-being of children with serious illness. The current study aimed to evaluate the feasibility and satisfaction of a novel resilience promoting intervention, the Promoting Resilience in Stress Management Intervention for Parents (PRISM-P) among parents of adolescents and young adults with Type 1 diabetes or cancer. Secondary analyses explored the effect of the PRISM-P on parent-reported resilience and distress. Method: The PRISM-P includes 4 short skills-based modules, delivered in either 2 or 4 separate, individual sessions. English-speaking parents of adolescents with cancer or Type 1 diabetes were eligible. Feasibility was conservatively defined as a completion rate of 80%; satisfaction was qualitatively evaluated based upon parent feedback regarding intervention content, timing, and format. Resilience and distress were assessed pre- and postintervention with the Connor Davidson Resilience Scale and the Kessler-6 Psychological Distress Scale. Results: Twelve of 24 caregivers of youth with diabetes (50%) and 13 of 15 caregivers of youth with cancer (87%) agreed to participate. Nine of 12 (75%) and 9 of 13 (64%) completed all PRISM-P modules, respectively. Among those who completed the intervention, qualitative satisfaction was high. Parent-reported resilience and distress scores improved after the intervention. Effect sizes for both groups indicated a moderate intervention effect. Discussion: Ultimately, the PRISM-P intervention was well accepted and impactful among parents who completed it. However, attrition rates were higher than anticipated, suggesting alternative or less time-intensive formats may be more feasible.

C1 [Yi-Frazier, Joyce P.; Fladeboe, Kaitlyn; Klein, Victoria; Eaton, Lauren; Wharton, Claire; Rosenberg, Abby R.] Seattle Childrens Res Inst, Ctr Clin & Translat Res, Seattle, WA USA.

[McCauley, Elizabeth] Seattle Childrens Res Inst, Ctr Child Hlth & Behav, Seattle, WA USA.

[McCauley, Elizabeth; Rosenberg, Abby R.] Univ Washington, Sch Med, Dept Psychiat & Behav Med, Seattle, WA 98195 USA.

[Rosenberg, Abby R.] Univ Washington, Sch Med, Dept Pediat, Div Pediat Hematol Oncol, Seattle, WA 98195 USA.

RP Rosenberg, AR (reprint author), Seattle Childrens Hosp Canc & Blood Disorders Ctr, 4800 Sand Point Way NE,M-S MB 8-501, Seattle, WA 98115 USA.

EM abby.rosenberg@seattlechildrens.org

FU Seattle Children's Hospital's Center for Clinical and Translational

Research (Pilot Grant Program); National Institutes of Health Center for

Translational Science of the University of Washington Institute of

Translational Health Science [UL1 TR000423]; St. Baldrick's Foundation;

CureSearch for Children's Cancer; National Center for Advancing

Translational Sciences of the National Institutes of Health

[KL2TR000421]

FX This research was supported by Seattle Children's Hospital's Center for

Clinical and Translational Research (Pilot Grant Program to Joyce P.

Yi-Frazier and Abby R. Rosenberg) and in part by the National Institutes

of Health Center for Translational Science Awards Grant UL1 TR000423 of

the University of Washington Institute of Translational Health Science.

In addition, Abby R. Rosenberg was supported by the St. Baldrick's

Foundation, CureSearch for Children's Cancer, and the National Center

for Advancing Translational Sciences of the National Institutes of

Health (KL2TR000421).

CR Barakat LP, 2006, J PEDIATR PSYCHOL, V31, P413, DOI 10.1093/jpepsy/jsj058

Bradshaw BG, 2007, DIABETES EDUCATOR, V33, P650, DOI 10.1177/0145721707303809

Bruwer B, 2008, COMPR PSYCHIAT, V49, P195, DOI 10.1016/j.comppsych.2007.09.002

Campbell-Sills L, 2006, BEHAV RES THER, V44, P585, DOI 10.1016/j.brat.2005.05.001

Carver CS, 2005, CANCER, V104, P2602, DOI 10.1002/cncr.21247

Connor KM, 2007, PSYCHIAT RES, V151, P249, DOI 10.1016/j.psychres.2006.11.008

Connor KM, 2003, DEPRESS ANXIETY, V18, P76, DOI 10.1002/da.10113

Davidson J. R., 2006, US PSYCH MENT HLTH C

Eilertsen ME, 2016, ACTA PAEDIATR, V105, pE30, DOI 10.1111/apa.13232

Ewald H, 2014, J PUBLIC HEALTH-UK, V36, P476, DOI 10.1093/pubmed/fdt108

Fergus S, 2005, ANNU REV PUBL HEALTH, V26, P399, DOI 10.1146/annurev.publhealth.26.021304.144357

Fisher L, 2000, J FAM PRACTICE, V49, P561

Fjorback LO, 2011, ACTA PSYCHIAT SCAND, V124, P102, DOI 10.1111/j.1600-0447.2011.01704.x

Folkman S, 1997, SOC SCI MED, V45, P1207, DOI 10.1016/S0277-9536(97)00040-3

Folkman S, 2000, PSYCHO-ONCOL, V9, P11, DOI 10.1002/(SICI)1099-1611(200001/02)9:1<11::AID-PON424>3.3.CO;2-Q

Fraser E, 2009, PSYCHOL HEALTH MED, V14, P573, DOI 10.1080/13548500903193820

Green JG, 2010, INT J METH PSYCH RES, V19, P23, DOI 10.1002/mpr.314

Henry M, 2010, PSYCHO-ONCOLOGY, V19, P1340, DOI 10.1002/pon.1764

Hilliard ME, 2015, J PEDIATR PSYCHOL, V40, P835, DOI 10.1093/jpepsy/jsv072

Hsieh HF, 2005, QUAL HEALTH RES, V15, P1277, DOI 10.1177/1049732305276687

IBM Corp, 2010, IBM SPSS STAT WIND V

Jacobsen PB, 2012, J CLIN ONCOL, V30, P1151, DOI 10.1200/JCO.2011.41.4078

Jang M, 2015, J PEDIATR NURS, V30, P877, DOI 10.1016/j.pedn.2015.05.004

Karlson CW, 2009, J PEDIATR PSYCHOL, V34, P782, DOI 10.1093/jpepsy/jsn122

Kazak AE, 2015, ACTA ONCOL, V54, P574, DOI 10.3109/0284186X.2014.995774

Kearney JA, 2015, PEDIATR BLOOD CANCER, V62, pS632, DOI 10.1002/pbc.25761

Kessler RC, 2003, ARCH GEN PSYCHIAT, V60, P184, DOI 10.1001/archpsyc.60.2.184

Kessler RC, 2002, PSYCHOL MED, V32, P959, DOI 10.1017/S0033291702006074

Kessler RC, 2010, INT J METH PSYCH RES, V19, P4, DOI 10.1002/mpr.310

Kim S., 2016, J APPL GERONTOLOGY

Landolt MA, 2005, J AM ACAD CHILD PSY, V44, P682, DOI 10.1097/01-chi.0000161645.98022.35

Landolt MA, 2002, J PEDIATR PSYCHOL, V27, P647, DOI 10.1093/jpepsy/27.7.647

Lyon ME, 2009, PEDIATRICS, V123, pE199, DOI 10.1542/peds.2008-2379

McCarty C. A., 2011, DEPRESSION RES TREAT

McMillan Susan C, 2003, Oncol Nurs Forum, V30, P123, DOI 10.1188/03.ONF.123-129

Miller KA, 2017, PEDIATR BLOOD CANCER, V64, P358, DOI 10.1002/pbc.26209

PERRIN EC, 1993, J DEV BEHAV PEDIATR, V14, P94

Rew Lynn, 2003, J Pediatr Nurs, V18, P379, DOI 10.1016/S0882-5963(03)00162-3

Robinson KE, 2007, J PEDIATR PSYCHOL, V32, P400, DOI 10.1093/jpepsy/Jsl038

Rohan JM, 2015, J PEDIATR PSYCHOL, V40, P956, DOI 10.1093/jpepsy/jsv061

Rosenberg AR, 2016, J PEDIATR PSYCHOL, V41, P506, DOI 10.1093/jpepsy/jsw018

Rosenberg AR, 2015, J PEDIATR PSYCHOL, V40, P992, DOI 10.1093/jpepsy/jsv004

Rosenberg AR, 2014, PEDIATR BLOOD CANCER, V61, P552, DOI 10.1002/pbc.24854

Rosenberg AR, 2013, JAMA PEDIATR, V167, P537, DOI 10.1001/jamapediatrics.2013.628

Sahler OJZ, 2013, J CLIN ONCOL, V31, P1329, DOI 10.1200/JCO.2011.39.1870

Saltzman WR, 2016, FAM PROCESS, V55, P647, DOI 10.1111/famp.12250

Saltzman WR, 2016, FAM PROCESS, V55, P633, DOI 10.1111/famp.12238

Sottile PD, 2016, CRIT CARE MED, V44, pE721, DOI 10.1097/CCM.0000000000001673

Southwick SM, 2014, EUR J PSYCHOTRAUMATO, V5, DOI 10.3402/ejpt.v5.25338

Southwick SM, 2012, SCIENCE, V338, P79, DOI 10.1126/science.1222942

Svoren BM, 2003, PEDIATRICS, V112, P914, DOI 10.1542/peds.112.4.914

Tan L, 2013, CLIN CHILD PSYCHOL P, V18, P300, DOI 10.1177/1359104512455182

Van Schoors M, 2015, J PEDIATR PSYCHOL, V40, P856, DOI 10.1093/jpepsy/jsv055

Walsh F, 2003, FAM PROCESS, V42, P1, DOI 10.1111/j.1545-5300.2003.00001.x

Walsh F, 2016, STRENGTHENING FAMILY

NR 55

TC 4

Z9 4

U1 1

U2 13

PU EDUCATIONAL PUBLISHING FOUNDATION-AMERICAN PSYCHOLOGICAL ASSOC

PI WASHINGTON

PA 750 FIRST ST, NE, WASHINGTON, DC 20002-4242 USA

SN 1091-7527

EI 1939-0602

J9 FAM SYST HEALTH

JI Fam. Syst. Health

PD SEP

PY 2017

VL 35

IS 3

BP 341

EP 351

DI 10.1037/fsh0000281

PG 11

WC Health Care Sciences & Services; Family Studies; Public, Environmental &

Occupational Health

SC Health Care Sciences & Services; Family Studies; Public, Environmental &

Occupational Health

GA FK9LW

UT WOS:000413833700008

PM 28541057

DA 2019-01-08

ER

PT J

AU Walls, ML

Sittner, KJ

Aronson, BD

Forsberg, AK

Whitbeck, LB

al'Absi, M

AF Walls, Melissa L.

Sittner, Kelley J.

Aronson, Benjamin D.

Forsberg, Angie K.

Whitbeck, Les B.

al'Absi, Mustafa

TI Stress Exposure and Physical, Mental, and Behavioral Health among

American Indian Adults with Type 2 Diabetes

SO INTERNATIONAL JOURNAL OF ENVIRONMENTAL RESEARCH AND PUBLIC HEALTH

LA English

DT Article

DE American Indian; diabetes; stress; Native American

ID COMORBIDITY SURVEY REPLICATION; DSM-IV DISORDERS; SCALE; LIFE;

PREVALENCE; DISTRESS; NONADHERENCE; DEPRESSION; EVENTS; CARE

AB American Indian ( AI) communities experience disproportionate exposure to stressors and health inequities including type 2 diabetes. Yet, we know little about the role of psychosocial stressors for AI diabetes-related health outcomes. We investigated associations between a range of stressors and psychological, behavioral, and physical health for AIs with diabetes. This community-based participatory research with 5 AI tribes includes 192 AI adult type 2 diabetes patients recruited from clinical records at tribal clinics. Data are from computer-assisted interviews and medical charts. We found consistent bivariate relationships between chronic to discrete stressors and mental and behavioral health outcomes; several remained even after accounting for participant age, gender, and income. Fewer stressors were linked to physical health. We also document a dose-response relationship between stress accumulation and worse health. Findings underscore the importance of considering a broad range of stressors for comprehensive assessment of stress burden and diabetes. Policies and practices aimed at reducing stress exposure and promoting tools for stress management may be mechanisms for optimal health for AI diabetes patients.

C1 [Walls, Melissa L.; Forsberg, Angie K.; al'Absi, Mustafa] Univ Minnesota, Sch Med, Dept Family Med & Biobehav Hlth, Duluth Campus,1035 Univ Dr,235 SMed, Duluth, MN 55812 USA.

[Sittner, Kelley J.] Oklahoma State Univ, Dept Sociol, Stillwater, OK 74078 USA.

[Aronson, Benjamin D.] Ohio Northern Univ, Dept Pharm Practice, Ada, OH 45810 USA.

[Whitbeck, Les B.] Univ Nebraska, Dept Sociol, Lincoln, NE 68588 USA.

RP Walls, ML (reprint author), Univ Minnesota, Sch Med, Dept Family Med & Biobehav Hlth, Duluth Campus,1035 Univ Dr,235 SMed, Duluth, MN 55812 USA.

EM mlwalls@d.umn.edu; kelley.sittner@okstate.edu; b-aronson.1@onu.edu;

aforsber@d.umn.edu; lwhitbeck2@unl.edu; malabsi@d.umn.edu

OI Aronson, Benjamin/0000-0001-9739-6221

FU National Institute of Diabetes and Digestive and Kidney Diseases of the

National Institutes of Health [DK091250]

FX We gratefully acknowledge the dedicated Community Research Council and

Clinical Project Members of the Gathering for Health team: Sidnee

Kellar, Rose Barber, Robert Miller, Tweed Shuman, Lorraine Smith, Sandy

Zeznanski, Patty Subera, Tracy Martin, Geraldine Whiteman, Lisa Perry,

Trisha Prentice, Alexis Mason, Charity Prentice-Pemberton, Kathy Dudley,

Romona Nelson, Eileen Miller, Geraldine Brun, Murphy Thomas, Mary

Sikora-Petersen, Tina Handeland, GayeAnn Allen, Frances Whitfield,

Phillip Chapman, Sr., Hope Williams, Betty Jo Graveen, Daniel Chapman,

Jr., Doris Isham, Stan Day, Jane Villebrun, Beverly Steel, Muriel

Deegan, Peggy Connor, Michael Connor, Ray E. Villebrun, Sr., Pam Hughes,

Cindy McDougall, Melanie McMichael, Robert Thompson, and Sandra Kier.

Research reported in this manuscript is supported by the National

Institute of Diabetes and Digestive and Kidney Diseases of the National

Institutes of Health under Award Number DK091250 (M. Walls, PI). The

content is solely the responsibility of the authors and does not

necessarily represent the official views of the National Institutes of

Health.

CR Acton KJ, 2003, JAMA-J AM MED ASSOC, V290, P1571

Aikens JE, 2012, DIABETES CARE, V35, P2472, DOI 10.2337/dc12-0181

Benyshek D C, 2001, Med Anthropol, V20, P25

Chae DH, 2009, AM J PUBLIC HEALTH, V99, pS144, DOI 10.2105/AJPH.2007.126003

de Koning L, 2007, EUR HEART J, V28, P850, DOI 10.1093/eurheartj/ehm026

DOHRENWEND BS, 1978, J HEALTH SOC BEHAV, V19, P205, DOI 10.2307/2136536

Egede LE, 2014, ANN PHARMACOTHER, V48, P562, DOI 10.1177/1060028014526362

Elm J., 2017, SOURCES STR IN PRESS

Fisher L, 2014, DIABETIC MED, V31, P764, DOI 10.1111/dme.12428

Fisher L, 2008, ANN FAM MED, V6, P246, DOI 10.1370/afm.842

Fisher L, 2010, DIABETES CARE, V33, P23, DOI 10.2337/dc09-1238

Gonzalez JS, 2008, DIABETES CARE, V31, P2398, DOI 10.2337/dc08-1341

Hill DL, 2006, ARCH PSYCHIAT NURS, V20, P210, DOI 10.1016/j.apnu.2006.04.003

HOLMES TH, 1967, J PSYCHOSOM RES, V11, P213, DOI 10.1016/0022-3999(67)90010-4

Jiang LH, 2008, DIABETES CARE, V31, P427, DOI 10.2337/dc07-2044

Kahn JR, 2006, J HEALTH SOC BEHAV, V47, P17, DOI 10.1177/002214650604700102

Kessler RC, 2005, ARCH GEN PSYCHIAT, V62, P617, DOI 10.1001/archpsyc.62.6.617

Kessler RC, 2005, ARCH GEN PSYCHIAT, V62, P593, DOI 10.1001/archpsyc.62.6.593

KOHN PM, 1992, J BEHAV MED, V15, P221, DOI 10.1007/BF00848327

LEE ET, 1992, DIABETES CARE, V15, P1620, DOI 10.2337/diacare.15.11.1620

MORISKY DE, 1986, MED CARE, V24, P67, DOI 10.1097/00005650-198601000-00007

O'Connell J, 2010, DIABETES CARE, V33, P1463, DOI 10.2337/dc09-1652

PEARLIN LI, 1981, J HEALTH SOC BEHAV, V22, P337, DOI 10.2307/2136676

Pearlin LI, 1997, J HEALTH SOC BEHAV, V38, P223, DOI 10.2307/2955368

Scarton L.J., 2016, HEALTH EDUC BEHAV, V44, P70

Shields CG, 1994, FAM SYST MED, V12, P361, DOI [10. 1037/h0089289, DOI 10.1037/H0089289]

Spitzer RL, 1999, JAMA-J AM MED ASSOC, V282, P1737, DOI 10.1001/jama.282.18.1737

STAHN RM, 1993, DIABETES CARE, V16, P244, DOI 10.2337/diacare.16.1.244

Sue DW, 2007, AM PSYCHOL, V62, P271, DOI 10.1037/0003-066X.62.4.271

SURWIT RS, 1992, DIABETES CARE, V15, P1413, DOI 10.2337/diacare.15.10.1413

Tiedt J.A., 2014, J THEORY CONSTR TEST, V18, P22

Toobert DJ, 2000, DIABETES CARE, V23, P943, DOI 10.2337/diacare.23.7.943

TOOBERT DJ, 1994, HDB PSYCHOL DIABETES, P351

Turner J., 2009, UNDERSTANDING HLTH D, P3

Turner RJ, 2003, J HEALTH SOC BEHAV, V44, P488, DOI 10.2307/1519795

TURNER RJ, 1995, AM SOCIOL REV, V60, P104, DOI 10.2307/2096348

Walders-Abramson N, 2014, J PEDIATR-US, V165, P504, DOI 10.1016/j.jpeds.2014.05.020

Walters KL, 2002, AM J PUBLIC HEALTH, V92, P520, DOI 10.2105/AJPH.92.4.520

Wheaton B., 1999, HDB STUDY MENTAL HLT, P171

Wheaton B., 1994, STRESS MENTAL HLTH C, P77, DOI DOI 10.1007/978-1-4899-1106-3

Xu J, 2016, NATL VITAL STAT REP, V64, P2

NR 41

TC 0

Z9 0

U1 3

U2 9

PU MDPI

PI BASEL

PA ST ALBAN-ANLAGE 66, CH-4052 BASEL, SWITZERLAND

SN 1660-4601

J9 INT J ENV RES PUB HE

JI Int. J. Environ. Res. Public Health

PD SEP

PY 2017

VL 14

IS 9

AR 1074

DI 10.3390/ijerph14091074

PG 11

WC Environmental Sciences; Public, Environmental & Occupational Health

SC Environmental Sciences & Ecology; Public, Environmental & Occupational

Health

GA FH9YO

UT WOS:000411574400126

PM 28926940

OA DOAJ Gold, Green Published

DA 2019-01-08

ER

PT J

AU Darawad, MW

Hammad, S

Samarkandi, OA

Hamdan-Mansour, AM

Khalil, AA

AF Darawad, Muhammad W.

Hammad, Sawsan

Samarkandi, Osama A.

Hamdan-Mansour, Ayman M.

Khalil, Amani A.

TI Evaluating the Psychometric Properties of the Arabic Version of the

Diabetes Distress Scale

SO JOURNAL OF PSYCHOSOCIAL NURSING AND MENTAL HEALTH SERVICES

LA English

DT Article

ID SELF-MANAGEMENT PRACTICES; EMOTIONAL DISTRESS; LONGITUDINAL

RELATIONSHIP; DEPRESSIVE SYMPTOMS; GLYCEMIC CONTROL; PROBLEM AREAS;

RISK-FACTORS; ADULTS; PREVALENCE; HEALTH

AB The current study evaluated the psychometric properties of the Arabic version of the Diabetes Distress Scale (DDS-A) among Arab patients with diabetes mellitus (DM) using a descriptive cross-sectional design. Participants' DDS-A total scores significantly correlated with depressive symptoms (r = 0.288, p = 0.000) as well as two subscales, emotional burden (r = 0.276, p = 0.000) and regimen distress (r = 0.265, p = 0.000). Participants' DDS-A scores had significant negative correlations with income, DM self-management, and knowledge (r = -0.184, p = 0.008; r = -0.310, p = 0.000; r = -0.174, p = 0.003, respectively) and a positive correlation with HbA1c level (r = 0.153, p = 0.018). Factor analysis revealed a four-factor solution that retained all items and explained a variance of 65.59%. Cronbach's alpha was 0.822 for the total scale and 0.778 to 0.881 for the subscales, indicating a high internal consistency. The DDS-A was found to be a psychometrically sound measure to evaluate DM-related distress among Arab patients.

C1 [Darawad, Muhammad W.; Hammad, Sawsan; Hamdan-Mansour, Ayman M.; Khalil, Amani A.] Univ Jordan, Sch Nursing, Amman 11942, Jordan.

[Samarkandi, Osama A.] King Saud Univ, Prince Sultan bin Abdulaziz Coll Emergency Med Se, Riyadh, Saudi Arabia.

RP Darawad, MW (reprint author), Univ Jordan, Sch Nursing, Amman 11942, Jordan.

EM m.darawad@ju.edu.jo

RI Samarkandi, Osama/J-1482-2017

OI Samarkandi, Osama/0000-0003-2698-7176

FU University of Jordan

FX The authors thank the University of Jordan for funding this study, as

well as the patients and directors of nursing at the participating

hospitals who granted access to participants in their organizations.

CR Ajlouni K, 2008, J DIABETES COMPLICAT, V22, P317, DOI 10.1016/j.jdiacomp.2007.01.004

Al-Amer RM, 2011, J DIABETES COMPLICAT, V25, P247, DOI 10.1016/j.jdiacomp.2011.03.001

Bani-issa WA, 2014, J TRANSCULT NURS, V25, P42, DOI 10.1177/1043659613503873

Baradaran HR, 2013, INT J PREVENTIVE MED, V4, P580

Boutayeb A., 2012, OPEN J EPIDEMIOL, V2, P55, DOI DOI 10.4236/0JEPI.2012.22009

Darawad MW, 2018, CLIN NURS RES, V27, P890, DOI 10.1177/1054773816683504

Darawad MW, 2017, IRAN J PUBLIC HEALTH, V46, P771

de Villiers L., 2015, INT J RES NURSING, V6, P30, DOI [10.3844/ijrnsp.2015.30.41, DOI 10.3844/IJRNSP.2015.30.41]

Department of Statistics, 2012, JORD ANN CENS

DUNN SM, 1986, DIABETES CARE, V9, P480, DOI 10.2337/diacare.9.5.480

Ehrmann D, 2015, DIABETIC MED, V32, P1264, DOI 10.1111/dme.12861

Fisher L, 2008, DIABETIC MED, V25, P1096, DOI 10.1111/j.1464-5491.2008.02533.x

Garcia AA, 2001, DIABETES CARE, V24, P16, DOI 10.2337/diacare.24.1.16

Gazmararian JA, 2009, DIABETES EDUCATOR, V35, P778, DOI 10.1177/0145721709338527

Gorsuch R, 1983, FACTOR ANAL

Graue M, 2012, INT J NURS STUD, V49, P174, DOI 10.1016/j.ijnurstu.2011.08.007

GUILLEMIN F, 1993, J CLIN EPIDEMIOL, V46, P1417, DOI 10.1016/0895-4356(93)90142-N

Hammad S, 2015, IRAN J PUBLIC HEALTH, V44, P1482

Herschbach P, 1997, HEALTH PSYCHOL, V16, P171, DOI 10.1037/0278-6133.16.2.171

International Diabetes Federation, 2012, DIABETES ATLAS

Karlsen B, 2012, J ADV NURS, V68, P391, DOI 10.1111/j.1365-2648.2011.05751.x

Kong Lulu, 2013, J Biomed Res, V27, P380, DOI 10.7555/JBR.27.20120112

Nunnally JC, 1994, PSYCHOMETRIC THEORY

Pandit AU, 2014, J EPIDEMIOL COMMUN H, V68, P557, DOI 10.1136/jech-2013-203063

Polit D.F., 2014, ESSENTIALS NURSING R

Polonsky WH, 2005, DIABETES CARE, V28, P626, DOI 10.2337/diacare.28.3.626

RADLOFF L S, 1977, Applied Psychological Measurement, V1, P385, DOI 10.1177/014662167700100306

Rogvi S, 2012, DIABETIC MED, V29, pe382, DOI 10.1111/j.1464-5491.2012.03703.x

Schmitt A, 2013, HEALTH QUAL LIFE OUT, V11, DOI 10.1186/1477-7525-11-138

Shakibazadeh E, 2011, IRAN J PUBLIC HEALTH, V40, P146

Shaw JE, 2010, DIABETES RES CLIN PR, V87, P4, DOI 10.1016/j.diabres.2009.10.007

Snoek FJ, 2011, DIABETES CARE, V34, P601, DOI 10.2337/dc10-1552

Stankovic Z, 2013, VOJNOSANIT PREGL, V70, P1138, DOI 10.2298/VSP1312138S

Strandberg RB, 2015, DIABETIC MED, V32, P1304, DOI 10.1111/dme.12781

Tabachnick B. G., 2007, USING MULTIVARIATE S

Ting RZW, 2011, DIABETES CARE, V34, P1094, DOI 10.2337/dc10-1612

van Bastelaar KMP, 2010, DIABETIC MED, V27, P798, DOI 10.1111/j.1464-5491.2010.03025.x

Ware JE, 1998, J CLIN EPIDEMIOL, V51, P903, DOI 10.1016/S0895-4356(98)00081-X

Welch GW, 1997, DIABETES CARE, V20, P760, DOI 10.2337/diacare.20.5.760

Whiting DR, 2011, DIABETES RES CLIN PR, V94, P311, DOI 10.1016/j.diabres.2011.10.029

Yacoub MI, 2014, INT NURS REV, V61, P255, DOI 10.1111/inr.12090

Yacoub MI, 2015, J CONTIN EDUC NURS, V46, P128, DOI [10.3928/00220124-20150220-16, 10.3928/00220124-20150126-02]

Zulman DM, 2012, PATIENT EDUC COUNS, V87, P74, DOI 10.1016/j.pec.2011.07.013

NR 43

TC 0

Z9 0

U1 0

U2 5

PU SLACK INC

PI THOROFARE

PA 6900 GROVE RD, THOROFARE, NJ 08086 USA

SN 0279-3695

EI 1938-2413

J9 J PSYCHOSOC NURS MEN

JI J. Psychosoc. Nurs. Ment. Health Serv.

PD SEP

PY 2017

VL 55

IS 9

BP 43

EP 51

DI 10.3928/02793695-20170818-12

PG 9

WC Nursing

SC Nursing

GA FG6WR

UT WOS:000410539300006

PM 28850650

DA 2019-01-08

ER

PT J

AU Hessler, DM

Fisher, L

Polonsky, WH

Masharani, U

Strycker, LA

Peters, AL

Blumer, I

Bowyer, V

AF Hessler, D. M.

Fisher, L.

Polonsky, W. H.

Masharani, U.

Strycker, L. A.

Peters, A. L.

Blumer, I.

Bowyer, V.

TI Diabetes distress is linked with worsening diabetes management over time

in adults with Type 1 diabetes

SO DIABETIC MEDICINE

LA English

DT Article

ID POOR SELF-CARE; EMOTIONAL DISTRESS; GLYCEMIC CONTROL; DEPRESSIVE

SYMPTOMS; REGIMEN DISTRESS; ASSOCIATIONS; MELLITUS; HBA(1C); TRIAL

AB AimTo determine the cross-sectional and longitudinal associations between diabetes distress and diabetes management.

MethodsIn a non-interventional study, 224 adults with Type 1 diabetes were assessed for diabetes distress, missed insulin boluses, hypoglycaemic episodes, and HbA(1c) at baseline and 9 months.

ResultsAt baseline, greater distress was associated with higher HbA(1c) and a greater percentage of missed insulin boluses. Longitudinally, elevated baseline distress was related to increased missed insulin boluses, and decreases in distress were associated with decreases in HbA(1c). In supplementary analyses, neither depression symptoms nor a diagnosis of major depressive disorder was associated with missed insulin boluses, HbA(1c) or hypoglycaemic episodes in cross-sectional or longitudinal analyses.

ConclusionsSignificant cross-sectional and longitudinal associations were found between diabetes distress and management; in contrast, no parallel associations were found for major depressive disorder or depression symptoms. Findings suggest that elevated distress may lead to more missed insulin boluses over time, suggesting a potential intervention target. The covarying association between distress and HbA(1c) points to the complex and likely interactive associations between these constructs. Findings highlight the need to address distress as an integral part of diabetes management in routine care.

C1 [Hessler, D. M.; Fisher, L.; Bowyer, V.] Univ Calif San Francisco, Dept Family & Community Med, San Francisco, CA 94143 USA.

[Polonsky, W. H.] Univ Calif San Francisco, Behav Diabet Inst, San Francisco, CA 94143 USA.

[Masharani, U.] Univ Calif San Francisco, Dept Med, San Francisco, CA USA.

[Strycker, L. A.] Oregon Res Inst, Eugene, OR 97403 USA.

[Peters, A. L.] Univ Southern Calif, Los Angeles, CA USA.

[Blumer, I.] Charles H Best Diabet Ctr, Whitby, ON, Canada.

RP Hessler, DM (reprint author), Univ Calif San Francisco, Dept Family & Community Med, San Francisco, CA 94143 USA.

EM Danielle.Hessler@ucsf.edu

FU NIH [DK094863]

FX This research was funded by NIH Grant DK094863.

CR Aikens JE, 2012, DIABETES CARE, V35, P2472, DOI 10.2337/dc12-0181

BENJAMINI Y, 1995, J R STAT SOC B, V57, P289

Fisher L, 2014, DIABETIC MED, V31, P764, DOI 10.1111/dme.12428

Fisher L, 2015, J DIABETES COMPLICAT, V29, P572, DOI 10.1016/j.jdiacomp.2015.01.012

Fisher L, 2013, DIABETES CARE, V36, P2551, DOI 10.2337/dc12-2493

Fisher L, 2010, DIABETES CARE, V33, P23, DOI 10.2337/dc09-1238

Gonzalez JS, 2008, DIABETOLOGIA, V51, P1822, DOI 10.1007/s00125-008-1113-x

Gonzalez JS, 2016, DIABETES CARE, V39, P2182, DOI 10.2337/dc16-1657

Gonzalez JS, 2011, DIABETES CARE, V34, P236, DOI 10.2337/dc10-1970

Hermanns N, 2015, DIABETES CARE, V38, P551, DOI 10.2337/dc14-1416

Hessler D, 2015, DIABETES RES CLIN PR, V108, P360, DOI 10.1016/j.diabres.2014.12.017

Hessler D, 2014, DIABETES CARE, V37, P617, DOI 10.2337/dc13-0762

Hislop AL, 2008, DIABETIC MED, V25, P91, DOI 10.1111/j.1464-5491.2007.02310.x

Joensen LE, 2013, DIABETIC MED, V30, P1132, DOI 10.1111/dme.12241

Kroenke K, 2002, PSYCHIAT ANN, V32, P509, DOI 10.3928/0048-5713-20020901-06

Lloyd CE, 2010, DIABETIC MED, V27, P234, DOI 10.1111/j.1464-5491.2009.02896.x

Ogbera A, 2011, J DIABETES, V3, P348, DOI 10.1111/j.1753-0407.2011.00156.x

Rohde P, 1997, AM J PSYCHIAT, V154, P1593, DOI 10.1176/ajp.154.11.1593

Snoek FJ, 2015, LANCET DIABETES ENDO, V3, P450, DOI 10.1016/S2213-8587(15)00135-7

Strandberg RB, 2015, DIABETIC MED, V32, P1304, DOI 10.1111/dme.12781

Strandberg RB, 2014, J PSYCHOSOM RES, V77, P174, DOI 10.1016/j.jpsychores.2014.06.015

Sturt J, 2015, INT DIAB NURS, V12, P40, DOI DOI 10.1179/2057332415Y.0000000004

van Bastelaar KMP, 2010, DIABETIC MED, V27, P798, DOI 10.1111/j.1464-5491.2010.03025.x

Weinger K, 2001, PATIENT EDUC COUNS, V42, P123, DOI 10.1016/S0738-3991(00)00098-7

NR 24

TC 8

Z9 8

U1 0

U2 3

PU WILEY

PI HOBOKEN

PA 111 RIVER ST, HOBOKEN 07030-5774, NJ USA

SN 0742-3071

EI 1464-5491

J9 DIABETIC MED

JI Diabetic Med.

PD SEP

PY 2017

VL 34

IS 9

BP 1228

EP 1234

DI 10.1111/dme.13381

PG 7

WC Endocrinology & Metabolism

SC Endocrinology & Metabolism

GA FD9CA

UT WOS:000407819200007

PM 28498610

DA 2019-01-08

ER

PT J

AU Sudhir, PM

AF Sudhir, Paulomi M.

TI Advances in psychological interventions for lifestyle disorders:

overview of interventions in cardiovascular disorder and type 2 diabetes

mellitus

SO CURRENT OPINION IN PSYCHIATRY

LA English

DT Review

DE cardiovascular disorders; psychological interventions; self-management;

type 2 diabetes

ID RANDOMIZED CONTROLLED-TRIAL; CORONARY-HEART-DISEASE;

COGNITIVE-BEHAVIORAL THERAPY; PHYSICAL-ACTIVITY; GLYCEMIC CONTROL;

SELF-REGULATION; CLINICAL-TRIAL; ADULTS; DEPRESSION; MANAGEMENT

AB Purpose of reviewThe present review examines the recent advances in psychological interventions for two major lifestyle disorders in adults namely, type 2 diabetes mellitus and cardiovascular disorders. The review summarizes findings from studies carried out between the years 2015 and 2017.Recent findingsThe effectiveness of psychological interventions in the management of lifestyle disorders has been examined with respect to adaptation, self-care, adherence, negative emotions and improving quality of life. There is an increasing recognition that psychological interventions are important for prevention of lifestyle disorders and promotion of health. Key psychological interventions include self-management and educational interventions based on learning and motivational principles, patient empowerment, cognitive behaviour therapy, behavioural skills and coaching. Recent developments also include the use of information technology to deliver these interventions through internet, mobile applications and text messages. Another significant development is that of mindfulness-based interventions within the third-generation behaviour therapy approaches to reduce distress and increase acceptance. In addition, family and couples interventions have also been emphasised as necessary in maintenance of healthy behaviours.SummaryStudies examining psychological interventions in cardiovascular and type 2 diabetes mellitus support the efficacy of these interventions in bringing about changes in biochemical / physiological parameters and in psychological outcomes such as self-efficacy, knowledge, quality of life and a sense of empowerment.

C1 [Sudhir, Paulomi M.] Natl Inst Mental Hlth, Dept Clin Psychol, Neurosci, Bangalore, Karnataka, India.

RP Sudhir, PM (reprint author), Natl Inst Mental Hlth & Neurosci, Dept Clin Psychol, Bangalore, Karnataka, India.

EM paulomi.sudhir@gmail.com

CR Abdi S, 2015, BMC NUTR, V1, P37

Abughosh SM, 2016, J MANAG CARE SPEC PH, V22, P63, DOI 10.18553/jmcp.2016.22.1.63

Alsaleh E, 2016, BMC PUBLIC HEALTH, V16, DOI 10.1186/s12889-016-3313-5

Bardideh K, 2017, GLOBAL J HLTH SCI, V9, P163

Blumenthal JA, 2016, CIRCULATION, V133, P1341, DOI 10.1161/CIRCULATIONAHA.115.018926

Chamany S, 2015, AM J PREV MED, V49, P832, DOI 10.1016/j.amepre.2015.04.016

Chapman A, 2016, BMC HEALTH SERV RES, V16, DOI 10.1186/s12913-016-1358-x

Chrvala CA, 2016, PATIENT EDUC COUNS, V99, P926, DOI 10.1016/j.pec.2015.11.003

de Groot Mary, 2010, Diabetes Spectr, V23, P15, DOI 10.2337/diaspect.23.1.15

de Hoog N, 2016, HEALTH EDUC RES, V31, P350, DOI 10.1093/her/cyv100

Doering LV, 2016, EUR J CARDIOVASC NUR, V15, P417, DOI 10.1177/1474515115592292

Ebert DD, 2017, DIABETIC MED, V34, P99, DOI 10.1111/dme.13173

Fard AD, 2016, IJHCS, P271

Figueroa C, 2016, PSYCHOLOGY, V07, P374

Fitzpatrick SL, 2016, DIABETES CARE, V39, P2149, DOI 10.2337/dc16-0941

Friis AM, 2016, DIABETES CARE, V39, P1963, DOI 10.2337/dc16-0416

Gomez-Pardo E, 2016, J AM COLL CARDIOL, V67, P476, DOI 10.1016/j.jacc.2015.10.033

Haenen S, 2016, J PSYCHOSOM RES, V85, P44, DOI 10.1016/j.jpsychores.2016.04.006

Hofmann M, 2016, J DIABETES RES, DOI 10.1155/2016/1476384

Holmen H, 2016, BMJ OPEN DIAB RES CA, V4, DOI 10.1136/bmjdrc-2016-000193

Hu J, 2016, DIABETES EDUCATOR, V42, P299, DOI 10.1177/0145721716636961

Islam SMS, 2015, DIABETES CARE, V38, pE112, DOI 10.2337/dc15-0505

Johnston N, 2016, AM HEART J, V178, P85, DOI 10.1016/j.ahj.2016.05.005

Kaltman S, 2016, DIABETES EDUCATOR, V42, P87, DOI 10.1177/0145721715617536

Kashani Mariam, 2016, Glob J Health Sci, V8, P55119, DOI 10.5539/gjhs.v8n9p322

Kleinman NJ, 2016, DIABETES CARE, V39, pE169, DOI 10.2337/dc16-0869

Lv JF, 2016, INT HEART J, V57, P167, DOI 10.1536/ihj.15-259

Mayberry LS, 2016, J DIABETES RES, DOI 10.1155/2016/7586385

McClintock HFD, 2016, AM J HEALTH BEHAV, V40, P503, DOI 10.5993/AJHB.40.4.12

Mendis S, 2011, GLOBAL ATLAS CARDIOV

Nanditha A, 2016, DIABETES CARE, V39, P472, DOI 10.2337/dc15-1536

Nelson Lyndsay A, 2016, JMIR Hum Factors, V3, pe13, DOI 10.2196/humanfactors.5177

Norlund F, 2017, EUR J PREV CARDIOL

Olson EA, 2015, J BEHAV MED, V38, P886, DOI 10.1007/s10865-015-9660-3

Onyechi KCN, 2016, MEDICINE, V95, DOI 10.1097/MD.0000000000004444

Park LG, 2016, PROG CARDIOVASC DIS, V58, P639, DOI 10.1016/j.pcad.2016.03.002

Pibernik-Okanovic M, 2015, TRIALS, V16, DOI 10.1186/s13063-015-0833-8

Redfern J, 2016, PLOS ONE, V11, DOI 10.1371/journal.pone.0163929

Rees G, 2017, BMJ OPEN DIABETES RE, V5

Rondags SMPA, 2016, DIABETES CARE, V39, P2190, DOI 10.2337/dc16-1614

Schneider KL, 2016, BEHAV THER, V47, P198, DOI 10.1016/j.beth.2015.10.005

Shayeghian Z, 2016, PLOS ONE, V11, DOI 10.1371/journal.pone.0166599

Sherwood A, 2017, AM HEART J, V183, P85, DOI 10.1016/j.ahj.2016.10.011

Silfee V, 2016, PSYCHOL HEALTH MED, V21, P715, DOI 10.1080/13548506.2016.1139144

Sultan S, 2016, PAK HEART J, V49, P9

Trief PM, 2016, DIABETES CARE, V39, P2165, DOI 10.2337/dc16-0035

Tully PJ, 2016, TRIALS, V17, DOI 10.1186/s13063-015-1109-z

Uchendu C, 2017, DIABETIC MED, V34, P328, DOI 10.1111/dme.13195

Wilczynska M, 2016, CONTEMP CLIN TRIALS, V49, P116, DOI 10.1016/j.cct.2016.06.013

Winter SJ, 2016, PROG CARDIOVASC DIS, V58, P605, DOI 10.1016/j.pcad.2016.02.005

Wolever RQ, 2016, BMJ OPEN DIAB RES CA, V4, DOI 10.1136/bmjdrc-2016-000201

Wu Y, 2017, JMIR MHEALTH UHEALTH, V5, DOI 10.2196/mhealth.6522

Younge JO, 2015, PLOS ONE, V10, DOI 10.1371/journal.pone.0143843

NR 53

TC 2

Z9 2

U1 5

U2 32

PU LIPPINCOTT WILLIAMS & WILKINS

PI PHILADELPHIA

PA TWO COMMERCE SQ, 2001 MARKET ST, PHILADELPHIA, PA 19103 USA

SN 0951-7367

EI 1473-6578

J9 CURR OPIN PSYCHIATR

JI Curr. Opin. Psychiatr.

PD SEP

PY 2017

VL 30

IS 5

BP 346

EP 351

DI 10.1097/YCO.0000000000000348

PG 6

WC Psychiatry

SC Psychiatry

GA FB9OD

UT WOS:000406467700005

PM 28682800

DA 2019-01-08

ER

PT J

AU Murri, MB

Mamberto, S

Briatore, L

Mazzucchelli, C

Amore, M

Cordera, R

AF Murri, Martino Belvederi

Mamberto, Sara

Briatore, Lucia

Mazzucchelli, Chiara

Amore, Mario

Cordera, Renzo

TI The interplay between diabetes, depression and affective temperaments: A

structural equation model

SO JOURNAL OF AFFECTIVE DISORDERS

LA English

DT Article

DE Depression; Diabetes; Distress; Temperament; Personality; Glycemic

control

ID MEDICATION ADHERENCE; RISK-FACTOR; ASSOCIATION; METAANALYSIS; SYMPTOMS;

DISTRESS; MELLITUS; INVARIANCE; GENDER; ISSUES

AB Background: Diabetes and depression are reciprocally linked, but few studies modeled their interplay considering the influence of affective temperaments (AT) and demographic factors.

Methods: Participants with type 1 and type 2 diabetes (T1DM and T2DM, n = 279) recruited from Diabetes Units were assessed with the Beck Depression Inventory (BDI), Temperament Evaluation of Memphis, Pisa, Paris and San Diego-autoquestionnaire version (TEMPS-A), Morisky Medication Adherence Scale (MMAS), Diabetes Distress Scale (DDS) and Cumulative Illness Rating Scales (CIRS). Glycosylated hemoglobin levels (HBA1C) was used as index of glycemic control. The bi-directional association between glycemic control, depression and candidate mediators was examined with Structural Equation Modeling, testing the impact of moderator variables (AT, diabetes type, age and gender) with multigroup comparison.

Results: The association between HBA1C and depressive symptoms was mediated by diabetes-related distress,, while there was no definite evidence of depression influencing HBA1C through changes of adherence, tiredness, appetite, alcohol intake or smoking. Among individuals with AT, distress was unrelated to HBA1C and had a higher impact on depression; adherence was inversely association with HBA1C. Moreover, physical comorbidities impacted on depression. While diabetes type had a moderation role, age and gender did not affect the model.

Limitations: Cross sectional design, lack of objective measures of diet and physical activity.

Conclusions: Glycemic control seem to influence the severity of depressive symptoms, but the reciprocal association seems non-significant. AT and diabetes type may shape this relationship influencing distress and adherence to medications. Findings may aid interventions aimed at improving patients' care and quality of life.

C1 [Murri, Martino Belvederi; Amore, Mario] Univ Genoa, Sect Psychiat, Dept Neurosci Rehabil Ophthalmol Genet Maternal &, Largo Rosanna Benzi 10, I-16132 Genoa, Italy.

[Murri, Martino Belvederi] Kings Coll London, Inst Psychiat Psychol & Neurosci, Dept Psychol Med, London, England.

[Mamberto, Sara; Cordera, Renzo] Univ Genoa, Dept Internal Med, Genoa, Italy.

[Briatore, Lucia] Pietra Ligure Hosp, ASL 2 Savonese, Internal Med Unit, Genoa, Italy.

[Mazzucchelli, Chiara] Osped Imperia, ASL 1 Imperiese, Diabet Unit, Imperia, Italy.

RP Murri, MB (reprint author), Univ Genoa, Sect Psychiat, Dept Neurosci Rehabil Ophthalmol Genet Maternal &, Largo Rosanna Benzi 10, I-16132 Genoa, Italy.

EM martino.belvederi@gmail.com

RI Belvederi Murri, Martino/G-8781-2011

OI Belvederi Murri, Martino/0000-0002-7262-3528; cordera,

renzo/0000-0002-5280-9691

CR Akiskal HS, 2005, J AFFECT DISORDERS, V85, P1, DOI 10.1016/j.jad.2004.12.003

Alexopoulos GS, 2011, INT J GERIATR PSYCH, V26, P1109, DOI 10.1002/gps.2672

Almeida OP, 2016, MATURITAS, V86, P3, DOI 10.1016/j.maturitas.2016.01.003

Bartoli F, 2016, INT J GERIATR PSYCH, V31, P829, DOI 10.1002/gps.4397

Beck AT, 1996, J PERS ASSESS, V67, P588, DOI 10.1207/s15327752jpa6703_13

Buchberger B, 2016, PSYCHONEUROENDOCRINO, V70, P70, DOI 10.1016/j.psyneuen.2016.04.019

Bullock HE, 1994, STRUCT EQU MODELING, V1, P253, DOI 10.1080/10705519409539977

Byrne BM, 2004, STRUCT EQU MODELING, V11, P272, DOI 10.1207/s15328007sem1102_8

Chen SG, 2016, ENDOCRINE, V53, P35, DOI 10.1007/s12020-016-0869-x

Chiu CJ, 2010, SOC PSYCH PSYCH EPID, V45, P67, DOI 10.1007/s00127-009-0043-3

Demakakos P, 2014, PSYCHOSOM MED, V76, P555, DOI 10.1097/PSY.0000000000000082

Ehrmann D, 2015, DIABETIC MED, V32, P1264, DOI 10.1111/dme.12861

Eliasson B, 2003, PROG CARDIOVASC DIS, V45, P405, DOI 10.1053/pcad.2003.00103

Fernandez-Aranda F, 2014, PLOS ONE, V9, DOI 10.1371/journal.pone.0104534

Gaskin J., 2011, MULTIGROUP MODERATIO, P25

Gois C, 2012, J AFFECT DISORDERS, V143, P1, DOI 10.1016/j.jad.2012.05.028

Gois C, 2012, J AFFECT DISORDERS, V142, pS67, DOI 10.1016/S0165-0327(12)70010-1

Gois C, 2011, J AFFECT DISORDERS, V134, P52, DOI 10.1016/j.jad.2011.05.021

Hair J., 2010, MULTIVARIATE DATA AN

Hall PA, 2009, J PSYCHOSOM RES, V67, P297, DOI 10.1016/j.jpsychores.2009.05.015

Hasan SS, 2013, ASIAN J PSYCHIATR, V6, P460, DOI 10.1016/j.ajp.2013.09.008

Hayes A. F., 2013, INTRO MEDIATION MODE

Hu LT, 1999, STRUCT EQU MODELING, V6, P1, DOI 10.1080/10705519909540118

Kaikkonen JE, 2015, SCAND J CLIN LAB INV, V75, P133, DOI 10.3109/00365513.2014.992945

Knol MJ, 2006, DIABETOLOGIA, V49, P837, DOI 10.1007/s00125-006-0159-x

Lasserre A. M., 2016, MOL PSYCHIAT

Mezuk B, 2015, PSYCHOSOM MED, V77, P559, DOI 10.1097/PSY.0000000000000182

MILLER MD, 1992, PSYCHIAT RES, V41, P237, DOI 10.1016/0165-1781(92)90005-N

Molfenter T. D., 2014, GEN MED LOS ANGEL, P2

MORISKY DE, 1986, MED CARE, V24, P67, DOI 10.1097/00005650-198601000-00007

Murri MB, 2014, PSYCHONEUROENDOCRINO, V41, P46, DOI 10.1016/j.psyneuen.2013.12.004

Nau DP, 2007, GENDER MED, V4, P205, DOI 10.1016/S1550-8579(07)80041-6

Nouwen A, 2010, DIABETOLOGIA, V53, P2480, DOI 10.1007/s00125-010-1874-x

Pietraszek A, 2010, NUTR METAB CARDIOVAS, V20, P366, DOI 10.1016/j.numecd.2010.05.001

Polonsky WH, 2005, DIABETES CARE, V28, P626, DOI 10.2337/diacare.28.3.626

Preti A, 2013, J AFFECT DISORDERS, V151, P995, DOI [10.1010/j.jad.2013.08.025, 10.1016/j.jad.2013.08.025]

Shamsi A, 2014, J DIABETES METAB DIS, V13, DOI 10.1186/s40200-014-0096-z

Shi LZ, 2010, PHARMACOECONOMICS, V28, P1097, DOI 10.2165/11537400-000000000-00000

Tabak AG, 2014, LANCET DIABETES ENDO, V2, P236, DOI 10.1016/S2213-8587(13)70139-6

van Dooren FEP, 2016, BMC PSYCHIATRY, V16, DOI 10.1186/s12888-016-0722-z

van Dooren FEP, 2013, PLOS ONE, V8, DOI 10.1371/journal.pone.0057058

Zhang YY, 2015, J DIABETES, V7, P800, DOI 10.1111/1753-0407.12238

NR 42

TC 0

Z9 0

U1 1

U2 19

PU ELSEVIER SCIENCE BV

PI AMSTERDAM

PA PO BOX 211, 1000 AE AMSTERDAM, NETHERLANDS

SN 0165-0327

EI 1573-2517

J9 J AFFECT DISORDERS

JI J. Affect. Disord.

PD SEP

PY 2017

VL 219

BP 64

EP 71

DI 10.1016/j.jad.2017.05.018

PG 8

WC Clinical Neurology; Psychiatry

SC Neurosciences & Neurology; Psychiatry

GA FB9MQ

UT WOS:000406463600009

PM 28525822

DA 2019-01-08

ER

PT J

AU Silveira, MSVM

Bovi, TG

Oliveira, PF

Pavin, EJ

Fisher, L

AF Silveira, M. S. V. M.

Bovi, T. G.

Oliveira, P. F.

Pavin, E. J.

Fisher, L.

TI Translation and cultural adaptation into Brazilian culture of type 1

diabetes distress scale

SO DIABETOLOGY & METABOLIC SYNDROME

LA English

DT Article

DE Diabetes mellitus type 1; Emotional distress; Translating

ID EMOTIONAL DISTRESS; GLYCEMIC CONTROL; YOUNG-ADULTS; DEPRESSION;

PREVALENCE

AB Background: Diabetes related distress is common in type 1 diabetes patients (T1D). High levels of diabetes distress are related to poor metabolic control. An instrument to evaluate diabetes distress in T1D patients is "type 1 diabetes scale-T1DDS". The aim of this study was to translate and culturally adapt the T1DDS into Brazilian culture.

Methods: T1DDS scale was translated into Portuguese. Back translation was performed and evaluated by a specialists committee. Pre-test was performed with 40 T1D outpatients at State University of Campinas hospital. Internal consistency, external consistency and re-test were performed.

Results: 72% women, mean age: 32, 1 +/- 9, 7 years, mean diabetes duration: 15, 8 +/- 9, 1 years, mean scholarity: 11, 5 +/- 3, 6, glycosylated hemoglobin mean: 9 +/- 2%. Internal consistency: Cronbach alpha of T1DDS Brazilian version was 0.93. External consistency: Spearman's coefficient between T1DDS and PAID, Brazilian version, was 0.7781; ( p < 0.0001).

Conclusions: The T1DDS Brazilian version is a reliable tool to evaluate diabetes distress in T1D patients in the Brazilian Population. This tool can be useful in clinical care and to identify patiens at risk and in need for psychosocial intervention.

C1 [Silveira, M. S. V. M.; Bovi, T. G.] Univ Estadual Campinas, Fac Med Sci, Internal Med Postgrad Program, Campinas, SP, Brazil.

[Oliveira, P. F.] Univ Estadual Campinas, Fac Med Sci, Stat Res Ctr, Campinas, SP, Brazil.

[Silveira, M. S. V. M.; Pavin, E. J.] Univ Estadual Campinas, Fac Med Sci, Dept Internal Med, Endocrinol Div, Rua Vital Brasil 251, BR-13083970 Campinas, SP, Brazil.

[Fisher, L.] Univ Calif San Francisco, Dept Family & Community Med, San Francisco, CA 94143 USA.

RP Silveira, MSVM (reprint author), Univ Estadual Campinas, Fac Med Sci, Internal Med Postgrad Program, Campinas, SP, Brazil.; Silveira, MSVM (reprint author), Univ Estadual Campinas, Fac Med Sci, Dept Internal Med, Endocrinol Div, Rua Vital Brasil 251, BR-13083970 Campinas, SP, Brazil.

EM msvms@uol.com.br

CR [Anonymous], 2002, SAS SYST WIND STAT A

Bachle C, 2015, PSYCHONEUROENDOCRINO, V55, P48, DOI 10.1016/j.psyneuen.2015.01.026

Balfe M, 2013, BMC ENDOCR DISORD, V13, DOI 10.1186/1472-6823-13-25

Beaton DE, 2000, SPINE, V25, P3186, DOI 10.1097/00007632-200012150-00014

Bernstein CM, 2013, CLIN PEDIATR, V52, P10, DOI 10.1177/0009922812459950

Bot M, 2013, DIABETIC MED, V30, pE115, DOI 10.1111/dme.12082

Conover W., 1999, PRACTICAL NONPARAMET

Fisher L, 2016, DIABETIC MED, V33, P1590, DOI 10.1111/dme.12973

Fisher L, 2014, DIABETIC MED, V31, P764, DOI 10.1111/dme.12428

Fisher L, 2007, DIABETES CARE, V30, P542, DOI 10.2337/dc06-1614

Fisher L, 2015, J DIABETES COMPLICAT, V29, P572, DOI 10.1016/j.jdiacomp.2015.01.012

Fisher L, 2012, PATIENT EDUC COUNS, V86, P372, DOI 10.1016/j.pec.2011.07.002

Fisher L, 2010, DIABETES CARE, V33, P23, DOI 10.2337/dc09-1238

Gomes MB, 2012, DIABETES RES CLIN PR, V97, P63, DOI 10.1016/j.diabres.2012.02.008

Gross CC, 2007, DIABETES RES CLIN PR, V76, P455, DOI 10.1016/j.diabres.2006.09.022

Lustman PJ, 2000, DIABETES CARE, V23, P934, DOI 10.2337/diacare.23.7.934

Nicolucci A, 2013, DIABETIC MED, V30, P767, DOI 10.1111/dme.12245

Nunnally JC, 1967, PSYCHOMETRIC THEORY

Strandberg RB, 2014, J PSYCHOSOM RES, V77, P174, DOI 10.1016/j.jpsychores.2014.06.015

The Diabetes Control and Complications Trial Research Group, 1993, NEW ENGL J MED, V329, P977

NR 20

TC 0

Z9 0

U1 0

U2 4

PU BIOMED CENTRAL LTD

PI LONDON

PA 236 GRAYS INN RD, FLOOR 6, LONDON WC1X 8HL, ENGLAND

SN 1758-5996

J9 DIABETOL METAB SYNDR

JI Diabetol. Metab. Syndr.

PD AUG 7

PY 2017

VL 9

AR 61

DI 10.1186/s13098-017-0260-y

PG 6

WC Endocrinology & Metabolism

SC Endocrinology & Metabolism

GA FD7MQ

UT WOS:000407710600001

PM 28794803

OA DOAJ Gold, Green Published

DA 2019-01-08

ER

PT J

AU Burke, LA

Rosenfeld, AG

Daya, MR

Vuckovic, KM

Zegre-Hemsey, JK

Diaz, MF

Santos, JTD

Mirzaei, S

DeVon, HA

AF Burke, Larisa A.

Rosenfeld, Anne G.

Daya, Mohamud R.

Vuckovic, Karen M.

Zegre-Hemsey, Jessica K.

Diaz, Maria Felix

Santos, Josemare Tosta Daiube

Mirzaei, Sahereh

DeVon, Holli A.

TI Impact of comorbidities by age on symptom presentation for suspected

acute coronary syndromes in the emergency department

SO EUROPEAN JOURNAL OF CARDIOVASCULAR NURSING

LA English

DT Article

DE Acute coronary syndrome; comorbidities; symptoms; age; cardiovascular

conditions; emergency medicine

ID AMIS PLUS REGISTRY; INDEX; ADULTS; WOMEN; SEX; QUESTIONNAIRE; SERVICES;

OUTCOMES; DISEASE; STROKE

AB Background: It is estimated half of acute coronary syndrome (ACS) patients have one or more associated comorbid conditions.

Aims: Aims were to: 1) examine the prevalence of comorbid conditions in patients presenting to the emergency department with symptoms suggestive of ACS; 2) determine if comorbid conditions influence ACS symptoms; and 3) determine if comorbid conditions predict the likelihood of receiving an ACS diagnosis.

Methods: A total of 1064 patients admitted to five emergency departments were enrolled in this prospective study. Symptoms were measured on presentation to the emergency department. The Charlson Comorbidity Index (CCI) was used to evaluate group differences in comorbidity burden across demographic traits, risk factors, clinical presentation, and diagnosis.

Results: The most prominent comorbid conditions were prior myocardial infarction, diabetes without target organ damage, and chronic lung disease. In younger ACS patients, higher CCI predicted less chest pain, chest discomfort, unusual fatigue and a lower number of symptoms. In older ACS patients, higher CCI predicted more chest discomfort, upper back pain, abrupt symptom onset, and greater symptom distress. For younger non-ACS patients, higher CCI predicted less chest pain and symptom distress. Higher CCI was associated with a greater likelihood of receiving an ACS diagnosis for younger but not older patients with suspected ACS.

Conclusions: Younger patients with ACS and higher number of comorbidities report less chest pain, putting them at higher risk for delayed diagnosis and treatment since chest pain is a hallmark symptom for ACS.

C1 [Burke, Larisa A.; Vuckovic, Karen M.; Diaz, Maria Felix; Santos, Josemare Tosta Daiube; Mirzaei, Sahereh; DeVon, Holli A.] Univ Illinois, Coll Nursing, Dept Biobehav Sci, 845 South Damen Ave M-C 802, Chicago, IL 60612 USA.

[Rosenfeld, Anne G.] Univ Arizona, Coll Nursing, Biobehav Hlth Sci Div, Tucson, AZ 85721 USA.

[Daya, Mohamud R.] Oregon Hlth & Sci Univ, Dept Emergency Med, Portland, OR 97201 USA.

[Zegre-Hemsey, Jessica K.] Univ N Carolina, Sch Nursing, Chapel Hill, NC USA.

RP Burke, LA (reprint author), Univ Illinois, Coll Nursing, Dept Biobehav Sci, 845 South Damen Ave M-C 802, Chicago, IL 60612 USA.

EM laburke@uic.edu

OI Zegre-Hemsey, Jessica/0000-0002-5650-0464; DeVon,

Holli/0000-0002-4526-9631

FU National Institute of Nursing Research [R01NR012012]

FX This work was supported by the National Institute of Nursing Research

(grant number R01NR012012).

CR Alfredsson J, 2016, CLIN GERIATR MED, V32, P291, DOI 10.1016/j.cger.2016.01.009

Bourassa MG, 2000, AM J CARDIOL, V85, P548, DOI 10.1016/S0002-9149(99)90809-3

Boyd CM, 2011, J AM GERIATR SOC, V59, P797, DOI 10.1111/j.1532-5415.2011.03391.x

Canto JG, 2012, JAMA-J AM MED ASSOC, V307, P813, DOI 10.1001/jama.2012.199

Chen Han-Yang, 2013, Clin Epidemiol, V5, P439, DOI 10.2147/CLEP.S49485

Chen SY, 2014, BMC HEALTH SERV RES, V14, DOI 10.1186/1472-6963-14-80

de Groot V, 2003, J CLIN EPIDEMIOL, V56, P221, DOI 10.1016/S0895-4356(02)00585-1

DeVon HA, 2003, NURS RES, V52, P108, DOI 10.1097/00006199-200303000-00007

DeVon HA, 2008, AM J CRIT CARE, V17, P14

DeVon HA, 2017, J CARDIOVASC NURS, V32, P383, DOI 10.1097/JCN.0000000000000351

DeVon HA, 2014, J AM HEART ASSOC, V3, DOI 10.1161/JAHA.113.000586

DeVon HA, 2013, J EMERG NURS, V39, P6, DOI 10.1016/j.jen.2012.08.012

DeVon HA, 2010, RES NURS HEALTH, V33, P386, DOI 10.1002/nur.20395

Fassa AA, 2010, CARDIOVASC MED, V13, P155

Francisco AR, 2010, REV PORT CARDIOL, V29, P7

Goldstein LB, 2004, STROKE, V35, P1941, DOI 10.1161/01.STR.0000135225.80898.1c

Hess EP, 2012, CIRC-CARDIOVASC QUAL, V5, P251, DOI 10.1161/CIRCOUTCOMES.111.964791

HLATKY MA, 1989, AM J CARDIOL, V64, P651, DOI 10.1016/0002-9149(89)90496-7

Hollander JE, 2004, ACAD EMERG MED, V11, P1331, DOI 10.1197/j.aem.2004.08.033

Katz DA, 2008, ANN EMERG MED, V51, P561, DOI 10.1016/j.annemergmed.2007.05.016

Krantz DS, 2006, J WOMENS HEALTH, V15, P1214, DOI 10.1089/jwh.2006.15.1214

Maeder MT, 2014, HEART, V100, P268, DOI 10.1136/heartjnl-2013-305104

McManus David D, 2012, Clin Epidemiol, V4, P115, DOI 10.2147/CLEP.S30883

Oka RK, 1996, AM J CARDIOL, V77, P159, DOI 10.1016/S0002-9149(96)90588-3

Radovanovic D, 2007, HEART, V93, P1369, DOI 10.1136/hrt.2006.106781

Radovanovic D, 2014, HEART, V100, P288, DOI 10.1136/heartjnl-2013-304588

RICKHAM PP, 1964, BRIT MED J, V2, P173

Rosengren A, 2004, EUR HEART J, V25, P663, DOI 10.1016/j.ehj.2004.02.023

Sanchis J, 2011, MAYO CLIN PROC, V86, P291, DOI 10.4065/mcp.2010.0702

Sangha O, 2003, ARTHRIT RHEUM-ARTHR, V49, P156, DOI 10.1002/art.10993

Schafer I, 2012, BMC HEALTH SERV RES, V12, DOI 10.1186/1472-6963-12-89

Scheuermeyer FX, 2014, CAN J EMERG MED, V16, P106, DOI 10.2310/8000.2013.130938

Schmidt M, 2012, BRIT MED J, V344, DOI 10.1136/bmj.e356

Tessier A, 2008, ARCH PHYS MED REHAB, V89, P1276, DOI 10.1016/j.apmr.2007.11.049

Than M, 2012, J AM COLL CARDIOL, V59, P2091, DOI 10.1016/j.jacc.2012.02.035

Radovanovic CAT, 2014, REV LAT-AM ENFERM, V22, P547, DOI 10.1590/0104-1169.3345.2450

NR 36

TC 1

Z9 1

U1 1

U2 5

PU SAGE PUBLICATIONS LTD

PI LONDON

PA 1 OLIVERS YARD, 55 CITY ROAD, LONDON EC1Y 1SP, ENGLAND

SN 1474-5151

EI 1873-1953

J9 EUR J CARDIOVASC NUR

JI Eur. J. Cardiovasc. Nurs.

PD AUG

PY 2017

VL 16

IS 6

BP 511

EP 521

DI 10.1177/1474515117693891

PG 11

WC Cardiac & Cardiovascular Systems; Nursing

SC Cardiovascular System & Cardiology; Nursing

GA FH8ZY

UT WOS:000411496500008

PM 28198635

OA Green Accepted

DA 2019-01-08

ER

PT J

AU Matsunaga, S

Tanaka, S

Fujihara, K

Horikawa, C

Iimuro, S

Kitaoka, M

Sato, A

Nakamura, J

Haneda, M

Shimano, H

Akanuma, Y

Ohashi, Y

Sone, H

AF Matsunaga, Satoshi

Tanaka, Shiro

Fujihara, Kazuya

Horikawa, Chika

Iimuro, Satoshi

Kitaoka, Masafumi

Sato, Asako

Nakamura, Jiro

Haneda, Masakazu

Shimano, Hitoshi

Akanuma, Yasuo

Ohashi, Yasuo

Sone, Hirohito

TI Association between all-cause mortality arid severity of depressive

symptoms in patients with type 2 diabetes: Analysis from the Japan

Diabetes Complications Study (JDCS)

SO JOURNAL OF PSYCHOSOMATIC RESEARCH

LA English

DT Article

DE Diabetes mellitus, type 2; Depressive disorder; Death; Hypoglycemia

ID SEVERE HYPOGLYCEMIA; COMORBID DEPRESSION; EXCESS MORTALITY; SLEEP

DURATION; METAANALYSIS; RISK; COHORT; ADULTS; COMMUNITY; DISTRESS

AB Objective: The aims of this study are to confirm whether the excess mortality caused by depressive symptoms is independent of severe hypoglycemia in patients with type 2 diabetes mellitus (T2DM) and to evaluate the association between all-cause mortality and degrees of severity of depressive symptoms in Japanese patients with T2DM.

Methods: A total of 1160 Japanese patients with T2DM were eligible for this analysis. Participants were followed prospectively for 3 years and their depressive states were evaluated at baseline by the Center for Epidemiologic Studies Depression Scale (CES-D). Cox proportional hazards model was used to evaluate the relative risk of all cause mortality and was adjusted by possible confounding factors, including severe hypoglycemia, all of which are known as risk factors for both depression and mortality.

Results: After adjustment for severe hypoglycemia, each 5-point increase in the CES-D score was significantly associated with excess all-cause mortality (hazard ratio 1.69 [95% CI 1.26-2.17]). The spline curve of HRs for mortality according to total CES-D scores showed that mortality risk was slightly increased at lower scores but was sharply elevated at higher scores.

Conclusion: A high score on the CES-D at baseline was significantly associated with all-cause mortality in patients with T2DM after adjusting for confounders including severe hypoglycemia. However, only a small effect on mortality risk was found at relatively lower levels of depressive symptoms in this population. Further research is needed to confirm this relationship between the severity of depressive symptoms and mortality in patients with T2DM.

C1 [Matsunaga, Satoshi; Fujihara, Kazuya; Sone, Hirohito] Niigata Univ, Dept Hematol Endocrinol & Metab, Fac Med, Niigata, Japan.

[Tanaka, Shiro] Kyoto Univ, Grad Sch Med, Dept Pharmacoepidemiol, Kyoto, Japan.

[Horikawa, Chika] Univ Niigata Prefecture, Fac Human Life Studies, Dept Hlth & Nutr, Niigata, Japan.

[Iimuro, Satoshi] Teikyo Univ, Teikyo Acad Res Ctr, Tokyo, Japan.

[Kitaoka, Masafumi] Showa Gen Hosp, Div Endocrinol & Metab, Tokyo, Japan.

[Sato, Asako] Tokyo Womens Med Univ, Clin Lab, Tokyo, Japan.

[Nakamura, Jiro] Aichi Med Univ, Dept Internal Med, Div Diabet, Sch Med, Nagakute, Aichi, Japan.

[Haneda, Masakazu] Asahikawa Med Univ, Dept Med, Div Metab & Biosyst Sci, Asahikawa, Hokkaido, Japan.

[Shimano, Hitoshi] Univ Tsukuba, Fac Med, Div Endocrinol & Metab, Dept Internal Med, Tsukuba, Ibaraki, Japan.

[Akanuma, Yasuo] Asahi Life Fdn, Inst Adult Dis, Tokyo, Japan.

[Ohashi, Yasuo] Chuo Univ, Dept Integrated Sci & Engn Sustainable Soc, Tokyo, Japan.

RP Sone, H (reprint author), Niigata Univ, Dept Hematol Endocrinol & Metab, Chuo Ku, 757 Asahimachi Dori, Niigata 9518510, Japan.

EM sone@med.niigata-u.ac.jp

FU Ministry of Health, Labor and Welfare

FX This study was supported by grants from the Ministry of Health, Labor

and Welfare. The sponsor had no role in the design and conduct of the

study.

CR Anderson RJ, 2001, DIABETES CARE, V24, P1069, DOI 10.2337/diacare.24.6.1069

BAECKE JAH, 1982, AM J CLIN NUTR, V36, P936

Bogner HR, 2012, ANN FAM MED, V10, P15, DOI 10.1370/afm.1344

Bonds DE, 2010, BRIT MED J, V340, DOI 10.1136/bmj.b4909

Bush DE, 2001, AM J CARDIOL, V88, P337, DOI 10.1016/S0002-9149(01)01675-7

Cappuccio FP, 2010, SLEEP, V33, P585, DOI 10.1093/sleep/33.5.585

Cooper C, 2015, AM J PSYCHIAT, V172, P323, DOI 10.1176/appi.ajp.2014.14070878

Cuijpers P, 2002, J AFFECT DISORDERS, V72, P227, DOI 10.1016/S0165-0327(01)00413-X

Cuijpers P, 2014, AM J PSYCHIAT, V171, P453, DOI 10.1176/appi.ajp.2013.13030325

Cuijpers P, 2013, BRIT J PSYCHIAT, V202, P22, DOI 10.1192/bjp.bp.112.112169

Da Silva MA, 2014, J SLEEP RES, V23, P94, DOI 10.1111/jsr.12077

Egede LE, 2005, DIABETES CARE, V28, P1339, DOI 10.2337/diacare.28.6.1339

Fisher L, 2007, DIABETES CARE, V30, P542, DOI 10.2337/dc06-1614

Forsblom CM, 1998, DIABETOLOGIA, V41, P1253, DOI 10.1007/s001250051062

Goto A, 2013, BMJ-BRIT MED J, V347, DOI 10.1136/bmj.f4533

Ishizawa K, 2016, J DIABETES COMPLICAT, V30, P597, DOI 10.1016/j.jdiacomp.2016.02.004

Katon W, 2009, PSYCHOSOM MED, V71, P965, DOI 10.1097/PSY.0b013e3181bd8f55

Katon WJ, 2013, ANN FAM MED, V11, P245, DOI 10.1370/afm.1501

Katon WJ, 2005, DIABETES CARE, V28, P2668, DOI 10.2337/diacare.28.11.2668

Kimbro LB, 2014, J AM GERIATR SOC, V62, P1017, DOI 10.1111/jgs.12833

Markowitz SM, 2011, PSYCHOSOMATICS, V52, P1, DOI 10.1016/j.psym.2010.11.007

Park M, 2013, GEN HOSP PSYCHIAT, V35, P217, DOI 10.1016/j.genhosppsych.2013.01.006

RADLOFF L S, 1977, Applied Psychological Measurement, V1, P385, DOI 10.1177/014662167700100306

Russ TC, 2012, BRIT MED J, V345, DOI 10.1136/bmj.e4933

Shima S, 1985, CLIN PSYCHIATR, V27, P717, DOI DOI 10.11477/MF.1405203967

Sullivan MD, 2012, DIABETES CARE, V35, P1708, DOI 10.2337/dc11-1791

Tanaka S, 2014, INT J EPIDEMIOL, V43, P1054, DOI 10.1093/ije/dyt057

Tesfaye S, 2010, DIABETES CARE, V33, P2285, DOI 10.2337/dc10-1303

Wada K, 2007, AM J IND MED, V50, P8, DOI 10.1002/ajim.20403

White J, 2015, PSYCHOL MED, V45, P2771, DOI 10.1017/S0033291715000732

Whitlock G, 2009, LANCET, V373, P1083, DOI 10.1016/S0140-6736(09)60318-4

Zhang YY, 2015, BMC PSYCHIATRY, V15, DOI 10.1186/s12888-015-0580-0

Zoungas S, 2010, NEW ENGL J MED, V363, P1410, DOI 10.1056/NEJMoa1003795

NR 33

TC 1

Z9 1

U1 1

U2 8

PU PERGAMON-ELSEVIER SCIENCE LTD

PI OXFORD

PA THE BOULEVARD, LANGFORD LANE, KIDLINGTON, OXFORD OX5 1GB, ENGLAND

SN 0022-3999

EI 1879-1360

J9 J PSYCHOSOM RES

JI J. Psychosomat. Res.

PD AUG

PY 2017

VL 99

BP 34

EP 39

DI 10.1016/j.jpsychores.2017.05.020

PG 6

WC Psychiatry

SC Psychiatry

GA FB7ZA

UT WOS:000406357300006

PM 28712428

DA 2019-01-08

ER

PT J

AU Wong, EM

Afshar, R

Qian, H

Zhang, M

Elliott, TG

Tang, TS

AF Wong, Evelyn M.

Afshar, Rowshanak

Qian, Hong

Zhang, Mira

Elliott, Thomas G.

Tang, Tricia S.

TI Diabetes Distress, Depression and Glycemic Control in a Canadian-Based

Specialty Care Setting

SO CANADIAN JOURNAL OF DIABETES

LA English

DT Article

DE depression; diabetes distress; type 2 diabetes mellitus; psychosocial

outcomes; tertiary care setting

ID EMOTIONAL DISTRESS; CLINICAL DEPRESSION; TYPE-2; ASSOCIATIONS;

PREVALENCE; AMERICANS; ANXIETY

AB Objectives: The objectives of this study were to determine rates of diabetes distress and depression in patients with type 2 diabetes in a tertiary care setting, to examine the relationship among glycemic control, diabetes distress and depression, and to identify predictors of diabetes distress and depression on the basis of demographic and clinical characteristics.

Methods: We recruited 148 adults with type 2 diabetes who were presenting to a specialty diabetes clinic in Vancouver, British Columbia, Canada. Participants completed a questionnaire measuring diabetes distress, depressive symptoms and demographic backgrounds. The Diabetes Distress Scale was used to assess overall distress as well as 4 distinct distress dimensions, including emotional burden, physician-related, regimen-related and interpersonal distress. The Personal Health Questionnaire-9 was used to assess depressive symptoms. Glycated hemoglobin (A1C) data were also collected.

Results: The prevalence of diabetes distress and depression was 39% and 12% in our population, respectively. A1C levels emerged as a significant predictor of emotional burden (p=0.03) and regimen-related distress (p=0.01); higher A1C levels were associated with increased distress regarding emotional functioning and regimen adherence. A1C levels (p=0.02) and education levels (p=0.03) emerged as predictors of physician-related distress, with higher A1C levels associated with decreased distress regarding confidence in physicians.

Conclusions: Our findings reveal that the rate of diabetes distress for patients in a tertiary care setting is high. Furthermore, diabetes distress, particularly emotion- and self-care-related distress, plays a significant role in glycemic control, whereas depression does not. Routine screening for diabetes distress as part of an initial specialty clinic evaluation should be explored. (C) 2017 Canadian Diabetes Association.

C1 [Wong, Evelyn M.] Univ Toronto, Dept Med, Div Endocrinol, Toronto, ON, Canada.

[Afshar, Rowshanak; Tang, Tricia S.] Univ British Columbia, Dept Med, Div Endocrinol, Vancouver, BC, Canada.

[Qian, Hong] St Pauls Hosp, Ctr Hlth Evaluat & Outcome Sci, Vancouver, BC, Canada.

[Zhang, Mira; Elliott, Thomas G.] BC Diabet, Vancouver, BC, Canada.

RP Wong, EM (reprint author), Toronto Gen Hosp, 200 Elizabeth St,Eaton North 7th Floor,Room 234, Toronto, ON M5G 2C4, Canada.

EM evelyn.mm.wong@gmail.com

FU BC Diabetes

FX Thanks to Paige Kendall, Alex Dang and Jas Sidhu for data entry. This

work was supported by BC Diabetes.

CR Aikens JE, 2012, DIABETES CARE, V35, P2472, DOI 10.2337/dc12-0181

Anderson RJ, 2001, DIABETES CARE, V24, P1069, DOI 10.2337/diacare.24.6.1069

Black SA, 1999, DIABETES CARE, V22, P56, DOI 10.2337/diacare.22.1.56

Delahanty LM, 2007, DIABETIC MED, V24, P48, DOI 10.1111/j.1464-5491.2007.02028.x

Fisher L, 2009, DIABETIC MED, V26, P622, DOI 10.1111/j.1464-5491.2009.02730.x

Fisher L, 2007, DIABETES CARE, V30, P542, DOI 10.2337/dc06-1614

Fisher L, 2012, DIABETES CARE, V35, P259, DOI 10.2337/dc11-1572

Fisher L, 2010, DIABETES CARE, V33, P1034, DOI 10.2337/dc09-2175

Fisher L, 2010, DIABETES CARE, V33, P23, DOI 10.2337/dc09-1238

Fleer J, 2013, DIABETIC MED, V30, P88, DOI 10.1111/dme.12001

Gariepy G, 2013, J PSYCHOSOM RES, V75, P147, DOI 10.1016/j.jpsychores.2013.05.009

Gregg EW, 2000, ARCH INTERN MED, V160, P174, DOI 10.1001/archinte.160.2.174

Hermanns N, 2006, DIABETOLOGIA, V49, P469, DOI 10.1007/s00125-005-0094-2

KESSLER RC, 1993, J AFFECT DISORDERS, V29, P85, DOI 10.1016/0165-0327(93)90026-G

Kokoszka A, 2009, EUR PSYCHIAT, V24, P425, DOI 10.1016/j.eurpsy.2009.04.002

Lustman PJ, 2000, DIABETES CARE, V23, P934, DOI 10.2337/diacare.23.7.934

Nichols GA, 2003, DIABETES CARE, V26, P744, DOI 10.2337/diacare.26.3.744

Nolen-Hoeksema S, 2001, CURR DIR PSYCHOL SCI, V10, P173, DOI 10.1111/1467-8721.00142

Pouwer F, 2003, DIABETOLOGIA, V46, P892, DOI 10.1007/s00125-003-1124-6

Pouwer F, 2009, NAT REV ENDOCRINOL, V5, P665, DOI 10.1038/nrendo.2009.214

Rogvi S, 2012, DIABETIC MED, V29, pe382, DOI 10.1111/j.1464-5491.2012.03703.x

Spencer MS, 2006, J HEALTH CARE POOR U, V17, P88, DOI 10.1353/hpu.2006.0095

Wu SFV, 2011, INT J NURS STUD, V48, P1376, DOI 10.1016/j.ijnurstu.2011.04.008

Zhang CX, 2008, DIABETES RES CLIN PR, V79, P523, DOI 10.1016/j.diabres.2007.10.014

NR 24

TC 4

Z9 4

U1 2

U2 8

PU ELSEVIER SCIENCE BV

PI AMSTERDAM

PA PO BOX 211, 1000 AE AMSTERDAM, NETHERLANDS

SN 1499-2671

J9 CAN J DIABETES

JI Can. J. Diabetes

PD AUG

PY 2017

VL 41

IS 4

BP 362

EP 365

DI 10.1016/j.jcjd.2016.11.006

PG 4

WC Endocrinology & Metabolism

SC Endocrinology & Metabolism

GA FB3BQ

UT WOS:000406018000005

PM 28462795

DA 2019-01-08

ER

PT J

AU Bai, JW

Lovblom, LE

Cardinez, M

Weisman, A

Farooqi, MA

Halpern, EM

Boulet, G

Eldelekli, D

Lovshin, JA

Lytvyn, Y

Keenan, HA

Brent, MH

Paul, N

Bril, V

Cherney, DZI

Perkins, BA

AF Bai, Johnny-Wei

Lovblom, Leif E.

Cardinez, Marina

Weisman, Alanna

Farooqi, Mohammed A.

Halpern, Elise M.

Boulet, Genevieve

Eldelekli, Devrim

Lovshin, Julie A.

Lytvyn, Yuliya

Keenan, Hillary A.

Brent, Michael H.

Paul, Narinder

Bril, Vera

Cherney, David Z. I.

Perkins, Bruce A.

TI Neuropathy and presence of emotional distress and depression in

longstanding diabetes: Results from the Canadian study of longevity in.

type 1 diabetes

SO JOURNAL OF DIABETES AND ITS COMPLICATIONS

LA English

DT Article

DE Type 1 diabetes; Complications; Neuropathy; Depression; Distress;

Psychology

ID PERIPHERAL NEUROPATHY; GLYCEMIC CONTROL; SELF-CARE; COMPLICATIONS;

SYMPTOMS; ADULTS; SCALE; METAANALYSIS; DISEASE; INTERVENTIONS

AB Aim: To determine the association of neuropathy and other complications with emotional distress and depression among patients with longstanding type 1 diabetes (T1DM).

Methods: Canadians with >= 50 years of T1DM completed a questionnaire including assessment of distress and depression by the Problem Areas in Diabetes Scale (PAID) and Geriatric Depression Scale (GDS), respectively. Complications were determined using the Michigan Neuropathy Screening Instrument (Questionnaire Component), fundoscopy reports, renal function tests, and self-reported peripheral-(PVD) and cardiovascular (CVD) disease. Associations were analyzed by Poisson regression.

Results: Among 323 participants, 137 (42.4%) had neuropathy, 113 (36.5%) nephropathy, 207 (69.5%) retinopathy, 95 (29.4%) CVD, and 31 (9.8%) PVD. The neuropathy subgroup had higher prevalence of distress (13 (9.5%) vs. 6 (3.3%), p = 0.029) and depression (34 (24.9%) vs. 12 (6.5%), p < 0.001). Adjusting for diabetes complications, neuropathy was associated with higher PAID (adjusted RR 1.44 (95% CI 1.14-1.82), p = 0.003) and GDS scores (adjusted RR1.57 (1.18-2.11), p = 0.002). Independent of potential confounders, neuropathy remained associated with higher PAID (adjusted RR 1.39 (1.10-1.76), p = 0.006) and GDS scores (adjusted RR 137 (1.03-1.83), p = 0.032). Associations with neuropathy were not fully explained by neuropathic pain.

Conclusion: Compared to other complications, neuropathy had the greatest association with distress and depression in longstanding T1DM, independent of pain. Strategies beyond pain management are needed to improve quality of life in diabetic neuropathy. (C) 2017 Published by Elsevier Inc.

C1 [Bai, Johnny-Wei; Lovblom, Leif E.; Cardinez, Marina; Weisman, Alanna; Farooqi, Mohammed A.; Halpern, Elise M.; Boulet, Genevieve; Eldelekli, Devrim; Lovshin, Julie A.; Perkins, Bruce A.] Mt Sinai Hosp, Lunenfeld Tanenbaum Res Inst, Toronto, ON, Canada.

[Lytvyn, Yuliya; Cherney, David Z. I.] Univ Toronto, Dept Med, Div Nephrol, Toronto, ON, Canada.

[Keenan, Hillary A.] Joslin Diabet Ctr, Div Res, 1 Joslin Pl, Boston, MA 02215 USA.

[Brent, Michael H.] Univ Toronto, Dept Med, Dept Ophthalmol & Vis Sci, Toronto, ON, Canada.

[Paul, Narinder] Univ Hlth Network, Div Cardiothorac Radiol, Joint Dept Med Imaging, Toronto, ON, Canada.

[Bril, Vera] Univ Toronto, Univ Hlth Network, Ellen & Martin Prosserman Ctr Neuromuscular Dis, Div Neurol,Krembil Neurosci Ctr,Dept Med, Toronto, ON, Canada.

[Perkins, Bruce A.] Univ Toronto, Dept Med, Div Endocrinol & Metab, Toronto, ON, Canada.

RP Perkins, BA (reprint author), Univ Toronto, Leadership Sinai Ctr Diabet, Mt Sinai Hosp, Endocrinol & Metab, L5-210,60 Murray St,Mail Box 16, Toronto, ON M5T 3L9, Canada.

EM BPerkins@mtsinai.on.ca

RI Perkins, Bruce/L-3726-2017

OI Farooqi, Mohammed/0000-0001-5282-4034; Lovblom, Leif

Erik/0000-0002-6774-8924

FU JDRF Canada (Juvenile Diabetes Research Foundation) [17-2013-312]; JDRF

Canadian Clinical Trial Network; Medtronic; Boehringer Ingelheim;

AstraZeneca; Merck

FX We are grateful to the support of JDRF Canada (Juvenile Diabetes

Research Foundation operating grant 17-2013-312), its Canadian Clinical

Trial Network and to the Canadian Diabetes Association for their

non-financial support with study advertisement. We greatly appreciate

our distinguished study participants, who have inspired our team and

spent considerable time participating in this study. A.W. has received a

post -doctoral fellowship award from the JDRF Canadian Clinical Trial

Network. B.A.P has received speaker honoraria from Medtronic Inc.,

Johnson and Johnson, Roche, Glaxo Smith Kline Canada, Novo Nordisk and

Sanofi; has received research grant support from Medtronic and

Boehringer Ingelheim; and serves as a consultant for Neurometrix.

D.Z.I.C. has received speaker honoraria from Janssen, AstraZeneca,

Boehringer Ingelheim, Lilly and Merck and has received research grant

support from AstraZeneca, Merck and Boehringer Ingelheim.

CR Aalto AM, 2000, EUR J PUBLIC HEALTH, V10, P68, DOI 10.1093/eurpub/10.1.68

Allison P. D., 1999, LOGISTIC REGRESSION

American Psychiatric, 2013, DIAGN STAT MAN MENT

Anderson RJ, 2001, DIABETES CARE, V24, P1069, DOI 10.2337/diacare.24.6.1069

Bai JW, 2016, CARDIOVASC DIABETOL, V15, DOI 10.1186/s12933-015-0318-9

Bain SC, 2003, DIABETIC MED, V20, P808, DOI 10.1046/j.1464-5491.2003.01029.x

Barnard KD, 2006, DIABETIC MED, V23, P445, DOI 10.1111/j.1464-5491.2006.01814.x

Chew BH, 2014, WORLD J DIABETES, V5, P796, DOI 10.4239/wjd.v5.i6.796

Conradsson M, 2013, AGING MENT HEALTH, V17, P638, DOI 10.1080/13607863.2012.758231

D'Amato C, 2016, DIAB VASC DIS RES

de Groot M, 2001, PSYCHOSOM MED, V63, P619, DOI 10.1097/00006842-200107000-00015

de Groot M, 2012, CURR DIABETES REP, V12, P157, DOI 10.1007/s11892-012-0261-z

Dhaliwal Ruban, 2014, Diabetes Spectr, V27, P9, DOI 10.2337/diaspect.27.1.9

Fisher L, 2015, J DIABETES COMPLICAT, V29, P572, DOI 10.1016/j.jdiacomp.2015.01.012

Fisher L, 2010, DIABETES CARE, V33, P23, DOI 10.2337/dc09-1238

Gore M, 2005, J PAIN SYMPTOM MANAG, V30, P374, DOI 10.1016/j.jpainsymman.2005.04.009

Herman WH, 2012, DIABETIC MED, V29, P937, DOI 10.1111/j.1464-5491.2012.03644.x

Ishizawa K, 2016, J DIABETES COMPLICAT, V30, P597, DOI 10.1016/j.jdiacomp.2016.02.004

Levey AS, 1999, ANN INTERN MED, V130, P461, DOI 10.7326/0003-4819-130-6-199903160-00002

Lloyd CE, 2010, DIABETIC MED, V27, P234, DOI 10.1111/j.1464-5491.2009.02896.x

Lyness JM, 1997, ARCH INTERN MED, V157, P449, DOI 10.1001/archinte.157.4.449

Marc LG, 2008, AM J GERIAT PSYCHIAT, V16, P914, DOI 10.1097/JGP.0b013e318186bd67

Nicolucci A, 2013, DIABETIC MED, V30, P767, DOI 10.1111/dme.12245

Pallayova Maria, 2014, Diabetes Spectr, V27, P143, DOI 10.2337/diaspect.27.2.143

Prior SL, 2011, DIABETES RES CLIN PR, V91, pE71, DOI 10.1016/j.diabres.2010.12.007

Robinson DJ, 2013, CAN J DIABETES, V37, pS87, DOI 10.1016/j.jcjd.2013.01.026

Selvarajah D, 2014, DIABETES VASC DIS RE, V11, P218, DOI 10.1177/1479164114522135

SHEIKH J I, 1986, Clinical Gerontologist, V5, P165

Sherifali D, 2015, DIABETIC MED, V32, P1404, DOI 10.1111/dme.12780

Snoek FJ, 2015, LANCET DIABETES ENDO, V3, P450, DOI 10.1016/S2213-8587(15)00135-7

Sun JK, 2011, DIABETES CARE, V34, P968, DOI 10.2337/dc10-1675

Tsujii S, 2012, DIABETIC MED, V29, P1451, DOI 10.1111/j.1464-5491.2012.03647.x

TURKINGTON RW, 1980, JAMA-J AM MED ASSOC, V243, P1147, DOI 10.1001/jama.243.11.1147

Van Acker K, 2009, DIABETES METAB, V35, P206, DOI 10.1016/j.diabet.2008.11.004

van Bastelaar KMP, 2010, DIABETIC MED, V27, P798, DOI 10.1111/j.1464-5491.2010.03025.x

Vileikyte L, 2005, DIABETES CARE, V28, P2378, DOI 10.2337/diacare.28.10.2378

Weintraub D, 2006, AM J GERIAT PSYCHIAT, V14, P169, DOI 10.1097/01.JGP.0000192488.66049.4b

Weisman A, 2016, DIABETES CARE, V39, pE67, DOI 10.2337/dc16-0102

Welch GW, 1997, DIABETES CARE, V20, P760, DOI 10.2337/diacare.20.5.760

YESAVAGE JA, 1983, J PSYCHIATR RES, V17, P37, DOI 10.1016/0022-3956(82)90033-4

NR 40

TC 3

Z9 3

U1 4

U2 5

PU ELSEVIER SCIENCE INC

PI NEW YORK

PA 360 PARK AVE SOUTH, NEW YORK, NY 10010-1710 USA

SN 1056-8727

EI 1873-460X

J9 J DIABETES COMPLICAT

JI J. Diabetes Complications

PD AUG

PY 2017

VL 31

IS 8

BP 1318

EP 1324

DI 10.1016/j.jdiacomp.2017.05.002

PG 7

WC Endocrinology & Metabolism

SC Endocrinology & Metabolism

GA FB3ZL

UT WOS:000406081200014

PM 28599823

DA 2019-01-08

ER

PT J

AU Hermanns, N

Ehrmann, D

Schall, S

Maier, B

Haak, T

Kulzer, B

AF Hermanns, N.

Ehrmann, D.

Schall, S.

Maier, B.

Haak, T.

Kulzer, B.

TI The effect of an education programme (MEDIAS 2 BSC) of non-intensive

insulin treatment regimens for people with Type2 diabetes: a randomized,

multi-centre trial

SO DIABETIC MEDICINE

LA English

DT Article

ID SELF-MANAGEMENT EDUCATION; GLYCEMIC CONTROL; THERAPY; ADULTS;

HYPOGLYCEMIA; METAANALYSIS; ASSOCIATION; DISTRESS; SCALE

AB AimsA self-management oriented education programme (MEDIAS2BSC) for people with Type2 diabetes who are on a non-intensive insulin treatment regimen was developed. In a randomized, multi-centre trial, the effect of MEDIAS2BSC was compared with an established education programme that acted as a control group.

MethodsThe primary outcome was the impact of MEDIAS2BSC on glycaemic control. Secondary outcomes included the incidence of severe hypoglycaemia, hypoglycaemia unawareness, diabetes-related distress, diabetes knowledge, quality of life and self-care behaviour.

ResultsIn total, 182 participants were randomized to the control group or MEDIAS2BSC [median age 64.0 (interquartile range 58.0-68.5) vs. 63.5 (57.0-70.0)years; HbA(1c) 62.812.7mmol/mol vs. 63.714.0mmol/mol; 7.9%1.2% vs. 8.0%1.3%]. After a 6-month follow-up, there was a mean decrease in HbA(1c) of 3.5mmol/mol (0.32%) in the control group and 6.7mmol/mol (0.61%) in MEDIAS2BSC. After adjusting for baseline differences and study centre, the mean difference between the groups was -3.3mmol/mol [95% confidence interval (CI) -0.54 to -5.90mmol/mol] [-0.30% (95% CI -0.05 to -0.54)] in favour of MEDIAS2BSC (P=0.018). There were no increases in severe hypoglycaemia or hypoglycaemia unawareness. The education programmes had no significant effects on psychosocial outcome variables.

ConclusionMEDIAS2BSC was more effective in lowering HbA(1c) than the control condition. MEDIAS2BSC is a safe educational tool that improves glycaemic control without increasing the risk for hypoglycaemia. (Clinical Trials Registry No; NCT 02748239)

C1 [Hermanns, N.; Ehrmann, D.; Schall, S.; Maier, B.; Haak, T.; Kulzer, B.] Diabet Acad Mergentheim FIDAM, Res Inst, Bad Mergentheim, Germany.

[Hermanns, N.; Maier, B.; Haak, T.; Kulzer, B.] Diabet Ctr Mergentheim, Bad Mergentheim, Germany.

[Hermanns, N.; Ehrmann, D.; Kulzer, B.] Otto Friedrich Univ Bamberg, Dept Clin Psychol, Bamberg, Germany.

RP Hermanns, N (reprint author), Diabet Acad Mergentheim FIDAM, Res Inst, Bad Mergentheim, Germany.; Hermanns, N (reprint author), Diabet Ctr Mergentheim, Bad Mergentheim, Germany.; Hermanns, N (reprint author), Otto Friedrich Univ Bamberg, Dept Clin Psychol, Bamberg, Germany.

EM hermanns@diabetes-zentrum.de

OI Ehrmann, Dominic/0000-0002-5794-5596

CR Anderson RM, 2005, PATIENT EDUC COUNS, V57, P153, DOI 10.1016/j.pec.2004.05.008

Berger M, 1989, STRUCTURED TREATMENT

Bundesaerztekammer (BAEK) Kassenaerztliche Bundesvereinigung (KBV) Arbeitsgemeinschaft der Wissenschaftlichen Medizinischen Fachgesellschaften (AWMF), 2013, NAT GUID THERP TYP 2

Chrvala CA, 2016, PATIENT EDUC COUNS, V99, P926, DOI 10.1016/j.pec.2015.11.003

CLARKE WL, 1995, DIABETES CARE, V18, P517, DOI 10.2337/diacare.18.4.517

Deakin T, 2005, COCHRANE DB SYST REV, DOI 10.1002/14651858.CD003417.pub2

Delahanty LM, 2007, DIABETIC MED, V24, P48, DOI 10.1111/j.1464-5491.2007.02028.x

Didjurgeit U, 2011, STRUCTURED TREATMENT

Greiner W, 2007, HLTH EC EVALUATION, P403

Hautzinger M, 1993, GERMAN VERSION CTR E

Hermanns N, 2007, DIABETES-METAB RES, V23, P528, DOI 10.1002/dmrr.710

Hermanns N, 2013, DIABETES RES CLIN PR, V102, P149, DOI 10.1016/j.diabres.2013.10.009

Hermanns N, 2012, PATIENT EDUC COUNS, V86, P226, DOI 10.1016/j.pec.2011.05.017

Hermanns N, 2010, HEALTH QUAL LIFE OUT, V8, DOI 10.1186/1477-7525-8-113

Holman RR, 2007, NEW ENGL J MED, V357, P1716, DOI 10.1056/NEJMoa075392

Holman RR, 2009, NEW ENGL J MED, V361, P1736, DOI 10.1056/NEJMoa0905479

Kulzer B, 2007, DIABETIC MED, V24, P415, DOI 10.1111/j.1464-5491.2007.02089.x

Kulzer B, 2015, MEDIAS 2 MORE DIABET

Muhlhauser I, 2002, DIABETOLOGIA, V45, P1723, DOI 10.1007/s00125-002-0987-2

Norris SL, 2002, AM J PREV MED, V22, P15, DOI 10.1016/S0749-3797(02)00423-3

Norris SL, 2002, DIABETES CARE, V25, P1159, DOI 10.2337/diacare.25.7.1159

Peyrot M, 2012, DIABETES OBES METAB, V14, P1081, DOI 10.1111/j.1463-1326.2012.01636.x

Peyrot M, 2010, DIABETES CARE, V33, P240, DOI 10.2337/dc09-1348

POLONSKY WH, 1995, DIABETES CARE, V18, P754, DOI 10.2337/diacare.18.6.754

Polonsky WH, 2005, DIABETES CARE, V28, P2543, DOI 10.2337/diacare.28.10.2543

Steinsbekk A, 2012, BMC HEALTH SERV RES, V12, DOI 10.1186/1472-6963-12-213

Stratton IM, 2000, BRIT MED J, V321, P405, DOI 10.1136/bmj.321.7258.405

Toobert DJ, 2000, DIABETES CARE, V23, P943, DOI 10.2337/diacare.23.7.943

UK Prospective Diabetes Study (UKPDS) Group, 1998, LANCET, V352, P837, DOI DOI 10.1016/S0140-6736(98)07019-6

Welch GW, 1997, DIABETES CARE, V20, P760, DOI 10.2337/diacare.20.5.760

NR 30

TC 1

Z9 1

U1 1

U2 3

PU WILEY

PI HOBOKEN

PA 111 RIVER ST, HOBOKEN 07030-5774, NJ USA

SN 0742-3071

EI 1464-5491

J9 DIABETIC MED

JI Diabetic Med.

PD AUG

PY 2017

VL 34

IS 8

BP 1084

EP 1091

DI 10.1111/dme.13346

PG 8

WC Endocrinology & Metabolism

SC Endocrinology & Metabolism

GA FA7CK

UT WOS:000405602000009

PM 28257159

DA 2019-01-08

ER

PT J

AU Al Sayah, F

Qiu, WY

Xie, F

Johnson, JA

AF Al Sayah, Fatima

Qiu, Weiyu

Xie, Feng

Johnson, Jeffrey A.

TI Comparative performance of the EQ-5D-5L and SF-6D index scores in adults

with type 2 diabetes

SO QUALITY OF LIFE RESEARCH

LA English

DT Article

DE EQ-5D-5L; SF-6D; Discriminative validity; Responsiveness; Diabetes

ID QUALITY-OF-LIFE; EUROQOL-5 DIMENSION EQ-5D; FUNCTIONAL DISABILITY;

GENERAL-POPULATION; PATIENT GROUPS; HEALTH; VALIDITY; RESPONSIVENESS;

QUESTIONNAIRE; INSTRUMENTS

AB To explore the comparative performance including discriminative and longitudinal validity of EQ-5D-5L and SF-6D index scores in adults with type 2 diabetes.

Data from an on-going cohort study of adults with type 2 diabetes in Alberta, Canada, were used. Known-groups approach was used to examine discriminative validity. Correlation and agreement indices and scatter and Bland-Altman plots were used to examine the relationship between the two measures. Longitudinal validity was explored using Wilcoxon signed-rank test, effect size, and standardized response mean.

In 1832 participants at baseline (age 64.3, standard deviation 10.6 years; 45% female), median EQ-5D-5L score was 0.85 [interquartile range (IQR) 0.17], and floor and ceiling effects of 0.1 and 16.1%, respectively; median SF-6D score was 0.72 (IQR 0.24), and floor and ceiling effects of 0.1 and 3.2%, respectively. EQ-5D-5L and SF-6D index scores were significantly correlated with an overall Spearman correlation coefficient of 0.73, and an ICC of 0.62 (95% CI 0.42-0.74). Both EQ-5D-5L and SF-6D scores demonstrated statistically significant differences in self-reported chronic conditions, depressive symptoms, and diabetes-related distress, and were able to detect changes in depressive symptoms and diabetes distress across all change groups.

Both EQ-5D-5L and SF-6D index scores provide valid measurement in this patient population. Considerable overlap between the measures means it is not necessary to include both in surveys, however, the advantages and disadvantages of each should be considered.

C1 [Al Sayah, Fatima; Qiu, Weiyu] Univ Alberta, Sch Publ Hlth, Edmonton, AB, Canada.

[Xie, Feng] McMaster Univ, Dept Clin Epidemiol & Biostat, Hamilton, ON, Canada.

[Xie, Feng] St Josephs Healthcare Hamilton, Father Sean OSullivan Res Ctr, Hamilton, ON, Canada.

[Xie, Feng] Program Hlth Econ & Outcome Measures, Hamilton, ON, Canada.

[Johnson, Jeffrey A.] Univ Alberta, Sch Publ Hlth, Li Ka Shing Ctr Hlth Res Innovat 2 040, Edmonton, AB T6G 2E1, Canada.

RP Johnson, JA (reprint author), Univ Alberta, Sch Publ Hlth, Li Ka Shing Ctr Hlth Res Innovat 2 040, Edmonton, AB T6G 2E1, Canada.

EM jeff.johnson@ualberta.ca

OI Johnson, Jeffrey/0000-0001-8290-2857

FU Alberta Health; CIHR - CIHR Institute of Nutrition, Metabolism and

Diabetes (INMD) [OTG- 88588]

FX This work was supported by grant from Alberta Health, and a CIHR Team

Grant to the Alliance for Canadian Health Outcomes Research in Diabetes

(reference #: OTG- 88588), sponsored by the CIHR Institute of Nutrition,

Metabolism and Diabetes (INMD).

CR Agborsangaya CB, 2014, HEALTH QUAL LIFE OUT, V12, DOI 10.1186/1477-7525-12-74

Al Sayah Fatima, 2015, Can J Diabetes, V39 Suppl 3, pS113, DOI 10.1016/j.jcjd.2015.05.005

Biering K, 2015, CLIN EPIDEMIOL, V7, P91, DOI 10.2147/CLEP.S72247

Brazier J, 2004, HEALTH ECON, V13, P873, DOI 10.1002/hec.866

Brazier J, 1999, HEALTH TECHNOL ASSES, V3, P1

Brazier JE, 2004, MED CARE, V42, P851, DOI 10.1097/01.mlr.0000135827.18610.0d

Bryan Stirling, 2005, Eur J Health Econ, V6, P253, DOI 10.1007/s10198-005-0299-9

Cicchetti D., 1994, PSYCHOL ASSESSMENTS, V6, P284, DOI [10.1037/1040-3590.6.4.284, DOI 10.1037/1040-3590.6.4.284]

Cohen J, 1988, STAT POWER ANAL BEHA

De Smedt D, 2014, VALUE HEALTH, V17, P84, DOI 10.1016/j.jval.2013.10.010

Drummond M, 2001, ANN MED, V33, P344, DOI 10.3109/07853890109002088

Drummond M. F., 1997, METHODS EC EVALUATIO

Egede LE, 2004, DIABETES CARE, V27, P421, DOI 10.2337/diacare.27.2.421

Fritz CO, 2012, J EXP PSYCHOL GEN, V141, P2, DOI 10.1037/a0024338

Garratt A., 2002, BMJ-BRIT MED J, V324, P1471

Giavarina D, 2015, BIOCHEM MEDICA, V25, P141, DOI 10.11613/BM.2015.015

Gregg EW, 2002, DIABETES CARE, V25, P61, DOI 10.2337/diacare.25.1.61

Herdman M, 2011, QUAL LIFE RES, V20, P1727, DOI 10.1007/s11136-011-9903-x

Janssen MF, 2013, QUAL LIFE RES, V22, P1717, DOI 10.1007/s11136-012-0322-4

Kontodimopoulos N, 2012, EUR J HEALTH ECON, V13, P111, DOI 10.1007/s10198-010-0290-y

Kontodimopoulos N, 2009, QUAL LIFE RES, V18, P87, DOI 10.1007/s11136-008-9420-8

Kroenke K, 2009, J AFFECT DISORDERS, V114, P163, DOI 10.1016/j.jad.2008.06.026

Maddigan SL, 2006, AM J PUBLIC HEALTH, V96, P1649, DOI 10.2105/AJPH.2005.067728

Maddigan SL, 2005, QUAL LIFE RES, V14, P1311, DOI 10.1007/s11136-004-6640-4

Manea L, 2012, CAN MED ASSOC J, V184, pE191, DOI 10.1503/cmaj.110829

Marrero D, 2014, QUAL LIFE RES, V23, P75, DOI 10.1007/s11136-013-0436-3

McGuire BE, 2010, DIABETOLOGIA, V53, P66, DOI 10.1007/s00125-009-1559-5

Mukaka MM, 2012, MALAWI MED J, V24, P69

Mulhern B, 2014, HEALTH QUAL LIFE OUT, V12, DOI 10.1186/1477-7525-12-42

Norman GR, 2003, MED CARE, V41, P582, DOI 10.1097/00005650-200305000-00004

Obradovic M, 2013, HEALTH QUAL LIFE OUT, V11, DOI 10.1186/1477-7525-11-110

Pattanaphesaj J, 2015, HEALTH QUAL LIFE OUT, V13, DOI 10.1186/s12955-014-0203-3

Petrou S, 2005, HEALTH ECON, V14, P1169, DOI 10.1002/hec.1006

Revicki DA., 2002, STAT METHODS QUALITY, P103

Royston P, 2011, J STAT SOFTW, V45, P1

Shaw JE, 2010, DIABETES RES CLIN PR, V87, P4, DOI 10.1016/j.diabres.2009.10.007

Simon Pickard A, 2005, QUAL LIFE RES, V14, P207, DOI 10.1007/s11136-004-3928-3

van Stel HF, 2006, HEALTH QUAL LIFE OUT, V4, DOI 10.1186/1477-7525-4-20

Wee HL, 2007, VALUE HEALTH, V10, P256, DOI 10.1111/j.1524-4733.2007.00174.x

Whitehurst DGT, 2011, MED DECIS MAKING, V31, pE34, DOI 10.1177/0272989X11421529

Wu J, 2014, HEALTH QUAL LIFE OUT, V12, DOI 10.1186/s12955-014-0156-6

Xie F, 2007, ARTHRIT RHEUM-ARTHR, V57, P1043, DOI 10.1002/art.22883

Xie F, 2016, MED CARE, V54, P98, DOI 10.1097/MLR.0000000000000447

Yordanova S, 2014, BIOTECHNOL BIOTEC EQ, V28, P769, DOI 10.1080/13102818.2014.935572

NR 44

TC 2

Z9 2

U1 1

U2 6

PU SPRINGER

PI DORDRECHT

PA VAN GODEWIJCKSTRAAT 30, 3311 GZ DORDRECHT, NETHERLANDS

SN 0962-9343

EI 1573-2649

J9 QUAL LIFE RES

JI Qual. Life Res.

PD AUG

PY 2017

VL 26

IS 8

BP 2057

EP 2066

DI 10.1007/s11136-017-1559-8

PG 10

WC Health Care Sciences & Services; Health Policy & Services; Public,

Environmental & Occupational Health

SC Health Care Sciences & Services; Public, Environmental & Occupational

Health

GA FA8XC

UT WOS:000405728800007

PM 28364183

DA 2019-01-08

ER

PT J

AU Ehrmann, D

Schmitt, A

Reimer, A

Haak, T

Kulzer, B

Hermanns, N

AF Ehrmann, Dominic

Schmitt, Andreas

Reimer, Andre

Haak, Thomas

Kulzer, Bernhard

Hermanns, Norbert

TI The affective and somatic side of depression: subtypes of depressive

symptoms show diametrically opposed associations with glycemic control

in people with type 1 diabetes

SO ACTA DIABETOLOGICA

LA English

DT Article

DE Depressive symptoms; Glycemic control; Subtypes; Psychosocial aspects

ID CO-MORBID DEPRESSION; ADULTS; METAANALYSIS; DISTRESS; CLUSTERS;

MELLITUS; PREVALENCE; TRIAL; PHQ-9

AB Aims While depression has been linked to serious adverse outcomes in diabetes, associations with glycemic control are not conclusive. Inconsistencies could be due to the complex symptomatology of depression. Aim of this study was to analyze the associations of depressive subtypes with glycemic control in people with type 1 and type 2 diabetes.

Methods Patients completed the Center for Epidemiological Studies-Depression scale which comprises affective, somatic, and anhedonic symptoms. These subtypes were analyzed in a joint linear regression analysis with glycemic control as a dependent variable. Subtype scores were calculated as mean item scores. Separate analyses for people with type 1 and type 2 diabetes were conducted. All analyses were controlled for demographic and medical confounders.

Results The sample comprised 604 patients with type 1 and 382 patients with type 2 diabetes. In people with type 1 diabetes, the somatic and affective subtype showed diametrically opposed associations with glycemic control (somatic: beta =+0.23, p < .05; affective: beta = -0.23, p < .05). Anhedonia was not significantly associated with glycemic control. In people with type 2 diabetes, none of the depressive subtypes was significantly associated with glycemic control.

Conclusions For people with type 1 diabetes, the distinction of subtypes offered a detailed picture of the associations of depressive symptoms with glycemic control. However, due to the cross-sectional design, inferences about the direction of these associations cannot be made. In clinical practice, instead of focusing on overall depression, healthcare providers should examine the nature of depressive symptoms and how they might be related to having diabetes.

C1 [Ehrmann, Dominic; Schmitt, Andreas; Reimer, Andre; Haak, Thomas; Kulzer, Bernhard; Hermanns, Norbert] Diabet Acad Mergentheim FIDAM, Res Inst, Johann Hammer Str 24, D-97980 Bad Mergentheim, Germany.

[Ehrmann, Dominic; Kulzer, Bernhard; Hermanns, Norbert] Otto Friedrich Univ Bamberg, Dept Clin Psychol & Psychotherapy, Markuspl 3, D-96047 Bamberg, Germany.

[Schmitt, Andreas; Reimer, Andre; Haak, Thomas; Kulzer, Bernhard; Hermanns, Norbert] Diabet Clin Mergentheim, Theodor Klotzbuecher Str 12, D-97980 Bad Mergentheim, Germany.

RP Ehrmann, D (reprint author), Diabet Acad Mergentheim FIDAM, Res Inst, Johann Hammer Str 24, D-97980 Bad Mergentheim, Germany.; Ehrmann, D (reprint author), Otto Friedrich Univ Bamberg, Dept Clin Psychol & Psychotherapy, Markuspl 3, D-96047 Bamberg, Germany.

EM ehrmann@fidam.de

OI Schmitt, Andreas/0000-0002-5913-1457; Ehrmann,

Dominic/0000-0002-5794-5596

CR Aikens JE, 2009, DIABETES CARE, V32, P1177, DOI 10.2337/dc09-0071

Ali S, 2006, DIABETIC MED, V23, P1165, DOI 10.1111/j.1464-5491.2006.01943.x

Asuzu CC, 2017, J DIABETES COMPLICAT, V31, P169, DOI 10.1016/j.jdiacomp.2016.09.013

Bachle C, 2015, PSYCHONEUROENDOCRINO, V55, P48, DOI 10.1016/j.psyneuen.2015.01.026

Barnard KD, 2006, DIABETIC MED, V23, P445, DOI 10.1111/j.1464-5491.2006.01814.x

Baumeister H, 2014, DIABETIC MED, V31, P773, DOI 10.1111/dme.12452

Black SA, 2003, DIABETES CARE, V26, P2822, DOI 10.2337/diacare.26.10.2822

Bot M, 2013, DIABETIC MED, V30, pE115, DOI 10.1111/dme.12082

de Groot M, 1999, J PSYCHOSOM RES, V46, P425, DOI 10.1016/S0022-3999(99)00014-8

de Jonge P, 2011, J PSYCHOSOM RES, V71, P59, DOI 10.1016/j.jpsychores.2010.12.013

Denic-Roberts H, 2016, DIABETES RES CLIN PR, V119, P1, DOI 10.1016/j.diabres.2016.06.013

Ehrmann D, 2016, PLOS ONE, V11, DOI 10.1371/journal.pone.0147581

Fisher L, 2010, DIABETES CARE, V33, P23, DOI 10.2337/dc09-1238

Georgiades A, 2007, PSYCHOSOM MED, V69, P235, DOI 10.1097/PSY.0b013e318042588d

Gonzalez JS, 2008, DIABETIC MED, V25, P1102, DOI 10.1111/j.1464-5491.2008.02535.x

Gonzalez JS, 2016, DIABETES CARE, V39, P2182, DOI 10.2337/dc16-1657

Hawkins MAW, 2014, PSYCHOSOM MED, V76, P38, DOI 10.1097/PSY.0000000000000023

Hermanns N, 2015, DIABETES CARE, V38, P551, DOI 10.2337/dc14-1416

Hermanns N, 2012, PATIENT EDUC COUNS, V86, P226, DOI 10.1016/j.pec.2011.05.017

Katon W, 2008, J GEN INTERN MED, V23, P1571, DOI 10.1007/s11606-008-0731-9

King DE, 2003, DIABETES CARE, V26, P1535, DOI 10.2337/diacare.26.5.1535

Lustman PJ, 2000, DIABETES CARE, V23, P934, DOI 10.2337/diacare.23.7.934

Lux V, 2010, PSYCHOL MED, V40, P1679, DOI 10.1017/S0033291709992157

Manea L, 2015, GEN HOSP PSYCHIAT, V37, P67, DOI 10.1016/j.genhosppsych.2014.09.009

McGrady ME, 2009, DIABETES CARE, V32, P804, DOI 10.2337/dc08-2111

Melin EO, 2013, EUR J ENDOCRINOL, V168, P861, DOI 10.1530/EJE-13-0137

Nefs G, 2012, J PSYCHIATR RES, V46, P549, DOI 10.1016/j.jpsychires.2012.01.013

Nefs G, 2015, PSYCHOSOMATICS, V56, P662, DOI 10.1016/j.psym.2015.06.006

Petrak F, 2015, DIABETES CARE, V38, P767, DOI 10.2337/dc14-1599

POLONSKY WH, 1995, DIABETES CARE, V18, P754, DOI 10.2337/diacare.18.6.754

RADLOFF L S, 1977, Applied Psychological Measurement, V1, P385, DOI 10.1177/014662167700100306

Reutrakul S, 2016, SLEEP MED, V23, P26, DOI 10.1016/j.sleep.2016.03.019

Schmitt A, 2015, J BEHAV MED, V38, P556, DOI 10.1007/s10865-014-9604-3

Schram MT, 2009, CURR DIABETES REV, V5, P112, DOI 10.2174/157339909788166828

Shafer AB, 2006, J CLIN PSYCHOL, V62, P123, DOI 10.1002/jclp.20213

Stewart JC, 2012, CIRCULATION, V126, P410, DOI 10.1161/CIRCULATIONAHA.112.094946

Stewart JC, 2009, BRAIN BEHAV IMMUN, V23, P936, DOI 10.1016/j.bbi.2009.04.011

Surwit RS, 2005, DIABETES RES CLIN PR, V69, P78, DOI 10.1016/j.diabres.2004.11.002

van Bastelaar KMP, 2010, DIABETIC MED, V27, P798, DOI 10.1111/j.1464-5491.2010.03025.x

Van der Feltz-Cornelis CM, 2010, GEN HOSP PSYCHIAT, V32, P380, DOI 10.1016/j.genhosppsych.2010.03.011

van Dooren FEP, 2013, PLOS ONE, V8, DOI 10.1371/journal.pone.0057058

van Steenbergen-Weijenburg KM, 2010, BMC HEALTH SERV RES, V10, DOI 10.1186/1472-6963-10-235

NR 42

TC 2

Z9 2

U1 0

U2 1

PU SPRINGER-VERLAG ITALIA SRL

PI MILAN

PA VIA DECEMBRIO, 28, MILAN, 20137, ITALY

SN 0940-5429

EI 1432-5233

J9 ACTA DIABETOL

JI Acta Diabetol.

PD AUG

PY 2017

VL 54

IS 8

BP 749

EP 756

DI 10.1007/s00592-017-1006-x

PG 8

WC Endocrinology & Metabolism

SC Endocrinology & Metabolism

GA FA2UX

UT WOS:000405297400004

PM 28555338

DA 2019-01-08

ER

PT J

AU Rautio, N

Varanka-Ruuska, T

Vaaramo, E

Palaniswamy, S

Nedelec, R

Miettunen, J

Karppinen, J

Auvinen, J

Jarvelin, MR

Keinanen-Kiukaanniemi, S

Sebert, S

Ala-Mursula, L

AF Rautio, Nina

Varanka-Ruuska, Tuulia

Vaaramo, Eeva

Palaniswamy, Saranya

Nedelec, Rozenn

Miettunen, Jouko

Karppinen, Jaro

Auvinen, Juha

Jarvelin, Marjo-Riitta

Keinanen-Kiukaanniemi, Sirkka

Sebert, Sylvain

Ala-Mursula, Leena

TI Accumulated exposure to unemployment is related to impaired glucose

metabolism in middle-aged men: A follow-up of the Northern Finland Birth

Cohort 1966

SO PRIMARY CARE DIABETES

LA English

DT Article

DE Type 2 diabetes; Pre-diabetes; Unemployment

ID DIABETES-MELLITUS; PSYCHOLOGICAL DISTRESS; RISK-FACTORS; LIFE-STYLE;

TYPE-2; HEALTH; TOLERANCE; WOMEN; WORK; POPULATION

AB Aims: We explored whether registered unemployment is associated with impaired glucose metabolism in general population.

Methods: Based on Northern Finland Birth Cohort 1966 at 46 years, we analyzed the oral glucose tolerance tests of 1970 men and 2544 women in relation to their preceding three-year employment records in three categories of unemployment exposure: no (employed), low (<= 1-year) and high exposure (>1-year).

Results: Among men, pre-diabetes was found in 19.2% of those with no unemployment, 23.0% with low and 27.0% with high exposure, the corresponding figures for screen-detected type 2 diabetes were 3.8%, 3.8% and 9.2% (p < 0.01). Among women, analogous figures for pre-diabetes were 10.0%, 12.6% and 16.2% and for screen-detected type 2 diabetes 1.7%, 3.4% and 3.6% (p < 0.01). Men with high exposure to unemployment had a higher risk for pre-diabetes (OR 1.61, CI 95% 1.03-2.51) and screen-detected type 2 diabetes (OR 2.58 95% CI 1.23-5.44) than employed men, after adjustment for education, smoking, alcohol intake, physical activity and body mass index. Among women, associations were attenuated in the adjusted models.

Conclusions: High exposure to unemployment may predispose to type 2 diabetes in middle-aged men. For clinicians, awareness of the patient's unemployment status may be helpful in recognizing undiagnosed cases. (C) 2017 The Authors. Published by Elsevier Ltd on behalf of Primary Care Diabetes Europe.

C1 [Rautio, Nina; Varanka-Ruuska, Tuulia; Vaaramo, Eeva; Palaniswamy, Saranya; Nedelec, Rozenn; Miettunen, Jouko; Karppinen, Jaro; Auvinen, Juha; Jarvelin, Marjo-Riitta; Keinanen-Kiukaanniemi, Sirkka; Sebert, Sylvain; Ala-Mursula, Leena] Univ Oulu, Ctr Life Course Hlth Res, POB 5000, FIN-90014 Oulu, Finland.

[Rautio, Nina; Auvinen, Juha; Jarvelin, Marjo-Riitta; Keinanen-Kiukaanniemi, Sirkka] Oulu Univ Hosp, Unit Primary Hlth Care, OYS, POB 20, Oulu 90029, Finland.

[Varanka-Ruuska, Tuulia] Kallio Primary Hlth Care Unit, Kirkkotie 4, Ylivieska 84100, Finland.

[Miettunen, Jouko; Karppinen, Jaro; Keinanen-Kiukaanniemi, Sirkka] Oulu Univ Hosp, Med Res Ctr Oulu, POB 5000, Oulu 90014, Finland.

[Miettunen, Jouko; Karppinen, Jaro; Keinanen-Kiukaanniemi, Sirkka] Univ Oulu, POB 5000, Oulu 90014, Finland.

[Karppinen, Jaro] Finnish Inst Occupat Hlth, Aapistie 1, Oulu 90220, Finland.

[Jarvelin, Marjo-Riitta] Imperial Coll London, MRC PHE Ctr Environm & Hlth, Praed St Wing,St Marys Campus, London W2 1PG, England.

[Palaniswamy, Saranya; Nedelec, Rozenn; Jarvelin, Marjo-Riitta; Sebert, Sylvain] Univ Oulu, Bioctr Oulu, POB 5000, Oulu 90014, Finland.

[Sebert, Sylvain] Imperial Coll London, Dept Genom, South Kensington Campus, London SW7, England.

RP Rautio, N (reprint author), Univ Oulu, Ctr Life Course Hlth Res, POB 5000, FIN-90014 Oulu, Finland.

EM nina.rautio@oulu.fi; tuulia.varanka-ruuska@oulu.fi;

eeva.vaaramo@oulu.fi; saranya.palaniswamy@oulu.fi;

rozenn.nedelec@oulu.fi; jouko.miettunen@oulu.fi; jaro.karppinen@oulu.fi;

juha.auvinen@oulu.fi; sirkka.keinanen-kiukaanniemi@oulu.fi;

sylvain.sebert@oulu.fi; leena.ala-mursula@oulu.fi

OI Sebert, Sylvain/0000-0001-6681-6983

FU University of Oulu [24000692]; Oulu University Hospital [24301140]; ERDF

European Regional Development Fund [539/2010 A31592]; Academy of Finland

[268336]; European Union's Horizon 2020 research and innovation program

for the DynaHEALTH action [633595]

FX NFBC1966 received financial support from University of Oulu [Grant no.

24000692]; Oulu University Hospital [Grant no. 24301140] and ERDF

European Regional Development Fund [Grant no. 539/2010 A31592]. This

project has received funding from the Academy of Finland [#268336] and

the European Union's Horizon 2020 research and innovation program [under

grant agreement No. 633595] for the DynaHEALTH action.

CR [Anonymous], 2015, UNEMPLOYMENT STAT

[Anonymous], 2015, SECURITY EVENT DISAB

BARTLEY M, 1994, J EPIDEMIOL COMMUN H, V48, P333, DOI 10.1136/jech.48.4.333

Centers for Disease Control and Prevention, 2011, DISTR AG DIAGN DIAB

Chung HR, 2009, NUTR RES PRACT, V3, P286, DOI 10.4162/nrp.2009.3.4.286

Eriksson AK, 2008, DIABETIC MED, V25, P834, DOI 10.1111/j.1464-5491.2008.02463.x

Eriksson AK, 2013, DIABETES CARE, V36, P2683, DOI 10.2337/dc12-1738

Heianza Y, 2013, DIABETES CARE, V36, P4007, DOI 10.2337/dc13-1048

Heraclides A, 2009, DIABETES CARE, V32, P2230, DOI 10.2337/dc09-0132

JAHODA M, 1981, AM PSYCHOL, V36, P184, DOI 10.1037/0003-066X.36.2.184

Knowler WC, 2002, NEW ENGL J MED, V346, P393

Laakso M, 2007, TYPE 2 DIABETES PRIN, P1

Lundin A, 2010, J EPIDEMIOL COMMUN H, V64, P22, DOI 10.1136/jech.2008.079269

Martikainen PT, 1998, AM J PUBLIC HEALTH, V88, P1859, DOI 10.2105/AJPH.88.12.1859

McEwen BS, 1998, NEW ENGL J MED, V338, P171, DOI 10.1056/NEJM199801153380307

Morikawa Y, 2005, SCAND J WORK ENV HEA, V31, P179, DOI 10.5271/sjweh.867

Muller G, 2013, BMJ OPEN, V3, DOI 10.1136/bmjopen-2013-002601

Novak M, 2013, DIABETIC MED, V30, pE8, DOI 10.1111/dme.12037

Nyberg ST, 2014, DIABETES CARE, V37, P2268, DOI 10.2337/dc13-2936

Rantakallio P, 1988, Paediatr Perinat Epidemiol, V2, P59, DOI 10.1111/j.1365-3016.1988.tb00180.x

Saaristo TE, 2008, BMC PUBLIC HEALTH, V8, DOI 10.1186/1471-2458-8-423

Sabanayagam C, 2009, ASIA-PAC J PUBLIC HE, V21, P385, DOI 10.1177/1010539509343958

Seuring T, 2015, PHARMACOECONOMICS, V33, P811, DOI 10.1007/s40273-015-0268-9

Stumvoll M., 2007, TYPE 2 DIABETES PRIN, P13

Tabak AG, 2012, LANCET, V379, P2279, DOI 10.1016/S0140-6736(12)60283-9

Toshihiro M, 2008, DIABETIC MED, V25, P1211, DOI 10.1111/j.1464-5491.2008.02566.x

Tuomilehto J, 2001, NEW ENGL J MED, V344, P1343, DOI 10.1056/NEJM200105033441801

van der Noordt M, 2014, OCCUP ENVIRON MED, V71, P730, DOI 10.1136/oemed-2013-101891

Virtanen M, 2014, DIABETES CARE, V37, P2091, DOI 10.2337/dc13-2725

World Health Organization, 1999, 992 WORLD HLTH ORG

NR 30

TC 1

Z9 1

U1 0

U2 2

PU ELSEVIER SCI LTD

PI OXFORD

PA THE BOULEVARD, LANGFORD LANE, KIDLINGTON, OXFORD OX5 1GB, OXON, ENGLAND

SN 1751-9918

EI 1878-0210

J9 PRIM CARE DIABETES

JI Prim. Care Diabetes

PD AUG

PY 2017

VL 11

IS 4

BP 365

EP 372

DI 10.1016/j.pcd.2017.03.010

PG 8

WC Endocrinology & Metabolism; Primary Health Care

SC Endocrinology & Metabolism; General & Internal Medicine

GA FA0VR

UT WOS:000405155200007

PM 28456438

OA Other Gold, Green Published

DA 2019-01-08

ER

PT J

AU Bennich, BB

Roder, ME

Overgaard, D

Egerod, I

Munch, L

Knop, FK

Vilsboll, T

Konradsen, H

AF Bennich, Birgitte B.

Roder, Michael E.

Overgaard, Dorthe

Egerod, Ingrid

Munch, Lene

Knop, Filip K.

Vilsboll, Tina

Konradsen, Hanne

TI Supportive and non-supportive interactions in families with a type 2

diabetes patient: an integrative review

SO DIABETOLOGY & METABOLIC SYNDROME

LA English

DT Review

DE Family; Interaction; Literature review; Support; Type 2 diabetes;

Involvement

ID QUALITATIVE CONTENT-ANALYSIS; CROSS-NATIONAL BENCHMARKING;

SELF-MANAGEMENT BEHAVIORS; SOCIAL-CONTROL; CHRONIC ILLNESS; NEEDS 2ND;

INTERVENTIONS; ADHERENCE; HEALTH; ADULTS

AB Background: Type 2 diabetes and its management affect the patient and the close family potentially causing either psychological distress or increased sense of responsibility and collaboration in these families. Interactions between patient and family play an important role in maintaining lifestyle changes and diabetes self-management. The purpose of this integrative review was to summarise and assess published studies on the intra-family perspective of supportive and non-supportive interactions in families with a type 2 diabetes patient.

Methods: Included in the review were published qualitative and quantitative studies that examined the intra-family perspective on supportive and non-supportive interactions. We searched the literature from 2000 to 2016 and the search strategy comprised the following databases: Cochrane, PubMed, CINAHL, Web of Science, PsycINFO and Psyc-ARTICLES as well as hand searching of reference lists. Quality assessment, data extraction and analysis were undertaken on all included studies.

Results: We identified five eligible research papers. Employing content analysis three categories describing interactions were refined: Impact of practical action, impact of emotional involvement, and impact of communication content. Supportive interactions included encouraging communication and family collaboration in managing diet, medications, and blood glucose checking. Non-supportive interactions were visible irritation, nagging behaviour and refusing to share the burden of living with diabetes.

Conclusion: The findings stress the importance of including both patient and family in clinical practice to target diabetes self-management adherence and well-being of the whole family. The majority of self-management occurs within the family environment. Therefore, the intra-family perspective of supportive and non-supportive interactions should be understood and addressed as the family members are interdependent and affected by each other. Future research assessing the impact of professional support and the family function will have the potential to improve the daily life and well-being of patients with type 2 diabetes as well as the whole family.

C1 [Bennich, Birgitte B.; Roder, Michael E.; Munch, Lene; Knop, Filip K.; Vilsboll, Tina] Univ Copenhagen, Gentofte Hosp, Ctr Diabet Res, Hellerup, Denmark.

[Bennich, Birgitte B.; Overgaard, Dorthe; Munch, Lene] Metropolitan Univ Coll, Inst Nursing, Copenhagen N, Denmark.

[Egerod, Ingrid] Copenhagen Univ Hosp, Intens Care Unit, Rigshosp, Copenhagen, Denmark.

[Egerod, Ingrid] Univ Copenhagen, Fac Hlth & Med Sci, Copenhagen, Denmark.

[Knop, Filip K.; Vilsboll, Tina] Univ Copenhagen, Fac Hlth & Med Sci, Dept Clin Med, Copenhagen, Denmark.

[Knop, Filip K.] Univ Copenhagen, Fac Hlth & Med Sci, NNF Ctr Basic Metab Res, Copenhagen, Denmark.

[Vilsboll, Tina] Univ Copenhagen, Steno Diabet Ctr Copenhagen, Copenhagen, Denmark.

[Konradsen, Hanne] Karolinska Inst, NVS, Dept Neurobiol Care Sci & Soc, Alfred Nobels Alle 23, S-14152 Hundinge, Sweden.

RP Konradsen, H (reprint author), Karolinska Inst, NVS, Dept Neurobiol Care Sci & Soc, Alfred Nobels Alle 23, S-14152 Hundinge, Sweden.

EM hanne.konradsen@ki.se

OI Konradsen, Hanne/0000-0002-7477-125X; Egerod, Ingrid/0000-0002-9576-4390

FU Research Foundation in the Capital Region of Denmark; Jascha Foundation

FX The authors disclosed receipt of the following financial support for the

research, authorship, and/or publication of this article: Financial

support for this research was provided by the Research Foundation in the

Capital Region of Denmark and the Jascha Foundation.

CR August KJ, 2013, J FAM PSYCHOL, V27, P712, DOI 10.1037/a0034181

August KJ, 2010, SOC SCI MED, V71, P1831, DOI 10.1016/j.socscimed.2010.08.022

Baig AA, 2015, ANN NY ACAD SCI, V1353, P89, DOI 10.1111/nyas.12844

Bailey CJ, 2011, INT J CLIN PRACT, V65, P314, DOI 10.1111/j.1742-1241.2010.02544.x

Burns KK, 2013, DIABETIC MED, V30, P778, DOI 10.1111/dme.12239

Chesla CA, 2010, J FAM NURS, V16, P355, DOI 10.1177/1074840710383145

Costa V., 2012, EUROPEAN DIABETES NU, V9, P81

Cowell JM, 2012, J SCH NURS, V28, P326, DOI 10.1177/1059840512458666

Cramer JA, 2008, INT J CLIN PRACT, V62, P76, DOI 10.1111/j.1742-1241.2007.01630.x

Daley B. J., 2010, HUMAN RESOURCES DEVE, V9, P357, DOI [DOI 10.1177/1534484310379101, 10.1177/1534484310379101]

Dalton JM, 2014, SELF CARE DEPEND CAR, V21, P12

de Lima Santos Aliny, 2014, Invest. educ. enferm, V32, P260, DOI 10.17533/udea.iee.v32n2a09

Denham SA, 2011, DIABETES EDUCATOR, V37, P528, DOI 10.1177/0145721711411312

Dimitraki G, 2014, INT J BEHAV MED, V21, P230, DOI 10.1007/s12529-013-9296-z

Franks MM, 2006, J FAM PSYCHOL, V20, P311, DOI 10.1037/0893-3200.20.2.311

GLASGOW RE, 1988, DIABETES CARE, V11, P377, DOI 10.2337/diacare.11.5.377

Glasgow RE, 2014, TRANSL BEHAV MED, V4, P26, DOI 10.1007/s13142-013-0232-1

Graneheim UH, 2004, NURS EDUC TODAY, V24, P105, DOI 10.1016/j.nedt.2003.10.001

Gunn KL, 2012, HEALTH SOC CARE COMM, V20, P591, DOI 10.1111/j.1365-2524.2012.01073.x

Hartmann M, 2010, PSYCHOTHER PSYCHOSOM, V79, P136, DOI 10.1159/000286958

Hornsten A, 2004, J ADV NURS, V47, P174, DOI 10.1111/j.1365-2648.2004.03076.x

Houston-Barrett RA, 2014, J MARITAL FAM THER, V40, P92, DOI 10.1111/j.1752-0606.2012.00322.x

Hsieh HF, 2005, QUAL HEALTH RES, V15, P1277, DOI 10.1177/1049732305276687

International Diabetes Federation, IDF DIABETES ATLAS

Joanna Briggs Institute reviewers' manual, 2011, REV MAN

Karlsen B, 2012, J ADV NURS, V68, P391, DOI 10.1111/j.1365-2648.2011.05751.x

Kaufman G, 1998, J MARRIAGE FAM, V60, P924, DOI 10.2307/353635

Kelly JB, 2007, FAM PROCESS, V46, P35, DOI 10.1111/j.1545-5300.2006.00190.x

Kerr ME, 2000, PRIMER BOWEN THEORY

King DK, 2010, DIABETES CARE, V33, P751, DOI 10.2337/dc09-1746

Kylma Jari, 2009, Palliat Support Care, V7, P365, DOI 10.1017/S1478951509990307

Laranjo L, 2015, EUR J GEN PRACT, V21, P103, DOI 10.3109/13814788.2014.1000855

Leong A, 2014, BMC MED, V12, DOI 10.1186/1741-7015-12-12

Lerman I, 2005, ARCH MED RES, V36, P300, DOI 10.1016/j.arcmed.2004.12.001

Lewinski AA, 2015, CHRONIC ILLN

Martire LM, 2010, ANN BEHAV MED, V40, P325, DOI 10.1007/s12160-010-9216-2

Mayberry LS, 2015, J BEHAV MED, V38, P363, DOI 10.1007/s10865-014-9611-4

Mayberry LS, 2014, PATIENT EDUC COUNS, V97, P418, DOI 10.1016/j.pec.2014.09.011

Menke A, 2015, JAMA-J AM MED ASSOC, V314, P1021, DOI 10.1001/jama.2015.10029

Miller TA, 2013, DIABETES METAB SYNDR, V6, P421, DOI 10.2147/DMSO.S36368

Nam S, 2011, DIABETES RES CLIN PR, V93, P1, DOI 10.1016/j.diabres.2011.02.002

National Collaborating Centre for Methods and Tools: Critical Appraisal Skills Programme (CASP), 2014, CRIT APPR SKILLS PRO

Nicolucci A, 2013, DIABETIC MED, V30, P767, DOI 10.1111/dme.12245

Oftedal B., 2014, EUR DIABETES NURS, V11, P43, DOI DOI 10.1002/EDN.247

Okun MA, 2007, BASIC APPL SOC PSYCH, V29, P185, DOI 10.1080/01973530701332245

Orvik E., 2010, EUR DIABETES NURS, V7, P63

Ostlund U, 2014, J FAM NURS, V20, P259, DOI 10.1177/1074840714542962

Peyrot M, 2005, DIABETIC MED, V22, P1379, DOI 10.1111/j.1464-5491.2005.01644.x

Rosland AM, 2010, CHRONIC ILLN, V6, P22, DOI 10.1177/1742395309354608

Rosland AM, 2008, J GEN INTERN MED, V23, P1992, DOI 10.1007/s11606-008-0814-7

Rubin RR, 2005, AM J MED, V118, P27, DOI 10.1016/j.amjmed.2005.04.012

Sandberg J, 2006, J COUPLE RELATSH THE, V5, P23, DOI 10.1300/J398v05n02_02

Stephens MAP, 2013, HEALTH PSYCHOL, V32, P1029, DOI 10.1037/a0030018

Stephens MAP, 2009, HEALTH PSYCHOL, V28, P48, DOI 10.1037/a0012385

Torenholt R, 2014, DIABETIC MED, V31, P15, DOI 10.1111/dme.12290

Torge C. J., 2013, QUALITATIVE STUDIES, V4, P100

Trief P., 2003, FAMILIES SYSTEMS HLT, V21, P57, DOI [DOI 10.1037/H0089502, 10.1037/h0089502]

Trief P, 2011, FAM SYST HEALTH, V29, P253, DOI 10.1037/a0024564

Tucker JS, 2001, J APPL SOC PSYCHOL, V31, P467, DOI 10.1111/j.1559-1816.2001.tb02051.x

Whittemore R, 2005, J ADV NURS, V52, P546, DOI 10.1111/j.1365-2648.2005.03621.x

Wright DDB, 2012, NURSES FAMILIES GUID

Wright L. M., 1999, J FAMILY NURSING, V5, P259, DOI DOI 10.1177/107484079900500302

Zoffmann V, 2005, QUAL HEALTH RES, V15, P750, DOI 10.1177/1049732304273888

NR 63

TC 5

Z9 5

U1 1

U2 5

PU BIOMED CENTRAL LTD

PI LONDON

PA 236 GRAYS INN RD, FLOOR 6, LONDON WC1X 8HL, ENGLAND

SN 1758-5996

J9 DIABETOL METAB SYNDR

JI Diabetol. Metab. Syndr.

PD JUL 21

PY 2017

VL 9

AR 57

DI 10.1186/s13098-017-0256-7

PG 9

WC Endocrinology & Metabolism

SC Endocrinology & Metabolism

GA FC1HV

UT WOS:000406588900001

PM 28736580

OA DOAJ Gold, Green Published

DA 2019-01-08

ER

PT J

AU Paula, JS

Braga, LD

Moreira, RO

Kupfer, R

AF Paula, Juliana Santos

Braga, Leticia Dinis

Moreira, Rodrigo Oliveira

Kupfer, Rosane

TI Correlation between parameters of self-monitoring of blood glucose and

the perception of health-related quality of life in patients with type 1

diabetes mellitus

SO ARCHIVES OF ENDOCRINOLOGY METABOLISM

LA English

DT Article

DE Type 1 diabetes mellitus; hypoglycemia; self-monitoring of blood

glucose; quality of life

ID GLYCEMIC CONTROL; SCALE; HYPOGLYCEMIA; DISTRESS; COHORT; AGENTS; TRIAL

AB Objective: The aim of this study was to evaluate how different parameters of short-term glycemic control would correlate with the perception of health-related quality of life (HRQoL) in patients with type 1 diabetes mellitus (T1D). Subjects and methods: A total of 50 T1D patients aged 18 to 50 years were evaluated with the questionnaires Problem Areas in Diabetes (PAID) scale and Diabetes Quality of Life (DQOL) measure after 30 days of self-monitoring of blood glucose (SMBG). Glycemic control was evaluated using glycated hemoglobin (HbA(1c)), mean glucose levels (MGL) in the prior month's data from SMBG (Accu-Check 360 degrees), number of hypoglycemic episodes (< 70 mg/dL and < 50 mg/dL), and glycemic variability (GV). Results: PAID correlated positively with MGL (r = 0.52; p < 0.001) and HbA(1c) (r = 0.36; p < 0.0097), but not with GV (r = 0.17; p = 0.23) or number of hypoglycemic episodes (r = 0.15; p = 0.17 for glucose < 70 mg/dL and r = 0.02; p = 0.85 for glucose < 50 mg/dL). After multiple linear regression, only MGL remained independently related to PAID scores. DQOL scores had a positive correlation with MGL (r = 0.45; p = 0.001), but not with HbA(1c) (r = 0.23; p = 0.09), GV (r = 0.20; p = 0.16), or number of hypoglycemic episodes (r = 0.06 p = 0.68). Conclusion: In T1D patients, MGL, but not HbA(1c) or number hypoglycemic episodes, was the glycemic control parameter that best correlated with short-term perception of HRQoL.

C1 [Paula, Juliana Santos; Braga, Leticia Dinis; Moreira, Rodrigo Oliveira; Kupfer, Rosane] Inst Estadual Diabet & Endocrinol Luiz Capriglion, Serv Diabet, Rio De Janeiro, RJ, Brazil.

RP Moreira, RO (reprint author), Inst Estadual Diabet & Endocrinol, Rua Moncorvo Filho 90, BR-20211340 Rio De Janeiro, RJ, Brazil.

EM rom_br@yahoo.com

CR Alcubierre N, 2014, HEALTH QUAL LIFE OUT, V12, DOI 10.1186/s12955-014-0131-2

[Anonymous], 2015, DIABETES ATLAS

CAREY MP, 1991, J BEHAV MED, V14, P43, DOI 10.1007/BF00844767

de Souza ACCB, 2015, HEALTH QUAL LIFE OUT, V13, DOI 10.1186/s12955-015-0396-0

DCCT Res Grp, 1988, DIABETES CARE, V11, P725

Felicio JS, 2015, DIABETOL METAB SYNDR, V7, DOI 10.1186/s13098-015-0081-9

Fenwick EK, 2011, BRIT J OPHTHALMOL, V95, P774, DOI 10.1136/bjo.2010.191312

Gross CC, 2007, DIABETES RES CLIN PR, V76, P455, DOI 10.1016/j.diabres.2006.09.022

Gusmai LD, 2015, REV ESC ENFERM USP, V49, P832, DOI 10.1590/S0080-623420150000500019

Hermanns N, 2015, HEALTH QUAL LIFE OUT, V13, DOI 10.1186/s12955-015-0279-4

Johnson SR, 2013, DIABETIC MED, V30, P1126, DOI 10.1111/dme.12247

Jones A, 2016, PRIM CARE DIABETES, V10, P83, DOI 10.1016/j.pcd.2015.03.008

Kuznetsov L, 2015, DIABETES-METAB RES, V31, P69, DOI 10.1002/dmrr.2559

Kuznetsov L, 2014, DIABETES RES CLIN PR, V104, P281, DOI 10.1016/j.diabres.2013.12.029

Maia Frederico F.R., 2004, Arq Bras Endocrinol Metab, V48, P261, DOI 10.1590/S0004-27302004000200009

Matziou V, 2011, J DIABETES, V3, P82, DOI 10.1111/j.1753-0407.2010.00106.x

Papelbaum M, 2010, DIABETES RES CLIN PR, V89, P227, DOI 10.1016/j.diabres.2010.05.024

Polonsky WH, 2005, DIABETES CARE, V28, P626, DOI 10.2337/diacare.28.3.626

*/* S 81, 2014, DIABETES CARE S1, V37, pS8, DOI DOI 10.2337/DC14-S081

Schunk M, 2015, J DIABETES COMPLICAT, V29, P203, DOI 10.1016/j.jdiacomp.2014.11.010

Sheu WHH, 2012, DIABETES RES CLIN PR, V96, P141, DOI 10.1016/j.diabres.2011.12.027

Siegelaar SE, 2010, ENDOCR REV, V31, P171, DOI 10.1210/er.2009-0021

Testa MA, 1996, NEW ENGL J MED, V334, P835, DOI 10.1056/NEJM199603283341306

Aguiar CCT, 2008, ARQ BRAS ENDOCRINOL, V52, P931, DOI 10.1590/S0004-27302008000600004

Vanstone Meredith, 2015, Ont Health Technol Assess Ser, V15, P1

WHOQL Group, 1997, PROGR MENT HLTH

Wikblad K, 1996, QUAL LIFE RES, V5, P123, DOI 10.1007/BF00435977

NR 27

TC 1

Z9 1

U1 0

U2 1

PU SBEM-SOC BRASIL ENDOCRINOLOGIA & METABOLOGIA

PI RIO DE JANEIRO, RJ

PA RUA HUMAITA, 85 CJ 501, RIO DE JANEIRO, RJ, 22261-000, BRAZIL

SN 2359-3997

EI 2359-4292

J9 ARCH ENDOCRIN METAB

JI Arch. Endocrinol. Metab.

PD JUL-AUG

PY 2017

VL 61

IS 4

BP 343

EP 347

DI 10.1590/2359-3997000000222

PG 5

WC Endocrinology & Metabolism

SC Endocrinology & Metabolism

GA FN4UW

UT WOS:000416003200007

PM 27901179

OA DOAJ Gold

DA 2019-01-08

ER

PT J

AU Deeb, A

AF Deeb, Asma

TI Challenges of Diabetes Management in Toddlers

SO DIABETES TECHNOLOGY & THERAPEUTICS

LA English

DT Review

DE Toddlers; Hypoglycemia; Glucose monitoring; Insulin pumps; Psychology

ID SUBCUTANEOUS INSULIN INFUSION; YOUNG-CHILDREN; GLYCEMIC CONTROL; PUMP

THERAPY; MELLITUS; HYPOGLYCEMIA; ADOLESCENTS; ASSOCIATION; CHILDHOOD;

COMPLICATIONS

AB Toddlers with type 1 diabetes have distinctive combination of challenges to their families and healthcare providers. A major factor is the difficulty in achieving metabolic control without risking hypoglycemia. The rising incidence of type 1 diabetes in toddlers increases the magnitude of the problem and creates a greater need for providing specialized service to cater for this age group's unique need. Type 1 has a specific disease nature in younger children and its clinical presentation mimics common childhood diseases. Symptoms and signs in the newly presented toddler with diabetes might be unspecific causing diagnosis to be missed or delayed. With the low cognitive ability and immature communication, toddlers might not be able to express their ill-feeling resulting from hypoglycemia or hyperglycemia. Hypoglycemia fear is common, which aggravates stress and reduces adherence to strict metabolic control. Nocturnal hypoglycemia is more common in toddlers and can be undiagnosed unless continuous glucose monitoring is used. Insulin administration and adjustment can be challenging due to pain, fear, refusal, and frequent intercurrent illness. Glucose monitoring is crucial. However, it can be distressing to the child and difficult to adhere to due to commitment or financial reasons. Insulin pump therapy is proven to be an effective and a safe method of treatment for toddlers, but it requires intensive training, resources, and long-term support. Provision of multidisciplinary team with special expertise in managing toddlers with diabetes is essential. Providing more physiological insulin regimes and customized technology is required to improve treatment compliance and diabetes control.

C1 [Deeb, Asma] Mafraq Hosp, Paediat Endocrinol Dept, Abu Dhabi 2951, U Arab Emirates.

RP Deeb, A (reprint author), Mafraq Hosp, Paediat Endocrinol Dept, Abu Dhabi 2951, U Arab Emirates.

EM adeeb@mafraqhospital.ae

CR Abul-Ainine AA, 2014, PEDIATR DIABETES, V15, P319, DOI 10.1111/pedi.12087

Adolfsson P, 2017, PEDIATR DIABETES, V18, P255, DOI 10.1111/pedi.12491

Alharfi IM, 2014, PEDIATR EMERG CARE, V30, P485, DOI 10.1097/PEC.0000000000000167

AMAN J, 1988, ACTA PAEDIATR SCAND, V77, P548, DOI 10.1111/j.1651-2227.1988.tb10698.x

American DA, 2014, DIABETES CARE S1, V37, pS14, DOI DOI 10.2337/DC14-S014

Anderson B, 1997, J PEDIATR-US, V130, P257, DOI 10.1016/S0022-3476(97)70352-4

Anderson BJ, 2001, J PEDIATR ENDOCR MET, V14, P645

[Anonymous], 2016, NICE GUID DIAB TYP 1

Bessaoud K, 2006, DIABETIC MED, V23, P857, DOI 10.1111/j.1464-5491.2006.01925.x

Braun M, 2016, J DIABETES RES, DOI 10.1155/2016/5490258

Ceriello A, 2013, DIABETES CARE, V36, pS272, DOI 10.2337/dcS13-2030

Chiang JL, 2014, DIABETES CARE, V37, P2034, DOI 10.2337/dc14-1140

Cryer PE, 2002, DIABETOLOGIA, V45, P937, DOI 10.1007/s00125-002-0822-9

Daneman D, 1999, Paediatr Child Health, V4, P57

Davis EA, 1998, ARCH DIS CHILD, V78, P111, DOI 10.1136/adc.78.2.111

Deiss D, 2001, DIABETIC MED, V18, P337, DOI 10.1046/j.1464-5491.2001.00429-3.x

Desrocher M, 2004, CHILD NEUROPSYCHOL, V10, P36, DOI 10.1076/chin.10.1.36.26241

Diabetes Australia, INF PAR KIDS AG 0 2

Doyle Boland EA, 2005, DIABETES ED, V31, P584

Doyle FJ, 2014, DIABETES CARE, V37, P1191, DOI 10.2337/dc13-2108

DRAELOS MT, 1995, AM J MED, V98, P135, DOI 10.1016/S0002-9343(99)80397-0

Flanagan SE, 2014, CELL METAB, V19, P146, DOI 10.1016/j.cmet.2013.11.021

Frank M, 1986, CLIN DIABETES, V4, P121

Fuld K, 2010, DIABETES TECHNOL THE, V12, pS67, DOI 10.1089/dia.2009.0182

Gardner SG, 1997, BMJ-BRIT MED J, V315, P712

Garg SK, 2017, DIABETES TECHNOL THE, V19, P155, DOI 10.1089/dia.2016.0421

Gillespie KM, 2004, LANCET, V364, P1699, DOI 10.1016/S0140-6736(04)17357-1

HATTON DL, 1995, J ADV NURS, V22, P569, DOI 10.1046/j.1365-2648.1995.22030569.x

HELLER SR, 1991, DIABETES, V40, P223, DOI 10.2337/diabetes.40.2.223

Jones TW, 1998, NEW ENGL J MED, V338, P1657, DOI 10.1056/NEJM199806043382303

Katte JC, 2015, PAN AFR MED J, V24, P293

Kaufman FR, 2002, J PEDIATR-US, V141, P625, DOI 10.1067/mpd.2002.129175

Komulainen J, 1999, DIABETES CARE, V22, P1950, DOI 10.2337/diacare.22.12.1950

Kushion W, 1991, Diabetes Educ, V17, P107, DOI 10.1177/014572179101700217

Lang EG, 2017, PEDIATR DIABETES, V18, P26, DOI 10.1111/pedi.12357

Littom J, 2002, J PEDIATR-US, V141, P490, DOI 10.1067/mpd.2002.127500

Mack-Fogg JE, 2005, PEDIATR DIABETES, V6, P17, DOI 10.1111/j.1399-543X.2005.00090.x

Muhammad J, 1999, ARCH DIS CHILD, V80, P367, DOI 10.1136/adc.80.4.367

Nordfeldt S, 1999, ACTA PAEDIATR, V88, P1184, DOI 10.1080/080352599750030275

Northam EA, 1998, DIABETES CARE, V21, P379, DOI 10.2337/diacare.21.3.379

Patton SR, 2006, J DEV BEHAV PEDIATR, V27, P202, DOI 10.1097/00004703-200606000-00004

Patton Susana R, 2017, J Diabetes Sci Technol, V11, P87, DOI 10.1177/1932296816658901

Patton SR, 2017, DIABETES TECHNOL THE, V19, P103, DOI 10.1089/dia.2016.0389

Paul P, 2005, ARCH DIS CHILD, V90, pA19

Pearson ER, 2006, NEW ENGL J MED, V355, P467, DOI 10.1056/NEJMoa061759

Peters A., 2013, AM DIABETES ASS JDRF

Porter PA, 1999, J PEDIAT, V130, P366

Raine JE, 2006, PRACTICAL ENDOCRINOL, P1

Rewers M, 2004, TYPE 1 DIABETES CELL

Rubio-Cabezas O, 2013, HORM RES PAEDIAT, V80, P137, DOI 10.1159/000354219

Rutledge KS, 1997, PEDIATRICS, V100, P968, DOI 10.1542/peds.100.6.968

Ryan CM, 1999, J PEDIAT, V117, P32

Silverstein J, 2005, DIABETES CARE, V28, P186, DOI 10.2337/diacare.28.1.186

The Diabetes Control and Complications Trial Research Group, 1993, NEW ENGL J MED, V329, P977

Tsalikian Eva, 2012, Pediatr Diabetes, V13, P301, DOI 10.1111/j.1399-5448.2011.00837.x

Villafuerte QB, 2017, ENDOCRINOL DIABETES, V64, P198

Virk SA, 2016, J CLIN ENDOCR METAB, V101, P3257, DOI 10.1210/jc.2015-3604

Voltarelli JC, 2007, JAMA-J AM MED ASSOC, V297, P1568, DOI 10.1001/jama.297.14.1568

WYSOCKI T, 1989, DIABETES CARE, V12, P524, DOI 10.2337/diacare.12.8.524

NR 59

TC 1

Z9 1

U1 0

U2 2

PU MARY ANN LIEBERT, INC

PI NEW ROCHELLE

PA 140 HUGUENOT STREET, 3RD FL, NEW ROCHELLE, NY 10801 USA

SN 1520-9156

EI 1557-8593

J9 DIABETES TECHNOL THE

JI Diabetes Technol. Ther.

PD JUL

PY 2017

VL 19

IS 7

BP 383

EP 390

DI 10.1089/dia.2017.0130

PG 8

WC Endocrinology & Metabolism

SC Endocrinology & Metabolism

GA FB3JE

UT WOS:000406038000001

PM 28686045

DA 2019-01-08

ER

PT J

AU Gomez, AM

Carrillo, LFM

Correa, CMA

Velandia, OMM

Sepulveda, MAR

Herrera, JLS

Carrillo, DCH

AF Gomez, Ana Maria

Marin Carrillo, Lisseth Fernanda

Arevalo Correa, Carol M.

Munoz Velandia, Oscar Mauricio

Rondon Sepulveda, Martin Alonso

Silva Herrera, Jaime Luis

Henao Carrillo, Diana Cristina

TI Maternal-Fetal Outcomes in 34 Pregnant Women with Type 1 Diabetes in

Sensor-Augmented Insulin Pump Therapy

SO DIABETES TECHNOLOGY & THERAPEUTICS

LA English

DT Article

DE Sensor-augmented pump therapy-Low-glucose suspension; Continuous glucose

monitoring; Pregnancy; Type 1 diabetes mellitus; Severe hypoglycemia and

maternal-fetal outcomes

ID INFUSION; HYPOGLYCEMIA; KETOACIDOSIS; DELIVERY; CARE

AB Background: Pregnancy in women with type 1 diabetes (T1D) is associated with increased risk of maternal complications and neonatal morbidity and mortality. Optimizing glycemic control improves these outcomes.

Objective: To describe the experience of using sensor-augmented insulin pump therapy (SAPT) and SAPT + low-glucose suspension (LGS) on pregnant women with T1D, including neonatal and maternal outcomes.

Methods: A prospective observational study was conducted in women with T1D who started SAPT and SAPT + LGS before or during pregnancy at the San Ignacio University Hospital Diabetes Center in Bogota',Colombia. The main indication was severe hypoglycemia (SH) and poor glycemic control. Glycated hemoglobin (A1c), hypoglycemia, and maternal and fetal outcomes were assessed.

Results: Thirty-four pregnant women with T1D on SAPT and SAPT + LGS were included. Sixteen patients started therapy during pregnancy at a mean gestational age of 17.6 +/- 8.3 weeks. Mean preconceptional A1c was 8.24%+/- 2.02%. Absolute reduction ofA1c level from prepregnancy to third trimesterwas -1.63% (P<0.0001), with a significant clinical and statistical reduction in both groups, women who initiated SAPT before or during pregnancy. 52.9% of patients in second trimester and 66.6% in third trimester achieved A1c <6.5%, respectively. 91.1% underwent cesarean section. The main reasons were iterative cesarean (30%), fetal distress (20%), and preeclampsia (16%). The median gestational age at delivery was 37 weeks and 15 pregnancies resulted in preterm delivery. There was neither maternal-fetal mortality nor severe hypoglycemic episodes. Two patients had diabetic ketoacidosis.

Conclusions: In pregnant patients with T1D and high risk of hypoglycemia, SAPT and SAPT + LGS should be considered as a therapeutic alternative for A1c reduction with a low risk of SH. However, additional studies are required to evaluate the efficacy and safety of this therapy during pregnancy.

C1 [Gomez, Ana Maria; Marin Carrillo, Lisseth Fernanda; Arevalo Correa, Carol M.; Henao Carrillo, Diana Cristina] Pontificia Univ Javeriana, Hosp Univ San Ignacio, Div Endocrinol, Bogota, Colombia.

[Munoz Velandia, Oscar Mauricio] Pontificia Univ Javeriana, Hosp Univ San Ignacio, Dept Internal Med, Bogota, Colombia.

[Munoz Velandia, Oscar Mauricio; Rondon Sepulveda, Martin Alonso] Pontificia Univ Javeriana, Hosp Univ San Ignacio, Dept Clin Epidemiol & Stat, Bogota, Colombia.

[Silva Herrera, Jaime Luis] Pontificia Univ Javeriana, Hosp Univ San Ignacio, Dept Gynecol & Obstet, Bogota, Colombia.

RP Gomez, AM (reprint author), Hosp Univ San Ignacio, Endocrinol Unit, Carrera 7 40-62, Bogota 110231, Colombia.

EM amgomezm5@gmail.com

OI Henao-Carrillo, Diana Cristina/0000-0002-1353-148X; SILVA HERRERA, JAIME

LUIS/0000-0001-6822-0374; Gomez-Medina, Ana Maria/0000-0002-8907-3470

FU Novo Nordisk; Elli Lilly; MSD; Novartis; Medtronic; Abbott

FX The authors declared the following potential conflicts of interest with

respect to research, authorship, and/or publication of this article:

A.M.G. reports speaker fees from Novo Nordisk, Elli Lilly, MSD,

Novartis, and Medtronic and research grants from Medtronic, Novartis,

Novo Nordisk, and Abbott. C.M.A. receives fees for insulin pump patient

training with Medtronic. D.C.H.C. reports speaker fees from Novo Nordisk

and research grants from Novo Nordisk. No other potential conflicts are

reported.

CR Bergenstal RM, 2013, DIABETES TECHNOL THE, V15, P198, DOI 10.1089/dia.2013.0051

Bruttomesso D, 2011, DIABETES METAB, V37, P426, DOI 10.1016/j.diabet.2011.02.002

Chamberlain JJ, 2016, ANN INTERN MED, V164, P542, DOI 10.7326/M15-3016

Dalfra MG, 2016, J MATERN-FETAL NEO M, V29, P2889, DOI 10.3109/14767058.2015.1107903

Evers IN, 2004, BRIT MED J, V328, P915, DOI 10.1136/bmj.38043.583160.EE

Farrar D, 2016, COCHRANE DB SYST REV, DOI 10.1002/14651858.CD005542.pub3

Fresa R, 2013, DIABETES TECHNOL THE, V15, P328, DOI 10.1089/dia.2012.0260

Medina AMG, 2011, AV DIABETOL, V27, P143, DOI 10.1016/j.avdiab.2011.08.003

Kallas-Koeman MM, 2014, DIABETOLOGIA, V57, P681, DOI 10.1007/s00125-014-3163-6

Maresh MJA, 2015, DIABETES CARE, V38, P34, DOI 10.2337/dc14-1755

Gomez AM, 2017, DIABETES TECHNOL THE, V19, P109, DOI 10.1089/dia.2016.0332

Mukhopadhyay A, 2007, AM J OBSTET GYNECOL, V197, P447, DOI 10.1016/j.ajog.2007.03.062

Neff KJ, 2014, IRISH J MED SCI, V183, P397, DOI 10.1007/s11845-013-1027-6

Nielsen LR, 2008, DIABETES CARE, V31, P9, DOI 10.2337/dc07-1066

Petrovski G, 2011, DIABETES TECHNOL THE, V13, P1109, DOI 10.1089/dia.2011.0081

Polsky S, 2016, POSTGRAD MED, V128, P418, DOI 10.1080/00325481.2016.1159910

Stewart ZA, 2016, NEW ENGL J MED, V375, P644, DOI 10.1056/NEJMoa1602494

NR 17

TC 2

Z9 2

U1 1

U2 1

PU MARY ANN LIEBERT, INC

PI NEW ROCHELLE

PA 140 HUGUENOT STREET, 3RD FL, NEW ROCHELLE, NY 10801 USA

SN 1520-9156

EI 1557-8593

J9 DIABETES TECHNOL THE

JI Diabetes Technol. Ther.

PD JUL

PY 2017

VL 19

IS 7

BP 417

EP 422

DI 10.1089/dia.2017.0030

PG 6

WC Endocrinology & Metabolism

SC Endocrinology & Metabolism

GA FB3JE

UT WOS:000406038000006

PM 28731823

DA 2019-01-08

ER

PT J

AU Dahal, KA

Premji, S

Patel, AB

Williamson, T

Peng, MK

Metcalfe, A

AF Dahal, Kamala Adhikari

Premji, Shahirose

Patel, Alka B.

Williamson, Tyler

Peng, Mingkai

Metcalfe, Amy

TI Variation in Maternal Co-morbidities and Obstetric Interventions across

Area-Level Socio-economic Status: A Cross-Sectional Study

SO PAEDIATRIC AND PERINATAL EPIDEMIOLOGY

LA English

DT Article

DE area-level socio-economic status; maternal co-morbidities; obstetric

interventions; prolonged hospital stay

ID PSYCHOLOGICAL DISTRESS; ADMINISTRATIVE DATA; MULTILEVEL ANALYSIS;

CESAREAN-SECTIONS; OUTCOMES; HEALTH; COMORBIDITY; ASSOCIATION;

PREGNANCY; CANADA

AB Background: Multiple studies indicate a significant association between area-level socio-economic status (SES) and adverse maternal health outcomes; however, the impact of area-level SES on maternal co-morbidities and obstetric interventions has not been examined.

Objective: To examine the variation in maternal co-morbidities and obstetric interventions across area-level SES.

Methods: This study used data from the Discharge Abstract Database that comprised birth data in Alberta between 2005-2007 (n = 120 285). Co-morbidities and obstetric interventions were identified using validated case-definitions. Material deprivation index was obtained for each dissemination area through linkage of hospitalisation and census data. Multilevel logistic regression was used to analyse the data adjusting for potential confounding variables.

Results: The prevalence of any co-morbidity varied across area-level SES. Drug abuse odds ratio (OR) 2.5 (95% confidence interval (CI) 1.8, 3.5), pre-existing diabetes OR 1.7 (95% CI 1.1, 2.6), and prolonged hospital stay OR 1.5 (95% CI 1.4, 1.6) were significantly more likely to occur in the most deprived areas compared to the least deprived areas. In contrast, caesarean delivery OR 0.9 (95% CI 0.8, 0.9) was less likely to occur in the most deprived areas compared to the least deprived areas. Area-level deprivation explained area-level variance of drug abuse, HIV, and other mental diseases only.

Conclusion: Many co-morbidities and obstetric interventions vary at the area-level, but only some are associated with area-level SES, and few of them vary due to the area-level SES. This indicates that other area-level factors, in addition to area-level SES, need to be considered when investigating maternal health and use of health interventions.

C1 [Dahal, Kamala Adhikari; Premji, Shahirose; Patel, Alka B.; Williamson, Tyler; Peng, Mingkai; Metcalfe, Amy] Univ Calgary, Dept Community Hlth Sci, Calgary, AB, Canada.

[Premji, Shahirose] Univ Calgary, Fac Nursing, Calgary, AB, Canada.

[Patel, Alka B.] Alberta Hlth Serv, Appl Res & Evaluat Serv Primary Hlth Care, Calgary, AB, Canada.

[Metcalfe, Amy] Univ Calgary, Dept Obstet & Gynecol, Calgary, AB, Canada.

RP Metcalfe, A (reprint author), Foothills Med Ctr, Calgary, AB, Canada.

EM amy.metcalfe@albertahealthservices.ca

CR Alberta Reproductive Health Report Working Group, 2011, ALB REPR HLTH PREGN

Alberta Services Services, 2016, US PAMP DEPR IND ALB

[Anonymous], 2013, OBSTET GYNECOL, V122, P957, DOI 10.1097/AOG.0b013e3182a603bb

Auger N, 2011, BMC PREGNANCY CHILDB, V11, DOI 10.1186/1471-2393-11-67

BARROS FC, 1991, LANCET, V338, P167, DOI 10.1016/0140-6736(91)90149-J

Comstock EE, 2016, J ASSOC NURSE AIDS C, V27, P98, DOI 10.1016/j.jana.2015.08.006

Fairley L, 2011, BMC PUBLIC HEALTH, V11, DOI 10.1186/1471-2458-11-330

Fiest KM, 2014, BMC PSYCHIATRY, V14, DOI 10.1186/s12888-014-0289-5

Fridman M, 2014, AM J PUBLIC HEALTH, V104, pS49, DOI 10.2105/AJPH.2013.301583

GOULD JB, 1989, NEW ENGL J MED, V321, P233, DOI 10.1056/NEJM198907273210406

*HLTH CAN, 2000, PER HLTH IND CAN RES

Hu WS, 2016, CLIN EXP OBSTET GYN, V43, P212

Joseph KS, 2010, J OBSTET GYNAECOL CA, V32, P837, DOI 10.1016/S1701-2163(16)34655-2

KAWACHI I, 2003, NEIGHBORHOOD HLTH

KESSLER RC, 1979, J HEALTH SOC BEHAV, V20, P259, DOI 10.2307/2136450

Leeb Kira, 2005, Healthc Policy, V1, P48

Marrie RA, 2013, BMC NEUROL, V13, DOI 10.1186/1471-2377-13-16

Merlo J, 2009, J EPIDEMIOL COMMUN H, V63, P1043, DOI 10.1136/jech.2009.088310

Merlo J, 2006, J EPIDEMIOL COMMUN H, V60, P290, DOI 10.1136/jech.2004.029454

Metcalfe A, 2015, BJOG-INT J OBSTET GY, V122, P1748, DOI 10.1111/1471-0528.13254

Metcalfe A, 2016, BMJ OPEN, V6, DOI 10.1136/bmjopen-2016-012007

Metcalfe A, 2011, PAEDIATR PERINAT EP, V25, P236, DOI 10.1111/j.1365-3016.2011.01192.x

Nagahawatte NT, 2008, ANN NY ACAD SCI, V1136, P80, DOI 10.1196/annals.1425.016

Pampalon R, 2000, Chronic Dis Can, V21, P104

Regidor E, 2004, J EPIDEMIOL COMMUN H, V58, P900, DOI 10.1136/jech.2004.023036

Roos NP, 1997, MILBANK Q, V75, P89, DOI 10.1111/1468-0009.00045

Saint-Jacques N, 2014, INT J EQUITY HEALTH, V13, DOI 10.1186/s12939-014-0094-2

Southern DA, 2005, MED CARE, V43, P1116, DOI 10.1097/01.mlr.0000182517.57235.6d

Statistics Canada, TABL 102 4516 LIV BI

Statistics Canada, 2016, 2011 NAT HOUS SURV C

Statistics Canada, POP GROWTH AG STRUCT

Tough S, 2006, CLIN MED RES, V4, P97, DOI 10.3121/cmr.4.2.97

Wen SW, 2005, CAN MED ASSOC J, V173, P759, DOI 10.1503/cmaj.045156

Yang SM, 2015, MATERN CHILD HLTH J, V19, P1142, DOI 10.1007/s10995-014-1623-8

Zhao YJ, 2013, INT J EQUITY HEALTH, V12, DOI 10.1186/1475-9276-12-79

NR 35

TC 0

Z9 0

U1 0

U2 1

PU WILEY

PI HOBOKEN

PA 111 RIVER ST, HOBOKEN 07030-5774, NJ USA

SN 0269-5022

EI 1365-3016

J9 PAEDIATR PERINAT EP

JI Paediatr. Perinat. Epidemiol.

PD JUL

PY 2017

VL 31

IS 4

BP 274

EP 283

DI 10.1111/ppe.12370

PG 10

WC Public, Environmental & Occupational Health; Obstetrics & Gynecology;

Pediatrics

SC Public, Environmental & Occupational Health; Obstetrics & Gynecology;

Pediatrics

GA FB0HY

UT WOS:000405827300005

PM 28590511

DA 2019-01-08

ER

PT J

AU Corathers, SD

Mara, CA

Chundi, PK

Kichler, JC

AF Corathers, Sarah D.

Mara, Constance A.

Chundi, Pavan K.

Kichler, Jessica C.

TI Psychosocial Patient-Reported Outcomes in Pediatric and Adolescent

Diabetes: a Review and Case Example

SO CURRENT DIABETES REPORTS

LA English

DT Review

DE Psychosocial; Patient-reported outcome (PRO); Adolescent; Diabetes

ID QUALITY-OF-LIFE; ROUTINE CLINICAL-PRACTICE; SELF-CARE;

METABOLIC-CONTROL; PSYCHOMETRIC PROPERTIES; POSITION STATEMENT;

CONTROLLED-TRIAL; SOCIAL SUPPORT; SCREENING TOOL; TYPE-1

AB Purpose of Review The purpose of this review is to define psychosocial patient-reported outcomes (PROs) relevant to pediatric and adolescent diabetes populations. Potential domains for PROs include a spectrum of emotional, behavioral, social, physical, overall health, and/or care management areas. A literature review of potential PRO measures, selection criteria, and implementation strategies including a case example will be presented.

Recent Findings Among the pediatric, adolescent, and emerging adult populations, research indicates a relative higher risk for distress, depression, anxiety, and eating disorders as compared to peers without diabetes. Use of PRO measures can expand providers' focus beyond glycemic control, or simply hemoglobin A1c, to better appreciate the impact of diabetes on the whole child/adolescent, and provide services that address patients' individually identified needs, which are most salient to them.

Summary Successful selection and implementation of psychosocial PRO measures should be designed to include pathways for real-time provider interaction with the patient and respective PRO data to guide clinical care.

C1 [Corathers, Sarah D.] Cincinnati Childrens Hosp Med Ctr, Div Endocrinol, 3333 Burnet Ave,MLC 7012, Cincinnati, OH 45229 USA.

[Corathers, Sarah D.; Chundi, Pavan K.] Cincinnati Childrens Hosp Med Ctr, James M Anderson Ctr Hlth Syst Excellence, Cincinnati, OH 45229 USA.

[Mara, Constance A.; Kichler, Jessica C.] Cincinnati Childrens Hosp Med Ctr, Div Behav Med & Clin Psychol, Cincinnati, OH 45229 USA.

RP Corathers, SD (reprint author), Cincinnati Childrens Hosp Med Ctr, Div Endocrinol, 3333 Burnet Ave,MLC 7012, Cincinnati, OH 45229 USA.; Corathers, SD (reprint author), Cincinnati Childrens Hosp Med Ctr, James M Anderson Ctr Hlth Syst Excellence, Cincinnati, OH 45229 USA.

EM sarah.corathers@cchmc.org; constance.mara@cchmc.org;

pavan.chundi@cchmc.org; jessica.kichler@cchmc.org

CR Allgaier AK, 2012, J PSYCHOSOM RES, V73, P369, DOI 10.1016/j.jpsychores.2012.08.016

Almario CV, 2016, AM J GASTROENTEROL, V111, P1546, DOI 10.1038/ajg.2016.305

[Anonymous], 2017, DIABETES CARE S1, V40, pS4

Berwick DM, 2016, JAMA-J AM MED ASSOC, V315, P1329, DOI 10.1001/jama.2016.1509

BROWNLEEDUFFECK M, 1987, J CONSULT CLIN PSYCH, V55, P139, DOI 10.1037//0022-006X.55.2.139

Chiang JL, 2014, DIABETES CARE, V37, P2034, DOI 10.2337/dc14-1140

Corathers SD, 2013, PEDIATRICS, V132, pE1395, DOI 10.1542/peds.2013-0681

COX DJ, 1987, DIABETES CARE, V10, P617, DOI 10.2337/diacare.10.5.617

de Wit M, 2010, PEDIATR DIABETES, V11, P175, DOI 10.1111/j.1399-5448.2009.00543.x

De Wit M, 2008, DIABETES CARE, V31, P1521, DOI 10.2337/dc08-0394

Delamater AM, 2014, PEDIATR DIABETES, V15, P232, DOI 10.1111/pedi.12191

Forrest CB, 2014, QUAL LIFE RES, V23, P1221, DOI 10.1007/s11136-013-0581-8

Garvey KC, 2016, DIABETES CARE

GREEN LB, 1990, J PEDIATR PSYCHOL, V15, P633, DOI 10.1093/jpepsy/15.5.633

Greenhalgh J, 2005, SOC SCI MED, V60, P833, DOI 10.1016/j.socscimed.2004.06.022

Hilliard ME, 2013, DIABETES CARE, V36, P1891, DOI 10.2337/dc12-1708

Hilliard ME, 2011, DIABETES RES CLIN PR, V94, P39, DOI 10.1016/j.diabres.2011.05.027

Hood KK, 2007, DIABETES CARE, V30, P1764, DOI 10.2337/dc06-2358

Iturralde E, 2016, HLTH PSYCHOL

Kamps JL, 2005, J PEDIATR PSYCHOL, V30, P287, DOI 10.1093/jpepsy/jsi038

Kovacs M, 2003, CHILDRENS DEPRESSION

Kroenke K, 2001, J GEN INTERN MED, V16, P606, DOI 10.1046/j.1525-1497.2001.016009606.x

La Greca AM, 2002, J PEDIATR PSYCHOL, V27, P665, DOI 10.1093/jpepsy/27.8.665

Langley GJ, 2009, IMPROVEMENT GUIDE PR, pxxi

Liese AD, 2006, PEDIATRICS, V118, P1510, DOI 10.1542/peds.2006-0690

Lohr K, 2009, QUAL LIFE RES, V18, P99, DOI 10.1007/s11136-008-9413-7

Markowitz JT, 2010, DIABETES CARE, V33, P495, DOI 10.2337/dc09-1890

MELAMED S, 1992, BEHAV MED, V18, P53, DOI 10.1080/08964289.1992.9935172

Modi AC, 2012, PEDIATRICS, V129, pE473, DOI 10.1542/peds.2011-1635

Mulvaney SA, 2011, DIABETES RES CLIN PR, V94, P77, DOI 10.1016/j.diabres.2011.06.010

Owens JA, 2000, SLEEP, V23, P1043

Owens JA, 2000, ARCH PEDIAT ADOL MED, V154, P549, DOI 10.1001/archpedi.154.6.549

Peters A, 2011, DIABETES CARE, V34, P2477, DOI 10.2337/dc11-1723

POLONSKY WH, 1995, DIABETES CARE, V18, P754, DOI 10.2337/diacare.18.6.754

Polonsky WH, 2005, DIABETES CARE, V28, P626, DOI 10.2337/diacare.28.3.626

PROCIDANO ME, 1983, AM J COMMUN PSYCHOL, V11, P1, DOI 10.1007/BF00898416

Pyatak EA, 2017, J ADOLESCENT HEALTH, V60, P212, DOI 10.1016/j.jadohealth.2016.09.004

Rodbard HW, 2017, DIABETES CARE, V40, P171, DOI 10.2337/dc16-1353

Rose M, 2009, QUAL LIFE RES, V18, P125, DOI 10.1007/s11136-008-9436-0

RUBIN RR, 1989, DIABETES CARE, V12, P673, DOI 10.2337/diacare.12.10.673

Santana MJ, 2015, QUAL LIFE RES, V24, P1707, DOI 10.1007/s11136-014-0903-5

SCHAFER LC, 1986, DIABETES CARE, V9, P179, DOI 10.2337/diacare.9.2.179

Schmitt A, 2013, HEALTH QUAL LIFE OUT, V11, DOI 10.1186/1477-7525-11-138

Sharifi A, 2016, DIABETES TECHNOL THE, V18, P772, DOI 10.1089/dia.2016.0288

Snyder CF, 2012, QUAL LIFE RES, V21, P1305, DOI 10.1007/s11136-011-0054-x

Spitzer RL, 2006, ARCH INTERN MED, V166, P1092, DOI 10.1001/archinte.166.10.1092

Streisand R, 2001, J PEDIATR PSYCHOL, V26, P155, DOI 10.1093/jpepsy/26.3.155

Tanenbaum ML, 2017, DIABETES CARE, V40, P181, DOI 10.2337/dc16-1536

Toobert DJ, 2000, DIABETES CARE, V23, P943, DOI 10.2337/diacare.23.7.943

van der Ven NCW, 2003, DIABETES CARE, V26, P713, DOI 10.2337/diacare.26.3.713

Varni James W, 2004, Health Qual Life Outcomes, V2, P55, DOI 10.1186/1477-7525-2-55

Varni JW, 2015, J PEDIATR PSYCHOL, V40, P591, DOI 10.1093/jpepsy/jsv005

Varni JW, 2013, QUAL LIFE RES, V22, P2169, DOI 10.1007/s11136-012-0339-8

Varni JW, 2001, MED CARE, V39, P800, DOI 10.1097/00005650-200108000-00006

Tran VT, 2014, BMC MED, V12, DOI 10.1186/1741-7015-12-109

Weissberg-Benchell Jill, 2016, J Diabetes Sci Technol, V10, P799, DOI 10.1177/1932296815619637

Welch GW, 1997, DIABETES CARE, V20, P760, DOI 10.2337/diacare.20.5.760

Wood JR, 2013, DIABETES CARE, V36, P2035, DOI 10.2337/dc12-1959

Young-Hyman D, 2016, DIABETES CARE, V39, P2126, DOI 10.2337/dc16-2053

NR 59

TC 2

Z9 2

U1 1

U2 15

PU CURRENT MEDICINE GROUP

PI PHILADELPHIA

PA 400 MARKET STREET, STE 700, PHILADELPHIA, PA 19106 USA

SN 1534-4827

EI 1539-0829

J9 CURR DIABETES REP

JI Curr. Diabetes Rep.

PD JUL

PY 2017

VL 17

IS 7

AR 45

DI 10.1007/s11892-017-0872-5

PG 10

WC Endocrinology & Metabolism

SC Endocrinology & Metabolism

GA EZ2KM

UT WOS:000404538200001

PM 28508255

DA 2019-01-08

ER

PT J

AU Swoboda, CM

Miller, CK

Wills, CE

AF Swoboda, Christine M.

Miller, Carla K.

Wills, Celia E.

TI Impact of a goal setting and decision support telephone coaching

intervention on diet, psychosocial, and decision outcomes among people

with type 2 diabetes

SO PATIENT EDUCATION AND COUNSELING

LA English

DT Article

DE Type 2 diabetes mellitus; Decision support; Goal setting; Self-efficacy;

Telephone coaching; Randomized controlled trial

ID PHYSICAL-ACTIVITY; RANDOMIZED-TRIAL; SELF-MANAGEMENT; DISTRESS SCALE;

MULTIPLE; BEHAVIOR; ADULTS; CARE; COACTION; SINGLE

AB Objective: Evaluate a 16-week decision support and goal-setting intervention to compare diet quality, decision, and diabetes-related outcomes to a control group.

Methods: Adults with type 2 diabetes (n = 54) were randomly assigned to an intervention or control group. Intervention group participants completed one in-person motivational interviewing and decision support session followed by seven biweekly telephone coaching calls. Participants reported previous goal attempts and set diet-and/or physical activity-related goals during coaching calls. Control group participants received information about local health care resources on the same contact schedule.

Results: There was a significant difference between groups for diabetes empowerment (p = 0.045). A significant increase in diet quality, diabetes self-efficacy, and diabetes empowerment, and a significant decrease in diabetes distress and depressive symptoms (all p <= 0.05) occurred in the intervention group. Decision confidence to achieve diet-related goals significantly improved from baseline to week 8 but then declined at study end (both p <= 0.05).

Conclusions: Setting specific diet-related goals may promote dietary change, and telephone coaching can improve psychosocial outcomes related to diabetes self-management.

Practice implications: Informed shared decision making can facilitate progressively challenging yet attainable goals tailored to individuals' lifestyle. Decision coaching may empower patients to improve self-management practices and reduce distress. (C) 2017 Elsevier B.V. All rights reserved.

C1 [Swoboda, Christine M.] Ohio State Univ, Dept Human Sci, Human, Nutr, Columbus, OH 43210 USA.

[Miller, Carla K.] Ohio State Univ, Human Nutr, Dept Human Sci, 1787 Neil Ave,325 Campbell Hall, Columbus, OH 43210 USA.

[Wills, Celia E.] Ohio State Univ, Coll Nursing, Columbus, OH 43210 USA.

[Swoboda, Christine M.] Ohio State Univ, Dept Family Med, Columbus, OH 43210 USA.

RP Miller, CK (reprint author), Ohio State Univ, Human Nutr, Dept Human Sci, 1787 Neil Ave,325 Campbell Hall, Columbus, OH 43210 USA.

EM miller.4453@osu.edu

OI Miller, Carla/0000-0002-1002-7288; Swoboda,

Christine/0000-0002-2085-2126

FU Ohio State University Department of Human Sciences

FX Funding for this research was provided by The Ohio State University

Department of Human Sciences, which had no role in study design,

analyses or interpretation.

CR Alden Dana L, 2013, Hawaii J Med Public Health, V72, P396

American Diabetes Association, 2017, Diabetes Care, V40, pS75

American Diabetes Association, 2017, Diabetes Care, V40, pS33

American Diabetes Association, 2017, Diabetes Care, V40, pS48

Anderson AR, 2010, DIABETES SPECTRUM, V23, P97, DOI [10.2337/diaspect.23.2.97, DOI 10.2337/DIASPECT.23.2.97]

Anderson RM, 2003, DIABETES CARE, V26, P1641, DOI 10.2337/diacare.26.5.1641-a

Bailey RA, 2016, BMC HEALTH SERV RES, V16, DOI 10.1186/s12913-016-1262-4

Baldoni N.R., 2016, PRIM CARE DIABETES

BANDURA A, 1977, PSYCHOL REV, V84, P191, DOI 10.1037/0033-295X.84.2.191

Bovend'Eerdt TJH, 2009, CLIN REHABIL, V23, P352, DOI 10.1177/0269215508101741

Corser W, 2007, DIABETES EDUCATOR, V33, P700, DOI 10.1177/0145721707304086

Fisher L, 2008, DIABETIC MED, V25, P1096, DOI 10.1111/j.1464-5491.2008.02533.x

Fisher L, 2007, DIABETES CARE, V30, P542, DOI 10.2337/dc06-1614

Fisher L, 2012, DIABETES CARE, V35, P259, DOI 10.2337/dc11-1572

Fisher L, 2010, DIABETES CARE, V33, P23, DOI 10.2337/dc09-1238

Glasgow RE, 1996, DIABETES CARE, V19, P835, DOI 10.2337/diacare.19.8.835

Graham I, 1995, USER MANUAL PREPARAT

Guenther PM, 2013, J ACAD NUTR DIET, V113, P569, DOI 10.1016/j.jand.2012.12.016

Guenther PM, 2010, J NUTR, V2014, P399, DOI DOI 10.3945/

Gunathilake Waruna, 2013, J Diabetes Sci Technol, V7, P653

HolmesRovner M, 1996, MED DECIS MAKING, V16, P58, DOI 10.1177/0272989X9601600114

Horner NK, 2002, AM J CLIN NUTR, V76, P766

Johnson SS, 2014, HEALTH PSYCHOL, V33, P475, DOI 10.1037/a0034215

Kroenke K, 2009, J AFFECT DISORDERS, V114, P163, DOI 10.1016/j.jad.2008.06.026

Lafata JE, 2013, PATIENT EDUC COUNS, V92, P94, DOI 10.1016/j.pec.2013.01.016

Lakerveld J, 2008, BMC MED RES METHODOL, V8, DOI 10.1186/1471-2288-8-17

Locke E. A., 1990, THEORY GOAL SETTING

Lorig K, 2009, DIABETES EDUCATOR, V35, P641, DOI 10.1177/0145721709335006

Mathers N, 2012, BMJ OPEN, V2, DOI 10.1136/bmjopen-2012-001469

Miller CK, 2014, CURR DIABETES REP, V14, DOI 10.1007/s11892-014-0509-x

Mullan RJ, 2009, ARCH INTERN MED, V169, P1560, DOI 10.1001/archinternmed.2009.293

Naik AD, 2011, ARCH INTERN MED, V171, P453, DOI 10.1001/archinternmed.2011.70

O' Connor A. M, 1995, USER MANUAL DECISION

O'Connor AM, 1998, PATIENT EDUC COUNS, V33, P267, DOI 10.1016/S0738-3991(98)00026-3

OCONNOR AM, 1995, MED DECIS MAKING, V15, P25, DOI 10.1177/0272989X9501500105

OConnor AM, 2000, USER MANUAL STAGE DE

Paiva AL, 2012, PREV MED, V54, P331, DOI 10.1016/j.ypmed.2012.02.017

Polonsky WH, 2005, DIABETES CARE, V28, P626, DOI 10.2337/diacare.28.3.626

Price AE, 2013, J AGING PHYS ACTIV, V21, P348, DOI 10.1123/japa.21.3.348

Stacey D, 2014, COCHRANE DB SYST REV, DOI 10.1002/14651858.CD001431.pub4

Sweet SN, 2010, INT J ENV RES PUB HE, V7, P1720, DOI 10.3390/ijerph7041720

Swoboda CM, 2016, DIABETES EDUCATOR, V42, P429, DOI 10.1177/0145721716650043

Tanenbaum ML, 2016, J DIABETES COMPLICAT, V30, P1060, DOI 10.1016/j.jdiacomp.2016.04.023

Volk R., 2012, 2012 IPDAS BACKGROUN

Wills Celia E, 2003, Health Expect, V6, P149, DOI 10.1046/j.1369-6513.2003.00220.x

NR 45

TC 3

Z9 3

U1 4

U2 24

PU ELSEVIER IRELAND LTD

PI CLARE

PA ELSEVIER HOUSE, BROOKVALE PLAZA, EAST PARK SHANNON, CO, CLARE, 00000,

IRELAND

SN 0738-3991

J9 PATIENT EDUC COUNS

JI Patient Educ. Couns.

PD JUL

PY 2017

VL 100

IS 7

BP 1367

EP 1373

DI 10.1016/j.pec.2017.02.007

PG 7

WC Public, Environmental & Occupational Health; Social Sciences,

Interdisciplinary

SC Public, Environmental & Occupational Health; Social Sciences - Other

Topics

GA FA2LI

UT WOS:000405272500016

PM 28215827

DA 2019-01-08

ER

PT J

AU Chiu, CJ

Tseng, YH

Hsu, YC

Wu, ST

AF Chiu, Ching-Ju

Tseng, Yu-Hsuan

Hsu, Yu-Ching

Wu, Shang-Te

TI Depressive symptom trajectories in the first 10 years of diabetes

diagnosis: antecedent factors and link with future disability in Taiwan

SO SOCIAL PSYCHIATRY AND PSYCHIATRIC EPIDEMIOLOGY

LA English

DT Article

DE Diabetes; Depressive symptoms; Long-term trajectories; Middle-aged and

older adults

ID COMORBID DEPRESSION; SELF-CARE; MEDICATION ADHERENCE; ADULTS;

PREVALENCE; MELLITUS; METAANALYSIS; COMMUNITY; IMPACT

AB ion, less This study identified depressive symptom trajectories in the years after diabetes diagnosis, examined factors that predict the probability for people following a specific trajectory, and investigated how the trajectories are associated with subsequent disability.

We drew data from a nationally representative survey in Taiwan to identify adults aged 50 and older diagnosed with diabetes; 487 patients newly diagnosed with diabetes during 1996-2007 were included. Time axis was set to zero when diabetes was first reported in any given wave in the survey, and data related to depressive symptoms after that were recorded. We used group-based semi-parametric mixture models to identify trajectories of depressive symptoms and multinomial logistic regressions to examine factors associated with the trajectories.

Older adults with newly diagnosis of diabetes in Taiwan follow different trajectories of depressive symptoms over time. Being female, lower educated, not married/partnered, with lower self-rated health, hospitalizations, more limitations in physical funct regular exercise before diagnosis, and not regularly using anti-diabetic medication at the beginning of their diagnosis were factors associated with increasing or high stable depressive symptom trajectories. Those who experienced high depressive symptoms were more likely than individuals with stable depressive symptoms to have physical limitations in the last follow-up interview.

Depressive symptom trajectories after diabetes diagnosis were associated with select sociodemographic, health, and lifestyle factors before diagnosis, and also predicted subsequent disability. Risk groups identified in the present study may be used for personalized diabetes care that prevents diabetes-related distress and future disability.

C1 [Chiu, Ching-Ju] Natl Cheng Kung Univ, Inst Gerontol, Coll Med, 1 Univ Rd, Tainan 70101, Taiwan.

[Tseng, Yu-Hsuan] Natl Cheng Kung Univ, Dept Occupat Therapy, Coll Med, Tainan 70101, Taiwan.

[Hsu, Yu-Ching] Tainan Hosp, Dept Chinese Med, Minist Hlth & Welf, Tainan 70043, Taiwan.

[Wu, Shang-Te] Kao Gen Hosp, Dept Neurol, Tainan 70054, Taiwan.

RP Chiu, CJ (reprint author), Natl Cheng Kung Univ, Inst Gerontol, Coll Med, 1 Univ Rd, Tainan 70101, Taiwan.

EM cjchiu@mail.ncku.edu.tw

FU Ministry of Science and Technology [MOST 102-2314-B-006-075]

FX This work was supported by a Grant from the Ministry of Science and

Technology (PI: Ching-Ju Chiu, MOST 102-2314-B-006-075).

CR Ali S, 2006, DIABETIC MED, V23, P1165, DOI 10.1111/j.1464-5491.2006.01943.x

Anderson RJ, 2001, DIABETES CARE, V24, P1069, DOI 10.2337/diacare.24.6.1069

Chiu CJ, 2011, DIABETES RES CLIN PR, V91, P413, DOI 10.1016/j.diabres.2010.12.003

Ciechanowski PS, 2000, ARCH INTERN MED, V160, P3278, DOI 10.1001/archinte.160.21.3278

Ciechanowski PS, 2003, GEN HOSP PSYCHIAT, V25, P246, DOI 10.1016/S0163-8343(03)00055-0

Collins MM, 2009, DIABETIC MED, V26, P153, DOI 10.1111/j.1464-5491.2008.02648.x

D'Unger AV, 1998, AM J SOCIOL, V103, P1593, DOI 10.1086/231402

de Jonge P, 2006, DIABETOLOGIA, V49, P2627, DOI 10.1007/s00125-006-0442-x

Egede LE, 2002, DIABETES CARE, V25, P464, DOI 10.2337/diacare.25.3.464

Goldman N, 2003, J CLIN EPIDEMIOL, V56, P148, DOI 10.1016/S0895(02)00580-2

Gonzalez JS, 2007, DIABETES CARE, V30, P2222, DOI 10.2337/dc07-0158

Jones BL, 2007, SOCIOL METHOD RES, V35, P542, DOI 10.1177/0049124106292364

Katon WJ, 2005, DIABETES CARE, V28, P2668, DOI 10.2337/diacare.28.11.2668

Khan MA, 2014, JCPSP-J COLL PHYSICI, V24, P770, DOI 10.2014/JCPSP.770771

Limongi F, 2014, DIABETES METAB, V40, P373, DOI 10.1016/j.diabet.2014.05.001

Lin EHB, 2004, DIABETES CARE, V27, P2154, DOI 10.2337/diacare.27.9.2154

Lipscombe C, 2015, J AFFECT DISORDERS, V188, P160, DOI 10.1016/j.jad.2015.08.003

Lloyd CE, 2000, DIABETIC MED, V17, P198, DOI 10.1046/j.1464-5491.2000.00260.x

Maraldi C, 2007, ARCH INTERN MED, V167, P1137, DOI 10.1001/archinte.167.11.1137

NAGI SZ, 1976, MILBANK FUND Q, V54, P439, DOI 10.2307/3349677

Nagin D, 2005, GROUP BASED MODELING

Nagin DS, 1999, PSYCHOL METHODS, V4, P139, DOI 10.1037//1082-989X.4.2.139

Nefs G, 2012, DIABETOLOGIA, V55, P608, DOI 10.1007/s00125-011-2411-2

Nouwen A, 2010, DIABETOLOGIA, V53, P2480, DOI 10.1007/s00125-010-1874-x

POPKIN MK, 1988, ARCH GEN PSYCHIAT, V45, P64

RADLOFF L S, 1977, Applied Psychological Measurement, V1, P385, DOI 10.1177/014662167700100306

Schmitz N, 2013, J PSYCHOSOM RES, V74, P128, DOI 10.1016/j.jpsychores.2012.09.021

Shaw JL, 2013, J COMMUN HEALTH, V38, P86, DOI 10.1007/s10900-012-9585-5

Thomasouli MA, 2013, SLEEP BREATH, V17, P925, DOI 10.1007/s11325-013-0806-7

Tsai YF, 2005, INT J GERIATR PSYCH, V20, P315, DOI 10.1002/gps.1281

Tseng CH, 2006, DIABETOLOGIA, V49, P1755, DOI 10.1007/s00125-006-0314-4

NR 31

TC 1

Z9 1

U1 3

U2 7

PU SPRINGER HEIDELBERG

PI HEIDELBERG

PA TIERGARTENSTRASSE 17, D-69121 HEIDELBERG, GERMANY

SN 0933-7954

EI 1433-9285

J9 SOC PSYCH PSYCH EPID

JI Soc. Psychiatry Psychiatr. Epidemiol.

PD JUL

PY 2017

VL 52

IS 7

BP 829

EP 836

DI 10.1007/s00127-016-1314-4

PG 8

WC Psychiatry

SC Psychiatry

GA FA3AV

UT WOS:000405314200008

PM 27878580

DA 2019-01-08

ER

PT J

AU Mashitani, T

Hayashino, Y

Okamura, S

Kitatani, M

Furuya, M

Iburi, T

Tsujii, S

Ishii, H

AF Mashitani, Tsuyoshi

Hayashino, Yasuaki

Okamura, Shintaro

Kitatani, Masako

Furuya, Miyuki

Iburi, Tadao

Tsujii, Satoru

Ishii, Hitoshi

CA Diabetes Distress Care Registry Te

TI Association between dipstick hematuria and decline in estimated

glomerular filtration rate among Japanese patients with type 2 diabetes:

A prospective cohort study [Diabetes Distress and Care Registry at Tenri

(DDCRT 14)]

SO JOURNAL OF DIABETES AND ITS COMPLICATIONS

LA English

DT Article

DE Diabetes; Hematuria; eGFR decline; Diabetic nephropathy; Albuminuria

ID STAGE RENAL-DISEASE; MICROSCOPIC HEMATURIA; CLINICAL-FEATURES; OVERT

PROTEINURIA; KIDNEY-FUNCTION; NEPHROPATHY; RISK; PROGRESSION;

RETINOPATHY; PREVALENCE

AB Aims: To assess the association between dipstick hematuria and estimated glomerular filtration rate (eGFR) decline in Japanese patients with type 2 diabetes.

Methods: Longitudinal data were obtained from 3068 Japanese patients with type 2 diabetes. To assess the independent association between dipstick hematuria and eGFR decline, we used Cox proportional hazard model adjusted for potential confounders.

Results: Median follow-up period was 699.7 days. Mean age, body mass index (BMI), and HbAlc level were 65.7 years, 24.6 kg/m(2), and 7.5% (58.1 mmol/mol), respectively. Positive dipstick hematuria was significantly associated with baseline eGFR and severity of albuminuria (p < 0.001). The multivariable-adjusted hazard ratio for eGFR decline in patients with dipstick hematuria compared with those without dipstick hematuria was 2.19 [95% confidence interval (CI): 1.22-3.91]; this association remained significant even after the exclusion of patients who did not have diabetic retinopathy (hazard ratio: 239; 95% CI: 1.13-5.04).

Conclusion: Positive dipstick hematuria was associated with severity of albuminuria and renal function. A significant association was found between dipstick hematuria and increased risk of eGFR decline among patients with type 2 diabetes. Therefore, our results suggest that dipstick hematuria is perhaps indicative of more severe diabetic nephropathy. (C) 2017 Elsevier Inc. All rights reserved.

C1 [Mashitani, Tsuyoshi; Ishii, Hitoshi] Nara Med Univ, Dept Diabetol, Kashihara, Nara 6348521, Japan.

[Mashitani, Tsuyoshi] Nara Med Univ, Dept Internal Med 3, Kashihara, Nara 6348521, Japan.

[Hayashino, Yasuaki; Okamura, Shintaro; Kitatani, Masako; Furuya, Miyuki; Iburi, Tadao; Tsujii, Satoru] Tenri Hosp, Dept Endocrinol, Tenri, Nara 6328552, Japan.

RP Mashitani, T (reprint author), Nara Med Univ, Dept Diabetol, 840 Shijo Cho, Kashihara, Nara 6348552, Japan.

EM mashiyan@gmail.com

OI Tsujii, Satoru/0000-0002-1834-6515

FU Manpei Suzuki Diabetes Foundation; JSPS KAKENHI [25460641]

FX This study was partially supported by the Manpei Suzuki Diabetes

Foundation and JSPS KAKENHI (grant 25460641).

CR Adler AI, 2003, KIDNEY INT, V63, P225, DOI 10.1046/j.1523-1755.2003.00712.x

Akimoto T, 2008, NEPHRON CLIN PRACT, V109, pC119, DOI 10.1159/000145454

American Diabetes A, 2011, DIABETES CARE S1, V34, pS11

Cohen RA, 2003, NEW ENGL J MED, V348, P2330, DOI 10.1056/NEJMcp012694

Collins Allan J, 2014, Am J Kidney Dis, V63, pA7, DOI 10.1053/j.ajkd.2013.11.001

Coresh J, 2014, JAMA-J AM MED ASSOC, V311, P2518, DOI 10.1001/jama.2014.6634

DEFARIA JBL, 1988, CLIN NEPHROL, V30, P117

DOSEMECI M, 1990, AM J EPIDEMIOL, V132, P746, DOI 10.1093/oxfordjournals.aje.a115716

Eckardt KU, 2013, LANCET, V382, P158, DOI 10.1016/S0140-6736(13)60439-0

Gale DP, 2013, PEDIATR NEPHROL, V28, P1183, DOI 10.1007/s00467-012-2399-y

GALL MA, 1993, DIABETOLOGIA, V36, P1071, DOI 10.1007/BF02374501

Goto M, 2009, NEPHROL DIAL TRANSPL, V24, P3068, DOI 10.1093/ndt/gfp273

Greene T, 2014, AM J KIDNEY DIS, V64, P867, DOI 10.1053/j.ajkd.2014.08.019

Hayashino Y, 2016, ACTA DIABETOL, V53, P599, DOI 10.1007/s00592-015-0825-x

Hayashino Y, 2014, DIABETES CARE, V37, P2947, DOI 10.2337/dc14-1357

Iseki K, 1996, KIDNEY INT, V49, P800, DOI 10.1038/ki.1996.111

Kido R, 2010, AM J TRANSPLANT, V10, P1597, DOI 10.1111/j.1600-6143.2010.03077.x

Liang S, 2013, PLOS ONE, V8, DOI 10.1371/journal.pone.0064184

Mashitani T, 2015, ACTA DIABETOL, V52, P639, DOI 10.1007/s00592-015-0725-0

Mashitani T, 2014, DIABETES CARE, V37, P252, DOI 10.2337/dc13-0407

Matsuo S, 2009, AM J KIDNEY DIS, V53, P982, DOI 10.1053/j.ajkd.2008.12.034

Mohanram A, 2004, KIDNEY INT, V66, P1131, DOI 10.1111/j.1523-1755.2004.00863.x

Molitch Mark E, 2004, Diabetes Care, V27 Suppl 1, pS79

Moriya T, 2013, DIABETES CARE, V36, P2803, DOI 10.2337/dc12-2327

Oh SW, 2012, DIABETES RES CLIN PR, V97, P418, DOI 10.1016/j.diabres.2012.03.016

Okada T, 2013, NEPHROLOGY, V18, P563, DOI 10.1111/nep.12104

Palmer SC, 2011, AM J KIDNEY DIS, V58, P335, DOI 10.1053/j.ajkd.2011.06.006

Pham TT, 2007, AM J NEPHROL, V27, P322, DOI 10.1159/000102598

Sakata M, 2013, DIABETES RES CLIN PR, V100, pE39, DOI 10.1016/j.diabres.2013.01.031

SAMAK MJ, 2003, CIRCULATION, V108, P2154, DOI DOI 10.1161/01.CIR.0000095676.90936.80

Seino Y, 2010, J DIABETES INVEST, V1, P212, DOI 10.1111/j.2040-1124.2010.00074.x

Sheen YJ, 2014, WORLD J DIABETES, V5, P835, DOI 10.4239/wjd.v5.i6.835

Shen FC, 2012, DIABETIC MED, V29, P1178, DOI 10.1111/j.1464-5491.2012.03608.x

Shimizu M, 2013, DIABETES CARE, V36, P3655, DOI 10.2337/dc13-0298

Tone A, 2005, DIABETES RES CLIN PR, V69, P237, DOI 10.1016/j.diabres.2005.02.009

Vivante A, 2011, JAMA-J AM MED ASSOC, V306, P729, DOI 10.1001/jama.2011.1141

Yokoyama H, 2009, CLIN J AM SOC NEPHRO, V4, P1432, DOI 10.2215/CJN.06511208

Yuste C, 2015, PLOS ONE, V10, DOI 10.1371/journal.pone.0128575

Zoppini G, 2012, CLIN J AM SOC NEPHRO, V7, P401, DOI 10.2215/CJN.07650711

NR 39

TC 0

Z9 0

U1 1

U2 3

PU ELSEVIER SCIENCE INC

PI NEW YORK

PA 360 PARK AVE SOUTH, NEW YORK, NY 10010-1710 USA

SN 1056-8727

EI 1873-460X

J9 J DIABETES COMPLICAT

JI J. Diabetes Complications

PD JUL

PY 2017

VL 31

IS 7

BP 1079

EP 1084

DI 10.1016/j.jdiacomp.2017.04.016

PG 6

WC Endocrinology & Metabolism

SC Endocrinology & Metabolism

GA EY6JK

UT WOS:000404088700003

PM 28499960

DA 2019-01-08

ER

PT J

AU Secinti, E

Thompson, EJ

Richards, M

Gaysina, D

AF Secinti, Ekin

Thompson, Ellen J.

Richards, Marcus

Gaysina, Darya

TI Research Review: Childhood chronic physical illness and adult emotional

health - a systematic review and meta-analysis

SO JOURNAL OF CHILD PSYCHOLOGY AND PSYCHIATRY

LA English

DT Review

DE Depression; anxiety; chronic disorders; meta-analysis; paediatrics

ID QUALITY-OF-LIFE; ACUTE LYMPHOBLASTIC-LEUKEMIA; LONG-TERM SURVIVORS;

YOUNG-ADULTS; PSYCHOLOGICAL DISTRESS; POSTTRAUMATIC STRESS; PSYCHOSOCIAL

OUTCOMES; PSYCHIATRIC-DISORDERS; ADOLESCENT SURVIVORS; DEPRESSIVE

SYMPTOMS

AB Background: Childhood chronic physical illness is associated with a greater vulnerability for emotional problems (i.e. depression and anxiety) in childhood. However, little is known about life-long effects of childhood chronic physical illness on mental health. The present study aims to systematically review evidence for associations between eight chronic physical illnesses with childhood onset (arthritis, asthma, cancer, chronic renal failure, congenital heart disease, cystic fibrosis, type 1 diabetes, and epilepsy) and adult emotional problems. Methods: A database search of MEDLINE, PsycARTICLES, PsycINFO, and ScienceDirect was undertaken, and random effects meta-analyses were used to synthesise evidence from eligible studies. Results: In total, 37 studies were eligible for the systematic review (n = 45,733) and of these, 34 studies were included in the meta-analyses (n = 45,358). There were overall associations between childhood chronic physical illness and adult depression (OR = 1.31; 95% CI [1.12, 1.54]) and anxiety(OR = 1.47; 95% CI [1.13, 1.92]). Separate meta-analyses for childhood asthma, type 1 diabetes and cancer were also conducted, with cancer being significantly associated with adult depression (OR = 1.19; 95% CI [1.00, 1.42]). Conclusions: The effects of childhood chronic physical illness on the risk of emotional problems persist beyond childhood and adolescence. Mental health prevention and intervention strategies targeting children with chronic physical illnesses can have long-term benefits.

C1 [Secinti, Ekin; Thompson, Ellen J.; Gaysina, Darya] Univ Sussex, Sch Psychol, EDGE Lab, Pevensey 1, Brighton BN1 9QH, E Sussex, England.

[Secinti, Ekin] Indiana Univ Purdue Univ, Dept Psychol, Indianapolis, IN 46205 USA.

[Richards, Marcus] UCL, MRC, Unit Lifelong Hlth & Ageing, London, England.

RP Gaysina, D (reprint author), Univ Sussex, Sch Psychol, EDGE Lab, Pevensey 1, Brighton BN1 9QH, E Sussex, England.

EM D.Gaysina@sussex.ac.uk

OI Secinti, Ekin/0000-0001-7794-8500

FU Medical Research Council [MC_UU_12019/3]

CR Alwash RH, 2000, SEIZURE-EUR J EPILEP, V9, P412, DOI 10.1053/seiz.2000.0427

Baca CB, 2015, QUAL LIFE RES, V24, P1379, DOI 10.1007/s11136-014-0867-5

Baldin E, 2015, EPILEPSIA, V56, P1623, DOI 10.1111/epi.13123

BLAIR C, 1994, THORAX, V49, P798, DOI 10.1136/thx.49.8.798

BLANZ BJ, 1993, DIABETES CARE, V16, P1579, DOI 10.2337/diacare.16.12.1579

Borenstein M., 2009, INTRO METAANALYSIS

Brinkman TM, 2013, BRIT J CANCER, V109, P1373, DOI 10.1038/bjc.2013.428

Brown RT, 2003, J TRAUMA STRESS, V16, P309, DOI 10.1023/A:1024465415620

Chaney JM, 1999, J PEDIATR PSYCHOL, V24, P259, DOI 10.1093/jpepsy/24.3.259

Chen MH, 2014, J AFFECT DISORDERS, V156, P232, DOI 10.1016/j.jad.2013.10.053

Eddington AR, 2010, J ASTHMA, V47, P14, DOI 10.3109/02770900903427003

Fedele DA, 2009, J ASTHMA, V46, P835, DOI 10.3109/02770900903184229

Ferro MA, 2016, J ASTHMA, V53, P282, DOI 10.3109/02770903.2015.1104694

Ferro MA, 2015, HEALTH PSYCHOL, V34, P820, DOI 10.1037/hea0000226

Field AP, 2010, BRIT J MATH STAT PSY, V63, P665, DOI 10.1348/000711010X502733

Gianinazzi ME, 2013, PSYCHO-ONCOLOGY, V22, P2051, DOI 10.1002/pon.3249

Gledhill J, 2000, ARCH DIS CHILD, V83, P104, DOI 10.1136/adc.83.2.104

Gunn ME, 2013, J CANCER SURVIV, V7, P404, DOI 10.1007/s11764-013-0285-7

Harila MJ, 2011, J PEDIAT HEMATOL ONC, V33, P194, DOI 10.1097/MPH.0b013e3181ff0e2e

Higgins JPT, 2003, BRIT MED J, V327, P557, DOI 10.1136/bmj.327.7414.557

Huurre TM, 2002, EUR CHILD ADOLES PSY, V11, P85, DOI 10.1007/s007870200015

Kamibeppu K, 2010, J CANCER SURVIV, V4, P303, DOI 10.1007/s11764-010-0124-z

Kazak AE, 2010, J CLIN ONCOL, V28, P2002, DOI 10.1200/JCO.2009.25.9564

Kessler RC, 2007, CURR OPIN PSYCHIATR, V20, P359, DOI 10.1097/YCO.0b013e32816ebc8c

Kokkonen ER, 2001, NORD J PSYCHIAT, V55, P237

Kremer A. L., 2016, INT J ADOLESCENT MED

Lupien SJ, 2009, NAT REV NEUROSCI, V10, P434, DOI 10.1038/nrn2639

Ly TT, 2011, DIABETES CARE, V34, P2192, DOI 10.2337/dc11-0697

Mackie E, 2000, LANCET, V355, P1310, DOI 10.1016/S0140-6736(00)02112-7

Maunsell E, 2006, J CLIN ONCOL, V24, P2527, DOI 10.1200/JCO.200503.9297

Michel G, 2010, J CLIN ONCOL, V28, P1740, DOI 10.1200/JCO.2009.23.4534

Muller J, 2013, INT J CARDIOL, V165, P142, DOI 10.1016/j.ijcard.2011.08.005

Musliner KL, 2016, J AFFECT DISORDERS, V192, P199, DOI 10.1016/j.jad.2015.12.030

Northam EA, 2010, DIABETES CARE, V33, P1430, DOI 10.2337/dc09-2232

Palladino DK, 2013, J PEDIATR PSYCHOL, V38, P506, DOI 10.1093/jpepsy/jst002

Perrin JM, 2007, JAMA-J AM MED ASSOC, V297, P2755, DOI 10.1001/jama.297.24.2755

Pike NA, 2012, J CARDIOVASC NURS, V27, P539, DOI 10.1097/JCN.0b013e31822ce5f6

Pinquart M, 2011, J PEDIATR PSYCHOL, V36, P1003, DOI 10.1093/jpepsy/jsr042

Pinquart M, 2011, J PEDIATR PSYCHOL, V36, P375, DOI 10.1093/jpepsy/jsq104

Pinquart M, 2011, ACTA PAEDIATR, V100, P1069, DOI 10.1111/j.1651-2227.2011.02223.x

PLESS IB, 1993, PEDIATRICS, V91, P1131

Purdy Jana, 2013, Yale Journal of Biology and Medicine, V86, P15

R Core Team, 2013, R LANG ENV STAT COMP

Schwartz L, 2006, J PEDIATR PSYCHOL, V31, P356, DOI 10.1093/jpepsy/jsj018

Seitz DCM, 2010, EUR J CANCER, V46, P1596, DOI 10.1016/j.ejca.2010.03.001

Servitzoglou M, 2008, SUPPORT CARE CANCER, V16, P29, DOI 10.1007/s00520-007-0278-z

Sivertsen B, 2014, BMC ENDOCR DISORD, V14, DOI 10.1186/1472-6823-14-83

Stam H, 2006, PSYCHO-ONCOL, V15, P31, DOI 10.1002/pon.920

STEIN REK, 1993, J PEDIATR-US, V122, P342, DOI 10.1016/S0022-3476(05)83414-6

Stroup DF, 2000, JAMA-J AM MED ASSOC, V283, P2008, DOI 10.1001/jama.283.15.2008

Sundberg KK, 2010, PEDIATR BLOOD CANCER, V55, P337, DOI 10.1002/pbc.22492

TEBBI CK, 1990, DIABETES CARE, V13, P98, DOI 10.2337/diacare.13.2.98

TETA MJ, 1986, J CHRON DIS, V39, P751, DOI 10.1016/0021-9681(86)90158-X

Tluczek A, 2014, J CYST FIBROS, V13, P227, DOI 10.1016/j.jcf.2013.10.001

van der Lee JH, 2007, JAMA-J AM MED ASSOC, V297, P2741, DOI 10.1001/jama.297.24.2741

Verhoof E. J. A., 2014, CHILD ADOLESCENT PSY, V8, P1

von Elm E, 2007, BRIT MED J, V335, P806

Zahn-Waxler C, 2000, DEV PSYCHOPATHOL, V12, P443, DOI 10.1017/S0954579400003102

Zeltzer LK, 1997, J CLIN ONCOL, V15, P547, DOI 10.1200/JCO.1997.15.2.547

Zeltzer LK, 2008, CANCER EPIDEM BIOMAR, V17, P435, DOI 10.1158/1055-9965.EPI-07-2541

NR 60

TC 4

Z9 4

U1 2

U2 25

PU WILEY

PI HOBOKEN

PA 111 RIVER ST, HOBOKEN 07030-5774, NJ USA

SN 0021-9630

EI 1469-7610

J9 J CHILD PSYCHOL PSYC

JI J. Child Psychol. Psychiatry

PD JUL

PY 2017

VL 58

IS 7

BP 753

EP 769

DI 10.1111/jcpp.12727

PG 17

WC Psychology, Developmental; Psychiatry; Psychology

SC Psychology; Psychiatry

GA EX7CG

UT WOS:000403404000002

PM 28449285

OA Bronze

DA 2019-01-08

ER

PT J

AU Tan, ML

Tan, CS

Griva, K

Lee, YS

Lee, J

Tai, ES

Khoo, EY

Wee, HL

AF Tan, Maudrene L.

Tan, Chuen S.

Griva, Konstadina

Lee, Yung S.

Lee, Jeannette

Tai, E. S.

Khoo, Eric Y.

Wee, Hwee-Lin

TI Factors associated with diabetes-related distress over time among

patients with T2DM in a tertiary hospital in Singapore

SO BMC ENDOCRINE DISORDERS

LA English

DT Article

DE Diabetes-related distress; Diabetes; Longitudinal; Excessive daytime

sleepiness

ID OBSTRUCTIVE SLEEP-APNEA; QUALITY-OF-LIFE; PSYCHOLOGICAL DISTRESS;

DEPRESSIVE SYMPTOMS; DAYTIME SLEEPINESS; TYPE-2; MELLITUS; ADULTS;

QUESTIONNAIRES; POPULATION

AB Background: Persistent diabetes-related distress (DRD) is experienced by patients with Type 2 Diabetes Mellitus. Knowing factors associated with persistent DRD will aid clinicians in prioritising interventions efforts.

Methods: A total of 216 patients were recruited from a tertiary hospital in Singapore, an Asian city state, and followed for 1.5 years (2011-2014). Data was collected by self-completed questionnaires assessing DRD (measured by the Problem Areas in Diabetes score) and other psychosocial aspects such as social support, presenteeism, depression, health-related quality of life (HRQoL) and excessive daytime sleepiness (EDS) at three time points. Clinical data (body-mass-index and glycated haemoglobin) was obtained from medical records. Change score was calculated for each clinical and psychosocial variable to capture changes in these variables from baseline. Generalized Linear Model with Generalized Estimating Equation method was used to assess whether baseline and change scores in clinical and psychosocial are associated with DRD over time.

Results: Complete data was available for 73 patients, with mean age 44 (SD 12.5) years and 67% males. Persistent DRD was experienced by 210% of the patients. In the final model, baseline HRQoL (OR = 0.56, p < 0.05) and change score of EDS (OR = 1.22, p < 0.05) was significantly associated with DRD over time.

Conclusions: EDS might be a surrogate marker for persistent DRD and should be explored in larger samples of population to confirm the findings from this study.

C1 [Tan, Maudrene L.; Tan, Chuen S.; Lee, Jeannette; Tai, E. S.; Wee, Hwee-Lin] Natl Univ Singapore, Saw Swee Hock Sch Publ Hlth, Singapore, Singapore.

[Griva, Konstadina] Natl Univ Singapore, Dept Psychol, Singapore, Singapore.

[Lee, Yung S.] Natl Univ Singapore, Yong Loo Lin Sch Med, Dept Pediat, Singapore, Singapore.

[Lee, Yung S.] Natl Univ Hlth Syst, Khoo Teck Puat Natl Univ Childrens Med Inst, Div Paediat Endocrinol & Diabet, Singapore, Singapore.

[Lee, Yung S.] Agcy Sci Technol & Res, Singapore Inst Clin Sci, Singapore, Singapore.

[Tai, E. S.; Khoo, Eric Y.] Natl Univ Hlth Syst, Dept Med, Singapore, Singapore.

[Wee, Hwee-Lin] Natl Univ Singapore, Dept Pharm, 10 Kent Ridge Crescent, Singapore 119260, Singapore.

RP Wee, HL (reprint author), Natl Univ Singapore, Saw Swee Hock Sch Publ Hlth, Singapore, Singapore.; Wee, HL (reprint author), Natl Univ Singapore, Dept Pharm, 10 Kent Ridge Crescent, Singapore 119260, Singapore.

EM phawhl@nus.edu.sg

RI Wee, Hwee-Lin/D-4126-2012; Griva, Konstadina/A-6549-2018; Lee, Yung

Seng/F-2169-2014

OI Wee, Hwee-Lin/0000-0002-7150-1801; Lee, Yung Seng/0000-0002-1253-0557;

Tan, Chuen Seng/0000-0002-6513-2309

FU Ministry of Education Singapore Academic Research Fund [FY2011-FRC3-007]

FX This work was supported by the Ministry of Education Singapore Academic

Research Fund Tier 1 (Grant No.: FY2011-FRC3-007).

CR Bradley C, 1999, QUAL LIFE RES, V8, P79, DOI 10.1023/A:1026485130100

Burns RJ, 2015, DIABETIC MED, V32, P1272, DOI 10.1111/dme.12860

Chasens Eileen R, 2008, Issues Ment Health Nurs, V29, P1134, DOI 10.1080/01612840802319878

Co MA, 2015, J DIABETES COMPLICAT, V29, P378, DOI 10.1016/j.jdiacomp.2015.01.009

Dixon JB, 2007, OBESITY, V15, P2504, DOI 10.1038/oby.2007.297

Ehrmann D, 2015, DIABETIC MED, V32, P1264, DOI 10.1111/dme.12861

Eriksson AK, 2008, DIABETIC MED, V25, P834, DOI 10.1111/j.1464-5491.2008.02463.x

FIFE BL, 1994, SOC SCI MED, V38, P309, DOI 10.1016/0277-9536(94)90400-6

Fisher L, 2008, DIABETIC MED, V25, P1096, DOI 10.1111/j.1464-5491.2008.02533.x

Fisher L, 2009, DIABETIC MED, V26, P622, DOI 10.1111/j.1464-5491.2009.02730.x

Hermanns N, 2006, DIABETOLOGIA, V49, P469, DOI 10.1007/s00125-005-0094-2

JOHNS MW, 1991, SLEEP, V14, P540, DOI 10.1093/sleep/14.6.540

Karlsen B, 2012, J ADV NURS, V68, P391, DOI 10.1111/j.1365-2648.2011.05751.x

Keskin A, 2015, CHINESE MED J-PEKING, V128, P3292, DOI 10.4103/0366-6999.171415

Kessler RC, 2002, PSYCHOL MED, V32, P959, DOI 10.1017/S0033291702006074

Koutsourelakis I, 2008, J SLEEP RES, V17, P437, DOI 10.1111/j.1365-2869.2008.00667.x

Lipscombe C, 2015, J AFFECT DISORDERS, V188, P160, DOI 10.1016/j.jad.2015.08.003

Lopes LA, 2005, DIABETES CARE, V28, P2633, DOI 10.2337/diacare.28.11.2633

Medeiros C, 2013, ARQ BRAS ENDOCRINOL, V57, P425, DOI 10.1590/S0004-27302013000600003

Pamidi S, 2011, SLEEP MED, V12, P827, DOI 10.1016/j.sleep.2011.08.003

POLONSKY WH, 1995, DIABETES CARE, V18, P754, DOI 10.2337/diacare.18.6.754

REILLY MC, 1993, PHARMACOECONOMICS, V4, P353, DOI 10.2165/00019053-199304050-00006

Resnick HE, 2003, DIABETES CARE, V26, P702, DOI 10.2337/diacare.26.3.702

Stewart A., 1992, MEASURING FUNCTIONIN

Tan LSM, 2015, QUAL LIFE RES, V24, P153, DOI 10.1007/s11136-014-0747-z

WHOQOL Group, 1998, WHOQOL BREF INTR ADM

Young-Hyman D, 2016, DIABETES CARE, V39, P2126, DOI 10.2337/dc16-2053

NR 27

TC 0

Z9 0

U1 1

U2 7

PU BIOMED CENTRAL LTD

PI LONDON

PA 236 GRAYS INN RD, FLOOR 6, LONDON WC1X 8HL, ENGLAND

SN 1472-6823

J9 BMC ENDOCR DISORD

JI BMC Endocr. Disord.

PD JUN 23

PY 2017

VL 17

AR 36

DI 10.1186/s.12902-017-0184-4

PG 6

WC Endocrinology & Metabolism

SC Endocrinology & Metabolism

GA EZ0QD

UT WOS:000404409700001

PM 28645273

OA DOAJ Gold

DA 2019-01-08

ER

PT J

AU Yoda, T

Yokoyama, K

Suzuki, H

Hirao, T

AF Yoda, Takeshi

Yokoyama, Katsunori

Suzuki, Hiromi

Hirao, Tomohiro

TI Relationship Between Long-term Flooding and Serious Mental Illness After

the 2011 Flood in Thailand

SO DISASTER MEDICINE AND PUBLIC HEALTH PREPAREDNESS

LA English

DT Article

DE flood victims; serious mental illness; psychological distress; risk

factors

ID EPIDEMIOLOGIC EVIDENCE; HEALTH; IMPACTS

AB Objective: The present study aimed to evaluate the long-term psychological effects of the 2011 flood in Thailand on flood victims and unaffected residents, taking into account risk factors such as floodwater levels in the house, period of flooding, and disruption to essential services.

Methods: Surveys were conducted in Salaya and Nakhon Chaisri subdistrict, Nakhonpatom Province, from May to June 2012. Approximately 400 households were selected. Measures of sociodemographic variables, exposure to flooding, incidents (eg, disruption of services), and outcome variables (Kessler 10) were recorded.

Results: A total of 407 respondents completed the survey. The results of the univariate analysis showed that possible serious mental illness was approximately 1.5 times higher in flood victims than in unaffected respondents. After adjustment for potential confounding factors, significant associations were found for disruption of essential services and hypertension or diabetes with possible serious mental illness.

Conclusions: Disruption of essential services and chronic illness may affect psychological distress among flood victims. Public health agencies should develop and evaluate strategies to improve risk communication and psychological support for flood victims.

C1 [Yoda, Takeshi; Yokoyama, Katsunori; Suzuki, Hiromi; Hirao, Tomohiro] Kagawa Univ, Dept Publ Hlth, Fac Med, 1750-1 Ikenobe, Miki, Kagawa 7610793, Japan.

RP Yoda, T (reprint author), Kagawa Univ, Dept Publ Hlth, Fac Med, 1750-1 Ikenobe, Miki, Kagawa 7610793, Japan.

EM tyoda@med.kagawa-u.ac.jp

FU MIU memorial foundation [H23]

FX We thank Orada Anotaipaiboon and Piyachatr Tragoolvongse for their

assistance in the questionnaire research and Orawan Quansri for her

friendly support. This research was supported by funding from the MIU

memorial foundation grant number H23.

CR Ahern M, 2005, EPIDEMIOL REV, V27, P36, DOI 10.1093/epirev/mxi004

Ginexi EM, 2000, AM J COMMUN PSYCHOL, V28, P495, DOI 10.1023/A:1005188515149

Kessler RC, 2002, PSYCHOL MED, V32, P959, DOI 10.1017/S0033291702006074

Mason V, 2010, PSYCHOL HEALTH MED, V15, P61, DOI 10.1080/13548500903483478

Ohl CA, 2000, BRIT MED J, V321, P1167, DOI 10.1136/bmj.321.7270.1167

Paranjothy S, 2011, BMC PUBLIC HEALTH, V11, DOI 10.1186/1471-2458-11-145

Piyaphanee W, 2012, SE ASIAN J TROP MED, V43, P1193

Sakurai K, 2011, PSYCHIAT CLIN NEUROS, V65, P434, DOI 10.1111/j.1440-1819.2011.02236.x

Bich TH, 2011, GLOBAL HEALTH ACTION, V4, DOI 10.3402/gha.v4i0.6356

Wisitwong A, 2010, NURS HEALTH SCI, V12, P4, DOI 10.1111/j.1442-2018.2009.00504.x

NR 10

TC 0

Z9 0

U1 1

U2 2

PU CAMBRIDGE UNIV PRESS

PI NEW YORK

PA 32 AVENUE OF THE AMERICAS, NEW YORK, NY 10013-2473 USA

SN 1935-7893

EI 1938-744X

J9 DISASTER MED PUBLIC

JI Dis. Med. Public Health Prep.

PD JUN

PY 2017

VL 11

IS 3

BP 300

EP 304

DI 10.1017/dmp.2016.148

PG 5

WC Public, Environmental & Occupational Health

SC Public, Environmental & Occupational Health

GA FA7WM

UT WOS:000405658100005

PM 27927268

DA 2019-01-08

ER

PT J

AU Roberts, DP

Ward, BM

Russell, DJ

O'Sullivan, BG

AF Roberts, Diane Patricia

Ward, Bernadette Maree

Russell, Deborah Jane

O'Sullivan, Belinda Gabrielle

TI Accessibility and outcomes from a rural diabetes nurse-educator led

self-management program

SO AUSTRALIAN JOURNAL OF ADVANCED NURSING

LA English

DT Article

DE access; diabetes nurse educator; diabetes self-management; health

outcomes; rural; primary health care

ID RESIDENTS

AB Objective

To investigate factors associated with access to, and health outcomes of, a diabetes nurse-educator led self-management program for rural Australians with diabetes.

Design

Retrospective cohort study

Setting

A rural community-health service with a dispersed catchment of 10,000 population.

Subjects

Clients diagnosed with type 2 diabetes mellitus and referred to the program between April 2008 and December 2012.

Intervention

A diabetes self-management program comprising an initial assessment, a group education session, and 3, 6, and 12 month clinical reviews.

Main outcome measures

Program attendance after referral; and achievement of management goals for HbA1c, BMI, total cholesterol, quality of life and psychological distress.

Results

Ninety-four percent (n= 219/232) of referred clients attended at least once. Multivariate logistic regression showed that attending at least once was significantly associated with living within 25km of the service. Non-smokers/former smokers, general practitioner-referred clients and those with diabetes management plans were significantly more likely to attend three or more sessions. At 12 months clients showed significant improvements in cholesterol, BMI, quality of life and psychological distress.

Conclusion

This study demonstrates that diabetes nurse-educator led self-management programs which adapt to their rural contexts - including geographically dispersed catchment populations and resource constraints - provide highly accessible services meeting the needs of most. Nevertheless, some groups (cigarette smokers, those living furthest from the service) may remain marginalised and less able to access services. Improvements in health outcomes for these clients may require further adaptation of models of care to better target their health care needs.

C1 [Roberts, Diane Patricia; Ward, Bernadette Maree; Russell, Deborah Jane] Monash Univ, Monash Rural Hlth, Ctr Res Excellence CRE Rural & Remote Primary Hlt, POB 666, Bendigo, Vic, Australia.

[O'Sullivan, Belinda Gabrielle] Monash Univ, Monash Rural Hlth, POB 666, Bendigo, Vic, Australia.

RP Roberts, DP (reprint author), Monash Univ, Monash Rural Hlth, Ctr Res Excellence CRE Rural & Remote Primary Hlt, POB 666, Bendigo, Vic, Australia.

EM diane.p.roberts@monash.edu; bernadette.ward@monash.edu;

deborah.russell@monash.edu; belinda.osullivan@monash.edu

FU Australian Government Department of Health and Ageing

FX The research reported in this paper was a project of the Australian

Primary Health Care Research Institute which was supported by a grant

from the Australian Government Department of Health and Ageing.

CR Arcury TA, 2005, HEALTH SERV RES, V40, P135, DOI 10.1111/j.1475-6773.2005.00346.x

Australian Bureau of Statistics Microdata, 2011, AUSTR HLTH SURV COR

Australian Institute for Primary Care & Ageing, 2012, IMM CLIENT OUTC COMM

Australian Institute of Health and Welfare, 2008, IND CHRON DIS THEIR

Australian Institute of Health and Welfare, 2012, AUSTR HLTH 2012

Australian Institute of Health and Welfare, 2009, KEY IND PROGR CHRON

Colagiuri R., 2009, NATL EVIDENCE BASED

Hawthorne G, 2013, AUST NZ J PUBL HEAL, V37, P7

Kessler RC, 2002, PSYCHOL MED, V32, P959, DOI 10.1017/S0033291702006074

McGrail MR, 2015, BMC HEALTH SERV RES, V15, DOI 10.1186/s12913-015-0880-6

National Health and Medical Research Council, 2001, NAT EV BAS GUID MAN

Primary Health Care Research and Information Service, 2012, NAT PERF IND DIV GEN

Russell DJ, 2013, AUST J RURAL HEALTH, V21, P61, DOI 10.1111/ajr.12023

Travaglia J, 2009, CLIN AUDIT COMPREHEN

Vos T, 2015, LANCET, V386, P743, DOI 10.1016/S0140-6736(15)60692-4

WHO, 2014, GLOBAL STATUS REPORT ON VIOLENCE PREVENTION 2014, P1

World Health Organisation: Obesity: Preventing and Managing a Global Epidemic Geneva: World Health Organisation, 2000, TECHNICAL REPORT SER, V894

NR 17

TC 0

Z9 0

U1 1

U2 5

PU AUSTRALIAN NURSING FEDERATION

PI KINGSTON

PA PO BOX 4239, KINGSTON, ACT 2604, AUSTRALIA

SN 0813-0531

EI 1447-4328

J9 AUST J ADV NURS

JI Aust. J. Adv. Nurs.

PD JUN-AUG

PY 2017

VL 34

IS 4

BP 26

EP 33

PG 8

WC Nursing

SC Nursing

GA FA0LG

UT WOS:000405125400003

DA 2019-01-08

ER

PT J

AU Li, DN

Elliott, T

Klein, G

Ur, E

Tang, TS

AF Li, Danni

Elliott, Tom

Klein, Gerri

Ur, Ehud

Tang, Tricia S.

TI Diabetes Nurse Case Management in a Canadian Tertiary Care Setting:

Results of a Randomized Controlled Trial

SO CANADIAN JOURNAL OF DIABETES

LA English

DT Article

DE diabetes nurse case manager; randomized controlled trial; tertiary care

setting; diabetes distress; psychosocial outcomes

ID CARDIOVASCULAR RISK-FACTORS; SELF-CARE; GLYCEMIC CONTROL; DISEASE;

DISTRESS; DEPRESSION; PROGRAMS; VALIDITY; SCALE; LIFE

AB Objectives: To examine the effects of a 6-month nurse case manager (NCM) intervention compared to standard care (SC) on glycemic control and diabetes distress in a Canadian tertiary-care setting.

Methods: We recruited 140 adults with type 2 diabetes and glycated hemoglobin (AlC) levels >8% (64 mmol/mol) from 2 tertiary care facilities and randomized them to: 1) a 6-month NCM intervention in addition to SC or 2) SC by the primary endocrinologists. Assessments were conducted at baseline and at 6 months. Primary outcomes included AlC levels and diabetes distress scores (DDS). Secondary outcomes included body mass index, blood pressure, diabetes-related behaviour measures, depressive symptoms, self-motivation and perception of support.

Results: At the 6-month follow up, the NCM group experienced larger reductions in AlC levels of -0.73% compared to the SC group (p=0.027; n=134). The NCM group also showed an additional reduction of -0.40 (26% reduction) in DDS compared to those in the SC group (p=0.001; n=134). The NCM group had lower blood pressure, ate more fruit and vegetables, exercised more, checked their feet more frequently, were more motivated, were less depressed and perceived more support. There were no changes and no group differences in terms of body mass index, medication compliance or frequency of testing.

Conclusions: Compared to SC, NCM intervention was more effective in improving glycemic control and reducing diabetes distress. It is, therefore, a viable adjunct to standard diabetes care in the tertiary care setting, particularly for patients at high risk and with poor control. (C) 2017 Canadian Diabetes Association.

C1 [Li, Danni; Elliott, Tom; Klein, Gerri; Ur, Ehud; Tang, Tricia S.] Univ British Columbia, Dept Med, Div Endocrinol, Vancouver, BC, Canada.

[Elliott, Tom; Klein, Gerri] BC Diabet, Vancouver, BC, Canada.

RP Tang, TS (reprint author), Univ British Columbia, 2775 Laurel St,Suite 10211, Vancouver, BC V5Z 1M9, Canada.

EM tricia.tang@vch.ca

FU British Columbia Endocrine Research Foundation (Sanofi-Aventis Canada);

Sanofi; NovoNordisk; Boehringer-Ingelheim; Astra-Zeneca; Merck

FX This study was supported by the British Columbia Endocrine Research

Foundation (itself supported by a grant from Sanofi-Aventis Canada).

Clinical trial registry #NCT01659294, clinicaltrials.gov. Dr. Tom

Elliott has received funding for giving lectures and symposia and

attending advisory boards for the following companies: Sanofi,

NovoNordisk, Boehringer-Ingelheim and Astra-Zeneca. Dr. Ehud Ur has

received funding for giving lectures and symposia and attending advisory

boards for the following companies: Sanofi, NovoNordisk,

Boehringer-Ingelheim, Merck and Astra-Zeneca.

CR Arsie MP, 2000, ACTA DIABETOL, V37, P1, DOI 10.1007/s005920070028

Bernard S, 2013, UNDERSTAND YOUR DIAB

Braga MFB, 2010, CAN J CARDIOL, V26, P297, DOI 10.1016/S0828-282X(10)70393-7

Couch Carl, 2003, Proc (Bayl Univ Med Cent), V16, P336

Drabik A, 2012, DIABETES RES CLIN PR, V95, P194, DOI 10.1016/j.diabres.2011.09.015

Fanning EL, 2004, DIABETES CARE, V27, P1638, DOI 10.2337/diacare.27.7.1638

Fisher L, 2010, DIABETES CARE, V33, P23, DOI 10.2337/dc09-1238

Funnell M, 2004, CLIN DIABETES, V223, P123

Gabbay RA, 2013, J DIABETES, V5, P349, DOI 10.1111/1753-0407.12030

Hibbard JH, 2004, HEALTH SERV RES, V39, P1005, DOI 10.1111/j.1475-6773.2004.00269.x

Ishani A, 2011, DIABETES CARE, V34, P1689, DOI 10.2337/dc10-2121

Joo JY, 2012, PROF CASE MANAG, V17, P72, DOI 10.1097/NCM.0b013e318243d473

Kroenke K, 2001, J GEN INTERN MED, V16, P606, DOI 10.1046/j.1525-1497.2001.016009606.x

Leyva B, 2011, ETHNIC DIS, V21, P322

MORISKY DE, 1986, MED CARE, V24, P67, DOI 10.1097/00005650-198601000-00007

Norris SL, 2002, AM J PREV MED, V22, P15, DOI 10.1016/S0749-3797(02)00423-3

Pandit AU, 2014, J EPIDEMIOL COMMUN H, V68, P557, DOI 10.1136/jech-2013-203063

Paula A, 2011, CONTEMP NURSE, V39, P157, DOI 10.5172/conu.2011.157

Pimouguet C, 2011, CAN MED ASSOC J, V183, pE115, DOI 10.1503/cmaj.091786

Polonsky WH, 2005, DIABETES CARE, V28, P626, DOI 10.2337/diacare.28.3.626

Saxena S, 2007, J AMBUL CARE MANAG, V30, P218

Shojania KG, 2006, JAMA-J AM MED ASSOC, V296, P427, DOI 10.1001/jama.296.4.427

Spitzer RL, 1999, JAMA-J AM MED ASSOC, V282, P1737, DOI 10.1001/jama.282.18.1737

Sutherland D, 2009, J CLIN NURS, V18, P2978, DOI 10.1111/j.1365-2702.2009.02900.x

Tang TS, 2008, DIABETES EDUCATOR, V34, P266, DOI 10.1177/0145721708315680

The Diabetes Control and Complications Trial Research Group, 1993, NEW ENGL J MED, V329, P977, DOI [10.1056/NEJM199309303291401, DOI 10.1056/NEJM199309303291401]

Thompson DM, 1999, CAN MED ASSOC J, V161, P959

Toobert DJ, 2000, DIABETES CARE, V23, P943, DOI 10.2337/diacare.23.7.943

Welch G, 2010, DIABETES RES CLIN PR, V88, P1, DOI 10.1016/j.diabres.2009.12.026

NR 29

TC 0

Z9 0

U1 1

U2 17

PU ELSEVIER SCIENCE BV

PI AMSTERDAM

PA PO BOX 211, 1000 AE AMSTERDAM, NETHERLANDS

SN 1499-2671

J9 CAN J DIABETES

JI Can. J. Diabetes

PD JUN

PY 2017

VL 41

IS 3

BP 297

EP 304

DI 10.1016/j.jcjd.2016.10.012

PG 8

WC Endocrinology & Metabolism

SC Endocrinology & Metabolism

GA EY9LG

UT WOS:000404319500007

PM 28318938

DA 2019-01-08

ER

PT J

AU Daniels, J

Turner-Cobb, JM

AF Daniels, J.

Turner-Cobb, J. M.

TI Adjuvant psychological therapy in long-term endocrine conditions

SO CLINICAL ENDOCRINOLOGY

LA English

DT Article

DE adjuvant; long-term endocrinological conditions; psychological; therapy

ID QUALITY-OF-LIFE; ADRENAL INSUFFICIENCY; HEALTH ANXIETY; STRESS; DISEASE;

MODEL; DEPRESSION; CHALLENGE; BEHAVIOR

AB Consideration of psychological distress in long-term endocrine conditions is of vital importance given the prevalence of anxiety and depression in such disorders. Poor mental health can lead to compromised self-care, higher utilization of health services, lower rates of adherence, reduced quality of life and ultimately poorer outcomes. Adjuvant psychological therapy offers an effective resource to reduce distress in-endocrine conditions. While the vast majority of work in this area has focused on-psychological screening and intervention in diabetes, identification and recognition of psychological distress are equally important in other endocrinological conditions, with supportive evidence in polycystic ovary syndrome and Addison's disease. Referral pathways and recommendations set out by UK guidelines and the Department of Health mandate requires greater attention across a wider range of long-term endocrine conditions to facilitate improved quality of life and health outcome.

C1 [Daniels, J.] Univ Bath, Dept Psychol, Bath, Avon, England.

[Turner-Cobb, J. M.] Bournemouth Univ, Res Ctr Behav Change, Dept Psychol, Bournemouth, Dorset, England.

RP Daniels, J (reprint author), Univ Bath, Dept Psychol, Bath, Avon, England.

EM j.daniels@bath.ac.uk

CR Aulinas A, 2014, EXPERT REV PHARM OUT, V14, P873, DOI 10.1586/14737167.2014.963559

Bleicken B, 2010, CLIN ENDOCRINOL, V72, P297, DOI 10.1111/j.1365-2265.2009.03596.x

Care Quality Commission, 2016, MY DIAB MY CAR

Daniels J, 2017, PSYCHOL PSYCHOTHER-T, V90, P502, DOI 10.1111/papt.12118

Daniels J, 2017, BEHAV COGN PSYCHOTH, V45, P419, DOI 10.1017/S1352465817000182

Department of Health, 2013, MAND GOV NHS ENGL AP

Department of Health, 2011, TALK THER 4 YEAR PLA

ENGEL GL, 1977, SCIENCE, V196, P129, DOI 10.1126/science.847460

European Commission, STAT MENT HLTH EUR U

Fischer S, 2016, PSYCHONEUROENDOCRINO, V63, P68, DOI 10.1016/j.psyneuen.2015.09.018

Golden SH, 2007, CURR DIABETES REV, V3, P252, DOI 10.2174/157339907782330021

Henningsen P, 2015, J PSYCHOSOM RES, V79, P362, DOI 10.1016/j.jpsychores.2015.09.003

Kaye JM, 2006, CLIN ENDOCRINOL, V65, P282, DOI 10.1111/j.1365-2265.2006.02589.x

Kroenke K, 2001, J GEN INTERN MED, V16, P606, DOI 10.1046/j.1525-1497.2001.016009606.x

LEVENTHAL H, 1983, HEALTH EDUC QUART, V10, P3, DOI 10.1177/109019818301000101

Lutgendorf SK, 2003, BRAIN BEHAV IMMUN, V17, P225, DOI 10.1016/S0889-1591(03)00033-3

McEwen BS, 1998, ANN NY ACAD SCI, V840, P33, DOI 10.1111/j.1749-6632.1998.tb09546.x

Mental Health Task Force, 2016, 5 YEAR FORW VIEW MEN

Morrison V, 2016, INTRO HLTH PSYCHOL

National Institute for Care and health Excellence, 2015, TYP 1 DIAB AD DIAGN

Naylor C., 2012, LONG TERM CONDITIONS

Naylor S, 2016, BRINGING TOGETHER PH

Nishikawa T, 2014, INTERNAL MED, V53, P169, DOI 10.2169/internalmedicine.53.1527

Power M, 1998, SOC SCI MED, V46, P1569

Press MJ, 2017, NEW ENGL J MED, V376, P405, DOI 10.1056/NEJMp1614134

Rofey DL, 2009, J PEDIATR PSYCHOL, V34, P156, DOI 10.1093/jpepsy/jsn057

Sage S, 2008, CBT CHRONIC ILLNESS

Salkovskis PM, 2002, PSYCHOL MED, V32, P843, DOI 10.1017/S0033291702005822

Sepa A, 2005, DIABETES CARE, V28, P290, DOI 10.2337/diacare.28.2.290

Sonino N, 2004, PSYCHOTHER PSYCHOSOM, V73, P78, DOI 10.1159/000075538

Sonino N, 2015, J ROY COLL PHYS EDIN, V45, P55, DOI [10.4997/JRCPE.2015.413, 10.4997/JRCPE.2015.113]

Spitzer RL, 2006, ARCH INTERN MED, V166, P1092, DOI 10.1001/archinte.166.10.1092

Sterling P, 1988, HDB LIFE STRESS COGN, P629, DOI [10.1016/0005-7967(90)90076-U, DOI 10.1016/0005-7967(90)90076-U]

Tyrer P, 2014, LANCET, V383, P219, DOI 10.1016/S0140-6736(13)61905-4

Tyrer P, 2011, J PSYCHOSOM RES, V71, P392, DOI 10.1016/j.jpsychores.2011.07.004

Uchendu C, 2017, DIABETIC MED, V34, P328, DOI 10.1111/dme.13195

WARE JE, 1992, MED CARE, V30, P473, DOI 10.1097/00005650-199206000-00002

White CA, 2001, COGNITIVE BEHAV THER

World Health Organization, 2017, EUR MENT HLTH ACT PL

ZIGMOND AS, 1983, ACTA PSYCHIAT SCAND, V67, P361, DOI 10.1111/j.1600-0447.1983.tb09716.x

NR 40

TC 1

Z9 1

U1 1

U2 1

PU WILEY

PI HOBOKEN

PA 111 RIVER ST, HOBOKEN 07030-5774, NJ USA

SN 0300-0664

EI 1365-2265

J9 CLIN ENDOCRINOL

JI Clin. Endocrinol.

PD JUN

PY 2017

VL 86

IS 6

BP 772

EP 777

DI 10.1111/cen.13341

PG 6

WC Endocrinology & Metabolism

SC Endocrinology & Metabolism

GA EY1GM

UT WOS:000403714100002

PM 28370206

DA 2019-01-08

ER

PT J

AU Zhou, HH

Zhu, JY

Liu, L

Li, F

Fish, AF

Chen, T

Lou, QQ

AF Zhou, Huanhuan

Zhu, Junya

Liu, Lin

Li, Fan

Fish, Anne F.

Chen, Tao

Lou, Qingqing

TI Diabetes-related distress and its associated factors among patients with

type 2 diabetes mellitus in China

SO PSYCHIATRY RESEARCH

LA English

DT Article

DE Chinese; Diabetes; Diabetes-related distress; Self-efficacy; Sleep

timep, Insulin, Physical activity

ID QUALITY-OF-LIFE; EMOTIONAL DISTRESS; GLYCEMIC CONTROL; SLEEP QUALITY;

INSULIN THERAPY; PROBLEM AREAS; DEPRESSION; SCALE; CARE; ADULTS

AB Diabetes-related distress is one of the psychological disorders affecting patients with diabetes, yet there are few studies about diabetes-related distress in Chinese patients. To assess the level of psychological distress and examine its associated factors, we conducted a cross-sectional analysis of patients with type 2 diabetes mellitus from a Chinese tertiary hospital. The Diabetes Distress Scale (DDS) and the General Self-Efficacy Scale (GSES) were administered. There were 210 (57.85%) patients with little or no diabetes-related distress, 84 (23.14%) with moderate diabetes-related distress and 69 (19.01%) with high diabetes-related distress. Stepwise multiple linear regression showed that sleep time was significantly related to the DDS total score and the subscale scores of emotional burden (EB) (beta=-0.190, -0.379), respectively. GSES was associated with the DDS total score (beta=-0.128) and the EB score (beta=-0.153). Oral medication plus insulin was significantly related to regimen-related distress (RD) (beta=0.137), physician-related distress (PD) (beta=0.152) and interpersonal distress (ID) (beta=0.103). Physical activity (beta=-0.185) and making meal plan with health care professionals(HCP) (beta=-0.169) were associated with RD. The prevalence of diabetes-related distress among patients with type 2 diabetes mellitus was high in China. DDS and EB were associated with poorer sleep time and lower self-efficacy. Interventions to improve sleep are needed. Qualitative and longitudinal studies are required to understand why type 2 diabetic patients are not getting enough sleep.

C1 [Zhou, Huanhuan; Liu, Lin; Li, Fan; Lou, Qingqing] Nanjing Univ Chinese Med, Affiliated Hosp Integrated Tradit Chinese & Weste, Nanjing, Jiangsu, Peoples R China.

[Zhou, Huanhuan; Liu, Lin; Li, Fan; Lou, Qingqing] Jiangsu Prov Acad Tradit Chinese Med, Nanjing, Jiangsu, Peoples R China.

[Zhou, Huanhuan] Nanjing Univ Chinese Med, Nursing Coll, Nanjing, Jiangsu, Peoples R China.

[Zhu, Junya] Johns Hopkins Bloomberg Sch Publ Hlth, Dept Hlth Policy & Management, 624 North Broadway, Baltimore, MD USA.

[Fish, Anne F.] Univ Missouri, Coll Nursing, ISP Program, St Louis, MO 63121 USA.

[Chen, Tao] Univ Liverpool Liverpool Sch Trop Med, Dept Clin Sci, Pembroke Pl, Liverpool L3 5QA, Merseyside, England.

RP Lou, QQ (reprint author), Jiangsu Prov Acad Tradit Chinese Med, Nanjing, Jiangsu, Peoples R China.

EM lqq188@yahoo.com

CR Aikens JE, 2012, DIABETES CARE, V35, P2472, DOI 10.2337/dc12-0181

Ali MK, 2013, NEW ENGL J MED, V368, P1613, DOI 10.1056/NEJMsa1213829

American Diabetes Association, 2016, DIABETES CARE S1, V39, pS13, DOI DOI 10.2337/DC16-S005

Brieler JA, 2016, FAM PRACT, V33, P30, DOI 10.1093/fampra/cmv100

Chew BH, 2016, PLOS ONE, V11, DOI 10.1371/journal.pone.0152095

Delahanty LM, 2007, DIABETIC MED, V24, P48, DOI 10.1111/j.1464-5491.2007.02028.x

Demirci H, 2010, DAN MED BULL, V57

Fenwick EK, 2016, J HLTH PSYCHOL

Fisher L, 2012, DIABETES CARE, V35, P259, DOI 10.2337/dc11-1572

Fisher L, 2010, DIABETES CARE, V33, P23, DOI 10.2337/dc09-1238

Graue M, 2012, INT J NURS STUD, V49, P174, DOI 10.1016/j.ijnurstu.2011.08.007

Hayashino Y, 2012, DIABETES RES CLIN PR, V97, P405, DOI 10.1016/j.diabres.2012.04.005

Hessler DM, 2011, PATIENT EDUC COUNS, V85, P154, DOI 10.1016/j.pec.2010.10.030

Holman RR, 2009, NEW ENGL J MED, V361, P1736, DOI 10.1056/NEJMoa0905479

Karlsen B, 2014, INT J NURS STUD, V51, P438, DOI 10.1016/j.ijnurstu.2013.06.016

KAWADA T, 2016, AM J PUBLIC HLTH, V106

Kong Lulu, 2013, J Biomed Res, V27, P380, DOI 10.7555/JBR.27.20120112

Luyster FS, 2011, DIABETES EDUCATOR, V37, P347, DOI 10.1177/0145721711400663

Makine C, 2009, DIABETIC MED, V26, P28, DOI 10.1111/j.1464-5491.2008.02606.x

Munhoz TN, 2016, J AFFECT DISORDERS, V192, P226, DOI 10.1016/j.jad.2015.12.038

Najafian J, 2013, NIGER J CLIN PRACT, V16, P59, DOI 10.4103/1119-3077.106756

Nicolucci A, 2013, DIABETIC MED, V30, P767, DOI 10.1111/dme.12245

Ogbera A, 2011, J DIABETES, V3, P348, DOI 10.1111/j.1753-0407.2011.00156.x

Park Chan Young, 2015, Osong Public Health Res Perspect, V6, P224, DOI 10.1016/j.phrp.2015.05.004

Polinski JM, 2013, DIABETES EDUCATOR, V39, P53, DOI 10.1177/0145721712467696

Polonsky WH, 2005, DIABETES CARE, V28, P626, DOI 10.2337/diacare.28.3.626

Reutrakul S, 2015, CHRONOBIOL INT, V32, P1469, DOI 10.3109/07420528.2015.1105812

Schwarzer R, 1997, WORLD PSYCHOL, V3, P177

Stankovic Z, 2013, VOJNOSANIT PREGL, V70, P1138, DOI 10.2298/VSP1312138S

Ting RZW, 2011, DIABETES CARE, V34, P1094, DOI 10.2337/dc10-1612

Trief PM, 2001, DIABETES CARE, V24, P1384, DOI 10.2337/diacare.24.8.1384

Wardian J, 2014, SOC WORK HEALTH CARE, V53, P364, DOI 10.1080/00981389.2014.884038

Wong S, 2011, DIABETIC MED, V28, P206, DOI 10.1111/j.1464-5491.2010.03195.x

Yang Q., 2010, J NURSING CHINA, V17, P8

Zhang J, 2013, NEUROPSYCH DIS TREAT, V9, P1289, DOI 10.2147/NDT.S49798

ZHANG JX, 1995, PSYCHOLOGIA, V38, P174

Zhang P, 2016, BMC FAM PRACT, V17, DOI 10.1186/s12875-016-0435-x

Zhao J, 2016, EUR REV MED PHARMACO, V20, P3087

NR 38

TC 4

Z9 4

U1 2

U2 19

PU ELSEVIER IRELAND LTD

PI CLARE

PA ELSEVIER HOUSE, BROOKVALE PLAZA, EAST PARK SHANNON, CO, CLARE, 00000,

IRELAND

SN 0165-1781

J9 PSYCHIAT RES

JI Psychiatry Res.

PD JUN

PY 2017

VL 252

BP 45

EP 50

DI 10.1016/j.psychres.2017.02.049

PG 6

WC Psychiatry

SC Psychiatry

GA EX3FX

UT WOS:000403119100007

PM 28246057

DA 2019-01-08

ER

PT J

AU McCarthy, MM

Whittemore, R

Gholson, G

Grey, M

AF McCarthy, Margaret M.

Whittemore, Robin

Gholson, Georica

Grey, Margaret

TI Self-management of physical activity in adults with type 1 diabetes

SO APPLIED NURSING RESEARCH

LA English

DT Article

ID CARDIOVASCULAR-DISEASE; BARRIERS; RISK; MELLITUS; CARE; ASSOCIATION;

DEPRESSION; MORTALITY; STATEMENT; DISTRESS

C1 [McCarthy, Margaret M.] NYU, Coll Nursing, 433 First Ave 404, New York, NY 10010 USA.

[Whittemore, Robin; Grey, Margaret] Yale Sch Nursing, Orange, CT USA.

[Gholson, Georica] Kimberly M Brooks & Associates, Bowie, MD USA.

RP McCarthy, MM (reprint author), NYU, Coll Nursing, 433 First Ave 404, New York, NY 10010 USA.

EM mmm529@nyu.edu

FU Multidisciplinary Behavioral Research Training in Type 1 Diabetes [MPI1

T32 DK 097718-01]; Sigma Theta Tau International, Delta Mu Chapter

FX Multidisciplinary Behavioral Research Training in Type 1 Diabetes Grant

\# MPI1 T32 DK 097718-01 and the Sigma Theta Tau International, Delta Mu

Chapter.

CR American Diabetes Association, 2016, STAT DIAB

American Diabetes Association, 2016, DIABETES CARE S1, V39, pS60, DOI DOI 10.2337/DC16-S011

Ashrafian H, 2016, OBES SURG, V26, P1697, DOI 10.1007/s11695-015-1999-6

Balfe M, 2014, HEALTH PLACE, V26, P180, DOI 10.1016/j.healthplace.2013.12.016

Bohn B, 2015, DIABETES CARE, V38, P1536, DOI 10.2337/dc15-0030

Brazeau AS, 2012, DIABETES METAB, V38, P164, DOI 10.1016/j.diabet.2011.10.005

Brazeau AS, 2008, DIABETES CARE, V31, P2108, DOI 10.2337/dc08-0720

Cerin E., 2016, MED SCI SPORTS EXERC

CHARLSON ME, 1987, J CHRON DIS, V40, P373, DOI 10.1016/0021-9681(87)90171-8

Clemes SA, 2012, MED SCI SPORT EXER, V44, P1097, DOI 10.1249/MSS.0b013e318242a377

Colberg SR, 2016, DIABETES CARE, V39, P2065, DOI 10.2337/dc16-1728

Corson K, 2004, AM J MANAG CARE, V10, P839

Craig CL, 2003, MED SCI SPORT EXER, V35, P1381, DOI 10.1249/01.MSS.0000078924.61453.FB

de Ferranti SD, 2014, DIABETES CARE, V37, P2843, DOI 10.2337/dc14-1720

Duca L, 2013, CURR DIABETES REP, V13, P350, DOI 10.1007/s11892-013-0380-1

ENGEL GL, 1977, SCIENCE, V196, P129, DOI 10.1126/science.847460

Faul F, 2007, BEHAV RES METHODS, V39, P175, DOI 10.3758/BF03193146

Galassetti P, 2013, COMPR PHYSIOL, V3, P1309, DOI 10.1002/cphy.c110040

Gonder-Frederick LA, 2011, DIABETES CARE, V34, P801, DOI 10.2337/dc10-1343

Halford J, 2010, DIABETES TECHNOL THE, V12, P201, DOI 10.1089/dia.2009.0121

Harris PA, 2009, J BIOMED INFORM, V42, P377, DOI 10.1016/j.jbi.2008.08.010

Kroenke K, 2001, J GEN INTERN MED, V16, P606, DOI 10.1046/j.1525-1497.2001.016009606.x

Lloyd CE, 2010, DIABETIC MED, V27, P234, DOI 10.1111/j.1464-5491.2009.02896.x

Martyn-Nemeth P, 2016, J DIABETES COMPLICAT, V30, P167, DOI 10.1016/j.jdiacomp.2015.09.003

McCarthy M, 2015, NURS RES, V64, P320, DOI 10.1097/NNR.0000000000000098

McCarthy MM, 2016, DIABETES EDUCATOR, V42, P108, DOI 10.1177/0145721715620021

Moon D. H., 2016, J SPORTS SCI, P1

Mozaffarian D, 2016, CIRCULATION, V133, pE38, DOI 10.1161/CIR.0000000000000350

Mulcahy K, 2003, DIABETES EDUCATOR, V29, P768, DOI 10.1177/014572170302900509

Mulcahy K, 2003, DIABETES ED, V29, P773, DOI DOI 10.1177/014572170302900509

Pambianco G, 2006, DIABETES, V55, P1463, DOI 10.2337/db05-1423

Peterson JC, 2012, ANN RHEUM DIS, V71, P635, DOI 10.1136/annrheumdis-2011-200473

Pinsker J. E., 2016, CANADIAN J DIABETES

Polonsky WH, 2005, DIABETES CARE, V28, P626, DOI 10.2337/diacare.28.3.626

Prime Group for JDRF, 2015, TYP 1 DIAB FACTS

Roy T, 2012, J AFFECT DISORDERS, V142, pS8, DOI 10.1016/S0165-0327(12)70004-6

Schneider PL, 2004, MED SCI SPORT EXER, V36, P331, DOI 10.1249/01.MSS.0000113486.60548.E9

Secrest AM, 2010, DIABETES, V59, P3216, DOI 10.2337/db10-0862

Snell-Bergeon JK, 2012, J CARDIOVASC TRANSL, V5, P446, DOI 10.1007/s12265-012-9363-x

Thomas N, 2004, POSTGRAD MED J, V80, P287, DOI 10.1136/pgmj.2003.010553

Tielemans SMAJ, 2013, DIABETOLOGIA, V56, P82, DOI 10.1007/s00125-012-2743-6

Tudor-Locke C, 2008, MED SCI SPORT EXER, V40, pS537, DOI 10.1249/MSS.0b013e31817c7133

Tudor-Locke C, 2009, RES Q EXERCISE SPORT, V80, P175, DOI 10.1080/02701367.2009.10599551

Wasserman LI, 2006, SPAN J PSYCHOL, V9, P75, DOI 10.1017/S1138741600005990

Weinger K, 2005, DIABETES CARE, V28, P1346, DOI 10.2337/diacare.28.6.1346

NR 45

TC 1

Z9 1

U1 2

U2 5

PU W B SAUNDERS CO-ELSEVIER INC

PI PHILADELPHIA

PA 1600 JOHN F KENNEDY BOULEVARD, STE 1800, PHILADELPHIA, PA 19103-2899 USA

SN 0897-1897

EI 1532-8201

J9 APPL NURS RES

JI Appl. Nurs. Res.

PD JUN

PY 2017

VL 35

BP 18

EP 23

DI 10.1016/j.apnr.2017.02.010

PG 6

WC Nursing

SC Nursing

GA EW8QZ

UT WOS:000402784500004

PM 28532721

DA 2019-01-08

ER

PT J

AU Montgomery, J

Lu, J

Ratliff, S

Mezuk, B

AF Montgomery, Joshua

Lu, Juan

Ratliff, Scott

Mezuk, Briana

TI Food Insecurity and Depression Among Adults With Diabetes: Results From

the National Health and Nutrition Examination Survey (NHANES)

SO DIABETES EDUCATOR

LA English

DT Article

ID LOW-INCOME PATIENTS; NEIGHBORHOOD CHARACTERISTICS; SOCIAL DETERMINANTS;

DISTRESS; MELLITUS; SAFETY; COSTS; RISK

AB Purpose While both food insecurity and depression have been linked to risk of type 2 diabetes, little is known about the relationship between food insecurity and depression among adults with diabetes.

Research Design and Methods Cross-sectional analyses of the National Health and Nutrition Examination Survey (2011-2014), a nationally representative, population-based survey. Analytic sample was limited to adults aged 20 with diabetes determined by either fasting plasma glucose (126 mg/dL) or self-report (n = 1724) and adults age 20 with prediabetes determined by fasting plasma glucose (100-125 mg/dL) or self-report (n = 2004). Food insecurity was measured using the US Food Security Survey Module. Depression was assessed using the Patient Health Questionnaire-9 (PHQ-9). Logistic regression was used to assess the relationship between food insecurity and depression while accounting for sociodemographic characteristics and health behaviors.

Results Approximately 10% of individuals with diabetes and 8.5% of individuals with prediabetes had severe food insecurity in the past year; an additional 20.3% of individuals with diabetes and 14.3% of those with prediabetes had mild food insecurity. Among individuals with diabetes, both mild and severe food insecurity were associated with elevated odds of depression These relationships were similar in magnitude among individuals with prediabetes.

Conclusions Food insecurity is significantly associated with depressive symptoms in people with diabetes and prediabetes. Results point to the need to address economic issues in conjunction with psychosocial issues for comprehensive diabetes care.

C1 [Montgomery, Joshua; Lu, Juan; Ratliff, Scott; Mezuk, Briana] Virginia Commonwealth Univ, Sch Med, Div Epidemiol, Dept Family Med & Populat Hlth, POB 980212, Richmond, VA 23298 USA.

[Ratliff, Scott; Mezuk, Briana] Univ Michigan, Dept Epidemiol, Ann Arbor, MI 48109 USA.

[Mezuk, Briana] Univ Michigan, Inst Social Res, Ann Arbor, MI USA.

RP Mezuk, B (reprint author), Virginia Commonwealth Univ, Sch Med, Div Epidemiol, Dept Family Med & Populat Hlth, POB 980212, Richmond, VA 23298 USA.

EM bmezuk@vcu.edu

FU America Diabetes Association [1-16-ICTS-082]; National Center for Health

Statistics; [K01-MH093642]; [P60-MD002249]; [R25-GM089614]

FX B. Mezuk is supported by K01-MH093642, B. Mezuk and S. Ratliff are

supported by P60-MD002249, and J. Montgomery was supported by

R25-GM089614. Additional support provided by the America Diabetes

Association (1-16-ICTS-082). The National Health and Nutrition

Examination Survey (NHANES) is supported by the National Center for

Health Statistics. The sponsors had no role in the design,

interpretation, or decision to publish this study. There is no conflict

of interest to report.

CR American DA, 2014, DIABETES CARE S1, V37, pS14, DOI DOI 10.2337/DC14-S014

American Diabetes Association, 2016, DIABETES CARE S1, V39, pS59

ANDERSON SA, 1990, J NUTR, V120, P1559, DOI 10.1093/jn/120.suppl_11.1555

Armstrong T, 2006, J PUBLIC HEALTH-HEID, V14, P66, DOI 10.1007/s10389-006-0024-x

Bickel G, 2000, GUIDE MEASURING HOUS

Centers for Disease Control and Prevention, 2012, NAT HLTH NUTR EX SUR

Christine PJ, 2015, JAMA INTERN MED, V175, P1311, DOI 10.1001/jamainternmed.2015.2691

Chung WT, 2012, J URBAN HEALTH, V89, P407, DOI 10.1007/s11524-011-9633-y

Drewnowski A, 2005, J NUTR, V135, P900

Drewnowski A, 2004, AM J CLIN NUTR, V79, P6

Garg A, 2016, JAMA-J AM MED ASSOC, V316, P813, DOI 10.1001/jama.2016.9282

Gucciardi E., 2014, Current Nutrition Reports, V3, P324

Hallahan B, 2016, BRIT J PSYCHIAT, V209, P192, DOI 10.1192/bjp.bp.114.160242

Hamelin AM, 1999, J NUTR, V129, p525S, DOI 10.1093/jn/129.2.525S

Hosmer DW, 2000, INTRO LOGISTIC REGRE

Katon WJ, 2004, ARCH GEN PSYCHIAT, V61, P1042, DOI 10.1001/archpsyc.61.10.1042

Kim D, 2008, EPIDEMIOL REV, V30, P101, DOI 10.1093/epirev/mxn009

Kroenke K, 2001, J GEN INTERN MED, V16, P606, DOI 10.1046/j.1525-1497.2001.016009606.x

Lewis JH, 2016, BMC FAM PRACT, V17, DOI 10.1186/s12875-016-0526-8

Lopez A., 2012, Diabetes Spectrum, V25, P14, DOI 10.2337/diaspect.25.1.14

LUSTMAN PJ, 1987, COMPR PSYCHIAT, V28, P22, DOI 10.1016/0010-440X(87)90040-X

Mezuk B, 2008, DIABETES CARE, V31, P2383, DOI 10.2337/dc08-0985

Mezuk B, 2013, HEALTH PLACE, V23, P63, DOI 10.1016/j.healthplace.2013.05.004

Parker ED, 2010, ANN EPIDEMIOL, V20, P364, DOI 10.1016/j.annepidem.2010.02.009

Sanchez-Villegas A, 2012, PUBLIC HEALTH NUTR, V15, P424, DOI 10.1017/S1368980011001856

Sanchez-Villegas A, 2011, PLOS ONE, V6, DOI 10.1371/journal.pone.0016268

Seligman HK, 2007, J GEN INTERN MED, V22, P1018, DOI 10.1007/s11606-007-0192-6

Seligman HK, 2012, DIABETES CARE, V35, P233, DOI 10.2337/dc11-1627

Seligman HK, 2011, ARCH INTERN MED, V171, P1204, DOI 10.1001/archinternmed.2011.287

Seligman HK, 2010, J NUTR, V140, P304, DOI 10.3945/jn.109.112573

Siefert K, 2001, WOMEN HEALTH, V32, P159, DOI 10.1300/J013v32n01_08

Silverman J, 2015, J GEN INTERN MED, V30, P1476, DOI 10.1007/s11606-015-3351-1

Singh A, 2013, ERR173

Snoek FJ, 2015, LANCET DIABETES ENDO, V3, P450, DOI 10.1016/S2213-8587(15)00135-7

Stuff JE, 2004, J NUTR, V134, P2330

Tennant C, 2002, AUST NZ J PSYCHIAT, V36, P173, DOI 10.1046/j.1440-1614.2002.01007.x

Weissman Judith F, 2015, NCHS Data Brief, P1

Whitaker RC, 2006, PEDIATRICS, V118, pE859, DOI 10.1542/peds.2006-0239

NR 38

TC 2

Z9 2

U1 0

U2 7

PU SAGE PUBLICATIONS INC

PI THOUSAND OAKS

PA 2455 TELLER RD, THOUSAND OAKS, CA 91320 USA

SN 0145-7217

EI 1554-6063

J9 DIABETES EDUCATOR

JI Diabetes Educ.

PD JUN

PY 2017

VL 43

IS 3

BP 260

EP 271

DI 10.1177/0145721717699890

PG 12

WC Endocrinology & Metabolism; Public, Environmental & Occupational Health

SC Endocrinology & Metabolism; Public, Environmental & Occupational Health

GA EV4SU

UT WOS:000401753100003

PM 28436293

OA Green Accepted

DA 2019-01-08

ER

PT J

AU McEwen, MM

Pasvogel, A

Murdaugh, C

Hepworth, J

AF McEwen, Marylyn Morris

Pasvogel, Alice

Murdaugh, Carolyn

Hepworth, Joseph

TI Effects of a Family-based Diabetes Intervention on Behavioral and

Biological Outcomes for Mexican American Adults

SO DIABETES EDUCATOR

LA English

DT Article

ID SELF-MANAGEMENT EDUCATION; GLYCEMIC CONTROL; CARE BEHAVIORS; STARR

COUNTY; TYPE-2; HEALTH; DISTRESS; SUPPORT; MEMBERS; SCALE

AB Purpose The purpose of the study was to investigate the effects of a family-based self-management support intervention for adults with type 2 diabetes (T2DM).

Methods Using a 2-group, experimental repeated measures design, 157 dyads (participant with T2DM and family member) were randomly assigned to an intervention (education, social support, home visits, and telephone calls) or a wait list control group. Data were collected at baseline, postintervention (3 months), and 6 months postintervention. A series of 2 x 3 repeated measures ANOVAs were used to test the hypotheses with interaction contrasts to assess immediate and sustained intervention effects.

Results Significant changes over time were reported in diet self-management, exercise self-management, total self-management, diabetes self-efficacy for general health and total diabetes self-efficacy, physician distress, regimen distress, interpersonal distress, and total distress. There were likewise sustained effects for diet self-management, total self-management, diabetes self-efficacy for general health, total self-efficacy, physician distress, regimen distress, and interpersonal distress.

Conclusions Results support and extend prior research documenting the value of culturally relevant family-based interventions to improve diabetes self-management and substantiate the need for intensive, longer, tailored interventions to achieve glycemic control.

C1 [McEwen, Marylyn Morris; Pasvogel, Alice; Murdaugh, Carolyn; Hepworth, Joseph] Univ Arizona, Coll Nursing, Community & Hlth Syst Sci Div, 1305 N Martin Ave,Room 411 POB 201203, Tucson, AZ 85721 USA.

RP McEwen, MM (reprint author), Univ Arizona, Coll Nursing, Community & Hlth Syst Sci Div, 1305 N Martin Ave,Room 411 POB 201203, Tucson, AZ 85721 USA.

EM marylynm@email.arizona.edu

FU National Institute for Minority Health and Health Disparities

[R01MD005837]

FX Grant Support: R01MD005837, National Institute for Minority Health and

Health Disparities.

CR Agency for Healthcare Research and Quality, 2011, 2010 NAT HEALTHC DIS

American Diabetes Association, 2016, LIV DIAB COMPL

American Diabetes Association, 2014, DIAB HISP ALL AR NOT

[Anonymous], 2016, DIABETES CARE S1, V39, pS11

Arizona Department of Health Services, 2011, AR DIAB BURD REP

Baig AA, 2015, ANN NY ACAD SCI, V1353, P89, DOI 10.1111/nyas.12844

Berry E, 2015, POSTGRAD MED J, V91, P278, DOI 10.1136/postgradmedj-2014-133017

Block G, 2000, AM J PREV MED, V18, P284, DOI 10.1016/S0749-3797(00)00119-7

Boren SA, 2007, DIABETES EDUCATOR, V33, P1053, DOI 10.1177/0145721707309809

Brown SA, 2016, PATIENT EDUC COUNS, V99, P1558, DOI 10.1016/j.pec.2016.03.020

Brown SA, 2014, DIABETES EDUCATOR, V40, P476, DOI 10.1177/0145721714531336

Burns KK, 2013, DIABETIC MED, V30, P778, DOI 10.1111/dme.12239

Carter JS, 1996, DIABETES CARE, V19, P764, DOI 10.2337/diacare.19.7.764

Centers for Disease Control and Prevention, 2014, NAT DIAB STAT REP ES

Ortiz LGC, 2016, CLIN NURS RES, V25, P120, DOI 10.1177/1054773815586542

Creamer J, 2016, DIABETIC MED, V33, P169, DOI 10.1111/dme.12865

CUELLAR I, 1995, HISPANIC J BEHAV SCI, V17, P275, DOI 10.1177/07399863950173001

Danaei G, 2011, LANCET, V378, P31, DOI 10.1016/S0140-6736(11)60679-X

Dominguez K, 2015, MMWR-MORBID MORTAL W, V64, P469

Fisher L, 2013, DIABETES CARE, V36, P2551, DOI 10.2337/dc12-2493

Fortmann AL, 2011, HEALTH PSYCHOL, V30, P251, DOI 10.1037/a0022850

Garcia AA, 2001, DIABETES CARE, V24, P16, DOI 10.2337/diacare.24.1.16

Garcia-Solano B, 2015, SAGE OPEN MED, V3

Pereira MG, 2014, FAM SYST HEALTH, V32, P198, DOI 10.1037/fsh0000029

Haas L, 2013, DIABETES CARE, V36, pS100, DOI 10.2337/dc13-S100

Hagstromer M, 2006, PUBLIC HEALTH NUTR, V9, P755, DOI 10.1079/PHN2005898

Hu FB, 2011, DIABETES CARE, V34, P1249, DOI 10.2337/dc11-0442

Hu J, 2016, DIABETES EDUCATOR, V42, P299, DOI 10.1177/0145721716636961

Hu J, 2013, DIABETES EDUCATOR, V39, P494, DOI 10.1177/0145721713486200

McEwen MM, 2014, DIABETES EDUCATOR, V40, P488, DOI 10.1177/0145721714528996

National Alliance for Hispanic Health, 2010, STAT DIAB HISP

Polonsky WH, 2005, DIABETES CARE, V28, P626, DOI 10.2337/diacare.28.3.626

Powers MA, 2015, DIABETES EDUCATOR, V41, P417, DOI 10.1177/0145721715588904

Rosland AM, 2010, CHRONIC ILLN, V6, P22, DOI 10.1177/1742395309354608

Stark CS, 2013, DIABETES CARE, V36, P2271, DOI DOI 10.2337/DC12-2258

Stratton IM, 2000, BRIT MED J, V321, P405, DOI 10.1136/bmj.321.7258.405

Sturt J, 2015, INT DIAB NURS, V12, P40, DOI DOI 10.1179/2057332415Y.0000000004

Toobert DJ, 2000, DIABETES CARE, V23, P943, DOI 10.2337/diacare.23.7.943

Trief PM, 2016, DIABETES CARE, V39, P2165, DOI 10.2337/dc16-0035

Vaccaro J. A., 2014, J NUTR HLTH, V2, P1, DOI DOI 10.12691/JNH-2-1-1

Vincent D, 2013, DIABETES EDUCATOR, V39, P222, DOI 10.1177/0145721713475842

Weiss BD, 2005, ANN FAM MED, V3, P514, DOI 10.1370/afm.405

NR 42

TC 4

Z9 4

U1 0

U2 2

PU SAGE PUBLICATIONS INC

PI THOUSAND OAKS

PA 2455 TELLER RD, THOUSAND OAKS, CA 91320 USA

SN 0145-7217

EI 1554-6063

J9 DIABETES EDUCATOR

JI Diabetes Educ.

PD JUN

PY 2017

VL 43

IS 3

BP 272

EP 285

DI 10.1177/0145721717706031

PG 14

WC Endocrinology & Metabolism; Public, Environmental & Occupational Health

SC Endocrinology & Metabolism; Public, Environmental & Occupational Health

GA EV4SU

UT WOS:000401753100004

PM 28447545

DA 2019-01-08

ER

PT J

AU Tully, C

Shneider, C

Monaghan, M

Hilliard, ME

Streisand, R

AF Tully, Carrie

Shneider, Caitlin

Monaghan, Maureen

Hilliard, Marisa E.

Streisand, Randi

TI Peer Coaching Interventions for Parents of Children with Type 1 Diabetes

SO CURRENT DIABETES REPORTS

LA English

DT Review

DE Diabetes; Peer mentor; Parent coach; Parents; Child; Psychosocial

ID FAMILY SYSTEMS THERAPY; RANDOMIZED-TRIAL; SOCIAL SUPPORT;

YOUNG-CHILDREN; PSYCHOLOGICAL DISTRESS; SELF-MANAGEMENT; EMPOWER

PARENTS; OUTCOMES; ADOLESCENTS; MOTHERS

AB Purpose of Review Peer support is a promising model of providing psychosocial support to parents of children with type 1 diabetes. This review seeks to discuss the findings of the existing literature in peer coaching as it relates to parents and diabetes as well as to identify gaps in knowledge for future intervention development and implementation.

Recent Findings Peer support programs vary widely with regard to recruitment, training, and delivery protocols. Across most programs, ongoing support and supervision are provided to peer coaches. Despite inconsistent effects on psychosocial and child health outcomes, parent coaching is consistently a highly acceptable and feasible intervention with parents of children with T1D. Current evidence supports use of parent coaching as part of a multicomponent intervention or program to increase patient satisfaction, but more research is needed to determine if it can stand alone as an active mechanism for behavior change.

Summary The use of peer coach interventions for parents of young children with diabetes is feasible to implement and highly acceptable. However, more research is needed to understand the enduring impact for target parents and peer coaches alike, as well as impact on child outcomes.

C1 [Tully, Carrie; Shneider, Caitlin; Monaghan, Maureen; Streisand, Randi] Childrens Natl Med Ctr, Ctr Translat Sci, 111 N Michigan Ave NW, Washington, DC 20010 USA.

[Monaghan, Maureen; Streisand, Randi] George Washington Univ, Sch Med & Hlth Sci, 2300 Eye St NW, Washington, DC 20037 USA.

[Hilliard, Marisa E.] Baylor Coll Med, 1102 Bates Ave,Suite 940, Houston, TX 77030 USA.

[Hilliard, Marisa E.] Texas Childrens Hosp, 1102 Bates Ave,Suite 940, Houston, TX 77030 USA.

RP Tully, C (reprint author), Childrens Natl Med Ctr, Ctr Translat Sci, 111 N Michigan Ave NW, Washington, DC 20010 USA.

EM ctully1@childrensnational.org; ceshneider@childrensnational.org;

mmonagha@childrensnational.org; marisa.hilliard@bcm.edu;

rstreis@childrensnational.org

OI Hilliard, Marisa/0000-0002-8813-629X

FU National Institute of Diabetes and Digestive and Kidney Diseases

(NIDDK)/National Institutes of Health (NIH) [R01DK102561-02]; NIDDK [DP3

DK103998-01, K12DK097696, K23DK099250]

FX Randi Streisand, Marisa Hilliard, Maureen Monaghan, Carrie Tully, and

Caitlin Shneider report grants from the National Institute of Diabetes

and Digestive and Kidney Diseases (NIDDK)/National Institutes of Health

(NIH): R01DK102561-02.; Randi Streisand and Carrie Tully report

additional grant support from NIDDK: DP3 DK103998-01. Marisa Hilliard

reports additional grant support from NIDDK: K12DK097696 (PI: B

Anderson). Maureen Monaghan reports additional grant support NIDDK:

K23DK099250.

CR Anderson BJ, 1999, DIABETES CARE, V22, P713, DOI 10.2337/diacare.22.5.713

Channon S, 2016, DIABETES EDUCATOR, V42, P462, DOI 10.1177/0145721716644673

Dale JR, 2012, DIABETIC MED, V29, P1361, DOI 10.1111/j.1464-5491.2012.03749.x

Dennis CL, 2003, INT J NURS STUD, V40, P321, DOI 10.1016/S0020-7489(02)00092-5

Donegan A, 2016, INFLAMM BOWEL DIS, V22, P1151, DOI 10.1097/MIB.0000000000000733

Flores G, 2009, PEDIATRICS, V124, P1522, DOI 10.1542/peds.2009-0230

Freeman Kurt A, 2013, J Diabetes Sci Technol, V7, P727

Grey Margaret, 2004, Curr Diab Rep, V4, P126, DOI 10.1007/s11892-004-0068-7

Grey M, 2013, DIABETES CARE, V36, P2475, DOI 10.2337/dc12-2199

Hebert M., 2008, PSYCHOL SERV, V5, P216, DOI [10.1037/1541-1559.5.3.216, DOI 10.1037/1541-1559.5.3.216]

Heisler M, 2010, ANN INTERN MED, V153, P507, DOI 10.7326/0003-4819-153-8-201010190-00007

Hilliard ME, 2016, AM PSYCHOL, V71, P590, DOI 10.1037/a0040359

Horsch A, 2007, J TRAUMA STRESS, V20, P881, DOI 10.1002/jts.20247

Ireys HT, 2001, ARCH PEDIAT ADOL MED, V155, P771, DOI 10.1001/archpedi.155.7.771

Jaser SS, 2014, MATERN CHILD HLTH J, V18, P101, DOI 10.1007/s10995-013-1239-4

Jaser SS, 2010, J PEDIATR PSYCHOL, V35, P738, DOI 10.1093/jpepsy/jsp098

Lorig KR, 1999, MED CARE, V37, P5, DOI 10.1097/00005650-199901000-00003

Lorig KR, 2015, FRONT PUBLIC HLTH, V2, P253

Maas-van Schaaijk NM, 2013, J PEDIATR PSYCHOL, V38, P30, DOI 10.1093/jpepsy/jss096

Mackey Eleanor Race, 2016, Clin Pract Pediatr Psychol, V4, P35

Mellin AE, 2004, J PEDIATR PSYCHOL, V29, P221, DOI 10.1093/jpepsy/jsh023

Monaghan M, 2015, HEALTH PSYCHOL, V34, P794, DOI 10.1037/hea0000204

Monaghan M, 2011, PATIENT EDUC COUNS, V82, P271, DOI 10.1016/j.pec.2010.04.007

Moskowitz D, 2013, J GEN INTERN MED, V28, P938, DOI 10.1007/s11606-013-2367-7

Rearick EM, 2011, DIABETES EDUCATOR, V37, P508, DOI 10.1177/0145721711412979

Shilling V, 2013, DEV MED CHILD NEUROL, V55, P602, DOI 10.1111/dmcn.12091

Streisand R, 2005, J PEDIATR PSYCHOL, V30, P513, DOI 10.1093/jpepsy/jsi076

Streisand R, 2008, PATIENT EDUC COUNS, V73, P333, DOI 10.1016/j.pec.2008.06.014

Sullivan-Bolyai S, 2004, DIABETES EDUCATOR, V30, P476, DOI 10.1177/014572170403000319

Sullivan-Bolyai S, 2003, J Pediatr Nurs, V18, P21, DOI 10.1053/jpdn.2003.4

Sullivan-Bolyai S, 2011, MCN-AM J MATERN-CHIL, V36, P224, DOI 10.1097/NMC.0b013e3182183bf5

Sullivan-Bolyai S, 2011, DIABETES EDUCATOR, V37, P35, DOI 10.1177/0145721710392248

Sullivan-Bolyai S, 2010, DIABETES EDUCATOR, V36, P88, DOI 10.1177/0145721709352384

Tang T, 2011, DIABETES SPECTR, V24, P85, DOI DOI 10.2337/DIASPECT.24.2.85

Tang TS, 2011, PEER LEADER MANUAL

Whittemore R, 2012, DIABETES EDUCATOR, V38, P562, DOI 10.1177/0145721712445216

Williams LB, 2009, DIABETIC MED, V26, P908, DOI 10.1111/j.1464-5491.2009.02794.x

Wysocki T, 2008, BEHAV THER, V39, P33, DOI 10.1016/j.beth.2007.04.001

Wysocki T, 2007, DIABETES CARE, V30, P555, DOI 10.2337/dc06-1613

NR 39

TC 3

Z9 3

U1 3

U2 12

PU CURRENT MEDICINE GROUP

PI PHILADELPHIA

PA 400 MARKET STREET, STE 700, PHILADELPHIA, PA 19106 USA

SN 1534-4827

EI 1539-0829

J9 CURR DIABETES REP

JI Curr. Diabetes Rep.

PD JUN

PY 2017

VL 17

IS 6

AR 39

DI 10.1007/s11892-017-0870-7

PG 10

WC Endocrinology & Metabolism

SC Endocrinology & Metabolism

GA EV4DA

UT WOS:000401707300004

PM 28434144

DA 2019-01-08

ER

PT J

AU Linetzky, B

Jiang, DF

Funnell, MM

Curtis, BH

Polonsky, WH

AF Linetzky, Bruno

Jiang, Dingfeng

Funnell, Martha M.

Curtis, Bradley H.

Polonsky, William H.

TI Exploring the role of the patient-physician relationship on insulin

adherence and clinical outcomes in type 2 diabetes: Insights from the

MOSAIc study

SO JOURNAL OF DIABETES

LA English

DT Article

DE medication adherence; patient-physician relationships; type 2 diabetes

ID INTERPERSONAL PROCESSES; HEALTH-CARE; BARRIERS; COMMUNICATION;

ATTITUDES; ASSOCIATION; PERSPECTIVE; CONCORDANCE; MANAGEMENT; LANGUAGE

AB Background: The 2-year prospective MOSAIc (Multinational Observational Study assessing Insulin use: understanding the challenges associated with progression of therapy) study is investigating whether patient-, physician-, and health system-related factors affect outcomes in patients with type 2 diabetes (T2D). This baseline subanalysis investigated how aspects of the patientphysician relationship are associated with diabetes-related distress, insulin adherence, and glycemic control.

Methods: Patients with T2D taking insulin for >= 3 months were recruited at primary care and specialty practice sites in 18 countries. Physicians provided usual care. Clinical history and most recent HbA1c values were collected; patients were surveyed regarding their perception of physician interactions, diabetes-related distress level, and insulin adherence.

Results: The analysis population comprised 4341 patients. Four (of six) domains showed a significant relationship with total diabetes-related distress (P < 0.01). Poor insulin adherence was associated with greater diabetes-related distress (adjusted odds ratio [aOR] 1.14; 95% confidence interval [CI] 1.06-1.22), higher Discrimination (aOR 1.13; 95% CI 1.02-1.27) and Hurried Communication (aOR 1.35; 95% CI 1.20-1.53) scores, and a lower Explained Results score (aOR 0.86; 95% CI 0.77-0.97). Poor insulin adherence was associated with a 0.43% increase in HbA1c, whereas a 1-unit increase in total diabetes-related distress and Hurried Communication scores was associated with a 0.171% and 0.145% increase in HbA1c, respectively.

Conclusions: Patients distressed about living with T2D, and dissatisfied with aspects of their interactions with physicians, exhibited poor insulin adherence. Perceived physician inattention and lack of engagement (and diabetes-related distress) directly affect insulin adherence and glycemic control.

C1 [Linetzky, Bruno] Eli Lilly & Co, Tronador 4890,Piso 12,C1430DNN, Buenos Aires, DF, Argentina.

[Jiang, Dingfeng; Curtis, Bradley H.] Eli Lilly & Co, Indianapolis, IN 46285 USA.

[Funnell, Martha M.] Univ Michigan, Med Sch, Ann Arbor, MI USA.

[Polonsky, William H.] Behav Diabet Inst, San Diego, CA USA.

[Polonsky, William H.] Univ Calif San Diego, San Diego, CA 92103 USA.

RP Linetzky, B (reprint author), Eli Lilly & Co, Tronador 4890,Piso 12,C1430DNN, Buenos Aires, DF, Argentina.

EM linetzky_bruno@lilly.com

FU Eli Lilly and Company

FX The MOSAIc study was sponsored by Eli Lilly and Company. Medical writing

assistance was provided by Lucy Rutherford and Andrew Sakko (inVentiv

Health Clinical, Sydney, NSW, Australia) and editorial assistance was

provided by Jeanne Claypoole (inVentiv Health Clinical, Princeton, NJ,

USA), sponsored by Eli Lilly and Company.

CR Forum for Injection Technique, 2015, UK INJECTION TECHNIQ

GREENFIELD S, 1988, J GEN INTERN MED, V3, P448, DOI 10.1007/BF02595921

Heisler M, 2002, J GEN INTERN MED, V17, P243, DOI 10.1046/j.1525-1497.2002.10905.x

Holt RIG, 2013, DIABETIC MED, V30, P789, DOI 10.1111/dme.12242

Inzucchi SE, 2015, DIABETOLOGIA, V58, P429, DOI 10.1007/s00125-014-3460-0

Karter AJ, 2010, DIABETES CARE, V33, P733, DOI 10.2337/dc09-1184

Kerse N, 2004, ANN FAM MED, V2, P455, DOI 10.1370/afm.139

Lee YY, 2009, SOC SCI MED, V68, P1060, DOI 10.1016/j.socscimed.2008.12.033

Mayberry LS, 2014, DIABETES CARE, V37, P1246, DOI 10.2337/dc13-1828

Nam Soohyun, 2014, J Contemp Diabetes Res, V1, P1

Napoles AM, 2009, HEALTH SERV RES, V44, P1326, DOI 10.1111/j.1475-6773.2009.00965.x

Peyrot M, 2012, DIABETIC MED, V29, P682, DOI 10.1111/j.1464-5491.2012.03605.x

Piette JD, 2005, ARCH INTERN MED, V165, P1749, DOI 10.1001/archinte.165.15.1749

Piette JD, 2003, J GEN INTERN MED, V18, P624, DOI 10.1046/j.1525-1497.2003.31968.x

Polinski JM, 2015, BMC ENDOCR DISORD, V15, DOI 10.1186/s12902-015-0044-z

Polinski JM, 2012, BMC ENDOCR DISORD, V12, DOI 10.1186/1472-6823-12-20

Polonsky WH, 2005, DIABETES CARE, V28, P626, DOI 10.2337/diacare.28.3.626

Polonsky WH, 2015, P EUR ASS STUD DIAB

Ratanawongsa N, 2013, JAMA INTERN MED, V173, P210, DOI 10.1001/jamainternmed.2013.1216

Rubin RR, 2006, DIABETES CARE, V29, P1249, DOI 10.2337/dc05-2494

Schmittdiel JA, 2009, J WOMENS HEALTH, V18, P2065, DOI 10.1089/jwh.2009.1406

Stewart AL, 2007, HEALTH SERV RES, V42, P1235, DOI 10.1111/j.1475-6773.2006.00637.x

Traylor AH, 2010, J GEN INTERN MED, V25, P1172, DOI 10.1007/s11606-010-1424-8

Weinger K, 2010, DIABETES CARE, V33, P450, DOI 10.2337/dc09-2132

NR 24

TC 8

Z9 8

U1 1

U2 2

PU WILEY

PI HOBOKEN

PA 111 RIVER ST, HOBOKEN 07030-5774, NJ USA

SN 1753-0393

EI 1753-0407

J9 J DIABETES

JI J. Diabetes

PD JUN

PY 2017

VL 9

IS 6

BP 596

EP 605

DI 10.1111/1753-0407.12443

PG 10

WC Endocrinology & Metabolism

SC Endocrinology & Metabolism

GA EV6AZ

UT WOS:000401853200008

PM 27368146

OA Other Gold

DA 2019-01-08

ER

PT J

AU Polonsky, WH

Hessler, D

Ruedy, KJ

Beck, RW

AF Polonsky, William H.

Hessler, Danielle

Ruedy, Katrina J.

Beck, Roy W.

CA DIAMOND Study Grp

TI The Impact of Continuous Glucose Monitoring on Markers of Quality of

Life in Adults With Type 1 Diabetes: Further Findings From the DIAMOND

Randomized Clinical Trial

SO DIABETES CARE

LA English

DT Article

ID GLYCEMIC CONTROL; HYPOGLYCEMIA; MULTICENTER; INJECTIONS; AWARENESS;

DISTRESS

AB OBJECTIVE

Continuous glucose monitoring (CGM) improves glycemic control, but data are inconclusive about its influence on quality of life (QOL). We investigated the impact of 24 weeks of CGM use on QOL in adults with type 1 diabetes (T1D) who use multiple daily insulin injections.

RESEARCH DESIGN AND METHODS DIAMOND

(Multiple Daily Injections and Continuous Glucose Monitoring in Diabetes) was a prospective randomized trial that assessed CGM versus self-monitoring of blood glucose (SMBG) only in 158 adults with poorly controlled T1D. At baseline and study end, participants completed QOL measures that assessed overall well-being (WHO-5), health status (EQ-5D-5L), diabetes distress (DDS), hypoglycemic fear (worry subscale of the HFS-II), and hypoglycemic confidence (HCS). At study end, CGM participants completed the CGM Satisfaction Survey. Linear regression analyses compared treatment group changes in QOL outcomes over time. Associations between CGM satisfaction and change in QOL outcomes and in glycemic control indices were assessed.

RESULTS

The CGM group demonstrated a greater increase in hypoglycemic confidence (P = 0.01) and a greater decrease in diabetes distress (P = 0.01) than the SMBG group. No significant group differences in well-being, health status, or hypoglycemic fear were observed. CGM satisfaction was not significantly associated with glycemic changes but was associated with reductions in diabetes distress (P < 0.001) and hypoglycemic fear (P = 0.02) and increases in hypoglycemic confidence (P < 0.001) and well-being (P = 0.01).

CONCLUSIONS

CGM contributes to significant improvement in diabetes-specific QOL (i.e., diabetes distress, hypoglycemic confidence) in adults with T1D, but not with QOL measures not specific to diabetes (i.e., well-being, health status). CGM satisfaction was associated with most of the QOL outcomes but not with glycemic outcomes.

C1 [Polonsky, William H.] Univ Calif San Diego, San Diego, CA 92103 USA.

[Polonsky, William H.] Behav Diabet Inst, San Diego, CA 92121 USA.

[Hessler, Danielle] Univ Calif San Francisco, San Francisco, CA 94143 USA.

[Ruedy, Katrina J.; Beck, Roy W.] Jaeb Ctr Hlth Res, Tampa, FL USA.

RP Polonsky, WH (reprint author), Univ Calif San Diego, San Diego, CA 92103 USA.; Polonsky, WH (reprint author), Behav Diabet Inst, San Diego, CA 92121 USA.

EM whp@behavioraldiabetes.org

FU Dexcom, Inc.; Dexcom and Abbott Diabetes Care

FX Dexcom, Inc. provided funding for the study to the Behavioral Diabetes

Institute and to the Jaeb Center for Health Research. W.H.P. reports

consulting fees from Dexcom and Abbott Diabetes Care. R.W.B. reports

that his institution has received supplies for research studies from

Dexcom and Abbott Diabetes Care. No other potential conflicts of

interest relevant to this article were reported.

CR Bailey Timothy S, 2015, J Diabetes Sci Technol, V9, P209, DOI 10.1177/1932296814559746

Beck RW, 2017, JAMA-J AM MED ASSOC, V317, P371, DOI 10.1001/jama.2016.19975

Beck RW, 2010, DIABETES CARE, V33, P2175, DOI 10.2337/dc10-0331

BENJAMINI Y, 1995, J R STAT SOC B, V57, P289

Bolinder J, 2016, LANCET, V388, P2254, DOI 10.1016/S0140-6736(16)31535-5

Clarke P, 2002, MED DECIS MAKING, V22, P340, DOI 10.1177/027298902400448902

Fisher L, 2010, DIABETES CARE, V33, P1034, DOI 10.2337/dc09-2175

Hajos TRS, 2013, DIABETIC MED, V30, pE63, DOI 10.1111/dme.12040

Hirsch IB, 2010, DIABETES TECHNOL THE, V12, P679, DOI 10.1089/dia.2010.0015

Irvin A, 1994, HDB PSYCHOL DIABETES, P133

Little SA, 2014, DIABETES CARE, V37, P2114, DOI 10.2337/dc14-0030

Pickup JC, 2015, DIABETES CARE, V38, P544, DOI 10.2337/dc14-1855

Polonsky WH, 2015, J DIABETES COMPLICAT, V29, P1171, DOI 10.1016/j.jdiacomp.2015.08.002

Polonsky WH, 2005, DIABETES CARE, V28, P626, DOI 10.2337/diacare.28.3.626

Polonsky WH, 2017, DIABETES TECHNOL THE, V19, P131, DOI 10.1089/dia.2016.0366

Polonsky William H, 2016, J Diabetes Sci Technol, V10, P892, DOI 10.1177/1932296816643542

Polonsky WH, 2013, DIABETES TECHNOL THE, V15, P295, DOI 10.1089/dia.2012.0298

Riveline JP, 2012, DIABETES CARE, V35, P965, DOI 10.2337/dc11-2021

Sturt J, 2015, INT DIAB NURS, V12, P40, DOI DOI 10.1179/2057332415Y.0000000004

van Beers CAJ, 2016, LANCET DIABETES ENDO, V4, P893, DOI 10.1016/S2213-8587(16)30193-0

Vigersky RA, 2012, DIABETES CARE, V35, P32, DOI 10.2337/dc11-1438

NR 21

TC 29

Z9 29

U1 2

U2 15

PU AMER DIABETES ASSOC

PI ALEXANDRIA

PA 1701 N BEAUREGARD ST, ALEXANDRIA, VA 22311-1717 USA

SN 0149-5992

EI 1935-5548

J9 DIABETES CARE

JI Diabetes Care

PD JUN 1

PY 2017

VL 40

IS 6

BP 736

EP 741

DI 10.2337/dc17-0133

PG 6

WC Endocrinology & Metabolism

SC Endocrinology & Metabolism

GA EV4AC

UT WOS:000401699500008

PM 28389582

OA Bronze

HC Y

HP N

DA 2019-01-08

ER

PT J

AU Nieuwesteeg, A

Hartman, E

Emons, W

van Bakel, H

Aanstoot, HJ

van Mil, E

Pouwer, F

AF Nieuwesteeg, A.

Hartman, E.

Emons, W.

van Bakel, H.

Aanstoot, H. -J.

van Mil, E.

Pouwer, F.

TI Paediatric parenting stress in fathers and mothers of young children

with Type 1 diabetes: a longitudinal study

SO DIABETIC MEDICINE

LA English

DT Article

ID PATERNAL INVOLVEMENT; DISEASE MANAGEMENT; CHRONIC ILLNESS; ASSOCIATIONS;

MULTICENTER; CAREGIVERS; INVENTORY; AMERICAN; MELLITUS; OUTCOMES

AB AimTo compare levels of paediatric parenting stress in the fathers and mothers of young children with Type 1 diabetes and study the variation in this stress over time.

MethodsOne hundred and twelve parents (56 mothers and 56 fathers) of young children (0-7 years) with Type 1 diabetes participated in this study. They completed the Pediatric Inventory for Parents to assess paediatric parenting stress (frequency and difficulty scores on the Communication, Emotional Distress, Medical Care and Role Functioning subscales and Total Score); 44 mothers (79%) and 31 fathers (55%) completed the questionnaire again, 1 year later. Independent and paired sample t-tests were used to examine the differences between fathers and mothers and the changes over time. Cohen's d effect sizes were also calculated.

ResultsMothers scored significantly higher than fathers on the stress subscales for Communication frequency and difficulty, Emotional Distress frequency and difficulty, Medical Care frequency and Total Score frequency and difficulty (d ranged from -0.44 to -0.56). Furthermore, fathers reported a decrease in Medical Care frequency (d = 0.10) and an increase in Emotional Distress difficulty (d = -0.32) and Total Score difficulty (d = -0.29), whereas mothers reported a decrease in Emotional Distress frequency, Medical Care frequency and Total Score frequency (d ranged from 0.31 to 0.66) over a 1-year period.

ConclusionsThese results show that within families with a young child with Type 1 diabetes, the burden of care increases in fathers and decreases in mothers, suggesting that fathers assume more responsibility for care of their child with Type 1 diabetes as the child grows.

C1 [Nieuwesteeg, A.; Hartman, E.; Emons, W.; Pouwer, F.] Tilburg Univ, Dept Med & Clin Psychol, Ctr Res Psychol Somat Dis CoRPS, Tilburg, Netherlands.

[Emons, W.] Tilburg Univ, Dept Methodol & Stat, Tilburg, Netherlands.

[van Bakel, H.] Tilburg Univ, Dept Tranzo, Sci Ctr Care & Welf, Tilburg, Netherlands.

[van Mil, E.] Jeroen Bosch Hosp, Kidz & Ko, sHertogenbosch, Netherlands.

RP Hartman, E (reprint author), Tilburg Univ, Dept Med & Clin Psychol, Ctr Res Psychol Somat Dis CoRPS, Tilburg, Netherlands.

EM e.e.hartman@tilburguniversity.edu

CR [Anonymous], 1995, LANCET, V1, P1

Brown RT, 2008, J PEDIATR PSYCHOL, V33, P408, DOI 10.1093/jpepsy/jsm079

Cohen BH., 1988, STAT POWER ANAL BEHA

Cousino MK, 2013, J PEDIATR PSYCHOL, V38, P809, DOI [10.1093/jpepsy/jsto49, 10.1093/jpepsy/jst049]

Cunningham NR, 2011, J PEDIATR PSYCHOL, V36, P196, DOI 10.1093/jpepsy/jsq071

Dashiff Carol, 2008, J Pediatr Nurs, V23, P101, DOI 10.1016/j.pedn.2007.08.007

Eccleston C, 2015, COCHRANE DB SYST REV, V4

Gavin L, 2006, J PEDIATR PSYCHOL, V31, P481, DOI 10.1093/jpepsy/jsj043

Goldbeck L, 2006, QUAL LIFE RES, V15, P1121, DOI 10.1007/s11136-006-0068-y

Hansen JA, 2012, FAM SYST HEALTH, V30, P47, DOI 10.1037/a0027519

Hilliard ME, 2011, CHILD CARE HLTH DEV, V37, P224, DOI 10.1111/j.1365-2214.2010.01162.x

Hullmann SE, 2010, J CLIN PSYCHOL MED S, V17, P357, DOI 10.1007/s10880-010-9213-4

Iwamoto V, 2008, J PEDIAT, V84, P91, DOI [10.2223/JPED.1738, 10.1590/S0021-75572008000100015]

Lewin A. B., 2005, FAMILIES SYSTEMS HLT, V23, P56, DOI DOI 10.1037/1091-7527.23.1.56

Mitchell SJ, 2009, FAM SYST HEALTH, V27, P314, DOI 10.1037/a0018191

Monaghan M, 2011, PATIENT EDUC COUNS, V82, P271, DOI 10.1016/j.pec.2010.04.007

Monaghan MC, 2009, FAM SYST HEALTH, V27, P28, DOI 10.1037/a0014770

Mueller-Godeffroy E, 2009, DIABETIC MED, V26, P493, DOI 10.1111/j.1464-5491.2009.02707.x

Nieuwesteeg A, 2014, BMC PEDIATR, V14, DOI 10.1186/1471-2431-14-145

Nieuwesteeg AM, 2016, EUR J PEDIATR, V175, P329, DOI 10.1007/s00431-015-2631-4

Patterson CC, 2009, LANCET, V373, P2027, DOI 10.1016/S0140-6736(09)60568-7

Patton SR, 2011, J CLIN PSYCHOL MED S, V18, P345, DOI 10.1007/s10880-011-9256-1

Romano MC, 2007, SOC INDIC RES, V84, P97, DOI 10.1007/s11205-006-9077-z

Silverstein J, 2005, DIABETES CARE, V28, P186, DOI 10.2337/diacare.28.1.186

Stallwood Lynda, 2005, J Pediatr Health Care, V19, P293, DOI 10.1016/j.pedhc.2005.04.003

Streisand R, 2005, J PEDIATR PSYCHOL, V30, P513, DOI 10.1093/jpepsy/jsi076

Streisand R, 2001, J PEDIATR PSYCHOL, V26, P155, DOI 10.1093/jpepsy/26.3.155

Streisand R, 2008, PATIENT EDUC COUNS, V73, P333, DOI 10.1016/j.pec.2008.06.014

Sullivan-Bolyai S, 2006, MCN-AM J MATERN-CHIL, V31, P24, DOI 10.1097/00005721-200601000-00007

Svavarsdottir EK, 2003, J NURS SCHOLARSHIP, V35, P351, DOI 10.1111/j.1547-5069.2003.00351.x

Vrijmoet-Wiersma CMJ, 2010, PSYCHO-ONCOLOGY, V19, P368, DOI 10.1002/pon.1571

Whittemore R, 2012, DIABETES EDUCATOR, V38, P562, DOI 10.1177/0145721712445216

Wu YP, 2010, DIABETES RES CLIN PR, V89, P121, DOI 10.1016/j.diabres.2010.04.010

NR 33

TC 6

Z9 6

U1 0

U2 13

PU WILEY

PI HOBOKEN

PA 111 RIVER ST, HOBOKEN 07030-5774, NJ USA

SN 0742-3071

EI 1464-5491

J9 DIABETIC MED

JI Diabetic Med.

PD JUN

PY 2017

VL 34

IS 6

BP 821

EP 827

DI 10.1111/dme.13300

PG 7

WC Endocrinology & Metabolism

SC Endocrinology & Metabolism

GA EU9CJ

UT WOS:000401334700012

PM 27973688

DA 2019-01-08

ER

PT J

AU Stoop, CH

Nefs, G

Pop, VJ

Pouwer, F

AF Stoop, Corinne H.

Nefs, Giesje

Pop, Victor J.

Pouwer, Francois

TI Screening for and subsequent participation in a trial for depression and

anxiety in people with type 2 diabetes treated in primary care: Who do

we reach?

SO PRIMARY CARE DIABETES

LA English

DT Article

DE Diabetes; Depression; Anxiety; Trial participation; Screening

ID RANDOMIZED CONTROLLED-TRIAL; CO-MORBID DEPRESSION; ILL ELDERLY-PATIENTS;

EMOTIONAL DISTRESS; PROSPECTIVE COHORT; METAANALYSIS; PREVALENCE;

DISEASE; INTERVENTION; MELLITUS

AB Aims: This study investigated (factors related to) (a) the response to a screening procedure for depression and anxiety in people with type 2 diabetes in primary care, and (b) participation in a subsequent randomised controlled trial targeting depressive or anxiety symptoms.

Methods: People with type 2 diabetes (n=1837) received a screening questionnaire assessing depressive symptoms (PHQ-9) and anxiety symptoms (GAD-7). Eligible persons who scored above the cut-off score (PHQ-9 >= 7 or GAD-7 >= 8) were offered to participate in the trial.

Results: In total, 798 people (43%) returned the screening questionnaire. Non-responders were more often female (53% vs 44%, p <0.001), had higher LDL cholesterol levels (Cohen's d = 0.17, p = 0.001) and a higher albumin/creatinine ratio (Cohen's d = 0.08, p = 0.01). In total, 130 people (18%) reported elevated depressive or anxiety symptoms. Twenty-seven persons agreed to participate in the trial. Factors related to participation were a high education level, a higher level of diabetes distress and a history of psychological problems.

Conclusions: Using screening as recruitment resulted in a small number of participants in a treatment trial for anxiety and depression. Research is needed to investigate whether screening is also followed by a low uptake of treatment in primary care outside a RCT setting. (C) 2017 Primary Care Diabetes Europe. Published by Elsevier Ltd. All rights reserved.

C1 [Stoop, Corinne H.; Nefs, Giesje; Pop, Victor J.; Pouwer, Francois] Tilburg Univ, Dept Med & Clin Psychol, Ctr Res Psychol & Somat Disorders CoRPS, POB 90153, NL-5000 LE Tilburg, Netherlands.

[Stoop, Corinne H.] PoZOB, POB 312, NL-5500 AH Veldhoven, Netherlands.

RP Nefs, G (reprint author), Tilburg Univ, Dept Med & Clin Psychol, Ctr Res Psychol & Somat Disorders CoRPS, POB 90153, NL-5000 LE Tilburg, Netherlands.

EM g.m.nefs@tilburguniversity.edu

RI Nefs, Giesje/C-9992-2018

FU ZonMw The Netherlands Organisation for Health Research and Development,

the Hague, the Netherlands [300020015]; CoRPS, Tilburg University

FX This study has been funded by ZonMw The Netherlands Organisation for

Health Research and Development, the Hague, the Netherlands, grant

number 300020015, and CoRPS, Tilburg University. The funding sources

were not involved in the design, data collection, analysis and

interpretation of data or in the writing of this article.

CR Ajdukovic D., 2012, INT J ENDOCRINOL, V2012

Ali S, 2006, DIABETIC MED, V23, P1165, DOI 10.1111/j.1464-5491.2006.01943.x

Amer Diabet Assoc, 2010, DIABETES CARE, V33, pS11, DOI 10.2337/dc10-S011

[Anonymous], 2005, IDF CLIN GUID TASK F

Baas KD, 2009, BRIT J PSYCHIAT, V194, P399, DOI 10.1192/bjp.bp.107.046052

Black SA, 2003, DIABETES CARE, V26, P2822, DOI 10.2337/diacare.26.10.2822

Cohen J, 1988, STAT POWER ANAL BEHA

Cuijpers P, 2009, BRIT J GEN PRACT, V59, P120, DOI 10.3399/bjgp09X395139

Fleer J, 2013, DIABETIC MED, V30, P88, DOI 10.1111/dme.12001

Hermanns N, 2006, DIABETOLOGIA, V49, P469, DOI 10.1007/s00125-005-0094-2

Ismail K, 2004, LANCET, V363, P1589, DOI 10.1016/S0140-6736(04)16202-8

Katon WJ, 2004, ARCH GEN PSYCHIAT, V61, P1042, DOI 10.1001/archpsyc.61.10.1042

Koopmans B, 2009, DIABETOLOGIA, V52, P2056, DOI 10.1007/s00125-009-1460-2

Kroenke K, 2001, J GEN INTERN MED, V16, P606, DOI 10.1046/j.1525-1497.2001.016009606.x

Kroenke K, 2007, ANN INTERN MED, V146, P317, DOI 10.7326/0003-4819-146-5-200703060-00004

Kroenke K, 2009, PSYCHOSOMATICS, V50, P613, DOI 10.1176/appi.psy.50.6.613

Lamers F, 2006, BMC PUBLIC HEALTH, V6, DOI 10.1186/1471-2458-6-161

Lamers F, 2008, J CLIN EPIDEMIOL, V61, P679, DOI 10.1016/j.jclinepi.2007.07.018

Lin EHB, 2010, DIABETES CARE, V33, P264, DOI 10.2337/dc09-1068

Manea L., 2012, CMAJ, V184

National Collaborating Centre for Mental Health National Institute for Health and Clinical Excellence, 2010, DEPR AD CHRON PHYS H

Nefs G, 2012, DIABETOLOGIA, V55, P608, DOI 10.1007/s00125-011-2411-2

Nouwen A., 2010, DIABETOLOGIA, P1

Piette JD, 2011, MED CARE, V49, P641, DOI 10.1097/MLR.0b013e318215d0c9

Pouwer F, 2006, PATIENT EDUC COUNS, V60, P235, DOI 10.1016/j.pec.2005.01.009

Pouwer F, 2011, DIABETOLOGIA, V54, P741, DOI 10.1007/s00125-010-2033-0

Pouwer F, 2010, DIABETIC MED, V27, P217, DOI 10.1111/j.1464-5491.2009.02903.x

Pouwer F, 2009, NAT REV ENDOCRINOL, V5, P665, DOI 10.1038/nrendo.2009.214

Schram MT, 2009, CURR DIABETES REV, V5, P112, DOI 10.2174/157339909788166828

Sluiter AC, 2012, HUISARTS WET, V55, P1

Smith KJ, 2013, J PSYCHOSOM RES, V74, P89, DOI 10.1016/j.jpsychores.2012.11.013

Snoek FJ, 2000, DIABETES CARE, V23, P1305, DOI 10.2337/diacare.23.9.1305

Snoek FJ, 2011, DIABETES CARE, V34, P601, DOI 10.2337/dc10-1552

Stoop CH, 2015, J AFFECT DISORDERS, V184, P269, DOI 10.1016/j.jad.2015.05.063

Stoop CH, 2014, DIABETIC MED, V31, P1252, DOI 10.1111/dme.12472

Stoop CH, 2011, BMC FAM PRACT, V12, DOI 10.1186/1471-2296-12-139

Thomas AJ, 2004, J AFFECT DISORDERS, V79, P81, DOI 10.1016/S0165-0327(02)00349-x

Thombs BD, 2012, CAN MED ASSOC J, V184, P413, DOI 10.1503/cmaj.111035

Van der Feltz-Cornelis CM, 2010, GEN HOSP PSYCHIAT, V32, P380, DOI 10.1016/j.genhosppsych.2010.03.011

van Dooren FEP, 2013, PLOS ONE, V8, DOI 10.1371/journal.pone.0057058

VANVLIET IM, 2000, MINI INT NEUROPSYCHI

Yu MK, 2013, NEPHRON CLIN PRACT, V124, P106, DOI 10.1159/000355551

NR 42

TC 2

Z9 2

U1 1

U2 1

PU ELSEVIER SCI LTD

PI OXFORD

PA THE BOULEVARD, LANGFORD LANE, KIDLINGTON, OXFORD OX5 1GB, OXON, ENGLAND

SN 1751-9918

EI 1878-0210

J9 PRIM CARE DIABETES

JI Prim. Care Diabetes

PD JUN

PY 2017

VL 11

IS 3

BP 273

EP 280

DI 10.1016/j.pcd.2017.02.006

PG 8

WC Endocrinology & Metabolism; Primary Health Care

SC Endocrinology & Metabolism; General & Internal Medicine

GA EU7JJ

UT WOS:000401210800008

PM 28330680

DA 2019-01-08

ER

PT J

AU Carmichael, AG

Hulswit, BB

Moe, EJ

Jayaratne, TE

Yashar, BM

AF Carmichael, Alicia G.

Hulswit, Bailey B.

Moe, Emily J.

Jayaratne, Toby Epstein

Yashar, Beverly M.

TI A Qualitative Study of Anticipated Decision Making around Type 2

Diabetes Genetic Testing: the Role of Scientifically Concordant and

Discordant Expectations

SO JOURNAL OF GENETIC COUNSELING

LA English

DT Article

DE Diabetes; Genetic testing; Decision-making; Qualitative research

ID RANDOMIZED CONTROLLED-TRIAL; RISK-FACTORS; INTERVENTIONS; METAANALYSIS;

PREVENTION; DISORDERS; FRAMEWORK; ATTITUDES

AB Type 2 diabetes mellitus (T2DM) genetic testing is undergoing clinical trials to measure the efficacy of genetic counseling for behavior-based risk reduction. The expectations patients bring to the testing process may play an important role in individual outcomes. We conducted a qualitative exploration of anticipated decision-making and expectations around T2DM genetic testing. Semi-structured interviews were completed with Mexican Americans (n = 34), non-Hispanic Black Americans (n = 39), and non-Hispanic White Americans (n = 39) at risk for T2DM. Transcripts were analyzed for themes. Most participants would accept T2DM genetic testing in order to motivate risk-reducing behaviors or apprise family members of their risk. Participants who would decline testing wished to avoid emotional distress or believed the test would not reveal new risk information. Non-Hispanic Whites and those with college education declined genetic testing more often than other groups. Those without college education were more likely to have testing expectations that were discordant with current science, such as conflating genetic testing with common 'blood tests.' Understanding expectations and decision-making factors around T2DM genetic testing will better prepare healthcare professionals to counsel their patients. This may lead to a higher efficacy of T2DM genetic testing and counseling.

C1 [Carmichael, Alicia G.] Univ Michigan, Inst Social Res, BioSocial Methods Collaborat, Res Ctr Grp Dynam, 426 Thompson St, Ann Arbor, MI 48109 USA.

[Hulswit, Bailey B.] Wayne State Univ, Ctr Mol Med & Genet, Detroit, MI USA.

[Moe, Emily J.] Childrens Hosp Wisconsin, Genet & Genom Program, Milwaukee, WI 53201 USA.

[Jayaratne, Toby Epstein] Univ Michigan, Sch Publ Hlth, Dept Hlth Behav & Hlth Educ, Ann Arbor, MI 48109 USA.

[Yashar, Beverly M.] Univ Michigan, Dept Human Genet, Ann Arbor, MI 48109 USA.

RP Carmichael, AG (reprint author), Univ Michigan, Inst Social Res, BioSocial Methods Collaborat, Res Ctr Grp Dynam, 426 Thompson St, Ann Arbor, MI 48109 USA.

EM almagior@umich.edu

FU National Institute of Diabetes and Digestive and Kidney Diseases of the

National Institutes of Health [R01DK083347]; Michigan Center for

Diabetes Translational Research from the National Institute of Diabetes

and Digestive and Kidney Diseases [P30DK092926]

FX The authors thank Hannah M. Curtis and Alix H. Bernholtz for assistance

with qualitative coding, and Ji Qi for help with data management. This

project was funded by the National Institute of Diabetes and Digestive

and Kidney Diseases of the National Institutes of Health under award

number R01DK083347 (to T.E.J.). Additional support was provided by the

Michigan Center for Diabetes Translational Research under award number

P30DK092926 from the National Institute of Diabetes and Digestive and

Kidney Diseases. The content of this publication is solely the

responsibility of the authors and does not necessarily represent the

official views of the National Institutes of Health.

CR Almgren P, 2011, DIABETOLOGIA, V54, P2811, DOI 10.1007/s00125-011-2267-5

[Anonymous], 2015, DIABETES CARE, V38, pS5, DOI 10.2337/dc15-S004

Becker F, 2011, EUR J HUM GENET, V19, pS6, DOI 10.1038/ejhg.2010.249

Cameron LD, 2008, BRIT J HEALTH PSYCH, V13, P215, DOI 10.1348/135910708X288800

Cardona-Morrell M, 2010, BMC PUBLIC HEALTH, V10, P1

Centre d'Etudes, 2012, VAL PRED RISK EQ TYP

Christensen KD, 2010, PUBLIC HEALTH GENOM, V13, P467, DOI 10.1159/000293287

de Miguel-Yanes JM, 2011, DIABETES CARE, V34, P121, DOI 10.2337/dc10-1265

Grant David, 2013, GEN RISK HLTH COACH

Grant RW, 2009, DIABETOLOGIA, V52, P2299, DOI 10.1007/s00125-009-1512-7

Grant RW, 2013, DIABETES CARE, V36, P13, DOI 10.2337/dc12-0884

Haga SB, 2013, GENET TEST MOL BIOMA, V17, P327, DOI 10.1089/gtmb.2012.0350

Hsieh HF, 2005, QUAL HEALTH RES, V15, P1277, DOI 10.1177/1049732305276687

IDF. International Diabetes Federation, 2013, IDF DIABETES ATLAS

Klitzman RL, 2010, J GENET COUNS, V19, P430, DOI 10.1007/s10897-010-9307-z

Kuczmarski MF, 2001, J AM DIET ASSOC, V101, P28, DOI 10.1016/S0002-8223(01)00008-6

Lanie Angela D, 2004, J Genet Couns, V13, P305, DOI 10.1023/B:JOGC.0000035524.66944.6d

Lea DH, 2011, PUBLIC HEALTH GENOM, V14, P279, DOI 10.1159/000294191

Lombard M., 2002, HUMAN COMMUNICATION, V28, P1468

MACQUEEN, 1998, CULTURAL ANTHR METHO, V10, P31, DOI DOI 10.1177/1525822X980100020301

Markowitz SM, 2011, DIABETES CARE, V34, P568, DOI 10.2337/dc10-1960

McAndrew LM, 2008, BRIT J HEALTH PSYCH, V13, P195, DOI 10.1348/135910708X295604

Meigs JB, 2008, NEW ENGL J MED, V359, P2208, DOI 10.1056/NEJMoa0804742

Mills R, 2015, J GENET COUNS, V24, P58, DOI 10.1007/s10897-014-9736-1

Nishigaki M, 2014, J GENET COUNS, V23, P762, DOI 10.1007/s10897-013-9680-5

Nishigaki M, 2007, DIABETES CARE, V30, P3033, DOI 10.2337/dc07-0688

Nishigaki Masakazu, 2011, Adv Prev Med, V2011, P365132, DOI 10.4061/2011/365132

O'Daniel JM, 2010, J GENET COUNS, V19, P315, DOI 10.1007/s10897-010-9302-4

Persky S, 2007, GENET MED, V9, P727, DOI 10.1097/GIM.0b013e318159a344

Proove Bioscience Inc., 2015, STUD IMP GEN TEST CL

Schellenberg ES, 2013, ANN INTERN MED, V159, P543, DOI 10.7326/0003-4819-159-8-201310150-00007

Tessaro I., 2005, PREVENTING CHRONIC D, V2, P1

U. S. Department of Health and Human Services, 2008, U0036 US DEP HLTH HU

Vassy JL, 2013, J COMMUN GENET, V4, P263, DOI 10.1007/s12687-013-0140-6

Vassy JL, 2012, MED DECIS MAKING, V32, P606, DOI 10.1177/0272989X11431608

Voils CI, 2015, J GEN INTERN MED, V30, P1591, DOI 10.1007/s11606-015-3315-5

Waxler JL, 2012, J GENET COUNS, V21, P684, DOI 10.1007/s10897-012-9486-x

Webb TL, 2006, PSYCHOL BULL, V132, P249, DOI 10.1037/0033-2909.132.2.249

WHO Department of Health Statistics and Information Systems, 2013, GHE YLL GLOB 2000 20

Wright C. F., 2009, HUM GENET, V127, P125

Yates T, 2009, POSTGRAD MED J, V85, P475, DOI 10.1136/pgmj.2008.076166

NR 41

TC 1

Z9 1

U1 1

U2 3

PU SPRINGER

PI NEW YORK

PA 233 SPRING ST, NEW YORK, NY 10013 USA

SN 1059-7700

EI 1573-3599

J9 J GENET COUNS

JI J. Genet. Couns.

PD JUN

PY 2017

VL 26

IS 3

BP 469

EP 479

DI 10.1007/s10897-016-9999-9

PG 11

WC Genetics & Heredity; Health Policy & Services; Social Sciences,

Biomedical

SC Genetics & Heredity; Health Care Sciences & Services; Biomedical Social

Sciences

GA ET8RQ

UT WOS:000400568900009

PM 27465809

DA 2019-01-08

ER

PT J

AU Terracciano, A

Stephan, Y

Luchetti, M

Albanese, E

Sutin, AR

AF Terracciano, Antonio

Stephan, Yannick

Luchetti, Martina

Albanese, Emiliano

Sutin, Angelina R.

TI Personality traits and risk of cognitive impairment and dementia

SO JOURNAL OF PSYCHIATRIC RESEARCH

LA English

DT Article

DE Personality; Dementia; Cognitive impairment; Prospective study

ID ALZHEIMERS-DISEASE; 5-FACTOR MODEL; PSYCHOLOGICAL DISTRESS; FOLLOW-UP;

HEALTH; METAANALYSIS; DECLINE; OLDER; RETIREMENT; MECHANISMS

AB We investigated the association between five factor model personality traits (neuroticism, extraversion, openness, agreeableness, and conscientiousness) and risk of dementia, cognitive impairment not dementia (CIND), and conversion from CIND to dementia in a large national cohort. Participants from the Health and Retirement Study (N > 10,000) completed a personality scale in 2006-2008 and their cognitive status was tracked for up to 8 years using the modified Telephone Interview for Cognitive Status (TICSm). Adjusting for age, sex, education, race, and ethnicity, lower conscientiousness and agreeableness and higher neuroticism were independently associated with increased risk of dementia. These associations remained significant after adjusting for other risk factors for dementia, including income, wealth, smoking, physical inactivity, obesity, diabetes, hypertension, and blood biomarkers. These associations were not modified by age, sex, race, ethnicity, and education, suggesting that the associations of personality with risk of dementia were similar across demographic groups. Neuroticism and conscientiousness were also associated with risk of CIND. Low conscientiousness predicted conversion from CIND to dementia. Using brief assessments of personality and cognition, we found robust evidence that personality is associated with risk of cognitive impairment and dementia in a large national sample. (C) 2017 Elsevier Ltd. All rights reserved.

C1 [Terracciano, Antonio; Luchetti, Martina; Sutin, Angelina R.] Florida State Univ, Coll Med, 1115 West Call St, Tallahassee, FL 32306 USA.

[Stephan, Yannick] Univ Montpellier, Montpellier, France.

[Albanese, Emiliano] Univ Geneva, Dept Psychiat, Geneva, Switzerland.

RP Terracciano, A (reprint author), Florida State Univ, Coll Med, Dept Geriatr, 1115 West Call St, Tallahassee, FL 32306 USA.

EM antonio.terracciano@med.fsu.edu

RI Luchetti, Martina/A-4530-2017

OI Luchetti, Martina/0000-0002-7370-8443

FU NIA NIH HHS [R01 AG053297, R03 AG051960]

CR Baumgart M, 2015, ALZHEIMERS DEMENT, V11, P718, DOI 10.1016/j.jalz.2015.05.016

Booth T, 2014, HEALTH PSYCHOL, V33, P1477, DOI 10.1037/hea0000012

Clark D. O., 2013, ALZ DIS ASSOC DIS, V27, P372

Crimmins EM, 2011, J GERONTOL B-PSYCHOL, V66, P162, DOI 10.1093/geronb/gbr048

Davydow DS, 2015, PSYCHOSOM MED, V77, P200, DOI 10.1097/PSY.0000000000000136

Duberstein PR, 2011, PSYCHOL AGING, V26, P351, DOI 10.1037/a0021377

Duchek JM, 2007, J GERONTOL B-PSYCHOL, V62, pP353, DOI 10.1093/geronb/62.6.P353

Duron E, 2014, FRONT AGING NEUROSCI, V6, DOI 10.3389/fnagi.2014.00195

Friedman HS, 2000, J PERS, V68, P1089, DOI 10.1111/1467-6494.00127

Hampson SE, 2012, ANNU REV PSYCHOL, V63, P315, DOI 10.1146/annurev-psych-120710-100419

Heneka MT, 2015, LANCET NEUROL, V14, P388, DOI 10.1016/S1474-4422(15)70016-5

Jackson J, 2011, NEUROBIOL AGING, V32, P2162, DOI 10.1016/j.neurobiolaging.2009.12.009

Johansson L, 2014, NEUROLOGY, V83, P1538, DOI 10.1212/WNL.0000000000000907

Jokela M, 2014, HEALTH PSYCHOL, V33, P1618, DOI 10.1037/hea0000003

Kolanowski A, 2011, J AM GERIATR SOC, V59, P1032, DOI 10.1111/j.1532-5415.2011.03449.x

Lachman M. E., 1997, MIDLIFE DEV INVENTOR

Lai MKP, 2010, NEUROREPORT, V21, P1111, DOI 10.1097/WNR.0b013e3283407204

Luchetti M, 2016, J GERONTOL B-PSYCHOL, V71, P591, DOI 10.1093/geronb/gbu184

Luchetti M, 2014, PSYCHONEUROENDOCRINO, V50, P181, DOI 10.1016/j.psyneuen.2014.08.014

MCCRAE RR, 1992, J PERS, V60, P175, DOI 10.1111/j.1467-6494.1992.tb00970.x

Orfei MD, 2010, AM J GERIAT PSYCHIAT, V18, P1133, DOI 10.1097/JGP.0b013e3181dd1c50

Pocnet C, 2011, CAN J PSYCHIAT, V56, P408, DOI 10.1177/070674371105600704

Prince M., 2014, DEMENTIA RISK REDUCT

Ramakers IHGB, 2015, J ALZHEIMERS DIS, V46, P805, DOI 10.3233/JAD-142493

Rankin KP, 2005, J NEUROL NEUROSUR PS, V76, P632, DOI 10.1136/jnnp.2004.042879

Rapp MA, 2008, AM J GERIAT PSYCHIAT, V16, P168, DOI 10.1097/JGP.0b013e31816029ec

Ritchie K, 2001, NEUROLOGY, V56, P37, DOI 10.1212/WNL.56.1.37

Saczynski JS, 2015, AM J MED, V128, P739, DOI 10.1016/j.amjmed.2015.01.007

Skogen JC, 2015, BMC GERIATR, V15, DOI 10.1186/s12877-015-0020-5

Stephan Y., 2016, J GERONTOLOGY B

Sutin AR, 2016, J RES PERS, V63, P22, DOI 10.1016/j.jrp.2016.05.001

Sutin AR, 2011, J PERS SOC PSYCHOL, V101, P579, DOI 10.1037/a0024286

Terracciano A, 2004, ADDICTION, V99, P472, DOI 10.1111/j.1360-0443.2004.00687.x

Terracciano A, 2014, ALZHEIMERS DEMENT, V10, P179, DOI 10.1016/j.jalz.2013.03.002

Terracciano A, 2013, NEUROBIOL AGING, V34, P1045, DOI 10.1016/j.neurobiolaging.2012.08.008

Terracciano A, 2011, PSYCHOSOM MED, V73, P638, DOI 10.1097/PSY.0b013e3182306a4f

Wilson RS, 2007, ARCH GEN PSYCHIAT, V64, P1204, DOI 10.1001/archpsyc.64.10.1204

Wilson RS, 2005, NEUROLOGY, V64, P380, DOI 10.1212/01.WNL.0000149525.53525.E7

Yaffe K, 2013, BMJ-BRIT MED J, V347, DOI 10.1136/bmj.f7051

NR 39

TC 16

Z9 16

U1 0

U2 14

PU PERGAMON-ELSEVIER SCIENCE LTD

PI OXFORD

PA THE BOULEVARD, LANGFORD LANE, KIDLINGTON, OXFORD OX5 1GB, ENGLAND

SN 0022-3956

EI 1879-1379

J9 J PSYCHIATR RES

JI J. Psychiatr. Res.

PD JUN

PY 2017

VL 89

BP 22

EP 27

DI 10.1016/j.jpsychires.2017.01.011

PG 6

WC Psychiatry

SC Psychiatry

GA ES9AD

UT WOS:000399847400004

PM 28153642

DA 2019-01-08

ER

PT J

AU Lee, SH

Shin, DS

Kim, JR

Kim, H

AF Lee, Sun Hee

Shin, Dong-Soo

Kim, Jong Ran

Kim, Hyunjung

TI Factors associated with mortality risk in critical care patients treated

with veno-arterial extracorporeal membrane oxygenation

SO HEART & LUNG

LA English

DT Article

DE Extracorporeal membrane oxygenation; Mortality; Intensive care unit;

Risk factors; Simplified acute physiology score

ID RESPIRATORY-DISTRESS-SYNDROME; ADULT PATIENTS; FAILURE; SUPPORT; SCORE;

ECMO; COMPLICATIONS; EXPERIENCE; OUTCOMES

AB Objectives: To identify factors associated with mortality in patients treated with veno-arterial extracorporeal membrane oxygenation (VA-ECMO) and to validate the discrimination of the risk assessment tools to predict mortality.

Background: VA-ECMO is a rescue therapy for patients with life-threatening cardiac failure, but mortality remains high.

Methods: In this retrospective study, we reviewed the medical records of adult patients who underwent VA-ECMO in an intensive care unit of a university hospital, between 2009 and 2013.

Results: VA-ECMO was performed in 89 patients, with a median duration of 116 h. The survival rate until hospital discharge was 27%. The pre-ECMO simplified acute physiology score (SAPS) II and diabetes mellitus were significant predictors of hospital mortality. The optimal prognostic SAPS II score was 81 overall, 80 in patients with diabetes, and 84 in those without diabetes.

Conclusions: Our findings indicate that high pre-ECMO SAPS II score and diabetes are risk factors for mortality in patients who undergo VA-ECMO. (C) 2017 Elsevier Inc. All rights reserved.

C1 [Lee, Sun Hee; Kim, Jong Ran] Hallym Univ, Sacred Heart Hosp, Dept Nursing, 22 Gwanpyeong Ro 170 Beon Gil, Anyang Si 14068, Gyeonggi Do, South Korea.

[Shin, Dong-Soo; Kim, Hyunjung] Hallym Univ, Res Inst Nursing Sci, Div Nursing, 1 Hallymdaehak Gil, Chunchon 24252, Gangwon Do, South Korea.

RP Kim, H (reprint author), Hallym Univ, Res Inst Nursing Sci, Div Nursing, 1 Hallymdaehak Gil, Chunchon 24252, Gangwon Do, South Korea.

EM hjkim97@hallym.ac.kr

CR Aubron C, 2013, CRIT CARE, V17, DOI 10.1186/cc12681

Aziz F, 2014, ANN VASC SURG, V28, P178, DOI 10.1016/j.avsg.2013.03.011

Beurtheret S, 2012, EUR J CARDIO-THORAC, V41, P691, DOI 10.1093/ejcts/ezr082

Brogan TV, 2009, INTENS CARE MED, V35, P2105, DOI 10.1007/s00134-009-1661-7

Choi H, 1992, KOREAN J ANESTHEOL, V25, P424

Das Evcimen N, 2007, PHARMACOL RES, V55, P498, DOI 10.1016/j.phrs.2007.04.016

DELONG ER, 1988, BIOMETRICS, V44, P837, DOI 10.2307/2531595

Enger TB, 2014, CRIT CARE, V18, DOI 10.1186/cc13824

Guttendorf J, 2014, AM J CRIT CARE, V23, P365, DOI 10.4037/ajcc2014115

Huh JW, 2015, KOREAN ACAD TUBERC R, V78, P149

Kim KI, 2015, SCAND J TRAUMA RESUS, V23, DOI 10.1186/s13049-015-0135-x

KNAUS WA, 1985, CRIT CARE MED, V13, P818, DOI 10.1097/00003246-198510000-00009

Kumar TKS, 2010, J THORAC CARDIOV SUR, V140, P330, DOI 10.1016/j.jtcvs.2010.02.034

Lafc G, 2014, HEART LUNG CIRC, V23, P10, DOI 10.1016/j.hlc.2013.08.009

Lan CC, 2010, ARTIF ORGANS, V34, pE59, DOI 10.1111/j.1525-1594.2009.00909.x

Lin CY, 2007, ANN THORAC SURG, V84, P1256, DOI 10.1016/j.athoracsur.2007.05.045

Lindskov C, 2013, ACTA ANAESTH SCAND, V57, P303, DOI 10.1111/aas.12050

Paden ML, 2013, ASAIO J, V59, P202, DOI 10.1097/MAT.0b013e3182904a52

Pappalardo F, 2013, INTENS CARE MED, V39, P275, DOI 10.1007/s00134-012-2747-1

Peigh G, 2015, J SURG RES, V198, P273, DOI 10.1016/j.jss.2015.04.044

Prakash P, 2006, J INDIAN ACAD CLIN M, V7, P202

Roch A, 2014, INTENS CARE MED, V40, P74, DOI 10.1007/s00134-013-3135-1

Schmidt M, 2013, INTENS CARE MED, V39, P1704, DOI 10.1007/s00134-013-3037-2

ZAPOL WM, 1979, JAMA-J AM MED ASSOC, V242, P2193, DOI 10.1001/jama.242.20.2193

NR 24

TC 2

Z9 2

U1 1

U2 2

PU MOSBY-ELSEVIER

PI NEW YORK

PA 360 PARK AVENUE SOUTH, NEW YORK, NY 10010-1710 USA

SN 0147-9563

EI 1527-3288

J9 HEART LUNG

JI Heart Lung

PD MAY-JUN

PY 2017

VL 46

IS 3

BP 137

EP 142

DI 10.1016/j.hrtlng.2017.02.003

PG 6

WC Cardiac & Cardiovascular Systems; Nursing; Respiratory System

SC Cardiovascular System & Cardiology; Nursing; Respiratory System

GA EX4IF

UT WOS:000403197500002

PM 28318620

DA 2019-01-08

ER

PT J

AU Wang, RH

Hsu, HC

Kao, CC

Yang, YM

Lee, YJ

Shin, SJ

AF Wang, Ruey-Hsia

Hsu, Hui-Chun

Kao, Chia-Chan

Yang, Yung-Mei

Lee, Yau-Jiunn

Shin, Shyi-Jang

TI Associations of changes in psychosocial factors and their interactions

with diabetes distress in patients with type 2 diabetes: a longitudinal

study

SO JOURNAL OF ADVANCED NURSING

LA English

DT Article

DE diabetes distress; interaction; nurses; patient empowerment; prospective

study; resilience; self-efficacy; self-management; social support; type

2 diabetes

ID SELF-MANAGEMENT PRACTICES; HEALTH LITERACY; PSYCHOLOGICAL DISTRESS;

EMOTIONAL DISTRESS; PERCEIVED SUPPORT; SOCIAL SUPPORT; EMPOWERMENT;

RESILIENCE; OUTCOMES; DEPRESSION

AB Aim. To assess the associations of changes in self-management behaviours, diabetes self-efficacy, resilience, social support, patient empowerment and their interactions with changes in diabetes distress in patients with type 2 diabetes mellitus.

Background. Many patients with type 2 diabetes mellitus experience diabetes distress. Few longitudinal studies have investigated the associations of changes in various psychosocial factors with changes in diabetes distress in patients with type 2 diabetes mellitus.

Design. This study adopted a longitudinal design. Data were collected at baseline and 12 months later.

Methods. Overall, 304 patients with type 2 diabetes were recruited from four hospitals in southern Taiwan by convenience sampling. A self-report questionnaire and medical record were used to collect demographic data, clinical indicators, self-management behaviours, diabetes self-efficacy, resilience, social support, patient empowerment, and diabetes distress. Data were collected from February 2014-March 2015.

Results/Findings. An increase in resilience or diabetes self-efficacy significantly associated with a decrease in diabetes distress, whereas an increase in patient empowerment significantly associated with an increase in diabetes distress. The interactions between increase in patient empowerment and increase in self-management behaviours significantly associated with decrease in diabetes distress.

Conclusion. Nurses could endeavour to improve the diabetes self-efficacy and resilience to reducing diabetes distress. Arbitrarily empowering patients may increase diabetes distress. Increasing self-care management behaviours and patient empowerment might need to be simultaneously addressed to reduce the diabetes distress in patients with type 2 diabetes.

C1 [Wang, Ruey-Hsia; Yang, Yung-Mei] Kaohsiung Med Univ, Coll Nursing, Kaohsiung, Taiwan.

[Hsu, Hui-Chun] Lees Endocrinol Clin, Dept Diabet Management, Pingtung, Taiwan.

[Kao, Chia-Chan] I Shou Univ, Dept Healthcare Adm, Kaohsiung, Taiwan.

[Lee, Yau-Jiunn] Lees Endocrinol Clin, Pingtung, Taiwan.

[Shin, Shyi-Jang] Kaohsiung Med Univ Hosp, Dept Internal Med, Div Endocrinol & Metab, Kaohsiung, Taiwan.

RP Wang, RH (reprint author), Kaohsiung Med Univ, Coll Nursing, Kaohsiung, Taiwan.

EM wrhsia@kmu.edu.tw

FU National Science Council, Taiwan [NSC-102-2628-B-037-012-MY3]

FX Studying funding was supported by the National Science Council, Taiwan.

(NSC-102-2628-B-037-012-MY3).

CR Aikens JE, 2012, DIABETES CARE, V35, P2472, DOI 10.2337/dc12-0181

American Diabetes Association, 2016, DIABETES CARE S1, V39, pS4, DOI DOI 10.2337/DC16-S003

Anderson RM, 2010, PATIENT EDUC COUNS, V79, P277, DOI 10.1016/j.pec.2009.07.025

Bandura A., 1997, SELF EFFICACY EXERCI

Brugge D, 2009, BMC PUBLIC HEALTH, V9, DOI 10.1186/1471-2458-9-354

Campbell-Sills L, 2007, J TRAUMA STRESS, V20, P1019, DOI 10.1002/jts.20271

Cochran J, 2008, DIABETES EDUCATOR, V34, P815, DOI 10.1177/0145721708323640

Fisher L, 2008, DIABETIC MED, V25, P1096, DOI 10.1111/j.1464-5491.2008.02533.x

Fisher L, 2014, DIABETIC MED, V31, P764, DOI 10.1111/dme.12428

Fisher L, 2009, DIABETIC MED, V26, P622, DOI 10.1111/j.1464-5491.2009.02730.x

Hsu HC, 2013, J NURS RES, V21, P212, DOI 10.1097/01.jnr.0000432048.31921.e2

International Diabetes Federation, 2015, IDF DIAB ATL 7

Karlsen B, 2014, INT J NURS STUD, V51, P438, DOI 10.1016/j.ijnurstu.2013.06.016

Karlsen B, 2012, J ADV NURS, V68, P391, DOI 10.1111/j.1365-2648.2011.05751.x

Kim G, 2015, J AGING HEALTH, V27, P320, DOI 10.1177/0898264314549662

Lee YJ, 2016, PATIENT EDUC COUNS, V99, P287, DOI 10.1016/j.pec.2015.08.021

Li JH, 2015, ASIAN J PSYCHIATR, V14, P22, DOI 10.1016/j.ajp.2015.01.011

Livneh H., 2007, COPING CHRONIC ILLNE

Londono AMM, 2015, PATIENT EDUC COUNS, V98, P908, DOI 10.1016/j.pec.2015.03.003

Min JA, 2013, SUPPORT CARE CANCER, V21, P2469, DOI 10.1007/s00520-013-1807-6

[Ministry of Health and Welfare Retrieved from], 2014, PREV DIAB YEARS

Ministry of Health and Welfare, 2015, ANN STAT REP DEATH

National Health Insurance (NHI), 2013, RES DAT ROC

Padesky CA, 2012, CLIN PSYCHOL PSYCHOT, V19, P283, DOI 10.1002/cpp.1795

Pandit AU, 2014, J EPIDEMIOL COMMUN H, V68, P557, DOI 10.1136/jech-2013-203063

POLONSKY WH, 1995, DIABETES CARE, V18, P754, DOI 10.2337/diacare.18.6.754

Pouwer F, 2010, DIABETIC MED, V27, P217, DOI 10.1111/j.1464-5491.2009.02903.x

Rose V, 2009, PATIENT EDUC COUNS, V77, P260, DOI 10.1016/j.pec.2009.03.026

Rossi MC, 2015, PATIENT EDUC COUNS, V98, P1142, DOI 10.1016/j.pec.2015.05.012

Schulz PJ, 2013, PATIENT EDUC COUNS, V90, P4, DOI 10.1016/j.pec.2012.09.006

Sigurdardottir AK, 2008, SCAND J CARING SCI, V22, P284, DOI 10.1111/j.1471-6712.2007.00506.x

Simpson G, 2013, CLIN REHABIL, V27, P367, DOI 10.1177/0269215512457961

Su D, 2012, ARCH GERONTOL GERIAT, V55, P564, DOI 10.1016/j.archger.2012.06.006

Taylor S, 2006, HLTH PSYCHOL

Tian XH, 2016, GEN HOSP PSYCHIAT, V39, P86, DOI 10.1016/j.genhosppsych.2015.12.004

Wang WR, 2013, QUAL LIFE RES, V22, P1965, DOI 10.1007/s11136-012-0345-x

Wardian J, 2014, SOC WORK HEALTH CARE, V53, P364, DOI 10.1080/00981389.2014.884038

WHO, 2014, GLOB STAT REP NONC D

Yang J, 2009, J CLIN NURS, V18, P1295, DOI 10.1111/j.1365-2702.2008.02703.x

Zulman DM, 2012, PATIENT EDUC COUNS, V87, P74, DOI 10.1016/j.pec.2011.07.013

NR 40

TC 5

Z9 5

U1 2

U2 6

PU WILEY

PI HOBOKEN

PA 111 RIVER ST, HOBOKEN 07030-5774, NJ USA

SN 0309-2402

EI 1365-2648

J9 J ADV NURS

JI J. Adv. Nurs.

PD MAY

PY 2017

VL 73

IS 5

BP 1137

EP 1146

DI 10.1111/jan.13201

PG 10

WC Nursing

SC Nursing

GA EX3CQ

UT WOS:000403110000013

PM 27862194

DA 2019-01-08

ER

PT J

AU Polonsky, WH

Capehorn, M

Belton, A

Down, S

Alzaid, A

Gamerman, V

Nagel, F

Lee, J

Edelman, S

AF Polonsky, William H.

Capehorn, Matthew

Belton, Anne

Down, Susan

Alzaid, Aus

Gamerman, Victoria

Nagel, Friedericke

Lee, Jisoo

Edelman, Steven

TI Physician-patient communication at diagnosis of type 2 diabetes and its

links to patient outcomes: New results from the global IntroDia (R)

study

SO DIABETES RESEARCH AND CLINICAL PRACTICE

LA English

DT Article

DE Type 2 diabetes; Physician-patient communication; Patient survey;

Psychological well-being; Patient-reported outcomes

ID SELF-CARE; PROVIDER COMMUNICATION; ATTITUDES; MANAGEMENT; SCALE; NEEDS;

PERCEPTIONS; WISHES

AB Aims: To investigate patient experiences during the diagnosis of type 2 diabetes mellitus (T2DM), focusing on how physician-patient communication at diagnosis influences patients' psychosocial stress and subsequent self-management and outcomes.

Methods: We surveyed adults with T2DM in 26 countries in a large cross-national study of physician-patient communication during early T2DM treatment (IntroDia (R)). The self-report questionnaire assessed retrospectively patient experiences during diagnosis conversations (focusing on 43 possible conversational elements, and communication quality) and potential effects on patient-reported outcomes.

Results: Data from 3628 people with T2DM who had been prescribed oral treatment at diagnosis were analysed. Exploratory factor analyses of the conversational elements yielded four coherent, meaningful factors: Encouraging (Cronbach's alpha = 0.86); Collaborative (alpha = 0.88); Recommending Other Resources (alpha = 0.75); and Discouraging (alpha = 0.72). Patient-perceived communication quality (PPCQ) at diagnosis was positively associated with Encouraging (beta = +1.764, p < 0.001) and Collaborative (beta = +0.347, p < 0.001), negatively associated with Discouraging (beta = -1.181, beta < 0.001) and not associated with Recommending Other Resources (beta = +0.087, p = 0.096), using a stable path model. PPCQ was associated with less current diabetes distress, greater current well-being and better current self-care. Conversation elements comprising factors associated with better PPCQ (Encouraging and Collaborative) were recalled more frequently by patients than elements associated with poor PPCQ (Discouraging).

Conclusions: Better physician-patient communication at T2DM diagnosis may contribute to subsequent greater patient well-being and self-care, and may be enhanced by greater physician use of Collaborative and Encouraging conversation elements. (C) 2017 Elsevier B.V. All rights reserved.

C1 [Polonsky, William H.] Univ Calif San Diego, Dept Psychiat, 9500 Gilman Dr, La Jolla, CA 92093 USA.

[Polonsky, William H.] Behav Diabet Inst, 5405 Oberlin Dr,Suite 100, San Diego, CA 92121 USA.

[Capehorn, Matthew] Rotherham Inst Obes, Rotherham S65 1DA, S Yorkshire, England.

[Capehorn, Matthew] Clifton Med Ctr, Rotherham S65 1DA, S Yorkshire, England.

[Belton, Anne] UHN, Michener Inst Educ, 222 St Patrick St, Toronto, ON M5T 1V4, Canada.

[Down, Susan] Somerset Partnership NHS Fdn Trust, Mallard Court, 2nd Floor,Express Pk,Bristol Rd, Bridgwater TA6 4RN, England.

[Alzaid, Aus] Prince Sultan Mil Med City, POB 7897, Riyadh 11159, Saudi Arabia.

[Gamerman, Victoria] Boehringer Ingelheim Pharmaceut Inc, 900 Ridgebury Rd,POB 368, Ridgefield, CT 06877 USA.

[Nagel, Friedericke; Lee, Jisoo] Boehringer Ingelheim Pharma GmbH & Co KG, Binger Str 173, D-55216 Ingelheim, Germany.

[Edelman, Steven] Univ Calif San Diego, Div Endocrinol & Metab, 9500 Gilman Dr, La Jolla, CA 92093 USA.

[Edelman, Steven] Vet Affairs Med Ctr, 3350 La Jolla Village Dr, La Jolla, CA 92161 USA.

RP Polonsky, WH (reprint author), 5405 Oberlin Dr,Suite 100, San Diego, CA 92121 USA.

EM whp@behavioraldiabetes.org

FU Boehringer Ingelheim & Eli Lilly and Company Diabetes Alliance

FX This study was sponsored by the Boehringer Ingelheim & Eli Lilly and

Company Diabetes Alliance.

CR Adriaanse MC, 2002, DIABETIC MED, V19, P406

Aikens JE, 2005, DIABETES EDUCATOR, V31, P681, DOI 10.1177/0145721705280829

Anderson LA, PSYCHOL REP

Beeney LJ, 1996, PATIENT EDUC COUNS, V29, P109, DOI 10.1016/0738-3991(96)00939-1

Bundesmann R, 2011, PATIENT EDUC COUNS, V85, P143, DOI 10.1016/j.pec.2010.09.025

Capehorn M, 2017, CHALLENGES FACED PHY

Cattell RB, 1952, FACTOR ANAL

Council of American Survey Research Organizations, CASRO COD STAND ETH

Crofton C, 1998, AHCPR PUB

Dietrich UC, 1996, PATIENT EDUC COUNS, V29, P13, DOI 10.1016/0738-3991(96)00930-5

European Pharmaceutical Market Research Association, EPHMRA CODE COND

Glasgow RE, 2005, DIABETES CARE, V28, P2655, DOI 10.2337/diacare.28.11.2655

Hajos TRS, 2013, DIABETIC MED, V30, pE63, DOI 10.1111/dme.12040

Hays RD, 1999, MED CARE, V37, pMS22, DOI 10.1097/00005650-199903001-00003

Heisler M, 2003, J GEN INTERN MED, V18, P893, DOI 10.1046/j.1525-1497.2003.21132.x

International Chamber of Commerce/ European Society for Opinion and Marketing Research, ICC ESOMAR INT CODE

KAISER HF, 1960, EDUC PSYCHOL MEAS, V20, P141, DOI 10.1177/001316446002000116

Lawson VL, 2008, DIABETIC MED, V25, P482, DOI 10.1111/j.1464-5491.2008.02394.x

Ohtaki S, 2003, FAM PRACT, V20, P276, DOI 10.1093/fampra/cmg308

Parry O, 2004, FAM PRACT, V21, P131, DOI 10.1093/fampra/cmh203

Peel E, 2004, PATIENT EDUC COUNS, V53, P269, DOI 10.1016/j.pec.2003.07.010

Peyrot M, 2005, DIABETIC MED, V22, P1379, DOI 10.1111/j.1464-5491.2005.01644.x

Peyrot M, 2013, DIABETES RES CLIN PR, V99, P174, DOI 10.1016/j.diabres.2012.11.016

PibernikOkanovic M, 1996, DIABETES RES CLIN PR, V34, P99, DOI 10.1016/S0168-8227(96)01342-3

Piette JD, 2003, J GEN INTERN MED, V18, P624, DOI 10.1046/j.1525-1497.2003.31968.x

Polonsky WH, 2005, DIABETES CARE, V28, P626, DOI 10.2337/diacare.28.3.626

Polonsky WH, 2010, DIABETES EDUCATOR, V36, P828, DOI 10.1177/0145721710378539

Ratanawongsa N, 2013, JAMA INTERN MED, V173, P210, DOI 10.1001/jamainternmed.2013.1216

Sastre MTM, 2011, HEALTH COMMUN, V26, P649, DOI 10.1080/10410236.2011.561919

Stewart AL, 2007, HEALTH SERV RES, V42, P1235, DOI 10.1111/j.1475-6773.2006.00637.x

Thom DH, 1999, MED CARE, V37, P510, DOI 10.1097/00005650-199905000-00010

Thoolen BJ, 2006, DIABETES CARE, V29, P2257, DOI 10.2337/dc06-0617

Toobert DJ, 2000, DIABETES CARE, V23, P943, DOI 10.2337/diacare.23.7.943

World Health Organization (n.d), PROC TRANSL AD INSTR

NR 34

TC 3

Z9 3

U1 2

U2 2

PU ELSEVIER IRELAND LTD

PI CLARE

PA ELSEVIER HOUSE, BROOKVALE PLAZA, EAST PARK SHANNON, CO, CLARE, 00000,

IRELAND

SN 0168-8227

EI 1872-8227

J9 DIABETES RES CLIN PR

JI Diabetes Res. Clin. Pract.

PD MAY

PY 2017

VL 127

BP 265

EP 274

DI 10.1016/j.diabres.2017.03.016

PG 10

WC Endocrinology & Metabolism

SC Endocrinology & Metabolism

GA EW4JT

UT WOS:000402468500030

PM 28407552

DA 2019-01-08

ER

PT J

AU Holland-Carter, L

Tuerk, PW

Wadden, TA

Fujioka, KN

Becker, LE

Miller-Kovach, K

Hollander, PL

Garvey, WT

Weiss, D

Rubino, DM

Kushner, RF

Malcolm, RJ

Raum, WJ

Hermayer, KL

Veliko, JL

Rost, SL

Sora, ND

Salyer, JL

O'Neil, PM

AF Holland-Carter, Lauren

Tuerk, Peter W.

Wadden, Thomas A.

Fujioka, Ken N.

Becker, Lynne E.

Miller-Kovach, Karen

Hollander, Priscilla L.

Garvey, W. Timothy

Weiss, Daniel

Rubino, Domenica M.

Kushner, Robert F.

Malcolm, Robert J.

Raum, William J.

Hermayer, Kathie L.

Veliko, Jan L.

Rost, Stephanie L.

Sora, Nicoleta D.

Salyer, Jonny L.

O'Neil, Patrick M.

TI Impact on psychosocial outcomes of a nationally available weight

management program tailored for individuals with type 2 diabetes:

Results of a randomized controlled trial

SO JOURNAL OF DIABETES AND ITS COMPLICATIONS

LA English

DT Article

DE Diabetes; Weight loss; Psychosocial; Diabetes distress; Commercial

weight loss programs; Quality of life

ID QUALITY-OF-LIFE; GLYCEMIC CONTROL; DISTRESS SCALE; HEALTH SURVEY;

RISK-FACTORS; DEPRESSION; ADULTS; METAANALYSIS; OVERWEIGHT; SYMPTOMS

AB Aims: Type 2 diabetes mellitus (T2DM) can substantially decrease quality of life (QOL). This study examined the effects on QOL-relevant psychosocial measures of a widely available commercial weight loss program enhanced for individuals with T2DM.

Methods: A year-long multi-site randomized clinical trial compared the Weight Watchers (WW) approach, supplemented with phone and email counseling with a certified diabetes educator (CDE), to brief standard diabetes nutrition counseling and education (Standard Care; SC). Participants were 400 women and 163 men (N = 279 WW; 284 SC) with T2DM [mean ( +/- SD) HbA(1c) 8.32 +/- 1%; BMI = 37.1 +/- 5.7 kg/m(2); age = 55.1 +/- 9.1 years]. Psychosocial outcomes were assessed at baseline, month 6, and month 12 using a diabetes specific psychosocial measure (Diabetes Distress Scale [DDS]), Impact of Weight on Quality of Life-Lite scale (IWQOL), a generic QOL measure (SF-36), and a depression screen (PHQ-9).

Results: WW participants showed significantly greater improvements than did SC participants on all DDS subscales and total score and on IWQOL total score and physical function, sex life and work domains (all ps < .05). There was no significant treatment effect on SF-36 scores or PHQ-9.

Conclusions: WW enhanced for individuals with T2DM was superior to SC in improving psychosocial outcomes most specific to T2DM and obesity. Available commercial WL programs, combined with scalable complementary program-specific diabetes counseling, may have benefits that extend to diabetes-related distress and weight-relevant QOL. (C) 2017 The Authors. Published by Elsevier Inc.

C1 [Holland-Carter, Lauren; Tuerk, Peter W.; Becker, Lynne E.; Malcolm, Robert J.; O'Neil, Patrick M.] Med Univ South Carolina, Dept Psychiat & Behav Sci, Weight Management Ctr, 67 President St,Suite 410 South, Charleston, SC 29425 USA.

[Tuerk, Peter W.] Ralph H Johnson VA Med Ctr, Mental Hlth Serv, 109 Bee St, Charleston, SC 29401 USA.

[Wadden, Thomas A.] Univ Penn, Perelman Sch Med, Dept Psychiat, Ctr Weight & Eating Disorders, 3535 Market St,Suite 3029, Philadelphia, PA 19104 USA.

[Fujioka, Ken N.] Scripps Clin Res, 11025 N Torrey Pines Rd,Suite 200, La Jolla, CA 92037 USA.

[Miller-Kovach, Karen; Veliko, Jan L.; Rost, Stephanie L.] Weight Watchers Int, 11 Madison Ave 17th Floor, New York, NY 10010 USA.

[Hollander, Priscilla L.] Baylor Endocrine Ctr, 3600 Gaston Ave,Wadley Tower,Suite 656, Dallas, TX 75246 USA.

[Garvey, W. Timothy] Univ Alabama Birmingham, Dept Nutr Sci, 1675 Univ Blvd, Birmingham, AL 35294 USA.

[Garvey, W. Timothy] Birmingham Vet Adm Med Ctr, Birmingham, AL USA.

[Weiss, Daniel] Your Diabet Endocrine Nutr Grp, 8300 Tyler Blvd Suite 102, Mentor, OH 44060 USA.

[Rubino, Domenica M.] Washington Ctr Weight Management & Res, 2800 S Shirlington Rd, Arlington, VA 22206 USA.

[Kushner, Robert F.] Northwestern Univ, Feinberg Sch Med, Rubloff Bldg 10th Floor,750 N Lake Shore Dr, Chicago, IL 60611 USA.

[Raum, William J.] Oregon Weight Loss Surg, 1040 NW 22nd Ave,Suite 500, Portland, OR 97210 USA.

[Hermayer, Kathie L.; Sora, Nicoleta D.] Med Univ South Carolina, Div Endocrinol Diabet & Med Genet, Coll Med, Clin Sci Bldg,8th Floor,96 Jonathan Lucas St, Charleston, SC 29425 USA.

[Salyer, Jonny L.] Lovelace Sci Resources, 2441 Ridgecrest Dr,SE, Albuquerque, NM 87108 USA.

RP O'Neil, PM (reprint author), Med Univ South Carolina, Weight Management Ctr, 67 President St,Suite 410 South, Charleston, SC 29425 USA.

EM holland@musc.edu; tuerk@musc.edu; wadden@mail.med.upenn.edu;

fujiolca.ken@scrippshealth.org; lynne.e.becker@gmail.com;

kmillerkovach@gmail.com; priscilh@baylorhealth.edu; garveyt@uab.edu;

drdanweiss@ameritech.net; drubino@wtmgmt.com; rkushner@northwestern.edu;

wraum@oregonwls.com; hermayer@musc.edu; jvelikocde@yahoo.com;

schoemers@gmail.com; sora@musc.edu; oneilp@musc.edu

OI Rubino, Domenica/0000-0002-3072-2819

FU Weight Watchers International (WWI)

FX This work was supported by a grant from Weight Watchers International

(WWI) to the Medical University of South Carolina (MUSC).

CR ADA, 2016, DIABETES CARE, V39, P2126

Ali MK, 2013, NEW ENGL J MED, V368, P1613, DOI 10.1056/NEJMsa1213829

Bjorner JB, 2013, VALUE HEALTH, V16, P993, DOI 10.1016/j.jval.2013.06.022

Buchwald H, 2009, AM J MED, V122, P248, DOI 10.1016/j.amjmed.2008.09.041

Centers for Disease Control and Prevention (CDC), 2014, NAT DIAB STAT REP ES

Crosby RD, 2004, J CLIN EPIDEMIOL, V57, P1153, DOI 10.1016/j.jclinepi.2004.04.004

Dall TM, 2014, DIABETES CARE, V37, P3172, DOI 10.2337/dc14-1036

Egede LE, 2002, DIABETES CARE, V25, P464, DOI 10.2337/diacare.25.3.464

Engel SG, 2003, OBES RES, V11, P1207, DOI 10.1038/oby.2003.166

Fisher L, 2012, DIABETES CARE, V35, P259, DOI 10.2337/dc11-1572

Fisher L, 2010, DIABETES CARE, V33, P1034, DOI 10.2337/dc09-2175

Foster GD, 2013, NUTR DIABETES, V3, DOI 10.1038/nutd.2013.3

Fujioka K, 2010, DIABETES OBES METAB, V12, P186, DOI 10.1111/j.1463-1326.2009.01155.x

GONDERFREDERICK LA, 1989, HEALTH PSYCHOL, V8, P45, DOI 10.1037//0278-6133.8.1.45

Gonzalez JS, 2011, PSYCHOLOGICAL CO-MORBIDITIES OF PHYSICAL ILLNESS: A BEHAVIORAL MEDICINE PERSPECTIVE, P73, DOI 10.1007/978-1-4419-0029-6_2

Greenberg I, 2009, J AM COLL NUTR, V28, P159, DOI 10.1080/07315724.2009.10719767

Hays RD, 2001, ANN MED, V33, P350, DOI 10.3109/07853890109002089

Joensen LE, 2013, DIABETIC MED, V30, P1132, DOI 10.1111/dme.12241

Kolotkin AL, 2001, OBESITY, V9, P564

Kolotkin RL, 2002, QUAL LIFE RES, V11, P157, DOI 10.1023/A:1015081805439

Kolotkin RL, 2001, OBES RES, V9, P102, DOI 10.1038/oby.2001.13

Kroenke K, 2002, PSYCHIAT ANN, V32, P509, DOI 10.3928/0048-5713-20020901-06

Lustman PJ, 2005, J DIABETES COMPLICAT, V19, P113, DOI 10.1016/j.jdiacomp.2004.01.002

Maruish ME, 2011, USERS MANUAL SF 36V2

Menke A, 2015, JAMA-J AM MED ASSOC, V314, P1021, DOI 10.1001/jama.2015.10029

Mezuk B, 2013, HEALTH PSYCHOL, V32, P254, DOI 10.1037/a0029014

Norris SL, 2004, AM J MED, V117, P762, DOI 10.1016/j.amjmed.2004.05.024

O'Neil PM, 2016, OBESITY, V24, P2269, DOI 10.1002/oby.21616

O'Neil PM, 2012, OBESITY, V20, P1426, DOI 10.1038/oby.2012.66

POLONSKY WH, 1995, DIABETES CARE, V18, P754, DOI 10.2337/diacare.18.6.754

Polonsky WH, 2005, DIABETES CARE, V28, P626, DOI 10.2337/diacare.28.3.626

Robbins JM, 2008, DIABETES CARE, V31, P655, DOI [10.2337/dc07-1871, 10.2337/dc07-187]

Rock CL, 2014, DIABETES CARE, V37, P1573, DOI 10.2337/dc13-2900

Steed L, 2003, PATIENT EDUC COUNS, V51, P5, DOI 10.1016/S0738-3991(02)00213-6

VanderDoes FEE, 1996, DIABETES CARE, V19, P204, DOI 10.2337/diacare.19.3.204

Vettor R, 2005, DIABETES CARE, V28, P942, DOI 10.2337/diacare.28.4.942

Wadden TA, 2014, DIABETES CARE, V37, P1544, DOI 10.2337/dc13-1928

WARE JE, 1992, MED CARE, V30, P473, DOI 10.1097/00005650-199206000-00002

White R, 2004, OBESITY, V12, P949

Williamson DA, 2009, ARCH INTERN MED, V169, P163, DOI 10.1001/archinternmed.2008.544

Winchester RJ, 2016, J DIABETES COMPLICAT, V30, P312, DOI 10.1016/j.jdiacomp.2015.11.010

Wing RR, 2011, DIABETES CARE, V34, P1481, DOI 10.2337/dc10-2415

NR 42

TC 1

Z9 1

U1 0

U2 6

PU ELSEVIER SCIENCE INC

PI NEW YORK

PA 360 PARK AVE SOUTH, NEW YORK, NY 10010-1710 USA

SN 1056-8727

EI 1873-460X

J9 J DIABETES COMPLICAT

JI J. Diabetes Complications

PD MAY

PY 2017

VL 31

IS 5

BP 891

EP 897

DI 10.1016/j.jdiacomp.2017.01.022

PG 7

WC Endocrinology & Metabolism

SC Endocrinology & Metabolism

GA EV3UC

UT WOS:000401683600020

PM 28319001

OA Other Gold

DA 2019-01-08

ER

PT J

AU Dennick, K

Sturt, J

Speight, J

AF Dennick, Kathryn

Sturt, Jackie

Speight, Jane

TI What is diabetes distress and how can we measure it? A narrative review

and conceptual model

SO JOURNAL OF DIABETES AND ITS COMPLICATIONS

LA English

DT Review

DE Diabetes distress; Patient-reported outcome measures; Validity;

Narrative review; Conceptual model

ID QUALITY-OF-LIFE; EMOTIONAL DISTRESS; PROBLEM AREAS; SELF-CARE;

PERCEPTION QUESTIONNAIRE; PSYCHOMETRIC VALIDATION; PROFESSIONAL SUPPORT;

SYMPTOM CHECKLIST; GLYCEMIC CONTROL; CHRONIC ILLNESS

AB Background: Diabetes distress is the negative emotional impact of living with diabetes. It has tangible clinical importance, being associated with sub-optimal self-care and glycemic control. Diabetes distress has been operationalized in various ways and several measures exist. Measurement clarity is needed for both scientific and clinical reasons.

Objectives: To clarify the conceptualization and operationalization of diabetes distress, identify and distinguish relevant measures, and evaluate their appropriateness for this purpose.

Results: Six measures were identified: Problem Areas in Diabetes (PAID) scale, Diabetes Distress Scale (DDS); Type I Diabetes Distress Scale (T1-DDS), Diabetes-specific Quality of Life Scale Revised (DSQoLs-R) 'Burden and Restrictions-Daily Hassles' sub-scale, Well-being Questionnaire 28 (W-BQ 28) 'Diabetes Well-being' sub-scale, and Illness Perceptions Questionnaire Revised (IPQ-R) 'Emotional Representations' sub-scale. Across these measures a broad spectrum of diabetes distress is captured, including distress associated with treatment regimen, food/eating, future/complications, hypoglycemia, social/interpersonal relationships, and healthcare professionals. No single measure appears fully comprehensive. Limited detail of the qualitative work informing scale design is reported, raising concerns about content validity.

Conclusions: Across the available measures diabetes distress is seemingly comprehensively assessed and measures should be considered in terms of their focus and scope to ensure the foci of interventions are appropriately targeted. (C) 2017 Elsevier Inc. All rights reserved.

C1 [Dennick, Kathryn; Sturt, Jackie] Kings Coll London, Florence Nightingale Fac Nursing & Midwifery, 57 Waterloo Rd, London SE1 8WA, England.

[Speight, Jane] Deakin Univ, Sch Psychol, 1 Gheringhap St, Geelong, Vic 3220, Australia.

[Speight, Jane] Australian Ctr Behav Res Diabet, Diabet Victoria, 570 Elizabeth St, Melbourne, Vic 3000, Australia.

[Speight, Jane] AHP Res, 16 Walden Way, Hornchurch, Essex, England.

[Dennick, Kathryn] UCL, Res Dept Clin Educ & Hlth Psychol, Room 209,Hunter St Hlth Ctr,8 Hunter St, London WC1N 1BN, England.

RP Dennick, K (reprint author), Kings Coll London, Florence Nightingale Fac Nursing & Midwifery, 57 Waterloo Rd, London SE1 8WA, England.; Dennick, K (reprint author), UCL, Res Dept Clin Educ & Hlth Psychol, Room 209,Hunter St Hlth Ctr,8 Hunter St, London WC1N 1BN, England.

EM kdennick@ucl.ac.uk

OI Speight, Jane/0000-0002-1204-6896

FU Florence Nightingale Faculty of Nursing and Midwifery; King's College

London; Diabetes Victoria; Deakin University

FX This research did not receive any specific grant from funding agencies

in the public, commercial, or not-for-profit sectors.JSt and KD were

supported by core funding provided by Florence Nightingale Faculty of

Nursing and Midwifery, King's College London. JSp is supported by the

core funding provided to the Australian Centre for Behavioural Research

in Diabetes by Diabetes Victoria and Deakin University.

CR Aikens JE, 2012, DIABETES CARE, V35, P2472, DOI 10.2337/dc12-0181

American Diabetes Association, 2003, Diabetes Care, V26 Suppl 1, pS25

Arbuckle RA, 2009, VALUE HEALTH, V12, P1168, DOI 10.1111/j.1524-4733.2009.00571.x

Baek RN, 2014, ANN BEHAV MED, V48, P145, DOI 10.1007/s12160-013-9585-4

Balfe M, 2013, BMC ENDOCR DISORD, V13, DOI 10.1186/1472-6823-13-25

Bott U, 1998, DIABETES CARE, V21, P757, DOI 10.2337/diacare.21.5.757

Boyer JG, 1997, MED CARE, V35, P440, DOI 10.1097/00005650-199705000-00003

Bradley C, 1999, QUAL LIFE RES, V8, P79, DOI 10.1023/A:1026485130100

Bradley G, 2000, DIABETES CARE, V23, P875

CAREY MP, 1991, J BEHAV MED, V14, P43, DOI 10.1007/BF00844767

Cooke D, 2013, DIABETES CARE, V36, P1117, DOI 10.2337/dc12-0076

COX DJ, 1987, DIABETES CARE, V10, P617, DOI 10.2337/diacare.10.5.617

DCCT Res Grp, 1988, DIABETES CARE, V11, P725

Delahanty LM, 2007, DIABETIC MED, V24, P48, DOI 10.1111/j.1464-5491.2007.02028.x

Dennidc K, 2016, INT DIABETES NURS

DURAN G, 1995, PSYCHOL REP, V76, P515, DOI 10.2466/pr0.1995.76.2.515

Eigenmann CA, 2009, DIABETIC MED, V26, P425, DOI 10.1111/j.1464-5491.2009.02697.x

El Achhab Y, 2008, DIABETES RES CLIN PR, V80, P171, DOI 10.1016/j.diabres.2007.12.020

Esbitt SA, 2013, SCREENING DEPRESSION, P27

European Medicines Agency Committee for Medicinal Products for Human Use, REFL PAP REG GUID US

Fenwick EK, 2016, J HLTH PSYCHOL

Fisher L, 2008, DIABETIC MED, V25, P1096, DOI 10.1111/j.1464-5491.2008.02533.x

Fisher L, 2014, DIABETIC MED, V31, P764, DOI 10.1111/dme.12428

Fisher L, 2014, 74 SCI SESS AM DIAB

Fisher L, 2008, ANN FAM MED, V6, P246, DOI 10.1370/afm.842

Fisher L, 2007, DIABETES CARE, V30, P542, DOI 10.2337/dc06-1614

Fisher L, 2015, J DIABETES COMPLICAT, V29, P572, DOI 10.1016/j.jdiacomp.2015.01.012

Fisher L, 2010, DIABETES CARE, V33, P23, DOI 10.2337/dc09-1238

Fonda Stephanie J, 2009, J Diabetes Sci Technol, V3, P117

Furler J, 2008, BMC HEALTH SERV RES, V8, DOI 10.1186/1472-6963-8-214

Garratt AM, 2002, DIABETIC MED, V19, P1, DOI 10.1046/j.1464-5491.2002.00650.x

Gonzalez JS, 2008, DIABETOLOGIA, V51, P1822, DOI 10.1007/s00125-008-1113-x

Gonzalez JS, 2015, HEALTH PSYCHOL, V34, P505, DOI 10.1037/hea0000131

Gonzalez JS, 2011, DIABETES CARE, V34, P236, DOI 10.2337/dc10-1970

GROOTENHUIS PA, 1994, DIABETIC MED, V11, P253, DOI 10.1111/j.1464-5491.1994.tb00268.x

Hagger V, 2016, CURR DIABETES REP, V16, DOI 10.1007/s11892-015-0694-2

Henry SL, 2013, J HEALTH PSYCHOL, V18, P1550, DOI 10.1177/1359105312465913

Herschbach P, 1997, HEALTH PSYCHOL, V16, P171, DOI 10.1037/0278-6133.16.2.171

Hirsch A, 2000, QUAL LIFE RES, V9, P207, DOI 10.1023/A:1008959810698

Johnson L. N., 2013, THESIS

Kempf K, 2013, BMC ENDOCR DISORD, V13, DOI 10.1186/1472-6823-13-57

Kokoszka A, 2009, EUR PSYCHIAT, V24, P425, DOI 10.1016/j.eurpsy.2009.04.002

Luscombe F A, 2000, Value Health, V3 Suppl 1, P15

Mannucci E, 1996, DIABETES NUTR METAB, V9, P89

Markowitz JT, 2012, DIABETIC MED, V29, P526, DOI 10.1111/j.1464-5491.2011.03434.x

McGuire BE, 2010, DIABETOLOGIA, V53, P66, DOI 10.1007/s00125-009-1559-5

Meadows K, 1996, QUAL LIFE RES, V5, P242, DOI 10.1007/BF00434746

Moss-Morris R, 2002, PSYCHOL HEALTH, V17, P1, DOI 10.1080/08870440290001494

Peeples M, 2007, DIABETES EDUCATOR, V33, P794, DOI 10.1177/0145721707307615

Peyrot M, 2005, DIABETES CARE, V28, P53, DOI 10.2337/diacare.28.1.53

POLONSKY WH, 1995, DIABETES CARE, V18, P754, DOI 10.2337/diacare.18.6.754

Polonsky WH, 2005, DIABETES CARE, V28, P626, DOI 10.2337/diacare.28.3.626

Polonsky WH, 2000, DIABETES SPECTRUM, V13, P36

Polonsky WH, 2016, DIABETES TECHNOL THE, V18, P292, DOI 10.1089/dia.2015.0451

Rubin RR, 2010, HEALTH QUAL LIFE OUT, V8, DOI 10.1186/1477-7525-8-32

Schmitt A, 2015, DIABET MED

Schmitt A, 2015, J BEHAV MED, V38, P556, DOI 10.1007/s10865-014-9604-3

Snoek FJ, 2015, LANCET DIABETES ENDO, V3, P450, DOI 10.1016/S2213-8587(15)00135-7

Speight J, 2012, DIABETIC MED, V29, pE345, DOI 10.1111/j.1464-5491.2012.03702.x

Speight J, 2009, DIABETIC MED, V26, P315, DOI 10.1111/j.1464-5491.2009.02682.x

Speight J, 2011, BEHALF DIABETES MILE

Speight J, 2002, DIAB UK PROF C

Stoop CH, 2014, DIABETIC MED, V31, P1252, DOI 10.1111/dme.12472

Strandberg RB, 2014, J PSYCHOSOM RES, V77, P174, DOI 10.1016/j.jpsychores.2014.06.015

Sturt J, 2015, INT DIABETES NURS, P1

Sturt J, 2015, CURR DIABETES REP, V15, DOI 10.1007/s11892-015-0660-z

Talbot F, 1997, J BEHAV MED, V20, P291, DOI 10.1023/A:1025508928696

Tanenbaum ML, 2016, J DIABETES COMPLICAT, V30, P1060, DOI 10.1016/j.jdiacomp.2016.04.023

Tang TS, 2008, DIABETES EDUCATOR, V34, P266, DOI 10.1177/0145721708315680

Taylor EP, 2005, DIABETES-METAB RES, V21, P264, DOI 10.1002/dmrr.524

Thorne SE, 2001, PATIENT EDUC COUNS, V42, P81, DOI 10.1016/S0738-3991(00)00095-1

US Department of Health and Human Services & Food and Drug Administration, 2009, GUID IND PAT REP OUT

Watkins K, 2004, PHARMACOECONOMICS, V22, P1109, DOI 10.2165/00019053-200422170-00002

Weinger K, 2001, PATIENT EDUC COUNS, V42, P123, DOI 10.1016/S0738-3991(00)00098-7

Weinman J, 1996, PSYCHOL HEALTH, V11, P431, DOI 10.1080/08870449608400270

Weissberg-Benchell J, 2011, PEDIATR DIABETES, V12, P341, DOI 10.1111/j.1399-5448.2010.00720.x

Welch GW, 1997, DIABETES CARE, V20, P760, DOI 10.2337/diacare.20.5.760

Wild D, 2007, PATIENT EDUC COUNS, V68, P10, DOI 10.1016/j.pec.2007.05.003

Zagarins SE, 2012, J BEHAV MED, V35, P299, DOI 10.1007/s10865-011-9359-z

NR 79

TC 4

Z9 4

U1 2

U2 11

PU ELSEVIER SCIENCE INC

PI NEW YORK

PA 360 PARK AVE SOUTH, NEW YORK, NY 10010-1710 USA

SN 1056-8727

EI 1873-460X

J9 J DIABETES COMPLICAT

JI J. Diabetes Complications

PD MAY

PY 2017

VL 31

IS 5

BP 898

EP 911

DI 10.1016/j.jdiacomp.2016.12.018

PG 14

WC Endocrinology & Metabolism

SC Endocrinology & Metabolism

GA EV3UC

UT WOS:000401683600021

PM 28274681

DA 2019-01-08

ER

PT J

AU Rechenberg, K

Grey, M

Sadler, L

AF Rechenberg, Kaitlyn

Grey, Margaret

Sadler, Lois

TI Stress and Posttraumatic Stress in Mothers of Children With Type 1

Diabetes

SO JOURNAL OF FAMILY NURSING

LA English

DT Article

DE stress; posttraumatic stress; chronic illness; maternal attachment; type

1 diabetes

ID PSYCHOLOGICAL DISTRESS; PATERNAL INVOLVEMENT; DEPRESSIVE SYMPTOMS;

YOUNG-CHILDREN; PARENTS; ADJUSTMENT; ADOLESCENTS; ASSOCIATION; DISORDER;

FATHERS

AB The onset of acute and chronic illness in children frequently triggers episodes of stress and posttraumatic stress symptoms (PTSS) in mothers. Mothers of children with type 1 diabetes (T1D) consistently report high levels of stress and PTSS. The purpose of this integrative review was to review and synthesize the published empirical research. The Preferred Reporting Items for Systematic Reviews and Meta-Analyses (PRISMA) guidelines were used to conduct this integrative literature review. A total of 19 studies were identified from a sample of 128. Stress and PTSS were prevalent in mothers of youth with T1D. While PTSS was most severe at disease onset, symptoms often persisted 1 to 5 years after diagnosis. The diagnosis of T1D in a child was traumatic for mothers. Stress and PTSS in mothers adversely affected children's health. Management of stress symptoms in mothers may lead to improved behavioral and metabolic outcomes in children.

C1 [Rechenberg, Kaitlyn; Grey, Margaret; Sadler, Lois] Yale Sch Nursing, 400 West Campus Dr, Orange, CT 06577 USA.

RP Rechenberg, K (reprint author), Yale Sch Nursing, 400 West Campus Dr, Orange, CT 06577 USA.

EM Kaitlyn.rechenberg@yale.edu

FU NCATS NIH HHS [UL1 TR001863]; NIDDK NIH HHS [T32 DK097718]; NINR NIH HHS

[P30 NR008999]

CR American Psychiatric Association, 2013, DIAGN STAT MAN MENT

American Psychiatric Association, 1994, DIAGN STAT MAN MENT

[Anonymous], 2012, DIABETES CARE, V35, pS11, DOI [10.2337/dc12-s011, 10.2337/dc35-S011]

Athaseri S., 2010, THAI J NURSING RES, V12, P55

Balluffi Andrew, 2004, Pediatr Crit Care Med, V5, P547, DOI 10.1097/01.PCC.0000137354.19807.44

Barakat LP, 2006, J PEDIATR PSYCHOL, V31, P413, DOI 10.1093/jpepsy/jsj058

Berg CA, 2007, J PEDIATR PSYCHOL, V32, P995, DOI 10.1093/jpepsy/jsm043

Beveridge RM, 2006, J PEDIATR PSYCHOL, V31, P818, DOI 10.1093/jpepsy/jsj094

Bodenmann G, 2005, COUPLES COPING STRES

Butler JM, 2007, J PEDIATR PSYCHOL, V32, P1227, DOI 10.1093/jpepsy/jsm065

Cameron FJ, 2007, DIABETES CARE, V30, P2716, DOI 10.2337/dc07-0603

Chaney JM, 1997, J PEDIATR PSYCHOL, V22, P229

Chiang JL, 2014, DIABETES CARE, V37, P2034, DOI 10.2337/dc14-1140

Critical Apprasial Skills Program (CASP), 2004, APPR TOOL QUAL RES

Genuth S, 2001, J PEDIATR-US, V139, P804, DOI 10.1067/mpd.2001.118887

Hall E, 2006, J PEDIATR PSYCHOL, V31, P403, DOI 10.1093/jpepsy/jsj016

Hannonen R, 2015, ACTA PAEDIATR, V104, P1144, DOI 10.1111/apa.13144

Hansen JA, 2012, FAM SYST HEALTH, V30, P47, DOI 10.1037/a0027519

Helgeson VS, 2012, J PEDIATR PSYCHOL, V37, P467, DOI 10.1093/jpepsy/jsr110

Herzer M, 2010, J PEDIATR PSYCHOL, V35, P415, DOI 10.1093/jpepsy/jsp063

Higgins JPT, 2011, BMJ-BRIT MED J, V343, DOI 10.1136/bmj.d5928

Hoff A. L., 2005, FAMILIES SYSTEMS HLT, V23, P329, DOI DOI 10.1037/1091-7527.23.3.329

Hood KK, 2006, DIABETES CARE, V29, P1389, DOI 10.2337/dc06-0087

Horsch A, 2007, J TRAUMA STRESS, V20, P881, DOI 10.1002/jts.20247

Horsch A, 2014, J HEALTH PSYCHOL, V19, P987, DOI 10.1177/1359105313482169

Horsch A, 2012, BEHAV COGN PSYCHOTH, V40, P400, DOI 10.1017/S1352465812000112

Horton TV, 2001, REHABIL PSYCHOL, V46, P382, DOI 10.1037/0090-5550.46.4.382

Jaser SS, 2008, J PEDIATR PSYCHOL, V33, P509, DOI 10.1093/jpepsy/jsm104

Jaser SS, 2009, CHILD HEALTH CARE, V38, P91, DOI 10.1080/02739610902813229

KAGER VA, 1992, J PEDIATR PSYCHOL, V17, P491, DOI 10.1093/jpepsy/17.4.491

Kazak AE, 2004, J PEDIATR PSYCHOL, V29, P211, DOI 10.1093/jpepsy/jsh022

Koizumi S, 1992, J Pediatr Nurs, V7, P154

Kokkonen J, 1997, NORD J PSYCHIAT, V51, P165, DOI 10.3109/08039489709109091

Landolt MA, 2005, J AM ACAD CHILD PSY, V44, P682, DOI 10.1097/01-chi.0000161645.98022.35

Landolt MA, 2002, J PEDIATR PSYCHOL, V27, P647, DOI 10.1093/jpepsy/27.7.647

Landolt MA, 2012, J CHILD PSYCHOL PSYC, V53, P767, DOI 10.1111/j.1469-7610.2011.02520.x

Levine B., 2014, MILITARY BEHAV HLTH, V2, P59, DOI [10.1080/21635781.2013.845070, DOI 10.1080/21635781.2013.845070]

Lewin A. B., 2005, FAMILIES SYSTEMS HLT, V23, P56, DOI DOI 10.1037/1091-7527.23.1.56

Liese AD, 2006, PEDIATRICS, V118, P1510, DOI 10.1542/peds.2006-0690

Maahs DM, 2010, ENDOCRIN METAB CLIN, V39, P481, DOI 10.1016/j.ecl.2010.05.011

Mitchell SJ, 2009, FAM SYST HEALTH, V27, P314, DOI 10.1037/a0018191

Moher D, 2009, ANN INTERN MED, V151, P264, DOI 10.7326/0003-4819-151-4-200908180-00135

National Institutes of Health & National Heart Lung and Blood Institute, 2014, QUAL ASS TOOL OBS CO

Pisula E, 2010, EUR J MED RES, V15, P115

Stewart M J, 1994, Can J Nurs Res, V26, P61

Streisand R, 2008, PATIENT EDUC COUNS, V73, P333, DOI 10.1016/j.pec.2008.06.014

Streisand R, 2010, MATERN CHILD HLTH J, V14, P612, DOI 10.1007/s10995-009-0497-7

Sullivan-Bolyai S, 2003, J Pediatr Nurs, V18, P21, DOI 10.1053/jpdn.2003.4

Sullivan-Bolyai S, 2016, J FAM NURS, V22, P579, DOI 10.1177/1074840716676589

Whittemore R, 2005, J ADV NURS, V52, P546, DOI 10.1111/j.1365-2648.2005.03621.x

Whittemore R, 2012, DIABETES EDUCATOR, V38, P562, DOI 10.1177/0145721712445216

Wood JR, 2013, DIABETES CARE, V36, P2035, DOI 10.2337/dc12-1959

Wysocki T, 2006, J PEDIATR PSYCHOL, V31, P501, DOI 10.1093/jpepsy/jsj042

NR 53

TC 0

Z9 0

U1 1

U2 6

PU SAGE PUBLICATIONS INC

PI THOUSAND OAKS

PA 2455 TELLER RD, THOUSAND OAKS, CA 91320 USA

SN 1074-8407

EI 1552-549X

J9 J FAM NURS

JI J. Fam. Nurs.

PD MAY

PY 2017

VL 23

IS 2

BP 201

EP 225

DI 10.1177/1074840716687543

PG 25

WC Family Studies; Nursing

SC Family Studies; Nursing

GA EV1OX

UT WOS:000401517000003

PM 28795899

OA Green Accepted

DA 2019-01-08

ER

PT J

AU Kuniss, N

Rechtacek, T

Kloos, C

Muller, UA

Roth, J

Burghardt, K

Kramer, G

AF Kuniss, N.

Rechtacek, T.

Kloos, C.

Mueller, U. A.

Roth, J.

Burghardt, K.

Kramer, G.

TI Diabetes-related burden and distress in people with diabetes mellitus at

primary care level in Germany

SO ACTA DIABETOLOGICA

LA English

DT Article

DE Diabetes-related distress; PAID questionnaire; Type 2 diabetes

ID EMOTIONAL DISTRESS; PROBLEM AREAS; SELF-MANAGEMENT; DEPRESSION;

ASSOCIATION; SCALE; HYPOGLYCEMIA; ADHERENCE; UTILITY; DUTCH

AB Aims The importance of diabetes-related distress for the treatment of diabetes is emphasised in national and international guidelines recommending routinely screening for psychosocial problems. Data of investigations regarding diabetes-related distress on primary care are rare in Germany though most people with diabetes are treated without insulin therapy at primary care level.

Methods Three hundred and forty-five people with diabetes mellitus type 2 (DM2, n = 336, 229 without and 107 with insulin therapy) and type 1 (DM1, n = 9) were interviewed with the PAID questionnaire in the period from 1 October 2015 to 31 December 2015 in a general practice. A PAID score >= 40 (range 0-100) was considered as high diabetes-related distress.

Results The mean PAID score of all participants was 3.9 +/- 7.0 (DM2 without insulin 2.7 +/- 6.3, DM2 with insulin therapy 6.0 +/- 8.0, DM1 6.8 +/- 4.9) and far below the threshold of 40 points. Only 1.2% of all responders showed high diabetes-related distress (score >= 40). People on insulin therapy with HbA1c > 7.5% and with diagnosed depression prior to the study scored significantly higher. Furthermore, there are weak correlations between the PAID score and HbA1c (r = 0.253, p < 0.001), duration of diabetes (r = 0.169, p = 0.002), insulin dosage (r = 0.283, p < 0.001) and age (r = -0.129, p = 0.016).

Conclusions Only 1.2% of our outpatients with diabetes on primary care level showed high diabetes-related distress. Higher rates in the current literature are probably due to not investigating on primary care level. Guidelines should consider this.

C1 [Kuniss, N.; Kloos, C.; Mueller, U. A.; Roth, J.; Burghardt, K.; Kramer, G.] Jena Univ Hosp, Dept Internal Med Endocrinol & Metab Disorder 3, Klinikum 1, D-07740 Jena, Germany.

RP Kuniss, N (reprint author), Jena Univ Hosp, Dept Internal Med Endocrinol & Metab Disorder 3, Klinikum 1, D-07740 Jena, Germany.

EM nadine.kuniss@med.uni-jena.de

CR American Diabetes Association, 2015, DIABETES CARE, V38

Bohm BO, EVIDENCE BASED GUIDE

Bundesarztekammer (BAK) Kassenarztliche Bundesvereinigung (KBV) Arbeitsgemeinschaft der Wissenschaftlichen Medizinischen Fachgesellschaften (AWMF), 2013, NAT VERS LEITL THER, DOI 10.6101/AXQ/0UU213

Delahanty LM, 2007, DIABETIC MED, V24, P48, DOI 10.1111/j.1464-5491.2007.02028.x

German Diabetes Association (DDG), 2015, GUID REC TREATM I CE

Gonzalez JS, 2015, HEALTH PSYCHOL, V34, P505, DOI 10.1037/hea0000131

Hagen B, 2015, QUALITATSBERICHT 201

Hermanns N, 2006, DIABETOLOGIA, V49, P469, DOI 10.1007/s00125-005-0094-2

Hermanns N, 2015, DIABETES CARE, V38, P551, DOI 10.2337/dc14-1416

International Diabetes Federation Guideline Development Group, 2014, DIABETES RES CLIN PR, V104, P1, DOI DOI 10.1016/J.DIABRES.2012.10.001

Joensen LE, 2013, DIABETES RES CLIN PR, V101, P278, DOI 10.1016/j.diabres.2013.07.001

Kovacs BK, 2013, DIABETIC MED, V30, P778, DOI DOI 10.1111/DME.12239

Kulzer BAC, 2013, S2 GUIDELINE PSYCHOS

Kuniss N, 2016, EXP CLIN ENDOCR DIAB, V124, P307, DOI 10.1055/s-0042-103685

Kuniss N, 2016, ACTA DIABETOL

Nicolucci A, 2013, DIABETIC MED, V30, P767, DOI 10.1111/dme.12245

Petrak F, 2011, DIABETES STOFFWECH H, V20, P7

Pettersson B, 2011, DIABETES RES CLIN PR, V92, P19, DOI 10.1016/j.diabres.2010.12.005

POLONSKY WH, 1995, DIABETES CARE, V18, P754, DOI 10.2337/diacare.18.6.754

Pouwer F, 2005, DIABETES RES CLIN PR, V70, P166, DOI 10.1016/j.diabres.2005.03.031

Reddy J, 2013, PSYCHOSOMATICS, V54, P44, DOI 10.1016/j.psym.2012.08.004

Schmitt A, 2015, J BEHAV MED, V38, P556, DOI 10.1007/s10865-014-9604-3

Seaquist ER, 2013, DIABETES CARE, V36, P1384, DOI 10.2337/dc12-2480

Snoek FJ, 2000, DIABETES CARE, V23, P1305, DOI 10.2337/diacare.23.9.1305

Stoop CH, 2014, DIABETIC MED, V31, P1252, DOI 10.1111/dme.12472

The Diabetes Control and Complications Trial Research Group, 1993, NEW ENGL J MED, V329, P977, DOI [10.1056/NEJM199309303291401, DOI 10.1056/NEJM199309303291401]

Wardian J, 2014, SOC WORK HEALTH CARE, V53, P364, DOI 10.1080/00981389.2014.884038

Welch GW, 1997, DIABETES CARE, V20, P760, DOI 10.2337/diacare.20.5.760

Wild D, 2007, PATIENT EDUC COUNS, V68, P10, DOI 10.1016/j.pec.2007.05.003

NR 29

TC 3

Z9 3

U1 1

U2 12

PU SPRINGER-VERLAG ITALIA SRL

PI MILAN

PA VIA DECEMBRIO, 28, MILAN, 20137, ITALY

SN 0940-5429

EI 1432-5233

J9 ACTA DIABETOL

JI Acta Diabetol.

PD MAY

PY 2017

VL 54

IS 5

BP 471

EP 478

DI 10.1007/s00592-017-0972-3

PG 8

WC Endocrinology & Metabolism

SC Endocrinology & Metabolism

GA ER2DD

UT WOS:000398603300007

PM 28210870

DA 2019-01-08

ER

PT J

AU Chew, BH

Vos, RC

Ghazali, SS

Shamsuddin, NH

Fernandez, A

Mukhtar, F

Ismail, M

Ahad, AM

Sundram, NN

Ali, SZM

Rutten, GEHM

AF Chew, Boon-How

Vos, Rimke C.

Ghazali, Sazlina Shariff

Shamsuddin, Nurainul Hana

Fernandez, Aaron

Mukhtar, Firdaus

Ismail, Mastura

Ahad, Azainorsuzila Mohd

Sundram, Narayanan N.

Ali, Siti Zubaidah Mohd

Rutten, Guy E. H. M.

TI The effectiveness of a value-based EMOtion-cognition-Focused educatIonal

programme to reduce diabetes-related distress in Malay adults with Type

2 diabetes (VEMOFIT): study protocol for a cluster randomised controlled

trial

SO BMC ENDOCRINE DISORDERS

LA English

DT Article

DE Diabetes-related distress; Self-care; Depression; Diabetes education;

Quality of life; Type 2 diabetes mellitus; HbA1c; Blood pressure;

LDL-cholesterol

ID SELF-MANAGEMENT BEHAVIORS; QUALITY-OF-LIFE; PRIMARY-CARE; ILLNESS

REPRESENTATIONS; GLYCEMIC CONTROL; TREATMENT FIDELITY; ANXIETY

DISORDERS; HEALTH-CARE; DEPRESSION; OUTCOMES

AB Background: Type 2 diabetes mellitus (T2DM) patients experience many psychosocial problems related to their diabetes. These often lead to emotional disorders such as distress, stress, anxiety and depression, resulting in decreased self-care, quality of life and disease control. The purpose of the current study is to evaluate the effectiveness of a brief value-based emotion-focused educational programme in adults with T2DM on diabetes-related distress (DRD), depressive symptoms, illness perceptions, quality of life, diabetes self-efficacy, self-care and clinical outcomes.

Methods: A cluster randomised controlled trial will be conducted in 10 public health clinics in Malaysia, all providing diabetes care according to national clinical practice guidelines. Patients' inclusion criteria: Malay, >= 18 years with T2DM for at least 2 years, on regular follow-up with one of three biomarkers HbA1c, systolic blood pressure and LDL-cholesterol sub-optimally controlled, and with a mean 17-item Diabetes Distress Scale (DDS-17) score >= 3. The intervention consists of four sessions and one booster over a period of 4 months that provide information and skills to assist patients in having proper perceptions of their T2DM including an understanding of the treatment targets, understanding and managing their emotions and goal-setting. The comparator is an attention-control group with three meetings over a similar period. With an estimated intra-cluster correlation coefficient. of 0.015, a cluster size of 20 and 20% non-completion, the trial will need to enroll 198 patients. Primary outcome: the between groups difference in proportion of patients achieving a mean DDS-17 score < 3 (non-significant distress) at 6 months post-intervention. Secondary outcomes will be the differences in the above mentioned variables between groups.

Discussion: We hypothesize that primary and secondary outcomes will improve significantly after the intervention compared to the comparator group. The results of this study can contribute to better care for T2DM patients with DRD.

C1 [Chew, Boon-How; Ghazali, Sazlina Shariff; Shamsuddin, Nurainul Hana] Univ Putra Malaysia, Fac Med & Hlth Sci, Dept Family Med, Serdang 43400, Selangor, Malaysia.

[Chew, Boon-How; Vos, Rimke C.; Rutten, Guy E. H. M.] Univ Med Ctr Utrecht, Julius Ctr Hlth Sci & Primary Care, Dept Gen Practice, Huispost Str 6-131,POB 855003508 GA, Utrecht, Netherlands.

[Fernandez, Aaron; Mukhtar, Firdaus] Univ Putra Malaysia, Dept Psychiat, Fac Med & Hlth Sci, Serdang 43400, Selangor, Malaysia.

[Ismail, Mastura] Hlth Clin Seremban 2, Jalan S2,A2,Seremban 2, Seremban 27300, Negeri Sembilan, Malaysia.

[Ahad, Azainorsuzila Mohd] Hlth Clin Port Dickson, KM 1,Jalan Seremban Port Dickson, Port Dickson 71000, Negeri Sembilan, Malaysia.

[Sundram, Narayanan N.] Hlth Clin Nilai, Port Dickson 71000, Negeri Sembilan, Malaysia.

[Ali, Siti Zubaidah Mohd] Hlth Clin Seremban, Jalan Rasah, Seremban 70300, Negeri Sembilan, Malaysia.

RP Chew, BH (reprint author), Univ Putra Malaysia, Fac Med & Hlth Sci, Dept Family Med, Serdang 43400, Selangor, Malaysia.; Chew, BH (reprint author), Univ Med Ctr Utrecht, Julius Ctr Hlth Sci & Primary Care, Dept Gen Practice, Huispost Str 6-131,POB 855003508 GA, Utrecht, Netherlands.

EM chewboonhow@gmail.com

RI Chew, Boon How/B-2385-2010

OI Chew, Boon How/0000-0002-8627-6248; Fernandez, Aaron/0000-0002-9834-472X

FU Malaysian MOH-NIH Research Grant (MRG)

FX The trial is funded by the Malaysian MOH-NIH Research Grant (MRG). This

funding source had no role in the design of this study and will not have

any role during its execution, analyses, interpretation of the data, or

decision to submit results. MRG can be contacted at the Secretariat

National Institutes of Health (NIH) Ministry of Health Malaysia, c/o

Institut Pengurusan Kesihatan Jalan Rumah Sakit Bangsar 59000 Kuala

Lumpur; telephone: 03 - 2287 4032/2282 0491/2282 9085/2282 9082; fax :

03 2287 4030/2282 8072, and email: nihsec@nih.gov.my.

CR Adams G, 2004, J CLIN EPIDEMIOL, V57, P785, DOI 10.1016/j.jclinepi.2003.12.013

Aikens JE, 2012, DIABETES CARE, V35, P2472, DOI 10.2337/dc12-0181

Anderson RJ, 2002, INT J PSYCHIAT MED, V32, P235, DOI 10.2190/KLGD-4H8D-4RYL-TWQ8

[Anonymous], 2017, DIABETES CARE, V40, pS1, DOI 10.2337/dc17-S001

August KJ, 2010, SOC SCI MED, V71, P1831, DOI 10.1016/j.socscimed.2010.08.022

BANDURA A, 1991, ORGAN BEHAV HUM DEC, V50, P248, DOI 10.1016/0749-5978(91)90022-L

Barnes L, 2004, NZ MED J, V117, P743

Bellg AJ, 2004, HEALTH PSYCHOL, V23, P443, DOI 10.1037/0278-6133.23.5.443

Borrelli B, 2005, J CONSULT CLIN PSYCH, V73, P852, DOI 10.1037/0022-006X.73.5.852

Boutron I, 2008, ANN INTERN MED, V148, P295, DOI 10.7326/0003-4819-148-4-200802190-00008

Bradshaw BG, 2007, DIABETES EDUCATOR, V33, P650, DOI 10.1177/0145721707303809

Broadbent E, 2006, J PSYCHOSOM RES, V60, P631, DOI 10.1016/j.jpsychores.2005.10.020

Cameron LDLH, 2003, SELF REGULATION HLTH

Campbell NC, 2007, BRIT MED J, V334, P455, DOI 10.1136/bmj.39108.379965.BE

Chan AW, 2013, BMJ-BRIT MED J, V346, DOI 10.1136/bmj.e7586

Chew BH, 2016, PRIM CARE DIABETES, V10, P383, DOI 10.1016/j.pcd.2016.07.003

Chew BH, 2016, PLOS ONE, V11, DOI 10.1371/journal.pone.0152095

Chew BH, 2014, WORLD J DIABETES, V5, P796, DOI 10.4239/wjd.v5.i6.796

Collins J, 2004, RADIOGRAPHICS, V24, P1483, DOI 10.1148/rg.245045020

Das-Munshi J, 2007, PSYCHOSOM MED, V69, P543, DOI 10.1097/PSY.0b013e3180cc3062

De Maeseneer J, 2008, BRIT J GEN PRACT, V58, P806, DOI 10.3399/bjgp08X342697

Deakin T, 2005, COCHRANE DB SYST REV, DOI 10.1002/14651858.CD003417.pub2

Delahanty LM, 2007, DIABETIC MED, V24, P48, DOI 10.1111/j.1464-5491.2007.02028.x

DiMatteo MR, 2004, HEALTH PSYCHOL, V23, P207, DOI 10.1037/0278-6133.23.2.207

Dimitraki G, 2014, INT J BEHAV MED, V21, P230, DOI 10.1007/s12529-013-9296-z

Duangdao KM, 2008, J BEHAV MED, V31, P291, DOI 10.1007/s10865-008-9155-6

Fisher L, 2008, DIABETIC MED, V25, P1096, DOI 10.1111/j.1464-5491.2008.02533.x

Fisher L, 2014, DIABETIC MED, V31, P764, DOI 10.1111/dme.12428

Fisher L, 2002, DIABETES CARE, V25, P1564, DOI 10.2337/diacare.25.9.1564

Fisher L, 2013, DIABETES CARE, V36, P2551, DOI 10.2337/dc12-2493

Fisher L, 2012, DIABETES CARE, V35, P259, DOI 10.2337/dc11-1572

Fisher L, 2010, DIABETES CARE, V33, P23, DOI 10.2337/dc09-1238

Franks MM, 2012, FAM SYST HEALTH, V30, P154, DOI 10.1037/a0028614

Gask L, 2011, CHRONIC ILLN, V7, P239, DOI 10.1177/1742395311403636

Glasgow RE, 2014, TRANSL BEHAV MED, V4, P26, DOI 10.1007/s13142-013-0232-1

Harkness E, 2010, DIABETES CARE, V33, P926, DOI 10.2337/dc09-1519

Harper A, 1998, PSYCHOL MED, V28, P551

Harvey JN, 2009, DIABETIC MED, V26, P5, DOI 10.1111/j.1464-5491.2008.02628.x

Hayes E, 2008, J AM ACAD NURSE PRAC, V20, P155, DOI 10.1111/j.1745-7599.2007.00297.x

Health Quality Ontario, 2009, Ont Health Technol Assess Ser, V9, P1

Hermanns N, 2005, DIABETIC MED, V22, P293, DOI 10.1111/j.1464-5491.2005.01414.x

Ismail K, 2004, LANCET, V363, P1589, DOI 10.1016/S0140-6736(04)16202-8

Kasteleyn MJ, 2015, DIABETIC MED, V32, P1617, DOI 10.1111/dme.12743

Kaur G, 2013, BMC FAM PRACT, V14, DOI 10.1186/1471-2296-14-69

Kroenke K, 2001, J GEN INTERN MED, V16, P606, DOI 10.1046/j.1525-1497.2001.016009606.x

Lau R, 2015, BMJ OPEN, V5, DOI 10.1136/bmjopen-2015-009993

Lewis F M, 1997, Oncol Nurs Forum, V24, P1695

Li CY, 2009, INT J PUBLIC HEALTH, V54, P43, DOI 10.1007/s00038-009-0006-1

Lloyd C, 2005, DIABETES SPECTR, V18, P121, DOI [DOI 10.2337/DIASPECT.18.2.121, 10.2337/diaspect.18.2.121]

Low LL, 2016, PLOS ONE, V11, DOI 10.1371/journal.pone.0147127

Lynch CP, 2012, DIABETES EDUCATOR, V38, P427, DOI 10.1177/0145721712440335

Ministry of Health Malaysia, 2015, CLIN PRACT GUID MAN

Murata GH, 2003, DIABETOLOGIA, V46, P1170, DOI 10.1007/s00125-003-1161-1

Myers DG, 2000, AM PSYCHOL, V55, P56, DOI 10.1037//0003-066X.55.1.56

Naik AD, 2012, BMC GERIATR, V12, DOI 10.1186/1471-2318-12-37

Nicolucci A, 2013, DIABETIC MED, V30, P767, DOI 10.1111/dme.12245

Ogbera A, 2011, J DIABETES, V3, P348, DOI 10.1111/j.1753-0407.2011.00156.x

Paschalides C, 2004, J PSYCHOSOM RES, V57, P557, DOI 10.1016/j.jpsychores.2004.03.006

Polonsky WH, 2005, DIABETES CARE, V28, P626, DOI 10.2337/diacare.28.3.626

Pouwer F, 2009, NAT REV ENDOCRINOL, V5, P665, DOI 10.1038/nrendo.2009.214

RADLOFF L S, 1977, Applied Psychological Measurement, V1, P385, DOI 10.1177/014662167700100306

Ramli AS, 2016, BMC FAM PRACT, V17, DOI 10.1186/s12875-016-0557-1

Rasmussen NH, 2013, PRIM CARE DIABETES, V7, P213, DOI 10.1016/j.pcd.2013.04.006

Reuben E, 2009, CAN WE TEACH EMOTION

Rosland AM, 2012, J BEHAV MED, V35, P221, DOI 10.1007/s10865-011-9354-4

Russell S. S., 2006, UROLOGIC NURSING, V26, P370

Rutten GEHM, 2014, LANCET DIABETES ENDO, V2, P191, DOI 10.1016/S2213-8587(13)70122-0

Searle A, 2007, J PSYCHOSOM RES, V63, P175, DOI 10.1016/j.jpyschores.2007.02.006

Sharoni SKA, 2012, NURS HEALTH SCI, V14, P38, DOI 10.1111/j.1442-2018.2011.00658.x

Sherina M S, 2012, Med J Malaysia, V67, P309

Skevington SM, 2004, QUAL LIFE RES, V13, P299, DOI 10.1023/B:QURE.0000018486.91360.00

Skinner TC, 2014, DIABETIC MED, V31, P1260, DOI 10.1111/dme.12484

Sturt J, 2015, INT DIAB NURS, V12, P40, DOI DOI 10.1179/2057332415Y.0000000004

Sturt Jackie, 2006, BMC Fam Pract, V7, P70, DOI 10.1186/1471-2296-7-70

Toobert DJ, 2000, DIABETES CARE, V23, P943, DOI 10.2337/diacare.23.7.943

van der Bijl J, 1999, J ADV NURS, V30, P352, DOI 10.1046/j.1365-2648.1999.01077.x

Walker RJ, 2012, GEN HOSP PSYCHIAT, V34, P598, DOI 10.1016/j.genhosppsych.2012.07.005

Wallace AS, 2009, PATIENT EDUC COUNS, V75, P328, DOI 10.1016/j.pec.2008.12.017

Whiting DR, 2011, DIABETES RES CLIN PR, V94, P311, DOI 10.1016/j.diabres.2011.10.029

WHO Consultation, 1999, 992 WHO

Willaing I, 2013, DIABETIC MED, V30, pe139, DOI 10.1111/dme.12121

Wu SFV, 2008, INT J NURS STUD, V45, P534, DOI 10.1016/j.ijnurstu.2006.08.020

Xu Y, 2008, J TRANSCULT NURS, V19, P234, DOI 10.1177/1043659608319239

Yi-Frazier JP, 2010, STRESS HEALTH, V26, P51, DOI 10.1002/smi.1258

Zeh P, 2012, DIABETIC MED, V29, P1237, DOI 10.1111/j.1464-5491.2012.03701.x

NR 85

TC 3

Z9 3

U1 1

U2 7

PU BIOMED CENTRAL LTD

PI LONDON

PA 236 GRAYS INN RD, FLOOR 6, LONDON WC1X 8HL, ENGLAND

SN 1472-6823

J9 BMC ENDOCR DISORD

JI BMC Endocr. Disord.

PD APR 4

PY 2017

VL 17

AR 22

DI 10.1186/s12902-017-0172-8

PG 13

WC Endocrinology & Metabolism

SC Endocrinology & Metabolism

GA EQ6VZ

UT WOS:000398223200001

PM 28376921

OA DOAJ Gold, Green Published

DA 2019-01-08

ER

PT J

AU Mutai, H

Furukawa, T

Houri, A

Suzuki, A

Hanihara, T

AF Mutai, Hitoshi

Furukawa, Tomomi

Houri, Ayumi

Suzuki, Akihito

Hanihara, Tokiji

TI Factors associated with multidimensional aspect of post-stroke fatigue

in acute stroke period

SO ASIAN JOURNAL OF PSYCHIATRY

LA English

DT Article

DE Post-stroke fatigue; Stroke; Anxiety; Depression

ID DEPRESSIVE SYMPTOMS; INVENTORY MFI; ACUTE-PHASE; INFARCTS; ANXIETY;

DISEASE; COHORT; RISK

AB Background: Post-stroke fatigue (PSF) is a frequent and distressing consequence of stroke, and can be both acute and long lasting. We aimed to investigate multidimensional aspects of acute PSF and to determine the clinical factors relevant to acute PSF.

Methods: We collected data of 101 patients admitted to the hospital for acute stroke. PSF was assessed using the Multidimensional Fatigue Inventory within 2 weeks of stroke. Measures included Mini-Mental State Examination, Hospital Anxiety and Depression Scale, and Functional Independence Measure. Stroke character, lesion location, and clinical variables that potentially influence PSF were also collected.

Results: The prevalence of pathological fatigue is 56.4% within 2 weeks of stroke. Binary logistic regression analysis revealed that anxiety was the only predictor for presence of PSF (OR = 1.32, 95% CI: 1.13-1.53, P < 0.001). Multivariate stepwise regression analysis showed anxiety, right lesion side, thalamus, and/or brainstem were independently associated with general fatigue, right lesion side, depression, diabetes mellitus, and anxiety with physical fatigue, depression with reduced activity, depression, and BMI with reduced motivation, depression, and diabetes mellitus with mental fatigue.

Conclusions: PSF was highly prevalent in the acute phase, and specific factors including lesion location (right side lesion, thalamic and brainstem lesion), anxiety, and depression were independently associated with multidimensional aspects of PSF. Further study is needed to elucidate how specific structural lesions and anxiety symptoms relate to the development of early fatigue following stroke. (C) 2016 Elsevier B.V. All rights reserved.

C1 [Mutai, Hitoshi; Furukawa, Tomomi; Houri, Ayumi; Suzuki, Akihito] Azumino Red Cross Hosp, Dept Rehabil, 5685 Toyoshina, Azumino, Nagano 3998292, Japan.

[Mutai, Hitoshi; Hanihara, Tokiji] Shinshu Univ, Sch Med, Sch Hlth Sci, 3-1-1 Asahi, Matsumoto, Nagano 3908621, Japan.

[Hanihara, Tokiji] Nagano Prefectural Mental Wellness Ctr Komagane, 2901 Shimodaira, Komagane, Nagano 3994101, Japan.

RP Hanihara, T (reprint author), Nagano Prefectural Mental Wellness Ctr, Komagane, Japan.

EM qhaniha@shinshu-u.ac.jp

CR Astrom M, 1996, STROKE, V27, P270, DOI 10.1161/01.STR.27.2.270

Burton CA Campbell, 2013, INT J STROKE, V8, P545, DOI [doi:10.1111/j.1747-4949.2012.00906.x, DOI 10.1111/J.1747-4949.2012.00906.X]

Chaudhuri A, 2004, LANCET, V363, P978, DOI 10.1016/S0140-6736(04)15794-2

CHINO N, 1994, JPN J REHABIL MED, V31, P119, DOI DOI 10.2490/JJRM1963.31.119

Choi-Kwon S, 2005, CEREBROVASC DIS, V19, P84, DOI 10.1159/000082784

Choi-Kwon S, 2011, INT J STROKE, V6, P328, DOI 10.1111/j.1747-4949.2011.00624.x

Christensen D, 2008, CEREBROVASC DIS, V26, P134, DOI 10.1159/000139660

Duncan F, 2015, STROKE, V46, P1052, DOI 10.1161/STROKEAHA.114.008079

Elbers RG, 2012, PARKINSONISM RELAT D, V18, P532, DOI 10.1016/j.parkreldis.2012.01.024

FOLSTEIN MF, 1975, J PSYCHIAT RES, V12, P189, DOI 10.1016/0022-3956(75)90026-6

Glader EL, 2002, STROKE, V33, P1327, DOI 10.1161/01.STR.0000014248.28711.D6

GRANGER CV, 1993, AM J PHYS MED REHAB, V72, P84, DOI 10.1097/00002060-199304000-00005

Ingles JL, 1999, ARCH PHYS MED REHAB, V80, P173, DOI 10.1016/S0003-9993(99)90116-8

Kutlubaev MA, 2013, CEREBROVASC DIS EXTR, V3, P26, DOI 10.1159/000347113

Lerdal A, 2013, NEUROLOGY, V81, P1581, DOI 10.1212/WNL.0b013e3182a9f471

Lerdal A, 2011, DISABIL REHABIL, V33, P334, DOI 10.3109/09638288.2010.490867

Manes F, 1999, J NERV MENT DIS, V187, P707, DOI 10.1097/00005053-199912000-00001

Naess H, 2010, J NEUROL, V257, P1446, DOI 10.1007/s00415-010-5539-y

Radman N, 2012, NEUROLOGY, V79, P1422, DOI 10.1212/WNL.0b013e31826d5f3a

Sagen U, 2010, GEN HOSP PSYCHIAT, V32, P80, DOI 10.1016/j.genhosppsych.2009.10.001

Schepers VP, 2006, ARCH PHYS MED REHAB, V87, P184, DOI 10.1016/j.apmr.2005.10.005

SMETS EMA, 1995, J PSYCHOSOM RES, V39, P315, DOI 10.1016/0022-3999(94)00125-O

Snaphaan L, 2011, EUR J NEUROL, V18, P611, DOI 10.1111/j.1468-1331.2010.03217.x

Staub F, 2001, CEREBROVASC DIS, V12, P75, DOI 10.1159/000047685

Tang WK, 2010, J NEUROL, V257, P178, DOI 10.1007/s00415-009-5284-2

van der Werf SP, 2001, EUR NEUROL, V45, P28, DOI 10.1159/000052085

Whyte EM, 2002, BIOL PSYCHIAT, V52, P253, DOI 10.1016/S0006-3223(02)01424-5

Wu SM, 2015, STROKE, V46, P893, DOI 10.1161/STROKEAHA.114.006647

Wu SM, 2014, STROKE, V45, P1778, DOI 10.1161/STROKEAHA.113.004584

NR 30

TC 2

Z9 2

U1 1

U2 7

PU ELSEVIER SCIENCE BV

PI AMSTERDAM

PA PO BOX 211, 1000 AE AMSTERDAM, NETHERLANDS

SN 1876-2018

EI 1876-2026

J9 ASIAN J PSYCHIATR

JI Asian J. Psychiatr.

PD APR

PY 2017

VL 26

BP 1

EP 5

DI 10.1016/j.ajp.2016.12.015

PG 5

WC Psychiatry

SC Psychiatry

GA EY8ZO

UT WOS:000404288300002

PM 28483068

DA 2019-01-08

ER

PT J

AU Clarke, TK

Obsteter, J

Hall, LS

Hayward, C

Thomson, PA

Smith, BH

Padmanabhan, S

Hocking, LJ

Deary, IJ

Porteous, DJ

McIntosh, AM

AF Clarke, Toni-Kim

Obsteter, Jana

Hall, Lynsey S.

Hayward, Caroline

Thomson, Pippa A.

Smith, Blair H.

Padmanabhan, Sandosh

Hocking, Lynne J.

Deary, Ian J.

Porteous, David J.

McIntosh, Andrew M.

TI Investigating Shared Aetiology Between Type 2 Diabetes and Major

Depressive Disorder in a Population Based Cohort

SO AMERICAN JOURNAL OF MEDICAL GENETICS PART B-NEUROPSYCHIATRIC GENETICS

LA English

DT Article

DE depression; type 2 diabetes; genetics; polygenic

ID GENETIC ARCHITECTURE; ASSOCIATION; EPIDEMIOLOGY; METAANALYSIS; HEALTH;

TWIN

AB Type II diabetes (T2D) and major depressive disorder (MDD) are often co-morbid. The reasons for this co-morbidity are unclear. Some studies have highlighted the importance of environmental factors and a causal relationship between T2D and MDD has also been postulated. In the present study we set out to investigate the shared aetiology between T2D and MDD using Mendelian randomization in a population based sample, Generation Scotland: the Scottish Family Health Study (N = 21,516). Eleven SNPs found to be associated with T2D were tested for association with MDD and psychological distress (General Health Questionnaire scores). We also assessed causality and genetic overlap between T2D and MDD using polygenic risk scores (PRS) assembled from the largest available GWAS summary statistics to date. No single T2D risk SNP was associated with MDD in the MR analyses and we did not find consistent evidence of genetic overlap between MDD and T2D in the PRS analyses. Linkage disequilibrium score regression analyses supported these findings as no genetic correlation was observed between T2D and MDD(rG = 0.0278 (S.E. 0.11), P-value = 0.79). As suggested by previous studies, T2D and MDD covariance may be better explained by environmental factors. Future studies would benefit from analyses in larger cohorts where stratifying by sex and looking more closely at MDD cases demonstrating metabolic dysregulation is possible. (C) 2016 The Authors. American Journal of Medical Genetics Part B: Neuropsychiatric Genetics Published by Wiley Periodicals, Inc.

C1 [Clarke, Toni-Kim; Obsteter, Jana; McIntosh, Andrew M.] Univ Edinburgh, Royal Edinburgh Hosp, Div Psychiat, Edinburgh EH10 5HF, Midlothian, Scotland.

[Hall, Lynsey S.] Newcastle Univ, Inst Genet Med, Newcastle Upon Tyne, Tyne & Wear, England.

[Hayward, Caroline; Thomson, Pippa A.] Univ Edinburgh, Inst Genet & Mol Med, Western Gen Hosp, Ctr Genom & Expt Med, Edinburgh, Midlothian, Scotland.

[Hayward, Caroline; Porteous, David J.] Univ Edinburgh, MRC Inst Genet & Mol Med, MRC Human Genet, Edinburgh, Midlothian, Scotland.

[Thomson, Pippa A.; Deary, Ian J.; Porteous, David J.; McIntosh, Andrew M.] Univ Edinburgh, Ctr Cognit Ageing & Cognit Epidemiol, Edinburgh, Midlothian, Scotland.

[Smith, Blair H.] Univ Dundee, Div Populat Hlth Sci, Dundee, Scotland.

[Padmanabhan, Sandosh] Univ Glasgow, Inst Cardiovasc & Med Sci, Glasgow, Lanark, Scotland.

[Hocking, Lynne J.] Univ Aberdeen, Inst Med Sci, Aberdeen, Scotland.

[Deary, Ian J.] Univ Edinburgh, Dept Psychol, Edinburgh, Midlothian, Scotland.

RP Clarke, TK (reprint author), Univ Edinburgh, Royal Edinburgh Hosp, Div Psychiat, Edinburgh EH10 5HF, Midlothian, Scotland.

EM toni.clarke@ed.ac.uk

RI Deary, Ian/C-6297-2009; McIntosh, Andrew/B-9379-2008

OI Deary, Ian/0000-0002-1733-263X; McIntosh, Andrew/0000-0002-0198-4588

FU Wellcome Trust [104036/Z/14/Z]; Scottish Government Health Department,

Chief Scientist Office [CZD/16/6]; Dr. Mortimer and Theresa Sackler

Foundation; Wellcome Trust [104036/Z/14/Z]; Scottish Government Health

Department, Chief Scientist Office [CZD/16/6]; Dr. Mortimer and Theresa

Sackler Foundation; Chief Scientist Office [CZD/16/6/4]; Medical

Research Council [1292844, MR/K026992/1, MC_PC_U127561128]

FX Grant sponsor: Wellcome Trust Through a Strategic Award; Grant number:

104036/Z/14/Z; Grant sponsor: Scottish Government Health Department,

Chief Scientist Office; Grant number: CZD/16/6; Grant sponsor: Dr.

Mortimer and Theresa Sackler Foundation.

CR Anderson RJ, 2001, DIABETES CARE, V24, P1069, DOI 10.2337/diacare.24.6.1069

Anwar H, 2011, DIABETIC MED, V28, P1514, DOI 10.1111/j.1464-5491.2011.03432.x

Bulik-Sullivan BK, 2015, NAT GENET, V47, P291, DOI 10.1038/ng.3211

Cai N, 2015, NATURE, V523, P588, DOI 10.1038/nature14659

Ferrari AJ, 2013, PLOS MED, V10, DOI 10.1371/journal.pmed.1001547

First M. B, 1997, STRUCTURED CLIN INTE

GOLDBERG DP, 1979, PSYCHOL MED, V9, P139, DOI 10.1017/S0033291700021644

Golden SH, 2008, JAMA-J AM MED ASSOC, V299, P2751, DOI 10.1001/jama.299.23.2751

Goodwin Guy M, 2006, Dialogues Clin Neurosci, V8, P259

Kan C, 2016, MOL PSYCHIATR, V21, P903, DOI 10.1038/mp.2016.28

Kessler RC, 2013, ANNU REV PUBL HEALTH, V34, P119, DOI 10.1146/annurev-publhealth-031912-114409

Lee SH, 2013, NAT GENET, V45, P984, DOI 10.1038/ng.2711

Mahajan A, 2014, NAT GENET, V46, P234, DOI 10.1038/ng.2897

Mezuk B, 2015, PSYCHOSOM MED, V77, P559, DOI 10.1097/PSY.0000000000000182

Morris AP, 2012, NAT GENET, V44, P981, DOI 10.1038/ng.2383

Nakagawa S, 2013, METHODS ECOL EVOL, V4, P133, DOI 10.1111/j.2041-210x.2012.00261.x

Purcell S, 2007, AM J HUM GENET, V81, P559, DOI 10.1086/519795

Purcell SM, 2009, NATURE, V460, P748, DOI 10.1038/nature08185

Samaan Z, 2015, TRANSL PSYCHIAT, V5, DOI 10.1038/tp.2015.113

Scherrer JF, 2011, TWIN RES HUM GENET, V14, P169, DOI 10.1375/twin.14.2.169

Schmitz N, 2016, MOL PSYCHIAT

Sharma S, 2013, INT J OBESITY, V37, P382, DOI 10.1038/ijo.2012.48

Smith BH, 2006, BMC MED GENET, V7, DOI 10.1186/1471-2350-7-74

Smith GD, 2003, INT J EPIDEMIOL, V32, P1, DOI 10.1093/ije/dyg070

Sullivan PF, 2013, MOL PSYCHIATR, V18, P497, DOI 10.1038/mp.2012.21

NR 25

TC 5

Z9 5

U1 1

U2 4

PU WILEY

PI HOBOKEN

PA 111 RIVER ST, HOBOKEN 07030-5774, NJ USA

SN 1552-4841

EI 1552-485X

J9 AM J MED GENET B

JI Am. J. Med. Genet. B

PD APR

PY 2017

VL 174

IS 3

BP 227

EP 234

DI 10.1002/ajmg.b.32478

PG 8

WC Genetics & Heredity; Psychiatry

SC Genetics & Heredity; Psychiatry

GA EV1PF

UT WOS:000401517800005

PM 27480393

OA Green Published, Other Gold

DA 2019-01-08

ER

PT J

AU Indelicato, L

Dauriz, M

Santi, L

Bonora, F

Negri, C

Cacciatori, V

Targher, G

Trento, M

Bonora, E

AF Indelicato, L.

Dauriz, M.

Santi, L.

Bonora, F.

Negri, C.

Cacciatori, V.

Targher, G.

Trento, M.

Bonora, E.

TI Psychological distress, self-efficacy and glycemic control in type 2

diabetes

SO NUTRITION METABOLISM AND CARDIOVASCULAR DISEASES

LA English

DT Article

DE Type 2 diabetes; Self-efficacy; Glycemic control; Anxiety; Depression

ID ANXIETY DISORDERS; RISK-FACTORS; DEPRESSION; ADULTS; PREVALENCE;

METAANALYSIS; SYMPTOMS; ASSOCIATION; ADHERENCE; CARE

AB Aim: To investigate the association of glycemic control with depression, anxiety, self-efficacy and other diabetes-specific psychological measures in a cohort of adult patients with type 2 diabetes (T2D) free of severe chronic diabetes-related complications.

Methods and Results: In 172 T2D outpatients consecutively recruited at the Diabetes Center of Verona City Hospital, we performed a standard medical assessment and completed the Beck Depression Inventory-II (BDI-II), the Beck Anxiety Inventory (BAI) and the Multidimensional Diabetes Questionnaire (MDQ) Age, body mass index (BMI) and glycosylated hemoglobin (HbA1c) were (median [IQR]): 64.0 [58.0-69.0] years, 31.0 [28.0-34.4] kg/m(2), and 7.3 [6.7 -8.0] %, respectively. The overall prevalence of anxiety and depression was 14.5% and 18.6%, respectively. Higher levels of HbA1c were significantly (p < 0.001) associated with a number of MDQ dimensions, such as higher perceived interference with daily activities (Spearman's rho coefficient = 0.33), higher perceived diabetes severity (rho = 0.28) and lower self-efficacy (rho = -0.27), but not with depression or anxiety. These three variables were also independent predictors of higher HbA1c levels, when entered in a multivariable stepwise-forward regression model that also included age, BMI, diabetes duration and diabetes-specific social support as covariates.

Conclusion: Lower self-efficacy and higher diabetes distress were closely associated with poorer glycemic control. No direct association between HbA1c and clinical psychological symptoms was detected. These results highlight that a number of diabetes-specific psychological variables may play a role amidst psychological distress and glycemic control. Further studies are needed to elucidate the relevance of diabetes distress and self-efficacy to the achievement of individual glycemic targets. (C) 2017 The Italian Society of Diabetology, the Italian Society for the Study of Atherosclerosis, the Italian Society of Human Nutrition, and the Department of Clinical Medicine and Surgery, Federico II University. Published by Elsevier B.V. All rights reserved.

C1 [Indelicato, L.; Dauriz, M.; Santi, L.; Bonora, F.; Negri, C.; Cacciatori, V.; Targher, G.; Bonora, E.] Univ Verona, Dept Med, Div Endocrinol Diabet & Metab, Piazzale Stefani 1, I-37126 Verona, Italy.

[Indelicato, L.; Dauriz, M.; Santi, L.; Bonora, F.; Negri, C.; Cacciatori, V.; Targher, G.; Bonora, E.] Hosp Trust Verona, Piazzale Stefani 1, I-37126 Verona, Italy.

[Trento, M.] Univ Turin, Dept Med Sci, Lab Clin Pedag, Turin, Italy.

RP Indelicato, L; Dauriz, M (reprint author), Univ Verona, Dept Med, Div Endocrinol Diabet & Metab, Piazzale Stefani 1, I-37126 Verona, Italy.; Indelicato, L; Dauriz, M (reprint author), Hosp Trust Verona, Piazzale Stefani 1, I-37126 Verona, Italy.

EM liliana.indelicato@univr.it; marco.dauriz@univr.it

RI Dauriz, Marco/S-5843-2016

OI Dauriz, Marco/0000-0002-5542-5941; TRENTO, Marina/0000-0003-3764-3539

FU Fondazione Diabete Ricerca (Fo.Di.Ri, Rome, Italy)

FX This study was supported by Fondazione Diabete Ricerca (Fo.Di.Ri, Rome,

Italy). The support of the administrative and clinical personnel of the

Verona Diabetes Center (University and General Hospital of Verona,

Verona, Italy) is gratefully acknowledged.

CR Ali S, 2006, DIABETIC MED, V23, P1165, DOI 10.1111/j.1464-5491.2006.01943.x

Anderson RJ, 2002, INT J PSYCHIAT MED, V32, P235, DOI 10.2190/KLGD-4H8D-4RYL-TWQ8

Anderson RJ, 2001, DIABETES CARE, V24, P1069, DOI 10.2337/diacare.24.6.1069

Arshad AR, 2016, J PAK MED ASSOC, V66, P425

Beck AT, MANUAL BECK ANXIETY

Beck AT, 1996, MANUAL BECK DEPRESSI

Bonora E, 2004, DIABETES, V53, P1782, DOI 10.2337/diabetes.53.7.1782

Cherrington A, 2010, J BEHAV MED, V33, P81, DOI 10.1007/s10865-009-9233-4

Chlebowy DO, 2006, DIABETES EDUCATOR, V32, P777, DOI 10.1177/0145721706291760

Ciechanowski PS, 2000, ARCH INTERN MED, V160, P3278, DOI 10.1001/archinte.160.21.3278

Collins MM, 2009, DIABETIC MED, V26, P153, DOI 10.1111/j.1464-5491.2008.02648.x

de Groot M, 2001, PSYCHOSOM MED, V63, P619, DOI 10.1097/00006842-200107000-00015

Fisher L, 2008, DIABETIC MED, V25, P1096, DOI 10.1111/j.1464-5491.2008.02533.x

Fisher L, 2000, DIABETES CARE, V23, P267, DOI 10.2337/diacare.23.3.267

Fisher L, 2007, DIABETES CARE, V30, P542, DOI 10.2337/dc06-1614

Fisher L, 2010, DIABETES CARE, V33, P23, DOI 10.2337/dc09-1238

Georgiades A, 2007, PSYCHOSOM MED, V69, P235, DOI 10.1097/PSY.0b013e318042588d

Ghisi M, BDI 2 BECK DEPRESSIO

Gonzalez JS, 2008, DIABETES CARE, V31, P2398, DOI 10.2337/dc08-1341

Grigsby AB, 2002, J PSYCHOSOM RES, V53, P1053, DOI 10.1016/S0022-3999(02)00417-8

Henderson AS, 1998, PSYCHOL MED, V28, P1321, DOI 10.1017/S0033291798007570

Hermanns N, 2005, DIABETIC MED, V22, P293, DOI 10.1111/j.1464-5491.2005.01414.x

Lazzari D, 2009, PSICOTER COGN COMPOR, V15, P171

LUDLOW AP, 1995, CANADIAN J DIABETES, V19, P10

Lustman PJ, 2000, DIABETES CARE, V23, P934, DOI 10.2337/diacare.23.7.934

Peyrot M, 1999, J HEALTH SOC BEHAV, V40, P141, DOI 10.2307/2676370

Rees G, 2016, JAMA OPHTHALMOL, V134, P1007, DOI 10.1001/jamaophthalmol.2016.2213

Sacco WP, 2005, HEALTH PSYCHOL, V24, P630, DOI 10.1037/0278-6133.6.630

Talbot F, 1997, J BEHAV MED, V20, P291, DOI 10.1023/A:1025508928696

Trento M, 2012, ACTA DIABETOL, V49, P199, DOI 10.1007/s00592-011-0275-z

Williams JW, 2004, ANN INTERN MED, V140, P1015, DOI 10.7326/0003-4819-140-12-200406150-00012

Young-Hyman D, 2016, DIABETES CARE, V39, P2126, DOI 10.2337/dc16-2053

NR 32

TC 2

Z9 2

U1 2

U2 12

PU ELSEVIER SCI LTD

PI OXFORD

PA THE BOULEVARD, LANGFORD LANE, KIDLINGTON, OXFORD OX5 1GB, OXON, ENGLAND

SN 0939-4753

EI 1590-3729

J9 NUTR METAB CARDIOVAS

JI Nutr. Metab. Carbiovasc. Dis.

PD APR

PY 2017

VL 27

IS 4

BP 300

EP 306

DI 10.1016/j.numecd.2017.01.006

PG 7

WC Cardiac & Cardiovascular Systems; Endocrinology & Metabolism; Nutrition

& Dietetics

SC Cardiovascular System & Cardiology; Endocrinology & Metabolism;

Nutrition & Dietetics

GA ET7GA

UT WOS:000400461300003

PM 28274728

OA Green Published

DA 2019-01-08

ER

PT J

AU Sandberg, J

Yorgason, JB

Fisher, L

Weinstock, RS

Hessler, D

Dimmock, J

Trief, PM

AF Sandberg, Jonathan

Yorgason, Jeremy B.

Fisher, Lawrence

Weinstock, Ruth S.

Hessler, Danielle

Dimmock, Jacqueline

Trief, Paula M.

TI Does Length of Relationship or Gender Predict Response to Behavioral

Diabetes Intervention? Generating Hypotheses From Secondary Analysis of

a Randomized Trial

SO DIABETES EDUCATOR

LA English

DT Article

ID MARITAL QUALITY; OLDER-ADULTS; COUPLES; HEALTH; MANAGEMENT; ADHERENCE;

DISTRESS; MARRIAGE; OUTCOMES; SPOUSES

AB Purpose

The purpose of the study was to determine, through secondary analysis, whether the length of a couple's relationship and the participants' gender are associated with glycemic response to a type 2 diabetes (T2D) behavioral couples-based intervention.

Methods

A randomized trial was conducted to test the impact of a couples-level, telephone-based behavioral intervention on hemoglobin A1C in patients with T2D. One hundred and four patients and partners participated in the couples intervention arm, and 94 individuals participated in the individual arm. A1C levels were measured at baseline and 1-year follow-up.

Results

Results of the regression analysis showed that for men with T2D (n = 35) in the couples intervention group, longer length of relationship was associated with lower A1C at 1-year follow-up, after controlling for baseline A1C, diabetes duration, and income. Length of relationship was not significantly related to follow-up glycemic measures for women or men with T2D in the individual intervention.

Conclusions

Study findings suggests that for men with T2D in a couples- - based intervention, those in longer relationships may be more likely to benefit from the intervention. More research is needed to better understand factors that contribute to successful couples- based behavioral approaches to help adults with T2D improve their glycemic control.

C1 [Sandberg, Jonathan; Yorgason, Jeremy B.] Brigham Young Univ, Sch Family Life, 266 TLRB, Provo, UT 84602 USA.

[Weinstock, Ruth S.; Dimmock, Jacqueline; Trief, Paula M.] SUNY Upstate Med Univ, Syracuse, NY 13210 USA.

[Fisher, Lawrence; Hessler, Danielle] Univ Calif San Francisco, San Francisco, CA 94143 USA.

RP Sandberg, J (reprint author), Brigham Young Univ, Sch Family Life, 266 TLRB, Provo, UT 84602 USA.

EM jgs@byu.edu

OI Yorgason, Jeremy/0000-0002-4208-7306

FU National Institutes of Health (NIDDK) [1R18DK080867-01A2]

FX Funding for this study was provided by the National Institutes of Health

(NIDDK) No. 1R18DK080867-01A2.

CR Bandura A, 1978, SOCIAL LEARNING THEO

Casagrande SS, 2013, DIABETES CARE, V36, P2271, DOI 10.2337/dc12-2258

Clark CD, 2008, DISS ABSTR INT, V68, P4619

Donato S, 2015, J SOC PERS RELAT, V32, P580, DOI 10.1177/0265407514541071

Fisher L, 2000, J FAM PRACTICE, V49, P561

Fisher L, 2000, FAM PROCESS, V39, P403, DOI 10.1111/j.1545-5300.2000.39402.x

Franks MM, 2012, FAM SYST HEALTH, V30, P154, DOI 10.1037/a0028614

Franks MM, 2010, FAM RELAT, V59, P599, DOI 10.1111/j.1741-3729.2010.00626.x

HAMPSON SE, 1995, DIABETES EDUCATOR, V21, P300, DOI 10.1177/014572179502100407

Hemphill RC, 2013, PSYCHOL HEALTH, V28, P139, DOI 10.1080/08870446.2012.685740

Iida M, 2010, PERS SOC PSYCHOL B, V36, P780, DOI 10.1177/0146167210369897

Kang CM, 2010, INT J NURS STUD, V47, P1363, DOI 10.1016/j.ijnurstu.2010.03.009

Kelley H. H., 1978, INTERPERSONAL RELATI

Keogh KM, 2011, AM J MANAG CARE, V17, P105

Kiecolt-Glaser JK, 2001, PSYCHOL BULL, V127, P472, DOI 10.1037//0033-2909.127.4.472

Landis M, 2013, GEROPSYCH, V26, P39, DOI 10.1024/1662-9647/a000077

LEVENSON RW, 1994, J PERS SOC PSYCHOL, V67, P56, DOI 10.1037//0022-3514.67.1.56

Lin WF, 2016, J HAPPINESS STUD, V17, P757, DOI 10.1007/s10902-015-9621-0

Lister Z, 2013, CONTEMP FAM THER, V35, P613, DOI 10.1007/s10591-013-9250-x

Martire LM, 2004, HEALTH PSYCHOL, V23, P599, DOI 10.1037/0278-6133.23.6.599

Martire LM, 2014, HEALTH PSYCHOL, V33, P501, DOI 10.1037/hea0000103

Padula C, 1996, WESTERN J NURS RES, V18, P675, DOI 10.1177/019394599601800605

Pasupathi M, 1999, J NONVERBAL BEHAV, V23, P173, DOI 10.1023/A:1021439627043

Rentscher KE, 2013, J FAM PSYCHOL, V27, P691, DOI 10.1037/a0034184

Robles TF, 2014, CURR DIR PSYCHOL SCI, V23, P427, DOI 10.1177/0963721414549043

Rohrbaugh MJ, 2008, J CONSULT CLIN PSYCH, V76, P781, DOI 10.1037/a0013238

Sandberg J, 2006, J COUPLE RELATSH THE, V5, P23, DOI 10.1300/J398v05n02_02

Seidel AJ, 2012, FAM RELAT, V61, P698, DOI 10.1111/j.1741-3729.2012.00719.x

Trief PM, 2016, DIABETES CARE, V39, P2165, DOI 10.2337/dc16-0035

Trief PM, 2011, TRANSL BEHAV MED, V1, P461, DOI 10.1007/s13142-011-0057-8

Trief PM, 2004, ANN BEHAV MED, V27, P148, DOI 10.1207/s15324796abm2703_2

Trief PM, 2001, DIABETES CARE, V24, P1384, DOI 10.2337/diacare.24.8.1384

United Kingdom Prospective Diabetes Study Group, 1998, LANCET, V352, P837

Whisman MA, 2014, HEALTH PSYCHOL, V33, P832, DOI 10.1037/hea0000064

Yang W, 2013, DIABETES CARE, V36, P1033, DOI DOI 10.2337/DC12-2625

NR 35

TC 0

Z9 0

U1 0

U2 2

PU SAGE PUBLICATIONS INC

PI THOUSAND OAKS

PA 2455 TELLER RD, THOUSAND OAKS, CA 91320 USA

SN 0145-7217

EI 1554-6063

J9 DIABETES EDUCATOR

JI Diabetes Educ.

PD APR

PY 2017

VL 43

IS 2

BP 216

EP 222

DI 10.1177/0145721717691147

PG 7

WC Endocrinology & Metabolism; Public, Environmental & Occupational Health

SC Endocrinology & Metabolism; Public, Environmental & Occupational Health

GA EQ3NX

UT WOS:000397980900008

PM 28162037

OA Green Accepted

DA 2019-01-08

ER

PT J

AU Billionnet, C

Mitanchez, D

Weill, A

Nizard, J

Alla, F

Hartemann, A

Jacqueminet, S

AF Billionnet, Cecile

Mitanchez, Delphine

Weill, Alain

Nizard, Jacky

Alla, Francois

Hartemann, Agnes

Jacqueminet, Sophie

TI Gestational diabetes and adverse perinatal outcomes from 716,152 births

in France in 2012

SO DIABETOLOGIA

LA English

DT Article

DE Cardiac malformation; Gestational diabetes mellitus; Macrosomia;

Perinatal death; Pre-eclampsia; Preterm

ID GLUCOSE-TOLERANT WOMEN; IMMORTAL TIME BIAS; BODY-MASS INDEX;

INTERNATIONAL ASSOCIATION; CONGENITAL-MALFORMATIONS; ATLANTIC-DIP;

PREGNANCY; MELLITUS; HYPERGLYCEMIA; RISK

AB Aims/hypothesis The aim of this study was to assess the risk of adverse perinatal outcomes in gestational diabetes mellitus (GDM) in a large national cohort.

Methods All deliveries taking place after 22 weeks in France in 2012 were included by extracting data from the hospital discharge database and the national health insurance system. The diabetic status of mothers was determined by the use of glucose-lowering agents and by hospital diagnosis. Outcomes were analysed according to the type of diabetes and, in the GDM group, whether or not diabetes was insulin-treated.

Results The cohort of 796,346 deliveries involved 57,629 (7.24%) mothers with GDM. Mother-infant linkage was obtained for 705,198 deliveries. The risks of adverse outcomes were much lower with GDM than with pregestational diabetes. After limiting the analysis to deliveries after 28 weeks to reduce immortal time bias, the risks of preterm birth (OR 1.3 [95% CI 1.3, 1.4]), Caesarean section (OR 1.4 [95% CI 1.4, 1.4]), pre-eclampsia/eclampsia (OR 1.7 [95% CI 1.6, 1.7]), macrosomia (OR 1.8 [95% CI 1.7, 1.8]), respiratory distress (OR 1.1 [95% CI 1.0, 1.3]), birth trauma (OR 1.3 [95% CI 1.1, 1.5]) and cardiac malformations (OR 1.3 [95% CI 1.1, 1.4]) were increased in women with GDM compared with the non-diabetic population. Higher risks were observed in women with insulin-treated GDMthan those with diet-treated GDM. After limiting the analysis to term deliveries, an increased risk of perinatal mortality was observed. After excluding women suspected to have undiagnosed pregestational diabetes, the risk remained moderately increased only for those with diet-treated GDM (OR 1.3 [95% CI 1.0, 1.6]).

Conclusions/interpretation GDM is associated with a moderately increased risk of adverse perinatal outcomes, which is higher in insulin-treated GDM than in non-insulintreated GDM for most outcomes.

C1 [Billionnet, Cecile; Weill, Alain; Alla, Francois] Natl Hlth Insurance, Dept Publ Hlth Studies, Div Stat Strateg Res & Dev, Paris, France.

[Mitanchez, Delphine] Armand Trousseau Hosp, AP HP, Dept Perinatol, Div Neonatol, Paris, France.

[Mitanchez, Delphine; Nizard, Jacky; Hartemann, Agnes] UPMC Univ Paris 06, Sorbonne Univ, Paris, France.

[Nizard, Jacky] Grp Hosp Pitie Salpetriere, Dept Obstet & Gynaecol, APHP CNRS UMR 7222, Inserm U1150, Paris, France.

[Hartemann, Agnes; Jacqueminet, Sophie] Hop La Pitie Salpetriere, AP HP, Inst Cardiometab & Nutr, Paris, France.

[Hartemann, Agnes; Jacqueminet, Sophie] Hop La Pitie Salpetriere, Diabet & Metab Dis Dept, 83 Blvd Hop, F-75013 Paris, France.

RP Jacqueminet, S (reprint author), Hop La Pitie Salpetriere, AP HP, Inst Cardiometab & Nutr, Paris, France.; Jacqueminet, S (reprint author), Hop La Pitie Salpetriere, Diabet & Metab Dis Dept, 83 Blvd Hop, F-75013 Paris, France.

EM sophie.jacqueminet@aphp.fr

OI alla, francois/0000-0002-5793-7190

CR [Anonymous], 2010, Diabetes Metab, V36, P695, DOI 10.1016/j.diabet.2010.11.019

Becquet O, 2015, BMJ OPEN, V5, DOI 10.1136/bmjopen-2015-008192

Catalano PM, 2011, AM J OBSTET GYNECOL, V204, P479, DOI 10.1016/j.ajog.2010.11.039

Charles MA, 2008, OBESITY, V16, P2180, DOI [10.1038/oby.2008.285, 10.1038/oby.2008.286]

Chu SY, 2007, DIABETES CARE, V30, P2070, DOI 10.2337/dc06-2559a

Cundy T, 2000, DIABETIC MED, V17, P33, DOI 10.1046/j.1464-5491.2000.00215.x

Dennedy MC, 2012, J CLIN ENDOCR METAB, V97, pE608, DOI 10.1210/jc.2011-2674

Fadl HE, 2010, DIABETIC MED, V27, P436, DOI 10.1111/j.1464-5491.2010.02978.x

Feig DS, 2014, DIABETES CARE, V37, P1590, DOI 10.2337/dc13-2717

Fong A, 2014, J DIABETES COMPLICAT, V28, P29, DOI 10.1016/j.jdiacomp.2013.08.009

Garcia-Patterson A, 2004, DIABETOLOGIA, V47, P509, DOI 10.1007/s00125-004-1337-3

Hutcheon JA, 2013, EPIDEMIOLOGY, V24, P787, DOI 10.1097/EDE.0b013e3182a6d9aa

Lepercq J, 2010, J GYNECOL OBST BIO R, V39, pS141, DOI 10.1016/S0368-2315(10)70042-9

Metzger BE, 2008, NEW ENGL J MED, V358, P1991, DOI 10.1056/NEJMoa0707943

Metzger BE, 2010, DIABETES CARE, V33, P676, DOI 10.2337/dc09-1848

Mitanchez D, 2010, DIABETES METAB, V36, P617, DOI 10.1016/j.diabet.2010.11.013

Mitanchez D, 2014, J PEDIATR-US, V164, P445, DOI 10.1016/j.jpeds.2013.10.076

Moses RG, 2016, AUST NZ J OBSTET GYN, V56, P341, DOI 10.1111/ajo.12447

Moulis G, 2015, REV MED INTERNE, V36, P411, DOI 10.1016/j.revmed.2014.11.009

O'Sullivan EP, 2011, DIABETOLOGIA, V54, P1670, DOI 10.1007/s00125-011-2150-4

Ogunleye OK, 2016, J MATERN-FETAL NEO M, V30, P1

Owens LA, 2010, DIABETES CARE, V33, P577, DOI 10.2337/dc09-0911

Oyen N, 2016, CIRCULATION, V133, P2243, DOI 10.1161/CIRCULATIONAHA.115.017465

Pierron A, 2015, REV EPIDEMIOL SANTE, V63, P237, DOI 10.1016/j.respe.2015.05.001

Piper JM, 2002, SEMIN PERINATOL, V26, P206, DOI 10.1053/sper.2002.33969

Quantin C, 2014, J GYNECOL OBST BIO R, V43, P680, DOI 10.1016/j.jgyn.2013.09.004

Reece EA, 2012, CURR DIABETES REP, V12, P24, DOI 10.1007/s11892-011-0251-6

Rosenstein MG, 2012, AM J OBSTET GYNECOL, V206

Schaefer-Graf UM, 2000, AM J OBSTET GYNECOL, V182, P313, DOI 10.1016/S0002-9378(00)70217-1

Suissa S, 2008, AM J EPIDEMIOL, V167, P492, DOI 10.1093/aje/kwm324

NR 30

TC 27

Z9 28

U1 0

U2 10

PU SPRINGER

PI NEW YORK

PA 233 SPRING ST, NEW YORK, NY 10013 USA

SN 0012-186X

EI 1432-0428

J9 DIABETOLOGIA

JI Diabetologia

PD APR

PY 2017

VL 60

IS 4

BP 636

EP 644

DI 10.1007/s00125-017-4206-6

PG 9

WC Endocrinology & Metabolism

SC Endocrinology & Metabolism

GA EQ4KS

UT WOS:000398046400005

PM 28197657

OA Other Gold

DA 2019-01-08

ER

PT J

AU Li, C

Xu, D

Hu, MY

Tan, YF

Zhang, P

Li, GC

Chen, L

AF Li, Chen

Xu, Duo

Hu, Mingyue

Tan, Yongfei

Zhang, Ping

Li, Guichen

Chen, Li

TI A systematic review and meta-analysis of randomized controlled trials of

cognitive behavior therapy for patients with diabetes and depression

SO JOURNAL OF PSYCHOSOMATIC RESEARCH

LA English

DT Review

DE Diabetes mellitus; Depression; Cognitive behavior therapy; Meta-analysis

ID QUALITY-OF-LIFE; MOTIVATIONAL ENHANCEMENT THERAPY; GLYCEMIC CONTROL;

TYPE-1; SYMPTOMS; ANXIETY; INTERVENTION; PREVALENCE; MELLITUS; ADULTS

AB Objective: The aim of this meta-analysis was to systematically examine the efficacy of cognitive behavior therapy (CBT) for diabetic patients who have comorbid depression and to identify which aspects can be improved through intervention.

Methods: A systematic literature review was performed using multiple databases. The inclusion criteria included randomized controlled trials (RCTs) of CBT that were conducted with diabetes patients with clinically relevant depression. Review Manager version 5.3 was used to obtain pooled results.

Results: Ten RCTs, with a total sample size of 998 participants, met the inclusion criteria. Compared with control groups, the CBT groups had statistically significant, long-term improvements in depression (standardized mean differences [SMD] = -0.65, 95% confidence interval [CI] (-0.98 to -031), P = 0.0002), quality of life (SMD = 0.29, 95%CI (0.08 to 0.51), P = 0.007), fasting glucose (SMD = 0.21, 95%CI (0.04 to 037), P = 0.01) and anxiety (SMD = -0.49, 95%CI (-0.88 to -0.10), P = 0.01). No improvements were found in glycemic control or in diabetes-related distress.

Conclusions: The results of this meta-analysis showed that CBT can be effective in reducing depression symptoms and fasting glucose in diabetes patients with comorbid depression as well as in improving quality of life and anxiety in the long-term. The results showed that CBT can serve as a promising treatment alternative for diabetes patients with comorbid depression. (C) 2017 Elsevier Inc. All rights reserved.

C1 [Li, Chen; Xu, Duo; Hu, Mingyue; Zhang, Ping; Li, Guichen; Chen, Li] Jilin Univ, Sch Nursing, 965 Xinjiang St, Changchun 130021, Jilin, Peoples R China.

[Tan, Yongfei] Jilin Univ, Coll Basic Med Sci, 126 Xinmin St, Changchun 130021, Jilin, Peoples R China.

RP Chen, L (reprint author), Jilin Univ, Sch Nursing, 965 Xinjiang St, Changchun 130021, Jilin, Peoples R China.

EM chen_care@126.com

CR Amsberg S, 2009, PATIENT EDUC COUNS, V77, P72, DOI 10.1016/j.pec.2009.01.015

Barnard KD, 2006, DIABETIC MED, V23, P445, DOI 10.1111/j.1464-5491.2006.01814.x

Baumeister H, 2014, DIABETIC MED, V31, P773, DOI 10.1111/dme.12452

Baumeister H., 2012, PSYCHOL PHARM INTERV

Beck A. T., 1976, COGNITIVE THERAPY EM

Bogner HR, 2016, J GEN INTERN MED, V31, P594, DOI 10.1007/s11606-016-3675-5

Bot M, 2010, DIABETIC MED, V27, P1295, DOI 10.1111/j.1464-5491.2010.03119.x

Browne JL, 2015, DIABETIC MED, V32, P133, DOI 10.1111/dme.12566

Carper MM, 2014, J BEHAV MED, V37, P501, DOI 10.1007/s10865-013-9505-x

Chapman A., 2015, FRONT PUBLIC HLTH, V3

COHEN J, 1962, J ABNORM PSYCHOL, V65, P145, DOI 10.1037/h0045186

Cuijpers P, 2008, J BEHAV MED, V31, P169, DOI 10.1007/s10865-007-9144-1

Dabelea D, 2014, JAMA-J AM MED ASSOC, V311, P1778, DOI 10.1001/jama.2014.3201

de Groot M, 2001, PSYCHOSOM MED, V63, P619, DOI 10.1097/00006842-200107000-00015

DOBSON KS, 1989, J CONSULT CLIN PSYCH, V57, P414, DOI 10.1037/0022-006X.57.3.414

Egede LE, 2005, CURR DIABETES REV, V1, P235, DOI 10.2174/157339905774574356

Egede LE, 2010, DIABETES RES CLIN PR, V87, P302, DOI 10.1016/j.diabres.2010.01.024

Ekers D, 2008, PSYCHOL MED, V38, P611, DOI 10.1017/S0033291707001614

Fisher L, 2010, DIABETES CARE, V33, P23, DOI 10.2337/dc09-1238

Ghasemzadeh H., 2001, COGNITIVE BEHAV THER

Grey M, 2002, J PSYCHOSOM RES, V53, P907, DOI 10.1016/S0022-3999(02)00312-4

Herder C, 2013, DIABETES CARE, V36, P3663, DOI 10.2337/dc13-0382

Hermanns N, 2015, DIABETES CARE, V38, P551, DOI 10.2337/dc14-1416

Higgins J, 2008, COCHRANE HDB SYSTEMA, P187, DOI DOI 10.1002/9780470712184

Higgins JPT, 2003, BRIT MED J, V327, P557, DOI 10.1136/bmj.327.7414.557

Hind D, 2014, BMC PSYCHIATRY, V14, DOI 10.1186/1471-244X-14-5

Hofmann SG, 2012, COGNITIVE THER RES, V36, P427, DOI 10.1007/s10608-012-9476-1

Huang CY, 2016, QUAL LIFE RES, V25, P1275, DOI 10.1007/s11136-015-1165-6

Huang YG, 2013, EURASIP J WIREL COMM, DOI 10.1186/1687-1499-2013-92

International Diabetes Federation, 2013, IDF DIABETES ATLAS

Ismail K, 2008, ANN INTERN MED, V149, P708, DOI 10.7326/0003-4819-149-10-200811180-00005

Johansson R, 2012, EXPERT REV NEUROTHER, V12, P861, DOI [10.1586/ern.12.63, 10.1586/ERN.12.63]

Johnson B, 2013, DIABETIC MED, V30, P199, DOI 10.1111/j.1464-5491.2012.03721.x

Katon WJ, 2004, ARCH GEN PSYCHIAT, V61, P1042, DOI 10.1001/archpsyc.61.10.1042

Koffel EA, 2015, SLEEP MED REV, V19, P6, DOI 10.1016/j.smrv.2014.05.001

Kokoszka A, 2009, EUR PSYCHIAT, V24, P425, DOI 10.1016/j.eurpsy.2009.04.002

Kovacs M, 1997, DIABETES CARE, V20, P36, DOI 10.2337/diacare.20.1.36

Lawrence JM, 2006, PEDIATRICS, V117, P1348, DOI 10.1542/peds.2005-1398

Lenze EJ, 2000, AM J PSYCHIAT, V157, P722, DOI 10.1176/appi.ajp.157.5.722

Li Liuzhi, 2008, INT J NURS, V27, P295

Lustman PJ, 2005, J DIABETES COMPLICAT, V19, P113, DOI 10.1016/j.jdiacomp.2004.01.002

Lustman PJ, 1998, ANN INTERN MED, V129, P613, DOI 10.7326/0003-4819-129-8-199810150-00005

De la Roca-Chiapas JM, 2013, REV INVEST CLIN, V65, P209

Markowitz SM, 2011, PSYCHOSOMATICS, V52, P1, DOI 10.1016/j.psym.2010.11.007

Marks I., 2007, HANDS ON HELP COMPUT

Matthews EE, 2013, SLEEP MED REV, V17, P453, DOI 10.1016/j.smrv.2013.01.001

Mosaku K, 2008, J NATL MED ASSOC, V100, P73, DOI 10.1016/S0027-9684(15)31178-0

Nefs G, 2012, DIABETOLOGIA, V55, P608, DOI 10.1007/s00125-011-2411-2

Paschalides C, 2004, J PSYCHOSOM RES, V57, P557, DOI 10.1016/j.jpsychores.2004.03.006

Peinemann F, 2013, COCHRANE DB SYST REV, DOI [10.1002/14651858.CD008216.pub4, 10.1371/journal.pmed.1000097, 10.1136/bmj.c869, 10.1136/bmj.b2535, 10.1016/j.jclinepi.2010.02.006, 10.1002/14651858.CD008242.pub3, 10.1016/j.jclinepi.2010.03.004]

Penckofer SM, 2012, ANN BEHAV MED, V44, P192, DOI 10.1007/s12160-012-9377-2

Petrak F, 2015, DIABETES CARE, V38, P767, DOI 10.2337/dc14-1599

Piette JD, 2011, MED CARE, V49, P641, DOI 10.1097/MLR.0b013e318215d0c9

Pignone M, 2008, CLIN DIABETES, V26, P15

Safren SA, 2014, DIABETES CARE, V37, P625, DOI [10.2337/dc13-0816, 10.2337/dc16-er06]

Schram MT, 2009, CURR DIABETES REV, V5, P112, DOI 10.2174/157339909788166828

Sharif Farkhondeh, 2014, Iran J Nurs Midwifery Res, V19, P529

Simos G., 2014, COGNITIVE BEHAV THER

Siu AL, 2016, JAMA-J AM MED ASSOC, V315, P380, DOI 10.1001/jama.2015.18392

Spek V, 2007, PSYCHOL MED, V37, P319, DOI 10.1017/S0033291706008944

Tarrier Nicholas, 2010, Clin Schizophr Relat Psychoses, V4, P176, DOI 10.3371/CSRP.4.3.4

Titov N, 2015, BEHAV THER, V46, P193, DOI 10.1016/j.beth.2014.09.008

Tovote KA, 2014, DIABETES CARE, V37, P2427, DOI 10.2337/dc13-2918

Uchendu C., 2016, DIABET MED

van Bastelaar KMP, 2011, DIABETES CARE, V34, P320, DOI 10.2337/dc10-1248

Van der Feltz-Cornelis CM, 2010, GEN HOSP PSYCHIAT, V32, P380, DOI 10.1016/j.genhosppsych.2010.03.011

van Dooren FEP, 2013, PLOS ONE, V8, DOI 10.1371/journal.pone.0057058

Wagner B, 2014, J AFFECT DISORDERS, V152, P113, DOI 10.1016/j.jad.2013.06.032

Zagarins SE, 2012, J BEHAV MED, V35, P299, DOI 10.1007/s10865-011-9359-z

Zhang Junqing, 2014, CHINA J POSTGRAD MED, V31, P58

Zheng AS Y., 2016, AUST NZ J OBSTET GYN

NR 71

TC 2

Z9 2

U1 6

U2 24

PU PERGAMON-ELSEVIER SCIENCE LTD

PI OXFORD

PA THE BOULEVARD, LANGFORD LANE, KIDLINGTON, OXFORD OX5 1GB, ENGLAND

SN 0022-3999

EI 1879-1360

J9 J PSYCHOSOM RES

JI J. Psychosomat. Res.

PD APR

PY 2017

VL 95

BP 44

EP 54

DI 10.1016/j.jpsychores.2017.02.006

PG 11

WC Psychiatry

SC Psychiatry

GA EP4OZ

UT WOS:000397361100007

PM 28314548

DA 2019-01-08

ER

PT J

AU Wang, RH

AF Wang, Ruey-Hsia

TI Psychological Care for Patients With Diabetes - Present and Future

SO JOURNAL OF NURSING RESEARCH

LA English

DT Article

ID DISTRESS; PEOPLE; OUTCOMES; DEPRESSION.; ATTITUDES; WISHES; TRIAL; 2ND

C1 [Wang, Ruey-Hsia] Kaohsiung Med Univ, Coll Nursing, Kaohsiung, Taiwan.

[Wang, Ruey-Hsia] Kaohsiung Med Univ Hosp, Dept Med Res, Kaohsiung, Taiwan.

RP Wang, RH (reprint author), 100 Shin Chuan 1st Rd, Kaohsiung 80708, Taiwan.

EM wrhsia@kmu.edu.tw

CR Aikens JE, 2012, DIABETES CARE, V35, P2472, DOI 10.2337/dc12-0181

American Diabetes Association, 2017, DIABETES CARE S1, V40, pS4

Chapman Anna, 2015, Front Public Health, V3, P252, DOI 10.3389/fpubh.2015.00252

Chapman A, 2016, BMC HEALTH SERV RES, V16, DOI 10.1186/s12913-016-1358-x

Dalsgaard EM, 2014, DIABETOLOGIA, V57, P710, DOI 10.1007/s00125-014-3165-4

DuBois Christina M, 2016, Prim Care Companion CNS Disord, V18, DOI 10.4088/PCC.15m01902

Fisher L, 2014, DIABETIC MED, V31, P764, DOI 10.1111/dme.12428

Fisher L, 2010, DIABETES CARE, V33, P23, DOI 10.2337/dc09-1238

Friis AM, 2016, DIABETES CARE, V39, P1963, DOI 10.2337/dc16-0416

Gonzalez JS, 2015, HEALTH PSYCHOL, V34, P505, DOI 10.1037/hea0000131

Hsu HC, 2013, J NURS RES, V21, P212, DOI 10.1097/01.jnr.0000432048.31921.e2

Kok JLA, 2015, INT J NURS STUD, V52, P1625, DOI 10.1016/j.ijnurstu.2015.05.012

Mommersteeg PMC, 2013, DIABETIC MED, V30, pE208, DOI 10.1111/dme.12193

Nicolucci A, 2013, DIABETIC MED, V30, P767, DOI 10.1111/dme.12245

Pandit AU, 2014, J EPIDEMIOL COMMUN H, V68, P557, DOI 10.1136/jech-2013-203063

POLONSKY WH, 1995, DIABETES CARE, V18, P754, DOI 10.2337/diacare.18.6.754

Rotella F, 2013, DIABETES RES CLIN PR, V99, P98, DOI 10.1016/j.diabres.2012.11.022

Stuckey HL, 2014, DIABETES CARE, V37, P2466, DOI 10.2337/dc13-2536

Tanenbaum ML, 2016, J DIABETES COMPLICAT, V30, P1060, DOI 10.1016/j.jdiacomp.2016.04.023

van Dijk-de Vries A., 2016, INT J NURS STUD, V63, P58, DOI [10.1016/j.ijnurstu.2016.08.018, DOI 10.1016/J.IJNURSTU.2016.08.018]

Wang RH, 2017, J ADV NURS, V73, P1137, DOI 10.1111/jan.13201

Wang RH, 2011, J ADV NURS, V67, P2658, DOI 10.1111/j.1365-2648.2011.05701.x

NR 22

TC 1

Z9 1

U1 0

U2 4

PU LIPPINCOTT WILLIAMS & WILKINS

PI PHILADELPHIA

PA TWO COMMERCE SQ, 2001 MARKET ST, PHILADELPHIA, PA 19103 USA

SN 1682-3141

J9 J NURS RES

JI J. Nurs. Res.

PD APR

PY 2017

VL 25

IS 2

BP 86

EP 89

DI 10.1097/jnr.0000000000000207

PG 4

WC Nursing

SC Nursing

GA EN3ZX

UT WOS:000395947700001

PM 28277388

DA 2019-01-08

ER

PT J

AU Green, MJ

Espie, CA

Popham, F

Robertson, T

Benzeval, M

AF Green, Michael J.

Espie, Colin A.

Popham, Frank

Robertson, Tony

Benzeval, Michaela

TI Insomnia symptoms as a cause of type 2 diabetes Incidence: a 20 year

cohort study

SO BMC PSYCHIATRY

LA English

DT Article

DE Insomnia; Sleep; Type 2 Diabetes; Longitudinal; Confounding; Causal

Effects; Marginal Structural Models

ID MARGINAL STRUCTURAL MODELS; COLLIDER-STRATIFICATION BIAS;

SOCIOECONOMIC-STATUS; PROPENSITY SCORE; HEALTH BEHAVIORS; SLEEP; RISK;

QUALITY; METAANALYSIS; DISTRESS

AB Background: Insomnia symptoms are associated with type 2 diabetes incidence but are also associated with a range of potential time-varying covariates which may confound and/or mediate associations. We aimed to assess whether cumulative exposure to insomnia symptoms has a causal effect on type 2 diabetes incidence.

Methods: A prospective cohort study in the West of Scotland, following respondents for 20 years from age 36. 996 respondents were free of diabetes at baseline and had valid data from up to four follow-up visits. Type 2 diabetes was assessed at the final visit by self-report, taking diabetic medication, or blood-test (HbA(1c) >= 6.5% or 48 mmol/mol). Effects of cumulative insomnia exposure on type 2 diabetes incidence were estimated with traditional regression and marginal structural models, adjusting for time-dependent confounding (smoking, diet, physical inactivity, obesity, heavy drinking, psychiatric distress) as well as for gender and baseline occupational class.

Results: Traditional regression yielded an odds ratio (OR) of 1.34 (95% CI: 1.06-1.70) for type 2 diabetes incidence for each additional survey wave in which insomnia was reported. Marginal structural models adjusted for prior covariates (assuming concurrently measured covariates were potential mediators), reduced this OR to 1.20 (95% CI: 0.98-1.46), and when concurrent covariates were also included (viewing them as potential confounders) this dropped further to 1.08 (95% CI: 0.85-1.37).

Conclusions: The association between cumulative experience of insomnia and type 2 diabetes incidence appeared confounded. Evidence for a residual causal effect depended on assumptions as to whether concurrently measured covariates were confounders or mediators.

C1 [Green, Michael J.; Popham, Frank] Univ Glasgow, MRC CSO Social & Publ Hlth Sci Unit, 200 Renfield St, Glasgow G2 3QB, Lanark, Scotland.

[Espie, Colin A.] Univ Oxford, Sleep & Circadian Neurosci Inst, Nuffield Dept Clin Neurosci, Oxford OX3 9DU, England.

[Robertson, Tony] Univ Stirling, Fac Hlth Sci & Sport, Stirling FK9 4LA, Scotland.

[Benzeval, Michaela] Univ Essex, Inst Social & Econ Res, Colchester CO4 3SQ, Essex, England.

[Benzeval, Michaela] Univ Glasgow, Inst Hlth & Wellbeing, 1 Lilybank Gardens, Glasgow G12 8RZ, Lanark, Scotland.

RP Green, MJ (reprint author), Univ Glasgow, MRC CSO Social & Publ Hlth Sci Unit, 200 Renfield St, Glasgow G2 3QB, Lanark, Scotland.

EM michael.green@glasgow.ac.uk

OI Green, Michael/0000-0003-3193-2452; Robertson, Tony/0000-0002-1962-5874;

Popham, Frank/0000-0002-7263-7954

FU UK Medical Research Council [MC_UU_12017/13, MC_A540_53462]; Chief

Scientist Office, Scottish Government Health Directorates [SPHSU 13];

University of Essex; UK Economic and Social Research Council

FX MJG and FP are supported by the UK Medical Research Council

(MC_UU_12017/13) and the Chief Scientist Office, Scottish Government

Health Directorates (SPHSU 13). MB has support from the University of

Essex and the UK Economic and Social Research Council. The Twenty-07

Study is funded by the UK Medical Research Council (MC_A540_53462).

CR Agardh EE, 2004, DIABETES CARE, V27, P716, DOI 10.2337/diacare.27.3.716

Ali MS, 2013, EUR J EPIDEMIOL, V28, P291, DOI 10.1007/s10654-013-9766-2

Anothaisintawee T, 2016, SLEEP MED REV, V30, P11, DOI 10.1016/j.smrv.2015.10.002

Asparouhov T., 2010, TECHNICAL REPORT

Austin PC, 2011, MULTIVAR BEHAV RES, V46, P399, DOI 10.1080/00273171.2011.568786

Benzeval M, 2009, INT J EPIDEMIOL, V38, P1215, DOI 10.1093/ije/dyn213

BUYSSE DJ, 1989, PSYCHIAT RES, V28, P193, DOI 10.1016/0165-1781(89)90047-4

Cappuccio FP, 2010, DIABETES CARE, V33, P414, DOI 10.2337/dc09-1124

Cole SR, 2008, AM J EPIDEMIOL, V168, P656, DOI 10.1093/aje/kwn164

Connolly V, 2000, J EPIDEMIOL COMMUN H, V54, P173, DOI 10.1136/jech.54.3.173

Demakakos P, 2012, EUR J EPIDEMIOL, V27, P367, DOI 10.1007/s10654-012-9688-4

Der G, 1998, MRC MED SOCIOLOGY UN, V60

Galobardes B, 2006, J EPIDEMIOL COMMUN H, V60, P7, DOI 10.1136/jech.2004.023531

Goldberg D., 1988, USERS GUIDE GEN HLTH

Green MJ, 2014, BMC PSYCHIATRY, V14, DOI 10.1186/1471-244X-14-152

Greenland S, 2003, EPIDEMIOLOGY, V14, P300, DOI 10.1097/00001648-200305000-00009

Kaufman JS, 2010, EPIDEMIOLOGY, V21, P490, DOI 10.1097/EDE.0b013e3181e00730

Lidfeldt J, 2007, AM J EPIDEMIOL, V165, P882, DOI 10.1093/aje/kwk078

Lucassen EA, 2012, ANN NY ACAD SCI, V1264, P110, DOI 10.1111/j.1749-6632.2012.06655.x

Maty SC, 2005, INT J EPIDEMIOL, V34, P1274, DOI 10.1093/ije/dyi167

Mommersteeg PMC, 2012, BMC PUBLIC HEALTH, V12, DOI 10.1186/1471-2458-12-1109

Moodie EEM, 2008, INT J BIOSTAT, V4, P13

Morin CM, 1999, SLEEP, V22, P1134, DOI 10.1093/sleep/22.8.1134

Muthen L. K., 1998, MPLUS USERS GUIDE

Office of Population Censuses and Surveys, 1980, CLASS OCC

Peuhkuri K, 2012, NUTR RES, V32, P309, DOI 10.1016/j.nutres.2012.03.009

PHILLIPS BA, 1995, ARCH INTERN MED, V155, P734, DOI 10.1001/archinte.155.7.734

Robins JM, 2000, EPIDEMIOLOGY, V11, P550, DOI 10.1097/00001648-200009000-00011

Royal Colleges of Physicians P.a.G.P., 1995, ALC HEART PERSP SENS

Schmid SM, 2015, LANCET DIABETES ENDO, V3, P52, DOI 10.1016/S2213-8587(14)70012-9

Schmid SM, 2011, SLEEP, V34, P371, DOI 10.1093/sleep/34.3.371

Seaman S, 2011, WORKING PAPER, V27

Seaman SR, 2012, BIOMETRICS, V68, P129, DOI 10.1111/j.1541-0420.2011.01666.x

Sherrill DL, 1998, ARCH INTERN MED, V158, P1894, DOI 10.1001/archinte.158.17.1894

Spiegel K, 1999, LANCET, V354, P1435, DOI 10.1016/S0140-6736(99)01376-8

Strine TW, 2005, SLEEP MED, V6, P23, DOI 10.1016/j.sleep.2004.06.003

Suarez D, 2011, EPIDEMIOLOGY, V22, P586, DOI 10.1097/EDE.0b013e31821d0507

Tanaka T, 2012, EUR J PUBLIC HEALTH, V22, P310, DOI 10.1093/eurpub/ckr050

Vgontzas AN, 2009, DIABETES CARE, V32, P1980, DOI 10.2337/dc09-0284

Williams ED, 2010, DIABETOLOGIA, V53, P2538, DOI 10.1007/s00125-010-1888-4

NR 40

TC 3

Z9 3

U1 1

U2 7

PU BIOMED CENTRAL LTD

PI LONDON

PA 236 GRAYS INN RD, FLOOR 6, LONDON WC1X 8HL, ENGLAND

SN 1471-244X

J9 BMC PSYCHIATRY

JI BMC Psychiatry

PD MAR 16

PY 2017

VL 17

AR 94

DI 10.1186/s12888-017-1268-4

PG 8

WC Psychiatry

SC Psychiatry

GA EO5WT

UT WOS:000396764400001

PM 28302102

OA DOAJ Gold, Green Published

DA 2019-01-08

ER

PT J

AU Conti, C

Mennitto, C

Di Francesco, G

Fraticelli, F

Vitacolonna, E

Fulcheri, M

AF Conti, Chiara

Mennitto, Chiara

Di Francesco, Giulia

Fraticelli, Federica

Vitacolonna, Ester

Fulcheri, Mario

TI Clinical Characteristics of Diabetes Mellitus and Suicide Risk

SO FRONTIERS IN PSYCHIATRY

LA English

DT Review

DE diabetes mellitus; suicidal behavior; suicidal ideation; suicide

attempt; depression

ID CO-MORBID DEPRESSION; IDEATION; ADULTS; PREVALENCE; ADOLESCENTS;

DISORDERS; REGIMEN; HEALTH

AB Background: Diabetes mellitus (DM) is a chronic illness with impaired health-related quality of life and a high risk of psychiatric disorders. We carried out a systematic review analyzing the relationship between DM and suicide by providing a qualitative data synthesis of the studies.

Methods: We conducted, in accordance with Preferred Reporting Items for Systematic Reviews and Meta-Analyses guidelines, a systematic search of the literature in PubMed, Scopus, ISI Web of Science, PsycINFO, Google Scholar, and ScienceDirect. Search terms were "suicid*" combined with the Boolean "AND" operator with "diabetes."

Results: The initial search identified 568 citations. A total of 17 research reports met the predefined inclusion criteria and were analyzed. DM was found to be significantly associated with a marked increase in suicidal behaviors and suicidal ideation (SI), especially in patients with depressive symptoms. Insulin therapy, DM of long duration, and unsatisfactory glycemic control were identified as risk factors for SI in Type 1 (T1DM) and Type 2 (T2DM).

Conclusion: Health-care professionals need to be aware of the higher suicidal risk in patient subgroups based on the clinical characteristics of DM; thus, patients with these characteristics warrant special attention. In this regard, clinical management should include efforts to manage emotional distress in DM care.

C1 [Conti, Chiara; Mennitto, Chiara; Di Francesco, Giulia; Fulcheri, Mario] Univ G Annunzio Chieti Pescara, Dept Psychol Hlth & Territorial Sci, Chieti, Italy.

[Fraticelli, Federica; Vitacolonna, Ester] Univ G Annunzio Chieti Pescara, Dept Med & Aging, Chieti, Italy.

RP Conti, C (reprint author), Univ G Annunzio Chieti Pescara, Dept Psychol Hlth & Territorial Sci, Chieti, Italy.

EM cconti@unich.it

OI Fulcheri, Mario/0000-0002-2389-9800

CR Ali S, 2006, DIABETIC MED, V23, P1165, DOI 10.1111/j.1464-5491.2006.01943.x

Amer NRY, 2014, ISSUES MENT HEALTH N, V35, P864, DOI 10.3109/01612840.2014.917752

[Anonymous], 2016, DIABETES CARE S1, pS1, DOI DOI 10.2337/DC16-S001

Ausili Davide, 2014, Prof Inferm, V67, P180, DOI 10.7429/pi.2014.673180

Avci D, 2013, RENAL FAILURE, V35, P863, DOI 10.3109/0886022X.2013.801299

Bai YM, 2013, J AFFECT DISORDERS, V150, P57, DOI 10.1016/j.jad.2013.02.019

Barnard KD, 2006, DIABETIC MED, V23, P445, DOI 10.1111/j.1464-5491.2006.01814.x

Ceretta LB, 2012, ACTA DIABETOL, V49, pS227, DOI 10.1007/s00592-012-0435-9

Chung JH, 2014, J PSYCHOSOM RES, V77, P457, DOI 10.1016/j.jpsychores.2014.08.008

Conti C, 2016, FRONT PSYCHIATRY, V7, DOI 10.3389/fpsyt.2016.00113

Corathers SD, 2013, PEDIATRICS, V132, pE1395, DOI 10.1542/peds.2013-0681

Davis WA, 2015, INTERN MED J, V45, P976, DOI 10.1111/imj.12853

Fornaro M, 2010, WORLD J BIOL PSYCHIA, V11, P991, DOI 10.3109/15622975.2010.491126

Fuller-Thomson Esme, 2009, Diabetes Res Clin Pract, V83, pe9, DOI 10.1016/j.diabres.2008.10.004

Gois C, 2012, J AFFECT DISORDERS, V142, pS67, DOI 10.1016/S0165-0327(12)70010-1

Goldston DB, 1997, J AM ACAD CHILD PSY, V36, P1528, DOI 10.1016/S0890-8567(09)66561-8

Han SJ, 2013, DIABETES RES CLIN PR, V101, pE14, DOI 10.1016/j.diabres.2013.06.012

Handley TE, 2016, DIABETIC MED, V33, P1582, DOI 10.1111/dme.13022

Lee HY, 2014, J PSYCHOSOM RES, V76, P275, DOI [10.1016/j.jpsychores2014.02.003, 10.1016/j.jpsychores.2014.02.003]

Liberati Alessandro, 2009, J Clin Epidemiol, V62, pe1, DOI 10.1016/j.jclinepi.2009.06.006

Lofman S, 2012, J PSYCHOSOM RES, V73, P268, DOI 10.1016/j.jpsychores.2012.08.002

Martino M, 2012, CURR NEUROPHARMACOL, V10, P97, DOI 10.2174/157015912800604542

Mueller N, 2007, MOL PSYCHIATR, V12, P988, DOI 10.1038/sj.mp.4002006

Myers AK, 2013, PSYCHONEUROENDOCRINO, V38, P2810, DOI 10.1016/j.psyneuen.2013.06.013

Pompili M, 2009, PSYCHOSOMATICS, V50, P16, DOI 10.1176/appi.psy.50.1.16

Pouwer F, 2013, ENDOCRIN METAB CLIN, V42, P529, DOI 10.1016/j.ecl.2013.05.002

Radobuljac MD, 2009, PEDIATR DIABETES, V10, P424, DOI 10.1111/j.1399-5448.2009.00501.x

Roy A, 2010, PSYCHIAT RES, V179, P53, DOI 10.1016/j.psychres.2010.06.004

Sarkar P, 2006, Indian J Psychiatry, V48, P78, DOI 10.4103/0019-5545.31594

van Dooren FEP, 2013, PLOS ONE, V8, DOI 10.1371/journal.pone.0057058

Westling S, 2004, PSYCHIAT RES, V129, P249, DOI 10.1016/j.psychres.2004.09.004

World Health Organization, 2012, PUBL HLTH ACT PREV S

NR 32

TC 3

Z9 3

U1 0

U2 9

PU FRONTIERS MEDIA SA

PI LAUSANNE

PA AVENUE DU TRIBUNAL FEDERAL 34, LAUSANNE, CH-1015, SWITZERLAND

SN 1664-0640

J9 FRONT PSYCHIATRY

JI Front. Psychiatry

PD MAR 13

PY 2017

VL 8

AR 40

DI 10.3389/fpsyt.2017.00040

PG 7

WC Psychiatry

SC Psychiatry

GA EN4PP

UT WOS:000395989700001

PM 28348533

OA DOAJ Gold, Green Published

DA 2019-01-08

ER

PT J

AU Delaney, G

Newlyn, N

Pamplona, E

Hocking, SL

Glastras, SJ

McGrath, RT

Fulcher, GR

AF Delaney, Grace

Newlyn, Neroli

Pamplona, Elline

Hocking, Samantha L.

Glastras, Sarah J.

McGrath, Rachel T.

Fulcher, Gregory R.

TI Identification of Patients With Diabetes Who Benefit Most From a Health

Coaching Program in Chronic Disease Management, Sydney, Australia, 2013

SO PREVENTING CHRONIC DISEASE

LA English

DT Article

ID QUALITY-OF-LIFE; RANDOMIZED CONTROLLED-TRIAL; SELF-MANAGEMENT; DISTRESS

SCALE; GLYCEMIC CONTROL; EMPOWERMENT; MELLITUS; PEOPLE; INTERVENTIONS;

COMPLICATIONS

AB Introduction

Chronic disease management programs (CDMPs) that include health coaching can facilitate and coordinate diabetes management. The aim of this study was to assess changes in patients' general knowledge of diabetes, self-reported health status, diabetes distress, body mass index (BMI), and glycemic control after enrollment in a face-to-face CDMP group health coaching session (with telephone follow-up) compared with participation in telephone-only health coaching, during a 12-month period.

Methods

Patients with diabetes were enrolled in a health coaching program at Royal North Shore Hospital, Sydney, Australia, in 2013. Questionnaires were administered at baseline and at 3, 6, and 12 months, and the results were compared with baseline. Glycemic control, measured with glycated hemoglobin A(1c) (HbA(1c)) and BMI, were measured at baseline and 12 months.

Results

Overall, 238 patients attended a face-to-face CDMP session with telephone follow-up (n = 178) or participated in telephone-only health coaching (n = 60). We found no change in BMI in either group; however, HbA(1c) levels in patients with baseline above the current recommended target (>7%) decreased significantly from 8.5% (standard deviation [SD], 1.0%) to 7.9% (SD, 1.0%) (P = .03). Patients with the lowest self-reported health status at baseline improved from 4.4 (SD, 0.5) to 3.7 (SD, 0.9) (P = .001). Diabetes knowledge improved in all patients (24.4 [SD, 2.4] to 25.2 [SD, 2.4]; P <.001), and diabetes distress decreased among those with the highest levels of distress at baseline (3.0 [SD, 0.4] vs 3.8 [SD, 0.6]; P = .003).

Conclusion

Diabetes health coaching programs can improve glycemic control and reduce diabetes distress in patients with high levels of these at baseline.

C1 [Delaney, Grace; Newlyn, Neroli; Pamplona, Elline; Hocking, Samantha L.; Glastras, Sarah J.; McGrath, Rachel T.; Fulcher, Gregory R.] Royal North Shore Hosp, Dept Diabet Endocrinol & Metab, Level 3,Acute Serv Bldg, Sydney, NSW 2065, Australia.

[Hocking, Samantha L.; McGrath, Rachel T.; Fulcher, Gregory R.] Univ Sydney, Royal North Shore Hosp, Northern Clin Sch, St Leonards, NSW 2065, Australia.

[Hocking, Samantha L.] Univ Sydney, Charles Perkins Ctr, Sydney, NSW, Australia.

[Glastras, Sarah J.; McGrath, Rachel T.] Univ Sydney, Kolling Inst Med Res, Royal North Shore Hosp, St Leonards, NSW 2065, Australia.

RP McGrath, RT (reprint author), Royal North Shore Hosp, Dept Diabet Endocrinol & Metab, Level 3,Acute Serv Bldg, Sydney, NSW 2065, Australia.

EM rachel.mcgrath@sydney.edu.au

OI McGrath, Rachel/0000-0003-3453-058X; Hocking,

Samantha/0000-0002-2514-9392; Glastras, Sarah/0000-0002-9317-1348

CR Alberti KGMM, 1998, DIABETIC MED, V15, P539, DOI 10.1002/(SICI)1096-9136(199807)15:7<539::AID-DIA668>3.0.CO;2-S

ANDERSON RM, 1995, DIABETES CARE, V18, P943, DOI 10.2337/diacare.18.7.943

Beverly EA, 2013, DIABETES CARE, V36, P1501, DOI 10.2337/dc12-2110

Davis TME, 2012, DIABETES CARE, V35, P2005, DOI 10.2337/dc12-0225

Dennis SM, 2013, AUST HEALTH REV, V37, P381, DOI 10.1071/AH13005

Fisher L, 2012, DIABETES CARE, V35, P259, DOI 10.2337/dc11-1572

Fisher L, 2010, DIABETES CARE, V33, P23, DOI 10.2337/dc09-1238

Forlani G, 2006, J ENDOCRINOL INVEST, V29, P405, DOI 10.1007/BF03344123

Glasgow RE, 2001, DIABETES SPECTRUM, V14, P33, DOI DOI 10.2337/DIASPECT.14.1.33

HealthStats NSW, SOC IND AR SEIFA LOC

Hill-Briggs F, 2005, DIABETES CARE, V28, P443, DOI 10.2337/diacare.28.2.443

Keech A, 2005, LANCET, V366, P1849, DOI 10.1016/S0140-6736(05)67667-2

Kueh YC, 2015, HEALTH QUAL LIFE OUT, V13, DOI 10.1186/s12955-015-0303-8

Lai PSM, 2012, BMC MED RES METHODOL, V12, DOI 10.1186/1471-2288-12-18

Norris SL, 2002, DIABETES CARE, V25, P1159, DOI 10.2337/diacare.25.7.1159

NSW Agency for Clinical Innovation, NSW CHRON DIS MAN PR

Pibernik-Okanovic M, 2004, PATIENT EDUC COUNS, V52, P193, DOI 10.1016/S0738-3991(03)00038-7

Polonsky WH, 2005, DIABETES CARE, V28, P626, DOI 10.2337/diacare.28.3.626

Rubin RR, 1999, DIABETES-METAB RES, V15, P205, DOI 10.1002/(SICI)1520-7560(199905/06)15:3<205::AID-DMRR29>3.0.CO;2-O

Schmitt A, 2016, DIABETIC MED, V33, P835, DOI 10.1111/dme.12887

Snoek FJ, 2015, LANCET DIABETES ENDO, V3, P450, DOI 10.1016/S2213-8587(15)00135-7

Steed L, 2003, PATIENT EDUC COUNS, V51, P5, DOI 10.1016/S0738-3991(02)00213-6

The Australian Government Department of Health, CHRON DIS MAN

Trento M, 2004, DIABETES CARE, V27, P670, DOI 10.2337/diacare.27.3.670

University of Adelaide, 2011, N W AD HLTH STUD HLT

Wattana C, 2007, NURS HEALTH SCI, V9, P135, DOI 10.1111/j.1442-2018.2007.00315.x

Wayne N, 2015, J MED INTERNET RES, V17, DOI 10.2196/jmir.4871

Wong-Rieger D, 2013, CAN J DIABETES, V37, P41, DOI 10.1016/j.jcjd.2013.01.001

NR 28

TC 3

Z9 3

U1 2

U2 5

PU CENTERS DISEASE CONTROL

PI ATLANTA

PA 1600 CLIFTON RD, ATLANTA, GA 30333 USA

SN 1545-1151

J9 PREV CHRONIC DIS

JI Prev. Chronic Dis.

PD MAR

PY 2017

VL 14

AR E21

DI 10.5888/pcd14.160504

PG 8

WC Public, Environmental & Occupational Health

SC Public, Environmental & Occupational Health

GA EW4IQ

UT WOS:000402465600002

PM 28253473

OA Green Published, Bronze

DA 2019-01-08

ER

PT J

AU Cummings, DM

Lutes, LD

Littlewood, K

Solar, C

Hambidge, B

Gatlin, P

AF Cummings, Doyle M.

Lutes, Lesley D.

Littlewood, Kerry

Solar, Chelsey

Hambidge, Bertha

Gatlin, Peggy

TI IMPACT OF DISTRESS REDUCTION ON BEHAVIORAL CORRELATES AND A1C IN AFRICAN

AMERICAN WOMEN WITH UNCONTROLLED TYPE 2 DIABETES: RESULTS FROM EMPOWER

SO ETHNICITY & DISEASE

LA English

DT Article

DE Diabetes-related Distress; A1C; Glycemic Control; Self-Care;

Self-Efficacy

ID GLYCEMIC CONTROL; MEDICATION ADHERENCE; DEPRESSIVE SYMPTOMS; SCALE;

TRIAL; MELLITUS; PROGRAM; STRESS

AB Objective: Symptoms of emotional distress related to diabetes have been associated with inadequate self-care behaviors, medication non-adherence, and poor glycemic control that may predispose patients to premature death. African American women, in whom diabetes is more common and social support is often insufficient, may be at particularly high risk. The objective of this study was to examine the impact of lowering diabetes-related emotional distress on glycemic control and associated behavioral correlates in rural African American women with uncontrolled type 2 diabetes (T2D).

Design: Post-hoc analysis of prospective, randomized, controlled trial.

Setting: Rural communities in the southeastern United States.

Patients: 129 rural middle-aged African American women with uncontrolled type 2 diabetes (T2D)(A1C >= 7.0).

Primary Independent Variable: Diabetesrelated distress.

Main Outcome Measures: Changes from baseline to 12-month follow-up in diabetesrelated distress, and associated changes in medication adherence, self-care activities, self-efficacy, and glycemic control (A1C).

Results: Patients with a reduction in diabetes- related distress (n= 79) had significantly greater improvement in A1C, medication adherence, self-care activities, and self-efficacy compared with those in whom diabetes distress worsened or was unchanged (n= 50). Changes in distress were also significantly and inversely correlated with improvements in medication adherence, self-care activities, and self-efficacy.

Conclusions: Among rural African American women, reductions in diabetes-related distress may be associated with lower A1C and improvements in self-efficacy, self-care behaviors, and medication adherence.

C1 [Cummings, Doyle M.; Hambidge, Bertha; Gatlin, Peggy] East Carolina Univ, Dept Family Med, Greenville, NC USA.

[Cummings, Doyle M.; Hambidge, Bertha; Gatlin, Peggy] East Carolina Univ, Dept Publ Hlth, Greenville, NC USA.

[Cummings, Doyle M.; Hambidge, Bertha; Gatlin, Peggy] East Carolina Univ, Ctr Hlth Dispar, Greenville, NC USA.

[Lutes, Lesley D.] Univ British Columbia, Dept Psychol, Vancouver, BC, Canada.

[Littlewood, Kerry] Univ S Florida, Sch Social Work, Tampa, FL USA.

[Solar, Chelsey] East Carolina Univ, Dept Psychol, Greenville, NC 27858 USA.

RP Cummings, DM (reprint author), East Carolina Univ, Brody Sch Med, Berbecker Distinguished Prof Rural Med, Dept Family Med, 101 Heart Dr,MS 654, Greenville, NC 27834 USA.

EM cummingsd@ecu.edu

OI CUMMINGS, DOYLE/0000-0002-8237-7885

FU Bristol-Myers Squibb Foundation's Together on Diabetes Initiative

FX The authors acknowledge the generous financial support of Bristol-Myers

Squibb Foundation's Together on Diabetes Initiative that funded this

study. The authors also acknowledge the substantial contributions of our

peer advisors: Ms. J. Royal-Burgess; Ms. J. Jordan; Ms. F. Parker;, Ms.

E. Roberson-Morning; and Ms. S. Taylor; as well as staff member Ms. T.

Kono; research assistants: Ms. J. King; Mr. K. Quinn; Mr. E. Soto; Ms.

L. Vines; and Ms. S. Williamson; our clinical lab assistants Mr. B.

Gilpin; Ms. T. Davis; Ms. B. Whitehurst; Ms. N. Johnson; and Ms. J.

Sanderson; our Research Division supporters: Ms. C. Kennedy and Ms. W.

Wynne; and our community participants.

CR American Diabetes Association, 2016, DIABETES CARE S1, V39, pS52, DOI DOI 10.2337/DC16-S010

Anderson RM, 2003, DIABETES CARE, V26, P1641, DOI 10.2337/diacare.26.5.1641-a

Cummings DM, 2016, DIABETES CARE, V39, P101, DOI 10.2337/dc15-1174

Cummings DM, 2014, ANN PHARMACOTHER, V48, P970, DOI 10.1177/1060028014536532

Cummings DM, 2013, CONTEMP CLIN TRIALS, V36, P147, DOI 10.1016/j.cct.2013.06.006

Ehrmann D, 2015, DIABETIC MED, V32, P1264, DOI 10.1111/dme.12861

Faulenbach M, 2012, DIABETIC MED, V29, P128, DOI 10.1111/j.1464-5491.2011.03431.x

Fisher L, 2008, ANN FAM MED, V6, P246, DOI 10.1370/afm.842

Fisher L, 2013, DIABETES CARE, V36, P2551, DOI 10.2337/dc12-2493

Fisher L, 2010, DIABETES CARE, V33, P1034, DOI 10.2337/dc09-2175

Fisher L, 2010, DIABETES CARE, V33, P23, DOI 10.2337/dc09-1238

Hermanns N, 2015, DIABETES CARE, V38, P551, DOI 10.2337/dc14-1416

Hessler D, 2014, DIABETES CARE, V37, P617, DOI 10.2337/dc13-0762

Littlewood K, 2015, ETHNIC DIS, V25, P193

Lorig K, 1996, OUTCOME MEASURES HLT

Morisky DE, 2008, J CLIN HYPERTENS, V10, P348, DOI 10.1111/j.1751-7176.2008.07572.x

Polonsky WH, 2005, DIABETES CARE, V28, P626, DOI 10.2337/diacare.28.3.626

Toobert DJ, 2000, DIABETES CARE, V23, P943, DOI 10.2337/diacare.23.7.943

NR 18

TC 0

Z9 0

U1 0

U2 6

PU INT SOC HYPERTENSION BLACKS-ISHIB

PI ATLANTA

PA 100 AUBURN AVE NE STE 401, ATLANTA, GA 30303-2527 USA

SN 1049-510X

EI 1945-0826

J9 ETHNIC DIS

JI Ethn. Dis.

PD SPR

PY 2017

VL 27

IS 2

BP 155

EP 160

DI 10.18865/ed.27.2.155

PG 6

WC Public, Environmental & Occupational Health

SC Public, Environmental & Occupational Health

GA ES8UC

UT WOS:000399831400012

PM 28439186

OA Green Published, Bronze

DA 2019-01-08

ER

PT J

AU Ohira, T

Nakano, H

Nagai, M

Yumiya, Y

Zhang, W

Uemura, M

Sakai, A

Hashimoto, S

AF Ohira, Tetsuya

Nakano, Hironori

Nagai, Masato

Yumiya, Yui

Zhang, Wen

Uemura, Mayu

Sakai, Akira

Hashimoto, Shigeatsu

CA Fukushima Hlth Management Survey

TI Changes in Cardiovascular Risk Factors After the Great East Japan

Earthquake: A Review of the Comprehensive Health Check in the Fukushima

Health Management Survey

SO ASIA-PACIFIC JOURNAL OF PUBLIC HEALTH

LA English

DT Review

DE epidemiology; non-communicable diseases; population studies; social

determinants of health; psychological/behavioral medicine

ID POWER-PLANT ACCIDENT; PSYCHOLOGICAL DISTRESS; EVACUATION; DISASTER;

HYPERTENSION; RESIDENTS; RADIATION; DISEASE

AB The Great East Japan Earthquake occurred on March 11, 2011, and was followed by a nuclear accident at the Fukushima Daiichi Nuclear Power Plant. The government ordered a mandatory evacuation from the high radioactive concentration area in Fukushima. This evacuation may have forced many evacuees to change specific aspects of their lifestyles such as diet and physical activity, which in turn may lead to future incidence of lifestyle diseases such as cardiovascular diseases (CVDs). To address this concern, the association between the evacuation and changes in CVD risk factors before and after the disaster was examined in the Fukushima Health Management Survey. In the present study, we reviewed the results of longitudinal studies in the Fukushima Health Management Survey. The proportion of overweight/obese people and those with hypertension, diabetes mellitus, dyslipidemia, liver dysfunction, atrial fibrillation, and polycythemia increased after the disaster. Furthermore, the evacuation was associated with an increase of these cardiovascular risk factors. Therefore, evacuees may be more disposed to CVDs such as myocardial infarction and stroke after the disaster. The prevention of future CVDs among evacuees from Fukushima requires ongoing preventive programs for obesity, hypertension, diabetes mellitus, and dyslipidemia, in collaboration with local governments and communities.

C1 [Ohira, Tetsuya; Nakano, Hironori; Nagai, Masato; Yumiya, Yui; Zhang, Wen; Uemura, Mayu; Sakai, Akira; Hashimoto, Shigeatsu] Fukushima Med Univ, Fukushima, Japan.

[Nagai, Masato] Tohoku Univ, Sendai, Miyagi, Japan.

RP Ohira, T (reprint author), Fukushima Med Univ, Sch Med, Dept Epidemiol, Hikarigaoka 1, Fukushima 9601295, Japan.

EM teoohira@fmu.ac.jp

FU National Health Fund for Children and Adults Affected by the Nuclear

Incident

FX The author(s) disclosed receipt of the following financial support for

the research, authorship, and/or publication of this article: This work

was supported by the National Health Fund for Children and Adults

Affected by the Nuclear Incident for the research, authorship, and/or

publication of this article.

CR Aoki T, 2012, EUR HEART J, V33, P2796, DOI 10.1093/eurheartj/ehs288

Batelaan NM, 2016, BRIT J PSYCHIAT, V208, P223, DOI 10.1192/bjp.bp.114.156554

Hasegawa A, 2015, LANCET, V386, P479, DOI 10.1016/S0140-6736(15)61106-0

Hashimoto S, 2017, J ATHEROSCLER THROMB, V24, P327, DOI 10.5551/jat.35824

Kario K, 1998, J Epidemiol, V8, P131

Kario K, 2012, CIRC J, V76, P553, DOI 10.1253/circj.CJ-11-1510

Kitamura T, 2013, NEW ENGL J MED, V369, P2165, DOI 10.1056/NEJMc1306058

Kunii Y, 2016, PLOS ONE, V11, DOI 10.1371/journal.pone.0158821

Nagai Masato, 2016, Nihon Koshu Eisei Zasshi, V63, P3, DOI 10.11236/jph.63.1_3

Nishina M, 2011, J ATHEROSCLER THROMB, V18, P396, DOI 10.5551/jat.6692

Noda H, 2005, J AM COLL CARDIOL, V46, P1761, DOI 10.1016/j.jacc.2005.07.038

Nozaki E, 2013, INT HEART J, V54, P247, DOI 10.1536/ihj.54.247

Oe M, 2016, PSYCHIAT CLIN NEUROS, V70, P245, DOI 10.1111/pcn.12387

Ohira T, 2016, HYPERTENSION, V68, P558, DOI 10.1161/HYPERTENSIONAHA.116.07499

Ohira T, 2016, AM J PREV MED, V50, P553, DOI 10.1016/j.amepre.2015.10.008

Ohira T, 2010, J EPIDEMIOL, V20, P185, DOI 10.2188/jea.JE20100011

Sakai A, 2014, BMC PUBLIC HEALTH, V14, DOI 10.1186/1471-2458-14-1318

Satoh H, 2015, J DIABETES RES, V2015, DOI DOI 10.1155/2015/627390

Satoh H, 2016, INTERNAL MED, V55, P1967, DOI 10.2169/internalmedicine.55.6030

Suzuki H, 2015, INT J CARDIOL, V198, P102, DOI 10.1016/j.ijcard.2015.06.151

Suzuki Y, 2015, B WORLD HEALTH ORGAN, V93, P598, DOI 10.2471/BLT.14.146498

Swerdel JN, 2014, J AM HEART ASSOC, V3, DOI 10.1161/JAHA.114.001354

Takahashi A, J EPIDEMIOL, DOI [10.1016/j.je.2016.05.006, DOI 10.1016/J.JE.2016.05.006]

Yabe Hirooki, 2014, Fukushima J Med Sci, V60, P57, DOI 10.5387/fms.2014-1

Yasumura S, 2012, J EPIDEMIOL, V22, P375, DOI 10.2188/jea.JE20120105

Yoshimura E, 2016, BMC GERIATR, V16, DOI 10.1186/s12877-016-0203-8

NR 26

TC 7

Z9 8

U1 2

U2 7

PU SAGE PUBLICATIONS INC

PI THOUSAND OAKS

PA 2455 TELLER RD, THOUSAND OAKS, CA 91320 USA

SN 1010-5395

EI 1941-2479

J9 ASIA-PAC J PUBLIC HE

JI Asia-Pac. J. Public Health

PD MAR

PY 2017

VL 29

SU 2

BP 47S

EP 55S

DI 10.1177/1010539517695436

PG 9

WC Public, Environmental & Occupational Health

SC Public, Environmental & Occupational Health

GA ER7ZD

UT WOS:000399033600007

PM 28330394

DA 2019-01-08

ER

PT J

AU Iturralde, E

Weissberg-Benchell, J

Hood, KK

AF Iturralde, Esti

Weissberg-Benchell, Jill

Hood, Korey K.

TI Avoidant Coping and Diabetes-Related Distress: Pathways to Adolescents'

Type 1 Diabetes Outcomes

SO HEALTH PSYCHOLOGY

LA English

DT Article

DE Type 1 diabetes; adolescents; diabetes distress; avoidant coping;

longitudinal mediation

ID METABOLIC-CONTROL; GLYCEMIC CONTROL; COMMITMENT THERAPY; LONGITUDINAL

DATA; SELF-CARE; CHILDREN; INTERVENTIONS; ADAPTATION; TRIAL; ASSOCIATION

AB Objective: Adolescents with Type 1 diabetes (T1D) are vulnerable to diabetes-related distress and often struggle to complete self-management tasks needed to maintain blood glucose values in target range. One way that youth with T1D handle problems is through avoidant coping. The current study examined cross-time associations between avoidant coping style and diabetes outcomes and tested the possible mediating role of diabetes-related distress. Method: Adolescents with T1D (N = 264) were assessed 4 times over 1 year to measure avoidant coping style, diabetes-related distress, adherence (on the basis of glucometer data and self-report), and glycemic control (hemoglobin A1c). Mediation and direct effects were tested across time using time-lagged autoregressive path models, making use of the repeated measurement of all constructs. Results: The hypothesized mediation effect was found for all 3 diabetes outcomes. Higher levels of avoidant coping style were associated with greater diabetes-related distress at the subsequent time point, which was related in turn to fewer blood glucose checks, less frequent self-care behaviors, and poorer glycemic control (higher A1c) at the next assessment. Conclusions: In the context of diabetes, an avoidant coping style may contribute to greater diabetes-specific distress followed by deterioration in self-management and glycemic control over time. Maladaptive coping styles are modifiable factors that offer an entry point into intervention before further difficulties can take hold.

C1 [Iturralde, Esti; Hood, Korey K.] Stanford Univ, Sch Med, Dept Pediat, 780 Welch Rd,MC 5776, Palo Alto, CA 94304 USA.

[Weissberg-Benchell, Jill] Northwestern Univ, Feinberg Sch Med, Ann & Robert H Lurie Childrens Hosp Chicago, Chicago, IL 60611 USA.

[Hood, Korey K.] Stanford Univ, Sch Med, Dept Psychiat & Behav Sci, Palo Alto, CA USA.

RP Hood, KK (reprint author), Stanford Univ, Sch Med, Dept Pediat, 780 Welch Rd,MC 5776, Palo Alto, CA 94304 USA.

EM kkhood@stanford.edu

OI Iturralde, Esti/0000-0001-6837-4998

FU National Institutes of Health [NIH R01 DK 090030]

FX We are grateful to the adolescents who generously participated in the

study. We also thank the following staff members who worked tirelessly

to enroll and retain participants: Sarah Hanes, Aneta Jedraszko, Paige

Staudenmaier, and Sarah Woods. This research was supported by (National

Institutes of Health) NIH R01 DK 090030 (Jill Weissberg-Benchell, Korey

K. Hood, PIs).

CR American Diabetes Association, 2015, Diabetes Care, V38 Suppl, pS33, DOI 10.2337/dc15-S009

Cole DA, 2003, J ABNORM PSYCHOL, V112, P558, DOI 10.1037/0021-843X.112.4.558

Corathers SD, 2013, PEDIATRICS, V132, pE1395, DOI 10.1542/peds.2013-0681

D'Zurilla T., 2002, SOCIAL PROBLEM SOLVI

Davidson M, 2004, APPL NURS RES, V17, P72, DOI 10.1016/j.apnr.2004.02.006

DELAMATER AM, 1987, J DEV BEHAV PEDIATR, V8, P136

Graue M, 2004, DIABETES CARE, V27, P1313, DOI 10.2337/diacare.27.6.1313

Grey M, 2000, J PEDIATR-US, V137, P107, DOI 10.1067/mpd.2000.106568

GREY M, 1991, NURS RES, V40, P144

Hadlandsmyth K, 2013, INT J BEHAV CONSULTA, V7, P12, DOI DOI 10.1037/H0100960

Hagger V, 2016, CURR DIABETES REP, V16, DOI 10.1007/s11892-015-0694-2

HANSON CL, 1989, J CONSULT CLIN PSYCH, V57, P644, DOI 10.1037//0022-006X.57.5.644

Hayes SC, 2006, BEHAV RES THER, V44, P1, DOI 10.1016/j.brat.2005.06.006

Hill-Briggs F, 2006, J BEHAV MED, V29, P69, DOI 10.1007/s10865-005-9037-0

Hilliard ME, 2011, DIABETES RES CLIN PR, V94, P39, DOI 10.1016/j.diabres.2011.05.027

Hood KK, 2006, DIABETES CARE, V29, P1389, DOI 10.2337/dc06-0087

Hood KK, 2010, DIABETES CARE, V33, P1658, DOI 10.2337/dc09-2268

Hood KK, 2009, PEDIATRICS, V124, pE1171, DOI 10.1542/peds.2009-0207

Hu LT, 1999, STRUCT EQU MODELING, V6, P1, DOI 10.1080/10705519909540118

Huang IC, 2014, QUAL LIFE RES, V23, P747, DOI 10.1007/s11136-013-0607-2

Jaser SS, 2011, CHILD CARE HLTH DEV, V37, P335, DOI 10.1111/j.1365-2214.2010.01184.x

Jaser SS, 2014, J PEDIATR PSYCHOL, V39, P306, DOI 10.1093/jpepsy/jst081

Jaser SS, 2012, ANN BEHAV MED, V43, P311, DOI 10.1007/s12160-012-9343-z

Miller KM, 2015, DIABETES CARE, V38, P971, DOI 10.2337/dc15-0078

Mortensen HB, 1997, DIABETES CARE, V20, P714, DOI 10.2337/diacare.20.5.714

Murphy HR, 2006, DIABETIC MED, V23, P935, DOI 10.1111/j.1464-5491.2005.01816.x

Muthen L. K., 2012, MPLUS USERS GUIDE

POLONSKY WH, 1995, DIABETES CARE, V18, P754, DOI 10.2337/diacare.18.6.754

REID GJ, 1994, J DEV BEHAV PEDIATR, V15, P327

Seiffge-Krenke I, 2003, J ADOLESCENT HEALTH, V33, P180, DOI 10.1016/S1054-139X(02)00707-3

Selig JP, 2009, RES HUM DEV, V6, P144, DOI 10.1080/15427600902911247

Serlachius AS, 2016, J HEALTH PSYCHOL, V21, P1157, DOI 10.1177/1359105314547940

Shrout PE, 2002, PSYCHOL METHODS, V7, P422, DOI 10.1037//1082-989X.7.4.422

Siu AL, 2016, PEDIATRICS, V137, DOI 10.1542/peds.2015-4467

Swain J, 2015, J CONTEXT BEHAV SCI, V4, P73, DOI 10.1016/j.jcbs.2015.02.001

Taylor SE, 2007, ANNU REV CLIN PSYCHO, V3, P377, DOI 10.1146/annurev.clinpsy.3.022806.091520

The Diabetes Control and Complications Trial/Epidemiology of Diabetes Interventions and Complications Research Group, 2000, NEW ENGL J MED, V342L, P381, DOI DOI 10.1056/NEJM200002103420603

The Diabetes Control and Complications Trial Research Group, 1993, NEW ENGL J MED, V329, P977

Wade SL, 2012, HEALTH PSYCHOL, V31, P767, DOI 10.1037/a0028440

Weinger K, 2005, DIABETES CARE, V28, P1346, DOI 10.2337/diacare.28.6.1346

Weissberg-Benchell J, 2016, CONTEMP CLIN TRIALS, V49, P78, DOI 10.1016/j.cct.2016.05.006

Weissberg-Benchell J, 2011, PEDIATR DIABETES, V12, P341, DOI 10.1111/j.1399-5448.2010.00720.x

WEISSBERGBENCHELL J, 1995, DIABETES CARE, V18, P77, DOI 10.2337/diacare.18.1.77

Whittemore R, 2010, NURS OUTLOOK, V58, P242, DOI 10.1016/j.outlook.2010.05.001

Wicksell RK, 2015, CURR OPIN PSYCHOL, V2, P1, DOI 10.1016/j.copsyc.2014.12.029

Wysocki T, 2008, J PEDIATR PSYCHOL, V33, P875, DOI 10.1093/jpepsy/jsn024

Wysocki Tim, 2006, Curr Diab Rep, V6, P117, DOI 10.1007/s11892-006-0022-y

NR 47

TC 10

Z9 10

U1 2

U2 11

PU AMER PSYCHOLOGICAL ASSOC

PI WASHINGTON

PA 750 FIRST ST NE, WASHINGTON, DC 20002-4242 USA

SN 0278-6133

EI 1930-7810

J9 HEALTH PSYCHOL

JI Health Psychol.

PD MAR

PY 2017

VL 36

IS 3

BP 236

EP 244

DI 10.1037/hea0000445

PG 9

WC Psychology, Clinical; Psychology

SC Psychology

GA EN4UN

UT WOS:000396002500006

PM 27808528

DA 2019-01-08

ER

PT J

AU Siaw, MYL

Tai, BWB

Lee, JYC

AF Siaw, Melanie Yee Lee

Tai, Bik-Wai Bilvick

Lee, Joyce Yu-Chia

TI Psychometric properties of the Chinese version of the Problem Areas in

Diabetes scale (SG-PAID-C) among high-risk polypharmacy patients with

uncontrolled type 2 diabetes in Singapore

SO JOURNAL OF DIABETES INVESTIGATION

LA English

DT Article

DE Quality of life; Type 2 diabetes mellitus; Validation studies

ID EMOTIONAL DISTRESS; VALIDITY; QUESTIONNAIRE; RELIABILITY; DEPRESSION;

ANXIETY; STRESS; HEALTH

AB Aims/Introduction: Undetected diabetes distress is a cause of concern. However, the lack of a validated questionnaire is a barrier to screening for diabetes distress. The aim of the present study was to examine the validity and reliability of the Chinese version of the Problem Areas in Diabetes scale (SG-PAID-C), and its association with sociodemographic and clinical parameters in patients with type 2 diabetes.

Materials and Methods: This cross-sectional study was carried out in four outpatient healthcare institutions in Singapore. Chinese-speaking patients with uncontrolled type 2 diabetes, polypharmacy, and multiple comorbidities were administered the SG-PAID-C and European Quality of Life-5 Dimensions questionnaires as quality of life measures. The factorial construct, convergent validity and internal consistency of SG-PAID-C were evaluated.

Results: The exploratory factor analysis resulted in a three-factor structure of SG-PAID-C with subscales on emotional-and management-related problem (11 items), ability to cope with diabetes problem (3 items) and support-related problem (2 items). The findings also showed good model fit in the confirmatory factor analysis, and provided support for the construct and convergent validity of SG-PAID-C. Overall, the internal consistency of SG-PAID-C was good (Cronbach's alpha = 0.900). Sex and duration of diabetes were positively associated with the 16-item SG-PAID-C, whereas age and type of antidiabetic agents were inversely associated with the 16-item SG-PAID-C.

Conclusions: The 16-item SG-PAID-C is a valid and reliable instrument for use among patients with uncontrolled type 2 diabetes in Singapore. Future studies on its clinical utility should be carried out.

C1 [Siaw, Melanie Yee Lee; Lee, Joyce Yu-Chia] Natl Univ Singapore, Dept Pharm, Fac Sci, Singapore, Singapore.

[Tai, Bik-Wai Bilvick] Caritas Bianchi Coll Careers, Sch Hlth Sci, Caritas Inst Higher Educ, Hong Kong, Hong Kong, Peoples R China.

RP Lee, JYC (reprint author), Natl Univ Singapore, Dept Pharm, Fac Sci, Singapore, Singapore.; Tai, BWB (reprint author), Caritas Bianchi Coll Careers, Sch Hlth Sci, Caritas Inst Higher Educ, Hong Kong, Hong Kong, Peoples R China.

EM btai@cihe.edu.hk; phalycj@nus.edu.sg

OI Siaw, Melanie/0000-0003-0546-0287; Tai, Bik-Wai

Bilvick/0000-0002-4017-334X

FU Health Service Research Competitive Research Grant from Singapore

Ministry of Health [HSRG/11MAY/016]

FX The authors thank Yii-Jen Lew, Elaine Tan and Soo Chung Chan for their

support in the study. This study was funded by a Health Service Research

Competitive Research Grant from Singapore Ministry of Health (grant

number: HSRG/11MAY/016).

CR Amsberg S, 2008, INT J NURS STUD, V45, P1319, DOI 10.1016/j.ijnurstu.2007.09.010

Ascher-Svanum H, 2015, DIABETES THER, V6, P303, DOI 10.1007/s13300-015-0118-y

Delahanty LM, 2007, DIABETIC MED, V24, P48, DOI 10.1111/j.1464-5491.2007.02028.x

Denneboom W, 2006, BRIT J GEN PRACT, V56, P504

Dong YR, 2013, SPRINGERPLUS, V2, DOI 10.1186/2193-1801-2-222

Field A., 2009, DISCOVERING STAT USI

Fisher L, 2009, DIABETIC MED, V26, P622, DOI 10.1111/j.1464-5491.2009.02730.x

Glasgow RE, 1999, DIABETES CARE, V22, P832, DOI 10.2337/diacare.22.5.832

Gross CC, 2007, DIABETES RES CLIN PR, V76, P455, DOI 10.1016/j.diabres.2006.09.022

Herschbach P, 1997, HEALTH PSYCHOL, V16, P171, DOI 10.1037/0278-6133.16.2.171

Huang MF, 2010, DIABETES CARE, V33, P38, DOI 10.2337/dc09-0768

Huis v EM, 2011, CARDIOVASC PSYCHIAT, V2011

International Diabetes Federation, 2015, IDF DIABETES ATLAS

Lee KH, 2015, ANN OCCUP ENVIRON ME, V27, DOI 10.1186/s40557-015-0075-y

Lim MCC, 2008, ANN ACAD MED SINGAP, V37, P753

Luo N, 2003, ANN ACAD MED SINGAP, V32, P685

Luo N, 2014, PHARMACOECONOMICS, V32, P495, DOI 10.1007/s40273-014-0142-1

Marengoni A, 2011, AGEING RES REV, V10, P430, DOI 10.1016/j.arr.2011.03.003

Miller ST, 2008, BMC PUBLIC HEALTH, V8, DOI 10.1186/1471-2458-8-70

Nunnally JC, 1994, PSYCHOMETRIC THEORY

Papathanasiou A, 2014, J PSYCHIATR MENT HLT, V21, P345, DOI 10.1111/j.1365-2850.2012.01875.x

Phan TP, 2014, BMJ OPEN DIAB RES CA, V2, DOI 10.1136/bmjdrc-2013-000012

POLONSKY WH, 1995, DIABETES CARE, V18, P754, DOI 10.2337/diacare.18.6.754

Polonsky WH, 2005, DIABETES CARE, V28, P2543, DOI 10.2337/diacare.28.10.2543

Polonsky WH, 2005, DIABETES CARE, V28, P626, DOI 10.2337/diacare.28.3.626

Pouwer F, 2006, PATIENT EDUC COUNS, V60, P235, DOI 10.1016/j.pec.2005.01.009

Ramachandran A, 2012, WORLD J DIABETES, V3, P110, DOI 10.4239/wjd.v3.i6.110

Schmitt A, 2016, DIABETIC MED, V33, P835, DOI 10.1111/dme.12887

Sigurdardottir AK, 2008, INT J NURS STUD, V45, P526, DOI 10.1016/j.ijnurstu.2006.10.008

Snoek FJ, 2000, DIABETES CARE, V23, P1305, DOI 10.2337/diacare.23.9.1305

Sperlich S, 2015, SOC SCI MED, V131, P58, DOI 10.1016/j.socscimed.2015.03.001

Tavakol M, 2011, INT J MED EDUC, V2, P53, DOI 10.5116/ijme.4dfb.8dfd

Tol Azar, 2015, J Educ Health Promot, V4, P9, DOI 10.4103/2277-9531.151903

Vathsala A, 2007, ANN ACAD MED SINGAP, V36, P157

Venkataraman K, 2015, PLOS ONE, V10, DOI 10.1371/journal.pone.0136759

West Christina, 2002, Br J Community Nurs, V7, P606

Zimmet P, 2001, NATURE, V414, P782, DOI 10.1038/414782a

NR 37

TC 1

Z9 1

U1 0

U2 4

PU WILEY

PI HOBOKEN

PA 111 RIVER ST, HOBOKEN 07030-5774, NJ USA

SN 2040-1124

J9 J DIABETES INVEST

JI J. Diabetes Investig.

PD MAR

PY 2017

VL 8

IS 2

BP 235

EP 242

DI 10.1111/jdi.12556

PG 8

WC Endocrinology & Metabolism

SC Endocrinology & Metabolism

GA EQ4PB

UT WOS:000398059100016

PM 27461266

OA DOAJ Gold, Green Published

DA 2019-01-08

ER

PT J

AU Uchendu, C

Blake, H

AF Uchendu, C.

Blake, H.

TI Effectiveness of cognitive-behavioural therapy on glycaemic control and

psychological outcomes in adults with diabetes mellitus: a systematic

review and meta-analysis of randomized controlled trials

SO DIABETIC MEDICINE

LA English

DT Review

ID GROUP TRAINING CBGT; PANIC DISORDER; DEPRESSION; INTERVENTION; ANXIETY;

PREVALENCE; CBT; PSYCHOTHERAPY; MANAGEMENT; LIFE

AB Aim Diabetes is a chronic progressive condition presenting physical, social and psychological challenges that increase the risk of comorbid mental health problems. Cognitive-behavioural therapy (CBT) is effective in treating a variety of psychological disorders, and may potentially improve glycaemic control and psychological outcomes in diabetes. This systematic review and meta-analysis aims to establish the effectiveness of CBT on glycaemic control and comorbid diabetes-related distress, depression, anxiety and quality of life in the short, medium and longer term among adults with diabetes.

Method An electronic search was conducted in PubMed, Embase, MEDLINE, PsycINFO, CINAHL, Web of Knowledge, Cochrane Central Register of Controlled Trials and references in reviews. Twelve randomized controlled trials (RCTs) were identified that evaluated the effectiveness of CBT on at least one of: glycaemic control, diabetes-related distress, anxiety, depression or quality of life in adults with Type 1 or Type 2 diabetes. The Cochrane Risk of Bias Tool and Review Manager version 5.3 were used for risk of bias assessment and meta-analysis, respectively.

Results CBT is effective in reducing short-term and medium-term glycaemic control, although no significant effect was found for long-term glycaemic control. CBT improved short-and medium-term anxiety and depression, and long-term depression. Mixed results were found for diabetes-related distress and quality of life.

Conclusion CBT is beneficial in improving depression for adults with diabetes. It may have benefits for improving glycaemic control and other aspects of psychological health, although the findings are inconclusive.

C1 [Uchendu, C.; Blake, H.] Univ Nottingham, Sch Hlth Sci, Nottingham, England.

RP Uchendu, C (reprint author), Univ Nottingham, Sch Hlth Sci, Nottingham, England.

EM ntxcou@nottingham.ac.uk

CR Alam R, 2009, PATIENT EDUC COUNS, V75, P25, DOI 10.1016/j.pec.2008.08.026

Ali Naseer, 2013, J Assoc Physicians India, V61, P619

Amsberg S, 2009, PATIENT EDUC COUNS, V77, P72, DOI 10.1016/j.pec.2009.01.015

Anderson RJ, 2001, DIABETES CARE, V24, P1069, DOI 10.2337/diacare.24.6.1069

Andrews G, 2011, AUST NZ J PSYCHIAT, V45, P337, DOI 10.3109/00048674.2010.538840

[Anonymous], 2015, LANCET DIABETES ENDO, V3, P393

Balhara Yatan Pal Singh, 2011, Indian J Endocrinol Metab, V15, P274, DOI 10.4103/2230-8210.85579

Barnard KD, 2006, DIABETIC MED, V23, P445, DOI 10.1111/j.1464-5491.2006.01814.x

Beltman MW, 2010, BRIT J PSYCHIAT, V197, P11, DOI 10.1192/bjp.bp.109.064675

Bergstrom J, 2010, BMC PSYCHIATRY, V10, DOI 10.1186/1471-244X-10-54

Borkovec TD, 2001, J CLIN PSYCHIAT, V62, P37

Butler AC, 2006, CLIN PSYCHOL REV, V26, P17, DOI 10.1016/j.cpr.2005.07.003

Chen SM, 2012, INT J NURS STUD, V49, P637, DOI 10.1016/j.ijnurstu.2011.11.011

Chew BH, 2014, WORLD J DIABETES, V5, P796, DOI 10.4239/wjd.v5.i6.796

COHEN J, 1992, PSYCHOL BULL, V112, P155, DOI 10.1037//0033-2909.112.1.155

Davazdahemamy M, 2009, IRAN J ENDOCRIN META, V11, P385

Davazdahemamy MH, 2012, IRAN J CLIN PSYCHOL, V1

Duval S, 2000, BIOMETRICS, V56, P455, DOI 10.1111/j.0006-341X.2000.00455.x

Elliott S, 2012, PRACT DIABETES, V29, P67, DOI 10.1002/pdi.1661

Fairburn CG, 2003, BEHAV RES THER, V41, P509, DOI 10.1016/S0005-7967(02)00088-8

Fisher L, 2007, DIABETES CARE, V30, P542, DOI 10.2337/dc06-1614

Gois C, 2014, CLIN PSYCHOL PSYCHOT, V21, P39, DOI 10.1002/cpp.1817

Gois CJ, 2012, ACTA DIABETOL, V49, pS33, DOI 10.1007/s00592-010-0191-7

Granath Jens, 2006, Cognitive Behaviour Therapy, V35, P3, DOI 10.1080/16506070500401292

Grigsby AB, 2002, J PSYCHOSOM RES, V53, P1053, DOI 10.1016/S0022-3999(02)00417-8

Henry JL, 1997, PSYCHOL HEALTH MED, V2, P109

Higgins JP, 2011, COCHRANE HDB SYSTEMA

Higgins JPT, 2011, BMJ-BRIT MED J, V343, DOI 10.1136/bmj.d5928

Hofmann SG, 2008, J CLIN PSYCHIAT, V69, P621

Ismail K, 2004, LANCET, V363, P1589, DOI 10.1016/S0140-6736(04)16202-8

Katon WJ, 2008, AM J MED, V121, P8, DOI 10.1016/j.amjmed.2008.09.008

Kiropoulos LA, 2008, J ANXIETY DISORD, V22, P1273, DOI 10.1016/j.janxdis.2008.01.008

Kroenke K, 2003, INT J METH PSYCH RES, V12, P34, DOI 10.1002/mpr.140

Llorente MD, 2007, PSYCHIAT DISORDERS D

Lustman PJ, 1998, ANN INTERN MED, V129, P613, DOI 10.7326/0003-4819-129-8-199810150-00005

National Institute for Health and Care Excellence (NICE), 2007, DEPR MAN DEPR PRIM S

Paschalides C, 2004, J PSYCHOSOM RES, V57, P557, DOI 10.1016/j.jpsychores.2004.03.006

Penckofer SM, 2012, ANN BEHAV MED, V44, P192, DOI 10.1007/s12160-012-9377-2

Piette JD, 2011, MED CARE, V49, P641, DOI 10.1097/MLR.0b013e318215d0c9

Polit D.F., 2014, ESSENTIALS NURSING R

Safren SA, 2014, DIABETES CARE, V37, P625, DOI [10.2337/dc13-0816, 10.2337/dc16-er06]

Simon GE, 2010, PSYCHIAT SERV, V61, P684, DOI 10.1176/ps.2010.61.7.684

Simos G., 2014, COGNITIVE BEHAV THER

Snoek FJ, 2008, DIABETIC MED, V25, P1337, DOI 10.1111/j.1464-5491.2008.02595.x

Snoek F. J., 1999, DIABETES SPECTRUM, V12, P147

Snoek FJ, 2001, PATIENT EDUC COUNS, V45, P143, DOI 10.1016/S0738-3991(01)00113-6

Turner J., 2010, J DIABETES NURSING, V14, P86

van Bastelaar KMP, 2011, DIABETES CARE, V34, P320, DOI 10.2337/dc10-1248

van der Ven NCW, 2005, DIABETIC MED, V22, P1619

Wampold BE, 2002, J AFFECT DISORDERS, V68, P159, DOI 10.1016/S0165-0327(00)00287-1

Wang MY, 2008, J CLIN NURS, V17, P2524, DOI 10.1111/j.1365-2702.2008.02301.x

Weinger K, 2011, ARCH INTERN MED, V171, P1990, DOI 10.1001/archinternmed.2011.502

Welschen LMC, 2013, J BEHAV MED, V36, P556, DOI 10.1007/s10865-012-9451-z

Welschen LMC, 2007, BMC PUBLIC HEALTH, V7, DOI 10.1186/1471-2458-7-74

Westbrook D., 2008, INTRO COGNITIVE BEHA

Winkley K, 2006, BRIT MED J, V333, P65, DOI 10.1136/bmj.38874.652569.55

NR 56

TC 8

Z9 9

U1 4

U2 21

PU WILEY

PI HOBOKEN

PA 111 RIVER ST, HOBOKEN 07030-5774, NJ USA

SN 0742-3071

EI 1464-5491

J9 DIABETIC MED

JI Diabetic Med.

PD MAR

PY 2017

VL 34

IS 3

BP 328

EP 339

DI 10.1111/dme.13195

PG 12

WC Endocrinology & Metabolism

SC Endocrinology & Metabolism

GA EP5FK

UT WOS:000397404200004

PM 27472405

DA 2019-01-08

ER

PT J

AU Schmitt, A

Reimer, A

Hermanns, N

Kulzer, B

Ehrmann, D

Krichbaum, M

Huber, J

Haak, T

AF Schmitt, Andreas

Reimer, Andre

Hermanns, Norbert

Kulzer, Bernhard

Ehrmann, Dominic

Krichbaum, Michael

Huber, Jorg

Haak, Thomas

TI Depression is linked to hyperglycaemia via suboptimal diabetes

self-management: A cross-sectional mediation analysis

SO JOURNAL OF PSYCHOSOMATIC RESEARCH

LA English

DT Article

DE Depressive symptoms; Mood disorder; HbA(1c); Hyperglycaemia Diabetes

self-care; Mediation

ID CO-MORBID DEPRESSION; GLYCEMIC CONTROL; CLINICAL DEPRESSION; SYMPTOMS;

DISTRESS; METAANALYSIS; TYPE-1; ADULTS; ASSOCIATION; PREVALENCE

AB Objective: To analyse if the association between depressive symptoms and hyperglycaemia is mediated by diabetes self-management.

Methods: 430 people with diabetes (57.7% type 1, 42.3% type 2) were cross-sectionally assessed using validated self-report scales for depressive symptoms (Center for Epidemiologic Studies Depression Scale (CES-D)) and diabetes self-management (Diabetes Self-Management Questionnaire (DSMQ)); HbA(1c) was analysed simultaneously in a central laboratory. Structural equation modelling was used to test if the association between depressive symptoms and hyperglycaemia (HbA(1c)) was mediated by suboptimal self-management in people with type 1 and type 2 diabetes.

Results: The hypothesised model of depressive symptoms, diabetes self-management and hyperglycaemia fit the data well for both diabetes types (SRMR <= 0.04, TLI >= 0.99, CFI > 0.99, RMSEA <= 0.02 for both models). In both the type I and type 2 diabetes group, higher depressive symptoms were associated with lower self-management (P < 0.001) and lower self-management was associated with higher HbAi, (P < 0.001). Results indicated that the association between depressive symptoms and hyperglycaemia was significantly mediated by suboptimal diabetes self-management in both type 1 and type 2 diabetes patients (P < 0.001). Significant direct associations between depressive symptoms and hyperglycaemia, not mediated by self-management, could not be observed.

Conclusions: This study provides good evidence supporting that depression is linked to hyperglycaemia via suboptimal diabetes self-management in both major diabetes types.(C) 2016 Elsevier Inc All rights reserved.

C1 [Schmitt, Andreas; Reimer, Andre; Hermanns, Norbert; Kulzer, Bernhard; Ehrmann, Dominic; Krichbaum, Michael; Haak, Thomas] Res Inst Diabet Acad Mergentheim FIDAM, Johann Hammer Str 24, D-97980 Bad Mergentheim, Germany.

[Schmitt, Andreas; Reimer, Andre; Hermanns, Norbert; Kulzer, Bernhard; Ehrmann, Dominic; Krichbaum, Michael; Haak, Thomas] Diabet Ctr Mergentheim DZM, Theodor Klotzbucher Str 12, D-97980 Bad Mergentheim, Germany.

[Schmitt, Andreas; Reimer, Andre; Hermanns, Norbert; Kulzer, Bernhard] German Ctr Diabet Res DZD, D-85764 Munich, Germany.

[Hermanns, Norbert; Kulzer, Bernhard] Otto Friedrich Univ Bamberg, Dept Psychol, Markuspl 3, D-96047 Bamberg, Germany.

[Huber, Jorg] Univ Brighton, Ctr Hlth Res, Falmer BN1 9PH, England.

RP Schmitt, A (reprint author), Diabet Acad Mergentheim FIDAM, Res Inst, Theodor Klotzbuecher Str 12, D-97980 Bad Mergentheim, Germany.

EM schmitt@diabetes-zentrum.de; reimer@diabetes-zentrum.de;

hermanns@diabetes-zentrum.de; kulzer@diabetes-zentrum.de;

ehrmann@diabetes-zentrum.de; krichbaum@diabetes-zentrum.de;

J.Huber@brighton.ac.uk; haak@diabetes-zentrum.de

OI Ehrmann, Dominic/0000-0002-5794-5596; Krichbaum,

Michael/0000-0002-5937-1266; Schmitt, Andreas/0000-0002-5913-1457

FU Federal Ministry of Education and Research [01G11107]; German Center for

Diabetes Research (DZD) [82DZD01101]

FX This work was supported by the "Kompetenznetz Diabetes mellitus

(Competence Network for Diabetes mellitus)" of the Federal Ministry of

Education and Research [grant number 01G11107] and the German Center for

Diabetes Research (DZD) [grant number 82DZD01101].

CR Aikens JE, 2009, DIABETES CARE, V32, P1177, DOI 10.2337/dc09-0071

Ali S, 2006, DIABETIC MED, V23, P1165, DOI 10.1111/j.1464-5491.2006.01943.x

Anderson RJ, 2001, DIABETES CARE, V24, P1069, DOI 10.2337/diacare.24.6.1069

Barnard KD, 2006, DIABETIC MED, V23, P445, DOI 10.1111/j.1464-5491.2006.01814.x

Black SA, 2003, DIABETES CARE, V26, P2822, DOI 10.2337/diacare.26.10.2822

Chiu CJ, 2010, SOC PSYCH PSYCH EPID, V45, P67, DOI 10.1007/s00127-009-0043-3

Clouse RE, 2003, PSYCHOSOM MED, V65, P376, DOI 10.1097/01.PSY.0000041624.96580.1F

COHEN J, 1992, PSYCHOL BULL, V112, P155, DOI 10.1037//0033-2909.112.1.155

Cole DA, 2007, PSYCHOL METHODS, V12, P381, DOI 10.1037/1082-989X.12.4.381

Dirmaier J, 2010, PSYCHOTHER PSYCHOSOM, V79, P172, DOI 10.1159/000296135

Egede LE, 2005, CURR DIABETES REV, V1, P235, DOI 10.2174/157339905774574356

Egede LE, 2010, DIABETES EDUCATOR, V36, P276, DOI 10.1177/0145721710361389

Fisher L, 2007, DIABETES CARE, V30, P542, DOI 10.2337/dc06-1614

Fisher L, 2010, DIABETES CARE, V33, P1034, DOI 10.2337/dc09-2175

Fisher L, 2010, DIABETES CARE, V33, P23, DOI 10.2337/dc09-1238

Gohel MG, 2013, J DIABETES METAB DIS, V12, DOI 10.1186/2251-6581-12-56

Gonzalez JS, 2007, DIABETES CARE, V30, P2222, DOI 10.2337/dc07-0158

Gonzalez JS, 2008, DIABETES CARE, V31, P2398, DOI 10.2337/dc08-1341

Hautzinger M, 2012, ADS ALLGEMEINE DEPRE

Hofmann M, 2013, PLOS ONE, V8, DOI 10.1371/journal.pone.0079809

Hu LT, 1998, PSYCHOL METHODS, V3, P424, DOI 10.1037//1082-989X.3.4.424

Kenny D. A., 2016, MEDIATION

Laake JPS, 2014, DIABETES CARE, V37, P2186, DOI 10.2337/dc13-2522

Lewinsohn PM, 1997, PSYCHOL AGING, V12, P277, DOI 10.1037//0882-7974.12.2.277

Lin EHB, 2010, DIABETES CARE, V33, P264, DOI 10.2337/dc09-1068

Lustman PJ, 2005, PSYCHOSOM MED, V67, P195, DOI 10.1097/01.psy.0000155670.88919.ad

Lustman PJ, 2000, DIABETES CARE, V23, P934, DOI 10.2337/diacare.23.7.934

McGrady ME, 2009, DIABETES CARE, V32, P804, DOI 10.2337/dc08-2111

Osborn CY, 2010, DIABETES TECHNOL THE, V12, P913, DOI 10.1089/dia.2010.0058

Park M, 2013, GEN HOSP PSYCHIAT, V35, P217, DOI 10.1016/j.genhosppsych.2013.01.006

Pibernik-Okanovic M, 2008, DIABETIC MED, V25, P1252, DOI 10.1111/j.1464-5491.2008.02553.x

Richardson LK, 2008, GEN HOSP PSYCHIAT, V30, P509, DOI 10.1016/j.genhosppsych.2008.07.001

Schmitt A., 2014, P 50 EASD ANN M SEP

Schmitt A., 2013, DIABETOLOGIE STOF S1, V8, P77

Schmitt A, 2016, PLOS ONE, V11, DOI 10.1371/journal.pone.0150774

Schmitt A, 2015, J BEHAV MED, V38, P556, DOI 10.1007/s10865-014-9604-3

Schmitt A, 2013, HEALTH QUAL LIFE OUT, V11, DOI 10.1186/1477-7525-11-138

Stuart MJ, 2012, NEUROSCI BIOBEHAV R, V36, P658, DOI 10.1016/j.neubiorev.2011.10.001

Templeton GF, 2011, COMMUN ASSOC INF SYS, V28, P41

TOOBERT DJ, 1994, HDB PSYCHOL DIABETES, P351

van Bastelaar KMP, 2010, DIABETIC MED, V27, P798, DOI 10.1111/j.1464-5491.2010.03025.x

NR 41

TC 9

Z9 10

U1 0

U2 9

PU PERGAMON-ELSEVIER SCIENCE LTD

PI OXFORD

PA THE BOULEVARD, LANGFORD LANE, KIDLINGTON, OXFORD OX5 1GB, ENGLAND

SN 0022-3999

EI 1879-1360

J9 J PSYCHOSOM RES

JI J. Psychosomat. Res.

PD MAR

PY 2017

VL 94

BP 17

EP 23

DI 10.1016/jjpsychores.2016.12.015

PG 7

WC Psychiatry

SC Psychiatry

GA EL4AK

UT WOS:000394562100003

PM 28183398

DA 2019-01-08

ER

PT J

AU Yoong, RKL

Mooppil, N

Khoo, EYH

Newman, SP

Lee, VYW

Kang, AWC

Griva, K

AF Yoong, Russell K. L.

Mooppil, Nandakumar

Khoo, Eric Y. H.

Newman, Stanton P.

Lee, Vanessa Y. W.

Kang, Augustine W. C.

Griva, Konstadina

TI Prevalence and determinants of anxiety and depression in end stage renal

disease (ESRD). A comparison between ESRD patients with and without

coexisting diabetes mellitus

SO JOURNAL OF PSYCHOSOMATIC RESEARCH

LA English

DT Article

DE Anxiety; Depression; Diabetes mellitus; End-stage renal disease

ID PSYCHOLOGICAL DISTRESS; PERITONEAL-DIALYSIS; SYMPTOMS; METAANALYSIS;

INTERVENTION; HEMODIALYSIS; MORTALITY; OUTCOMES; ADULTS

AB Objective: To compare anxiety and/or depressive symptoms between patients with end-stage renal disease with and without comorbid diabetes and identify factors associated with symptoms of distress in this population.

Methods: Data from two studies (conducted between 2010 and 2014) were pooled. A total of 526 patients on hemodialysis (68.8% with diabetes) completed the Hospital Anxiety and Depression Scale (HADS). Elevated symptoms were defined as HADS-Anxiety or HADS-Depression >= 8. Univariate and multivariate logistic regressions were used to estimate associations between diabetic status, and other socio-demographic and clinical factors with baseline clinical anxiety and depression.

Results: A total of 233 (45.4%) reported elevated anxiety symptoms and 256 (49.9%) reported elevated depressive symptoms sufficient for caseness. Rates were not different between patients with and without diabetes. Risk for clinical depression was higher in patients who were single/unpartnered (OR = 1.828), Chinese vs. Malay (OR = 2.05), or had lower albumin levels (OR = 0.932). None of the parameters were associated with anxiety caseness.

Conclusion: Sociocultural factors rather than comorbid burden may help identify patients at risk for depression. The high rates of anxiety and depression underlie the importance for monitoring and intervention in dialysis care. (C) 2017 Elsevier Inc.All rights reserved.

C1 [Yoong, Russell K. L.; Lee, Vanessa Y. W.; Kang, Augustine W. C.; Griva, Konstadina] Natl Univ Singapore, Dept Psychol, Singapore, Singapore.

[Mooppil, Nandakumar] Natl Kidney Fdn Singapore, Singapore, Singapore.

[Khoo, Eric Y. H.] Natl Univ Singapore, Yong Loo Lin Sch Med, Dept Med, Singapore, Singapore.

[Khoo, Eric Y. H.] Natl Univ Hlth Syst, Univ Med Cluster, Div Endocrinol, Singapore, Singapore.

[Newman, Stanton P.] City Univ London, London, England.

RP Griva, K (reprint author), Natl Univ Singapore, Dept Psychol, Singapore, Singapore.

EM psygk@nus.edu.sg

RI Griva, Konstadina/A-6549-2018

FU Venerable Yen Pei National Kidney Foundation Research Fund

[NKFRC2008/07/24, NKFRC2013/01/20]; Ministry of Education - NUS Academic

Research Fund [FY2007-FRC5-006]

FX This research was supported by grants from the Venerable Yen Pei

National Kidney Foundation Research Fund (NKFRC2008/07/24 and

NKFRC2013/01/20) and the Ministry of Education - NUS Academic Research

Fund (FY2007-FRC5-006). The authors also thank Dr. Tonia Griva, the NKF

Health Care Professionals, Senior Renal Nurse Clinicians and Medical

Social Workers from the Care and Counselling department and all

respondents for their support in the programme.

CR Alavi NM, 2009, TRANSPL P, V41, P3693, DOI 10.1016/j.transproceed.2009.06.217

Anderson RJ, 2001, DIABETES CARE, V24, P1069, DOI 10.2337/diacare.24.6.1069

BENTELSPACHER CE, 1994, FAM SOC-J CONTEMP H, V75, P287, DOI 10.1177/104438949407500504

Bjelland I, 2002, J PSYCHOSOM RES, V52, P69, DOI 10.1016/S0022-3999(01)00296-3

Blake M., 1992, GROWING OLD MALAY CO, DOI [10.1080/21650993.1993.9755647., DOI 10.1080/21650993.1993.9755647]

Bossola M, 2010, GEN HOSP PSYCHIAT, V32, P125, DOI 10.1016/j.genhosppsych.2009.10.009

CHARLSON ME, 1987, J CHRON DIS, V40, P373, DOI 10.1016/0021-9681(87)90171-8

Cheung E., 1986, PSYCHOL CHINESE PEOP, P171

Dickens C, 2002, PSYCHOSOM MED, V64, P52, DOI 10.1097/00006842-200201000-00008

Feng L, 2013, AM J GERIAT PSYCHIAT, V21, P570, DOI 10.1016/j.jagp.2012.12.020

Fortin M, 2006, ANN FAM MED, V4, P417, DOI 10.1370/afm.528

Gould CE, 2016, INT J GERIATR PSYCH, V31, P1105, DOI 10.1002/gps.4532

Griva K, 2015, BMJ OPEN, V5, DOI 10.1136/bmjopen-2014-007253

Griva K, 2011, BMC NEPHROL, V12, DOI 10.1186/1471-2369-12-4

Griva K, 2010, J PSYCHOSOM RES, V69, P363, DOI 10.1016/j.jpsychores.2010.04.008

Griva K, 2014, PSYCHOSOM MED, V76, pA84

Hudson JL, 2015, CARDIO RENAL CLIN CH, P227, DOI [10.1007/978-3-319-09162-4_22, DOI 10.1007/978-3-319-09162-4_22]

Katon WJ, 2003, BIOL PSYCHIAT, V54, P216, DOI 10.1016/S0006-3223(03)00273-7

National Registry of Diseases Office, 2014, TRENDS CHRON KIDN FA

NEFF JA, 1993, J COMMUNITY PSYCHOL, V21, P3, DOI 10.1002/1520-6629(199301)21:1<3::AID-JCOP2290210102>3.0.CO;2-9

Palmer S, 2013, KIDNEY INT, V84, P179, DOI 10.1038/ki.2013.77

Rutledge T, 2006, J AM COLL CARDIOL, V48, P1527, DOI 10.1016/j.jacc.2006.06.055

Salsberg E, 2015, US NEPHROLOGY WORKFO

Tanvir S., 2013, PREVALENCE DEPRESSIO

Turkistani I, 2014, RENAL FAILURE, V36, P1510, DOI 10.3109/0886022X.2014.949761

van Dijk S, 2013, J PSYCHOSOM RES, V74, P511, DOI 10.1016/j.jpsychores.2013.03.001

van Dooren FEP, 2013, PLOS ONE, V8, DOI 10.1371/journal.pone.0057058

Willingham F, 2012, J REN CARE, V38, P40, DOI 10.1111/j.1755-6686.2012.00283.x

Ye XQ, 2008, J PSYCHOSOM RES, V65, P157, DOI 10.1016/j.jpsychores.2008.04.007

ZIGMOND AS, 1983, ACTA PSYCHIAT SCAND, V67, P361, DOI 10.1111/j.1600-0447.1983.tb09716.x

NR 30

TC 6

Z9 6

U1 0

U2 7

PU PERGAMON-ELSEVIER SCIENCE LTD

PI OXFORD

PA THE BOULEVARD, LANGFORD LANE, KIDLINGTON, OXFORD OX5 1GB, ENGLAND

SN 0022-3999

EI 1879-1360

J9 J PSYCHOSOM RES

JI J. Psychosomat. Res.

PD MAR

PY 2017

VL 94

BP 68

EP 72

DI 10.1016/jjpsychores.2017.01.009

PG 5

WC Psychiatry

SC Psychiatry

GA EL4AK

UT WOS:000394562100010

PM 28183405

DA 2019-01-08

ER

PT J

AU Garvey, KC

Foster, NC

Agarwal, S

DiMeglio, LA

Anderson, BJ

Corathers, SD

Desimone, ME

Libman, IM

Lyons, SK

Peters, AL

Raymond, JK

Laffel, LM

AF Garvey, Katharine C.

Foster, Nicole C.

Agarwal, Shivani

DiMeglio, Linda A.

Anderson, Barbara J.

Corathers, Sarah D.

Desimone, Marisa E.

Libman, Ingrid M.

Lyons, Sarah K.

Peters, Anne L.

Raymond, Jennifer K.

Laffel, Lori M.

TI Health Care Transition Preparation and Experiences in a US National

Sample of Young Adults With Type 1 Diabetes

SO DIABETES CARE

LA English

DT Article

ID EMERGING ADULTS; ADOLESCENTS; MELLITUS; SYSTEMS; DISTRESS; PEOPLE;

COHORT; YOUTH

AB OBJECTIVE

Young adults with type 1 diabetes transitioning from pediatric to adult care are at risk for adverse outcomes. We developed a survey to evaluate transition experiences in two groups of young adults with type 1 diabetes, before (PEDS) and after (ADULT) transition to adult care.

RESEARCH DESIGN AND METHODS

We fielded an electronic survey to young adults (18 to < 30 years) at 60 T1D Exchange Clinic Registry centers.

RESULTS

Surveys were completed by 602 young adults, 303 in the PEDS group (60% female, age 20 6 2 years) and 299 in the ADULT group (62% female, age 24 6 3 years). In the PEDS group, mean anticipated transition age was 22 62 years; 64% remained in pediatric care because of emotional attachment to the provider. The ADULT group transitioned at age 19 6 2 years, mainly after pediatric provider recommendation. More than 80% of respondents reported receiving counseling on type 1 diabetes self-management and screening tests from pediatric providers, but less than half (43% PEDS and 33% ADULT) reported discussing reproductive health. In the PEDS group, half had discussed transfer with pediatric providers. Of the ADULT participants, 63% received an adult provider referral, and 66% felt mostly/ completely prepared to transition. ADULT participants with fewer pretransition pediatric visits orwho felt unprepared for transition had increased odds of gaps > 6 months between pediatric and adult care. Receipt of transition preparation counseling was not associated with self-reported hemoglobin A1c < 7.0% in either group.

CONCLUSIONS

These results support the need for intensive efforts to integrate transition preparation counseling and care coordination into pediatric type 1 diabetes care.

C1 [Garvey, Katharine C.] Boston Childrens Hosp, Boston, MA USA.

[Foster, Nicole C.] Jaeb Ctr Hlth Res, Tampa, FL 33647 USA.

[Agarwal, Shivani] Univ Penn, Perelman Sch Med, Philadelphia, PA 19104 USA.

[DiMeglio, Linda A.] Indiana Univ Sch Med, Indianapolis, IN 46202 USA.

[Anderson, Barbara J.; Lyons, Sarah K.] Baylor Coll Med, Houston, TX 77030 USA.

[Corathers, Sarah D.] Cincinnati Childrens Hosp Med Ctr, Cincinnati, OH 45229 USA.

[Corathers, Sarah D.] Univ Cincinnati, Cincinnati, OH USA.

[Desimone, Marisa E.] SUNY Upstate Med Univ, Syracuse, NY 13210 USA.

[Libman, Ingrid M.] UPMC, Childrens Hosp Pittsburgh, Pittsburgh, PA USA.

[Peters, Anne L.] Univ Southern Calif, Keck Sch Med, Los Angeles, CA USA.

[Raymond, Jennifer K.] Barbara Davis Ctr Diabet, Aurora, CO USA.

[Laffel, Lori M.] Joslin Diabet Ctr, Boston, MA 02215 USA.

RP Foster, NC (reprint author), Jaeb Ctr Hlth Res, Tampa, FL 33647 USA.

EM t1dstats@jaeb.org

OI Lyons, Sarah/0000-0002-3686-0112

FU Leona M. and Harry B. Helmsley Charitable Trust; National Institute for

Diabetes and Digestive and Kidney Diseases [K23-DK-102655,

K12-DK-094721]

FX Funding was provided by The Leona M. and Harry B. Helmsley Charitable

Trust. K.C.G.'s work on this study was also supported by the National

Institute for Diabetes and Digestive and Kidney Diseases (K23-DK-102655

and K12-DK-094721).

CR Agarwal S, 2016, PEDIAT DIABETES, DOI [10.1111/pedi.12436, DOI 10.1111/PEDI.12436]

Arnett JJ, 2000, AM PSYCHOL, V55, P469, DOI 10.1037/0003-066X.55.5.469

Beck RW, 2012, J CLIN ENDOCR METAB, V97, P4383, DOI 10.1210/jc.2012-1561

BLUM RW, 1993, J ADOLESCENT HEALTH, V14, P570, DOI 10.1016/1054-139X(93)90143-D

Bryden KS, 2003, DIABETES CARE, V26, P1052, DOI 10.2337/diacare.26.4.1052

Bryden KS, 2001, DIABETES CARE, V24, P1536, DOI 10.2337/diacare.24.9.1536

Busse FP, 2007, HORM RES, V67, P132, DOI 10.1159/000096583

Cadario F, 2009, CLIN ENDOCRINOL, V71, P346, DOI 10.1111/j.1365-2265.2008.03467.x

Charron-Prochownik D, 2006, DIABETES EDUCATOR, V32, P235, DOI 10.1177/0145721706286895

Cooley WC, 2011, PEDIATRICS, V128, P182, DOI DOI 10.1542/PEDS.2011-0969

Fernandes SM, 2014, J PEDIATR REHAB MED, V7, P43, DOI 10.3233/PRM-140269

Garvey KC, 2012, DIABETES CARE, V35, P1716, DOI 10.2337/dc11-2434

Helgeson VS, 2013, DIABETIC MED, V30, P610, DOI 10.1111/dme.12067

Hilliard ME, 2014, DIABETES CARE, V37, P346, DOI 10.2337/dc13-1346

Hislop AL, 2008, DIABETIC MED, V25, P91, DOI 10.1111/j.1464-5491.2007.02310.x

Holmes-Walker DJ, 2007, DIABETIC MED, V24, P764, DOI 10.1111/j.1464-5491.2007.02152.x

Jacobson AM, 1997, J PEDIATR-US, V131, P727, DOI 10.1016/S0022-3476(97)70101-X

Kipps S, 2002, DIABETIC MED, V19, P649, DOI 10.1046/j.1464-5491.2002.00757.x

La Greca AM, 1988, 9 ANN SESS SOC BEH M, pA42

LAGRECA AM, 2003, HDB PEDIAT PSYCHOL, P119

Laing SP, 2005, DIABETES CARE, V28, P1618, DOI 10.2337/diacare.28.7.1618

Laing SP, 1999, DIABETIC MED, V16, P459, DOI 10.1046/j.1464-5491.1999.00075.x

Lotstein DS, 2013, PEDIATRICS, V131, pE1062, DOI 10.1542/peds.2012-1450

Lotstein DS, 2010, PEDIATRICS, V126, pS129, DOI 10.1542/peds.2010-1466F

Nakhla M, 2009, PEDIATRICS, V124, pE1134, DOI 10.1542/peds.2009-0041

Pacaud D, 2005, CAN J DIABETES, V29, P13, DOI DOI 10.2337/DC07-1249

Peters A, 2011, DIABETES CARE, V34, P2477, DOI 10.2337/dc11-1723

POLONSKY WH, 1995, DIABETES CARE, V18, P754, DOI 10.2337/diacare.18.6.754

Raymond Jennifer K, 2013, Diabetes Manag (Lond), V3, DOI 10.2217/dmt.13.27

Ritholz MD, 2014, DIABETES EDUCATOR, V40, P40, DOI 10.1177/0145721713513177

Sawicki GS, 2011, PEDIATRICS, V128, pE521, DOI 10.1542/peds.2010-3017

Van Walleghem N, 2008, DIABETES CARE, V31, P1529, DOI 10.2337/dc07-2247

Waitzfelder B, 2011, PEDIATRICS, V128, P531, DOI 10.1542/peds.2010-3641

Weinger K, 2005, DIABETES CARE, V28, P1346, DOI 10.2337/diacare.28.6.1346

Weissberg-Benchell J, 2007, DIABETES CARE, V30, P2441, DOI 10.2337/dc07-1249

Welch GW, 1997, DIABETES CARE, V20, P760, DOI 10.2337/diacare.20.5.760

Wills CJ, 2003, BRIT MED J, V327, P260, DOI 10.1136/bmj.327.7409.260

NR 37

TC 8

Z9 8

U1 1

U2 5

PU AMER DIABETES ASSOC

PI ALEXANDRIA

PA 1701 N BEAUREGARD ST, ALEXANDRIA, VA 22311-1717 USA

SN 0149-5992

EI 1935-5548

J9 DIABETES CARE

JI Diabetes Care

PD MAR

PY 2017

VL 40

IS 3

BP 317

EP 324

DI 10.2337/dc16-1729

PG 8

WC Endocrinology & Metabolism

SC Endocrinology & Metabolism

GA EM7UZ

UT WOS:000395519100010

PM 28007779

OA Green Published, Bronze

DA 2019-01-08

ER

PT J

AU Paika, V

Andreoulakis, E

Ntountoulaki, E

Papaioannou, D

Kotsis, K

Siafaka, V

Fountoulakis, KN

Pargament, KI

Carvalho, AF

Hyphantis, T

AF Paika, Vassiliki

Andreoulakis, Elias

Ntountoulaki, Elisavet

Papaioannou, Dimitra

Kotsis, Konstantinos

Siafaka, Vassiliki

Fountoulakis, Konstantinos N.

Pargament, Kenneth I.

Carvalho, Andre F.

Hyphantis, Thomas

CA ASSERT-DEP Study Grp

TI The Greek-Orthodox version of the Brief Religious Coping (B-RCOPE)

instrument: psychometric properties in three samples and associations

with mental disorders, suicidality, illness perceptions, and quality of

life

SO ANNALS OF GENERAL PSYCHIATRY

LA English

DT Article

DE Religious coping; Religiousness; Mental disorder; Depression; Anxiety;

Suicidal risk; Illness perceptions; Quality of life; Psychometric

properties; Chronic illness

ID ILL ELDERLY-PATIENTS; PSYCHOLOGICAL DISTRESS; EMERGENCY-DEPARTMENT;

OLDER-ADULTS; SPIRITUAL STRUGGLE; INITIAL VALIDATION; MEDICAL

CONDITIONS; ANXIETY DISORDERS; LIKERT VARIABLES; ADVANCED CANCER

AB Background: The B-RCOPE is a brief measure assessing religious coping. We aimed to assess the psychometric properties of its Greek version in people with and without long-term conditions (LTCs). Associations between religious coping and mental illness, suicidality, illness perceptions, and quality of life were also investigated.

Methods: The B-RCOPE was administered to 351 patients with diabetes, chronic pulmonary obstructive disease (COPD), and rheumatic diseases attending either the emergency department (N = 74) or specialty clinics (N = 302) and 127 people without LTCs. Diagnosis of mental disorders was established by the MINI. Associations with depressive symptom severity (PHQ-9), suicidal risk (RASS), illness perceptions (B-IPQ), and health-related quality of life (WHOQOL-BREF) were also investigated.

Results: The Greek version of B-RCOPE showed a coherent two-dimensional factor structure with remarkable stability across the three samples corresponding to the positive (PRC) and negative (NRC) religious coping dimensions. Cronbach's alphas were 0.91-0.96 and 0.77-0.92 for the PRC and NRC dimensions, respectively. Furthermore, NRC was associated with poorer mental health, greater depressive symptom severity and suicidality, and impaired HRQoL. In patients with LTCs, PRC correlated with lower perceived illness timeline, while NRC was associated with greater perceived illness consequences, lower perceived treatment control, greater illness concern, and lower illness comprehensibility.

Conclusions: These findings indicate that the Greek-Orthodox B-RCOPE version may reliably assess religious coping. In addition, negative religious coping (i.e., religious struggle) is associated with adverse illness perceptions, and thus may detrimentally impact adaptation to medical illness. These findings deserve replication in prospective studies.

C1 [Paika, Vassiliki; Ntountoulaki, Elisavet; Papaioannou, Dimitra; Kotsis, Konstantinos; Hyphantis, Thomas] Univ Ioannina, Fac Med, Sch Hlth Sci, Dept Psychiat, GR-45110 Ioannina, Greece.

[Andreoulakis, Elias; Fountoulakis, Konstantinos N.] Aristotle Univ Thessaloniki, Sch Med, Dept Psychiat 3, Thessaloniki, Greece.

[Siafaka, Vassiliki] Technol Educ Inst Epirus, Dept Speech & Language Therapy, Ioannina, Greece.

[Pargament, Kenneth I.] Bowling Green State Univ, Dept Psychol, Bowling Green, OH 43403 USA.

[Carvalho, Andre F.] Univ Fed Ceara, Fac Med, Dept Clin Med, Fortaleza, Ceara, Brazil.

[Carvalho, Andre F.] Univ Fed Ceara, Fac Med, Translat Psychiat Res Grp, Fortaleza, Ceara, Brazil.

RP Hyphantis, T (reprint author), Univ Ioannina, Fac Med, Sch Hlth Sci, Dept Psychiat, GR-45110 Ioannina, Greece.

EM tyfantis@cc.uoi.gr

RI Fountoulakis, Konstantinos/N-6990-2017

OI Andreoulakis, Elias/0000-0001-7067-9467; Hyphantis,

Thomas/0000-0002-7647-4312

FU European Economic Area (EEA) Financial Mechanism [EEA GR07/3767];

National funds, program "Dissimilarity, Inequality and Social

Integration" [132324/I4-25/8/2015]; Conselho Nacional de Desenvolvimento

Cientifico e Tecnologico (CNPq; Brazil)

FX The project "ASSessing and Enhancing Resilience To DEPression in people

with long term medical conditions in the era of the current Greek social

and financial crisis (ASSERT-DEP)" has been co-financed by the European

Economic Area (EEA) Financial Mechanism 2009-2014 (EEA GR07/3767) and

National funds as part of the program "Dissimilarity, Inequality and

Social Integration" (Grant number: 132324/I4-25/8/2015). The funding

source had no involvement in study design; in the collection, analysis,

and interpretation of data; in the writing of the report; and in the

decision to submit the article for publication.; AFC is supported by a

research scholarship award from the Conselho Nacional de Desenvolvimento

Cientifico e Tecnologico (CNPq; Brazil).

CR Abiary M, 2010, CONT PSYCHOLY, V93, P81

Ai AL, 2009, PSYCHOL RELIG SPIRIT, V1, P112, DOI 10.1037/a0015775

Ai AL, 2010, J HEALTH PSYCHOL, V15, P186, DOI 10.1177/1359105309345556

Al-Hadethe A, 2014, J RELIG HLTH J RELIG, V55, P16

ALTMAN DG, 1991, PRACTICAL STAT MED R, P285

Ano GG, 2005, J CLIN PSYCHOL, V61, P461, DOI 10.1002/jclp.20049

Bay PS, 2008, J RELIG HEALTH, V47, P57, DOI 10.1007/s10943-007-9131-4

Berger D, 2015, SPAN J PSYCHOL, V19, pE3

Broadbent E, 2006, J PSYCHOSOM RES, V60, P631, DOI 10.1016/j.jpsychores.2005.10.020

Broadbent E, 2015, PSYCHOL HEALTH, V30, P1361, DOI 10.1080/08870446.2015.1070851

CHARLSON ME, 1987, J CHRON DIS, V40, P373, DOI 10.1016/0021-9681(87)90171-8

Ford D, 2010, CHEST, V138, P59, DOI 10.1378/chest.09-2124

Fountoulakis KN, 2012, J AFFECT DISORDERS, V138, P449, DOI 10.1016/j.jad.2011.12.045

Freitas TH, 2015, REV BRAS PSIQUIATR, V37, P219, DOI 10.1590/1516-4446-2014-1507

Ginieri-Coccossis M, 2012, Psychiatriki, V23, P130

Goulia P, 2012, J PSYCHOSOM RES, V72, P120, DOI 10.1016/j.jpsychores.2011.11.011

Hill PC, 2003, AM PSYCHOL, V58, P64, DOI 10.1037/0003-066X.58.1.64

Hyphantis T, 2015, J AFFECT DISORDERS, V176, P155, DOI 10.1016/j.jad.2015.01.062

Hyphantis T, 2014, COMPR PSYCHIAT, V55, P1950, DOI 10.1016/j.comppsych.2014.08.042

Hyphantis T, 2011, ARTHRIT CARE RES, V63, P1313, DOI 10.1002/acr.20505

Ibrahim N, 2012, ASIAN SOCIAL SCI, V8, P23, DOI [10.5539/ass.v8n9p23, DOI 10.5539/ASS.V8N9P23]

Ironson G, 2016, J GEN INTERN MED, V31, P1068, DOI 10.1007/s11606-016-3668-4

Khalaf DR, 2015, J RELIG HEALTH, V54, P1438, DOI 10.1007/s10943-014-9981-5

Koenig HG, 2010, RELIGIONS, V1, P78, DOI 10.3390/rel1010078

Koenig HG, 2009, CAN J PSYCHIAT, V54, P283, DOI 10.1177/070674370905400502

Koenig HG, 1998, J NERV MENT DIS, V186, P513, DOI 10.1097/00005053-199809000-00001

Lawrence RE, 2016, ARCH SUICIDE RES, V20, P1, DOI 10.1080/13811118.2015.1004494

Leventhal H., 1984, HDB PSYCHOL HLTH, V4, P219

Lorenzo-Seva U, 2006, BEHAV RES METHODS, V38, P88, DOI 10.3758/BF03192753

Lorenzo-Seva U, 1999, MULTIVAR BEHAV RES, V34, P347, DOI 10.1207/S15327906MBR3403_3

Lorenzo-Seva U, 2013, APPL PSYCH MEAS, V37, P497, DOI 10.1177/0146621613487794

Moreira-Almeida A, 2006, REV BRAS PSIQUIATR, V28, P242, DOI 10.1590/S1516-44462006000300018

MUTHEN B, 1985, BRIT J MATH STAT PSY, V38, P171, DOI 10.1111/j.2044-8317.1985.tb00832.x

MUTHEN B, 1992, BRIT J MATH STAT PSY, V45, P19, DOI 10.1111/j.2044-8317.1992.tb00975.x

Ng GC, 2016, J RELIG HLTH, V10, P1

Ntountoulaki E, 2016, COMPR PSYCHIAT, V69, P193, DOI 10.1016/j.comppsych.2016.06.010

Papadimitriou GN, 2004, GREEK TRANSLATION MI

Pargament K. I., 1997, PSYCHOL RELIG COPING

Pargament K. I., 2005, INT J PSYCHOL RELIG, V15, P179, DOI [10.1207/s15327582ijpr1503_1, DOI 10.1207/S15327582IJPR1503_1]

Pargament K, 2011, RELIGIONS, V2, P51, DOI 10.3390/rel2010051

Pargament KI, 2000, J CLIN PSYCHOL, V56, P519, DOI 10.1002/(SICI)1097-4679(200004)56:4<519::AID-JCLP6>3.3.CO;2-T

Pargament KI, 1998, J SCI STUD RELIG, V37, P710, DOI 10.2307/1388152

Pargament KI, 2004, SOUTH MED J, V97, P1201, DOI 10.1097/01.SMJ.0000146508.14898.E2

Pargament KI, 2004, J HEALTH PSYCHOL, V9, P713, DOI 10.1177/1359105304045366

Pargament KI, 2001, ARCH INTERN MED, V161, P1881, DOI 10.1001/archinte.161.15.1881

Pargament KI, 2012, RELIG COPING CURRENT

Pearce MJ, 2006, J HEALTH PSYCHOL, V11, P743, DOI 10.1177/1359105306066629

Petrie KJ, 2007, CURR OPIN PSYCHIATR, V20, P163, DOI 10.1097/YCO.0b013e328014a871

Piderman KM, 2007, AM J ADDICTION, V16, P232, DOI 10.1080/10550490701375616

Ramirez SP, 2012, J PSYCHOSOM RES, V72, P129, DOI 10.1016/j.jpsychores.2011.11.012

Rosen E., 1958, TRANSLATION ADAPTATI

Rosmarin DH, 2013, PSYCHIAT RES, V210, P182, DOI 10.1016/j.psychres.2013.03.023

Rosmarin DH, 2009, J CLIN PSYCHOL, V65, P670, DOI 10.1002/jclp.20574

Sareen J, 2005, ARCH GEN PSYCHIAT, V62, P1249, DOI 10.1001/archpsyc.62.11.1249

Schanowitz JY, 2006, J BEHAV MED, V29, P191, DOI 10.1007/s10865-005-9034-3

Sheehan DV, 1998, J CLIN PSYCHIAT, V59, P34, DOI 10.4088/JCP.09m05305whi

Shreve-Neiger AK, 2004, CLIN PSYCHOL REV, V24, P379, DOI 10.1016/j.cpr.2004.02.003

Simonsen E., 1990, J PERSONALITY DISORD, V4, P290

Talik EB, 2013, J RELIG HEALTH, V52, P143, DOI 10.1007/s10943-011-9464-x

Tarakeshwar N, 2006, J PALLIAT MED, V9, P646, DOI 10.1089/jpm.2006.9.646

Tarakeshwar N, 2003, J COMMUNITY PSYCHOL, V31, P607, DOI 10.1002/jcop.10071

Terwee CB, 2007, J CLIN EPIDEMIOL, V60, P34, DOI 10.1016/j.jclinepi.2006.03.012

Thune-Boyle IC, 2006, SOC SCI MED, V63, P151, DOI 10.1016/j.socscimed.2005.11.055

Timmerman ME, 2011, PSYCHOL METHODS, V16, P209, DOI 10.1037/a0023353

Trevino KM, 2014, PSYCHO-ONCOLOGY, V23, P936, DOI 10.1002/pon.3505

NR 65

TC 1

Z9 1

U1 0

U2 7

PU BIOMED CENTRAL LTD

PI LONDON

PA 236 GRAYS INN RD, FLOOR 6, LONDON WC1X 8HL, ENGLAND

SN 1744-859X

J9 ANN GEN PSYCHIATR

JI Ann. Gen. Psychiatr.

PD FEB 16

PY 2017

VL 16

AR 13

DI 10.1186/s12991-017-0136-4

PG 13

WC Psychiatry

SC Psychiatry

GA EL2RF

UT WOS:000394466400001

PM 28239407

OA DOAJ Gold, Green Published

DA 2019-01-08

ER

PT J

AU Joensen, LE

Willaing, I

Holt, RIG

Wens, J

Skovlund, S

Peyrot, M

AF Joensen, Lene E.

Willaing, Ingrid

Holt, Richard I. G.

Wens, Johan

Skovlund, Soren

Peyrot, Mark

TI Household composition and psychological health: Results of the Second

Diabetes Attitudes, Wishes and Needs (DAWN2) study

SO JOURNAL OF DIABETES AND ITS COMPLICATIONS

LA English

DT Article

DE Diabetes; Psychological health; Household composition; Social network;

Social support

ID QUALITY-OF-LIFE; MARITAL-STATUS; GLYCEMIC CONTROL; BARRIERS; NETWORK;

SUPPORT

AB Aims: 1) To explore the effect of household composition on the psychological health of adults with diabetes by comparing those living with other adult(s) including a partner with those living with neither partner nor other adult(s); 2) to examine potential mediation of social support in the association between household composition and psychological health.

Methods: The study is part of the DAWN2 study conducted in 17 countries. The population comprised 8596 people with diabetes (PWD). Multiple regression models (linear and binary) were applied.

Results: People living with 'other adult(s) but no partner' experienced significantly lower well-being, higher diabetes distress and worried more frequently about hypoglycaemic events than those with a partner or those not co-habiting with another adult. However, participants living with 'other adult(s) but no partner' were more empowered compared to the other household composition groups. The association between household composition and psychological health was not mediated by diabetes-specific social support.

Conclusions: The study indicates the psychological vulnerability of respondents living without a partner but with other adult(s). Appropriate support interventions must be developed and tested in order to enhance psychological health in people with diabetes living with other adults such as adult children, but with no partner. (C) 2017 Elsevier Inc. All rights reserved.

C1 [Joensen, Lene E.; Willaing, Ingrid] Steno Diabet Ctr, Gentofte, Denmark.

[Holt, Richard I. G.] Univ South Hampton, Southampton, Hants, England.

[Wens, Johan] Univ Antwerp, Antwerp, Belgium.

[Skovlund, Soren] Novo Nordisk, Copenhagen, Denmark.

[Peyrot, Mark] Loyola Univ Maryland, Baltimore, MD USA.

RP Joensen, LE (reprint author), Niels Steensens Vej 6, DK-2820 Gentofte, Denmark.

EM ljoe@steno.dk

OI Holt, Richard/0000-0001-8911-6744; Wens, Johan/0000-0002-0229-9064;

Joensen, Lene Eide/0000-0002-4790-8759; Willaing,

Ingrid/0000-0002-3082-8816

FU Novo Nordisk A/S

FX The DAWN2 study is funded by Novo Nordisk A/S.

CR August KJ, 2010, SOC SCI MED, V71, P1831, DOI 10.1016/j.socscimed.2010.08.022

Bech P, 1996, PSYCHOTHER PSYCHOSOM, V65, P183, DOI 10.1159/000289073

Bei kman LF, 1979, AM J EPIDEMIOL, V109, P186

Berkman LF, 2000, SOC SCI MED, V51, P843, DOI 10.1016/S0277-9536(00)00065-4

Burns KK, 2013, DIABETIC MED, V30, P778, DOI 10.1111/dme.12239

Due P, 1999, SOC SCI MED, V48, P661, DOI 10.1016/S0277-9536(98)00381-5

Egede LE, 2012, CURR PSYCHIAT REP, V14, P15, DOI 10.1007/s11920-011-0240-0

Eller M, 2008, INT J PUBLIC HEALTH, V53, P188, DOI 10.1007/s00038-008-7091-4

Fisher L, 2010, DIABETES CARE, V33, P23, DOI 10.2337/dc09-1238

GOVE WR, 1983, J HEALTH SOC BEHAV, V24, P122, DOI 10.2307/2136639

Hart HE, 2003, QUAL LIFE RES, V12, P1089, DOI 10.1023/A:1026197119569

Holt RIG, 2013, DIABETIC MED, V30, P789, DOI 10.1111/dme.12242

HOUSE JS, 1988, SCIENCE, V241, P540, DOI 10.1126/science.3399889

Kawachi I, 2001, J URBAN HEALTH, V78, P458, DOI 10.1093/jurban/78.3.458

Leonard Barbara J, 2005, J Pediatr Nurs, V20, P405, DOI 10.1016/j.pedn.2005.03.010

LLOYD CE, 1993, DIABETES RES CLIN PR, V21, P187, DOI 10.1016/0168-8227(93)90068-G

McGuire BE, 2010, DIABETOLOGIA, V53, P66, DOI 10.1007/s00125-009-1559-5

Metayer C, 1996, EUR J EPIDEMIOL, V12, P573, DOI 10.1007/BF00499455

Nicolucci A, 2013, DIABETIC MED, V30, P767, DOI 10.1111/dme.12245

Nilsson PM, 2005, SCAND J PUBLIC HEALT, V33, P412, DOI 10.1080/14034940510005905

Peyrot M, 2005, DIABETIC MED, V22, P1379, DOI 10.1111/j.1464-5491.2005.01644.x

Peyrot M, 1999, J HEALTH SOC BEHAV, V40, P141, DOI 10.2307/2676370

Peyrot M, 2013, DIABETES RES CLIN PR, V99, P174, DOI 10.1016/j.diabres.2012.11.016

SHAMOON H, 1993, NEW ENGL J MED, V329, P977

Skovlund S. E., 2005, DIABETES SPECTRUM, V18, P136, DOI DOI 10.2337/DIASPECT.18.3.136

Weinger K, 2001, J ADOLESCENT HEALTH, V29, P330, DOI 10.1016/S1054-139X(01)00270-1

WIKBY A, 1991, DIABETES RES CLIN PR, V13, P53, DOI 10.1016/0168-8227(91)90033-A

NR 27

TC 0

Z9 0

U1 0

U2 1

PU ELSEVIER SCIENCE INC

PI NEW YORK

PA 360 PARK AVE SOUTH, NEW YORK, NY 10010-1710 USA

SN 1056-8727

EI 1873-460X

J9 J DIABETES COMPLICAT

JI J. Diabetes Complications

PD FEB

PY 2017

VL 31

IS 2

BP 340

EP 346

DI 10.1016/j.jdiacomp.2016.07.006

PG 7

WC Endocrinology & Metabolism

SC Endocrinology & Metabolism

GA ES3NA

UT WOS:000399435100008

PM 27491452

DA 2019-01-08

ER

PT J

AU Dunning, T

Speight, J

Bennett, C

AF Dunning, Trisha

Speight, Jane

Bennett, Craig

TI Language, the "Diabetes Restricted Code/Dialect," and What It Means for

People With Diabetes and Clinicians

SO DIABETES EDUCATOR

LA English

DT Article

ID POSITION STATEMENTS

AB Purpose

The purpose of the paper is to explore the notion of a diabetes language restricted code or "dialect" and its effect on people with diabetes. Language is a complex phenomenon comprising verbal and nonverbal components used to communicate in human interactions.

Methods

Information was collected from a literature review, during clinical consultations, and from an email survey.

Results

Language and the way clinicians use language affect motivation, behaviors, and outcomes of people with diabetes. Language is influenced by culture, experience, and familiarity with words and their use and is idiosyncratic. "Diabetes" is a particular restricted code or dialect that people with diabetes gradually learn to speak, usually after they are diagnosed with diabetes. The diabetes dialect contains many metric and target words and very few positive, encouraging words, and it is often discriminatory, negative, judgmental, labelling, distressing, and stigmatizing: for example, victim, sufferer, and lifestyle disease.

Conclusion

Language codes/dialects can compound the already high levels of emotional distress and self-care burden associated with living with diabetes and can affect outcomes. The information presented in this article will be useful for clinicians caring for people with diabetes and will be helpful for professionals who develop information for people with diabetes and those who create policies and guidelines.

C1 [Dunning, Trisha] Deakin Univ, Sch Nursing & Midwifery, Geelong, Vic, Australia.

[Dunning, Trisha] Barwon Hlth, Geelong, Vic, Australia.

[Speight, Jane] Deakin Univ, Sch Psychol, Burwood, Vic, Australia.

[Speight, Jane] AHP Res, Hornchurch, Essex, England.

[Speight, Jane; Bennett, Craig] Diabet Victoria, Melbourne, Vic, Australia.

RP Dunning, T (reprint author), Deakin Univ, Kitchener House,Ryrie St,POB 281, Geelong, Vic 3220, Australia.; Dunning, T (reprint author), C Univ Hosp Geelong, Barwon Hlth, Kitchener House,Ryrie St,POB 281, Geelong, Vic 3220, Australia.

EM trisha.dunning@barwonhealth.org.au

OI Speight, Jane/0000-0002-1204-6896

CR Fain JA, 2003, DIABETES EDUCATOR, V29, P880, DOI 10.1177/014572170302900601

Fain JA, 2002, DIABETES EDUCATOR, V28, P650, DOI 10.1177/014572170202800501

Fain JA, 2012, DIABETES EDUCATOR, V38, P14, DOI 10.1177/0145721711435220

Institute of Medicine Committee on Standards for Developing Trustworthy Clinical Practice Guidelines, 2011, CLIN PRACTICE GUIDEL

NR 4

TC 3

Z9 3

U1 0

U2 2

PU SAGE PUBLICATIONS INC

PI THOUSAND OAKS

PA 2455 TELLER RD, THOUSAND OAKS, CA 91320 USA

SN 0145-7217

EI 1554-6063

J9 DIABETES EDUCATOR

JI Diabetes Educ.

PD FEB

PY 2017

VL 43

IS 1

BP 18

EP 26

DI 10.1177/0145721716683449

PG 9

WC Endocrinology & Metabolism; Public, Environmental & Occupational Health

SC Endocrinology & Metabolism; Public, Environmental & Occupational Health

GA EL8VB

UT WOS:000394897300002

PM 28072923

OA Bronze

DA 2019-01-08

ER

PT J

AU Powers, MA

Richter, SA

Ackard, DM

Craft, C

AF Powers, Margaret A.

Richter, Sara A.

Ackard, Diann M.

Craft, Cheryl

TI Diabetes Distress Among Persons With Type 1 Diabetes Associations With

Disordered Eating, Depression, and Other Psychological Health Concerns

SO DIABETES EDUCATOR

LA English

DT Article

ID EDUCATION; CARE; SIMULATION; ADOLESCENTS; MANAGEMENT; VIGNETTE;

CHILDREN; PARENTS; INTERVENTION; OUTCOMES

AB Purpose

The purpose of this study is to evaluate associations between diabetes distress and a range of psychological health behaviors and concerns among persons with type 1 diabetes for the benefit of enhancing early identification and intervention of at-risk individuals.

Methods

Persons with type 1 diabetes (n = 268; 57.1% female, 91.0% white, 76.8% < 18 years of age, average A1C 8.4%) completed the 2-item Diabetes Distress Screening Scale (DDS2) and a battery of psychometrically sound instruments measuring satisfaction with life, self-esteem, self-efficacy, depression, perfectionism, body image satisfaction, dietary restraint and eating, and shape and weight concerns. Each subscale score was compared within age groups (< 18 years vs = 18 years) between groups (diabetes distress level [low, moderate, high]) using analysis of variance (with Bonferroni correction or the Kruskal-Wallis test if the variables were not normally distributed).

Results

For both age groups, high diabetes distress was independently associated with greater A1C values, higher depression scores and eating, and shape and weight concerns than those with low or moderate distress. For patients < 18 years of age, those with high diabetes distress scored lower on measures of satisfaction with life, self-esteem, and self-efficacy and higher on dietary restraint and several areas of perfectionism than those with low or moderate distress.

Conclusions

Individuals with type 1 diabetes who have high diabetes distress also report higher A1C values and poorer psychological health concerns. A brief diabetes distress questionnaire can help to identify those who need additional screening, education and support, and treatment for overall health and well-being.

C1 [Powers, Margaret A.] Int Diabet Ctr Pk Nicollet, 3800 Pk Nicollet Blvd, Minneapolis, MN 55416 USA.

[Powers, Margaret A.; Richter, Sara A.; Craft, Cheryl] Hlth Partners Inst, Minneapolis, MN USA.

[Richter, Sara A.] Profess Data Analysts Inc, Minneapolis, MN USA.

[Ackard, Diann M.] Off Diann M Ackard, Minneapolis, MN USA.

[Ackard, Diann M.] Pk Nicollet Melrose Ctr, Minneapolis, MN USA.

RP Powers, MA (reprint author), Int Diabet Ctr Pk Nicollet, 3800 Pk Nicollet Blvd, Minneapolis, MN 55416 USA.

EM margaret.powers@parknicollet.com

FU Park Nicollet Foundation

FX This work was supported by the Park Nicollet Foundation. The study is

registered through Clinical Trials (NCT00948311;

https://clinicaltrials.gov).

CR Ayling K, 2015, PSYCHOL HEALTH, V30, P85, DOI 10.1080/08870446.2014.953528

Berlin KS, 2002, DIABETES EDUCATOR, V28, P809, DOI 10.1177/014572170202800521

Bova C, 2016, NURS RES IN PRESS

Cant R, 2013, INT J NURS PRACT, V19, P163, DOI 10.1111/ijn.12053

Grant RW, 2009, J AM BOARD FAM MED, V22, P513, DOI 10.3122/jabfm.2009.05.080232

Greenhalgh Trisha, 2006, Psychol Health Med, V11, P432, DOI 10.1080/13548500500429379

Hsieh HF, 2005, QUAL HEALTH RES, V15, P1277, DOI 10.1177/1049732305276687

Hughes P, 2015, DIABETES EDUCATOR, V41, P452, DOI 10.1177/0145721715587743

Jiwa M, 2012, DIABETES RES CLIN PR, V95, P326, DOI 10.1016/j.diabres.2011.11.004

Johnson M, 2005, HEALTH EXPECT, V8, P324, DOI 10.1111/j.1369-7625.2005.00344.x

Lange K, 2014, PEDIATR DIABETES, V15, P77, DOI 10.1111/pedi.12187

Levett-Jones Tracy, 2014, Nurse Educ Today, V34, pe58, DOI 10.1016/j.nedt.2013.09.020

Maguire LL, 2015, DIABETES EDUCATOR, V41, P678, DOI 10.1177/0145721715607982

Martin D, 2012, PEDIATR DIABETES, V13, P20, DOI 10.1111/j.1399-5448.2012.00909.x

Ramchandani N, 2016, PATIENT EDUC COUNS, V99, P1362, DOI 10.1016/j.pec.2016.03.019

Silverstein J, 2005, DIABETES CARE, V28, P186, DOI 10.2337/diacare.28.1.186

Simon ACR, 2015, DIABETIC MED, V32, P69, DOI 10.1111/dme.12586

Sullivan-Bolyai S, 2015, DIABETES EDUCATOR, V41, P537, DOI 10.1177/0145721715598383

Sullivan-Bolyai S, 2014, ADV NURS SCI, V37, P340, DOI 10.1097/ANS.0000000000000050

Sullivan-Bolyai S, 2012, DIABETES EDUCATOR, V38, P50, DOI 10.1177/0145721711432457

Williamson C, 2014, POSTGRAD MED, V126, P145, DOI 10.3810/pgm.2014.05.2764

Willis DG, 2016, WESTERN J NURS RES, V38, P1185, DOI 10.1177/0193945916645499

Woodring B C, 2000, Pediatr Nurs, V26, P505

Wysocki T, 2008, J PEDIATR PSYCHOL, V33, P875, DOI 10.1093/jpepsy/jsn024

NR 24

TC 1

Z9 1

U1 3

U2 16

PU SAGE PUBLICATIONS INC

PI THOUSAND OAKS

PA 2455 TELLER RD, THOUSAND OAKS, CA 91320 USA

SN 0145-7217

EI 1554-6063

J9 DIABETES EDUCATOR

JI Diabetes Educ.

PD FEB

PY 2017

VL 43

IS 1

BP 105

EP 113

DI 10.1177/0145721716680888

PG 9

WC Endocrinology & Metabolism; Public, Environmental & Occupational Health

SC Endocrinology & Metabolism; Public, Environmental & Occupational Health

GA EL8VB

UT WOS:000394897300010

PM 27932687

DA 2019-01-08

ER

PT J

AU Jimenez, K

Vargas, C

Garcia, K

Guzman, H

Angulo, M

Billimek, J

AF Jimenez, Krystal

Vargas, Cristina

Garcia, Karla

Guzman, Herlinda

Angulo, Marco

Billimek, John

TI Evaluating the Validity and Reliability of the Beliefs About Medicines

Questionnaire in Low-Income, Spanish-Speaking Patients With Diabetes in

the United States

SO DIABETES EDUCATOR

LA English

DT Article

ID MENTAL-HEALTH ISSUES; POSITION STATEMENT; EMOTIONAL DISTRESS; GLYCEMIC

CONTROL; EDE-Q; TYPE-1; ASSOCIATION; SCALE; CARE; ADOLESCENTS

AB Purpose

The purpose of this study was to examine the reliability and validity of a Spanish version of the Beliefs about Medicines Questionnaire (BMQ) as a measure to evaluate beliefs about medications and to differentiate adherent from nonadherent patients among low-income Latino patients with diabetes in the United States.

Methods

Seventy-three patients were administered the BMQ and surveyed for evidence of medication nonadherence. Internal consistency of the BMQ was assessed by Cronbach's alpha along with performing a confirmatory factor analysis. Criterion validity was assessed by comparing mean scores on 3 subscales of the BMQ (General Overuse, General Harm, and Specific NecessityConcerns difference score) between adherent patients and patients reporting nonadherence for 3 different reasons (unintentional nonadherence, cost-related nonadherence, and nonadherence due to reasons other than cost) using independent samples t tests.

Results

The BMQ is a reliable instrument to examine beliefs about medications in this Spanish-speaking population. Construct validity testing shows nearly identical factor loading as the original construct map. General Overuse scores were significantly more negative for patients reporting each reason for nonadherence compared with their adherent counterparts. Necessity-Concerns difference scores were significantly more negative for patients reporting nonadherence for reasons other than cost compared with those who did not report this reason for nonadherence.

Conclusion

The Spanish version of the BMQ is appropriate to assess beliefs about medications in Latino patients with type 2 diabetes in the United States and may help identify patients who become nonadherent to medications for reasons other than out-of-pocket costs.

C1 [Jimenez, Krystal; Vargas, Cristina; Garcia, Karla; Guzman, Herlinda; Billimek, John] Univ Calif Irvine, Hlth Policy Res Inst, 100 Theory,Suite 110, Irvine, CA 92697 USA.

[Billimek, John] Univ Calif Irvine, Dept Med, Div Gen Internal Med & Primary Care, Sch Med, Irvine, CA 92697 USA.

[Billimek, John] Univ Calif Irvine, Dept Family Med, Sch Med, Irvine, CA 92697 USA.

RP Billimek, J (reprint author), Univ Calif Irvine, Hlth Policy Res Inst, 100 Theory,Suite 110, Irvine, CA 92697 USA.

EM jbillime@uci.edu

FU National Center for Research Resources; National Center for Advancing

Translational Sciences, National Institutes of Health (NIH) [UL1

TR001414]

FX The project described was supported by the National Center for Research

Resources and the National Center for Advancing Translational Sciences,

National Institutes of Health (NIH), through Grant UL1 TR001414. The

content is solely the responsibility of the authors and does not

necessarily represent the official views of the NIH. All authors have no

conflicts of interest to report. Dr Billimek had full access to all of

the data in the study and takes responsibility for the integrity of the

data and the accuracy of the data analysis.

CR Balfe M, 2013, BMC ENDOCR DISORD, V13, DOI 10.1186/1472-6823-13-25

Bernstein CM, 2013, CLIN PEDIATR, V52, P10, DOI 10.1177/0009922812459950

Colton P., 2009, Diabetes Spectrum, V22, P138, DOI 10.2337/diaspect.22.3.138

Daneman D., 2002, Diabetes Spectrum, V15, P83, DOI 10.2337/diaspect.15.2.83

de Wit M, 2014, J CLIN PSYCHOL MED S, V21, P190, DOI 10.1007/s10880-014-9395-2

Delamater AM, 2014, PEDIATR DIABETES, V15, P232, DOI 10.1111/pedi.12191

DIENER E, 1985, J PERS ASSESS, V49, P71, DOI 10.1207/s15327752jpa4901_13

Ducat L, 2015, DIABETES CARE, V38, P333, DOI 10.2337/dc14-1383

Esbitt SA, 2013, SCREENING DEPRESSION, P27

Fairburn C. G., 2008, COGNITIVE BEHAV THER, P308

Fisher L, 2008, ANN FAM MED, V6, P246, DOI 10.1370/afm.842

Fisher L, 2007, DIABETES CARE, V30, P542, DOI 10.2337/dc06-1614

Fisher L, 2015, J DIABETES COMPLICAT, V29, P572, DOI 10.1016/j.jdiacomp.2015.01.012

Fisher L, 2012, DIABETES CARE, V35, P259, DOI 10.2337/dc11-1572

Fisher L, 2010, DIABETES CARE, V33, P23, DOI 10.2337/dc09-1238

FROST RO, 1990, COGNITIVE THER RES, V14, P449, DOI 10.1007/BF01172967

Goebel-Fabbri AE, 2008, DIABETES CARE, V31, P415, DOI 10.2337/dc07-2026

Goebel-Fabbri AE, 2011, DIABETES CARE, V34, P545, DOI 10.2337/dc10-1547

Hackorth NJ, 2013, AUST PSYCHOL, V48, P360, DOI [DOI 10.1111/AP.12007, 10.1111/ap.12007]

Jones JM, 2000, BMJ-BRIT MED J, V320, P1563, DOI 10.1136/bmj.320.7249.1563

Marrero DG, 2013, DIABETES CARE, V36, P463, DOI 10.2337/dc12-2305

Mond JM, 2006, BEHAV RES THER, V44, P53, DOI 10.1016/j.brat.2004.12.003

Pavot W., 1993, PSYCHOL ASSESSMENT, V5, P164, DOI DOI 10.1037/1040-3590.5.2.164

Polonsky WH, 2005, DIABETES CARE, V28, P626, DOI 10.2337/diacare.28.3.626

Powers MA, J HLTH PSYCHOL

Powers MA, 2016, DIABETES CARE, V39, P2101, DOI 10.2337/dc16-2085

Powers MA, 2016, EAT DISORD, V24, P271, DOI 10.1080/10640266.2015.1090866

Powers MA, 2015, DIABETES CARE, V38, P1372, DOI 10.2337/dc15-0730

Powers MA, 2013, DIABETES EDUCATOR, V39, P387, DOI 10.1177/0145721713482737

RADLOFF L S, 1977, Applied Psychological Measurement, V1, P385, DOI 10.1177/014662167700100306

Reas DL, 2012, EAT DISORD, V20, P437, DOI 10.1080/10640266.2012.715523

Robinson DJ, 2013, CAN J DIABETES, V37, pS87, DOI 10.1016/j.jcjd.2013.01.026

Rose JS, 2013, J EAT DISORD, V1, DOI 10.1186/2050-2974-1-42

Rosenberg M, 1965, SOC ADOLESCENT SELF

Schwarzer R, 2005, SELF EFFICACY BELIEF, P139

Schwarzer R, 1995, MEASURES HLTH PSYCHO, P35, DOI DOI 10.1037/T00393-000

Schwarzer R., 1993, MEASUREMENT PERCEIVE

Strandberg RB, 2014, J PSYCHOSOM RES, V77, P174, DOI 10.1016/j.jpsychores.2014.06.015

van Bastelaar KMP, 2010, DIABETIC MED, V27, P798, DOI 10.1111/j.1464-5491.2010.03025.x

Young-Hyman D, 2016, DIABETES CARE, V39, P2126, DOI 10.2337/dc16-2053

Young-Hyman DL, 2010, DIABETES CARE, V33, P683, DOI 10.2337/dc08-1077

NR 41

TC 0

Z9 1

U1 0

U2 2

PU SAGE PUBLICATIONS INC

PI THOUSAND OAKS

PA 2455 TELLER RD, THOUSAND OAKS, CA 91320 USA

SN 0145-7217

EI 1554-6063

J9 DIABETES EDUCATOR

JI Diabetes Educ.

PD FEB

PY 2017

VL 43

IS 1

BP 114

EP 124

DI 10.1177/0145721716675740

PG 11

WC Endocrinology & Metabolism; Public, Environmental & Occupational Health

SC Endocrinology & Metabolism; Public, Environmental & Occupational Health

GA EL8VB

UT WOS:000394897300011

PM 27831521

OA Green Accepted

DA 2019-01-08

ER

PT J

AU Kreider, KE

AF Kreider, Kathryn Evans

TI Diabetes Distress or Major Depressive Disorder? A Practical Approach to

Diagnosing and Treating Psychological Comorbidities of Diabetes

SO DIABETES THERAPY

LA English

DT Article

DE Chronic care management; Depression; Diabetes distress; Major

depressive; disorder

ID GLYCEMIC CONTROL; TRIAL; CARE; EPIDEMIOLOGY; METAANALYSIS; SYMPTOMS;

THERAPY; PEOPLE; ADULTS; TYPE-1

AB The presence of major depressive disorder (MDD) in people with diabetes may be up to three times more common than in the general population. People with diabetes and major depressive disorder have worse health outcomes and higher mortality rates. Diabetes distress refers to an emotional state where people experience feelings such as stress, guilt, or denial that arise from living with diabetes and the burden of self-management. Diabetes distress has also been linked to worse health outcomes. There are multiple treatment options for MDD including pharmacotherapy and cognitive behavioral approaches. Providers treating patients with diabetes must be aware of the frequent comorbidity of diabetes, diabetes distress, and depression and manage patients using a multidisciplinary team approach. This article discusses the epidemiology, pathophysiology, and bi-directional relationship of diabetes and depression and provides a practical, patient-centered approach to diagnosis and management.

C1 [Kreider, Kathryn Evans] Duke Univ, Sch Nursing, Durham, NC 27708 USA.

[Kreider, Kathryn Evans] Duke Univ, Med Ctr, Durham, NC 27706 USA.

RP Kreider, KE (reprint author), Duke Univ, Sch Nursing, Durham, NC 27708 USA.; Kreider, KE (reprint author), Duke Univ, Med Ctr, Durham, NC 27706 USA.

EM kathryn.evans@duke.edu

CR Aikens JE, 2008, DIABETIC MED, V25, P1324, DOI 10.1111/j.1464-5491.2008.02590.x

American Diabetes Association, 2017, DIABETES CARE S1, V40, pS39

American Psychiatric Association, 2013, DIAGN STAT MAN MENT

Anderson RJ, 2001, DIABETES CARE, V24, P1069, DOI 10.2337/diacare.24.6.1069

de Vries M, 2016, AM J HEALTH BEHAV, V40, P503

Egede LE, 2009, GEN HOSP PSYCHIAT, V31, P422, DOI 10.1016/j.genhosppsych.2009.06.007

Fisher L, 2013, DIABETES CARE, V36, P2551, DOI 10.2337/dc12-2493

Fisher L, 2012, DIABETES CARE, V35, P259, DOI 10.2337/dc11-1572

Fisher L, 2010, DIABETES CARE, V33, P1034, DOI 10.2337/dc09-2175

Fisher L, 2010, DIABETES CARE, V33, P23, DOI 10.2337/dc09-1238

Fournier JC, 2010, JAMA-J AM MED ASSOC, V303, P47, DOI 10.1001/jama.2009.1943

Gonzalez JS, 2011, DIABETES CARE, V34, P236, DOI 10.2337/dc10-1970

Hermanns N, 2013, PRIM CARE DIABETES, V7, P1, DOI 10.1016/j.pcd.2012.11.002

Hollander P, 2013, DIABETES CARE, V36, P4022, DOI 10.2337/dc13-0234

Holt RG, 2014, CURRENT DIABETES REP, V14, P1

Huang Y, 2013, INT J ADV MANUF TECH, V2013, P1

Knol MJ, 2007, PSYCHOSOM MED, V69, P300, DOI 10.1097/PSY.0b013e31805f48b9

Lin EHB, 2010, DIABETES CARE, V33, P264, DOI 10.2337/dc09-1068

Lloyd CE, 2010, DIABETIC MED, V27, P234, DOI 10.1111/j.1464-5491.2009.02896.x

Lloyd CE, 2012, J AFFECT DISORDERS, V142, pS22, DOI 10.1016/S0165-0327(12)70005-8

Lustman PJ, 2000, DIABETES CARE, V23, P934, DOI 10.2337/diacare.23.7.934

Markowitz SM, 2011, PSYCHOSOMATICS, V52, P1, DOI 10.1016/j.psym.2010.11.007

McGuire BE, 2010, DIABETOLOGIA, V53, P66, DOI 10.1007/s00125-009-1559-5

McIntyre Roger S, 2007, Ann Clin Psychiatry, V19, P257, DOI 10.1080/10401230701653377

National Collaborating Centre for Mental Health (Great Britain) and Royal College of Psychiatrists, 2011, COMM MENT HLTH DIS I

O'Connor PJ, 2012, DIABETES CARE, V35, P1479, DOI 10.2337/dc11-1868

POLONSKY WH, 1995, DIABETES CARE, V18, P754, DOI 10.2337/diacare.18.6.754

Rossom RC, 2017, GEN HOSP PSYCHIAT, V44, P77, DOI 10.1016/j.genhosppsych.2016.05.006

Roy T, 2012, DIABETIC MED, V29, P164, DOI 10.1111/j.1464-5491.2011.03401.x

Roy T, 2012, J AFFECT DISORDERS, V142, pS8, DOI 10.1016/S0165-0327(12)70004-6

Rustad JK, 2011, PSYCHONEUROENDOCRINO, V36, P1276, DOI 10.1016/j.psyneuen.2011.03.005

Safren SA, 2014, DIABETES CARE, V37, P625, DOI [10.2337/dc13-0816, 10.2337/dc16-er06]

Schram MT, 2009, CURR DIABETES REV, V5, P112, DOI 10.2174/157339909788166828

Talbot F, 2000, DIABETES CARE, V23, P1556, DOI 10.2337/diacare.23.10.1556

Van der Feltz-Cornelis CM, 2010, GEN HOSP PSYCHIAT, V32, P380, DOI 10.1016/j.genhosppsych.2010.03.011

van Dooren FEP, 2013, PLOS ONE, V8, DOI 10.1371/journal.pone.0057058

Ye Z, 2011, PLOS ONE, V6, DOI 10.1371/journal.pone.0021551

NR 37

TC 5

Z9 6

U1 1

U2 7

PU SPRINGER HEIDELBERG

PI HEIDELBERG

PA TIERGARTENSTRASSE 17, D-69121 HEIDELBERG, GERMANY

SN 1869-6953

EI 1869-6961

J9 DIABETES THER

JI Diabetes Ther.

PD FEB

PY 2017

VL 8

IS 1

BP 1

EP 7

DI 10.1007/s13300-017-0231-1

PG 7

WC Endocrinology & Metabolism

SC Endocrinology & Metabolism

GA EL0WO

UT WOS:000394342700001

PM 28160185

OA DOAJ Gold, Green Published

DA 2019-01-08

ER

PT J

AU Palar, K

Napoles, T

Hufstedler, LL

Seligman, H

Hecht, FM

Madsen, K

Ryle, M

Pitchford, S

Frongillo, EA

Weiser, SD

AF Palar, Kartika

Napoles, Tessa

Hufstedler, Lee L.

Seligman, Hilary

Hecht, Fredrick M.

Madsen, Kimberly

Ryle, Mark

Pitchford, Simon

Frongillo, Edward A.

Weiser, Sheri D.

TI Comprehensive and Medically Appropriate Food Support Is Associated with

Improved HIV and Diabetes Health

SO JOURNAL OF URBAN HEALTH-BULLETIN OF THE NEW YORK ACADEMY OF MEDICINE

LA English

DT Article

DE Food; Nutrition; HIV; Diabetes; Community-based; Food security; Food

support; Food-assistance; Intervention; Medically tailored

ID ANTIRETROVIRAL THERAPY; INFECTED INDIVIDUALS; SAN-FRANCISCO;

SOCIOECONOMIC DISPARITIES; CARE UTILIZATION; GLYCEMIC CONTROL;

SELF-MANAGEMENT; CHRONIC DISEASE; UNITED-STATES; DIET-QUALITY

AB Food insecurity is associated with negative chronic health outcomes, yet few studies have examined how providing medically appropriate food assistance to food-insecure individuals may improve health outcomes in resource-rich settings. We evaluated a communitybased food support intervention in the San Francisco Bay Area for people living with HIV and/or type 2 diabetes mellitus (T2DM) to determine the feasibility, acceptability, and potential impact of the intervention on nutritional, mental health, disease management, healthcare utilization, and physical health outcomes. The 6-month intervention provided meals and snacks designed to comprise 100% of daily energy requirements and meet nutritional guidelines for a healthy diet. We assessed paired outcomes at baseline and 6 months using validated measures. Paired t tests and McNemar exact tests were used with continuous and dichotomous outcomes, respectively, to compare pre-post changes. Fifty-two participants (out of 72 initiators) had both baseline and follow-up assessments, including 23 with HIV, 24 with T2DM, and 7 with both HIVand T2DM. Median food pick-up adherence was 93%. Comparing baseline to follow-up, very low food security decreased from 59.6% to 11.5% (p < 0.0001). Frequency of consumption of fats (p= 0.003) decreased, while frequency increased for fruits and vegetables (p = 0.011). Among people with diabetes, frequency of sugar consumption decreased (p = 0.006). We also observed decreased depressive symptoms (p = 0.028) and binge drinking (p =0.008). At follow-up, fewer participants sacrificed food for healthcare (p = 0.007) or prescriptions (p = 0.046), or sacrificed healthcare for food (p = 0.029). Among people with HIV, 95% adherence to antiretroviral therapy increased from 47 to 70% (p = 0.046). Among people with T2DM, diabetes distress (p < 0.001), and perceived diabetes self-management (p = 0.007) improved. Comprehensive, medically appropriate food support is feasible and may improve multiple health outcomes for food-insecure individuals living with chronic health conditions. Future studies should formally test the impact of medically appropriate food support interventions for food-insecure populations through rigorous, randomized controlled designs.

C1 [Palar, Kartika; Napoles, Tessa; Hufstedler, Lee L.; Hecht, Fredrick M.; Weiser, Sheri D.] Univ Calif San Francisco, San Francisco Gen Hosp, Dept Med, Div HIV Infect Dis & Global Med, San Francisco, CA 94143 USA.

[Seligman, Hilary] UCSF, San Francisco Gen Hosp, Dept Med, Div Gen Internal Med, San Francisco, CA USA.

[Madsen, Kimberly; Ryle, Mark] Project Open Hand, San Francisco, CA USA.

[Pitchford, Simon] Homebridge Inc, San Francisco, CA USA.

[Frongillo, Edward A.] Univ South Carolina, Dept Hlth Promot Educ & Behav, Columbia, SC 29208 USA.

[Weiser, Sheri D.] UCSF, Ctr AIDS Prevent Studies, San Francisco, CA USA.

[Hufstedler, Lee L.] Univ Calif Berkeley, Sch Publ Hlth, Univ Calif Berkeley Univ Calif San Francisco Join, Berkeley, CA 94720 USA.

RP Palar, K (reprint author), Univ Calif San Francisco, San Francisco Gen Hosp, Dept Med, Div HIV Infect Dis & Global Med, San Francisco, CA 94143 USA.

EM Kartika.Palar@ucsf.edu

FU Project Open Hand (POH); Burke Global Health; NIH/NIDDK [K01DK107335];

NIH/NIMH [R01MH095683]

FX Project Open Hand (POH) provided funding for this study as an external

evaluation, but had no role in data collection or analysis, nor in

scientific interpretation of the data. Funding from Burke Global Health

supported student and intern participation in data collection. The

authors acknowledge the following sources of salary support: NIH/NIDDK

K01DK107335 (Dr. Palar) and NIH/NIMH R01MH095683 (Dr. Weiser).

CR Amorosa V, 2005, JAIDS-J ACQ IMM DEF, V39, P557

[Anonymous], 2016, FOOD MED ACH TRIPL A

[Anonymous], 2015, CAL 2015 STAT HLTH P

Bangsberg DR, 2001, AIDS, V15, P1181, DOI 10.1097/00002030-200106150-00015

Berger BE, 2001, RES NURS HEALTH, V24, P518, DOI 10.1002/nur.10011

Bickel G, 2000, GUIDE MEASURING HOUS

Bradley KA, 2007, ALCOHOL CLIN EXP RES, V31, P1208, DOI 10.1111/j.1530-0277.2007.00403.x

Bunn JY, 2007, AIDS EDUC PREV, V19, P198, DOI 10.1521/aeap.2007.19.3.198

Cantrell RA, 2008, JAIDS-J ACQ IMM DEF, V49, P190, DOI 10.1097/QAI.0b013e31818455d2

Coleman-Jensen A, 2016, ERR215 US DEP AGR EC

Crum-Cianflone N, 2010, PLOS ONE, V5, DOI 10.1371/journal.pone.0010106

Darmon N, 2015, NUTR REV, V73, P643, DOI 10.1093/nutrit/nuv027

DiSantis KI, 2013, AM J PUBLIC HEALTH, V103, P516, DOI 10.2105/AJPH.2012.301149

Drewnowski A., 2012, AM J PUBLIC HEALTH, V102, P74, DOI DOI 10.2105/AJPH.2012.300660

Ellwood M, 2014, FOOD IS MED OPPORTUN

GARCIA DOP, 2002, J ACQ IMMUN DEF SYND, V30, P105, DOI DOI 10.1097/00042560-200205010-00014

Gurvey J, 2013, J PRIM CARE COMMUNIT, V4, P311, DOI 10.1177/2150131913490737

Habicht JP, 1999, INT J EPIDEMIOL, V28, P10, DOI 10.1093/ije/28.1.10

Ivers L., 2015, FOOD INSECURITY PUBL

Kalichman S, 2010, J URBAN HEALTH, V87, P1

Kroenke K, 2001, J GEN INTERN MED, V16, P606, DOI 10.1046/j.1525-1497.2001.016009606.x

Li R, 2013, AM J MANAG CARE, V19, P421

Martin KS, 2013, AM J PREV MED, V45, P569, DOI 10.1016/j.amepre.2013.06.012

Martinez H, 2014, AIDS BEHAV, V18, pS566, DOI 10.1007/s10461-014-0786-z

McMahon JH, 2011, JAIDS-J ACQ IMM DEF, V58, P60, DOI 10.1097/QAI.0b013e318227f8dd

Nelson K, 1998, JAMA-J AM MED ASSOC, V279, P1211, DOI 10.1001/jama.279.15.1211

Palar K, 2016, AIDS, V30, P1457, DOI 10.1097/QAD.0000000000001095

Palar K, 2015, AIDS CARE, V27, P409, DOI 10.1080/09540121.2014.983041

Polonsky WH, 2005, DIABETES CARE, V28, P626, DOI 10.2337/diacare.28.3.626

Seligman HK, 2007, J GEN INTERN MED, V22, P1018, DOI 10.1007/s11606-007-0192-6

Seligman HK, 2015, HEALTH AFFAIR, V34, P1956, DOI 10.1377/hlthaff.2015.0641

Seligman HK, 2012, DIABETES CARE, V35, P233, DOI 10.2337/dc11-1627

Seligman HK, 2010, J HEALTH CARE POOR U, V21, P1227, DOI 10.1353/hpu.2010.0921

Seligman HK, 2010, NEW ENGL J MED, V363, P6, DOI 10.1056/NEJMp1000072

Seligman HK, 2010, J NUTR, V140, P304, DOI 10.3945/jn.109.112573

Shannon K, 2011, AIDS, V25, P2037, DOI 10.1097/QAD.0b013e32834b35c9

Smith R, 2008, AIDS CARE, V20, P1266, DOI 10.1080/09540120801926977

Thompson FE, 2004, PUBLIC HEALTH NUTR, V7, P1097, DOI 10.1079/PHN2004642

Trenkamp B, 2007, SOCIAL SECURITY B, V67

Vogenthaler NS, 2012, AIDS BEHAV, P1

Wallston KA, 2007, J BEHAV MED, V30, P395, DOI 10.1007/s10865-007-9110-y

Wang EA, 2011, J GEN INTERN MED, V26, P1012, DOI 10.1007/s11606-011-1723-8

Weinfield NS, 2014, HUNGER AM 2014 NATL

Weiser S. D., 2015, FOOD INSECURITY PUBL, P23

Weiser SD, 2013, AIDS, V27, P2953, DOI 10.1097/01.aids.0000432538.70088.a3

Weiser SD, 2013, J GEN INTERN MED, V28, P91, DOI 10.1007/s11606-012-2176-4

Weiser SD, 2012, AIDS, V26, P67, DOI 10.1097/QAD.0b013e32834cad37

Weiser SD, 2011, AM J CLIN NUTR, V94, p1729S, DOI 10.3945/ajcn.111.012070

Weiser SD, 2009, JAIDS-J ACQ IMM DEF, V52, P342, DOI 10.1097/QAI.0b013e3181b627c2

Weiser SD, 2009, J GEN INTERN MED, V24, P14, DOI 10.1007/s11606-008-0824-5

Whittle HJ, 2015, SOC SCI MED, V143, P154, DOI 10.1016/j.socscimed.2015.08.027

NR 51

TC 5

Z9 5

U1 2

U2 10

PU SPRINGER

PI NEW YORK

PA 233 SPRING ST, NEW YORK, NY 10013 USA

SN 1099-3460

EI 1468-2869

J9 J URBAN HEALTH

JI J. Urban Health

PD FEB

PY 2017

VL 94

IS 1

BP 87

EP 99

DI 10.1007/s11524-016-0129-7

PG 13

WC Public, Environmental & Occupational Health; Medicine, General &

Internal

SC Public, Environmental & Occupational Health; General & Internal Medicine

GA EP5GD

UT WOS:000397406100009

PM 28097614

OA Green Published

DA 2019-01-08

ER

PT J

AU Sidhu, R

Tang, TS

AF Sidhu, Rawel

Tang, Tricia S.

TI Diabetes Distress and Depression in South Asian Canadians with Type 2

Diabetes

SO CANADIAN JOURNAL OF DIABETES

LA English

DT Article

DE depression; diabetes distress; South Asia; type 2 diabetes mellitus

AB Objectives: South Asians are disproportionately affected by diabetes compared to some other ethnic groups in Canada. Although depression and diabetes distress are psychological issues well studied in the general population of those with diabetes, they have not been investigated in South Asian Canadians with type 2 diabetes. We sought to identify the rates of depression and diabetes distress in South Asian adults with type 2 diabetes and to explore the relationship among glycemic control, depression and diabetes distress.

Methods: We recruited 41 South Asian adults with type 2 diabetes for this study. Glycated hemoglobin (A1C) levels were collected via venous puncture. We utilized the Diabetes Distress Scale to assess total diabetes distress and its subscales (emotional distress, interpersonal distress, regimen-related distress and physician distress) and the Personal Health Questionnaire-9 to assess depressive symptoms.

Results: The rate of depression was 15%, and the rate of total diabetes distress was 52.5%. Although neither measure was found to be correlated with A1C levels, depression had a moderate positive correlation with total diabetes distress (r=0.696; p< 0.001); subscales of regimen distress and emotional burden emerged as the strongest correlates.

Conclusions: This is the first study to report that diabetes distress is a serious concern for South Asian Canadians with type 2 diabetes. Given that depression and diabetes distress are linked, studies recruiting a larger and more diverse sample of South Asian Canadians should be conducted to better understand the psychological issues that may impact diabetes self-management in this community. (C) 2016 Canadian Diabetes Association.

C1 [Sidhu, Rawel] Univ British Columbia, Fac Sci, Dept Med, Vancouver, BC, Canada.

[Tang, Tricia S.] Univ British Columbia, Div Endocrinol, Vancouver, BC, Canada.

RP Sidhu, R (reprint author), 7675 Prince Albert St, Vancouver, BC V5X 3Z3, Canada.

EM rawel95@gmail.com

FU Vancouver General Hospital University of British Columbia Foundation

Grant; Azad and Yasmin Shamji family

FX This study was supported by a Vancouver General Hospital University of

British Columbia Foundation Grant and was financially sponsored by the

Azad and Yasmin Shamji family.

CR Ali S, 2006, DIABETIC MED, V23, P1165, DOI 10.1111/j.1464-5491.2006.01943.x

Ali S, 2009, POSTGRAD MED J, V85, P238, DOI 10.1136/pgmj.2008.074641

Aujla N, 2010, DIABETIC MED, V27, P896, DOI 10.1111/j.1464-5491.2010.03042.x

Aujla N, 2009, PLOS ONE, V411, pe7755

Canadian Diabetes Association Clinical Practice Guidelines Expert Committee, 2013, DIAB MENT HLTH

Clayton D, 2013, CAN J DIABETES, V37, pS69, DOI 10.1016/j.jcjd.2013.01.022

Creatore MI, 2010, CAN MED ASSOC J, V182, P781, DOI 10.1503/cmaj.091551

Ell K, 2015, GEN HOSP PSYCHIAT, V37, P414, DOI 10.1016/j.genhosppsych.2015.05.010

Fisher L, 2007, DIABETES CARE, V30, P2746, DOI 10.2337/dc07-1166

Fisher L, 2010, DIABETES CARE, V33, P1034, DOI 10.2337/dc09-2175

Fisher L, 2010, DIABETES CARE, V33, P23, DOI 10.2337/dc09-1238

Hessler D, 2015, DIABETES RES CLIN PR, V108, P360, DOI 10.1016/j.diabres.2014.12.017

Imran SA, 2013, CAN J DIABETES, V37, pS31, DOI 10.1016/j.jcjd.2013.01.016

Islam MR, 2013, INT J MED BIOMED RES, V2, P113, DOI DOI 10.14194/IJMBR.224

Ke C, 2015, DIABETIC MED, V32, P487, DOI 10.1111/dme.12657

Leyva B, 2011, ETHNIC DIS, V21, P322

Pandit AU, 2014, J EPIDEMIOL COMMUN H, V68, P557, DOI 10.1136/jech-2013-203063

Pinkington FB, 2010, CAN J DIABETES, V342, P119

RAPHAEL D, 2003, LEADERSHIP HLTH SERV, V16, P10

Rastogi PP, 2014, ASIAN J PSYCHIAT, V71, P15

Shah BR, 2008, J PUBLIC HEALTH-UK, V30, P327, DOI 10.1093/pubmed/fdn042

Statistics Canada, 2011, IMM ETHN DIV CAN

Tang T, 2014, CAN J DIABETES, V385, pS31

Williams ED, 2015, PSYCHOL MED, V45, P1861, DOI 10.1017/S0033291714002967

NR 24

TC 3

Z9 3

U1 1

U2 11

PU ELSEVIER SCIENCE BV

PI AMSTERDAM

PA PO BOX 211, 1000 AE AMSTERDAM, NETHERLANDS

SN 1499-2671

J9 CAN J DIABETES

JI Can. J. Diabetes

PD FEB

PY 2017

VL 41

IS 1

BP 69

EP 72

DI 10.1016/j.jcjd.2016.07.008

PG 4

WC Endocrinology & Metabolism

SC Endocrinology & Metabolism

GA EO7VY

UT WOS:000396899400011

PM 27745846

DA 2019-01-08

ER

PT J

AU Strandberg, RB

Graue, M

Wentzel-Larsen, T

Peyrot, M

Wahl, AK

Rokne, B

AF Strandberg, Ragnhild B.

Graue, Marit

Wentzel-Larsen, Tore

Peyrot, Mark

Wahl, Astrid K.

Rokne, Berit

TI The relationships among fear of hypoglycaemia, diabetes-related quality

of life and psychological well-being in Norwegian adults with Type 1

diabetes

SO DIABETES RESEARCH AND CLINICAL PRACTICE

LA English

DT Article

DE Diabetes-related quality of life; Fear of hypoglycaemia; Psychological

well-being; Type 1 diabetes; Mediation analysis

ID DEPRESSIVE SYMPTOMS; DISTRESS; PERCEPTIONS; MEDIATION; SUBSCALE;

VERSION; ISSUES; IMPACT

AB Aims: To examine the associations among fear of hypoglycaemia, diabetes- related quality of life and psychological well- being, and determine whether diabetes- related quality of life is a mediator of the relationship between fear of hypoglycaemia and psychological wellbeing in adults with Type 1 diabetes.

Methods: A total of 235 of 319 invited adults (8-69 years) with Type 1 diabetes agreed to participate. Hierarchical linear regression was applied to 188 individuals with complete data. Mediation analysis was used to determine whether diabetes- related quality of life mediated the relationship between fear of hypoglycaemia and psychological well-being.

Results: Fear of hypoglycaemia was significantly associated with diabetes-related quality of life and psychological well-being. The behaviour and worry components of fear of hypoglycaemia were significantly associated with diabetes-related quality of life (behaviour component: unstandardised coefficient = -0.04, p < 0.001, standardised coefficient = -0.40; worry component: unstandardised coefficient = -0.01, p = 0.041, standardised coefficient = -0.18). Fear of hypoglycaemia worry had a significant independent association with psychological well-being (unstandardised coefficient = -0.28, p = 0.009, standardised coefficient = -0.25), whereas fear of hypoglycaemia behaviour did not. Diabetes-related quality of life mediated approximately half of the association between fear of hypoglycaemia worry and psychological well-being.

Conclusions: Hypoglycaemic episodes can have serious consequences, and assessing fear of hypoglycaemia might help health care providers offer suitable care strategies to individuals with Type 1 diabetes. (C) 2016 Elsevier Ireland Ltd. All rights reserved.

C1 [Strandberg, Ragnhild B.; Graue, Marit; Peyrot, Mark] Bergen Univ Coll, Ctr Evidence Based Practice, POB 7030, N-5020 Bergen, Norway.

[Strandberg, Ragnhild B.; Rokne, Berit] Univ Bergen, Fac Med & Dent, Dept Global Publ Hlth & Primary Care, Bergen, Norway.

[Graue, Marit] Haukeland Hosp, Dept Paediat, Bergen, Norway.

[Wentzel-Larsen, Tore] Norwegian Ctr Violence & Traumat Stress Studies, Oslo, Norway.

[Wentzel-Larsen, Tore] Ctr Child & Adolescent Mental Hlth, Eastern & Southern Norway, Oslo, Norway.

[Wentzel-Larsen, Tore] Haukeland Hosp, Clin Res Ctr, Bergen, Norway.

[Peyrot, Mark] Loyola Univ Maryland, Dept Sociol, Baltimore, MD USA.

[Wahl, Astrid K.] Univ Oslo, Fac Med, Inst Hlth & Soc, Oslo, Norway.

[Rokne, Berit] Haukeland Hosp, Dept Res & Dev, Bergen, Norway.

RP Strandberg, RB (reprint author), Bergen Univ Coll, Ctr Evidence Based Practice, POB 7030, N-5020 Bergen, Norway.

EM Ragnhild.Bjarkoy.Strandberg@hib.no

FU Bergen University College

FX The data collection was funded by Bergen University College. The funding

source had no involvement in the study design, the data collection,

analysis, interpretation and writing, or in the decision to submit the

article for publication.

CR Anderbro T, 2008, PATIENT EDUC COUNS, V73, P127, DOI 10.1016/j.pec.2008.03.022

Bech P, 2003, INT J METH PSYCH RES, V12, P85, DOI 10.1002/mpr.145

Bohme P, 2013, DIABETES METAB, V39, P63, DOI 10.1016/j.diabet.2012.10.006

Bradley C, 2002, DIABETES METAB RES, V18, pS64, DOI 10.1002/dmrr.279

Bradley C, 1999, QUAL LIFE RES, V8, P79, DOI 10.1023/A:1026485130100

Burns RJ, 2015, DIABETIC MED, V32, P1272, DOI 10.1111/dme.12860

Buuren S, 2011, R J STAT SOFTWARE, V45, P3, DOI DOI 10.18637/JSS.V045.I03

Cryer PE, 2003, DIABETES CARE, V26, P1902, DOI 10.2337/diacare.26.6.1902

Ducat L, 2015, DIABETES CARE, V38, P333, DOI 10.2337/dc14-1383

Ehrmann D, 2015, DIABETIC MED, V32, P1264, DOI 10.1111/dme.12861

Fisher L, 2015, J DIABETES COMPLICAT, V29, P572, DOI 10.1016/j.jdiacomp.2015.01.012

Fisher L, 2010, DIABETES CARE, V33, P23, DOI 10.2337/dc09-1238

Gonder-Frederick LA, 2013, DIABETIC MED, V30, P603, DOI 10.1111/dme.12129

Gonder-Frederick LA, 2011, DIABETES CARE, V34, P801, DOI 10.2337/dc10-1343

Graue M., 2013, Norsk Epidemiologi, V23, P75

Hendrieckx C, 2014, DIABETES RES CLIN PR, V103, P430, DOI 10.1016/j.diabres.2013.12.005

Hessler D, 2014, DIABETES CARE, V37, P617, DOI 10.2337/dc13-0762

Hofmann M, 2013, PLOS ONE, V8, DOI 10.1371/journal.pone.0079809

Huang H, 2012, PSYCHOSOMATICS, V53, P21, DOI 10.1016/j.psym.2011.07.001

Imai K, 2010, PSYCHOL METHODS, V15, P309, DOI 10.1037/a0020761

Iversen MM, 2013, QUAL LIFE RES, V22, P2809, DOI 10.1007/s11136-013-0413-x

MacDonald IA, 2007, HYPOGLYCEMIA

Martyn-Nemeth P, 2016, J DIABETES COMPLICAT, V30, P167, DOI 10.1016/j.jdiacomp.2015.09.003

Maxwell SE, 2007, PSYCHOL METHODS, V12, P23, DOI 10.1037/1082-989X.12.1.23

Nefs G, 2015, DIABETIC MED, V32, P1289, DOI 10.1111/dme.12739

Park M, 2013, GEN HOSP PSYCHIAT, V35, P217, DOI 10.1016/j.genhosppsych.2013.01.006

Peyrot M, 2010, DIABETES CARE, V33, P240, DOI 10.2337/dc09-1348

Shi LZ, 2014, HEALTH QUAL LIFE OUT, V12, DOI 10.1186/s12955-014-0167-3

Shiu ATY, 2002, J ADV NURS, V39, P155

Strandberg RB, 2014, J PSYCHOSOM RES, V77, P174, DOI 10.1016/j.jpsychores.2014.06.015

Tingley D, 2014, J STAT SOFTW, P59

Topp CW, 2015, PSYCHOTHER PSYCHOSOM, V84, DOI 10.1159/000376585

Vallis M, 2014, CURR DIABETES REV, V10, P364, DOI 10.2174/1573399810666141113115026

Van Buuren S, 2012, FLEXIBLE IMPUTATION

WHO, THE WHO 5 WEBS

NR 35

TC 3

Z9 3

U1 0

U2 5

PU ELSEVIER IRELAND LTD

PI CLARE

PA ELSEVIER HOUSE, BROOKVALE PLAZA, EAST PARK SHANNON, CO, CLARE, 00000,

IRELAND

SN 0168-8227

EI 1872-8227

J9 DIABETES RES CLIN PR

JI Diabetes Res. Clin. Pract.

PD FEB

PY 2017

VL 124

BP 11

EP 19

DI 10.1016/j.diabres.2016.12.018

PG 9

WC Endocrinology & Metabolism

SC Endocrinology & Metabolism

GA EO7NZ

UT WOS:000396878700002

PM 28081448

DA 2019-01-08

ER

PT J

AU Maiorino, MI

Bellastella, G

Castaldo, F

Petrizzo, M

Giugliano, D

Esposito, K

AF Maiorino, M. I.

Bellastella, G.

Castaldo, F.

Petrizzo, M.

Giugliano, D.

Esposito, K.

TI Sexual function in young women with type 1 diabetes: the METRO study

SO JOURNAL OF ENDOCRINOLOGICAL INVESTIGATION

LA English

DT Article

DE Female sexual dysfunction; Type 1 diabetes; Female sexual function

index; Multiple daily insulin injections; Continuous subcutaneous

insulin infusion

ID QUALITY-OF-LIFE; UNITED-STATES; TREATMENT SATISFACTION; INDEX FSFI;

DYSFUNCTION; FEMALE; PREVALENCE; HEALTH; DISTRESS; ADULTS

AB Purpose The aim of this study was to evaluate the prevalence and risk factors associated with female sexual dysfunction (FSD) in young women with type 1 diabetes treated with different intensive insulin regimens.

Methods Type 1 diabetic women aged 18-35 years were included in this study if they had stable couple relationship and no oral contraceptive use. All women were asked to complete the Female Sexual Function Index (FSFI) and other validated multiple-choice questionnaires assessing sexual-related distress (Female Sexual Distress Scale, FSDS), quality of life (SF-36 Health Survey), physical activity (International Physical Activity Questionnaire), depressive symptoms (Zung Self-Rating Depression Scale, SRDS) and diabetes-related problems (Diabetes Integration Scale ATT-19). FSD was diagnosed according to a FSFI score higher than 26.55 and a FSDS score lower than 15.

Results The overall prevalence of FSD in diabetic and control women was 20 and 15 %, respectively (P = 0.446). Compared with the continuous subcutaneous insulin infusion group and control women, diabetic women on multiple daily injections (MDI) had lower global FSFI score (P = 0.007), FSDS score (P = 0.045) and domains such as arousal (P = 0.006), lubrication and satisfaction scores (P < 0.001 for both). In the multiple regression analysis, only the mental component summary (P = 0.047) and the SRDS score (P = 0.042) were independent predictors of FSFI score in the overall diabetic women.

Conclusion Young women with type 1 diabetes wearing an insulin pump show a prevalence of sexual dysfunction similar to that of healthy age-matched women, but sexual function was significantly impaired in diabetic women on MDI therapy. Depression and the mental health status were independent predictors for FSD in diabetic women.

C1 [Maiorino, M. I.; Bellastella, G.; Castaldo, F.; Giugliano, D.; Esposito, K.] Univ Naples 2, Diabet Unit, Piazza L Miraglia 2, I-80138 Naples, Italy.

[Petrizzo, M.; Esposito, K.] Univ Naples 2, Dept Med Surg Neurol Metab Sci & Aging, Diabet Unit, Piazza L Miraglia 2, I-80138 Naples, Italy.

RP Maiorino, MI (reprint author), Univ Naples 2, Diabet Unit, Piazza L Miraglia 2, I-80138 Naples, Italy.

EM mariaida.maiorino@unina2.it

CR Abu Ali RM, 2008, DIABETES CARE, V31, P1580, DOI 10.2337/dc08-0081

American Diabetes Association, 2016, DIABETES CARE S1, V39, pS52, DOI DOI 10.2337/DC16-S010

Baser RE, 2012, CANCER, V15, P4606

Bultrini A, 2004, J SEX MED, V1, P337, DOI 10.1111/j.1743-6109.04048.x

Cherubini V, 2014, ACTA DIABETOL, V51, P43, DOI 10.1007/s00592-013-0466-x

Ciocca G, 2015, J ENDOCRINOL INVEST, V38, P999, DOI 10.1007/s40618-015-0286-7

Ciocca G, 2015, ACTA DIABETOL, V52, P513, DOI 10.1007/s00592-014-0686-8

Cortelazzi D, 2013, ACTA DIABETOL, V50, P911, DOI 10.1007/s00592-013-0482-x

Derogatis LR, 2002, J SEX MARITAL THER, V28, P317, DOI 10.1080/00926230290001448

Dimitropoulos K, 2012, J SEX MED, V9, P1374, DOI 10.1111/j.1743-6109.2012.02664.x

Enzlin P, 2003, DIABETES CARE, V26, P409, DOI 10.2337/diacare.26.2.409

Enzlin P, 2009, DIABETES CARE, V32, P780, DOI 10.2337/dc08-1164

Esposito K, 2008, INT J IMPOT RES, V20, P358, DOI 10.1038/ijir.2008.9

Esposito K, 2007, INT J IMPOT RES, V19, P353, DOI 10.1038/sj.ijir.3901548

Esposito K, 2010, INT J IMPOT RES, V22, P179, DOI 10.1038/ijir.2010.6

IPAQ Research Committee, GUID DAT PROC AN INT

Isidori AM, 2010, J SEX MED, V7, P1139, DOI 10.1111/j.1743-6109.2009.01635.x

KOKKONEN J, 1995, NORD J PSYCHIAT, V49, P175, DOI 10.3109/08039489509011903

Laumann EO, 2005, INT J IMPOT RES, V17, P39, DOI 10.1038/sj.ijir.3901250

Laumann EO, 1999, JAMA-J AM MED ASSOC, V281, P537, DOI 10.1001/jama.281.6.537

Lindau ST, 2007, NEW ENGL J MED, V357, P762, DOI 10.1056/NEJMoa067423

Maiorino MI, 2014, ENDOCRINE, V46, P256, DOI 10.1007/s12020-013-0060-6

Maiorino MI, 2014, DIABET METAB SYND OB, V7, P95, DOI 10.2147/DMSO.S36455

Maseroli E, 2016, J ENDOCRINOL INVEST, V39, P401, DOI 10.1007/s40618-015-0378-4

McCabe MP, 2016, J SEX MED, V13, P135, DOI 10.1016/j.jsxm.2015.12.019

Meston CM, 2002, J SEX MARITAL THER, V28, P155, DOI 10.1080/00926230252851276

Nicolucci A, 2008, DIABETIC MED, V25, P213, DOI 10.1111/j.1464-5491.2007.02346.x

Peters A, 2011, DIABETES CARE, V34, P2477, DOI 10.2337/dc11-1723

Pontiroli AE, 2013, J SEX MED, V10, P1044, DOI 10.1111/jsm.12065

Rosen R, 2000, J SEX MARITAL THER, V26, P191, DOI 10.1080/009262300278597

Salonia A, 2006, DIABETES CARE, V29, P312, DOI 10.2337/diacare.29.02.06.dc05-1067

Shiften JL, 2008, OBSTET GYNECOL, V112, P970, DOI 10.1097/AOG.0b013e3181898cdb

Tagliabue M, 2011, J SEX MED, V8, P1694, DOI 10.1111/j.1743-6109.2011.02262.x

The Diabetes Control and Complications Trial Research Group, 1993, NEW ENGL J MED, V329, P977, DOI [10.1056/NEJM199309303291401, DOI 10.1056/NEJM199309303291401]

WARE JE, 1992, MED CARE, V30, P473, DOI 10.1097/00005650-199206000-00002

Welch G., 1994, HDB PSYCHOL DIABETES, P223

ZUNG WWK, 1965, ARCH GEN PSYCHIAT, V12, P63

NR 37

TC 6

Z9 6

U1 1

U2 6

PU SPRINGER

PI NEW YORK

PA 233 SPRING ST, NEW YORK, NY 10013 USA

SN 1720-8386

J9 J ENDOCRINOL INVEST

JI J. Endocrinol. Invest.

PD FEB

PY 2017

VL 40

IS 2

BP 169

EP 177

DI 10.1007/s40618-016-0542-5

PG 9

WC Endocrinology & Metabolism

SC Endocrinology & Metabolism

GA EO7OR

UT WOS:000396880500007

PM 27613610

DA 2019-01-08

ER

PT J

AU Cheng, L

Sit, JWH

Choi, KC

Chair, SY

Li, XM

He, XL

AF Cheng, Li

Sit, Janet W. H.

Choi, Kai-chow

Chair, Sek-ying

Li, Xiaomei

He, Xiao-le

TI Effectiveness of Interactive Self-Management Interventions in

Individuals With Poorly Controlled Type 2 Diabetes: A Meta-Analysis of

Randomized Controlled Trials

SO WORLDVIEWS ON EVIDENCE-BASED NURSING

LA English

DT Article

DE meta-analysis; interactive; self-management; intervention; type 2

diabetes; poor control

ID IMPROVE GLYCEMIC CONTROL; BEHAVIORAL INTERVENTION; GENERAL-PRACTICE;

CLINICAL-TRIAL; BLOOD-GLUCOSE; ADULTS; SUPPORT; EDUCATION; CARE;

AMERICANS

AB Aims: To identify, assess, and summarize available scientific evidence on the effectiveness of interactive self-management interventions on glycemic control and patient-centered outcomes in individuals with poorly controlled type 2 diabetes.

Methods: Major English and Chinese electronic databases including Medline, EMBASE, Cochrane Central Register of Controlled Trials, China National Knowledge Infrastructure, and WanFang Data were searched to identify randomized controlled trials that reported the effectiveness of interactive self-management interventions in individuals with poorly controlled type 2 diabetes (glycated hemoglobin [HbA1c] >= 7.5% or 58 mmol/mol), from inception to June 2015. Data extraction and risk-of-bias assessment were performed by two reviewers independently. Meta-analysis was performed using Review Manager 5.3.

Results: A total of 16 trials with 3,545 participants were included in the meta-analysis. Interactive self-management interventions could have a beneficial effect in individuals with poorly controlled type 2 diabetes in reducing HbA1c (mean difference: -0.43%, 95% CI: -0.67% to -0.18%), improving diabetes knowledge (standardized mean difference [SMD]: 0.30, 95% CI: 0.03 to 0.58), enhancing self-efficacy (SMD: 0.29, 95% CI: 0.14 to 0.44), and reducing diabetes-related distress (SMD: -0.21, 95% CI: -0.39 to -0.04). Self-management interventions supported with theory and structured curriculum showed desirable results in glycemic control. The behavioral change techniques, including providing feedback on performance, problem-solving, and action planning, were associated with a significant reduction in HbA1c.

Linking Evidence to Action: Individuals with poorly controlled type 2 diabetes could benefit from interactive self-management interventions. Interventions targeting patients with poorly controlled diabetes, those who are at the greatest risk of developing complications, should be prioritized. Our findings indicate that providing feedback on performance, problem-solving, and action planning are promising behavioral change techniques specifically for individuals with poor glycemic control.

C1 [Cheng, Li; Sit, Janet W. H.; Choi, Kai-chow; Chair, Sek-ying] Chinese Univ Hong Kong, Nethersole Sch Nursing, Fac Med, Shatin, Hong Kong, Peoples R China.

[Li, Xiaomei; He, Xiao-le] Xi An Jiao Tong Univ, Sch Nursing, Xian, Peoples R China.

RP Cheng, L (reprint author), Chinese Univ Hong Kong, Nethersole Sch Nursing, Room 821,Esther Lee Bldg, Shatin, Hong Kong, Peoples R China.

EM lilycheng@link.cuhk.edu.hk

RI Chair, Sek Ying/G-9965-2016; Sit, Janet/A-5159-2015; Choi, Kai

Chow/F-1398-2018

OI Chair, Sek Ying/0000-0003-2387-7035; Sit, Janet/0000-0002-4714-5915;

Choi, Kai Chow/0000-0001-7157-8668

CR AgursCollins TD, 1997, DIABETES CARE, V20, P1503, DOI 10.2337/diacare.20.10.1503

American Diabetes Association, 2016, DIABETES CARE S1, V39, pS4, DOI DOI 10.2337/DC16-S003

Baek RN, 2014, ANN BEHAV MED, V48, P145, DOI 10.1007/s12160-013-9585-4

Blackberry ID, 2013, BMJ-BRIT MED J, V347, DOI 10.1136/bmj.f5272

Bolen SD, 2014, J GEN INTERN MED, V29, P1166, DOI 10.1007/s11606-014-2855-4

Cattaneo LB, 2010, AM PSYCHOL, V65, P646, DOI 10.1037/a0018854

Cotter AP, 2014, J DIABETES COMPLICAT, V28, P243, DOI 10.1016/j.jdiacomp.2013.07.003

Duke SAS, 2009, COCHRANE DB SYST REV, DOI 10.1002/14651858.CD005268.pub2

Farmer A, 2012, BMC FAM PRACT, V13, DOI 10.1186/1471-2296-13-30

Frosch DL, 2011, ARCH INTERN MED, V171, P2011, DOI 10.1001/archinternmed.2011.497

Heinrich E, 2010, EUR DIAB NURSING, V7, P71, DOI DOI 10.1002/EDN.160

Higgins J. P., 2008, COCHRANE HDB SYSTEMA, V5

International Diabetes Federation Guideline Development Group, 2014, DIABETES RES CLIN PR, V104, P1

Ismail K, 2004, LANCET, V363, P1589, DOI 10.1016/S0140-6736(04)16202-8

Kim MT, 2009, DIABETES EDUCATOR, V35, P986, DOI 10.1177/0145721709345774

Krein SL, 2004, AM J MED, V116, P732, DOI 10.1016/j.amjmed.2003.11.028

[李蒙 Li Meng], 2014, [中华护理杂志, Chinese Journal of Nursing], V49, P6

Liang X, 2011, DIABETIC MED, V28, P455, DOI 10.1111/j.1464-5491.2010.03180.x

Litwak L, 2013, DIABETOL METAB SYNDR, V5, DOI 10.1186/1758-5996-5-57

Minet L, 2010, PATIENT EDUC COUNS, V80, P29, DOI 10.1016/j.pec.2009.09.033

Moher D, 2009, ANN INTERN MED, V151, P264, DOI 10.7326/0003-4819-151-4-200908180-00135

Monasta L, 2011, OBES REV, V12, pe107, DOI 10.1111/j.1467-789X.2010.00774.x

Naik AD, 2011, ARCH INTERN MED, V171, P453, DOI 10.1001/archinternmed.2011.70

Norris SL, 2001, DIABETES CARE, V24, P561, DOI 10.2337/diacare.24.3.561

Pal K, 2014, DIABETES CARE, V37, P1759, DOI 10.2337/dc13-1386

Philis-Tsimikas A, 2011, DIABETES CARE, V34, P1926, DOI 10.2337/dc10-2081

Powers MA, 2015, DIABETES EDUCATOR, V41, P417, DOI 10.1177/0145721715588904

Quinn CC, 2011, DIABETES CARE, V34, P1934, DOI 10.2337/dc11-0366

Rickheim PL, 2002, DIABETES CARE, V25, P269, DOI 10.2337/diacare.25.2.269

Riddle MC, 2013, DIABETES OBES METAB, V15, P819, DOI 10.1111/dom.12096

Schwedes U, 2002, DIABETES CARE, V25, P1928, DOI 10.2337/diacare.25.11.1928

Sherifali D, 2015, DIABETIC MED, V32, P1404, DOI 10.1111/dme.12780

Slingerland AS, 2013, DIABETES CARE, V36, P3054, DOI 10.2337/dc12-1865

Speight J, 2009, DIABETIC MED, V26, P315, DOI 10.1111/j.1464-5491.2009.02682.x

Tang PC, 2013, J AM MED INFORM ASSN, V20, P526, DOI 10.1136/amiajnl-2012-001263

Tshiananga JKT, 2012, DIABETES EDUCATOR, V38, P108, DOI 10.1177/0145721711423978

Tuomi T, 2014, LANCET, V383, P1084, DOI 10.1016/S0140-6736(13)62219-9

Urrechaga E, 2012, AM J CLIN PATHOL, V138, P448, DOI 10.1309/AJCPVYW9QZ9EVFXI

Walker EA, 2011, DIABETES CARE, V34, P2, DOI 10.2337/dc10-1005

Welch G, 2015, DIABETES CARE, V38, P561, DOI 10.2337/dc14-1412

Welch G, 2011, DIABETES RES CLIN PR, V91, P54, DOI 10.1016/j.diabres.2010.09.036

Welch G, 2010, DIABETES RES CLIN PR, V88, P1, DOI 10.1016/j.diabres.2009.12.026

Williams ED, 2012, BMC PUBLIC HEALTH, V12, DOI 10.1186/1471-2458-12-602

Woodcock AJ, 2001, QUAL LIFE RES, V10, P661, DOI 10.1023/A:1013837709224

NR 44

TC 7

Z9 7

U1 5

U2 20

PU WILEY

PI HOBOKEN

PA 111 RIVER ST, HOBOKEN 07030-5774, NJ USA

SN 1545-102X

EI 1741-6787

J9 WORLDV EVID-BASED NU

JI Worldviews Evid.-Based Nurs.

PD FEB

PY 2017

VL 14

IS 1

BP 65

EP 73

DI 10.1111/wvn.12191

PG 9

WC Nursing

SC Nursing

GA EP2KI

UT WOS:000397211500008

PM 27984672

DA 2019-01-08

ER

PT J

AU Tanenbaum, ML

Hanes, SJ

Miller, KM

Naranjo, D

Bensen, R

Hood, KK

AF Tanenbaum, Molly L.

Hanes, Sarah J.

Miller, Kellee M.

Naranjo, Diana

Bensen, Rachel

Hood, Korey K.

TI Diabetes Device Use in Adults With Type 1 Diabetes: Barriers to Uptake

and Potential Intervention Targets

SO DIABETES CARE

LA English

DT Article

ID SUBCUTANEOUS INSULIN INFUSION; EXCHANGE CLINIC REGISTRY; PUMP THERAPY;

PSYCHOSOCIAL FACTORS; GLYCEMIC CONTROL; SELF-MANAGEMENT; GLUCOSE; CARE;

TECHNOLOGY; TRANSITION

AB OBJECTIVE Diabetes devices (insulin pumps, continuous glucose monitors [CGMs]) are associated with benefits for glycemic control, yet uptake of these devices continues to be low. Some barriers to device uptake may be modifiable through psychosocial intervention, but little is known aboutwhich barriers andwhich patients to target.

RESEARCH DESIGN AND METHODS We surveyed 1,503 adult T1D Exchange participants (mean age 35.3 [SD 14.8] years, mean diagnosis duration 20.4 [SD 12.5] years) to investigate barriers to device uptake, understand profiles of device users versus nonusers, and explore differences by age and sex. Scales used were the Diabetes Distress Scale, Technology Use Attitudes (General and Diabetes-Specific), and Barriers to Device Use and Reasons for Discontinuing Devices.

RESULTS Most commonly endorsed modifiable barriers were related to the hassle of wearing devices (47%) and disliking devices on one's body (35%). CGM users (37%) were older than nonusers (mean 38.3 vs. 33.5 years), had diabetes for longer (22.9 vs. 18.8 years), had more positive technology attitudes (22.6-26.0 vs. 21.4-24.8), and reported fewer barriers to using diabetes technology than nonusers (3.3 vs. 4.3). The youngest age-group (18-25 years) had the lowest CGM (26% vs. 40-48%) and insulin pump (64% vs. 69-77%) uptake, highest diabetes distress (2.2 vs. 1.8-2.1), and highest HbA1c levels (8.3% [67 mmol/ mol] vs. 7.2- 7.4% [ 55-57 mmol/ mol]).

CONCLUSIONS Efforts to increase device use need to target physical barriers to wearing devices. Because young adults had the lowest device uptake rates, highest distress, and highest HbA1c compared with older age- groups, they should be the focus of future interventions to increase device use.

C1 [Tanenbaum, Molly L.; Hanes, Sarah J.; Bensen, Rachel; Hood, Korey K.] Stanford Univ, Sch Med, Dept Pediat, Palo Alto, CA 94304 USA.

[Miller, Kellee M.] Jaeb Ctr Hlth Res, Tampa, FL USA.

[Naranjo, Diana] Stanford Univ, Sch Med, Dept Psychiat & Behav Sci, Palo Alto, CA 94304 USA.

RP Hood, KK (reprint author), Stanford Univ, Sch Med, Dept Pediat, Palo Alto, CA 94304 USA.

EM kkhood@stanford.edu

FU Leona M. and Harry B. Helmsley Charitable Trust

FX This study was made possible with the support of The Leona M. and Harry

B. Helmsley Charitable Trust.

CR Amer Assoc Diabetes Educ, 2003, DIABETES EDUCATOR, V29, P97

[Anonymous], 2016, THE DIABETES TIMES

Barnard KD, 2015, DIABETES TECHNOL THE, V17, P311, DOI 10.1089/dia.2014.0316

BENJAMINI Y, 1995, J R STAT SOC B, V57, P289

Bode BW, 2002, DIABETES-METAB RES, V18, pS14, DOI 10.1002/dmrr.205

Bruttomesso D, 2009, DIABETES-METAB RES, V25, P99, DOI 10.1002/dmrr.931

Cortina S, 2010, PEDIATR DIABETES, V11, P337, DOI 10.1111/j.1399-5448.2009.00593.x

de Vries L, 2011, PEDIATR DIABETES, V12, P506, DOI 10.1111/j.1399-5448.2010.00701.x

Deis D, 2006, DIABETES CARE, V29, P2730, DOI 10.2337/dc06-1134

Fisher L, 2015, J DIABETES COMPLICAT, V29, P572, DOI 10.1016/j.jdiacomp.2015.01.012

Floyd BD, 2017, PEDIATR DIABETES, V18, P204, DOI 10.1111/pedi.12373

Garvey K, 2011, DIABETES SPECTRUM, V24, P22, DOI [10.2337/diaspect.24.1.22, DOI 10.2337/DIASPECT.24.1.22]

Garvey KC, 2012, CURR DIABETES REP, V12, P533, DOI 10.1007/s11892-012-0311-6

Hermanides J, 2011, DIABETES CARE, V34, pS197, DOI 10.2337/dc11-s219

Hill-Briggs F, 2007, DIABETES EDUCATOR, V33, P1032, DOI 10.1177/0145721707308412

HILLBRIGGS F, 2007, DIABETES ED, V33, P1051

Klonoff DC, 2005, DIABETES CARE, V28, P1231, DOI 10.2337/diacare.28.5.1231

Liberman A, 2014, DIABETES TECHNOL THE, V16, pS110, DOI 10.1089/dia.2014.1513

Miller KM, 2015, DIABETES CARE, V38, P971, DOI 10.2337/dc15-0078

Modi AC, 2012, PEDIATRICS, V129, pE473, DOI 10.1542/peds.2011-1635

Norgaard K, 2013, DIABETES TECHNOL THE, V15, P273, DOI 10.1089/dia.2012.0288

Peters A, 2011, DIABETES CARE, V34, P2477, DOI 10.2337/dc11-1723

Phillip M, 2007, DIABETES CARE, V30, P1653, DOI 10.2337/dc07-9922

PICKUP JC, 1978, BRIT MED J, V1, P204, DOI 10.1136/bmj.1.6107.204

Raymond Jennifer K, 2015, Diabetes Spectr, V28, P68, DOI 10.2337/diaspect.28.1.68

Ritholz MD, 2007, DIABETES CARE, V30, P549, DOI 10.2337/dc06-1755

Ronsin O, 2005, DIABETES METAB, V31, P90, DOI 10.1016/S1262-3636(07)70173-8

Rubin Richard R, 2009, J Diabetes Sci Technol, V3, P1402

Scheiner G., 2009, DIABETES ED S2, V35, p42S

Scheiner G., 2009, DIABETES ED S2, V35, p28S

Scheiner G, 2009, DIABETES EDUCATOR, V35, p29S, DOI 10.1177/0145721709333493

Shalitin S, 2010, DIABETIC MED, V27, P339, DOI 10.1111/j.1464-5491.2009.02925.x

Tamborlane WV, 2008, NEW ENGL J MED, V359, P1464, DOI 10.1056/NEJMoa0805017

TAMBORLANE WV, 1979, NEW ENGL J MED, V300, P573, DOI 10.1056/NEJM197903153001101

Weissberg-Benchell J, 2007, DIABETES CARE, V30, P2441, DOI 10.2337/dc07-1249

Wong JC, 2014, DIABETES CARE, V37, P2702, DOI 10.2337/dc14-0303

NR 36

TC 18

Z9 19

U1 0

U2 4

PU AMER DIABETES ASSOC

PI ALEXANDRIA

PA 1701 N BEAUREGARD ST, ALEXANDRIA, VA 22311-1717 USA

SN 0149-5992

EI 1935-5548

J9 DIABETES CARE

JI Diabetes Care

PD FEB

PY 2017

VL 40

IS 2

BP 181

EP 187

DI 10.2337/dc16-1536

PG 7

WC Endocrinology & Metabolism

SC Endocrinology & Metabolism

GA EL2ZL

UT WOS:000394489300015

PM 27899489

OA Bronze

DA 2019-01-08

ER

PT J

AU Rumburg, TM

Lord, JH

Savin, KL

Jaser, SS

AF Rumburg, Tamara M.

Lord, Jadienne H.

Savin, Kimberly L.

Jaser, Sarah S.

TI Maternal diabetes distress is linked to maternal depressive symptoms and

adolescents' glycemic control

SO PEDIATRIC DIABETES

LA English

DT Article

DE adolescents; diabetes distress; family functioning; parenting; type 1

diabetes

ID TYPE-1; VALIDITY; SCALE; YOUTH

AB Objective: Mothers of youth with type 1 diabetes experience increased levels of stress and depression related to the burden of diabetes management, but the concept of diabetes distress, or distress linked specifically to diabetes and its management, has not been applied to mothers. The current study examined diabetes distress in relation to maternal depressive symptoms and adolescents' glycemic control.

Research Design and Methods: Mothers of youth with type 1 diabetes (age: 10-16) completed a measure of depressive symptoms and diabetes distress as part of a screening questionnaire. Adolescents' glycosylated hemoglobin (HbA1c) was obtained from medical records.

Results: Mothers' diabetes distress was strongly related to maternal depressive symptoms, and relationship-related diabetes distress was significantly associated with adolescents' HbA1c. In multivariate analyses, maternal depression was the only significant predictor of glycemic control.

Conclusions: Given the links between mothers' diabetes distress, maternal depressive symptoms, and adolescents' glycemic control, diabetes distress may be important to consider when targeting both maternal and adolescent adjustment to type 1 diabetes.

C1 [Rumburg, Tamara M.; Lord, Jadienne H.; Savin, Kimberly L.; Jaser, Sarah S.] Vanderbilt Univ, Dept Pediat, 2200 Childrens Way, DOT 11136, Nashville, TN 37232 USA.

RP Jaser, SS (reprint author), Vanderbilt Univ, Dept Pediat, 2200 Childrens Way, DOT 11136, Nashville, TN 37232 USA.

EM sarah.jaser@vanderbilt.edu

OI Savin, Kimberly/0000-0003-1658-1228

FU Center for Diabetes Translation Research from the National Institute of

Diabetes and Digestive and Kidney Diseases [P30 DK092986]

FX Funding for this study was provided by the Center for Diabetes

Translation Research (P30 DK092986) from the National Institute of

Diabetes and Digestive and Kidney Diseases.

CR American Diabetes, 2015, CLIN DIABETES, V33, P97, DOI DOI 10.2337/DIACLIN.33.2.97

Fisher L, 2013, DIABETES CARE, V36, P2551, DOI 10.2337/dc12-2493

Fisher L, 2012, DIABETES CARE, V35, P259, DOI 10.2337/dc11-1572

Fisher L, 2010, DIABETES CARE, V33, P23, DOI 10.2337/dc09-1238

Gonzalez JS, 2011, DIABETES CARE, V34, P236, DOI 10.2337/dc10-1970

Hessler D., 2012, SCI SESS AM DIAB ASS

Kroenke K, 2001, J GEN INTERN MED, V16, P606, DOI 10.1046/j.1525-1497.2001.016009606.x

Maas-van Schaaijk NM, 2013, J PEDIATR PSYCHOL, V38, P30, DOI 10.1093/jpepsy/jss096

Mackey ER, 2014, HEALTH PSYCHOL, V33, P783, DOI 10.1037/hea0000066

Polonsky WH, 2005, DIABETES CARE, V28, P626, DOI 10.2337/diacare.28.3.626

Weissberg-Benchell J, 2011, PEDIATR DIABETES, V12, P341, DOI 10.1111/j.1399-5448.2010.00720.x

Whittemore R, 2012, DIABETES EDUCATOR, V38, P562, DOI 10.1177/0145721712445216

Wood JR, 2013, DIABETES CARE, V36, P2035, DOI 10.2337/dc12-1959

Zamora I., 2014, AUTISM RES TREAT, V2014

NR 14

TC 4

Z9 4

U1 2

U2 3

PU WILEY-BLACKWELL

PI HOBOKEN

PA 111 RIVER ST, HOBOKEN 07030-5774, NJ USA

SN 1399-543X

EI 1399-5448

J9 PEDIATR DIABETES

JI Pediatr. Diabetes

PD FEB

PY 2017

VL 18

IS 1

BP 67

EP 70

DI 10.1111/pedi.12350

PG 4

WC Endocrinology & Metabolism; Pediatrics

SC Endocrinology & Metabolism; Pediatrics

GA EK4OY

UT WOS:000393907700009

PM 26712240

OA Green Accepted

DA 2019-01-08

ER

PT J

AU Polonsky, WH

Fisher, L

Hessler, D

Edelman, SV

AF Polonsky, William H.

Fisher, Lawrence

Hessler, Danielle

Edelman, Steven V.

TI Investigating Hypoglycemic Confidence in Type 1 and Type 2 Diabetes

SO DIABETES TECHNOLOGY & THERAPEUTICS

LA English

DT Article

DE Hypoglycemia; Confidence; Type 1 diabetes; Type 2 diabetes

ID ADULTS; FEAR; DISTRESS; IMPACT

AB Background: While research designed to understand the concerns about hypoglycemia among patients with diabetes has been primarily focused on the investigation of fear and anxiety, recent evidence suggests a potentially important and conceptually distinct element-hypoglycemic confidence. To investigate this dimension, we developed the Hypoglycemic Confidence Scale (HCS); herein, we describe the construction and validation of the HCS and examine how key patient factors are associated with hypoglycemic confidence.

Methods: Items were developed from interviews with six type 1 diabetes (T1D) adults, six insulin-using type 2 diabetes (T2D) adults, and seven diabetes healthcare professionals, resulting in nine self-report items. Separate exploratory factor analyses (EFAs) were conducted with T1D adults (N=326), with T2D adults using both basal and prandial insulins (T2D-BP, N=145) and with T2D adults using only basal insulin (T2D-BO, N=82). Construct validity for the HCS was established with overall well-being (World Health Organization-5), diabetes distress (Diabetes Distress Scale), global anxiety (Generalized Anxiety Disorder), hypoglycemic fear (Hypoglycemic Fear Survey-II), and glycemic control (self-reported A1C). Hierarchical regression analyses examined the unique contribution of HCS scores, independent of hypoglycemic fear, on key psychosocial constructs and A1C.

Results: EFAs of the nine HCS items yielded a single factor solution for each of the three subject samples, accounting for 50.8%, 65.1%, and 73.7% of the variance for the T1D, T2D-BP, and T2D-BO groups, respectively. Construct validity was established by significant correlations with criterion variables. The HCS was associated with well-being and diabetes distress in the T1D (in both cases, P < 0.001) and T2D-BP groups (in both cases, P < .05) and for self-reported A1C in the T2D-BP group (P < .05) independent of hypoglycemic fear.

Conclusions: Hypoglycemic confidence is a unique dimension of patient experience, different from hypoglycemic fear, and is deserving of further study. The HCS is a reliable valid measure of hypoglycemic confidence for adults with T1D and insulin-using T2D.

C1 [Polonsky, William H.] Univ Calif San Diego, Dept Psychiat, San Diego, CA 92103 USA.

[Polonsky, William H.] Behav Diabet Inst, San Diego, CA USA.

[Fisher, Lawrence; Hessler, Danielle] Univ Calif San Francisco, Dept Family & Community Med, San Francisco, CA 94143 USA.

[Edelman, Steven V.] Univ Calif San Diego, Div Endocrinol & Metab, San Diego, CA 92103 USA.

[Edelman, Steven V.] Vet Affairs Med Ctr, San Diego, CA 92161 USA.

RP Polonsky, WH (reprint author), POB 2148, Del Mar, CA 92014 USA.

EM whp@behavioraldiabetes.org

FU Dexcom

FX This investigator-initiated study was supported by Dexcom.

CR COX DJ, 1987, DIABETES CARE, V10, P617, DOI 10.2337/diacare.10.5.617

Domgaard M, 2015, POSTGRAD MED, V127, P438, DOI 10.1080/00325481.2015.1045815

Fidler Carrie, 2011, J Med Econ, V14, P646, DOI 10.3111/13696998.2011.610852

Fisher L, 2015, J DIABETES COMPLICAT, V29, P572, DOI 10.1016/j.jdiacomp.2015.01.012

Gonder-Frederick L., 2013, DIABETIC HYPOGLYCEMI, V5, P3

Hajos TRS, 2013, DIABETIC MED, V30, pE63, DOI 10.1111/dme.12040

Irvine A, 1994, HDB PSYCHOL DIABETES

Polonsky WH, 2015, J DIABETES COMPLICAT, V29, P1171, DOI 10.1016/j.jdiacomp.2015.08.002

Polonsky WH, 2005, DIABETES CARE, V28, P626, DOI 10.2337/diacare.28.3.626

Sheeran P, 2016, HEALTH PSYCHOL, V35, P1178, DOI 10.1037/hea0000387

Spitzer RL, 2006, ARCH INTERN MED, V166, P1092, DOI 10.1001/archinte.166.10.1092

Wild D, 2007, PATIENT EDUC COUNS, V68, P10, DOI 10.1016/j.pec.2007.05.003

Wong ND, 2013, DIABETES VASC DIS RE, V10, P505, DOI 10.1177/1479164113496828

NR 13

TC 6

Z9 6

U1 0

U2 3

PU MARY ANN LIEBERT, INC

PI NEW ROCHELLE

PA 140 HUGUENOT STREET, 3RD FL, NEW ROCHELLE, NY 10801 USA

SN 1520-9156

EI 1557-8593

J9 DIABETES TECHNOL THE

JI Diabetes Technol. Ther.

PD FEB

PY 2017

VL 19

IS 2

BP 131

EP 136

DI 10.1089/dia.2016.0366

PG 6

WC Endocrinology & Metabolism

SC Endocrinology & Metabolism

GA EI9JG

UT WOS:000392823500012

PM 27997217

DA 2019-01-08

ER

PT J

AU Shin, JK

Poltavskiy, E

Kim, TN

Hasan, A

Bang, HJ

AF Shin, Jinah K.

Poltavskiy, Eduard

Kim, Tae Nyun

Hasan, Abdullah

Bang, Heejung

TI Help-seeking behaviors for serious psychological distress among

individuals with diabetes mellitus: The California Health Interview

Survey, 2011-2012

SO PRIMARY CARE DIABETES

LA English

DT Article

DE Diabetes mellitus; Help-seeking behaviors; Perceived needs; Serious

psychological distress

ID COMORBID DEPRESSION; PERCEIVED NEED; SUICIDAL IDEATION; ADULTS; CARE;

PREVALENCE; METAANALYSIS; ASSOCIATION; POPULATION; SERVICES

AB Aim: To investigate patterns of and factors associated with help-seeking behaviors among individuals with diabetes mellitus (DM) and/or serious psychological distress (SPD).

Methods: The analysis was conducted with the California Health Interview Survey (CHIS) 2011-2012 of 40,803 adults. Logistic regression was used to examine the associations between the multiple facilitating/preventing factors and outcome, guided by the Andersen's Health Care Utilization Model.

Results: The prevalence of DM and SPD were 10.9% and 3.4%, respectively, among participants in the survey. The participants with DM were more likely to experience SPD than those without DM (OR 1.46, 95% CI = 1.11-1.91, p = 0.006). Participants with combined DM and SPD, the most underserved, were less likely to perceive the need for mental health services and less likely to seek help, compared to those with only SPD. Need factors (SPD status and perceived need) were significantly associated with help-seeking behaviors for mental health, along with predisposing factors (age, gender, obesity, race, and employment), and enabling factors (insurance, English proficiency).

Conclusions: Perceptions about need for seeking help seem to play an important role in receiving mental health services in addition to other predisposing or enabling factors. Identification of these factors may improve clinical outcomes related to DM and SPD. (C) 2016 Primary Care Diabetes Europe. Published by Elsevier Ltd. All rights reserved.

C1 [Shin, Jinah K.; Hasan, Abdullah] Elmhurst Hosp New York, Dept Psychiat, 79-01 Broadway, Elmhurst, NY 11373 USA.

[Poltavskiy, Eduard] Univ Calif Davis, Sch Med, Dept Publ Hlth Sci, Grad Grp Epidemiol, Davis, CA 95616 USA.

[Kim, Tae Nyun] Inje Univ, Haeundae Paik Hosp, Sch Med, Dept Internal Med,Div Endocrinol & Metab, Busan, South Korea.

[Bang, Heejung] Univ Calif Davis, Sch Med, Dept Publ Hlth Sci, Div Biostat, Davis, CA 95616 USA.

EM jshin124@hotmail.com

FU National Center for Advancing Translational Sciences, National

Institutes of Health [UL1 TR000002]

FX H. Bang was partly supported by the National Center for Advancing

Translational Sciences, National Institutes of Health, through grant

number UL1 TR000002. The authors declare that they have no conflict of

interest.

CR Ali S, 2006, DIABETIC MED, V23, P1165, DOI 10.1111/j.1464-5491.2006.01943.x

ANDERSEN RM, 1995, J HEALTH SOC BEHAV, V36, P1, DOI 10.2307/2137284

Andersen RM, 2001, CHANGING US HLTH CAR, P3

Anderson RJ, 2001, DIABETES CARE, V24, P1069, DOI 10.2337/diacare.24.6.1069

California Health Interview Survey, 2014, CHIS 2011 2012 METH

Chung JH, 2014, J PSYCHOSOM RES, V77, P457, DOI 10.1016/j.jpsychores.2014.08.008

Codony M, 2009, PSYCHIAT SERV, V60, P1051, DOI 10.1176/appi.ps.60.8.1051

de Groot M, 2001, PSYCHOSOM MED, V63, P619, DOI 10.1097/00006842-200107000-00015

Dhingra SS, 2010, PSYCHIAT SERV, V61, P524, DOI 10.1176/appi.ps.61.5.524

Dismuke CE, 2011, GEN HOSP PSYCHIAT, V33, P311, DOI 10.1016/j.genhosppsych.2011.03.014

Edlund MJ, 2006, SOC PSYCH PSYCH EPID, V41, P480, DOI 10.1007/s00127-006-0047-1

Egede LE, 2002, DIABETES CARE, V25, P464, DOI 10.2337/diacare.25.3.464

Egede LE, 2010, DIABETES RES CLIN PR, V87, P302, DOI 10.1016/j.diabres.2010.01.024

Grigsby AB, 2002, J PSYCHOSOM RES, V53, P1053, DOI 10.1016/S0022-3999(02)00417-8

Han SJ, 2013, DIABETES RES CLIN PR, V101, pE14, DOI 10.1016/j.diabres.2013.06.012

Hochhausen L., 2009, COMMUNITY MENT HLTH, V47, P14

Huang CJ, 2011, GEN HOSP PSYCHIAT, V33, P8, DOI 10.1016/j.genhosppsych.2010.10.008

Katon WJ, 2005, DIABETES CARE, V28, P2668, DOI 10.2337/diacare.28.11.2668

Kessler RC, 2003, ARCH GEN PSYCHIAT, V60, P184, DOI 10.1001/archpsyc.60.2.184

Kessler RC, 2002, PSYCHOL MED, V32, P959, DOI 10.1017/S0033291702006074

Li C, 2007, DIABETES CARE, V33, P1061, DOI DOI 10.2337/DC09-1515]

Li CY, 2009, INT J PUBLIC HEALTH, V54, P43, DOI 10.1007/s00038-009-0006-1

Meadows G, 2002, PSYCHOL MED, V32, P299, DOI 10.1017/S0033291701004913

Mechanic D, 2002, HEALTH AFFAIR, V21, P137, DOI 10.1377/hlthaff.21.3.137

Mojtabai R, 2002, ARCH GEN PSYCHIAT, V59, P77, DOI 10.1001/archpsyc.59.1.77

Shin JK, 2012, DIABETES RES CLIN PR, V95, P406, DOI 10.1016/j.diabres.2011.10.043

Subramaniam M, 2009, GEN HOSP PSYCHIAT, V31, P220, DOI 10.1016/j.genhosppsych.2009.01.001

Surood S, 2010, CAN J PUBLIC HEALTH, V101, P176

Vamos EP, 2009, PSYCHOSOM MED, V71, P501, DOI 10.1097/PSY.0b013e3181a5a7ad

Zhang XP, 2005, AM J EPIDEMIOL, V161, P652, DOI 10.1093/aje/kwi089

NR 30

TC 0

Z9 0

U1 1

U2 7

PU ELSEVIER SCI LTD

PI OXFORD

PA THE BOULEVARD, LANGFORD LANE, KIDLINGTON, OXFORD OX5 1GB, OXON, ENGLAND

SN 1751-9918

EI 1878-0210

J9 PRIM CARE DIABETES

JI Prim. Care Diabetes

PD FEB

PY 2017

VL 11

IS 1

BP 63

EP 70

DI 10.1016/j.pcd.2016.07.007

PG 8

WC Endocrinology & Metabolism; Primary Health Care

SC Endocrinology & Metabolism; General & Internal Medicine

GA EI7LK

UT WOS:000392679200009

PM 27492797

OA Green Accepted

DA 2019-01-08

ER

PT J

AU Huang, SY

Huang, WC

Chen, YC

Tsai, CY

Lee, IK

AF Huang, Shi-Yu

Huang, Wen-Chi

Chen, Yi-Chun

Tsai, Ching-Yen

Lee, Ing-Kit

TI Increased Mortality in Seasonal H3N2 Patients Compared with those with

Pandemic 2009 H1N1 in Taiwan, 2009-2010

SO AMERICAN JOURNAL OF TROPICAL MEDICINE AND HYGIENE

LA English

DT Article

ID VIRUS-INFECTION; INFLUENZA INFECTION; ADULT PATIENTS; CALIFORNIA;

THERAPY; HUMANS

AB We conducted a retrospective study to compare clinical and laboratory findings between 1) severe influenza A and mild influenza A and 2) pandemic 2009 H1N1 (pdm09 A/H1) and seasonal H3N2 (A/H3) from 2009 to 2010. A total of 526 (mean age, 13.6 years; 447 pdm09 A/H1, 79 seasonal A/H3) patients were included, 41 (7.8%) with severe influenza (mean age, 28.1 years; 26 pdm09 A/H1, 15 seasonal A/H3). Influenza-associated complications were pneumonia (75.6%), meningoencephalitis (14.6%), acute kidney injury (14.6%), and acute respiratory distress syndrome (12.2%). Patients with seasonal A/H3 were significantly less likely to experience sore throat (P < 0.001), malaise (P < 0.001), and muscle pain (P < 0.001); they were significantly more likely to have hypertension (P < 0.001), diabetes mellitus (P = 0.001), and chronic obstructive pulmonary disease (P < 0.001), delayed hospital presentation (P = 0.001), delayed oseltamivir treatment (P < 0.001), and higher in-hospital mortality (P = 0.02) than patients with pdm09 A/H1. Further comparison between severe pdm09 A/H1 and severe seasonal A/H3 revealed that severe seasonal A/H3 patients (median age, 71 years) were significantly older than patients with severe pdm09 A/H1 (median age, 7 years) (P < 0.001). Comparison between severe influenza and mild influenza, regardless of influenza A subtypes, by multivariate analysis, found that tachypnea (odds ratio [OR] = 44.3,95% confidence interval [CI] = 15.7-124.6) and delayed oseltamivir therapy >= 48 hours after illness onset (OR = 3.7, 95% CI = 1.3-10.5) were independent risk factors for severe influenza. The findings of this study will improve the understanding of the clinical differences between pdm09 A/H1 and seasonal A/H3, and of influenza-associated complications and predictors for severe outcomes that can help to direct clinicians toward the most effective management of influenza patients to reduce the preventable mortality and morbidity.

C1 [Huang, Shi-Yu] Kaohsiung Chang Gung Mem Hosp, Dept Emergency Med, Kaohsiung, Taiwan.

[Huang, Wen-Chi; Chen, Yi-Chun; Tsai, Ching-Yen; Lee, Ing-Kit] Kaohsiung Chang Gung Mem Hosp, Dept Internal Med, Div Infect Dis, Kaohsiung 833, Taiwan.

[Lee, Ing-Kit] Chang Gung Univ, Coll Med, Taoyuan, Taiwan.

RP Lee, IK (reprint author), Kaohsiung Chang Gung Mem Hosp, Dept Internal Med, Div Infect Dis, Kaohsiung 833, Taiwan.

EM sarawah@ms28.hinet.net; heteyland@cgmh.org.tw; sonice83@cgmh.org.tw;

greenswallow822@yahoo.com.tw; leee@cgmh.org.tw

FU Kaohsiung Chang Gung Memorial Hospital, Kaohsiung, Taiwan [CMRPG8E0691]

FX This work was partly supported by a grant from the Kaohsiung Chang Gung

Memorial Hospital (CMRPG8E0691), Kaohsiung, Taiwan.

CR [Anonymous], 2009, Wkly Epidemiol Rec, V84, P185

Bautista E, 2010, NEW ENGL J MED, V362, P1708, DOI 10.1056/NEJMra1000449

Centers for Disease Control and Prevention (CDC), 2009, MMWR Morb Mortal Wkly Rep, V58, P467

Centers for Disease Control and Prevention (CDC), 2009, MMWR Morb Mortal Wkly Rep, V58, P536

Chan KH, 2008, J CLIN VIROL, V42, P65, DOI 10.1016/j.jcv.2007.12.003

Chang YS, 2010, MED J AUSTRALIA, V192, P90

Chowell G, 2009, NEW ENGL J MED, V361, P674, DOI 10.1056/NEJMoa0904023

Dawood FS, 2009, NEW ENGL J MED, V360, P2605, DOI 10.1056/NEJMoa0903810

Finkelman BS, 2007, PLOS ONE, V2, DOI 10.1371/journal.pone.0001296

Glaser CA, 2012, CLIN INFECT DIS, V55, P514, DOI 10.1093/cid/cis454

Gordon A, 2010, CLIN INFECT DIS, V50, P1462, DOI 10.1086/652647

Kelly HA, 2009, MED J AUSTRALIA, V191, P146

Lee IK, 2012, INFLUENZA OTHER RESP, V6, pe152, DOI 10.1111/j.1750-2659.2012.00410.x

Lemaitre M, 2012, PLOS ONE, V7, DOI 10.1371/journal.pone.0045051

Ling LM, 2010, CLIN INFECT DIS, V50, P963, DOI 10.1086/651083

Martin S, 2017, FRONT MED, V4, DOI 10.3389/fmed.2017.00020

Mauad T, 2010, AM J RESP CRIT CARE, V181, P72, DOI 10.1164/rccm.200909-1420OC

McGeer A, 2007, CLIN INFECT DIS, V45, P1568, DOI 10.1086/523584

Miller MA, 2009, NEW ENGL J MED, V360, P2595, DOI 10.1056/NEJMp0903906

Monto AS, 2009, CLIN INFECT DIS, V48, pS20, DOI 10.1086/591853

Monto AS, 2000, ARCH INTERN MED, V160, P3243, DOI 10.1001/archinte.160.21.3243

Opal SM, 2005, CLIN INFECT DIS, V41, pS504, DOI 10.1086/432007

Simonsen L, 2013, PLOS MED, V10, DOI 10.1371/journal.pmed.1001558

Sivadon-Tardy V, 2009, CLIN INFECT DIS, V48, P48, DOI 10.1086/594124

STANLEY TV, 1994, NEW ZEAL MED J, V107, P382

Taiwan CDC, 2009, GLOB INFL A H1N1 PAN

Tang JWT, 2010, ARCH INTERN MED, V170, P861, DOI 10.1001/archinternmed.2010.108

Thompson WW, 2003, JAMA-J AM MED ASSOC, V289, P179, DOI 10.1001/jama.289.2.179

Venkatesan A, 2013, CLIN INFECT DIS, V57, P1114, DOI 10.1093/cid/cit458

WELLS DL, 1991, JAMA-J AM MED ASSOC, V265, P478, DOI 10.1001/jama.265.4.478

*WHO, 2009, INFL LIK ILLN US MEX

World Health Organization, 2009, CDC PROT REAL TIM RT

World Health Organization, INFL UPD

World Health Organization, 2010, PAND H1N1 2009 UPD 1

World Health Organization, 2009, CLIN MAN HUM INF PAN

NR 35

TC 0

Z9 0

U1 1

U2 1

PU AMER SOC TROP MED & HYGIENE

PI MCLEAN

PA 8000 WESTPARK DR, STE 130, MCLEAN, VA 22101 USA

SN 0002-9637

EI 1476-1645

J9 AM J TROP MED HYG

JI Am. J. Trop. Med. Hyg.

PY 2017

VL 97

IS 6

BP 1945

EP 1951

DI 10.4269/ajtmh.17-0172

PG 7

WC Public, Environmental & Occupational Health; Tropical Medicine

SC Public, Environmental & Occupational Health; Tropical Medicine

GA FT5ZI

UT WOS:000423231800056

PM 29016325

DA 2019-01-08

ER

PT J

AU Alam, U

Fawwad, A

Shaheen, F

Tahir, B

Basit, A

Malik, RA

AF Alam, Uazman

Fawwad, Asher

Shaheen, Fariha

Tahir, Bilal

Basit, Abdul

Malik, Rayaz A.

TI Improvement in Neuropathy Specific Quality of Life in Patients with

Diabetes after Vitamin D Supplementation

SO JOURNAL OF DIABETES RESEARCH

LA English

DT Article

ID PERIPHERAL NEUROPATHY; D DEFICIENCY; PAIN; ASSOCIATION; PREVALENCE;

PREGABALIN; MELLITUS; HEALTH; SLEEP; RISK

AB Objective. To assess the effect of vitamin D supplementation on neuropathy specific quality of life (NeuroQoL) in patients with painful diabetic neuropathy. Methods. This prospective, open label study was conducted between June 2012 and April 2013. Patients with symptomatic diabetic neuropathy were given a single dose of 600,000 IU intramuscular vitamin D, and NeuroQol was assessed at baseline and at five follow-up visits every 4 weeks. Results. Of 143 participants, 41.3% were vitamin D deficient (vitamin D < 20 ng/ml). Treatment with vitamin D resulted in a significant increase in 25(OH)D (P < 0.0001) and a significant improvement in the NeuroQoL subscale score for emotional distress (P = 0.04), with no significant change in the other NeuroQoL domains of painful symptoms and paresthesia, loss of temperature and touch sensation, unsteadiness, limitation in daily activities, and interpersonal problems. There was a significant reduction in patient perception about foot problems on QoL of "quite a lot" (P < 0.05) and "very much" (P < 0.0001) with a significant reduction in the baseline response of having a "poor" QoL from 5.2% to 0.7% (P < 0.0001) and an increase in the response of an "excellent QoL" from 1.5% to 7.4% (P < 0.0001). Conclusion. Vitamin D is effective in improving quality of life in patients with painful diabetic neuropathy.

C1 [Alam, Uazman] Univ Liverpool, Dept Eye & Vis Sci, Inst Ageing & Chron Dis, Liverpool, Merseyside, England.

[Alam, Uazman] Aintree Univ Hosp NHS Fdn Trust, Liverpool, Merseyside, England.

[Alam, Uazman] Univ Manchester, Div Diabet Endocrinol & Gastroenterol, Sch Med Sci, Manchester, Lancs, England.

[Fawwad, Asher; Shaheen, Fariha; Tahir, Bilal; Basit, Abdul] Baqai Inst Diabetol & Endocrinol, Karachi, Pakistan.

[Fawwad, Asher] Baqai Med Univ, Karachi, Pakistan.

[Malik, Rayaz A.] Weill Cornell Med Qatar, Doha, Qatar.

[Malik, Rayaz A.] Univ Manchester, Inst Cardiovasc Sci, Manchester, Lancs, England.

[Malik, Rayaz A.] Cent Manchester Hosp Fdn Trust, Manchester, Lancs, England.

[Malik, Rayaz A.] Manchester Metropolitan Univ, Manchester, Lancs, England.

RP Malik, RA (reprint author), Weill Cornell Med Qatar, Doha, Qatar.; Malik, RA (reprint author), Univ Manchester, Inst Cardiovasc Sci, Manchester, Lancs, England.; Malik, RA (reprint author), Cent Manchester Hosp Fdn Trust, Manchester, Lancs, England.; Malik, RA (reprint author), Manchester Metropolitan Univ, Manchester, Lancs, England.

EM ram2045@qatar-med.cornell.edu

OI Malik, Rayaz/0000-0002-7188-8903; Alam, Uazman/0000-0002-3190-1122

CR Abbott CA, 2011, DIABETES CARE, V34, P2220, DOI 10.2337/dc11-1108

Alam U, 2012, DIABETIC MED, V29, P1343, DOI 10.1111/j.1464-5491.2012.03692.x

Alam U., 2016, J DIABETES RES, V2016, DOI DOI 10.1155/2016/6156217.NUTRES.2015.11.008

Alam U, 2016, DIABETES THER, V7, P11, DOI 10.1007/s13300-016-0159-x

Alam U, 2014, J DIABETES COMPLICAT, V28, P66, DOI 10.1016/j.jdiacomp.2013.09.003

Ali A, 2013, PAK J MED SCI, V29, P899, DOI 10.12669/pjms.294.3704

Almurdhi MM, 2016, DIABETES CARE, V39, P441, DOI 10.2337/dc15-0995

Arabi A, 2010, NAT REV ENDOCRINOL, V6, P550, DOI 10.1038/nrendo.2010.146

Backonja M, 1998, JAMA-J AM MED ASSOC, V280, P1831, DOI 10.1001/jama.280.21.1831

Basit A, 2016, BMJ OPEN DIAB RES CA, V4, DOI 10.1136/bmjdrc-2015-000148

Benbow SJ, 1998, QJM-MON J ASSOC PHYS, V91, P733, DOI 10.1093/qjmed/91.11.733

Bondor C. I., 2016, J DIABETES RES, V2016, DOI DOI 10.1155/2016/5439521

Boyd Amanda, 2011, J Diabetes Sci Technol, V5, P714

Boyle J, 2012, DIABETES CARE, V35, P2451, DOI 10.2337/dc12-0656

Celikbilek A, 2015, ACTA NEUROL BELG, V115, P47, DOI 10.1007/s13760-014-0304-0

Galer BS, 2000, DIABETES RES CLIN PR, V47, P123, DOI 10.1016/S0168-8227(99)00112-6

Gore M, 2005, J PAIN SYMPTOM MANAG, V30, P374, DOI 10.1016/j.jpainsymman.2005.04.009

He R, 2017, DIABETES-METAB RES, V33, DOI 10.1002/dmrr.2820

Huang W, 2013, CLIN J PAIN, V29, P341, DOI 10.1097/AJP.0b013e318255655d

Humble MB, 2010, J PHOTOCH PHOTOBIO B, V101, P142, DOI 10.1016/j.jphotobiol.2010.08.003

Jawaid SA, 2006, PAK J MED SCI, V22, P97

Lee P, 2008, ARCH INTERN MED, V168, P771, DOI 10.1001/archinte.168.7.771

Lv WS, 2015, J ENDOCRINOL INVEST, V38, P513, DOI 10.1007/s40618-014-0210-6

Marchettini P, 2016, EUR J PAIN, V20, P472, DOI 10.1002/ejp.763

Moore A, 2014, JAMA-J AM MED ASSOC, V312, P182, DOI 10.1001/jama.2014.6336

Rubin RR, 1999, DIABETES-METAB RES, V15, P205, DOI 10.1002/(SICI)1520-7560(199905/06)15:3<205::AID-DMRR29>3.0.CO;2-O

Sadosky A, 2013, DIABETES METAB SYNDR, V6, P79, DOI 10.2147/DMSO.S37415

Shehab D, 2015, MED PRIN PRACT, V24, P250, DOI 10.1159/000375304

Shehab D, 2012, DIABETIC MED, V29, P43, DOI 10.1111/j.1464-5491.2011.03510.x

Sheikh A, 2012, PLOS ONE, V7, DOI 10.1371/journal.pone.0033452

Skalli S, 2012, EUR J INTERN MED, V23, pE67, DOI 10.1016/j.ejim.2011.11.008

Smith SC, 2012, DIABETES RES CLIN PR, V96, P261, DOI 10.1016/j.diabres.2011.11.013

Soderstrom LH, 2012, DIABETIC MED, V29, P50, DOI 10.1111/j.1464-5491.2011.03379.x

Van Acker K, 2009, DIABETES METAB, V35, P206, DOI 10.1016/j.diabet.2008.11.004

Veresiu AI, 2015, J DIABETES COMPLICAT, V29, P644, DOI 10.1016/j.jdiacomp.2015.04.001

Vileikyte L, 2005, DIABETES CARE, V28, P2378, DOI 10.2337/diacare.28.10.2378

Vileikyte L, 2003, DIABETES CARE, V26, P2549, DOI 10.2337/diacare.26.9.2549

Vinik A, 2013, CLIN THER, V35, P612, DOI 10.1016/j.clinthera.2013.03.008

Wang DD, 2014, PLOS ONE, V9, DOI 10.1371/journal.pone.0106935

NR 39

TC 0

Z9 0

U1 1

U2 2

PU HINDAWI LTD

PI LONDON

PA ADAM HOUSE, 3RD FLR, 1 FITZROY SQ, LONDON, W1T 5HF, ENGLAND

SN 2314-6745

EI 2314-6753

J9 J DIABETES RES

JI J. Diabetes Res.

PY 2017

AR 7928083

DI 10.1155/2017/7928083

PG 7

WC Endocrinology & Metabolism; Medicine, Research & Experimental

SC Endocrinology & Metabolism; Research & Experimental Medicine

GA FS0XD

UT WOS:000419496400001

PM 29445752

OA DOAJ Gold, Green Published

DA 2019-01-08

ER

PT J

AU Eilander, MMA

Snoek, FJ

Rotteveel, J

Aanstoot, HJ

Bakker-van Waarde, WM

Houdijk, ECAM

Nuboer, R

Winterdijk, P

de Wit, M

AF Eilander, Minke M. A.

Snoek, Frank J.

Rotteveel, Joost

Aanstoot, Henk-Jan

Bakker-van Waarde, Willie M.

Houdijk, Euphemia C. A. M.

Nuboer, Roos

Winterdijk, Per

de Wit, Maartje

TI Parental Diabetes Behaviors and Distress Are Related to Glycemic Control

in Youth with Type 1 Diabetes: Longitudinal Data from the DINO Study

SO JOURNAL OF DIABETES RESEARCH

LA English

DT Article

ID QUALITY-OF-LIFE; MATERNAL DEPRESSIVE SYMPTOMS; METABOLIC-CONTROL;

PSYCHOLOGICAL DISTRESS; ADOLESCENTS; CHILDREN; MOTHERS; CARE;

RESPONSIBILITY; DISCREPANCIES

AB Objective. To evaluate (1) the longitudinal relationship between parental well-being and glycemic control in youth with type 1 diabetes and (2) if youth's problem behavior, diabetes parenting behavior, and parental diabetes-distress influence this relationship. Research Design and Methods. Parents of youth 8-15 yrs (at baseline) (N = 174) participating in the DINO study completed questionnaires at three time waves (1 yr interval). Using generalized estimating equations, the relationship between parental well-being (WHO-5) and youth's HbA1c was examined. Second, relationships between WHO-5, Strength and Difficulties Questionnaire (SDQ), Diabetes Family Behavior Checklist (DFBC), Problem Areas In Diabetes-Parent Revised (PAID-Pr) scores, and HbA1c were analyzed. Results. Low well-being was reported by 32% of parents. No relationship was found between parents' WHO-5 scores and youth's HbA1c (beta = -0 052, p = 0 650). WHO-5 related to SDQ (beta = -0 219, p < 0 01), DFBC unsupportive scale (beta = -0 174, p < 0 01), and PAID-Pr (beta = -0 666, p < 0 01). Both DFBC scales (supportive beta = -0 259, p = 0 01; unsupportive beta = 0 383, p = 0 017), PAID-Pr (beta = 0 276, p < 0 01), and SDQ (beta = 0 424, p < 0 01) related to HbA1c.Conclusions. Over time, reduced parental well-being relates to increased problem behavior in youth, unsupportive parenting, and parental distress, which negatively associate with HbA1c. More unsupportive diabetes parenting and distress relate to youth's problem behavior.

C1 [Eilander, Minke M. A.; Snoek, Frank J.; de Wit, Maartje] Vrije Univ Amsterdam, Med Ctr, Dept Med Psychol, De Boelenlaan 1117, NL-1081 HV Amsterdam, Netherlands.

[Eilander, Minke M. A.; Snoek, Frank J.; Rotteveel, Joost; de Wit, Maartje] Amsterdam Publ Hlth Res Inst, Dept Epidemiol & Biostat, Boechorststr 7, NL-1081 BT Amsterdam, Netherlands.

[Eilander, Minke M. A.; Aanstoot, Henk-Jan; Winterdijk, Per] Diabeter, Ctr Pediat & Adolescent Diabet Care & Res, Blaak 6, NL-3011 TA Rotterdam, Netherlands.

[Snoek, Frank J.] Acad Med Ctr, Dept Med Psychol, Meibergdreef 9, NL-1100 DD Amsterdam, Netherlands.

[Rotteveel, Joost] Vrije Univ Amsterdam, Med Ctr, Dept Pediat, De Boelelaan 1118, NL-1081 HV Amsterdam, Netherlands.

[Bakker-van Waarde, Willie M.] Univ Groningen, Univ Med Ctr Groningen, Dept Pediat, Hanzepl 1, NL-9713 GZ Groningen, Netherlands.

[Houdijk, Euphemia C. A. M.] Juliana Childrens Hosp, Haga Hosp, Dept Pediat, Els Borst Eilerspl 275, NL-2545 AA The Hague, Netherlands.

[Nuboer, Roos] Meander Med Ctr, Dept Pediat, Maatweg 3, NL-3813 TZ Amersfoort, Netherlands.

RP Eilander, MMA (reprint author), Vrije Univ Amsterdam, Med Ctr, Dept Med Psychol, De Boelenlaan 1117, NL-1081 HV Amsterdam, Netherlands.; Eilander, MMA (reprint author), Amsterdam Publ Hlth Res Inst, Dept Epidemiol & Biostat, Boechorststr 7, NL-1081 BT Amsterdam, Netherlands.; Eilander, MMA (reprint author), Diabeter, Ctr Pediat & Adolescent Diabet Care & Res, Blaak 6, NL-3011 TA Rotterdam, Netherlands.

EM m.eilander@vumc.nl

FU Dutch Diabetes Foundation [2009.80.103]

FX Minke M. A. Eilander collected and researched the data, wrote the

manuscript, and is a guarantor of the work. Frank J. Snoek, Joost

Rotteveel, Henk-Jan Aanstoot, Willie M. Bakker-van Waarde, Euphemia C.

A. M. Houdijk, Per Winterdijk, and Maartje de Wit reviewed and edited

the manuscript. The authors would like to thank Professor Jos Twisk of

the Department of Epidemiology and Biostatistics of the VU University

Medical Center for his advisory role in the statistical analyses. This

work was supported by the Dutch Diabetes Foundation (Grant no.

2009.80.103).

CR ANDERSON BL, 1991, ADV CHILD HLTH PSYCH

Armstrong B, 2011, J PEDIATR PSYCHOL, V36, P1052, DOI 10.1093/jpepsy/jsr039

Bech P, 2003, INT J METH PSYCH RES, V12, P85, DOI 10.1002/mpr.145

CBS, 2017, WELZ REL GEZ LEEFST

De Wit M, 2008, DIABETES CARE, V31, P1521, DOI 10.2337/dc08-0394

Delamater AM, 2014, PEDIATR DIABETES, V15, P232, DOI 10.1111/pedi.12191

Driscoll KA, 2016, CURR DIABETES REP, V16, DOI 10.1007/s11892-016-0762-2

Duke DC, 2008, J PEDIATR PSYCHOL, V33, P719, DOI 10.1093/jpepsy/jsn012

Eccleston C, 2015, COCHRANE DB SYST REV, V4, DOI DOI 10.1002/14651858.CD009660.PUB3

Eilander M. M. A., 2016, J DIABETES RES, V2016

Eilander M, 2016, PEDIATR DIABETES, V17, P112, DOI 10.1111/pedi.12237

Eilander MMA, 2015, BMC PEDIATR, V15, DOI 10.1186/s12887-015-0400-1

Hannonen R, 2015, ACTA PAEDIATR, V104, P1144, DOI 10.1111/apa.13144

Hassan K, 2006, J PEDIATR-US, V149, P526, DOI 10.1016/j.jpeds.2006.05.039

Helgeson VS, 2009, J PEDIATR PSYCHOL, V34, P254, DOI 10.1093/jpepsy/jsn079

Hoey H, 2009, PEDIATR DIABETES, V10, P9, DOI 10.1111/j.1399-5448.2009.00609.x

Jaser SS, 2008, J PEDIATR PSYCHOL, V33, P509, DOI 10.1093/jpepsy/jsm104

Jaser SS, 2014, MATERN CHILD HLTH J, V18, P101, DOI 10.1007/s10995-013-1239-4

Laffel LMB, 2003, J PEDIATR-US, V142, P409, DOI 10.1067/mpd.2003.138

Law GU, 2013, J PSYCHOSOM RES, V74, P334, DOI 10.1016/j.jpsychores.2012.12.009

Lohan A, 2015, CHILD CARE HLTH DEV, V41, P803, DOI 10.1111/cch.12278

Lopez-Perez B, 2015, J EXP CHILD PSYCHOL, V139, P249, DOI 10.1016/j.jecp.2015.06.006

Lowe B, 2004, J AFFECT DISORDERS, V78, P131, DOI 10.1016/S0165-0327(02)00237-9

Maas-van Schaaijk NM, 2013, J PEDIATR PSYCHOL, V38, P30, DOI 10.1093/jpepsy/jss096

Mackey ER, 2014, HEALTH PSYCHOL, V33, P783, DOI 10.1037/hea0000066

Markowitz JT, 2012, DIABETIC MED, V29, P526, DOI 10.1111/j.1464-5491.2011.03434.x

Rumburg TM, 2017, PEDIATR DIABETES, V18, P67, DOI 10.1111/pedi.12350

SCHAFER LC, 1986, DIABETES CARE, V9, P179, DOI 10.2337/diacare.9.2.179

Topp CW, 2015, PSYCHOTHER PSYCHOSOM, V84, DOI 10.1159/000376585

van Widenfelt BM, 2003, EUR CHILD ADOLES PSY, V12, P281, DOI 10.1007/s00787-003-0341-3

Verweij A., 2008, SCHOLING OPLEIDING I

Wiebe DJ, 2014, J PEDIATR PSYCHOL, V39, P532, DOI 10.1093/jpepsy/jsu006

Wu YP, 2013, DIABETIC MED, V30, P596, DOI 10.1111/dme.12117

Wysocki T, 2007, DIABETES CARE, V30, P555, DOI 10.2337/dc06-1613

Young MT, 2014, CURR DIABETES REP, V14, DOI 10.1007/s11892-014-0546-5

NR 35

TC 0

Z9 0

U1 2

U2 3

PU HINDAWI LTD

PI LONDON

PA ADAM HOUSE, 3RD FLR, 1 FITZROY SQ, LONDON, W1T 5HF, ENGLAND

SN 2314-6745

EI 2314-6753

J9 J DIABETES RES

JI J. Diabetes Res.

PY 2017

AR 1462064

DI 10.1155/2017/1462064

PG 7

WC Endocrinology & Metabolism; Medicine, Research & Experimental

SC Endocrinology & Metabolism; Research & Experimental Medicine

GA FR2OS

UT WOS:000418907200001

PM 29376080

OA DOAJ Gold, Green Published

DA 2019-01-08

ER

PT J

AU Berry, E

Davies, M

Dempster, M

AF Berry, Emma

Davies, Mark

Dempster, Martin

TI Illness perception clusters and relationship quality are associated with

diabetes distress in adults with Type 2 diabetes

SO PSYCHOLOGY HEALTH & MEDICINE

LA English

DT Article

DE Type 2 diabetes mellitus; emotional adjustment; illness perceptions;

interpersonal relations

ID DEPRESSIVE SYMPTOMS; EMOTIONAL DISTRESS; SCALE; REPRESENTATIONS;

ADJUSTMENT; ADHERENCE; MELLITUS; TRIAL

AB This report aims to augment what is already known about emotional distress in Type 2 diabetes, by assessing the predictive value of illness perception clusters and relationship quality on four subcategories of Diabetes Distress.162 individuals with Type 2 diabetesresponded to a postal questionnaireassessing demographics, depression, diabetes distress, illness perceptions and relationship quality. Long-term blood glucose was retrievedfrom participants' General Practitioner. Three illness perception clusters emerged from the data,capturing three subgroups of participants sharing similar illness perception schemas. Regression analyses were performed across eachdiabetes distress subscale, with demographics, illness perception clusters, and relationship variables entered into three blocks.Covariates explained 51.1% of the variance in emotional burden, 41% of the variance in regimen-related distress, 20% of the variance in interpersonal distress, and 8.6% of the variance in physician-related distress. Cluster membership was strongly associated with emotional burden, regimen-related distress, and to a lesser degree interpersonal distress, but was not associated with physician-related distress. Relationship quality most strongly predicted regimen-related distress. Illness perception schemas and interpersonal issuesinfluence emotional adjustment in diabetes. This study provides direction for the content of a novel approach to identifying and reducing diabetes distress in people with Type 2 diabetes.

C1 [Berry, Emma; Dempster, Martin] Queens Univ Belfast, Sch Psychol, Belfast, Antrim, North Ireland.

[Davies, Mark] Belfast City Hosp, Clin Psychol, Belfast, Antrim, North Ireland.

RP Berry, E (reprint author), Queens Univ Belfast, Sch Psychol, Belfast, Antrim, North Ireland.

EM eberry05@qub.ac.uk

OI , Martin/0000-0001-9499-3186

FU Queen's University Belfast; Department of Education and Learning

FX This work was supported by Queen's University Belfast; and the

Department of Education and Learning.

CR Beck AT, 1996, BECK DEPRESSION INVE

Burns RJ, 2015, DIABETIC MED, V32, P1272, DOI 10.1111/dme.12860

BUSBY DM, 1995, J MARITAL FAM THER, V21, P289, DOI 10.1111/j.1752-0606.1995.tb00163.x

Dempster M, 2011, DIABETIC MED, V28, P487, DOI 10.1111/j.1464-5491.2010.03214.x

Ebrahimi H, 2016, PRIM CARE DIABETES, V10, P129, DOI 10.1016/j.pcd.2015.09.003

Fisher L, 2014, DIABETIC MED, V31, P764, DOI 10.1111/dme.12428

Fisher L, 2007, DIABETES CARE, V30, P542, DOI 10.2337/dc06-1614

Fisher L, 2012, DIABETES CARE, V35, P259, DOI 10.2337/dc11-1572

Fisher L, 2010, DIABETES CARE, V33, P23, DOI 10.2337/dc09-1238

Garcia-Huidobro D, 2011, FAM PRACT, V28, P4, DOI 10.1093/fampra/cmq069

Gonzalez JS, 2015, HEALTH PSYCHOL, V34, P505, DOI 10.1037/hea0000131

Hosoya T, 2012, INTERNAL MED, V51, P263, DOI 10.2169/internalmedicine.51.5768

Hudson JL, 2014, J PSYCHOSOM RES, V76, P265, DOI 10.1016/j.jpsychores.2014.02.004

Islam MR, 2013, INT J MED BIOMED RES, V2, P113, DOI DOI 10.14194/IJMBR.224

Johnson MD, 2015, BRIT J HEALTH PSYCH, V20, P360, DOI 10.1111/bjhp.12105

Karlsen B, 2014, INT J NURS STUD, V51, P438, DOI 10.1016/j.ijnurstu.2013.06.016

Miller D, 2005, J NUTR EDUC BEHAV, V37, P226, DOI 10.1016/S1499-4046(06)60276-5

Moss-Morris R, 2002, PSYCHOL HEALTH, V17, P1, DOI 10.1080/08870440290001494

Ngcobo M., 2008, EUR DIABETES NURSING, V11, P133, DOI [10.4314/ajpsy.v11i2.30266, DOI 10.1002/EDN.72]

NICE National Institute for Health and Clinical Excellence, 2015, CLIN GUID NG28 TYP 2

Polonsky WH, 2005, DIABETES CARE, V28, P626, DOI 10.2337/diacare.28.3.626

Skinner TC, 2011, PSYCHOL HEALTH, V26, P321, DOI 10.1080/08870440903411039

Strandberg RB, 2015, DIABETIC MED, V32, P1304, DOI 10.1111/dme.12781

NR 23

TC 2

Z9 2

U1 1

U2 4

PU ROUTLEDGE JOURNALS, TAYLOR & FRANCIS LTD

PI ABINGDON

PA 2-4 PARK SQUARE, MILTON PARK, ABINGDON OX14 4RN, OXON, ENGLAND

SN 1354-8506

EI 1465-3966

J9 PSYCHOL HEALTH MED

JI Psychol. Health Med.

PY 2017

VL 22

IS 9

BP 1118

EP 1126

DI 10.1080/13548506.2017.1281976

PG 9

WC Public, Environmental & Occupational Health

SC Public, Environmental & Occupational Health

GA FB6FU

UT WOS:000406237700011

PM 28103702

OA Green Published

DA 2019-01-08

ER

PT J

AU Ippolito, MM

Lyles, CR

Prendergast, K

Marshall, MB

Waxman, E

Seligman, HK

AF Ippolito, Matthew M.

Lyles, Courtney R.

Prendergast, Kimberly

Marshall, Michelle Berger

Waxman, Elaine

Seligman, Hilary Kessler

TI Food insecurity and diabetes self-management among food pantry clients

SO PUBLIC HEALTH NUTRITION

LA English

DT Article

DE Food security; Food pantries; Diabetes self-management

ID LOW-INCOME PATIENTS; GLYCEMIC CONTROL; MEDICATION ADHERENCE;

MENTAL-HEALTH; SOCIOECONOMIC-STATUS; WELFARE RECIPIENTS; HEMOGLOBIN

A(1C); DEPRESSION; DISTRESS; INSUFFICIENCY

AB Objective: To examine the association between level of food security and diabetes self-management among food pantry clients, which is largely not possible using clinic-based sampling methods.

Design: Cross-sectional descriptive study.

Setting: Community-based food pantries in California, Ohio and Texas, USA, from March 2012 through March 2014.

Subjects: Convenience sample of adults with diabetes queuing at pantries (n 1237; 83 % response). Sampled adults were stratified as food secure, low food secure or very low food secure. We used point-of-care glycated Hb (HbA(1c)) testing to determine glycaemic control and captured diabetes self-management using validated survey items.

Results: The sample was 70 % female, 55 % Latino/Hispanic, 25 % white and 10 % black/African American, with a mean age of 56 years. Eighty-four per cent were food insecure, one-half of whom had very low food security. Mean HbA(1c) was 8.1 % and did not vary significantly by food security status. In adjusted models, very-low-food-secure participants, compared with both low-food-secure and food-secure participants, had poorer diabetes self-efficacy, greater diabetes distress, greater medication non-adherence, higher prevalence of severe hypoglycaemic episodes, higher prevalence of depressive symptoms, more medication affordability challenges, and more food and medicine or health supply trade-offs.

Conclusions: Few studies of the health impact of food security have been able to examine very low food security. In a food pantry sample with high rates of food insecurity, we found that diabetes self-management becomes increasingly difficult as food security worsens. The efficacy of interventions to improve diabetes self-management may increase if food security is simultaneously addressed.

C1 [Ippolito, Matthew M.] Johns Hopkins Univ, Sch Med, Dept Med, 1830 East Monument St,Room 450B, Baltimore, MD 21287 USA.

[Lyles, Courtney R.; Seligman, Hilary Kessler] Univ Calif San Francisco, Dept Med, San Francisco, CA 94143 USA.

[Lyles, Courtney R.; Seligman, Hilary Kessler] San Francisco Gen Hosp, Ctr Vulnerable Populat, San Francisco, CA 94110 USA.

[Prendergast, Kimberly; Marshall, Michelle Berger] Feeding Amer, Chicago, IL USA.

[Waxman, Elaine] Urban Inst, Washington, DC 20037 USA.

RP Ippolito, MM (reprint author), Johns Hopkins Univ, Sch Med, Dept Med, 1830 East Monument St,Room 450B, Baltimore, MD 21287 USA.

EM mippolito@jhu.edu

FU NIH/NCRR/OD UCSF-CTSI [KL2 RR024130]; AHRQ Career Development Award

[R00HS022408]; The Bristol-Myers Squibb Foundation; National Institute

of General Medical Sciences of the National Institutes of Health

[T32GM066691]

FX This work was supported by NIH/NCRR/OD UCSF-CTSI Grant Number KL2

RR024130, AHRQ Career Development Award R00HS022408, and The

Bristol-Myers Squibb Foundation. Research reported in this publication

was supported by the National Institute of General Medical Sciences of

the National Institutes of Health under award number T32GM066691. The

content is solely the responsibility of the authors and does not

necessarily represent the official views of the National Institutes of

Health. Funders had no role in the design, analysis or writing of this

article.

CR Agardh E, 2011, INT J EPIDEMIOL, V40, P804, DOI 10.1093/ije/dyr029

Anater AS, 2011, J HUNGER ENVIRON NUT, V6, P27, DOI 10.1080/19320248.2011.549368

Anderson RJ, 2001, DIABETES CARE, V24, P1069, DOI 10.2337/diacare.24.6.1069

Baronberg S, 2013, PREV CHRONIC DIS, V10, DOI 10.5888/pcd10.130113

Berkowitz SA, 2013, DIABETES CARE, V36, P3093, DOI 10.2337/dc13-0570

Bogner HR, 2012, ANN FAM MED, V10, P15, DOI 10.1370/afm.1344

Bunnell R, 2012, J COMMUN HEALTH, V37, P1081, DOI 10.1007/s10900-012-9542-3

Centers for Disease Control and Prevention, 2014, MEAN MED AIC AD DIAG

Coleman-Jensen Alisha, 2012, 141 US DEP AGR EC RE

Corcoran M, 2001, WELFARE WORK WELLBEI, P159

County Health Rankings and Roadmaps, 2016, HLTH FOOD IN FOOD BA

Dammann KW, 2009, J NUTR EDUC BEHAV, V41, P242, DOI [10.1016/j.jneb.2008.07.003, 10.1016/j.neb.2008.07.003]

Drewnowski A, 2005, J NUTR, V135, P900

Fisher L, 2008, ANN FAM MED, V6, P246, DOI 10.1370/afm.842

Fisher L, 2010, DIABETES CARE, V33, P1034, DOI 10.2337/dc09-2175

Fisher L, 2010, DIABETES CARE, V33, P23, DOI 10.2337/dc09-1238

Gaskin DJ, 2014, AM J PUBLIC HEALTH, V104, P2147, DOI 10.2105/AJPH.2013.301420

Glasgow RE, 2005, ANN FAM MED, V3, P73, DOI 10.1370/afm.261

Gross R, 2005, J GEN INTERN MED, V20, P460, DOI 10.1111/j.1525-1497.2005.30003.x

Hanson KL, 2012, J HEALTH CARE POOR U, V23, P1174, DOI 10.1353/hpu.2012.0116

Heflin CM, 2005, SOC SCI MED, V61, P1971, DOI 10.1016/j.socscimed.2005.04.014

Herman DR, 2008, AM J PUBLIC HEALTH, V98, P98, DOI 10.2105/AJPH.2005.079418

Johnston-Brooks CH, 2002, PSYCHOSOM MED, V64, P43, DOI 10.1097/00006842-200201000-00007

KAVANAGH DJ, 1993, J BEHAV MED, V16, P509, DOI 10.1007/BF00844820

Krapek K, 2004, ANN PHARMACOTHER, V38, P1357, DOI 10.1345/aph.1D612

Lustman PJ, 2000, DIABETES CARE, V23, P934, DOI 10.2337/diacare.23.7.934

Lyles CR, 2013, DIABETES CARE, V36, P1448, DOI 10.2337/dc12-1961

MORISKY DE, 1986, MED CARE, V24, P67, DOI 10.1097/00005650-198601000-00007

National Research Council, 2006, FOOD INS HUMG US ASS

Polonsky WH, 2005, DIABETES CARE, V28, P626, DOI 10.2337/diacare.28.3.626

Robbins JM, 2005, DIABETES RES CLIN PR, V68, P230, DOI 10.1016/j.diabres.2004.09.007

Sarkar U, 2006, DIABETES CARE, V29, P823, DOI 10.2337/diacare.29.04.06.dc05-1615

Seligman HK, 2007, J GEN INTERN MED, V22, P1018, DOI 10.1007/s11606-007-0192-6

Seligman HK, 2015, HEALTH AFFAIR, V34, P1956, DOI 10.1377/hlthaff.2015.0641

Seligman HK, 2014, HEALTH AFFAIR, V33, P116, DOI 10.1377/hlthaff.2013.0096

Seligman HK, 2012, DIABETES CARE, V35, P233, DOI 10.2337/dc11-1627

Seligman HK, 2011, ARCH INTERN MED, V171, P1204, DOI 10.1001/archinternmed.2011.287

Seligman HK, 2010, J HEALTH CARE POOR U, V21, P1227, DOI 10.1353/hpu.2010.0921

Seligman HK, 2010, NEW ENGL J MED, V363, P6, DOI 10.1056/NEJMp1000072

Siefert K, 2004, J HEALTH SOC BEHAV, V45, P171, DOI 10.1177/002214650404500204

Siefert K, 2001, WOMEN HEALTH, V32, P159, DOI 10.1300/J013v32n01_08

Silverman J, 2015, J GEN INTERN MED, V30, P1476, DOI 10.1007/s11606-015-3351-1

Talbot F, 1997, J BEHAV MED, V20, P291, DOI 10.1023/A:1025508928696

Wang EA, 2011, J GEN INTERN MED, V26, P1012, DOI 10.1007/s11606-011-1723-8

Whitaker RC, 2006, PEDIATRICS, V118, pE859, DOI 10.1542/peds.2006-0239

NR 45

TC 4

Z9 4

U1 0

U2 6

PU CAMBRIDGE UNIV PRESS

PI CAMBRIDGE

PA EDINBURGH BLDG, SHAFTESBURY RD, CB2 8RU CAMBRIDGE, ENGLAND

SN 1368-9800

EI 1475-2727

J9 PUBLIC HEALTH NUTR

JI Public Health Nutr.

PD JAN

PY 2017

VL 20

IS 1

BP 183

EP 189

DI 10.1017/S1368980016001786

PG 7

WC Public, Environmental & Occupational Health; Nutrition & Dietetics

SC Public, Environmental & Occupational Health; Nutrition & Dietetics

GA FA1LN

UT WOS:000405201100020

PM 27406399

OA Bronze

DA 2019-01-08

ER

PT J

AU Otieno, CFF

Kanu, JE

Karari, EM

Okech-Helu, V

Joshi, MD

Mutai, K

AF Otieno, C. F. Frederick

Kanu, Joseph E.

Karari, Emma M.

Okech-Helu, Violet

Joshi, Mark D.

Mutai, Kenn

TI Glucose-lowering therapies, adequacy of metabolic control, and their

relationship with comorbid depression in outpatients with type 2

diabetes in a tertiary hospital in Kenya

SO DIABETES METABOLIC SYNDROME AND OBESITY-TARGETS AND THERAPY

LA English

DT Article

DE type 2 diabetes; comorbid depression; insulin therapy and poor glycemic

control

ID GLYCEMIC CONTROL; MEDICATION ADHERENCE; CLINICAL DEPRESSION; SELF-CARE;

DISTRESS; MELLITUS; METAANALYSIS; MANAGEMENT; SYMPTOMS; ADULTS

AB Background: Depression and diabetes mellitus are important comorbid conditions with serious health consequences. When depression and diabetes are comorbid, depression negatively affects self-management activities of diabetes with serious consequences. Relationship between treatment regimens of diabetes, the adequacy of glycemic control, and occurrence of comorbid depression is not known among our patients.

Patients and methods: This was a cross-sectional descriptive study at the outpatient diabetes clinic of the Kenyatta National Hospital where 220 ambulatory patients with type 2 diabetes on follow-up were systematically sampled. Sociodemographic data and clinical information were documented. The Patient Health Questionnaire-9 (PHQ-9) was used to assess depression. Ethylenediaminetetraacetic acid-anticoagulated blood was used for glycated hemoglobin (HbA(1C)) assay on automated system, COBAS INTEGRA machine.

Results: Two hundred twenty patients with type 2 diabetes were enrolled. The prevalence of comorbid depression by PHQ-9 was 32.3% (95% confidence interval: 26.4%-38.6%). The majority, 69.5%, had poor glycemic control, HbA(1C)> 7.0%, mean HbA(1C) was 8.9%+/- 2.4%. Half, 50.4%, of the study subjects were on insulin-containing regimens. Over 8% (84.5%) of the participants with comorbid depression had poor glycemic control, which worsened with increasing severity of depression. There was significant correlation between comorbid depression and poor glycemic control, which is more consistent in the insulin-treated patients. However, patients on oral agents only, both with and without comorbid depression, were similar in their glycemic control.

Conclusion: Among our type 2 diabetic population with comorbid depression, a large proportion had poor glycemic control, which worsened with increasing severity of depression. The insulin treatment increased the odds of comorbid depression and poor glycemic control in patients. It is justifiable to screen for comorbid depression in patients with type 2 diabetes who are in poor glycemic control, especially the insulin-treated, and then provide specific and appropriate interventions that are necessary to optimize their metabolic outcomes.

C1 [Otieno, C. F. Frederick; Kanu, Joseph E.; Karari, Emma M.; Joshi, Mark D.] Univ Nairobi, Dept Clin Med & Therapeut, Nairobi, Kenya.

[Okech-Helu, Violet; Mutai, Kenn] Kenyatta Natl Hosp, Nairobi, Kenya.

RP Otieno, CFF (reprint author), Univ Nairobi, Dept Clin Med & Therapeut, Nairobi, Kenya.

EM cfotieno@gmail.com

CR Aikens JE, 2008, DIABETIC MED, V25, P1324, DOI 10.1111/j.1464-5491.2008.02590.x

Aikens JE, 2009, DIABETES CARE, V32, P1177, DOI 10.2337/dc09-0071

Albright TL, 2001, FAM MED, V33, P354

American Diabetes Association, 2015, DIABETES CARE S1, V38, pS1, DOI DOI 10.2337/DC15-S001

Ascher-Svanum H, 2015, DIABETES THER, V6, P303, DOI 10.1007/s13300-015-0118-y

Bogner HR, 2012, ANN FAM MED, V10, P15, DOI 10.1370/afm.1344

Chao Jingdong, 2005, Res Social Adm Pharm, V1, P508, DOI 10.1016/j.sapharm.2005.09.002

Cochran WG., 1977, SAMPLING TECHNIQUES

DiMatteo MR, 2000, ARCH INTERN MED, V160, P2101, DOI 10.1001/archinte.160.14.2101

DSouza MS, 2015, GSTF J NURSING HLTH, V2, P29

Fisher L, 2007, DIABETES CARE, V30, P542, DOI 10.2337/dc06-1614

Fisher L, 2013, DIABETES CARE, V36, P2551, DOI 10.2337/dc12-2493

Fisher L, 2012, DIABETES CARE, V35, P259, DOI 10.2337/dc11-1572

Fisher L, 2010, DIABETES CARE, V33, P1034, DOI 10.2337/dc09-2175

Fisher L, 2010, DIABETES CARE, V33, P23, DOI 10.2337/dc09-1238

Georgiades A, 2007, PSYCHOSOM MED, V69, P235, DOI 10.1097/PSY.0b013e318042588d

Gonzalez JS, 2007, DIABETES CARE, V30, P2222, DOI 10.2337/dc07-0158

Gonzalez JS, 2008, DIABETES CARE, V31, P2398, DOI 10.2337/dc08-1341

Gramer JA, 2005, DIABETES CARE, V28, P78

Huang YF, 2013, BMC PSYCHIATRY, V13, DOI 10.1186/1471-244X-13-260

International Obesity Task Force of WHO 2000, 2004, INT J OBESITY, V28, P152

Inzucchi SE, 2012, DIABETES CARE, V35, P1364, DOI 10.2337/dc12-0413

James PA, 2014, JAMA-J AM MED ASSOC, V311, P507, DOI 10.1001/jama.2013.284427

Katon W, 2009, PSYCHOSOM MED, V71, P965, DOI 10.1097/PSY.0b013e3181bd8f55

Lee HJ, 2009, J AM ACAD NURSE PRAC, V21, P214, DOI 10.1111/j.1745-7599.2009.00396.x

Levy J, 1998, DIABETIC MED, V15, P290, DOI 10.1002/(SICI)1096-9136(199804)15:4<290::AID-DIA570>3.0.CO;2-M

Lin EHB, 2004, DIABETES CARE, V27, P2154, DOI 10.2337/diacare.27.9.2154

Lustman PJ, 2000, DIABETES CARE, V23, P934, DOI 10.2337/diacare.23.7.934

Manea L, 2012, CAN MED ASSOC J, V184, pE191, DOI 10.1503/cmaj.110829

Markowitz SM, 2011, PSYCHOSOMATICS, V52, P1, DOI 10.1016/j.psym.2010.11.007

Niraula K, 2013, BMC PSYCHIATRY, V13, DOI 10.1186/1471-244X-13-309

Otieno C F, 2005, East Afr Med J, V82, pS184

Otieno C F, 2003, East Afr Med J, V80, P406

Paul SK, 2015, CARDIOVASC DIABETOL, V14, DOI 10.1186/s12933-015-0260-x

Peyrot M, 2005, DIABETES CARE, V28, P2673, DOI 10.2337/diacare.28.11.2673

Peyrot M, 2010, DIABETES CARE, V33, P240, DOI 10.2337/dc09-1348

Snoek FJ, 2015, LANCET DIABETES ENDO, V3, P450, DOI 10.1016/S2213-8587(15)00135-7

Tan MY, 2008, PATIENT EDUC COUNS, V72, P252, DOI 10.1016/j.pec.2008.03.017

Teklay G, 2013, J MED SCI, V13, P578

U. K. Prospective Diabetes Study Group, 1995, DIABETES, V44, P1249

Vijan S, 2005, J GEN INTERN MED, V20, P479, DOI 10.1111/j.1525-1497.2005.0117.x

Zafar A, 2010, PRIM CARE DIABETES, V4, P203, DOI 10.1016/j.pcd.2010.07.003

NR 42

TC 1

Z9 1

U1 0

U2 0

PU DOVE MEDICAL PRESS LTD

PI ALBANY

PA PO BOX 300-008, ALBANY, AUCKLAND 0752, NEW ZEALAND

SN 1178-7007

J9 DIABET METAB SYND OB

JI Diabetes Metab. Syndr. Obes.

PY 2017

VL 10

DI 10.2147/DMSO.S124473

PG 9

WC Endocrinology & Metabolism

SC Endocrinology & Metabolism

GA ET7YH

UT WOS:000400514900001

PM 28496345

OA DOAJ Gold, Green Published

DA 2019-01-08

ER

PT J

AU Asuzu, CC

Walker, RJ

Williams, JS

Egede, LE

AF Asuzu, Christopher C.

Walker, Rebekah J.

Williams, Joni Strom

Egede, Leonard E.

TI Pathways for the relationship between diabetes distress, depression,

fatalism and glycemic control in adults with type 2 diabetes

SO JOURNAL OF DIABETES AND ITS COMPLICATIONS

LA English

DT Article

DE Diabetes; Depression; Diabetes distress; Fatalism; HbA1c

ID SELF-CARE; SOCIAL DETERMINANTS; MEDICATION ADHERENCE; METAANALYSIS;

HEALTH; OUTCOMES; SCALE; PREVALENCE; MANAGEMENT; VALIDITY

AB Background: The aim of this study was to examine the mechanism by which depressive symptoms, diabetes distress, and diabetes fatalism together influence diabetes outcomes using structured equation modeling.

Methods: 615 adults with type 2 diabetes were recruited from two primary care clinics in the southeastern United States. Psychosocial factors found to be associated with diabetes outcomes were measured using validated questionnaires. Structured equation modeling (SEM) was used to investigate the relationship between diabetes fatalism, depressive symptoms, diabetes distress, self-care and glycemic control.

Results: The final model (chi(2)(903) = 24,088.91, p < 0.0001, R-2 = 0.93, RMSEA = 0.05 and CFI = 0.90) showed that higher diabetes distress was directly significantly related to a decreased self-care (r = -0.69, p < 0.001) and increased HbA1c (r = 0.69, p < 0.001). There was no significant direct association between depressive symptoms or fatalism, and glycemic control or self-care. There was, however, an indirect association between increased depressive symptoms and increased fatalism, explained through the direct association with diabetes distress in that higher depressive symptoms (0.76, p < 0.001) and higher fatalism (0.11, p < 0.001) were significantly associated with higher diabetes distress.

Conclusion: Diabetes distretc-serves as a pathway through which depressive symptoms and fatalism impact both glycemic control and self-care. In addition, pathways between diabetes distress and both self-care behaviors and glycemic control in patients with type 2 diabetes remained separate, suggesting the need to address both psychological and behavioral factors in standard diabetes care, rather than focusing on psyChological care primarily through support for self-management and treatment of depression. (C) 2017 Elsevier Inc. All rights reserved.

C1 [Asuzu, Christopher C.] Med Univ South Carolina, Coll Med, Charleston, SC USA.

[Walker, Rebekah J.; Williams, Joni Strom; Egede, Leonard E.] Med Univ South Carolina, Ctr Hlth Dispar Res, Charleston, SC 29425 USA.

[Walker, Rebekah J.; Williams, Joni Strom; Egede, Leonard E.] Ralph H Johnson VAMC, Charleston VA HSR&D COIN, HEROIC, Charleston, SC USA.

[Walker, Rebekah J.; Williams, Joni Strom; Egede, Leonard E.] Med Univ South Carolina, Dept Med, Div Gen Internal Med & Geriatr, Charleston, SC USA.

RP Egede, LE (reprint author), Med Univ South Carolina, Ctr Hlth Dispar Res, 135 Rutledge Ave,Room 280, Charleston, SC 29425 USA.

EM egedel@musc.edu

FU National Institute of Diabetes and Digestive and Kidney Disease

[K24DK093699]

FX This study was supported by grant K24DK093699 from The National

Institute of Diabetes and Digestive and Kidney Disease (PI: Leonard

Egede).

CR Ali S, 2006, DIABETIC MED, V23, P1165, DOI 10.1111/j.1464-5491.2006.01943.x

Anderson RJ, 2001, DIABETES CARE, V24, P1069, DOI 10.2337/diacare.24.6.1069

Centers for Disease Control and Prevention, 2015, NAT DIAB STAT REP ES

Chailson M. E., 1987, J CHRON DIS, V40, P373

Costello AB, 2005, PRACTICAL ASSESSMENT, V10, P1, DOI DOI 10.1316/S0782-051X03024046-9

de Groot M, 2001, PSYCHOSOM MED, V63, P619, DOI 10.1097/00006842-200107000-00015

Egede LE, 2003, DIABETES EDUCATOR, V29, P105, DOI 10.1177/014572170302900115

Egede LE, 2010, J GEN INTERN MED, V25, P61, DOI 10.1007/s11606-009-1168-5

Fisher L, 2014, DIABETIC MED, V31, P764, DOI 10.1111/dme.12428

Fisher L, 2008, ANN FAMILY MED, V6

Fisher L, 2013, DIABETES CARE, V36, P2551, DOI 10.2337/dc12-2493

Fisher L, 2010, DIABETES CARE, V33, P23, DOI 10.2337/dc09-1238

Foundations of Care, 2014, DIABETES CARE S1, V38, pS20

Gilbody S, 2007, J GEN INTERN MED, V22, P1596, DOI 10.1007/s11606-007-0333-y

Gonzalez JS, 2008, DIABETIC MED, V25, P1102, DOI 10.1111/j.1464-5491.2008.02535.x

Hooper D., 2008, EJBRM, V6, P53, DOI DOI 10.1016/J.INFBEH.2006.11.010

Ismail K, 2004, LANCET, V363, P1589, DOI 10.1016/S0140-6736(04)16202-8

Kroenke K, 2001, J GEN INTERN MED, V16, P606, DOI 10.1046/j.1525-1497.2001.016009606.x

Lin EHB, 2004, DIABETES CARE, V27, P2154, DOI 10.2337/diacare.27.9.2154

Lustman PJ, 2000, DIABETES CARE, V23, P934, DOI 10.2337/diacare.23.7.934

Marmot M, 2014, AM J PUBLIC HEALTH, V104, pS517, DOI 10.2105/AJPH.2014.302200

MORISKY DE, 1986, MED CARE, V24, P67, DOI 10.1097/00005650-198601000-00007

National Center for Health Statistics, 2004, SURV QUEST NAT HLTH

Polonsky WH, 2005, DIABETES CARE, V28, P626, DOI 10.2337/diacare.28.3.626

Schram MT, 2009, CURR DIABETES REV, V5, P112, DOI 10.2174/157339909788166828

Schreiber JB, 2008, RES SOC ADM PHARM, V4, P83, DOI 10.1016/j.sapharm.2007.04.003

Schumacker RE, 2010, BEGINNERS GUIDE STRU

Toobert DJ, 2000, DIABETES CARE, V23, P943, DOI 10.2337/diacare.23.7.943

Walker RJ, 2014, GEN HOSP PSYCHIAT, V36, P662, DOI 10.1016/j.genhosppsych.2014.06.011

Walker RJ, 2014, BMC ENDOCR DISORD, V14, DOI 10.1186/1472-6823-14-82

Walker RJ, 2014, ENDOCRINE, V47, P29, DOI 10.1007/s12020-014-0195-0

Walker RJ, 2012, GEN HOSP PSYCHIAT, V34, P598, DOI 10.1016/j.genhosppsych.2012.07.005

World Health Organization, 2015, CONCEPTUAL FRAMEWORK

Zulman DM, 2012, PATIENT EDUC COUNS, V87, P74, DOI 10.1016/j.pec.2011.07.013

NR 34

TC 4

Z9 4

U1 0

U2 4

PU ELSEVIER SCIENCE INC

PI NEW YORK

PA 360 PARK AVE SOUTH, NEW YORK, NY 10010-1710 USA

SN 1056-8727

EI 1873-460X

J9 J DIABETES COMPLICAT

JI J. Diabetes Complications

PD JAN

PY 2017

VL 31

IS 1

BP 169

EP 174

DI 10.1016/j.jdiacomp.2016.09.013

PG 6

WC Endocrinology & Metabolism

SC Endocrinology & Metabolism

GA ES3MZ

UT WOS:000399435000025

PM 27746088

OA Green Accepted

DA 2019-01-08

ER

PT J

AU Chernyak, N

Julich, F

Kasperidus, J

Stephan, A

Begun, A

Kaltheuner, M

Icks, A

AF Chernyak, Nadja

Juelich, Fabian

Kasperidus, Julia

Stephan, Astrid

Begun, Alexander

Kaltheuner, Matthias

Icks, Andrea

TI Time cost of diabetes: Development of a questionnaire to assess time

spent on diabetes self-care

SO JOURNAL OF DIABETES AND ITS COMPLICATIONS

LA English

DT Article

DE Patient time; Time measurement; Recall questionnaire; Self-care;

Diabetes

ID HEALTH STATE VALUATIONS; SILENCE; LEISURE; INCOME; SCALE

AB Background: Methods to measure patient time spent on health-related activities are currently not well elaborated or standardized.

Aim: The purpose of this study was to develop a recall questionnaire measuring patient time devoted to diabetes self-care and to examine its feasibility and validity under field conditions.

Methods: The initial questionnaire was developed on the basis of instruments frequently used to assess self-care behavior in patients with diabetes, evaluated in two focus groups with patients with type 2 diabetes (N = 15) and tested in a random sample of patients with type 2 diabetes (N = 178). To assess the validity of the questionnaire, four hypotheses about expected differences in self-care time across various patient sub-groups were tested.

Results: The final questionnaire includes thirteen items estimating time spent on regular diabetes-related activities undertaken in the previous seven days. 78% of respondents completed the questionnaire without item non-response or other evident problems. As hypothesized, respondents receiving insulin treatment, those with poor self-rated health and those with diabetes-related emotional distress (PAID-5 score >= 8) reported spending more time on diabetes self-care than the rest of the sample. Contrary to our assumption, no differences in time spent on diabetes self-care between employed and retired individuals were detected by the questionnaire.

Conclusion: The recall questionnaire measuring patient time devoted to a broad range of regular diabetes self-care activities was developed and its feasibility was proved under field conditions. Ideally, the questionnaire should be further validated within a variety of populations. Exploration of the convergent validity between the recall method and prospective diary may be also useful. (C) 2017 Elsevier Inc. All rights reserved.

C1 [Chernyak, Nadja; Juelich, Fabian; Kasperidus, Julia; Stephan, Astrid; Icks, Andrea] Heinrich Heine Univ Dusseldorf, Fac Med, Inst Hlth Serv Res & Hlth Econ, Mooren Str 5, D-40225 Dusseldorf, Germany.

[Begun, Alexander; Icks, Andrea] Heinrich Heine Univ Dusseldorf, German Diabet Ctr, Inst Biometr & Epidemiol, Aufm Hennekamp 65, D-40225 Dusseldorf, Germany.

[Icks, Andrea] German Ctr Diabet Res DZD eV, Partner Dusseldorf, Dusseldorf, Germany.

RP Chernyak, N (reprint author), Heinrich Heine Univ Dusseldorf, Fac Med, Inst Hlth Serv Res & Hlth Econ, Mooren Str 5, D-40225 Dusseldorf, Germany.

EM Nadja.Chernyak@uni-duesseldorf.de; fabian-juelich@gmx.de;

juliakasperidus@gmx.de; astrid.stephan@uni-duesseldorf.de;

Alexander.Begun@ddz.uni-duesseldorf.de; m.kaltheuner@web.de;

Andrea.lcks@uni-duesseldorf.de

FU German Research Foundation (DFG) [701 010 607]; German Federal Ministry

of Education and Research (BMBF)

FX The study was funded by the German Research Foundation (DFG, 701 010

607) and supported in part by a grant from the German Federal Ministry

of Education and Research (BMBF) to the German Center for Diabetes

Research (DZD e.V.)

CR Brouwer WBF, 2003, HEALTH ECON, V12, P629, DOI 10.1002/hec.764

Brouwer WBF, 2009, MED DECIS MAKING, V29, P503, DOI 10.1177/0272989X09336161

Ettner SL, 2009, HEALTH ECON, V18, P645, DOI 10.1002/hec.1394

Gold MR, 1996, COST EFFECTIVENESS H

Jowsey T, 2012, BMC PUBLIC HEALTH, V12, DOI 10.1186/1471-2458-12-1044

JUSTER FT, 1991, J ECON LIT, V29, P471

Kamble S, 2013, MED DECIS MAKING, V33, P215, DOI 10.1177/0272989X12464824

McEwen LN, 2011, DIABETES CARE, V34, P1180, DOI 10.2337/dc10-2038

McGuire BE, 2010, DIABETOLOGIA, V53, P66, DOI 10.1007/s00125-009-1559-5

Russell LB, 2005, J FAM PRACTICE, V54, P52

Russell LB, 2009, MED CARE, V47, pS89, DOI 10.1097/MLR.0b013e31819bc077

Safford MM, 2005, J AM BOARD FAM PRACT, V18, P262, DOI 10.3122/jabfm.18.4.262

Sendi P, 2005, HEALTH ECON, V14, P643, DOI 10.1002/hec.971

Streiner DL, 2003, HLTH MEASUREMENT SCA

Terwee CB, 2007, J CLIN EPIDEMIOL, V60, P34, DOI 10.1016/j.jclinepi.2006.03.012

Toobert DJ, 2000, DIABETES CARE, V23, P943, DOI 10.2337/diacare.23.7.943

van der Ven NCW, 2003, DIABETES CARE, V26, P713, DOI 10.2337/diacare.26.3.713

Weinger K, 2005, DIABETES CARE, V28, P1346, DOI 10.2337/diacare.28.6.1346

Yen L, 2013, CHRONIC ILLN, V9, P268, DOI 10.1177/1742395313476720

Yen LE, 2013, J DIABETES METAB DIS, V12, DOI 10.1186/2251-6581-12-33

NR 20

TC 0

Z9 0

U1 1

U2 1

PU ELSEVIER SCIENCE INC

PI NEW YORK

PA 360 PARK AVE SOUTH, NEW YORK, NY 10010-1710 USA

SN 1056-8727

EI 1873-460X

J9 J DIABETES COMPLICAT

JI J. Diabetes Complications

PD JAN

PY 2017

VL 31

IS 1

BP 260

EP 266

DI 10.1016/j.jdiacomp.2016.06.016

PG 7

WC Endocrinology & Metabolism

SC Endocrinology & Metabolism

GA ES3MZ

UT WOS:000399435000040

PM 27411888

DA 2019-01-08

ER

PT J

AU Schmidt, CB

van Loon, BJP

Torensma, B

Snoek, FJ

Honig, A

AF Schmidt, Charlotte B.

van Loon, Bert Jan Potter

Torensma, Bart

Snoek, Frank J.

Honig, Adriaan

TI Ethnic Minorities with Diabetes Differ in Depressive and Anxiety

Symptoms and Diabetes-Distress

SO JOURNAL OF DIABETES RESEARCH

LA English

DT Article

DE Objective. To determine the association between ethnicity;

diabetes-distress; and depressive and anxiety symptoms in adult;

outpatients with diabetes. Research Design and Methods.

Diabetes-distress (Problem Areas in Diabetes Scale; PAID5); depressive

and; anxiety symptoms ( Extended Kessler-10; EK10); and quality of life

(Short-Form12; SF12) were assessed in an ethnic diverse diabetes;

outpatient population of a teaching hospital in Amsterdam. Descent of

one's parents and self-classified ethnicity were obtained to; define

ethnicity. HbA1c; clinical data; and socioeconomic status were derived

from the medical charts. Based on established cut-offs; for PAID5; and

EK10; scores; emotional distress was dichotomized for the purpose of

logistic regression analyses. Results. Of 1007; consecutive patients

approached; 575 participated. Forty-nine percent were of non-Dutch

ethnicity and 24.7% had type 1 diabetes.; Diabetes-distress was reported

by 12.5% of the native Dutch patients and by 22.0%; 34.5%; and 42.6% of

the Surinamese; Turkish; and Moroccan patients; respectively. Prevalence

of depressive symptoms was 9.4% in native Dutch patients and 20.4%;

34.5%; and; 27.3% in the other groups mentioned. Diabetes-distress and

Moroccan origin were significantly associated (OR=3.60; p < .01) as;

well as depressive symptoms and Turkish origin (OR=4.23; p =.04).

Conclusions. Different ethnic minorities with diabetes vary; in their

vulnerability for emotional distress; warranting clinical attention.

Future research should elucidate explanatory factors and; opportunities

for tailored interventions.

ID EMOTIONAL DISTRESS; RISK-FACTORS; SOCIOECONOMIC-STATUS; COMORBID

DEPRESSION; MENTAL-DISORDERS; SECONDARY CARE; PREVALENCE; POPULATION;

MOROCCAN; TURKISH

AB Objective. To determine the association between ethnicity, diabetes-distress, and depressive and anxiety symptoms in adult outpatients with diabetes. Research Design and Methods. Diabetes-distress (Problem Areas in Diabetes Scale, PAID5), depressive and anxiety symptoms (Extended Kessler-10, EK10), and quality of life (Short-Form12, SF12) were assessed in an ethnic diverse diabetes outpatient population of a teaching hospital in Amsterdam. Descent of one's parents and self-classified ethnicity were obtained to define ethnicity. HbA1c, clinical data, and socioeconomic status were derived from the medical charts. Based on established cut-offs for PAID5- and EK10-scores, emotional distress was dichotomized for the purpose of logistic regression analyses. Results. Of 1007 consecutive patients approached, 575 participated. Forty-nine percent were of non-Dutch ethnicity and 24.7% had type 1 diabetes. Diabetes-distress was reported by 12.5% of the native Dutch patients and by 22.0%, 34.5%, and 42.6% of the Surinamese, Turkish, and Moroccan patients, respectively. Prevalence of depressive symptoms was 9.4% in native Dutch patients and 20.4%, 34.5%, and 27.3% in the other groups mentioned. Diabetes- distress and Moroccan origin were significantly associated (OR = 3.60, p < .01) as well as depressive symptoms and Turkish origin ( OR = 4.23, p =.04). Conclusions. Different ethnic minorities with diabetes vary in their vulnerability for emotional distress, warranting clinical attention. Future research should elucidate explanatory factors and opportunities for tailored interventions.

C1 [Schmidt, Charlotte B.; Honig, Adriaan] OLVG, Dept Psychiat, Amsterdam, Netherlands.

[Schmidt, Charlotte B.; Snoek, Frank J.] Amsterdam Publ Hlth Res Inst, Amsterdam, Netherlands.

[van Loon, Bert Jan Potter] OLVG, Dept Internal Med, Amsterdam, Netherlands.

[Torensma, Bart] OLVG, Dept Epidemiol & Biostat, Amsterdam, Netherlands.

[Snoek, Frank J.] AMC, Dept Med Psychol, Amsterdam, Netherlands.

[Snoek, Frank J.] VU Univ Med Ctr VUMC, Dept Med Psychol, Amsterdam, Netherlands.

[Honig, Adriaan] Vrije Univ Amsterdam Med Ctr, Dept Psychiat, Amsterdam, Netherlands.

RP Schmidt, CB (reprint author), OLVG, Dept Psychiat, Amsterdam, Netherlands.; Schmidt, CB (reprint author), Amsterdam Publ Hlth Res Inst, Amsterdam, Netherlands.

EM c.schmidt@olvg.nl

OI Schmidt, Charlotte/0000-0002-7852-4264

FU Innovation Fund of OLVG-W

FX The authors thank all people that participated in this study and Saskia

Gorter-de Vries, Trudy Nieuwint, Nabila Chaara, Patrick Bonestroo, Leni

Telleman, Renee Elsas, Inge de Vlieger, and Evert van der Poest Clement

(all from OLVG) for valuable contributions. The authors also thank the

personnel of the Internal Medicine Outpatient Department for assistance

in data collection. This study was supported by the Innovation Fund of

OLVG-W.

CR Al-Krenawi A, 2005, ISRAEL J PSYCHIAT, V42, P71

Ali S, 2009, POSTGRAD MED J, V85, P238, DOI 10.1136/pgmj.2008.074641

[Anonymous], 1985, INT DEMOGRAPHICS, V4, P1

[Anonymous], 2014, AOF FONDS HUIS ACHT

Bambra C, 2010, J EPIDEMIOL COMMUN H, V64, P284, DOI 10.1136/jech.2008.082743

BARON RM, 1986, J PERS SOC PSYCHOL, V51, P1173, DOI 10.1037//0022-3514.51.6.1173

Baumeister H, 2011, PSYCHOTHER PSYCHOSOM, V80, P275, DOI 10.1159/000323404

Bhopal R, 2004, J EPIDEMIOL COMMUN H, V58, P441, DOI 10.1136/jech.2003.013466

Brazier J, 2014, HEALTH TECHNOL ASSES, V18, P1, DOI 10.3310/hta18340

Cinnirella M, 1999, BRIT J MED PSYCHOL, V72, P505, DOI 10.1348/000711299160202

Danesh J, 1999, BRIT MED J, V318, P843, DOI 10.1136/bmj.318.7187.843

de Wit MAS, 2008, SOC PSYCH PSYCH EPID, V43, P905, DOI 10.1007/s00127-008-0382-5

Donker T, 2010, PSYCHIAT RES, V176, P45, DOI 10.1016/j.psychres.2009.01.012

Everson SA, 2002, J PSYCHOSOM RES, V53, P891, DOI 10.1016/S0022-3999(02)00303-3

Fisher L, 2009, DIABETIC MED, V26, P622, DOI 10.1111/j.1464-5491.2009.02730.x

Fisher L, 2007, DIABETES CARE, V30, P542, DOI 10.2337/dc06-1614

Fisher L, 2010, DIABETES CARE, V33, P23, DOI 10.2337/dc09-1238

Gandek B, 1998, J CLIN EPIDEMIOL, V51, P1171, DOI 10.1016/S0895-4356(98)00109-7

Grigsby AB, 2002, J PSYCHOSOM RES, V53, P1053, DOI 10.1016/S0022-3999(02)00417-8

Hair J., 1995, MULTIVARIATE DATA AN

Hans E, 2013, J CONSULT CLIN PSYCH, V81, P75, DOI 10.1037/a0031080

Haverkamp GLG, 2015, PLOS ONE, V10, DOI 10.1371/journal.pone.0130961

Ince BU, 2014, BMC PSYCHIATRY, V14, DOI 10.1186/s12888-014-0252-5

Kamperman A. M., 2003, TIJDSCHR PSYCHIAT, V45, P315

Karter AJ, 2002, JAMA-J AM MED ASSOC, V287, P2519, DOI 10.1001/jama.287.19.2519

Katon WJ, 2005, DIABETES CARE, V28, P2668, DOI 10.2337/diacare.28.11.2668

Lanting LC, 2005, DIABETES CARE, V28, P2280, DOI 10.2337/diacare.28.9.2280

Li C, 2008, DIABETIC MED, V25, P878, DOI 10.1111/j.1464-5491.2008.02477.x

Loewenthal KM, 2001, BRIT J MED PSYCHOL, V74, P293, DOI 10.1348/000711201160993

Maurischat C, 2008, PSYCHOL SCI Q, V50, P7

McGuire BE, 2010, DIABETOLOGIA, V53, P66, DOI 10.1007/s00125-009-1559-5

Muntaner C, 2004, EPIDEMIOL REV, V26, P53, DOI 10.1093/epirev/mxh001

Peduzzi P, 1996, J CLIN EPIDEMIOL, V49, P1373, DOI 10.1016/S0895-4356(96)00236-3

Peyrot M, 2014, CURR MED RES OPIN, V30, P2241, DOI 10.1185/03007995.2014.947023

Pouwer F, 2013, DIABETIC MED, V30, pE25, DOI 10.1111/dme.12031

Pouwer F, 2010, DIABETIC MED, V27, P217, DOI 10.1111/j.1464-5491.2009.02903.x

Roy T, 2012, J AFFECT DISORDERS, V142, pS8, DOI 10.1016/S0165-0327(12)70004-6

Snoek FJ, 2000, DIABETES CARE, V23, P1305, DOI 10.2337/diacare.23.9.1305

Snoek FJ, 2015, LANCET DIABETES ENDO, V3, P450, DOI 10.1016/S2213-8587(15)00135-7

Snoek FJ, 2012, DIABETES CARE, V35, P2128, DOI 10.2337/dc11-1326

Snoek FJ, 2011, DIABETES CARE, V34, P601, DOI 10.2337/dc10-1552

Sobel M. E., 1982, SOCIOL METHODOL, P290, DOI DOI 10.2307/270723

Stoop CH, 2014, DIABETIC MED, V31, P1252, DOI 10.1111/dme.12472

Stronks K, 2009, ETHNIC HEALTH, V14, P255, DOI 10.1080/13557850802509206

Uitewaal PJM, 2004, PREV MED, V39, P1068, DOI 10.1016/j.ypmed.2004.04.009

Ujcic-Voortman JK, 2009, EUR J PUBLIC HEALTH, V19, P511, DOI 10.1093/eurpub/ckp096

Unsal A, 2011, INT J NURS PRACT, V17, P411, DOI 10.1111/j.1440-172X.2011.01949.x

van Bastelaar KMP, 2010, DIABETIC MED, V27, P798, DOI 10.1111/j.1464-5491.2010.03025.x

van der Wurff FB, 2004, J AFFECT DISORDERS, V83, P33, DOI 10.1016/j.jad.2004.04.009

Ware J, 1995, SF 12 SCORE SF 12 PH

Yuan Y, 2005, MULTIPLE IMPUTATION

Zimmerman FJ, 2005, HEALTH ECON, V14, P1197, DOI 10.1002/hec.1011

NR 52

TC 2

Z9 2

U1 0

U2 2

PU HINDAWI LTD

PI LONDON

PA ADAM HOUSE, 3RD FLR, 1 FITZROY SQ, LONDON, WIT 5HE, ENGLAND

SN 2314-6745

EI 2314-6753

J9 J DIABETES RES

JI J. Diabetes Res.

PY 2017

AR 1204237

DI 10.1155/2017/1204237

PG 11

WC Endocrinology & Metabolism; Medicine, Research & Experimental

SC Endocrinology & Metabolism; Research & Experimental Medicine

GA EQ9IH

UT WOS:000398397600001

PM 28373992

OA DOAJ Gold, Green Published

DA 2019-01-08

ER

PT J

AU Feng, XQ

Astell-Burt, T

AF Feng, Xiaoqi

Astell-Burt, Thomas

TI Impact of a type 2 diabetes diagnosis on mental health, quality of life,

and social contacts: a longitudinal study

SO BMJ OPEN DIABETES RESEARCH & CARE

LA English

DT Article

ID PSYCHOLOGICAL DISTRESS; ETHNIC-DIFFERENCES; SCREENING SCALES;

RISK-FACTOR; DEPRESSION; METAANALYSIS; MELLITUS; STIGMA; ADULTS;

PREVENTION

AB Aims: The aim was to examine whether a type 2 diabetes mellitus (T2DM) diagnosis increases the odds of psychological distress, a worsening in overall quality of life, and a potential reduction in social contacts.

Method: Longitudinal data were obtained from the 45 and Up Study (baseline 2006-2008; 3.4 +/- 0.95 years follow-up time). Fixed effects logistic and negative binomial regression models were fitted on a complete case on outcome sample that did not report T2DM at baseline (N=26 344), adjusted for time-varying confounders. The key exposure was doctor-diagnosed T2DM at follow-up. Outcome variables examined included the Kessler Psychological Distress Scale, self-rated quality of life, and four indicators of social contacts.

Results: A modest increase in the odds of psychological distress associated with T2DM diagnosis (OR= 1.30) was not statistically significant (95% CI 0.75 to 2.25). A T2DM diagnosis was associated with a fivefold increase in the odds of a participant reporting that their quality of life had become significantly poorer (OR 5.49, 95% CI 1.26 to 23.88). T2DM diagnosis was also associated with a reduction in times spent with friends and family (RR 0.88, 95% CI 0.82 to 0.95), contacts by telephone (RR 0.95, 95% CI 0.87 to 1.02), attendance at social clubs or religious groups (RR 0.82, 95% CI 0.73 to 0.91), and the number of people nearby but outside the home that participants felt they could rely on (RR 0.92, 95% CI 0.86 to 0.98).

Conclusions: A T2DM diagnosis can have important impacts on quality of life and on social contacts, which may have negative impacts on mental health and T2DM management in the longer term.

C1 [Feng, Xiaoqi; Astell-Burt, Thomas] Univ Wollongong, Fac Social Sci, Sch Hlth & Soc, Populat Wellbeing & Environm Res Lab PowerLab, Wollongong, NSW, Australia.

[Feng, Xiaoqi; Astell-Burt, Thomas] Univ Wollongong, Fac Social Sci, Early Start Res Inst, Wollongong, NSW, Australia.

[Feng, Xiaoqi; Astell-Burt, Thomas] Univ Wollongong, Illawarra Hlth & Med Res Inst, Wollongong, NSW, Australia.

RP Feng, XQ (reprint author), Univ Wollongong, Fac Social Sci, Sch Hlth & Soc, Populat Wellbeing & Environm Res Lab PowerLab, Wollongong, NSW, Australia.; Feng, XQ (reprint author), Univ Wollongong, Fac Social Sci, Early Start Res Inst, Wollongong, NSW, Australia.; Feng, XQ (reprint author), Univ Wollongong, Illawarra Hlth & Med Res Inst, Wollongong, NSW, Australia.

EM xfeng@uow.edu.au

OI Feng, Xiaoqi/0000-0002-3421-220X; Astell-Burt,

Thomas/0000-0002-1498-4851

FU National Health and Medical Research Council [1101065]; Vanguard grant

from the National Heart Foundation of Australia [101460]; National Heart

Foundation of Australia Postdoctoral Fellowship [100948]

FX This work was supported by a project grant from the National Health and

Medical Research Council (grant number #1101065) and a Vanguard grant

from the National Heart Foundation of Australia (grant number #101460).

Dr Feng's contribution was also supported by a National Heart Foundation

of Australia Postdoctoral Fellowship: grant number #100948.

CR Ali S, 2006, DIABETIC MED, V23, P1165, DOI 10.1111/j.1464-5491.2006.01943.x

Allison P. D., 2005, FIXED EFFECTS REGRES

*AUSTR BUR STAT, 2003, INF PAP US KESSL PSY

Banks E, 2008, INT J EPIDEMIOL, V37, P941, DOI 10.1093/ije/dym184

Barkow K, 2001, EUR ARCH PSY CLIN N, V251, P21, DOI 10.1007/BF03035122

Browne JL, 2013, DIABETIC MED, V30, P620, DOI 10.1111/dme.12078

Browne JL, 2013, BMJ OPEN, V3, DOI 10.1136/bmjopen-2013-003384

Chapple A, 2004, BRIT MED J, V328, P1470, DOI 10.1136/bmj.38111.639734.7C

Chen L, 2012, NAT REV ENDOCRINOL, V8, P228, DOI 10.1038/nrendo.2011.183

Colagiuri S, 2012, DIABETES OBES METAB, V14, P3, DOI 10.1111/j.1463-1326.2011.01506.x

Colagiuri S, 2014, MED J AUSTRALIA, V201, P501, DOI 10.5694/mja14.01307

Diener E, 2006, AM PSYCHOL, V61, P305, DOI 10.1037/0003-066X.61.4.305

Ducat L, 2014, JAMA-J AM MED ASSOC, V312, P691, DOI 10.1001/jama.2014.8040

Everson SA, 2002, J PSYCHOSOM RES, V53, P891, DOI 10.1016/S0022-3999(02)00303-3

Feng XQ, 2013, BMJ OPEN, V3, DOI 10.1136/bmjopen-2013-002713

Furukawa TA, 2003, PSYCHOL MED, V33, P357, DOI 10.1017/S0033291702006700

Gonzalez JS, 2007, DIABETES CARE, V30, P2222, DOI 10.2337/dc07-0158

Gunasekara FI, 2014, INT J EPIDEMIOL, V43, P264, DOI 10.1093/ije/dyt221

Harris MI, 1999, DIABETES CARE, V22, P403, DOI 10.2337/diacare.22.3.403

Jimenez DE, 2013, J AM GERIATR SOC, V61, P18, DOI 10.1111/jgs.12063

Johnson Joy L, 2007, Proc Am Thorac Soc, V4, P680, DOI 10.1513/pats.200706-084SD

Jutagir DR, 2016, ETHNIC HEALTH, V21, P411, DOI 10.1080/13557858.2015.1066494

Katon WJ, 2005, DIABETES CARE, V28, P2668, DOI 10.2337/diacare.28.11.2668

Kawachi I., 2008, SOCIAL CAPITAL HLTH

Kessler RC, 2002, PSYCHOL MED, V32, P959, DOI 10.1017/S0033291702006074

Knol MJ, 2006, DIABETOLOGIA, V49, P837, DOI 10.1007/s00125-006-0159-x

KOENIG HG, 1993, PSYCHOSOMATICS, V34, P61, DOI 10.1016/S0033-3182(93)71928-3

Kuh D., 2004, LIFE COURSE APPROACH

Marrero D, 2014, QUAL LIFE RES, V23, P75, DOI 10.1007/s11136-013-0436-3

Mezuk B, 2008, DIABETES CARE, V31, P2383, DOI 10.2337/dc08-0985

Narayan KMV, 2012, HEALTH AFFAIR, V31, P84, DOI 10.1377/hlthaff.2011.1040

Nouwen A, 2010, DIABETOLOGIA, V53, P2480, DOI 10.1007/s00125-010-1874-x

Room R, 2005, DRUG ALCOHOL REV, V24, P143, DOI 10.1080/0959230500102434

ROSENBAUM M, 1990, LEARNED RESOURCEFULN, P3, DOI DOI 10.1016/S0005-7894(80)80040-2

Schabert J, 2013, PATIENT, V6, P1, DOI 10.1007/s40271-012-0001-0

Semenkovich K, 2015, DRUGS, V75, P577, DOI 10.1007/s40265-015-0347-4

Shaw J, 2012, DIABETES SILENT PAND

Skaff MM, 2009, HEALTH PSYCHOL, V28, P265, DOI 10.1037/a0014429

Speight J, 2016, MED J AUSTRALIA, V205, P149, DOI 10.5694/mja16.00556

Stewart-Brown S, 2011, J EPIDEMIOL COMMU S2, V11, DOI [10.1136/jech.2011.143586.86, DOI 10.1136/JECH.2011.143586.86]

Zacks S, 2006, J CLIN GASTROENTEROL, V40, P220, DOI 10.1097/00004836-200603000-00009

Zimmet PZ, 2014, LANCET DIABETES ENDO, V2, P56, DOI 10.1016/S2213-8587(13)70112-8

NR 42

TC 0

Z9 0

U1 2

U2 7

PU BMJ PUBLISHING GROUP

PI LONDON

PA BRITISH MED ASSOC HOUSE, TAVISTOCK SQUARE, LONDON WC1H 9JR, ENGLAND

SN 2052-4897

J9 BMJ OPEN DIAB RES CA

JI BMJ Open Diab. Res. Care

PD JAN

PY 2017

VL 5

IS 1

DI 10.1136/bmjdrc-2016-000198

PG 5

WC Endocrinology & Metabolism

SC Endocrinology & Metabolism

GA EO9VX

UT WOS:000397037300001

PM 28243446

OA DOAJ Gold, Green Published

DA 2019-01-08

ER

PT J

AU Rees, G

O'Hare, F

Saeed, M

Sudholz, B

Sturrock, BA

Xie, J

Speight, J

Lamoureux, EL

AF Rees, Gwyneth

O'Hare, Fleur

Saeed, Marian

Sudholz, Bronwyn

Sturrock, Bonnie A.

Xie, Jing

Speight, Jane

Lamoureux, Ecosse L.

TI Problem-solving therapy for adults with diabetic retinopathy and

diabetesspecific distress: a pilot randomized controlled trial

SO BMJ OPEN DIABETES RESEARCH & CARE

LA English

DT Article

ID GLYCEMIC CONTROL; DEPRESSION; ASSOCIATION; CARE; COMPLICATIONS;

METAANALYSIS; TYPE-1; SCALE

AB Objective: To provide preliminary evidence for the impact of problem-solving therapy for diabetes ( PST-D) in adults with diabetic retinopathy ( DR) and diabetes distress. Research design and methods: In a pilot randomized controlled trial, 40 participants with DR and diabetes distress were allocated to the PST-D or control groups. Diabetes distress ( DDS), depressive symptoms ( PHQ-9), self-care activities ( SDSCA), and HbA1c were assessed at baseline, and 3 and 6-month follow-ups. Results: At the 6-month follow-up, the PST-D group showed significant improvements relative to the control group, in `regimen-related distress' ( PST-D: - 1.3 +/- 1.4; control: - 0.4 +/- 1.1), depressive symptoms (PST-D: - 4.3 +/- 6.1; control: - 0.3 +/- 4.6), and HbA1c (PST-D: -1.2%+/- 1.01; control: 0.2%+/- 1.2%) (all p<0.05). In multiple regression analysis, adjusting for baseline values and sociodemographic factors, PST- D was associated with significant improvement in `regimenrelated distress', depressive symptoms, and HbA1c at the 6-month follow-up (p<0.05). Conclusions: PST- D is a promising intervention for improving psychological outcomes and glycemic control. A fully powered study is required to confirm these findings and examine mechanisms of change in HbA1c. Trial registration number: ACTRN12616001010482; results.

C1 [Rees, Gwyneth; O'Hare, Fleur; Saeed, Marian; Sturrock, Bonnie A.; Xie, Jing; Lamoureux, Ecosse L.] Univ Melbourne, Royal Victorian Eye & Ear Hosp, Ctr Eye Res Australia, Melbourne, Vic, Australia.

[Sudholz, Bronwyn] Deakin Univ, IPAN, Sch Exercise & Nutr Sci, Geelong, Vic, Australia.

[Sudholz, Bronwyn; Speight, Jane] Deakin Univ, Sch Psychol, Geelong, Vic, Australia.

[Speight, Jane] Australian Ctr Behav Res Diabet, Diabet Victoria, Melbourne, Vic, Australia.

[Speight, Jane] AHP Res, Hornchurch, England.

[Lamoureux, Ecosse L.] Natl Univ Singapore, Singapore Eye Res Inst, Singapore, Singapore.

[Lamoureux, Ecosse L.] Duke NUS Med Sch, Singapore, Singapore.

RP Rees, G (reprint author), Univ Melbourne, Royal Victorian Eye & Ear Hosp, Ctr Eye Res Australia, Melbourne, Vic, Australia.

EM grees@unimelb.edu.au

OI Speight, Jane/0000-0002-1204-6896

FU Australian Research Council [LP0884108]

FX Australian Research Council (grant number LP0884108).

CR Brooke P, 1999, INT J GERIATR PSYCH, V14, P936, DOI 10.1002/(SICI)1099-1166(199911)14:11<936::AID-GPS39>3.0.CO;2-1

Chew EY, 2010, NEW ENGL J MED, V363, P233, DOI 10.1056/NEJMoa1001288

de Groot M, 2001, PSYCHOSOM MED, V63, P619, DOI 10.1097/00006842-200107000-00015

Early Treatment Diabetic Retinopathy Study Research Group, 1991, OPHTHALMOLOGY, V98, P786

Fenwick EK, 2016, J HLTH PSYCHOL

Fisher L, 2008, ANN FAM MED, V6, P246, DOI 10.1370/afm.842

Fisher L, 2012, PATIENT EDUC COUNS, V86, P372, DOI 10.1016/j.pec.2011.07.002

Fisher L, 2012, DIABETES CARE, V35, P259, DOI 10.2337/dc11-1572

Fisher L, 2010, DIABETES CARE, V33, P23, DOI 10.2337/dc09-1238

Harkness E, 2010, DIABETES CARE, V33, P926, DOI 10.2337/dc09-1519

Hegel M, 1999, PROBLEM SOLVING TREA

Hessler D, 2014, DIABETES CARE, V37, P617, DOI 10.2337/dc13-0762

Kinder LS, 2006, J GEN INTERN MED, V21, P1036, DOI 10.1111/j.1525-1497.2006.00552.x

Kroenke K, 2001, J GEN INTERN MED, V16, P606, DOI 10.1046/j.1525-1497.2001.016009606.x

Lipscombe C, 2016, J DIABETES, V8, P269, DOI 10.1111/1753-0407.12298

Markowitz SM, 2011, PSYCHOSOMATICS, V52, P1, DOI 10.1016/j.psym.2010.11.007

Nicolucci A, 2013, DIABETIC MED, V30, P767, DOI 10.1111/dme.12245

Roy MS, 2007, PSYCHOSOM MED, V69, P537, DOI 10.1097/PSY.0b013e3180df84e2

Sturt J, 2015, INT DIAB NURS, V12, P40, DOI DOI 10.1179/2057332415Y.0000000004

Toobert DJ, 2000, DIABETES CARE, V23, P943, DOI 10.2337/diacare.23.7.943

van Bastelaar KMP, 2010, DIABETIC MED, V27, P798, DOI 10.1111/j.1464-5491.2010.03025.x

NR 21

TC 0

Z9 0

U1 2

U2 6

PU BMJ PUBLISHING GROUP

PI LONDON

PA BRITISH MED ASSOC HOUSE, TAVISTOCK SQUARE, LONDON WC1H 9JR, ENGLAND

SN 2052-4897

J9 BMJ OPEN DIAB RES CA

JI BMJ Open Diab. Res. Care

PD JAN

PY 2017

VL 5

IS 1

DI 10.1136/bmjdrc-2016-000307

PG 7

WC Endocrinology & Metabolism

SC Endocrinology & Metabolism

GA EO9VX

UT WOS:000397037300010

PM 28243448

OA DOAJ Gold, Green Published

DA 2019-01-08

ER

PT J

AU Shibuta, T

Waki, K

Tomizawa, N

Igarashi, A

Yamamoto-Mitani, N

Yamaguchi, S

Fujita, H

Kimura, S

Fujiu, K

Waki, H

Izumida, Y

Sasako, T

Kobayashi, M

Suzuki, R

Yamauchi, T

Kadowaki, T

Ohe, K

AF Shibuta, Tomomi

Waki, Kayo

Tomizawa, Nobuko

Igarashi, Ayumi

Yamamoto-Mitani, Noriko

Yamaguchi, Satoko

Fujita, Hideo

Kimura, Shigeko

Fujiu, Katsuhito

Waki, Hironori

Izumida, Yoshihiko

Sasako, Takayoshi

Kobayashi, Masatoshi

Suzuki, Ryo

Yamauchi, Toshimasa

Kadowaki, Takashi

Ohe, Kazuhiko

TI Willingness of patients with diabetes to use an ICT-based

self-management tool: a cross-sectional study

SO BMJ OPEN DIABETES RESEARCH & CARE

LA English

DT Article

ID GLYCEMIC CONTROL; MICROVASCULAR COMPLICATIONS; PROBLEM AREAS; CARE;

SCALE; DISTRESS; MELLITUS; ASSOCIATION; OLDER

AB Objectives: To examine the prevalence of the willingness of patients with diabetes to use a selfmanagement tool based on information and communication technology (ICT) such as personal computers, smartphones, and mobile phones; and to examine the patient characteristics associated with that willingness. Research design and methods: We conducted a cross-sectional interview survey of 312 adults with diabetes at a university hospital in an urban area in Japan. Participants were classified into 2 groups: those who were willing to use an ICT-based self-management tool and those who were unwilling. Multiple logistic regression analysis was used to identify factors associated with the willingness, including clinical and social factors, current use of ICT, self-management practices, self-efficacy, and diabetes-related emotional distress. Results: The mean age of the 312 participants was 66.3 years (SD=11.5) and 198 (63%) were male. Most of the participants (93%) had type 2 diabetes. Although only 51 (16%) currently used ICT-based selfmanagement tools, a total of 157 (50%) expressed the willingness to use such a tool. Factors associated with the willingness included: not having nephropathy (OR=2.02, 95% CI 1.14 to 3.58); outpatient visits once a month or more (vs less than once a month, OR=2.13, 95% CI 1.13 to 3.99); current use of personal computers and/or smartphones (OR=4.91, 95% CI 2.69 to 8.98); and having greater diabetesrelated emotional distress (OR=1.10, 95% CI 1.01 to 1.20). Conclusions: Approximately half of the patients showed interest in using an ICT-based selfmanagement tool. Willing patients may expect ICTbased self-management tools to complement outpatient visits and to make self-management easier. Starting with patients who display the willingness factors might optimize programs based on such tools.

C1 [Shibuta, Tomomi; Igarashi, Ayumi; Yamamoto-Mitani, Noriko] Univ Tokyo, Grad Sch Med, Sch Hlth Sci & Nursing, Tokyo, Japan.

[Waki, Kayo; Tomizawa, Nobuko; Yamaguchi, Satoko; Fujita, Hideo; Kimura, Shigeko; Fujiu, Katsuhito] Univ Tokyo, Grad Sch Med, Dept Ubiquitous Hlth Informat, Tokyo, Japan.

[Waki, Kayo; Waki, Hironori; Izumida, Yoshihiko; Sasako, Takayoshi; Kobayashi, Masatoshi; Suzuki, Ryo; Yamauchi, Toshimasa; Kadowaki, Takashi] Univ Tokyo, Grad Sch Med, Dept Diabet & Metab Dis, Tokyo, Japan.

[Ohe, Kazuhiko] Univ Tokyo, Grad Sch Med, Dept Biomed Informat, Tokyo, Japan.

RP Waki, K (reprint author), Univ Tokyo, Grad Sch Med, Dept Ubiquitous Hlth Informat, Tokyo, Japan.; Waki, K (reprint author), Univ Tokyo, Grad Sch Med, Dept Diabet & Metab Dis, Tokyo, Japan.

EM kwaki-tky@umin.ac.jp

OI Shibuta, Tomomi/0000-0001-8867-4656

FU NTT DOCOMO; Japan Society for Promotion of Science KAKENHI grant

[26460858]

FX This study was funded by NTT DOCOMO and was partly supported by Japan

Society for Promotion of Science KAKENHI grant numbers 26460858 to KW.

CR Akao A., 2011, Journal of the Japan Diabetes Society, V54, P128

Akimoto M, 2004, PSYCHOSOMATICS, V45, P343, DOI 10.1176/appi.psy.45.4.343

Amante DJ, 2014, DIABETES TECHNOL THE, V16, P784, DOI 10.1089/dia.2014.0078

Arnhold M, 2014, J MED INTERNET RES, V16, P34, DOI 10.2196/jmir.2968

Coyle ME, 2013, AUST HEALTH REV, V37, P513, DOI 10.1071/AH13060

Daitoku Masuko, 2006, Journal of the Japan Diabetes Society, V49, P1

Franks MM, 2012, AGING MENT HEALTH, V16, P902, DOI 10.1080/13607863.2012.674486

Hayashino Y, 2012, DIABETES RES CLIN PR, V97, P405, DOI 10.1016/j.diabres.2012.04.005

International Diabetes Federation, 2015, IDF DIABETES ATLAS

Jennings CA, 2014, PREV MED, V60, P33, DOI 10.1016/j.ypmed.2013.12.011

Liang X, 2011, DIABETIC MED, V28, P455, DOI 10.1111/j.1464-5491.2010.03180.x

Lim S, 2011, DIABETES METAB J, V35, P50, DOI 10.4093/dmj.2011.35.1.50

Lim S, 2011, DIABETES CARE, V34, P308, DOI 10.2337/dc10-1447

Morrison F, 2011, ARCH INTERN MED, V171, P1542, DOI 10.1001/archinternmed.2011.400

Nijland N, 2011, J MED INTERNET RES, V13, DOI 10.2196/jmir.1603

OHKUBO Y, 1995, DIABETES RES CLIN PR, V28, P103, DOI 10.1016/0168-8227(95)01064-K

Ostbye T, 2005, ANN FAM MED, V3, P209, DOI 10.1370/afm.310

Peduzzi P, 1996, J CLIN EPIDEMIOL, V49, P1373, DOI 10.1016/S0895-4356(96)00236-3

POLONSKY WH, 1995, DIABETES CARE, V18, P754, DOI 10.2337/diacare.18.6.754

PROCHASKA JO, 1992, AM PSYCHOL, V47, P1102, DOI 10.1037//0003-066X.47.9.1102

Ramadas A, 2011, INT J MED INFORM, V80, P389, DOI 10.1016/j.ijmedinf.2011.02.002

Sarkar U, 2011, J AM MED INFORM ASSN, V18, P318, DOI [10.1136/jamia.2010.006015, 10.1136/amiajnl.2010.006015]

Stratton IM, 2000, BRIT MED J, V321, P405, DOI 10.1136/bmj.321.7258.405

Tang PC, 2013, J AM MED INFORM ASSN, V20, P526, DOI 10.1136/amiajnl-2012-001263

Toobert DJ, 2000, DIABETES CARE, V23, P943, DOI 10.2337/diacare.23.7.943

Waki Kayo, 2014, J Diabetes Sci Technol, V8, P209

Welch GW, 1997, DIABETES CARE, V20, P760, DOI 10.2337/diacare.20.5.760

Zickuhr K., 2010, GENERATIONS 2010

NR 30

TC 1

Z9 1

U1 2

U2 5

PU BMJ PUBLISHING GROUP

PI LONDON

PA BRITISH MED ASSOC HOUSE, TAVISTOCK SQUARE, LONDON WC1H 9JR, ENGLAND

SN 2052-4897

J9 BMJ OPEN DIAB RES CA

JI BMJ Open Diab. Res. Care

PD JAN

PY 2017

VL 5

IS 1

DI 10.1136/bmjdrc-2016-000322

PG 7

WC Endocrinology & Metabolism

SC Endocrinology & Metabolism

GA EO9VX

UT WOS:000397037300014

PM 28243450

OA DOAJ Gold, Green Published

DA 2019-01-08

ER

PT J

AU Gerlanc, NM

Cai, J

Tkacz, J

Bolge, SC

Brady, BL

AF Gerlanc, Nicole M.

Cai, Jennifer

Tkacz, Joseph

Bolge, Susan C.

Brady, Brenna L.

TI The association of weight loss with patient experience and outcomes in a

population of patients with type 2 diabetes mellitus prescribed

canagliflozin

SO DIABETES METABOLIC SYNDROME AND OBESITY-TARGETS AND THERAPY

LA English

DT Article

DE T2DM; patient outcomes; diabetes self-management; SGLT2 inhibitor;

weight management

ID QUALITY-OF-LIFE; TREATMENT SATISFACTION; CENTERED APPROACH; GLYCEMIC

CONTROL; HEALTH; CARE; MANAGEMENT; COMPLICATIONS; EFFICACY; ADULTS

AB Objective: Type 2 diabetes mellitus (T2DM) is a chronic condition complicated by being overweight or obese. This study used a patient survey to assess health, satisfaction, and diabetes self-management in relation to weight management.

Methods: A survey including the Current Health Satisfaction Questionnaire, Diabetes Distress Scale, and Diabetes Treatment Satisfaction Questionnaire was administered using an online platform to a sample of 205 patients with T2DM prescribed canagliflozin. Patients were placed into 5 groups based on their self-reported weight change since initiation of canagliflozin: Lost >10 lbs, Lost 5-10 lbs, Lost <5 lbs, No Change, and Gained Weight. One-way ANOVAs, Kruskall-Wallis tests, and multivariable regression were used to explore differences between weight loss groups.

Results: The majority of patients (66.8%) reported losing weight. Compared to other groups, patients who lost >10 lbs were more likely to be engaged in a weight loss program for at least 6 months. Patients in the Lost >10 lbs and Lost 5-10 lbs groups reported the greatest satisfaction with canagliflozin (p<0.05 for both). Multivariable analyses controlling for patient demographic and treatment characteristics revealed that losing >10 lbs was associated with reduced diabetes distress, improved A1c and blood glucose levels, and decreased perceived frequency of hyperglycemia (p<0.05).

Conclusion: Increased positive patient outcomes, engagement in diabetes self-management, and medication satisfaction were observed among patients who reported weight loss. These findings suggest that a T2DM regimen that includes canagliflozin as part of a weight loss regimen can help improve patient outcomes and experiences with T2DM.

C1 [Gerlanc, Nicole M.; Tkacz, Joseph; Brady, Brenna L.] Hlth Analyt LLC, 9200 Rumsey Rd,Suite 215, Columbia, MD 21045 USA.

[Cai, Jennifer; Bolge, Susan C.] Janssen Sci Affairs LLC, Titusville, NJ USA.

RP Brady, BL (reprint author), Hlth Analyt LLC, 9200 Rumsey Rd,Suite 215, Columbia, MD 21045 USA.

EM bbrady@healthanalytic.com

FU Janssen Scientific Affairs, LLC

FX Janssen Scientific Affairs, LLC, funded this study.

CR A. American Diabetes, 2013, DIABETES CARE, V36, P1033, DOI DOI 10.2337/DC12-2625

American Association of Diabetes Educators, AADE7 SELFC BEH

American Diabetes Association, 2016, CLIN DIABETES, V34, P3, DOI DOI 10.2337/DIACLIN.34.1.3

Bash LD, 2008, ARCH INTERN MED, V168, P2440, DOI 10.1001/archinte.168.22.2440

Beckman JA, 2002, JAMA-J AM MED ASSOC, V287, P2570, DOI 10.1001/jama.287.19.2570

Berwick DM, 2008, HEALTH AFFAIR, V27, P759, DOI 10.1377/hlthaff.27.3.759

Bradley C., 1994, HDB PSYCHOL DIABETES

Centers for Disease Control and Prevention, 2014, NAT DIAB STAT REP ES

Fu AZ, 2009, DIABETES CARE, V32, P2187, DOI 10.2337/dc09-1128

Funnell MM, 2004, CLIN DIABETES, V22, P123, DOI DOI 10.2337/DIACLIN.22.3.123

Goldney RD, 2004, DIABETES CARE, V27, P1066, DOI 10.2337/diacare.27.5.1066

Guisasola FA, 2008, DIABETES OBES METAB, V10, P25, DOI 10.1111/j.1463-1326.2008.00882.x

Hibbard JH, 2013, HEALTH AFFAIR, V32, P207, DOI 10.1377/hlthaff.2012.1061

Huang ES, 2007, DIABETES CARE, V30, P2478, DOI 10.2337/dc07-0499

Idris I, 2009, DIABETES OBES METAB, V11, P79, DOI 10.1111/j.1463-1326.2008.00982.x

Inzucchi SE, 2012, DIABETES CARE, V35, P1364, DOI 10.2337/dc12-0413

Kempen JH, 2004, ARCH OPHTHALMOL-CHIC, V122, P552

Klein S, 2004, DIABETES CARE, V27, P2067, DOI 10.2337/diacare.27.8.2067

Krentz AJ, 2005, DRUGS, V65, P385, DOI 10.2165/00003495-200565030-00005

Lau DCW, 2013, CAN J DIABETES, V37, P128, DOI 10.1016/j.jcjd.2013.03.023

Look ARG, 2007, DIABETES CARE, V30, P1374, DOI DOI 10.2337/DC07-0048]

Norris SL, 2004, ARCH INTERN MED, V164, P1395, DOI 10.1001/archinte.164.13.1395

Pi-Sunyer FX, 2009, POSTGRAD MED, V121, P94, DOI 10.3810/pgm.2009.09.2056

Polonsky WH, 2005, DIABETES CARE, V28, P626, DOI 10.2337/diacare.28.3.626

Redekop WK, 2002, DIABETES CARE, V25, P458, DOI 10.2337/diacare.25.3.458

Rothman K J, 1990, Epidemiology, V1, P43, DOI 10.1097/00001648-199001000-00010

Schernthaner G, 2013, DIABETES CARE, V36, P2508, DOI 10.2337/dc12-2491

Shetty Sharashchandra, 2005, J Manag Care Pharm, V11, P559

Stenlof K, 2013, DIABETES OBES METAB, V15, P372, DOI 10.1111/dom.12054

Stenlof K, 2014, CURR MED RES OPIN, V30, P163, DOI 10.1185/03007995.2013.850066

Stratton IM, 2000, BRIT MED J, V321, P405, DOI 10.1136/bmj.321.7258.405

Sullivan PW, 2005, DIABETES CARE, V28, P1599, DOI 10.2337/diacare.28.7.1599

Tchicaya A, 2015, HEALTH QUAL LIFE OUT, V13, DOI 10.1186/s12955-015-0348-8

Traina S, 2014, POSTGRAD MED, V126, P7, DOI 10.3810/pgm.2014.05.2752

Traina SB, 2015, DIABETES THER, V6, P611, DOI 10.1007/s13300-015-0145-8

Traina SB, 2015, PATIENT-RELAT OUTCOM, V6, P103, DOI 10.2147/PROM.S79368

Wandell PE, 2005, SCAND J PRIM HEALTH, V23, P68, DOI 10.1080/02813430510015296

Watkins KW, 2000, DIABETES CARE, V23, P1511, DOI 10.2337/diacare.23.10.1511

Zhuo XH, 2013, AM J PREV MED, V45, P253, DOI 10.1016/j.amepre.2013.04.017

NR 39

TC 2

Z9 2

U1 0

U2 0

PU DOVE MEDICAL PRESS LTD

PI ALBANY

PA PO BOX 300-008, ALBANY, AUCKLAND 0752, NEW ZEALAND

SN 1178-7007

J9 DIABET METAB SYND OB

JI Diabetes Metab. Syndr. Obes.

PY 2017

VL 10

BP 89

EP 99

DI 10.2147/DMSO.S129824

PG 11

WC Endocrinology & Metabolism

SC Endocrinology & Metabolism

GA EO6NZ

UT WOS:000396811100001

PM 28360528

OA DOAJ Gold, Green Published

DA 2019-01-08

ER

PT J

AU Mina, TH

Lahti, M

Drake, AJ

Raikkonen, K

Minnis, H

Denison, FC

Norman, JE

Reynolds, RM

AF Mina, T. H.

Lahti, M.

Drake, A. J.

Raikkonen, K.

Minnis, H.

Denison, F. C.

Norman, J. E.

Reynolds, R. M.

TI Prenatal exposure to very severe maternal obesity is associated with

adverse neuropsychiatric outcomes in children

SO PSYCHOLOGICAL MEDICINE

LA English

DT Article

DE Affective; attention deficit; externalizing; hyperactivity;

neurodevelopmental; obesity; prenatal; sleep

ID BODY-MASS INDEX; AUTISM SPECTRUM DISORDERS; DIFFICULTIES QUESTIONNAIRE;

PRESCHOOL-CHILDREN; MENTAL-DISORDERS; UNITED-STATES; PREGNANCY;

BEHAVIOR; SYMPTOMS; COHORT

AB Background. Prenatal maternal obesity has been linked to adverse childhood neuropsychiatric outcomes, including increased symptoms of attention deficit hyperactivity disorder (ADHD), internalizing and externalizing problems, affective disorders and neurodevelopmental problems but few studies have studied neuropsychiatric outcomes among offspring born to very severely obese women or assessed potential familial confounding by maternal psychological distress.

Method. We evaluated neuropsychiatric symptoms in 112 children aged 3-5 years whose mothers had participated in a longitudinal study of obesity in pregnancy (50 very severe obesity, BMI >= 40 kg/m(2), obese class III and 62 lean, BMI 18.5-25 kg/m(2)). The mothers completed the Conners' Hyperactivity Scale, Early Symptomatic Syndrome Eliciting Neurodevelopmental Clinical Examination Questionnaire (ESSENCE-Q), Child's Sleep Habits Questionnaire (CSHQ), Strengths and Difficulties Questionnaire (SDQ), and Child Behavior Checklist (CBCL) to assess child neuropsychiatric symptoms. Covariates included child's sex, age, birthweight, gestational age, socioeconomic deprivation levels, maternal age, parity, smoking status during pregnancy, gestational diabetes and maternal concurrent symptoms of anxiety and depression assessed using State Anxiety of Spielberger State-Trait Anxiety Index (STAI) and General Health Questionnaire (GHQ), respectively.

Results. Children exposed to prenatal maternal very severe obesity had significantly higher scores in the Conners' Hyperactivity Scale; ESSENCE-Q; total sleep problems in CSHQ; hyperactivity, conduct problems and total difficulties scales of the SDQ; higher externalizing and total problems, anxious/depressed, aggressive behaviour and other problem syndrome scores and higher DSM-oriented affective, anxiety and ADHD problems in CBCL. Prenatal maternal very severe obesity remained a significant predictor of child neuropsychiatric problems across multiple scales independent of demographic factors, prenatal factors and maternal concurrent symptoms of anxiety and depression.

Conclusions. Prenatal maternal very severe obesity is a strong predictor of increased neuropsychiatric problems in early childhood.

C1 [Mina, T. H.; Lahti, M.; Drake, A. J.; Reynolds, R. M.] Univ Edinburgh, Univ BHF Ctr Cardiovasc Sci, Queens Med Res Inst, 47 Little France, Edinburgh EH16 4TJ, Midlothian, Scotland.

[Mina, T. H.; Denison, F. C.; Norman, J. E.; Reynolds, R. M.] Univ Edinburgh, Tommys Ctr Maternal & Fetal Hlth, Queens Med Res Inst, Edinburgh EH16 4TJ, Midlothian, Scotland.

[Lahti, M.; Raikkonen, K.] Univ Helsinki, Inst Behav Sci, Helsinki, Finland.

[Minnis, H.] Univ Glasgow, Inst Hlth & Wellbeing, Glasgow, Lanark, Scotland.

[Denison, F. C.; Norman, J. E.] Univ Edinburgh, MRC Ctr Reprod Hlth, Queens Med Res Inst, Edinburgh, Midlothian, Scotland.

RP Reynolds, RM (reprint author), Univ Edinburgh, Univ BHF Ctr Cardiovasc Sci, Queens Med Res Inst, 47 Little France, Edinburgh EH16 4TJ, Midlothian, Scotland.

EM r.reynolds@ed.ac.uk

RI Reynolds, Rebecca/C-3044-2008

OI Raikkonen, Katri/0000-0003-3124-3470; Denison,

Fiona/0000-0003-0371-2014; Lahti-Pulkkinen, Marius/0000-0002-5244-5525

FU Tommy's the Baby Charity; University of Edinburgh, Scotland; Academy of

Finland; University of Helsinki Funds; Scottish Senior Clinical

Fellowship [SCD/09]; British Heart Foundation; Medical Research Council

[G0501904]

FX We acknowledge funding from Tommy's the Baby Charity. THM is funded by

Principal Development Scholarship, Charles Darwin Scholarship and Global

Research Scholarship, University of Edinburgh, Scotland. M.L. is funded

by the Academy of Finland and University of Helsinki Funds. A.J.D. was

supported by Scottish Senior Clinical Fellowship (SCD/09). T.H.M.,

A.J.D. and R.M.R. acknowledge the support of the British Heart

Foundation.

CR Achenbach T., 2000, MANUAL ASEBA PRESCHO

Antoniou EE, 2014, BMJ OPEN, V4, DOI 10.1136/bmjopen-2014-005974

Atladottir HO, 2015, EUR CHILD ADOLES PSY, V24, P173, DOI 10.1007/s00787-014-0553-8

Bilbo SD, 2012, FRONT NEUROENDOCRIN, V33, P267, DOI 10.1016/j.yfrne.2012.08.006

Boyle CA, 2011, PEDIATRICS, V127, P1034, DOI 10.1542/peds.2010-2989

Brion MJ, 2011, PEDIATRICS, V127, pE202, DOI 10.1542/peds.2010-0651

Brown AS, 2014, MOL PSYCHIATR, V19, P259, DOI 10.1038/mp.2012.197

Buescher AVS, 2014, JAMA PEDIATR, V168, P721, DOI 10.1001/jamapediatrics.2014.210

Buss C, 2012, PLOS ONE, V7, DOI 10.1371/journal.pone.0037758

Caspi A, 1996, ARCH GEN PSYCHIAT, V53, P1033

Chen Q, 2014, INT J EPIDEMIOL, V43, P83, DOI 10.1093/ije/dyt152

Chu SY, 2009, MATERN CHILD HLTH J, V13, P614, DOI 10.1007/s10995-008-0388-3

Croft S, 2015, PEDIATRICS, V135, pE1210, DOI 10.1542/peds.2014-2920

Dunn GA, 2011, HORM BEHAV, V59, P290, DOI 10.1016/j.yhbeh.2010.05.004

Erhart M, 2008, EUR CHILD ADOLES PSY, V17, P106, DOI 10.1007/s00787-008-1012-1

Fisher SC, 2013, PREV MED, V56, P372, DOI 10.1016/j.ypmed.2013.02.015

Gardner RM, 2015, INT J EPIDEMIOL, V44, P870, DOI 10.1093/ije/dyv081

Gillberg C, 2010, RES DEV DISABIL, V31, P1543, DOI 10.1016/j.ridd.2010.06.002

Goodlin-Jones BL, 2008, J DEV BEHAV PEDIATR, V29, P82, DOI 10.1097/DBP.0b013e318163c39a

Goodman R, 1997, J CHILD PSYCHOL PSYC, V38, P581, DOI 10.1111/j.1469-7610.1997.tb01545.x

Goodman SH, 2011, CLIN CHILD FAM PSYCH, V14, P1, DOI 10.1007/s10567-010-0080-1

Heinrich A, 2015, BEHAV GENET, V45, P529, DOI 10.1007/s10519-015-9721-y

Heslehurst N, 2010, INT J OBESITY, V34, P420, DOI 10.1038/ijo.2009.250

Jo H, 2015, PEDIATRICS, V135, pE1198, DOI 10.1542/peds.2014-3058

Krakowiak P, 2012, PEDIATRICS, V129, pE1121, DOI 10.1542/peds.2011-2583

Le HH, 2014, EUR CHILD ADOLES PSY, V23, P587, DOI 10.1007/s00787-013-0477-8

Li YM, 2016, J AUTISM DEV DISORD, V46, P95, DOI 10.1007/s10803-015-2549-8

Lovejoy MC, 2000, CLIN PSYCHOL REV, V20, P561, DOI 10.1016/S0272-7358(98)00100-7

Madigan S, 2016, PSYCHOL BULL, V142, P367, DOI 10.1037/bul0000029

McLoone P, 2004, CARSTAIRS SCORES SCO

Mina TH, 2015, PSYCHOL MED, V45, P3133, DOI 10.1017/S0033291715001087

Molyneaux E, 2014, OBSTET GYNECOL, V123, P857, DOI 10.1097/AOG.0000000000000170

Norman JE, 2011, P NUTR SOC, V70, P450, DOI 10.1017/S0029665111003077

Parade SH, 2016, CHILD DEV, V87, P86, DOI 10.1111/cdev.12484

Peng XL, 2013, PLOS ONE, V8, DOI 10.1371/journal.pone.0079476

Pihlakoski L, 2006, EUR CHILD ADOLES PSY, V15, P409, DOI 10.1007/s00787-006-0548-1

Pugh SJ, 2016, BJOG-INT J OBSTET GY, V123, P2094, DOI 10.1111/1471-0528.13909

Raikkonen K, 2015, PSYCHOL MED, V45, P3217, DOI 10.1017/S003329171500121X

Reising MM, 2013, J CHILD FAM STUD, V22, P335, DOI 10.1007/s10826-012-9582-4

Rescorla LA, 2011, J CLIN CHILD ADOLESC, V40, P456, DOI 10.1080/15374416.2011.563472

Reynolds RM, 2013, BMJ-BRIT MED J, V347, DOI 10.1136/bmj.f4539

Robinson M, 2013, J DEV ORIG HLTH DIS, V4, P42, DOI 10.1017/S2040174412000578

Rodriguez A, 2008, INT J OBESITY, V32, P550, DOI 10.1038/sj.ijo.0803741

Rodriguez A, 2010, J CHILD PSYCHOL PSYC, V51, P134, DOI 10.1111/j.1469-7610.2009.02133.x

Simpkin CT, 2014, J SLEEP RES, V23, P397, DOI 10.1111/jsr.12142

Van Batenburg-Eddes T, 2013, J CHILD PSYCHOL PSYC, V54, P591, DOI 10.1111/jcpp.12023

Van den Bergh BRH, 2005, NEUROSCI BIOBEHAV R, V29, P259, DOI 10.1016/j.neubiorev.2004.10.010

van der Heijden KB, 2013, CHRONOBIOL INT, V30, P919, DOI 10.3109/07420528.2013.790041

Van Lieshout RJ, 2013, CHILD PSYCHIAT HUM D, V44, P382, DOI 10.1007/s10578-012-0332-z

Yap MBH, 2015, J AFFECT DISORDERS, V175, P424, DOI 10.1016/j.jad.2015.01.050

NR 50

TC 13

Z9 13

U1 8

U2 33

PU CAMBRIDGE UNIV PRESS

PI NEW YORK

PA 32 AVENUE OF THE AMERICAS, NEW YORK, NY 10013-2473 USA

SN 0033-2917

EI 1469-8978

J9 PSYCHOL MED

JI Psychol. Med.

PD JAN

PY 2017

VL 47

IS 2

BP 353

EP 362

DI 10.1017/S0033291716002452

PG 10

WC Psychology, Clinical; Psychiatry; Psychology

SC Psychology; Psychiatry

GA EG8PU

UT WOS:000391320100014

PM 27776561

DA 2019-01-08

ER

PT J

AU Ebert, DD

Nobis, S

Lehr, D

Baumeister, H

Riper, H

Auerbach, RP

Snoek, F

Cuijpers, P

Berking, M

AF Ebert, D. D.

Nobis, S.

Lehr, D.

Baumeister, H.

Riper, H.

Auerbach, R. P.

Snoek, F.

Cuijpers, P.

Berking, M.

TI The 6-month effectiveness of Internet-based guided self-help for

depression in adults with Type 1 and 2 diabetes mellitus

SO DIABETIC MEDICINE

LA English

DT Article

ID RANDOMIZED CONTROLLED-TRIAL; COGNITIVE-BEHAVIOR THERAPY; WEB-BASED

INTERVENTION; MOBILE PHONE SUPPORT; COMORBID DEPRESSION; MAJOR

DEPRESSION; METAANALYSIS; CARE; EFFICACY; ACCEPTANCE

AB Aim The aim of this research is to examine the 6-month effects of an Internet-based guided self-help intervention for comorbid depressive symptoms in people with diabetes.

Methods Participants (n = 260) with Type 1 or 2 diabetes and elevated depressive symptoms [Center for Epidemiological Studies Depression Scale (CES-D) >= 23] were randomly assigned to a guided Internet-based self-help intervention or a control condition (treatment as usual + online psychoeducation about depression). The primary outcome was a change in depressive symptom severity (CES-D) from baseline to 6-month follow-up. The secondary outcomes included numbers of people achieving treatment response (reliable change of depressive symptoms) and remission (CES-D = 16), as well as the effects on glycaemic control, diabetes-related emotional distress and diabetes acceptance. Repeated measures analysis of variance examined between-group differences using intent-to-treat principles.

Results Both conditions showed improvements in depression severity: intervention condition, d = 1.48 [95% confidence interval (95% CI): 1.21 to 1.76]; control condition d = 0.55 (95% CI: 0.30 to 0.80). Changes were significantly greater in the intervention condition with a large between-group effect size (d = 0.83, 95% CI: 0.57 to 1.08). Accordingly, effects on response [relative risk (RR) = 2.60 (95% CI: 2.01 to 3.36), P < 0.001] and remission [RR = 3.36 (95% CI: 2.98 to 5.44), P < 0.001] were in favour of the intervention group, as were differences in change in diabetes emotional distress (d = 0.50, 95% CI: 0.04 to 0.54), and physical and mental functioning [Short Form Health Survey (SF-12) Physical d = 0.27 (95% CI: 0.01 to 0.51) and SF-12 Mental d = 0.68 (95% CI: 0.11 to 0.40)]. The intervention group was not superior with regard to glycaemic control, diabetes self-management and diabetes acceptance.

Conclusions The trial indicates that Internet-based guided self-help treatments for depression in people with diabetes can have sustained effects on depressive symptoms, well-being and emotional distress associated with diabetes.

C1 [Ebert, D. D.; Berking, M.] Friedrich Alexander Univ Erlangen Nuremberg, Dept Clin Psychol & Psychotherapy, Erlangen, Germany.

[Ebert, D. D.; Nobis, S.; Lehr, D.; Riper, H.; Cuijpers, P.; Berking, M.] Leuphana Univ, Div Online Hlth Trainings, Innovat Incubator, Luneburg, Germany.

[Ebert, D. D.] Harvard Univ, Dept Hlth Care Policy, Boston, MA 02115 USA.

[Baumeister, H.] Univ Ulm, Inst Psychol & Educ, Dept Clin Psychol & Psychotherapy, Ulm, Germany.

[Riper, H.; Cuijpers, P.] Vrije Univ Amsterdam, Dept Clin Psychol, Amsterdam, Netherlands.

[Auerbach, R. P.] Harvard Med Sch, McLean Hosp, Dept Psychiat, Belmont, MA USA.

[Snoek, F.] Vrije Univ Amsterdam, Med Ctr, Dept Med Psychol, Amsterdam, Netherlands.

[Snoek, F.] Acad Med Ctr, Amsterdam, Netherlands.

[Snoek, F.] Vrije Univ Amsterdam, Med Ctr, Inst Hlth & Care Res EMGO, Amsterdam, Netherlands.

RP Ebert, DD (reprint author), Friedrich Alexander Univ Erlangen Nuremberg, Dept Clin Psychol & Psychotherapy, Erlangen, Germany.

EM David.Ebert@fau.de

OI Ebert, David Daniel/0000-0001-6820-0146

FU BARMER GEK; European Union [EU EFRE: ZW6-80119999, CCI 2007DE161PR001]

FX The BARMER GEK and the European Union funded this study (EU EFRE:

ZW6-80119999, CCI 2007DE161PR001).

CR Anderson RJ, 2001, DIABETES CARE, V24, P1069, DOI 10.2337/diacare.24.6.1069

[Anonymous], 2017, DIABET MED, V34, P99

Baumeister H, 2014, DIABETES RES CLIN PR, V105, P30, DOI 10.1016/j.diabres.2014.04.031

Baumeister H, 2012, COCHRANE DB SYST REV, V12, DOI DOI 10.1002/14651858

Bjelland I, 2002, J PSYCHOSOM RES, V52, P69, DOI 10.1016/S0022-3999(01)00296-3

Cuijpers P, 2014, J AFFECT DISORDERS, V159, P118, DOI 10.1016/j.jad.2014.02.026

Donker T, 2009, BMC MED, V7, DOI 10.1186/1741-7015-7-79

Ebert DD, 2015, HEALTH PSYCHOL, V34, P1240, DOI 10.1037/hea0000277

Ebert DD, 2015, PLOS ONE, V10, DOI 10.1371/journal.pone.0119895

Gandek B, 1998, J CLIN EPIDEMIOL, V51, P1171, DOI 10.1016/S0895-4356(98)00109-7

Gonzalez JS, 2008, DIABETES CARE, V31, P2398, DOI 10.2337/dc08-1341

Hautzinger M, 1993, ALLGEMEINE DEPRESSIO

Huang YF, 2013, BMC PSYCHIATRY, V13, DOI 10.1186/1471-244X-13-260

Hutter N, 2010, DIABETOLOGIA, V53, P2470, DOI 10.1007/s00125-010-1873-y

JACOBSON NS, 1991, J CONSULT CLIN PSYCH, V59, P12, DOI 10.1037//0022-006X.59.1.12

Katon WJ, 2005, DIABETES CARE, V28, P2668, DOI 10.2337/diacare.28.11.2668

Katon WJ, 2004, MED CARE, V42, P1222, DOI 10.1097/00005650-200412000-00009

Lamers F, 2010, PSYCHOTHER PSYCHOSOM, V79, P217, DOI 10.1159/000313690

Lee EH, 2014, HEALTH QUAL LIFE OUT, V12, DOI 10.1186/s12955-014-0142-z

Lustman PJ, 1998, GEN HOSP PSYCHIAT, V20, P302, DOI 10.1016/S0163-8343(98)00039-5

Lustman PJ, 1998, ANN INTERN MED, V129, P613, DOI 10.7326/0003-4819-129-8-199810150-00005

Markowitz SM, 2011, PSYCHOSOMATICS, V52, P1, DOI 10.1016/j.psym.2010.11.007

Nobis S, 2015, DIABETES CARE, V38, P776, DOI 10.2337/dc14-1728

Nobis S, 2013, BMC PSYCHIATRY, V13, DOI 10.1186/1471-244X-13-306

Nouwen A, 2010, DIABETOLOGIA, V53, P2480, DOI 10.1007/s00125-010-1874-x

Petrak F, 2015, LANCET DIABETES ENDO, V3, P472, DOI 10.1016/S2213-8587(15)00045-5

Safren SA, 2014, DIABETES CARE, V37, P625, DOI [10.2337/dc13-0816, 10.2337/dc16-er06]

Schmitt A, 2013, DIABETES STOFFWECH H, V22, P9

Schmitt A, 2013, HEALTH QUAL LIFE OUT, V11, DOI 10.1186/1477-7525-11-138

Sharif Farkhondeh, 2014, Iran J Nurs Midwifery Res, V19, P529

vansBastelaar KMP, 2011, DIABETES CARE, V34, P320

NR 31

TC 12

Z9 12

U1 3

U2 35

PU WILEY-BLACKWELL

PI HOBOKEN

PA 111 RIVER ST, HOBOKEN 07030-5774, NJ USA

SN 0742-3071

EI 1464-5491

J9 DIABETIC MED

JI Diabetic Med.

PD JAN

PY 2017

VL 34

IS 1

BP 99

EP 107

DI 10.1111/dme.13173

PG 9

WC Endocrinology & Metabolism

SC Endocrinology & Metabolism

GA EF9SF

UT WOS:000390669800013

PM 27334444

DA 2019-01-08

ER

PT J

AU Gibbons, E

Casey, AF

Brewster, KZ

AF Gibbons, Emma

Casey, Amanda Faith

Brewster, Keith Z.

TI Bariatric surgery and intellectual disability: Furthering evidence-based

practice

SO DISABILITY AND HEALTH JOURNAL

LA English

DT Review

DE Weight loss; Scoping review; Prader-Willi syndrome; Obesity

ID PRADER-WILLI-SYNDROME; BILIOPANCREATIC DIVERSION; COGNITIVE FUNCTION;

GASTRIC BYPASS; WEIGHT-LOSS; MORBID-OBESITY; GASTROPLASTY; MANAGEMENT;

PATIENT; SUPPORT

AB Background: Rates of morbid obesity are higher for individuals with intellectual disability (ID). Individuals with ID may find nutritional guidelines difficult to follow and many face personal and environmental barriers for physical activity. Bariatric surgery may reduce obesity related health comorbidities while promoting sustained weight loss in diverse populations. Yet no study has reviewed the feasibility of conducting bariatric surgery on individuals with ID.

Objectives: To conduct a scoping review of literature on bariatric procedures performed on individuals with D.

Methods: Authors searched electronic database via PubMED, Science Direct, Wiley and Medline (1975-2014). Extracted articles were evaluated independently following scoping reviews guidelines.

Results: Reviewers included sixteen studies. Nine surgical interventions were reported on 49 patients with ID. Studies followed either case report or case series design. The most common procedure patients received was biliopancreatic diversion (n = 24) followed by Roux en -Y Gastric Bypass (n = 12). Degree of weight loss was the primary outcome in each study. Excess weight loss (%EWL) ranged from 12% to 86%. Further benefits included improved quality of life, decreased psychological tension within family and resolution of sleep apnea, hypertension, respiratory distress and type II diabetes. Six studies included a post-operative follow-up period below two years.

Conclusions: Bariatric surgery may be a viable option to treat obesity in individuals with ID but there is no consensus which procedure is preferred and which associated interventions should be in place to warrant long lasting results. Further research featuring randomized control trials may be beneficial. (C) 2016 Elsevier Inc. All rights reserved.

C1 [Gibbons, Emma; Casey, Amanda Faith] St Francis Xavier Univ, Dept Human Kinet, POB 5000, Antigonish, NS B2G 2W5, Canada.

[Brewster, Keith Z.] Okanagan Bariatr Surg Ctr, Patient Serv, Kelowna, BC, Canada.

[Brewster, Keith Z.] Sch Hlth & Exercise Sci, Kelowna, BC, Canada.

RP Casey, AF (reprint author), St Francis Xavier Univ, Dept Human Kinet, POB 5000, Antigonish, NS B2G 2W5, Canada.

EM acasey@stfx.ca

CR Alosco ML, 2014, AM J SURG, V207, P870, DOI 10.1016/j.amjsurg.2013.05.018

ANDERSON AE, 1980, J PEDIATR SURG, V15, P876, DOI 10.1016/S0022-3468(80)80297-1

Antal SC, 1996, OBES SURG, V6, P58, DOI 10.1381/096089296765557286

Arksey H, 2005, INT J SOC RES METHOD, V8, P19, DOI DOI 10.1080/1364557032000119616

Bauchowitz AU, 2005, PSYCHOSOM MED, V67, P825, DOI 10.1097/01.psy.0000174173.32271.01

Bertoli S, 2006, NUTR METAB CARDIOVAS, V16, P100, DOI 10.1016/j.numecd.2005.05.007

Buchwald H, 2004, JAMA-J AM MED ASSOC, V292, P1724, DOI 10.1001/jama.292.14.1724

Buchwald H, 2009, AM J MED, V122, P248, DOI 10.1016/j.amjmed.2008.09.041

Casey AF, 2013, DISABIL HEALTH J, V6, P2, DOI 10.1016/j.dhjo.2012.09.002

Chelala E, 2014, SURG ENDOSC, V11, P268

Chow A, 2016, J OBES, V2016

de Almeida MQ, 2005, OBES SURG, V15, P901, DOI 10.1381/0960892054222885

DOUSEI T, 1992, OBES SURG, V2, P189, DOI 10.1381/096089292765560411

Fong AKW, 2012, OBES SURG, V22, P1742, DOI 10.1007/s11695-012-0725-x

FONKALSRUD EW, 1981, J PEDIATR SURG, V16, P888, DOI 10.1016/S0022-3468(81)80841-X

Grugni G, 2000, OBES SURG, V10, P179, DOI 10.1381/096089200321668631

Heinberg LJ, 2014, SURG OBES RELAT DIS, P1

Kobayashi J, 2003, OBES SURG, V13, P803, DOI 10.1381/096089203322509453

Laurent-Jaccard 17. A, 1991, OBES SURG, V1, P83

Levac D, 2010, IMPLEMENT SCI, V5, DOI 10.1186/1748-5908-5-69

Livhits M, 2011, OBES REV, V12, P142, DOI 10.1111/j.1467-789X.2010.00720.x

Marinari GM, 2001, OBES SURG, V11, P491, DOI 10.1381/096089201321209404

MASON EE, 1995, OBES SURG, V5, P23, DOI 10.1381/096089295765558114

Mechanick JI, 2013, SURG OBES RELAT DIS, V9, P159, DOI 10.1016/j.soard.2012.12.010

Musella M, 2014, J INVEST SURG, V27, P102, DOI 10.3109/08941939.2013.832824

Papavramidis ST, 2006, J PEDIATR SURG, V41, P1153, DOI 10.1016/j.jpedsurg.2006.01.065

Rimmer JH, 2010, J INTELL DISABIL RES, V54, P787, DOI 10.1111/j.1365-2788.2010.01305.x

Rimmer JH, 2012, LANCET, V380, P193, DOI 10.1016/S0140-6736(12)61028-9

Rimmer JH, 2005, AM J HEALTH PROMOT, V19, P327, DOI 10.4278/0890-1171-19.5.327

Spanos D, 2013, NUTR J, V12, DOI 10.1186/1475-2891-12-132

Spitznagel MB, 2013, SURG OBES RELAT DIS, V9, P765, DOI 10.1016/j.soard.2013.04.011

Spitznagel MB, 2013, SURG OBES RELAT DIS, V9, P580, DOI 10.1016/j.soard.2013.04.007

TOUQUET VLR, 1983, BRIT J SURG, V70, P180, DOI 10.1002/bjs.1800700314

Votruba Kristen, 2014, Curr Psychiatry Rep, V16, P448, DOI 10.1007/s11920-014-0448-x

Yamaki K, 2005, MENT RETARD, V43, P1, DOI 10.1352/0047-6765(2005)43<1:BWSAAW>2.0.CO;2

2013, SURG OBES RELAT DIS, V9, P159, DOI DOI 10.1016/J.SOARD.2012.12.010

NR 36

TC 2

Z9 2

U1 1

U2 9

PU ELSEVIER SCIENCE INC

PI NEW YORK

PA 360 PARK AVE SOUTH, NEW YORK, NY 10010-1710 USA

SN 1936-6574

EI 1876-7583

J9 DISABIL HEALTH J

JI Disabil. Health J.

PD JAN

PY 2017

VL 10

IS 1

BP 3

EP 10

DI 10.1016/j.dhjo.2016.09.005

PG 8

WC Health Care Sciences & Services; Health Policy & Services; Public,

Environmental & Occupational Health; Rehabilitation

SC Health Care Sciences & Services; Public, Environmental & Occupational

Health; Rehabilitation

GA EF4KB

UT WOS:000390293100002

PM 27720223

DA 2019-01-08

ER

PT J

AU Kampling, H

Petrak, F

Farin, E

Kulzer, B

Herpertz, S

Mittag, O

AF Kampling, Hanna

Petrak, Frank

Farin, Erik

Kulzer, Bernd

Herpertz, Stephan

Mittag, Oskar

TI Trajectories of depression in adults with newly diagnosed type 1

diabetes: results from the German Multicenter Diabetes Cohort Study

SO DIABETOLOGIA

LA English

DT Article

DE Adults; Depression; Growth mixture modelling; Longitudinal data; Onset

cohort; Psychological outcomes; Type 1 diabetes

ID GLYCEMIC CONTROL; COMORBID DEPRESSION; PREVALENCE; SYMPTOMS;

INDIVIDUALS; DISORDERS; DISTRESS; THERAPY; TRIAL; CARE

AB There is a paucity of longitudinal data on type 1 diabetes and depression, especially in adults. The present study prospectively analysed trajectories of depressive symptoms in adults during the first 5 years of living with type 1 diabetes. We aimed to identify distinct trajectories of depressive symptoms and to examine how they affect diabetes outcome.

We reanalysed data from a prospective multicentre observational cohort study including 313 adults with newly diagnosed type 1 diabetes. At baseline and in annual postal surveys over 5 consecutive years, we gathered patient characteristics and behavioural and psychosocial data (e.g. Symptom Checklist-90-R [SCL-90-R]). Medical data (e.g. HbA(1c) levels) was obtained from the treating physicians. We applied growth mixture modelling (GMM) to identify distinct trajectories of depression over time.

Five years after diagnosis, 7.8% (n = 20) of patients were moderately depressed and 10.2% (n = 26) were severely depressed. GMM statistics identified three possible models of trajectories (class 1, 'no depressive symptoms'; class 2, 'worsening depressive symptoms that improve after 2 years'; class 3, 'worsening depressive symptoms'). Severity of depression symptoms at baseline (subscale of the SCL-90-R questionnaire) significantly predicted membership of classes 2 and 3 vs class 1. After 5 years, higher HbA(1c) values were detected in class 3 patients (mean = 8.2%, 66 mmol/mol) compared with class 1 and class 2 (both: mean = 7.2%, 55 mmol/mol).

We identified distinct trajectories of depressive symptoms that are also relevant for diabetes outcome. Patients with worsening depressive symptoms over time exhibited poor glycaemic control after the first 5 years of living with diabetes. They also exhibited a reduced quality of life and increased diabetes-related distress.

C1 [Kampling, Hanna; Farin, Erik; Mittag, Oskar] Univ Freiburg, Sect Hlth Care Res & Rehabil Res, Med Ctr, Fac Med, Hugstetterstr 49, D-79106 Freiburg, Germany.

[Petrak, Frank; Herpertz, Stephan] Ruhr Univ Bochum, LWL Univ Clin Bochum, Dept Psychosomat Med & Psychotherapy, Bochum, Germany.

[Petrak, Frank] Ctr Psychotherapy Wiesbaden, Wiesbaden, Germany.

[Kulzer, Bernd] Diabet Ctr Mergentheim, Bad Mergentheim, Germany.

[Kulzer, Bernd] Res Inst Diabet Acad Mergentheim, Bad Mergentheim, Germany.

[Kulzer, Bernd] Otto Friedrich Univ Bamberg, Dept Clin Psychol & Psychotherapy, Bamberg, Germany.

RP Kampling, H (reprint author), Univ Freiburg, Sect Hlth Care Res & Rehabil Res, Med Ctr, Fac Med, Hugstetterstr 49, D-79106 Freiburg, Germany.

EM hanna.kampling@uniklinik-freiburg.de

OI Kampling, Hanna/0000-0002-5213-0179

FU German Diabetes Association (DDG); Johannes Gutenberg-University Mainz;

Novo Nordisk and Berlin-Chemie

FX The study was funded by the German Diabetes Association (DDG), Johannes

Gutenberg-University Mainz, Novo Nordisk and Berlin-Chemie (all German

institutions).

CR American Psychiatric Association, 1994, DIAGN STAT MAN MENT

Anderson RJ, 2001, DIABETES CARE, V24, P1069, DOI 10.2337/diacare.24.6.1069

AYMANNS P, 1992, KREBSERKRANKUNG FAMI

Barnard KD, 2006, DIABETIC MED, V23, P445, DOI 10.1111/j.1464-5491.2006.01814.x

Baumeister H, 2012, COCHRANE DB SYST REV, DOI 10.1002/14651858.CD008381.pub2

Berge Line Iden, 2013, BMC Res Notes, V6, P543, DOI 10.1186/1756-0500-6-543

Bot M, 2013, DIABETIC MED, V30, pE115, DOI 10.1111/dme.12082

Maia ACCD, 2014, REV ASSOC MED BRAS, V60, P59, DOI 10.1590/1806-9282.60.01.013

de Ornelas Maia Ana Claudia C, 2013, Clin Pract Epidemiol Ment Health, V9, P136, DOI 10.2174/1745017901309010136

Derogatis L. R., 1995, SYMPTOM CHECKLISTE D

Gendelman N, 2009, DIABETES CARE, V32, P575, DOI 10.2337/dc08-1835

Golden SH, 2008, JAMA-J AM MED ASSOC, V299, P2751, DOI 10.1001/jama.299.23.2751

Hirsch A, 2000, QUAL LIFE RES, V9, P207, DOI 10.1023/A:1008959810698

Korczak DJ, 2011, DIABETOLOGIA, V54, P2483, DOI 10.1007/s00125-011-2240-3

Lloyd CE, 2010, DIABETIC MED, V27, P234, DOI 10.1111/j.1464-5491.2009.02896.x

Luyckx JRK, 2015, HEALTH PSYCHOL, V34, P1058, DOI 10.1037/hea0000212

Margraf J, 2015, DIAGNOSTIC INTERVIEW

Melin EO, 2013, EUR J ENDOCRINOL, V168, P861, DOI 10.1530/EJE-13-0137

Moulton CD, 2015, LANCET DIABETES ENDO, V3, P461, DOI 10.1016/S2213-8587(15)00134-5

Nefs G, 2015, DIABETIC MED, V32, P1289, DOI 10.1111/dme.12739

Nefs G, 2012, DIABETOLOGIA, V55, P608, DOI 10.1007/s00125-011-2411-2

Petrak F, 2003, DIABETES-METAB RES, V19, P216, DOI 10.1002/dmrr.374

Petrak F, 2015, LANCET DIABETES ENDO, V3, P472, DOI 10.1016/S2213-8587(15)00045-5

Petrak F, 2015, DIABETES CARE, V38, P767, DOI 10.2337/dc14-1599

Pouwer F, 2010, DIABETIC MED, V27, P217, DOI 10.1111/j.1464-5491.2009.02903.x

Roy MS, 2007, PSYCHOSOM MED, V69, P537, DOI 10.1097/PSY.0b013e3180df84e2

Roy T, 2012, J AFFECT DISORDERS, V142, pS8, DOI 10.1016/S0165-0327(12)70004-6

Strandberg RB, 2014, J PSYCHOSOM RES, V77, P174, DOI 10.1016/j.jpsychores.2014.06.015

Tanenbaum ML, 2012, DIABETES EDUCATOR, V38, P695, DOI 10.1177/0145721712452795

Tovote KA, 2014, DIABETES CARE, V37, P2427, DOI 10.2337/dc13-2918

Trief PM, 2014, DIABETES CARE, V37, P1563, DOI 10.2337/dc13-1867

Uher R, 2010, PSYCHOL MED, V40, P1367, DOI 10.1017/S0033291709991528

Van der Feltz-Cornelis CM, 2010, GEN HOSP PSYCHIAT, V32, P380, DOI 10.1016/j.genhosppsych.2010.03.011

Waadt S, 1995, DIABETES PSYCHOL DIA, P17

Zoffmann V, 2014, DIABETIC MED, V31, P493, DOI 10.1111/dme.12363

NR 35

TC 4

Z9 4

U1 1

U2 22

PU SPRINGER

PI NEW YORK

PA 233 SPRING ST, NEW YORK, NY 10013 USA

SN 0012-186X

EI 1432-0428

J9 DIABETOLOGIA

JI Diabetologia

PD JAN

PY 2017

VL 60

IS 1

BP 60

EP 68

DI 10.1007/s00125-016-4123-0

PG 9

WC Endocrinology & Metabolism

SC Endocrinology & Metabolism

GA EE5GQ

UT WOS:000389634000009

PM 27787619

OA Bronze

DA 2019-01-08

ER

PT J

AU Due-Christensen, M

Hommel, E

Ridderstrale, M

AF Due-Christensen, M.

Hommel, E.

Ridderstrale, M.

TI Potential positive impact of group-based diabetes dialogue meetings on

diabetes distress and glucose control in people with type 1 diabetes

SO PATIENT EDUCATION AND COUNSELING

LA English

DT Article

DE Patient education; Patient participation; Peer support; Disease specific

distress; Type 1 diabetes; Chronic disease

ID RANDOMIZED CONTROLLED-TRIAL; GLYCEMIC CONTROL; SELF-MANAGEMENT; PEER

SUPPORT; EDUCATION; OUTCOMES; INTERVENTION; BEHAVIOR; THERAPY; BURDEN

AB Objective: To evaluate the effect of group-based diabetes dialogue meetings (DDMs) on diabetes distress, perceived competence and glycaemic control.

Methods: Patients with type 1 diabetes (T1D) were invited to DDMs with peers and healthcare professionals. The impact of participation was evaluated by change in diabetes distress measured by Problem Areas in Diabetes (PAID), diabetes competence measured by Perceived Competence in Diabetes (PCD), change in HbA1c before and one year after the DDMs.

Results: 120 patients with T1D participated in at least one DDM: 75% female, mean age 50 years (range 21-76), mean diabetes duration 23 years (range 0-64); 39% of all participants had a baseline PAID score >= 33, indicating high levels of distress. After one year, both PAID (from 30.4 +/- 16.6 to 27.4 +/- 17.1; n = 81, p = 0.03), and mean HbA1c (61.6 +/- 10.2 to 58.8 +/- 9.9; n = 120, p < 0.0001) had improved significantly. PCD showed no change. Meanwhile, the benefit from participating was rated high with a median of four out of five and the major gain being the possibility to share experiences with peers.

Conclusion: Group-based DDMs were highly appreciated by participants and associated with significant improvements in diabetes distress and HbA1c.

Practice implications: DDMs target a large group of patients using few staff resources. (C) 2016 Elsevier Ireland Ltd. All rights reserved.

C1 [Due-Christensen, M.; Hommel, E.; Ridderstrale, M.] Steno Diabet Ctr, Niels Steensens Vej 6, D-2820 Gentofte, Denmark.

[Due-Christensen, M.] Kings Coll London, Fac Nursing & Midwifery, London, England.

RP Due-Christensen, M (reprint author), Steno Diabet Ctr, Niels Steensens Vej 6, D-2820 Gentofte, Denmark.

EM Mtdc@steno.dk

OI Due-Christensen, Mette/0000-0003-2604-3730; Hommel,

Eva/0000-0001-7570-5919

FU Steno Diabetes Center

FX We would like to thank the people with type 1 diabetes who took part in

the study and Anette Hougaard and Anne Skouboe who helped facilitate the

meetings. We also thank Caroline Horness for helping with data

management in regards to the surveys. Steno Diabetes Center funded the

research.

CR Amsberg S, 2009, PATIENT EDUC COUNS, V77, P72, DOI 10.1016/j.pec.2009.01.015

Bandura A, 2001, ANNU REV PSYCHOL, V52, P1, DOI 10.1146/annurev.psych.52.1.1

Berry E, 2015, POSTGRAD MED J, V91, P278, DOI 10.1136/postgradmedj-2014-133017

Braun V., 2006, QUALITATIVE RES PSYC, V3, P77, DOI DOI 10.1191/1478088706QP0630A

Colagiuri R, 2009, DIABETIC MED, V26, P442, DOI 10.1111/j.1464-5491.2009.02700.x

Craig P, 2008, BRIT MED J, V337, DOI 10.1136/bmj.a1655

Dale JR, 2012, DIABETIC MED, V29, P1361, DOI 10.1111/j.1464-5491.2012.03749.x

Dennis CL, 2003, INT J NURS STUD, V40, P321, DOI 10.1016/S0020-7489(02)00092-5

Due-Christensen M, 2012, DIABETIC MED, V29, P251, DOI 10.1111/j.1464-5491.2011.03521.x

Fisher EB, 2007, DIABETES ED, V33, P6

Fisher L, 2010, DIABETES CARE, V33, P23, DOI 10.2337/dc09-1238

Funnell MM, 2007, DIABETES SPECTR, V20, P221, DOI DOI 10.2337/DIASPECT.20.4.221

Haas L, 2014, DIABETES CARE, V37, pS144, DOI 10.2337/dc14-S144

Hermanns N, 2006, DIABETOLOGIA, V49, P469, DOI 10.1007/s00125-005-0094-2

Hermanns N., 2015, DIABETES CARE

Hermanns N, 2013, DIABETES RES CLIN PR, V102, P149, DOI 10.1016/j.diabres.2013.10.009

Hopkins D, 2012, DIABETES CARE, V35, P1638, DOI 10.2337/dc11-1579

Joensen LE, 2016, PRIM CARE DIABETES, V10, P41, DOI 10.1016/j.pcd.2015.06.007

Joensen LE, 2013, DIABETES RES CLIN PR, V101, P278, DOI 10.1016/j.diabres.2013.07.001

Krichbaum K, 2003, DIABETES EDUCATOR, V29, P653, DOI 10.1177/014572170302900411

Luyckx K, 2015, PSYCHOL HEALTH MED, V20, P77, DOI 10.1080/13548506.2014.902482

POLONSKY WH, 1995, DIABETES CARE, V18, P754, DOI 10.2337/diacare.18.6.754

Rane K, 2011, DIABETES RES CLIN PR, V93, P371, DOI 10.1016/j.diabres.2011.05.009

Schmitt A., 2014, J BEHAV MED, P1

Simmons D, 2012, DIABETES MANAGEMENT, V3, P61

Strandberg R. Bjarkoy, 2015, DIABETES MED, V130, P4

Sturt J, 2015, CURR DIABETES REP, V15, P1, DOI DOI 10.1007/S11892-015-0660-Z

Zoffmann V, 2014, DIABETIC MED, V31, P493, DOI 10.1111/dme.12363

Zoffmann V., 2015, DIABETIC MED, V123, P9

Zoffmann V, 2006, PATIENT EDUC COUNS, V64, P78, DOI 10.1016/j.pec.2005.11.017

NR 30

TC 1

Z9 1

U1 0

U2 7

PU ELSEVIER IRELAND LTD

PI CLARE

PA ELSEVIER HOUSE, BROOKVALE PLAZA, EAST PARK SHANNON, CO, CLARE, 00000,

IRELAND

SN 0738-3991

J9 PATIENT EDUC COUNS

JI Patient Educ. Couns.

PD DEC

PY 2016

VL 99

IS 12

BP 1978

EP 1983

DI 10.1016/j.pec.2016.07.023

PG 6

WC Public, Environmental & Occupational Health; Social Sciences,

Interdisciplinary

SC Public, Environmental & Occupational Health; Social Sciences - Other

Topics

GA EG7JM

UT WOS:000391223200008

PM 27444233

DA 2019-01-08

ER

PT J

AU Trief, PM

Fisher, L

Sandberg, J

Cibula, DA

Dimmock, J

Hessler, DM

Forken, P

Weinstock, RS

AF Trief, Paula M.

Fisher, Lawrence

Sandberg, Jonathan

Cibula, Donald A.

Dimmock, Jacqueline

Hessler, Danielle M.

Forken, Patricia

Weinstock, Ruth S.

TI Health and Psychosocial Outcomes of a Telephonic Couples Behavior Change

Intervention in Patients With Poorly Controlled Type 2 Diabetes: A

Randomized Clinical Trial

SO DIABETES CARE

LA English

DT Article

ID SELF-MANAGEMENT EDUCATION; SUPPORT PROJECT; CHRONIC ILLNESS; FAMILY;

INTERDEPENDENCE; METAANALYSIS; MEDICINE; CONTEXT; ADULTS

AB OBJECTIVE

To compare glycemic control and secondary outcomes of a 4-month telephonic couples behavioral intervention to individual intervention, and to education, for adults with type 2 diabetes.

RESEARCH DESIGN AND METHODS

A randomized trial with the following three arms: couples calls (CC) (n = 104); individual calls (IC) (n = 94); and diabetes education (DE) (n = 82). All arms had self-management education (two calls). CC and IC had 10 additional behavior change calls. CC addressed collaboration and relationships/communication. Participants consisted of 280 couples, among whom one partner had type 2 diabetes and an A1C level >= 7.5%. Blinded assessments occurred at 4, 8, and 12 months. The primary outcome was change in A1C; and secondary outcomes were BMI, waist circumference, blood pressure, depressive symptoms, diabetes self-efficacy, and diabetes distress.

RESULTS

Patients had a mean age of 56.8 years; 61.6% were male, and 30.4% were minorities. The baseline mean A1C level was 9.1%. Intention-to-treat analyses found significant A1C reductions for all (12 months: CC -0.47%, IC -0.52%, DE -0.57%), with no differences between arms. Preplanned within-arm analyses were stratified by baseline A1C tertiles: lowest tertile (7.5-8.2%), no change from baseline; middle tertile (8.3-9.2%), only CC led to significantly lower A1C level; and highest tertile (>= 9.3%), significant improvement for all interventions. For BMI, CC showed significant improvement, and CC and DE led to decreased waist circumference. The IC group showed greater blood pressure improvement. Results for secondary psychosocial outcomes favored the CC group.

CONCLUSIONS

In adults with poorly controlled type 2 diabetes, a collaborative couples intervention resulted in significant, lasting improvement in A1C levels, obesity measures, and some psychosocial outcomes. For those with exceedingly high A1C levels, education alone was beneficial, but additional intervention is needed to achieve glycemic targets.

C1 [Trief, Paula M.; Dimmock, Jacqueline; Forken, Patricia] SUNY Upstate Med Univ, Dept Psychiat & Behav Neurosci, Syracuse, NY 13210 USA.

[Trief, Paula M.; Weinstock, Ruth S.] SUNY Upstate Med Univ, Dept Med, Syracuse, NY 13210 USA.

[Fisher, Lawrence; Hessler, Danielle M.] Univ Calif San Francisco, Dept Family Med, San Francisco, CA 94143 USA.

[Sandberg, Jonathan] Brigham Young Univ, Sch Family Life, Provo, UT 84602 USA.

[Cibula, Donald A.] SUNY Upstate Med Univ, Dept Publ Hlth & Prevent Med, Syracuse, NY 13210 USA.

RP Trief, PM (reprint author), SUNY Upstate Med Univ, Dept Psychiat & Behav Neurosci, Syracuse, NY 13210 USA.; Trief, PM (reprint author), SUNY Upstate Med Univ, Dept Med, Syracuse, NY 13210 USA.

EM triefp@upstate.edu

OI Trief, Paula/0000-0002-2827-5722

FU National Institutes of Health (NIH) [1R18-DK-080867-01A2]; NIH

FX This study was supported by National Institutes of Health (NIH) grant

1R18-DK-080867-01A2. The first year of the study was funded by an NIH

Diversity Fellowship Supplement.

CR Agency for Healthcare Research and Quality, 2014, BEH PROGR DIAB MELL

Bandura A, 1978, SOCIAL LEARNING THEO

BLACK DR, 1990, HEALTH PSYCHOL, V9, P330, DOI 10.1037//0278-6133.9.3.330

Campbell TA, 2002, EFFECTIVENESS RES MA, P311

Chamany S, 2015, AM J PREV MED, V49, P832, DOI 10.1016/j.amepre.2015.04.016

DEVOOGD JN, 1993, J MUSCULOSKELET PAIN, V1, P273

Duncan I, 2009, DIABETES EDUCATOR, V35, P752, DOI 10.1177/0145721709343609

Eakin EG, 2007, AM J PREV MED, V32, P419, DOI 10.1016/j.amepre.2007.01.004

Fisher L, 2000, J FAM PRACTICE, V49, P561

Glasgow RE, 1999, AM J PUBLIC HEALTH, V89, P1322, DOI 10.2105/AJPH.89.9.1322

Gonder-Frederick LA, 2002, J CONSULT CLIN PSYCH, V70, P611, DOI 10.1037//0022-006X.70.3.611

Haas L, 2012, DIABETES CARE S1, V37, pS144

Kang CM, 2010, INT J NURS STUD, V47, P1363, DOI 10.1016/j.ijnurstu.2010.03.009

Kelley H. H., 1978, INTERPERSONAL RELATI

Keogh KM, 2011, AM J MANAG CARE, V17, P105

Kiecolt-Glaser JK, 2001, PSYCHOL BULL, V127, P472, DOI 10.1037//0033-2909.127.4.472

Kroenke K, 2009, J AFFECT DISORDERS, V114, P163, DOI 10.1016/j.jad.2008.06.026

Laurenceau JP, 2004, J CONSULT CLIN PSYCH, V72, P933, DOI 10.1037/0022-006X.72.6.933

Lewis MA, 2006, SOC SCI MED, V62, P1369, DOI 10.1016/j.socscimed.2005.08.006

Li R, 2014, MMWR-MORBID MORTAL W, V63, P1045

Lorig K, 2009, DIABETES EDUCATOR, V35, P641, DOI 10.1177/0145721709335006

Marrero DG, 2013, DIABETES CARE, V36, P463, DOI 10.2337/dc12-2305

Martire LM, 2004, HEALTH PSYCHOL, V23, P599, DOI 10.1037/0278-6133.23.6.599

Martire LM, 2014, HEALTH PSYCHOL, V33, P501, DOI 10.1037/hea0000103

NATHAN DM, 1984, NEW ENGL J MED, V310, P341, DOI 10.1056/NEJM198402093100602

Norris SL, 2001, DIABETES CARE, V24, P561, DOI 10.2337/diacare.24.3.561

Palmer C. A., 2000, PSYCHOL COUPLES ILLN, P311

PIANTADOSI S, 1997, CLIN TRIALS METHODOL

Polonsky WH, 2005, DIABETES CARE, V28, P626, DOI 10.2337/diacare.28.3.626

Powers MA, 2015, DIABETES CARE, V38, P1372, DOI 10.2337/dc15-0730

Rook KS, 2011, J SOC PERS RELAT, V28, P772, DOI 10.1177/0265407510391335

Rusbult CE, 2003, ANNU REV PSYCHOL, V54, P351, DOI 10.1146/annurev.psych.54.101601.145059

Stark CS, 2013, DIABETES CARE, V36, P2271, DOI DOI 10.2337/DC12-2258

Steinsbekk A, 2012, BMC HEALTH SERV RES, V12, DOI 10.1186/1472-6963-12-213

Stokols D, 1996, AM J HEALTH PROMOT, V10, P282, DOI 10.4278/0890-1171-10.4.282

Torenholt R, 2014, DIABETIC MED, V31, P15, DOI 10.1111/dme.12290

Trief P. M., 2006, FAMILIES SYSTEMS HLT, V24, P318, DOI [10.1037/1091-7527.24.3.318, DOI 10.1037/1091-7527.24.3.318]

Trief P, 2011, FAM SYST HEALTH, V29, P253, DOI 10.1037/a0024564

Trief PM, 2011, TRANSL BEHAV MED, V1, P461, DOI 10.1007/s13142-011-0057-8

Trief PM, 2001, DIABETES CARE, V24, P1384, DOI 10.2337/diacare.24.8.1384

UK Prospective Diabetes Study (UKPDS) Group, 1998, LANCET, V352, P837, DOI DOI 10.1016/S0140-6736(98)07019-6

Yang W, 2013, DIABETES CARE, V36, P1033, DOI DOI 10.2337/DC12-2625

NR 42

TC 12

Z9 12

U1 0

U2 4

PU AMER DIABETES ASSOC

PI ALEXANDRIA

PA 1701 N BEAUREGARD ST, ALEXANDRIA, VA 22311-1717 USA

SN 0149-5992

EI 1935-5548

J9 DIABETES CARE

JI Diabetes Care

PD DEC

PY 2016

VL 39

IS 12

BP 2165

EP 2173

DI 10.2337/dc16-0035

PG 9

WC Endocrinology & Metabolism

SC Endocrinology & Metabolism

GA EG1IF

UT WOS:000390785000021

PM 27456837

OA Bronze, Green Published

DA 2019-01-08

ER

PT J

AU Gonzalez, JS

Kane, NS

Binko, DH

Shapira, A

Hoogendoorn, CJ

AF Gonzalez, Jeffrey S.

Kane, Naomi S.

Binko, Deborah H.

Shapira, Amit

Hoogendoorn, Claire J.

TI Tangled Up in Blue: Unraveling the Links Between Emotional Distress and

Treatment Adherence in Type 2 Diabetes

SO DIABETES CARE

LA English

DT Article

ID RATING-SCALE MADRS; MEDICATION ADHERENCE; DEPRESSIVE SYMPTOMS;

SELF-CARE; CLINICAL DEPRESSION; SOMATIC SYMPTOMS; GLYCEMIC CONTROL;

ADULTS; METAANALYSIS; OUTCOMES

AB OBJECTIVE

We conducted comprehensive assessments of emotional distress to examine relations with diabetes medication adherence over time.

RESEARCH DESIGN AND METHODS

Ethnically and socioeconomically diverse adults treated for type 2 diabetes completed validated self-reports (SRs) for diabetes distress and depression, were administered semistructured depression interviews, and provided blood samples for A1C. Medication adherence among 104 participants was electronically monitored (EM) over the subsequent 3 months; validated SRs of medication adherence were also obtained. Hierarchical linear regression evaluated independent effects of diabetes distress and depression on adherence.

RESULTS

Mean +/- SD 3-month medication adherence was 76.1% +/- 25.7% for EM and 83.7% +/- 21.9% for SR. Higher levels of SR (P < 0.001) and interview-based (P < 0.05) depressive symptom severity (P < 0.05) and diabetes-related distress (P < 0.01) showed a significant bivariate association with EM and SR nonadherence. Regression models showed baseline diabetes distress was a significant independent predictor of EM (beta = -0.29; P = 0.001) and SR adherence (beta = -0.24; P < 0.02) at follow-up. SR depression was an independent predictor of EM and SR adherence and reduced the effects of diabetes distress to nonsignificance. Subsequent models indicated this effect was driven by somatic rather than cognitive-affective symptoms of depression. Results were consistent but weaker for interview-based depressive symptoms.

CONCLUSIONS

Findings support diabetes-related distress and depression symptom severity as risk factors for type 2 diabetes medication nonadherence. Somatic symptoms captured by depression measures, but not cognitive-affective symptoms, independently predict nonadherence and should be further investigated as a potential link between emotional distress and nonadherence.

C1 [Gonzalez, Jeffrey S.; Kane, Naomi S.; Binko, Deborah H.; Shapira, Amit; Hoogendoorn, Claire J.] Yeshiva Univ, Ferkauf Grad Sch Psychol, Bronx, NY USA.

[Gonzalez, Jeffrey S.] Albert Einstein Coll Med, Dept Med Endocrinol, Bronx, NY 10467 USA.

[Gonzalez, Jeffrey S.] Albert Einstein Coll Med, Dept Epidemiol & Populat Hlth, Bronx, NY 10467 USA.

RP Gonzalez, JS (reprint author), Yeshiva Univ, Ferkauf Grad Sch Psychol, Bronx, NY USA.; Gonzalez, JS (reprint author), Albert Einstein Coll Med, Dept Med Endocrinol, Bronx, NY 10467 USA.; Gonzalez, JS (reprint author), Albert Einstein Coll Med, Dept Epidemiol & Populat Hlth, Bronx, NY 10467 USA.

EM jeffrey.gonzalez@einstein.yu.edu

FU Albert Einstein Diabetes Research Center; National Institutes of Health

(NIH) [P60 DK020541]; National Institute of Diabetes and Digestive and

Kidney Diseases [R18 DK09872, R01 DK104845, P30 DK11022]; Drs. David and

Jane Willner Bloomgarden Family Fellowship Fund

FX This work was partially supported by a pilot and feasibility grant from

the Albert Einstein Diabetes Research Center, which was supported at the

time of data collection by National Institutes of Health (NIH) grant P60

DK020541. J.S.G. is additionally supported by grants R18 DK09872, R01

DK104845, and P30 DK11022 from the National Institute of Diabetes and

Digestive and Kidney Diseases. C.J.H. is supported by The Drs. David and

Jane Willner Bloomgarden Family Fellowship Fund.

CR Aikens JE, 2012, DIABETES CARE, V35, P2472, DOI 10.2337/dc12-0181

Ali S, 2006, DIABETIC MED, V23, P1165, DOI 10.1111/j.1464-5491.2006.01943.x

American Psychiatric Association, 2013, DIAGN STAT MAN MENT

Azevedo RD, 2014, PSYCHOL MED, V44, P2689, DOI 10.1017/S0033291714000063

Baek RN, 2014, ANN BEHAV MED, V48, P145, DOI 10.1007/s12160-013-9585-4

de Groot M, 2001, PSYCHOSOM MED, V63, P619, DOI 10.1097/00006842-200107000-00015

Fisher L, 2008, DIABETIC MED, V25, P1096, DOI 10.1111/j.1464-5491.2008.02533.x

Fisher L, 2014, DIABETIC MED, V31, P764, DOI 10.1111/dme.12428

Fisher L, 2007, DIABETES CARE, V30, P542, DOI 10.2337/dc06-1614

Fisher L, 2012, DIABETES CARE, V35, P259, DOI 10.2337/dc11-1572

Fisher L, 2010, DIABETES CARE, V33, P1034, DOI 10.2337/dc09-2175

Gonzalez JS, 2008, DIABETOLOGIA, V51, P1822, DOI 10.1007/s00125-008-1113-x

Gonzalez JS, 2007, DIABETES CARE, V30, P2222, DOI 10.2337/dc07-0158

Gonzalez JS, 2015, HEALTH PSYCHOL, V34, P505, DOI 10.1037/hea0000131

Gonzalez JS, 2013, DIABETES CARE, V36, P831, DOI 10.2337/dc12-0410

Gonzalez JS, 2011, DIABETES CARE, V34, P236, DOI 10.2337/dc10-1970

Gonzalez JS, 2008, DIABETES CARE, V31, P2398, DOI 10.2337/dc08-1341

Hofmann M, 2013, PLOS ONE, V8

Hwang B, 2015, PSYCHOSOM MED, V77, P798, DOI 10.1097/PSY.0000000000000221

Kroenke K, 2001, J GEN INTERN MED, V16, P606, DOI 10.1046/j.1525-1497.2001.016009606.x

Lin EHB, 2006, ANN FAM MED, V4, P46, DOI 10.1370/afm.423

Lu M, 2008, AIDS BEHAV, V12, P86, DOI 10.1007/s10461-007-9261-4

Lustman PJ, 2000, DIABETES CARE, V23, P934, DOI 10.2337/diacare.23.7.934

McIntyre RS, 2006, J PSYCHOSOM RES, V60, P279, DOI 10.1016/j.jpsychores.2005.09.010

Michal M, 2013, PLOS ONE, V8, DOI 10.1371/journal.pone.0072014

MONTGOMERY SA, 1979, BRIT J PSYCHIAT, V134, P382, DOI 10.1192/bjp.134.4.382

Muller MJ, 2003, J AFFECT DISORDERS, V77, P255, DOI 10.1016/S0165-0327(02)00120-9

Nicolucci A, 2013, DIABETIC MED, V30, P767, DOI 10.1111/dme.12245

Polonsky WH, 2005, DIABETES CARE, V28, P626, DOI 10.2337/diacare.28.3.626

Reijnders JSAM, 2010, J PSYCHOSOM RES, V68, P561, DOI 10.1016/j.jpsychores.2009.10.006

Roest AM, 2016, HEALTH PSYCHOL, V35, P413, DOI 10.1037/hea0000256

Roy T, 2012, DIABETIC MED, V29, P164, DOI 10.1111/j.1464-5491.2011.03401.x

Sheehan DV, 1998, J CLIN PSYCHIAT, V59, P34, DOI 10.4088/JCP.09m05305whi

Snoek FJ, 2015, LANCET DIABETES ENDO, V3, P450, DOI 10.1016/S2213-8587(15)00135-7

Svanborg P, 2001, J AFFECT DISORDERS, V64, P203, DOI 10.1016/S0165-0327(00)00242-1

Tanenbaum ML, 2013, J AFFECT DISORDERS, V150, P533, DOI 10.1016/j.jad.2013.01.029

Twist K, 2013, PSYCHOSOM MED, V75, P791, DOI 10.1097/PSY.0b013e3182a2b108

Williams JBW, 2008, BRIT J PSYCHIAT, V192, P52, DOI 10.1192/bjp.bp.106.032532

Wiltink J, 2014, PLOS ONE, V9, DOI 10.1371/journal.pone.0105499

NR 39

TC 7

Z9 7

U1 2

U2 13

PU AMER DIABETES ASSOC

PI ALEXANDRIA

PA 1701 N BEAUREGARD ST, ALEXANDRIA, VA 22311-1717 USA

SN 0149-5992

EI 1935-5548

J9 DIABETES CARE

JI Diabetes Care

PD DEC

PY 2016

VL 39

IS 12

BP 2182

EP 2189

DI 10.2337/dc16-1657

PG 8

WC Endocrinology & Metabolism

SC Endocrinology & Metabolism

GA EG1IF

UT WOS:000390785000023

PM 27797932

OA Green Published, Bronze

DA 2019-01-08

ER

PT J

AU Rondags, SMPA

de Wit, M

Twisk, JW

Snoek, FJ

AF Rondags, Stefanie M. P. A.

de Wit, Maartje

Twisk, Jos W.

Snoek, Frank J.

TI Effectiveness of HypoAware, a Brief Partly Web-Based Psychoeducational

Intervention for Adults With Type 1 and Insulin-Treated Type 2 Diabetes

and Problematic Hypoglycemia: A Cluster Randomized Controlled Trial

SO DIABETES CARE

LA English

DT Article

ID AWARENESS; FREQUENCY; DUTCH; RISK

AB OBJECTIVE

To establish the effectiveness of a brief, partly web-based group intervention, HypoAware, in patients with problematic hypoglycemia, in terms of the reduction of severe hypoglycemic episodes, impaired hypoglycemia awareness, and worries.

RESEARCH DESIGN AND METHODS

A two-arm, cluster randomized controlled trial was conducted in insulin-treated patients with problematic hypoglycemia in eight Dutch clinics comparing HypoAware with care as usual. Primary outcomes included self-reported severe hypoglycemia, impaired hypoglycemia awareness (Gold score), and worries and distress about hypoglycemia (Hypoglycemia Fear Survey-II/Problem Areas In Diabetes scale hypo-item), assessed at baseline, and at 2, 4, and 6 months of follow-up. We used t tests, nonparametric tests, and intention-to-treat generalized estimation equation (GEE) analyses with linear, logistic, and Poisson or negative binomial models.

RESULTS

We enrolled 137 participants. Adjusted GEE analyses over four time points showed a nonsignificant 33% fewer episodes of severe hypoglycemia in the HypoAware group compared with the control group (relative risk [RR] 0.67 [95% CI 0.39-1.16], P = 0.150); a significant reduced odds of impaired awareness (odds ratio 0.38 [95% CI 0.15-0.95], P = 0.038), a trend toward 20% fewer worries about hypoglycemia (RR 0.80 [95% CI 0.64-1.01], P = 0.059), and a significant 30% less hypo-distress (RR 0.70 [95% CI 0.56-0.88], P = 0.002). Over the 6-month study duration, participants experienced a median of 2.5 events of severe hypoglycemia (interquartile range [IQR] 1-10) in the control condition versus 1 event (IQR 0-6.5) in the HypoAware group (P = 0.030). There was no significant change in HbA(1c) level within and between both groups.

CONCLUSIONS

HypoAware resulted in fewer severe hypoglycemic episodes, significantly improved hypoglycemia awareness, and less hypo-distress in comparison with usual care, and deserves further dissemination.

C1 [Rondags, Stefanie M. P. A.; de Wit, Maartje; Snoek, Frank J.] Vrije Univ Amsterdam, Med Ctr, Dept Med Psychol, Amsterdam, Netherlands.

[Rondags, Stefanie M. P. A.; de Wit, Maartje; Snoek, Frank J.] Vrije Univ Amsterdam, Med Ctr, EMGO Inst Hlth & Care Res, Amsterdam, Netherlands.

[Twisk, Jos W.] Vrije Univ Amsterdam, Med Ctr, Dept Epidemiol & Biostat, Amsterdam, Netherlands.

[Snoek, Frank J.] Acad Med Ctr, Dept Med Psychol, Amsterdam, Netherlands.

RP Snoek, FJ (reprint author), Vrije Univ Amsterdam, Med Ctr, Dept Med Psychol, Amsterdam, Netherlands.; Snoek, FJ (reprint author), Vrije Univ Amsterdam, Med Ctr, EMGO Inst Hlth & Care Res, Amsterdam, Netherlands.; Snoek, FJ (reprint author), Acad Med Ctr, Dept Med Psychol, Amsterdam, Netherlands.

EM fj.snoek@vumc.nl

FU ZonMw, Dutch Organisation for Health Research and Development

[837001406]; Novo Nordisk; Agis Achmea; VU University Medical Center

FX The study and manuscript preparation are funded by ZonMw (837001406),

the Dutch Organisation for Health Research and Development. The authors

have received unrestricted cofunding from Novo Nordisk, Agis Achmea (a

Dutch health care insurer), and VU University Medical Center.

CR Broers S, 2002, DIABETIC MED, V19, P157, DOI 10.1046/j.1464-5491.2002.00682.x

Cox DJ, 2001, DIABETES CARE, V24, P637, DOI 10.2337/diacare.24.4.637

Cox DJ, 2006, DIABETES SPECTRUM, V19, P43, DOI DOI 10.2337/DIASPECT.19.1.43

Cryer PE, 2008, DIABETES, V57, P3169, DOI 10.2337/db08-1084

DCCT Res Grp, 1991, AM J MED, V90, P450

GOLD AE, 1994, DIABETES CARE, V17, P697, DOI 10.2337/diacare.17.7.697

Gonder-Frederick LA, 2011, DIABETES CARE, V34, P801, DOI 10.2337/dc10-1343

GonderFrederick L, 1997, DIABETES CARE, V20, P661, DOI 10.2337/diacare.20.4.661

Gruden G, 2012, DIABETES CARE, V35, P1598, DOI 10.2337/dc11-1531

Ha WC, 2012, DIABETES METAB J, V36, P280, DOI 10.4093/dmj.2012.36.4.280

Heller SR, 2007, DIABETOLOGIA, V50, P1140, DOI 10.1007/s00125-007-0599-y

Herdman M, 2011, QUAL LIFE RES, V20, P1727, DOI 10.1007/s11136-011-9903-x

McGuire BE, 2010, DIABETOLOGIA, V53, P66, DOI 10.1007/s00125-009-1559-5

Oyer DS, 2013, CURR DIABETES REV, V9, P195, DOI 10.2174/15733998113099990059

Pedersen-Bjergaard U, 2004, DIABETES-METAB RES, V20, P479, DOI 10.1002/dmrr.482

Pedersen-Bjergaard U, 2003, DIABETES-METAB RES, V19, P232, DOI 10.1002/dmrr.377

Rondags SMPA, 2016, DIABETIC MED, V33, P184, DOI 10.1111/dme.12876

Rondags SMPA, 2015, BMC ENDOCR DISORD, V15, DOI 10.1186/s12902-015-0035-0

Schachinger H, 2005, J BEHAV MED, V28, P587, DOI 10.1007/s10865-005-9026-3

Seaquist ER, 2013, DIABETES CARE, V36, P1384, DOI 10.2337/dc12-2480

Snoek FJ, 2014, HYPOGLYCAEMIA CLIN D, P323

Twisk J. W. R, 2006, APPL MULTILEVEL ANAL

Unger J, 2011, POSTGRAD MED, V123, P81, DOI 10.3810/pgm.2011.07.2307

van Beers CAJ, 2016, LANCET DIABETES ENDO, V4, P893, DOI 10.1016/S2213-8587(16)30193-0

van der Ven NCW, 2003, DIABETES CARE, V26, P713, DOI 10.2337/diacare.26.3.713

Yeoh E, 2015, DIABETES CARE, V38, P1592, DOI 10.2337/dc15-0102

Zammitt NN, 2005, DIABETES CARE, V28, P2948, DOI 10.2337/diacare.28.12.2948

ZIGMOND AS, 1983, ACTA PSYCHIAT SCAND, V67, P361, DOI 10.1111/j.1600-0447.1983.tb09716.x

NR 28

TC 13

Z9 13

U1 0

U2 3

PU AMER DIABETES ASSOC

PI ALEXANDRIA

PA 1701 N BEAUREGARD ST, ALEXANDRIA, VA 22311-1717 USA

SN 0149-5992

EI 1935-5548

J9 DIABETES CARE

JI Diabetes Care

PD DEC

PY 2016

VL 39

IS 12

BP 2190

EP 2196

DI 10.2337/dc16-1614

PG 7

WC Endocrinology & Metabolism

SC Endocrinology & Metabolism

GA EG1IF

UT WOS:000390785000024

PM 27797931

OA Bronze

DA 2019-01-08

ER

PT J

AU Nyklicek, I

van Son, J

Pop, VJ

Denollet, J

Pouwer, F

AF Nyklicek, Ivan

van Son, Jenny

Pop, Victor J.

Denollet, Johan

Pouwer, Francois

TI Does Mindfulness-Based Cognitive Therapy benefit all people with

diabetes and comorbid emotional complaints equally? Moderators in the

DiaMind trial

SO JOURNAL OF PSYCHOSOMATIC RESEARCH

LA English

DT Article

DE Diabetes; Mindfulness-Based Cognitive Therapy; Moderator; Personality;

Randomized trial

ID RANDOMIZED CONTROLLED-TRIAL; QUALITY-OF-LIFE; NEGATIVE AFFECTIVITY;

BEHAVIORAL THERAPY; STRESS REDUCTION; D PERSONALITY; DEPRESSION;

ANXIETY; INTERVENTION; DISTRESS

AB Objectives: Research has shown the effectiveness of mindfulness-based interventions for a variety of emotional problems in different samples, but it is unknown which factors influence this effectiveness. Therefore, the aim of the current study was: which factors (demographic, personality, and baseline levels of mindfulness skills) moderate the effectiveness of Mindfulness-Based Cognitive Therapy (MBCT)?

Methods: Outpatients with diabetes (type I or type 2; N = 139) and an elevated level of emotional distress participated in the Diabetes and Mindfulness (DiaMind) trial. They were randomized into MBCT (N = 70) or a control group (N = 69) that received treatment as usual and that was offered the intervention 6 months later. Primary outcomes were anxiety, depressive symptoms, and perceived stress at post-intervention and at 6-month follow-up.

Results: Mixed models analyses showed that sex, extraversion, and baseline acting with awareness were significant moderators of effectiveness. In the MBCT group, women showed larger decreases in anxiety and depression across time (large effects) compared to men (medium to small effects). For extraversion divided into quartiles, the three lowest quartiles generally exhibited large decreases in symptoms, whereas the high extraversion group showed medium (perceived stress) to small (depression) decreases.

Conclusion: MBCT seems to be effective to decrease symptoms of anxiety, depression, and perceived stress for a broad range of person characteristics in patients with diabetes. However, men and those high in extraversion showed considerably lower effectiveness compared to the other groups. The small effect in high extraverts may be due to the large dropout in this subgroup. (C) 2016 Elsevier Inc. All rights reserved.

C1 [Nyklicek, Ivan; van Son, Jenny; Pop, Victor J.; Denollet, Johan; Pouwer, Francois] Tilburg Univ, Ctr Res Psychol Somat Dis CoRPS, Dept Med & Clin Psychol, Tilburg, Netherlands.

RP Nyklicek, I (reprint author), Tilburg Univ, Ctr Res Psychol Somat Dis CoRPS, Dept Med & Clin Psychol, Tilburg, Netherlands.; Nyklicek, I (reprint author), P Box 90153, NL-5000 LE Tilburg, Netherlands.

EM I.Nyklicek@tilburguniversity.edu

FU Dutch Diabetes Research Foundation or 'Diabetesfonds' [2008.13.005];

Tilburg University, Center of Research on Psychology in Somatic diseases

(CoRPS)

FX The study is supported by grants from 1) the Dutch Diabetes Research

Foundation or 'Diabetesfonds' (project number 2008.13.005, awarded to

Dr. I. Nyklicek) and 2) Tilburg University, Center of Research on

Psychology in Somatic diseases (CoRPS).

CR Arch JJ, 2013, BEHAV RES THER, V51, P434, DOI 10.1016/j.brat.2013.04.004

ASENDORPF JB, 1993, EMOTION INHIBITION H, P80

Baer RA, 2006, ASSESSMENT, V13, P27, DOI 10.1177/1073191105283504

Baer RA, 2008, ASSESSMENT, V15, P329, DOI 10.1177/1073191107313003

Beck AT, 1979, COGNITIVE THERAPY DE

Bishop SR, 2004, CLIN PSYCHOL-SCI PR, V11, P230, DOI 10.1093/clipsy/bph077

Blom MBJ, 2007, J AFFECT DISORDERS, V104, P119, DOI 10.1016/j.jad.2007.03.010

Brown KW, 2003, J PERS SOC PSYCHOL, V84, P822, DOI 10.1037/0022-3514.84.4.822

COHEN S, 1983, J HEALTH SOC BEHAV, V24, P385, DOI 10.2307/2136404

de Vibe M, 2015, MINDFULNESS, V6, P281, DOI 10.1007/s12671-013-0258-y

Denollet J, 2005, PSYCHOSOM MED, V67, P89, DOI 10.1097/01.psy.0000149256.81953.49

Eysenck HJ, 1991, MANUAL EYSENCK PERSO

Grossman P, 2008, J PSYCHOSOM RES, V64, P405, DOI 10.1016/j.jpsychores.2008.02.001

Hoekstra H.A., 2003, HANDLEIDING NEO PERS

Hofmann SG, 2010, J CONSULT CLIN PSYCH, V78, P169, DOI 10.1037/a0018555

Kabat-Zinn J., 1990, FULL CATASTROPHE LIV

KABATZINN J, 1982, GEN HOSP PSYCHIAT, V4, P33, DOI 10.1016/0163-8343(82)90026-3

Kadengye DT, 2012, BEHAV RES METHODS, V44, P516, DOI 10.3758/s13428-011-0157-x

Khoury B, 2013, CLIN PSYCHOL REV, V33, P763, DOI 10.1016/j.cpr.2013.05.005

Kiekens G, 2015, EUR PSYCHIAT, V30, P743, DOI 10.1016/j.eurpsy.2015.06.007

Kraemer HC, 2002, ARCH GEN PSYCHIAT, V59, P877, DOI 10.1001/archpsyc.59.10.877

Kupper N, 2014, INT J BEHAV MED, V21, P496, DOI 10.1007/s12529-013-9350-x

Nyklicek I, 2008, ANN BEHAV MED, V35, P331, DOI 10.1007/s12160-008-9030-2

Nyklicek I, 2014, J BEHAV MED, V37, P135, DOI 10.1007/s10865-012-9475-4

Nyklicek I, 2009, PSYCHOL ASSESSMENT, V21, P32, DOI 10.1037/a0014418

Okumura Y, 2014, J AFFECT DISORDERS, V164, P155, DOI 10.1016/j.jad.2014.04.023

Pouwer F, 2003, DIABETOLOGIA, V46, P892, DOI 10.1007/s00125-003-1124-6

Quilty LC, 2008, J AFFECT DISORDERS, V108, P241, DOI 10.1016/j.jad.2007.10.022

SANDERMAN R, 1991, PSYCHOL REP, V69, P1091, DOI 10.2466/PR0.69.8.1091-1096

Segal Z., 2002, MINDFULNESS BASED CO

Shapiro SL, 2011, J CLIN PSYCHOL, V67, P267, DOI 10.1002/jclp.20761

Spek V, 2008, J AFFECT DISORDERS, V105, P137, DOI 10.1016/j.jad.2007.05.001

Spinhoven P, 1997, PSYCHOL MED, V27, P363, DOI 10.1017/S0033291796004382

van Son J, 2014, J PSYCHOSOM RES, V77, P81, DOI 10.1016/j.jpsychores.2014.03.013

van Son J, 2013, DIABETES CARE, V36, P823, DOI 10.2337/dc12-1477

van Son J, 2011, BMC PUBLIC HEALTH, V11, DOI 10.1186/1471-2458-11-131

WATSON D, 1989, PSYCHOL REV, V96, P234, DOI 10.1037//0033-295X.96.2.234

ZIGMOND AS, 1983, ACTA PSYCHIAT SCAND, V67, P361, DOI 10.1111/j.1600-0447.1983.tb09716.x

NR 38

TC 5

Z9 5

U1 3

U2 26

PU PERGAMON-ELSEVIER SCIENCE LTD

PI OXFORD

PA THE BOULEVARD, LANGFORD LANE, KIDLINGTON, OXFORD OX5 1GB, ENGLAND

SN 0022-3999

EI 1879-1360

J9 J PSYCHOSOM RES

JI J. Psychosomat. Res.

PD DEC

PY 2016

VL 91

BP 40

EP 47

DI 10.1016/j.jpsychores.2016.10.009

PG 8

WC Psychiatry

SC Psychiatry

GA EE6PY

UT WOS:000389735900007

PM 27894461

DA 2019-01-08

ER

PT J

AU Robinson, EM

Weaver, P

Chen, R

Streisand, R

Holmes, CS

AF Robinson, Elizabeth M.

Weaver, Patrick

Chen, Rusan

Streisand, Randi

Holmes, Clarissa S.

TI A Model of Parental Distress and Factors That Mediate Its Link With

Parental Monitoring of Youth Diabetes Care, Adherence, and Glycemic

Control

SO HEALTH PSYCHOLOGY

LA English

DT Article

DE type 1 diabetes; adherence; adolescence; parental monitoring; parental

distress

ID MATERNAL DEPRESSIVE SYMPTOMS; METABOLIC-CONTROL; SELF-EFFICACY;

YOUNG-CHILDREN; ILLNESS MANAGEMENT; REGIMEN ADHERENCE; FAMILY CONFLICT;

HEALTH OUTCOMES; DISEASE CARE; FIT INDEXES

AB Objective: Parental monitoring of adolescents' diabetes self-care is associated with better adherence and glycemic control (A1c). A number of parent-level factors are associated with higher levels of parental monitoring, including lower levels of parental distress (depressive symptoms, stress, anxiety), as well as higher levels of parental self-efficacy for diabetes management and authoritative parenting. Often studied in isolation, these factors may be best considered simultaneously as they are interrelated and are associated with parental monitoring and youth adherence. Method: Structural equation modeling with a cross-sectional sample of 257 parent/youth (aged 11-14) dyads: (a) examined a broad model of parental factors (i.e., parental distress, parental diabetes self-efficacy, authoritative parenting), and (b) assessed their relation to parental monitoring, youth adherence, and A1c. Post hoc analyses of variance (ANOVAs) evaluated clinical implications of daily parental monitoring. Results: Parental distress was not related directly to parental monitoring. Instead less distress related indirectly to more monitoring via higher parental self-efficacy and more authoritative parenting which, in turn, related to better adherence and A1c. Higher parental self-efficacy also related directly to better youth adherence and then to better A1c. Clinically, more parental monitoring related to more daily blood glucose checks and to better A1c (8.48% vs. 9.17%). Conclusions: A broad model of parent-level factors revealed more parental distress was linked only indirectly to less monitoring via lower parental self-efficacy and less authoritative parenting. Behaviorally, more parental monitoring related to better adherence and to clinically better A1c in adolescents. Further study of parent-level factors that relate to parental distress and monitoring of adherence appears warranted.

C1 [Robinson, Elizabeth M.] Virginia Commonwealth Univ, Dept Psychol, Richmond, VA 23284 USA.

[Weaver, Patrick] Eastern Michigan Univ, Dept Psychol, Ypsilanti, MI 48197 USA.

[Chen, Rusan] Georgetown Univ, Ctr New Designs Learning & Scholarship, Washington, DC 20057 USA.

[Streisand, Randi] Childrens Natl Med Ctr, Dept Psychiat & Behav Sci, Div Behav Med & Clin Psychol, Washington, DC 20010 USA.

[Streisand, Randi] George Washington Univ, Dept Pediat, Washington, DC 20052 USA.

[Holmes, Clarissa S.] Virginia Commonwealth Univ, Dept Pediat, Richmond, VA 23284 USA.

[Holmes, Clarissa S.] Georgetown Univ, Dept Psychiat, Washington, DC 20057 USA.

RP Robinson, EM (reprint author), Childrens Hosp Philadelphia, 3401 Civ Ctr Blvd,Room 11400-03, Philadelphia, PA 19104 USA.

EM robinson.elizabeth.m@gmail.com

FU National Institute of Diabetes and Digestive and Kidney Diseases at the

National Institutes of Health [5R01DK070917]

FX This work was supported by the National Institute of Diabetes and

Digestive and Kidney Diseases at the National Institutes of Health

(Grant 5R01DK070917) awarded to CH.

CR ABIDIN RR, 1992, J CLIN CHILD PSYCHOL, V21, P407, DOI 10.1207/s15374424jccp2104_12

AMIEL SA, 1986, NEW ENGL J MED, V315, P215, DOI 10.1056/NEJM198607243150402

Anderson BJ, 2002, DIABETIC MED, V19, P635, DOI 10.1046/j.1464-5491.2002.00752.x

BANDURA A, 1977, PSYCHOL REV, V84, P191, DOI 10.1037/0033-295X.84.2.191

BAUMRIND D, 1967, GENET PSYCHOL MONOGR, V75, P43

Beck AT, 1996, MANUAL BECK DEPRESSI

BELSKY J, 1984, CHILD DEV, V55, P83, DOI 10.1111/j.1467-8624.1984.tb00275.x

BLAND JM, 1995, BRIT MED J, V310, P170, DOI 10.1136/bmj.310.6973.170

Carcone AI, 2012, J DEV BEHAV PEDIATR, V33, P343, DOI 10.1097/DBP.0b013e31824eaac8

Center for Disease Control and Prevention, 2011, NAT DIAB FACT SHEET

Chiang JL, 2014, DIABETES CARE, V37, P2034, DOI 10.2337/dc14-1140

Clarke WL, 1998, J PEDIATR ENDOCR MET, V11, P189

Collins LM, 2001, PSYCHOL METHODS, V6, P330, DOI 10.1037//1082-989X.6.4.330

Driscoll KA, 2010, J PEDIATR PSYCHOL, V35, P814, DOI 10.1093/jpepsy/jsp138

Eckshtain D, 2010, J PEDIATR PSYCHOL, V35, P426, DOI 10.1093/jpepsy/jsp068

Ellis DA, 2008, J ADOLESCENT HEALTH, V42, P146, DOI 10.1016/j.jadohealth.2007.08.012

Ellis DA, 2007, J PEDIATR PSYCHOL, V32, P907, DOI 10.1093/jpepsy/jsm009

EVANS CL, 1987, J PSYCHOSOM RES, V31, P367, DOI 10.1016/0022-3999(87)90057-2

GILLIS JS, 1993, PSYCHOL REP, V72, P1355, DOI 10.2466/pr0.1993.72.3c.1355

Glatz T, 2015, J FAM PSYCHOL, V29, P427, DOI 10.1037/fam0000076

Greene MS, 2010, DIABETES EDUCATOR, V36, P326, DOI 10.1177/0145721710361270

Grey M, 2001, J ADOLESCENT HEALTH, V28, P377, DOI 10.1016/S1054-139X(00)00211-1

Helgeson VS, 2012, J PEDIATR PSYCHOL, V37, P467, DOI 10.1093/jpepsy/jsr110

Helgeson VS, 2009, J PEDIATR PSYCHOL, V34, P254, DOI 10.1093/jpepsy/jsn079

Hilliard ME, 2013, HEALTH PSYCHOL, V32, P388, DOI 10.1037/a0027811

Hilliard ME, 2011, ARCH PEDIAT ADOL MED, V165, P624, DOI 10.1001/archpediatrics.2011.86

Hollingshead A. A., 1975, 4 FACTOR INDEX UNPUB

Holmbeck GN, 2002, J PEDIATR PSYCHOL, V27, P5, DOI 10.1093/jpepsy/27.1.5

Holmes CS, 2006, J PEDIATR PSYCHOL, V31, P770, DOI 10.1093/jpepsy/jsj083

Holmes CS, 2014, DIABETES CARE, V37, P1535, DOI 10.2337/dc13-1053

Horsch A, 2007, J TRAUMA STRESS, V20, P881, DOI 10.1002/jts.20247

Hu LT, 1999, STRUCT EQU MODELING, V6, P1, DOI 10.1080/10705519909540118

Hu LT, 1998, PSYCHOL METHODS, V3, P424, DOI 10.1037//1082-989X.3.4.424

Iannotti RJ, 2006, J DEV BEHAV PEDIATR, V27, P98, DOI 10.1097/00004703-200604000-00003

Iannotti RJ, 2006, DIABETES CARE, V29, P2263, DOI 10.2337/dc06-0685

IRVINE AA, 1992, HEALTH PSYCHOL, V11, P135, DOI 10.1037/0278-6133.11.2.135

Jaser SS, 2008, J PEDIATR PSYCHOL, V33, P509, DOI 10.1093/jpepsy/jsm104

Jaser SS, 2010, J PEDIATR PSYCHOL, V35, P738, DOI 10.1093/jpepsy/jsp098

JOHNSON SB, 1986, HEALTH PSYCHOL, V5, P545, DOI 10.1037/0278-6133.5.6.545

JOHNSON SB, 1992, J PEDIATR PSYCHOL, V17, P537, DOI 10.1093/jpepsy/17.5.537

Johnson SR, 2013, DIABETOLOGIA, V56, P2392, DOI 10.1007/s00125-013-3007-9

Jones TL, 2005, CLIN PSYCHOL REV, V25, P341, DOI 10.1016/j.cpr.2004.12.004

KENNY DA, 1995, PERS RELATIONSHIP, V2, P67, DOI 10.1111/j.1475-6811.1995.tb00078.x

King PS, 2014, HEALTH PSYCHOL, V33, P424, DOI 10.1037/a0032804

Kline R. B., 2011, PRINCIPLES PRACTICE

Leonard B J, 1998, J Pediatr Nurs, V13, P224, DOI 10.1016/S0882-5963(98)80049-3

MacCallum RC, 2000, ANNU REV PSYCHOL, V51, P201, DOI 10.1146/annurev.psych.51.1.201

Mackey ER, 2014, HEALTH PSYCHOL, V33, P783, DOI 10.1037/hea0000066

MacKinnon D., 2008, INTRO STAT MEDIATION

Marrero DG, 1997, DIABETES EDUCATOR, V23, P281, DOI 10.1177/014572179702300306

McGrady ME, 2009, DIABETES CARE, V32, P804, DOI 10.2337/dc08-2111

Mlynarczyk Susan M, 2013, Pediatr Nurs, V39, P181

Monaghan M, 2012, J CLIN PSYCHOL MED S, V19, P255, DOI 10.1007/s10880-011-9284-x

Monaghan MC, 2009, FAM SYST HEALTH, V27, P28, DOI 10.1037/a0014770

Muthen L. K., 1998, MPLUS USERS GUIDE

Patton SR, 2007, PEDIATR DIABETES, V8, P362, DOI 10.1111/j.1399-5448.2007.00242.x

Patton SR, 2011, J CLIN PSYCHOL MED S, V18, P345, DOI 10.1007/s10880-011-9256-1

Rausch JR, 2012, DIABETES CARE, V35, P1219, DOI 10.2337/dc11-2163

Snorer M, 2011, DIABETES CARE, V34, P1735, DOI 10.2337/dc10-1602

STEINBERG L, 1994, CHILD DEV, V65, P754, DOI 10.1111/j.1467-8624.1994.tb00781.x

Streisand R, 2005, J PEDIATR PSYCHOL, V30, P513, DOI 10.1093/jpepsy/jsi076

Streisand R, 2001, J PEDIATR PSYCHOL, V26, P155, DOI 10.1093/jpepsy/26.3.155

Whittemore R, 2012, DIABETES EDUCATOR, V38, P562, DOI 10.1177/0145721712445216

Wiebe DJ, 2011, J PEDIATR PSYCHOL, V36, P837, DOI 10.1093/jpepsy/jsr002

Wiebe DJ, 2010, J PEDIATR PSYCHOL, V35, P1152, DOI 10.1093/jpepsy/jsq012

Wiebe DJ, 2005, J PEDIATR PSYCHOL, V30, P167, DOI 10.1093/jpepsy/jsi004

WYSOCKI T, 1992, DIABETES CARE, V15, P43, DOI 10.2337/diacare.15.1.43

NR 67

TC 3

Z9 3

U1 0

U2 18

PU AMER PSYCHOLOGICAL ASSOC

PI WASHINGTON

PA 750 FIRST ST NE, WASHINGTON, DC 20002-4242 USA

SN 0278-6133

EI 1930-7810

J9 HEALTH PSYCHOL

JI Health Psychol.

PD DEC

PY 2016

VL 35

IS 12

BP 1373

EP 1382

DI 10.1037/hea0000406

PG 10

WC Psychology, Clinical; Psychology

SC Psychology

GA ED3MD

UT WOS:000388752300010

PM 27513476

OA Green Accepted

DA 2019-01-08

ER

PT J

AU Das, A

AF Das, Aniruddha

TI Psychosocial distress and inflammation: Which way does causality flow?

SO SOCIAL SCIENCE & MEDICINE

LA English

DT Article

DE Inflammation; Psychosocial distress; Older adults; NSHAP

ID C-REACTIVE PROTEIN; NATIONAL SOCIAL-LIFE; MARGINAL STRUCTURAL MODELS;

TYPE-2 DIABETES-MELLITUS; CORONARY-HEART-DISEASE; AGING PROJECT;

CARDIOVASCULAR-DISEASE; OLDER-ADULTS; DEPRESSIVE SYMPTOMS; METABOLIC

SYNDROME

AB Objectives: This study queried causal direction in linkages of inflammation with psychosocial distress.

Methods: Data were from the 2005-2006 and 2010-2011 waves of the U.S. National Social Life, Health, and Aging Project. Inflammation was indicated by C-reactive protein, and distress by depression, anxiety, as well as stress. Autoregressive cross-lagged panel models were used to examine causal direction.

Results: Rather than being an outcome of psychosocial distress, inflammation was a predictor of it. Linkages were gender differentiated, with inflammation seeming to induce depression among men but stress among women.

Discussion: Contrary to previous literature, inflammation may not be a mechanism through which psychosocial distress gets "under the skin" to cause cardiovascular and metabolic issues. Rather, it may be a node through which social pathologies and life events influence both mental health and physiological problems. (C) 2016 Elsevier Ltd. All rights reserved.

C1 [Das, Aniruddha] McGill Univ, Dept Sociol, Room 712,Leacock Bldg,855 Sherbrooke St West, Montreal, PQ H3A 2T7, Canada.

RP Das, A (reprint author), McGill Univ, Dept Sociol, Room 712,Leacock Bldg,855 Sherbrooke St West, Montreal, PQ H3A 2T7, Canada.

EM aniruddha.das@mcgill.ca

CR Baune BT, 2012, PSYCHONEUROENDOCRINO, V37, P1521, DOI 10.1016/j.psyneuen.2012.02.006

Berk M, 2013, BMC MED, V11, DOI 10.1186/1741-7015-11-200

Bierhaus A, 2003, P NATL ACAD SCI USA, V100, P1920, DOI 10.1073/pnas.0438019100

Capuron L, 2011, PHARMACOL THERAPEUT, V130, P226, DOI 10.1016/j.pharmthera.2011.01.014

Carpenter LL, 2010, NEUROPSYCHOPHARMACOL, V35, P2617, DOI 10.1038/npp.2010.159

CARR D, 2002, AGEING INT, V0027

Chiu HM, 2008, AM J GASTROENTEROL, V103, P2317, DOI 10.1111/j.1572-0241.2008.01952.x

COHEN S, 1983, J HEALTH SOC BEHAV, V24, P385, DOI 10.2307/2136404

Cole SR, 2005, AM J EPIDEMIOL, V162, P471, DOI 10.1093/aje/kwi216

Cole SR, 2008, AM J EPIDEMIOL, V168, P656, DOI 10.1093/aje/kwn164

Copeland WE, 2012, BIOL PSYCHIAT, V71, P15, DOI 10.1016/j.biopsych.2011.09.023

Cuijpers P, 2014, AM J PSYCHIAT, V171, P453, DOI 10.1176/appi.ajp.2013.13030325

CURRAN PJ, 2000, MULT APPL BK SER, P1

Danesh J, 2000, BRIT MED J, V321, P199, DOI 10.1136/bmj.321.7255.199

Deverts DJ, 2010, PSYCHOSOM MED, V72, P734, DOI 10.1097/PSY.0b013e3181ec4b98

Duivis HE, 2011, AM J PSYCHIAT, V168, P913, DOI 10.1176/appi.ajp.2011.10081163

Elwert F, 2006, AM SOCIOL REV, V71, P16, DOI 10.1177/000312240607100102

Ford DE, 2004, ARCH INTERN MED, V164, P1010, DOI 10.1001/archinte.164.9.1010

Fuente-Martin E, 2013, REV ENDOCR METAB DIS, V14, P331, DOI 10.1007/s11154-013-9263-7

Gimeno D, 2009, PSYCHOL MED, V39, P413, DOI 10.1017/S0033291708003723

Glassman AH, 2007, BIOL PSYCHIAT, V62, P280, DOI 10.1016/j.biopsych.2007.05.032

Gouin JP, 2012, ANN BEHAV MED, V44, P287, DOI 10.1007/s12160-012-9386-1

Gouin JP, 2012, HEALTH PSYCHOL, V31, P264, DOI 10.1037/a0025536

Grundy SM, 2004, ARTERIOSCL THROM VAS, V24, pE13, DOI 10.1161/01.ATV.0000111245.75752.C6

Haroon E, 2012, NEUROPSYCHOPHARMACOL, V37, P137, DOI 10.1038/npp.2011.205

Hayward MD, 2004, DEMOGRAPHY, V41, P87, DOI 10.1353/dem.2004.0005

Hernan MA, 2000, EPIDEMIOLOGY, V11, P561, DOI 10.1097/00001648-200009000-00012

Kautzky-Willer A, 2016, ENDOCR REV, V37, P278, DOI 10.1210/er.2015-1137

Kiecolt-Glaser JK, 2015, AM J PSYCHIAT, V172, P1075, DOI 10.1176/appi.ajp.2015.15020152

Kiecolt-Glaser JK, 2010, NEUROSCI BIOBEHAV R, V35, P33, DOI 10.1016/j.neubiorev.2009.09.003

Kiecolt-Glaser JK, 2003, P NATL ACAD SCI USA, V100, P9090, DOI 10.1073/pnas.1531903100

KiecoltGlaser JK, 1997, PSYCHOSOM MED, V59, P339, DOI 10.1097/00006842-199707000-00001

Lagraauw HM, 2015, BRAIN BEHAV IMMUN, V50, P18, DOI 10.1016/j.bbi.2015.08.007

Lancaster GI, 2014, TRENDS IMMUNOL, V35, P262, DOI 10.1016/j.it.2014.02.008

Leonard B, 2012, NEUROSCI BIOBEHAV R, V36, P764, DOI 10.1016/j.neubiorev.2011.12.005

Lykken D, 1996, PSYCHOL SCI, V7, P186, DOI 10.1111/j.1467-9280.1996.tb00355.x

Matthews KA, 2010, BRAIN BEHAV IMMUN, V24, P96, DOI 10.1016/j.bbi.2009.08.005

McDade TW, 2013, BRAIN BEHAV IMMUN, V31, P23, DOI 10.1016/j.bbi.2012.08.010

McDade TW, 2012, P NATL ACAD SCI USA, V109, P17281, DOI 10.1073/pnas.1202244109

McDade TW, 2009, BIODEMOGR SOC BIOL, V55, P159, DOI 10.1080/19485560903382478

McDade TW, 2006, PSYCHOSOM MED, V68, P376, DOI 10.1097/01.psy.0000221371.43607.64

McDade TW, 2004, CLIN CHEM, V50, P652, DOI 10.1373/clinchem.2003.029488

Meier-Ewert HK, 2004, J AM COLL CARDIOL, V43, P678, DOI 10.1016/j.jacc.2003.07.050

Melamed S, 2006, PSYCHOL BULL, V132, P327, DOI 10.1037/0033-2909.132.3.327

Milaneschi Y, 2009, BIOL PSYCHIAT, V65, P973, DOI [10.1016/j.biopsych.2008.11.01, 10.1016/j.biopsych.2008.11.011]

Miller AH, 2013, DEPRESS ANXIETY, V30, P297, DOI 10.1002/da.22084

Miller AH, 2009, BIOL PSYCHIAT, V65, P732, DOI 10.1016/j.biopsych.2008.11.029

Mukamal KJ, 2006, ALCOHOL RES HEALTH, V29, P199

Muthen L. K., 1998, MPLUS USERS GUIDE

O'Muircheartaigh C., 2007, NSHAP NATL SOCIAL LI

O'Muircheartaigh C, 2014, J GERONTOL B-PSYCHOL, V69, pS15, DOI 10.1093/geronb/gbu053

Owen N, 2003, BRAIN BEHAV IMMUN, V17, P286, DOI 10.1016/S0889-1591(03)00058-8

Pace TWW, 2007, BRAIN BEHAV IMMUN, V21, P9, DOI 10.1016/j.bbi.2006.08.009

Phelan JC, 2010, J HEALTH SOC BEHAV, V51, pS28, DOI 10.1177/0022146510383498

Pradhan AD, 2001, JAMA-J AM MED ASSOC, V286, P327, DOI 10.1001/jama.286.3.327

Qato DM, 2009, J GERONTOL B-PSYCHOL, V64, pI86, DOI 10.1093/geronb/gbp036

RADLOFF L S, 1977, Applied Psychological Measurement, V1, P385, DOI 10.1177/014662167700100306

Raison CL, 2013, BRAIN BEHAV IMMUN, V31, P1, DOI 10.1016/j.bbi.2013.04.009

Raison CL, 2011, CURR PSYCHIAT REP, V13, P467, DOI 10.1007/s11920-011-0232-0

Raison CL, 2006, TRENDS IMMUNOL, V27, P24, DOI 10.1016/j.it.2005.11.006

Ridker PM, 2003, CIRCULATION, V107, P391, DOI 10.1161/01.CIR.0000055014.62083.05

Schnittker J, 2008, AM J SOCIOL, V114, pS233, DOI 10.1086/592424

Schultze-Florey CR, 2012, BRAIN BEHAV IMMUN, V26, P1066, DOI 10.1016/j.bbi.2012.06.009

Shaffer JA, 2011, PSYCHOSOM MED, V73, P370, DOI 10.1097/PSY.0b013e31821deafd

Shelton RC, 2010, PROG NEUROBIOL, V91, P275, DOI 10.1016/j.pneurobio.2010.04.004

Shiovitz-Ezra S, 2009, J GERONTOL B-PSYCHOL, V64, pI30, DOI 10.1093/geronb/gbn020

Simanek AM, 2014, PSYCHONEUROENDOCRINO, V50, P139, DOI 10.1016/j.psyneuen.2014.08.002

Slavich GM, 2014, PSYCHOL BULL, V140, P774, DOI 10.1037/a0035302

Slopen N, 2015, PSYCHONEUROENDOCRINO, V51, P403, DOI 10.1016/j.psyneuen.2014.10.016

Smith S, 2009, J GERONTOL B-PSYCHOL, V64, pI20, DOI 10.1093/geronb/gbn013

Stata Corp, 2014, STATA REL 14 0

Stetler C, 2011, PSYCHOSOM MED, V73, P114, DOI 10.1097/PSY.0b013e31820ad12b

Valkanova V, 2013, J AFFECT DISORDERS, V150, P736, DOI 10.1016/j.jad.2013.06.004

WEINSTEIN M, 2007, BIOSOCIAL SURVEYS

Williams SR, 2009, J GERONTOL B-PSYCHOL, V64, pI67, DOI 10.1093/geronb/gbn015

Williams SR, 2009, J GERONTOL B-PSYCHOL, V64, pI131, DOI 10.1093/geronb/gbn022

Willson AE, 2007, AM J SOCIOL, V112, P1886, DOI 10.1086/512712

Yudkin JS, 2003, INT J OBESITY, V27, pS25, DOI 10.1038/sj.ijo.0802496

ZIGMOND AS, 1983, ACTA PSYCHIAT SCAND, V67, P361, DOI 10.1111/j.1600-0447.1983.tb09716.x

NR 79

TC 18

Z9 18

U1 0

U2 18

PU PERGAMON-ELSEVIER SCIENCE LTD

PI OXFORD

PA THE BOULEVARD, LANGFORD LANE, KIDLINGTON, OXFORD OX5 1GB, ENGLAND

SN 0277-9536

J9 SOC SCI MED

JI Soc. Sci. Med.

PD DEC

PY 2016

VL 170

BP 1

EP 8

DI 10.1016/j.socscimed.2016.10.001

PG 8

WC Public, Environmental & Occupational Health; Social Sciences, Biomedical

SC Public, Environmental & Occupational Health; Biomedical Social Sciences

GA ED8FZ

UT WOS:000389108700001

PM 27728857

DA 2019-01-08

ER

PT J

AU Han, Y

Zheng, YL

Wu, AM

Liu, HB

Su, JB

Lu, XY

Han, YW

Ji, NL

Ji, JH

Shi, Y

AF Han, Yun

Zheng, Yan-li

Wu, Ai-min

Liu, Hong-bin

Su, Jian-bin

Lu, Xiao-yan

Han, Yu-wen

Ji, Jin-long

Ji, Ju-hua

Shi, Yue

TI Effects of management in gestational diabetes mellitus with normal

prepregnancy body mass index on pregnancy outcomes and placental

ultrastructures: a prospective cohort study

SO ENDOCRINE

LA English

DT Article

DE Gestational diabetes mellitus; Pregnancy outcome; Placenta;

Ultrastructure; Management

ID WEIGHT-GAIN; DIAGNOSTIC-CRITERIA; NEONATAL OUTCOMES; WOMEN;

RECOMMENDATIONS; ASSOCIATION; GLUCOSE; TRIAL; BIRTH

AB A great quantity of gestational diabetes mellitus with normal prepregnancy body mass index have emerged with the new criteria of gestational diabetes mellitus in China based on the International Diabetes in Pregnancy Consensus group criteria, and understanding placental changes and how they affect outcomes are necessary in order to develop effective management approach. The aim of this study was to prospectively explore the effect of active management starting from the late second trimester in gestational diabetes mellitus women with normal prepregnancy body mass index on pregnancy outcomes and placental ultrastructures, and to provide scientific evidences for optimizing the management of gestational diabetes mellitus in China. Gestational diabetes mellitus women with normal prepregnancy body mass index in the same period of this prospective cohort study were divided into intervention group (n = 51) and control group (n = 55). The intervention group was managed rigorously, while the control group received conventional prenatal cares. The glucose profile, gestational weight gain and pregnancy outcomes were followed up and placental ultrastructures were observed and recorded by transmission electron microscopy. The blood glucose level and gestational weight gain in intervention group were significantly better controlled than those in control group (P < 0.01). The incidences of fetal distress, cesarean section and large for gestational age were significantly lower in intervention group than in control group (P < 0.05). There was a significant reduction in the incidence of abnormal placental ultrastructure in the intervention group (P < 0.01). After adjustment for confounding factors, the undesirable glycemic control and conventional management were related to abnormal placental ultrastructure (P < 0.05). Meanwhile, the undesirable glycemic control, abnormal placental ultrastructure and conventional management made sense in the incidence of fetal distress (P < 0.05), and the target glycemic control, recommend weight gain and active management were associated with reductions in the prevalence of cesarean delivery and large for gestational age (P < 0.05). The active management of gestational diabetes mellitus women with normal prepregnancy body mass index can improve pregnancy outcomes and placental ultrastructures, and the abnormal placental ultrastructure might be closely associated with the undesirable glycemic control and adverse pregnancy outcomes.

C1 [Han, Yun; Zheng, Yan-li; Wu, Ai-min; Lu, Xiao-yan; Han, Yu-wen; Ji, Jin-long] Nantong Univ, Affiliated Hosp 2, Dept Obstet & Gynecol, 6 North Hai Er Xiang Rd, Nantong 226001, Peoples R China.

[Liu, Hong-bin] Nantong Univ, Affiliated Hosp 2, Dept Pathol, 6 North Hai Er Xiang Rd, Nantong 226001, Peoples R China.

[Su, Jian-bin] Nantong Univ, Affiliated Hosp 2, Dept Endocrinol, 6 North Hai Er Xiang Rd, Nantong 226001, Peoples R China.

[Ji, Ju-hua] Nantong Univ, Affiliated Hosp 2, Dept Pediat, 6 North Hai Er Xiang Rd, Nantong 226001, Peoples R China.

[Shi, Yue] Johns Hopkins Med, 1013 N Wolfe St, Baltimore, MD 21205 USA.

RP Zheng, YL (reprint author), Nantong Univ, Affiliated Hosp 2, Dept Obstet & Gynecol, 6 North Hai Er Xiang Rd, Nantong 226001, Peoples R China.

EM gaoshan1189@sina.com

OI Su, Jian-bin/0000-0002-2138-0263

FU National Natural Science Foundation of China [81501258]; Maternal and

Children Health Research Project of Jiangsu Province [F201329]; Jiangsu

Provincial Commission of Health and Family Planning Youth Research

Project [Q201512]

FX The study was partly supported by National Natural Science Foundation of

China (81501258), Maternal and Children Health Research Project of

Jiangsu Province (F201329) and Jiangsu Provincial Commission of Health

and Family Planning Youth Research Project (Q201512).

CR Abenhaim Haim A., 2007, Archives of Gynecology and Obstetrics, V275, P39, DOI 10.1007/s00404-006-0219-y

ALOKAIL MS, 1994, ENDOCR J, V41, P355, DOI 10.1507/endocrj.41.355

Berggren EK, 2015, AM J PERINAT, V32, P251, DOI 10.1055/s-0034-1383848

Black MH, 2013, DIABETES CARE, V36, P56, DOI 10.2337/dc12-0741

Blomberg M, 2011, OBSTET GYNECOL, V117, P1065, DOI 10.1097/AOG.0b013e318214f1d1

Bonomo M, 2005, DIABETIC MED, V22, P1536, DOI 10.1111/j.1464-5491.2005.01690.x

Casey BM, 2015, AM J PERINAT, V32, P93, DOI 10.1055/s-0034-1374815

Derbyshire E, 2006, J HUM NUTR DIET, V19, P267, DOI 10.1111/j.1365-277X.2006.00705.x

Dzakpasu S, 2014, BMC PREGNANCY CHILDB, V14, DOI 10.1186/1471-2393-14-106

Gabbay-Benziv R., 2014, CLIN OBSTET GYNAECOL, V29, P150, DOI [10.1016/j.bpobgyn.2014.04.025, DOI 10.1016/J.BPOBGYN.2014.04.025]

Gasim Turki, 2012, Oman Med J, V27, P140, DOI 10.5001/omj.2012.29

Getahun D, 2007, AM J OBSTET GYNECOL, V196, pe1

Greuter MJE, 2012, BMC PREGNANCY CHILDB, V12, DOI 10.1186/1471-2393-12-58

Hadar E, 2009, J PERINAT MED, V37, P447, DOI 10.1515/JPM.2009.114

Hartling L, 2014, DIABETIC MED, V31, P319, DOI 10.1111/dme.12357

Institute of Medicine, 2009, WEIGHT GAIN PREGNANC

JONES CJP, 1976, OBSTET GYNECOL, V48, P274

Landon MB, 2009, NEW ENGL J MED, V361, P1339, DOI 10.1056/NEJMoa0902430

Langer O, 2005, AM J OBSTET GYNECOL, V192, P989, DOI 10.1016/j.ajog.2004.11.039

Langer O, 2010, J MATERN-FETAL NEO M, V23, P196, DOI 10.3109/14767050903550634

McGiveron A, 2015, J HUM NUTR DIET, V28, P29, DOI 10.1111/jhn.12240

Metzger BE, 2010, DIABETES CARE, V33, P676, DOI 10.2337/dc09-1848

Shen Cai-xia, 2011, Chinese Journal of Pathology, V40, P856, DOI 10.3760/cma.j.issn.0529-5807.2011.12.018

Strom-Roum EM, 2013, ACTA OBSTET GYN SCAN, V92, P783, DOI 10.1111/aogs.12104

Turok DK, 2003, AM FAM PHYSICIAN, V68, P1767

Yang HX, 2012, CHINESE MED J-PEKING, V125, P1212, DOI 10.3760/cma.j.issn.0366-6999.2012.07.004

Yang Yan-dong, 2012, Zhonghua Fu Chan Ke Za Zhi, V47, P646

Zhu WW, 2013, DIABETES CARE, V36, P586, DOI 10.2337/dc12-1157

NR 28

TC 0

Z9 0

U1 0

U2 7

PU SPRINGER

PI NEW YORK

PA 233 SPRING ST, NEW YORK, NY 10013 USA

SN 1355-008X

EI 1559-0100

J9 ENDOCRINE

JI Endocrine

PD DEC

PY 2016

VL 54

IS 3

BP 691

EP 699

DI 10.1007/s12020-016-1064-9

PG 9

WC Endocrinology & Metabolism

SC Endocrinology & Metabolism

GA EC5TA

UT WOS:000388197900014

PM 27481362

DA 2019-01-08

ER

PT J

AU Kurajoh, M

Kadoya, M

Morimoto, A

Naka, M

Miyoshi, A

Kanzaki, A

Kakutani-Hatayama, M

Hamamoto, K

Shoji, T

Moriwaki, Y

Yamamoto, T

Inaba, M

Namba, M

Koyama, H

AF Kurajoh, Masafumi

Kadoya, Manabu

Morimoto, Akiko

Naka, Mariko

Miyoshi, Akio

Kanzaki, Akinori

Kakutani-Hatayama, Mild

Hamamoto, Kae

Shoji, Takuhito

Moriwaki, Yuji

Yamamoto, Tetsuya

Inaba, Masaaki

Namba, Mitsuyoshi

Koyama, Hidenori

TI Plasma leptin concentration is associated with fatigue severity in

patients with cardiovascular risk factors - HSCAA study

SO PSYCHONEUROENDOCRINOLOGY

LA English

DT Article

DE Fatigue; Leptin; Cardiovascular risk factor

ID OBSTRUCTIVE SLEEP-APNEA; PSYCHOLOGICAL DISTRESS; DEPRESSIVE SYMPTOMS;

AUTONOMIC FUNCTION; CHOLESTEROL LEVELS; DOPAMINE NEURONS; ALCOHOL;

QUALITY; BRAIN; DISEASE

AB Fatigue induced by complex dysfunctions of the central nervous system is frequently complained by patients with cardiovascular risk factors. Although leptin is considered to regulate the central nervous system, there are no reports regarding its association with fatigue in those patients. This cross-sectional study included 347 patients with cardiovascular risk factors. Fatigue score and plasma leptin concentration were measured. In addition, abdominal fat accumulation, systemic inflammation, sleep condition, and functions of hypothalamus-pituitary axis and autonomic system were estimated. Plasma leptin concentration (natural logarithm transformed) was significantly and positively (r= 0.222, p < 0.001) associated with fatigue score, and significantly (p <0.001) higher in the moderately-fatigued group (2.32 +/- 10.75 ng/ml, mean +/- SD, n = 52) than in the normally-fatigued group (1.85 +/- 1.02 ng/ml, mean +/- SD, n=295). Multiple logistic regression analysis showed that plasma leptin concentration was significantly and independently associated with a moderately-fatigued condition independent of other factors, including age, gender, presence of diabetes, hypertension, dyslipidemia, alcohol consumption habit, urinary free cortisol, serum high-sensitive CRP concentration, visceral and subcutaneous fat area, apnea/hypopnea index, sleep efficiency, and heart rate variability. Hyperleptinemia may contribute to fatigue severity in patients with cardiovascular risk factors. (C) 2016 Elsevier Ltd. All rights reserved.

C1 [Kurajoh, Masafumi; Kadoya, Manabu; Morimoto, Akiko; Naka, Mariko; Miyoshi, Akio; Kanzaki, Akinori; Kakutani-Hatayama, Mild; Hamamoto, Kae; Shoji, Takuhito; Moriwaki, Yuji; Yamamoto, Tetsuya; Namba, Mitsuyoshi; Koyama, Hidenori] Hyogo Coll Med, Div Diabet Endocrinol & Metab, Dept Internal Med, 1-1 Mukogawa Cho, Nishinomiya, Hyogo 6638501, Japan.

[Kurajoh, Masafumi; Inaba, Masaaki] Osaka City Univ, Grad Sch Med, Dept Endocrinol Metab & Mol Med, Osaka, Japan.

RP Kurajoh, M (reprint author), Hyogo Coll Med, Div Diabet Endocrinol & Metab, Dept Internal Med, 1-1 Mukogawa Cho, Nishinomiya, Hyogo 6638501, Japan.

EM mkurajoh@hyo-med.ac.jp

CR Atmaca M, 2002, NEUROPSYCHOBIOLOGY, V46, P176, DOI 10.1159/000067809

BAILEY SP, 1993, INT J SPORTS MED, V14, P330, DOI 10.1055/s-2007-1021187

Bardwell WA, 2003, AM J PSYCHIAT, V160, P350, DOI 10.1176/appi.ajp.160.2.350

Berridge KC, 1998, BRAIN RES REV, V28, P309, DOI 10.1016/S0165-0173(98)00019-8

Brewerton TD, 2000, PSYCHONEUROENDOCRINO, V25, P649, DOI 10.1016/S0306-4530(00)00016-0

Bultmann U, 2002, J OCCUP ENVIRON MED, V44, P116

Bunevicius A, 2012, PSYCHOSOM MED, V74, P848, DOI 10.1097/PSY.0b013e318268e7f0

Burguera B, 2000, NEUROENDOCRINOLOGY, V71, P187, DOI 10.1159/000054536

Calapi G, 1999, J CLIN INVEST, V104, P975, DOI 10.1172/JCI5867

Camm AJ, 1996, EUR HEART J, V17, P354

Cho HJ, 2009, BIOL PSYCHIAT, V66, P871, DOI 10.1016/j.biopsych.2009.06.008

Cleare AJ, 2003, ENDOCR REV, V24, P236, DOI 10.1210/er.2001-0014

Dawson D, 1997, NATURE, V388, P235, DOI 10.1038/40775

Drivsholm T, 2005, DIABETOLOGIA, V48, P210, DOI 10.1007/s00125-004-1625-y

Eckert ED, 1998, J CLIN ENDOCR METAB, V83, P791, DOI 10.1210/jc.83.3.791

Farr OM, 2015, METABOLISM, V64, P114, DOI 10.1016/j.metabol.2014.07.004

Fujii H, 2013, J RENAL NUTR, V23, P340, DOI 10.1053/j.jrn.2012.12.008

Holliday EB, 2016, RADIOTHER ONCOL, V118, P105, DOI 10.1016/j.radonc.2015.12.015

Hommel JD, 2006, NEURON, V51, P801, DOI 10.1016/j.neuron.2006.08.023

Jarosz PA, 2014, WOMEN HEALTH ISS, V24, pE447, DOI 10.1016/j.whi.2014.04.005

Jow GM, 2006, J AFFECT DISORDERS, V90, P21, DOI 10.1016/j.jad.2005.09.015

Kadoya M, 2015, ATHEROSCLEROSIS, V238, P409, DOI 10.1016/j.atherosclerosis.2014.12.032

Kadoya M, 2014, PLOS ONE, V9, DOI 10.1371/journal.pone.0105977

King AC, 2011, ARCH GEN PSYCHIAT, V68, P389, DOI 10.1001/archgenpsychiatry.2011.26

Koyama H, 2010, CLIN J AM SOC NEPHRO, V5, P659, DOI 10.2215/CJN.08151109

Kuppuswamy A, 2015, J NEUROL NEUROSUR PS, V86, P902, DOI 10.1136/jnnp-2015-310431

Kurajoh M, 2015, CARDIOVASC DIABETOL, V14, DOI 10.1186/s12933-015-0280-6

Leinninger GM, 2009, CELL METAB, V10, P89, DOI 10.1016/j.cmet.2009.06.011

Licinio J, 1997, NAT MED, V3, P575, DOI 10.1038/nm0597-575

Lieb W, 2009, JAMA-J AM MED ASSOC, V302, P2565, DOI 10.1001/jama.2009.1836

Lim WJ, 2005, ARCH INTERN MED, V165, P910, DOI 10.1001/archinte.165.8.910

Loppenthin K, 2015, CLIN RHEUMATOL, V34, P2029, DOI 10.1007/s10067-015-2875-4

Macdonald JH, 2012, AM J KIDNEY DIS, V60, P930, DOI 10.1053/j.ajkd.2012.06.021

Matsuo S, 2009, AM J KIDNEY DIS, V53, P982, DOI 10.1053/j.ajkd.2008.12.034

McKenzie R, 1998, JAMA-J AM MED ASSOC, V280, P1061, DOI 10.1001/jama.280.12.1061

MORRISS R, 1993, BRIT MED J, V306, P1161, DOI 10.1136/bmj.306.6886.1161

Neu D, 2007, NEUROPSYCHOBIOLOGY, V56, P40, DOI 10.1159/000110727

Nicolas JM, 2001, ALCOHOL CLIN EXP RES, V25, P83

PAWLIKOWSKA T, 1994, BRIT MED J, V308, P763, DOI 10.1136/bmj.308.6931.763

PERSSON LO, 1980, PSYCHOPHARMACOLOGY, V68, P295, DOI 10.1007/BF00428119

Piche T, 2002, GUT, V51, P434, DOI 10.1136/gut.51.3.434

Piche T, 2007, EUR J GASTROEN HEPAT, V19, P237, DOI 10.1097/01.meg.0000252627.50302.b4

Rizo C, 2011, J AFFECT DISORDERS, V130, P290, DOI 10.1016/j.jad.2010.07.027

Saad MF, 1997, J CLIN ENDOCR METAB, V82, P579, DOI 10.1210/jc.82.2.579

Santos-Alvarez J, 1999, CELL IMMUNOL, V194, P6, DOI 10.1006/cimm.1999.1490

Schrieks IC, 2015, PLOS ONE, V10, DOI 10.1371/journal.pone.0126421

Schwartz MW, 1997, DIABETES CARE, V20, P1476, DOI 10.2337/diacare.20.9.1476

Stringer EA, 2013, J TRANSL MED, V11, DOI 10.1186/1479-5876-11-93

Stubbs B, 2016, PSYCHONEUROENDOCRINO, V63, P144, DOI 10.1016/j.psyneuen.2015.09.026

Tobler PN, 2005, SCIENCE, V307, P1642, DOI 10.1126/science.1105370

Wiese JG, 2000, ANN INTERN MED, V132, P897, DOI 10.7326/0003-4819-132-11-200006060-00008

WILSON WM, 1992, EXP PHYSIOL, V77, P921, DOI 10.1113/expphysiol.1992.sp003660

Woolley J, 2004, J PSYCHOSOM RES, V56, P203, DOI 10.1016/S0022-3999(03)00077-1

Wu MN, 2015, BMC PULM MED, V15, DOI 10.1186/s12890-015-0112-1

Zhuang XX, 1999, NEUROPSYCHOPHARMACOL, V21, pS52, DOI 10.1038/sj.npp.1395371

Zupancic ML, 2011, PSYCHOSOM MED, V73, P407, DOI 10.1097/PSY.0b013e31821a196f

NR 56

TC 2

Z9 2

U1 1

U2 4

PU PERGAMON-ELSEVIER SCIENCE LTD

PI OXFORD

PA THE BOULEVARD, LANGFORD LANE, KIDLINGTON, OXFORD OX5 1GB, ENGLAND

SN 0306-4530

J9 PSYCHONEUROENDOCRINO

JI Psychoneuroendocrinology

PD DEC

PY 2016

VL 74

BP 7

EP 12

DI 10.1016/j.psyneuen.2016.08.016

PG 6

WC Endocrinology & Metabolism; Neurosciences; Psychiatry

SC Endocrinology & Metabolism; Neurosciences & Neurology; Psychiatry

GA EB6VW

UT WOS:000387524700002

PM 27567116

DA 2019-01-08

ER

PT J

AU Lasaite, L

Dobrovolskiene, R

Danyte, E

Stankute, I

Razanskaite-Virbickiene, D

Schwitzgebel, V

Marciulionyte, D

Verkauskiene, R

AF Lasaite, Lina

Dobrovolskiene, Rimante

Danyte, Evalda

Stankute, Ingrida

Razanskaite-Virbickiene, Dovile

Schwitzgebel, Valerie

Marciulionyte, Dalia

Verkauskiene, Rasa

TI Diabetes distress in males and females with type 1 diabetes in

adolescence and emerging adulthood

SO JOURNAL OF DIABETES AND ITS COMPLICATIONS

LA English

DT Article

DE Diabetes distress; Type 1 diabetes; Adolescence; Emerging adulthood;

Gender-specific differences

ID PROBLEM-AREAS; EMOTIONAL DISTRESS; GLYCEMIC CONTROL; GLYCOSYLATED

HEMOGLOBIN; METABOLIC-CONTROL; DEPRESSION; SCALE; COMPLICATIONS;

CHILDREN; HEALTH

AB Background: Age and gender are important factors in the adjustment and psychological well-being of patients with chronic physical illness.

Aim: To explore the gender and age differences in diabetes distress between adolescents and emerging adults with type 1 diabetes (T1D).

Subjects and methods: Diabetes distress was compared in 255 adolescents and 283 emerging adults with T1D using Problem Areas in Diabetes scale.

Results: High diabetes distress level was found in 22.8% of participants. Lack of confidence in self-care (6.0 vs 3.0,p = 0.002), negative emotional consequences (10.0 vs 6.0,p = 0.004), and overall score (18.75 vs 11.25, p = 0.002) were higher in adult than in adolescent males, when adjusted for age at T1D onset. Negative emotional consequences (13.0 vs 10.0, p = 0.005) and overall score (25.0 vs 20.0, p = 0.016) were higher in adult compared to adolescent females, when adjusted for age at T1D onset. Lack of confidence in self-care (6.0 vs 3.0,p = 0.002), negative emotional consequences (10.0 vs 6.0,p = 0.015), and overall score (20.0 vs 11.2, p = 0.005) were higher in adolescent females compared to males, when adjusted for age at T1D onset. Negative emotional consequences score was higher in adult females compared to males (13.0 vs 10.0, p = 0.029), when adjusted for age at T1D onset.

In conclusion, our findings show that patients with TID have greater burden of diabetes distress in emerging adulthood than in adolescence and add to evidence suggesting the importance of addressing diabetes distress in clinical care and the necessity of wider picture beyond the physical manifestation of diabetes to be taken into consideration. (C) 2016 Elsevier Inc. All rights reserved.

C1 [Lasaite, Lina; Danyte, Evalda; Razanskaite-Virbickiene, Dovile; Marciulionyte, Dalia; Verkauskiene, Rasa] Lithuanian Univ Hlth Sci, Inst Endocrinol, Kaunas, Lithuania.

[Dobrovolskiene, Rimante; Stankute, Ingrida] Hosp Lithuanian Univ Hlth Sci Kauno Klinikos, Dept Endocrinol, Kaunas, Lithuania.

[Schwitzgebel, Valerie] Univ Hosp Geneva, Dept Child & Adolescent, Geneva, Switzerland.

[Schwitzgebel, Valerie] Univ Geneva, Diabet Ctr, Fac Med, Geneva, Switzerland.

RP Lasaite, L (reprint author), Lithuanian Univ Hlth Sci, Inst Endocrinol, Kaunas, Lithuania.

EM linlasla@yahoo.com

FU Lithuanian Research Council Lithuanian Swiss program "Research and

Development" [CH-3-SMM-01/09]; Swiss National Science Foundation

[CR33I3_140655]

FX This study was supported by a grant of Lithuanian Research Council

Lithuanian Swiss program "Research and Development", CH-3-SMM-01/09, and

the Swiss National Science Foundation (grant No. CR33I3_140655 to VMS).

CR Amsberg S, 2008, INT J NURS STUD, V45, P1319, DOI 10.1016/j.ijnurstu.2007.09.010

Arnett J. J., 2007, HDB SOCIALIZATION TH, P208, DOI DOI 10.1093/ACPROF:OSO/9780199757176.001.0001

Arnett JJ, 2000, AM PSYCHOL, V55, P469, DOI 10.1037/0003-066X.55.5.469

Balfe M, 2013, BMC ENDOCR DISORD, V13, DOI 10.1186/1472-6823-13-25

Barry CM, 2009, J ADULT DEV, V16, P209, DOI 10.1007/s10804-009-9067-x

BERN SL, 1974, J CONSULT CLIN PSYCH, V42, P155

Byrne DG, 2007, J ADOLESCENCE, V30, P393, DOI 10.1016/j.adolescence.2006.04.004

de Ridder D, 2008, LANCET, V372, P246, DOI 10.1016/S0140-6736(08)61078-8

DELAMATER AM, 1987, J DEV BEHAV PEDIATR, V8, P136

Eriksson AK, 2008, DIABETIC MED, V25, P834, DOI 10.1111/j.1464-5491.2008.02463.x

Fisher L, 2014, DIABETIC MED, V31, P764, DOI 10.1111/dme.12428

Fisher L, 2008, ANN FAM MED, V6, P246, DOI 10.1370/afm.842

Fisher L, 2007, DIABETES CARE, V30, P542, DOI 10.2337/dc06-1614

Fisher L, 2015, J DIABETES COMPLICAT, V29, P572, DOI 10.1016/j.jdiacomp.2015.01.012

Fisher L, 2012, DIABETES CARE, V35, P259, DOI 10.2337/dc11-1572

Fisher L, 2010, DIABETES CARE, V33, P23, DOI 10.2337/dc09-1238

GAFVELS C, 1993, DIABETIC MED, V10, P768, DOI 10.1111/j.1464-5491.1993.tb00162.x

Gendelman N, 2009, DIABETES CARE, V32, P575, DOI 10.2337/dc08-1835

GORTMAKER SL, 1990, PEDIATRICS, V85, P267

Graber JA, 2004, HDB ADOLESCENT PSYCH, V2

Hagger V, 2016, CURR DIABETES REP, V16, DOI 10.1007/s11892-015-0694-2

Helgeson VS, 2014, J PSYCHOSOM RES, V77, P484, DOI 10.1016/j.jpsychores.2014.09.013

Helgeson VS, 2010, HEALTH PSYCHOL, V29, P153, DOI 10.1037/a0018163

Hermanns N, 2006, DIABETOLOGIA, V49, P469, DOI 10.1007/s00125-005-0094-2

Hessler D. M., 2010, DIABETES S1, V59, P1435

Hessler DM, 2011, PATIENT EDUC COUNS, V85, P154, DOI 10.1016/j.pec.2010.10.030

Holmbeck G. N., 2006, BEHAV EMOTIONAL DISO, P21

Holt RIG, 2014, DIABETES CARE, V37, P2067, DOI 10.2337/dc13-2134

Huang MF, 2010, DIABETES CARE, V33, P38, DOI 10.2337/dc09-0768

Joensen LE, 2013, DIABETIC MED, V30, P1132, DOI 10.1111/dme.12241

Johnson JG, 2002, ARCH GEN PSYCHIAT, V59, P741, DOI 10.1001/archpsyc.59.8.741

Jones JM, 2000, BMJ-BRIT MED J, V320, P1563, DOI 10.1136/bmj.320.7249.1563

Kendler KS, 2005, AM J PSYCHIAT, V162, P250, DOI 10.1176/appi.ajp.162.2.250

Kessler RC, 2004, MAC FDN MEN, P227

Lasaite L, 2016, J DIABETES COMPLICAT, V30, P133, DOI 10.1016/j.jdiacomp.2015.09.012

Luecken L. J, 2010, HDB ADULT RESILIENCE, P238

Luyckx K, 2009, DIABETES CARE, V32, P797, DOI 10.2337/dc08-1990

MANN NP, 1982, ARCH DIS CHILD, V57, P434, DOI 10.1136/adc.57.6.434

Matthews S, 1999, SOC SCI MED, V48, P49, DOI 10.1016/S0277-9536(98)00288-3

METSCH J, 1995, INT J BEHAV MED, V2, P104, DOI 10.1207/s15327558ijbm0202_2

Miller ST, 2008, BMC PUBLIC HEALTH, V8, DOI 10.1186/1471-2458-8-70

Northam EA, 2005, DIABETIC MED, V22, P152, DOI 10.1111/j.1464-5491.2004.01370.x

Pintaudi B, 2015, J PSYCHOSOM RES, V79, P348, DOI 10.1016/j.jpsychores.2015.08.010

POLONSKY WH, 1995, DIABETES CARE, V18, P754, DOI 10.2337/diacare.18.6.754

Reddy J, 2013, PSYCHOSOMATICS, V54, P44, DOI 10.1016/j.psym.2012.08.004

Rubin RR, 1999, DIABETES-METAB RES, V15, P205, DOI 10.1002/(SICI)1520-7560(199905/06)15:3<205::AID-DMRR29>3.0.CO;2-O

Rydall AC, 1997, NEW ENGL J MED, V336, P1849, DOI 10.1056/NEJM199706263362601

Schmitt A, 2016, DIABETIC MED, V33, P835, DOI 10.1111/dme.12887

Schmitt A, 2015, J BEHAV MED, V38, P556, DOI 10.1007/s10865-014-9604-3

Schultz CJ, 1999, DIABETES CARE, V22, P495, DOI 10.2337/diacare.22.3.495

Simmons JH, 2013, DIABETES CARE, V36, P3573, DOI 10.2337/dc12-2643

Snoek FJ, 2000, DIABETES CARE, V23, P1305, DOI 10.2337/diacare.23.9.1305

Snoek FJ, 2015, LANCET DIABETES ENDO, V3, P450, DOI 10.1016/S2213-8587(15)00135-7

Snoek FJ, 2012, DIABETES CARE, V35, P2128, DOI 10.2337/dc11-1326

Stone AA, 2010, P NATL ACAD SCI USA, V107, P9985, DOI 10.1073/pnas.1003744107

Strandberg RB, 2014, J PSYCHOSOM RES, V77, P174, DOI 10.1016/j.jpsychores.2014.06.015

van Bastelaar KMP, 2010, DIABETIC MED, V27, P798, DOI 10.1111/j.1464-5491.2010.03025.x

Welch GW, 1997, DIABETES CARE, V20, P760, DOI 10.2337/diacare.20.5.760

*WHO, 1989, REPR HLTH AD STRAT A, P1

Wolpert HA, 2001, DIABETES CARE, V24, P1513, DOI 10.2337/diacare.24.9.1513

Zoffmann V, 2014, DIABETIC MED, V31, P493, DOI 10.1111/dme.12363

NR 61

TC 7

Z9 8

U1 2

U2 5

PU ELSEVIER SCIENCE INC

PI NEW YORK

PA 360 PARK AVE SOUTH, NEW YORK, NY 10010-1710 USA

SN 1056-8727

EI 1873-460X

J9 J DIABETES COMPLICAT

JI J. Diabetes Complications

PD NOV-DEC

PY 2016

VL 30

IS 8

BP 1500

EP 1505

DI 10.1016/j.jdiacomp.2016.08.013

PG 6

WC Endocrinology & Metabolism

SC Endocrinology & Metabolism

GA ES3MY

UT WOS:000399434900016

PM 27613444

DA 2019-01-08

ER

PT J

AU Pelizza, L

Pupo, S

AF Pelizza, Lorenzo

Pupo, Simona

TI Brittle diabetes: Psychopathology and personality

SO JOURNAL OF DIABETES AND ITS COMPLICATIONS

LA English

DT Article

DE Brittle diabetes; Glycaemic instability; Personality disorder;

Psychopathology; Assessment

ID HYPOGLYCEMIA; PREVALENCE; NEUROPATHY; MELLITUS

AB The term "brittle" is used to describe an uncommon subgroup of patients with type I diabetes whose lives are disrupted by severe glycaemic instability with repeated and prolonged hospitalization. Psychosocial problems are the major perceived underlying causes of brittle diabetes. Aim of this study is a systematic psychopathological and personological assessment of patients with brittle diabetes in comparison with subjects without brittle diabetes, using specific parameters of general psychopathology and personality disorders following the multi-axial format of the current DSM-IV-TR (Diagnostic and Statistical manual of Mental Disorders - IV Edition - Text Revised) diagnostic criteria for mental disorders. Patients comprised 42 subjects with brittle diabetes and a case-control group of 42 subjects with stable diabetes, matched for age, gender, years of education, and diabetes duration. General psychopathology and the DSM-IV-TR personality disorders were assessed using the Symptom Checklist-90-Revised (SCL-90-R) and the Structured Clinical Interview for axis II personality Disorders (SCID-II). The comparison for SCL-90-R parameters revealed no differences in all primary symptom dimensions and in the three global distress indices between the two groups. However, patients with brittle diabetes showed higher percentages in borderline, histrionic, and narcissistic personality disorder. In this study, patients with brittle diabetes show no differences in terms of global severity of psychopathological distress and specific symptoms of axis I DSM-IV-TR psychiatric diagnoses in comparison with subjects without brittle diabetes. Differently, individuals with brittle diabetes are more frequently affected by specific DSM-IV-TR cluster B personality disorders. (C) 2016 Elsevier Inc. All rights reserved.

C1 [Pelizza, Lorenzo] Reggio Emilia Mental Hlth Dept, Guastalla Psychiat Outpatients Serv, Via Salvo DAcquisto 7, I-42016 Guastalla, RE, Italy.

[Pupo, Simona] Guastalla Civil Hosp, Reggio Emilia Hlth Care Serv, Intens Care Unit, Via Donatori Sangue 1, I-42016 Guastalla, RE, Italy.

RP Pelizza, L (reprint author), CSM Guastalla, Via Salvo DAcquisto 7, I-42016 Guastalla, RE, Italy.

EM anolino@yahoo.it

RI Pelizza, Lorenzo/W-4951-2017

OI Pelizza, Lorenzo/0000-0003-4746-2061

CR American PsychiatricAssociation, 2001, DIAGNOSTIC STAT MANU

Bertuzzi F, 2007, CURR MED CHEM, V14, P1739

BHATNAGAR D, 1988, BRIT J HOSP MED, V40, P140

Brosig B, 2001, PSYCHOTHER PSYCH MED, V51, P232, DOI 10.1055/s-2001-14301

Cartwright A, 2011, QJM-INT J MED, V104, P575, DOI 10.1093/qjmed/hcr010

Conti L, 1999, REPERTORIO SCALE VAL

Derogatis L., 1977, SCL 90 R ADM SCORING

Dutour A, 1996, PSYCHONEUROENDOCRINO, V21, P525, DOI 10.1016/S0306-4530(96)00014-5

First M. B, 1997, STRUCTURED CLIN INTE

Gill G, 1999, DIABETES METAB, V25, P308

Gill G, 1986, Diabet Med, V3, P284

GILL GV, 1985, DIABETOLOGIA, V28, P579, DOI 10.1007/BF00281992

Gill GV, 1996, QJM-MON J ASSOC PHYS, V89, P839, DOI 10.1093/qjmed/89.11.839

GILL GV, 1992, J ROY SOC MED, V85, P259

HEPBURN DA, 1990, DIABETIC MED, V7, P711, DOI 10.1111/j.1464-5491.1990.tb01475.x

KENT LA, 1994, LANCET, V344, P778, DOI 10.1016/S0140-6736(94)92340-X

Leweke F, 2004, PRAX KINDERPSYCHOL K, V53, P347

PICKUP J, 1983, DIABETES CARE, V6, P279, DOI 10.2337/diacare.6.3.279

Schade D S, 1995, Adv Endocrinol Metab, V6, P289

STEEL JM, 1987, BRIT MED J, V294, P859, DOI 10.1136/bmj.294.6576.859

TATTERSALL R, 1985, BRIT MED J, V291, P555, DOI 10.1136/bmj.291.6495.555

TATTERSALL R, 1991, BRIT MED J, V302, P1240, DOI 10.1136/bmj.302.6787.1240

TATTERSALL R, 1977, CLIN ENDOCRINOL META, V6, P403, DOI 10.1016/S0300-595X(77)80045-5

Tattersall RB, 1997, DIABETIC MED, V14, P99, DOI 10.1002/(SICI)1096-9136(199702)14:2<99::AID-DIA320>3.3.CO;2-9

TATTERSALL RB, 1981, BRIT J PSYCHIAT, V139, P485, DOI 10.1192/bjp.139.6.485

Vantyghem MC, 2006, ANN ENDOCRINOL-PARIS, V67, P287, DOI 10.1016/S0003-4266(06)72600-2

Voulgari C, 2012, DIABETES TECHNOL THE, V14, P835, DOI 10.1089/dia.2012.0058

NR 27

TC 1

Z9 1

U1 1

U2 3

PU ELSEVIER SCIENCE INC

PI NEW YORK

PA 360 PARK AVE SOUTH, NEW YORK, NY 10010-1710 USA

SN 1056-8727

EI 1873-460X

J9 J DIABETES COMPLICAT

JI J. Diabetes Complications

PD NOV-DEC

PY 2016

VL 30

IS 8

BP 1544

EP 1547

DI 10.1016/j.jdiacomp.2016.07.028

PG 4

WC Endocrinology & Metabolism

SC Endocrinology & Metabolism

GA ES3MY

UT WOS:000399434900024

PM 27524279

DA 2019-01-08

ER

PT J

AU Stenberg, U

Haaland-Overby, M

Fredriksen, K

Westermann, KF

Kvisvik, T

AF Stenberg, Una

Haaland-Overby, Mette

Fredriksen, Kari

Westermann, Karl Fredrik

Kvisvik, Toril

TI A scoping review of the literature on benefits and challenges of

participating in patient education programs aimed at promoting

self-management for people living with chronic illness

SO PATIENT EDUCATION AND COUNSELING

LA English

DT Review

DE Patient education; Patient participation; Disease management; Self

efficacy; Chronic disease

ID RANDOMIZED CONTROLLED-TRIAL; TYPE-2 DIABETES MELLITUS.; CHRONIC DISEASE;

MENTAL-ILLNESS; PRIMARY-CARE; DECISION-MAKING; SOCIAL SUPPORT;

HEALTH-STATUS; SHORT-FORM; FOLLOW-UP

AB Objective: To give a comprehensive overview of benefits and challenges from participating in group based patient education programs that are carried out by health care professionals and lay participants, aimed at promoting self-management for people living with chronic illness.

Methods: We searched 8 literature databases. Full text articles meeting the inclusion criteria were retrieved and reviewed. Arksey and O'Malley's framework for scoping studies guided the review process and thematic analysis was undertaken to synthesize extracted data.

Results: Of the 5935 titles identified, 47 articles were included in this review. The participants experienced the programs as beneficial according to less symptom distress and greater awareness of their own health, improved self-management strategies, peer support, learning and hope.

Conclusion: A substantial evidence base supports the conclusion that group based self-management patient education programs in different ways have been experienced as beneficial, but more research is needed.

Practice implications: The insights gained from this review can enable researchers, health care professionals, and participants to understand the complexity in evaluating self-management patient education programs, and constitute a basis for a more standardized and systematic evaluation. The results may also encourage health care professionals in planning and carrying out programs in cooperation with lay participants. (C) 2016 Elsevier Ireland Ltd. All rights reserved.

C1 [Stenberg, Una; Haaland-Overby, Mette; Westermann, Karl Fredrik] Oslo Univ Hosp, Norwegian Natl Advisory Unit Learning & Mastery H, Postboks 4959, N-0424 Oslo, Norway.

[Stenberg, Una] Oslo Univ Hosp, Ctr Shared Decis Making & Collaborat Care Res, Postboks 4959, N-0424 Oslo, Norway.

[Fredriksen, Kari] Stavanger Univ Hosp, Learning & Mastery Ctr, Postboks 8100, N-4068 Stavanger, Norway.

[Kvisvik, Toril] More & Romsdal Hosp, Patient Educ Resource Ctr, Herman Dohlens Vei 1, N-6508 Kristiansand, Norway.

RP Stenberg, U (reprint author), Oslo Univ Hosp, Norwegian Natl Advisory Unit Learning & Mastery H, Postboks 4959, N-0424 Oslo, Norway.

EM una.stenberg@mestring.no; mette.haaland-overby@gmail.com;

kari.fredriksen@sus.no; karl.fredrik.westermann@gmail.com;

toril.kvisvik@helse-mr.no

FU Norwegian National Advisory Unit on Learning and Mastery in Health, Oslo

University Hospital, Norway

FX This work was funded by Norwegian National Advisory Unit on Learning and

Mastery in Health, Oslo University Hospital, Norway. We would like to

thank all our colleagues for daily support in this work, with special

thanks to Andre Vagan for reading abstracts and Ann Britt Sandvin Olsson

for reading and giving us feedback on the manuscript. Thanks to Bente

Schjodt-Osmo for quality assurance of literature searches and Margaret

Forbes for language editing.

CR AARONSON NK, 1993, J NATL CANCER I, V85, P365, DOI 10.1093/jnci/85.5.365

Abraham C, 2009, PSYCHOL HEALTH, V24, P1153, DOI 10.1080/08870440802521110

Ackerman IN, 2012, J RHEUMATOL, V39, P1047, DOI 10.3899/jrheum.111358

Albert Hanne B, 2003, Ugeskr Laeger, V165, P1875

Altuntas YE, 2012, EUR J CANCER CARE, V21, P776, DOI 10.1111/j.1365-2354.2012.01360.x

Ambrosio L, 2015, J CLIN NURS, V24, P2357, DOI 10.1111/jocn.12827

Andenaes R, 2012, HEALTH QUAL LIFE OUT, V10, DOI 10.1186/1477-7525-10-95

Anderson RM, 2000, DIABETES CARE, V23, P739, DOI 10.2337/diacare.23.6.739

Antonovsky A., 1987, UNRAVELING MYSTERY H

Arksey H.L.O.M., 2005, INT J SOC RES METHOD, V8, P13

Attridge M, 2014, COCHRANE DB SYST REV, V9, DOI DOI 10.1002/14651858.CD006424.PUB3

Austevoll-Dahlgren A.N., 2011, EFFECT GROUP ED PATI

Barlow J, 2009, PSYCHOL HEALTH, V24, P1167, DOI 10.1080/08870440802040277

Beattie J, 2013, PSYCHIATR REHABIL J, V36, P306, DOI 10.1037/prj0000031

BERGNER M, 1981, MED CARE, V19, P787, DOI 10.1097/00005650-198108000-00001

Bodenheimer T, 2002, JAMA-J AM MED ASSOC, V288, P2469, DOI 10.1001/jama.288.19.2469

Bonsaksen T, 2015, J HEALTH PSYCHOL, V20, P942, DOI 10.1177/1359105313504235

Bonsaksen T, 2014, PSYCHOL HEALTH, V29, P350, DOI 10.1080/08870446.2013.856432

Bonsaksen T, 2014, INT J CHRONIC OBSTR, V9, P41, DOI 10.2147/COPD.S52700

Borge L, 2012, SYKEPLEIEN FORSKNING, V7, P14

Bradley C, 1999, QUAL LIFE RES, V8, P79, DOI 10.1023/A:1026485130100

Brady TJ, 2013, PREV CHRONIC DIS, V10, DOI 10.5888/pcd10.120112

Brashers DE, 1999, HEALTH COMMUN, V11, P97, DOI 10.1207/s15327027hc1102_1

Broadbent E, 2006, J PSYCHOSOM RES, V60, P631, DOI 10.1016/j.jpsychores.2005.10.020

Brooks R, 1996, HEALTH POLICY, V37, P53, DOI 10.1016/0168-8510(96)00822-6

Brown JPR, 2011, COCHRANE DB SYST REV, DOI 10.1002/14651858.CD008895.pub2

Cade JE, 2009, DIABETIC MED, V26, P1048, DOI 10.1111/j.1464-5491.2009.02808.x

Carmack CL, 2011, CANCER-AM CANCER SOC, V117, P4993, DOI 10.1002/cncr.26110

CARVER CS, 1989, J PERS SOC PSYCHOL, V56, P267, DOI 10.1037/0022-3514.56.2.267

Chin WW, 1999, SMALL GR RES, V30, P751, DOI 10.1177/104649649903000605

Christensen KS, 2005, FAM PRACT, V22, P448, DOI 10.1093/fampra/cmi025

Cohen S, 1997, JAMA-J AM MED ASSOC, V277, P1940

Cook JA, 2010, PSYCHIATR REHABIL J, V34, P113, DOI 10.2975/34.2.2010.113.120

Cook JA, 2009, PSYCHIAT SERV, V60, P246, DOI 10.1176/ps.2009.60.2.246

Cooper LA, 2000, GEN HOSP PSYCHIAT, V22, P163

Corbin J. M., 1988, UNENDING WORK CARE M

Craig P, 2008, BRIT MED J, V337, DOI 10.1136/bmj.a1655

Deakin T, 2005, COCHRANE DB SYST REV, DOI 10.1002/14651858.CD003417.pub2

Derogatis LR, 2001, BSI 18 ADM SCORING P

Elg E.J.M., 2012, J SERV MANAGE, V23, P16

Fagermoen MS, 2015, J MULTIDISCIP HEALTH, V8, P157, DOI 10.2147/JMDH.S77763

Fisher L, 2008, ANN FAM MED, V6, P246, DOI 10.1370/afm.842

Gandek B, 1998, J CLIN EPIDEMIOL, V51, P1171, DOI 10.1016/S0895-4356(98)00109-7

Giffort DA, 1995, CONSTRUCTION SCALE M

Goldberg D., 1988, USERS GUIDE GEN HLTH

Goldsmith MR, 2007, J EPIDEMIOL COMMUN H, V61, P262, DOI 10.1136/jech.2006.046110

Green CA, 2013, PSYCHIAT SERV, V64, P1211, DOI 10.1176/appi.ps.201200546

Harrison M, 2011, PATIENT EDUC COUNS, V85, pE39, DOI 10.1016/j.pec.2010.10.026

Hibbard JH, 2005, HEALTH SERV RES, V40, P1918, DOI 10.1111/j.1475-6773.2005.00438.x

Jarvis J, 2010, DIABETES OBES METAB, V12, P12, DOI 10.1111/j.1463-1326.2009.01098.x

JOHNSTON M, 1995, MEASURES HLTH PSYCHO

Johnston S, 2012, BMC FAM PRACT, V13, DOI 10.1186/1471-2296-13-65

Jones N, 2013, PSYCHIATR REHABIL J, V36, P209, DOI 10.1037/prj0000008

JONES PW, 2003, ST GEORGES RESP QUES

Joosten EAG, 2008, PSYCHOTHER PSYCHOSOM, V77, P219, DOI 10.1159/000126073

Jormfeldt Henrika, 2012, Issues Ment Health Nurs, V33, P209, DOI 10.3109/01612840.2011.653041

Gagliardino JJ, 2013, DIABETES-METAB RES, V29, P152, DOI 10.1002/dmrr.2368

Kroenke K, 2009, J AFFECT DISORDERS, V114, P163, DOI 10.1016/j.jad.2008.06.026

Kroon FP, 2014, COCHRANE DB SYST REV, V1, DOI [DOI 10.1002/14651858.CD008963.PUB2, 10.1002/14651858.CD008963.pub2]

Levac D, 2010, IMPLEMENT SCI, V5, DOI 10.1186/1748-5908-5-69

Lomundal BK, 2012, INT J CHRONIC OBSTR, V7, P87, DOI 10.2147/COPD.S27352

LORIG K, 1989, ARTHRITIS RHEUM, V32, P37, DOI 10.1002/anr.1780320107

Lorig K, 1996, OUTCOME MEASURES HLT

Lorig K, 2014, COMMUNITY MENT HLT J, V50, P96, DOI 10.1007/s10597-013-9615-5

Lorig K, 2009, DIABETES EDUCATOR, V35, P641, DOI 10.1177/0145721709335006

Lorig KR, 2003, ANN BEHAV MED, V26, P1, DOI 10.1207/S15324796ABM2601_01

Lucksted A, 2013, ACTA PSYCHIAT SCAND, V127, P279, DOI 10.1111/j.1600-0447.2012.01901.x

Ludman E, 2003, PSYCHOL MED, V33, P1061, DOI 10.1017/S003329170300816X

Mahler C, 2010, J EVAL CLIN PRACT, V16, P574, DOI 10.1111/j.1365-2753.2009.01169.x

MAYS N., 2001, STUDYING ORG DELIVER, P188

Mehlsen M, 2015, PATIENT EDUC COUNS, V98, P677, DOI 10.1016/j.pec.2015.01.008

MELZACK R, 1987, PAIN, V30, P191, DOI 10.1016/0304-3959(87)91074-8

Mustafa M, 2013, COCHRANE DB SYST REV, V6

Nossum R, 2013, SCAND J PUBLIC HEALT, V41, P429, DOI 10.1177/1403494813480279

Nunez DE, 2009, WORLDV EVID-BASED NU, V6, P130, DOI 10.1111/j.1741-6787.2009.00157.x

Osborne RH, 2013, BMC PUBLIC HEALTH, V13, DOI 10.1186/1471-2458-13-658

Osborne RH, 2011, NURS CLIN N AM, V46, P255, DOI 10.1016/j.cnur.2011.05.010

Pace R, 2012, INT J NURS STUD, V49, P47, DOI 10.1016/j.ijnurstu.2011.07.002

Packer TL, 2012, PATIENT EDUC COUNS, V87, P93, DOI 10.1016/j.pec.2011.09.007

Pallaveshi L, 2014, COMMUNITY MENT HLT J, V50, P388, DOI 10.1007/s10597-013-9612-8

Parahoo K., 2013, COCHRANE DB SYST REV, V12, DOI DOI 10.1002/14651858.CD008529.PUB3

PATRICK DL, 1995, SPINE, V20, P1899, DOI 10.1097/00007632-199509000-00011

Pettman TL, 2008, INT J BEHAV NUTR PHY, V5, DOI 10.1186/1479-5868-5-53

Pham MT, 2014, RES SYNTH METHODS, V5, P371, DOI 10.1002/jrsm.1123

Pickett SA, 2012, COMMUNITY MENT HLT J, V48, P420, DOI 10.1007/s10597-012-9507-0

Pickett SA, 2010, PSYCHIATR REHABIL J, V34, P96, DOI 10.2975/34.2.2010.96.103

Polonsky WH, 2005, DIABETES CARE, V28, P626, DOI 10.2337/diacare.28.3.626

Quinones AR, 2014, PATIENT EDUC COUNS, V95, P3, DOI 10.1016/j.pec.2013.12.021

RADLOFF L S, 1977, Applied Psychological Measurement, V1, P385, DOI 10.1177/014662167700100306

Radloff L. S., 1986, COMMUNITY SURVEYS PS, P177

Rise MB, 2014, INT J SOC PSYCHIATR, V60, P387, DOI 10.1177/0020764013491739

Rise MB, 2013, PLOS ONE, V8, DOI 10.1371/journal.pone.0064009

Rogers A, 2009, CHRONIC ILLN, V5, P305, DOI 10.1177/1742395309350384

Rogers ES, 1997, PSYCHIATR SERV, V48, P1042

ROLAND M, 1983, SPINE, V8, P141, DOI 10.1097/00007632-198303000-00004

ROSENBERG M, 1995, AM SOCIOL REV, V60, P141, DOI 10.2307/2096350

Rosenberg M, 1965, SOC ADOLESCENT SELF

Rygg LO, 2012, PATIENT EDUC COUNS, V86, P98, DOI 10.1016/j.pec.2011.04.008

Sallinen M, 2011, PATIENT EDUC COUNS, V85, pE126, DOI 10.1016/j.pec.2011.02.011

Sandelowski M, 2012, J MIX METHOD RES, V6, P317, DOI 10.1177/1558689811427913

SARASON IG, 1982, J PERS, V50, P331, DOI 10.1111/j.1467-6494.1982.tb00754.x

Sharma S, 2013, PATIENT EDUC COUNS, V90, P38, DOI 10.1016/j.pec.2012.09.002

Shaya FT, 2014, J EPIDEMIOL COMMUN H, V68, P326, DOI 10.1136/jech-2013-203274

SHERBOURNE CD, 1991, SOC SCI MED, V32, P705, DOI 10.1016/0277-9536(91)90150-B

Smeulders ESTF, 2010, PATIENT EDUC COUNS, V81, P214, DOI 10.1016/j.pec.2010.01.003

Snaith RP, 1994, HOSP ANXIETY DEPRESS

SNYDER CR, 1991, J PERS SOC PSYCHOL, V60, P570, DOI 10.1037/0022-3514.60.4.570

Sohanpal R, 2012, CHRON RESP DIS, V9, P163, DOI 10.1177/1479972312444630

Spielberger C. D., 1983, STAI MANUAL STAIT TR

Steigman PJ, 2014, J NERV MENT DIS, V202, P193, DOI 10.1097/NMD.0000000000000098

Steinsbekk A, 2012, BMC HEALTH SERV RES, V12, DOI 10.1186/1472-6963-12-213

Stewart A., 1992, MEASURING FUNCTIONIN

Sullivan MJL, 1995, PSYCHOL ASSESSMENT, V7, P524, DOI 10.1037/1040-3590.7.4.524

Taylor SJC, 2012, BRIT J GEN PRACT, V62, DOI 10.3399/bjgp12X656829

Tjelta M, 2013, NORDISK SYGEPLEJEFOR, V3, P193

Van Metre Laura, 2011, Issues Ment Health Nurs, V32, P408, DOI 10.3109/01612840.2011.553771

Ware JE, 2001, SCORE INTERPRET SING

Webel AR, 2010, AIDS CARE, V22, P1029, DOI 10.1080/09540120903214389

Webel AR, 2009, J ASSOC NURSE AIDS C, V20, P458, DOI 10.1016/j.jana.2009.05.003

Whitfield K, 2006, HEALTH QUAL LIFE OUT, V4, DOI 10.1186/1477-7525-4-19

World Health Organization, GLOBAL HLTH AGING

Wu CJ, 2012, INT NURS REV, V59, P345, DOI 10.1111/j.1466-7657.2012.00998.x

Zangi HA, 2011, PATIENT EDUC COUNS, V85, P419, DOI 10.1016/j.pec.2010.12.032

NR 123

TC 11

Z9 11

U1 0

U2 20

PU ELSEVIER IRELAND LTD

PI CLARE

PA ELSEVIER HOUSE, BROOKVALE PLAZA, EAST PARK SHANNON, CO, CLARE, 00000,

IRELAND

SN 0738-3991

J9 PATIENT EDUC COUNS

JI Patient Educ. Couns.

PD NOV

PY 2016

VL 99

IS 11

BP 1759

EP 1771

DI 10.1016/j.pec.2016.07.027

PG 13

WC Public, Environmental & Occupational Health; Social Sciences,

Interdisciplinary

SC Public, Environmental & Occupational Health; Social Sciences - Other

Topics

GA EG7JC

UT WOS:000391222100003

PM 27461944

DA 2019-01-08

ER

PT J

AU Bolge, SC

Flores, NM

Phan, JH

AF Bolge, Susan C.

Flores, Natalia M.

Phan, Jennifer H.

TI The Burden of Poor Mental Well-being Among Patients With Type 2 Diabetes

Mellitus: Examining Health Care Resource Use and Work Productivity Loss

SO JOURNAL OF OCCUPATIONAL AND ENVIRONMENTAL MEDICINE

LA English

DT Article

ID DEPRESSIVE SYMPTOMS; SELF-CARE; UNITED-STATES; SOCIOECONOMIC

DISPARITIES; EMOTIONAL DISTRESS; MAJOR DEPRESSION; COSTS; DISABILITY;

ADHERENCE; RISK

AB Objective:The aim of this study was to evaluate the association of mental well-being with outcomes among patients with type 2 diabetes mellitus (T2DM).Methods:Seven thousand eight hundred fifty-two adults with T2DM were identified from a national, Internet-based study. Mental well-being [SF-36v2 mental component summary (MCS)] was categorized as good (MCS 50), poor (40 MCS < 50), and very poor (MCS < 40). Outcomes included past 6 months of health care resource use and lost productivity (Work Productivity and Activity Impairment questionnaire).Results:Respondents with very poor/poor versus good mental well-being were more likely to visit the emergency room (27%/18% vs 11%, P<0.001) or be hospitalized (19%/14% vs 9%, P<0.001). Among labor force participants, those with very poor/poor versus good mental well-being experienced greater overall work impairment (43.7/26.0 vs 10.7, P<0.001).Conclusions:Greater resource use and work productivity impairment associated with poorer mental well-being among patients with T2DM has cost implications.

C1 [Bolge, Susan C.; Phan, Jennifer H.] Janssen Sci Affairs LLC, Raritan, NJ USA.

[Flores, Natalia M.] Kantar Hlth, Foster City, CA USA.

[Phan, Jennifer H.] Anthem Inc, New York, NY USA.

RP Flores, NM (reprint author), 393 Vintage Pk Dr, Foster City, CA 94404 USA.

EM Natalia.Flores@KantarHealth.com

FU Janssen Scientific Affairs, LLC.

FX The study was funded by Janssen Scientific Affairs, LLC.

CR Aikens JE, 2012, DIABETES CARE, V35, P2472, DOI 10.2337/dc12-0181

Alegria M, 2008, PSYCHIAT SERV, V59, P1264, DOI 10.1176/appi.ps.59.11.1264

Amer Diabet Assoc, 2013, DIABETES CARE, V36, P1033, DOI 10.2337/dc12-2625

Black SA, 2003, DIABETES CARE, V26, P2822, DOI 10.2337/diacare.26.10.2822

Bolge SC, 2010, MENOPAUSE, V17, P80, DOI 10.1097/gme.0b013e3181b4c286

Bosmans JE, 2012, BMC HEALTH SERV RES, V12, DOI 10.1186/1472-6963-12-46

Braveman PA, 2010, AM J PUBLIC HEALTH, V100, pS186, DOI 10.2105/AJPH.2009.166082

Brazier J, 2002, J HEALTH ECON, V21, P271, DOI 10.1016/S0167-6296(01)00130-8

Browne JL, 2013, BMJ OPEN, V3, DOI 10.1136/bmjopen-2013-003384

Center for Financing Access and Cost Trends Agency for Healthcare Research and Quality: Medical Expenditure Panel Survey, 2012, TABL 5 HOSP INP SERV

CHARLSON ME, 1987, J CHRON DIS, V40, P373, DOI 10.1016/0021-9681(87)90171-8

Ciechanowski PS, 2000, ARCH INTERN MED, V160, P3278, DOI 10.1001/archinte.160.21.3278

Ciechanowski PS, 2003, GEN HOSP PSYCHIAT, V25, P246, DOI 10.1016/S0163-8343(03)00055-0

Control CfD Prevention, 2014, NAT DIAB STAT REP ES

Davydow DS, 2013, J GEN INTERN MED, V28, P921, DOI 10.1007/s11606-013-2336-1

Davydow DS, 2011, PSYCHOSOMATICS, V52, P117, DOI 10.1016/j.psym.2010.12.020

DiBonaventura Marco DaCosta, 2010, J Med Econ, V13, P709, DOI 10.3111/13696998.2010.535576

Egede LE, 2004, DIABETES CARE, V27, P1751, DOI 10.2337/diacare.27.7.1751

Egede LE, 2004, DIABETES CARE, V27, P421, DOI 10.2337/diacare.27.2.421

Finkelstein EA, 2011, J OCCUP ENVIRON MED, V53, P1025, DOI 10.1097/JOM.0b013e318229aae4

Flores NM, 2015, VALUE HEALTH, V3, pA110

Gonzalez HM, 2010, J PSYCHIATR RES, V44, P1043, DOI 10.1016/j.jpsychires.2010.03.017

Gonzalez HM, 2010, ARCH GEN PSYCHIAT, V67, P37, DOI 10.1001/archgenpsychiatry.2009.168

Gonzalez JS, 2008, DIABETIC MED, V25, P1102, DOI 10.1111/j.1464-5491.2008.02535.x

Gonzalez JS, 2007, DIABETES CARE, V30, P2222, DOI 10.2337/dc07-0158

Grandy S, 2008, INT J CLIN PRACT, V62, P562, DOI 10.1111/j.1742-1241.2008.01703.x

Huang H, 2012, PSYCHOSOMATICS, V53, P21, DOI 10.1016/j.psym.2011.07.001

Hudson JL, 2014, J PSYCHOSOM RES, V76, P265, DOI 10.1016/j.jpsychores.2014.02.004

Karlsen B, 2012, J ADV NURS, V68, P391, DOI 10.1111/j.1365-2648.2011.05751.x

Katon WJ, 2003, BIOL PSYCHIAT, V54, P216, DOI 10.1016/S0006-3223(03)00273-7

Nouwen A, 2010, DIABETOLOGIA, V53, P2480, DOI 10.1007/s00125-010-1874-x

Nozaki T, 2009, BIOPSYCHOSOC MED, V3, DOI 10.1186/1751-0759-3-4

Ogbera A, 2011, J DIABETES, V3, P348, DOI 10.1111/j.1753-0407.2011.00156.x

Pampel FC, 2010, ANNU REV SOCIOL, V36, P349, DOI 10.1146/annurev.soc.012809.102529

Park H, 2004, J CLIN EPIDEMIOL, V57, P978, DOI 10.1016/j.jclinepi.2004.01.015

REILLY MC, 1993, PHARMACOECONOMICS, V4, P353, DOI 10.2165/00019053-199304050-00006

Shim RS, 2011, J AM BOARD FAM MED, V24, P33, DOI 10.3122/jabfm.2011.01.100121

Simon GE, 2005, GEN HOSP PSYCHIAT, V27, P344, DOI 10.1016/j.genhosppsych.2005.04.008

Simon GE, 2007, ARCH GEN PSYCHIAT, V64, P65, DOI 10.1001/archpsyc.64.1.65

Strine T.W., 2015, PSYCHIAT SERV

US Bureau of Labor Statistics, 2013, BLS REP HIGHL WOM EA

van Dooren FEP, 2013, PLOS ONE, V8, DOI 10.1371/journal.pone.0057058

WARE JE, 1992, MED CARE, V30, P473, DOI 10.1097/00005650-199206000-00002

Ware JE, 2008, USERS MANUAL SF 36V2

Zulman DM, 2010, J GEN INTERN MED, V25, P408, DOI 10.1007/s11606-009-1232-1

NR 45

TC 1

Z9 1

U1 0

U2 1

PU LIPPINCOTT WILLIAMS & WILKINS

PI PHILADELPHIA

PA TWO COMMERCE SQ, 2001 MARKET ST, PHILADELPHIA, PA 19103 USA

SN 1076-2752

EI 1536-5948

J9 J OCCUP ENVIRON MED

JI J. Occup. Environ. Med.

PD NOV

PY 2016

VL 58

IS 11

BP 1121

EP 1126

DI 10.1097/JOM.0000000000000874

PG 6

WC Public, Environmental & Occupational Health

SC Public, Environmental & Occupational Health

GA EB3OD

UT WOS:000387273800016

PM 27820762

OA Green Published

DA 2019-01-08

ER

PT J

AU Friis, AM

Johnson, MH

Cutfield, RG

Consedine, NS

AF Friis, Anna M.

Johnson, Malcolm H.

Cutfield, Richard G.

Consedine, Nathan S.

TI Kindness Matters: A Randomized Controlled Trial of a Mindful

Self-Compassion Intervention Improves Depression, Distress, and HbA(1c)

Among Patients With Diabetes

SO DIABETES CARE

LA English

DT Article

ID GLYCEMIC CONTROL; METAANALYSIS; STRESS; HEALTH; INTERLEUKIN-6;

MANAGEMENT; THERAPY; PROGRAM; ANXIETY; VALUES

AB OBJECTIVE

Mood difficulties are common among patients with diabetes and are linked to poor blood glucose control and increased complications. Evidence on psychological treatments that improve both mood and metabolic outcomes is limited. Greater self-compassion predicts better mental and physical health in both healthy and chronically ill populations. Thus, the purpose of this randomized controlled trial (RCT) was to evaluate the effects of self-compassion training on mood and metabolic outcomes among patients with diabetes.

RESEARCH DESIGN AND METHODS

This RCT tested the effects of a standardized 8-week mindful self-compassion (MSC) program (n = 32) relative to a wait-list control condition (n = 31) among patients with type 1 and type 2 diabetes. Measures of self-compassion, depressive symptoms, diabetes-specific distress, and HbA(1c) were taken at baseline (preintervention), at week 8 (postintervention), and at 3-month follow-up.

RESULTS

Repeated-measures ANOVA using intention to treat showed that MSC training increased self-compassion and produced statistically and clinically significant reductions in depression and diabetes distress in the intervention group, with results maintained at 3-month follow-up. MSC participants also averaged a clinically and statistically meaningful decrease in HbA(1c) between baseline and follow-up of >10 mmol/mol (nearly 1%). There were no overall changes for the wait-list control group.

CONCLUSIONS

This initial report suggests that learning to be kinder to oneself (rather than being harshly self-critical) may have both emotional and metabolic benefits among patients with diabetes.

C1 [Friis, Anna M.; Johnson, Malcolm H.; Consedine, Nathan S.] Univ Auckland, Auckland, New Zealand.

[Cutfield, Richard G.] Waitemata Dist Hlth Board, Auckland, New Zealand.

RP Friis, AM (reprint author), Univ Auckland, Auckland, New Zealand.

EM a.friis@auckland.ac.nz

FU New Zealand Society for the Study of Diabetes; New Zealand Diabetes

Foundation

FX This study was made possible with the support of the New Zealand Society

for the Study of Diabetes and the New Zealand Diabetes Foundation.

CR Anderson RJ, 2001, DIABETES CARE, V24, P1069, DOI 10.2337/diacare.24.6.1069

Basco M., 1998, DIABETES SPECTRUM, V11, P43

Breines JG, 2014, BRAIN BEHAV IMMUN, V37, P109, DOI 10.1016/j.bbi.2013.11.006

Derr R, 2003, DIABETES CARE, V26, P2728, DOI 10.2337/diacare.26.10.2728

Dodds SE, 2015, SUPPORT CARE CANCER, V23, P3599, DOI 10.1007/s00520-015-2888-1

Fisher L, 2008, DIABETIC MED, V25, P1096, DOI 10.1111/j.1464-5491.2008.02533.x

Fisher L, 2013, DIABETES CARE, V36, P2551, DOI 10.2337/dc12-2493

Friis AM, 2015, DIABETIC MED, V32, P1634, DOI 10.1111/dme.12774

Gilbert P, 2005, COMPASSION CONCEPTUA, P263

Gregg JA, 2007, J CONSULT CLIN PSYCH, V75, P336, DOI 10.1037/0022-006X.75.2.336

Harkness E, 2010, DIABETES CARE, V33, P926, DOI 10.2337/dc09-1519

Hermanns N, 2015, DIABETES CARE, V38, P551, DOI 10.2337/dc14-1416

Kristiansen OP, 2005, DIABETES, V54, pS114, DOI 10.2337/diabetes.54.suppl_2.S114

Lenters-Westra E, 2014, NETH J MED, V72, P462

Lowe B, 2004, MED CARE, V42, P1194

Lustman PJ, 2005, J DIABETES COMPLICAT, V19, P113, DOI 10.1016/j.jdiacomp.2004.01.002

Neff KD, 2015, MINDFULNESS, V7, P1

Neff KD, 2003, SELF IDENTITY, V2, P223, DOI 10.1080/15298860390209035

Neff KD, 2013, J CLIN PSYCHOL, V69, P28, DOI 10.1002/jclp.21923

Noordali F, 2017, J HEALTH PSYCHOL, V22, P965, DOI 10.1177/1359105315620293

Pauley G, 2010, PSYCHOL PSYCHOTHER-T, V83, P129, DOI 10.1348/147608309X471000

Polonsky WH, 2005, DIABETES CARE, V28, P626, DOI 10.2337/diacare.28.3.626

Porges SW, 2007, BIOL PSYCHOL, V74, P116, DOI 10.1016/j.biopsycho.2006.06.009

Rockliff H., 2008, J CLIN NEUROPSYCHOLO, V5, P132

Rosenzweig S, 2007, ALTERN THER HEALTH M, V13, P36

Roy T, 2012, J AFFECT DISORDERS, V142, pS8, DOI 10.1016/S0165-0327(12)70004-6

Safren SA, 2014, DIABETES CARE, V37, P625, DOI [10.2337/dc13-0816, 10.2337/dc16-er06]

Slavich GM, 2014, PSYCHOL BULL, V140, P774, DOI 10.1037/a0035302

Snoek FJ, 2015, LANCET DIABETES ENDO, V3, P450, DOI 10.1016/S2213-8587(15)00135-7

Surwit RS, 2002, DIABETES CARE, V25, P30, DOI 10.2337/diacare.25.1.30

Terry ML, 2013, PERS SOC PSYCHOL B, V39, P911, DOI 10.1177/0146167213488213

Tovote KA, 2014, DIABETES CARE, V37, P2427, DOI 10.2337/dc13-2918

van Bastelaar KMP, 2011, DIABETES CARE, V34, P320, DOI 10.2337/dc10-1248

Van der Feltz-Cornelis CM, 2010, GEN HOSP PSYCHIAT, V32, P380, DOI 10.1016/j.genhosppsych.2010.03.011

van Son J, 2013, DIABETES CARE, V36, P823, DOI 10.2337/dc12-1477

van Steenbergen-Weijenburg KM, 2010, BMC HEALTH SERV RES, V10, DOI 10.1186/1472-6963-10-235

Winkley K, 2006, BRIT MED J, V333, P65, DOI 10.1136/bmj.38874.652569.55

Young Laura A, 2009, Pract Diabetes Int, V26, P222, DOI 10.1002/pdi.1380

Zessin U, 2015, APPL PSYCHOL-HLTH WE, V7, P340, DOI 10.1111/aphw.12051

NR 39

TC 24

Z9 24

U1 5

U2 31

PU AMER DIABETES ASSOC

PI ALEXANDRIA

PA 1701 N BEAUREGARD ST, ALEXANDRIA, VA 22311-1717 USA

SN 0149-5992

EI 1935-5548

J9 DIABETES CARE

JI Diabetes Care

PD NOV

PY 2016

VL 39

IS 11

BP 1963

EP 1971

DI 10.2337/dc16-0416

PG 9

WC Endocrinology & Metabolism

SC Endocrinology & Metabolism

GA EA1BZ

UT WOS:000386328800027

PM 27335319

OA Bronze

DA 2019-01-08

ER

PT J

AU Handley, TE

Ventura, AD

Browne, JL

Rich, J

Attia, JR

Reddy, P

Pouwer, F

Speight, J

AF Handley, T. E.

Ventura, A. D.

Browne, J. L.

Rich, J.

Attia, J. R.

Reddy, P.

Pouwer, F.

Speight, J.

TI Suicidal ideation reported by adults with Type 1 or Type 2 diabetes:

results from Diabetes MILESAustralia

SO DIABETIC MEDICINE

LA English

DT Article

ID CO-MORBID DEPRESSION; QUALITY-OF-LIFE; GENERAL-POPULATION; AUSTRALIAN

ADULTS; HEALTH OUTCOMES; NATIONAL-SURVEY; PRIMARY-CARE; RISK-FACTORS;

PREVALENCE; DISTRESS

AB AimsTo examine the prevalence and correlates of suicidal ideation (SI) in a community-based sample of adults with Type 1 or Type 2 diabetes.

MethodsParticipants were 3338 adults aged 18-70 years with Type 1 diabetes (n = 1376) or Type 2 diabetes (non-insulin: n = 1238; insulin: n = 724) from a national survey administered to a random sample registered with the National Diabetes Services Scheme. Depression and SI were assessed using the Patient Health Questionnaire, and diabetes-specific distress with the Problem Areas In Diabetes scale. Separate logistic regression analyses by diabetes type/treatment were used to determine relative contribution to SI.

ResultsOverall, we observed a SI rate of 14% in our sample. Participants with Type 2 diabetes using insulin reported more frequent depressive symptoms, and were more likely to report recent SI (19%) compared with those with either Type 1 diabetes or Type 2 diabetes not using insulin (14 and 12%, respectively). After controlling for depression, there was little difference in the prevalence of SI between diabetes types/treatments, but higher diabetes-specific distress significantly increased the odds of SI.

ConclusionsAs SI is a significant risk factor for a suicide attempt, the findings have implications for healthcare professionals, pointing to the importance of adequate screening and action plans for appropriate follow-up of those reporting depression. Our findings are also indicative of the psychological toll of diabetes more generally, and the need to integrate physical and mental healthcare for people with diabetes.

C1 [Handley, T. E.; Rich, J.; Attia, J. R.; Reddy, P.] Univ Newcastle, Sch Med & Publ Hlth, Callaghan, NSW, Australia.

[Handley, T. E.] Univ New S Wales, Natl Drug & Alcohol Res Ctr, Randwick, NSW, Australia.

[Ventura, A. D.; Browne, J. L.; Speight, J.] Australian Ctr Behav Res Diabet, Diabet Victoria, Melbourne, Vic, Australia.

[Ventura, A. D.; Browne, J. L.; Speight, J.] Deakin Univ, Sch Psychol, Ctr Social & Early Emot Dev, Burwood, Vic, Australia.

[Attia, J. R.] Hunter Med Res Inst, New Lambton, NSW, Australia.

[Pouwer, F.] Tilburg Univ, CoRPS, Dept Med & Clin Psychol, NL-5000 LE Tilburg, Netherlands.

[Speight, J.] AHP Res, Hornchurch, England.

RP Handley, TE (reprint author), Univ Newcastle, Sch Med & Publ Hlth, Callaghan, NSW, Australia.; Handley, TE (reprint author), Univ New S Wales, Natl Drug & Alcohol Res Ctr, Randwick, NSW, Australia.

EM tonelle.handley@newcastle.edu.au

OI Speight, Jane/0000-0002-1204-6896

FU National Diabetes Services Scheme (NDSS) Strategic Development Grant;

Sanofi Aventis

FX The Diabetes MILES-Australia 2011 Survey was funded by a National

Diabetes Services Scheme (NDSS) Strategic Development Grant. The NDSS is

an initiative of the Australian Government administered by Diabetes

Australia. In addition, Diabetes MILES-Australia received sponsorship

from Sanofi Aventis to support the development of the study website

http://www.diabetesMILES.org and related activities.

CR Ali S, 2006, DIABETIC MED, V23, P1165, DOI 10.1111/j.1464-5491.2006.01943.x

American Psychiatric Association, 2013, DIAGN STAT MAN MENT

Barnard KD, 2006, DIABETIC MED, V23, P445, DOI 10.1111/j.1464-5491.2006.01814.x

Bauer AM, 2013, J GEN INTERN MED, V28, P363, DOI 10.1007/s11606-012-2194-2

Beyondblue, 2013, BEYONDBL GUID MAN DE

Black SA, 2003, DIABETES CARE, V26, P2822, DOI 10.2337/diacare.26.10.2822

Browne JL, 2015, DIABETES RES CLIN PR, V108, pE5, DOI 10.1016/j.diabres.2015.01.021

Ceretta LB, 2012, ACTA DIABETOL, V49, pS227, DOI 10.1007/s00592-012-0435-9

Engum A, 2005, DIABETES CARE, V28, P1904, DOI 10.2337/diacare.28.8.1904

Fuller-Thomson Esme, 2009, Diabetes Res Clin Pract, V83, pe9, DOI 10.1016/j.diabres.2008.10.004

Gonzalez JS, 2008, DIABETES CARE, V31, P2398, DOI 10.2337/dc08-1341

Goodwin RD, 2003, PSYCHOSOM MED, V65, P501, DOI 10.1097/01.PSY.0000041544.14277.EC

Han SJ, 2013, DIABETES RES CLIN PR, V101, pE14, DOI 10.1016/j.diabres.2013.06.012

Handley TE, 2014, AM J GERIAT PSYCHIAT, V22, P1325, DOI 10.1016/j.jagp.2013.05.009

Handley TE, 2012, BMC PSYCHIATRY, V12, DOI 10.1186/1471-244X-12-105

Handley TE, 2012, SOC PSYCH PSYCH EPID, V47, P1281, DOI 10.1007/s00127-011-0436-y

Hintikka J, 2009, SOC PSYCH PSYCH EPID, V44, P29, DOI 10.1007/s00127-008-0401-6

Johnston AK, 2009, AUST NZ J PSYCHIAT, V43, P635, DOI 10.1080/00048670902970874

Katon WJ, 2004, ARCH GEN PSYCHIAT, V61, P1042, DOI 10.1001/archpsyc.61.10.1042

Kessler RC, 1999, ARCH GEN PSYCHIAT, V56, P617, DOI 10.1001/archpsyc.56.7.617

Kroenke K, 2001, J GEN INTERN MED, V16, P606, DOI 10.1046/j.1525-1497.2001.016009606.x

Ladwig KH, 2008, PSYCHIAT RES, V161, P248, DOI 10.1016/j.psychres.2008.03.011

LEMPERS JD, 1989, CHILD DEV, V60, P25, DOI 10.1111/j.1467-8624.1989.tb02692.x

Lin EHB, 2009, ANN FAM MED, V7, P414, DOI 10.1370/afm.998

ORCHARD TJ, 1991, DIABETES CARE, V14, P55

Organisation for Economic Co-operation and Development, 2011, HLTH AT A GLANC

Polonsky WH, 2005, DIABETES CARE, V28, P626, DOI 10.2337/diacare.28.3.626

Pompili M, 2009, PSYCHOSOMATICS, V50, P16, DOI 10.1176/appi.psy.50.1.16

Pouwer F, 2001, DIABETES CARE, V24, P1929, DOI 10.2337/diacare.24.11.1929

Pouwer F, 2013, ENDOCRIN METAB CLIN, V42, P529, DOI 10.1016/j.ecl.2013.05.002

Roy T, 2012, J AFFECTIVE DISORD S, V142, pS8

Snoek FJ, 2015, LANCET DIABETES ENDO, V3, P450, DOI 10.1016/S2213-8587(15)00135-7

Speight J, 2012, BMC PUBLIC HEALTH, V12, DOI 10.1186/1471-2458-12-120

Talbot F, 2000, DIABETES CARE, V23, P1556, DOI 10.2337/diacare.23.10.1556

Tang TS, 2008, DIABETES EDUCATOR, V34, P266, DOI 10.1177/0145721708315680

ten Have M, 2009, CAN J PSYCHIAT, V54, P824

vansDam HA, 2005, PATIENT EDUC COUNS, V59, P1

vansDooren FE, 2013, PLOS ONE, V8

Wu SF, 2014, DEPRESS RES TREAT

NR 39

TC 13

Z9 13

U1 1

U2 9

PU WILEY-BLACKWELL

PI HOBOKEN

PA 111 RIVER ST, HOBOKEN 07030-5774, NJ USA

SN 0742-3071

EI 1464-5491

J9 DIABETIC MED

JI Diabetic Med.

PD NOV

PY 2016

VL 33

IS 11

BP 1582

EP 1589

DI 10.1111/dme.13022

PG 8

WC Endocrinology & Metabolism

SC Endocrinology & Metabolism

GA DZ7FB

UT WOS:000386029200021

PM 26525943

DA 2019-01-08

ER

PT J

AU Fisher, L

Hessler, DM

Polonsky, WH

Masharani, U

Peters, AL

Blumer, I

Strycker, LA

AF Fisher, L.

Hessler, D. M.

Polonsky, W. H.

Masharani, U.

Peters, A. L.

Blumer, I.

Strycker, L. A.

TI Prevalence of depression in Type 1 diabetes and the problem of

over-diagnosis

SO DIABETIC MEDICINE

LA English

DT Article

ID EXCHANGE CLINIC REGISTRY; DISTRESS; ADULTS; SEVERITY; VALIDITY;

OUTCOMES; PHQ-9

AB AimsTo determine the prevalence of depression and diabetes distress in adults with Type 1 diabetes and the rate of false-positives when compared with rates of major depressive disorder.

MethodsThe sample consisted of 368 individuals with Type 1 diabetes, aged 19 years. Individuals completed: the eight-item Patient Health Questionnaire depression scale (PHQ8), which was coded using four scoring criteria (scores 10, 12 and 15, and Diagnostic and Statistical Manual of Mental Disorders 5 (DSM) algorithm scores); the Type 1 Diabetes Distress Scale; and the Structured Clinical Interview for DSM Disorders (SCID) to assess major depressive disorder.

ResultsThe prevalence rates of depression according to the eight-item Patient Health Questionnaire were: score10, 11.4%; score 12, 7.1%; score 15, 3.8%; and positive algorithm result, 4.6%. The prevalence of major depressive disorder was 3.5%; and the prevalence of at least moderate diabetes distress was 42.1%. Depending on the criterion used, the false-positive rate when using the Patient Health Questionnaire compared with the results when using the SCID varied from 52 to 71%. Of those classified as depressed on the PHQ-8 or Structured Clinical Interview for DSM Disorders, between 92.3 and 96.2% also reported elevated diabetes distress. No significant association was found between any group classed as having depression according to the PHQ8 or the SCID and HbA(1c) concentration. Depression was significantly associated with more other life stress, more complications and a lower level of education.

ConclusionsWe found an unexpectedly low rate of current depression and major depressive disorder in this diverse sample of adults with Type 1 diabetes, and a very high rate of false-positive results using the Patient Health Questionnaire. Considering the high prevalence of diabetes distress, much of what has been considered depression in adults with Type 1 diabetes may be attributed to the emotional distress associated with managing a demanding chronic disease and other life stressors and not necessarily to underlying psychopathology.

C1 [Fisher, L.; Hessler, D. M.] UCSF, Dept Family & Community Med, San Francisco, CA 94143 USA.

[Polonsky, W. H.] Behav Diabet Inst, San Diego, CA USA.

[Masharani, U.] UCSF, Dept Med, San Francisco, CA USA.

[Peters, A. L.] Univ So Calif, Los Angeles, CA USA.

[Blumer, I.] Charles H Best Diabet Ctr, Whitby, ON, Canada.

[Strycker, L. A.] Oregon Res Inst, Eugene, OR 97403 USA.

RP Fisher, L (reprint author), UCSF, Dept Family & Community Med, San Francisco, CA 94143 USA.

EM fisherl@fcm.ucsf.edu

OI Peters, Anne/0000-0003-0520-0776

FU National Institutes of Health [DK094863]

FX This research was funded by National Institutes of Health grant

DK094863.

CR Aikens JE, 2012, DIABETES CARE, V35, P2472, DOI 10.2337/dc12-0181

Anderson RJ, 2001, DIABETES CARE, V24, P1069, DOI 10.2337/diacare.24.6.1069

Andreoulakis E, 2012, HIPPOKRATIA, V16, P205

Barnard KD, 2006, DIABETIC MED, V23, P445, DOI 10.1111/j.1464-5491.2006.01814.x

Beck RW, 2012, J CLIN ENDOCR METAB, V97, P4383, DOI 10.1210/jc.2012-1561

Biometric Research Department, STRUCT CLIN INT DSM

Black SA, 2003, DIABETES CARE, V26, P2822, DOI 10.2337/diacare.26.10.2822

Centers for Disease Control and Prevention, 2010, MMWR-MORBID MORTAL W, V59, P1229

COYNE JC, 1994, PSYCHOL BULL, V116, P29, DOI 10.1037/0033-2909.116.1.29

Fisher L, 2014, DIABETIC MED, V31, P764, DOI 10.1111/dme.12428

Fisher L, 2007, DIABETES CARE, V30, P542, DOI 10.2337/dc06-1614

Fisher L, 2015, J DIABETES COMPLICAT, V29, P572, DOI 10.1016/j.jdiacomp.2015.01.012

Fisher L, 2012, DIABETES CARE, V35, P259, DOI 10.2337/dc11-1572

Fisher L, 2010, DIABETES CARE, V33, P23, DOI 10.2337/dc09-1238

Gonzalez JS, 2011, DIABETES CARE, V34, P236, DOI 10.2337/dc10-1970

Harris L.R., 2010, PRACTICAL ASSESSMENT, V15, P1

Katon W, 2010, DEPRESSION DIABETES

Kroenke K, 2002, PSYCHIAT ANN, V32, P509, DOI 10.3928/0048-5713-20020901-06

Kroenke K, 2002, PSYCHOSOM MED, V64, P258, DOI 10.1097/00006842-200203000-00008

Kroenke K, 2001, J GEN INTERN MED, V16, P606, DOI 10.1046/j.1525-1497.2001.016009606.x

Li CY, 2008, DIABETES CARE, V31, P105, DOI 10.2337/dc07-1154

PEARLIN LI, 1981, J HEALTH SOC BEHAV, V22, P337, DOI 10.2307/2136676

Rohde P, 1997, AM J PSYCHIAT, V154, P1593, DOI 10.1176/ajp.154.11.1593

Roy T, 2012, DIABETIC MED, V29, P164, DOI 10.1111/j.1464-5491.2011.03401.x

Roy T, 2012, J AFFECT DISORDERS, V142, pS8, DOI 10.1016/S0165-0327(12)70004-6

Snoek FJ, 2015, LANCET DIABETES ENDO, V858, P135

Sullivan MD, 2012, DIABETES CARE, V35, P1708, DOI 10.2337/dc11-1791

Trief PM, 2014, DIABETES CARE, V37, P1563, DOI 10.2337/dc13-1867

NR 28

TC 14

Z9 15

U1 1

U2 9

PU WILEY-BLACKWELL

PI HOBOKEN

PA 111 RIVER ST, HOBOKEN 07030-5774, NJ USA

SN 0742-3071

EI 1464-5491

J9 DIABETIC MED

JI Diabetic Med.

PD NOV

PY 2016

VL 33

IS 11

BP 1590

EP 1597

DI 10.1111/dme.12973

PG 8

WC Endocrinology & Metabolism

SC Endocrinology & Metabolism

GA DZ7FB

UT WOS:000386029200022

PM 26433004

DA 2019-01-08

ER

PT J

AU van Dijk-de Vries, A

van Bokhoven, MA

de Jong, S

Metsemakers, JFM

Verhaak, PPM

van der Weijden, T

van Eijk, JTM

AF van Dijk-de Vries, Anneke

van Bokhoven, Marloes A.

de Jong, Sabine

Metsemakers, Job F. M.

Verhaak, Peter P. M.

van der Weijden, Trudy

van Eijk, Jacques Th. M.

TI Patients' readiness to receive psychosocial care during nurse-led

routine diabetes consultations in primary care: A mixed methods study

SO INTERNATIONAL JOURNAL OF NURSING STUDIES

LA English

DT Article

DE Diabetes mellitus; Family practices; Mixed methods; Primary care

nursing; Psychosocial; Self-management

ID SELF-MANAGEMENT SUPPORT; QUALITATIVE EVALUATION; DEPRESSION; PEOPLE;

ILLNESS

AB Background: Patients with type 2 diabetes mellitus face several emotional and social consequences of their chronic illness in their everyday life. Symptoms of distress and depression are prevalent. For providing psychosocial self-management support, nurses in primary care were trained to identify patients with psychosocial problems during routine medically-shaped diabetes consultations. However, detection rates appeared to be strikingly low.

Objectives: Our study aimed to examine patients' readiness to discuss psychosocial problems with nurses during diabetes consultations.

Design: A mixed methods design was used in which qualitative data collection was followed up by quantitative data collection.

Setting: Diabetes care in a regional group of family practices in the south of the Netherlands.

Participants: Type 2 diabetes patients with psychosocial problems, determined by a self-administered questionnaire.

Methods: First, in-depth interviews (n=12) were conducted about patients' experiences with routine diabetes consultations and their perspective on a biopsychosocial care approach. Based on a qualitative content analysis, a structured questionnaire was designed to further explore the findings among a larger group of patients. This questionnaire was completed by 205 patients. The questionnaire included 14 items measuring patients' agreement with statements about diabetes care and the role of the nurse to focus on patients' emotional and social functioning.

Results: The interviews showed that patients view a diabetes consultation primarily as a biomedical check-up, and do not perceive discussion of psychosocial well-being as an integral part of diabetes management. More than 90% of the sample showed a positive attitude towards current diabetes consultations. Patients' intentions and perceived needs regarding a biopsychosocial care approach of the nurse were variable. Younger patients seemed more open to discussing psychosocial problems with the nurse than patients over 65. Patients' openness to discussing psychosocial problems was not significantly (p < 0.05) associated with the nurses being trained in the biopsychosocial self-management approach.

Conclusion: Patients see primary care nurses primarily as specialists regarding the biomedical management of diabetes. Although patients seemed to support the ideal of integrated care, they did not expect a discussion about psychosocial problems in diabetes consultations. The incorporation of systematic detection of patients with psychosocial problems in diabetes care requires endeavours to make patients acquainted with the new role of the nurse. (C) 2016 Elsevier Ltd. All rights reserved.

C1 [van Dijk-de Vries, Anneke; van Bokhoven, Marloes A.; de Jong, Sabine; Metsemakers, Job F. M.; van der Weijden, Trudy] Maastricht Univ, CAPHRI Sch Publ Hlth & Primary Care, Dept Family Med, Maastricht, Netherlands.

[van Dijk-de Vries, Anneke; van Eijk, Jacques Th. M.] Maastricht Univ, CAPHRI Sch Publ Hlth & Primary Care, Dept Social Med, Maastricht, Netherlands.

[Verhaak, Peter P. M.] Netherlands Inst Hlth Serv Res, Utrecht, Netherlands.

[Verhaak, Peter P. M.] Univ Groningen, Univ Med Ctr Groningen, Dept Gen Practice, Groningen, Netherlands.

RP van Dijk-de Vries, A (reprint author), Maastricht Univ, CAPHRI Sch Publ Hlth & Primary Care, Dept Family Med, Maastricht, Netherlands.; van Dijk-de Vries, A (reprint author), Maastricht Univ, CAPHRI Sch Publ Hlth & Primary Care, Dept Social Med, Maastricht, Netherlands.

EM anneke.vandijk@maastrichtuniversity.nl;

loes.vanbokhoven@maastrichtuniversity.nl; sabinedejong88@gmail.com;

job.metsemakers@maastrichtuniversity.nl; p.verhaak@nivel.nl;

trudy.vanderweijden@maastrichtuniversity.nl;

j.vaneijk@maastrichtuniversity.nl

FU Dutch Diabetes Research Foundation (Diabetes Fonds) [2010.13.1366];

'Annadal Foundation' in Maastricht

FX We wish to thank all patients who participated in this study. We

received funding from the Dutch Diabetes Research Foundation (Diabetes

Fonds) under grant no. 2010.13.1366 (Voice of the Patient programme),

and a financial contribution from the 'Annadal Foundation' in

Maastricht, an independent financial support fund for healthcare.

CR Alderson S.L., 2014, BMJ OPEN, V4

Alderson SL, 2014, BMC FAM PRACT, V15, DOI 10.1186/1471-2296-15-37

Barlow J, 2002, PATIENT EDUC COUNS, V48, P177, DOI 10.1016/S0738-3991(02)00032-0

Bentley M., 2016, J ADV NURS

Bodenheimer T, 2002, JAMA-J AM MED ASSOC, V288, P1775, DOI 10.1001/jama.288.14.1775

Braam C, 2009, J OCCUP REHABIL, V19, P231, DOI 10.1007/s10926-009-9178-z

Caracelli V. J., 1989, EDUC EVAL POLICY AN, V11, P255, DOI DOI 10.3102/01623737011003255

Chew-Graham CA, 2013, BMC FAM PRACT, V14, DOI 10.1186/1471-2296-14-103

EDMONDS W. A., 2013, APPL REFERENCE GUIDE

Egede LE, 2010, DIABETES RES CLIN PR, V87, P302, DOI 10.1016/j.diabres.2010.01.024

Elissen A, 2013, BMC HEALTH SERV RES, V13, DOI 10.1186/1472-6963-13-117

Fisher L, 2010, DIABETES CARE, V33, P23, DOI 10.2337/dc09-1238

Franek J, 2013, Ont Health Technol Assess Ser, V13, P1

Graneheim UH, 2004, NURS EDUC TODAY, V24, P105, DOI 10.1016/j.nedt.2003.10.001

Grund J, 2012, J PRIM CARE COMMUNIT, V3, P230, DOI 10.1177/2150131911435263

Halcomb EJ, 2013, BMC FAM PRACT, V14, DOI 10.1186/1471-2296-14-26

Heiligers PJM, 2012, KENNISVRAAG PRAKTIJK

Hinder S, 2012, BMC HEALTH SERV RES, V12, DOI 10.1186/1472-6963-12-83

IBM Corp, 2012, IBM SPSS STAT WIND V

IDF Clinical Guidelines Task Force, 2012, GLOB GUID TYP 2 DIAB

Jakimowicz S, 2015, J CLIN NURS, V24, P19, DOI 10.1111/jocn.12676

Knowles SE, 2015, BMC FAM PRACT, V16, DOI 10.1186/s12875-015-0246-5

Laurant M, 2005, COCHRANE DB SYST REV, DOI 10.1002/14651858.CD001271.pub2

Lorig KR, 2003, ANN BEHAV MED, V26, P1, DOI 10.1207/S15324796ABM2601_01

Mulder BC, 2015, PATIENT EDUC COUNS, V98, P156, DOI 10.1016/j.pec.2014.11.006

Netherlands Diabetes Federation, 2007, NDF CAR STAND TRANSP

Pearce MJ, 2013, J AM ASSOC NURSE PRA, V25, P578, DOI 10.1002/2327-6924.12035

Pouwer F, 2009, NAT REV ENDOCRINOL, V5, P665, DOI 10.1038/nrendo.2009.214

Raaijmakers LGM, 2013, BMC FAM PRACT, V14, DOI 10.1186/1471-2296-14-114

Struijs J., 2012, BUNDL PAYM DIAB CAR

The Dutch College of General Practitioners (NHG), 2011, COR VAL GEN PRACT FA

Townsend A, 2006, CHRONIC ILLN, V2, P185, DOI 10.1179/174592006X129518

Van de Laar FA, 2013, HUISARTS WET, V56, P512

van Dijk-de Vries A, 2015, BMJ OPEN, V5, DOI 10.1136/bmjopen-2014-007014

van Dijk-de Vries A, 2013, BMC FAM PRACT, V14, DOI 10.1186/1471-2296-14-77

NR 35

TC 1

Z9 1

U1 0

U2 12

PU PERGAMON-ELSEVIER SCIENCE LTD

PI OXFORD

PA THE BOULEVARD, LANGFORD LANE, KIDLINGTON, OXFORD OX5 1GB, ENGLAND

SN 0020-7489

EI 1873-491X

J9 INT J NURS STUD

JI Int. J. Nurs. Stud.

PD NOV

PY 2016

VL 63

BP 58

EP 64

DI 10.1016/j.ijnurstu.2016.08.018

PG 7

WC Nursing

SC Nursing

GA DZ1LS

UT WOS:000385600500008

PM 27597730

DA 2019-01-08

ER

PT J

AU Fatani, SH

Saleh, SAK

Adly, HM

Abdulkhaliq, AA

AF Fatani, Sameer H.

Saleh, Saleh A. K.

Adly, Heba M.

Abdulkhaliq, Altaf A.

TI Trace Element Alterations in the Hair of Diabetic and Obese Women

SO BIOLOGICAL TRACE ELEMENT RESEARCH

LA English

DT Article

DE Hair; Trace elements; Obesity; Diabetes mellitus; Women; Saudi Arabia

ID BODY-MASS INDEXES; BIOLOGICAL SAMPLES; MELLITUS PATIENTS; FEMALE HAIR;

COPPER; IRON; ZINC; MAGNESIUM; SELENIUM; ADOLESCENTS

AB Alterations in the trace element content can induce metabolic disorders as these elements are involved in the regulation of metabolism. Obesity increases the likelihood of various diseases, particularly cardiovascular disease and type 2 diabetes, and is more prevalent in Saudi Arabia, especially in women. This study explored the potential of alterations in hair trace elements as long-term markers in diabetic and/or obese Saudi females. In total, 65 diabetic obese women, 47 non-diabetic obese women, and 70 normal-weight women were recruited. Clinical and familial history and anthropometric variables were recorded. Hair Se, Zn, Cu, Mn, and Fe levels were analyzed. Fasting blood sugar (FBS), glycated hemoglobin (HbA1c), and lipid profile were analyzed. Our findings revealed a marked decrease of hair Zn, Mn, and Fe and elevated Se and Cu levels in obese women. In addition, Zn and Fe levels were decreased in diabetic women. Thus, the metabolic distress occurring in obesity and hyperglycemia may affect trace element status by increasing the excretion and decreasing the bioavailability of trace elements or redistributing them among various pools. Hair trace elements can serve as important long-term markers for metabolic disorders; however, larger prospective studies are warranted to validate their diagnostic and follow-up utilities.

C1 [Fatani, Sameer H.; Saleh, Saleh A. K.; Adly, Heba M.; Abdulkhaliq, Altaf A.] Umm Al Qura Univ, Dept Biochem, Fac Med, POB 715, Mecca 21955, Saudi Arabia.

RP Saleh, SAK (reprint author), Umm Al Qura Univ, Dept Biochem, Fac Med, POB 715, Mecca 21955, Saudi Arabia.

EM sameer_fatani2003@yahoo.co.uk; Saleh-A-S@hotmail.com;

Hebamadly@hotmail.com; dr.altaf@gmail.com

FU Institute of Scientific Research and Revival of Islamic Culture, Umm

Al-Qura University, Makkah, Saudi Arabia [43209016]

FX The authors thank the Institute of Scientific Research and Revival of

Islamic Culture, Umm Al-Qura University, Makkah, Saudi Arabia, for

funding this project (Project number 43209016).

CR Afridi HI, 2008, DIABETES RES CLIN PR, V80, P280, DOI 10.1016/j.diabres.2007.12.021

Al-Nozha MM, 2005, SAUDI MED J, V26, P824

Al-Othaimeen A. I., 2007, Eastern Mediterranean Health Journal, V13, P441

Al-Saleh E, 2006, BIOL TRACE ELEM RES, V113, P113, DOI 10.1385/BTER:113:2:113

Alzaid AA, 1997, ANN SAUDI MED, V17, P154, DOI 10.5144/0256-4947.1997.154

Anetor J I, 2002, Nutr Health, V16, P291

Boosalis MG, 2008, NUTR CLIN PRACT, V23, P152, DOI 10.1177/0884533608314532

Chojnacka K, 2010, ENVIRON TOXICOL PHAR, V3, P52

CLOUET P, 1986, BIOCHEM J, V239, P103, DOI 10.1042/bj2390103

Dambal SS, 2011, IJABPT, V2, P280

Griffith H, 1995, COMPLETE GUIDE VITAM, P100

Groop LC, 1999, DIABETES OBES METAB, V1, pS1

Institute for Health Metrics and Evaluation, 2013, GBD COMP CAUS DIS IN

Kahn BB, 2000, J CLIN INVEST, V106, P473, DOI 10.1172/JCI10842

Kazi TG, 2008, BIOL TRACE ELEM RES, V122, P1, DOI 10.1007/s12011-007-8062-y

KORC M, 1983, AM J PHYSIOL, V245, pG628

Lima SCVC, 2006, BIOL TRACE ELEM RES, V114, P19, DOI 10.1385/BTER:114:1:19

Mehra R, 2005, J SCI IND RES INDIA, V64, P119

Nead KG, 2004, PEDIATRICS, V114, P104, DOI 10.1542/peds.114.1.104

Neymotin F, 2011, OBESITY, V19, P191, DOI 10.1038/oby.2010.112

Ozturk P, 2014, J TRACE ELEM MED BIO, V28, P266, DOI 10.1016/j.jtemb.2014.03.003

Pinhas-Hamiel O, 2003, INT J OBESITY, V27, P416, DOI [10.1038/sj.ijo.0802224, 10.1038/sj.ijo.802224]

Rashed MN, 2011, J TRACE ELEM MED BIO, V25, P181, DOI 10.1016/j.jtemb.2011.07.001

REGAN L, 1993, ANNU REV BIOPH BIOM, V22, P257, DOI 10.1146/annurev.bb.22.060193.001353

Rungby J, 2010, DIABETOLOGIA, V53, P1549, DOI 10.1007/s00125-010-1793-x

Skalnaya MG, 2007, J TRACE ELEM MED BIO, V21, P59, DOI 10.1016/j.jtemb.2007.09.019

Steinbrenner H, 2011, J CLIN BIOCHEM NUTR, V48, P40, DOI 10.3164/jcbn.11-002FR

Stranges S, 2010, BMC PUBLIC HEALTH, V10, DOI 10.1186/1471-2458-10-564

Tungtrongchitr Rungsunn, 2003, Journal of the Medical Association of Thailand, V86, P543

Viktorinova A, 2009, METABOLISM, V58, P1477, DOI 10.1016/j.metabol.2009.04.035

Wang CT, 2005, CLIN CHEM LAB MED, V43, P389, DOI 10.1515/CCLM.2005.070

Wang CT, 2005, J HEALTH SCI, V51, P70, DOI 10.1248/jhs.51.70

WANG CT, 1994, EUR J CLIN CHEM CLIN, V32, P107

Warsy A S, 1999, East Mediterr Health J, V5, P1236

WHO, 2014, GLOBAL STATUS REPORT ON VIOLENCE PREVENTION 2014, P1

Wiernsperger N, 2010, DIABETOL METAB SYNDR, V2, DOI 10.1186/1758-5996-2-70

Zargar AH, 2002, SAUDI MED J, V23, P539

NR 37

TC 2

Z9 2

U1 2

U2 8

PU HUMANA PRESS INC

PI TOTOWA

PA 999 RIVERVIEW DRIVE SUITE 208, TOTOWA, NJ 07512 USA

SN 0163-4984

EI 1559-0720

J9 BIOL TRACE ELEM RES

JI Biol. Trace Elem. Res.

PD NOV

PY 2016

VL 174

IS 1

BP 32

EP 39

DI 10.1007/s12011-016-0691-6

PG 8

WC Biochemistry & Molecular Biology; Endocrinology & Metabolism

SC Biochemistry & Molecular Biology; Endocrinology & Metabolism

GA DY5IG

UT WOS:000385132300005

PM 27075547

DA 2019-01-08

ER

PT J

AU Wagner, JA

Bermudez-Millan, A

Damio, G

Segura-Perez, S

Chhabra, J

Vergara, C

Feinn, R

Perez-Escamilla, R

AF Wagner, Julie Ann

Bermudez-Millan, Angela

Damio, Grace

Segura-Perez, Sofia

Chhabra, Jyoti

Vergara, Cunegundo

Feinn, Richard

Perez-Escamilla, Rafael

TI A randomized, controlled trial of a stress management intervention for

Latinos with type 2 diabetes delivered by community health workers:

Outcomes for psychological wellbeing, glycemic control, and cortisol

SO DIABETES RESEARCH AND CLINICAL PRACTICE

LA English

DT Article

DE Diabetes; Latinos; Community health workers; Depression; Anxiety

ID DEPRESSION; IMPLEMENTATION; METAANALYSIS; PREVALENCE; PEOPLE; ADULTS

AB Aims: To test the efficacy of a community health worker (CHW) delivered stress management (SM) intervention on psychosocial, glycemic, and cortisol outcomes among U.S. Latinos with type 2 diabetes.

Methods: A randomized, controlled trial compared CHW-delivered diabetes education (DE; one group session) to DE plus CHW-delivered SM (DE + SM; 8 group sessions). Psychosocial variables and urinary cortisol were measured at baseline and posttreatment. HbA1c was measured at baseline, posttreatment, and 3-month follow-up.

Results: In intent to treat analysis, compared to DE (n = 46), DE + SM (n = 61) showed significantly improved symptoms of depression, anxiety, and self-reported health status. There were no significant group effects for HbA1c, diabetes distress, or urinary cortisol. However, there was a dose response effect for HbA1c and diabetes distress; increasing attendance at SM sessions was associated with greater improvements in HbA1c and diabetes distress.

Conclusions: This is the first randomized, controlled trial demonstrating that CHWs can improve psychological symptoms and self-reported health among Latinos with type 2 diabetes. Efforts to increase intervention attendance may improve HbA1c and diabetes distress. (C) 2016 Elsevier Ireland Ltd. All rights reserved.

C1 [Wagner, Julie Ann; Bermudez-Millan, Angela] UConn Hlth, 263 Farmington Ave, Farmington, CT 06030 USA.

[Damio, Grace; Segura-Perez, Sofia] Hispan Hlth Council, Hartford, CT USA.

[Chhabra, Jyoti; Vergara, Cunegundo] Hartford Hosp, Hartford, CT 06115 USA.

[Feinn, Richard] Quinnipiac Univ, Sch Med, Hamden, CT USA.

[Perez-Escamilla, Rafael] Yale Sch Publ Hlth, New Haven, CT USA.

RP Wagner, JA (reprint author), UConn Hlth, 263 Farmington Ave, Farmington, CT 06030 USA.

EM juwagner@uchc.edu

FU NIH Minority Health and Health Disparities Institute [5 R01 MD005879];

Yale Center for Clinical Investigation through Clinical and Translation

Science Award from the National Center for Advancing Translational

Sciences, a component of NIH [UL1-TR-000142]

FX CALMS-D was funded by the NIH Minority Health and Health Disparities

Institute (Grant No. 5 R01 MD005879 to J.W. and R.P.-E., principal

investigators). R.P.-E. received funding support for this publication

from the Yale Center for Clinical Investigation through Clinical and

Translation Science Award grant UL1-TR-000142 from the National Center

for Advancing Translational Sciences, a component of NIH. The content is

solely the responsibility of the authors and does not necessarily

represent the official views of the National Center on Minority Health

and Health Disparities or the National Institutes of Health (NIH).

CR Al-Zoairy R, 2013, CURR DIABETES REV, V9, P362, DOI 10.2174/15733998113099990067

Anderson RJ, 2001, DIABETES CARE, V24, P1069, DOI 10.2337/diacare.24.6.1069

Bermudez-Millan A, 2008, NOTICIA SALUD OFFICI, V3, P4

Bermudez-Millan A, 2015, J HLTH PSYCHOL

Brown SA, 2005, DIABETES CARE, V28, P527, DOI 10.2337/diacare.28.3.527

Cella D, 2010, J CLIN EPIDEMIOL, V63, P1179, DOI 10.1016/j.jclinepi.2010.04.011

Center for Disease Control and Prevention, 2011, NAT DIAB FACT SHEET

Chapman DJ, 2012, ADV NUTR, V3, P95, DOI 10.3945/an.111.001016

Chapman DJ, 2010, J HUM LACT, V26, P314, DOI 10.1177/0890334410369481

de Vibe M, 2013, BMC MED EDUC, V13, DOI 10.1186/1472-6920-13-107

Egede LE, 2010, DIABETES RES CLIN PR, V87, P302, DOI 10.1016/j.diabres.2010.01.024

Feathers JT, 2007, DIABETES EDUCATOR, V33, P509, DOI 10.1177/0145721707301371

Fisher L, 2010, DIABETES CARE, V33, P23, DOI 10.2337/dc09-1238

Global Health Workforce Alliance World Health Organization, 2010, COUNTR CAS STUD REC

Grigsby AB, 2002, J PSYCHOSOM RES, V53, P1053, DOI 10.1016/S0022-3999(02)00417-8

Harris PA, 2009, J BIOMED INFORM, V42, P377, DOI 10.1016/j.jbi.2008.08.010

International Diabetes Federation, 2015, DIABETES ATLAS

International Labour Organization, 2008, INT STAND CLASS OCC

Little TV, 2014, CURR DIABETES REP, V14

McGee DL, 1999, AM J EPIDEMIOL, V149, P41

McGuire BE, 2010, DIABETOLOGIA, V53, P66, DOI 10.1007/s00125-009-1559-5

Naranjo D, 2012, CURR DIABETES REP, V12, P729, DOI 10.1007/s11892-012-0319-y

Pallas SW, 2013, AM J PUBLIC HEALTH, V103, pE74, DOI 10.2105/AJPH.2012.301102

Palmas W, 2015, J GEN INTERN MED, V30, P1004, DOI 10.1007/s11606-015-3247-0

Perez-Escamilla R., 2010, J HISP HIGH EDUC, V9, P43, DOI DOI 10.1177/1538192709349917

Perez-Escamilla R, 2015, DIABETES CARE, V38, P197, DOI 10.2337/dc14-0327

Roman LA, 2009, ARCH WOMEN MENT HLTH, V12, P379, DOI 10.1007/s00737-009-0083-4

Segura-Perez S, 2011, IMPACT PROMOTORES SA

Shah M, 2013, CURR DIABETES REP, V13, P163, DOI 10.1007/s11892-012-0359-3

Snijders A.B., 2012, MULTILEVEL ANAL INTR

UKPDS Study Group, 1998, LANCET, V352, P837, DOI DOI 10.1016/S0140-6736(98)07019-6

van Son J, 2014, J PSYCHOSOM RES, V77, P81, DOI 10.1016/j.jpsychores.2014.03.013

Vincent D, 2008, NURS RES, V57, P101, DOI 10.1097/01.NNR.0000313484.18670.ab

Wagner J, 2015, INT J BEHAV MED, V5, P2

Wagner J, 2015, CURR DIABETES REP, V15

Wagner J, 2015, TRANSL BEHAV MED, V5, P415, DOI 10.1007/s13142-015-0332-1

Weber B, 2000, EXP CLIN ENDOCR DIAB, V108, P187, DOI 10.1055/s-2000-7742

Welch G, 2007, ETHNIC DIS, V17, P541

Wennerstrom A, 2015, J AMBUL CARE MANAG, V38, P263, DOI 10.1097/JAC.0000000000000087

World Health Organization, 2007, TASK SHIFT TACKL HLT

WULSIN L, 2002, PRIM CARE COMPANION, V4, P191, DOI DOI 10.4088/PCC.V04N0504

NR 41

TC 13

Z9 14

U1 0

U2 12

PU ELSEVIER IRELAND LTD

PI CLARE

PA ELSEVIER HOUSE, BROOKVALE PLAZA, EAST PARK SHANNON, CO, CLARE, 00000,

IRELAND

SN 0168-8227

EI 1872-8227

J9 DIABETES RES CLIN PR

JI Diabetes Res. Clin. Pract.

PD OCT

PY 2016

VL 120

BP 162

EP 170

DI 10.1016/j.diabres.2016.07.022

PG 9

WC Endocrinology & Metabolism

SC Endocrinology & Metabolism

GA EA6PK

UT WOS:000386750900022

PM 27568646

OA Green Accepted

DA 2019-01-08

ER

PT J

AU Jaser, SS

Lord, JH

Simmons, JH

Malow, BA

AF Jaser, Sarah S.

Lord, Jadienne H.

Simmons, Jill H.

Malow, Beth A.

TI Brief report: Sleep disturbances in young children with type 1 diabetes

SO DIABETES RESEARCH AND CLINICAL PRACTICE

LA English

DT Article

DE Sleep; Psychological stress; Type 1 diabetes mellitus; Parenting

ID BEHAVIORS; PARENTS; YOUTH

AB This multi-method study, including actigraphy, sleep diaries, and questionnaires, indicated significant sleep disturbances in young children with type 1 diabetes (age 3-5) and insufficient sleep duration in children and their parents. Results provide initial support for sleep as a potential target to improve both diabetes outcomes and parental distress. (C) 2016 Elsevier Ireland Ltd. All rights reserved.

C1 [Jaser, Sarah S.; Lord, Jadienne H.; Simmons, Jill H.; Malow, Beth A.] Vanderbilt Univ, 221 Kirkland Hall, Nashville, TN 37235 USA.

RP Jaser, SS (reprint author), Vanderbilt Univ, Dept Pediat, 2200 Childrens Way,DOT 11136, Nashville, TN 37232 USA.

EM sarah.jaser@vanderbilt.edu

FU CTSA Award from the National Center for Advancing Translational Sciences

[UL1TR000455]

FX This study was supported by CTSA Award UL1TR000455 from the National

Center for Advancing Translational Sciences.

CR Adkins KW, 2012, BEHAV SLEEP MED, V10, P138, DOI 10.1080/15402002.2011.596598

American Diabetes Association, 2016, DIABETES CARE S1, V39, pS1 , DOI DOI 10.2337/DC13-S011

Donga E, 2010, DIABETES CARE, V33, P1573, DOI 10.2337/dc09-2317

Goodlin-Jones BL, 2008, J DEV BEHAV PEDIATR, V29, P82, DOI 10.1097/DBP.0b013e318163c39a

Meltzer LJ, 2008, J PEDIATR PSYCHOL, V33, P279, DOI 10.1093/jpepsy/jsm118

Miller KM, 2015, DIABETES CARE, V38, P971, DOI 10.2337/dc15-0078

Mindell JA, 2006, SLEEP, V29, P1263

Monaghan M, 2012, CHILD HEALTH CARE, V41, P246, DOI 10.1080/02739615.2012.685385

Monaghan MC, 2009, FAM SYST HEALTH, V27, P28, DOI 10.1037/a0014770

National Sleep Foundation, MUCH SLEEP DO WE REA

Owens JA, 2000, SLEEP, V23, P1043

Perfect MM, 2012, SLEEP, V35, P81, DOI 10.5665/sleep.1590

Smaldone A, 2011, J PEDIATR HEALTH CAR, V25, P87, DOI 10.1016/j.pedhc.2009.09.003

Vehik K, 2007, DIABETES CARE, V30, P503, DOI 10.2337/dc06-1837

NR 14

TC 3

Z9 3

U1 0

U2 4

PU ELSEVIER IRELAND LTD

PI CLARE

PA ELSEVIER HOUSE, BROOKVALE PLAZA, EAST PARK SHANNON, CO, CLARE, 00000,

IRELAND

SN 0168-8227

EI 1872-8227

J9 DIABETES RES CLIN PR

JI Diabetes Res. Clin. Pract.

PD OCT

PY 2016

VL 120

BP 232

EP 234

DI 10.1016/j.diabres.2016.08.012

PG 3

WC Endocrinology & Metabolism

SC Endocrinology & Metabolism

GA EA6PK

UT WOS:000386750900033

PM 27599087

OA Green Accepted

DA 2019-01-08

ER

PT J

AU Aghili, R

Polonsky, WH

Valojerdi, AE

Malek, M

Keshtkar, AA

Esteghamati, A

Heyman, M

Khamseh, ME

AF Aghili, Rokhsareh

Polonsky, William H.

Valojerdi, Ameneh Ebrahim

Malek, Mojtaba

Keshtkar, Abbas Ali

Esteghamati, Alireza

Heyman, Mark

Khamseh, Mohammad Ebrahim

TI Type 2 Diabetes: Model of Factors Associated with Glycemic Control

SO CANADIAN JOURNAL OF DIABETES

LA English

DT Article

DE diabetes mellitus; distress; glycemic control; psychosocial; type 2

diabetes

ID QUALITY-OF-LIFE; SELF-MANAGEMENT; DEPRESSIVE SYMPTOMS; EMOTIONAL

DISTRESS; CLINICAL DEPRESSION; SOCIAL SUPPORT; CARE; PATIENT; HEALTH;

METAANALYSIS

AB Objectives: The purpose of this study was to evaluate the related factors and their intercorrelated impacts on glycemic control in people with type 2 diabetes mellitus.

Methods: Patients with type 2 diabetes were recruited for this study during their regular clinic visits at a major medical centre in Iran. Glycated hemoglobin (A1C) levels were used as the indicator of glycemic control. Regression analysis was used to determine the relationships between glycemic control and demographics, self-care behaviours, resources and affective variables. Moreover, the associations between diabetes related distress and measured variables were tested.

Results: Three hundred eighty people with type 2 diabetes completed the study. The mean duration of diabetes was 8.94 +/- 6.57 years, and the mean A1C levels were 7.78%+/- 1.7%. Diabetes-related distress was significantly associated with A1C levels, controlling for all other variables (p=0.01). On the other hand, depression (p<0.001), self-management (p<0.001), anxiety (p<0.001) and patient-physician relationship (p=0.023) were significantly associated with diabetes-related distress.

Conclusions: Diabetes-related distress was found to be associated with glycemic control in people with type 2 diabetes, whereas age, depression, anxiety, self-management and family and social support may affect glycemic control indirectly through diabetes-related distress. Thus, it is important to assess and, if appropriate, treat people with diabetes for diabetes-related distress in order to identify and help them overcome barriers to optimal glycemic control. (C) 2016 Canadian Diabetes Association.

C1 [Aghili, Rokhsareh; Valojerdi, Ameneh Ebrahim; Khamseh, Mohammad Ebrahim] Iran Univ Med Sci, Inst Endocrinol & Metab, Endocrine Res Ctr, Tehran, Iran.

[Polonsky, William H.] Behav Diabet Inst, San Diego, CA USA.

[Malek, Mojtaba] Iran Univ Med Sci, Inst Endocrinol & Metab, Res Ctr Prevent Cardiovasc Dis, Tehran, Iran.

[Keshtkar, Abbas Ali] Univ Tehran Med Sci, Endocrinol & Metab Res Inst, Osteoporosis Res Ctr, Tehran, Iran.

[Esteghamati, Alireza] Univ Tehran Med Sci, Vali Asr Hosp, Endocrinol & Metab Res Ctr EMRC, Tehran, Iran.

[Heyman, Mark] UCSD Sch Med, Dept Psychiat, San Diego, CA USA.

RP Khamseh, ME (reprint author), Inst Endocrinol & Metab, 10 Firouzeh St,South Vali Asr Ave,Vali Asr Sq, Tehran, Iran.

EM khamseh.m@iums.ac.ir

RI Khamseh, Mohammad Ebrahim/L-9391-2018

FU Iran University of Medical Sciences [93-02-122-24800]

FX This study was funded and supported by Iran University of Medical

Sciences, grant number 93-02-122-24800. The authors thank all the people

who participated in this study.

CR Aghili R, 2013, INT NURS REV, V60, P267, DOI 10.1111/inr.12007

Aikens JE, 2012, DIABETES CARE, V35, P2472, DOI 10.2337/dc12-0181

[Anonymous], 2015, DIABETES CARE S, V38, pS1

Baradaran HR, 2013, INT J PREVENTIVE MED, V4, P580

Brown JB, 2002, FAM PRACT, V19, P344, DOI 10.1093/fampra/19.4.344

COYNE JC, 1994, PSYCHOL BULL, V116, P29, DOI 10.1037/0033-2909.116.1.29

Davis RE, 2005, CURR MED RES OPIN, V21, P1477, DOI 10.1185/030079905X61929

Delahanty LM, 2007, DIABETIC MED, V24, P48, DOI 10.1111/j.1464-5491.2007.02028.x

DiMatteo MR, 2004, HEALTH PSYCHOL, V23, P207, DOI 10.1037/0278-6133.23.2.207

Farshchi A, 2014, J DIABETES METAB DIS, V13, DOI 10.1186/2251-6581-13-42

Fisher L, 2007, DIABETES CARE, V30, P2746, DOI 10.2337/dc07-1166

Fisher L, 2007, DIABETES CARE, V30, P542, DOI 10.2337/dc06-1614

Fisher L, 2012, DIABETES CARE, V35, P259, DOI 10.2337/dc11-1572

Fisher L, 2010, DIABETES CARE, V33, P1034, DOI 10.2337/dc09-2175

Fitzgerald JT, 1998, DIABETES CARE, V21, P706, DOI 10.2337/diacare.21.5.706

Georgiades A, 2007, PSYCHOSOM MED, V69, P235, DOI 10.1097/PSY.0b013e318042588d

Glasgow RE, 2005, DIABETES CARE, V28, P2655, DOI 10.2337/diacare.28.11.2655

Golden SH, 2008, JAMA-J AM MED ASSOC, V299, P2751, DOI 10.1001/jama.299.23.2751

Gonzalez JS, 2008, DIABETIC MED, V25, P1102, DOI 10.1111/j.1464-5491.2008.02535.x

Gonzalez JS, 2011, DIABETES CARE, V34, P236, DOI 10.2337/dc10-1970

Gonzalez JS, 2008, DIABETES CARE, V31, P2398, DOI 10.2337/dc08-1341

Hermanns N, 2015, DIABETES CARE, V38, P551, DOI 10.2337/dc14-1416

Hessler D, 2015, DIABETES RES CLIN PR, V108, P360, DOI 10.1016/j.diabres.2014.12.017

Jones A, 2016, PRIM CARE DIABETES, V10, P83, DOI 10.1016/j.pcd.2015.03.008

Katon WJ, 2010, INT J GERIATR PSYCH, V25, P466, DOI 10.1002/gps.2363

KAVIANI H, 2008, TEHRAN U MED J, V66, P136

Khamseh ME, 2011, BMC PSYCHIATRY, V11, DOI 10.1186/1471-244X-11-61

Khodapanahi MK, 2010, J FAM RES, V5, P423

Lustman PJ, 2000, DIABETES CARE, V23, P934, DOI 10.2337/diacare.23.7.934

Nam S, 2011, DIABETES RES CLIN PR, V93, P1, DOI 10.1016/j.diabres.2011.02.002

Norris SL, 2001, DIABETES CARE, V24, P561, DOI 10.2337/diacare.24.3.561

Peyrot M, 1999, J HEALTH SOC BEHAV, V40, P141, DOI 10.2307/2676370

Peyrot M, 2012, HEALTH QUAL LIFE OUT, V10, DOI 10.1186/1477-7525-10-125

POLONSKY WH, 1995, DIABETES CARE, V18, P754, DOI 10.2337/diacare.18.6.754

Polonsky WH, 2005, DIABETES CARE, V28, P2543, DOI 10.2337/diacare.28.10.2543

Polonsky WH, 2005, DIABETES CARE, V28, P626, DOI 10.2337/diacare.28.3.626

Polonsky William H, 2002, Curr Diab Rep, V2, P153, DOI 10.1007/s11892-002-0075-5

Robinson DJ, 2013, CAN J DIABETES, V37, pS87, DOI 10.1016/j.jcjd.2013.01.026

Rosland AM, 2008, J GEN INTERN MED, V23, P1992, DOI 10.1007/s11606-008-0814-7

Stark CS, 2013, DIABETES CARE, V36, P2271, DOI DOI 10.2337/DC12-2258

Strandberg RB, 2014, J PSYCHOSOM RES, V77, P174, DOI 10.1016/j.jpsychores.2014.06.015

Tong WT, 2015, BMJ OPEN, V5, DOI 10.1136/bmjopen-2014-006407

Vasheghani-Farahani Ali, 2011, Asian J Sports Med, V2, P106

Wang RH, 2011, J ADV NURS, V67, P2658, DOI 10.1111/j.1365-2648.2011.05701.x

Wardian J, 2014, SOC WORK HEALTH CARE, V53, P364, DOI 10.1080/00981389.2014.884038

Weinger K, 2010, DIABETES CARE, V33, P450, DOI 10.2337/dc09-2132

Zhang CX, 2009, DIABETIC MED, V26, P1282, DOI 10.1111/j.1464-5491.2009.02840.x

NR 47

TC 12

Z9 12

U1 0

U2 14

PU ELSEVIER SCIENCE BV

PI AMSTERDAM

PA PO BOX 211, 1000 AE AMSTERDAM, NETHERLANDS

SN 1499-2671

J9 CAN J DIABETES

JI Can. J. Diabetes

PD OCT

PY 2016

VL 40

IS 5

BP 424

EP 430

DI 10.1016/j.jcjd.2016.02.014

PG 7

WC Endocrinology & Metabolism

SC Endocrinology & Metabolism

GA EC6ML

UT WOS:000388250300013

PM 27291886

DA 2019-01-08

ER

PT J

AU Cheng, L

Sit, JWH

Leung, DYP

Li, XM

AF Cheng, Li

Sit, Janet W. H.

Leung, Doris Y. P.

Li, Xiaomei

TI The Association Between Self-Management Barriers and Self-Efficacy in

Chinese Patients with Type 2 Diabetes: The Mediating Role of Appraisal

SO WORLDVIEWS ON EVIDENCE-BASED NURSING

LA English

DT Article

DE diabetes; self-management barriers; appraisal; self-efficacy; mediating

effect; knowledge; decision making

ID EMOTIONAL DISTRESS; PERCEIVED BARRIERS; BEHAVIOR-CHANGE; LOW-INCOME;

STRESS; MODEL; PEOPLE; PERFORMANCE; DEPRESSION; ADHERENCE

AB Background: Patients with higher levels of self-management barriers are more likely to exhibit a lower level of self-efficacy. However, the theoretically meaningful mechanisms underlying the association between the two variables have not yet been established.

Aims: Informed by the Transactional Model of Stress and Coping, this study aimed to examine the potential role of diabetes appraisal on the association of self-management barriers and self-efficacy in patients with type 2 diabetes mellitus.

Methods: This article presents the secondary data analyses of a multicenter, cross-sectional study. A sample of 346 adults with type 2 diabetes was interviewed, using the Personal Diabetes Questionnaire, the Appraisal of Diabetes scale, the Diabetes Empowerment Scale-Short Form, and the Summary of Diabetes Self-Care Activities. Structure equation modeling was performed with 10,000 bootstrap samples using Mplus 7.

Results: The hypothesized model provided a good fit to the data (chi(2) = 22.975, df = 33; p = .1144; CFI = 0.989; SRMR = 0.036; RMSEA = 0.042). The mediating effect of diabetes appraisal on the association of self-management barriers and self-efficacy was significant (beta = -0.521; 95% CI: -0.865, -0.283), explaining 44.82% of the total effect of barriers on self-efficacy. Significant associations were also detected between diet knowledge and diabetes appraisal (beta = 0.148, p = .047).

Linking Evidence to Action: Diabetes appraisal plays a mediating role in the association between self-management barriers and self-efficacy in patients with type 2 diabetes. Reflecting on patients' appraisal of diabetes can help to develop evidence-based and patient-centered interventions. Interventions that enhance individuals' positive appraisal of diabetes have the potential to buffer the negative effects of self-management barriers on self-efficacy.

C1 [Cheng, Li; Sit, Janet W. H.; Leung, Doris Y. P.] Chinese Univ Hong Kong, Fac Med, Nethersole Sch Nursing, Shatin, Hong Kong, Peoples R China.

[Li, Xiaomei] Xi An Jiao Tong Univ, Fac Med, Sch Nursing, Xian, Peoples R China.

RP Cheng, L (reprint author), Chinese Univ Hong Kong, Nethersole Sch Nursing, Room 806,Esther Lee Bldg, Shatin, Hong Kong, Peoples R China.

EM lilycheng@link.cuhk.edu.hk

RI Leung, Doris/K-4878-2014; Sit, Janet/A-5159-2015

OI Leung, Doris/0000-0003-0138-8839; Sit, Janet/0000-0002-4714-5915

CR Abubakari A-R, 2011, J NURS HEALTHC CHRON, V3, P245, DOI DOI 10.1111/J.1752-9824.2011.01098.X

Ahola AJ, 2013, DIABETIC MED, V30, P413, DOI 10.1111/dme.12105

Anderson RM, 2003, DIABETES CARE, V26, P1641, DOI 10.2337/diacare.26.5.1641-a

Arbuckle J. L., 2013, IBM SPSS AMOS 22 USE

Arnsten AFT, 2009, NAT REV NEUROSCI, V10, P410, DOI 10.1038/nrn2648

Baek RN, 2014, ANN BEHAV MED, V48, P145, DOI 10.1007/s12160-013-9585-4

BANDURA A, 1977, PSYCHOL REV, V84, P191, DOI 10.1037/0033-295X.84.2.191

Baumann LC, 2012, NURSE PRACT, V37, P32, DOI DOI 10.1097/01.NPR.0000411104.12617.64

Breland JY, 2013, DIABETES CARE, V36, P2895, DOI 10.2337/dc12-1632

Burner Elizabeth, 2013, J Diabetes Sci Technol, V7, P111

CAREY MP, 1991, J BEHAV MED, V14, P43, DOI 10.1007/BF00844767

Carpenter R. D., 2008, THESIS

Carpenter R, 2012, J HEALTH CARE POOR U, V23, P726, DOI 10.1353/hpu.2012.0049

Cattaneo LB, 2010, AM PSYCHOL, V65, P646, DOI 10.1037/a0018854

Chen X P, 2011, J MANAGE, P796

Cheng L, 2014, CHIN J NUR, V49, P749

Cheng L, 2016, PATIENT PREFER ADHER, V10, DOI 10.2147/PPA.S94275

[程丽 Cheng Li], 2013, [中华护理杂志, Chinese Journal of Nursing], V48, P707

Enders CK, 2001, EDUC PSYCHOL MEAS, V61, P713, DOI 10.1177/00131640121971482

Garland E, 2009, EXPLORE-NY, V5, P37, DOI 10.1016/j.explore.2008.10.001

Ho AYK, 2010, NURS HEALTH SCI, V12, P259, DOI 10.1111/j.1442-2018.2010.00517.x

[胡贝贝 Hu Beibei], 2011, [中华护理杂志, Chinese Journal of Nursing], V46, P225

Hu LT, 1999, STRUCT EQU MODELING, V6, P1, DOI 10.1080/10705519909540118

JanzenClaude JA, 2014, J HEALTH PSYCHOL, V19, P312, DOI DOI 10.1177/1359105312470157

Jung SY, 2015, ASIAN NURS RES, V9, P73, DOI 10.1016/j.anr.2014.11.002

Kidd LA, 2014, HEALTH EXPECT, V17, P565, DOI 10.1111/j.1369-7625.2012.00785.x

Kuo CC, 2014, WORLDV EVID-BASED NU, V11, P301, DOI 10.1111/wvn.12066

Lazarus R. S., 1987, EUROPEAN J PERSONALI, V1, P141, DOI DOI 10.1002/PER.2410010304

Li Y., 2011, MOD HOSP, V11, P148

Makine C, 2009, DIABETIC MED, V26, P28, DOI 10.1111/j.1464-5491.2008.02606.x

Mansyur CL, 2013, J BEHAV MED, V36, P75, DOI 10.1007/s10865-012-9403-7

Marcy Todd R, 2011, Diabetes Ther, V2, P9, DOI 10.1007/s13300-010-0012-6

McEwen BS, 2011, ANNU REV MED, V62, P431, DOI 10.1146/annurev-med-052209-100430

McGuire AM, 2014, COLLEGIAN, V21, P301, DOI 10.1016/j.colegn.2013.07.001

Mika A, 2012, BEHAV NEUROSCI, V126, P605, DOI 10.1037/a0029642

Nagelkerk J, 2006, J ADV NURS, V54, P151, DOI 10.1111/j.1365-2648.2006.03799.x

Nam S, 2011, DIABETES RES CLIN PR, V93, P1, DOI 10.1016/j.diabres.2011.02.002

Nicholls AR, 2012, PSYCHOL SPORT EXERC, V13, P263, DOI 10.1016/j.psychsport.2011.12.003

Prochaska JO, 1997, AM J HEALTH PROMOT, V12, P38, DOI 10.4278/0890-1171-12.1.38

Samuel-Hodge CD, 2000, DIABETES CARE, V23, P928, DOI 10.2337/diacare.23.7.928

Shah BM, 2012, STRESS HEALTH, V28, P111, DOI 10.1002/smi.1410

Shansky RM, 2013, FRONT HUM NEUROSCI, V7, DOI 10.3389/fnhum.2013.00123

Sharma M., 2011, THEORETICAL FDN HLTH

Shrout PE, 2002, PSYCHOL METHODS, V7, P422, DOI 10.1037//1082-989X.7.4.422

Smalls BL, 2012, GEN HOSP PSYCHIAT, V34, P385, DOI 10.1016/j.genhosppsych.2012.03.018

Stetson B, 2011, DIABETES RES CLIN PR, V91, P321, DOI 10.1016/j.diabres.2010.12.002

Strandberg RB, 2014, J PSYCHOSOM RES, V77, P174, DOI 10.1016/j.jpsychores.2014.06.015

Toobert DJ, 2000, DIABETES CARE, V23, P943, DOI 10.2337/diacare.23.7.943

NR 48

TC 2

Z9 3

U1 3

U2 15

PU WILEY

PI HOBOKEN

PA 111 RIVER ST, HOBOKEN 07030-5774, NJ USA

SN 1545-102X

EI 1741-6787

J9 WORLDV EVID-BASED NU

JI Worldviews Evid.-Based Nurs.

PD OCT

PY 2016

VL 13

IS 5

BP 356

EP 362

DI 10.1111/wvn.12159

PG 7

WC Nursing

SC Nursing

GA EC7HL

UT WOS:000388307700004

PM 27074413

DA 2019-01-08

ER

PT J

AU Bajaj, HS

Venn, K

Ye, CL

Aronson, R

AF Bajaj, Harpreet S.

Venn, Karri

Ye, Chenglin

Aronson, Ronnie

TI Randomized Trial of Long-Acting Insulin Glargine Titration Web Tool

(LTHome) Versus Enhanced Usual Therapy of Glargine Titration (INNOVATE

Trial)

SO DIABETES TECHNOLOGY & THERAPEUTICS

LA English

DT Article

ID TYPE-2 DIABETES-MELLITUS; GLUCOSE-LOWERING DRUGS; SELF-TITRATION;

HYPOGLYCEMIA; DETEMIR; 52-WEEK; PEOPLE

AB Background: Basal insulin titration in the real world is often unsuccessful. LTHome, a web tool, applies a rules engine-based algorithm providing insulin titration advice directly to the patient. Methods: This pilot, randomized trial evaluates basal insulin glargine titration by LTHome compared to enhanced usual therapy ([EUT]diabetes education program) over 12 weeks. Important inclusion criteria: 18-75 years, type 2 diabetes, computer literacy, and HbA1c >7.0%. Trial protocol was approved by ethics board. Results: We randomized 139 subjects. The achievement of primary composite outcome (four out of seven fasting plasma glucose [FPG] within 5-7.2mmol/L + mean for three consecutive FPG within 5-7.2mmol/L + no severe hypoglycemia) was 15% in LTHome versus 41% in EUT (noninferiority not met, P-value=0.92). Other outcomes were similar between the LTHome and EUT arms: alternate composite outcome achievement (last five FPG mean within the range of 5-7.2mmol/L + no hypoglycemia, 47% and 51%, P=0.73); A1c reduction (-1.0% and -1.1%, P=0.66); proportion achieving A1c 7% (14% and 20%, P=0.36); and hypoglycemia incidence (31% and 37%, P=0.4), respectively. Patient satisfaction score improvements were greater in LTHome versus EUT (change in fear of hypoglycemia score P=0.04 and change in diabetes distress score P=0.04). The mean number of additional healthcare provider visits was 0.13 for LTHome and 1.22 for EUT (P<0.01). Conclusion: INNOVATE trial suggests clinical utility of LTHome compared to EUT in real-life settings. Further research is needed to evaluate the efficacy and safety of automated insulin titration algorithms.

C1 [Bajaj, Harpreet S.; Venn, Karri; Aronson, Ronnie] LMC Diabet & Endocrinol, Toronto, ON, Canada.

[Bajaj, Harpreet S.] Mt Sinai Hosp, Leadership Sinai Ctr Diabet, Toronto, ON, Canada.

[Ye, Chenglin] Hlth Qual Ontario, Toronto, ON, Canada.

RP Bajaj, HS (reprint author), 2130 North Pk Dr,Suite 238, Brampton, ON L6S 0C9, Canada.

EM harpreet.bajaj@lmc.ca

OI Bajaj, Harpreet/0000-0002-1461-1465

FU AstraZeneca; Eli Lilly; Janssen; Merck; Novo Nordisk; Pfizer; Regeneron;

Sanofi; Takeda; Abbott; Bayer; Boehringer Ingelheim; BD;

GlaxoSmithKline; Medtronic; Amgen

FX H.B.: Advisory Panel or Consultant: AstraZeneca, Boehringer Ingelheim,

Eli Lilly, Janssen, Merck, Sanofi. Research Support; AstraZeneca, Eli

Lilly, Janssen, Merck, Novo Nordisk, Pfizer, Regeneron, Sanofi, Takeda.

Speaker's Bureau or fees; Abbott, AstraZeneca, Bayer, Boehringer

Ingelheim, Eli Lilly, Janssen, Merck, Novo Nordisk, Sanofi. No competing

financial interests exist for authors K.V. and C.Y; R.A.: Funding

(Grants/Honoraria): Sanofi, Takeda, BD, GlaxoSmithKline.

Research/Clinical Trials: BD, Takeda, Sanofi, Merck, Janssen, Medtronic,

Boehringer Ingelheim, Regeneron, Andromeda, Tolerx, Bristol-Myers

Squibb, AstraZeneca, Novo Nordisk, Novartis, Eli Lilly, ConjuChem,

Abbott, Piramal, Diartis, Roche, Abbott, Quintiles, ICON, Medpace, Essem

Research. Speaker/Consulting Fees: Novo Nordisk, Janssen, Sanofi,

Medtronic, Bristol-Myers Squibb, AstraZeneca, Takeda, Amgen.

CR American Diabetes Association, 2015, DIABETES CARE S1, V38, pS1, DOI DOI 10.2337/DC15-S001

Anderson RM, 2005, DIABETES EDUCATOR, V31, P513, DOI 10.1177/0145721705278947

[Anonymous], 2008, THESIS

Boussageon R, 2011, BMJ-BRIT MED J, V343

Bradley C, 2007, HEALTH QUAL LIFE OUT, V5, DOI 10.1186/1477-7525-5-57

Briscoe VJ, 2006, CLIN DIABETES, V24, P115, DOI DOI 10.2337/DIACLIN.24.3.115

Canadian Diabetes Association, 2013, CANADIAN J DIABETES, V37, pA3

COX DJ, 1987, DIABETES CARE, V10, P617, DOI 10.2337/diacare.10.5.617

Cryer PE, 2002, DIABETOLOGIA, V45, P937, DOI 10.1007/s00125-002-0822-9

DCCT Res Grp, 1991, AM J MED, V90, P450

Finkelstein J., 2012, EVIDENCE REPORTS TEC, V206

Gerstein HC, 2006, DIABETIC MED, V23, P736, DOI 10.1111/j.1464-5491.2006.01881.x

Hajos TRS, 2013, DIABETIC MED, V30, pE63, DOI 10.1111/dme.12040

Harris S, 2008, CAN FAM PHYSICIAN, V54, P550

Hollander P, 2008, CLIN THER, V30, P1976, DOI 10.1016/j.clinthera.2008.11.001

Kostev K, 2015, DIABETES METAB SYNDR, V14, P45

Lyles CR, 2014, CURR DIABETES REP, V14, DOI 10.1007/s11892-014-0534-9

Morrison F, 2011, ARCH INTERN MED, V171, P1542, DOI 10.1001/archinternmed.2011.400

Nicola N, 2005, DIABETES CARE, V28, P2948

Polonsky WH, 2005, DIABETES CARE, V28, P626, DOI 10.2337/diacare.28.3.626

Roek MGA, 2009, BMC FAM PRACT, V10, DOI 10.1186/1471-2296-10-40

Rosenstock J, 2008, DIABETOLOGIA, V51, P408, DOI 10.1007/s00125-007-0911-x

Seaquist ER, 2012, DIABETES CARE, V35, P409, DOI 10.2337/dc11-0996

Simon ACR, 2014, J MED INTERNET RES, V16, P63, DOI 10.2196/jmir.3198

Simon ACR, 2013, ARTIF INTELL MED, V59, P23, DOI 10.1016/j.artmed.2013.04.009

Strange Poul, 2007, J Diabetes Sci Technol, V1, P540

Swinnen SG, 2010, DIABETES CARE, V33, P1176, DOI 10.2337/dc09-2294

NR 27

TC 7

Z9 7

U1 0

U2 3

PU MARY ANN LIEBERT, INC

PI NEW ROCHELLE

PA 140 HUGUENOT STREET, 3RD FL, NEW ROCHELLE, NY 10801 USA

SN 1520-9156

EI 1557-8593

J9 DIABETES TECHNOL THE

JI Diabetes Technol. Ther.

PD OCT

PY 2016

VL 18

IS 10

BP 610

EP 615

DI 10.1089/dia.2016.0182

PG 6

WC Endocrinology & Metabolism

SC Endocrinology & Metabolism

GA EA3BM

UT WOS:000386473000003

PM 27652718

DA 2019-01-08

ER

PT J

AU Polonsky, WH

Hessler, D

Layne, JE

Zisser, H

AF Polonsky, William H.

Hessler, Danielle

Layne, Jennifer E.

Zisser, Howard

TI Impact of the Omnipod((R)) Insulin Management System on Quality of Life:

A Survey of Current Users

SO DIABETES TECHNOLOGY & THERAPEUTICS

LA English

DT Article

DE Continuous subcutaneous insulin infusion; Quality of life; Type 1

diabetes; Glycemic control

ID RANDOMIZED-TRIAL; ADULTS; INFUSION; INJECTION; DISTRESS; THERAPY; TIME

AB Background: Few recent studies have examined the impact of continuous subcutaneous insulin infusion systems on patient-reported quality of life (QOL). We explored QOL changes resulting from treatment with the Omnipod((R)) Insulin Management System (Insulet Corp., Billerica, MA). Methods: One thousand two hundred forty-five adults (>18 years) with type 1 diabetes and current Omnipod users completed an online questionnaire examining perceived changes in QOL and glycemic control since Omnipod initiation. The QOL dimensions included overall well-being (World Health Organization-5, modified to examine changes retrospectively), diabetes distress (Type 1 Diabetes Distress Scale, current distress and a modified retrospective version), and psychosocial impact (two subscales from the Diabetes Technology Impact Measure, perceived control over diabetes, and hypoglycemic safety). Regression analyses examined associations between demographics, key psychological factors, and perceived change in glycemic control with the QOL dimensions. Results: Broad QOL and clinical benefits associated with Omnipod use were common. The majority reported positive changes in the following: overall well-being (53.5%), perceived control over diabetes (72.5%), hypoglycemic safety (50.6%), and diabetes distress (69.6%). Worsening in any of these areas was uncommon. In addition, 64.2% of patients reported glycemic improvement post-Omnipod initiation, while 35.2% reported a decrease in severe hypoglycemic episodes. Trust in one's Omnipod, perceived improvement in glycemic control, and reductions in severe hypoglycemia independently predicted benefits in all QOL measures (all P<0.001). Conclusions: These findings suggest that Omnipod users perceived substantial QOL benefits from the device; benefits are more apparent in those who trust the device and have noted positive changes in glycemic control.

C1 [Polonsky, William H.] Univ Calif San Diego, San Diego, CA 92103 USA.

[Polonsky, William H.] Behav Diabet Inst, POB 2148, San Diego, CA 92014 USA.

[Hessler, Danielle] Univ Calif San Francisco, San Francisco, CA 94143 USA.

[Layne, Jennifer E.] Insulet Corp, Billerica, MA USA.

[Zisser, Howard] Univ Calif Santa Barbara, Santa Barbara, CA 93106 USA.

RP Polonsky, WH (reprint author), Behav Diabet Inst, POB 2148, San Diego, CA 92014 USA.

EM whp@behavioraldiabetes.org

FU Insulet Corporation

FX This investigator-initiated study was supported by Insulet Corporation.

CR DeVries JH, 2002, DIABETES CARE, V25, P2074, DOI 10.2337/diacare.25.11.2074

Fisher L, 2016, J DIABETES COMPLICAT, V30, P1123, DOI 10.1016/j.jdiacomp.2016.03.032

Fisher L, 2015, J DIABETES COMPLICAT, V29, P572, DOI 10.1016/j.jdiacomp.2015.01.012

Hajos TRS, 2013, DIABETIC MED, V30, pE63, DOI 10.1111/dme.12040

Hoogma R. P., 2005, DIABETIC MED, V23, P141

John O. P., 1999, HDB PERSONALITY THEO, V2, P102, DOI DOI 10.1525/FQ.1998.51.4.04A00260

Lau C Y, 2004, J Postgrad Med, V50, P189

Lebenthal Y, 2012, DIABETES TECHNOL THE, V14, P411, DOI 10.1089/dia.2011.0228

Nicolucci A, 2008, DIABETIC MED, V25, P213, DOI 10.1111/j.1464-5491.2007.02346.x

Peyrot M, 2011, DIABETES TECHNOL THE, V13, P471, DOI 10.1089/dia.2010.0167

Polonsky WH, 2013, DIABETES TECHNOL THE, V15, P295, DOI 10.1089/dia.2012.0298

Tsui E, 2001, DIABETES CARE, V24, P1722, DOI 10.2337/diacare.24.10.1722

NR 12

TC 1

Z9 1

U1 0

U2 4

PU MARY ANN LIEBERT, INC

PI NEW ROCHELLE

PA 140 HUGUENOT STREET, 3RD FL, NEW ROCHELLE, NY 10801 USA

SN 1520-9156

EI 1557-8593

J9 DIABETES TECHNOL THE

JI Diabetes Technol. Ther.

PD OCT

PY 2016

VL 18

IS 10

BP 664

EP 670

DI 10.1089/dia.2016.0239

PG 7

WC Endocrinology & Metabolism

SC Endocrinology & Metabolism

GA EA3BM

UT WOS:000386473000010

PM 27673352

DA 2019-01-08

ER

PT J

AU Wang, SB

Li, B

Ungvari, GS

Ng, CH

Chiu, HFK

Kou, CG

Liu, YW

Tao, YC

Wu, YH

Fu, YL

Qi, Y

Yu, YQ

Xiang, YT

AF Wang, Shibin

Li, Bo

Ungvari, Gabor S.

Ng, Chee H.

Chiu, Helen F. K.

Kou, Changgui

Liu, Yawen

Tao, Yuchun

Wu, Yanhua

Fu, Yingli

Qi, Yue

Yu, Yaqin

Xiang, Yu-Tao

TI Poor mental health status and its associations with demographic

characteristics and chronic diseases in Chinese elderly

SO SOCIAL PSYCHIATRY AND PSYCHIATRIC EPIDEMIOLOGY

LA English

DT Article

DE Mental health; Chronic disease; Chinese elderly

ID LIFE-STYLE; HONG-KONG; PSYCHOLOGICAL DISTRESS; PREVALENCE; POPULATION;

DISORDERS; MULTIMORBIDITY; DEPRESSION; CARE; CHALLENGES

AB Although poor mental health is associated with significant personal and societal burden, it is rarely reported in older Chinese populations. This study examined the mental health status of a large representative sample of Chinese elderly in relation to socio-demographic characteristics, lifestyle, and chronic diseases.

Multistage stratified cluster sampling was used in this cross-sectional study. A total of 4115 people aged between 60 and 79 years were selected and interviewed with standardized assessment tools. The 12-item General Health Questionnaire (GHQ-12) was used to measure general mental health with the total score of aeyen4 as the threshold for poor mental health status.

The adjusted percentage of poor mental health status in the whole sample was 23.8 %; 18.5 % in men and 28.9 % in women. Multivariate logistic regression analysis revealed that female gender, widowed/separated marital status, rural abode, low income, poor diet, lack of physical exercise, and multi-morbidity were independently associated with poor mental health. The percentage of poor mental health status was significantly higher in patients with anemia, diabetes, hyperlipidemia, cataract/glaucoma, ischemic heart disease, cerebrovascular diseases, nasopharyngitis, chronic gastroenteritis/peptic ulcer, liver diseases, cholecystitis/gallstone, arthritis, or chronic low back pain.

Given the high rate of poor mental health status among older Chinese population, policy makers and health professionals in China should address the mental health burden of its aging population.

C1 [Wang, Shibin; Li, Bo; Kou, Changgui; Liu, Yawen; Tao, Yuchun; Wu, Yanhua; Fu, Yingli; Qi, Yue; Yu, Yaqin] Jilin Univ, Sch Publ Hlth, Dept Epidemiol & Biostat, Changchun, Peoples R China.

[Wang, Shibin; Xiang, Yu-Tao] Univ Macau, Fac Hlth Sci, Unit Psychiat, Ave Univ,3-F,Bldg E12, Taipa, Macau, Peoples R China.

[Ungvari, Gabor S.] Univ Notre Dame Australia, Marian Ctr, Perth, WA, Australia.

[Ungvari, Gabor S.] Univ Western Australia, Sch Psychiat & Clin Neurosci, Perth, WA, Australia.

[Ng, Chee H.] Univ Melbourne, Dept Psychiat, Melbourne, Vic, Australia.

[Chiu, Helen F. K.] Chinese Univ Hong Kong, Dept Psychiat, Hong Kong, Hong Kong, Peoples R China.

RP Li, B (reprint author), Jilin Univ, Sch Publ Hlth, Dept Epidemiol & Biostat, Changchun, Peoples R China.; Xiang, YT (reprint author), Univ Macau, Fac Hlth Sci, Unit Psychiat, Ave Univ,3-F,Bldg E12, Taipa, Macau, Peoples R China.

EM li_bo@jlu.edu.cn; xyutly@gmail.com

RI liu, yawen/J-2471-2014

FU Scientific Research Foundation of the Health Bureau of Jilin Province,

China [2011Z116]

FX This study was funded by the Scientific Research Foundation of the

Health Bureau of Jilin Province, China (Grant Number: 2011Z116).

CR Agborsangaya CB, 2013, QUAL LIFE RES, V22, P791, DOI 10.1007/s11136-012-0214-7

Boey KW, 2005, AGING MENT HEALTH, V9, P555, DOI 10.1080/13607860500193351

Bowling A, 2007, AGING MENT HEALTH, V11, P301, DOI 10.1080/13607860600963752

Chiu HFK, 2004, ACTA PSYCHIAT SCAND, V109, P299, DOI 10.1046/j.1600-0447.2003.00263.x

Cornah D. D., 2006, CHEERS UNDERSTANDING

Edmans J, 2013, AGE AGEING, V42, P747, DOI 10.1093/ageing/aft054

Feng ZL, 2012, HEALTH AFFAIR, V31, P2764, DOI 10.1377/hlthaff.2012.0535

do Nascimento KKF, 2015, INT J GERIATR PSYCH, V30, P1171, DOI 10.1002/gps.4271

Fone DL, 2006, HEALTH PLACE, V12, P332, DOI 10.1016/j.healthplace.2005.02.002

Forsman AK, 2011, SCAND J PUBLIC HEALT, V39, P757, DOI 10.1177/1403494811418281

Fortin M, 2012, ANN FAM MED, V10, P142, DOI 10.1370/afm.1337

Fu R, 2016, PLOS ONE, V11, DOI 10.1371/journal.pone.0148990

Gale CR, 2016, J EPIDEMIOL COMMUN H, V70, P813, DOI 10.1136/jech-2015-206479

Gomez-Pinilla F, 2008, NAT REV NEUROSCI, V9, P568, DOI 10.1038/nrn2421

Gorstein J, 2007, INDICATORS METHODS C

Hassanzadeh J, 2018, AM J MENS HEALTH, V12, P96, DOI 10.1177/1557988316630720

Jokela M, 2013, PSYCHOL MED, V43, P2037, DOI 10.1017/S0033291712003042

KATON W, 1992, MED CARE, V30, P67, DOI 10.1097/00005650-199201000-00007

Li KQ, 2008, ASIAN J PSYCHIATR, V1, P51, DOI 10.1016/j.ajp.2008.09.010

Lopresti AL, 2013, J AFFECT DISORDERS, V148, P12, DOI 10.1016/j.jad.2013.01.014

Ma X, 2008, PSYCHOL MED, V38, P1723, DOI 10.1017/S0033291708003164

Mui A. C., 2010, CHINA J SOCIAL WORK, V3, P111, DOI [10.1080/17525098.2010.492634, DOI 10.1080/17525098.2010.492634]

Naimi TS, 2016, SELECTION BIASES OBS, DOI [10.1111/add.13451, DOI 10.1111/ADD.13451]

National Bureau of Statistics of China, 2013, CHIN STAT ABSTR

Paul Constanca, 2006, Psychol Health Med, V11, P221

Phillips MR, 2009, LANCET, V373, P2041, DOI 10.1016/S0140-6736(09)60660-7

Qu B, 2012, POPUL HEALTH MANAG, V15, P181, DOI 10.1089/pop.2011.0033

Resnick Barbara, 2003, J Community Health Nurs, V20, P135, DOI 10.1207/S15327655JCHN2003_01

Schneiderman N, 2005, ANNU REV CLIN PSYCHO, V1, P607, DOI 10.1146/annurev.clinpsy.1.102803.144141

Schoenborn Charlotte A, 2010, Vital Health Stat 10, P1

Schuckit MA, 2009, LANCET, V373, P492, DOI 10.1016/S0140-6736(09)60009-X

Su D, 2012, ARCH GERONTOL GERIAT, V55, P564, DOI 10.1016/j.archger.2012.06.006

Tian T, 2015, IRAN J PUBLIC HEALTH, V44, P1084

Walsh R, 2011, AM PSYCHOL, V66, P579, DOI 10.1037/a0021769

Wang SB, 2015, PUBLIC HEALTH, V129, P1539, DOI 10.1016/j.puhe.2015.06.013

Wang SB, 2015, ASIA-PAC J PUBLIC HE, V27, P394, DOI 10.1177/1010539514551200

WHO, 2004, LANCET, V363, P902

Wong TS, 2014, AGING MENT HEALTH, V18, P588, DOI 10.1080/13607863.2013.856862

Xiang YT, 2012, LANCET, V380, P1715, DOI 10.1016/S0140-6736(11)60893-3

Xiang YT, 2009, ALCOHOL CLIN EXP RES, V33, P1111, DOI 10.1111/j.1530-0277.2009.00933.x

Yamamoto N, 2014, J PHYSIOL ANTHROPOL, V33, DOI 10.1186/1880-6805-33-32

Yang Ting-zhong, 2003, Zhonghua Liu Xing Bing Xue Za Zhi, V24, P769

Zhu H, 2010, J POPUL AGEING, V3, P161, DOI 10.1007/s12062-011-9034-8

NR 43

TC 5

Z9 5

U1 4

U2 24

PU SPRINGER HEIDELBERG

PI HEIDELBERG

PA TIERGARTENSTRASSE 17, D-69121 HEIDELBERG, GERMANY

SN 0933-7954

EI 1433-9285

J9 SOC PSYCH PSYCH EPID

JI Soc. Psychiatry Psychiatr. Epidemiol.

PD OCT

PY 2016

VL 51

IS 10

BP 1449

EP 1455

DI 10.1007/s00127-016-1271-y

PG 7

WC Psychiatry

SC Psychiatry

GA DY5SZ

UT WOS:000385164700010

PM 27539269

DA 2019-01-08

ER

PT J

AU Deschenes, SS

Burns, RJ

Graham, E

Schmitz, N

AF Deschenes, Sonya S.

Burns, Rachel J.

Graham, Eva

Schmitz, Norbert

TI Prediabetes, depressive and anxiety symptoms, and risk of type 2

diabetes: A community-based cohort study

SO JOURNAL OF PSYCHOSOMATIC RESEARCH

LA English

DT Article

DE Anxiety; Cohort study; Depression; Prediabetes; Type 2 diabetes

ID IMPAIRED GLUCOSE-TOLERANCE; WHITEHALL II; LIFE-STYLE; PSYCHOLOGICAL

DISTRESS; CARDIOVASCULAR-DISEASE; COST-EFFECTIVENESS; PHYSICAL-ACTIVITY;

FASTING GLUCOSE; MELLITUS; HEALTH

AB Objective: To examine the potential synergistic associations between prediabetes, depressive and anxiety symptoms, and the risk of incident type 2 diabetes.

Methods: Data were from the Emotional Well-Being, Metabolic Factors and Health Status (EMHS) study and included 2486 adults between 40 and 69 years without diabetes at baseline. Hemoglobin Alc levels and measures of depressive and anxiety symptoms were collected at baseline and mutually exclusive groups were formed based on the presence/absence of prediabetes and high/low depressive and anxiety symptoms. A follow-up telephone interview conducted approximately 4.6 years later inquired about new diabetes diagnoses.

Results: 86 participants developed diabetes during the follow-up period. After accounting for sociodemographic, lifestyle, and metabolic characteristics, participants with prediabetes and elevated depressive symptoms had an increased risk of developing diabetes compared to those without prediabetes and with low depressive symptoms (OR = 10.65, 95% CI = 4.60, 24.66). The joint effect of prediabetes and depressive symptoms on diabetes risk was synergistic (Synergy Index = 2.57, 95% CI = 1.02, 6.49). Similar results were found for participants with prediabetes and high symptoms of anxiety (OR = 8.95, 95% CI = 3.54, 22.63), however the joint effect of prediabetes and anxiety symptoms did not significantly exceed additive risk after adjusting for covariates (Synergy Index = 239, 95% CI = 0.83, 6.87).

Conclusion: The combination of prediabetes and depressive or anxiety symptoms was associated with an increased risk of developing diabetes. This study underscores the importance of mental health in the progression from prediabetes to type 2 diabetes. (C) 2016 Elsevier Inc. All rights reserved.

C1 [Deschenes, Sonya S.; Burns, Rachel J.; Schmitz, Norbert] McGill Univ, Dept Psychiat, Montreal, PQ, Canada.

[Deschenes, Sonya S.; Burns, Rachel J.; Graham, Eva; Schmitz, Norbert] Douglas Mental Hlth Univ Inst, 6875 Boul Lasalle,Frank B Common Pavil,F2117-2, Montreal, PQ H4H 1R3, Canada.

[Graham, Eva; Schmitz, Norbert] McGill Univ, Dept Epidemiol Biostat & Occupat Hlth, Montreal, PQ, Canada.

RP Deschenes, SS (reprint author), Douglas Mental Hlth Univ Inst, 6875 Boul Lasalle,Frank B Common Pavil,F2117-2, Montreal, PQ H4H 1R3, Canada.

EM sonya.deschenes@mail.mcgill.ca

RI Schmitz, Norbert/A-5177-2010; Deschenes, Sonya/G-6341-2017

OI Schmitz, Norbert/0000-0001-7777-6323; Burns, Rachel/0000-0002-5337-7878

FU Operating Grant from the Canadian Institutes of Health Research

[MOP-130552]; Fonds de recherche du Quebec - Sante, Canada; fellowship

from the Canadian Institutes of Health Research [201411MFE-338860

FRN-142923]

FX This work was supported by an Operating Grant from the Canadian

Institutes of Health Research (MOP-130552). Sonya Deschenes is supported

by a fellowship from the Fonds de recherche du Quebec - Sante, Canada

and Rachel Burns is supported by a fellowship from the Canadian

Institutes of Health Research (201411MFE-338860 FRN-142923). The funding

agencies had no role in the design and conduct of the study, the

collection, analysis and interpretation of the data, in the writing of

the report, or in the decision to submit the article for publication.

CR Association. A. D., 2010, DIABETES CARE S1, V33, pS62

Au B, 2014, J PSYCHOSOM RES, V77, P180, DOI 10.1016/j.jpsychores.2014.07.012

Awadalla P, 2013, INT J EPIDEMIOL, V42, P1285, DOI 10.1093/ije/dys160

Bassuk SS, 2005, J APPL PHYSIOL, V99, P1193, DOI 10.1152/japplphysiol.00160.2005

Bonora E, 2011, DIABETES CARE, V34, P1038, DOI 10.2337/dc10-1180

Bray GA, 2009, LANCET, V374, P1677, DOI 10.1016/S0140-6736(09)61457-4

Committee IR, 2005, GUIDELINES DATA PROC

Da Silva MA, 2012, EUR J EPIDEMIOL, V27, P537, DOI 10.1007/s10654-012-9692-8

Deschenes SS, 2015, J PSYCHOSOM RES, V78, P137, DOI 10.1016/j.jpsychores.2014.11.023

Deuschle M, 2013, CURR OPIN PSYCHIATR, V26, P60, DOI 10.1097/YCO.0b013e32835a4206

Eaton WW, 2007, INT REV PSYCHIATR, V19, P497, DOI 10.1080/09540260701564906

Eddy DM, 2005, ANN INTERN MED, V143, P251, DOI 10.7326/0003-4819-143-4-200508160-00006

Engum A, 2007, J PSYCHOSOM RES, V62, P31, DOI 10.1016/j.jpsychores.2006.07.009

Eriksson AK, 2008, DIABETIC MED, V25, P834, DOI 10.1111/j.1464-5491.2008.02463.x

Heraclides AM, 2012, OBESITY, V20, P428, DOI 10.1038/oby.2011.95

Herman WH, 2005, ANN INTERN MED, V142, P323, DOI 10.7326/0003-4819-142-5-200503010-00007

HOSMER DW, 1992, EPIDEMIOLOGY, V3, P452, DOI 10.1097/00001648-199209000-00012

Hu FB, 2001, NEW ENGL J MED, V345, P790, DOI 10.1056/NEJMoa010492

Kessler RC, 2003, JAMA-J AM MED ASSOC, V289, P3095, DOI 10.1001/jama.289.23.3095

Knol MJ, 2006, DIABETOLOGIA, V49, P837, DOI 10.1007/s00125-006-0159-x

Kroenke K, 2001, J GEN INTERN MED, V16, P606, DOI 10.1046/j.1525-1497.2001.016009606.x

Lamers F, 2008, J CLIN EPIDEMIOL, V61, P679, DOI 10.1016/j.jclinepi.2007.07.018

Mainous AG, 2014, BMJ OPEN, V4, DOI 10.1136/bmjopen-2014-005002

Manea L, 2012, CAN MED ASSOC J, V184, pE191, DOI 10.1503/cmaj.110829

Meurs M, 2016, PSYCHOSOM MED, V78, P233, DOI 10.1097/PSY.0000000000000255

Mezuk B, 2008, DIABETES CARE, V31, P2383, DOI 10.2337/dc08-0985

Nathan DM, 2007, DIABETES CARE, V30, P753, DOI 10.2337/dc07-9920

Pouwer F, 2003, DIABETOLOGIA, V46, P892, DOI 10.1007/s00125-003-1124-6

Pouwer F, 2010, DISCOV MED, V9, P112

Rosmond R, 2000, J INTERN MED, V247, P188, DOI 10.1046/j.1365-2796.2000.00603.x

Schmitz N, 2016, MOL PSYCHIAT

Spitzer RL, 2006, ARCH INTERN MED, V166, P1092, DOI 10.1001/archinte.166.10.1092

Strine TW, 2008, GEN HOSP PSYCHIAT, V30, P127, DOI 10.1016/j.genhosppsych.2007.12.008

Tabak AG, 2012, LANCET, V379, P2279, DOI 10.1016/S0140-6736(12)60283-9

Toshihiro M, 2008, DIABETIC MED, V25, P1211, DOI 10.1111/j.1464-5491.2008.02566.x

Tully PJ, 2013, PSYCHOL HEALTH MED, V18, P627, DOI 10.1080/13548506.2012.749355

Tuomilehto J, 2001, NEW ENGL J MED, V344, P1343, DOI 10.1056/NEJM200105033441801

Virtanen M, 2014, DIABETES CARE, V37, P2091, DOI 10.2337/dc13-2725

Vos T, 2015, LANCET, V386, P743, DOI 10.1016/S0140-6736(15)60692-4

Whiting DR, 2011, DIABETES RES CLIN PR, V94, P311, DOI 10.1016/j.diabres.2011.10.029

Zhang XP, 2010, DIABETES CARE, V33, P1665, DOI 10.2337/dc09-1939

NR 41

TC 9

Z9 9

U1 0

U2 12

PU PERGAMON-ELSEVIER SCIENCE LTD

PI OXFORD

PA THE BOULEVARD, LANGFORD LANE, KIDLINGTON, OXFORD OX5 1GB, ENGLAND

SN 0022-3999

EI 1879-1360

J9 J PSYCHOSOM RES

JI J. Psychosomat. Res.

PD OCT

PY 2016

VL 89

BP 85

EP 90

DI 10.1016/j.jpsychores.2016.08.011

PG 6

WC Psychiatry

SC Psychiatry

GA DX8HU

UT WOS:000384629500014

PM 27663115

DA 2019-01-08

ER

PT J

AU Coccaro, EF

Drossos, T

Phillipson, L

AF Coccaro, Emil F.

Drossos, Tina

Phillipson, Louis

TI HbA(1c) levels as a function of emotional regulation and emotional

intelligence in patients with type 2 diabetes

SO PRIMARY CARE DIABETES

LA English

DT Article

DE T2D; HbA(1c); Emotional regulation; Emotional intelligence

ID GLYCEMIC CONTROL; INSULIN-RESISTANCE; GLUCOSE-LEVELS; DEPRESSION;

ADULTS; HYPERGLYCEMIA; METAANALYSIS; ADHERENCE; DISTRESS; MELLITUS

AB Aims: Understanding the role of emotion in glycemic control may be critical for the long-term treatment of patients with type 2 diabetes (T2D). In this study we investigated the relationship between measures of emotional regulation and emotional intelligence and HbA(1c) levels in adult patients with T2 diabetes.

Methods: 100 adult patients with T2 diabetes completed assessments of emotional regulation (i.e., affect intensity/lability) and emotional intelligence and were then correlated with HbA(1c) levels with several relevant covariates.

Results: HbA(1c) levels were significantly associated with affect intensity (AI: r = .24, p = .018) and with emotional intelligence (EI: r = -.29, p = .004), but not affect lability. These results were the same even after adding income, state depression scores, insulin-dependent status, serum cholesterol, diabetes literacy and self-care as covariates (AI: beta = .33, p = .001; EI: beta = -.31, p = .002). Diabetes self-care, but not diabetes literacy, was also associated with HbA(1c) levels (beta = -.29, p = .003).

Conclusions: These data suggest that aspects of emotional regulation and emotional intelligence play a role in glycemic control in adult patients with T2 diabetes and do so even in the context of several variables relevant to diabetes. If so, interventions that can reduce affect intensity and/or increase emotional intelligence may represent a new strategy in the glycemic control of adult patients with T2 diabetes. (C) 2016 Primary Care Diabetes Europe. Published by Elsevier Ltd. All rights reserved.

C1 [Coccaro, Emil F.; Drossos, Tina] Univ Chicago, Pritzker Sch Med, Dept Psychiat & Behav Neurosci, Clin Neurosci Res Unit, Chicago, IL 60637 USA.

[Phillipson, Louis] Univ Chicago, Pritzker Sch Med, Endocrinol Sect, Kovler Diabet Ctr,Dept Med, Chicago, IL 60637 USA.

RP Coccaro, EF (reprint author), Univ Chicago, Dept Psychiat & Behav Neurosci, Clin Neurosci Res Unit, 5841 South Maryland Ave, Chicago, IL 60637 USA.

EM ecoccaro@bsd.uchicago.edu

FU University of Chicago's Institute for Translational Medicine (ITM);

Kovler Diabetes Center at the University of Chicago

FX This work was supported in part by a pilot grant from the University of

Chicago's Institute for Translational Medicine (ITM; Drs. Drossos and

Coccaro) and from the Kovler Diabetes Center at the University of

Chicago (Dr. Phillipson).

CR Ali S, 2006, DIABETIC MED, V23, P1165, DOI 10.1111/j.1464-5491.2006.01943.x

Arigo D, 2015, CHRONIC ILLN, V11, P33, DOI 10.1177/1742395314531990

Bailey CJ, 2011, INT J CLIN PRACT, V65, P314, DOI 10.1111/j.1742-1241.2010.02544.x

Beck A, 1993, BECK ANXIETY INVENTO

Beck AT, 1996, BECK DEPRESSION INVE

Coccaro EF, 2011, BIOL PSYCHIAT, V69, P1153, DOI 10.1016/j.biopsych.2011.02.032

COHEN S, 1983, J HEALTH SOC BEHAV, V24, P385, DOI 10.2307/2136404

Cukierman-Yaffe T, 2009, DIABETES CARE, V32, P221, DOI 10.2337/dc08-1153

Egede LE, 2010, DIABETES RES CLIN PR, V87, P302, DOI 10.1016/j.diabres.2010.01.024

Erus G, 2015, DIABETES CARE, V38, P97, DOI 10.2337/dc14-1196

GONDERFREDERICK LA, 1989, HEALTH PSYCHOL, V8, P45, DOI 10.1037//0278-6133.8.1.45

Gonzalez JS, 2015, HEALTH PSYCHOL, V34, P505, DOI 10.1037/hea0000131

Gross J. J., 1998, REV GEN PSYCHOL, V2, P271, DOI DOI 10.1037/1089-2680.2.3.271

HARVEY PD, 1989, J CLIN PSYCHOL, V45, P786, DOI 10.1002/1097-4679(198909)45:5<786::AID-JCLP2270450515>3.0.CO;2-P

Hughes AE, 2012, J PEDIATR PSYCHOL, V37, P925, DOI 10.1093/jpepsy/jss062

Karachan T.F., 2009, TURK KLIN J MED SCI, V1, P16

Kruyt ND, 2012, DIABETES TECHNOL THE, V14, P311, DOI 10.1089/dia.2011.0171

Kumar R, 2008, AM J GERIAT PSYCHIAT, V16, P989, DOI 10.1097/JGP.0b013e31818b40fc

LARSEN RJ, 1987, J RES PERS, V21, P1, DOI 10.1016/0092-6566(87)90023-7

Lin EHB, 2008, J PSYCHOSOM RES, V65, P571, DOI 10.1016/j.jpsychores.2008.06.007

Markowitz SM, 2011, PSYCHOSOMATICS, V52, P1, DOI 10.1016/j.psym.2010.11.007

Morris JK, 2014, NEUROBIOL AGING, V35, DOI 10.1016/j.neurobiolaging.2014.04.018

Nath CR, 2001, DIABETES EDUCATOR, V27, P857, DOI 10.1177/014572170102700611

Nef G., 2012, J PSYCHIATR RES, V46, P549

Ng TP, 2014, J ALZHEIMERS DIS, V41, P61, DOI 10.3233/JAD-131901

ORZI F, 1988, J CEREBR BLOOD F MET, V8, P346, DOI 10.1038/jcbfm.1988.70

Padala PR, 2008, DIABETES RES CLIN PR, V79, P37, DOI 10.1016/j.diabres.2007.06.012

Penckofer S, 2012, DIABETES TECHNOL THE, V14, P303, DOI 10.1089/dia.2011.0191

Ravona-Springer R, 2012, J ALZHEIMERS DIS, V30, P299, DOI 10.3233/JAD-2012-120106

SALOVEY P, 1995, EMOTION DISCLOSURE H, P125, DOI DOI 10.1037/10182-006

Sanz CM, 2013, DIABETES CARE, V36, P1512, DOI 10.2337/dc12-1017

Schopf V, 2013, FRONT HUM NEUROSCI, V7, DOI 10.3389/fnhum.2013.00212

Shen BJ, 2008, DIABETES CARE, V31, P1293, DOI 10.2337/dc07-1945

Siddiqui S, 2014, DIABETES METAB SYND, V8, P62, DOI 10.1016/j.dsx.2013.06.010

Stankovic Z, 2011, PSYCHIAT DANUB, V23, P34

van Harten B, 2007, AGE AGEING, V36, P164, DOI 10.1093/ageing/afl180

Weinger K, 2005, DIABETES CARE, V28, P1346, DOI 10.2337/diacare.28.6.1346

WHO, 2012, GLOBAL STATUS REPORT

Winkley K, 2006, BRIT MED J, V333, P65, DOI 10.1136/bmj.38874.652569.55

Zagarins SE, 2012, J BEHAV MED, V35, P299, DOI 10.1007/s10865-011-9359-z

NR 40

TC 1

Z9 1

U1 1

U2 6

PU ELSEVIER SCI LTD

PI OXFORD

PA THE BOULEVARD, LANGFORD LANE, KIDLINGTON, OXFORD OX5 1GB, OXON, ENGLAND

SN 1751-9918

EI 1878-0210

J9 PRIM CARE DIABETES

JI Prim. Care Diabetes

PD OCT

PY 2016

VL 10

IS 5

BP 334

EP 341

DI 10.1016/j.pcd.2016.05.006

PG 8

WC Endocrinology & Metabolism; Primary Health Care

SC Endocrinology & Metabolism; General & Internal Medicine

GA DW8ZQ

UT WOS:000383944400004

PM 27344533

DA 2019-01-08

ER

PT J

AU Barnacle, M

Strand, MA

Werremeyer, A

Maack, B

Petry, N

AF Barnacle, Mykell

Strand, Mark A.

Werremeyer, Amy

Maack, Brody

Petry, Natasha

TI Depression Screening in Diabetes Care to Improve Outcomes: Are We

Meeting the Challenge?

SO DIABETES EDUCATOR

LA English

DT Article

ID BIDIRECTIONAL RELATIONSHIP; SOCIOECONOMIC-STATUS; GLYCEMIC CONTROL;

POPULATION; DISTRESS; COHORT; PHQ-9; RISK

AB Purpose Evidence-based guidelines recommend regular depression screening among individuals with type 2 diabetes mellitus (T2DM). The purpose of this study is to examine depression screening patterns among a primary care population with T2DM, through use of the Patient Health Questionnaire-9 (PHQ-9).

Methods In total, 1817 patients with T2DM were analyzed through a retrospective observational study at 2 sites, a regional health center and a federally qualified health center (FQHC). The T2DM sample was divided into those with and without a major depressive disorder (MDD) diagnosis. Depression screening rates and depression severity were assessed through the PHQ-9.

Results Both sites had higher rates of PHQ-9 screening among individuals with a history of MDD (64.82%) vs those without MDD (11.39%). Individuals from the FQHC without a history of depression had a higher mean PHQ-9 score (10.11) than those with a previous MDD diagnosis at both RHS and FQHC (7.16 and 9.85, respectively).

Conclusions Depression screening rates among individuals with diabetes and no history of MDD were remarkably low. Patients with diabetes but no MDD diagnosis who sought health care at a FQHC clinic had more depressive symptoms than those with a history of MDD at both sites. Individuals with diabetes and who have a MDD diagnosis are much more likely to receive regular depression screening than those without MDD, which leaves a substantial proportion of patients with undetected depression. Depression screening must be enhanced for all individuals with diabetes, particularly for low-income individuals and those without a previous diagnosis of MDD.

C1 [Barnacle, Mykell] North Dakota State Univ, Sch Nursing, POB 6050, Fargo, ND 58108 USA.

[Strand, Mark A.; Werremeyer, Amy; Maack, Brody; Petry, Natasha] North Dakota State Univ, Dept Pharm Practice, Fargo, ND 58108 USA.

[Strand, Mark A.] North Dakota State Univ, Masters Publ Hlth Program, Fargo, ND 58108 USA.

RP Barnacle, M (reprint author), North Dakota State Univ, Sch Nursing, POB 6050, Fargo, ND 58108 USA.

EM Mykell.barnacle@ndsu.edu

FU Dean of the College of Health Professions, North Dakota State University

FX Partial funding for this research was provided by the Dean of the

College of Health Professions, North Dakota State University.

CR American Diabetes Association, 2013, DIABETES CARE S1, V36, pS11, DOI DOI 10.2337/DC13-S011

Ascher-Svanum H, 2015, DIABETES THER, V6, P303, DOI 10.1007/s13300-015-0118-y

Bhautesh DJ, 2013, PLOS ONE, V8

Bowser DM, 2009, DIABETES EDUCATOR, V35, P966, DOI 10.1177/0145721709345164

Brieler JA, 2016, FAM PRACT, V33, P30, DOI 10.1093/fampra/cmv100

Center for Disease Control and Prevention, 2011, NAT DIAB FACT SHEET

Chen PC, 2013, DIABETES CARE, V36, P376, DOI 10.2337/dc12-0473

Ducat L, 2014, JAMA-J AM MED ASSOC, V312, P691, DOI 10.1001/jama.2014.8040

Egede LE, 2007, J GEN INTERN MED, V22, P701, DOI 10.1007/s11606-007-0170-z

Everson SA, 2002, J PSYCHOSOM RES, V53, P891, DOI 10.1016/S0022-3999(02)00303-3

Kosidou K, 2011, J AFFECT DISORDERS, V134, P160, DOI 10.1016/j.jad.2011.05.024

Kroenke K, 2001, J GEN INTERN MED, V16, P606, DOI 10.1046/j.1525-1497.2001.016009606.x

National Center for Health Statistics, 2011, HLTH US 2011 SPEC FE

Renn BN, 2011, CLIN PSYCHOL REV, V31, P1239, DOI 10.1016/j.cpr.2011.08.001

Rotella F, 2013, J CLIN PSYCHIAT, V74, P32, DOI 10.4088/JCP.12r07922

Siu AL, 2016, JAMA-J AM MED ASSOC, V315, P380, DOI 10.1001/jama.2015.18392

Strand M. A., 2014, International Journal of Social Ecology and Sustainable Development (IJSESD), V5, P31, DOI 10.4018/ijsesd.2014010104

Tanenbaum ML, 2013, J AFFECT DISORDERS, V150, P533, DOI 10.1016/j.jad.2013.01.029

Trangle M, ICSI I CLIN SYST IMP

Urbine TF, 2012, JNP-J NURSE PRACT, V8, P231, DOI 10.1016/j.nurpra.2011.09.003

van Steenbergen-Weijenburg KM, 2010, BMC HEALTH SERV RES, V10, DOI 10.1186/1472-6963-10-235

Werremeyer A, 2016, J PRIM CARE COMMUNIT, V7, P130, DOI 10.1177/2150131915627423

NR 22

TC 3

Z9 3

U1 0

U2 5

PU SAGE PUBLICATIONS INC

PI THOUSAND OAKS

PA 2455 TELLER RD, THOUSAND OAKS, CA 91320 USA

SN 0145-7217

EI 1554-6063

J9 DIABETES EDUCATOR

JI Diabetes Educ.

PD OCT

PY 2016

VL 42

IS 5

BP 646

EP 651

DI 10.1177/0145721716662917

PG 6

WC Endocrinology & Metabolism; Public, Environmental & Occupational Health

SC Endocrinology & Metabolism; Public, Environmental & Occupational Health

GA DW1KW

UT WOS:000383403100011

PM 27558266

DA 2019-01-08

ER

PT J

AU Wahl, AK

Osborne, RH

Langeland, E

Wentzel-Larsen, T

Mengshoel, AM

Ribu, L

Peersen, K

Elsworth, GR

Nolte, S

AF Wahl, Astrid K.

Osborne, Richard H.

Langeland, Eva

Wentzel-Larsen, Tore

Mengshoel, Anne Marit

Ribu, Lis

Peersen, Kari

Elsworth, Gerald R.

Nolte, Sandra

TI Making robust decisions about the impact of health education programs:

Psychometric evaluation of the Health Education Impact Questionnaire

(heiQ) in diverse patient groups in Norway

SO PATIENT EDUCATION AND COUNSELING

LA English

DT Article

DE Health Education Impact Questionnaire (heiQ); Self-management; Norway;

Chronic disease; Confirmatory factor analysis (CFA)

ID SELF-MANAGEMENT INTERVENTIONS; CHRONIC DISEASE; MEASUREMENT INVARIANCE;

OUTCOMES; EMPOWERMENT; ADAPTATION; PSORIASIS; SEVERITY; VALIDITY; PEOPLE

AB Objective: To undertake a rigorous psychometric evaluation of the widely used eight-scale heiQ version 2.0 (evaluating immediate effects of self-management interventions) in diverse patient groups in Norway.

Methods: Cross-sectional survey data were collected from 1019 Norwegians. Data were extracted from studies among people with musculoskeletal disorders (n = 516), psoriasis (n = 254), heart disease (n = 97), and Type 2 diabetes (n = 152). To investigate the factorial validity of the Norwegian heiQ, confirmatory factor analyses (CFA) were carried out using Mplus.

Results: One-factor model fit, without modifications, was acceptable for the Emotional distress scale. Only one correlated residual was required to be fitted in each of the other scales to achieve satisfactory model fit. The postulated highly restricted full eight-factor model (no cross-loadings, no correlated residuals) showed good fit to the data. Internal consistency was acceptable for most scales (0.72-0.90) but low for Self-monitoring and insight.

Conclusion: This study of the Norwegian heiQ replicates the factor structure of the original Australian heiQ, using robust and highly restricted CFA procedures, demonstrating a clean independent clusters model structure. (C) 2016 Elsevier Ireland Ltd. All rights reserved.

C1 [Wahl, Astrid K.; Mengshoel, Anne Marit] Univ Oslo, Inst Hlth & Soc, Dept Hlth Sci, POB 1089, N-0317 Oslo, Norway.

[Osborne, Richard H.; Elsworth, Gerald R.; Nolte, Sandra] Deakin Univ, Sch Hlth & Social Dev, Hlth Syst Improvement Unit, Geelong, Vic, Australia.

[Langeland, Eva] Bergen Univ Coll, Fac Hlth & Social Sci, Bergen, Norway.

[Wentzel-Larsen, Tore] Ctr Child & Adolescent Mental Hlth Eastern & Sout, Oslo, Norway.

[Wentzel-Larsen, Tore] Norwegian Ctr Violence & Traumat Stress Studies, Oslo, Norway.

[Ribu, Lis] Oslo & Akershus Univ Coll Appl Sci, Fac Hlth Sci, Dept Nursing, Oslo, Norway.

[Peersen, Kari] Vestfold Hosp Trust, Tonsberg, Norway.

[Nolte, Sandra] Charite, Ctr Internal Med & Dermatol, Dept Psychosomat Med, Berlin, Germany.

RP Wahl, AK (reprint author), Univ Oslo, Inst Hlth & Soc, Dept Hlth Sci, POB 1089, N-0317 Oslo, Norway.

EM a.k.wahl@medisin.uio.no; richard.osborne@deakin.edu.au;

langeland@hib.no; tore.wentzel-larsen@r-bup.no;

a.m.mengshoel@medisin.uio.no; lis.ribu@hioa.no; kari.peersen@siv.no;

gerald.elsworth@deakin.edu.au; sandra.nolte@charite.de

RI Nolte, Sandra/B-7498-2008

OI Nolte, Sandra/0000-0001-6185-9423; Osborne, Richard/0000-0002-9081-2699;

Langeland, Eva/0000-0003-3594-2877

CR Abegunde DO, 2007, LANCET, V370, P1929, DOI 10.1016/S0140-6736(07)61696-1

[Anonymous], 2014, WHITE PAPER MELD ST

Browne M. W., 1993, TESTING STRUCTURAL E

Brunet J, 2015, QUAL LIFE RES, V24, P2375, DOI 10.1007/s11136-015-0972-0

Buchbinder R, 2011, ARTHRITIS RES THER, V13, DOI 10.1186/ar3468

Elsworth G. R., 2015, SAGE OPEN MED

Epstein J, 2015, J CLIN EPIDEMIOL, V68, P360, DOI 10.1016/j.jclinepi.2013.07.013

Holmen H, 2014, JMIR MHEALTH UHEALTH, V2, DOI 10.2196/mhealth.3882

Hu LT, 1999, STRUCT EQU MODELING, V6, P1, DOI 10.1080/10705519909540118

Jordan JE, 2007, MED J AUSTRALIA, V186, P84

Larsen MH, 2014, BRIT J DERMATOL, V171, P1458, DOI 10.1111/bjd.13363

Maunsell E, 2014, CANCER-AM CANCER SOC, V120, P3228, DOI 10.1002/cncr.28847

McDonald R. P., 1999, TEST THEORY UNIFIED

Morita R, 2013, JPN J NURS SCI, V10, P255, DOI 10.1111/j.1742-7924.2012.00224.x

Newman S, 2004, LANCET, V364, P1523, DOI 10.1016/S0140-6736(04)17277-2

Nolte E., 2014, CONCEPTS APPROACHES

Nolte S, 2007, PATIENT EDUC COUNS, V65, P351, DOI 10.1016/j.pec.2006.08.016

Nolte S, 2013, QUAL LIFE RES, V22, P1805, DOI 10.1007/s11136-012-0302-8

Nolte S, 2013, QUAL LIFE RES, V22, P1655, DOI 10.1007/s11136-012-0317-1

Nolte S, 2009, J CLIN EPIDEMIOL, V62, P1173, DOI 10.1016/j.jclinepi.2009.01.021

Osborne RH, 2007, PATIENT EDUC COUNS, V66, P192, DOI 10.1016/j.pec.2006.12.002

Osborne RH, 2011, NURS CLIN N AM, V46, P255, DOI 10.1016/j.cnur.2011.05.010

Richardson G, 2008, J EPIDEMIOL COMMUN H, V62, P361, DOI 10.1136/jech.2006.057430

Schuler M, 2014, QUAL LIFE RES, V23, P2531, DOI 10.1007/s11136-014-0693-9

Schuler M, 2014, HEALTH QUAL LIFE OUT, V12, DOI 10.1186/1477-7525-12-56

Schuler M, 2013, QUAL LIFE RES, V22, P1391, DOI 10.1007/s11136-012-0268-6

Torbjornsen A, 2014, JMIR MHEALTH UHEALTH, V2, DOI 10.2196/mhealth.3535

Wahl AK, 2015, ACTA DERM-VENEREOL, V95, P317, DOI 10.2340/00015555-1939

Warsi A, 2004, ARCH INTERN MED, V164, P1641, DOI 10.1001/archinte.164.15.1641

NR 29

TC 6

Z9 6

U1 0

U2 9

PU ELSEVIER IRELAND LTD

PI CLARE

PA ELSEVIER HOUSE, BROOKVALE PLAZA, EAST PARK SHANNON, CO, CLARE, 00000,

IRELAND

SN 0738-3991

J9 PATIENT EDUC COUNS

JI Patient Educ. Couns.

PD OCT

PY 2016

VL 99

IS 10

BP 1733

EP 1738

DI 10.1016/j.pec.2016.05.001

PG 6

WC Public, Environmental & Occupational Health; Social Sciences,

Interdisciplinary

SC Public, Environmental & Occupational Health; Social Sciences - Other

Topics

GA DW0VQ

UT WOS:000383361500026

PM 27211224

DA 2019-01-08

ER

PT J

AU Kuniss, N

Kramer, G

Muller, N

Kloos, C

Wolf, G

Muller, UA

AF Kuniss, Nadine

Kramer, Guido

Mueller, Nicolle

Kloos, Christof

Wolf, Gunter

Mueller, Ulrich Alfons

TI History of mild hypoglycaemia does not affect the prevalence of

diabetes-related distress in people with diabetes

SO ACTA DIABETOLOGICA

LA English

DT Article

DE Diabetes-related distress; Mild hypoglycaemia; PAID questionnaire

ID QUALITY-OF-LIFE; INSULIN THERAPY; TYPE-1; IMPACT; ADULTS; EVENTS; FEAR;

ASSOCIATION; MANAGEMENT; AWARENESS

AB The occurrence of hypoglycaemia is assumed to be associated with increased diabetes-related distress. We investigated the association of mild hypoglycaemia (MH) with diabetes-related distress in a large outpatient cohort with diabetes type 1 (DM1) and type 2 (DM2).

In a cross-sectional study, we recorded MH and simultaneously assessed diabetes-related distress with the PAID questionnaire in 783 people with diabetes [female 43.8 %, age 63.7 years, duration of diabetes 17.3 years, HbA1c 7.0 % (53 mmol/mol)] in an university outpatient department for metabolic diseases over a period of three months. Participants with and without MH were compared.

People with DM1 (n = 191) had 1.09 MH per week. Diabetes-related distress was not different comparing people with DM1 with (n = 125) and without (n = 66) MH (PAID score 18.4 +/- 16.2 vs. 16.6 +/- 15.0, p = 0.449). The frequency of MH per week in people with DM2 on oral antidiabetic therapy (n = 182) and with insulin therapy (n = 410) is low (0.03 vs. 0.1 episodes). People with DM2 on insulin therapy with (n = 72) and without (n = 338) MH have a comparable PAID score (17.9 +/- 13.3 vs. 16.8 +/- 14.9, p = 0.552). 14.4 % of those with DM1 as well as 12.5 % of those with DM2 on insulin feel hypoglycaemic events to be a "somewhat serious problem" or "serious problem".

In an outpatient setting, MH is not associated with increased diabetes-related distress or burden in people with DM1 or DM2.

C1 [Kuniss, Nadine; Kramer, Guido; Mueller, Nicolle; Kloos, Christof; Wolf, Gunter; Mueller, Ulrich Alfons] Jena Univ Hosp, Div Endocrinol & Metab Dis, Dept Internal Med 3, Bachstr 18, D-07743 Jena, Germany.

RP Kuniss, N (reprint author), Jena Univ Hosp, Div Endocrinol & Metab Dis, Dept Internal Med 3, Bachstr 18, D-07743 Jena, Germany.

EM nadine.kuniss@med.uni-jena.de

CR Bohme P, 2013, DIABETES METAB, V39, P63, DOI 10.1016/j.diabet.2012.10.006

Brod M, 2013, DIABETES OBES METAB, V15, P546, DOI 10.1111/dom.12070

Davis RE, 2005, CURR MED RES OPIN, V21, P1477, DOI 10.1185/030079905X61929

Evans M, 2013, HEALTH QUAL LIFE OUT, V11, DOI 10.1186/1477-7525-11-90
[truncated: 1,296,783 more chars]
