# Supplementary material for: Characterizing Hotspots and Frontier Landscapes of Diabetes-Specific Distress from 2000 to 2018: A Bibliometric Study
Source: Biomed Res Int. 2020 Jan 15;2020:8691451. doi: 10.1155/2020/8691451 (PMC6985931; doi:10.1155/2020/8691451)
Supplement: Supplementary Materials — S1-1: raw citing literature and cited references. S1-2: raw citing literature and cited references (1). S1-3: raw citing literatures and cited references. S2: the number of publications by year. S3: country/region distribution. S4: institutes distribution. S5: authors distribution. S6: journals distribution. [file 8691451.f1.zip › 8691451.f1/S1-2 Raw citing literatures and cited references.doc]

FN Clarivate Analytics Web of Science

VR 1.0

PT J

AU Piane, GM

Smith, TC

AF Piane, Gina M.

Smith, Tyler C.

TI Building an Evidence Base for the Co-Occurrence of Chronic Disease and

Psychiatric Distress and Impairment

SO PREVENTING CHRONIC DISEASE

LA English

DT Article

ID NATIONAL-COMORBIDITY-SURVEY; MENTAL-HEALTH-SERVICES; DSM-IV DISORDERS;

SURVEY REPLICATION; UNITED-STATES; PRIMARY-CARE; PREVALENCE; LIFETIME;

POPULATION; DEPLOYMENT

AB Introduction Mental disorders and chronic diseases have been reported to independently affect half of the US population. The objective of this study was to evaluate the comorbid nature of these conditions.

Methods We analyzed data from 39,954 participants from the 2009 California Health Interview Survey who reported both psychological distress and impairment, on the basis of the Kessler 6 and the Sheehan Disability Scale, and 1 or more of 4 chronic diseases (type 2 diabetes, high blood pressure, asthma, heart disease). Weighted and nonweighted multivariable logistic regression were used to investigate the association between psychological distress and impairment and chronic disease, after adjusting for sex, age, race, current smoking, binge drinking in the previous year, moderate physical activity, and body mass index.

Results After controlling for covariates in the model, we found a significant dose-response relationship between reported chronic diseases and psychiatric distress and impairment that ranged from 1.50 for 1 reported chronic disease to 4.68 for 4 reported chronic diseases.

Conclusion The growing chronic disease burden should be understood clinically in the context of mental health conditions. Further research is needed to identify ways to integrate mental health and chronic disease prevention in primary care.

C1 [Piane, Gina M.] Natl Univ, Sch Hlth & Human Serv, Dept Community Hlth, San Diego, CA 92123 USA.

[Smith, Tyler C.] Natl Univ, San Diego, CA 92123 USA.

RP Piane, GM (reprint author), Natl Univ, Sch Hlth & Human Serv, Dept Community Hlth, 3678 Aero Court, San Diego, CA 92123 USA.

EM GPiane@nu.edu

CR [Anonymous], 2009, LANCET, V373, P185, DOI 10.1016/S0140-6736(09)60048-9

[Anonymous], 2011, CAL HLTH INT SURV 20

Boyko EJ, 2010, DIABETES CARE, V33, P1771, DOI 10.2337/dc10-0296

Breslau J, 2005, PSYCHOL MED, V35, P317, DOI 10.1017/S0033291704003514

*COMM CROSS QUAL C, 2006, AD MENT HLTH ADD DIS

Committee on Quality of Care in America, 2001, CROSSING QUALITY CHA

Demyttenaere K, 2004, JAMA-J AM MED ASSOC, V291, P2581

Fabricatore AN, 2004, CLIN DERMATOL, V22, P332, DOI 10.1016/j.clindermatol.2004.01.006

Granado NS, 2009, HYPERTENSION, V54, P966, DOI 10.1161/HYPERTENSIONAHA.109.132555

Hambrick JP, 2004, J ANXIETY DISORD, V18, P825, DOI 10.1016/j.janxdis.2003.10.004

Johnson-Lawrence V, 2012, PSYCHIAT SERV, V63, P1137, DOI 10.1176/appi.ps.201100365

Kessler RC, 2002, PSYCHOL MED, V32, P959, DOI 10.1017/S0033291702006074

KESSLER RC, 1995, ARCH GEN PSYCHIAT, V52, P1048, DOI 10.1001/archpsyc.1995.03950240066012

Kessler RC, 2005, ARCH GEN PSYCHIAT, V62, P617, DOI 10.1001/archpsyc.62.6.617

Kessler RC, 2005, ARCH GEN PSYCHIAT, V62, P593, DOI 10.1001/archpsyc.62.6.593

KESSLER RC, 1994, ARCH GEN PSYCHIAT, V51, P8

Kessler RC, 2003, JAMA-J AM MED ASSOC, V289, P3095, DOI 10.1001/jama.289.23.3095

Kim G, 2012, INT J GERIATR PSYCH, V27, P1070, DOI 10.1002/gps.2825

King H, 1998, DIABETES CARE, V21, P1414, DOI 10.2337/diacare.21.9.1414

Kung Hsiang-Ching, 2008, Natl Vital Stat Rep, V56, P1

Mascie-Taylor CGN, 2003, SCIENCE, V302, P1921, DOI 10.1126/science.1092488

Pfeiffer PN, 2011, PSYCHIAT SERV, V62, P422, DOI 10.1176/ps.62.4.pss6204_0422

Pratt KM, 2012, PROF PSYCHOL-RES PR, V43, P281, DOI 10.1037/a0028122

Reeves William C., 2011, Morbidity and Mortality Weekly Report, V60, P1

Remington Patrick L, 2011, MMWR Suppl, V60, P70

Satcher D, 2010, PREV CHRONIC DIS, V7

Scott KM, 2008, INT J OBESITY, V32, P192, DOI 10.1038/sj.ijo.0803701

Seelig AD, 2010, SLEEP, V33, P1615, DOI 10.1093/sleep/33.12.1615

Sheehan DV, 1983, ANXIETY DIS

Zivin K, 2010, MED CARE, V48, P843, DOI 10.1097/MLR.0b013e3181e5792b

NR 30

TC 5

Z9 5

U1 0

U2 4

PU CENTERS DISEASE CONTROL

PI ATLANTA

PA 1600 CLIFTON RD, ATLANTA, GA 30333 USA

SN 1545-1151

J9 PREV CHRONIC DIS

JI Prev. Chronic Dis.

PD OCT

PY 2014

VL 11

AR E188

DI 10.5888/pcd11.140211

PG 11

WC Public, Environmental & Occupational Health

SC Public, Environmental & Occupational Health

GA AR3XM

UT WOS:000343522500017

PM 25340360

OA Green Published, Bronze

DA 2019-01-08

ER

PT J

AU Jansen, HJ

Vervoort, GMM

De Haan, AFJ

Netten, PM

de Grauw, WJ

Tack, CJ

AF Jansen, Henry J.

Vervoort, Gerald M. M.

De Haan, Anton F. J.

Netten, Paetrick M.

de Grauw, Wim J.

Tack, Cees J.

TI Diabetes-Related Distress, Insulin Dose, and Age Contribute to

Insulin-Associated Weight Gain in Patients With Type 2 Diabetes: Results

of a Prospective Study

SO DIABETES CARE

LA English

DT Article

ID PREDICTING BODY DENSITY; PROBLEM AREAS; GENERALIZED EQUATIONS;

DEPRESSIVE SYMPTOMS; EMOTIONAL DISTRESS; MELLITUS; METFORMIN; VALIDITY;

THERAPY

AB OBJECTIVE

The determinants of insulin-associated weight gain in type 2 diabetes mellitus (T2DM) are partly unknown. Therefore, we conducted a prospective study to identify predictors of insulin-associated weight gain.

RESEARCH DESIGN AND METHODS

In patients with T2DM, we assessed physical activity by accelerometry and measured diabetes-related distress by questionnaires before and 6 and 12 months after starting insulin therapy. Glycemic control (HbA(1c)) and insulin dose were monitored.

RESULTS

After 12 months of insulin therapy, mean body weight had increased by 3.0 +/- 2.5 kg (P < 0.001). The drop in HbA(1c) was correlated with insulin-associated weight gain. With the use of a multiple linear regression model, a cluster of variables was identified that significantly related to weight gain. Diabetes-related distress, initial insulin dose, and the increase of insulin dose during the course of the study as well as age appeared to be important predictors of weight gain after initiation of insulin therapy. Physical activity (measured as MET) decreased from 1.40 +/- 0.04 at baseline to 1.32 +/- 0.04 MET (P < 0.05) but was not significantly related to weight changes.

CONCLUSIONS

Diabetes-related distress, initial and titration of insulin dose, and age all significantly predict insulin-associated weight gain. After the initiation of insulin therapy, physical activity decreased significantly, but this did not determine weight gain over the first 12 months. Our study findings may have clinical implications.

C1 [Jansen, Henry J.; Vervoort, Gerald M. M.; Tack, Cees J.] Radboud Univ Nijmegen Med Ctr, Dept Internal Med, Nijmegen, Netherlands.

[De Haan, Anton F. J.] Radboud Univ Nijmegen, Dept Hlth Evidence, Biostat Grp, Nijmegen, Netherlands.

[Netten, Paetrick M.] Jeroen Bosch Hosp, Dept Internal Med, Shertogenbosch, Netherlands.

[de Grauw, Wim J.] Radboud Univ Nijmegen Med Ctr, Dept Primary & Community Care, Nijmegen, Netherlands.

[de Grauw, Wim J.] Family Practice Berghem, Berghem, Netherlands.

RP Jansen, HJ (reprint author), Radboud Univ Nijmegen Med Ctr, Dept Internal Med, Nijmegen, Netherlands.

EM h.jansen@jbz.nl

RI de Haan, Antonius/L-4344-2015; Tack, Cees/A-2368-2014; Vervoort,

G.M.M./L-4718-2015; Grauw, W.J.C./L-4334-2015

CR Balkau B, 2014, DIABETES CARE, V37, P2108, DOI 10.2337/dc13-3010

Bouma M, 2006, Ned Tijdschr Geneeskd, V150, P2251

Brumpton B, 2013, INT J OBESITY, V37, P1268, DOI 10.1038/ijo.2012.204

DEFRONZO RA, 1981, INT J OBESITY, V5, P93

Ekelund U, 2005, AM J CLIN NUTR, V81, P964

Forman-Hoffman VL, 2007, J GERONTOL B-PSYCHOL, V62, pS43, DOI 10.1093/geronb/62.1.S43

Fruin ML, 2004, MED SCI SPORT EXER, V36, P1063, DOI 10.1249/01.MSS.0000128144.91337.38

Heller S, 2004, DIABETES RES CLIN PR, V65, pS23, DOI 10.1016/j.diabres.2004.07.005

Holman RR, 2007, NEW ENGL J MED, V357, P1716, DOI 10.1056/NEJMoa075392

Hosoya T, 2012, INTERNAL MED, V51, P263, DOI 10.2169/internalmedicine.51.5768

JACKSON AS, 1978, BRIT J NUTR, V40, P497, DOI 10.1079/BJN19780152

JACKSON AS, 1980, MED SCI SPORT EXER, V12, P175

Jansen HJ, 2011, ENDOCRINE, V39, P190, DOI 10.1007/s12020-010-9423-4

Jansen HJ, 2010, NETH J MED, V68, P359

Miller ST, 2008, BMC PUBLIC HEALTH, V8, DOI 10.1186/1471-2458-8-70

Rotella F, 2013, DIABETES RES CLIN PR, V99, P98, DOI 10.1016/j.diabres.2012.11.022

Ryan M, 2008, DIABETES CARE, V31, P448, DOI 10.2337/dc07-1171

Salle A, 2005, BRIT J NUTR, V94, P931, DOI 10.1079/BJN20051592

Snoek FJ, 2000, DIABETES CARE, V23, P1305, DOI 10.2337/diacare.23.9.1305

St-Arnaud-McKenzie D, 2010, J GERONTOL A-BIOL, V65, P1362, DOI 10.1093/gerona/glq150

Thompson F. E, 2008, NUTR PREVENTION TREA, V2, P3

Turner RC, 1999, JAMA-J AM MED ASSOC, V281, P2005, DOI 10.1001/jama.281.21.2005

Verbeke G, 1997, LINEAR MIXED MODELS

Welch G, 2003, DIABETIC MED, V20, P69, DOI 10.1046/j.1464-5491.2003.00832.x

Westenbrink S, 2012, NEVO KWALITEISHANDBO, P1

Wolfe R R, 2000, Curr Opin Clin Nutr Metab Care, V3, P67, DOI 10.1097/00075197-200001000-00011

Yki-Jarvinen H, 2006, DIABETOLOGIA, V49, P442, DOI 10.1007/s00125-005-0132-0

NR 27

TC 15

Z9 16

U1 1

U2 6

PU AMER DIABETES ASSOC

PI ALEXANDRIA

PA 1701 N BEAUREGARD ST, ALEXANDRIA, VA 22311-1717 USA

SN 0149-5992

EI 1935-5548

J9 DIABETES CARE

JI Diabetes Care

PD OCT

PY 2014

VL 37

IS 10

BP 2710

EP 2717

DI 10.2337/dc13-1205

PG 8

WC Endocrinology & Metabolism

SC Endocrinology & Metabolism

GA AR4UM

UT WOS:000343582400023

PM 25011948

OA Bronze

DA 2019-01-08

ER

PT J

AU Reeve, R

Church, J

Haas, M

Bradford, W

Viney, R

AF Reeve, Rebecca

Church, Jody

Haas, Marion

Bradford, Wylie

Viney, Rosalie

TI Factors that drive the gap in diabetes rates between Aboriginal and

non-Aboriginal people in non-remote NSW

SO AUSTRALIAN AND NEW ZEALAND JOURNAL OF PUBLIC HEALTH

LA English

DT Article

DE Aboriginal; diabetes; non-remote; risk factors

ID ANTHROPOMETRIC INDEXES; AUSTRALIANS; POPULATIONS; PREVALENCE; RISK

AB Objective: To identify factors underpinning the gap in diabetes rates between Aboriginal and non-Aboriginal people in non-remote NSW. This will indicate appropriate target areas for policy and for monitoring progress towards reducing the gap.

Methods: Data from the 2004-05 National Health Survey and National Aboriginal and Torres Strait Islander Health Survey were used to estimate differences in self-reported diabetes rates and risk/prevention factors between Aboriginal and non-Aboriginal people in non-remote NSW. Logistic regression models were used to investigate the contribution of each factor to predicting the probability of diabetes.

Results: Risk factors for diabetes are more prevalent and diabetes rates 2.5 to 4 times higher in Aboriginal compared to non-Aboriginal adults in non-remote NSW. The odds of (known) diabetes for both groups are significantly higher for older people, those with low levels of education and those who are overweight or obese. In the Aboriginal sample, the odds of diabetes are significantly higher for people reporting forced removal of their relatives.

Conclusions: Differences in BMI and education appear to be driving the diabetes gap, together with onset at younger ages in the Aboriginal population. Psychological distress, indicated by removal of relatives, may contribute to increased risk of diabetes in the Aboriginal population.

Implications: The results imply that improved nutrition and exercise, capacity to access and act upon health care information and early intervention are required to reduce the diabetes gap. Current strategies appear to be appropriately aligned with the evidence; however, further research is required to determine whether implementation methods are effective.

C1 [Reeve, Rebecca; Church, Jody; Haas, Marion; Viney, Rosalie] Univ Technol Sydney, Ctr Hlth Econ Res & Evaluat, Broadway, NSW 2007, Australia.

[Bradford, Wylie] Macquarie Univ, N Ryde, NSW 2109, Australia.

RP Reeve, R (reprint author), Univ Technol Sydney, Ctr Hlth Econ Res & Evaluat, POB 123, Broadway, NSW 2007, Australia.

EM rebecca.reeve@chere.uts.edu.au

RI Haas, Marion/H-2732-2017

OI Bradford, Wylie/0000-0002-7478-2290; Reeve, Rebecca/0000-0003-1022-8052

FU UTS Faculty of Business Research Grant; National Health and Medical

Research Council [571926]

FX This study was approved by the ethics committees of the University of

Technology, Sydney and the Aboriginal Health and Medical Research

Council. We are grateful for advice provided by the study's Aboriginal

Reference Group, in particular Debra Fernando and Wylie Bradford (who is

also a co-author of the paper), and for comments from Richard Norman and

Patsy Kenny on a previous draft and the anonymous reviewers. The work

was supported by a UTS Faculty of Business Research Grant and a National

Health and Medical Research Council Capacity Building Grant (571926).

CR Aboriginal and Torres Strait Islander Healing Foundation Development Team, 2009, AB TORR STRAIT ISL H

Aboriginal Health and Medical Research Council of NSW, 2009, 10 OUT 10 DEADL HLTH

Australian Bureau of Statistics, 2010, 4720 0 NAT AB TORR S

Australian Bureau of Statistics, 2006, 4715 0 55 002 NAT AB

Australian Bureau of Statistics, 2008, 4820 0 55 001 DIAB A

Australian Bureau of Statistics, 2006, 4715 0 NAT AB TORR S

Australian Bureau of Statistics, 2006, 2039 0 55 001 SOC IN

Australian Indigenous HealthInfoNet, 2007, REV DIAB IND PEOPL

Australian Institute of Health and Welfare, 2009, MEAS SOC EM WELLB AB

Bombay A, 2009, INT J INDIG HEALTH, V5, P6

Brunner E, 2006, SOCIAL DETERMINANTS, P6

Cunningham J, 2008, ETHNIC HEALTH, V13, P23, DOI 10.1080/13557850701803130

Cunningham J, 2010, AUST NZ J PUBL HEAL, V34, pS18, DOI 10.1111/j.1753-6405.2010.00547.x

Daniel M, 1999, DIABETES CARE, V22, P1993, DOI 10.2337/diacare.22.12.1993

Daniel M, 2002, DIABETES RES CLIN PR, V57, P23, DOI 10.1016/S0168-8227(02)00006-2

Dea K, 2008, DIABETES RES CLIN PR, V80, P483

Diabetes Australia, 2010, CAUS TYP 2 DIAB

Gracey M, 2007, ASIA PAC J CLIN NUTR, V16, P688

Kelly K., 2009, 10 COOP RES CTR AB H

Kennedy B, 2004, P 12 BIENN C POP SOC

Li M, 2010, DIABETES RES CLIN PR, V87, P401, DOI 10.1016/j.diabres.2009.12.004

Lloyd C, 2005, DIABETES SPECTR, V18, P121, DOI [DOI 10.2337/DIASPECT.18.2.121, 10.2337/diaspect.18.2.121]

MCCARTY DJ, 1996, RISE RISE DIABETES A

Minges KE, 2011, DIABETES RES CLIN PR, V93, P139, DOI 10.1016/j.diabres.2011.06.012

National Health and Medical Research Centre, 2001, NAT EV BAS GUID MA 2

NSW Health, 2012, NSW STAT KNOCK CHALL

NSW Health, 2008, NAT PARTN AGR CLOS G

Nutbeam D, 1993, NATL ACTION PLAN DIA

Rowley KG, 1997, CLIN EXP PHARMACOL P, V24, P776, DOI 10.1111/j.1440-1681.1997.tb02131.x

Silburn S. R., 2006, FAMILY MATTERS, V75, P10

Wang Z, 2004, INT J OBESITY, V28, P1580, DOI 10.1038/sj.ijo.0802771

Wang ZM, 2007, EUR J CARDIOV PREV R, V14, P172, DOI 10.1097/01.hjr.0000220580.34763.fb

World Health Organization (WHO), 2000, WHO TECH REP SER, V894, P1

NR 33

TC 1

Z9 1

U1 1

U2 11

PU WILEY-BLACKWELL

PI HOBOKEN

PA 111 RIVER ST, HOBOKEN 07030-5774, NJ USA

SN 1326-0200

EI 1753-6405

J9 AUST NZ J PUBL HEAL

JI Aust. N. Z. Publ. Health

PD OCT

PY 2014

VL 38

IS 5

SI SI

BP 459

EP 465

DI 10.1111/1753-6405.12211

PG 7

WC Public, Environmental & Occupational Health

SC Public, Environmental & Occupational Health

GA AQ4KL

UT WOS:000342765100014

PM 25168646

DA 2019-01-08

ER

PT J

AU Loprinzi, PD

Codey, K

AF Loprinzi, Paul D.

Codey, Kathleen

TI Influence of visual acuity on anxiety, panic and depression disorders

among young and middle age adults in the United States

SO JOURNAL OF AFFECTIVE DISORDERS

LA English

DT Article

DE Epidemiology; Psychological distress; Vision

ID PHYSICAL-ACTIVITY; OLDER-ADULTS; VISION; IMPAIRMENT; SYMPTOMS; LIFE

AB Background: Previous research, albeit limited, has demonstrated an association of visual acuity with depression and anxiety. However, these studies are limited in that they have focused on older adults, used a convenient sample, and/or used a subjective assessment of visual function. As a result, the purpose of this study was to examine the association of objectively-measured visual acuity with depression and anxiety (and panic disorder) among a national sample of young- and middle-age U.S. adults (20-39 years).

Methods: Using data from the 2003-2004 NI-LANES (n=602), the presence of anxiety, depression, and panic disorders was assessed from a diagnostic interview. Visual acuity was assessed from a vision exam using the Nidek Auto Lensmeter Model (LM-990A) and expressed as LogMAR units.

Results: After adjusting for age, gender, race-ethnicity, body mass index, mean arterial pressure, cotinine, diabetes, and physical activity, visual acuity was not associated with panic disorder (p=0.71) or depression disorder (p=0.20), but for every 0.1 LogMAR Unit change in vision, participants had a 14% (OR=1.14; p=0.04) higher odds of having an anxiety disorder.

Limitations: The main limitation of this study was the cross-sectional design.

Conclusion: Young- and middle-age U.S. adults with worse visual function are at increased odds of having an anxiety disorder. Strategies to prevent and treat anxiety among those with worse visual function are needed. (C) 2014 Elsevier B.V. All rights reserved.

C1 [Loprinzi, Paul D.; Codey, Kathleen] Bellarmine Univ, Donna & Allan Lansing Sch Nursing & Hlth Sci, Louisville, KY 40205 USA.

RP Loprinzi, PD (reprint author), Bellarmine Univ, Donna & Allan Lansing Sch Nursing & Hlth Sci, Louisville, KY 40205 USA.

EM ploprinzi@bellarmine.edu

CR Bernabei V, 2011, AGING MENT HEALTH, V15, P467, DOI 10.1080/13607863.2011.562483

Boerner K, 2004, J GERONTOL B-PSYCHOL, V59, pP35, DOI 10.1093/geronb/59.1.P35

Bull FC, 2009, J PHYS ACT HEALTH, V6, P790, DOI 10.1123/jpah.6.6.790

Crewe JM, 2011, CLIN EXP OPHTHALMOL, V39, P336, DOI 10.1111/j.1442-9071.2010.02466.x

de Mello MT, 2013, J AFFECT DISORDERS, V149, P241, DOI 10.1016/j.jad.2013.01.035

Grue EV, 2009, SCAND J CARING SCI, V23, P635, DOI 10.1111/j.1471-6712.2008.00654.x

Han JN, 1996, J PSYCHOSOM RES, V41, P481, DOI 10.1016/S0022-3999(96)00220-6

Hassan SE, 2002, OPTOMETRY VISION SCI, V79, P697, DOI 10.1097/00006324-200211000-00007

Holladay JT, 1997, J REFRACT SURG, V13, P388

Karlsson JS, 1998, J VISUAL IMPAIR BLIN, V92, P483

Kempen GIJM, 2012, QUAL LIFE RES, V21, P1405, DOI 10.1007/s11136-011-0061-y

Kulaksizoglu IB, 2007, CNS DRUGS, V21, P473, DOI 10.2165/00023210-200721060-00004

LACEY JI, 1954, SCIENCE, V120, P1045, DOI 10.1126/science.120.3130.1045

Loprinzi P.D., 2014, J PHYS ACT IN PRESS

Loprinzi P.D., 2014, AM J HLTH P IN PRESS

Loprinzi PD, 2013, MAYO CLIN PROC, V88, P690, DOI 10.1016/j.mayocp.2013.04.008

Monteso P, 2012, J PSYCHIATR MENT HLT, V19, P426, DOI 10.1111/j.1365-2850.2011.01798.x

National Health and Nutrition Examination Survey, 2006, COMP INT DIAGN INT

Richman J, 2010, ARCH OPHTHALMOL-CHIC, V128, P1576, DOI 10.1001/archophthalmol.2010.275

Robertson N, 2006, DIABETIC MED, V23, P1110, DOI 10.1111/j.1464-5491.2006.01970.x

Scilley K, 2002, OPHTHALMOLOGY, V109, P1235, DOI 10.1016/S0161-6420(02)01060-6

Velayudhan A, 2010, Ind Psychiatry J, V19, P41, DOI 10.4103/0972-6748.77636

Vitale S, 2006, JAMA-J AM MED ASSOC, V295, P2158, DOI 10.1001/jama.295.18.2158

NR 23

TC 9

Z9 9

U1 0

U2 7

PU ELSEVIER SCIENCE BV

PI AMSTERDAM

PA PO BOX 211, 1000 AE AMSTERDAM, NETHERLANDS

SN 0165-0327

EI 1573-2517

J9 J AFFECT DISORDERS

JI J. Affect. Disord.

PD OCT 1

PY 2014

VL 167

BP 8

EP 11

DI 10.1016/j.jad.2014.05.052

PG 4

WC Clinical Neurology; Psychiatry

SC Neurosciences & Neurology; Psychiatry

GA AO4UA

UT WOS:000341335700002

PM 25082107

DA 2019-01-08

ER

PT J

AU Persson, M

Fadl, H

AF Persson, M.

Fadl, H.

TI Perinatal outcome in relation to fetal sex in offspring to mothers with

pre-gestational and gestational diabetes-a population-based study

SO DIABETIC MEDICINE

LA English

DT Article

ID RESPIRATORY-DISTRESS SYNDROME; CONGENITAL-ANOMALIES; NEONATAL OUTCOMES;

PREGNANCY; WOMEN; MELLITUS; MALFORMATIONS; INFANTS; GLUCOSE; EMBRYOS

AB Aim The objective of the present study was to investigate if perinatal outcome differs with fetal sex in pregnancies with maternal Type 1 diabetes, Type 2 diabetes or gestational diabetes.

Methods This was a population-based cohort study, with data from the Medical Birth Registry in Sweden throughout the period 1998-2007. Singleton pregnancies with maternal Type 1 diabetes (n = 4092), Type 2 diabetes (n = 412) and gestational diabetes (n = 8602) were identified based on the International Classification of Diseases, 10th edition code. For comparison, 905 565 pregnancies without diabetes were included. The primary outcome was a composite outcome, consisting of any of the following diagnoses: perinatal mortality rate, major malformation, preterm delivery, acute respiratory disorders and neonatal hypoglycaemia. Logistic regression was used to obtain odds ratios for adverse outcomes in male offspring within the diabetic and reference cohorts, respectively.

Results In pregnancies with diabetes, maternal characteristics did not differ with fetal sex, except for a higher rate of Caesarean delivery in male offspring of women with Type 1 diabetes. Male infants to mothers with Type 1 diabetes and gestational diabetes had significantly increased odds of respiratory disorders [adjusted odds ratio (confidence interval) Type 1 diabetes: 1.50 (1.12-2.02); gestational diabetes: 1.81 (1.27-2.57)]. Male infants to mothers with gestational diabetes also had significantly increased odds of major malformations [adjusted odds ratio: 1.44 (1.07-1.93)]. In offspring of mothers with Type 2 diabetes, odds ratios of most outcomes were higher in male infants; however, not significantly different from female infants. In pregnancies without diabetes, male infants had significantly higher odds of all adverse outcomes, except perinatal mortality rate.

Conclusion The risk of adverse perinatal outcome in offspring of mothers with Type 1 diabetes and gestational diabetes did not differ by sex, except for a higher risk in male infants for respiratory disorders. The risk of major malformations was also significantly increased in male offspring to mothers with gestational diabetes. In offspring of mothers with Type 2 diabetes, no significant differences between sexes were found.

C1 [Persson, M.] Karolinska Inst, Karolinska Univ Hosp, Dept Med, Clin Epidemiol Unit, Stockholm, Sweden.

[Fadl, H.] Univ Orebro, Dept Obstet & Gynecol, Sch Hlth & Med Sci, SE-70182 Orebro, Sweden.

RP Persson, M (reprint author), Karolinska Inst, Karolinska Univ Hosp, Dept Med, Clin Epidemiol Unit, Stockholm, Sweden.

EM Martina.Persson@ki.se

FU Stockholm City Council

FX This work was supported by unrestricted grants from the Stockholm City

Council.

CR Anderberg E, 2007, ACTA OBSTET GYN SCAN, V86, P1432, DOI 10.1080/00016340701532444

[Anonymous], 2003, SUMM CONT QUAL

Bracero LA, 1996, GYNECOL OBSTET INVES, V41, P10, DOI 10.1159/000292026

Chaabane S, 2013, CURR DRUG SAF, V8, P128, DOI 10.2174/15748863112079990011

Di Renzo GC, 2007, GENDER MED, V4, P19, DOI 10.1016/S1550-8579(07)80004-0

Eidem I, 2010, ACTA OBSTET GYN SCAN, V89, P1403, DOI 10.3109/00016349.2010.518594

ERIKSSON UJ, 1993, DIABETES, V42, P411, DOI 10.2337/diabetes.42.3.411

Evers IM, 2009, DIABETES CARE, V32, P1194, DOI 10.2337/dc09-0367

Fadl HE, 2010, DIABETIC MED, V27, P436, DOI 10.1111/j.1464-5491.2010.02978.x

Garcia-Patterson A, 2011, DIABETIC MED, V28, P436, DOI 10.1111/j.1464-5491.2011.03227.x

Ghidini A, 2005, BJOG-INT J OBSTET GY, V112, P140, DOI 10.1111/j.1471-0528.2004.00308.x

GREENOUGH A, 1987, ACTA PAEDIATR SCAND, V76, P54, DOI 10.1111/j.1651-2227.1987.tb10414.x

HANSON U, 1990, DIABETOLOGIA, V33, P100, DOI 10.1007/BF00401047

Hogberg U, 1997, ACTA OBSTET GYN SCAN, V76, P907, DOI 10.3109/00016349709034900

Jensen DM, 2004, DIABETES CARE, V27, P2819, DOI 10.2337/diacare.27.12.2819

KHOURY MJ, 1985, AM J OBSTET GYNECOL, V151, P777, DOI 10.1016/0002-9378(85)90518-6

Kraemer S, 2000, BRIT MED J, V321, P1609, DOI 10.1136/bmj.321.7276.1609

Lisi A, 2005, AM J MED GENET A, V134A, P49, DOI 10.1002/ajmg.a.30514

Newham JJ, 2013, DIABETIC MED, V30, P1442, DOI 10.1111/dme.12293

OLVER RE, 1977, J PHYSL, V273, P58

Ostlund I, 2004, ACTA OBSTET GYN SCAN, V83, P46, DOI 10.1111/j.1600-0412.2004.00267.x

PAPAGEORGIOU AN, 1981, PEDIATRICS, V67, P614

Perez-Crespo M, 2005, MOL REPROD DEV, V72, P502, DOI 10.1002/mrd.20366

Persson B, 2005, FRONT DIABETES, V17, P288

Persson M, 2012, BJOG-INT J OBSTET GY, V119, P565, DOI 10.1111/j.1471-0528.2012.03277.x

Persson M, 2012, BMJ OPEN, V2, DOI 10.1136/bmjopen-2011-000601

Persson M, 2009, DIABETES CARE, V32, P2005, DOI 10.2337/dc09-0656

Tennant PWG, 2011, BIRTH DEFECTS RES A, V91, P894, DOI 10.1002/bdra.22846

TIFFIN GJ, 1991, J REPROD FERTIL, V93, P125

Tundidor D, 2012, GENDER MED, V9, P411, DOI 10.1016/j.genm.2012.09.002

Vatten LJ, 2004, EARLY HUM DEV, V76, P47, DOI 10.1016/j.earlhumdev.2003.10.006

NR 31

TC 13

Z9 13

U1 0

U2 2

PU WILEY-BLACKWELL

PI HOBOKEN

PA 111 RIVER ST, HOBOKEN 07030-5774, NJ USA

SN 0742-3071

EI 1464-5491

J9 DIABETIC MED

JI Diabetic Med.

PD SEP

PY 2014

VL 31

IS 9

BP 1047

EP 1054

DI 10.1111/dme.12479

PG 8

WC Endocrinology & Metabolism

SC Endocrinology & Metabolism

GA AS1AN

UT WOS:000344008700006

PM 24773081

DA 2019-01-08

ER

PT J

AU Tovote, KA

Fleer, J

Snippe, E

Peeters, ACTM

Emmelkamp, PMG

Sanderman, R

Links, TP

Schroevers, MJ

AF Tovote, K. Annika

Fleer, Joke

Snippe, Evelien

Peeters, Anita C. T. M.

Emmelkamp, Paul M. G.

Sanderman, Robbert

Links, Thera P.

Schroevers, Maya J.

TI Individual Mindfulness-Based Cognitive Therapy and Cognitive Behavior

Therapy for Treating Depressive Symptoms in Patients With Diabetes:

Results of a Randomized Controlled Trial

SO DIABETES CARE

LA English

DT Article

ID PRIMARY-CARE PATIENTS; TREATMENT PREFERENCES; MENTAL-HEALTH;

METAANALYSIS; INTERVENTION; OUTPATIENTS; DISTRESS; MELLITUS; ANXIETY;

FUTURE

AB OBJECTIVE

Depression is a common comorbidity of diabetes, undesirably affecting patients' physical and mental functioning. Psychological interventions are effective treatments for depression in the general population as well as in patients with a chronic disease. The aim of this study was to assess the efficacy of individual mindfulness-based cognitive therapy (MBCT) and individual cognitive behavior therapy (CBT) in comparison with awaiting-list control condition for treating depressive symptoms in adults with type 1 or type 2 diabetes.

RESEARCH DESIGN AND METHODS

In this randomized controlled trial, 94 outpatients with diabetes and comorbid depressive symptoms (i.e., Beck Depression Inventory-II [ BDI-II] >= 14) were randomized to MBCT (n = 31), CBT (n = 32), or waiting list (n = 31). All participants completed written questionnaires and interviews at pre- and postmeasurement (3 months later). Primary outcome measure was severity of depressive symptoms (BDI-II and Toronto Hamilton Depression Rating Scale). Anxiety (Generalized Anxiety Disorder 7), well-being (Well-Being Index), diabetes-related distress (Problem Areas In Diabetes), and HbA(1c) levels were assessed as secondary outcomes.

RESULTS

Results showed that participants receiving MBCT and CBT reported significantly greater reductions in depressive symptoms compared with patients in the waiting-list control condition (respectively, P = 0.004 and P < 0.001; d = 0.80 and 1.00; clinically relevant improvement 26% and 29% vs. 4%). Both interventions also had significant positive effects on anxiety, well-being, and diabetes-related distress. No significant effect was found on HbA(1c) values.

CONCLUSIONS

Both individual MBCT and CBT are effective in improving a range of psychological symptoms in individuals with type 1 and type 2 diabetes.

C1 [Tovote, K. Annika; Fleer, Joke; Snippe, Evelien; Sanderman, Robbert; Schroevers, Maya J.] Univ Groningen, Univ Med Ctr Groningen, Dept Hlth Psychol, Groningen, Netherlands.

[Peeters, Anita C. T. M.] Hosp Rivierenland Tiel, Dept Internal Med, Tiel, Netherlands.

[Emmelkamp, Paul M. G.] Univ Amsterdam, Dept Clin Psychol, NL-1018 WB Amsterdam, Netherlands.

[Emmelkamp, Paul M. G.] King Abdulaziz Univ, Ctr Social & Humanities Res, Jeddah 21413, Saudi Arabia.

[Sanderman, Robbert] Univ Twente, Dept Psychol Hlth & Technol, NL-7500 AE Enschede, Netherlands.

[Links, Thera P.] Univ Groningen, Univ Med Ctr Groningen, Dept Endocrinol, Groningen, Netherlands.

RP Schroevers, MJ (reprint author), Univ Groningen, Univ Med Ctr Groningen, Dept Hlth Psychol, Groningen, Netherlands.

EM m.j.schroevers@umcg.nl

RI Emmelkamp, Paul/G-3274-2011

OI Emmelkamp, Paul/0000-0001-6066-5512

FU University of Groningen

FX This study was financed by the University of Groningen.

CR Anderson RJ, 2001, DIABETES CARE, V24, P1069, DOI 10.2337/diacare.24.6.1069

Bech P., 2004, QUALITY LIFE NEWSLET, V32, P15

Beck AT, 1996, J PERS ASSESS, V67, P588, DOI 10.1207/s15327752jpa6703_13

Beck AT, 1979, COGNITIVE THERAPY DE

Beltman MW, 2010, BRIT J PSYCHIAT, V197, P11, DOI 10.1192/bjp.bp.109.064675

Butler AC, 2006, CLIN PSYCHOL REV, V26, P17, DOI 10.1016/j.cpr.2005.07.003

Chiesa A, 2011, PSYCHIAT RES, V187, P441, DOI 10.1016/j.psychres.2010.08.011

Coelho HF, 2007, J CONSULT CLIN PSYCH, V75, P1000, DOI 10.1037/0022-006X.75.6.1000

Cohen J, 1977, STAT POWER ANAL BEHA

Cohen J, 2003, METHODOLOGICAL ISSUE, P427

Dwight-Johnson M, 2000, J GEN INTERN MED, V15, P527, DOI 10.1046/j.1525-1497.2000.08035.x

Egede LE, 2010, DIABETES RES CLIN PR, V87, P302, DOI 10.1016/j.diabres.2010.01.024

Galante J, 2013, J RES NURS, V18, P133, DOI 10.1177/1744987112466087

Griffiths K, 2009, J HEALTH PSYCHOL, V14, P675, DOI 10.1177/1359105309104911

Harkness E, 2010, DIABETES CARE, V33, P926, DOI 10.2337/dc09-1519

Hofmann SG, 2010, J CONSULT CLIN PSYCH, V78, P169, DOI 10.1037/a0018555

JACOBSON NS, 1991, J CONSULT CLIN PSYCH, V59, P12, DOI 10.1037//0022-006X.59.1.12

Kabat-Zinn J, 2003, CLIN PSYCHOL-SCI PR, V10, P144, DOI 10.1093/clipsy/bpg016

Katon WJ, 2008, AM J MED, V121, P8, DOI 10.1016/j.amjmed.2008.09.008

Khoury B, 2013, CLIN PSYCHOL REV, V33, P763, DOI 10.1016/j.cpr.2013.05.005

Lamers F, 2010, PSYCHOTHER PSYCHOSOM, V79, P217, DOI 10.1159/000313690

Lang AJ, 2005, J BEHAV MED, V28, P581, DOI 10.1007/s10865-005-9019-2

Lau MA, 2012, MINDFULNESS, V3, P318, DOI 10.1007/s12671-012-0108-3

Lustman PJ, 1998, ANN INTERN MED, V129, P613, DOI 10.7326/0003-4819-129-8-199810150-00005

Manicavasgar V, 2011, J AFFECT DISORDERS, V130, P138, DOI 10.1016/j.jad.2010.09.027

McIntyre RS, 2005, CAN MED ASSOC J, V173, P1327, DOI 10.1503/cmaj.050786

Penckofer SM, 2012, ANN BEHAV MED, V44, P192, DOI 10.1007/s12160-012-9377-2

POLONSKY WH, 1995, DIABETES CARE, V18, P754, DOI 10.2337/diacare.18.6.754

Safren SA, 2014, DIABETES CARE, V37, P625, DOI [10.2337/dc13-0816, 10.2337/dc16-er06]

Schroevers MJ, 2013, MINDFULNESS	0814

Segal Z., 2002, MINDFULNESS BASED CO

Spitzer RL, 2006, ARCH INTERN MED, V166, P1092, DOI 10.1001/archinte.166.10.1092

Strong V, 2008, LANCET, V372, P40, DOI 10.1016/S0140-6736(08)60991-5

Swift JK, 2011, J CLIN PSYCHOL, V67, P155, DOI 10.1002/jclp.20759

Tolin DF, 2010, CLIN PSYCHOL REV, V30, P710, DOI 10.1016/j.cpr.2010.05.003

Tovote K Annika, 2013, BMC Psychol, V1, P17, DOI 10.1186/2050-7283-1-17

van Bastelaar KMP, 2011, DIABETES CARE, V34, P320, DOI 10.2337/dc10-1248

Van der Feltz-Cornelis CM, 2010, GEN HOSP PSYCHIAT, V32, P380, DOI 10.1016/j.genhosppsych.2010.03.011

van Son J, 2013, DIABETES CARE, V36, P823, DOI 10.2337/dc12-1477

Welch GW, 1997, DIABETES CARE, V20, P760, DOI 10.2337/diacare.20.5.760

NR 40

TC 46

Z9 49

U1 4

U2 77

PU AMER DIABETES ASSOC

PI ALEXANDRIA

PA 1701 N BEAUREGARD ST, ALEXANDRIA, VA 22311-1717 USA

SN 0149-5992

EI 1935-5548

J9 DIABETES CARE

JI Diabetes Care

PD SEP

PY 2014

VL 37

IS 9

BP 2427

EP 2434

DI 10.2337/dc13-2918

PG 8

WC Endocrinology & Metabolism

SC Endocrinology & Metabolism

GA AN8HX

UT WOS:000340846300014

PM 24898301

OA Bronze

DA 2019-01-08

ER

PT J

AU Hayashino, Y

Mashitani, T

Tsujii, S

Ishii, H

AF Hayashino, Yasuaki

Mashitani, Tsuyoshi

Tsujii, Satoru

Ishii, Hitoshi

CA Diabet Distress Care Registry

TI Elevated Levels of hs-CRP Are Associated With High Prevalence of

Depression in Japanese Patients With Type 2 Diabetes: The Diabetes

Distress and Care Registry at Tenri (DDCRT 6)

SO DIABETES CARE

LA English

DT Article

ID C-REACTIVE PROTEIN; RANDOMIZED CONTROLLED-TRIALS; GLYCEMIC CONTROL;

METAANALYSIS; INFLAMMATION; ADULTS; RISK; SYMPTOMS; CYTOKINE; EXERCISE

AB OBJECTIVE

Because of the absence of data on the direct association between inflammation and depression in patients with diabetes, we examined the association between hs-CRP levels and the high prevalence of depression in adult patients with type 2 diabetes.

RESEARCH DESIGN AND METHODS

Cross-sectional data were obtained from 3,573 patients with type 2 diabetes recruited from a Japanese diabetes registry. A multiple logistic regression analysis adjusted for potential confounders was used to assess independent associations between hs-CRP levels and major depression, as defined by the Patient Health Questionnaire-9.

RESULTS

Mean age, BMI, and HbA(1c) levels were 66.0 years, 24.6 kg/m(2), and 7.4% (57.8 mmol/mol), respectively, and 122 patients (3.4%) suffered from major depression. In the age-and sex-adjusted model, the odds ratio (OR) for major depression was 1.86 (95% CI 1.01-3.42; P = 0.045) in the highest CRP quintile compared with that in the 3rd CRP quintile; however, this association disappeared after adjustment for other possible confounders (OR 1.58 [95% CI 0.85-2.94]; P = 0.148). Among patients with a BMI of >= 25 kg/m(2), a significant association was observed between the highest hs-CRP quintile and major depression (multivariable-adjusted OR 2.69 [95% CI 1.09-7.08]; P = 0.032).

CONCLUSIONS

We observed a significant positive association between high hs-CRP levels and depression in patients with diabetes who had a high BMI.

C1 [Hayashino, Yasuaki; Mashitani, Tsuyoshi; Tsujii, Satoru; Ishii, Hitoshi; Diabet Distress Care Registry] Tenri Hosp, Dept Endocrinol, Tenri, Nara 632, Japan.

RP Hayashino, Y (reprint author), Tenri Hosp, Dept Endocrinol, Tenri, Nara 632, Japan.

EM hayasino-y@umin.net

OI Tsujii, Satoru/0000-0002-1834-6515; Hayashino,

Yasuaki/0000-0002-2932-8478

FU Manpei Suzuki Diabetes Foundation; JSPS KAKENHI [25460641]

FX This study was partially supported by the Manpei Suzuki Diabetes

Foundation and JSPS KAKENHI (grant 25460641).

CR Anderson RJ, 2001, DIABETES CARE, V24, P1069, DOI 10.2337/diacare.24.6.1069

Bertoni AG, 2010, DIABETES CARE, V33, P804, DOI 10.2337/dc09-1679

Brummett BH, 2010, PSYCHOSOM MED, V72, P333, DOI 10.1097/PSY.0b013e3181d2f104

Champaneri S, 2010, CURR DIABETES REP, V10, P396, DOI 10.1007/s11892-010-0148-9

Craig CL, 2003, MED SCI SPORT EXER, V35, P1381, DOI 10.1249/01.MSS.0000078924.61453.FB

Dantzer R, 2007, BRAIN BEHAV IMMUN, V21, P153, DOI 10.1016/j.bbi.2006.09.006

de Groot M, 2001, PSYCHOSOM MED, V63, P619, DOI 10.1097/00006842-200107000-00015

Dentino AN, 1999, J AM GERIATR SOC, V47, P6, DOI 10.1111/j.1532-5415.1999.tb01894.x

DOSEMECI M, 1990, AM J EPIDEMIOL, V132, P746, DOI 10.1093/oxfordjournals.aje.a115716

Doyle TA, 2013, J PSYCHOSOM RES, V75, P419, DOI 10.1016/j.jpsychores.2013.08.006

Elovainio M, 2009, PSYCHOSOM MED, V71, P423, DOI 10.1097/PSY.0b013e31819e333a

Gellis ZD, 2012, AM J CARDIOL, V110, P1219, DOI 10.1016/j.amjcard.2012.06.021

Gimeno D, 2009, PSYCHOL MED, V39, P413, DOI 10.1017/S0033291708003723

Golden SH, 2008, JAMA-J AM MED ASSOC, V299, P2751, DOI 10.1001/jama.299.23.2751

Gonzalez JS, 2008, DIABETES CARE, V31, P2398, DOI 10.2337/dc08-1341

Hayashino Y, 2014, METABOLISM, V63, P431, DOI 10.1016/j.metabol.2013.08.018

Hayashino Y, 2012, DIABETES RES CLIN PR, V98, P349, DOI 10.1016/j.diabres.2012.10.004

Hayashino Y, 2012, DIABETES RES CLIN PR, V97, P405, DOI 10.1016/j.diabres.2012.04.005

Hood KK, 2012, DIABETES CARE, V35, P2443, DOI 10.2337/dc11-2329

HOTAMISLIGIL GS, 1993, SCIENCE, V259, P87, DOI 10.1126/science.7678183

Kobrosly R, 2010, NEUROTOXICOLOGY, V31, P126, DOI 10.1016/j.neuro.2009.10.005

Kroenke K, 2001, J GEN INTERN MED, V16, P606, DOI 10.1046/j.1525-1497.2001.016009606.x

Maes M, 1999, NEUROPSYCHOBIOLOGY, V39, P1, DOI 10.1159/000026552

Mashitani T, 2013, DIABETES RES CLIN PR, V100, P189, DOI 10.1016/j.diabres.2013.03.006

NHS National Institute for Health and Clinical Excellence (NICE), 2004, TYP 1 DIAB DIAGN MAN, V15

Pickup JC, 2004, DIABETES CARE, V27, P813, DOI 10.2337/diacare.27.3.813

Pradhan AD, 2001, JAMA-J AM MED ASSOC, V286, P327, DOI 10.1001/jama.286.3.327

Raison CL, 2006, TRENDS IMMUNOL, V27, P24, DOI 10.1016/j.it.2005.11.006

Reichenberg A, 2001, ARCH GEN PSYCHIAT, V58, P445, DOI 10.1001/archpsyc.58.5.445

Schellenberg ES, 2013, ANN INTERN MED, V159, P543, DOI 10.7326/0003-4819-159-8-201310150-00007

Seino Y, 2010, J DIABETES INVEST, V1, P212, DOI 10.1111/j.2040-1124.2010.00074.x

SPITZER RL, 1992, ARCH GEN PSYCHIAT, V49, P624

Spitzer RL, 1986, BIOMETRICS RES DEV, P4

Spranger J, 2003, DIABETES, V52, P812, DOI 10.2337/diabetes.52.3.812

Stewart JC, 2009, BRAIN BEHAV IMMUN, V23, P936, DOI 10.1016/j.bbi.2009.04.011

Tsujii S, 2012, DIABETIC MED, V29, P1451, DOI 10.1111/j.1464-5491.2012.03647.x

Umpierre D, 2011, JAMA-J AM MED ASSOC, V305, P1790, DOI 10.1001/jama.2011.576

van Dooren FEP, 2013, PLOS ONE, V8, DOI 10.1371/journal.pone.0057058

van Steenbergen-Weijenburg KM, 2010, BMC HEALTH SERV RES, V10, DOI 10.1186/1472-6963-10-235

Wang X, 2013, DIABETES CARE, V36, P166, DOI 10.2337/dc12-0702

Williams LS, 2005, STROKE, V36, P635, DOI 10.1161/01.STR.0000155688.18207.33

NR 41

TC 19

Z9 19

U1 1

U2 8

PU AMER DIABETES ASSOC

PI ALEXANDRIA

PA 1701 N BEAUREGARD ST, ALEXANDRIA, VA 22311-1717 USA

SN 0149-5992

EI 1935-5548

J9 DIABETES CARE

JI Diabetes Care

PD SEP

PY 2014

VL 37

IS 9

BP 2459

EP 2465

DI 10.2337/dc13-2312

PG 7

WC Endocrinology & Metabolism

SC Endocrinology & Metabolism

GA AN8HX

UT WOS:000340846300018

PM 24969579

OA Bronze

DA 2019-01-08

ER

PT J

AU Stuckey, HL

Mullan-Jensen, CB

Reach, G

Burns, KK

Piana, N

Vallis, M

Wens, J

Willaing, I

Skovlund, SE

Peyrot, M

AF Stuckey, Heather L.

Mullan-Jensen, Christine B.

Reach, Gerard

Burns, Katharina Kovacs

Piana, Natalia

Vallis, Michael

Wens, Johan

Willaing, Ingrid

Skovlund, Soren E.

Peyrot, Mark

TI Personal Accounts of the Negative and Adaptive Psychosocial Experiences

of People With Diabetes in the Second Diabetes Attitudes, Wishes and

Needs (DAWN2) Study

SO DIABETES CARE

LA English

DT Article

ID RANDOMIZED-CONTROLLED-TRIAL; GLYCEMIC CONTROL; PSYCHOLOGICAL DISTRESS;

CARE; DEPRESSION; OUTCOMES; HEALTH; MANAGEMENT; MELLITUS; INTERVENTION

AB OBJECTIVE

To identify the psychosocial experiences of diabetes, including negative accounts of diabetes and adaptive ways of coping from the perspective of the person with diabetes.

RESEARCH DESIGN AND METHODS

Participants were 8,596 adults (1,368 with type 1 diabetes and 7,228 with type 2 diabetes) in the second Diabetes Attitudes, Wishes and Needs (DAWN2) study. Qualitative data were responses to open-ended survey questions about successes, challenges, and wishes for improvement in living with diabetes and about impactful experiences. Emergent coding developed with multinational collaborators identified thematic content about psychosocial aspects. The kappa measure of interrater reliability was 0.72.

RESULTS

Analysis identified two negative psychosocial themes: 1) anxiety/fear, worry about hypoglycemia and complications of diabetes, depression, and negative moods/hopelessness and 2) discrimination at work and public misunderstanding about diabetes. Two psychosocial themes demonstrated adaptive ways of coping with diabetes: 1) having a positive outlook and sense of resilience in the midst of having diabetes and 2) receiving psychosocial support through caring and compassionate family, friends, health care professionals, and other people with diabetes.

CONCLUSIONS

The personal accounts give insight into the psychosocial experiences and coping strategies of people with diabetes and can inform efforts to meet those needs and capitalize on strengths.

C1 [Stuckey, Heather L.] Penn State Univ, Dept Med, Coll Med, Hershey, PA 17033 USA.

[Mullan-Jensen, Christine B.; Skovlund, Soren E.] Novo Nordisk AS, Publ Hlth Evidence & Insights, Global Publ Affairs, Copenhagen, Denmark.

[Reach, Gerard] Univ Paris 13, Sorbonne Paris Cite, Dept Endocrinol Diabet & Metab Dis, Avicenne Hosp, Bobigny, France.

[Reach, Gerard] Univ Paris 13, Sorbonne Paris Cite, Ctr Rech Nutr Humaine Ile De France, Bobigny, France.

[Burns, Katharina Kovacs] Univ Alberta, Edmonton Clin Hlth Acad, Interdisciplinary Hlth Res Acad, Edmonton, AB, Canada.

[Piana, Natalia] Univ Perugia, Hlth Lifestyle Inst, Ctr Univ Ric Interdipartimentale Attivita Motoria, I-06100 Perugia, Italy.

[Vallis, Michael] Dalhousie Univ, Dept Family Med, Halifax, NS, Canada.

[Vallis, Michael] Dalhousie Univ, Dept Psychiat, Halifax, NS, Canada.

[Wens, Johan] Univ Antwerp, Dept Med & Hlth Sci Primary & Interdisciplinary C, B-2020 Antwerp, Belgium.

[Willaing, Ingrid] Steno Diabet Ctr, Steno Hlth Promot Ctr, DK-2820 Gentofte, Denmark.

[Peyrot, Mark] Loyola Univ Maryland, Dept Sociol, Baltimore, MD USA.

RP Stuckey, HL (reprint author), Penn State Univ, Dept Med, Coll Med, Hershey, PA 17033 USA.

EM hstuckey@hmc.psu.edu

OI Vallis, Michael/0000-0002-0165-5936; Wens, Johan/0000-0002-0229-9064;

Willaing, Ingrid/0000-0002-3082-8816

FU Novo Nordisk; Novo Nordisk A/S; Eli Lilly; Sanofi; Merck Serono;

Novartis; Bristol-Myers Squibb; Janssen; Abbott; LifeScan; Roche

Diagnostics; Bayer Diagnostics; Dexcom; AbbVie; Pfizer; BD; Merck;

Bayer; Bristol-Myers Squibb-Amylin; Roche-Genentech; Tethys

FX Novo Nordisk funded the DAWN2 study. H. L. S. received funding from Novo

Nordisk for her role in developing the coding scheme and

training/supervising the coders. C.B.M.-J.'s salary is paid by Novo

Nordisk A/S. G.R. has received travel reimbursement from Novo Nordisk to

attend the International DAWN2 Publication Planning Committee meetings;

received speaking fees from Novo Nordisk, Eli Lilly, Sanofi, Merck

Serono, Novartis, Bristol-Myers Squibb, Janssen, Abbott, LifeScan, Roche

Diagnostics, Bayer Diagnostics, and Dexcom; and acted as an advisory

board member for LifeScan. K. K. B. has received travel reimbursement

from Novo Nordisk to attend the International DAWN2 Publication Planning

Committee meetings. M. V. has research funding from AbbVie and Pfizer

and receives speaking fees from Novo Nordisk, AbbVie, Sanofi, BD, Merck,

and Bayer. J.W. has acted as an advisory board member for Eli Lilly,

Bristol-Myers Squibb, AstraZeneca, and Novo Nordisk. I. W. is employed

by Steno Diabetes Center A/S, a research hospital working in the Danish

National Health Service and owned by Novo Nordisk A/S. S. E. S. is an

employee of Novo Nordisk A/S. M. P. has recently received research

grants and/or consulting fees from Bristol-Myers Squibb-Amylin, Eli

Lilly, Novo Nordisk, Roche-Genentech, and Tethys; has received speaking

honoraria from Novo Nordisk; has participated in advisory panels for

Novo Nordisk and Roche; and has received financial support from Novo

Nordisk for his participation as principal investigator for the DAWN2

study. No other potential conflicts of interest relevant to this article

were reported.

CR Ando M, 2005, PSYCHOL REP, V96, P129

Assal J P, 1981, Journ Annu Diabetol Hotel Dieu, P223

Bogner HR, 2012, ANN FAM MED, V10, P15, DOI 10.1370/afm.1344

Brands AMA, 2007, J INT NEUROPSYCH SOC, V13, P288, DOI 10.1017/S1355617707070312

Braun V., 2006, QUALITATIVE RES PSYC, V3, P77, DOI DOI 10.1191/1478088706QP0630A

Chen SM, 2012, INT J NURS STUD, V49, P637, DOI 10.1016/j.ijnurstu.2011.11.011

Egede LE, 2012, CURR PSYCHIAT REP, V14, P15, DOI 10.1007/s11920-011-0240-0

Fisher EB, 2012, J AFFECT DISORDERS, V142, pS56, DOI 10.1016/S0165-0327(12)70009-5

Fisher L, 2010, DIABETES CARE, V33, P1034, DOI 10.2337/dc09-2175

Fortmann AL, 2011, HEALTH PSYCHOL, V30, P251, DOI 10.1037/a0022850

Gask L, 2011, CHRONIC ILLN, V7, P239, DOI 10.1177/1742395311403636

Gonzalez JS, 2008, DIABETES CARE, V31, P2398, DOI 10.2337/dc08-1341

Hojat M, 2011, ACAD MED, V86, P359, DOI 10.1097/ACM.0b013e3182086fe1

Karlsen B, 2012, J ADV NURS, V68, P391, DOI 10.1111/j.1365-2648.2011.05751.x

Kim SS, 2004, EVAL HEALTH PROF, V27, P237, DOI 10.1177/0163278704267037

LANDIS JR, 1977, BIOMETRICS, V33, P159, DOI 10.2307/2529310

Leventhal H, 1997, TREATMENT COMPLIANCE, P21

Lustman PJ, 2005, J DIABETES COMPLICAT, V19, P113, DOI 10.1016/j.jdiacomp.2004.01.002

Mirzaei M, 2013, BMC HEALTH SERV RES, V13, DOI 10.1186/1472-6963-13-251

Mosnier-Pudar H, 2009, DIABETES METAB, V35, P220, DOI 10.1016/j.diabet.2009.02.001

Nicolucci A, 2013, DIABETIC MED, V30, P767, DOI 10.1111/dme.12245

Peyrot M, 2006, DIABETES CARE, V29, P1256, DOI 10.2337/dc05-2444

Peyrot M, 2005, DIABETIC MED, V22, P1379, DOI 10.1111/j.1464-5491.2005.01644.x

Peyrot M, 2013, DIABETES RES CLIN PR, V99, P174, DOI 10.1016/j.diabres.2012.11.016

Polonsky William H, 2002, Curr Diab Rep, V2, P153, DOI 10.1007/s11892-002-0075-5

Porojan M, 2009, Rom J Intern Med, V47, P409

Robertson SM, 2012, PSYCHOSOMATICS, V53, P1, DOI 10.1016/j.psym.2011.09.008

Rubin RR, 2006, DIABETES CARE, V29, P1249, DOI 10.2337/dc05-2494

Saatci E, 2010, HEALTH QUAL LIFE OUT, V8, DOI 10.1186/1477-7525-8-67

Schunk M, 2012, DIABETIC MED, V29, P646, DOI 10.1111/j.1464-5491.2011.03465.x

Shubrook JH, 2011, J AM OSTEOPATH ASSOC, V111, P531

Sultan S, 2003, DIABETES METAB, V29, pS21

Sultan S, 2011, PSYCHOL HEALTH, V26, P101, DOI 10.1080/08870441003703226

Tuncay T, 2008, HEALTH QUAL LIFE OUT, V6, DOI 10.1186/1477-7525-6-79

Weinger K, 2011, ARCH INTERN MED, V171, P1990, DOI 10.1001/archinternmed.2011.502

NR 35

TC 35

Z9 35

U1 0

U2 27

PU AMER DIABETES ASSOC

PI ALEXANDRIA

PA 1701 N BEAUREGARD ST, ALEXANDRIA, VA 22311-1717 USA

SN 0149-5992

EI 1935-5548

J9 DIABETES CARE

JI Diabetes Care

PD SEP

PY 2014

VL 37

IS 9

BP 2466

EP 2474

DI 10.2337/dc13-2536

PG 9

WC Endocrinology & Metabolism

SC Endocrinology & Metabolism

GA AN8HX

UT WOS:000340846300019

PM 24973437

OA Bronze

DA 2019-01-08

ER

PT J

AU Strandberg, RB

Graue, M

Wentzel-Larsen, T

Peyrot, M

Rokne, B

AF Strandberg, Ragnhild Bjarkoy

Graue, Mark

Wentzel-Larsen, Tore

Peyrot, Mark

Rokne, Berk

TI Relationships of diabetes-specific emotional distress, depression,

anxiety, and overall well-being with HbA(1c) in adult persons with type

1 diabetes

SO JOURNAL OF PSYCHOSOMATIC RESEARCH

LA English

DT Article

DE Depression; Anxiety; Diabetes-specific emotional distress; HbA(1c); Type

1 diabetes

ID PROBLEM AREAS; HOSPITAL ANXIETY; GLYCEMIC CONTROL; SELF-CARE; CLINICAL

DEPRESSION; NEEDS 2ND; SCALE; VALIDITY; HEALTH; ASSOCIATION

AB Objective: Emotional problems are common in adults with diabetes, and knowledge about how different indicators of emotional problems are related with glycemic control is required. The aim was to examine the relationships of diabetes-specific emotional distress, depression, anxiety, and overall well-being with glycosylated hemoglobin (HbA(1c)).

Methods: Of the 319 adults with type 1 diabetes attending the endocrinology outpatient clinic at a university hospital in Norway, 235 (74%) completed the Diabetes Distress Scale, the Problem Areas in Diabetes Survey, the Hospital Anxiety and Depression Scale, and the World Health Organization-Five Well-Being Index. Blood samples were taken at the time of data collection to determine HbA(1c). Regression analyses examined associations of diabetes-specific emotional distress, anxiety, depression, and overall well-being with HbA(1c). The relationship between diabetes-specific emotional distress and HbA(1c) was tested for nonlinearity.

Results: Diabetes-specific emotional distress was related to glycemic control (DDS total: unstandardized coefficient = 0.038, P < .001; PAID total: coefficient = 0.021, P = .007), but depression, anxiety, and overall well-being were not. On the DDS, only regimen-related distress was independently related to HbA(1c) (coefficient = 0.056, P < .001). A difference of 0.5 standard deviation of baseline regimen distress is associated with a difference of 0.6 in HbA(1c). No significant nonlineatity was detected in the relationship between diabetes-specific distress and HbA(1c).

Conclusions: To stimulate adequate care strategies, health personnel should acknowledge depression and diabetes-specific emotional distress as different conditions in clinical consultations. Addressing diabetes-specific emotional distress, in particular regimen distress, in clinical consultation might improve glycemic control. 2014 The Authors. Published by Elsevier Inc.

C1 [Strandberg, Ragnhild Bjarkoy; Graue, Mark] Bergen Univ Coll, Ctr Evidence Based Practice, N-5020 Bergen, Norway.

[Strandberg, Ragnhild Bjarkoy; Rokne, Berk] Univ Bergen, Fac Med & Dent, Dept Global Publ Hlth & Primary Care, N-5020 Bergen, Norway.

[Graue, Mark] Haukeland Hosp, Dept Paediat, N-5021 Bergen, Norway.

[Wentzel-Larsen, Tore] Norwegian Ctr Violence & Traumat Stress Studies, Oslo, Norway.

[Wentzel-Larsen, Tore] Ctr Child & Adolescent Mental Hlth, Oslo, Norway.

[Wentzel-Larsen, Tore] Haukeland Hosp, Clin Res Ctr, Oslo, Norway.

[Peyrot, Mark] Loyola Univ, Dept Sociol, Baltimore, MD USA.

[Rokne, Berk] Haukeland Hosp, Dept Res & Dev, Oslo, Norway.

RP Strandberg, RB (reprint author), Bergen Univ Coll, Ctr Evidence Based Practice, POB 7030, N-5020 Bergen, Norway.

EM rob@hib.no

CR A. American Diabetes, 2013, DIABETES CARE, V36, P1033, DOI DOI 10.2337/DC12-2625

Aikens JE, 2012, DIABETES CARE, V35, P2472, DOI 10.2337/dc12-0181

Ali MK, 2013, NEW ENGL J MED, V368, P1613, DOI 10.1056/NEJMsa1213829

American Diabetes Association, 2014, DIABETES CARE S1, V37, pS14, DOI DOI 10.2337/DC14-S014

ATKINSON MA, 1994, NEW ENGL J MED, V331, P1428

Atkinson MA, 2014, LANCET, V383, P69, DOI 10.1016/S0140-6736(13)60591-7

Bech P, 2003, INT J METH PSYCH RES, V12, P85, DOI 10.1002/mpr.145

Beverly EA, 2012, DIABETES CARE, V35, P1466, DOI [10.2337/dc11-2422, 10.2337/dc12-0661]

Bjelland I, 2002, J PSYCHOSOM RES, V52, P69, DOI 10.1016/S0022-3999(01)00296-3

Bluestone JA, 2010, NATURE, V464, P1293, DOI 10.1038/nature08933

Cohen J., 1969, STAT POWER ANAL BEHA

Cooper John G, 2013, Tidsskr Nor Laegeforen, V133, P2257, DOI 10.4045/tidsskr.13.0153

Coyne JC, 2012, J PSYCHOSOM RES, V72, P173, DOI 10.1016/j.jpsychores.2011.12.003

de Wit M, 2007, DIABETES CARE, V30, P2003, DOI 10.2337/dc07-0447

Doyle F, 2012, J PSYCHOSOM RES, V73, P74, DOI 10.1016/j.jpsychores.2012.04.003

Fayers P. M., 2000, QUALITY LIFE ASSESSM

Fisher L, 2014, DIABETIC MED, V31, P764, DOI 10.1111/dme.12428

Fisher L, 2007, DIABETES CARE, V30, P542, DOI 10.2337/dc06-1614

Fisher L, 2012, DIABETES CARE, V35, P259, DOI 10.2337/dc11-1572

Fisher L, 2010, DIABETES CARE, V33, P1034, DOI 10.2337/dc09-2175

Fisher L, 2010, DIABETES CARE, V33, P23, DOI 10.2337/dc09-1238

Gonzalez JS, 2008, DIABETOLOGIA, V51, P1822, DOI 10.1007/s00125-008-1113-x

Gonzalez JS, 2011, DIABETES CARE, V34, P236, DOI 10.2337/dc10-1970

Graue M, 2012, INT J NURS STUD, V49, P174, DOI 10.1016/j.ijnurstu.2011.08.007

Harrell F, 2001, REGRESSION MODELING

Hendrieckx C, 2014, DIABETES RES CLIN PR, V103, P430, DOI 10.1016/j.diabres.2013.12.005

Hermanns N, 2006, DIABETOLOGIA, V49, P469, DOI 10.1007/s00125-005-0094-2

Hessler D, 2014, DIABETES CARE, V37, P617, DOI 10.2337/dc13-0762

Holt RIG, 2013, DIABETIC MED, V30, P789, DOI 10.1111/dme.12242

Hudson JL, 2014, J PSYCHOSOM RES, V76, P265, DOI 10.1016/j.jpsychores.2014.02.004

Joensen LE, 2013, DIABETIC MED, V30, P1132, DOI 10.1111/dme.12241

Lloyd CE, 2010, DIABETIC MED, V27, P234, DOI 10.1111/j.1464-5491.2009.02896.x

Moskowitz JT, 2008, HEALTH PSYCHOL, V27, pS73, DOI 10.1037/0278-6133.27.1.S73

Mykletun A, 2001, BRIT J PSYCHIAT, V179, P540, DOI 10.1192/bjp.179.6.540

Nicolucci A, 2013, DIABETIC MED, V30, P767, DOI 10.1111/dme.12245

Peyrot M, 1999, J HEALTH SOC BEHAV, V40, P141, DOI 10.2307/2676370

Peyrot M, 2008, DIABETES TECHNOL THE, V10, P461, DOI 10.1089/dia.2008.0031

POLONSKY WH, 1995, DIABETES CARE, V18, P754, DOI 10.2337/diacare.18.6.754

Polonsky WH, 2005, DIABETES CARE, V28, P626, DOI 10.2337/diacare.28.3.626

Pouwer F, 2006, PATIENT EDUC COUNS, V60, P235, DOI 10.1016/j.pec.2005.01.009

Reddy J, 2013, PSYCHOSOMATICS, V54, P44, DOI 10.1016/j.psym.2012.08.004

Robertson SM, 2012, PSYCHOSOMATICS, V53, P1, DOI 10.1016/j.psym.2011.09.008

Rubin RR, 1999, DIABETES-METAB RES, V15, P205, DOI 10.1002/(SICI)1520-7560(199905/06)15:3<205::AID-DMRR29>3.0.CO;2-O

Snaith R Philip, 2003, Health Qual Life Outcomes, V1, P29, DOI 10.1186/1477-7525-1-29

Snoek FJ, 2000, DIABETES CARE, V23, P1305, DOI 10.2337/diacare.23.9.1305

Snoek FJ, 2012, DIABETES CARE, V35, P2128, DOI 10.2337/dc11-1326

The Diabetes Control and Complications Trial Research Group, 1993, NEW ENGL J MED, V329, P977, DOI [10.1056/NEJM199309303291401, DOI 10.1056/NEJM199309303291401]

WATSON D, 1995, J ABNORM PSYCHOL, V104, P3, DOI 10.1037/0021-843X.104.1.3

Welch G, 2003, DIABETIC MED, V20, P69, DOI 10.1046/j.1464-5491.2003.00832.x

Wild S, 2004, DIABETES CARE, V27, P1047, DOI 10.2337/diacare.27.5.1047

Wilkinson A, 2014, INT J NURS STUD, V51, P111, DOI 10.1016/j.ijnurstu.2013.01.006

ZIGMOND AS, 1983, ACTA PSYCHIAT SCAND, V67, P361, DOI 10.1111/j.1600-0447.1983.tb09716.x

NR 52

TC 54

Z9 55

U1 0

U2 30

PU PERGAMON-ELSEVIER SCIENCE LTD

PI OXFORD

PA THE BOULEVARD, LANGFORD LANE, KIDLINGTON, OXFORD OX5 1GB, ENGLAND

SN 0022-3999

EI 1879-1360

J9 J PSYCHOSOM RES

JI J. Psychosomat. Res.

PD SEP

PY 2014

VL 77

IS 3

BP 174

EP 179

DI 10.1016/j.jpsychores.2014.06.015

PG 6

WC Psychiatry

SC Psychiatry

GA AO2DU

UT WOS:000341126500003

PM 25149027

OA Other Gold, Green Published

DA 2019-01-08

ER

PT J

AU Jokela, M

Hakulinen, C

Singh-Manoux, A

Kivimaki, M

AF Jokela, M.

Hakulinen, C.

Singh-Manoux, A.

Kivimaeki, M.

TI Personality change associated with chronic diseases: pooled analysis of

four prospective cohort studies

SO PSYCHOLOGICAL MEDICINE

LA English

DT Article

DE Chronic disease; longitudinal studies; meta-analyses; morbidity;

personality development

ID MENTAL-HEALTH; LIFE-SPAN; PSYCHOLOGICAL ADJUSTMENT; POSTTRAUMATIC

GROWTH; HEART-DISEASE; METAANALYSIS; MORTALITY; CONSCIENTIOUSNESS;

SATISFACTION; DEPRESSION

AB Background. Common chronic conditions, such as heart disease and cancer, are associated with increased psychological distress, functional limitations and shortened life expectancy, but whether these diseases alter aspects of personality remains unclear.

Method. To examine whether the onset of heart disease, stroke, diabetes, cancer, hypertension, arthritis and respiratory disease is associated with subsequent changes in personality traits of the five-factor model, we pooled data from the Health and Retirement Study, the Midlife in the United States Survey, and the graduate and sibling samples of the Wisconsin Longitudinal Study for an individual-participant meta-analysis (total n=17 493; mean age at baseline 55.8 years).

Results. After adjustment for age, we observed consistent decreases in extraversion [-0.25 T-scores per one disease; 95% confidence interval (CI) -0.40 to -0.10], emotional stability (-0.40, 95% CI -0.61 to -0.19), conscientiousness (-0.44, 95% CI -0.57 to -0.30) and openness to experience (-0.25, 95% CI -0.37 to -0.13) but not in agreeableness (-0.05, 95% CI -0.19 to 0.08) after the onset of chronic diseases. The onset of each additional chronic disease accelerated the average age-related personality change by 2.5 years in decreasing extraversion, 5.5 years in decreasing conscientiousness, and 1.6 years in decreasing openness to experience, and attenuated the increasing levels of emotional stability by 1.9 years. Co-morbid conditions were associated with larger changes than single diseases, suggesting a dose-response association between morbidity and personality change.

Conclusions. These results support the hypothesis that chronic diseases influence personality development in adulthood.

C1 [Jokela, M.; Hakulinen, C.] Univ Helsinki, Inst Behav Sci, FIN-00014 Helsinki, Finland.

[Jokela, M.] Univ Cambridge, Dept Psychol, Cambridge, England.

[Singh-Manoux, A.; Kivimaeki, M.] UCL, Res Dept Epidemiol & Publ Hlth, London, England.

[Singh-Manoux, A.] INSERM, U1018, Ctr Res Epidemiol & Populat Hlth, Villejuif, France.

RP Jokela, M (reprint author), Univ Helsinki, Inst Behav Sci, Siltavuorenpenger 1A,POB 9, FIN-00014 Helsinki, Finland.

EM markus.jokela@helsinki.fi

RI Singh-Manoux, Archana/F-6804-2013; Jokela, Markus/A-4669-2009

OI Singh-Manoux, Archana/0000-0002-1244-5037; Jokela,

Markus/0000-0003-0117-0012; Kivimaki, Mika/0000-0002-4699-5627;

Hakulinen, Christian/0000-0001-8629-9960

FU National Institute on Aging [AG-9775, AG-21079]; Vilas Estate Trust;

National Science Foundation; Spencer Foundation; Graduate School of the

University of Wisconsin-Madison; Academy of Finland [124322, 124271,

132944]; Medical Research Council [MRC K013351]; US National Institutes

of Health (NIH) [R01HL036310, R01AG034454]; Finnish Work Environment

Fund; Research Foundation of the University of Helsinki; Finnish

Cultural Foundation; National Institute on Aging, NIH [R01AG034454,

R01AG013196]; Economic and Social Research Council [ES/J023299/1];

Medical Research Council [MR/K013351/1]

FX The authors wish to gratefully thank the original collectors of the

data, and the ESDS (Economic and Social Data Service;

http://www.esds.ac.uk/) and the Inter-university Consortium for

Political and Social Research (http://www.icpsr.umich.edu) for making

the data available. The research uses data from the Wisconsin

Longitudinal Study (WLS) of the University of Wisconsin-Madison. Since

1991, the WLS has been supported principally by the National Institute

on Aging (AG-9775 and AG-21079), with additional support from the Vilas

Estate Trust, the National Science Foundation, the Spencer Foundation

and the Graduate School of the University of Wisconsin-Madison. A public

use file of data from the WLS is available from the Wisconsin

Longitudinal Study, University of Wisconsin-Madison, 1180 Observatory

Drive, Madison, Wisconsin 53706 (and

http://www.ssc.wisc.edu/wlsresearch/data/). The interpretations,

opinions and inferences based on the data are solely the responsibility

of the authors.; This research was supported by the Academy of Finland

(grant no. 124322, 124271 and 132944); the Medical Research Council (MRC

K013351); the US National Institutes of Health (NIH) (R01HL036310;

R01AG034454); and the Finnish Work Environment Fund. C. H. was

financially supported by the Research Foundation of the University of

Helsinki and the Finnish Cultural Foundation; A. S.-M. was supported by

the National Institute on Aging, NIH (R01AG013196; R01AG034454). M. K.

is an Economic and Social Research Council Professor.

CR Affleck G, 2001, J CONSULT CLIN PSYCH, V69, P587, DOI 10.1037//0022-006X.69.4.587

Baird BM, 2010, SOC INDIC RES, V99, P183, DOI 10.1007/s11205-010-9584-9

Barskova T, 2009, DISABIL REHABIL, V31, P1709, DOI 10.1080/09638280902738441

Bogg T, 2004, PSYCHOL BULL, V130, P887, DOI 10.1037/0033-2909-130.6.887

Bostock L, 2009, J CLIN PSYCHOL MED S, V16, P281, DOI 10.1007/s10880-009-9175-6

Carver CS, 2010, ANNU REV PSYCHOL, V61, P679, DOI 10.1146/annurev.psych.093008.100352

Cho HJ, 2013, PSYCHOL MED, V43, P1773, DOI 10.1017/S0033291712002437

Clark AE, 2008, ECON J, V118, pF222, DOI 10.1111/j.1468-0297.2008.02150.x

Crimmins EM, 2004, ANNU REV PUBL HEALTH, V25, P79, DOI 10.1146/annurev.publhealth.25.102802.124401

Davydov DM, 2010, CLIN PSYCHOL REV, V30, P479, DOI 10.1016/j.cpr.2010.03.003

de Ridder D, 2008, LANCET, V372, P246, DOI 10.1016/S0140-6736(08)61078-8

Evers AWM, 2011, NAT REV RHEUMATOL, V7, P409, DOI 10.1038/nrrheum.2011.80

Franzen-Dahlin A, 2006, SCAND J CARING SCI, V20, P412, DOI 10.1111/j.1471-6712.2006.00422.x

Gao SJ, 2000, AM J GERIAT PSYCHIAT, V8, P209, DOI 10.1176/appi.ajgp.8.3.209

Go AS, 2013, CIRCULATION, V127, P143, DOI 10.1161/CIR.0b013e318282ab8f

Headey B, 2010, SOC INDIC RES, V97, P7, DOI 10.1007/s11205-009-9559-x

Hulbert-Williams N, 2012, PSYCHO-ONCOLOGY, V21, P857, DOI 10.1002/pon.1980

Jokela M, 2014, MOL PSYCHIATR, V19, P910, DOI 10.1038/mp.2013.162

Jokela M, 2013, PSYCHOL MED, V43, P2037, DOI 10.1017/S0033291712003042

Jokela M, 2013, OBES REV, V14, P315, DOI 10.1111/obr.12007

Jokela M, 2014, HEALTH PSYCHOL, V33, P1618, DOI 10.1037/hea0000003

Jokela M, 2013, AM J EPIDEMIOL, V178, P667, DOI 10.1093/aje/kwt170

Juster FT, 1995, J HUM RESOUR, V30, pS7, DOI 10.2307/146277

KATZ PP, 1995, ARTHRITIS RHEUM, V38, P49, DOI 10.1002/art.1780380108

Kivimaki M, 2014, MOL PSYCHIATR, V19, P149, DOI 10.1038/mp.2013.35

Lucas RE, 2011, J PERS SOC PSYCHOL, V101, P847, DOI 10.1037/a0024298

Martin LR, 2007, HEALTH PSYCHOL, V26, P428, DOI 10.1037/0278-6133.26.4.428

McCathie HCF, 2002, EUR RESPIR J, V19, P47, DOI 10.1183/09031936.02.00240702

MENDELSOHN GA, 1995, J PERS, V63, P233, DOI 10.1111/j.1467-6494.1995.tb00809.x

NELSON L, 1993, NEUROPSYCHOLOGY, V7, P553

Obonsawin MC, 2007, J NEUROL NEUROSUR PS, V78, P1239, DOI 10.1136/jnnp.2004.052654

Osterberg L, 2005, NEW ENGL J MED, V353, P487, DOI 10.1056/NEJMra050100

Penley JA, 2002, J BEHAV MED, V25, P551, DOI 10.1023/A:1020641400589

Powdthavee N, 2009, SOC SCI MED, V69, P1834, DOI 10.1016/j.socscimed.2009.09.023

Prince M, 2007, LANCET, V370, P859, DOI 10.1016/S0140-6736(07)61238-0

Raison CL, 2013, MOL PSYCHIATR, V18, P15, DOI 10.1038/mp.2012.2

Reich M, 2008, CURR OPIN ONCOL, V20, P353, DOI 10.1097/CCO.0b013e3282fc734b

Remer-Osborn J, 1998, TOP STROKE REHABIL, V5, P45

Roberts BW, 2006, PSYCHOL BULL, V132, P1, DOI 10.1037/0033-2909.132.1.1

Robins Wahlin T-B, 2010, INT J GERIATR PSYCH, V26, P1019

Saulsman LM, 2004, CLIN PSYCHOL REV, V23, P1055, DOI 10.1016/j.cpr.2002.09.001

Sharpe L, 2006, SOC SCI MED, V62, P1153, DOI 10.1016/j.socscimed.2005.07.010

Sheikh AI, 2004, J CLIN PSYCHOL MED S, V11, P265, DOI 10.1023/B:JOCS.0000045346.76242.73

Stanton AL, 2007, ANNU REV PSYCHOL, V58, P565, DOI 10.1146/annurev.psych.58.110405.085615

Stone J, 2004, J NEUROL NEUROSUR PS, V75, P1708, DOI 10.1136/jnnp.2004.037887

Strong K, 2005, LANCET, V366, P1578, DOI 10.1016/S0140-6736(05)67341-2

Wang HD, 2012, LANCET, V380, P2071, DOI 10.1016/S0140-6736(12)61719-X

WOLLMERING E, 2007, WISCONSIN LONGITUDIN

Wortman J, 2012, PSYCHOL AGING, V27, P867, DOI 10.1037/a0029322

Wu S, 2001, J HEALTH ECON, V20, P495, DOI 10.1016/S0167-6296(01)00084-4

NR 50

TC 48

Z9 48

U1 0

U2 50

PU CAMBRIDGE UNIV PRESS

PI NEW YORK

PA 32 AVENUE OF THE AMERICAS, NEW YORK, NY 10013-2473 USA

SN 0033-2917

EI 1469-8978

J9 PSYCHOL MED

JI Psychol. Med.

PD SEP

PY 2014

VL 44

IS 12

BP 2629

EP 2640

DI 10.1017/S0033291714000257

PG 12

WC Psychology, Clinical; Psychiatry; Psychology

SC Psychology; Psychiatry

GA AN2GV

UT WOS:000340403400016

PM 25055176

DA 2019-01-08

ER

PT J

AU Bani-Issa, W

Almomani, F

Eldeirawi, K

AF Bani-Issa, Wegdan

Almomani, Fidaa

Eldeirawi, Kamal

TI Urinary incontinence among adult women with diabetes in Jordan:

epidemiology, correlates and perceived impact on emotional and social

well-being

SO JOURNAL OF CLINICAL NURSING

LA English

DT Article

DE correlates; diabetes; impact on emotional and social well-being; stress

incontinence; urge incontinence; women's health

ID QUALITY-OF-LIFE; RISK-FACTORS; PREVALENCE; HEALTH

AB Aims and objectives. To assess the epidemiology and correlates of urinary incontinence and its perceived impact on the emotional and social well-being of Jordanian women.

Background. Diabetes is a chronic disease with rising prevalence in women worldwide. Although urinary incontinence is a complication of diabetes, it is not commonly assessed with limited nurses' knowledge available on its prevalence, correlates and its perceived impact on lives of women.

Design. A cross-sectional survey design using probability sampling approach was used to collect information on incontinence.

Methods. A total of 1011 adult women, 20-65 years old, attending primary healthcare centres in Jordan were interviewed using a standardised incontinence questionnaire. Questionnaire included items to assess the perceived impact of incontinence on social and emotional well-being of women. The study outcome included weekly or more any, stress and urge incontinence.

Results. Of 435 (43%) diabetic and 576 (57%) nondiabetic women, a total of 676 (66.8%) women reported incontinence. The prevalence of weekly or more any, urge and stress incontinence was significantly higher in diabetic than in nondiabetic women, 31.5, 13.8 and 20% versus 18.2, 5.9 and 14.2%, respectively. After adjusting for age, body mass index, parity and history of urinary tract infections, in multiple logistic regression analyses, diabetes was significantly associated with any (OR: 1.99; 95% CI: 1.44-2.74) urge (OR: 2.23; 95% CI: 1.38-3.61) and stress incontinence (OR: 1.54; CI: 1.07-2.22). Obesity and age were found to be significant correlates for urge incontinence in diabetics. Women with incontinence perceived incontinence as bothersome condition with negative impact on social well-being.

Conclusions. Incontinence is a common and distressing problem in diabetic women, especially for those who are older and obese. Prospective cohort studies are needed to assess how nurse-led interventions may impact the negative consequences of incontinence on women well-being.

Relevance to clinical practice. Nurses are in the right position to assess urinary incontinence in diabetic women. Proper communications, culturally sensitive screening and tailored interventions may assist nurses in providing care and support to alleviate the impact of incontinence on women well-being.

C1 [Bani-Issa, Wegdan] Univ Sharjah, Dept Nursing, Coll Hlth Sci, Sharjah, U Arab Emirates.

[Almomani, Fidaa] Jordan Univ Sci & Technol, Fac Appl Med Sci, Dept Allied Hlth & Occupat Therapy, Irbid, Jordan.

[Eldeirawi, Kamal] Univ Illinois, Coll Nursing, Dept Hlth Syst Sci, Chicago, IL USA.

RP Bani-Issa, W (reprint author), Univ Sharjah, Dept Nursing, Coll Hlth Sci, POB 27272, Sharjah, U Arab Emirates.

EM wbaniissa@sharjah.ac.ae

OI Baniissa, Wegdan/0000-0001-8551-9052

FU Jordan University of Science and Technology Jordan [20120064]

FX This research received a grant from Jordan University of Science and

Technology Jordan: Grant number 20120064. The grant covered expenses

associated with data collection process and data analysis and

interpretation.

CR Abrams P, 2003, UROLOGY, V61, P37, DOI 10.1016/S0090-4295(02)02243-4

Albers-Heitner CP, 2011, SCAND J CARING SCI, V25, P303, DOI 10.1111/j.1471-6712.2010.00827.x

Alnsour M, 2012, PREVENTING CHRONIC D, V9, P1

Altaweel W, 2012, NEUROUROL URODYNAM, V31, P642, DOI 10.1002/nau.22201

American Diabetes Association, 2012, STAND MED CAR DIAB

Andersson G, 2010, J TRANSCULT NURS, V20, P296

Brown J, 2006, DIABETES SELF MANAGE

Brown JS, 2009, J UROLOGY, V181, P13, DOI 10.1016/j.juro.2008.10.078

Brown JS, 2006, DIABETES CARE, V29, P1307, DOI 10.2337/dc05-2463

Brown JS, 2006, DIABETES CARE, V29, P385, DOI 10.2337/diacare.29.02.06.dc05-1781

Brown JS, 2005, DIABETES CARE, V28, P177, DOI 10.2337/diacare.28.1.177

Danforth N, 2009, J UROLOGY, V181, P193

Department of Statistics [Jordan] and ICF Macro, 2010, JORD POP FAM HLTH SU

Ebbesen H, 2007, ACTA OBESTETRIC GYNO, V86, P1256

Gil KM, 2009, HEALTH QUAL LIFE OUT, V7, DOI 10.1186/1477-7525-7-8

Hanzaree Zahirah, 2010, Nurs Stand, V25, P41

Howard Faith, 2010, Br J Nurs, V19, P748

International Diabetes Federation (IDF), 2012, DIAB GLOB BURD

Izci Y, 2009, INT UROGYNECOL J, V20, P947, DOI 10.1007/s00192-009-0888-8

Jackson SL, 2005, DIABETES CARE, V28, P1730, DOI 10.2337/diacare.28.7.1730

Lewis M, 2005, AM J OBSTET GYNECOL, V193, P2154

Lifford KL, 2005, J AM GERIATR SOC, V53, P1851, DOI 10.1111/j.1532-5415.2005.53565.x

McCleary-Sills J, 2012, WOMENS DEMAND REPROD

National Health and Nutrition Examination Survey (NHANES), 2008, DOC COD FREQ KIDN CO

National Heart Lung and Blood Institute, 2012, CALC YOUR BODY MASS

National Institute of Health (NIH), 2012, UR INC NEUR DIS NICE

National Kidney and Urologic Diseases Information Clearinghouse, 2010, UR INC WOM

Nazarko L, 2010, BRIT J HEALTHCARE AS, V4, P353

Nazarko Linda, 2013, Br J Community Nurs, V18, P62

Nojomi M, 2008, J RES MED SCI, V13, P22

Phalen S, 2009, J UROLOGY S, V182, pS14

Phalen S, 2009, DIABETES CARE, V32, P1391

Polit DF, 2010, ESSENTIALS NURSING R

Rizk D E, 1999, Int Urogynecol J Pelvic Floor Dysfunct, V10, P160, DOI 10.1007/s001920050038

Rohr G, 2005, SCAND J PRIM HEALTH, V23, P203, DOI 10.1080/02813430500362803

Shakhatreh FMN, 2005, SAUDI MED J, V26, P830

Thuroff JW, 2011, EUR UROL, V59, P387, DOI 10.1016/j.eururo.2010.11.021

World Health Organization, 2011, NONC DIS COUNTRY PRO

Yuan HB, 2010, HEALTH SOC CARE COMM, V18, P82, DOI 10.1111/j.1365-2524.2009.00876.x

NR 39

TC 5

Z9 5

U1 1

U2 3

PU WILEY-BLACKWELL

PI HOBOKEN

PA 111 RIVER ST, HOBOKEN 07030-5774, NJ USA

SN 0962-1067

EI 1365-2702

J9 J CLIN NURS

JI J. Clin. Nurs.

PD SEP

PY 2014

VL 23

IS 17-18

BP 2451

EP 2460

DI 10.1111/jocn.12392

PG 10

WC Nursing

SC Nursing

GA AL9AI

UT WOS:000339431300009

PM 24112089

DA 2019-01-08

ER

PT J

AU Mackey, ER

Struemph, K

Powell, PW

Chen, R

Streisand, R

Holmes, CS

AF Mackey, Eleanor R.

Struemph, Kari

Powell, Priscilla W.

Chen, Rusan

Streisand, Randi

Holmes, Clarissa S.

TI Maternal Depressive Symptoms and Disease Care Status in Youth With Type

1 Diabetes

SO HEALTH PSYCHOLOGY

LA English

DT Article

DE diabetes; maternal depression; adolescents; disease care; adherence

ID METABOLIC-CONTROL; GLYCEMIC CONTROL; FAMILY CONFLICT; PSYCHOLOGICAL

DISTRESS; REGIMEN ADHERENCE; RISK-FACTORS; FIT INDEXES; ADOLESCENTS;

MANAGEMENT; CHILDREN

AB Objective: The current study assessed relations among maternal depressive symptoms, poorer youth diabetes adherence, and glycemic control. Specifically, hypothesized mediating links of lowered expectations of parental involvement, less parental monitoring, and more conflict were examined. Method: Participants included 225 mothers and their young adolescents, aged 11-14 years (M = 12.73 years, SD = 1.2) diagnosed with T1D. Maternal depressive symptoms and outcome expectancies for maternal involvement were evaluated with self-report questionnaires. Multisource, parent/youth, and multimethod assessment of adherence, parental monitoring, and conflict were evaluated during a baseline assessment from a larger randomized clinical trial. Results: The first hypothesized structural equation model demonstrated a good fit and indicated that more maternal depressive symptoms were directly associated with less parental monitoring and more conflict, which in turn each were associated with poorer adherence and glycemic control. Although higher involvement expectancies were associated with more monitoring and less conflict, they were not associated with other model variables. A second alternative model also fit the data well; poorer youth adherence was associated with more conflict that in turn related to maternal depressive symptoms. Conclusions: Two models were tested by which maternal depressive symptoms and poorer youth adherence were interrelated via less monitoring and more conflict. Follow-up longitudinal evaluation can best characterize the full extent of these relations.

C1 [Mackey, Eleanor R.; Streisand, Randi] Childrens Natl Hlth Syst, Washington, DC USA.

[Struemph, Kari; Powell, Priscilla W.; Holmes, Clarissa S.] VA Commonwealth Univ, Dept Psychol, Richmond, VA 23284 USA.

[Chen, Rusan] Georgetown Univ, Ctr New Designs Learning & Scholarship, Washington, DC 20057 USA.

[Holmes, Clarissa S.] Georgetown Univ, Washington, DC 20057 USA.

RP Holmes, CS (reprint author), VA Commonwealth Univ, 806 West Franklin St,POB 842018, Richmond, VA 23284 USA.

EM cholmes@richmond.edu

OI Mackey, Eleanor/0000-0001-8490-6798

FU NIH/NIDDK [5R01DK070917-04]

FX This work was supported by NIH/NIDDK 5R01DK070917-04 awarded to CH. The

authors gratefully acknowledge the assistance of Elizabeth Robinson with

manuscript preparations.

CR Anderson BJ, 2009, FAM SYST HEALTH, V27, P141, DOI 10.1037/a0015759

Anderson BJ, 2002, DIABETIC MED, V19, P635, DOI 10.1046/j.1464-5491.2002.00752.x

Bandura A., 1997, SELF EFFICACY EXERCI

Beck AT, 1996, MANUAL BECK DEPRESSI

Center for Disease Control and Prevention, 2011, NAT DIAB FACT SHEET

Collins LM, 2001, PSYCHOL METHODS, V6, P330, DOI 10.1037//1082-989X.6.4.330

Cunningham NR, 2011, J PEDIATR PSYCHOL, V36, P196, DOI 10.1093/jpepsy/jsq071

Driscoll KA, 2010, J PEDIATR PSYCHOL, V35, P814, DOI 10.1093/jpepsy/jsp138

Dubowitz H, 2007, PEDIATRICS, V119, P435, DOI 10.1542/peds.2006-2010

Eckshtain D, 2010, J PEDIATR PSYCHOL, V35, P426, DOI 10.1093/jpepsy/jsp068

Ellis DH, 2008, FORTUNE, V157, P42

Ellis DA, 2007, J PEDIATR PSYCHOL, V32, P907, DOI 10.1093/jpepsy/jsm009

Ertel KA, 2011, J WOMENS HEALTH, V20, P1609, DOI 10.1089/jwh.2010.2657

Helgeson VS, 2009, J PEDIATR PSYCHOL, V34, P254, DOI 10.1093/jpepsy/jsn079

Hilliard ME, 2013, HEALTH PSYCHOL, V32, P388, DOI 10.1037/a0027811

Hollingshead A. A., 1975, 4 FACTOR INDEX UNPUB

Holmes CS, 2006, J PEDIATR PSYCHOL, V31, P770, DOI 10.1093/jpepsy/jsj083

Hood KK, 2007, DIABETES CARE, V30, P1764, DOI 10.2337/dc06-2358

Horsch A, 2007, J TRAUMA STRESS, V20, P881, DOI 10.1002/jts.20247

Horwitz SM, 2007, J WOMENS HEALTH, V16, P678, DOI 10.1089/jwh.2006.0185

Hu LT, 1999, STRUCT EQU MODELING, V6, P1, DOI 10.1080/10705519909540118

Hu LT, 1998, PSYCHOL METHODS, V3, P424, DOI 10.1037//1082-989X.3.4.424

Iannotti RJ, 2006, J DEV BEHAV PEDIATR, V27, P98, DOI 10.1097/00004703-200604000-00003

Iannotti RJ, 2006, DIABETES CARE, V29, P2263, DOI 10.2337/dc06-0685

Jaser SS, 2010, J PEDIATR PSYCHOL, V35, P738, DOI 10.1093/jpepsy/jsp098

JOHNSON SB, 1986, HEALTH PSYCHOL, V5, P545, DOI 10.1037/0278-6133.5.6.545

Kline R. B., 2011, PRINCIPLES PRACTICE

MacCallum RC, 2000, ANNU REV PSYCHOL, V51, P201, DOI 10.1146/annurev.psych.51.1.201

Moos R. H., 2002, FAMILY ENV SCALE MAN

Muthen B, 1998, MPLUS USERS GUIDE

Quittner AL, 2008, J PEDIATR PSYCHOL, V33, P916, DOI 10.1093/jpepsy/jsm064

Schilling Lynne S, 2006, J Pediatr Nurs, V21, P412, DOI 10.1016/j.pedn.2006.01.034

Swift EE, 2006, ANN BEHAV MED, V32, P39, DOI 10.1207/s15324796abm3201_5

Wiebe DJ, 2011, J PEDIATR PSYCHOL, V36, P837, DOI 10.1093/jpepsy/jsr002

Williams LB, 2009, DIABETIC MED, V26, P908, DOI 10.1111/j.1464-5491.2009.02794.x

Wissow LS, 2008, PEDIATRICS, V121, P266, DOI 10.1542/peds.2007-0418

WYSOCKI T, 1992, DIABETES CARE, V15, P43, DOI 10.2337/diacare.15.1.43

Wysocki T, 2006, J PEDIATR PSYCHOL, V31, P928, DOI 10.1093/jpepsy/jsj098

NR 38

TC 9

Z9 9

U1 0

U2 20

PU AMER PSYCHOLOGICAL ASSOC

PI WASHINGTON

PA 750 FIRST ST NE, WASHINGTON, DC 20002-4242 USA

SN 0278-6133

EI 1930-7810

J9 HEALTH PSYCHOL

JI Health Psychol.

PD AUG

PY 2014

VL 33

IS 8

BP 783

EP 791

DI 10.1037/hea0000066

PG 9

WC Psychology, Clinical; Psychology

SC Psychology

GA AS3HA

UT WOS:000344167100004

PM 24799001

OA Green Accepted

DA 2019-01-08

ER

PT J

AU Johnson, MD

Anderson, JR

Walker, A

Wilcox, A

Lewis, VL

Robbins, DC

AF Johnson, Matthew D.

Anderson, Jared R.

Walker, Ann

Wilcox, Allison

Lewis, Virginia L.

Robbins, David C.

TI Spousal Protective Buffering and Type 2 Diabetes Outcomes

SO HEALTH PSYCHOLOGY

LA English

DT Article

DE couple coping; dietary adherence; exercise; illness appraisal; type 2

diabetes

ID CHRONIC ILLNESS; SELF-CARE; SUPPORT; QUESTIONNAIRE; SATISFACTION;

BEHAVIORS; DISTRESS

AB Objective: Guided by the developmental-contextual model of couples coping with chronic illness (Berg & Upchurch, 2007), the purpose of this study is to explore moderated associations between spousal protective buffering and illness outcomes for partners diagnosed with type 2 diabetes (dietary adherence, frequency of exercise, and HbA(1c) level). Patient diabetes appraisals (distress, control, self-efficacy, and consequences) were explored as potential moderators. Methods: Participants were 117 married couples in which one member had been diagnosed with type 2 diabetes. Data were gathered from spouses and patients through a survey instrument and analyzed with path analysis. Results: Protective buffering was associated with fewer days of exercise when patients reported low diabetes distress and diabetes consequences. Additionally, protective buffering was associated with higher HbA1c when patients reported high diabetes control. Conclusions: Protective buffering did not exhibit a uniform association with the type 2 diabetes outcomes. Rather, the association between spousal protective buffering and patient illness adjustment was dependent on patient appraisal of the illness. These findings contribute a nuanced addition to the literature documenting the role of couple coping in chronic illness management and also provide impetus for further, longitudinal investigation of the ways healthy spouses cope with partner illness.

C1 [Johnson, Matthew D.] Univ Alberta, Dept Human Ecol, Edmonton, AB T6G 2N1, Canada.

[Anderson, Jared R.] Kansas State Univ, Sch Family Studies & Human Serv, Manhattan, KS 66506 USA.

[Walker, Ann; Wilcox, Allison; Lewis, Virginia L.; Robbins, David C.] Univ Kansas Med Ctr, KU Diabet Inst, Kansas City, KS USA.

RP Johnson, MD (reprint author), Univ Alberta, 302 Human Ecol Bldg, Edmonton, AB T6G 2N1, Canada.

EM matt.johnson@ualberta.ca

CR Berg CA, 2007, PSYCHOL BULL, V133, P920, DOI 10.1037/0033-2909.133.6.920

Buunk A. P., 1996, GEDRAG GEZONDHEID, V24, P304

Coyne JC, 1994, J FAMILY PSYCHOL, V8, P43, DOI DOI 10.1037/0893-3200.8.1.43

De Ridder DTD, 2005, PSYCHOL HEALTH, V20, P497, DOI 10.1080/14768320500098699

Franks MM, 2012, AGING MENT HEALTH, V16, P902, DOI 10.1080/13607863.2012.674486

Hagedoorn M, 2000, HEALTH PSYCHOL, V19, P274, DOI 10.1037//0278-6133.19.3.274

King DK, 2010, DIABETES CARE, V33, P751, DOI 10.2337/dc09-1746

Moss-Morris R, 2002, PSYCHOL HEALTH, V17, P1, DOI 10.1080/08870440290001494

Muthen L. K., 1998, MPLUS USERS GUIDE

Nouwen A, 2011, HEALTH PSYCHOL, V30, P771, DOI 10.1037/a0024500

POLONSKY WH, 1995, DIABETES CARE, V18, P754, DOI 10.2337/diacare.18.6.754

Preacher KJ, 2006, J EDUC BEHAV STAT, V31, P437, DOI 10.3102/10769986031004437

Rosland AM, 2012, J BEHAV MED, V35, P221, DOI 10.1007/s10865-011-9354-4

Schokker MC, 2010, J FAM PSYCHOL, V24, P578, DOI 10.1037/a0021009

Talbot F, 1997, J BEHAV MED, V20, P291, DOI 10.1023/A:1025508928696

Toobert DJ, 2000, DIABETES CARE, V23, P943, DOI 10.2337/diacare.23.7.943

NR 16

TC 7

Z9 7

U1 0

U2 11

PU AMER PSYCHOLOGICAL ASSOC

PI WASHINGTON

PA 750 FIRST ST NE, WASHINGTON, DC 20002-4242 USA

SN 0278-6133

EI 1930-7810

J9 HEALTH PSYCHOL

JI Health Psychol.

PD AUG

PY 2014

VL 33

IS 8

BP 841

EP 844

DI 10.1037/hea0000054

PG 4

WC Psychology, Clinical; Psychology

SC Psychology

GA AS3HA

UT WOS:000344167100010

PM 24447192

DA 2019-01-08

ER

PT J

AU Staniute, M

Bunevicius, A

Brozaitiene, J

Bunevicius, R

AF Staniute, Margarita

Bunevicius, Adomas

Brozaitiene, Julija

Bunevicius, Robertas

TI Relationship of health-related quality of life with fatigue and exercise

capacity in patients with coronary artery disease

SO EUROPEAN JOURNAL OF CARDIOVASCULAR NURSING

LA English

DT Article

DE Health-related quality of life; fatigue; exercise capacity; coronary

artery disease

ID CHRONIC HEART-FAILURE; MYOCARDIAL-INFARCTION; ANXIETY DISORDERS;

DEPRESSION; ASSOCIATION; PERSONALITY; ANGINA; OUTPATIENTS; PREDICTOR;

SYMPTOMS

AB Objective: The study objective was to evaluate the relationship of health-related quality of life (HRQoL) with fatigue and exercise capacity in coronary artery disease (CAD) patients.

Methods: A total of 1072 consecutive CAD patients on admission to a cardiac rehabilitation program were evaluated for HRQoL (36-item Short Form Medical Outcome Questionnaire; SF-36), body mass index, clinical characteristics (New York Heart Association (NYHA) class, angina pectoris class, coronary interventions, treatment with beta blockers, hypertension and diabetes), symptoms of depression and anxiety (Hospital Anxiety and Depression Scale), fatigue (Multidimensional Fatigue Inventory-20; MFI-20), and exercise capacity (bicycle ergometer test).

Results: In univariate regression analyses lower scores on all SF-36 domains were associated with greater scores on all MFI-20 subscales. Exercise capacity was associated with all SF-36 domains, except for social functioning and mental health domains. In multivariate regression analyses, after adjusting for age, gender, body mass index, NYHA class, angina pectoris class, hypertension, diabetes, coronary interventions, treatment with betablockers, and symptoms of depression and anxiety, greater limitation due to physical and due to emotional problems, poor social functioning, decreased energy/vitality, worse general health perception, reduced mental component summary and lower global SF-36 score were independently associated with higher MFI-20 general fatigue score. Reduced physical functioning, greater pain, and reduced physical component summary SF-36 scores were associated with greater MFI-20 physical fatigue score. Lower SF-36 mental health score was associated, with greater MFI-20 mental fatigue score.

Conclusion: In CAD patients undergoing rehabilitation, poor HRQoL is associated with greater fatigue and decreased exercise capacity independently from mental distress and CAD severity score.

C1 [Staniute, Margarita; Bunevicius, Adomas; Brozaitiene, Julija; Bunevicius, Robertas] Lithuanian Univ Hlth Sci, Behav Med Inst, LT-00135 Palanga, Lithuania.

RP Staniute, M (reprint author), Lithuanian Univ Hlth Sci, Behav Med Inst, Vyduno 4, LT-00135 Palanga, Lithuania.

EM mstaniute@ktl.mii.lt

OI Bunevicius, Adomas/0000-0003-0446-6898

FU Research Council of Lithuania [LIG-04/2011]

FX This research was funded by a grant (No. LIG-04/2011) from the Research

Council of Lithuania.

CR Alsen P, 2010, J CARDIOVASC NURS, V25, pE1, DOI 10.1097/JCN.0b013e3181c6dcfd

Bender R, 2001, J CLIN EPIDEMIOL, V54, P343, DOI 10.1016/S0895-4356(00)00314-0

Bunevicius A, 2007, DEPRESS ANXIETY, V24, P455, DOI 10.1002/da.20274

Bunevicius A, 2013, HEALTH QUAL LIFE OUT, V11, DOI 10.1186/1477-7525-11-37

Bunevicius A, 2012, PSYCHOSOM MED, V74, P848, DOI 10.1097/PSY.0b013e318268e7f0

Bunevicius A, 2012, J PSYCHOSOM RES, V72, P22, DOI 10.1016/j.jpsychores.2011.10.006

Bunevicius A, 2011, GEN HOSP PSYCHIAT, V33, P527, DOI 10.1016/j.genhosppsych.2011.06.009

Bunevicius A, 2011, AM HEART J, V162, P310, DOI 10.1016/j.ahj.2011.05.003

Bunevicius R, 2013, INT J CARDIOL, V164, P246, DOI 10.1016/j.ijcard.2012.06.076

Buneviciute J, 2013, J HEALTH PSYCHOL, V18, P1493, DOI 10.1177/1359105312465909

CAMPEAU L, 1976, CIRCULATION, V54, P522, DOI 10.1161/circ.54.3.947585

Chen LH, 2010, J CLIN NURS, V19, P1588, DOI 10.1111/j.1365-2702.2010.03218.x

Deaton C, 2011, EUR J CARDIOVASC NUR, V10, P75, DOI 10.1016/j.ejcnurse.2010.08.007

Evangelista Lorraine S, 2008, Prog Cardiovasc Nurs, V23, P12, DOI 10.1111/j.1751-7117.2008.07275.x

Fini A, 2009, REV LAT-AM ENFERM, V17, P557, DOI 10.1590/S0104-11692009000400019

Fletcher GF, 2001, CIRCULATION, V104, P1694, DOI 10.1161/hc3901.095960

Flynn KE, 2012, AM HEART J, V163, P88, DOI 10.1016/j.ahj.2011.09.027

Johnson NP, 2008, AM J CARDIOL, V102, P1028, DOI 10.1016/j.amjcard.2008.06.017

Juenger J, 2002, HEART, V87, P235, DOI 10.1136/heart.87.3.235

Kent LK, 2009, HARVARD REV PSYCHIAT, V17, P377, DOI 10.3109/10673220903463333

Kimble LP, 2011, J ADV NURS, V67, P1000, DOI 10.1111/j.1365-2648.2010.05564.x

Lavoie KL, 2004, AM HEART J, V148, P621, DOI 10.1016/j.ahj.2004.04.009

MARK DB, 1991, NEW ENGL J MED, V325, P849, DOI 10.1056/NEJM199109193251204

Middel B, 2011, J EVAL CLIN PRACT, V17, P452, DOI 10.1111/j.1365-2753.2010.01449.x

Myers J, 2006, J CARD FAIL, V12, P439, DOI 10.1016/j.cardfail.2006.04.004

Peric VM, 2006, ANN THORAC SURG, V81, P2115, DOI 10.1016/j.athoracsur.2006.01.033

Saeed T., 2011, Eastern Mediterranean Health Journal, V17, P46

Sarkar U, 2007, PSYCHOSOM MED, V69, P306, DOI 10.1097/PSY.0b013e3180514d57

Silverman MN, 2010, PM&R, V2, P338, DOI 10.1016/j.pmrj.2010.04.008

SMETS EMA, 1995, J PSYCHOSOM RES, V39, P315, DOI 10.1016/0022-3999(94)00125-O

Smith ORF, 2008, EUR J CARDIOV PREV R, V15, P583, DOI 10.1097/HJR.0b013e3283083e17

Smith ORF, 2010, EUR J HEART FAIL, V12, P841, DOI 10.1093/eurjhf/hfq075

Smith ORF, 2009, J PSYCHOSOM RES, V66, P195, DOI 10.1016/j.jpsychores.2008.10.021

Stafford L, 2007, J PSYCHOSOM RES, V62, P401, DOI 10.1016/j.jpsychores.2006.12.009

Staniute M, 2013, J CARDIOVASC NURS, V28, P83, DOI 10.1097/JCN.0b013e318233e69d

The Criteria Committee of the New York Heart Association, 1994, NOM CRIT DIAGN DIS H, P253

Thombs BD, 2006, J GEN INTERN MED, V21, P30, DOI 10.1111/j.1525-1497.2005.00269x

WARE JE, 1992, MED CARE, V30, P473, DOI 10.1097/00005650-199206000-00002

ZIGMOND AS, 1983, ACTA PSYCHIAT SCAND, V67, P361, DOI 10.1111/j.1600-0447.1983.tb09716.x

NR 39

TC 13

Z9 13

U1 2

U2 26

PU SAGE PUBLICATIONS LTD

PI LONDON

PA 1 OLIVERS YARD, 55 CITY ROAD, LONDON EC1Y 1SP, ENGLAND

SN 1474-5151

EI 1873-1953

J9 EUR J CARDIOVASC NUR

JI Eur. J. Cardiovasc. Nurs.

PD AUG

PY 2014

VL 13

IS 4

BP 338

EP 344

DI 10.1177/1474515113496942

PG 7

WC Cardiac & Cardiovascular Systems; Nursing

SC Cardiovascular System & Cardiology; Nursing

GA AQ1YB

UT WOS:000342579700007

PM 23818215

DA 2019-01-08

ER

PT J

AU Martyn-Nemeth, P

Quinn, L

Hacker, E

Park, H

Kujath, AS

AF Martyn-Nemeth, Pamela

Quinn, Laurie

Hacker, Eileen

Park, Hanjong

Kujath, Amber S.

TI Diabetes distress may adversely affect the eating styles of women with

type 1 diabetes

SO ACTA DIABETOLOGICA

LA English

DT Article

DE Diabetes mellitus type 1; Diabetes distress; Eating style; Fear of

hypoglycemia

ID BEHAVIOR; QUESTIONNAIRE; HYPOGLYCEMIA; SCALE

AB The aim of this study was to determine the relationships among diabetes distress, fear of hypoglycemia, and eating styles in women with type 1 diabetes (T1DM). Fifteen women (mean age 37 +/- A 13.5 years) with T1DM completed surveys measuring diabetes distress, fear of hypoglycemia (FOH), and eating style. Height, weight, and A1C were obtained, and open-ended comments regarding hypoglycemic concerns and experiences were recorded. Diabetes distress was positively associated with A1C (r = .655, p = .008). High levels of external (73 %), emotional (47 %), and restrained (53 %) eating styles were reported. Emotional and external eating styles were positively associated with diabetes distress (r = .575 and r = .622; p < .05). Those with poorer glycemic control (A1C > 7 %; 53 mmol/mol) had higher levels of restrained eating behavior (F = 10.69, p = .006) and greater interpersonal distress (F = 5.916, p = .03) than those with better glycemic control (A1C < 7 %; 53 mmol/mol). A nonlinear relationship was identified between interpersonal distress and FOH (behavior subscale, p = .0383) indicating that fewer behavioral approaches were employed to avoid hypoglycemia at higher distress levels. The women in this sample were emotionally burdened by their disease. Distress was associated with eating styles linked with overeating and poor glycemic control. High levels of emotional and external eating styles may have important clinical implications for those with T1DM.

C1 [Martyn-Nemeth, Pamela; Quinn, Laurie; Hacker, Eileen; Park, Hanjong] Univ Illinois, Coll Nursing, Dept Biobehav Hlth Sci, Chicago, IL 60607 USA.

[Kujath, Amber S.] Rush Univ, Coll Nursing, Dept Adult Hlth & Gerontol Nursing, Chicago, IL 60612 USA.

RP Martyn-Nemeth, P (reprint author), Univ Illinois, Coll Nursing, Dept Biobehav Hlth Sci, Chicago, IL 60607 USA.

EM pmartyn@uic.edu

OI Park, HanJong/0000-0003-0896-654X

FU UIC Center for Reducing Risks in Vulnerable Populations [NIH P30 NR

09-014-04]; UIC College of Nursing Internal Research Support Program

(IRSP)

FX This study was supported in part by the UIC Center for Reducing Risks in

Vulnerable Populations Grant NIH P30 NR 09-014-04 and by the UIC College

of Nursing Internal Research Support Program (IRSP). The authors wish to

acknowledge Dr. Chang Park for statistical consultation. The authors

thank Kevin Grandfield, Publication Manager for the UIC Department of

Biobehavioral Health Science for editorial assistance.

CR Adam TC, 2007, PHYSIOL BEHAV, V91, P449, DOI 10.1016/j.physbeh.2007.04.011

Anderbro T, 2010, DIABETIC MED, V27, P1151, DOI 10.1111/j.1464-5491.2010.03078.x

Cryer PE, 2002, DIABETOLOGIA, V45, P937, DOI 10.1007/s00125-002-0822-9

Fisher L, 2012, DIABETES CARE, V35, P259, DOI 10.2337/dc11-1572

Gonder-Frederick LA, 2011, DIABETES CARE, V34, P801, DOI 10.2337/dc10-1343

Lloyd CE, 2010, DIABETIC MED, V27, P234, DOI 10.1111/j.1464-5491.2009.02896.x

Ouwens MA, 2003, APPETITE, V40, P291, DOI 10.1016/S0195-6663(03)00006-0

POLONSKY WH, 1995, DIABETES CARE, V18, P754, DOI 10.2337/diacare.18.6.754

Polonsky WH, 2005, DIABETES CARE, V28, P626, DOI 10.2337/diacare.28.3.626

Solli O, 2010, HEALTH QUAL LIFE OUT, V8, DOI 10.1186/1477-7525-8-18

van de Laar FA, 2006, DIABETIC MED, V23, P788, DOI 10.1111/j.1464-5491.2006.01885.x

VANSTRIEN T, 1986, INT J EAT DISORDER, V5, P295, DOI 10.1002/1098-108X(198602)5:2<295::AID-EAT2260050209>3.0.CO;2-T

WARDLE J, 1987, J PSYCHOSOM RES, V31, P161, DOI 10.1016/0022-3999(87)90072-9

NR 13

TC 14

Z9 14

U1 0

U2 11

PU SPRINGER-VERLAG ITALIA SRL

PI MILAN

PA VIA DECEMBRIO, 28, MILAN, 20137, ITALY

SN 0940-5429

EI 1432-5233

J9 ACTA DIABETOL

JI Acta Diabetol.

PD AUG

PY 2014

VL 51

IS 4

BP 683

EP 686

DI 10.1007/s00592-014-0575-1

PG 4

WC Endocrinology & Metabolism

SC Endocrinology & Metabolism

GA AN3RW

UT WOS:000340507200023

PM 24615054

OA Green Accepted

DA 2019-01-08

ER

PT J

AU Virtanen, M

Ferrie, JE

Tabak, AG

Akbaraly, TN

Vahtera, J

Singh-Manoux, A

Kivimaki, M

AF Virtanen, Marianna

Ferrie, Jane E.

Tabak, Adam G.

Akbaraly, Tasnime N.

Vahtera, Jussi

Singh-Manoux, Archana

Kivimaeki, Mika

TI Psychological Distress and Incidence of Type 2 Diabetes in High-Risk and

Low-Risk Populations: The Whitehall II Cohort Study

SO DIABETES CARE

LA English

DT Article

ID WORK-RELATED STRESS; PSYCHOSOCIAL FACTORS; MELLITUS; DEPRESSION;

METAANALYSIS; ASSOCIATION; DIAGNOSIS; OBESITY; WOMEN; MEN

AB OBJECTIVE

We examined whether psychological distress predicts incident type 2 diabetes and if the association differs between populations at higher or lower risk of type 2 diabetes.

RESEARCH DESIGN AND METHODS

This was a prospective cohort of 5,932 diabetes-free adults (4,189 men and 1,743 women, mean age 54.6 years) with three 5-year data cycles (1991-2009): a total of 13,207 person-observations. Participants were classified into four groups according to their prediabetes status and Framingham Offspring Type 2 Diabetes Risk Score: normoglycemia with a risk score of 0-9, normoglycemia with a risk score of 10-19, prediabetes with a risk score of 10-19, and prediabetes with a risk score of >19. Psychological distress was assessed by the General Health Questionnaire. Incident type 2 diabetes was ascertained by 2-h oral glucose tolerance test, doctor diagnosis, or use of antihyperglycemic medication at the 5-year follow-up for each data cycle. Adjustments were made for age, sex, ethnicity, socioeconomic status, antidepressant use, smoking, and physical activity.

RESULTS

Among participants with normoglycemia and among those with prediabetes combined with a low risk score, psychological distress did not predict type 2 diabetes. Diabetes incidence in these groups varied between 1.6 and 15.6%. Among participants with prediabetes and a high risk score, 40.9% of those with psychological distress compared with 28.5% of those without distress developed diabetes during the follow-up. The corresponding adjusted odds ratio for psychological distress was 2.07 (95% CI 1.19-3.62).

CONCLUSIONS

These data suggest that psychological distress is associated with an accelerated progression to manifest diabetes in a subpopulation with advanced prediabetes.

C1 [Virtanen, Marianna; Vahtera, Jussi] Finnish Inst Occupat Hlth, Helsinki, Finland.

[Ferrie, Jane E.; Tabak, Adam G.; Singh-Manoux, Archana; Kivimaeki, Mika] UCL, Dept Epidemiol & Publ Hlth, London, England.

[Ferrie, Jane E.] Univ Bristol, Sch Community & Social Med, Bristol, Avon, England.

[Tabak, Adam G.] Semmelweis Univ, Fac Med, Dept Med 1, H-1085 Budapest, Hungary.

[Akbaraly, Tasnime N.] INSERM, U1061, Montpellier, France.

[Vahtera, Jussi] Univ Turku, Turku, Finland.

[Vahtera, Jussi] Turku Univ Hosp, FIN-20520 Turku, Finland.

[Singh-Manoux, Archana] INSERM, U1018, Paris, France.

RP Virtanen, M (reprint author), Finnish Inst Occupat Hlth, Helsinki, Finland.

EM marianna.virtanen@ttl.fi

RI Singh-Manoux, Archana/F-6804-2013; AKBARALY, Tasnime/H-1389-2018

OI Singh-Manoux, Archana/0000-0002-1244-5037; AKBARALY,

Tasnime/0000-0002-2150-4190; Vahtera, Jussi/0000-0002-6036-061X;

Kivimaki, Mika/0000-0002-4699-5627; Tabak, Adam/0000-0002-6234-3936

FU Medical Research Council [K013351]; British Heart Foundation; National

Heart, Lung, and Blood Institute; National Institutes of Health (NIH)

[R01-HL-036310]; National Institute on Aging, NIH [R01-AG-013196,

R01-AG-034454]; Academy of Finland [258598, 265174]; National Excellence

Program [TAMOP 4.2.4.A/1-11-1-2012-0001]; European Union; European

Social Fund; National Heart, Lung, and Blood Institute [R01-HL-036310];

Languedoc-Roussillon Region (Chercheur d'Avenir Grant); NIH

[R01-HL-036310, R01-AG-034454]; professorial fellowship from the

Economic and Social Research Council; British Heart Foundation

[RG/13/2/30098]; Economic and Social Research Council [ES/J023299/1];

Medical Research Council [MR/K013351/1]

FX The Whitehall II Study is supported by grants from the Medical Research

Council (K013351); the British Heart Foundation; the National Heart,

Lung, and Blood Institute; National Institutes of Health (NIH)

(R01-HL-036310); and the National Institute on Aging, NIH (R01-AG-013196

and R01-AG-034454). M.V. is supported by the Academy of Finland (258598

and 265174); A.G.T. by the TAMOP 4.2.4.A/1-11-1-2012-0001 National

Excellence Program (a research fellowship cofinanced by the European

Union and the European Social Fund); T.N.A. by the National Heart, Lung,

and Blood Institute (R01-HL-036310) and by the Languedoc-Roussillon

Region (Chercheur d'Avenir Grant 2011); and A.S.-M. by the National

Institute on Aging, NIH (R01-AG-013196 and R01-AG-034454). M.K. is

supported by the Medical Research Council (K013351), the NIH

(R01-HL-036310 and R01-AG-034454), and a professorial fellowship from

the Economic and Social Research Council.

CR Alberti KGMM, 1998, DIABETIC MED, V15, P539, DOI 10.1002/(SICI)1096-9136(199807)15:7<539::AID-DIA668>3.0.CO;2-S

*AM DIAB ASS, 2010, DIABETES CARE S1, V33, pS62, DOI DOI 10.2337/DC10-S062

Aroda VR, 2008, J CLIN ENDOCR METAB, V93, P3259, DOI 10.1210/jc.2008-1091

Brotman DJ, 2007, LANCET, V370, P1089, DOI 10.1016/S0140-6736(07)61305-1

Brunner EJ, 1997, DIABETOLOGIA, V40, P1341, DOI 10.1007/s001250050830

Brunner EJ, 2013, NAT REV ENDOCRINOL, V9, P449, DOI 10.1038/nrendo.2013.124

Chida Y, 2008, DIABETOLOGIA, V51, P2168, DOI 10.1007/s00125-008-1154-1

Cosgrove MP, 2012, OCCUP MED-OXFORD, V62, P167, DOI 10.1093/occmed/kqs002

Eriksson AK, 2013, DIABETES CARE, V36, P2683, DOI 10.2337/dc12-1738

Fonseca VA, 2007, CLIN CORNERSTONE, V8, P19

Fonseca Vivian A, 2007, Clin Cornerstone, V8, P10

Goldberg D, 1972, DETECTION PSYCHIAT I

Golden SH, 2008, JAMA-J AM MED ASSOC, V299, P2751, DOI 10.1001/jama.299.23.2751

Gonzalez JS, 2008, DIABETES CARE, V31, P2398, DOI 10.2337/dc08-1341

Head J, 2013, PSYCHOL MED, V43, P2649, DOI 10.1017/S0033291713000342

Heraclides A, 2009, DIABETES CARE, V32, P2230, DOI 10.2337/dc09-0132

Heraclides AM, 2012, OBESITY, V20, P428, DOI 10.1038/oby.2011.95

Kawakami N, 1999, J EPIDEMIOL COMMUN H, V53, P359, DOI 10.1136/jech.53.6.359

Knol MJ, 2006, DIABETOLOGIA, V49, P837, DOI 10.1007/s00125-006-0159-x

Kumari M, 2004, ARCH INTERN MED, V164, P1873, DOI 10.1001/archinte.164.17.1873

Lindstrom J, 2010, HORM METAB RES, V42, pS37, DOI 10.1055/s-0029-1240975

Marmot M, 2005, INT J EPIDEMIOL, V34, P251, DOI 10.1093/ije/dyh372

McEwen BS, 1998, NEW ENGL J MED, V338, P171, DOI 10.1056/NEJM199801153380307

Mezuk B, 2008, DIABETES CARE, V31, P2383, DOI 10.2337/dc08-0985

Mommersteeg PMC, 2012, BMC PUBLIC HEALTH, V12, DOI 10.1186/1471-2458-12-1109

Norberg M, 2007, DIABETES RES CLIN PR, V76, P368, DOI 10.1016/j.diabres.2006.09.002

Pouwer F, 2010, DISCOV MED, V9, P112

Ryden L, 2013, EUR HEART J, V34, P3035, DOI 10.1093/eurheartj/eht108

Stringhini S, 2010, JAMA-J AM MED ASSOC, V303, P1159, DOI 10.1001/jama.2010.297

Tabak AG, 2012, LANCET, V379, P2279, DOI 10.1016/S0140-6736(12)60283-9

Tabak AG, 2009, LANCET, V373, P2215, DOI 10.1016/S0140-6736(09)60619-X

Toshihiro M, 2008, DIABETIC MED, V25, P1211, DOI 10.1111/j.1464-5491.2008.02566.x

Wilson PWF, 2007, ARCH INTERN MED, V167, P1068, DOI 10.1001/archinte.167.10.1068

NR 33

TC 22

Z9 22

U1 0

U2 20

PU AMER DIABETES ASSOC

PI ALEXANDRIA

PA 1701 N BEAUREGARD ST, ALEXANDRIA, VA 22311-1717 USA

SN 0149-5992

EI 1935-5548

J9 DIABETES CARE

JI Diabetes Care

PD AUG

PY 2014

VL 37

IS 8

BP 2091

EP 2097

DI 10.2337/dc13-2725

PG 7

WC Endocrinology & Metabolism

SC Endocrinology & Metabolism

GA AN3MI

UT WOS:000340491800020

PM 24784831

OA Green Published, Bronze

DA 2019-01-08

ER

PT J

AU Korda, RJ

Paige, E

Yiengprugsawan, V

Latz, I

Friel, S

AF Korda, Rosemary J.

Paige, Ellie

Yiengprugsawan, Vasoontara

Latz, Isabel

Friel, Sharon

TI Income-related inequalities in chronic conditions, physical functioning

and psychological distress among older people in Australia:

cross-sectional findings from the 45 and up study

SO BMC PUBLIC HEALTH

LA English

DT Article

ID SOCIOECONOMIC INEQUALITIES; HEALTH INEQUALITIES; DISABILITY FINDINGS;

PREVALENCE; ENGLAND; COHORT; LEVEL; RISK; AGE; PARTICIPANTS

AB Background: The burden of chronic disease continues to rise as populations age. There is relatively little published on the socioeconomic distribution of this burden in older people. This study quantifies absolute and relative income-related inequalities in prevalence of chronic diseases, severe physical functioning limitation and high psychological distress in mid-age and older people in Australia.

Methods: Cross-sectional study of 208,450 participants in the 45 and Up Study, a population-based cohort of men and women aged 45-106 years from New South Wales, Australia. Chronic conditions included self-reported heart disease, diabetes, Parkinson's disease, cancer and osteoarthritis; physical functioning limitation (severe/not) was measured using Medical Outcomes Study measures and psychological distress (high/not) using the Kessler Psychological Distress Scale. For each outcome, prevalence was estimated in relation to annual household income (6 categories). Prevalence differences (PDs) and ratios (PRs) were generated, comparing the lowest income category (<$20,000) to the highest (>=$70,000), using Poisson regression with robust standard errors, weighted for age, sex and region of residence. Analyses were stratified by age group (45-64, 65-79 and >= 80 years) and sex and adjusted for age and country of birth.

Results: With few exceptions, there were income gradients in the prevalence of chronic conditions among all age-sex groups, with prevalence decreasing with increasing income. Of the chronic diseases, PDs were highest for diabetes (ranging between 5.69% and 10.36% across age-sex groups) and in women, also for osteoarthritis (5.72% to 8.14%); PRs were highest for osteoarthritis in men aged 45-64 years (4.01), otherwise they were highest for diabetes (1.78 to 3.43). Inequalities were very high for both physical functioning limitation and psychological distress, particularly among those aged 45-64 (PDs between 18.67% and 29.23% and PRs between 4.63 and 16.51). Absolute and relative inequalities tended to decrease with age, but remained relatively high for diabetes and physical functioning in the elderly (>= 80 years).

Conclusions: Significant inequalities in the prevalence of chronic conditions, physical functioning and psychological distress persist into old age. The additional health burden placed on those who are already disadvantaged is likely to become an increasingly important issue in an ageing population.

C1 [Korda, Rosemary J.; Paige, Ellie; Yiengprugsawan, Vasoontara; Friel, Sharon] Australian Natl Univ, Natl Ctr Epidemiol & Populat Hlth, Canberra, ACT 0200, Australia.

[Latz, Isabel] Univ Calif Los Angeles, Sch Publ Hlth, Los Angeles, CA 90024 USA.

RP Korda, RJ (reprint author), Australian Natl Univ, Natl Ctr Epidemiol & Populat Hlth, GPO Box 4, Canberra, ACT 0200, Australia.

EM Rosemary.Korda@anu.edu.au

OI Yiengprugsawan, Vasoontara/0000-0001-9101-4704; Friel,

Sharon/0000-0002-8345-5435

CR ABS (Australian Bureau of Statistics), 2008, POP PROJ AUSTR 2006

Australian Bureau of Statistics, 2006, 2039 0 INF PAP INTR

Australian Bureau of Statistics, 2006, 2006 CENS DAT CANB A

Australian Institute of Health and Welfare, 2012, AUSTR HLTH 2012

Australian Institute of Health and Welfare, 2012, 4817055001 AUSTR I H

Avendano M, 2008, STROKE, V39, P1533, DOI 10.1161/STROKEAHA.107.490383

Banks E, 2008, INT J EPIDEMIOL, V37, P941, DOI 10.1093/ije/dym184

Banks E, 2011, PUBLIC HEALTH NUTR, V14, P34, DOI 10.1017/S1368980010000674

Banks E, 2010, MED J AUSTRALIA, V193, pS62

Beaglehole R, 2011, LANCET, V377, P1438, DOI 10.1016/S0140-6736(11)60393-0

Beckett M, 2000, J HEALTH SOC BEHAV, V41, P106, DOI 10.2307/2676363

Berkman CS, 1998, J AGING HEALTH, V10, P81, DOI 10.1177/089826439801000105

BOULT C, 1994, J GERONTOL, V49, pM28, DOI 10.1093/geronj/49.1.M28

Breeze E, 2001, AM J PUBLIC HEALTH, V91, P277, DOI 10.2105/AJPH.91.2.277

Byles JE, 2013, DEPRESS ANXIETY

Chittleborough CR, 2011, INT J PUBLIC HEALTH, V56, P71, DOI 10.1007/s00038-010-0197-5

COAG Reform Council, 2013, HEALTHC 2011 12 COMP

Dalstra JAA, 2005, INT J EPIDEMIOL, V34, P316, DOI 10.1093/ije/dyh386

Di Cesare M, 2013, LANCET, V381, P585, DOI 10.1016/S0140-6736(12)61851-0

Espelt A, 2013, GAC SANIT, V27, P494, DOI 10.1016/j.gaceta.2013.03.002

Lima-Costa MF, 2012, AM J PUBLIC HEALTH, V102, P1535, DOI 10.2105/AJPH.2012.300765

Glover J, 2004, AUSTR NZ HLTH POLICY, V1, P8, DOI DOI 10.1186/1743-8462-1-8

Holden L, 2010, AUST NZ J PUBL HEAL, V34, P304, DOI 10.1111/j.1753-6405.2010.00531.x

Huisman M, 2003, SOC SCI MED, V57, P861, DOI 10.1016/S0277-9536(02)00454-9

Institute for Health Metrics and Evaluation, 2013, SEATTL IHME

Jan S, 2012, MED J AUSTRALIA, V196, P29, DOI 10.5694/mja11.11105

Kempen GIJM, 1997, AM J PUBLIC HEALTH, V87, P38, DOI 10.2105/AJPH.87.1.38

Korda RJ, 2012, MED J AUSTRALIA, V197, P631, DOI 10.5694/mja12.11035

Korda RJ, 2009, AUST NZ J PUBL HEAL, V33, P458, DOI 10.1111/j.1753-6405.2009.00430.x

Koster A, 2006, BMC PUBLIC HEALTH, V6, DOI 10.1186/1471-2458-6-244

Mackenbach JP, 2011, J EPIDEMIOL COMMUN H, V65, P412, DOI 10.1136/jech.2010.112680

Marmot M, 2002, HEALTH AFFAIR, V21, P31, DOI 10.1377/hlthaff.21.2.31

McMunn A, 2009, AGE AGEING, V38, P181, DOI 10.1093/ageing/afn236

Mealing NM, 2010, BMC MED RES METHODOL, V10, DOI 10.1186/1471-2288-10-26

Mishra GD, 2004, SOC SCI MED, V58, P1585, DOI 10.1016/S0277-9536(03)00368-X

Nagel G, 2008, BMC PUBLIC HEALTH, V8, DOI 10.1186/1471-2458-8-384

Ponsonby AL, 1996, AUST NZ J PUBL HEAL, V20, P54, DOI 10.1111/j.1467-842X.1996.tb01336.x

Rai D, 2013, BRIT J PSYCHIAT, V202, P195, DOI 10.1192/bjp.bp.112.112482

Ramsay SE, 2008, ANN EPIDEMIOL, V18, P896, DOI 10.1016/j.annepidem.2008.09.006

Savva GM, 2013, J AM GERIATR SOC, V61, pS291, DOI 10.1111/jgs.12196

Stringhini S, 2011, PLOS MED, V8, DOI 10.1371/journal.pmed.1000419

Sulander T, 2006, INT J EPIDEMIOL, V35, P1255, DOI 10.1093/ije/dyl183

Taylor M, 2009, COMMUN SER, P76

Thorslund M, 1994, J Aging Health, V6, P51, DOI 10.1177/089826439400600104

Tsimbos C, 2010, INT J PUBLIC HEALTH, V55, P5, DOI 10.1007/s00038-009-0083-1

Turrell G, 2006, HLTH INEQUALITIES AU, V2

Ware JE, SF 36 HLTH SURV UPD

WHO, 2008, CLOS GAP GEN HLTH EQ

Wilson KCM, 1999, BRIT J PSYCHIAT, V175, P549, DOI 10.1192/bjp.175.6.549

Zou GY, 2004, AM J EPIDEMIOL, V159, P702, DOI 10.1093/aje/kwh090

NR 50

TC 13

Z9 13

U1 1

U2 18

PU BIOMED CENTRAL LTD

PI LONDON

PA 236 GRAYS INN RD, FLOOR 6, LONDON WC1X 8HL, ENGLAND

SN 1471-2458

J9 BMC PUBLIC HEALTH

JI BMC Public Health

PD JUL 22

PY 2014

VL 14

AR 741

DI 10.1186/1471-2458-14-741

PG 10

WC Public, Environmental & Occupational Health

SC Public, Environmental & Occupational Health

GA AP0EF

UT WOS:000341733400001

PM 25048295

OA DOAJ Gold, Green Published

DA 2019-01-08

ER

PT J

AU Perera, J

Kirthinanda, DS

Wijeratne, S

Wickramarachchi, TK

AF Perera, Jennifer

Kirthinanda, Dinoo S.

Wijeratne, Sujani

Wickramarachchi, Thanuja K.

TI Descriptive cross sectional study on prevalence, perceptions,

predisposing factors and health seeking behaviour of women with stress

urinary incontinence

SO BMC WOMENS HEALTH

LA English

DT Article

DE Stress urinary incontinence; Prevalence; Risk factors; Health seeking

behaviour

ID MIDDLE-AGED WOMEN; KNOWLEDGE; DISTRESS; YOUNG

AB Background: Stress urinary incontinence (SUI) leads to considerable physical and psychological morbidity. The highest prevalence reported was found in Caucasian Americans (range 23% -67%) and the lowest in Singaporean females (4.8%). The study assessed the prevalence, perceptions, predisposing factors and health seeking behaviour of women with SUI in an Asian setting which may have different sociocultural implications.

Methods: 400 consecutive women > 20 years of age attending the outpatient department of a tertiary care hospital in Sri Lanka, for non-urinary conditions were studied over a 3 week period using an interviewer administered questionnaire. SUI was diagnosed on clinical history alone when leakage of urine occurred either with coughing, sneezing, walking or lifting heavy objects. The severity was graded using the Finnish Gynaecological Society's Urinary Incontinence Severity Score (UISS). Data were analysed using SPSS version 20. Odds ratios were calculated using univariate and multivariate analysis.

Results: Ninety three (23.33%) had SUI and only 12 (12.9%) had sought treatment. The prevalence among women > 50 years of age was 34.71% (n = 121) compared to 18.28% (n = 279) in those = 50 years. 25 (26.88%) had mild SUI, 66 (70.97%) moderate and 2 (2.15%) severe as per UISS. SUI was perceived as an illness by 210 (52.5%). SUI was significantly associated with pregnancy, parity, vaginal delivery, complicated labour, diabetes mellitus, chronic cough, constipation and faecal incontinence (p < 0.05). Among those affected main reasons for not seeking medical advice included; being embarrassed (n = 27, 33.33%), not knowing that it is remediable (n = 23, 28.40%), perceiving SUI to be a normal consequence of childbirth (n = 19, 23.46%) and having to attend to needs of the family (n = 12, 14.81%). None who had been pregnant (n = 313) had received advice on postnatal pelvic floor exercises. SUI interfered with social activities (71;76.34%), sexual function (21; 22.58%) and resulted in despair (67; 72.09%). It was associated with clinically diagnosed candidiasis (50; 53.76%) and soreness in the perineal region (49; 52.69%).

Conclusions: SUI is a common and neglected gynaecological problem with poor healthcare seeking behaviour. Community based education may help to minimize the occurrence and improve the quality of life of those affected.

C1 [Perera, Jennifer; Kirthinanda, Dinoo S.; Wijeratne, Sujani; Wickramarachchi, Thanuja K.] Univ Colombo, Fac Med, Dept Microbiol, Colombo, Sri Lanka.

RP Perera, J (reprint author), Univ Colombo, Fac Med, Dept Microbiol, POB 271, Colombo, Sri Lanka.

EM jennifer_perera55@yahoo.com

CR Abrams P, 2003, UROLOGY, V61, P37, DOI 10.1016/S0090-4295(02)02243-4

CHAN CJ, 1991, AGE AGEING, V20, P262

Ebbesen MH, 2007, ACTA OBSTET GYN SCAN, V86, P1256, DOI 10.1080/00016340701625347

EL-Hefnawy AS, 2011, MATURITAS, V68, P374, DOI 10.1016/j.maturitas.2011.01.005

FOLDSPANG A, 1992, J EPIDEMIOL COMMUN H, V46, P595, DOI 10.1136/jech.46.6.595

Fultz NH, 2000, J AGING HEALTH, V12, P459, DOI 10.1177/089826430001200401

Gleason JL, 2013, INT UROGYNECOL J, V24, P295, DOI 10.1007/s00192-012-1829-5

Gomman H M, 2001, J Egypt Public Health Assoc, V76, P313

Hahnestad YS, 2004, BRIT MED J, V329, P889, DOI 10.1136/bmj.329.7471.889

Hannestad YS, 2000, J CLIN EPIDEMIOL, V53, P1150, DOI 10.1016/S0895-4356(00)00232-8

Hemachandra NN, 2009, SOC SCI MED, V69, P1395, DOI 10.1016/j.socscimed.2009.08.019

HOLST K, 1988, NEW ZEAL MED J, V101, P756

Hunskaar S, 2000, INT UROGYNECOL J, V11, P301, DOI 10.1007/s001920070021

Kirss F, 2013, SPRINGERPLUS, V2, DOI 10.1186/2193-1801-2-524

Knarr J, 2010, JCOM, V17, P519

Makinen J, 1992, FINN MED J, V47, P2373

MITTENESS LS, 1990, J AM GERIATR SOC, V38, P374, DOI 10.1111/j.1532-5415.1990.tb03525.x

Mouritsen L, 1998, ACTA OBSTET GYN SCAN, V77, P361

NORTON PA, 1988, BRIT MED J, V297, P1187, DOI 10.1136/bmj.297.6657.1187

Novielli Karen D., 2003, International Urology and Nephrology, V35, P423, DOI 10.1023/B:UROL.0000022868.73066.9a

Peyrat L, 2002, BJU INT, V89, P61, DOI 10.1046/j.1464-410X.2002.02546.x

REKERS H, 1992, EUR J OBSTET GYN R B, V43, P229, DOI 10.1016/0028-2243(92)90178-2

Reymert Jannike, 1994, Scandinavian Journal of Primary Health Care, V12, P180, DOI 10.3109/02813439409003696

Samuelsson E, 2000, ACTA OBSTET GYN SCAN, V79, P208, DOI 10.1034/j.1600-0412.2000.079003208.x

Shaw C, 2006, BJU INT, V97, P752, DOI 10.1111/j.1464-410X.2006.06071.x

SHUMAKER SA, 1994, QUAL LIFE RES, V3, P291, DOI 10.1007/BF00451721

St John W, 2013, J WOUND OSTOMY CONT, V40, P524, DOI 10.1097/WON.0b013e3182a2198a

Swanson JG, 2005, CAN FAM PHYSICIAN, V51, P85

Thomas S, 2000, Nurs Stand, V14, P43

Wan XJ, 2014, J CLIN NURS, V23, P2170, DOI 10.1111/jocn.12482

Whitford HM, 2007, MIDWIFERY, V23, P204, DOI 10.1016/j.midw.2006.06.006

NR 31

TC 18

Z9 19

U1 2

U2 23

PU BMC

PI LONDON

PA CAMPUS, 4 CRINAN ST, LONDON N1 9XW, ENGLAND

SN 1472-6874

J9 BMC WOMENS HEALTH

JI BMC Womens Health

PD JUL 2

PY 2014

VL 14

AR 78

DI 10.1186/1472-6874-14-78

PG 7

WC Public, Environmental & Occupational Health; Obstetrics & Gynecology

SC Public, Environmental & Occupational Health; Obstetrics & Gynecology

GA AL2MU

UT WOS:000338960500001

PM 24985068

OA DOAJ Gold, Green Published

DA 2019-01-08

ER

PT J

AU Kapoor, S

Thorn, BE

AF Kapoor, S.

Thorn, B. E.

TI Healthcare use and prescription of opioids in rural residents with pain

SO RURAL AND REMOTE HEALTH

LA English

DT Article

DE chronic pain; depression; healthcare utilization; psychosocial factors;

USA

ID LOW-BACK-PAIN; INDUCED HYPERALGESIA; CHRONIC DISEASE; NONMEDICAL USE;

MENTAL-HEALTH; MEDICAL USE; THERAPY; VALIDATION; DEPRESSION; ABUSE

AB Introduction: Chronic pain is a major public health problem. Increased healthcare utilization by individuals with pain puts enormous burden on financial and health resources. There is extremely limited understanding of psychosocial factors that affect healthcare use and prescription of opioids in individuals who experience heightened healthcare disparities associated with being African-American, having low income, and with rural residency. Health disparities research indicates that rural residency and low socioeconomic status are associated with greater self-reported pain levels. It is logical to expect then that this would be associated with increased needs for health services. However, at the same time, these very variables function as barriers in accessing health care. This disparity between greater need and limited access in turn creates greater distress. Further complicating the picture is the rapidly emerging concern about the misuse of prescription opioids in rural areas. As a result, empirical inquiry has started focusing on the variables influencing the likelihood of receiving opioid prescriptions in rural areas. The understanding of psychosocial factors affecting healthcare use and prescription of opioids in individuals who experience heightened healthcare disparities associated with being African-American, low-income, and living in rural areas remains extremely limited. The primary aim of this study was to examine the demographic and psychosocial variables that affect health services use in a rural, low-income population with chronic pain. Secondarily, the influence of these same variables on receiving prescription for opioids was examined.

Methods: Healthcare use during a 3 month period, prescription analgesics, as well as medical comorbidities were obtained from the medical records of 64 patients with chronic pain. The participants were enrolling in an upcoming psychosocial intervention offered at two rural federally qualified health centers in a south-eastern state in the USA. For the present study, these participants consented to have their medical records reviewed for the 3 months prior to beginning the intervention protocol. Additionally, the pre-treatment (baseline) assessments were used in the present analyses. Demographic information, including age, sex, and education level, as well as measures of pain intensity, depressive symptoms, pain-related disability, and pain catastrophizing were collected.

Results: The participants were rural residents in medically underserved counties, primarily female (73.4%) and African-American (67.2%), and approximately 77% reported annual household income of less than $13,000. A majority had medical comorbidities, including diabetes mellitus (46.89%), cardiovascular disorders (29.7%), chronic renal disorder (14.1%), and asthma (6.3%). Approximately 30% had a diagnosis of depression. Demographic variables such as age, sex, and ethnicity did not influence the healthcare use or prescription of opioids. Depressive symptoms uniquely influenced health services use, with higher scores predicting greater health services utilization. In addition, those with a diagnosis of depression (per medical records) and those with a higher number of medical comorbidities were more likely to receive prescription opioids.

Conclusions: This study adds to the current understanding of the factors affecting healthcare use and prescription of opioids in low-income individuals living in rural areas with chronic pain receiving treatment at federally qualified health centers. Since healthcare use was predicted by depressive symptoms and the prescription of opioids by a clinical diagnosis of depression, screening for depression is advised as part of the standard care of patients with pain, ideally with follow-up assessments and treatment of depression as necessary. Furthermore, making psychosocial interventions more available at rural healthcare centers may help in lowering psychological distress, which may have the ultimate effect of reducing opioid prescriptions for this subset of patients.

C1 [Kapoor, S.; Thorn, B. E.] Univ Alabama, Tuscaloosa, AL 35487 USA.

RP Kapoor, S (reprint author), Univ Alabama, Tuscaloosa, AL 35487 USA.

FU National Institute of Nursing Research; National Institute of Mental

Health [NR010112]

FX This research was supported by the National Institute of Nursing

Research and the National Institute of Mental Health, NR010112. The

authors have no conflicts of interest to report. The authors thank all

of the patients and medical staff who made this research possible. The

authors also acknowledge Drs Jennifer Gardner and Melissa Day for their

earlier contributions to this research project.

CR Adam P, 2012, JOURNAL OF RURAL HEA, V29, P205

Andersen T. E., 2011, SOCIAL SCIENCE AND M, V74, P1461

[Anonymous], 2009, CALL REV CHRON PAIN

Ballantyne JC, 2003, NEW ENGL J MED, V349, P1943, DOI 10.1056/NEJMra025411

Becker A, 2010, SPINE, V35, P1714, DOI 10.1097/BRS.0b013e3181cd656f

Bennett MI, 2005, J PAIN, V6, P149, DOI 10.1016/j.jpain.2004.11.007

Blanchard EB, 1985, MANAGEMENT OF CHRONI

Blyth FM, 2004, PAIN, V111, P51, DOI 10.1016/j.pain.2004.05.020

Board on Health Sciences Policy, 2011, RELIEVING PAIN IN AM

Breckenridge J, 2003, J PAIN, V4, P344, DOI 10.1016/S1526-5900(03)00638-2

Cicero TJ, 2007, PHARMACOEPIDEM DR S, V16, P827, DOI 10.1002/pds.1452

Cimmino MA, 2011, BEST PRACT RES CL RH, V25, P173, DOI 10.1016/j.berh.2010.01.012

Cleeland C. S., 1994, Annals Academy of Medicine Singapore, V23, P129

Cranford JA, 2013, ADDICT BEHAV, V38, P2768, DOI 10.1016/j.addbeh.2013.06.015

Dhalla IA, 2011, BRITISH MEDICAL JOUR, V343, P1

Dobscha SK, 2013, CLIN J PAIN, V29, P102, DOI 10.1097/AJP.0b013e3182490bdb

Ehde D, 2005, EDUCATION INTERVENTI

Elliott AM, 1999, LANCET, V354, P1248, DOI 10.1016/S0140-6736(99)03057-3

Engel CC, 1996, PAIN, V65, P197, DOI 10.1016/0304-3959(95)00164-6

Grabe HJ, 2009, SOC PSYCH PSYCH EPID, V44, P835, DOI 10.1007/s00127-009-0005-9

Grattan A, 2012, ANN FAM MED, V10, P304, DOI 10.1370/afm.1371

Griffiths C, 2007, BRIT MED J, V334, P1254, DOI 10.1136/bmj.39227.698785.47

Groenewald C, 2013, J PAIN, V14, pS39, DOI 10.1016/j.jpain.2013.01.452

Hoffman Pamela K, 2002, J Community Health Nurs, V19, P213, DOI 10.1207/153276502320948445

Hojsted J, 2013, PAIN, V154, P2677, DOI 10.1016/j.pain.2013.07.046

Johnson JL, 2013, CEPHALALGIA, V33, P52, DOI 10.1177/0333102412467512

Kalso E, 2012, ABC OF PAIN, V218, P103

Katz C, 2013, DRUG ALCOHOL DEPEN, V132, P107, DOI 10.1016/j.drugalcdep.2013.01.010

Kissin I, 2013, J PAIN RES, V6, P513, DOI [10.2147/JPR.S47182, 10.2147/JPR.S47162]

Kuhajda MC, 2010, TRANSLATIONAL BEHAVI, V1, P216

Larrison CR, 2011, PSYCHIAT SERV, V62, P1255, DOI 10.1176/ps.62.11.pss6211_1255

Laxmaiah M, 2012, PAIN PHYSICIAN, V15, pES9

Lee JL, 2013, HEALTH PSYCHOL, V32, P320, DOI 10.1037/a0027806

Lee M, 2011, PAIN PHYSICIAN, V14, P145

Leo RJ, 2013, ESSENTIALS OF PALLIA

McCabe SE, 2013, PAIN, V154, P708, DOI 10.1016/j.pain.2013.01.011

Merrill JO, 2012, GEN HOSP PSYCHIAT, V34, P581, DOI 10.1016/j.genhosppsych.2012.06.018

Miller D, 2004, J WOMENS HEALTH, V13, P217, DOI 10.1089/154099904322966209

Nelson JA, 2010, HOME HLTH CARE MAN P, V22, P339, DOI 10.1177/1084822309353552

Okie S, 2010, NEW ENGL J MED, V363, P1981, DOI 10.1056/NEJMp1011512

RADLOFF L S, 1977, Applied Psychological Measurement, V1, P385, DOI 10.1177/014662167700100306

Rost K, 2002, MED CARE RES REV, V59, P231, DOI 10.1177/1077558702059003001

Stroud MW, 2004, J PAIN, V5, P257, DOI 10.1016/j.jpain.2004.04.002

Sullivan MD, 2013, PAIN, V154, pS94, DOI 10.1016/j.pain.2013.09.009

Sullivan MD, 2005, PAIN, V119, P95, DOI 10.1016/j.pain.2005.09.020

Sullivan MJL, 1995, PSYCHOL ASSESSMENT, V7, P524, DOI 10.1037/1040-3590.7.4.524

Tan G, 2004, J PAIN, V5, P133, DOI 10.1016/j.jpain.2003.12.005

Thorn BE, 2011, PAIN, V152, P2710, DOI 10.1016/j.pain.2011.07.007

Toblin RL, 2011, PAIN, V152, P1249, DOI 10.1016/j.pain.2010.12.036

Tollefson Joanne, 2006, Aust J Rural Health, V14, P134, DOI 10.1111/j.1440-1584.2006.00780.x

Trevino CM, 2013, J PAIN, V14, P424, DOI 10.1016/j.jpain.2012.12.016

Turk D, 2002, CLINICAL JOURNAL OF, V18, P257

TURK DC, 1994, BEHAV RES THER, V32, P9, DOI 10.1016/0005-7967(94)90078-7

NR 53

TC 4

Z9 4

U1 0

U2 14

PU AUSTRALIAN RURAL HEALTH EDUC NETWORK

PI DEAKIN WEST

PA PO BOX 242, DEAKIN WEST, ACT 2600, AUSTRALIA

SN 1445-6354

J9 RURAL REMOTE HEALTH

JI Rural Remote Health

PD JUL-SEP

PY 2014

VL 14

IS 3

AR 2879

PG 12

WC Public, Environmental & Occupational Health

SC Public, Environmental & Occupational Health

GA AR3WZ

UT WOS:000343521000039

PM 25204693

DA 2019-01-08

ER

PT J

AU Selvarajah, D

Cash, T

Sankar, A

Thomas, L

Davies, J

Cachia, E

Gandhi, R

Wilkinson, ID

Wilkinson, N

Emery, CJ

Tesfaye, S

AF Selvarajah, Dinesh

Cash, Thomas

Sankar, Adhithya

Thomas, Lloyd

Davies, Jennifer

Cachia, Elaine

Gandhi, Rajiv

Wilkinson, Iain D.

Wilkinson, Nicholas

Emery, Celia J.

Tesfaye, Solomon

TI The contributors of emotional distress in painful diabetic neuropathy

SO DIABETES & VASCULAR DISEASE RESEARCH

LA English

DT Article

DE Painful diabetic neuropathy; emotional distress; depression; anxiety

ID LOW-BACK-PAIN; PERIPHERAL NEUROPATHY; PRIMARY-CARE; DEPRESSION SCALE;

HOSPITAL ANXIETY; VALIDATION; PREDICTORS; DISABILITY; DULOXETINE;

COMMUNITY

AB Aims: To examine the contribution of demographic, social, clinical and psychological factors to emotional distress in patients with painful diabetic neuropathy (DN).

Methods: In total, 142 patients with confirmed painful DN underwent detailed clinical and self-assessment measures (Neuropathic Pain Scale, Hospital Anxiety and Depression Scale, Pain Acceptance Questionnaire and Pain Catastrophizing Scale).

Results: The prevalence of emotional distress was 51.4% in this cohort. Age, sex, marital status, employment history, pain intensity, duration of diabetes and the presence of diabetic and non-diabetic complications were significantly correlated to anxiety and depressive symptom scores. Multiple regression analysis confirmed that the presence of catastrophic thinking was an independent contributor to greater symptoms of anxiety and depression. Being young, single and unemployed significantly contributed to greater anxiety symptoms. Pain-related restriction of quality of life was associated with greater depression symptom scores.

Conclusions: This study found a high prevalence of emotional distress in patients with painful DN. It highlights that the differing independent contributors to anxiety and depressive symptoms are based on an individual's circumstances and experience. We conclude by highlighting the importance of adopting a holistic approach to pain management, incorporating interventions to increase psychological flexibility alongside conventional pharmacological treatments to improve emotional distress in painful DN.

C1 [Selvarajah, Dinesh; Cash, Thomas; Sankar, Adhithya; Thomas, Lloyd; Davies, Jennifer; Cachia, Elaine; Wilkinson, Iain D.] Univ Sheffield, Sch Med, Dept Human Metab, Sheffield S10 2JF, S Yorkshire, England.

[Gandhi, Rajiv; Emery, Celia J.; Tesfaye, Solomon] Sheffield Teaching Hosp NHS Fdn Trust, Royal Hallamshire Hosp, Diabet Res Dept, Sheffield, S Yorkshire, England.

[Wilkinson, Nicholas] Sheffield Hlth & Social Care NHS Fdn Trust, Improving Access Psychol Therapies Sheffield IAPT, Sheffield, S Yorkshire, England.

RP Selvarajah, D (reprint author), Univ Sheffield, Sch Med, Dept Human Metab, Beech Hill Rd, Sheffield S10 2JF, S Yorkshire, England.

EM d.selvarajah@sheffield.ac.uk

OI Tesfaye, Solomon/0000-0003-1190-1472; Wilkinson,

Iain/0000-0003-2703-6890

FU Diabetes UK; Juvenile Diabetes Research Foundation grant

FX This research was supported by a Diabetes UK and Juvenile Diabetes

Research Foundation grant.

CR Abbott CA, 2002, DIABETIC MED, V19, P377, DOI 10.1046/j.1464-5491.2002.00698.x

Bair MJ, 2003, ARCH INTERN MED, V163, P2433, DOI 10.1001/archinte.163.20.2433

BARON RM, 1986, J PERS SOC PSYCHOL, V51, P1173, DOI 10.1037//0022-3514.51.6.1173

Boothby J, 1999, PSYCHOSOCIAL FACTORS, P334

Collins MM, 2009, DIABETIC MED, V26, P153, DOI 10.1111/j.1464-5491.2008.02648.x

Daousi C, 2004, DIABETIC MED, V21, P976, DOI 10.1111/j.1464-5491.2004.01271.x

Davies M, 2006, DIABETES CARE, V29, P1518, DOI 10.2337/dc05-2228

de Groot M, 2001, PSYCHOSOM MED, V63, P619, DOI 10.1097/00006842-200107000-00015

Fishbain DA, 2008, CURR MED RES OPIN, V24, P3105, DOI 10.1185/03007990802429627

Galer BS, 2000, DIABETES RES CLIN PR, V47, P123, DOI 10.1016/S0168-8227(99)00112-6

Galer BS, 1997, NEUROLOGY, V48, P332, DOI 10.1212/WNL.48.2.332

Gore M, 2011, PAIN PRACT, V11, P167, DOI 10.1111/j.1533-2500.2010.00415.x

Gureje O, 1998, JAMA-J AM MED ASSOC, V280, P147, DOI 10.1001/jama.280.2.147

Herrmann C, 1997, J PSYCHOSOM RES, V42, P17, DOI 10.1016/S0022-3999(96)00216-4

Index of Multiple Deprivation (IMD), 2004, WHICH IS US QUAL LIF

Jensen Troels S, 2006, Diab Vasc Dis Res, V3, P108, DOI 10.3132/dvdr.2006.013

Kleinbaum D., 2007, APPL REGRESSION ANAL

McCracken LM, 2004, PAIN, V107, P159, DOI 10.1016/j.pain.2003.10.012

McWilliams LA, 2003, PAIN, V106, P127, DOI 10.1016/S0304-3959(03)00301-4

Means-Christensen AJ, 2008, DEPRESS ANXIETY, V25, P593, DOI 10.1002/da.20342

Mrtikainen P, 2002, INT J EPIDEMIOL, V31, P1091

Ohayon MM, 2010, J PSYCHIATR RES, V44, P454, DOI 10.1016/j.jpsychires.2009.10.013

Rosemann T, 2007, ARTHRIT RHEUM-ARTHR, V57, P415, DOI 10.1002/art.22624

Selvarajah D, 2010, DIABETES CARE, V33, P128, DOI 10.2337/dc09-1029

Sullivan MJL, 1995, PSYCHOL ASSESSMENT, V7, P524, DOI 10.1037/1040-3590.7.4.524

Sullivan MJL, 2005, PAIN, V113, P310, DOI 10.1016/j.pain.2004.11.003

Tesfaye S, 2010, DIABETES CARE, V33, P2285, DOI 10.2337/dc10-1303

Thorn BE, 2002, COGN BEHAV PRACT, V9, P127, DOI 10.1016/S1077-7229(02)80006-2

Tsai PF, 2005, J ADV NURS, V51, P158, DOI 10.1111/j.1365-2648.2005.03481.x

Turk DC, 1996, BIOPSYCHOSOCIAL PERS, P3

Turk DC, 2010, MAYO CLIN PROC, V85, pS42, DOI 10.4065/mcp.2009.0648

Turner JA, 2000, PAIN, V85, P115, DOI 10.1016/S0304-3959(99)00259-6

Vinik Etta J, 2005, Diabetes Technol Ther, V7, P497, DOI 10.1089/dia.2005.7.497

VLAEYEN JWS, 1995, PAIN, V62, P363, DOI 10.1016/0304-3959(94)00279-N

Vlaeyen JWS, 1999, PAIN, V82, P297, DOI 10.1016/S0304-3959(99)00054-8

Williamson GM, 1998, REHABIL PSYCHOL, V43, P327

ZIGMOND AS, 1983, ACTA PSYCHIAT SCAND, V67, P361, DOI 10.1111/j.1600-0447.1983.tb09716.x

NR 37

TC 17

Z9 17

U1 0

U2 12

PU SAGE PUBLICATIONS LTD

PI LONDON

PA 1 OLIVERS YARD, 55 CITY ROAD, LONDON EC1Y 1SP, ENGLAND

SN 1479-1641

EI 1752-8984

J9 DIABETES VASC DIS RE

JI Diabetes Vasc. Dis. Res.

PD JUL

PY 2014

VL 11

IS 4

BP 218

EP 225

DI 10.1177/1479164114522135

PG 8

WC Endocrinology & Metabolism; Peripheral Vascular Disease

SC Endocrinology & Metabolism; Cardiovascular System & Cardiology

GA AN6RW

UT WOS:000340725200001

PM 24821753

DA 2019-01-08

ER

PT J

AU Abdallah, M

Raza, R

Abdallah, T

McCord, D

Khoueiry, G

AF Abdallah, Mokhtar

Raza, Rehan

Abdallah, Tarek

McCord, Donald

Khoueiry, Georges

TI A rare case of silent transmural myocardial infarction with diffuse ST

elevations complicated by concomitant severe hyperkalemia

SO HEART & LUNG

LA English

DT Article

DE Hyperkalemia; ST elevation; Myocardial infarction

ID DIABETIC-KETOACIDOSIS; SEGMENT ELEVATION; PSEUDOINFARCTION PATTERN;

CORONARY STENOSES; ISCHEMIA; OUTCOMES

AB It is well described that certain group of patients do not display the typical symptoms of myocardial infarction (MI). Elderly patients, diabetics and those with previous coronary artery bypass graft surgery are at high risk for silent MI. The diagnosis of Acute MI in the emergency room (ER) is mainly based on the electrocardiogram (EKG) findings of ST elevations or new onset left bundle branch block which is supported by the clinical presentation and positive biomarkers when present. The diagnoses can sometimes become challenging when the patient is asymptomatic and has coincidental finding of hyperkalemia with diffuse ST segment elevations simulating that seen with electrolyte disturbance. Despite the well known pseudoinfarction pattern of hyperkalemia, acute MI should be ruled out first. A high index of suspicion is needed, especially in high risk patients. We think that in rare clinical situation when the diagnosis is in doubt, MI should be ruled out, as time has a high impact on patient mortality. An urgent bedside echocardiogram is very beneficial in excluding regional wall motion abnormalities and preventing any delay in destination therapy for transmural MI. We present a 67 years old female with history of diabetes and chronic kidney disease sent by her nephrologist to the ER for severe hyperkalemia (Potassium 7.2 milliequivalent/L). She was found to have ST elevations on EKG despite having no chest pain or distress. On cardiac catheterization she had a total occlusion of the proximal left circumflex artery, with a filling defect consistent with large thrombus. (C) 2014 Elsevier Inc. All rights reserved.

C1 [McCord, Donald; Khoueiry, Georges] SIUH, Dept Med, Div Cardiol, New York, NY USA.

[Abdallah, Mokhtar; Raza, Rehan] SIUH, Dept Med, New York, NY USA.

[Abdallah, Tarek] SIUH, Dept Med, Div Pulm, New York, NY USA.

RP Khoueiry, G (reprint author), Dartmouth Hitchcock Med Ctr, 1 Med Ctr Dr, Lebanon, NH 03756 USA.

EM Khoueiry_gm@hotmail.com

CR Cho JY, 2012, AM J CARDIOL, V109, P337, DOI 10.1016/j.amjcard.2011.09.017

Cosson E, 2003, DIABETES CARE, V26, P1313, DOI 10.2337/diacare.26.4.1313

Cosson E, 2003, DIABETES METAB, V29, P470, DOI 10.1016/S1262-3636(07)70060-5

Erne P, 2007, JAMA-J AM MED ASSOC, V297, P1985, DOI 10.1001/jama.297.18.1985

Gibson CM, 2001, CIRCULATION, V104, P2632, DOI 10.1161/circ.104.22.2632

KAMIMURA M, 1992, HOSP PRACT, V27, P28

LEVINE HD, 1956, CIRCULATION, V13, P29, DOI 10.1161/01.CIR.13.1.29

Moulik PK, 2002, BRIT MED J, V325, P1346, DOI 10.1136/bmj.325.7376.1346

Sims DB, 2005, CIRCULATION, V111, pE295, DOI 10.1161/01.CIR.0000165127.41028.D1

Sweterlitsch EM, 1996, AM HEART J, V132, P1086, DOI 10.1016/S0002-8703(96)90037-X

Wang K, 2003, NEW ENGL J MED, V349, P2128, DOI 10.1056/NEJMra022580

Wang KY, 2004, NEW ENGL J MED, V351, P593, DOI 10.1056/NEJMicm970566

NR 12

TC 1

Z9 1

U1 0

U2 2

PU MOSBY-ELSEVIER

PI NEW YORK

PA 360 PARK AVENUE SOUTH, NEW YORK, NY 10010-1710 USA

SN 0147-9563

EI 1527-3288

J9 HEART LUNG

JI Heart Lung

PD JUL-AUG

PY 2014

VL 43

IS 4

BP 286

EP 288

DI 10.1016/j.hrtlng.2014.04.017

PG 3

WC Cardiac & Cardiovascular Systems; Nursing; Respiratory System

SC Cardiovascular System & Cardiology; Nursing; Respiratory System

GA AL2RJ

UT WOS:000338972500007

PM 24856226

DA 2019-01-08

ER

PT J

AU Baumeister, H

Nowoczin, L

Lin, J

Seifferth, H

Seufert, J

Laubner, K

Ebert, DD

AF Baumeister, H.

Nowoczin, L.

Lin, J.

Seifferth, H.

Seufert, J.

Laubner, K.

Ebert, D. D.

TI Impact of an acceptance facilitating intervention on diabetes patients'

acceptance of Internet-based interventions for depression: A randomized

controlled trial

SO DIABETES RESEARCH AND CLINICAL PRACTICE

LA English

DT Article

DE Diabetes; Depression; Psychotherapy; Acceptance; E-health

ID COMORBID MENTAL-DISORDERS; PSYCHOLOGICAL TREATMENTS;

INFORMATION-TECHNOLOGY; HEALTH; METAANALYSIS; CARE; ADULTS;

ACCEPTABILITY; POPULATION; INTENTION

AB Aims: To (1) determine diabetes patients' acceptance of Internet-based interventions (IBIs) for depression, to (2) examine the effectiveness of an acceptance facilitating intervention (AFI) and to (3) explore subgroup specific effects.

Methods: 141 diabetes patients from two inpatient rehabilitation units and one outpatient clinic in Germany were randomly allocated to an intervention (IG) and a no-intervention control group (CG). The IG received an AFI consisting of a personal information session before filling-out a questionnaire on patients' acceptance of IBIs, predictors of acceptance (performance expectancy, effort expectancy, social influence, facilitating conditions, and Internet anxiety) as well as sociodemographic, depression-related and diabetes-related variables. The CG filled out the questionnaire immediately. Patients' acceptance of IBIs was measured with a four-item scale (sum-score ranging from 4 to 20).

Results: The CG showed a low (50.7%) to medium (40.8%) acceptance with only 8.5% of all diabetes patients reporting a high acceptance of IBIs for depression. The AFI had no significant effect on acceptance (IG: M = 10.55, SD = 4.69, n = 70; KG: M = 9.65, SD = 4.27, n = 71; d = 0.20 [95%-CI: -0.13; 0.53]) and the predictors of acceptance. Yet, subgroup analyses yielded a trend for depressed, diabetes-related distressed, female and younger (<59) participants and for those who do not frequently use the Internet to profit from the AFI.

Conclusion: Diabetes patients show a rather low acceptance toward IBIs for depression. Findings indicate that the AFI is likely to be effective in the subgroup of depressed, diabetes-related distressed, female or younger diabetes patients, but not in the whole target population. Hence, AFIs might need to be tailored to the specific needs of subpopulations. (C) 2014 Elsevier Ireland Ltd. All rights reserved.

C1 [Baumeister, H.; Nowoczin, L.; Lin, J.; Seifferth, H.] Univ Freiburg, Inst Psychol, Dept Rehabil Psychol & Psychotherapy, D-79085 Freiburg, Germany.

[Baumeister, H.; Lin, J.] Univ Freiburg, Fac Med, D-79085 Freiburg, Germany.

[Seufert, J.; Laubner, K.] Univ Med Ctr Freiburg, Freiburg, Germany.

[Ebert, D. D.] Leuphana Univ, Div Hlth Trainings Online, Luneburg, Germany.

[Ebert, D. D.] Univ Marburg, Dept Psychol Clin Psychol & Psychotherapy, D-35032 Marburg, Germany.

RP Baumeister, H (reprint author), Univ Freiburg, Inst Psychol, Dept Rehabil Psychol & Psychotherapy, Engelbergerstr 41, D-79085 Freiburg, Germany.

EM baumeister@psychologie.uni-freiburg.de

FU German statutory pension insurance scheme

FX This study was partly funded by the German statutory pension insurance

scheme (project: Internet-based health interventions: state of research

and implementation possibilities for the medical rehabilitation).

CR Andrews G, 2010, PLOS ONE, V5, DOI 10.1371/journal.pone.0013196

Baumeister H, CLIN J PAIN IN PRESS

Baumeister H, 2012, COCHRANE DB SYST REV, V12, DOI DOI 10.1002/14651858

Baumeister H, 2008, KLIN VERHALTENSMED R, V82, P254

Baumeister H, 2007, SOC PSYCH PSYCH EPID, V42, P537, DOI 10.1007/s00127-007-0204-1

Baumeister H, 2012, CURR OPIN PSYCHIATR, V25, P405, DOI 10.1097/YCO.0b013e3283556c63

Baumeister H, 2012, J AFFECT DISORDERS, V139, P240, DOI 10.1016/j.jad.2011.05.025

Baumeister H, 2011, PSYCHOTHER PSYCHOSOM, V80, P275, DOI 10.1159/000323404

Baumeister H, 2010, J CLIN EPIDEMIOL, V63, P558, DOI 10.1016/j.jclinepi.2009.08.009

Bennett GG, 2009, ANNU REV PUBL HEALTH, V30, P273, DOI 10.1146/annurev.publhealth.031308.100235

Bhattacherjee A, 2009, BEHAV INFORM TECHNOL, V28, P389, DOI 10.1080/01449290802121230

Blankers M, 2010, J MED INTERNET RES, V12, DOI 10.2196/jmir.1448

Brouwer W, 2009, HEALTH EDUC, V109, P460, DOI 10.1108/09654280911001149

Brouwer W, 2008, J MED INTERNET RES, V10, DOI 10.2196/jmir.956

Celik V, 2013, COMPUT EDUC, V60, P148, DOI 10.1016/j.compedu.2012.06.008

Corrigan P. W., 2002, AM J PSYCHIAT REHABI, V6, P312, DOI [10.1080/10973430208408441, DOI 10.1080/10973430208408441]

Cranen K, 2012, HEALTH EXPECT, V15, P339, DOI 10.1111/j.1369-7625.2011.00668.x

Crutzen R, 2011, HEALTH EDUC BEHAV, V38, P49, DOI 10.1177/1090198110372878

Dattakumar Ambica, 2012, Stud Health Technol Inform, V178, P33

de Groot M, 2001, PSYCHOSOM MED, V63, P619, DOI 10.1097/00006842-200107000-00015

Donkin L, 2011, J MED INTERNET RES, V13, DOI 10.2196/jmir.1772

Gun SY, 2011, AUSTRALAS PSYCHIATRY, V19, P259, DOI 10.3109/10398562.2011.562295

Haase R, 2012, J MED INTERNET RES, V14, DOI 10.2196/jmir.2133

Hermanns N, 2006, DIABETOLOGIA, V49, P469, DOI 10.1007/s00125-005-0094-2

Holden RJ, 2010, J BIOMED INFORM, V43, P159, DOI 10.1016/j.jbi.2009.07.002

Hutter N, 2010, DIABETOLOGIA, V53, P2470, DOI 10.1007/s00125-010-1873-y

Jimison Holly, 2008, Evid Rep Technol Assess (Full Rep), P1

Kaltenthaler E, 2008, PSYCHOL MED, V38, P1521, DOI 10.1017/S0033291707002607

Kim J, 2012, J MED INTERNET RES, V14, DOI 10.2196/jmir.2143

Kroenke K, 2009, J AFFECT DISORDERS, V114, P163, DOI 10.1016/j.jad.2008.06.026

Lin EHB, 2008, J PSYCHOSOM RES, V65, P571, DOI 10.1016/j.jpsychores.2008.06.007

Lin J, 2013, REHABILITATION, V52, P155, DOI 10.1055/s-0033-1343491

Mitchell N, 2007, BEHAV COGN PSYCHOTH, V35, P421, DOI 10.1017/S1352465807003700

Mohr DC, 2010, ANN BEHAV MED, V40, P89, DOI 10.1007/s12160-010-9203-7

Moxham C, 2012, HEALTH SERV MANAG RE, V25, P129, DOI 10.1258/hsmr.2012.012018

NICE, DEPR AD CHRON PHYS H

Nijland N, 2011, J MED INTERNET RES, V13, DOI 10.2196/jmir.1603

Nobis S, 2013, BMC PSYCHIATRY, V13, DOI 10.1186/1471-244X-13-306

Nowoczin L., 2013, AKZEPTANZ INTERNETBA

Or CKL, 2011, J AM MED INFORM ASSN, V18, P51, DOI 10.1136/jamia.2010.007336

Park M, 2013, GEN HOSP PSYCHIAT, V35, P217, DOI 10.1016/j.genhosppsych.2013.01.006

POLONSKY WH, 1995, DIABETES CARE, V18, P754, DOI 10.2337/diacare.18.6.754

Richards D, 2012, CLIN PSYCHOL REV, V32, P329, DOI 10.1016/j.cpr.2012.02.004

Smith KLW, 2013, HEALTH N Y, V5, P1561, DOI DOI 10.4236/HEALTH.2013.510212

Taiwo Ayankunle Adegbite, 2013, Journal of Theoretical and Applied Information Technology, V49, P48

Thoolen B, 2007, DIABETES EDUCATOR, V33, P465, DOI 10.1177/0145721707301491

van Bastelaar KMP, 2011, DIABETES CARE, V34, P320, DOI 10.2337/dc10-1248

van Gemert-Pijnen JEWC, 2011, J MED INTERNET RES, V13, DOI 10.2196/jmir.1672

Venkatesh V, 2003, MIS QUART, V27, P425

Waller R, 2009, PSYCHOL MED, V39, P705, DOI 10.1017/S0033291708004224

Wilson EV, 2004, J AM MED INFORM ASSN, V11, P241, DOI 10.1197/jamia.1475

Wootton BM, 2011, PLOS ONE, V6, DOI 10.1371/journal.pone.0020548

NR 52

TC 30

Z9 31

U1 0

U2 25

PU ELSEVIER IRELAND LTD

PI CLARE

PA ELSEVIER HOUSE, BROOKVALE PLAZA, EAST PARK SHANNON, CO, CLARE, 00000,

IRELAND

SN 0168-8227

EI 1872-8227

J9 DIABETES RES CLIN PR

JI Diabetes Res. Clin. Pract.

PD JUL

PY 2014

VL 105

IS 1

BP 30

EP 39

DI 10.1016/j.diabres.2014.04.031

PG 10

WC Endocrinology & Metabolism

SC Endocrinology & Metabolism

GA AK8YT

UT WOS:000338715400007

PM 24862240

DA 2019-01-08

ER

PT J

AU Hernandez, R

Ruggiero, L

Riley, BB

Wang, YM

Chavez, N

Quinn, LT

Gerber, BS

Choi, YK

AF Hernandez, Rosalba

Ruggiero, Laurie

Riley, Barth B.

Wang, Yamin

Chavez, Noel

Quinn, Lauretta T.

Gerber, Ben S.

Choi, Young-Ku

TI Correlates of Self-Care in Low-Income African American and Latino

Patients With Diabetes

SO HEALTH PSYCHOLOGY

LA English

DT Article

DE diabetes self-care; minorities; biopsychosocial factors; correlates;

Type 2 diabetes

ID GLYCEMIC CONTROL; RACIAL/ETHNIC DIFFERENCES; PHYSICAL-ACTIVITY;

BLOOD-GLUCOSE; UNITED-STATES; ETHNIC-GROUPS; MANAGEMENT; ADHERENCE;

ASSOCIATION; DISPARITIES

AB Objective: This study aimed to examine diabetes self-care (DSC) patterns in low-income African American and Latino patients with Type 2 diabetes, and identify patient-related, biomedical/disease-related, and psychosocial correlates of DSC. Method: We performed cross-sectional analysis of survey data from African Americans and Latinos aged >= 18 years with Type 2 diabetes (n = 250) participating in a diabetes self-management intervention at 4 primary care clinics. The Summary of Diabetes Self-Care Activities captured the subcomponents of healthy eating, physical activity, blood sugar testing, foot care, and smoking. Correlates included patient-related attributes, biomedical/disease-related factors, and psychosocial constructs, with their multivariable influence assessed with a 3-step model building procedure using regression techniques. Results: Baseline characteristics were as follows: mean age of 53 years (SD = 12.4); 69% female; 53% African American; 74% with incomes below $20,000; and 60% with less than a high school education. DSC performance levels were highest for foot care (4.5/7 days) and lowest for physical activity (2.5/7 days). Across racial/ethnic subgroups, diabetes-related distress was the strongest correlate for DSC when measured as a composite score. Psychosocial factors accounted for 14% to 33% of variance in self-care areas for both racial/ethnic groups. Patient characteristics were more salient correlates in Hispanic/Latinos when examining the self-care subscales, particularly those requiring monetary resources. Conclusions: Important information is provided on specific DSC patterns in a sample of ethnic/racial minorities with Type 2 diabetes. Significant correlates found may help with identification and intervention of patients who may benefit from strategies to increase self-care adherence.

C1 [Hernandez, Rosalba] Northwestern Univ, Dept Prevent Med, Feinberg Sch Med, Chicago, IL 60611 USA.

[Hernandez, Rosalba; Ruggiero, Laurie; Wang, Yamin; Chavez, Noel; Gerber, Ben S.] Univ Illinois, Sch Publ Hlth, Inst Hlth Res & Policy & Community Hlth Sci, Chicago, IL USA.

[Riley, Barth B.] Univ Illinois, Coll Nursing, Dept Hlth Syst Sci, Chicago, IL USA.

[Quinn, Lauretta T.] Univ Illinois, Coll Nursing, Dept Biobehav Hlth Sci, Chicago, IL USA.

[Gerber, Ben S.] Jesse Brown VA Med Ctr, Chicago, IL USA.

[Choi, Young-Ku] Univ Illinois, Sch Publ Hlth, Inst Hlth Res & Policy & Epidemiol & Biostat, Chicago, IL USA.

RP Hernandez, R (reprint author), Northwestern Univ, Dept Prevent Med, Feinberg Sch Med, 680 N Lake Shore Dr, Chicago, IL 60611 USA.

EM rosalba.hernandez@northwestern.edu

OI Gerber, Ben/0000-0003-4367-6396

FU National Institute of Nursing Research [5RO1 NR010313]; National

Institute of Health (NIH) [5T32 HL069771-10]

FX Research reported in this publication was supported by the National

Institute of Nursing Research under Award Number 5RO1 NR010313 (Laurie

Ruggiero, Principal Investigator). Rosalba Hernandez was supported by

the National Institute of Health (NIH) under Award Number 5T32

HL069771-10 (Martha L. Daviglus, Principal Investigator). The content

and views expressed in this article are those of the authors and do not

necessarily reflect the position or policy of the National Institute of

Nursing Research, the Department of Veterans Affairs, the United States

government, or the National Institutes of Health. Rosalba Hernandez

researched data, wrote, and reviewed/edited the manuscript. Laurie

Ruggiero contributed to conceptualization of study, interpretation of

data, discussion, and reviewed/edited the manuscript. Barth B. Riley and

Young-Ku Choi researched data, contributed to the design of analytical

methods, and reviewed/edited the manuscript. Yamin Wang conducted the

data analysis and reviewed/edited the manuscript. Noel Chavez, Lauretta

T. Quinn, and Ben S. Gerber contributed to conceptualization of study,

provided recommendations on study methods, and reviewed/edited the

manuscript. Rosalba Hernandez, Laurie Ruggiero, and Yamin Wang had full

access to the data and serve as guarantors, taking responsibility for

the integrity of the data and accuracy of data analysis. The authors

would like to thank all members of the research team and the

administrators, staff, and patients of Access Community Health Network

for their efforts and support of this project.

CR Anderson RM, 2003, DIABETES CARE, V26, P1641, DOI 10.2337/diacare.26.5.1641-a

Asche C, 2011, CLIN THER, V33, P74, DOI 10.1016/j.clinthera.2011.01.019

Belza Basia, 2004, Prev Chronic Dis, V1, pA09

Boule NG, 2001, JAMA-J AM MED ASSOC, V286, P1218, DOI 10.1001/jama.286.10.1218

Centers for Disease Control and Prevention, 2005, TRENDS LEIS TIM PHYS, V54, P991

Choi S, 2011, ETHNIC DIS, V21, P183

Concha JB, 2009, DIABETES EDUCATOR, V35, P941, DOI 10.1177/0145721709344125

Delamater AM, 2006, CLIN DIABETES, V24, P71, DOI [10.2337/diaclin.24.2.71, DOI 10.2337/DIACLIN.24.2.71]

Dunn MZ, 2008, J TRANSCULT NURS, V19, P40, DOI 10.1177/1043659607309138

Federal Interagency Forum on Aging-Related Statics, 2010, OLD AM 2010 KEY IND

Harris MI, 1998, DIABETES CARE, V21, pC11, DOI 10.2337/diacare.21.3.C11

Hayes C, 2008, J AM DIET ASSOC, V108, pS19, DOI 10.1016/j.jada.2008.01.016

HILL AB, 1965, P ROY SOC MED, V58, P295

Huang ES, 2009, DIABETES CARE, V32, P311, DOI 10.2337/dc08-1307

Jette AM, 1998, GERONTOLOGIST, V38, P412, DOI 10.1093/geront/38.4.412

Kirk JK, 2007, ETHNIC DIS, V17, P135

Levine DA, 2009, ETHNIC DIS, V19, P97

Little R.J.A, 1987, STAT ANAL MISSING DA

Lynch EB, 2012, HEALTH EDUC RES, V27, P814, DOI 10.1093/her/cys058

Mier Nelda, 2007, Prev Chronic Dis, V4, pA24

National Glycohemoglobin Standardization Program, 2013, HARM HEM ALC TEST

Norris SL, 2002, DIABETES CARE, V25, P1159, DOI 10.2337/diacare.25.7.1159

Nwasuruba C, 2007, J GEN INTERN MED, V22, P115, DOI 10.1007/s11606-007-0120-9

Orzech KM, 2013, HEALTH EDUC BEHAV, V40, P56, DOI 10.1177/1090198112436970

Parada H, 2012, DIABETES EDUCATOR, V38, P552, DOI 10.1177/0145721712445215

Polonsky WH, 2005, DIABETES CARE, V28, P626, DOI 10.2337/diacare.28.3.626

Ruggiero L, 1997, DIABETES CARE, V20, P568, DOI 10.2337/diacare.20.4.568

Sallis JF, 2000, MED SCI SPORT EXER, V32, P1598

Schectman JM, 2002, DIABETES CARE, V25, P1015, DOI 10.2337/diacare.25.6.1015

Sigal RJ, 2006, DIABETES CARE, V29, P1433, DOI 10.2337/dc06-9910

Snowling NJ, 2006, DIABETES CARE, V29, P2518, DOI 10.2337/dc06-1317

St John A, 2006, CLIN CHIM ACTA, V365, P257, DOI 10.1016/j.cca.2005.09.003

Thackeray R, 2004, DIABETES EDUCATOR, V30, P665, DOI 10.1177/014572170403000418

Toobert DJ, 2000, DIABETES CARE, V23, P943, DOI 10.2337/diacare.23.7.943

Trief PM, 2013, ETHNIC HEALTH, V18, P83, DOI 10.1080/13557858.2012.700915

Vincent D, 2008, NURS RES, V57, P101, DOI 10.1097/01.NNR.0000313484.18670.ab

WILSON W, 1986, DIABETES CARE, V9, P614, DOI 10.2337/diacare.9.6.614

NR 37

TC 5

Z9 5

U1 1

U2 27

PU AMER PSYCHOLOGICAL ASSOC

PI WASHINGTON

PA 750 FIRST ST NE, WASHINGTON, DC 20002-4242 USA

SN 0278-6133

EI 1930-7810

J9 HEALTH PSYCHOL

JI Health Psychol.

PD JUL

PY 2014

VL 33

IS 7

BP 597

EP 607

DI 10.1037/hea0000043

PG 11

WC Psychology, Clinical; Psychology

SC Psychology

GA AK5CS

UT WOS:000338442500003

PM 24364373

OA Green Accepted

DA 2019-01-08

ER

PT J

AU van Son, J

Nyklicek, I

Pop, VJ

Blonk, MC

Erdtsieck, RJ

Pouwer, F

AF van Son, Jenny

Nyklicek, Ivan

Pop, Victor J.

Blonk, Marion C.

Erdtsieck, Ronald J.

Pouwer, Francois

TI Mindfulness-based cognitive therapy for people with diabetes and

emotional problems: Long-term follow-up findings from the DiaMind

randomized controlled trial

SO JOURNAL OF PSYCHOSOMATIC RESEARCH

LA English

DT Article

DE Anxiety; Comorbidity; Diabetes; Depressive symptoms; Mindfulness-based

cognitive therapy; Randomized controlled trial

ID QUALITY-OF-LIFE; DEPRESSION; DISTRESS; INTERVENTION; ANXIETY; ADULTS;

OUTPATIENTS; PREVALENCE; MELLITUS.; SYMPTOMS

AB Objective: The DiaMind trial showed beneficial immediate effects of mindfulness-based cognitive therapy (MBCT) on emotional distress, but not on diabetes distress and HbA(1c). The aim of the present report was to examine if the effects would be sustained after six month follow-up.

Methods: In the DiaMind trial, 139 outpatients with diabetes (type-I or type-II) and a lowered level of emotional well-being were randomized into MBCT (n = 70) or a waiting list with treatment as usual (TAU: n = 69). Primary outcomes were perceived stress, anxiety and depressive symptoms, and diabetes distress. Secondary outcomes were, among others, health status, and glycemic control (HbA(1c)).

Results: Compared to TAU, MBCT showed sustained reductions at follow-up in perceived stress (p < .001, d = .76), anxiety (p < .001, assessed by HADS d = .83; assessed by POMS d = .92), and HADS depressive symptoms (p = .004, d = .51), but not POMS depressive symptoms when using Bonferroni correction for multiple testing (p = .016, d = .48). No significant between-group effect was found on diabetes distress and HbA(1c).

Conclusion: This study showed sustained benefits of MBCT six months after the intervention on emotional distress in people with diabetes and a lowered level of emotional well-being. (C) 2014 Elsevier Inc. All rights reserved.

C1 [van Son, Jenny; Nyklicek, Ivan; Pop, Victor J.; Pouwer, Francois] Tilburg Univ, Dept Med & Clin Psychol, Ctr Res Psychol Somat Dis CoRPS, NL-5000 LE Tilburg, Netherlands.

[Blonk, Marion C.] Catharina Hosp, Dept Internal Med, Eindhoven, Netherlands.

[Erdtsieck, Ronald J.] Maxima Med Ctr, Dept Internal Med, Eindhoven, Netherlands.

RP Nyklicek, I (reprint author), POB 90153, NL-5000 LE Tilburg, Netherlands.

EM I.Nyklicek@tilburguniversity.edu

FU Dutch Diabetes Research Foundation (Diabetesfonds) [2008.13.005]

FX The study is supported by a grant from the Dutch Diabetes Research

Foundation (Diabetesfonds) (project number 2008.13.005, awarded to Dr I.

Nyklicek).

CR American Diabetes Association, 2013, DIABETES CARE, V36, pS11

Baer RA, 2006, ASSESSMENT, V13, P27, DOI 10.1177/1073191105283504

Baumeister H, 2012, COCHRANE DB SYST REV, V12

Branstrom R, 2012, INT J BEHAV MED, V19, P535, DOI 10.1007/s12529-011-9192-3

Cohen J, 1977, STAT POWER ANAL BEHA

Cohen S, 1988, SOC PSYCHOL HEALTH, V1, P31

Egede LE, 2005, DIABETES CARE, V28, P1339, DOI 10.2337/diacare.28.6.1339

Fisher L, 2008, DIABETIC MED, V25, P1096, DOI 10.1111/j.1464-5491.2008.02533.x

Franck E, 2008, PSYCHOL BELG, V48, P25, DOI 10.5334/pb-48-1-25

Gregg JA, 2007, J CONSULT CLIN PSYCH, V75, P336, DOI 10.1037/0022-006X.75.2.336

Grigsby AB, 2002, J PSYCHOSOM RES, V53, P1053, DOI 10.1016/S0022-3999(02)00417-8

Hartmann M, 2012, DIABETES CARE, V35, P945, DOI 10.2337/dc11-1343

Hofmann SG, 2010, J CONSULT CLIN PSYCH, V78, P169, DOI 10.1037/a0018555

Jenkinson C, 1997, J PUBLIC HEALTH MED, V19, P179, DOI 10.1093/oxfordjournals.pubmed.a024606

Lustman PJ, 2000, DIABETES CARE, V23, P934, DOI 10.2337/diacare.23.7.934

Makine C, 2009, DIABETIC MED, V26, P28, DOI 10.1111/j.1464-5491.2008.02606.x

Mirowsky J, 2002, J HEALTH SOC BEHAV, V43, P152, DOI 10.2307/3090194

Morone NE, 2008, PAIN, V134, P310, DOI 10.1016/j.pain.2007.04.038

Pbert L, 2012, THORAX, V67, P769, DOI 10.1136/thoraxjnl-2011-200253

POLONSKY WH, 1995, DIABETES CARE, V18, P754, DOI 10.2337/diacare.18.6.754

Pouwer F, 2010, DIABETIC MED, V27, P217, DOI 10.1111/j.1464-5491.2009.02903.x

Schram MT, 2009, CURR DIABETES REV, V5, P112, DOI 10.2174/157339909788166828

Segal Z., 2002, MINDFULNESS BASED CO

van Dooren FEP, 2013, PLOS ONE, V8, DOI 10.1371/journal.pone.0057058

van Son J, 2013, DIABETES CARE, V36, P823, DOI 10.2337/dc12-1477

van Son J, 2011, BMC PUBLIC HEALTH, V11, DOI 10.1186/1471-2458-11-131

Vollestad J, 2011, BEHAV RES THER, V49, P281, DOI 10.1016/j.brat.2011.01.007

Wald F., 1990, NED T PSYCHOL, V45, P86

Zangi HA, 2012, ANN RHEUM DIS, V71, P911, DOI 10.1136/annrheumdis-2011-200351

ZIGMOND AS, 1983, ACTA PSYCHIAT SCAND, V67, P361, DOI 10.1111/j.1600-0447.1983.tb09716.x

NR 30

TC 25

Z9 25

U1 4

U2 56

PU PERGAMON-ELSEVIER SCIENCE LTD

PI OXFORD

PA THE BOULEVARD, LANGFORD LANE, KIDLINGTON, OXFORD OX5 1GB, ENGLAND

SN 0022-3999

EI 1879-1360

J9 J PSYCHOSOM RES

JI J. Psychosomat. Res.

PD JUL

PY 2014

VL 77

IS 1

BP 81

EP 84

DI 10.1016/j.jpsychores.2014.03.013

PG 4

WC Psychiatry

SC Psychiatry

GA AJ4KK

UT WOS:000337645000013

PM 24913347

OA Bronze

DA 2019-01-08

ER

PT J

AU Fisher, L

Gonzalez, JS

Polonsky, WH

AF Fisher, L.

Gonzalez, J. S.

Polonsky, W. H.

TI The confusing tale of depression and distress in patients with diabetes:

a call for greater clarity and precision

SO DIABETIC MEDICINE

LA English

DT Review

ID GLYCEMIC CONTROL; SELF-CARE; BIDIRECTIONAL ASSOCIATION; MEDICATION

ADHERENCE; CLINICAL DEPRESSION; MAJOR DEPRESSION; CONTROLLED-TRIAL;

METAANALYSIS; MANAGEMENT; SYMPTOMS

AB Studies have identified significant linkages between depression and diabetes, with depression associated with poor self-management behaviour, poor clinical outcomes and high rates of mortality. However, findings are not consistent across studies, yielding confusing and contradictory results about these relationships. We suggest that there has been a failure to define and measure depression' in a consistent manner. Because the diagnosis of depression is symptom-based only, without reference to source or content, the context of diabetes is not considered when addressing the emotional distress experienced by individuals struggling with diabetes. To reduce this confusion, we suggest that an underlying construct of emotional distress' be considered as a core construct to link diabetes-related distress, subclinical depression, elevated depression symptoms and major depressive disorder (MDD). We view emotional distress as a single, continuous dimension that has two primary characteristics: content and severity; that the primary content of emotional distress among these individuals include diabetes and its management, other life stresses and other contributors; and that both the content and severity of distress be addressed directly in clinical care. We suggest further that all patients, even those whose emotional distress rises to the level of MDD or anxiety disorders, can benefit from consideration of the content of distress to direct care effectively, and we suggest strategies for integrating the emotional side of diabetes into regular diabetes care. This approach can reduce confusion between depression and distress so that appropriate and targeted patient-centred interventions can occur.

C1 [Fisher, L.] Univ Calif San Francisco, San Francisco, CA 94143 USA.

[Gonzalez, J. S.] Yeshiva Univ, Ferkauf Grad Sch Psychol, Bronx, NY USA.

[Gonzalez, J. S.] Albert Einstein Coll Med, Bronx, NY 10467 USA.

[Polonsky, W. H.] Univ Calif San Diego, San Diego, CA 92103 USA.

[Polonsky, W. H.] Behav Diabet Inst, San Diego, CA USA.

RP Fisher, L (reprint author), Univ Calif San Francisco, San Francisco, CA 94143 USA.

EM fisherl@fcm.ucsf.edu

FU National Institutes of Health (NIH) [DK061937, DK094863, DK020541,

DK098742]

FX This article was supported in part by National Institutes of Health

(NIH) grants DK061937, DK094863, DK020541 and DK098742.

CR Aikens JE, 2012, DIABETES CARE, V35, P2472, DOI 10.2337/dc12-0181

Anderson RJ, 2001, DIABETES CARE, V24, P1069, DOI 10.2337/diacare.24.6.1069

Black SA, 2003, DIABETES CARE, V26, P2822, DOI 10.2337/diacare.26.10.2822

Bogner HR, 2012, ANN FAM MED, V10, P15, DOI 10.1370/afm.1344

Chan JCN, 2009, DIABETES CARE, V32, P227, DOI 10.2337/dc08-0435

Cole J, 2008, BRIT J PSYCHIAT, V192, P83, DOI 10.1192/bjp.bp.107.039826

COYNE JC, 1994, PSYCHOL BULL, V116, P29, DOI 10.1037/0033-2909.116.1.29

de Groot M, 2001, PSYCHOSOM MED, V63, P619, DOI 10.1097/00006842-200107000-00015

Esbitt S. A., 2013, SCREENING DEPRESSION

Fisher EB, 2012, J AFFECT DISORDERS, V142S1, P556

Fisher L, 2008, DIABETIC MED, V25, P1096, DOI 10.1111/j.1464-5491.2008.02533.x

Fisher L, 2011, PATIENT EDUC COUNS, V86, P372

Fisher L, 2007, DIABETES CARE, V30, P542, DOI 10.2337/dc06-1614

Fisher L, 2013, DIABETES CARE, V36, P2551, DOI 10.2337/dc12-2493

Fisher L, 2010, DIABETES CARE, V33, P1034, DOI 10.2337/dc09-2175

Fisher L, 2010, DIABETES CARE, V33, P23, DOI 10.2337/dc09-1238

Gask L, 2011, CHRONIC ILLN, V7, P239, DOI 10.1177/1742395311403636

Georgiades A, 2007, PSYCHOSOM MED, V69, P235, DOI 10.1097/PSY.0b013e318042588d

Goldberg D, 2011, WORLD PSYCHIATRY, V10, P226

Golden SH, 2008, JAMA-J AM MED ASSOC, V299, P2751, DOI 10.1001/jama.299.23.2751

Gonzalez JS, 2008, DIABETIC MED, V25, P1102, DOI 10.1111/j.1464-5491.2008.02535.x

Gonzalez JS, 2010, DIABETOLOGIA, V53, P2241, DOI 10.1007/s00125-010-1821-x

Gonzalez JS, 2007, DIABETES CARE, V30, P2222, DOI 10.2337/dc07-0158

Gonzalez JS, 2011, DIABETES CARE, V34, P236, DOI 10.2337/dc10-1970

Gonzalez JS, 2008, DIABETES CARE, V31, P2398, DOI 10.2337/dc08-1341

Katon W, 2010, DEPRESSION DIABETES

Katon WJ, 2010, NEW ENGL J MED, V363, P2622

Li C, 2008, DIABETIC MED, V25, P878, DOI 10.1111/j.1464-5491.2008.02477.x

Lin EHB, 2006, ANN FAM MED, V4, P46, DOI 10.1370/afm.423

Lustman PJ, 2000, DIABETES CARE, V23, P618, DOI 10.2337/diacare.23.5.618

Mezuk B, 2013, HEALTH PSYCHOL, V32, P254, DOI 10.1037/a0029014

Nouwen A, 2011, DIABETES CARE, V34, P752, DOI 10.2337/dc10-1414

Pan A, 2010, ARCH INTERN MED, V170, P1884, DOI 10.1001/archinternmed.2010.356

Polonsky WH, 2005, DIABETES CARE, V28, P626, DOI 10.2337/diacare.28.3.626

Pouwer F, 2003, DIABETOLOGIA, V46, P892, DOI 10.1007/s00125-003-1124-6

Radloff LS, 1977, APPL PSYCH MEAS, V1, P385, DOI DOI 10.1177/014662167700100306

Roy T, 2012, DIABETIC MED, V29, P164, DOI 10.1111/j.1464-5491.2011.03401.x

Spitzer RL, 1999, JAMA-J AM MED ASSOC, V282, P1737, DOI 10.1001/jama.282.18.1737

Sullivan MD, 2012, DIABETES CARE, V35, P1708, DOI 10.2337/dc11-1791

Tanenbaum ML, 2012, DIABETES EDUCATOR, V38, P695, DOI 10.1177/0145721712452795

Trief PM, 2007, DIABETES CARE, V30, P1266, DOI 10.2337/dc06-2476

Twist K, 2013, PSYCHOSOM MED, V75, P803

WAKEFIELD JC, 1992, AM PSYCHOL, V47, P373, DOI 10.1037//0003-066X.47.3.373

Wakefield JC, 2013, WORLD PSYCHIATRY, V12, P44, DOI 10.1002/wps.20015

Wakefield JC, 2012, COMPR PSYCHIAT, V53, P130, DOI 10.1016/j.comppsych.2011.03.001

Welch GW, 1997, DIABETES CARE, V20, P760, DOI 10.2337/diacare.20.5.760

Williams JW, 2004, ANN INTERN MED, V140, P1015, DOI 10.7326/0003-4819-140-12-200406150-00012

Wittchen HU, 1994, J PSYCHIATR RES, V28, P57

NR 48

TC 123

Z9 125

U1 6

U2 44

PU WILEY-BLACKWELL

PI HOBOKEN

PA 111 RIVER ST, HOBOKEN 07030-5774, NJ USA

SN 0742-3071

EI 1464-5491

J9 DIABETIC MED

JI Diabetic Med.

PD JUL

PY 2014

VL 31

IS 7

BP 764

EP 772

DI 10.1111/dme.12428

PG 9

WC Endocrinology & Metabolism

SC Endocrinology & Metabolism

GA AJ4DL

UT WOS:000337621100004

PM 24606397

OA Green Accepted

HC Y

HP N

DA 2019-01-08

ER

PT J

AU Pengpid, S

Peltzer, K

Skaal, L

AF Pengpid, Supa

Peltzer, Karl

Skaal, Linda

TI Efficacy of a church-based lifestyle intervention programme to control

high normal blood pressure and/or high normal blood glucose in church

members: a randomized controlled trial in Pretoria, South Africa

SO BMC PUBLIC HEALTH

LA English

DT Article

ID DIABETES PREVENTION PROGRAM; NEW-ZEALAND; RISK; HEALTH; TRANSLATION

AB Background: In persons 15 years and above in South Africa the prevalence of pre-diabetes and diabetes has been estimated at 9.1% and 9.6%, respectively, and the prevalence of systolic prehypertension and hypertension, 38.2% and 24.6%, respectively. Elevated blood glucose and elevated blood pressure are prototype of preventable chronic cardiovascular disease risk factors. Lifestyle interventions have been shown to control high normal blood pressure and/or high normal blood glucose.

Methods/Design: This study proposes to evaluate the efficacy of a community (church)-based lifestyle intervention programme to control high normal blood pressure and/or high normal blood glucose in church members in a randomized controlled trial in Gauteng, South Africa. The objectives are to: (1) measure non-communicable diseases profile, including hypertension and diabetes, health behaviours, weight management and psychological distress of church members; (2) measure the reduction of blood glucose and blood pressure levels after the intervention; (3) prevent the development of impaired glucose tolerance; (4) compare health behaviours, weight management and psychological distress, blood glucose and blood pressure levels between intervention and control groups, and within group during 6, 12, 24 and 36 months during and post intervention. The study will use a group-randomized design, recruiting 300 church members from 12 churches. Churches will be randomly assigned to experimental and control conditions.

Discussion: Lifestyle interventions may prevent from the development of high blood pressure and/or diabetes. The findings will impact public health and will enable the health ministry to formulate policy related to lifestyle interventions to control blood pressure and glucose.

C1 [Pengpid, Supa] Univ Limpopo, Dept Res Dev & Innovat, ZA-0727 Sovenga, Turfloop, South Africa.

[Pengpid, Supa; Peltzer, Karl] Mahidol Univ, ASEAN Inst Hlth Dev, Phutthamonthon 73170, Nakhonpathom, Thailand.

[Peltzer, Karl] Univ Limpopo, Dept Psychol, Sovenga, Turfloop, South Africa.

[Peltzer, Karl] Human Sci Res Council, HIV AIDS TB & STIs HAST, ZA-0001 Pretoria, South Africa.

[Skaal, Linda] Univ Limpopo, Dept Publ Hlth, ZA-0727 Sovenga, Turfloop, South Africa.

RP Pengpid, S (reprint author), Univ Limpopo, Dept Res Dev & Innovat, ZA-0727 Sovenga, Turfloop, South Africa.

EM supaprom@yahoo.com

FU Directorate General for Development Cooperation (DGDC) through the

Flemish Interuniversity Council (VLIR-UOS)

FX The trial is funded by the Directorate General for Development

Cooperation (DGDC) through the Flemish Interuniversity Council

(VLIR-UOS). Brian Oldenburg and Pelvikki Absetz are thanked for their

input in the development of previous versions of the protocol. We also

acknowledge input from Pindile Mntla, Busi Ntuli-Ngcobo, Mashudu Manafe,

Christophe De Block, Marc Van Sprundel.

CR Absetz P, 2011, GOAL LIFESTYLE IMPLE

Ackermann RT, 2008, AM J PREV MED, V35, P357, DOI 10.1016/j.amepre.2008.06.035

Adams G, 2004, J CLIN EPIDEMIOL, V57, P785, DOI 10.1016/j.jclinepi.2003.12.013

Anderson A, 2002, INT REV MISSION, V91, P523

Anderson A, 2000, ZION PENTECOST SPIRI

Anderson Allan H, 1992, THESIS U S AFRICA

[Anonymous], 2001, SAJCN, V14

Boltri JM, 2011, J NATL MED ASSOC, V103, P194, DOI 10.1016/S0027-9684(15)30301-1

Brown N, 2012, DIABETES RES CLIN PR, V98, P369, DOI 10.1016/j.diabres.2012.09.005

Campbell MK, 2004, BMJ-BRIT MED J, V328, P702, DOI 10.1136/bmj.328.7441.702

Dagogo-Jack S, 2010, MED PRIN PRACT, V19, P167, DOI 10.1159/000285280

Egan BM, 2010, CARDIOL CLIN, V28, P561, DOI 10.1016/j.ccl.2010.07.008

Funk KL, 2008, HEALTH PROMOT PRACT, V9, P271, DOI 10.1177/1524839906289035

Glasgow RE, 2003, AM J PUBLIC HEALTH, V93, P1261, DOI 10.2105/AJPH.93.8.1261

Hedayati SS, 2011, KIDNEY INT, V79, P1061, DOI 10.1038/ki.2011.46

Knowler WC, 2002, NEW ENGL J MED, V346, P393

Lambert E. V., 2001, SAJCN - South African Journal of Clinical Nutrition, V14, pS12

Lancaster KJ, 2014, AM HEART J, V167, P301, DOI 10.1016/j.ahj.2013.10.026

Lien LF, 2007, HYPERTENSION, V50, P609, DOI 10.1161/HYPERTENSIONAHA.107.089458

Lindstrom J, 2013, DIABETOLOGIA, V56, P284, DOI 10.1007/s00125-012-2752-5

Makrilakis K, 2011, DIABETES METAB, V37, P144, DOI 10.1016/j.diabet.2010.09.006

Marquez-Celedonio FG, 2009, REV ESP CARDIOL, V62, P86, DOI 10.1016/S0300-8932(09)70025-9

Maruthur NM, 2009, CIRCULATION, V119, P2026, DOI 10.1161/CIRCULATIONAHA.108.809491

NATHAN DM, 1997, LANCET S1, V350, pS14, DOI DOI 10.1016/S0140-6736(97)90021-0

Oettingen G., 2000, INT J EDUC RES, V33, P705, DOI DOI 10.1016/S0883-0355(00)00046-X

Oexmann Mary Joan, 2000, Ethnicity and Disease, V10, P17

Orozco LJ, 2008, COCHRANE DB SYST REV, DOI 10.1002/14651858.CD003054.pub3

Papadopoulos DP, 2008, HYPERTENS RES, V31, P1681, DOI 10.1291/hypres.31.1681

Peltzer K, 1999, J RELIG AFR, V29, P387

Petrella RJ, 2011, TRIALS, V12, DOI 10.1186/1745-6215-12-45

Schulz KF, 2010, PLOS MED, V7, DOI 10.1371/journal.pmed.1000251

Schwarzer R, 1995, SELF EFFICACY CHANGI, P259, DOI DOI 10.1017/CBO9780511527692.011

Seedat Y K, 2011, S Afr Med J, V102, P57

Shisana O LD, 2013, SANHANES 1 TEAM S AF

Simmons D, 1998, DIABETIC MED, V15, P136, DOI 10.1002/(SICI)1096-9136(199802)15:2<136::AID-DIA530>3.3.CO;2-G

Simmons D, 2004, DIABETIC MED, V21, P122, DOI 10.1111/j.1464-5491.2004.01020.x

Thomas GN, 2010, CURR DIABETES REV, V6, P378, DOI 10.2174/157339910793499092

Tuomilehto J, 2001, NEW ENGL J MED, V344, P1343, DOI 10.1056/NEJM200105033441801

Uutela A, 2004, J HEALTH PSYCHOL, V9, P73, DOI 10.1177/1359105304036103

WHO, 2011, HYP FACT SHEET

Yoon U, 2012, METABOLISM

NR 41

TC 4

Z9 4

U1 2

U2 23

PU BMC

PI LONDON

PA CAMPUS, 4 CRINAN ST, LONDON N1 9XW, ENGLAND

SN 1471-2458

J9 BMC PUBLIC HEALTH

JI BMC Public Health

PD JUN 6

PY 2014

VL 14

AR 568

DI 10.1186/1471-2458-14-568

PG 8

WC Public, Environmental & Occupational Health

SC Public, Environmental & Occupational Health

GA AL2OF

UT WOS:000338964200004

PM 24906450

OA DOAJ Gold, Green Published

DA 2019-01-08

ER

PT J

AU Kopf, S

Oikonomou, D

Hartmann, M

Feier, F

Faude-Lang, V

Morcos, M

Haring, HU

Herzog, W

Bierhaus, A

Humpert, PM

Nawroth, PP

AF Kopf, S.

Oikonomou, D.

Hartmann, M.

Feier, F.

Faude-Lang, V.

Morcos, M.

Haering, H. -U.

Herzog, W.

Bierhaus, A.

Humpert, P. M.

Nawroth, P. P.

TI Effects of Stress Reduction on Cardiovascular Risk Factors in Type 2

Diabetes Patients with Early Kidney Disease - Results of a Randomized

Controlled Trial (HEIDIS)

SO EXPERIMENTAL AND CLINICAL ENDOCRINOLOGY & DIABETES

LA English

DT Article

DE type 2 diabetes; psychosocial stress; MBSR; diabetic kidney disease;

cardiovascular risk

ID CORONARY-HEART-DISEASE; BLOOD-GLUCOSE CONTROL; LONG-TERM; CAROTID

ATHEROSCLEROSIS; MYOCARDIAL-INFARCTION; PSYCHOSOCIAL STRESS; EMOTIONAL

DISTRESS; GLYCEMIC CONTROL; MINDFULNESS; MORTALITY

AB Objective: Current guidelines for the treatment of type 2 diabetes focus on pharmacological treatment of glucose and cardio-vascular risk factors. The aim of this prospective randomized controlled intervention study was to examine the effects of a psychosocial intervention on clinical endpoints and risk factors in patients with type 2 diabetes and early diabetic kidney disease.

Methods: 110 patients were randomized to receive an 8-week mindfulness-based stress reduction (MBSR) training (n = 53) compared to standard care (n = 57). The study was carried out open-labelled and randomization was performed computer-generated in a 1:1 ratio. Primary outcome of the study was the change in urinary albumin excretion (albumin-creatinine-ratio, ACR); secondary outcomes were metabolic parameters, intima media thickness (IMT), psychosocial parameters and cardiovascular events.

Results: 89 patients (42 in control group and 47 in intervention group) were analysed after 3 years of follow-up. After 1 year, the intervention group showed a reduction of ACR from 44 [16/80] to 39 [20/71] mg/g, while controls increased from 47 [16/120] to 59 [19/128] mg/g (p = 0.05). Parallel to the reduction of stress levels after 1 year, the intervention-group additionally showed reduced catecholamine levels (p < 0.05), improved 24 h-mean arterial (p < 0.05) and maximum systolic blood pressure (p < 0.01), as well as a reduction in IMT (p < 0.01). However, these effects were lost after 2 and 3 years of follow-up.

Conclusions: This is the first study to show that a psychosocial intervention improves cardiovascular risk factors in high risk type 2 diabetes patients.

C1 [Kopf, S.; Oikonomou, D.; Feier, F.; Morcos, M.; Bierhaus, A.; Humpert, P. M.; Nawroth, P. P.] Heidelberg Univ, Dept Med & Clin Chem 1, D-69120 Heidelberg, Germany.

[Hartmann, M.; Faude-Lang, V.; Herzog, W.] Heidelberg Univ, Dept Med & Psychosomat 2, D-69120 Heidelberg, Germany.

[Faude-Lang, V.] Univ Hamburg Eppendorf, Dept Psychosomat Med & Psychotherapy, Hamburg, Germany.

[Morcos, M.; Humpert, P. M.] Stoffwechselzentrum Rhein Pfalz, Mannheim, Germany.

[Haering, H. -U.] Univ Tubingen, Dept Med & Clin Chem 4, Tubingen, Germany.

RP Kopf, S (reprint author), Heidelberg Univ, Dept Med & Clin Chem 1, Neuenheimer Feld 410, D-69120 Heidelberg, Germany.

EM Stefan.Kopf@med.uni-heidelberg.de

FU Manfred Lautenschlager Foundation; Dietmar Hopp Foundation; Deutsches

Zentrum fur Diabetesforschund (DZD); Institute for Diabetes Research and

Metabolic Diseases; Federal Ministry of Education and Research (BMBF)

FX This study was supported by the Manfred Lautenschlager Foundation and

Dietmar Hopp Foundation (both to P. Nawroth) and the Deutsches Zentrum

fur Diabetesforschund (DZD) in cooperation with the Institute for

Diabetes Research and Metabolic Diseases (to H-U. Haring, University of

Tubingen, Germany). The Manfred Lautenschlager Foundation is a privately

sourced foundation to this project. The funding was used to support

physicians and study nurses as well as laboratory materials. The Dietmar

Hopp Foundation is a privately sourced foundation supporting part of

this study with laboratory materials. The Deutsches Zentrum fur

Diabetesforschung is funded by the Federal Ministry of Education and

Research (BMBF). The funders had no role in study design, data

collection and analysis, decision to publish, or preparation of the

manuscript.

CR ADAMS DO, 1994, PSYCHOSOM MED, V56, P316, DOI 10.1097/00006842-199407000-00007

Berry JD, 2012, NEW ENGL J MED, V366, P321, DOI 10.1056/NEJMoa1012848

Bierhaus A, 2006, ANESTHESIOL CLIN N A, V24, P325, DOI 10.1016/j.atc.2006.01.001

Bierhaus A, 2003, P NATL ACAD SCI USA, V100, P1920, DOI 10.1073/pnas.0438019100

Blumenthal JA, 2005, JAMA-J AM MED ASSOC, V293, P1626, DOI 10.1001/jama.293.13.1626

Brand S, 2012, NEUROPSYCHOBIOLOGY, V65, P109, DOI 10.1159/000330362

Cao JJ, 2008, ATHEROSCLEROSIS, V197, P806, DOI 10.1016/j.atherosclerosis.2007.07.029

Castillo-Richmond A, 2000, STROKE, V31, P568, DOI 10.1161/01.STR.31.3.568

Colhoun HM, 2004, LANCET, V364, P685, DOI 10.1016/S0140-6736(04)16895-5

Crouse JR, 2007, JAMA-J AM MED ASSOC, V297, P1344, DOI 10.1001/jama.297.12.1344

Danesh J, 2004, NEW ENGL J MED, V350, P1387, DOI 10.1056/NEJMoa032804

de Groot E, 1998, J AM COLL CARDIOL, V31, P1561, DOI 10.1016/S0735-1097(98)00170-3

Denollet J, 2001, CIRCULATION, V104, P2018, DOI 10.1161/hc4201.097940

Deutsche Diabetes Gesellschaft. (DDG), 2006, GUID DIAGN TREATM FO

Duckworth W, 2009, NEW ENGL J MED, V360, P129, DOI 10.1056/NEJMoa0808431

Eurich DT, 2004, DIABETES CARE, V27, P1330, DOI 10.2337/diacare.27.6.1330

Faude-Lang V, 2010, PSYCHOTHER PSYCH MED, V60, P185, DOI 10.1055/s-0030-1248420

Fields JZ, 2002, AM J CARDIOL, V89, P952, DOI 10.1016/S0002-9149(02)02245-2

Fujihara K, 2012, EXP CLIN ENDOCR DIAB, V120, P560, DOI 10.1055/s-0032-1314873

Gaede P, 2008, NEW ENGL J MED, V358, P580, DOI 10.1056/NEJMoa0706245

Gerstein HC, 2008, NEW ENGL J MED, V358, P2545, DOI 10.1056/NEJMoa0802743

Goldstein David S, 2008, Endocr Regul, V42, P111

Haffner SM, 1998, NEW ENGL J MED, V339, P229, DOI 10.1056/NEJM199807233390404

Hamer M, 2008, J AM COLL CARDIOL, V52, P2156, DOI 10.1016/j.jacc.2008.08.057

Hartmann M, 2012, DIABETES CARE, V35, P945, DOI 10.2337/dc11-1343

Hu ZW, 1999, J PHARMACOL EXP THER, V290, P28

Humpert PM, 2007, HORM METAB RES, V39, P899, DOI 10.1055/s-2007-993155

Kabat-Zinn J., 1990, FULL CATASTROPHE LIV

Levey AS, 2009, ANN INTERN MED, V150, P604, DOI 10.7326/0003-4819-150-9-200905050-00006

Lin EHB, 2010, DIABETES CARE, V33, P264, DOI 10.2337/dc09-1068

Lowe B, 2004, J AFFECT DISORDERS, V78, P131, DOI 10.1016/S0165-0327(02)00237-9

Ludwig DS, 2008, JAMA-J AM MED ASSOC, V300, P1350, DOI 10.1001/jama.300.11.1350

McEwen BS, 2000, NEUROPSYCHOPHARMACOL, V22, P108, DOI 10.1016/S0893-133X(99)00129-3

Nawroth PP, 2010, EXP CLIN ENDOCR DIAB, V118, P1, DOI 10.1055/s-0029-1246117

Ninomiya T, 2009, J AM SOC NEPHROL, V20, P1813, DOI 10.1681/ASN.2008121270

Parving HH, 2001, NEW ENGL J MED, V345, P870, DOI 10.1056/NEJMoa011489

Patel A, 2008, NEW ENGL J MED, V358, P2560, DOI 10.1056/NEJMoa0802987

Rosenkranz MA, 2013, BRAIN BEHAV IMMUN, V27, P174, DOI 10.1016/j.bbi.2012.10.013

Rosenzweig S, 2010, J PSYCHOSOM RES, V68, P29, DOI 10.1016/j.jpsychores.2009.03.010

Rozanski A, 1999, CIRCULATION, V99, P2192, DOI 10.1161/01.CIR.99.16.2192

RUBIN RR, 1992, DIABETES CARE, V15, P1640, DOI 10.2337/diacare.15.11.1640

Samann A, 2012, EXP CLIN ENDOCR DIAB, V120, P335, DOI 10.1055/s-0031-1299705

Surwit RS, 2002, DIABETES CARE, V25, P30, DOI 10.2337/diacare.25.1.30

Thayer JF, 2010, INT J CARDIOL, V141, P122, DOI 10.1016/j.ijcard.2009.09.543

van Son J, 2013, DIABETES CARE, V36, P823, DOI 10.2337/dc12-1477

von Eynatten M, 2009, DIABETES, V58, P2093, DOI 10.2337/db09-0204

Walton KG, 2002, BEHAV MED, V28, P106, DOI 10.1080/08964280209596049

Ward D, 2002, J VASC RES, V39, P1, DOI 10.1159/000048988

Yang WY, 2010, NEW ENGL J MED, V362, P1090, DOI 10.1056/NEJMoa0908292

Yang Z, 2013, EXP CLIN ENDOCR DIAB, V121, P372, DOI 10.1055/s-0033-1341520

Yusuf S, 2004, LANCET, V364, P937, DOI 10.1016/S0140-6736(04)17018-9

1998, LANCET, V352, P854

2000, LANCET, V355, P253

NR 53

TC 12

Z9 12

U1 5

U2 31

PU JOHANN AMBROSIUS BARTH VERLAG MEDIZINVERLAGE HEIDELBERG GMBH

PI STUTTGART

PA RUEDIGERSTR 14, D-70469 STUTTGART, GERMANY

SN 0947-7349

EI 1439-3646

J9 EXP CLIN ENDOCR DIAB

JI Exp. Clin. Endocrinol. Diabet.

PD JUN

PY 2014

VL 122

IS 6

BP 341

EP 349

DI 10.1055/s-0034-1372583

PG 9

WC Endocrinology & Metabolism

SC Endocrinology & Metabolism

GA AW4RQ

UT WOS:000346268700005

PM 24798861

DA 2019-01-08

ER

PT J

AU Byles, JE

Robinson, I

Banks, E

Gibson, R

Leigh, L

Rodgers, B

Curryer, C

Jorm, L

AF Byles, Julie E.

Robinson, Ian

Banks, Emily

Gibson, Richard

Leigh, Lucy

Rodgers, Bryan

Curryer, Cassie

Jorm, Louisa

TI PSYCHOLOGICAL DISTRESS AND COMORBID PHYSICAL CONDITIONS: DISEASE OR

DISABILITY?

SO DEPRESSION AND ANXIETY

LA English

DT Article

DE psychological distress; mental health; comorbidity; chronic disease;

socioeconomic factors; patient care planning; disability

ID PERCEIVED SOCIAL SUPPORT; COMMUNITY-HEALTH SURVEY; FUNCTIONAL

DISABILITY; DEPRESSIVE SYMPTOMS; INTERVIEW SURVEY; HEART-DISEASE;

RISK-FACTOR; US ADULTS; LATE-LIFE; ILLNESS

AB Background: The relationship between comorbidity and psychological distress is well documented, however the mechanism of this association is unclear. We aim to assess the extent to which the association between common chronic conditions and high scores on the Kessler Psychological Distress Scale (K10) measure of psychological distress vary according to comorbid conditions, disability, and sociodemographic circumstances. Methods: Analysis of self-reported cross-sectional data from the New South Wales 45 and Up Study, Australia, for 236,508 participants aged 45 years and over, using logistic regression modeling. Results: Self-reported heart attack/angina, other heart disease, stroke, and diabetes were all significantly associated with higher risk of high/very high K10 scores. These associations were attenuated, but remained statistically significant, when comorbidity, disability, and sociodemographic factors were added to the model. Men reporting needing help for daily tasks were nine times as likely to report high/very high K10 scores as those without this need, and women reporting needing help were seven times more likely to have high/very high K10 scores. Conclusions: Heart attack/angina, other heart disease, stroke, and diabetes are all significantly associated with psychological distress. However, these effects are partly explained by other comorbid conditions, limitations on physical functioning, and sociodemographic factors. These findings highlight the importance of developing public health policies that encompass psychological, physiological, and social domains, and provide crucial insights for clinicians in identifying and supporting those people at risk of psychological distress. Depression and Anxiety 31: 524-532, 2014. (C) 2013 Wiley Periodicals, Inc.

C1 [Byles, Julie E.; Gibson, Richard; Leigh, Lucy; Curryer, Cassie] Univ Newcastle, Res Ctr Gender Hlth & Ageing, Fac Hlth & Med, Newcastle, NSW 2308, Australia.

[Byles, Julie E.] Univ Newcastle, World Hlth Org WHO Collaborating Ctr Internatl Lo, Newcastle, NSW 2308, Australia.

[Byles, Julie E.] HMRI, Publ Hlth Capac Bldg Grp, Newcastle, NSW, Australia.

[Byles, Julie E.] AAG, Melbourne, Vic, Australia.

[Robinson, Ian] Univ Newcastle, Acad & Global Relat Div, Newcastle, NSW 2308, Australia.

[Banks, Emily; Jorm, Louisa] Univ Technol Sydney, Sax Inst, Sydney, NSW 2007, Australia.

[Banks, Emily] Australian Natl Univ, Natl Ctr Epidemiol & Populat Hlth, Canberra, ACT, Australia.

[Rodgers, Bryan] Australian Natl Univ, Australian Demog & Social Res Inst, Canberra, ACT, Australia.

[Rodgers, Bryan] Australian Natl Univ, Coll Arts & Social Sci, Canberra, ACT, Australia.

[Jorm, Louisa] Univ Western Sydney, Dept Populat Hlth, Sydney, NSW, Australia.

RP Byles, JE (reprint author), Univ Newcastle, Res Ctr Gender Hlth & Ageing, Fac Hlth & Med, HMRI Bldg, Newcastle, NSW 2308, Australia.

EM julie.byles@newcastle.edu.au

RI Byles, Julie/C-5062-2008

OI Byles, Julie/0000-0002-3984-6877

CR AKAIKE H, 1987, PSYCHOMETRIKA, V52, P317, DOI 10.1007/BF02294359

Australian Bureau of Statistics, 2009, NAT HLTH SURV 2007 2

Australian Bureau of Statistics, 2008, NAT SURV MENT HLTH W

Balon R, 2006, DEPRESS ANXIETY, V23, P377, DOI 10.1002/da.20217

Bambara JK, 2011, DISABIL REHABIL, V33, P1, DOI 10.3109/09638288.2010.481026

Banks E, 2001, J Epidemiol Biostat, V6, P357

Banks E, 2010, MED J AUSTRALIA, V193, P62

Banks E, 2008, INT J EPIDEMIOL, V37, P941, DOI 10.1093/ije/dym184

Barnett K, 2012, LANCET, V380, P37, DOI 10.1016/S0140-6736(12)60240-2

Cairney J, 2007, CAN J PSYCHIAT, V52, P111, DOI 10.1177/070674370705200209

Carney RA, 2002, STROKE, V33, P5, DOI 10.1161/str.33.1.5

Centre for Epidemiology and Research, 2009, 2008 REP AD HLTH NEW

Cunningham J, 2012, BMC PUBLIC HEALTH, V12, DOI 10.1186/1471-2458-12-95

Department of Health and Ageing, 2001, DEP HLTH AG OCC PAP, V14

Drapeau A., 2012, MENTAL ILLNESSES UND, P105, DOI DOI 10.5772/1235

Dunlop DD, 2004, MED CARE, V42, P502, DOI 10.1097/01.mlr.0000127997.51128.81

Egede LE, 2012, CURR PSYCHIAT REP, V14, P15, DOI 10.1007/s11920-011-0240-0

Fenton WS, 2006, CURR OPIN PSYCHIATR, V19, P421, DOI 10.1097/01.yco.0000228765.33356.9f

Fortin M, 2006, ANN FAM MED, V4, P417, DOI 10.1370/afm.528

Greenbaum JN, 2012, J ARTHROPLASTY, V27, P1310, DOI 10.1016/j.arth.2011.10.031

Hagger MS, 2003, PSYCHOL HEALTH, V18, P141, DOI 10.1080/088704403100081321

Hamer M, 2008, J AM COLL CARDIOL, V52, P2156, DOI 10.1016/j.jacc.2008.08.057

Hamer M, 2009, J PSYCHOSOM RES, V66, P255, DOI 10.1016/j.jpsychores.2008.11.002

Holden L, 2010, AUST NZ J PUBL HEAL, V34, P304, DOI 10.1111/j.1753-6405.2010.00531.x

Hough E S, 1999, Health Care Women Int, V20, P349

Huang CQ, 2010, AGEING RES REV, V9, P131, DOI 10.1016/j.arr.2009.05.005

Kessler RC, 2002, PSYCHOL MED, V32, P959, DOI 10.1017/S0033291702006074

Leventhal H, 1998, PSYCHOL HEALTH, V13, P717, DOI 10.1080/08870449808407425

Leventhal H, 2012, SELF REGULATION HLTH, P42

Lewis G, 2012, BRIT MED J, V345, DOI 10.1136/bmj.e5177

May M, 2002, STROKE, V33, P7, DOI 10.1161/hs0102.100529

Mirowsky J, 2002, J HEALTH SOC BEHAV, V43, P152, DOI 10.2307/3090194

Mommersteeg PMC, 2012, BMC PUBLIC HEALTH, V12, DOI 10.1186/1471-2458-12-1109

Moon L, 2006, SOCIOECONOMIC INEQUA

Moore JL, 2009, EPILEPSIA, V50, P1077, DOI 10.1111/j.1528-1167.2008.01996.x

Ormel J, 2002, J GERONTOL B-PSYCHOL, V57, pP338, DOI 10.1093/geronb/57.4.P338

Quail JM, 2011, CAN J AGING, V30, P591, DOI 10.1017/S0714980811000493

Rasul F, 2005, J EPIDEMIOL COMMUN H, V59, P140, DOI 10.1136/jech.2004.019786

Ridner SH, 2004, J ADV NURS, V45, P536, DOI 10.1046/j.1365-2648.2003.02938.x

Russ TC, 2012, BRIT MED J, V345, DOI 10.1136/bmj.e4933

Schmitz N, 2008, J PSYCHOSOM RES, V65, P165, DOI 10.1016/j.jpsychores.2008.03.004

Schmitz N, 2009, PSYCHOSOMATICS, V50, P42, DOI 10.1176/appi.psy.50.1.42

Serrano CV, 2011, VASC HEALTH RISK MAN, V7, P159, DOI 10.2147/VHRM.S10783

Shih M, 2006, J GEN INTERN MED, V21, P1160, DOI 10.1111/j.1525-1497.2006.00573.x

Strine TW, 2005, EPILEPSIA, V46, P1133, DOI 10.1111/j.1528-1167.2005.01605.x

Valderas JM, 2009, ANN FAM MED, V7, P357, DOI 10.1370/afm.983

WARE JE, 1994, SF 36 PHYSL MENTAL H

Yang Y, 2006, J HEALTH SOC BEHAV, V47, P355, DOI 10.1177/002214650604700404

Zeiss AM, 1996, PSYCHOL AGING, V11, P572, DOI 10.1037/0882-7974.11.4.572

NR 49

TC 16

Z9 16

U1 0

U2 16

PU WILEY-BLACKWELL

PI HOBOKEN

PA 111 RIVER ST, HOBOKEN 07030-5774, NJ USA

SN 1091-4269

EI 1520-6394

J9 DEPRESS ANXIETY

JI Depress. Anxiety

PD JUN

PY 2014

VL 31

IS 6

BP 524

EP 532

DI 10.1002/da.22162

PG 9

WC Psychology, Clinical; Psychiatry; Psychology

SC Psychology; Psychiatry

GA AL3XZ

UT WOS:000339066600008

PM 23922120

DA 2019-01-08

ER

PT J

AU Sorkin, DH

Mavandadi, S

Rook, KS

Biegler, KA

Kilgore, D

Dow, E

Ngo-Metzger, Q

AF Sorkin, Dara H.

Mavandadi, Shahrzad

Rook, Karen S.

Biegler, Kelly A.

Kilgore, David

Dow, Emily

Ngo-Metzger, Quyen

TI Dyadic Collaboration in Shared Health Behavior Change: The Effects of a

Randomized Trial to Test a Lifestyle Intervention for High-Risk Latinas

SO HEALTH PSYCHOLOGY

LA English

DT Article

DE dyadic behavioral lifestyle intervention; social support and social

control; type 2 diabetes control and prevention; weight loss; Hispanic;

Latina

ID DIABETES PREVENTION PROGRAM; WEIGHT-LOSS INTERVENTION; MEXICAN-AMERICAN

WOMEN; SOCIAL SUPPORT; PHYSICAL-ACTIVITY; CHRONIC ILLNESS; PSYCHOLOGICAL

DISTRESS; GLYCEMIC LOAD; US ADULTS; FAMILY

AB Objective: This study sought to evaluate the feasibility of a pilot, dyad-based lifestyle intervention, the Unidas por la Vida program, for improving weight loss and dietary intake among high-risk Mexican American mothers who have Type 2 diabetes and their overweight/obese adult daughters. Method: Mother-daughter dyads (N = 89) were recruited from two federally qualified health centers and randomly assigned to either the Unidas intervention or to the control condition. The 16-week Unidas intervention consisted of the following: (a) four group meetings, (b) eight home visits, and (c) booster telephone calls by a lifestyle community coach. The control condition consisted of educational materials mailed to participants' homes. Participants completed surveys at T1 (baseline) and T2 (16 weeks) that assessed various demographic, social network involvement, and dietary variables. Results: Unidas participants lost significantly more weight at T2 (p < .003) compared with the control participants. Furthermore, intervention participants also were more likely to be eating foods with lower glycemic load (p < .001) and less saturated fat (p = .004) at T2. Unidas participants also reported a significant increase in health-related social support and social control (persuasion control only) and a decrease in undermining. Conclusions: The Unidas program promoted weight loss and improved dietary intake, as well as changes in diet-related involvement of participants' social networks. The results from this study demonstrate that interventions that draw upon multiple people who share a health-risk have the potential to foster significant changes in lifestyle behaviors and in social network members' health-related involvement. Future research that builds on these findings is needed to elucidate the specific dyadic and social network processes that may drive health behavior change.

C1 [Sorkin, Dara H.; Biegler, Kelly A.] Univ Calif Irvine, Div Gen Internal Med, Irvine, CA USA.

[Sorkin, Dara H.; Biegler, Kelly A.] Univ Calif Irvine, Primary Care & Hlth Policy Res Inst, Irvine, CA USA.

[Mavandadi, Shahrzad] Univ Penn, Perelman Sch Med, Philadelphia VA Med Center;, Mental Illness Res Educ & Clin Ctr,Dept Psychiat, Philadelphia, PA 19104 USA.

[Rook, Karen S.] Univ Calif Irvine, Dept Psychol & Social Behav, Irvine, CA USA.

[Kilgore, David; Dow, Emily] Univ Calif Irvine, Div Family Med, Irvine, CA USA.

[Ngo-Metzger, Quyen] Agcy Healthcare Res & Qual, Ctr Primary Care Prevent & Clin Partnership, US Prevent Serv Task Force Program, US Dept Hlth & Human Serv, Rockville, MD USA.

RP Sorkin, DH (reprint author), UCI Hlth Policy Res Inst, Suite 110, Irvine, CA 92617 USA.

EM dsorkin@uci.edu

FU National Center for Research Resources; National Center for Advancing

Translational Sciences, National Institutes of Health [UL1 TR000153,

R34DK083500, K01DK078939]

FX We thank Carolina Vilchis and Grada Christensen for their contributions.

We would also like to thank Karen McGlinn, Executive Director, Share Our

Selves Medical Clinic, for her unwavering support. The project described

was supported by the National Center for Research Resources and the

National Center for Advancing Translational Sciences, National

Institutes of Health, through Grants UL1 TR000153, R34DK083500, and

K01DK078939. The views expressed in this publication are solely the

opinions of the authors and do not necessarily reflect the official

policies of U.S. Department of Veterans Affairs, Department of Health

and Human Services, the National Institutes of Health, or the Agency for

Healthcare Research and Quality, nor does mention of the department or

agency names imply endorsement by the U.S. government.

CR American Diabetes Association, 2013, CREAT YOUR PLAT

August KJ, 2010, SOC SCI MED, V71, P1831, DOI 10.1016/j.socscimed.2010.08.022

Berg Judith A, 2002, Health Care Women Int, V23, P894, DOI 10.1080/07399330290112399

Berkman LF, 2000, SOC WORK HEALTH CARE, V31, P3, DOI 10.1300/J010v31n02_02

Berkman LF, 2000, SOC SCI MED, V51, P843, DOI 10.1016/S0277-9536(00)00065-4

Bishop J, 2013, FAM COMMUNITY HEALTH, V36, P158, DOI 10.1097/FCH.0b013e318282b2d3

BLOCK G, 1986, AM J EPIDEMIOL, V124, P453, DOI 10.1093/oxfordjournals.aje.a114416

Block G, 2000, AM J PREV MED, V18, P284, DOI 10.1016/S0749-3797(00)00119-7

Block G., 2006, PREVENTING CHRONIC D, V3, P1

Campbell L, 2002, PERS RELATIONSHIP, V9, P327, DOI 10.1111/1475-6811.00023

Center for Disease Control and Prevention, 2011, NAT DIAB FACT SHEET

Chang VW, 2003, AM J PREV MED, V24, P332, DOI 10.1016/S0749-3797(03)00020-5

Cleland V, 2013, OBES REV, V14, P197, DOI 10.1111/j.1467-789X.2012.01058.x

Coleman KJ, 2010, DIABETES EDUCATOR, V36, P784, DOI 10.1177/0145721710377360

Corsino L, 2012, ETHNIC DIS, V22, P51

COUSINS JH, 1992, PUBLIC HEALTH REP, V107, P549

Everson-Hock ES, 2013, PREV MED, V56, P265, DOI 10.1016/j.ypmed.2013.02.023

Flegal KM, 2012, JAMA-J AM MED ASSOC, V307, P491, DOI 10.1001/jama.2012.39

Flegal KM, 2010, JAMA-J AM MED ASSOC, V303, P235, DOI 10.1001/jama.2009.2014

Franks MM, 2006, J FAM PSYCHOL, V20, P311, DOI 10.1037/0893-3200.20.2.311

Gallant MP, 2003, HEALTH EDUC BEHAV, V30, P170, DOI 10.1177/1090198102251030

Garcia-Maas L D, 1999, J Transcult Nurs, V10, P213, DOI 10.1177/104365969901000312

Gonder-Frederick LA, 2002, J CONSULT CLIN PSYCH, V70, P611, DOI 10.1037//0022-006X.70.3.611

Gorin AA, 2008, INT J OBESITY, V32, P1678, DOI 10.1038/ijo.2008.150

Greaves CJ, 2011, BMC PUBLIC HEALTH, V11, DOI 10.1186/1471-2458-11-119

Henry SL, 2013, J HEALTH PSYCHOL, V18, P1550, DOI 10.1177/1359105312465913

Herman WH, 2003, DIABETES CARE, V26, P36

Hogan BE, 2002, CLIN PSYCHOL REV, V22, P381, DOI 10.1016/S0272-7358(01)00102-7

Johnson M, 2013, DIABETIC MED, V30, P3, DOI 10.1111/dme.12018

Kenny D. A., 2006, DYADIC DATA ANAL

Knowler WC, 2002, NEW ENGL J MED, V346, P393

Lewis MA, 1999, HEALTH PSYCHOL, V18, P63, DOI 10.1037/0278-6133.18.1.63

Lindberg NM, 2013, J OBES, DOI 10.1155/2013/542736

Lindberg NM, 2012, J IMMIGR MINOR HEALT, V14, P1030, DOI 10.1007/s10903-012-9616-4

Livesey G, 2013, AM J CLIN NUTR, V97, P584, DOI 10.3945/ajcn.112.041467

Marquez B, 2013, J ACAD NUTR DIET, V113, P680, DOI 10.1016/j.jand.2013.01.020

Martire LM, 2004, HEALTH PSYCHOL, V23, P599, DOI 10.1037/0278-6133.23.6.599

Martire LM, 2013, ANN BEHAV MED, V45, P213, DOI 10.1007/s12160-012-9442-x

McLean N, 2003, INT J OBESITY, V27, P987, DOI 10.1038/sj.ijo.0802383

Melanson KJ, 2012, NUTR J, V11, DOI 10.1186/1475-2891-11-57

Narayan KMV, 2007, DIABETES CARE, V30, P1562, DOI 10.2337/dc06-2544

O'Brien MJ, 2013, DIABETES CARE, V36, P355, DOI 10.2337/dc12-0548

Perez LG, 2013, AM J PREV MED, V44, P550, DOI 10.1016/j.amepre.2013.01.016

Ritzwoller DP, 2013, J GEN INTERN MED, V28, P1581, DOI 10.1007/s11606-013-2492-3

ROOK KS, 1990, PSYCHOL AGING, V5, P327, DOI 10.1037/0882-7974.5.3.327

Rook KS., 2011, HDB STRESS SCI BIOL, P123

ROSENTHAL B, 1980, INT J OBESITY, V4, P165

SALLIS JF, 1987, PREV MED, V16, P825, DOI 10.1016/0091-7435(87)90022-3

Seeman TE, 2000, AM J HEALTH PROMOT, V14, P362, DOI 10.4278/0890-1171-14.6.362

Sorkin DH, 2013, J HEALTH CARE POOR U, V24, P116, DOI 10.1353/hpu.2013.0103

Stephens MAP, 2013, HEALTH PSYCHOL, V32, P1029, DOI 10.1037/a0030018

Stephens MAP, 2009, HEALTH PSYCHOL, V28, P48, DOI 10.1037/a0012385

TEUFELSHONE NI, 2005, PREVENTING CHRONIC D, V2, P1

Tucker JS, 2006, HEALTH PSYCHOL, V25, P715, DOI 10.1037/0278-6133.25.6.715

Tucker JS, 2006, PERS SOC PSYCHOL B, V32, P1143, DOI 10.1177/0146167206289728

US Department of Health and Human Services, WHAT AR FED QUAL HLT

Waters LA, 2011, J PHYS ACT HEALTH, V8, P85, DOI 10.1123/jpah.8.1.85

Willett W, 2002, AM J CLIN NUTR, V76, p274S

NR 58

TC 30

Z9 30

U1 2

U2 28

PU AMER PSYCHOLOGICAL ASSOC

PI WASHINGTON

PA 750 FIRST ST NE, WASHINGTON, DC 20002-4242 USA

SN 0278-6133

EI 1930-7810

J9 HEALTH PSYCHOL

JI Health Psychol.

PD JUN

PY 2014

VL 33

IS 6

SI SI

BP 566

EP 575

DI 10.1037/hea0000063

PG 10

WC Psychology, Clinical; Psychology

SC Psychology

GA AK5CQ

UT WOS:000338442300008

PM 24884910

OA Green Published

DA 2019-01-08

ER

PT J

AU Schrepf, A

Markon, K

Lutgendorf, SK

AF Schrepf, Andrew

Markon, Kristian

Lutgendorf, Susan K.

TI From Childhood Trauma to Elevated C-Reactive Protein in Adulthood: The

Role of Anxiety and Emotional Eating

SO PSYCHOSOMATIC MEDICINE

LA English

DT Article

DE C-reactive protein; inflammation; emotional eating; childhood trauma;

anxiety; stress; cardiovascular disease

ID RISK-FACTORS; PSYCHOMETRIC PROPERTIES; PSYCHOLOGICAL STRESS;

GENDER-DIFFERENCES; SEXUAL-ABUSE; LIFE-SPAN; DRUG-USE; DEPRESSION;

INFLAMMATION; OBESITY

AB Objective: Childhood trauma is known to be related to inflammatory processes in adulthood, but underlying psychological/behavioral mechanisms have not been fully characterized. To investigate associations between childhood trauma and inflammation (indexed by C-reactive protein [CRP]), we used a structural equation modeling approach on a subsample of the Midlife in the United States biomarker project. Methods: Participants included 687 men and women without history of cancer, diabetes, cardiovascular disease, or strokewho completed a physical examination and extensive questionnaires and provided blood. To test for sex differences, we held as many parameters invariant across sexes as possible while still retaining good model fit. Results: Tests of direct and indirect effects revealed that childhood trauma was significantly associated with elevated CRP, via elevated body mass index (BMI; p < .001). This relationship was mediated by a broad latent measure of distress, which was associated with using food as a coping mechanism. Men and women differed in reported levels of physical abuse, sexual abuse, and physical neglect. Compared with men, women showed a stronger association between BMI and CRP, whereas men had a stronger association between use of food to cope and elevated BMI. Conclusions: Our results are consistent with a model in which childhood trauma is associated with elevated CRP, a relationship associated with stress reactivity and compensatory emotional eating. Men and women may experience trauma in qualitatively distinct patterns but share many vulnerabilities, which can lead to elevated health risks. Emotional eating may be an important target for intervention in this population.

C1 [Schrepf, Andrew; Markon, Kristian; Lutgendorf, Susan K.] Univ Iowa, Dept Psychol, Iowa City, IA 52242 USA.

[Lutgendorf, Susan K.] Univ Iowa, Dept Obstet & Gynecol, Iowa City, IA 52242 USA.

[Lutgendorf, Susan K.] Univ Iowa, Dept Urol, Iowa City, IA 52242 USA.

[Lutgendorf, Susan K.] Univ Iowa, Holden Comprehens Canc Ctr, Iowa City, IA 52242 USA.

RP Lutgendorf, SK (reprint author), Univ Iowa, Dept Psychol, E11 Seashore Hall, Iowa City, IA 52242 USA.

EM susan-lutgendorf@uiowa.edu

FU National Institutes of Health [CA140933]; National Institute on Aging

[P01-AG020166]; John D. and Catherine T. MacArthur Foundation Research

Network on Successful Midlife Development

FX This manuscript was supported, in part, by the National Institutes of

Health (Grant CA140933; awarded to S. L.) and by the National Institute

on Aging (Grant P01-AG020166). The original study was supported, in

part, by the John D. and Catherine T. MacArthur Foundation Research

Network on Successful Midlife Development. The authors have no conflicts

of interest.

CR Allen B, 2007, J TRAUMA STRESS, V20, P587, DOI 10.1002/jts.20195

Anda RF, 2006, EUR ARCH PSY CLIN N, V256, P174, DOI 10.1007/s00406-005-0624-4

Bader K, 2007, J SLEEP RES, V16, P285, DOI 10.1111/j.1365-2869.2007.00608.x

Bernstein D, 1998, MANUAL CHILDHOOD TRA

Bornstein SR, 2006, MOL PSYCHIATR, V11, P892, DOI 10.1038/sj.mp.4001873

Briere J, 2003, CHILD ABUSE NEGLECT, V27, P1205, DOI 10.1016/j.chiabu.2003.09.008

Calabro P, 2005, J AM COLL CARDIOL, V46, P1112, DOI 10.1016/j.jacc.2005.06.017

Carpenter LL, 2011, PSYCHOPHARMACOLOGY, V214, P367, DOI 10.1007/s00213-010-2007-4

Center for Disease Control, 2013, ADV CHILDH EXP ACE S

Cheung GW, 2002, STRUCT EQU MODELING, V9, P233, DOI 10.1207/S15328007SEM0902_5

COHEN S, 1983, J HEALTH SOC BEHAV, V24, P385, DOI 10.2307/2136404

Copeland WE, 2012, PSYCHOL MED, V42, P2641, DOI 10.1017/S0033291712000554

Cortes AM, 2005, CHILD ABUSE NEGLECT, V29, P905, DOI 10.1016/j.chiabu.2004.12.010

D'Argenio A, 2009, PHYSIOL BEHAV, V98, P543, DOI 10.1016/j.physbeh.2009.08.010

Danese A, 2014, MOL PSYCHIATR, V19, P544, DOI 10.1038/mp.2013.54

Danese A, 2009, ARCH PEDIAT ADOL MED, V163, P1135, DOI 10.1001/archpediatrics.2009.214

Dannlowski U, 2012, BIOL PSYCHIAT, V71, P286, DOI 10.1016/j.biopsych.2011.10.021

de Beurs E, 1999, PSYCHOL MED, V29, P583, DOI 10.1017/S0033291799008351

DeSantis SM, 2011, DEPRESS ANXIETY, V28, P383, DOI 10.1002/da.20795

Fagundes CP, 2013, BRAIN BEHAV IMMUN, V27, P8, DOI 10.1016/j.bbi.2012.06.014

FAMULARO R, 1992, J AM ACAD CHILD PSY, V31, P863, DOI 10.1097/00004583-199209000-00013

Felitti VJ, 1998, AM J PREV MED, V14, P245, DOI 10.1016/S0749-3797(98)00017-8

FINKELHOR D, 1990, CHILD ABUSE NEGLECT, V14, P19, DOI 10.1016/0145-2134(90)90077-7

Fresco DM, 2001, PSYCHOL MED, V31, P1025

Geliebter Allan, 2003, Eat Behav, V3, P341, DOI 10.1016/S1471-0153(02)00100-9

Goossens L, 2009, EUR EAT DISORD REV, V17, P68, DOI 10.1002/erv.892

Greenfield EA, 2009, SOC SCI MED, V68, P791, DOI 10.1016/j.socscimed.2008.12.004

Gunstad J, 2006, PSYCHIAT RES, V142, P31, DOI 10.1016/j.psychres.2005.11.007

Harrington M, 2011, CAN J PSYCHIAT, V56, P686, DOI 10.1177/070674371105601107

Heffner JL, 2011, AM J ADDICTION, V20, P307, DOI 10.1111/j.1521-0391.2011.00141.x

Heim C, 2001, BIOL PSYCHIAT, V49, P1023, DOI 10.1016/S0006-3223(01)01157-X

Heim C, 2009, FRONT BEHAV NEUROSCI, V3, DOI 10.3389/neuro.08.041.2009

Kenardy Justin, 2003, Eat Behav, V4, P149, DOI 10.1016/S1471-0153(03)00019-9

Khera A, 2009, J CLIN ENDOCR METAB, V94, P3251, DOI 10.1210/jc.2008-2406

Kiecolt-Glaser JK, 2011, PSYCHOSOM MED, V73, P16, DOI 10.1097/PSY.0b013e31820573b6

Klaassens ER, 2009, PROG NEURO-PSYCHOPH, V33, P889, DOI 10.1016/j.pnpbp.2009.04.011

Kling KC, 1997, PSYCHOL AGING, V12, P288, DOI 10.1037/0882-7974.12.2.288

Koenig W, 1999, CIRCULATION, V99, P237, DOI 10.1161/01.CIR.99.2.237

Love GD, 2010, J AGING HEALTH, V22, P1059, DOI 10.1177/0898264310374355

McCauley J, 1997, JAMA-J AM MED ASSOC, V277, P1362, DOI 10.1001/jama.277.17.1362

Merikangas KR, 1998, ADDICT BEHAV, V23, P893, DOI 10.1016/S0306-4603(98)00076-8

Midei AJ, 2011, OBES REV, V12, pe159, DOI 10.1111/j.1467-789X.2010.00823.x

Miller GE, 2012, BIOL PSYCHIAT, V72, P34, DOI 10.1016/j.biopsych.2012.02.034

Miller GE, 2011, PSYCHOL BULL, V137, P959, DOI 10.1037/a0024768

Miller GE, 2010, PSYCHOL SCI, V21, P848, DOI 10.1177/0956797610370161

Morozink JA, 2010, HEALTH PSYCHOL, V29, P626, DOI 10.1037/a0021360

Oliver G, 2000, PSYCHOSOM MED, V62, P853, DOI 10.1097/00006842-200011000-00016

Otte C, 2005, BIOL PSYCHIAT, V57, P27, DOI 10.1016/j.biopsych.2004.10.009

Pasquali R, 2006, ANN NY ACAD SCI, V1083, P111, DOI 10.1196/annals.1367.009

Patton GC, 1998, AM J PUBLIC HEALTH, V88, P1518, DOI 10.2105/AJPH.88.10.1518

Pervanidou P, 2012, METABOLISM, V61, P611, DOI 10.1016/j.metabol.2011.10.005

PLINER P, 1990, PERS SOC PSYCHOL B, V16, P263, DOI 10.1177/0146167290162007

Ridker PM, 1998, CIRCULATION, V98, P731, DOI 10.1161/01.CIR.98.8.731

Rodgers CS, 2004, CHILD ABUSE NEGLECT, V28, P575, DOI 10.1016/j.chiabu.2004.01.002

Rooks C, 2012, PSYCHOSOM MED, V74, P146, DOI 10.1097/PSY.0b013e318240a7d8

Scher CD, 2001, J TRAUMA STRESS, V14, P843, DOI 10.1023/A:1013058625719

Slopen N, 2010, PSYCHOSOM MED, V72, P694, DOI 10.1097/PSY.0b013e3181e9c16f

Strine TW, 2005, SOC PSYCH PSYCH EPID, V40, P432, DOI 10.1007/s00127-005-0914-1

Torres SJ, 2007, NUTRITION, V23, P887, DOI 10.1016/j.nut.2007.08.008

Tsenkova V, 2013, APPETITE, V69, P151, DOI 10.1016/j.appet.2013.05.020

VANSTRIEN T, 1986, INT J OBESITY, V10, P29

Visser M, 1999, JAMA-J AM MED ASSOC, V282, P2131, DOI 10.1001/jama.282.22.2131

Vythilingam M, 2002, AM J PSYCHIAT, V159, P2072, DOI 10.1176/appi.ajp.159.12.2072

Waller G, 1998, INT J EAT DISORDER, V23, P419, DOI 10.1002/(SICI)1098-108X(199805)23:4<419::AID-EAT9>3.0.CO;2-L

WATSON D, 1995, J ABNORM PSYCHOL, V104, P3, DOI 10.1037/0021-843X.104.1.3

NR 65

TC 14

Z9 14

U1 2

U2 33

PU LIPPINCOTT WILLIAMS & WILKINS

PI PHILADELPHIA

PA TWO COMMERCE SQ, 2001 MARKET ST, PHILADELPHIA, PA 19103 USA

SN 0033-3174

EI 1534-7796

J9 PSYCHOSOM MED

JI Psychosom. Med.

PD JUN

PY 2014

VL 76

IS 5

BP 327

EP 336

DI 10.1097/PSY.0000000000000072

PG 10

WC Psychiatry; Psychology; Psychology, Multidisciplinary

SC Psychiatry; Psychology

GA AK0TQ

UT WOS:000338128800002

PM 24933013

OA Green Accepted

DA 2019-01-08

ER

PT J

AU Tang, TS

Funnell, M

Sinco, B

Piatt, G

Palmisano, G

Spencer, MS

Kieffer, EC

Heisler, M

AF Tang, Tricia S.

Funnell, Martha

Sinco, Brandy

Piatt, Gretchen

Palmisano, Gloria

Spencer, Michael S.

Kieffer, Edith C.

Heisler, Michele

TI Comparative Effectiveness of Peer Leaders and Community Health Workers

in Diabetes Self-management Support: Results of a Randomized Controlled

Trial

SO DIABETES CARE

LA English

DT Article

ID LIFE-STYLE INTERVENTION; MEXICAN-AMERICANS; LATINO ADULTS;

AFRICAN-AMERICAN; GLYCEMIC CONTROL; EDUCATION; OUTCOMES; PROGRAM;

PARTNERSHIPS; METAANALYSIS

AB OBJECTIVE

To compare a peer leader (PL) versus a community health worker (CHW) telephone outreach intervention in sustaining improvements in HbA(1c) over 12 months after a 6-month diabetes self-management education (DSME) program.

RESEARCH DESIGN AND METHODS

One hundred and sixteen Latino adults with type 2 diabetes were recruited from a federally qualified health center and randomized to 1) a 6-month DSME program followed by 12 months of weekly group sessions delivered by PLs with telephone outreach to those unable to attend or 2) a 6-month DSME program followed by 12 months of monthly telephone outreach delivered by CHWs. The primary outcome was HbA(1c). Secondary outcomes were cardiovascular disease risk factors, diabetes distress, and diabetes social support. Assessments were conducted at baseline, 6, 12, and 18 months.

RESULTS

After DSME, the PL group achieved a reduction in mean HbA(1c) (8.2-7.5% or 66-58 mmol/mol, P < 0.0001) that was maintained at 18 months (-0.6% or -6.6 mmol/mol from baseline [P = 0.009]). The CHW group also showed a reduction in HbA(1c) (7.8 vs. 7.3% or 62 vs. 56 mmol/mol, P = 0.0004) post-6 month DSME; however, it was attenuated at 18 months (-0.3% or -3.3 mmol/mol from baseline, within-group P = 0.234). Only the PL group maintained improvements achieved in blood pressure at 18 months. At the 18-month follow-up, both groups maintained improvements in waist circumference, diabetes support, and diabetes distress, with no significant differences between groups.

CONCLUSIONS

Both low-cost maintenance programs led by either a PL or a CHW maintained improvements in key patient-reported diabetes outcomes, but the PL intervention may have additional benefit in sustaining clinical improvements beyond 12 months.

C1 [Tang, Tricia S.] Univ British Columbia, Dept Med, Vancouver, BC, Canada.

[Funnell, Martha; Piatt, Gretchen] Univ Michigan, Dept Med Educ, Ann Arbor, MI 48109 USA.

[Sinco, Brandy; Spencer, Michael S.; Kieffer, Edith C.] Univ Michigan, Sch Social Work, Ann Arbor, MI 48109 USA.

[Palmisano, Gloria] Community Hlth & Social Serv Ctr, Detroit, MI USA.

[Heisler, Michele] Univ Michigan, Dept Internal Med, Ann Arbor, MI 48109 USA.

[Heisler, Michele] Ann Arbor VA Ctr Clin Management Res, Ann Arbor, MI USA.

RP Tang, TS (reprint author), Univ British Columbia, Dept Med, Vancouver, BC, Canada.

EM tricia.tang@vch.ca

RI Albertin, Paloma/X-1093-2018; Heisler, Michele/B-5774-2015

OI Heisler, Michele/0000-0002-6889-2063

FU American Academy of Family Physicians Foundation [P30DK092926]; National

Institute of Diabetes and Digestive and Kidney Diseases

[R18DK0785501A1]; Centers for Disease Control and Prevention

[U50/CCU417409]

FX This research was supported by a Peers for Progress grant from the

American Academy of Family Physicians Foundation (grant P30DK092926)

(Michigan Center for Diabetes Translational Research), by the National

Institute of Diabetes and Digestive and Kidney Diseases (grant

R18DK0785501A1), and by the Centers for Disease Control and Prevention

(cooperative agreement no. U50/CCU417409). The funding sources had no

role in the study design; data collection; administration of the

interventions; analysis, interpretation, or reporting of data; or

decision to submit the findings for publication.

CR ANDERSON RM, 2002, PRACTICAL PSYCHOL DI, P3

Arsie MP, 2000, ACTA DIABETOL, V37, P1, DOI 10.1007/s005920070028

Baig AA, 2012, DIABETES EDUCATOR, V38, P733, DOI 10.1177/0145721712455700

Barrera M, 2002, AM J COMMUN PSYCHOL, V30, P637, DOI 10.1023/A:1016369114780

Brown HS, 2012, PREV CHRONIC DIS, V9, DOI 10.5888/pcd9.120074

Brown SA, 2005, DIABETES CARE, V28, P527, DOI 10.2337/diacare.28.3.527

Brown SA, 2002, DIABETES CARE, V25, P259, DOI 10.2337/diacare.25.2.259

Diggle P, 2002, ANAL LONGITUDINAL DA

Emmons KM, 2001, AM J PREV MED, V20, P68, DOI 10.1016/S0749-3797(00)00254-3

Feathers JT, 2007, DIABETES EDUCATOR, V33, P509, DOI 10.1177/0145721707301371

Feathers JT, 2005, AM J PUBLIC HEALTH, V95, P1552, DOI 10.2105/AJPH.2005.066134

Fedder DO, 2003, ETHNIC DIS, V13, P22

Funnel MM, 2004, MED MANAGEMENT TYPE, P124

Gary TL, 2003, DIABETES EDUCATOR, V29, P488, DOI 10.1177/014572170302900313

Glasgow RE, 2012, PATIENT EDUC COUNS, V87, P81, DOI 10.1016/j.pec.2011.07.024

Haas L, 2012, DIABETES CARE, V35, P2393, DOI 10.2337/dc12-1707

Heisler M, 2009, AM J PREV MED, V37, pS270, DOI 10.1016/j.amepre.2009.08.016

Israel B. A., 2005, METHODS COMMUNITY BA

Lawlor MS, 2013, AM J PREV MED, V44, pS381, DOI 10.1016/j.amepre.2012.12.016

LIANG KY, 1986, BIOMETRIKA, V73, P13, DOI 10.1093/biomet/73.1.13

Lorig K, 2008, DIABETES CARE, V31, P408, DOI 10.2337/dc07-1313

Norris SL, 2002, DIABETES CARE, V25, P1159, DOI 10.2337/diacare.25.7.1159

Norris SL, 2001, DIABETES CARE, V24, P561, DOI 10.2337/diacare.24.3.561

Philis-Tsimikas A, 2011, DIABETES CARE, V34, P1926, DOI 10.2337/dc10-2081

Polonsky WH, 2005, DIABETES CARE, V28, P626, DOI 10.2337/diacare.28.3.626

ROGERS EJ, 1993, CLIN CHEM, V39, P860

Rosal MC, 2011, DIABETES CARE, V34, P838, DOI 10.2337/dc10-1981

Rothschild SK, 2012, CONTEMP CLIN TRIALS, V33, P369, DOI 10.1016/j.cct.2011.10.013

Schulz KF, 2010, ANN INTERN MED, V152, P726, DOI 10.7326/0003-4819-152-11-201006010-00232

Shah M, 2013, CURR DIABETES REP, V13, P163, DOI 10.1007/s11892-012-0359-3

Siminerio L, 2013, DIABETES EDUCATOR, V39, P705, DOI 10.1177/0145721713492570

Sixta CS, 2008, DIABETES EDUCATOR, V34, P299, DOI 10.1177/0145721708314490

Spencer MS, 2011, AM J PUBLIC HEALTH, V101, P2253, DOI 10.2105/AJPH.2010.300106

Steed L, 2003, PATIENT EDUC COUNS, V51, P5, DOI 10.1016/S0738-3991(02)00213-6

Tang TS, 2012, DIABETES RES CLIN PR, V95, P85, DOI 10.1016/j.diabres.2011.04.003

Tang TS, 2011, PATIENT EDUC COUNS, V85, P160, DOI 10.1016/j.pec.2010.12.013

Trento M, 2002, DIABETOLOGIA, V45, P1231, DOI 10.1007/s00125-002-0904-8

Turner RC, 1998, LANCET, V352, P837

United States Census Bureau, 2010, AM FACTFINDER

West B. T., 2007, LINEAR MIXED MODELS

NR 40

TC 55

Z9 56

U1 2

U2 28

PU AMER DIABETES ASSOC

PI ALEXANDRIA

PA 1701 N BEAUREGARD ST, ALEXANDRIA, VA 22311-1717 USA

SN 0149-5992

EI 1935-5548

J9 DIABETES CARE

JI Diabetes Care

PD JUN

PY 2014

VL 37

IS 6

BP 1525

EP 1534

DI 10.2337/dc13-2161

PG 10

WC Endocrinology & Metabolism

SC Endocrinology & Metabolism

GA AJ5RX

UT WOS:000337746100026

PM 24722495

OA Green Published, Other Gold

DA 2019-01-08

ER

PT J

AU Shim, U

Oh, JY

Lee, H

Sung, YA

Kim, HN

Kim, HL

AF Shim, Unjin

Oh, Jee-Young

Lee, Hyejin

Sung, Yeon-Ah

Kim, Han-Na

Kim, Hyung-Lae

TI Association between extraversion personality and abnormal glucose

regulation in young Korean women

SO ACTA DIABETOLOGICA

LA English

DT Article

DE Five-factor model; Extraversion personality; Abnormal glucose regulation

ID ALL-CAUSE MORTALITY; GLYCEMIC CONTROL; OLDER-ADULTS; STRESS RESPONSES;

SEX-DIFFERENCES; 5-FACTOR MODEL; METAANALYSIS; POPULATION; OPENNESS;

ABILITY

AB Depression and psychological distress are known to be associated with diabetes development as well as the disease progression including glycemic control and chronic complication, but relationship of personality with diabetes is controversial. We examined whether personality trait and the presence of abnormal glucose regulation (AGR; diabetes and pre-diabetes) are associated in young women. A total of 1,617 young women aged 19-39 years without previously diagnosed diabetes were participated voluntarily. Personality trait was assessed by self-reported questionnaire using the five-factor model (neuroticism, extraversion, openness to experience, agreeableness and conscientiousness) consisting of five-point scale ranging from 'strongly disagreeable' to 'strongly agreeable.' Glucose tolerance status was assessed by standard 75-g oral glucose tolerance test. One hundred and eleven women were newly diagnosed with AGR (6.9 %). Among five factors, only extraversion trait was significantly associated with AGR. Multiple linear regression analysis showed significant negative association between extraversion trait and 2-h post-load glucose after adjustment for age, BMI, systolic blood pressure, triglycerides, HDL cholesterol and family history of diabetes (beta = -0.16, P = 0.026). Multiple logistic regression showed extraversion trait having a significant association with the presence of AGR after adjustment for the same covariates (OR 0.97, 95 % CI 0.95-0.99, P = 0.011). The frequency of AGR was significantly increased according to the decrease in extraversion score (P for trend with exact test = 0.047). In conclusion, extraversion may be an important personality trait having a beneficial effect on decreasing the risk of AGR.

C1 [Shim, Unjin] Ewha Womans Univ, Dept Internal Med, Seoul Seonam Hosp, Med Ctr, Seoul, South Korea.

[Oh, Jee-Young; Lee, Hyejin; Sung, Yeon-Ah] Ewha Womans Univ, Mokdong Hosp, Sch Med, Dept Internal Med, Seoul 158710, South Korea.

[Kim, Han-Na; Kim, Hyung-Lae] Ewha Womans Univ, Sch Med, Dept Biochem, Seoul, South Korea.

RP Oh, JY (reprint author), Ewha Womans Univ, Mokdong Hosp, Sch Med, Dept Internal Med, 911-1 Mokdong, Seoul 158710, South Korea.

EM jyoh@ewha.ac.kr

FU Ewha Global Top5 Grant of Ewha Womans University; Basic Science Research

Program through the National Research Foundation of Korea (NRF) -

Ministry of Education, Science, and Technology [2010-0026606]

FX This work was supported by the Ewha Global Top5 Grant 2013 of Ewha

Womans University and the Basic Science Research Program through the

National Research Foundation of Korea (NRF), funded by the Ministry of

Education, Science, and Technology (2010-0026606).

CR Ahn CK, 1997, KOREAN J COUNSELING, V9, P443

American Diabetes Association: Diagnosis and classification of diabetes mellitus, 2012, DIABETES CARE S1, V35, pS64, DOI [10.2337/dc12-s064, DOI 10.2337/DC12-S064]

Anderson RJ, 2002, INT J PSYCHIAT MED, V32, P235, DOI 10.2190/KLGD-4H8D-4RYL-TWQ8

Anderson RJ, 2001, DIABETES CARE, V24, P1069, DOI 10.2337/diacare.24.6.1069

Barrett-Connor E, 2013, BEST PRACT RES CL EN, V27, P481, DOI 10.1016/j.beem.2013.05.013

Barrick MR, 1998, J APPL PSYCHOL, V83, P377, DOI 10.1037/0021-9010.83.3.377

Bogg T, 2004, PSYCHOL BULL, V130, P887, DOI 10.1037/0033-2909-130.6.887

Chan O, 2003, Minerva Endocrinol, V28, P87

Costa PT, 1992, REVISED NEO PERSONAL

Eriksson AK, 2012, DIABETES RES CLIN PR, V95, P145, DOI 10.1016/j.diabres.2011.10.003

Fisher L, 2008, DIABETIC MED, V25, P1096, DOI 10.1111/j.1464-5491.2008.02533.x

Golden SH, 2006, PSYCHONEUROENDOCRINO, V31, P325, DOI 10.1016/j.psyneuen.2005.08.008

Hill PL, 2011, HEALTH PSYCHOL, V30, P797, DOI 10.1037/a0023860

Hogan MJ, 2012, PSYCHOL AGING, V27, P849, DOI 10.1037/a0029066

Iwasa H, 2008, AM J GERIAT PSYCHIAT, V16, P399, DOI 10.1097/JGP.0b013e3181662ac9

Jokela M, 2013, OBES REV, V14, P315, DOI 10.1111/obr.12007

Jokela M, 2013, HLTH PSYCHOL

Jonassaint CR, 2009, INT J PSYCHOPHYSIOL, V74, P274, DOI 10.1016/j.ijpsycho.2009.09.012

Kiecolt-Glaser JK, 2003, P NATL ACAD SCI USA, V100, P9090, DOI 10.1073/pnas.1531903100

Kim DJ, 2011, DIABETES METAB J, V35, P303, DOI 10.4093/dmj.2011.35.4.303

Lane JD, 2000, DIABETES CARE, V23, P1321, DOI 10.2337/diacare.23.9.1321

Lebovitz HE, 1999, CLIN CHEM, V45, P1339

Rhee J, 2013, SPRINGERPLUS, V2, DOI 10.1186/2193-1801-2-16

Robertson SM, 2012, PSYCHOSOMATICS, V53, P1, DOI 10.1016/j.psym.2011.09.008

Rosmond R, 2003, MED SCI MONITOR, V9, P35

Rostambeigi N, 2010, DIABETES RES CLIN PR, V88, P117, DOI 10.1016/j.diabres.2009.12.025

Schneider TR, 2012, STRESS HEALTH, V28, P102, DOI 10.1002/smi.1409

Skinner TC, 2002, HEALTH PSYCHOL, V21, P61, DOI 10.1037//0278-6133.21.1.61

Stilley CS, 2004, ANN BEHAV MED, V27, P117, DOI 10.1207/s15324796abm2702_6

Suarez EC, 2006, HEALTH PSYCHOL, V25, P484, DOI 10.1037/0278-6133.25.4.484

Sutin AR, 2010, BIOL PSYCHOL, V84, P186, DOI 10.1016/j.biopsycho.2010.01.012

Ter Horst GJ, 2009, PHYSIOL BEHAV, V97, P239, DOI 10.1016/j.physbeh.2009.02.036

Terracciano A, 2009, PSYCHOSOM MED, V71, P682, DOI 10.1097/PSY.0b013e3181a2925b

Terry DP, 2013, J CLIN EXP NEUROPSYC, V35, P509, DOI 10.1080/13803395.2013.795932

Tornroos M, 2013, J OCCUP HEALTH PSYCH, V18, P492, DOI 10.1037/a0033987

Vollrath ME, 2007, DIABETIC MED, V24, P1028, DOI 10.1111/j.1464-5491.2007.02215.x

Waller D, 2013, DIABETES CARE, V36, P2968, DOI 10.2337/dc12-1743

Weiss A, 2005, PSYCHOSOM MED, V67, P724, DOI 10.1097/01.psy.0000181272.58103.18

Wilson RS, 2005, PSYCHOSOM MED, V67, P841, DOI 10.1097/01.psy.0000190615.20656.83

Zimmet P, 2001, NATURE, V414, P782, DOI 10.1038/414782a

NR 40

TC 2

Z9 2

U1 0

U2 11

PU SPRINGER-VERLAG ITALIA SRL

PI MILAN

PA VIA DECEMBRIO, 28, MILAN, 20137, ITALY

SN 0940-5429

EI 1432-5233

J9 ACTA DIABETOL

JI Acta Diabetol.

PD JUN

PY 2014

VL 51

IS 3

BP 421

EP 427

DI 10.1007/s00592-013-0529-z

PG 7

WC Endocrinology & Metabolism

SC Endocrinology & Metabolism

GA AH8PL

UT WOS:000336400100010

PM 24212719

DA 2019-01-08

ER

PT J

AU Fisher, L

Hessler, D

Masharani, U

Strycker, L

AF Fisher, L.

Hessler, D.

Masharani, U.

Strycker, L.

TI Impact of baseline patient characteristics on interventions to reduce

diabetes distress: the role of personal conscientiousness and diabetes

self-efficacy

SO DIABETIC MEDICINE

LA English

DT Article

ID PRIMARY-CARE PATIENTS; OLDER-ADULTS; SCALE; DEPRESSION; OUTCOMES;

THERAPY

AB AimsTo improve patient-centred care by determining the impact of baseline levels of conscientiousness and diabetes self-efficacy on the outcomes of efficacious interventions to reduce diabetes distress and improve disease management.

MethodsAdults with Type 2 diabetes with diabetes distress and self-care problems (N=392) were randomized to one of three distress reduction interventions: computer-assisted self-management; computer-assisted self-management plus problem-solving therapy; and health education. The baseline assessment included conscientiousness and self-efficacy, demographics, diabetes status, regimen distress, emotional burden, medication adherence, diet and physical activity. Changes in regimen distress, emotional burden and self-care between baseline and 12months were recorded and ancova models assessed how conscientiousness and self-efficacy qualified the significant improvements in distress and management outcomes.

ResultsParticipants with high baseline conscientiousness displayed significantly larger improvements in medication adherence and emotional burden than participants with low baseline conscientiousness. Participants with high baseline self-efficacy showed greater improvements in diet, physical activity and regimen distress than participants with low baseline self-efficacy. The impact of conscientiousness and self-efficacy were independent of each other and occurred across all three intervention groups. A significant interaction indicated that those with both high self-efficacy and high conscientiousness at baseline had the biggest improvement in physical activity by 12months.

ConclusionsBoth broad personal traits and disease-specific expectations qualify the outcomes of efficacious interventions. These findings reinforce the need to change from a one-size-fits-all approach to diabetes interventions to an approach that crafts clinical interventions in ways that fit the personal traits and skills of individual people.

What's new?

<list list-type="bulleted" id="dme12403-list-0001"> The traits and beliefs that people with diabetes bring with them to clinical interventions influence the outcomes of interventions, even those previously shown to be efficacious. Conscientiousness, a personal trait, and diabetes self-efficacy, a set of beliefs and expectations about management, are independent predictors of the success of interventions to improve management and reduce distress.

C1 [Fisher, L.; Hessler, D.] Univ Calif San Francisco, Dept Family & Community Med, San Francisco, CA 94143 USA.

[Masharani, U.] Univ Calif San Francisco, Dept Med, San Francisco, CA USA.

[Strycker, L.] Oregon Res Inst, Eugene, OR 97403 USA.

RP Fisher, L (reprint author), Univ Calif San Francisco, Dept Family & Community Med, San Francisco, CA 94143 USA.

EM fisherl@fcm.ucsf.edu

FU National Institute of Diabetes, Digestive and Kidney Disease [DK 061937]

FX This research was funded by the National Institute of Diabetes,

Digestive and Kidney Disease (DK 061937).

CR Allan JL, 2013, ANN BEHAV MED, V46, P114, DOI 10.1007/s12160-013-9483-9

Arean P, 2008, GERONTOLOGIST, V48, P311, DOI 10.1093/geront/48.3.311

Bandura A., 1986, SOCIAL FDN THOUGHT A

Benet-Martinez V, 1998, J PERS SOC PSYCHOL, V75, P729, DOI 10.1037//0022-3514.75.3.729

Bogg T, 2013, ANN BEHAV MED, V45, P278, DOI 10.1007/s12160-012-9454-6

Chapman BP, 2013, AM J GERIAT PSYCHIAT, V21, P737, DOI 10.1016/j.jagp.2012.11.013

Chiolero A, 2009, FRONTIERS PUBLIC HLT, V1, P8

Costa Jr P. T., 1992, NEO PERSONALITY INVE

Duncan T., 2006, INTRO LATENT VARIABL

Fisher L, 2009, DIABETIC MED, V26, P622, DOI 10.1111/j.1464-5491.2009.02730.x

Fisher L, 2008, ANN FAM MED, V6, P246, DOI 10.1370/afm.842

Fisher L, 2013, DIABETES CARE, V36, P2551, DOI 10.2337/dc12-2493

Fisher L, 2012, DIABETES CARE, V35, P259, DOI 10.2337/dc11-1572

Fisher L, 2010, DIABETES CARE, V33, P23, DOI 10.2337/dc09-1238

Fry PS, 2009, J HEALTH PSYCHOL, V14, P513, DOI 10.1177/1359105309103571

Gao J, 2013, BMC FAM PRAC, V24, P500

Glasgow RE, 2010, J GEN INTERN MED, V25, P1315, DOI 10.1007/s11606-010-1480-0

Kroenke K, 2001, J GEN INTERN MED, V16, P606, DOI 10.1046/j.1525-1497.2001.016009606.x

Krousel-Wood M, 2005, J MED SCI, V330, P182

Lorig K, 1996, OUTCOME MEASURES HLT

Masharani U, 2007, DIABETES DEMYSTIFIED

Nezu AM, 2004, BEHAV THER, V35, P1, DOI 10.1016/S0005-7894(04)80002-9

Polonsky WH, 2005, DIABETES CARE, V28, P626, DOI 10.2337/diacare.28.3.626

Schafer J., 1999, NORM USERS GUIDE VER

Stewart AL, 2001, MED SCI SPORT EXER, V33, P1126

Thompson F. E., 1998, European Journal of Clinical Nutrition, V52, pS63

Toobert DJ, 2000, DIABETES CARE, V23, P943, DOI 10.2337/diacare.23.7.943

Triad Group, 2010, DIABETES CARE, V33, P940

Tsjii S., 2012, DIABETIC MED, V29, P1451

Vollrath ME, 2007, DIABETIC MED, V24, P1028, DOI 10.1111/j.1464-5491.2007.02215.x

NR 30

TC 11

Z9 13

U1 4

U2 29

PU WILEY-BLACKWELL

PI HOBOKEN

PA 111 RIVER ST, HOBOKEN 07030-5774, NJ USA

SN 0742-3071

EI 1464-5491

J9 DIABETIC MED

JI Diabetic Med.

PD JUN

PY 2014

VL 31

IS 6

BP 739

EP 746

DI 10.1111/dme.12403

PG 8

WC Endocrinology & Metabolism

SC Endocrinology & Metabolism

GA AH2KD

UT WOS:000335949000017

PM 24494593

OA Green Accepted

DA 2019-01-08

ER

PT J

AU van der Pols-Vijlbrief, R

Dekker, JM

Stehouwer, CD

de Boer, MR

Nijpels, G

Snoek, FJ

Adriaanse, MC

AF van der Pols-Vijlbrief, R.

Dekker, J. M.

Stehouwer, C. D.

de Boer, M. R.

Nijpels, G.

Snoek, F. J.

Adriaanse, M. C.

TI Symptom burden and its association with change in glucose metabolism

status over a 7-year period: the Hoorn Study

SO DIABETIC MEDICINE

LA English

DT Article

ID QUALITY-OF-LIFE; PATIENT-REPORTED OUTCOMES; DIABETES-MELLITUS;

POPULATION; PARTICIPANTS; PREVALENCE; DIAGNOSIS; DISTRESS

AB Aims To study symptom burden among older people and its associations with change in glucose metabolism status over a 7-year period.

Methods We conducted a prospective population-based cohort study among 397 older people. We used the revised Diabetes Symptom Checklist to assess symptom burden. Glucose metabolism status was determined using an oral glucose tolerance test. Analyses were adjusted for multiple confounders, including cardiovascular risk and risk of depression (Center for Epidemiological Studies Depression Scale score 16).

Results Revised Diabetes Symptom Checklist total scores (range 0-100) increased slightly over time among people with normal glucose metabolism (mean difference 1.04; P = 0.04) and those with impaired glucose metabolism (1.96; P = 0.01), but not among people with Type 2 diabetes (0.46; P = 0.55). These associations between symptom burden and glucose status were attenuated after full adjustment for multiple confounders and remained statistically significant for those with impaired glucose status. Linear mixed models showed significant mean differences in revised Diabetes Symptom Checklist total scores over time when comparing people with Type 2 diabetes with those with normal or impaired glucose metabolism, but not when comparing subjects with impaired vs normal glucose metabolism; these results did not alter after full adjustment.

Conclusions Symptom burden increased gradually over time in the people with impaired glucose metabolism and those with normal glucose metabolism, but not in patients with Type 2 diabetes over a 7-year follow-up period.

What's new?

This is the first study investigating diabetes-related symptom burden among older people and its associations with a change in glucose metabolism status over a 7-year period. The results indicate that symptom burden increases gradually over time among the participants with impaired glucose and those with normal glucose status, but not among patients with Type 2 diabetes. Prospective studies are needed in larger populations, including data on comorbidities and information about diabetes treatment, given the potential of treatment to reduce diabetes complications and subsequent symptom burden .

C1 [van der Pols-Vijlbrief, R.; de Boer, M. R.; Adriaanse, M. C.] Vrije Univ Amsterdam, Fac Earth & Life Sci, Dept Hlth Sci, Amsterdam, Netherlands.

[van der Pols-Vijlbrief, R.; Dekker, J. M.; de Boer, M. R.; Nijpels, G.; Snoek, F. J.; Adriaanse, M. C.] Vrije Univ Amsterdam Med Ctr, EMGO Inst Hlth & Care Res, Amsterdam, Netherlands.

[Dekker, J. M.; de Boer, M. R.] Vrije Univ Amsterdam Med Ctr, Dept Epidemiol & Biostat, Amsterdam, Netherlands.

[Dekker, J. M.; Nijpels, G.] Vrije Univ Amsterdam Med Ctr, Dept Gen Practice, Amsterdam, Netherlands.

[Stehouwer, C. D.] Maastricht Univ Med Ctr, Dept Internal Med, Maastricht, Netherlands.

[Stehouwer, C. D.] Maastricht Univ Med Ctr, Cardiovasc Res Inst, Maastricht, Netherlands.

[Snoek, F. J.] Vrije Univ Amsterdam Med Ctr, Dept Med Psychol, Amsterdam, Netherlands.

RP van der Pols-Vijlbrief, R (reprint author), Vrije Univ Amsterdam, Fac Earth & Life Sci, Dept Hlth Sci, Amsterdam, Netherlands.

EM rachel.vijlbrief@vu.nl

OI de Boer, Michiel/0000-0001-9240-0199

FU Dutch Diabetes Research Foundation [2009.13.011]

FX The present study was funded by the Dutch Diabetes Research Foundation

in the 'Stem van de Patient programme', project number: 2009.13.011.

CR Adriaanse MC, 2008, DIABETES CARE, V31, P2268, DOI 10.2337/dc08-1074

Adriaanse MC, 2005, QUAL LIFE RES, V14, P1501, DOI 10.1007/s11136-004-0028-3

Alberti KGMM, 1998, DIABETIC MED, V15, P539, DOI 10.1002/(SICI)1096-9136(199807)15:7<539::AID-DIA668>3.0.CO;2-S

Ali MK, 2012, DIABETIC MED, V29, pE67, DOI 10.1111/j.1464-5491.2011.03532.x

Amer Diabet Assoc, 2008, DIABETES CARE, V31, pS55, DOI 10.2337/dc08-S055

Arbuckle RA, 2009, VALUE HEALTH, V12, P1168, DOI 10.1111/j.1524-4733.2009.00571.x

Black SA, 2002, AM J PUBLIC HEALTH, V92, P543, DOI 10.2105/AJPH.92.4.543

Campbell RK, 2009, J AM PHARM ASSOC, V49, pS3, DOI 10.1331/JAPhA.2009.09077

de Vegt F, 2001, JAMA-J AM MED ASSOC, V285, P2109, DOI 10.1001/jama.285.16.2109

Dueck AC, 2007, VALUE HEALTH, V10, pS64, DOI 10.1111/j.1524-4733.2007.00268.x

Florez H, 2010, POSTGRAD MED, V122, P112, DOI 10.3810/pgm.2010.03.2128

Gavin JR, 1999, DIABETES CARE, V22, pS5

Hajos TRS, 2012, QUAL LIFE RES, V21, P1359, DOI 10.1007/s11136-011-0051-0

Henry RMA, 2003, CIRCULATION, V107, P2089, DOI 10.1161/01.CIR.0000065222.34933.FC

Koopman RJ, 2005, ANN FAM MED, V3, P60, DOI 10.1370/afm.214

MOOY JM, 1995, DIABETES CARE, V18, P1270, DOI 10.2337/diacare.18.9.1270

Petrie KJ, 2003, J PSYCHOSOM RES, V54, P401, DOI 10.1016/S0022-3999(02)00461-0

Peyrot M, 2011, DIABETES TECHNOL THE, V13, P471, DOI 10.1089/dia.2010.0167

Pischke CR, 2006, AM J CARDIOL, V97, P1267, DOI 10.1016/j.amjcard.2005.11.051

RADLOFF L S, 1977, Applied Psychological Measurement, V1, P385, DOI 10.1177/014662167700100306

Rubin RR, 1999, DIABETES-METAB RES, V15, P205, DOI 10.1002/(SICI)1520-7560(199905/06)15:3<205::AID-DMRR29>3.0.CO;2-O

Schram MT, 2009, CURR DIABETES REV, V5, P112, DOI 10.2174/157339909788166828

Shafer AB, 2006, J CLIN PSYCHOL, V62, P123, DOI 10.1002/jclp.20213

Wild S, 2004, DIABETES CARE, V27, P1047, DOI 10.2337/diacare.27.5.1047

NR 24

TC 1

Z9 1

U1 0

U2 6

PU WILEY

PI HOBOKEN

PA 111 RIVER ST, HOBOKEN 07030-5774, NJ USA

SN 0742-3071

EI 1464-5491

J9 DIABETIC MED

JI Diabetic Med.

PD JUN

PY 2014

VL 31

IS 6

BP 747

EP 753

DI 10.1111/dme.12406

PG 7

WC Endocrinology & Metabolism

SC Endocrinology & Metabolism

GA AH2KD

UT WOS:000335949000018

PM 24494697

DA 2019-01-08

ER

PT J

AU Lacey, CJ

Salzberg, MR

D'Souza, WJ

AF Lacey, Cameron J.

Salzberg, Michael R.

D'Souza, Wendyl J.

TI Risk factors for psychological distress in community-treated epilepsy

SO EPILEPSY & BEHAVIOR

LA English

DT Article

DE Epilepsy; Psychiatric comorbidity; Community; Risk factors

ID PSYCHIATRIC COMORBIDITY; ANTIEPILEPTIC DRUGS; DEPRESSION; ADULTS;

POPULATION; ANXIETY; PREDICTORS; DIAGNOSES; STRESS

AB The study aimed to determine risk factors for psychological distress in a community-treated sample of patients with epilepsy. This study investigated the Tasmanian Epilepsy Register participants. Participants included were as follows: aged 13 years and over, able to complete the individual computer-assisted participant interview, and diagnosed with epilepsy following an epilepsy specialist review of the diagnostic epilepsy interview, which was interpreted using standardized diagnostic guidelines. Psychological distress was assessed with the Kessler-10 questionnaire. Risk factors were grouped into four domains: sociodemographic factors, disease-related factors, psychological factors, and treatment-related factors. High or very high levels of psychological distress were reported by 22% of the participants, with 7.8% having very high distress. The regression model showed that psychological distress was significantly associated with female gender (F = 18.1, p < 0.001), diabetes mellitus (F= 8.7, p = 0.003), intellectual disability (F= 7.1, p = 0.06), and not receiving phenytoin (F= 5.1, p = 0.02). While the model was significant (F = 5.78, p < 0.001), only 11% of the variance of the K-10 score was explained by these factors (adjusted R-squared = 0.11). This study identifies female gender and comorbid medical conditions as risk factors for psychological distress and the use of phenytoin as a protective factor. The few factors identified and the limited variance explained suggest that a focus on epilepsy-related variables is unlikely to explain key influences underlying psychiatric comorbidity in patients with epilepsy. (C) 2014 Elsevier Inc. All rights reserved.

C1 [Lacey, Cameron J.; Salzberg, Michael R.] Univ Melbourne, St Vincents Hosp Melbourne, Dept Psychiat, Melbourne, Vic 3065, Australia.

[Lacey, Cameron J.; D'Souza, Wendyl J.] Univ Melbourne, St Vincents Hosp Melbourne, Dept Med, Fitzroy, Vic 3065, Australia.

RP Lacey, CJ (reprint author), Univ Otago, Dept Psychol Med, POB 4345, Christchurch, New Zealand.

EM cameron.lacey@otago.ac.nz

OI D'Souza, Wendyl/0000-0002-1750-5131

FU Lundbeck Neuropsychiatry Research Fellowship; Menzies Research Institute

NHMRC Capacity Building Grant; NHMRC Health Professional Fellowship; The

Booth Estate Launceston; The Royal Hobart Hospital Research Foundation;

GSK Neurology; Clifford Craig Medical Research Trust, North West

Tasmania

FX Cameron Lacey has received support from a Lundbeck Neuropsychiatry

Research Fellowship. Wendyl D'Souza has received support from a Menzies

Research Institute NHMRC Capacity Building Grant and an NHMRC Health

Professional Fellowship. The Tasmanian Epilepsy Register was supported

by The Booth Estate Launceston, The Royal Hobart Hospital Research

Foundation, GSK Neurology, and the Clifford Craig Medical Research

Trust, North West Tasmania. We also thank our research assistant Pam

McDonald for her considerable efforts in participant liaison, data

processing, and management.

CR Adams SJ, 2008, BRIT J PSYCHIAT, V192, P464, DOI 10.1192/bjp.bp.107.046664

Australian Bureau of Statistics, 2012, INF PAP US KESSL PSY

BABOR TF, 2001, WHOMSDMSB016A

Barry JJ, 2008, EPILEPSY BEHAV, V13, pS1, DOI 10.1016/j.yebeh.20008.04.005

Brummett BH, 2007, PSYCHOSOM MED, V69, P621, DOI 10.1097/PSY.0b013e31814b8de6

Christensen J, 2007, LANCET NEUROL, V6, P693, DOI 10.1016/S1474-4422(07)70175-8

Clarke DM, 2009, MED J AUSTRALIA, V190, pS54

Cook MJ, 2013, LANCET NEUROL, V12, P563, DOI 10.1016/S1474-4422(13)70075-9

Cramer JA, 2003, EPILEPSIA, V44, P1578, DOI 10.1111/j.0013-9580.2003.28403.x

D'Souza WJ, 2010, EPILEPSY RES, V91, P20, DOI 10.1016/j.eplepsyres.2010.06.003

D'Souza WJ, 2008, EPIDEMIOLOGY EPILEPS

Ettinger A, 2004, NEUROLOGY, V63, P1008, DOI 10.1212/01.WNL.0000138430.11829.61

Ettinger AB, 2006, PSYCHIAT ISSUES EPIL

Ettinger AB, 2006, NEUROLOGY, V67, P1916, DOI 10.1212/01.wnl.0000247045.85646.c0

Foulds J, 2013, INT J ALCOHOL DRUG R, V2, P59, DOI 10.7895/ijadr.v2i1.73

Gaitatzis A, 2004, ACTA NEUROL SCAND, V110, P207, DOI 10.1111/j.1600-0404.2004.00324.x

Goodwin R, 2006, AM PSYCHIAT PUBLISHI, P33

Hermann BP, 2000, EPILEPSIA S2, V41, P31

Hitiris N, 2007, EPILEPSY RES, V75, P192, DOI 10.1016/j.eplepsyres.2007.06.003

Hoppe C, 2011, NAT REV NEUROL, V7, P462, DOI 10.1038/nrneurol.2011.104

Kanner AM, 2009, NEUROLOGY, V72, P793, DOI 10.1212/01.wnl.0000343850.85763.9c

Karouni M, 2010, EUR J CLIN PHARMACOL, V66, P1151, DOI 10.1007/s00228-010-0861-y

Kendler KS, 2002, AM J PSYCHIAT, V159, P1133, DOI 10.1176/appi.ajp.159.7.1133

Kessler RC, 2012, MOL PSYCHIATR, V17, P748, DOI 10.1038/mp.2011.56

Kessler RC, 2002, PSYCHOL MED, V32, P959, DOI 10.1017/S0033291702006074

Lacey CJ, 2009, EPILEPSIA, V50, P1991, DOI 10.1111/j.1528-1167.2009.02165.x

Mensah SA, 2006, EPILEPSY BEHAV, V8, P213, DOI 10.1016/j.yebeh.2005.09.014

OTTMAN R, 1990, EPILEPSIA, V31, P110, DOI 10.1111/j.1528-1157.1990.tb05368.x

Ottman R, 2011, EPILEPSIA, V52, P308, DOI 10.1111/j.1528-1167.2010.02927.x

Petrovski S, 2010, NEUROLOGY, V75, P1015, DOI 10.1212/WNL.0b013e3181f25b16

REUTENS DC, 1992, EPILEPSIA, V33, P1065, DOI 10.1111/j.1528-1157.1992.tb01760.x

RUTGERS MJ, 1986, EPILEPSIA, V27, P734, DOI 10.1111/j.1528-1157.1986.tb03603.x

Statistics ABo, 2001, INF PAP CENS POP HOU

Stefanello S, 2010, EPILEPSIA, V51, P1120, DOI 10.1111/j.1528-1167.2009.02386.x

Taylor RS, 2011, EPILEPSIA, V52, P2168, DOI 10.1111/j.1528-1167.2011.03213.x

Thapar A, 2009, EPILEPSY BEHAV, V14, P134, DOI 10.1016/j.yebeh.2008.09.004

Trewin D, 2005, AUSTR STANDARD GEOGR

VICTOROFF J, 1994, NEUROPSY NEUROPSY BE, V7, P87

Vink D, 2008, J AFFECT DISORDERS, V106, P29, DOI 10.1016/j.jad.2007.06.005

Weintraub D, 2007, EPILEPSY BEHAV, V10, P105, DOI 10.1016/j.yebeh.2006.08.008

NR 40

TC 5

Z9 5

U1 0

U2 6

PU ACADEMIC PRESS INC ELSEVIER SCIENCE

PI SAN DIEGO

PA 525 B ST, STE 1900, SAN DIEGO, CA 92101-4495 USA

SN 1525-5050

EI 1525-5069

J9 EPILEPSY BEHAV

JI Epilepsy Behav.

PD JUN

PY 2014

VL 35

BP 1

EP 5

DI 10.1016/j.yebeh.2014.03.025

PG 5

WC Behavioral Sciences; Clinical Neurology; Psychiatry

SC Behavioral Sciences; Neurosciences & Neurology; Psychiatry

GA AH8YQ

UT WOS:000336424500001

PM 24785427

DA 2019-01-08

ER

PT J

AU De Smedt, D

Clays, E

Annemans, L

Pardaens, S

Kotseva, K

De Bacquer, D

AF De Smedt, Delphine

Clays, Els

Annemans, Lieven

Pardaens, Sofie

Kotseva, Kornelia

De Bacquer, Dirk

TI Risk factor awareness in a coronary population and the association with

health-related quality of life outcomes

SO INTERNATIONAL JOURNAL OF PUBLIC HEALTH

LA English

DT Article

DE Coronary heart disease; Awareness; Health-related quality of life

ID ACUTE MYOCARDIAL-INFARCTION; HEART-DISEASE PATIENTS; HYPERTENSION;

MORTALITY; KNOWLEDGE; PREVALENCE; PERCEPTION; DEPRESSION; ADHERENCE

AB The purpose of this study was to investigate the risk factor level awareness in coronary patients and to assess its associations with health-related quality of life (HRQoL)/psychological distress.

Data on 8,743 coronary patients from 22 European countries, interviewed and examined at least 6 months after their acute event was available.

81.7 % of patients indicated to be aware of their own blood pressure level, whereas only 46.6 % of patients indicated to be aware of their cholesterol level. Furthermore, 43.7 % of patients were aware of their blood glucose level, whereas in diabetes patients blood glucose level awareness reached 81.8 %. Risk factor level awareness was significantly associated with HRQoL/psychological distress, with patients being unaware of their risk factor levels having worse outcomes. The relationship between awareness and HRQoL/psychological distress seemed to be partly mediated by the attempt of patients to adopt a healthier behaviour.

Health care workers should be encouraged to inform their patients about the importance of their coronary risk factors, the actual level and their personal target.

C1 [De Smedt, Delphine; Clays, Els; Annemans, Lieven; De Bacquer, Dirk] Univ Ghent, Dept Publ Hlth, B-9000 Ghent, Belgium.

[Annemans, Lieven] Vrije Univ Brussel, Fac Med & Pharm, Brussels, Belgium.

[Annemans, Lieven] I CHER Interuniv Ctr Hlth Econ, Ghent, Belgium.

[Pardaens, Sofie] Univ Ghent, Dept Internal Med, B-9000 Ghent, Belgium.

[Kotseva, Kornelia] Univ London Imperial Coll Sci Technol & Med, Dept Cardiovasc Med, Natl Heart & Lung Inst, London, England.

RP De Smedt, D (reprint author), Univ Ghent, Dept Publ Hlth, Pintelaan 185,Blok A-2, B-9000 Ghent, Belgium.

EM delphine.desmedt@ugent.be

FU AstraZeneca; Bristol-Myers Squibb; GlaxoSmithKline; Pfizer;

Sanofi-Aventis; Servier; Merck/Schering-Plough; Novartis

FX The EUROASPIRE survey was supported by the aid of unrestricted

educational grants, which were given to the European Society of

Cardiology by the following companies: main sponsors: AstraZeneca,

Bristol-Myers Squibb, GlaxoSmithKline, Pfizer, Sanofi-Aventis, and

Servier; sponsors: Merck/Schering-Plough and Novartis.

CR Alm-Roijer Carin, 2004, Eur J Cardiovasc Nurs, V3, P321

Alves E, 2012, INT J PUBLIC HEALTH, V57, P837, DOI 10.1007/s00038-012-0340-6

Baroletti S, 2010, CIRCULATION, V121, P1455, DOI 10.1161/CIRCULATIONAHA.109.904003

BRODY DS, 1989, J GEN INTERN MED, V4, P506, DOI 10.1007/BF02599549

Calvillo J, 2015, HEALTH EXPECT, V18, P643, DOI 10.1111/hex.12089

Cheng S, 2005, J HUM HYPERTENS, V19, P769, DOI 10.1038/sj.jhh.1001895

Cheng Susan, 2005, Prev Cardiol, V8, P11, DOI 10.1111/j.1520-037X.2005.3939.x

De Smedt D, 2014, EUR J PREV CARDIOL, V21, P796, DOI 10.1177/2047487312473846

De Smedt D, 2013, INT J CARDIOL, V167, P2294, DOI 10.1016/j.ijcard.2012.06.025

French DP, 2005, BRIT J HEALTH PSYCH, V10, P167, DOI 10.1348/13590705X26722

Grover SA, 2007, ARCH INTERN MED, V167, P2296, DOI 10.1001/archinte.167.21.2296

Karthik Shishir, 2006, Interact Cardiovasc Thorac Surg, V5, P268, DOI 10.1510/icvts.2005.120956

Korhonen PE, 2011, J HYPERTENS, V29, P2070, DOI 10.1097/HJH.0b013e32834bbca7

Kotseva K, 2009, EUR J CARDIOV PREV R, V16, P121, DOI 10.1097/HJR.0b013e3283294b1d

Kurdyak PA, 2011, J EVAL CLIN PRACT, V17, P444, DOI 10.1111/j.1365-2753.2010.01446.x

Lau-Walker MO, 2009, J CLIN NURS, V18, P63, DOI 10.1111/j.1365-2702.2008.02386.x

Leynen F, 2006, EUR J CARDIOV PREV R, V13, P261, DOI 10.1097/01.hjr.0000194420.62379.de

Ludt S, 2011, PLOS ONE, V6, DOI 10.1371/journal.pone.0029334

Macedo ME, 2005, J HYPERTENS, V23, P1661, DOI 10.1097/01.hjh.0000179908.51187.de

Mena-Martin FJ, 2003, J HYPERTENS, V21, P1283, DOI 10.1097/01.hjh.0000059086.43904.17

Nash IS, 2003, ARCH INTERN MED, V163, P1597, DOI 10.1001/archinte.163.13.1597

Perk J, 2012, EUR HEART J, V33, P1635, DOI 10.1093/eurheartj/ehs092

Rabin R, 2013, USER GUIDE BASIC INF

Rasmussen JN, 2007, JAMA-J AM MED ASSOC, V297, P177, DOI 10.1001/jama.297.2.177

Redfern J, 2007, MED J AUSTRALIA, V186, P21

Small N, 2013, BMC HEALTH SERV RES, V13, DOI 10.1186/1472-6963-13-263

Spertus JA, 2002, CIRCULATION, V106, P43, DOI 10.1161/01.CIR.0000020688.24874.90

Stafford L, 2009, J PSYCHOSOM RES, V66, P211, DOI 10.1016/j.jpsychores.2008.09.005

Thombs BD, 2008, EUR J CARDIOV PREV R, V15, P479, DOI 10.1097/HJR.0b013e328300b717

Ware J., 2002, SCORE VERSION 2 SF 1

ZIGMOND AS, 1983, ACTA PSYCHIAT SCAND, V67, P361, DOI 10.1111/j.1600-0447.1983.tb09716.x

NR 31

TC 1

Z9 1

U1 0

U2 4

PU SPRINGER BASEL AG

PI BASEL

PA PICASSOPLATZ 4, BASEL, 4052, SWITZERLAND

SN 1661-8556

EI 1661-8564

J9 INT J PUBLIC HEALTH

JI Int. J. Public Health

PD JUN

PY 2014

VL 59

IS 3

BP 475

EP 483

DI 10.1007/s00038-014-0551-0

PG 9

WC Public, Environmental & Occupational Health

SC Public, Environmental & Occupational Health

GA AH3NP

UT WOS:000336031100007

PM 24770848

DA 2019-01-08

ER

PT J

AU Bergmann, N

Gyntelberg, F

Faber, J

AF Bergmann, N.

Gyntelberg, F.

Faber, J.

TI The appraisal of chronic stress and the development of the metabolic

syndrome: a systematic review of prospective cohort studies

SO ENDOCRINE CONNECTIONS

LA English

DT Review

DE metabolic syndrome; adiposity; hypertension; dyslipidemia; type 2

diabetes mellitus; psychological stress; job stress

AB Chronic psychosocial stress has been proposed as a risk factor for the development of the metabolic syndrome (MES). This review gives a systematic overview of prospective cohort studies investigating chronic psychosocial stress as a risk factor for incident MES and the individual elements of MES. Thirty-nine studies were included. An association between chronic psychosocial stress and the development of MES was generally supported. Regarding the four elements of MES: i) weight gain: the prospective studies supported etiological roles for relationship stress, perceived stress, and distress, while the studies on work-related stress (WS) showed conflicting results; ii) dyslipidemi: too few studies on psychosocial stress as a risk factor for dyslipidemia were available to draw a conclusion; however, a trend toward a positive association was present; iii) type 2 diabetes mellitus (DM2): prospective studies supported perceived stress and distress as risk factors for the development of DM2 among men, but not among women, while WS was generally not supported as a risk factor among neither men nor women; iv) hypertension: marital stress and perceived stress might have an influence on blood pressure (BP), while no association was found regarding distress. Evaluating WS the results were equivocal and indicated that different types of WS affected the BP differently between men and women. In conclusion, a longitudinal association between chronic psychosocial stress and the development of MES seems present. However, the number of studies with sufficient quality is limited and the design of the studies is substantially heterogeneous.

C1 [Bergmann, N.; Faber, J.] Herlev Univ Hosp, Dept Med O, Endocrine Unit, DK-2730 Herlev, Denmark.

[Gyntelberg, F.] Natl Res Ctr Working Environm, Copenhagen, Denmark.

[Faber, J.] Univ Copenhagen, Fac Hlth Sci, Copenhagen, Denmark.

RP Bergmann, N (reprint author), Herlev Univ Hosp, Dept Med O, Endocrine Unit, DK-2730 Herlev, Denmark.

EM n.c.bergmann@hotmail.com

OI Bergmann, natasha/0000-0002-6797-8041

CR Allard KO, 2011, J OCCUP ENVIRON MED, V53, P1364, DOI 10.1097/JOM.0b013e3182363bda

Baker B, 2000, ARCH INTERN MED, V160, P3453, DOI 10.1001/archinte.160.22.3453

Bech P., 2012, CLIN PSYCHOMETRICS

Berset M, 2011, SCAND J WORK ENV HEA, V37, P45, DOI 10.5271/sjweh.3089

Bjorntorp P, 1999, DIABETES-METAB RES, V15, P427

Block JP, 2009, AM J EPIDEMIOL, V170, P181, DOI 10.1093/aje/kwp104

Brunner EJ, 2007, AM J EPIDEMIOL, V165, P828, DOI 10.1093/aje/kwk058

Brunner EJ, 2013, NAT REV ENDOCRINOL, V9, P449, DOI 10.1038/nrendo.2013.124

Chandola T, 2006, BRIT MED J, V332, P521, DOI 10.1136/bmj.38693.435301.80

Choi B, 2014, J INTERN MED, V275, P438, DOI 10.1111/joim.12173

COHEN S, 1983, J HEALTH SOC BEHAV, V24, P385, DOI 10.2307/2136404

Cosgrove MP, 2012, OCCUP MED-OXFORD, V62, P167, DOI 10.1093/occmed/kqs002

Dweck JS, 2014, APPETITE, V72, P106, DOI 10.1016/j.appet.2013.10.001

Edwards EM, 2012, J OCCUP ENVIRON MED, V54, P1447, DOI 10.1097/JOM.0b013e3182783f27

Eek F, 2009, SCAND J PUBLIC HEALT, V37, P532, DOI 10.1177/1403494809104359

Eriksson AK, 2008, DIABETIC MED, V25, P834, DOI 10.1111/j.1464-5491.2008.02463.x

Eriksson AK, 2013, DIABETES CARE, V36, P2683, DOI 10.2337/dc12-1738

Fauvel JP, 2003, HYPERTENSION, V42, P1112, DOI 10.1161/01.HYP.0000102862.93418.EE

Fowler-Brown AG, 2009, OBESITY, V17, P2106, DOI 10.1038/oby.2009.130

Gerace TA, 1996, PREV MED, V25, P593, DOI 10.1006/pmed.1996.0094

Gilbert-Ouimet M, 2012, J PSYCHOSOM RES, V72, P26, DOI 10.1016/j.jpsychores.2011.07.002

Gilbert-Ouimet M, 2014, SCAND J WORK ENV HEA, V40, P109, DOI 10.5271/sjweh.3390

Gimeno D, 2010, OCCUP ENVIRON MED, V67, P256, DOI 10.1136/oem.2009.047324

Goldberg DP, 1988, USERSS GUIDE GEN HLT

Goldstein DS, 2002, STRESS, V5, P54, DOI 10.1080/10253890290012345

Goodwin JE, 2012, PEDIATR NEPHROL, V27, P1059, DOI 10.1007/s00467-011-1928-4

Guimont C, 2006, AM J PUBLIC HEALTH, V96, P1436, DOI 10.2105/AJPH.2004.057679

Heraclides AM, 2012, OBESITY, V20, P428, DOI 10.1038/oby.2011.95

Heslop P, 2002, SOC SCI MED, V54, P1589, DOI 10.1016/S0277-9536(01)00138-1

Holmes ME, 2010, OBES REV, V11, P492, DOI 10.1111/j.1467-789X.2009.00680.x

Ishizaki M, 2008, SCAND J WORK ENV HEA, V34, P288, DOI 10.5271/sjweh.1267

Karasek R, 1998, J Occup Health Psychol, V3, P322, DOI 10.1037/1076-8998.3.4.322

Kato M, 2009, ENDOCR J, V56, P459, DOI 10.1507/endocrj.K09E-003

Kawakami N, 1999, J EPIDEMIOL COMMUN H, V53, P359, DOI 10.1136/jech.53.6.359

Kivimaki M, 2006, INT J OBESITY, V30, P982, DOI 10.1038/sj.ijo.0803229

Kivimaki M, 2002, BRIT MED J, V325, P857, DOI 10.1136/bmj.325.7369.857

Kivimaki M, 2007, AM J HYPERTENS, V20, P1146, DOI 10.1016/j.amjhyper.2007.06.006

Korkeila M, 1998, INT J OBESITY, V22, P949, DOI 10.1038/sj.ijo.0800694

Kouvonen A, 2006, BMC PUBLIC HEALTH, V6, DOI 10.1186/1471-2458-6-24

Kouvonen A, 2011, AM J PUBLIC HEALTH, V101, P1474, DOI 10.2105/AJPH.2010.300115

Kroenke CH, 2007, AM J EPIDEMIOL, V165, P175, DOI 10.1093/aje/kwj355

Kumari M, 2004, ARCH INTERN MED, V164, P1873, DOI 10.1001/archinte.164.17.1873

Lallukka T, 2008, EUR J EPIDEMIOL, V23, P303, DOI 10.1007/s10654-008-9233-7

Landsbergis P, 2000, OCCUP MED, V15, P163

Landsbergis PA, 2013, AM J PUBLIC HEALTH, V103, pE61, DOI 10.2105/AJPH.2012.301153

Levenstein S, 2001, ARCH INTERN MED, V161, P1341, DOI 10.1001/archinte.161.10.1341

Liberati A, 2009, BMJ-BRIT MED J, V339, DOI 10.1136/bmj.b2700

Macfarlane DP, 2008, J ENDOCRINOL, V197, P189, DOI 10.1677/JOE-08-0054

Magee CA, 2010, J OCCUP ENVIRON MED, V52, P977, DOI 10.1097/JOM.0b013e3181f26e72

Markovitz JH, 2004, ANN BEHAV MED, V28, P4, DOI 10.1207/s15324796abm2801_2

Matud MP, 2004, PERS INDIV DIFFER, V37, P1401, DOI 10.1016/j.paid.2004.01.010

Mente A, 2010, J AM COLL CARDIOL, V55, P2390, DOI 10.1016/j.jacc.2009.12.053

Mommersteeg PMC, 2012, BMC PUBLIC HEALTH, V12, DOI 10.1186/1471-2458-12-1109

Mottillo S, 2010, J AM COLL CARDIOL, V56, P1113, DOI 10.1016/j.jacc.2010.05.034

Myers MG, 1999, AM J HYPERTENS, V12, P1149, DOI 10.1016/S0895-7061(99)00199-5

NIAURA R, 1992, BIOL PSYCHOL, V34, P1, DOI 10.1016/0301-0511(92)90022-M

Novak M, 2013, DIABETIC MED, V30, pE8, DOI 10.1111/dme.12037

Nyberg ST, 2012, J INTERN MED, V272, P65, DOI 10.1111/j.1365-2796.2011.02482.x

Ohlin B, 2007, J HYPERTENS, V25, P525

Oksanen T, 2012, J HYPERTENS, V30, P1129, DOI 10.1097/HJH.0b013e32835377ed

Pouwer F, 2010, DISCOV MED, V9, P112

Puustinen PJ, 2011, PSYCHOSOM MED, V73, P158, DOI 10.1097/PSY.0b013e3182037315

Quist HG, 2013, BMC PUBLIC HEALTH, V13, DOI 10.1186/1471-2458-13-43

Raikkonen K, 2007, DIABETES CARE, V30, P872, DOI 10.2337/dc06-1857

Rod NH, 2009, J INTERN MED, V266, P467, DOI 10.1111/j.1365-2796.2009.02124.x

Roos E, 2013, ARCH ENVIRON OCCUP H, V68, P166, DOI 10.1080/19338244.2012.686931

Rosmond R, 2005, PSYCHONEUROENDOCRINO, V30, P1, DOI 10.1016/j.psyneuen.2004.05.007

Schnall PL, 1998, PSYCHOSOM MED, V60, P697, DOI 10.1097/00006842-199811000-00007

Schubert C, 2009, BIOL PSYCHOL, V80, P325, DOI 10.1016/j.biopsycho.2008.11.005

Siegrist J, 2004, SOC SCI MED, V58, P1483, DOI 10.1016/S0277-9536(03)00351-4

Smith PM, 2012, OCCUP MED-OXFORD, V62, P413, DOI 10.1093/occmed/kqs128

Sparrenberger F, 2009, J HUM HYPERTENS, V23, P12, DOI 10.1038/jhh.2008.74

Tobe SW, 2007, AM J HYPERTENS, V20, P148, DOI 10.1016/j.amjhyper.2006.07.011

Toker S, 2012, J OCCUP HEALTH PSYCH, V17, P259, DOI 10.1037/a0028401

Toshihiro M, 2008, DIABETIC MED, V25, P1211, DOI 10.1111/j.1464-5491.2008.02566.x

Troxel WM, 2005, ARCH INTERN MED, V165, P1022, DOI 10.1001/archinte.165.9.1022

van Holland BJ, 2012, INT ARCH OCC ENV HEA, V85, P849, DOI 10.1007/s00420-011-0727-3

Wardle J, 2011, OBESITY, V19, P771, DOI 10.1038/oby.2010.241

Whisman MA, 2012, HEALTH PSYCHOL, V31, P80, DOI 10.1037/a0025671

Williams ED, 2013, ANN BEHAV MED, V46, P62, DOI 10.1007/s12160-013-9473-y

Zellner DA, 2006, PHYSIOL BEHAV, V87, P789, DOI 10.1016/j.physbeh.2006.01.014

NR 81

TC 58

Z9 58

U1 4

U2 15

PU BIOSCIENTIFICA LTD

PI BRISTOL

PA EURO HOUSE, 22 APEX COURT WOODLANDS, BRADLEY STOKE, BRISTOL BS32 4JT,

ENGLAND

SN 2049-3614

J9 ENDOCR CONNECT

JI Endocr. Connect.

PD JUN 1

PY 2014

VL 3

IS 2

BP R55

EP R80

DI 10.1530/EC-14-0031

PG 26

WC Endocrinology & Metabolism

SC Endocrinology & Metabolism

GA V44UM

UT WOS:000209773900003

PM 24743684

OA DOAJ Gold, Green Published

DA 2019-01-08

ER

PT J

AU Molina, Y

Lehavot, K

Beadnell, B

Simoni, J

AF Molina, Yamile

Lehavot, Keren

Beadnell, Blair

Simoni, Jane

TI Racial Disparities in Health Behaviors and Conditions Among Lesbian and

Bisexual Women: The Role of Internalized Stigma

SO LGBT HEALTH

LA English

DT Article

DE bisexual; health disparities; lesbian/gay; physical health;

race/ethnicity; women

ID FEMINIST IDENTITY DEVELOPMENT; ACTIVITY QUESTIONNAIRE IPAQ; BODY-FAT

DISTRIBUTION; SEXUAL ORIENTATION; AFRICAN-AMERICAN; PHYSICAL-ACTIVITY;

US ADULTS; GAY MEN; PSYCHOLOGICAL DISTRESS; VEGETABLE INTAKE

AB There are documented disparities in physical health behaviors and conditions, such as physical activity and obesity, with regard to both race/ethnicity and sexual orientation. However, physical health disparities for lesbian and bisexual (LB) women who are also racial minorities are relatively unexplored. Minority stressors, such as internalized stigma, may account for disparities in such multiply marginalized populations. We sought to (1) characterize inequalities among non-Hispanic white and African American LB women and (2) examine the roles of internalized sexism and homophobia in disparities. Data on health behaviors (diet, physical activity); physical health (hypertension, diabetes, overweight/obesity); internalized sexism; and internalized homophobia were collected via a web-based survey. Recruitment ads were sent electronically to over 200 listservs, online groups, and organizations serving the lesbian, gay, and bisexual community in all 50 U.S. states. The analytic sample consisted of 954 white and 75 African American LB women. African American participants were more likely than white participants to report low fruit/vegetable intake and physical activity, a higher body mass index, and a history of diabetes and hypertension. There were no racial differences in internalized homophobia, but African American women reported higher levels of internalized sexism. Internalized sexism partially mediated racial disparities in physical activity and diabetes, but not in the other outcomes. Findings suggest that African American LB women may be at greater risk than their white counterparts for poor health and that internalized sexism may be a mediator of racial differences for certain behaviors and conditions.

C1 [Molina, Yamile] Fred Hutchinson Canc Res Ctr, Seattle, WA 98109 USA.

[Molina, Yamile] Univ Illinois, Epidemiol & Biostat Div, Chicago, IL USA.

[Molina, Yamile] Univ Washington, Dept Hlth Serv, Seattle, WA 98195 USA.

[Lehavot, Keren] Univ Washington, Dept Psychiat & Behav Sci, Seattle, WA 98195 USA.

[Beadnell, Blair] Univ Washington, Sch Social Work, Seattle, WA 98195 USA.

[Simoni, Jane] Univ Washington, Dept Psychol, Seattle, WA 98195 USA.

[Lehavot, Keren] VA Puget Sound Hlth Care Syst, Seattle, WA USA.

RP Molina, Y (reprint author), Fred Hutchinson Canc Res Ctr, 1100 Fairview Ave N,M3-B232, Seattle, WA 98109 USA.

EM ymolina@fhcrc.org

OI Simoni, Jane/0000-0002-8711-1576

FU Centers for Disease Control Grant for Public Health Research

Dissertation [R36 CD000996]; National Cancer Institute Supplement to

Promote Diversity in Health-Related Research [P50CA148143];

Biobehavioral Cancer Prevention and Control Training Program at the

University of Washington [R25CA92408]

FX This research was supported by a Centers for Disease Control Grant for

Public Health Research Dissertation (R36 CD000996) award to K.L.

Manuscript preparation was supported by funding to Y.M. through a

National Cancer Institute Supplement to Promote Diversity in

Health-Related Research (P50CA148143) and a postdoctoral fellowship

through the Biobehavioral Cancer Prevention and Control Training Program

at the University of Washington (R25CA92408).

CR Aaron DJ, 2001, AM J PUBLIC HEALTH, V91, P972, DOI 10.2105/AJPH.91.6.972

Acevedo-Garcia D, 2003, AM J PUBLIC HEALTH, V93, P214

Adler NE, 2008, ANNU REV PUBL HEALTH, V29, P235, DOI 10.1146/annurev.publhealth.29.020907.090852

Amadio DM, 2006, ADDICT BEHAV, V31, P1153, DOI 10.1016/j.addbeh.2005.08.013

[Anonymous], 2010, BIENN REP DIR NAT I

Baker AH, 2003, APPETITE, V40, P269, DOI 10.1016/S0195-6663(03)00014-X

Balsam KF, 2011, CULT DIVERS ETHN MIN, V17, P163, DOI 10.1037/a0023244

Bird CE, 1999, SOC SCI MED, V48, P745, DOI 10.1016/S0277-9536(98)00402-X

Block G, 2000, AM J PREV MED, V18, P284, DOI 10.1016/S0749-3797(00)00119-7

Borrell C, 2010, J WOMENS HEALTH, V19, P741, DOI 10.1089/jwh.2009.1594

Bowen DJ, 2008, OBESITY, V16, P221, DOI 10.1038/oby.2007.34

Brancati FL, 2000, JAMA-J AM MED ASSOC, V283, P2253, DOI 10.1001/jama.283.17.2253

Braveman P, 2006, ANNU REV PUBL HEALTH, V27, P167, DOI 10.1146/annurev.publhealth.27.021405.102103

Bridges SK, 2003, J MULTICULT COUNS D, V31, P113, DOI 10.1002/j.2161-1912.2003.tb00537.x

Burgess D., 2007, J LGBT HLTH RES, V3, P1, DOI DOI 10.1080/15574090802226626

Butler C, 2002, J NATL MED ASSOC, V94, P143

Calle EE, 1999, NEW ENGL J MED, V341, P1097, DOI 10.1056/NEJM199910073411501

Case P, 2004, J WOMENS HEALTH, V13, P1033, DOI 10.1089/jwh.2004.13.1033

Chambers EC, 2004, J NATL MED ASSOC, V96, P1594

Conron KJ, 2010, AM J PUBLIC HEALTH, V100, P1953, DOI 10.2105/AJPH.2009.174169

Dilley JA, 2010, AM J PUBLIC HEALTH, V100, P460, DOI 10.2105/AJPH.2007.130336

Dube EM, 1999, DEV PSYCHOL, V35, P1389, DOI 10.1037//0012-1649.35.6.1389

Fischer AR, 2000, PSYCHOL WOMEN QUART, V24, P15, DOI 10.1111/j.1471-6402.2000.tb01018.x

Gorman BK, 2006, J HEALTH SOC BEHAV, V47, P95, DOI 10.1177/002214650604700201

Greene B, 1997, J Lesbian Stud, V1, P109, DOI 10.1300/J155v01n01_09

Hagstromer M, 2006, PUBLIC HEALTH NUTR, V9, P755, DOI 10.1079/PHN2005898

Hallal PC, 2004, MED SCI SPORT EXER, V36, P556, DOI 10.1249/01.MSS.0000117161.66394.07

Hancock A. -M., 2004, POLITICS DISGUST PUB

Hatzenbuehler ML, 2010, AM J PUBLIC HEALTH, V100, P452, DOI 10.2105/AJPH.2009.168815

Hayes AF, 2009, COMMUN MONOGR, V76, P408, DOI 10.1080/03637750903310360

Herek GM, 1998, J GAY LESBIAN MED AS, V2, P17, DOI DOI 10.1016/0145-2134(92)90011-F

Herek GM, 2002, HEALTH PSYCHOL, V22, P595

Hertz RP, 2005, ARCH INTERN MED, V165, P2098, DOI 10.1001/archinte.165.18.2098

Hill SA, 2002, SEX ROLES, V47, P493, DOI 10.1023/A:1022026303937

Institute of Medicine (US) Committee on Lesbian Gay Bisexual and Transgender Health Issues and Research Gaps and Opportunities, 2011, HLTH LESB GAY BISEX

Jones CP, 2000, AM J PUBLIC HEALTH, V90, P1212, DOI 10.2105/AJPH.90.8.1212

Kant AK, 2007, AM J PREV MED, V32, P264, DOI 10.1016/j.amepre.2006.12.011

Katznelson Ira, 2005, AFFIRMATIVE ACTION W

Kertzner RM, 2009, AM J ORTHOPSYCHIAT, V79, P500, DOI 10.1037/a0016848

Keyhani S, 2008, HYPERTENSION, V51, P1149, DOI 10.1161/HYPERTENSIONAHA.107.107342

Kirkpatrick SI, 2012, J ACAD NUTR DIET, V112, P624, DOI 10.1016/j.jand.2011.11.012

Krane V, 2004, SEX ROLES, V50, P315, DOI 10.1023/B:SERS.0000018888.48437.4f

Krieger N, 2004, EPIDEMIOL REV, V26, P92, DOI 10.1093/epirev/mxh009

Krieger N, 1997, ANNU REV PUBL HEALTH, V18, P341, DOI 10.1146/annurev.publhealth.18.1.341

Laditka SB, 2002, J WOMEN AGING, V14, P163, DOI 10.1300/J074v14n01_11

LaVeist T. A., 2002, RACE ETHNICITY HLTH

Lehman P., 2000, THESIS FLORIDA I TEC

Lewis RJ, 2003, J SOC CLIN PSYCHOL, V22, P716, DOI 10.1521/jscp.22.6.716.22932

LINK BG, 1995, J HEALTH SOC BEHAV, V35, P80, DOI 10.2307/2626958

Mak WWS, 2007, SOC SCI MED, V65, P245, DOI 10.1016/j.socscimed.2007.03.015

Martin J. L., 1992, SUMMARY MEASUR UNPUB

Massey D., 2007, CATEGORICALLY UNEQUA

Mays VM, 2002, AM J PUBLIC HEALTH, V92, P632, DOI 10.2105/AJPH.92.4.632

McCollum Marianne, 2005, Gend Med, V2, P246, DOI 10.1016/S1550-8579(05)80054-3

McKay Becky, 2011, Medical Reference Services Quarterly, V30, P393, DOI 10.1080/02763869.2011.608971

Mensah GA, 2005, CIRCULATION, V111, P1233, DOI 10.1161/01.CIR.0000158136.76824.04

MEYER IH, 1995, J HEALTH SOC BEHAV, V36, P38, DOI 10.2307/2137286

Moradi B, 2002, COUNS PSYCHOL, V30, P66, DOI 10.1177/0011000002301004

Moradi B, 2010, COUNS PSYCHOL, V38, P397, DOI 10.1177/0011000009335263

National Heart Lung and Blood institute National Institute of Diabetes and Digestive and Kidney Diseases (U.S.), 1998, CLIN GUID ID EV TREA

Ogden CL, 2009, AM J CLIN NUTR, V89, P1001, DOI 10.3945/ajcn.2009.27592

Packard GM, 2002, AM NIGHTMARE HIST JI

Piggot M, 2004, THESIS SWINBURNE U T

Preacher KJ, 2008, BEHAV RES MET, V40, P87

Ransdell LB, 1998, MED SCI SPORT EXER, V30, P1608, DOI 10.1097/00005768-199811000-00009

Roberts SA, 2003, WOMEN HEALTH ISS, V13, P167, DOI 10.1016/S1049-3867(03)00041-0

Sartore ML, 2009, QUEST, V61, P289, DOI 10.1080/00336297.2009.10483617

SCHAFER JL, 2002, PSYCHOL METHODS, V7, P142, DOI DOI 10.1037/1082-989X.7.2.147

Sekayi D, 2003, J NEGRO EDUC, V72, P467, DOI DOI 10.2307/3211197

Szymanski DA, 2005, J COUNS DEV, V83, P355, DOI 10.1002/j.1556-6678.2005.tb00355.x

Szymanski DM, 2008, COUNS PSYCHOL, V36, P525, DOI 10.1177/0011000007309489

Szymanski DM, 2009, J COUNS PSYCHOL, V56, P110, DOI 10.1037/a0013317

Szymanski DM, 2005, COUNS PSYCHOL, V33, P729, DOI 10.1177/0011000005278408

Thomas AJ, 2008, CULT DIVERS ETHN MIN, V14, P307, DOI 10.1037/1099-9809.14.4.307

Troiano RP, 2008, MED SCI SPORT EXER, V40, P181, DOI 10.1249/mss.0b013e31815a51b3

Tull ES, 2005, J NATL MED ASSOC, V97, P206

Tull ES, 2007, ETHNIC DIS, V17, P731

U. S. Department of Health and Human Services, 2011, HLTH PEOPL 2020 OBJ

Valanis BG, 2000, ARCH FAM MED, V9, P843, DOI 10.1001/archfami.9.9.843

Veenstra G, 2013, SOC SCI MED, V89, P16, DOI 10.1016/j.socscimed.2013.04.014

Watters JL, 2007, PUBLIC HEALTH NUTR, V10, P701, DOI 10.1017/S1368980007662284

West CM, 1995, PSYCHOTHER, V32, P458, DOI 10.1037/0033-3204.32.3.458

Whitson HE, 2010, J GERONTOL A-BIOL, V65, P1325, DOI 10.1093/gerona/glq139

Williams DR, 2009, J BEHAV MED, V32, P20, DOI 10.1007/s10865-008-9185-0

Williams DR, 2005, HEALTH AFFAIR, V24, P325, DOI 10.1377/hlthaff.24.2.325

Williams DR, 2002, AM J PUBLIC HEALTH, V92, P588, DOI 10.2105/AJPH.92.4.588

Wilson P.A., 2007, HLTH SEXUAL MINORITI, P607

Wolin KY, 2008, J PHYS ACT HEALTH, V5, P746, DOI 10.1123/jpah.5.5.746

Zhang Q, 2009, ETHNIC HEALTH, V14, P439, DOI 10.1080/13557850802699155

NR 89

TC 10

Z9 10

U1 3

U2 11

PU MARY ANN LIEBERT, INC

PI NEW ROCHELLE

PA 140 HUGUENOT STREET, 3RD FL, NEW ROCHELLE, NY 10801 USA

SN 2325-8292

EI 2325-8306

J9 LGBT HEALTH

JI LGBT Health

PD JUN

PY 2014

VL 1

IS 2

BP 131

EP 139

DI 10.1089/lgbt.2013.0007

PG 9

WC Public, Environmental & Occupational Health

SC Public, Environmental & Occupational Health

GA CR9PV

UT WOS:000361689800012

PM 25364769

OA Green Accepted

DA 2019-01-08

ER

PT J

AU Pandit, AU

Bailey, SC

Curtis, LM

Seligman, HK

Davis, TC

Parker, RM

Schillinger, D

DeWalt, D

Fleming, D

Mohr, DC

Wolf, MS

AF Pandit, Anjali U.

Bailey, Stacy C.

Curtis, Laura M.

Seligman, Hilary K.

Davis, Terry C.

Parker, Ruth M.

Schillinger, Dean

DeWalt, Darren

Fleming, David

Mohr, David C.

Wolf, Michael S.

TI Disease-related distress, self-care and clinical outcomes among

low-income patients with diabetes

SO JOURNAL OF EPIDEMIOLOGY AND COMMUNITY HEALTH

LA English

DT Article

ID DEPRESSION; HEALTH; ADHERENCE; SCALE

AB Background The purpose of this study is to determine the prevalence of diabetes distress and its relationship with health behaviours and clinical outcomes in low-income patients.

Methods Secondary analyses were conducted using baseline data from a clinical trial evaluating a diabetes self-management intervention. Interviews were conducted with 666 participants receiving care at nine safety net clinics in Missouri. Distress was measured using the Diabetes Distress Scale, and outcomes included medication adherence, physical activity, nutrition and clinical biomarkers (haemoglobin A1C (HbA1C), blood pressure, low-density lipoprotein (LDL) cholesterol).

Results In a sample of 666 participants, 14.1% and 27.3% of patients were identified as highly and moderately distressed, respectively, with higher rates among younger, female and lower income patients. When compared with moderately and no distress groups, highly distressed patients were less adherent to medications (20.7% vs 29.9% vs 39.4%, p<0.001) and had higher HbA1C values (9.3% (SD=2.0) vs 8.2% (SD=1.8) vs 7.8% (SD=1.7), p<0.001), diastolic blood pressure (81.8 (SD=9.4) vs 80.2 (9.7) vs 78.9 (SD=8.8), p=0.02) and LDL cholesterol (104.6 (SD=42.4) vs 97.2 (34.3) vs 95.5 (37.9)) In multivariable analyses, high and moderate distress were associated with lower medication adherence (OR=0.44; 0.27 to 0.23, p=0.001) and (OR=0.58; 0.42 to 0.79; p=0.001), respectively, and higher HbA1C in only the highly distressed group (B=1.3; 0.81 to 1.85; p<0.001) compared with the no distress group.

Conclusions Diabetes distress is prevalent and linked to poorer adherence to health behaviours and glycemic control in a sample of patients receiving care from low-income clinics.

C1 [Pandit, Anjali U.; Bailey, Stacy C.; Curtis, Laura M.; Wolf, Michael S.] Feinberg Sch Med, Hlth Literacy & Learning Program, Dept Gen Internal Med, Chicago, IL USA.

[Seligman, Hilary K.; Schillinger, Dean] Univ Calif San Francisco, Div Gen Internal Med, San Francisco, CA 94143 USA.

[Davis, Terry C.] Louisiana State Univ, Hlth Sci Ctr, Dept Med & Pediat, Shreveport, LA 71105 USA.

[Parker, Ruth M.] Emory Univ, Sch Med, Div Gen Med, Atlanta, GA USA.

[DeWalt, Darren] Univ N Carolina, Sch Med, Div Gen Internal Med, Chapel Hill, NC USA.

[Fleming, David] Univ Missouri, Sch Med, Dept Med, Columbia, MO 65212 USA.

[Mohr, David C.] Northwestern Univ, Dept Prevent Med, Dept Psychiat & Behav Sci, Chicago, IL 60611 USA.

RP Pandit, AU (reprint author), Northwestern Univ, Div Gen Internal Med, Hlth Literacy & Learning Program HeLP, Feinberg Sch Med, 750 N Lake Shore,Dr 10th Floor, Chicago, IL 60611 USA.

EM a-pandit@northwestern.edu

OI DeWalt, Darren/0000-0003-2270-751X; Mohr, David/0000-0002-5443-7596;

Curtis, Laura/0000-0003-2380-2201

FU Missouri Foundation for Health

FX This paper was supported by the Missouri Foundation for Health.

CR American Association for Public Opinion, 2004, STAND DEF FIN DISP C

Bailey Stacy Cooper, 2009, Med Care, V47, P707, DOI 10.1097/MLR.0b013e318195fd02

Baker DW, 1999, PATIENT EDUC COUNS, V38, P33, DOI 10.1016/S0738-3991(98)00116-5

Bener A, 2011, ONCOL REV, V5, P5, DOI 10.1007/s12156-010-0061-0

Centers for Disease Control and Prevention, 2006, BEH RISK FACT SURV F

Ciechanowski PS, 2000, ARCH INTERN MED, V160, P3278, DOI 10.1001/archinte.160.21.3278

Fisher L, 2009, DIABETIC MED, V26, P622, DOI 10.1111/j.1464-5491.2009.02730.x

Fisher L, 2012, DIABETES CARE, V35, P259, DOI 10.2337/dc11-1572

Fisher L, 2010, DIABETES CARE, V33, P1034, DOI 10.2337/dc09-2175

Fisher L, 2010, DIABETES CARE, V33, P23, DOI 10.2337/dc09-1238

Hays RD, 2009, QUAL LIFE RES, V18, P873, DOI 10.1007/s11136-009-9496-9

Jimenez-Garcia R, 2012, PRIM CARE DIABETES, V6, P149, DOI 10.1016/j.pcd.2011.10.002

Lopez-Quintero C, 2010, J AM DIET ASSOC, V110, pS62, DOI 10.1016/j.jada.2010.03.009

MacKinnon DP, 2009, CURR DIR PSYCHOL SCI, V18, P16, DOI 10.1111/j.1467-8721.2009.01598.x

MORISKY DE, 1986, MED CARE, V24, P67, DOI 10.1097/00005650-198601000-00007

Moussavi S, 2007, LANCET, V370, P851, DOI 10.1016/S0140-6736(07)61415-9

Polonsky WH, 2005, DIABETES CARE, V28, P626, DOI 10.2337/diacare.28.3.626

Slean GR, 2012, MED CARE, V50, pS69, DOI 10.1097/MLR.0b013e3182641127

Tol A, 2012, J DIABETES METAB DIS, V11, DOI 10.1186/2251-6581-11-20

U.S. Preventive Services Task Force, 2010, 1005145 US PREV SERV

Wolf MS, 2011, ARCH INTERN MED, V171, P300, DOI 10.1001/archinternmed.2011.39

NR 21

TC 29

Z9 29

U1 6

U2 28

PU BMJ PUBLISHING GROUP

PI LONDON

PA BRITISH MED ASSOC HOUSE, TAVISTOCK SQUARE, LONDON WC1H 9JR, ENGLAND

SN 0143-005X

EI 1470-2738

J9 J EPIDEMIOL COMMUN H

JI J. Epidemiol. Community Health

PD JUN

PY 2014

VL 68

IS 6

BP 557

EP 564

DI 10.1136/jech-2013-203063

PG 8

WC Public, Environmental & Occupational Health

SC Public, Environmental & Occupational Health

GA AG4AV

UT WOS:000335362000012

PM 24489044

DA 2019-01-08

ER

PT J

AU Salam, RA

Das, JK

Bhutta, ZA

AF Salam, Rehana A.

Das, Jai K.

Bhutta, Zulfiqar A.

TI Impact of intrauterine growth restriction on long-term health

SO CURRENT OPINION IN CLINICAL NUTRITION AND METABOLIC CARE

LA English

DT Review

DE catch-up growth; intrauterine growth restriction; metabolic diseases;

prenatal programming

ID FOR-GESTATIONAL-AGE; INSULIN-RESISTANCE; FETAL-GROWTH; METABOLIC

SYNDROME; POSTNATAL LIFE; CHILDREN BORN; PRETERM BIRTH; LEPTIN LEVELS;

RETARDATION; IUGR

AB Purpose of reviewIntrauterine growth restriction (IUGR) is responsible for the higher rates of fetal, perinatal, and neonatal morbidity and mortality. This review details the IUGR risk factors, its short and long-term sequel, the mechanism underlying the long-term consequences, and the strategies to tackle IUGR burden.Recent findingsShort-term consequences of IUGR involve metabolic, thermal, and hematological disturbances leading to morbidities. Long term consequences due to changes in the fetal nutritional environment is associated with increased risk of developing metabolic syndrome and cardiovascular disease, systolic hypertension, obesity, insulin resistance, and diabetes type II in adulthood. There are no effective therapies to reverse IUGR, and antenatal management is aimed at determining the ideal time and mode of delivery. In order to prevent complications associated with IUGR, it is important to first detect the condition and institute appropriate surveillance to assess fetal well-being coupled with suitable intervention in case of fetal distress.SummaryReliable prediction of IUGR may be achieved by combining clinical risk factors with Doppler abnormalities, fetal growth, and biomarkers. If this can be achieved, there is potential to reduce future perinatal morbidity, mortality and long-term consequences, but steps geared toward the prevention of IUGR are of unparalleled importance.

C1 [Salam, Rehana A.; Das, Jai K.; Bhutta, Zulfiqar A.] Aga Khan Univ, Div Woman & Child Hlth, Karachi 74800, Pakistan.

[Bhutta, Zulfiqar A.] Hosp Sick Children, Program Global Pediat Res, Toronto, ON M5G 1X8, Canada.

RP Bhutta, ZA (reprint author), Aga Khan Univ, Ctr Excellence Women & Child Hlth, Karachi 74800, Pakistan.

EM zulfiqar.bhutta@aku.edu

CR Abitbol CL, 2012, NAT REV NEPHROL, V8, P265, DOI 10.1038/nrneph.2012.38

Arcangeli T, 2012, ULTRASOUND OBST GYN, V40, P267, DOI 10.1002/uog.11112

Bacchetta J, 2008, ARCH PEDIATRIE, V15, P1212, DOI 10.1016/j.arcped.2008.04.013

Bacchetta J, 2009, KIDNEY INT, V76, P445, DOI 10.1038/ki.2009.201

Baschat AA, 2011, ULTRASOUND OBST GYN, V37, P501, DOI 10.1002/uog.9008

Bjarnegard N, 2013, ULTRASOUND OBST GYN, V41, P177, DOI 10.1002/uog.12314

Black RE, 2013, LANCET, V382, P427, DOI 10.1016/S0140-6736(13)60937-X

Boiko J, 2005, INT J OBESITY, V29, P565, DOI 10.1038/sj.ijo.0802901

Briana DD, 2009, EUR J ENDOCRINOL, V160, P337, DOI 10.1530/EJE-08-0621

Chan PYL, 2010, INT J PEDIAT, V2010, P1

Cianfarani S, 2012, INT J OBESITY, V36, P1270, DOI 10.1038/ijo.2012.54

Conde-Agudelo A, 2013, BJOG-INT J OBSTET GY, V120, P681, DOI 10.1111/1471-0528.12172

Crume T. L., 2013, OBESITY, V22, P608

Dessi A, 2012, J MATERN-FETAL NEO M, V25, P13, DOI 10.3109/14767058.2012.714639

Fowden AL, 2005, EARLY HUM DEV, V81, P723, DOI 10.1016/j.earlhumdev.2005.06.007

Fowden AL, 2004, REPRODUCTION, V127, P515, DOI 10.1530/rep.1.00033

Gatford KL, 2013, CLIN OBSTET GYNECOL, V56, P520, DOI 10.1097/GRF.0b013e31829e5b29

Gerhardt A, 2013, J THROMB THROMBOLYS, V37, P107

Geva R, 2006, PEDIATRICS, V118, P91, DOI 10.1542/peds.2005-2343

Grivell R, 2009, BEST PRACT RES CL OB, V23, P795, DOI 10.1016/j.bpobgyn.2009.06.004

Ibanez L, 2008, J CLIN ENDOCR METAB, V93, P2079, DOI 10.1210/jc.2007-2850

Imdad A, 2011, BMC PUBLIC HEALTH, V11, pS22, DOI DOI 10.1186/1471-2458-11-S3-S20.PUBMED:21501438

Jaquet D, 2005, DIABETOLOGIA, V48, P849, DOI 10.1007/s00125-005-1724-4

Jaquet D, 2000, J CLIN ENDOCR METAB, V85, P1401, DOI 10.1210/jc.85.4.1401

Javaid MK, 2005, CALCIFIED TISSUE INT, V76, P341, DOI 10.1007/s00223-004-1128-3

Kallen B, 2013, EUR RESPIR J, V41, P671, DOI 10.1183/09031936.00041912

Koklu E, 2007, PEDIATR RES, V62, P704, DOI 10.1203/PDR.0b013e318157caaa

Lindqvist PG, 2005, ULTRASOUND OBST GYN, V25, P258, DOI 10.1002/uog.1806

Longo S, 2013, J MATERN-FETAL NEO M, V26, P222, DOI 10.3109/14767058.2012.715006

Lopez-Bermejo A, 2006, CLIN ENDOCRINOL, V64, P479, DOI 10.1111/j.1365-2265.2006.02496.x

Mandruzzato G, 2008, J PERINAT MED, V36, P277, DOI 10.1515/JPM.2008.050

Martinez-Cordero C, 2006, ARCH MED RES, V37, P490, DOI 10.1016/j.arcmed.2005.11.004

McMillen IC, 2005, PHYSIOL REV, V85, P571, DOI 10.1152/physrev.00053.2003

McMillen IC, 2004, P NUTR SOC, V63, P405, DOI 10.1079/PNS2004370

Miller J, 2008, SEMIN PERINATOL, V32, P274, DOI 10.1053/j.semperi.2008.04.010

Neitzke U, 2008, PLACENTA, V29, P246, DOI 10.1016/j.placenta.2007.11.014

Ong KK, 2004, EUR J ENDOCRINOL, V151, pU131, DOI 10.1530/eje.0.151U131

Phillips DIW, 2001, CLIN EXP PHARMACOL P, V28, P967, DOI 10.1046/j.1440-1681.2001.03558.x

Pike K, 2012, SEM FET NEON MED

Roberts D, 2006, COCHRANE DB SYST REV, DOI 10.1002/14651858.CD004454.pub2

Roberts DJ, 2008, J CLIN PATHOL, V61, P1254, DOI 10.1136/jcp.2008.055236

Rosenberg A, 2008, SEM PER

Saenger P, 2007, ENDOCR REV, V28, P219, DOI 10.1210/er.2006-0039

Salam RA, 2013, EXPERT REV OBSTET GY, V8, P559

Schreuder M, 2006, CLIN COURSE PEDIAT I, P112

Singh S, 2013, INT J REPROD CONTRAC, V2, P566

Smith GCS, 2007, LANCET, V370, P1715, DOI 10.1016/S0140-6736(07)61723-1

Srinivas SK, 2009, J PERINATOL, V29, P680, DOI 10.1038/jp.2009.83

Stocker CJ, 2005, P NUTR SOC, V64, P143, DOI 10.1079/PNS2005417

Suhag A, 2013, CURR OBSTET GYNECOL, V2, P1

Sukrat B, 2013, BIOMED RES INT, V2013, P1

Teeninga N, 2008, NEPHROL DIAL TRANSPL, V23, P1615, DOI 10.1093/ndt/gfm829

Tinnion R, 2013, ARCH DIS CHILD, DOI 10.1136/archdischild-2013-304615

Valuniene M, 2007, MED LITH, V43, P784

Von Beckerath A-K, 2012, AM J OBSTET GYNECOL, V208, P130

Wojcik W, 2013, PSYCHOL MED, V43, P1, DOI 10.1017/S0033291712000682

Yajnik CS, 2002, J CLIN ENDOCR METAB, V87, P5575, DOI 10.1210/jc.2002-020434

Yuen BSJ, 2003, FASEB J, V17, P1102, DOI 10.1096/fj.02-0756fje

Zanardo V, 2013, HYPERTENS RES, V36, P440, DOI 10.1038/hr.2012.219

NR 59

TC 47

Z9 52

U1 0

U2 20

PU LIPPINCOTT WILLIAMS & WILKINS

PI PHILADELPHIA

PA 530 WALNUT ST, PHILADELPHIA, PA 19106-3621 USA

SN 1363-1950

EI 1473-6519

J9 CURR OPIN CLIN NUTR

JI Curr. Opin. Clin. Nutr. Metab. Care

PD MAY

PY 2014

VL 17

IS 3

BP 249

EP 254

DI 10.1097/MCO.0000000000000051

PG 6

WC Endocrinology & Metabolism; Nutrition & Dietetics

SC Endocrinology & Metabolism; Nutrition & Dietetics

GA AG5FM

UT WOS:000335444900008

PM 24613859

DA 2019-01-08

ER

PT J

AU Elgart, JF

Caporale, JE

Asteazaran, S

De La Fuente, JL

Camilluci, C

Brown, JB

Gonzalez, CD

Gagliardino, JJ

AF Elgart, Jorge F.

Caporale, Joaquin E.

Asteazaran, Santiago

De La Fuente, Jorge L.

Camilluci, Cecilia

Brown, Jonathan B.

Gonzalez, Claudio D.

Gagliardino, Juan J.

TI Association between socioeconomic status, type 2 diabetes and its

chronic complications in Argentina

SO DIABETES RESEARCH AND CLINICAL PRACTICE

LA English

DT Article

DE Socioeconomic status; Type 2 diabetes; Chronic complications; Diabetes

costs in Argentina

ID GLUCOSE CONTROL; MARITAL-STATUS; HEALTH; CARE; IMPACT; POPULATION;

PREVALENCE; MELLITUS; DISEASE; COSTS

AB Aim: To compare the socioeconomic status (SES) of people with type 2 diabetes (T2DM) in Argentina (Cordoba) with and without major chronic complications of diabetes, with that recorded in persons without diabetes matched by age and gender.

Methods: For this descriptive and analytic case-control study, potential candidates were identified from the electronic records of one institution of the Social Security System of the city of Cordoba. We identified and recruited 387 persons each with T2DM with or without chronic complications and 774 gender-and age-matched persons without T2DM (recruitment rate, 83%). Data were obtained by telephone interviews and supplemented with data from the institution's records. Group comparisons were performed with parametric or non-parametric tests as appropriate. We used ordinary least squares to regress household income and the difference between income and household expenses on diabetes status, age, sex, education and body mass index.

Results: Persons with T2DM, particularly those with complications, reported fewer years of general education (13.6 +/- 4.2 years vs. 12.2 +/- 4.4 years), a lower percentage of full time jobs (43.0% vs. 26.9%), lower salaries and monthly household income among those with full-time jobs (> 5000 ARG$: 52.6% vs. 24.5%), and a higher propensity to spend more money than they earned (expenditure/income ratio >= 1: 10.2% vs. 16.0%). The percentage of unmarried people was also higher among people with type 2 diabetes (7.0% vs. 10.9%).

Conclusion: T2DM and the development of its complications are each positively associated with lower SES and greater economic distress in Argentina. (C) 2014 Elsevier Ireland Ltd. All rights reserved.

C1 [Elgart, Jorge F.; Caporale, Joaquin E.; Asteazaran, Santiago; Brown, Jonathan B.; Gagliardino, Juan J.] Natl Univ La Plata, Natl Res Council, PAHO WHO Collaborating Ctr Diabet, CENEXA Ctr Expt & Appl Endocrinol,Sch Med, RA-1900 La Plata, Argentina.

[De La Fuente, Jorge L.; Camilluci, Cecilia] Hosp Privado, Ctr Med Cordoba, Cordoba, Argentina.

[Gonzalez, Claudio D.] Univ Buenos Aires, Sch Med, Dept Pharmacol, RA-1053 Buenos Aires, DF, Argentina.

RP Elgart, JF (reprint author), CENEXA UNLP CONICET LA PLATA, Fac Ciencias Med UNLP, 60 & 120, RA-1900 La Plata, Argentina.

EM jelgart@cenexa.org

OI Gonzalez, Claudio/0000-0002-3772-8850; Elgart, Jorge/0000-0002-6101-1219

FU Novo Nordisk International

FX This study was partially supported with an unrestricted grant provided

by Novo Nordisk International. The authors thank the authorities at

Hospital Privado - Centro Medico de Cordoba, Argentina, Enzo Rucci for

informatic support and Adriana Di Maggio for careful manuscript editing

prior to submission.

CR ADVANCE Collaborative Group, 2008, NEW ENGL J MED, V358, P2560, DOI [10.1056/NEJMoa0802987, DOI 10.1056/NEJM0A0802987]

Agardh E, 2011, INT J EPIDEMIOL, V40, P804, DOI 10.1093/ije/dyr029

American Diabetes Association, DIABETES CARE

[Anonymous], 2008, DIABETES EDUCATOR, V34, P451, DOI 10.1177/0145721708316627

Artazcoz L, GAC SANIT

August KJ, 2010, SOC SCI MED, V71, P1831, DOI 10.1016/j.socscimed.2010.08.022

Brown AF, 2004, EPIDEMIOL REV, V26, P63, DOI 10.1093/epirev/mxh002

Chaikiat A, 2012, HEALTH PLACE, V18, P877, DOI 10.1016/j.healthplace.2012.03.003

Chen RL, 2012, PLOS ONE, V7, DOI 10.1371/journal.pone.0050957

Cohen S, 2004, AM PSYCHOL, V59, P676, DOI 10.1037/0003-066X.59.8.676

Commendatore V, 2013, INT J CLIN PRACT, V67, P1261, DOI 10.1111/ijcp.12208

Duckworth W, 2009, NEW ENGL J MED, V360, P129, DOI 10.1056/NEJMoa0808431

Elovainio M, 2011, AM J EPIDEMIOL, V174, P779, DOI 10.1093/aje/kwr149

Fano V, 2013, EUR J PUBLIC HLT JAN

Ferrante D, 2011, REV ARGENT SALUD PUB, V2, P34

Fletcher J, 2008, J HEALTH ECON, V27, P794, DOI 10.1016/j.jhealeco.2007.10.010

Gaede P, 2003, NEW ENGL J MED, V348, P383, DOI 10.1056/NEJMoa021778

Gagliardino JJ, 2013, DIABETIC MED, V30, P1102, DOI 10.1111/dme.12230

Gagliardino JJ, 2012, DIABETES METAB, V38, P128, DOI 10.1016/j.diabet.2011.09.002

Gagliardino JJ, PAN AM J PUBLIC HLTH

Guariguata L, 2013, DIABETES RES CLIN PR

Hex N, 2012, DIABETIC MED, V29, P855, DOI 10.1111/j.1464-5491.2012.03698.x

Holman RR, 2008, NEW ENGL J MED, V359, P1577, DOI 10.1056/NEJMoa0806470

Hsu CC, 2012, DIABETES CARE, V35, P2286, DOI 10.2337/dc11-2052

International Diabetes Federation, 2010, DIAB SOC EC IMP STUD

Johnson NJ, 2000, ANN EPIDEMIOL, V10, P224, DOI 10.1016/S1047-2797(99)00052-6

Gagliardino JJ, 2013, DIABETES-METAB RES, V29, P152, DOI 10.1002/dmrr.2368

Kanjilal S, 1971, ARCH INTERN MED

Larranaga I, 2005, DIABETIC MED, V22, P1047, DOI 10.1111/j.1464-5491.2005.01598.x

Lavigne JE, 2003, PHARMACOECONOMICS, V21, P1123, DOI 10.2165/00019053-200321150-00006

Li YP, 2010, DIABETES, V59, P2400, DOI 10.2337/db10-0385

Maier W, 2013, DIABETIC MED, V30, pE78, DOI 10.1111/dme.12062

Morsanutto A, 2006, J DIABETES COMPLICAT, V20, P163, DOI 10.1016/j.jdiacomp.2005.06.011

OLIVERA EM, 1991, DIABETES CARE, V14, P593, DOI 10.2337/diacare.14.7.593

Pan American Health Organization/World Health Organization (PAHO/WHO), 2012, HLTH SIT AM BAS IND

Shaw K, 2011, DIABETES EDUCATOR, V37, P813, DOI 10.1177/0145721711424618

Shen J, 2012, CARDIOL RES PRACT, DOI 10.1155/2012/178675

Trento M, 2004, DIABETES CARE, V27, P670, DOI 10.2337/diacare.27.3.670

UK Prospective Diabetes Study Group, BMJ

Venermo M., 2013, BMJ OPEN, V8, P3

Williams R, 2002, DIABETOLOGIA, V45, pS13, DOI 10.1007/s00125-002-0859-9

Wilper AP, 2008, ANN INTERN MED, V149, P170, DOI 10.7326/0003-4819-149-3-200808050-00006

Wolf G, 2011, NEPHROL DIAL TRANSPL, V26, P4017, DOI 10.1093/ndt/gfr185

Zhang H, 2013, DIABETIC MED, V30, pE157, DOI 10.1111/dme.12156

Zimmet P, 2001, NATURE, V414, P782, DOI 10.1038/414782a

NR 45

TC 7

Z9 7

U1 0

U2 18

PU ELSEVIER IRELAND LTD

PI CLARE

PA ELSEVIER HOUSE, BROOKVALE PLAZA, EAST PARK SHANNON, CO, CLARE, 00000,

IRELAND

SN 0168-8227

EI 1872-8227

J9 DIABETES RES CLIN PR

JI Diabetes Res. Clin. Pract.

PD MAY

PY 2014

VL 104

IS 2

BP 241

EP 247

DI 10.1016/j.diabres.2014.02.010

PG 7

WC Endocrinology & Metabolism

SC Endocrinology & Metabolism

GA AF6SV

UT WOS:000334845800013

PM 24629409

DA 2019-01-08

ER

PT J

AU Zdunczyk, B

Sendela, J

Szypowska, A

AF Zdunczyk, Beata

Sendela, Joanna

Szypowska, Agnieszka

TI High prevalence of depressive symptoms in wellcontrolled adolescents

with type 1 diabetes treated with continuous subcutaneous insulin

infusion

SO DIABETES-METABOLISM RESEARCH AND REVIEWS

LA English

DT Article

DE quality of life; glycated haemoglobin; insulin pump; depression;

adolescent

ID QUALITY-OF-LIFE; GLYCEMIC CONTROL; CHILDRENS DEPRESSION;

METABOLIC-CONTROL; FAMILY CONFLICT; MEDIATING ROLE; MANAGEMENT; YOUTH;

INVENTORY; STRESS

AB BackgroundResearchers report co-morbid depressive symptoms in children with poorly controlled type 1 diabetes (T1D). However, excellent diabetes control may be associated with emotional distress as well. The aim of this study was to evaluate the prevalence of depressive symptoms in T1D children with HbA(1c) <7.5% and 7.5% treated with insulin pump.

MethodsA total of 372 children (186 girls) with a mean age of 14.22.0years and mean diabetes duration of 5.33.5years were included. All subjects were treated for T1D for a minimum of 1year. Children filled out a Polish version of the Children's Depression Inventory (CDI) and answered questions in the Quality of Life Questionnaire. Statistical analysis was made using Student's t-test, Mann-Whitney U-test, Fisher's exact test and Spearman's rank correlation.

ResultsEighteen per cent (31/177) of participants with HbA(1c) <7.5% and 21% (41/195) with HbA(1c) 7.5% reported depressive symptoms (CDI scores 13). Adolescents with HbA(1c) <7.5% and CDI13 had higher total insulin dose (p=0.039) and longer diabetes duration (p=0.043) than subjects with CDI<13. There was no difference in prevalence of depressive symptoms among patients with HbA(1c) 7.5% and HbA(1c) <7.5% (odds ratio [1.25 95% confidence interval (CI) 0.75-2.11], p=0.432). In both groups, adolescents with CDI13 had worse quality of life than those with CDI<13 (p<0.0001).

ConclusionsIn both groups, one in five adolescents with either good or poor glycemic control had depressive symptoms compared with <7% in subjects without diabetes. Long-lasting T1D significantly increased the risk of depressive symptoms in well-controlled youth. Depressive symptoms strongly affected the quality of life regardless of diabetes control. Copyright (c) 2013 John Wiley & Sons, Ltd.

C1 [Zdunczyk, Beata; Sendela, Joanna; Szypowska, Agnieszka] Med Univ Warsaw, Dept Pediat, PL-01184 Warsaw, Poland.

RP Szypowska, A (reprint author), Med Univ Warsaw, Dept Pediat, Dzialdowska 1, PL-01184 Warsaw, Poland.

EM agnieszka.szypowska@gmail.com

FU Medical University of Warsaw

FX We declare that we have no conflict of interest. The study was fully

sponsored by the Medical University of Warsaw.

CR Alsaleh FM, 2012, J CLIN PHARM THER, V37, P140, DOI 10.1111/j.1365-2710.2011.01283.x

Berg CA, 2011, J PEDIATR PSYCHOL, V36, P329, DOI 10.1093/jpepsy/jsq088

Bot M, 2013, DIABETIC MED, V30, pE115, DOI 10.1111/dme.12082

Colton PA, 2013, PEDIATR DIABETES, V14, P372, DOI 10.1111/pedi.12016

de Ornelas Maia AC, 2013, CLIN PRACT EPIDEMIOL, V12, P136

de Wit M, 2011, PEDIATR DIABETES, V12, P172, DOI 10.1111/j.1399-5448.2010.00673.x

Diabet Control Complications Trial Res Grp, 1996, DIABETES CARE, V19, P195

Gonder-Frederick Linda, 2011, Diabetes Manag (Lond), V1, P627

Goossens L, 2010, EUR CHILD ADOLES PSY, V19, P587, DOI 10.1007/s00787-010-0089-5

Grey M, 2002, J PSYCHOSOM RES, V53, P907, DOI 10.1016/S0022-3999(02)00312-4

Hains AA, 2006, DIABETES CARE, V29, P818, DOI 10.2337/diacare.29.04.06.dc05-1828

Hassan K, 2006, J PEDIATR-US, V149, P526, DOI 10.1016/j.jpeds.2006.05.039

Helgeson VS, 2012, J PEDIATR PSYCHOL, V37, P467, DOI 10.1093/jpepsy/jsr110

Hood KK, 2006, DIABETES CARE, V29, P1389, DOI 10.2337/dc06-0087

Ingersoll G M, 1991, Diabetes Educ, V17, P114, DOI 10.1177/014572179101700219

Johnson B, 2013, DIABETIC MED, V30, P199, DOI 10.1111/j.1464-5491.2012.03721.x

Kakleas K, 2009, DIABETES METAB, V35, P339, DOI 10.1016/j.diabet.2009.05.002

Korbel CD, 2007, CHILD HEALTH CARE, V36, P83, DOI 10.1080/02739610701316936

Kordonouri O, 2011, DIABETES RES CLIN PR, V93, pS118, DOI 10.1016/S0168-8227(11)70027-4

Kovac M, 2003, CHILDRENS DEPRESSION

KOVACS M, 1985, PSYCHOPHARMACOL BULL, V21, P995

Laffel LMB, 2003, DIABETES CARE, V26, P3067, DOI 10.2337/diacare.26.11.3067

Massengale Jill, 2005, Issues Ment Health Nurs, V26, P137, DOI 10.1080/01612840590901590

Matziou V, 2011, J DIABETES, V3, P82, DOI 10.1111/j.1753-0407.2010.00106.x

Rewers M, 2009, PEDIATR DIABETES, V10, P71, DOI 10.1111/j.1399-5448.2009.00582.x

Santiprabhob J, 2012, SE ASIAN J TROP MED, V43, P172

Seiffge-Krenke I, 2003, J ADOLESCENT HEALTH, V33, P180, DOI 10.1016/S1054-139X(02)00707-3

SMUCKER MR, 1986, J ABNORM CHILD PSYCH, V14, P25, DOI 10.1007/BF00917219

Timbremont B, 2004, J CLIN CHILD ADOLESC, V33, P149, DOI 10.1207/S15374424JCCP3301_14

Weissberg-Benchell J, 2009, J PEDIATR PSYCHOL, V34, P977, DOI 10.1093/jpepsy/jsp003

Williams LB, 2009, DIABETIC MED, V26, P908, DOI 10.1111/j.1464-5491.2009.02794.x

NR 31

TC 8

Z9 8

U1 0

U2 14

PU WILEY-BLACKWELL

PI HOBOKEN

PA 111 RIVER ST, HOBOKEN 07030-5774, NJ USA

SN 1520-7560

J9 DIABETES-METAB RES

JI Diabetes-Metab. Res. Rev.

PD MAY

PY 2014

VL 30

IS 4

BP 333

EP 338

DI 10.1002/dmrr.2499

PG 6

WC Endocrinology & Metabolism

SC Endocrinology & Metabolism

GA AE4BE

UT WOS:000333922200009

PM 24323887

DA 2019-01-08

ER

PT J

AU Papathanasiou, A

Koutsovasilis, A

Shea, S

Philalithis, A

Papavasiliou, S

Melidonis, A

Lionis, C

AF Papathanasiou, A.

Koutsovasilis, A.

Shea, S.

Philalithis, A.

Papavasiliou, S.

Melidonis, A.

Lionis, C.

TI The Problem Areas in Diabetes ( PAID) scale: psychometric evaluation

survey in a Greek sample with type 2 diabetes

SO JOURNAL OF PSYCHIATRIC AND MENTAL HEALTH NURSING

LA English

DT Article

DE diabetes mellitus; Problem Areas in Diabetes scale; quality of life

ID QUALITY-OF-LIFE; SF-36 HEALTH SURVEY; EMOTIONAL DISTRESS; OUTCOME

MEASURES; PREVALENCE; MELLITUS; VERSION; RESPONSIVENESS; PROJECTIONS;

VALIDATION

AB Accessible summary<list list-type="custom">

center dot Several instruments have been developed for the assessment of emotional distress in patients with diabetes. The Problem Areas in Diabetes Scale (PAID) is a brief self-report scale that evaluates diabetes-related distress.

center dot There is a lack of validated instruments for the evaluation of psychological aspects in patients with diabetes in Greek language.

center dot The current study was conducted to translate and adapt the PAID scale in Greek language and to evaluate the psychometric properties in two different study populations of patients with diabetes.

AbstractThe aim of this study was to translate the Problem Areas in Diabetes (PAID) scale into Greek, adapt it culturally to Greece and determine its psychometric properties. The translation process included two forward translations, reconciliation, backward translation and pre-testing steps. The validation incorporated the exploration of internal consistency (Cronbach's alpha), test-retest reliability (interclass correlation coefficient), construct validity (exploratory factor analysis) and responsiveness (Spearman correlation coefficient). Participants included 101 consecutive patients from a rural primary healthcare centre and 101 patients from an urban hospital. All patients completed the PAID scale and the Short Form-36 (SF-36) version 2. Internal consistency considered good (Cronbach's alpha=0.948). Interclass correlation coefficient was 0.942 (95% CI 0.915-0.961). Factor analysis yielded three factors: Diabetes-related emotional problems' (51.79% variance, Cronbach's alpha=0.910), Food-related problems' (9.55% variance, Cronbach's alpha=0.824) and Social support-related problems' (5.96% variance, Cronbach's alpha=0.704). Scree plot test and conceptual congruency of items supported a three-factor solution. Total PAID showed a negative correlation with both SF-36 mental component summary (r=-0.733, P<0.0001) and SF-36 physical component summary (r=-0.594, P<0.0001). Our findings indicate that the Greek version of the PAID questionnaire is reliable and valid for patients with diabetes mellitus in Greece.

C1 [Papathanasiou, A.] Hlth Med Ctr Stylida, Lamia, Greece.

[Papathanasiou, A.; Koutsovasilis, A.; Melidonis, A.] Tzaneio Gen Hosp Piraeus, Ctr Diabet, Piraeus, Greece.

[Shea, S.] Univ Crete, Fac Med, Clin Social & Family Med, Iraklion, Greece.

[Papathanasiou, A.; Philalithis, A.; Lionis, C.] Univ Crete, Sch Med, Clin Social & Family Med, Iraklion, Greece.

[Papavasiliou, S.] Univ Hosp Heraklion, Dept Endocrinol Diabet Mellitus & Dis Metab, Iraklion, Greece.

RP Papathanasiou, A (reprint author), Rue Beau Sejour 14, CH-1003 Lausanne, Switzerland.

EM ath_papathanasiou@yahoo.com

CR Alonso J, 2004, QUAL LIFE RES, V13, P283, DOI 10.1023/B:QURE.0000018472.46236.05

Amsberg S, 2008, INT J NURS STUD, V45, P1319, DOI 10.1016/j.ijnurstu.2007.09.010

Anagnostopoulos F, 2005, QUAL LIFE RES, V14, P1959, DOI 10.1007/s11136-005-3866-8

Anderson RJ, 2001, DIABETES CARE, V24, P1069, DOI 10.2337/diacare.24.6.1069

Anderson RM, 1997, DIABETES CARE, V20, P299, DOI 10.2337/diacare.20.3.299

Bowling A, 2002, RES METHODS HLTH INV

Bradley C, 2002, DIABETES METAB RES, V18, pS64, DOI 10.1002/dmrr.279

de Vet HCW, 2001, INT J TECHNOL ASSESS, V17, P479

Delahanty LM, 2007, DIABETIC MED, V24, P48, DOI 10.1111/j.1464-5491.2007.02028.x

DeVellis RF, 1991, SCALE DEV THEORY APP

Fisher EB, 2007, DIABETES EDUCATOR, V33, P1080, DOI 10.1177/0145721707309808

Garratt AM, 2002, DIABETIC MED, V19, P1, DOI 10.1046/j.1464-5491.2002.00650.x

GLASGOW RE, 1992, DIABETES CARE, V15, P1423, DOI 10.2337/diacare.15.10.1423

Glasgow RE, 1997, DIABETES CARE, V20, P562, DOI 10.2337/diacare.20.4.562

Gonder-Frederick LA, 2002, J CONSULT CLIN PSYCH, V70, P611, DOI 10.1037//0022-006X.70.3.611

Gross CC, 2007, DIABETES RES CLIN PR, V76, P455, DOI 10.1016/j.diabres.2006.09.022

Ishii H. W. G., 1999, DIABETES S1, V48, pA139

JACOBSON AM, 1997, SEMIN CLIN NEUROPSYC, V2, P82

KAISER HF, 1960, EDUC PSYCHOL MEAS, V20, P141, DOI 10.1177/001316446002000116

King H, 1998, DIABETES CARE, V21, P1414, DOI 10.2337/diacare.21.9.1414

Lionis C, 2008, Prim Care Diabetes, V2, P97, DOI 10.1016/j.pcd.2008.03.003

Lionis C, 2001, DIABETIC MED, V18, P768, DOI 10.1046/j.1464-5491.2001.00526.x

Lionis CD, 1996, FAM PRACT, V13, P18, DOI 10.1093/fampra/13.1.18

Medical Outcomes Trust, 1997, MED OUTCOMES TRUST B, V5, P3

Miller S. T., 2008, BMC PUBLIC HEALTH, V8, P70

Papathanasiou A, 2005, ARCH HELL MED, V22, P336

POLONSKY WH, 1995, DIABETES CARE, V18, P754, DOI 10.2337/diacare.18.6.754

Polonsky WH, 2005, DIABETES CARE, V28, P626, DOI 10.2337/diacare.28.3.626

Polonsky WH, 2000, DIABETES SPECTRUM, V13, P36

Rubin R, 2000, DIABETES SPECTR, V13, P21

Rubin RR, 1999, DIABETES-METAB RES, V15, P205, DOI 10.1002/(SICI)1520-7560(199905/06)15:3<205::AID-DMRR29>3.0.CO;2-O

Rubin RR, 2001, J CLIN PSYCHOL, V57, P457, DOI 10.1002/jclp.1041

Sen S. S., 1999, AM J HEALTH-SYST PH, V56, P1965

Sen S. S., 1999, AM J HEALTH-SYST PH, V56, P1971

Sigurdardottir AK, 2008, INT J NURS STUD, V45, P526, DOI 10.1016/j.ijnurstu.2006.10.008

Snoek FJ, 2000, DIABETES CARE, V23, P1305, DOI 10.2337/diacare.23.9.1305

Spencer MS, 2006, J HEALTH CARE POOR U, V17, P88, DOI 10.1353/hpu.2006.0095

WARE JE, 1992, MED CARE, V30, P473, DOI 10.1097/00005650-199206000-00002

Ware JE, 2000, SPINE, V25, P3130, DOI 10.1097/00007632-200012150-00008

Watkins K, 2004, PHARMACOECONOMICS, V22, P1109, DOI 10.2165/00019053-200422170-00002

Welch G, 2003, DIABETIC MED, V20, P69, DOI 10.1046/j.1464-5491.2003.00832.x

Welch GW, 1997, DIABETES CARE, V20, P760, DOI 10.2337/diacare.20.5.760

Wild S, 2004, DIABETES CARE, V27, P1047, DOI 10.2337/diacare.27.5.1047

World Health Organization, 1999, DEF DIAGN CLASS DI 1

NR 44

TC 5

Z9 8

U1 0

U2 18

PU WILEY-BLACKWELL

PI HOBOKEN

PA 111 RIVER ST, HOBOKEN 07030-5774, NJ USA

SN 1351-0126

EI 1365-2850

J9 J PSYCHIATR MENT HLT

JI J. Psychiatr. Ment. Health Nurs.

PD MAY

PY 2014

VL 21

IS 4

BP 345

EP 353

DI 10.1111/j.1365-2850.2012.01875.x

PG 9

WC Nursing; Psychiatry

SC Nursing; Psychiatry

GA AD8TL

UT WOS:000333538400008

PM 22340071

DA 2019-01-08

ER

PT J

AU Dalsgaard, EM

Vestergaard, M

Skriver, MV

Maindal, HT

Lauritzen, T

Borch-Johnsen, K

Witte, D

Sandbaek, A

AF Dalsgaard, Else-Marie

Vestergaard, Mogens

Skriver, Mette V.

Maindal, Helle T.

Lauritzen, Torsten

Borch-Johnsen, Knut

Witte, Daniel

Sandbaek, Annelli

TI Psychological distress, cardiovascular complications and mortality among

people with screen-detected type 2 diabetes: follow-up of the

ADDITION-Denmark trial

SO DIABETOLOGIA

LA English

DT Article

DE All-cause mortality; Cardiovascular risk factors.; Complications;

General practice; Mental health; Morbidity; Type 2 diabetes mellitus

ID ALL-CAUSE MORTALITY; GLYCEMIC CONTROL; MENTAL-HEALTH; GENERAL-PRACTICE;

DEPRESSION; RISK; METAANALYSIS; ASSOCIATION; MELLITUS; OUTCOMES

AB Aim/hypothesis The aim of this study was to examine the association between psychological distress and the risk of cardiovascular disease (CVD) events and all-cause mortality in patients with screen-detected type 2 diabetes mellitus. In addition, we explored whether or not metabolic control and medication adherence could explain part of this association.

Methods A follow-up study was performed including 1,533 patients aged 40-69 years with screen-detected type 2 diabetes mellitus identified in general practice during 2001-2006 in the Denmark arm of the ADDITION (Anglo-Danish-Dutch Study of Intensive Treatment in People with Screen-Detected Diabetes in Primary Care) study. Mental health was measured at baseline with the Mental Health Inventory 5 (MHI-5). Psychological distress was defined as an MHI-5 score of <= 68 (18.2% of the population). CVD risk factors were measured at baseline and repeated at the follow-up examination. Information on death, hospital discharge diagnosis, and antihypertensive and lipid-lowering drug treatment was obtained from national registers. Cox proportional regression was used to estimate HRs for the association between psychological distress, CVD events and all-cause mortality. Age- and sex-adjusted risk difference analyses were performed to estimate differences in meeting treatment targets.

Results Patients with psychological distress had a 1.8-fold higher mortality rate (HR 1.76, 95% CI 1.23, 2.53) and a 1.7-fold higher risk of having a CVD event (HR: 1.69, 95% CI 1.05, 2.70) compared with those with an MHI-5 score of > 68. Overall, psychological distress was not associated with the ability to meet treatment targets for HbA(1c) levels, cholesterol levels or BP, or to redeem antihypertensive or lipid-lowering drug treatment.

Conslusions/interpretation In people detected and treated early in the diabetes disease trajectory, those with psychological distress at the time of diagnosis had a higher risk of CVD events and death than those without psychological distress.

C1 [Dalsgaard, Else-Marie; Vestergaard, Mogens; Lauritzen, Torsten; Sandbaek, Annelli] Aarhus Univ, Dept Publ Hlth, Sect Gen Practice, DK-8000 Aarhus, Denmark.

[Vestergaard, Mogens] Aarhus Univ, Res Unit Gen Practice, DK-8000 Aarhus, Denmark.

[Skriver, Mette V.; Maindal, Helle T.] Aarhus Univ, Sect Hlth Promot & Hlth Serv, Dept Publ Hlth, DK-8000 Aarhus, Denmark.

[Borch-Johnsen, Knut] Holbaek Cent Hosp, Holbaek, Region Zealand, Denmark.

[Witte, Daniel] Ctr Rech Publ Sante, Strassen, Luxembourg.

RP Dalsgaard, EM (reprint author), Aarhus Univ, Dept Publ Hlth, Sect Gen Practice, Bartholins Alle 2, DK-8000 Aarhus, Denmark.

EM emdl@alm.au.dk

RI Vestergaard, Mogens/M-9333-2014; Maindal, Helle Terkildsen/N-8916-2016;

Witte, Daniel/C-1722-2008

OI Vestergaard, Mogens/0000-0001-8830-2174; Maindal, Helle

Terkildsen/0000-0003-0525-7254; Witte, Daniel/0000-0002-0769-2922;

Dalsgaard, Else-Marie/0000-0003-2115-7480; Skriver,

Mette/0000-0002-7017-8221

FU National Health Services in the county of Copenhagen in Denmark;

National Health Services in the county of Aarhus in Denmark; National

Health Services in the county of Ringkobing in Denmark; National Health

Services in the county of Ribe Denmark; Danish Council for Strategic

Research; Danish Research Foundation for General Practice; Novo Nordisk

Foundation; Danish Centre for Evaluation and Health Technology

Assessment; Danish Fund of the National Board of Health; Danish Medical

Research Council; Aarhus University Research Foundation; Novo Nordisk

AS; Novo Nordisk Scandinavia AB; Novo Nordisk UK; ASTRA Denmark; Pfizer

Denmark; Glaxo SmithKline; Pharma Denmark; Servier Denmark A/S; HemoCue

Denmark A/S

FX ADDITION-Denmark was supported by the National Health Services in the

counties of Copenhagen, Aarhus, Ringkobing, Ribe and South Jutland in

Denmark, The Danish Council for Strategic Research, the Danish Research

Foundation for General Practice, Novo Nordisk Foundation, the Danish

Centre for Evaluation and Health Technology Assessment, the Danish Fund

of the National Board of Health, the Danish Medical Research Council,

and the Aarhus University Research Foundation. The trial has been given

unrestricted grants from Novo Nordisk AS, Novo Nordisk Scandinavia AB,

Novo Nordisk UK, ASTRA Denmark, Pfizer Denmark, Glaxo SmithKline Pharma

Denmark, Servier Denmark A/S and HemoCue Denmark A/S. Parts of the

grants from Novo Nordisk Foundation and Novo Nordisk were transferred to

the other centres.

CR Adriaanse MC, 2005, QUAL LIFE RES, V14, P1501, DOI 10.1007/s11136-004-0028-3

Alberti KGMM, 1998, DIABETIC MED, V15, P539, DOI 10.1002/(SICI)1096-9136(199807)15:7<539::AID-DIA668>3.0.CO;2-S

Ali S, 2006, DIABETIC MED, V23, P1165, DOI 10.1111/j.1464-5491.2006.01943.x

[Anonymous], 1991, IDA INT DAT ARB HOV

Arroyo C, 2004, DIABETES CARE, V27, P129, DOI 10.2337/diacare.27.1.129

Bek T, 2009, ACTA OPHTHALMOL, V87, P270, DOI 10.1111/j.1755-3768.2008.01207.x

BERWICK DM, 1991, MED CARE, V29, P169, DOI 10.1097/00005650-199102000-00008

CHARLSON ME, 1987, J CHRON DIS, V40, P373, DOI 10.1016/0021-9681(87)90171-8

Christensen J, 2004, DIABETOLOGIA, V47, P1566, DOI 10.1007/s00125-004-1496-2

Dalsgaard EM, 2009, DIABETIC MED, V26, P518, DOI 10.1111/j.1464-5491.2009.02715.x

de Jonge P, 2010, NEUROSCI BIOBEHAV R, V35, P84, DOI 10.1016/j.neubiorev.2009.11.025

Gonzalez JS, 2008, DIABETES CARE, V31, P2398, DOI 10.2337/dc08-1341

Griffin SJ, 2011, LANCET, V378, P156, DOI 10.1016/S0140-6736(11)60698-3

Hamer M, 2008, J AM COLL CARDIOL, V52, P2156, DOI 10.1016/j.jacc.2008.08.057

Hamer M, 2010, PSYCHOSOM MED, V72, P882, DOI 10.1097/PSY.0b013e3181f6696e

Katon W, 2009, PSYCHOSOM MED, V71, P965, DOI 10.1097/PSY.0b013e3181bd8f55

Kelly MJ, 2008, BMC PSYCHIATRY, V8, DOI 10.1186/1471-244X-8-10

Khalil CA, 2012, EUR J PREV CARDIOL, V19, P374, DOI 10.1177/1741826711409324

Kildemoes HW, 2011, SCAND J PUBLIC HEALT, V39, P38, DOI 10.1177/1403494810394717

Lauritzen T, 2000, INT J OBESITY, V24, pS6, DOI 10.1038/sj.ijo.0801420

Lauritzen T, 2012, PRIM CARE DIABETES, V6, P193, DOI 10.1016/j.pcd.2012.04.005

Lin EHB, 2009, ANN FAM MED, V7, P414, DOI 10.1370/afm.998

Lustman PJ, 2005, J DIABETES COMPLICAT, V19, P113, DOI 10.1016/j.jdiacomp.2004.01.002

Lustman PJ, 2000, DIABETES CARE, V23, P934, DOI 10.2337/diacare.23.7.934

Lynge E, 2011, SCAND J PUBLIC HEALT, V39, P30, DOI 10.1177/1403494811401482

MCHORNEY CA, 1995, MED CARE, V33, P15, DOI 10.1097/00005650-199501000-00002

Meijer A, 2013, BMC MED, V11, DOI 10.1186/1741-7015-11-130

Pan A, 2011, ARCH GEN PSYCHIAT, V68, P42, DOI 10.1001/archgenpsychiatry.2010.176

Pan A, 2010, ARCH INTERN MED, V170, P1884, DOI 10.1001/archinternmed.2010.356

Park M, 2013, GEN HOSP PSYCHIAT, V35, P217, DOI 10.1016/j.genhosppsych.2013.01.006

Park P, 2008, BMC PUBLIC HEALTH, V8, DOI 10.1186/1471-2458-8-350

Pedersen CB, 2011, SCAND J PUBLIC HEALT, V39, P22, DOI 10.1177/1403494810387965

Pouwer F, 2010, DIABETIC MED, V27, P217, DOI 10.1111/j.1464-5491.2009.02903.x

Pouwer F, 2013, ENDOCRIN METAB CLIN, V42, P529, DOI 10.1016/j.ecl.2013.05.002

Rumpf HJ, 2001, PSYCHIAT RES, V105, P243, DOI 10.1016/S0165-1781(01)00329-8

Russ TC, 2012, BRIT MED J, V345, DOI 10.1136/bmj.e4933

Sandbaek A, 2008, DIABETOLOGIA, V51, P1127, DOI 10.1007/s00125-008-1013-0

Simmons RK, 2012, LANCET, V380, P1741, DOI 10.1016/S0140-6736(12)61422-6

Smith KJ, 2013, J PSYCHOSOM RES, V74, P89, DOI 10.1016/j.jpsychores.2012.11.013

Spijkerman AMW, 2002, DIABETES CARE, V25, P1784, DOI 10.2337/diacare.25.10.1784

Sullivan MD, 2012, DIABETES CARE, V35, P1708, DOI 10.2337/dc11-1791

Surtees PG, 2008, NEUROLOGY, V70, P788, DOI 10.1212/01.wnl.0000304109.18563.81

van Dooren FEP, 2013, PLOS ONE, V8, DOI 10.1371/journal.pone.0057058

Ware JE, 1998, J CLIN EPIDEMIOL, V51, P903, DOI 10.1016/S0895-4356(98)00081-X

WARE JE, 1992, MED CARE, V30, P473, DOI 10.1097/00005650-199206000-00002

NR 45

TC 12

Z9 12

U1 1

U2 21

PU SPRINGER

PI NEW YORK

PA 233 SPRING ST, NEW YORK, NY 10013 USA

SN 0012-186X

EI 1432-0428

J9 DIABETOLOGIA

JI Diabetologia

PD APR

PY 2014

VL 57

IS 4

BP 710

EP 717

DI 10.1007/s00125-014-3165-4

PG 8

WC Endocrinology & Metabolism

SC Endocrinology & Metabolism

GA AC5ZX

UT WOS:000332600700009

PM 24442448

DA 2019-01-08

ER

PT J

AU Zoffmann, V

Vistisen, D

Due-Christensen, M

AF Zoffmann, V.

Vistisen, D.

Due-Christensen, M.

TI A cross-sectional study of glycaemic control, complications and

psychosocial functioning among 18- to 35-year-old adults with Type 1

diabetes

SO DIABETIC MEDICINE

LA English

DT Article

ID YOUNG-ADULTS; SELF-DETERMINATION; CARE; EXPERIENCES; TRANSITION;

DEPRESSION; DISTRESS; DISEASE; LIFE

AB Aims

To describe the level of glycaemic control, complications and psychosocial functioning and the relationships between these variables in the under-researched group of younger adults with Type1 diabetes.

Methods

Local electronic health records provided data on age, gender, disease duration, HbA(1c) and complications for 710 younger adults (18-35years) with Type1 diabetes. A questionnaire with wide-ranging psychometric scales was used to measure various aspects of psychosocial functioning: the burden of diabetes-related problems, well-being, self-esteem, perceived competence in managing diabetes, perceived autonomy support from health professionals and self-management motivations. Furthermore, patients reported weekly self-monitored blood glucose measurements and insulin administration. Associations between HbA(1c), complication and psychosocial indicators were tested using linear and logistic regression models, adjusted stepwise for confounders, including age, gender, diabetes duration, continuous subcutaneous insulin infusion, smoking and BMI.

Results

In total, 406 (57%) participants responded. The responders had a mean age of 27.1 (5.1) years, a mean diabetes duration of 13.5 (7.9) years and an HbA(1c) of 66mmol/mol (8.2%), with similar values for both genders (P=0.87). Complications were observed among women more commonly than among men (31.6 vs. 18.8%, P<0.01), and high distress levels were more prevalent among women compared with men (51.2 vs. 31.9%, P<0.0001). Except for perceived autonomy support, the psychosocial variables were all associated with HbA(1c) (P<0.001).

Conclusions

The high prevalence of poor glycaemic control, early complications and psychosocial distress require health-promoting interventions tailored to the interrelated clinical and psychosocial needs of younger adults with Type1 diabetes.

C1 [Zoffmann, V.; Due-Christensen, M.] Steno Diabet Ctr, Patient Care Ctr, DK-2820 Gentofte, Denmark.

[Zoffmann, V.] Oslo Univ Hosp, NKLMS, Oslo, Norway.

[Vistisen, D.] Steno Diabet Ctr, DK-2820 Gentofte, Denmark.

RP Zoffmann, V (reprint author), Steno Diabet Ctr, Patient Care Ctr, DK-2820 Gentofte, Denmark.

EM vzof@steno.dk

OI Vistisen, Dorte/0000-0001-5045-5351; Due-Christensen,

Mette/0000-0003-2604-3730

FU Steno Diabetes Center

FX This study was supported by grants from the Steno Diabetes Center.

CR BENJAMINI Y, 1995, J R STAT SOC B, V57, P289

Bowman GS, 2001, J ADV NURS, V34, P256, DOI 10.1046/j.1365-2648.2001.01752.x

Brierley S, 2012, DIABETIC MED, V29, P677, DOI 10.1111/j.1464-5491.2012.03601.x

Bryden KS, 2003, DIABETES CARE, V26, P1052, DOI 10.2337/diacare.26.4.1052

Costacou T, 2011, AM J KIDNEY DIS, V58, P565, DOI 10.1053/j.ajkd.2011.05.025

DeVries JH, 2004, DIABETIC MED, V21, P1263, DOI 10.1111/j.1464-5491.2004.01386.x

Due-Christensen M, 2012, DIABETIC MED, V29, P251, DOI 10.1111/j.1464-5491.2011.03521.x

Franciosi M, 2013, NUTR METAB CARDIOVAS, V23, P606, DOI 10.1016/j.numecd.2012.03.002

Franconi Flavia, 2012, Endocrine Metabolic & Immune Disorders-Drug Targets, V12, P179

Garvey KC, 2012, DIABETES CARE, V35, P1716, DOI 10.2337/dc11-2434

Gonzalez JS, 2011, DIABETES CARE, V34, P236, DOI 10.2337/dc10-1970

Hansen MV, 2009, DIABETES RES CLIN PR, V85, P183, DOI 10.1016/j.diabres.2009.04.022

Harjutsalo V, 2011, BMJ-BRIT MED J, V343, DOI 10.1136/bmj.d5364

Hermanns N, 2006, DIABETOLOGIA, V49, P469, DOI 10.1007/s00125-005-0094-2

Hislop AL, 2008, DIABETIC MED, V25, P91, DOI 10.1111/j.1464-5491.2007.02310.x

Holmes-Walker DJ, 2007, DIABETIC MED, V24, P764, DOI 10.1111/j.1464-5491.2007.02152.x

JACOBSON AM, 1991, DIABETES CARE, V14, P599, DOI 10.2337/diacare.14.7.599

Kipps S, 2002, DIABETIC MED, V19, P649, DOI 10.1046/j.1464-5491.2002.00757.x

Levesque CS, 2007, HEALTH EDUC RES, V22, P691, DOI 10.1093/her/cyl148

Markowitz JT, 2012, DIABETIC MED, V29, P522, DOI 10.1111/j.1464-5491.2011.03537.x

Marrero DG, 2013, DIABETES CARE, V36, P463, DOI 10.2337/dc12-2305

Peveler RC, 2005, DIABETES CARE, V28, P84, DOI 10.2337/diacare.28.1.84

Ryan RM, 2006, J PERS, V74, P1557, DOI 10.1111/j.1467-6494.2006.00420.x

Saunders SA, 2004, QJM-INT J MED, V97, P575, DOI 10.1093/qjmed/hch098

Snoek FJ, 2000, DIABETES CARE, V23, P1305, DOI 10.2337/diacare.23.9.1305

Williams GC, 2000, SELF DETERMINATION H

Wisman PP, 2012, EUR J VASC ENDOVASC, V43, P441, DOI 10.1016/j.ejvs.2012.01.006

Zoffmann V, 2005, QUAL HEALTH RES, V15, P750, DOI 10.1177/1049732304273888

Zoffmann V, 2006, PATIENT EDUC COUNS, V64, P78, DOI 10.1016/j.pec.2005.11.017

Zoffmann V, 2012, QUAL HEALTH RES, V22, P103, DOI 10.1177/1049732311420735

NR 30

TC 17

Z9 17

U1 0

U2 12

PU WILEY

PI HOBOKEN

PA 111 RIVER ST, HOBOKEN 07030-5774, NJ USA

SN 0742-3071

EI 1464-5491

J9 DIABETIC MED

JI Diabetic Med.

PD APR

PY 2014

VL 31

IS 4

BP 493

EP 499

DI 10.1111/dme.12363

PG 7

WC Endocrinology & Metabolism

SC Endocrinology & Metabolism

GA AC8BQ

UT WOS:000332758400019

PM 24236961

DA 2019-01-08

ER

PT J

AU Lee, HY

Hahm, MI

Lee, SG

AF Lee, Hoo-Yeon

Hahm, Myung-Il

Lee, Sang Gyu

TI Risk of suicidal ideation in diabetes varies by diabetes regimen,

diabetes duration, and HbA1c level

SO JOURNAL OF PSYCHOSOMATIC RESEARCH

LA English

DT Article

DE Suicidal ideation; Diabetes; Depression; Insulin; HbA1c

ID SOUTH-KOREA; DEPRESSIVE SYMPTOMS; PHYSICAL ILLNESS; GLYCEMIC CONTROL;

ECONOMIC-CRISIS; ADULTS; ASSOCIATION; PREVALENCE; JAPAN; AGE

AB Objective: To investigate patient subgroups based on the clinical characteristics of diabetes to evaluate risk factors for suicidal ideation using a large population-based sample in South Korea.

Methods: Data from the Fifth Korea National Health and Nutrition Examination Survey, a cross-sectional, nationally representative survey, were analyzed. The participants were 9159 subjects aged >= 40 years. We defined patients with diabetes based on self-reported physician-diagnosed diabetes. We evaluated clinical risk factors for suicidal ideation according to diabetes regimen, diabetes duration, and glycated hemoglobin (HbA1c) level compared with no diabetes. Given the complex sample design and unequal weights, we analyzed weighted percentages and used survey logistic regression.

Results: Diabetes per se was not associated with suicidal ideation. However, suicidal ideation was significantly more prevalent among patients who had injected insulin, had a duration of diabetes >= 5 years and had HbA1c levels >= 6.5 compared with those without diabetes. Depressive symptoms were the most prominent predictor of suicidal ideation.

Conclusions: Insulin therapy, diabetes of long duration, and unsatisfactory glycemic control were identified as risk factors for suicidal ideation; thus, patients with these characteristics warrant special attention. Our findings suggest the need to integrate efforts to manage emotional distress into diabetes care. (C) 2014 Elsevier Inc All rights reserved.

C1 [Lee, Hoo-Yeon] Dankook Univ, Coll Med, Dept Social Med, Cheonan Si 330714, Choongnam, South Korea.

[Hahm, Myung-Il] Soonchunhyang Univ, Coll Med Sci, Dept Hlth Adm & Management, Asan 336745, Choongnam, South Korea.

[Lee, Sang Gyu] Yonsei Univ, Grad Sch Publ Hlth, Seoul 120749, South Korea.

RP Lee, SG (reprint author), Yonsei Univ, Grad Sch Publ Hlth, 50 Yonsei Ro, Seoul 120749, South Korea.

EM LEEVAN@yuhs.ac

FU Dankook University

FX The present research was conducted by the research fund of Dankook

University in 2012.

CR Aikens JE, 2008, DIABETIC MED, V25, P1324, DOI 10.1111/j.1464-5491.2008.02590.x

Aikens JE, 2009, DIABETES CARE, V32, P1177, DOI 10.2337/dc09-0071

Berge LI, 2012, DIABETIC MED, V29, P509, DOI 10.1111/j.1464-5491.2011.03530.x

Bogner HR, 2007, DIABETES CARE, V30, P3005, DOI 10.2337/dc07-0974

Bot M, 2013, DIABETIC MED, V30, pE115, DOI 10.1111/dme.12082

Chang SS, 2009, SOC SCI MED, V68, P1322, DOI 10.1016/j.socscimed.2009.01.010

Chen YY, 2012, EPIDEMIOL REV, V34, P129, DOI 10.1093/epirev/mxr025

Chin YR, 2011, INT J PUBLIC HEALTH, V56, P429, DOI 10.1007/s00038-011-0245-9

Crump C, 2013, PSYCHOL MED, P1

Maia ACCD, 2012, COMPR PSYCHIAT, V53, P1169, DOI 10.1016/j.comppsych.2012.03.011

Dombrovski AY, 2008, AM J GERIAT PSYCHIAT, V16, P905, DOI 10.1097/JGP.0b013e3181860034

Fisher L, 2010, DIABETES CARE, V33, P23, DOI 10.2337/dc09-1238

Gallo JJ, 2013, BMJ-BRIT MED J, P346

Golden SH, 2007, PSYCHOSOM MED, V69, P529, DOI 10.1097/PSY.0b013e3180f61c5c

Gonzalez JS, 2011, DIABETES CARE, V34, P236, DOI 10.2337/dc10-1970

Han S, 2013, J PUBLIC HEALTH-UK, V35, P228, DOI 10.1093/pubmed/fds097

Han SJ, 2013, DIABETES RES CLIN PR, V101, pE14, DOI 10.1016/j.diabres.2013.06.012

Harrison KE, 2010, INT PSYCHOGERIATR, V22, P445, DOI 10.1017/S1041610209991463

Hawton K, 2009, LANCET, V373, P1372, DOI 10.1016/S0140-6736(09)60372-X

Holt RIG, 2009, DIABETIC MED, V26, P641, DOI 10.1111/j.1464-5491.2009.02742.x

Hong JH, 2011, WORLD PSYCHIATRY, V10, P40

Kageyama T, 2012, PSYCHIAT CLIN NEUROS, V66, P105, DOI 10.1111/j.1440-1819.2011.02313.x

Kim MH, 2010, SOC SCI MED, V70, P1254, DOI 10.1016/j.socscimed.2010.01.004

Kim SY, 2011, CRISIS, V32, P5, DOI 10.1027/0227-5910/a000046

Kim Yangho, 2013, J Prev Med Public Health, V46, P96, DOI 10.3961/jpmph.2013.46.2.96

Kim YR, 2011, INT PSYCHOGERIATR, V23, P979, DOI 10.1017/S1041610211000263

Ko SH, 2011, DIABETES METAB J, V35, P431, DOI 10.4093/dmj.2011.35.5.431

Korea Centers for Disease Control and Prevention, 2012, 5 KOR NAT HLTH NUTR

Kposowa AJ, 2000, J EPIDEMIOL COMMUN H, V54, P254, DOI 10.1136/jech.54.4.254

Lee HY, 2013, PSYCHIAT RES, V210, P323, DOI 10.1016/j.psychres.2013.05.012

Levy TB, 2011, ARCH GERONTOL GERIAT, V52, P115, DOI 10.1016/j.archger.2010.02.012

Li CY, 2008, DIABETES CARE, V31, P105, DOI 10.2337/dc07-1154

Lofman S, 2012, J PSYCHOSOM RES, V73, P268, DOI 10.1016/j.jpsychores.2012.08.002

Moon SS, 2012, INT J SOC PSYCHIATR, V58, P657, DOI 10.1177/0020764011433626

Myers AK, 2013, PSYCHONEUROENDOCRINO, V38, P2810, DOI 10.1016/j.psyneuen.2013.06.013

O'Connor E, 2013, ANN INTERN MED, V158, P741, DOI 10.7326/0003-4819-158-10-201305210-00642

Park SM, 2010, J PSYCHOSOM RES, V69, P389, DOI 10.1016/j.jpsychores.2010.03.002

Purselle DC, 2009, PSYCHIAT RES, V167, P258, DOI 10.1016/j.psychres.2008.02.003

Quan H, 2002, SOC PSYCH PSYCH EPID, V37, P190, DOI 10.1007/s001270200014

Taylor P, 2003, AGEING SOC, V23, P25, DOI 10.1017/S0144686X02001022

Tektonidou MG, 2011, ARTHRIT CARE RES, V63, P1322, DOI 10.1002/acr.20516

Trento M, 2012, ACTA DIABETOL, V49, P199, DOI 10.1007/s00592-011-0275-z

van Bastelaar KMP, 2011, DIABETES CARE, V34, P320, DOI 10.2337/dc10-1248

NR 43

TC 6

Z9 6

U1 1

U2 9

PU PERGAMON-ELSEVIER SCIENCE LTD

PI OXFORD

PA THE BOULEVARD, LANGFORD LANE, KIDLINGTON, OXFORD OX5 1GB, ENGLAND

SN 0022-3999

EI 1879-1360

J9 J PSYCHOSOM RES

JI J. Psychosomat. Res.

PD APR

PY 2014

VL 76

IS 4

BP 275

EP 279

DI 10.1016/j.jpsychores2014.02.003

PG 5

WC Psychiatry

SC Psychiatry

GA AD6NX

UT WOS:000333378600002

PM 24630176

DA 2019-01-08

ER

PT J

AU de Vries, L

van der Heijden, AAWA

van 't Riet, E

Baan, CA

Kostense, PJ

Rijken, M

Rutten, GEHM

Nijpels, G

AF de Vries, Lianne

van der Heijden, Amber A. W. A.

van 't Riet, Esther

Baan, Caroline A.

Kostense, Piet J.

Rijken, Mieke

Rutten, Guy E. H. M.

Nijpels, Giel

TI Peer support to decrease diabetes-related distress in patients with type

2 diabetes mellitus: design of a randomised controlled trial

SO BMC ENDOCRINE DISORDERS

LA English

DT Article

DE Peer support; Diabetes mellitus type 2; Distress; Randomised controlled

trial

ID SELF-MANAGEMENT; SOCIAL SUPPORT; PROBLEM AREAS; CARE; SCALE; OUTCOMES

AB Background: Many type 2 diabetes mellitus patients face difficulties self-managing their illness, which can lead to high levels of diabetes-related distress. Diabetes distress may be decreased by peer support, as peers understand and have dealt with similar problems, and can help motivate each other. A recent systematic review concluded that evidence of benefits of peer support in patients with type 2 diabetes mellitus is too inconsistent due to weak theoretical foundation of the interventions. This study describes the design of a trial evaluating the effectiveness of a group-based, peer support programme with a strong theoretical foundation on diabetes-related distress in type 2 diabetes patients.

Methods: This is a parallel group randomised controlled trial of a six session group-based peer support intervention, delivered by peer leaders and group psychotherapists, compared with one educational meeting on diabetes. At least 152 patients with a type 2 diabetes duration of three years or more and between 50 and 70 years of age, recruited via their general practitioner, will be randomised to receive the peer support intervention or one educational meeting. The intervention is developed in line with three key stages of research development of the Medical Research Council framework. The primary outcome measure for this study is diabetes-related distress. Secondary outcomes include self-management behaviour, well-being and health-related quality of life. Perceived social support is a process measure. Outcomes will be measured one month before, and 6, and 12 months after the intervention by means of self-reported questionnaires. Analysis will be on an intention-to-treat basis.

Discussion: This article contains a description of the design of a study that will investigate the effect of a group-based, peer support intervention on diabetes-related distress in type 2 diabetes patients. The intervention was developed in recognition of the limited evidence, and the importance of a theoretical foundation and its implementation. Findings will contribute to knowledge in the field of peer support and patient-important outcomes in type 2 diabetes patients.

C1 [de Vries, Lianne; van der Heijden, Amber A. W. A.; Nijpels, Giel] Vrije Univ Amsterdam, Med Ctr, Dept Gen Practice & Elderly Care Med, Amsterdam, Netherlands.

[van 't Riet, Esther; Kostense, Piet J.] Vrije Univ Amsterdam, Med Ctr, Dept Epidemiol & Biostat, Amsterdam, Netherlands.

[de Vries, Lianne; van der Heijden, Amber A. W. A.; van 't Riet, Esther; Kostense, Piet J.; Nijpels, Giel] Vrije Univ Amsterdam, Med Ctr, EMGO Inst Hlth & Care Res, Amsterdam, Netherlands.

[Baan, Caroline A.] Natl Inst Publ Hlth & Environm, RIVM, NL-3720 BA Bilthoven, Netherlands.

[Rijken, Mieke] NIVEL, Netherlands Inst Hlth Serv Res, Utrecht, Netherlands.

[Rutten, Guy E. H. M.] Univ Med Ctr Utrecht, Julius Ctr Hlth Sci & Primary Care, Utrecht, Netherlands.

RP de Vries, L (reprint author), Vrije Univ Amsterdam, Med Ctr, Dept Gen Practice & Elderly Care Med, Amsterdam, Netherlands.

EM li.devries@vumc.nl

FU Dutch Diabetes Research Foundation

FX This study is funded by the Dutch Diabetes Research Foundation.

CR Barrera M, 2002, AM J COMMUN PSYCHOL, V30, P637, DOI 10.1023/A:1016369114780

Bech P, 1996, PSYCHOTHER PSYCHOSOM, V65, P183, DOI 10.1159/000289073

Boothrovd R. I., 2010, FAM PRACT S1, V27, pi62

Brownson CA, 2009, PATIENT, V2, P5, DOI 10.2165/01312067-200902010-00002

Burleson B. R., 1994, COMMUNICATION SOCIAL, pxi

Carper MM, 2013, J BEHAV MED, P1

Cleveringa FGW, 2010, DIABETIC MED, V27, P442, DOI 10.1111/j.1464-5491.2010.02968.x

Dale JR, 2012, DIABETIC MED, V29, P1361, DOI 10.1111/j.1464-5491.2012.03749.x

Dennis CL, 2003, INT J NURS STUD, V40, P321, DOI 10.1016/S0020-7489(02)00092-5

Fisher L, 2013, DIABETES CARE, V36, P2551, DOI 10.2337/dc12-2493

Heisler M., 2006, BUILDING PEER SUPPOR

Heisler M., 2007, DIABETES SPECTRUM, V20, P214, DOI DOI 10.2337/DIASPECT.20.4.214

Heisler M, 2010, ANN INTERN MED, V153, P507, DOI 10.7326/0003-4819-153-8-201010190-00007

Heisler M, 2010, FAM PRACT, V27, pI23, DOI 10.1093/fampra/cmp003

Medical Research Council, 2000, FRAM DEV EV RCTS COM, P18

Polonsky WH, 2005, DIABETES CARE, V28, P626, DOI 10.2337/diacare.28.3.626

Snoek FJ, 2000, DIABETES CARE, V23, P1305, DOI 10.2337/diacare.23.9.1305

Toobert DJ, 2000, DIABETES CARE, V23, P943, DOI 10.2337/diacare.23.7.943

van Dam HA, 2005, PATIENT EDUC COUNS, V59, P1, DOI 10.1016/j.pec.2004.11.001

Vinicor F, 1998, DIABETES CARE, V21, pC15, DOI 10.2337/diacare.21.3.C15

Welch G, 2003, DIABETIC MED, V20, P69, DOI 10.1046/j.1464-5491.2003.00832.x

Whiting DR, 2011, DIABETES RES CLIN PR, V94, P311, DOI 10.1016/j.diabres.2011.10.029

Wills T. A., 2000, SOCIAL SUPPORT MEASU, P86

NR 23

TC 8

Z9 8

U1 2

U2 23

PU BIOMED CENTRAL LTD

PI LONDON

PA 236 GRAYS INN RD, FLOOR 6, LONDON WC1X 8HL, ENGLAND

SN 1472-6823

J9 BMC ENDOCR DISORD

JI BMC Endocr. Disord.

PD MAR 4

PY 2014

VL 14

AR 21

DI 10.1186/1472-6823-14-21

PG 6

WC Endocrinology & Metabolism

SC Endocrinology & Metabolism

GA AF3QW

UT WOS:000334628200002

PM 24593296

OA DOAJ Gold, Green Published

DA 2019-01-08

ER

PT J

AU Kulzer, B

Seitz, L

Kern, W

AF Kulzer, B.

Seitz, L.

Kern, W.

TI Real-world Patient-reported Rates of Non-severe Hypoglycaemic Events in

Germany

SO EXPERIMENTAL AND CLINICAL ENDOCRINOLOGY & DIABETES

LA English

DT Article

DE hypoglycaemia; insulin therapy; diabetes-related distress; hypoglycaemia

awareness

ID DIABETES MANAGEMENT; TYPE-1; FREQUENCY; RISK; AWARENESS; PRODUCTIVITY;

EDUCATION; IMPACT

AB Aims: Hypoglycaemia is a common side effect of insulin therapy in diabetes patients, with negative physical and emotional impacts. Despite this, there are few studies investigating the frequency of non-severe hypoglycaemic events from the perspective of patients in the real-world setting. We investigated self-reported NSHE frequency and levels of hypoglycaemia awareness in Germany.

Methods: Respondents > 15 years with Type 1 or insulin-treated Type 2 diabetes (receiving basal only, basal-bolus or other insulin regimens) were recruited using online panels to complete <= 4 questionnaires. Questionnaires collected demographics, non-severe hypoglycaemic event rates and patient-reported level of hypoglycaemia awareness. Non-severe hypoglycaemic event rates are reported as respondent-week records and calculated using data from all respondents completing at least one questionnaire.

Results: A total of 1 771 respondent-week records were obtained from 614 participants. Mean non-severe hypoglycaemic event rates per respondent-week were 1.6 for Type 1 and 0.6-0.8 for Type 2, with estimated annual rates of 83 and 31-42 respectively. Two-thirds of Type 1 (65 %) and Type 2 (61-72 %) respondents reported impaired levels of awareness or unawareness of hypoglycaemic events (inability or impaired ability to recognise the symptoms of hypoglycaemia). Respon dents' self-reported hypoglycaemia-awareness was significantly associated with the proportion of asymptomatic non-severe hypoglycaemic events; respondents classified as being unaware of hypoglycaemia had a higher proportion of asymptomatic non-severe hypoglycaemic events than aware respondents.

Conclusion: Non-severe hypoglycaemic events are common in people with Type 1 or insulin-treated Type 2 diabetes in the real-world setting in Germany but may still be underestimated due to an inability to recognise the symptoms of hypoglycaemia.

C1 [Kulzer, B.] Forschungsinst Diabet Akad, Bad Mergentheim, Germany.

[Seitz, L.] Novo Nordisk Pharma GmbH, D-55127 Mainz, Germany.

[Kern, W.] Endokrinologikum, Ulm, Germany.

RP Seitz, L (reprint author), Novo Nordisk Pharma GmbH, Brucknerstr 1, D-55127 Mainz, Germany.

EM lsiz@novonordisk.com

CR Akram K, 2006, DIABETIC MED, V23, P750, DOI 10.1111/j.1464-5491.2006.01880.x

Brod M, 2011, VALUE HEALTH, V14, P665, DOI 10.1016/j.jval.2011.02.001

Childs BP, 2005, DIABETES CARE, V28, P1245

(CHMP) CfMPfHU, 2012, GUID CLIN INV MED PR

CLARKE WL, 1995, DIABETES CARE, V18, P517, DOI 10.2337/diacare.18.4.517

Davis RE, 2005, CURR MED RES OPIN, V21, P1477, DOI 10.1185/030079905X61929

Donnelly LA, 2005, DIABETIC MED, V22, P749, DOI 10.1111/j.1464-5491.2005.01501.x

EphMRA, 1997, COD COND

ESOMAR, 2007, ICC ESOMAR INT COD M

GOLD AE, 1994, DIABETES CARE, V17, P697, DOI 10.2337/diacare.17.7.697

Heller SR, 2007, DIABETOLOGIA, V50, P1140, DOI 10.1007/s00125-007-0599-y

Hoi-Hansen T, 2010, J DIABETES COMPLICAT, V24, P392, DOI 10.1016/j.jdiacomp.2009.07.006

IDF Clinical Guidelines Task Force, 2012, GLOB GUID TYP 2 DIAB

Internet World Stats, 2012, INT PEN RAT

Janssen MMJ, 2000, DIABETES METAB RES, V16, P157, DOI 10.1002/1520-7560(0000)9999:9999<::AID-DMRR104>3.0.CO;2-U

Kristensen PL, 2012, DIABETES RES CLIN PR, V96, P17, DOI 10.1016/j.diabres.2011.10.046

Kulzer B, 2013, DIABETOL STOFFWECHS, V8, P107, DOI 10.1055/s-0033-1335312

Leiter LA, 2005, CAN J DIABETES, V29, P1

Lundkvist Jonas, 2005, Eur J Health Econ, V6, P197, DOI 10.1007/s10198-005-0276-3

Miller CD, 2001, ARCH INTERN MED, V161, P1653, DOI 10.1001/archinte.161.13.1653

Ostenson CG, 2014, DIABETIC MED, V31, P92, DOI 10.1111/dme.12261

Pedersen-Bjergaard U, 2004, DIABETES-METAB RES, V20, P479, DOI 10.1002/dmrr.482

Pedersen-Bjergaard U, 2003, DIABETES-METAB RES, V19, P232, DOI 10.1002/dmrr.377

PRAMMING S, 1991, DIABETIC MED, V8, P217, DOI 10.1111/j.1464-5491.1991.tb01575.x

Rathmann W, 2013, DTSCH ARZTEBL INT, V110, P331, DOI 10.3238/arztebl.2013.0331

The DCCT Research Group, 1997, DIABETES, V46, P271, DOI 10.2337/diabetes. 46.2.271

Turner RC, 1998, LANCET, V352, P837

Wild D, 2007, PATIENT EDUC COUNS, V68, P10, DOI 10.1016/j.pec.2007.05.003

NR 28

TC 13

Z9 13

U1 0

U2 0

PU JOHANN AMBROSIUS BARTH VERLAG MEDIZINVERLAGE HEIDELBERG GMBH

PI STUTTGART

PA RUEDIGERSTR 14, D-70469 STUTTGART, GERMANY

SN 0947-7349

EI 1439-3646

J9 EXP CLIN ENDOCR DIAB

JI Exp. Clin. Endocrinol. Diabet.

PD MAR

PY 2014

VL 122

IS 3

BP 167

EP 172

DI 10.1055/s-0033-1363688

PG 6

WC Endocrinology & Metabolism

SC Endocrinology & Metabolism

GA AY2FF

UT WOS:000347403300240

PM 24643694

DA 2019-01-08

ER

PT J

AU Hessler, D

Fisher, L

Glasgow, RE

Strycker, LA

Dickinson, LM

Arean, PA

Masharani, U

AF Hessler, Danielle

Fisher, Lawrence

Glasgow, Russell E.

Strycker, Lisa A.

Dickinson, L. Miriam

Arean, Patricia A.

Masharani, Umesh

TI Reductions in Regimen Distress Are Associated With Improved Management

and Glycemic Control Over Time

SO DIABETES CARE

LA English

DT Article

ID DIABETES DISTRESS; DEPRESSIVE SYMPTOMS; CLINICAL DEPRESSION; OLDER

ADULTS; OUTCOMES; METAANALYSIS; ADHERENCE; STRESS; THERAPY; HUMANS

AB OBJECTIVECross-sectional and longitudinal associations among regimen distress (RD), self-management, and glycemic control were undertaken to explore mechanisms of operation among these variables.RESEARCH DESIGN AND METHODSIn a behavioral randomized control trial (RCT) to reduce RD, 392 adults with type 2 diabetes were assessed for RD, diet, exercise, medication adherence, and HbA(1c) at baseline and at 4 and 12 months. Associations among RD, self-management, and HbA(1c) were examined in cross-sectional analyses at baseline, in prospective analyses using baseline values to predict change over time, and in time-varying analyses.RESULTSAt baseline, greater RD and poorer medication adherence were independently associated with higher HbA(1c) (P = 0.05 and P < 0.001, respectively), and greater RD was associated with poorer medication adherence (P = 0.03). No consistent pattern of significant prospective associations was found. Significant time-varying findings showed that decreases in RD were associated with improvements in medication adherence (P < 0.01), physical activity (P < 0.001), and HbA(1c) (P = 0.02) over time following intervention. Changes in self-management were not associated with changes in HbA(1c) over time.CONCLUSIONSIn the context of an RCT to reduce distress, RD, self-management, and HbA(1c) were interrelated in cross-sectional and time-varying analyses. Decreases in RD were associated with improvements in both self-management and HbA(1c) over 12 months. Findings point to the complex and likely multifaceted pathways of association among these key constructs, with results indicating significant linkages between RD and both self-management and glycemic control over time.

C1 [Hessler, Danielle; Fisher, Lawrence; Arean, Patricia A.; Masharani, Umesh] Univ Calif San Francisco, San Francisco, CA 94143 USA.

[Glasgow, Russell E.] NCI, Div Canc Control & Populat Sci, Rockville, MD USA.

[Strycker, Lisa A.] Oregon Res Inst, Eugene, OR 97403 USA.

[Dickinson, L. Miriam] Univ Colorado, Aurora, CO USA.

RP Hessler, D (reprint author), Univ Calif San Francisco, San Francisco, CA 94143 USA.

EM hesslerd@fcm.ucsf.edu

FU National Institute of Diabetes and Digestive and Kidney Diseases

[DK-061937]

FX This research was funded by National Institute of Diabetes and Digestive

and Kidney Diseases grant DK-061937.

CR Aikens JE, 2012, DIABETES CARE, V35, P2472, DOI 10.2337/dc12-0181

Arean P, 2008, GERONTOLOGIST, V48, P311, DOI 10.1093/geront/48.3.311

Bocchieri LE, 2002, J PSYCHOSOM RES, V52, P155, DOI 10.1016/S0022-3999(01)00241-0

Chapman B. P., 2006, J GERONTOL B-PSYCHOL, V61, P362

Fisher L, 2008, ANN FAM MED, V6, P246, DOI 10.1370/afm.842

Fisher L, 2007, DIABETES CARE, V30, P542, DOI 10.2337/dc06-1614

Fisher L, 2013, DIABETES CARE, V36, P2551, DOI 10.2337/dc12-2493

Fisher L, 2010, DIABETES CARE, V33, P1034, DOI 10.2337/dc09-2175

Fisher L, 2010, DIABETES CARE, V33, P23, DOI 10.2337/dc09-1238

Glasgow RE, 2011, J MED INTERNET RES, V13, P70, DOI 10.2196/jmir.1391

Glasgow RE, 2010, J GEN INTERN MED, V25, P1315, DOI 10.1007/s11606-010-1480-0

Golden SH, 2008, JAMA-J AM MED ASSOC, V299, P2751, DOI 10.1001/jama.299.23.2751

Gonzalez JS, 2011, DIABETES CARE, V34, P236, DOI 10.2337/dc10-1970

Goodwin RD, 2006, J HEALTH PSYCHOL, V11, P643, DOI 10.1177/1359105306066610

HANSON CL, 1987, DIABETES CARE, V10, P752, DOI 10.2337/diacare.10.6.752

Harada ND, 2001, MED SCI SPORT EXER, V33, P962, DOI 10.1097/00005768-200106000-00016

Hedeker D, 2006, LONGITUDINAL DATA AN, P69

Kroenke K, 2001, J GEN INTERN MED, V16, P606, DOI 10.1046/j.1525-1497.2001.016009606.x

Krousel-Wood M, 2005, AM J MED SCI, V330, P128, DOI 10.1097/00000441-200509000-00006

Lloyd C, 2005, DIABETES SPECTR, V18, P121, DOI [DOI 10.2337/DIASPECT.18.2.121, 10.2337/diaspect.18.2.121]

Masharani U, 2007, DIABETES DEMYSTIFIED

Mezuk B, 2008, DIABETES CARE, V31, P2383, DOI 10.2337/dc08-0985

Miller GE, 2007, PSYCHOL BULL, V133, P25, DOI 10.1037/0033-2909.133.1.25

Neuhaus JM, 1998, BIOMETRICS, V54, P638, DOI 10.2307/3109770

Nezu AM, 2004, BEHAV THER, V35, P1, DOI 10.1016/S0005-7894(04)80002-9

NORM, 1999, NORM MULT IMP INC MU

Peyrot M, 1999, J HEALTH SOC BEHAV, V40, P141, DOI 10.2307/2676370

Peyrot MF, 2001, DIABETES CARE, V24, P1703, DOI 10.2337/diacare.24.10.1703

Polonsky WH, 2005, DIABETES CARE, V28, P626, DOI 10.2337/diacare.28.3.626

Rhee MK, 2005, DIABETES EDUCATOR, V31, P240, DOI 10.1177/0145721705274927

Skaff MM, 2009, HEALTH PSYCHOL, V28, P265, DOI 10.1037/a0014429

Steptoe A, 2007, BRAIN BEHAV IMMUN, V21, P901, DOI 10.1016/j.bbi.2007.03.011

Stewart AL, 2001, MED SCI SPORT EXER, V33, P1126

Thompson F. E., 1998, European Journal of Clinical Nutrition, V52, pS63

Toobert DJ, 2000, DIABETES CARE, V23, P943, DOI 10.2337/diacare.23.7.943

Tsujii S, 2012, DIABETIC MED, V29, P1451, DOI 10.1111/j.1464-5491.2012.03647.x

Umpierre D, 2011, JAMA-J AM MED ASSOC, V305, P1790, DOI 10.1001/jama.2011.576

Zwarenstein M, 2008, BRIT MED J, V337, DOI 10.1136/bmj.a2390

NR 38

TC 36

Z9 39

U1 0

U2 11

PU AMER DIABETES ASSOC

PI ALEXANDRIA

PA 1701 N BEAUREGARD ST, ALEXANDRIA, VA 22311-1717 USA

SN 0149-5992

EI 1935-5548

J9 DIABETES CARE

JI Diabetes Care

PD MAR

PY 2014

VL 37

IS 3

BP 617

EP 624

DI 10.2337/dc13-0762

PG 8

WC Endocrinology & Metabolism

SC Endocrinology & Metabolism

GA AB3RT

UT WOS:000331708600014

PM 24170750

OA Green Published, Other Gold

DA 2019-01-08

ER

PT J

AU Hendrieckx, C

Halliday, JA

Bowden, JP

Colman, PG

Cohen, N

Jenkins, A

Speight, J

AF Hendrieckx, C.

Halliday, J. A.

Bowden, J. P.

Colman, P. G.

Cohen, N.

Jenkins, A.

Speight, J.

TI Severe hypoglycaemia and its association with psychological well-being

in Australian adults with type 1 diabetes attending specialist tertiary

clinics

SO DIABETES RESEARCH AND CLINICAL PRACTICE

LA English

DT Article

DE Type 1 diabetes; Severe hypoglycaemia; Impaired awareness of

hypoglycaemia; Well-being; Fear of hypoglycaemia

ID IMPAIRED AWARENESS; MANAGEMENT; FEAR; EDUCATION; IMPACT; LIFE

AB Aim: To investigate severe hypoglycaemia (SH) in adults with type 1 diabetes and its associations with impaired awareness of hypoglycaemia (IAH), clinical, psychological and sociodemographic factors.

Methods: Attendees of three specialist diabetes clinics in Melbourne, Australia completed questions about frequency of SH in the past six months; impaired awareness of hypoglycaemia (Gold score); and measures of general emotional well-being (WHO-5), diabetes-specific positive well-being (subscale of W-BQ28), diabetes-related distress (PAID) and fear of hypoglycaemia (HFS).

Results: Of 422 participants (mean +/- SD age 37.5 +/- 15.0 years; 54% women), 78 (18.5%) reported at least one SH event and 86 (20.5%) had IAH. SH and IAH frequencies were similar at all clinics. In total, 194 SH events were reported, with 10 people experiencing 40% of events. Compared with those without SH, participants with SH had longer diabetes duration, were younger at diabetes onset and more likely to have IAH (p < 0.01). Those with SH had greater fear of hypoglycaemia and diabetes-related distress, poorer general emotional well-being, and lower diabetes-specific positive well-being, (p < 0.01). There were no associations with age, gender, insulin regimen or HbA1c.

Conclusions: This study has identified that SH and IAH in Australian adults with type 1 diabetes exist at similar levels to those reported in US and European research. SH was significantly associated with IAH and fear of hypoglycaemia.

Assessment of hypoglycaemia, IAH and psychological well-being as part of a routine diabetes clinic visit was well accepted by attendees and enabled identification of those who may benefit from medical, educational or therapeutic interventions. (C) 2013 Elsevier Ireland Ltd. All rights reserved.

C1 [Hendrieckx, C.; Halliday, J. A.; Speight, J.] Diabet Australia Vic, Australian Ctr Behav Res Diabet, Melbourne, Vic, Australia.

[Hendrieckx, C.; Halliday, J. A.; Speight, J.] Deakin Univ, Sch Psychol, Ctr Mental Hlth & Well Being Res, Burwood, Vic, Australia.

[Bowden, J. P.; Colman, P. G.] Royal Melbourne Hosp, Dept Endocrinol & Diabet, Melbourne, Vic, Australia.

[Cohen, N.] Baker IDI Heart & Diabet Inst, Melbourne, Vic, Australia.

[Jenkins, A.] Univ Melbourne, St Vincents Hosp, Dept Med, Melbourne, Vic, Australia.

[Speight, J.] AHP Res, Hornchurch, Essex, England.

RP Hendrieckx, C (reprint author), Australian Ctr Behav Res Diabet, 570 Elizabeth St, Melbourne, Vic 3000, Australia.

EM chendrieckx@acbrd.org.au

RI Jenkins, Alicia/N-2482-2015

OI Cohen, Neale/0000-0002-4441-9511; Speight, Jane/0000-0002-1204-6896

FU Diabetes Australia-Vic; Deakin University

FX We thank Elizabeth Maclean for data collection at Baker IDI, Daniel

Calandro for data collection assistance at SVH, Lucy Morrish for data

collection assistance at RMH, and Laura Smith and Anna Scovelle for

biomedical data input and quality control of the survey data. We thank

the participants for their contribution to this project. We also thank

Diabetes Australia-Vic and Deakin University for their financial

support.

CR Anderbro T, 2010, DIABETIC MED, V27, P1151, DOI 10.1111/j.1464-5491.2010.03078.x

Barendse S, 2012, DIABETIC MED, V29, P293, DOI 10.1111/j.1464-5491.2011.03416.x

Bech P., 2004, QUALITY LIFE NEWSLET, V32, P15

Bohme P, 2013, DIABETES METAB, V39, P63, DOI 10.1016/j.diabet.2012.10.006

Choudhary P, 2011, POSTGRAD MED J, V87, P298, DOI 10.1136/pgmj.2008.068197

Cox D, 2008, DIABETES CARE, V31, P1527, DOI 10.2337/dc07-1956

COX DJ, 1987, DIABETES CARE, V10, P617, DOI 10.2337/diacare.10.5.617

Cox DJ, 2001, DIABETES CARE, V24, P637, DOI 10.2337/diacare.24.4.637

Cryer PE, 2002, DIABETOLOGIA, V45, P937, DOI 10.1007/s00125-002-0822-9

Cryer PE, 2009, J CLIN ENDOCR METAB, V94, P709, DOI 10.1210/jc.2008-1410

Frier BM, 2008, DIABETES-METAB RES, V24, P87, DOI 10.1002/dmrr.796

Geddes J, 2008, DIABETIC MED, V25, P501, DOI 10.1111/j.1464-5491.2008.02413.x

GOLD AE, 1994, DIABETES CARE, V17, P697, DOI 10.2337/diacare.17.7.697

Gonder-Frederick LA, 2011, DIABETES CARE, V34, P801, DOI 10.2337/dc10-1343

Graveling Alex J, 2009, Prim Care Diabetes, V3, P131, DOI 10.1016/j.pcd.2009.08.007

Heller SR, 2007, DIABETOLOGIA, V50, P1140, DOI 10.1007/s00125-007-0599-y

Hendrieckx C, 2012, ANN SCI M AUSTR DIAB, Va274, P159

Hermanns N, 2007, DIABETES-METAB RES, V23, P528, DOI 10.1002/dmrr.710

Hermanns Norbert, 2010, Diabetes Care, V33, pe36, DOI 10.2337/dc09-1656

Jorgensen HV, 2003, DIABETES CARE, V26, P1106, DOI 10.2337/diacare.26.4.1106

Kristensen PL, 2012, DIABETES RES CLIN PR, V96, P17, DOI 10.1016/j.diabres.2011.10.046

Leiter LA, 2005, CAN J DIABETES, V29, P186

Little S, 2012, BMC ENDOCR DISORD, V12, DOI 10.1186/1472-6823-12-33

Misso ML, 2010, COCHRANE DB SYST REV, DOI 10.1002/14651858.CD005103.pub2

O'Connell SM, 2011, DIABETES CARE, V34, P2379, DOI 10.2337/dc11-0748

Pedersen-Bjergaard U, 2003, DIABETES-METAB RES, V19, P232, DOI 10.1002/dmrr.377

Snoek FJ, 2011, DIABETES CARE, V34, P601, DOI 10.2337/dc10-1552

Speight J, 2000, P BRIT PSYCHOL SOC, V8, P21

Speight J, 2011, DIABETES MILES AUSTR

Tu E, 2010, INT J CARDIOL, V138, P91, DOI 10.1016/j.ijcard.2008.06.021

Welch GW, 1997, DIABETES CARE, V20, P760, DOI 10.2337/diacare.20.5.760

Wild D, 2007, PATIENT EDUC COUNS, V68, P10, DOI 10.1016/j.pec.2007.05.003

NR 32

TC 35

Z9 37

U1 0

U2 11

PU ELSEVIER IRELAND LTD

PI CLARE

PA ELSEVIER HOUSE, BROOKVALE PLAZA, EAST PARK SHANNON, CO, CLARE, 00000,

IRELAND

SN 0168-8227

EI 1872-8227

J9 DIABETES RES CLIN PR

JI Diabetes Res. Clin. Pract.

PD MAR

PY 2014

VL 103

IS 3

BP 430

EP 436

DI 10.1016/j.diabres.2013.12.005

PG 7

WC Endocrinology & Metabolism

SC Endocrinology & Metabolism

GA AF3AC

UT WOS:000334582700028

PM 24513121

DA 2019-01-08

ER

PT J

AU Aikens, JE

Zivin, K

Trivedi, R

Piette, JD

AF Aikens, James E.

Zivin, Kara

Trivedi, Ranak

Piette, John D.

TI Diabetes self-management support using mHealth and enhanced informal

caregiving

SO JOURNAL OF DIABETES AND ITS COMPLICATIONS

LA English

DT Article

DE Diabetes; mHealth; Primary care; Care management

ID MEDICATION ADHERENCE; PREDICTIVE-VALIDITY; HEALTH LITERACY; QUESTIONS;

DISTRESS; MELLITUS; OUTCOMES; FAMILY; SCALE

AB Objective: To characterize diabetes patient engagement and clinician notifications for an mHealth interactive voice response (IVR) service.

Design: Observational study.

Methods: For three to six months, VA patients with diabetes received weekly IVR calls assessing health status and self-care along with tailored education. Patients could enroll with an informal caregiver who received suggestions on self-management support. Notifications were issued to clinicians when patients reported significant problems.

Results: Patients (n = 303) participated for a total of 5684 patient-weeks, during which 84% of calls were completed. The odds of call completion decreased over time (AOR = 0.96, p < 0.001), and were lower among unmarried patients (AOR = 0.67, p = 0.038) and those who had difficulties with health literacy (AOR = 0.67, p = 0.039), diabetes-related distress (AOR = 0.30, p = 0.018), or medication nonadherence (AOR = 0.57, p = 0.002). Twenty-one clinician notifications were triggered per 100 patient-weeks. The odds of notification were higher during the early weeks of the program (AOR = 0.95, p < 0.001) and among patients who were older (AOR = 1.03, p = 0.004) or more physically impaired (AOR = 0.97, p < 0.001).

Conclusions: By providing information that is reliable, valid, and actionable, IVR-based mHealth services may increase access to between-visit monitoring and diabetes self-management support. The system detects abnormal glycemia and blood pressure levels that might otherwise go unreported, although thresholds for clinician notifications might require adjustment to avoid overloading clinicians. Patient engagement might be enhanced by addressing health literacy and psychological distress. (C) 2014 Elsevier Inc. All rights reserved.

C1 [Aikens, James E.] Univ Michigan, Dept Family Med, Ann Arbor, MI 48104 USA.

[Zivin, Kara] Univ Michigan, Dept Psychiat, Ann Arbor, MI 48109 USA.

[Zivin, Kara; Piette, John D.] Ann Arbor Dept Vet Affairs Ctr Clin Management Re, Ann Arbor, MI USA.

[Zivin, Kara] Univ Michigan, Inst Social Res, Ann Arbor, MI USA.

[Trivedi, Ranak] VA Palo Alto Hlth Care Syst, Ctr Innovat Implementat, Palo Alto, CA USA.

[Trivedi, Ranak] Stanford Univ, Dept Psychiat & Behav Sci, Palo Alto, CA 94304 USA.

[Piette, John D.] Univ Michigan, Sch Publ Hlth, Dept Hlth Behav & Hlth Educ, Ann Arbor, MI 48109 USA.

RP Aikens, JE (reprint author), Univ Michigan, Dept Family Med, 1018 Fuller St, Ann Arbor, MI 48104 USA.

EM aikensj@umich.edu

OI Zivin, Kara/0000-0001-8246-6864

FU VA Career Development Awards [CD2 07-206-1, CDA-09-206]; Michigan

Diabetes Translational Research Center [P30DK092926]; National Institute

of Diabetes and Digestive and Kidney Diseases [R18DK088294]

FX JDP is a VA Senior Research Career Scientist. KZ and RT are supported by

VA Career Development Awards (grant # CD2 07-206-1 to K.Z. and grant #

CDA-09-206 to R.T.). Other financial support came from the Michigan

Diabetes Translational Research Center (grant # P30DK092926) and grant #

R18DK088294 from the National Institute of Diabetes and Digestive and

Kidney Diseases. The views expressed in this manuscript are those of the

authors and do not necessarily represent the opinions of the Department

of Veterans Affairs.

CR Aikens JE, 2012, DIABETES CARE, V35, P2472, DOI 10.2337/dc12-0181

Aikens JE, 2009, DIABETES CARE, V32, P19, DOI 10.2337/dc08-1533

Alberti KGMM, 1998, DIABETIC MED, V15, P539, DOI 10.1002/(SICI)1096-9136(199807)15:7<539::AID-DIA668>3.0.CO;2-S

Anderson Robert M, 2009, Ther Patient Educ, V1, P3

Armour TA, 2005, DIABETIC MED, V22, P1295, DOI 10.1111/j.1464-5491.2005.01618.x

Attkisson C C, 1982, Eval Program Plann, V5, P233, DOI 10.1016/0149-7189(82)90074-X

Chew LD, 2004, FAM MED, V36, P588

Chew LD, 2008, J GEN INTERN MED, V23, P561, DOI 10.1007/s11606-008-0520-5

Irwin M, 1999, ARCH INTERN MED, V159, P1701, DOI 10.1001/archinte.159.15.1701

Kawachi I, 2001, J URBAN HEALTH, V78, P458, DOI 10.1093/jurban/78.3.458

Kohout F J, 1993, J Aging Health, V5, P179, DOI 10.1177/089826439300500202

MORISKY DE, 1986, MED CARE, V24, P67, DOI 10.1097/00005650-198601000-00007

Morisky DE, 2008, J CLIN HYPERTENS, V10, P348, DOI 10.1111/j.1751-7176.2008.07572.x

Piette J. D., 2013, J MED INTERNET RES, V15

Piette JD, 1997, DIABETES EDUCATOR, V23, P672, DOI 10.1177/014572179702300607

Piette JD, MED CARE IN PRESS

Piette JD, 2007, DIABETES CARE, V30, P2425, DOI 10.2337/dc07-1046

Piette JD, 2013, MED CARE, V51, P216, DOI 10.1097/MLR.0b013e318277ebf8

Polonsky WH, 2005, DIABETES CARE, V28, P626, DOI 10.2337/diacare.28.3.626

Robles TF, 2003, PHYSIOL BEHAV, V79, P409, DOI 10.1016/S0031-9384(03)00160-4

Rosland AM, 2013, FAM SYST HEALTH, V31, P119, DOI 10.1037/a0031535

Schwartz S, 1998, Health Manag Technol, V19, P41

StataCorp, 2011, STAT STAT SOFTW REL

Talley RC, 2007, AM J PUBLIC HEALTH, V97, P224, DOI 10.2105/AJPH.2004.059337

Ware JE, 1996, MED CARE, V34, P220, DOI 10.1097/00005650-199603000-00003

NR 25

TC 22

Z9 25

U1 1

U2 26

PU ELSEVIER SCIENCE INC

PI NEW YORK

PA 360 PARK AVE SOUTH, NEW YORK, NY 10010-1710 USA

SN 1056-8727

EI 1873-460X

J9 J DIABETES COMPLICAT

JI J. Diabetes Complications

PD MAR-APR

PY 2014

VL 28

IS 2

BP 171

EP 176

DI 10.1016/j.jdiacomp.2013.11.008

PG 6

WC Endocrinology & Metabolism

SC Endocrinology & Metabolism

GA AD8VD

UT WOS:000333542900014

PM 24374137

OA Green Accepted

DA 2019-01-08

ER

PT J

AU Karlsen, B

Bru, E

AF Karlsen, Bjorg

Bru, Edvin

TI The relationship between diabetes-related distress and clinical

variables and perceived support among adults with type 2 diabetes: A

prospective study

SO INTERNATIONAL JOURNAL OF NURSING STUDIES

LA English

DT Article

DE Adults with type 2 diabetes; Clinical variables; Diabetes-related

distress; Healthcare professionals; Prospective longitudinal study;

Social support

ID SOCIAL SUPPORT; EMOTIONAL DISTRESS; PROBLEM AREAS; GLYCEMIC CONTROL;

SCALE; DEPRESSION; MANAGEMENT; VALIDITY; PAID; PERSPECTIVES

AB Background: Diabetes-related distress is a prevalent emotional state experienced among people living with type 2 diabetes. It has predominantly been studied in relation to diabetes management and metabolic control and to some extent in relation to perceived social support. Little is known about the relative prospective contribution of clinical variables and perceived support on diabetes-related distress.

Objective: To investigate the predictive influence of clinical variables and perceived support from health care professionals and family on diabetes-related distress.

Design: The study has a prospective, longitudinal design.

Settings: The participants were recruited from seven general practitioners in the south western part of Norway and from members of the Norwegian Diabetes Association.

Participants: The sample comprised 296 people with type 2 diabetes. Inclusion criteria were: adults (30-70 years) diagnosed with type 2 diabetes who were willing and able to complete a questionnaire written in Norwegian.

Methods: Data were collected by postal questionnaires assessing perceived support from health care professionals and family and diabetes-related distress measured by the Problem Areas in Diabetes Scale at two time points separated by an interval of one year (October 2008 and 2009). Clinical data were collected by self-report. The follow-up group, for which both assessment data were available, constituted our sample comprising 296 adults.

Results: Descriptive results and findings from correlations and prospective multivariate associations indicate small changes in diabetes-related distress over a period of one year. In total, multiple regression analyses showed that clinical variables were very weak predictors of diabetes-related distress, whereas perceived social support emerged as statistically significant although a moderate predictor of distress. Among the support variables, only 'constructive support from health care professionals' and 'non-supportive family behaviour' accounted for changes in diabetes-related distress.

Conclusions: Findings suggest that diabetes-related distress among adults with type 2 diabetes is relatively stable over time and may be difficult to alter. Health care professionals should therefore be aware that stimulating people to change is challenging and complex. Moreover, they should devote more attention to non-clinical factors such as social support when addressing diabetes-related distress. (C) 2013 Elsevier Ltd. All rights reserved.

C1 [Karlsen, Bjorg; Bru, Edvin] Univ Stavanger, Fac Social Sci, Dept Hlth Studies, N-4036 Stavanger, Norway.

[Bru, Edvin] Univ Stavanger, Norwegian Ctr Learning Environm & Behav Res Educ, N-4036 Stavanger, Norway.

RP Karlsen, B (reprint author), Univ Stavanger, Fac Social Sci, Dept Hlth Studies, N-4036 Stavanger, Norway.

EM bjorg.karlsen@uis.no

OI Bru, Edvin/0000-0003-2004-6957

FU Norwegian Diabetes Association

FX The study was supported by grants from the Norwegian Diabetes

Association.

CR Birkeland K. I., 2009, NASJONALE KLIN RETNI

CARVER CS, 1989, J PERS SOC PSYCHOL, V56, P267, DOI 10.1037/0022-3514.56.2.267

Caspi A, 2005, ANNU REV PSYCHOL, V56, P453, DOI 10.1146/annurev.psych.55.090902.141913

COBB S, 1976, PSYCHOSOM MED, V38, P300, DOI 10.1097/00006842-197609000-00003

COHEN S, 1985, PSYCHOL BULL, V98, P310, DOI 10.1037//0033-2909.98.2.310

COLEMAN JS, 1988, AM J SOCIOL, V94, pS95, DOI 10.1086/228943

Costa P. T., 1996, HDB COPING THEORY RE, P44

COX DJ, 1992, J CONSULT CLIN PSYCH, V60, P628, DOI 10.1037//0022-006X.60.4.628

de Groot Mary, 2010, Diabetes Spectr, V23, P15, DOI 10.2337/diaspect.23.1.15

DECI EL, 1991, EDUC PSYCHOL-US, V26, P325, DOI 10.1207/s15326985ep2603&4_6

Delahanty LM, 2007, DIABETIC MED, V24, P48, DOI 10.1111/j.1464-5491.2007.02028.x

Fisher L, 2009, DIABETIC MED, V26, P622, DOI 10.1111/j.1464-5491.2009.02730.x

Fisher L, 2001, DIABETES CARE, V24, P1751, DOI 10.2337/diacare.24.10.1751

Fisher L, 2010, DIABETES CARE, V33, P1034, DOI 10.2337/dc09-2175

Ford ME, 1998, J NATL MED ASSOC, V90, P425

Graue M, 2012, INT J NURS STUD, V49, P174, DOI 10.1016/j.ijnurstu.2011.08.007

Hewitt PL, 1996, HDB COPING THEORY RE, P410

Hunt LM, 1998, WESTERN J NURS RES, V20, P656, DOI 10.1177/019394599802000602

Jenum Anne Karen, 2008, Prim Care Diabetes, V2, P203, DOI 10.1016/j.pcd.2008.06.003

Karlsen B., 2004, PSYCHOL HEALTH MED, V9, P53

Karlsen B, 2012, J ADV NURS, V68, P391, DOI 10.1111/j.1365-2648.2011.05751.x

Kunitz SJ, 2004, BRIT MED BULL, V69, P61, DOI 10.1093/bmb/ldh015

Lazarus R. S, 1984, STRESS APPRAISAL COP

Livneh H., 2007, COPING CHRONIC ILLNE

Lloyd C, 2005, DIABETES SPECTR, V18, P121, DOI [DOI 10.2337/DIASPECT.18.2.121, 10.2337/diaspect.18.2.121]

Norusis M. J., 2011, PASW STAT 18 GUID DA

Oftedal B, 2011, J NURSING HEALTHCARE, V3, P12

Papelbaum M, 2010, DIABETES RES CLIN PR, V89, P227, DOI 10.1016/j.diabres.2010.05.024

Pedhazur E. J., 1982, MULTIPLE REGRESSIONS

Peyrot M, 2005, DIABETIC MED, V22, P1379, DOI 10.1111/j.1464-5491.2005.01644.x

POLONSKY WH, 1995, DIABETES CARE, V18, P754, DOI 10.2337/diacare.18.6.754

Polonsky WH, 2005, DIABETES CARE, V28, P626, DOI 10.2337/diacare.28.3.626

Sacco WP, 2006, INTERPERSONAL, COGNITIVE, AND SOCIAL NATURE OF DEPRESSION, P101

Sacco WP, 1999, INTERACTIONAL NATURE OF DEPRESSION, P329, DOI 10.1037/10311-012

SCHAFER LC, 1986, DIABETES CARE, V9, P179, DOI 10.2337/diacare.9.2.179

Schreurs KMG, 1997, CLIN PSYCHOL REV, V17, P89, DOI 10.1016/S0272-7358(96)00050-5

Sigurdardottir AK, 2008, INT J NURS STUD, V45, P526, DOI 10.1016/j.ijnurstu.2006.10.008

Skinner Chas T., 2000, PSYCHOL DIABETES CAR

Snoek FJ, 2000, DIABETES CARE, V23, P1305, DOI 10.2337/diacare.23.9.1305

Stene Lars Christian, 2004, Tidsskr Nor Laegeforen, V124, P1511

STEWART M, 2000, CHRONIC CONDITIONS C

Tang TS, 2008, DIABETES EDUCATOR, V34, P266, DOI 10.1177/0145721708315680

Taylor S, 2006, HLTH PSYCHOL

Thorne SE, 2001, PATIENT EDUC COUNS, V42, P81, DOI 10.1016/S0738-3991(00)00095-1

Trief PM, 1998, DIABETES CARE, V21, P241, DOI 10.2337/diacare.21.2.241

Welch G, 2003, DIABETIC MED, V20, P69, DOI 10.1046/j.1464-5491.2003.00832.x

Welch G, 2007, ETHNIC DIS, V17, P541

Welch GW, 1997, DIABETES CARE, V20, P760, DOI 10.2337/diacare.20.5.760

Yang J, 2009, J CLIN NURS, V18, P1295, DOI 10.1111/j.1365-2702.2008.02703.x

NR 49

TC 18

Z9 20

U1 0

U2 29

PU PERGAMON-ELSEVIER SCIENCE LTD

PI OXFORD

PA THE BOULEVARD, LANGFORD LANE, KIDLINGTON, OXFORD OX5 1GB, ENGLAND

SN 0020-7489

EI 1873-491X

J9 INT J NURS STUD

JI Int. J. Nurs. Stud.

PD MAR

PY 2014

VL 51

IS 3

BP 438

EP 447

DI 10.1016/j.ijnurstu.2013.06.016

PG 10

WC Nursing

SC Nursing

GA AC8YB

UT WOS:000332819900012

PM 23891535

DA 2019-01-08

ER

PT J

AU Rassart, J

Luyckx, K

Moons, P

Weets, I

AF Rassart, Jessica

Luyckx, Koen

Moons, Philip

Weets, Ilse

TI Personality and self-esteem in emerging adults with Type 1 diabetes

SO JOURNAL OF PSYCHOSOMATIC RESEARCH

LA English

DT Article

DE Big Five; Diabetes Type 1; Insulin pump; Personality; Self-esteem; Sex

differences

ID QUALITY-OF-LIFE; GENDER-DIFFERENCES; GLYCEMIC CONTROL; YOUNG-ADULTS;

PSYCHOLOGICAL ADJUSTMENT; CHRONIC ILLNESS; HEALTH-STATUS; ADOLESCENTS;

CARE; DEPRESSION

AB Objective: The present study examined (1) mean-level differences in self-esteem and Big Five personality traits between individuals with and without diabetes; and (2) demographic, clinical, and psychological correlates of patients' self-esteem and Big Five.

Research design and methods: A total of 478 emerging adults with Type 1 diabetes (18-35 years old) were selected from the Belgian Diabetes Registry and completed questionnaires on personality, self-esteem, and diabetes-related distress. The control group consisted of 341 healthy participants who were matched (1:1) on sex and age with the patient group.

Results: First, mean-level differences between patients and controls differed according to patients' sex and illness duration. Women with diabetes reported lower self-esteem and were less extraverted and emotionally stable as compared to female controls. In contrast, men with diabetes reported higher self-esteem and were more agreeable but less emotionally stable as compared to male controls. Furthermore, whereas both patients with shorter and longer illness duration were less extraverted and emotionally stable as compared to controls, only patients with longer illness duration reported heightened agreeableness. Second, self-esteem and Big Five were found to relate to patients' sex and (to a lesser extent) age and illness duration. Finally, patients reporting elevated diabetes-related distress reported lower self-esteem, and were less agreeable and emotionally stable as compared to patients not reporting such distress.

Conclusions: Patients' personality and self-esteem might be important targets for future prevention and intervention efforts. The present findings can assist healthcare professionals in identifying those patients who might benefit the most from such programs. (C) 2013 Elsevier Inc. All rights reserved.

C1 [Rassart, Jessica; Luyckx, Koen; Moons, Philip] Katholieke Univ Leuven, B-3000 Louvain, Belgium.

[Moons, Philip] Katholieke Univ Leuven Hosp, Louvain, Belgium.

[Moons, Philip] Copenhagen Univ Hosp, Ctr Heart, Copenhagen, Denmark.

[Weets, Ilse] Free Univ Brussels, Brussels, Belgium.

RP Rassart, J (reprint author), Katholieke Univ Leuven, Dept Psychol, Tiensestr 102, B-3000 Louvain, Belgium.

EM Jessica.Rassart@ppw.kuleuven.be

OI Moons, Philip/0000-0002-8609-4516

CR Abraido-Lanza AF, 1997, AM J COMMUN PSYCHOL, V25, P601, DOI 10.1023/A:1024682800238

Arnett JJ, 2000, AM PSYCHOL, V55, P469, DOI 10.1037/0003-066X.55.5.469

Auryan S, 2008, DIABETES OBES METAB, V10, P1135, DOI 10.1111/j.1463-1326.2008.00896.x

Austin S, 2011, J HEALTH PSYCHOL, V16, P917, DOI 10.1177/1359105310396392

Boeger A, 1996, Z KINDER JUG-PSYCH, V24, P231

Branje SJT, 2004, J PERS SOC PSYCHOL, V86, P615, DOI 10.1037/0022-3514.86.4.615

Brickman AL, 1996, PSYCHOSOMATICS, V37, P459, DOI 10.1016/S0033-3182(96)71534-7

Bryden KS, 2003, DIABETES CARE, V26, P1052, DOI 10.2337/diacare.26.4.1052

Bury M, 1982, Sociol Health Illn, V4, P167, DOI 10.1111/1467-9566.ep11339939

Chapman B. P., DEV PSYCHOL IN PRESS

Costa PT, 1992, REVISED NEO PERSONAL

Daneman D., 2002, Diabetes Spectrum, V15, P83, DOI 10.2337/diaspect.15.2.83

Enzlin P, 2002, PATIENT EDUC COUNS, V48, P139, DOI 10.1016/S0738-3991(02)00009-5

Erol RY, 2011, J PERS SOC PSYCHOL, V101, P607, DOI 10.1037/a0024299

Ferro MA, 2013, HEALTH PSYCHOL, V32, P839, DOI 10.1037/a0031861

Friedman HS, 2008, BRAIN BEHAV IMMUN, V22, P668, DOI 10.1016/j.bbi.2007.09.004

Fritschi C, 2010, J PSYCHOSOM RES, V69, P33, DOI 10.1016/j.jpsychores.2010.01.021

Gendelman N, 2009, DIABETES CARE, V32, P575, DOI 10.2337/dc08-1835

HAUSER ST, 1983, ARCH GEN PSYCHIAT, V40, P325

Helgeson VS, 2007, J PEDIATR PSYCHOL, V32, P794, DOI 10.1093/jpepsy/jsm020

Hermanns N, 2006, DIABETOLOGIA, V49, P469, DOI 10.1007/s00125-005-0094-2

Hirose M, 2012, CURR DIABETES REP, V12, P711, DOI 10.1007/s11892-012-0313-4

Hocking MC, 2005, CLIN CHILD FAM PSYCH, V8, P221, DOI 10.1007/s10567-005-6667-2

Johnston-Brooks CH, 2002, PSYCHOSOM MED, V64, P43, DOI 10.1097/00006842-200201000-00007

Jylha P, 2006, DEPRESS ANXIETY, V23, P281, DOI 10.1002/da.20167

Korbel CD, 2007, CHILD HEALTH CARE, V36, P83, DOI 10.1080/02739610701316936

Lawson VL, 2010, BRIT J HEALTH PSYCH, V15, P623, DOI 10.1348/135910709X478664

Linley PA, 2004, J TRAUMA STRESS, V17, P11, DOI 10.1023/B:JOTS.0000014671.27856.7e

Lorig KR, 2001, MED CARE, V39, P1217, DOI 10.1097/00005650-200111000-00008

Luyckx K, 2009, DIABETES CARE, V32, P797, DOI 10.2337/dc08-1990

McAdams D.P., 2009, ANNU REV CLIN PSYCHO, V61, P517

McCrae R. R., 1999, HDB PERSONALITY THEO, P139

Pek Hatice, 2002, J Pediatr Nurs, V17, P279, DOI 10.1053/jpdn.2002.126710

Peters A, 2011, DIABETES CARE, V34, P2477, DOI 10.2337/dc11-1723

POLONSKY WH, 1995, DIABETES CARE, V18, P754, DOI 10.2337/diacare.18.6.754

Rassart J, 2013, PSYCHOL HEALTH, V28, P319, DOI 10.1080/08870446.2012.729836

Roberts B. W., 2008, HDB PERSONALITY THEO, P375

Rosenberg M, 1965, SOC ADOLESCENT SELF

Sawyer SM, 2003, J PAEDIATR CHILD H, V39, P2, DOI 10.1046/j.1440-1754.2003.00081.x

Schneider S, 2007, DIABETES CARE, V30, P1107, DOI 10.2337/dc06-2197

Seiffge-Krenke I., 2001, DIABETIC ADOLESCENTS

SEIGEL WM, 1990, J ADOLESCENT HEALTH, V11, P501, DOI 10.1016/0197-0070(90)90110-N

Sparud-Lundin C, 2010, J ADV NURS, V66, P128, DOI 10.1111/j.1365-2648.2009.05166.x

Specht J, 2011, J PERS SOC PSYCHOL, V101, P862, DOI 10.1037/a0024950

Spijkerman AMW, 2002, EUR J CLIN INVEST, V32, P924, DOI 10.1046/j.1365-2362.2002.01090.x

Stahl A, 2011, DIABETOLOGIA, V54, pS41

Tackett JL, 2006, CLIN PSYCHOL REV, V26, P584, DOI 10.1016/j.cpr.2006.04.003

Tamres LK, 2002, PERS SOC PSYCHOL REV, V6, P2, DOI 10.1207/S15327957PSPR0601_1

Tang TZ, 2009, ARCH GEN PSYCHIAT, V66, P1322, DOI 10.1001/archgenpsychiatry.2009.166

Taylor MD, 2003, DIABETIC MED, V20, P135, DOI 10.1046/j.1464-5491.2003.00887.x

THOMPSON RJ, 1994, J PEDIATR PSYCHOL, V19, P189, DOI 10.1093/jpepsy/19.2.189

Unden AL, 2008, GENDER MED, V5, P162, DOI 10.1016/j.genm.2008.05.003

van Bastelaar KMP, 2010, DIABETIC MED, V27, P798, DOI 10.1111/j.1464-5491.2010.03025.x

Van De yen MOM, 2011, J PSYCHOSOM RES, V71, P166

Vandewalle CL, 1997, DIABETES CARE, V20, P1556, DOI 10.2337/diacare.20.10.1556

Varni JW, 2009, PEDIATR DIABETES, V10, P321, DOI 10.1111/j.1399-5448.2008.00482.x

Vermulst A. A., 2005, QBF QUICK BIG 5 PERS

Vlachioti E, 2010, J DIABETES, V2, P104, DOI 10.1111/j.1753-0407.2010.00067.x

Vollrath ME, 2007, DIABETIC MED, V24, P1028, DOI 10.1111/j.1464-5491.2007.02215.x

Weissberg-Benchell J, 2003, DIABETES CARE, V26, P1079, DOI 10.2337/diacare.26.4.1079

Weissberg-Benchell J, 2007, DIABETES CARE, V30, P2441, DOI 10.2337/dc07-1249

Wennick A, 2007, J ADV NURS, V60, P299, DOI 10.1111/j.1365-2648.2007.04411.x

Wheeler K, 2012, J CHILD ADOL PS NURS, V25, P66, DOI 10.1111/j.1744-6171.2012.00329.x

Williams SJ, 2000, SOCIOL HEALTH ILL, V22, P40, DOI 10.1111/1467-9566.00191

NR 64

TC 7

Z9 7

U1 4

U2 16

PU PERGAMON-ELSEVIER SCIENCE LTD

PI OXFORD

PA THE BOULEVARD, LANGFORD LANE, KIDLINGTON, OXFORD OX5 1GB, ENGLAND

SN 0022-3999

EI 1879-1360

J9 J PSYCHOSOM RES

JI J. Psychosomat. Res.

PD FEB

PY 2014

VL 76

IS 2

BP 139

EP 145

DI 10.1016/j.jpsychores.2013.11.015

PG 7

WC Psychiatry

SC Psychiatry

GA AB2BJ

UT WOS:000331597700009

PM 24439690

DA 2019-01-08

ER

PT J

AU Nakaya, N

Kogure, M

Saito-Nakaya, K

Tomata, Y

Sone, T

Kakizaki, M

Tsuji, I

AF Nakaya, Naoki

Kogure, Mana

Saito-Nakaya, Kumi

Tomata, Yasutake

Sone, Toshimasa

Kakizaki, Masako

Tsuji, Ichiro

TI The association between self-reported history of physical diseases and

psychological distress in a community-dwelling Japanese population: the

Ohsaki Cohort 2006 Study

SO EUROPEAN JOURNAL OF PUBLIC HEALTH

LA English

DT Article

ID SOCIAL SUPPORT; SCREENING SCALES; DEPRESSION; METAANALYSIS; CANCER;

QUESTIONNAIRE; DISABILITY; OUTCOMES; ILLNESS; DENMARK

AB Background: Patients with physical disease are known to suffer considerable psychological distress. Social support may confound the association between physical disease and psychological distress. Population-based epidemiological studies have not been conducted on the association between history of physical disease, psychological distress and social support. Methods: Using cross-sectional data from 2006, we studied 43 487 community-dwelling people aged epsilon 40 years living in Japan. We examined the association between 13 self-reported histories of physical disease and psychological distress evaluated using the Kessler 6-item psychological distress scale (K6), defined as epsilon 13 points out of 24. To investigate the association, we performed multiple logistic regression analyses adjusted for age, gender, social support and possible confounders. Social support, as the interaction between physical disease and psychological depression, was tested through the addition of cross-product terms to the multivariate-adjusted model. Results: The following histories of physical disease were found significantly and positively associated with psychological distress: cancer, diabetes mellitus, hyperlipidemia, hypertension, myocardial infarction, stroke, gastric or duodenal ulcer, liver disease, arthritis, osteoporosis, kidney disease and fall or fracture (odds ratio, 1.2-2.3). Social support did not modify the association between most histories of physical disease and psychological distress. Conclusions: Subjects with a history of physical disease were significantly and positively associated with psychological distress, and social support did not modify this association for most physical diseases. Even after patients have left hospital following treatment for physical disease, they require continuous monitoring for psychological distress by doctors and paramedics.

C1 [Nakaya, Naoki] Tohoku Univ, Tohoku Med Megabank Org, Dept Prevent Med & Epidemiol, Sendai, Miyagi 9808573, Japan.

[Nakaya, Naoki; Kogure, Mana; Tomata, Yasutake; Sone, Toshimasa; Kakizaki, Masako; Tsuji, Ichiro] Tohoku Univ, Grad Sch Med, Dept Publ Hlth & Forens Med, Div Epidemiol, Sendai, Miyagi 9808573, Japan.

[Nakaya, Naoki] Kamakura Womens Univ, Dept Nutr & Dietet, Fac Family & Consumer Sci, Kamakura, Kanagawa, Japan.

[Saito-Nakaya, Kumi] Tohoku Univ, Grad Sch Med, Dept Behav Med, Sendai, Miyagi 9808573, Japan.

RP Nakaya, N (reprint author), Tohoku Univ, Tohoku Med Megabank Org, Dept Prevent Med & Epidemiol, 2-1 Seiryo, Sendai, Miyagi 9808573, Japan.

EM nakaya-thk@umin.ac.jp

OI Kakizaki, Masako/0000-0001-6030-8953

FU Japan Society for Challenging Exploratory Research [23650420]

FX Supported by grants from the Japan Society for Challenging Exploratory

Research (No. 23650420).

CR Anderson RJ, 2001, DIABETES CARE, V24, P1069, DOI 10.2337/diacare.24.6.1069

AROMAA A, 1994, ACTA PSYCHIAT SCAND, V89, P77, DOI 10.1111/j.1600-0447.1994.tb05807.x

BARNETT PA, 1988, PSYCHOL BULL, V104, P97, DOI 10.1037/0033-2909.104.1.97

Berkman L.F., 2000, SOCIAL EPIDEMIOLOGY

Bower JE, 2000, J CLIN ONCOL, V18, P743, DOI 10.1200/JCO.2000.18.4.743

Brummett BH, 2003, INT J BEHAV MED, V10, P239, DOI 10.1207/S15327558IJBM1003_04

Ciesla JA, 2001, AM J PSYCHIAT, V158, P725, DOI 10.1176/appi.ajp.158.5.725

Dalton SO, 2009, J CLIN ONCOL, V27, P1440, DOI 10.1200/JCO.2008.20.5526

Dalton SO, 2008, EUR J CANCER, V44, P2074, DOI 10.1016/j.ejca.2008.06.018

Dickens C, 2002, PSYCHOSOM MED, V64, P52, DOI 10.1097/00006842-200201000-00008

Furukawa TA, 2003, PSYCHOL MED, V33, P357, DOI 10.1017/S0033291702006700

Hammar N, 1998, OCCUP ENVIRON MED, V55, P548, DOI 10.1136/oem.55.8.548

Hjollund NH, 2007, HEALTH QUAL LIFE OUT, V5, DOI 10.1186/1477-7525-5-12

Hozawa A, 2009, AM J CLIN NUTR, V90, P1390, DOI 10.3945/ajcn.2009.28214

Kessler RC, 2003, ARCH GEN PSYCHIAT, V60, P184, DOI 10.1001/archpsyc.60.2.184

Kessler RC, 2002, PSYCHOL MED, V32, P959, DOI 10.1017/S0033291702006074

Koizumi Y, 2005, PSYCHIAT CLIN NEUROS, V59, P563, DOI 10.1111/j.1440-1819.2005.01415.x

Kurimoto Ayumi, 2011, Nihon Ronen Igakkai Zasshi, V48, P149

Kuriyama S, 2009, J EPIDEMIOL, V19, P294, DOI 10.2188/jea.JE20080076

Lee SJ, 2007, AM J PUBLIC HEALTH, V97, P1820, DOI 10.2105/AJPH.2005.084871

Lenze EJ, 2001, AM J GERIAT PSYCHIAT, V9, P113, DOI 10.1176/appi.ajgp.9.2.113

Lett HS, 2005, PSYCHOSOM MED, V67, P869, DOI 10.1097/01.psy.0000188393.73571.0a

MURAOKA Y, 1996, NIPPON RONEN SEISHIN, V7, P397

Nausheen B, 2009, J PSYCHOSOM RES, V67, P403, DOI 10.1016/j.jpsychores.2008.12.012

Ogawa K, 2003, PUBLIC HEALTH NUTR, V6, P147, DOI 10.1079/PHN2002411

Oldehinkel AJ, 2011, NEUROSCI BIOBEHAV R, V35, P1757, DOI 10.1016/j.neubiorev.2010.10.013

Picavet HSJ, 1997, AM J PUBLIC HEALTH, V87, P1680, DOI 10.2105/AJPH.87.10.1680

SARASON IG, 1983, J PERS SOC PSYCHOL, V44, P127, DOI 10.1037/0022-3514.44.1.127

STEWART AL, 1989, JAMA-J AM MED ASSOC, V262, P907, DOI 10.1001/jama.262.7.907

Symister P, 2003, HEALTH PSYCHOL, V22, P123, DOI 10.1037/0278-6133.22.2.123

NR 30

TC 14

Z9 14

U1 0

U2 6

PU OXFORD UNIV PRESS

PI OXFORD

PA GREAT CLARENDON ST, OXFORD OX2 6DP, ENGLAND

SN 1101-1262

EI 1464-360X

J9 EUR J PUBLIC HEALTH

JI Eur. J. Public Health

PD FEB

PY 2014

VL 24

IS 1

BP 45

EP 49

DI 10.1093/eurpub/ckt017

PG 5

WC Public, Environmental & Occupational Health

SC Public, Environmental & Occupational Health

GA AA1EC

UT WOS:000330838200012

PM 23444426

OA Bronze

DA 2019-01-08

ER

PT J

AU Kasteleyn, MJ

Gorter, KJ

Stellato, RK

Rijken, M

Nijpels, G

Rutten, GEHM

AF Kasteleyn, Marise J.

Gorter, Kees J.

Stellato, Rebecca K.

Rijken, Mieke

Nijpels, Giel

Rutten, Guy E. H. M.

TI Tailored support for type 2 diabetes patients with an acute coronary

event after discharge from hospital - design and development of a

randomised controlled trial

SO DIABETOLOGY & METABOLIC SYNDROME

LA English

DT Article

DE Type 2 diabetes; Acute Coronary Event (ACE); Distress; Self-management;

Self-efficacy; Quality of life; Depression; Primary care; Illness

representations

ID QUALITY-OF-LIFE; ACUTE MYOCARDIAL-INFARCTION; CHANGING ILLNESS

PERCEPTIONS; CARDIOVASCULAR-DISEASE RISK; CARDIAC REHABILITATION;

EMOTIONAL DISTRESS; SELF-MANAGEMENT; ARTERY-DISEASE; HEALTH-STATUS;

PROBLEM AREAS

AB Background: Type 2 diabetes mellitus patients with an acute coronary event (ACE) experience decreased quality of life and increased distress. According to the American Diabetes Association, discharge from the hospital is a time of increased distress for all patients. Tailored support specific to diabetes is scarce in that period. We developed an intervention based on Bandura's Social Cognitive Theory, Leventhal's Common Sense Model, and results of focus groups. The aim of this study is to evaluate the effectiveness of the intervention to reduce distress in type 2 diabetes patients who experienced a first ACE.

Methods: Randomised controlled trial. Two hundred patients are recruited in thirteen hospitals. A diabetes nurse visits the patients in the intervention group (n = 100) at home within three weeks after discharge from hospital, and again after two weeks and two months. The control group (n = 100) receives a consultation by telephone. The primary outcome is diabetes-related distress, measured with the Problem Areas in Diabetes (PAID) questionnaire. Secondary outcomes are well-being, health status, anxiety, depression, HbA1c, blood pressure and lipids. Mediating variables are self-management, self-efficacy and illness representations. Outcomes are measured with questionnaires directly after discharge from hospital and five months later. Biomedical variables are obtained from the records from the primary care physician and the hospital. Differences between groups in change over time are analysed according to the intention-to-treat principle. The Holm-Bonferroni correction is used to adjust for multiplicity.

Discussion: Type 2 diabetes patients who experience a first ACE need tailored support after discharge from the hospital. This trial will provide evidence on the effectiveness of a supportive intervention in reducing distress in these patients.

C1 [Kasteleyn, Marise J.; Gorter, Kees J.; Stellato, Rebecca K.; Rutten, Guy E. H. M.] Univ Med Ctr Utrecht, Julius Ctr Hlth Sci & Primary Care, NL-3508 GA Utrecht, Netherlands.

[Rijken, Mieke] NIVEL, Netherlands Inst Hlth Serv Res, Utrecht, Netherlands.

[Nijpels, Giel] Vrije Univ Amsterdam, Med Ctr, EMGO Inst, Amsterdam, Netherlands.

RP Kasteleyn, MJ (reprint author), Univ Med Ctr Utrecht, Julius Ctr Hlth Sci & Primary Care, STR 6-131,POB 85500, NL-3508 GA Utrecht, Netherlands.

EM m.j.kasteleyn@umcutrecht.nl

OI Kasteleyn, Marise/0000-0001-7751-7516

FU Dutch Diabetes Research Foundation [2009.70]

FX This study is funded by the Dutch Diabetes Research Foundation (no:

2009.70.). We would like to thank Professor K.C.B. Roes for his

statistical support.

CR Aikens JE, 2012, DIABETES CARE, V35, P2472, DOI 10.2337/dc12-0181

Bandura A, 1986, SELF EFFICACY SOCIAL

Bech P, 1996, PSYCHOTHER PSYCHOSOM, V65, P183, DOI 10.1159/000289073

Beck CA, 2001, AM HEART J, V142, P271, DOI 10.1067/mhj.2001.116758

Beswick AD, 2005, J ADV NURS, V49, P538, DOI 10.1111/j.1365-2648.2004.03327.x

Borm GF, 2007, J CLIN EPIDEMIOL, V60, P1234, DOI 10.1016/j.jclinepi.2007.02.006

Boter H, 2003, BRIT MED J, V327, P284, DOI 10.1136/bmj.327.7409.284

Buunk A. P., 1996, GEDRAG GEZONDHEID, V24, P304

Chen SM, 2012, INT J NURS STUD, V49, P637, DOI 10.1016/j.ijnurstu.2011.11.011

Coffey JT, 2002, DIABETES CARE, V25, P2238, DOI 10.2337/diacare.25.12.2238

COHEN J, 1992, PSYCHOL BULL, V112, P155, DOI 10.1037//0033-2909.112.1.155

de Raaij EJ, 2012, MANUAL THER, V17, P330, DOI 10.1016/j.math.2012.03.001

De Ridder DTD, 2005, PSYCHOL HEALTH, V20, P497, DOI 10.1080/14768320500098699

Fisher L, 2007, DIABETES CARE, V30, P542, DOI 10.2337/dc06-1614

Fisher L, 2013, DIABETES CARE, V36, P2551, DOI 10.2337/dc12-2493

Fonda Stephanie J, 2009, J Diabetes Sci Technol, V3, P117

Franklin K, 2004, ARCH INTERN MED, V164, P1457, DOI 10.1001/archinte.164.13.1457

French DP, 2006, J PSYCHOSOM RES, V61, P757, DOI 10.1016/j.jpsychores.2006.07.029

Gabbay RA, 2006, DIABETES RES CLIN PR, V71, P28, DOI 10.1016/j.diabres.2005.05.002

Groenwold RHH, 2012, AM J EPIDEMIOL, V175, P210, DOI 10.1093/aje/kwr302

Hagger MS, 2003, PSYCHOL HEALTH, V18, P141, DOI 10.1080/088704403100081321

Hasdai D, 2002, EUR HEART J, V23, P1190, DOI 10.1053/euhj.2002.3193

HOLM S, 1979, SCAND J STAT, V6, P65

Hunt Caralise W, 2012, Home Healthc Nurse, V30, P255, DOI 10.1097/NHH.0b013e31824c28d2

Huxley R, 2006, BMJ-BRIT MED J, V332, P73, DOI 10.1136/bmj.38678.389583.7C

Keogh KM, 2011, AM J MANAG CARE, V17, P105

Keogh KM, 2007, BMC FAM PRACT, V8, DOI 10.1186/1471-2296-8-36

Leventhal H, 2008, HDB PSYCHOL HLTH, VVI, P219

Malmberg K, 2000, CIRCULATION, V102, P1014, DOI 10.1161/01.CIR.102.9.1014

Marks R, 2005, HEALTH PROMOT PRACT, V6, P37, DOI 10.1177/1524839904266790

Marks R, 2005, HEALTH PROMOT PRACT, V6, P148, DOI 10.1177/1524839904266792

Mazzone T, 2008, LANCET, V371, P1800, DOI 10.1016/S0140-6736(08)60768-0

Miketic JK, 2011, J CARDIOVASC NURS, V26, P210, DOI 10.1097/JCN.0b013e31820017dc

Miller W. R., 2002, MOTIVATIONAL INTERVI

Moss-Morris R, 2007, CLIN J PAIN, V23, P558, DOI 10.1097/AJP.0b013e318093fcab

Nv Ven, 2003, DIABETES CARE, V26, P713

Peterson Pamela N, 2006, BMC Cardiovasc Disord, V6, P41, DOI 10.1186/1471-2261-6-41

Petrie KJ, 2007, CURR OPIN PSYCHIATR, V20, P163, DOI 10.1097/YCO.0b013e328014a871

Petrie KJ, 2002, PSYCHOSOM MED, V64, P580, DOI 10.1097/00006842-200207000-00007

POLONSKY WH, 1995, DIABETES CARE, V18, P754, DOI 10.2337/diacare.18.6.754

Preis SR, 2009, CIRCULATION, V120, P212, DOI 10.1161/CIRCULATIONAHA.108.846519

Rabin R, 2001, ANN MED, V33, P337, DOI 10.3109/07853890109002087

Rollnick S., 2008, MOTIVATIONAL INTERVI

Rubin RR, 1999, DIABETES-METAB RES, V15, P205, DOI 10.1002/(SICI)1520-7560(199905/06)15:3<205::AID-DMRR29>3.0.CO;2-O

Ryden L, 2007, EUR HEART J, V28, P88, DOI [10.1093/eurheartj/ehl260, 10.1093/eurheartj/ehm124]

Sarkar U, 2008, PATIENT EDUC COUNS, V70, P102, DOI 10.1016/j.pec.2007.09.008

Schuz B, 2012, PSYCHOL HEALTH, V27, P13, DOI 10.1080/08870446.2010.541908

Snoek FJ, 2000, DIABETES CARE, V23, P1305, DOI 10.2337/diacare.23.9.1305

Spinhoven P, 1997, PSYCHOL MED, V27, P363, DOI 10.1017/S0033291796004382

STRECHER VJ, 1986, HEALTH EDUC QUART, V13, P73, DOI 10.1177/109019818601300108

Toobert DJ, 2000, DIABETES CARE, V23, P943, DOI 10.2337/diacare.23.7.943

Trief P., 2003, FAMILIES SYSTEMS HLT, V21, P57, DOI [DOI 10.1037/H0089502, 10.1037/h0089502]

Uchmanowicz I, 2013, ACTA DIABETOL, V50, P217, DOI 10.1007/s00592-011-0280-2

van Jaarsveld CHM, 2001, J AM GERIATR SOC, V49, P1052, DOI 10.1046/j.1532-5415.2001.49208.x

Vandelanotte C., 2005, J PHYS ACT HEALTH, V2, P63, DOI [10.1123/jpah.2.1.63, DOI 10.1123/JPAH.2.1.63]

Verges B, 2004, DIABETIC MED, V21, P889, DOI 10.1111/j.1464-5491.2004.01262.x

Vickers AJ, 2001, BMJ-BRIT MED J, V323, P1123, DOI 10.1136/bmj.323.7321.1123

Watkins KW, 2000, DIABETES CARE, V23, P1511, DOI 10.2337/diacare.23.10.1511

Welch G, 2003, DIABETIC MED, V20, P69, DOI 10.1046/j.1464-5491.2003.00832.x

Welch G, 1994, HDB PSYCHOL DIABETES, P400

Wermeling PR, 2012, CARDIOVASC DIABETOL, V11, DOI 10.1186/1475-2840-11-121

Zulman DM, 2012, PATIENT EDUC COUNS, V87, P74, DOI 10.1016/j.pec.2011.07.013

NR 62

TC 4

Z9 4

U1 1

U2 16

PU BIOMED CENTRAL LTD

PI LONDON

PA 236 GRAYS INN RD, FLOOR 6, LONDON WC1X 8HL, ENGLAND

SN 1758-5996

J9 DIABETOL METAB SYNDR

JI Diabetol. Metab. Syndr.

PD JAN 18

PY 2014

VL 6

AR 5

DI 10.1186/1758-5996-6-5

PG 8

WC Endocrinology & Metabolism

SC Endocrinology & Metabolism

GA AB9FP

UT WOS:000332096400001

PM 24438342

OA DOAJ Gold, Green Published

DA 2019-01-08

ER

PT J

AU Dismuke, CE

Hernandez-Tejada, MA

Egede, LE

AF Dismuke, Clara E.

Hernandez-Tejada, Melba A.

Egede, Leonard E.

TI RELATIONSHIP OF SERIOUS PSYCHOLOGICAL DISTRESS TO QUALITY OF LIFE IN

ADULTS WITH DIABETES

SO INTERNATIONAL JOURNAL OF PSYCHIATRY IN MEDICINE

LA English

DT Article

DE serious psychological distress; quality of life; diabetes; SF-12

ID FACTOR SURVEILLANCE SYSTEM; MAJOR DEPRESSIVE DISORDER; US ADULTS;

UNITED-STATES; RISK-FACTORS; PREVALENCE; VALIDITY; SCALES

AB Objective: To examine the association between serious psychological distress (SPD) and the Physical and Mental Health components of Quality of Life (QOL) while controlling for depression in a national sample of adults with diabetes. Methods: SPD was assessed in 1,659 adults with diabetes who participated in the 2007 Medical Care Expenditure Survey (MEPS). SPD was measured by the 6-item Kessler scale. Depression was assessed with the PHQ-2 screen. Quality of life was measured with the physical (PCS) and mental (MCS) components of the SF-12. We used multiple linear regression to assess the relationship between SPD and quality of life while controlling for relevant covariates and depression screen results to assess the independent effect of SPD on QOL above and beyond the effect of depression. Results: Among US adults with diabetes, 9% had SPD and 15.4% screened positive for depression. Among those with SPD, 85.8% had depression and among those with depression, 50.5% had SPD. In the adjusted model for socio-demographic factors and comorbidities, SPD was significantly associated with lower PCS scores (-5.51 95% CI -7.55; -3.45) and MCS scores (-18.99 95% CI -20.81; -17.18). In the adjusted model that also controlled for depression, SPD was still significantly associated with lower PCS scores (-3.03 95% CI -5.63; -0.43) and MCS scores (-9.46 95% CI -11.67; -7.24). Conclusions: Among U.S. adults, SPD is associated with significantly diminished QOL above and beyond the effects of depression. Targeted interventions to mitigate the adverse effects of SPD are needed, independent of programs to address depression.

C1 [Dismuke, Clara E.; Egede, Leonard E.] Ralph H Johnson VA Med Ctr, Charleston, SC USA.

[Dismuke, Clara E.; Hernandez-Tejada, Melba A.; Egede, Leonard E.] Med Univ S Carolina, Charleston, SC 29425 USA.

RP Egede, LE (reprint author), Med Univ S Carolina, Ctr Hlth Dispar Res, 135 Rutledge Ave,Room 280G,POB 250593, Charleston, SC 29425 USA.

EM egedel@musc.edu

FU National Institute for Diabetes, Digestive and Kidney Diseases [K24

DK093699]

FX Dr. Egede is supported by grant #K24 DK093699 funded by the National

Institute for Diabetes, Digestive and Kidney Diseases. The manuscript

represents the views of the authors and not those of the VA or NIH. Dr.

Leonard E. Egede is the guarantor of this work and, as such, had full

access to all the data in the study and takes responsibility for the

integrity of the data and the accuracy of the data analysis.

CR [Anonymous], MED EXP PAN SURV 200

BRUCE ML, 1994, SOC PSYCH PSYCH EPID, V29, P165

Center for Disease Control and Prevention, 2011, NAT DIAB FACT SHEET

Croft JB, 2009, INT J PUBLIC HEALTH, V54, P4, DOI 10.1007/s00038-009-0017-y

Egede L., 2004, MED CLIN N AM, V90, P627

Egede LE, 2011, CURRENT PSYCHIAT REP, V14, P15

Egede LE, 2007, GEN HOSP PSYCHIAT, V29, P409, DOI 10.1016/j.genhosppsych.2007.06.002

Egede LE, 2010, GEN HOSP PSYCHIAT, V32, P563, DOI 10.1016/j.genhosppsych.2010.08.002

Egede LE, 2010, DIABETES RES CLIN PR, V87, P302, DOI 10.1016/j.diabres.2010.01.024

Elliott JO, 2009, SEIZURE-EUR J EPILEP, V18, P332, DOI 10.1016/j.seizure.2008.11.003

Gonzalez JS, 2011, DIABETES CARE, V34, P236, DOI 10.2337/dc10-1970

Kessler RC, 2002, PSYCHOL MED, V32, P959, DOI 10.1017/S0033291702006074

Kroenke K, 2003, MED CARE, V41, P1284, DOI 10.1097/01.MLR.0000093487.78664.3C

Li C, 2007, DIABETES CARE, V33, P1061, DOI DOI 10.2337/DC09-1515]

Li CY, 2009, INT J PUBLIC HEALTH, V54, P43, DOI 10.1007/s00038-009-0006-1

Lincoln KD, 2011, RES SOCIAL WORK PRAC, V21, P278, DOI 10.1177/1049731510386122

McGuire LC, 2009, INT J PUBLIC HEALTH, V54, P84, DOI 10.1007/s00038-009-0011-4

McKnight-Eily LR, 2009, INT J PUBLIC HEALTH, V54, P111, DOI 10.1007/s00038-009-0015-0

Okoro CA, 2009, INT J PUBLIC HEALTH, V54, P52, DOI 10.1007/s00038-009-0077-z

Oraka E, 2010, CHEST, V137, P609, DOI 10.1378/chest.09-1777

Pearson WS, 2009, INT J PUBLIC HEALTH, V54, P23, DOI 10.1007/s00038-009-0003-4

Penninx BWJH, 1998, J NATL CANCER I, V90, P1888, DOI 10.1093/jnci/90.24.1888

Pratt Laura A, 2007, Adv Data, P1

Rubin R, 2000, DIABETES SPECTR, V13, P21

Shih M, 2008, QUAL LIFE RES, V17, P521, DOI 10.1007/s11136-008-9330-9

Shih M, 2006, J GEN INTERN MED, V21, P1160, DOI 10.1111/j.1525-1497.2006.00573.x

Shin JK, 2012, DIABETES RES CLIN PR, V95, P406, DOI 10.1016/j.diabres.2011.10.043

Strine TW, 2009, INT J PUBLIC HEALTH, V54, P9, DOI 10.1007/s00038-009-0001-6

Ware JE, 1996, MED CARE, V34, P220, DOI 10.1097/00005650-199603000-00003

NR 29

TC 5

Z9 5

U1 0

U2 6

PU BAYWOOD PUBL CO INC

PI AMITYVILLE

PA 26 AUSTIN AVE, PO BOX 337, AMITYVILLE, NY 11701 USA

SN 0091-2174

EI 1541-3527

J9 INT J PSYCHIAT MED

JI Int. J. Psychiatr. Med.

PY 2014

VL 48

IS 2

BP 135

EP 146

DI 10.2190/PM.48.2.f

PG 12

WC Psychiatry

SC Psychiatry

GA AU5XU

UT WOS:000345677700006

PM 25377154

DA 2019-01-08

ER

PT J

AU Kofahl, C

Dogan, M

Dogan, G

Mnich, E

von dem Knesebeck, O

AF Kofahl, Christopher

Dogan, Mustafa

Dogan, Gulsun

Mnich, Eva

von dem Knesebeck, Olaf

TI Quality of life of Turkish type 2 diabetics in Germany and Turkey - a

comparison between Hamburg and Istanbul

SO ETHNICITY & HEALTH

LA English

DT Article

DE quality of life; WHOQOL; migrants; diabetes; Turkey; Germany; SES;

education; environment; IADL; functional limitations; social support;

cross-sectional comparative study

ID PSYCHOMETRIC PROPERTIES; PSYCHOLOGICAL DISTRESS; CARE SERVICES; HEALTH;

IMMIGRANTS; MIGRANTS; MELLITUS; SWEDEN; WHOQOL; DETERMINANTS

AB Objectives. The analyses address the following research questions: (1) Do Turkish diabetics in Germany and Turkey differ in terms of quality of life? (2) If yes, can these differences (in part) be explained by social factors (age, gender, education, household size), functional limitations and availability of support? (3) Are social factors, functional limitations and availability of support differently associated with quality of life among Turkish diabetics in Germany and Turkey?

Design. For this comparative cross-sectional study, 111 patients with type 2 diabetes were personally interviewed in Istanbul (Turkey) and 294 Turkish patients in Hamburg (Germany). For quality of life measurement we have used the Turkish version of the WHOQOL-Bref-26. Sociodemographics included age, sex, education and householdsize. Health related functional limitations were assessed on the basis of an index of (instrumental) activities of daily living including the availability of help. Statistical analyses were conducted on group comparisons with Chi-square-and T-tests as well as linear regressions.

Results. There are no significant differences between Turkish diabetics in Germany and Turkey in the physical and the psychological dimensions of the WHOQOL-Bref. However, in the WHOQOL-domains 'social QoL' and ` environmental QoL' Turkish diabetics living in Hamburg have a significantly better quality of life than their counterparts in Istanbul. These differences cannot be explained by individual sociodemographic factors, functional limitations and availability of support. Furthermore, we found much stronger positive associations between education and quality of life in Istanbul than in Hamburg.

Conclusion. Beyond strong similarities between the two samples in sociodemographics, physical and mental health the social and environmental quality of life was significantly assessed better by the Turkish diabetics living in Hamburg. This is most likely an effect of public investment in social security, infrastructure and health care which is also influencing the decision as to where to spend life in retirement.

C1 [Kofahl, Christopher; Mnich, Eva; von dem Knesebeck, Olaf] Univ Med Ctr Hamburg Eppendorf, Dept Med Sociol, Hamburg, Germany.

[Dogan, Mustafa; Dogan, Gulsun] Afyon Kocatepe Univ, Tip Fak, Guz Hastaliklari Anabilim Dali, Afyon, Turkey.

RP Kofahl, C (reprint author), Univ Med Ctr Hamburg Eppendorf, Dept Med Sociol, Hamburg, Germany.

EM kofahl@uke.de

FU German Federal Ministry of Education and Research [01GX0749]; project

'Health literacy of Turkish immigrants with diabetes 2 related to course

of disease, health care, socioeconomic status and integration'

[02/2008-09/2011]

FX The study in Hamburg was undertaken as part of the project 'Health

literacy of Turkish immigrants with diabetes 2 related to course of

disease, health care, socioeconomic status and integration'

(02/2008-09/2011). The project was funded by the German Federal Ministry

of Education and Research [01GX0749]. We also like to thank the two

anonymous reviewers for their engaged comments and helpful advice in the

progress of this paper.

CR Akbiyik DI, 2008, EUR PSYCHIAT, V23, pS4, DOI 10.1016/S0924-9338(08)70055-5

BARON RM, 1986, J PERS SOC PSYCHOL, V51, P1173, DOI 10.1037//0022-3514.51.6.1173

Baykara-Krumme H, 2006, ALTWERDEN DEUTSCHLAN, P447

Bayram N, 2007, QUAL LIFE RES, V16, P1319, DOI 10.1007/s11136-007-9249-6

Bien B, 2013, EUR J PUBLIC HEALTH, V23, P1032, DOI 10.1093/eurpub/cks190

Bradley DE, 2010, J AGING HEALTH, V22, P882, DOI 10.1177/0898264310368430

Chu Nancy, 2010, J Cult Divers, V17, P105

DESTATIS, 2012, BEV ERW BEV MIGR ERG, V2.2

DESTATIS, 2013, DESTATIS FACHSERIE

Di Rosa M, 2011, GEROPSYCH, V24, P5, DOI 10.1024/1662-9647/a000031

Dundar P., 2002, HIPPOKRATIA, V6, P37

Eisenman DP, 2003, JAMA-J AM MED ASSOC, V290, P627, DOI 10.1001/jama.290.5.627

Eser E., 1999, PSIKIYATRI PSIKOLOJI, V7, P23

Fassaert T, 2011, INT J SOC PSYCHIATR, V57, P132, DOI 10.1177/0020764009103647

Harper A, 1998, PSYCHOL MED, V28, P551

Hohne A., 2008, ETABLIERUNG WEITEREN, V55, P103

Kocken PL, 2008, PATIENT EDUC COUNS, V70, P25, DOI 10.1016/j.pec.2007.09.016

Kofahl C, 2013, GESUNDHEITSWESEN, V75, P803, DOI 10.1055/s-0033-1334936

Koochek A, 2007, HEALTH QUAL LIFE OUT, V5, DOI 10.1186/1477-7525-5-60

Laube H, 2001, DIABETES STOFFWECH H, V10, P51

Gonzalez-Castro JL, 2011, INT J SOC PSYCHIATR, V57, P30, DOI 10.1177/0020764010347336

Mladovsky P, 2012, HEALTH POLICY, V105, P1, DOI 10.1016/j.healthpol.2012.01.007

Nielsen SS, 2009, BMC HEALTH SERV RES, V9, DOI 10.1186/1472-6963-9-210

Norredam M, 2010, EUR J PUBLIC HEALTH, V20, P555, DOI 10.1093/eurpub/ckp195

Ozcan V., 2006, LEBENSSITUATION GESU, P7

Parmakerli-Czemmel B, 2007, DIABETOL STOFFWECHS, V2, P46, DOI 10.1055/s-2007-960524

Power M, 1998, SOC SCI MED, V46, P1569

Satman I, 2002, DIABETES CARE, V25, P1551, DOI 10.2337/diacare.25.9.1551

Sin MK, 2011, J GERONTOL NURS, V37, P43, DOI 10.3928/00989134-20100930-02

Skevington SM, 2004, QUAL LIFE RES, V13, P299, DOI 10.1023/B:QURE.0000018486.91360.00

Sujoldzic A, 2006, COLLEGIUM ANTROPOL, V30, P703

Taloyan M, 2008, NORD J PSYCHIAT, V62, P392, DOI 10.1080/08039480801984263

Tinghog P, 2010, INT J SOC PSYCHIATR, V56, P74, DOI 10.1177/0020764008096163

Wandell PE, 2003, DIABETES METAB, V29, P435, DOI 10.1016/S1262-3636(07)70057-5

WHO, 1948, OFF REC WHO

NR 35

TC 4

Z9 4

U1 1

U2 13

PU ROUTLEDGE JOURNALS, TAYLOR & FRANCIS LTD

PI ABINGDON

PA 2-4 PARK SQUARE, MILTON PARK, ABINGDON OX14 4RN, OXON, ENGLAND

SN 1355-7858

EI 1465-3419

J9 ETHNIC HEALTH

JI Ethn. Health

PY 2014

VL 19

IS 6

BP 617

EP 630

DI 10.1080/13557858.2014.885932

PG 14

WC Ethnic Studies; Public, Environmental & Occupational Health

SC Ethnic Studies; Public, Environmental & Occupational Health

GA AP7VH

UT WOS:000342285000003

PM 24517236

DA 2019-01-08

ER

PT J

AU Brewer, C

de Jong, C

Williams, J

AF Brewer, Colin

de Jong, Catherine

Williams, Jonathan

TI Rapid opiate detoxification and antagonist induction under general

anaesthesia or intravenous sedation is humane, sometimes essential and

should always be an option. Three illustrative case reports involving

diabetes and epilepsy and a review of the literature

SO JOURNAL OF PSYCHOPHARMACOLOGY

LA English

DT Review

DE Naltrexone; heroin; opiate; detoxification; antagonist; sedation;

anaesthesia; diabetes; epilepsy; withdrawal

ID RANDOMIZED CONTROLLED-TRIAL; NALTREXONE-ACCELERATED DETOXIFICATION;

ULTRARAPID OPIOID DETOXIFICATION; ABSTINENCE RATES; HEROIN-ADDICTS;

PRECIPITATED WITHDRAWAL; MAINTENANCE TREATMENT; DEPENDENCE; METHADONE;

MORTALITY

AB When abstinence is an appropriate goal, controlled studies and systematic reviews confirm that rapid, antagonist-precipitated opiate withdrawal procedures are the most effective and cost effective methods of initiating abstinence, and naltrexone (NTX) maintenance. While 'rapid' withdrawal, better conceptualised as Rapid Antagonist Induction (RAI), can often be humanely achieved with modest sedation levels, we present three case histories to support our argument that for some patients, general anaesthesia (GA), or techniques of intravenous sedation (IVS) that approach GA, are essential for safety and success. This includes patients with intercurrent disease (e.g. epilepsy or insulin-dependent diabetes) but also those with severe withdrawal phobia after previous distressing experiences. We discuss the history of the procedure. The dangers of RAI under GA or IVS in experienced hands have been exaggerated and the appropriate expertise should be more easily available. Patients and clinicians readily accept risks of major surgery for the excessive intake of food that causes most obesity. Similar risk-acceptance exists in cosmetic surgery and obstetrics. The increasing use and effectiveness of long-acting implants or depot-injections of NTX for relapse-prevention have largely solved compliance problems that undermined the potential of oral NTX. Their ability to prevent opiate overdose in abstinent, non-tolerant patients also strengthens arguments both for offering RAI as a therapeutic option and for reducing psychological, professional and practical barriers to using it.

C1 [Brewer, Colin] Stapleford Ctr, London SW1W 9NP, England.

[Williams, Jonathan] Brighton & Sussex Univ Hosp, Brighton, E Sussex, England.

RP Brewer, C (reprint author), Stapleford Ctr, 25a Eccleston St, London SW1W 9NP, England.

EM brewerismo@gmail.com

CR Ali R, 2003, DRUG ALCOHOL REV, V22, P425, DOI 10.1080/09595230310001613949

Arbous MS, 2005, ANESTHESIOLOGY, V102, P257, DOI 10.1097/00000542-200502000-00005

Armstrong J, 2003, ACAD EMERG MED, V10, P860, DOI 10.1197/aemj.10.8.860

Arnold-Reed Diane E, 2005, J Opioid Manag, V1, P17

Bainbridge D, 2012, LANCET, V380, P1075, DOI 10.1016/S0140-6736(12)60990-8

Ballantyne GH, 2008, OBES SURG, V18, P660, DOI 10.1007/s11695-007-9357-y

Beaini AY, 2000, ADDICT BIOL, V5, P451, DOI 10.1111/j.1369-1600.2000.tb00215.x

Bell JR, 1999, MED J AUSTRALIA, V171, P26

BLACHLY P, 1975, DEV FIELD DRUG ABUSE, P327

BREWER C, 1988, BRIT J PSYCHIAT, V153, P340, DOI 10.1192/bjp.153.3.340

Brewer C, 1997, ADDICT BIOL, V2, P291, DOI 10.1080/13556219772589

Brewer C, 1998, LANCET, V351, P218, DOI 10.1016/S0140-6736(05)78171-X

Brewer C, 2000, CNS DRUGS, V14, P329, DOI 10.2165/00023210-200014050-00001

Brewer C, 1999, REV ESP DROGODEPENDE, V24, P426

Brewer C, 2009, CONT NEUROSCIENCE SE

Brewer C, 2003, ADICCIONES, V4, P299

Brewer C, 1989, WELLINGTON HOSP JUL, P4

Carreno J. E., 1997, ACT 24 JORN NAC SOC, P607

Carreno JE, 2002, ADDICT BIOL, V7, P243, DOI 10.1080/135562102200120479

Carreno JE, 2003, ADDICT BIOL, V8, P429, DOI 10.1080/13556210310001646402

Chan K Y, 1996, J Clin Forensic Med, V3, P87, DOI 10.1016/S1353-1131(96)90012-7

CHARNEY DS, 1982, ARCH GEN PSYCHIAT, V39, P1327

Collins ED, 2005, JAMA-J AM MED ASSOC, V294, P903, DOI 10.1001/jama.294.8.903

Cornish JW, 1997, J SUBST ABUSE TREAT, V14, P529, DOI 10.1016/S0740-5472(97)00020-2

Currie J, 2000, RAPID INDUCTION ONTO

Day E, 2011, J SUBST ABUSE TREAT, V40, P56, DOI 10.1016/j.jsat.2010.08.007

De Jong CAJ, 2005, ADDICTION, V100, P206, DOI 10.1111/j.1360-0443.2004.00959.x

de Jong CJ, 2006, JAMA-J AM MED ASSOC, V295, P887, DOI 10.1001/jama.295.8.887-a

De Jong CJ, 2005, ADDICTION, V100, P1742, DOI 10.1111/j.1360-0443.2005.01267.x

De Jong CAJ, 2007, AM J ADDICTION, V16, P124, DOI 10.1080/10550490601184472

Digiusto E, 2004, ADDICTION, V99, P450, DOI 10.1111/j.1360-0443.2004.00654.x

Eklund C, 1996, WITHDRAWAL METHADONE, P9

Escalante-Tattersfield T, 2008, SURG OBES RELAT DIS, V4, P126, DOI 10.1016/j.soard.2007.12.015

FISHBAIN DA, 1988, J CLIN PSYCHOPHARM, V8, P441, DOI 10.1097/00004714-198812000-00018

Floch-Tudal C, 2000, ANN MED INTERNE, V151, pB30

Foster J, 2003, ADDICT BIOL, V8, P211, DOI 10.1080/1355621031000117446

Foster J, 1999, ADDICT BIOL, V4, P232

GERRA G, 1995, J SUBST ABUSE TREAT, V12, P35, DOI 10.1016/0740-5472(94)00077-8

GOLZ J, 2000, SUCHTTHERAPIE, V1, P166

GOSSOP M, 1991, BRIT J PSYCHIAT, V158, P697, DOI 10.1192/bjp.158.5.697

Gossop M, 2000, BRIT J PSYCHIAT, V177, P262, DOI 10.1192/bjp.177.3.262

Gowing L, 2010, COCHRANE DB SYST REV, DOI 10.1002/14651858.CD002022.pub3

Greenberg M, 2000, J ADDICT DIS, V19, P53, DOI 10.1300/J069v19n04_05

Hamilton RJ, 2002, ACAD EMERG MED, V9, P63, DOI 10.1111/j.1553-2712.2002.tb01169.x

Hankins GDV, 2006, SEMIN PERINATOL, V30, P276, DOI 10.1053/j.semperi.2006.07.009

Home Office Drug Strategy, 2010, RED DEM RESTR SUPPL

Horspool MJ, 2008, EUR ADDICT RES, V14, P179, DOI 10.1159/000141641

Hulse GK, 2009, ARCH GEN PSYCHIAT, V66, P1108, DOI 10.1001/archgenpsychiatry.2009.130

Hulse GK, 2005, DRUG ALCOHOL DEPEN, V79, P351, DOI 10.1016/j.drugalcdep.2005.02.009

Hulse GK, 2004, ADDICT BIOL, V9, P59, DOI 10.1080/13556210410001674103

Hulse GK, 2003, ADDICT BIOL, V8, P337, DOI 10.1080/13556210310001602257

Jorgensen CH, 2011, ALCOHOL CLIN EXP RES, V35, P1749, DOI 10.1111/j.1530-0277.2011.01523.x

KOSTEN TA, 1989, AM J DRUG ALCOHOL AB, V15, P237, DOI 10.3109/00952998908993405

Krabbe PFM, 2003, ADDICT BIOL, V8, P351, DOI 10.1080/13556210310001602275

Krupitsky EM, 2004, J SUBST ABUSE TREAT, V26, P285, DOI 10.1016/j.jsat.2004.02.002

Krupitsky E, 2012, ARCH GEN PSYCHIAT, V69, P973, DOI 10.1001/archgenpsychiatry.2012.1a

Kunoe N, 2009, BRIT J PSYCHIAT, V194, P541, DOI 10.1192/bjp.bp.108.055319

Laheij RJF, 2000, JAMA-J AM MED ASSOC, V283, P1143, DOI 10.1001/jama.283.9.1143

LEGARDA JJ, 1994, DRUG ALCOHOL DEPEN, V35, P91, DOI 10.1016/0376-8716(94)90115-5

Li G, 2009, ANESTHESIOLOGY, V110, P759, DOI 10.1097/ALN.0b013e31819b5bdc

LOIMER N, 1989, J PSYCHIAT RES, V23, P81, DOI 10.1016/0022-3956(89)90020-4

Madlung-Kratzer E, 2009, ADDICTION, V104, P1549, DOI 10.1111/j.1360-0443.2009.02653.x

McGregor C, 2002, DRUG ALCOHOL DEPEN, V68, P5, DOI 10.1016/S0376-8716(02)00077-7

McKeganey N, 2004, DRUG-EDUC PREV POLIC, V11, P423, DOI 10.1080/09687630410001723229

MILBY JB, 1986, AM J PSYCHIAT, V143, P739

MUNOZ E, 1988, PLAST RECONSTR SURG, V82, P116, DOI 10.1097/00006534-198882010-00021

National Institute for Clinical Excellence. (NICE), 2008, DRUG MIS OP DET NICE, P122

Oliver P, 2005, ADDICTION, V100, P560, DOI 10.1111/j.1360-0443.2005.01020.x

Pfab R, 1999, J TOXICOL-CLIN TOXIC, V37, P43, DOI 10.1081/CLT-100102407

RESNICK RB, 1977, CLIN PHARMACOL THER, V21, P409

SENFT RA, 1991, J SUBST ABUSE TREAT, V8, P257, DOI 10.1016/0740-5472(91)90048-F

Seoane A, 1997, BRIT J PSYCHIAT, V171, P340, DOI 10.1192/bjp.171.4.340

Shanahan MD, 2005, ADDICT BEHAV, V20

Sigmon SC, 2012, AM J DRUG ALCOHOL AB, V38, P187, DOI 10.3109/00952990.2011.653426

Stella L, 2005, LIFE SCI, V77, P2717, DOI 10.1016/j.lfs.2005.05.036

Strang J, 2005, BRIT J GEN PRACT, V55, P444

Strang J, 2003, BRIT MED J, V326, P959, DOI 10.1136/bmj.326.7396.959

Tait RJ, 2008, J SUBST ABUSE TREAT, V35, P116, DOI 10.1016/j.jsat.2007.08.014

Waal Helge, 2003, Tidsskr Nor Laegeforen, V123, P1660

Weiss RD, 2011, ARCH GEN PSYCHIAT, V68, P1238, DOI 10.1001/archgenpsychiatry.2011.121

NR 80

TC 0

Z9 0

U1 1

U2 2

PU SAGE PUBLICATIONS LTD

PI LONDON

PA 1 OLIVERS YARD, 55 CITY ROAD, LONDON EC1Y 1SP, ENGLAND

SN 0269-8811

EI 1461-7285

J9 J PSYCHOPHARMACOL

JI J. Psychopharmacol.

PD JAN

PY 2014

VL 28

IS 1

BP 67

EP 75

DI 10.1177/0269881113504835

PG 9

WC Clinical Neurology; Neurosciences; Pharmacology & Pharmacy; Psychiatry

SC Neurosciences & Neurology; Pharmacology & Pharmacy; Psychiatry

GA AJ8RJ

UT WOS:000337974400009

PM 24043724

DA 2019-01-08

ER

PT J

AU Wycherley, TP

Clifton, PM

Noakes, M

Brinkworth, GD

AF Wycherley, Thomas Philip

Clifton, Peter Marshall

Noakes, Manny

Brinkworth, Grant David

TI Weight loss on a structured hypocaloric diet with or without exercise

improves emotional distress and quality of life in overweight and obese

patients with type 2 diabetes

SO JOURNAL OF DIABETES INVESTIGATION

LA English

DT Article

DE Lifestyle intervention; Well-being; Caloric restriction

ID HIGH-PROTEIN DIET; LOW-CARBOHYDRATE; BODY-COMPOSITION; MELLITUS; IMPACT

AB Aims/IntroductionTo evaluate the effects of a caloric restricted weight loss program with or without supervised resistance exercise training (EX) on diabetes-related emotional distress and quality of life (QOL) in overweight and obese patients with type2 diabetes.

Materials and MethodsIn a parallel design, 106 men and women with type2 diabetes were randomized to a prescriptive 16-week caloric restricted diet (D; 6,000-7,000kJ/day), with (n=65) or without (n=41) EX (three times per week). Bodyweight, glycated hemoglobin, diabetes-specific emotional distress (Problem Areas in Diabetes [PAID] questionnaire) and QOL (Diabetes-39 [D-39] questionnaire) was assessed pre- and post-intervention.

ResultsA total of 84 participants completed the study (D n=33, D+EX n=51). Weight loss was significantly greater in D+EX compared with D (-11.45.8 vs -8.8 +/- 5.8kg, P=0.04 timexdiet). Overall, there were significant improvements in glycated hemoglobin, PAID total score and the D-39 dimensions of diabetes control', anxiety and worry', sexual functioning', energy and mobility', overall rating of QOL' and severity of diabetes' (P0.01 for time). The D-39 dimension, social burden', did not change (P=0.07 for time). There was no difference between groups in the response for any of these variables (P0.10).

ConclusionA structured caloric restricted diet with or without EX improves emotional distress and QOL in overweight and obese patients with type 2 diabetes. This trial was registered with Australian New Zealand Clinical Trials Registry (; ACTR No: ACTRN12608000206325).

C1 [Wycherley, Thomas Philip] Univ S Australia, Div Hlth Sci, Sansom Inst Hlth Res, Adelaide, SA 5001, Australia.

[Clifton, Peter Marshall] Baker IDI Heart & Diabet Inst, Adelaide, SA, Australia.

[Noakes, Manny; Brinkworth, Grant David] Commonwealth Sci & Ind Res Org, Anim Food & Hlth Sci, Preventat Hlth Flagship, Adelaide, SA, Australia.

RP Brinkworth, GD (reprint author), Commonwealth Sci & Ind Res Org, Anim Food & Hlth Sci, Preventat Hlth Flagship, Adelaide, SA, Australia.

EM grant.brinkworth@csiro.au

RI Biguzzi, Felipe/E-4724-2015; noakes, manny/H-2813-2013; Clifton,

Peter/F-4446-2013

OI Clifton, Peter/0000-0002-6411-626X; Brinkworth,

Grant/0000-0001-9017-8395

FU Heart Foundation; Diabetes Australia Research Trust

FX This work was supported with project grants from the Heart Foundation

and the Diabetes Australia Research Trust. George Weston Foods donated

food for this study. None of the funding agencies played a role in the

conception, design or conduct of the study, collection, management,

analysis and interpretation of the data; or preparation, review and

approval of the manuscript.

CR Boyer JG, 1997, MED CARE, V35, P440, DOI 10.1097/00005650-199705000-00003

Bradley C, 2002, DIABETES METAB RES, V18, pS64, DOI 10.1002/dmrr.279

Colberg Sheri R, 2010, Diabetes Care, V33, pe147, DOI 10.2337/dc10-9990

Davis NJ, 2012, DIABETES EDUCATOR, V38, P250, DOI 10.1177/0145721711436132

Fine JT, 1999, JAMA-J AM MED ASSOC, V282, P2136, DOI 10.1001/jama.282.22.2136

Fontaine KR, 1999, QUAL LIFE RES, V8, P275, DOI 10.1023/A:1008835602894

Garratt AM, 2002, DIABETIC MED, V19, P1, DOI 10.1046/j.1464-5491.2002.00650.x

Gillison FB, 2009, SOC SCI MED, V68, P1700, DOI 10.1016/j.socscimed.2009.02.028

Halyburton AK, 2007, AM J CLIN NUTR, V86, P580

Huang IC, 2008, VALUE HEALTH, V11, P450, DOI 10.1111/j.1524-4733.2007.00261.x

KAPLAN RM, 1987, J GEN INTERN MED, V2, P220, DOI 10.1007/BF02596443

Kaukua JK, 2004, INT J OBESITY, V28, P600, DOI 10.1038/sj.ijo.0802591

POLONSKY WH, 1995, DIABETES CARE, V18, P754, DOI 10.2337/diacare.18.6.754

Rubin RR, 1999, DIABETES-METAB RES, V15, P205, DOI 10.1002/(SICI)1520-7560(199905/06)15:3<205::AID-DMRR29>3.0.CO;2-O

Scully D, 1998, BRIT J SPORT MED, V32, P111, DOI 10.1136/bjsm.32.2.111

Thomson RL, 2010, FERTIL STERIL, V94, P1812, DOI 10.1016/j.fertnstert.2009.11.001

Wadden TA, 1996, AM J CLIN NUTR, V63, P461, DOI 10.1093/ajcn/63.3.461

Watkins K, 2004, PHARMACOECONOMICS, V22, P1109, DOI 10.2165/00019053-200422170-00002

Welch G, 2003, DIABETIC MED, V20, P69, DOI 10.1046/j.1464-5491.2003.00832.x

Williamson DA, 2009, ARCH INTERN MED, V169, P163, DOI 10.1001/archinternmed.2008.544

Wycherley TP, 2010, DIABETES OBES METAB, V12, P1097, DOI 10.1111/j.1463-1326.2010.01307.x

Wycherley TP, 2010, DIABETES CARE, V33, P969, DOI 10.2337/dc09-1974

NR 22

TC 11

Z9 12

U1 1

U2 16

PU WILEY

PI HOBOKEN

PA 111 RIVER ST, HOBOKEN 07030-5774, NJ USA

SN 2040-1124

J9 J DIABETES INVEST

JI J. Diabetes Investig.

PD JAN

PY 2014

VL 5

IS 1

BP 94

EP 98

DI 10.1111/jdi.12120

PG 5

WC Endocrinology & Metabolism

SC Endocrinology & Metabolism

GA AA6HC

UT WOS:000331198400017

PM 24843744

OA DOAJ Gold, Green Published

DA 2019-01-08

ER

PT J

AU Miller, JL

Linville, TD

Dykens, EM

AF Miller, Jennifer L.

Linville, Tiffany D.

Dykens, Elisabeth M.

TI Effects of metformin in children and adolescents with Prader-Willi

syndrome and early-onset morbid obesity: a pilot study

SO JOURNAL OF PEDIATRIC ENDOCRINOLOGY & METABOLISM

LA English

DT Article

DE metformin; obesity; Prader-Willi syndrome

ID BODY-MASS INDEX; INSULIN SENSITIVITY; METABOLIC SYNDROME; SYNDROME PWS;

DIABETES-MELLITUS; BRAIN; RESISTANCE; HYPERPHAGIA; DYSFUNCTION;

PREVENTION

AB Prader-Willi syndrome (PWS) is one of the most commonly recognized causes of early-onset childhood obesity. Individuals with PWS have significant hyperphagia and decreased recognition of satiety. The exact etiology of the hyperphagia remains unknown and, therefore, untreatable. We conducted a pilot, open-label study of response to metformin in 21 children with PWS and six with early morbid obesity (EMO). Participants had significant insulin resistance and glucose intolerance on oral glucose tolerance testing (OGTT) and were started on metformin for these biochemical findings. We administered the Hyperphagia Questionnaire to parents of patients before and after starting metformin treatment. Both the PWS and EMO groups showed significant improvements in food-related distress, anxiety, and ability to be redirected away from food on the Hyperphagia Questionnaire. In the PWS group, improvements were predominantly seen in females. Within the PWS group, responders to metformin had higher 2-h glucose levels on OGTT (7.48 mmol/L vs. 4.235 mmol/L; p=0.003) and higher fasting insulin levels (116 pmol/L vs. 53.5 pmol/L; p=0.04). Additionally, parents of 5/13 individuals with PWS and 5/6 with EMO reported that their child was able to feel full while on metformin (for many this was the first time they had ever described a feeling of fullness). Metformin may improve sense of satiety and decrease anxiety about food in some individuals with PWS and EMO. Positive response to metformin may depend on the degree of hyperinsulinism and glucose intolerance. Nonetheless, the results of this pilot study bear further investigation.

C1 [Miller, Jennifer L.] Univ Florida, Dept Pediat, Div Endocrinol, Gainesville, FL 32610 USA.

[Linville, Tiffany D.] Univ Florida, Dept Pediat, Gainesville, FL USA.

[Dykens, Elisabeth M.] Vanderbilt Univ, Dept Psychol, Nashville, TN 37240 USA.

RP Miller, JL (reprint author), Univ Florida, Dept Pediat, Div Endocrinol, Gainesville, FL 32610 USA.

EM millejl@peds.ufl.edu

FU NIDDK NIH HHS [K23 DK081203]

CR Adam CL, 2010, INT J OBESITY, V34, P980, DOI 10.1038/ijo.2010.28

Adler-Wailes DC, 2013, J CLIN ENDOCR METAB, V98, P1676, DOI 10.1210/jc.2012-3817

Aubert G, 2011, METABOLISM, V60, P327, DOI 10.1016/j.metabol.2010.02.007

Banks WA, 2012, ENDOCRINOLOGY, V153, P4111, DOI 10.1210/en.2012-1435

Bizzarri C, 2010, EUR J ENDOCRINOL, V162, P499, DOI 10.1530/EJE-09-1033

Butler MG, 2008, AM J MED GENET A, V146A, P854, DOI 10.1002/ajmg.a.32249

Cassidy SB, 2009, EUR J HUM GENET, V17, P3, DOI 10.1038/ejhg.2008.165

Chan NN, 1998, J ROY SOC MED, V91, P598, DOI 10.1177/014107689809101116

Davies W, 2008, FRONT NEUROENDOCRIN, V29, P413, DOI 10.1016/j.yfrne.2007.12.001

Debette S, 2010, ANN NEUROL, V68, P136, DOI 10.1002/ana.22062

Dykens EM, 2007, OBESITY, V15, P1816, DOI 10.1038/oby.2007.216

Freemark M, 2001, PEDIATRICS, V107, DOI 10.1542/peds.107.4.e55

Garfield AS, 2012, ENDOCRINOLOGY, V153, P4600, DOI 10.1210/en.2012-1282

Goldstone AP, 2006, PROG BRAIN RES, V153, P57, DOI 10.1016/S0079-6123(06)53033-1

Haqq AM, 2008, CLIN ENDOCRINOL, V69, P911, DOI 10.1111/j.1365-2265.2008.03385.x

Haqq AM, 2007, CLIN ENDOCRINOL, V67, P944, DOI 10.1111/j.1365-2265.2007.02991.x

Haqq AM, 2011, J CLIN ENDOCR METAB, V96, pE225, DOI 10.1210/jc.2010-1733

Hirsch HJ, 2009, J CLIN ENDOCR METAB, V94, P2262, DOI 10.1210/jc.2008-2760

Holsen LM, 2012, INT J OBESITY, V36, P638, DOI 10.1038/ijo.2011.204

Holsen LM, 2009, INT J OBESITY, V33, P273, DOI 10.1038/ijo.2008.255

Holsen LM, 2006, OBESITY, V14, P1028, DOI 10.1038/oby.2006.118

Karmi A, 2010, DIABETES, V59, P2171, DOI 10.2337/db09-0138

Kennedy L, 2006, INT J OBESITY, V30, P382, DOI 10.1038/sj.ijo.0803115

Kim F, 2008, ARTERIOSCL THROM VAS, V28, P1982, DOI 10.1161/ATVBAHA.108.169722

Li JJ, 2012, PHARMACOL BIOCHEM BE, V101, P564, DOI 10.1016/j.pbb.2012.03.002

Luchsinger JA, 2010, J ALZHEIMERS DIS, V20, P723, DOI 10.3233/JAD-2010-091687

Lv WS, 2012, BRAIN RES, V1444, P11, DOI 10.1016/j.brainres.2012.01.028

McNeilly AD, 2011, BEHAV BRAIN RES, V217, P134, DOI 10.1016/j.bbr.2010.10.017

Miller J, 2006, J PEDIATR-US, V149, P192, DOI 10.1016/j.jpeds.2006.04.013

Miller JL, 2007, J NEUROL NEUROSUR PS, V78, P615, DOI 10.1136/jnnp.2006.099044

Newall H, 2012, INT CLIN PSYCHOPHARM, V27, P69, DOI 10.1097/YIC.0b013e32834d0a5b

PIMENTEL GD, 2012, METABOLISM, V62, P171

Proto C, 2007, METABOLISM, V56, P1076, DOI 10.1016/j.metabol.2007.03.016

Ring LE, 2010, J CLIN INVEST, V120, P2931, DOI 10.1172/JCI41985

Roth CL, 2010, J PEDIATR ENDOCR MET, V23, P1299, DOI 10.1515/jpem.2010.204

Schmidt F, 2012, EXP CLIN ENDOCR DIAB, V120, P579, DOI 10.1055/s-0032-1306330

Schrander-Stumpel CTRM, 2004, AM J MED GENET A, V124A, P333, DOI 10.1002/ajmg.a.20371

Segura B, 2010, BMC NEUROL, V10, DOI 10.1186/1471-2377-10-64

Stark R, 2013, MOL CELL ENDOCRINOL, V366, P215, DOI 10.1016/j.mce.2012.06.012

Thompson JA, 2011, SEMIN REPROD MED, V29, P211, DOI 10.1055/s-0031-1275522

Viollet B, 2011, HANDB EXP PHARMACOL, V203, P303, DOI 10.1007/978-3-642-17214-4_13

Yau PL, 2010, DIABETOLOGIA, V53, P2298, DOI 10.1007/s00125-010-1857-y

Yau PL, 2012, PEDIATRICS, V130, pE856, DOI 10.1542/peds.2012-0324

Zhang YY, 2012, MOL CELL BIOCHEM, V368, P69, DOI 10.1007/s11010-012-1344-5

NR 44

TC 8

Z9 9

U1 3

U2 24

PU WALTER DE GRUYTER GMBH

PI BERLIN

PA GENTHINER STRASSE 13, D-10785 BERLIN, GERMANY

SN 0334-018X

EI 2191-0251

J9 J PEDIATR ENDOCR MET

JI J. Pediatr. Endocrinol. Metab.

PD JAN

PY 2014

VL 27

IS 1-2

BP 23

EP 29

DI 10.1515/jpem-2013-0116

PG 7

WC Endocrinology & Metabolism; Pediatrics

SC Endocrinology & Metabolism; Pediatrics

GA 296EW

UT WOS:000330168900006

PM 23893676

OA Green Accepted

DA 2019-01-08

ER

PT J

AU Rogers, EA

Hessler, DM

Bodenheimer, TS

Ghorob, A

Vittinghoff, E

Thom, DH

AF Rogers, Elizabeth A.

Hessler, Danielle M.

Bodenheimer, Thomas S.

Ghorob, Amireh

Vittinghoff, Eric

Thom, David H.

TI Diabetes Peer Coaching Do "Better Patients" Make Better Coaches?

SO DIABETES EDUCATOR

LA English

DT Article

ID RANDOMIZED CONTROLLED-TRIAL; SELF-MANAGEMENT; SCREENING INSTRUMENT;

GLYCEMIC CONTROL; DISTRESS SCALE; RISK-FACTORS; ADHERENCE; SUPPORT; CARE

AB Purpose The purpose of this study was to identify characteristics of peer coaches associated with improvement in diabetes control among low-income patients with type 2 diabetes.

Methods Low-income patients with type 2 diabetes who spoke English or Spanish from 6 urban clinics in San Francisco, California, were invited to participate in the study. Twenty participants received training and provided peer coaching to 109 patients over a 6-month peer coaching intervention. Primary outcome was average change in patient glycosylated hemoglobin (A1C). Characteristics of peer coaches included age, gender, years with diabetes, A1C, body mass index (BMI), levels of diabetes-related distress, self-efficacy in diabetes self-management, and depression.

Results Patient improvement in A1C was associated with having a coach with a lower sense of self-efficacy in diabetes management (P < .001), higher level of diabetes-related distress (P = .01), and lower depression score (P = .03).

Conclusions Coach characteristics are associated with patient success in improving A1C. Better levels of coach diabetes self-efficacy and distress were not helpful and, in fact, were associated with less improvement in patient A1C, suggesting that some coach uncertainty about his or her own diabetes might foster improved patient self-management. These coach characteristics should be considered when recruiting peer coaches.

C1 [Rogers, Elizabeth A.] Univ Calif San Francisco, Div Gen Internal Med, San Francisco, CA 94143 USA.

[Rogers, Elizabeth A.] Univ Calif San Francisco, Div Gen Pediat, San Francisco, CA 94143 USA.

[Rogers, Elizabeth A.] Univ Minnesota, Div Gen Internal Med, Minneapolis, MN 55414 USA.

[Rogers, Elizabeth A.] Div Gen Pediat & Adolescent Hlth, Minneapolis, MN USA.

[Hessler, Danielle M.; Bodenheimer, Thomas S.; Ghorob, Amireh; Thom, David H.] Univ Calif San Francisco, Dept Family & Community Med, San Francisco, CA 94143 USA.

[Vittinghoff, Eric] Univ Calif San Francisco, Dept Epidemiol & Biostat, San Francisco, CA 94143 USA.

RP Rogers, EA (reprint author), Univ Minnesota, Div Gen Internal Med, Appl Clin Res Program, Room 166,717 Delaware St SE, Minneapolis, MN 55414 USA.

EM earogers@umn.edu

RI Rogers, Elizabeth/I-2042-2015

OI Rogers, Elizabeth/0000-0002-4819-7178

FU American Academy of Family Physicians; NRSA [T32 HP19025]

FX The randomized controlled trial generating the cohort in this study was

funded by the American Academy of Family Physicians. At the time of the

study, E. Rogers was a UCSF primary care research fellow funded by NRSA

grant T32 HP19025.

CR Bodenheimer T, 2007, JAMA-J AM MED ASSOC, V298, P2048, DOI 10.1001/jama.298.16.jrr70000

Center for Disease Control and Prevention, 2011, NAT DIAB FACT SHEET

Clark SE., 2001, PEER VERSUS COPING M

CUNNINGHAM CE, 1993, J CONSULT CLIN PSYCH, V61, P871, DOI 10.1037//0022-006X.61.5.871

Dale JR, 2012, DIABETIC MED, V29, P1361, DOI 10.1111/j.1464-5491.2012.03749.x

Fernandez A, 2011, J GEN INTERN MED, V26, P170, DOI 10.1007/s11606-010-1507-6

Fisher L, 2008, ANN FAM MED, V6, P246, DOI 10.1370/afm.842

Fisher L, 2012, DIABETES CARE, V35, P259, DOI 10.2337/dc11-1572

Ghorob A, 2011, BMC PUBLIC HEALTH, V11, DOI 10.1186/1471-2458-11-208

Heisler M, 2010, ANN INTERN MED, V153, P507, DOI 10.7326/0003-4819-153-8-201010190-00007

Gagliardino JJ, 2013, DIABETES-METAB RES, V29, P152, DOI 10.1002/dmrr.2368

Kroenke K, 2009, J AFFECT DISORDERS, V114, P163, DOI 10.1016/j.jad.2008.06.026

Long JA, 2012, ANN INTERN MED, V156, P416, DOI 10.7326/0003-4819-156-6-201203200-00004

Lorig K, 2008, DIABETES CARE, V31, P408, DOI 10.2337/dc07-1313

Philis-Tsimikas A, 2011, DIABETES CARE, V34, P1926, DOI 10.2337/dc10-2081

Polonsky WH, 2005, DIABETES CARE, V28, P626, DOI 10.2337/diacare.28.3.626

SCHUNK DH, 1991, EDUC PSYCHOL, V26, P207, DOI 10.1207/s15326985ep2603&4_2

Thom DH, 2013, ANN FAM MED, V11, P137, DOI 10.1370/afm.1443

Thornton RLJ, 2011, PATIENT EDUC COUNS, V85, pE201, DOI 10.1016/j.pec.2011.07.015

Traylor AH, 2010, DIABETES CARE, V33, P520, DOI 10.2337/dc09-0760

van der Wulp I, 2012, DIABETIC MED, V29, pe390, DOI 10.1111/j.1464-5491.2012.03629.x

van Steenbergen-Weijenburg KM, 2010, BMC HEALTH SERV RES, V10, DOI 10.1186/1472-6963-10-235

Wallston KA, 2007, J BEHAV MED, V30, P395, DOI 10.1007/s10865-007-9110-y

Wu CJ, 2012, INT NURS REV, V59, P345, DOI 10.1111/j.1466-7657.2012.00998.x

NR 24

TC 10

Z9 10

U1 0

U2 12

PU SAGE PUBLICATIONS INC

PI THOUSAND OAKS

PA 2455 TELLER RD, THOUSAND OAKS, CA 91320 USA

SN 0145-7217

EI 1554-6063

J9 DIABETES EDUCATOR

JI Diabetes Educ.

PD JAN

PY 2014

VL 40

IS 1

BP 107

EP 115

DI 10.1177/0145721713513178

PG 9

WC Endocrinology & Metabolism; Public, Environmental & Occupational Health

SC Endocrinology & Metabolism; Public, Environmental & Occupational Health

GA 291YU

UT WOS:000329869600009

PM 24258250

DA 2019-01-08

ER

PT J

AU Goedendorp, MM

Tack, CJ

Steggink, E

Bloot, L

Bazelmans, E

Knoop, H

AF Goedendorp, Martine M.

Tack, Cees J.

Steggink, Elles

Bloot, Lotte

Bazelmans, Ellen

Knoop, Hans

TI Chronic Fatigue in Type 1 Diabetes: Highly Prevalent but Not Explained

by Hyperglycemia or Glucose Variability

SO DIABETES CARE

LA English

DT Article

ID RANDOMIZED CONTROLLED-TRIAL; COGNITIVE-BEHAVIOR THERAPY;

RHEUMATOID-ARTHRITIS; SYMPTOM CHECKLIST; PHYSICAL-ACTIVITY;

SELF-MANAGEMENT; BREAST-CANCER; PRIMARY-CARE; DEPRESSION; DISTRESS

AB OBJECTIVEFatigue is a classical symptom of hyperglycemia, but the relationship between chronic fatigue and diabetes has not been systematically studied. We investigated prevalence, impact, and potential determinants of chronic fatigue in patients with type 1 diabetes mellitus (T1DM).RESEARCH DESIGN AND METHODSOut of 324 randomly selected T1DM outpatients, 214 participated in this cross-sectional observational study. Participants were compared with age- and sex-matched population-based controls. Chronic fatigue, functional impairments, current health status, comorbidity, diabetes-related factors, and fatigue-related cognitions and behaviors were assessed with questionnaires, and HbA(1c) values and comorbidity were assessed with medical records. Sixty-six patients underwent continuous glucose monitoring combined with an electronic fatigue diary for 5 days. Acute fatigue and four glucose parameters were determined: mean, variability, and relative time spent in hypoglycemia and hyperglycemia.RESULTST1DM patients were significantly more often chronically fatigued (40%; 95% CI 34-47%) compared with matched controls (7%; 95% CI 3-10%; P < 0.001). Chronically fatigued patients had significantly more functional impairments. Fatigue was the most troublesome symptom. Age, depression, pain, sleeping problems, low self-efficacy concerning fatigue, and physical inactivity were significantly associated with chronic fatigue. Chronically fatigued patients spent slightly less time in hypoglycemia (proportion 0.07 0.06 vs. 0.12 +/- 0.10; P = 0.025). Glucose parameters were not related to acute fatigue.CONCLUSIONSChronic fatigue is highly prevalent and clinically relevant in T1DM. Its significant relationship with cognitive behavioral variables and weak association with blood glucose levels suggests that behavioral interventions could be helpful in managing chronic fatigue in T1DM.

C1 [Goedendorp, Martine M.; Bloot, Lotte; Knoop, Hans] Radboud Univ Nijmegen, Med Ctr, Expert Ctr Chron Fatigue, NL-6525 ED Nijmegen, Netherlands.

[Tack, Cees J.] Radboud Univ Nijmegen, Med Ctr, Dept Internal Med, NL-6525 ED Nijmegen, Netherlands.

[Steggink, Elles] Wageningen Univ, Div Human Nutr, NL-6700 AP Wageningen, Netherlands.

[Bazelmans, Ellen] Radboud Univ Nijmegen, Med Ctr, Dept Med Psychol, NL-6525 ED Nijmegen, Netherlands.

RP Knoop, H (reprint author), Radboud Univ Nijmegen, Med Ctr, Expert Ctr Chron Fatigue, NL-6525 ED Nijmegen, Netherlands.

EM hans.knoop@radboudumc.nl

RI Tack, Cees/A-2368-2014; Knoop, Hans/D-3264-2009

OI Knoop, Hans/0000-0001-7763-3517

FU Dutch Diabetes Research Foundation

FX This study was supported by the Dutch Diabetes Research Foundation.

CR Aaronson NK, 1998, J CLIN EPIDEMIOL, V51, P1055, DOI 10.1016/S0895-4356(98)00097-3

Alberts M, 1997, Ned Tijdschr Geneeskd, V141, P1526

Anderson RJ, 2001, DIABETES CARE, V24, P1069, DOI 10.2337/diacare.24.6.1069

Arbuckle RA, 2009, VALUE HEALTH, V12, P1168, DOI 10.1111/j.1524-4733.2009.00571.x

Beck AT, 1997, BEHAV RES THER, V35, P785, DOI 10.1016/S0005-7967(97)00025-9

Beck AT, 1997, ASSESSMENT, V4, P211, DOI 10.1177/107319119700400301

BERGNER M, 1981, MED CARE, V19, P787, DOI 10.1097/00005650-198108000-00001

CHARLSON ME, 1987, J CHRON DIS, V40, P373, DOI 10.1016/0021-9681(87)90171-8

Craig CL, 2003, MED SCI SPORT EXER, V35, P1381, DOI 10.1249/01.MSS.0000078924.61453.FB

Drivsholm T, 2005, DIABETOLOGIA, V48, P210, DOI 10.1007/s00125-004-1625-y

Fritschi C, 2010, J PSYCHOSOM RES, V69, P33, DOI 10.1016/j.jpsychores.2010.01.021

FUKUDA K, 1994, ANN INTERN MED, V121, P953, DOI 10.7326/0003-4819-121-12-199412150-00009

Goedendorp MM, 2010, ONCOLOGIST, V15, P1122, DOI 10.1634/theoncologist.2010-0092

GROOTENHUIS PA, 1994, DIABETIC MED, V11, P253, DOI 10.1111/j.1464-5491.1994.tb00268.x

Hewlett S, 2011, RHEUMATOLOGY, V50, P1004, DOI 10.1093/rheumatology/keq282

Hewlett S, 2011, ANN RHEUM DIS, V70, P1060, DOI 10.1136/ard.2010.144691

Jacobsen PB, 2004, J CONSULT CLIN PSYCH, V72, P355, DOI 10.1037/0022-006X.72.2.355

Jacobsen PB, 1999, CANC RES THER CONTRO, V8, P155

Kalkman JS, 2007, J PSYCHOSOM RES, V62, P571, DOI 10.1016/j.jpsychores.2006.11.014

Kalkman JS, 2005, J NEUROL NEUROSUR PS, V76, P1406, DOI 10.1136/jnnp.2004.050005

King P, 1998, DIABETES CARE, V21, P341, DOI 10.2337/diacare.21.3.341

Lasselin J, 2012, PSYCHONEUROENDOCRINO, V37, P1468, DOI 10.1016/j.psyneuen.2012.01.016

MONTOYE HJ, 1983, MED SCI SPORT EXER, V15, P403

POLONSKY WH, 1995, DIABETES CARE, V18, P754, DOI 10.2337/diacare.18.6.754

Prins JB, 2001, LANCET, V357, P841, DOI 10.1016/S0140-6736(00)04198-2

RAY C, 1993, SOC SCI MED, V37, P385, DOI 10.1016/0277-9536(93)90268-9

Reeves WC, 2003, BMC HEALTH SERV RES, V3, DOI 10.1186/1472-6963-3-25

Repping-Wuts H, 2007, J CLIN NURS, V16, P377, DOI 10.1111/j.1365-2702.2007.02082.x

Servaes P, 2002, ANN ONCOL, V13, P589, DOI 10.1093/annonc/mdf082

Siegelaar SE, 2010, ENDOCR REV, V31, P171, DOI 10.1210/er.2009-0021

Snoek FJ, 2000, DIABETES CARE, V23, P1305, DOI 10.2337/diacare.23.9.1305

Sommerfield AJ, 2004, DIABETES CARE, V27, P2335, DOI 10.2337/diacare.27.10.2335

van der Ven NCW, 2003, DIABETES CARE, V26, P713, DOI 10.2337/diacare.26.3.713

van Hoogmoed D, 2010, RHEUMATOLOGY, V49, P1294, DOI 10.1093/rheumatology/keq043

Vercoulen JHMM, 1996, ARCH NEUROL-CHICAGO, V53, P642, DOI 10.1001/archneur.1996.00550070080014

VERCOULEN JHMM, 1994, J PSYCHOSOM RES, V38, P383, DOI 10.1016/0022-3999(94)90099-X

Vercoulen JHMM, 1999, GEDRAGSTHERAPIE, V32, P131

Warren RE, 2003, DIABETES-METAB RES, V19, P408, DOI 10.1002/dmrr.396

Weijman I, 2005, DIABETES RES CLIN PR, V68, P56, DOI 10.1016/j.diabres.2004.08.004

NR 39

TC 29

Z9 29

U1 4

U2 34

PU AMER DIABETES ASSOC

PI ALEXANDRIA

PA 1701 N BEAUREGARD ST, ALEXANDRIA, VA 22311-1717 USA

SN 0149-5992

EI 1935-5548

J9 DIABETES CARE

JI Diabetes Care

PD JAN

PY 2014

VL 37

IS 1

BP 73

EP 80

DI 10.2337/dc13-0515

PG 8

WC Endocrinology & Metabolism

SC Endocrinology & Metabolism

GA 275KV

UT WOS:000328676000024

PM 23949561

OA Other Gold

DA 2019-01-08

ER

PT J

AU Hajos, TRS

Polonsky, WH

Pouwer, F

Gonder-Frederick, L

Snoek, FJ

AF Hajos, Tibor R. S.

Polonsky, William H.

Pouwer, Frans

Gonder-Frederick, Linda

Snoek, Frank J.

TI Toward Defining a Cutoff Score for Elevated Fear of Hypoglycemia on the

Hypoglycemia Fear Survey Worry Subscale in Patients With Type 2 Diabetes

SO DIABETES CARE

LA English

DT Article

ID QUALITY-OF-LIFE; HEALTH-RELATED QUALITY; GLYCEMIC CONTROL; DISTRESS;

MELLITUS; MANAGEMENT; SCALE; STATE; CARE

AB OBJECTIVETo determine a cutoff score for clinically meaningful fear of hypoglycemia (FoH) on the Hypoglycemia Fear Survey Worry subscale (HFS-W).RESEARCH DESIGN AND METHODSData on the HFS-W, history of hypoglycemia, emotional well-being (World Health Organization-5 well-being index), and distress about diabetes symptoms (Diabetes Symptom Checklist-Revised) were available from Dutch patients with type 2 diabetes who were treated with oral medication or insulin (n = 1,530). Four criteria were applied to define a threshold for clinically meaningful FoH: 1) modal score distribution (MD criterion), 2) scores 2 SDs above the mean (SD criterion), 3) concurrent validity with severe hypoglycemia and suboptimal well-being (CV criterion), and 4) an elevated score (3) on more than one HFS-W item (elevated item endorsement [EI criterion]). Associations between the outcomes of these approaches and a history of severe hypoglycemia and suboptimal well-being were studied.RESULTSOf the 1,530 patients, 19% had a HFS-W score of 0 (MD criterion), and 5% reported elevated FoH (HFS-W mean + 2 SD; SD criterion). Patients with severe hypoglycemia reported higher HFS-W scores than those without (25 20 vs. 15 +/- 17; P < 0.001). Patients with suboptimal well-being reported higher HFS-W scores than those with satisfactory well-being (20 +/- 18 vs. 13 +/- 15; P < 0.001, CV criterion). Elevated FoH (defined by the EI criterion) was seen in 26% of patients. The SD and EI criteria were the strongest associated with history of severe hypoglycemia. The EI criterion was the strongest associated with suboptimal well-being.CONCLUSIONSAlthough no definite cutoff score has been determined, the EI criterion may be most indicative of clinically relevant FoH in this exploratory study. Further testing of the clinical relevance of this criterion is needed.

C1 [Hajos, Tibor R. S.; Snoek, Frank J.] Vrije Univ Amsterdam, Med Ctr, EMGO Inst, Diabet Psychol Res Grp,Dept Med Psychol, Amsterdam, Netherlands.

[Polonsky, William H.] Behav Diabet Inst, San Diego, CA USA.

[Pouwer, Frans] Tilburg Univ, Ctr Res Psychol Somat Dis CoRPS, NL-5000 LE Tilburg, Netherlands.

[Gonder-Frederick, Linda] Univ Virginia, Behav Med Ctr, Charlottesville, VA USA.

RP Snoek, FJ (reprint author), Vrije Univ Amsterdam, Med Ctr, EMGO Inst, Diabet Psychol Res Grp,Dept Med Psychol, Amsterdam, Netherlands.

EM fj.snoek@vumc.nl

FU EMGO+ Institute, Amsterdam

FX This work was financially supported by a travel grant from the EMGO+

Institute, Amsterdam.

CR Amiel SA, 2008, DIABETIC MED, V25, P245, DOI 10.1111/j.1464-5491.2007.02341.x

Arbuckle RA, 2009, VALUE HEALTH, V12, P1168, DOI 10.1111/j.1524-4733.2009.00571.x

Barendse S, 2012, DIABETIC MED, V29, P293, DOI 10.1111/j.1464-5491.2011.03416.x

Bech P, 2003, INT J METH PSYCH RES, V12, P85, DOI 10.1002/mpr.145

COX DJ, 1987, DIABETES CARE, V10, P617, DOI 10.2337/diacare.10.5.617

Cryer PE, 2003, DIABETES CARE, V26, P1902, DOI 10.2337/diacare.26.6.1902

Cryer PE, 1999, DIABETES-METAB RES, V15, P42, DOI 10.1002/(SICI)1520-7560(199901/02)15:1<42::AID-DMRR1>3.0.CO;2-B

Gold AE, 1997, DIABETIC MED, V14, P309

Gonder-Frederick LA, 2011, DIABETES CARE, V34, P801, DOI 10.2337/dc10-1343

Hajos TRS, 2013, DIABETIC MED, V30, pE63, DOI 10.1111/dme.12040

Hajos TRS, 2012, QUAL LIFE RES, V21, P1359, DOI 10.1007/s11136-011-0051-0

Hajos TRS, 2011, DIABETIC MED, V28, P1096, DOI 10.1111/j.1464-5491.2011.03329.x

Heller SR, 2007, DIABETOLOGIA, V50, P1140, DOI 10.1007/s00125-007-0599-y

Makoul G, 2007, PATIENT EDUC COUNS, V67, P333, DOI 10.1016/j.pec.2007.05.005

Marrett Elizabeth, 2011, BMC Res Notes, V4, P251, DOI 10.1186/1756-0500-4-251

Nixon R, 2011, DIABETES TECHNOL THE, V13, P93, DOI 10.1089/dia.2010.0192

Pedersen-Bjergaard U, 2003, DIABETES-METAB RES, V19, P232, DOI 10.1002/dmrr.377

POLONSKY WH, 1992, HEALTH PSYCHOL, V11, P199, DOI 10.1037/0278-6133.11.3.199

Rubin DB, 1987, MULTIPLE IMPUTATION

Schafer JL, 2002, PSYCHOL METHODS, V7, P147, DOI 10.1037//1082-989X.7.2.147

Sheu WHH, 2012, DIABETES RES CLIN PR, V96, P141, DOI 10.1016/j.diabres.2011.12.027

Snoek FJ, 2000, DIABETES CARE, V23, P1305, DOI 10.2337/diacare.23.9.1305

Snoek FJ, 1996, GEDRAG GEZOND, V5, P287

Stargardt T, 2009, HEALTH QUAL LIFE OUT, V7, DOI 10.1186/1477-7525-7-91

ter Braak EWMT, 2000, DIABETES CARE, V23, P1467, DOI 10.2337/diacare.23.10.1467

Wild D, 2007, PATIENT EDUC COUNS, V68, P10, DOI 10.1016/j.pec.2007.05.003

NR 26

TC 17

Z9 17

U1 0

U2 11

PU AMER DIABETES ASSOC

PI ALEXANDRIA

PA 1701 N BEAUREGARD ST, ALEXANDRIA, VA 22311-1717 USA

SN 0149-5992

EI 1935-5548

J9 DIABETES CARE

JI Diabetes Care

PD JAN

PY 2014

VL 37

IS 1

BP 102

EP 108

DI 10.2337/dc13-0971

PG 7

WC Endocrinology & Metabolism

SC Endocrinology & Metabolism

GA 275KV

UT WOS:000328676000028

PM 23990513

OA Other Gold

DA 2019-01-08

ER

PT J

AU Mashitani, T

Hayashino, Y

Okamura, S

Tsujii, S

Ishii, H

AF Mashitani, Tsuyoshi

Hayashino, Yasuaki

Okamura, Shintarou

Tsujii, Satoru

Ishii, Hitoshi

TI Correlations Between Serum Bilirubin Levels and Diabetic Nephropathy

Progression Among Japanese Type 2 Diabetic Patients: A Prospective

Cohort Study (Diabetes Distress and Care Registry at Tenri [DDCRT 5])

SO DIABETES CARE

LA English

DT Article

ID CARDIOVASCULAR-DISEASE; OXIDATIVE STRESS; GLYCEMIC CONTROL;

GILBERT-SYNDROME; KIDNEY-FUNCTION; HEART-DISEASE; ALBUMINURIA; RISK;

COMPLICATIONS; ASSOCIATION

AB OBJECTIVETo assess the correlations between serum bilirubin levels and diabetic nephropathy development and progression in type 2 diabetic patients.RESEARCH DESIGN AND METHODSLongitudinal data were obtained from 2,511 type 2 diabetic patients registered in a Japanese diabetes registry. To assess the independent correlations between serum bilirubin levels and either the development or progression of diabetic nephropathy, we used logistic regression analysis adjusted for potential confounders.RESULTSThe median follow-up period was 503.4 days (range 238-777). The mean patient age, BMI, and HbA(1c) level was 65.2 years, 24.7 kg/m(2), and 7.5% (58.5 mmol/mol), respectively. Baseline serum bilirubin levels were significantly associated with the urinary albumin-creatinine ratio at baseline (P < 0.001) and 1 year after registration (P < 0.001). Multivariable adjusted odds ratios for progression from microalbuminuria to macroalbuminuria for the second, third, and fourth quartile of serum bilirubin levels were 0.89 (95% CI 0.49-1.58), 0.93 (0.47-1.83), and 0.33 (0.13-0.84), respectively, showing a statistically significant linear trend across categories (P = 0.032). However, this trend disappeared after adjustment for hemoglobin levels.CONCLUSIONSSerum bilirubin levels were associated with diabetic nephropathy progression in type 2 diabetic patients independent of possible confounders. Serum bilirubin levels might be the link in the correlation between hemoglobin levels and nephropathy progression.

C1 [Mashitani, Tsuyoshi; Hayashino, Yasuaki; Okamura, Shintarou; Tsujii, Satoru; Ishii, Hitoshi] Tenri Hosp, Diabet Distress & Care Registry, Tenri Study Grp, Dept Endocrinol, Tenri, Nara 632, Japan.

RP Mashitani, T (reprint author), Tenri Hosp, Diabet Distress & Care Registry, Tenri Study Grp, Dept Endocrinol, Tenri, Nara 632, Japan.

EM mashiyan@gmail.com

OI Hayashino, Yasuaki/0000-0002-2932-8478; Tsujii,

Satoru/0000-0002-1834-6515

FU Manpei Suzuki Diabetes Foundation; Japan Society for the Promotion of

Science KAKENHI [25460641]

FX This study was partially supported by the Manpei Suzuki Diabetes

Foundation and Japan Society for the Promotion of Science KAKENHI (Grant

25460641, Grant-in-Aid for Scientific Research).

CR Adler AI, 2003, KIDNEY INT, V63, P225, DOI 10.1046/j.1523-1755.2003.00712.x

[Anonymous], 2008, KIDNEY INT, V74, P1197

Babazono T, 2006, DIABETOLOGIA, V49, P1387, DOI 10.1007/s00125-006-0247-y

BAYNES JW, 1991, DIABETES, V40, P405, DOI 10.2337/diabetes.40.4.405

Brenner BM, 2001, NEW ENGL J MED, V345, P861, DOI 10.1056/NEJMoa011161

Caramori ML, 2000, DIABETES, V49, P1399, DOI 10.2337/diabetes.49.9.1399

Djousse L, 2001, AM J CARDIOL, V87, P1196, DOI 10.1016/S0002-9149(01)01494-1

Djousse L, 2001, AM J CARDIOL, V87, pA7

Djousse L, 2001, AM J CARDIOL, V87, pA4

DOSEMECI M, 1990, AM J EPIDEMIOL, V132, P746, DOI 10.1093/oxfordjournals.aje.a115716

Endler G, 2003, CLIN CHEM, V49, P1201, DOI 10.1373/49.7.1201

Fujii M, 2010, KIDNEY INT, V78, P905, DOI 10.1038/ki.2010.265

Gerstein HC, 2001, JAMA-J AM MED ASSOC, V286, P421, DOI 10.1001/jama.286.4.421

Hayashino Y, 2012, DIABETES RES CLIN PR, V97, P405, DOI 10.1016/j.diabres.2012.04.005

Inoguchi T, 2007, JAMA-J AM MED ASSOC, V298, P1398, DOI 10.1001/jama.298.12.1398-b

Katayama S, 2011, DIABETOLOGIA, V54, P1025, DOI 10.1007/s00125-010-2025-0

Lewis EJ, 2001, NEW ENGL J MED, V345, P851, DOI 10.1056/NEJMoa011303

Lin JP, 2006, CIRCULATION, V114, P1476, DOI 10.1161/CIRCULATIONAHA.106.633206

Madsen-Bouterse SA, 2008, REV ENDOCR METAB DIS, V9, P315, DOI 10.1007/s11154-008-9090-4

Makino H, 2007, DIABETES CARE, V30, P1577, DOI 10.2337/dc06-1998

Matsuo S, 2009, AM J KIDNEY DIS, V53, P982, DOI 10.1053/j.ajkd.2008.12.034

Molitch Mark E, 2004, Diabetes Care, V27 Suppl 1, pS79

Riphagen IJ, 2012, AM SOC NEPHR 45 ANN

Ritz E, 1999, NEW ENGL J MED, V341, P1127, DOI 10.1056/NEJM199910073411506

Sahai A, 1999, KIDNEY INT, V56, P1277, DOI 10.1046/j.1523-1755.1999.00703.x

Seino Y, 2010, J DIABETES INVEST, V1, P1, DOI 10.1111/j.2040-1124.2010.00005.x

STOCKER R, 1987, SCIENCE, V235, P1043, DOI 10.1126/science.3029864

Targher G, 2009, CLIN CHEM LAB MED, V47, P1055, DOI 10.1515/CCLM.2009.244

Tsujii S, 2012, DIABETIC MED, V29, P1451, DOI 10.1111/j.1464-5491.2012.03647.x

Vitek L, 2002, ATHEROSCLEROSIS, V160, P449, DOI 10.1016/S0021-9150(01)00601-3

Weir MR, 2007, CLIN J AM SOC NEPHRO, V2, P581, DOI 10.2215/CJN.03190906

Yasuda M, 2011, OPHTHALMOLOGY, V118, P1423, DOI 10.1016/j.ophtha.2010.12.009

NR 32

TC 25

Z9 28

U1 0

U2 7

PU AMER DIABETES ASSOC

PI ALEXANDRIA

PA 1701 N BEAUREGARD ST, ALEXANDRIA, VA 22311-1717 USA

SN 0149-5992

EI 1935-5548

J9 DIABETES CARE

JI Diabetes Care

PD JAN

PY 2014

VL 37

IS 1

BP 252

EP 258

DI 10.2337/dc13-0407

PG 7

WC Endocrinology & Metabolism

SC Endocrinology & Metabolism

GA 275KV

UT WOS:000328676000047

PM 24009299

OA Other Gold

DA 2019-01-08

ER

PT J

AU Hermanns, N

Kulzer, B

Ehrmann, D

Bergis-Jurgan, N

Haak, T

AF Hermanns, Norbert

Kulzer, Bernhard

Ehrmann, Dominic

Bergis-Jurgan, Nikola

Haak, Thomas

TI The effect of a diabetes education programme (PRIMAS) for people with

type 1 diabetes: Results of a randomized trial

SO DIABETES RESEARCH AND CLINICAL PRACTICE

LA English

DT Article

DE Type 1 diabetes; Diabetes self-management education; Diabetes education

programme; Intensive insulin treatment

ID SELF-MANAGEMENT EDUCATION; INSULIN-TREATMENT; HYPOGLYCEMIA; MELLITUS;

SCALE; METAANALYSIS; DISTRESS; THERAPY; DISEASE; ADULTS

AB Objective: In a randomized, multi-centre trial, the efficacy of a self-management-oriented education programme (PRIMAS) for people with type 1 diabetes was compared with an established education programme as control group (CG). Primary outcome was the effect on glycaemic control in a 6-month follow-up. Secondary outcomes were the impact on emotional aspects, self-management related aspects and hypoglycaemia problems.

Methods: The study was conducted in an outpatient setting. 160 participants were randomized. Baseline characteristics in PRIMAS and CG were similar (age 45.1 +/- 13.5 vs. 45.9 +/- 13.1 years, p = .716; diabetes duration 18.8 +/- 12.3 vs. 19.8 +/- 13.4 years, p = .615; BMI 26.5 +/- 4.6 vs. 27.5 +/- 5.0 kg/m(2), p = .236; HbA1c 8.3 +/- 1.1 vs. 8.1 +/- 1.0%, p = .236).

Results: At follow-up there was a significant 0.4 percentage points greater reduction of HbA1c in PRIMAS compared to CG (Delta -0.4 +/- 1.0% vs. Delta 0.0 +/- 0.6%; p = .012). Also, diabetes-related distress (Delta -0.3 +/- 0.7 vs. -0.1 +/- 0.4, p = .032) and dissatisfaction with diabetes treatment (Delta -3.3 +/- 6.9 vs. -1.9 +/- 5.6, p = .024) decreased more in PRIMAS. Diabetes empowerment (Delta 2.6 +/- 5.9 vs. 0.8 +/- 5.1, p = .037) and diabetes self-efficacy (Delta 1.4 +/- 3.6 vs. 0.2 +/- 4.0, p = .013) increased in PRIMAS. Incidence of severe hypoglycemia, hypoglycemia awareness, diabetes knowledge, and self-care behaviour improved in both groups with no significant differences between groups.

Conclusion: PRIMAS is more effective in lowering HbA1c than a previously established education programmes and also showed superiority in reducing diabetes-related distress and increasing diabetes empowerment, diabetes self-efficacy and satisfaction with insulin therapy. (C) 2013 Elsevier Ireland Ltd. All rights reserved.

C1 [Hermanns, Norbert; Kulzer, Bernhard; Ehrmann, Dominic; Bergis-Jurgan, Nikola; Haak, Thomas] Res Inst Diabet Acad Mergentheim FIDAM, D-97980 Bad Mergentheim, Germany.

[Hermanns, Norbert; Kulzer, Bernhard] Otto Friedrich Univ Bamberg, D-96047 Bamberg, Germany.

[Hermanns, Norbert; Kulzer, Bernhard; Haak, Thomas] Diabet Ctr Mergentheim, D-97980 Bad Mergentheim, Germany.

RP Hermanns, N (reprint author), Theodor Klotzbuecher Str 12, D-97980 Bad Mergentheim, Germany.

EM hermanns@diabetes-zentrum.de

OI Ehrmann, Dominic/0000-0002-5794-5596

FU Berlin Chemie AG/Menarini Diagnostics, Germany

FX The development and evaluation was supported by an unrestricted grant of

Berlin Chemie AG/Menarini Diagnostics, Germany. The funders had no role

in the conceptualisation of PRIMAS, study design, data collection and

analysis, decision to publish, or preparation of the manuscript.

CR Anderson R, 2000, NAT HIST, V109, P23

Bohm BO, 2012, THERAPIE DIABETES ME

Bragd J, 2003, DIABETIC MED, V20, P216, DOI 10.1046/j.1464-5491.2003.00902.x

Centers for Disease Control and Prevention (CDC), NAT DIAB FACTSH

CLARKE WL, 1995, DIABETES CARE, V18, P517, DOI 10.2337/diacare.18.4.517

DAFNE Study Group, 2002, BMJ-BRIT MED J, V325, DOI DOI 10.1136/BMJ.325.7367.746

Davies MJ, 2008, BRIT MED J, V336, P491, DOI 10.1136/bmj.39474.922025.BE

*DIAB CONTR COMPL, 1997, DIABETES, V46, P271, DOI DOI 10.2337/DIABETES.46.2.271

Ehrmann D, 2011, DIABETOL STOFFWECHS, V6, P62

Ellis SE, 2004, PATIENT EDUC COUNS, V52, P97, DOI 10.1016/S0738-3991(03)00016-8

Funnell MM, 2011, DIABETES CARE, V34, pS89, DOI 10.2337/dc11-S089

Hautzinger M, 1993, ALLGEMEINE DEPRESSIO

Hermanns N, 2007, DIABETES-METAB RES, V23, P528, DOI 10.1002/dmrr.710

Hopkins D, 2012, DIABETES CARE, V35, P1638, DOI 10.2337/dc11-1579

IDF Clinical Guidelines Task Force, 2005, GLOB GUID TYP 2 DIAB

Institute for Clinical Excellence, 2003, GUID US PAT DEUC MOD

International Diabetes Federation, IDF DIABETES ATLAS

Jorgens V, 2009, BEHANDLUNGS SCHULUNG, V5

Kempe J, 2002, DIABETES STOFFWECH H, V11, P144

KULZER B, 1995, VERHALTENSTHERAPIE, V5, pA72

MUHLHAUSER I, 1983, DIABETOLOGIA, V25, P470

MUHLHAUSER I, 1987, DIABETOLOGIA, V30, P681, DOI 10.1007/BF00296989

Nathan DM, 2005, NEW ENGL J MED, V353, P2643

Norris SL, 2002, DIABETES CARE, V25, P1159, DOI 10.2337/diacare.25.7.1159

PIEBER TR, 1995, DIABETES CARE, V18, P625, DOI 10.2337/diacare.18.5.625

Polonsky WH, 2005, DIABETES CARE, V28, P626, DOI 10.2337/diacare.28.3.626

Pouwer F, 2003, DIABETOLOGIA, V46, P892, DOI 10.1007/s00125-003-1124-6

REICHARD P, 1991, J INTERN MED, V230, P101, DOI 10.1111/j.1365-2796.1991.tb00415.x

Roy T, 2012, J AFFECT DISORDERS, V142, P8

Snoek FJ, 2000, DIABETES CARE, V23, P1305, DOI 10.2337/diacare.23.9.1305

The Diabetes Control and Complications Trial Research Group, 1993, NEW ENGL J MED, V329, P977, DOI [10.1056/NEJM199309303291401, DOI 10.1056/NEJM199309303291401]

Toobert DJ, 2000, DIABETES CARE, V23, P943, DOI 10.2337/diacare.23.7.943

NR 32

TC 33

Z9 33

U1 0

U2 16

PU ELSEVIER IRELAND LTD

PI CLARE

PA ELSEVIER HOUSE, BROOKVALE PLAZA, EAST PARK SHANNON, CO, CLARE, 00000,

IRELAND

SN 0168-8227

EI 1872-8227

J9 DIABETES RES CLIN PR

JI Diabetes Res. Clin. Pract.

PD DEC

PY 2013

VL 102

IS 3

BP 149

EP 157

DI 10.1016/j.diabres.2013.10.009

PG 9

WC Endocrinology & Metabolism

SC Endocrinology & Metabolism

GA 272UX

UT WOS:000328488100003

PM 24210673

DA 2019-01-08

ER

PT J

AU Pasek, RC

Gannon, M

AF Pasek, Raymond C.

Gannon, Maureen

TI Advancements and challenges in generating accurate animal models of

gestational diabetes mellitus

SO AMERICAN JOURNAL OF PHYSIOLOGY-ENDOCRINOLOGY AND METABOLISM

LA English

DT Review

DE gestational diabetes mellitus; pregnancy; beta-cell; hyperplasia;

hypertrophy; glucose-stimulated insulin secretion

ID PANCREATIC BETA-CELL; INSULIN-SECRETION; GROWTH-HORMONE;

TRANSCRIPTION-FACTOR-7-LIKE-2 TCF7L2; TRANSCRIPTION FACTOR; PROLACTIN

RECEPTOR; GENE POLYMORPHISMS; EMBRYO DEVELOPMENT; GLUCOSE-TOLERANCE;

HUMAN-PREGNANCY

AB The maintenance of glucose homeostasis during pregnancy is critical to the health and well-being of both the mother and the developing fetus. Strikingly, approximately 7% of human pregnancies are characterized by insufficient insulin production or signaling, resulting in gestational diabetes mellitus (GDM). In addition to the acute health concerns of hyperglycemia, women diagnosed with GDM during pregnancy have an increased incidence of complications during pregnancy as well as an increased risk of developing type 2 diabetes (T2D) later in life. Furthermore, children born to mothers diagnosed with GDM have increased incidence of perinatal complications, including hypoglycemia, respiratory distress syndrome, and macrosomia, as well as an increased risk of being obese or developing T2D as adults. No single environmental or genetic factor is solely responsible for the disease; instead, a variety of risk factors, including weight, ethnicity, genetics, and family history, contribute to the likelihood of developing GDM, making the generation of animal models that fully recapitulate the disease difficult. Here, we discuss and critique the various animal models that have been generated to better understand the etiology of diabetes during pregnancy and its physiological impacts on both the mother and the fetus. Strategies utilized are diverse in nature and include the use of surgical manipulation, pharmacological treatment, nutritional manipulation, and genetic approaches in a variety of animal models. Continued development of animal models of GDM is essential for understanding the consequences of this disease as well as providing insights into potential treatments and preventative measures.

C1 [Gannon, Maureen] Tennessee Valley Healthcare Syst, Dept Vet Affairs, Nashville, TN USA.

[Pasek, Raymond C.; Gannon, Maureen] Vanderbilt Univ, Dept Med, Med Ctr, Div Diabet Endocrinol & Metab, Nashville, TN 37232 USA.

[Gannon, Maureen] Vanderbilt Univ, Dept Cell & Dev Biol, Med Ctr, Nashville, TN 37232 USA.

[Gannon, Maureen] Vanderbilt Univ, Dept Mol Physiol & Biophys, Med Ctr, Nashville, TN 37232 USA.

RP Gannon, M (reprint author), Vanderbilt Univ, Dept Med, Med Ctr, 2213 Garland Ave,MRB 4 7435, Nashville, TN 37232 USA.

EM Maureen.gannon@vanderbilt.edu

OI Pasek, Raymond/0000-0002-1817-8697

FU Vanderbilt Molecular Endocrinology Training Program [5 T32 DK-07563];

Veterans Administration [1BX000990-01A1]; Juvenile Diabetes Research

Foundation [1-2011-592]

FX R. C. Pasek was supported by the Vanderbilt Molecular Endocrinology

Training Program (Grant 5 T32 DK-07563). M. Gannon was supported by a

grant from the Veterans Administration (1BX000990-01A1) and the Juvenile

Diabetes Research Foundation (1-2011-592).

CR Aerts L, 1998, DIABETES RES CLIN PR, V41, P9, DOI 10.1016/S0168-8227(98)00063-1

American Diabetes Association, 2004, Diabetes Care, V27 Suppl 1, pS88

ARISON RN, 1967, DIABETES, V16, P51, DOI 10.2337/diab.16.1.51

Brelje TC, 2004, ENDOCRINOLOGY, V145, P4162, DOI 10.1210/en.2004-0201

BRUCKMANN G, 1947, J BIOL CHEM, V168, P241

Butler AE, 2010, DIABETOLOGIA, V53, P2167, DOI 10.1007/s00125-010-1809-6

Cardinal JW, 1998, ENDOCRINOLOGY, V139, P2885, DOI 10.1210/en.139.6.2885

Carlson AJ, 1911, AM J PHYSIOL, V27, P323

Chen H, 1996, CELL, V84, P491, DOI 10.1016/S0092-8674(00)81294-5

Cheung NW, 2003, DIABETES CARE, V26, P2005, DOI 10.2337/diacare.26.7.2005

COHEN G, 1974, J BIOL CHEM, V249, P2447

Cuthbert FP, 1936, AM J PHYSIOL, V115, P480

Cypryk Katarzyna, 2008, Endokrynol Pol, V59, P393

Demirci C, 2012, DIABETES, V61, P1143, DOI 10.2337/db11-1154

DIAMOND MP, 1989, J REPROD FERTIL, V86, P1

DICKINSON JE, 1990, AM J OBSTET GYNECOL, V163, P1831, DOI 10.1016/0002-9378(90)90759-Z

DICKINSON JE, 1991, AM J OBSTET GYNECOL, V165, P1673, DOI 10.1016/0002-9378(91)90013-H

DOOLEY SL, 1991, DIABETES, V40, P25, DOI 10.2337/diab.40.2.S25

Dunn JS, 1943, LANCET, V1, P484

EZEKWE MO, 1984, J ANIM SCI, V59, P974

Ford SP, 2009, AM J PHYSIOL-REG I, V297, pR835, DOI 10.1152/ajpregu.00072.2009

Frias JL, 2007, AM J MED GENET A, V143A, P2904, DOI 10.1002/ajmg.a.32071

Fujinaka Y, 2007, J BIOL CHEM, V282, P30707, DOI 10.1074/jbc.M702607200

GAUGUIER D, 1990, DIABETES, V39, P734, DOI 10.2337/diabetes.39.6.734

GEWOLB IH, 1982, PEDIATR RES, V16, P869, DOI 10.1203/00006450-198210000-00013

Giachini FRC, 2008, J ANAT, V212, P31, DOI 10.1111/j.1469-7580.2007.00839.x

GILLMAN J, 1958, BRIT MED J, V2, P1260, DOI 10.1136/bmj.2.5107.1260

Gilmartin Amanda Bird Hoffert, 2008, Rev Obstet Gynecol, V1, P129

Gloyn AL, 2006, HUM MUTAT, V27, P220, DOI 10.1002/humu.20292

GORUS FK, 1982, BIOCHEM J, V208, P513, DOI 10.1042/bj2080513

GRIFFITHS M, 1948, AUST J EXP BIOL MED, V26, P339, DOI 10.1038/icb.1948.35

Gupta RK, 2007, GENE DEV, V21, P756, DOI 10.1101/gad.1535507

Gupta RK, 2005, J CLIN INVEST, V115, P1006, DOI 10.1172/JCI22365

Harder T, 2001, GYNECOL OBSTET INVES, V51, P160, DOI 10.1159/000052916

Higashide S, 1992, Nihon Geka Hokan, V61, P311

HOFTIEZER V, 1973, DIABETOLOGIA, V9, P178, DOI 10.1007/BF01219780

Holemans K, 2004, AM J OBSTET GYNECOL, V190, P858, DOI 10.1016/j.ajog.2003.09.025

Huang C, 2009, ENDOCRINOLOGY, V150, P1618, DOI 10.1210/en.2008-1003

HUMMEL KP, 1966, SCIENCE, V153, P1127, DOI 10.1126/science.153.3740.1127

JARRETT I. G., 1956, AUSTRALIAN JOUR EXPTL BIOL AND MED SCI, V34, P133, DOI 10.1038/icb.1956.16

JAWERBAUM A, 1993, PROSTAGLANDINS, V45, P487, DOI 10.1016/0090-6980(93)90124-P

Johansson M, 2006, ENDOCRINOLOGY, V147, P2315, DOI 10.1210/en.2005-0997

JONES N, 1957, Proc Soc Study Fertil, V9, P51

Karnik SK, 2007, SCIENCE, V318, P806, DOI 10.1126/science.1146812

Karnik SK, 2005, P NATL ACAD SCI USA, V102, P14659, DOI 10.1073/pnas.0503484102

KATO R, 1970, JPN J PHARMACOL, V20, P554, DOI 10.1254/jjp.20.554

KAUFMANN RC, 1981, AM J OBSTET GYNECOL, V141, P479, DOI 10.1016/S0002-9378(15)33263-4

Kelly PA, 2002, MOL CELL ENDOCRINOL, V197, P127, DOI 10.1016/S0303-7207(02)00286-1

KEMNITZ JW, 1984, DIABETOLOGIA, V26, P60

Kilpatrick DC, 2002, BBA-GEN SUBJECTS, V1572, P401, DOI 10.1016/S0304-4165(02)00321-5

Kim H, 2010, NAT MED, V16, P804, DOI 10.1038/nm.2173

Kim S. Y., 2004, PREV CHRONIC DIS, V9, pE88

Kivimaki M, 2010, DIABETES CARE, V33, P2611, DOI 10.2337/dc10.1187

Kobayashi K, 2004, ARTIF ORGANS, V28, P1035, DOI 10.1111/j.1525-1594.2004.00002.x

Le TN, 2013, GENET TEST MOL BIOMA, V17, P567, DOI 10.1089/gtmb.2013.0009

LENICH CM, 1991, J LIPID RES, V32, P431

LENZEN S, 1988, DIABETOLOGIA, V31, P337, DOI 10.1007/BF02341500

Liang CY, 2010, METABOLISM, V59, P943, DOI 10.1016/j.metabol.2009.10.015

Long H, 2013, CURR DIABETES REP, V13, P43, DOI 10.1007/s11892-012-0330-3

Lopez-Soldado Iliana, 2003, Exp Diabesity Res, V4, P107, DOI 10.1155/EDR.2003.107

Markowitz J, 1927, AM J PHYSIOL, V79, P553

MARTIN ME, 1995, BIOSCIENCE REP, V15, P15, DOI 10.1007/BF01200211

Megia A, 2004, J CLIN ENDOCR METAB, V89, P5081, DOI 10.1210/jc.2004-0211

Metzger BE, 1998, DIABETES CARE, V21, pB161

Metzger BE, 2010, DIABETES CARE, V33, P676, DOI 10.2337/dc09-1848

MIODOVNIK M, 1989, AM J OBSTET GYNECOL, V160, P1239, DOI 10.1016/0002-9378(89)90203-2

Miura A, 2006, J BIOL CHEM, V281, P5246, DOI 10.1074/jbc.M507496200

Moehn S, 2012, J NUTR, V142, p2231S, DOI 10.3945/jn.112.159061

Moore MC, 2011, J APPL PHYSIOL, V110, P458, DOI 10.1152/japplphysiol.00768.2010

Morisset J, 2000, PANCREAS, V21, P321, DOI 10.1097/00006676-200010000-00015

Murata M, 1999, BIOCHEM PHARMACOL, V57, P881, DOI 10.1016/S0006-2952(98)00370-0

Nebigil CG, 2000, P NATL ACAD SCI USA, V97, P9508, DOI 10.1073/pnas.97.17.9508

OSULLIVAN JB, 1973, AM J OBSTET GYNECOL, V116, P895

Pan A, 2012, DIABETOLOGIA, V55, P63, DOI 10.1007/s00125-011-2268-4

PANG K, 1994, P NATL ACAD SCI USA, V91, P9559, DOI 10.1073/pnas.91.20.9559

PARSONS JA, 1995, ENDOCRINOLOGY, V136, P2013, DOI 10.1210/en.136.5.2013

PARSONS JA, 1992, ENDOCRINOLOGY, V130, P1459, DOI 10.1210/en.130.3.1459

Perera HK, 2006, GENE EXPR PATTERNS, V6, P971, DOI 10.1016/j.modgep.2006.04.001

Perkins JM, 2007, CLIN DIABETES, V25, P57, DOI DOI 10.2337/DIACLIN.25.2.57

PHELPS RL, 1981, AM J OBSTET GYNECOL, V140, P730, DOI 10.1016/0002-9378(81)90731-6

Plank JL, 2011, ENDOCRINOLOGY, V152, P4589, DOI 10.1210/en.2010-1462

RAMSAY TG, 1994, AM J PHYSIOL-REG I, V267, pR1391

REYNOLDS WA, 1974, DIABETES, V23, P777, DOI 10.2337/diab.23.9.777

Rieck S, 2010, TRENDS ENDOCRIN MET, V21, P151, DOI 10.1016/j.tem.2009.11.001

Robitaille J, 2008, GENET MED, V10, P240, DOI 10.1097/GIM.0b013e31816b10

ROSSINI AA, 1978, ENDOCRINOLOGY, V103, P1518, DOI 10.1210/endo-103-4-1518

RYAN EA, 1993, DIABETES, V42, P316, DOI 10.2337/diabetes.42.2.316

Savitz DA, 2008, BJOG-INT J OBSTET GY, V115, P969, DOI 10.1111/j.1471-0528.2008.01763.x

SCHEIN PS, 1974, CANCER, V34, P993, DOI 10.1002/1097-0142(197410)34:4<993::AID-CNCR2820340404>3.0.CO;2-T

SCHEIN PS, 1968, CANCER RES, V28, P1501

SCHNEDL WJ, 1994, DIABETES, V43, P1326, DOI 10.2337/diabetes.43.11.1326

Schraenen A, 2010, DIABETOLOGIA, V53, P2589, DOI 10.1007/s00125-010-1913-7

Segar EM, 2009, CLIN SCI, V117, P129, DOI 10.1042/CS20080550

Shaat N, 2007, DIABETOLOGIA, V50, P972, DOI 10.1007/s00125-007-0623-2

Shaat N, 2006, DIABETOLOGIA, V49, P1545, DOI 10.1007/s00125-006-0258-8

Shaat N, 2005, DIABETOLOGIA, V48, P2544, DOI 10.1007/s00125-005-0035-0

Sharma A, 1999, DIABETES, V48, P507, DOI 10.2337/diabetes.48.3.507

Sivan Eyal, 2003, Curr Diab Rep, V3, P319, DOI 10.1007/s11892-003-0024-y

Szkudelski T, 2001, PHYSIOL RES, V50, P537

Tein MSC, 1998, EXP PHYSIOL, V83, P165, DOI 10.1113/expphysiol.1998.sp004099

TSAI MY, 1982, BIOCHIM BIOPHYS ACTA, V712, P395, DOI 10.1016/0005-2760(82)90358-7

VANASSCHE FA, 1978, BRIT J OBSTET GYNAEC, V85, P818

VERCHEVAL M, 1990, DIABETOLOGIA, V33, P187, DOI 10.1007/BF00404794

Waguri M, 1997, DIABETES, V46, P1281, DOI 10.2337/diabetes.46.8.1281

Wang RN, 1996, DIABETOLOGIA, V39, P548, DOI 10.1007/s001250050477

Watanabe RM, 2007, DIABETES, V56, P1481, DOI 10.2337/db06-1682

Weinhaus AJ, 2007, J ENDOCRINOL, V193, P367, DOI 10.1677/JOE-07-0043

Xavier GD, 2012, DIABETOLOGIA, V55, P2667, DOI 10.1007/s00125-012-2600-7

Yamagata K, 1996, NATURE, V384, P458, DOI 10.1038/384458a0

Yamashita H, 2003, PEDIATR RES, V53, P411, DOI 10.1203/01.PDR.0000049667.58071.7D

Yamashita H, 2001, ENDOCRINOLOGY, V142, P2888, DOI 10.1210/en.142.7.2888

Zabihi S, 2008, TOXICOL SCI, V105, P166, DOI 10.1093/toxsci/kfn101

Zhang HJ, 2006, MOL ENDOCRINOL, V20, P1853, DOI 10.1210/me.2006-0056

Zhang H, 2010, DIABETES, V59, P143, DOI 10.2337/db09-0050

NR 114

TC 18

Z9 19

U1 0

U2 24

PU AMER PHYSIOLOGICAL SOC

PI BETHESDA

PA 9650 ROCKVILLE PIKE, BETHESDA, MD 20814 USA

SN 0193-1849

EI 1522-1555

J9 AM J PHYSIOL-ENDOC M

JI Am. J. Physiol.-Endocrinol. Metab.

PD DEC

PY 2013

VL 305

IS 11

BP E1327

EP E1338

DI 10.1152/ajpendo.00425.2013

PG 12

WC Endocrinology & Metabolism; Physiology

SC Endocrinology & Metabolism; Physiology

GA 268IX

UT WOS:000328164700001

PM 24085033

OA Green Published

DA 2019-01-08

ER

PT J

AU Singh, H

Murphy, HR

Hendrieckx, C

Ritterband, L

Speight, J

AF Singh, Harsimran

Murphy, Helen R.

Hendrieckx, Christel

Ritterband, Lee

Speight, Jane

TI The Challenges and Future Considerations Regarding Pregnancy-Related

Outcomes in Women with Pre-Existing Diabetes

SO CURRENT DIABETES REPORTS

LA English

DT Article

DE Pregnancy; Pre-existing diabetes; Congenital malformations;

Preconception counseling; Gestational; Prepregnancy care; Contraception;

Glycemic control; High-risk pregnancy; Distress; Fetal outcomes;

Stillbirth; Neonatal death; Newborn; Motherhood

ID PRE-CONCEPTION CARE; PRECONCEPTION CARE; PREPREGNANCY CARE;

CONGENITAL-MALFORMATIONS; METABOLIC-CONTROL; GLYCEMIC CONTROL; TYPE-1;

MELLITUS; RISK; HYPOGLYCEMIA

AB Ineffective management of blood glucose levels during preconception and pregnancy has been associated with severe maternal and fetal complications in women with pre-existing diabetes. Studies have demonstrated that preconception counseling and pre-pregnancy care can dramatically reduce these risks. However, pregnancy-related outcomes in women with diabetes continue to be less than ideal. This review highlights and discusses a variety of patient, provider, and organizational factors that can contribute to these suboptimal outcomes. Based on the findings of studies reviewed and authors' clinical and research experiences, recommendations have been proposed focusing on various aspects of care provided, including improved accessibility to effective preconception and pregnancy-related care and better organized clinic consultations that are sensitive to women's diabetes and pregnancy needs.

C1 [Singh, Harsimran; Ritterband, Lee] Univ Virginia, Sch Med, Dept Psychiat & Neurobehav Sci, Div Behav Hlth & Technol, Charlottesville, VA 22903 USA.

[Murphy, Helen R.] Addenbrookes Hosp, Metab Res Labs, Cambridge CB2 0QQ, England.

[Murphy, Helen R.] Addenbrookes Hosp, NIHR Cambridge Biomed Ctr, Inst Metab Sci, Cambridge CB2 0QQ, England.

[Hendrieckx, Christel; Speight, Jane] Australian Ctr Behav Res Diabet, Diabet Australia Vic, Melbourne, Vic 3000, Australia.

[Hendrieckx, Christel; Speight, Jane] Deakin Univ, Ctr Mental Hlth & Wellbeing Res, Sch Psychol, Burwood, Australia.

[Speight, Jane] AHP Res, Uxbridge, Middx, England.

RP Singh, H (reprint author), Univ Virginia, Sch Med, Dept Psychiat & Neurobehav Sci, Div Behav Hlth & Technol, 310 Old Ivy Way,Suite 102, Charlottesville, VA 22903 USA.

EM hs9dc@virginia.edu; hm386@medschl.cam.ac.uk; chendrieckx@acbrd.org.au;

lr5b@virginia.edu; jspeight@acbrd.org.au

RI Ritterband, Lee/G-7810-2011

OI Ritterband, Lee/0000-0001-7624-5213; Speight, Jane/0000-0002-1204-6896

FU National Institute for Health Research; NIH/NIDDK; National Institute

for Health Research [PDF/01/036, CDF-2013-06-035]

FX Helen R. Murphy is funded by a research fellowship supported by the

National Institute for Health Research. The views expressed in this

publication are those of the authors and not necessarily those of the

NHS, the National Institute for Health Research or the Department of

Health. Lee Ritterband has been awarded an R21 grant by NIH/NIDDK to

develop and test the feasibility of an Internet intervention to assist

women with type 1 diabetes contemplating pregnancy better regulate their

blood glucose levels in preparation for pregnancy. She is a part equity

owner of BeHealth Solutions, LLC, a company who licenses software from

UVA and makes this software and services for evidence-based clinical

research and who are working to expand public access to proven eHealth

interventions. This software includes a platform to build and host

Internet interventions as well as 2 interventions developed by Dr.

Ritterband and his team (1 for pediatric encopresis and 1 for adults

with insomnia). Although none of this is explicitly discussed in this

paper, within the Recommendation section, mention is made of accessing

care via the Internet. Dr. Ritterband's wife, Dawn Ritterband, provides

consultation to BeHealth Solutions, LLC.

CR *AM DIAB ASS, 2003, DIABETES CARE S1, V26, pS91

American Diabetes Association, 2004, Diabetes Care, V27 Suppl 1, pS76

American Diabetes Association, 2013, DIABETES CARE S1, V36, pS11, DOI DOI 10.2337/DC13-S011

Balsells M, 2009, J CLIN ENDOCR METAB, V94, P4284, DOI 10.1210/jc.2009-1231

Bell R, 2008, BJOG-INT J OBSTET GY, V115, P445, DOI 10.1111/j.1471-0528.2007.01644.x

Bell R, 2012, DIABETOLOGIA

Berg M, 2000, J PSYCHOSOM OBST GYN, V21, P39, DOI 10.3109/01674820009075607

Berg M, 2009, BMC PREGNANCY CHILDB, V3, P27

Berg Marie, 2003, J Perinat Educ, V12, P1

Berg M, 2005, J PERINAT EDUC, V14, P23, DOI 10.1624/105812405X57552

Bernasko J, 2004, OBSTET GYNECOL SURV, V59, P628, DOI 10.1097/00006254-200408000-00024

Boulot P, 2003, DIABETES CARE, V26, P2990

Brindley BG, 2004, GROWTH GENET HORM, V20, P49

Casele HL, 1998, ARCH INTERN MED, V158, P1321, DOI 10.1001/archinte.158.12.1321

Charron-Prochownik D, 2008, DIABETES CARE, V31, P1327, DOI 10.2337/dc07-1266

CHEW EY, 1995, DIABETES CARE, V18, P631, DOI 10.2337/diacare.18.5.631

Chuang CH, 2005, WOMEN HEALTH ISS, V15, P167, DOI 10.1016/j.whi.2005.04.002

Colstrup M, 2013, J MATERN FETAL NEONA

de Valk HW, 2011, BEST PRACT RES CL OB, V25, P65, DOI 10.1016/j.bpobgyn.2010.10.002

Delamater AM, 2001, DIABETES CARE, V24, P1286, DOI 10.2337/diacare.24.7.1286

Downs JS, 2010, DIABETES EDUCATOR, V36, P911, DOI 10.1177/0145721710383586

Ekbom P, 2000, DIABETOLOGIA, V43, P927, DOI 10.1007/s001250051471

Evers IM, 2002, DIABETES CARE, V25, P554, DOI 10.2337/diacare.25.3.554

Evers IN, 2004, BRIT MED J, V328, P915, DOI 10.1136/bmj.38043.583160.EE

Feig DS, 2002, LANCET, V359, P1690, DOI 10.1016/S0140-6736(02)08599-9

Finer LB, 2006, PERSPECT SEX REPRO H, V38, P90, DOI 10.1363/3809006

GARNER P, 1995, LANCET, V346, P157, DOI 10.1016/S0140-6736(95)91213-4

Gupton A, 2001, J Obstet Gynecol Neonatal Nurs, V30, P192, DOI 10.1111/j.1552-6909.2001.tb01535.x

Hatmaker DD, 1998, J PERINAT NEONAT NUR, V12, P1

Holing EV, 1998, DIABETES CARE, V21, P889, DOI 10.2337/diacare.21.6.889

JANZ NK, 1995, DIABETES CARE, V18, P157, DOI 10.2337/diacare.18.2.157

Jensen DM, 2004, DIABETES CARE, V27, P2819, DOI 10.2337/diacare.27.12.2819

Jovanovic L, 2001, DIABETES CARE, V24, P1130, DOI 10.2337/diacare.24.7.1130

Kendrick JM, 2004, J PERINAT NEONAT NUR, V18, P14, DOI 10.1097/00005237-200401000-00003

Kendrick JM, 2004, J PERINATAL NEONATAL, V18, P26

Kim C, 2005, AM J OBSTET GYNECOL, V192, P227, DOI 10.1016/j.ajog.2004.06.105

King R, 2009, MIDWIFERY, V25, P126, DOI 10.1016/j.midw.2007.01.016

Kinsley B, 2007, CLIN THER, V29, pS153, DOI 10.1016/j.clinthera.2007.12.015

Kitzmiller JL, 1996, DIABETES CARE, V19, P514, DOI 10.2337/diacare.19.5.514

Kitzmiller JL, 2008, DIABETES CARE, V31, P1060, DOI 10.2337/dc08-9020

Kitzmiller JL, 2010, BIRTH DEFECTS RES A, V88, P791, DOI 10.1002/bdra.20734

Korenbrot Carol C, 2002, Matern Child Health J, V6, P75, DOI 10.1023/A:1015460106832

Lain KY, 2007, CLIN OBSTET GYNECOL, V50, P938, DOI 10.1097/GRF.0b013e31815a5494

Lambert K, 2013, DIABETES OBES METAB, DOI [10.1111/dom.12098, DOI 10.1111/D0M.12098]

Langer N, 1998, J Matern Fetal Med, V7, P257, DOI 10.1002/(SICI)1520-6661(199811/12)7:6<257::AID-MFM1>3.0.CO;2-H

Lapolla A, 2008, ACTA DIABETOL, V45, P61, DOI 10.1007/s00592-008-0024-0

Lavender T, 2010, MIDWIFERY, V26, P589, DOI 10.1016/j.midw.2009.01.003

Lee-Parritz A, 2012, OBSTET GYNECOL SURV, V67, P168, DOI 10.1097/OGX.0b013e31824bb538

Lusignan S, 2005, BMC FAM PRACT, V22

Macintosh MCM, 2006, BRIT MED J, V333, P177, DOI 10.1136/bmj.38856.692986.AE

Makda SI, 2013, DIABETIC MED, V30, P457, DOI 10.1111/dme.12051

McCorry NK, 2012, J MIDWIFERY WOM HEAL, V57, P396, DOI 10.1111/j.1542-2011.2011.00143.x

Meltzer SJ, 2010, DIABETES CARE, V33, P2713, DOI [10.2337/dc10-1744, 10.2337/dc10.1744]

Murphy HR, 2012, DIABETOLOGIA, V55, P282, DOI 10.1007/s00125-011-2363-6

Murphy HR, 2011, DIABETIC MED, V28, P1060, DOI 10.1111/j.1464-5491.2011.03333.x

Murphy HR, 2010, DIABETIC MED, V27, P92, DOI 10.1111/j.1464-5491.2009.02890.x

Murphy HR, 2007, DIABETES CARE, V30, P2785, DOI 10.2337/dc0-0500

Murphy HR, 2010, DIABETES CARE, V33, P2514, DOI 10.2337/dc10.1113

Negrato CA, 2012, DIABETOL METAB SYNDR, V4, DOI 10.1186/1758-5996-4-41

Neithercott T., REAL LIFE STORIES DI

Nielsen LR, 2008, DIABETES CARE, V31, P9, DOI 10.2337/dc07-1066

Owens MD, 2006, MATERN CHILD HLTH J, V10, pS137, DOI 10.1007/s10995-006-0098-7

Pearson DWM, 2007, BJOG-INT J OBSTET GY, V114, P104, DOI 10.1111/j.1471-0528.2006.01145.x

Penney GC, 2003, BJOG-INT J OBSTET GY, V110, P315, DOI 10.1016/S1470-0328(03)02967-7

Rasmussen B, 2013, BMC PREGNANCY CHILDB, V13, DOI 10.1186/1471-2393-13-54

Ray JG, 2001, QJM-INT J MED, V94, P435, DOI 10.1093/qjmed/94.8.435

Ringholm L, 2012, DIABETIC MED, V29, P558, DOI 10.1111/j.1464-5491.2012.03604.x

Ringholm L, 2011, DIABETIC HYPOGLYCEMI, V3, P3

Ritterband LM, 2003, PROF PSYCHOL-RES PR, V34, P527, DOI 10.1037/0735-7028.34.5.527

Romano AM, 2007, J PERINAT EDUC, V16, P18, DOI 10.1624/105812407X244903

ROSENN BM, 1995, OBSTET GYNECOL, V85, P417, DOI 10.1016/0029-7844(94)00415-A

Rosenn BM, 1996, OBSTET GYNECOL, V87, P568, DOI 10.1016/0029-7844(95)00495-5

Schwarz EB, 2012, J GEN INTERN MED, V27, P196, DOI 10.1007/s11606-011-1875-6

Sparud-Lundin C, 2011, BMC MED INFORM DECIS, V7, P49

Stenhouse E, 2011, MIDWIFERY, V27, P120, DOI 10.1016/j.midw.2009.06.004

Temple R, 2010, BEST PRACT RES CL EN, V24, P591, DOI 10.1016/j.beem.2010.05.011

Tripathi A, 2010, DIABETES CARE, V33, P586, DOI 10.2337/dc09-1585

Trujillo AI, 2007, DIABETES SPECTRUM, V20, P94

Vargas Roberto, 2010, Rev Obstet Gynecol, V3, P92

Wahabi HA, 2010, BMC PREGNANCY CHILDB, V14, P63

Zhu HD, 2012, AUST NZ J OBSTET GYN, V52, P593, DOI 10.1111/ajo.12010

1990, DIABET MED, V7, P360

1995, INT J GYNAECOL OBSTE, V50, P201

NR 83

TC 11

Z9 12

U1 2

U2 12

PU CURRENT MEDICINE GROUP

PI PHILADELPHIA

PA 400 MARKET STREET, STE 700, PHILADELPHIA, PA 19106 USA

SN 1534-4827

EI 1539-0829

J9 CURR DIABETES REP

JI Curr. Diabetes Rep.

PD DEC

PY 2013

VL 13

IS 6

BP 869

EP 876

DI 10.1007/s11892-013-0417-5

PG 8

WC Endocrinology & Metabolism

SC Endocrinology & Metabolism

GA 241SR

UT WOS:000326188500012

PM 24013963

OA Green Accepted

DA 2019-01-08

ER

PT J

AU Lloyd, CE

Gill, P

Stone, M

AF Lloyd, Cathy E.

Gill, Paramjit

Stone, Margaret

TI Psychological Care in a National Health Service: Challenges for People

with Diabetes

SO CURRENT DIABETES REPORTS

LA English

DT Article

DE Diabetes mellitus; Depression; Diabetes-related emotional distress;

Primary care; Screening; Guidelines; Psychological care; National health

service

ID COMORBID DEPRESSION; PREVALENCE; DISTRESS; TYPE-1; ADULTS

AB Recently, there has been a growing interest in psychological problems in people with diabetes and a concomitant increasing concern that these often go unreported and, thus, unidentified and treated. This has serious implications for both the self-management of diabetes and the individual's quality of life. In this review article we consider the question of screening for depression in people with diabetes within a national health service in the UK. The inadequacies of psychological care for patients with diabetes are discussed, in particular with regard to the importance of distinguishing between depressive symptoms and emotional distress related to having diabetes. Criteria for assessing the validity of screening for depression are discussed, together with national and international recommendations, with particular emphasis on current practice. The screening strategy currently recommended for implementation in primary care in the UK is outlined. The need for rigorous evaluation of screening initiatives is highlighted and a key conclusion is that case-finding alone is unlikely to be effective in terms of improving patient outcomes unless considered and applied in the context of overall case management. This review highlights the barriers and challenges to optimizing care for patients with co-morbid diabetes and depression, and outlines the therapies currently available in the UK, which might be disseminated in other countries.

C1 [Lloyd, Cathy E.] Open Univ, Fac Hlth & Social Care, Milton Keynes MK7 6AA, Bucks, England.

[Gill, Paramjit] Univ Birmingham, Coll Med & Dent Sci, Sch Hlth & Populat Sci, Birmingham B15 2TT, W Midlands, England.

[Stone, Margaret] Univ Leicester, Leicester Gen Hosp, Diabet Res Unit, Leicester LE5 4PW, Leics, England.

RP Lloyd, CE (reprint author), Open Univ, Fac Hlth & Social Care, Milton Keynes MK7 6AA, Bucks, England.

EM Cathy.Lloyd@open.ac.uk

OI Lloyd, Cathy/0000-0002-8863-3069

CR *AM DIAB ASS, 2011, DIABETES CARE S1, V34, pS11, DOI DOI 10.2337/DC11-S011

Anderson RJ, 2001, DIABETES CARE, V24, P1069, DOI 10.2337/diacare.24.6.1069

Barnard KD, 2006, DIABETIC MED, V23, P445, DOI 10.1111/j.1464-5491.2006.01814.x

BECK AT, 1988, CLIN PSYCHOL REV, V8, P77, DOI 10.1016/0272-7358(88)90050-5

Doran T, 2006, NEW ENGL J MED, V355, P375, DOI 10.1056/NEJMsa055505

Esbitt S. A., 2013, SCREENING DEPRESSION

Hood K, 2013, SCREENING DEPRESSION

*INT DIAB FED CLIN, 2005, GLOB GUID TYP 2 DIAB

Kroenke K, 2001, J GEN INTERN MED, V16, P606, DOI 10.1046/j.1525-1497.2001.016009606.x

Lloyd CE, 2010, DIABETIC MED, V27, P234, DOI 10.1111/j.1464-5491.2009.02896.x

Lloyd CE, 2010, DEPRESSION DIABETES

Lloyd CE, 2013, SCREENING DEPRESSION

Lustman PJ, 2000, DIABETES CARE, V23, P618, DOI 10.2337/diacare.23.5.618

National Institute for Health and Care Excellence, NICE CLIN GUID 90 DE

National Institute for Health and Care Excellence, 2009, CLIN GUID 90 DEPR TR

National Institutes of Health and Clinical Excellence, 2011, QUAL STAND PROGR COS

POLONSKY WH, 1995, DIABETES CARE, V18, P754, DOI 10.2337/diacare.18.6.754

Polonsky WH, 2005, DIABETES CARE, V28, P626, DOI 10.2337/diacare.28.3.626

Pouwer F, 2006, PATIENT EDUC COUNS, V60, P235, DOI 10.1016/j.pec.2005.01.009

Pouwer F, 2010, DIABETIC MED, V27, P217, DOI 10.1111/j.1464-5491.2009.02903.x

Pouwer F, 2009, NAT REV ENDOCRINOL, V5, P665, DOI 10.1038/nrendo.2009.214

RADLOFF L S, 1977, Applied Psychological Measurement, V1, P385, DOI 10.1177/014662167700100306

Spitzer RL, 1999, JAMA-J AM MED ASSOC, V282, P1737, DOI 10.1001/jama.282.18.1737

NR 23

TC 2

Z9 2

U1 0

U2 6

PU CURRENT MEDICINE GROUP

PI PHILADELPHIA

PA 400 MARKET STREET, STE 700, PHILADELPHIA, PA 19106 USA

SN 1534-4827

EI 1539-0829

J9 CURR DIABETES REP

JI Curr. Diabetes Rep.

PD DEC

PY 2013

VL 13

IS 6

BP 894

EP 899

DI 10.1007/s11892-013-0416-6

PG 6

WC Endocrinology & Metabolism

SC Endocrinology & Metabolism

GA 241SR

UT WOS:000326188500015

PM 24013962

DA 2019-01-08

ER

PT J

AU Christodoulou, C

Douzenis, A

Mommersteeg, PMC

Rallidis, L

Poulios, A

Efstathiou, V

Bouras, G

Varounis, C

Korkoliakou, P

Palios, J

Kremastinos, DT

Lykouras, L

AF Christodoulou, Christos

Douzenis, Athanasios

Mommersteeg, Paula M. C.

Rallidis, Loukianos

Poulios, Antonis

Efstathiou, Vasiliki

Bouras, Georgios

Varounis, Christos

Korkoliakou, Panagiota

Palios, John

Kremastinos, Dimitrios Th

Lykouras, Lefteris

TI A case-control validation of Type D personality in Greek patients with

stable coronary heart disease

SO ANNALS OF GENERAL PSYCHIATRY

LA English

DT Article

DE Coronary heart disease; Type D personality; DS14; Validity; HADS

ID D DISTRESSED PERSONALITY; UNHEALTHY LIFE-STYLE; D SCALE DS14;

PSYCHOLOGICAL DISTRESS; MYOCARDIAL-INFARCTION; SOCIAL INHIBITION;

CARDIAC PATIENTS; HEALTHY CONTROLS; RISK-FACTORS; PROGNOSIS

AB Background: Type D personality has been associated with a variety of emotional and social difficulties as well as with poor prognosis in patients with established coronary heart disease (CHD). We examined the psychometric properties and validity of the Type D Scale-14 (DS14) and the prevalence of Type D personality among Greek patients with CHD while taking into account demographic; clinical, such as diabetes mellitus, hypertension, and hypercholesterolemia; as well as psychological variables such as depression, anxiety, and psychological stress.

Methods: Ninety-six patients with stable coronary heart disease and 80 healthy participants from the general population completed the Greek version of the DS14 and the Hospital Anxiety and Depression Scale (HADS).

Results: Cronbach's a coefficient for the negative affectivity (NA) and social inhibition (SI) subscales was 0.83 and 0.72 for the CHD and 0.88 and 0.76 for the control group, respectively. Internal-structural validity was assessed by a factor analysis (two-factor solution), and the factor structure of the original DS14 was replicated. Using the standardized cutoff point of NA >= 10 and SI >= 10, instead of the median scores, in order to have compatible results with the majority of studies, the prevalence of Type D personality was 51% for the CHD patients and 13% for the control group. Higher NA and SI were connected with higher anxiety, depression, and total psychological stress. Finally, more patients with CHD and Type D personality than those without were diagnosed with type 2 diabetes; however, no differences were observed in hypertension or hypercholesterolemia.

Conclusions: These results indicate that the Type D construct is reliable and valid in a Greek population. The prevalence of Type D personality was higher in patients with stable coronary heart disease than in people from the general population. The DS14 subscales were positively correlated with higher anxiety, depression, and total psychological stress. Regarding other CHD risk factors, only diabetes mellitus was found more frequently in CHD patients with Type D personality.

C1 [Christodoulou, Christos; Douzenis, Athanasios; Poulios, Antonis; Efstathiou, Vasiliki; Bouras, Georgios; Korkoliakou, Panagiota; Lykouras, Lefteris] Univ Athens, Sch Med, Dept Psychiat 2, Attikon Gen Hosp, Athens 12462, Greece.

[Mommersteeg, Paula M. C.] Tilburg Univ, Dept Med & Clin Psychol, CoRPS Ctr Res Psychol Somat Dis, NL-5000 LE Tilburg, Netherlands.

[Rallidis, Loukianos; Varounis, Christos; Palios, John; Kremastinos, Dimitrios Th] Univ Athens, Sch Med, Dept Cardiol 2, Attikon Gen Hosp, Athens 12462, Greece.

RP Christodoulou, C (reprint author), Univ Athens, Sch Med, Dept Psychiat 2, Attikon Gen Hosp, Athens 12462, Greece.

EM christo.christodoulou@gmail.com

RI Efstathiou, Vasiliki/I-3398-2014

OI Efstathiou, Vasiliki/0000-0003-2303-5997; , Douzenis/0000-0002-5321-7276

CR Albus C, 2004, EUR J CARDIOV PREV R, V11, P75, DOI 10.1097/01.hjr.0000116623.84388.6c

Aquarius AE, 2005, AM J CARDIOL, V96, P996, DOI 10.1016/j.amjcard.2005.05.059

Chida Y, 2009, J AM COLL CARDIOL, V53, P936, DOI 10.1016/j.jacc.2008.11.044

Coyne J. C., 2011, PSYCHOSOM MED, V73, P563

Denollet J, 2000, CIRCULATION, V102, P630, DOI 10.1161/01.CIR.102.6.630

Denollet J, 2006, EUR HEART J, V27, P171, DOI 10.1093/eurheartj/ehi616

Denollet J, 2005, PSYCHOSOM MED, V67, P89, DOI 10.1097/01.psy.0000149256.81953.49

Denollet J, 2001, J PSYCHOSOM RES, V51, P465, DOI 10.1016/S0022-3999(01)00230-6

Denollet J, 2000, J PSYCHOSOM RES, V49, P255, DOI 10.1016/S0022-3999(00)00177-X

Denollet J, 1998, CIRCULATION, V97, P167, DOI 10.1161/01.CIR.97.2.167

Denollet J, 1996, LANCET, V347, P417, DOI 10.1016/S0140-6736(96)90007-0

Denollet J, 2010, CIRC-CARDIOVASC QUAL, V3, P546, DOI 10.1161/CIRCOUTCOMES.109.934406

Frasure-Smith N, 2008, ARCH GEN PSYCHIAT, V65, P62, DOI 10.1001/archgenpsychiatry.2007.4

Grande G, 2004, PSYCHOTHER PSYCH MED, V54, P413, DOI 10.1088/s-2004-828376

Grande G, 2011, PSYCHOSOM MED, V73, P548, DOI 10.1097/PSY.0b013e318227a9bc

Hemingway H, 1999, BMJ-BRIT MED J, V318, P1460, DOI 10.1136/bmj.318.7196.1460

Kupper N, 2013, INT J CARDIOL, V166, P327, DOI 10.1016/j.ijcard.2011.10.084

Lim HE, 2011, J KOREAN MED SCI, V26, P116, DOI 10.3346/jkms.2011.26.1.116

Michopoulos Ioannis, 2008, Ann Gen Psychiatry, V7, P4, DOI 10.1186/1744-859X-7-4

Mommersteeg PMC, 2010, BMC PUBLIC HEALTH, V10, DOI 10.1186/1471-2458-10-714

Pedersen SS, 2004, J PSYCHOSOM RES, V57, P265, DOI 10.1016/S0022-3999(03)00614-7

Pedersen SS, 2004, PSYCHOSOM MED, V66, P714, DOI 10.1097/01.psy.0000132874.82202.21

Pedersen SS, 2004, J AM COLL CARDIOL, V44, P997, DOI 10.1016/j.jacc.2004.05.064

Pelle AJ, 2010, CIRC-HEART FAIL, V3, P261, DOI 10.1161/CIRCHEARTFAILURE.109.871483

Rallidis LS, 2011, EXPERT OPIN PHARMACO, V12, P1481, DOI 10.1517/14656566.2011.591379

Rozanski A, 1999, CIRCULATION, V99, P2192, DOI 10.1161/01.CIR.99.16.2192

Sogaro Elena, 2010, Monaldi Arch Chest Dis, V74, P181

Spindler H, 2009, INT J BEHAV MED, V16, P98, DOI 10.1007/s12529-009-9037-5

Svansdottir E, 2013, EUR J PREV CARDIOL, V20, P322, DOI 10.1177/2047487312441723

Svansdottir E, 2012, J BEHAV MED, V35, P155, DOI 10.1007/s10865-011-9337-5

Tziallas Dimitrios, 2011, BMC Res Notes, V4, P105, DOI 10.1186/1756-0500-4-105

Whitehead DL, 2007, J PSYCHOSOM RES, V62, P419, DOI 10.1016/j.jpsychores.2006.11.005

Williams L, 2008, J PSYCHOSOM RES, V64, P63, DOI 10.1016/j.jpsychores.2007.06.008

Yu DSF, 2010, J PSYCHOSOM RES, V69, P111, DOI 10.1016/j.jpsychores.2010.01.014

Yu XN, 2008, J PSYCHOSOM RES, V65, P595, DOI 10.1016/j.jpsychores.2008.06.009

Zigmond AS, 1983, PSYCHIAT SCAND, V76, P361, DOI DOI 10.1111/J.1600-0447.1983.TB09716.X

NR 36

TC 6

Z9 6

U1 0

U2 9

PU BIOMED CENTRAL LTD

PI LONDON

PA 236 GRAYS INN RD, FLOOR 6, LONDON WC1X 8HL, ENGLAND

SN 1744-859X

J9 ANN GEN PSYCHIATR

JI Ann. Gen. Psychiatr.

PD NOV 27

PY 2013

VL 12

AR 38

DI 10.1186/1744-859X-12-38

PG 7

WC Psychiatry

SC Psychiatry

GA 268ET

UT WOS:000328152600001

PM 24283252

OA DOAJ Gold, Green Published

DA 2019-01-08

ER

PT J

AU Nobis, S

Lehr, D

Ebert, DD

Berking, M

Heber, E

Baumeister, H

Becker, A

Snoek, F

Riper, H

AF Nobis, Stephanie

Lehr, Dirk

Ebert, David Daniel

Berking, Matthias

Heber, Elena

Baumeister, Harald

Becker, Annette

Snoek, Frank

Riper, Heleen

TI Efficacy and cost-effectiveness of a web-based intervention with mobile

phone support to treat depressive symptoms in adults with diabetes

mellitus type 1 and type 2: design of a randomised controlled trial

SO BMC PSYCHIATRY

LA English

DT Article

DE Diabetes; Depression; Guided web-based self-help; Internet; Randomised

controlled trial; Efficacy; Cost-effectiveness

ID COGNITIVE-BEHAVIORAL THERAPY; EFFECTIVENESS ACCEPTABILITY CURVES;

COMORBID MENTAL-DISORDERS; STATE WORRY QUESTIONNAIRE; SCALE-SHORT-FORM;

SELF-HELP; PSYCHOLOGICAL TREATMENTS; CLINICAL-SIGNIFICANCE; PROBLEM

DRINKING; GLYCEMIC CONTROL

AB Background: A diagnosis of diabetes mellitus types 1 or 2 doubles the odds of a comorbid depressive disorder. The combined diseases have a wide range of adverse outcomes, such as a lower quality of life, poorer diabetes outcomes and increased healthcare utilisation. Diabetes patients with depression can be treated effectively with psychotherapy, but access to psychological care is limited. In this study we will examine the efficacy and cost-effectiveness of a newly developed web-based intervention (GET. ON Mood Enhancer Diabetes) for people with diabetes and comorbid depressive symptoms.

Methods/Design: A two-arm randomised controlled trial will be conducted. Adults with diabetes (type 1 or type 2) with increased depression scores (> 22 on the German version of the Center for Epidemiological Studies Depression Scale (CES-D)) will be included. Eligible participants will be recruited through advertisement in diabetes patient journals and via a large-scale German health insurance company. The participants will be randomly assigned to either a 6-week minimally guided web-based self-help program or an online psychoeducation program on depression. The study will include 260 participants, which will enable us to detect a statistically significant difference with a group effect size of d = 0.35 at a power of 80% and a significance level of p = 0.05. The primary outcome measure will be the level of depression as assessed by the CES-D. The secondary outcome measures will be: diabetes-specific emotional distress, glycaemic control, self-management behaviour and the participants' satisfaction with the intervention. Online self-assessments will be collected at baseline and after a 2 months period, with additional follow-up measurements 6 and 12 months after randomisation. The data will be analysed on an intention-to-treat basis and per protocol. In addition, we will conduct an economic evaluation from a societal perspective.

Discussion: If this intervention is shown to be cost-effective, it has considerable potential for implementing psychological care for large numbers of people with diabetes and comorbid depression in routine practice and improve health outcomes.

C1 [Nobis, Stephanie; Lehr, Dirk; Ebert, David Daniel; Berking, Matthias; Heber, Elena; Riper, Heleen] Univ Luneburg, Div Online Hlth Training, D-21335 Luneburg, Germany.

[Lehr, Dirk; Ebert, David Daniel; Berking, Matthias] Univ Marburg, Dept Clin Psychol & Psychotherapy, Marburg, Germany.

[Baumeister, Harald] Univ Freiburg, D-79106 Freiburg, Germany.

[Becker, Annette] Univ Marburg, Dept Gen Med Family Med, Marburg, Germany.

[Snoek, Frank] Vrije Univ Amsterdam Med Ctr, Dept Med Psychol, Amsterdam, Netherlands.

[Riper, Heleen] Vrije Univ Amsterdam, Dept Clin Psychol, Amsterdam, Netherlands.

[Riper, Heleen] Vrije Univ Amsterdam Med Ctr, Inst Hlth & Care Res EMGO, Amsterdam, Netherlands.

RP Nobis, S (reprint author), Univ Luneburg, Div Online Hlth Training, D-21335 Luneburg, Germany.

EM nobis@inkubator.leuphana.de

RI Emchi, Karma/Q-1952-2016

OI Ebert, David Daniel/0000-0001-6820-0146

FU European Union [EFRE: CCI 2007DE161PR001]

FX We would like to acknowledge the European Union for funding this project

(EFRE: CCI 2007DE161PR001) and the BARMER GEK for supporting the

recruitment of this study.

CR Aguilera A, 2011, PROF PSYCHOL-RES PR, V42, P472, DOI 10.1037/a0025499

Anderson RJ, 2001, DIABETES CARE, V24, P1069, DOI 10.2337/diacare.24.6.1069

Andersson G, 2008, BRIT J PSYCHIAT, V193, P270, DOI 10.1192/bjp.bp.108.054080

Andersson Gerhard, 2009, Cognitive Behaviour Therapy, V38, P196, DOI 10.1080/16506070903318960

Andrews G, 2001, AUST NZ J PUBL HEAL, V25, P494, DOI 10.1111/j.1467-842X.2001.tb00310.x

Andrews G, 2010, PLOS ONE, V5, DOI 10.1371/journal.pone.0013196

[Anonymous], 1990, HLTH POLICY, V16, P199

Attkisson C C, 1982, Eval Program Plann, V5, P233, DOI 10.1016/0149-7189(82)90074-X

Baglioni C, 2011, J AFFECT DISORDERS, V135, P10, DOI 10.1016/j.jad.2011.01.011

Barak A, 2008, J TECHNOL HUMAN SERV, V26, P109, DOI 10.1080/15228830802094429

Barney LJ, 2006, AUST NZ J PSYCHIAT, V40, P51, DOI 10.1080/j.1440-1614.2006.01741.x

Baumeister H, 2012, COCHRANE DB SYST REV, V12, DOI DOI 10.1002/14651858

Baumeister H, 2011, PSYCHOTHER PSYCHOSOM, V80, P275, DOI 10.1159/000323404

Berle D, 2011, CLIN PSYCHOL PSYCHOT, V18, P339, DOI 10.1002/cpp.724

BLACK WC, 1990, MED DECIS MAKING, V10, P212, DOI 10.1177/0272989X9001000308

Brazier J, 1998, J CLIN EPIDEMIOL, V51, P1115, DOI 10.1016/S0895-4356(98)00103-6

Bundesarztekammer Kassenarztliche Bundesvereinigung Arbeitsgemeinschaft der Wissenschaftlichen Medizinische Fachgesellschaften, 2011, PAT NAT VERS UN DEPR

Cepoiu M, 2008, J GEN INTERN MED, V23, P25, DOI 10.1007/s11606-007-0428-5

Christensen H, 2006, PSYCHOL MED, V36, P1737, DOI 10.1017/S0033291706008695

Ciechanowski PS, 2000, ARCH INTERN MED, V160, P3278, DOI 10.1001/archinte.160.21.3278

Clarke G, 2005, J MED INTERNET RES, V7, DOI 10.2196/jmir.7.2.e16

Cohen J, 1988, STAT POWER ANAL BEHA

Coventry PA, 2011, BMC FAM PRACT, V12, DOI 10.1186/1471-2296-12-10

Cuijpers P, 2010, PSYCHOL MED, V40, P1943, DOI 10.1017/S0033291710000772

Cuijpers P, 2007, CLIN PSYCHOL REV, V27, P318, DOI 10.1016/j.cpr.2006.11.001

Cuijpers P, 2007, EUR PSYCHIAT, V22, P9, DOI 10.1016/j.eurpsy.2006.11.001

D'Zurilla T. J., 1990, PSYCHOL ASSESSMENT, V2, P156, DOI DOI 10.1037/1040-3590.2.2.156

de Groot M, 2001, PSYCHOSOM MED, V63, P619, DOI 10.1097/00006842-200107000-00015

de Jongh T, 2012, COCHRANE DB SYST REV, DOI 10.1002/14651858.CD007459.pub2

Delamater AM, 2006, CLIN DIABETES, V24, P71, DOI [10.2337/diaclin.24.2.71, DOI 10.2337/DIACLIN.24.2.71]

DiMatteo MR, 2000, ARCH INTERN MED, V160, P2101, DOI 10.1001/archinte.160.14.2101

Dong C, 2004, INT J OBESITY, V28, P790, DOI 10.1038/sj.ijo.0802626

Donker T, 2009, BMC MED, V7, DOI 10.1186/1741-7015-7-79

Drummond MF, 2007, METHODS EC EVALUATIO

Egede LE, 2010, DIABETES RES CLIN PR, V87, P302, DOI 10.1016/j.diabres.2010.01.024

Ehrmann D, 2010, DIABETOL STOFFWECHS, V5, pFV14

Elhai JD, 2008, PSYCHIAT RES, V159, P320, DOI 10.1016/j.psychres.2007.04.020

Eysenbach G, 2011, J MED INTERNET RES, V13, DOI 10.2196/jmir.1923

Fenwick E, 2004, HEALTH ECON, V13, P405, DOI 10.1002/hec.903

Fenwick E, 2001, HEALTH ECON, V10, P779, DOI 10.1002/hec.635

Ferrer-Roca O, 2004, J TELEMED TELECARE, V10, P282, DOI 10.1258/1357633042026341

First M., 1996, USER GUIDE STRUCTURE

FISCHER EH, 1970, J CONSULT CLIN PSYCH, V35, P79, DOI 10.1037/h0029636

Fogg BJ, 2003, MORGAN KAUFMANN SERI

Gahr A, 2011, DIABETOLOGIE STOFFWE, V6

Ginter E, 2013, ADV EXP MED BIOL, V771, P42

Gonzalez JS, 2008, DIABETES CARE, V31, P2398, DOI 10.2337/dc08-1341

Gregg JA, 2007, J CONSULT CLIN PSYCH, V75, P336, DOI 10.1037/0022-006X.75.2.336

Hakkaart-Van Roijen L., 2002, MANUAL TRIMBOS IMTA

Halle M, EVIDENZBASIERTE LEIT

Harrison V, 2011, J MENT HEALTH, V20, P509, DOI 10.3109/09638237.2011.608746

Hautzinger M, 2006, BDI 2 BECK DEPRESSIO

Hautzinger M, 1993, ALLGEMEINE DEPRESSIO

HERRMANN C, 1994, DIAGNOSTICA, V40, P143

Hutter N, 2010, DIABETOLOGIA, V53, P2470, DOI 10.1007/s00125-010-1873-y

International Diabetes Federation, 2012, IDF DIAB ATL

JACOBSON NS, 1991, J CONSULT CLIN PSYCH, V59, P12, DOI 10.1037//0022-006X.59.1.12

Kaltenthaler E, 2006, HEALTH TECHNOL ASSES, V10, P1, DOI 10.3310/hta10330

Kanter JW, 2007, J PSYCHOPATHOL BEHAV, V29, P191, DOI 10.1007/s10862-006-9038-5

Katon W, 2008, J GEN INTERN MED, V23, P1571, DOI 10.1007/s11606-008-0731-9

Katon WJ, 2005, DIABETES CARE, V28, P2668, DOI 10.2337/diacare.28.11.2668

Katon WJ, 2004, MED CARE, V42, P1222, DOI 10.1097/00005650-200412000-00009

Kay-Lambkin FJ, 2009, ADDICTION, V104, P378, DOI 10.1111/j.1360-0443.2008.02444.x

Krauth C, 2010, GESUNDH QUAL, V15, P251, DOI 10.1055/s-0029-1245680

Ladwig I, VERHALTENST IN PRESS

Lambert MJ, 2009, PSYCHOTHER RES, V19, P493, DOI 10.1080/10503300902849483

Lehnert T, 2011, PSYCHIAT PRAX, V38, P369, DOI 10.1055/s-0031-1276934

Leone T, 2012, GLOBALIZATION HEALTH, V8, DOI 10.1186/1744-8603-8-39

Little R J, 2002, STAT ANAL MISSING DA

Lokkerbol J, 2013, AM J GERIAT IN PRESS

Manos RC, 2011, BEHAV THER, V42, P726, DOI 10.1016/j.beth.2011.04.004

Meyer B, 2009, J MED INTERNET RES, V11, DOI 10.2196/jmir.1151

MEYER TJ, 1990, BEHAV RES THER, V28, P487, DOI 10.1016/0005-7967(90)90135-6

Mitchell AJ, 2009, LANCET, V374, P609, DOI 10.1016/S0140-6736(09)60879-5

Mitchell J., 2010, ELECT J APPL PSYCHOL, V6, P30, DOI DOI 10.7790/EJAP.V6I2.230

Morin CM, 2006, SLEEP, V29, P1398, DOI 10.1093/sleep/29.11.1398

Papelbaum M, 2011, DIABETOL METAB SYNDR, V3, DOI 10.1186/1758-5996-3-26

Peinemann F, 2013, COCHRANE DB SYST REV, DOI [10.1002/14651858.CD008216.pub4, 10.1371/journal.pmed.1000097, 10.1136/bmj.c869, 10.1136/bmj.b2535, 10.1016/j.jclinepi.2010.02.006, 10.1002/14651858.CD008242.pub3, 10.1016/j.jclinepi.2010.03.004]

Petrak F, 2009, CURR OPIN PSYCHIATR, V22, P211, DOI 10.1097/YCO.0b013e3283207b45

Piette JD, 2004, AM J MANAG CARE, V10, P152

POLONSKY WH, 1995, DIABETES CARE, V18, P754, DOI 10.2337/diacare.18.6.754

Prochaska JO, 1997, AM J HEALTH PROMOT, V12, P38, DOI 10.4278/0890-1171-12.1.38

RADLOFF L S, 1977, Applied Psychological Measurement, V1, P385, DOI 10.1177/014662167700100306

Riper H, 2011, J MED INTERNET RES, V13, DOI 10.2196/jmir.1691

Riper H, 2009, ALCOHOL CLIN EXP RES, V33, P1401, DOI 10.1111/j.1530-0277.2009.00970.x

Rohde P, 1997, AM J PSYCHIAT, V154, P1593, DOI 10.1176/ajp.154.11.1593

Ruwaard J, 2012, PLOS ONE, V7, DOI 10.1371/journal.pone.0040089

Schafer JL, 2000, MONOGRAPHS STAT APPL, V72

SCHMIDT J, 1989, PSYCHOTHER PSYCH MED, V39, P248

Schmitt A, 2013, HEALTH QUAL LIFE OUT, V11, DOI 10.1186/1477-7525-11-138

Schram MT, 2009, CURR DIABETES REV, V5, P112, DOI 10.2174/157339909788166828

Schulte D, 2005, Z KL PSYCH PSYCHOTH, V34, P176, DOI 10.1026/1616-3443.34.3.176

Simon GE, 2005, GEN HOSP PSYCHIAT, V27, P344, DOI 10.1016/j.genhosppsych.2005.04.008

Simon GE, 2007, ARCH GEN PSYCHIAT, V64, P65, DOI 10.1001/archpsyc.64.1.65

Smit F, 2011, J MED INTERNET RES, V13, DOI 10.2196/jmir.1694

Spek V, 2007, PSYCHOL MED, V37, P319, DOI 10.1017/S0033291706008944

van Bastelaar K, 2011, PATIENT EDUC COUNS, V84, P49, DOI 10.1016/j.pec.2010.06.013

van Bastelaar KM, 2008, BMC PSYCHIATRY, V8, DOI 10.1186/1471-244X-8-9

van Bastelaar KMP, 2012, J MED INTERNET RES, V14, DOI 10.2196/jmir.1657

van Bastelaar KMP, 2011, DIABETES CARE, V34, P320, DOI 10.2337/dc10-1248

van Eimeren B, 2012, 76 PROZENT DTSCH ONL

Wangberg Silje C, 2008, Patient Prefer Adherence, V2, P57

Ware JE, 1996, MED CARE, V34, P220, DOI 10.1097/00005650-199603000-00003

Warmerdam L, 2010, J MED INTERNET RES, V12, DOI 10.2196/jmir.1436

ZIGMOND AS, 1983, ACTA PSYCHIAT SCAND, V67, P361, DOI 10.1111/j.1600-0447.1983.tb09716.x

NR 105

TC 17

Z9 17

U1 4

U2 49

PU BMC

PI LONDON

PA CAMPUS, 4 CRINAN ST, LONDON N1 9XW, ENGLAND

SN 1471-244X

J9 BMC PSYCHIATRY

JI BMC Psychiatry

PD NOV 15

PY 2013

VL 13

AR 306

DI 10.1186/1471-244X-13-306

PG 10

WC Psychiatry

SC Psychiatry

GA 281YA

UT WOS:000329135700001

PM 24238346

OA DOAJ Gold, Green Published

DA 2019-01-08

ER

PT J

AU Hayashino, Y

Tsujii, S

Ishii, H

AF Hayashino, Y.

Tsujii, S.

Ishii, H.

CA Tenri Study Grp

TI High Frequency of Non-nocturnal Hypoglycemia was Associated with Poor

Sleep Quality Measure by Pittsburg Sleep Quality Index in Patients with

Diabetes Receiving Insulin Therapy: Diabetes Distress and Care Registry

at Tenri (DDCRT 4)

SO EXPERIMENTAL AND CLINICAL ENDOCRINOLOGY & DIABETES

LA English

DT Article

DE diabetes; autoimmunity; hypoglycemia; sleep

ID YOUNG-CHILDREN; MELLITUS; DEPRESSION; DURATION; IMPACT

AB Aims: To investigate the temporal association between frequency of non-nocturnal hypoglycemia and sleep quality among patients with diabetes receiving insulin therapy.

Methods: We used data from 1 513 patients with diabetes receiving insulin therapy. We estimated the relative risks (RR) of the frequency of non-nocturnal hypoglycemia for poor sleep quality measured by the Pittsburgh Sleep Quality Index.

Results: The average age and HbA1c value of the patients were 63.7 years and 7.8%, respectively. Compared with poor sleep quality in patients without any type of non-nocturnal disabling hypoglycemia (NNDH), the multivariablea-djusted RR values for poor sleep quality were 1.30 (95 % confidence interval [CI], 1.06-1.61) and 1.37 (95 % CI, 0.96-1.95) in patients who experienced 1-4 and >= 5 episodes of NNDH, respectively (p = 0.004). Experiencing non-nocturnal severe hypoglycemia (NNSH) once in the past 90 days significantly increased the risk of poor sleep quality by 1.54 episodes (95 % CI, 1.16-2.05; p = 0.003). By adding the presence of depression as a variable to the multivariable-adjusted model, these associations were attenuated because we did not observe any significant association between NNDH and poor sleep quality (p = 0.178). However, a significant association between NNSH and poor sleep quality was observed (RR = 1.43; 95 % CI, 1.09-1.90; p = 0.011).

Conclusions: A high frequency of non-nocturnal hypoglycemia was associated with poor sleep quality in patients with diabetes receiving insulin therapy. Our data also suggested that the association of NNDH, and not NNSH, was mediated by comorbid depression.

C1 [Hayashino, Y.; Tsujii, S.; Ishii, H.] Tenri Hosp, Dept Endocrinol, Tenri, Nara 6328552, Japan.

[Tsujii, S.] Tenri Hosp, Ctr Diabet, Tenri, Nara 6328552, Japan.

RP Hayashino, Y (reprint author), Tenri Hosp, Dept Endocrinol, Mishima Cho 200, Tenri, Nara 6328552, Japan.

EM hayasino-y@umin.net

OI Hayashino, Yasuaki/0000-0002-2932-8478; Tsujii,

Satoru/0000-0002-1834-6515

FU Manpei Suzuki Diabetes Foundation

FX The Manpei Suzuki Diabetes Foundation supported this study.

CR Ayas NT, 2003, DIABETES CARE, V26, P380, DOI 10.2337/diacare.26.2.380

Batal O, 2011, LUNG, V189, P141, DOI 10.1007/s00408-010-9277-9

Brod Meryl, 2012, J Med Econ, V15, P77, DOI 10.3111/13696998.2011.624144

BUYSSE DJ, 1989, PSYCHIAT RES, V28, P193, DOI 10.1016/0165-1781(89)90047-4

CUZICK J, 1985, STAT MED, V4, P87, DOI 10.1002/sim.4780040112

Doi Y, 1998, JAPANESE J PSYCHIAT, V13, P755

Gottlieb DJ, 2005, ARCH INTERN MED, V165, P863, DOI 10.1001/archinte.165.8.863

Green AJ, 2012, DIABETES RES CLIN PR, V96, P313, DOI 10.1016/j.diabres.2012.01.002

Guillod L, 2007, DIABETES METAB, V33, P360, DOI 10.1016/j.diabet.2007.03.007

Hayashino Y, 2012, DIABET MED

HAYASHINO Y, 2012, DIABETES RES CLIN PR

Jauch-Chara K, 2007, DIABETES, V56, P1938, DOI 10.2337/db07-0044

Mashitani T, 2013, DIABETES RES CLIN PR, V100, P189, DOI 10.1016/j.diabres.2013.03.006

Matyka KA, 2000, J PEDIATR-US, V137, P233, DOI 10.1067/mpd.2000.107186

McHale M, 2008, PSYCHOSOM MED, V70, P869, DOI 10.1097/PSY.0b013e318186dea9

Park MJ, 2012, METABOLISM, V61, P229, DOI 10.1016/j.metabol.2011.06.013

Porter PA, 1997, J PEDIATR-US, V130, P366, DOI 10.1016/S0022-3476(97)70197-5

Randloff LS, 1977, APPL PSYCH MEAS, V3, P385

Resnick HE, 2003, DIABETES CARE, V26, P702, DOI 10.2337/diacare.26.3.702

Rothman KJ, 1998, MODERN EPIDEMIOLOGY

Schultes Bernd, 2007, PLoS Med, V4, pe69, DOI 10.1371/journal.pmed.0040069

Skomro RP, 2001, SLEEP MED, V2, P417, DOI 10.1016/S1389-9457(01)00110-1

Yaggi HK, 2006, DIABETES CARE, V29, P657, DOI 10.2337/diacare.29.03.06.dc05-0879

NR 23

TC 6

Z9 6

U1 0

U2 2

PU JOHANN AMBROSIUS BARTH VERLAG MEDIZINVERLAGE HEIDELBERG GMBH

PI STUTTGART

PA RUEDIGERSTR 14, D-70469 STUTTGART, GERMANY

SN 0947-7349

EI 1439-3646

J9 EXP CLIN ENDOCR DIAB

JI Exp. Clin. Endocrinol. Diabet.

PD NOV

PY 2013

VL 121

IS 10

BP 628

EP 634

DI 10.1055/s-0033-1355424

PG 7

WC Endocrinology & Metabolism

SC Endocrinology & Metabolism

GA 267TV

UT WOS:000328122000009

PM 24277485

DA 2019-01-08

ER

PT J

AU Jacob, MM

Gonzales, KL

Calhoun, D

Beals, J

Muller, CJ

Goldberg, J

Nelson, L

Welty, TK

Howard, BV

AF Jacob, Michelle M.

Gonzales, Kelly L.

Calhoun, Darren

Beals, Janette

Muller, Clemma Jacobsen

Goldberg, Jack

Nelson, Lonnie

Welty, Thomas K.

Howard, Barbara V.

TI Psychological trauma symptoms and Type 2 diabetes prevalence, glucose

control, and treatment modality among American Indians in the Strong

Heart Family Study

SO JOURNAL OF DIABETES AND ITS COMPLICATIONS

LA English

DT Article

DE Diabetes treatment modality; American Indians; Psychological trauma

symptoms

ID HEALTH-CARE PROVIDERS; RESERVATION COMMUNITIES; CARDIOVASCULAR-DISEASE;

DEPRESSIVE SYMPTOMS; EMOTIONAL DISTRESS; MENTAL-DISORDERS;

STRESS-DISORDER; OLDER-ADULTS; ASSOCIATION; RISK

AB Aims: The aims of this paper are to examine the relationship between psychological trauma symptoms and Type 2 diabetes prevalence, glucose control, and treatment modality among 3776 American Indians in Phase V of the Strong Heart Family Study.

Methods: This cross-sectional analysis measured psychological trauma symptoms using the National Anxiety Disorder Screening Day instrument, diabetes by American Diabetes Association criteria, and treatment modality by four categories: no medication, oral medication only, insulin only, or both oral medication and insulin. We used binary logistic regression to evaluate the association between psychological trauma symptoms and diabetes prevalence. We used ordinary least squares regression to evaluate the association between psychological trauma symptoms and glucose control. We used binary logistic regression to model the association of psychological trauma symptoms with treatment modality.

Results: Neither diabetes prevalence (22%-31%; p = 0.19) nor control (8.0-8.6; p = 0.25) varied significantly by psychological trauma symptoms categories. However, diabetes treatment modality was associated with psychological trauma symptoms categories, as people with greater burden used either no medication, or both oral and insulin medications (odds ratio = 3.1, p < 0.001).

Conclusions: The positive relationship between treatment modality and psychological trauma symptoms suggests future research investigate patient and provider treatment decision making. (C) 2013 Elsevier Inc. All rights reserved.

C1 [Jacob, Michelle M.] Univ San Diego, Dept Ethn Studies, San Diego, CA 92110 USA.

[Jacob, Michelle M.] Heritage Univ, Ctr Native Hlth & Culture, San Diego, CA 92110 USA.

[Gonzales, Kelly L.] Portland State Univ, Coll Urban & Publ Affairs, Sch Community Hlth, Portland, OR 97201 USA.

[Calhoun, Darren] MedStar Hlth Res Inst, Phoenix, AZ 85016 USA.

[Beals, Janette] Univ Colorado, Ctr Amer Indian Hlth, Aurora, CO 80045 USA.

[Beals, Janette] Univ Colorado, Ctr Alaska Native Hlth, Aurora, CO 80045 USA.

[Muller, Clemma Jacobsen] Univ Washington, Seattle, WA 98101 USA.

[Goldberg, Jack] Univ Washington, VET Registry, Seattle VA ERIC Res Prof Epidemiol, Seattle, WA 98101 USA.

[Nelson, Lonnie] Univ Washington, Sch Publ Hlth, Dept Hlth Serv, Seattle, WA 98101 USA.

[Welty, Thomas K.] Missouri Breaks Res Inc, Timber Lake, SD 57656 USA.

[Howard, Barbara V.] MedStar Hlth Res Inst, Hyattsville, MD USA.

[Howard, Barbara V.] Georgetown Univ, Ctr Translat Sci, Hyattsville, MD USA.

[Howard, Barbara V.] Howard Univ, Ctr Translat Sci, Hyattsville, MD USA.

RP Jacob, MM (reprint author), Univ San Diego, Dept Ethn Studies, 5998 Alcala Pk,Maher 206, San Diego, CA 92110 USA.

EM mjacob@sandiego.edu

OI Muller, Clemma/0000-0001-5379-8747

FU Native Elder Research Center [AG015292]; Heritage University Center for

Native Health & Culture Faculty Fellowship; National Heart, Lung and

Blood Institute [U01HL-41642, U01HL-41652, U01HL-41654]

FX Native Elder Research Center AG015292; Heritage University Center for

Native Health & Culture Faculty Fellowship.; National Heart, Lung and

Blood Institute U01HL-41642, U01HL-41652 and U01HL-41654.

CR Anderson RJ, 2001, DIABETES CARE, V24, P1069, DOI 10.2337/diacare.24.6.1069

Beals J, 2005, ARCH GEN PSYCHIAT, V62, P99, DOI 10.1001/archpsyc.62.1.99

Boyko EJ, 2010, DIABETES CARE, V33, P1771, DOI 10.2337/dc10-0296

Calhoun D, 2009, J DIABETES COMPLICAT, V24, P217

Centers for Disease Control and Prevention, 2007, NAT DIAB FACT SHEET

Clar C, 2010, HEALTH TECHNOL ASSES, V14, P1, DOI 10.3310/hta14120

Dedert EA, 2010, ANN BEHAV MED, V39, P61, DOI 10.1007/s12160-010-9165-9

Delahanty LM, 2007, DIABETIC MED, V24, P48, DOI 10.1111/j.1464-5491.2007.02028.x

Everson-Rose SA, 2004, DIABETES CARE, V27, P2856, DOI 10.2337/diacare.27.12.2856

Garroutte EM, 2006, J GEN INTERN MED, V21, P111, DOI 10.1111/j.1525-1497.2006.00321.x

Garroutte EM, 2004, SOC SCI MED, V59, P2233, DOI 10.1016/j.socscimed.2004.03.032

Garroutte EM, 2008, SOC SCI MED, V67, P546, DOI 10.1016/j.socscimed.2008.04.015

Goodwin RD, 2005, PREV MED, V40, P570, DOI 10.1016/j.ypmed.2004.07.013

Jiang LH, 2008, DIABETES CARE, V31, P427, DOI 10.2337/dc07-2044

Jiang L, 2007, DIABETES CARE, V30, P2228, DOI 10.2337/dc07-0097

LEE ET, 1990, AM J EPIDEMIOL, V132, P1141, DOI 10.1093/oxfordjournals.aje.a115757

Lin EHB, 2008, J PSYCHOSOM RES, V65, P571, DOI 10.1016/j.jpsychores.2008.06.007

Lin PI, 2010, SCHIZOPHR RES, V123, P234, DOI 10.1016/j.schres.2010.08.022

Lustman PJ, 2007, PSYCHOSOM MED, V69, P297, DOI 10.1097/PSY.0b013e318060cc2d

Lustman PJ, 2002, J PSYCHOSOM RES, V53, P917, DOI 10.1016/S0022-3999(02)00416-6

Marshall RD, 2001, AM J PSYCHIAT, V158, P1467, DOI 10.1176/appi.ajp.158.9.1467

O'Connell J, 2010, DIABETES CARE, V33, P1463, DOI 10.2337/dc09-1652

RADLOFF L S, 1977, Applied Psychological Measurement, V1, P385, DOI 10.1177/014662167700100306

Ratanawongsa N, 2012, DIABETES EDUCATOR, V38, P94, DOI 10.1177/0145721711432649

Ritsher JB, 2002, PSYCHIAT RES, V111, P199, DOI 10.1016/S0165-1781(02)00135-X

Roubideaux Y., 2010, PRIORITIES REFORMING

van Bastelaar KMP, 2010, DIABETIC MED, V27, P798, DOI 10.1111/j.1464-5491.2010.03025.x

Wang H, 2010, DIABETES-METAB RES, V26, P378, DOI 10.1002/dmrr.1089

WELTY TK, 1995, AM J EPIDEMIOL, V142, P269, DOI 10.1093/oxfordjournals.aje.a117633

NR 29

TC 5

Z9 5

U1 0

U2 12

PU ELSEVIER SCIENCE INC

PI NEW YORK

PA 360 PARK AVE SOUTH, NEW YORK, NY 10010-1710 USA

SN 1056-8727

EI 1873-460X

J9 J DIABETES COMPLICAT

JI J. Diabetes Complications

PD NOV-DEC

PY 2013

VL 27

IS 6

BP 553

EP 557

DI 10.1016/j.jdiacomp.2013.07.008

PG 5

WC Endocrinology & Metabolism

SC Endocrinology & Metabolism

GA 257UZ

UT WOS:000327414600004

PM 24051029

OA Green Accepted

DA 2019-01-08

ER

PT J

AU Brumby, S

Chandrasekara, A

Kremer, P

Torres, S

McCoombe, S

Lewandowski, P

AF Brumby, Susan

Chandrasekara, Ananda

Kremer, Peter

Torres, Susan

McCoombe, Scott

Lewandowski, Paul

TI The effect of physical activity on psychological distress, cortisol and

obesity: results of the farming fit intervention program

SO BMC PUBLIC HEALTH

LA English

DT Article

DE Obesity; Cortisol; Mental health; Rural; Farmer

ID METABOLIC SYNDROME; DEPRESSION; EXERCISE; STRESS

AB Background: Rural and regional Australians have a higher likelihood of mental illness throughout their lifetime than people living in major cities, although the underlying reasons are not yet well defined. Additionally, rural populations experience more lifestyle associated co-morbidities including obesity, diabetes and cardiovascular disease. Research conducted by the National Centre for Farmer Health between 2004 and 2009 revealed a positive correlation between obesity and psychological distress among the farming community. Chronic stress is known to overstimulate the regulation of the hypothalamic-pituitary-adrenal (HPA) axis and cortisol secretion which are associated with abdominal adiposity. Increasing physical activity may normalise cortisol secretion and thereby positively impact both physical and mental health. This paper assesses the effects of increasing physical activity on obesity, health behaviors and mental health in Victorian farming men and women.

Methods: Farming Fit was a six month quasi-experimental (convenience sample) longitudinal design control-intervention study. Overweight or obese (BMI >= 25 kg/m(2)) farm men (n = 43) and women (n = 29) were recruited with demographic, health behaviors, anthropometric, blood pressure and biochemistry data collected at baseline and at a six months. Salivary cortisol and depression anxiety stress scale results were collected at baseline, three and six months. The intervention group (n = 37) received a personalized exercise program and regular phone coaching to promote physical activity. Results: The intervention group showed significant reductions in body weight and waist circumference.

Results indicated that following the six month exercise program, the intervention group were 2.64 +/- 0.65 kg lighter (p < 0.001), had reduced waist circumference by 2.01 +/- 0.86 cm (p = 0.02) and BMI by 0.97 +/- 0.22 kg/m(2) (p < 0.001) relative to the control group.

Conclusion: Increasing physical activity altered measures of obesity in farm men and women but did not affect mental health measures or cortisol secretion levels.

C1 [Brumby, Susan; Chandrasekara, Ananda; McCoombe, Scott] Western Dist Hlth Serv, Natl Ctr Farmer Hlth, Hamilton, Vic 3300, Australia.

[Brumby, Susan; Chandrasekara, Ananda; McCoombe, Scott; Lewandowski, Paul] Deakin Univ, Sch Med, Geelong, Vic 3216, Australia.

[Kremer, Peter] Deakin Univ, Sch Exercise & Nutr Sci, Geelong, Vic 3216, Australia.

[Torres, Susan] Deakin Univ, Sch Exercise & Nutr Sci, Burwood, Vic 3125, Australia.

RP Brumby, S (reprint author), Western Dist Hlth Serv, Natl Ctr Farmer Hlth, POB 283, Hamilton, Vic 3300, Australia.

EM susan.brumby@deakin.edu.au

RI Mashamba-Thompson, Tivani/B-6087-2014

OI Kremer, Peter/0000-0003-2476-1958

FU beyondblue; Victorian Department of Primary Industries

FX We acknowledge the funding agencies involved in this study: beyondblue

and the Victorian Department of Primary Industries. We would also like

to thank and acknowledge the contributions of Jason Thomas, Hannah

Simkin, Tracey Hatherell, Cate Mercer-Grant, Wayne Munro, April

Brinkmann, Jo Williams, Bianca Todd, SFF health professionals and the

farm men and women participating.

CR Alston M, 2004, AGR HUM VALUES, V21, P37, DOI 10.1023/B:AHUM.0000014019.84085.59

*AUSTR BUR STAT, 2009, NAT HLTH SURV SUMM R, V2012

Australian Bureau of Statistics, 2007, NAT SURV MENT HLTH W

Australian Bureau of Statistics, 2010, CAUS DEATH AUSTR 200

Australian Institute of Health and Welfare Australia's Health, 2010, 12 AIHW

Bjorntorp P, 2000, BLOOD PRESSURE, V9, P71, DOI 10.1080/08037050050151762

Booth ML, 2003, MED SCI SPORTS EXERC, V195, P3508

Brumby S, 2009, RURAL REMOTE HEALTH, V9, P1

BRUMBY S, 2012, 22 WORLD C GLOB WELL

Brumby S, 2011, BMC PUBLIC HEALTH, V11, DOI 10.1186/1471-2458-11-362

Brumby Susan, 2011, BMC Res Notes, V4, P89, DOI 10.1186/1756-0500-4-89

Collins P, 2012, INT J ENV RES PUB HE, V9, P3030, DOI 10.3390/ijerph9093030

Dallman MF, 2003, P NATL ACAD SCI USA, V100, P11696, DOI 10.1073/pnas.1934666100

Dunbar JA, 2008, DIABETES CARE, V31, P2368, DOI 10.2337/dc08-0175

EGGER G, 2005, PHYS ACTIVITY GUIDEL

International Physical Activity Questionnaire, 2005, GUID DAT PROC AN INT

Jones AP, 1998, J PUBLIC HEALTH MED, V20, P312, DOI 10.1093/oxfordjournals.pubmed.a024774

Keppel G., 1991, DESIGN ANAL RES HDB

LOVIBOND PF, 1995, BEHAV RES THER, V33, P335, DOI 10.1016/0005-7967(94)00075-U

Lovibond SH, 1995, MANUAL DEPRESSION AN

MACHADORODRIGUE.AM, 2012, URBAN RURAL CONTRAST

Mathers CD, 2001, B WORLD HEALTH ORGAN, V79, P1076

Physical Activity Guidelines Advisory Committee, 2008, PHYS ACT GUID ADV CO

SCHWARZ L, 1992, SPORTS MED, V13, P25, DOI 10.2165/00007256-199213010-00003

Vaughan C, 2009, BMC PUBLIC HEALTH, V9, DOI 10.1186/1471-2458-9-273

Wu T, 2009, OBES REV, V10, P313, DOI 10.1111/j.1467-789X.2008.00547.x

NR 26

TC 7

Z9 7

U1 1

U2 37

PU BIOMED CENTRAL LTD

PI LONDON

PA 236 GRAYS INN RD, FLOOR 6, LONDON WC1X 8HL, ENGLAND

SN 1471-2458

J9 BMC PUBLIC HEALTH

JI BMC Public Health

PD OCT 28

PY 2013

VL 13

AR UNSP 1018

DI 10.1186/1471-2458-13-1018

PG 6

WC Public, Environmental & Occupational Health

SC Public, Environmental & Occupational Health

GA 284BH

UT WOS:000329290500002

PM 24165391

OA DOAJ Gold, Green Published

DA 2019-01-08

ER

PT J

AU Gucciardi, E

Fortugno, M

Senchuk, A

Beanlands, H

McCay, E

Peel, EE

AF Gucciardi, Enza

Fortugno, Mariella

Senchuk, Andrea

Beanlands, Heather

McCay, Elizabeth

Peel, Elizabeth E.

TI Self-monitoring of blood glucose in Black Caribbean and South Asian

Canadians with non-insulin treated Type 2 diabetes mellitus: a

qualitative study of patients' perspectives

SO BMC ENDOCRINE DISORDERS

LA English

DT Article

DE Self-monitoring of blood glucose; Diabetes mellitus; Caribbean; South

Asian

ID GLYCEMIC CONTROL; HEALTH BELIEFS; TEST STRIPS; ORIGIN; METAANALYSIS;

INDIVIDUALS; PERCEPTIONS; MANAGEMENT; PAKISTANI; EFFICACY

AB Background: To examine the views and current practice of SMBG among Black Caribbean and South Asian individuals with non-insulin treated Type 2 diabetes mellitus.

Methods: Twelve participants completed semi-structured interviews that were guided by the Health Belief Model and analyzed using thematic network analysis.

Results: The frequency of monitoring among participants varied from several times a day to once per week. Most participants expressed similar experiences regarding their views and practices of SMBG. Minor differences across gender and culture were observed. All participants understood the benefits, but not all viewed SMBG as beneficial to their personal diabetes management. SMBG can facilitate a better understanding and maintenance of self-care behaviours. However, it can trigger both positive and negative emotional responses, such as a sense of disappointment when high readings are not anticipated, resulting in emotional distress. Health care professionals play a key role in the way SMBG is perceived and used by patients.

Conclusion: While the majority of participants value SMBG as a self-management tool, barriers exist that impede its practice, particularly its cost. How individuals cope with these barriers is integral to understanding why some patients adopt SMBG more than others.

C1 [Gucciardi, Enza; Fortugno, Mariella; Senchuk, Andrea] Ryerson Univ, Sch Nutr, Toronto, ON M5B 2K3, Canada.

[Beanlands, Heather; McCay, Elizabeth] Ryerson Univ, Daphne Cockwell Sch Nursing, Toronto, ON M5B 2K3, Canada.

[Peel, Elizabeth E.] Aston Univ, Sch Life & Hlth Sci, Birmingham B4 7ET, W Midlands, England.

RP Gucciardi, E (reprint author), Ryerson Univ, Sch Nutr, 350 Victoria St, Toronto, ON M5B 2K3, Canada.

EM egucciar@ryerson.ca

FU SSHRC Institutional Grant (SIG) Research, Ryerson University; Faculty of

Community Services Seed Grant, Ryerson University

FX SSHRC Institutional Grant (SIG) Research, Ryerson University; Faculty of

Community Services Seed Grant, Ryerson University.

CR Allemann S, 2009, CURR MED RES OPIN, V25, P2903, DOI 10.1185/03007990903364665

[Anonymous], 2007, INT J TECHNOL ASSESS, V23, P146

Attride-Stirling J, 2001, QUALITATIVE RES, V1, P385, DOI [DOI 10.1177/146879410100100307, 10.1177/146879410100100307]

Barnett AH, 2008, DIABETES OBES METAB, V10, P1239, DOI 10.1111/j.1463-1326.2008.00894.x

Bhopal R, 1999, BRIT MED J, V319, P215, DOI 10.1136/bmj.319.7204.215

Cameron C, 2010, CAN J DIABETES, V34, P34, DOI 10.1016/S1499-2671(10)41008-4

Cameron C, 2010, CAN MED ASSOC J, V182, P28, DOI 10.1503/cmaj.090765

*CAN DIAB ASS, 2008, CAN J DIABETES S1, V32, P1

Canadian Agency for Drugs and Technologies in Health, 2009, GAP AN KEY MESS PRES

Canadian Diabetes Association, 2013, SELF MON BLOOD GLUC

Canadian Optimal Medication Prescribing and Use Service, 2008, SYST REV US BLOOD GL

Creatore MI, 2010, CAN MED ASSOC J, V182, P781, DOI 10.1503/cmaj.091551

DiMatteo MR, 2007, MED CARE, V45, P521, DOI 10.1097/MLR.0b013e318032937e

Ezenwaka CE, 2012, ARCH PHYSIOL BIOCHEM, V118, P16, DOI 10.3109/13813455.2011.625950

Funnell MM, 2007, DIABETES EDUCATOR, V33, P1012, DOI 10.1177/0145721707308630

Gerrald KR, 2010, CLIN DIABETES, V28, P121

Gomersall T, 2011, QUAL HEALTH RES, V21, P853, DOI 10.1177/1049732311402096

Gomes T, 2010, CAN MED ASSOC J, V182, P35, DOI 10.1503/cmaj.091017

Harvey JN, 2009, DIABETIC MED, V26, P5, DOI 10.1111/j.1464-5491.2008.02628.x

Hjelm KG, 2005, J ADV NURS, V50, P47, DOI 10.1111/j.1365-2648.2004.03348.x

Karter AJ, 2000, DIABETES CARE, V23, P477, DOI 10.2337/diacare.23.4.477

King H, 1998, DIABETES CARE, V21, P1414, DOI 10.2337/diacare.21.9.1414

Latter C, 2011, CAN J DIABETES, V35, P31, DOI 10.1016/S1499-2671(11)51006-8

Lawton J, 2005, BRIT MED J, V330, P1247, DOI 10.1136/bmj.38460.642789.E0

Malanda UL, 2012, COCHRANE DB SYST REV, V1, P1, DOI DOI 10.1002/14651858.CD005060

McAndrew L, 2007, DIABETES EDUCATOR, V33, P991, DOI 10.1177/0145721707309807

McGeoch G, 2007, DIABETES-METAB RES, V23, P423, DOI 10.1002/dmrr.749

Miller D, 2011, CAN J DIABETES, V35, P317, DOI 10.1016/S1499-2671(11)54003-1

Nyomba BLG, 2002, DIABETES CARE, V25, P1244, DOI 10.2337/diacare.25.7.1244-a

O'Kane MJ, 2008, BRIT MED J, V336, P1174, DOI 10.1136/bmj.39534.571644.BE

Peel E, 2004, BRIT J GEN PRACT, V54, P183

Peel E, 2007, BRIT MED J, V335, P493, DOI 10.1136/bmj.39302.444572.DE

Poolsup N, 2008, DIABETES TECHNOL THE, V10, pS51, DOI 10.1089/dia.2008.0001

Rosenstock I. M., 1997, HLTH BEHAV HLTH ED T

SMBG International Working Group, 2008, Diabetes Res Clin Pract, V82, pe15, DOI 10.1016/j.diabres.2008.08.021

St John A, 2010, J DIABETES COMPLICAT, V24, P129, DOI 10.1016/j.jdiacomp.2009.01.002

Tan MY, 2004, DIABETES RES CLIN PR, V66, P71, DOI 10.1016/j.diabres.2004.02.021

The British Heart Foundation Statistics Website, 2008, ETHN DIFF PREV DIAB

Towfigh A, 2008, AM J MANAG CARE, V14, P468

Williams AS, 2004, DIABETES ACTION NETW, P19

Woo V, 2010, CAN J DIABETES, V34, P19, DOI 10.1016/S1499-2671(10)41003-5

Yi-Frazier JP, 2010, STRESS HEALTH, V26, P51, DOI 10.1002/smi.1258

NR 42

TC 7

Z9 7

U1 1

U2 19

PU BIOMED CENTRAL LTD

PI LONDON

PA 236 GRAYS INN RD, FLOOR 6, LONDON WC1X 8HL, ENGLAND

SN 1472-6823

J9 BMC ENDOCR DISORD

JI BMC Endocr. Disord.

PD OCT 14

PY 2013

VL 13

AR 46

DI 10.1186/1472-6823-13-46

PG 10

WC Endocrinology & Metabolism

SC Endocrinology & Metabolism

GA 258GM

UT WOS:000327447300001

PM 24119213

OA DOAJ Gold, Green Published

DA 2019-01-08

ER

PT J

AU Stephens, MAP

Franks, MM

Rook, KS

Iida, M

Hemphill, RC

Salem, JK

AF Stephens, Mary Ann Parris

Franks, Melissa M.

Rook, Karen S.

Iida, Masumi

Hemphill, Rachel C.

Salem, James K.

TI Spouses' Attempts to Regulate Day-to-Day Dietary Adherence Among

Patients With Type 2 Diabetes

SO HEALTH PSYCHOLOGY

LA English

DT Article

DE social support; social control; dietary adherence; daily diary

assessments; married couples

ID ECOLOGICAL MOMENTARY ASSESSMENT; SOCIAL-CONTROL; HEALTH BEHAVIORS;

PSYCHOLOGICAL DISTRESS; SELF-MANAGEMENT; CHRONIC ILLNESS; SUPPORT;

MELLITUS; IMPACT; ADULTS

AB Objective: To investigate daily dietary adherence and diabetes-specific distress among older adults with type 2 diabetes mellitus (T2DM) as a function of spouses' diet-related support and diet-related control (persuasion and pressure) and whether these daily processes differ among couples who do and do not appraise responsibility for managing T2DM as shared. Methods: End-of-day diaries were completed by 126 couples in which one partner had T2DM (patient) and the other did not (spouse). Using electronic diary methods, each partner independently recorded data for 24 consecutive days (patients recorded their day's dietary adherence and diabetes-specific distress; spouses recorded their day's involvement in patients' dietary management). To assess dietary adherence, patients reported the extent to which they followed dietary recommendations that day with items from the Summary of Diabetes Self-Care Activities Measure. To assess diabetes-specific distress, patients reported the extent to which they worried about diabetes that day using items from the Problem Areas in Diabetes (PAID) scale. Results: Multilevel modeling revealed that, relative to the prior day, spouses' diet-related support was associated with increases in patients' adherence whereas diet-related persuasion and pressure were associated with decreases in adherence; spouses' pressure was associated with increases in patients' diabetes-specific distress. When partners appraised responsibility for managing T2DM as shared, support was associated with decreases in diabetes-specific distress; pressure was associated with decreases in adherence. Conclusions: Our findings offer insight into partners' day-to-day disease-related interactions and identify those that are likely to be beneficial versus detrimental for patients' physical and psychological health.

C1 [Stephens, Mary Ann Parris; Iida, Masumi; Hemphill, Rachel C.] Kent State Univ, Dept Psychol, Kent, OH 44242 USA.

[Franks, Melissa M.] Purdue Univ, Dept Human Dev & Family Studies, W Lafayette, IN 47907 USA.

[Rook, Karen S.] Univ Calif Irvine, Dept Psychol & Social Behav, Irvine, CA 92717 USA.

[Salem, James K.] Summa Hlth Syst, Dept Diabet & Metab, Akron, OH USA.

RP Stephens, MAP (reprint author), Kent State Univ, Dept Psychol, POB 5190, Kent, OH 44242 USA.

EM mstephen@kent.edu

FU National Institute on Aging [R01 AG24833]

FX This research, entitled "Mind-Body Interactions in Management of Type 2

Diabetes," was supported by grant R01 AG24833 from the National

Institute on Aging.

CR Berg CA, 2007, PSYCHOL BULL, V133, P920, DOI 10.1037/0033-2909.133.6.920

Berg CA, 2009, J FAM PSYCHOL, V23, P521, DOI 10.1037/a0015556

Brehm S. S., 1981, PSYCHOL REACTANCE TH

Cain AE, 2009, J PSYCHIATR RES, V43, P987, DOI 10.1016/j.jpsychires.2009.01.014

Center for Disease Control and Prevention, 2011, NAT DIAB FACT SHEET

Centers for Disease Control and Prevention, 2010, DIAB SUCC OPP POP BA

Clemes SA, 2009, MED SCI SPORT EXER, V41, P674, DOI 10.1249/MSS.0b013e31818cae32

CONNELL CM, 1994, HEALTH PSYCHOL, V13, P263, DOI 10.1037//0278-6133.13.3.263

Cranford JA, 2006, PERS SOC PSYCHOL B, V32, P917, DOI 10.1177/0146167206287721

DiMatteo MR, 2004, HEALTH PSYCHOL, V23, P207, DOI 10.1037/0278-6133.23.2.207

Dunbar-Jacob J, 2001, HANDBOOK OF HEALTH PSYCHOLOGY, P571

Fekete E, 2009, PSYCHOL HEALTH, V24, P501, DOI 10.1080/08870440801894674

Fisher L, 2008, ANN FAM MED, V6, P246, DOI 10.1370/afm.842

Franks MM, 2006, J FAM PSYCHOL, V20, P311, DOI 10.1037/0893-3200.20.2.311

Gallant MP, 2003, HEALTH EDUC BEHAV, V30, P170, DOI 10.1177/1090198102251030

GARAYSEVILA ME, 1995, J DIABETES COMPLICAT, V9, P81, DOI 10.1016/1056-8727(94)00021-F

GLASGOW RE, 1988, DIABETES CARE, V11, P377, DOI 10.2337/diacare.11.5.377

Gonder-Frederick LA, 2002, J CONSULT CLIN PSYCH, V70, P611, DOI 10.1037//0022-006X.70.3.611

GOODALL TA, 1991, HEALTH PSYCHOL, V10, P1, DOI 10.1037/0278-6133.10.1.1

Green AJ, 2007, INT J CLIN PRACT, V61, P1791, DOI 10.1111/j.1742-1241.2007.01588.x

HAMMOND GS, 1992, DIABETES CARE, V15, P469, DOI 10.2337/diacare.15.4.469

Helgeson VS, 2004, J SOC PERS RELAT, V21, P53, DOI 10.1177/0265407504039840

Iida M, 2010, PERS SOC PSYCHOL B, V36, P780, DOI 10.1177/0146167210369897

KAPLAN RM, 1987, HEALTH PSYCHOL, V6, P387, DOI 10.1037/0278-6133.6.5.387

Lewis MA, 1999, HEALTH PSYCHOL, V18, P63, DOI 10.1037/0278-6133.18.1.63

Lewis MA, 2007, J APPL SOC PSYCHOL, V37, P298, DOI 10.1111/j.0021-9029.2007.00161.x

LITTLEFIELD CH, 1990, HEALTH PSYCHOL, V9, P737, DOI 10.1037//0278-6133.9.6.737

POLONSKY WH, 1995, DIABETES CARE, V18, P754, DOI 10.2337/diacare.18.6.754

ROOK KS, 1990, PSYCHOL AGING, V5, P327, DOI 10.1037/0882-7974.5.3.327

Shiffman S, 2008, ANNU REV CLIN PSYCHO, V4, P1, DOI 10.1146/annurev.clinpsy.3.022806.091415

Skinner TC, 2000, J PEDIATR PSYCHOL, V25, P257, DOI 10.1093/jpepsy/25.4.257

Stephens MAP, 2010, FAM SYST HEALTH, V28, P199, DOI 10.1037/a0020513

Stephens MAP, 2009, HEALTH PSYCHOL, V28, P48, DOI 10.1037/a0012385

Thompson A, 1999, J MARRIAGE FAM, V61, P38, DOI 10.2307/353881

Thorpe CT, 2008, J BEHAV MED, V31, P93, DOI 10.1007/s10865-007-9125-4

Toobert DJ, 2000, DIABETES CARE, V23, P943, DOI 10.2337/diacare.23.7.943

Trief P., 2003, FAMILIES SYSTEMS HLT, V21, P57, DOI [DOI 10.1037/H0089502, 10.1037/h0089502]

Tucker JS, 2006, HEALTH PSYCHOL, V25, P715, DOI 10.1037/0278-6133.25.6.715

Tucker JS, 2001, J APPL SOC PSYCHOL, V31, P467, DOI 10.1111/j.1559-1816.2001.tb02051.x

UMBERSON D, 1992, SOC SCI MED, V34, P907, DOI 10.1016/0277-9536(92)90259-S

Westmaas JL, 2002, HEALTH PSYCHOL, V21, P368, DOI 10.1037//0278-6133.21.4.368

Wild S, 2004, DIABETES CARE, V27, P1047, DOI 10.2337/diacare.27.5.1047

Williams K. E., 2002, PSYCHOL HEALTH MED, V7, P127, DOI [10.1080/13548500120116076, DOI 10.1080/13548500120116076]

Woodcock A, 2001, PATIENT EDUC COUNS, V42, P257, DOI 10.1016/S0738-3991(00)00131-2

NR 44

TC 53

Z9 53

U1 0

U2 38

PU AMER PSYCHOLOGICAL ASSOC

PI WASHINGTON

PA 750 FIRST ST NE, WASHINGTON, DC 20002-4242 USA

SN 0278-6133

EI 1930-7810

J9 HEALTH PSYCHOL

JI Health Psychol.

PD OCT

PY 2013

VL 32

IS 10

BP 1029

EP 1037

DI 10.1037/a0030018

PG 9

WC Psychology, Clinical; Psychology

SC Psychology

GA 232GW

UT WOS:000325481800001

PM 23025302

DA 2019-01-08

ER

PT J

AU Rasmussen, NH

Smith, SA

Maxson, JA

Bernard, ME

Cha, SS

Agerter, DC

Shah, ND

AF Rasmussen, Norman H.

Smith, Steven A.

Maxson, Julie A.

Bernard, Matthew E.

Cha, Stephen S.

Agerter, David C.

Shah, Nilay D.

TI Association of HbA(1c) with emotion regulation, intolerance of

uncertainty, and purpose in life in type 2 diabetes mellitus

SO PRIMARY CARE DIABETES

LA English

DT Article

DE Emotion regulation; HbA(1c); Intolerance of uncertainty; Primary care;

Purpose in life satisfaction

ID GLYCEMIC CONTROL; PRIMARY-CARE; VALIDATION; DEPRESSION; SCALE;

INTERVENTIONS; METAANALYSIS; POPULATION; MORBIDITY; EVENTS

AB Background: The extant literature lacks breadth on psychological variables associated with health outcome for type 2 diabetes mellitus (T2DM). This investigation extends the scope of psychological information by reporting on previously unpublished factors.

Objective: To investigate if intolerance of uncertainty, emotion regulation, or purpose in life differentiate T2DM adults with sustained high HbA(1c) (HH) vs. sustained acceptable HbA(1c) (AH).

Subjects and methods: Cross-sectional observational study. Adult patients with diagnosed T2DM meeting inclusionary criteria for AR, HH, or a nondiabetic reference group (NDR) were randomly selected and invited to participate. Patients who consented and participated resulted in a final sample of 312 subgrouped as follows: HH (n = 108); AH (n = 98); and NDR (n = 106). Data sources included a survey, self-report questionnaires, and electronic medical record (EMR).

Results: HH individuals with T2DM reported lower purpose in life satisfaction (p = 0.005) compared to the NDR group. The effect size for this finding is in the small-to-medium range using Cohen's guidelines for estimating clinical relevance. The HH-AH comparison on purpose in life was nonsignificant. The emotion regulation and intolerance of uncertainty comparisons across the three groups were not significant.

Conclusions: The present study determined that lower purpose in life satisfaction is associated with higher HbA(1c). In a T2DM patient with sustained high HbA(1c), the primary care clinician is encouraged to consider screening for purpose in life satisfaction by asking a single question such as "Do the things you do in your life seem important and worthwhile?" The patient's response will assist the clinician in determining if meaning or purpose in life distress may be interferring with diabetes self-care. If this is the case, the clinician can shift the conversation to the value of behavioral and emotional health counseling. (C) 2013 Primary Care Diabetes Europe. Published by Elsevier Ltd. All rights reserved.

C1 [Rasmussen, Norman H.] Mayo Clin, Div Integrated Behav Healthcare, Dept Psychiat & Psychol, Rochester, MN 55905 USA.

[Smith, Steven A.] Mayo Clin, Div Endocrinol Diabet Metab & Nutr, Rochester, MN 55905 USA.

[Rasmussen, Norman H.; Maxson, Julie A.; Bernard, Matthew E.; Agerter, David C.] Mayo Clin, Dept Family Med, Rochester, MN 55905 USA.

[Cha, Stephen S.] Mayo Clin, Div Biomed Stat & Informat, Rochester, MN 55905 USA.

[Shah, Nilay D.] Mayo Clin, Div Hlth Care Policy & Res, Rochester, MN 55905 USA.

RP Rasmussen, NH (reprint author), Mayo Clin, Div Integrated Behav Healthcare, Dept Family Med, 200 1st St SW, Rochester, MN 55905 USA.

EM rasmussen.norman@mayo.edu

FU Mayo Clinic Department of Family Medicine

FX Internal institutional discretionary research monies, allocated through

the Mayo Clinic Department of Family Medicine, funded this study. Robert

C. Colligan, PhD, Emeritus Member, Department of Psychiatry and

Psychology, Mayo Clinic, made several suggestions that improved the

clarity and scientific accuracy of the manuscript.

CR American Psychiatric Association, 1994, DIAGN STAT MAN MENT

Angstman KB, 2012, J PRIM CARE COMMUNIT, V3, P155, DOI 10.1177/2150131911423824

Arroll B, 2010, ANN FAM MED, V8, P348, DOI 10.1370/afm.1139

Bloomgarden Zachary T, 2009, Diabetes Care, V32, pe141, DOI 10.2337/dc09-zb12

Carleton RN, 2007, BEHAV RES THER, V45, P2307, DOI 10.1016/j.brat.2007.04.006

Carleton RN, 2007, J ANXIETY DISORD, V21, P105, DOI 10.1016/j.janxdis.2006.03.014

CHARLSON ME, 1987, J CHRON DIS, V40, P373, DOI 10.1016/0021-9681(87)90171-8

Cherrington A, 2010, J BEHAV MED, V33, P81, DOI 10.1007/s10865-009-9233-4

Ciechanowski PS, 2006, MED CARE, V44, P283, DOI 10.1097/01.mlr.0000199695.03840.0d

Cohen J, 1988, STAT POWER ANAL BEHA

Currie CJ, 2010, LANCET, V375, P481, DOI 10.1016/S0140-6736(09)61969-3

de Groot Mary, 2010, Diabetes Spectr, V23, P15, DOI 10.2337/diaspect.23.1.15

de Groot V, 2003, J CLIN EPIDEMIOL, V56, P221, DOI 10.1016/S0895-4356(02)00585-1

Duangdao KM, 2008, J BEHAV MED, V31, P291, DOI 10.1007/s10865-008-9155-6

Dugas MJ, 1997, COGNITIVE THER RES, V21, P593, DOI 10.1023/A:1021890322153

Fowler GC, 2010, SOUTH MED J, V103, P911, DOI 10.1097/SMJ.0b013e3181eb34b2

Garnefski N, 2001, PERS INDIV DIFFER, V30, P1311, DOI 10.1016/S0191-8869(00)00113-6

Grant P, 2010, PRIM CARE DIABETES, V4, P69, DOI 10.1016/j.pcd.2010.02.001

Gratz KL, 2004, J PSYCHOPATHOL BEHAV, V26, P41, DOI 10.1023/B:JOBA.0000007455.08539.94

Hall PA, 2009, J PSYCHOSOM RES, V67, P297, DOI 10.1016/j.jpsychores.2009.05.015

Huntley AL, 2012, ANN FAM MED, V10, P134, DOI 10.1370/afm.1363

Imayama I, 2011, HEALTH QUAL LIFE OUT, V9, DOI 10.1186/1477-7525-9-115

Ismail K, 2004, LANCET, V363, P1589, DOI 10.1016/S0140-6736(04)16202-8

Ismail K., 2009, PSYCHIATRY, V6, P203

Katon WJ, 2008, AM J MED, V121, P8, DOI 10.1016/j.amjmed.2008.09.008

KAZIS LE, 1989, MED CARE, V27, pS178, DOI 10.1097/00005650-198903001-00015

Keshen Aaron, 2006, Am J Psychother, V60, P285

Kraemer HC, 2001, AM J PSYCHIAT, V158, P848, DOI 10.1176/appi.ajp.158.987.848

Martin A, 2006, GEN HOSP PSYCHIAT, V28, P71, DOI 10.1016/j.genhosppsych.2005.07.003

Martins RK, 2009, CLIN PSYCHOL REV, V29, P283, DOI 10.1016/j.cpr.2009.02.001

Nouwen A, 2011, HEALTH PSYCHOL, V30, P771, DOI 10.1037/a0024500

Osborn CY, 2011, ANN BEHAV MED, V41, P300, DOI 10.1007/s12160-010-9241-1

Padala PR, 2008, DIABETES RES CLIN PR, V79, P37, DOI 10.1016/j.diabres.2007.06.012

Pani LN, 2008, DIABETES CARE, V31, P1991, DOI 10.2337/dc08-0577

Peyrot M, 2007, DIABETES CARE, V30, P2433, DOI 10.2337/dc07-1222

Rasmussen Norman H, 2010, Ment Health Fam Med, V7, P197

Rasmussen Norman H, 2008, Ment Health Fam Med, V5, P139

Reich JW, 2006, ARTHRIT RHEUM-ARTHR, V55, P86, DOI 10.1002/art.21700

Renn BN, 2011, CLIN PSYCHOL REV, V31, P1239, DOI 10.1016/j.cpr.2011.08.001

Ryder R., 1999, SPM HDB HLTH ASSESSM, P179

Scheier MF, 2006, J BEHAV MED, V29, P291, DOI 10.1007/s10865-005-9044-1

Seery MD, 2011, CURR DIR PSYCHOL SCI, V20, P390, DOI 10.1177/0963721411424740

Weinger K, 2011, ARCH INTERN MED, V171, P1990, DOI 10.1001/archinternmed.2011.502

Zoegemfiss K.Y., 2011, PATIENT, V4, P241

NR 44

TC 7

Z9 9

U1 2

U2 12

PU ELSEVIER SCI LTD

PI OXFORD

PA THE BOULEVARD, LANGFORD LANE, KIDLINGTON, OXFORD OX5 1GB, OXON, ENGLAND

SN 1751-9918

EI 1878-0210

J9 PRIM CARE DIABETES

JI Prim. Care Diabetes

PD OCT

PY 2013

VL 7

IS 3

BP 213

EP 221

DI 10.1016/j.pcd.2013.04.006

PG 9

WC Endocrinology & Metabolism; Primary Health Care

SC Endocrinology & Metabolism; General & Internal Medicine

GA 225LZ

UT WOS:000324965700006

PM 23685023

DA 2019-01-08

ER

PT J

AU Tanenbaum, ML

Ritholz, MD

Binko, DH

Baek, RN

Shreck, MSE

Gonzalez, JS

AF Tanenbaum, Molly L.

Ritholz, Marilyn D.

Binko, Deborah H.

Baek, Rachel N.

Shreck, M. S. Erica

Gonzalez, Jeffrey S.

TI Probing for depression and finding diabetes: a mixed-methods analysis of

depression interviews with adults treated for type 2 diabetes

SO JOURNAL OF AFFECTIVE DISORDERS

LA English

DT Article

DE Diabetes; Depression; Diabetes-related distress; Screening; Comorbidity

ID MONTGOMERY-ASBERG DEPRESSION; COMORBID DEPRESSION; DISTRESS SCALE;

SELF-CARE; METAANALYSIS; ASSOCIATION; SYMPTOMS; PEOPLE; TEMPERAMENT;

PREVALENCE

AB Background: Depression has increased prevalence and consistently predicts poor health outcomes among patients with diabetes. The impact of stressors related to diabetes and its treatment on depression assessment is infrequently considered.

Methods: We used mixed methods to evaluate depressive symptoms in adults with type 2 diabetes. We categorized responses related to diabetes and its treatment during interviews (n=70) using the Montgomery-Asberg Depression Rating Scale (MADRS) and administered questionnaires to measure diabetes related distress and depressive symptoms.

Results: Participants (M age=56, SD=7; 67% female; 64% Black; 21% Latino) had mild depression on average (MADRS M=10, SD=9). Half of those with symptoms spontaneously mentioned diabetes context; 61% said diabetes contributed to their symptoms when questioned directly. Qualitative themes included: overlapping symptoms of diabetes and depression; burden of diabetes treatment; emotional impact of diabetes; and the bidirectional influence of depression and diabetes. Diabetes was mentioned more often at higher levels of depression severity (r=.38, p=.001). Higher HbA1c was associated with mentioning diabetes as a context for depressive symptoms (r=.32, p=.007). Insulin-users mentioned diabetes more often than those on oral medications only (p=.005).

Limitations: MADRS is not a traditional qualitative interview so themes may not provide an exhaustive view of the role of diabetes context in depression assessment.

Conclusions and clinical implications: The burden of type 2 diabetes and its treatment often provide an explanatory context for depressive symptoms assessed by structured clinical interviews, the gold standard of depression assessment. Diabetes context may influence accuracy of assessment and should inform intervention planning for those needing treatment. (C) 2013 Elsevier B.V. All rights reserved.

C1 [Tanenbaum, Molly L.; Binko, Deborah H.; Baek, Rachel N.; Shreck, M. S. Erica; Gonzalez, Jeffrey S.] Yeshiva Univ, Ferkauf Grad Sch Psychol, Bronx, NY 10461 USA.

[Ritholz, Marilyn D.] Joslin Diabet Ctr, Boston, MA 02215 USA.

[Ritholz, Marilyn D.] Harvard Univ, Sch Med, Boston, MA USA.

[Ritholz, Marilyn D.] Boston Childrens Hosp, Boston, MA USA.

[Gonzalez, Jeffrey S.] Albert Einstein Coll Med, Diabet Res Ctr, Bronx, NY 10467 USA.

RP Gonzalez, JS (reprint author), Yeshiva Univ, Ferkauf Grad Sch Psychol, 1300 Morris Pk Ave, Bronx, NY 10461 USA.

EM jeffrey.gonzalez@einstein.yu.edu

OI Tanenbaum, Molly/0000-0003-4222-4224

FU National Institute of Diabetes and Digestive and Kidney Diseases (NIDDK)

[DK-020541]

FX This study was partially supported by Grant DK-020541 from the National

Institute of Diabetes and Digestive and Kidney Diseases (NIDDK).

CR Aikens JE, 2008, DIABETIC MED, V25, P1324, DOI 10.1111/j.1464-5491.2008.02590.x

American Psychiatric Association, DIAGN STAT MAN MENT

American Psychiatric Association, 2000, DIAGN STAT MAN MENT

Anderson RJ, 2001, DIABETES CARE, V24, P1069, DOI 10.2337/diacare.24.6.1069

Beverly EA, 2012, J GEN INTERN MED, V27, P1180, DOI 10.1007/s11606-012-2070-0

Beverly EA, 2011, DIABETES CARE, V34, P1086, DOI 10.2337/dc10-2298

Black SA, 2003, DIABETES CARE, V26, P2822, DOI 10.2337/diacare.26.10.2822

Cohen J, 1988, STAT POWER ANAL BEHA

de Groot M, 2001, PSYCHOSOM MED, V63, P619, DOI 10.1097/00006842-200107000-00015

Egede LE, 2005, DIABETES CARE, V28, P1339, DOI 10.2337/diacare.28.6.1339

Egede LE, 2005, PSYCHOSOM MED, V67, P46, DOI 10.1097/01.psy.0000149260.82006.fb

Fisher EB, 2012, J AFFECT DISORDERS, V142, pS56, DOI 10.1016/S0165-0327(12)70009-5

Fisher L, 2008, DIABETIC MED, V25, P1096, DOI 10.1111/j.1464-5491.2008.02533.x

Fisher L, 2007, DIABETES CARE, V30, P542, DOI 10.2337/dc06-1614

Fisher L, 2012, DIABETES CARE, V35, P259, DOI 10.2337/dc11-1572

Fisher L, 2010, DIABETES CARE, V33, P23, DOI 10.2337/dc09-1238

Gask L, 2011, CHRONIC ILLN, V7, P239, DOI 10.1177/1742395311403636

Gois C, 2012, J AFFECT DISORDERS, V143, P1, DOI 10.1016/j.jad.2012.05.028

Gois C, 2012, J AFFECT DISORDERS, V142, pS67, DOI 10.1016/S0165-0327(12)70010-1

Gois C, 2011, J AFFECT DISORDERS, V134, P52, DOI 10.1016/j.jad.2011.05.021

Golden SH, 2008, JAMA-J AM MED ASSOC, V299, P2751, DOI 10.1001/jama.299.23.2751

Gonzalez JS, 2008, DIABETOLOGIA, V51, P1822, DOI 10.1007/s00125-008-1113-x

Gonzalez JS, 2007, DIABETES CARE, V30, P2222, DOI 10.2337/dc07-0158

Gonzalez JS, 2011, DIABETES CARE, V34, P236, DOI 10.2337/dc10-1970

Gonzalez JS, 2008, DIABETES CARE, V31, P2398, DOI 10.2337/dc08-1341

Holt RIG, 2012, J AFFECT DISORDERS, V142, pS72, DOI 10.1016/S0165-0327(12)70011-3

Katon WJ, 2005, DIABETES CARE, V28, P2668, DOI 10.2337/diacare.28.11.2668

Kim NS, 2012, MED DECIS MAKING, V32, P820, DOI 10.1177/0272989X12443417

Knol MJ, 2007, PSYCHOSOM MED, V69, P300, DOI 10.1097/PSY.0b013e31805f48b9

Kokanovic R., 2012, DEPRESSION AMBIVALEN

Ludman EJ, 2004, GEN HOSP PSYCHIAT, V26, P430, DOI 10.1016/j.genhosppsych.2004.08.010

Lustman PJ, 2000, DIABETES CARE, V23, P934, DOI 10.2337/diacare.23.7.934

McPherson S, 2009, SOC SCI MED, V69, P1137, DOI 10.1016/j.socscimed.2009.05.032

Mitchell AJ, 2011, J AFFECT DISORDERS, V130, P26, DOI 10.1016/j.jad.2010.07.028

MONTGOMERY SA, 1979, BRIT J PSYCHIAT, V134, P382, DOI 10.1192/bjp.134.4.382

Patton M.Q., 1999, HEALTH SERV RES, V34, P1139

POLONSKY WH, 1995, DIABETES CARE, V18, P754, DOI 10.2337/diacare.18.6.754

Polonsky WH, 2005, DIABETES CARE, V28, P626, DOI 10.2337/diacare.28.3.626

Pope C., 2000, QUALITATIVE RES HLTH

RADLOFF L S, 1977, Applied Psychological Measurement, V1, P385, DOI 10.1177/014662167700100306

Ritholz MD, 2011, CURR DIABETES REP, V11, P494, DOI 10.1007/s11892-011-0226-7

Roy T, 2012, DIABETIC MED, V29, P164, DOI 10.1111/j.1464-5491.2011.03401.x

Rubin RR, 2001, J CLIN PSYCHOL, V57, P457, DOI 10.1002/jclp.1041

Russell Cynthia K, 2003, Evid Based Nurs, V6, P36

Sheehan DV, 1998, J CLIN PSYCHIAT, V59, P34, DOI 10.4088/JCP.09m05305whi

SNAITH RP, 1986, BRIT J PSYCHIAT, V148, P599, DOI 10.1192/bjp.148.5.599

Svanborg P, 2001, J AFFECT DISORDERS, V64, P203, DOI 10.1016/S0165-0327(00)00242-1

Tanenbaum ML, 2012, DIABETES EDUCATOR, V38, P695, DOI 10.1177/0145721712452795

van den Boogaard TM, 2011, SOC SCI MED, V73, P1733, DOI 10.1016/j.socscimed.2011.09.027

Vileikyte L, 2009, DIABETOLOGIA, V52, P1265, DOI 10.1007/s00125-009-1363-2

Wittink MN, 2008, QUAL HEALTH RES, V18, P1174, DOI 10.1177/1049732308321737

NR 51

TC 5

Z9 5

U1 0

U2 23

PU ELSEVIER SCIENCE BV

PI AMSTERDAM

PA PO BOX 211, 1000 AE AMSTERDAM, NETHERLANDS

SN 0165-0327

EI 1573-2517

J9 J AFFECT DISORDERS

JI J. Affect. Disord.

PD SEP 5

PY 2013

VL 150

IS 2

BP 533

EP 539

DI 10.1016/j.jad.2013.01.029

PG 7

WC Clinical Neurology; Psychiatry

SC Neurosciences & Neurology; Psychiatry

GA 206YQ

UT WOS:000323563300051

PM 23453278

OA Green Accepted

DA 2019-01-08

ER

PT J

AU Hsu, HC

Chang, YH

Lee, PJ

Chen, SY

Hsieh, CH

Lee, YJ

Wang, RH

AF Hsu, Hui-Chun

Chang, Yu-Hung

Lee, Pei-Ju

Chen, Shi-Yu

Hsieh, Chang-Hsun

Lee, Yau-Jiunn

Wang, Ruey-Hsia

TI Developing and Psychometric Testing of a Short-Form Problem Areas in

Diabetes Scale in Chinese Patients

SO JOURNAL OF NURSING RESEARCH

LA English

DT Article

DE diabetes; Problem Areas in Diabetes; emotional distress

ID EMOTIONAL DISTRESS; DEPRESSION; VERSION; VALIDITY; CARE

AB Background: The 20-item Problem Areas in Diabetes (PAID) scale is widely used to measure diabetes-related emotional distress. The short-form PAID scale is helpful for the rapid screening of diabetes-related emotional distress in clinical settings.

Purpose: This study developed and examined the psychometric properties of a short-form Chinese-version PAID (SFPAID- C) scale.

Methods: The Chinese-version 20-item PAID (PAID-C) scale was administered to 855 patients with type 2 diabetes mellitus. Item analysis, exploratory factor analysis, and confirmatory factor analysis were then applied to develop the SF-PAID-C and evaluate its construct validity. The correlations between SFPAID- C and the latest HbA1c close to the measurement of PAID-C (baseline HbA1c) 3 months and 12 months later were used to examine the concurrent and predictive validity of the SF-PAID-C. Receiver operating characteristic curve analysis was used to examine the sensitivity and specificity of the SF-PAID-C. Cronbach's alpha was used to assess internal consistency. Test-retest on 24 patients was used to examine the stability of the SF-PAID-C.

Results: An 8-item SF-PAID-C was developed. The SF-PAID-C significantly correlated with the PAID-C (r =.941, p < .001), baseline HbA1c (r = .148, p < .001), 3-month HbA1c (r = .147, p < .001), and 12-month HbA1c (r = .142, p < .001). The sensitivity and specificity of the SF-PAID-C were 93.2% and 94.2%, respectively. The Cronbach's alpha and test-retest reliability of the SF-PAID-C were .85 and.93, respectively.

Conclusions/Implications for Practice: The SF-PAID-C is a reliable and valid scale that can be used to screen for diabetes-related emotional problem in Chinese patients with type 2 diabetes mellitus in clinical settings.

C1 [Hsu, Hui-Chun; Chang, Yu-Hung; Lee, Pei-Ju; Lee, Yau-Jiunn] Lees Endocrinol Clin, Pingtung, Taiwan.

[Hsu, Hui-Chun] Kaohsiung Med Univ, Sch Nursing, Kaohsiung, Taiwan.

[Chen, Shi-Yu; Hsieh, Chang-Hsun] Triserv Gen Hosp, Div Endocrinol & Metab, Taipei, Taiwan.

[Wang, Ruey-Hsia] Kaohsiung Med Univ, Coll Nursing, Kaohsiung, Taiwan.

RP Wang, RH (reprint author), 100 Shih Chuan 1st Rd, Kaohsiung 80708, Taiwan.

EM wrhsia@kmu.edu.tw

RI Chang, Yu Hung/F-8176-2011

OI Chang, Yu Hung/0000-0001-6116-1893

CR Amer Diabet Assoc, 2013, DIABETES CARE, V36, pS11, DOI 10.2337/dc13-S011

Amsberg S, 2008, INT J NURS STUD, V45, P1319, DOI 10.1016/j.ijnurstu.2007.09.010

Bai YL, 2008, INT J NURS STUD, V45, P571, DOI 10.1016/.i.ijnurstu.2006.07.026

Biggerstaff BJ, 2000, STAT MED, V19, P649, DOI 10.1002/(SICI)1097-0258(20000315)19:5<649::AID-SIM371>3.0.CO;2-H

Chawla A, 2010, DIABETES EDUCATOR, V36, P337, DOI 10.1177/0145721709354607

Cheng ST, 2006, INT J GERIATR PSYCH, V21, P333, DOI 10.1002/gps.1467

Department of Health Executive Yuan Taiwan ROC, 2012, 2011 CAUS DEATH STAT

Eigenmann CA, 2009, DIABETIC MED, V26, P425, DOI 10.1111/j.1464-5491.2009.02697.x

Ely JJ, 2011, RURAL REMOTE HEALTH, V11

Eom YS, 2011, DIABETES METAB J, V35, P182, DOI 10.4093/dmj.2011.35.2.182

FERKETICH S, 1991, RES NURS HEALTH, V14, P165, DOI 10.1002/nur.4770140211

Hermanns N, 2006, DIABETOLOGIA, V49, P469, DOI 10.1007/s00125-005-0094-2

Hu LT, 1999, STRUCT EQU MODELING, V6, P1, DOI 10.1080/10705519909540118

Huang MF, 2010, DIABETES CARE, V33, P38, DOI 10.2337/dc09-0768

Karlsen B, 2012, J ADV NURS, V68, P391, DOI 10.1111/j.1365-2648.2011.05751.x

Kline R. B., 2011, PRINCIPLES PRACTICE

Lustman PJ, 1998, SEMIN CLIN NEUROPSYC, V3, P102

McGuire BE, 2010, DIABETOLOGIA, V53, P66, DOI 10.1007/s00125-009-1559-5

Nozaki T, 2009, BIOPSYCHOSOC MED, V3, DOI 10.1186/1751-0759-3-4

Ogbera A, 2011, J DIABETES, V3, P348, DOI 10.1111/j.1753-0407.2011.00156.x

POLONSKY WH, 1995, DIABETES CARE, V18, P754, DOI 10.2337/diacare.18.6.754

RINDSKOPF D, 1988, MULTIVAR BEHAV RES, V23, P51, DOI 10.1207/s15327906mbr2301_3

Rosner B, 2006, FUNDAMENTALS BIOSTAT

Sigurdardottir AK, 2008, INT J NURS STUD, V45, P526, DOI 10.1016/j.ijnurstu.2006.10.008

Snoek FJ, 2000, DIABETES CARE, V23, P1305, DOI 10.2337/diacare.23.9.1305

Tien KJ, 2008, DIABETES RES CLIN PR, V79, P276, DOI 10.1016/j.diabres.2007.08.015

Ullman JB, 2006, J PERS ASSESS, V87, P35, DOI 10.1207/s15327752jpa8701_03

Wang RH, 2011, J ADV NURS, V67, P2658, DOI 10.1111/j.1365-2648.2011.05701.x

NR 28

TC 8

Z9 9

U1 0

U2 11

PU LIPPINCOTT WILLIAMS & WILKINS

PI PHILADELPHIA

PA TWO COMMERCE SQ, 2001 MARKET ST, PHILADELPHIA, PA 19103 USA

SN 1682-3141

J9 J NURS RES

JI J. Nurs. Res.

PD SEP

PY 2013

VL 21

IS 3

BP 212

EP 217

DI 10.1097/01.jnr.0000432048.31921.e2

PG 6

WC Nursing

SC Nursing

GA 300MY

UT WOS:000330469200009

PM 23958611

OA Bronze

DA 2019-01-08

ER

PT J

AU Wagner, JA

Tennen, H

Feinn, R

Finan, PH

AF Wagner, Julie A.

Tennen, Howard

Feinn, Richard

Finan, Patrick H.

TI RACIAL DISCRIMINATION AND METABOLIC CONTROL IN WOMEN WITH TYPE 2

DIABETES

SO ETHNICITY & DISEASE

LA English

DT Article

DE Discrimination; Racism; Diabetes; Glycemic Control; Women

ID STRESSFUL LIFE EVENTS; PERCEIVED DISCRIMINATION; INSULIN-RESISTANCE;

HEALTH; DEPRESSION; ASSOCIATION; EXPERIENCES; CARDIA; ADULTS; CARE

AB Purpose: We investigated whether self-reported racial discrimination was associated with insulin resistance (IR) and glycosylated hemoglobin (A1c) in women with type 2 diabetes in the United States, after controlling for covariates.

Methods: Seventy-seven Black and White women with type 2 diabetes completed the Experiences of Discrimination Scale, which assesses self-reported lifetime frequency of racially motivated discrimination. Participants provided fasting blood samples for assessment of glucose and insulin for determination of IR and A1c. Covariates included age, education, waist circumference, diabetes distress, and stressful life events.

Results: In unadjusted regression analysis discrimination was significantly associated with IR. There was a trend for a race by discrimination interaction, with a weaker effect for Blacks than Whites. Follow up analysis showed that discrimination was significantly associated with IR in both Blacks and Whites, even after adjustment, as was waist circumference. In unadjusted regression analysis, discrimination was significantly associated with A1c. There was a significant race by discrimination interaction. Follow up analysis showed that discrimination was not significantly associated with A1c among Blacks, but was among Whites, even after adjustment, as was diabetes distress and insulin use.

Conclusions: Racial discrimination is associated with insulin resistance in Black and White women with diabetes, and with A1c in White women with diabetes.

C1 [Wagner, Julie A.; Tennen, Howard] Univ Connecticut, Ctr Hlth, Farmington, CT 06410 USA.

[Finan, Patrick H.] Johns Hopkins Univ, Sch Med, Baltimore, MD 21218 USA.

RP Wagner, JA (reprint author), Univ Connecticut, Ctr Hlth, Div Behav Sci & Community Hlth MC3910, 263 Farmington Ave, Farmington, CT 06410 USA.

EM juwagner@uchc.edu

FU NIDDK [5R21DK074468]; University of Connecticut Health Center General

Clinical Research Center [M01RR006192]

FX Supported by grants from NIDDK 5R21DK074468 and the University of

Connecticut Health Center General Clinical Research Center (M01RR006192)

CR Aikens JE, 1997, DIABETES CARE, V20, P1111, DOI 10.2337/diacare.20.7.1111

Bacha F, 2012, PEDIATR DIABETES, V13, P259, DOI 10.1111/j.1399-5448.2011.00820.x

Barnes LL, 2008, AM J PUBLIC HEALTH, V98, P1241, DOI 10.2105/AJPH.2007.114397

Borrell LN, 2013, ETHNIC HEALTH, V18, P227, DOI 10.1080/13557858.2012.713092

Cunningham TJ, 2012, SOC SCI MED, V75, P922, DOI 10.1016/j.socscimed.2012.04.027

De Vogli R, 2007, J PSYCHOSOM RES, V63, P413, DOI 10.1016/j.jpsychores.2007.04.006

Donga E, 2010, J CLIN ENDOCR METAB, V95, P2963, DOI 10.1210/jc.2009-2430

ESPOSITODELPUENTE A, 1994, INT J OBESITY, V18, P766

Gibbons FX, 2012, J PERS SOC PSYCHOL, V102, P1089, DOI 10.1037/a0027404

Grandner MA, 2012, BEHAV SLEEP MED, V10, P235, DOI 10.1080/15402002.2012.654548

Hausmann LRM, 2008, MED CARE, V46, P905, DOI 10.1097/MLR.0b013e3181792562

Helgeson VS, 2010, HEALTH PSYCHOL, V29, P153, DOI 10.1037/a0018163

Hickson DA, 2012, ANN BEHAV MED, V43, P4, DOI 10.1007/s12160-011-9334-5

HOLMES TH, 1967, J PSYCHOSOM RES, V11, P213, DOI 10.1016/0022-3999(67)90010-4

Jamieson JP, 2013, PSYCHOL SCI, V24, P131, DOI 10.1177/0956797612448194

Johnson Portia, 2012, J Natl Black Nurses Assoc, V23, P34

Kan C, 2013, DIABETES CARE, V36, P480, DOI 10.2337/dc12-1442

Krieger N, 2005, SOC SCI MED, V61, P1576, DOI 10.1016/j.socscimed.2005.03.006

Lustman PJ, 2000, DIABETES CARE, V23, P934, DOI 10.2337/diacare.23.7.934

MATTHEWS DR, 1985, DIABETOLOGIA, V28, P412, DOI 10.1007/BF00280883

Miyazaki Y, 2002, AM J PHYSIOL-ENDOC M, V283, P1135

Pascoe AE, 2009, PSYCHOL BULL, V135, P531

POLONSKY WH, 1995, DIABETES CARE, V18, P754, DOI 10.2337/diacare.18.6.754

Pyykkonen AJ, 2010, DIABETES CARE, V33, P378, DOI 10.2337/dc09-1027

SURWIT RS, 1992, DIABETES CARE, V15, P1413, DOI 10.2337/diacare.15.10.1413

Wagner J, EXPOSURE DI IN PRESS

Wagner J, 2007, DIABETES CARE, V30, P364, DOI 10.2337/dc06-1756

Wagner JA, 2013, STRESS HEALTH, V29, P214, DOI 10.1002/smi.2449

Wagner JA, 2011, J NATL MED ASSOC, V103, P224, DOI 10.1016/S0027-9684(15)30298-4

Williams DR, 2012, AM J PUBLIC HEALTH, V102, P975, DOI 10.2105/AJPH.2012.300702

NR 30

TC 8

Z9 8

U1 0

U2 8

PU INT SOC HYPERTENSION BLACKS-ISHIB

PI ATLANTA

PA 100 AUBURN AVE NE STE 401, ATLANTA, GA 30303-2527 USA

SN 1049-510X

EI 1945-0826

J9 ETHNIC DIS

JI Ethn. Dis.

PD FAL

PY 2013

VL 23

IS 4

BP 421

EP 427

PG 7

WC Public, Environmental & Occupational Health

SC Public, Environmental & Occupational Health

GA 280HN

UT WOS:000329019400005

PM 24392603

DA 2019-01-08

ER

PT J

AU Joensen, LE

Almdal, TP

Willaing, I

AF Joensen, Lene E.

Almdal, Thomas P.

Willaing, Ingrid

TI Type 1 diabetes and living without a partner: Psychological and social

aspects, self-management behaviour, and glycaemic control

SO DIABETES RESEARCH AND CLINICAL PRACTICE

LA English

DT Article

DE Type 1 diabetes; Cohabitation; Social support; Diabetes distress;

Glycaemic control

ID QUALITY-OF-LIFE; MARITAL-STATUS; CHRONIC ILLNESS; EMOTIONAL DISTRESS;

GENDER-DIFFERENCES; METABOLIC-CONTROL; HEALTH SURVEY; SCALE; MELLITUS;

SUPPORT

AB Aims: (1) To investigate the association between cohabitation status and psychological aspects of living with diabetes (diabetes distress, diabetes empowerment, quality of life), self-management behaviours, and glycaemic control and (2) to explore whether potential associations are mediated by social support.

Methods: Cross-sectional survey of 2419 adult outpatients with type 1 diabetes from a specialized diabetes clinic in Denmark. Stepwise multiple regression gender-stratified analyses assessed the association between cohabitation status and the variables of interest and the influence of social support.

Results: Significant associations existed between living without a partner and low quality of life, low diabetes empowerment and HbA1c for both men and women. For women, living without a partner was significantly associated with higher diabetes distress and poor self-management behaviours. All associations were mediated by social support to varying degrees.

Conclusions: Social network and social support are related to important diabetes outcomes in type 1 diabetes. Living without a partner indicates a need for support to prevent poorer diabetes outcomes. Women appear more susceptible to living without a partner in terms of psychosocial diabetes outcomes and glycaemic control. Generally, social support is a mediator in the association between cohabitation status and diabetes outcomes, but social support, as well as cohabitation status, are also independently associated with poorer diabetes outcomes. Assessment of cohabitation status as an indicator of psychosocial capabilities and glycaemic control may be useful in diabetes care and support. (C) 2013 Elsevier Ireland Ltd. All rights reserved.

C1 [Joensen, Lene E.; Willaing, Ingrid] Steno Diabet Ctr, Steno Hlth Promot Ctr, DK-2820 Gentofte, Denmark.

[Almdal, Thomas P.] Gentofte Univ Hosp, Gentofte, Denmark.

RP Joensen, LE (reprint author), Niels Steensens Vej 8, DK-2820 Gentofte, Denmark.

EM ljoe@steno.dk; almdal@dadlnet.dk; iwtp@steno.dk

OI Joensen, Lene Eide/0000-0002-4790-8759; Willaing,

Ingrid/0000-0002-3082-8816

FU Novo Foundation; Novo Nordisk A/S

FX L.J. and I.W. are employed by Steno Diabetes Center A/S, a research

hospital working in the Danish National Health Service and owned by Novo

Nordisk A/S. Steno Diabetes Center receives part of its core funding

from unrestricted grants from the Novo Foundation and Novo Nordisk A/S.

CR Anderson R, 2000, NAT HIST, V109, P23

Anderson RM, 2003, DIABETES CARE, V26, P1641, DOI 10.2337/diacare.26.5.1641-a

ANTONUCCI TC, 1987, SEX ROLES, V17, P737, DOI 10.1007/BF00287685

August KJ, 2010, SOC SCI MED, V71, P1831, DOI 10.1016/j.socscimed.2010.08.022

BERKMAN LF, 1979, AM J EPIDEMIOL, V109, P186, DOI 10.1093/oxfordjournals.aje.a112674

Christensen AI, 2012, SCAND J PUBLIC HEALT, V40, P391, DOI 10.1177/1403494812451412

de Oliveira IR, 2010, INT J NURS PRACT, V16, P106, DOI 10.1111/j.1440-172X.2009.01806.x

Due P, 1999, SOC SCI MED, V48, P661, DOI 10.1016/S0277-9536(98)00381-5

Eller M, 2008, INT J PUBLIC HEALTH, V53, P188, DOI 10.1007/s00038-008-7091-4

Enzlin P, 2002, PATIENT EDUC COUNS, V48, P139, DOI 10.1016/S0738-3991(02)00009-5

Fisher L, 2007, DIABETES CARE, V30, P542, DOI 10.2337/dc06-1614

Fisher L, 2012, DIABETES CARE, V35, P259, DOI 10.2337/dc11-1572

Fitzgerald JT, 1996, EVAL HEALTH PROF, V19, P208, DOI 10.1177/016327879601900205

Funnell MM, 2010, FAM PRACT, V27, pI17, DOI 10.1093/fampra/cmp027

Gallant MP, 2003, HEALTH EDUC BEHAV, V30, P170, DOI 10.1177/1090198102251030

Gandek B, 1998, J CLIN EPIDEMIOL, V51, P1171, DOI 10.1016/S0895-4356(98)00109-7

GOVE WR, 1983, J HEALTH SOC BEHAV, V24, P122, DOI 10.2307/2136639

Harkness E, 2010, DIABETES CARE, V33, P926, DOI 10.2337/dc09-1519

Hart HE, 2003, QUAL LIFE RES, V12, P1089, DOI 10.1023/A:1026197119569

Hermanns N, 2006, DIABETOLOGIA, V49, P469, DOI 10.1007/s00125-005-0094-2

HOUSE JS, 1988, SCIENCE, V241, P540, DOI 10.1126/science.3399889

Joensen LE, 2013, DIABETIC MED, V30, P1132, DOI 10.1111/dme.12241

LLOYD CE, 1993, DIABETES RES CLIN PR, V21, P187, DOI 10.1016/0168-8227(93)90068-G

Lloyd CE, 1999, DIABETES RES CLIN PR, V44, P9, DOI 10.1016/S0168-8227(99)00004-2

Manzoli L, 2007, SOC SCI MED, V64, P77, DOI 10.1016/j.socscimed.2006.08.031

Metayer C, 1996, EUR J EPIDEMIOL, V12, P573, DOI 10.1007/BF00499455

Nilsson PM, 2005, SCAND J PUBLIC HEALT, V33, P412, DOI 10.1080/14034940510005905

PARKERSON GR, 1993, MED CARE, V31, P629, DOI 10.1097/00005650-199307000-00005

Peyrot M, 2005, DIABETIC MED, V22, P1379, DOI 10.1111/j.1464-5491.2005.01644.x

Peyrot M, 1999, J HEALTH SOC BEHAV, V40, P141, DOI 10.2307/2676370

Polonsky WH, 2005, DIABETES CARE, V28, P626, DOI 10.2337/diacare.28.3.626

Rosland AM, 2012, J BEHAV MED, V35, P221, DOI 10.1007/s10865-011-9354-4

Rothman JGS, 1998, MODERN EPIDEMIOLOGY, P115

Schiotz ML, 2012, DIABETIC MED, V29, P654, DOI 10.1111/j.1464-5491.2011.03485.x

Shaban C, 2009, DIABETES RES CLIN PR, V85, pE26, DOI 10.1016/j.diabres.2009.05.006

Snoek FJ, 2000, DIABETES CARE, V23, P1305, DOI 10.2337/diacare.23.9.1305

Solli O, 2010, HEALTH QUAL LIFE OUT, V8, DOI 10.1186/1477-7525-8-18

Toobert DJ, 2000, DIABETES CARE, V23, P943, DOI 10.2337/diacare.23.7.943

van Bastelaar KMP, 2010, DIABETIC MED, V27, P798, DOI 10.1111/j.1464-5491.2010.03025.x

Weinger K, 2001, PATIENT EDUC COUNS, V42, P123, DOI 10.1016/S0738-3991(00)00098-7

WIKBY A, 1991, DIABETES RES CLIN PR, V13, P53, DOI 10.1016/0168-8227(91)90033-A

WIKBY A, 1993, QUAL LIFE RES, V2, P281, DOI 10.1007/BF00434799

Wysocki Tim, 2006, Curr Diab Rep, V6, P117, DOI 10.1007/s11892-006-0022-y

Zulman DM, 2012, PATIENT EDUC COUNS, V87, P74, DOI 10.1016/j.pec.2011.07.013

NR 44

TC 16

Z9 16

U1 1

U2 14

PU ELSEVIER IRELAND LTD

PI CLARE

PA ELSEVIER HOUSE, BROOKVALE PLAZA, EAST PARK SHANNON, CO, CLARE, 00000,

IRELAND

SN 0168-8227

EI 1872-8227

J9 DIABETES RES CLIN PR

JI Diabetes Res. Clin. Pract.

PD SEP

PY 2013

VL 101

IS 3

BP 278

EP 285

DI 10.1016/j.diabres.2013.07.001

PG 8

WC Endocrinology & Metabolism

SC Endocrinology & Metabolism

GA 231NW

UT WOS:000325424800009

PM 23891305

DA 2019-01-08

ER

PT J

AU Choi, SE

Rush, EB

Henry, SL

AF Choi, Sarah E.

Rush, Elizabeth B.

Henry, Shayna L.

TI Negative Emotions and Risk for Type 2 Diabetes Among Korean Immigrants

SO DIABETES EDUCATOR

LA English

DT Article

ID BODY-MASS INDEX; ASIAN-AMERICANS; WEIGHT-GAIN; DEPRESSION; PREVALENCE;

ADULTS; POPULATIONS; DISTRESS; ANXIETY; OBESITY

AB Purpose The purpose of this study is to examine the relationship between negative emotions and bio-behavioral risk factors among Korean immigrants at risk for type 2 diabetes (T2DM).

Methods Data were collected from 148 Korean immigrant adults who are at risk for T2DM as defined by having family history of T2DM in first-degree relatives, body mass index greater than 23, or history of gestational diabetes in women. Participants completed questionnaires and underwent biological measures. Negative emotions included feeling nervous, hopeless, restless, anxious, and stressed as well as depressive symptoms.

Results High percentages of participants had T2DM risk factors including overweight, greater than normal waist to hip ratio, and blood glucose readings that are indicative of T2DM. Feeling stressed was the most commonly reported negative emotion (66%), followed by feeling anxious (51%), restless (38%), nervous (30%), and hopeless (13%). Experience of negative emotions was significantly related to behavioral risk factors; higher levels of experiencing negative emotions were related to increased soda intake and a decreased likelihood of doing at least 10 minutes of moderate exercise. Stress and anxiety were each negatively related to moderate exercise, and depressive symptoms were negatively related to both moderate and vigorous exercise. No significant relationship was found between negative emotions and biological risk factors.

Conclusions Findings suggest that negative emotions, individually and taken together, may be related to T2DM risk behaviors in high-risk Korean immigrants. Behavioral interventions to prevent T2DM in this population should consider assessing and addressing negative emotions.

C1 [Choi, Sarah E.] Univ Calif Irvine, Coll Hlth Sci, Program Nursing Sci, Irvine, CA 92697 USA.

[Rush, Elizabeth B.; Henry, Shayna L.] Univ Calif Irvine, Sch Social Ecol, Dept Psychol & Social Behav, Irvine, CA 92697 USA.

RP Choi, SE (reprint author), Univ Calif Irvine, Coll Hlth Sci, Program Nursing Sci, 100B Berk Hall, Irvine, CA 92697 USA.

EM sechoi@uci.edu

FU National Center for Research Resources; National Center for Advancing

Translational Sciences, National Institute of Health [UL1 TR000153, 8KL2

TR000147]

FX This study was supported by a grant from the National Center for

Research Resources and the National Center for Advancing Translational

Sciences, National Institute of Health, through Grant UL1 TR000153 and

8KL2 TR000147 (Dr Choi).

CR Al-Amer RM, 2011, J DIABETES COMPLICAT, V25, P247, DOI 10.1016/j.jdiacomp.2011.03.001

American Diabetes A, 2012, DIABETES CARE S1, V35, pS11, DOI DOI 10.2337/DC12-S011

Bailey RC, 2013, DRUG ALCOHOL REV, V32, P162, DOI 10.1111/j.1465-3362.2012.00500.x

Barba C, 2004, LANCET, V363, P157

Barnes PM, 2008, HLTH CHARACTERISTICS, V394

Barzi F, 2005, ANN EPIDEMIOL, V15, P405, DOI 10.1016/j.annepidem.2005.01.005

Bouwman V, 2010, PLOS ONE, V5, DOI 10.1371/journal.pone.0009971

Breslau J, 2006, SOC PSYCH PSYCH EPID, V41, P943, DOI 10.1007/s00127-006-0119-2

BRISLIN RW, 1970, J CROSS CULT PSYCHOL, V1, P185, DOI 10.1177/135910457000100301

California Health Interview Survey, 2009, CHIS 2005 AD PUBL US

Center for Disease Control and Prevention, 2011, NAT DIAB FACT SHEET

CHAN JM, 1994, DIABETES CARE, V17, P961, DOI 10.2337/diacare.17.9.961

Cho MJ, 1998, PSYCHIAT RES, V81, P341, DOI 10.1016/S0165-1781(98)00122-X

Cho MJ, 1998, J NERV MENT DIS, V186, P304, DOI 10.1097/00005053-199805000-00007

Choi SE, 2011, J IMMIGR MINOR HEALT, V13, P803, DOI 10.1007/s10903-010-9397-6

Choi SE, 2011, NURS RES, V60, P240, DOI 10.1097/NNR.0b013e31822232a0

Clark J. L. D., 1988, STUDIES 2 LANGUAGE A, V10, P129, DOI [10.1017/S0272263100007270, DOI 10.1017/S0272263100007270]

COLDITZ GA, 1995, ANN INTERN MED, V122, P481, DOI 10.7326/0003-4819-122-7-199504010-00001

Daniels MC, 2006, J APPL GERONTOL, V25, p60S, DOI 10.1177/0733464805285483

*DPP RES GROUP, 2002, DIABETES CARE, V25, P2165, DOI DOI 10.2337/DIACARE.25.12.2165

Engum A, 2007, J PSYCHOSOM RES, V62, P31, DOI 10.1016/j.jpsychores.2006.07.009

Fisher L, 2008, DIABETIC MED, V25, P1096, DOI 10.1111/j.1464-5491.2008.02533.x

Fisher L, 2007, DIABETES CARE, V30, P542, DOI 10.2337/dc06-1614

Franks MM, 2012, AGING MENT HEALTH, V16, P902, DOI 10.1080/13607863.2012.674486

Gaitan-Sierra C, 2011, HEALTH PSYCHOL, V30, P793, DOI 10.1037/a0025582

Gonzalez JS, 2008, DIABETIC MED, V25, P1102, DOI 10.1111/j.1464-5491.2008.02535.x

Han HR, 2002, NURS RES, V51, P309, DOI 10.1097/00006199-200209000-00007

Hawkley LC, 2009, HEALTH PSYCHOL, V28, P354, DOI 10.1037/a0014400

HURH WM, 1990, J NERV MENT DIS, V178, P703, DOI 10.1097/00005053-199011000-00006

Huxley R, 2008, J HYPERTENS, V26, P169, DOI 10.1097/HJH.0b013e3282f16ad3

Katon WJ, 2010, INT J GERIATR PSYCH, V25, P466, DOI 10.1002/gps.2363

Kiecolt-Glaser JK, 2002, ANNU REV PSYCHOL, V53, P83, DOI 10.1146/annurev.psych.53.100901.135217

Kim MT, 2005, ARCH PSYCHIAT NURS, V19, P217, DOI 10.1016/j.apnu.2005.07.004

KUO WH, 1984, J NERV MENT DIS, V172, P449, DOI 10.1097/00005053-198408000-00002

Lasser K, 2000, JAMA-J AM MED ASSOC, V284, P2606, DOI 10.1001/jama.284.20.2606

Laugero KD, 2010, APPETITE, V56, P194

McNeely MJ, 2004, DIABETES CARE, V27, P66, DOI 10.2337/diacare.27.1.66

Noh S, 1998, CROSS CULTURAL RES J, V32, P358, DOI DOI 10.1177/106939719803200403

Paddison CAM, 2011, BRIT J HEALTH PSYCH, V16, P213, DOI 10.1348/135910710X495366

Park SY, 2008, ARCH PSYCHIAT NURS, V22, P12, DOI 10.1016/j.apnu.2007.06.011

Perna FM, 2008, HEALTH PSYCHOL, V27, P275, DOI 10.1037/0278-6133.27.2.275

Prochaska J, 2011, AM J LIFESTYLE MED, V5, P208, DOI DOI 10.1177/1559827610391883

RADLOFF L S, 1977, Applied Psychological Measurement, V1, P385, DOI 10.1177/014662167700100306

Rajpathak SN, 2010, ETHNIC DIS, V20, P225

Schwerdtfeger A, 2010, J SPORT EXERCISE PSY, V32, P674, DOI 10.1123/jsep.32.5.674

Sepulveda A, 2010, SPAN J PSYCHOL, V13, P364, DOI 10.1017/S1138741600003929

Shai I, 2006, DIABETES CARE, V29, P1585, DOI 10.2337/dc06-0057

Shin HS, 2007, INT J NURS STUD, V44, P415, DOI 10.1016/j.ijnurstu.2006.04.007

Shiwaku K, 2005, DIABETES RES CLIN PR, V69, P52, DOI 10.1016/j.diabres.2004.11.001

Simon GE, 2008, GEN HOSP PSYCHIAT, V30, P32, DOI 10.1016/j.genhosppsych.2007.09.001

Skaff MM, 2009, HEALTH PSYCHOL, V28, P265, DOI 10.1037/a0014429

Steptoe A, 2009, J PERS, V77, P1747, DOI 10.1111/j.1467-6494.2009.00599.x

[U. S. Dietary Guidelines Advisory Committee U. S. Department of Agriculture U. S. Department of Health and Human Services], 2010, DIET GUID AM 2010

Wismer B, 1999, KOREAN AM COMMUNITY

Yeomans MR, 2009, APPETITE, V52, P290, DOI 10.1016/j.appet.2008.10.006

Yun WJ, 2012, BMC PUBLIC HEALTH, V12, DOI 10.1186/1471-2458-12-724

NR 56

TC 1

Z9 2

U1 0

U2 14

PU SAGE PUBLICATIONS INC

PI THOUSAND OAKS

PA 2455 TELLER RD, THOUSAND OAKS, CA 91320 USA

SN 0145-7217

J9 DIABETES EDUCATOR

JI Diabetes Educ.

PD SEP

PY 2013

VL 39

IS 5

BP 679

EP 688

DI 10.1177/0145721713492566

PG 10

WC Endocrinology & Metabolism; Public, Environmental & Occupational Health

SC Endocrinology & Metabolism; Public, Environmental & Occupational Health

GA 223TF

UT WOS:000324832200011

PM 23793726

OA Green Accepted

DA 2019-01-08

ER

PT J

AU Siminerio, L

Ruppert, KM

Gabbay, RA

AF Siminerio, Linda

Ruppert, Kristine M.

Gabbay, Robert A.

TI Who Can Provide Diabetes Self-Management Support in Primary Care?

Findings From a Randomized Controlled Trial

SO DIABETES EDUCATOR

LA English

DT Article

ID EMPOWERMENT; EDUCATION; ADULTS; SCALE

AB Purpose The purpose of this comparative effectiveness study is to compare diabetes self-management support (DSMS) approaches and determine who can be most effective in helping patients maintain/improve clinical outcomes, self-care behaviors, distress, and satisfaction following diabetes self-management education (DSME) delivered in primary care.

Methods After receiving DSME, 141 participants were randomized to receive DSMS delivered by a trained supporter: educator, peer, practice staff, or usual education during a 6-month follow-up period. DSMS groups were compared to determine which supporter helped participants to maintain/improve A1C, blood pressure, lipids, weight, self-care, and distress. DSMS satisfaction was also examined.

Results There was a significant improvement in A1C, empowerment, aspects of self-care, and distress following DSME at 6 weeks. Those in the educator DSMS group best sustained improved A1C while those in the other DSMS groups maintained glycemic improvements but began to show trends toward worsening. No significant differences or clear trends were seen in other clinical, behavioral, or psychosocial outcomes. The Program Reinforcement Impacts Self-Management (PRISM) study demonstrates that following DSME, participants maintained improved glycemia, lipid, weight, and self-care behaviors and reductions in distress throughout the delivery of DSMS interventions regardless of DSMS supporter. All of the participants reported satisfaction with DSMS.

Conclusions These findings reaffirm the critical role of educators but suggest that others may serve as DSMS supporters. Results suggest that DSME delivered in primary care is effective and multiple DSMS agents are reasonable. As patient-centered self-management approaches are being explored in primary care, delivery of DSME and DSMS becomes paramount.

C1 [Siminerio, Linda] Univ Pittsburgh, Dept Med, Pittsburgh, PA 15203 USA.

[Ruppert, Kristine M.] Univ Pittsburgh, Grad Sch Publ Hlth, Pittsburgh, PA 15203 USA.

[Gabbay, Robert A.] Penn State Coll Med, Hershey, PA USA.

RP Siminerio, L (reprint author), Univ Pittsburgh, Dept Med, Quantum 1 Bldg,2 Hot Met St,2nd Fl,Rm 275, Pittsburgh, PA 15203 USA.

EM simineriol@upmc.edu

OI Ruppert, Kristine/0000-0002-3008-6584

FU Air Force Surgeon General's Office [FA7014-10-2-0005]

FX This research study is sponsored by the Air Force Surgeon General's

Office under agreement number FA7014-10-2-0005. The views and

conclusions contained herein are those of the authors and should not be

interpreted as necessarily representing the official policies or

endorsements, either expressed or implied, of the Air Force Surgeon

General's Office or the US Government.

CR American Academy of Family Physicians American Academy of Paediatrics American College of Physicians American Osteopathic Association, 2007, JOINT PRINC PAT CTR

Anderson R, 2000, NAT HIST, V109, P23

ANDERSON RM, 1995, DIABETES CARE, V18, P943, DOI 10.2337/diacare.18.7.943

Anderson RM, 2003, DIABETES CARE, V26, P1641, DOI 10.2337/diacare.26.5.1641-a

Haas L, 2013, DIABETES CARE, V36, pS100, DOI 10.2337/dc13-S100

Heisler M, 2010, ANN INTERN MED, V153, P507, DOI 10.7326/0003-4819-153-8-201010190-00007

JANES GR, 1995, DIABETES AM, V95, P541

Kocher R, 2010, ANN INTERN MED, V153, P536, DOI 10.7326/0003-4819-153-8-201010190-00274

Lorig KR, 2001, MED CARE, V39, P1217, DOI 10.1097/00005650-200111000-00008

National Committee for Quality Assurance, PHYS PRACT CONN PAT

Norris SL, 2002, DIABETES CARE, V25, P1159, DOI 10.2337/diacare.25.7.1159

Norris SL, 2001, DIABETES CARE, V24, P561, DOI 10.2337/diacare.24.3.561

Peyrot M, 2007, DIABETES EDUCATOR, V33, P818, DOI 10.1177/0145721707307614

Piette JD, 2001, DIABETES CARE, V24, P202, DOI 10.2337/diacare.24.2.202

POLONSKY WH, 1995, DIABETES CARE, V18, P754, DOI 10.2337/diacare.18.6.754

Tang T, 2011, DIABETES SPECTR, V24, P85, DOI DOI 10.2337/DIASPECT.24.2.85

Tang Tricia S., 2011, PEER LEAD MAN

Toobert DJ, 2000, DIABETES CARE, V23, P943, DOI 10.2337/diacare.23.7.943

Walker EA, 2011, DIABETES CARE, V34, P2, DOI 10.2337/dc10-1005

NR 19

TC 29

Z9 31

U1 1

U2 11

PU SAGE PUBLICATIONS INC

PI THOUSAND OAKS

PA 2455 TELLER RD, THOUSAND OAKS, CA 91320 USA

SN 0145-7217

J9 DIABETES EDUCATOR

JI Diabetes Educ.

PD SEP

PY 2013

VL 39

IS 5

BP 705

EP 713

DI 10.1177/0145721713492570

PG 9

WC Endocrinology & Metabolism; Public, Environmental & Occupational Health

SC Endocrinology & Metabolism; Public, Environmental & Occupational Health

GA 223TF

UT WOS:000324832200014

PM 23782622

DA 2019-01-08

ER

PT J

AU Pouwer, F

Nefs, G

Nouwen, A

AF Pouwer, Francois

Nefs, Giesje

Nouwen, Arie

TI Adverse Effects of Depression on Glycemic Control and Health Outcomes in

People with Diabetes A Review

SO ENDOCRINOLOGY AND METABOLISM CLINICS OF NORTH AMERICA

LA English

DT Review

DE Type 1 diabetes; Type 2 diabetes; Depression; Mortality; Cardiovascular

disease; Microvascular disease; Review

ID EDID RESEARCH CONSORTIUM; MINDFULNESS-BASED INTERVENTION;

CORONARY-HEART-DISEASE; CO-MORBID DEPRESSION; EMOTIONAL DISTRESS;

PROSPECTIVE COHORT; EUROPEAN DEPRESSION; INCREASED RISK; COMORBID

DEPRESSION; INSULIN THERAPY

AB In the past decades, important advances have been achieved in the psychological aspects of diabetes. This article reviews the associations between diabetes, depression, and adverse health outcomes. The article provides an update on the literature regarding the prevalence of depression in diabetes, discusses the impact of depression on diabetes self-care and glycemic control in people with diabetes, and summarizes the results of longitudinal studies that have investigated depression as a risk factor for adverse health outcomes.

C1 [Pouwer, Francois; Nefs, Giesje] Tilburg Univ, Ctr Res Psychol Somat Dis CoRPS, Dept Med & Clin Psychol, NL-5000 LE Tilburg, Netherlands.

[Nouwen, Arie] Middlesex Univ, Sch Hlth & Social Sci, London NW4 4BT, England.

RP Pouwer, F (reprint author), Tilburg Univ, Ctr Res Psychol Somat Dis CoRPS, Dept Med & Clin Psychol, Warandelaan 2,POB 90153, NL-5000 LE Tilburg, Netherlands.

EM f.pouwer@tilburguniversity.edu

RI Nefs, Giesje/C-9992-2018

CR Aikens JE, 2012, DIABETES CARE, V35, P2472, DOI 10.2337/dc12-0181

Aikens JE, 2009, DIABETES CARE, V32, P1177, DOI 10.2337/dc09-0071

American Psychiatric Association, 2000, DIAGN STAT MAN MENT

Barnard KD, 2006, DIABETIC MED, V23, P445, DOI 10.1111/j.1464-5491.2006.01814.x

Baumeister H, 2012, COCHRANE DB SYST REV, V12, DOI DOI 10.1002/14651858

Bogner HR, 2007, DIABETES CARE, V30, P3005, DOI 10.2337/dc07-0974

Bot M, 2013, DIABETIC MED, V30, pE115, DOI 10.1111/dme.12082

Cappuccio FP, 2010, DIABETES CARE, V33, P414, DOI 10.2337/dc09-1124

Champaneri S, 2010, CURR DIABETES REP, V10, P396, DOI 10.1007/s11892-010-0148-9

Clouse RE, 2003, PSYCHOSOM MED, V65, P376, DOI 10.1097/01.PSY.0000041624.96580.1F

Colton PA, 2013, PEDIATR DIABETES, V14, P372, DOI 10.1111/pedi.12016

Cosgrove MP, 2008, OCCUP MED-OXFORD, V58, P7, DOI 10.1093/occmed/kqm105

de Groot M, 2001, PSYCHOSOM MED, V63, P619, DOI 10.1097/00006842-200107000-00015

de Jonge P, 2010, NEUROSCI BIOBEHAV R, V35, P84, DOI 10.1016/j.neubiorev.2009.11.025

Fisher L, 2010, DIABETES CARE, V33, P23, DOI 10.2337/dc09-1238

Gendelman N, 2009, DIABETES CARE, V32, P575, DOI 10.2337/dc08-1835

Georgiades A, 2007, PSYCHOSOM MED, V69, P235, DOI 10.1097/PSY.0b013e318042588d

Gonzalez JS, 2010, DIABETOLOGIA, V53, P2241, DOI 10.1007/s00125-010-1821-x

Gonzalez JS, 2008, DIABETES CARE, V31, P2398, DOI 10.2337/dc08-1341

Gregg EW, 2000, DIABETES CARE, V23, P1272, DOI 10.2337/diacare.23.9.1272

Howren MB, 2009, PSYCHOSOM MED, V71, P171, DOI 10.1097/PSY.0b013e3181907c1b

Johnson B, 2013, DIABETIC MED, V30, P199, DOI 10.1111/j.1464-5491.2012.03721.x

Katon W, 2012, ARCH GEN PSYCHIAT, V69, P410, DOI 10.1001/archgenpsychiatry.2011.154

Katon W, 2009, PSYCHOSOM MED, V71, P965, DOI 10.1097/PSY.0b013e3181bd8f55

Katon WJ, 2010, J GEN INTERN MED, V25, P423, DOI 10.1007/s11606-009-1248-6

Knol MJ, 2006, DIABETOLOGIA, V49, P837, DOI 10.1007/s00125-006-0159-x

Koopmans B, 2009, FAM PRACT, V26, P171, DOI 10.1093/fampra/cmp016

Kop WJ, 2010, PSYCHOSOM MED, V72, P626, DOI 10.1097/PSY.0b013e3181eadd2b

Korczak DJ, 2011, DIABETOLOGIA, V54, P2483, DOI 10.1007/s00125-011-2240-3

Li J, 2012, PLOS ONE, V7, DOI 10.1371/journal.pone.0043508

Lin EHB, 2010, DIABETES CARE, V33, P264, DOI 10.2337/dc09-1068

Lu FP, 2009, PLOS ONE, V4, DOI 10.1371/journal.pone.0004144

Lustman PJ, 2000, DIABETES CARE, V23, P934, DOI 10.2337/diacare.23.7.934

MacQueen G, 2011, MOL PSYCHIATR, V16, P252, DOI 10.1038/mp.2010.80

Makine C, 2009, DIABETIC MED, V26, P28, DOI 10.1111/j.1464-5491.2008.02606.x

Mezuk B, 2008, DIABETES CARE, V31, P2383, DOI 10.2337/dc08-0985

Mommersteeg PMC, 2013, DIABETIC MED, V30, pE208, DOI 10.1111/dme.12193

Mommersteeg PMC, 2012, BMC PUBLIC HEALTH, V12, DOI 10.1186/1471-2458-12-1109

Moussavi S, 2007, LANCET, V370, P851, DOI 10.1016/S0140-6736(07)61415-9

Nefs G, 2012, J PSYCHIATR RES, V46, P549, DOI 10.1016/j.jpsychires.2012.01.013

Nouwen A, 2010, DIABETOLOGIA, V53, P2480, DOI 10.1007/s00125-010-1874-x

Nouwen A, 2011, DIABETES CARE, V34, P752, DOI 10.2337/dc10-1414

Novak M, 2013, DIABETIC MED, V30, pE8, DOI 10.1111/dme.12037

Peyrot M, 2005, DIABETES CARE, V28, P2673, DOI 10.2337/diacare.28.11.2673

Pouwer F, 2003, DIABETOLOGIA, V46, P892, DOI 10.1007/s00125-003-1124-6

Pouwer F, 2005, DIABETIC MED, V22, P1465, DOI 10.1111/j.1464-5491.2005.01661.x

Pouwer F, 2010, DIABETIC MED, V27, P217, DOI 10.1111/j.1464-5491.2009.02903.x

Pouwer F, 2010, DISCOV MED, V9, P112

Roglic G, 2005, DIABETES CARE, V28, P2130, DOI 10.2337/diacare.28.9.2130

Rotella F, 2013, J CLIN PSYCHIAT, V74, P32, DOI 10.4088/JCP.12r07922

Roy T, 2012, J AFFECT DISORDERS, V142, pS8, DOI 10.1016/S0165-0327(12)70004-6

Scherrer JF, 2011, DIABETES CARE, V34, P1729, DOI 10.2337/dc11-0031

Sieu N, 2011, GEN HOSP PSYCHIAT, V33, P429, DOI 10.1016/j.genhosppsych.2011.05.021

Stuart MJ, 2012, NEUROSCI BIOBEHAV R, V36, P658, DOI 10.1016/j.neubiorev.2011.10.001

Thombs Brett D, 2008, Diabetes Care, V31, pe55, DOI 10.2337/dc08-0446

Turner RC, 1999, JAMA-J AM MED ASSOC, V281, P2005, DOI 10.1001/jama.281.21.2005

van Bastelaar KMP, 2011, DIABETES CARE, V34, P320, DOI 10.2337/dc10-1248

van Bastelaar KM, 2008, BMC PSYCHIATRY, V19, P9

Van der Feltz-Cornelis CM, 2010, GEN HOSP PSYCHIAT, V32, P380, DOI 10.1016/j.genhosppsych.2010.03.011

van Dooren FEP, 2013, PLOS ONE, V8, DOI 10.1371/journal.pone.0057058

van Son J, 2013, DIABETES CARE, V36, P823, DOI 10.2337/dc12-1477

van Son J, 2011, BMC PUBLIC HEALTH, V11, DOI 10.1186/1471-2458-11-131

Virk J, 2010, PLOS ONE, V5, DOI 10.1371/journal.pone.0011523

Wagner JA, 2012, INT J BEHAV MED, V19, P503, DOI 10.1007/s12529-011-9190-5

Whooley MA, 2008, JAMA-J AM MED ASSOC, V300, P2379, DOI 10.1001/jama.2008.711

Wild S, 2004, DIABETES CARE, V27, P1047, DOI 10.2337/diacare.27.5.1047

Williams LH, 2011, J DIABETES COMPLICAT, V25, P175, DOI 10.1016/j.jdiacomp.2010.07.002

Williams LH, 2010, AM J MED, V123, P748, DOI 10.1016/j.amjmed.2010.01.023

Williams R, 2002, DIABETOLOGIA, V45, pS13, DOI 10.1007/s00125-002-0859-9

Willis T., PHARM RATIONALIS SIV

Young V, 2013, DIABETIC MED, V30, P189, DOI 10.1111/j.1464-5491.2012.03771.x

NR 71

TC 43

Z9 44

U1 2

U2 35

PU W B SAUNDERS CO-ELSEVIER INC

PI PHILADELPHIA

PA 1600 JOHN F KENNEDY BOULEVARD, STE 1800, PHILADELPHIA, PA 19103-2899 USA

SN 0889-8529

J9 ENDOCRIN METAB CLIN

JI Endocrinol. Metabol. Clin. North Amer.

PD SEP

PY 2013

VL 42

IS 3

BP 529

EP +

DI 10.1016/j.ecl.2013.05.002

PG 17

WC Endocrinology & Metabolism

SC Endocrinology & Metabolism

GA 228MO

UT WOS:000325191100009

PM 24011885

DA 2019-01-08

ER

PT J

AU Fisher, L

Hessler, D

Glasgow, RE

Arean, PA

Masharani, U

Naranjo, D

Strycker, LA

AF Fisher, Lawrence

Hessler, Danielle

Glasgow, Russell E.

Arean, Patricia A.

Masharani, Umesh

Naranjo, Diana

Strycker, Lisa A.

TI REDEEM: A Pragmatic Trial to Reduce Diabetes Distress

SO DIABETES CARE

LA English

DT Article

ID RANDOMIZED CONTROLLED-TRIAL; MANAGEMENT SUPPORT PROGRAM;

SELF-MANAGEMENT; GLYCEMIC CONTROL; DEPRESSIVE SYMPTOMS; INTERVENTION;

OUTCOMES; SCALE; RECRUITMENT; THERAPY

AB OBJECTIVE To compare three interventions to reduce diabetes distress (DD) and improve self-management among non-clinically depressed adults with type 2 diabetes mellitus (T2DM).RESEARCH DESIGN AND METHODS In REDEEM, 392 adults with T2DM and DD were randomized to computer-assisted self-management (CASM), CASM plus DD-specific problem solving (CAPS), or a computer-administered minimal supportive intervention. Primary outcomes were Diabetes Distress Scale (DDS) total, the Emotional Burden (EB) and Regimen Distress (RD) DDS subscales, and diet, exercise, and medication adherence.RESULTS Significant and clinically meaningful reductions in DD (DDS, EB, and RD) and self-management behaviors occurred in all three conditions (P < 0.001), with no significant between-group differences. There was, however, a significant group x baseline distress interaction (P < 0.02), in which patients with high baseline RD in the CAPS condition displayed significantly larger RD reductions than those in the other two conditions. RD generated the most distress and displayed the greatest distress reduction as a result of intervention. The pace of DD reduction varied by patient age: older patients demonstrated significant reductions in DD early in the intervention, whereas younger adults displayed similar reductions later. Reductions in DD were accompanied by significant improvements in healthy eating, physical activity, and medication adherence, although not by change in HbA(1c).CONCLUSIONS DD is malleable and highly responsive to intervention. Interventions that enhance self-management also reduce DD significantly, but DD-specific interventions may be necessary for patients with high initial levels of DD. Future research should identify the minimal, most cost-effective interventions to reduce DD and improve self-management.

C1 [Fisher, Lawrence; Hessler, Danielle; Arean, Patricia A.; Masharani, Umesh; Naranjo, Diana] Univ Calif San Francisco, San Francisco, CA 94143 USA.

[Glasgow, Russell E.] NCI, Div Canc Control & Populat Sci, Rockville, MD USA.

[Strycker, Lisa A.] Oregon Res Inst, Eugene, OR 97403 USA.

RP Fisher, L (reprint author), Univ Calif San Francisco, San Francisco, CA 94143 USA.

EM fisherl@fcm.ucsf.edu

FU National Institute of Diabetes and Digestive and Kidney Diseases (NIDDK)

[DK061937]

FX This research was funded by National Institute of Diabetes and Digestive

and Kidney Diseases (NIDDK) Grant DK061937.

CR Arean P, 2008, GERONTOLOGIST, V48, P311, DOI 10.1093/geront/48.3.311

Fisher L, 2009, DIABETIC MED, V26, P622, DOI 10.1111/j.1464-5491.2009.02730.x

Fisher L, 2008, ANN FAM MED, V6, P246, DOI 10.1370/afm.842

Fisher L, 2012, PATIENT EDUC COUNS, V86, P372, DOI 10.1016/j.pec.2011.07.002

Fisher L, 2012, DIABETES CARE, V35, P259, DOI 10.2337/dc11-1572

Fisher L, 2010, DIABETES CARE, V33, P1034, DOI 10.2337/dc09-2175

Fisher L, 2010, DIABETES CARE, V33, P23, DOI 10.2337/dc09-1238

Fredrickson BL, 2005, COGNITION EMOTION, V19, P313, DOI 10.1080/02699930441000238

Gierisch JM, 2010, AM J PREV MED, V39, P334, DOI 10.1016/j.amepre.2010.05.020

Glasgow RE, 2010, J INTERNET RES, V13, P9

Glasgow RE, 2006, J CHRONIC ILLNESS, V2, P270

Glasgow RE, 2012, PATIENT EDUC COUNS, V87, P81, DOI 10.1016/j.pec.2011.07.024

Glasgow RE, 2010, J GEN INTERN MED, V25, P1315, DOI 10.1007/s11606-010-1480-0

Glasgow RE, 2010, ANN BEHAV MED, V40, P40, DOI 10.1007/s12160-010-9189-1

Glasgow RE, 2009, ANN BEHAV MED, V38, P46, DOI 10.1007/s12160-009-9134-3

Gonzalez JS, 2011, DIABETES CARE, V34, P236, DOI 10.2337/dc10-1970

Hartmann M, 2012, DIABETES CARE, V35, P945, DOI 10.2337/dc11-1343

Hessler DM, 2011, PATIENT EDUC COUNS, V85, P154, DOI 10.1016/j.pec.2010.10.030

Kroenke K, 2001, J GEN INTERN MED, V16, P606, DOI 10.1046/j.1525-1497.2001.016009606.x

Krousel-Wood M, 2005, J MED SCI, V330, P182

Lazarus R. S, 1984, STRESS APPRAISAL COP

Lorig K, 2008, DIABETES CARE, V31, P408, DOI 10.2337/dc07-1313

Masharani U, 2007, DIABETES DE MYSTIFIE

Moskowitz J. T., 2010, HDB ADULT RESILIENCE, P465

Nezu AM, 2004, BEHAV THER, V35, P1, DOI 10.1016/S0005-7894(04)80002-9

NEZU AM, 2006, PROMOTING TREATMENT, P135

Polonsky WH, 2005, DIABETES CARE, V28, P626, DOI 10.2337/diacare.28.3.626

Reich JW, 2003, REV GEN PSYCHOL, V7, P66, DOI 10.1037/1089-2680.7.1.66

Schafer JL, 1999, NORM USERS GUIDE MUL, P1

Stewart AL, 2001, MED SCI SPORT EXER, V33, P1126

Thompson F. E., 1998, European Journal of Clinical Nutrition, V52, pS63

Toobert DJ, 2010, AM J HEALTH BEHAV, V34, P680

Toobert DJ, 2000, DIABETES CARE, V23, P943, DOI 10.2337/diacare.23.7.943

Tsujii S, 2012, DIABETIC MED, V29, P1451, DOI 10.1111/j.1464-5491.2012.03647.x

van Son J, 2011, BMC PUBLIC HEALTH, V11, DOI 10.1186/1471-2458-11-131

Welch G, 2011, DIABETES RES CLIN PR, V91, P54, DOI 10.1016/j.diabres.2010.09.036

Winkley K, 2006, BRIT MED J, V55, P1

Zagarins SE, 2012, J BEHAV MED, V35, P299, DOI 10.1007/s10865-011-9359-z

Zwarenstein M, 2008, BRIT MED J, V337, DOI 10.1136/bmj.a2390

NR 39

TC 84

Z9 87

U1 2

U2 22

PU AMER DIABETES ASSOC

PI ALEXANDRIA

PA 1701 N BEAUREGARD ST, ALEXANDRIA, VA 22311-1717 USA

SN 0149-5992

J9 DIABETES CARE

JI Diabetes Care

PD SEP

PY 2013

VL 36

IS 9

BP 2551

EP 2558

DI 10.2337/dc12-2493

PG 8

WC Endocrinology & Metabolism

SC Endocrinology & Metabolism

GA 205CZ

UT WOS:000323420200038

PM 23735726

OA Other Gold, Green Published

DA 2019-01-08

ER

PT J

AU Eriksson, AK

van den Donk, M

Hilding, A

Ostenson, CG

AF Eriksson, Anna-Karin

van den Donk, Maureen

Hilding, Agneta

Ostenson, Claes-Goran

TI Work Stress, Sense of Coherence, and Risk of Type 2 Diabetes in a

Prospective Study of Middle-Aged Swedish Men and Women

SO DIABETES CARE

LA English

DT Article

ID JOB DECISION LATITUDE; WHITE-COLLAR WORKERS; CARDIOVASCULAR-DISEASE;

UNITED-STATES; HEALTH; POPULATION; MELLITUS; DEMANDS; GENDER; STRAIN

AB OBJECTIVE To investigate the prospective influence of work stress on type 2 diabetes (T2D).RESEARCH DESIGN AND METHODS This population-based cohort included 3,205 women and 2,227 men, aged 35-56 years, with baseline normal glucose tolerance measured with oral glucose tolerance test. At follow-up 8-10 years later, T2D was diagnosed in 60 women and 111 men. Work stress factors evaluated by questionnaire (i.e., demands, decision latitude, job strain, shift work, overtime work, and also sense of coherence) were studied in association with T2D. Odds ratios (ORs) and 95% CIs adjusted for age, education, BMI, physical activity, smoking, family history of diabetes, and psychological distress were calculated.RESULTS In women, low decision latitude was associated with T2D on its own (OR 2.4 [95% CI 1.1-5.2]) and combined with high demands: job strain (OR 4.2 [2.0-8.7]), adjusted for all available potential confounders. Also, shift work increased the risk of T2D in women (OR 2.2 [1.0-4.7]) when adjusted for age, education, and psychological distress, although this risk was diluted after multifactor adjustment (OR 1.9 [0.8-4.4]). In men, high work demands and high strain decreased the risk of T2D (OR 0.5 [0.3-0.9]) for both measures, as did an active job (high demands and high decision latitude, OR 0.4 [0.2-0.9]).CONCLUSIONS Work stress and shift work may contribute to the development of T2D in women. In men, the risk was decreased by high work demands, high strain, and an active job.

C1 [Eriksson, Anna-Karin] Karolinska Inst, Clin Epidemiol Unit, Dept Med, Stockholm, Sweden.

[Eriksson, Anna-Karin; Hilding, Agneta; Ostenson, Claes-Goran] Karolinska Inst, Dept Mol Med & Surg, Endocrine & Diabet Unit, Stockholm, Sweden.

[Eriksson, Anna-Karin] Karolinska Inst, Div Social Med, Dept Publ Hlth Sci, Stockholm, Sweden.

[van den Donk, Maureen] Dutch Coll Gen Practitioners, Utrecht, Netherlands.

RP Eriksson, AK (reprint author), Karolinska Inst, Clin Epidemiol Unit, Dept Med, Stockholm, Sweden.

EM anna-karin.eriksson.2@ki.se

FU Stockholm County Council; Swedish Council of Working Life and Social

Research; Novo Nordisk Scandinavia; Swedish Diabetes Association

FX This study was supported by grants from Stockholm County Council,

Swedish Council of Working Life and Social Research, Novo Nordisk

Scandinavia, and Swedish Diabetes Association. No other potential

conflicts of interest relevant to this article were reported.

CR Agardh EE, 2003, DIABETES CARE, V26, P719, DOI 10.2337/diacare.26.3.719

Antonovsky A, 1987, UNRAVELING MYSTERY H

Belkic KL, 2004, SCAND J WORK ENV HEA, V30, P85, DOI 10.5271/sjweh.769

Berntsson L, 2006, J EPIDEMIOL COMMUN H, V60, P1070, DOI 10.1136/jech.2005.042192

Cosgrove MP, 2012, OCCUP MED-OXFORD, V62, P167, DOI 10.1093/occmed/kqs002

Eriksson AK, 2008, DIABETIC MED, V25, P834, DOI 10.1111/j.1464-5491.2008.02463.x

Eriksson AK, 2012, EUR J EPIDEMIOL, V27, P153, DOI 10.1007/s10654-011-9630-1

Evertsson M, 2004, J MARRIAGE FAM, V66, P1272, DOI 10.1111/j.0022-2445.2004.00092.x

FRANKENHAEUSER M, 1989, J ORGAN BEHAV, V10, P321, DOI 10.1002/job.4030100404

Heraclides A, 2009, DIABETES CARE, V32, P2230, DOI 10.2337/dc09-0132

KARASEK R, 1981, AM J PUBLIC HEALTH, V71, P694, DOI 10.2105/AJPH.71.7.694

Karasek R, 1990, HLTH WORK STRESS PRO

KARASEK RA, 1979, ADMIN SCI QUART, V24, P285, DOI 10.2307/2392498

KARASEK RA, 1988, AM J PUBLIC HEALTH, V78, P910, DOI 10.2105/AJPH.78.8.910

Kawakami N, 2004, DIABETES CARE, V27, P282, DOI 10.2337/diacare.27.1.282

Kawakami N, 1999, J EPIDEMIOL COMMUN H, V53, P359, DOI 10.1136/jech.53.6.359

KERSTEDT T, 2009, SLEEP MED CLIN, V4, P257, DOI DOI 10.1016/J.JSMC.2009.03.001

Kivimaki M, 2011, PLOS MED, V8

Knutsson A, 2003, OCCUP MED-OXFORD, V53, P103, DOI 10.1093/occmed/kqg048

Kouvonen AM, 2008, BMC PUBLIC HEALTH, V8, DOI 10.1186/1471-2458-8-46

Kroenke CH, 2007, AM J EPIDEMIOL, V165, P175, DOI 10.1093/aje/kwj355

Kumari M, 2004, ARCH INTERN MED, V164, P1873, DOI 10.1001/archinte.164.17.1873

LUNDBERG O, 1995, EUROPEAN J PUBLIC HL, V5, P56, DOI DOI 10.1093/EURPUB/5.1.56

Lundberg U, 2005, PSYCHONEUROENDOCRINO, V30, P1017, DOI 10.1016/j.psyneuen.2005.03.014

Lundberg U, 1999, J Occup Health Psychol, V4, P142, DOI 10.1037//1076-8998.4.2.142

LUNDBERG U, 1994, SCAND J PSYCHOL, V35, P315, DOI 10.1111/j.1467-9450.1994.tb00956.x

Mallon L, 2005, DIABETES CARE, V28, P2762, DOI 10.2337/diacare.28.11.2762

Nakanishi N, 2001, OCCUP ENVIRON MED, V58, P569, DOI 10.1136/oem.58.9.569

Norberg M, 2007, DIABETES RES CLIN PR, V76, P368, DOI 10.1016/j.diabres.2006.09.002

Pan A., 2011, PLOS MED, V8

Rosmond R, 2000, J INTERN MED, V247, P188, DOI 10.1046/j.1365-2796.2000.00603.x

World Health Organization, 1999, DEF DIAGN CLASS DI 1

NR 32

TC 49

Z9 50

U1 0

U2 29

PU AMER DIABETES ASSOC

PI ALEXANDRIA

PA 1701 N BEAUREGARD ST, ALEXANDRIA, VA 22311-1717 USA

SN 0149-5992

J9 DIABETES CARE

JI Diabetes Care

PD SEP

PY 2013

VL 36

IS 9

BP 2683

EP 2689

DI 10.2337/dc12-1738

PG 7

WC Endocrinology & Metabolism

SC Endocrinology & Metabolism

GA 205CZ

UT WOS:000323420200057

PM 23637356

OA Other Gold, Green Published

DA 2019-01-08

ER

PT J

AU Gabbay, RA

Anel-Tiangco, RM

Dellasega, C

Mauger, DT

Adelman, A

Van Horn, DHA

AF Gabbay, Robert A.

Anel-Tiangco, Raquel M.

Dellasega, Cheryl

Mauger, David T.

Adelman, Alan

Van Horn, Deborah H. A.

TI Diabetes nurse case management and motivational interviewing for change

(DYNAMIC): Results of a 2-year randomized controlled pragmatic trial

SO JOURNAL OF DIABETES

LA English

DT Article

DE behavior change; motivational interviewing; nurse case management

ID GLYCEMIC CONTROL; SELF-MANAGEMENT; OLDER-ADULTS; PRIMARY-CARE;

DOUBLE-BLIND; MELLITUS; DISEASE; SCALE; QUESTIONNAIRE; INTERVENTION

AB Background: The aim of the present study was to determine whether the addition of nurse case managers (NCMs) trained in motivational interviewing (MI) to usual care would result in improved outcomes in high-risk type 2 diabetes patients.

Methods: A 2-year randomized controlled pragmatic trial randomized 545 patients to usual care control (n = 313) or those who received the intervention (n = 232) with additional practice-embedded NCM care, including MI-guided behavior change counseling. The NCMs received intensive MI training with ongoing fidelity assessment.

Results: Systolic blood pressure (SBP) was better in the intervention than usual care group (131 +/- 15 vs. 135 +/- 18 mmHg, respectively; P < 0.05). Improvements were seen in both the control and intervention groups in terms of HbA1c (from 9.1% to 8.0% and from 8.8% to 7.8%, respectively), low-density lipoprotein (LDL; from 127 to 100 mg/dL and from 128 to 102 mg/dL, respectively), and diastolic blood pressure (from 78 to 74 mmHg and from 80 to 74 mmHg, respectively). Depression symptom scores were better in the intervention group. The reduction in diabetes-related distress approached statistical significance.

Conclusions: The NCMs and MI improved SBP and complications screening. The large decrease in HbA1C and LDL in the control group may have obscured any further intervention effect. Although nurses prompted providers for medication titration, strategies to reduce provider clinical inertia may also be needed.

C1 [Gabbay, Robert A.] Penn State Hershey Diabet Inst, Hershey, PA USA.

[Gabbay, Robert A.; Anel-Tiangco, Raquel M.] Penn State Milton S Hershey Med Ctr, Div Endocrinol Diabet & Metab, Hershey, PA USA.

[Dellasega, Cheryl] Penn State Coll Med, Dept Humanities & Womens Studies, Hershey, PA 17033 USA.

[Mauger, David T.] Penn State Coll Med, Dept Publ Hlth Sci, Hershey, PA 17033 USA.

[Adelman, Alan] Penn State Hershey Med Grp Fishburn Rd, Hershey, PA USA.

[Van Horn, Deborah H. A.] Van Horn Consulting, W Deptford, NJ USA.

RP Gabbay, RA (reprint author), Penn State Coll Med, Div Endocrinol Diabet & Metab, 500 Univ Dr,H044, Hershey, PA 17033 USA.

EM rgabbay@hmc.psu.edu

FU National Institutes of Health; National Institute of Diabetes and

Digestive and Kidney Diseases [R18-DK067495]

FX This study was supported by a grant from the National Institutes of

Health and National Institute of Diabetes and Digestive and Kidney

Diseases (grant R18-DK067495). The authors thank Kendra DURDOCK, Nancy

MARTINEZ-KING, and David GEORGE (Reading Hospital) for their

contributions in conducting the study. R. A. G. is the guarantor of this

work and, as such, had full access to all the data in the study and

takes responsibility for the integrity of the data and the accuracy of

the data analysis.

CR Anderson DR, 2010, J GEN INTERN MED, V25, P1116, DOI 10.1007/s11606-010-1419-5

Bojadzievski, 2011, DIABETES CARE, V34, P1047

Bradley C, 1999, DIABETES CARE, V22, P530, DOI 10.2337/diacare.22.3.530

Bradley C, 1999, QUAL LIFE RES, V8, P79, DOI 10.1023/A:1026485130100

Bricker PL, 2010, J CONTIN EDUC HEALTH, V30, P114, DOI 10.1002/chp.20067

Brug J, 2007, J NUTR EDUC BEHAV, V39, P8, DOI 10.1016/j.jneb.2006.08.010

Channon SJ, 2007, DIABETES CARE, V30, P1390, DOI 10.2337/dc06-2260

Clark Marie, 2008, Prim Care Diabetes, V2, P113, DOI 10.1016/j.pcd.2008.04.004

Davidson MB, 2003, DIABETES CARE, V26, P2281, DOI 10.2337/diacare.26.8.2281

Dellasega C, 2012, DIABETES RES CLIN PR, V95, P37, DOI 10.1016/j.diabres.2011.08.011

Echeverry D, 2009, DIABETES CARE, V32, P2156, DOI 10.2337/dc09-0785

Ford ES, 2008, DIABETES CARE, V31, P102, DOI 10.2337/dc07-0565

Fox KM, 2003, LANCET, V362, P782

Gabbay RA, 2006, DIABETES RES CLIN PR, V71, P28, DOI 10.1016/j.diabres.2005.05.002

Gerstein HC, 2000, LANCET, V355, P253

Glettler E, 1996, J Case Manag, V5, P121

Greaves CJ, 2008, BRIT J GEN PRACT, V58, P535, DOI 10.3399/bjgp08X319648

Hampson SE, 2000, DIABETES CARE, V23, P943

Hawkins SY, 2010, RES THEOR NURS PRACT, V24, P217, DOI 10.1891/1541-6577.24.4.217

Katon WJ, 2003, NEW ENGL J MED, V363, P2611

Khan L, 2009, DIABETES TECHNOL THE, V11, P255, DOI 10.1089/dia.2008.0057

Lane C, 2005, PATIENT EDUC COUNS, V56, P166, DOI 10.1016/j.pec.2004.01.003

Lewinsohn PM, 1997, PSYCHOL AGING, V12, P277, DOI 10.1037//0882-7974.12.2.277

Lustman PJ, 2006, ARCH GEN PSYCHIAT, V63, P521, DOI 10.1001/archpsyc.63.5.521

Martins RK, 2009, CLIN PSYCHOL REV, V29, P283, DOI 10.1016/j.cpr.2009.02.001

Miller WR, 2001, BEHAV COGN PSYCHOTH, V29, P457, DOI 10.1017/S1352465801004064

Minet LKR, 2011, DIABETOLOGIA, V54, P1620, DOI 10.1007/s00125-011-2120-x

National Committee on Quality Assurance, 2008, STAND GUID PHYS PRAC

Norris SL, 2002, AM J PREV MED, V22, P15, DOI 10.1016/S0749-3797(02)00423-3

RADLOFF L S, 1977, Applied Psychological Measurement, V1, P385, DOI 10.1177/014662167700100306

Renders CM, 2001, DIABETES CARE, V24, P1821, DOI 10.2337/diacare.24.10.1821

Rollnick S., 2008, MOTIVATIONAL INTERVI

Rubak S, 2005, BRIT J GEN PRACT, V55, P305

Rubak S, 2011, SCAND J PRIM HEALTH, V29, P92, DOI 10.3109/02813432.2011.554271

Shah BR, 2005, DIABETES CARE, V28, P600, DOI 10.2337/diacare.28.3.600

Shojania KG, 2006, JAMA-J AM MED ASSOC, V296, P427, DOI 10.1001/jama.296.4.427

Smith DE, 1997, DIABETES CARE, V20, P52, DOI 10.2337/diacare.20.1.52

Viner RM, 2003, DIABETIC MED, V20, P739, DOI 10.1046/j.1464-5491.2003.00995.x

Welch G, 2011, DIABETES RES CLIN PR, V91, P54, DOI 10.1016/j.diabres.2010.09.036

Welch G, 2010, DIABETES RES CLIN PR, V88, P1, DOI 10.1016/j.diabres.2009.12.026

Welch GW, 1997, DIABETES CARE, V20, P760, DOI 10.2337/diacare.20.5.760

NR 41

TC 31

Z9 32

U1 5

U2 50

PU WILEY-BLACKWELL

PI HOBOKEN

PA 111 RIVER ST, HOBOKEN 07030-5774, NJ USA

SN 1753-0393

J9 J DIABETES

JI J. Diabetes

PD SEP

PY 2013

VL 5

IS 3

BP 349

EP 357

DI 10.1111/1753-0407.12030

PG 9

WC Endocrinology & Metabolism

SC Endocrinology & Metabolism

GA 195NZ

UT WOS:000322711300020

PM 23368423

OA Green Accepted

DA 2019-01-08

ER

PT J

AU Rees, G

Lamoureux, EL

Nicolaou, TE

Hodgson, LAB

Weinman, J

Speight, J

AF Rees, G.

Lamoureux, E. L.

Nicolaou, T. E.

Hodgson, L. A. B.

Weinman, J.

Speight, J.

TI Feedback of personal retinal images appears to have a motivational

impact in people with non-proliferative diabetic retinopathy and

suboptimal HbA(1c): findings of a pilot study

SO DIABETIC MEDICINE

LA English

DT Article

ID TRIAL; RISK; CARE

AB Aim To conduct a pilot study to explore the potential impact of visual feedback of personal retinal images on diabetes outcomes.

Methods Twenty-five participants with non-proliferative diabetic retinopathy and suboptimal HbA(1c) (>53mmol/mol; >7%) were randomized to receive visual feedback of their own retinal images or to a control group. At baseline and 3-month follow-up, HbA(1c), standard measures of beliefs, diabetes-related distress and self-care activities were assessed.

Results In unadjusted models, relative to controls, the intervention group showed significantly greater improvement in HbA(1c) at 3-month follow-up (-0.6% vs. +0.3%, P<0.01), as well as enhanced motivation to improve blood glucose management (P<0.05).

Conclusions This small pilot study provides preliminary evidence that visual feedback of personal retinal images may offer a practical educational strategy for clinicians in eye care services to improve diabetes outcomes in non-target compliant patients. A fully powered randomized controlled trial is required to confirm these findings and determine the optimal use of feedback to produce sustained effects.

C1 [Rees, G.; Lamoureux, E. L.; Nicolaou, T. E.; Hodgson, L. A. B.] Univ Melbourne, Royal Victorian Eye & Ear Hosp, Ctr Eye Res Australia, Melbourne, Vic 3010, Australia.

[Lamoureux, E. L.] Natl Univ Singapore, Singapore Eye Res Inst, Singapore 117548, Singapore.

[Weinman, J.] Kings Coll London, Inst Psychiat, London, England.

[Speight, J.] Diabet Australia Vic, Australian Ctr Behav Res Diabet, Melbourne, Vic, Australia.

[Speight, J.] Deakin Univ, Sch Psychol, Ctr Mental Hlth & Wellbeing Res, Burwood, Vic, Australia.

RP Rees, G (reprint author), Univ Melbourne, Royal Victorian Eye & Ear Hosp, Ctr Eye Res Australia, Melbourne, Vic 3010, Australia.

EM grees@unimelb.edu.au

OI Speight, Jane/0000-0002-1204-6896; Weinman, John

Alfred/0000-0002-6786-0166

CR Broadbent E, 2006, J PSYCHOSOM RES, V60, P631, DOI 10.1016/j.jpsychores.2005.10.020

*ETDRS, 1991, OPHTHALMOLOGY, V98, P786

Fisher L, 2008, ANN FAM MED, V6, P246, DOI 10.1370/afm.842

Funnel MM, 2009, DIABETES CARE, V32, pS87, DOI 10.2337/dc09-S087

Hollands G. J., 2010, COCHRANE DATABASE SY, V2010, P1

Lamoureux EL, 2012, CLIN EXP OPHTHALMOL, V40, P73, DOI 10.1111/j.1442-9071.2011.02697.x

Milne S, 2000, J APPL SOC PSYCHOL, V30, P106, DOI 10.1111/j.1559-1816.2000.tb02308.x

Nathan DM, 2008, ARCH OPHTHALMOL-CHIC, V126, P1707, DOI 10.1001/archopht.126.12.1707

Scanlon PH, 2012, DIABETIC MED, V29, P836, DOI 10.1111/j.1464-5491.2012.03630.x

Shahab L, 2011, HEALTH PSYCHOL, V30, P49, DOI 10.1037/a0021821

Stratton IM, 2001, DIABETOLOGIA, V44, P156, DOI 10.1007/s001250051594

Toobert DJ, 2000, DIABETES CARE, V23, P943, DOI 10.2337/diacare.23.7.943

NR 12

TC 5

Z9 5

U1 0

U2 1

PU WILEY-BLACKWELL

PI HOBOKEN

PA 111 RIVER ST, HOBOKEN 07030-5774, NJ USA

SN 0742-3071

J9 DIABETIC MED

JI Diabetic Med.

PD SEP

PY 2013

VL 30

IS 9

BP 1122

EP 1125

DI 10.1111/dme.12192

PG 4

WC Endocrinology & Metabolism

SC Endocrinology & Metabolism

GA 202DZ

UT WOS:000323195000017

PM 23601012

DA 2019-01-08

ER

PT J

AU Joensen, LE

Tapager, I

Willaing, I

AF Joensen, L. E.

Tapager, I.

Willaing, I.

TI Diabetes distress in Type1 diabetesa new measurement fit for purpose

SO DIABETIC MEDICINE

LA English

DT Article

ID QUALITY-OF-LIFE; PROBLEM-AREAS; EMOTIONAL DISTRESS; SCALE; HEALTH;

DEPRESSION; VALIDITY; VERSION; QUESTIONNAIRES; RELIABILITY

AB Aims To examine and assess (1) the internal consistency and validity of a Danish translation of the Diabetes Distress Scale (DDS17) among adults with Type1 diabetes mellitus, (2) the properties of the brief version of DDS17 and (3) cut points for the DDS17.

Methods Cross-sectional survey of 2419 adults with Type1 diabetes mellitus. Validated scales and questions measured diabetes distress (DDS17), diabetes empowerment (DES-SF), healthy diet, physical activity, self-rated health (SF-12) and quality of life (WHOQoL-BREF). An electronic patient record provided information about glycaemic control (HbA(1c)). We examined internal consistency, construct validity, subscale construction and cut points for determining clinically relevant diabetes distress, as well as two- and four-item brief versions of the DDS17.

Results The DDS17 had a Cronbach's alpha of 0.92; subscale alphas were 0.83-0.89. A four-factor division into subscales was appropriate and the division corresponded closely to the four domains described in the development of the DDS17. The DDS17 correlated well with relevant measures; higher distress was correlated with low diabetes empowerment, low quality of life, unhealthy diet, not being physically active, poor glycaemic control and low scores on the mental component score of the SF-12. A two-item brief scale performed as well as a four-item version. The relationship between DDS17 and outcomes did not suggest distinct cut points for DDS17.

Conclusions Our results provide support for the use of this Danish adaptation of the DDS17 for assessing psychosocial distress among adults with Type1 diabetes mellitus.

C1 [Joensen, L. E.; Willaing, I.] Steno Diabet Ctr, Steno Hlth Promot Ctr, DK-2820 Gentofte, Denmark.

[Tapager, I.] Incentive, Holte, Denmark.

RP Joensen, LE (reprint author), Steno Diabet Ctr, Steno Hlth Promot Ctr, DK-2820 Gentofte, Denmark.

EM ljoe@steno.dk

OI Joensen, Lene Eide/0000-0002-4790-8759; Willaing,

Ingrid/0000-0002-3082-8816

FU Novo Nordisk; Novo Nordisk Foundation; Capital Region of Denmark

FX The Steno Diabetes Center is an independent academic institution owned

by Novo Nordisk and financed partly by Novo Nordisk, partly by the Novo

Nordisk Foundation and partly by the Capital Region of Denmark.

CR Amsberg S, 2008, INT J NURS STUD, V45, P1319, DOI 10.1016/j.ijnurstu.2007.09.010

Anderson R, 2000, NAT HIST, V109, P23

Anderson RM, 2003, DIABETES CARE, V26, P1641, DOI 10.2337/diacare.26.5.1641-a

Egede LE, 2012, CURR PSYCHIAT REP, V14, P15, DOI 10.1007/s11920-011-0240-0

Fisher L, 2008, ANN FAM MED, V6, P246, DOI 10.1370/afm.842

Fisher L, 2007, DIABETES CARE, V30, P542, DOI 10.2337/dc06-1614

Fisher L, 2012, DIABETES CARE, V35, P259, DOI 10.2337/dc11-1572

Gandek B, 1998, J CLIN EPIDEMIOL, V51, P1171, DOI 10.1016/S0895-4356(98)00109-7

Gonzalez JS, 2011, DIABETES CARE, V34, P236, DOI 10.2337/dc10-1970

Graue M, 2012, INT J NURS STUD, V49, P174, DOI 10.1016/j.ijnurstu.2011.08.007

Harper A, 1998, PSYCHOL MED, V28, P551

Hatcher L, 1994, DEV MEASUREMENT MODE, P249

Hatcher L, 1994, STEPS CONDUCTION EXP, P79

Hermanns N, 2006, DIABETOLOGIA, V49, P469, DOI 10.1007/s00125-005-0094-2

Huis In 't Veld EM, 2011, CARDIOVASC PSYCHIAT, V2011, DOI [10.1155/2011/315086, DOI 10.1155/2011/315086]

Maindal HT, 2012, EUR J PUBLIC HEALTH, V22, P31, DOI 10.1093/eurpub/ckq188

Papathanasiou A, 2012, J PSYCHIATR MENT HLT, DOI [10.1111/j.1356-2850.2012.01875.x, DOI 10.1111/J.1356-2850.2012.01875.X.[]

Peyrot M, 2005, DIABETIC MED, V22, P1379, DOI 10.1111/j.1464-5491.2005.01644.x

POLONSKY WH, 1995, DIABETES CARE, V18, P754, DOI 10.2337/diacare.18.6.754

Polonsky WH, 2005, DIABETES CARE, V28, P626, DOI 10.2337/diacare.28.3.626

Pouwer F, 2006, PATIENT EDUC COUNS, V60, P235, DOI 10.1016/j.pec.2005.01.009

SAS/STAT, SAS STAT R 9 2 US GU

Sigurdardottir AK, 2008, INT J NURS STUD, V45, P526, DOI 10.1016/j.ijnurstu.2006.10.008

Snoek FJ, 2000, DIABETES CARE, V23, P1305, DOI 10.2337/diacare.23.9.1305

Snoek FJ, 2011, DIABETES CARE, V34, P601, DOI 10.2337/dc10-1552

Streiner D, 2009, SELECTING ITEMS HLTH

Terwee CB, 2007, J CLIN EPIDEMIOL, V60, P34, DOI 10.1016/j.jclinepi.2006.03.012

Ting RZW, 2011, DIABETES CARE, V34, P1094, DOI 10.2337/dc10-1612

Toobert DJ, 2000, DIABETES CARE, V23, P943, DOI 10.2337/diacare.23.7.943

Wang RH, 2011, J ADV NURS, V67, P2658, DOI 10.1111/j.1365-2648.2011.05701.x

NR 30

TC 25

Z9 28

U1 0

U2 13

PU WILEY-BLACKWELL

PI HOBOKEN

PA 111 RIVER ST, HOBOKEN 07030-5774, NJ USA

SN 0742-3071

J9 DIABETIC MED

JI Diabetic Med.

PD SEP

PY 2013

VL 30

IS 9

BP 1132

EP 1139

DI 10.1111/dme.12241

PG 8

WC Endocrinology & Metabolism

SC Endocrinology & Metabolism

GA 202DZ

UT WOS:000323195000019

PM 23701311

DA 2019-01-08

ER

PT J

AU Al-Nsour, M

Zindah, M

Belbeisi, A

Rolle, IV

Walke, H

Strine, T

Perry, GS

Jarrar, B

Mokdad, A

AF Al-Nsour, Mohannad

Zindah, Meyasser

Belbeisi, Adel

Rolle, Italia V.

Walke, Henry

Strine, Tara

Perry, Geraldine S.

Jarrar, Bassam

Mokdad, Ali

TI Frequent Mental Distress, Chronic Conditions, and Adverse Health

Behaviors in the Behavioral Risk Factor Surveillance Survey, Jordan,

2007

SO PREVENTING CHRONIC DISEASE

LA English

DT Article

ID QUALITY-OF-LIFE; HEART-DISEASE; ADULTS; DEPRESSION; ATTITUDES; OLDER

AB Introduction

Recent evidence indicates that chronic diseases and mental illness are associated. In the Middle Eastern country of Jordan, chronic diseases and frequent mental distress (FMD) are increasing; however, the capacity for mental health care is limited. The objective of this study was to determine the association between FMD, chronic conditions, and adverse health behaviors in Jordan.

Methods

The third cycle of the Jordan Behavioral Risk Factor Surveillance Survey (2007) served as the data source for this study. The sample consisted of 3,612 noninstitutionalized Jordanian adults aged 18 years or older. Logistic regression was used to obtain odds ratios for the association between chronic conditions, health behaviors, and FMD adjusted for age, sex, marital status, education, income, and employment.

Results

In the adjusted models, people with hypertension (adjusted odds ratio [AOR], 2.0; 95% confidence interval [CI], 1.6 2.7), high cholesterol (AOR, 2.3; 95% CI, 1.6-3.2), diabetes (AOR, 1.6; 95% CI, 1.1-2.4), and asthma (AOR, 2.2; 95% CI, 1.5-3.1) and smokers (AOR, 1.5; 95% CI, 1.1-2.0) were more likely to have FMD than people without each of these conditions. Adults who reported vigorous physical activity were less likely to have FMD (AOR, 0.6; 95% CI, 0.4-0.9) than their less active counterparts.

Conclusions

In Jordan, FMD was associated with several chronic conditions. As a result, we suggest additional research to examine the complex relationship between FMD and chronic conditions. More doctors in the primary health care system should be trained in mental health.

C1 [Al-Nsour, Mohannad] Eastern Mediterranean Publ Hlth Network, Amman, Jordan.

[Zindah, Meyasser; Belbeisi, Adel] Jordan Minist Hlth, Amman, Jordan.

[Rolle, Italia V.; Walke, Henry; Strine, Tara; Perry, Geraldine S.; Jarrar, Bassam] Ctr Dis Control & Prevent, Atlanta, GA USA.

[Mokdad, Ali] Univ Washington, Seattle, WA 98195 USA.

RP Al-Nsour, M (reprint author), Eastern Mediterranean Publ Hlth Network, Amman, Jordan.

EM mohannadnsour973@yahoo.com

CR Al-Krenawi A, 2004, INT J SOC PSYCHIATR, V50, P102, DOI 10.1177/0020764004040957

Al-Krenawi A, 2000, COMMUNITY MENT HLT J, V36, P501, DOI 10.1023/A:1001963714338

Al-Nsour M, 2012, PREV CHRONIC DIS, V9, DOI 10.5888/pcd9.110077

Al-Safi Saafan A, 2005, Eur J Cardiovasc Nurs, V4, P286, DOI 10.1016/j.ejcnurse.2005.03.004

Bahou Y, 2004, East Mediterr Health J, V10, P138

Belbeisi A., 2006, Morbidity and Mortality Weekly Report, V55, P653

Belbeisi A, 2009, INT J PUBLIC HEALTH, V54, P106, DOI 10.1007/s00038-009-0014-1

Caldwell JC, 2001, B WORLD HEALTH ORGAN, V79, P159

Centers for Disease Control and Prevention, 2000, MEAS HLTH DAYS POP A

Chapman Daniel P, 2005, Prev Chronic Dis, V2, pA14

Cook J, 1998, JAMA-J AM MED ASSOC, V279, P1772

Haddad LG, 2002, INT J NURS STUD, V39, P793, DOI 10.1016/S0020-7489(02)00016-0

Hamid Hamada, 2004, Transcult Psychiatry, V41, P487, DOI 10.1177/1363461504047931

Hammoudeh AJ, 2006, INT J CARDIOL, V110, P179, DOI 10.1002/j.ijcard.2005.08.005

Jaddou HY, 2003, SAUDI MED J, V24, P472

Khasawneh NF, 2005, SAUDI MED J, V26, P215

Kulwicki A D, 2001, J Cult Divers, V8, P34

Lopez AD, 1998, NAT MED, V4, P1241, DOI 10.1038/3218

McGuire LC, 2007, AM J GERIAT PSYCHIAT, V15, P754, DOI 10.1097/JGP.0b013e3180986125

Mohit A, 1999, East Mediterr Health J, V5, P231

Moussavi S, 2007, LANCET, V370, P851, DOI 10.1016/S0140-6736(07)61415-9

Nasir LS, 2005, J AM BOARD FAM PRACT, V18, P125, DOI 10.3122/jabfm.18.2.125

National Mental Health Team, 2010, NAT REP MENT HLTH SY

Prince M, 2007, LANCET, V370, P859, DOI 10.1016/S0140-6736(07)61238-0

Rohrer J, 2007, J EVAL CLIN PRACT, V13, P435, DOI 10.1111/j.1365-2753.2006.00737.x

Strine TW, 2005, AM J PREV MED, V28, P182, DOI 10.1016/j.ampre.2004.10.002

Strine TW, 2004, PREV MED, V39, P191, DOI 10.1016/j.ypmed.2004.01.022

Strine TW, 2004, CHEST, V126, P1849, DOI 10.1378/chest.126.6.1849

World Health Organization, 2011, MENT HLTH ATL 2011

Zahran H. S., 2004, Morbidity and Mortality Weekly Report, V53, P963

NR 30

TC 2

Z9 2

U1 0

U2 1

PU CENTERS DISEASE CONTROL

PI ATLANTA

PA 1600 CLIFTON RD, ATLANTA, GA 30333 USA

SN 1545-1151

J9 PREV CHRONIC DIS

JI Prev. Chronic Dis.

PD AUG

PY 2013

VL 10

AR UNSP 130030

DI 10.5888/pcd10.130030

PG 7

WC Public, Environmental & Occupational Health

SC Public, Environmental & Occupational Health

GA 285KJ

UT WOS:000329392600014

OA Green Published, Bronze

DA 2019-01-08

ER

PT J

AU Gariepy, G

Smith, KJ

Schmitz, N

AF Gariepy, Genevieve

Smith, Kimberley J.

Schmitz, Norbert

TI Diabetes distress and neighborhood characteristics in people with type 2

diabetes

SO JOURNAL OF PSYCHOSOMATIC RESEARCH

LA English

DT Article

DE Diabetes; Diabetes, type 2; Distress; Stress; Neighborhood; Epidemiology

ID DEPRESSIVE SYMPTOMS; GLYCEMIC CONTROL; CLINICAL DEPRESSION;

OLDER-ADULTS; HEALTH; DISORDER; ASSOCIATIONS; ENVIRONMENT; OUTCOMES;

SMOKING

AB Objective: Diabetes-specific distress is an important psychological issue in people with diabetes. The neighborhood environment has the potential to be an important factor for diabetes distress. This study investigates the associations between neighborhood characteristics and diabetes distress in adults with type 2 diabetes.

Methods: We used cross-sectional data from a community-based sample of 578 adults with type 2 diabetes from Quebec, Canada. Information on perceived neighborhood characteristics and diabetes distress was collected from phone interviews. We used factor analysis to combine questionnaire items into neighborhood factors. Information on neighborhood deprivation was derived from census data. We performed linear regressions for diabetes distress and specific domains of diabetes distress (emotional, regimen-related, physician-related and interpersonal distress), adjusting for individual-level variables.

Results: Factorial analysis uncovered 3 important neighborhood constructs: perceived order (social and physical order), culture (social and cultural environment) and access (access to services and facilities). After adjusting for individual-level confounders, neighborhood order was significantly associated with diabetes distress and all specific domains of distress; neighborhood culture was specifically associated with regimen-related distress; and neighborhood access was specifically associated with physician-related distress. The objective measure of neighborhood material deprivation was associated with regimen-related distress.

Conclusions: Neighborhood characteristics are associated with diabetes distress in people with type 2 diabetes. Clinicians should consider the neighborhood environment reported by their patients with diabetes when assessing and addressing diabetes-specific distress. (C) 2013 Elsevier Inc. All rights reserved.

C1 [Gariepy, Genevieve; Schmitz, Norbert] McGill Univ, Dept Epidemiol & Biostat, Montreal, PQ H4H 1R3, Canada.

[Gariepy, Genevieve; Smith, Kimberley J.; Schmitz, Norbert] Douglas Mental Hlth Univ Inst, Montreal, PQ, Canada.

[Smith, Kimberley J.; Schmitz, Norbert] McGill Univ, Dept Psychiat, Montreal, PQ H4H 1R3, Canada.

RP Gariepy, G (reprint author), McGill Univ, Douglas Mental Hlth Univ Inst, 6875 LaSalle Blvd, Montreal, PQ H4H 1R3, Canada.

EM genevieve.gariepy@mail.mcgill.ca

RI Schmitz, Norbert/A-5177-2010

OI Schmitz, Norbert/0000-0001-7777-6323; Smith,

Kimberley/0000-0002-1323-627X; Gariepy, Genevieve/0000-0001-9209-4240

FU Canadian Institutes of Health; Canadian Diabetes Association

FX GG, KS and NS were supported by funding from the Canadian Institutes of

Health. The Diabetes Health Study is funded by the Canadian Diabetes

Association.

CR Aikens JE, 2012, DIABETES CARE, V35, P2472, DOI 10.2337/dc12-0181

[Anonymous], 2008, CANADIAN J DIABETES

Bandura A, 2004, HEALTH EDUC BEHAV, V31, P143, DOI 10.1177/1090198104263660

Billimek J, 2012, J GEN INTERN MED, V27, P292, DOI 10.1007/s11606-011-1882-7

Browne JL, 2013, DIABETIC MED, V30, P620, DOI 10.1111/dme.12078

Casciano R, 2012, HEALTH PLACE, V18, P180, DOI 10.1016/j.healthplace.2011.09.002

Chaikiat A, 2012, HEALTH PLACE, V18, P877, DOI 10.1016/j.healthplace.2012.03.003

CURRY SJ, 1993, AM J PREV MED, V9, P78

Deshpande AD, 2008, PHYS THER, V88, P1254, DOI 10.2522/ptj.20080020

Echeverria SE, 2004, J URBAN HEALTH, V81, P682, DOI 10.1093/jurban/jth151

Fiori KL, 2013, GERONTOLOGY, V59, P40, DOI 10.1159/000339747

Fisher JTM L, 2009, DIABETIC MED, V26, P622

Fisher L, 2008, DIABETIC MED, V25, P1096, DOI 10.1111/j.1464-5491.2008.02533.x

Fisher L, 2007, DIABETES CARE, V30, P542, DOI 10.2337/dc06-1614

Fisher L, 2012, DIABETES CARE, V35, P259, DOI 10.2337/dc11-1572

Fisher L, 2010, DIABETES CARE, V33, P1034, DOI 10.2337/dc09-2175

Fisher L, 2010, DIABETES CARE, V33, P23, DOI 10.2337/dc09-1238

Gary TL, 2008, DIABETES CARE, V31, P273, DOI 10.2337/dc07-1111

Gary-Webb TL, 2011, BMC PUBLIC HEALTH, V11, P7

Gonzalez JS, 2011, DIABETES CARE, V34, P236, DOI 10.2337/dc10-1970

Hoehner CM, 2005, AM J PREV MED, V28, P105, DOI 10.1016/j.amepre.2004.10.023

Karasek D, 2012, AM J PUBLIC HEALTH, V102, P343, DOI 10.2105/AJPH.2011.300364

Kim J, 2010, SOC SCI RES, V39, P260, DOI 10.1016/j.ssresearch.2009.08.007

Kneck A, 2012, J ADV NURS, V68, P2486, DOI 10.1111/j.1365-2648.2012.05947.x

Mair C, 2008, J EPIDEMIOL COMMUN H, V62, P940, DOI 10.1136/jech.2007.066605

Mair C, 2010, HEALTH PLACE, V16, P811, DOI 10.1016/j.healthplace.2010.04.006

Mansfield ED, 2012, INT J BEHAV NUTR PHY, P9

Martin KR, 2010, ARTHRIT CARE RES, V62, P1602, DOI 10.1002/acr.20267

Mathew R., 2012, BMC FAM PRACT, V13

Moore LV, 2012, AM J EPIDEMIOL, V175, P1037, DOI 10.1093/aje/kwr445

Mujahid MS, 2007, AM J EPIDEMIOL

Murrock CJ, 2013, WOMEN HEALTH, V53, P173, DOI 10.1080/03630242.2012.753979

Owen N, 2004, AM J PREV MED, V27, P67, DOI 10.1016/j.amepre.2004.03.006

Pampalon R., 2009, CHRONIC DIS CAN, V29, P174

Polonsky WH, 2005, DIABETES CARE, V28, P626, DOI 10.2337/diacare.28.3.626

Ross CE, 1999, URBAN AFF REV, V34, P412, DOI 10.1177/10780879922184004

Sampson RJ, 2004, SOC PSYCHOL QUART, V67, P319, DOI 10.1177/019027250406700401

Schmitz MF, 2012, AGE AGEING, V41, P388, DOI 10.1093/ageing/afr163

Schmitz N, 2009, DIABETES CARE, V32, P1998, DOI 10.2337/dc09-0838

Stafford M, 2008, HEALTH PLACE, V14, P394, DOI 10.1016/j.healthplace.2007.08.006

Steptoe A, 2001, ANN BEHAV MED, V23, P177, DOI 10.1207/S15324796ABM2303_5

Tsujii S, 2012, DIABETIC MED, V29, P1451, DOI 10.1111/j.1464-5491.2012.03647.x

Wen M, 2006, SOC SCI MED, V63, P2575, DOI 10.1016/j.socscimed.2006.06.025

WETHINGTON E, 1986, J HEALTH SOC BEHAV, V27, P78, DOI 10.2307/2136504

Wilkerson A, 2012, ENVIRON BEHAV, V44, P595, DOI 10.1177/0013916511402058

Zagarins SE, 2012, J BEHAV MED, V35, P299, DOI 10.1007/s10865-011-9359-z

NR 46

TC 7

Z9 7

U1 1

U2 27

PU PERGAMON-ELSEVIER SCIENCE LTD

PI OXFORD

PA THE BOULEVARD, LANGFORD LANE, KIDLINGTON, OXFORD OX5 1GB, ENGLAND

SN 0022-3999

J9 J PSYCHOSOM RES

JI J. Psychosomat. Res.

PD AUG

PY 2013

VL 75

IS 2

BP 147

EP 152

DI 10.1016/j.jpsychores.2013.05.009

PG 6

WC Psychiatry

SC Psychiatry

GA 204UQ

UT WOS:000323397100008

PM 23915771

DA 2019-01-08

ER

PT J

AU Wagner, JA

Tennen, H

Finan, PH

Ghuman, N

Burg, MM

AF Wagner, Julie A.

Tennen, Howard

Finan, Patrick H.

Ghuman, Nimrta

Burg, Matthew M.

TI Self-reported Racial Discrimination and Endothelial Reactivity to Acute

Stress in Women

SO STRESS AND HEALTH

LA English

DT Article

DE endothelial function; racism; discrimination; diabetes; stress;

reactivity

ID CORONARY-ARTERY-DISEASE; MENTAL STRESS; PERCEIVED DISCRIMINATION;

SOCIOECONOMIC-STATUS; CARDIOVASCULAR REACTIVITY; ETHNIC DISCRIMINATION;

HEALTH DISPARITIES; AFRICAN-AMERICANS; HEART-DISEASE; DYSFUNCTION

AB This study investigated the effect of self-reported racial discrimination on endothelial responses to acute laboratory mental stress among post-menopausal women. One-hundred thirteen women (n = 94 self-identified as White and n = 19 self-identified as racial/ethnic minority), 43% with type 2 diabetes, reported lifetime experiences of racial/ ethnic discrimination. Repeated assessments of flow-mediated dilation were performed at baseline, immediately after 5 min of mental arithmetic and at 20-min recovery. Both White and racial/ethnic minority women reported lifetime discrimination, with rates significantly higher among minorities. Self-reported lifetime discrimination was associated with attenuated flow-mediated dilation at recovery. Confounding variables, including clinical characteristics, mood, personality traits, other life stressors and general distress, did not better account for the effect of racial discrimination. Neither race/ethnicity nor diabetes status moderated the effect. The perceived stressfulness of the mental arithmetic was not associated with the endothelial response. In conclusion, self-reported lifetime discrimination is associated with attenuated endothelial recovery from acute mental stress. Elucidating the effects of discrimination and the biological mechanisms through which it affects the vasculature may suggest interventions to improve health. Copyright (C) 2012 John Wiley & Sons, Ltd.

C1 [Wagner, Julie A.] Univ Connecticut, Ctr Hlth, Div Behav Sci & Community Hlth, Farmington, CT 06030 USA.

[Tennen, Howard] Univ Connecticut, Ctr Hlth, Dept Community Med, Farmington, CT 06030 USA.

[Finan, Patrick H.] Johns Hopkins Univ, Baltimore, MD USA.

[Ghuman, Nimrta] Univ Connecticut, Ctr Hlth, Calhoun Cardiol Ctr, Farmington, CT 06030 USA.

[Burg, Matthew M.] Columbia Univ, Columbia, NY USA.

RP Wagner, JA (reprint author), Univ Connecticut, Ctr Hlth, Div Behav Sci & Community Hlth, 263 Farmington Ave, Farmington, CT 06030 USA.

EM juwagner@uchc.edu

FU University of Connecticut Center for Interdisciplinary Research in

Women's Health; American Heart Association; American Diabetes

Association; University of Connecticut Health Center General Clinical

Research Center [M01RR006192]

FX This study was supported by grants from the University of Connecticut

Center for Interdisciplinary Research in Women's Health, the American

Heart Association, the American Diabetes Association and the University

of Connecticut Health Center General Clinical Research Center

(M01RR006192).

CR ANDRESEN EM, 1994, AM J PREV MED, V10, P77

Blumenthal JA, 2005, JAMA-J AM MED ASSOC, V293, P1626, DOI 10.1001/jama.293.13.1626

Borrell LN, 2006, SOC SCI MED, V63, P1415, DOI 10.1016/j.socscimed.2006.04.008

Brion MJ, 2005, PSYCHOPHYSIOLOGY, V42, P282, DOI 10.1111/j.1469-8986.2005.00284.x

Broudy R, 2007, J BEHAV MED, V30, P31, DOI 10.1007/s10865-006-9081-4

Carlson ED, 2005, RES NURS HEALTH, V28, P306, DOI 10.1002/nur.20084

Chan SY, 2003, J AM COLL CARDIOL, V42, P1037, DOI 10.1016/S0735-1097(03)00927-6

Clark R, 2006, HEALTH PSYCHOL, V25, P20, DOI 10.1037/0278-6133.25.1.20

COHEN S, 1983, J HEALTH SOC BEHAV, V24, P385, DOI 10.2307/2136404

Cooper DC, 2010, ANN BEHAV MED, V39, P222, DOI 10.1007/s12160-010-9181-9

Corretti MC, 2002, J AM COLL CARDIOL, V39, P257, DOI 10.1016/S0735-1097(01)01746-6

Costa P. T., 1992, NEO PI R PROFESSIONA

Fogel J, 2003, PSYCHO-ONCOL, V12, P107, DOI 10.1002/pon.617

Fujishiro K, 2009, SOC SCI MED, V68, P840, DOI 10.1016/j.socscimed.2008.12.007

Ghiadoni L, 2000, CIRCULATION, V102, P2473, DOI 10.1161/01.CIR.102.20.2473

Gottdiener JS, 2003, AM J CARDIOL, V92, P687, DOI 10.1016/S0002-9149(03)00823-3

Gu K, 1999, JAMA-J AM MED ASSOC, V281, P1291, DOI 10.1001/jama.281.14.1291

Hausmann LRM, 2008, MED CARE, V46, P905, DOI 10.1097/MLR.0b013e3181792562

Hogue C. J. R., 2000, MINORITY HLTH AM FIN

HOLMES TH, 1967, J PSYCHOSOM RES, V11, P213, DOI 10.1016/0022-3999(67)90010-4

Jackson JS, 1997, PERCEPTIONS DISCRIMI

Jones Camara Phyllis, 2002, PHYLON, V50, P7, DOI DOI 10.2307/4149999

Krieger N, 2005, SOC SCI MED, V61, P1576, DOI 10.1016/j.socscimed.2005.03.006

Lepore SJ, 2006, ANN BEHAV MED, V31, P120, DOI 10.1207/s15324796abm3102_3

Lewis TT, 2006, PSYCHOSOM MED, V68, P362, DOI 10.1097/01.psy.0000221360.94700.16

Lewis TT, 2010, BRAIN BEHAV IMMUN, V24, P438, DOI 10.1016/j.bbi.2009.11.011

McEwen BS, 1998, NEW ENGL J MED, V338, P171, DOI 10.1056/NEJM199801153380307

Merritt MM, 2006, HEALTH PSYCHOL, V25, P364, DOI 10.1037/0278-6133.25.3.364

RADLOFF L S, 1977, Applied Psychological Measurement, V1, P385, DOI 10.1177/014662167700100306

Richman LS, 2008, ANN BEHAV MED, V35, P105, DOI 10.1007/s12160-007-9013-8

Richman LS, 2007, HEALTH PSYCHOL, V26, P675, DOI 10.1037/0278-6133.26.6.675

ROSS R, 1986, NEW ENGL J MED, V314, P488, DOI 10.1056/NEJM198602203140806

Salomon K, 2008, HEALTH PSYCHOL, V27, P473, DOI 10.1037/0278-6133.27.4.473

Schwartz AR, 2003, PSYCHOSOM MED, V65, P22, DOI 10.1097/01.PSY.0000046075.79922.61

Shavers VL, 2007, J NATL MED ASSOC, V99, P1013

Soufer R, 1998, P NATL ACAD SCI USA, V95, P6454, DOI 10.1073/pnas.95.11.6454

Szanton SL, 2011, INT J BEHAV MED

Tomfohr L, 2010, PSYCHOSOM MED, V72, P266, DOI 10.1097/PSY.0b013e3181d0d8b2

Wagner JA, 2006, PSYCHOSOM MED, V68, P80, DOI 10.1097/01.psy.0000195868.68122.9e

Wagner J, 2007, DIABETES CARE, V30, P364, DOI 10.2337/dc06-1756

Watson JM, 2002, J WOMEN HEALTH GEN-B, V11, P441, DOI 10.1089/15246090260137617

WEISSMAN MM, 1977, AM J EPIDEMIOL, V106, P203, DOI 10.1093/oxfordjournals.aje.a112455

White JB, 2004, ORGAN BEHAV HUM DEC, V94, P102, DOI 10.1016/j.obhdp.2004.03.005

Williams DR, 2009, J BEHAV MED, V32, P20, DOI 10.1007/s10865-008-9185-0

WILLIAMS DR, 1997, J HLTH PSYCHOL, V0002

Zhang HR, 2012, BASIC RES CARDIOL, V107, DOI 10.1007/s00395-011-0237-1

NR 46

TC 11

Z9 11

U1 0

U2 14

PU WILEY

PI HOBOKEN

PA 111 RIVER ST, HOBOKEN 07030-5774, NJ USA

SN 1532-3005

EI 1532-2998

J9 STRESS HEALTH

JI Stress Health

PD AUG

PY 2013

VL 29

IS 3

BP 214

EP 221

DI 10.1002/smi.2449

PG 8

WC Psychology, Applied; Psychiatry; Psychology

SC Psychology; Psychiatry

GA 193WJ

UT WOS:000322591900006

PM 22962001

DA 2019-01-08

ER

PT J

AU Chesla, CA

Chun, KM

Kwan, CML

Mullan, JT

Kwong, Y

Hsu, L

Huang, P

Strycker, LA

Shum, T

To, D

Kao, R

Waters, CM

AF Chesla, Catherine A.

Chun, Kevin M.

Kwan, Christine M. L.

Mullan, Joseph T.

Kwong, Yulanda

Hsu, Lydia

Huang, Peggy

Strycker, Lisa A.

Shum, Tina

To, Diana

Kao, Rudy

Waters, Catherine M.

TI Testing the efficacy of culturally adapted coping skills training for

Chinese American immigrants with type 2 diabetes using community-based

participatory research

SO RESEARCH IN NURSING & HEALTH

LA English

DT Article

DE Asian; Chinese; community-based participatory research; diabetes;

distress; intervention; quality of life

ID QUALITY-OF-LIFE; SELF-MANAGEMENT EDUCATION; DISEASE MANAGEMENT; GLYCEMIC

CONTROL; DEPRESSIVE SYMPTOMS; PACIFIC ISLANDERS; COLLEGE-STUDENTS;

DISTRESS SCALE; METAANALYSIS; INTERVENTIONS

AB Chinese Americans demonstrate greater prevalence of diabetes than non-Hispanic whites and find standard diabetes care disregards their cultural health beliefs. Academic researchers and Chinatown agencies collaborated to culturally adapt and test an efficacious cognitive-behavioral intervention using community-based participatory research. Using a delayed-treatment repeated-measures design, 145 adult Chinese immigrants with Type 2 diabetes completed treatment. Immediate benefits of treatment were evident in the improvement (p<.05) in diabetes self-efficacy, diabetes knowledge, bicultural efficacy, family emotional and instrumental support, diabetes quality of life, and diabetes distress. Prolonged benefits were evident in all changed variables 2 months post-intervention. The CBPR approach enabled the development of a culturally acceptable, efficacious behavioral intervention, and provides a model for working with communities that demonstrate health disparities. (c) 2013 Wiley Periodicals, Inc. Res Nurs Health 36: 359-372, 2013

C1 [Chesla, Catherine A.; Kwan, Christine M. L.] Univ Calif San Francisco, Dept Family Hlth Care Nursing, San Francisco, CA 94143 USA.

[Chun, Kevin M.] Univ San Francisco, Dept Psychol, San Francisco, CA 94117 USA.

[Mullan, Joseph T.] Univ Calif San Francisco, Dept Social & Behav Sci, San Francisco, CA 94143 USA.

[Kwong, Yulanda] Donaldina Cameron House, Social Serv Minist, San Francisco, CA USA.

[Shum, Tina; To, Diana] Donaldina Cameron House, San Francisco, CA USA.

[Hsu, Lydia] North East Med Serv, Hlth Counseling Serv Dept, San Francisco, CA USA.

[Huang, Peggy] Univ Calif San Francisco, Ctr Diabet, San Francisco, CA 94143 USA.

[Strycker, Lisa A.] Oregon Res Inst, Eugene, OR 97403 USA.

[Kao, Rudy] North East Med Serv, San Francisco, CA USA.

[Waters, Catherine M.] Univ Calif San Francisco, Dept Community Hlth Syst, San Francisco, CA 94143 USA.

RP Chesla, CA (reprint author), Univ Calif San Francisco, Dept Family Hlth Care Nursing, 2 Koret Way,Box 0606, San Francisco, CA 94143 USA.

FU National Institute of Nursing Research [1R01NR010693]

FX This project was supported by the National Institute of Nursing Research

(1R01NR010693). We thank the following organizations and individuals:

Doreen Der-McLeod and Monica Walters of Donaldina Cameron House; Daniel

Chan and Edna Tam of North East Medical Services; Anita Au-Yeung and

Doris Hung of University of California San Francisco; Eunice Lew and all

study participants; members of our Community Advisory Board. We thank

Margaret Grey of Yale University for her ongoing consultation. An

earlier version of this work was presented in the American Diabetes

Association Convention in Philadelphia in 2012.

CR [Anonymous], 2009, SPSS STAT WIND VERS

Bacallao Martica L, 2005, J Prim Prev, V26, P485, DOI 10.1007/s10935-005-0008-6

Bandura A, 2004, HEALTH EDUC BEHAV, V31, P143, DOI 10.1177/1090198104263660

Castro FG, 2010, ANNU REV CLIN PSYCHO, V6, P213, DOI 10.1146/annurev-clinpsy-033109-132032

Chesla CA, 2004, DIABETES CARE, V27, P2850, DOI 10.2337/diacare.27.12.2850

Chesla CA, 2009, DIABETES CARE, V32, P1812, DOI 10.2337/dc09-0278

Chun KM, 2011, SOC SCI MED, V72, P256, DOI 10.1016/j.socscimed.2010.11.010

Chun KM, 2004, PSYCHOL HEALTH, V19, P767, DOI 10.1080/08870440410001722958

Cochran J, 2008, DIABETES EDUCATOR, V34, P815, DOI 10.1177/0145721708323640

Coyne JC, 1994, J FAMILY PSYCHOL, V8, P43, DOI DOI 10.1037/0893-3200.8.1.43

David EJR, 2009, J COUNS PSYCHOL, V56, P211, DOI 10.1037/a0015419

Ellis SE, 2004, PATIENT EDUC COUNS, V52, P97, DOI 10.1016/S0738-3991(03)00016-8

Fisher L, 2004, J FAM PSYCHOL, V18, P302, DOI 10.1037/0893-3200.18.2.302

Fisher L, 2000, BEHAV MED, V26, P53, DOI 10.1080/08964280009595752

Fisher L, 2012, DIABETES CARE, V35, P259, DOI 10.2337/dc11-1572

Fisher L, 2010, DIABETES CARE, V33, P1034, DOI 10.2337/dc09-2175

Fitzgerald JT, 1998, DIABETES CARE, V21, P706, DOI 10.2337/diacare.21.5.706

Fitzgerald JT, 1996, EVAL HEALTH PROF, V19, P209, DOI DOI 10.1177/016327879601900205

Funnell MM, 2012, DIABETES CARE, V35, pS101, DOI [10.2337/dc12-s101, 10.2337/dc12-S089]

Gary TL, 2003, DIABETES EDUCATOR, V29, P488, DOI 10.1177/014572170302900313

Grey M, 2000, J PEDIATR-US, V137, P107, DOI 10.1067/mpd.2000.106568

Grey M, 2011, NURS RES, V60, P173, DOI 10.1097/NNR.0b013e3182159c8f

Grey M, 2009, RES NURS HEALTH, V32, P405, DOI 10.1002/nur.20336

Grzywacz JG, 2006, HISPANIC J BEHAV SCI, V28, P404, DOI 10.1177/0739986306290645

Guo XH, 2009, PSYCHOL ASSESSMENT, V21, P22, DOI 10.1037/a0014495

Gupta LS, 2011, DIABETES CARE, V34, P1791, DOI 10.2337/dc11-0088

Hodges J, 2007, BEHAV RES THER, V45, P901, DOI 10.1016/j.brat.2006.08.015

Hox J. J., 2010, MULTILEVEL ANAL TECH

Hsu WC, 2006, DIABETES CARE, V29, P415, DOI 10.2337/diacare.29.02.06.dc05-1915

Huang MF, 2009, J NURS SCHOLARSHIP, V41, P385, DOI 10.1111/j.1547-5069.2009.01306.x

Ismail K, 2004, LANCET, V363, P1589, DOI 10.1016/S0140-6736(04)16202-8

Israel B. A., 2003, COMMUNITY BASED PART, P53

Iwamasa G. Y, 2006, COGNITIVE BEHAV THER

Jack Jr L., 2004, DIABETES MED, V22, P1295

JACOBSON AM, 1994, DIABETES CARE, V17, P267, DOI 10.2337/diacare.17.4.267

King GL, 2012, DIABETES CARE, V35, P1181, DOI 10.2337/dc12-0210

Kwan CML, 2011, ASIAN AM J PSYCHOL, V2, P115, DOI 10.1037/a0024184

LAFROMBOISE T, 1993, PSYCHOL BULL, V114, P395, DOI 10.1037/0033-2909.114.3.395

LaFromboise TD, 2010, CULT DIVERS ETHN MIN, V16, P68, DOI 10.1037/a0016181

McCoach DB, 2006, J EDUC PSYCHOL, V98, P14, DOI 10.1037/0022-0663.98.1.14

Minet L, 2009, PATIENT EDUC COUNS, V80, P29

Muller I, 2011, J TELEMED TELECARE, V17, P177, DOI 10.1258/jtt.2010.100709

Nam S, 2012, J CARDIOVASC NURS, V27, P505, DOI 10.1097/JCN.0b013e31822375a5

NICOS, 2004, CHIN COMM HLTH REP S

Norris SL, 2002, DIABETES CARE, V25, P1159, DOI 10.2337/diacare.25.7.1159

Plack K, 2010, CURR OPIN PSYCHIATR, V23, P131, DOI 10.1097/YCO.0b013e3283366555

Polonsky WH, 2005, DIABETES CARE, V28, P626, DOI 10.2337/diacare.28.3.626

RADLOFF L S, 1977, Applied Psychological Measurement, V1, P385, DOI 10.1177/014662167700100306

RUBIN RR, 1991, DIABETES CARE, V14, P335, DOI 10.2337/diacare.14.4.335

Saudek C. D., 2010, DIABETES ED, V19, DOI 2010.1177/014572179301900308

Schafer JL, 2002, PSYCHOL METHODS, V7, P147, DOI 10.1037//1082-989X.7.2.147

Sigurdardottir AK, 2007, PATIENT EDUC COUNS, V67, P21, DOI 10.1016/j.pec.2007.03.007

Singer J. D., 2003, APPL LONGITUDINAL DA

Smith TB, 2011, J CLIN PSYCHOL, V67, P166, DOI 10.1002/jclp.20757

SUINN RM, 1987, EDUC PSYCHOL MEAS, V47, P401, DOI 10.1177/0013164487472012

Trief PM, 2004, ANN BEHAV MED, V27, P148, DOI 10.1207/s15324796abm2703_2

Tschann JM, 1999, J MARRIAGE FAM, V61, P269, DOI 10.2307/353747

U. S. Census Bureau, 2010, AS PAC AM HER MONTH

Wallerstein N., 2003, COMMUNITY BASED PART, P27

Wang CY, 1999, DIABETES EDUCATOR, V25, P738, DOI 10.1177/014572179902500506

Wei MF, 2010, J COUNS PSYCHOL, V57, P411, DOI 10.1037/a0020790

Ying Y. W, 2009, HDB MENTAL HLTH ACCU, P45, DOI DOI 10.1007/978-1-60327-437-1_3

Zhong XF, 2011, SE ASIAN J TROP MED, V42, P185

NR 63

TC 15

Z9 15

U1 3

U2 35

PU WILEY-BLACKWELL

PI HOBOKEN

PA 111 RIVER ST, HOBOKEN 07030-5774, NJ USA

SN 0160-6891

J9 RES NURS HEALTH

JI Res. Nurs. Health

PD AUG

PY 2013

VL 36

IS 4

BP 359

EP 372

DI 10.1002/nur.21543

PG 14

WC Nursing

SC Nursing

GA 185XJ

UT WOS:000322003900005

PM 23606271

DA 2019-01-08

ER

PT J

AU Balfe, M

Doyle, F

Smith, D

Sreenan, S

Brugha, R

Hevey, D

Conroy, R

AF Balfe, Myles

Doyle, Frank

Smith, Diarmuid

Sreenan, Seamus

Brugha, Ruairi

Hevey, David

Conroy, Ronan

TI What's distressing about having type 1 diabetes? A qualitative study of

young adults' perspectives

SO BMC ENDOCRINE DISORDERS

LA English

DT Article

DE Young adult; Distress; Qualitative; Type 1 diabetes; Emerging adulthood

ID PSYCHOLOGICAL DISTRESS; PSYCHIATRIC MORBIDITY; DEPRESSION; PREVALENCE;

CARE; EXPLORATION; EXPERIENCES; TRANSITION; SUPPORT; ANXIETY

AB Background: Diabetes distress is a general term that refers to the emotional burdens, anxieties, frustrations, stressors and worries that stem from managing a severe, complex condition like Type 1 diabetes. To date there has been limited research on diabetes-related distress in younger people with Type 1 diabetes. This qualitative study aimed to identify causes of diabetes distress in a sample of young adults with Type 1 diabetes.

Methods: Semi-structured interviews with 35 individuals with Type 1 diabetes (23-30 years of age).

Results: This study found diabetes related-distress to be common in a sample of young adults with Type 1 diabetes in the second phase of young adulthood (23-30 years of age). Diabetes distress was triggered by multiple factors, the most common of which were: self-consciousness/stigma, day-to-day diabetes management difficulties, having to fight the healthcare system, concerns about the future and apprehension about pregnancy. A number of factors appeared to moderate distress in this group, including having opportunities to talk to healthcare professionals, attending diabetes education programmes and joining peer support groups. Young adults felt that having opportunities to talk to healthcare professionals about diabetes distress should be a component of standard diabetes care.

Conclusions: Some aspects of living with diabetes frequently distress young adults with Type 1 diabetes who are in their twenties. Clinicians should facilitate young adults' attendance at diabetes education programmes, provide them with opportunities to talk about their diabetes-related frustrations and difficulties and, where possible, assist in the development of peer-support networks for young adults with diabetes.

C1 [Balfe, Myles; Brugha, Ruairi; Conroy, Ronan] Royal Coll Surgeons Ireland, Dept Epidemiol & Publ Hlth Med, Dublin 2, Ireland.

[Balfe, Myles] Natl Univ Ireland Univ Coll Cork, Dept Sociol, Cork, Ireland.

[Doyle, Frank] Royal Coll Surgeons Ireland, Dept Psychol, Dublin 2, Ireland.

[Smith, Diarmuid] Beaumont Hosp, Dept Endocrinol, Dublin 9, Ireland.

[Sreenan, Seamus] Connolly Hosp, Dept Endocrinol, Dublin, Ireland.

[Hevey, David] Univ Dublin Trinity Coll, Dept Psychol, Dublin 2, Ireland.

RP Balfe, M (reprint author), Royal Coll Surgeons Ireland, Dept Epidemiol & Publ Hlth Med, Dublin 2, Ireland.

EM mylesbalfe@rcsi.ie

RI Doyle, Frank/C-2750-2012; Conroy, Ronan/C-6416-2008

OI Doyle, Frank/0000-0002-3785-7433; Conroy, Ronan/0000-0001-5983-8682;

Hevey, David/0000-0003-2844-0449

FU Irish Health Research Board, Diabetes Ireland; MRCG

FX The authors would like to sincerely thank the Irish Health Research

Board, Diabetes Ireland and the MRCG for funding this study, and the

staff of Connolly and Beaumont Hospitals in Dublin for providing advice

on various aspects of this project. They would like to thank the three

article reviewers and the journal editorial staff for their extremely

helpful and thorough feedback on the article. And finally they would

like to thank the young adults who took part in the study.

CR Anderson B, 2010, DIABETES SPECTR, V23, P32

Anderson BJ, 2004, PATIENT EDUC COUNS, V53, P347, DOI 10.1016/j.pec.2003.03.001

Anderson RJ, 2001, DIABETES CARE, V24, P1069, DOI 10.2337/diacare.24.6.1069

Arnett JJ, 2000, YOUTH SOC, V31, P267, DOI 10.1177/0044118X00031003001

Arnett JJ, 2000, AM PSYCHOL, V55, P469, DOI 10.1037/0003-066X.55.5.469

Braun V., 2006, QUALITATIVE RES PSYC, V3, P77, DOI DOI 10.1191/1478088706QP0630A

Bryden KS, 2003, DIABETES CARE, V26, P1052, DOI 10.2337/diacare.26.4.1052

Bryden KS, 2001, DIABETES CARE, V24, P1536, DOI 10.2337/diacare.24.9.1536

Cabassa LJ, 2008, SOC SCI MED, V66, P2413, DOI 10.1016/j.socscimed.2008.01.054

Cameron F, 2010, DIABETES CARE, V33, P1470

Cameron F, 2012, DIABETES MANAGE, V2, P1

DAFNE Study Group, 2002, BMJ-BRIT MED J, V325, DOI DOI 10.1136/BMJ.325.7367.746

Dantzer C, 2003, CLIN PSYCHOL REV, V23, P787, DOI 10.1016/S0272-7358(03)00069-2

Duke D, 2011, AM J LIFESTYLE MED, V5, P85

Egede LE, 2012, CURR PSYCHIAT REP, V14, P15, DOI 10.1007/s11920-011-0240-0

Esbitt SA, 2013, SCREENING DEPRESSION, P27

Farmer AJ, 2005, DIABETES CARE, V28, P2697, DOI 10.2337/diacare.28.11.2697

Fisher L, 2012, DIABETES CARE, V35, P259, DOI 10.2337/dc11-1572

Garvey K, 2011, DIABETES SPECTRUM, V24, P22, DOI [10.2337/diaspect.24.1.22, DOI 10.2337/DIASPECT.24.1.22]

Gask L, 2011, CHRONIC ILLN, V7, P239, DOI 10.1177/1742395311403636

Gendelman N, 2009, DIABETES CARE, V32, P575, DOI 10.2337/dc08-1835

Gonzalez JS, 2011, DIABETES CARE, V34, P236, DOI 10.2337/dc10-1970

Grigsby AB, 2002, J PSYCHOSOM RES, V53, P1053, DOI 10.1016/S0022-3999(02)00417-8

Guest G, 2006, FIELD METHOD, V18, P59, DOI 10.1177/1525822X05279903

Haghighat R, 2001, BRIT J PSYCHIAT, V178, P207, DOI 10.1192/bjp.178.3.207

Hillege S, 2011, ARCH PSY NURSING, V25, pe7

Hislop AL, 2008, DIABETIC MED, V25, P91, DOI 10.1111/j.1464-5491.2007.02310.x

Hood KK, 2006, DIABETES CARE, V29, P1389, DOI 10.2337/dc06-0087

Insabella G, 2007, PEDIATR DIABETES, V8, P228, DOI 10.1111/j.1399-5448.2007.00266.x

Iwasaki Y, 2005, SOC SCI MED, V60, P977, DOI 10.1016/j.socscimed.2004.06.032

Kay C, 2009, J HEALTH PSYCHOL, V14, P242, DOI 10.1177/1359105308100208

Kovacs M, 1997, DIABETES CARE, V20, P36, DOI 10.2337/diacare.20.1.36

Lewis V, 2011, DIABETES SPECTRUM, V24, P4

Markowitz JT, 2012, DIABETIC MED, V29, P522, DOI 10.1111/j.1464-5491.2011.03537.x

Marshall MN, 1996, FAM PRACT, V13, P522, DOI 10.1093/fampra/13.6.522

MAYOU R, 1991, PSYCHOL MED, V21, P639, DOI 10.1017/S0033291700022273

McGuire BE, 2010, DIABETOLOGIA, V53, P66, DOI 10.1007/s00125-009-1559-5

Monaghan M., 2010, Diabetes Spectrum, V23, P25, DOI 10.2337/diaspect.23.1.25

Morse JM, 2000, QUAL HEALTH RES, V10, P3, DOI 10.1177/104973200129118183

Northam EA, 2005, DIABETIC MED, V22, P152, DOI 10.1111/j.1464-5491.2004.01370.x

Peters A, 2011, DIABETES CARE, V34, P2477, DOI 10.2337/dc11-1723

Polonsky WH, 2005, DIABETES CARE, V28, P626, DOI 10.2337/diacare.28.3.626

Schabert J, 2013, PATIENT, V6, P1, DOI 10.1007/s40271-012-0001-0

Scott L, 2005, CAN J DIABETES, V29, P203

Steinsdottir F, 2012, LAEKNABLADID, V94, P823

Watts S, 2010, PSYCHOL HEALTH, V25, P491, DOI 10.1080/08870440802688588

Weissberg-Benchell J, 2007, DIABETES CARE, V30, P2441, DOI 10.2337/dc07-1249

Wolpert HA, 2001, DIABETES CARE, V24, P1513, DOI 10.2337/diacare.24.9.1513

NR 48

TC 27

Z9 29

U1 1

U2 36

PU BIOMED CENTRAL LTD

PI LONDON

PA 236 GRAYS INN RD, FLOOR 6, LONDON WC1X 8HL, ENGLAND

SN 1472-6823

J9 BMC ENDOCR DISORD

JI BMC Endocr. Disord.

PD JUL 25

PY 2013

VL 13

AR 25

DI 10.1186/1472-6823-13-25

PG 14

WC Endocrinology & Metabolism

SC Endocrinology & Metabolism

GA 194AN

UT WOS:000322602700001

PM 23885644

OA DOAJ Gold, Green Published

DA 2019-01-08

ER

PT J

AU Gubhaju, L

McNamara, BJ

Banks, E

Joshy, G

Raphael, B

Williamson, A

Eades, SJ

AF Gubhaju, Lina

McNamara, Bridgette J.

Banks, Emily

Joshy, Grace

Raphael, Beverley

Williamson, Anna

Eades, Sandra J.

TI The overall health and risk factor profile of Australian Aboriginal and

Torres Strait Islander participants from the 45 and up study

SO BMC PUBLIC HEALTH

LA English

DT Article

DE Aboriginal Australians; Torres Strait Islanders; 45 and Up study

ID COHORT

AB Background: Despite large disparities in health outcomes between Aboriginal and non-Aboriginal Australians, detailed evidence on the health and lifestyle characteristics of older Aboriginal Australians is lacking. The aim of this study is to quantify socio-demographic and health risk factors and mental and physical health status among Aboriginal participants from the 45 and Up Study and to compare these with non-Aboriginal participants from the study.

Methods: The 45 and Up Study is a large-scale study of individuals aged 45 years and older from the general population of New South Wales, Australia responding to a baseline questionnaire distributed from 2006-2008. Odds ratios (OR) and 95% confidence intervals (CI) of self-reported responses from the baseline questionnaire for Aboriginal versus non-Aboriginal participants relating to socio-demographic factors, health risk factors, current and past medical and surgical history, physical disability, functional health limitations and levels of current psychological distress were calculated using unconditional logistic regression, with adjustments for age and sex.

Results: Overall, 1939 of 266,661 45 and Up Study participants examined in this study identified as Aboriginal and/or Torres Strait Islander (0.7%). Compared to non-Aboriginal participants, Aboriginal participants were significantly more likely to be: younger (mean age 58 versus 63 years); without formal educational qualifications (age-and sex-adjusted OR = 6.2, 95% CI 5.3-7.3); of unemployed (3.7, 2.9-4.6) or disabled (4.6, 3.9-5.3) work status; and with a household income < $ 20,000/year versus >= $ 70,000/year (5.8, 5.0-6.9). Following additional adjustment for income and education, Aboriginal participants were significantly more likely than non-Aboriginal participants to: be current smokers (2.4, 2.0-2.8), be obese (2.1, 1.8-2.5), have ever been diagnosed with certain medical conditions (especially: diabetes [2.1, 1.8-2.4]; depression [1.6, 1.4-1.8] and stroke [1.8, 1.4-2.3]), have care-giving responsibilities (1.8, 1.5-2.2); have a major physical disability (2.6, 2.2-3.1); have severe physical functional limitation (2.9, 2.4-3.4) and have very high levels of psychological distress (2.4, 2.0-3.0).

Conclusions: Aboriginal participants from the 45 and Up Study experience greater levels of disadvantage and have greater health needs (including physical disability and psychological distress) compared to non-Aboriginal participants. The study highlights the need to address the social determinants of health in Australia and to provide appropriate mental health services and disability support for older Aboriginal people.

C1 [Gubhaju, Lina; McNamara, Bridgette J.] Preventat Hlth, Baker IDI Heart & Diabet Inst, Melbourne, Vic 3004, Australia.

[Banks, Emily; Joshy, Grace] Australian Natl Univ, Natl Ctr Epidemiol & Populat Hlth, Canberra, ACT 0200, Australia.

[Raphael, Beverley] Australian Natl Univ, Canberra, ACT 0200, Australia.

[Banks, Emily; Williamson, Anna] Sax Inst, Sydney, NSW 1240, Australia.

[Eades, Sandra J.] Univ Sydney, Sch Publ Hlth, Sydney, NSW 2006, Australia.

RP Eades, SJ (reprint author), Univ Sydney, Sch Publ Hlth, A27 Edward Ford Bldg, Sydney, NSW 2006, Australia.

EM Sandra.Eades@sydney.edu.au

OI Eades, Sandra/0000-0001-8629-3390

CR ABS, 2010, DEM SOC EC CHAR OV L

ABS, 2006, INTR SOC IND

AIHW, 2004, RUR REG REM HLTH GUI

Andrews G, 2001, AUST NZ J PUBL HEAL, V25, P494, DOI 10.1111/j.1467-842X.2001.tb00310.x

Australian Bureau of Statistics, 2006, NAT AB TORR STRAIT I

Australian Bureau of Statistics [ABS], 2001, INF PAP US KESSL PSY

Australian Institute of Health and Welfare (AIHW), 2011, HLTH WELF AUSTR AB T

Australian Institute of Health and Welfare (AIHW), 2003, ACT AUSTR SURV GUID

Banks E, 2008, INT J EPIDEMIOL, V37, P941, DOI 10.1093/ije/dym184

Centre for Epidemiology and Research, 2010, 2006 2009 REP AD AB

Cunningham J, 2006, BMC PUBLIC HEALTH, V6, DOI 10.1186/1471-2458-6-8

Hays R. D., 1995, USERS MANUAL MED OUT

Investigators S, 2010, BMC PUBLIC HEALTH, V10, P287

Mathews JD, 1998, MED J AUSTRALIA, V169, P625

Mealing NM, 2010, BMC MED RES METHODOL, V10, DOI 10.1186/1471-2288-10-26

*NHMRC, 2003, DIET GUID AUSTR AD

Ranzijn R, 2010, J HEALTH PSYCHOL, V15, P716, DOI 10.1177/1359105310368181

Ross CE, 1996, J HEALTH SOC BEHAV, V37, P104, DOI 10.2307/2137234

Sayers S, 2009, BMC INT HEALTH HUM R, V9, DOI 10.1186/1472-698X-9-23

Stewart A., 1992, MEASURING FUNCTIONIN

SWAN P, 1995, WAYS FORWARD NATL AB

Vos T, 2007, BURDEN DIS INJURY AB

Vos T, 2009, INT J EPIDEMIOL, V38, P470, DOI 10.1093/ije/dyn240

World Health Organization, 2000, WHO TECHN REP SER

NR 24

TC 16

Z9 16

U1 2

U2 24

PU BIOMED CENTRAL LTD

PI LONDON

PA 236 GRAYS INN RD, FLOOR 6, LONDON WC1X 8HL, ENGLAND

SN 1471-2458

J9 BMC PUBLIC HEALTH

JI BMC Public Health

PD JUL 17

PY 2013

VL 13

AR 661

DI 10.1186/1471-2458-13-661

PG 14

WC Public, Environmental & Occupational Health

SC Public, Environmental & Occupational Health

GA 186GW

UT WOS:000322031100001

PM 23866062

OA DOAJ Gold, Green Published

DA 2019-01-08

ER

PT J

AU Beverly, EA

Fitzgerald, SM

Brooks, KM

Hultgren, BA

Ganda, OP

Munshi, M

Weinger, K

AF Beverly, Elizabeth A.

Fitzgerald, Shane M.

Brooks, Kelly M.

Hultgren, Brittney A.

Ganda, Om P.

Munshi, Medha

Weinger, Katie

TI Impact of Reinforcement of Diabetes Self-Care on Poorly Controlled

Diabetes A Randomized Controlled Trial

SO DIABETES EDUCATOR

LA English

DT Article

ID QUALITY-OF-LIFE; GLYCEMIC CONTROL; COMPLICATIONS TRIAL; INTENSIVE

TREATMENT; PROBLEM AREAS; ADULTS; INTERVENTIONS; IMPLEMENTATION;

QUESTIONNAIRE; METAANALYSIS

AB Purpose The purpose of the study was to assess the value of reinforcing diabetes self-management for improving glycemia and self-care among adults with type 2 diabetes who had at least 3 hours of prior diabetes education.

Methods In this randomized controlled trial, 134 participants (75% white, 51% female, 59 9 years old, 13 8 years with diabetes, A1C = 8.4% +/- 1.2%) were randomized to either a group map-based program (intervention) or group education on cholesterol and blood pressure (control). Participants were assessed for A1C levels, diabetes self-care behaviors (3-day pedometer readings, 6-minute walk test, blood glucose checks, frequency of self-care), and psychosocial factors (distress, frustration, quality of life) at baseline, 3, 6, and 12 months post intervention and health literacy at baseline.

Results Groups did not differ on baseline characteristics including A1C levels, health literacy, or self-care; however, the intervention group had more years of education than controls. Intervention arm participants modestly improved A1C levels at 3 months post intervention but did not maintain that improvement at 6 and 12 months while control patients did not improve A1C levels at any time during follow-up. Importantly, frequency of self-reported self-care, diabetes quality of life, diabetes-related distress, and frustration with diabetes self-care improved in both groups over time.

Conclusions Reinforcing self-care with diabetes education for patients who have not met glycemic targets helps improve A1C and could be considered a necessary component of ongoing diabetes care. The best method to accomplish reinforcement needs to be established.

C1 [Beverly, Elizabeth A.; Fitzgerald, Shane M.; Brooks, Kelly M.; Hultgren, Brittney A.; Ganda, Om P.; Munshi, Medha; Weinger, Katie] Joslin Diabet Ctr, Boston, MA 02215 USA.

[Beverly, Elizabeth A.; Ganda, Om P.; Munshi, Medha; Weinger, Katie] Harvard Univ, Sch Med, Boston, MA USA.

RP Weinger, K (reprint author), Joslin Diabet Ctr, 1 Joslin Pl, Boston, MA 02215 USA.

EM Katie.Weinger@joslin.harvard.edu

RI Albertin, Paloma/X-1093-2018

FU American Diabetes Association (ADA) [7-08-CR-62]; Diabetes and

Endocrinology Research Core NIH [P30 DK36836]; NIH [T32 DK007260]

FX This work was supported by the American Diabetes Association (ADA) grant

7-08-CR-62, the Diabetes and Endocrinology Research Core NIH P30

DK36836, and the NIH Training Grant No. T32 DK007260. Bayer HealthCare

LLC (Tarrytown, New York) contributed glucose meters and test strips.

ADA and these companies had no role in the conduct of the study or

preparation of this manuscript..

CR AHERN J, 1993, DIABETES EDUCATOR, V19, P509, DOI 10.1177/014572179301900606

Bradley C, 1994, HDB PSYCHOL DIABETES

Conn VS, 2007, DIABETOLOGIA, V50, P913, DOI 10.1007/s00125-007-0625-0

Davidson MB, 2002, DIABETES CARE, V25, P2116, DOI 10.2337/diacare.25.11.2116

DCCT Res Grp, 1988, DIABETES CARE, V11, P725

Deakin T, 2005, COCHRANE DB SYST REV, DOI 10.1002/14651858.CD003417.pub2

Derogatis LR, 2000, BSI 18 BRIEF SYMPT I

Dyer PH, 1998, DIABETIC MED, V15, P339, DOI 10.1002/(SICI)1096-9136(199804)15:4<339::AID-DIA577>3.0.CO;2-E

Fitzmaurice G. M., 2011, APPL LONGITUDINAL AN

Gafarian CT, 1999, DIABETES EDUCATOR, V25, P585, DOI 10.1177/014572179902500411

Genuth S, 1999, DIABETES CARE, V22, P99

HAMBURG BA, 1983, DIABETES CARE, V6, P409, DOI 10.2337/diacare.6.4.409

Ismail K, 2004, LANCET, V363, P1589, DOI 10.1016/S0140-6736(04)16202-8

JACOBSON AM, 1994, DIABETES CARE, V17, P267, DOI 10.2337/diacare.17.4.267

JACOBSON AM, 1991, DIABETES CARE, V14, P599, DOI 10.2337/diacare.14.7.599

Karter AJ, 2001, AM J MED, V111, P1, DOI 10.1016/S0002-9343(01)00742-2

Khan H, 2011, PRIM CARE DIABETES, V5, P251, DOI 10.1016/j.pcd.2011.07.001

Little R J, 2002, STAT ANAL MISSING DA

Lorenz RA, 1996, DIABETES CARE, V19, P648, DOI 10.2337/diacare.19.6.648

Maiorana A, 2002, DIABETES RES CLIN PR, V56, P115, DOI 10.1016/S0168-8227(01)00368-0

Norris SL, 2002, DIABETES CARE, V25, P1159, DOI 10.2337/diacare.25.7.1159

Norris SL, 2001, DIABETES CARE, V24, P561, DOI 10.2337/diacare.24.3.561

NURSS JR, 2001, TEST FUNCTIONAL HLTH

Peyrot M, 1999, J HEALTH SOC BEHAV, V40, P141, DOI 10.2307/2676370

Pi-Sunyer FX, 1999, DIABETES CARE, V22, P191, DOI 10.2337/diacare.22.2.191

POLONSKY WH, 1995, DIABETES CARE, V18, P754, DOI 10.2337/diacare.18.6.754

Rubin RR, 2001, J CLIN PSYCHOL, V57, P457, DOI 10.1002/jclp.1041

SHAMOON H, 1995, DIABETES CARE, V18, P361

SHAMOON H, 1993, NEW ENGL J MED, V329, P977

Sperl-Hillen J, 2011, ARCH INTERN MED, V171, P2001, DOI 10.1001/archinternmed.2011.507

van der Ven NCW, 2003, DIABETES CARE, V26, P713, DOI 10.2337/diacare.26.3.713

Wdowik MJ, 1997, DIABETES EDUCATOR, V23, P558, DOI 10.1177/014572179702300507

Weinger K, 2005, DIABETES CARE, V28, P1346, DOI 10.2337/diacare.28.6.1346

Weinger K, 2001, PATIENT EDUC COUNS, V42, P123, DOI 10.1016/S0738-3991(00)00098-7

Weinger K, 2002, PRINCIPLES DIABETES, P639

Weinger K, 2000, DIABETES S1, V48, pA318

Weinger K, 2009, COMPLETE NURSES GUID

Weinger K, 2011, ARCH INTERN MED, V171, P1990, DOI 10.1001/archinternmed.2011.502

Welch G, 2003, DIABETIC MED, V20, P69, DOI 10.1046/j.1464-5491.2003.00832.x

Welch GW, 1997, DIABETES CARE, V20, P760, DOI 10.2337/diacare.20.5.760

NR 40

TC 17

Z9 17

U1 0

U2 10

PU SAGE PUBLICATIONS INC

PI THOUSAND OAKS

PA 2455 TELLER RD, THOUSAND OAKS, CA 91320 USA

SN 0145-7217

EI 1554-6063

J9 DIABETES EDUCATOR

JI Diabetes Educ.

PD JUL

PY 2013

VL 39

IS 4

BP 504

EP 514

DI 10.1177/0145721713486837

PG 11

WC Endocrinology & Metabolism; Public, Environmental & Occupational Health

SC Endocrinology & Metabolism; Public, Environmental & Occupational Health

GA 245EE

UT WOS:000326442300008

PM 23640303

OA Green Accepted

DA 2019-01-08

ER

PT J

AU Ronda, MCM

Dijkhorst-Oei, LT

Gorter, KJ

Beulens, JWJ

Rutten, GEHM

AF Ronda, Maaike C. M.

Dijkhorst-Oei, Lioe-Ting

Gorter, Kees J.

Beulens, Joline W. J.

Rutten, Guy E. H. M.

TI Differences Between Diabetes Patients Who Are Interested or Not in the

Use of a Patient Web Portal

SO DIABETES TECHNOLOGY & THERAPEUTICS

LA English

DT Article

ID PILOT RANDOMIZED-TRIAL; COLLABORATIVE CARE; DIGITAL DIVIDE; HEALTH

RECORD; DISPARITIES; DISTRESS; VALIDITY; ADOPTION; OUTCOMES; OLDER

AB Objective: A patient Web portal allows patients to access their personal health record through the Internet. It may improve diabetes outcomes, but the adoption is unsatisfactory. We examined the differences between patients with and without a login in order to optimize its use.

Patients and Methods: A survey was conducted among patients from 62 general practices and one outpatient clinic that all use a diabetes Web portal. Between November 2011 and March 2012 questionnaires were sent to 1,500 patients with and 3,000 patients without a login. Patient groups were stratified according to type of diabetes. Demographic and diabetes-related variables were analyzed with multivariable regression analysis.

Results: The total response rate was 67%. Fewer than 50% of the patients did request a login. Among 128 patients with type 1 diabetes mellitus, those with a login (89.8%) were younger and more frequently treated by an internist. In 1,262 patients with type 2 diabetes mellitus, fewer patients had a log-in (41.0%), and the likelihood of having a login was independently associated with younger age, male gender, higher educational level, treatment by an internist, longer duration of diabetes, and polypharmacy (all P < 0.001).

Conclusions: Patients with type 1 diabetes request a login more frequently than patients with type 2 diabetes, and patients with a login are strikingly different than patients without. The healthcare provider seems to play an important role in patients' Web utilization. Simply promoting use of electronic healthcare methods does not make sense. It is important to address disparities between patient groups to optimize the use of a Web portal.

C1 [Ronda, Maaike C. M.; Gorter, Kees J.; Beulens, Joline W. J.; Rutten, Guy E. H. M.] Univ Med Ctr Utrecht, Julius Ctr Hlth Sci & Primary Care, NL-3508 GA Utrecht, Netherlands.

[Dijkhorst-Oei, Lioe-Ting] Meander Med Ctr, Amersfoort, Netherlands.

RP Ronda, MCM (reprint author), Univ Med Ctr Utrecht, Julius Ctr Hlth Sci & Primary Care, STR 6-131,POB 85500, NL-3508 GA Utrecht, Netherlands.

EM m.c.m.ronda@umcutrecht.nl

RI Ronda, Maaike/L-2208-2014

OI Ronda, Maaike/0000-0002-6829-6982

FU Diabetes Fund; Netherlands Organization for Scientific Research in

Diabetes [2010.13.1369]

FX This study was supported by The Diabetes Fund, The Netherlands

Organization for Scientific Research in Diabetes (grant 2010.13.1369).

CR Ancker JS, 2011, J GEN INTERN MED, V26, P1117, DOI 10.1007/s11606-011-1749-y

Andreassen HK, 2007, BMC PUBLIC HEALTH, V7, DOI 10.1186/1471-2458-7-53

Baan Caroline A, 2009, Ned Tijdschr Geneeskd, V153, pA580

Bond GE, 2007, DIABETES TECHNOL THE, V9, P52, DOI 10.1089/dia.2006.0057

Bradley C., 1994, HDB PSYCHOL DIABETES, P111

Brooks R, 1996, HEALTH POLICY, V37, P53, DOI 10.1016/0168-8510(96)00822-6

*CBS, US INF COMM TECHN IC

Cho AH, 2010, TELEMED J E-HEALTH, V16, P595, DOI 10.1089/tmj.2009.0164

Delbanco T, 2012, ANN INTERN MED, V157, P461, DOI 10.7326/0003-4819-157-7-201210020-00002

Donders ART, 2006, J CLIN EPIDEMIOL, V59, P1087, DOI 10.1016/j.jclinepi.2006.01.014

Fitzgerald JT, 1998, DIABETES CARE, V21, P706, DOI 10.2337/diacare.21.5.706

Goel MS, 2011, J AM MED INFORM ASSN, V18, pI8, DOI 10.1136/amiajnl-2011-000473

Goel MS, 2011, J GEN INTERN MED, V26, P1112, DOI 10.1007/s11606-011-1728-3

Gorter KJ, 2011, PATIENT EDUC COUNS, V84, P56, DOI 10.1016/j.pec.2010.06.019

Greenhalgh T, 2010, BMJ-BRIT MED J, V341, DOI 10.1136/bmj.c5814

Holbrook A, 2009, CAN MED ASSOC J, V181, P37, DOI 10.1503/cmaj.081272

Lin CT, 2005, J MED INTERNET RES, V7, P13, DOI 10.2196/jmir.7.4.e47

McCarrier KP, 2009, DIABETES TECHNOL THE, V11, P211, DOI 10.1089/dia.2008.0063

McMahon GT, 2005, DIABETES CARE, V28, P1624, DOI 10.2337/diacare.28.7.1624

Oh H, 2005, J MED INTERNET RES, V7

Osborn CY, 2010, CURR DIABETES REP, V10, P422, DOI 10.1007/s11892-010-0151-1

POLONSKY WH, 1995, DIABETES CARE, V18, P754, DOI 10.2337/diacare.18.6.754

Ralston JD, 2009, DIABETES CARE, V32, P234, DOI 10.2337/dc08-1220

Rubin DB, 1987, MULTIPLE IMPUTATION

RUTTEN GEH, 2006, HUISARTS WET, V49, P137

Sarkar U, 2011, J AM MED INFORM ASSN, V18, P318, DOI [10.1136/jamia.2010.006015, 10.1136/amiajnl.2010.006015]

Sarkar U, 2010, J HEALTH COMMUN, V15, P183, DOI 10.1080/10810730.2010.499988

*SCP, 2012, STAT

Shaw JE, 2010, DIABETES RES CLIN PR, V87, P4, DOI 10.1016/j.diabres.2009.10.007

Shea S, 2009, J AM MED INFORM ASSN, V16, P446, DOI 10.1197/jamia.M3157

Snoek FJ, 2000, DIABETES CARE, V23, P1305, DOI 10.2337/diacare.23.9.1305

STUIJS JN, 2009, 260014001 RIJKS VOLK

van der Bijl J, 1999, J ADV NURS, V30, P352, DOI 10.1046/j.1365-2648.1999.01077.x

Weingart SN, 2006, J AM MED INFORM ASSN, V13, P91, DOI 10.1197/jamia.M1833

Weppner WG, 2010, DIABETES CARE, V33, P2314, DOI 10.2337/dc10-1124

Witry Matthew J, 2010, Perspect Health Inf Manag, V7, p1d

Yamin CK, 2011, ARCH INTERN MED, V171, P568, DOI 10.1001/archinternmed.2011.34

NR 37

TC 13

Z9 13

U1 0

U2 11

PU MARY ANN LIEBERT, INC

PI NEW ROCHELLE

PA 140 HUGUENOT STREET, 3RD FL, NEW ROCHELLE, NY 10801 USA

SN 1520-9156

EI 1557-8593

J9 DIABETES TECHNOL THE

JI Diabetes Technol. Ther.

PD JUL

PY 2013

VL 15

IS 7

BP 556

EP 563

DI 10.1089/dia.2013.0023

PG 8

WC Endocrinology & Metabolism

SC Endocrinology & Metabolism

GA 180RD

UT WOS:000321611700006

PM 23777369

OA Green Published

DA 2019-01-08

ER

PT J

AU Nicolucci, A

Burns, KK

Holt, RIG

Comaschi, M

Hermanns, N

Ishii, H

Kokoszka, A

Pouwer, F

Skovlund, SE

Stuckey, H

Tarkun, I

Vallis, M

Wens, J

Peyrot, M

AF Nicolucci, A.

Burns, K. Kovacs

Holt, R. I. G.

Comaschi, M.

Hermanns, N.

Ishii, H.

Kokoszka, A.

Pouwer, F.

Skovlund, S. E.

Stuckey, H.

Tarkun, I.

Vallis, M.

Wens, J.

Peyrot, M.

CA DAWN2 Study Grp

TI Diabetes Attitudes, Wishes and Needs second study (DAWN2 (TM)):

Cross-national benchmarking of diabetes-related psychosocial outcomes

for people with diabetes

SO DIABETIC MEDICINE

LA English

DT Article

ID GLYCEMIC CONTROL; CARE; DISTRESS; BARRIERS; SUPPORT

AB Aims The second Diabetes Attitudes, Wishes and Needs (DAWN2) study aimed to assess psychosocial outcomes in people with diabetes across countries for benchmarking. Methods Surveys included new and adapted questions from validated questionnaires that assess health-related quality of life, self-management, attitudes/beliefs, social support and priorities for improving diabetes care. Questionnaires were conducted online, by telephone or in person. Results Participants were 8596 adults with diabetes across 17 countries. There were significant between-country differences for all benchmarking indicators; no one country's outcomes were consistently better or worse than others. The proportion with likely depression [WHO-5 Well-Being Index (WHO-5) score 28] was 13.8% (country range 6.5-24.1%). Diabetes-related distress [Problem Areas in Diabetes Scale5 (PAID-5) score 40] was reported by 44.6% of participants (17.2-67.6%). Overall quality of life was rated poor' or very poor' by 12.2% of participants (7.6-26.1%). Diabetes had a negative impact on all aspects investigated, ranging from 20.5% on relationship with family/friends to 62.2% on physical health. Approximately 40% of participants (18.6-64.9%) reported that their medication interfered with their ability to live a normal life. The availability of person-centred chronic illness care and support for active involvement was rated as low. Following self-care advice for medication and diet was most common, and least common for glucose monitoring and foot examination, with marked country variation. Only 48.8% of respondents had participated in diabetes educational programmes/activities to help manage their diabetes. Conclusions Cross-national benchmarking using psychometrically validated indicators can help identify areas for improvement and best practices to drive changes that improve outcomes for people with diabetes.

C1 [Nicolucci, A.] Ist Ric Farmacol Mario Negri, Consorzio Mario Negri Sud, I-66030 Santa Maria Imbaro, Italy.

[Burns, K. Kovacs] Univ Alberta, Edmonton Clin Hlth Acad, Edmonton, AB, Canada.

[Holt, R. I. G.] Univ Southampton, Southampton, Hants, England.

[Comaschi, M.] GVM Care & Res, Ist Clin Ligure Alta Specialita, Rapallo, Italy.

[Hermanns, N.] Diabet Zentrum Mergentheim, Diabet Res Inst, Bad Mergentheim, Germany.

[Ishii, H.] Tenri Hosp, Tenri, Nara 632, Japan.

[Kokoszka, A.] Med Univ Warsaw, Warsaw, Poland.

[Pouwer, F.] Tilburg Univ, NL-5000 LE Tilburg, Netherlands.

[Skovlund, S. E.] Novo Nordisk AS, Copenhagen, Denmark.

[Stuckey, H.] Penn State Coll Med, Hershey, PA USA.

[Tarkun, I.] Kocaeli Univ, Kocaeli, Turkey.

[Vallis, M.] Dalhousie Univ, Halifax, NS, Canada.

[Wens, J.] Univ Antwerp, B-2020 Antwerp, Belgium.

[Peyrot, M.] Loyola Univ Maryland, Baltimore, MD USA.

RP Nicolucci, A (reprint author), Ist Ric Farmacol Mario Negri, Consorzio Mario Negri Sud, I-66030 Santa Maria Imbaro, Italy.

EM nicolucci@negrisud.it

RI Wens, Johan/K-2705-2013; Kokoszka, Andrzej/O-7454-2018; Menendez Torre,

Edelmiro/G-1547-2013

OI Wens, Johan/0000-0002-0229-9064; Kokoszka, Andrzej/0000-0003-4518-5494;

Menendez Torre, Edelmiro/0000-0002-9548-7821; Vallis,

Michael/0000-0002-0165-5936; Holt, Richard/0000-0001-8911-6744

FU Novo Nordisk A/S; Novo Nordisk

FX The DAWN2 study is funded by Novo Nordisk A/S. Medical writing and

editorial support in the preparation of this manuscript, which was

funded by Novo Nordisk A/S, was provided by Bioscript Medical Ltd. In

collaboration with national, regional and global partners, including the

International Diabetes Federation, International Alliance of Patients'

Organizations and the international Steno Diabetes Center, the DAWN2

survey working group, the DAWN2 International Publication Planning

Committee (IPPC) and Harris Interactive, Novo Nordisk has assisted with

the planning and designing of the DAWN2 study. Data collection was

independently performed by Harris Interactive, who were funded by Novo

Nordisk. Data analysis and publication preparation were performed by

members of the DAWN2 IPPC and authors.

CR Anderson RM, 2010, PATIENT EDUC COUNS, V79, P277, DOI 10.1016/j.pec.2009.07.025

[Anonymous], 2004, PRACT DIABETES INT, V21, P201

Barnard KD, 2012, DIABETIC MED, V29, P1358, DOI 10.1111/j.1464-5491.2012.03727.x

Beaser RS, 2011, ENDOCR PRACT, V17, P880, DOI 10.4158/EP10398.OR

Beverly EA, 2011, DIABETES CARE, V34, P1086, DOI 10.2337/dc10-2298

Crosson JC, 2010, J AM BOARD FAM MED, V23, P171, DOI 10.3122/jabfm.2010.02.090125

De Berardis G, 2004, DIABETES CARE, V27, P398, DOI 10.2337/diacare.27.2.398

de Wit M, 2007, DIABETES CARE, V30, P2003, DOI 10.2337/dc07-0447

Fidler Carrie, 2011, J Med Econ, V14, P646, DOI 10.3111/13696998.2011.610852

Funnell MM, 2006, CLIN DIABETES, V24, P154

Holt RIG, 2013, DIABETIC MED, V30, P789, DOI 10.1111/dme.12242

International Alliance of Patients Organizations (IAPO), 2012, PAT CTR HEALTHC IND

Kaufman N, 2010, Int J Clin Pract Suppl, P41, DOI 10.1111/j.1742-1241.2009.02277.x

Khan Mohammad A, 2011, Diabetes Ther, V2, P178, DOI 10.1007/s13300-011-0007-y

Kim MY, 2012, DIABETES METAB J, V36, P452

Mansell K, 2011, RES SOC ADMIN PHARM, V7, P396, DOI 10.1016/j.sapharm.2010.10.004

Mayberry LS, 2012, DIABETES CARE, V35, P1239, DOI 10.2337/dc11-2103

Mosnier-Pudar H, 2010, DIABETES METAB, V36, P476, DOI 10.1016/j.diabet.2010.08.004

Mosnier-Pudar H, 2009, DIABETES METAB, V35, P220, DOI 10.1016/j.diabet.2009.02.001

Nam S, 2011, DIABETES RES CLIN PR, V93, P1, DOI 10.1016/j.diabres.2011.02.002

Peyrot M, 2005, DIABETIC MED, V22, P1379, DOI 10.1111/j.1464-5491.2005.01644.x

Peyrot M, 2013, DIABETES RES CLIN PR, V99, P174, DOI 10.1016/j.diabres.2012.11.016

Piya MK, 2010, BRIT J CLIN PHARMACO, V70, P631, DOI 10.1111/j.1365-2125.2010.03711.x

Reddy J, 2013, PSYCHOSOMATICS, V54, P44, DOI 10.1016/j.psym.2012.08.004

Singer J. D., 2003, APPL LONGITUDINAL DA

Skovlund S. E., 2005, DIABETES SPECTRUM, V18, P136, DOI DOI 10.2337/DIASPECT.18.3.136

Snijders T, 1999, MULTILEVEL ANAL INTR

Zagarins SE, 2012, J BEHAV MED, V35, P299, DOI 10.1007/s10865-011-9359-z

NR 28

TC 215

Z9 227

U1 6

U2 58

PU WILEY-BLACKWELL

PI HOBOKEN

PA 111 RIVER ST, HOBOKEN 07030-5774, NJ USA

SN 0742-3071

J9 DIABETIC MED

JI Diabetic Med.

PD JUL

PY 2013

VL 30

IS 7

BP 767

EP 777

DI 10.1111/dme.12245

PG 11

WC Endocrinology & Metabolism

SC Endocrinology & Metabolism

GA 169MR

UT WOS:000320784900002

PM 23711019

OA Bronze

HC Y

HP N

DA 2019-01-08

ER

PT J

AU Burns, KK

Nicolucci, A

Holt, RIG

Willaing, I

Hermanns, N

Kalra, S

Wens, J

Pouwer, F

Skovlund, SE

Peyrot, M

AF Burns, K. Kovacs

Nicolucci, A.

Holt, R. I. G.

Willaing, I.

Hermanns, N.

Kalra, S.

Wens, J.

Pouwer, F.

Skovlund, S. E.

Peyrot, M.

CA DAWN2 Study Grp

TI Diabetes Attitudes, Wishes and Needs second study (DAWN2 (TM)):

Cross-national benchmarking indicators for family members living with

people with diabetes

SO DIABETIC MEDICINE

LA English

DT Article

ID MELLITUS PATIENTS; TYPE-1; EXPERIENCE; PARENTS; SUPPORT; CARE;

ADOLESCENTS; CHILDREN; OUTCOMES; FATHERS

AB Aims The second Diabetes Attitudes, Wishes and Needs (DAWN2) study examined the experiences of family members of people with diabetes for benchmarking and identifying unmet needs or areas for improvement to assist family members and those with diabetes to effectively self-manage. Methods In total, 2057 family members of people with diabetes participated in an online, telephone or in-person survey designed to assess the impact of diabetes on family life, family support for people with diabetes and educational and community support. Results Supporting a relative with diabetes was perceived as a burden by 35.3% (range across countries 10.6-61.7%) of respondents. Over half of respondents [51.4% (22.5-76.0%)] rated their quality of life as good' or very good'. However, distress about the person with diabetes was high, with 61.3% (31.5-86.4%) worried about hypoglycaemia. The impact of diabetes on aspects of life was felt by 51.8% (46.9-58.6%). The greatest negative effect was on emotional well-being [44.6% (31.8-63.0%)], although depression was less common [11.6% (4.2-20.0%)]. Many respondents did not know how to help the person with diabetes [37.1% (17.5-53.0%)] and wanted to be more involved in their care [39.4% (15.5-61.7%)]. Participation in diabetes educational programmes was low [23.1% (9.4-43.3%)], although most of those who participated found them helpful [72.1% (42.1-90.3%)]. Conclusions Diabetes has a negative impact on family members of people with diabetes. DAWN2 provides benchmarking indicators of family members' psychosocial needs that will help identify the support required for, and from, them to improve the lives of people with diabetes and their families.

C1 [Burns, K. Kovacs] Univ Alberta, Edmonton Clin Hlth Acad, Edmonton, AB, Canada.

[Nicolucci, A.] Consorzio Mario Negri Sud, Chieti, Italy.

[Holt, R. I. G.] Univ Southampton, Fac Med, Southampton SO9 5NH, Hants, England.

[Willaing, I.] Steno Diabet Ctr, DK-2820 Gentofte, Denmark.

[Hermanns, N.] Diabet Zentrum Mergentheim, Diabet Res Inst, Bad Mergentheim, Germany.

[Kalra, S.] Bharti Hosp, Karnal, India.

[Kalra, S.] BRIDE, Karnal, India.

[Wens, J.] Univ Antwerp, B-2020 Antwerp, Belgium.

[Pouwer, F.] Tilburg Univ, NL-5000 LE Tilburg, Netherlands.

[Skovlund, S. E.] Novo Nordisk AS, Copenhagen, Denmark.

[Peyrot, M.] Loyola Univ Maryland, Baltimore, MD USA.

RP Burns, KK (reprint author), Univ Alberta, Edmonton Clin Hlth Acad, Edmonton, AB, Canada.

EM kathy.kovacsburns@ualberta.ca

RI Wens, Johan/K-2705-2013; Menendez Torre, Edelmiro/G-1547-2013; Kokoszka,

Andrzej/O-7454-2018

OI Wens, Johan/0000-0002-0229-9064; Menendez Torre,

Edelmiro/0000-0002-9548-7821; Kokoszka, Andrzej/0000-0003-4518-5494;

Holt, Richard/0000-0001-8911-6744; Willaing, Ingrid/0000-0002-3082-8816

FU Novo Nordisk A/S; Novo Nordisk

FX The DAWN2 study is funded by Novo Nordisk A/S. Medical writing and

editorial support in the preparation of this manuscript, which was

funded by Novo Nordisk A/S, was provided by Bioscript Medical Ltd. In

collaboration with national, regional and global partners, including the

International Diabetes Federation, International Alliance of Patient

Organizations and the international Steno Diabetes Center, the DAWN2

survey working group, the DAWN2 International Publication Planning

Committee (IPPC) and Harris Interactive, Novo Nordisk has assisted with

the planning and designing of the DAWN2 study. Data collection was

independently performed by Harris Interactive, who were funded by Novo

Nordisk. Data analysis and publication preparation were performed by

members of the DAWN2 IPPC and authors.

CR ANDERSON BJ, 1981, DIABETES CARE, V4, P586, DOI 10.2337/diacare.4.6.586

Anderson RM, 2003, DIABETES CARE, V26, P1641, DOI 10.2337/diacare.26.5.1641-a

[Anonymous], 2004, PRACT DIABETES INT, V21, P201

Awadalla AW, 2006, J NATL MED ASSOC, V98, P727

Barnard KD, 2012, DIABETIC MED, V29, P1358, DOI 10.1111/j.1464-5491.2012.03727.x

Beverly EA, 2007, J PSYCHOSOC NURS, V45, P25

Boman A, 2013, HEALTH SOC CARE COMM, V21, P263, DOI 10.1111/hsc.12013

Cameron FJ, 2008, DIABETIC MED, V25, P463, DOI 10.1111/j.1464-5491.2008.02399.x

De Berardis G, 2004, DIABETES CARE, V27, P398, DOI 10.2337/diacare.27.2.398

Escudero-Carretero MJ, 2007, HEALTH EXPECT, V10, P337, DOI 10.1111/j.1369-7625.2007.00452.x

Haugstvedt A, 2011, PEDIATR DIABETES, V12, P107, DOI 10.1111/j.1399-5448.2010.00661.x

International Alliance of Patients Organizations (IAPO), 2012, PAT CTR HEALTHC IND

International Diabetes Federation, 2004, DIABETES VOICE, V49, P1

Knottnerus JA, 2012, J CLIN EPIDEMIOL, V65, P581, DOI 10.1016/j.jclinepi.2012.03.007

Malerbi FEK, 2012, DIABETOL METAB SYNDR, V4, DOI 10.1186/1758-5996-4-48

Markowitz JT, 2012, DIABETIC MED, V29, P526, DOI 10.1111/j.1464-5491.2011.03434.x

Mayberry LS, 2012, DIABETES CARE, V35, P1239, DOI 10.2337/dc11-2103

Peyrot M, 2005, DIABETES CARE, V28, P2673, DOI 10.2337/diacare.28.11.2673

Peyrot M, 2005, DIABETIC MED, V22, P1379, DOI 10.1111/j.1464-5491.2005.01644.x

Peyrot M, 2013, DIABETES RES CLIN PR, V99, P174, DOI 10.1016/j.diabres.2012.11.016

Rintala TM, 2013, J FAM NURS, V19, P3, DOI 10.1177/1074840712471899

Rosland AM, 2010, CHRONIC ILLN, V6, P22, DOI 10.1177/1742395309354608

Roy T, 2012, DIABETIC MED, V29, P164, DOI 10.1111/j.1464-5491.2011.03401.x

Samuel-Hodge CD, 2013, J GEN INTERN MED, V28, P428, DOI 10.1007/s11606-012-2230-2

Sinclair AJ, 2010, DIABETIC MED, V27, P1055, DOI 10.1111/j.1464-5491.2010.03066.x

Singer J. D., 2003, APPL LONGITUDINAL DA

Skovlund S. E., 2005, DIABETES SPECTRUM, V18, P136, DOI DOI 10.2337/DIASPECT.18.3.136

Snijders T, 1999, MULTILEVEL ANAL INTR

Snoek FJ, 2011, DIABETES CARE, V34, P601, DOI 10.2337/dc10-1552

Stodberg R, 2007, J CLIN NURS, V16, P215, DOI 10.1111/j.1365-2702.2006.01797.x

Whittemore R, 2012, DIABETES EDUCATOR, V38, P562, DOI 10.1177/0145721712445216

World Health Organization (WHO), 2002, INN CAR CHRON COND B

NR 32

TC 67

Z9 68

U1 2

U2 17

PU WILEY

PI HOBOKEN

PA 111 RIVER ST, HOBOKEN 07030-5774, NJ USA

SN 0742-3071

EI 1464-5491

J9 DIABETIC MED

JI Diabetic Med.

PD JUL

PY 2013

VL 30

IS 7

BP 778

EP 788

DI 10.1111/dme.12239

PG 11

WC Endocrinology & Metabolism

SC Endocrinology & Metabolism

GA 169MR

UT WOS:000320784900003

PM 23701236

OA Bronze

DA 2019-01-08

ER

PT J

AU Kibbey, KJ

Speight, J

Wong, JLA

Smith, LA

Teede, HJ

AF Kibbey, K. J.

Speight, J.

Wong, J. L. A.

Smith, L. A.

Teede, H. J.

TI Diabetes care provision: barriers, enablers and service needs of young

adults with Type1 diabetes from a region of social disadvantage

SO DIABETIC MEDICINE

LA English

DT Article

ID ILLNESS PERCEPTION QUESTIONNAIRE; HOSPITAL ANXIETY; DEPRESSION SCALE;

EATING BEHAVIORS; OUTCOMES; EXPERIENCES; PREVALENCE; DISTRESS; PEOPLE;

ISSUES

AB Aims To determine the barriers to and enablers of engaging with specialist diabetes care and the service requirements of young adults with Type1 diabetes mellitus from a low socio-economic, multicultural region. Methods A cross-sectional survey targeted 357 young adults with Type1 diabetes, aged 18-30years. Participants completed questions about barriers/enablers to accessing diabetes care and service preferences, self-reported HbA1c, plus measures of diabetes-related distress (Problem Areas in Diabetes), depression/anxiety (Hospital Anxiety and Depression Scale), and illness perceptions (Brief Illness Perceptions Questionnaire). Results Eighty-six (24%) responses were received [55 (64%) female; mean +/- sd age 24 +/- 4years; diabetes duration 12 +/- 7years; HbA1c 68 +/- 16mmol/mol (8.4 +/- 1.5%)]. Logistical barriers to attending diabetes care were reported; for example, time constraints (30%), transportation (26%) and cost (21%). However, a previous unsatisfactory diabetes health experience' was cited as a barrier by 27%. Enablers were largely matched to overcoming these barriers. Over 90% preferred a multidisciplinary team environment, close to home, with after-hours appointment times. Forty per cent reported severe diabetes-related distress, 19% reported moderate-to-severe depressive symptoms and 50% reported moderate-to-severe anxiety. Conclusions Among these young adults with Type1 diabetes, glycaemic control was suboptimal and emotional distress common. They had identifiable logistical barriers to accessing and maintaining contact with diabetes care services, which can be addressed with flexible service provision. A substantial minority were discouraged by previous unsatisfactory experiences, suggesting health providers need to improve their interactions with young adults. This research will inform the design of life-stage-appropriate diabetes services targeting optimal engagement, access, attendance and ultimately improved healthcare outcomes in this vulnerable population.

C1 [Kibbey, K. J.; Wong, J. L. A.; Teede, H. J.] Southern Hlth, Diabet Unit, Melbourne, Vic, Australia.

[Speight, J.; Smith, L. A.] Diabet Australia Vic, Australian Ctr Behav Res Diabet, Melbourne, Vic, Australia.

[Speight, J.] Deakin Univ, Sch Psychol, Ctr Mental Hlth & Well Being Res, Burwood, Vic, Australia.

[Smith, L. A.] Deakin Univ, Sch Psychol, Melbourne, Vic, Australia.

[Wong, J. L. A.; Teede, H. J.] Monash Univ, Sch Publ Hlth & Preventat Med, Clayton, Vic 3800, Australia.

RP Teede, HJ (reprint author), Southern Hlth, Diabet Unit, Melbourne, Vic, Australia.

EM helena.teede@monash.edu

OI Speight, Jane/0000-0002-1204-6896; Teede, Helena/0000-0001-7609-577X

FU Novo Nordisk Regional Diabetes Support Scheme; Southern Health Diabetes

Unit

FX This work was partially funded by a grant from the Novo Nordisk Regional

Diabetes Support Scheme and by funds from the Southern Health Diabetes

Unit.

CR Ambler GR, 2006, J PAEDIATR CHILD H, V42, P403, DOI 10.1111/j.1440-1754.2006.00889.x

*AUSTR BUR STAT, 2006, HIGH YEAR SCH COMPL

Australian Bureau of Statistics, 2011, HOUS US INF TECHN AU

Bernstein CM, 2013, CLIN PEDIATR, V52, P10, DOI 10.1177/0009922812459950

Broadbent E, 2006, J PSYCHOSOM RES, V60, P631, DOI 10.1016/j.jpsychores.2005.10.020

Browne JL, 2013, DIABETIC MED, V30, P620, DOI 10.1111/dme.12078

Craig M, 2011, NATL EVIDENCE BASED

Crowley R, 2011, ARCH DIS CHILD, V96, P548, DOI 10.1136/adc.2010.202473

Dovey-Pearce G, 2005, HEALTH SOC CARE COMM, V13, P409, DOI 10.1111/j.1365-2524.2005.00577.x

Goyder EC, 2001, PUBLIC HEALTH, V115, P78, DOI 10.1038/sj.ph.1900730

Greene A, 2009, PEDIATR DIABETES, V10, P50, DOI 10.1111/j.1399-5448.2009.00615.x

Hargreaves Dougal S, 2011, J Clin Res Pediatr Endocrinol, V3, P43, DOI 10.4274/jcrpe.v3i2.10

Herrmann C, 1997, J PSYCHOSOM RES, V42, P17, DOI 10.1016/S0022-3999(96)00216-4

Ingerski LM, 2010, PEDIATR DIABETES, V11, P563, DOI 10.1111/j.1399-5448.2010.00645.x

Kipps S, 2002, DIABETIC MED, V19, P649, DOI 10.1046/j.1464-5491.2002.00757.x

Lloyd CE, 2000, DIABETIC MED, V17, P198, DOI 10.1046/j.1464-5491.2000.00260.x

Maslakpak MH, 2010, SCAND J CARING SCI, V24, P463, DOI 10.1111/j.1471-6712.2009.00736.x

Moss-Morris R, 2002, PSYCHOL HEALTH, V17, P1, DOI 10.1080/08870440290001494

National Collaborating Centre for Women's and Children's Health National Collaborating Centre for Chronic Conditions, 2004, TYPE1 DIAB DIAGN MAN

Neumark-Sztainer D, 2002, DIABETES CARE, V25, P1289, DOI 10.2337/diacare.25.8.1289

Perry L, 2012, J CLIN NURS, V21, P1955, DOI 10.1111/j.1365-2702.2011.04012.x

POLONSKY WH, 1995, DIABETES CARE, V18, P754, DOI 10.2337/diacare.18.6.754

Quick VM, 2012, EAT BEHAV, V13, P207, DOI 10.1016/j.eatbeh.2012.02.003

Scott L, 2005, CAN J DIABETES, V29, P203

Snoek FJ, 2000, DIABETES CARE, V23, P1305, DOI 10.2337/diacare.23.9.1305

Snow R, 2012, DIABETIC MED, V29, P257, DOI 10.1111/j.1464-5491.2011.03447.x

Speight J, 2011, DIABETES AUSTR LANGU

Speight J, 2011, DIABETES MILES AUSTR

Unger CC, 2011, RURAL REMOTE HEALTH, V11

Wills CJ, 2003, BRIT MED J, V327, P260, DOI 10.1136/bmj.327.7409.260

ZIGMOND AS, 1983, ACTA PSYCHIAT SCAND, V67, P361, DOI 10.1111/j.1600-0447.1983.tb09716.x

NR 31

TC 14

Z9 14

U1 0

U2 10

PU WILEY-BLACKWELL

PI HOBOKEN

PA 111 RIVER ST, HOBOKEN 07030-5774, NJ USA

SN 0742-3071

J9 DIABETIC MED

JI Diabetic Med.

PD JUL

PY 2013

VL 30

IS 7

BP 878

EP 884

DI 10.1111/dme.12227

PG 7

WC Endocrinology & Metabolism

SC Endocrinology & Metabolism

GA 169MR

UT WOS:000320784900016

PM 23659590

DA 2019-01-08

ER

PT J

AU Kavanagh, K

Flynn, DM

Jenkins, KA

Wilson, MD

Chilton, FH

AF Kavanagh, K.

Flynn, D. M.

Jenkins, K. A.

Wilson, M. D.

Chilton, F. H.

TI Stearidonic and gamma-linolenic acids in echium oil improves glucose

disposal in insulin resistant monkeys

SO PROSTAGLANDINS LEUKOTRIENES AND ESSENTIAL FATTY ACIDS

LA English

DT Article

DE Echium oil; Fish oil; Stearidonic acid; Polyunsaturated fatty acids;

Gamma-Linolenic acid; Diabetes

ID N-3 FATTY-ACIDS; RESPIRATORY-DISTRESS-SYNDROME; EICOSAPENTAENOIC ACID;

DOUBLE-BLIND; DIABETES-MELLITUS; CONTROLLED TRIAL; GENE-EXPRESSION;

DIETARY-INTAKE; BORAGE OIL; ALPHA

AB Echium oil (EO) contains stearidonic acid (18:4), a n-3 polyunsaturated fatty acids (PUFAs), and gammalinolenic acids (18:3), a n-6 PUFA that can be converted to long chain (LC)-PUFAs. We aimed to compare a safflower oil (SO)-enriched diet to EO- and fish oil (FO)-enriched diets on circulating and tissue PUFAs levels and glycemic, inflammatory, and cardiovascular health biomarkers in insulin resistant African green monkeys. In a Latin-square cross-over study, eight monkeys consumed matched diets for 6 weeks with 3-week washout periods. Monkeys consuming FO had significantly higher levels of n-3 LC-PUFAs and EO supplementation resulted in higher levels of circulating n-3 LC-PUFAs and a significant increase in dihomo-gamma linolenic acid (DGLA) in red blood cells and muscle. Glucose disposal was improved after EO consumption. These data suggest that PUFAs in EO supplementation have the capacity to alter circulating, RBC and muscle LC-PUFA levels and improve glucose tolerance in insulin-resistant monkeys. (C) 2013 Elsevier Ltd. All rights reserved.

C1 [Kavanagh, K.; Flynn, D. M.; Jenkins, K. A.; Wilson, M. D.] Wake Forest Sch Med, Dept Pathol, Wake Forest, NC USA.

[Chilton, F. H.] Wake Forest Sch Med, Dept Physiol & Pharmacol, Wake Forest, NC USA.

RP Kavanagh, K (reprint author), Wake Forest Univ Hlth Sci, Sect Comparat Med & Lipid Sci, Med Ctr Blvd, Winston Salem, NC 27127 USA.

EM kkavanag@wakehealth.edu

FU Wake Forest School of Medicine; [P50 AT002782]; [P40 RR019963];

[K01AG 033641]; [T32HL07115]

FX Sources of support: Grants P50 AT002782, P40 RR019963, K01AG 033641, and

T32HL07115 as well as Wake Forest School of Medicine.

CR Abraham E, 1996, CRIT CARE MED, V24, P10, DOI 10.1097/00003246-199601000-00005

Andreassi M, 1997, J INT MED RES, V25, P266, DOI 10.1177/030006059702500504

ARISAKA M, 1991, PROSTAG LEUKOTR ESS, V43, P197, DOI 10.1016/0952-3278(91)90169-6

Bosch J, 2012, NEW ENGL J MED, V367, P309, DOI 10.1056/NEJMoa1203859

Brown AL, 2012, ARTERIOSCL THROM VAS, V32, P2122, DOI 10.1161/ATVBAHA.112.253435

CHOPRA J, 1988, AM REV RESPIR DIS, V138, P915, DOI 10.1164/ajrccm/138.4.915

Djousse L, 2011, AM J CLIN NUTR, V94, P527, DOI 10.3945/ajcn.111.013334

Forrest LM, 2012, ATHEROSCLEROSIS, V220, P118, DOI 10.1016/j.atherosclerosis.2011.10.025

Giacco R., NUTR METAB CARDIOVAS, V17, P572

Henz BM, 1999, BRIT J DERMATOL, V140, P685

Hotamisligil GS, 1996, SCIENCE, V271, P665, DOI 10.1126/science.271.5249.665

HOTAMISLIGIL GS, 1994, P NATL ACAD SCI USA, V91, P4854, DOI 10.1073/pnas.91.11.4854

HUANG YS, 1991, BIOCHIM BIOPHYS ACTA, V1082, P319, DOI 10.1016/0005-2760(91)90208-Y

Iggman D, 2010, DIABETOLOGIA, V53, P850, DOI 10.1007/s00125-010-1669-0

James MJ, 2003, AM J CLIN NUTR, V77, P1140

JONES DA, 1990, J BIOL CHEM, V265, P20166

Kapoor R, 2006, CURR PHARM BIOTECHNO, V7, P531, DOI 10.2174/138920106779116874

Karlstrom BE, 2011, AM J CLIN NUTR, V94, P26, DOI 10.3945/ajcn.110.006221

Khamaisi M, 1999, ANTIOXID REDOX SIGN, V1, P523, DOI 10.1089/ars.1999.1.4-523

Kitessa S.M., 2011, BR J NUTR, P1

Knowler WC, 2002, NEW ENGL J MED, V346, P393

Krachler B, 2008, NUTR METAB CARDIOVAS, V18, P503, DOI 10.1016/j.numecd.2007.04.005

Lemke SL, 2010, AM J CLIN NUTR, V92, P766, DOI 10.3945/ajcn.2009.29072

MANTZIORIS E, 1994, AM J CLIN NUTR, V59, P1304

McAuley KA, 2002, DIABETES CARE, V25, P445, DOI 10.2337/diacare.25.3.445

METCALFE LD, 1966, ANAL CHEM, V38, P514, DOI 10.1021/ac60235a044

Meyer KA, 2001, DIABETES CARE, V24, P1528, DOI 10.2337/diacare.24.9.1528

Montori VM, 2000, DIABETES CARE, V23, P1407, DOI 10.2337/diacare.23.9.1407

Mori TA, 2003, FREE RADICAL BIO MED, V35, P772, DOI 10.1016/S0891-5849(03)00407-6

Pacht ER, 2003, CRIT CARE MED, V31, P491, DOI 10.1097/01.CCM.0000049952.96496.3E

Palombo JD, 1999, CRIT CARE MED, V27, P1908, DOI 10.1097/00003246-199909000-00032

Sergeant S, 2012, BRIT J NUTR, V107, P547, DOI 10.1017/S0007114511003230

Surette ME, 2004, J NUTR, V134, P1406

Surette ME, 2003, CLIN THER, V25, P948, DOI 10.1016/S0149-2918(03)80116-9

Takahashi Y, 2000, COMP BIOCHEM PHYS B, V127, P213, DOI 10.1016/S0305-0491(00)00254-6

Takai S, 2009, J ATHEROSCLER THROMB, V16, P480, DOI 10.5551/jat.No430

Villegas R, 2011, AM J CLIN NUTR, V94, P543, DOI 10.3945/ajcn.111.013193

Weaver KL, 2009, J BIOL CHEM, V284, P15400, DOI 10.1074/jbc.M109.004861

Whelan J, 2012, J NUTR, V142, p630S, DOI 10.3945/jn.111.149138

Wu JHY, 2012, BRIT J NUTR, V107, pS214, DOI 10.1017/S0007114512001602

YAMAZAKI K, 1992, BIOCHIM BIOPHYS ACTA, V1123, P18, DOI 10.1016/0005-2760(92)90166-S

Zhang P, 2008, J NUTR BIOCHEM, V19, P655, DOI 10.1016/j.jnutbio.2007.08.005

Zurier RB, 1996, ARTHRITIS RHEUM, V39, P1808, DOI 10.1002/art.1780391106

NR 43

TC 5

Z9 5

U1 0

U2 14

PU ELSEVIER SCI LTD

PI OXFORD

PA THE BOULEVARD, LANGFORD LANE, KIDLINGTON, OXFORD OX5 1GB, OXON, ENGLAND

SN 0952-3278

J9 PROSTAG LEUKOTR ESS

JI Prostaglandins Leukot. Essent. Fatty Acids

PD JUL

PY 2013

VL 89

IS 1

BP 39

EP 45

DI 10.1016/j.plefa.2013.04.003

PG 7

WC Biochemistry & Molecular Biology; Cell Biology; Endocrinology &

Metabolism

SC Biochemistry & Molecular Biology; Cell Biology; Endocrinology &

Metabolism

GA 173PO

UT WOS:000321091600005

PM 23664597

OA Green Accepted

DA 2019-01-08

ER

PT J

AU Beverly, EA

Fitzgerald, S

Sitnikov, L

Ganda, OP

Caballero, AE

Weinger, K

AF Beverly, Elizabeth A.

Fitzgerald, Shane

Sitnikov, Lilya

Ganda, Om P.

Caballero, A. Enrique

Weinger, Katie

TI Do Older Adults Aged 60-75 Years Benefit From Diabetes Behavioral

Interventions?

SO DIABETES CARE

LA English

DT Article

ID QUALITY-OF-LIFE; RANDOMIZED CONTROLLED-TRIALS; SELF-CARE; GLYCEMIC

CONTROL; EDUCATIONAL INTERVENTIONS; META-ANALYSIS; MANAGEMENT; MELLITUS;

METAANALYSIS; COMPLICATIONS

AB OBJECTIVE-In this secondary analysis, we examined whether older adults with diabetes (aged 60-75 years) could benefit from self-management interventions compared with younger adults. Seventy-one community-dwelling older adults and 151 younger adults were randomized to attend a structured behavioral group, an attention control group, or one-to-one education.

RESEARCH DESIGN AND METHODS-We measured AlC, self-care (3-day pedometer readings, blood glucose checks, and frequency of self-care), and psychosocial factors (quality of life, diabetes distress, frustration with self-care, depression, self-efficacy, and coping styles) at baseline and 3, 6, and 12 months postintervention.

RESULTS-Both older (age 67 +/- 5 years, AlC 8.7 +/- 0.8%, duration 20 +/- 12 years, 30% type 1 diabetes, 83% white, 41% female) and younger (age 47 +/- 9 years, AlC 9.2 +/- 1.2%, 18 +/- 12 years with diabetes, 59% type 1 diabetes, 82% white, 55% female) adults had improved AlC equally overtime. Importantly, older and younger adults in the group conditions improved more and maintained improvements at 12 months (older structured behavioral group change in AlC -0.72 +/- 1.4%, older control group -0.65 +/- 0.9%, younger behavioral group -0.55 +/- 1.2%, younger control group -0.43 +/- 1.7%). Furthermore, frequency of self-care, glucose checks, depressive symptoms, quality of life, distress, frustration with self-care, self-efficacy, and emotional coping improved in older and younger participants at follow-up.

CONCLUSIONS-The findings suggest that, compared with younger adults, older adults receive equal glycemic benefit from participating in self-management interventions. Moreover, older adults showed the greatest glycemic improvement in the two group conditions. Clinicians can safely recommend group diabetes interventions to community-dwelling older adults with poor glycemic control.

C1 [Beverly, Elizabeth A.; Fitzgerald, Shane; Sitnikov, Lilya; Ganda, Om P.; Caballero, A. Enrique; Weinger, Katie] Joslin Diabet Ctr, Boston, MA 02215 USA.

[Beverly, Elizabeth A.; Ganda, Om P.; Caballero, A. Enrique; Weinger, Katie] Harvard Univ, Sch Med, Boston, MA USA.

[Sitnikov, Lilya] Univ Vermont, Burlington, VT USA.

RP Weinger, K (reprint author), Joslin Diabet Ctr, Boston, MA 02215 USA.

EM katie.weinger@joslin.harvard.edu

FU National Institutes of Health (NIH) [R01-DK-060115]; Diabetes and

Endocrinology Research Core NIH [P30-DK-36836]; NIH [T32-DK-007260];

Merck; Abbott; GlaxoSmithKline; Bristol-Myers Squibb; AstraZeneca;

Daiichi-Sankyo

FX This work was supported by National Institutes of Health (NIH) Grant

R01-DK-060115 (to K.W.), Diabetes and Endocrinology Research Core NIH

Grant P30-DK-36836, and NIH Training Grant T32-DK-007260. The following

companies contributed glucose meters and test strips: Abbott

Laboratories (Abbott Park, IL), Life Scan (Milpitas, CA), and Roche

Diagnostics (Indianapolis, IN). O.P.G. has provided consulting services

to Abbott; received payment for lectures from Merck, Abbott,

GlaxoSmithKline, Bristol-Myers Squibb, AstraZeneca, and Daiichi-Sankyo;

and has stock options with Bristol-Myers Squibb. A.E.C. serves on the

advisory panels of the following companies: Eli Lilly and Company;

Amylin Pharmaceuticals, Inc.; Takeda Pharmaceuticals America, Inc;

sanofi-aventis; and Daiichi-Sankyo. No other potential conflicts of

interest relevant to this article were reported.

CR Blaum CS, 2002, J AM GERIATR SOC, V50, P581, DOI 10.1046/j.1532-5415.2002.50130.x

Bradley C, 1994, HDB PSYCHOL DIABETES

BROWN SA, 1988, NURS RES, V37, P223

BROWN SA, 1990, PATIENT EDUC COUNS, V16, P189, DOI 10.1016/0738-3991(90)90070-2

Center for Disease Control and Prevention, 2011, NAT DIAB FACT SHEET

Ciechanowski PS, 2000, ARCH INTERN MED, V160, P3278, DOI 10.1001/archinte.160.21.3278

COONROD BA, 1994, DIABETES CARE, V17, P852, DOI 10.2337/diacare.17.8.852

Deakin T, 2005, COCHRANE DB SYST REV, DOI 10.1002/14651858.CD003417.pub2

Derogatis L. R., 2000, BSI 18 BRIEF SYMPTOM

Espeland M, 2007, DIABETES CARE, V30, P1374

Funnell MM, 2012, DIABETES CARE, V35, pS101, DOI [10.2337/dc12-s101, 10.2337/dc12-S089]

GLASGOW RE, 1992, PATIENT EDUC COUNS, V19, P61, DOI 10.1016/0738-3991(92)90102-O

Glasgow RE, 1997, DIABETES CARE, V20, P562, DOI 10.2337/diacare.20.4.562

Ickowicz E, 2003, J AM GERIATR SOC, V51, pS265

Ismail K, 2004, LANCET, V363, P1589, DOI 10.1016/S0140-6736(04)16202-8

JACOBSON AM, 1994, DIABETES CARE, V17, P267, DOI 10.2337/diacare.17.4.267

Kerr EA, 2007, J GEN INTERN MED, V22, P1635, DOI 10.1007/s11606-007-0313-2

Krein SL, 2005, DIABETES CARE, V28, P65, DOI 10.2337/diacare.28.1.65

Little R J, 2002, STAT ANAL MISSING DA

Lundebjerg N, 2001, J AM GERIATR SOC, V49, P664

Miller CK, 2002, PREV MED, V34, P252, DOI 10.1006/pmed.2001.0985

Minet L, 2010, PATIENT EDUC COUNS, V80, P29, DOI 10.1016/j.pec.2009.09.033

Munshi M, 2006, DIABETES CARE, V29, P1794, DOI 10.2337/dc06-0506

PADGETT D, 1988, J CLIN EPIDEMIOL, V41, P1007, DOI 10.1016/0895-4356(88)90040-6

Peyrot M, 1999, J HEALTH SOC BEHAV, V40, P141, DOI 10.2307/2676370

PEYROT MF, 1992, DIABETES CARE, V15, P842, DOI 10.2337/diacare.15.7.842

Piven M L, 2001, J Gerontol Nurs, V27, P8

POLONSKY WH, 1995, DIABETES CARE, V18, P754, DOI 10.2337/diacare.18.6.754

Schoenberg NE, 2001, J AGING HEALTH, V13, P443, DOI 10.1177/089826430101300401

Stearne MR, 1998, BRIT MED J, V317, P720

Stratton IM, 2000, BRIT MED J, V321, P405, DOI 10.1136/bmj.321.7258.405

Suhl E, 2006, DIABETES SPECTR, V19, P234, DOI DOI 10.2337/DIASPECT.19.4.234

UK Prospective Diabetes Study (UKPDS) Group, 1998, LANCET, V352, P837, DOI DOI 10.1016/S0140-6736(98)07019-6

van der Ven NCW, 2003, DIABETES CARE, V26, P713, DOI 10.2337/diacare.26.3.713

Weinger K, 2005, DIABETES CARE, V28, P1346, DOI 10.2337/diacare.28.6.1346

Weinger K, 2001, PATIENT EDUC COUNS, V42, P123, DOI 10.1016/S0738-3991(00)00098-7

Weinger K, 2006, DIABETES SPECTRUM, V19, P194

Weinger K, 2011, ARCH INTERN MED, V171, P1990, DOI 10.1001/archinternmed.2011.502

Welch G, 2003, DIABETIC MED, V20, P69, DOI 10.1046/j.1464-5491.2003.00832.x

Zhang P, 2010, DIABETES RES CLIN PR, V87, P293, DOI 10.1016/j.diabres.2010.01.026

NR 40

TC 18

Z9 19

U1 2

U2 20

PU AMER DIABETES ASSOC

PI ALEXANDRIA

PA 1701 N BEAUREGARD ST, ALEXANDRIA, VA 22311-1717 USA

SN 0149-5992

EI 1935-5548

J9 DIABETES CARE

JI Diabetes Care

PD JUN

PY 2013

VL 36

IS 6

BP 1501

EP 1506

DI 10.2337/dc12-2110

PG 6

WC Endocrinology & Metabolism

SC Endocrinology & Metabolism

GA 178UJ

UT WOS:000321472600012

PM 23315603

OA Green Published

DA 2019-01-08

ER

PT J

AU Sachdev, PS

Levitan, C

Crawford, J

Sidhu, M

Slavin, M

Richmond, R

Kochan, N

Brodaty, H

Wen, W

Kang, K

Mather, KA

AF Sachdev, Perminder S.

Levitan, Charlene

Crawford, John

Sidhu, Mamta

Slavin, Melissa

Richmond, Robyn

Kochan, Nicole

Brodaty, Henry

Wen, Wei

Kang, Kristan

Mather, Karen A.

CA Sydney Centenarian Study Team

TI The Sydney Centenarian Study: methodology and profile of centenarians

and near-centenarians

SO INTERNATIONAL PSYCHOGERIATRICS

LA English

DT Article

DE centenarians; aging; longevity; dementia; MRI; methodology; genetics

ID ALZHEIMERS-DISEASE; BAYER-ACTIVITIES; DEMENTIA; SCALE; PREVALENCE; LIFE;

AGE; QUESTIONNAIRE; LONGEVITY; STRENGTH

AB Background: The study of exceptionally long-living individuals can inform us about the determinants of successful aging. There are few population-based studies of centenarians and near-centenarians internationally, but none in Australia.

Methods: Individuals 95 years and older were recruited from seven electoral districts in Sydney using the electoral roll, Medicare lists, and multiple other strategies to obtain a representative sample. Physical and mental health and cognitive status were assessed using standard instruments in multiple sessions, with assessments individually adapted. An informant was interviewed, and participants were invited to donate a blood sample, undergo an MRI scan, and enrol into the brain donation program.

Results: Preliminary data on the first 200 participants are reported. Mean age was 97.4 years (range 95-106), with 29.5% being men, and 58.5% living in a private dwelling. Rates of heart disease and diabetes were lower than in octogenarians, but hearing and visual deficits were common. The mean mini-mental state examination (MMSE) score was 21.1, with men performing better. Rates of psychological distress were low and satisfaction with life high (mean 5.91 out of a maximum of 7); 54% scored <24 on MMSE; 39.5% were impaired on both MMSE and a functional measure; and 20% had previous diagnosis of dementia.

Conclusions: This is a preliminary report describing the methodology of the study. It provides further evidence that dementia is not inevitable at this age and independent living is common. The study provides an excellent resource to determine the genetic and environmental contributions to long and successful cognitive aging.

C1 [Sachdev, Perminder S.; Levitan, Charlene; Crawford, John; Sidhu, Mamta; Slavin, Melissa; Kochan, Nicole; Brodaty, Henry; Wen, Wei; Kang, Kristan; Mather, Karen A.; Sydney Centenarian Study Team] Univ New S Wales, UNSW Med, Sch Psychiat, Brain & Ageing Res Program,Ctr Hlth Brain Ageing, Kensington, NSW 2052, Australia.

[Sachdev, Perminder S.; Kochan, Nicole; Wen, Wei; Mather, Karen A.] Prince Wales Hosp, Inst Neuropsychiat, Randwick, NSW 2031, Australia.

[Sachdev, Perminder S.; Slavin, Melissa; Brodaty, Henry] Univ New S Wales, Sch Psychiat, Primary Dementia Collaborat Res Ctr, UNSW Med, Kensington, NSW 2052, Australia.

[Richmond, Robyn] Univ New S Wales, Sch Publ Hlth & Community Med, UNSW Med, Kensington, NSW 2052, Australia.

[Brodaty, Henry] Prince Wales Hosp, Acad Dept Old Age Psychiat, Randwick, NSW 2031, Australia.

RP Sachdev, PS (reprint author), Prince Wales Hosp, UNSW Sch Psychiat, NPI, Euroa Ctr, Barker St, Randwick, NSW 2031, Australia.

EM p.sachdev@unsw.edu.au

RI Karen, Mather/O-9795-2016

OI Karen, Mather/0000-0003-4143-8941; Richmond, Robyn/0000-0003-2897-4109;

Sachdev, Perminder/0000-0002-9595-3220

FU National Health & Medical Research Council of Australia [401184, 630593,

568969]; University of New South Wales Gold Star Award

FX Many research assistants and administrative assistants contributed to

data collection. DNA samples were extracted by Genetic Repositories

Australia, an Enabling Facility, which is supported by a National Health

& Medical Research Council of Australia grant no. 401184. The APOE

genotyping was performed by Drs Arezoo Assareh and Karen A. Mather under

the supervision of Professor Peter Schofield and Dr John Kwok at the

Neuroscience Research Australia (NeuRA), Sydney, Australia. Angie

Russell and Kate Crosbie assisted in manuscript preparation. We thank

the participants for their enthusiastic support. This study was

supported by the National Health & Medical Research Council of Australia

(project grant ID 630593 and program grant ID 568969) and the University

of New South Wales Gold Star Award to Melissa Slavin.

CR Australian Bureau of Statistics, 1991, CENS POP HOUS

Australian Bureau of Statistics, 2001, CENS POP HOUS

Australian Bureau of Statistics, 2006, CENS POP HOUS

Benton AL, 1967, CORTEX, V3, P32, DOI DOI 10.1016/S0010-9452(67)80005-4

Blansjaar BA, 2000, INT J GERIATR PSYCH, V15, P219, DOI 10.1002/(SICI)1099-1166(200003)15:3<219::AID-GPS97>3.0.CO;2-N

BOHANNON RW, 1995, PERCEPT MOTOR SKILL, V80, P163, DOI 10.2466/pms.1995.80.1.163

Brandt J, 2001, HOPKINS VERBAL LEARN

Brodaty H, 2002, J AM GERIATR SOC, V50, P530, DOI 10.1046/j.1532-5415.2002.50122.x

Cherbuin N, 2009, DEMENT GERIATR COGN, V28, P47, DOI 10.1159/000229025

DIENER E, 1985, J PERS ASSESS, V49, P71, DOI 10.1207/s15327752jpa4901_13

Esiri MM, 2000, ANN NY ACAD SCI, V903, P239, DOI 10.1111/j.1749-6632.2000.tb06373.x

Folquitto JC, 2007, REV BRAS PSIQUIATR, V29, P350, DOI 10.1590/S1516-44462006005000037

Frederiksen H, 2006, ANN EPIDEMIOL, V16, P554, DOI 10.1016/j.annepidem.2005.10.006

GARDNER RM, 1986, AM REV RESPIR DIS, V134, P625

Gondo Y, 2008, ANNU REV GERONTOL GE, V27, P129

Halliday G, 2002, ACTA NEUROPATHOL, V104, P72, DOI 10.1007/s00401-002-0529-5

Hindmarch I, 1998, DEMENT GERIATR COGN, V9, P20, DOI 10.1159/000051195

Jeune B, 2006, J GERONTOL A-BIOL, V61, P707, DOI 10.1093/gerona/61.7.707

JORM AF, 1989, INT J GERIATR PSYCH, V4, P35, DOI 10.1002/gps.930040109

Kaplan E, 2001, BOSTON NAMING TEST

KATZ S, 1970, GERONTOLOGIST, V10, P20, DOI 10.1093/geront/10.1_Part_1.20

LAWTON MP, 1969, GERONTOLOGIST, V9, P179, DOI 10.1093/geront/9.3_Part_1.179

Maguire EA, 2003, BRAIN, V126, P1511, DOI 10.1093/brain/awg157

Mathuranath PS, 2000, NEUROLOGY, V55, P1613

MORRIS JC, 1993, NEUROLOGY, V43, P2412, DOI 10.1212/WNL.43.11.2412-a

Nelson HE, 1991, NATL ADULT READING T

Perls T, 2004, EXP GERONTOL, V39, P1587, DOI 10.1016/j.exger.2004.08.015

PODSIADLO D, 1991, J AM GERIATR SOC, V39, P142, DOI 10.1111/j.1532-5415.1991.tb01616.x

Richmond RL, 2008, MED J AUSTRALIA, V188, P720

Ricker J. H., 1994, ASSESSMENT, V1, P51

RITCHIE K, 1995, BRIT J PSYCHIAT, V166, P229, DOI 10.1192/bjp.166.2.229

Robine JM, 2003, RES PERSPECT LONGEV, P145

ROSEN WG, 1984, AM J PSYCHIAT, V141, P1356

Sachdev PS, 2010, INT PSYCHOGERIATR, V22, P1248, DOI 10.1017/S1041610210001067

Sachdev PS, 2012, ASIAN J GERONTOL GER, V7, P44

Samuelsson SM, 1997, INT J AGING HUM DEV, V45, P223, DOI 10.2190/XKG9-YP7Y-QJTK-BGPG

SHEIKH J I, 1986, Clinical Gerontologist, V5, P165

Thomassen R, 1998, NEUROLOGY, V50, P283, DOI 10.1212/WNL.50.1.283

Valenzuela MJ, 2007, PSYCHOL MED, V37, P1015, DOI 10.1017/S003329170600938X

Ware JE, 1996, MED CARE, V34, P220, DOI 10.1097/00005650-199603000-00003

Wechsler D., 1997, WECHSLER MEMORY SCAL

Wechsler D., 1997, WECHSLER ADULT INTEL

Wen W, 2009, HUM BRAIN MAPP, V30, P1155, DOI 10.1002/hbm.20586

Willis S. L., 1993, TEST MANUAL EVERYDAY

Wimo A, 2003, BRAIN AGING, V3, P48

NR 45

TC 17

Z9 19

U1 0

U2 19

PU CAMBRIDGE UNIV PRESS

PI NEW YORK

PA 32 AVENUE OF THE AMERICAS, NEW YORK, NY 10013-2473 USA

SN 1041-6102

J9 INT PSYCHOGERIATR

JI Int. Psychogeriatr.

PD JUN

PY 2013

VL 25

IS 6

BP 993

EP 1005

DI 10.1017/S1041610213000197

PG 13

WC Psychology, Clinical; Geriatrics & Gerontology; Gerontology; Psychiatry;

Psychology

SC Psychology; Geriatrics & Gerontology; Psychiatry

GA 175VH

UT WOS:000321261200015

PM 23510643

DA 2019-01-08

ER

PT J

AU Hodge, AM

Flicker, L

O'Dea, K

English, DR

Giles, GG

AF Hodge, Allison M.

Flicker, Leon

O'Dea, Kerin

English, Dallas R.

Giles, Graham G.

TI Diabetes and ageing in the Melbourne Collaborative Cohort Study (MCCS)

SO DIABETES RESEARCH AND CLINICAL PRACTICE

LA English

DT Article

DE Prospective study; Epidemiology; Type 2 diabetes; Healthy ageing

ID DEPRESSIVE SYMPTOMS; AGE 70; WOMEN; OLDER; POPULATION; SURVIVAL;

MIDLIFE; SIZE; RISK; MEN

AB Aims: To examine the association between diabetes, and ageing 12 years later.

Methods: Subjects reporting angina, cancer, heart attack or stroke at baseline (1990-1994) were excluded. Diabetes at baseline was identified by self-report or elevated plasma glucose (>= 7.0 mmol/l fasting or >= 11.1 mmol/l non-fasting). 6431 eligible men and women had survived to age 70 years at follow-up (2003-2007), and 5704 with complete data were included in these analyses. Those without ischemic heart disease, stroke, and cancer, no perceived major difficulty with physical functioning, and no evidence of psychological distress were considered to have aged successfully, irrespective of diabetes at follow-up (n = 1271). Logistic regression was used to examine the independent association between baseline diabetes and successful ageing.

Results: At baseline 216 eligible people were identified with diabetes. This was inversely associated with successful ageing at follow-up independent of smoking, physical activity, alcohol use and obesity (OR 0.65, 95% 0.42-0.99), Of the people with diabetes at follow-up, only 12.5% did not have another condition characteristic of usual ageing, compared with 22.7% of people who did not have diabetes.

Conclusions: Diabetes is strongly associated with factors characterising less successful ageing, suggesting that the impact of diabetes may be larger than currently estimated. (C) 2013 Elsevier Ireland Ltd. All rights reserved.

C1 [Hodge, Allison M.; English, Dallas R.; Giles, Graham G.] Canc Council Victoria, Canc Epidemiol Ctr, Carlton, Vic 3053, Australia.

[Flicker, Leon] Western Australian Inst Med Res, Western Australian Ctr Hlth & Ageing, Perth, WA, Australia.

[Flicker, Leon] Univ Western Australia, Sch Med & Pharmacol, Nedlands, WA 6009, Australia.

[Flicker, Leon] Royal Perth Hosp, Dept Geriatr Med, Perth, WA, Australia.

[O'Dea, Kerin] Univ S Australia, Sansom Inst Hlth Res, Adelaide, SA 5001, Australia.

[English, Dallas R.] Univ Melbourne, Ctr MEGA Epidemiol, Sch Populat Hlth, Melbourne, Vic 3010, Australia.

RP Hodge, AM (reprint author), Canc Council Victoria, Canc Epidemiol Ctr, 1 Rathdowne St, Carlton, Vic 3053, Australia.

EM allison.hodge@cancervic.org.au

RI O'Dea, Kerin/B-6916-2009

OI Giles, Graham/0000-0003-4946-9099; Hodge, Allison/0000-0001-5464-2197;

English, Dallas/0000-0001-7828-8188

FU VicHealth; Cancer Council Victoria; National Health and Medical Research

Council; NHMRC [520316]

FX This work was funded by VicHealth, The Cancer Council Victoria and the

National Health and Medical Research Council. These funding sources had

no input into study design, or collection, analysis and interpretation

of the data.; AH was funded by NHMRC grant ID 520316.

CR AIHW, DIAB PREV AUSTR DET

Australian Government, 2010, AUSTR 2050 FUT CHALL

Begg S, 2007, BURDEN DIS INJURY AU

Bouwman V, 2010, PLOS ONE, V5, DOI 10.1371/journal.pone.0009971

Collins MM, 2009, DIABETIC MED, V26, P153, DOI 10.1111/j.1464-5491.2008.02648.x

Gandek B, 1998, J CLIN EPIDEMIOL, V51, P1171, DOI 10.1016/S0895-4356(98)00109-7

Giles G. G., 2002, Nutrition and lifestyle: opportunities for cancer prevention. European Conference on Nutrition and Cancer held in Lyon, France on 21-24 June, 2003, P69

Glymour MM, 2008, MODERN EPIDEMIOLOGY, P183

Golden SH, 2008, JAMA-J AM MED ASSOC, V299, P2751, DOI 10.1001/jama.299.23.2751

Hodge A, 2010, AUSTR DIAB SOC AUSTR

Hubbard RE, 2010, DIABETIC MED, V27, P603, DOI 10.1111/j.1464-5491.2010.02977.x

Kessler RC, 2002, PSYCHOL MED, V32, P959, DOI 10.1017/S0033291702006074

Klijs B, 2011, PLOS ONE, V6, DOI 10.1371/journal.pone.0025325

Knol MJ, 2007, PSYCHOSOM MED, V69, P300, DOI 10.1097/PSY.0b013e31805f48b9

Lu FP, 2009, PLOS ONE, V4, DOI 10.1371/journal.pone.0004144

MacInnis RJ, 2004, CANCER EPIDEM BIOMAR, V13, P553

Magliano DJ, 2009, AUST NZ J PUBL HEAL, V33, P540, DOI 10.1111/j.1753-6405.2009.00450.x

Montross LP, 2006, AM J GERIAT PSYCHIAT, V14, P43, DOI 10.1097/01.JGP.0000192489.43179.31

Nooyens ACJ, 2010, DIABETES CARE, V33, P1964, DOI 10.2337/dc09-2038

Rowe JW, 1997, GERONTOLOGIST, V37, P433, DOI 10.1093/geront/37.4.433

Strawbridge WJ, 2002, GERONTOLOGIST, V42, P727, DOI 10.1093/geront/42.6.727

Sun Q, 2010, ARCH INTERN MED, V170, P194, DOI 10.1001/archinternmed.2009.503

Sun Q, 2009, BRIT MED J, V339, DOI 10.1136/bmj.b3796

van Dongen E, 2001, ARCH INTERN MED, V161

Volpato S, 2010, CURR DIABETES REV, V6, P134, DOI 10.2174/157339910791162961

WARE JE, 1995, MED CARE, V33, pAS264, DOI 10.1097/00005650-199501001-00005

Yen CH, 2010, ARCH GERONTOL GERIAT, V50, pS53, DOI 10.1016/S0167-4943(10)70014-5

NR 27

TC 6

Z9 6

U1 0

U2 10

PU ELSEVIER IRELAND LTD

PI CLARE

PA ELSEVIER HOUSE, BROOKVALE PLAZA, EAST PARK SHANNON, CO, CLARE, 00000,

IRELAND

SN 0168-8227

J9 DIABETES RES CLIN PR

JI Diabetes Res. Clin. Pract.

PD JUN

PY 2013

VL 100

IS 3

BP 398

EP 403

DI 10.1016/j.diabres.2013.03.024

PG 6

WC Endocrinology & Metabolism

SC Endocrinology & Metabolism

GA 166WV

UT WOS:000320591300019

PM 23582874

DA 2019-01-08

ER

PT J

AU Mashitani, T

Hayashino, Y

Okamura, S

Kitatani, M

Furuya, M

Matsunaga, S

Kuwata, H

Tsujii, S

Ishii, H

AF Mashitani, Tsuyoshi

Hayashino, Yasuaki

Okamura, Shintarou

Kitatani, Masako

Furuya, Miyuki

Matsunaga, Satoshi

Kuwata, Hirohito

Tsujii, Satoru

Ishii, Hitoshi

CA Tenri Study Grp

TI Patient-reported adherence to insulin regimen is associated with

glycemic control among Japanese patients with type 2 diabetes: Diabetes

Distress and Care Registry at Tenri (DDCRT 3)

SO DIABETES RESEARCH AND CLINICAL PRACTICE

LA English

DT Article

DE Type 2 diabetes; Insulin adherence; Number of injections; Glycemic

control

ID MICROVASCULAR COMPLICATIONS; THERAPY; MELLITUS; WOMEN; BEHAVIORS;

OMISSION; WELL

AB Aims: We investigated the association between self-reported adherence to an insulin regimen and glycemic control in Japanese patients with type 2 diabetes.

Methods: Data from 1441 patients with type 2 diabetes who were treated with insulin were obtained from a diabetes registry in Japan. We obtained information on self-reported adherence to an insulin regimen. Relative risk regression analysis was employed to assess the independent association of various demographic factors with good glycemic control (HbA1c <7.0% [53 mmol/mol]) while adjusting for possible confounders.

Results: The mean age, body mass index, and number of daily insulin injections of participants were 65.4 years, 24.7 kg/m(2), and 2.3, respectively. Of all patients, 70.6% reported high adherence to their insulin regimen. Compared with participants with higher adherence, the crude relative risk of good glycemic control was 0.82 (95% CI, 0.67-1.00) for those with middle adherence and 0.64 (95% CI, 0.31-1.31) for those with lower adherence (P = 0.029 for trend). Subgroup analysis confirmed this association in patients below 65 years old, but not in those 65 years old and over.

Conclusions: A higher adherence to a daily insulin regimen was associated with a greater likelihood of good glycemic control in Japanese type 2 diabetes patients. This association was not seen in patients of 65 years old or over. Self-reported adherence to an insulin regimen may prove useful in titrating insulin dose in patients in the younger age group, but requires further investigation. (C) 2013 Elsevier Ireland Ltd. All rights reserved.

C1 [Mashitani, Tsuyoshi; Hayashino, Yasuaki; Okamura, Shintarou; Kitatani, Masako; Furuya, Miyuki; Matsunaga, Satoshi; Kuwata, Hirohito; Tsujii, Satoru; Ishii, Hitoshi] Tenri Hosp, Dept Endocrinol, Tenri, Nara 6328552, Japan.

[Tsujii, Satoru] Tenri Hosp, Ctr Diabet, Tenri, Nara 6328552, Japan.

RP Hayashino, Y (reprint author), Tenri Hosp, Dept Endocrinol, 200 Mishima Cho, Tenri, Nara 6328552, Japan.

EM hayasino-y@umin.net

OI Hayashino, Yasuaki/0000-0002-2932-8478; Tsujii,

Satoru/0000-0002-1834-6515

FU Manpei Suzuki Diabetes Foundation

FX Grant support: The Manpei Suzuki Diabetes Foundation supported this

study.

CR ANDERSON EJ, 1993, J AM DIET ASSOC, V93, P768, DOI 10.1016/0002-8223(93)91750-K

Briesacher BA, 2008, PHARMACOTHERAPY, V28, P437, DOI 10.1592/phco.28.4.437

Cramer JA, 2005, DIABETES CARE, V28, P78, DOI 10.2337/diacare.28.1.78

Dhatt GS, 2005, CLIN CHIM ACTA, V358, P81, DOI 10.1016/j.cccn.2005.02.009

Donnelly LA, 2007, QJM-INT J MED, V100, P345, DOI 10.1093/qjmed/hcm031

GLASGOW RE, 1987, J CHRON DIS, V40, P399, DOI 10.1016/0021-9681(87)90173-1

Goebel-Fabbri AE, 2008, DIABETES CARE, V31, P415, DOI 10.2337/dc07-2026

Hayashino Y, 2012, DIABETES RES CLIN PR, V97, P405, DOI 10.1016/j.diabres.2012.04.005

Mollema ED, 2001, J PSYCHOSOM RES, V51, P665, DOI 10.1016/S0022-3999(01)00229-X

Morris AD, 1997, LANCET, V350, P1505, DOI 10.1016/S0140-6736(97)06234-X

Nathan DM, 2009, DIABETES CARE, V32, P193, DOI 10.2337/dc08-9025

OHKUBO Y, 1995, DIABETES RES CLIN PR, V28, P103, DOI 10.1016/0168-8227(95)01064-K

Peyrot M, 2010, DIABETES CARE, V33, P240, DOI 10.2337/dc09-1348

POLONSKY WH, 1994, DIABETES CARE, V17, P1178, DOI 10.2337/diacare.17.10.1178

Rubin Richard R, 2005, Am J Med, V118 Suppl 5A, p27S

Rubin RR, 2009, DIABETES EDUCATOR, V35, P1014, DOI 10.1177/0145721709345773

Rydall AC, 1997, NEW ENGL J MED, V336, P1849, DOI 10.1056/NEJM199706263362601

SCHAFER LC, 1986, DIABETES CARE, V9, P179, DOI 10.2337/diacare.9.2.179

StataCorp, 2007, STAT 10 BAS REF MAN

Tsujii S, 2012, DIABETIC MED, V29, P1451, DOI 10.1111/j.1464-5491.2012.03647.x

UK Prospective Diabetes Study Group, 1995, DIABETES, V44, P1249

NR 21

TC 18

Z9 18

U1 1

U2 5

PU ELSEVIER IRELAND LTD

PI CLARE

PA ELSEVIER HOUSE, BROOKVALE PLAZA, EAST PARK SHANNON, CO, CLARE, 00000,

IRELAND

SN 0168-8227

J9 DIABETES RES CLIN PR

JI Diabetes Res. Clin. Pract.

PD MAY

PY 2013

VL 100

IS 2

BP 189

EP 194

DI 10.1016/j.diabres.2013.03.006

PG 6

WC Endocrinology & Metabolism

SC Endocrinology & Metabolism

GA 166WR

UT WOS:000320590900014

PM 23522915

DA 2019-01-08

ER

PT J

AU Okumura, Y

Ito, H

AF Okumura, Yasuyuki

Ito, Hiroto

TI Out-of-pocket expenditure burdens in patients with cardiovascular

conditions and psychological distress: a nationwide cross-sectional

study

SO GENERAL HOSPITAL PSYCHIATRY

LA English

DT Article

DE Burden of disease; Resource allocation; Mental health policy; Equity in

health

ID CORONARY-HEART-DISEASE; PHYSICAL CONDITIONS; MENTAL-HEALTH; RISK-FACTOR;

REPRESENTATIVE SAMPLE; DEPRESSION; POPULATION; DISABILITY; JAPAN; CARE

AB Objectives: This study aimed to estimate the prevalence of psychological distress in individuals with and without cardiovascular risks and events [cardiovascular disease (CVD) conditions] and the incremental effects of psychological distress on the out-of-pocket health care expenditure burdens.

Methods: We used data from the Comprehensive Survey of Living Conditions 2007, a nationally representative cross-sectional survey in Japan. Psychological distress assessed by the K6 scale, the presence of treated CVD conditions and out-of-pocket health care expenditures as a share of household consumption expenditures were self-reported by 20,763 individuals living alone and aged between 20 and 59 years.

Results: Individuals with obesity [adjusted odds ratio (AOR), 4.3], stroke (AOR, 3.2), ischemic heart disease (AOR, 2.3), hyperlipidemia (AOR, 1.8) or diabetes (AOR, 1.7) were more likely than those without to have serious psychological distress (SPD). With the exception of ischemic heart disease, less than half of CVD patients comorbid with SPD received treatment for mental illness. Patients comorbid with SPD and obesity (AOR, 6.1), SPD and ischemic heart disease (AOR, 3.4), and SPD and hypertension (AOR, 2.6) had higher out-of-pocket burdens than patients with only CVD conditions.

Conclusions: Our findings suggest the need for physicians to identify and manage SPD in patients with CVD conditions and for policymakers to find solutions to reduce the high out-of-pocket burdens among these patients. (C) 2013 Elsevier Inc. All rights reserved.

C1 [Okumura, Yasuyuki; Ito, Hiroto] Natl Ctr Neurol & Psychiat, Dept Social Psychiat, Natl Inst Mental Hlth, Kodaira, Tokyo 1878553, Japan.

RP Okumura, Y (reprint author), Natl Ctr Neurol & Psychiat, Dept Social Psychiat, Natl Inst Mental Hlth, Kodaira, Tokyo 1878553, Japan.

EM yokumura@ncnp.go.jp

OI Okumura, Yasuyuki/0000-0001-5851-8919

FU Comprehensive Research on Disability Health and Welfare (Ministry of

Health, Labour, and Welfare) [H21-KOKORO-IPPAN-007]

FX Funding for analyzing and writing this article was supported by

Comprehensive Research on Disability Health and Welfare (Ministry of

Health, Labour, and Welfare, H21-KOKORO-IPPAN-007).

CR Banthin JS, 2006, JAMA-J AM MED ASSOC, V296, P2712, DOI 10.1001/jama.296.22.2712

Barth J, 2004, PSYCHOSOM MED, V66, P802, DOI 10.1097/01.psy.0000146332.53619.b2

Blough DK, 2000, HLTH SERV OUTCOMES R, V1, P185, DOI DOI 10.1023/A:1012597123667

Bureau Statistics, 2005, POP CENS JAP

Bureau Statistics, 2007, POP CENS JAP

Centres for Disease Control and Prevention, 2004, MMWR-MORBID MORTAL W, V53, P1089

Chittleborough CR, 2011, INT J PUBLIC HEALTH, V56, P71, DOI 10.1007/s00038-010-0197-5

Dobson A. J., 2008, INTRO GEN LINEAR MOD

Fan AZ, 2009, INT J PUBLIC HEALTH, V54, P100, DOI 10.1007/s00038-009-1313-2

Ferketich AK, 2005, EUR HEART J, V26, P1923, DOI 10.1093/eurheartj/ehi329

Furukawa TA, 2008, INT J METH PSYCH RES, V17, P152, DOI 10.1002/mpr.257

Gehi A, 2005, ARCH INTERN MED, V165, P2508, DOI 10.1001/archinte.165.21.2508

Inoue A, 2010, J OCCUP HEALTH, V52, P227, DOI 10.1539/joh.O10002

Kessler RC, 2002, PSYCHOL MED, V32, P959, DOI 10.1017/S0033291702006074

Kessler RC, 2003, ARCH GEN PSYCHIAT, V60, P1117, DOI 10.1001/archpsyc.60.11.1117

Li CY, 2009, INT J PUBLIC HEALTH, V54, P43, DOI 10.1007/s00038-009-0006-1

Lichtman JH, 2008, CIRCULATION, V118, P1768, DOI 10.1161/CIRCULATIONAHA.108.190769

Long J. S., 1997, REGRESSION MODELS CA

Mathers CD, 2006, PLOS MED, V3, DOI 10.1371/journal.pmed.0030442

Merikangas KR, 2007, ARCH GEN PSYCHIAT, V64, P1180, DOI 10.1001/archpsyc.64.10.1180

Mitchell AJ, 2009, LANCET, V374, P609, DOI 10.1016/S0140-6736(09)60879-5

Organisation for Economic Co-operation and Development, 2011, GOV GLANC 2011

R Development Core Team, 2012, R LANG ENV STAT COMP

Sareen J, 2006, ARCH INTERN MED, V166, P2109, DOI 10.1001/archinte.166.19.2109

Schmitz N, 2007, PSYCHOSOM MED, V69, P332, DOI 10.1097/PSY.0b013e31804259e0

Schwarz EB, 2009, OBSTET GYNECOL, V113, P974, DOI 10.1097/01.AOG.0000346884.67796.ca

Scott KM, 2007, J AFFECT DISORDERS, V103, P113, DOI 10.1016/j.jad.2007.01.015

Shibuya K, 2002, BRIT MED J, V324, P16, DOI 10.1136/bmj.324.7328.16

Shibuya K, 2011, LANCET, V378, P1265, DOI 10.1016/S0140-6736(11)61098-2

Shih M, 2008, QUAL LIFE RES, V17, P521, DOI 10.1007/s11136-008-9330-9

Statistics and Information Department Minister's Secretariat Ministry of Health Labour and Welfare, 2009, COMPR SURV LIV COND

Stein MB, 2006, PSYCHOL MED, V36, P587, DOI 10.1017/S0033291706007239

Suliman S, J NERV MENT DIS, V198, P8

Sullivan M, 2002, ARCH INTERN MED, V162, P1860, DOI 10.1001/archinte.162.16.1860

Sullivan PW, 2007, OBESITY, V15, P3150, DOI 10.1038/oby.2007.375

Xu K, 2003, LANCET, V362, P111, DOI 10.1016/S0140-6736(03)13861-5

Xu K, 2003, HOUSEHOLD HLTH SYSTE

Zhao GX, 2009, INT J PUBLIC HEALTH, V54, P30, DOI 10.1007/s00038-009-0004-3

NR 38

TC 8

Z9 8

U1 0

U2 10

PU ELSEVIER SCIENCE INC

PI NEW YORK

PA 360 PARK AVE SOUTH, NEW YORK, NY 10010-1710 USA

SN 0163-8343

J9 GEN HOSP PSYCHIAT

JI Gen. Hosp. Psych.

PD MAY-JUN

PY 2013

VL 35

IS 3

BP 233

EP 238

DI 10.1016/j.genhosppsych.2012.12.013

PG 6

WC Psychiatry

SC Psychiatry

GA 145DZ

UT WOS:000318995800004

PM 23391611

DA 2019-01-08

ER

PT J

AU Van Haltren, K

Malhotra, A

AF Van Haltren, Karen

Malhotra, Atul

TI Characteristics of infants admitted with hypoglycemia to a neonatal unit

SO JOURNAL OF PEDIATRIC ENDOCRINOLOGY & METABOLISM

LA English

DT Article

DE diabetes; hypoglycemia; neonate

ID OUTCOMES; INJURY

AB Background: Neonatal hypoglycemia is a common symptom in early infant life. The currently available literature identifies the risk factors but not the degree to which each factor lends itself to the development or severity of an ensuing hypoglycemia.

Methods: A retrospective electronic chart review of near-term and term infants (>35 weeks' gestation) admitted to a tertiary-level neonatal unit over 16 months was carried out from the initiation of electronic record keeping.

Results: Ninety-five infants admitted with hypoglycemia to the unit were identified with complete records available. Fifty-eight percent of the infants were males, and the median gestation was 38 weeks. Twenty-two percent of the infants were premature, whereas 18% were macrosomic. Maternal diabetes was present in 27% infants. Comorbidities were common in this cohort, with approximately two-thirds of infants having multiple morbidities. The majority of which were jaundice and infection, whereas the minor ones were respiratory distress, initial lactic acidosis, feeding issues, and temperature instability.

Conclusions: Neonatal hypoglycemia continues to be a significant morbidity in near-term and term infants. The presence of multiple morbidities is common in the setting of hypoglycemia and is the main determinant of the length of stay in the neonatal unit in this study population.

C1 [Malhotra, Atul] Monash Med Ctr, Melbourne, Vic 3168, Australia.

[Van Haltren, Karen; Malhotra, Atul] Monash Univ, Dept Paediat, Melbourne, Vic 3004, Australia.

RP Malhotra, A (reprint author), Monash Med Ctr, 246 Clayton Rd, Melbourne, Vic 3168, Australia.

EM atul.malhotra@southernhealth.org.au

OI Malhotra, Atul/0000-0001-9664-4182

CR Adamkin DH, 2011, PEDIATRICS, V127, P575, DOI 10.1542/peds.2010-3851

Burns CM, 2008, PEDIATRICS, V122, P65, DOI 10.1542/peds.2007-2822

Cornblath M, 2000, PEDIATRICS, V105, P1141, DOI 10.1542/peds.105.5.1141

Flores-le Roux JA, 2012, DIABETES RES CLIN PR, V97, P217, DOI 10.1016/j.diabres.2012.03.011

Harris DL, 2012, J PEDIATR-US, V161, P787, DOI 10.1016/j.jpeds.2012.05.022

Hay WW, 2009, J PEDIATR-US, V155, P612, DOI 10.1016/j.jpeds.2009.06.044

Stanley CA, 1999, NEW ENGL J MED, V18, P127

Tam EWY, 2008, PEDIATRICS, V122, P507, DOI 10.1542/peds.2007-2002

NR 8

TC 7

Z9 7

U1 0

U2 4

PU WALTER DE GRUYTER GMBH

PI BERLIN

PA GENTHINER STRASSE 13, D-10785 BERLIN, GERMANY

SN 0334-018X

J9 J PEDIATR ENDOCR MET

JI J. Pediatr. Endocrinol. Metab.

PD MAY

PY 2013

VL 26

IS 5-6

BP 525

EP 529

DI 10.1515/jpem-2013-0009

PG 5

WC Endocrinology & Metabolism; Pediatrics

SC Endocrinology & Metabolism; Pediatrics

GA 138HU

UT WOS:000318500400017

PM 23443262

DA 2019-01-08

ER

PT J

AU Negrato, CA

Gomes, MB

AF Negrato, Carlos Antonio

Gomes, Marilia Brito

TI RETRACTED: Historical facts of screening and diagnosing diabetes in

pregnancy (Retracted article. See vol. 6, 59, 2014)

SO DIABETOLOGY & METABOLIC SYNDROME

LA English

DT Review; Retracted Publication

DE History of diabetes; Diabetes in pregnancy; Screening diabetes in

pregnancy; Diagnosing diabetes in pregnancy

ID INTERNATIONAL WORKSHOP-CONFERENCE; CARBOHYDRATE-METABOLISM; PREDIABETIC

STATE; GLUCOSE TOLERANCE; MELLITUS; RECOMMENDATIONS; HYPERGLYCEMIA;

CRITERIA

AB Diabetes is the most common metabolic disorder affecting pregnancy. Its prevalence seems to be growing in parallel with the epidemics of overweight and obesity. Recognizing and treating diabetes or any degree of glucose intolerance in pregnancy results in lowering maternal and fetal complications. These patients present higher risk for excessive weight gain, preeclampsia, cesarean sections, a high risk of developing type 2 diabetes and cardiovascular disease in the future. Infants born to these mothers are at higher risk for macrosomia and birth trauma, and after delivery, these infants have a higher risk of developing hypoglycemia, hypocalcemia, hyperbilirubinemia, respiratory distress syndrome, polycythemia and subsequent obesity and type 2 diabetes. Despite several international workshops and a lot of research there is still no unique approach to diagnose and treat diabetes in pregnancy. Who, when and how to screen and diagnose diabetes in pregnancy has been debated in the literature for so many decades and this debate seems to be endless. We present the evolution that screening and diagnosing diabetes in pregnancy has had over time. Besides many evidence of the benefits these procedures bring, health care providers still often prefer to use alternate criteria for this purpose. The myriad of maternal and fetal complications that could be avoided with an appropriate and simple screening procedure are ignored. Robust clinical trials such as the Hyperglycemia and Adverse Pregnancy Outcomes (HAPO) study have shown how harmful can even slightly altered blood glucose levels be, but it has been found a resistance in the adoption of the new criteria proposed after this and other trials by many diabetes organizations. These organizations state that these new criteria would increase the incidence of diabetes in pregnancy, would imply in longer term follow-up of these patients and would pose an economic problem; they also state that alerting too many people in order to benefit a relatively few potential diabetics would arise psychologic ill-effects. We think that health care providers should look for an uniformity in the screening and diagnosing diabetes in pregnancy based on evidence based medicine and not on specialists consensus.

C1 [Negrato, Carlos Antonio] Baurus Diabet Assoc, Dept Internal Med, BR-17012433 Sao Paulo, Brazil.

[Gomes, Marilia Brito] State Univ Hosp Rio de Janeiro, Dept Internal Med, Diabet Unit, Rio De Janeiro, Brazil.

RP Negrato, CA (reprint author), Baurus Diabet Assoc, Dept Internal Med, BR-17012433 Sao Paulo, Brazil.

EM carlosnegrato@uol.com.br

RI Negrato, Carlos/F-8363-2013

CR Alberti KGMM, 1998, DIABETIC MED, V15, P539, DOI 10.1002/(SICI)1096-9136(199807)15:7<539::AID-DIA668>3.0.CO;2-S

American Diabetes Association, 2009, DIABETES CARE, V32, P62

American Diabetes Association (ADA), 2011, DIABETES CARE S1, V34, pS62, DOI DOI 10.2337/DC11-S062

[Anonymous], 1985, Diabetes, V34 Suppl 2, P1

[Anonymous], 1883, COMPT RENDUS SOC BIO, V5, P193

Bennewitz HG, 1824, THESIS U BERLIN

Brasil. Ministerio da Saude, 2010, GEST ALT RISC MAN TE, P302

CARPENTER MW, 1982, AM J OBSTET GYNECOL, V144, P768, DOI 10.1016/0002-9378(82)90349-0

CARRINGTON ER, 1957, OBSTET GYNECOL, V9, P664, DOI 10.1097/00006250-195706000-00008

COLLIER E, 1991, DIABETES, V40, P197, DOI 10.2337/diabetes.40.2.197

CONN JW, 1961, AM J MED, V31, P839, DOI 10.1016/0002-9343(61)90023-7

Dubreuil G, 1920, COMPT RENDUS SOC BIO, V133, P1490

Duncan JM, 1882, T OBSTET SOC LONDON, V24, P256

FREINKEL N, 1980, DIABETES CARE, V3, P399, DOI 10.2337/diacare.3.3.399

Graham G, 1924, Proc R Soc Med, V17, P102

Hadden DR, 1975, CARBOHYD METABOL, P19

HARRIS M, 1979, DIABETES, V28, P1039

HOET JP, 1954, DIABETES, V3, P1

Hofmeister Ztschr F, 1877, HOFMEISTER Z F, V1, P101

JACKSON W. P. U., 1953, S AFRICAN MED JOUR, V27, P795

JACKSON WPU, 1960, DIABETES, V9, P373, DOI 10.2337/diab.9.5.373

Jovanovic L, 2001, JAMA-J AM MED ASSOC, V286, P2516, DOI 10.1001/jama.286.20.2516

Knopp RH, 2002, DIABETES CARE, V25, P943, DOI 10.2337/diacare.25.5.943

Lambie CG, 1926, J OBSTET GYN BR EMP, V33, P563

Lecorche, 1885, ANN GYNECOL OBST, P257

Lever JCW, 1847, GUYS HOSP REPORT, P3

Mestman JH, 2002, ENDOCRINOLOGIST, V12, DOI 10.1097/00019616-200205000-00010

Metzger BE, 1998, DIABETES CARE, V21, pB161

Metzger BE, 2008, NEW ENGL J MED, V358, P1991, DOI 10.1056/NEJMoa0707943

Metzger BE, 2007, DIABETES CARE, V30, pS251, DOI 10.2337/dc07-s225

Metzger BE, 2010, DIABETES CARE, V33, P676, DOI 10.2337/dc09-1848

MILLER HC, 1945, NEW ENGL J MED, V233, P376, DOI 10.1056/NEJM194509272331302

MOSS JM, 1951, ANN INTERN MED, V34, P678, DOI 10.7326/0003-4819-34-3-678

Negrato CA, 2010, DIABETOL METAB SYNDR, V2, DOI 10.1186/1758-5996-2-27

OSULLIVAN JB, 1964, DIABETES, V13, P278

PEDERSEN J, 1956, LANCET, V270, P607

Professional Practice Committee/American Diabetes Association, 2004, DIABETES CARE S1, V27, pS5

Reichelt Angela J., 2002, Arq Bras Endocrinol Metab, V46, P574, DOI 10.1590/S0004-27302002000500012

Reveno WS, 1923, JAMA-J AM MED ASSOC, V81, P2001

Skipper E, 1933, Q J MED, V2, P353

UNGER RH, 1958, DIABETES, V7, P455, DOI 10.2337/diab.7.6.455

Von Noorden C, 1917, ZUCKERKRANKHEIT, V4

Walker A, 1927, P ROY SOC MED, Vxxi, P13

WHITE P, 1949, AM J MED, V7, P609, DOI 10.1016/0002-9343(49)90382-4

WILKERSON HLC, 1957, DIABETES, V6, P324, DOI 10.2337/diab.6.4.324

Williams JW, 1909, AM J MED SCI, V137, P1

NR 46

TC 6

Z9 7

U1 2

U2 17

PU BIOMED CENTRAL LTD

PI LONDON

PA 236 GRAYS INN RD, FLOOR 6, LONDON WC1X 8HL, ENGLAND

SN 1758-5996

J9 DIABETOL METAB SYNDR

JI Diabetol. Metab. Syndr.

PD MAY 1

PY 2013

VL 5

AR 22

DI 10.1186/1758-5996-5-22

PG 8

WC Endocrinology & Metabolism

SC Endocrinology & Metabolism

GA 137EC

UT WOS:000318417100001

PM 23634949

OA DOAJ Gold, Green Published

DA 2019-01-08

ER

PT J

AU Wu, YP

Hilliard, ME

Rausch, J

Dolan, LM

Hood, KK

AF Wu, Y. P.

Hilliard, M. E.

Rausch, J.

Dolan, L. M.

Hood, K. K.

TI Family involvement with the diabetes regimen in young people: the role

of adolescent depressive symptoms

SO DIABETIC MEDICINE

LA English

DT Article

ID GLYCEMIC CONTROL; HEALTH OUTCOMES; CHILDREN; ADHERENCE; RESPONSIBILITY;

MANAGEMENT; MELLITUS; CARE; INTERVENTION; ASSOCIATION

AB Aims In young people with Type1 diabetes, depressive symptoms and shared responsibility for management of diabetes impact upon diabetes management and control. However, the simultaneous effects of both depressive symptoms and parental involvement on diabetes self-care and glycaemic control have not been examined. Thus, the aim of the current study was to examine the relationships between parental involvement and adolescent depressive symptoms in predicting blood glucose monitoring and glycaemic control. Methods One hundred and fifty young people with Type1 diabetes (mean age 15.3years) and their parents completed responsibility sharing and depressive symptom assessments, meter assessment of blood glucose monitoring and HbA1c at baseline and then 6, 12 and 18months. Results Parental involvement affected HbA1c through blood glucose monitoring only at low levels of adolescent depressive symptoms (score 6), which made up only 20% of the sample. In the presence of more depressive symptoms, parental involvement no longer was related to HbA1c through blood glucose monitoring. This was the relationship in the majority of the sample (80%). Conclusions While most young people in this sample are not showing evidence of high levels of depressive symptoms, even modest levels of distress interfere with parental involvement in diabetes management. By addressing adolescent depressive symptoms, interventions promoting parental involvement in these families may be more effective.

C1 [Wu, Y. P.; Hilliard, M. E.] Univ Cincinnati, Coll Med, Ctr Adherence Promot & Self Management, Cincinnati, OH USA.

[Wu, Y. P.; Hilliard, M. E.; Rausch, J.] Univ Cincinnati, Coll Med, Cincinnati, OH USA.

[Dolan, L. M.] Univ Cincinnati, Coll Med, Div Endocrinol, Cincinnati, OH USA.

[Dolan, L. M.] Univ Cincinnati, Coll Med, Dept Pediat, Cincinnati, OH USA.

[Hood, K. K.] Univ Calif San Francisco, Dept Pediat, Madison Clin Pediat Diabet, Div Endocrinol, San Francisco, CA 94143 USA.

RP Hood, KK (reprint author), Univ Calif San Francisco, Dept Pediat, Madison Clin Pediat Diabet, Div Endocrinol, San Francisco, CA 94143 USA.

EM hoodk@peds.ucsf.edu

RI Rausch, Joseph/E-8107-2018

OI Hilliard, Marisa/0000-0002-8813-629X

FU National Institutes of Health [K23, DK077340, T32HD068223]

FX This research was supported by a career development award from the

National Institutes of Health to KKH (K23, DK077340) and a training

grant from the National Institutes of Health supporting YPW

(T32HD068223).

CR Anderson B, 1997, J PEDIATR-US, V130, P257, DOI 10.1016/S0022-3476(97)70352-4

Anderson BJ, 2009, FAM SYST HEALTH, V27, P141, DOI 10.1037/a0015759

ANDERSON BJ, 1990, J PEDIATR PSYCHOL, V15, P477, DOI 10.1093/jpepsy/15.4.477

Berg CA, 2007, J PEDIATR PSYCHOL, V32, P995, DOI 10.1093/jpepsy/jsm043

COLE DA, 1993, J FAMILY PSYCHOL, V7, P119, DOI DOI 10.1037/0893-3200.7.1.119

Dantzer C, 2003, CLIN PSYCHOL REV, V23, P787, DOI 10.1016/S0272-7358(03)00069-2

Dumenci L, 1996, MULTIVAR BEHAV RES, V31, P313, DOI 10.1207/s15327906mbr3103_3

Ellis DA, 2007, J PEDIATR PSYCHOL, V32, P907, DOI 10.1093/jpepsy/jsm009

Grey M, 2002, J PSYCHOSOM RES, V53, P907, DOI 10.1016/S0022-3999(02)00312-4

Guilfoyle SM, 2011, PEDIATR DIABETES, V12, P560, DOI 10.1111/j.1399-5448.2010.00735.x

Helgeson VS, 2008, J PEDIATR PSYCHOL, V33, P497, DOI 10.1093/jpepsy/jsm081

Helgeson VS, 2011, PEDIATR DIABETES, V12, P25, DOI 10.1111/j.1399-5448.2010.00663.x

Hilliard ME, 2011, DIABETES RES CLIN PR, V94, P39, DOI 10.1016/j.diabres.2011.05.027

Hood KK, 2006, DIABETES CARE, V29, P1389, DOI 10.2337/dc06-0087

Hood KK, 2007, DIABETES SPECTR, V20, P251

JACOBSON AM, 1994, PSYCHOSOM MED, V56, P401, DOI 10.1097/00006842-199409000-00004

Kovacs M., 1992, CHILDRENS DEPRESSION

Kurt MD, 2002, CLIN PSYCHOL REV, V22, P247

Laffel LMB, 2003, J PEDIATR-US, V142, P409, DOI 10.1067/mpd.2003.138

LAGRECA AM, 1995, J PEDIATR PSYCHOL, V20, P449, DOI 10.1093/jpepsy/20.4.449

McGrady Meghan E, 2010, Diabetes Res Clin Pract, V88, pe35, DOI 10.1016/j.diabres.2010.03.025

McGrady ME, 2009, DIABETES CARE, V32, P804, DOI 10.2337/dc08-2111

Morris AD, 1997, LANCET, V350, P1505, DOI 10.1016/S0140-6736(97)06234-X

Nansel TR, 2009, PEDIATR DIABETES, V10, P105, DOI 10.1111/j.1399-5448.2008.00448.x

Palmer DL, 2004, J PEDIATR PSYCHOL, V29, P35, DOI 10.1093/jpepsy/jsh005

Preacher KJ, 2004, BEHAV RES METH INS C, V36, P717, DOI 10.3758/BF03206553

Preacher KJ, 2007, MULTIVAR BEHAV RES, V42, P185, DOI 10.1080/00273170701341316

Rausch JR, 2012, DIABETES CARE, V35, P1219, DOI 10.2337/dc11-2163

Rice F, 2002, J CHILD PSYCHOL PSYC, V43, P65, DOI 10.1111/1469-7610.00004

Sheeber L, 1997, J ABNORM CHILD PSYCH, V25, P333, DOI 10.1023/A:1025768504415

Silverstein J, 2005, DIABETES CARE, V28, P186, DOI 10.2337/diacare.28.1.186

SPSS I, 2009, SPSS 17

Vesco AT, 2010, J PEDIATR PSYCHOL, V35, P1168, DOI 10.1093/jpepsy/jsq038

Whittemore Robin, 2002, Pediatr Diabetes, V3, P135, DOI 10.1034/j.1399-5448.2002.30303.x

Wysocki T, 2005, PEDIATR CLIN N AM, V52, P1755, DOI 10.1016/j.pcl.2005.07.003

NR 35

TC 8

Z9 8

U1 0

U2 14

PU WILEY-BLACKWELL

PI HOBOKEN

PA 111 RIVER ST, HOBOKEN 07030-5774, NJ USA

SN 0742-3071

J9 DIABETIC MED

JI Diabetic Med.

PD MAY

PY 2013

VL 30

IS 5

BP 596

EP 602

DI 10.1111/dme.12117

PG 7

WC Endocrinology & Metabolism

SC Endocrinology & Metabolism

GA 129SQ

UT WOS:000317863000019

PM 23320523

OA Green Accepted

DA 2019-01-08

ER

PT J

AU Browne, JL

Scibilia, R

Speight, J

AF Browne, J. L.

Scibilia, R.

Speight, J.

TI The needs, concerns, and characteristics of younger Australian adults

with Type 2 diabetes

SO DIABETIC MEDICINE

LA English

DT Article

ID IMPAIRED GLUCOSE-TOLERANCE; EMOTIONAL DISTRESS; SOCIAL STIGMA; PROBLEM

AREAS; MELLITUS; COMPLICATIONS; MANAGEMENT; SCALE; ADOLESCENTS;

EMPOWERMENT

AB Aims The mean age of onset of Type 2 diabetes mellitus is decreasing in Australia and internationally. We conducted an internet-based survey to improve our understanding of the emotional well-being and unmet needs of younger adults with Type 2 diabetes, and to inform service provision for this group. Methods A random sample of National Diabetes Services Scheme registrants (n=1,417) with Type 2 diabetes, aged 1839years, living in the Australian state of Victoria received an invitation to complete the online survey. The study was also advertised state-wide. The survey included validated scales (PAID-5: diabetes-related distress; WHO-5: general emotional well-being) and study-specific items. A total of 149 eligible respondents participated. Results Almost two-thirds (63%) of respondents reported severe-diabetes related distress; more than a quarter (27%) had impaired general emotional well-being. Most (82%) were overweight or obese (BMI25); most (77%) had at least one other co-morbidity. Lack of motivation, feeling burned out, and being time-poor were identified as top barriers to self-management. More than half (59%) of respondents had not participated in structured diabetes education. Respondents perceived that younger adults with Type 2 diabetes had different health-care needs than their older counterparts (68%), and that most Type 2 diabetes information/services were aimed at older adults (62%). Of a range of potential new services, respondents indicated greatest interest in an online forum specifically for younger adults with Type 2 diabetes. Conclusions Younger adults with Type 2 diabetes have impaired emotional well-being and physical health. Population-based research is needed to confirm the current findings, to further inform service delivery and optimise outcomes for this group.

C1 [Browne, J. L.; Speight, J.] Diabet Australia Vic, Australian Ctr Behav Res Diabet, Melbourne, Vic, Australia.

[Browne, J. L.; Speight, J.] Deakin Univ, Ctr Mental Hlth & Wellbeing Res, Sch Psychol, Burwood, Vic, Australia.

[Scibilia, R.] Diabet Australia Vic, Melbourne, Vic, Australia.

[Speight, J.] AHP Res, Hornchurch, Essex, England.

RP Browne, JL (reprint author), Diabet Australia Vic, Australian Ctr Behav Res Diabet, Melbourne, Vic, Australia.

EM jbrowne@acbrd.org.au

OI Speight, Jane/0000-0002-1204-6896

FU Centre for Mental Health and Wellbeing Research at Deakin University;

Diabetes Australia - Vic; Deakin University

FX The Australian Centre for Behavioural Research in Diabetes is a

partnership for better health between Diabetes Australia - Vic and

Deakin University. Both bodies provide core funding for the Centre,

which enabled this work to be undertaken. The Centre for Mental Health

and Wellbeing Research at Deakin University also provided a small grant

to support this work.

CR Anderson RM, 2003, DIABETES CARE, V26, P1641, DOI 10.2337/diacare.26.5.1641-a

*AUSTR BUR STAT, 2006, NAT AB TORR STRAIT I

Bech P., 2004, QUALITY LIFE NEWSLET, V32, P15

Benhalima K, 2011, PRIM CARE DIABETES, V5, P245, DOI 10.1016/j.pcd.2011.06.003

Benhalima K, 2011, PRIM CARE DIABETES, V5, P57, DOI 10.1016/j.pcd.2010.08.001

Berge LI, 2012, DIABETIC MED, V29, P509, DOI 10.1111/j.1464-5491.2011.03530.x

Broadbent E, 2011, DIABETES CARE, V34, P338, DOI 10.2337/dc10-1779

Delahanty LM, 2007, DIABETIC MED, V24, P48, DOI 10.1111/j.1464-5491.2007.02028.x

Drivsholm T, 2005, DIABETOLOGIA, V48, P210, DOI 10.1007/s00125-004-1625-y

Dunning T, 1999, J NURS HLTH CHRONIC, V16, P73, DOI DOI 10.1111/J.1752-9824.2009.01035.X

Dunstan DW, 2002, DIABETES CARE, V25, P829, DOI 10.2337/diacare.25.5.829

Feltbower RG, 2003, ARCH DIS CHILD, V88, P676, DOI 10.1136/adc.88.8.676

Fisher L, 2008, DIABETIC MED, V25, P1096, DOI 10.1111/j.1464-5491.2008.02533.x

GLATTHAAR C, 1985, MED J AUSTRALIA, V143, P436

Hessler DM, 2011, PATIENT EDUC COUNS, V85, P154, DOI 10.1016/j.pec.2010.10.030

Hillier TA, 2003, DIABETES CARE, V26, P2999, DOI 10.2337/diacare.26.11.2999

Koopman RJ, 2005, ANN FAM MED, V3, P60, DOI 10.1370/afm.214

Lammi N, 2007, DIABETOLOGIA, V50, P1393, DOI 10.1007/s00125-007-0690-4

McGrath NM, 1999, DIABETES RES CLIN PR, V43, P205, DOI 10.1016/S0168-8227(99)00003-0

McGuire BE, 2010, DIABETOLOGIA, V53, P66, DOI 10.1007/s00125-009-1559-5

Pinhas-Hamiel O, 2005, J PEDIATR-US, V146, P693, DOI 10.1016/j.jpeds.2004.12.042

Rhodes ET, 2012, DIABETIC MED, V29, P453, DOI 10.1111/j.1464-5491.2011.03542.x

Schabert J, 2013, PATIENT, V6, P1, DOI 10.1007/s40271-012-0001-0

Scott Adrian, 2004, N Z Med J, V117, pU1219

Senecal C, 2000, HEALTH PSYCHOL, V19, P452, DOI 10.1037//0278-6133.19.5.452

Shah AS, 2011, DIABETOLOGIA, V54, P722, DOI 10.1007/s00125-010-1974-7

Shiu ATY, 2003, J CLIN NURS, V12, P149, DOI 10.1046/j.1365-2702.2003.00735.x

Sillars BA, 2010, INTERN MED J, V40, P850, DOI 10.1111/j.1445-5994.2010.02372.x

Snoek FJ, 2000, DIABETES CARE, V23, P1305, DOI 10.2337/diacare.23.9.1305

Speight J, 2012, BMC PUBLIC HEALTH, V12, DOI 10.1186/1471-2458-12-120

Urbina EM, 2009, CIRCULATION, V119, P2913, DOI 10.1161/CIRCULATIONAHA.108.830380

van Dam HA, 2005, PATIENT EDUC COUNS, V59, P1, DOI 10.1016/j.pec.2004.11.001

Welch GW, 1997, DIABETES CARE, V20, P760, DOI 10.2337/diacare.20.5.760

NR 33

TC 24

Z9 25

U1 1

U2 26

PU WILEY-BLACKWELL

PI HOBOKEN

PA 111 RIVER ST, HOBOKEN 07030-5774, NJ USA

SN 0742-3071

J9 DIABETIC MED

JI Diabetic Med.

PD MAY

PY 2013

VL 30

IS 5

BP 620

EP 626

DI 10.1111/dme.12078

PG 7

WC Endocrinology & Metabolism

SC Endocrinology & Metabolism

GA 129SQ

UT WOS:000317863000023

PM 23181664

DA 2019-01-08

ER

PT J

AU Robertson, SM

Amspoker, AB

Cully, JA

Ross, EL

Naik, AD

AF Robertson, S. M.

Amspoker, A. B.

Cully, J. A.

Ross, E. L.

Naik, A. D.

TI Affective symptoms and change in diabetes self-efficacy and glycaemic

control

SO DIABETIC MEDICINE

LA English

DT Article

ID DEPRESSION; STRESS; DISTRESS; ADULTS; TRIAL; CARE

AB Aims To examine the role of baseline depression, anxiety and stress symptoms on post-intervention diabetes self-efficacy and glycaemic control (HbA1c). Methods The current study analysed data from patients (n=85) with treated but uncontrolled Type2 diabetes who participated in a comparative effectiveness study of two diabetes self-management interventions. Hierarchical linear regression was used to examine the relationships between baseline affective symptoms and post-intervention diabetes self-efficacy and the moderating effects of baseline affective symptoms on the relationship between changes in diabetes self-efficacy and post-intervention HbA1c. Results Baseline depression was inversely associated with post-intervention diabetes self-efficacy (P=0.0001) after adjusting for baseline characteristics including diabetes self-efficacy. In contrast, normalmild levels of stress were associated with higher post-intervention diabetes self-efficacy (P=0.04). Anxiety and stress symptoms significantly and independently moderated the relationship between changes in diabetes self-efficacy and post-intervention HbA1c (P=0.02 and P=0.03, respectively). Further evaluation of these interactions demonstrated that changes in diabetes self-efficacy were associated with lower post-intervention HbA1c, but only among those with higher baseline affective symptoms. Conclusions We found a moderating effect across affective symptoms on the relationship between diabetes self-efficacy changes and post-intervention HbA1c in the context of a self-management intervention. Results suggest that patients with poorly controlled diabetes who have higher levels of depression, anxiety and stress symptoms may derive greater benefits from self-management interventions known to improve diabetes self-efficacy.

C1 [Robertson, S. M.; Amspoker, A. B.; Cully, J. A.; Ross, E. L.; Naik, A. D.] Univ Houston, Michael E DeBakey Vet Affairs Med Ctr, Houston Hlth Serv Res &Development Ctr Excellence, Houston, TX 77004 USA.

[Robertson, S. M.; Amspoker, A. B.; Naik, A. D.] Baylor Coll Med, Dept Med, Houston, TX 77030 USA.

[Amspoker, A. B.; Cully, J. A.; Naik, A. D.] Baylor Coll Med, Menninger Dept Psychiat & Behav Sci, Houston, TX 77030 USA.

[Cully, J. A.; Naik, A. D.] Univ Houston, VA South Cent Mental Illness Res Educ & Clin Ctr, Houston, TX USA.

[Ross, E. L.] Univ Houston, Dept Psychol, Houston, TX USA.

RP Robertson, SM (reprint author), Univ Houston, Michael E DeBakey Vet Affairs Med Ctr, Houston Hlth Serv Res &Development Ctr Excellence, Houston, TX 77004 USA.

EM smr1@bcm.tmc.edu

FU Agency for Healthcare Research and Quality; Centers for Research and

Education on Therapeutics [U18HS016093]; Doris Duke Charitable

Foundation; Veterans Affairs Office of Academic Affiliations; National

Institute of Aging [5K23AG027144]

FX This manuscript is supported with resources and the use of facilities at

the Houston Veterans Affairs Health Services Research and Development

Center of Excellence (HEP90-020) at the Michael E. DeBakey Veterans

Affairs Medical Center, Houston, TX. The parent study from which the

data for the current analysis were derived was funded by the Agency for

Healthcare Research and Quality, Centers for Research and Education on

Therapeutics (U18HS016093) and a Doris Duke Charitable Foundation

Clinical Scientist Development Award (Naik). SMR is a Veterans Affairs

Health Services Research post-doc fellow supported by Veterans Affairs

Office of Academic Affiliations. ADN received support from a National

Institute of Aging Career Development Award (5K23AG027144).

CR Ali S, 2006, DIABETIC MED, V23, P1165, DOI 10.1111/j.1464-5491.2006.01943.x

Bandura A., 1994, ENCY HUMAN BEHAV, V4, P71, DOI DOI 10.1002/9780470479216.C0RPSY0836

Cherrington A, 2010, J BEHAV MED, V33, P81, DOI 10.1007/s10865-009-9233-4

Cohen J., 2003, APPL MULTIPLE REGRES

DEYO RA, 1992, J CLIN EPIDEMIOL, V45, P613, DOI 10.1016/0895-4356(92)90133-8

Fisher L, 2010, DIABETES CARE, V33, P1034, DOI 10.2337/dc09-2175

GARMEZY N, 1984, CHILD DEV, V55, P97, DOI 10.2307/1129837

Huang CJ, 2011, GEN HOSP PSYCHIAT, V33, P8, DOI 10.1016/j.genhosppsych.2010.10.008

Kapson HS, 2010, BEHAV THER, V41, P447, DOI 10.1016/j.beth.2009.10.001

Katon WJ, 2010, NEW ENGL J MED, V363, P2611, DOI 10.1056/NEJMoa1003955

Krichbaum K, 2003, DIABETES ED, V29, P82

LORIG K, 2001, PATIENT ED PRACTICAL

LOVIBOND PF, 1995, BEHAV RES THER, V33, P335, DOI 10.1016/0005-7967(94)00075-U

Marks R, 2005, HEALTH PROMOT PRACT, V6, P148, DOI 10.1177/1524839904266792

Mishali M, 2011, FAM PRACT, V28, P82, DOI 10.1093/fampra/cmq086

Naik AD, 2011, ARCH INTERN MED, V171, P453, DOI 10.1001/archinternmed.2011.70

Nelson KM, 2007, J GEN INTERN MED, V22, P442, DOI 10.1007/s11606-006-0053-8

PEYROT M, 1985, PSYCHOSOM MED, V47, P542, DOI 10.1097/00006842-198511000-00004

Peyrot M, 1999, J HEALTH SOC BEHAV, V40, P141, DOI 10.2307/2676370

POLONSKY WH, 1995, DIABETES CARE, V18, P754, DOI 10.2337/diacare.18.6.754

Polonsky WH, 2005, DIABETES CARE, V28, P626, DOI 10.2337/diacare.28.3.626

Pouwer F, 2010, DISCOV MED, V9, P112

Riazi A, 2004, DIABETES RES CLIN PR, V66, P237, DOI 10.1016/j.diabres.2004.04.001

Robertson SM, 2012, PSYCHOSOMATICS, V53, P1, DOI 10.1016/j.psym.2011.09.008

Rose V, 2009, PATIENT EDUC COUNS, V77, P260, DOI 10.1016/j.pec.2009.03.026

Sacco WP, 2010, DIABETES RES CLIN PR, V90, P141, DOI 10.1016/j.diabres.2010.06.026

Schneider S, 2010, J BEHAV MED, V33, P1, DOI 10.1007/s10865-009-9227-2

NR 27

TC 10

Z9 10

U1 2

U2 13

PU WILEY-BLACKWELL

PI HOBOKEN

PA 111 RIVER ST, HOBOKEN 07030-5774, NJ USA

SN 0742-3071

EI 1464-5491

J9 DIABETIC MED

JI Diabetic Med.

PD MAY

PY 2013

VL 30

IS 5

BP E189

EP E196

DI 10.1111/dme.12146

PG 8

WC Endocrinology & Metabolism

SC Endocrinology & Metabolism

GA 129SQ

UT WOS:000317863000006

PM 23350920

OA Green Accepted

DA 2019-01-08

ER

PT J

AU Forlani, G

Nuccitelli, C

Caselli, C

Moscatiello, S

Mazzotti, A

Centis, E

Marchesini, G

AF Forlani, Gabriele

Nuccitelli, Chiara

Caselli, Chiara

Moscatiello, Simona

Mazzotti, Arianna

Centis, Elena

Marchesini, Giulio

TI A psychological support program for individuals with Type 1 diabetes

SO ACTA DIABETOLOGICA

LA English

DT Article

DE Anxiety; Depression; Locus of control; Quality of life; Type 1 diabetes;

Well-being

ID QUALITY-OF-LIFE; DEPRESSIVE SYMPTOMS; COMORBID DEPRESSION;

FOOT-SYNDROME; GROUP CARE; PREVALENCE; ADULTS; ANXIETY; LOCUS; MELLITUS

AB We aimed to test the effects of a psychological support program on the psychological distress, mood, and quality of life of well-educated individuals with type 1 diabetes. A newly developed support program was offered to 60 patients with type 1 diabetes on intensive insulin treatment, previously enrolled in group-care educational programs. Thirty-three subjects participated (experimental group, in groups of 8-12 subjects); 22, who postponed their entry, were used as controls. The program consisted of 7 weekly work sessions of 2 hours chaired by a psychologist and covered aspects of daily living with diabetes using role-playing, metaplan, and problem solving. At baseline and approximately 6 months later, all participants completed a battery of questionnaires, and the differences between the experimental and the control group were analyzed by repeated-measurements ANOVA. In response to the psychological support program, subjects in the experimental group reduced their depressive mood (Beck Depression Inventory and depression scales of the Psychological Well-Being Index) and anxiety (Self-rating Anxiety Scale), improved disease-specific quality of life (Symptom and Well-Being scales of the Well-Being Enquiry for Diabetes), increased their internal and decreased their external locus of control. These changes were accompanied by a 0.3% decrease in glycosylated hemoglobin, whereas no significant changes were observed in the control group (ANOVA, P = 0.032). These results underline the importance of psychological aspects in individuals with type 1 diabetes; treating the psychological aspects related to the disease may be as important as medical control in order to improve living with diabetes.

C1 [Forlani, Gabriele; Nuccitelli, Chiara; Caselli, Chiara; Moscatiello, Simona; Mazzotti, Arianna; Centis, Elena; Marchesini, Giulio] Alma Mater Studiorum Univ, Unit Metab Dis & Clin Dietet, S Orsola Malpighi Hosp, I-40138 Bologna, Italy.

RP Marchesini, G (reprint author), Alma Mater Studiorum Univ, Unit Metab Dis & Clin Dietet, S Orsola Malpighi Hosp, Via Massarenti 9, I-40138 Bologna, Italy.

EM giulio.marchesini@unibo.it

OI Marchesini, Giulio/0000-0003-2407-9860

FU Novo Nordisk

FX The psychological support program described in this article received the

Italian DAWN award 2010 "Premio Roberto Sivieri", supported by Novo

Nordisk.

CR Amsberg S, 2009, PATIENT EDUC COUNS, V77, P72, DOI 10.1016/j.pec.2009.01.015

Anderson JA, 2003, COMMUNITY MENT HLT J, V39, P63, DOI 10.1023/A:1021225907821

Anderson RJ, 2001, DIABETES CARE, V24, P1069, DOI 10.2337/diacare.24.6.1069

BAGGIO A, 1997, INT J PAIN THERAPY, V7, P4

Beck A, 1978, DEPRESSION INVENTORY

BECK AT, 1961, ARCH GEN PSYCHIAT, V4, P561

Collins MM, 2009, DIABETIC MED, V26, P153, DOI 10.1111/j.1464-5491.2008.02648.x

Conti L., 2000, REPERTORIO SCALE VAL

Dupuy HJ., 1984, ASSESSMENT QUALITY L, P170

Eiber R, 1997, ENCEPHALE, V23, P351

El Achhab Y, 2008, DIABETES RES CLIN PR, V80, P171, DOI 10.1016/j.diabres.2007.12.020

Forlani G, 2006, J ENDOCRINOL INVEST, V29, P405, DOI 10.1007/BF03344123

Gendelman N, 2009, DIABETES CARE, V32, P575, DOI 10.2337/dc08-1835

GROSSI E, 2002, QUESTIONARIO PSYCHOL

Hart HE, 2003, QUAL LIFE RES, V12, P1089, DOI 10.1023/A:1026197119569

Hart HE, 2007, INDIAN J MED RES, V125, P203

Hislop AL, 2008, DIABETIC MED, V25, P91, DOI 10.1111/j.1464-5491.2007.02310.x

Li CY, 2008, DIABETES CARE, V31, P105, DOI 10.2337/dc07-1154

Lin EHB, 2010, DIABETES CARE, V33, P264, DOI 10.2337/dc09-1068

Lloyd CE, 2010, DIABETIC MED, V27, P234, DOI 10.1111/j.1464-5491.2009.02896.x

Lloyd CE, 2000, DIABETIC MED, V17, P198, DOI 10.1046/j.1464-5491.2000.00260.x

Lustman PJ, 1997, PSYCHOSOM MED, V59, P24, DOI 10.1097/00006842-199701000-00004

LUSTMAN PJ, 1992, DIABETES CARE, V15, P1631, DOI 10.2337/diacare.15.11.1631

Lustman PJ, 2002, PRACTICAL PSYCHOL DI, P219

Manini R, 2007, NUTR METAB CARDIOVAS, V17, P493, DOI 10.1016/j.numecd.2006.07.001

Mannucci E, 1996, DIABETES NUTR METAB, V9, P89

PEYROT M, 1994, DIABETES CARE, V17, P994, DOI 10.2337/diacare.17.9.994

Peyrot M, 2002, PRACTICAL PSYCHOL DI, P211

Pouwer F, 2010, DIABETIC MED, V27, P217, DOI 10.1111/j.1464-5491.2009.02903.x

Shaban C, 2009, DIABETES RES CLIN PR, V85, pe26

Shaban MC, 2006, DIABETIC MED, V23, P1381, DOI 10.1111/j.1464-5491.2006.02012.x

Shaw BF, 1985, HDB DEPRESSION TREAT, P372

Simson U, 2008, DIABETIC MED, V25, P206, DOI 10.1111/j.1464-5491.2007.02370.x

Simson U, 2008, PSYCHOTHER PSYCH MED, V58, P44, DOI 10.1055/s-2007-971001

Trento M, 2008, DIABETIC MED, V25, P86, DOI 10.1111/j.1464-5491.2007.02319.x

Trento M, 2006, DIABETES METAB, V32, P77, DOI 10.1016/S1262-3636(07)70250-1

WALLSTON BS, 1978, HEALTH EDUC QUART, V6, P107, DOI 10.1177/109019817800600107

Wild D, 2007, PATIENT EDUC COUNS, V68, P10, DOI 10.1016/j.pec.2007.05.003

Zhang XP, 2007, MED CARE, V45, P820, DOI 10.1097/MLR.0b013e3180618b55

ZUNG WWK, 1971, PSYCHOSOMATICS, V12, P371

NR 40

TC 4

Z9 4

U1 0

U2 3

PU SPRINGER

PI NEW YORK

PA 233 SPRING ST, NEW YORK, NY 10013 USA

SN 0940-5429

J9 ACTA DIABETOL

JI Acta Diabetol.

PD APR

PY 2013

VL 50

IS 2

BP 209

EP 216

DI 10.1007/s00592-011-0269-x

PG 8

WC Endocrinology & Metabolism

SC Endocrinology & Metabolism

GA 135LA

UT WOS:000318288400015

PM 21365339

DA 2019-01-08

ER

PT J

AU Jaremka, LM

Lindgren, ME

Kiecolt-Glaser, JK

AF Jaremka, Lisa M.

Lindgren, Monica E.

Kiecolt-Glaser, Janice K.

TI SYNERGISTIC RELATIONSHIPS AMONG STRESS, DEPRESSION, AND TROUBLED

RELATIONSHIPS: INSIGHTS FROM PSYCHONEUROIMMUNOLOGY

SO DEPRESSION AND ANXIETY

LA English

DT Review

DE biological markers; depression; cognition; life events; stress;

interpersonal relationships; psychoneuroimmunology; antidepressants

ID C-REACTIVE PROTEIN; CORONARY-HEART-DISEASE; RANDOMIZED CONTROLLED-TRIAL;

GINGIVAL CREVICULAR FLUID; ACUTE MENTAL STRESS; SUPPLEMENTATION LOWERS

INFLAMMATION; SOCIOECONOMIC-STATUS; PSYCHOLOGICAL STRESS; OLDER-ADULTS;

CARDIOVASCULAR-DISEASE

AB Stress and depression consistently elevate inflammation and are often experienced simultaneously, which is exemplified by people in troubled relationships. Troubled relationships also elevate inflammation, which may be partially explained by their ability to engender high levels of stress and depression. People who are stressed, depressed, or in troubled relationships are also at greater risk for health problems than their less distressed counterparts. Inflammation, a risk factor for a variety of age-related diseases including cardiovascular disease, Type II diabetes, metabolic syndrome, and frailty, may be one key mechanistic pathway linking distress to poor health. Obesity may further broaden the health implications of stress and depression; people who are stressed or depressed are often overweight, and adipose tissue is a major source of proinflammatory cytokines. Stress, depression, and troubled relationships may have synergistic inflammatory effects: loneliness, subclinical depression, and major depression enhance inflammatory responses to an acute stressful event. The relationship between distress and inflammation is bidirectional; depression enhances inflammation and inflammation promotes depression. Interesting questions emerge from this literature. For instance, some stressors may be more potent than others and thus may be more strongly linked to inflammation. In addition, it is possible that psychological and interpersonal resources may buffer the negative inflammatory effects of stress. Understanding the links among stress, depression, troubled relationships, and inflammation is an exciting area of research that may provide mechanistic insight into the links between distress and poor health.

C1 [Jaremka, Lisa M.; Lindgren, Monica E.; Kiecolt-Glaser, Janice K.] Ohio State Univ, Inst Behav Med Res, Coll Med, Columbus, OH 43210 USA.

[Kiecolt-Glaser, Janice K.] Ohio State Univ, Coll Med, Dept Psychiat, Columbus, OH 43210 USA.

RP Jaremka, LM (reprint author), Ohio State Univ, Inst Behav Med Res, Coll Med, 460 Med Ctr Dr, Columbus, OH 43210 USA.

EM lisa.jaremka@osumc.edu

RI Kiecolt-Glaser, Janice/A-3236-2009

OI Kiecolt-Glaser, Janice/0000-0003-4900-9578

FU American Cancer Society Postdoctoral Fellowship

[121911-PF-12-040-01-CPPB]; Ohio State University Comprehensive Cancer

Center Pelotonia Postdoctoral Fellowship; National Cancer Institute

Research Supplement to Promote Diversity in Health-Related Research

[CA126857]; NIH [CA126857, CA131029, CA154054, AG029562]

FX Contract grant sponsor: American Cancer Society Postdoctoral Fellowship;

Contract grant number: 121911-PF-12-040-01-CPPB; Contract grant sponsor:

Ohio State University Comprehensive Cancer Center Pelotonia Postdoctoral

Fellowship; Contract grant sponsor: National Cancer Institute Research

Supplement to Promote Diversity in Health-Related Research; Contract

grant number: CA126857; Contract grant sponsor: NIH; Contract grant

numbers: CA131029, CA126857, CA154054, and AG029562.

CR Aggarwal BB, 2006, BIOCHEM PHARMACOL, V72, P1605, DOI 10.1016/j.bcp.2006.06.029

Alley DE, 2006, BRAIN BEHAV IMMUN, V20, P498, DOI 10.1016/j.bbi.2005.10.003

Altemus M, 2001, J INVEST DERMATOL, V117, P309, DOI 10.1046/j.1523-1747.2001.01373.x

Appelhans BM, 2012, J ACAD NUTR DIET, V112, P693, DOI 10.1016/j.jand.2012.02.006

Appleton KM, 2010, AM J CLIN NUTR, V91, P757, DOI 10.3945/ajcn.2009.28313

Baum A, 1999, ANN NY ACAD SCI, V896, P131, DOI 10.1111/j.1749-6632.1999.tb08111.x

BAUMEISTER RF, 1995, PSYCHOL BULL, V117, P497, DOI 10.1037/0033-2909.117.3.497

Beach SRH, 1998, CLIN PSYCHOL REV, V18, P635, DOI 10.1016/S0272-7358(98)00023-3

Benoit D, 2001, LIFE SCI, V68, P1641, DOI 10.1016/S0024-3205(01)00963-8

Bierhaus A, 2003, P NATL ACAD SCI USA, V100, P1920, DOI 10.1073/pnas.0438019100

Bjorntorp P, 2001, Obes Rev, V2, P73, DOI 10.1046/j.1467-789x.2001.00027.x

BODNAR JC, 1994, PSYCHOL AGING, V9, P372

Bremmer MA, 2008, J AFFECT DISORDERS, V106, P249, DOI 10.1016/j.jad.2007.07.002

Brydon L, 2005, BRAIN BEHAV IMMUN, V19, P540, DOI 10.1016/j.bbi.2004.12.003

Buske-Kirschbaum A, 2007, BRAIN BEHAV IMMUN, V21, P92, DOI 10.1016/j.bbi.2006.03.006

Cacioppo JT, 2010, PSYCHOL AGING, V25, P453, DOI 10.1037/a0017216

Capuron L, 2004, BIOL PSYCHIAT, V56, P819, DOI 10.1016/j.biopsych.2004.02.009

Capuron L, 2002, NEUROPSYCHOPHARMACOL, V26, P643, DOI 10.1016/S0893-133X(01)00407-9

Capuron L, 2009, J AFFECT DISORDERS, V119, P181, DOI 10.1016/j.jad.2009.02.017

Carlson LE, 2003, PSYCHOSOM MED, V65, P571, DOI 10.1097/01.PSY.0000074003.35911.41

Carroll JE, 2011, BRAIN BEHAV IMMUN, V25, P232, DOI 10.1016/j.bbi.2010.09.024

Chandola T, 2008, EUR HEART J, V29, P640, DOI 10.1093/eurheartj/ehm584

COHEN S, 1983, J APPL SOC PSYCHOL, V13, P99, DOI 10.1111/j.1559-1816.1983.tb02325.x

COHEN S, 1991, NEW ENGL J MED, V325, P606, DOI 10.1056/NEJM199108293250903

Cohen S, 2006, PSYCHOSOM MED, V68, P414, DOI 10.1097/01.psy.0000221236.37158.b9

Cole SW, 2007, GENOME BIOL, V8, DOI 10.1186/gb-2007-8-9-r189

Corren J, 2011, CURR OPIN ALLERGY CL, V11, P565, DOI 10.1097/ACI.0b013e32834c3d30

Creswell JD, 2012, BRAIN BEHAV IMMUN, V26, P1095, DOI 10.1016/j.bbi.2012.07.006

Damjanovic AK, 2007, J IMMUNOL, V179, P4249, DOI 10.4049/jimmunol.179.6.4249

Danner M, 2003, PSYCHOSOM MED, V65, P347, DOI 10.1097/01.PSY.0000041542.29808.01

Dantzer R, 2008, NAT REV NEUROSCI, V9, P46, DOI 10.1038/nrn2297

Deinzer R, 2000, J CLIN PERIODONTOL, V27, P74, DOI 10.1034/j.1600-051x.2000.027001074.x

Deinzer R, 1999, J CLIN PERIODONTOL, V26, P1, DOI 10.1034/j.1600-051X.1999.260101.x

DellaGioia N, 2010, NEUROSCI BIOBEHAV R, V34, P130, DOI 10.1016/j.neubiorev.2009.07.014

Dentino AN, 1999, J AM GERIATR SOC, V47, P6, DOI 10.1111/j.1532-5415.1999.tb01894.x

DOBBIN JP, 1991, BRAIN BEHAV IMMUN, V5, P339, DOI 10.1016/0889-1591(91)90029-A

Dowlati Y, 2010, BIOL PSYCHIAT, V67, P446, DOI 10.1016/j.biopsych.2009.09.033

Eisenberger NI, 2010, BRAIN BEHAV IMMUN, V24, P558, DOI 10.1016/j.bbi.2009.12.009

Ershler WB, 2000, ANNU REV MED, V51, P245, DOI 10.1146/annurev.med.51.1.245

Fagundes CP, BRAIN BEHAV IN PRESS

FITZGERALD JE, 1995, ORAL MICROBIOL IMMUN, V10, P297, DOI 10.1111/j.1399-302X.1995.tb00158.x

Ford MB, 2010, J PERS SOC PSYCHOL, V98, P405, DOI 10.1037/a0017345

Foster PS, 1996, J EXP MED, V183, P195, DOI 10.1084/jem.183.1.195

Frasure-Smith N, 2007, BIOL PSYCHIAT, V62, P302, DOI 10.1016/j.biopsych.2006.09.029

Giannopoulou C, 2003, J CLIN PERIODONTOL, V30, P145, DOI 10.1034/j.1600-051X.2003.300201.x

Glaser R, 2003, ARCH GEN PSYCHIAT, V60, P1009, DOI 10.1001/archpsyc.60.10.1009

Gleeson M, 2011, NAT REV IMMUNOL, V11, P607, DOI 10.1038/nri3041

Goebel MU, 2000, PSYCHOSOM MED, V62, P591, DOI 10.1097/00006842-200007000-00019

Gruenewald TL, 2009, SOC SCI MED, V69, P451, DOI 10.1016/j.socscimed.2009.05.018

Guh DP, 2009, BMC PUBLIC HEALTH, V9, DOI 10.1186/1471-2458-9-88

Hackett RA, 2012, PSYCHONEUROENDOCRINO, V37, P1801, DOI 10.1016/j.psyneuen.2012.03.016

Hammen C, 2005, ANNU REV CLIN PSYCHO, V1, P293, DOI 10.1146/annurev.clinpsy.1.102803.143938

Hansson GK, 2005, NEW ENGL J MED, V352, P1685, DOI 10.1056/NEJMra043430

Harris TB, 1999, AM J MED, V106, P506, DOI 10.1016/S0002-9343(99)00066-2

Hawkley LC, 2010, ANN BEHAV MED, V40, P218, DOI 10.1007/s12160-010-9210-8

Heffner KL, 2004, J BEHAV MED, V27, P233, DOI 10.1023/B:JOBM.0000028497.79129.ad

Hemingway H, 2003, AM J CARDIOL, V92, P984, DOI 10.1016/S0002-9149(03)00985-8

Herring MP, 2012, ARCH INTERN MED, V172, P101, DOI 10.1001/archinternmed.2011.696

Hoffman BM, 2011, PSYCHOSOM MED, V73, P127, DOI 10.1097/PSY.0b013e31820433a5

Holt-Lunstad J, 2010, PLOS MED, V7, DOI 10.1371/journal.pmed.1000316

Hotamisligil GS, 2006, NATURE, V444, P860, DOI 10.1038/nature05485

Howren MB, 2009, PSYCHOSOM MED, V71, P171, DOI 10.1097/PSY.0b013e3181907c1b

Hugo FN, 2006, J PERIODONTOL, V77, P1008, DOI 10.1902/jop.2006.050037

Irwin MR, 2008, BRAIN BEHAV IMMUN, V22, P129, DOI 10.1016/j.bbi.2007.07.013

Irwin MR, 2007, BRAIN BEHAV IMMUN, V21, P374, DOI 10.1016/j.bbi.2007.01.010

Jaremka LM, PSYCHOL SCI IN PRESS

Jehn CF, 2006, CANCER, V107, P2723, DOI 10.1002/cncr22294

Johannsen A, 2007, J PERIODONTAL RES, V42, P546, DOI 10.1111/j.1600-0765.2007.00980.x

Johannsen A, 2006, J PERIODONTOL, V77, P1403, DOI 10.1902/jop.2006.050411

Jones SA, 2001, FASEB J, V15, P43, DOI 10.1096/fj.99-1003rev

Jousilahti P, 2003, J EPIDEMIOL COMMUN H, V57, P730, DOI 10.1136/jech.57.9.730

Joynt KE, 2003, BIOL PSYCHIAT, V54, P248, DOI 10.1016/S0006-3223(03)00568-7

Kaptoge S, 2010, LANCET, V375, P132, DOI 10.1016/S0140-6736(09)61717-7

Kiecolt-Glaser JK, 2012, BRAIN BEHAV IMMUN, V26, P988, DOI 10.1016/j.bbi.2012.05.011

Kiecolt-Glaser JK, 2011, BRAIN BEHAV IMMUN, V25, P1725, DOI 10.1016/j.bbi.2011.07.229

Kiecolt-Glaser JK, 2010, NEUROSCI BIOBEHAV R, V35, P33, DOI 10.1016/j.neubiorev.2009.09.003

Kiecolt-Glaser JK, 2010, PSYCHOSOM MED, V72, P365, DOI 10.1097/PSY.0b013e3181dbf489

Kiecolt-Glaser JK, 2010, PSYCHOSOM MED, V72, P113, DOI 10.1097/PSY.0b013e3181cb9377

Kiecolt-Glaser JK, 2005, ARCH GEN PSYCHIAT, V62, P1377, DOI 10.1001/archpsyc.62.12.1377

Kiecolt-Glaser JK, 2001, PSYCHOL BULL, V127, P472, DOI 10.1037//0033-2909.127.4.472

Kiecolt-Glaser JK, 2003, P NATL ACAD SCI USA, V100, P9090, DOI 10.1073/pnas.1531903100

Kiecolt-Glaser JK, 2002, J PSYCHOSOM RES, V53, P873, DOI 10.1016/S0022-3999(02)00309-4

Kiecolt-Glaser JK, 2002, ANNU REV PSYCHOL, V53, P83, DOI 10.1146/annurev.psych.53.100901.135217

Kiecolt-Glaser JK, 2001, CURR DIR PSYCHOL SCI, V10, P18, DOI 10.1111/1467-8721.00105

KIECOLTGLASER JK, 1991, PSYCHOSOM MED, V53, P345, DOI 10.1097/00006842-199107000-00001

Kling MA, 2007, BIOL PSYCHIAT, V62, P309, DOI 10.1016/j.biopsych.2006.09.033

Koster A, 2006, J GERONTOL A-BIOL, V61, P284, DOI 10.1093/gerona/61.3.284

Kuper H, 2003, J EPIDEMIOL COMMUN H, V57, P147, DOI 10.1136/jech.57.2.147

Lanquillon S, 2000, NEUROPSYCHOPHARMACOL, V22, P370, DOI 10.1016/S0893-133X(99)00134-7

Lavie CJ, 2009, J AM COLL CARDIOL, V53, P1925, DOI 10.1016/j.jacc.2008.12.068

LeResche L, 2002, PERIODONTOL 2000, V30, P91, DOI 10.1034/j.1600-0757.2002.03009.x

Lin PY, 2007, J CLIN PSYCHIAT, V68, P1056, DOI 10.4088/JCP.v68n0712

Loucks EB, 2006, AM J EPIDEMIOL, V163, P622, DOI 10.1093/aje/kwj076

Luppino FS, 2010, ARCH GEN PSYCHIAT, V67, P220, DOI 10.1001/archgenpsychiatry.2010.2

Lutgendorf SK, 1999, J GERONTOL A-BIOL, V54, pM434, DOI 10.1093/gerona/54.9.M434

Maes M, 2001, PSYCHOL MED, V31, P847, DOI 10.1017/S0033291701004007

Maes M, 1998, CYTOKINE, V10, P313, DOI 10.1006/cyto.1997.0290

MARKUS HR, 1991, PSYCHOL REV, V98, P224, DOI 10.1037/0033-295X.98.2.224

McDade TW, 2006, PSYCHOSOM MED, V68, P376, DOI 10.1097/01.psy.0000221371.43607.64

McNutt MD, 2012, NEUROPSYCHOPHARMACOL, V37, P1444, DOI 10.1038/npp.2011.330

Michelson D, 1996, NEW ENGL J MED, V335, P1176, DOI 10.1056/NEJM199610173351602

Miller AH, 2010, BRAIN BEHAV IMMUN, V24, P1, DOI 10.1016/j.bbi.2009.09.009

Miller AH, 2009, BIOL PSYCHIAT, V65, P732, DOI 10.1016/j.biopsych.2008.11.029

Miller GE, 2005, AM J CARDIOL, V95, P317, DOI 10.1016/j.amjcard.2004.09.026

Musselman DL, 2001, NEW ENGL J MED, V344, P961, DOI 10.1056/NEJM200103293441303

Musselman DL, 2001, AM J PSYCHIAT, V158, P1252, DOI 10.1176/appi.ajp.158.8.1252

O'Brien SM, 2006, BRIT J PSYCHIAT, V188, P449, DOI 10.1192/bjp.bp.105.011015

Orth-Gomer K, 2000, JAMA-J AM MED ASSOC, V284, P3008, DOI 10.1001/jama.284.23.3008

Owen N, 2003, BRAIN BEHAV IMMUN, V17, P286, DOI 10.1016/S0889-1591(03)00058-8

Ozbay Fatih, 2008, Curr Psychiatry Rep, V10, P304, DOI 10.1007/s11920-008-0049-7

Pace TWW, 2009, PSYCHONEUROENDOCRINO, V34, P87, DOI 10.1016/j.psyneuen.2008.08.011

Paik IH, 2000, BEHAV MED, V26, P139, DOI 10.1080/08964280009595761

Pan A, 2012, DIABETES CARE, V35, P1171, DOI 10.2337/dc11-2055

Penninx BWJH, 2003, BIOL PSYCHIAT, V54, P566, DOI 10.1016/S0006-3223(03)01811-5

Pollitt RA, 2008, J EPIDEMIOL COMMUN H, V62, P484, DOI 10.1136/jech.2006.054106

Pratt LA, 1996, CIRCULATION, V94, P3123, DOI 10.1161/01.CIR.94.12.3123

Ranjit N, 2007, CIRCULATION, V116, P2383, DOI 10.1161/CIRCULATIONAHA.107.706226

Rector NA, 1997, PERS INDIV DIFFER, V23, P799, DOI 10.1016/S0191-8869(97)00095-0

Reichenberg A, 2001, ARCH GEN PSYCHIAT, V58, P445, DOI 10.1001/archpsyc.58.5.445

Ridker PM, 2000, NEW ENGL J MED, V342, P836, DOI 10.1056/NEJM200003233421202

Schulz R, 2000, ARCH INTERN MED, V160, P1761, DOI 10.1001/archinte.160.12.1761

Shimbo D, 2006, HEART, V92, P1316, DOI 10.1136/hrt.2005.075861

Song C, 1999, PSYCHIAT RES, V85, P293, DOI 10.1016/S0165-1781(99)00012-8

Steptoe A, 1996, J PSYCHOSOM RES, V41, P171, DOI 10.1016/0022-3999(96)00095-5

Steptoe A, 2002, BRAIN BEHAV IMMUN, V16, P774, DOI 10.1016/S0889-1591(02)00030-2

Steptoe A, 2001, CLIN SCI, V101, P185, DOI 10.1042/CS20010038

Stewart JC, 2009, BRAIN BEHAV IMMUN, V23, P936, DOI 10.1016/j.bbi.2009.04.011

Strike PC, 2004, J PSYCHOSOM RES, V57, P189, DOI 10.1016/S0022-3999(03)00569-5

Theorell T, 1998, AM J PUBLIC HEALTH, V88, P382, DOI 10.2105/AJPH.88.3.382

Tiemeier H, 2003, EPIDEMIOLOGY, V14, P103, DOI 10.1097/00001648-200301000-00025

Tilg H, 2006, NAT REV IMMUNOL, V6, P772, DOI 10.1038/nri1937

Trayhurn P, 2004, BRIT J NUTR, V92, P347, DOI 10.1079/BJN20041213

Tyring S, 2006, LANCET, V367, P29, DOI 10.1016/S0140-6736(05)67763-X

Uchino BN, 1996, PSYCHOL BULL, V119, P488, DOI 10.1037/0033-2909.119.3.488

Vaccarino V, 2007, J AM COLL CARDIOL, V50, P2044, DOI 10.1016/j.jacc.2007.07.069

Vitaliano PP, 2005, PSYCHOSOM MED, V67, P930, DOI 10.1097/01.psy.0000188485.65153.7b

Vitaliano PP, 2002, PSYCHOSOM MED, V64, P418, DOI 10.1097/00006842-200205000-00006

Vogelzangs N, 2008, ARCH GEN PSYCHIAT, V65, P1386, DOI 10.1001/archpsyc.65.12.1386

von Kanel R, 2006, J AM GERIATR SOC, V54, P431, DOI 10.1111/j.1532-5415.2005.00642.x

von Kanel R, 2006, BRAIN BEHAV IMMUN, V20, P40, DOI 10.1016/j.bbi.2005.03.013

Weik U, 2008, PSYCHOSOM MED, V70, P906, DOI 10.1097/PSY.0b013e3181835bf3

Whisman MA, 2003, TREATING DIFFICULT COUPLES: HELPING CLIENTS WITH COEXISTING MENTAL AND RELATIONSHIP DISORDERS, P3

Whisman MA, 2010, HEALTH PSYCHOL, V29, P550, DOI 10.1037/a0020760

Wirtz PH, 2007, BRAIN BEHAV IMMUN, V21, P851, DOI 10.1016/j.bbi.2007.02.003

World Health Organisation, 2012, FACT SHEET OB OV

Wright CE, 2005, BRAIN BEHAV IMMUN, V19, P345, DOI 10.1016/j.bbi.2004.10.003

NR 146

TC 46

Z9 48

U1 3

U2 73

PU WILEY

PI HOBOKEN

PA 111 RIVER ST, HOBOKEN 07030-5774, NJ USA

SN 1091-4269

EI 1520-6394

J9 DEPRESS ANXIETY

JI Depress. Anxiety

PD APR

PY 2013

VL 30

IS 4

BP 288

EP 296

DI 10.1002/da.22078

PG 9

WC Psychology, Clinical; Psychiatry; Psychology

SC Psychology; Psychiatry

GA 126GJ

UT WOS:000317600800002

PM 23412999

OA Green Accepted

DA 2019-01-08

ER

PT J

AU Willaing, I

Rogvi, SA

Bogelund, M

Almdal, T

Schiotz, M

AF Willaing, I.

Rogvi, S. A.

Bogelund, M.

Almdal, T.

Schiotz, M.

TI Recall of HbA(1c) and self-management behaviours, patient activation,

perception of care and diabetes distress in Type2 diabetes

SO DIABETIC MEDICINE

LA English

DT Article

ID DANISH VERSION; SHORT-FORM; ADAPTATION; A1C

AB Aims The aim of this study was to investigate associations between HbA1c recall by patients with Type2 diabetes and self-management behaviours, patient activation, perception of care and diabetes distress. Methods In a cross-sectional survey, 993 patients with Type2 diabetes reported their latest HbA1c. Validated scales measured self-management behaviours, patient activation, perception of care and diabetes distress. Self-reported HbA1c was compared with latest measured HbA1c. Using logistic regressions, associations between HbA1c recall and the variables of interest were investigated. Results Sixty-four per cent of patients recalled their latest HbA1c. Poor HbA1c recall was significantly associated with unhealthy eating behaviour, low level of exercise, low level of patient activation, poor glycaemic control, not knowing target HbA1c level, low educational level and being female. There were no associations between recall of HbA1c and diabetes distress or perceived care. Conclusions Recall of HbA1c level may increase patient activation and health-promoting behaviours. However, low educational level may be a barrier for using HbA1c as a motivator for health-promoting behaviours. This study indicates a need for studies of causal relationships, as well as mechanisms and challenges in using patient awareness of HbA1c as a driver for health-promoting behaviours.

C1 [Willaing, I.; Rogvi, S. A.; Schiotz, M.] Steno Diabet Ctr, Steno Hlth Promot Ctr, DK-2820 Gentofte, Denmark.

[Bogelund, M.] Incent Partners, Holte, Denmark.

[Almdal, T.] Steno Diabet Ctr, Steno Patient Care Clin, DK-2820 Gentofte, Denmark.

RP Willaing, I (reprint author), Steno Diabet Ctr, Steno Hlth Promot Ctr, DK-2820 Gentofte, Denmark.

EM iwtp@steno.dk

OI Willaing, Ingrid/0000-0002-3082-8816

CR Beard E, 2010, PATIENT EDUC COUNS, V80, P227, DOI 10.1016/j.pec.2009.11.008

BLAND JM, 1995, BRIT MED J, V310, P170, DOI 10.1136/bmj.310.6973.170

Gugiu PC, 2009, CHRONIC ILLN, V5, P268, DOI 10.1177/1742395309348072

Guirguis LM, 2007, J AM PHARM ASSOC, V47, P29, DOI 10.1331/1544-3191.47.1.29.Guirguis

Harwell TS, 2002, DIABETES EDUCATOR, V28, P99, DOI 10.1177/014572170202800111

Heisler M, 2005, DIABETES CARE, V28, P816, DOI 10.2337/diacare.28.4.816

Hibbard JH, 2005, HEALTH SERV RES, V40, P1918, DOI 10.1111/j.1475-6773.2005.00438.x

Hibbard JH, 2007, HEALTH SERV RES, V42, P1443, DOI 10.1111/j.1475-6773.2006.00669.x

*IDF CLIN GUID TAS, 2005, GLOB GUID TYP 2 DIAB

Kumpatla Satyavani, 2010, Indian J Community Med, V35, P290, DOI 10.4103/0970-0218.66858

Maindal HT, 2012, EUR J PUBLIC HEALTH, V22, P31, DOI 10.1093/eurpub/ckq188

Maindal HT, 2009, BMC PUBLIC HEALTH, V9, DOI 10.1186/1471-2458-9-209

McGuire BE, 2009, DIABETOLOGIA, V53, P66

Mosen DM, 2007, J AMBUL CARE MANAG, V30, P21

Schiotz ML, 2012, DIABETIC MED, V29, P654, DOI 10.1111/j.1464-5491.2011.03485.x

Toobert DJ, 2000, DIABETES CARE, V23, P943, DOI 10.2337/diacare.23.7.943

Williams G., 2004, HDB DIABETES

NR 17

TC 13

Z9 13

U1 1

U2 15

PU WILEY-BLACKWELL

PI HOBOKEN

PA 111 RIVER ST, HOBOKEN 07030-5774, NJ USA

SN 0742-3071

J9 DIABETIC MED

JI Diabetic Med.

PD APR

PY 2013

VL 30

IS 4

BP e139

EP e142

DI 10.1111/dme.12121

PG 4

WC Endocrinology & Metabolism

SC Endocrinology & Metabolism

GA 110SG

UT WOS:000316465400003

PM 23331258

DA 2019-01-08

ER

PT J

AU Hermanns, N

Caputo, S

Dzida, G

Khunti, K

Meneghini, LF

Snoek, F

AF Hermanns, Norbert

Caputo, Salvatore

Dzida, Grzegorz

Khunti, Kamlesh

Meneghini, Luigi F.

Snoek, Frank

TI Screening, evaluation and management of depression in people with

diabetes in primary care

SO PRIMARY CARE DIABETES

LA English

DT Review

DE Diabetes mellitus; Depression; Diabetes distress; Screening; Primary

care; Cognitive behavioral therapy

ID PLACEBO-CONTROLLED TRIAL; CO-MORBID DEPRESSION; QUALITY-OF-LIFE;

RANDOMIZED CONTROLLED-TRIAL; MAJOR DEPRESSION; GLYCEMIC CONTROL;

SELF-CARE; COMORBID DEPRESSION; DOUBLE-BLIND; RISK-FACTOR

AB Family physicians are responsible for diagnosing and treating the majority of people with type 2 diabetes mellitus and co-morbid depression. As a result of the impact of co-morbid depression on patient self-care and treatment outcomes, screening for depression in the context of a structured approach to case management and patient follow up is recommended in people with diabetes and cardiovascular disease. This review summarizes the need for improved recognition and treatment of depression in diabetes; and makes expert recommendations with regard to integrating screening tools and therapies into a busy family or general medical practice setting. (C) 2012 Primary Care Diabetes Europe. Published by Elsevier Ltd. All rights reserved.

C1 [Hermanns, Norbert] FIDAM GmbH, Forschungsinst Diabet Akad Bad Mergentheim, Diabet Zentrum Mergentheim, D-97980 Bad Mergentheim, Germany.

[Caputo, Salvatore] Univ Cattolica Sacro Cuore, Policlin Gemelli, Serv Diabetol, I-00168 Rome, Italy.

[Dzida, Grzegorz] Med Univ Lublin, Dept Internal Dis, Lublin, Poland.

[Khunti, Kamlesh] Univ Leicester, Dept Hlth Sci, Leicester, Leics, England.

[Meneghini, Luigi F.] Univ Miami, Miller Sch Med, Miami, FL 33136 USA.

[Snoek, Frank] Vrije Univ Amsterdam, Med Ctr, Dept Med Psychol, Diabet Psychol Res Grp, Amsterdam, Netherlands.

RP Hermanns, N (reprint author), FIDAM GmbH, Forschungsinst Diabet Akad Bad Mergentheim, Diabet Zentrum Mergentheim, Theodor Klotzbucher Str 12, D-97980 Bad Mergentheim, Germany.

EM hermanns@diabetes-zentrum.de

OI Meneghini, Luigi/0000-0003-4539-2725; Khunti,

Kamlesh/0000-0003-2343-7099

FU Eli Lilly; Merck; MSD; Novo Nordisk; Sanofi Aventis; Takeda; Bioton;

Servier; NIHR CLAHRC; Boehringer-Ingelheim; Mannkind; Pfizer; Astra

Zeneca; Diabetes Research Foundation; Lifescan

FX SC has consulted for MSD and Novo Nordisk, and has received lecture fees

from Eli Lilly, Merck, MSD, Novo Nordisk, Sanofi Aventis and Takeda. GD

consults for Lifescan and Novo Nordisk; and has received-lecture fees

from Bioton, Eli Lilly, Novo Nordisk, Sanofi Aventis and Servier. NH

consults for Eli Lilly and Novo Nordisk; and has received lecture fees

and grants/grants pending with Berlin Chemie and Eli Lilly. KK has

received grants to his institution from NIHR CLAHRC, consults for

Bristol Myers Squibb, Eli Lilly, MSD, Novo Nordisk and Roche, and has

received lecture fees from Eli Lilly, MSD, Novartis, Novo Nordisk and

Sanofi Aventis. LFM consults for Novo Nordisk and Sanofi Aventis, and

has received grants of has grants pending from Boehringer-Ingelheim,

Mannkind, and Pfizer. FS consults for Eli Lilly, Novo Nordisk and Roche;

has grants/grants pending with Astra Zeneca, the Diabetes Research

Foundation, Lifescan and Novo Nordisk; and has received lecture fees

from Eli Lilly, Lifescan, Novartis and Novo Nordisk.

CR Ali S, 2006, DIABETIC MED, V23, P1165, DOI 10.1111/j.1464-5491.2006.01943.x

Ali S, 2010, DIABETES-METAB RES, V26, P75, DOI 10.1002/dmrr.1065

Anderson RJ, 2010, DIABETES CARE, V33, P485, DOI 10.2337/dc09-1466

[Anonymous], 2005, BRIT J GEN PRACTICE, V55, P665

Aujla N, 2010, DIABETIC MED, V27, P896, DOI 10.1111/j.1464-5491.2010.03042.x

Barnard KD, 2006, DIABETIC MED, V23, P445, DOI 10.1111/j.1464-5491.2006.01814.x

Bryan C, 2010, GEN HOSP PSYCHIAT, V32, P33, DOI 10.1016/j.genhosppsych.2009.07.009

Carnethon MR, 2003, AM J EPIDEMIOL, V158, P416, DOI 10.1093/aje/kwg172

Ciechanowski PS, 2000, ARCH INTERN MED, V160, P3278, DOI 10.1001/archinte.160.21.3278

Ciechanowski PS, 2003, GEN HOSP PSYCHIAT, V25, P246, DOI 10.1016/S0163-8343(03)00055-0

Croxford A.M., 2010, J UNDERGRADUATE RES, V3

Cuijpers P, 2004, ACTA PSYCHIAT SCAND, V109, P325, DOI 10.1111/j.1600-0447.2004.00301.x

Daly JM, 2009, J AM BOARD FAM MED, V22, P280, DOI 10.3122/jabfm.2009.03.080114

Davies MJ, 2008, BRIT MED J, V336, P491, DOI 10.1136/bmj.39474.922025.BE

de G.M., 2001, PSYCHOSOM MED, V63, P619

de J.P., 2009, PSYCHOSOMATICS, V50, P227

Delahanty LM, 2007, DIABETIC MED, V24, P48, DOI 10.1111/j.1464-5491.2007.02028.x

Demakakos P, 2010, DIABETES CARE, V33, P792, DOI 10.2337/dc09-1663

DiMatteo MR, 2000, ARCH INTERN MED, V160, P2101, DOI 10.1001/archinte.160.14.2101

Dirmaier J, 2010, PSYCHOTHER PSYCHOSOM, V79, P172, DOI 10.1159/000296135

Echeverry D, 2009, DIABETES CARE, V32, P2156, DOI 10.2337/dc09-0785

Egede LE, 2005, DIABETES CARE, V28, P1339, DOI 10.2337/diacare.28.6.1339

Egede LE, 2004, DIABETES CARE, V27, P1751, DOI 10.2337/diacare.27.7.1751

Egede LE, 2003, DIABETES CARE, V26, P104, DOI 10.2337/diacare.26.1.104

Egede LE, 2002, DIABETES CARE, V25, P464, DOI 10.2337/diacare.25.3.464

Egede LE, 2008, DIABETES TECHNOL THE, V10, P213, DOI 10.1089/dia.2007.0278

Fisher L, 2007, DIABETES CARE, V30, P542, DOI 10.2337/dc06-1614

Fisher L, 2010, DIABETES CARE, V33, P1034, DOI 10.2337/dc09-2175

Gallo JJ, 2005, AM J GERIAT PSYCHIAT, V13, P748, DOI 10.1176/appi.ajgp.13.9.748

Gazmararian JA, 2009, DIABETES EDUCATOR, V35, P778, DOI 10.1177/0145721709338527

Gill JM, 2008, INT J PSYCHIAT MED, V38, P203, DOI 10.2190/PM.38.2.g

Goldberg D, 2001, BRIT MED J, V323, P948

Golden SH, 2004, DIABETES CARE, V27, P429, DOI 10.2337/diacare.27.2.429

Golden SH, 2008, JAMA-J AM MED ASSOC, V299, P2751, DOI 10.1001/jama.299.23.2751

Goldney RD, 2004, DIABETES CARE, V27, P1066, DOI 10.2337/diacare.27.5.1066

Gonzalez JS, 2007, DIABETES CARE, V30, P2222, DOI 10.2337/dc07-0158

Gulseren L, 2005, ARCH MED RES, V36, P159, DOI 10.1016/j.arcmed.2004.12.018

Hermanns N, 2006, DIABETOLOGIA, V49, P469, DOI 10.1007/s00125-005-0094-2

Hermanns N, 2005, DIABETIC MED, V22, P293, DOI 10.1111/j.1464-5491.2005.01414.x

Jacobson AM, 1997, QUAL LIFE RES, V6, P11

Judd LL, 1997, J AFFECT DISORDERS, V45, P5, DOI 10.1016/S0165-0327(97)00055-4

Kalsekar ID, 2006, ANN PHARMACOTHER, V40, P605, DOI 10.1345/aph.1G606

Katon W, 2004, DIABETES CARE, V27, P914, DOI 10.2337/diacare.27.4.914

Katon W, 2002, J PSYCHOSOM RES, V53, P859, DOI 10.1016/S0022-3999(02)00313-6

Katon W, 2008, J GEN INTERN MED, V23, P1571, DOI 10.1007/s11606-008-0731-9

Katon W, 2009, PSYCHOSOMATICS, V50, P570, DOI 10.1176/appi.psy.50.6.570

Katon WJ, 2010, NEW ENGL J MED, V363, P2611, DOI 10.1056/NEJMoa1003955

Katon WJ, 2005, DIABETES CARE, V28, P2668, DOI 10.2337/diacare.28.11.2668

Katon WJ, 2004, MED CARE, V42, P1222, DOI 10.1097/00005650-200412000-00009

Khunti K, 2000, J ROY SOC MED, V93, P183, DOI 10.1177/014107680009300407

Knol MJ, 2006, DIABETOLOGIA, V49, P837, DOI 10.1007/s00125-006-0159-x

Li CY, 2009, DIABETES RES CLIN PR, V83, P268, DOI 10.1016/j.diabres.2008.11.006

Lin EHB, 2004, DIABETES CARE, V27, P2154, DOI 10.2337/diacare.27.9.2154

Lin EHB, 2009, ANN FAM MED, V7, P414, DOI 10.1370/afm.998

Lustman PJ, 2006, ARCH GEN PSYCHIAT, V63, P521, DOI 10.1001/archpsyc.63.5.521

Lustman PJ, 1997, PSYCHOSOM MED, V59, P241, DOI 10.1097/00006842-199705000-00007

Lustman PJ, 2000, DIABETES CARE, V23, P934, DOI 10.2337/diacare.23.7.934

Lustman PJ, 2000, DIABETES CARE, V23, P618, DOI 10.2337/diacare.23.5.618

Lustman PJ, 1998, ANN INTERN MED, V129, P613, DOI 10.7326/0003-4819-129-8-199810150-00005

McHale M, 2008, PSYCHOSOM MED, V70, P869, DOI 10.1097/PSY.0b013e318186dea9

Mezuk B, 2008, DIABETES CARE, V31, P2383, DOI 10.2337/dc08-0985

Munoz RF, 2010, ANNU REV CLIN PSYCHO, V6, P181, DOI 10.1146/annurev-clinpsy-033109-132040

Paile-Hyvarinen Maria, 2003, BMC Fam Pract, V4, P7, DOI 10.1186/1471-2296-4-7

Pan A, 2010, ARCH INTERN MED, V170, P1884, DOI 10.1001/archinternmed.2010.356

Simon GE, 2005, GEN HOSP PSYCHIAT, V27, P344, DOI 10.1016/j.genhosppsych.2005.04.008

Snoek FJ, 2012, DIABETES CARE, V35, P2128, DOI 10.2337/dc11-1326

Subramanian Deepak N, 2008, Qual Prim Care, V16, P341

Thomas J, 2003, DIABETES CARE, V26, P2311, DOI 10.2337/diacare.26.8.2311

Thoolen BJ, 2006, DIABETES CARE, V29, P2257, DOI 10.2337/dc06-0617

Vamos EP, 2009, PSYCHOSOM MED, V71, P501, DOI 10.1097/PSY.0b013e3181a5a7ad

van Bastelaar KM, 2008, BMC PSYCHIATRY, V8, DOI 10.1186/1471-244X-8-9

Van der Feltz-Cornelis CM, 2010, GEN HOSP PSYCHIAT, V32, P380, DOI 10.1016/j.genhosppsych.2010.03.011

Vileikyte L, 2005, DIABETES CARE, V28, P2378, DOI 10.2337/diacare.28.10.2378

Zhang XP, 2005, AM J EPIDEMIOL, V161, P652, DOI 10.1093/aje/kwi089

NR 74

TC 39

Z9 40

U1 1

U2 38

PU ELSEVIER SCI LTD

PI OXFORD

PA THE BOULEVARD, LANGFORD LANE, KIDLINGTON, OXFORD OX5 1GB, OXON, ENGLAND

SN 1751-9918

J9 PRIM CARE DIABETES

JI Prim. Care Diabetes

PD APR

PY 2013

VL 7

IS 1

BP 1

EP 10

DI 10.1016/j.pcd.2012.11.002

PG 10

WC Endocrinology & Metabolism; Primary Health Care

SC Endocrinology & Metabolism; General & Internal Medicine

GA 113DR

UT WOS:000316647200001

PM 23280258

OA Other Gold

DA 2019-01-08

ER

PT J

AU van Son, J

Nyklicek, I

Pop, VJ

Blonk, MC

Erdtsieck, RJ

Spooren, PF

Toorians, AW

Pouwer, F

AF van Son, Jenny

Nyklicek, Ivan

Pop, Victor J.

Blonk, Marion C.

Erdtsieck, Ronald J.

Spooren, Pieter F.

Toorians, Arno W.

Pouwer, Francois

TI The Effects of a Mindfulness-Based Intervention on Emotional Distress,

Quality of Life, and HbA(1c) in Outpatients With Diabetes (DiaMind)

SO DIABETES CARE

LA English

DT Article

ID STRESS-REDUCTION INTERVENTION; RANDOMIZED CONTROLLED-TRIALS; IMPROVE

GLYCEMIC CONTROL; PSYCHOLOGICAL INTERVENTIONS; COGNITIVE THERAPY;

DEPRESSION SCALE; HOSPITAL ANXIETY; ADULTS; METAANALYSIS; PREVALENCE

AB OBJECTIVE-Emotional distress is common in outpatients with diabetes, affecting; similar to 20-40% of the patients. The aim of this study was to determine the effectiveness of group therapy with Mindfulness-Based Cognitive Therapy (MBCT), relative to usual care, for patients with diabetes with regard to reducing emotional distress and improving health-related quality of life and glycemic control.

RESEARCH DESIGN AND METHODS-In the present randomized controlled trial, 139 outpatients with diabetes (type 1 or type 2) and low levels of emotional well-being were randomized to MBCT (n = 70) or a waiting list group (n = 69). Primary outcomes were perceived stress (Perceived Stress Scale), anxiety and depressive symptoms (Hospital Anxiety and Depression Scale), mood (Profiles of Mood States), and diabetes-specific distress (Problem Areas In Diabetes). Secondary outcomes were health-related quality of life (12-Item Short-Form Health Survey), and glycemic control (HbA(1c)). Assessments were conducted at baseline and at 4 and 8 weeks of follow-up.

RESULTS-Compared with control, MBCT was more effective in reducing stress (P < 0.001, Cohen d = 0.70), depressive symptoms (P = 0.006, d = 0.59), and anxiety (P = 0.019, d = 0.44). In addition, MBCT was more effective in improving quality of life (mental: P = 0.003, d = 0.55; physical: P = 0.032, d = 0.40). We found no significant effect on HbA1c or diabetes-specific distress, although patients with elevated diabetes distress in the MBCT group tended to show a decrease in diabetes distress (P = 0.07, d = 0.70) compared with the control group.

CONCLUSIONS-Compared with usual care, MBCT resulted in a reduction of emotional distress and an increase in health-related quality of life in diabetic patients who had lower levels of emotional well-being. Diabetes Care 36:823-830, 2013

C1 [van Son, Jenny; Nyklicek, Ivan; Pop, Victor J.; Pouwer, Francois] Tilburg Univ, Dept Med & Clin Psychol, Ctr Res Psychol Somat Dis, NL-5000 LE Tilburg, Netherlands.

[Blonk, Marion C.] Catharina Hosp, Dept Internal Med, Eindhoven, Netherlands.

[Erdtsieck, Ronald J.] Maxima Med Ctr, Dept Internal Med, Eindhoven, Netherlands.

[Spooren, Pieter F.] TweeSteden Hosp, Dept Internal Med, Tilburg, Netherlands.

[Toorians, Arno W.] St Anna Hosp, Dept Internal Med, Geldrop, Netherlands.

RP Nyklicek, I (reprint author), Tilburg Univ, Dept Med & Clin Psychol, Ctr Res Psychol Somat Dis, NL-5000 LE Tilburg, Netherlands.

EM i.nyklicek@tilburguniversity.edu

FU Dutch Diabetes Research Foundation or "Diabetesfonds" [2008.13.005];

Tilburg University, Center of Research on Psychology in Somatic diseases

FX The study is supported by grants from the Dutch Diabetes Research

Foundation or "Diabetesfonds" (project number 2008.13.005, awarded to

I.N.) and Tilburg University, Center of Research on Psychology in

Somatic diseases.

CR Alam R, 2009, PATIENT EDUC COUNS, V75, P25, DOI 10.1016/j.pec.2008.08.026

Bishop SR, 2004, CLIN PSYCHOL-SCI PR, V11, P230, DOI 10.1093/clipsy/bph077

Bjelland I, 2002, J PSYCHOSOM RES, V52, P69, DOI 10.1016/S0022-3999(01)00296-3

Bogner HR, 2007, DIABETES CARE, V30, P3005, DOI 10.2337/dc07-0974

Bohlmeijer E, 2010, J PSYCHOSOM RES, V68, P539, DOI 10.1016/j.jpsychores.2009.10.005

Branstrom R, 2010, ANN BEHAV MED, V39, P151, DOI 10.1007/s12160-010-9168-6

COHEN S, 1988, CLAR SYMP, P31

Egede LE, 2005, DIABETES CARE, V28, P1339, DOI 10.2337/diacare.28.6.1339

Fisher L, 2008, DIABETIC MED, V25, P1096, DOI 10.1111/j.1464-5491.2008.02533.x

Fjorback LO, 2011, ACTA PSYCHIAT SCAND, V124, P102, DOI 10.1111/j.1600-0447.2011.01704.x

Foley E, 2010, J CONSULT CLIN PSYCH, V78, P72, DOI 10.1037/a0017566

Gonzalez JS, 2008, DIABETES CARE, V31, P2398, DOI 10.2337/dc08-1341

Grigsby AB, 2002, J PSYCHOSOM RES, V53, P1053, DOI 10.1016/S0022-3999(02)00417-8

Hartmann M, 2012, DIABETES CARE, V35, P945, DOI 10.2337/dc11-1343

Hermanns N, 2006, DIABETOLOGIA, V49, P469, DOI 10.1007/s00125-005-0094-2

Hofmann SG, 2010, J CONSULT CLIN PSYCH, V78, P169, DOI 10.1037/a0018555

Ismail K, 2004, LANCET, V363, P1589, DOI 10.1016/S0140-6736(04)16202-8

JACOBSON NS, 1991, J CONSULT CLIN PSYCH, V59, P12, DOI 10.1037//0022-006X.59.1.12

Kabat-Zinn J., 1990, FULL CATASTROPHE LIV

Lustman PJ, 2000, DIABETES CARE, V23, P934, DOI 10.2337/diacare.23.7.934

Makine C, 2009, DIABETIC MED, V26, P28, DOI 10.1111/j.1464-5491.2008.02606.x

Mols F, 2009, QUAL LIFE RES, V18, P403, DOI 10.1007/s11136-009-9455-5

Morone NE, 2008, PAIN, V134, P310, DOI 10.1016/j.pain.2007.04.038

Nyklicek I, 2008, ANN BEHAV MED, V35, P331, DOI 10.1007/s12160-008-9030-2

POLONSKY WH, 1995, DIABETES CARE, V18, P754, DOI 10.2337/diacare.18.6.754

Pouwer F, 2006, PATIENT EDUC COUNS, V60, P235, DOI 10.1016/j.pec.2005.01.009

Pouwer F, 2010, DIABETIC MED, V27, P217, DOI 10.1111/j.1464-5491.2009.02903.x

Pouwer F, 2009, NAT REV ENDOCRINOL, V5, P665, DOI 10.1038/nrendo.2009.214

Pradhan EK, 2007, ARTHRIT RHEUM-ARTHR, V57, P1134, DOI 10.1002/art.23010

Rosenzweig S, 2007, ALTERN THER HEALTH M, V13, P36

Schram MT, 2009, CURR DIABETES REV, V5, P112, DOI 10.2174/157339909788166828

Segal Z., 2002, MINDFULNESS BASED CO

Snoek FJ, 2000, DIABETES CARE, V23, P1305, DOI 10.2337/diacare.23.9.1305

van Bastelaar KMP, 2011, DIABETES CARE, V34, P320, DOI 10.2337/dc10-1248

Van der Feltz-Cornelis CM, 2010, GEN HOSP PSYCHIAT, V32, P380, DOI 10.1016/j.genhosppsych.2010.03.011

van Son J, 2011, BMC PUBLIC HEALTH, V11, DOI 10.1186/1471-2458-11-131

Wald F., 1990, NED T PSYCHOL, V45, P86

Wang MY, 2008, J CLIN NURS, V17, P2524, DOI 10.1111/j.1365-2702.2008.02301.x

Winkley K, 2006, BRIT MED J, V333, P65, DOI 10.1136/bmj.38874.652569.55

ZIGMOND AS, 1983, ACTA PSYCHIAT SCAND, V67, P361, DOI 10.1111/j.1600-0447.1983.tb09716.x

NR 40

TC 60

Z9 60

U1 2

U2 72

PU AMER DIABETES ASSOC

PI ALEXANDRIA

PA 1701 N BEAUREGARD ST, ALEXANDRIA, VA 22311-1717 USA

SN 0149-5992

J9 DIABETES CARE

JI Diabetes Care

PD APR

PY 2013

VL 36

IS 4

BP 823

EP 830

DI 10.2337/dc12-1477

PG 8

WC Endocrinology & Metabolism

SC Endocrinology & Metabolism

GA 110RG

UT WOS:000316462400025

PM 23193218

OA Green Published, Other Gold

DA 2019-01-08

ER

PT J

AU D'Anna, R

Scilipoti, A

Giordano, D

Caruso, C

Cannata, ML

Interdonato, ML

Corrado, F

Di Benedetto, A

AF D'Anna, Rosario

Scilipoti, Angela

Giordano, Domenico

Caruso, Carmela

Cannata, Maria Letizia

Interdonato, Maria Lieta

Corrado, Francesco

Di Benedetto, Antonino

TI myo-Inositol Supplementation and Onset of Gestational Diabetes Mellitus

in Pregnant Women With a Family History of Type 2 Diabetes A

prospective, randomized, placebo-controlled study

SO DIABETES CARE

LA English

DT Article

ID POLYCYSTIC-OVARY-SYNDROME; METFORMIN; INOSITOL

AB OBJECTIVE-To check the hypothesis that myo-inositol supplementation may reduce gestational diabetesmellitus (GDM) onset in pregnant women with a family history of type 2 diabetes.

RESEARCH DESIGN AND METHODS-A 2-year, prospective, randomized, open-label, placebo-controlled study was carried out in pregnant outpatients with a parent with type 2 diabetes who were treated from the end of the first trimester with 2 g myo-inositol plus 200 mu g folic acid twice a day (n = 110) and in the placebo group (n = 110), who were only treated with 200 mu g folic acid twice a day. The main outcome measure was the incidence of GDM in both groups. Secondary outcome measures were as follows: the incidence of fetal macrosomia (>4,000 g), gestational hypertension, preterm delivery, caesarean section, shoulder dystocia, neonatal hypoglycemia, and neonatal distress respiratory syndrome. GDM diagnosis was performed according to the International Association of the Diabetes and Pregnancy Study Groups (IADPSG) recommendations.

RESULTS-Incidence of GDM was significantly reduced in the myo-inositol group compared with the placebo group: 6 vs. 15.3%, respectively (P = 0.04). In the myo-inositol group, a reduction of GDM risk occurrence was highlighted (odds ratio 0.35). A statistically significant reduction of fetal macrosomia in the myo-inositol group was also highlighted together with a significant reduction in mean fetal weight at delivery. In the other secondary outcome measures, there were no differences between groups.

CONCLUSIONS-myo-Inositol supplementation in pregnant women with a family history of type 2 diabetes may reduce GDM incidence and the delivery of macrosomia fetuses. Diabetes Care 36:854-857, 2013

C1 [D'Anna, Rosario; Scilipoti, Angela; Giordano, Domenico; Caruso, Carmela; Cannata, Maria Letizia; Interdonato, Maria Lieta; Corrado, Francesco] Univ Messina, Dept Gynecol & Obstet Sci, Messina, Italy.

[Di Benedetto, Antonino] Univ Messina, Dept Internal Med, Messina, Italy.

RP D'Anna, R (reprint author), Univ Messina, Dept Gynecol & Obstet Sci, Messina, Italy.

EM rosariodanna@tin.it

RI Di Benedetto, Antonino/H-9703-2012

OI Di Benedetto, Antonino/0000-0003-4093-5655

CR American College of Obstetricians and Gynecologists Committee on Practice Bulletins--Obstetrics, 2001, Obstet Gynecol, V98, P525

Corrado F, 2011, DIABETIC MED, V28, P972, DOI 10.1111/j.1464-5491.2011.03284.x

D'Anna R, 2012, GYNECOL ENDOCRINOL, V28, P440, DOI 10.3109/09513590.2011.633665

Davey RX, 2001, MED J AUSTRALIA, V174, P118

Genazzani AD, 2008, GYNECOL ENDOCRINOL, V24, P139, DOI 10.1080/09513590801893232

Giordano D, 2011, MENOPAUSE, V18, P102, DOI 10.1097/gme.0b013e3181e8e1b1

Glueck CJ, 2008, FERTIL STERIL, V89, P625, DOI 10.1016/j.fertnstert.2007.03.036

Haffner SM, 1997, DIABETES CARE, V20, P1087, DOI 10.2337/diacare.20.7.1087

Jenum AK, 2012, EUR J ENDOCRINOL, V166, P317, DOI 10.1530/EJE-11-0866

Khattab S, 2011, GYNECOL ENDOCRINOL, V27, P789, DOI 10.3109/09513590.2010.540600

Metzger BE, 2008, NEW ENGL J MED, V358, P1991, DOI 10.1056/NEJMoa0707943

Metzger BE, 2010, DIABETES CARE, V33, P676, DOI 10.2337/dc09-1848

Nestler JE, 1999, NEW ENGL J MED, V340, P1314, DOI 10.1056/NEJM199904293401703

Santamaria A, 2012, CLIMACTERIC, V15, P490, DOI 10.3109/13697137.2011.631063

Scioscia M, 2007, DIABETIC MED, V24, P1300, DOI 10.1111/j.1464-5491.2007.02267.x

Vanky E, 2010, J CLIN ENDOCR METAB, V95, pE448, DOI 10.1210/jc.2010-0853

NR 16

TC 54

Z9 54

U1 0

U2 15

PU AMER DIABETES ASSOC

PI ALEXANDRIA

PA 1701 N BEAUREGARD ST, ALEXANDRIA, VA 22311-1717 USA

SN 0149-5992

J9 DIABETES CARE

JI Diabetes Care

PD APR

PY 2013

VL 36

IS 4

BP 854

EP 857

DI 10.2337/dc12-1371

PG 4

WC Endocrinology & Metabolism

SC Endocrinology & Metabolism

GA 110RG

UT WOS:000316462400029

PM 23340885

OA Green Published, Other Gold

DA 2019-01-08

ER

PT J

AU Law, GU

Walsh, J

Queralt, V

Nouwen, A

AF Law, G. Urquhart

Walsh, Jennifer

Queralt, Victoria

Nouwen, Arie

TI Adolescent and parent diabetes distress in type 1 diabetes: The role of

self-efficacy, perceived consequences, family responsibility and

adolescent-parent discrepancies

SO JOURNAL OF PSYCHOSOMATIC RESEARCH

LA English

DT Article

DE Adolescent; Distress; Parent; Responsibility; Self-efficacy; Type 1

diabetes

ID QUALITY-OF-LIFE; ILLNESS REPRESENTATIONS; PSYCHOLOGICAL DISTRESS;

METABOLIC-CONTROL; GLYCEMIC CONTROL; EMOTIONAL DISTRESS; YOUNG-ADULTS;

MANAGEMENT; CARE; CHILDREN

AB Objective: To examine the association of adolescent and parent diabetes distress with perceived consequences, dietary self-efficacy, and discrepancies in diabetes family responsibility, in type 1 diabetes (T1D).

Methods: 203 adolescents with T1D, aged 12-18, and their parents completed self-report questionnaires cross-sectionally.

Results: Higher HbA1c, greater perceived negative consequences of diabetes, and reduced self-efficacy predicted adolescent diabetes distress. Higher HbA1c predicted parental diabetes distress, as did diabetes family responsibility disagreements when both family members claimed responsibility, and parents' perception of reduced adolescent self-efficacy.

Conclusion: Dietary self-efficacy and perceived negative consequence's of diabetes are important factors to consider in assessing and managing adolescent diabetes distress. Perceptions of family responsibility for self-care tasks and parental confidence in adolescents' self-management have implications for parental diabetes distress. Clinical implications support long-held recommendations of taking a family-perspective of T1D care. (C) 2012 Elsevier Inc. All rights reserved.

C1 [Law, G. Urquhart; Walsh, Jennifer; Queralt, Victoria; Nouwen, Arie] Univ Birmingham, Birmingham B15 2TT, W Midlands, England.

RP Law, GU (reprint author), Univ Birmingham, Sch Psychol, Edgbaston B15 2TT, England.

EM g.u.law@bham.ac.uk

OI Walsh, Jennifer/0000-0002-7122-2650

CR Anderson BJ, 2009, FAM SYST HEALTH, V27, P141, DOI 10.1037/a0015759

ANDERSON BJ, 1990, J PEDIATR PSYCHOL, V15, P477, DOI 10.1093/jpepsy/15.4.477

Butler JM, 2009, J FAM PSYCHOL, V23, P611, DOI 10.1037/a0016141

Butner J, 2009, DEV PSYCHOL, V45, P835, DOI 10.1037/a0015363

Copp C, 1998, DIABETIC MED, V15, P119

Cunningham NR, 2011, J PEDIATR PSYCHOL, V36, P196, DOI 10.1093/jpepsy/jsq071

Dashiff C. J., 2003, J FAMILY NURSING, V9, P166, DOI DOI 10.1177/1074840703009002004

de Wit M, 2007, HEALTH QUAL LIFE OUT, V5, DOI 10.1186/1477-7525-5-10

Dekovic M, 1997, J YOUTH ADOLESCENCE, V26, P253, DOI 10.1007/s10964-005-0001-7

Delamater A, 2012, PEDIAT DIABETES, P1, DOI DOI 10.1111/11399-5448.2012.00894.X

Eckshtain D, 2010, J PEDIATR PSYCHOL, V35, P426, DOI 10.1093/jpepsy/jsp068

Edgar KA, 2003, J PEDIATR PSYCHOL, V28, P485, DOI 10.1093/jpepsy/jsg039

Eiser JR, 2001, PSYCHOL HEALTH, V16, P99, DOI 10.1080/08870440108405492

Graue M, 2003, ACTA PAEDIATR, V92, P1190, DOI 10.1080/08035250310005657

Grey M, 1998, DIABETES CARE, V21, P909, DOI 10.2337/diacare.21.6.909

Guo J, 2011, J ADV NURS, V67, P2294, DOI 10.1111/j.1365-2648.2011.05697.x

Haugstvedt A, 2011, PEDIATR DIABETES, V12, P107, DOI 10.1111/j.1399-5448.2010.00661.x

Hislop AL, 2008, DIABETIC MED, V25, P91, DOI 10.1111/j.1464-5491.2007.02310.x

Iannotti RJ, 2006, J DEV BEHAV PEDIATR, V27, P98, DOI 10.1097/00004703-200604000-00003

Ivey JB, 2009, J PEDIATR HEALTH CAR, V23, P10, DOI 10.1016/j.pedhc.2007.12.008

Johnston-Brooks CH, 2002, PSYCHOSOM MED, V64, P43, DOI 10.1097/00006842-200201000-00007

Law GU, 2002, J ADOLESCENT HEALTH, V31, P381, DOI 10.1016/S1054-139X(02)00397-X

Leventhal H, 1980, MED PSYCHOL, P7

Miller VA, 2003, J PEDIATR PSYCHOL, V28, P265, DOI 10.1093/jpepsy/jsg014

Modi AC, 2012, PEDIATRICS, V129, pE473, DOI 10.1542/peds.2011-1635

Nouwen A, 2009, PSYCHOL HEALTH, V24, P1071, DOI 10.1080/08870440802254597

Ott J, 2000, CHILD HEALTH CARE, V29, P47, DOI 10.1207/S15326888CHC2901_4

Patterson S, 2011, J MENT HEALTH, V20, P328, DOI 10.3109/09638237.2011.556163

POLONSKY WH, 1995, DIABETES CARE, V18, P754, DOI 10.2337/diacare.18.6.754

Pomerantz EM, 2001, DEV PSYCHOL, V37, P174, DOI 10.1037//0012-1649.37.2.174

Schilling Lynne S, 2006, J Pediatr Nurs, V21, P412, DOI 10.1016/j.pedn.2006.01.034

Senecal C, 2000, HEALTH PSYCHOL, V19, P452, DOI 10.1037//0278-6133.19.5.452

Silverstein J, 2005, DIABETES CARE, V28, P186, DOI 10.2337/diacare.28.1.186

Skinner TC, 2000, J PEDIATR PSYCHOL, V25, P257, DOI 10.1093/jpepsy/25.4.257

Streisand R, 2005, J PEDIATR PSYCHOL, V30, P513, DOI 10.1093/jpepsy/jsi076

Weissberg-Benchell J, 2011, PEDIATR DIABETES, V12, P341, DOI 10.1111/j.1399-5448.2010.00720.x

Wiebe DJ, 2005, J PEDIATR PSYCHOL, V30, P167, DOI 10.1093/jpepsy/jsi004

Williams LB, 2009, DIABETIC MED, V26, P908, DOI 10.1111/j.1464-5491.2009.02794.x

Wysocki T, 2008, BEHAV THER, V39, P33, DOI 10.1016/j.beth.2007.04.001

NR 39

TC 18

Z9 19

U1 2

U2 44

PU PERGAMON-ELSEVIER SCIENCE LTD

PI OXFORD

PA THE BOULEVARD, LANGFORD LANE, KIDLINGTON, OXFORD OX5 1GB, ENGLAND

SN 0022-3999

J9 J PSYCHOSOM RES

JI J. Psychosomat. Res.

PD APR

PY 2013

VL 74

IS 4

BP 334

EP 339

DI 10.1016/j.jpsychores.2012.12.009

PG 6

WC Psychiatry

SC Psychiatry

GA 112IT

UT WOS:000316586600011

PM 23497836

DA 2019-01-08

ER

PT J

AU Hendricks, M

Monaghan, M

Soutor, S

Chen, RS

Holmes, CS

AF Hendricks, Melissa

Monaghan, Maureen

Soutor, Sari

Chen, Rusan

Holmes, Clarissa S.

TI A Profile of Self-Care Behaviors in Emerging Adults With Type 1 Diabetes

SO DIABETES EDUCATOR

LA English

DT Article

ID QUALITY-OF-LIFE; GLYCEMIC CONTROL; YOUNG-ADULTS; PSYCHOLOGICAL

ADJUSTMENT; TREATMENT ADHERENCE; INSULIN-TREATMENT; HEALTH-STATUS;

CHILDREN; ADOLESCENTS; PREDICTORS

AB Purpose

The purpose of this study is to characterize daily diabetes self-care behaviors and to evaluate associations among self-care behaviors, psychosocial adjustment, and glycemic control in an understudied sample of emerging adults with type 1 diabetes.

Methods

Forty-nine emerging adults (65% women; ages 18-26 years) completed 2 diabetes interviews to assess self-care behaviors and self-report measures of psychosocial adjustment. Glycemic control was assessed via hemoglobin A1C.

Results

Diabetes self-care behaviors varied widely and were largely suboptimal; only a small percentage of participants demonstrated self-care behaviors consistent with national and international recommendations. Psychosocial adjustment was within normal limits and was unrelated to frequency of self-care behaviors in this sample. Mean glycemic control (8.3%) was higher than the recommended A1C level (< 7.0%) for this age group. Use of intensive (eg, multiple daily injections or pump) insulin regimens was related to better glycemic control.

Conclusions

The majority of emerging adults in this sample did not engage in optimal daily diabetes self-care. Intensive insulin therapy was associated with better glycemic control without corresponding psychosocial distress. Diabetes care behaviors could be improved in this age group, and emerging adults may benefit from targeted education and behavioral support to enhance diabetes self-management and optimize health outcomes.

C1 [Hendricks, Melissa; Soutor, Sari; Holmes, Clarissa S.] Virginia Commonwealth Univ, Richmond, VA USA.

[Monaghan, Maureen] Childrens Natl Med Ctr, Washington, DC 20010 USA.

[Chen, Rusan; Holmes, Clarissa S.] Georgetown Univ, Washington, DC USA.

RP Hendricks, M (reprint author), Kennedy Krieger Inst, Dept Behav Psychol, 707 North Broadway, Baltimore, MD 21205 USA.

EM hendricksm@kennedykrieger.org

FU NIH/NIDDK [5R01DK070917-04]

FX This work was supported by NIH/NIDDK grant 5R01DK070917-04 awarded to

principal investigator Clarissa S. Holmes.

CR *AM DIAB ASS, 2010, DIABETES CARE S1, V33, pS11, DOI DOI 10.2337/DC10-S011

*AM DIAB ASS, 2004, DIABETES CARE S1, V27, pS58, DOI DOI 10.2337/DIACARE.27.2007.S58

Anderson RJ, 2002, INT J PSYCHIAT MED, V32, P235, DOI 10.2190/KLGD-4H8D-4RYL-TWQ8

[Anonymous], 2002, ALCNOW DISP HEM ALC

Arnett JJ, 2000, AM PSYCHOL, V55, P469, DOI 10.1037/0003-066X.55.5.469

Bangstad HJ, 2009, PEDIATR DIABETES, V10, P82, DOI 10.1111/j.1399-5448.2009.00578.x

Barnard KD, 2007, DIABETIC MED, V24, P607, DOI 10.1111/j.1464-5491.2007.02120.x

Bryden KS, 2003, DIABETES CARE, V26, P1052, DOI 10.2337/diacare.26.4.1052

Bryden KS, 2001, DIABETES CARE, V24, P1536, DOI 10.2337/diacare.24.9.1536

Chisholm V, 2007, J ADV NURS, V57, P482, DOI 10.1111/j.1365-2648.2006.04137.x

Copeland WE, 2009, ARCH GEN PSYCHIAT, V66, P764, DOI 10.1001/archgenpsychiatry.2009.85

DCCT Res Grp, 1988, DIABETES CARE, V11, P725

DCCT Res Grp, 1994, J PEDIATR-US, V125, P177, DOI 10.1016/S0022-3476(94)70190-3

DEROGATIS LR, 1983, PSYCHOL MED, V13, P595, DOI 10.1017/S0033291700048017

Ebbeling CB, 2007, JAMA-J AM MED ASSOC, V297, P2092, DOI 10.1001/jama.297.19.2092

Ellis DA, 2005, PEDIATRICS, V116, pE826, DOI 10.1542/peds.2005-0638

Hendricks MC, 2008, DIABETES, V57, pA524

Hislop AL, 2008, DIABETIC MED, V25, P91, DOI 10.1111/j.1464-5491.2007.02310.x

Hollingshead AB, 1975, 4 FACTOR INDEX SOCIA

Holmes CS, 2006, J PEDIATR PSYCHOL, V31, P770, DOI 10.1093/jpepsy/jsj083

Ingerski LM, 2010, PEDIATR DIABETES, V11, P563, DOI 10.1111/j.1399-5448.2010.00645.x

Insabella G, 2007, PEDIATR DIABETES, V8, P228, DOI 10.1111/j.1399-5448.2007.00266.x

Jacobson A, 1994, HDB PSYCHOL DIABETES, P64

JACOBSON AM, 1994, DIABETES CARE, V17, P267, DOI 10.2337/diacare.17.4.267

Jacobson AM, 1997, DIABETES CARE, V20, P811, DOI 10.2337/diacare.20.5.811

Johns C, 2008, DIABETES EDUCATOR, V34, P874, DOI 10.1177/0145721708322857

JOHNSON SB, 1986, HEALTH PSYCHOL, V5, P545, DOI 10.1037/0278-6133.5.6.545

Liese AD, 2006, PEDIATRICS, V118, P1510, DOI 10.1542/peds.2006-0690

Luyckx K, 2009, DIABETES CARE, V32, P797, DOI 10.2337/dc08-1990

McNally K, 2010, DIABETES CARE, V33, P1159, DOI 10.2337/dc09-2116

Northam EA, 2010, DIABETES CARE, V33, P1430, DOI 10.2337/dc09-2232

Overby NC, 2008, PEDIATR DIABETES, V9, P393, DOI 10.1111/j.1399-5448.2008.00381.x

Peters A, 2011, DIABETES CARE, V34, P2477, DOI 10.2337/dc11-1723

Pettiti DB, 2009, J PEDIAT, V155, P668

Ramchandani N, 2000, DIABETES EDUCATOR, V26, P656, DOI 10.1177/014572170002600413

Rewers A, 2002, JAMA-J AM MED ASSOC, V287, P2511, DOI 10.1001/jama.287.19.2511

Rewers M, 2009, PEDIATR DIABETES, V10, P71, DOI 10.1111/j.1399-5448.2009.00582.x

Rohlfing CL, 2002, DIABETES CARE, V25, P275, DOI 10.2337/diacare.25.2.275

Smart C, 2009, PEDIATR DIABETES, V10, P100, DOI 10.1111/j.1399-5448.2009.00572.x

Streisand R, 2002, J PEDIATR PSYCHOL, V27, P759, DOI 10.1093/jpepsy/27.8.759

Weissberg-Benchell J, 2007, DIABETES CARE, V30, P2441, DOI 10.2337/dc07-1249

Wu YP, 2010, DIABETES RES CLIN PR, V89, P121, DOI 10.1016/j.diabres.2010.04.010

WYSOCKI T, 1992, J DEV BEHAV PEDIATR, V13, P194

NR 43

TC 13

Z9 13

U1 0

U2 16

PU SAGE PUBLICATIONS INC

PI THOUSAND OAKS

PA 2455 TELLER RD, THOUSAND OAKS, CA 91320 USA

SN 0145-7217

EI 1554-6063

J9 DIABETES EDUCATOR

JI Diabetes Educ.

PD MAR-APR

PY 2013

VL 39

IS 2

BP 195

EP 203

DI 10.1177/0145721713475840

PG 9

WC Endocrinology & Metabolism; Public, Environmental & Occupational Health

SC Endocrinology & Metabolism; Public, Environmental & Occupational Health

GA 148DJ

UT WOS:000319222800005

PM 23396184

OA Green Accepted

DA 2019-01-08

ER

PT J

AU Xaverius, PK

Salas, J

AF Xaverius, Pamela K.

Salas, Joanne

TI Surveillance of Preconception Health Indicators in Behavioral Risk

Factor Surveillance System: Emerging Trends in the 21st Century

SO JOURNAL OF WOMENS HEALTH

LA English

DT Article

ID PREVENTIVE SERVICES; DIABETES-MELLITUS; CARE; WOMEN; ADULTS; BRFSS

AB Objectives: This article assesses emerging trends in the 21st century, if any, in preconception health indicators among women of reproductive age.

Methods: This is a secondary analysis of cross-sectional data from the Behavioral Risk Factor Surveillance System (BRFSS), 2003-2010. Subjects were a sample of noninstitutionalized, 18-44-year-old, nonpregnant, women in the United States (n = 547,177) grouped into two categories, 2003-2006 (n = 275,630) and 2007-2010 (n = 271,547). Overall crude and adjusted prevalence odds ratios were calculated for preconception indicators before 2006 and after 2006.

Results: Significant improvements were found for any and heavy alcohol use, smoking, social and emotional support, moderate/vigorous physical activity, and having had an influenza shot in the last year. In contrast, binge drinking, having a medical condition (i.e., diabetes, high blood pressure, asthma, or obesity), and self-reported health significantly worsened. No change was found for mental distress, HIV testing, and having a routine checkup.

Conclusions: As the 21st century unfolds, emerging trends suggest that we need to focus on educating women, providers, and public health advocates about improved health before pregnancy, especially for women with chronic conditions and those who binge drink alcohol.

C1 [Xaverius, Pamela K.] St Louis Univ, Sch Publ Hlth, St Louis, MO 63104 USA.

[Salas, Joanne] St Louis Univ, Sch Med, St Louis, MO 63104 USA.

RP Xaverius, PK (reprint author), St Louis Univ, Sch Publ Hlth, 3545 Lafayette,Room 474, St Louis, MO 63104 USA.

EM Pxaverius@slu.edu

OI Xaverius, Pamela/0000-0001-8824-2933

CR Ahluwalia IB, 2005, J WOMENS HEALTH, V14, P382, DOI 10.1089/jwh.2005.14.382

*AM AC PED AM COLL, 2002, GUID PER CAR

American College of Obstetricians and Gynecologists. Preconception Care Work Group, 2005, J OBSTET GYNECOL, V106, P665

Atrash H, 2008, CURR OPIN OBSTET GYN, V20, P581, DOI 10.1097/GCO.0b013e328317a27c

BLOOMBERG L, 1994, HEALTH EDUC QUART, V21, P447, DOI 10.1177/109019819402100407

Centers for Disease Control and Prevention, 1993, MMWR-MORBID MORTAL W, V44, P219

Centers for Disease Control and Prevention, 2007, BEH RISK FACT SURV S

Council of State and Territorial Epidemiologists (CSTE), 2009, COR STAT PREC HLTH H

Curtis M, 2006, MATERN CHILD HLTH J, V10, P553

de Weerd S, 2002, OBSTET GYNECOL, V99, P45, DOI 10.1016/S0029-7844(01)01573-3

Ettner SL, 1996, AM J PUBLIC HEALTH, V86, P1748, DOI 10.2105/AJPH.86.12.1748

Guide to community preventive services, 2012, PROM PHYS ACT BEH SO

Hayes D. K., 2011, PREV CHRONIC DIS, V8, P1

Institute of Medicine Committee to Study the Prevention of Low Birth-weight (US), 1985, PREV LOW BIRTHW

Jack B, 2008, WOMENS HLTH ISSUES S, V18S, pS18

Johnson Kay, 2006, Morbidity and Mortality Weekly Report, V55, P1

Kent H, 2008, J WOMENS HLTH ISS S6, V18, P567

Kieffer EC, 2006, DIABETES CARE, V29, P1788, DOI 10.2337/dc06-0199

LeBlanc ES, 2011, ANN INTERN MED, V155, P434, DOI 10.7326/0003-4819-155-7-201110040-00006

Moos MK, 2010, J WOMENS HEALTH, V19, P561, DOI 10.1089/jwh.2009.1411

Nelson DE, 2001, SOZ PRAVENTIV MED, V46, pS3

O'Connor EA, 2009, ANN INTERN MED, V151, P793, DOI 10.7326/0003-4819-151-11-200912010-00007

Pignone MP, 2001, AHRQ PUBLICATION

Ranji UR, 2011, WOMENS HLTH CARE CHA

Ray JG, 2001, QJM-INT J MED, V94, P435, DOI 10.1093/qjmed/94.8.435

Strine TW, 2004, CHEST, V126, P1849, DOI 10.1378/chest.126.6.1849

*TASK FORC COMM PR, 2002, AM J PREV MED, V22, P67, DOI DOI 10.1016/S0749-3797(02)00433-6

Thompson BK, 2008, J WOMENS HEALTH, V17, P723, DOI 10.1089/jwh.2008.0870

U.S. Department of Health Education and Welfare (PHS) HAS/BCHS, 1979, PRIM CAR EFF APPR CL

*US DEP HHS, 1991, HLTH PEOPL 2000 NAT

US Department of Health and Human Services, HLTH PEOPL 2020

*US PREV SERV TASK, 2004, ANN INTERN MED, V140, P555

NR 32

TC 13

Z9 14

U1 0

U2 17

PU MARY ANN LIEBERT INC

PI NEW ROCHELLE

PA 140 HUGUENOT STREET, 3RD FL, NEW ROCHELLE, NY 10801 USA

SN 1540-9996

J9 J WOMENS HEALTH

JI J. Womens Health

PD MAR

PY 2013

VL 22

IS 3

BP 203

EP 209

DI 10.1089/jwh.2012.3804

PG 7

WC Public, Environmental & Occupational Health; Medicine, General &

Internal; Obstetrics & Gynecology; Women's Studies

SC Public, Environmental & Occupational Health; General & Internal

Medicine; Obstetrics & Gynecology; Women's Studies

GA 105IU

UT WOS:000316061700126

PM 23428283

DA 2019-01-08

ER

PT J

AU Munshi, MN

Segal, AR

Suhl, E

Ryan, C

Sternthal, A

Giusti, J

Lee, Y

Fitzgerald, S

Staum, E

Bonsignor, P

DesRochers, L

McCartney, R

Weinger, K

AF Munshi, Medha N.

Segal, Alissa R.

Suhl, Emmy

Ryan, Courtney

Sternthal, Adrianne

Giusti, Judy

Lee, Yishan

Fitzgerald, Shane

Staum, Elizabeth

Bonsignor, Patricia

DesRochers, Laura

McCartney, Richard

Weinger, Katie

TI Assessment of Barriers to Improve Diabetes Management in Older Adults A

randomized controlled study

SO DIABETES CARE

LA English

DT Article

ID DYSFUNCTION; MELLITUS; VALIDITY

AB OBJECTIVE-To evaluate whether assessment of barriers to self-care and strategies to cope with these barriers in older adults with diabetes is superior to usual care with attention control. The American Diabetes Association guidelines recommend the assessment of age-specific barriers. However, the effect of such strategy on outcomes is unknown.

RESEARCH DESIGN AND METHODS-We randomized 100 subjects aged >= 69 years with poorly controlled diabetes (A1C >8%) in two groups. A geriatric diabetes team assessed barriers and developed strategies to help patients cope with barriers for an intervention group. The control group received equal amounts of attention time. The active intervention was performed for the first 6 months, followed by a "no-contact" period. Outcome measures included A1C, Tinetti test, 6-min walk test (6MWT), self-care frequency, and diabetes-related distress.

RESULTS-We assessed 100 patients (age 75 +/- 5 years, duration 21 +/- 13 years, 68% type 2 diabetes, 89% on insulin) over 12 months. After the active period, A1C decreased by -0.45% in the intervention group vs. -0.31% in the control group. At 12 months, A1C decreased further in the intervention group by -0.21% vs. 0% in control group (linear mixed-model, P < 0.03). The intervention group showed additional benefits in scores on measures of self-care (Self-Care Inventory-R), gait and balance (Tinetti), and endurance (6MWT) compared with the control group. Diabetes-related distress improved in both groups.

CONCLUSIONS-Only attention between clinic visits lowers diabetes-related distress in older adults. However, communication with an educator cognizant of patients' barriers improves glycemic control and self-care frequency, maintains functionality, and lowers distress in this population. Diabetes Care 36:543-549, 2013

C1 [Munshi, Medha N.; Segal, Alissa R.; Suhl, Emmy; Ryan, Courtney; Sternthal, Adrianne; Giusti, Judy; Lee, Yishan; Fitzgerald, Shane; Staum, Elizabeth; Bonsignor, Patricia; DesRochers, Laura; McCartney, Richard; Weinger, Katie] Joslin Diabet Ctr, Boston, MA 02215 USA.

[Munshi, Medha N.] Beth Israel Deaconess Med Ctr, Boston, MA 02215 USA.

[Munshi, Medha N.; Weinger, Katie] Harvard Univ, Sch Med, Boston, MA USA.

[Segal, Alissa R.] Massachusetts Coll Pharm Hlth Sci, Boston, MA USA.

RP Munshi, MN (reprint author), Joslin Diabet Ctr, Boston, MA 02215 USA.

EM mmunshi@bidmc.harvard.edu

FU American Diabetes Association [1-07-CR-40]; U.S. Department of Defense

Peer Reviewed Medical Research Program of the Office of the

Congressionally Directed Medical Research Programs [W81XWH-07-1-0282];

sanofi-aventis

FX The study was partly supported by a grant from a clinical research award

from the American Diabetes Association, 1-07-CR-40 (M.N.M.), and partly

from the U.S. Department of Defense Peer Reviewed Medical Research

Program of the Office of the Congressionally Directed Medical Research

Programs, W81XWH-07-1-0282 (M.N.M.).; M.N.M. received research funding

from sanofi-aventis. No other potential conflicts of interest relevant

to this article were reported.

CR Abrahams S, 2000, NEUROPSYCHOLOGIA, V38, P734, DOI 10.1016/S0028-3932(99)00146-3

Anker SD, 2011, LANCET, V378, P731, DOI 10.1016/S0140-6736(11)61229-4

[Anonymous], 2012, DIABETES CARE, V35, pS11, DOI [10.2337/dc12-s011, 10.2337/dc35-S011]

Araki A, 2009, GERIATR GERONTOL INT, V9, P105, DOI 10.1111/j.1447-0594.2008.00495.x

Bautmans Ivan, 2004, BMC Geriatr, V4, P6, DOI 10.1186/1471-2318-4-6

Ciechanowski PS, 2000, ARCH INTERN MED, V160, P3278, DOI 10.1001/archinte.160.21.3278

Drane DL, 2002, NEUROPSY NEUROPSY BE, V15, P39

FILLENBAUM GG, 1981, J GERONTOL, V36, P428, DOI 10.1093/geronj/36.4.428

Gregg EW, 2000, DIABETES CARE, V23, P1272, DOI 10.2337/diacare.23.9.1272

Huang ES, 2005, J AM GERIATR SOC, V53, P306, DOI 10.1111/j.1532-5415.2005.53119.x

Huang ES, 2009, AGING HLTH, V5, P207

Ickowicz E, 2003, J AM GERIATR SOC, V51, pS265

KATZ S, 1963, JAMA-J AM MED ASSOC, V185, P914, DOI 10.1001/jama.1963.03060120024016

LAWTON MP, 1969, GERONTOLOGIST, V9, P179, DOI 10.1093/geront/9.3_Part_1.179

Montorio I, 1996, Int Psychogeriatr, V8, P103, DOI 10.1017/S1041610296002505

Munshi M, 2006, DIABETES CARE, V29, P1794, DOI 10.2337/dc06-0506

National Diabetes fact sheet, 2011, NAT DIAB FACT SHEET

Nishiwaki Y, 2004, AM J EPIDEMIOL, V160, P797, DOI 10.1093/aje/kwh288

POLONSKY WH, 1995, DIABETES CARE, V18, P754, DOI 10.2337/diacare.18.6.754

POSNER BM, 1993, AM J PUBLIC HEALTH, V83, P972, DOI 10.2105/AJPH.83.7.972

Shea S, 2009, J AM MED INFORM ASSN, V16, P446, DOI 10.1197/jamia.M3157

TINETTI ME, 1986, J AM GERIATR SOC, V34, P119, DOI 10.1111/j.1532-5415.1986.tb05480.x

Weinger K, 2005, DIABETES CARE, V28, P1346, DOI 10.2337/diacare.28.6.1346

NR 23

TC 21

Z9 21

U1 0

U2 22

PU AMER DIABETES ASSOC

PI ALEXANDRIA

PA 1701 N BEAUREGARD ST, ALEXANDRIA, VA 22311-1717 USA

SN 0149-5992

EI 1935-5548

J9 DIABETES CARE

JI Diabetes Care

PD MAR

PY 2013

VL 36

IS 3

BP 543

EP 549

DI 10.2337/dc12-1303

PG 7

WC Endocrinology & Metabolism

SC Endocrinology & Metabolism

GA 103OU

UT WOS:000315928700018

PM 23193208

OA Other Gold, Green Published

DA 2019-01-08

ER

PT J

AU Choi, SE

Reed, PL

AF Choi, Sarah E.

Reed, Preston L.

TI Contributors to Depressive Symptoms Among Korean Immigrants With Type 2

Diabetes

SO NURSING RESEARCH

LA English

DT Article

DE depressive symptoms; gender differences; Korean immigrants;

quality-of-life; type 2 diabetes

ID QUALITY-OF-LIFE; PSYCHOSOCIAL FACTORS; CHINESE PATIENTS; PREVALENCE;

HEALTH; ADULTS; ASSOCIATION; ADHERENCE; AMERICANS; DISTRESS

AB Background: Patients with diabetes have a higher prevalence of depression than the general population. Korean immigrants with type 2 diabetes are understudied.

Objectives: The aim of this study was to identify the prevalence and correlates of depressive symptoms in Korean immigrants.

Methods: In this cross-sectional descriptive study, a community sample of 164 Korean immigrant adults with type 2 diabetes were assessed for depressive symptoms using the Center for Epidemiological Studies Depression scale. Predictors of depression were grouped into three categories: demographic (age, gender, education, English proficiency), clinical (duration of diabetes, comorbidities, insulin use), and psychosocial (general health, diabetes-related quality of life [QOL], family support).

Results: Approximately 56% of participants had Center for Epidemiological Studies Depression Scale scores of >= 16. Higher levels of depression were associated with greater impact of diabetes on QOL (b = 5.68, p = .001), worse overall health (b = -0.09, p = .012), and less family support (b = 4.02, p = .042). The relationship between depression and diabetes impact on QOL was stronger for men than women (b = 6.67, p = .020).

Discussion: Depressive symptoms are common among Korean immigrants with type 2 diabetes. Assessing diabetes-related QOL, general health, and family support may be of value in better understanding depressive symptoms among this population. Among Korean immigrant men with type 2 diabetes, specific attention should be paid to diabetes-related QOL.

C1 [Choi, Sarah E.] Univ Calif Irvine, Program Nursing Sci, Coll Hlth Sci, Irvine, CA 92697 USA.

[Reed, Preston L.] Univ Calif Irvine, Dept Psychol & Social Behav, Irvine, CA 92697 USA.

RP Choi, SE (reprint author), Univ Calif Irvine, Program Nursing Sci, Coll Hlth Sci, 100B Berk Hall, Irvine, CA 92697 USA.

EM sechoi@uci.edu

FU National Center for Research Resources; National Center for Advancing

Translational Sciences at the National Institutes of Health [UL1

TR000153, KL2 TR000147]

FX This study was supported by a grant from the National Center for

Research Resources and by the National Center for Advancing

Translational Sciences at the National Institutes of Health through

Grants UL1 TR000153 and KL2 TR000147 (Dr. Choi).

CR Ali S, 2010, DIABETES-METAB RES, V26, P75, DOI 10.1002/dmrr.1065

Anderson RJ, 2000, DIABETES, V49, pA64

Anderson RJ, 2001, DIABETES CARE, V24, P1069, DOI 10.2337/diacare.24.6.1069

Barnes P.M., 2008, ADV DATA, V394, P1

Black SA, 2003, DIABETES CARE, V26, P2822, DOI 10.2337/diacare.26.10.2822

BRISLIN RW, 1970, J CROSS CULT PSYCHOL, V1, P185, DOI 10.1177/135910457000100301

Center for Disease Control and Prevention, 2011, NAT DIAB FACT SHEET

Cheng AY, 1999, DIABETES CARE, V22, P1216, DOI 10.2337/diacare.22.7.1216

Choi S, 2008, DIABETES EDUCATOR, V34, P484, DOI 10.1177/0145721708316949

Choi SE, 2011, J IMMIGR MINOR HEALT, V13, P803, DOI 10.1007/s10903-010-9397-6

Ciechanowski PS, 2000, ARCH INTERN MED, V160, P3278, DOI 10.1001/archinte.160.21.3278

Clark J. L. D., 1988, STUDIES 2 LANGUAGE A, V10, P129, DOI [10.1017/S0272263100007270, DOI 10.1017/S0272263100007270]

DCCT Res Grp, 1988, DIABETES CARE, V11, P725

Fisher L, 2008, DIABETIC MED, V25, P1096, DOI 10.1111/j.1464-5491.2008.02533.x

Fisher L, 2001, DIABETES CARE, V24, P1751, DOI 10.2337/diacare.24.10.1751

Fisher L, 2007, DIABETES CARE, V30, P542, DOI 10.2337/dc06-1614

GLASGOW RE, 1988, DIABETES CARE, V11, P377, DOI 10.2337/diacare.11.5.377

Gonzalez JS, 2007, DIABETES CARE, V30, P2222, DOI 10.2337/dc07-0158

HURH WM, 1990, J NERV MENT DIS, V178, P703, DOI 10.1097/00005053-199011000-00006

Katon W, 2004, DIABETES CARE, V27, P914, DOI 10.2337/diacare.27.4.914

Kim MT, 2009, DIABETES EDUCATOR, V35, P986, DOI 10.1177/0145721709345774

KUO WH, 1984, J NERV MENT DIS, V172, P449, DOI 10.1097/00005053-198408000-00002

Lange LJ, 2005, J PSYCHOSOM RES, V58, P129, DOI 10.1016/j.jpsychores.2004.08.004

Li CY, 2009, DIABETES RES CLIN PR, V83, P268, DOI 10.1016/j.diabres.2008.11.006

Misra R, 2009, J DIABETES COMPLICAT, V23, P54, DOI 10.1016/j.jdiacomp.2007.11.003

Nichols GA, 2003, DIABETES CARE, V26, P744, DOI 10.2337/diacare.26.3.744

RADLOFF L S, 1977, Applied Psychological Measurement, V1, P385, DOI 10.1177/014662167700100306

Shin HS, 2007, INT J NURS STUD, V44, P415, DOI 10.1016/j.ijnurstu.2006.04.007

WARE JE, 1992, MED CARE, V30, P473, DOI 10.1097/00005650-199206000-00002

Zhang CX, 2008, DIABETES RES CLIN PR, V79, P523, DOI 10.1016/j.diabres.2007.10.014

Zhao W, 2006, PUBLIC HEALTH, V120, P696, DOI 10.1016/j.puhe.2006.04.012

NR 31

TC 2

Z9 2

U1 0

U2 10

PU LIPPINCOTT WILLIAMS & WILKINS

PI PHILADELPHIA

PA 530 WALNUT ST, PHILADELPHIA, PA 19106-3621 USA

SN 0029-6562

EI 1538-9847

J9 NURS RES

JI Nurs. Res.

PD MAR-APR

PY 2013

VL 62

IS 2

BP 115

EP 121

DI 10.1097/NNR.0b013e31827aec29

PG 7

WC Nursing

SC Nursing

GA 103WX

UT WOS:000315951300344

PM 23190692

OA Green Accepted

DA 2019-01-08

ER

PT J

AU Aikens, JE

Piette, JD

AF Aikens, J. E.

Piette, J. D.

TI Longitudinal association between medication adherence and glycaemic

control in Type2 diabetes

SO DIABETIC MEDICINE

LA English

DT Article

ID GLUCOSE CONTROL; CARE; OUTCOMES; HEALTH; DEPRESSION; CONCURRENT;

METFORMIN; VALIDITY; PROGRAM; A1C

AB Aim Despite the widespread assumption that adherence drives glycaemic control, there is little published support for this in Type2 diabetes. The study objective was to determine whether self-reported medication adherence predicts future glycaemic control in Type2 diabetes, after accounting for baseline control. Methods Medication adherence (4-item Morisky scale), glycaemic control (HbA1c%), and other variables were assessed in 287 adult primary care patients prescribed oral medication (40% also on insulin) for Type2 diabetes. Glycaemic control was reassessed 6months later. Regression analyses examined concurrent and future glycaemic control as a function of baseline medication adherence after adjustment for baseline glycaemia and other potential confounders. Results Only half of patients reported high adherence. Cross-sectional adjusted analysis replicated prior reports of an adherence-HbA1c association (P=0.011). Even after adjusting for baseline HbA1c, each one-point increase in baseline Morisky total score was associated with a 1.8mmol/mol (or 0.16%) increase in HbA1c measured 6months later. Additionally, baseline endorsement of forgetting to take medication was associated with a 4.7mmol/mol (or 0.43%) increase in 6-month HbA1c (P=0.005). This effect persisted after adjusting for psychological distress and did not vary by key demographic and medical features. Conclusions Even after stringent adjustment for baseline glycaemic control, self-reported adherence to diabetes medication predicts long-term glycaemic control. The Morisky scale is an easy-to-use clinical tool to identify patients whose glycaemic control will subsequently worsen, regardless of age, gender and psychological distress.

C1 [Aikens, J. E.] Univ Michigan, Dept Family Med, Ann Arbor, MI 48109 USA.

[Piette, J. D.] Univ Michigan, Dept Internal Med, Ann Arbor VA Ctr Clin Management Res, Ann Arbor, MI 48109 USA.

RP Aikens, JE (reprint author), Univ Michigan, Dept Family Med, Ann Arbor, MI 48109 USA.

EM aikensj@umich.edu

FU National Institute of Diabetes and Digestive and Kidney Diseases

(NIDDK), National Institutes of Health (NIH) [R01 DK066016, P60DK020572]

FX This research was supported by National Institutes of Health (NIH) grant

numbers R01 DK066016 and P60DK020572 (MDRTC), both from the National

Institute of Diabetes and Digestive and Kidney Diseases (NIDDK). JDP is

a VA Senior Research Career Scientist.

CR Adams AS, 2008, DIABETES CARE, V31, P916, DOI 10.2337/dc07-1924

Aikens J, 2012, DIABETES CARE

Aikens JE, 2008, DIABETIC MED, V25, P1324, DOI 10.1111/j.1464-5491.2008.02590.x

Aikens JE, 2009, DIABETES CARE, V32, P1177, DOI 10.2337/dc09-0071

Aikens JE, 2005, ANN FAM MED, V3, P23, DOI 10.1370/afm.238

Asche C, 2011, CLIN THER, V33, P74, DOI 10.1016/j.clinthera.2011.01.019

Blonde L, 2003, DIABETES OBES METAB, V5, P424, DOI 10.1046/j.1463-1326.2003.00297.x

Bosworth HB, 2011, AM HEART J, V162, P412, DOI 10.1016/j.ahj.2011.06.007

Cass AR, 1999, INT J PSYCHIAT MED, V29, P293, DOI 10.2190/CPYJ-2HBF-RGCN-64V2

Cramer JA, 2004, DIABETES CARE, V27, P1218, DOI 10.2337/diacare.27.5.1218

Donnelly LA, 2007, QJM-INT J MED, V100, P345, DOI 10.1093/qjmed/hcm031

Fitzgerald JT, 1996, EVAL HEALTH PROF, V19, P208, DOI 10.1177/016327879601900205

Garber MC, 2004, MED CARE, V42, P649, DOI 10.1097/01.mlr.0000129496.05898.02

George J, 2007, BRIT J CLIN PHARMACO, V63, P488, DOI 10.1111/j.1365-2125.2006.02800.x

Granger BB, 2011, CURR OPIN CARDIOL, V26, P279, DOI 10.1097/HCO.0b013e328347c150

Guillausseau PJ, 2003, DIABETES METAB, V29, P79, DOI 10.1016/S1262-3636(07)70011-3

Heisler M, 2007, ARCH INTERN MED, V167, P1853, DOI 10.1001/archinte.167.17.1853

Horswell RL, 2008, J HEALTH CARE POOR U, V19, P677, DOI 10.1353/hpu.0.0062

Kindmalm L, 2007, ACTA DIABETOL, V44, P209, DOI 10.1007/s00592-007-0006-7

Krapek K, 2004, ANN PHARMACOTHER, V38, P1357, DOI 10.1345/aph.1D612

Kroenke K, 2001, J GEN INTERN MED, V16, P606, DOI 10.1046/j.1525-1497.2001.016009606.x

Krousel-Wood M, 2009, AM J MANAG CARE, V15, P59

Lawrence DB, 2006, J MANAGE CARE PHARM, V12, P466

MORISKY DE, 1986, MED CARE, V24, P67, DOI 10.1097/00005650-198601000-00007

Parchman ML, 2007, DIABETES CARE, V30, P2849, DOI 10.2337/dc06-2516

Piette JD, 2001, DIABETES CARE, V24, P202, DOI 10.2337/diacare.24.2.202

Piette JD, 2004, MED CARE, V42, P102, DOI 10.1097/01.mlr.0000108742.26446.17

Pladevall M, 2004, DIABETES CARE, V27, P2800, DOI 10.2337/diacare.27.12.2800

POLONSKY WH, 1995, DIABETES CARE, V18, P754, DOI 10.2337/diacare.18.6.754

Possidente CJ, 2005, AM J HEALTH-SYST PH, V62, P1311

Rhee MK, 2005, DIABETES EDUCATOR, V31, P240, DOI 10.1177/0145721705274927

Rozenfeld Y, 2008, AM J MANAG CARE, V14, P71

Saini SD, 2009, AM J MANAG CARE, V15, pE22

2002, FED REG, V67, P6931

NR 34

TC 47

Z9 49

U1 0

U2 21

PU WILEY-BLACKWELL

PI HOBOKEN

PA 111 RIVER ST, HOBOKEN 07030-5774, NJ USA

SN 0742-3071

J9 DIABETIC MED

JI Diabetic Med.

PD MAR

PY 2013

VL 30

IS 3

BP 338

EP 344

DI 10.1111/dme.12046

PG 7

WC Endocrinology & Metabolism

SC Endocrinology & Metabolism

GA 095MT

UT WOS:000315339300020

PM 23075262

OA Green Accepted

DA 2019-01-08

ER

PT J

AU Peyrot, M

Burns, KK

Davies, M

Forbes, A

Hermanns, N

Holt, R

Kalra, S

Nicolucci, A

Pouwer, F

Wens, J

Willaing, I

Skovlund, SE

AF Peyrot, Mark

Burns, Katharina Kovacs

Davies, Melanie

Forbes, Angus

Hermanns, Norbert

Holt, Richard

Kalra, Sanjay

Nicolucci, Antonio

Pouwer, Frans

Wens, Johan

Willaing, Ingrid

Skovlund, Soren E.

CA Global DAWN2 Study Grp

TI Diabetes Attitudes Wishes and Needs 2 (DAWN2): A multinational,

multi-stakeholder study of psychosocial issues in diabetes and

person-centred diabetes care

SO DIABETES RESEARCH AND CLINICAL PRACTICE

LA English

DT Article

DE People with diabetes; Family members; Healthcare providers; Survey;

Person-centred care; Psychosocial; Self-management

ID SELF-MANAGEMENT EDUCATION; PATIENT ASSESSMENT; VALIDATION; HEALTH; SCALE

AB Aims: The Diabetes Attitudes Wishes and Needs 2 (DAWN2) study aims to provide a holistic assessment of diabetes care and management among people with diabetes (PWD), family members (FM), and healthcare professionals (HCPs) and explores potential drivers leading to active management.

Methods: DAWN2 survey over 16,000 individuals (similar to 9000 PWD, similar to 2000 FM of PWD, and similar to 5000 HCPs) in 17 countries across 4 continents. Respondents complete a group-specific questionnaire; items are designed to allow cross-group comparisons on common topics. The questionnaires comprise elements from the original DAWN study (2001), as well as psychometrically validated instruments and novel questions developed for this study to assess self-management, attitudes/beliefs, disease impact/burden, psychosocial distress, health-related quality of life, healthcare provision/receipt, social support and priorities for improvement in the future. The questionnaires are completed predominantly online or by telephone interview, supplemented by face-to-face interviews in countries with low internet access. In each country, recruitment ensures representation of the diabetes population in terms of geographical distribution, age, gender, education and disease status.

Discussion: DAWN2 aims to build on the original DAWN study to identify new avenues for improving diabetes care. This paper describes the study rationale, goals and methodology. (C) 2012 Elsevier Ireland Ltd. All rights reserved.

C1 [Peyrot, Mark] Loyola Univ Maryland, Dept Sociol, Baltimore, MD 21210 USA.

[Burns, Katharina Kovacs] Univ Alberta, Edmonton Clin Hlth Acad, Interdisciplinary Hlth Res Acad, Edmonton, AB, Canada.

[Davies, Melanie] Univ Leicester, Dept Cardiovasc Sci, Leicester LE1 5WW, Leics, England.

[Forbes, Angus] Kings Coll London, Florence Nightingale Sch Nursing & Midwifery, Dept Primary & Intermediate Care, London WC2R 2LS, England.

[Hermanns, Norbert] Diabet Acad Mergentheim FIDAM, Res Inst, Bad Mergentheim, Germany.

[Holt, Richard] Univ Southampton, Fac Med, Human Dev & Hlth Acad Unit, Southampton SO9 5NH, Hants, England.

[Kalra, Sanjay] Bharti Hosp, Karnal 132001, Haryana, India.

[Kalra, Sanjay] BRIDE, Karnal 132001, Haryana, India.

[Nicolucci, Antonio] Consorzio Mario Negri Sud, Dept Clin Pharmacol & Epidemiol, Chieti, Italy.

[Pouwer, Frans] Tilburg Univ, Dept Med & Clin Psychol, NL-5000 LE Tilburg, Netherlands.

[Wens, Johan] Univ Antwerp, Dept Med & Hlth Sci, Primary & Interdisciplinary Care Antwerp, B-2020 Antwerp, Belgium.

[Willaing, Ingrid] Steno Diabet Ctr, Steno Hlth Promot Ctr, DK-2820 Gentofte, Denmark.

[Skovlund, Soren E.] Novo Nordisk AS, Patient Res Advocacy & Support, Copenhagen, Denmark.

RP Peyrot, M (reprint author), Loyola Univ Maryland, Dept Sociol, 4501 North Charles St, Baltimore, MD 21210 USA.

EM mpeyrot@loyola.edu

RI Wens, Johan/K-2705-2013

OI Wens, Johan/0000-0002-0229-9064; Willaing, Ingrid/0000-0002-3082-8816;

Davies, Melanie/0000-0002-9987-9371; Holt, Richard/0000-0001-8911-6744;

Forbes, Angus/0000-0003-3331-755X

FU Novartis; Novo Nordisk; Sanofi-Aventis; Lilly; Pfizer; Merck Sharp and

Dohme; GlaxoSmithKline; Servier; Roche; Berlin Chemie; Eli Lilly;

Bristol Myers Squibb-Astra Zeneca; Bayer; Amylin; Genentech; MannKind;

Medtronic; Novo Nordisk A/S; National Institute for Health Research

[RP-PG-0606-1272, RP-DG-1210-10183]

FX Melanie Davies has acted as consultant, advisory board member and

speaker for Novartis, Novo Nordisk, SanofiAventis, Lilly, Merck Sharp

and Dohme, and Roche, and as a speaker for Servier. She has received

grants in support of investigator and investigator-initiated trials from

Novartis, Novo Nordisk, Sanofi-Aventis, Lilly, Pfizer, Merck Sharp and

Dohme, GlaxoSmithKline and Servier.; Norbert Hermanns is a member of the

International DAWN2 Advisory Board. He has received research grants in

the last year from Roche, Berlin Chemie, Eli Lilly and Sanofi Aventis.

He also received speaking honoraria from Novo, Germany, Lilly, Berlin

Chemie, Lifescan, Sanofi Aventis and Menarini Diagnostics.; Antonio

Nicolucci has received research grants in the last year from Novo

Nordisk, Eli Lilly, Sanofi Aventis, Merck Sharp and Dohme, Bristol Myers

Squibb-Astra Zeneca and Bayer.; Mark Peyrot has recently received

research grants and/ or consulting fees from Amylin, Genentech, Lilly,

MannKind, Medtronic, and Novo Nordisk. He has received speaking

honoraria from Novo Nordisk and has participated in advisory panels for

Novo Nordisk and Roche. He has received financial support from Novo

Nordisk for his participation as Principal Investigator for the DAWN2

study.; The DAWN2 study is funded by Novo Nordisk A/S. Editorial

assistance in the preparation of this manuscript, which was funded by

Novo Nordisk A/S was provided by Dr. Mark Warren of Bioscript Stirling

Ltd. The surveys were conducted by Harris Interactive Inc., an

independent research organisation. The authors wish to acknowledge Anna

Ginovker of Harris Interactive for directing the global fieldwork and

Bioscript Stirling Ltd. for providing assistance with obtaining ethical

approvals in several countries. Harris Interactive conducted the

surveys, and provided expertise in designing the questionnaires and

planning survey implementation, including sampling frames and

strategies, questionnaire administration strategies, weighting criteria,

etc.

CR American Diabetes Association, 2011, DIABETES CARE S1, V34

Anderson R, 2000, NAT HIST, V109, P23

Anderson RM, 2003, DIABETES CARE, V26, P1641, DOI 10.2337/diacare.26.5.1641-a

[Anonymous], 2008, DIABETES EDUCATOR, V34, P451, DOI 10.1177/0145721708316627

[Anonymous], 2011, QUAL STAND DIAB AD

[Anonymous], 2011, CLIN GUID 87 TYP 2 D

[Anonymous], 2011, IDF CHART RIGHTS RES

[Anonymous], 2004, PRACT DIABETES INT, V21, P201

[Anonymous], 2008, CLIN GUID 66 TYP 2 D

[Anonymous], 2007, INT COD MARK SOC RES

[Anonymous], 2011, COD STAND ETH SURV R

Bech P, 1996, PSYCHOTHER PSYCHOSOM, V65, P183, DOI 10.1159/000289073

Drab S, 2009, J AM PHARM ASSOC, V49, pE152, DOI 10.1331/JAPhA.2009.08181

Funnell MM, 2007, DIABETES EDUCATOR, V33, P45, DOI 10.1177/0145721706298308

Funnell MM, 2011, DIABETES CARE, V34, pS89, DOI 10.2337/dc11-S089

Glasgow RE, 2005, DIABETES CARE, V28, P2655, DOI 10.2337/diacare.28.11.2655

Glasgow RE, 2005, MED CARE, V43, P436, DOI 10.1097/01.mlr.0000160375.47920.8c

Harkness E, 2010, DIABETES CARE, V33, P926, DOI 10.2337/dc09-1519

Harper A, 1998, PSYCHOL MED, V28, P551

International Diabetes Federation, 2004, DIABETES VOICE, V49, P1

International Diabetes Federation Clinical Guidelines Task Force. Psychological care, 2005, PENT PROC EXTR ED DA, P19

International Society for Pharmacoepidemiology, 2007, GUID GOOD PHARM PRAC

Knottnerus JA, 2012, J CLIN EPIDEMIOL, V65, P581, DOI 10.1016/j.jclinepi.2012.03.007

McGuire BE, 2010, DIABETOLOGIA, V53, P66, DOI 10.1007/s00125-009-1559-5

National Collaborating Centre for Chronic Conditions, 2004, TYPE 1 DIAB AD NAT C

National Collaborating Centre for Chronic Conditions (UK), 2008, TYP 2 DIAB NAT CLIN

Parkin T, 2003, DIABETIC MED, V20, P909, DOI 10.1046/j.1464-5491.2003.01056.x

Petrak F, 2005, CURR DIABETES REV, V1, P255, DOI 10.2174/157339905774574329

Peyrot M, 2005, DIABETES CARE, V28, P2673, DOI 10.2337/diacare.28.11.2673

Peyrot M, 2005, DIABETIC MED, V22, P1379, DOI 10.1111/j.1464-5491.2005.01644.x

POLONSKY WH, 1995, DIABETES CARE, V18, P754, DOI 10.2337/diacare.18.6.754

SCHAFER LC, 1986, DIABETES CARE, V9, P179, DOI 10.2337/diacare.9.2.179

Skovlund S. E., 2005, DIABETES SPECTRUM, V18, P136, DOI DOI 10.2337/DIASPECT.18.3.136

Szende A, 2007, EUROQOL GROUP MONOGR

Toobert DJ, 2000, DIABETES CARE, V23, P943, DOI 10.2337/diacare.23.7.943

Uplinger N, 2009, DIABETES EDUCATOR, V35, P713, DOI 10.1177/0145721709340804

Welch G, 2003, DIABETIC MED, V20, P69, DOI 10.1046/j.1464-5491.2003.00832.x

Westberg SM, 2010, AM J PHARM EDUC, V74, DOI 10.5688/aj740349

World Health Organization (WHO), 2002, INN CAR CHRON COND B

NR 39

TC 96

Z9 104

U1 0

U2 22

PU ELSEVIER IRELAND LTD

PI CLARE

PA ELSEVIER HOUSE, BROOKVALE PLAZA, EAST PARK SHANNON, CO, CLARE, 00000,

IRELAND

SN 0168-8227

EI 1872-8227

J9 DIABETES RES CLIN PR

JI Diabetes Res. Clin. Pract.

PD FEB

PY 2013

VL 99

IS 2

BP 174

EP 184

DI 10.1016/j.diabres.2012.11.016

PG 11

WC Endocrinology & Metabolism

SC Endocrinology & Metabolism

GA 119UC

UT WOS:000317122400019

PM 23273515

OA Other Gold

DA 2019-01-08

ER

PT J

AU Gagliardino, JJ

Arrechea, V

Assad, D

Gagliardino, GG

Gonzalez, L

Lucero, S

Rizzuti, L

Zufriategui, Z

Clark, C

AF Jose Gagliardino, Juan

Arrechea, Viviana

Assad, Daniel

Gagliardino, Gabriel G.

Gonzalez, Lorena

Lucero, Soledad

Rizzuti, Liliana

Zufriategui, Zulma

Clark, Charles, Jr.

TI Type 2 diabetes patients educated by other patients perform at least as

well as patients trained by professionals

SO DIABETES-METABOLISM RESEARCH AND REVIEWS

LA English

DT Article

DE peer support; diabetes education; type 2 diabetes management; quality of

care; patient satisfaction; psychological impact

ID RANDOMIZED CONTROLLED-TRIAL; SELF-MANAGEMENT EDUCATION;

ECONOMIC-ANALYSIS; GLUCOSE CONTROL; PEER SUPPORT; PROGRAM; CARE; ADULTS;

INTERVENTIONS; DISTRESS

AB Background Diabetes education can improve the quality of care of people with diabetes, but many organizations are not equipped to manage its implementation. Involving people with diabetes in the education process can overcome the problem. Thus, we compared clinical, metabolic and psychological outcomes in people with type 2 diabetes 1year after attending a structured diabetes education programme implemented by professional educators versus the same programme implemented by trained peers with diabetes that also provided ongoing peer support. Methods People with type 2 diabetes (2575years) were randomly assigned to attend a 4-week structured diabetes education course delivered by professional educators (control) or previously trained peers (peer). Peers also received continuing psychological support, including examples on how to apply diabetes knowledge in daily life via weekly peer cellular phone calls and bimonthly face-to-face interviews in small groups (ten patients), using a structured questionnaire related to the patient's clinical, metabolic and psychological progress. Identical outcome data from both groups were used for follow-up. Results Both groups had a comparable positive effect on clinical, metabolic and psychological indicators immediately following the programme. Over the following year, peer-educated subjects had lower A1C and systolic blood pressure and showed higher adherence to physical activity and better control of hypoglycaemic episodes. Conclusion The non-inferiority of the peer outcomes and the mentioned improvements in this group suggest that volunteer trained peer educators and ongoing support can be successful. This approach provides an effective alternative method of education, especially in areas with limited availability of professionals and economic resources. Copyright (c) 2012 John Wiley & Sons, Ltd.

C1 [Jose Gagliardino, Juan; Gonzalez, Lorena; Clark, Charles, Jr.] UNLP, CENEXA Ctr Endocrinol Expt & Aplicada UNLPCONICET, Ctr Colaborador OPS OMS Diabet, Fac Ciencias Med, RA-1900 La Plata, Argentina.

[Arrechea, Viviana; Assad, Daniel; Gagliardino, Gabriel G.; Lucero, Soledad; Rizzuti, Liliana; Zufriategui, Zulma] Ctr Educ Terapeut Bernardo A Houssay, La Plata, Argentina.

RP Gagliardino, JJ (reprint author), UNLP, Fac Ciencias Med, CENEXA UNLP CONICET LA PLATA, 60 Y 120, RA-1900 La Plata, Argentina.

EM cenexa@speedy.com.ar

FU American Academy of Family Physicians Foundation

FX This study was partially supported by a grant provided by the American

Academy of Family Physicians Foundation for the Peers for Progress

Program. JJG is member of the Research career of CONICET. VA, DA, GGG,

SL, LR and ZZ are members of the Houssay Centre Education team. LG is a

fellow from the National University of La Plata, CC Jr is a research

advisor of CENEXA by this study. Thanks are due to A. Di Maggio for

assistance in editing the manuscript.

CR Am Diabetes Assoc, 2006, DIABETES CARE, V29, pS4

Anderson RM, 2005, ETHNIC DIS, V15, P671

Baksi AK, 2008, DIABETIC MED, V25, P1076, DOI 10.1111/j.1464-5491.2008.02542.x

Balducci S, 2012, DIABETES CARE, V35, P39, DOI 10.2337/dc11-1450

Balducci S, 2010, ARCH INTERN MED, V170, P1794, DOI 10.1001/archinternmed.2010.380

Barlow J, 2002, PATIENT EDUC COUNS, V48, P177, DOI 10.1016/S0738-3991(02)00032-0

Beckles GLA, 1998, DIABETES CARE, V21, P1432, DOI 10.2337/diacare.21.9.1432

Brown SA, 2005, DIABETES CARE, V28, P527, DOI 10.2337/diacare.28.3.527

Clark CM, 2010, FAM PRACT, V27, pI3, DOI 10.1093/fampra/cmq001

Cramer JA, 2004, DIABETES CARE, V27, P1218, DOI 10.2337/diacare.27.5.1218

Duke SAS, 2009, COCHRANE DB SYST REV, DOI 10.1002/14651858.CD005268.pub2

Fisher EB, 2010, FAM PRACT, V27, pI6, DOI 10.1093/fampra/cmp013

Fisher L, 2008, ANN FAM MED, V6, P246, DOI 10.1370/afm.842

Fisher L, 2007, DIABETES CARE, V30, P542, DOI 10.2337/dc06-1614

Fisher L, 2012, DIABETES CARE, V35, P259, DOI 10.2337/dc11-1572

Fisher L, 2010, DIABETES CARE, V33, P23, DOI 10.2337/dc09-1238

Forkan R, 2006, PHYS THER, V86, P401

Funnell MM, 2007, DIABETES CARE, V30, P1630, DOI 10.2337/dc07-9923

Gaede P, 2003, NEW ENGL J MED, V348, P383, DOI 10.1056/NEJMoa021778

Gagliardino J J, 2001, Rev Panam Salud Publica, V10, P309, DOI 10.1590/S1020-49892001001100003

Gagliardino JJ, 2006, DIABETES RES CLIN PR, V72, P284, DOI 10.1016/j.diabres.2006.02.004

Gagliardino JJ, 2001, DIABETES CARE, V24, P1001, DOI 10.2337/diacare.24.6.1001

Gary TL, 2003, DIABETES EDUCATOR, V29, P488, DOI 10.1177/014572170302900313

Gonzalez JS, 2011, DIABETES CARE, V34, P236, DOI 10.2337/dc10-1970

Gray A, 2000, BRIT MED J, V320, P1373, DOI 10.1136/bmj.320.7246.1373

Holman RR, 2008, NEW ENGL J MED, V359, P1577, DOI 10.1056/NEJMoa0806470

Home P, 2006, DIABETIC MED, V23, P579

Karwalajtys Tina, 2005, BMC Fam Pract, V6, P35, DOI 10.1186/1471-2296-6-35

Karwalajtys T, 2009, J COMMUN HEALTH, V34, P336, DOI 10.1007/s10900-009-9149-5

Klonoff DC, 2000, DIABETES CARE, V23, P390, DOI 10.2337/diacare.23.3.390

Li CY, 2010, DIABETES CARE, V33, P1061, DOI 10.2337/dc09-1515

LORIG K, 1985, ARTHRITIS RHEUM, V28, P680, DOI 10.1002/art.1780280612

Lorig KR, 1999, MED CARE, V37, P5, DOI 10.1097/00005650-199901000-00003

McGlynn EA, 2003, NEW ENGL J MED, V348, P2635, DOI 10.1056/NEJMsa022615

Morsanutto A, 2006, J DIABETES COMPLICAT, V20, P163, DOI 10.1016/j.jdiacomp.2005.06.011

Norris S, 2003, RACTICAL DIABETOLOGY, V22, P7

Norris SL, 2002, DIABETES CARE, V25, P1159, DOI 10.2337/diacare.25.7.1159

Norris SL, 2001, DIABETES CARE, V24, P561, DOI 10.2337/diacare.24.3.561

Odegard PS, 2007, DIABETES EDUCATOR, V33, P1014, DOI 10.1177/0145721707308407

Peyrot M, 2005, DIABETIC MED, V22, P1379, DOI 10.1111/j.1464-5491.2005.01644.x

Polonsky WH, 2003, DIABETES CARE, V26, P3048, DOI 10.2337/diacare.26.11.3048

Renders CM, 2003, DIABETIC MED, V20, P846, DOI 10.1046/j.1464-5491.2003.01009.x

Renders CM, 2001, DIABETES CARE, V24, P1821, DOI 10.2337/diacare.24.10.1821

Roter DL, 1998, MED CARE, V36, P1138, DOI 10.1097/00005650-199808000-00004

Sherman BW, 2011, AM J MANAG CARE, V17, P729

Snoek FJ, 2011, DIABETES CARE, V34, P601, DOI 10.2337/dc10-1552

Stearne MR, 1998, BRIT MED J, V317, P703

Trento M, 2004, DIABETES CARE, V27, P670, DOI 10.2337/diacare.27.3.670

Williams R, 2002, DIABETOLOGIA, V45, pS13, DOI 10.1007/s00125-002-0859-9

Zhang QY, 2011, AM J MANAG CARE, V17, P746

NR 50

TC 34

Z9 38

U1 4

U2 28

PU WILEY

PI HOBOKEN

PA 111 RIVER ST, HOBOKEN 07030-5774, NJ USA

SN 1520-7560

J9 DIABETES-METAB RES

JI Diabetes-Metab. Res. Rev.

PD FEB

PY 2013

VL 29

IS 2

BP 152

EP 160

DI 10.1002/dmrr.2368

PG 9

WC Endocrinology & Metabolism

SC Endocrinology & Metabolism

GA 086CK

UT WOS:000314660400008

PM 23166062

DA 2019-01-08

ER

PT J

AU Rintala, TM

Jaatinen, P

Paavilainen, E

Astedt-Kurki, P

AF Rintala, Tuula-Maria

Jaatinen, Pia

Paavilainen, Eija

Astedt-Kurki, Paivi

TI Interrelation Between Adult Persons With Diabetes and Their Family: A

Systematic Review of the Literature

SO JOURNAL OF FAMILY NURSING

LA English

DT Article

DE diabetes; diabetes self-management; adult person; family; family

relationships; literature review

ID SELF-MANAGEMENT BEHAVIORS; SOCIAL-ENVIRONMENTAL SUPPORT; PARTNERS

EXPRESSED EMOTION; EUROPEAN-AMERICAN PATIENTS; DISEASE MANAGEMENT;

GLYCEMIC CONTROL; SPOUSAL SUPPORT; CARE BEHAVIORS; MARITAL RELATIONSHIP;

AFRICAN-AMERICANS

AB Diabetes mellitus is a common chronic disease all over the world. Self-management plays a crucial role in diabetes management. The purpose of this systematic review was to summarize what is known about the interactions between adult persons with diabetes, their family, and diabetes self-management. MEDLINE, CINAHL, PSYCHINFO, LINDA, and MEDIC databases were searched for the years 2000 to 2011 and for English language articles, and the reference lists of the studies included were reviewed to capture additional studies. The findings indicate that family members have influence on the self-management of adult persons with diabetes. The support from family members plays a crucial role in maintaining lifestyle changes and optimizing diabetes management. Diabetes and its treatment also affect the life of family members in several ways, causing, for example, different types of psychological distress. More attention should be paid to family factors in diabetes management among adult persons.

C1 [Rintala, Tuula-Maria] Tampere Univ Appl Sci, Tampere, Finland.

[Jaatinen, Pia] Univ Tampere, Sch Med, FIN-33101 Tampere, Finland.

[Rintala, Tuula-Maria; Paavilainen, Eija; Astedt-Kurki, Paivi] Univ Tampere, Sch Hlth Sci, FIN-33101 Tampere, Finland.

RP Rintala, TM (reprint author), Tampere Univ Appl Sci, Kuntokatu 4, Tampere, Finland.

EM tuula-maria.rintala@uta.fi

FU University of Tampere; Finnish Diabetes Association

FX The authors disclosed receipt of the following financial support for the

research, authorship, and/or publication of this article: Financial

support for this research was provided by the University of Tampere and

the Finnish Diabetes Association.

CR Albright TL, 2001, FAM MED, V33, P354

Aljasem LI, 2001, DIABETES EDUCATOR, V27, P393, DOI 10.1177/014572170102700309

Anderson Barbara J, 2003, Curr Diab Rep, V3, P134, DOI 10.1007/s11892-003-0037-6

Anderson RM, 2005, PATIENT EDUC COUNS, V57, P153, DOI 10.1016/j.pec.2004.05.008

Armour TA, 2005, DIABETIC MED, V22, P1295, DOI 10.1111/j.1464-5491.2005.01618.x

Awadalla AW, 2006, J NATL MED ASSOC, V98, P727

Baanders AN, 2007, FAM COMMUNITY HEALTH, V30, P305, DOI 10.1097/01.FCH.0000290543.48576.cf

Beverly EA, 2007, J PSYCHOSOC NURS, V45, P25

Beverly EA, 2008, HEALTH EDUC BEHAV, V35, P707, DOI 10.1177/1090198107299787

Beverly EA, 2010, HEALTH EDUC RES, V25, P211, DOI 10.1093/her/cyn032

Cameron FJ, 2008, DIABETIC MED, V25, P463, DOI 10.1111/j.1464-5491.2008.02399.x

Chesla CA, 2005, QUAL HEALTH RES, V15, P240, DOI 10.1177/1049732304272050

Chesla CA, 2004, DIABETES CARE, V27, P2850, DOI 10.2337/diacare.27.12.2850

Chesla CA, 2003, FAM PROCESS, V42, P375, DOI 10.1111/j.1545-5300.2003.00375.x

Chesla CA, 2010, J FAM NURS, V16, P355, DOI 10.1177/1074840710383145

Chesla CA, 2009, DIABETES CARE, V32, P1812, DOI 10.2337/dc09-0278

Chlebowy DO, 2006, DIABETES EDUCATOR, V32, P777, DOI 10.1177/0145721706291760

Chlebowy DO, 2010, DIABETES EDUCATOR, V36, P897, DOI 10.1177/0145721710385579

Choi SE, 2009, DIABETES EDUCATOR, V35, P978, DOI 10.1177/0145721709349220

De Ridder DTD, 2005, PSYCHOL HEALTH, V20, P497, DOI 10.1080/14768320500098699

Denham SA, 2007, FAMILIES SYSTEMS HLT, V25, P36, DOI DOI 10.1037/1091-7527.25.1.36

Egede LE, 2010, DIABETES EDUCATOR, V36, P276, DOI 10.1177/0145721710361389

Fisher L, 2004, J FAM PSYCHOL, V18, P302, DOI 10.1037/0893-3200.18.2.302

Fisher L, 2002, DIABETES CARE, V25, P1564, DOI 10.2337/diacare.25.9.1564

Fisher L, 2000, DIABETES CARE, V23, P267, DOI 10.2337/diacare.23.3.267

Fisher L, 2000, FAM PROCESS, V39, P403, DOI 10.1111/j.1545-5300.2000.39402.x

Fortmann AL, 2011, HEALTH PSYCHOL, V30, P251, DOI 10.1037/a0022850

Funnell MM, 2004, CLIN DIABETES, V22, P123, DOI DOI 10.2337/DIACLIN.22.3.123

Gavin JR, 2010, DIABETES EDUCATOR, V36, p26S, DOI 10.1177/0145721710369637

Gerstle JF, 2001, J AM DIET ASSOC, V101, P918, DOI 10.1016/S0002-8223(01)00227-9

Gherman A, 2011, DIABETES EDUCATOR, V37, P392, DOI 10.1177/0145721711403012

Hagedoorn M, 2006, DIABETIC MED, V23, P271, DOI 10.1111/j.1464-5491.2006.01794.x

Hill-Briggs F, 2007, DIABETES EDUCATOR, V33, P1032, DOI 10.1177/0145721707308412

Jones RA, 2008, DIABETES EDUCATOR, V34, P318, DOI 10.1177/0145721708314485

Jorgensen HV, 2003, DIABETES CARE, V26, P1106, DOI 10.2337/diacare.26.4.1106

Kang CM, 2010, INT J NURS STUD, V47, P1363, DOI 10.1016/j.ijnurstu.2010.03.009

Keogh KM, 2007, BMC FAM PRACT, V8, DOI 10.1186/1471-2296-8-36

King DK, 2010, DIABETES CARE, V33, P751, DOI 10.2337/dc09-1746

Leonard BJ, 2005, J FAM NURS, V11, P102, DOI 10.1177/1074840705275152

Lohri-Posey B, 2006, FAM COMMUNITY HEALTH, V29, P214, DOI 10.1097/00003727-200607000-00008

Magarey J M, 2001, Int J Nurs Pract, V7, P376

Manoogian MM, 2010, J FAM COMMUN, V10, P40, DOI 10.1080/15267430903385826

McBroom LA, 2009, DIABETES EDUCATOR, V35, P428, DOI 10.1177/0145721709332814

McGowan J, 2005, J MED LIBR ASSOC, V93, P74

Minet L, 2010, PATIENT EDUC COUNS, V80, P29, DOI 10.1016/j.pec.2009.09.033

Nardacci EA, 2010, DIABETES EDUCATOR, V36, p4S, DOI 10.1177/0145721710362798

Needleman IG, 2002, J CLIN PERIODONTOL, V29, P6, DOI 10.1034/j.1600-051X.29.s3.15.x

Norris SL, 2002, DIABETES CARE, V25, P1159, DOI 10.2337/diacare.25.7.1159

Paddison C, 2010, EUR DIABETES NURS, V7, P29, DOI DOI 10.1002/EDN.152

Rees CA, 2010, DIABETES EDUCATOR, V36, P435, DOI 10.1177/0145721710364419

Rosland AM, 2008, J GEN INTERN MED, V23, P1992, DOI 10.1007/s11606-008-0814-7

Sabone MB, 2008, J FAM NURS, V14, P363, DOI 10.1177/1074840708323047

Searle A, 2007, J PSYCHOSOM RES, V63, P175, DOI 10.1016/j.jpyschores.2007.02.006

Song M, 2010, J CARDIOVASC NURS, V25, P93, DOI 10.1097/JCN.0b013e3181c5a364

Stephens MAP, 2010, FAM SYST HEALTH, V28, P199, DOI 10.1037/a0020513

Stodberg R, 2007, J CLIN NURS, V16, P215, DOI 10.1111/j.1365-2702.2006.01797.x

Tang TS, 2008, DIABETES EDUCATOR, V34, P266, DOI 10.1177/0145721708315680

Toljamo M, 2001, J CLIN NURS, V10, P618, DOI 10.1046/j.1365-2702.2001.00520.x

Trief P., 2003, FAMILIES SYSTEMS HLT, V21, P57, DOI [DOI 10.1037/H0089502, 10.1037/h0089502]

Trief P. M., 2006, FAMILIES SYSTEMS HLT, V24, P318, DOI [10.1037/1091-7527.24.3.318, DOI 10.1037/1091-7527.24.3.318]

Trief PM, 2004, ANN BEHAV MED, V27, P148, DOI 10.1207/s15324796abm2703_2

Trief PM, 2002, DIABETES CARE, V25, P1154, DOI 10.2337/diacare.25.7.1154

Trief PM, 2001, DIABETES CARE, V24, P1384, DOI 10.2337/diacare.24.8.1384

van Dam HA, 2005, PATIENT EDUC COUNS, V59, P1, DOI 10.1016/j.pec.2004.11.001

Watanabe K, 2010, INTERNAL MED, V49, P983, DOI 10.2169/internalmedicine.49.3230

Wearden AJ, 2000, J PSYCHOSOM RES, V49, P125, DOI 10.1016/S0022-3999(00)00141-0

Wearden AJ, 2006, BRIT J HEALTH PSYCH, V11, P1, DOI 10.1348/135910705X73676

Weiler DM, 2009, DIABETES EDUCATOR, V35, P285, DOI 10.1177/0145721708329545

Wen LK, 2004, DIABETES EDUCATOR, V30, P980, DOI 10.1177/014572170403000619

White P, 2007, DIABETIC MED, V24, P796, DOI 10.1111/j.1464-5491.2007.02271.x

White P, 2009, DIABETES EDUCATOR, V35, P810, DOI 10.1177/0145721709340930

Williams K. E., 2002, PSYCHOL HEALTH MED, V7, P127, DOI [10.1080/13548500120116076, DOI 10.1080/13548500120116076]

NR 72

TC 46

Z9 46

U1 0

U2 26

PU SAGE PUBLICATIONS INC

PI THOUSAND OAKS

PA 2455 TELLER RD, THOUSAND OAKS, CA 91320 USA

SN 1074-8407

EI 1552-549X

J9 J FAM NURS

JI J. Fam. Nurs.

PD FEB

PY 2013

VL 19

IS 1

BP 3

EP 28

DI 10.1177/1074840712471899

PG 26

WC Family Studies; Nursing

SC Family Studies; Nursing

GA 076XV

UT WOS:000313993400001

PM 23288886

DA 2019-01-08

ER

PT J

AU Hajos, TRS

Pouwer, F

Skovlund, SE

Den Oudsten, BL

Geelhoed-Duijvestijn, PHLM

Tack, CJ

Snoek, FJ

AF Hajos, T. R. S.

Pouwer, F.

Skovlund, S. E.

Den Oudsten, B. L.

Geelhoed-Duijvestijn, P. H. L. M.

Tack, C. J.

Snoek, F. J.

TI Psychometric and screening properties of the WHO-5 well-being index in

adult outpatients with Type 1 or Type 2 diabetes mellitus

SO DIABETIC MEDICINE

LA English

DT Article

ID PATIENT HEALTH QUESTIONNAIRE; QUALITY-OF-LIFE; COMORBID DEPRESSION;

VALIDITY; METAANALYSIS; PREVALENCE; VALIDATION; DISTRESS; CLINICS; PHQ-9

AB Objective The 5-item World Health Organization well-being index is a commonly used measure of emotional well-being, but research on psychometric properties in outpatients with diabetes is scarce. We examined psychometric and screening properties for depression of this index in a large sample of Dutch outpatients with diabetes. Methods Patients with Type 1 (n = 384) and Type 2 (n = 549) diabetes from three outpatient clinics completed the WHO-5 index, the nine-item Patient Health Questionnaire, the Problem Areas in Diabetes survey and the Short Form-12 health survey. Internal consistency of the WHO-5 index was determined by Cronbach's alpha. The factor structure was tested by confirmatory factor analysis. Concurrent validity was assessed by correlations with the Patient Health Questionnaire, Problem Areas in Diabetes and the Short Form-12 mental component scores. Sensitivity and specificity of the WHO-5 index as depression screener were tested against two existing Patient Health Questionnaire cut-off scores for depression using receiver operating characteristic curves. Results A one-factor structure of the WHO-5 index was verified by confirmatory factor analysis for patients with Type 1 and Type 2 diabetes. Moderate to strong correlations were observed between the WHO-5 index and the Patient Health Questionnaire scores, the Problem Areas in Diabetes scores and the Short Form-12 mental component scores (r = 0.550.69, P < 0.001). Receiver operating characteristic curves showed that a WHO-5 index cut-off of < 50 performed best as an indication for likely depression, with sensitivity compared with a Patient Health Questionnaire score = 10 and = 12 of 79% and 88%, respectively, and specificity of 88% and 76%, respectively. Conclusions The WHO-5 index is a short, psychometrically sound measure of emotional well-being that appears suitable for use as screening test for likely depression in outpatients with Type 1 and Type 2 diabetes.

C1 [Hajos, T. R. S.; Snoek, F. J.] Vrije Univ Amsterdam Med Ctr, EMGO Inst, Dept Med Psychol, Diabet Psychol Res Grp, Amsterdam, Netherlands.

[Pouwer, F.; Den Oudsten, B. L.] Tilburg Univ, Dept Med Psychol & Neuropsychol, CoRPS Ctr Res Psychol Somat Dis, NL-5000 LE Tilburg, Netherlands.

[Skovlund, S. E.] Novo Nordisk AS, DAWN Diabet Attitudes Wishes & Needs Programme, DK-2880 Bagsvaerd, Denmark.

[Den Oudsten, B. L.] St Elizabeth Hosp, Dept Educ & Res, Tilburg, Netherlands.

[Geelhoed-Duijvestijn, P. H. L. M.] Haaglanden Med Ctr, Dept Internal Med, The Hague, Netherlands.

[Tack, C. J.] Radboud Univ Nijmegen, Med Ctr, Dept Internal Med, NL-6525 ED Nijmegen, Netherlands.

RP Hajos, TRS (reprint author), Vrije Univ Amsterdam Med Ctr, EMGO Inst, Dept Med Psychol, Diabet Psychol Res Grp, Amsterdam, Netherlands.

EM t.hajos@vumc.nl

RI Tack, Cees/A-2368-2014

CR *AM DIAB ASS, 2010, DIABETES CARE S1, V33, pS11, DOI DOI 10.2337/DC10-S011

Anderson RJ, 2001, DIABETES CARE, V24, P1069, DOI 10.2337/diacare.24.6.1069

Awata S, 2007, PSYCHIAT CLIN NEUROS, V61, P112, DOI 10.1111/j.1440-1819.2007.01619.x

Barnard KD, 2006, DIABETIC MED, V23, P445, DOI 10.1111/j.1464-5491.2006.01814.x

de Groot M, 2001, PSYCHOSOM MED, V63, P619, DOI 10.1097/00006842-200107000-00015

de Vet HC, 2011, MEASUREMENT MED, P150

de Wit M, 2007, DIABETES CARE, V30, P2003, DOI 10.2337/dc07-0447

Egede LE, 2010, DIABETES TECHNOL THE, V12, P257, DOI 10.1089/dia.2009.0143

Gilbody S, 2007, J GEN INTERN MED, V22, P1596, DOI 10.1007/s11606-007-0333-y

Herzog W, 2009, STRUCT EQU MODELING, V16, P1, DOI 10.1080/10705510802561279

Home P, 2006, DIABETIC MED, V23, P579

Hu LT, 1999, STRUCT EQU MODELING, V6, P1, DOI 10.1080/10705519909540118

Kroenke K, 2001, J GEN INTERN MED, V16, P606, DOI 10.1046/j.1525-1497.2001.016009606.x

Lowe B, 2004, J AFFECT DISORDERS, V78, P131, DOI 10.1016/S0165-0327(02)00237-9

POLONSKY WH, 1995, DIABETES CARE, V18, P754, DOI 10.2337/diacare.18.6.754

Pouwer F, 2006, PATIENT EDUC COUNS, V60, P235, DOI 10.1016/j.pec.2005.01.009

Pouwer F, 2011, DIABETOLOGIA, V54, P741, DOI 10.1007/s00125-010-2033-0

Pouwer F, 2010, DIABETIC MED, V27, P217, DOI 10.1111/j.1464-5491.2009.02903.x

Pouwer F, 2009, NAT REV ENDOCRINOL, V5, P665, DOI 10.1038/nrendo.2009.214

Roy T, 2012, DIABETIC MED, V29, P164, DOI 10.1111/j.1464-5491.2011.03401.x

Rubin RR, 1999, DIABETES-METAB RES, V15, P205, DOI 10.1002/(SICI)1520-7560(199905/06)15:3<205::AID-DMRR29>3.0.CO;2-O

Snoek FJ, 2000, DIABETES CARE, V23, P1305, DOI 10.2337/diacare.23.9.1305

Snoek FJ, 2011, DIABETES CARE, V34, P601, DOI 10.2337/dc10-1552

Streiner DL, 2008, HLTH MEASUREMENT SCA

van Steenbergen-Weijenburg KM, 2010, BMC HEALTH SERV RES, V10, DOI 10.1186/1472-6963-10-235

WHO, 1998, WELLB MEAS HLTH CAR

Wittkampf KA, 2007, GEN HOSP PSYCHIAT, V29, P388, DOI 10.1016/j.genhosppsych.2007.06.004

NR 27

TC 50

Z9 55

U1 1

U2 20

PU WILEY-BLACKWELL

PI HOBOKEN

PA 111 RIVER ST, HOBOKEN 07030-5774, NJ USA

SN 0742-3071

J9 DIABETIC MED

JI Diabetic Med.

PD FEB

PY 2013

VL 30

IS 2

BP E63

EP E69

DI 10.1111/dme.12040

PG 7

WC Endocrinology & Metabolism

SC Endocrinology & Metabolism

GA 075HV

UT WOS:000313876500005

PM 23072401

DA 2019-01-08

ER

PT J

AU Sabourin, BC

Pursley, S

AF Sabourin, Brigitte C.

Pursley, Shannon

TI Psychosocial Issues in Diabetes Self-Management: Strategies for

Healthcare Providers

SO CANADIAN JOURNAL OF DIABETES

LA English

DT Article

DE behaviour modification; emotion management; motivational enhancement;

psychosocial; type 2 diabetes

ID RANDOMIZED CONTROLLED-TRIALS; COMORBID DEPRESSION; BEHAVIORAL-CHANGE;

GLYCEMIC CONTROL; INTERVENTIONS; MELLITUS; HYPOGLYCEMIA; METAANALYSIS;

ADULTS; FEAR

AB Education and training for diabetes mellitus self-management is widely available and an essential part of many diabetes centres. Nonetheless, the majority of individuals with diabetes do not adhere to optimal self-management recommendations. It is believed that psychosocial issues play an important role in individuals' ability to undertake the extensive behavioural demands involved in managing diabetes. The goal of the present article is to provide an overview of psychosocial issues and suggest strategies for healthcare providers in supporting patients with the challenges of diabetes self-management. First, motivational enhancement strategies have the potential to augment patients' own motivation to engage in health behaviours. Second, behavior modification principles can increase patients' self-efficacy and their experiences of success. Third, managing distressing emotions, including anxiety, depression, distress specifically related to diabetes care, and fear of hypoglycemia, can facilitate motivation and ability to undertake diabetes self-management efforts. Recognizing and addressing psychosocial challenges allows healthcare providers to better support their patients in the demanding tasks of diabetes self-management. (C) 2013 Canadian Diabetes Association

C1 [Sabourin, Brigitte C.] Dalhousie Univ, Queen Elizabeth II Hlth Sci Ctr, Halifax, NS, Canada.

[Pursley, Shannon] Burlington Psychol & Counselling Serv, Burlington, ON, Canada.

[Pursley, Shannon] Grandview Med Ctr, Cambridge, ON, Canada.

[Pursley, Shannon] Waterloo Wellington Self Management Program, Cambridge, ON, Canada.

RP Sabourin, BC (reprint author), Capital Dist Hlth Author, QEII Psychol, Room 4055,Abbie J Lane Bldg,5909 Vet Mem Lane, Halifax, NS B3H 2E2, Canada.

EM brigitte.sabourin@dal.ca

OI Sabourin, Brigitte/0000-0001-7600-9974

CR Anderson RJ, 2001, DIABETES CARE, V24, P1069, DOI 10.2337/diacare.24.6.1069

BANDURA A, 1977, PSYCHOL REV, V84, P191, DOI 10.1037/0033-295X.84.2.191

Boyle S, 2004, BEHAV RES THER, V42, P357, DOI 10.1016/j.brat.2003.11.006

Canadian Diabetes Association Clinical Practice Guidelines Committee: Canadian, 2008, CAN J DIABETES S1, V32, pS1, DOI DOI 10.1503/CMAJ.080554

COX DJ, 1987, DIABETES CARE, V10, P617, DOI 10.2337/diacare.10.5.617

COX DJ, 1990, DIABETES CARE, V13, P458, DOI 10.2337/diacare.13.4.458

Deakin T, 2005, COCHRANE DB SYST REV, DOI 10.1002/14651858.CD003417.pub2

Egede LE, 2002, DIABETES CARE, V25, P464, DOI 10.2337/diacare.25.3.464

Elliott TR, 2001, J CLIN PSYCHOL MED S, V8, P283, DOI 10.1023/A:1011920914079

Fisher L, 2007, DIABETES CARE, V30, P2746, DOI 10.2337/dc07-1166

Funnell MM, 2004, CLIN DIABETES, V22, P123, DOI DOI 10.2337/DIACLIN.22.3.123

Glasgow RE, 1999, DIABETES CARE, V22, P832, DOI 10.2337/diacare.22.5.832

Grigsby AB, 2002, J PSYCHOSOM RES, V53, P1053, DOI 10.1016/S0022-3999(02)00417-8

Ismail K, 2004, LANCET, V363, P1589, DOI 10.1016/S0140-6736(04)16202-8

Lehrer P. M., 2007, PRINCIPLES PRACTICES

Locke EA, 2002, AM PSYCHOL, V57, P705, DOI 10.1037//0003-066X.57.9.705

LUSTMAN PJ, 1992, DIABETES CARE, V15, P1631, DOI 10.2337/diacare.15.11.1631

Lustman PJ, 1998, ANN INTERN MED, V129, P613, DOI 10.7326/0003-4819-129-8-199810150-00005

Malemute CL, 2011, DIABETES EDUCATOR, V37, P549, DOI 10.1177/0145721711410718

Markowitz SM, 2011, PSYCHOSOMATICS, V52, P1, DOI 10.1016/j.psym.2010.11.007

Miller W. R., 2002, MOTIVATIONAL INTERVI

Minet L, 2010, PATIENT EDUC COUNS, V80, P29, DOI 10.1016/j.pec.2009.09.033

Nezu AM, 2004, BEHAV THER, V35, P1, DOI 10.1016/S0005-7894(04)80002-9

Peyrot M, 2005, DIABETIC MED, V22, P1379, DOI 10.1111/j.1464-5491.2005.01644.x

Peyrot M, 2007, DIABETES CARE, V30, P2433, DOI 10.2337/dc07-1222

POLONSKY WH, 1992, HEALTH PSYCHOL, V11, P199, DOI 10.1037/0278-6133.11.3.199

Rubin RR, 2001, J CLIN PSYCHOL, V57, P457, DOI 10.1002/jclp.1041

Sabourin BC, 2011, CAN J DIABETES, V35, P287, DOI 10.1016/S1499-2671(11)53013-8

Shiu ATY, 2002, J ADV NURS, V39, P155

Sultan S, 2001, J HEALTH PSYCHOL, V6, P731, DOI 10.1177/135910530100600616

SURWIT RS, 1992, DIABETES CARE, V15, P1413, DOI 10.2337/diacare.15.10.1413

Thoolen BJ, 2009, PSYCHOL HEALTH, V24, P237, DOI 10.1080/08870440701864504

Vallis M., 2010, FAM MED FOR VANC OCT

Vallis TM, 2001, CAN J DIABETES, V25, P31

Wild D, 2007, PATIENT EDUC COUNS, V68, P10, DOI 10.1016/j.pec.2007.05.003

Zhang XP, 2007, MED CARE, V45, P820, DOI 10.1097/MLR.0b013e3180618b55

NR 36

TC 4

Z9 6

U1 2

U2 14

PU CANADIAN DIABETES ASSOC

PI TORONTO

PA 1400-522 UNIVERSITY AVE, TORONTO, ONTARIO M5G 2R5, CANADA

SN 1499-2671

J9 CAN J DIABETES

JI Can. J. Diabetes

PD FEB

PY 2013

VL 37

IS 1

BP 36

EP 40

DI 10.1016/j.jcjd.2013.01.002

PG 5

WC Endocrinology & Metabolism

SC Endocrinology & Metabolism

GA AL8SE

UT WOS:000339408500007

PM 24070746

DA 2019-01-08

ER

PT J

AU Liu, Y

Croft, JB

Wheaton, AG

Perry, GS

Chapman, DP

Strine, TW

McKnight-Eily, LR

Presley-Cantrell, L

AF Liu, Yong

Croft, Janet B.

Wheaton, Anne G.

Perry, Geraldine S.

Chapman, Daniel P.

Strine, Tara W.

McKnight-Eily, Lela R.

Presley-Cantrell, Letitia

TI Association between perceived insufficient sleep, frequent mental

distress, obesity and chronic diseases among US adults, 2009 behavioral

risk factor surveillance system

SO BMC PUBLIC HEALTH

LA English

DT Article

DE Insufficient sleep; Chronic disease; Population-based study

ID CARDIOVASCULAR-DISEASE; MYOCARDIAL-INFARCTION; DURATION; POPULATION;

HYPERTENSION; DEPRIVATION; DEPRESSION; ANXIETY; HEALTH; ASTHMA

AB Background: Although evidence suggests that poor sleep is associated with chronic disease, little research has been conducted to assess the relationships between insufficient sleep, frequent mental distress (FMD >= 14 days during the past 30 days), obesity, and chronic disease including diabetes mellitus, coronary heart disease, stroke, high blood pressure, asthma, and arthritis.

Methods: Data from 375,653 US adults aged >= 18 years in the 2009 Behavioral Risk Factor Surveillance System were used to assess the relationships between insufficient sleep and chronic disease. The relationships were further examined using a multivariate logistic regression model after controlling for age, sex, race/ethnicity, education, and potential mediators (FMD and obesity).

Results: The overall prevalence of insufficient sleep during the past 30 days was 10.4% for all 30 days, 17.0% for 14-29 days, 42.0% for 1-13 days, and 30.6% for zero day. The positive relationships between insufficient sleep and each of the six chronic disease were significant (p < 0.0001) after adjustment for covariates and were modestly attenuated but not fully explained by FMD. The relationships between insufficient sleep and both diabetes and high blood pressure were also modestly attenuated but not fully explained by obesity.

Conclusions: Assessment of sleep quantity and quality and additional efforts to encourage optimal sleep and sleep health should be considered in routine medical examinations. Ongoing research designed to test treatments for obesity, mental distress, or various chronic diseases should also consider assessing the impact of these treatments on sleep health.

C1 [Liu, Yong; Croft, Janet B.; Wheaton, Anne G.; Perry, Geraldine S.; Chapman, Daniel P.; McKnight-Eily, Lela R.; Presley-Cantrell, Letitia] Ctr Dis Control & Prevent CDC, Div Populat Hlth, Natl Ctr Chron Dis Prevent & Hlth Promot, Atlanta, GA 30341 USA.

[Strine, Tara W.] Ctr Dis Control & Prevent CDC, Div Behav Surveillance, Off Surveillance Epidemiol & Lab Serv, Atlanta, GA 30345 USA.

RP Liu, Y (reprint author), Ctr Dis Control & Prevent CDC, Div Populat Hlth, Natl Ctr Chron Dis Prevent & Hlth Promot, 4770 Buford Highway NE,Mail Stop K-67, Atlanta, GA 30341 USA.

EM ikd8@cdc.gov

CR Anonymous, 2009, Morbidity and Mortality Weekly Report, V58, P1175

Arora VM, 2007, ARCH INTERN MED, V167, P1738, DOI 10.1001/archinte.167.16.1738

Axon RN, 2010, AM J HYPERTENS, V23, P30, DOI 10.1038/ajh.2009.199

Ayas NT, 2003, DIABETES CARE, V26, P380, DOI 10.2337/diacare.26.2.380

Ayas NT, 2003, ARCH INTERN MED, V163, P205, DOI 10.1001/archinte.163.2.205

BARON RM, 1986, J PERS SOC PSYCHOL, V51, P1173, DOI 10.1037//0022-3514.51.6.1173

BOWLIN SJ, 1993, J CLIN EPIDEMIOL, V46, P561, DOI 10.1016/0895-4356(93)90129-O

CATTERALL JR, 1986, THORAX, V41, P676, DOI 10.1136/thx.41.9.676

Centers for Disease Control and Prevention (CDC), 2009, BEH RISK FACT SURV S

Daniel LC, 2012, ANN ALLERG ASTHMA IM, V109, P41, DOI 10.1016/j.anai.2012.05.015

Davidson K, 2000, ARCH INTERN MED, V160, P1495, DOI 10.1001/archinte.160.10.1495

Engum A, 2007, J PSYCHOSOM RES, V62, P31, DOI 10.1016/j.jpsychores.2006.07.009

Gangwisch JE, 2006, HYPERTENSION, V47, P833, DOI 10.1161/01.HYP.0000217362.34748.e0

Gangwisch JE, 2005, SLEEP, V28, P1289, DOI 10.1093/sleep/28.10.1289

Gottlieb DJ, 2005, ARCH INTERN MED, V165, P863, DOI 10.1001/archinte.165.8.863

Grandner MA, 2011, J PUBLIC HEALTH-HEID, V19, P463, DOI 10.1007/s10389-011-0398-2

Haghighatdoost F, 2012, NUTRITION, V28, P1146, DOI 10.1016/j.nut.2012.04.015

Hamer M, 2008, J AM COLL CARDIOL, V52, P2156, DOI 10.1016/j.jacc.2008.08.057

Hanson MD, 2008, J PEDIATR PSYCHOL, V33, P312, DOI 10.1093/jpepsy/jsm120

HUNG J, 1990, LANCET, V336, P261, DOI 10.1016/0140-6736(90)91799-G

*I MED, 2006, SLEEP DIS SLEEP DEPR

Irwin MR, 2012, SLEEP, V35, P537, DOI 10.5665/sleep.1742

JACKSON C, 1992, AM J PUBLIC HEALTH, V82, P412, DOI 10.2105/AJPH.82.3.412

Kopec JA, 2004, CAN J PUBLIC HEALTH, V95, P361

Kripke DF, 2002, ARCH GEN PSYCHIAT, V59, P131, DOI 10.1001/archpsyc.59.2.131

Lavie P, 2000, BRIT MED J, V320, P479, DOI 10.1136/bmj.320.7233.479

Leander M, 2009, RESP MED, V103, P194, DOI 10.1016/j.rmed.2008.09.015

Luckhaupt Sara E., 2012, Morbidity and Mortality Weekly Report, V61, P281

Malhotra A, 2009, PROG CARDIOVASC DIS, V51, P279, DOI 10.1016/j.pcad.2008.10.004

Mallon L, 2005, DIABETES CARE, V28, P2762, DOI 10.2337/diacare.28.11.2762

McKnight-Eily L. R., 2008, Morbidity and Mortality Weekly Report, V57, P200

Merikanto I, 2012, CHRONOBIOL INT, V29, P311, DOI 10.3109/07420528.2012.655870

Mezick EJ, 2011, SLEEP MED REV, V15, P51, DOI 10.1016/j.smrv.2010.03.001

Mokdad Ali H, 2003, MMWR Recomm Rep, V52, P1

MOLDOFSKY H, 1976, PSYCHOSOM MED, V38, P35, DOI 10.1097/00006842-197601000-00006

Moriarty David G, 2003, Health Qual Life Outcomes, V1, P37, DOI 10.1186/1477-7525-1-37

*NAT HEART LUNG BL, 1998, NIH PUBL

Nelson D.E., 2001, INT J PUBLIC HEALTH, V46, P1, DOI DOI 10.1186/1471-2288-13-49

Patel SR, 2006, AM J EPIDEMIOL, V164, P947, DOI 10.1093/aje/kwj280

Patel SR, 2004, SLEEP, V27, P440, DOI 10.1093/sleep/27.3.440

Prasanna SJ, 2010, PLOS ONE, V5, DOI [10.1371/journal.pone.0009016, 10.1371/journal.pone.0014189]

Rothman K. J., 2008, MODERN EPIDEMIOLOGY, P51

Sabanayagam C, 2011, SLEEP MED, V12, P7, DOI 10.1016/j.sleep.2010.09.002

Shahar E, 2001, AM J RESP CRIT CARE, V163, P19, DOI 10.1164/ajrccm.163.1.2001008

Shen BJ, 2008, J AM COLL CARDIOL, V51, P113, DOI 10.1016/j.jacc.2007.09.033

Spiegel K, 1999, LANCET, V354, P1435, DOI 10.1016/S0140-6736(99)01376-8

Strine TW, 2005, SLEEP MED, V6, P23, DOI 10.1016/j.sleep.2004.06.003

Taheri S, 2006, ARCH DIS CHILD, V91, P881, DOI 10.1136/adc.2005.093013

van de Mheen HD, 1998, SOCIOL HEALTH ILL, V20, P754, DOI 10.1111/1467-9566.00128

van den Berg JF, 2009, J CLIN PSYCHIAT, V70, P1105, DOI 10.4088/JCP.08m04448

van Mill JG, 2010, J CLIN PSYCHIAT, V71, P239, DOI 10.4088/JCP.09m05218gry

Vishnu A, 2011, INT J ENDOCRINOL, DOI 10.1155/2011/789358

Yaggi HK, 2005, NEW ENGL J MED, V353, P2034, DOI 10.1056/NEJMoa043104

NR 53

TC 44

Z9 45

U1 0

U2 28

PU BIOMED CENTRAL LTD

PI LONDON

PA 236 GRAYS INN RD, FLOOR 6, LONDON WC1X 8HL, ENGLAND

SN 1471-2458

J9 BMC PUBLIC HEALTH

JI BMC Public Health

PD JAN 29

PY 2013

VL 13

AR 84

DI 10.1186/1471-2458-13-84

PG 8

WC Public, Environmental & Occupational Health

SC Public, Environmental & Occupational Health

GA 082LE

UT WOS:000314392500001

PM 23360346

OA DOAJ Gold, Green Published

DA 2019-01-08

ER

PT J

AU Hung, CH

Tzeng, JI

Chang, CN

Chen, YW

Cho, CY

Wang, JJ

AF Hung, Ching-Hsia

Tzeng, Jann-Inn

Chang, Che-Ning

Chen, Yu-Wen

Cho, Chia-Ying

Wang, Jhi-Joung

TI Treadmill Exercise Preconditioning Attenuates Lung Damage Caused by

Systemic Endotoxemia in Type 1 Diabetic Rats

SO JOURNAL OF DIABETES RESEARCH

LA English

DT Article

ID RESPIRATORY-DISTRESS-SYNDROME; PROTEIN RESPONSE; CONSCIOUS RATS; SHOCK;

INJURY; INSULIN; MELLITUS; INTERLEUKIN-6; HYPERTHERMIA; PRETREATMENT

AB Endotoxemia induces a series of inflammatory responses that may result in lung injury. However, heat shock protein72 (HSP72) has the potential to protect the lungs from damage. The objective of this study was to determine whether prior exercise conditioning could increase the expression of HSP72 in the lungs and attenuate lung damage in diabetic rats receiving lipopolysaccharide (LPS). Streptozotocin was used to induce diabetes in adult male Wistar rats. Rats were randomly assigned to sedentary or exercise groups. Rats in the exercise condition ran on a treadmill 5 days/week, 30-60 min/day, with an intensity of 1.0 mile/hour over a 3-week period. Rats received an intravenous infusion of LPS after 24 hrs from the last training session. Elevated lavage tumor necrosis factor-alpha (TNF-alpha) level in response to LPS was more marked in diabetic rats. HSP72 expression in lungs was significantly increased after exercise conditioning, but less pronounced in diabetic rats. After administration of LPS, exercised rats displayed higher survival rate as well as decreased lavage TNF-alpha level and lung edema in comparison to sedentary rats. Our findings suggest that exercise conditioning could attenuate the occurrence of inflammatory responses and lung damage, thereby reducing mortality rate in diabetic rats during endotoxemia.

C1 [Hung, Ching-Hsia; Chang, Che-Ning; Cho, Chia-Ying] Natl Cheng Kung Univ, Dept Phys Therapy, Tainan 701, Taiwan.

[Tzeng, Jann-Inn] Chia Nan Univ Pharm & Sci, Dept Food Sci & Technol, Tainan 717, Taiwan.

[Tzeng, Jann-Inn] Chi Mei Med Ctr, Dept Anesthesiol, Tainan 710, Taiwan.

[Chen, Yu-Wen] China Med Univ, Dept Phys Therapy, Taichung 404, Taiwan.

[Wang, Jhi-Joung] Chi Mei Med Ctr, Dept Med Res, Tainan 710, Taiwan.

RP Chen, YW (reprint author), China Med Univ, Dept Phys Therapy, Taichung 404, Taiwan.

EM cywhwok@mail.cmu.edu.tw

FU National Science Council of Taiwan [NSC 95-2314-B-006-072-MY3, NSC

101-2314-B-006-037-MY3]

FX Financial support for this study was provided by the National Science

Council of Taiwan (NSC 95-2314-B-006-072-MY3 and NSC

101-2314-B-006-037-MY3).

CR Atalay M, 2004, J APPL PHYSIOL, V97, P605, DOI 10.1152/japplphysiol.01183.2003

Bannerman DD, 2003, AM J PHYSIOL-LUNG C, V284, pL899, DOI 10.1152/ajplung.00338.2002

BOCZKOWSKI J, 1988, AM REV RESPIR DIS, V138, P260, DOI 10.1164/ajrccm/138.2.260

CARROLL P, 1982, DIABETES CARE, V5, P574, DOI 10.2337/diacare.5.6.574

Chang SL, 1999, DIABETOLOGIA, V42, P250, DOI 10.1007/s001250051146

Chatterjee S, 2007, J SURG RES, V140, P99, DOI 10.1016/j.jss.2006.09.002

Chen HI, 2007, MED SCI SPORT EXER, V39, P435, DOI 10.1249/mss.0b013e31802d11c8

Chen YW, 2007, BRIT J SPORT MED, V41, DOI 10.1136/bjsm.2006.033829

Dekker MJ, 2007, METABOLISM, V56, P332, DOI 10.1016/j.metabol.2006.10.015

Demirel HA, 1998, MED SCI SPORT EXER, V30, P1211, DOI 10.1097/00005768-199808000-00005

ENSOR JE, 1994, AM J PHYSIOL, V266, pC967

Fakioglu H, 2004, PHARMACOL RES, V49, P45, DOI 10.1016/j.phrs.2003.03.001

Freudenberg MA, 2008, IMMUNOBIOLOGY, V213, P193, DOI 10.1016/j.imbio.2007.11.008

Ganter MT, 2006, AM J PHYSIOL-LUNG C, V291, pL354, DOI 10.1152/ajplung.00405.2005

Geerlings SE, 1999, FEMS IMMUNOL MED MIC, V26, P259, DOI 10.1111/j.1574-695X.1999.tb01397.x

GLASS EJ, 1987, DIABETOLOGIA, V30, P228, DOI 10.1007/BF00270420

Goldhammer E, 2005, INT J CARDIOL, V100, P93, DOI 10.1016/j.ijcard.2004.08.073

Hawkins LD, 2004, CURR TOP MED CHEM, V4, P1147, DOI 10.2174/1568026043388123

HILLERDAL G, 1982, ACTA MED SCAND, V211, P221

Honiden S, 2009, CRIT CARE MED, V37, P2455, DOI 10.1097/CCM.0b013e3181a0fea5

Hung CH, 2005, SHOCK, V23, P426, DOI 10.1097/01.shk.0000159557.95285.96

Hung CH, 2004, NEUROSCI LETT, V364, P63, DOI 10.1016/j.neulet.2004.02.074

Hung CH, 2008, APPL PHYSIOL NUTR ME, V33, P976, DOI 10.1139/H08-081

Koh Y, 2001, INFLAMMATION, V25, P187, DOI 10.1023/A:1011040515262

Kregel KC, 2002, J APPL PHYSIOL, V92, P2177, DOI 10.1152/japplphysiol.01267.2001

Lepock JR, 2003, INT J HYPERTHER, V19, P252, DOI 10.1080/0265673031000065042

Martins JD, 2006, SHOCK, V25, P260, DOI 10.1097/01.shk.0000194042.18699.b4

Mehanna A, 2007, LIFE SCI, V81, P1301, DOI 10.1016/j.lfs.2007.09.006

Okamoto H, 1998, STROKE, V29, P1209, DOI 10.1161/01.STR.29.6.1209

Petersen AMW, 2005, J APPL PHYSIOL, V98, P1154, DOI 10.1152/japplphysiol.00164.2004

Rojas M, 2005, AM J PHYSIOL-LUNG C, V288, pL333, DOI 10.1152/ajplung.00334.2004

Rusko H, 1987, J SPORT SCI, V5, P273, DOI 10.1080/02640418708729782

Shimada H, 2006, SHOCK, V26, P311, DOI 10.1097/01.shk.0000224960.17274.6f.

Takao Y, 2005, ANESTH ANALG, V100, P810, DOI 10.1213/01.ANE.0000144775.19385.8C

Tang GJ, 1998, CRIT CARE MED, V26, P1558, DOI 10.1097/00003246-199809000-00024

Van Molle W, 2002, IMMUNITY, V16, P685, DOI 10.1016/S1074-7613(02)00310-2

Wong HR, 1997, SHOCK, V8, P213, DOI 10.1097/00024382-199709000-00010

Yang CH, 2009, RESUSCITATION, V80, P1204, DOI 10.1016/j.resuscitation.2009.06.017

NR 38

TC 6

Z9 6

U1 0

U2 5

PU HINDAWI PUBLISHING CORPORATION

PI NEW YORK

PA 410 PARK AVENUE, 15TH FLOOR, #287 PMB, NEW YORK, NY 10022 USA

SN 2314-6745

EI 2314-6753

J9 J DIABETES RES

JI J. Diabetes Res.

PY 2013

AR 527090

DI 10.1155/2013/527090

PG 10

WC Endocrinology & Metabolism; Medicine, Research & Experimental

SC Endocrinology & Metabolism; Research & Experimental Medicine

GA 276VZ

UT WOS:000328779100001

PM 24392457

OA DOAJ Gold, Green Published

DA 2019-01-08

ER

PT J

AU Zhang, J

Xu, CP

Wu, HX

Xue, XJ

Xu, ZJ

Li, Y

Gao, Q

Liu, QZ

AF Zhang, Jie

Xu, Cui-ping

Wu, Hong-xia

Xue, Xiu-juan

Xu, Ze-jun

Li, Yan

Gao, Qing

Liu, Qing-zhi

TI Comparative study of the influence of diabetes distress and depression

on treatment adherence in Chinese patients with type 2 diabetes: a

cross-sectional survey in the People's Republic of China

SO NEUROPSYCHIATRIC DISEASE AND TREATMENT

LA English

DT Article

DE distress; depression; type 2 diabetes; treatment; adherence; People's

Republic of China

ID CLINICAL DEPRESSION; EMOTIONAL-PROBLEMS; GLYCEMIC CONTROL; SCALE;

ADULTS; PREVALENCE; SYMPTOMS; ANXIETY; IMPACT

AB Background: The purpose of this study was to explore diabetes-related distress and depression and their influence on treatment adherence in Chinese patients with type 2 diabetes.

Methods: We surveyed 200 type 2 diabetic patients from two public hospitals using the Diabetes Distress Scale (DDS), Zung Self-rating Depression Scale, and Revised Treatment Adherence in Diabetes Questionnaire (RADQ). A multiple regression model was used to explore the relationship between diabetes distress, depression, and treatment adherence.

Results: In the 200 eligible patients, the incidence of depression and diabetes distress was approximately 24% and 64%, respectively. The mean score on the RADQ was 23.0 +/- 6.0. Multiple regression analysis showed that DDS scores (beta = 5.34, P = 0.000), age (beta = 0.15, P = 0.014), and family history (beta = 3.2, P = 0.016) had a positive correlation with depression. DDS scores (beta = -2.30, P = 0.000) and treatment methods (beta = -0.93, P = 0.012) were risk factors for poor treatment adherence, whereas age (beta = 0.089, P = 0.000) and cohabitation (beta = 0.93, P = 0.012) increased treatment adherence. The independent-samples t-test showed that depression also affected treatment adherence (t = 2.53, P < 0.05).

Conclusion: These findings suggest that the DDS is a predictor of depression and that diabetes distress plays a more important part than depression in treatment adherence. Screening for diabetes distress may be useful for primary prevention of psychologic problems; however, some form of specialized psychologic intervention should be incorporated to promote patient adherence with treatment.

C1 [Zhang, Jie] Shandong Med Coll, Jinan, Peoples R China.

[Xu, Cui-ping; Wu, Hong-xia; Xue, Xiu-juan; Gao, Qing] Shandong Univ, Qianfoshan Hosp, Jinan 250100, Shandong, Peoples R China.

[Xu, Ze-jun] Sichuan Prov Peoples Hosp, Chengdu, Sichuan, Peoples R China.

[Li, Yan] Shandong Univ Tradit Chinese Med, Jinan, Shandong, Peoples R China.

[Liu, Qing-zhi] Shandong Univ, QiLu Hosp, Jinan 250100, Shandong, Peoples R China.

RP Xu, CP (reprint author), Shandong Univ, Qianfoshan Hosp, Jinan 250100, Shandong, Peoples R China.

EM xucuiping775@sohu.com

FU Qianfoshan Hospital, Shandong Province

FX This study was supported by a grant from Qianfoshan Hospital, Shandong

Province. The authors thank the endocrinology nurses for their help with

data collection and Professor Ju Liu for assistance with preparation of

the manuscript.

CR Barefoot JC, 2000, PSYCHOSOM MED, V62, P790, DOI 10.1097/00006842-200011000-00008

Chen ZH, 2000, ACAD J KUNMING MED C, V4, P67

Ciechanowski PS, 2000, ARCH INTERN MED, V160, P3278, DOI 10.1001/archinte.160.21.3278

Egede LE, 2012, CURR PSYCHIAT REP, V14, P15, DOI 10.1007/s11920-011-0240-0

Fisher L, 2008, DIABETIC MED, V25, P1096, DOI 10.1111/j.1464-5491.2008.02533.x

Fisher L, 2008, ANN FAM MED, V6, P246, DOI 10.1370/afm.842

Fisher L, 2007, DIABETES CARE, V30, P542, DOI 10.2337/dc06-1614

Fisher L, 2012, DIABETES CARE, V35, P259, DOI 10.2337/dc11-1572

Fisher L, 2010, DIABETES CARE, V33, P1034, DOI 10.2337/dc09-2175

Fisher L, 2010, DIABETES CARE, V33, P23, DOI 10.2337/dc09-1238

GAVARD JA, 1993, DIABETES CARE, V16, P1167, DOI 10.2337/diacare.16.8.1167

Gonzalez JS, 2007, DIABETES CARE, V30, P2222, DOI 10.2337/dc07-0158

Graue M, 2012, INT J NURS STUD, V49, P174, DOI 10.1016/j.ijnurstu.2011.08.007

Grigsby AB, 2002, J PSYCHOSOM RES, V53, P1053, DOI 10.1016/S0022-3999(02)00417-8

Hermanns N, 2006, DIABETOLOGIA, V49, P469, DOI 10.1007/s00125-005-0094-2

Kalsekar ID, 2006, ANN PHARMACOTHER, V40, P605, DOI 10.1345/aph.1G606

Kokoszka A, 2009, EUR PSYCHIAT, V24, P425, DOI 10.1016/j.eurpsy.2009.04.002

Nichols GA, 2003, DIABETES CARE, V26, P744, DOI 10.2337/diacare.26.3.744

Odusan O, 2012, Afr J Med Med Sci, V41, P141

Passik SD, 2000, PSYCHOSOMATICS, V41, P121, DOI 10.1176/appi.psy.41.2.121

Polonsky WH, 2005, DIABETES CARE, V28, P626, DOI 10.2337/diacare.28.3.626

Pouwer F, 2005, DIABETES RES CLIN PR, V70, P166, DOI 10.1016/j.diabres.2005.03.031

Ting RZW, 2011, DIABETES CARE, V34, P1094, DOI 10.2337/dc10-1612

Yang Q., 2010, J NURSING CHINA, V17, P8

Zagarins SE, 2012, J BEHAV MED, V35, P299, DOI 10.1007/s10865-011-9359-z

ZUNG WWK, 1965, ARCH GEN PSYCHIAT, V12, P63

NR 26

TC 18

Z9 19

U1 0

U2 16

PU DOVE MEDICAL PRESS LTD

PI ALBANY

PA PO BOX 300-008, ALBANY, AUCKLAND 0752, NEW ZEALAND

SN 1176-6328

J9 NEUROPSYCH DIS TREAT

JI Neuropsychiatr. Dis. Treat.

PY 2013

VL 9

BP 1289

EP 1294

DI 10.2147/NDT.S49798

PG 6

WC Clinical Neurology; Psychiatry

SC Neurosciences & Neurology; Psychiatry

GA 208SN

UT WOS:000323699900001

PM 24039431

OA DOAJ Gold, Green Published

DA 2019-01-08

ER

PT J

AU Wang, L

Wang, KS

AF Wang, Liang

Wang, Ke-Sheng

TI Age Differences in the Associations of Behavioral and Psychosocial

Factors with Stroke

SO NEUROEPIDEMIOLOGY

LA English

DT Article

DE Stroke; Risk factor; Age difference; Severe psychological distress;

Smoking; Physical activity

ID AMERICAN-HEART-ASSOCIATION; BLACK-WHITE DIFFERENCES; SERIOUS

MENTAL-ILLNESS; RISK-FACTORS; PSYCHOLOGICAL DISTRESS; PHYSICAL-ACTIVITY;

1ST-EVER STROKE; ISCHEMIC-STROKE; SEX-DIFFERENCES; DISEASE

AB Background: Stroke remains a major public health burden. Few studies have focused on the age differences in the associations of behavioral and psychosocial factors with stroke while no study focusing on the effect of severe psychological distress (SPD) on stroke has been conducted. The aim of this study was to examine the age differences in these risk factors for stroke as young (18-44 years), middle aged (45 64 years), and elderly (65 years or older). Methods: A total of 1,258 adults with stroke and 39,985 controls were selected from the 2005 California Health Interview Survey. Multiple logistic regression analyses were used to estimate the associations of the factors with stroke at different ages. Results: The prevalence of SPD was 10% in cases and 3.6% in controls, respectively. Overall, current smoking, lack of physical activity, alcohol consumption, SPD, type II diabetes, male, older age, and unemployment were all associated with a higher prevalence of stroke. Practically, we found that smoking and SPD were associated with the prevalence of stroke in young adults, lack of physical activity was associated with the prevalence of stroke in middle-aged adults, and lack of physical activity and SPD were associated with the prevalence of stroke in the elderly. Conclusions: Appropriate intervention for reducing stroke and eliminating its disparities may be developed separately at each age. Copyright (C) 2013 S. Karger AG, Basel

C1 [Wang, Liang; Wang, Ke-Sheng] E Tennessee State Univ, Dept Biostat & Epidemiol, Coll Publ Hlth, Johnson City, TN 37614 USA.

RP Wang, L (reprint author), E Tennessee State Univ, Dept Biostat & Epidemiol, POB 70259, Johnson City, TN 37614 USA.

EM wangl2@etsu.edu

CR [Anonymous], 1996, 1995 HEART STROK FAC

Baggaley RF, 2007, TROP MED INT HEALTH, V12, P1225, DOI 10.1111/j.1365-3156.2007.01906.x

Brown RD, 1996, STROKE, V27, P373

Carney RA, 2002, STROKE, V33, P5, DOI 10.1161/str.33.1.5

Carson AP, 2009, ANN EPIDEMIOL, V19, P630, DOI 10.1016/j.annepidem.2009.04.008

Centers for Disease Control and Prevention (CDC), VIT STAT PUBL US DAT

Engstad T, 2000, STROKE, V31, P1602, DOI 10.1161/01.STR.31.7.1602

GILES WH, 1995, ARCH INTERN MED, V155, P1319, DOI 10.1001/archinte.155.12.1319

GILLUM RF, 1988, STROKE, V19, P1

Gillum RF, 1999, AM J EPIDEMIOL, V150, P1266

Hamer M, 2012, CAN MED ASSOC J, V184, P1461, DOI 10.1503/cmaj.111719

Hankey GJ, 1998, STROKE, V29, P2491, DOI 10.1161/01.STR.29.12.2491

Heidenreich PA, 2011, CIRCULATION, V123, P933, DOI 10.1161/CIR.0b013e31820a55f5

House A, 2001, STROKE, V32, P696, DOI 10.1161/01.STR.32.3.696

HOWARD G, 1994, STROKE, V25, P2120, DOI 10.1161/01.STR.25.11.2120

Jonas BS, 2000, PSYCHOSOM MED, V62, P463, DOI 10.1097/00006842-200007000-00001

Kessler RC, 2003, ARCH GEN PSYCHIAT, V60, P184, DOI 10.1001/archpsyc.60.2.184

Kessler RC, 2002, PSYCHOL MED, V32, P959, DOI 10.1017/S0033291702006074

Kissela B, 2004, STROKE, V35, P426, DOI 10.1161/01.STR.0000110982.74967.39

KITTNER SJ, 1990, JAMA-J AM MED ASSOC, V264, P1267, DOI 10.1001/jama.264.10.1267

Lee CD, 2003, STROKE, V34, P2475, DOI 10.1161/01.STR.0000091843.02517.9D

Liao YL, 2009, STROKE, V40, P3336, DOI 10.1161/STROKEAHA.109.561688

Lloyd-Jones D, 2010, CIRCULATION, V121, pE46, DOI 10.1161/CIRCULATIONAHA.109.192667

Lloyd-Jones DM, 2010, CIRCULATION, V121, P586, DOI 10.1161/CIRCULATIONAHA.109.192703

Lofmark U, 2007, NEUROEPIDEMIOLOGY, V28, P135, DOI 10.1159/000102141

Mathers CD, 2006, PLOS MED, V3, DOI 10.1371/journal.pmed.0030442

Myint PK, 2008, EUR J CARDIOV PREV R, V15, P663, DOI 10.1097/HJR.0b013e32830fe465

Nelson DE, 2001, SOZ PRAVENTIV MED, V46, pS3

OMAHONY PG, 1995, STROKE, V26, P1334, DOI 10.1161/01.STR.26.8.1334

Physical Activity Guidelines Advisory Committee, 2008, PHYS ACT GUID ADV CO

PLEIS JR, 2007, VITAL HLTH STAT, V10, P1

Reeves MJ, 2008, LANCET NEUROL, V7, P915, DOI 10.1016/S1474-4422(08)70193-5

Roger VL, 2012, CIRCULATION, V125, pE2, DOI 10.1161/CIR.0b013e31823ac046

Sacco RL, 1998, AM J EPIDEMIOL, V147, P259

Schiller JS, 2012, VITAL HLTH STAT, V10, P1

Simons LA, 1998, STROKE, V29, P1341, DOI 10.1161/01.STR.29.7.1341

Sims NR, 2010, BBA-MOL BASIS DIS, V1802, P80, DOI 10.1016/j.bbadis.2009.09.003

Suadicani P, 2011, J OCCUP ENVIRON MED, V53, P1388, DOI 10.1097/JOM.0b013e31823c149d

Swartz JA, 2006, J SUBST ABUSE TREAT, V31, P287, DOI 10.1016/j.jsat.2006.04.009

Tsadok MA, 2012, JAMA-J AM MED ASSOC, V307, P1952, DOI 10.1001/jama.2012.3490

Tuttolomondo A, 2008, NUTR METAB CARDIOVAS, V18, P152, DOI 10.1016/j.numecd.2007.02.003

Wendel-Vos GCW, 2004, INT J EPIDEMIOL, V33, P787, DOI 10.1093/ije/dyh168

You JS, 2008, STROKE, V39, pE42, DOI 10.1161/STROKEAHA.107.506717

Zhang Y, 2008, CIRCULATION, V118, P1577, DOI 10.1161/CIRCULATIONAHA.108.772285

NR 44

TC 1

Z9 2

U1 0

U2 3

PU KARGER

PI BASEL

PA ALLSCHWILERSTRASSE 10, CH-4009 BASEL, SWITZERLAND

SN 0251-5350

J9 NEUROEPIDEMIOLOGY

JI Neuroepidemiology

PY 2013

VL 41

IS 2

BP 94

EP 100

DI 10.1159/000350018

PG 7

WC Public, Environmental & Occupational Health; Clinical Neurology

SC Public, Environmental & Occupational Health; Neurosciences & Neurology

GA 198RQ

UT WOS:000322940400004

PM 23774713

DA 2019-01-08

ER

PT J

AU Saydah, SH

Imperatore, G

Beckles, GL

AF Saydah, Sharon H.

Imperatore, Giuseppina

Beckles, Gloria L.

TI Socioeconomic Status and Mortality Contribution of health care access

and psychological distress among US adults with diagnosed diabetes

SO DIABETES CARE

LA English

DT Article

ID ALL-CAUSE MORTALITY; EDUCATIONAL-LEVEL; RISK-FACTORS; LIFE-COURSE;

FOLLOW-UP; POSITION; INEQUALITIES; ASSOCIATION; DEPRESSION; BEHAVIORS

AB OBJECTIVE-Although several studies have examined the association between socioeconomic status (SES) and mortality in the general population, few have investigated this relationship among people with diabetes. This study sought to determine how risk of mortality associated with measures of SES among adults with diagnosed diabetes is mitigated by association with demographics, comorbidities, diabetes treatment, psychological distress, or health care access and utilization.

RESEARCH DESIGN AND METHODS-The study included 6,177 adults aged 25 years or older with diagnosed diabetes who participated in the National Health Interview Surveys (1997-2003) linked to mortality data (follow-up through 2006). SES was measured by education attained, financial wealth (either stocks/dividends or home ownership), and income-to-poverty ratio.

RESULTS-In unadjusted analysis, risk of death was significantly greater for people with lower levels of education and income-to-poverty ratio than for those at the highest levels. After adjusting for demographics, comorbidities, diabetes treatment and duration, health care access, and psychological distress variables, the association with greater risk of death remained significant only for people with the lowest level of education (relative hazard 1.52 [95% CI 1.04-2.23]). After multivariate adjustment, the risk of death was significantly greater for people without certain measures of financial wealth (e. g., stocks, home ownership) (1.56 [1.07-2.27]) than for those with them.

CONCLUSIONS-The findings suggest that after adjustments for demographics, health care access, and psychological distress, the level of education attained and financial wealth remain strong predictors of mortality risk among adults with diabetes. Diabetes Care 36: 49-55, 2013

C1 [Saydah, Sharon H.; Imperatore, Giuseppina; Beckles, Gloria L.] Ctr Dis Control & Prevent, Div Diabet Translat, Atlanta, GA 30329 USA.

RP Saydah, SH (reprint author), Ctr Dis Control & Prevent, Div Diabet Translat, Atlanta, GA 30329 USA.

EM ssaydah@cdc.gov

CR Braveman PA, 2005, JAMA-J AM MED ASSOC, V294, P2879, DOI 10.1001/jama.294.22.2879

Brown AF, 2004, EPIDEMIOL REV, V26, P63, DOI 10.1093/epirev/mxh002

Centers for Disease Control and Prevention National Center for Health Statistics, 2000, DES EST NAT HLTH INT

Chaturvedi N, 1998, BRIT MED J, V316, P100

Cubbin C, 2011, AM J HEALTH BEHAV, V35, P28

Dalaker J, 2009, CURRENT POPULATION R

Dowd JB, 2011, INT J EPIDEMIOL, V40, P183, DOI 10.1093/ije/dyq189

Dray-Spira R, 2010, DIABETES CARE, V33, P1200, DOI 10.2337/dc09-2094

Egede LE, 2005, DIABETES CARE, V28, P1339, DOI 10.2337/diacare.28.6.1339

Galobardes B, 2006, J EPIDEMIOL COMMUN H, V60, P95, DOI 10.1136/jech.2004.028092

Galobardes B, 2006, J EPIDEMIOL COMMUN H, V60, P7, DOI 10.1136/jech.2004.023531

Gnavi R, 2004, INT J EPIDEMIOL, V33, P864, DOI 10.1093/ije/dyh089

Hajat A, 2011, AM J EPIDEMIOL, V173, P192, DOI 10.1093/aje/kwq348

HUIE SAB, 2003, SOCIAL SCI Q, V84, P667, DOI DOI 10.1111/1540-6237.8403011

Katon W, 2008, J GEN INTERN MED, V23, P1571, DOI 10.1007/s11606-008-0731-9

Kessler RC, 2003, ARCH GEN PSYCHIAT, V60, P184, DOI 10.1001/archpsyc.60.2.184

Krieger N, 1997, ANNU REV PUBL HEALTH, V18, P341, DOI 10.1146/annurev.publhealth.18.1.341

Laaksonen M, 2008, EUR J PUBLIC HEALTH, V18, P38, DOI 10.1093/eurpub/ckm051

Lantz PM, 2010, SOC SCI MED, V70, P1558, DOI 10.1016/j.socscimed.2010.02.003

Lawlor DA, 2007, DIABETIC MED, V24, P892, DOI 10.1111/j.1464-5491.2007.02187.x

Lee ET, 2003, PARAMETRIC METHODS C, P246

Lipscombe LL, 2010, CAN MED ASSOC J, V182, pE1, DOI 10.1503/cmaj.090495

Lorant V, 2003, AM J EPIDEMIOL, V157, P98, DOI 10.1093/aje/kwf182

Macintyre S, 2003, J PUBLIC HEALTH MED, V25, P288, DOI 10.1093/pubmed/fdg089

Mackenbach JP, 2008, NEW ENGL J MED, V358, P2468, DOI 10.1056/NEJMsa0707519

McEwen LN, 2007, DIABETES CARE, V30, P1736, DOI 10.2337/dc07-0305

Miech RA, 2009, AM J PREV MED, V36, P126, DOI 10.1016/j.amepre.2008.09.041

National Center for Health Statistics Office of Analysis and Epidemiology The National Health Interview Survey, 1986, LINK MORT FIL 1986 2

Pollack CE, 2007, AM J PREV MED, V33, P250, DOI 10.1016/j.amerpe.2007.04.033

Saydah S, 2010, PUBLIC HEALTH REP, V125, P377, DOI 10.1177/003335491012500306

Schenker N., 2008, MULTIPLE IMPUTATION

Secrest AM, 2011, ANN EPIDEMIOL, V21, P367, DOI 10.1016/j.annepidem.2011.02.011

Smith GD, 1997, BMJ-BRIT MED J, V314, P547, DOI 10.1136/bmj.314.7080.547

Smith JP, 1999, J ECON PERSPECT, V13, P144

Stringhini S, 2010, JAMA-J AM MED ASSOC, V303, P1159, DOI 10.1001/jama.2010.297

Turrell G, 2007, J EPIDEMIOL COMMUN H, V61, P723, DOI 10.1136/jech.2007.050609

van Oort FVA, 2005, J EPIDEMIOL COMMUN H, V59, P214, DOI 10.1136/jech.2003.016493

Walker JJ, 2011, DIABETES CARE, V34, P1127, DOI 10.2337/dc10-1862

Weng C, 2000, DIABETIC MED, V17, P612, DOI 10.1046/j.1464-5491.2000.00352.x

Zhang XP, 2005, AM J EPIDEMIOL, V161, P652, DOI 10.1093/aje/kwi089

NR 40

TC 47

Z9 47

U1 0

U2 11

PU AMER DIABETES ASSOC

PI ALEXANDRIA

PA 1701 N BEAUREGARD ST, ALEXANDRIA, VA 22311-1717 USA

SN 0149-5992

J9 DIABETES CARE

JI Diabetes Care

PD JAN

PY 2013

VL 36

IS 1

BP 49

EP 55

DI 10.2337/dc11-1864

PG 7

WC Endocrinology & Metabolism

SC Endocrinology & Metabolism

GA 083LP

UT WOS:000314465700030

PM 22933434

OA Green Published, Other Gold

DA 2019-01-08

ER

PT J

AU Rodriguez-Penney, AT

Iudicello, JE

Riggs, PK

Doyle, K

Ellis, RJ

Letendre, SL

Grant, I

Woods, SP

AF Rodriguez-Penney, Alan T.

Iudicello, Jennifer E.

Riggs, Patricia K.

Doyle, Katie

Ellis, Ronald J.

Letendre, Scott L.

Grant, Igor

Woods, Steven Paul

CA HIV Neurobehav Res Program

TI Co-Morbidities in Persons Infected with HIV: Increased Burden with Older

Age and Negative Effects on Health-Related Quality of Life

SO AIDS PATIENT CARE AND STDS

LA English

DT Article

ID ACTIVE ANTIRETROVIRAL THERAPY; COMORBIDITY INDEX; NEUROCOGNITIVE

IMPAIRMENT; MEDICAL COMORBIDITIES; METABOLIC SYNDROME; AIDS; COHORT;

ADULTS; RISK; IMPACT

AB This study sought to determine the synergistic effects of age and HIV infection on medical co-morbidity burden, along with its clinical correlates and impact on health-related quality of life (HRQoL) across the lifespan in HIV. Participants included 262 individuals across four groups stratified by age (<= 40 and 50 years) and HIV serostatus. Medical co-morbidity burden was assessed using a modified version of the Charlson Co-morbidity Index (CCI). Multiple regression accounting for potentially confounding demographic, psychiatric, and medical factors revealed an interaction between age and HIV infection on the CCI, with the highest medical co-morbidity burden in the older HIV + cohort. Nearly half of the older HIV + group had at least one major medical co-morbidity, with the most prevalent being diabetes (17.8%), syndromic neurocognitive impairment (15.4%), and malignancy (12.2%). Affective distress and detectable plasma viral load were significantly associated with the CCI in the younger and older HIV-infected groups, respectively. Greater co-morbidity burden was uniquely associated with lower physical HRQoL across the lifespan. These findings highlight the prevalence and clinical impact of co-morbidities in older HIV-infected adults and underscore the importance of early detection and treatment efforts that might enhance HIV disease outcomes.

C1 [Rodriguez-Penney, Alan T.] Univ Puerto Rico, Sch Med, San Juan, PR 00936 USA.

[Iudicello, Jennifer E.; Riggs, Patricia K.; Doyle, Katie; Grant, Igor] Univ Calif San Diego, Dept Psychiat, San Diego, CA 92103 USA.

[Ellis, Ronald J.] Univ Calif San Diego, Dept Neurosci, San Diego, CA 92103 USA.

[Letendre, Scott L.] Univ Calif San Diego, Dept Med, San Diego, CA 92103 USA.

RP Woods, SP (reprint author), Univ Calif San Diego, Dept Psychiat 8231, 220 Dickinson St,Suite B, San Diego, CA 92103 USA.

EM spwoods@ucsd.edu

OI Ellis, Ronald/0000-0003-4931-752X

FU National Institutes of Health [R01-MH73419, T32-DA31098, P30-MH62512,

T35-AG026757, L30-DA034362]

FX The San Diego HIV Neurobehavioral Research Program (HNRP) group is

affiliated with the University of California, San Diego, the Naval

Hospital, San Diego, and the Veterans Affairs San Diego Healthcare

System, and includes: Director: Igor Grant, MD; Co-Directors: J. Hampton

Atkinson, MD, Ronald J. Ellis, MD, PhD, and J. Allen McCutchan, MD;

Center Manager: Thomas D. Marcotte, PhD; Jennifer Marquie-Beck, MPH;

Melanie Sherman; Neuromedical Component: Ronald J. Ellis, MD, PhD

(P.I.), J. Allen McCutchan, MD, Scott Letendre, MD, Edmund Capparelli,

PharmD, Rachel Schrier, PhD, Terry Alexander, RN, Debra Rosario, MPH,

Shannon LeBlanc; Neurobehavioral Component: Robert K. Heaton, PhD

(P.I.), Steven Paul Woods, PsyD, MarianaCherner, PhD, David J. Moore,

PhD, Matthew Dawson; Neuroimaging Component: Terry Jernigan, PhD (P.I.),

Christine Fennema-Notestine, PhD, Sarah L. Archibald, MA, John

Hesselink, MD, Jacopo Annese, PhD, Michael J. Taylor, PhD; Neurobiology

Component: Eliezer Masliah, MD (P.I.), Cristian Achim, MD, PhD, Ian

Everall, FRCPsych, FRCPath, PhD (Consultant); Neurovirology Component:

Douglas Richman, MD, (P.I.), David M. Smith, MD; International

Component: J. Allen McCutchan, MD, (P.I.); Developmental Component:

Cristian Achim, MD, PhD; (P.I.), Stuart Lipton, MD, PhD; Participant

Accrual and Retention Unit: J. Hampton Atkinson, MD (P.I.); Data

Management Unit: Anthony C. Gamst, PhD (P.I.), Clint Cushman (Data

Systems Manager); Statistics Unit: Ian Abramson, PhD (P.I.), Florin

Vaida, PhD, Reena Deutsch, PhD, Anya Umlauf, MS. The authors thank

Marizela Cameron, Erica Weber, and Nichole Duarte for their assistance

with study management. This research was supported by National

Institutes of Health grants R01-MH73419 and T32-DA31098 to Dr. Woods,

P30-MH62512 to Dr. Grant, T35-AG026757 to Dr. Dilip Jeste, and

L30-DA034362 to Dr. Iudicello.

CR Alencastro PR, 2011, AIDS PATIENT CARE ST, V25, P627, DOI 10.1089/apc.2010.0360

American Psychiatric Association, 1994, DIAGN STAT MAN MENT

Antinori A, 2007, NEUROLOGY, V69, P1789, DOI 10.1212/01.WNL.0000287431.88658.8b

Backus LI, 2011, AIDS PATIENT CARE ST, V25, P203, DOI 10.1089/apc.2010.0379

Bamford LP, 2010, AIDS, V24, P928, DOI 10.1097/QAD.0b013e328337b116

Barclay TR, 2007, HEALTH PSYCHOL, V26, P40, DOI 10.1037/0278-6133.26.1.40

Bing EG, 2000, QUAL LIFE RES, V9, P55, DOI 10.1023/A:1008919227665

Blanco JR, 2010, AIDS REV, V12, P218

Campsmith Michael L, 2003, Health Qual Life Outcomes, V1, P12, DOI 10.1186/1477-7525-1-12

Cederfjall C, 2001, AIDS PATIENT CARE ST, V15, P31, DOI 10.1089/108729101460083

Center for Disease Control and Prevention, 2009, HIV SURV REP 2008

Center for Disease Control and Prevention, 2011, HIV SURV REP 2010

CHARLSON ME, 1987, J CHRON DIS, V40, P373, DOI 10.1016/0021-9681(87)90171-8

Deeks SG, 2009, BRIT MED J, V338, DOI 10.1136/bmj.a3172

DEYO RA, 1992, J CLIN EPIDEMIOL, V45, P613, DOI 10.1016/0895-4356(92)90133-8

Doyle K, 2012, AIDS BEHAV, V16, P2309, DOI 10.1007/s10461-011-0121-x

Egger M, 2002, LANCET, V360, P119, DOI 10.1016/S0140-6736(02)09411-4

Ellis RJ, 1997, ARCH NEUROL-CHICAGO, V54, P416, DOI 10.1001/archneur.1997.00550160054016

Friis-Moller N, 2003, NEW ENGL J MED, V349, P1993

GARDNER ID, 1980, REV INFECT DIS, V2, P801

Goulet JL, 2007, CLIN INFECT DIS, V45, P1593, DOI 10.1086/523577

Grabar S, 2004, AIDS, V18, P2029, DOI 10.1097/00002030-200410210-00007

Grinspoon S, 2005, NEW ENGL J MED, V352, P48, DOI 10.1056/NEJMra041811

Grunfeld C, 2009, AIDS, V23, P1841, DOI 10.1097/QAD.0b013e32832d3b85

Guaraldi G, 2011, CLIN INFECT DIS, V53, P1120, DOI 10.1093/cid/cir627

Hall SF, 2006, J CLIN EPIDEMIOL, V59, P849, DOI 10.1016/j.jclinepi.2005.11.013

Hasse B, 2011, CLIN INFECT DIS, V53, P1130, DOI 10.1093/cid/cir626

Health and Human Services Agency, 2012, HIV AIDS EP REP 2012

Heaton RK, 2010, NEUROLOGY, V75, P2087, DOI 10.1212/WNL.0b013e318200d727

Heaton RK, 2004, J INT NEUROPSYCH SOC, V10, P317, DOI 10.1017/S1355617704102130

Heaton RK, 2011, J NEUROVIROL, V17, P3, DOI 10.1007/s13365-010-0006-1

Hernandez MD, 2011, CURR OPIN HIV AIDS, V6, P478, DOI 10.1097/COH.0b013e32834bd365

High KP, 2012, JAIDS-J ACQ IMM DEF, V60, pS1, DOI 10.1097/QAI.0b013e31825a3668

Hinkin CH, 2008, J ADDICT DIS, V27, P11, DOI 10.1300/J069v27n02_02

Holzemer WL, 1998, J ADV NURS, V28, P622, DOI 10.1046/j.1365-2648.1998.00800.x

Hughes AK, 2011, AIDS PATIENT CARE ST, V25, P539, DOI 10.1089/apc.2011.0026

Isomaa B, 2001, DIABETES CARE, V24, P683, DOI 10.2337/diacare.24.4.683

Iudicello JE, 2012, J CLIN EXP NEUROPSYC, V34, P476, DOI 10.1080/13803395.2011.651103

Jia HG, 2004, AIDS PATIENT CARE ST, V18, P594, DOI 10.1089/1087291042404666

Kamat R, 2012, ARCH CLIN NEUROPSYCH, V27, P520, DOI 10.1093/arclin/acs055

Kilbourne AM, 2001, J CLIN EPIDEMIOL, V54, pS22, DOI 10.1016/S0895-4356(01)00443-7

LAMPING DL, 1994, PSYCHOL HEALTH, V9, P31, DOI 10.1080/08870449408407458

LAWTON MP, 1969, GERONTOLOGIST, V9, P179, DOI 10.1093/geront/9.3_Part_1.179

Lohse N, 2011, JAIDS-J ACQ IMM DEF, V57, P334, DOI 10.1097/QAI.0b013e31821d34ed

McCutchan JA, 2012, NEUROLOGY, V78, P485, DOI 10.1212/WNL.0b013e3182478d64

McNair D. M, 1981, PROFILE MOOD STATES

Mocroft A, 2010, JAIDS-J ACQ IMM DEF, V55, P262, DOI 10.1097/QAI.0b013e3181e9be6b

Monroe AK, 2011, JAIDS-J ACQ IMM DEF, V58, P458, DOI 10.1097/QAI.0b013e31823801c4

Morgan EE, 2012, JAIDS-J ACQ IMM DEF, V61, P341, DOI 10.1097/QAI.0b013e31826bfc53

Neidig Judith L, 2003, J Assoc Nurses AIDS Care, V14, P30, DOI 10.1177/1055329002250992

Nokes KM, 2011, J ASSOC NURSE AIDS C, V22, P186, DOI 10.1016/j.jana.2010.10.006

OKeefe EA, 1996, QUAL LIFE RES, V5, P275, DOI 10.1007/BF00434749

Oursler KK, 2006, AIDS PATIENT CARE ST, V20, P782, DOI 10.1089/apc.2006.20.782

Oursler KK, 2011, AIDS PATIENT CARE ST, V25, P13, DOI 10.1089/apc.2010.0242

Palella FJ, 2006, JAIDS-J ACQ IMM DEF, V43, P27, DOI 10.1097/01.qai.0000233310.90484.16

Peters L, 2011, ANTIVIR THER, V16, P667, DOI 10.3851/IMP1815

PHILLIPS AN, 1991, J ACQ IMMUN DEF SYND, V4, P970

Reekie J, 2010, CANCER-AM CANCER SOC, V116, P5306, DOI 10.1002/cncr.25311

Schuster Randi Melissa, 2012, Neurobehav HIV Med, V2012, P15

Skiest DJ, 1996, AM J MED, V101, P605, DOI 10.1016/S0002-9343(96)00329-4

Sorensen K, 2012, BMC PUBLIC HEALTH, V12, DOI 10.1186/1471-2458-12-80

Tate D, 2003, AIDS PATIENT CARE ST, V17, P115, DOI 10.1089/108729103763807936

Thames AD, 2011, J CLIN EXP NEUROPSYC, V33, P200, DOI 10.1080/13803395.2010.499357

Tozzi V, 2005, AIDS RES HUM RETROV, V21, P706, DOI 10.1089/aid.2005.21.706

Tumbarello M, 2004, BMC INFECT DIS, V4, DOI 10.1186/1471-2334-4-46

Valcour V, 2004, NEUROLOGY, V63, P822, DOI 10.1212/01.WNL.0000134665.58343.8D

Vance D, 2011, J NEUROSCI NURS, V43, P215, DOI 10.1097/JNN.0b013e3182212a04

Wechsler D., 2001, WECHSLER TEST ADULT

Weiss JJ, 2010, AIDS PATIENT CARE ST, V24, P39, DOI 10.1089/apc.2009.0152

*WHO, 1998, COMP INT DIAGN INT C

Woods SP, 2004, J CLIN EXP NEUROPSYC, V26, P759, DOI 10.1080/13803390490509565

Woods SP, 2006, J CLIN EXP NEUROPSYC, V28, P1030, DOI 10.1080/13803390500350985

Woods SP, 2008, NEUROPSYCHOLOGY, V22, P110, DOI 10.1037/0894-4105.22.1.110

Woods SP, 2010, J CLIN EXP NEUROPSYC, V32, P398, DOI 10.1080/13803390903130737

Zavascki AP, 2007, J CLIN EPIDEMIOL, V60, P867, DOI 10.1016/j.jclinepi.2006.11.004

Zisook S, 1998, J CLIN PSYCHIAT, V59, P217, DOI 10.4088/JCP.v59n0502

NR 76

TC 108

Z9 109

U1 1

U2 21

PU MARY ANN LIEBERT, INC

PI NEW ROCHELLE

PA 140 HUGUENOT STREET, 3RD FL, NEW ROCHELLE, NY 10801 USA

SN 1087-2914

EI 1557-7449

J9 AIDS PATIENT CARE ST

JI Aids Patient Care STDS

PD JAN

PY 2013

VL 27

IS 1

BP 5

EP 16

DI 10.1089/apc.2012.0329

PG 12

WC Public, Environmental & Occupational Health; Infectious Diseases

SC Public, Environmental & Occupational Health; Infectious Diseases

GA 071HX

UT WOS:000313583400002

PM 23305257

OA Green Published

HC Y

HP N

DA 2019-01-08

ER

PT J

AU Faria, M

Pavin, EJ

Parisi, MCR

Lorena, SLS

Brunetto, SQ

Ramos, CD

Pavan, CR

Mesquita, MA

AF Faria, Mariza

Pavin, Elizabeth Joao

Ribeiro Parisi, Maria Candida

Silva Lorena, Sonia Leticia

Brunetto, Sergio Quirino

Ramos, Celso Dario

Pavan, Celia Regina

Mesquita, Maria Aparecida

TI Delayed Small Intestinal Transit in Patients with Long-Standing Type 1

Diabetes Mellitus: Investigation of the Relationships with Clinical

Features, Gastric Emptying, Psychological Distress, and Nutritional

Parameters

SO DIABETES TECHNOLOGY & THERAPEUTICS

LA English

DT Article

ID SMALL-BOWEL TRANSIT; BACTERIAL OVERGROWTH; OROCECAL TRANSIT;

GASTROINTESTINAL MOTILITY; AUTONOMIC NEUROPATHY; FUNCTIONAL DYSPEPSIA;

GUT TRANSIT; ANXIETY; DISORDERS; SYMPTOMS

AB Background: Studies on small intestinal transit in type 1 diabetes mellitus have reported contradictory results. This study assessed the orocecal transit time (OCTT) in a group of patients with type 1 diabetes mellitus and its relationships with gastrointestinal symptoms, glycemic control, chronic complications of diabetes, anthropometric indices, gastric emptying, small intestinal bacterial overgrowth (SIBO), and psychological distress.

Subjects and Methods: Twenty-eight patients with long-standing (> 10 years) type 1 diabetes mellitus (22 women, six men; mean age, 39 +/- 9 years) participated in the study. The lactulose hydrogen breath test was used to determine OCTT and the occurrence of SIBO. The presence of anxiety and depression was assessed by the Hospital Anxiety and Depression scale. Gastric emptying was measured by scintigraphy. Anthropometric indices included body mass index, percentage body fat, midarm circumference, and arm muscle area.

Results: There was a statistically significant increase in OCTT values in diabetes patients (79 +/- 41 min) in comparison with controls (54 +/- 17 min) (P = 0.01). Individual analysis showed that OCTT was above the upper limit (mean + 2 SD) in 30.8% of patients. All anthropometric parameters were significantly decreased (P < 0.05) in patients with prolonged OCTT in comparison with those with normal OCTT. In contrast, there was no statistically significant association between prolonged OCTT and gastrointestinal symptoms, peripheral neuropathy, diabetic retinopathy, glycated hemoglobin, delayed gastric emptying, SIBO, anxiety, or depression.

Conclusions: Small bowel transit may be delayed in about one-third of patients with long-standing type 1 diabetes mellitus. This abnormality seems to have a negative effect on nutritional status in these patients.

C1 [Faria, Mariza; Silva Lorena, Sonia Leticia; Pavan, Celia Regina; Mesquita, Maria Aparecida] Univ Estadual Campinas, Div Gastroenterol, Sao Paulo, Brazil.

[Pavin, Elizabeth Joao; Ribeiro Parisi, Maria Candida] Univ Estadual Campinas, Div Endocrinol, Dept Clin Med, Fac Med Sci, Sao Paulo, Brazil.

[Brunetto, Sergio Quirino; Ramos, Celso Dario] Univ Estadual Campinas, Nucl Med Serv, Dept Radiol, Sao Paulo, Brazil.

RP Mesquita, MA (reprint author), Univ Estadual Campinas, Dept Clin Med, Disciplina Gastroenterol, Rua Tessalia Vieira de Camargo,126 Cidade Univ Ze, BR-13083887 Campinas, SP, Brazil.

EM mesquita@fcm.unicamp.br

RI 5, INCT/H-4505-2013; Diabetes, Inct/I-2186-2013

CR BASILISCO G, 1989, DIGEST DIS SCI, V34, P509, DOI 10.1007/BF01536325

Botega NJ, 1995, REV SAUDE PUBL, V29, P355, DOI 10.1590/S0034-89101995000500004

Cuoco L, 2002, HEPATO-GASTROENTEROL, V49, P1582

DEBOER SY, 1993, DIGEST DIS SCI, V38, P2228, DOI 10.1007/BF01299901

DURNIN JVGA, 1974, BRIT J NUTR, V32, P77, DOI 10.1079/BJN19740060

FOLWACZNY C, 1995, Z GASTROENTEROL, V33, P517

Frisancho AR, 1990, ANTHROPOMETRIC STAND

Ghoshal UC, 2011, J NEUROGASTROENTEROL, V17, P312, DOI 10.5056/jnm.2011.17.3.312

Gorard DA, 1996, GUT, V39, P551, DOI 10.1136/gut.39.4.551

Grigsby AB, 2002, J PSYCHOSOM RES, V53, P1053, DOI 10.1016/S0022-3999(02)00417-8

GURNEY JM, 1973, AM J CLIN NUTR, V26, P912

IBER FL, 1993, DIGEST DIS SCI, V38, P45, DOI 10.1007/BF01296772

Zhao JB, 2006, WORLD J GASTROENTERO, V12, P2846, DOI 10.3748/wjg.v12.i18.2846

Kuo P, 2007, DRUGS, V67, P1671, DOI 10.2165/00003495-200767120-00003

Lewis SJ, 1997, SCAND J GASTROENTERO, V32, P920, DOI 10.3109/00365529709011203

Lloyd CE, 2000, DIABETIC MED, V17, P198, DOI 10.1046/j.1464-5491.2000.00260.x

Longstreth GF, 2006, GASTROENTEROLOGY, V130, P1480, DOI 10.1053/j.gastro.2005.11.061

Lorena SLS, 2002, J CLIN GASTROENTEROL, V35, P21, DOI 10.1097/01.MCG.0000017334.66204.F3

Lorena SLS, 2004, J CLIN GASTROENTEROL, V38, P230, DOI 10.1097/00004836-200403000-00007

MADSEN JL, 1992, DIGEST DIS SCI, V37, P1548

Ojetti V, 2009, EUR REV MED PHARMACO, V13, P419

Park, 2005, OBES RES, V13, P491

Punkkinen J, 2008, DIABETIC MED, V25, P570, DOI 10.1111/j.1464-5491.2008.02428.x

Rana SV, 2008, SCAND J GASTROENTERO, V43, P1030, DOI 10.1080/00365520801947074

Rana S, 2011, DIABETES TECHNOL THE, V13, P1115, DOI 10.1089/dia.2011.0078

Rayner CK, 2001, DIABETES CARE, V24, P371, DOI 10.2337/diacare.24.2.371

Read NW, 1991, GASTROINTESTINAL TRA, P97

RosaeSilva L, 1996, GUT, V39, P748, DOI 10.1136/gut.39.5.748

Russo A, 1996, DIABETOLOGIA, V39, P984

Sadik R, 2004, AM J GASTROENTEROL, V99, P2429, DOI 10.1111/j.1572-0241.2004.40406.x

Sadik R, 2003, SCAND J GASTROENTERO, V38, P36, DOI 10.1080/00365520310000410

SARNO S, 1993, ITAL J GASTROENTEROL, V25, P490

SCARPELLO JHB, 1976, BRIT MED J, V2, P1225, DOI 10.1136/bmj.2.6046.1225

Schvarcz E, 1996, DIABETIC MED, V13, P478

Shakil A, 2008, AM FAM PHYSICIAN, V77, P1697

SPENGLER U, 1989, PANCREAS, V4, P65, DOI 10.1097/00006676-198902000-00010

Toeller M, 2001, INT J OBESITY, V25, P1815, DOI 10.1038/sj.ijo.0801816

WERTH B, 1992, GUT, V33, P1199, DOI 10.1136/gut.33.9.1199

World Health Organization, 1998, OB PREV MAN GLOB EP

Xing JH, 2004, OBES RES, V12, P1723, DOI 10.1038/oby.2004.213

NR 40

TC 10

Z9 10

U1 0

U2 5

PU MARY ANN LIEBERT INC

PI NEW ROCHELLE

PA 140 HUGUENOT STREET, 3RD FL, NEW ROCHELLE, NY 10801 USA

SN 1520-9156

J9 DIABETES TECHNOL THE

JI Diabetes Technol. Ther.

PD JAN

PY 2013

VL 15

IS 1

BP 32

EP 38

DI 10.1089/dia.2012.0158

PG 7

WC Endocrinology & Metabolism

SC Endocrinology & Metabolism

GA 069FL

UT WOS:000313420600006

PM 23126582

DA 2019-01-08

ER

PT J

AU Smith, KJ

Gariepy, G

Pedneault, M

Beland, M

Clyde, M

Schmitz, N

AF Smith, Kimberley J.

Gariepy, Genevieve

Pedneault, Maxime

Beland, Melanie

Clyde, Matthew

Schmitz, Norbert

TI Exploring the Association of Psychological Status with Self-Rated

Diabetes Control: Results from the Montreal Evaluation of Diabetes

Treatment Study

SO PSYCHOSOMATICS

LA English

DT Article

ID DISTRESS SCALE; DEPRESSION; ANXIETY; METAANALYSIS; POPULATION; PHQ-9

AB Aims: There is an increasing interest in single-item self-rated indicators of perceived health and control status in people with chronic illnesses such as diabetes. However, self-rated measures can be associated with indicators of psychological status. The aim of this paper is to explore the association of anxiety, depression, and diabetes distress with self-rated diabetes control. Methods: Telephone interviews were conducted with 1,787 people with type 2 diabetes taking oral hypoglycemic medication. Diabetes control, health behaviors, and outcomes, anxiety, depression, and diabetes distress were assessed by standardized questionnaires. Self-reported diabetes control was modeled using logistic regression. Results: The best fit logistic regression model for self-rated poor diabetes control was a model that incorporated diabetes distress. When adjusted for age, sex, and all other health behaviors and outcomes, poor diabetes control was most associated with diabetes distress, physical inactivity, being overweight, and poor eating habits. Conclusions: Results from this study indicate that poor self-rated diabetes control shares the strongest associations with diabetes-specific distress along with perceptions of diabetes-specific healthcare behaviors and outcomes. (Psychosomatics 2013; 54:35-43)

C1 [Smith, Kimberley J.; Pedneault, Maxime; Beland, Melanie; Clyde, Matthew; Schmitz, Norbert] McGill Univ, Dept Psychiat, Montreal, PQ H4H 1R3, Canada.

[Smith, Kimberley J.; Gariepy, Genevieve; Pedneault, Maxime; Beland, Melanie; Clyde, Matthew; Schmitz, Norbert] McGill Univ, Douglas Mental Hlth Univ Inst, Montreal, PQ H4H 1R3, Canada.

[Gariepy, Genevieve; Schmitz, Norbert] McGill Univ, Dept Epidemiol & Biostat, Montreal, PQ H4H 1R3, Canada.

[Schmitz, Norbert] Montreal Diabet Res Ctr, Montreal, PQ, Canada.

RP Smith, KJ (reprint author), McGill Univ, Douglas Mental Hlth Univ Inst, Montreal, PQ H4H 1R3, Canada.

EM lcimberly.smith@douglas.mcgill.ca

RI Schmitz, Norbert/A-5177-2010

OI Schmitz, Norbert/0000-0001-7777-6323; Gariepy,

Genevieve/0000-0001-9209-4240; Smith, Kimberley/0000-0002-1323-627X

FU Canadian Institutes of Health Research (CIHR); BIP; Regie de l'assurance

maladies du Quebec (RAMQ)

FX The authors extend their thanks to the Canadian Institutes of Health

Research (CIHR) for funding this study. They thank the BIP and Regie de

l'assurance maladies du Quebec (RAMQ) for their support of this

research. They also thank all the study collaborators and all the

participants who kindly gave their time to help us with our research.

CR Brez S, 2008, CAN J DIABETES, V32, pS32

Fincke BG, 2005, J AMBUL CARE MANAG, V28, P262

Fisher L, 2008, DIABETIC MED, V25, P1096, DOI 10.1111/j.1464-5491.2008.02533.x

Fisher L, 2009, DIABETIC MED, V26, P622, DOI 10.1111/j.1464-5491.2009.02730.x

Fisher L, 2012, DIABETES CARE, V35, P259, DOI 10.2337/dc11-1572

Fisher L, 2010, DIABETES CARE, V33, P23, DOI 10.2337/dc09-1238

Gonzalez JS, 2008, DIABETES CARE, V31, P2398, DOI 10.2337/dc08-1341

Hayashino Y, 2012, DIABET MED

Katon W, 2007, GEN HOSP PSYCHIAT, V29, P147, DOI 10.1016/j.genhosppsych.2006.11.005

Kroenke K, 2001, J GEN INTERN MED, V16, P606, DOI 10.1046/j.1525-1497.2001.016009606.x

Lange LJ, 2005, J PSYCHOSOM RES, V58, P129, DOI 10.1016/j.jpsychores.2004.08.004

Leyva B, 2011, ETHNIC DIS, V21, P322

Lin EHB, 2010, DIABETES CARE, V33, P264, DOI 10.2337/dc09-1068

Lowe B, 2008, MED CARE, V46, P266, DOI 10.1097/MLR.0b013e318160d093

Manea L, 2012, CAN MED ASSOC J, V184, pE191, DOI 10.1503/cmaj.110829

Polonsky WH, 2005, DIABETES CARE, V28, P626, DOI 10.2337/diacare.28.3.626

Schmitz N, 2011, PSYCHOSOMATICS, V52, P167, DOI 10.1016/j.psym.2010.11.020

Schmitz N, 2009, DIABETES CARE, V32, P1998, DOI 10.2337/dc09-0838

Scott KM, 2007, J AFFECT DISORDERS, V103, P113, DOI 10.1016/j.jad.2007.01.015

Smith KJ, 2012, DIABETES RES CLIN PR, V95, P162, DOI 10.1016/j.diabres.2011.10.019

Stankovic Z, 2011, PSYCHIAT DANUB, V23, P34

Statistics Canada, 2003, CANADIAN COMMUNITY H

Wennberg P, 2012, BMJ OPEN, V2, DOI 10.1136/bmjopen-2011-000760

Wu SFV, 2011, INT J NURS STUD, V48, P1376, DOI 10.1016/j.ijnurstu.2011.04.008

Zagarins SE, 2012, J BEHAV MED, V35, P299, DOI 10.1007/s10865-011-9359-z

Zulman DM, 2012, PATIENT EDUC COUNS, V87, P74, DOI 10.1016/j.pec.2011.07.013

NR 26

TC 15

Z9 16

U1 1

U2 21

PU ELSEVIER SCIENCE INC

PI NEW YORK

PA 360 PARK AVE SOUTH, NEW YORK, NY 10010-1710 USA

SN 0033-3182

J9 PSYCHOSOMATICS

JI Psychosomatics

PD JAN-FEB

PY 2013

VL 54

IS 1

BP 35

EP 43

DI 10.1016/j.psym.2012.08.002

PG 9

WC Psychiatry; Psychology

SC Psychiatry; Psychology

GA 071QS

UT WOS:000313609900005

PM 23295005

DA 2019-01-08

ER

PT J

AU Reddy, J

Wilhelm, K

Campbell, L

AF Reddy, Jaya

Wilhelm, Kay

Campbell, Lesley

TI Putting PAID to Diabetes-Related Distress: The Potential Utility of the

Problem Areas in Diabetes (PAID) Scale in Patients with Diabetes

SO PSYCHOSOMATICS

LA English

DT Article

ID DEPRESSIVE SYMPTOMS; EMOTIONAL-PROBLEMS; GLYCEMIC CONTROL; PRIMARY-CARE;

SELF-CARE; ADULTS; QUESTIONNAIRE; EXPECTATIONS; VALIDATION; DISORDERS

AB Introduction: Distress and depression are commonly reported negative affects in people with diabetes (PWD), and may influence glycemic control. 'Distress' can be measured as general or diabetes-related. 'Depression' is considered through symptom severity or episodes of depressive disorder, both potentially derived by the Patient Health Questionnaire (PHQ-9). While current depressive disorder is found to affect the course of diabetes mellitus (DM), past depressive disorder may have a similar impact. Objectives: To test the relationships between DM-distress (measured by the Problem Areas in Diabetes instrument [PAID]) and glycemic control, while considering the effects of general distress; depression severity; and both current and previous depressive disorder. Methods: In a diabetic service, 184 type 1 (n = 51) and type 2 (n = 133) PWD completed the PAID, PHQ-9 depression scale from the Patient Health Questionnaire, Kessler-10 psychological distress (K10), and Short-Form Survey (SF-12) instruments. Glycosylated hemoglobin (HbA1c) levels measured on the day of recruitment were recorded from the case notes. Results: DM-related distress correlated positively with HbA1c, PHQ-9 depression, K10, and SF-12 MCS (mental component summary) scores, (all p < 0.01); and negatively with age(p < 0.01). Regression analysis revealed a significant association between PAID with PHQ-9 depression scores (p < 0.01) and with HbA1c (p < 0.01). PWD with past depressive disorder had higher PAID scores than those without (p < 0.05), and this was significant even after controlling for current depression scores (p < 0.05). Conclusions: In PWD, DM-specific distress measured by the PAID correlated significantly with impaired glycemic control. Both past and current depressions are independently associated with DM-distress. The PAID is discussed as a potential screening tool. (Psychosomatics 2013; 54:44-51)

C1 [Reddy, Jaya; Wilhelm, Kay] Univ New S Wales, Sch Psychiat, Fac Med, Randwick, NSW 2031, Australia.

[Reddy, Jaya; Wilhelm, Kay] Univ New S Wales, Black Dog Inst, Randwick, NSW 2031, Australia.

[Reddy, Jaya; Wilhelm, Kay] St Vincents Hosp, St Vincents Urban Mental Hlth Res Inst, Sydney, NSW 2010, Australia.

[Reddy, Jaya] Univ Kebangsaan Malaysia, Dept Psychiat, Kuala Lumpur, Malaysia.

[Campbell, Lesley] St Vincents Hosp, Diabet Serv, Sydney, NSW 2010, Australia.

[Campbell, Lesley] Univ New S Wales, Sch Med, Fac Med, Sydney, NSW 2052, Australia.

RP Reddy, J (reprint author), St Vincents Hosp, St Vincents Urban Mental Hlth Res Inst, Faces St, Sydney, NSW 2010, Australia.

EM jreddy68@yahoo.com

FU NHMRC [222708, 230802, 157209, 510137]; Centre for Mental Health, NSW

Department of Health

FX The study was supported by NHMRC Program grants 222708, 230802, 157209,

and 510137; an Infrastructure Grant from the Centre for Mental Health,

NSW Department of Health. The authors thank the Diabetes Services at St.

Vincent's and Prince of Wales Hospitals for access to their patients,

and the patients themselves, for their time, interest, and provision of

material.

CR Chawla A, 2010, DIABETES EDUCATOR, V36, P337, DOI 10.1177/0145721709354607

Delahanty LM, 2007, DIABETIC MED, V24, P48, DOI 10.1111/j.1464-5491.2007.02028.x

Diez-Quevedo C, 2001, PSYCHOSOM MED, V63, P679, DOI 10.1097/00006842-200107000-00021

Fisher L, 2007, DIABETES CARE, V30, P542, DOI 10.2337/dc06-1614

Fisher L, 2010, DIABETES CARE, V33, P23, DOI 10.2337/dc09-1238

Gonzalez JS, 2008, DIABETOLOGIA, V51, P1822, DOI 10.1007/s00125-008-1113-x

Hermanns N, 2006, DIABETOLOGIA, V49, P469, DOI 10.1007/s00125-005-0094-2

Jackson JL, 2001, ANN INTERN MED, V134, P889, DOI 10.7326/0003-4819-134-9_Part_2-200105011-00013

Karlsen B, 2004, PATIENT EDUC COUNS, V53, P299, DOI 10.1016/j.pec.2003.10.008

Kessler RC, 2002, PSYCHOL MED, V32, P959, DOI 10.1017/S0033291702006074

Kroenke K, 2001, J GEN INTERN MED, V16, P606, DOI 10.1046/j.1525-1497.2001.016009606.x

Kroenke K, 2010, GEN HOSP PSYCHIAT, V32, P345, DOI 10.1016/j.genhosppsych.2010.03.006

Lloyd CE, 2010, DIABETIC MED, V27, P234, DOI 10.1111/j.1464-5491.2009.02896.x

Lustman PJ, 1997, GEN HOSP PSYCHIAT, V19, P138, DOI 10.1016/S0163-8343(96)00170-3

Nakahara R, 2006, PSYCHOSOMATICS, V47, P240, DOI 10.1176/appi.psy.47.3.240

Nozaki T, 2009, BIOPSYCHOSOC MED, V3, DOI 10.1186/1751-0759-3-4

Perini SJ, 2006, J AFFECT DISORDERS, V90, P123, DOI 10.1016/j.jad.2005.10.011

Peyrot M, 1999, DIABETES CARE, V22, P448, DOI 10.2337/diacare.22.3.448

POLONSKY WH, 1995, DIABETES CARE, V18, P754, DOI 10.2337/diacare.18.6.754

Polonsky WH, 2005, DIABETES CARE, V28, P626, DOI 10.2337/diacare.28.3.626

Pouwer F, 2005, DIABETES RES CLIN PR, V70, P166, DOI 10.1016/j.diabres.2005.03.031

Rao JK, 2000, ARCH FAM MED, V9, P1148, DOI 10.1001/archfami.9.10.1148

Reddy JP, 2011, THESIS

Rubin RR, 2001, J CLIN PSYCHOL, V57, P457, DOI 10.1002/jclp.1041

Spitzer RL, 1999, JAMA-J AM MED ASSOC, V282, P1737, DOI 10.1001/jama.282.18.1737

Surwit Richard S., 1993, Annals of Behavioral Medicine, V15, P227

Wagner JA, 2007, DIABETIC MED, V24, P211, DOI 10.1111/j.1464-5491.2007.02044.x

Ware JE, 1996, MED CARE, V34, P220, DOI 10.1097/00005650-199603000-00003

Welch G, 2003, DIABETIC MED, V20, P69, DOI 10.1046/j.1464-5491.2003.00832.x

Welch GW, 1997, DIABETES CARE, V20, P760, DOI 10.2337/diacare.20.5.760

Wilhelm K, 2004, PSYCHOSOMATICS, V45, P461, DOI 10.1176/appi.psy.45.6.461

Yi JP, 2008, INT J BEHAV MED, V15, P167, DOI 10.1080/10705500802219481

NR 32

TC 27

Z9 29

U1 2

U2 23

PU ELSEVIER SCIENCE INC

PI NEW YORK

PA 360 PARK AVE SOUTH, NEW YORK, NY 10010-1710 USA

SN 0033-3182

J9 PSYCHOSOMATICS

JI Psychosomatics

PD JAN-FEB

PY 2013

VL 54

IS 1

BP 44

EP 51

DI 10.1016/j.psym.2012.08.004

PG 8

WC Psychiatry; Psychology

SC Psychiatry; Psychology

GA 071QS

UT WOS:000313609900006

PM 23295006

DA 2019-01-08

ER

PT J

AU Hilliard, ME

Wu, YP

Rausch, J

Dolan, LM

Hood, KK

AF Hilliard, Marisa E.

Wu, Yelena P.

Rausch, Joseph

Dolan, Lawrence M.

Hood, Korey K.

TI Predictors of Deteriorations in Diabetes Management and Control in

Adolescents With Type 1 Diabetes

SO JOURNAL OF ADOLESCENT HEALTH

LA English

DT Article

DE Type 1 diabetes; Depression; Distress; Family conflict; Blood glucose

monitoring; Glycemic control

ID GLYCEMIC CONTROL; FAMILY CONFLICT; DEPRESSIVE SYMPTOMS; ADHERENCE;

CHILDREN; IMPACT; TRAJECTORIES; ASSOCIATION; STATEMENT; ADULTHOOD

AB Purpose: Deteriorating type 1 diabetes management and control are common among adolescents; however, clinical evidence suggests that individual trajectories can vary. The aim of this study was to examine patterns and predictors of blood glucose monitoring (BGM) frequency and glycemic control (hemoglobin A1c).

Methods: Prospective data analysis spanning 18-24 months was conducted with 150 adolescent-parent pairs. Latent group-based trajectory modeling identified subgroups and determined medical, demographic, psychological, and family predictors of subgroup membership.

Results: Three subgroups emerged, representing diabetes management and control that are "meeting treatment targets" (40%; A1c at baseline = 7.4%, BGM frequency at baseline = 4.8 checks/day) and two levels "not meeting targets":"normatively similar" youth (40%; A1c = 9.2%, BGM frequency = 2.8 checks/day), and "high-risk" youth (20%; A1c = 11.2%, BGM frequency = 2.9 checks/day). Subgroup membership was maintained over 18-24 months. There was minimal change across time, although only one-third of adolescents met treatment targets. Older age, longer diabetes duration, ethnic minority status, unmarried caregiver status, insulin delivery via injections versus continuous subcutaneous insulin infusion, greater depressive symptoms, negative affect about BGM, and diabetes-specific family conflict each predicted membership in a subgroup with poorer diabetes management and control.

Conclusions: Among the nearly two-thirds of adolescents with management and control that do not meet treatment targets, modifiable and nonmodifiable factors may signal the need for prevention or intervention. Demographic and medical factors may call for proactive efforts to prevent deterioration, and psychological symptoms and family conflict signal opportunities for clinical intervention to promote improved diabetes management and control in adolescence. (C) 2013 Society for Adolescent Health and Medicine. All rights reserved.

C1 [Hood, Korey K.] Univ Calif San Francisco, Madison Clin Pediat Diabet, Div Endocrinol, Dept Pediat, San Francisco, CA 94143 USA.

[Hilliard, Marisa E.] Johns Hopkins Univ, Sch Med, Johns Hopkins Adherence Res Ctr, Dept Med, Baltimore, MD USA.

[Wu, Yelena P.; Rausch, Joseph] Cincinnati Childrens Hosp, Med Ctr, Ctr Promot Treatment Adherence & Self Management, Div Behav Med & Clin Psychol, Cincinnati, OH USA.

[Rausch, Joseph; Dolan, Lawrence M.] Univ Cincinnati, Coll Med, Dept Pediat, Cincinnati, OH USA.

[Dolan, Lawrence M.] Cincinnati Childrens Hosp, Med Ctr, Div Endocrinol, Cincinnati, OH USA.

RP Hood, KK (reprint author), Univ Calif San Francisco, Madison Clin Pediat Diabet, Div Endocrinol, Dept Pediat, 400 Parnassus Ave,4th Floor,UCSF Mail Box 0318, San Francisco, CA 94143 USA.

EM hoodk@peds.ucsf.edu

RI Rausch, Joseph/E-8107-2018

OI Hilliard, Marisa/0000-0002-8813-629X

FU National Institutes of Health [K23, DK077340]

FX This research was supported by a career development award from the

National Institutes of Health to K.K.H. (K23, DK077340).

CR AMIEL SA, 1986, NEW ENGL J MED, V315, P215, DOI 10.1056/NEJM198607243150402

Anderson B. J., 2004, DIABETES SPECTRUM, V17, P22, DOI [DOI 10.2337/DIASPECT.17.1.22, 10.2337/diaspect.17.1.22]

Anderson BJ, 2002, DIABETIC MED, V19, P635, DOI 10.1046/j.1464-5491.2002.00752.x

Anderson BJ, 1999, DIABETES CARE, V22, P713, DOI 10.2337/diacare.22.5.713

Barnard KD, 2008, PRACT DIABETES INT, V25, P194

Bryden KS, 2001, DIABETES CARE, V24, P1536, DOI 10.2337/diacare.24.9.1536

Butler DA, 2008, PEDIATR DIABETES, V9, P373, DOI 10.1111/j.1399-5448.2008.00370.x

David-Ferdon C, 2008, J CLIN CHILD ADOLESC, V37, P62, DOI 10.1080/15374410701817865

Genuth S, 2001, J PEDIATR-US, V139, P804, DOI 10.1067/mpd.2001.118887

Graffy J, 2009, PRIM HEALTH CARE RES, V10, P210

Grey M, 2002, J PSYCHOSOM RES, V53, P907, DOI 10.1016/S0022-3999(02)00312-4

Helgeson VS, 2011, PEDIATR DIABETES, V12, P25, DOI 10.1111/j.1399-5448.2010.00663.x

Helgeson VS, 2010, J PEDIATR PSYCHOL, V35, P1161, DOI 10.1093/jpepsy/jsq011

Helgeson VS, 2009, J PEDIATR PSYCHOL, V34, P254, DOI 10.1093/jpepsy/jsn079

Hood KK, 2004, DIABETES CARE, V27, P2610, DOI 10.2337/diacare.27.11.2610

Hood KK, 2007, DIABETES CARE, V30, P1764, DOI 10.2337/dc06-2358

Hood KK, 2011, PEDIATR DIABETES, V12, P718, DOI 10.1111/j.1399-5448.2011.00771.x

Hood KK, 2009, PEDIATRICS, V124, pE1171, DOI 10.1542/peds.2009-0207

JACOBSON AM, 1994, PSYCHOSOM MED, V56, P401, DOI 10.1097/00006842-199409000-00004

JOHNSON SB, 1992, J PEDIATR PSYCHOL, V17, P537, DOI 10.1093/jpepsy/17.5.537

Kazak AE, 2006, FAM SYST HEALTH, V24, P381, DOI DOI 10.1037/1091-7527.24.4.381

KOVACS M, 1992, J AM ACAD CHILD PSY, V31, P1112, DOI 10.1097/00004583-199211000-00020

KOVACS M, 1985, PSYCHOPHARMACOL BULL, V21, P995

Kovacs M, 1997, DIABETES CARE, V20, P45, DOI 10.2337/diacare.20.1.45

Lustman PJ, 2002, J PSYCHOSOM RES, V53, P917, DOI 10.1016/S0022-3999(02)00416-6

Luyckx K, 2009, DIABETES CARE, V32, P797, DOI 10.2337/dc08-1990

McGrady ME, 2009, DIABETES CARE, V32, P804, DOI 10.2337/dc08-2111

Moreland EC, 2006, ARCH INTERN MED, V166, P689, DOI 10.1001/archinte.166.6.689

Muthen L. K., 1998, MPLUS USERS GUIDE

Nagin D, 2005, GROUP BASED MODELING

Patino AM, 2005, J PEDIATR PSYCHOL, V30, P503, DOI 10.1093/jpepsy.jsi075

Petitti DB, 2009, J PEDIATR-US, V155, P668, DOI 10.1016/j.jpeds.2009.05.025

Phillip M, 2007, DIABETES CARE, V30, P1653, DOI 10.2337/dc07-9922

Rohan JM, 2011, PEDIATR DIABETES, V12, P611, DOI 10.1111/j.1399-5448.2010.00752.x

Silverstein J, 2005, DIABETES CARE, V28, P186, DOI 10.2337/diacare.28.1.186

Swift EE, 2006, ANN BEHAV MED, V32, P39, DOI 10.1207/s15324796abm3201_5

Wagner EH, 2001, JAMA-J AM MED ASSOC, V285, P182, DOI 10.1001/jama.285.2.182

Wysocki T, 2007, DIABETES CARE, V30, P555, DOI 10.2337/dc06-1613

NR 38

TC 73

Z9 73

U1 1

U2 41

PU ELSEVIER SCIENCE INC

PI NEW YORK

PA 360 PARK AVE SOUTH, NEW YORK, NY 10010-1710 USA

SN 1054-139X

EI 1879-1972

J9 J ADOLESCENT HEALTH

JI J. Adolesc. Health

PD JAN

PY 2013

VL 52

IS 1

BP 28

EP 34

DI 10.1016/j.jadohealth.2012.05.009

PG 7

WC Psychology, Developmental; Public, Environmental & Occupational Health;

Pediatrics

SC Psychology; Public, Environmental & Occupational Health; Pediatrics

GA 061LU

UT WOS:000312850500004

PM 23260831

OA Green Accepted

DA 2019-01-08

ER

PT J

AU Fleer, J

Tovote, KA

Keers, JC

Links, TP

Sanderman, R

Coyne, JC

Schroevers, MJ

AF Fleer, J.

Tovote, K. A.

Keers, J. C.

Links, T. P.

Sanderman, R.

Coyne, J. C.

Schroevers, M. J.

TI Screening for depression and diabetes-related distress in a diabetes

outpatient clinic

SO DIABETIC MEDICINE

LA English

DT Article

ID PLACEBO-CONTROLLED TRIAL; CO-MORBID DEPRESSION; GLYCEMIC CONTROL;

PROBLEM AREAS; DOUBLE-BLIND; PREVALENCE; ADULTS; SCALE; DISORDERS;

SERVICES

AB Aims To investigate: (1) the willingness of patients with diabetes to participate in a screening programme; (2) the extent to which patients with diabetes who screen positive endorse need for psychosocial care; (3) the rate of referral to psychosocial care during screening vs. usual care. Methods Four hundred and ninety-nine patients with diabetes were invited to complete the Center for Epidemiologic Studies Depression and the Problem Areas in Diabetes questionnaires. Patients screening positive on either instrument were invited for an interview. One year after screening was withdrawn, rates of referral to psychosocial care were assessed from physician reports of patient referrals. Results In total, 349/499 (70%) patients with diabetes completed the questionnaire. Patients who did not take up the screening were younger, smoked more often and had higher HbA1c values. No-shows for clinical appointments accounted for 74% of non-participation. Of the 104 (30% of 349) patients screening positive, 45 accepted an invitation for an interview. Finally, 36/104 (35%) would like a referral for psychological care. Seven per cent of patients were referred to psychological care during screening compared with 1% when screening was withdrawn. Conclusions Results raise questions as to whether screening is the most efficient way to identify patients with psychological problems. Many patients did not take up the screening, especially those with low adherence to diabetes care in general. Furthermore, few patients screening positive wanted to be referred. Screening should be evaluated in the context of consideration of alternative ways to identify at-risk patients, including providing resources to deal with patients with already known adjustment and adherence problems. Diabet. Med. 30, 88-94 (2013)

C1 [Fleer, J.; Tovote, K. A.; Sanderman, R.; Coyne, J. C.; Schroevers, M. J.] Univ Groningen, Dept Hlth Sci, Sect Hlth Psychol, Groningen, Netherlands.

[Fleer, J.; Tovote, K. A.; Keers, J. C.; Links, T. P.; Sanderman, R.; Coyne, J. C.; Schroevers, M. J.] Univ Groningen, Univ Med Ctr Groningen, NL-9713 AV Groningen, Netherlands.

[Keers, J. C.; Links, T. P.] Univ Groningen, Univ Med Ctr Groningen, Dept Endocrinol, Groningen, Netherlands.

[Keers, J. C.] Martini Hosp Groningen, Res Inst, Groningen, Netherlands.

[Coyne, J. C.] Univ Penn, Dept Psychiat, Perelman Sch Med, Philadelphia, PA 19104 USA.

RP Fleer, J (reprint author), Univ Groningen, Dept Hlth Sci, Sect Hlth Psychol, Groningen, Netherlands.

EM j.fleer@umcg.nl

FU Dutch Diabetes Foundation [2006.13.007]

FX This study was supported by the Dutch Diabetes Foundation (2006.13.007).

CR Ali S, 2006, DIABETIC MED, V23, P1165, DOI 10.1111/j.1464-5491.2006.01943.x

Amer Diabet Assoc, 2009, DIABETES CARE, V32, pS13, DOI 10.2337/dc09-S013

Barnard KD, 2006, DIABETIC MED, V23, P445, DOI 10.1111/j.1464-5491.2006.01814.x

Beekman ATF, 1997, PSYCHOL MED, V27, P231, DOI 10.1017/S0033291796003510

Bot M, 2010, DIABETIC MED, V27, P1295, DOI 10.1111/j.1464-5491.2010.03119.x

Brown JSL, 2004, BRIT J PSYCHIAT, V185, P511, DOI 10.1192/bjp.185.6.511

de Wit M, 2010, PEDIATR DIABETES, V11, P175, DOI 10.1111/j.1399-5448.2009.00543.x

FECHNERBATES S, 1994, J CONSULT CLIN PSYCH, V62, P550, DOI 10.1037//0022-006X.62.3.550

Forsell Y, 2006, PSYCHIAT SERV, V57, P114, DOI 10.1176/appi.ps.57.1.114

Garssen B, 2008, PSYCHO-ONCOLOGY, V17, P726, DOI 10.1002/pon.1362

Garssen B, 2010, J NATL CANCER I, V102, P507, DOI 10.1093/jnci/djq047

Gilbody S, 2010, SCREENING DEPRESSION, P335

Gottlieb SS, 2004, J AM COLL CARDIOL, V43, P1542, DOI 10.1016/j.jacc.2003.10.064

Holland W, 2005, SCREENING DIS PREVEN

International Diabetes Federation, 2005, GLOB GUID TYP 2 DIAB

Katon WJ, 2004, ARCH GEN PSYCHIAT, V61, P1042, DOI 10.1001/archpsyc.61.10.1042

Korhonen T, 2007, PSYCHOL MED, V37, P705, DOI 10.1017/S0033291706009639

Li CY, 2010, DIABETES CARE, V33, P1061, DOI 10.2337/dc09-1515

Lustman PJ, 1997, PSYCHOSOM MED, V59, P241, DOI 10.1097/00006842-199705000-00007

Lustman PJ, 2000, DIABETES CARE, V23, P934, DOI 10.2337/diacare.23.7.934

Lustman PJ, 2000, DIABETES CARE, V23, P618, DOI 10.2337/diacare.23.5.618

Lustman PJ, 1998, ANN INTERN MED, V129, P613, DOI 10.7326/0003-4819-129-8-199810150-00005

Luttik MLA, 2011, EUR J CARDIOVASC NUR, V10, P228, DOI 10.1016/j.ejcnurse.2010.08.005

Nefs G, 2012, DIABETOLOGIA, V55, P608, DOI 10.1007/s00125-011-2411-2

Palmer SC, 2012, CANCER-AM CANCER SOC, V118, P2735, DOI 10.1002/cncr.26603

PEYROT M, 1994, DIABETES EDUCATOR, V20, P143, DOI 10.1177/014572179402000210

Pouwer F, 2006, PATIENT EDUC COUNS, V60, P235, DOI 10.1016/j.pec.2005.01.009

Pouwer F, 2011, DIABETOLOGIA, V54, P741, DOI 10.1007/s00125-010-2033-0

Pouwer F, 2009, NAT REV ENDOCRINOL, V5, P665, DOI 10.1038/nrendo.2009.214

RADLOFF L S, 1977, Applied Psychological Measurement, V1, P385, DOI 10.1177/014662167700100306

Roy T, 2012, DIABETIC MED, V29, P164, DOI 10.1111/j.1464-5491.2011.03401.x

Rubin R. R., 1993, DIABETES ED, V19, P210, DOI [DOI 10.1177/014572179301900308, 10.1177/014572179301900308]

Snoek FJ, 2000, DIABETES CARE, V23, P1305, DOI 10.2337/diacare.23.9.1305

Snoek FJ, 2011, DIABETES CARE, V34, P601, DOI 10.2337/dc10-1552

Tuinman MA, 2008, CANCER-AM CANCER SOC, V113, P870, DOI 10.1002/cncr.23622

van Scheppingen C, 2011, PSYCHO-ONCOLOGY, V20, P655, DOI 10.1002/pon.1939

Welch GW, 1997, DIABETES CARE, V20, P760, DOI 10.2337/diacare.20.5.760

NR 37

TC 19

Z9 19

U1 0

U2 22

PU WILEY

PI HOBOKEN

PA 111 RIVER ST, HOBOKEN 07030-5774, NJ USA

SN 0742-3071

EI 1464-5491

J9 DIABETIC MED

JI Diabetic Med.

PD JAN

PY 2013

VL 30

IS 1

BP 88

EP 94

DI 10.1111/dme.12001

PG 7

WC Endocrinology & Metabolism

SC Endocrinology & Metabolism

GA 057GD

UT WOS:000312550100017

PM 22924587

DA 2019-01-08

ER

PT J

AU Pouwer, F

Wijnhoven, HAH

Ujcic-Voortman, JK

de Wit, M

Schram, MT

Baan, CA

Snoek, FJ

AF Pouwer, F.

Wijnhoven, H. A. H.

Ujcic-Voortman, J. K.

de Wit, M.

Schram, M. T.

Baan, C. A.

Snoek, F. J.

TI Ethnic aspects of emotional distress in patients with diabetes - the

Amsterdam Health Monitor Study

SO DIABETIC MEDICINE

LA English

DT Article

ID CO-MORBID DEPRESSION; PSYCHOLOGICAL DISTRESS; RISK-FACTORS; PREVALENCE;

METAANALYSIS; NETHERLANDS; MELLITUS; ANXIETY; MOROCCAN; TURKISH

AB Aims Depression and anxiety are relatively common in patients with diabetes, but it is unclear whether migrant patients with diabetes are at increased risk for emotional distress. We determined levels of emotional distress in patients with diabetes with a Turkish, Moroccan or Dutch ethnic background and compare distress levels with healthy control subjects. Among patients with diabetes, we examined demographic and clinical correlates of higher levels of emotional distress. Methods Cross-sectional data were collected within the framework of the population-based Amsterdam Health Monitor Survey. Adult participants were interviewed to assess demographics, presence of chronic disease(s) and ethnic background. Emotional distress was determined with the Kessler psychological distress scale. Blood was drawn to determine HbA1c, glucose, HDL and total cholesterol. Anthropometrics and blood pressure were assessed during a medical examination. Results The total sample comprised of 1736 participants. The prevalence of emotional distress was significantly higher in participants with diabetes (31%) compared with healthy participants (19%). Increased levels of emotional distress were reported by 38% of the Turkish, 35% of the native Dutch and 29% of the Moroccan patients with diabetes. Among patients with diabetes, the presence of two or more co-morbid chronic diseases was most strongly associated with higher levels of emotional distress, whereas glycaemic control, cholesterol, blood pressure or waist circumference were not. Conclusions Emotional distress affects approximately one third of adult patients with diabetes living in Amsterdam. Having multiple co-morbid diseases seems related to more emotional distress among these patients, while ethnicity and diabetes-related characteristics are not. Diabet. Med. 30, e25-e31 (2013)

C1 [Pouwer, F.] Tilburg Univ, Ctr Res Psychol Somat Dis, Dept Med & Clin Psychol, NL-5000 LE Tilburg, Netherlands.

[Wijnhoven, H. A. H.] Vrije Univ Amsterdam, Dept Hlth Sci, Amsterdam, Netherlands.

[Wijnhoven, H. A. H.; Baan, C. A.; Snoek, F. J.] Vrije Univ Amsterdam, EMGO Inst Hlth & Care Res, Amsterdam, Netherlands.

[Ujcic-Voortman, J. K.; de Wit, M.] Amsterdam Publ Hlth Serv GGD, Amsterdam, Netherlands.

[Schram, M. T.] Maastricht Univ, Dept Internal Med, Med Ctr, Maastricht, Netherlands.

[Schram, M. T.; Baan, C. A.] Ctr Prevent & Hlth Serv Res, Natl Inst Publ Hlth & Environm, Bilthoven, Netherlands.

[Snoek, F. J.] Vrije Univ Amsterdam, Dept Med Psychol, Med Ctr, Amsterdam, Netherlands.

RP Pouwer, F (reprint author), Tilburg Univ, Ctr Res Psychol Somat Dis, Dept Med & Clin Psychol, NL-5000 LE Tilburg, Netherlands.

EM f.pouwer@tilburguniversity.edu

CR Adriaanse MC, 2008, DIABETIC MED, V25, P843, DOI 10.1111/j.1464-5491.2008.02464.x

Agyemang C, 2006, J HYPERTENS, V24, P2169, DOI 10.1097/01.hjh.0000249693.73618.c9

Ali S, 2006, DIABETIC MED, V23, P1165, DOI 10.1111/j.1464-5491.2006.01943.x

Barnard KD, 2006, DIABETIC MED, V23, P445, DOI 10.1111/j.1464-5491.2006.01814.x

de Groot M, 2001, PSYCHOSOM MED, V63, P619, DOI 10.1097/00006842-200107000-00015

Fassaert T, 2009, INT J METH PSYCH RES, V18, P159, DOI 10.1002/mpr.288

Fassaert T, 2010, GEN HOSP PSYCHIAT, V32, P368, DOI 10.1016/j.genhosppsych.2010.04.010

Grigsby AB, 2002, J PSYCHOSOM RES, V53, P1053, DOI 10.1016/S0022-3999(02)00417-8

Home P, 2006, DIABETIC MED, V23, P579

Ismail K, 2007, DIABETES CARE, V30, P1473, DOI 10.2337/dc06-2313

Kessler RC, 2002, PSYCHOL MED, V32, P959, DOI 10.1017/S0033291702006074

Knol MJ, 2007, PSYCHOSOM MED, V69, P300, DOI 10.1097/PSY.0b013e31805f48b9

Koopmans B, 2009, DIABETOLOGIA, V52, P2056, DOI 10.1007/s00125-009-1460-2

Koyvess-Masfety V, 2000, CAN J PSYCHIAT, V2005, P926

Mezuk B, 2008, DIABETES CARE, V31, P2383, DOI 10.2337/dc08-0985

Nefs G, 2012, DIABETOLOGIA, V55, P608, DOI 10.1007/s00125-011-2411-2

Nicolaas Han, BUITENLANDSE MIGRATI

Nouwen A, 2010, DIABETOLOGIA, V53, P2480, DOI 10.1007/s00125-010-1874-x

Pouwer F, 2003, DIABETOLOGIA, V46, P892, DOI 10.1007/s00125-003-1124-6

Pouwer F, 2006, PATIENT EDUC COUNS, V60, P235, DOI 10.1016/j.pec.2005.01.009

Pouwer F, 2010, DIABETIC MED, V27, P217, DOI 10.1111/j.1464-5491.2009.02903.x

Pouwer F, 2001, DIABETES CARE, V24, P1929, DOI 10.2337/diacare.24.11.1929

Pouwer F, 2009, NAT REV ENDOCRINOL, V5, P665, DOI 10.1038/nrendo.2009.214

Pouwer F, 2010, DISCOV MED, V9, P112

Schram MT, 2009, CURR DIABETES REV, V5, P112, DOI 10.2174/157339909788166828

Shankman SA, 2003, CLIN PSYCHOL REV, V23, P605, DOI 10.1016/S0272-7358(03)00038-2

Snoek FJ, 2011, DIABETES CARE, V34, P601, DOI 10.2337/dc10-1552

Uittewaal PJM, 2004, PREV MED, V39, P1068

Ujcic-Voortman JK, 2009, EUR J PUBLIC HEALTH, V19, P511, DOI 10.1093/eurpub/ckp096

van der Wurff FB, 2004, J AFFECT DISORDERS, V83, P33, DOI 10.1016/j.jad.2004.04.009

NR 30

TC 7

Z9 7

U1 0

U2 18

PU WILEY-BLACKWELL

PI HOBOKEN

PA 111 RIVER ST, HOBOKEN 07030-5774, NJ USA

SN 0742-3071

EI 1464-5491

J9 DIABETIC MED

JI Diabetic Med.

PD JAN

PY 2013

VL 30

IS 1

BP E25

EP E31

DI 10.1111/dme.12031

PG 7

WC Endocrinology & Metabolism

SC Endocrinology & Metabolism

GA 057GD

UT WOS:000312550100004

PM 23072362

DA 2019-01-08

ER

PT J

AU Kay, W

Inika, G

Jaya, R

Phillip, M

Lesley, C

Carol, DS

Kerrie, P

Peter, S

AF Kay, Wilhelm

Inika, Gillis

Jaya, Reddy

Phillip B, Mitchell

Lesley, Campbell

Carol, Dobson-Stone

Kerrie, Pierce

Peter R, Schofield

TI Association between serotonin transporter promoter polymorphisms and

psychological distress in a diabetic population

SO PSYCHIATRY RESEARCH

LA English

DT Article

DE Serotonin transporter; SLC6A4; 5-HTTLPR; rs25531; Anxiety; Depression

ID DEPRESSIVE SYMPTOMS; GENE POLYMORPHISM; LIFE EVENTS; 5-HTTLPR;

METAANALYSIS; PREVALENCE; STRESS; MODERATION; MELLITUS; DISEASE

AB Investigations into serotonin transporter and anxiety and depression have shown an association between stress, depression onset and genotype. We investigated the relationship between 5-HTTLPR genotype and depression and anxiety in a population with diabetes mellitus, a condition associated with high rates of stress and depression. Participants were classified according to 'S' and 'L' alleles as well as the modification of the single nucleotide polymorphism (SNP) rs25531. The 5-HTTLPR low-expression genotype group (S or L-G allele carriers) had significantly higher psychological distress (K10) scores (N=234, P=0.047). Subsequent analysis revealed that the effect of genotype was related to anxiety symptoms rather than depression symptoms. Furthermore, the main effect of genotype was not observed when the modification of the SNP polymorphism was not taken into account. Findings suggest that 5-HTTLPR/rs25531 genotype is associated with psychological distress in a sample of subjects with diabetes. Crown Copyright (C) 2012 Published by Elsevier Ireland Ltd. All rights reserved.

C1 [Kay, Wilhelm; Inika, Gillis; Jaya, Reddy; Phillip B, Mitchell] Univ New S Wales, Fac Med, Sch Psychiat, Randwick, NSW 2031, Australia.

[Kay, Wilhelm; Inika, Gillis; Jaya, Reddy; Phillip B, Mitchell] Univ New S Wales, Black Dog Inst, Randwick, NSW 2031, Australia.

[Kay, Wilhelm; Jaya, Reddy] St Vincents Hosp, Urban Mental Hlth & Wellbeing Inst, Sydney, NSW 2010, Australia.

[Jaya, Reddy] Univ Kebangsaan Malaysia, Dept Psychiat, Kuala Lumpur, Malaysia.

[Lesley, Campbell] Univ New S Wales, Fac Med, Sch Med, Sydney, NSW 2052, Australia.

[Carol, Dobson-Stone; Kerrie, Pierce; Peter R, Schofield] Neurosci Res Inst, Randwick, NSW 2031, Australia.

[Carol, Dobson-Stone; Peter R, Schofield] Univ New S Wales, Sch Med Sci, Sydney, NSW 2052, Australia.

RP Kay, W (reprint author), Univ NSW, Sch Psychiat, St Vincents Hosp, OBrien Ctr, Level 4,390 Victoria St, Darlinghurst, NSW 2010, Australia.

EM kwilhelm@stvincents.com.au

RI Pierce, Kerrie/O-7300-2018

OI Pierce, Kerrie/0000-0003-0636-4836; Schofield,

Peter/0000-0003-2967-9662; Mitchell, Philip/0000-0002-7954-5235

FU NHMRC [222708]; ARC [DP0774248]; Centre for Mental Health, NSW

Department of Health; [510216]

FX Adam Finch, Liesbeth Geerligs, Elaine Royal, Lucinda Wedgwood, Tara

Showyin and Jennifer Siegel were involved in data collection. Funding

from the NHMRC Programme Grant no. 222708, the Project Grant no. 510216,

the ARC Discovery Project Grant no. DP0774248, and an Infrastructure

Grant from the Centre for Mental Health, NSW Department of Health. We

thank Kevin Bird and Dusan Hadzi-Pavlovic for statistical input and

advice; the Diabetes Services at St Vincent's and Prince of Wales

Hospitals, Sydney for access to their patients and the patients

themselves, for their time, interest and provision of material for

genotyping.

CR Aikens JE, 2009, DIABETES CARE, V32, P1177, DOI 10.2337/dc09-0071

Ali S, 2006, DIABETIC MED, V23, P1165, DOI 10.1111/j.1464-5491.2006.01943.x

Anderson RJ, 2001, DIABETES CARE, V24, P1069, DOI 10.2337/diacare.24.6.1069

Andrews G, 2001, AUST NZ J PUBL HEAL, V25, P494, DOI 10.1111/j.1467-842X.2001.tb00310.x

Aujla N, 2010, DIABETIC MED, V27, P896, DOI 10.1111/j.1464-5491.2010.03042.x

Bull SJ, 2009, MOL PSYCHIATR, V14, P1095, DOI 10.1038/mp.2008.48

Caspi A, 2003, SCIENCE, V301, P386, DOI 10.1126/science.1083968

Caspi A, 2010, AM J PSYCHIAT, V167, P509, DOI 10.1176/appi.ajp.2010.09101452

Costa PT, 1992, REVISED NEO PERSONAL

DOHRENWEND BP, 1980, ARCH GEN PSYCHIAT, V37, P1229

Gonda X, 2007, PSYCHIAT RES, V149, P261, DOI 10.1016/j.psychres.2006.05.014

Grabe HJ, 2005, MOL PSYCHIATR, V10, P220, DOI 10.1038/sj.mp.4001555

Grafe K, 2004, DIAGNOSTICA, V50, P171, DOI 10.1026/0012-1924.50.4.171

Grigsby AB, 2002, J PSYCHOSOM RES, V53, P1053, DOI 10.1016/S0022-3999(02)00417-8

Hu XZ, 2006, AM J HUM GENET, V78, P815, DOI 10.1086/503850

Karg K, 2011, ARCH GEN PSYCHIAT, V68, P444, DOI 10.1001/archgenpsychiatry.2010.189

Kessler RC, 2002, PSYCHOL MED, V32, P959, DOI 10.1017/S0033291702006074

Knol MJ, 2006, DIABETOLOGIA, V49, P837, DOI 10.1007/s00125-006-0159-x

Kohen R, 2008, ARCH GEN PSYCHIAT, V65, P1296, DOI 10.1001/archpsyc.65.11.1296

Kroenke K, 2001, J GEN INTERN MED, V16, P606, DOI 10.1046/j.1525-1497.2001.016009606.x

LENZE EJ, 2005, AM J GERIATR PSYCHIA, V13, P428

Lesch KP, 1996, SCIENCE, V274, P1527, DOI 10.1126/science.274.5292.1527

Link B. G., 1980, MENTAL ILLNESS US EP, P114

Miyata S, 2007, BRAIN RES, V1147, P233, DOI 10.1016/j.brainres.2007.02.001

Mossner R, 2001, MOL PSYCHIATR, V6, P350, DOI 10.1038/sj.mp.4000849

Munafo MR, 2009, BIOL PSYCHIAT, V65, P211, DOI 10.1016/j.biopsych.2008.06.009

Nakamura M, 2000, MOL PSYCHIATR, V5, P32, DOI 10.1038/sj.mp.4000698

Nakatani D, 2005, AM HEART J, V150, P652, DOI 10.1016/j.ahj.2005.03.062

Otte C, 2007, AM J PSYCHIAT, V164, P1379, DOI 10.1176/appi.apj.2007.06.06101617

Pan A, 2008, J AFFECT DISORDERS, V109, P75, DOI 10.1016/j.jad.2007.11.002

Petrisic MS, 1997, J NEUROCHEM, V68, P2435

Ramasubbu R, 2006, J NEUROPSYCH CLIN N, V18, P96, DOI 10.1176/appi.neuropsych.18.1.96

Risch N, 2009, JAMA-J AM MED ASSOC, V301, P2462, DOI 10.1001/jama.2009.878

Rutter M, 2010, DEPRESS ANXIETY, V27, P1, DOI 10.1002/da.20641

Skinner TC, 2010, DIABETIC MED, V27, P965, DOI 10.1111/j.1464-5491.2010.03028.x

Spitzer RL, 1999, JAMA-J AM MED ASSOC, V282, P1737, DOI 10.1001/jama.282.18.1737

Szily E, 2008, J NEURAL TRANSM, V115, P819, DOI 10.1007/s00702-008-0029-4

Uher R, 2008, MOL PSYCHIATR, V13, P131, DOI 10.1038/sj.mp.4002067

Wendland JR, 2006, MOL PSYCHIATR, V11, P224, DOI 10.1038/sj.mp.4001789

Wilhelm K, 2006, BRIT J PSYCHIAT, V188, P210, DOI 10.1192/bjp.bp.105.009522

NR 40

TC 5

Z9 6

U1 0

U2 16

PU ELSEVIER IRELAND LTD

PI CLARE

PA ELSEVIER HOUSE, BROOKVALE PLAZA, EAST PARK SHANNON, CO, CLARE, 00000,

IRELAND

SN 0165-1781

J9 PSYCHIAT RES

JI Psychiatry Res.

PD DEC 30

PY 2012

VL 200

IS 2-3

BP 343

EP 348

DI 10.1016/j.psychres.2012.07.008

PG 6

WC Psychiatry

SC Psychiatry

GA 073TF

UT WOS:000313764700044

PM 22921508

DA 2019-01-08

ER

PT J

AU Mommersteeg, PMC

Herr, R

Zijlstra, WP

Schneider, S

Pouwer, F

AF Mommersteeg, Paula M. C.

Herr, Raphael

Zijlstra, Wobbe P.

Schneider, Sven

Pouwer, Francois

TI Higher levels of psychological distress are associated with a higher

risk of incident diabetes during 18 year follow-up: results from the

British household panel survey

SO BMC PUBLIC HEALTH

LA English

DT Article

DE Type 2 diabetes; Psychological distress; Prospective; Risk factor;

British household panel survey

ID PHYSICAL-ACTIVITY; DEPRESSIVE SYMPTOMS; HEART-DISEASE; TYPE-2; MELLITUS;

ADULTS; CARE; METAANALYSIS; MORTALITY; JAPANESE

AB Background: Reviews have shown that depression is a risk factor for the development of type 2 diabetes. However, there is limited evidence for general psychological distress to be associated with incident diabetes. The aim of the present study was to test whether persons who report higher levels of psychological distress are at increased risk to develop type 2 diabetes during 18 years follow up, adjusted for confounders.

Methods: A prospective analysis using data from 9,514 participants (41 years, SD=14; 44% men) of the British Household Panel Survey. The General Health Questionnaire 12 item version was used to assess general psychological distress, diabetes was measured by means of self-report. Cox proportional hazards regression models were used to calculate the multivariate-adjusted hazard ratio (HR) of incident diabetes during 18 years follow up, comparing participants with low versus high psychological distress at baseline (1991).

Results: A total of 472 participants developed diabetes 18 year follow up. Those with a high level of psychological distress had a 33% higher hazard of developing diabetes (HR=1.33, 95% CI 1.10-1.61), relative to those with a low level of psychological distress, adjusted for age, sex, education level and household income. After further adjustment for differences in level of energy, health status, health problems and activity level, higher psychological distress was no longer associated with incident diabetes (HR=1.10, 95% CI 0.91-1.34).

Conclusions: Higher levels of psychological distress are a risk factor for the development of diabetes during an 18 year follow up period. This association may be potentially mediated by low energy level and impaired health status.

C1 [Mommersteeg, Paula M. C.; Zijlstra, Wobbe P.; Pouwer, Francois] Tilburg Univ, Dept Med & Clin Psychol, Ctr Res Psychol Somat Dis, CoRPS, NL-5000 LE Tilburg, Netherlands.

[Herr, Raphael; Schneider, Sven] Med Fac Mannheim, Mannheim Inst Publ Hlth Social & Prevent Med, MIPH, Mannheim, Germany.

[Zijlstra, Wobbe P.] Tilburg Univ, Dept Methodol & Stat, NL-5000 LE Tilburg, Netherlands.

RP Mommersteeg, PMC (reprint author), Tilburg Univ, Dept Med & Clin Psychol, Ctr Res Psychol Somat Dis, CoRPS, NL-5000 LE Tilburg, Netherlands.

EM P.M.C.Mommersteeg@tilburguniversity.edu

FU Roche Diagnostics Deutschland GmbH, Mannheim, Germany

FX SvS was supported by a small grant from Roche Diagnostics Deutschland

GmbH, Mannheim, Germany, for a study on gestational diabetes. All other

authors: Nothing to declare.

CR Barr-Anderson DJ, 2011, AM J PREV MED, V40, P76, DOI 10.1016/j.amepre.2010.09.033

Brice J, 2010, BRIT HOUSEHOLD PANEL, VA

Champaneri S, 2010, CURR DIABETES REP, V10, P396, DOI 10.1007/s11892-010-0148-9

Dandona P, 2005, CIRCULATION, V111, P1448, DOI 10.1161/01.CIR.0000158483.13093.9D

Fisher L, 2011, CURR MED RES OPIN, V27, P39, DOI 10.1185/03007995.2011.619176

Gillett M, 2012, DIABETIC MED

Goldberg DP, 1997, PSYCHOL MED, V27, P191, DOI 10.1017/S0033291796004242

Golden SH, 2006, PSYCHONEUROENDOCRINO, V31, P325, DOI 10.1016/j.psyneuen.2005.08.008

Hahn D, 2006, PSYCHOSOC MED, V3, P9

Hamer M, 2010, PSYCHOSOM MED, V72, P882, DOI 10.1097/PSY.0b013e3181f6696e

Hankins M, 2008, BMC PUBLIC HEALTH, V8, DOI 10.1186/1471-2458-8-355

Home P, 2006, DIABETIC MED, V23, P579

Institute for Social and Economic Research British Household Panel Survey, 2010, WAV 1 18 1991 2009 C

Kato M, 2009, ENDOCR J, V56, P459, DOI 10.1507/endocrj.K09E-003

Klein M, 2005, SURVIVAL ANAL SELF L

Knol MJ, 2006, DIABETOLOGIA, V49, P837, DOI 10.1007/s00125-006-0159-x

Koopmans B, 2009, FAM PRACT, V26, P171, DOI 10.1093/fampra/cmp016

Kriegsman DMW, 1996, J CLIN EPIDEMIOL, V49, P1407, DOI 10.1016/S0895-4356(96)00274-0

Kumari M, 2004, ARCH INTERN MED, V164, P1873, DOI 10.1001/archinte.164.17.1873

Lysy Z, 2008, DIABETIC MED, V25, P1133, DOI 10.1111/j.1464-5491.2008.02545.x

Gonzalez ELM, 2009, J EPIDEMIOL COMMUN H, V63, P332, DOI 10.1136/jech.2008.080382

Mezuk B, 2008, DIABETES CARE, V31, P2383, DOI 10.2337/dc08-0985

Orozco LJ, 2008, COCHRANE DB SYST REV, V3, DOI DOI 10.02/1461051858

Pouwer F, 2011, DIABETOLOGIA, V54, P741, DOI 10.1007/s00125-010-2033-0

Pouwer F, 2010, DISCOV MED, V9, P112

Ridner SH, 2004, J ADV NURS, V45, P536, DOI 10.1046/j.1365-2648.2003.02938.x

Rod NH, 2009, J INTERN MED, V266, P467, DOI 10.1111/j.1365-2796.2009.02124.x

Sawatzky R, 2007, HEALTH QUAL LIFE OUT, V5, DOI 10.1186/1477-7525-5-68

Shirom A, 2010, PSYCHOSOM MED, V72, P727, DOI 10.1097/PSY.0b013e3181eeb643

Toshihiro M, 2008, DIABETIC MED, V25, P1211, DOI 10.1111/j.1464-5491.2008.02566.x

van der Heijden MMP, 2012, BMC PUBLIC HEALTH, V12, DOI 10.1186/1471-2458-12-331

Whooley MA, 2008, JAMA-J AM MED ASSOC, V300, P2379, DOI 10.1001/jama.2008.711

Willis T, 1675, PHARM RATIONALIS SIV

Yates T, 2012, BMC FAM PRACT, V13, DOI 10.1186/1471-2296-13-46

NR 34

TC 20

Z9 20

U1 1

U2 23

PU BIOMED CENTRAL LTD

PI LONDON

PA 236 GRAYS INN RD, FLOOR 6, LONDON WC1X 8HL, ENGLAND

SN 1471-2458

J9 BMC PUBLIC HEALTH

JI BMC Public Health

PD DEC 23

PY 2012

VL 12

AR 1109

DI 10.1186/1471-2458-12-1109

PG 10

WC Public, Environmental & Occupational Health

SC Public, Environmental & Occupational Health

GA 074RT

UT WOS:000313831600001

PM 23259455

OA DOAJ Gold, Green Published

DA 2019-01-08

ER

PT J

AU Gois, C

Akiskal, H

Akiskal, K

Figueira, ML

AF Gois, Carlos

Akiskal, Hagop

Akiskal, Kareen

Luisa Figueira, M.

TI Depressive temperament, distress, psychological adjustment and

depressive symptoms in type 2 diabetes

SO JOURNAL OF AFFECTIVE DISORDERS

LA English

DT Review

DE Depressive temperament; Depression; TEMPS-A; Type 2 diabetes; Diabetes

distress; Metabolic control

ID SELF-REPORTED DISTRESS; GLYCEMIC CONTROL; ANXIETY DISORDERS;

BIDIRECTIONAL ASSOCIATION; RISK-FACTOR; ADULTS; METAANALYSIS;

PERSONALITY; WOMEN; VALIDATION

AB Major depression, depressive symptoms, distress with diabetes and psychological adjustment to type 2 diabetes (T2DM) are somehow overlapping constructs with shared covariance. Nevertheless, diabetes distress remains the most prevalent long-lasting factor associated with hyperglycemia in T2DM. This ought to be taken on account when treatment is planned, namely when one is looking for metabolic control and emotional distress integrative care. Temperament or other long-term individual characteristics may support the interplay of distress, psychological adjustment and depressive states in T2DM. Depressive temperament may constitute a vulnerability factor to behavioral or biological T2DM outcome or even a potential risk factor to T2DM later incidence. (C) 2012 Elsevier B.V. All rights reserved.

C1 [Gois, Carlos; Luisa Figueira, M.] Univ Lisbon, Hosp Santa Maria, Fac Med, Dept Psychiat, P-1600 Lisbon, Portugal.

[Akiskal, Hagop; Akiskal, Kareen] Univ Calif San Diego, La Jolla, CA 92093 USA.

[Akiskal, Hagop; Akiskal, Kareen] Int Mood Ctr, La Jolla, CA USA.

RP Gois, C (reprint author), Univ Lisbon, Hosp Santa Maria, Fac Med, Dept Psychiat, Av Prof Egas Moniz, P-1600 Lisbon, Portugal.

EM carlosgois13@gmail.com

OI Gois, Carlos/0000-0003-4346-4844

CR Akiskal HS, 2005, J AFFECT DISORDERS, V85, P3, DOI 10.1016/j.jad.2004.12.001

AKISKAL HS, 1983, J AFFECT DISORDERS, V40, P801

Anderson RJ, 2001, DIABETES CARE, V24, P1069, DOI 10.2337/diacare.24.6.1069

Arroyo C, 2004, DIABETES CARE, V27, P129, DOI 10.2337/diacare.27.1.129

Brown LC, 2006, CAN MED ASSOC J, V175, P42, DOI 10.1503/cmaj.051429

Carnethon MR, 2003, AM J EPIDEMIOL, V158, P416, DOI 10.1093/aje/kwg172

Ciechanowski PS, 2000, ARCH INTERN MED, V160, P3278, DOI 10.1001/archinte.160.21.3278

CLARK LA, 1994, J ABNORM PSYCHOL, V103, P103, DOI 10.1037/0021-843X.103.1.103

COYNE JC, 1994, PSYCHOL BULL, V116, P29, DOI 10.1037/0033-2909.116.1.29

de Groot M, 2001, PSYCHOSOM MED, V63, P619, DOI 10.1097/00006842-200107000-00015

de Ridder D, 2008, LANCET, V372, P246, DOI 10.1016/S0140-6736(08)61078-8

Delahanty LM, 2007, DIABETIC MED, V24, P48, DOI 10.1111/j.1464-5491.2007.02028.x

Demakakos P, 2010, DIABETES CARE, V33, P792, DOI 10.2337/dc09-1663

Eaton WW, 1996, DIABETES CARE, V19, P1097, DOI 10.2337/diacare.19.10.1097

Engum A, 2005, DIABETES CARE, V28, P1904, DOI 10.2337/diacare.28.8.1904

FECHNERBATES S, 1994, J CONSULT CLIN PSYCH, V62, P550, DOI 10.1037//0022-006X.62.3.550

Figueira ML, 2008, J AFFECT DISORDERS, V111, P193, DOI 10.1016/j.jad.2008.03.001

Fisher L, 2008, DIABETIC MED, V25, P1096, DOI 10.1111/j.1464-5491.2008.02533.x

Fisher L, 2001, DIABETES CARE, V24, P1751, DOI 10.2337/diacare.24.10.1751

Fisher L., 2009, DIABETIC MED, V29, P622

Fisher L, 2007, DIABETES CARE, V30, P542, DOI 10.2337/dc06-1614

Gois C.J., 2010, ACTA DIABETOLOGY

Gois C, 2011, J AFFECT DISORDERS, V134, P52, DOI 10.1016/j.jad.2011.05.021

Golden SH, 2008, JAMA-J AM MED ASSOC, V299, P2751, DOI 10.1001/jama.299.23.2751

Gonzalez J.S., 2007, DIABETES CARE, V30, P222

Gonzalez J.S., 2011, DIABETES CARE, V4, P236

Ismail K, 2007, DIABETES CARE, V30, P1473, DOI 10.2337/dc06-2313

Katon Wayne J, 2011, Dialogues Clin Neurosci, V13, P7

Kawakami N, 1999, DIABETES CARE, V22, P1071, DOI 10.2337/diacare.22.7.1071

KENDLER KS, 1993, ARCH GEN PSYCHIAT, V50, P853

Knol MJ, 2006, DIABETOLOGIA, V49, P837, DOI 10.1007/s00125-006-0159-x

Lane JD, 2000, DIABETES CARE, V23, P1321, DOI 10.2337/diacare.23.9.1321

Lin EHB, 2004, DIABETES CARE, V27, P2154, DOI 10.2337/diacare.27.9.2154

LUSTMAN PJ, 1991, PSYCHOSOM MED, V53, P305, DOI 10.1097/00006842-199105000-00004

Lustman PJ, 2000, DIABETES CARE, V23, P934, DOI 10.2337/diacare.23.7.934

Mezuk B, 2008, DIABETES CARE, V31, P2383, DOI 10.2337/dc08-0985

Mezuk B, 2008, AM J PUBLIC HEALTH, V98, P1480, DOI 10.2105/AJPH.2007.126441

Nakahara R, 2006, PSYCHOSOMATICS, V47, P240, DOI 10.1176/appi.psy.47.3.240

Nichols GA, 2000, DIABETES CARE, V23, P273, DOI 10.2337/diacare.23.3.273

Pan A, 2010, ARCH INTERN MED, V170, P1884, DOI 10.1001/archinternmed.2010.356

Power F., 2005, DIABETES RES CLIN PR, V70, P166

Richardson LK, 2008, GEN HOSP PSYCHIAT, V30, P509, DOI 10.1016/j.genhosppsych.2008.07.001

Rihmer Z, 2010, CURR OPIN PSYCHIATR, V23, P12, DOI 10.1097/YCO.0b013e32833299d4

Rubin R.R., 2006, CLIN DIABETES, V21, P269

Sharpe L, 2006, SOC SCI MED, V62, P1153, DOI 10.1016/j.socscimed.2005.07.010

Stanton A.L., 2007, ANNU REV PSYCHOL, V58, P656

Tomassini A, 2009, INT J PSYCHIAT CLIN, V13, P31, DOI 10.1080/13651500802369482

Watson D, 2005, J RES PERS, V39, P46, DOI 10.1016/j.jrp.2004.09.006

Welch GW, 1997, DIABETES CARE, V20, P760, DOI 10.2337/diacare.20.5.760

NR 49

TC 6

Z9 6

U1 1

U2 30

PU ELSEVIER SCIENCE BV

PI AMSTERDAM

PA PO BOX 211, 1000 AE AMSTERDAM, NETHERLANDS

SN 0165-0327

J9 J AFFECT DISORDERS

JI J. Affect. Disord.

PD DEC 20

PY 2012

VL 143

IS 1-3

BP 1

EP 4

DI 10.1016/j.jad.2012.05.028

PG 4

WC Clinical Neurology; Psychiatry

SC Neurosciences & Neurology; Psychiatry

GA 038ZY

UT WOS:000311213800001

PM 22901331

DA 2019-01-08

ER

PT J

AU Schrier, AC

Ujcic-Voortman, JK

de Wit, MAS

Verhoeff, AP

Kupka, R

Dekker, J

Beekman, ATF

AF Schrier, Agnes C.

Ujcic-Voortman, Joanne K.

de Wit, Matty A. S.

Verhoeff, Arnoud P.

Kupka, Ralph

Dekker, Jack

Beekman, Aartjan T. F.

TI Ethnic differences in the association between cardiovascular risk

factors and psychological distress in a population study in the

Netherlands

SO BMC PUBLIC HEALTH

LA English

DT Article

DE Obesity; Cardiovascular; Depression; Ethnicity; Epidemiology

ID MAJOR DEPRESSIVE DISORDER; METABOLIC SYNDROME; GENERAL-POPULATION;

ANXIETY DISORDERS; SCREENING SCALES; NATIVE DUTCH; FOLLOW-UP; OBESITY;

SYMPTOMS; TURKISH

AB Background: There is growing body of evidence of an association between cardiovascular risk factors and depressive and anxiety symptoms. The purpose of this study was to investigate whether these associations are similar in ethnic minority groups.

Methods: A random urban population sample, aged 18+, stratified by ethnicity (484 native Dutch subjects, 383 Turkish-Dutch subjects, and 316 Moroccan-Dutch subjects), in Amsterdam, the Netherlands, was interviewed with the Kessler Psychological Distress scale (K10) in combination with measurements of several cardiovascular risk factors. The association of psychological distress (defined as a K10 score above cut-off of 20) with cardiovascular risk factors (obesity, abdominal obesity, hypertension, hypercholesterolemia, low HDL cholesterol levels or diabetes), ethnicity and their interaction was analyzed using logistic regression analyses, stratified by gender and adjusted for age.

Results: Cardiovascular risk factors were not significantly associated with psychological distress in any of the gender/ethnic groups, with the exception of a positive association of obesity and hypertension with psychological distress in native Dutch women and a negative association of hypertension and psychological distress in Turkish men. Interaction terms of cardiovascular risk factors and ethnicity were approaching significance only in the association of obesity with the K10 in women.

Conclusion: In this cross-sectional multi-ethnic adult population sample the majority of the investigated cardiovascular risk factors were not associated with psychological distress. The association of obesity with psychological distress varies by gender and ethnicity. Our findings indicate that the prevention of obesity and psychological distress calls for an integrated approach in native Dutch women, but not necessarily in Turkish-Dutch and Moroccan-Dutch women, in whom these problems may be targeted separately.

C1 [Schrier, Agnes C.; Kupka, Ralph] Altrecht Inst Mental Hlth Care, Utrecht, Netherlands.

[Ujcic-Voortman, Joanne K.; de Wit, Matty A. S.; Verhoeff, Arnoud P.] Publ Hlth Serv Amsterdam, Dept Epidemiol, Amsterdam, Netherlands.

[Verhoeff, Arnoud P.] Univ Amsterdam, Dept Sociol & Anthropol, Amsterdam, Netherlands.

[Kupka, Ralph; Beekman, Aartjan T. F.] Vrije Univ Amsterdam, Med Ctr, Dept Psychiat, Amsterdam, Netherlands.

[Dekker, Jack] Vrije Univ Amsterdam, Med Ctr, Dept Clin Psychol, Amsterdam, Netherlands.

[Dekker, Jack; Beekman, Aartjan T. F.] Vrije Univ Amsterdam, Med Ctr, EMGO Inst Hlth & Care Res, Amsterdam, Netherlands.

RP Schrier, AC (reprint author), Altrecht Inst Mental Hlth Care, Utrecht, Netherlands.

EM a.schrier@altrecht.nl

RI Verhoeff, Arnoud/F-7924-2013

FU Municipality of Amsterdam; National Institute of Public Health;

Environment in the Netherlands; Public Health Service Amsterdam;

Altrecht Institute for Mental Health

FX The data collection of the study was financed by the Municipality of

Amsterdam and the National Institute of Public Health and the

Environment in the Netherlands. The data analyses built on the work of

Mrs. Joanne Ujcic-Voortman, who is financed by the Public Health Service

Amsterdam. Mrs. A. C. Schrier was supported by Altrecht Institute for

Mental Health during data analysis and writing of the manuscript.

CR Agyemang C, 2006, J HYPERTENS, V24, P2169, DOI 10.1097/01.hjh.0000249693.73618.c9

Alberti KGMM, 2005, LANCET, V366, P1059, DOI 10.1016/S0140-6736(05)67402-8

Alonso J, 2004, INT J METH PSYCH RES, V18, P159

Andrews G, 2001, AUST NZ J PUBL HEAL, V25, P494, DOI 10.1111/j.1467-842X.2001.tb00310.x

Beydoun MA, 2010, J AFFECT DISORDERS, V123, P52, DOI 10.1016/j.jad.2009.09.021

Carpenter KM, 2000, AM J PUBLIC HEALTH, V90, P251, DOI 10.2105/AJPH.90.2.251

CBO Dutch Institute for Healthcare Improvement, 2006, DUTCH GUID CARD RISK

de Wit L, 2010, PSYCHIAT RES, V178, P230, DOI 10.1016/j.psychres.2009.04.015

de Wit MAS, 2008, SOC PSYCH PSYCH EPID, V43, P905, DOI 10.1007/s00127-008-0382-5

Delaney JAC, 2010, HYPERTENSION, V55, P408, DOI 10.1161/HYPERTENSIONAHA.109.139824

Donker T, 2010, PSYCHIAT RES, V176, P45, DOI 10.1016/j.psychres.2009.01.012

Dortland AKBV, 2010, ACTA PSYCHIAT SCAND, V122, P30, DOI 10.1111/j.1600-0447.2010.01565.x

Dortland AKBV, 2010, J CLIN PSYCHIAT, V71, P729, DOI 10.4088/JCP.08m04865blu

Fassaert T, 2008, INT J METH PSYCH RES, V18, P159

Gavin AR, 2010, PUBLIC HEALTH REP, V125, P698, DOI 10.1177/003335491012500512

Grabe S, 2006, PSYCHOL BULL, V132, P622, DOI 10.1037/0033-2909.132.4.622

Heo M, 2006, INT J OBESITY, V30, P513, DOI 10.1038/sj.ijo.0803122

Hildrum B, 2009, ACTA PSYCHIAT SCAND, V120, P14, DOI 10.1111/j.1600-0447.2008.01315.x

Jackson JS, 2010, AM J PUBLIC HEALTH, V100, P933, DOI 10.2105/AJPH.2008.143446

Kessler RC, 2002, PSYCHOL MED, V32, P959, DOI 10.1017/S0033291702006074

Keyes KM, 2011, SOC SCI MED, V72, P650, DOI 10.1016/j.socscimed.2010.12.005

Kinder LS, 2004, PSYCHOSOM MED, V66, P316, DOI 10.1097/01.psy.0000124755.91880.f4

Koponen H, 2008, J CLIN PSYCHIAT, V69, P178, DOI 10.4088/JCP.v69n0202

Levecque K, 2007, J AFFECT DISORDERS, V97, P229, DOI 10.1016/j.jad.2006.06.022

Luppino FS, 2011, PSYCHOSOM MED, V73, P257, DOI 10.1097/PSY.0b013e31820a59c0

Luppino FS, 2010, ARCH GEN PSYCHIAT, V67, P220, DOI 10.1001/archgenpsychiatry.2010.2

Marijnissen RM, 2010, J AM GERIATR SOC, V59, P67

Nicolaou M, 2008, PUBLIC HEALTH NUTR, V11, P1332, DOI 10.1017/S1368980008002966

Ozmen D, 2007, BMC PUBLIC HEALTH, V7, DOI 10.1186/1471-2458-7-80

Simon GE, 2006, ARCH GEN PSYCHIAT, V63, P824, DOI 10.1001/archpsyc.63.7.824

Skilton MR, 2007, BIOL PSYCHIAT, V62, P1251, DOI 10.1016/j.biopsych.2007.01.012

Slot J, 2006, MEASUREMENT ACTUAL Q

Tedders SH, 2011, J AFFECT DISORDERS, V135, P115, DOI 10.1016/j.jad.2011.06.045

Toker S, 2008, DEPRESS ANXIETY, V25, P661, DOI 10.1002/da.20379

Ujcic-Voortman JK, 2011, OBESITY FACTS, V4, P53, DOI 10.1159/000324555

Ujcic-Voortman JK, 2010, BMC PUBLIC HEALTH, V10, DOI 10.1186/1471-2458-10-740

Ujcic-Voortman JK, 2009, EUR J PUBLIC HEALTH, V19, P511, DOI 10.1093/eurpub/ckp096

Vogelzangs N, 2007, J GERONTOL A-BIOL, V62, P563, DOI 10.1093/gerona/62.5.563

Vogelzangs N, 2010, J CLIN PSYCHIAT, V71, P391, DOI 10.4088/JCP.08m04743blu

Vogelzangs N, 2008, ARCH GEN PSYCHIAT, V65, P1386, DOI 10.1001/archpsyc.65.12.1386

World Health Organization, 2000, 903 WHO

NR 41

TC 2

Z9 2

U1 1

U2 9

PU BIOMED CENTRAL LTD

PI LONDON

PA 236 GRAYS INN RD, FLOOR 6, LONDON WC1X 8HL, ENGLAND

SN 1471-2458

J9 BMC PUBLIC HEALTH

JI BMC Public Health

PD DEC 18

PY 2012

VL 12

AR 1090

DI 10.1186/1471-2458-12-1090

PG 7

WC Public, Environmental & Occupational Health

SC Public, Environmental & Occupational Health

GA 077OB

UT WOS:000314035900001

PM 23249268

OA DOAJ Gold, Green Published

DA 2019-01-08

ER

PT J

AU Mendenhall, E

Shivashankar, R

Tandon, N

Ali, MK

Narayan, KMV

Prabhakaran, D

AF Mendenhall, Emily

Shivashankar, Roopa

Tandon, Nikhil

Ali, Mohammed K.

Narayan, K. M. Venkat

Prabhakaran, Dorairaj

TI Stress and diabetes in socioeconomic context: A qualitative study of

urban Indians

SO SOCIAL SCIENCE & MEDICINE

LA English

DT Article

DE Social distress; Depression; Type 2 diabetes; Qualitative interviews;

Urban health; India

ID PRIMARY-CARE; DEVELOPING-COUNTRIES; CARDIOVASCULAR RISK; RURAL

EPIDEMIOLOGY; ANXIETY DISORDERS; DEPRESSION; PREVALENCE; MELLITUS;

PERSPECTIVES; METAANALYSIS

AB Type 2 diabetes has escalated in urban India in the past two decades. Historically a disease of the affluent, recent epidemiological evidence indicates rising diabetes incidence and prevalence in urban India's middle class and working poor. Although there is substantial qualitative data about people with diabetes from high-income countries, scant resources provide insight into diabetes experiences among those in India, and lower-income groups specifically. In this article, we use individual-level analysis of illness narratives to understand how people experience and understand diabetes across income groups in Delhi, India. We conducted in-depth qualitative interviews and administered the Hopkins Symptoms Check-List (HSCL-25) to evaluate depression among 59 people with diabetes in northeast Delhi between December 2011 and February 2012. We analyzed their responses to: 1) what caused your diabetes?: 2) what do you find most stressful in your daily life?: and 3) where do you seek diabetes care? We found few people held diabetes beliefs that were congruent with socio-spiritual or biomedical explanatory models, and higher income participants commonly cited "tension" as a contributor to diabetes. Stress associated with children's futures, financial security, and family dynamics were most commonly reported, but how these subjective stresses were realized in people's lives varied across income groups. Depression was most common among the poorest income group (55%) but was also reported among middle- (38%) and high-income (29%) participants. One-quarter of respondents reported diabetes distress, but only those from the low-income community reported co-occurring depression and these respondents often revealed poor access to diabetes care. These data suggest that lower-income populations not only have higher rates of depression but also may be more likely to delay health care and therefore develop diabetes complications. This research has many implications for public health care in India as diabetes prevalence shifts to affect lower income groups who concurrently experience higher rates of depression and poorer access to medical care. (C) 2012 Elsevier Ltd. All rights reserved.

C1 [Mendenhall, Emily] Univ Witwatersrand, Fac Hlth Sci, MRC Wits Dev Pathways Hlth Res Unit, ZA-2198 Johannesburg, South Africa.

[Mendenhall, Emily; Shivashankar, Roopa; Tandon, Nikhil; Ali, Mohammed K.; Narayan, K. M. Venkat; Prabhakaran, Dorairaj] Publ Hlth Fdn India, COE CARRS, New Delhi, India.

[Shivashankar, Roopa; Prabhakaran, Dorairaj] Ctr Chron Dis Control, New Delhi, India.

[Tandon, Nikhil] All Indian Inst Med Res, New Delhi, India.

[Ali, Mohammed K.; Narayan, K. M. Venkat] Emory Univ, Rollins Sch Publ Hlth, Atlanta, GA 30322 USA.

RP Mendenhall, E (reprint author), Univ Witwatersrand, Fac Hlth Sci, MRC Wits Dev Pathways Hlth Res Unit, ZA-2198 Johannesburg, South Africa.

EM emily.mendenhall@gmail.com

RI Narayan, K.M. Venkat/J-9819-2012

OI Narayan, K.M. Venkat/0000-0001-8621-5405; Prabhakaran,

Dorairaj/0000-0002-3172-834X; Mendenhall, Emily/0000-0002-5826-1321

FU Fogarty International Center of the National Institutes of Health

through the International Clinical Research Fellows Program at

Vanderbilt University [R24 TW007988]; American Relief and Recovery Act;

Fogarty International Center [D43 HD065249]; Eunice Kennedy Shriver

National Institute of Child Health & Human Development

FX We are grateful to the study participants who shared their time and

personal experiences with us. Planning this study would have been

difficult without the guidance from Lesley Jo Weaver regarding

qualitative diabetes research in Delhi, and conducting the study would

have been impossible without the hard work of Sneha Sharma and Allam

Ashraf. Thank you to John Millhauser and Sara Lewis for reviewing the

manuscript. The first author (EM) was supported by the Fogarty

International Center of the National Institutes of Health through the

International Clinical Research Fellows Program at Vanderbilt University

(R24 TW007988) and the American Relief and Recovery Act. The second

author (RS) was supported by a National Institutes of Health D43

Fellowship (D43 HD065249) from the Fogarty International Center and the

Eunice Kennedy Shriver National Institute of Child Health & Human

Development.

CR Ajay VS, 2008, DIABETIC MED, V25, P1187, DOI 10.1111/j.1464-5491.2008.02554.x

Bernard H. R., 1988, RES METHODS CULTURAL

Brown RA, 2009, ANN HUM BIOL, V36, P248, DOI 10.1080/03014460902832942

Chokkanathan S, 2009, SOC SCI MED, V68, P243, DOI [10.1016/j.socscimed.2008.10.008, 10.1016/j.socscimed.2008.40.008]

DALAL AK, 2000, PSYCHOL DEV SOC, V12, P67, DOI DOI 10.1177/097133360001200105

de Groot M, 2001, PSYCHOSOM MED, V63, P619, DOI 10.1097/00006842-200107000-00015

de Groot M, 2006, DIABETES CARE, V29, P549, DOI 10.2337/diacare.29.03.06.dc05-1396

Deepa Mohan, 2011, J Diabetes Sci Technol, V5, P918

Ebrahim S, 2010, PLOS MED, V7, DOI 10.1371/journal.pmed.1000268

Egede LE, 2010, DIABETES RES CLIN PR, V87, P302, DOI 10.1016/j.diabres.2010.01.024

Finkler K, 2004, SOC SCI MED, V59, P2037, DOI 10.1016/j.socscimed.2004.03.008

Golden SH, 2008, JAMA-J AM MED ASSOC, V299, P2751, DOI 10.1001/jama.299.23.2751

Golden SH, 2007, PSYCHOSOM MED, V69, P529, DOI 10.1097/PSY.0b013e3180f61c5c

Gupta R, 2007, BR J DIABETES VASC D, V7, P12, DOI DOI 10.1177/14746514070070010301)

Hunt LM, 1998, SOC SCI MED, V46, P959, DOI 10.1016/S0277-9536(97)10014-4

Katon W, 2010, CONTEMP CLIN TRIALS, V31, P312, DOI 10.1016/j.cct.2010.03.009

Kielmann Karina, 2002, Med Anthropol, V21, P157, DOI 10.1080/01459740212903

Kirmayer L. J., 1998, PSYCHOSOM MED, V3, P343

Kleinman A., 1980, PATIENTS HEALERS CON

Knol MJ, 2006, DIABETOLOGIA, V49, P837, DOI 10.1007/s00125-006-0159-x

Lieberman LS, 2003, ANNU REV NUTR, V23, P345, DOI 10.1146/annurev.nutr.23.011702.073212

Lin EHB, 2009, ANN FAM MED, V7, P414, DOI 10.1370/afm.998

Loewe R, 1998, SOC SCI MED, V47, P1267, DOI 10.1016/S0277-9536(98)00192-0

Lustman PJ, 2000, DIABETES CARE, V23, P934, DOI 10.2337/diacare.23.7.934

McDade TW, 2002, MED ANTHROPOL Q, V16, P123, DOI 10.1525/maq.2002.16.2.123

McGarvey S. T., 1989, HUMAN POPULATION BIO, P263

Mendenhall E, 2012, DIABETIC MED, V29, pE308, DOI 10.1111/j.1464-5491.2012.03708.x

Mendenhall E, 2010, MED ANTHROPOL Q, V24, P220, DOI 10.1111/j.1548-1387.2010.01098.x

Mezuk B, 2008, DIABETES CARE, V31, P2383, DOI 10.2337/dc08-0985

Mollica RF, 2004, MEASURING TRAUMA MEA

Nair M, 2012, BMC PUBLIC HEALTH, V12, DOI 10.1186/1471-2458-12-701

Nunnally JC, 1994, PSYCHOMETRIC THEORY

Patel V, 1998, BRIT J PSYCHIAT, V172, P533, DOI 10.1192/bjp.172.6.533

Patel V, 2003, B WORLD HEALTH ORGAN, V81, P609

Patel V, 2011, BRIT J PSYCHIAT, V199, P459, DOI 10.1192/bjp.bp.111.092155

Patel V, 2011, LANCET, V377, P413, DOI 10.1016/S0140-6736(10)61188-9

Patel V, 2010, LANCET, V376, P2086, DOI 10.1016/S0140-6736(10)61508-5

Poongothai S, 2010, DIABETES TECHNOL THE, V12, P989, DOI 10.1089/dia.2010.0081

Poongothai S, 2009, PLOS ONE, V4, DOI 10.1371/journal.pone.0007185

Popkin BM, 2012, NUTR REV, V70, P3, DOI 10.1111/j.1753-4887.2011.00456.x

Poss J, 2002, MED ANTHROPOL Q, V16, P360, DOI 10.1525/maq.2002.16.3.360

Rai M, 2009, INT J DIABETES DEV C, V29, P129, DOI 10.4103/0973-3930.54290

Ramachandram A, 2007, EPIDEMIOLOGY DIABETE

Raval A, 2010, INDIAN J MED RES, V132, P195

Reddy KS, 2007, P NATL ACAD SCI USA, V104, P16263, DOI 10.1073/pnas.0700933104

Renders CM, 2001, DIABETES CARE, V24, P1821, DOI 10.2337/diacare.24.10.1821

Sarkar S, 2008, STRESS HEALTH, V24, P23, DOI 10.1002/smi.1159

Shobhana R, 2003, J Assoc Physicians India, V51, P363

Sridhar G, 2000, DIABETES RES CLIN PR, V50, P981

Sridhar GR, 2007, DIABETES METAB SYND, V1, P181, DOI 10.1016/j.dsx.2007.05.006

Sridhar GR, 2002, CURR SCI INDIA, V83, P1556

Weaver LJ, 2011, ETHOS, V39, P211, DOI 10.1111/j.1548-1352.2011.01185.x

Zimmet P, 2001, NATURE, V414, P782, DOI 10.1038/414782a

NR 53

TC 27

Z9 27

U1 0

U2 42

PU PERGAMON-ELSEVIER SCIENCE LTD

PI OXFORD

PA THE BOULEVARD, LANGFORD LANE, KIDLINGTON, OXFORD OX5 1GB, ENGLAND

SN 0277-9536

J9 SOC SCI MED

JI Soc. Sci. Med.

PD DEC

PY 2012

VL 75

IS 12

BP 2522

EP 2529

DI 10.1016/j.socscimed.2012.09.040

PG 8

WC Public, Environmental & Occupational Health; Social Sciences, Biomedical

SC Public, Environmental & Occupational Health; Biomedical Social Sciences

GA 060DX

UT WOS:000312757800058

PM 23111063

OA Green Accepted

DA 2019-01-08

ER

PT J

AU Yorgason, JB

Roper, SO

Sandberg, JG

Berg, CA

AF Yorgason, Jeremy B.

Roper, Susanne O.

Sandberg, Jonathan G.

Berg, Cynthia A.

TI Stress Spillover of Health Symptoms From Healthy Spouses to Patient

Spouses in Older Married Couples Managing Both Diabetes and

Osteoarthritis

SO FAMILIES SYSTEMS & HEALTH

LA English

DT Article

DE health; coping with illness/disability; marriage; comorbid illnesses

ID STAGE RENAL-DISEASE; QUALITY-OF-LIFE; MARITAL SATISFACTION; CHRONIC

ILLNESS; FAMILY PROCESS; DIARY METHODS; CROSSOVER; SUPPORT; ASSOCIATION;

DISTRESS

AB Many studies examining illness within marriage have investigated how illness in one spouse influences the other spouse. In later-life marriages, where both spouses are more likely to have health challenges, there is an increased likelihood that health symptoms from both spouses affect each other. In the current study we examined how health symptoms in a "healthy" spouse may exacerbate health problems in a partner (the patient) who is managing multiple chronic illnesses. Surveys were collected across 14 days from 27 later-life couples where patients had both diabetes and osteoarthritis. Results indicated that higher healthy spouse symptoms were generally associated with higher patient symptoms, suggesting a spillover effect. Spouse reports of positive and negative mood were inversely linked with patient health outcomes. Spouse reports of higher positive marital interactions were surprisingly linked with higher patients' arthritis activity and activity limitations, possibly indicating a compensatory effect where marital interactions increase with symptoms. Daily spouse reports of positive marital interactions and mood were linked with patient health outcomes even after the spillover of health symptoms was taken into account.

C1 [Yorgason, Jeremy B.; Roper, Susanne O.; Sandberg, Jonathan G.] Brigham Young Univ, Sch Family Life, Provo, UT 84602 USA.

[Berg, Cynthia A.] Univ Utah, Dept Psychol, Salt Lake City, UT 84112 USA.

RP Yorgason, JB (reprint author), Brigham Young Univ, Sch Family Life, 2079 JFSB, Provo, UT 84602 USA.

EM Jeremy_yorgason@byu.edu

OI Yorgason, Jeremy/0000-0002-4208-7306

FU Marjorie Pay Hinckley Chair; Family Studies Center; Gerontology Center

at Brigham Young University

FX Funding for this study was provided by the Marjorie Pay Hinckley Chair,

the Family Studies Center, and the Gerontology Center at Brigham Young

University. We express appreciation to the couples who participated in

this study, and to the students who assisted with data collection.

CR Administration on Aging (AoA), 2009, PROF OLD AM 2009

Bayliss Elizabeth A, 2003, Ann Fam Med, V1, P15, DOI 10.1370/afm.4

Berg CA, 2008, PSYCHOL AGING, V23, P505, DOI 10.1037/a0012687

Berg CA, 2007, PSYCHOL BULL, V133, P920, DOI 10.1037/0033-2909.133.6.920

Bigatti SM, 2002, HEALTH PSYCHOL, V21, P157, DOI 10.1037//0278-6133.21.2.157

Bolger N, 2003, ANNU REV PSYCHOL, V54, P579, DOI 10.1146/annurev.psych.54.101601.145030

Bookwala J, 1996, PSYCHOL AGING, V11, P582, DOI 10.1037//0882-7974.11.4.582

BOOTH A, 1994, J MARRIAGE FAM, V56, P218, DOI 10.2307/352716

Burgener S, 2002, ALZ DIS ASSOC DIS, V16, P88, DOI 10.1097/01.WAD.0000015567.76518.5C

Center on an Aging Society, 2003, MULT CHRON COND CHAL

COYNE JC, 1991, J PERS SOC PSYCHOL, V61, P404, DOI 10.1037/0022-3514.61.3.404

Gleason MEJ, 2008, J PERS SOC PSYCHOL, V94, P824, DOI 10.1037/0022-3514.94.5.824

Gunthert K. C., 2012, HDB RES METHODS STUD

Hays R D, 1993, Health Econ, V2, P217, DOI 10.1002/hec.4730020305

Henry NJM, 2007, PSYCHOL AGING, V22, P428, DOI 10.1037/0882.7974.22.3.428

Iida M, 2010, PERS SOC PSYCHOL B, V36, P780, DOI 10.1177/0146167210369897

LARSEN RJ, 1991, J PERS, V59, P387, DOI 10.1111/j.1467-6494.1991.tb00254.x

Larson RW, 1999, J MARRIAGE FAM, V61, P5, DOI 10.2307/353879

Laurenceau JP, 2005, J FAM PSYCHOL, V19, P86, DOI 10.1037/0893-3200.19.1.86

Lawrence RC, 1998, ARTHRITIS RHEUM-US, V41, P778, DOI 10.1002/1529-0131(199805)41:5<778::AID-ART4>3.0.CO;2-V

Neff LA, 2005, J PERS SOC PSYCHOL, V88, P480, DOI 10.1037/0022-3514.88.3.480

Newton-John TRO, 2002, PAIN REV, V9, P7, DOI 10.1191/0968130202pr186ra

PASTOR PN, 2002, CHARTBOOK TRENDS HLT

Piazza JR, 2007, J GERONTOL B-PSYCHOL, V62, pP313, DOI 10.1093/geronb/62.6.P313

Pruchno R, 2009, J GERONTOL B-PSYCHOL, V64, P212, DOI 10.1093/geronb/gbp006

REISS D, 1986, ARCH GEN PSYCHIAT, V43, P795

REVENSON TA, 2005, COUPLES COPING STRES, P137, DOI DOI 10.1037/11031-007

Scheibe S, 2010, J GERONTOL B-PSYCHOL, V65, P135, DOI 10.1093/geronb/gbp132

SHAVELSON RJ, 1991, GEN THEORY PRIMER

Stephens MAP, 2006, REHABIL PSYCHOL, V51, P3, DOI 10.1037/0090-5550.51.1.3

Story TN, 2007, PSYCHOL AGING, V22, P719, DOI 10.1037/0882-7974.22.4.719

Turner-Musa J, 1999, SOC SCI MED, V48, P1333, DOI 10.1016/S0277-9536(98)00437-7

WATSON D, 1988, J PERS SOC PSYCHOL, V54, P1063, DOI 10.1037/0022-3514.54.6.1063

Westman M, 2001, HUM RELAT, V54, P717, DOI 10.1177/0018726701546002

Westman M, 1998, HUM RELAT, V51, P137, DOI 10.1177/001872679805100202

Westman M, 2008, J OCCUP HEALTH PSYCH, V13, P168, DOI 10.1037/1076-8998.13.2.168

Whittemore R, 2003, DIABETES EDUCATOR, V29, P847, DOI 10.1177/014572170302900514

Wray LA, 2006, J GERONTOL B-PSYCHOL, V61, pS290, DOI 10.1093/geronb/61.6.S290

Xu JQ, 2010, NATL VITAL STAT REPO, V58

Yorgason JB, 2008, RES AGING, V30, P623, DOI 10.1177/0164027508322570

Yorgason JB, 2006, FAM RELAT, V55, P613, DOI 10.1111/j.1741-3729.2006.00430.x

Yorgason JB, 2010, FAM SYST HEALTH, V28, P30, DOI 10.1037/a0019396

NR 42

TC 9

Z9 9

U1 1

U2 16

PU EDUCATIONAL PUBLISHING FOUNDATION-AMERICAN PSYCHOLOGICAL ASSOC

PI WASHINGTON

PA 750 FIRST ST, NE, WASHINGTON, DC 20002-4242 USA

SN 1091-7527

J9 FAM SYST HEALTH

JI Fam. Syst. Health

PD DEC

PY 2012

VL 30

IS 4

BP 330

EP 343

DI 10.1037/a0030670

PG 14

WC Health Care Sciences & Services; Family Studies; Public, Environmental &

Occupational Health

SC Health Care Sciences & Services; Family Studies; Public, Environmental &

Occupational Health

GA 062LC

UT WOS:000312920300006

PM 23148979

DA 2019-01-08

ER

PT J

AU Sato, M

Yamazaki, Y

AF Sato, Miho

Yamazaki, Yoshihiko

TI Work-related factors associated with self-care and psychological health

among people with type 2 diabetes in Japan

SO NURSING & HEALTH SCIENCES

LA English

DT Article

DE employment; Japanese; psychological health; self-care; shift work; type

2 diabetes

ID CHRONIC ILLNESS; SOCIAL SUPPORT; MANAGEMENT; DEPRESSION; DISTRESS;

BEHAVIORS; EFFICACY; MELLITUS; ANXIETY; ADULTS

AB This study on individuals with type 2 diabetes living in Japan aimed to examine work-related factors that influence self-care and psychological health among people. A cross-sectional survey was conducted among 121 working adults with type 2 diabetes. A self-report questionnaire assessed demographics, work characteristics, self-disclosure of diabetes, support in the workplace, work-related difficulties due to diabetes, and workplace conformity. Dietary self-care, exercise, depression, and emotional distress were also evaluated. The results indicated statistically significant influence of working night shifts, self-disclosure of diabetes, and workplace conformity on dietary self-care. Work-related difficulties due to diabetes had negative effects on depression and emotional distress, and job control and support in the workplace were found to be correlated with emotional distress. These findings suggest that work-related factors have an impact on some forms of self-care activities and psychological health and that it is important to increase understanding of these issues and provide appropriate support for workers through education and counseling and adjustments in the workplace.

C1 [Sato, Miho] Hokkaido Univ, Dept Fundamental Nursing, Fac Hlth Sci, Kita Ku, Sapporo, Hokkaido 0600812, Japan.

[Yamazaki, Yoshihiko] Univ Tokyo, Dept Hlth Sociol, Grad Sch Hlth Sci & Nursing, Tokyo, Japan.

RP Sato, M (reprint author), Hokkaido Univ, Dept Fundamental Nursing, Fac Hlth Sci, Kita Ku, Kita 12,Nishi 5, Sapporo, Hokkaido 0600812, Japan.

EM m_sato@med.hokudai.ac.jp

RI Sato, Miho/D-8722-2012

FU Japan Society for the Promotion of Science

FX This study was conducted with research support from Young Scientists

(B), from the Japan Society for the Promotion of Science Grants-in-Aid

for Scientific Research, fiscal year 2007-2008. The authors would like

to thank the study's participants for their involvement in this research

project. We also wish to express our appreciation to the members of the

Department of Health Sociology, University of Tokyo, Japan, for their

support.

CR Anderson RJ, 2001, DIABETES CARE, V24, P1069, DOI 10.2337/diacare.24.6.1069

Atkinson G, 2008, SPORTS MED, V38, P671, DOI 10.2165/00007256-200838080-00005

Daitoku Masuko, 2006, Journal of the Japan Diabetes Society, V49, P1

Delahanty LM, 2007, DIABETIC MED, V24, P48, DOI 10.1111/j.1464-5491.2007.02028.x

Detaille SI, 2006, PATIENT EDUC COUNS, V64, P183, DOI 10.1016/j.pec.2005.12.015

Fisher L, 2008, DIABETIC MED, V25, P1096, DOI 10.1111/j.1464-5491.2008.02533.x

Fisher L, 2009, DIABETIC MED, V26, P622, DOI 10.1111/j.1464-5491.2009.02730.x

Glasgow RE, 1999, ANN BEHAV MED, V21, P159, DOI 10.1007/BF02908297

Gotay CC, 2004, HEALTH PLACE, V10, P153, DOI 10.1016/S1353-8292(03)00055-8

Harvey JN, 2009, DIABETIC MED, V26, P5, DOI 10.1111/j.1464-5491.2008.02628.x

Hatta H, 1998, SHINSHIN IGAKU, V38, P310

ISHIYAMA FI, 1994, INT J ADV COUNS, V17, P167, DOI 10.1007/BF01407732

Kusuba Y, 2000, J JAPAN ACAD DIAB ED, V4, P32

Ministry of Health Labor and Welfare (MHLW), 2007, OV NAT HLTH NUTR SUR

Ministry of Labour, 1999, SAG KANR SHIKK NO YO, P351

Morimoto A., 2010, JMAJ - Japan Medical Association Journal, V53, P36

Mulcahy K, 2003, DIABETES EDUCATOR, V29, P804

Munir F, 2005, SOC SCI MED, V60, P1397, DOI 10.1016/j.socscimed.2004.07.012

PADGETT DL, 1996, DIABETES SPECTRUM, V9, P13

Rosland AM, 2008, J GEN INTERN MED, V23, P1992, DOI 10.1007/s11606-008-0814-7

Rubin RR, 1999, DIABETES-METAB RES, V15, P205, DOI 10.1002/(SICI)1520-7560(199905/06)15:3<205::AID-DMRR29>3.0.CO;2-O

Sacco WP, 2006, J BEHAV MED, V29, P523, DOI 10.1007/s1086-006-9072-5

Sarkar U, 2006, DIABETES CARE, V29, P823, DOI 10.2337/diacare.29.04.06.dc05-1615

Sharoni SKA, 2012, NURS HEALTH SCI, V14, P38, DOI 10.1111/j.1442-2018.2011.00658.x

Sugimoto H, 2005, J JPN SOC HLTH ED PR, V13, P11

Tellez-Zenteno JF, 2002, ARCH MED RES, V33, P53, DOI 10.1016/S0188-4409(01)00349-6

Tokyo Metropolitan Government Institution of Labor, 1993, TYUS DANS NO SYOK SE, P77

TOOBERT DJ, 1991, J BEHAV MED, V14, P71, DOI 10.1007/BF00844769

Toobert DJ, 2000, DIABETES CARE, V23, P943, DOI 10.2337/diacare.23.7.943

Trief PM, 1999, DIABETES CARE, V22, P569, DOI 10.2337/diacare.22.4.569

Uehata T, 1991, 1 I IND MED, P38

van Hooff MLM, 2011, WORK STRESS, V25, P55, DOI 10.1080/02678373.2011.570941

Weijman I, 2005, PATIENT EDUC COUNS, V59, P87, DOI 10.1016/j.pec.2004.10.004

ZIGMOND AS, 1983, ACTA PSYCHIAT SCAND, V67, P361, DOI 10.1111/j.1600-0447.1983.tb09716.x

NR 34

TC 5

Z9 5

U1 1

U2 20

PU WILEY-BLACKWELL

PI HOBOKEN

PA 111 RIVER ST, HOBOKEN 07030-5774, NJ USA

SN 1441-0745

J9 NURS HEALTH SCI

JI Nurs. Health Sci.

PD DEC

PY 2012

VL 14

IS 4

SI SI

BP 520

EP 527

DI 10.1111/j.1442-2018.2012.00729.x

PG 8

WC Nursing

SC Nursing

GA 045IL

UT WOS:000311689100015

PM 22957813

DA 2019-01-08

ER

PT J

AU Jortberg, BT

Miller, BF

Gabbay, RA

Sparling, K

Dickinson, WP

AF Jortberg, Bonnie T.

Miller, Benjamin F.

Gabbay, Robert A.

Sparling, Kerri

Dickinson, W. Perry

TI Patient-Centered Medical Home: How It Affects Psychosocial Outcomes for

Diabetes

SO CURRENT DIABETES REPORTS

LA English

DT Article

DE Patient-centered medical home; Diabetes; Psychosocial outcomes; Mental

health; Behavioral health; Team approach; Care management; Care

coordination; Payment reform

ID MENTAL-HEALTH-CARE; UNITED-STATES; DISORDERS; DISTRESS; DEPRESSION;

SERVICES; DELIVERY; SYSTEM; COST; TIME

AB Fragmentation of the current U.S. health care system and the increased prevalence of chronic diseases in the U.S. have led to the recognition that new models of care are needed. Chronic disease management, including diabetes, is often accompanied by a myriad of associated psychosocial issues that need to be addressed as part of a comprehensive treatment plan. Diabetes care should be aligned with comprehensive whole-person health care. The patient-centered medical home (PCMH) has emerged as a model for enhanced primary care that focuses on comprehensive integrated care. PCMH demonstration projects have shown improvements in quality of care, patient experience, care coordination, access to care, and quality measures for diabetes. Key PCMH transformative features associated with psychosocial issues related to diabetes reviewed in this article include integration of mental and behavioral health, care management/coordination, payment reform, advanced access, and putting the patient at the center of health care. This article also reviews the evidence supporting comprehensive and integrated care for addressing psychosocial issues associated with diabetes in the medical home.

C1 [Jortberg, Bonnie T.; Miller, Benjamin F.; Dickinson, W. Perry] Univ Colorado, Sch Med, Dept Family Med, Aurora, CO 80045 USA.

[Gabbay, Robert A.] Penn State Coll Med, Div Endocrinol Diabet & Metab, Penn State Inst Diabet, Hershey, PA 17036 USA.

[Sparling, Kerri] Patient Advocate, Coventry, RI 02816 USA.

RP Jortberg, BT (reprint author), Univ Colorado, Sch Med, Dept Family Med, Mail Stop F496,AO1,12631 E 17th Ave,Room 3519, Aurora, CO 80045 USA.

EM bonnie.jortberg@ucdenver.edu; Benjamin.miller@ucdenver.edu;

rgabbay@psu.edu; kerri@sixuntilme.com; perry.dickinson@ucdenver.edu

OI Miller, Benjamin/0000-0003-1647-0122

FU Animas Corporation; Juvenile Diabetes Research Foundation; NIDDK

FX Conflicts of interest: B. T. Jortberg, none; B. F. Miller, none; R. A.

Gabbay, none. K. Sparling: has been a consultant for Animas Corporation

and Lifespan Corporation; has received honoraria (contracted speaker)

and consulting fees (TV commercial) from Animas Corporation; has

received payment for development of educational presentations, including

service on speakers' bureaus from the Juvenile Diabetes Research

Foundation; has received travel/accommodations expenses covered or

reimbursed from Animas Corporation and Juvenile Diabetes Research

Foundation; is a columnist for Web page for dLIFE, diatribe, Juvenile

Diabetes Research Foundation, and Animas Corporation (columnist for Web

page and videos); has been a consultant in documentary for Novo Nordisk;

has contract writing for WEGOHealth and CVS; has speaking engagements

for Johnson & Johnson; and has been a consultant for Roche. W. P.

Dickinson has received grant support from NIDDK.

CR American Academy of Family Physicians (AAFP) American Academy of Pediatrics ( AAP) American College of Physicians (ACP) American Osteopathic Association (AOA), 2007, JOINT PRINC PAT CTR

Blount A., 2003, FAMILIES SYSTEMS HLT, V21, P121, DOI DOI 10.1037/1091-7527.21.2.121

Blumenthal D, 2010, NEW ENGL J MED, V362, P382, DOI 10.1056/NEJMp0912825

Bojadzievski T, 2011, DIABETES CARE, V34, P1047, DOI 10.2337/dc10-1671

Butler M, 2008, AHRQ PUBLICATION, V09-E003

Ciechanowski P, 2010, DIABETES CARE, V33, P539, DOI 10.2337/dc09-1298

Coleman K, 2009, HEALTH AFFAIR, V28, P75, DOI 10.1377/hlthaff.28.1.75

Collins C, 2010, EVOLVING MODELS BEHA

Crabtree Benjamin F, 2010, Ann Fam Med, V8 Suppl 1, pS80, DOI 10.1370/afm.1107

Craven MA, 2006, CAN J PSYCHIAT, V51, p7S

Cunningham PJ, 2009, HEALTH AFFAIR, V28, pW490, DOI 10.1377/hlthaff.28.3.w490

DEGRUY F, 1996, PRIMARY CARE AM HLTH

Delamater AM, 2001, DIABETES CARE, V24, P1286, DOI 10.2337/diacare.24.7.1286

Delamater AM, 2006, CLIN DIABETES, V24, P71, DOI [10.2337/diaclin.24.2.71, DOI 10.2337/DIACLIN.24.2.71]

Dickinson WP, 2010, FAM SYST HEALTH, V28, P348, DOI 10.1037/a0021866

Fisher L, 2008, DIABETIC MED, V25, P1096, DOI 10.1111/j.1464-5491.2008.02533.x

Fisher L, 2007, DIABETES CARE, V30, P542, DOI 10.2337/dc06-1614

FRIES JF, 1993, NEW ENGL J MED, V329, P321, DOI 10.1056/NEJM199307293290506

Glasgow RE, 2001, DIABETES SPECTRUM, V14, P33, DOI DOI 10.2337/DIASPECT.14.1.33

Glasgow RE, 2012, HEALTH AFFAIR, V31, P497, DOI 10.1377/hlthaff.2010.1295

Golden SH, 2008, JAMA-J AM MED ASSOC, V299, P2751, DOI 10.1001/jama.299.23.2751

Gonder-Frederick LA, 2002, J CONSULT CLIN PSYCH, V70, P611, DOI 10.1037//0022-006X.70.3.611

Goodson JD, 2010, ANN INTERN MED, V152, P742, DOI 10.7326/0003-4819-152-11-201006010-00249

Green CA, 2010, ADM POLICY MENT HLTH, V37, P327, DOI 10.1007/s10488-009-0239-6

Grumbach K, 2012, OUTCOMES IMPLEMENTIN

Institute of Medicine, 2006, COMM CROSS QUAL CHAS

Institute of Medicine, 2001, CROSS QUAL CHASM NEW

Kathol RG, 2010, PSYCHOSOM MED, V72, P511, DOI 10.1097/PSY.0b013e3181e2c4a0

Kessler RC, 2005, NEW ENGL J MED, V352, P2515, DOI 10.1056/NEJMsa043266

Leff B, 2009, AM J MANAG CARE, V15, P555

Lowes L, 2005, J ADV NURS, V50, P253, DOI 10.1111/j.1365-2648.2005.03388.x

Lustman P, 1995, PSYCHOSOCIAL ASPECTS

Mauch D., 2008, HHS PUB

McCarthy D, 2008, N DAKOTA EXPERIENCE

McClellan M, 2010, HEALTH AFFAIR, V29, P982, DOI 10.1377/hlthaff.2010.0194

Miller BF, 2011, BRIT J GEN PRACT, V61, P471, DOI 10.3399/bjgp11X583470

Miller BF, 2011, FAM SYST HEALTH, V29, P144, DOI 10.1037/a0023993

Miller BF, 2011, AHRQ PUBLICATION

Moussavi S, 2007, LANCET, V370, P851, DOI 10.1016/S0140-6736(07)61415-9

Nutting PA, 2011, HEALTH AFFAIR, V30, P439, DOI 10.1377/hlthaff.2010.0159

Office of the Congressional Budget, 2004, AN LIT DIS MAN PROGR

Paulus RA, 2008, HEALTH AFFAIR, V27, P1235, DOI 10.1377/hlthaff.27.5.1235

Peek C. J., 2008, COLLABORATIVE MED CA

Petterson SM, 2008, AM FAM PHYSICIAN, V77, P757

Polonsky WH, 2005, DIABETES CARE, V28, P626, DOI 10.2337/diacare.28.3.626

REGIER DA, 1993, ARCH GEN PSYCHIAT, V50, P85

Reid RJ, 2010, HEALTH AFFAIR, V29, P835, DOI 10.1377/hlthaff.2010.0158

Reid RJ, 2009, AM J MANAG CARE, V15, pE71

Shahady EJ, 2011, J PARTICIPATORY 0404, V4, pe18

Shojania KG, 2006, JAMA-J AM MED ASSOC, V296, P427, DOI 10.1001/jama.296.4.427

Sia C, 2004, PEDIATRICS, V113, P1473

Starfield B, 2005, MILBANK Q, V83, P457, DOI 10.1111/j.1468-0009.2005.00409.x

Steiner BD, 2008, ANN FAM MED, V6, P361, DOI 10.1370/afm.866

Tapp H, 2012, J AM BOARD FAM MED, V25, P245, DOI 10.3122/jabfm.2012.02.110243

Tsai AC, 2005, AM J MANAG CARE, V11, P478

Unutzer Jurgen, 2012, Am J Public Health, V102, pe41, DOI 10.2105/AJPH.2011.300555

Wang PS, 2005, ARCH GEN PSYCHIAT, V62, P629, DOI 10.1001/archpsyc.62.6.629

Weber V, 2008, J GEN INTERN MED, V23, P379, DOI 10.1007/s11606-007-0439-2

Williams JW, 2012, AHRQ PUBLICATION

Yarnall KSH, 2003, AM J PUBLIC HEALTH, V93, P635, DOI 10.2105/AJPH.93.4.635

NR 60

TC 17

Z9 17

U1 0

U2 13

PU CURRENT MEDICINE GROUP

PI PHILADELPHIA

PA 400 MARKET STREET, STE 700, PHILADELPHIA, PA 19106 USA

SN 1534-4827

EI 1539-0829

J9 CURR DIABETES REP

JI Curr. Diabetes Rep.

PD DEC

PY 2012

VL 12

IS 6

BP 721

EP 728

DI 10.1007/s11892-012-0316-1

PG 8

WC Endocrinology & Metabolism

SC Endocrinology & Metabolism

GA 032HP

UT WOS:000310706500011

PM 22961115

DA 2019-01-08

ER

PT J

AU Naranjo, D

Hessler, DM

Deol, R

Chesla, CA

AF Naranjo, Diana

Hessler, Danielle M.

Deol, Rupinder

Chesla, Catherine A.

TI Health and Psychosocial Outcomes in US Adult Patients with Diabetes from

Diverse Ethnicities

SO CURRENT DIABETES REPORTS

LA English

DT Article

DE Diabetes; Psychosocial; Health; Distress; Depression; Culture;

Diversity; Ethnicity; African American; Latino; Asian; American Indian

ID OLDER MEXICAN-AMERICANS; NUTRITION EXAMINATION SURVEY; IMPAIRED

GLUCOSE-TOLERANCE; URBAN AFRICAN-AMERICANS; 3RD NATIONAL-HEALTH; STAGE

RENAL-DISEASE; QUALITY-OF-CARE; GLYCEMIC CONTROL; DEPRESSIVE SYMPTOMS;

UNITED-STATES

AB Within the United States, diabetes is a serious public health concern and patients with diabetes are more likely to experience clinical depression, psychological distress, and depressive symptoms than those without. Negative psychosocial factors are associated with poorer diabetes management and glycemic control. Overall, both the rates of diabetes and related psychological distress are greater for persons of diverse ethnicities than for non-Latino whites, and have reached epidemic proportions in certain groups. The following article will provide an overview across ethnicities of the rates of diabetes, health outcomes, psychosocial outcomes, and unique cultural and linguistic challenges that contribute to disparities within US diabetes patients of diverse ethnicities. Using this information, our hope is that health care practitioners and researchers alike can better respond to the psychosocial needs of ethnically diverse patients.

C1 [Naranjo, Diana] Univ Calif San Francisco, Dept Pediat, San Francisco, CA 94143 USA.

[Hessler, Danielle M.] Univ Calif San Francisco, Dept Family & Community Med, San Francisco, CA 94143 USA.

[Deol, Rupinder; Chesla, Catherine A.] Univ Calif San Francisco, Dept Family Hlth Care Nursing, San Francisco, CA 94143 USA.

RP Naranjo, D (reprint author), Univ Calif San Francisco, Dept Pediat, 400 Parnassus Ave,4th Floor,MailBox 0318, San Francisco, CA 94143 USA.

EM NaranjoD@peds.ucsf.edu

CR Anderson RJ, 2000, DIABETES, V49, pA64

Baines D., 1992, HLTH BEHAV RES MINOR, P230

Barnes Patricia M, 2008, Adv Data, P1

Bell RA, 2005, DIABETES CARE, V28, P823, DOI 10.2337/diacare.28.4.823

Black SA, 1999, AM J PUBLIC HEALTH, V89, P546, DOI 10.2105/AJPH.89.4.546

Black SA, 1998, J GERONTOL A-BIOL, V53, pM188, DOI 10.1093/gerona/53A.3.M188

Black SA, 2002, AM J PUBLIC HEALTH, V92, P543, DOI 10.2105/AJPH.92.4.543

Blazer DG, 2002, J PSYCHOSOM RES, V53, P913, DOI 10.1016/S0022-3999(02)00314-8

Braginsky Nafanua, 2011, Hawaii Med J, V70, P196

Brown AF, 2005, DIABETES CARE, V28, P2864, DOI 10.2337/diacare.28.12.2864

Brown SA, 2000, DIABETES EDUCATOR, V26, P425, DOI 10.1177/014572170002600310

Caban A, 2006, DIABETES EDUCATOR, V32, P584, DOI 10.1177/0145721706290435

Carter JS, 1996, DIABETES CARE, V19, P764, DOI 10.2337/diacare.19.7.764

Carter JS, 1996, ANN INTERN MED, V125, P221, DOI 10.7326/0003-4819-125-3-199608010-00011

Carter-Edwards L, 2004, DIABETES EDUCATOR, V30, P493, DOI 10.1177/014572170403000321

CDC, 2011, PREV DIAB HISP 6 US

CDC NCHS, 2000, STAT CFDCAPANCFH

Centers for Disease Control and Prevention (CDC), 2011, NAT DIAB FACT SHEET

Chesla CA, 2000, DIABETES CARE, V23, P1780, DOI 10.2337/diacare.23.12.1780

Chesla CA, 2005, QUAL HEALTH RES, V15, P240, DOI 10.1177/1049732304272050

Chesla CA, 2004, DIABETES CARE, V27, P2850, DOI 10.2337/diacare.27.12.2850

Chesla CA, 2009, DIABETES CARE, V32, P1812, DOI 10.2337/dc09-0278

Chun KM, 2011, SOC SCI MED, V72, P256, DOI 10.1016/j.socscimed.2010.11.010

Chun KM, 2004, PSYCHOL HEALTH, V19, P767, DOI 10.1080/08870440410001722958

Ciechanowski PS, 2000, ARCH INTERN MED, V160, P3278, DOI 10.1001/archinte.160.21.3278

Concha JB, 2009, DIABETES EDUCATOR, V35, P941, DOI 10.1177/0145721709344125

COWIE CC, 1989, NEW ENGL J MED, V321, P1074, DOI 10.1056/NEJM198910193211603

Davidson Mayer B., 2001, Ethnicity and Disease, V11, P148

de Groot M, 2006, DIABETES CARE, V29, P549, DOI 10.2337/diacare.29.03.06.dc05-1396

De Groot M, 2001, DIABETES CARE, V24, P407, DOI 10.2337/diacare.24.2.407

Deshpande AD, 2008, PHYS THER, V88, P1254, DOI 10.2522/ptj.20080020

Devlin H, 2006, HEALTH PROMOT PRACT, V7, P47, DOI 10.1177/1524839905275395

DHHS, 2000, HLTH PEOPL 2010 OBJ

Egede LE, 2006, DIABETES CARE, V29, P131, DOI 10.2337/diacare.29.1.131

Egede LE, 2002, DIABETES EDUCATOR, V28, P258, DOI 10.1177/014572170202800211

El-Kebbi IM, 2003, ARCH INTERN MED, V163, P69, DOI 10.1001/archinte.163.1.69

Everson SA, 2002, J PSYCHOSOM RES, V53, P891, DOI 10.1016/S0022-3999(02)00303-3

Eyler AA, 1999, SOC SCI MED, V49, P781, DOI 10.1016/S0277-9536(99)00137-9

Fernandez A, 2004, J GEN INTERN MED, V19, P167, DOI 10.1111/j.1525-1497.2004.30266.x

Fisher L, 2008, DIABETIC MED, V25, P1096, DOI 10.1111/j.1464-5491.2008.02533.x

Fisher L, 2001, DIABETES CARE, V24, P1751, DOI 10.2337/diacare.24.10.1751

Fisher L, 2007, DIABETES CARE, V30, P542, DOI 10.2337/dc06-1614

Fisher L, 2010, DIABETES CARE, V33, P23, DOI 10.2337/dc09-1238

Gamble VN, 1997, AM J PUBLIC HEALTH, V87, P1773, DOI 10.2105/AJPH.87.11.1773

Garduno-Diaz SD, 2012, DIABETES-METAB RES, V28, P6, DOI 10.1002/dmrr.1219

Gleeson-Kreig J, 2002, PUBLIC HEALTH NURS, V19, P215, DOI 10.1046/j.0737-1209.2002.19310.x

Golden SH, 2008, JAMA-J AM MED ASSOC, V299, P2751, DOI 10.1001/jama.299.23.2751

Gonzalez HM, 2001, J AM GERIATR SOC, V49, P948, DOI 10.1046/j.1532-5415.2001.49186.x

Gross R, 2005, J GEN INTERN MED, V20, P460, DOI 10.1111/j.1525-1497.2005.30003.x

Harris MI, 1998, DIABETES CARE, V21, P518, DOI 10.2337/diacare.21.4.518

Hausmann LRM, 2010, PATIENT PREFER ADHER, V4, P291, DOI 10.2147/PPA.S12353

Hennessy CH, 1999, DIABETES EDUCATOR, V25, P747, DOI 10.1177/014572179902500507

Hessler DM, 2012, J HEALTH PSYCHOL, V17, P535, DOI 10.1177/1359105311422120

Hessler DM, 2010, PATIENT EDUC COUNS, V85, P154

Hill-Briggs F, 2002, J GEN INTERN MED, V17, P412, DOI 10.1046/j.1525-1497.2002.11002.x

Hoeman SP, 1996, WESTERN J NURS RES, V18, P518, DOI 10.1177/019394599601800504

Hsu WC, 2006, DIABETES CARE, V29, P415, DOI 10.2337/diacare.29.02.06.dc05-1915

Ingram M, 2007, DIABETES EDUCATOR, V33, p172S, DOI 10.1177/0145721707304170

Jacobs EA, 2006, J GEN INTERN MED, V21, P642, DOI 10.1111/j.1525-1497.2006.00485.x

Jiang LH, 2008, DIABETES CARE, V31, P427, DOI 10.2337/dc07-2044

Jiang LH, 2009, QUAL LIFE RES, V18, P709, DOI 10.1007/s11136-009-9495-x

Johnson JA, 1996, MED CARE, V34, P97, DOI 10.1097/00005650-199602000-00001

Katon W, 2009, PSYCHOSOM MED, V71, P965, DOI 10.1097/PSY.0b013e3181bd8f55

Katon WJ, 2005, DIABETES CARE, V28, P2668, DOI 10.2337/diacare.28.11.2668

Katon WJ, 2005, ARCH GEN PSYCHIAT, V62, P1313, DOI 10.1001/archpsyc.62.12.1313

KENNY SJ, 1995, DIABETES AM, P47

King GL, 2012, DIABETES CARE, V35, P1181, DOI 10.2337/dc12-0210

KING H, 1993, DIABETES CARE, V16, P157, DOI 10.2337/diacare.16.1.157

Kirk JK, 2006, DIABETES CARE, V29, P2130, DOI 10.2337/dc05-1973

KISSEBAH AH, 1982, J CLIN ENDOCR METAB, V54, P254, DOI 10.1210/jcem-54-2-254

KNOWLER WC, 1993, DIABETES CARE, V16, P216, DOI 10.2337/diacare.16.1.216

Kogan SM, 2007, ETHNIC DIS, V17, P106

Kozak DL, 1997, OMEGA-J DEATH DYING, V35, P347, DOI 10.2190/C47K-EGNU-DC36-97DG

Lanting LC, 2005, DIABETES CARE, V28, P2280, DOI 10.2337/diacare.28.9.2280

Lawton J, 2007, SOCIOL HEALTH ILL, V29, P891, DOI 10.1111/j.1467-9566.2007.01036.x

Lee CMY, 2007, ASIA PAC J CLIN NUTR, V16, P187

Lee ET, 2000, DIABETES CARE, V23, P181, DOI 10.2337/diacare.23.2.181

Lee S, 2006, DIABETES RES CLIN PR, V74, P282, DOI 10.1016/j.diabres.2006.03.026

Li CY, 2008, DIABETES CARE, V31, P105, DOI 10.2337/dc07-1154

Lujan J, 2007, DIABETES EDUCATOR, V33, P660, DOI 10.1177/0145721707304080

Lynch EB, 2012, HEALTH EDUC RES, V27, P814, DOI 10.1093/her/cys058

Macaden L, 2006, INT J OLDER PEOPLE N, V1, P177, DOI 10.1111/j.1748-3743.2006.00026.x

Mau MK, 2003, J NATL MED ASSOC, V95, P955

McBean AM, 2004, DIABETES CARE, V27, P2317, DOI 10.2337/diacare.27.10.2317

Meetoo Danny, 2005, Br J Nurs, V14, P154

Melkus GD, 2009, DIABETES EDUCATOR, V35, P293, DOI 10.1177/0145721708327532

Miller P, 1987, Am Indian Alsk Native Ment Health Res, V1, P24

Moffet HH, 2009, INT J EPIDEMIOL, V38, P38, DOI 10.1093/ije/dyn040

MUNETA B, 1993, DIABETES CARE, V16, P346, DOI 10.2337/diacare.16.1.346

Munoz RF, 2010, J MED INTERNET RES, V12, DOI 10.2196/jmir.1463

Naranjo D, 2012, J IMMIGRANT HLTH, DOI [10.1007/s10903-012-9689-0, DOI 10.1007/S10903-012-9689-0]

Naranjo DM, 2011, ANN FAM MED, V9, P115, DOI 10.1370/afm.1212

Narayan KMV, 2003, JAMA-J AM MED ASSOC, V290, P1884, DOI 10.1001/jama.290.14.1884

Naylor J L, 2003, Int J Circumpolar Health, V62, P363

NEWMAN JM, 1993, DIABETES CARE, V16, P297, DOI 10.2337/diacare.16.1.297

Novins DK, 2004, MED CARE, V42, P670, DOI 10.1097/01.mlr.0000129902.29132.a6

O'Connell J, 2010, DIABETES CARE, V33, P1463, DOI 10.2337/dc09-1652

Oza-Frank R, 2009, DIABETES CARE, V32, P1644, DOI 10.2337/dc09-0573

Peek ME, 2007, MED CARE RES REV, V64, p101S, DOI 10.1177/1077558707305409

Perez-Stable EJ, 1997, MED CARE, V35, P1212, DOI 10.1097/00005650-199712000-00005

Pieroni A, 2007, J ETHNOPHARMACOL, V113, P100, DOI 10.1016/j.jep.2007.05.009

Piette JD, 2000, MED CARE, V38, P218, DOI 10.1097/00005650-200002000-00011

Pistulka GM, 2012, QUAL HEALTH RES, V22, P825, DOI 10.1177/1049732312438778

RALEIGH VS, 1997, HLTH TRENDS, V28, P122

Robbins JM, 2000, J EPIDEMIOL COMMUN H, V54, P839, DOI 10.1136/jech.54.11.839

Sahota PKC, 2008, J CLIN PSYCHIAT, V69, P800, DOI 10.4088/JCP.v69n0513

Samuel-Hodge CD, 2000, DIABETES CARE, V23, P928, DOI 10.2337/diacare.23.7.928

Schenker Y, 2010, PATIENT EDUC COUNS, V81, P222, DOI 10.1016/j.pec.2010.02.005

SORLIE PD, 1993, JAMA-J AM MED ASSOC, V270, P2464, DOI 10.1001/jama.270.20.2464

Spencer MS, 2006, J HEALTH CARE POOR U, V17, P88, DOI 10.1353/hpu.2006.0095

Stone M, 2005, FAM PRACT, V22, P647, DOI 10.1093/fampra/cmi069

Talbot F, 2000, DIABETES CARE, V23, P1556, DOI 10.2337/diacare.23.10.1556

Taylor Christopher, 2004, Prev Chronic Dis, V1, pA06

Teufel-Shone Nicolette I, 2005, Prev Chronic Dis, V2, pA20

Tillman L. C., 2002, ED RES, V31, P3, DOI DOI 10.3102/0013189X031009003

Timmins CL, 2002, J MIDWIFERY WOM HEAL, V47, P80, DOI 10.1016/S1526-9523(02)00218-0

Tuomilehto J, 2003, DIABETES CARE, V26, P1770

Turner RC, 1998, LANCET, V352, P854

Wagner J, 2007, DIABETES RES CLIN PR, V75, P119, DOI 10.1016/j.diabres.2006.05.004

Wagner J, 2007, DIABETES CARE, V30, P364, DOI 10.2337/dc06-1756

Wang CY, 2005, NURS RES, V54, P347, DOI 10.1097/00006199-200509000-00009

Washington Gail, 2009, J Health Hum Serv Adm, V32, P305

Wen LK, 2004, DIABETES EDUCATOR, V30, P980, DOI 10.1177/014572170403000619

Young BA, 2003, DIABETES CARE, V26, P2392, DOI 10.2337/diacare.26.8.2392

NR 124

TC 14

Z9 14

U1 0

U2 16

PU CURRENT MEDICINE GROUP

PI PHILADELPHIA

PA 400 MARKET STREET, STE 700, PHILADELPHIA, PA 19106 USA

SN 1534-4827

J9 CURR DIABETES REP

JI Curr. Diabetes Rep.

PD DEC

PY 2012

VL 12

IS 6

BP 729

EP 738

DI 10.1007/s11892-012-0319-y

PG 10

WC Endocrinology & Metabolism

SC Endocrinology & Metabolism

GA 032HP

UT WOS:000310706500012

PM 22961116

DA 2019-01-08

ER

PT J

AU Aikens, JE

AF Aikens, James E.

TI Prospective Associations Between Emotional Distress and Poor Outcomes in

Type 2 Diabetes

SO DIABETES CARE

LA English

DT Article

ID QUALITY-OF-LIFE; HEALTH-RISK BEHAVIORS; PRIMARY-CARE PATIENTS; GLYCEMIC

CONTROL; SELF-CARE; DEPRESSIVE SYMPTOMS; MEDICATION ADHERENCE; CLINICAL

DEPRESSION; MULTIPLE-SCLEROSIS; VALIDITY

AB OBJECTIVE-Cross-sectional studies link both depressive symptoms (DS) and diabetes-related distress (DRD) to diabetes self-management and/or glycemic control. However, longitudinal studies of these variables are rare, and their results are somewhat conflicting. The study objective was to compare DS and DRD as longitudinal predictors of medication adherence, self-care behavior, and glycemic control in type 2 diabetes.

RESEARCH DESIGN AND METHODS-Primary care patients with type 2 diabetes reported DS, DRD, and other variables at baseline were studied. Medication adherence, self-care behaviors (diet, physical activity, and glucose testing), and glycemic control (HbA(1c)) were assessed 6 months later (n = 253). Cross-sectional and longitudinal regression analyses were used to model behavioral and medical outcomes as a function of baseline confounders, DS, and DRD.

RESULTS-Adjusted cross-sectional and longitudinal analyses yielded very similar results. In the latter, only DS were significantly associated with future diet behavior (P = 0.049), physical activity (P = 0.001), and glucose testing (P = 0.018). In contrast, only DRD predicted future glycemic control (P < 0.001) and medication adherence (P = 0.011).

CONCLUSIONS-Distress-outcome associations seem to vary by type of distress under consideration. Only DS predicts future lifestyle-oriented self-management behaviors. In contrast, only DRD predicts glycemic control, perhaps by decreasing medication adherence. Clinical assessment and intervention should encompass both types of distress, unless the goal is to narrowly target a highly specific outcome.

C1 Univ Michigan, Dept Family Med, Ann Arbor, MI 48109 USA.

RP Aikens, JE (reprint author), Univ Michigan, Dept Family Med, Ann Arbor, MI 48109 USA.

EM aikensj@umich.edu

FU National Institutes of Health from the National Institute of Diabetes

and Digestive and Kidney Diseases [R01-DK-066016, P60-DK-020572]

FX This research was supported by National Institutes of Health grants

R01-DK-066016 and P60-DK-020572 (to Michigan Diabetes Research and

Training Center), both from the National Institute of Diabetes and

Digestive and Kidney Diseases.

CR Aikens JE, 2008, DIABETIC MED, V25, P1324, DOI 10.1111/j.1464-5491.2008.02590.x

Aikens JE, 2009, DIABETES CARE, V32, P1177, DOI 10.2337/dc09-0071

Aikens JE, 2005, ANN FAM MED, V3, P23, DOI 10.1370/afm.238

Campayo A, 2011, CURR PSYCHIAT REP, V13, P26, DOI 10.1007/s11920-010-0165-z

Cass AR, 1999, INT J PSYCHIAT MED, V29, P293, DOI 10.2190/CPYJ-2HBF-RGCN-64V2

Delahanty LM, 2007, DIABETIC MED, V24, P48, DOI 10.1111/j.1464-5491.2007.02028.x

Fisher L, 2007, DIABETES CARE, V30, P542, DOI 10.2337/dc06-1614

Fisher L, 2010, DIABETES CARE, V33, P1034, DOI 10.2337/dc09-2175

Fisher L, 2010, DIABETES CARE, V33, P23, DOI 10.2337/dc09-1238

Fitzgerald JT, 1996, EVAL HEALTH PROF, V19, P208, DOI 10.1177/016327879601900205

Golden-Kreutz DM, 2004, PSYCHO-ONCOLOGY, V13, P211, DOI 10.1002/pon.736

Gonzalez JS, 2008, DIABETIC MED, V25, P1102, DOI 10.1111/j.1464-5491.2008.02535.x

Gonzalez JS, 2008, DIABETOLOGIA, V51, P1822, DOI 10.1007/s00125-008-1113-x

Gonzalez JS, 2011, DIABETES CARE, V34, P236, DOI 10.2337/dc10-1970

Graue M, 2012, INT J NURS STUD, V49, P174, DOI 10.1016/j.ijnurstu.2011.08.007

Janssens ACJW, 2004, J CLIN EPIDEMIOL, V57, P180, DOI 10.1016/S0895-4356(03)00260-9

Janssens ACJW, 2003, ACTA NEUROL SCAND, V108, P389, DOI 10.1034/j.1600-0404.2003.00166.x

Kandasamy A, 2011, INDIAN J CANCER, V48, P55, DOI 10.4103/0019-509X.75828

Katon WJ, 2010, NEW ENGL J MED, V363, P2611, DOI 10.1056/NEJMoa1003955

Katon WJ, 2010, INT J GERIATR PSYCH, V25, P466, DOI 10.1002/gps.2363

Krapek K, 2004, ANN PHARMACOTHER, V38, P1357, DOI 10.1345/aph.1D612

Kroenke K, 2001, J GEN INTERN MED, V16, P606, DOI 10.1046/j.1525-1497.2001.016009606.x

Lustman PJ, 2005, J DIABETES COMPLICAT, V19, P113, DOI 10.1016/j.jdiacomp.2004.01.002

Lustman PJ, 2000, DIABETES CARE, V23, P934, DOI 10.2337/diacare.23.7.934

Mann DM, 2009, J BEHAV MED, V32, P278, DOI 10.1007/s10865-009-9202-y

Markowitz SM, 2011, PSYCHOSOMATICS, V52, P1, DOI 10.1016/j.psym.2010.11.007

MORISKY DE, 1986, MED CARE, V24, P67, DOI 10.1097/00005650-198601000-00007

Nouwen A, 2010, DIABETOLOGIA, V53, P2480, DOI 10.1007/s00125-010-1874-x

POLONSKY WH, 1995, DIABETES CARE, V18, P754, DOI 10.2337/diacare.18.6.754

Polonsky WH, 2005, DIABETES CARE, V28, P626, DOI 10.2337/diacare.28.3.626

Strine TW, 2005, AM J PUBLIC HEALTH, V95, P2042, DOI 10.2105/AJPH.2005.066225

Toobert DJ, 2000, DIABETES CARE, V23, P943, DOI 10.2337/diacare.23.7.943

van Bastelaar KMP, 2010, DIABETIC MED, V27, P798, DOI 10.1111/j.1464-5491.2010.03025.x

van Bastelaar K, 2011, PATIENT EDUC COUNS, V84, P49, DOI 10.1016/j.pec.2010.06.013

Wang RH, 2011, J ADV NURS, V67, P2658, DOI 10.1111/j.1365-2648.2011.05701.x

Weinger K, 2005, DIABETES CARE, V28, P1346, DOI 10.2337/diacare.28.6.1346

2002, FED REG, V67, P6931

NR 37

TC 86

Z9 86

U1 1

U2 33

PU AMER DIABETES ASSOC

PI ALEXANDRIA

PA 1701 N BEAUREGARD ST, ALEXANDRIA, VA 22311-1717 USA

SN 0149-5992

J9 DIABETES CARE

JI Diabetes Care

PD DEC

PY 2012

VL 35

IS 12

BP 2472

EP 2478

DI 10.2337/dc12-0181

PG 7

WC Endocrinology & Metabolism

SC Endocrinology & Metabolism

GA 041UF

UT WOS:000311426000027

PM 23033244

OA Other Gold, Green Published

DA 2019-01-08

ER

PT J

AU Byrne, M

Newell, J

Coffey, N

O' Hara, MC

Cooke, D

Dinneen, SF

AF Byrne, M.

Newell, J.

Coffey, N.

O' Hara, M. C.

Cooke, D.

Dinneen, S. F.

TI Predictors of quality of life gains among people with type 1 diabetes

participating in the Dose Adjustment for Normal Eating (DAFNE)

structured education programme

SO DIABETES RESEARCH AND CLINICAL PRACTICE

LA English

DT Article

DE Diabetes mellitus type 1; Quality of life; Predictors; Self-management

programmes; DSQOLS

ID PROBLEM AREAS; MULTIPLE IMPUTATION; HOSPITAL ANXIETY; DEPRESSION SCALE;

MANAGEMENT; HEALTH; DISTRESS; VALIDITY; OUTCOMES; FREEDOM

AB Aims: To examine predictors of quality of life gains among people with type 1 diabetes following the Dose Adjustment for Normal Eating (DAFNE) self-management training programme.

Methods: Clinical and questionnaire data were collected from 437 patients from 6 hospital centres before, and 18 months post-DAFNE intervention. Glycated haemoglobin (HbA(1c)), weight, height, and blood pressure levels were recorded by clinicians during clinic appointments. Questionnaires included the Diabetes-Specific Quality of Life Scale (DSQOLS), the Problem Areas in Diabetes Scale (PAID) and the Hospital Anxiety and Depression Scale (HADS). Basic demographics were recorded at baseline. Linear mixed models were fitted to identify predictors of change in quality of life at an 18 month follow-up assessment.

Results: Patients with high levels of diabetes-related distress experienced greatest improvement in DSQOLS quality of life scores (p = 0.001). Patients with poor glycaemic control (higher levels of HbA(1c); p = 0.03) and those with high levels of anxiety (p = 0.001) experienced the greatest reductions in diabetes-related distress.

Conclusions: Patients with higher baseline levels of anxiety, higher levels of diabetes-related distress and higher baseline levels of HbA(1c) are most likely to experience quality of life gain from participation in self-management programmes such as DAFNE. (C) 2012 Elsevier Ireland Ltd. All rights reserved.

C1 [Byrne, M.] Natl Univ Ireland, Sch Psychol, Galway, Ireland.

[Newell, J.] Natl Univ Ireland, HRB Clin Res Facil, Galway, Ireland.

[Newell, J.; Coffey, N.] Natl Univ Ireland, Sch Math Stat & Appl Math, Galway, Ireland.

[O' Hara, M. C.; Dinneen, S. F.] Natl Univ Ireland, Sch Med, Galway, Ireland.

[Cooke, D.] UCL, Dept Epidemiol & Publ Hlth, London WC1E 6BT, England.

RP Byrne, M (reprint author), Natl Univ Ireland, Sch Psychol, Galway, Ireland.

EM molly.byrne@nuigalway.ie

RI Bargary, Norma/J-7728-2013

OI Bargary, Norma/0000-0003-4372-0019; Byrne, Molly/0000-0001-8900-4320;

Newell, John/0000-0001-7685-6655; Dinneen, Sean/0000-0002-6636-0493

FU Health Research Board through a health services research award

[HS-2005-25]

FX This study was supported by the Health Research Board through a health

services research award (HS-2005-25).

CR Amiel S, 2002, BRIT MED J, V325, P746

Barnard J, 1999, BIOMETRIKA, V86, P948, DOI 10.1093/biomet/86.4.948

Bjelland I, 2002, J PSYCHOSOM RES, V52, P69, DOI 10.1016/S0022-3999(01)00296-3

Bott U, 1998, DIABETES CARE, V21, P757, DOI 10.2337/diacare.21.5.757

Cochran J, 2008, DIABETES EDUCATOR, V34, P815, DOI 10.1177/0145721708323640

Colagiuri R, 2009, DIABETIC MED, V26, P442, DOI 10.1111/j.1464-5491.2009.02700.x

Debaty I, 2008, DIABETES METAB, V34, P507, DOI 10.1016/j.diabet.2008.03.007

Dinneen SF, 2009, TRIALS, V10, DOI [10.1186/745-6215-10-88, DOI 10.1186/745-6215-10-88]

Glasgow RE, 1997, DIABETES CARE, V20, P562, DOI 10.2337/diacare.20.4.562

Hart HE, 2005, QUAL LIFE RES, V14, P1, DOI 10.1007/s11136-004-0782-2

Hlatky MA, 2010, AM HEART J, V159, DOI 10.1016/j.ahj.2009.11.004

King G, 2001, AM POLIT SCI REV, V95, P49

Maddigan SL, 2005, QUAL LIFE RES, V14, P1311, DOI 10.1007/s11136-004-6640-4

McGuire BE, 2010, DIABETOLOGIA, V53, P66, DOI 10.1007/s00125-009-1559-5

Oliver L, 2009, PRACT DIAB INT, V26, P371, DOI DOI 10.1002/PDI.1424

Peyrot M, 2005, DIABETIC MED, V22, P1379, DOI 10.1111/j.1464-5491.2005.01644.x

POLONSKY WH, 1995, DIABETES CARE, V18, P754, DOI 10.2337/diacare.18.6.754

Redekop WK, 2002, DIABETES CARE, V25, P458, DOI 10.2337/diacare.25.3.458

Rubin RR, 1999, DIABETES-METAB RES, V15, P205, DOI 10.1002/(SICI)1520-7560(199905/06)15:3<205::AID-DMRR29>3.0.CO;2-O

Rubin RR, 2001, J CLIN PSYCHOL, V57, P457, DOI 10.1002/jclp.1041

Snoek FJ, 2000, DIABETES CARE, V23, P1305, DOI 10.2337/diacare.23.9.1305

Speight J, 2010, DIABETES RES CLIN PR, V89, P22, DOI 10.1016/j.diabres.2010.03.017

Tuncay T, 2008, HEALTH QUAL LIFE OUT, V6, DOI 10.1186/1477-7525-6-79

Welch G, 2003, DIABETIC MED, V20, P69, DOI 10.1046/j.1464-5491.2003.00832.x

Welch GW, 1997, DIABETES CARE, V20, P760, DOI 10.2337/diacare.20.5.760

ZIGMOND AS, 1983, ACTA PSYCHIAT SCAND, V67, P361, DOI 10.1111/j.1600-0447.1983.tb09716.x

NR 26

TC 15

Z9 16

U1 0

U2 20

PU ELSEVIER IRELAND LTD

PI CLARE

PA ELSEVIER HOUSE, BROOKVALE PLAZA, EAST PARK SHANNON, CO, CLARE, 00000,

IRELAND

SN 0168-8227

J9 DIABETES RES CLIN PR

JI Diabetes Res. Clin. Pract.

PD NOV

PY 2012

VL 98

IS 2

BP 243

EP 248

DI 10.1016/j.diabres.2012.09.017

PG 6

WC Endocrinology & Metabolism

SC Endocrinology & Metabolism

GA 049CA

UT WOS:000311958400011

PM 23018180

DA 2019-01-08

ER

PT J

AU Hughes, LC

Keith, SE

Byars, A

Wiginton, KL

AF Hughes, Lynne C.

Keith, Susan E.

Byars, Allyn

Wiginton, Kristin L.

TI Cognitive Mapping in Persons Newly Diagnosed With Type 2 Diabetes

SO DIABETES EDUCATOR

LA English

DT Article

ID EMOTIONAL DISTRESS; SELF-MANAGEMENT; ADHERENCE; ILLNESS; EXPERIENCES;

DEPRESSION; ATTITUDES; BELIEFS; WOMEN

AB Purpose

The purpose of this study was to determine the issues and perceptions of persons newly diagnosed (within last 3 months) with type 2 diabetes.

Methods

In this qualitative study, 16 adults newly diagnosed with type 2 diabetes were recruited, who completed a demographic, lifestyle, and diabetes management questionnaire and then were interviewed using cognitive mapping. Participants were asked to write words or phrases that came to mind when thinking about living with diabetes. From these words, common concepts were identified, grouped into clusters, and reduced into categories. Associations were sought between demographics and categories.

Results

A total of 260 Post-It notes were written, from which 123 concepts were identified and grouped into 24 clusters. The most frequent concept was eat, and the 3 most frequent clusters were food, negative emotions, and diabetes and its complications. The 9 categories, in order of frequency, were as follows: (1) need to (self-management), (2) concerns, (3) "I'm sick", (4) emotional distress, (5) loss, (6) the no's (limitations), (7) fears and complications, (8) have to (medical management), and (9) coping. Persons with a college education and a later interview date since diagnosis were associated with the concepts in the need to and have to categories. Concepts in the I'm sick category were associated with lower income and Hispanic ethnicity.

Conclusions

Diabetes educators should consider this holistic representation of the issues and perceptions reflected in the common concepts, clusters, and categories expressed by persons newly diagnosed with diabetes.

C1 [Hughes, Lynne C.] Rocky Mt Univ Hlth Profess, Provo, UT USA.

[Keith, Susan E.; Byars, Allyn] Angelo State Univ, San Angelo, TX 76909 USA.

[Wiginton, Kristin L.] Texas Womans Univ, Denton, TX 76204 USA.

RP Hughes, LC (reprint author), Univ Incarnate Word, Sch Phys Therapy, 4301 Broadway,CPO 412, San Antonio, TX 78209 USA.

EM lchpt@yahoo.com

RI Albertin, Paloma/X-1093-2018

CR American Association of Diabetes Educators, 2007, DIABETES ED, V33, P45

Beeney LJ, 1996, PATIENT EDUC COUNS, V29, P109, DOI 10.1016/0738-3991(96)00939-1

Briesacher BA, 2008, PHARMACOTHERAPY, V28, P437, DOI 10.1592/phco.28.4.437

CABAN A, 2008, DIABETES SPECTRUM, V21, P120, DOI DOI 10.2337/DIASPECT.21.2.120

Center for Disease Control and Prevention, 2011, NATIONAL DIABETES FA

Dansereau DF, 2002, J SUBST ABUSE TREAT, V22, P219, DOI 10.1016/S0740-5472(02)00235-0

Everett J, 1998, J DIABETES NURS, V2, P170

Fisher L, 2007, DIABETES CARE, V30, P542, DOI 10.2337/dc06-1614

Fisher L, 2010, DIABETES CARE, V33, P23, DOI 10.2337/dc09-1238

FITZGERALD JT, 1995, DIABETES EDUCATOR, V21, P523, DOI 10.1177/014572179502100605

Gillibrand W, 2001, J ADV NURS, V34, P501, DOI 10.1046/j.1365-2648.2001.01779.x

Grams GD, 1996, CAN MED ASSOC J, V155, P1563

Haynes RB, 2002, JAMA-J AM MED ASSOC, V288, P2880, DOI 10.1001/jama.288.22.2880

Heuer L, 2006, J COMMUN HEALTH NURS, V23, P49, DOI 10.1207/s15327655jchn2301_5

IRVINE AA, 1989, PATIENT EDUC COUNS, V13, P3, DOI 10.1016/0738-3991(89)90065-7

Koch T, 2000, J CLIN NURS, V9, P247, DOI 10.1046/j.1365-2702.2000.00341.x

Koch T, 1999, J CLIN NURS, V8, P712, DOI 10.1046/j.1365-2702.1999.00320.x

Kralik D, 2001, J ADV NURS, V33, P594, DOI 10.1046/j.1365-2648.2001.01704.x

Krug L M, 1991, Diabetes Educ, V17, P185, DOI 10.1177/014572179101700314

Lohri-Posey B, 2006, FAM COMMUNITY HEALTH, V29, P214, DOI 10.1097/00003727-200607000-00008

Lustman PJ, 2000, DIABETES CARE, V23, P934, DOI 10.2337/diacare.23.7.934

McCord E C, 1995, Fam Med, V27, P267

Mier N, 2007, PREV CHRONIC DIS

Nathan DM, 2008, DIABETES CARE, V31, P1473, DOI 10.2337/dc08-0545

Oomen JS, 1999, DIABETES EDUCATOR, V25, P220, DOI 10.1177/014572179902500207

Peel E, 2004, PATIENT EDUC COUNS, V53, P269, DOI 10.1016/j.pec.2003.07.010

Penckofer S, 2007, DIABETES EDUCATOR, V33, P680, DOI 10.1177/0145721707304079

Quackenbush PA, 1996, DIABETES EDUCATOR, V22, P231, DOI 10.1177/014572179602200306

Satterfield DL, 2003, J PUBLIC HEALTH MAN, pS56

Shenolikar RA, 2006, J NATL MED ASSOC, V98, P1071

Shewchuk RM, 2004, AM J HEALTH BEHAV, V28, P43, DOI 10.5993/AJHB.28.1.5

Snoek FJ, 2000, DIABETES CARE, V23, P1305, DOI 10.2337/diacare.23.9.1305

Spencer MS, 2006, J HEALTH CARE POOR U, V17, P88, DOI 10.1353/hpu.2006.0095

SWIFT CS, 1995, DIABETES EDUCATOR, V21, P533, DOI 10.1177/014572179502100607

US Census Bureau, MEAN EARNINGS BY HIG

von Goeler DS, 2003, DIABETES EDUCATOR, V29, P663, DOI 10.1177/014572170302900412

WIERENGA ME, 1994, DIABETES EDUCATOR, V20, P138, DOI 10.1177/014572179402000209

Wiginton KL, 1999, HEALTH EDUC BEHAV, V26, P443, DOI 10.1177/109019819902600403

Wiginton KL, 1997, J AM COLL HEALTH, V45, P175, DOI 10.1080/07448481.1997.9936879

NR 39

TC 0

Z9 0

U1 1

U2 13

PU SAGE PUBLICATIONS INC

PI THOUSAND OAKS

PA 2455 TELLER RD, THOUSAND OAKS, CA 91320 USA

SN 0145-7217

J9 DIABETES EDUCATOR

JI Diabetes Educ.

PD NOV-DEC

PY 2012

VL 38

IS 6

BP 845

EP 854

DI 10.1177/0145721712460100

PG 10

WC Endocrinology & Metabolism; Public, Environmental & Occupational Health

SC Endocrinology & Metabolism; Public, Environmental & Occupational Health

GA 038WQ

UT WOS:000311205200008

PM 22986638

DA 2019-01-08

ER

PT J

AU Snoek, FJ

Kersch, NYA

Eldrup, E

Harman-Boehm, I

Hermanns, N

Kokoszka, A

Matthews, DR

McGuire, BE

Pibernik-Okanovic, M

Singer, J

de Wit, M

Skovlund, SE

AF Snoek, Frank J.

Kersch, Nancy Y. A.

Eldrup, Ebbe

Harman-Boehm, Ilana

Hermanns, Norbert

Kokoszka, Andrzej

Matthews, David R.

McGuire, Brian E.

Pibernik-Okanovic, Mirjana

Singer, Joelle

de Wit, Maartje

Skovlund, Soren E.

TI Monitoring of Individual Needs in Diabetes (MIND)-2 Follow-up data from

the cross-national Diabetes Attitudes, Wishes, and Needs (DAWN) MIND

study

SO DIABETES CARE

LA English

DT Article

ID RANDOMIZED CONTROLLED-TRIAL; QUALITY-OF-LIFE; CARE; DEPRESSION;

VALIDITY; METAANALYSIS; DISTRESS; ADULTS; SCALE

AB OBJECTIVE-To test the effects of implementing computer-assisted Monitoring of Individual Needs in Diabetes (MIND) in routine diabetes care on psychological status and glycemic control, identify predictors of poor psychological outcomes, and evaluate care providers' experiences.

RESEARCH DESIGN AND METHODS-The MIND procedure was implemented as part of the annual review in diabetes clinics across eight countries in a prospective observational study with a 1-year follow-up. MIND encompasses well-being (World Health Organization Five Well-Being Index [WHO-5]), diabetes-related distress (Problem Areas in Diabetes [PAID]), a Life Event Inventory, and the patient's agenda for their consultation. Medical data and agreed case- management actions were retrieved from the charts.

RESULTS-Of the total 1,567 patients, 891 patients (57%) were monitored at a 1-year follow-up. Twenty-eight percent of the patients screened positive for depression and/or diabetes distress at baseline and considered cases, 17% of whom were receiving psychological care. Cases were significantly more often female and had type 2 diabetes and worse glycemic control compared with noncases. Clinically relevant improvements in WHO-5 and PAID were observed over time in cases, irrespective of referral (effects sizes 0.59 and 0.48, respectively; P < 0.0001). Glycemic control did not change. Female sex, life events, and concomitant chronic diseases were predictors of poor psychological outcomes. MIND was well received by patients and staff.

CONCLUSIONS-MIND appears suitable for screening and discussion of emotional distress as part of the annual review. Broader dissemination in diabetes care is recommendable, but sustainability will depend on reimbursement and availability of support services.

C1 [Snoek, Frank J.; Kersch, Nancy Y. A.; de Wit, Maartje] Vrije Univ Amsterdam, Med Ctr, Amsterdam, Netherlands.

[Eldrup, Ebbe] Steno Diabet Ctr, Copenhagen, Denmark.

[Harman-Boehm, Ilana] Soroka Univ, Med Ctr, Beer Sheva, Israel.

[Hermanns, Norbert] Mergentheim Diabet Zentrum, Mergentheim, Germany.

[Kokoszka, Andrzej] Med Univ Warsaw, Dept Psychiat, Warsaw, Poland.

[Kokoszka, Andrzej] Diabet Outpatients Clin Ochota, Warsaw, Poland.

[Matthews, David R.] Oxford Ctr Diabet Endocrinol & Metab, Oxford, England.

[McGuire, Brian E.] Natl Univ Ireland, Sch Psychol, Galway, Ireland.

[McGuire, Brian E.] Univ Coll Hosp, Galway, Ireland.

[Pibernik-Okanovic, Mirjana] Vuk Vrhovac Univ Clin, Zagreb, Croatia.

[Singer, Joelle] Rabin Med Ctr, Petah Tiqwa, Israel.

[Skovlund, Soren E.] Diabet Attitudes Wishes & Needs Program, Bagsvaerd, Denmark.

RP Snoek, FJ (reprint author), Vrije Univ Amsterdam, Med Ctr, Amsterdam, Netherlands.

EM fj.snoek@vumc.nl

RI Kokoszka, Andrzej/O-7454-2018

OI Kokoszka, Andrzej/0000-0003-4518-5494

FU Novo Nordisk; National Institute for Health Research [NF-SI-0508-10272]

FX This study was supported by an unrestricted research grant from Novo

Nordisk. No other potential conflicts of interest relevant to this

article were reported.

CR Ali S, 2006, DIABETIC MED, V23, P1165, DOI 10.1111/j.1464-5491.2006.01943.x

American Diabetes Association, 2006, DIABETES CARE S1, V29, pS4

Awata S, 2007, PSYCHIAT CLIN NEUROS, V61, P112, DOI 10.1111/j.1440-1819.2007.01619.x

Bower P, 2005, BRIT J PSYCHIAT, V186, P11, DOI 10.1192/bjp.186.1.11

Cohen J, 1988, STAT POWER ANAL BEHA

de Wit M, 2010, PEDIATR DIABETES, V11, P175, DOI 10.1111/j.1399-5448.2009.00543.x

De Wit M, 2008, DIABETES CARE, V31, P1521, DOI 10.2337/dc08-0394

Gilbody S, 2006, BRIT MED J, V332, P1027, DOI 10.1136/bmj.332.7548.1027

HOLMES TH, 1967, J PSYCHOSOM RES, V11, P213, DOI 10.1016/0022-3999(67)90010-4

Home P, 2006, DIABETIC MED, V23, P579

Katon WJ, 2010, NEW ENGL J MED, V363, P2611, DOI 10.1056/NEJMoa1003955

Li CY, 2010, DIABETES CARE, V33, P1061, DOI 10.2337/dc09-1515

Lowe B, 2004, J AFFECT DISORDERS, V78, P131, DOI 10.1016/S0165-0327(02)00237-9

McGuire BE, 2010, DIABETOLOGIA, V53, P66, DOI 10.1007/s00125-009-1559-5

Nefs G, 2012, DIABETOLOGIA, V55, P608, DOI 10.1007/s00125-011-2411-2

Peyrot M, 2005, DIABETIC MED, V22, P1379, DOI 10.1111/j.1464-5491.2005.01644.x

Pouwer F, 2011, DIABETOLOGIA, V54, P741, DOI 10.1007/s00125-010-2033-0

Pouwer F, 2001, DIABETES CARE, V24, P1929, DOI 10.2337/diacare.24.11.1929

Rogers E. M., 1995, DIFFUSION INNOVATION

Snoek FJ, 2000, DIABETES CARE, V23, P1305, DOI 10.2337/diacare.23.9.1305

Snoek FJ, 2011, DIABETES CARE, V34, P601, DOI 10.2337/dc10-1552

van Bastelaar KMP, 2011, DIABETES CARE, V34, P320, DOI 10.2337/dc10-1248

Winkley K, 2006, BRIT MED J, V333, P65, DOI 10.1136/bmj.38874.652569.55

NR 23

TC 21

Z9 21

U1 2

U2 20

PU AMER DIABETES ASSOC

PI ALEXANDRIA

PA 1701 N BEAUREGARD ST, ALEXANDRIA, VA 22311-1717 USA

SN 0149-5992

J9 DIABETES CARE

JI Diabetes Care

PD NOV

PY 2012

VL 35

IS 11

BP 2128

EP 2132

DI 10.2337/dc11-1326

PG 5

WC Endocrinology & Metabolism

SC Endocrinology & Metabolism

GA 041TM

UT WOS:000311424100009

PM 22837364

OA Other Gold, Green Published

DA 2019-01-08

ER

PT J

AU McMahon, GT

Fonda, SJ

Gomes, HE

Alexis, G

Conlin, PR

AF McMahon, Graham T.

Fonda, Stephanie J.

Gomes, Helen E.

Alexis, George

Conlin, Paul R.

TI A Randomized Comparison of Online- and Telephone-Based Care Management

with Internet Training Alone in Adult Patients with Poorly Controlled

Type 2 Diabetes

SO DIABETES TECHNOLOGY & THERAPEUTICS

LA English

DT Article

ID NURSE CASE-MANAGEMENT; CONTROLLED-TRIAL; GLYCEMIC CONTROL; PROBLEM

AREAS; HEALTH; VETERANS; QUALITY; INTERVENTIONS; EDUCATION; MELLITUS

AB Aims: Care management may improve the quality of diabetes care by enhancing contact between high-risk patients and their providers. This prospective, longitudinal, randomized trial sought to investigate whether telephone or online care management improves diabetes-related outcomes over time compared with usual care supplemented with Internet access and training.

Subjects and Methods: One hundred fifty-one adult subjects with type 2 diabetes mellitus and an elevated hemoglobin A1c (A1c) level (>= 8.5%) were randomly assigned to online care management (n = 51), telephone-based care management (n = 51), or Web training (n = 49) groups. Online and telephone participants interacted with a care manager through a diabetes education and care management Web site and by telephone, respectively. The Web training group was provided with online diabetes self-management resources but no care management support. The primary outcome measure was A1c measured every 3 months for a year.

Results: A1c declined significantly and substantially in all groups over 12 months. A1c declined linearly at a rate of 0.32% (P < 0.0001) per quarter for the online group, 0.36% (P < 0.0001) for the telephone group, and 0.41% for the Web training group (P < 0.0001). The rate of change over time did not differ significantly among groups. The groups converged at 12 months with average absolute A1c difference of -1.5%. The number of interactions with care providers was not significantly associated with the change in A1c. Blood pressure, weight, lipid levels, and diabetes distress did not differ among groups over time.

Conclusions: Online, telephone-based care management, and Web training for diabetes patients with elevated A1c were each associated with a substantial improvement in A1c over a 1-year period. Internet access and training alone may be as effective as care management in patients with poorly controlled diabetes.

C1 [McMahon, Graham T.; Conlin, Paul R.] Brigham & Womens Hosp, Div Endocrinol Diabet & Hypertens, Boston, MA 02115 USA.

[McMahon, Graham T.; Fonda, Stephanie J.; Gomes, Helen E.; Alexis, George; Conlin, Paul R.] Vet Affairs Boston Healthcare Syst, Med Serv, Boston, MA USA.

[McMahon, Graham T.; Conlin, Paul R.] Harvard Univ, Sch Med, Boston, MA USA.

RP McMahon, GT (reprint author), Brigham & Womens Hosp, Div Endocrinol Diabet & Hypertens, 221 Longwood Ave,RF-291, Boston, MA 02115 USA.

EM gmcmahon@partners.org

RI McMahon, Graham/D-3740-2009

OI McMahon, Graham/0000-0003-4288-6535

FU VA Health Services Research and Development [TEL-02-100]; National

Institutes of Health [K24 DK063214]; Department of the Army Cooperative

Agreement [DAMD 17-98-2-8017]

FX The authors would like to acknowledge the generous participation of

patients and the collaborative support of providers within the VA Boston

Healthcare System. The study was supported by grants from VA Health

Services Research and Development (TEL-02-100), National Institutes of

Health (K24 DK063214), and the Department of the Army Cooperative

Agreement (DAMD 17-98-2-8017).

CR [Anonymous], 2012, DIABETES CARE, V35, pS11, DOI [10.2337/dc12-s011, 10.2337/dc35-S011]

Aubert RE, 1998, ANN INTERN MED, V129, P605, DOI 10.7326/0003-4819-129-8-199810150-00004

Heisler M, 2010, ANN INTERN MED, V153, P507, DOI 10.7326/0003-4819-153-8-201010190-00007

Ishani A, 2011, DIABETES CARE, V34, P1689, DOI 10.2337/dc10-2121

Izquierdo RE, 2003, DIABETES CARE, V26, P1002, DOI 10.2337/diacare.26.4.1002

Kaufman N, 2010, Int J Clin Pract Suppl, P41, DOI 10.1111/j.1742-1241.2009.02277.x

Kaufman Neal D, 2011, J Diabetes Sci Technol, V5, P798

Klonoff DC, 2000, DIABETES CARE, V23, P390, DOI 10.2337/diacare.23.3.390

Kok PE, 2011, J CLIN NURS, V20, P2997, DOI 10.1111/j.1365-2702.2011.03743.x

Krein SL, 2004, AM J MED, V116, P732, DOI 10.1016/j.amjmed.2003.11.028

Lorig K, 2010, DIABETES CARE, V33, P1275, DOI 10.2337/dc09-2153

Lynch CP, 2010, DIABETES TECHNOL THE, V12, P427, DOI 10.1089/dia.2009.0171

McMahon GT, 2005, DIABETES CARE, V28, P1624, DOI 10.2337/diacare.28.7.1624

Piette JD, 2001, DIABETES CARE, V24, P202, DOI 10.2337/diacare.24.2.202

Polonsky WH, 2003, DIABETES CARE, V26, P3048, DOI 10.2337/diacare.26.11.3048

Raji A, 2002, ARCH INTERN MED, V162, P1301, DOI 10.1001/archinte.162.11.1301

Ramadas A, 2011, INT J MED INFORM, V80, P389, DOI 10.1016/j.ijmedinf.2011.02.002

Samoocha David, 2010, J Med Internet Res, V12, pe23, DOI 10.2196/jmir.1286

Shea S, 2009, J AM MED INFORM ASSN, V16, P446, DOI 10.1197/jamia.M3157

Shojania KG, 2006, JAMA-J AM MED ASSOC, V296, P427, DOI 10.1001/jama.296.4.427

Sidorov J, 2002, DIABETES CARE, V25, P684, DOI 10.2337/diacare.25.4.684

Solomon M, 2012, J MED INTERNET RES, V14, DOI 10.2196/jmir.1924

Steffens B, 2000, AM J MANAG CARE, V6, pS697

Trivedi AN, 2011, MED CARE, V49, P560, DOI 10.1097/MLR.0b013e31820fb0f6

Vouri SM, 2011, J MANAGE CARE PHARM, V17, P304, DOI 10.18553/jmcp.2011.17.4.304

Wakefield BJ, 2011, TELEMED E-HEALTH, V17, P254, DOI 10.1089/tmj.2010.0176

Wang YC, 2011, LANCET, V378, P815, DOI 10.1016/S0140-6736(11)60814-3

Welch G, 2003, DIABETIC MED, V20, P69, DOI 10.1046/j.1464-5491.2003.00832.x

Welch GW, 1997, DIABETES CARE, V20, P760, DOI 10.2337/diacare.20.5.760

Zhang P, 2010, DIABETES RES CLIN PR, V87, P293, DOI 10.1016/j.diabres.2010.01.026

NR 30

TC 17

Z9 18

U1 2

U2 18

PU MARY ANN LIEBERT, INC

PI NEW ROCHELLE

PA 140 HUGUENOT STREET, 3RD FL, NEW ROCHELLE, NY 10801 USA

SN 1520-9156

EI 1557-8593

J9 DIABETES TECHNOL THE

JI Diabetes Technol. Ther.

PD NOV

PY 2012

VL 14

IS 11

BP 1060

EP 1067

DI 10.1089/dia.2012.0137

PG 8

WC Endocrinology & Metabolism

SC Endocrinology & Metabolism

GA 030LX

UT WOS:000310573300015

PM 22953754

OA Green Published

DA 2019-01-08

ER

PT J

AU Tsujii, S

Hayashino, Y

Ishii, H

AF Tsujii, S.

Hayashino, Y.

Ishii, H.

CA Tenri Study Grp

TI Diabetes distress, but not depressive symptoms, is associated with

glycaemic control among Japanese patients with Type 2 diabetes: Diabetes

Distress and Care Registry at Tenri (DDCRT 1)

SO DIABETIC MEDICINE

LA English

DT Article

ID PROBLEM AREAS; COMORBID DEPRESSION; EMOTIONAL DISTRESS; ADULTS;

MELLITUS; SCALE

AB Aims To investigate the association between glycaemic control, diabetes distress and depressive symptoms among Japanese patients with Type 2 diabetes. Methods Cross-sectional data from 3305 patients with Type 2 diabetes were obtained from a baseline assessment of a diabetes registry at a general hospital in Japan. The Centre for Epidemiologic Studies Depression scale and Problem Areas in Diabetes scale were used to measure depressive symptoms and diabetes-related distress, respectively. Modified Poisson regression analysis was used to estimate the relative risks for poor glycaemic control across the quartiles of Centre for Epidemiologic Studies Depression scale and Problem Areas in Diabetes scale scores. Results The average age of the participants was 64.9 years and the average HbA1c level was 58.1 mmol/mol (7.5%). Clinically significant levels of depressive symptoms (Centre for Epidemiologic Studies Depression scale scores = 16) were reported by 27.8% of participants. These scores significantly correlated with Problem Areas in Diabetes scale scores (r = 0.4354, P < 0.0001). Diabetes distress, but not depressive symptoms, was significantly associated with higher HbA1c levels. The relative risks for poor glycaemic control (HbA1c= 64 mmol/mol; 8.0%), when adjusted for age, sex, BMI, type of diabetes therapy and duration of diabetes, was 67% higher among patients with Problem Areas in Diabetes scale scores in the highest quartile (= 26.25) compared with those in the lowest quartile (03.75). Conclusion A significant association between glycaemic control and diabetes-related distress, but not depressive symptoms, was observed in Japanese patients with Type 2 diabetes.

C1 [Tsujii, S.] Tenri Hosp, Ctr Diabet, Tenri, Nara 632, Japan.

[Hayashino, Y.] Kyoto Univ, Sch Med, Dept Epidemiol & Healthcare Res, Kyoto 606, Japan.

[Ishii, H.] Tenri Hosp, Dept Endocrinol, Tenri, Nara 632, Japan.

RP Tsujii, S (reprint author), Tenri Hosp, Ctr Diabet, Tenri, Nara 632, Japan.

EM stsujii@tenriyorozu.jp

OI Hayashino, Yasuaki/0000-0002-2932-8478; Tsujii,

Satoru/0000-0002-1834-6515

FU Manpei Suzuki Diabetes Foundation

FX The Manpei Suzuki Diabetes Foundation supported this study. The funding

source had no role in the design and conduct of the study, the

collection, analysis and interpretation of the data, or in the

preparation, review or approval of the manuscript.

CR Anderson RJ, 2001, DIABETES CARE, V24, P1069, DOI 10.2337/diacare.24.6.1069

Dhatt GS, 2005, CLIN CHIM ACTA, V358, P81, DOI 10.1016/j.cccn.2005.02.009

Fisher L, 2008, DIABETIC MED, V25, P1096, DOI 10.1111/j.1464-5491.2008.02533.x

Fisher L, 2010, DIABETES CARE, V33, P23, DOI 10.2337/dc09-1238

Georgiades A, 2007, PSYCHOSOM MED, V69, P235, DOI 10.1097/PSY.0b013e318042588d

Golden SH, 2008, JAMA-J AM MED ASSOC, V299, P2751, DOI 10.1001/jama.299.23.2751

Hermanns N, 2006, DIABETOLOGIA, V49, P469, DOI 10.1007/s00125-005-0094-2

Huang MF, 2010, DIABETES CARE, V33, P38, DOI 10.2337/dc09-0768

Ishii H, 1999, DIABETES S, V48

Katon WJ, 2005, DIABETES CARE, V28, P2668, DOI 10.2337/diacare.28.11.2668

Lloyd CE, 2010, DIABETIC MED, V27, P234, DOI 10.1111/j.1464-5491.2009.02896.x

McHale M, 2008, PSYCHOSOM MED, V70, P869, DOI 10.1097/PSY.0b013e318186dea9

Nichols GA, 2000, DIABETES CARE, V23, P273, DOI 10.2337/diacare.23.3.273

POLONSKY WH, 1995, DIABETES CARE, V18, P754, DOI 10.2337/diacare.18.6.754

Randloff LA, 1977, APPL PSYCH MEAS, V1, P385

Rubin RR, 1999, DIABETES-METAB RES, V15, P205, DOI 10.1002/(SICI)1520-7560(199905/06)15:3<205::AID-DMRR29>3.0.CO;2-O

Snoek FJ, 2000, DIABETES CARE, V23, P1305, DOI 10.2337/diacare.23.9.1305

van Bastelaar KMP, 2010, DIABETIC MED, V27, P798, DOI 10.1111/j.1464-5491.2010.03025.x

Welch G, 2003, DIABETIC MED, V20, P69, DOI 10.1046/j.1464-5491.2003.00832.x

Welch GW, 1997, DIABETES CARE, V20, P760, DOI 10.2337/diacare.20.5.760

Zou GY, 2004, AM J EPIDEMIOL, V159, P702, DOI 10.1093/aje/kwh090

NR 21

TC 33

Z9 34

U1 1

U2 18

PU WILEY-BLACKWELL

PI HOBOKEN

PA 111 RIVER ST, HOBOKEN 07030-5774, NJ USA

SN 0742-3071

J9 DIABETIC MED

JI Diabetic Med.

PD NOV

PY 2012

VL 29

IS 11

BP 1451

EP 1455

DI 10.1111/j.1464-5491.2012.03647.x

PG 5

WC Endocrinology & Metabolism

SC Endocrinology & Metabolism

GA 017QE

UT WOS:000309604600021

PM 22416679

DA 2019-01-08

ER

PT J

AU Nefs, G

Bot, M

Browne, JL

Speight, J

Pouwer, F

AF Nefs, Giesje

Bot, Mariska

Browne, Jessica L.

Speight, Jane

Pouwer, Francois

TI Diabetes MILES - The Netherlands: rationale, design and sample

characteristics of a national survey examining the psychosocial aspects

of living with diabetes in Dutch adults

SO BMC PUBLIC HEALTH

LA English

DT Article

DE Diabetes; Psychology; Self-care; Well-being; National survey

ID QUALITY-OF-LIFE; D DISTRESSED PERSONALITY; POOR GLYCEMIC CONTROL; SEXUAL

DYSFUNCTION; SOCIAL SUPPORT; SELF-CARE; PSYCHOMETRIC PROPERTIES;

NEGATIVE AFFECTIVITY; COMORBID DEPRESSION; PROBLEM AREAS

AB Background: As the number of people with diabetes is increasing rapidly worldwide, a more thorough understanding of the psychosocial aspects of living with this condition has become an important health care priority. While our knowledge has grown substantially over the past two decades with respect to the physical, emotional and social difficulties that people with diabetes may encounter, many important issues remain to be elucidated. Under the umbrella of the Diabetes MILES (Management and Impact for Long-term Empowerment and Success) Study International Collaborative, Diabetes MILES - The Netherlands aims to examine how Dutch adults with diabetes manage their condition and how it affects their lives. Topics of special interest in Diabetes MILES The Netherlands include subtypes of depression, Type D personality, mindfulness, sleep and sexual functioning.

Methods/design: Diabetes MILES - The Netherlands was designed as a national online observational study among adults with diabetes. In addition to a main set of self-report measures, the survey consisted of five complementary modules to which participants were allocated randomly. From September to October 2011, a total of 3,960 individuals with diabetes (40% type 1, 53% type 2) completed the battery of questionnaires covering a broad range of topics, including general health, self-management, emotional well-being and contact with health care providers. People with self-reported type 1 diabetes (specifically those on insulin pump therapy) were over-represented, as were those using insulin among respondents with self-reported type 2 diabetes. People from ethnic minorities were under-represented. The sex distribution was fairly equal in the total sample, participants spanned a broad age range (19-90 years), and diabetes duration ranged from recent diagnosis to living with the condition for over fifty years.

Discussion: The Diabetes MILES Study enables detailed investigation of the psychosocial aspects of living with diabetes and an opportunity to put these findings in an international context. With several papers planned resulting from a pooled Australian-Dutch dataset and data collections planned in other countries, the Diabetes MILES Study International Collaborative will contribute substantially to identifying potentially unmet needs of those living with diabetes and to inform clinical research and care across the globe.

C1 [Nefs, Giesje; Bot, Mariska; Pouwer, Francois] Tilburg Univ, Dept Med & Clin Psychol, Ctr Res Psychol Somat Dis CoRPS, NL-5000 LE Tilburg, Netherlands.

[Bot, Mariska] Vrije Univ Amsterdam, Med Ctr, Dept Psychiat, Amsterdam, Netherlands.

[Bot, Mariska] GGZ Geest, Amsterdam, Netherlands.

[Browne, Jessica L.; Speight, Jane] Diabet Australia Vic, Australian Ctr Behav Res Diabet, Melbourne, Vic, Australia.

[Browne, Jessica L.; Speight, Jane] Deakin Univ, Sch Psychol, Ctr Mental Hlth & Wellbeing Res, Burwood, Vic, Australia.

[Speight, Jane] AHP Res, Hornchurch, Essex, England.

RP Pouwer, F (reprint author), Tilburg Univ, Dept Med & Clin Psychol, Ctr Res Psychol Somat Dis CoRPS, POB 90153, NL-5000 LE Tilburg, Netherlands.

EM f.pouwer@uvt.nl

RI Nefs, Giesje/C-9992-2018

OI Speight, Jane/0000-0002-1204-6896

FU Dutch Association for Diabetes Research (Nederlandse Vereniging voor

Diabetes Onderzoek)/Lilly Diabetes

FX This study was supported by the Prof. dr. J. Terpstra Young Investigator

Award 2010 from the Dutch Association for Diabetes Research (Nederlandse

Vereniging voor Diabetes Onderzoek)/Lilly Diabetes to GN. The funding

source had no role in the design, data collection, analysis or

interpretation of the study, or in the decision to submit the manuscript

for publication. In particular, we thank all people with diabetes who

participated in Diabetes MILES - The Netherlands. We also thank the

Dutch Diabetes Association and the Dutch Diabetes Research Foundation

for their kind and very valuable assistance in the recruitment of

participants. We gratefully acknowledge the technical support of SpITs,

the IT service of the School of Social and Behavioural Sciences of

Tilburg University that hosted both the web application and the website.

Finally, we thank Dr Christel Hendrieckx and Elizabeth Holmes-Truscott

of The Australian Centre for Behavioural Research in Diabetes and prof.

dr. Johannes Ruige of Ghent University for their expert advice during

the data cleaning process.

CR Anderson RJ, 2001, DIABETES CARE, V24, P1069, DOI 10.2337/diacare.24.6.1069

Barone MTU, 2011, DIABETES RES CLIN PR, V91, P129, DOI 10.1016/j.diabres.2010.07.011

Bhasin S, 2007, LANCET, V369, P597, DOI 10.1016/S0140-6736(07)60280-3

Black SA, 2003, DIABETES CARE, V26, P2822, DOI 10.2337/diacare.26.10.2822

Bohlmeijer E, 2011, ASSESSMENT, V18, P308, DOI 10.1177/1073191111408231

Bradley C, 1999, QUAL LIFE RES, V8, P79, DOI 10.1023/A:1026485130100

Broadbent E, 2006, J PSYCHOSOM RES, V60, P631, DOI 10.1016/j.jpsychores.2005.10.020

BUYSSE DJ, 1989, PSYCHIAT RES, V28, P193, DOI 10.1016/0165-1781(89)90047-4

Craig CL, 2003, MED SCI SPORT EXER, V35, P1381, DOI 10.1249/01.MSS.0000078924.61453.FB

Daneman D, 2006, LANCET, V367, P847, DOI 10.1016/S0140-6736(06)68341-4

de Jonge P, 2011, J PSYCHOSOM RES, V71, P59, DOI 10.1016/j.jpsychores.2010.12.013

Denollet J, 2008, J INTERN MED, V263, P203, DOI 10.1111/j.1365-2796.2007.01870.x

Denollet J, 2005, PSYCHOSOM MED, V67, P89, DOI 10.1097/01.psy.0000149256.81953.49

Denollet J, 2010, CIRC-CARDIOVASC QUAL, V3, P546, DOI 10.1161/CIRCOUTCOMES.109.934406

Diabetes Fonds, 2011, DIAB CIJF

DiMatteo MR, 2004, HEALTH PSYCHOL, V23, P207, DOI 10.1037/0278-6133.23.2.207

Egede LE, 2005, DIABETES CARE, V28, P1339, DOI 10.2337/diacare.28.6.1339

Egede LE, 2002, DIABETES CARE, V25, P464, DOI 10.2337/diacare.25.3.464

Einvik G, 2011, EUR J CARDIOV PREV R, V18, P504, DOI 10.1177/1741826710389383

Frasure-Smith N, 2010, POSTGRAD MED J, V86, P193, DOI 10.1136/hrt.2009.186957

Funnell MM, 2004, CLIN DIABETES, V22, P123, DOI DOI 10.2337/DIACLIN.22.3.123

Giraldi A, 2010, J SEX RES, V47, P199, DOI 10.1080/00224491003632834

Glasgow RE, 1999, DIABETES CARE, V22, P832, DOI 10.2337/diacare.22.5.832

Gonder-Frederick LA, 2011, DIABETES CARE, V34, P801, DOI 10.2337/dc10-1343

Gonzalez JS, 2007, DIABETES CARE, V30, P2222, DOI 10.2337/dc07-0158

Gonzalez JS, 2008, DIABETES CARE, V31, P2398, DOI 10.2337/dc08-1341

Gregg EW, 2000, DIABETES CARE, V23, P1272, DOI 10.2337/diacare.23.9.1272

Gregg JA, 2007, J CONSULT CLIN PSYCH, V75, P336, DOI 10.1037/0022-006X.75.2.336

Grigsby AB, 2002, J PSYCHOSOM RES, V53, P1053, DOI 10.1016/S0022-3999(02)00417-8

Hausteiner C, 2010, PSYCHOSOM MED, V72, P163, DOI 10.1097/PSY.0b013e3181cb8bae

Heine RJ, 2004, HDB DIABETES MELLITU

Hirschfeld RMA, 2000, AM J PSYCHIAT, V157, P1873, DOI 10.1176/appi.ajp.157.11.1873

International Diabetes Federation, 2011, IDF DIAB ATL

JOHNS MW, 1991, SLEEP, V14, P540, DOI 10.1093/sleep/14.6.540

Kroenke K, 2001, J GEN INTERN MED, V16, P606, DOI 10.1046/j.1525-1497.2001.016009606.x

Lamers SMA, 2011, J CLIN PSYCHOL, V67, P99, DOI 10.1002/jclp.20741

Lin EH, 2009, DIABETES CARE, V33, P264

Ludwig DS, 2008, JAMA-J AM MED ASSOC, V300, P1350, DOI 10.1001/jama.300.11.1350

Lustman PJ, 2000, DIABETES CARE, V23, P934, DOI 10.2337/diacare.23.7.934

Malavige LS, 2009, J SEX MED, V6, P1232, DOI 10.1111/j.1743-6109.2008.01168.x

Marshall SM, 2006, BRIT MED J, V333, P475, DOI 10.1136/bmj.38922.650521.80

Matza LS, 2009, ANN PHARMACOTHER, V43, P1621, DOI 10.1345/aph.1M174

Michielsen HJ, 2004, EUR J PSYCHOL ASSESS, V20, P39, DOI 10.1027//1015-5759.20.1.39

Mols F, 2010, GEN HOSP PSYCHIAT, V32, P66, DOI 10.1016/j.genhosppsych.2009.09.010

Mommersteeg PMC, 2010, BMC PUBLIC HEALTH, V10, DOI 10.1186/1471-2458-10-714

Nefs G, 2012, J PSYCHIATR RES, V46, P549, DOI 10.1016/j.jpsychires.2012.01.013

Nefs G, 2012, J PSYCHOSOM RES, V72, P251, DOI 10.1016/j.jpsychores.2012.01.006

Pelle AJ, 2010, INT J CARDIOL, V142, P65, DOI 10.1016/j.ijcard.2008.12.086

Penson DF, 2009, J SEX MED, V6, P1969, DOI 10.1111/j.1743-6109.2009.01292.x

Peyrot M, 2005, DIABETES CARE, V28, P2673, DOI 10.2337/diacare.28.11.2673

Poortvliet MC, 2007, DIABETES NEDERLAND O

Resnick HE, 2003, DIABETES CARE, V26, P702, DOI 10.2337/diacare.26.3.702

Roglic G, 2005, DIABETES CARE, V28, P2130, DOI 10.2337/diacare.28.9.2130

Rosenzweig S, 2007, ALTERN THER HEALTH M, V13, P36

Rubin RR, 1999, DIABETES-METAB RES, V15, P205, DOI 10.1002/(SICI)1520-7560(199905/06)15:3<205::AID-DMRR29>3.0.CO;2-O

RUSH AJ, 1986, PSYCHIAT RES, V18, P65

Skomro RP, 2001, SLEEP MED, V2, P417, DOI 10.1016/S1389-9457(01)00110-1

Snoek FJ, 2000, DIABETES CARE, V23, P1305, DOI 10.2337/diacare.23.9.1305

Snoek FJ, 2011, DIABETES CARE, V34, P601, DOI 10.2337/dc10-1552

Snoek FJ, 2007, HEALTH QUAL LIFE OUT, V5, DOI 10.1186/1477-7525-5-69

SPANIER GB, 1976, J MARRIAGE FAM, V38, P15, DOI 10.2307/350547

Speight J, 2012, BMC PUBLIC HEALTH, V12, DOI 10.1186/1471-2458-12-120

Spitzer RL, 2006, ARCH INTERN MED, V166, P1092, DOI 10.1001/archinte.166.10.1092

Statline-Centraal Bureau voor de Statistiek, 2011, ICT GEBR PERS NAAR P

Tao B, 2010, PLOS ONE, V5, DOI 10.1371/journal.pone.0011501

The Diabetes Control and Complications Trial Research Group, 1993, N ENGL J MED, V329, P977

Toobert DJ, 2000, DIABETES CARE, V23, P943, DOI 10.2337/diacare.23.7.943

van der Ven NCW, 2005, PATIENT EDUC COUNS, V56, P313, DOI 10.1016/j.pec.2004.03.005

VANSTRIEN T, 1986, INT J EAT DISORDER, V5, P295, DOI 10.1002/1098-108X(198602)5:2<295::AID-EAT2260050209>3.0.CO;2-T

Vileikyte L, 2003, DIABETES CARE, V26, P2549, DOI 10.2337/diacare.26.9.2549

Weinger K, 2005, DIABETES CARE, V28, P1346, DOI 10.2337/diacare.28.6.1346

Welch GW, 1997, DIABETES CARE, V20, P760, DOI 10.2337/diacare.20.5.760

Wild S, 2004, DIABETES CARE, V27, P1047, DOI 10.2337/diacare.27.5.1047

Williams L, 2008, J PSYCHOSOM RES, V64, P63, DOI 10.1016/j.jpsychores.2007.06.008

Williams L, 2011, PSYCHOL HEALTH, V26, P703, DOI 10.1080/08870446.2010.488265

Williams R, 2002, DIABETOLOGIA, V45, pS13, DOI 10.1007/s00125-002-0859-9

ZIMET GD, 1988, J PERS ASSESS, V52, P30, DOI 10.1207/s15327752jpa5201_2

Zimmet P, 2001, NATURE, V414, P782, DOI 10.1038/414782a

1998, LANCET, V352, P837

1998, BMJ, V317, P703

NR 80

TC 13

Z9 13

U1 2

U2 25

PU BIOMED CENTRAL LTD

PI LONDON

PA 236 GRAYS INN RD, FLOOR 6, LONDON WC1X 8HL, ENGLAND

SN 1471-2458

J9 BMC PUBLIC HEALTH

JI BMC Public Health

PD OCT 30

PY 2012

VL 12

AR 925

DI 10.1186/1471-2458-12-925

PG 11

WC Public, Environmental & Occupational Health

SC Public, Environmental & Occupational Health

GA 082CM

UT WOS:000314369300001

PM 23110382

OA DOAJ Gold, Green Published

DA 2019-01-08

ER

PT J

AU Hajos, TRS

Pouwer, F

de Grooth, R

Holleman, F

Twisk, JWR

Diamant, M

Snoek, FJ

AF Hajos, T. R. S.

Pouwer, F.

de Grooth, R.

Holleman, F.

Twisk, J. W. R.

Diamant, M.

Snoek, F. J.

TI The longitudinal association between glycaemic control and

health-related quality of life following insulin therapy optimisation in

type 2 diabetes patients. A prospective observational study in secondary

care

SO QUALITY OF LIFE RESEARCH

LA English

DT Article

DE Type 2 diabetes mellitus; Insulin glargine; Glycaemic control; Quality

of life; Emotional well-being

ID SYMPTOM CHECKLIST; CONTROLLED TRIAL; MELLITUS; COMPLICATIONS;

HYPOGLYCEMIA; DEPRESSION; MANAGEMENT; DISTRESS; RISK

AB Purpose To test whether improvement in glycosylated haemoglobin (HbA(1c)) as a marker of glycaemic control, following intensifying insulin therapy, is associated with improvements in HRQoL.

Methods Dutch sub-optimally controlled (HbA(1c) > 7%) type 2 diabetes patients (N = 447, mean age 59 +/- 11) initiated insulin glargine therapy. Data were collected at baseline, 3 and 6 months, and included HbA1c and measures of HRQoL: diabetes symptom distress (Diabetes Symptom Checklist-revised; DSC-r), fear of hypoglycaemia (Hypoglycaemia Fear Survey; HFS-w) and emotional well-being (WHO-5 wellbeing index).

Results HbA(1c) decreased from 8.8 +/- 1.4% to 8.0 +/- 1.2% and 7.7 +/- 1.3% at 3 and 6 months follow-up, respectively (P < 0.001), DSC-r score improved from 17.7 +/- 14.7 to 14.3 +/- 13.3 and 13.6 +/- 13.3 (P < 0.001). HFS-w score did not significantly change. WHO-5 score increased from 56 +/- 23 to 62 +/- 23 and 65 +/- 22 P < 0.001). A modest, significant association was found between HbA(1c) and WHO-5 score (B = -1.8, 95% CI: -2.7 to -0.8) and HbA1c and DSC-r score (B = 1.0, 95% CI: 0.4 to 1.6). No such association was found for HFS-w score.

Conclusions An association between improvement in HbA(1c) by means of optimising insulin therapy and improvement in HRQoL in type 2 diabetes patients has been observed. A weak, yet significant longitudinal association was found between improved HbA(1c) and emotional well-being and diabetes symptom distress.

C1 [Hajos, T. R. S.; Snoek, F. J.] VU Univ Med Ctr VUMC, Dept Med Psychol, Amsterdam, Netherlands.

[Hajos, T. R. S.; Twisk, J. W. R.; Snoek, F. J.] Vrije Univ Amsterdam Med Ctr, EMGO Res Inst, NL-1081 BT Amsterdam, Netherlands.

[Pouwer, F.] Tilburg Univ, Ctr Res Psychol Somat Dis CoRPS, NL-5000 LE Tilburg, Netherlands.

[de Grooth, R.] Sanofi Aventis, Gouda, Netherlands.

[Holleman, F.] Univ Amsterdam, Acad Med Ctr, Dept Internal Med, NL-1105 AZ Amsterdam, Netherlands.

[Twisk, J. W. R.] Vrije Univ Amsterdam Med Ctr, Dept Clin Epidemiol & Biostat, NL-1081 BT Amsterdam, Netherlands.

[Diamant, M.] Vrije Univ Amsterdam Med Ctr, Ctr Diabet, Dept Internal Med, NL-1081 BT Amsterdam, Netherlands.

RP Hajos, TRS (reprint author), Vrije Univ Amsterdam Med Ctr, EMGO Res Inst, Boechorststr 7, NL-1081 BT Amsterdam, Netherlands.

EM t.hajos@vumc.nl

FU Sanofi-Aventis

FX This study was financially supported by an unrestricted grant of

Sanofi-Aventis.

CR Amiel S, 2002, BRIT MED J, V325, P746

Arbuckle R. A., 2009, VALUE HLTH

Arbuckle RA, 2009, VALUE HEALTH, V12, P1168, DOI 10.1111/j.1524-4733.2009.00571.x

Bech P, 2003, INT J METH PSYCH RES, V12, P85, DOI 10.1002/mpr.145

COX DJ, 1987, DIABETES CARE, V10, P617, DOI 10.2337/diacare.10.5.617

de Sonnaville JJJ, 1998, DIABETES CARE, V21, P919, DOI 10.2337/diacare.21.6.919

Dluhy RG, 2008, NEW ENGL J MED, V358, P2630, DOI 10.1056/NEJMe0804182

GOLDSTEIN DE, 1995, DIABETES CARE, V18, P896, DOI 10.2337/diacare.18.6.896

Gonder-Frederick LA, 1997, SEMIN CLIN NEUROPSYC, V2, P57

GROOTENHUIS PA, 1994, DIABETIC MED, V11, P253, DOI 10.1111/j.1464-5491.1994.tb00268.x

Heisler M, 2005, DIABETES CARE, V28, P816, DOI 10.2337/diacare.28.4.816

Heller SR, 2007, DIABETOLOGIA, V50, P1140, DOI 10.1007/s00125-007-0599-y

Horvath K, 2007, COCHRANE DB SYST REV

Hunt SM, 1997, QUAL LIFE RES, V6, P205

King H, 1998, DIABETES CARE, V21, P1414, DOI 10.2337/diacare.21.9.1414

Lloyd A, 2001, Value Health, V4, P392, DOI 10.1046/j.1524-4733.2001.45029.x

Lustman PJ, 2000, DIABETES CARE, V23, P934, DOI 10.2337/diacare.23.7.934

Pouwer F, 2001, DIABETIC MED, V18, P595, DOI 10.1046/j.1464-5491.2001.00499.x

Rubin DB, 1987, MULTIPLE IMPUTATION

Rubin Richard R, 2010, Pediatr Endocrinol Rev, V7 Suppl 3, P405

Rubin RR, 1999, DIABETES-METAB RES, V15, P205, DOI 10.1002/(SICI)1520-7560(199905/06)15:3<205::AID-DMRR29>3.0.CO;2-O

Schafer JL, 2002, PSYCHOL METHODS, V7, P147, DOI 10.1037//1082-989X.7.2.147

Swinnen SG, 2010, DIABETES CARE, V33, P1176, DOI 10.2337/dc09-2294

Testa MA, 1998, JAMA-J AM MED ASSOC, V280, P1490, DOI 10.1001/jama.280.17.1490

Turner R, 1996, ANN INTERN MED, V124, P136, DOI 10.7326/0003-4819-124-1_Part_2-199601011-00011

Turner RC, 1998, LANCET, V352, P837

UK Prospective Diabetes Study Group, 1999, DIABETES CARE, V22, P1125, DOI DOI 10.2337/DIACARE.22.7.1125

VanderDoes FEE, 1996, DIABETES CARE, V19, P204, DOI 10.2337/diacare.19.3.204

WEINBERGER M, 1994, MED CARE, V32, P1173, DOI 10.1097/00005650-199412000-00002

2010, DIABETES CARE S1, V33, pS11

NR 30

TC 16

Z9 17

U1 0

U2 2

PU SPRINGER

PI DORDRECHT

PA VAN GODEWIJCKSTRAAT 30, 3311 GZ DORDRECHT, NETHERLANDS

SN 0962-9343

J9 QUAL LIFE RES

JI Qual. Life Res.

PD OCT

PY 2012

VL 21

IS 8

BP 1359

EP 1365

DI 10.1007/s11136-011-0051-0

PG 7

WC Health Care Sciences & Services; Health Policy & Services; Public,

Environmental & Occupational Health

SC Health Care Sciences & Services; Public, Environmental & Occupational

Health

GA 089LR

UT WOS:000314912500006

PM 22065281

OA Other Gold, Green Published

DA 2019-01-08

ER

PT J

AU Chan, HL

Lin, CK

Chau, YL

Chang, CM

AF Chan, Hsiang-Lin

Lin, Chih-Kuang

Chau, Yeuk-Lun

Chang, Chia-Ming

TI The impact of depression on self-care activities and health care

utilization among people with diabetes in Taiwan

SO DIABETES RESEARCH AND CLINICAL PRACTICE

LA English

DT Article

DE Diabetes; Depression; Self-care; Health care utilization

ID COMPLICATIONS; SYMPTOMS; METAANALYSIS; INDIVIDUALS; ASSOCIATION;

DISTRESS; ASIA

AB Using cross-sectional analyses of a nationally representative community sample (n = 1260), we found that in people with diabetes, depression was associated with lower rates of reducing or quitting smoking and alcohol, less exercise, less regular lifestyle, but more health care utilization and a higher rate of foot care. (C) 2012 Elsevier Ireland Ltd. All rights reserved.

C1 Chang Gung Mem Hosp, Dept Psychiat, Linkou, Taiwan.

Chang Gung Univ, Coll Med, Tao Yuan, Taiwan.

RP Chang, CM (reprint author), Chang Gung Mem Hosp, Dept Psychiat, 5 Fusing St, Gueishan Township 333, Taoyuan County, Taiwan.

EM cmchang58@yahoo.com.tw

FU [CMRPG370411]

FX This study was supported by a grant from CMRPG370411 to CM Chang. We

thank the Bureau of Health Promotion for providing HPKAP data for

analysis.

CR Bogner HR, 2007, DIABETES CARE, V30, P3005, DOI 10.2337/dc07-0974

de Groot M, 2001, PSYCHOSOM MED, V63, P619, DOI 10.1097/00006842-200107000-00015

Egede LE, 2004, DIABETES CARE, V27, P1751, DOI 10.2337/diacare.27.7.1751

Egede LE, 2009, GEN HOSP PSYCHIAT, V31, P422, DOI 10.1016/j.genhosppsych.2009.06.007

Gonzalez JS, 2008, DIABETOLOGIA, V51, P1822, DOI 10.1007/s00125-008-1113-x

Gonzalez JS, 2008, DIABETES CARE, V31, P2398, DOI 10.2337/dc08-1341

Katon WJ, 2005, DIABETES CARE, V28, P2668, DOI 10.2337/diacare.28.11.2668

Lee S, 2006, DIABETES RES CLIN PR, V74, P282, DOI 10.1016/j.diabres.2006.03.026

Lee Y, 2000, Chang Gung Med J, V23, P688

Lin EHB, 2004, DIABETES CARE, V27, P2154, DOI 10.2337/diacare.27.9.2154

Lin EHB, 2010, DIABETES CARE, V33, P264, DOI 10.2337/dc09-1068

Lloyd CE, 2010, DIABETIC MED, V27, P234, DOI 10.1111/j.1464-5491.2009.02896.x

Lustman PJ, 2000, DIABETES CARE, V23, P934, DOI 10.2337/diacare.23.7.934

Mohamed M, 2008, CURR MED RES OPIN, V24, P507, DOI 10.1185/030079908X261131

Papelbaum M, 2010, DIABETES RES CLIN PR, V89, P227, DOI 10.1016/j.diabres.2010.05.024

Simon GE, 2005, GEN HOSP PSYCHIAT, V27, P344, DOI 10.1016/j.genhosppsych.2005.04.008

Yoon KH, 2006, LANCET, V368, P1681, DOI 10.1016/S0140-6736(06)69703-1

Yoshida S, 2009, PSYCHIAT CLIN NEUROS, V63, P65, DOI 10.1111/j.1440-1819.2008.01889.x

NR 18

TC 6

Z9 6

U1 0

U2 3

PU ELSEVIER IRELAND LTD

PI CLARE

PA ELSEVIER HOUSE, BROOKVALE PLAZA, EAST PARK SHANNON, CO, CLARE, 00000,

IRELAND

SN 0168-8227

J9 DIABETES RES CLIN PR

JI Diabetes Res. Clin. Pract.

PD OCT

PY 2012

VL 98

IS 1

BP E4

EP E7

DI 10.1016/j.diabres.2012.06.003

PG 4

WC Endocrinology & Metabolism

SC Endocrinology & Metabolism

GA 036KZ

UT WOS:000311026100002

PM 22749685

DA 2019-01-08

ER

PT J

AU Testa, MA

Gill, J

Su, M

Turner, RR

Blonde, L

Simonson, DC

AF Testa, Marcia A.

Gill, Jasvinder

Su, Max

Turner, Ralph R.

Blonde, Lawrence

Simonson, Donald C.

TI Comparative Effectiveness of Basal-Bolus Versus Premix Analog Insulin on

Glycemic Variability and Patient-Centered Outcomes during Insulin

Intensification in Type 1 and Type 2 Diabetes: A Randomized, Controlled,

Crossover Trial

SO JOURNAL OF CLINICAL ENDOCRINOLOGY & METABOLISM

LA English

DT Article

ID QUALITY-OF-LIFE; MULTIPLE DAILY INJECTIONS; GLUCOSE VARIABILITY;

PLASMA-GLUCOSE; ANTIHYPERTENSIVE THERAPY; HYPERTENSIVE PATIENTS;

CLINICAL-TRIAL; BLOOD-GLUCOSE; MELLITUS; ASSOCIATION

AB Context: In patients with diabetes, intraday glucose variability might predict health outcomes independently from glycosylated hemoglobin (HbA(1c)).

Objective: Our objective was to evaluate patient satisfaction (PS), quality of life (QoL), glycemic control, and variability during insulin intensification to HbA(1c) below 7.0%.

Patients, Design, and Setting: Eighty-two type 1 and 306 insulin-treated type 2 diabetes patients (47% male; age 54 +/- 11 yr; HbA(1c) = 7.8 +/- 0.7%) participated in this multicenter, randomized, crossover trial at 52 U.S. centers.

Interventions: Interventions included insulin glargine plus premeal glulisine (n = 192) vs. twice-daily premix 75/25 or 70/30 analog insulin (n = 196) for 12 wk and crossed to the alternate arm for 12 wk.

Main Outcome Measures: Main outcome measures included PS and QoL questionnaires, 3-d continuous glucose monitoring (CGM), and HbA(1c) every 4-8 wk.

Results: Mean +/- SE HbA(1c) change was -0.39 +/- 0.09% for glargine-glulisine and -0.05 +/- 0.09% for premix (P < 0.0001). The PS net benefit scale (0-100) improved from 51.1 to 60.5 +/- 1.2 for glargine-glulisine and worsened to 45.4 +/- 1.2 for premix (P < 0.0001). The PS regimen acceptance scale was comparable (P = 0.33). Overall QoL favored glargine-glulisine (P < 0.001), as did perceived health (P < 0.0001), symptom distress (P < 0.0001), general health perceptions (P < 0.01), and psychosocial (P < 0.02). CGM daily glucose mean, daily glucose SD (glycemic variability), and percent time over 140 mg/dl were lower for glargine-glulisine by 13.1 +/- 2.7 mg/dl, 5.9 +/- 1.4 mg/dl, and 7.3 +/- 1.6%, respectively (all P < 0.0001), with no difference in CGM percent time below 70 mg/dl (P = 0.09). Symptomatic hypoglycemia rates were comparable. HbA(1c), mean CGM daily glucose, and glycemic variability were independent predictors of PS net benefit.

Conclusions: Patient satisfaction was impacted more positively by improved QoL, reduced glucose variability, and better glycemic control with a basal-bolus regimen than negatively by the burden of additional injections, thereby facilitating insulin intensification and the ability to achieve HbA(1c) below 7.0%. (J Clin Endocrinol Metab 97: 3504-3514, 2012)

C1 [Testa, Marcia A.] Harvard Univ, Sch Publ Hlth, Dept Biostat, Boston, MA 02115 USA.

[Simonson, Donald C.] Harvard Univ, Brigham & Womens Hosp, Sch Med, Div Endocrinol Diabet & Hypertens, Boston, MA 02115 USA.

[Gill, Jasvinder] Sanofi Aventis US, Bridgewater, NJ 08807 USA.

[Su, Max; Turner, Ralph R.] Phase V Technol, Wellesley Hills, MA 02481 USA.

[Blonde, Lawrence] Ochsner Med Ctr, Dept Endocrinol Diabet & Metab Dis, Ochsner Diabet Clin Res Unit, New Orleans, LA 70121 USA.

RP Testa, MA (reprint author), Harvard Univ, Sch Publ Hlth, Dept Biostat, 655 Huntington Ave, Boston, MA 02115 USA.

EM testa@hsph.harvard.edu

FU sanofi-aventis, U.S.; sanofi-aventis; Amylin Pharmaceuticals; Boehringer

Ingelheim; Eli Lilly and Co.; AstraZeneca LP; Bristol-Myers Squibb Co.;

Daiichi-Sankyo; GlaxoSmithKline; Lifescan; Mann-Kind Corp.; Merck Co.;

Novartis Pharmaceuticals Corp.; Novo Nordisk; Pfizer; Roche

Pharmaceuticals; Harvard School of Public Health

FX The clinical trial was funded by sanofi-aventis, U.S., which had no role

in the data collection, management, analysis, and manuscript preparation

except for the contributions of one of the co-authors, J.G., who is an

employee of sanofi-aventis and also served as the clinical trial medical

monitor.; L.B. has received consulting fees and research grants from

sanofi-aventis, in addition to consulting and research grants from

Amylin Pharmaceuticals, Boehringer Ingelheim, Eli Lilly and Co.,

AstraZeneca LP, Bristol-Myers Squibb Co., Daiichi-Sankyo,

GlaxoSmithKline, Lifescan, Mann-Kind Corp., Merck & Co., Novartis

Pharmaceuticals Corp., Novo Nordisk, Pfizer, and Roche Pharmaceuticals.

M.A.T., M.S., R.R.T., and D.C.S. have nothing to declare. The survey

design and statistical analyses were supported by unrestricted funds

from the Harvard School of Public Health.

CR *AM DIAB ASS, 2010, DIABETES CARE S1, V33, pS11, DOI DOI 10.2337/DC10-S011

Anderson R. B., 1994, DRUG INF J, V28, P89

ANDERSON RB, 1995, DRUG INF J, V29, pS1689

Blonde L, 2009, DIABETES OBES METAB, V11, P623, DOI 10.1111/j.1463-1326.2009.01060.x

Bode BW, 2007, CLIN THER, V29, pS135, DOI 10.1016/j.clinthera.2007.12.013

Borg R, 2011, DIABETOLOGIA, V54, P69, DOI 10.1007/s00125-010-1918-2

Cameron Fergus J, 2010, Pediatr Endocrinol Rev, V7 Suppl 3, P432

Danne T, 2009, DIABETOLOGIA, V52, P1496, DOI 10.1007/s00125-009-1408-6

Herman WH, 2005, DIABETES CARE, V28, P1568, DOI 10.2337/diacare.28.7.1568

Hermanns N, 2007, DIABETOLOGIA, V50, P930, DOI 10.1007/s00125-007-0643-y

Hirsch IB, 2005, J DIABETES COMPLICAT, V19, P178, DOI 10.1016/j.jdiacomp.2004.10.001

Johnson EL, 2010, J FAM PRACTICE, V59, pE1

Kilpatrick ES, 2007, DIABETOLOGIA, V50, P2553, DOI 10.1007/s00125-007-0820-z

McDonnell CM, 2007, DIABETES CARE, V30, P2211, DOI 10.2337/dc07-0328

Monnier L, 2006, JAMA-J AM MED ASSOC, V295, P1681, DOI 10.1001/jama.295.14.1681

Raskin P, 2003, DIABETES CARE, V26, P2598, DOI 10.2337/diacare.26.9.2598

Riddle MC, 2003, DIABETES CARE, V26, P3080, DOI 10.2337/diacare.26.11.3080

Rodbard D, 2009, DIABETES TECHNOL THE, V11, P551, DOI 10.1089/dia.2009.0015

Rodbard D, 2009, DIABETES TECHNOL THE, V11, pS55, DOI 10.1089/dia.2008.0132

Rodbard HW, 2009, ENDOCR PRACT, V15, P540, DOI 10.4158/EP.15.6.540

SHAMOON H, 1993, NEW ENGL J MED, V329, P977

Stratton IM, 2000, BRIT MED J, V321, P405, DOI 10.1136/bmj.321.7258.405

Temelkova-Kurktschiev TS, 2000, DIABETES CARE, V23, P1830, DOI 10.2337/diacare.23.12.1830

Testa M, 2003, DIABETES S1, V52, pA420

Testa M, 2000, DIABETES SPECTRUM, V13, P29

Testa M A, 2000, Med Care, V38, pII166

Testa MA, 1998, DIABETES CARE, V21, pC44, DOI 10.2337/diacare.21.3.C44

Testa MA, 2000, DIABETES, V49, pA73

TESTA MA, 1991, AM J HYPERTENS, V4, P363, DOI 10.1093/ajh/4.4.363

TESTA MA, 1993, NEW ENGL J MED, V328, P907, DOI 10.1056/NEJM199304013281302

Testa MA, 1998, JAMA-J AM MED ASSOC, V280, P1490, DOI 10.1001/jama.280.17.1490

Testa MA, 1998, J HYPERTENS, V16, P1839, DOI 10.1097/00004872-199816120-00018

Testa MA, 1996, NEW ENGL J MED, V334, P835, DOI 10.1056/NEJM199603283341306

TESTA MA, 1988, POSTGRAD MED J, V64, P50

Testa MA, 2007, DIABETES CARE, V30, P1399, DOI 10.2337/dc06-1497

TURNER RC, 1995, DIABETES, V44, P1249

Wei M, 2000, CIRCULATION, V101, P2047, DOI 10.1161/01.CIR.101.17.2047

Weintrob N, 2003, PEDIATRICS, V112, P559, DOI 10.1542/peds.112.3.559

Zambanini A, 1999, DIABETES RES CLIN PR, V46, P239, DOI 10.1016/S0168-8227(99)00099-6

NR 39

TC 25

Z9 25

U1 0

U2 3

PU ENDOCRINE SOC

PI CHEVY CHASE

PA 8401 CONNECTICUT AVE, SUITE 900, CHEVY CHASE, MD 20815-5817 USA

SN 0021-972X

J9 J CLIN ENDOCR METAB

JI J. Clin. Endocrinol. Metab.

PD OCT 1

PY 2012

VL 97

IS 10

BP 3504

EP 3514

DI 10.1210/jc.2012-1763

PG 11

WC Endocrinology & Metabolism

SC Endocrinology & Metabolism

GA 018MH

UT WOS:000309664400044

PM 22851487

OA Bronze

DA 2019-01-08

ER

PT J

AU Atlantis, E

AF Atlantis, Evan

TI Excess burden of type 1 and type 2 diabetes due to psychopathology

SO JOURNAL OF AFFECTIVE DISORDERS

LA English

DT Article

DE Diabetes; Psychological distress; Depression; Anxiety; Antidepressants;

Anti-anxiety medications

ID CO-MORBID DEPRESSION; PSYCHOLOGICAL DISTRESS; COMORBID DEPRESSION;

PROSPECTIVE COHORT; HEALTH-CARE; PREVALENCE; ANTIDEPRESSANTS;

METAANALYSIS; MELLITUS; TRENDS

AB Background: Whether the prevalence of type 1 or 2 diabetes is rising among people with psychopathology is uncertain. This study investigates changes in the prevalence of type 1 and type 2 diabetes associated with psychopathology in the adult Australian population from 2001 to 2008.

Methods: Data analysed were from 48,359 participants aged >= 25 years from the 2001, 2004-05 or 2007-08 National Health Surveys. Lifetime diagnosis of type 1 and type 2 diabetes was determined by self-report. Psychopathology status was determined with the 10-item Kessler Psychological Distress Scale (using scores >= 30), contemporaneous use of antidepressants or anti-anxiety medications, or both.

Results: Overall, the prevalence of type 1 diabetes remained stable whereas the prevalence of type 2 diabetes increased by 36% between 2001 and 2008. On average, odds ratios with 95% confidence intervals (95% CI) for type 1 and type 2 diabetes ranged from 1.43 (0.98, 2.10) to 2.44 (1.63, 3.64) and 1.32 (1.13, 1.53) to 1.67 (1.39, 2.02) for people with compared to those without psychopathology by any definition independent of socio-demographic covariates, consistently over the 8-year period. After further adjustments for lifestyle covariates, the strength of these odds ratios were attenuated and ranged from 1.32 (0.90, 1.95) to 2.24 (1.49, 3.36) and 1.16 (0.99, 1.36) to 1.51 (1.24, 1.83) for type 1 and type 2 diabetes.

Limitations: Data were self-report from serial surveys.

Conclusions: The prevalence of both type 1 and type 2 diabetes was consistently higher for people with psychopathology from 2001 to 2008. Clinicians should consider routinely screening patients with diabetes for psychopathology and vice versa, as well as lifestyle risk factors, to inform practice for more effective management and prevention planning. (C) 2012 Elsevier B. V. All rights reserved.

C1 [Atlantis, Evan] Univ Western Sydney, Family & Community Hlth Res Grp, Sch Nursing & Midwifery, Penrith, NSW 2751, Australia.

[Atlantis, Evan] Univ Adelaide, Sch Med, Adelaide, SA, Australia.

RP Atlantis, E (reprint author), Univ Western Sydney, Family & Community Hlth Res Grp, Sch Nursing & Midwifery, Bldg 17,Room 17-1-14,Campbelltown Campus,Locked B, Penrith, NSW 2751, Australia.

EM e.atlantis@uws.edu.au

CR Ali S, 2006, DIABETIC MED, V23, P1165, DOI 10.1111/j.1464-5491.2006.01943.x

American Diabetes Association, 2011, DIABETES CARE S1, V34, pS62, DOI [10.2337/dc11-S062, DOI 10.2337/DC11-S062]

Andrews G, 2001, AUST NZ J PUBL HEAL, V25, P494, DOI 10.1111/j.1467-842X.2001.tb00310.x

Atlantis E, 2008, INT J OBESITY, V32, P715, DOI 10.1038/sj.ijo.0803762

Atlantis E, 2009, OBES REV, V10, P543, DOI 10.1111/j.1467-789X.2009.00590.x

Atlantis E, 2012, SOC PSYCH PSYCH EPID, V47, P871, DOI 10.1007/s00127-011-0394-4

Atlantis E, 2012, AUST NZ J PSYCHIAT, V46, P445, DOI 10.1177/0004867411433218

Atlantis E, 2012, SOC PSYCH PSYCH EPID, V47, P615, DOI 10.1007/s00127-011-0365-9

Atlantis E, 2010, INT J GERIATR PSYCH, V25, P688, DOI 10.1002/gps.2409

Atlantis E, 2009, BRIT MED J, V339, DOI 10.1136/bmj.b3868

Australian Bureau of Statistics A National Health Survey, 2001, 990 AUSTR BUR STAT A

Australian Bureau of Statistics B National Health Survey, 2005, 1778 AUSTR BUR STAT

Australian Bureau of Statistics C National Health Survey, 2007, 4324 AUSTR BUR STAT

Australian Institute of Health and Welfare, 2009, AUSTR I HLTH WELF DI

Barbui C, 1999, SOC PSYCH PSYCH EPID, V34, P152, DOI 10.1007/s001270050127

Barnard KD, 2006, DIABETIC MED, V23, P445, DOI 10.1111/j.1464-5491.2006.01814.x

Barr ELM, 2007, CIRCULATION, V116, P151, DOI 10.1161/CIRCULATIONAHA.106.685628

Brhlikova P, 2011, J ROY SOC MED, V104, P25, DOI 10.1258/jrsm.2010.100080

Chiu CJ, 2010, SOC PSYCH PSYCH EPID, V45, P67, DOI 10.1007/s00127-009-0043-3

Coles-Rutishauser I., 2001, EVALUATION SHORT DIE

Davydow DS, 2011, PSYCHOSOMATICS, V52, P117, DOI 10.1016/j.psym.2010.12.020

Glasziou P, 2008, BRIT MED J, V336, P1472, DOI 10.1136/bmj.39590.732037.47

Goldney RD, 2010, AUST NZ J PSYCHIAT, V44, P901, DOI 10.3109/00048674.2010.490520

Gonzalez JS, 2008, DIABETES CARE, V31, P2398, DOI 10.2337/dc08-1341

GREY M, 1995, DIABETES CARE, V18, P1330, DOI 10.2337/diacare.18.10.1330

Harman JS, 2009, PSYCHIAT SERV, V60, P611, DOI 10.1176/appi.ps.60.5.611

Hilliard ME, 2011, DIABETES RES CLIN PR, V94, P39, DOI 10.1016/j.diabres.2011.05.027

Hutter N, 2010, DIABETOLOGIA, V53, P2470, DOI 10.1007/s00125-010-1873-y

International Diabetes Federation, 2011, IDF DIAB ATL

Katon WJ, 2010, NEW ENGL J MED, V363, P2611, DOI 10.1056/NEJMoa1003955

Kessler RC, 2002, PSYCHOL MED, V32, P959, DOI 10.1017/S0033291702006074

Kovacs M, 1997, DIABETES CARE, V20, P45, DOI 10.2337/diacare.20.1.45

Lin EHB, 2010, DIABETES CARE, V33, P264, DOI 10.2337/dc09-1068

Lustman PJ, 2000, DIABETES CARE, V23, P934, DOI 10.2337/diacare.23.7.934

Manderbacka K, 2011, PHARMACOEPIDEM DR S, V20, P338, DOI 10.1002/pds.2072

Mant A, 2004, MED J AUSTRALIA, V181, pS21

McFall M, 2010, JAMA-J AM MED ASSOC, V304, P2485, DOI 10.1001/jama.2010.1769

Meijer WEE, 2004, EUR J CLIN PHARMACOL, V60, P57, DOI 10.1007/s00228-004-0726-3

Mezuk B, 2008, DIABETES CARE, V31, P2383, DOI 10.2337/dc08-0985

Moore M, 2009, BRIT MED J, V339, DOI 10.1136/bmj.b3999

Northam EA, 2005, DIABETIC MED, V22, P152, DOI 10.1111/j.1464-5491.2004.01370.x

Nouwen A, 2010, DIABETOLOGIA, V53, P2480, DOI 10.1007/s00125-010-1874-x

Petersen M, 2008, DIABETES CARE, V31, P596, DOI 10.2337/dc08-9017

Pouwer F, 2010, DIABETIC MED, V27, P217, DOI 10.1111/j.1464-5491.2009.02903.x

Schram MT, 2009, CURR DIABETES REV, V5, P112, DOI 10.2174/157339909788166828

Simon GE, 2010, GEN HOSP PSYCHIAT, V32, P583, DOI 10.1016/j.genhosppsych.2010.09.010

Stewart SM, 2005, PEDIATRICS, V115, P1315, DOI 10.1542/peds.2004-1717

Van der Feltz-Cornelis CM, 2010, GEN HOSP PSYCHIAT, V32, P380, DOI 10.1016/j.genhosppsych.2010.03.011

Wilkins Tricia Lee, 2011, Int Clin Psychopharmacol, V26, P159, DOI 10.1097/YIC.0b013e328342ce31

World Health Organization, 2008, GLOB BURD DIS 2004 U

NR 50

TC 7

Z9 7

U1 0

U2 4

PU ELSEVIER SCIENCE BV

PI AMSTERDAM

PA PO BOX 211, 1000 AE AMSTERDAM, NETHERLANDS

SN 0165-0327

J9 J AFFECT DISORDERS

JI J. Affect. Disord.

PD OCT

PY 2012

VL 142

SU S

BP S36

EP S41

DI 10.1016/S0165-0327(12)70007-1

PG 6

WC Clinical Neurology; Psychiatry

SC Neurosciences & Neurology; Psychiatry

GA 017GJ

UT WOS:000309577800006

PM 23062855

DA 2019-01-08

ER

PT J

AU Rogvi, S

Tapager, I

Almdal, TP

Schiotz, ML

Willaing, I

AF Rogvi, S.

Tapager, I.

Almdal, T. P.

Schiotz, M. L.

Willaing, I.

TI Patient factors and glycaemic control - associations and explanatory

power

SO DIABETIC MEDICINE

LA English

DT Article

ID DIABETIC-PATIENTS; CHRONIC ILLNESS; PRIMARY-CARE; DANISH VERSION;

KNOWLEDGE; ADAPTATION; MANAGEMENT; PREDICTORS; ACTIVATION; OUTCOMES

AB Diabet. Med. 29, e382e389 (2012) Abstract Aims To investigate the association between glycaemic control and patient socio-demographics, activation level, diabetes-related distress, assessment of care, knowledge of target HbA1c, and self-management behaviours, and to determine to what extent these factors explain the variance in HbA1c in a large Danish population of patients with Type 2 diabetes. Methods Cross-sectional survey and record review of 2045 patients from a specialist diabetes clinic. Validated scales measured patient activation, self-management behaviours, diabetes-related emotional distress, and perceived care. The electronic patient record provided information about HbA1c, medication, body mass index, and duration of diabetes. Data were analysed using multiple linear regression models with stepwise addition of covariates. Results The response rate was 54% (n = 1081). Good glycaemic control was significantly associated with older age, higher education, higher patient activation, lower diabetes-related emotional distress, better diet and exercise behaviours, lower body mass index, shorter duration of disease and knowledge of HbA1c targets (P < 0.05 for all). Patient socio-demographics, behaviour; perceptions of care and diabetes distress accounted for 14% of the total variance in HbA1c levels (P = 0.0134), but the variance explained was higher for respondents treated with medications other than insulin. Conclusions Our study emphasizes the complex relationships between patient activation, distress and behaviour, specific treatment modalities and glycaemic control. Knowledge of treatment goals, achieving patient activation in coping with diabetes, and lowering disease-related emotional stress are important patient education goals. However, the large unexplained component of HbA1c variance highlights the need for more research to understand the mechanisms of glycaemic control.

C1 [Rogvi, S.; Schiotz, M. L.; Willaing, I.] Steno Diabet Ctr, Steno Hlth Promot Ctr, DK-2820 Gentofte, Denmark.

[Tapager, I.] Incent Partners, Holte, Denmark.

[Almdal, T. P.] Steno Diabet Ctr, Steno Patient Care Clin, DK-2820 Gentofte, Denmark.

RP Willaing, I (reprint author), Steno Diabet Ctr, Steno Hlth Promot Ctr, DK-2820 Gentofte, Denmark.

EM iwtp@steno.dk

CR *AM DIAB ASS, 2011, DIABETES CARE S1, V34, pS11, DOI DOI 10.2337/DC11-S011

Bains SS, 2011, DIABETES TECHNOL THE, V13, P335, DOI 10.1089/dia.2010.0160

Bebb C, 2005, DIABETIC MED, V22, P1364, DOI 10.1111/j.1464-5491.2005.01662.x

Benoit SR, 2005, BMC PUBLIC HEALTH, V5, DOI 10.1186/1471-2458-5-36

Berikai P, 2007, DIABETES CARE, V30, P1587, DOI 10.2337/dc06-2026

BLAND JM, 1995, BRIT MED J, V310, P170, DOI 10.1136/bmj.310.6973.170

Bodenheimer T, 2002, JAMA-J AM MED ASSOC, V288, P1775, DOI 10.1001/jama.288.14.1775

Chiu C, 2010, PREVENTING CHRONIC D, V7, P1

Daly JM, 2009, J AM BOARD FAM MED, V22, P280, DOI 10.3122/jabfm.2009.03.080114

Delahanty LM, 2007, DIABETIC MED, V24, P48, DOI 10.1111/j.1464-5491.2007.02028.x

Glasgow RE, 2005, DIABETES CARE, V28, P2655, DOI 10.2337/diacare.28.11.2655

Gugiu PC, 2009, CHRONIC ILLN, V5, P268, DOI 10.1177/1742395309348072

He X, 2007, INT NURS REV, V54, P280, DOI 10.1111/j.1466-7657.2007.00570.x

Hermanns N, 2006, DIABETOLOGIA, V49, P469, DOI 10.1007/s00125-005-0094-2

Hibbard JH, 2004, HEALTH SERV RES, V39, P1005, DOI 10.1111/j.1475-6773.2004.00269.x

Hojat M, 2011, ACAD MED, V86, P359, DOI 10.1097/ACM.0b013e3182086fe1

Huang ES, 2011, DIABETES CARE, V34, P1329, DOI 10.2337/dc10-2377

Khattab M, 2010, J DIABETES COMPLICAT, V24, P84, DOI 10.1016/j.jdiacomp.2008.12.008

Maindal HT, 2012, EUR J PUBLIC HEALTH, V22, P31, DOI 10.1093/eurpub/ckq188

Maindal HT, 2009, BMC PUBLIC HEALTH, V9, DOI 10.1186/1471-2458-9-209

McGuire BE, 2009, DIABETOLOGIA, V53, P66

Nichols GA, 2000, DIABETES CARE, V23, P273, DOI 10.2337/diacare.23.3.273

Ozcelik F, 2010, POL ARCH MED WEWN, V120, P399

Papelbaum M, 2010, DIABETES RES CLIN PR, V89, P227, DOI 10.1016/j.diabres.2010.05.024

Remmers C, 2009, J AMBUL CARE MANAG, V32, P320, DOI 10.1097/JAC.0b013e3181ba6e77

Rhee MK, 2005, DIABETES EDUCATOR, V31, P240, DOI 10.1177/0145721705274927

Schiotz M, 2012, PATIENT EDUC COUNS, V87, P217, DOI 10.1016/j.pec.2011.09.003

Schmittdiel J, 2008, J GEN INTERN MED, V23, P77, DOI 10.1007/s11606-007-0452-5

Snoek FJ, 2011, DIABETES CARE, V34, P601, DOI 10.2337/dc10-1552

Toobert DJ, 2000, DIABETES CARE, V23, P943, DOI 10.2337/diacare.23.7.943

NR 30

TC 31

Z9 32

U1 0

U2 16

PU WILEY-BLACKWELL

PI HOBOKEN

PA 111 RIVER ST, HOBOKEN 07030-5774, NJ USA

SN 0742-3071

J9 DIABETIC MED

JI Diabetic Med.

PD OCT

PY 2012

VL 29

IS 10

BP e382

EP e389

DI 10.1111/j.1464-5491.2012.03703.x

PG 8

WC Endocrinology & Metabolism

SC Endocrinology & Metabolism

GA 008MF

UT WOS:000308960400005

PM 22540962

DA 2019-01-08

ER

PT J

AU van der Wulp, I

de Leeuw, JRJ

Gorter, KJ

Rutten, GEHM

AF van der Wulp, I.

de Leeuw, J. R. J.

Gorter, K. J.

Rutten, G. E. H. M.

TI Effectiveness of peer-led self-management coaching for patients recently

diagnosed with Type 2 diabetes mellitus in primary care: a randomized

controlled trial

SO DIABETIC MEDICINE

LA English

DT Article

ID QUALITY-OF-LIFE; MARITAL RELATIONSHIP; CHRONIC ILLNESS; PROBLEM AREAS;

SCALE; INTERVENTION; ADAPTATION; DISTRESS; VALIDITY; PROGRAM

AB Diabet. Med. 29, e390e397 (2012) Summary Aims To study the effectiveness of a peer-led self-management coaching intervention in recently diagnosed patients with Type 2 diabetes. Methods Randomized controlled trial of recently diagnosed patients with Type 2 diabetes from 54 participating general practices. The intervention group received three home visits by an experienced peer (expert patient) who adhered to the recommended treatment and lifestyle guidelines. Together with their expert patient, participants set feasible goals and these were evaluated in the next visit. Participants in the control group received care as usual. At baseline, 3 months and 6 months post-intervention, participants completed a questionnaire measuring changes in self-efficacy, coping, physical activity, dietary habits, psychological well-being, depressive symptoms and diabetes related distress. Results In total, 327 patients were eligible for inclusion in the study of which 133 consented to participate. In participating patients, self-efficacy, coping and saturated fat intake improved significantly over time. Analyses of participants with low self-efficacy at baseline (25th percentile: 44) revealed a significant time X group difference, F = 3.71; P = 0.03. Participants who reported low psychological well-being at baseline increased substantially throughout the study (F = 23.84; P < 0.01) but no significant time X group differences were found. Conclusions A peer-led self-management coaching programme for recently diagnosed patients with Type 2 diabetes improved self-efficacy of patients experiencing low self-efficacy shortly after diagnosis.

C1 [van der Wulp, I.; de Leeuw, J. R. J.; Gorter, K. J.; Rutten, G. E. H. M.] UMC Utrecht, Julius Ctr Hlth Sci & Primary Care, Utrecht, Netherlands.

RP van der Wulp, I (reprint author), UMC Utrecht, Julius Ctr Hlth Sci & Primary Care, Utrecht, Netherlands.

EM i.vanderwulp-2@umcutrecht.nl

FU Dutch Diabetes Research Foundation [2005.13.017]

FX We thank all patients and expert patients for participating in this

study. This study was supported by a grant of the Dutch Diabetes

Research Foundation, grant number: 2005.13.017.

CR Bandura A., 1986, SOCIAL FDN THOUGHT A, P391

Barlow J, 2002, PATIENT EDUC COUNS, V48, P177, DOI 10.1016/S0738-3991(02)00032-0

Cade JE, 2009, DIABETIC MED, V26, P1048, DOI 10.1111/j.1464-5491.2009.02808.x

Chaveepojnkamjorn W, 2009, SE ASIAN J TROP MED, V40, P169

Dale J, 2009, PATIENT EDUC COUNS, V75, P91, DOI 10.1016/j.pec.2008.09.014

Dennis CL, 2003, INT J NURS STUD, V40, P321, DOI 10.1016/S0020-7489(02)00092-5

Field A, 2005, DISCOVERING STAT USI, P428

Glasgow RE, 2003, PREV MED, V36, P410, DOI 10.1016/S0091-7435(02)00056-7

Heijmans M, 1999, PSYCHOL HEALTH, V14, P451, DOI 10.1080/08870449908407340

Kalo I, 2003, DIABETOLOGIA, V46, pA88

Kotani Kazuhiko, 2004, Aust J Rural Health, V12, P251, DOI 10.1111/j.1440-1854.2004.00626.x

Lorig K, 2009, DIABETES EDUCATOR, V35, P641, DOI 10.1177/0145721709335006

Newman S, 2004, LANCET, V364, P1523, DOI 10.1016/S0140-6736(04)17277-2

Paul GM, 2007, BMC FAM PRACT, V8, DOI 10.1186/1471-2296-8-45

Pieper B A, 1990, Diabetes Educ, V16, P108, DOI 10.1177/014572179001600206

POLONSKY WH, 1995, DIABETES CARE, V18, P754, DOI 10.2337/diacare.18.6.754

RADLOFF L S, 1977, Applied Psychological Measurement, V1, P385, DOI 10.1177/014662167700100306

RUTTEN GEH, 2006, HUISARTS WET, V49, P137

Snoek FJ, 2000, DIABETES CARE, V23, P1305, DOI 10.2337/diacare.23.9.1305

Thoolen B., 2008, HLTH PSYCHOL REV, V2, P65, DOI [10.1080/17437190802311361, DOI 10.1080/17437190802311361]

Thoolen B, 2007, DIABETES CARE, V30, P2832, DOI 10.2337/dc07-0777

Trief PM, 2002, DIABETES CARE, V25, P1154, DOI 10.2337/diacare.25.7.1154

Trief PM, 2001, DIABETES CARE, V24, P1384, DOI 10.2337/diacare.24.8.1384

van Assema P, 2001, J HUM NUTR DIET, V14, P377, DOI 10.1046/j.1365-277X.2001.00310.x

van Assema P, 1992, Eur J Cancer Prev, V1, P375, DOI 10.1097/00008469-199208000-00006

van der Bijl J, 1999, J ADV NURS, V30, P352, DOI 10.1046/j.1365-2648.1999.01077.x

VanWormer JJ, 2004, DIABETES EDUCATOR, V30, P404, DOI 10.1177/014572170403000309

WASHBURN RA, 1993, J CLIN EPIDEMIOL, V46, P153, DOI 10.1016/0895-4356(93)90053-4

Welch G, 1994, HDB PSYCHOL DIABETES, P391

Welch GW, 1997, DIABETES CARE, V20, P760, DOI 10.2337/diacare.20.5.760

WHO Collaborating Center for Mental Health, WHO 5 WELL BEING IND

NR 31

TC 27

Z9 27

U1 0

U2 28

PU WILEY

PI HOBOKEN

PA 111 RIVER ST, HOBOKEN 07030-5774, NJ USA

SN 0742-3071

EI 1464-5491

J9 DIABETIC MED

JI Diabetic Med.

PD OCT

PY 2012

VL 29

IS 10

BP e390

EP e397

DI 10.1111/j.1464-5491.2012.03629.x

PG 8

WC Endocrinology & Metabolism

SC Endocrinology & Metabolism

GA 008MF

UT WOS:000308960400006

PM 22414198

DA 2019-01-08

ER

PT J

AU Slean, GR

Jacobs, EA

Lahiff, M

Fisher, L

Fernandez, A

AF Slean, Geraldine Rosaura

Jacobs, Elizabeth A.

Lahiff, Maureen

Fisher, Lawrence

Fernandez, Alicia

TI Aspects of Culturally Competent Care are Associated With Less Emotional

Burden Among Patients With Diabetes

SO MEDICAL CARE

LA English

DT Article

DE emotional burden; diabetes distress; doctor-patient relationship;

doctor-patient communication; culturally competent care; trust;

minority; Hispanic/Latino; African American

ID DEPRESSIVE SYMPTOMS; DISTRESS; COMMUNICATION; RACE/ETHNICITY; LANGUAGE

AB Background: Diabetes distress is associated with poor diabetes self-management and worse clinical outcomes. Whether there is an association between patient experience of health care and degree of emotional burden (EB) of diabetes distress is not known.

Objective: To investigate whether aspects of culturally competent care are associated with the EB of diabetes distress.

Design: Cross-sectional survey consisting of face-to-face interviews.

Subjects: A total of 502 ethnically diverse patients with diabetes receiving care in safety-net clinics in 2 cities.

Measures: The main outcome measure was high EB on the Diabetes Distress Scale (mean score >= 3). The predictors were 3 domains (Doctor Communication-Positive Behaviors, Trust, and Doctor Communication-Health Promotion) of the Consumer Assessment of Healthcare Providers and Systems-Cultural Competence (Consumer Assessments of Healthcare Providers and Systems' Cultural Competence Item Set) instrument.

Results: Of 502 patients, 263 (52%) reported high EB. In adjusted logistic regressions controlling for sociodemographic and clinical factors, patient report of optimal Doctor Communication-Positive Behaviors (adjusted odds ratio, 0.46; 95% confidence interval, 0.39-0.54), and optimal Trust (adjusted odds ratio, 0.65; 95% confidence interval, 0.54-0.78) were associated with lower EB. Doctor Communication-Health Promotion Communication was not associated with EB.

Discussion: Patient report of better doctor communication behavior and higher trust in physician are inversely associated with high EB among patients with diabetes. Further research should determine whether interventions improving patient physician communication and trust can lower the EB of diabetes.

C1 [Slean, Geraldine Rosaura] Univ Calif San Francisco, Joint Med Program, San Francisco, CA 94143 USA.

[Slean, Geraldine Rosaura] Univ Calif Berkeley, Joint Med Program, Berkeley, CA 94720 USA.

[Jacobs, Elizabeth A.] Univ Wisconsin, Sch Med & Publ Hlth, Dept Med, Madison, WI 53706 USA.

[Jacobs, Elizabeth A.] Univ Wisconsin, Sch Med & Publ Hlth, Dept Populat Hlth Sci, Madison, WI 53706 USA.

[Lahiff, Maureen] Univ Calif Berkeley, Sch Publ Hlth, Berkeley, CA 94720 USA.

[Fisher, Lawrence] Univ Calif San Francisco, Dept Family & Community Med & Psychiat, San Francisco, CA 94143 USA.

[Fernandez, Alicia] Univ Calif San Francisco, Dept Internal Med, San Francisco Gen Hosp, San Francisco, CA 94143 USA.

RP Fernandez, A (reprint author), Univ Calif San Francisco, Dept Internal Med, San Francisco Gen Hosp, POB 1364, San Francisco, CA 94143 USA.

EM afernandez@medsfgh.ucsf.edu

OI Slean, Geraldine/0000-0003-2859-5723

FU Russell Sage Foundation; Commonwealth Fund; Arnold P. Gold Foundation;

UCSF Dean's Summer Research Fellowship Program; UCB-UCSF Smith Thesis

Research Fellowship Program

FX Supported by Russell Sage Foundation, Commonwealth Fund, Arnold P. Gold

Foundation, UCSF Dean's Summer Research Fellowship Program, and UCB-UCSF

Smith Thesis Research Fellowship Program.

CR Aikens JE, 2005, DIABETES EDUCATOR, V31, P681, DOI 10.1177/0145721705280829

Doescher MP, 2000, ARCH FAM MED, V9, P1156, DOI 10.1001/archfami.9.10.1156

Fernandez A, 2004, J GEN INTERN MED, V19, P167, DOI 10.1111/j.1525-1497.2004.30266.x

Fernandez A, 2011, J GEN INTERN MED, V26, P170, DOI 10.1007/s11606-010-1507-6

Fisher L, 2008, DIABETIC MED, V25, P1096, DOI 10.1111/j.1464-5491.2008.02533.x

Fisher L, 2009, DIABETIC MED, V26, P622, DOI 10.1111/j.1464-5491.2009.02730.x

Fisher L, 2008, ANN FAM MED, V6, P246, DOI 10.1370/afm.842

Fisher L, 2007, DIABETES CARE, V30, P542, DOI 10.2337/dc06-1614

Fisher L, 2010, DIABETES CARE, V33, P23, DOI 10.2337/dc09-1238

Johnson RL, 2004, AM J PUBLIC HEALTH, V94, P2084, DOI 10.2105/AJPH.94.12.2084

Kroenke K, 2001, J GEN INTERN MED, V16, P606, DOI 10.1046/j.1525-1497.2001.016009606.x

Morales LS, 1999, J GEN INTERN MED, V14, P409, DOI 10.1046/j.1525-1497.1999.06198.x

National Quality Forum (NQF), 2009, COMPR FRAM PREF PRAC

Peek ME, 2009, J GEN INTERN MED, V24, P1135, DOI 10.1007/s11606-009-1047-0

Pibernik-Okanovic M, 2008, DIABETIC MED, V25, P1252, DOI 10.1111/j.1464-5491.2008.02553.x

Polonsky WH, 2005, DIABETES CARE, V28, P626, DOI 10.2337/diacare.28.3.626

Pouwer F, 2005, DIABETES RES CLIN PR, V70, P166, DOI 10.1016/j.diabres.2005.03.031

Pouwer F, 2009, NAT REV ENDOCRINOL, V5, P665, DOI 10.1038/nrendo.2009.214

Rabi DM, 2006, BMC HEALTH SERV RES, V6, DOI 10.1186/1472-6963-6-124

Rivadeneyra R, 2000, AM J MED, V108, P470, DOI 10.1016/S0002-9343(99)00445-3

Schenker Y, 2010, PATIENT EDUC COUNS, V81, P222, DOI 10.1016/j.pec.2010.02.005

Schenker Y, 2009, J GEN INTERN MED, V24, P550, DOI 10.1007/s11606-009-0937-5

Spencer MS, 2006, J HEALTH CARE POOR U, V17, P88, DOI 10.1353/hpu.2006.0095

Spitzer RL, 1999, JAMA-J AM MED ASSOC, V282, P1737, DOI 10.1001/jama.282.18.1737

Stern RJ, 2012, MED CARE, V50, pS49, DOI 10.1097/MLR.0b013e31826410fb

Swenson SL, 2008, MED CARE, V46, P257, DOI 10.1097/MLR.0b013e31816080e9

Weech-Maldonado R, 2003, HEALTH SERV RES, V38, P789, DOI 10.1111/1475-6773.00147

Weech-Maldonado R, 2012, MED CARE, V50, pS22, DOI 10.1097/MLR.0b013e318263134b

Zweifler J, 2010, INT J EQUITY HEALTH, V9, DOI 10.1186/1475-9276-9-4

NR 29

TC 12

Z9 12

U1 0

U2 15

PU LIPPINCOTT WILLIAMS & WILKINS

PI PHILADELPHIA

PA 530 WALNUT ST, PHILADELPHIA, PA 19106-3621 USA

SN 0025-7079

J9 MED CARE

JI Med. Care

PD SEP

PY 2012

VL 50

IS 9

SU 2

BP S69

EP S73

DI 10.1097/MLR.0b013e3182641127

PG 5

WC Health Care Sciences & Services; Health Policy & Services; Public,

Environmental & Occupational Health

SC Health Care Sciences & Services; Public, Environmental & Occupational

Health

GA 080IO

UT WOS:000314235300011

PM 22895234

OA Bronze

DA 2019-01-08

ER

PT J

AU Cleator, J

Abbott, J

Judd, P

Sutton, C

Wilding, JPH

AF Cleator, J.

Abbott, J.

Judd, P.

Sutton, C.

Wilding, J. P. H.

TI Night eating syndrome: implications for severe obesity

SO NUTRITION & DIABETES

LA English

DT Review

DE obesity; night eating; sleep; circadian rhythm

ID OBSTRUCTIVE SLEEP-APNEA; BODY-MASS INDEX; WEIGHT-LOSS;

GENERAL-POPULATION; BARIATRIC SURGERY; METABOLIC SYNDROME;

BULIMIA-NERVOSA; MORBIDLY OBESE; ENERGY-INTAKE; FOOD-INTAKE

AB Night eating syndrome (NES) was first identified in 1955 by Stunkard, a psychiatrist specialising in eating disorders (ED). Over the last 20 years considerable progress has been made in defining NES as a significant clinical entity in its own right and it has now been accepted for inclusion in the fifth edition of the Diagnostic and Statistical Manual of Mental Disorders (DSM-5) due for publication in 2013. NES is considered a dysfunction of circadian rhythm with a disassociation between eating and sleeping. Core criteria include a daily pattern of eating with a significantly increased intake in the evening and/or night time, as manifested by one or both of the following: at least 25% of food intake is consumed after the evening meal or at least two episodes of nocturnal eating per week. An important recent addition to core criteria includes the presence of significant distress and/or impairment in functioning. Stunkard's team recommend further investigation on the pathogenesis of NES, in particular its relationship with traumatic life events, psychiatric comorbidity, the age of onset of NES and course of NES over time. The relationship between NES and other ED also requires further clarification as night-eaters exhibit some features of other ED; previous guidance to separate NES from other ED may have hindered earlier characterisation of NES. Evidence from European and American studies suggests NES features strongly in populations with severe obesity. The complex interplay between depression, impaired sleep and obesity-related comorbidity in severely obese individuals makes understanding NES in this context even more difficult. This review examines evidence to date on the characterisation of NES and concludes by examining the applicability of current NES criteria to individuals with severe obesity. Nutrition and Diabetes (2012) 2, e44; doi:10.1038/nutd.2012.16; published online 10 September 2012

C1 [Cleator, J.; Wilding, J. P. H.] Univ Liverpool, Dept Obes & Endocrinol, Liverpool L69 3BX, Merseyside, England.

[Abbott, J.] Univ Cent Lancashire, Sch Psychol, Preston PR1 2HE, Lancs, England.

[Judd, P.; Sutton, C.] Univ Cent Lancashire, Sch Hlth, Preston PR1 2HE, Lancs, England.

RP Cleator, J (reprint author), Univ Manchester, Sch Nursing Midwifery & Social Work, Jean McFarlane Bldg,Oxford Rd, Manchester M13 9PL, Lancs, England.

EM jacqueline.cleator@manchester.ac.uk

RI Wilding, John/A-7106-2008

OI Wilding, John/0000-0003-2839-8404; Sutton, Chris/0000-0002-6406-1318;

Cleator, Jacqueline/0000-0002-0313-101X

FU Roche Products Limited

FX Professor Wilding received an educational grant from Roche Products

Limited to support the study fees of Jacqueline Cleator while she

conducted research into NES at the University of Liverpool, UK.

Professors Abbott and Judd and Dr Sutton have no relevant conflict of

interest to declare.

CR Adami GF, 2002, NUTRITION, V18, P587, DOI 10.1016/S0899-9007(02)00761-X

Adami GF, 1999, INT J EAT DISORDER, V25, P335, DOI 10.1002/(SICI)1098-108X(199904)25:3<335::AID-EAT12>3.0.CO;2-1

Allison K. C., 2004, OVERCOMING NIGHT EAT

Allison KC, 2005, J CLIN ENDOCR METAB, V90, P6214, DOI 10.1210/jc.2005-1018

Allison KC, 2005, J CONSULT CLIN PSYCH, V73, P1107, DOI 10.1037/0022-006X.73.6.1107

Allison Kelly C, 2008, Eat Behav, V9, P62, DOI 10.1016/j.eatbeh.2007.03.007

Allison KC, 2007, BEHAV RES THER, V45, P2874, DOI 10.1016/j.brat.2007.05.007

Allison Kelly C, 2010, Am J Psychother, V64, P91

Allison KC, 2010, INT J EAT DISORDER, V43, P241, DOI 10.1002/eat.20693

Allison Kelly C, 2008, Eat Behav, V9, P398, DOI 10.1016/j.eatbeh.2008.04.004

Allison KC, 2006, OBESITY, V14, p77S, DOI 10.1038/oby.2006.286

Andersen GS, 2004, INT J OBESITY, V28, P1338, DOI 10.1038/sj.ijo.0802731

Aronoff NJ, 2001, J AM DIET ASSOC, V101, P102, DOI 10.1016/S0002-8223(01)00022-0

Atlantis E, 2008, INT J OBESITY, V32, P881, DOI 10.1038/ijo.2008.54

Birketvedt GS, 1999, JAMA-J AM MED ASSOC, V282, P657, DOI 10.1001/jama.282.7.657

Birketvedt GS, 2002, AM J PHYSIOL-ENDOC M, V282, pE366, DOI 10.1152/ajpendo.00251.2001

Bixler EO, 2005, J CLIN ENDOCR METAB, V90, P4510, DOI 10.1210/jc.2005-0035

Blaine B, 2008, J HEALTH PSYCHOL, V13, P1190, DOI 10.1177/1359105308095977

Boston RC, 2008, AM J CLIN NUTR, V87, P1672

Bower B, 2010, J SLEEP RES, V19, P323, DOI 10.1111/j.1365-2869.2009.00816.x

Busetto L, 2005, OBES SURG, V15, P195, DOI 10.1381/0960892053268327

Calugi S, 2009, INT J OBESITY, V33, P899, DOI 10.1038/ijo.2009.105

Ceru-Bjork C, 2001, INT J OBESITY, V25, P365, DOI 10.1038/sj.ijo.0801552

Chaput JP, 2005, PHYSIOL BEHAV, V86, P224, DOI 10.1016/j.physbeh.2005.07.014

Cizza G, 2005, SLEEP, V28, P1217, DOI 10.1093/sleep/28.10.1217

Colles SL, 2007, INT J OBESITY, V31, P1722, DOI 10.1038/sj.ijo.0803664

Colles SL, 2008, OBESITY, V16, P615, DOI 10.1038/oby.2007.101

Colles SL, 2008, OBESITY, V16, P608, DOI 10.1038/oby.2007.99

Cooper-Kazaz R, 2012, J CLIN PSYCHOPHARM, V32, P143, DOI 10.1097/JCP.0b013e31823f9051

Coughlin SR, 2004, EUR HEART J, V25, P735, DOI 10.1016/j.ehj.2004.02.021

Dalgleish T, 2001, PERS INDIV DIFFER, V31, P453, DOI 10.1016/S0191-8869(00)00150-1

Dalle GR, 2011, INT J EAT DISORDER, V44, P150, DOI DOI 10.1002/EAT.20786

de Zwaan M, 2006, INT J EAT DISORDER, V39, P224, DOI 10.1002/eat.20246

Devlin MJ, 2004, OBES SURG, V14, P1252, DOI 10.1381/0960892042386940

Dixon JB, 2003, ARCH INTERN MED, V163, P2058, DOI 10.1001/archinte.163.17.2058

Drevets WC, 2001, CURR OPIN NEUROBIOL, V11, P240, DOI 10.1016/S0959-4388(00)00203-8

EHLERS CL, 1988, ARCH GEN PSYCHIAT, V45, P948

FAIRBURN CG, 1990, AM J PSYCHIAT, V147, P401

Friedman S, 2004, CAN J PSYCHIAT, V49, P790

Friedman S, 2006, APPETITE, V47, P119, DOI 10.1016/j.appet.2006.03.159

Gangwisch JE, 2005, SLEEP, V28, P1289, DOI 10.1093/sleep/28.10.1289

Garaulet M, 2011, PLOS ONE, V6, DOI 10.1371/journal.pone.0017435

Geliebter A, 2002, APPETITE, V39, P175, DOI 10.1006/appe.2001.0472

Gluck ME, 2001, OBES RES, V9, P264, DOI 10.1038/oby.2001.31

Goel N, 2009, J BIOL RHYTHM, V24, P85, DOI 10.1177/0748730408328914

Goldschmidt AB, 2008, INT J EAT DISORDER, V41, P153, DOI 10.1002/eat.20481

Goncalves MD, 2009, EUR EAT DISORD REV, V17, P184, DOI 10.1002/erv.918

GREENO CG, 1995, INT J EAT DISORDER, V18, P343, DOI 10.1002/1098-108X(199512)18:4<343::AID-EAT2260180407>3.0.CO;2-P

Grilo CM, 2004, BEHAV RES THER, V42, P397, DOI 10.1016/S0005-7967(03)00148-7

Hasler G, 2004, SLEEP, V27, P661, DOI 10.1093/sleep/27.4.661

HEALEY ES, 1981, PSYCHOSOM MED, V43, P439

Hildebrandt T, 2006, EUR EAT DISORD REV, V14, P17, DOI 10.1002/erv.667

Huang CJ, 2010, INT J OBESITY, V34, P720, DOI 10.1038/ijo.2009.285

International Classification of Sleep Disorders, 2005, DIAGN COD MAN

Jennings JR, 2007, SLEEP, V30, P219, DOI 10.1093/sleep/30.2.219

Karp JF, 2005, J CLIN PSYCHIAT, V66, P591, DOI 10.4088/JCP.v66n0508

Knutson KL, 2006, ARCH INTERN MED, V166, P1768, DOI 10.1001/archinte.166.16.1768

Kretsch MJ, 1999, J AM DIET ASSOC, V99, P300, DOI 10.1016/S0002-8223(99)00078-4

KULDAU JM, 1986, INT J EAT DISORDER, V5, P143, DOI 10.1002/1098-108X(198601)5:1<143::AID-EAT2260050114>3.0.CO;2-T

Lamerz A, 2005, J CHILD PSYCHOL PSYC, V46, P385, DOI 10.1111/j.1469-7610.2004.00363.x

Latner JD, 2007, BEHAV RES THER, V45, P2203, DOI 10.1016/j.brat.2006.12.002

Latner JD, 2004, OBES RES, V12, P956, DOI 10.1038/oby.2004.117

Leskela US, 2004, J NERV MENT DIS, V192, P373, DOI 10.1097/01.nmd.0000126705.15497.c9

LICHTMAN SW, 1992, NEW ENGL J MED, V327, P1893, DOI 10.1056/NEJM199212313272701

Lundgren JD, 2006, AM J PSYCHIAT, V163, P156, DOI 10.1176/appi.ajp.163.1.156

Lundgren JD, 2008, PSYCHIAT RES-NEUROIM, V162, P214, DOI 10.1016/j.pscychresns.2007.07.006

Lundgren JD, 2006, INT J EAT DISORDER, V39, P516, DOI 10.1002/eat.20269

Lundgren JD, 2011, PSYCHIAT RES, V186, P103, DOI 10.1016/j.psychres.2010.08.008

Lundgren JD, 2010, PSYCHIAT RES, V175, P233, DOI 10.1016/j.psychres.2008.10.027

Lundgren Jennifer D, 2008, Eat Behav, V9, P343, DOI 10.1016/j.eatbeh.2007.12.004

Marshall HM, 2004, INT J EAT DISORDER, V35, P217, DOI 10.1002/eat.10241

Miyaoka T, 2003, INT CLIN PSYCHOPHARM, V18, P175, DOI 10.1097/01.yic.0000068440.56680.8e

Morse SA, 2006, DIABETES CARE, V29, P1800, DOI 10.2337/dc06-0315

Napolitano MA, 2001, INT J EAT DISORDER, V30, P193, DOI 10.1002/eat.1072

Nordin M, 2005, J OCCUP HEALTH PSYCH, V10, P54, DOI 10.1037/1076-8998.10.1.54

O'Brien Bridget, 2005, Ir Med J, V98, P250

O'Reardon JP, 2006, AM J PSYCHIAT, V163, P893, DOI 10.1176/appi.ajp.163.5.893

O'Reardon JP, 2004, OBES RES, V12, P1789, DOI 10.1038/oby.2004.222

O'Reardon JP, 2004, INT J EAT DISORDER, V35, P16, DOI 10.1002/eat.10224

Olbrich K, 2009, EUR EAT DISORD REV, V17, P120, DOI 10.1002/erv.908

Palmese LB, 2011, SCHIZOPHR RES, V133, P238, DOI 10.1016/j.schres.2011.07.030

Patel SR, 2008, INT J OBESITY, V32, P1825, DOI 10.1038/ijo.2008.198

Pawlow LA, 2003, INT J OBESITY, V27, P970, DOI 10.1038/sj.ijo.0802320

Powers PS, 1999, INT J EAT DISORDER, V25, P293, DOI 10.1002/(SICI)1098-108X(199904)25:3<293::AID-EAT7>3.0.CO;2-G

RAND CSW, 1993, INT J OBESITY, V17, P657

RAND CSW, 1986, INT J EAT DISORDER, V5, P75, DOI 10.1002/1098-108X(198601)5:1<75::AID-EAT2260050107>3.0.CO;2-V

Rand CSW, 1997, INT J EAT DISORDER, V22, P65

Rogers NL, 2006, SLEEP, V29, P814, DOI 10.1093/sleep/29.6.814

Root TL, 2010, EAT BEHAV, V11, P92, DOI 10.1016/j.eatbeh.2009.10.004

Rosmond R, 2004, MED HYPOTHESES, V62, P976, DOI 10.1016/j.mehy.2003.12.030

Sassaroli Sandra, 2009, Eat Disord, V17, P140, DOI 10.1080/10640260802714597

SPAGGIARI MC, 1994, SLEEP, V17, P339, DOI 10.1093/sleep/17.4.339

Striegel-Moore RH, 2006, OBESITY, V14, P139, DOI 10.1038/oby.2006.17

Striegel-Moore RH, 2005, INT J EAT DISORDER, V37, P200, DOI 10.1002/eat.20128

Striegel-Moore RH, 2008, INT J EAT DISORDER, V41, P411, DOI 10.1002/eat.20514

Striegel-Moore RH, 2010, INT J EAT DISORDER, V43, P520, DOI 10.1002/eat.20735

Stunkard A, 2010, EAT WEIGHT DISORD-ST, V15, pE2, DOI 10.1007/BF03325274

Stunkard A, 1996, INT J OBESITY, V20, P1

STUNKARD AJ, 1955, AM J MED, V19, P78, DOI 10.1016/0002-9343(55)90276-X

Stunkard AJ, 2003, INT J OBESITY, V27, P1, DOI 10.1038/sj.ijo.0802186

Stunkard A, 2008, AM J PSYCHIAT, V165, P424, DOI 10.1176/appi.ajp.2007.07081351

Taheri S, 2006, ARCH DIS CHILD, V91, P881, DOI 10.1136/adc.2005.093013

Taheri S, 2004, PLOS MED, V1, P210, DOI 10.1371/journal.pmed.0010062

Takeda Eiji, 2004, J Med Invest, V51, P139, DOI 10.2152/jmi.51.139

Telch CF, 1998, INT J EAT DISORDER, V24, P313, DOI 10.1002/(SICI)1098-108X(199811)24:3<313::AID-EAT9>3.0.CO;2-P

Tholin S, 2009, OBESITY, V17, P1050, DOI 10.1038/oby.2008.676

Tzischinsky O, 2004, EUR EAT DISORD REV, V12, P101, DOI 10.1002/erv.538

Vetrugno R, 2006, SLEEP, V29, P949, DOI 10.1093/sleep/29.7.949

Vorona RD, 2005, ARCH INTERN MED, V165, P25, DOI 10.1001/archinte.165.1.25

Whitaker RC, 1997, NEW ENGL J MED, V337, P869, DOI 10.1056/NEJM199709253371301

Winkelman JW, 2003, SLEEP MED, V4, P243, DOI 10.1016/S1389-9457(03)00060-1

Youngstedt SD, 2004, SLEEP MED REV, V8, P159, DOI 10.1016/j.smrv.2003.10.002

NR 112

TC 22

Z9 22

U1 0

U2 23

PU NATURE PUBLISHING GROUP

PI LONDON

PA MACMILLAN BUILDING, 4 CRINAN ST, LONDON N1 9XW, ENGLAND

SN 2044-4052

J9 NUTR DIABETES

JI Nutr. Diabetes

PD SEP

PY 2012

VL 2

AR e44

DI 10.1038/nutd.2012.16

PG 10

WC Endocrinology & Metabolism; Nutrition & Dietetics

SC Endocrinology & Metabolism; Nutrition & Dietetics

GA 084GW

UT WOS:000314527700002

PM 23446659

OA DOAJ Gold, Green Published

DA 2019-01-08

ER

PT J

AU Fritschi, C

Quinn, L

Hacker, ED

Penckofer, SM

Wang, E

Foreman, M

Ferrans, CE

AF Fritschi, Cynthia

Quinn, Laurie

Hacker, Eileen D.

Penckofer, Sue M.

Wang, Edward

Foreman, Marquis

Ferrans, Carol E.

TI Fatigue in Women With Type 2 Diabetes

SO DIABETES EDUCATOR

LA English

DT Article

ID PHYSICAL-ACTIVITY; GLYCEMIC CONTROL; AFRICAN-AMERICANS; BLOOD-PRESSURE;

SYMPTOMS; GLUCOSE; ADULTS; PEOPLE; SAMPLE; RISK

AB Purpose

The purpose of this study was to explore the relationship between fatigue and physiological, psychological, and lifestyle phenomena in women with type 2 diabetes (T2DM) in order to establish the magnitude and correlates of fatigue in women with T2DM and explore the interrelationships between fatigue and specific diabetes-related factors that may be associated with increased levels of fatigue. These factors included physiological factors (glucose control, diabetes symptoms), psychological factors (diabetes emotional distress, depressive symptoms in general), and lifestyle factors (body mass index, physical activity).

Methods

A cross-sectional, descriptive design was used. Women who reported conditions known to cause fatigue were excluded. Physiological measures included fasting blood glucose (FBG), hemoglobin A1C (A1C), glucose variability, and body mass index (BMI). Women completed questionnaires about health, fatigue levels, diabetes symptoms, diabetes emotional distress, depressive symptoms, physical activity, and current diabetes self-care practices. A subset of the women wore a Medtronic Gold CGM sensor for 3 days for assessment of glucose variability.

Results

Eighty-three women aged 40 to 65 years with T2DM completed the study. Fatigue was significantly related to diabetes symptoms, diabetes emotional distress, depressive symptoms, higher BMI, and reduced physical activity. There was no relationship between fatigue and FBG or A1C. The strongest explanatory factors for fatigue were diabetes symptoms, depressive symptoms, and BMI, which accounted for 48% of the variance in fatigue scores. Glucose variability was not significantly associated with fatigue in these women.

Conclusions

Fatigue is a persistent clinical complaint among women with T2DM and may signal the presence of physiological, psychological, and lifestyle-related phenomena that could undermine diabetes health outcomes.

C1 [Fritschi, Cynthia; Quinn, Laurie; Hacker, Eileen D.; Wang, Edward; Ferrans, Carol E.] Univ Illinois, Coll Nursing, Dept Biobehav Hlth Sci, Chicago, IL 60612 USA.

[Penckofer, Sue M.] Loyola Univ, Marcella Niehoff Sch Nursing, Chicago, IL 60611 USA.

[Foreman, Marquis] Rush Univ, Coll Nursing, Chicago, IL 60612 USA.

RP Fritschi, C (reprint author), Univ Illinois, Coll Nursing, Dept Biobehav Hlth Sci, MC 802,845 S Damen Ave, Chicago, IL 60612 USA.

EM Fritschi@uic.edu

OI Fritschi, Cynthia/0000-0001-5447-8315

FU National Institutes of Health/National Institute for Nursing research

[F31 NR009751]

FX This work was supported in part by the National Institutes of

Health/National Institute for Nursing research F31 NR009751. The authors

thank Kevin Grandfield, Publication Manager for the UIC Department of

Biobehavioral Health Science, for editorial assistance.

CR Amer Diabet Assoc, 2008, DIABETES CARE, V31, pS55, DOI 10.2337/dc08-S055

Anderson RJ, 2001, DIABETES CARE, V24, P1069, DOI 10.2337/diacare.24.6.1069

Bonora E, 2001, DIABETES CARE, V24, P2023, DOI 10.2337/diacare.24.12.2023

Bonora E, 2001, DIABETOLOGIA, V44, P2107, DOI 10.1007/s001250100020

Bulpitt CJ, 1998, DIABETES CARE, V21, P111, DOI 10.2337/diacare.21.1.111

Casalenuovo GA, 2002, FATIGUE DIABETES MEL

Cavelti-Weder Claudia, 2011, Diabetes Care, V34, pe158, DOI 10.2337/dc11-1196

Colberg SR, 2010, MED SCI SPORT EXER, V42, P2282, DOI 10.1249/MSS.0b013e3181eeb61c

de Sonnaville JJJ, 1998, DIABETES CARE, V21, P919, DOI 10.2337/diacare.21.6.919

Deshpande AD, 2005, DIABETES CARE, V28, P1012, DOI 10.2337/diacare.28.5.1012

Drivsholm T, 2005, DIABETOLOGIA, V48, P210, DOI 10.1007/s00125-004-1625-y

Fritschi C, 2010, J PSYCHOSOM RES, V69, P33, DOI 10.1016/j.jpsychores.2010.01.021

Fritschi C, 2010, DIABETES EDUCATOR, V36, P250, DOI 10.1177/0145721709355835

GROOTENHUIS PA, 1994, DIABETIC MED, V11, P253, DOI 10.1111/j.1464-5491.1994.tb00268.x

Gross T M, 2000, Diabetes Technol Ther, V2 Suppl 1, pS19

Hays LM, 1999, DIABETES CARE, V22, P706, DOI 10.2337/diacare.22.5.706

Hume C, 2010, DIABETES EDUCATOR, V36, P495, DOI 10.1177/0145721710368326

Kennedy G, 2005, FREE RADICAL BIO MED, V39, P584, DOI 10.1016/j.freeradbiomed.2005-04.020

Kovatchev B., 2003, Journal of Applied Research, V3, P449

Lim K, 2005, PREV MED, V40, P33, DOI 10.1016/j.ypmed.2004.04.046

Lim WJ, 2005, ARCH INTERN MED, V165, P910, DOI 10.1001/archinte.165.8.910

Lin Jin-Mann S, 2009, Popul Health Metr, V7, P18, DOI 10.1186/1478-7954-7-18

McArdle W. D., 2001, EXERCISE PHYSL, P752

McDonnell C M, 2005, Diabetes Technol Ther, V7, P253, DOI 10.1089/dia.2005.7.253

McNair D, 1992, MANUAL PROFILE MOOD

Morrato EH, 2007, DIABETES CARE, V30, P203, DOI 10.2337/dc06-1128

Nielsen ABS, 2011, SCAND J PRIM HEALTH, V29, P157, DOI 10.3109/02813432.2011.585542

Pearte CA, 2004, ETHNIC DIS, V14, P198

Plotnikoff RC, 2006, MED SCI SPORT EXER, V38, P1526, DOI 10.1249/01.mss.0000228937.86539.95

Polonsky WH, 2005, DIABETES CARE, V28, P626, DOI 10.2337/diacare.28.3.626

Puetz TW, 2006, SPORTS MED, V36, P767, DOI 10.2165/00007256-200636090-00004

REGENSTEINER JG, 1995, MED SCI SPORT EXER, V27, P874

Resnick HE, 2006, J CLIN SLEEP MED, V2, P163

Sachedina N, 2003, DIABETIC MED, V20, P1012, DOI 10.1046/j.1464-5491.2003.01037.x

Sallis JF, 1999, RES Q EXERCISE SPORT, V70, P127, DOI 10.1080/02701367.1999.10608030

SALLIS JF, 1985, AM J EPIDEMIOL, V121, P91, DOI 10.1093/oxfordjournals.aje.a113987

Schwarz R, 2003, ONKOLOGIE, V26, P140, DOI 10.1159/000069834

SMETS EMA, 1995, J PSYCHOSOM RES, V39, P315, DOI 10.1016/0022-3999(94)00125-O

Sommerfield AJ, 2004, DIABETES CARE, V27, P2335, DOI 10.2337/diacare.27.10.2335

St John A, 2006, CLIN CHIM ACTA, V365, P257, DOI 10.1016/j.cca.2005.09.003

STEWART AL, 1994, J CLIN EPIDEMIOL, V47, P719, DOI 10.1016/0895-4356(94)90169-4

Tamborlane WV, 2005, PEDIATR DIABETES, V6, P13

Theorell-Haglow J, 2006, SLEEP, V29, P751, DOI 10.1093/sleep/29.6.751

Thomas LE, 2008, DIABETES TECHNOL THE, V10, P102, DOI 10.1089/dia.2007.0244

Thomas N, 2004, POSTGRAD MED J, V80, P287, DOI 10.1136/pgmj.2003.010553

Van der Does FEE, 1998, DIABETES CARE, V21, P2085, DOI 10.2337/diacare.21.12.2085

VanderDoes FEE, 1996, DIABETES CARE, V19, P204, DOI 10.2337/diacare.19.3.204

Warren RE, 2003, DIABETES-METAB RES, V19, P408, DOI 10.1002/dmrr.396

Weijman I, 2004, J OCCUP ENVIRON MED, V46, P828, DOI 10.1097/01.jom.0000135605.62330.ca

Weijman I, 2003, OCCUP ENVIRON MED, V60, P93

Wenzel J, 2005, DIABETES EDUCATOR, V31, P98, DOI 10.1177/0145721704273242

Wijeratne C, 2007, J PSYCHOSOM RES, V62, P153, DOI 10.1016/j.jpsychores.2006.09.011

Wood FG, 2004, J ADV NURS, V45, P190, DOI 10.1046/j.1365-2648.2003.02880.x

NR 53

TC 18

Z9 18

U1 0

U2 11

PU SAGE PUBLICATIONS INC

PI THOUSAND OAKS

PA 2455 TELLER RD, THOUSAND OAKS, CA 91320 USA

SN 0145-7217

EI 1554-6063

J9 DIABETES EDUCATOR

JI Diabetes Educ.

PD SEP-OCT

PY 2012

VL 38

IS 5

BP 662

EP 672

DI 10.1177/0145721712450925

PG 11

WC Endocrinology & Metabolism; Public, Environmental & Occupational Health

SC Endocrinology & Metabolism; Public, Environmental & Occupational Health

GA 038WM

UT WOS:000311204800006

PM 22713262

OA Green Accepted

DA 2019-01-08

ER

PT J

AU Cha, E

Clark, PC

Reilly, CM

Higgins, M

Lobb, M

Smith, AL

Dunbar, SB

AF Cha, EunSeok

Clark, Patricia C.

Reilly, Carolyn Miller

Higgins, Melinda

Lobb, Maureen

Smith, Andrew L.

Dunbar, Sandra B.

TI Educational Needs for Improving Self-Care in Heart Failure Patients With

Diabetes

SO DIABETES EDUCATOR

LA English

DT Article

ID 6-MINUTE WALK; HYPERTENSION; DEPRESSION; MELLITUS; OUTCOMES;

DYSFUNCTION; MECHANISMS; MANAGEMENT; MORBIDITY; MORTALITY

AB Purpose

To explore the need for self-monitoring and self-care education in heart failure patients with diabetes (HF-DM patients) by describing cognitive and affective factors to provide guidance in developing effective self-management education.

Methods

A cross-sectional correlation design was employed using baseline patient data from a study testing a 12-week patient and family dyad intervention to improve dietary and medication-taking self-management behaviors in HF patients. Data from 116 participants recruited from metropolitan Atlanta area were used. Demographic and comorbidities, physical function, psychological distress, relationship with health care provider, self-efficacy (medication taking and low sodium diet), and behavioral outcomes (medications, dietary habits) were assessed. Descriptive statistics and a series of chi-square tests, t tests, or Mann-Whitney tests were performed to compare HF patients with and without DM.

Results

HF-DM patients were older and heavier, had more comorbidities, and took more daily medications than HF patients. High self-efficacy on medication and low-sodium diet was reported in both groups with no significant difference. Although HF-DM patients took more daily medications than HF, both groups exhibited high HF medication-taking behaviors. The HF-DM patients consumed significantly lower total sugar than HF patients but clinically higher levels of sodium.

Conclusions

Diabetes educators need to be aware of potential conflicts of treatment regimens to manage 2 chronic diseases. Special and integrated diabetes self-management education programs that incorporate principles of HF self-management should be developed to improve self-management behavior in HF-DM patients.

C1 [Cha, EunSeok; Reilly, Carolyn Miller; Higgins, Melinda; Lobb, Maureen; Dunbar, Sandra B.] Emory Univ, Nell Hodgson Woodruff Sch Nursing, Atlanta, GA 30322 USA.

[Clark, Patricia C.] Georgia State Univ, Byrdine F Lewis Sch Nursing & Hlth Profess, Atlanta, GA 30303 USA.

[Smith, Andrew L.] Emory Univ, Sch Med, Atlanta, GA USA.

RP Cha, E (reprint author), Emory Univ, Nell Hodgson Woodruff Sch Nursing, 1520 Clifton Rd NE, Atlanta, GA 30322 USA.

EM echa5@emory.edu

RI Higgins, Melinda/B-6459-2013; Albertin, Paloma/X-1093-2018

OI Higgins, Melinda/0000-0001-6579-5885; Reilly,

Carolyn/0000-0003-1641-1747; cha, eunseok/0000-0001-6115-567X

FU Family Partnership Intervention for Heart Failure [R01 R08800]; National

Institute of Nursing Research [NIH 07/01/04-11/30/09]; PHS grant from

the General Clinical Research Center program, National Institutes of

Health, National Center for Research Resources [M01 RR0039]; PHS Grant

from the Clinical and Translational Science Award program, National

Institutes of Health, National Center for Research Resources [UL1

RR025008]; Nitromed; Atlanta Veteran's Administration Medical Center

FX This study was supported by A Family Partnership Intervention for Heart

Failure (R01 R08800), National Institute of Nursing Research, NIH

07/01/04-11/30/09, PI: S. Dunbar. The study was supported in part by PHS

grant M01 RR0039 from the General Clinical Research Center program,

National Institutes of Health, National Center for Research Resources,

and by PHS Grant UL1 RR025008 from the Clinical and Translational

Science Award program, National Institutes of Health, National Center

for Research Resources. The study was supported by Nitromed through an

unrestricted educational grant. We also acknowledge the Atlanta

Veteran's Administration Medical Center for their support.

CR Aguilar D, 2009, J AM COLL CARDIOL, V54, P422, DOI 10.1016/j.jacc.2009.04.049

*AM DIAB ASS, 2011, DIABETES CARE S1, V34, pS11, DOI DOI 10.2337/DC11-S011

American Diabetes Association, TOTAL PREVALENCE OF

American Heart Association, DIET AND LIFESTYLE R

ANDREN L, 1989, AM J MED, V87, pS39, DOI 10.1016/0002-9343(89)90494-4

Ashrafian H, 2007, CIRCULATION, V116, P434, DOI 10.1161/CIRCULATIONAHA.107.702795

Beck AT, 1996, J PERS ASSESS, V67, P588, DOI 10.1207/s15327752jpa6703_13

BITTNER V, 1993, JAMA-J AM MED ASSOC, V270, P1702, DOI 10.1001/jama.270.14.1702

Bodenheimer T, 2002, JAMA-J AM MED ASSOC, V288, P1775, DOI 10.1001/jama.288.14.1775

Boyle JP, 2010, POPULATION HLTH METR, V8

Brunzell JD, 2008, DIABETES CARE, V31, P811, DOI 10.2337/dc08-9018

Center for Disease Control and Prevention, 2009, OVERWEIGHT AND OBESI

CHARLSON ME, 1987, J CHRON DIS, V40, P373, DOI 10.1016/0021-9681(87)90171-8

Doran GT, 1981, MANAG REV, V71, P35

Dunbar SB, 2008, J CARDIOVASC NURS, V23, P258, DOI 10.1097/01.JCN.0000305093.20012.b8

Fujita T, 2007, NEPHROL DIAL TRANSPL, V22, P3102, DOI 10.1093/ndt/gfm409

Gerstein HC, 2011, NEW ENGL J MED, V364, P818, DOI 10.1056/NEJMoa1006524

Giamouzis G, 2010, J CARD FAIL, V16, P207, DOI 10.1016/j.cardfail.2009.12.007

Greenberg BH, 2007, AM HEART J, V154, DOI 10.1016/j.ahj.2007.05.001

Greenberg BH, 2007, AM HEART J, V154, P277

Gress TW, 2000, NEW ENGL J MED, V342, P905, DOI 10.1056/NEJM200003303421301

GUTIERREZ RC, 1999, CAN J CARDIOVASC NUR, V10, P29

GUYATT GH, 1984, THORAX, V39, P818, DOI 10.1136/thx.39.11.818

HealthyPeople. Gov, 2017, HLTH PEOPL 2020

Hill J. M., 2011, THESIS

Hunt SA, 2009, CIRCULATION, V119, pE391, DOI 10.1161/CIRCULATIONAHA.109.192065

Kamalesh M, 2005, INT J CARDIOL, V99, P125, DOI 10.1016/j.ijcard.2004.05.049

Koelling TM, 2005, CIRCULATION, V111, P179, DOI 10.1161/01.CIR.0000151811.53450.B8

Lin EHB, 2006, ANN FAM MED, V4, P46, DOI 10.1370/afm.423

Lorig K, 2006, LIVING A HEALTHY LIF

Masoudi FA, 2007, AM J CARDIOL, V99, p113B, DOI 10.1016/j.amjcard.2006.11.013

McCance K, 2010, PATHOPHYSIOLOGY THE

Melander O, 2000, HYPERTENSION, V35, P827, DOI 10.1161/01.HYP.35.3.827

Morgan AL, 2006, J CARD FAIL, V12, P54, DOI 10.1016/j.cardfail.2005.08.004

*NAT CTR HLTH STAT, 2007, HEALTH

Nichols GA, 2004, DIABETES CARE, V27, P1879, DOI 10.2337/diacare.27.8.1879

Orzano AJ, 2004, J AM BOARD FAM PRACT, V17, P359, DOI 10.3122/jabfm.17.5.359

Reilly CM, 2009, J CARDIOVASC NURS, V24, P500, DOI 10.1097/JCN.0b013e3181aff0b0

Resnick B, 2004, MIDDLE RANGE THEORIE, P183

Riegel B, 2002, PATIENT EDUC COUNS, V46, P287, DOI 10.1016/S0738-3991(01)00165-3

Shah MR, 2001, AM J CARDIOL, V88, P987, DOI 10.1016/S0002-9149(01)01975-0

Tahrani AA, 2007, BRIT MED J, V335, P508, DOI 10.1136/bmj.39255.669444.AE

Tang WHW, 2007, DIABETES OBES METAB, V9, P447, DOI 10.1111/j.1463-1326.2006.00616.x

U. S. Department of Agriculture U. S. Department of Health and Human Services, 2010, DIETARY GUIDELINES F

Williams GC, 1996, J PERS SOC PSYCHOL, V70, P767, DOI 10.1037/0022-3514.70.4.767

Williams GC, 1998, DIABETES CARE, V21, P1644, DOI 10.2337/diacare.21.10.1644

NR 46

TC 10

Z9 10

U1 0

U2 23

PU SAGE PUBLICATIONS INC

PI THOUSAND OAKS

PA 2455 TELLER RD, THOUSAND OAKS, CA 91320 USA

SN 0145-7217

J9 DIABETES EDUCATOR

JI Diabetes Educ.

PD SEP-OCT

PY 2012

VL 38

IS 5

BP 673

EP 684

DI 10.1177/0145721712450923

PG 12

WC Endocrinology & Metabolism; Public, Environmental & Occupational Health

SC Endocrinology & Metabolism; Public, Environmental & Occupational Health

GA 038WM

UT WOS:000311204800007

PM 22722611

OA Green Accepted

DA 2019-01-08

ER

PT J

AU Tanenbaum, ML

Gonzalez, JS

AF Tanenbaum, Molly L.

Gonzalez, Jeffrey S.

TI The Influence of Diabetes on a Clinician-Rated Assessment of Depression

in Adults With Type 1 Diabetes

SO DIABETES EDUCATOR

LA English

DT Article

ID MONTGOMERY-ASBERG DEPRESSION; CO-MORBID DEPRESSION; QUALITATIVE

RESEARCH; COMORBID DEPRESSION; PROSPECTIVE COHORT; FOOT ULCERS;

PREVALENCE; DISTRESS; METAANALYSIS; ASSOCIATION

AB Purpose

The purpose of the study was to examine the role of diabetes and diabetes-related distress within clinician-administered depression interviews of adults with type 1 diabetes.

Methods

This mixed-methods study coded responses to a structured clinical interview of depressive symptom severity administered to adults with type 1 diabetes (n = 34; 65% female; 56% white, 38% African American, 27% Hispanic). Pearson correlations and t tests assessed relationships between interview-based and self-reported ratings of diabetes-related distress and depression.

Results

Among participants endorsing depressive symptoms in the interview, 73% mentioned diabetes as a contributing factor. Themes emerged relating to (1) a link between diabetes symptoms and distress, including problems with appetite, sleep, concentration, and social relationships; (2) overlapping symptoms between diabetes and depression; and (3) the perceived interconnectedness of mood and blood glucose levels. Clinician-assessed depression ratings were strongly associated with self-reported ratings of depression and self-reported diabetes-related distress. Interview-based diabetes-related distress was significantly associated with self-reported diabetes-related distress. Those with a diagnosis of major depressive disorder (44%) reported more diabetes-related distress.

Conclusions

Results suggest that diabetes may influence the evaluation of depression, even in standardized clinical interviews administered by trained professionals, the gold standard of assessment. Findings highlight a need for improved conceptualization and measurement of distress in individuals with diabetes to distinguish between symptoms caused by illness burden and those indicating a psychiatric disorder.

C1 [Tanenbaum, Molly L.; Gonzalez, Jeffrey S.] Yeshiva Univ, Ferkauf Grad Sch Psychol, Bronx, NY 10461 USA.

[Gonzalez, Jeffrey S.] Albert Einstein Coll Med, Diabet Res Ctr, Bronx, NY 10467 USA.

RP Gonzalez, JS (reprint author), Yeshiva Univ, Ferkauf Grad Sch Psychol, 1300 Morris Pk Ave, Bronx, NY 10461 USA.

EM jeffrey.gonzalez@einstein.yu.edu

FU Einstein Diabetes Research Center; NIDDK [DK020541]

FX This project was supported by a pilot and feasibility grant awarded to

JSG by the Einstein Diabetes Research Center (supported by NIDDK

DK020541). We are grateful to Persis Commissariat, BA, and Sabrina A.

Esbitt, MA, for assisting with analyzing the qualitative data for this

project.

CR Aikens JE, 2008, DIABETIC MED, V25, P1324, DOI 10.1111/j.1464-5491.2008.02590.x

Ali S, 2006, DIABETIC MED, V23, P1165, DOI 10.1111/j.1464-5491.2006.01943.x

Ali S, 2009, POSTGRAD MED J, V85, P238, DOI 10.1136/pgmj.2008.074641

American Psychiatric Association, 2000, DIAGN STAT MAN MENT

Anderson RJ, 2001, DIABETES CARE, V24, P1069, DOI 10.2337/diacare.24.6.1069

Anderson RM, 1998, DIABETES CARE, V21, P1387, DOI 10.2337/diacare.21.9.1387

Barnard KD, 2006, DIABETIC MED, V23, P445, DOI 10.1111/j.1464-5491.2006.01814.x

Black SA, 2003, DIABETES CARE, V26, P2822, DOI 10.2337/diacare.26.10.2822

Braun V., 2006, QUALITATIVE RES PSYC, V3, P77, DOI DOI 10.1191/1478088706QP0630A

Cabassa LJ, 2008, SOC SCI MED, V66, P2413, DOI 10.1016/j.socscimed.2008.01.054

Castro FG, 2010, J MIX METHOD RES, V4, P342, DOI 10.1177/1558689810382916

Cohen J, 1988, STAT POWER ANAL BEHA

COYNE JC, 1994, PSYCHOL BULL, V116, P29, DOI 10.1037/0033-2909.116.1.29

de Groot M, 2001, PSYCHOSOM MED, V63, P619, DOI 10.1097/00006842-200107000-00015

Egede LE, 2005, DIABETES CARE, V28, P1339, DOI 10.2337/diacare.28.6.1339

Egede LE, 2005, PSYCHOSOM MED, V67, P46, DOI 10.1097/01.psy.0000149260.82006.fb

Fisher L, 2008, DIABETIC MED, V25, P1096, DOI 10.1111/j.1464-5491.2008.02533.x

Fisher L, 2008, ANN FAM MED, V6, P246, DOI 10.1370/afm.842

Fisher L, 2007, DIABETES CARE, V30, P542, DOI 10.2337/dc06-1614

Fisher L, 2010, DIABETES CARE, V33, P23, DOI 10.2337/dc09-1238

Folkman S, 2004, ANNU REV PSYCHOL, V55, P745, DOI 10.1146/annurev.psych.55.090902.141456

Gask L, 2011, CHRONIC ILLN, V7, P239, DOI 10.1177/1742395311403636

Gonzalez JS, 2010, DIABETOLOGIA, V53, P2241, DOI 10.1007/s00125-010-1821-x

Gonzalez JS, 2007, DIABETES CARE, V30, P2222, DOI 10.2337/dc07-0158

Gonzalez JS, 2011, DIABETES CARE, V34, P236, DOI 10.2337/dc10-1970

Gonzalez Jeffrey S, 2010, Epidemiol Psichiatr Soc, V19, P110

Gonzalez JS, 2008, DIABETES CARE, V31, P2398, DOI 10.2337/dc08-1341

Katon WJ, 2005, DIABETES CARE, V28, P2668, DOI 10.2337/diacare.28.11.2668

Kim NS, MED DECIS MAKING

Lin EHB, 2010, DIABETES CARE, V33, P264, DOI 10.2337/dc09-1068

Lustman PJ, 2000, DIABETES CARE, V23, P934, DOI 10.2337/diacare.23.7.934

Markowitz SM, 2011, PSYCHOSOMATICS, V52, P1, DOI 10.1016/j.psym.2010.11.007

MONTGOMERY SA, 1979, BRIT J PSYCHIAT, V134, P382, DOI 10.1192/bjp.134.4.382

Polonsky WH, 2005, DIABETES CARE, V28, P626, DOI 10.2337/diacare.28.3.626

RADLOFF L S, 1977, Applied Psychological Measurement, V1, P385, DOI 10.1177/014662167700100306

Ritholz MD, 2011, CURR DIABETES REP, V11, P494, DOI 10.1007/s11892-011-0226-7

Roy T, 2012, DIABETIC MED, V29, P164, DOI 10.1111/j.1464-5491.2011.03401.x

Sheehan DV, 1998, J CLIN PSYCHIAT, V59, P34, DOI 10.4088/JCP.09m05305whi

SNAITH RP, 1986, BRIT J PSYCHIAT, V148, P599, DOI 10.1192/bjp.148.5.599

Svanborg P, 2001, J AFFECT DISORDERS, V64, P203, DOI 10.1016/S0165-0327(00)00242-1

Vileikyte L, 2005, DIABETES CARE, V28, P2378, DOI 10.2337/diacare.28.10.2378

Vileikyte L, 2009, DIABETOLOGIA, V52, P1265, DOI 10.1007/s00125-009-1363-2

Williams LH, 2010, AM J MED, V123, P748, DOI 10.1016/j.amjmed.2010.01.023

Yoshikawa H, 2008, DEV PSYCHOL, V44, P344, DOI 10.1037/0012-1649.44.2.344

Zhang XP, 2005, AM J EPIDEMIOL, V161, P652, DOI 10.1093/aje/kwi089

NR 45

TC 9

Z9 9

U1 0

U2 11

PU SAGE PUBLICATIONS INC

PI THOUSAND OAKS

PA 2455 TELLER RD, THOUSAND OAKS, CA 91320 USA

SN 0145-7217

J9 DIABETES EDUCATOR

JI Diabetes Educ.

PD SEP-OCT

PY 2012

VL 38

IS 5

BP 695

EP 704

DI 10.1177/0145721712452795

PG 10

WC Endocrinology & Metabolism; Public, Environmental & Occupational Health

SC Endocrinology & Metabolism; Public, Environmental & Occupational Health

GA 038WM

UT WOS:000311204800009

PM 22814358

DA 2019-01-08

ER

PT J

AU Hayashino, Y

Okamura, S

Matsunaga, S

Tsujii, S

Ishii, H

AF Hayashino, Yasuaki

Okamura, Shintaro

Matsunaga, Satoshi

Tsujii, Satoru

Ishii, Hitoshi

CA Tenri Cohort Study Grp

TI The association between Problem Areas in Diabetes Scale scores and

glycemic control is modified by types of diabetes therapy: Diabetes

Distress and Care Registry in Tenri (DDCRT 2)

SO DIABETES RESEARCH AND CLINICAL PRACTICE

LA English

DT Article

DE Diabetes distress; Glycemic control; Diabetes registry

ID QUALITY-OF-LIFE; MELLITUS; ADULTS

AB Aim: To evaluate the joint association of Problem Areas in Diabetes (PAID) Scale scores and glycemic control with diabetes therapy.

Methods: We used 3479 patients' data with type 1 and type 2. Modified Poisson regression analysis was used to estimate relative risks (RRs) for poor glycemic control (HbA1c >= 7.0%) across quartiles of PAID scores.

Results: Compared with the 1st quartile of PAID score, multivariable-adjusted RRs for poor glycemic control were 0.99 (95% CI, 0.87-1.11), 1.05 (95% CI, 0.93-1.18), and 1.12 (95% CI, 1.00-1.27), respectively, for 2nd to 4th quartiles, and we observed significant trend (p for trend = 0.03). We observed significant interaction of PAID score and HbA1c with diabetes therapy (p = 0.0469). In patients receiving diet only therapy, the RRs for poor glycemic control were 1.38 (95% CI, 0.93-2.05), 1.18 (95% CI, 0.51-5.13), and 1.81 (95% CI, 1.16-2.79), respectively for 2nd to 4th quartiles of PAID score compared with the 1st quartile (p for trend = 0.025); while we did not observe significant association between PAID and poor glycemic control in patients receiving medication therapy.

Conclusions: Diabetes distress measured by PAID survey was associated with poor glycemic control, and this association was modified by diabetes therapy. (C) 2012 Elsevier Ireland Ltd. All rights reserved.

C1 [Hayashino, Yasuaki] Kyoto Univ, Grad Sch Med & Publ Hlth, Dept Healthcare Epidemiol, Sakyo Ku, Kyoto 6068501, Japan.

[Okamura, Shintaro; Matsunaga, Satoshi; Ishii, Hitoshi] Tenri Hosp, Dept Endocrinol, Tenri, Nara 632, Japan.

[Tsujii, Satoru] Tenri Hosp, Ctr Diabet, Tenri, Nara 632, Japan.

RP Hayashino, Y (reprint author), Kyoto Univ, Grad Sch Med & Publ Hlth, Dept Healthcare Epidemiol, Sakyo Ku, Yoshida Konoe Cho, Kyoto 6068501, Japan.

EM hayasino-y@umin.net

OI Tsujii, Satoru/0000-0002-1834-6515; Hayashino,

Yasuaki/0000-0002-2932-8478

FU Manpei Suzuki Diabetes Foundation

FX This study was supported by the Manpei Suzuki Diabetes Foundation. The

funding source had no role in the design and conduct of the study; in

the collection, analysis, and interpretation of the data; or in the

preparation, review, or approval of the manuscript.

CR American Diabetes Association, 2011, DIABETES CARE S1, V35, pS4

Dhatt GS, 2005, CLIN CHIM ACTA, V358, P81, DOI 10.1016/j.cccn.2005.02.009

Hajos TR, 2011, QUAL LIFE RES

Huang MF, 2010, DIABETES CARE, V33, P38, DOI 10.2337/dc09-0768

Ishii H., 2000, Journal of the Japan Diabetes Society, V43, P13

McNutt LA, 2003, AM J EPIDEMIOL, V157, P940, DOI 10.1093/aje/kwg074

Nichols GA, 2000, DIABETES CARE, V23, P273, DOI 10.2337/diacare.23.3.273

POLONSKY WH, 1995, DIABETES CARE, V18, P754, DOI 10.2337/diacare.18.6.754

Testa MA, 1998, JAMA-J AM MED ASSOC, V280, P1490, DOI 10.1001/jama.280.17.1490

UK Prospective Diabetes Study Group, 1999, DIABETES CARE, V22, P1125, DOI DOI 10.2337/DIACARE.22.7.1125

VanderDoes FEE, 1996, DIABETES CARE, V19, P204, DOI 10.2337/diacare.19.3.204

WEINBERGER M, 1994, MED CARE, V32, P1173, DOI 10.1097/00005650-199412000-00002

Weinger K, 2005, DIABETES CARE, V28, P1346, DOI 10.2337/diacare.28.6.1346

Welch GW, 1997, DIABETES CARE, V20, P760, DOI 10.2337/diacare.20.5.760

Zou GY, 2004, AM J EPIDEMIOL, V159, P702, DOI 10.1093/aje/kwh090

NR 15

TC 16

Z9 16

U1 0

U2 5

PU ELSEVIER IRELAND LTD

PI CLARE

PA ELSEVIER HOUSE, BROOKVALE PLAZA, EAST PARK SHANNON, CO, CLARE, 00000,

IRELAND

SN 0168-8227

J9 DIABETES RES CLIN PR

JI Diabetes Res. Clin. Pract.

PD SEP

PY 2012

VL 97

IS 3

BP 405

EP 410

DI 10.1016/j.diabres.2012.04.005

PG 6

WC Endocrinology & Metabolism

SC Endocrinology & Metabolism

GA 013FU

UT WOS:000309292100012

PM 22554998

DA 2019-01-08

ER

PT J

AU Speight, J

Conn, J

Dunning, T

Skinner, TC

AF Speight, J.

Conn, J.

Dunning, T.

Skinner, T. C.

CA Diabet Australia

TI Diabetes Australia position statement. A new language for diabetes:

Improving communications with and about people with diabetes

SO DIABETES RESEARCH AND CLINICAL PRACTICE

LA English

DT Article

DE Language; Diabetes; Communication; Adherence; Control

ID SELF-MANAGEMENT; CHRONIC ILLNESS; CARE; TYPE-2; ADHERENCE; OUTCOMES;

RECOMMENDATIONS; EXPERIENCES; SUPPORT; TRIAL

AB Diabetes is the fastest growing chronic condition in Australia, affecting 1.7 million Australians, requiring daily self-care, and known to reduce quantity and quality of life. On average, people with diabetes experience greater emotional distress than those without diabetes. One source of distress can be the language used to refer to diabetes, its management and the person with diabetes. The way verbal and written language is used reflects and shapes people's thoughts, beliefs and behaviours. Language has the power to persuade, change or reinforce beliefs and stereotypes - for better or worse. Words do more than reflect people's reality: they create reality and affect how people view the world and their diabetes. Language needs to engage people with diabetes and support their self-care efforts. Importantly, language that de-motivates or induces fear, guilt or distress needs to be avoided and countered. Diabetes Australia believes optimal communication increases the motivation, health and well-being of people with diabetes, and that careless or negative language can be de-motivating, is often inaccurate, and can be harmful. Diabetes Australia developed this position statement to encourage greater awareness of the language surrounding diabetes and provide recommendations for more careful and positive language use. (C) 2012 Elsevier Ireland Ltd. All rights reserved.

C1 [Speight, J.] Australian Ctr Behav Res Diabet, Melbourne, Vic 3000, Australia.

[Speight, J.] Deakin Univ, Sch Psychol, Ctr Mental Hlth & Wellbeing Res, Burwood, Australia.

[Speight, J.] AHP Res, Uxbridge, Middx, England.

[Conn, J.] Royal Melbourne Hosp, Dept Endocrinol & Diabet, Parkville, Vic 3050, Australia.

[Dunning, T.] Deakin Univ, Fac Hlth, Sch Nursing, Geelong, Vic 3217, Australia.

[Dunning, T.] Geelong Hosp, Geelong, Vic, Australia.

[Skinner, T. C.] Univ Tasmania, Rural Clin Sch, Burnie, Tas 7320, Australia.

RP Speight, J (reprint author), Australian Ctr Behav Res Diabet, 570 Elizabeth St, Melbourne, Vic 3000, Australia.

EM jspeight@acbrd.org.au

OI Skinner, Timothy/0000-0002-0018-6963; Speight, Jane/0000-0002-1204-6896

FU Australian Centre for Behavioural Research in Diabetes

FX We thank Elizabeth Holmes-Truscott and Jennifer Halliday (The Australian

Centre for Behavioural Research in Diabetes) for their support of the

Working Group. We thank Dr Jessica Browne, Virginia Hagger, Dr Christel

Hendrieckx and Renza Scibilia for their valuable comments on an earlier

draft.

CR Anderson RM, 2000, DIABETES EDUCATOR, V26, P597, DOI 10.1177/014572170002600405

Bissell P, 2004, SOC SCI MED, V58, P851, DOI 10.1016/S0277-9536(03)00259-4

Boroditsky L, 2003, ENCY COGNITIVE SCI, P917, DOI DOI 10.1002/0470018860.500567

Broom D, 2004, SOC SCI MED, V58, P2371, DOI 10.1016/j.socscimed.2003.09.002

Fisher EB, 2012, HEALTH AFFAIR, V31, P130, DOI 10.1377/hlthaff.2011.0914

Fleischman S, 1999, J MED HUMANITIES, V20, P3

Funnell MM, 2000, JAMA-J AM MED ASSOC, V284, P1709, DOI 10.1001/jama.284.13.1709

Gallivan J, 2007, SOC MARK Q, V13, P65, DOI 10.1080/15245000701678453

Glasgow RE, 1999, DIABETES CARE, V22, P2090, DOI 10.2337/diacare.22.12.2090

Greenfield C, 2011, AUST J RURAL HEALTH, V19, P15, DOI 10.1111/j.1440-1584.2010.01171.x

HARDIN C, 1993, SOC COGNITION, V11, P277, DOI 10.1521/soco.1993.11.3.277

Hawthorne K, 2011, DIABETIC MED, V28, P1103, DOI 10.1111/j.1464-5491.2011.03292.x

Hayashino Y, 2011, EXP CLIN ENDOCR DIAB, V119, P276, DOI 10.1055/s-0030-1265213

Heisler M, 2003, J GEN INTERN MED, V18, P893, DOI 10.1046/j.1525-1497.2003.21132.x

Heisler M, 2002, J GEN INTERN MED, V17, P243, DOI 10.1046/j.1525-1497.2002.10905.x

Khan H, 2011, PRIM CARE DIABETES, V5, P251, DOI 10.1016/j.pcd.2011.07.001

Khattab M, 2010, J DIABETES COMPLICAT, V24, P84, DOI 10.1016/j.jdiacomp.2008.12.008

Kollias Anastasios, 2009, Prim Care Diabetes, V3, P249, DOI 10.1016/j.pcd.2009.08.001

Murphy HR, 2010, DIABETIC MED, V27, P92, DOI 10.1111/j.1464-5491.2009.02890.x

Paterson B, 2001, J ADV NURS, V34, P574, DOI 10.1046/j.1365-2648.2001.01786.x

Polonsky WH, 2005, DIABETES CARE, V28, P2543, DOI 10.2337/diacare.28.10.2543

Roberson MH, 1992, QUALITATIVE HLTH RES, V2, P7

Schillinger D, 2009, DIABETES CARE, V32, P559, DOI 10.2337/dc08-0787

SHERBOURNE CD, 1992, J BEHAV MED, V15, P447, DOI 10.1007/BF00844941

Singh H, 2010, DIABETES, V59

Snow R, 2012, DIABETIC MED, V29, P257, DOI 10.1111/j.1464-5491.2011.03447.x

Street RL, 2009, PATIENT EDUC COUNS, V74, P295, DOI 10.1016/j.pec.2008.11.015

Stuckey HL, 2010, QUAL HEALTH RES, V20, P42, DOI 10.1177/1049732309355286

Swift PGF, 2010, PEDIATR DIABETES, V11, P271, DOI 10.1111/j.1399-5448.2009.00596.x

Thorne SE, 2000, INT J NURS STUD, V37, P303, DOI 10.1016/S0020-7489(00)00007-9

Turner RC, 1998, LANCET, V352, P837

Wild D, 2007, PATIENT EDUC COUNS, V68, P10, DOI 10.1016/j.pec.2007.05.003

Zhao G, 2008, DIABETIC MED, V25, P221, DOI 10.1111/j.1464-5491.2007.02332.x

NR 33

TC 16

Z9 17

U1 0

U2 11

PU ELSEVIER IRELAND LTD

PI CLARE

PA ELSEVIER HOUSE, BROOKVALE PLAZA, EAST PARK SHANNON, CO, CLARE, 00000,

IRELAND

SN 0168-8227

J9 DIABETES RES CLIN PR

JI Diabetes Res. Clin. Pract.

PD SEP

PY 2012

VL 97

IS 3

BP 425

EP 431

DI 10.1016/j.diabres.2012.03.015

PG 7

WC Endocrinology & Metabolism

SC Endocrinology & Metabolism

GA 013FU

UT WOS:000309292100015

PM 22513346

DA 2019-01-08

ER

PT J

AU Munshi, MN

Hayes, M

Iwata, I

Lee, Y

Weinger, K

AF Munshi, M. N.

Hayes, M.

Iwata, I.

Lee, Y.

Weinger, K.

TI Which aspects of executive dysfunction influence ability to manage

diabetes in older adults?

SO DIABETIC MEDICINE

LA English

DT Article

DE cognition; diabetes; elderly

ID COGNITIVE IMPAIRMENT; DYSEXECUTIVE SYMPTOMS; MELLITUS; TRAIL;

QUESTIONNAIRE; DECLINE; SAMPLE; WOMEN

AB Diabet. Med. 29, 1171-1177 (2012) Abstract Aims To examine whether different aspects of executive function as measured by different assessment tools are associated with glycaemic control and other clinical characteristics in older adults with Type 2 diabetes. Methods We performed a cross-sectional study of older adults aged >= 70 years with Type 2 diabetes at a tertiary care diabetes centre. The Dysexecutive Questionnaire was used to measure self-reported executive dysfunction. Objective tests of executive functions included a modified clock drawing test (Clock-in-a-Box), Trail Making Tests (parts A and B) and verbal fluency. Demographic and clinical information was collected using questionnaires and surveys. Glycaemic control was measured by HbA1c. Results We evaluated 145 patients [average age 77 +/- 5 years, diabetes duration 15 +/- 11 years, mean HbA1c 56 +/- 11 mmol/mol (7.3 +/- 1.1%)]. Poor performances on objective tests (low scores on Clock-in-a-Box and verbal fluency; and high scores on Trail Making Tests A and B) but not on the subjective test (the Dysexecutive Questionnaire), were associated with poor glycaemic control (r = -0.23, P < 0.005; r = -0.17, P < 0.04; r = 0.20, P < 0.01, r = 0.22, P < 0.008, r = -0.07, P < 0.42, respectively). In a multiple regression model (r2 = 0.39), high Dysexecutive Questionnaire scores were associated with higher diabetes-related distress (P < 0.0004), depressive symptoms (P < 0.004), number of falls (P < 0.009), fear of falling (P < 0.01), less years of education (P < 0.0007) and fewer medications (P < 0.001). Conclusions On the one hand, in older adults, executive dysfunction detected by objective tests is associated with poor glycaemic control and may be considered before prescribing complex treatment regimens. On the other hand, self-reported executive dysfunction is associated with risk and fear of falls, and more affective symptoms, which may indicate higher awareness of subtle deficits.

C1 [Munshi, M. N.; Lee, Y.; Weinger, K.] Joslin Diabet Ctr, Boston, MA 02215 USA.

[Munshi, M. N.] Beth Israel Deaconess Med Ctr, Boston, MA 02215 USA.

[Munshi, M. N.; Weinger, K.] Harvard Univ, Sch Med, Boston, MA USA.

[Hayes, M.] Univ Calif San Francisco, San Francisco, CA 94143 USA.

[Iwata, I.] Duke Univ, Med Ctr, Durham, NC USA.

RP Munshi, MN (reprint author), 1 Joslin Pl, Boston, MA 02215 USA.

EM medha.munshi@joslin.harvard.edu

FU American Diabetes Association [1-07-CR-40]; Department of Defense PRMRP

of the Office of the Congressionally Directed Medical Research Programs

[W81XWH-07-1-0282]; Joslin Diabetes Center Clinical Research Center

FX The study was partly supported by a grant from a clinical research award

from American Diabetes Association, 1-07-CR-40 (MM), Department of

Defense PRMRP of the Office of the Congressionally Directed Medical

Research Programs, W81XWH-07-1-0282 (MM), and the Joslin Diabetes Center

Clinical Research Center.

CR Abbatecola AM, 2004, J AM GERIATR SOC, V52, P1713, DOI 10.1111/j.1532-5415.2004.52466.x

Abrahams S, 2000, NEUROPSYCHOLOGIA, V38, P734, DOI 10.1016/S0028-3932(99)00146-3

Amieva H, 2003, BRAIN COGNITION, V53, P129, DOI 10.1016/S0278-2626(03)00094-0

Arvanitakis Z, 2004, ARCH NEUROL-CHICAGO, V61, P661, DOI 10.1001/archneur.61.5.661

Burgess PW, 1998, J INT NEUROPSYCH SOC, V4, P547

Chan RCK, 2001, BRIT J PSYCHOL, V92, P551, DOI 10.1348/000712601162338

Chaytor N, 2007, BRAIN INJURY, V21, P615, DOI 10.1080/02699050701426949

CORRIGAN JD, 1987, J CLIN PSYCHOL, V43, P402, DOI 10.1002/1097-4679(198707)43:4<402::AID-JCLP2270430411>3.0.CO;2-E

Drane DL, 2002, NEUROPSY NEUROPSY BE, V15, P39

Fontbonne A, 2001, DIABETES CARE, V24, P366, DOI 10.2337/diacare.24.2.366

Gerstorf D, 2008, AGING NEUROPSYCHOL C, V15, P424, DOI 10.1080/13825580701640374

Grande L., 2005, J INT NEUROPSYCH SOC, V11, P9

Gregg EW, 2000, ARCH INTERN MED, V160, P174, DOI 10.1001/archinte.160.2.174

Ickowicz E, 2003, J AM GERIATR SOC, V51, pS265

KATZ S, 1963, JAMA-J AM MED ASSOC, V185, P914, DOI 10.1001/jama.1963.03060120024016

LAWTON MP, 1969, GERONTOLOGIST, V9, P179, DOI 10.1093/geront/9.3_Part_1.179

Mitchell RLC, 2007, NEUROPSYCHOLOGIA, V45, P617, DOI 10.1016/j.neuropsychologia.2006.06.030

Montorio I, 1996, Int Psychogeriatr, V8, P103, DOI 10.1017/S1041610296002505

Munshi M, 2006, DIABETES CARE, V29, P1794, DOI 10.2337/dc06-0506

Nguyen HT, 2010, J AM GERIATR SOC, V58, P1123, DOI 10.1111/j.1532-5415.2010.02857.x

POLONSKY WH, 1995, DIABETES CARE, V18, P754, DOI 10.2337/diacare.18.6.754

SCHMIDT R, 1992, ARCH NEUROL-CHICAGO, V49, P825, DOI 10.1001/archneur.1992.00530320049011

Sinclair AJ, 2000, DIABETES RES CLIN PR, V50, P203, DOI 10.1016/S0168-8227(00)00195-9

Spreen O, 1991, COMPENDIUM NEUROPSYC

Stewart R, 1999, DIABETIC MED, V16, P93, DOI 10.1046/j.1464-5491.1999.00027.x

Thabit H, 2009, DIABETES RES CLIN PR, V86, P208, DOI 10.1016/j.diabres.2009.09.004

Tombaugh TN, 2004, ARCH CLIN NEUROPSYCH, V19, P203, DOI 10.1016/S0887-6177(03)00039-8

van Harten B, 2007, EUR NEUROL, V57, P70, DOI 10.1159/000098054

Yaffe K, 2004, NEUROLOGY, V63, P658, DOI 10.1212/01.WNL.0000134666.64593.BA

NR 29

TC 13

Z9 17

U1 0

U2 17

PU WILEY

PI HOBOKEN

PA 111 RIVER ST, HOBOKEN 07030-5774, NJ USA

SN 0742-3071

EI 1464-5491

J9 DIABETIC MED

JI Diabetic Med.

PD SEP

PY 2012

VL 29

IS 9

BP 1171

EP 1177

DI 10.1111/j.1464-5491.2012.03606.x

PG 7

WC Endocrinology & Metabolism

SC Endocrinology & Metabolism

GA 988EC

UT WOS:000307470200029

PM 22340082

DA 2019-01-08

ER

PT J

AU Tanda, R

Salsberry, PJ

AF Tanda, Rika

Salsberry, Pamela J.

TI Integrating Risks for Type 2 Diabetes Across Childhood: A Life Course

Perspective

SO JOURNAL OF PEDIATRIC NURSING-NURSING CARE OF CHILDREN & FAMILIES

LA English

DT Article

DE Type 2 diabetes; Childhood obesity; Life course model; Cumulative risks

ID INTRAUTERINE GROWTH RESTRICTION; BODY-MASS INDEX; INSULIN-RESISTANCE;

SOCIOECONOMIC-STATUS; BIRTH-WEIGHT; OVERWEIGHT ADOLESCENTS;

PSYCHOLOGICAL DISTRESS; DEVELOPMENTAL ORIGINS; PROSPECTIVE COHORT;

METABOLIC SYNDROME

AB Type 2 diabetes (T2DM) emerged among children, due in large measure to a strong physiological link between increased weight states and T2DM. In this article, cumulative risk factors for T2DM across childhood and its underlying mechanisms are reviewed. The points of intervention for T2DM should occur throughout childhood. The use of Halfon and Hochstein's framework enables practitioners and researchers in the nursing field to better understand a child's individual risk for T2DM. Only with this long view will prevention and interventions be successful in stemming the tide of the "twin epidemic" threatening children worldwide. (C) 2012 Elsevier Inc. All rights reserved.

C1 [Tanda, Rika; Salsberry, Pamela J.] Ohio State Univ, Coll Nursing, Columbus, OH 43210 USA.

RP Tanda, R (reprint author), Ohio State Univ, Coll Nursing, Columbus, OH 43210 USA.

EM tanda.3@buckeyemail.osu.edu

CR Armstrong J, 2002, LANCET, V359, P2003, DOI 10.1016/S0140-6736(02)08837-2

Baker Elizabeth A, 2006, Prev Chronic Dis, V3, pA76

BARKER DJP, 1995, BRIT MED J, V311, P171, DOI 10.1136/bmj.311.6998.171

Bell JF, 2008, AM J PREV MED, V35, P547, DOI 10.1016/j.amepre.2008.07.006

Bender BG, 2007, PEDIATRICS, V120, P805, DOI 10.1542/peds.2007-0500

Bertram CE, 2002, REPRODUCTION, V124, P459, DOI 10.1530/rep.0.1240459

Black PH, 2006, MED HYPOTHESES, V67, P879, DOI 10.1016/j.mehy.2006.04.008

Bohringer A, 2008, PSYCHONEUROENDOCRINO, V33, P1394, DOI 10.1016/j.psyneuen.2008.08.002

Boney CM, 2005, PEDIATRICS, V115, pE290, DOI 10.1542/peds.2004-1808

Brufani C, 2009, EUR J ENDOCRINOL, V160, P769, DOI 10.1530/EJE-08-0878

Cottrell EC, 2008, PHYSIOL BEHAV, V94, P17, DOI 10.1016/j.physbeh.2007.11.017

Dabelea D, 2007, JAMA-J AM MED ASSOC, V297, P2716

Danielzik S, 2004, INT J OBESITY, V28, P1494, DOI 10.1038/sj.ijo.0802756

Davison KK, 2005, J PEDIATR-US, V147, P436, DOI 10.1016/j.jpeds.2005.05.002

De Boo HA, 2006, AUST NZ J OBSTET GYN, V46, P4, DOI 10.1111/j.1479-828X.2006.00506.x

Devaskar SU, 2007, REV ENDOCR METAB DIS, V8, P105, DOI 10.1007/s11154-007-9050-4

Diego MA, 2006, PSYCHOSOM MED, V68, P747, DOI 10.1097/01.psy.0000238212.21598.7b

Doyle AC, 2007, OBESITY, V15, P145, DOI 10.1038/oby.2007.515

Elenkov IJ, 2008, NEUROCHEM INT, V52, P40, DOI 10.1016/j.neuint.2007.06.037

FEMANDEZTWINN DS, 2006, PHYSIOL BEHAV, V88, P234

Franco M, 2008, AM J PREV MED, V35, P561, DOI 10.1016/j.amepre.2008.07.003

Gibson LY, 2007, MED J AUSTRALIA, V186, P591

Gluckman PD, 2008, NEW ENGL J MED, V359, P61, DOI 10.1056/NEJMra0708473

Goran MI, 2001, DIABETES, V50, P2444, DOI 10.2337/diabetes.50.11.2444

Goran MI, 2006, INT J PEDIATR OBES, V1, P139, DOI 10.1080/17477160600780423

Gordon-Larsen P, 2006, PEDIATRICS, V117, P417, DOI 10.1542/peds.2005-0058

Gray L, 2008, EUR J PUBLIC HEALTH, V18, P616, DOI 10.1093/eurpub/ckn044

HALES CN, 1992, DIABETOLOGIA, V35, P595, DOI 10.1007/BF00400248

Halfon N, 2002, MILBANK Q, V80, P433, DOI 10.1111/1468-0009.00019

Halfon N., 2005, ZERO 3

Harshaw C, 2008, DEV REV, V28, P541, DOI 10.1016/j.dr.2008.08.001

Hemphill E, 2008, AM J HEALTH PROMOT, V22, P426, DOI 10.4278/ajhp.22.6.426

Ievers-Landis CE, 2008, J DEV BEHAV PEDIATR, V29, P243, DOI 10.1097/DBP.0b013e31816d923d

Jenkins SK, 2005, COMPR CHILD ADOLES N, V28, P175, DOI 10.1080/01460860500227580

Kajantie E, 2008, HORM-INT J ENDOCRINO, V7, P101, DOI 10.1007/BF03401501

Kaplowitz PB, 2008, PEDIATRICS, V121, pS208, DOI 10.1542/peds.2007-1813F

Khashan AS, 2008, PSYCHOSOM MED, V70, P688, DOI 10.1097/PSY.0b013e318177940d

Lee JM, 2008, ARCH PEDIAT ADOL MED, V162, P682, DOI 10.1001/archpedi.162.7.682

Lesage J, 2004, J ENDOCRINOL, V181, P291, DOI 10.1677/joe.0.1810291

Li FZ, 2009, AM J HEALTH PROMOT, V23, P203, DOI 10.4278/ajhp.071214133

Li J, 2008, ACTA PAEDIATR, V97, P221, DOI 10.1111/j.1651-2227.2007.00602.x

Libbey HP, 2008, OBESITY, V16, pS24, DOI 10.1038/oby.2008.455

Liese AD, 2006, PEDIATRICS, V118, P1510, DOI 10.1542/peds.2006-0690

Lillycrop KA, 2005, J NUTR, V135, P1382

Lobel M, 2008, HEALTH PSYCHOL, V27, P604, DOI 10.1037/a0013242

Lumeng JC, 2007, PEDIATRICS, V120, P1020, DOI 10.1542/peds.2006-3295

Lutfiyya MN, 2008, J AM BOARD FAM MED, V21, P191, DOI 10.3122/jabfm.2008.03.070207

Maffeis C, 2008, J CLIN ENDOCR METAB, V93, P2122, DOI 10.1210/jc.2007-2089

Martyn-Nemeth P, 2009, RES NURS HEALTH, V32, P96, DOI 10.1002/nur.20304

Mayer-Davis EJ, 2008, J AM DIET ASSOC, V108, pS45, DOI 10.1016/j.jada.2008.01.018

Mayer-Davis EJ, 2008, DIABETES CARE, V31, P470, DOI 10.2337/dc071321

Miralles O, 2006, OBESITY, V14, P1371, DOI 10.1038/oby.2006.155

Moran A, 2008, CIRCULATION, V117, P2361, DOI 10.1161/CIRCULATIONAHA.107.704569

O'Regan D, 2004, AM J PHYSIOL-ENDOC M, V287, pE863, DOI 10.1152/ajpendo.00137.2004

Ogden CL, 2002, JAMA-J AM MED ASSOC, V288, P1728, DOI 10.1001/jama.288.14.1728

Ogden CL, 2008, JAMA-J AM MED ASSOC, V299, P2401, DOI 10.1001/jama.299.20.2401

Owen CG, 2006, AM J CLIN NUTR, V84, P1043

Park JH, 2008, J CLIN INVEST, V118, P2316, DOI [10.1172/JC133655, 10.1172/JCI33655]

Precht DH, 2007, ACTA OBSTET GYN SCAN, V86, P266, DOI 10.1080/00016340601088406

Reagan LP, 2007, CURR OPIN PHARMACOL, V7, P633, DOI 10.1016/j.coph.2007.10.012

Salsberry PJ, 2005, PEDIATRICS, V116, P1329, DOI 10.1542/peds.2004-2583

Sharma AJ, 2008, AM J EPIDEMIOL, V168, P995, DOI 10.1093/aje/kwn223

Sherwood NE, 2009, PREV CHRONIC DIS, V6, DOI 10.1177/1479972308095936

Sinclair KD, 2007, P NATL ACAD SCI USA, V104, P19351, DOI 10.1073/pnas.0707258104

Smyth S, 2006, NAT MED, V12, P75, DOI 10.1038/nm0106-75

Spence JC, 2008, INT J PEDIATR OBES, V3, P109, DOI 10.1080/17477160701875007

Swallen KC, 2005, PEDIATRICS, V115, P340, DOI 10.1542/peds.2004-0678

Swanson JM, 2009, SEMIN REPROD MED, V27, P391, DOI 10.1055/s-0029-1237427

Taksali SE, 2008, DIABETES, V57, P367, DOI 10.2337/db07-0932

Taveras EM, 2008, J ALLERGY CLIN IMMUN, V121, P1161, DOI 10.1016/j.jaci.2008.03.021

Taveras EM, 2004, PEDIATRICS, V114, pE577, DOI 10.1542/peds.2004-0801

Taylor SE, 2007, ANNU REV CLIN PSYCHO, V3, P377, DOI 10.1146/annurev.clinpsy.3.022806.091520

Teegarden SL, 2008, PHYSIOL BEHAV, V93, P713, DOI 10.1016/j.physbeh.2007.11.030

Tsiotra PC, 2006, ANN NY ACAD SCI, V1083, P63, DOI 10.1196/annals.1367.007

Valsamakis G, 2006, ANN NY ACAD SCI, V1092, P138, DOI 10.1196/annals.1365.012

van Dijk CE, 2009, PEDIATRICS, V123, P102, DOI 10.1542/peds.2007-3382

Vickers MH, 2005, ENDOCRINOLOGY, V146, P4211, DOI 10.1210/en.2005-0581

Voss LD, 2008, CHILD CARE HLTH DEV, V34, P470, DOI 10.1111/j.1365-2214.2008.00827.x

Weiss R, 2005, DIABETES CARE, V28, P902, DOI 10.2337/diacare.28.4.902

Weyermann M, 2007, EPIDEMIOLOGY, V18, P722, DOI 10.1097/EDE.0b013e3181567ed4

*WHO, 2007, PREV EXC BOD WEIGHT

Woo JG, 2008, PEDIATRICS, V121, pE458, DOI 10.1542/peds.2007-1446

Xekouki P, 2007, OBESITY, V15, P860, DOI 10.1038/oby.2007.600

Zeller MH, 2007, OBESITY, V15, P126, DOI 10.1038/oby.2007.517

NR 84

TC 4

Z9 4

U1 0

U2 19

PU ELSEVIER SCIENCE INC

PI NEW YORK

PA 360 PARK AVE SOUTH, NEW YORK, NY 10010-1710 USA

SN 0882-5963

J9 J PEDIATR NURS

JI J. Pediatr. Nurs.

PD AUG

PY 2012

VL 27

IS 4

BP 310

EP 318

DI 10.1016/j.pedn.2011.03.014

PG 9

WC Nursing; Pediatrics

SC Nursing; Pediatrics

GA 045OO

UT WOS:000311706400006

PM 22703677

DA 2019-01-08

ER

PT J

AU Hopkins, D

Lawrence, I

Mansell, P

Thompson, G

Amiel, S

Campbell, M

Heller, S

AF Hopkins, David

Lawrence, Ian

Mansell, Peter

Thompson, Gillian

Amiel, Stephanie

Campbell, Michael

Heller, Simon

TI Improved Biomedical and Psychological Outcomes 1 Year After Structured

Education in Flexible Insulin Therapy for People With Type 1 Diabetes

The U.K. DAFNE experience

SO DIABETES CARE

LA English

DT Article

ID ENABLE DIETARY FREEDOM; SEVERE HYPOGLYCEMIA; HOSPITAL ANXIETY;

DEPRESSION SCALE; PREVALENCE; SYMPTOMS; DISTRESS; VALIDITY; PROGRAM;

IDDM

AB OBJECTIVE-DAFNE (Dose Adjustment For Normal Eating), a structured education program in flexible insulin therapy, has been widely adopted in the U.K. after validation in a randomized trial. To determine benefits in routine practice, we collected biomedical and psychological data from all participants attending during a 12-month period.

RESEARCH DESIGN AND METHODS-HbA(1c), weight, self-reported hypoglycemia awareness, severe hypoglycemia frequency, PAID (Problem Areas In Diabetes), HADS (Hospital Anxiety and Depression Scale), and EuroQol Group 5-Dimension Self-Report Questionnaire scores were recorded prior to DAFNE and after 1 year.

RESULTS-Complete baseline and follow-up HbA(1c) data were available for 639 (54.9%) of 1,1.63 attendees. HbA(1c) fell from 8.51 +/- 1.41 (mean +/- SD) to 8.24 +/- 1.29% (difference 0.27 [95% CI 0.16-0.38]; P < 0.001), with a greater mean fall of 0.44% from baseline HbA(1c) >8.5%. Severe hypoglycemia rate fell from 1.7 +/- 8.5 to 0.6 +/- 3.7 episodes per person per year (1.1 [0.7-1.4]) and hypoglycemia recognition improved in 43% of those reporting unawareness. Baseline psychological distress was evident, with a PAID score of 25.2 and HADS scores of 5.3 (anxiety) and 4.8 (depression), falling to 16.7 (8.5 [6.6-10.4]), 4.6 (0.7 [0.4-1.0]), and 4.2 (0.6 [0.3-0.8]), respectively (all P < 0.001 at 1 year). Clinically relevant anxiety and depression (HADS >= 8) fell from 24.4 to 18.0% and 20.9 to 15.5%, respectively.

CONCLUTIONS-A structured education program delivered in routine clinical practice not only improves HbA(1c) while reducing severe hypoglycemia rate and restoring hypoglycemia awareness but also reduces psychological distress and improves perceived well-being.

C1 [Hopkins, David] Kings Coll Hosp Natl Hlth Serv Fdn Trust, Dept Diabet Med, London, England.

[Lawrence, Ian] Univ Hosp Leicester Natl Hlth Serv Trust, Dept Diabet, Leicester, Leics, England.

[Mansell, Peter] Univ Nottingham, Sch Biomed Sci, Nottingham NG7 2RD, England.

[Thompson, Gillian] Northumbria Healthcare Fdn Trust, Cent DAFNE Off, Northumbria, England.

[Amiel, Stephanie] Kings Coll London, Sch Med, Diabetes Res Grp, London WC2R 2LS, England.

[Campbell, Michael] Univ Sheffield, Sch Hlth & Related Res, Sheffield, S Yorkshire, England.

[Heller, Simon] Univ Sheffield, Acad Unit Diabet Endocrinol & Metab, Sheffield, S Yorkshire, England.

RP Hopkins, D (reprint author), Kings Coll Hosp Natl Hlth Serv Fdn Trust, Dept Diabet Med, London, England.

EM dhopkins3@nhs.net

OI Campbell, Michael/0000-0003-3529-2739; Heller, Simon/0000-0002-2425-9565

FU U.K. National Institute for Health Research; U.K. DAFNE Collaborative;

National Institute for Health Research [RP-PG-0606-1184]

FX This study forms part of a broader program of research entitled

"Improving Management of Type 1 Diabetes in the U.K.: The DAFNE Program

as a Research Test Bed" funded by the U.K. National Institute for Health

Research. G.T. is employed as the national director of the DAFNE program

and funded by the U.K. DAFNE Collaborative.

CR Amiel S, 2002, BRIT MED J, V325, P746

Bjelland I, 2002, J PSYCHOSOM RES, V52, P69, DOI 10.1016/S0022-3999(01)00296-3

Bott S, 1997, DIABETOLOGIA, V40, P926, DOI 10.1007/s001250050769

Bradley C, 2001, LANCET, V357, P7, DOI 10.1016/S0140-6736(00)03562-5

Campbell MJ, 2007, MED STAT TXB HLTH SC, P278

CRANSTON I, 1994, LANCET, V344, P283, DOI 10.1016/S0140-6736(94)91336-6

DAGOGOJACK S, 1994, DIABETES, V43, P1426, DOI 10.2337/diabetes.43.12.1426

FANELLI CG, 1993, DIABETES, V42, P1683, DOI 10.2337/diabetes.42.11.1683

Hermanns N, 2006, DIABETOLOGIA, V49, P469, DOI 10.1007/s00125-005-0094-2

Lloyd CE, 2000, DIABETIC MED, V17, P198, DOI 10.1046/j.1464-5491.2000.00260.x

MUHLHAUSER I, 1983, DIABETOLOGIA, V25, P470

PIEBER TR, 1995, DIABETES CARE, V18, P625, DOI 10.2337/diacare.18.5.625

POLONSKY WH, 1995, DIABETES CARE, V18, P754, DOI 10.2337/diacare.18.6.754

Samann A, 2005, DIABETOLOGIA, V48, P1965, DOI 10.1007/s00125-005-1905-1

Shaban MC, 2006, DIABETIC MED, V23, P1381, DOI 10.1111/j.1464-5491.2006.02012.x

Snoek FJ, 2000, DIABETES CARE, V23, P1305, DOI 10.2337/diacare.23.9.1305

The EuroQol Group, 1990, HLTH POLICY, V16, P199, DOI DOI 10.1016/0168-8510(90)90421-9

ZIGMOND AS, 1983, ACTA PSYCHIAT SCAND, V67, P361, DOI 10.1111/j.1600-0447.1983.tb09716.x

NR 18

TC 91

Z9 93

U1 0

U2 14

PU AMER DIABETES ASSOC

PI ALEXANDRIA

PA 1701 N BEAUREGARD ST, ALEXANDRIA, VA 22311-1717 USA

SN 0149-5992

J9 DIABETES CARE

JI Diabetes Care

PD AUG

PY 2012

VL 35

IS 8

BP 1638

EP 1642

DI 10.2337/dc11-1579

PG 5

WC Endocrinology & Metabolism

SC Endocrinology & Metabolism

GA 980KD

UT WOS:000306891200003

PM 22619082

OA Other Gold, Green Published

DA 2019-01-08

ER

PT J

AU Webb, RT

Kontopantelis, E

Doran, T

Qin, P

Creed, F

Kapur, N

AF Webb, Roger T.

Kontopantelis, Evangelos

Doran, Tim

Qin, Ping

Creed, Francis

Kapur, Nav

TI Risk of self-harm in physically ill patients in UK primary care

SO JOURNAL OF PSYCHOSOMATIC RESEARCH

LA English

DT Article

DE Self-harm; Suicidal behaviour; Physical illness; Depression; Primary

care; Epidemiology

ID PRACTICE RESEARCH DATABASE; SUICIDAL-BEHAVIOR; MEDICAL ILLNESS;

ASSOCIATION; COHORT; IDEATION; EPILEPSY; DISEASES; LIFE

AB Objective: To examine self-harm risk across the adult age range in patients diagnosed with various physical illnesses using the General Practice Research Database - a broadly representative sample of all people registered with a family practice in the United Kingdom.

Methods: We conducted a large nested case-control study sampled from the whole primary care cohort. During 2001-2008 we studied 2306 cases of self-harm and 46,120 age and gender-matched controls without such an episode recorded. Relative risks were estimated against reference patients with none of the examined physical illnesses. Additionally, we assessed confounding by recorded depression, effect modification by gender and multi-morbidity effects.

Results: Risk was significantly elevated in relation to any of the physical illnesses (male OR 1.35, 95% CI 1.18-1.54; female OR 1.62,95% CI 1.40-1.86). For both genders combined, risk was raised with each specific illness. Effects sizes were consistently larger in women. Adjustment for recorded depression explained much of the elevated risk, but not so in women with asthma, back pain, diabetes, epilepsy or hypertension. Raised risk was seen in younger adults and during middle age, but not among older people. There was a dose-response relationship with increasing number of physical illnesses, and in women this was independent of depression.

Conclusion: Heightened risk was seen with a variety of physical illnesses. The findings indicate a need for tackling psychological distress and reducing self-harm risk in physically ill patients who attend primary healthcare services for non-psychiatric reasons, particularly so for women and younger and middle aged adults. (c) 2012 Elsevier Inc. All rights reserved.

C1 [Webb, Roger T.; Kapur, Nav] Univ Manchester, Ctr Suicide Prevent, Ctr Mental Hlth & Risk, Manchester M13 9PL, Lancs, England.

[Kontopantelis, Evangelos; Doran, Tim] Univ Manchester, Hlth Sci Primary Care Res Grp, Manchester M13 9PL, Lancs, England.

[Qin, Ping] Univ Aarhus, Natl Ctr Register Based Res, DK-8000 Aarhus C, Denmark.

[Creed, Francis] Univ Manchester, Mental Hlth & Neurodegenerat Res Grp, Manchester M13 9PL, Lancs, England.

RP Webb, RT (reprint author), Univ Manchester, Ctr Suicide Prevent, Ctr Mental Hlth & Risk, Room 2-311,Jean McFarlane Bldg,Oxford Rd, Manchester M13 9PL, Lancs, England.

EM roger.webb@manchester.ac.uk

RI Research Datalink, Clinical Practice/H-2477-2013; CPRD, CPRD/B-9594-2017

OI Kontopantelis, Evangelos/0000-0001-6450-5815; Webb,

Roger/0000-0001-8532-2647

FU National Institute for Health Research [CDF-2011-04-016]; Department of

Health [CDF-2011-04-016]; Medical Research Council [MC_PC_13042,

MR/K006665/1]

CR ASHLEY MJ, 1977, ARCH INTERN MED, V137, P883, DOI 10.1001/archinte.137.7.883

Barry D, 1996, STAT MED, V15, P2603

Bergen H, 2010, BRIT J PSYCHIAT, V197, P493, DOI 10.1192/bjp.bp.110.077651

Brady J, 2006, ALCOHOL ALCOHOLISM, V41, P473, DOI 10.1093/alcalc/agl060

Christensen J, 2007, LANCET NEUROL, V6, P693, DOI 10.1016/S1474-4422(07)70175-8

Clayton D., 1993, STAT MODELS EPIDEMIO

Conwell Y, 2002, BIOL PSYCHIAT, V52, P193, DOI 10.1016/S0006-3223(02)01347-1

Cooper J, 2005, AM J PSYCHIAT, V162, P297, DOI 10.1176/appi.ajp.162.2.297

Cukrowicz KC, 2011, PSYCHOL AGING, V26, P331, DOI 10.1037/a0021836

Dennis M, 2009, SOC PSYCH PSYCH EPID, V44, P608, DOI 10.1007/s00127-008-0474-2

Doran T, 2011, BRIT MED J, V342, DOI 10.1136/bmj.d3590

Druss B, 2000, ARCH INTERN MED, V160, P1522, DOI 10.1001/archinte.160.10.1522

Goodwin RD, 2003, SOC SCI MED, V56, P1783, DOI 10.1016/S0277-9536(02)00174-0

Grabbe L, 1997, AM J PUBLIC HEALTH, V87, P434, DOI 10.2105/AJPH.87.3.434

Hammad TA, 2008, PHARMACOEPIDEM DR S, V17, P1197, DOI 10.1002/pds.1672

Haw C, 2005, SOC PSYCH PSYCH EPID, V40, P964, DOI 10.1007/s00127-005-0981-3

Hawton K, 2008, SUICIDE LIFE-THREAT, V38, P650, DOI 10.1521/suli.2008.38.6.650

Hendin H, 1999, J CLIN PSYCHIAT, V60, P46

Hennessy S, 1999, AM J EPIDEMIOL, V149, P195

Ilgen MA, 2010, SUICIDE LIFE-THREAT, V40, P597, DOI 10.1521/suli.2010.40.6.597

Juurlink DN, 2004, ARCH INTERN MED, V164, P1179, DOI 10.1001/archinte.164.11.1179

Kaplan MS, 2007, AM J ORTHOPSYCHIAT, V77, P56, DOI 10.1037/0002-9432.77.1.56

Khan NF, 2010, BRIT J GEN PRACT, V60, DOI 10.3399/bjgp10X483562

Khan NF, 2010, BMC FAM PRACT, V11, DOI 10.1186/1471-2296-11-1

LUBIN JH, 1984, BIOMETRICS, V40, P63, DOI 10.2307/2530744

MacLean J, 2011, J AFFECT DISORDERS, V130, P245, DOI 10.1016/j.jad.2010.10.028

Marusic A, 2006, SUICIDE LIFE-THREAT, V36, P323, DOI 10.1521/suli.2006.36.3.323

McCormick A, 1995, MORBIDITY STAT GEN P, V3

Nilsson L, 1997, EPILEPSIA, V38, P1062, DOI 10.1111/j.1528-1157.1997.tb01194.x

Quan H, 2002, SOC PSYCH PSYCH EPID, V37, P190, DOI 10.1007/s001270200014

RIFKIN A, 1992, POSTGRAD MED, V92, P147

Scott KM, 2010, PSYCHOSOM MED, V72, P712, DOI 10.1097/PSY.0b013e3181e3333d

Venkoba Rao A, 1990, Crisis, V11, P48

Viilo KM, 2005, PSYCHOSOM MED, V67, P241, DOI 10.1097/01.psy.0000151488.71789.7f

Waern M, 2002, BMJ-BRIT MED J, V324, P1355, DOI 10.1136/bmj.324.7350.1355

Walley T, 1997, LANCET, V350, P1097, DOI 10.1016/S0140-6736(97)04248-7

Webb RT, 2012, ARCH GEN PSYCHIAT, V69, P256, DOI 10.1001/archgenpsychiatry.2011.1561

NR 37

TC 13

Z9 13

U1 0

U2 11

PU PERGAMON-ELSEVIER SCIENCE LTD

PI OXFORD

PA THE BOULEVARD, LANGFORD LANE, KIDLINGTON, OXFORD OX5 1GB, ENGLAND

SN 0022-3999

EI 1879-1360

J9 J PSYCHOSOM RES

JI J. Psychosomat. Res.

PD AUG

PY 2012

VL 73

IS 2

BP 92

EP 97

DI 10.1016/j.jpsychores.2012.05.010

PG 6

WC Psychiatry

SC Psychiatry

GA 977BZ

UT WOS:000306632700003

PM 22789410

DA 2019-01-08

ER

PT J

AU Wermeling, PR

van den Donk, M

Gorter, KJ

Beulens, JWJ

Rutten, GEHM

AF Wermeling, Paulien R.

van den Donk, Maureen

Gorter, Kees J.

Beulens, Joline W. J.

Rutten, Guy E. H. M.

TI Frequency of Monitoring Diabetes in Primary Care: What Do

Well-Controlled Patients Prefer?

SO CANADIAN JOURNAL OF DIABETES

LA English

DT Article

DE diabetes mellitus type 2; general practitioners; monitoring frequency;

patient preference; primary health care

AB Objective: To describe the preferences and the associated patients' characteristics of well-controlled type 2 diabetes patients for the diabetes monitoring frequency in primary care.

Methods: Cross-sectional study with 233 participating general practitioners across the Netherlands. Eligible patients were between 40 and 80 years, diagnosed with type 2 diabetes for more than 1 year not on insulin, and with glycated hemoglobin (A1C) <= 7.5%, systolic blood pressure <= 145 mm Hg and total cholesterol <= 5.2 mmol/L. Participants were asked whether they had a preference for 3-monthly or 6-monthly monitoring or no preference.

Results: From 2215 patients, 747 patients (33.7%) preferred 3-monthly and 677 (30.6%) 6-monthly monitoring by either the general practitioner or the practice nurse. The former group consisted of less smokers, felt less healthy, reported more diabetes-related distress, had the highest reported frequency of hyperglycemic episodes and used more oral blood glucose lowering drugs compared to the other patients. Those preferring 6-monthly monitoring were least satisfied with diabetes treatment, reported the lowest frequency of hyperglycemic episodes and used less oral blood glucose lowering drugs compared to the other patients.

Conclusion: A preference for more frequent monitoring was associated with a worse disease status, whereas a preference for less frequent monitoring tended to be associated with the opposite. Patients seem to have logical preferences that need to be accounted for in diabetes care. (C) 2012 Canadian Diabetes Association

C1 [Wermeling, Paulien R.; van den Donk, Maureen; Gorter, Kees J.; Beulens, Joline W. J.; Rutten, Guy E. H. M.] Univ Med Ctr Utrecht, Julius Ctr Hlth Sci & Primary Care, NL-3508 GA Utrecht, Netherlands.

RP Wermeling, PR (reprint author), Univ Med Ctr Utrecht, Julius Ctr Hlth Sci & Primary Care, STR 6-131,POB 85500, NL-3508 GA Utrecht, Netherlands.

EM P.Wermeling@umcutrecht.nl

FU ZonMw, the Netherlands organisation for health research and development

[80-82310-98-09058]

FX This study was supported by ZonMw, the Netherlands organisation for

health research and development (grant 80-82310-98-09058).

CR *AM DIAB ASS, 2011, DIABETES CARE S1, V34, pS11, DOI DOI 10.2337/DC11-S011

Bradley C., 1994, HDB PSYCHOL DIABETES, P111

Brooks R, 1996, HEALTH POLICY, V37, P53, DOI 10.1016/0168-8510(96)00822-6

Burgers JS, 2002, DIABETES CARE, V25, P1933, DOI 10.2337/diacare.25.11.1933

Canadian Diabetes Association Clinical Practice Guidelines Expert Committee, 2008, CAN J DIABETES, V32, pS32

Cleveringa FGW, 2010, DIABETIC MED, V27, P442, DOI 10.1111/j.1464-5491.2010.02968.x

Cleveringa FGW, 2008, DIABETES CARE, V31, P2273, DOI 10.2337/dc08-0312

Coulter A, 2010, BMJ-BRIT MED J, V341, DOI 10.1136/bmj.c4989

DeSalvo KB, 2003, INT J QUAL HEALTH C, V15, P399, DOI 10.1093/intqhc/mzg067

Gorter KJ, 2010, DIABETIC MED, V27, P85, DOI 10.1111/j.1464-5491.2009.02886.x

Gorter KJ, 2010, PATIENT EDUC COUNS, V84, P56

Griffin SJ, 2011, LANCET, V378, P156, DOI 10.1016/S0140-6736(11)60698-3

Hakkaart-van Roijen L., 2007, MANUAL SHORT FORM HL

Joosten EAG, 2008, PSYCHOTHER PSYCHOSOM, V77, P219, DOI 10.1159/000126073

Kleefstra N, 2010, NETH J MED, V68, P311

Lamers LM, 2006, HEALTH ECON, V15, P1121, DOI 10.1002/hec.1124

Parchman ML, 2010, ANN FAM MED, V8, P410, DOI 10.1370/afm.1161

POLONSKY WH, 1995, DIABETES CARE, V18, P754, DOI 10.2337/diacare.18.6.754

Rijksinstituut voor Volksgezondheid en Milieu (RIVM), 2012, DRIE JAAR INT BEK DI

Rubin DB, 1987, MULTIPLE IMPUTATION

RUTTEN GEH, 2006, HUISARTS WET, V49, P137

Say R, 2006, PATIENT EDUC COUNS, V60, P102, DOI 10.1016/j.pec.2005.02.003

Schwartz LM, 1999, J GEN INTERN MED, V14, P230, DOI 10.1046/j.1525-1497.1999.00322.x

Statistics Netherlands (CBS), 2000, STAND ALL IND 10 24, V10, P24

Stone MA, 2010, DIABETES RES CLIN PR, V87, P252, DOI 10.1016/j.diabres.2009.10.020

Underwood M, 2008, BMJ-BRIT MED J, V336, P138, DOI 10.1136/bmj.39399.656331.25

van Bruggen R, 2008, BR J GEN PRACT, V58, P339

van den Donk M, 2010, QUAL LIFE RES, V19, P509, DOI 10.1007/s11136-010-9604-x

WARE JE, 1992, MED CARE, V30, P473, DOI 10.1097/00005650-199206000-00002

Welch HG, 1999, J GEN INTERN MED, V14, P223, DOI 10.1046/j.1525-1497.1999.00321.x

Wendel-Vos GCW, 2003, J CLIN EPIDEMIOL, V56, P1163, DOI 10.1016/S0895-4356(03)00220-8

Wermeling PR, 2010, BMC FAM PRACT, V11, DOI 10.1186/1471-2296-11-35

NR 32

TC 2

Z9 2

U1 0

U2 5

PU ELSEVIER SCIENCE BV

PI AMSTERDAM

PA PO BOX 211, 1000 AE AMSTERDAM, NETHERLANDS

SN 1499-2671

J9 CAN J DIABETES

JI Can. J. Diabetes

PD AUG

PY 2012

VL 36

IS 4

BP 187

EP 192

DI 10.1016/j.jcjd.2012.07.012

PG 6

WC Endocrinology & Metabolism

SC Endocrinology & Metabolism

GA V33EQ

UT WOS:000209002500012

DA 2019-01-08

ER

PT J

AU Vallis, M

AF Vallis, Michael

TI A New Dawn for Diabetes in Canada

SO CANADIAN JOURNAL OF DIABETES

LA English

DT Article

DE depression; diabetes distress; knowledge translation; psychosocial

AB This paper is intended to highlight the need for diabetes healthcare professionals to better address psychosocial issues in diabetes by summarizing selective evidence that identifies the important psychosocial needs and issues of those living with diabetes. The main purpose of this paper is to draw the attention of Canadian diabetes professionals to a specific international psychosocial initiative called the Diabetes Attitudes, Wishes and Needs (DAWN) program. The DAWN program began with the DAWN study, a large-scale psychosocial study conducted at an international level but not including Canada. This is a relevant study for 2 reasons. First, the results of DAWN can guide Canadian diabetes healthcare providers by virtue of the fact that multiple knowledge translation tools have been developed and can be directly accessed by Canadian providers. Second, DAWN2, a follow-up study, has recently been launched and Canada is one of 18 participating countries. Increasing the awareness of Canadian diabetes healthcare providers about the results of DAWN and the launch of DAWN2 can further advance knowledge translation for the management of psychosocial factors in diabetes. (C) 2012 Canadian Diabetes Association

C1 [Vallis, Michael] Capital Hlth, Diabet Management Ctr, Halifax, NS, Canada.

[Vallis, Michael] Dalhousie Univ, Halifax, NS, Canada.

RP Vallis, M (reprint author), Diabet Management Ctr, 7071 Bayers Rd,Suite 165, Halifax, NS B3L 2C2, Canada.

EM tvallis@dal.ca

OI Vallis, Michael/0000-0002-0165-5936

FU Novo Nordisk

FX MV is the Canadian lead, and sits on the Global Advisory Board of the

Novo Nordisk funded DAWN2 study. In this capacity he receives honoraria

and travel expenses to present at professional venues regarding the DAWN

studies. No funding was provided for the preparation of this paper.

CR Alberti G, 2002, PRACT DIABETES INT, V19, P22, DOI DOI 10.1002/PDI.305/PDF

Crosson JC, 2010, J AM BOARD FAM MED, V23, P171, DOI 10.3122/jabfm.2010.02.090125

Crow S, 2001, INT J EAT DISORDER, V30, P222, DOI 10.1002/eat.1077

de Wit M, 2012, PEDIAT DIABETES

Egede LE, 2005, DIABETES CARE, V28, P1339, DOI 10.2337/diacare.28.6.1339

Egede LE, 2004, DIABETES CARE, V27, P421, DOI 10.2337/diacare.27.2.421

Fisher L, 2008, DIABETIC MED, V25, P1096, DOI 10.1111/j.1464-5491.2008.02533.x

Fisher L, 2009, DIABETES CARE, V33, P23

Fisher L, 2007, DIABETES CARE, V30, P2746, DOI 10.2337/dc07-1166

Fisher L, 2007, DIABETES CARE, V30, P542, DOI 10.2337/dc06-1614

Fisher L, 2010, DIABETES CARE, V33, P1034, DOI 10.2337/dc09-2175

Fisher L, 2010, DIABETES CARE, V33, P23, DOI 10.2337/dc09-1238

Glasgow RE, 1999, DIABETES CARE, V22, P832, DOI 10.2337/diacare.22.5.832

Grigsby AB, 2004, J PSYCHOSOM RES, V27, P1053

Group DS, 2006, PRACT DIABETES INT, V23, P313

Group DS, 2002, PRACT DIABETES INT, V19, P187

Group DS, 2004, PRACT DIABETES INT, V21, P201

Jones JM, 2000, BMJ-BRIT MED J, V320, P1563, DOI 10.1136/bmj.320.7249.1563

Lipscombe LL, 2007, LANCET, V369, P750, DOI 10.1016/S0140-6736(07)60361-4

Mezuk B, 2008, DIABETES CARE, V31, P2383, DOI 10.2337/dc08-0985

Moussavi S, 2007, LANCET, V370, P851, DOI 10.1016/S0140-6736(07)61415-9

Petrak F, 2005, CURR DIABETES REV, V1, P255, DOI 10.2174/157339905774574329

Peyrot M, 2005, DIABETIC MED, V22, P1379, DOI 10.1111/j.1464-5491.2005.01644.x

Polonsky WH, 2005, DIABETES CARE, V28, P626, DOI 10.2337/diacare.28.3.626

Skovlund S. E., 2005, DIABETES SPECTRUM, V18, P136, DOI DOI 10.2337/DIASPECT.18.3.136

Snoek FJ, 2011, DIABETES CARE, V34, P601, DOI 10.2337/dc10-1552

Soewondo P, 2010, MED J INDONES, V19, P235

van Bastelaar KMP, 2011, DIABETES CARE, V34, P320, DOI 10.2337/dc10-1248

Welch GW, 1997, DIABETES CARE, V20, P760, DOI 10.2337/diacare.20.5.760

NR 29

TC 1

Z9 1

U1 0

U2 2

PU ELSEVIER SCIENCE BV

PI AMSTERDAM

PA PO BOX 211, 1000 AE AMSTERDAM, NETHERLANDS

SN 1499-2671

J9 CAN J DIABETES

JI Can. J. Diabetes

PD AUG

PY 2012

VL 36

IS 4

BP 210

EP 213

DI 10.1016/j.jcjd.2012.06.006

PG 4

WC Endocrinology & Metabolism

SC Endocrinology & Metabolism

GA V33EQ

UT WOS:000209002500016

DA 2019-01-08

ER

PT J

AU Gill, RG

Bishop, NH

AF Gill, Ronald G.

Bishop, Nicholas H.

TI Clinical islet transplantation: where immunity and metabolism intersect?

SO CURRENT OPINION IN ENDOCRINOLOGY DIABETES AND OBESITY

LA English

DT Review

DE autoimmunity; islet transplantation; metabolism; type 1 diabetes

ID PROLONGS ALLOGRAFT SURVIVAL; TYPE-1 DIABETIC-PATIENTS; EARLY GRAFT

FAILURE; BETA-CELL FUNCTION; AMYLOID DEPOSITION; IMMUNOSUPPRESSIVE

REGIMEN; PANCREATIC-ISLETS; THERAPY; BLOCKADE; MICE

AB Purpose of review

The dramatic results of the Edmonton Protocol in 2000 triggered tremendous excitement over the application of pancreatic islet transplantation as a viable approach to achieving consistent insulin independence in type 1 diabetic patients. However, this optimism in the field was tempered by follow-up studies showing frequent attrition of graft function commonly requiring a return to exogenous insulin therapy within 1-3 years after transplant. The purpose of this review is to put these initial studies in perspective and to highlight progress and challenges in this important field.

Recent findings

Recent clinical and experimental findings demonstrate a progressive improvement in the function and durability of islet allografts. Induction therapies targeting T lymphocytes and costimulatory pathways have been highly effective at promoting islet transplant function. It is also apparent that islet injury associated with metabolic distress provides a nonimmune barrier to islet transplant outcomes.

Summary

Newer therapeutic interventions show great promise for attenuating the adaptive immune response to islet allografts. Also, clarifying the mechanisms of metabolic-related tissue distress may provide additional potential targets for improving islet graft outcomes.

C1 [Gill, Ronald G.] Univ Colorado, Dept Surg & Immunol, Colorado Ctr Transplantat Care Res & Educ CCTCARE, Aurora, CO 80045 USA.

RP Gill, RG (reprint author), Univ Colorado, Dept Surg & Immunol, Colorado Ctr Transplantat Care Res & Educ CCTCARE, 1775 Aurora Ct,Box F-528, Aurora, CO 80045 USA.

EM Ronald.Gill@ucdenver.edu

FU Juvenile Diabetes Research Foundation [4-2008-812]

FX This work was supported in part by Juvenile Diabetes Research Foundation

grant 4-2008-812.

CR Ablamunits V, 2012, DIABETES, V61, P145, DOI 10.2337/db11-1033

Badell IR, 2012, AM J TRANSPLANT, V12, P126, DOI 10.1111/j.1600-6143.2011.03736.x

Badell IR, 2010, J CLIN INVEST, V120, P4520, DOI 10.1172/JCI43895

Badell IR, 2012, AM J TRANSPLANT

Bellin MD, 2011, TRANSPLANTATION, V91, P376

Bussiere CT, 2006, DIABETOLOGIA, V49, P2341, DOI 10.1007/s00125-006-0374-5

Cai K, 2011, DIABETOLOGIA, V54, P617, DOI 10.1007/s00125-010-1972-9

Cantaluppi V, 2006, AM J TRANSPLANT, V6, P2601, DOI 10.1111/j.1600-6143.2006.01534.x

Cantley J, 2010, DIABETES OBES METAB, V12, P159, DOI 10.1111/j.1463-1326.2010.01276.x

Davalli AM, 1996, DIABETES, V45, P1161, DOI 10.2337/diabetes.45.9.1161

Donath MY, 2008, DIABETES CARE, V31, pS161, DOI 10.2337/dc08-s243

Emamaullee JA, 2006, DIABETES, V55, P1907, DOI 10.2337/db05-1254

Gao Q, 2010, AM J TRANSPLANT, V10, P1588, DOI 10.1111/j.1600-6143.2010.03151.x

Grossman EJ, 2010, PLOS ONE, V5, pe8759

Hering BJ, 2004, AM J TRANSPLANT, V4, P390, DOI 10.1046/j.1600-6143.2003.00351.x

Hull RL, 2004, J CLIN ENDOCR METAB, V89, P3629, DOI 10.1210/jc.2004-0405

Huurman VAL, 2008, PLOS ONE, V3, DOI 10.1371/journal.pone.0002435

Johansson H, 2006, AM J TRANSPLANT, V6, P305, DOI 10.1111/j.1600-6143.2005.01186.x

Koulmanda M, 2012, AM J TRANSPLANT

Kruger B, 2010, EUR J IMMUNOL, V40, P2914, DOI 10.1002/eji.201040601

Larsen CM, 2007, NEW ENGL J MED, V356, P1517, DOI 10.1056/NEJMoa065213

Masters SL, 2010, NAT IMMUNOL, V11, P897, DOI 10.1038/ni.1935

Matsuoka N, 2010, J CLIN INVEST, V120, P735, DOI 10.1172/JCI41360

McCall M, 2012, AM J TRANSPLANT, V12, P322, DOI 10.1111/j.1600-6143.2011.03796.x

Nicolls MR, 2000, J IMMUNOL, V164, P3627, DOI 10.4049/jimmunol.164.7.3627

Nir T, 2007, J CLIN INVEST, V117, P2553, DOI 10.1172/JCI32959

Ozmen L, 2002, DIABETES, V51, P1779, DOI 10.2337/diabetes.51.6.1779

Posselt AM, 2010, AM J TRANSPLANT, V10, P1870, DOI 10.1111/j.1600-6143.2010.03073.x

Posselt AM, 2010, TRANSPLANTATION, V90, P1595, DOI 10.1097/TP.0b013e3181fe1377

Potter KJ, 2010, P NATL ACAD SCI USA, V107, P4305, DOI 10.1073/pnas.0909024107

Roelen DL, 2009, CLIN EXP IMMUNOL, V156, P141, DOI 10.1111/j.1365-2249.2008.03812.x

Roep BO, 1999, DIABETES, V48, P484, DOI 10.2337/diabetes.48.3.484

Ryan EA, 2002, DIABETES, V51, P2148, DOI 10.2337/diabetes.51.7.2148

Ryan EA, 2005, DIABETES, V54, P2060, DOI 10.2337/diabetes.54.7.2060

Shapiro AMJ, 2006, NEW ENGL J MED, V355, P1318, DOI 10.1056/NEJMoa061267

Shapiro AMJ, 2000, NEW ENGL J MED, V343, P230, DOI 10.1056/NEJM200007273430401

Steer SA, 2006, PLOS MED, V3, P253, DOI 10.1371/journal.pmed.0030017

Sutherland DER, 2010, TRANSPLANTATION, V86, P1799

Tanemura M, 2011, AM J TRANSPLANT, V12, P102

Turgeon NA, 2010, AM J TRANSPLANT, V10, P2082, DOI 10.1111/j.1600-6143.2010.03212.x

Udayasankar J, 2009, DIABETOLOGIA, V52, P145, DOI 10.1007/s00125-008-1185-7

Westermark GT, 2008, NEW ENGL J MED, V359, P977, DOI 10.1056/NEJMc0802893

Westermark GT, 2012, TRANSPLANTATION, V93, P219, DOI 10.1097/TP.0b013e31823e46ef

Westwell-Roper C, 2011, J IMMUNOL, V187, P2755, DOI 10.4049/jimmunol.1002854

Zhang N, 2006, DIABETES, V55, P2429, DOI 10.2337/db06-0173

Zheng XX, 2003, IMMUNITY, V19, P503, DOI 10.1016/S1074-7613(03)00259-0

NR 46

TC 8

Z9 8

U1 0

U2 6

PU LIPPINCOTT WILLIAMS & WILKINS

PI PHILADELPHIA

PA 530 WALNUT ST, PHILADELPHIA, PA 19106-3621 USA

SN 1752-296X

J9 CURR OPIN ENDOCRINOL

JI Curr. Opin. Endocrinol. Diabetes Obes.

PD AUG

PY 2012

VL 19

IS 4

BP 249

EP 254

DI 10.1097/MED.0b013e328355a2ec

PG 6

WC Endocrinology & Metabolism

SC Endocrinology & Metabolism

GA 970FX

UT WOS:000306114600001

PM 22647539

DA 2019-01-08

ER

PT J

AU Roberts, MH

Mapel, DW

AF Roberts, Melissa H.

Mapel, Douglas W.

TI Limited Lung Function: Impact of Reduced Peak Expiratory Flow on Health

Status, Health-Care Utilization, and Expected Survival in Older Adults

SO AMERICAN JOURNAL OF EPIDEMIOLOGY

LA English

DT Article

DE aged; health services; mortality; peak expiratory flow rate; respiratory

function tests

ID OBSTRUCTIVE PULMONARY-DISEASE; ELDERLY POPULATION; COGNITIVE FUNCTION;

MORTALITY; SPIROMETRY; PREDICTOR; QUALITY; SAMPLE; PERFORMANCE;

DISABILITY

AB The authors examined whether peak expiratory flow (PEF) is a valid measure of health status in older adults. Survey and test data from the 2006 and 2008 cycles of the Health and Retirement Study, a longitudinal study of US adults over age 50 years (with biennial surveys initiated in 1992), were used to develop predicted PEF regression models and to examine relations between low PEF values and other clinical factors. Low PEF (80 of predicted value) was prevalent among persons with chronic conditions, including frequent pain, obstructive lung disease, heart disease, diabetes, and psychological distress. Persons with higher physical disability scores had substantially higher adjusted odds of having low PEF, on par with those for conditions known to be associated with poor health (cancer, heart disease, and stroke). In a multivariate regression model for difficulty with mobility, PEF remained an independent factor (odds ratio (OR) 1.69, 95 confidence interval (CI): 1.53, 1.86). Persons with low PEF in 2006 were more likely to be hospitalized (OR 1.26, 95 CI: 1.10, 1.43) within the subsequent 2 years and to estimate their chances of surviving for 10 or more years at less than 50 (OR 1.69, 95 CI: 1.24, 2.30). PEF is a valid measure of health status in older persons, and low PEF is an independent predictor of hospitalization and poor subjective mortality assessment.

C1 [Roberts, Melissa H.; Mapel, Douglas W.] LCF Res, Albuquerque, NM 87106 USA.

RP Roberts, MH (reprint author), LCF Res, 2309 Renard Pl SE,Suite 103, Albuquerque, NM 87106 USA.

EM melissa.roberts@lcfresearch.org

FU National Institute on Aging [U01AG009740]

FX The Health and Retirement Study was sponsored by the National Institute

on Aging (grant U01AG009740). No financial support was received for the

current analysis.

CR Allen S, 2008, CLIN RESPIR J, V2, P208, DOI 10.1111/j.1752-699X.2008.00063.x

[Anonymous], 2007, NIH PUBL

Bellia V, 2003, EUR RESPIR J, V21, p21S, DOI 10.1183/09031936.03.00402303

Bellia V, 2000, AM J RESP CRIT CARE, V161, P1094, DOI 10.1164/ajrccm.161.4.9810093

CARVALHAESNETO N, 1995, AM J RESP CRIT CARE, V152, P1611, DOI 10.1164/ajrccm.152.5.7582303

COOK NR, 1995, J GERONTOL A-BIOL, V50, pM317, DOI 10.1093/gerona/50A.6.M317

COOK NR, 1991, AM J EPIDEMIOL, V133, P784, DOI 10.1093/oxfordjournals.aje.a115957

COOK NR, 1989, AM J EPIDEMIOL, V130, P66, DOI 10.1093/oxfordjournals.aje.a115324

Cooper R, 2010, BMJ-BRIT MED J, V341, DOI 10.1136/bmj.c4467

Coultas DB, 2001, AM J RESP CRIT CARE, V164, P372, DOI 10.1164/ajrccm.164.3.2004029

Eisner MD, 2008, AM J EPIDEMIOL, V167, P1090, DOI 10.1093/aje/kwn025

Fonda SJ, 2004, DOCUMENTATION PHYS F

Fragoso CAV, 2008, J AM GERIATR SOC, V56, P1014, DOI 10.1111/j.1532-5415.2008.01687.x

Fragoso CAV, 2007, J GERONTOL A-BIOL, V62, P1147

Hankinson JL, 1999, AM J RESP CRIT CARE, V159, P179, DOI 10.1164/ajrccm.159.1.9712108

Hansen EF, 2001, AM J RESP CRIT CARE, V163, P690, DOI 10.1164/ajrccm.163.3.2006120

Hegewald MJ, 2007, CHEST, V131, P1494, DOI 10.1378/chest.06-2707

Hurd MD, 2002, ECON J, V112, P966, DOI 10.1111/1468-0297.00065

Klein BEK, 2001, DIABETES CARE, V24, P1967, DOI 10.2337/diacare.24.11.1967

Klein R, 2005, AM J OPHTHALMOL, V140, P129, DOI 10.1016/j.ajo.2004.12.049

Miller MR, 2009, RESP MED, V103, P442, DOI 10.1016/j.rmed.2008.09.016

Miller MR, 2005, EUR RESPIR J, V26, P319, DOI 10.1183/09031936.05.00034805

Newman AB, 2006, JAMA-J AM MED ASSOC, V295, P2018, DOI 10.1001/jama.295.17.2018

NUNN AJ, 1989, BRIT MED J, V298, P1068, DOI 10.1136/bmj.298.6680.1068

Pezzoli L, 2003, AGE AGEING, V32, P43, DOI 10.1093/ageing/32.1.43

Quanjer PH, 1997, EUR RESPIR J, V10, pS2

Salm M, 2010, CAN J ECON, V43, P1040, DOI 10.1111/j.1540-5982.2010.01605.x

Schunemann HJ, 2000, CHEST, V118, P656, DOI 10.1378/chest.118.3.656

Sin DD, 2005, CHEST, V127, P1952, DOI 10.1378/chest.127.6.1952

Steffick D. E., 2000, DOCUMENTATION AFFECT

Studenski S, 2011, JAMA-J AM MED ASSOC, V305, P50, DOI 10.1001/jama.2010.1923

Tilvis R, 1997, BRIT MED J, V314, P605, DOI 10.1136/bmj.314.7080.605

TVERSKY A, 1974, SCIENCE, V185, P1124, DOI 10.1126/science.185.4157.1124

Young RP, 2007, EUR RESPIR J, V30, P616, DOI 10.1183/09031936.00021707

NR 34

TC 16

Z9 16

U1 0

U2 5

PU OXFORD UNIV PRESS INC

PI CARY

PA JOURNALS DEPT, 2001 EVANS RD, CARY, NC 27513 USA

SN 0002-9262

J9 AM J EPIDEMIOL

JI Am. J. Epidemiol.

PD JUL 15

PY 2012

VL 176

IS 2

BP 127

EP 134

DI 10.1093/aje/kwr503

PG 8

WC Public, Environmental & Occupational Health

SC Public, Environmental & Occupational Health

GA 974CD

UT WOS:000306406500007

PM 22759722

OA Bronze, Green Published

DA 2019-01-08

ER

PT J

AU Whittemore, R

Jaser, S

Chao, A

Jang, M

Grey, M

AF Whittemore, Robin

Jaser, Sarah

Chao, Ariana

Jang, Myoungock

Grey, Margaret

TI Psychological Experience of Parents of Children With Type 1 Diabetes A

Systematic Mixed-Studies Review

SO DIABETES EDUCATOR

LA English

DT Review

ID POSTTRAUMATIC-STRESS-DISORDER; YOUNG-CHILDREN; GLYCEMIC CONTROL;

PSYCHOSOCIAL ADJUSTMENT; DEPRESSIVE SYMPTOMS; METABOLIC-CONTROL;

CONTROLLED-TRIAL; FAMILY; MELLITUS; MOTHERS

AB Purpose

The purpose of this review is to describe the prevalence of psychological distress in parents of children with type 1 diabetes (T1DM), the relationship between parental psychological distress and health outcomes, and parents' psychological experience of having a child with T1DM. Clinical and research implications are presented.

Method

A systematic mixed-studies review was undertaken to review the quantitative and qualitative research on the parental experience of having a child with T1DM. A total of 34 articles met the inclusion criteria and were included in the review.

Results

The prevalence of parental psychological distress across all studies ranged from 10% to 74%, with an average of 33.5% of parents reporting distress at diagnosis and 19% of parents reporting distress 1 to 4 years after diagnosis. Parental psychological distress in parents of children with T1DM, regardless of how it was defined, was associated with higher child self-report of stress and depressive symptoms, more problematic child behavior, and lower child self-report of quality of life. Parental psychological distress also had negative effects on diabetes management. Themes of the qualitative synthesis indicated that parents perceived T1DM as a difficult diagnosis that contributed to significant family disruption. Adjustment occurred over time; however, ongoing stress was experienced.

Conclusions

Screening for psychological distress in parents of children with T1DM is indicated, and preventive interventions are needed.

C1 [Whittemore, Robin; Jaser, Sarah; Chao, Ariana; Jang, Myoungock; Grey, Margaret] Yale Univ, Sch Nursing, New Haven, CT 06536 USA.

RP Whittemore, R (reprint author), Yale Univ, Sch Nursing, 100 Church St S, New Haven, CT 06536 USA.

EM robin.whittemore@yale.edu

FU [NIH/NR04009]; [RC1NR011594]

FX Special acknowledgements to Heather Jacobs for her assistance in the

preparation of this manuscript. Funding sources: NIH/NR04009;

RC1NR011594.

CR ANDERSON BJ, 1991, ADVANCES IN CHILD HEALTH PSYCHOLOGY, P167

Anderson BJ, 1999, DIABETES CARE, V22, P713, DOI 10.2337/diacare.22.5.713

Armour TA, 2005, DIABETIC MED, V22, P1295, DOI 10.1111/j.1464-5491.2005.01618.x

Auslander W F, 1993, Health Soc Work, V18, P101

Beardslee WR, 1998, J AM ACAD CHILD PSY, V37, P1134, DOI 10.1097/00004583-199811000-00012

Bowes S, 2009, J ADV NURS, V65, P992, DOI 10.1111/j.1365-2648.2009.04963.x

Buckloh LM, 2008, DIABETES CARE, V31, P1516, DOI 10.2337/dc07-2349

CADMAN D, 1991, PEDIATRICS, V87, P884

Cameron FJ, 2007, DIABETES CARE, V30, P2716, DOI 10.2337/dc07-0603

Cameron LD, 2007, J PEDIATR PSYCHOL, V32, P733, DOI 10.1093/jpepsy/jslo53

Chaney JM, 1997, J PEDIATR PSYCHOL, V22, P229

Eckshtain D, 2010, J PEDIATR PSYCHOL, V35, P426, DOI 10.1093/jpepsy/jsp068

Edmonds-Myles S, 2010, DIABETES EDUCATOR, V36, P318, DOI 10.1177/0145721709349219

Ellis DA, 2007, J CONSULT CLIN PSYCH, V75, P168, DOI 10.1037/0022-006X.75.1.168

Faulkner M S, 1996, J Pediatr Nurs, V11, P82, DOI 10.1016/S0882-5963(96)80065-0

Forsander GA, 2000, ACTA PAEDIATR, V89, P1462, DOI 10.1080/080352500456651

Golderberg D., 1988, USERS GUIDE GEN HLTH

Grey M, 2009, RES NURS HEALTH, V32, P405, DOI 10.1002/nur.20336

HATTON DL, 1995, J ADV NURS, V22, P569, DOI 10.1046/j.1365-2648.1995.22030569.x

Hilliard ME, 2011, CHILD CARE HLTH DEV, V37, P224, DOI 10.1111/j.1365-2214.2010.01162.x

Hoff A. L., 2005, FAMILIES SYSTEMS HLT, V23, P329, DOI DOI 10.1037/1091-7527.23.3.329

Jaser SS, 2008, J PEDIATR PSYCHOL, V33, P509, DOI 10.1093/jpepsy/jsm104

Jaser SS, 2010, J PEDIATR PSYCHOL, V35, P738, DOI 10.1093/jpepsy/jsp098

Jaser SS, 2009, CHILD HEALTH CARE, V38, P91, DOI 10.1080/02739610902813229

Kerr LK, 2001, WESTERN J MED, V175, P349, DOI 10.1136/ewjm.175.5.349

KOVACS M, 1990, J CONSULT CLIN PSYCH, V58, P189, DOI 10.1037//0022-006X.58.2.189

Laffel LMB, 2003, J PEDIATR-US, V142, P409, DOI 10.1067/mpd.2003.138

Landolt MA, 2005, J AM ACAD CHILD PSY, V44, P682, DOI 10.1097/01-chi.0000161645.98022.35

Landolt MA, 2002, J PEDIATR PSYCHOL, V27, P647, DOI 10.1093/jpepsy/27.7.647

Lowes L, 2005, J ADV NURS, V50, P253, DOI 10.1111/j.1365-2648.2005.03388.x

Lowes L, 2004, DIABETIC MED, V21, P531, DOI 10.1111/j.1464-5491.2004.01193.x

Marshall M, 2009, J CLIN NURS, V18, P1703, DOI 10.1111/j.1365-2702.2008.02737.x

McBroom LA, 2009, DIABETES EDUCATOR, V35, P428, DOI 10.1177/0145721709332814

Mellin AE, 2004, J PEDIATR PSYCHOL, V29, P221, DOI 10.1093/jpepsy/jsh023

Miles M., 1994, QUALITATIVE DATA ANA

Mitchell SJ, 2009, FAM SYST HEALTH, V27, P314, DOI 10.1037/a0018191

Monaghan MC, 2009, FAM SYST HEALTH, V27, P28, DOI 10.1037/a0014770

Mullins LL, 2004, CHILD HEALTH CARE, V33, P21, DOI 10.1207/s15326888chc3301_2

Northam E, 1996, J PEDIATR PSYCHOL, V21, P699, DOI 10.1093/jpepsy/21.5.699

Olson AL, 2006, PEDIATRICS, V118, P207, DOI 10.1542/peds.2005-2346

Pai ALH, 2008, J PEDIATR PSYCHOL, V33, P50, DOI 10.1093/jpepsy/jsm053

Pluye P, 2009, INT J NURS STUD, V46, P529, DOI 10.1016/j.ijnurstu.2009.01.009

Powers SW, 2002, DIABETES CARE, V25, P313, DOI 10.2337/diacare.25.2.313

RADLOFF L S, 1977, Applied Psychological Measurement, V1, P385, DOI 10.1177/014662167700100306

Rodrigue J R, 1994, Child Health Care, V23, P181, DOI 10.1207/s15326888chc2303_3

Seppanen S M, 1999, Nurs Health Sci, V1, P63, DOI 10.1046/j.1442-2018.1999.00009.x

Smaldone A, 2011, J PEDIATR HEALTH CAR, V25, P87, DOI 10.1016/j.pedhc.2009.09.003

Spielberger C. D., 1983, MANUAL STATE TRAIT A

Stallwood Lynda, 2005, J Pediatr Health Care, V19, P293, DOI 10.1016/j.pedhc.2005.04.003

Streisand R, 2005, J PEDIATR PSYCHOL, V30, P513, DOI 10.1093/jpepsy/jsi076

Streisand R, 2001, J PEDIATR PSYCHOL, V26, P155, DOI 10.1093/jpepsy/26.3.155

Streisand R, 2008, PATIENT EDUC COUNS, V73, P333, DOI 10.1016/j.pec.2008.06.014

Sullivan-Bolyai S, 2006, MCN-AM J MATERN-CHIL, V31, P24, DOI 10.1097/00005721-200601000-00007

Sullivan-Bolyai S, 2004, DIABETES EDUCATOR, V30, P476, DOI 10.1177/014572170403000319

Sullivan-Bolyai S, 2003, J Pediatr Nurs, V18, P21, DOI 10.1053/jpdn.2003.4

Sullivan-Bolyai Susan, 2002, J Spec Pediatr Nurs, V7, P93, DOI 10.1111/j.1744-6155.2002.tb00158.x

Sullivan-Bolyai S, 2010, DIABETES EDUCATOR, V36, P88, DOI 10.1177/0145721709352384

Viner R, 1996, ARCH DIS CHILD, V74, P418, DOI 10.1136/adc.74.5.418

Weinzimer SA, 2006, CLIN DIABETES, P505

Wennick A, 2006, J FAM NURS, V12, P368, DOI 10.1177/1074840706296724

Whittemore R, 2005, J ADV NURS, V52, P546, DOI 10.1111/j.1365-2648.2005.03621.x

Wood JJ, 2003, J CHILD PSYCHOL PSYC, V44, P134, DOI 10.1111/1469-7610.00106

Wysocki T, 2000, J PEDIATR PSYCHOL, V25, P23, DOI 10.1093/jpepsy/25.1.23

NR 63

TC 83

Z9 86

U1 4

U2 38

PU SAGE PUBLICATIONS INC

PI THOUSAND OAKS

PA 2455 TELLER RD, THOUSAND OAKS, CA 91320 USA

SN 0145-7217

J9 DIABETES EDUCATOR

JI Diabetes Educ.

PD JUL-AUG

PY 2012

VL 38

IS 4

BP 562

EP 579

DI 10.1177/0145721712445216

PG 18

WC Endocrinology & Metabolism; Public, Environmental & Occupational Health

SC Endocrinology & Metabolism; Public, Environmental & Occupational Health

GA 976AX

UT WOS:000306554000010

PM 22581804

OA Green Accepted

DA 2019-01-08

ER

PT J

AU Tsai, CH

Wu, JS

Chang, YF

Lu, FH

Yang, YC

Chang, CJ

AF Tsai, Chung-Hung

Wu, Jin-Shang

Chang, Yin-Fan

Lu, Feng-Hwa

Yang, Yi-Ching

Chang, Chih-Jen

TI The relationship between psychiatric symptoms and glycemic status in a

Chinese population

SO JOURNAL OF PSYCHIATRIC RESEARCH

LA English

DT Article

DE Brief symptoms rating scale; Depression; Anxiety; Diabetes mellitus;

Pre-diabetes; Hyperglycemia

ID TYPE-2 DIABETES-MELLITUS; DEPRESSIVE SYMPTOMS; INSULIN-RESISTANCE;

RISK-FACTOR; BIDIRECTIONAL ASSOCIATION; GLUCOSE-METABOLISM; FASTING

GLUCOSE; SLEEP DURATION; WOMEN; HOSTILITY

AB With the exception of depression and anxiety, there has been no study designed to evaluate the association between other psychiatric symptoms and Type 2 diabetes.

The aim of this study was to investigate the relationship between different psychiatric symptoms and diabetes as well as pre-diabetes (Pre-DM) in a Chinese population. Totally, 9561 participants without a history of diabetes, depression, psychosis, use of hypnotics, and abnormal thyroid function were enrolled. Psychiatric symptoms were measured by Brief Symptoms Rating Scale questionnaire, which consists of three global indices [General Severity Index (GSI), Total Number of Positive Symptoms (PST), and Positive Symptom Distress Index (PSDI)] and ten subscales, including somatization, obsession, interpersonal sensitivity, depression, anxiety, hostility, phobia, paranoid ideation, psychoticism and additional symptoms. Different glycemic statuses included normal glucose tolerance (NGT), Pre-DM, and newly-diagnosed diabetes (NDD) group. GSI, somatization, hostility, phobia, psychoticism, and additional symptoms were the factors positively associated with NDD as well as pre-DM in an age-adjusted model. After adjustments for age, gender, body mass index, educational level, hypertension, plasma triglycerides and creatinine, smoking, alcohol use, regular exercise, marital status, and family history of diabetes mellitus, the following psychiatric symptoms were independently related to both NDD and pre-DM: GSI, PST, somatization, obsession, interpersonal sensitivity, depression, anxiety, hostility, phobia, psychoticism, and additional symptoms. In addition to depression and anxiety, global indices of psychiatric symptoms and other subscales, including somatization, obsession, interpersonal sensitivity, hostility, phobia, psychoticism and additional symptoms, may have an impact on both diabetes and Pre-DM. (C) 2012 Elsevier Ltd. All rights reserved.

C1 [Tsai, Chung-Hung; Wu, Jin-Shang; Chang, Yin-Fan; Lu, Feng-Hwa; Yang, Yi-Ching; Chang, Chih-Jen] Natl Cheng Kung Univ Hosp, Dept Family Med, Tainan 70428, Taiwan.

[Wu, Jin-Shang; Lu, Feng-Hwa; Yang, Yi-Ching; Chang, Chih-Jen] Natl Cheng Kung Univ, Coll Med, Dept Family Med, Tainan 70101, Taiwan.

RP Yang, YC (reprint author), Natl Cheng Kung Univ Hosp, Dept Family Med, 138 Sheng Li Rd, Tainan 70428, Taiwan.

EM yiching@mail.ncku.edu.tw; em75210@email.nck.edu.tw

OI Yang, Yi-Ching/0000-0003-1391-8040

FU Department of Family Medicine, National Cheng-Kung University Hospital

[NCKUHFM-100-003]

FX This study was supported by the grants from Department of Family

Medicine, National Cheng-Kung University Hospital (NCKUHFM-100-003).

CR *AM DIAB ASS, 2009, DIABETES CARE S1, V32, pS13, DOI DOI 10.2337/DC09-S013

Appels A, 2000, PSYCHOSOM MED, V62, P601, DOI 10.1097/00006842-200009000-00001

Arroyo C, 2004, DIABETES CARE, V27, P129, DOI 10.2337/diacare.27.1.129

Aujla N, 2009, PLOS ONE, V4, DOI 10.1371/journal.pone.0007755

Ayas NT, 2003, DIABETES CARE, V26, P380, DOI 10.2337/diacare.26.2.380

Bjorntorp P, 2001, SCAND CARDIOVASC J, V35, P172, DOI 10.1080/140174301750305045

Bouwman V, 2010, PLOS ONE, V5, DOI 10.1371/journal.pone.0009971

Brown ES, 2004, BIOL PSYCHIAT, V55, P1, DOI 10.1016/S0006-3223(03)00473-6

Campayo A, 2010, AM J PSYCHIAT, V167, P580, DOI 10.1176/appi.ajp.2009.09010038

Carnethon MR, 2007, ARCH INTERN MED, V167, P802, DOI 10.1001/archinte.167.8.802

Carnethon MR, 2003, AM J EPIDEMIOL, V158, P416, DOI 10.1093/aje/kwg172

de Vegt F, 1999, DIABETOLOGIA, V42, P926, DOI 10.1007/s001250051249

Eaton WW, 1996, DIABETES CARE, V19, P1097, DOI 10.2337/diacare.19.10.1097

Engum A, 2007, J PSYCHOSOM RES, V62, P31, DOI 10.1016/j.jpsychores.2006.07.009

Eriksson AK, 2008, DIABETIC MED, V25, P834, DOI 10.1111/j.1464-5491.2008.02463.x

Everson-Rose SA, 2004, DIABETES CARE, V27, P2856, DOI 10.2337/diacare.27.12.2856

Georgiades A, 2009, PSYCHOSOM MED, V71, P642, DOI 10.1097/PSY.0b013e3181acee3a

Golden SH, 2004, DIABETES CARE, V27, P429, DOI 10.2337/diacare.27.2.429

Golden SH, 2008, JAMA-J AM MED ASSOC, V299, P2751, DOI 10.1001/jama.299.23.2751

Gonzalez JS, 2007, DIABETES CARE, V30, P2222, DOI 10.2337/dc07-0158

Grigsby AB, 2002, J PSYCHOSOM RES, V53, P1053, DOI 10.1016/S0022-3999(02)00417-8

Haupt DW, 2001, J CLIN PSYCHIAT, V62, P15

Haupt DW, 2001, J CLIN PSYCHIAT S27, V62, P40

Hotamisligil GS, 2006, NATURE, V444, P860, DOI 10.1038/nature05485

Huxley R, 2006, BMJ-BRIT MED J, V332, P73, DOI 10.1136/bmj.38678.389583.7C

Jiang LH, 2008, DIABETES CARE, V31, P427, DOI 10.2337/dc07-2044

Kato M, 2009, ENDOCR J, V56, P459, DOI 10.1507/endocrj.K09E-003

KAWAKAMI N, 1995, IND HEALTH, V33, P153, DOI 10.2486/indhealth.33.153

Kawakami N, 1999, DIABETES CARE, V22, P1071, DOI 10.2337/diacare.22.7.1071

Kivimaki M, 2010, DIABETES CARE, V33, P2611, DOI 10.2337/dc10.1187

Knol MJ, 2006, DIABETOLOGIA, V49, P837, DOI 10.1007/s00125-006-0159-x

Kumari M, 2004, ARCH INTERN MED, V164, P1873, DOI 10.1001/archinte.164.17.1873

Lee M B, 1990, J Formos Med Assoc, V89, P1081

Lin LS, 1995, CHINESE PSYCHIAT, V9, P65

Mallon L, 2005, DIABETES CARE, V28, P2762, DOI 10.2337/diacare.28.11.2762

Mezuk B, 2008, DIABETES CARE, V31, P2383, DOI 10.2337/dc08-0985

Narita K, 2008, PSYCHONEUROENDOCRINO, V33, P305, DOI 10.1016/j.psyneuen.2007.11.013

Olson MM, 2010, INT J PSYCHIAT MED, V40, P289, DOI 10.2190/PM.40.3.e

Palinkas LA, 2004, DIABETIC MED, V21, P1185, DOI 10.1111/j.1464-5491.2004.01315.x

Pan A, 2010, ARCH INTERN MED, V170, P1884, DOI 10.1001/archinternmed.2010.356

Pouwer F, 2010, DISCOV MED, V9, P112

Powers A. C., 2001, HARRISONS PRINCIPLES, P2109

Raikkonen K, 1996, METABOLISM, V45, P1533, DOI 10.1016/S0026-0495(96)90184-5

Rief W, 1998, PSYCHOSOM MED, V60, P198, DOI 10.1097/00006842-199803000-00016

Rod NH, 2009, J INTERN MED, V266, P467, DOI 10.1111/j.1365-2796.2009.02124.x

Rosmond Roland, 2003, Med Sci Monit, V9, pRA35

SCHERWITZ LW, 1992, AM J EPIDEMIOL, V136, P136, DOI 10.1093/oxfordjournals.aje.a116480

Shen BJ, 2008, DIABETES CARE, V31, P1293, DOI 10.2337/dc07-1945

Smith TW, 2001, HANDBOOK OF HEALTH PSYCHOLOGY, P139

Surwit RS, 2002, DIABETES CARE, V25, P835, DOI 10.2337/diacare.25.5.835

van den Akker M, 2004, ACTA PSYCHIAT SCAND, V110, P178

Young EA, 2004, BIOL PSYCHIAT, V56, P113, DOI 10.1016/j.biopsych.2004.03.017

Zhang JP, 2006, PSYCHOSOM MED, V68, P718, DOI 10.1097/01.psy.0000228343.89466.11

NR 53

TC 2

Z9 3

U1 1

U2 13

PU PERGAMON-ELSEVIER SCIENCE LTD

PI OXFORD

PA THE BOULEVARD, LANGFORD LANE, KIDLINGTON, OXFORD OX5 1GB, ENGLAND

SN 0022-3956

EI 1879-1379

J9 J PSYCHIATR RES

JI J. Psychiatr. Res.

PD JUL

PY 2012

VL 46

IS 7

BP 927

EP 932

DI 10.1016/j.jpsychires.2012.04.003

PG 6

WC Psychiatry

SC Psychiatry

GA 975UD

UT WOS:000306536100013

PM 22608774

DA 2019-01-08

ER

PT J

AU Mols, F

Oerlemans, S

Denollet, J

Roukema, JA

van de Poll-Franse, LV

AF Mols, Floortje

Oerlemans, Simone

Denollet, Johan

Roukema, Jan-Anne

van de Poll-Franse, Lonneke V.

TI Type D personality is associated with increased comorbidity burden and

health care utilization among 3080 cancer survivors

SO GENERAL HOSPITAL PSYCHIATRY

LA English

DT Article

DE Cancer; Comorbidity; Health care utilization; Type D personality

ID QUALITY-OF-LIFE; CORONARY-HEART-DISEASE; MYOCARDIAL-INFARCTION;

DISTRESSED PERSONALITY; DEPRESSION; QUESTIONNAIRE; MECHANISMS;

POPULATION; MORTALITY; SYMPTOMS

AB Objective: Cancer survivors often report comorbid diseases, but there are individual differences in risk. Type D personality is a general propensity to psychological distress that is related to poor cardiovascular outcomes. In this study, we examined whether type D was also related to comorbidity burden and health care utilization among cancer survivors.

Methods: Individuals diagnosed with endometrial cancer or colorectal cancer between 1998 and 2007, or with lymphoma or multiple myeloma between 1999 and 2008 as registered in the Eindhoven Cancer Registry, received the Self-Administered Comorbidity Questionnaire, questions on health care utilization and the Type D personality scale; 69% (n=3080) responded.

Results: Nineteen percent of survivors had a type D personality. Over a 12-month period, type D survivors significantly more often reported osteoarthritis, back pain, and depression than non type D survivors. Also, type D survivors more often reported to feel bothered by high blood pressure, osteoarthritis, heart disease, depression, diabetes and lung disease during daily activities. Type D survivors more often visited their general practitioner than non type D survivors (P<.001), also in relation to cancer (0 visits: 54% vs. 60%; 1-5: 28% vs. 22%; >5: 9% vs. 5%; P<.001), as well as their specialist (0 visits: 6% vs. 7%; 1-5 visits: 59% vs. 64%; >5 visits: 30% vs. 23%; P<.01).

Conclusion: Type D personality is a vulnerability factor that may help to identify subgroups of cancer survivors who are at an increased risk for comorbidity burden and increased health care utilization. (C) 2012 Elsevier Inc. All rights reserved.

C1 [Mols, Floortje; Oerlemans, Simone; Denollet, Johan; Roukema, Jan-Anne; van de Poll-Franse, Lonneke V.] Tilburg Univ, Dept Med Psychol & Neuropsychol, CoRPS Ctr Res Psychol Somat Dis, NL-5000 LE Tilburg, Netherlands.

[Mols, Floortje; Oerlemans, Simone; van de Poll-Franse, Lonneke V.] Eindhoven Canc Registry, Ctr Comprehens Canc S, Eindhoven, Netherlands.

[Roukema, Jan-Anne] St Elizabeth Hosp, Dept Surg, Tilburg, Netherlands.

RP Mols, F (reprint author), Tilburg Univ, Dept Med Psychol & Neuropsychol, CoRPS Ctr Res Psychol Somat Dis, POB 90153, NL-5000 LE Tilburg, Netherlands.

EM F.Mols@uvt.nl

FU Netherlands Organization for Scientific Research (The Hague, The

Netherlands) [451-10-041]; Netherlands Organization for Scientific

Research [480-08-009]

FX The present research was supported by a VENI grant (#451-10-041) from

the Netherlands Organization for Scientific Research (The Hague, The

Netherlands) awarded to Floortje Mols. The data collection of this study

was funded by an Investment Subsidy (#480-08-009) of the Netherlands

Organization for Scientific Research awarded to Lonneke van de

Poll-Franse for setting up the PROFILES registry. These funding agencies

had no further role in study design; in the collection, analysis and

interpretation of data; in the writing of the report; and in the

decision to submit the article for publication.

CR Aquarius AE, 2005, AM J CARDIOL, V96, P996, DOI 10.1016/j.amjcard.2005.05.059

Babyak MA, 2004, PSYCHOSOM MED, V66, P411, DOI 10.1097/01.psy.0000127692.23278.a9

Bartels H, 2009, OTOL NEUROT IN PRESS

Brostrom A, 2007, J SLEEP RES, V16, P439, DOI 10.1111/j.1365-2869.2007.00620.x

Chapman BP, 2007, EUR J PERSONALITY, V21, P911, DOI 10.1002/per.645

COLDITZ GA, 1986, AM J EPIDEMIOL, V123, P894, DOI 10.1093/oxfordjournals.aje.a114319

de Valck CFJ, 2007, AUDIOLOGICAL MED, V5, P169

DENOLLET J, 1995, PSYCHOSOM MED, V57, P582, DOI 10.1097/00006842-199511000-00011

Denollet J, 2000, CIRCULATION, V102, P630, DOI 10.1161/01.CIR.102.6.630

Denollet J, 2005, PSYCHOSOM MED, V67, P89, DOI 10.1097/01.psy.0000149256.81953.49

Denollet J, 2009, PSYCHOL MED, V39, P749, DOI 10.1017/S0033291708004157

Denollet J, 1996, LANCET, V347, P417, DOI 10.1016/S0140-6736(96)90007-0

Doyle F, 2011, GEN HOSP PSYCHIAT, V33, P224, DOI 10.1016/j.genhosppsych.2011.03.008

Ganz PA, 2003, EUR J CANCER, V39, P2136, DOI 10.1016/S0959-8049(03)00489-1

Janssen-Heijnen MLG LW, 2005, RESULTS 50 YEARS CAN

Jellesma FC, 2008, J DEV BEHAV PEDIAT

Katz JN, 1996, MED CARE, V34, P73, DOI 10.1097/00005650-199601000-00006

Kupper N, 2007, J PERS ASSESS, V89, P265, DOI 10.1080/00223890701629797

Lang K, 2009, BMC HEALTH SERV RES, V9, P227

Li X, 2011, ANN ONCOL

LINET MS, 1989, J CLIN EPIDEMIOL, V42, P1207, DOI 10.1016/0895-4356(89)90119-4

Louwman WJ, 2010, BRIT J CANCER, V103, P1742, DOI 10.1038/sj.bjc.6605949

Martens EJ, 2008, PSYCHOL MED, V38, P257, DOI 10.1017/S0033291707001377

Meulenbroek LFP, 2010, J PSYCHOSOM RES, V68, P379, DOI 10.1016/j.jpsychores.2009.10.002

Mols F, 2011, J AFFECT DISORD

Mols F, 2007, INT J CANCER, V121, P871, DOI 10.1002/ijc.22739

Mols F, 2012, J AFFECT DISORDERS, V136, P26, DOI 10.1016/j.jad.2011.08.034

Mols F, 2010, EUR J CANCER, V46, P573, DOI 10.1016/j.ejca.2009.09.016

Mols F, 2010, GEN HOSP PSYCHIAT, V32, P66, DOI 10.1016/j.genhosppsych.2009.09.010

Mols F, 2010, HEALTH QUAL LIFE OUT, V8, DOI 10.1186/1477-7525-8-9

Pedersen SS, 2004, J PSYCHOSOM RES, V57, P265, DOI 10.1016/S0022-3999(03)00614-7

Pedersen SS, 2003, EUR J CARDIOV PREV R, V10, P241, DOI 10.1097/01.hjr.0000085246.65733.06

Pedersen SS, 2006, AM HEART J, V151, p367e1

Pedersen SS, 2011, AM J CARDIOL, V108, P69, DOI 10.1016/j.amjcard.2011.02.344

Pelle AJ, 2009, INT J CARDIOL

Sangha O, 2003, ARTHRIT RHEUM-ARTHR, V49, P156, DOI 10.1002/art.10993

Schiffer AA, 2007, HEART, V93, P814, DOI 10.1136/hrt.2006.102822

Shun SC, 2011, ONCOL NURS FORUM, V38, P378, DOI 10.1188/11.ONF.E221-E228

*SIGN KANK, 2004, KANK NED TRENDS PROG

Thomas G, 2006, FOLIA PHONIATR LOGO, V58, P250, DOI 10.1159/000093182

UICC, 1992, TNM ATL ILL GUID TNM, P141

van de Poll-Franse LV, 2011, EUR J CANC

van Duijn C, 2002, MAANDSTATISTIEK BEVO, V50, P32

Williams L, 2008, J PSYCHOSOM RES, V64, P63, DOI 10.1016/j.jpsychores.2007.06.008

Williams L, 2011, J PSYCHOSOM RES, V70, P141, DOI 10.1016/j.jpsychores.2010.07.015

NR 45

TC 22

Z9 22

U1 0

U2 11

PU ELSEVIER SCIENCE INC

PI NEW YORK

PA 360 PARK AVE SOUTH, NEW YORK, NY 10010-1710 USA

SN 0163-8343

J9 GEN HOSP PSYCHIAT

JI Gen. Hosp. Psych.

PD JUL-AUG

PY 2012

VL 34

IS 4

BP 352

EP 359

DI 10.1016/j.genhosppsych.2012.01.014

PG 8

WC Psychiatry

SC Psychiatry

GA 972TI

UT WOS:000306302700005

PM 22459997

OA Bronze

DA 2019-01-08

ER

PT J

AU Beverly, EA

Ganda, OP

Ritholz, MD

Lee, Y

Brooks, KM

Lewis-Schroeder, NF

Hirose, M

Weinger, K

AF Beverly, Elizabeth A.

Ganda, Om P.

Ritholz, Marilyn D.

Lee, Yishan

Brooks, Kelly M.

Lewis-Schroeder, Nina F.

Hirose, Masakazu

Weinger, Katie

TI Look Who's (Not) Talking Diabetic patients' willingness to discuss

self-care with physicians

SO DIABETES CARE

LA English

DT Article

ID QUALITY-OF-LIFE; GLYCEMIC CONTROL; PROVIDER COMMUNICATION; DEPRESSIVE

SYMPTOMS; HEALTH-CARE; ADULTS; COMPLICATIONS; MANAGEMENT; METAANALYSIS;

PREVALENCE

AB OBJECTIVE-Nearly one-half of diabetic patients have glycated hemoglobin A(1c) (HbA(1c)) levels above recommended targets. Effective physician patient communication improves glycemia and diabetes self-care; however, communication gaps may exist that prevent patients from discussing self-care problems with treatment providers.

RESEARCH DESIGN AND METHODS-We assessed diabetic patients' (n = 316, 85% white, 51% female, 71% type 2 diabetes, 59 +/- 11 years old, 16 +/- 3 years education, 19 +/- 13 years diabetes duration, and HbA(1c) = 7.9 +/- 1.4%) HbA(1c), frequency of self-care, diabetes-related distress, depressive and anxiety symptoms, coping styles, diabetes quality of life, and self-care communication in the treatment relationship. Multivariate logistic regression models examined the main and interaction effects of health and psychosocial factors associated with patients' reluctance to discuss self-care.

RESULTS-Patients reported positive relationships with their doctors and valued honest communication; however, 30% of patients were reluctant to discuss self-care. Reluctant patients reported less frequent self-care (P = 0.05), lower diabetes quality of life (P = 0.002), and more diabetes-related distress (P = 0.001), depressive symptoms (P < 0.001), and anxiety symptoms (P = 0.001). Patients who reported elevated depressive symptoms, although not necessarily major depression, were more likely to be reluctant to discuss self-care (odds ratio [OR] 1.66 for 10-point change in t score; P < 0.001), whereas patients who were older (OR 0.78 for 10-year change; P = 0.05) and those who used more self-controlled coping styles (OR 0.78 for 10-point change; P = 0.007) were less likely to be reluctant.

CONCLUSIONS-Awareness of elevated depressive symptoms is important in clinical practice given that these patients may be more reluctant to discuss self-care. Interventions and evidence-based approaches are needed to improve both depressive symptoms and physician-patient communication about self-care.

C1 [Beverly, Elizabeth A.; Ganda, Om P.; Ritholz, Marilyn D.; Lee, Yishan; Brooks, Kelly M.; Lewis-Schroeder, Nina F.; Hirose, Masakazu; Weinger, Katie] Joslin Diabet Ctr, Boston, MA 02215 USA.

[Beverly, Elizabeth A.; Ganda, Om P.; Ritholz, Marilyn D.; Lewis-Schroeder, Nina F.; Hirose, Masakazu; Weinger, Katie] Harvard Univ, Sch Med, Boston, MA USA.

[Ritholz, Marilyn D.] Childrens Hosp, Boston, MA 02115 USA.

RP Weinger, K (reprint author), Joslin Diabet Ctr, Boston, MA 02215 USA.

EM katie.weinger@joslin.harvard.edu

FU Kathleen P. Welsh Fund; National Institutes of Health (NIH) Grant

[R01-DK-60115]; American Diabetes Association Grant [7-08-CR-62];

Diabetes and Endocrinology Research Core NIH [P30-DK-36836]; NIH

Training Grant [T32-DK-007260]; Merck; Abbott; GlaxoSmithKline;

Bristol-Myers Squibb; AstraZeneca; Daiichi Sankyo

FX This work was supported by the Kathleen P. Welsh Fund, National

Institutes of Health (NIH) Grant R01-DK-60115, American Diabetes

Association Grant 7-08-CR-62, the Diabetes and Endocrinology Research

Core NIH P30-DK-36836, and NIH Training Grant T32-DK-007260.; O.P.G. has

provided consulting services to Abbott; received payment for lectures

from Merck, Abbott, GlaxoSmithKline, Bristol-Myers Squibb, AstraZeneca,

and Daiichi Sankyo; and has stock options with BMS. No other potential

conflicts of interest relevant to this article were reported.

CR Aikens JE, 2005, DIABETES EDUCATOR, V31, P681, DOI 10.1177/0145721705280829

*AM DIAB ASS, 2011, DIABETES CARE S1, V34, pS11, DOI DOI 10.2337/DC11-S011

Anderson RJ, 2001, DIABETES CARE, V24, P1069, DOI 10.2337/diacare.24.6.1069

Bastiaens H, 2007, PATIENT EDUC COUNS, V68, P33, DOI 10.1016/j.pec.2007.03.025

Beck A. T., 1976, COGNITIVE THERAPY EM

Bennett IM, 2009, ARCH WOMEN MENT HLTH, V12, P301, DOI 10.1007/s00737-009-0103-4

Berry LL, 2008, ANN FAM MED, V6, P6, DOI 10.1370/afm.757

Blaum CS, 2002, J AM GERIATR SOC, V50, P581, DOI 10.1046/j.1532-5415.2002.50130.x

Bradley C, 1994, HDB PSYCHOL DIABETES, P65

Bundesmann R, 2011, PATIENT EDUC COUNS, V85, P143, DOI 10.1016/j.pec.2010.09.025

Burns DD, 1980, FEELING GOOD NEW MOO

Ciechanowski P, 2006, SOC SCI MED, V63, P3067, DOI 10.1016/j.socscimed.2006.08.002

Derogatis L. R., 2000, BSI 18 BRIEF SYMPTOM

Fisher E. B., 2007, DIABETES ED, V33, P1104

Fisher EB, 2007, DIABETES EDUCATOR, V33, P1080, DOI 10.1177/0145721707309808

Fisher L, 2010, DIABETES CARE, V33, P1034, DOI 10.2337/dc09-2175

Gonzalez JS, 2008, DIABETES CARE, V31, P2398, DOI 10.2337/dc08-1341

Heisler M, 2007, J GERONTOL A-BIOL, V62, P1435, DOI 10.1093/gerona/62.12.1435

Hoerger TJ, 2008, DIABETES CARE, V31, P81, DOI 10.2337/dc07-1.572

JACOBSON AM, 1994, DIABETES CARE, V17, P267, DOI 10.2337/diacare.17.4.267

Kokanovic R, 2007, J GEN INTERN MED, V22, P459, DOI 10.1007/s11606-007-0143-2

Lin EHB, 2004, DIABETES CARE, V27, P2154, DOI 10.2337/diacare.27.9.2154

Lin EHB, 2009, ANN FAM MED, V7, P414, DOI 10.1370/afm.998

Lin EHB, 2010, DIABETES CARE, V33, P264, DOI 10.2337/dc09-1068

Lustman PJ, 2000, DIABETES CARE, V23, P934, DOI 10.2337/diacare.23.7.934

Nathan DM, 2005, NEW ENGL J MED, V353, P2643

Peters S, 2009, J GEN INTERN MED, V24, P443, DOI 10.1007/s11606-008-0872-x

Peyrot M, 2005, DIABETIC MED, V22, P1379, DOI 10.1111/j.1464-5491.2005.01644.x

Peyrot M, 1999, J HEALTH SOC BEHAV, V40, P141, DOI 10.2307/2676370

Piette JD, 2003, J GEN INTERN MED, V18, P624, DOI 10.1046/j.1525-1497.2003.31968.x

POLONSKY WH, 1995, DIABETES CARE, V18, P754, DOI 10.2337/diacare.18.6.754

Ritholz MD, 2011, DIABETES EDUCATOR, V37, P794, DOI 10.1177/0145721711423320

Rogers AE, 2000, BRIT MED J, V321, P605, DOI 10.1136/bmj.321.7261.605

Schenker Y, 2009, J GEN INTERN MED, V24, P550, DOI 10.1007/s11606-009-0937-5

SHAMOON H, 1993, NEW ENGL J MED, V329, P977

Speight J, 2009, DIABETIC MED, V26, P315, DOI 10.1111/j.1464-5491.2009.02682.x

Swenson SL, 2008, MED CARE, V46, P257, DOI 10.1097/MLR.0b013e31816080e9

Turner RC, 1998, LANCET, V352, P837

Weinger K, 2005, DIABETES CARE, V28, P1346, DOI 10.2337/diacare.28.6.1346

Welch GW, 1997, DIABETES CARE, V20, P760, DOI 10.2337/diacare.20.5.760

Wolff JL, 2002, ARCH INTERN MED, V162, P2269, DOI 10.1001/archinte.162.20.2269

NR 41

TC 40

Z9 42

U1 0

U2 15

PU AMER DIABETES ASSOC

PI ALEXANDRIA

PA 1701 N BEAUREGARD ST, ALEXANDRIA, VA 22311-1717 USA

SN 0149-5992

J9 DIABETES CARE

JI Diabetes Care

PD JUL

PY 2012

VL 35

IS 7

BP 1466

EP 1472

DI 10.2337/dc11-2422

PG 7

WC Endocrinology & Metabolism

SC Endocrinology & Metabolism

GA 962MJ

UT WOS:000305548900012

PM 22826455

OA Green Published, Other Gold

DA 2019-01-08

ER

PT J

AU Denny, CH

Floyd, RL

Green, PP

Hayes, DK

AF Denny, Clark H.

Floyd, R. Louise

Green, Patricia P.

Hayes, Donald K.

TI Racial and Ethnic Disparities in Preconception Risk Factors and

Preconception Care

SO JOURNAL OF WOMENS HEALTH

LA English

DT Article

ID PREGESTATIONAL DIABETES-MELLITUS; FREQUENT MENTAL DISTRESS; RANDOMIZED

CONTROLLED-TRIAL; UNITED-STATES; BIRTH OUTCOMES; HEALTH-CARE; PERINATAL

OUTCOMES; PREGNANCY OUTCOMES; REPRODUCTIVE-AGE; PRETERM BIRTH

AB Objective: At-risk drinking, cigarette smoking, obesity, diabetes, and frequent mental distress, as well as their co-occurrence in childbearing aged women, are risk factors for adverse pregnancy outcomes. This study estimated the prevalence of these five risk factors individually and in combination among nonpregnant women aged 18-44 years by demographic and psychosocial characteristics, with a focus on racial and ethnic disparities.

Methods: Data from the 2008 Behavioral Risk Factor Surveillance System (BRFSS) on nonpregnant women aged 18-44 years (n = 54,612) were used to estimate the prevalences of five risk factors, pairs of co-occurring risk factors, and multiple risk factors for poor pregnancy outcomes.

Results: The majority of women had at least one risk factor, and 18.7% had two or more risk factors. Having two or more risk factors was highest among women who were American Indian and Alaska Native (34.4%), had less than a high school education (28.7%), were unable to work (50.1%), were unmarried (23.3%), and reported sometimes, rarely, or never receiving sufficient social and emotional support (32.8%). The most prevalent pair of co-occurring risk factors was at-risk drinking and smoking (5.7%).

Conclusions: The high proportion of women of childbearing age with preconception risk factors highlights the need for preconception care. The common occurrence of multiple risk factors suggests the importance of developing screening tools and interventions that address risk factors that can lead to poor pregnancy outcomes. Increased attention should be given to high-risk subgroups.

C1 [Denny, Clark H.; Floyd, R. Louise; Green, Patricia P.] Ctr Dis Control & Prevent, Fetal Alcohol Syndrome Prevent Team, Div Birth Defects & Dev Disabil, Natl Ctr Birth Defects & Dev Disabil, Atlanta, GA 30333 USA.

[Hayes, Donald K.] Ctr Dis Control & Prevent, Appl Sci Branch, Div Reprod Hlth, Natl Ctr Chron Dis Prevent & Hlth Promot, Atlanta, GA 30333 USA.

RP Denny, CH (reprint author), Ctr Dis Control & Prevent, Fetal Alcohol Syndrome Prevent Team, Div Birth Defects & Dev Disabil, Natl Ctr Birth Defects & Dev Disabil, 1600 Clifton Rd NE,Mailstop E-86, Atlanta, GA 30333 USA.

EM cdenny@cdc.gov

CR Ahluwalia IB, 2005, J WOMENS HEALTH, V14, P382, DOI 10.1089/jwh.2005.14.382

Ahluwalia IB, 2004, OBSTET GYNECOL, V104, P477, DOI 10.1097/01.AOG.0000137920.58741.26

Alder J, 2007, J MATERN-FETAL NEO M, V20, P189, DOI 10.1080/14767050701209560

Alexander GR, 2008, MATERN CHILD HLTH J, V12, pS5, DOI 10.1007/s10995-007-0295-z

Allaire AD, 1998, EUR J OBSTET GYN R B, V78, P163, DOI 10.1016/S0301-2115(98)00062-1

American Academy of Pediatrics, 2000, PEDIATRICS, V106, P358

American Diabetes Association, 2011, DIABETES CARE S1, V34, pS11, DOI DOI 10.2337/DC11-S011

Anderson JE, 2006, MATERN CHILD HLTH J, V10, pS101, DOI 10.1007/s10995-006-0093-z

Andresen EM, 2001, QUAL LIFE RES, V10, P609, DOI 10.1023/A:1013187903591

Atrash H, 2008, CURR OPIN OBSTET GYN, V20, P581, DOI 10.1097/GCO.0b013e328317a27c

Atrash HK, 2006, MATERN CHILD HLTH J, V10, pS3, DOI 10.1007/s10995-006-0100-4

Berg C J, 2001, Matern Child Health J, V5, P75, DOI 10.1023/A:1011344914802

Blumberg Stephen J, 2011, Natl Health Stat Report, P1

Castor ML, 2006, AM J PUBLIC HEALTH, V96, P1478, DOI 10.2105/AJPH.2004.053942

*CDC, 2002, MMWR-MORBID MORTAL W, V51, P1

Centers for Disease Control and Prevention (CDC), 1998, MMWR Morb Mortal Wkly Rep, V47, P326

Centers for Disease Control and Prevention (CDC), 2009, MMWR Morb Mortal Wkly Rep, V58, P1227

Committee on Obstetric Practice, 2009, Obstet Gynecol, V113, P1419, DOI 10.1097/AOG.0b013e3181ac06b6

Correa A, 2008, AM J OBSTET GYNECOL, V199, DOI 10.1016/j.ajog.2008.06.028

Cowie CC, 2006, DIABETES CARE, V29, P1263, DOI 10.2337/dc06-0062

Delnevo CD, 2008, AM J EPIDEMIOL, V167, P15, DOI 10.1093/aje/kwm313

Dominguez TP, 2008, HEALTH PSYCHOL, V27, P194, DOI 10.1037/0278-6133.27.2.194

Dorfman S. F., 2008, TREATING TOBACCO USE

Elsinga J, 2008, WOMEN HEALTH ISS, V18, pS117, DOI 10.1016/j.whi.2008.09.003

Finer LB, 2006, PERSPECT SEX REPRO H, V38, P90, DOI 10.1363/3809006

Floyd RL, 2007, AM J PREV MED, V32, P1, DOI 10.1016/j.amepre.2006.08.028

Floyd RL, 1999, AM J PREV MED, V17, P101, DOI 10.1016/S0749-3797(99)00059-8

Galindo A, 2006, J PERINAT MED, V34, P323, DOI 10.1515/JPM.2006.052

Gennaro S, 2003, JOGNN, V32, P668, DOI 10.1177/0884217503257484

Giscombe CL, 2005, PSYCHOL BULL, V131, P662, DOI 10.1037/0033-2909.131.5.662

Glynn LM, 2007, PEPTIDES, V28, P1155, DOI 10.1016/j.peptides.2007.04.005

Harelick L, 2011, WOMEN HEALTH ISS, V21, P272, DOI 10.1016/j.whi.2011.03.006

Heron Melonie, 2009, Natl Vital Stat Rep, V57, P1

Hessol NA, 2005, PEDIATRICS, V115, pE44, DOI 10.1542/peds.2004-0478

Hessol NA, 1998, OBSTET GYNECOL, V92, P814, DOI 10.1016/S0029-7844(98)00310-X

Holtzman D, 2004, COMMUNITY-BASED HEALTH RESEARCH: ISSUES AND METHODS, P115

Hulsey T M, 2001, J Obstet Gynecol Neonatal Nurs, V30, P275, DOI 10.1111/j.1552-6909.2001.tb01545.x

Jack BW, 2008, AM J OBSTET GYNECOL, V199, pS266, DOI 10.1016/j.ajog.2008.07.067

Johnson Kay, 2006, Morbidity and Mortality Weekly Report, V55, P1

Kanny D, 2010, JAMA-J AM MED ASSOC, V304, P2474

Keppel KG, 2007, AM J EPIDEMIOL, V166, P97, DOI 10.1093/aje/kwm044

Livingood WC, 2010, MATERN CHILD HLTH J, V14, P382, DOI 10.1007/s10995-009-0471-4

Lu Michael C, 2003, Matern Child Health J, V7, P13, DOI 10.1023/A:1022537516969

Lu MC, 2010, ETHNIC DIS, V20, P62

Mateja WA, 2012, J WOMENS HEALTH, V21, P26, DOI 10.1089/jwh.2010.2582

Mathews T J, 2010, Natl Vital Stat Rep, V58, P1

Merrill RM, 2009, PREV CHRONIC DIS, V6

Mokdad Ali H, 2003, MMWR Recomm Rep, V52, P1

Moore LL, 2000, EPIDEMIOLOGY, V11, P689, DOI 10.1097/00001648-200011000-00013

Moos MK, 2008, AM J OBSTET GYNECOL, V199, pS280, DOI 10.1016/j.ajog.2008.08.060

Moriarty David G, 2003, Health Qual Life Outcomes, V1, P37, DOI 10.1186/1477-7525-1-37

Moriarty DG, 2009, AM J PREV MED, V36, P497, DOI 10.1016/j.amepre.2009.01.038

National Research Council and Institute of Medicine Board on Children Youth and Families, 2007, INFL PREGN WEIGHT MA

Nelson DE, 2010, ADDICTION, V105, P1589, DOI 10.1111/j.1360-0443.2010.03007.x

Nelson DE, 2001, SOZ PRAVENTIV MED, V46, pS3

Newschaffer CJ, 1998, VALIDATION BEHAV RIS

Okoro C, 2007, OCCUP MED-OXFORD, V57, P217, DOI 10.1093/occmed/kql177

PAGEL MD, 1990, SOC SCI MED, V30, P597, DOI 10.1016/0277-9536(90)90158-O

Posner SF, 2006, MATERN CHILD HLTH J, V10, pS197, DOI 10.1007/s10995-006-0107-x

Ramos RG, 2008, AM J PUBLIC HEALTH, V98, P1122, DOI 10.2105/AJPH.2007.120055

Rao AK, 2006, AM J OBSTET GYNECOL, V195, P834, DOI 10.1016/j.ajog.2006.06.079

Rosenbaum S, 2008, WOMEN HEALTH ISS, V18, pS26, DOI 10.1016/j.whi.2008.07.013

Salihu Hamisu M, 2003, Matern Child Health J, V7, P219, DOI 10.1023/A:1027319517405

Sanders Lorraine B, 2009, Policy Polit Nurs Pract, V10, P129, DOI 10.1177/1527154409338494

Sarwer DB, 2006, J WOMENS HEALTH, V15, P720, DOI 10.1089/jwh.2006.15.720

Schempf AH, 2007, AM J PUBLIC HEALTH, V97, P1255, DOI 10.2105/AJPH.2006.093708

Sheffield JS, 2002, OBSTET GYNECOL, V100, P925, DOI 10.1016/S0029-7844(02)02242-1

Sibai BM, 2000, AM J OBSTET GYNECOL, V182, P364, DOI 10.1016/S0002-9378(00)70225-0

Sibai BM, 2000, AM J OBSTET GYNECOL, V183, P1520, DOI 10.1067/mob.2000.107621

Siega-Riz AM, 2006, MATERN CHILD HLTH J, V10, pS153, DOI 10.1007/s10995-006-0115-x

Spring B, 2004, J CONSULT CLIN PSYCH, V72, P785, DOI 10.1037/0022-006X.72.5.785

STEER RA, 1992, J CLIN EPIDEMIOL, V45, P1093, DOI 10.1016/0895-4356(92)90149-H

Strine TW, 2004, AM J PREV MED, V26, P213, DOI 10.1016/j.amepre.2003.11.002

Toet J, 2006, QUAL LIFE RES, V15, P179, DOI 10.1007/s11136-005-8484-y

Tsai J, 2010, PREV SCI, V11, P197, DOI 10.1007/s11121-009-0158-5

Tucker MJ, 2007, AM J PUBLIC HEALTH, V97, P247, DOI 10.2105/AJPH.2005.072975

Weisman CS, 2011, WOMEN HEALTH ISS, V21, P265, DOI 10.1016/j.whi.2011.03.007

Xaverius PK, 2009, J WOMENS HEALTH, V18, P49, DOI 10.1089/jwh.2007.0629

NR 78

TC 18

Z9 18

U1 1

U2 25

PU MARY ANN LIEBERT, INC

PI NEW ROCHELLE

PA 140 HUGUENOT STREET, 3RD FL, NEW ROCHELLE, NY 10801 USA

SN 1540-9996

EI 1931-843X

J9 J WOMENS HEALTH

JI J. Womens Health

PD JUL

PY 2012

VL 21

IS 7

BP 720

EP 729

DI 10.1089/jwh.2011.3259

PG 10

WC Public, Environmental & Occupational Health; Medicine, General &

Internal; Obstetrics & Gynecology; Women's Studies

SC Public, Environmental & Occupational Health; General & Internal

Medicine; Obstetrics & Gynecology; Women's Studies

GA 969SP

UT WOS:000306078900004

PM 22559934

DA 2019-01-08

ER

PT J

AU Ali, MK

Feeney, P

Hire, D

Simmons, DL

O'Connor, PJ

Ganz-Lord, F

Goff, D

Zhang, P

Anderson, RT

Narayan, KMV

Sullivan, MD

AF Ali, M. K.

Feeney, P.

Hire, D.

Simmons, D. L.

O'Connor, P. J.

Ganz-Lord, F.

Goff, D., Jr.

Zhang, P.

Anderson, R. T.

Narayan, K. M. V.

Sullivan, M. D.

TI Glycaemia and correlates of patient-reported outcomes in ACCORD trial

participants

SO DIABETIC MEDICINE

LA English

DT Article

DE ACCORD study; cardiovascular disease; health-related quality of life;

patient-reported outcomes; Type 2 diabetes

ID QUALITY-OF-LIFE; RANDOMIZED CONTROLLED-TRIAL; TYPE-2 DIABETES-MELLITUS;

TREATMENT SATISFACTION; HEALTH; ADULTS; CARE

AB Diabet. Med. 29, e67e74 (2012) Abstract Aims Post-hoc evaluation of relationships between first-year change in glycaemic control (HbA1c) and change in patient-reported outcomes among ACCORD health-related quality of life (HRQoL) substudy participants. Methods Data from 2053 glycaemia-trial subjects were analysed. We assessed physical and mental health status (36-Item Short Form Health Survey, Version-2), symptom count and severity (Diabetes Symptoms Distress Checklist) and treatment satisfaction (Diabetes Treatment Satisfaction Questionnaire). Linear mixed models were used to test relationships between 1-year changes in HbA1c and patient reported outcomes sequentially adjusting for correlates (baseline characteristics, baseline patient reported outcomes, treatment assignment, frequency of clinical contact and post-randomization weight change plus new complications). Results Poorer baseline control of HbA1c and cardiovascular disease risk factors predicted greater one-year improvements in treatment satisfaction. Similarly, poorer baseline patient reported outcome scores all individually predicted greater 1-year improvement in that same outcome. Accounting for baseline and post-randomization characteristics and treatment arm, 1-year change in HbA1c was unrelated to changes in overall physical or mental health; however, every one percentage-point (10.9 mmol/mol) reduction in HbA1c was associated with lower symptom count (beta = 0.599; P = 0.012), lower symptom distress (beta = 0.051; P = 0.001), and higher treatment satisfaction (beta = -2.514; P < 0.001). Conclusions Independent of all relevant covariates, better glycaemic control over 1 year was associated with reduced patient-reported diabetes symptoms and symptom distress, and increased treatment satisfaction, but not overall physical and mental health. Further investigation is required to understand the specific psychosocial mechanisms that affect how patients value health and treatments.

C1 [Ali, M. K.; Narayan, K. M. V.] Emory Univ, Rollins Sch Publ Hlth, Atlanta, GA 30322 USA.

[Feeney, P.; Hire, D.; Sullivan, M. D.] Wake Forest Univ Hlth Sci, Winston Salem, NC USA.

[Feeney, P.] Stat Collaborat Inc, Washington, DC USA.

[Simmons, D. L.] Cent Arkansas Vet Healthcare Syst, Little Rock, AR USA.

[Simmons, D. L.] Univ Arkansas Med Sci, Little Rock, AR 72205 USA.

[O'Connor, P. J.] Hlth Partners Res Fdn, Minneapolis, MN USA.

[Ganz-Lord, F.] Cornell Univ, Weill Cornell Med Coll, Ithaca, NY 14853 USA.

[Goff, D., Jr.] Wake Forest Univ, Sch Med, Winston Salem, NC 27109 USA.

[Zhang, P.] US Ctr Dis Control, Atlanta, GA USA.

[Anderson, R. T.] Penn State Univ, Coll Med, Hershey, PA USA.

[Sullivan, M. D.] Univ Washington, Sch Med, Seattle, WA USA.

RP Ali, MK (reprint author), Emory Univ, Rollins Sch Publ Hlth, 1518 Clifton Rd NE,CNR 7th Floor, Atlanta, GA 30322 USA.

EM mkali@emory.edu

RI Narayan, K.M. Venkat/J-9819-2012

OI Narayan, K.M. Venkat/0000-0001-8621-5405

CR Adriaanse MC, 2008, DIABETES CARE, V31, P2268, DOI 10.2337/dc08-1074

ANDERSON RM, 1995, DIABETES CARE, V18, P943, DOI 10.2337/diacare.18.7.943

Anderson RT, 2011, DIABETES CARE, V34, P807, DOI 10.2337/dc10-1926

Buse JB, 2007, AM J CARDIOL, V99, p21I, DOI 10.1016/j.amjcard.2007.03.003

De Wit M, 2008, DIABETES CARE, V31, P1521, DOI 10.2337/dc08-0394

Diabet Control Complications Trial Res Grp, 1996, DIABETES CARE, V19, P195

Fincke BG, 2005, J AMBUL CARE MANAG, V28, P262

Gerstein HC, 2008, NEW ENGL J MED, V358, P2545, DOI 10.1056/NEJMoa0802743

Glasgow RE, 1997, DIABETES CARE, V20, P562, DOI 10.2337/diacare.20.4.562

Goddijn PPM, 1999, DIABETIC MED, V16, P23, DOI 10.1046/j.1464-5491.1999.00002.x

Gore M, 2005, J PAIN SYMPTOM MANAG, V30, P374, DOI 10.1016/j.jpainsymman.2005.04.009

Hermansen K, 2007, DRUG SAFETY, V30, P1127, DOI 10.2165/00002018-200730120-00005

Janssen PGH, 2009, BRIT J GEN PRACT, V59, P43, DOI 10.3399/bjgp09X394851

Nicolucci A, 2009, NUTR METAB CARDIOVAS, V19, P45, DOI 10.1016/j.numecd.2007.12.005

PEYROT M, 1994, DIABETES CARE, V17, P994, DOI 10.2337/diacare.17.9.994

Peyrot M, 2009, DIABETES CARE, V32, P1411, DOI 10.2337/dc08-2256

Pitale S, 2005, J DIABETES COMPLICAT, V19, P207, DOI 10.1016/j.jdiacomp.2004.12.002

Pouwer F, 2001, DIABETES CARE, V24, P1929, DOI 10.2337/diacare.24.11.1929

Redekop WK, 2002, DIABETES CARE, V25, P458, DOI 10.2337/diacare.25.3.458

Rejeski WJ, 2006, OBESITY, V14, P870, DOI 10.1038/oby.2006.101

Rubin RR, 1999, DIABETES-METAB RES, V15, P205, DOI 10.1002/(SICI)1520-7560(199905/06)15:3<205::AID-DMRR29>3.0.CO;2-O

Speight J, 2009, DIABETIC MED, V26, P315, DOI 10.1111/j.1464-5491.2009.02682.x

Sullivan MD, 2007, AM J CARDIOL, V99, p90I, DOI 10.1016/j.amjcard.2007.03.027

Tabaei BP, 2004, QUAL LIFE RES, V13, P1153, DOI 10.1023/B:QURE.0000031336.81580.52

Testa MA, 1998, JAMA-J AM MED ASSOC, V280, P1490, DOI 10.1001/jama.280.17.1490

Testa MA, 1996, NEW ENGL J MED, V334, P835, DOI 10.1056/NEJM199603283341306

UK Prospective Diabetes Study Grp, 1999, DIABETES CARE, V22, P1125

Unden AL, 2008, GENDER MED, V5, P162, DOI 10.1016/j.genm.2008.05.003

VanderDoes FEE, 1996, DIABETES CARE, V19, P204, DOI 10.2337/diacare.19.3.204

WEINBERGER M, 1994, MED CARE, V32, P1173, DOI 10.1097/00005650-199412000-00002

Wexler DJ, 2006, DIABETOLOGIA, V49, P1489, DOI 10.1007/s00125-006-0249-9

NR 31

TC 10

Z9 10

U1 0

U2 4

PU WILEY

PI HOBOKEN

PA 111 RIVER ST, HOBOKEN 07030-5774, NJ USA

SN 0742-3071

EI 1464-5491

J9 DIABETIC MED

JI Diabetic Med.

PD JUL

PY 2012

VL 29

IS 7

BP E67

EP E74

DI 10.1111/j.1464-5491.2011.03532.x

PG 8

WC Endocrinology & Metabolism

SC Endocrinology & Metabolism

GA 961IQ

UT WOS:000305458500010

PM 22141437

DA 2019-01-08

ER

PT J

AU Jimenez-Garcia, R

Huedo, MAM

Hernandez-Barrera, V

de Andres, AL

Martinez, D

Jimenez-Trujillo, I

Carrasco-Garrido, P

AF Jimenez-Garcia, Rodrigo

Martinez Huedo, Maria Angeles

Hernandez-Barrera, Valentin

Lopez de Andres, Ana

Martinez, David

Jimenez-Trujillo, Isabel

Carrasco-Garrido, Pilar

TI Psychological distress and mental disorders among Spanish diabetic

adults: A case-control study

SO PRIMARY CARE DIABETES

LA English

DT Article

DE Anxiety; Depression; Diabetes; Mental disorders; Psychological distress

ID QUALITY-OF-LIFE; COMORBID DEPRESSION; US ADULTS; HEALTH; CARE;

DISABILITY; MELLITUS; ANXIETY; PREVALENCE; ATTITUDES

AB Aim: To compare the prevalence of psychological distress and mental disorders between diabetes and non-diabetes sufferers and to identify associated factors.

Methods: Case-control study based on data from the 2006 to 2007 Spanish National Health Survey. We identified 2193 type 2 diabetic adults. Non-diabetic controls were 1:1 matched by age-and-sex. The presence of a mental disorder was considered if subjects answered yes to the questions: "Have you suffered depression and/or anxiety over the previous 12 months? AND "Has your medical doctor confirmed the diagnosis?". The 12-item General Health Questionnaire was used to measure psychological distress. Independent covariables included socio-demographics and heath related variables.

Results: Prevalence of mental disorders was 18.6% among diabetics and 16.4% among controls (adjusted OR 1.17 CI 95% 1.01-1.38). 26% of diabetes and 18.9% of the non-diabetic suffered psychological distress (adjusted OR 1.51 CI 95% 1.25-1.83). Among diabetics variables associated with suffering a mental disorder and psychological distress were: female sex, younger age, worse self rated health, comorbidity, GP visit in the last 4 weeks and ER attendance in last year.

Conclusions: Diabetic adults have significantly higher prevalence of diagnosed mental disorders and psychological distress than non-diabetic subjects. Programs targeted at preventing, monitoring and controlling these mental health problems at primary care should be implemented. (C) 2011 Primary Care Diabetes Europe. Published by Elsevier Ltd. All rights reserved.

C1 [Jimenez-Garcia, Rodrigo; Martinez Huedo, Maria Angeles; Hernandez-Barrera, Valentin; Lopez de Andres, Ana; Jimenez-Trujillo, Isabel; Carrasco-Garrido, Pilar] Univ Rey Juan Carlos, Prevent Med & Publ Hlth Teaching & Res Unit, Dept Hlth Sci, Madrid, Spain.

[Martinez, David] Univ Complutense, Dept Prevent Med, E-28040 Madrid, Spain.

RP Jimenez-Garcia, R (reprint author), Fac Ciencias Salud, Unidad Med Preventiva, Avda Atenas S-N, Madrid 28922, Spain.

EM rodrigo.jimenez@urjc.es

FU ISCIII [ETES: PI09/90515]

FX The work was supported by a Grant from the ISCIII (ETES: PI09/90515).

CR American Diabetes Association, 2009, DIABETES CARE S1, V32, pS26

Anderson RJ, 2002, INT J PSYCHIAT MED, V32, P235, DOI 10.2190/KLGD-4H8D-4RYL-TWQ8

Anderson RJ, 2001, DIABETES CARE, V24, P1069, DOI 10.2337/diacare.24.6.1069

Badia X, 1999, MED CLIN-BARCELONA, V112, P79

CALLAHAN CM, 1992, J GEN INTERN MED, V7, P26, DOI 10.1007/BF02599097

Collins MM, 2009, DIABETIC MED, V26, P153, DOI 10.1111/j.1464-5491.2008.02648.x

Das-Munshi J, 2007, PSYCHOSOM MED, V69, P543, DOI 10.1097/PSY.0b013e3180cc3062

de Jonge P, 2006, DIABETOLOGIA, V49, P2627, DOI 10.1007/s00125-006-0442-x

Egede LE, 2004, DIABETES CARE, V27, P421, DOI 10.2337/diacare.27.2.421

Egede LE, 2002, DIABETES CARE, V25, P464, DOI 10.2337/diacare.25.3.464

Egede LE, 2009, GEN HOSP PSYCHIAT, V31, P422, DOI 10.1016/j.genhosppsych.2009.06.007

Faresjo T, 2010, INT J ENV RES PUB HE, V7, P325, DOI 10.3390/ijerph7010325

Fisher L, 2007, DIABETES CARE, V30, P542, DOI 10.2337/dc06-1614

Frayne SM, 2005, ARCH INTERN MED, V165, P2631, DOI 10.1001/archinte.165.22.2631

Gispert R, 2003, SOC PSYCH PSYCH EPID, V38, P677, DOI 10.1007/s00127-003-0692-6

Goldberg D., 1988, USERS GUIDE GEN HLTH

Grigsby AB, 2002, J PSYCHOSOM RES, V53, P1053, DOI 10.1016/S0022-3999(02)00417-8

Hervas A, 2007, AN SIST SANIT NAVAR, V30, P45, DOI 10.4321/s1137-66272007000100005

Huerta JM, 2009, REV ESP CARDIOL, V62, P143, DOI 10.1016/S0300-8932(09)70156-3

*INE, ENC NAC SAL ESP 2006

Katon WJ, 2008, AM J MED, V121, P8, DOI 10.1016/j.amjmed.2008.09.008

Kruse J, 2003, DIABETES CARE, V26, P1841, DOI 10.2337/diacare.26.6.1841

Li C, 2008, DIABETIC MED, V25, P878, DOI 10.1111/j.1464-5491.2008.02477.x

Lin EHB, 2004, DIABETES CARE, V27, P2154, DOI 10.2337/diacare.27.9.2154

Lin EHB, 2008, J PSYCHOSOM RES, V65, P571, DOI 10.1016/j.jpsychores.2008.06.007

MUNOZ PE, 1979, ARCH NEUROBIOL, V42, P139

Petersen M, 2008, DIABETES CARE, V31, P596, DOI 10.2337/dc08-9017

Ruiz-Ramos Miguel, 2006, Gac Sanit, V20 Suppl 1, P15, DOI 10.1157/13086022

Sanchez-Lopez Mdel P, 2008, PSICOTHEMA, V20, P839

Sanchez-Villegas A., 2008, BMC PSYCHIATRY, V17, P38

Schieman S, 2002, J AGING HEALTH, V14, P260, DOI 10.1177/089826430201400205

Shao WA, 1997, J FAM PRACTICE, V44, P161

Snoek FJ, 2002, DIABETIC MED, V19, P265, DOI 10.1046/j.1464-5491.2002.00678.x

Sturmer T, 2000, J Epidemiol Biostat, V5, P125

Unden AL, 2008, GENDER MED, V5, P162, DOI 10.1016/j.genm.2008.05.003

Von Korff M, 2005, PSYCHOSOM MED, V67, P233, DOI 10.1097/01.psy.0000155662.82621.50

NR 36

TC 8

Z9 8

U1 4

U2 15

PU ELSEVIER SCI LTD

PI OXFORD

PA THE BOULEVARD, LANGFORD LANE, KIDLINGTON, OXFORD OX5 1GB, OXON, ENGLAND

SN 1751-9918

J9 PRIM CARE DIABETES

JI Prim. Care Diabetes

PD JUL

PY 2012

VL 6

IS 2

BP 149

EP 156

DI 10.1016/j.pcd.2011.10.002

PG 8

WC Endocrinology & Metabolism; Primary Health Care

SC Endocrinology & Metabolism; General & Internal Medicine

GA 960HJ

UT WOS:000305378300009

PM 22047940

DA 2019-01-08

ER

PT J

AU Franks, MM

Sahin, ZS

Seidel, AJ

Shields, CG

Oates, SK

Boushey, CJ

AF Franks, Melissa M.

Sahin, Z. Seda

Seidel, Amber J.

Shields, Cleveland G.

Oates, Shannon K.

Boushey, Carol J.

TI Table for Two: Diabetes Distress and Diet-Related Interactions of

Married Patients With Diabetes and Their Spouses

SO FAMILIES SYSTEMS & HEALTH

LA English

DT Article

DE marriage; shared meals; diabetes distress

ID SOCIAL-CONTROL; SELF-CARE; CHRONIC ILLNESS; MEDICATION ADHERENCE;

EMOTIONAL DISTRESS; EATING PATTERNS; OLDER PATIENTS; MARITAL-STATUS;

TYPE-2; HEALTH

AB In adjusting to chronic illness, patients often negotiate new or altered daily routines within a dynamic family context. Yet, the responses of family members to the disease and its management are understudied. The authors investigated patients with Type 2 diabetes and their spouses (N = 55 couples) and examined the association of diet-related interactions (i.e., diet-related support, diet-related pressure, and frequency of sharing meals together) with each partner's adjustment to the illness context (i.e., diabetes distress). All spouses (100%) reported providing some type of diet-related support to their partners with diabetes in the past month, and many reported exerting pressure to improve their partners' diet choices (60%). In addition, many couples (64%) indicated that they frequently shared meals together in the past month. For spouses, their provision of diet-related pressure was associated (positively) with their diabetes distress. Frequently sharing meals was associated with less diabetes distress among patients, even after controlling for their glycemic control and diet adherence. Findings reveal that spouses of patients with diabetes are actively involved in illness management with their partners, and these activities are associated with their own diabetes distress and with that of their ill partners.

C1 [Franks, Melissa M.; Sahin, Z. Seda; Seidel, Amber J.; Shields, Cleveland G.] Purdue Univ, Dept Human Dev & Family Studies, W Lafayette, IN 47906 USA.

[Oates, Shannon K.] Indiana Univ Hlth Arnett, Div Endocrinol & Metab, Lafayette, IN USA.

[Boushey, Carol J.] Univ Hawaii, Ctr Canc, Program Epidemiol, Honolulu, HI 96822 USA.

RP Franks, MM (reprint author), Purdue Univ, Dept Human Dev & Family Studies, W Lafayette, IN 47906 USA.

EM mmfranks@purdue.edu

OI Shields, Cleveland/0000-0003-3709-6969

FU Regenstrief Center for Healthcare Engineering; Purdue Research

Foundation; Purdue University

FX Support for this project was provided by the Regenstrief Center for

Healthcare Engineering, Purdue Research Foundation, and Agricultural

Research Programs, Purdue University.

CR American Diabetes Association, 2008, DIABETES CARE, V31, pS61, DOI DOI 10.2337/DC08-5061

Anderson B. J., 1993, DEV ASPECTS HLTH COM, P77

[Anonymous], 2012, DIABETES CARE, V35, pS11, DOI [10.2337/dc12-s011, 10.2337/dc35-S011]

August KJ, 2011, J HEALTH PSYCHOL, V16, P1109, DOI 10.1177/1359105311401670

August KJ, 2010, SOC SCI MED, V71, P1831, DOI 10.1016/j.socscimed.2010.08.022

Berg CA, 2007, PSYCHOL BULL, V133, P920, DOI 10.1037/0033-2909.133.6.920

Beverly EA, 2008, HEALTH EDUC BEHAV, V35, P707, DOI 10.1177/1090198107299787

Center for Disease Control and Prevention, 2011, NAT DIAB FACT SHEET

Delahanty LM, 2007, DIABETIC MED, V24, P48, DOI 10.1111/j.1464-5491.2007.02028.x

Delormier T, 2009, SOCIOL HEALTH ILL, V31, P215, DOI 10.1111/j.1467-9566.2008.01128.x

Fiese BH, 2006, NEW DIR CHILD ADOLES, V111, P67, DOI 10.1002/cad.155

Fisher L, 2008, DIABETIC MED, V25, P1096, DOI 10.1111/j.1464-5491.2008.02533.x

Fisher L, 2002, DIABETES CARE, V25, P1564, DOI 10.2337/diacare.25.9.1564

Fisher L, 2009, DIABETIC MED, V26, P622, DOI 10.1111/j.1464-5491.2009.02730.x

Fisher L, 2000, J FAM PRACTICE, V49, P561

Fisher L, 2007, DIABETES CARE, V30, P542, DOI 10.2337/dc06-1614

Franks M. M., 2010, 63 ANN M GER SOC AM

Franks MM, 2010, FAM RELAT, V59, P599, DOI 10.1111/j.1741-3729.2010.00626.x

Franks MM, 2006, J FAM PSYCHOL, V20, P311, DOI 10.1037/0893-3200.20.2.311

Fulikerson JA, 2008, J AM DIET ASSOC, V108, P706, DOI 10.1016/j.jada.2008.01.005

Fulkerson JA, 2007, J CONSULT CLIN PSYCH, V75, P181, DOI 10.1037/0022-006X.75.1.181

Gillman MW, 2000, ARCH FAM MED, V9, P235, DOI 10.1001/archfami.9.3.235

Gonder-Frederick LA, 2002, J CONSULT CLIN PSYCH, V70, P611, DOI 10.1037//0022-006X.70.3.611

Gonzalez JS, 2007, DIABETES CARE, V30, P2222, DOI 10.2337/dc07-0158

Halter J. B., 1999, DIABETES MELLITUS IN, P991

Huang ES, 2005, J AM GERIATR SOC, V53, P306, DOI 10.1111/j.1532-5415.2005.53119.x

Lin EHB, 2004, DIABETES CARE, V27, P2154, DOI 10.2337/diacare.27.9.2154

Magkos F, 2009, ANNU REV NUTR, V29, P223, DOI 10.1146/annurev-nutr-080508-141200

Miller D, 2005, J NUTR EDUC BEHAV, V37, P226, DOI 10.1016/S1499-4046(06)60276-5

NORTON R, 1983, J MARRIAGE FAM, V45, P141, DOI 10.2307/351302

Paisley J, 2008, J NUTR EDUC BEHAV, V40, P80, DOI 10.1016/j.jneb.2007.04.374

Penckofer S, 2007, DIABETES EDUCATOR, V33, P680, DOI 10.1177/0145721707304079

Peyrot M, 2005, DIABETIC MED, V22, P1379, DOI 10.1111/j.1464-5491.2005.01644.x

POLONSKY WH, 1995, DIABETES CARE, V18, P754, DOI 10.2337/diacare.18.6.754

Polonsky WH, 2005, DIABETES CARE, V28, P626, DOI 10.2337/diacare.28.3.626

Rook KS, 2011, J SOC PERS RELAT, V28, P772, DOI 10.1177/0265407510391335

Savoca M, 2001, J NUTR EDUC, V33, P224, DOI 10.1016/S1499-4046(06)60035-3

Schafer RB, 1999, SOC SCI MED, V48, P787, DOI 10.1016/S0277-9536(98)00377-3

Shaw BA, 2006, J COMMUN HEALTH, V31, P393, DOI 10.1007/s10900-006-9018-4

Snoek FJ, 2000, DIABETES CARE, V23, P1305, DOI 10.2337/diacare.23.9.1305

Stephens MAP, 2010, FAM SYST HEALTH, V28, P199, DOI 10.1037/a0020513

Stephens MAP, 2009, HEALTH PSYCHOL, V28, P48, DOI 10.1037/a0012385

Tang TS, 2008, DIABETES EDUCATOR, V34, P266, DOI 10.1177/0145721708315680

Toobert DJ, 2000, DIABETES CARE, V23, P943, DOI 10.2337/diacare.23.7.943

Trief P., 2003, FAMILIES SYSTEMS HLT, V21, P57, DOI [DOI 10.1037/H0089502, 10.1037/h0089502]

Trief P. M., 2006, FAMILIES SYSTEMS HLT, V24, P318, DOI [10.1037/1091-7527.24.3.318, DOI 10.1037/1091-7527.24.3.318]

Tucker JS, 2001, J APPL SOC PSYCHOL, V31, P467, DOI 10.1111/j.1559-1816.2001.tb02051.x

UMBERSON D, 1992, SOC SCI MED, V34, P907, DOI 10.1016/0277-9536(92)90259-S

Wansink B, 2007, ENVIRON BEHAV, V39, P106, DOI 10.1177/0013916506295573

NR 49

TC 18

Z9 18

U1 0

U2 18

PU EDUCATIONAL PUBLISHING FOUNDATION-AMERICAN PSYCHOLOGICAL ASSOC

PI WASHINGTON

PA 750 FIRST ST, NE, WASHINGTON, DC 20002-4242 USA

SN 1091-7527

EI 1939-0602

J9 FAM SYST HEALTH

JI Fam. Syst. Health

PD JUN

PY 2012

VL 30

IS 2

BP 154

EP 165

DI 10.1037/a0028614

PG 12

WC Health Care Sciences & Services; Family Studies; Public, Environmental &

Occupational Health

SC Health Care Sciences & Services; Family Studies; Public, Environmental &

Occupational Health

GA 962NG

UT WOS:000305551200006

PM 22709327

DA 2019-01-08

ER

PT J

AU Nadimpalli, SB

Hutchinson, MK

AF Nadimpalli, Sarah B.

Hutchinson, M. Katherine

TI An Integrative Review of Relationships Between Discrimination and Asian

American Health

SO JOURNAL OF NURSING SCHOLARSHIP

LA English

DT Article

DE Nursing; discrimination; racism; health outcomes; Asian American; health

disparities

ID MEXICAN-ORIGIN ADULTS; RACIAL-DISCRIMINATION; PERCEIVED DISCRIMINATION;

ETHNIC-IDENTITY; UNITED-STATES; PSYCHOLOGICAL DISTRESS; DEPRESSIVE

SYMPTOMS; FILIPINO AMERICANS; KOREAN IMMIGRANTS; PHYSICAL HEALTH

AB Purpose: Many ethnic minorities in the United States experience disproportionate rates of adverse health outcomes or health disparities. Factors such as socioeconomic status do not fully explain how these disparities are generated and maintained. Research has demonstrated that chronic experiences of discrimination are harmful to the health of African Americans and Latinos. However, there is a dearth of research examining Asian Americans experiences with discrimination and health disparities. The purpose of this integrative review was to summarize the current literature examining discrimination and the mental and physical health of Asian Americans. Design and Methods: Combinations of search terms related to discrimination, health, and Asian Americans were used to search five electronic databases. Inclusion criteria were primary research studies, published in English between 1980 and 2011, Asian American adults, and discrimination examined in relationship to a physical or mental health outcome. The search initially yielded 489 results; 14 quantitative studies met inclusion criteria. Findings and Conclusions: Quantitative studies in this review revealed several significant associations between discrimination and health outcomes in Asian Americans. Discrimination was significantly associated with depressive symptoms in seven studies. Three studies found associations between discrimination and physical health, including cardiovascular disease, respiratory conditions, obesity, and diabetes. Although the literature was limited by self-reported data, cross-sectional designs, and inconsistent definitions and measurement of discrimination, the findings suggest that discrimination is a significant contributor to poorer health and health disparities for Asian Americans. The findings clearly demonstrate the need for further nursing research in this area to inform evidence-based practice and social policy. Clinical Relevance: Patient care providers can recognize discrimination as a significant stressor or purveyor of illness and explore ways to facilitate coping and resilience with their Asian American patients. Community-based participatory research approaches can be implemented by clinicians, academicians, and Asian American community partners to address the issue of discrimination and Asian American health outcomes.

C1 [Nadimpalli, Sarah B.; Hutchinson, M. Katherine] NYU, Coll Nursing, New York, NY 10003 USA.

RP Nadimpalli, SB (reprint author), NYU, Coll Nursing, 726 Broadway,10th Floor, New York, NY 10003 USA.

EM sb3354@nyu.edu

OI Hutchinson, M.Katherine/0000-0001-8270-9491

CR Bernstein KS, 2011, COMMUNITY MENT HLT J, V47, P24, DOI 10.1007/s10597-009-9261-0

Creswell J, 2009, RES DESIGN QUALITATI

de Castro AB, 2008, AM J PUBLIC HEALTH, V98, P520, DOI 10.2105/AJPH.2007.110163

Finch BK, 2000, J HEALTH SOC BEHAV, V41, P295, DOI 10.2307/2676322

Flores E, 2008, HISPANIC J BEHAV SCI, V30, P401, DOI 10.1177/0739986308323056

Gee GC, 2006, AM J PUBLIC HEALTH, V96, P677, DOI 10.2105/AJPH.2004.060442

Gee GC, 2002, AM J PUBLIC HEALTH, V92, P615, DOI 10.2105/AJPH.92.4.615

Gee GC, 2008, AM J PUBLIC HEALTH, V98, P493, DOI 10.2105/AJPH.2007.114025

Gee GC, 2007, AM J PUBLIC HEALTH, V97, P1275, DOI 10.2105/AJPH.2006.091827

Gee GC, 2007, SOC SCI MED, V64, P1984, DOI 10.1016/j.socscimed.2007.02.013

Hahm HC, 2010, WOMEN HEALTH ISS, V20, P350, DOI 10.1016/j.whi.2010.05.002

Jones CP, 2000, AM J PUBLIC HEALTH, V90, P1212, DOI 10.2105/AJPH.90.8.1212

Krieger N, 1996, AM J PUBLIC HEALTH, V86, P1370, DOI 10.2105/AJPH.86.10.1370

Krieger N, 1999, INT J HEALTH SERV, V29, P295, DOI 10.2190/M11W-VWXE-KQM9-G97Q

Lam BT, 2007, AM J ORTHOPSYCHIAT, V77, P370, DOI 10.1037/0002-9432.77.3.370

Lee MY, 2004, J MARITAL FAM THER, V30, P497, DOI 10.1111/j.1752-0606.2004.tb01258.x

Lee RM, 2005, J COUNS PSYCHOL, V52, P36, DOI 10.1037/0022-0167-52.1.36

Mossakowski KN, 2003, J HEALTH SOC BEHAV, V44, P318, DOI 10.2307/1519782

Mossakowski KN, 2007, SOC PSYCHOL QUART, V70, P290, DOI 10.1177/019027250707000307

New York City Commission on Human Rights, 2003, DISCR MUSL AR S AS N

Oh YJ, 2002, J SOC PSYCHOL, V142, P511, DOI 10.1080/00224540209603915

Okihiro G, 2001, COLUMBIA GUIDE ASIAN

Paradies Y, 2006, INT J EPIDEMIOL, V35, P888, DOI 10.1093/ije/dyl056

Piette JD, 2006, PATIENT EDUC COUNS, V60, P41, DOI 10.1016/j.pec.2004.12.001

Polit D. F., 2012, NURSING RES GENERATI

Ryan AM, 2006, J HEALTH CARE POOR U, V17, P116, DOI 10.1353/hpu.2006.0079

Trinh-Shevrin C., 2009, ASIAN AM COMMUNITIES, P162

U.S. Census Bureau, 2005, AM COMM SURV 2004

U. S. Department of Health and Human Services, 2001, MENT HLTH CULT RAC E

US Bureau of the Census, 2007, AM COMM AS 2004

US Department of Health and Human Services, 1999, MENT HLTH REP SURG G

von Elm E, 2007, PLOS MED, V4, P1623, DOI 10.1371/journal.pmed.0040296

Whittemore R, 2005, J ADV NURS, V52, P546, DOI 10.1111/j.1365-2648.2005.03621.x

Williams D R, 1997, J Health Psychol, V2, P335, DOI 10.1177/135910539700200305

World Health Organization, 1998, HLTH PROM GLOSS

Yip T, 2008, DEV PSYCHOL, V44, P787, DOI 10.1037/0012-1649.44.3.787

Yoo HC, 2009, SOC SCI MED, V68, P726, DOI 10.1016/j.socscimed.2008.11.013

NR 37

TC 17

Z9 18

U1 2

U2 30

PU WILEY

PI HOBOKEN

PA 111 RIVER ST, HOBOKEN 07030-5774, NJ USA

SN 1527-6546

EI 1547-5069

J9 J NURS SCHOLARSHIP

JI J. Nurs. Scholarsh.

PD JUN

PY 2012

VL 44

IS 2

BP 127

EP 135

DI 10.1111/j.1547-5069.2012.01448.x

PG 9

WC Nursing

SC Nursing

GA 950PD

UT WOS:000304660300004

PM 22551064

DA 2019-01-08

ER

PT J

AU Brumby, S

Chandrasekara, A

McCoombe, S

Kremer, P

Lewandowski, P

AF Brumby, Susan

Chandrasekara, Ananda

McCoombe, Scott

Kremer, Peter

Lewandowski, Paul

TI Cardiovascular risk factors and psychological distress in Australian

farming communities

SO AUSTRALIAN JOURNAL OF RURAL HEALTH

LA English

DT Article

DE obesity; mental health; farmer; cardiovascular disease

ID METABOLIC SYNDROME; PHYSICAL-ACTIVITY; LIFE-STYLE; OBESITY; DISEASE;

POPULATION; OVERWEIGHT; FARMERS; BURDEN; HEALTH

AB Objective: To examine the prevalence of cardiovascular disease (CVD) risk factors, psychological distress and associations between physical and mental health parameters within a cohort of the Australian farming community. Design: Cross-sectional descriptive study. Setting: Farming communities across Australia. Participants: Data of men (n = 957) and women (n = 835) farmers from 97 locations across Australia were stratified into categories based on National Cholesterol Education Program guidelines. Main outcome measure(s): Prevalence of and interrelationship between overweight, obesity, dyslipidaemia, hypertension, diabetes risk and psychological distress. Results: There was a higher prevalence of overweight (42.5%, 95% confidence interval (CI), 34.250.8), obesity (21.8%, 95% CI, 18.325.3), abdominal adiposity (38.4% 95% CI, 24.552.5), hypertension (54.0%, 95% CI, 34.473.5) and diabetes risk (25.3%, 95% CI, 17.736.7) in the farming cohort compared with national data. There was also a positive significant association between the prevalence of psychological distress and obesity, abdominal adiposity, body fat percentage and metabolic syndrome in older (age = 50 years) participants. Conclusions: This study group of farming men and women exhibited an increased prevalence of CVD risk factors and co-morbidities. The findings indicate a positive association between psychological distress and risk for developing CVD, particularly in the older farmers. If the younger cohort were to maintain elevated rates of psychological distress, then it is foreseeable that the next generation of farmers could experience poorer physical health than their predecessors.

C1 [Brumby, Susan; Chandrasekara, Ananda; McCoombe, Scott] Natl Ctr Farmer Hlth, Western Dist Hlth Serv, Hamilton, Vic 3300, Australia.

[Brumby, Susan; Chandrasekara, Ananda; McCoombe, Scott; Lewandowski, Paul] Deakin Univ, Sch Med, Geelong, Vic 3217, Australia.

[Kremer, Peter] Deakin Univ, Sch Psychol, Geelong, Vic 3217, Australia.

RP Brumby, S (reprint author), Natl Ctr Farmer Hlth, Western Dist Hlth Serv, Hamilton, Vic 3300, Australia.

EM susan.brumby@wdhs.net

OI Chandrasekara, Ananda/0000-0003-0947-6083; Kremer,

Peter/0000-0003-2476-1958

CR Alston M, 2004, AGR HUM VALUES, V21, P37, DOI 10.1023/B:AHUM.0000014019.84085.59

Australian Bureau of Statistics, 2011, AUSTR DEM STAT 3101

Australian Government Department of Agriculture Fisheries and Forestry, 2008, NAT RUR ADV COUNC AN

Australian Institute of Health and Welfare, 2008, DIAB AUSTR FACTS 200, P58

Bjorntorp P, 2007, BR J NUTR S1, V83, P49

BRACKBILL RM, 1994, AM J EPIDEMIOL, V139, P1055, DOI 10.1093/oxfordjournals.aje.a116949

Brumby SA, 2009, RURAL REMOTE HEALTH, V9

Brumby S, 2011, BMC PUBLIC HEALTH, V11, DOI 10.1186/1471-2458-11-362

Brumby Susan, 2011, BMC Res Notes, V4, P89, DOI 10.1186/1756-0500-4-89

CAMACHO TC, 1991, AM J EPIDEMIOL, V134, P220, DOI 10.1093/oxfordjournals.aje.a116074

Cameron AJ, 2003, MED J AUSTRALIA, V178, P427

Dallman MF, 2003, P NATL ACAD SCI USA, V100, P11696, DOI 10.1073/pnas.1934666100

Department for Environment Food and Rural Affairs, 2009, SERV CORD OR PROGR S, P48

Dunstan D, 2001, DIABESITY ASS DISORD

Ezzati M, 2002, LANCET, V360, P1347, DOI 10.1016/S0140-6736(02)11403-6

Frager L, 2000, HLTH SAFETY AUSTR FA

Janus ED, 2007, MED J AUSTRALIA, V187, P147

JOHNSON JV, 1989, SCAND J WORK ENV HEA, V15, P271, DOI 10.5271/sjweh.1852

Kessler RC, 2002, PSYCHOL MED, V32, P959, DOI 10.1017/S0033291702006074

Kousar R, 2008, METABOLISM, V57, P1502, DOI 10.1016/j.metabol.2008.06.003

Lorenzo C, 2007, DIABETES CARE, V30, P8, DOI 10.2337/dc06-1414

Mathers CD, 2001, B WORLD HEALTH ORGAN, V79, P1076

Miller K, 2008, AUST J RURAL HEALTH, V16, P327, DOI 10.1111/j.1440-1584.2008.01011.x

Rautiainen Risto H, 2005, J Agromedicine, V10, P21

SCHWARZ L, 1992, SPORTS MED, V13, P25, DOI 10.2165/00007256-199213010-00003

NR 25

TC 11

Z9 12

U1 0

U2 20

PU WILEY-BLACKWELL

PI HOBOKEN

PA 111 RIVER ST, HOBOKEN 07030-5774, NJ USA

SN 1038-5282

J9 AUST J RURAL HEALTH

JI Aust. J. Rural Health

PD JUN

PY 2012

VL 20

IS 3

BP 131

EP 137

DI 10.1111/j.1440-1584.2012.01273.x

PG 7

WC Public, Environmental & Occupational Health; Nursing

SC Public, Environmental & Occupational Health; Nursing

GA 946XH

UT WOS:000304390300007

PM 22620477

DA 2019-01-08

ER

PT J

AU Woudenberg, YJC

Lucas, C

Latour, C

Reimer, WJMSO

AF Woudenberg, Y. J. C.

Lucas, C.

Latour, C.

Reimer, W. J. M. Scholte Op

TI Acceptance of insulin therapy: a long shot? Psychological insulin

resistance in primary care

SO DIABETIC MEDICINE

LA English

DT Article

DE insulin therapy; primary care; psychology; Type 2 diabetes

ID NATIONAL DIABETES ATTITUDES; NEEDS DAWN; COMPLICATIONS; DEPRESSION;

APPRAISAL; DISTRESS; BARRIERS; WISHES

AB Diabet. Med. 29, 796802 (2012) Abstract Aim To explore which factors are associated with psychological insulin resistance in insulin-naive patients with Type 2 diabetes in primary care. Methods A sample of 101 insulin-naive patients with Type 2 diabetes completed self-administered questionnaires including demographic and clinical characteristics, the Insulin Treatment Appraisal Scale and the Center for Epidemiological Studies Depression scale. Psychological insulin resistance was denoted by negative appraisal of insulin (Insulin Treatment Appraisal Scale). Results Thirty-nine per cent of the sample were unwilling to accept insulin therapy. Unwilling participants perceived taking insulin more often as a failure to control their diabetes with tablets or lifestyle compared with willing participants (59 vs. 33%), unwilling participants were more reluctant to accept the responsibilities of everyday management of insulin therapy (49 vs. 24%). Multiple linear regression analysis revealed that depression and objection to lifelong insulin therapy were independently associated with psychological insulin resistance. Conclusions In this study in primary care, depression and objection to lifelong insulin therapy are associated with psychological insulin resistance. Analysis of the objection to the indefiniteness of insulin therapy showed a sense of limitation of daily life and loss of independence that should not be underestimated. Insulin should be offered as a means to improve health as this might facilitate the acceptance of insulin therapy.

C1 [Latour, C.; Reimer, W. J. M. Scholte Op] Amsterdam Univ Appl Sci, Sch Nursing, NL-1000 CN Amsterdam, Netherlands.

[Woudenberg, Y. J. C.] Univ Amsterdam, Med Ctr Oost, Amsterdam, Netherlands.

[Lucas, C.] Univ Amsterdam, Dept Clin Epidemiol Biostat & Bioinformat, Amsterdam, Netherlands.

[Lucas, C.] Univ Amsterdam, Acad Med Ctr, NL-1105 AZ Amsterdam, Netherlands.

RP Reimer, WJMSO (reprint author), Amsterdam Univ Appl Sci, Sch Nursing, Room C211,POB 2557, NL-1000 CN Amsterdam, Netherlands.

EM w.j.m.scholte.op.reimer@hva.nl

CR Bogatean MP, 2004, PRACT DIABETES INT, V21, P247, DOI DOI 10.1002/PDI.670

Brod M, 2009, QUAL LIFE RES, V18, P23, DOI 10.1007/s11136-008-9419-1

Brunton Stephen A, 2006, Clin Cornerstone, V8 Suppl 2, pS19, DOI 10.1016/S1098-3597(06)80012-8

Fisher L, 2007, DIABETES CARE, V30, P542, DOI 10.2337/dc06-1614

Heijmans M, 2010, ONTWIKKELING ZORG CH

Larkin ME, 2008, DIABETES EDUCATOR, V34, P511, DOI 10.1177/0145721708317869

Makine C, 2009, DIABETIC MED, V26, P28, DOI 10.1111/j.1464-5491.2008.02606.x

Meulepas MA, 2007, FAM PRACT, V24, P20, DOI 10.1093/fampra/cml055

Morris JE, 2005, PRACTICAL DIABETES I, V22, P239, DOI DOI 10.1002/PDI.829

Noordman J, 2008, INZET DOKTERSASSISTE

Peyrot M, 2005, DIABETES CARE, V28, P2673, DOI 10.2337/diacare.28.11.2673

Peyrot M, 2005, DIABETIC MED, V22, P1379, DOI 10.1111/j.1464-5491.2005.01644.x

Polonsky WH, 2005, DIABETES CARE, V28, P2543, DOI 10.2337/diacare.28.10.2543

Polonsky W, 2007, DIABETES EDUCATOR, V33, p241S, DOI 10.1177/0145721707305701

RADLOFF L S, 1977, Applied Psychological Measurement, V1, P385, DOI 10.1177/014662167700100306

Rubino A, 2007, DIABETIC MED, V24, P1412, DOI 10.1111/j.1464-5491.2007.02279.x

Snoek FJ, 2007, HEALTH QUAL LIFE OUT, V5, DOI 10.1186/1477-7525-5-69

Steyerberg EW, 2009, CLIN PREDICTION MODE, P207

Stratton IM, 2000, BRIT MED J, V321, P405, DOI 10.1136/bmj.321.7258.405

ten Brinke R, 2008, PRIM CARE DIABETES, V2, P45, DOI 10.1016/j.pcd.2007.12.004

Turner RC, 1998, LANCET, V352, P837

Yu PC, 2010, DIABETES RES CLIN PR, V89, P1, DOI 10.1016/j.diabres.2009.12.009

NR 22

TC 23

Z9 23

U1 2

U2 8

PU WILEY-BLACKWELL

PI HOBOKEN

PA 111 RIVER ST, HOBOKEN 07030-5774, NJ USA

SN 0742-3071

J9 DIABETIC MED

JI Diabetic Med.

PD JUN

PY 2012

VL 29

IS 6

BP 796

EP 802

DI 10.1111/j.1464-5491.2011.03552.x

PG 7

WC Endocrinology & Metabolism

SC Endocrinology & Metabolism

GA 942YN

UT WOS:000304087700018

PM 22150962

DA 2019-01-08

ER

PT J

AU Atlantis, E

Sullivan, T

Sartorius, N

Almeida, OP

AF Atlantis, Evan

Sullivan, Thomas

Sartorius, Norman

Almeida, Osvaldo P.

TI Changes in the prevalence of psychological distress and use of

antidepressants or anti-anxiety medications associated with comorbid

chronic diseases in the adult Australian population, 2001-2008

SO AUSTRALIAN AND NEW ZEALAND JOURNAL OF PSYCHIATRY

LA English

DT Article

DE Psychological distress; depression; anxiety; antidepressants;

anti-anxiety medications; chronic disease; trends

ID COMMUNITY SAMPLE; MAJOR DEPRESSION; NATIONAL TRENDS; HEALTH; DISORDERS;

EFFICACY; PEOPLE; PUBLICATION; DISABILITY; WEIGHT

AB Objective: To investigate changes in the prevalence of psychological distress and use of antidepressants or anti-anxiety medications associated with comorbid chronic diseases in the adult Australian population from 2001 to 2008.

Methods: Participants were 48,359 adults aged >= 25 years from the 2001, 2004-05 or 2007-08 Australian National Health Surveys. Clinically significant psychological distress was determined with the 10-item Kessler Psychological Distress Scale (using scores >= 30). Contemporaneous use of antidepressants or anti-anxiety medications and the presence of chronic diseases including diabetes, cardiovascular disease, cancer, asthma and arthritis were by self-report.

Results: Overall, the prevalence of psychological distress was relatively stable (3.9 vs. 3.7%) between 2001 and 2008, but increased from 5.9 to 7.0% and 4.6 to 5.0% for people with diabetes and cardiovascular disease. In contrast, the use of antidepressants or anti-anxiety medications decreased from 7.3 to 4.8% across all disease categories. On average, the odds of psychological distress and use of antidepressants or anti-anxiety medications was 1.15 to 1.59-fold and 1.10 to 1.64-fold higher, respectively, for all chronic diseases after adjustments for socio-demographic and lifestyle variables. After further adjustment for all chronic disease predictors fitted jointly, these associations decreased in strength (percentage change in the log odds ratio) by 14 to 98%. Each additional chronic disease increased the odds of both psychological distress (1.34-fold higher) and use of antidepressants or anti-anxiety medications (1.40-fold higher) in a dose response manner.

Conclusions: Although the prevalence of psychological distress changed little for adult Australians between 2001 and 2008, it increased for people with diabetes and cardiovascular disease. Conversely, the reported use of antidepressants or anti-anxiety medications progressively decreased for people with, and without, chronic diseases. Overall, there was a persistently higher psychopathology burden associated with each and increasing number of comorbid chronic diseases.

C1 [Atlantis, Evan] Univ Adelaide, Sch Med, Adelaide, SA 5000, Australia.

[Atlantis, Evan] Royal Adelaide Hosp, Inst Med & Vet Sci, SA Hlth, Govt S Australia, Adelaide, SA 5000, Australia.

[Sullivan, Thomas] Univ Adelaide, Data Management & Anal Ctr, Discipline Publ Hlth, Adelaide, SA 5000, Australia.

[Sartorius, Norman] Assoc Improvement Mental Hlth Programmes, Geneva, Switzerland.

[Almeida, Osvaldo P.] Univ Western Australia, Sch Psychiat & Clin Neurosci, Perth, WA 6009, Australia.

[Almeida, Osvaldo P.] Univ Western Australia, Western Australian Ctr Hlth & Ageing, Med Res Ctr, Perth, WA 6009, Australia.

[Almeida, Osvaldo P.] Royal Perth Hosp, Dept Psychiat, Perth, WA, Australia.

RP Atlantis, E (reprint author), Univ Adelaide, Sch Med, Adelaide, SA 5000, Australia.

EM evan.atlantis@adelaide.edu.au

RI Almeida, Osvaldo/A-4925-2008

OI Almeida, Osvaldo/0000-0002-8689-6199; Atlantis,

Evan/0000-0001-5877-6141; Sullivan, Thomas/0000-0002-6930-5406

CR Andrews G, 2001, AUST NZ J PUBL HEAL, V25, P494, DOI 10.1111/j.1467-842X.2001.tb00310.x

Atlantis E, 2008, INT J OBESITY, V32, P715, DOI 10.1038/sj.ijo.0803762

Atlantis E, 2009, OBES REV, V10, P543, DOI 10.1111/j.1467-789X.2009.00590.x

Atlantis E, 2011, SOCIAL PSYCHIAT PSYC

Atlantis E, 2011, SOC PSYCH PSYCH EPID, V46, P1303, DOI 10.1007/s00127-010-0302-3

Australian Bureau of Statistics, 1778 AUSTR BUR STAT

Australian Bureau of Statistics, 2008, 2007 NAT SURV MENT H

Australian Bureau of Statistics, 4324 AUSTR BUR STAT

Australian Bureau of Statistics, 990 AUSTR BUR STAT

Australian Bureau of Statistics, 1998, AUSTR MEAS 1995

Barbui C, 1999, SOC PSYCH PSYCH EPID, V34, P152, DOI 10.1007/s001270050127

Brhlikova P, 2011, J ROY SOC MED, V104, P25, DOI 10.1258/jrsm.2010.100080

Britt HC, 2008, MED J AUSTRALIA, V189, P72

Caughey GE, 2010, AGE AGEING, V39, P488, DOI 10.1093/ageing/afq055

Clarke DM, 2009, MED J AUSTRALIA, V190, pS54

Coles-Rutishauser I., 2001, EVALUATION SHORT DIE

Danaei G, 2011, LANCET, V378, P31, DOI 10.1016/S0140-6736(11)60679-X

Danaei G, 2011, LANCET, V377, P568, DOI 10.1016/S0140-6736(10)62036-3

DiMatteo MR, 2000, ARCH INTERN MED, V160, P2101, DOI 10.1001/archinte.160.14.2101

Dunstan D, 2001, DIABESITY ASS DISORD

Egede LE, 2007, GEN HOSP PSYCHIAT, V29, P409, DOI 10.1016/j.genhosppsych.2007.06.002

Farzadfar F, 2011, LANCET, V377, P578, DOI 10.1016/S0140-6736(10)62038-7

Fletcher JR, 2008, MED J AUSTRALIA, V188, pS107

Goldney RD, 2010, AUST NZ J PSYCHIAT, V44, P901, DOI 10.3109/00048674.2010.490520

Grayson DA, 2000, J GERONTOL B-PSYCHOL, V55, pP273, DOI 10.1093/geronb/55.5.P273

Gunn J, 2010, SOCIAL PSYCHIAT PSYC, V47, P175

Harman JS, 2009, PSYCHIAT SERV, V60, P611, DOI 10.1176/appi.ps.60.5.611

Harris MG, 2011, AUST NZ J PSYCHIAT, V45, P249, DOI 10.3109/00048674.2010.531459

Holden L, 2010, AUST NZ J PUBL HEAL, V34, P304, DOI 10.1111/j.1753-6405.2010.00531.x

Hollingworth SA, 2010, AUST NZ J PSYCHIAT, V44, P513, DOI 10.3109/00048670903555138

Katon W, 2007, GEN HOSP PSYCHIAT, V29, P147, DOI 10.1016/j.genhosppsych.2006.11.005

Kessler RC, 2002, PSYCHOL MED, V32, P959, DOI 10.1017/S0033291702006074

Kirsch I, 2008, PLOS MED, V5, P260, DOI 10.1371/journal.pmed.0050045

Kosidou K, 2010, ACTA PSYCHIAT SCAND, V122, P47, DOI 10.1111/j.1600-0447.2009.01487.x

Licht CMM, 2008, ARCH GEN PSYCHIAT, V65, P1358, DOI 10.1001/archpsyc.65.12.1358

Mant A, 2004, MED J AUSTRALIA, V181, pS21

Mathew SJ, 2009, AM J PSYCHIAT, V166, P140, DOI 10.1176/appi.ajp.2008.08071102

Meijer WEE, 2004, EUR J CLIN PHARMACOL, V60, P57, DOI 10.1007/s00228-004-0726-3

Moore M, 2009, BRIT MED J, V339, DOI 10.1136/bmj.b3999

Moussavi S, 2007, LANCET, V370, P851, DOI 10.1016/S0140-6736(07)61415-9

Olfson M, 2004, J CLIN PSYCHIAT, V65, P1166, DOI 10.4088/JCP.v65n0903

Olfson M, 2008, ARCH GEN PSYCHIAT, V65, P94, DOI 10.1001/archgenpsychiatry.2007.5

Taylor D, 2011, BRIT J PSYCHIAT, V198, P179, DOI 10.1192/bjp.bp.110.077610

Turner EH, 2008, NEW ENGL J MED, V358, P252, DOI 10.1056/NEJMsa065779

Uijen Annemarie A, 2008, Eur J Gen Pract, V14 Suppl 1, P28, DOI 10.1080/13814780802436093

World Health Organization, 2008, GLOB BURD DIS 2004 U

NR 46

TC 21

Z9 21

U1 0

U2 4

PU SAGE PUBLICATIONS LTD

PI LONDON

PA 1 OLIVERS YARD, 55 CITY ROAD, LONDON EC1Y 1SP, ENGLAND

SN 0004-8674

J9 AUST NZ J PSYCHIAT

JI Aust. N. Z. J. Psych.

PD MAY

PY 2012

VL 46

IS 5

BP 445

EP 456

DI 10.1177/0004867411433218

PG 12

WC Psychiatry

SC Psychiatry

GA 944EI

UT WOS:000304181700008

PM 22535293

DA 2019-01-08

ER

PT J

AU Carcone, AI

Ellis, DA

Naar-King, S

AF Carcone, April Idalski

Ellis, Deborah A.

Naar-King, Sylvie

TI Linking Caregiver Strain to Diabetes Illness Management and Health

Outcomes in a Sample of Adolescents in Chronically Poor Metabolic

Control

SO JOURNAL OF DEVELOPMENTAL AND BEHAVIORAL PEDIATRICS

LA English

DT Article

DE caregiver strain; caregiver mental health symptoms; adherence; metabolic

control; diabetes

ID SOCIAL SUPPORT; GLYCEMIC CONTROL; PSYCHOLOGICAL DISTRESS; SELF-CARE;

FAMILY CAREGIVERS; PARENTAL BURDEN; MEDIATING ROLE; CHILDREN; YOUTH;

QUESTIONNAIRE

AB Objective: This study examined a multivariate model of the relationship between caregiver strain and adolescent diabetes illness outcomes in a sample of caregivers of adolescents in chronically poor metabolic control (hemoglobin A(1c) >= 8.0% at enrollment and for previous 12 mo). Caregiver mental health symptoms were hypothesized to mediate the relationship between caregiver strain and adolescent illness management behavior and metabolic control. Caregivers' perceptions of social support and their level of responsibility for diabetes care tasks were hypothesized to be directly related to caregiver strain and indirectly to caregiver mental health symptoms. Methods: One hundred forty-six caregiver-adolescent dyads completed baseline measures of caregiver strain, responsibility for diabetes care, social support, mental health symptoms, and illness management behavior. Adolescent metabolic control was also assessed. Results: Findings from structural equation modeling suggested that caregiver strain was directly and positively associated with caregiver mental health symptoms which mediated the relationship to adolescent diabetes management behavior and metabolic control. Caregivers' responsibility for diabetes care tasks was directly related to caregiver strain and indirectly to caregiver mental health, but caregivers' perception of social support was not. Conclusions: These findings suggest that caregiver strain is an important dimension of the caregiving context of diabetes. Clinicians and researchers should consider how caregiving strain, responsibility for illness management, and mental health symptoms might be impacting children's diabetes care behavior and diabetes health when working with and designing interventions for adolescents with diabetes. (J Dev Behav Pediatr 33:343-351, 2012)

C1 [Carcone, April Idalski; Ellis, Deborah A.; Naar-King, Sylvie] Wayne State Univ, Pediat Prevent Res Ctr, Carman & Ann Adams Dept Pediat, Detroit, MI 48202 USA.

RP Carcone, AI (reprint author), Wayne State Univ, Pediat Prevent Res Ctr, Carman & Ann Adams Dept Pediat, 4707 St Antoine St,Suite W534,Bin 502, Detroit, MI 48202 USA.

EM acarcone@med.wayne.edu

OI Idalski Carcone, April/0000-0002-4731-1995

FU National Institute of Diabetes, Digestive and Kidney Diseases [R01

DK59067]

FX This project was supported by grant #R01 DK59067 from the National

Institute of Diabetes, Digestive and Kidney Diseases.

CR AMIEL SA, 1986, NEW ENGL J MED, V315, P215, DOI 10.1056/NEJM198607243150402

ANDERSON BJ, 1990, J PEDIATR PSYCHOL, V15, P477, DOI 10.1093/jpepsy/15.4.477

Angold A, 1998, AM J PUBLIC HEALTH, V88, P75, DOI 10.2105/AJPH.88.1.75

[Anonymous], 2010, IBM SPSS AM COMP PRO

Arbuckle J., 2009, AMOS 18 USERS GUIDE

BAILLIE V, 1988, NURS RES, V37, P217

BARRERA M, 1986, AM J COMMUN PSYCHOL, V14, P413, DOI 10.1007/BF00922627

Bearman KJ, 2002, J PEDIATR PSYCHOL, V27, P417, DOI 10.1093/jpepsy/27.5.417

Brannan A. M., 2001, J CHILD FAM STUD, V10, P405, DOI DOI 10.1023/A:1016705306848

Brannan AM, 1997, J EMOT BEHAV DISORD, V5, P212, DOI 10.1177/106342669700500404

Brannan AM, 2003, J EMOT BEHAV DISORD, V11, P77, DOI 10.1177/106342660301100202

Bussing R, 2003, J AM ACAD CHILD PSY, V42, P842, DOI 10.1097/01.CHI.0000046876.27264.BF

Bussing R, 2003, J AM ACAD CHILD PSY, V42, P184, DOI 10.1097/01.CHI.0000037011.34553.A9

Butler DA, 2008, PEDIATR DIABETES, V9, P373, DOI 10.1111/j.1399-5448.2008.00370.x

Calderon C, 2011, J PEDIATR PSYCHOL, V36, P188, DOI 10.1093/jpepsy/jsq069

Canning RD, 1996, J PEDIATR PSYCHOL, V21, P735, DOI 10.1093/jpepsy/21.5.735

Carcone AI, 2011, J DEV BEHAV PEDIATR, V32, P581, DOI 10.1097/DBP.0b013e31822c1a27

Cooper C, 2003, J AM ACAD CHILD PSY, V42, P1370, DOI 10.1097/01.CHI.0000085751.71002.48

Cunningham NR, 2011, J PEDIATR PSYCHOL, V36, P196, DOI 10.1093/jpepsy/jsq071

Dashiff C. J., 2003, J FAMILY NURSING, V9, P166, DOI DOI 10.1177/1074840703009002004

DCCT Res Grp, 1994, J PEDIATR-US, V125, P177, DOI 10.1016/S0022-3476(94)70190-3

DELAMATER AM, 1991, DIABETES CARE, V14, P20, DOI 10.2337/diacare.14.1.20

Derogatis LR, 2004, BSI 18 MANUAL

Diabetes Technologies Inc, 2004, LAB TEST KIT ACCUBAS

Dovey-Pearce G, 2007, BRIT J HEALTH PSYCH, V12, P75, DOI 10.1348/135910706X98317

Forsander G, 1998, ACTA PAEDIATR, V87, P857, DOI 10.1080/080352598750013635

FREY MA, 1989, ADV NURS SCI, V12, P67, DOI 10.1097/00012272-198910000-00009

Frey MA, 2004, DIABETES EDUCATOR, V30, P647, DOI 10.1177/014572170403000416

Haller MJ, 2004, J PEDIATR-US, V144, P660, DOI 10.1016/j.jpeds.2003.12.042

Heflinger C. A., 2004, J FAMILY SOCIAL WORK, V8, P27, DOI DOI 10.1300/J039V08N01_

IBM Corporation, 2011, IBM SPSS STAT COMP P

Johnson SB, 2000, J PEDIATR PSYCHOL, V25, P171, DOI 10.1093/jpepsy/25.3.171

Johnson SB, 1990, NEUROPSYCHOLOGICAL B, P30

Kang E. J., 2005, J CHILD FAM STUD, V14, P43, DOI DOI 10.1007/S10826-005-1112-1

Kline R. B., 2005, PRINCIPLES PRACTICE

La Greca AM, 2002, J PEDIATR PSYCHOL, V27, P665, DOI 10.1093/jpepsy/27.8.665

Lee JM, 2006, DIABETES CARE, V29, P420, DOI 10.2337/diacare.29.02.06.dc05-2182

Leishman JM, 2010, INDIVIDUAL FAMILY PR

Levine BS, 2001, J PEDIATR-US, V139, P197, DOI 10.1067/mpd.2001.116283

Luescher JL, 1999, J CHILD NEUROL, V14, P642, DOI 10.1177/088307389901401004

McCabe Kristen M, 2003, Ment Health Serv Res, V5, P137, DOI 10.1023/A:1024439317884

MONTGOMERY RJV, 1985, FAM RELAT, V34, P19, DOI 10.2307/583753

Moran A, 1999, DIABETES, V48, P2039, DOI 10.2337/diabetes.48.10.2039

Morris AD, 1997, LANCET, V350, P1505, DOI 10.1016/S0140-6736(97)06234-X

Mullins LL, 2011, J PEDIATR PSYCHOL, V36, P249, DOI 10.1093/jpepsy/jsq080

Oh H, 2009, INT J DISABIL DEV ED, V56, P149, DOI 10.1080/10349120902868624

Palmer DL, 2004, J PEDIATR PSYCHOL, V29, P35, DOI 10.1093/jpepsy/jsh005

Patterson JM, 1997, J DEV BEHAV PEDIATR, V18, P383, DOI 10.1097/00004703-199712000-00003

Quittner AL, 2008, J PEDIATR PSYCHOL, V33, P916, DOI 10.1093/jpepsy/jsm064

Raina P, 2005, PEDIATRICS, V115, pE626, DOI 10.1542/peds.2004-1689

Ratner PA, 1998, ADV DESIGN NURSING R

Sales E, 2004, SOC WORK RES, V28, P211, DOI 10.1093/swr/28.4.211

Schilling LS, 2002, DIABETES EDUCATOR, V28, P796, DOI 10.1177/014572170202800520

Schoeder C. E., 2007, J CHILD FAM STUD, V16, P888, DOI DOI 10.1007/S10826-007-9133-6

Schreiber JB, 2006, J EDUC RES, V99, P323, DOI 10.3200/JOER.99.6.323-338

Seiffge-Krenke I, 2003, J ADOLESCENT HEALTH, V33, P180, DOI 10.1016/S1054-139X(02)00707-3

Serr J, 2007, 18 INT NURS RES C FO

Silverstein J, 2005, DIABETES CARE, V28, P186, DOI 10.2337/diacare.28.1.186

Thomas AM, 1997, J PEDIATR PSYCHOL, V22, P541, DOI 10.1093/jpepsy/22.4.541

THOMPSON EH, 1993, J GERONTOL, V48, pS245, DOI 10.1093/geronj/48.5.S245

Varekamp I, 1990, FAM SYST MED, V8, P291

Wade SL, 1998, PEDIATRICS, V102, P110, DOI 10.1542/peds.102.1.110

WEISSBERGBENCHELL J, 1995, DIABETES CARE, V18, P77, DOI 10.2337/diacare.18.1.77

Wysocki T, 1996, DIABETES CARE, V19, P119, DOI 10.2337/diacare.19.2.119

WYSOCKI T, 1992, J DEV BEHAV PEDIATR, V13, P194

Wysocki Tim, 2006, Curr Diab Rep, V6, P117, DOI 10.1007/s11892-006-0022-y

NR 66

TC 8

Z9 8

U1 0

U2 13

PU LIPPINCOTT WILLIAMS & WILKINS

PI PHILADELPHIA

PA TWO COMMERCE SQ, 2001 MARKET ST, PHILADELPHIA, PA 19103 USA

SN 0196-206X

EI 1536-7312

J9 J DEV BEHAV PEDIATR

JI J. Dev. Behav. Pediatr.

PD MAY

PY 2012

VL 33

IS 4

BP 343

EP 351

DI 10.1097/DBP.0b013e31824eaac8

PG 9

WC Behavioral Sciences; Psychology, Developmental; Pediatrics

SC Behavioral Sciences; Psychology; Pediatrics

GA 939PQ

UT WOS:000303825200008

PM 22566030

OA Green Accepted

DA 2019-01-08

ER

PT J

AU Hartmann, M

Kopf, S

Kircher, C

Faude-Lang, V

Djuric, Z

Augstein, F

Friederich, HC

Kieser, M

Bierhaus, A

Humpert, PM

Herzog, W

Nawroth, PP

AF Hartmann, Mechthild

Kopf, Stefan

Kircher, Claudia

Faude-Lang, Verena

Djuric, Zdenka

Augstein, Florian

Friederich, Hans-Christoph

Kieser, Meinhard

Bierhaus, Angelika

Humpert, Per M.

Herzog, Wolfgang

Nawroth, Peter P.

TI Sustained Effects of a Mindfulness-Based Stress-Reduction Intervention

in Type 2 Diabetic Patients Design and first results of a randomized

controlled trial (the Heidelberger Diabetes and Stress-Study)

SO DIABETES CARE

LA English

DT Article

ID METAANALYSIS; DEPRESSION; COMPLICATIONS; ACCEPTANCE

AB OBJECTIVE-To determine whether a mindfulness-based stress reduction (MBSR) intervention is effective for reducing psychosocial distress (i.e., depression, psychosocial stress) and the progression of nephropathy (i.e., albuminuria) and for improving the subjective health status of patients with type 2 diabetes.

RESEARCH DESIGN AND METHODS-Patients with type 2 diabetes and microalbuminuria were randomized to a mindfulness-based intervention (n = 53) or a treatment-as-usual control (n = 57) group. The study is designed to investigate long-term outcomes over a period of 5 years. We present data up to the first year of follow-up (FU).

RESULTS-At FU, the MBSR group showed lower levels of depression (d = 0.71) and improved health status (d = 0.54) compared with the control group. No significant differences in albuminuria were found. Per-protocol analysis also showed higher stress reduction in the intervention group (d = 0.64).

CONCLUSIONS-MBSR intervention achieved a prolonged reduction in psychosocial distress. The effects on albuminuria will be followed up further.

C1 [Hartmann, Mechthild; Faude-Lang, Verena; Friederich, Hans-Christoph; Herzog, Wolfgang] Heidelberg Univ, Dept Med & Psychosomat 2, Heidelberg, Germany.

[Kopf, Stefan; Kircher, Claudia; Djuric, Zdenka; Augstein, Florian; Bierhaus, Angelika; Humpert, Per M.; Nawroth, Peter P.] Heidelberg Univ, Dept Med & Clin Chem 1, Heidelberg, Germany.

[Faude-Lang, Verena] Univ Hamburg Eppendorf, Dept Psychosomat Med & Psychotherapy, Hamburg, Germany.

[Kieser, Meinhard] Heidelberg Univ, Inst Med Biometry, Heidelberg, Germany.

RP Hartmann, M (reprint author), Heidelberg Univ, Dept Med & Psychosomat 2, Heidelberg, Germany.

EM mechthild.hartmann@med.uni-heidelberg.de

FU Lautenschlager Stiftung; European Foundation for the Study of Diabetes

FX This study was supported by the Lautenschlager Stiftung (to P.P.N.) and

the European Foundation for the Study of Diabetes (to A.B.).

CR Anderson RJ, 2001, DIABETES CARE, V24, P1069, DOI 10.2337/diacare.24.6.1069

Bierhaus A, 2003, P NATL ACAD SCI USA, V100, P1920, DOI 10.1073/pnas.0438019100

Bierhaus A, 2009, DIABETOLOGIA, V52, P2251, DOI 10.1007/s00125-009-1458-9

Bohlmeijer E, 2010, J PSYCHOSOM RES, V68, P539, DOI 10.1016/j.jpsychores.2009.10.005

de Groot M, 2001, PSYCHOSOM MED, V63, P619, DOI 10.1097/00006842-200107000-00015

Faude-Lang V, 2010, PSYCHOTHER PSYCH MED, V60, P185, DOI 10.1055/s-0030-1248420

Gregg JA, 2007, J CONSULT CLIN PSYCH, V75, P336, DOI 10.1037/0022-006X.75.2.336

Grossman P, 2004, J PSYCHOSOM RES, V57, P35, DOI 10.1016/S0022-3999(03)00573-7

Kabat-Zinn J, 1990, FULL CATASTROPHY LIV

Lin EHB, 2010, DIABETES CARE, V33, P264, DOI 10.2337/dc09-1068

Pouwer F, 2010, DISCOV MED, V9, P112

Rosenzweig S, 2007, ALTERN THER HEALTH M, V13, P36

Spitzer RL, 2000, AM J OBSTET GYNECOL, V183, P759, DOI 10.1067/mob.2000.106580

Ware JE, 1998, J CLIN EPIDEMIOL, V51, P1167, DOI 10.1016/S0895-4356(98)00108-5

NR 14

TC 55

Z9 60

U1 3

U2 42

PU AMER DIABETES ASSOC

PI ALEXANDRIA

PA 1701 N BEAUREGARD ST, ALEXANDRIA, VA 22311-1717 USA

SN 0149-5992

EI 1935-5548

J9 DIABETES CARE

JI Diabetes Care

PD MAY

PY 2012

VL 35

IS 5

BP 945

EP 947

DI 10.2337/dc11-1343

PG 3

WC Endocrinology & Metabolism

SC Endocrinology & Metabolism

GA 931KN

UT WOS:000303218900003

PM 22338101

OA Green Published, Other Gold

DA 2019-01-08

ER

PT J

AU Schiotz, ML

Bogelund, M

Almdal, T

Jensen, BB

Willaing, I

AF Schiotz, M. L.

Bogelund, M.

Almdal, T.

Jensen, B. B.

Willaing, I.

TI Social support and self-management behaviour among patients with Type 2

diabetes

SO DIABETIC MEDICINE

LA English

DT Article

DE diabetes; psychosocial problems; self-management; social network

ID CHRONIC ILLNESS; ACTIVATION; FAMILY; CARE; ASSOCIATION; OUTCOMES;

NETWORK

AB Aims To investigate the relationship between structural and functional social support and patient activation, diabetes-related emotional distress, perceived diabetes care, self-management behaviour and HbA1c levels among patients with Type 2 diabetes. Methods Self-administered questionnaires were collected from 2572 patients with Type 2 diabetes. After adjusting for gender, age and education, Tobit and logistic regression models were used to examine associations between social network and patient activation, psychosocial problems, self-management behaviours and HbA1c levels. Results Frequent contact with friends was associated with more positive scores for activation, fewer psychosocial problems, more positive assessment of care and health-promoting self-management behaviours such as frequent exercising and frequent foot examinations. Frequent contact with family was associated with more positive assessments of care. Living with a partner was associated with lower prevalence of smoking, a higher frequency of foot examinations and higher HbA1c levels. A poor functional social network, measured as perceived lack of help in the event of severe illness, was associated with low patient activation, greater emotional distress, negative assessment of care, less health-promoting eating habits and less frequent foot examinations. Conclusions Good social support is significantly associated with health-promoting behaviours and well-being among patients with Type 2 diabetes. However, HbA1c levels are higher for cohabitant persons, indicating barriers for social support. Intervention research is needed to investigate the causal relationship between social networks and health-promoting behaviours. This knowledge should be used in clinical practice when targeting and designing education, support and care for patients with Type 2 diabetes.

C1 [Schiotz, M. L.; Almdal, T.; Jensen, B. B.; Willaing, I.] Steno Diabet Ctr, Steno Hlth Promot Ctr, DK-2820 Gentofte, Denmark.

[Bogelund, M.] Incent Partners, Holte, Denmark.

RP Willaing, I (reprint author), Steno Diabet Ctr, Steno Hlth Promot Ctr, Niels Steensens Vej 8, DK-2820 Gentofte, Denmark.

EM iwtp@steno.dk

OI Jensen, Bjarne Bruun/0000-0002-3403-8475; Willaing,

Ingrid/0000-0002-3082-8816

CR Bodenheimer T, 2002, JAMA-J AM MED ASSOC, V288, P1775, DOI 10.1001/jama.288.14.1775

Due P, 1999, SOC SCI MED, V48, P661, DOI 10.1016/S0277-9536(98)00381-5

Funnell MM, 2008, DIABETES CARE, V31, pS97, DOI 10.2337/dc08-S097

Gallant MP, 2007, RES AGING, V29, P375, DOI 10.1177/0164027507303169

Gallant MP, 2003, HEALTH EDUC BEHAV, V30, P170, DOI 10.1177/1090198102251030

Glasgow RE, 2005, DIABETES CARE, V28, P2655, DOI 10.2337/diacare.28.11.2655

Hermanns N, 2006, DIABETOLOGIA, V49, P469, DOI 10.1007/s00125-005-0094-2

Hibbard JH, 2004, HEALTH SERV RES, V39, P1005, DOI 10.1111/j.1475-6773.2004.00269.x

Hibbard JH, 2007, HEALTH SERV RES, V42, P1443, DOI 10.1111/j.1475-6773.2006.00669.x

Hibbard JH, 2007, J AMBUL CARE MANAG, V30, P2

IDF Clinical Guidelines Task Force, 2005, GLOB GUID TYP 2 DIAB

Jones RA, 2008, DIABETES EDUCATOR, V34, P318, DOI 10.1177/0145721708314485

KAPLAN RM, 1987, HEALTH PSYCHOL, V6, P387, DOI 10.1037/0278-6133.6.5.387

LEVY RL, 1983, SOC SCI MED, V17, P1329, DOI 10.1016/0277-9536(83)90192-2

Maindal HT, 2012, EUR J PUBLIC HEALTH, V22, P31, DOI 10.1093/eurpub/ckq188

Maindal HT, 2009, BMC PUBLIC HEALTH, V9, DOI 10.1186/1471-2458-9-209

McGuire BE, 2009, DIABETOLOGIA, V53, P66

Mosen DM, 2007, J AMBUL CARE MANAG, V30, P21

Nathan DM, 2008, DIABETES CARE, V31, P173, DOI 10.2337/dc08-9016

NICE, 2008, IMPL NICE GUID COMM

Rosland AM, 2010, CHRONIC ILLN, V6, P22, DOI 10.1177/1742395309354608

Rosland AM, 2008, J GEN INTERN MED, V23, P1992, DOI 10.1007/s11606-008-0814-7

SHERBOURNE CD, 1992, J BEHAV MED, V15, P447, DOI 10.1007/BF00844941

Snoek FJ, 2011, DIABETES CARE, V34, P601, DOI 10.2337/dc10-1552

Toobert DJ, 2000, DIABETES CARE, V23, P943, DOI 10.2337/diacare.23.7.943

Wooldridge JM, 2006, INTRO ECONOMETRICS M, P595

NR 26

TC 47

Z9 48

U1 1

U2 42

PU WILEY-BLACKWELL

PI MALDEN

PA COMMERCE PLACE, 350 MAIN ST, MALDEN 02148, MA USA

SN 0742-3071

J9 DIABETIC MED

JI Diabetic Med.

PD MAY

PY 2012

VL 29

IS 5

BP 654

EP 661

DI 10.1111/j.1464-5491.2011.03485.x

PG 8

WC Endocrinology & Metabolism

SC Endocrinology & Metabolism

GA 927LJ

UT WOS:000302908600016

PM 21992512

DA 2019-01-08

ER

PT J

AU Keen, AJA

Duncan, E

McKillop-Smith, A

Evans, ND

Gold, AE

AF Keen, A. J. A.

Duncan, E.

McKillop-Smith, A.

Evans, N. D.

Gold, A. E.

TI Dose Adjustment for Normal Eating (DAFNE) in routine clinical practice:

who benefits?

SO DIABETIC MEDICINE

LA English

DT Article

DE education; patient education; Type 1 diabetes

ID HOSPITAL ANXIETY; DEPRESSION SCALE

AB Aims To explore the effectiveness of Dose Adjustment for Normal Eating in routine clinical practice in the UK. Methods Participants were 124 adults with Type 1 diabetes who had completed a Dose Adjustment for Normal Eating course. Data were collected before the course and again 1 year later on a variety of biological, psychological and social measures. Results There were a range of significant benefits consistent with Dose Adjustment for Normal Eating aims, including: better control among those with baseline HbA1c= 81 mmol/mol (9.6%) (z = -2.8, P = 0.004); reduced number of participants reporting severe hypoglycaemia (chi(2) = 4.27, P = 0.039); total eradication of diabetic ketoacidosis (chi(2) = 4.17, P = 0.041) and lower diabetes-related distress (z = -4.5, P < 0.001). The most deprived of the clinic population were significantly under-represented (chi(2) = 17.8, P = 0.001) and the levels of clinical depression were unusually low. Conclusions These results indicate that Dose Adjustment for Normal Eating delivered in routine clinical practice is associated with a range of benefits and that certain clinical and psychosocial characteristics are associated with better outcomes.

C1 [Keen, A. J. A.; McKillop-Smith, A.; Evans, N. D.; Gold, A. E.] Univ Aberdeen, Ctr Diabet, NHS Grampian, Aberdeen, Scotland.

[Duncan, E.] Univ Aberdeen, Hlth Serv Res Unit, Aberdeen, Scotland.

RP Keen, AJA (reprint author), Woolmanhill Hosp, Ctr Diabet, Aberdeen AB25 1LD, Scotland.

EM a.keen@nhs.net

CR Barnard KD, 2006, DIABETIC MED, V23, P445, DOI 10.1111/j.1464-5491.2006.01814.x

Bjelland I, 2002, J PSYCHOSOM RES, V52, P69, DOI 10.1016/S0022-3999(01)00296-3

BLAND JM, 1994, BRIT MED J, V308, P1499, DOI 10.1136/bmj.308.6942.1499

Collins MM, 2009, DIABETIC MED, V26, P153, DOI 10.1111/j.1464-5491.2008.02648.x

DAFNE Study Group, BR MED J, V325, P746

Department of Health, 2010, GEN MOD LONG TERM CO

Department of Health, 2005, SELF CAR REAL CHOIC

Department of Health, 2005, SUPP PEOPL LONG TERM

Glasgow RE, 2007, ANNU REV PUBL HEALTH, V28, P413, DOI 10.1146/annurev.publhealth.28.021406.144145

Heller SR, 2009, BR J DIABETES VASC D, V9, P269

Hermanns N, 2006, DIABETOLOGIA, V49, P468

Jorde R, 2000, DIABETIC MED, V17, P451, DOI 10.1046/j.1464-5491.2000.00295.x

Newman S, 2004, LANCET, V364, P1523, DOI 10.1016/S0140-6736(04)17277-2

Oliver L, 2009, PRACT DIAB INT, V26, P371, DOI DOI 10.1002/PDI.1424

POLONSKY WH, 1995, DIABETES CARE, V18, P754, DOI 10.2337/diacare.18.6.754

Redman BK, 2007, CHRONIC ILLN, V3, P88, DOI 10.1177/1742395307079196

Rijken M, 2008, CARING PEOPLE CHRONI, V6, P116

Speight J, 2010, DIABETES RES CLIN PR, V89, P22, DOI 10.1016/j.diabres.2010.03.017

The EuroQol Group, 1990, HLTH POLICY, V16, P199, DOI DOI 10.1016/0168-8510(90)90421-9

Warsi A, 2004, ARCH INTERN MED, V164, P1641, DOI 10.1001/archinte.164.15.1641

WHO, 2008, 2008 2013 ACT PLAN G

Yach D, 2004, JAMA-J AM MED ASSOC, V291, P2616, DOI 10.1001/jama.291.21.2616

ZIGMOND AS, 1983, ACTA PSYCHIAT SCAND, V67, P361, DOI 10.1111/j.1600-0447.1983.tb09716.x

NR 23

TC 13

Z9 13

U1 0

U2 5

PU WILEY-BLACKWELL

PI MALDEN

PA COMMERCE PLACE, 350 MAIN ST, MALDEN 02148, MA USA

SN 0742-3071

J9 DIABETIC MED

JI Diabetic Med.

PD MAY

PY 2012

VL 29

IS 5

BP 670

EP 676

DI 10.1111/j.1464-5491.2011.03479.x

PG 7

WC Endocrinology & Metabolism

SC Endocrinology & Metabolism

GA 927LJ

UT WOS:000302908600018

PM 21988559

DA 2019-01-08

ER

PT J

AU Zulman, DM

Rosland, AM

Choi, H

Langa, KM

Heisler, M

AF Zulman, Donna M.

Rosland, Ann-Marie

Choi, HwaJung

Langa, Kenneth M.

Heisler, Michele

TI The influence of diabetes psychosocial attributes and self-management

practices on change in diabetes status

SO PATIENT EDUCATION AND COUNSELING

LA English

DT Article

DE Diabetes; Self-management; Self-efficacy; Diabetes distress

ID BLOOD-GLUCOSE CONTROL; GLYCEMIC CONTROL; CONTROLLED-TRIAL; PROBLEM

AREAS; CARE; COMPLICATIONS; KNOWLEDGE; EFFICACY; HEALTH; BEHAVIORS

AB Objective: To examine the influence of diabetes psychosocial attributes and self-management on glycemic control and diabetes status change.

Methods: Using data from the Health and Retirement Study, a nationally representative longitudinal study of U.S. adults >51 years, we examined cross-sectional relationships among diabetes psychosocial attributes (self-efficacy, risk awareness, care understanding, prioritization of diabetes, and emotional distress), self-management ratings, and glycemic control. We then explored whether self-management ratings and psychosocial attributes in 2003 predicted change in diabetes status in 2004.

Results: In multivariate analyses (N = 1834), all diabetes psychosocial attributes were associated with self-management ratings, with self-efficacy and diabetes distress having the strongest relationships (adj coeff = 8.1, p < 0.01 and -4.1, p < 0.01, respectively). Lower self-management ratings in 2003 were associated cross-sectionally with higher hemoglobin A1C (adj coeff = 0.16, p < 0.01), and with perceived worsening diabetes status in 2004 (adj OR = 1.36, p < 0.05), with much of this latter relationship explained by diabetes distress.

Conclusion: Psychosocial attributes, most notably diabetes-related emotional distress, contribute to difficulty with diabetes self-management, poor glycemic control, and worsening diabetes status over time.

Practice implications: Self-management and adherence interventions should target psychosocial attributes such as disease-related emotional distress. Published by Elsevier Ireland Ltd.

C1 [Zulman, Donna M.] VA Palo Alto Hlth Care Syst, Ctr Hlth Care Evaluat, Menlo Pk, CA 94025 USA.

[Zulman, Donna M.] Stanford Univ, Dept Internal Med, Div Gen Med Disciplines, Stanford, CA 94305 USA.

[Rosland, Ann-Marie; Choi, HwaJung; Langa, Kenneth M.; Heisler, Michele] Univ Michigan, Dept Internal Med, Ann Arbor, MI 48109 USA.

[Rosland, Ann-Marie; Langa, Kenneth M.; Heisler, Michele] Hlth Serv Res & Dev Ctr Excellence, Dept Vet Affairs, Ann Arbor, MI USA.

[Choi, HwaJung; Heisler, Michele] Univ Michigan, Robert Wood Johnson Fdn, Clin Scholars Program, Ann Arbor, MI 48109 USA.

[Heisler, Michele] Univ Michigan, Sch Publ Hlth, Dept Hlth Behav & Hlth Educ, Ann Arbor, MI 48109 USA.

RP Zulman, DM (reprint author), VA Palo Alto Hlth Care Syst, Ctr Hlth Care Evaluat, 795 Willow Rd,152-MPD, Menlo Pk, CA 94025 USA.

EM dzulman@stanford.edu

RI Northe, Hilda/I-1916-2012; Heisler, Michele/B-5774-2015; Rosland,

Annmarie/B-7750-2016

OI Northe, Hilda/0000-0002-8873-5014; Heisler, Michele/0000-0002-6889-2063;

FU The National Institute on Aging (NIA) [U01 AG09740]; Robert Wood Johnson

Foundation

FX The National Institute on Aging (NIA) provided funding for the Health

and Retirement Study (HRS) (U01 AG09740), data from which were used for

this analysis. The HRS is performed at the Institute for Social

Research, University of Michigan. Dr. Zulman was supported by the Robert

Wood Johnson Foundation Clinical Scholars Program and an associated VA

Advanced Fellowship.

CR ANDERSON RM, 1995, DIABETES CARE, V18, P943, DOI 10.2337/diacare.18.7.943

Anderson Robert M, 2009, Ther Patient Educ, V1, P3

DeSalvo KB, 2006, QUAL LIFE RES, V15, P191, DOI 10.1007/s11136-005-0887-2

Egede LE, 2005, CURR DIABETES REV, V1, P235, DOI 10.2174/157339905774574356

Fisher L, 2007, DIABETES CARE, V30, P542, DOI 10.2337/dc06-1614

Fisher L, 2010, DIABETES CARE, V33, P23, DOI 10.2337/dc09-1238

Fitzgerald JT, 1996, EVAL HEALTH PROF, V19, P208, DOI 10.1177/016327879601900205

Fonda Stephanie J, 2009, J Diabetes Sci Technol, V3, P117

Hayward RA, 1997, JAMA-J AM MED ASSOC, V278, P1663, DOI 10.1001/jama.278.20.1663

Heisler M, 2003, DIABETES CARE, V26, P738, DOI 10.2337/diacare.26.3.738

Heisler M, 2002, J GEN INTERN MED, V17, P243, DOI 10.1046/j.1525-1497.2002.10905.x

Heisler M, 2007, ARCH INTERN MED, V167, P1853, DOI 10.1001/archinte.167.17.1853

Hurley A C, 1992, Diabetes Educ, V18, P146, DOI 10.1177/014572179201800208

Jones H, 2003, DIABETES CARE, V26, P732, DOI 10.2337/diacare.26.3.732

Karter AJ, 2001, AM J MED, V111, P1, DOI 10.1016/S0002-9343(01)00742-2

Kerr EA, 2007, J GEN INTERN MED, V22, P1635, DOI 10.1007/s11606-007-0313-2

King DK, 2010, DIABETES CARE, V33, P751, DOI 10.2337/dc09-1746

Kingery P M, 1989, Health Educ, V20, P13

Klungel OH, 2008, PRIM CARE DIABETES, V2, P25, DOI 10.1016/j.pcd.2008.01.003

Lorig KR, 2006, MED CARE, V44, P964, DOI 10.1097/01.mlr.0000233678.80203.c1

Maddigan SL, 2004, DIABETES CARE, V27, P1306, DOI 10.2337/diacare.27.6.1306

McPherson ML, 2008, RES SOC ADM PHARM, V4, P37, DOI 10.1016/j.sapharm.2007.01.002

National Institute on Aging, 2011, HLTH RET STUD LONG S

Nelson KM, 2007, J GEN INTERN MED, V22, P442, DOI 10.1007/s11606-006-0053-8

Norris SL, 2001, DIABETES CARE, V24, P561, DOI 10.2337/diacare.24.3.561

Panja S, 2005, J INVEST MED, V53, P264, DOI 10.2310/6650.2005.53509

Persell SD, 2004, PREV MED, V39, P746, DOI 10.1016/j.ypmed.2004.02.045

POLONSKY WH, 1995, DIABETES CARE, V18, P754, DOI 10.2337/diacare.18.6.754

RADLOFF L S, 1977, Applied Psychological Measurement, V1, P385, DOI 10.1177/014662167700100306

Sarkar U, 2006, DIABETES CARE, V29, P823, DOI 10.2337/diacare.29.04.06.dc05-1615

Scollan-Koliopoulos M, 2010, DIABETES EDUCATOR, V36, P473, DOI 10.1177/0145721710362108

SHAMOON H, 1993, NEW ENGL J MED, V329, P977

Sousa Valmi D, 2005, Res Theory Nurs Pract, V19, P217, DOI 10.1891/rtnp.2005.19.3.217

Steffic D, 2000, DOCUMENTATION AFFECT

Stolar M, 2010, AM J MED, V123, pS3, DOI 10.1016/j.amjmed.2009.12.004

Trief PM, 2009, AGE AGEING, V38, P219, DOI 10.1093/ageing/afn299

Turner RC, 1998, LANCET, V352, P837

Weinger K, 2005, DIABETES CARE, V28, P1346, DOI 10.2337/diacare.28.6.1346

Welch G, 2003, DIABETIC MED, V20, P69, DOI 10.1046/j.1464-5491.2003.00832.x

Welch GW, 1997, DIABETES CARE, V20, P760, DOI 10.2337/diacare.20.5.760

NR 40

TC 26

Z9 27

U1 1

U2 10

PU ELSEVIER IRELAND LTD

PI CLARE

PA ELSEVIER HOUSE, BROOKVALE PLAZA, EAST PARK SHANNON, CO, CLARE, 00000,

IRELAND

SN 0738-3991

J9 PATIENT EDUC COUNS

JI Patient Educ. Couns.

PD APR

PY 2012

VL 87

IS 1

BP 74

EP 80

DI 10.1016/j.pec.2011.07.013

PG 7

WC Public, Environmental & Occupational Health; Social Sciences,

Interdisciplinary

SC Public, Environmental & Occupational Health; Social Sciences - Other

Topics

GA 930YX

UT WOS:000303182000013

PM 21840149

OA Green Accepted

DA 2019-01-08

ER

PT J

AU Dennedy, MC

Avalos, G

O'Reilly, MW

O'Sullivan, EP

Gaffney, G

Dunne, F

AF Dennedy, Michael Conall

Avalos, Gloria

O'Reilly, Michael W.

O'Sullivan, Eoin P.

Gaffney, Geraldine

Dunne, Fidelma

TI ATLANTIC-DIP: Raised Maternal Body Mass Index (BMI) Adversely Affects

Maternal and Fetal Outcomes in Glucose-Tolerant Women According to

International Association of Diabetes and Pregnancy Study Groups

(IADPSG) Criteria

SO JOURNAL OF CLINICAL ENDOCRINOLOGY & METABOLISM

LA English

DT Article

ID WEIGHT-GAIN; SHORT-TERM; OBESITY; IMPACT; METAANALYSIS; PREVALENCE;

MELLITUS

AB Context: Raised maternal body mass index (BMI) in association with hyperglycemia is associated with adverse pregnancy outcome. The contribution of raised BMI as an independent risk factor for adverse pregnancy outcome is of growing concern and increasing prevalence.

Objective: The aim of this study was to investigate the effects of raised maternal BMI on pregnancy outcome in glucose-tolerant women using the International Association of Diabetes and Pregnancy Study Groups criteria.

Participants and Setting: We studied a cohort of glucose-tolerant, pregnant women (n = 3656) who were attending antenatal obstetric clinics and were recruited to a universal screening program for gestational diabetes under the ATLANTIC-DIP partnership.

Design: We conducted a prospective observational study of pregnancy outcome. Maternal outcomes include glucose, delivery mode, pregnancy-induced hypertension, preeclampsia, antepartum hemorrhage, and postpartum hemorrhage. Fetal outcomes included birth weight, congenital malformation, fetal death, neonatal jaundice, hypoglycemia, and respiratory distress.

Results: Increasing maternal BMI was associated with adverse pregnancy outcomes: higher cesarean section rates, preeclampsia, pregnancy-induced hypertension, increased birth weight, and congenital malformation. The association of glucose with adverse pregnancy outcome was weak and did not interact with raised BMI. A BMI threshold of 28 kg/m(2) was associated with a significant rise in adverse pregnancy outcome.

Conclusions: Raised maternal BMI, within the overweight range, is associated with adverse pregnancy outcomes. These adverse effects of BMI occur independently of maternal glucose. It is apparent that pregnancy unmasks an underlying unhealthy metabolic milieu in obese and overweight women. (J Clin Endocrinol Metab 97: E608-E612, 2012)

C1 [Dennedy, Michael Conall] Natl Univ Ireland, Dept Med, Inst Clin Sci, Dept Endocrinol, Galway, Ireland.

[Gaffney, Geraldine] Natl Univ Ireland, Dept Obstet & Gynecol, Galway, Ireland.

RP Dennedy, MC (reprint author), Natl Univ Ireland, Dept Med, Inst Clin Sci, Dept Endocrinol, Galway, Ireland.

EM cdennedy@me.com

OI O'Reilly, Michael/0000-0002-9108-9105; Dennedy,

Michael/0000-0001-9325-6520

FU Health Research Board of Ireland

FX This work was supported by the Health Research Board of Ireland.

CR Catalano PM, 2006, BJOG-INT J OBSTET GY, V113, P1126, DOI 10.1111/j.1471-0528.2006.00989.x

Dennedy MC, 2010, BEST PRACT RES CL EN, V24, P573, DOI 10.1016/j.beem.2010.06.001

Fitzsimons KJ, 2010, SEMIN FETAL NEONAT M, V15, P100, DOI 10.1016/j.siny.2009.09.004

Heslehurst N, 2008, OBES REV, V9, P635, DOI 10.1111/j.1467-789X.2008.00511.x

Heslehurst N, 2007, BJOG-INT J OBSTET GY, V114, P334, DOI 10.1111/j.1471-0528.2006.01230.x

Heslehurst N, 2007, BJOG-INT J OBSTET GY, V114, P187, DOI 10.1111/j.1471-0528.2006.01180.x

Huda SS, 2010, SEMIN FETAL NEONAT M, V15, P70, DOI 10.1016/j.siny.2009.09.006

Jensen DM, 2005, DIABETES CARE, V28, P2118, DOI 10.2337/diacare.28.9.2118

Jensen DM, 2003, AM J OBSTET GYNECOL, V189, P239, DOI 10.1067/mob.2003.441

Lederman SA, 1997, OBSTET GYNECOL, V90, P483, DOI 10.1016/S0029-7844(97)00355-4

Lewis G, 2007, SAVING MOTHERS LIVES

Metzger BE, 2008, NEW ENGL J MED, V358, P1991, DOI 10.1056/NEJMoa0707943

Metzger BE, 2010, DIABETES CARE, V33, P676, DOI 10.2337/dc09-1848

Modder J., 2010, CMACE RCOG JOINT GUI

National Institute for Health and Clinical Excellence, 2010, DIET INT PHYS ACT IN

O'Reilly MW, 2011, EUR J ENDOCRINOL, V165, P953, DOI 10.1530/EJE-11-0663

O'Sullivan EP, 2011, DIABETOLOGIA, V54, P1670, DOI 10.1007/s00125-011-2150-4

Owens LA, 2010, DIABETES CARE, V33, P577, DOI 10.2337/dc09-0911

PEDERSEN J, 1952, Ugeskr Laeger, V114, P685

Stothard KJ, 2009, JAMA-J AM MED ASSOC, V301, P636, DOI 10.1001/jama.2009.113

NR 20

TC 30

Z9 31

U1 0

U2 3

PU ENDOCRINE SOC

PI CHEVY CHASE

PA 8401 CONNECTICUT AVE, SUITE 900, CHEVY CHASE, MD 20815-5817 USA

SN 0021-972X

J9 J CLIN ENDOCR METAB

JI J. Clin. Endocrinol. Metab.

PD APR

PY 2012

VL 97

IS 4

BP E608

EP E612

DI 10.1210/jc.2011-2674

PG 5

WC Endocrinology & Metabolism

SC Endocrinology & Metabolism

GA 925US

UT WOS:000302787800012

PM 22319044

OA Bronze

DA 2019-01-08

ER

PT J

AU Nefs, G

Pouwer, F

Pop, V

Denollet, J

AF Nefs, Giesje

Pouwer, Francois

Pop, Victor

Denollet, Johan

TI Type D (distressed) personality in primary care patients with type 2

diabetes: Validation and clinical correlates of the DS14 assessment

SO JOURNAL OF PSYCHOSOMATIC RESEARCH

LA English

DT Article

DE Clinical care; Personality; Psychology; Type 2 diabetes; Validation

ID CORONARY-HEART-DISEASE; NEGATIVE AFFECTIVITY; SOCIAL INHIBITION;

CARDIOVASCULAR-DISEASE; DEPRESSION SCALE; RISK-FACTORS; ANXIETY;

PREVALENCE; MECHANISMS; OUTCOMES

AB Objective: In cardiovascular research, Type D personality (high negative affectivity and social inhibition) has been associated with a more than 3-fold increased risk of adverse health outcomes. This study examined the validity and clinical correlates of the Type D construct as assessed by the Type D Scale-14 (DS14) in type 2 diabetes patients.

Methods: 1553 primary care patients with type 2 diabetes were assessed for demographic, clinical, lifestyle and psychological characteristics in 2007. A subgroup (n=1012) completed the DS14 again 1 year later.

Results: The two-factor model of the Type D construct was confirmed in exploratory and confirmatory factor analyses; results were stable across gender. The Negative Affectivity (NA) and Social Inhibition (SI) subscales had adequate reliability in both men and women, as measured by Cronbach's alpha (NA=0.87, SI=0.83), lambda2 (NA=0.87/0.88, SI=0.84), corrected item-total correlations (NA 0.47-0.77, SI 0.34-0.72) and mean inter-item correlations (NA=0.50/0.51, SI=0.42). One year test-retest reliability using intraclass correlation coefficients was 0.64/0.63 for NA and 0.73/0.65 for SI. Type D and non-Type D patients did not differ in vascular history or physiological risk factors, but Type D women had a more sedentary lifestyle (p=.003). Type D patients experienced less social support and more stressful life events, loneliness, and more depressed mood, anhedonia and anxiety (p<.001 for most variables). These differences were clinically significant (Cohen's d>0.60 for most variables).

Conclusion: Type D personality can be reliably assessed in primary care patients with type 2 diabetes, and is associated with increased loneliness, stress and emotional distress in these patients. (C) 2012 Elsevier Inc. All rights reserved.

C1 [Nefs, Giesje; Pouwer, Francois; Pop, Victor; Denollet, Johan] Tilburg Univ, Ctr Res Psychol Somat Dis CoRPS, Dept Med Psychol & Neuropsychol, NL-5000 LE Tilburg, Netherlands.

RP Denollet, J (reprint author), Tilburg Univ, Ctr Res Psychol Somat Dis CoRPS, Dept Med Psychol & Neuropsychol, POB 90153, NL-5000 LE Tilburg, Netherlands.

EM denollet@uvt.nl

RI Nefs, Giesje/C-9992-2018

FU Netherlands Organisation for Health Research and Development;

Netherlands Organisation for Scientific Research (The Hague, The

Netherlands)

FX This study was supported by a ZonMW grant from the Netherlands

Organisation for Health Research and Development and by a Vici grant

from the Netherlands Organisation for Scientific Research (The Hague,

The Netherlands). The funding sources had no role in the design, data

collection, analysis or interpretation of the study, or in the decision

to submit the article for publication. G.N; F.P., V.P. and J.D. wrote

the manuscript, researched data, reviewed/edited the manuscript and

contributed to the discussion.

CR Bergvik S, 2010, SCAND J PSYCHOL, V51, P334, DOI 10.1111/j.1467-9450.2009.00793.x

Black SA, 2003, DIABETES CARE, V26, P2822, DOI 10.2337/diacare.26.10.2822

Brown T. A., 2006, CONFIRMATORY FACTOR

Denollet J, 2006, EUR HEART J, V27, P171, DOI 10.1093/eurheartj/ehi616

Denollet J, 2005, PSYCHOSOM MED, V67, P89, DOI 10.1097/01.psy.0000149256.81953.49

Denollet J, 2010, CIRC-CARDIOVASC QUAL, V3, P546, DOI 10.1161/CIRCOUTCOMES.109.934406

Einvik G, 2011, EUR J CARDIOV PREV R, V18, P504, DOI 10.1177/1741826710389383

Emons WHM, 2007, J PSYCHOSOM RES, V63, P27, DOI 10.1016/j.jphyschores.2007.03.010

Hausteiner C, 2010, PSYCHOSOM MED, V72, P163, DOI 10.1097/PSY.0b013e3181cb8bae

Kubzansky LD, 2006, ANN BEHAV MED, V31, P21, DOI 10.1207/s15324796abm3101_5

Kupper N, 2007, J PERS ASSESS, V89, P265, DOI 10.1080/00223890701629797

Lin EHB, 2010, DIABETES CARE, V33, P264, DOI 10.2337/dc09-1068

Marshall SM, 2006, BRIT MED J, V333, P475, DOI 10.1136/bmj.38922.650521.80

Martens EJ, 2007, J PSYCHOSOM RES, V63, P545, DOI 10.1016/j.jpsychores.2007.06.005

McGraw KO, 1996, PSYCHOL METHODS, V1, P30, DOI 10.1037//1082-989X.1.1.30

Mols F, 2010, GEN HOSP PSYCHIAT, V32, P66, DOI 10.1016/j.genhosppsych.2009.09.010

Mols F, 2010, HEALTH QUAL LIFE OUT, V8, DOI 10.1186/1477-7525-8-9

Mommersteeg PMC, 2010, BMC PUBLIC HEALTH, V10, DOI 10.1186/1471-2458-10-714

Nefs G, 2010, BMC PUBLIC HEALTH, V10, DOI 10.1186/1471-2458-10-388

OHARA MW, 1986, ARCH GEN PSYCHIAT, V43, P569

Pedersen SS, 2007, EUR J CARDIOV PREV R, V14, P135, DOI 10.1097/HJR.0b013e328045c282

Pedersen SS, 2009, INT J BEHAV MED, V16, P108, DOI 10.1007/s12529-008-9022-4

Pelle AJ, 2010, INT J CARDIOL, V142, P65, DOI 10.1016/j.ijcard.2008.12.086

POP VJ, 1992, J AFFECT DISORDERS, V26, P105, DOI 10.1016/0165-0327(92)90041-4

Pouwer F, 2009, NAT REV ENDOCRINOL, V5, P665, DOI 10.1038/nrendo.2009.214

Roest AM, 2010, PSYCHOSOM MED, V72, P563, DOI 10.1097/PSY.0b013e3181dbff97

Roest AM, 2010, J AM COLL CARDIOL, V56, P38, DOI 10.1016/j.jacc.2010.03.034

Simson U, 2008, PSYCHOTHER PSYCH MED, V58, P44, DOI 10.1055/s-2007-971001

Smith LL, 1998, J PERS SOC PSYCHOL, V75, P1350, DOI 10.1037/0022-3514.75.5.1350

Spindler H, 2009, INT J BEHAV MED, V16, P98, DOI 10.1007/s12529-009-9037-5

Streiner DL, 2008, HLTH MEASUREMENT SCA

Svansdottir E, 2012, J BEHAV MED, V35, P155, DOI 10.1007/s10865-011-9337-5

Tuohy A, 2008, BRIT J CLIN PSYCHOL, V47, P153, DOI 10.1348/014466507X238608

Van der Kooy K, 2007, INT J GERIATR PSYCH, V22, P613, DOI 10.1002/gps.1723

Weir JP, 2005, J STRENGTH COND RES, V19, P231, DOI 10.1519/15184.1

Williams L, 2008, J PSYCHOSOM RES, V64, P63, DOI 10.1016/j.jpsychores.2007.06.008

Williams L, 2011, PSYCHOL HEALTH, V26, P703, DOI 10.1080/08870446.2010.488265

Yu DSF, 2010, J PSYCHOSOM RES, V69, P111, DOI 10.1016/j.jpsychores.2010.01.014

NR 38

TC 19

Z9 20

U1 1

U2 13

PU PERGAMON-ELSEVIER SCIENCE LTD

PI OXFORD

PA THE BOULEVARD, LANGFORD LANE, KIDLINGTON, OXFORD OX5 1GB, ENGLAND

SN 0022-3999

EI 1879-1360

J9 J PSYCHOSOM RES

JI J. Psychosomat. Res.

PD APR

PY 2012

VL 72

IS 4

BP 251

EP 257

DI 10.1016/j.jpsychores.2012.01.006

PG 7

WC Psychiatry

SC Psychiatry

GA 910FG

UT WOS:000301622000002

PM 22405217

DA 2019-01-08

ER

PT J

AU Atlantis, E

Shi, ZM

Penninx, BJWH

Wittert, GA

Taylor, A

Almeida, OP

AF Atlantis, Evan

Shi, Zumin

Penninx, Brenda J. W. H.

Wittert, Gary A.

Taylor, Anne

Almeida, Osvaldo P.

TI Chronic medical conditions mediate the association between depression

and cardiovascular disease mortality

SO SOCIAL PSYCHIATRY AND PSYCHIATRIC EPIDEMIOLOGY

LA English

DT Article

DE Depressive symptoms; Chronic diseases; Comorbidity; High blood pressure;

Diabetes; Heart disease; Death

ID CORONARY-HEART-DISEASE; HEALTHY AGING MELSHA; NATIONAL DEATH INDEX;

SCALE CES-D; MYOCARDIAL-INFARCTION; PSYCHOLOGICAL DISTRESS;

OLDER-ADULTS; ENDOTHELIAL FUNCTION; PHYSICAL ILLNESS; RANDOMIZED-TRIAL

AB To determine whether chronic medical conditions mediate the association between depression and cardiovascular disease (CVD) mortality.

Data analyzed were from 6,394 subjects aged 25-74 years who participated in extensive health examinations in the NHEFS conducted between 1971 and 1975 and follow-up studies to 1992. CVD mortality was the endpoint. Depression predictors were clinically significant depressive symptoms at baseline by the GWB-D, and/or at 1982-1984 by the CES-D ('baseline', 'new', or 'twice' depression). Chronic conditions were prevalent/incident high blood pressure, diabetes, and non-fatal CVD by examination and/or self-report. Mediation effects were assessed by stepwise adjustments of covariates and additive interactions in competing risks regression models (accounting for other mortality causes) and logit models.

Baseline, new, and twice depression were significant predictors of CVD mortality in competing-risks models adjusted for demographics (HRs 1.3, 1.4, and 2.0), but effects were progressively weakened and became non-significant after adjustment for lifestyle factors, prevalent and incident medical conditions, respectively. CVD mortality risk was 80% higher for depression plus incident non-fatal CVD than without (HR 4.0 vs. 3.2, additive interaction), and mediation effects of depression via chronic medical conditions (particularly via incident non-fatal CVD) increased the risk by 2-11% in logit models, independent of all covariates.

Several levels of evidence suggest that the association between depression and CVD mortality is partially mediated by prevalent/incident chronic medical conditions, as well as unhealthy lifestyle behaviors. Patients presenting with clinically significant depressive symptoms, particularly if persistent, should be assessed for both chronic conditions and lifestyle risk factors.

C1 [Atlantis, Evan; Wittert, Gary A.] Govt S Australia, S Australia Hlth, Royal Adelaide Hosp, Inst Med & Vet Sci, Adelaide, SA, Australia.

[Atlantis, Evan; Wittert, Gary A.] Univ Adelaide, Sch Med, Freemasons Fdn Ctr Mens Hlth, Adelaide, SA, Australia.

[Wittert, Gary A.] Univ Adelaide, Sch Med, Ctr Clin Res Excellence Nutr Physiol Intervent &, Adelaide, SA, Australia.

[Penninx, Brenda J. W. H.] Vrije Univ Amsterdam Med Ctr, Dept Psychiat, EMGO Inst Hlth & Care Res, Amsterdam, Netherlands.

[Almeida, Osvaldo P.] Univ Western Australia, Sch Psychiat & Clin Neurosci, Western Australian Ctr Hlth & Ageing, Med Res Ctr, Perth, WA 6009, Australia.

[Almeida, Osvaldo P.] Royal Perth Hosp, Dept Psychiat, Perth, WA, Australia.

RP Atlantis, E (reprint author), Govt S Australia, S Australia Hlth, Royal Adelaide Hosp, Inst Med & Vet Sci, Adelaide, SA, Australia.

EM evan.atlantis@adelaide.edu.au

RI Taylor, Anne/F-5708-2010; Almeida, Osvaldo/A-4925-2008; Shi,

Zumin/A-1093-2009; Atlantis, Evan/D-8338-2012

OI Taylor, Anne/0000-0002-4422-7974; Almeida, Osvaldo/0000-0002-8689-6199;

Shi, Zumin/0000-0002-3099-3299; Atlantis, Evan/0000-0001-5877-6141

CR Akbaraly TN, 2009, BRIT J PSYCHIAT, V195, P408, DOI 10.1192/bjp.bp.108.058925

Atlantis E, 2011, SOC PSYCH PSYCH EPID, V46, P1303, DOI 10.1007/s00127-010-0302-3

Atlantis E, 2011, INT J GERIATR PSYCH, V26, P341, DOI 10.1002/gps.2532

Atlantis E, 2010, INT J GERIATR PSYCH, V25, P688, DOI 10.1002/gps.2409

Bao AM, 2008, BRAIN RES REV, V57, P531, DOI 10.1016/j.brainresrev.2007.04.005

Barton DA, 2007, J HYPERTENS, V25, P2117, DOI 10.1097/HJH.0b013e32829baae7

Beekman ATF, 1997, PSYCHOL MED, V27, P231, DOI 10.1017/S0033291796003510

Berkman LF, 2003, JAMA-J AM MED ASSOC, V289, P3106

Buis ML, 2010, STATA J, V10, P11, DOI 10.1177/1536867X1001000104

Campayo A, 2010, AM J PSYCHIAT, V167, P580, DOI 10.1176/appi.ajp.2009.09010038

Centers for Disease Control and Prevention, 2009, NAT HLTH NUTR EX SUR

Dahl CAF, 2010, SOC PSYCH PSYCH EPID, V45, P309, DOI 10.1007/s00127-009-0069-6

de Jonge P, 2007, AM J PSYCHIAT, V164, P1371, DOI 10.1176/appi.apj.2007.06091492

Fazio A F, 1977, Vital Health Stat 2, P1

Ferketich AK, 2000, ARCH INTERN MED, V160, P1261, DOI 10.1001/archinte.160.9.1261

Gallo JJ, 2007, ANN INTERN MED, V146, P689, DOI 10.7326/0003-4819-146-10-200705150-00002

Gunn JM, 2012, SOC PSYCH PSYCH EPID, V47, P175, DOI 10.1007/s00127-010-0330-z

Haukkala A, 2009, ANN EPIDEMIOL, V19, P623, DOI 10.1016/j.annepidem.2009.01.010

Howren MB, 2009, PSYCHOSOM MED, V71, P171, DOI 10.1097/PSY.0b013e3181907c1b

Lewinsohn PM, 1997, PSYCHOL AGING, V12, P277, DOI 10.1037//0882-7974.12.2.277

Lloyd-Jones D, 2010, CIRCULATION, V121, pE46, DOI 10.1161/CIRCULATIONAHA.109.192667

Luppino FS, 2010, ARCH GEN PSYCHIAT, V67, P220, DOI 10.1001/archgenpsychiatry.2010.2

Mapppsy LKR, 2008, DIABETES CARE, V31, P880, DOI 10.2337/dc07-2215

Marzari C, 2005, J GERONTOL A-BIOL, V60, P85, DOI 10.1093/gerona/60.1.85

Mezuk B, 2008, DIABETES CARE, V31, P2383, DOI 10.2337/dc08-0985

Musselman DL, 2000, ARCH GEN PSYCHIAT, V57, P875, DOI 10.1001/archpsyc.57.9.875

Nabi H, 2008, ARTERIOSCL THROM VAS, V28, P1398, DOI 10.1161/ATVBAHA.108.167239

Nicholson A, 2005, PSYCHOSOM MED, V67, P522, DOI 10.1097/01.psy.0000171159.86446.9e

Nicholson A, 2006, EUR HEART J, V27, P2763, DOI 10.1093/eurheartj/ehl338

Parker G, 2009, BRIT J PSYCHIAT, V194, P1, DOI 10.1192/bjp.bp.108.054767

Pasco JA, 2008, BRIT J PSYCHIAT, V193, P322, DOI 10.1192/bip.bp.107.046706

Patten SB, 2009, GEN HOSP PSYCHIAT, V31, P571, DOI 10.1016/j.genhosppsych.2009.08.001

Patton GC, 1998, AM J PUBLIC HEALTH, V88, P1518, DOI 10.2105/AJPH.88.10.1518

Penninx BWJH, 2001, ARCH GEN PSYCHIAT, V58, P221, DOI 10.1001/archpsyc.58.3.221

Pizzi C, 2009, CLIN PHARMACOL THER, V86, P527, DOI 10.1038/clpt.2009.121

Pizzi C, 2008, EUR HEART J, V29, P1110, DOI 10.1093/eurheartj/ehn137

RADLOFF L S, 1977, Applied Psychological Measurement, V1, P385, DOI 10.1177/014662167700100306

Rasul F, 2007, PSYCHOL MED, V37, P1305, DOI 10.1017/S0033291707000402

Rasul F, 2005, J EPIDEMIOL COMMUN H, V59, P140, DOI 10.1136/jech.2004.019786

RICHEDWARDS JW, 1994, AM J EPIDEMIOL, V140, P1016, DOI 10.1093/oxfordjournals.aje.a117191

Sauer WH, 2003, CIRCULATION, V108, P32, DOI 10.1161/01.CIR.0000079172.43229.CD

STAMPFER MJ, 1984, AM J EPIDEMIOL, V119, P837, DOI 10.1093/oxfordjournals.aje.a113804

Strawbridge WJ, 2002, AM J EPIDEMIOL, V156, P328, DOI 10.1093/aje/kwf047

Thombs BD, 2008, JAMA-J AM MED ASSOC, V300, P2161, DOI 10.1001/jama.2008.667

Thurston RC, 2006, PSYCHOSOM MED, V68, P25, DOI 10.1097/01.psy.0000195883.68888.68

Van der Kooy K, 2007, INT J GERIATR PSYCH, V22, P613, DOI 10.1002/gps.1723

Whooley MA, 2008, JAMA-J AM MED ASSOC, V300, P2379, DOI 10.1001/jama.2008.711

Wouts L, 2008, ARCH GEN PSYCHIAT, V65, P596, DOI 10.1001/archpsyc.65.5.596

Ziegelstein RC, 2000, ARCH INTERN MED, V160, P1818, DOI 10.1001/archinte.160.12.1818

ZONDERMAN AB, 1989, JAMA-J AM MED ASSOC, V262, P1191, DOI 10.1001/jama.262.9.1191

NR 50

TC 18

Z9 18

U1 0

U2 15

PU SPRINGER HEIDELBERG

PI HEIDELBERG

PA TIERGARTENSTRASSE 17, D-69121 HEIDELBERG, GERMANY

SN 0933-7954

EI 1433-9285

J9 SOC PSYCH PSYCH EPID

JI Soc. Psychiatry Psychiatr. Epidemiol.

PD APR

PY 2012

VL 47

IS 4

BP 615

EP 625

DI 10.1007/s00127-011-0365-9

PG 11

WC Psychiatry

SC Psychiatry

GA 912HI

UT WOS:000301786400012

PM 21384119

DA 2019-01-08

ER

PT J

AU Eriksson, AK

Ekbom, A

Hilding, A

Ostenson, CG

AF Eriksson, Anna-Karin

Ekbom, Anders

Hilding, Agneta

Ostenson, Claes-Goran

TI The influence of non-response in a population-based cohort study on type

2 diabetes evaluated by the Swedish Prescribed Drug Register

SO EUROPEAN JOURNAL OF EPIDEMIOLOGY

LA English

DT Article

DE Cohort; Lifestyle; Non-participation; Screening; Selection bias; Type 2

diabetes

ID MIDDLE-AGED MEN; SCREENING-PROGRAM; FAMILY-HISTORY; GLUCOSE; SWEDEN;

WOMEN; LIFE; PRESCRIPTION; PREVALENCE; ATTENDANCE

AB Bias due to non-response in observational studies may lead to false risk estimates. We evaluated potential selective non-response in Stockholm Diabetes Prevention Program (SDPP) using a population-based drug register. A cohort of 12,952 men and 19,416 women, aged 35-56 years, was screened for diabetes and family history of diabetes (FHD). Response rate: 79% in men and 85% in women. Of the 4,209 men and 6,916 women that were invited, 70 and 68% participated in a baseline health examination including oral glucose tolerance test. A follow-up was performed in 79% of male and 70% of female baseline participants. We used data from the prescription register to estimate absolute risks and ORs for drug-treated diabetes in the non-response/non-participation groups. At both screening and baseline steps absolute risks of drug-treated diabetes were equal in non-participants and participants. Adjusted ORs were 0.9-1.0. At follow-up, absolute risks were higher among non-participants than participants, men 6.2/4.4% and women 2.6/1.6%, adjusted ORs 1.4 (0.9-2.3) and 1.5 (0.9-2.4), suggesting selective non-participation. Further analyses of FHD, smoking, physical activity, socioeconomic position and psychological distress demonstrated no previous false risk estimates for type 2 diabetes. However, for body mass index, there were indications of overestimation in women. We conclude that bias from non-response was not present at screening or baseline steps, suggesting that diabetes prevalence and risks may be estimated from a population-based cohort study with high attendance rate such as the SDPP. However, follow-up data should be treated with some caution, since the sample may have become biased.

C1 [Eriksson, Anna-Karin; Ekbom, Anders] Karolinska Univ Hosp, Dept Med, Clin Epidemiol Unit, Karolinska Inst, S-17176 Stockholm, Sweden.

[Hilding, Agneta; Ostenson, Claes-Goran] Karolinska Inst, Dept Mol Med & Surg, Endocrine & Diabet Unit, Stockholm, Sweden.

RP Eriksson, AK (reprint author), Karolinska Univ Hosp, Dept Med, Clin Epidemiol Unit, Karolinska Inst, T2 Eugeniahemmet, S-17176 Stockholm, Sweden.

EM anna-karin.eriksson@karolinska.se

FU Stockholm County Council; Swedish Council of Working Life and Social

Research; Novo Nordisk Scandinavia

FX This study was supported by grants from Stockholm County Council,

Swedish Council of Working Life and Social Research, and Novo Nordisk

Scandinavia. The authors thank the nurses, technicians and other staff

members at the health-care centres and the laboratory who carried out

the oral glucose tolerance tests and other measurements.

CR Agardh EE, 2007, INT J EPIDEMIOL, V36, P84, DOI 10.1093/ije/dyl269

Alberti KGMM, 1998, DIABETIC MED, V15, P539, DOI 10.1002/(SICI)1096-9136(199807)15:7<539::AID-DIA668>3.0.CO;2-S

BERGMAN U, 1984, ACTA MED SCAND, P15

Carlsson S, 1999, DIABETES CARE, V22, P1043, DOI 10.2337/diacare.22.7.1043

Cowie CC, 2006, DIABETES CARE, V29, P1263, DOI 10.2337/dc06-0062

Dalsgaard EM, 2009, DIABETIC MED, V26, P518, DOI 10.1111/j.1464-5491.2009.02715.x

Eriksson AK, 2008, DIABETIC MED, V25, P834, DOI 10.1111/j.1464-5491.2008.02463.x

FORREST RD, 1990, J INTERN MED, V228, P267, DOI 10.1111/j.1365-2796.1990.tb00230.x

Hilding A, 2006, DIABETOLOGIA, V49, P2589, DOI 10.1007/s00125-006-0402-5

Juel K, 2008, J PUBLIC HEALTH-UK, V30, P111, DOI 10.1093/pubmed/fdm072

Lagerlund M, 2000, EUR J CANCER PREV, V9, P25, DOI 10.1097/00008469-200002000-00004

Larsen IK, 2006, EUR J CANCER PREV, V15, P10, DOI 10.1097/01.cej.0000186636.27496.bb

Rodvall Y, 2005, EUR J CANCER PREV, V14, P459, DOI 10.1097/01.cej.0000178078.21910.da

Rothman KJ, 2008, MODERN EPIDEMIOLOGY

Sargeant LA, 2010, DIABETIC MED, V27, P995, DOI 10.1111/j.1464-5491.2010.03056.x

Stalhammar J, 2001, SCAND J PRIM HEALTH, V19, P163

Statistics Sweden, 2005, SWED SOC CLASS

WALINDER O, 1986, UPSALA J MED SCI, V91, P89, DOI 10.3109/03009738609178494

Wandell PE, 1997, EUR J CLIN PHARMACOL, V52, P249, DOI 10.1007/s002280050285

Wettermark B, 2007, PHARMACOEPIDEM DR S, V16, P726, DOI 10.1002/pds.1294

NR 20

TC 7

Z9 7

U1 0

U2 2

PU SPRINGER

PI DORDRECHT

PA VAN GODEWIJCKSTRAAT 30, 3311 GZ DORDRECHT, NETHERLANDS

SN 0393-2990

J9 EUR J EPIDEMIOL

JI Eur. J. Epidemiol.

PD MAR

PY 2012

VL 27

IS 3

BP 153

EP 162

DI 10.1007/s10654-011-9630-1

PG 10

WC Public, Environmental & Occupational Health

SC Public, Environmental & Occupational Health

GA 958EH

UT WOS:000305218800001

PM 22089424

DA 2019-01-08

ER

PT J

AU Dalfra, MG

Nicolucci, A

Bisson, T

Bonsembiante, B

Lapolla, A

AF Dalfra, M. G.

Nicolucci, A.

Bisson, T.

Bonsembiante, B.

Lapolla, A.

CA QLISG

TI Quality of life in pregnancy and post-partum: a study in diabetic

patients

SO QUALITY OF LIFE RESEARCH

LA English

DT Article

DE Quality of life; Pregnancy; Gestational diabetes; Type 1 diabetes

mellitus; Insulin therapy

ID EMOTIONAL ADJUSTMENT; DEPRESSION; MELLITUS; EXPERIENCES; DIAGNOSIS;

SYMPTOMS; IMPACT

AB Purpose We evaluated quality of life in pregnant women with diabetes followed up at Italian diabetes clinics.

Methods A total of 245 pregnant women (30 type 1 diabetes mellitus (T1DM), 176 gestational diabetes (GDM) and 39 controls) were asked to fill in a questionnaire including the SF-36 Health Survey and the Center for Epidemiological Studies-Depression (CES-D) Scale in third trimester of pregnancy and after delivery. GDM and T1DM also completed two diabetes-specific questionnaires (Diabetes-related stress and Diabetes health distress). Quality of life scores were compared between the groups with the Mann-Whitney U-test, mean changes in scores (after delivery to 3rd trimester) were compared between groups by ANCOVA.

Results Regarding the SF-36 scores in the third trimester of pregnancy, T1DM and GDM women had a better Standardised Physical Component score than controls (P < 0.0001, P = 0.009, respectively). GDM and T1DM pregnant women scored significantly lower for general health perception than controls (P = 0.009 and P = 0.001, respectively). T1DM patients had lower Standardised Mental Component scores than controls (P = 0.03). Compared with the third trimester of pregnancy, the severity of depressive symptoms increased significantly after delivery in both diabetic groups, but not in controls (P < 0.0001). Scores improved in all SF-36 areas in healthy and GDM women, while they all became worse in the T1DM group.

Conclusions Pregnancy is associated with a perception of poor general health in women with both T1DM and GDM. After delivery, significantly worse depressive symptoms were documented in both groups, while a generally worse physical and psychological well-being was only identified in women with T1DM. These findings have important implications for pregnancy follow-up.

C1 [Dalfra, M. G.; Bisson, T.; Bonsembiante, B.; Lapolla, A.] Univ Padua, DPT Sci Med & Chirurg, I-35128 Padua, Italy.

[Nicolucci, A.] Consorzio Mario Negri Sud, DPT Farmacol Clin & Epidemiol, Chieti, Italy.

RP Lapolla, A (reprint author), Univ Padua, DPT Sci Med & Chirurg, Via Giustiniani 2, I-35128 Padua, Italy.

EM annunziata.lapolla@unipd.it

OI VITACOLONNA, Ester/0000-0003-1756-661X

CR American Diabetes Association, 2004, Diabetes Care, V27 Suppl 1, pS76

Apolone G, 1998, J CLIN EPIDEMIOL, V51, P1025, DOI 10.1016/S0895-4356(98)00094-8

Bradley C, 2002, DIABETES METAB RES, V18, pS64, DOI 10.1002/dmrr.279

CARPENTER MW, 1982, AM J OBSTET GYNECOL, V144, P768, DOI 10.1016/0002-9378(82)90349-0

Chida Y, 2008, DIABETOLOGIA, V51, P2168, DOI 10.1007/s00125-008-1154-1

COMSTOCK GW, 1976, PSYCHOL MED, V6, P551

De Berardis G, 2002, DIABETES CARE, V25, P284, DOI 10.2337/diacare.25.2.284

DUNN SM, 1986, DIABETES CARE, V9, P480, DOI 10.2337/diacare.9.5.480

Franciosi M, 2001, DIABETES CARE, V24, P1870, DOI 10.2337/diacare.24.11.1870

GREENFIELD S, 1994, DIABETES CARE, V17, P32

Halkoaho A, 2010, EUR J OBSTET GYN R B, V148, P40, DOI 10.1016/j.ejogrb.2009.09.025

Horwitz SM, 2007, J WOMENS HEALTH, V16, P678, DOI 10.1089/jwh.2006.0185

Kamysheva E, 2008, BODY IMAGE, V5, P152, DOI 10.1016/j.bodyim.2007.12.005

Kim C, 2005, J WOMENS HEALTH, V14, P729, DOI 10.1089/jwh.2005.14.729

Kim C, 2002, DIABETES CARE, V25, P1862, DOI 10.2337/diacare.25.10.1862

Lacasse A, 2008, BJOG-INT J OBSTET GY, V115, P1484, DOI 10.1111/j.1471-0528.2008.01891.x

Langer N, 2000, DIABETES EDUCATOR, V26, P667, DOI 10.1177/014572170002600414

LANGER N, 1994, OBSTET GYNECOL, V84, P329

Metzger BE, 2007, DIABETES CARE, V30, pS251, DOI 10.2337/dc07-s225

Mezuk B, 2008, DIABETES CARE, V31, P2383, DOI 10.2337/dc08-0985

RADLOFF L S, 1977, Applied Psychological Measurement, V1, P385, DOI 10.1177/014662167700100306

Rumbold AR, 2002, AUST NZ J OBSTET GYN, V42, P131, DOI 10.1111/j.0004-8666.2002.00131.x

Samsa G, 1999, PHARMACOECONOMICS, V15, P141, DOI 10.2165/00019053-199915020-00003

SPIRITO A, 1989, OBSTET GYNECOL, V73, P562

Ware JE, 1993, SF 36 HLTH SURVEY MA

Ware JE, 1994, SF 36 PHYS MENTAL HL

NR 26

TC 34

Z9 35

U1 0

U2 12

PU SPRINGER

PI DORDRECHT

PA VAN GODEWIJCKSTRAAT 30, 3311 GZ DORDRECHT, NETHERLANDS

SN 0962-9343

J9 QUAL LIFE RES

JI Qual. Life Res.

PD MAR

PY 2012

VL 21

IS 2

BP 291

EP 298

DI 10.1007/s11136-011-9940-5

PG 8

WC Health Care Sciences & Services; Health Policy & Services; Public,

Environmental & Occupational Health

SC Health Care Sciences & Services; Public, Environmental & Occupational

Health

GA 933YX

UT WOS:000303404500011

PM 21633879

DA 2019-01-08

ER

PT J

AU Fisher, L

Hessler, D

Naranjo, D

Polonsky, W

AF Fisher, Lawrence

Hessler, Danielle

Naranjo, Diana

Polonsky, William

TI AASAP: A program to increase recruitment and retention in clinical

trials

SO PATIENT EDUCATION AND COUNSELING

LA English

DT Article

DE Recruitment; Clinical trials; Retention; Diabetes

ID SOCIAL-EXCHANGE; FOLLOW-UP; ATTRITION; RATES

AB Objective: To evaluate a theory based, subject-centered, staff/subject communication program, AASAP (anticipate, acknowledge, standardize, accept, plan), to increase recruitment and retention in RCTs.

Methods: AASAP was evaluated with logistical regression by comparing rates of recruitment (at telephone screening, baseline assessment, initial intervention) and intervention retention (over 16 weeks) before (-AASAP) and after (+AASAP) it was introduced to a 3-arm RCT to reduce disease distress among highly distressed subjects with type 2 diabetes.

Results: Included were 250 subjects in -AASAP and 338 in +AASAP. Significant improvement in recruitment occurred at each of the 3 recruitment stages: agreed at screening (OR = 2.52; p < 0.001), attended baseline assessment (OR = 1.91; p < 0.001), attended initial intervention (OR = 1.46; p < 0.03). Higher education and shorter diabetes duration predicted better recruitment in -AASAP (OR = 2.23: p < 0.001). but not in +AASAP. AASAP also improved intervention retention over 16 weeks (OR = 3.46; p < 0.05).

Conclusion: AASAP is a structured program of subject/staff communication that helps improve external validity by enhancing both subject recruitment and retention. Practical implications: AASAP can be taught to non-professional staff and can be adapted to a variety of health settings. It can also be used by clinicians to engage patients in programs of ongoing care. Published by Elsevier Ireland Ltd.

C1 [Fisher, Lawrence; Hessler, Danielle; Naranjo, Diana] Univ Calif San Francisco, Dept Family & Community Med, San Francisco, CA 94143 USA.

[Polonsky, William] Univ Calif San Diego, Behav Diabet Inst, San Diego, CA 92103 USA.

RP Hessler, D (reprint author), Univ Calif San Francisco, Dept Family & Community Med, San Francisco, CA 94143 USA.

EM fisherl@fcm.ucsf.edu; hesslerd@fcm.ucsf.edu

FU National Institute of Diabetes, Digestive and Kidney Disease [DK61937-7]

FX This research was funded by Grant DK61937-7 from the National Institute

of Diabetes, Digestive and Kidney Disease. The following medical groups

and education centers collaborated in this research: Alta Bates Diabetes

Education Center, Brown and Toland Medical Group, California Pacific

Diabetes Education Center, Hill Physicians Medical Group, University of

California, San Francisco Hospital and Clinics.

CR Bonell C, 2006, BRIT MED J, V333, P346, DOI 10.1136/bmj.333.7563.346

Bornhoft G., 2006, BIOMED CENTRAL MED R, V6, P1

EMERSON RM, 1976, ANNU REV SOCIOL, V2, P335, DOI 10.1146/annurev.so.02.080176.002003

FREEDMAN DS, 1980, SOCIOL METHOD RES, V9, P87, DOI 10.1177/004912418000900104

Froelicher Erika Sivarajan, 2003, J Cardiopulm Rehabil, V23, P269, DOI 10.1097/00008483-200307000-00004

Glasgow RE, 1996, DIABETES EDUCATOR, V22, P573, DOI 10.1177/014572179602200605

Gross D, 2001, RES NURS HEALTH, V24, P530, DOI 10.1002/nur.10010

Homans G. C., 1961, SOCIAL BEHAV ITS ELE

Lawler EJ, 2001, AM J SOCIOL, V107, P321, DOI 10.1086/324071

LeBlanc P, 2010, NURS RES, V17, P74

Marcellus Lenora, 2004, Can J Nurs Res, V36, P82

Meyers K, 2003, DRUG ALCOHOL DEPEN, V69, P73, DOI 10.1016/S0376-8716(02)00252-1

Miller W. R., 2002, MOTIVATIONAL INTERVI

POLIT DF, 1995, NURSING RES PRINCIPL

Rothwell PM, 2005, LANCET, V365, P82, DOI 10.1016/S0140-6736(04)17670-8

Schafer JL, 2002, PSYCHOL METHODS, V7, P147, DOI 10.1037//1082-989X.7.2.147

Scott CK, 2004, DRUG ALCOHOL DEPEN, V74, P21, DOI 10.1016/j.drugalcdep.2003.11.007

Toobert DJ, 2000, DIABETES CARE, V23, P943, DOI 10.2337/diacare.23.7.943

Woolard RH, 2004, ACAD EMERG MED, V11, P859, DOI 10.1197/j.aem.2003.09.021

NR 19

TC 20

Z9 20

U1 1

U2 6

PU ELSEVIER IRELAND LTD

PI CLARE

PA ELSEVIER HOUSE, BROOKVALE PLAZA, EAST PARK SHANNON, CO, CLARE, 00000,

IRELAND

SN 0738-3991

J9 PATIENT EDUC COUNS

JI Patient Educ. Couns.

PD MAR

PY 2012

VL 86

IS 3

BP 372

EP 377

DI 10.1016/j.pec.2011.07.002

PG 6

WC Public, Environmental & Occupational Health; Social Sciences,

Interdisciplinary

SC Public, Environmental & Occupational Health; Social Sciences - Other

Topics

GA 924EA

UT WOS:000302672700016

PM 21831557

OA Green Accepted

DA 2019-01-08

ER

PT J

AU Schmidt, CA

Bernaix, LW

Chiappetta, M

Carroll, E

Beland, A

AF Schmidt, Cynthia A.

Bernaix, Laura W.

Chiappetta, Maria

Carroll, Emily

Beland, Ann

TI In-Hospital Survival Skills Training for Type 1 Diabetes: Perceptions OF

Children AND Parents

SO MCN-THE AMERICAN JOURNAL OF MATERNAL-CHILD NURSING

LA English

DT Article

DE Children; Diabetes education; Parents; Type 1 diabetes; Self-care skills

ID QUALITATIVE DESCRIPTION; EDUCATION; CARE

AB Purpose: When a child is diagnosed with type 1 diabetes, parents and/or children must gain significant knowledge and learn specific skills to maintain health. Children in the United States who experience diabetic ketoacidosis (DKA) at the time of diagnosis typically spend 3 days in the hospital learning these life-saving skills. The purpose of this study was to gain knowledge concerning children's and parents' perceptions of this hospitalization period and of the initial education and support received from nurses.

Study Design and Methods: The study used qualitative descriptive methods. A child interview guide and written parent survey were used to elicit children's and parents' perceptions. Two outside experts reviewed these tools. Twenty children aged 8 to 15 years along with 25 parents participated. Reductionistic and constructionistic steps were used to analyze the qualitative data.

Results: During this hospitalization, children were most disturbed by the invasiveness of the required interventions while parents reported significant emotional distress related to the diagnosis. Children and parents alike felt the Survival Skills Training they received was effective. Education involving demonstration and return demonstration, supervision of skill performance, positive feedback from nurses about skill performance, and reassurance for long-term quality of life were nurse behaviors found to be helpful.

Clinical Implications: Implications for care include age-appropriate information in a variety of formats, minimizing the invasive nature of the treatment, providing opportunities for demonstration/return demonstration of skills, providing positive support and reassurance, and delivering concentrated instruction related to carbohydrate counting.

C1 [Schmidt, Cynthia A.; Bernaix, Laura W.] So Illinois Univ, Edwardsville, IL 62026 USA.

[Chiappetta, Maria; Beland, Ann] Childrens Mem Med Ctr, Chicago, IL USA.

[Carroll, Emily] Hlth Finders Collaborat, Northfield, MN USA.

RP Schmidt, CA (reprint author), So Illinois Univ, Edwardsville, IL 62026 USA.

EM caschmi@siue.edu

FU Children's Memorial Medical Center, Chicago, IL through the Shaw Faculty

FX This study was funded through the Shaw Faculty Collaborative Research

Grant program awarded by Children's Memorial Medical Center, Chicago,

IL.

CR *AM DIAB ASS, 1996, DIABETES CARE, V19, pS50, DOI DOI 10.1002/14651858.CD004099.PUB2

Beck JK, 2004, PEDIATRICS, V113, pE47, DOI 10.1542/peds.113.1.e47

Clar C, 2007, COCHRANE DB SYST REV, DOI 10.1002/14651858.CD004099.pub2

FIELD P, 1985, NURSING RES APPL QUA

Fisher MJ, 2011, J PEDIATR NURS, V26, P58, DOI 10.1016/j.pedn.2009.12.071

Habich Michele, 2006, Pediatr Nurs, V32, P113

Howe CJ, 2011, MCN-AM J MATERN-CHIL, V36, P25, DOI 10.1097/NMC.0b013e3181fc6093

Insel R., 2011, PREVENTION TYPE 1 DI

Jonsson L, 2010, BMC PEDIATR, V10, DOI 10.1186/1471-2431-10-36

Juvenile Diabetes Research Foundation, 2012, DIAB FACTS

KAZAK AE, 1996, FAMILIES SYSTEMS HLT, V14, P365, DOI DOI 10.1037/H0089795

Knafl K A, 1988, West J Nurs Res, V10, P195, DOI 10.1177/019394598801000207

Kortesluoma RL, 2003, J ADV NURS, V42, P434, DOI 10.1046/j.1365-2648.2003.02643.x

Krahenbuhl S, 2006, CHILD CARE HLTH DEV, V32, P321, DOI 10.1111/j.1365-2214.2006.00608.x

Rennick Janet E, 2004, Pediatr Crit Care Med, V5, P358, DOI 10.1097/01.PCC.0000128603.20501.0D

Sandelowski M, 2000, RES NURS HEALTH, V23, P334, DOI 10.1002/1098-240X(200008)23:4<334::AID-NUR9>3.0.CO;2-G

Sandelowski M, 2001, RES NURS HEALTH, V24, P230, DOI 10.1002/nur.1025

Sandelowski M, 2010, RES NURS HEALTH, V33, P77, DOI 10.1002/nur.20362

Silverstein J, 2005, DIABETES CARE, V28, P186, DOI 10.2337/diacare.28.1.186

Smaldone A, 2011, J PEDIATR HEALTH CAR, V25, P87, DOI 10.1016/j.pedhc.2009.09.003

Streisand R, 2008, PATIENT EDUC COUNS, V73, P333, DOI 10.1016/j.pec.2008.06.014

Svoren BM, 2003, PEDIATRICS, V112, P914, DOI 10.1542/peds.112.4.914

Swift PGF, 2007, PEDIATR DIABETES, V8, P103, DOI 10.1111/j.1399-5448.2007.00232.x

Wanzer MB, 2004, HEALTH COMMUN, V16, P363, DOI 10.1207/S15327027HC1603_6

NR 24

TC 3

Z9 4

U1 0

U2 17

PU LIPPINCOTT WILLIAMS & WILKINS

PI PHILADELPHIA

PA TWO COMMERCE SQ, 2001 MARKET ST, PHILADELPHIA, PA 19103 USA

SN 0361-929X

EI 1539-0683

J9 MCN-AM J MATERN-CHIL

JI MCN-Am. J. Matern.-Child Nurs.

PD MAR-APR

PY 2012

VL 37

IS 2

BP 88

EP 94

DI 10.1097/NMC.0b013e318244febc

PG 7

WC Nursing

SC Nursing

GA 906BE

UT WOS:000301319100005

PM 22357069

DA 2019-01-08

ER

PT J

AU Striegel, RH

Bedrosian, R

Wang, C

Schwartz, S

AF Striegel, Ruth H.

Bedrosian, Richard

Wang, Chun

Schwartz, Steven

TI Why Men Should Be Included in Research on Binge Eating: Results from a

Comparison of Psychosocial Impairment in Men and Women

SO INTERNATIONAL JOURNAL OF EATING DISORDERS

LA English

DT Article

DE eating disorders; binge eating; obesity; metabolic syndrome; work

productivity impairment; psychosocial impairment

ID GUIDED SELF-HELP; DISORDER SYMPTOMS; COMMUNITY SAMPLE; YOUNG-ADULTS;

COMORBIDITY; PREVALENCE; VALIDITY; REPLICATION; DEPRESSION; OVERWEIGHT

AB Objective: Prevalence of binge eating has been shown to be as common in men as in women, yet few studies have included men. Men are especially underrepresented in treatment studies, raising the question of whether men who binge eat experience less distress or impairment than women. This study compared demographic and clinical correlates of binge eating in a large employee sample of men and women.

Method: Cross-sectional data from 21,743 men and 24,608 women who participated in a health risk self assessment screening were used. Group differences in obesity, hypertension, dyslipidemia, Type 2 diabetes, depression, stress, sleep, sick days, work impairment, and non-work activity impairment were tested using chi-square tests (categorical variables) and independent sample t-tests (continuous variables).

Results: Effect size estimates indicate that men (n = 1,630) and women (n = 2,754) who binge eat experience comparable levels of clinical impairment. They also report substantially greater impairment when compared with men and women who do not binge eat.

Discussion: The underrepresentation of men in treatment-seeking samples does not appear to reflect lower levels of impairment in men versus women. Efforts are needed to raise awareness of the clinical significance of binge eating in men so that this group can receive appropriate screening and treatment services. (C) 2011 by Wiley Periodicals, Inc.

C1 [Striegel, Ruth H.] Wesleyan Univ, Dept Psychol, Middletown, CT 06459 USA.

[Bedrosian, Richard; Wang, Chun; Schwartz, Steven] HealthMedia Inc, Ann Arbor, MI USA.

RP Striegel, RH (reprint author), Wesleyan Univ, Dept Psychol, 207 High St, Middletown, CT 06459 USA.

EM rstriegel@wesleyan.edu

FU HealthMedia, Inc.

FX Dr. Striegel-Moore was compensated by HealthMedia, Inc. for her work on

this study. Drs. Bedrosian, Wang, and Schwartz are employees of

HealthMedia, Inc.

CR Bedrosian RC, 2011, INT J EAT DISORDER, V44, P639, DOI 10.1002/eat.20844

Brownley KA, 2007, INT J EAT DISORDER, V40, P337, DOI 10.1002/eat.20370

COHEN S, 1983, J HEALTH SOC BEHAV, V24, P385, DOI 10.2307/2136404

DeBar LL, 2011, PSYCHIAT SERV, V62, P367, DOI 10.1176/ps.62.4.pss6204_0367

Debar LL, 2009, CONTEMP CLIN TRIALS, V30, P326, DOI 10.1016/j.cct.2009.02.007

Dickerson JF, 2011, INT J EAT DISORDER, V44, P524, DOI 10.1002/eat.20842

Grilo CM, 2009, INT J EAT DISORDER, V42, P228, DOI 10.1002/eat.20599

Hudson JI, 2007, BIOL PSYCHIAT, V61, P348, DOI 10.1016/j.biopsych.2006.03.040

Hudson JI, 2010, AM J CLIN NUTR, V91, P1568, DOI 10.3945/ajcn.2010.29203

Kraemer HC, 2003, J AM ACAD CHILD PSY, V42, P1524, DOI 10.1097/01.chi.0000091507.46853.d1

Lewinsohn PM, 2002, INT J EAT DISORDER, V32, P426, DOI 10.1002/eat.10103

Marcus MD, 2009, INT J EAT DISORDER, V42, P739, DOI 10.1002/eat.20725

Mond JM, 2007, INT J EAT DISORDER, V40, P391, DOI 10.1002/eat.20380

Mond JM, 2009, OBESITY, V17, P1627, DOI 10.1038/oby.2009.27

Mond JM, 2009, J WOMENS HEALTH, V18, P1081, DOI 10.1089/jwh.2008.1174

Perez M, 2011, OBESITY SILVER SPRIN

RADLOFF LS, 1979, J ABNORM PSYCHOL, V88, P174, DOI 10.1037//0021-843X.88.2.174

REILLY MC, 1993, PHARMACOECONOMICS, V4, P353, DOI 10.2165/00019053-199304050-00006

Roberto CA, 2010, INT J EAT DISORDER, V43, P724, DOI 10.1002/eat.20770

Smith DE, 1998, ANN BEHAV MED, V20, P227, DOI 10.1007/BF02884965

Striegel-Moore RH, 2000, INT J EAT DISORDER, V27, P381

Striegel-Moore RH, 2010, J CONSULT CLIN PSYCH, V78, P312, DOI 10.1037/a0018915

Striegel-Moore RH, 2009, INT J EAT DISORDER, V42, P471, DOI 10.1002/eat.20625

Wilson GT, 2010, ARCH GEN PSYCHIAT, V67, P94, DOI 10.1001/archgenpsychiatry.2009.170

Wilson GT, 2009, INT J EAT DISORDER, V42, P603, DOI 10.1002/eat.20726

Wolfe BE, 2009, INT J EAT DISORDER, V42, P674, DOI 10.1002/eat.20728

Wonderlich SA, 2009, INT J EAT DISORDER, V42, P687, DOI 10.1002/eat.20719

NR 27

TC 74

Z9 75

U1 0

U2 24

PU WILEY-BLACKWELL

PI MALDEN

PA COMMERCE PLACE, 350 MAIN ST, MALDEN 02148, MA USA

SN 0276-3478

J9 INT J EAT DISORDER

JI Int. J. Eating Disord.

PD MAR

PY 2012

VL 45

IS 2

BP 233

EP 240

DI 10.1002/eat.20962

PG 8

WC Psychology, Clinical; Nutrition & Dietetics; Psychiatry; Psychology

SC Psychology; Nutrition & Dietetics; Psychiatry

GA 904WB

UT WOS:000301228500009

PM 22031213

DA 2019-01-08

ER

PT J

AU Shin, JK

Chiu, YL

Choi, S

Cho, S

Bang, H

AF Shin, Jinah K.

Chiu, Ya-Lin

Choi, Sarah

Cho, Soyeon

Bang, Heejung

TI Serious psychological distress, health risk behaviors, and diabetes care

among adults with type 2 diabetes: The California Health Interview

Survey 2007

SO DIABETES RESEARCH AND CLINICAL PRACTICE

LA English

DT Article

DE Diabetes care; Health risk behaviors; Physical activity; Serious

psychological distress; Smoking cigarettes; Type 2 diabetes

ID POOR GLYCEMIC CONTROL; DEPRESSIVE SYMPTOMS; SELF-CARE; METAANALYSIS;

PREVALENCE; ADHERENCE; MORTALITY; MELLITUS; SMOKING; ASSOCIATION

AB Aims: To examine the association between type 2 diabetes and serious psychological distress (SPD) and to assess if SPD was an independent risk factor for health risk behaviors and diabetes care among adults with diabetes.

Methods: Cross-sectional analysis was performed using the 2007 California Health Interview Survey with adults in California. Multiple logistic models were used to examine the associations between the multiple risk and outcome factors.

Results: Of the participants, 6.9% of adults had diagnoses of type 2 diabetes. SPD was present in 7.0% of adults with diabetes but only in 3.5% of adults without diabetes. Multiple logistic regression analysis revealed that participants with diabetes were significantly more likely than those without diabetes to experience SPD (OR = 1.81; 95% CI = [1.3-2.5], p = 0.0002), adjusting for other factors. In addition, participants with diabetes and SPD were more likely to report insufficient physical activity and more current cigarette smoking than participants without SPD. SPD among participants with diabetes, however, did not significantly affect good diabetes care.

Conclusions: The findings suggest that adults with diabetes are more likely to experience SPD than adults without diabetes. Further research is needed to explore the underlying mechanisms for this association among adults with both diabetes and SPD. (C) 2011 Elsevier Ireland Ltd. All rights reserved.

C1 [Shin, Jinah K.] Elmhurst Hosp Ctr, Dept Psychiat, Elmhurst, NY 11373 USA.

[Chiu, Ya-Lin] Cornell Univ, Weill Med Coll, Dept Publ Hlth, Div Biostat & Epidemiol, New York, NY 10065 USA.

[Choi, Sarah] Univ Calif Irvine, Nursing Sci Coll Hlth Sci, Irvine, CA 92697 USA.

[Cho, Soyeon] CUNY NYC Coll Technol, Human Serv Dept, Brookyn, NY 11201 USA.

[Bang, Heejung] Univ Calif Davis, Dept Publ Hlth Sci, Div Biostat, Davis, CA USA.

RP Shin, JK (reprint author), Elmhurst Hosp, Dept Psychiat, New York, NY 11373 USA.

EM JSHIN124@HOTMAIL.COM; yac2003@med.cornell.edu; sechoi@uci.edu;

scho@city-tech.cuny.edu; hbang@ucdavis.edu

CR Al-Delaimy WK, 2002, ARCH INTERN MED, V162, P273, DOI 10.1001/archinte.162.3.273

Al-Delaimy WK, 2001, DIABETES CARE, V24, P2043, DOI 10.2337/diacare.24.12.2043

*AM DIAB ASS, 2011, DIABETES CARE S1, V34, pS11, DOI DOI 10.2337/DC11-S011

Anderson RJ, 2002, INT J PSYCHIAT MED, V32, P235, DOI 10.2190/KLGD-4H8D-4RYL-TWQ8

Anderson RJ, 2001, DIABETES CARE, V24, P1069, DOI 10.2337/diacare.24.6.1069

[Anonymous], 2003, MMWR MORB MORTAL WKL, V53, P1089

California Health Interview Survey, 1 CHIS UCLA CTR HLTH

Center for Disease Control and Prevention, 2011, NAT DIAB FACT SHEET

Choi SE, 2011, J IMMIGR MINOR HEALT, V13, P803, DOI 10.1007/s10903-010-9397-6

Ciechanowski PS, 2000, ARCH INTERN MED, V160, P3278, DOI 10.1001/archinte.160.21.3278

Cosgrove MP, 2008, OCCUP MED-OXFORD, V58, P7, DOI 10.1093/occmed/kqm105

de Groot M, 2001, PSYCHOSOM MED, V63, P619, DOI 10.1097/00006842-200107000-00015

Golden SH, 2008, JAMA-J AM MED ASSOC, V299, P2751, DOI 10.1001/jama.299.23.2751

Grigsby AB, 2002, J PSYCHOSOM RES, V53, P1053, DOI 10.1016/S0022-3999(02)00417-8

Hamer M, 2010, PSYCHOSOM MED, V72, P882, DOI 10.1097/PSY.0b013e3181f6696e

Ismail K, 2007, DIABETES CARE, V30, P1473, DOI 10.2337/dc06-2313

Kessler RC, 2003, ARCH GEN PSYCHIAT, V60, P184, DOI 10.1001/archpsyc.60.2.184

Kessler RC, 2002, PSYCHOL MED, V32, P959, DOI 10.1017/S0033291702006074

Knol MJ, 2006, DIABETOLOGIA, V49, P837, DOI 10.1007/s00125-006-0159-x

Korff MV, 2005, PSYCHOSOM MED, V67, P233

Laaksonen DE, 2005, DIABETES, V54, P158, DOI 10.2337/diabetes.54.1.158

Li CY, 2009, INT J PUBLIC HEALTH, V54, P43, DOI 10.1007/s00038-009-0006-1

Lin EHB, 2004, DIABETES CARE, V27, P2154, DOI 10.2337/diacare.27.9.2154

Lustman PJ, 2000, DIABETES CARE, V23, P934, DOI 10.2337/diacare.23.7.934

Mezuk B, 2008, DIABETES CARE, V31, P2383, DOI 10.2337/dc08-0985

Narayan KMV, 2006, DIABETES CARE, V29, P2114, DOI 10.2337/dc06-1136

Norris SL, 2001, DIABETES CARE, V24, P561, DOI 10.2337/diacare.24.3.561

O'Connor PJ, 2009, ANN FAM MED, V7, P328, DOI 10.1370/afm.964

Park H, 2004, J CLIN EPIDEMIOL, V57, P978, DOI 10.1016/j.jclinepi.2004.01.015

Shih M, 2008, QUAL LIFE RES, V17, P521, DOI 10.1007/s11136-008-9330-9

Solberg LI, 2004, ANN FAM MED, V2, P26, DOI 10.1370/afm.36

Spangler JG, 2001, ADDICT BEHAV, V26, P21, DOI 10.1016/S0306-4603(00)00077-0

Thiebaut ACM, 2007, ANN INTERN MED, V146, P65, DOI 10.7326/0003-4819-146-1-200701020-00012

Wild S, 2004, DIABETES CARE, V27, P1047, DOI 10.2337/diacare.27.5.1047

NR 34

TC 13

Z9 14

U1 0

U2 12

PU ELSEVIER IRELAND LTD

PI CLARE

PA ELSEVIER HOUSE, BROOKVALE PLAZA, EAST PARK SHANNON, CO, CLARE, 00000,

IRELAND

SN 0168-8227

J9 DIABETES RES CLIN PR

JI Diabetes Res. Clin. Pract.

PD MAR

PY 2012

VL 95

IS 3

BP 406

EP 414

DI 10.1016/j.diabres.2011.10.043

PG 9

WC Endocrinology & Metabolism

SC Endocrinology & Metabolism

GA 897HG

UT WOS:000300638200022

PM 22169109

DA 2019-01-08

ER

PT J

AU Guilfoyle, SM

Denson, LA

Baldassano, RN

Hommel, KA

AF Guilfoyle, S. M.

Denson, L. A.

Baldassano, R. N.

Hommel, K. A.

TI Paediatric parenting stress in inflammatory bowel disease: application

of the Pediatric Inventory for Parents

SO CHILD CARE HEALTH AND DEVELOPMENT

LA English

DT Article

DE adolescence; inflammatory bowel disease; parenting distress; Pediatric

Inventory for Parents; sociodemographics

ID QUALITY-OF-LIFE; ULCERATIVE-COLITIS; CROHNS-DISEASE; MEDICATION

ADHERENCE; ACTIVITY INDEX; CHILDREN; ADOLESCENTS; HEALTH; OUTCOMES;

THERAPY

AB Background The psychosocial functioning of caregivers of adolescents managing inflammatory bowel disease (IBD) has been understudied; yet, poor caregiver functioning can place youth at risk for compromised disease management. The current study addressed this limitation by examining a sample of caregivers of adolescents with IBD. Study aims included (1) documenting rates of paediatric parenting stress; (2) identifying associated sociodemographic predictors of parenting stress; and (3) comparing previously published rates of parenting stress to those within other paediatric chronic conditions, including cancer, type 1 diabetes, obesity, sickle cell disease, bladder exstrophy.

Methods Caregivers of adolescents with an IBD diagnosis (Mage = 15.4 +/- 1.4, 44.4% female, 88.7% Caucasian) and receiving tertiary care within a gastroenterology clinic (n = 62) completed the Pediatric Inventory for Parents (PIP) as a measure of paediatric parenting stress with frequency and difficulty as PIP subscales. Paediatric gastroenterologists provided disease severity assessments.

Results Adolescents with IBD were experiencing relatively mild disease activity. Bivariate correlations revealed that PIP-difficulty was positively associated with Crohn's disease severity (r = 0.38, P < 0.01). Caregiver age was negatively associated with the frequency of parenting stress total (r = -0.25, P = 0.05) and communication scores (r = -0.25, P < 0.05). The frequency and difficulty of parenting stressors within the IBD sample were similar to rates within type 1 diabetes, but were significantly lower than rates identified in other paediatric chronic conditions.

Conclusions Caregivers of adolescents with IBD seem to experience low rates of parenting stress when their adolescents are receiving outpatient care and during phases of IBD relative inactivity. The sociodemographic characteristics of IBD families (i. e. primarily Caucasian, well-educated and higher socio-economic status) likely encourage greater access to financial and psychosocial resources, which may aid in promoting more optimal stress management.

C1 [Guilfoyle, S. M.; Hommel, K. A.] Cincinnati Childrens Hosp, Div Behav Med & Clin Psychol, Ctr Promot Treatment Adherence & Self Management, Med Ctr, Cincinnati, OH 45229 USA.

[Denson, L. A.] Cincinnati Childrens Hosp, Div Gastroenterol Hepatol & Nutr, Med Ctr, Cincinnati, OH 45229 USA.

[Denson, L. A.; Hommel, K. A.] Univ Cincinnati, Coll Med, Dept Pediat, Cincinnati, OH USA.

[Baldassano, R. N.] Childrens Hosp Philadelphia, Div Gastroenterol Hepatol & Nutr, Philadelphia, PA 19104 USA.

[Baldassano, R. N.] Univ Penn Sch Med, Dept Paediat, Philadelphia, PA USA.

RP Hommel, KA (reprint author), Cincinnati Childrens Hosp, Div Behav Med & Clin Psychol, Ctr Promot Treatment Adherence & Self Management, Med Ctr, 3333 Burnet Ave,MLC 7039, Cincinnati, OH 45229 USA.

EM kevin.hommel@cchmc.org

FU NIDDK [K23 DK079037]; PHS [P30 DK 078392]; Procter and Gamble

Pharmaceuticals; Prometheus Laboratories, Inc.; USPHS from the General

Clinical Research Centers, National Center for Research Resources, NIH

[M01 RR 08084]

FX Research is supported in part by NIDDK K23 DK079037, PHS Grant P30 DK

078392, Procter and Gamble Pharmaceuticals, Prometheus Laboratories,

Inc., USPHS Grant # M01 RR 08084 from the General Clinical Research

Centers Program, National Center for Research Resources, NIH.

CR Benchimol E. I., 2010, INFLAMMATOR IN PRESS, DOI [10.1002/ibd.21349, DOI 10.1002/IBD.21349]

Blanchard JF, 2001, AM J EPIDEMIOL, V154, P328, DOI 10.1093/aje/154.4.328

BURKE PM, 1994, CHILD PSYCHIAT HUM D, V25, P45, DOI 10.1007/BF02251099

COHEN J, 1992, PSYCHOL BULL, V112, P155, DOI 10.1037//0033-2909.112.1.155

ENGSTROM I, 1991, J AM ACAD CHILD PSY, V30, P904, DOI 10.1097/00004583-199111000-00007

Engstrom I, 1999, J PEDIATR GASTR NUTR, V28, pS28, DOI 10.1097/00005176-199904001-00004

Fanjiang G, 2007, J PEDIATR GASTR NUTR, V44, P312, DOI 10.1097/MPG.0b013e31802e98d4

Fredericks EM, 2007, AM J TRANSPLANT, V7, P1974, DOI 10.1111/j.1600-6143.2007.01878.x

GITLIN K, 1991, ADVANCES IN CHILD HEALTH PSYCHOLOGY, P54

Greenley RN, 2009, J PEDIATR PSYCHOL, V34, P129, DOI 10.1093/jpepsy/jsn056

Hollingshead A. A., 1975, 4 FACTOR INDEX UNPUB

Hommel KA, 2008, J PEDIATR PSYCHOL, V33, P867, DOI 10.1093/jpepsy/jsn022

Hyams J, 2005, J PEDIATR GASTR NUTR, V41, P416, DOI 10.1097/01.mpg.0000183350.46795.42

HYAMS JS, 1991, J PEDIATR GASTR NUTR, V12, P439, DOI 10.1097/00005176-199105000-00005

Kappelman MD, 2007, CLIN GASTROENTEROL H, V5, P1424, DOI 10.1016/j.cgh.2007.07.012

LICHTIGER S, 1994, NEW ENGL J MED, V330, P1841, DOI 10.1056/NEJM199406303302601

Logan DE, 2002, J PEDIATR PSYCHOL, V27, P475, DOI 10.1093/jpepsy/27.5.475

Mackner LM, 2005, INFLAMM BOWEL DIS, V11, P1006, DOI 10.1097/01.MIB.0000186409.15392.54

Mackner LM, 2005, AM J GASTROENTEROL, V100, P1386, DOI 10.1111/j.1572-0241.2005.41428.x

Mednick L, 2009, J UROLOGY, V181, P1312, DOI 10.1016/j.juro.2008.10.051

Mullins L, 2000, INT J REHABILITATION, V5, P177, DOI DOI 10.1023/A:1012999205376

Ohleyer Vanessa, 2007, J Child Health Care, V11, P132, DOI 10.1177/1367493507076065

Quittner A. L., 2003, HDB PEDIAT PSYCHOL, V3, P696

Streisand R, 2005, J PEDIATR PSYCHOL, V30, P513, DOI 10.1093/jpepsy/jsi076

Streisand R, 2001, J PEDIATR PSYCHOL, V26, P155, DOI 10.1093/jpepsy/26.3.155

van der Zaag-Loonen HJ, 2004, QUAL LIFE RES, V13, P1011, DOI 10.1023/B:QURE.0000025598.89003.0c

NR 26

TC 12

Z9 12

U1 1

U2 7

PU WILEY-BLACKWELL

PI MALDEN

PA COMMERCE PLACE, 350 MAIN ST, MALDEN 02148, MA USA

SN 0305-1862

J9 CHILD CARE HLTH DEV

JI Child Care Health Dev.

PD MAR

PY 2012

VL 38

IS 2

BP 273

EP 279

DI 10.1111/j.1365-2214.2010.01200.x

PG 7

WC Psychology, Developmental; Pediatrics

SC Psychology; Pediatrics

GA 885JV

UT WOS:000299776100015

PM 21299591

OA Green Accepted

DA 2019-01-08

ER

PT J

AU Paradies, YC

Cunningham, J

AF Paradies, Yin C.

Cunningham, Joan

TI The DRUID study: racism and self-assessed health status in an indigenous

population

SO BMC PUBLIC HEALTH

LA English

DT Article

ID AMERICAN YOUNG-ADULTS; BODY-MASS INDEX; PERCEIVED DISCRIMINATION;

DEPRESSIVE SYMPTOMS; MENTAL-HEALTH; ASIAN-AMERICANS; SUBSTANCE USE;

RACIAL/ETHNIC DISCRIMINATION; PSYCHOLOGICAL DISTRESS; ETHNIC

DISCRIMINATION

AB Background: There is now considerable evidence from around the world that racism is associated with both mental and physical ill-health. However, little is known about the mediating factors between racism and ill-health. This paper investigates relationships between racism and self-assessed mental and physical health among Indigenous Australians as well as potential mediators of these relationships.

Methods: A total of 164 adults in the Darwin Region Urban Indigenous Diabetes (DRUID) study completed a validated instrument assessing interpersonal racism and a separate item on discrimination-related stress. Self-assessed health status was measured using the SF-12. Stress, optimism, lack of control, social connections, cultural identity and reactions/responses to interpersonal racism were considered as mediators and moderators of the relationship between racism/discrimination and self-assessed health status.

Results: After adjusting for socio-demographic factors, interpersonal racism was significantly associated with the SF-12 mental (but not the physical) health component. Stress, lack of control and feeling powerless as a reaction to racism emerged as significant mediators of the relationship between racism and general mental health. Similar findings emerged for discrimination-related stress.

Conclusions: Racism/discrimination is significantly associated with poor general mental health among this indigenous population. The mediating factors between racism and mental health identified in this study suggest new approaches to ameliorating the detrimental effects of racism on health. In particular, the importance of reducing racism-related stress, enhancing general levels of mastery, and minimising negative social connections in order to ameliorate the negative consequences of racism.

C1 [Paradies, Yin C.] Univ Melbourne, McCaughey Ctr, Melbourne Sch Populat Hlth, Melbourne, Vic, Australia.

[Cunningham, Joan] Univ Melbourne, Sch Populat Hlth, Melbourne, Vic, Australia.

[Cunningham, Joan] Charles Darwin Univ, Menzies Sch Hlth Res, Darwin, NT 0909, Australia.

RP Paradies, YC (reprint author), Univ Melbourne, McCaughey Ctr, Melbourne Sch Populat Hlth, Melbourne, Vic, Australia.

EM yinp@unimelb.edu.au

RI Cunningham, Joan/E-6339-2012

OI Cunningham, Joan/0000-0002-1012-0909; Paradies, Yin/0000-0001-9927-7074

FU DRUID; Indigenous Steering Group; National Health and Medical Research

Council (NHMRC) [236207, 283310, 545200]; Australian Government

Department of Employment and Workplace Relations; Clive and Vera

Ramaciotti Foundation; Vincent Fairfax Family Foundation; International

Diabetes Institute; Bayer HealthCare; University of Melbourne

FX The authors gratefully acknowledge the support of DRUID study

participants, study staff, members of the Indigenous Steering Group, and

partner organisations. The DRUID Study was funded by the National Health

and Medical Research Council (NHMRC Project Grant #236207), with

additional support from the Australian Government Department of

Employment and Workplace Relations, the Clive and Vera Ramaciotti

Foundation, the Vincent Fairfax Family Foundation, the International

Diabetes Institute (AusDiab Partnership), and Bayer HealthCare. The

DRUID Study is an in-kind project of the Cooperative Research Centre for

Aboriginal Health (CRCAH). Ms Hannah Reich provided research assistance

in the drafting of this manuscript. The first author was supported by a

University of Melbourne McKenzie fellowship. The second author was

supported by an NHMRC Career Development Award (#283310) and an NHMRC

Research Fellowship (#545200).

CR Alvarez AN, 2010, J COUNS PSYCHOL, V57, P167, DOI 10.1037/a0019091

[Anonymous], 4704 0 HLTH WELFARE

Barnes LL, 2008, AM J PUBLIC HEALTH, V98, P1241, DOI 10.2105/AJPH.2007.114397

Bennett GG, 2005, AM J PUBLIC HEALTH, V95, P238, DOI 10.2105/AJPH.2004.037812

Bennett GG, 2004, AM BEHAV SCI, V47, P963, DOI 10.1177/0002764203261070

Berman G, 2010, ETHNIC RACIAL STUD, V33, P214, DOI 10.1080/01419870802302272

Borrell LN, 2006, SOC SCI MED, V63, P1415, DOI 10.1016/j.socscimed.2006.04.008

Brondolo E, 2010, HDB STRESS SCI

Brondolo E, 2011, AM J HYPERTENSION

Brondolo E, 2009, J BEHAV MED, V32, P64, DOI 10.1007/s10865-008-9193-0

Broudy R, 2007, J BEHAV MED, V30, P31, DOI 10.1007/s10865-006-9081-4

Caputo RK, 2003, SOC WORK HEALTH CARE, V37, P17, DOI 10.1300/J0101v37n02_02

Carver CS, CLIN PSYCHOL REV

Chae DH, 2008, AM J PUBLIC HEALTH, V98, P485, DOI 10.2105/AJPH.2006.102012

Chandola T, 2004, SOC SCI MED, V58, P1501, DOI 10.1016/S0277-9536(03)00352-6

Chen CY, 2009, CURR OPIN PSYCHIATR, V22, P287, DOI 10.1097/YCO.0b013e32832a2349

Choi Y, 2006, AM J ORTHOPSYCHIAT, V76, P86, DOI 10.1037/0002-9432.76.1.86

Cunningham J, 2006, BMC PUBLIC HEALTH, V6, DOI 10.1186/1471-2458-6-8

Deardorff J, 2003, J ABNORM CHILD PSYCH, V31, P205, DOI 10.1023/A:1022582410183

DeMaris A, 2002, SOCIOL METHOD RES, V31, P27, DOI 10.1177/0049124102031001002

DeSalvo KB, 2006, J GEN INTERN MED, V21, P267, DOI 10.1111/j.1525-1497.2005.0291.x

DeSantis AS, 2007, J ADOLESCENT HEALTH, V41, P3, DOI 10.1016/j.jadohealth.2007.03.006

Din-Dzietham R, 2004, SOC SCI MED, V58, P449, DOI 10.1016/S0277-9536(03)00211-9

Ender P, 2010, CAN I PERFORM MEDIAT

Franzini L, 2004, SOC SCI MED, V59, P1629, DOI 10.1016/j.socscimed.2004.02.014

Fritz MS, 2007, PSYCHOL SCI, V18, P233, DOI 10.1111/j.1467-9280.2007.01882.x

Gallaher G, 2009, BOOK OUR OWN BACKYAR

Gee GC, 2002, AM J PUBLIC HEALTH, V92, P615, DOI 10.2105/AJPH.92.4.615

Gee G, 2009, SOC SCI MED, V68, P1676, DOI 10.1016/j.socscimed.2009.02.002

Gee GC, 2008, AM J PUBLIC HEALTH, V98, P493, DOI 10.2105/AJPH.2007.114025

Gee GC, 2007, AM J PUBLIC HEALTH, V97, P933, DOI 10.2105/AJPH.2005.075739

Gibbons FX, 2007, DRUG ALCOHOL DEPEN, V88, pS27, DOI 10.1016/j.drugalcdep.2006.12.015

Gibbons FX, 2004, J PERS SOC PSYCHOL, V86, P517, DOI 10.1037/0022-3514.86.4.517

Gill SC, 2007, PSYCHIAT RES, V152, P63, DOI 10.1016/j.psychres.2006.11.005

Griffin JM, 2002, SOC SCI MED, V54, P783, DOI 10.1016/S0277-9536(01)00109-5

Guthrie BJ, 2002, NURS RES, V51, P183, DOI 10.1097/00006199-200205000-00007

Hagenaars AK, 1994, BOOK POVERTY STAT LA

Hair F. J., 2006, MULTIVARIATE DATA AN

Harrell CJP, 2011, DU BOIS REV, V8, P143, DOI 10.1017/S1742058X11000178

Harris R, 2006, SOC SCI MED, V63, P1428, DOI 10.1016/j.socscimed.2006.04.009

Heim D, J CROSS CULTURAL PSY

Banks KH, 2007, J BLACK PSYCHOL, V33, P331, DOI 10.1177/0095798407302540

Hunte HER, 2011, AM J EPIDEMIOL, V2011, P1

Idler EL, 1997, J HEALTH SOC BEHAV, V38, P21, DOI 10.2307/2955359

Jane-Llopis E, 2006, DRUG ALCOHOL REV, V25, P515, DOI 10.1080/09595230600944461

Juang LP, 2009, J PRIM PREV, V30, P475, DOI 10.1007/s10935-009-0177-9

Kaiser CR, 2006, LAW SOCIAL INQUIRY, V31, P801, DOI 10.1111/j.1747-4469.2006.00036.x

Krieger N, 1999, INT J HEALTH SERV, V29, P295, DOI 10.2190/M11W-VWXE-KQM9-G97Q

Krieger N, 2010, AM J PUBLIC HEALTH, V100, P1485, DOI 10.2105/AJPH.2009.159517

Landrine Hope, 2000, Ethnicity and Disease, V10, P195

Larson A, 2007, AUST NZ J PUBL HEAL, V31, P322, DOI 10.1111/j.1753-6405.2007.00079.x

Lewis TT, 2011, AM J EPIDEMIOL, V2011, P1

Lewis TT, 2011, BOOK DO CHRONIC EXPE

Liang CTH, 2007, J COUNS PSYCHOL, V54, P132, DOI 10.1037/0022-0167.542.132

Manson S, 1995, BOOK INDIAN SERVICES

MCCLELLAND GH, 1993, PSYCHOL BULL, V114, P376, DOI 10.1037//0033-2909.114.2.376

Mellor D, 2001, J ETHN MIGR STUD, V27, P473, DOI DOI 10.1080/13691830124387

Moradi B, 2004, J COUNS PSYCHOL, V51, P418, DOI 10.1037/0022-0167.51.4.418

Mossakowski KN, 2003, J HEALTH SOC BEHAV, V44, P318, DOI 10.2307/1519782

Nolh S, 2007, AM J PUBLIC HEALTH, V97, P1269, DOI 10.2105/AJPH.2005.085316

Okamoto J, 2009, AM J HEALTH BEHAV, V33, P718

Ong AD, 2008, J SOC CLIN PSYCHOL, V27, P105, DOI 10.1521/jscp.2008.27.2.105

Ong AD, 2009, J PERS SOC PSYCHOL, V96, P1259, DOI 10.1037/a0015335

Operario D, 2004, PSYCHOL HEALTH, V19, P237, DOI 10.1080/08870440310001638098

Otiniano AD, 2011, BOOK DISCRIMINATION

Paradies Y., 2008, INT ENCY PUBLIC HLTH, V5, P474

Paradies Y, SOCIAL PSYCHIAT PSYC

Paradies Y, 2009, BOOK BUILDING OUR ST

Paradies Y., 2009, YOUTH IDENTITY MIGRA, P207

Paradies Y, 2008, BOOK IMPACT RACISM I

Paradies Y, 2010, INT J PSYCHOL RES, V5

Paradies Y, 2008, INT J EQUITY HLTH, V7

Paradies Y, 2006, INT J EPIDEMIOL, V35, P888, DOI 10.1093/ije/dyl056

Paradies Y, 2009, ETHNIC RACIAL STUD, V32, P548, DOI 10.1080/01419870802065234

Pascoe EA, 2009, PSYCHOL BULL, V135, P531, DOI 10.1037/a0016059

Piette JD, 2006, PATIENT EDUC COUNS, V60, P41, DOI 10.1016/j.pec.2004.12.001

Pituch KA, 2008, MULTIVAR BEHAV RES, V43, P237, DOI 10.1080/00273170802034844

Preacher KJ, 2008, BEHAV RES METHODS, V40, P879, DOI 10.3758/BRM.40.3.879

Priest N, 2012, J EPIDEMIOL COMMUN H

Priest N, J EPIDEMIOL COMMUN H

Priest NC, 2011, MED J AUSTRALIA, V194, P546

Richman LS, 2008, ANN BEHAV MED, V35, P105, DOI 10.1007/s12160-007-9013-8

Rodriguez MS, 2004, EFFECTS PERCEIVED DI

Sanders-Phillips K, 2009, CLIN CHILD FAM PSYCH, V12, P174, DOI 10.1007/s10567-009-0053-4

SCHEIER MF, 1994, J PERS SOC PSYCHOL, V67, P1063, DOI 10.1037/0022-3514.67.6.1063

Sechrist GB, 2004, J PERS SOC PSYCHOL, V87, P111, DOI 10.1037/0022-3514.87.1.111

Sellers RM, 2003, J HEALTH SOC BEHAV, V44, P302, DOI 10.2307/1519781

Sellers SL, 2006, HLTH ED BEHAV

Shelton RC, 2009, ETHNIC DIS, V19, P251

Shorey HS, 2002, HISPANIC J BEHAV SCI, V24, P3, DOI 10.1177/0739986302024001001

Sibthorpe B, 2001, AM J PUBLIC HEALTH, V91, P1660, DOI 10.2105/AJPH.91.10.1660

Sohn L, 2008, MIL MED, V173, P331, DOI 10.7205/MILMED.173.4.331

Spatz C, 2008, RES METHODS IDEAS TE

Steering Committee for the Review of Government Service P, 2009, BOOK OV IND DIS KEY

Steffen PR, 2006, ETHNIC DIS, V16, P16

Sterne JAC, 2009, BMJ-BRIT MED J, V339, DOI 10.1136/bmj.b2393

Thomas KS, 2006, HEALTH PSYCHOL, V25, P635, DOI 10.1037/0278-6133.25.5.635

Todorova ILG, 2010, SOCIOL HEALTH ILL, V32, P843, DOI 10.1111/j.1467-9566.2010.01257.x

Tull ES, 2005, J NATL MED ASSOC, V97, P206

Ware JE, 2002, BOOK SCORE VERSION S

Williams DR, 2009, J BEHAV MED, V32, P20, DOI 10.1007/s10865-008-9185-0

Yoo HC, 2009, SOC SCI MED, V68, P726, DOI 10.1016/j.socscimed.2008.11.013

ZUBRICK SR, 2005, BOOK W AUSTR ABORIGI

NR 103

TC 19

Z9 19

U1 0

U2 18

PU BIOMED CENTRAL LTD

PI LONDON

PA 236 GRAYS INN RD, FLOOR 6, LONDON WC1X 8HL, ENGLAND

SN 1471-2458

J9 BMC PUBLIC HEALTH

JI BMC Public Health

PD FEB 14

PY 2012

VL 12

AR 131

DI 10.1186/1471-2458-12-131

PG 12

WC Public, Environmental & Occupational Health

SC Public, Environmental & Occupational Health

GA 908YU

UT WOS:000301529800001

PM 22333047

OA DOAJ Gold, Green Published

DA 2019-01-08

ER

PT J

AU Speight, J

Browne, JL

Holmes-Truscott, E

Hendrieckx, C

Pouwer, F

AF Speight, Jane

Browne, Jessica L.

Holmes-Truscott, Elizabeth

Hendrieckx, Christel

Pouwer, Frans

TI Diabetes MILES-Australia (management and impact for long-term

empowerment and success): methods and sample characteristics of a

national survey of the psychological aspects of living with type 1 or

type 2 diabetes in Australian adults

SO BMC PUBLIC HEALTH

LA English

DT Article

DE Diabetes; Psychology; National survey; Self-care; Quality of life;

Diabetes-related distress; Depression

ID QUALITY-OF-LIFE; CO-MORBID DEPRESSION; SOCIAL SUPPORT; NEEDS DAWN;

SELF-CARE; PREVALENCE; DISTRESS; ILLNESS; QUESTIONNAIRE; VALIDATION

AB Background: Successful management of diabetes requires attention to the behavioural, psychological and social aspects of this progressive condition. The Diabetes MILES (Management and Impact for Long-term Empowerment and Success) Study is an international collaborative. Diabetes MILES-Australia, the first Diabetes MILES initiative to be undertaken, was a national survey of adults living with type 1 or type 2 diabetes in Australia. The aim of this study was to gather data that will provide insights into how Australians manage their diabetes, the support they receive and the impact of diabetes on their lives, as well as to use the data to validate new diabetes outcome measures.

Methods/design: The survey was designed to include a core set of self-report measures, as well as modules specific to diabetes type or management regimens. Other measures or items were included in only half of the surveys. Cognitive debriefing interviews with 20 participants ensured the survey content was relevant and easily understood. In July 2011, the survey was posted to 15,000 adults (aged 18-70 years) with type 1 or type 2 diabetes selected randomly from the National Diabetes Services Scheme (NDSS) database. An online version of the survey was advertised nationally. A total of 3,338 eligible Australians took part; most (70.4%) completed the postal survey. Respondents of both diabetes types and genders, and of all ages, were adequately represented in both the postal and online survey sub-samples. More people with type 2 diabetes than type 1 diabetes took part in Diabetes MILES-Australia (58.8% versus 41.2%). Most respondents spoke English as their main language, were married/in a de facto relationship, had at least a high school education, were occupied in paid work, had an annual household income > $AUS40,000, and lived in metropolitan areas.

Discussion: A potential limitation of the study is the under-representation of respondents from culturally and linguistically diverse backgrounds (including Aboriginal and Torres Strait Islander origin). Diabetes MILES-Australia represents a major achievement in the study of diabetes in Australia, where for the first time, the focus is on psychosocial and behavioural aspects of this condition at a national level.

C1 [Speight, Jane; Browne, Jessica L.; Holmes-Truscott, Elizabeth; Hendrieckx, Christel] Australian Ctr Behav Res Diabet, Melbourne, Vic 3000, Australia.

[Speight, Jane; Browne, Jessica L.; Holmes-Truscott, Elizabeth; Hendrieckx, Christel] Deakin Univ, Sch Psychol, Ctr Mental Hlth & Wellbeing Res, Burwood, Vic 3125, Australia.

[Speight, Jane] AHP Res, Hornchurch, Essex, England.

[Pouwer, Frans] Tilburg Univ, Ctr Res Psychol Somat Dis CoRPS FSW, NL-5037 AB Tilburg, Netherlands.

RP Speight, J (reprint author), Australian Ctr Behav Res Diabet, 570 Elizabeth St, Melbourne, Vic 3000, Australia.

EM jspeight@acbrd.org.au

OI Holmes-Truscott, Elizabeth/0000-0001-9139-4663; Speight,

Jane/0000-0002-1204-6896

FU Diabetes Australia-Vic; Deakin University; sanofi aventis; National

Diabetes Services Scheme (NDSS) Strategic Development Grant

FX The Diabetes MILES-Australia 2011 Survey was funded by a National

Diabetes Services Scheme (NDSS) Strategic Development Grant. The NDSS is

an initiative of the Australia Government administered by Diabetes

Australia.; This funding supported the part-time employment of F HT and

JB as well as the conduct of the data collection (including

distribution, return and scanning of postal surveys; the development of

the online survey).; The contributions of JS and CH were supported by

the core funding provided by Diabetes Australia-Vic and Deakin

University to The Australian Centre for Behavioural Research in

Diabetes. In addition, Diabetes MILES-Australia received sponsorship

from sanofi aventis to support the development of the study website

http://www.diabetesMILES.org and miscellaneous activities.; We thank the

many people with diabetes who took part in the cognitive debriefing

interviews and the Diabetes MILES-Australia 2011 Survey.; We acknowledge

the Diabetes MILES-Australia Reference Group for their generous advice

throughout the study and for their continued collaboration on the

dissemination of findings. We thank Jennifer Halliday and Jasmin

Schabert from The Australian Centre for Behavioural Research in Diabetes

for their contributions to the cognitive debriefing interviews and

processing of return postal surveys. We also thank Luke Barisic from the

School of Psychology, Deakin University for programming the online

survey. Finally, we thank the Diabetes Australia-Vic mailroom

coordinators and volunteers for kindly undertaking the survey packaging

and distribution.

CR Alberti G, 2002, PRACT DIABETES INT, V19, P22, DOI DOI 10.1002/PDI.305/PDF

Ali S, 2006, DIABETIC MED, V23, P1165, DOI 10.1111/j.1464-5491.2006.01943.x

Amiel S, 2002, BRIT MED J, V325, P746

Anderson RM, 2003, DIABETES CARE, V26, P1641, DOI 10.2337/diacare.26.5.1641-a

Anderson RM, 2010, PATIENT EDUC COUNS, V79, P277, DOI 10.1016/j.pec.2009.07.025

Australian Bureau of Statistics (ABS), 2008, MIGR, P09

Australian Institute of Health and Welfare (AIHW), 1999, NAT HLTH DAT DICT VE

Barnard KD, 2006, DIABETIC MED, V23, P445, DOI 10.1111/j.1464-5491.2006.01814.x

Bech P., 2004, QUALITY LIFE NEWSLET, V32, P15

Bradley C, 2002, DIABETES METAB RES, V18, pS64, DOI 10.1002/dmrr.279

Broadbent E, 2006, J PSYCHOSOM RES, V60, P631, DOI 10.1016/j.jpsychores.2005.10.020

Broadbent E, 2011, DIABETES CARE, V34, P338, DOI 10.2337/dc10-1779

COX DJ, 1987, DIABETES CARE, V10, P617, DOI 10.2337/diacare.10.5.617

Craig CL, 2003, MED SCI SPORT EXER, V35, P1381, DOI 10.1249/01.MSS.0000078924.61453.FB

David M, 2011, BMJ OPEN, V1, P1

de Groot M, 2001, PSYCHOSOM MED, V63, P619, DOI 10.1097/00006842-200107000-00015

de Ridder D, 2004, J PSYCHOSOM RES, V56, P341, DOI 10.1016/S0022-3999(03)00034-5

Donald M, 2011, BMC PUBLIC HLTH

Dunstan DW, 2002, DIABETES CARE, V25, P829, DOI 10.2337/diacare.25.5.829

Fisher L, 2008, DIABETIC MED, V25, P1096, DOI 10.1111/j.1464-5491.2008.02533.x

Funnell MM, 2004, CLIN DIABETES, V22, P123, DOI DOI 10.2337/DIACLIN.22.3.123

Gale EAM, 2009, DIABETOLOGIA, V52, P559, DOI 10.1007/s00125-009-1275-1

GOLD AE, 1994, DIABETES CARE, V17, P697, DOI 10.2337/diacare.17.7.697

Herdman M, QUALITY LIFE RES

Horne R, 1999, J PSYCHOSOM RES, V47, P555, DOI 10.1016/S0022-3999(99)00057-4

Horne R, 1999, PSYCHOL HEALTH, V14, P1, DOI 10.1080/08870449908407311

International Wellbeing Group, 2006, PERS WELLB INDEX

Kroenke K, 2001, J GEN INTERN MED, V16, P606, DOI 10.1046/j.1525-1497.2001.016009606.x

LEMPERS JD, 1989, CHILD DEV, V60, P25, DOI 10.1111/j.1467-8624.1989.tb02692.x

McCormack LA, 2008, DIABETES EDUCATOR, V34, P707, DOI 10.1177/0145721708321021

Paddison C, 2010, EUR DIABETES NURS, V7, P29, DOI DOI 10.1002/EDN.152

Peyrot M, 2005, DIABETES CARE, V28, P2673, DOI 10.2337/diacare.28.11.2673

Peyrot M, 2005, DIABETIC MED, V22, P1379, DOI 10.1111/j.1464-5491.2005.01644.x

POLONSKY WH, 1995, DIABETES CARE, V18, P754, DOI 10.2337/diacare.18.6.754

Polonsky WH, 2005, DIABETES CARE, V28, P626, DOI 10.2337/diacare.28.3.626

Rosenberg M., 1965, SOC ADOLESCENT CHILD

Rubin RR, 2006, DIABETES CARE, V29, P1249, DOI 10.2337/dc05-2494

Rubin RR, 1999, DIABETES-METAB RES, V15, P205, DOI 10.1002/(SICI)1520-7560(199905/06)15:3<205::AID-DMRR29>3.0.CO;2-O

SCHEIER MF, 1994, J PERS SOC PSYCHOL, V67, P1063, DOI 10.1037/0022-3514.67.6.1063

Schwarzer R, 1995, MEASURES HLTH PSYCHO, P35, DOI DOI 10.1037/T00393-000

Skinner TC, 2000, J PEDIATR PSYCHOL, V25, P257, DOI 10.1093/jpepsy/25.4.257

Skovlund S. E., 2005, DIABETES SPECTRUM, V18, P136, DOI DOI 10.2337/DIASPECT.18.3.136

Snoek FJ, 2007, HEALTH QUAL LIFE OUT, V5, DOI 10.1186/1477-7525-5-69

Speight J, 2010, DIABETES RES CLIN PR, V89, P22, DOI 10.1016/j.diabres.2010.03.017

Speight J, 2010, DIABETIC MED, V27, P3

Speight J, 2000, P BRIT PSYCHOL SOC, V8, P21

Speight J, 2010, DIABETES	0303

Spitzer RL, 2006, ARCH INTERN MED, V166, P1092, DOI 10.1001/archinte.166.10.1092

Tang TS, 2008, DIABETES EDUCATOR, V34, P266, DOI 10.1177/0145721708315680

van Dam HA, 2005, PATIENT EDUC COUNS, V59, P1, DOI 10.1016/j.pec.2004.11.001

Wild S, 2004, DIABETES CARE, V27, P1047, DOI 10.2337/diacare.27.5.1047

Wilson P, 2010, BMJ, V340, pc3228

World Health Organization, 1983, HLTH ED SELF CAR POS

Yi JP, 2008, BRIT J HEALTH PSYCH, V13, P311, DOI 10.1348/135910707X186994

Zimmet PZ, 1999, DIABETOLOGIA, V42, P499, DOI 10.1007/s001250051188

NR 55

TC 34

Z9 34

U1 1

U2 22

PU BIOMED CENTRAL LTD

PI LONDON

PA 236 GRAYS INN RD, FLOOR 6, LONDON WC1X 8HL, ENGLAND

SN 1471-2458

J9 BMC PUBLIC HEALTH

JI BMC Public Health

PD FEB 12

PY 2012

VL 12

AR 120

DI 10.1186/1471-2458-12-120

PG 13

WC Public, Environmental & Occupational Health

SC Public, Environmental & Occupational Health

GA 921HV

UT WOS:000302469600001

PM 22325032

OA DOAJ Gold, Green Published

DA 2019-01-08

ER

PT J

AU Hermanns, N

Kulzer, B

Maier, B

Mahr, M

Haak, T

AF Hermanns, Norbert

Kulzer, Bernhard

Maier, Berthold

Mahr, Marina

Haak, Thomas

TI The effect of an education programme (MEDIAS 2 ICT) involving intensive

insulin treatment for people with type 2 diabetes

SO PATIENT EDUCATION AND COUNSELING

LA English

DT Article

DE Type 2 diabetes; Diabetes education; Self-management; Intensive insulin

treatment

ID EMOTIONAL DISTRESS; PROBLEM AREAS; MANAGEMENT; METAANALYSIS; ADULTS;

SCALE; DEPRESSION; DISEASE; RISK

AB Objective: In a randomized, multi-centre trial, the effect of an education programme (MEDIAS 2 ICT) involving intensive insulin treatment for people with type 2 diabetes was compared with an established education programme as an active comparator condition (ACC).

Methods: We investigated whether MEDIAS 2 ICT was non-inferior to ACC in overall glycaemic control. Secondary outcomes were the diabetes-related distress, diabetes knowledge, quality of life, self-care behavior, lipids, blood pressure and weight.

Results: 186 subjects were randomized. After a six month follow-up the mean HbA1c decrease was 0.37% (from 8.2 +/- 1.1% to 7.8 +/- 1.5%) in the ACC and 0.63% (from 8.5 +/- 1.5% to 7.9 +/- 1.2%) in MEDIAS 2 ICT. The mean difference between both groups was -0.26% (95% CI -0.63 to -0.14) in favor of MEDIAS 2 ICT. This result was within the predefined limit for non-inferiority. Diabetes-related distress was significantly more reduced in MEDIAS 2 ICT (-3.4 +/- 7.1) than in ACC (0.4 +/- 9.0; p = 0.31).

Conclusion: MEDIAS 2 ICT is as effective in lowering HbA1c as previously established education programmes, but showed superiority in reducing diabetes-related distress.

Practical implications: MEDIAS 2 ICT provides an alternative for education of people with type 2 diabetes treated by multiple injection therapy. (C) 2011 Elsevier Ireland Ltd. All rights reserved.

C1 [Hermanns, Norbert; Kulzer, Bernhard; Maier, Berthold; Mahr, Marina; Haak, Thomas] Diabet Acad Mergentheim FIDAM, Res Inst, Bad Mergentheim, Germany.

RP Hermanns, N (reprint author), Theodor Klotzbuecher Str 12, D-97980 Bad Mergentheim, Germany.

EM hermanns@diabetes-zentrum.de

RI Albertin, Paloma/X-1093-2018

FU Lilly, Germany

FX The conduct of this study was supported by an unrestricted grant from

Lilly, Germany.

CR Berger M, 1987, BEHANDLUNGS SCHULUNG

Bretzel RG, 2008, LANCET, V371, P1073, DOI 10.1016/S0140-6736(08)60485-7

BULLINGER M, 1995, SOC SCI MED, V41, P1359, DOI 10.1016/0277-9536(95)00115-N

Chodosh J, 2005, ANN INTERN MED, V143, P427, DOI 10.7326/0003-4819-143-6-200509200-00007

Cochran J, 2008, DIABETES EDUCATOR, V34, P815, DOI 10.1177/0145721708323640

CURRIE CJ, 2009, DIABETOLOGIA

Deakin T, 2005, COCHRANE DB SYST REV, DOI 10.1002/14651858.CD003417.pub2

Delahanty LM, 2007, DIABETIC MED, V24, P48, DOI 10.1111/j.1464-5491.2007.02028.x

Ellis SE, 2004, PATIENT EDUC COUNS, V52, P97, DOI 10.1016/S0738-3991(03)00016-8

Fisher L, 2007, DIABETES CARE, V30, P542, DOI 10.2337/dc06-1614

Grusser M, 2009, BEHANDLUNGS SCHULUNG

Hermanns N, 2005, DIABETIC MED, V22, P293, DOI 10.1111/j.1464-5491.2005.01414.x

Holman RR, 2009, NEW ENGL J MED, V361, P1736, DOI 10.1056/NEJMoa0905479

Kalfhaus J, 2000, DIABETES METAB, V26, P197

McCall WA, 1930, MEASUREMENT

Norris SL, 2002, AM J PREV MED, V22, P15, DOI 10.1016/S0749-3797(02)00423-3

Norris SL, 2002, DIABETES CARE, V25, P1159, DOI 10.2337/diacare.25.7.1159

Norris SL, 2001, DIABETES CARE, V24, P561, DOI 10.2337/diacare.24.3.561

Peyrot M, 1999, DIABETES CARE, V22, P448, DOI 10.2337/diacare.22.3.448

Peyrot M, 1997, DIABETES CARE, V20, P585, DOI 10.2337/diacare.20.4.585

POLONSKY WH, 1995, DIABETES CARE, V18, P754, DOI 10.2337/diacare.18.6.754

SAWICKI PT, 1995, DIABETIC MED, V12, P893, DOI 10.1111/j.1464-5491.1995.tb00392.x

Snoek FJ, 2000, DIABETES CARE, V23, P1305, DOI 10.2337/diacare.23.9.1305

Toobert DJ, 2000, DIABETES CARE, V23, P943, DOI 10.2337/diacare.23.7.943

Turner RC, 1998, LANCET, V352, P837

Welch GW, 1997, DIABETES CARE, V20, P760, DOI 10.2337/diacare.20.5.760

NR 26

TC 18

Z9 21

U1 0

U2 11

PU ELSEVIER IRELAND LTD

PI CLARE

PA ELSEVIER HOUSE, BROOKVALE PLAZA, EAST PARK SHANNON, CO, CLARE, 00000,

IRELAND

SN 0738-3991

J9 PATIENT EDUC COUNS

JI Patient Educ. Couns.

PD FEB

PY 2012

VL 86

IS 2

BP 226

EP 232

DI 10.1016/j.pec.2011.05.017

PG 7

WC Public, Environmental & Occupational Health; Social Sciences,

Interdisciplinary

SC Public, Environmental & Occupational Health; Social Sciences - Other

Topics

GA 904QN

UT WOS:000301213100014

PM 21715124

DA 2019-01-08

ER

PT J

AU Graue, M

Haugstvedt, A

Wentzel-Larsen, T

Iversen, MM

Karlsen, B

Rokne, B

AF Graue, Marit

Haugstvedt, Anne

Wentzel-Larsen, Tore

Iversen, Marjolein M.

Karlsen, Bjorg

Rokne, Berit

TI Diabetes-related emotional distress in adults: Reliability and validity

of the Norwegian versions of the Problem Areas in Diabetes Scale (PAID)

and the Diabetes Distress Scale (DDS)

SO INTERNATIONAL JOURNAL OF NURSING STUDIES

LA English

DT Article

DE Diabetes; Psychological factors; Psychosocial aspects; Psychometrics;

Reliability; Validity

ID PERFORMANCE-MEASURES; HOSPITAL ANXIETY; DEPRESSION; QUALITY; MANAGEMENT;

CARE

AB Background: Regular assessment of diabetes-related emotional distress is recommended to identify high-risk people with diabetes and to further prevent negative effects on self-management. Nevertheless, psychological problems are greatly under diagnosed. Translating and testing instruments for psychosocial assessment across languages, countries and cultures allow for further research collaboration and enhance the prospect of improving treatment and care.

Objectives: To examine the psychometric properties of the Norwegian versions of the Problem Areas in Diabetes Scale and the Diabetes Distress Scale.

Design: Cross-sectional survey design.

Settings: A sample comprising adults with diabetes (response rate 71%) completed the Problem Areas in Diabetes Scale and the Diabetes Distress Scale, which were translated into Norwegian with standard forward-backwards translation.

Participants: The study included 292 participants with type 1 (80%) and type 2 diabetes (20%) aged 18-69 years, 58% males, mean diabetes duration 17.3 years (11.6), mean HbA(1c) 8.2% (1.6).

Methods: We used exploratory factor analysis with principal axis factoring and varimax rotation to investigate the factor structure and performed confirmatory factor analysis to test the best fit of a priori-defined models. Convergent and discriminate validity were examined using the Short Form-36 Health Survey, Hospital Anxiety and Depression Scale and demographic and disease-related clinical variables. We explored reliability by internal consistency and test-retest analysis.

Results: Exploratory factor analysis supported a four-factor model for the Diabetes Distress Scale. Confirmatory factor analysis indicated that the data and the hypothesized model for the Diabetes Distress Scale fit acceptably but not for the Problem Areas in Diabetes Scale. Greater distress assessed with both instruments correlated moderately with lower health-related quality of life and greater anxiety and depression. The instruments discriminated between those having additional health conditions or disabilities, foot problems or neuropathy. Women and participants with higher HbA(1c) levels reported significantly higher diabetes-related emotional distress.

Conclusions: The Norwegian versions of the Problem Areas in Diabetes Scale and the Diabetes Distress Scale have satisfactory psychometric properties and can be used to map diabetes-related emotional distress for diagnostic or clinical use. The Diabetes Distress Scale also contributes to identifying sub-domains of distress and seems promising for use in clinical trials. (C) 2011 Elsevier Ltd. All rights reserved.

C1 [Graue, Marit; Haugstvedt, Anne; Iversen, Marjolein M.] Bergen Univ Coll, Dept Nursing, N-5020 Bergen, Norway.

[Graue, Marit] Haukeland Hosp, Childrens Clin, N-5021 Bergen, Norway.

[Haugstvedt, Anne; Rokne, Berit] Univ Bergen, Dept Publ Hlth & Primary Hlth Care, Bergen, Norway.

[Wentzel-Larsen, Tore] Haukeland Hosp, Clin Res Ctr, N-5021 Bergen, Norway.

[Iversen, Marjolein M.] Stavanger Univ Hosp, Endocrinol Sect, Dept Med, Stavanger, Norway.

[Karlsen, Bjorg] Univ Stavanger, Dept Hlth Studies, Stavanger, Norway.

[Rokne, Berit] Haukeland Hosp, Dept Res & Dev, N-5021 Bergen, Norway.

RP Graue, M (reprint author), Bergen Univ Coll, Dept Nursing, Postbox 7030, N-5020 Bergen, Norway.

EM marit.graue@hib.no

FU Bergen University College; Western Norway Health Authority Trust; Bergen

University College, University of Bergen; Stavanger University

FX Bergen University College and the Western Norway Health Authority Trust

kindly supported this research. We thank the patients for participating

and nurses at the endocrinology outpatient clinic, Ingvild Hernar, Kari

Horn, Elisabeth Iversen, Befit M. Tarlebo and Elin Irrborg, for

enthusiastic participating in data collection.; This study was supported

by the Bergen University College, University of Bergen and Stavanger

University as part of the research group DIABEST.

CR Bjelland I, 2002, J PSYCHOSOM RES, V52, P69, DOI 10.1016/S0022-3999(01)00296-3

Chawla A, 2010, DIABETES EDUCATOR, V36, P337, DOI 10.1177/0145721709354607

Collins MM, 2009, DIABETIC MED, V26, P153, DOI 10.1111/j.1464-5491.2008.02648.x

Cooper JG, 2009, DIABETES CARE, V32, P81, DOI 10.2337/dc08-0605

Eeg-Olofsson K, 2007, DIABETES CARE, V30, P496, DOI 10.2337/dc06-1406

Fayers P, 2007, ASSESSMENT ANAL INTE

Fisher L, 2008, DIABETIC MED, V25, P1096, DOI 10.1111/j.1464-5491.2008.02533.x

Fisher L, 2009, DIABETIC MED, V26, P622, DOI 10.1111/j.1464-5491.2009.02730.x

Fisher L, 2007, DIABETES CARE, V30, P542, DOI 10.2337/dc06-1614

Fisher L, 2010, DIABETES CARE, V33, P23, DOI 10.2337/dc09-1238

Glasgow RE, 2008, DIABETES CARE, V31, P1046, DOI 10.2337/dc07-1845

Goldney RD, 2004, DIABETES CARE, V27, P1066, DOI 10.2337/diacare.27.5.1066

GUILLEMIN F, 1993, J CLIN EPIDEMIOL, V46, P1417, DOI 10.1016/0895-4356(93)90142-N

Hermanns N, 2006, DIABETOLOGIA, V49, P469, DOI 10.1007/s00125-005-0094-2

Huang MF, 2010, DIABETES CARE, V33, P38, DOI 10.2337/dc09-0768

IDF, 2006, DIABETIC MED, V23, P579

Kartal A, 2011, INT J NURS STUD, V48, P227, DOI 10.1016/j.ijnurstu.2010.07.004

Lloyd CE, 2010, DIABETIC MED, V27, P234, DOI 10.1111/j.1464-5491.2009.02896.x

Lloyd CE, 2003, DIABETES-METAB RES, V19, P401, DOI 10.1002/dmrr.394

Loge JH, 1998, J CLIN EPIDEMIOL, V51, P1069, DOI 10.1016/S0895-4356(98)00098-5

O'Connor PJ, 2011, DIABETES CARE, V34, P1651, DOI 10.2337/dc11-0735

Peyrot M, 2005, DIABETIC MED, V22, P1379, DOI 10.1111/j.1464-5491.2005.01644.x

Peyrot M, 2007, DIABETES CARE, V30, P2433, DOI 10.2337/dc07-1222

POLONSKY WH, 1995, DIABETES CARE, V18, P754, DOI 10.2337/diacare.18.6.754

Polonsky WH, 2005, DIABETES CARE, V28, P626, DOI 10.2337/diacare.28.3.626

Pouwer F, 2006, PATIENT EDUC COUNS, V60, P235, DOI 10.1016/j.pec.2005.01.009

Sigurdardottir AK, 2008, INT J NURS STUD, V45, P526, DOI 10.1016/j.ijnurstu.2006.10.008

Snoek FJ, 2000, DIABETES CARE, V23, P1305, DOI 10.2337/diacare.23.9.1305

Ware J.E., 2000, SF36 HLTH SURVEY MAN

Welch G, 2003, DIABETIC MED, V20, P69, DOI 10.1046/j.1464-5491.2003.00832.x

Welch GW, 1997, DIABETES CARE, V20, P760, DOI 10.2337/diacare.20.5.760

ZIGMOND AS, 1983, ACTA PSYCHIAT SCAND, V67, P361, DOI 10.1111/j.1600-0447.1983.tb09716.x

NR 32

TC 32

Z9 37

U1 1

U2 25

PU PERGAMON-ELSEVIER SCIENCE LTD

PI OXFORD

PA THE BOULEVARD, LANGFORD LANE, KIDLINGTON, OXFORD OX5 1GB, ENGLAND

SN 0020-7489

EI 1873-491X

J9 INT J NURS STUD

JI Int. J. Nurs. Stud.

PD FEB

PY 2012

VL 49

IS 2

BP 174

EP 182

DI 10.1016/j.ijnurstu.2011.08.007

PG 9

WC Nursing

SC Nursing

GA 900AV

UT WOS:000300859900007

PM 21924422

DA 2019-01-08

ER

PT J

AU Seligman, HK

Jacobs, EA

Lopez, A

Tschann, J

Fernandez, A

AF Seligman, Hilary K.

Jacobs, Elizabeth A.

Lopez, Andrea

Tschann, Jeanne

Fernandez, Alicia

TI Food Insecurity and Glycemic Control Among Low-Income Patients With Type

2 Diabetes

SO DIABETES CARE

LA English

DT Article

ID NUTRITION EXAMINATION SURVEY; NATIONAL-HEALTH; MEDICATION ADHERENCE;

SELF-MANAGEMENT; CHRONIC DISEASE; HUNGER; DISTRESS; ADULTS; WOMEN;

HYPOGLYCEMIA

AB OBJECTIVE-To determine whether food insecurity-the inability to reliably afford safe and nutritious food-is associated with poor glycemic control and whether this association is mediated by difficulty following a healthy diet, diabetes self-efficacy, or emotional distress related to diabetes.

RESEARCH DESIGN AND METHODS-We used multivariable regression models to examine the association between food insecurity and poor glycemic control using a cross-sectional survey and chart review of 711 patients with diabetes in safety net health clinics. We then examined whether difficulty following a diabetic diet, self-efficacy, or emotional distress related to diabetes mediated the relationship between food insecurity and glycemic control.

RESULTS-The food insecurity prevalence in our sample was 46%. Food-insecure participants were significantly more likely than food-secure participants to have poor glycemic control, as defined by hemoglobin A(1c) >= 8.5% (42 vs. 33%; adjusted odds ratio 1.48 [95% CI 1.07-2.04]). Food-insecure participants were more likely to report difficulty affording a diabetic diet (64 vs. 49%, P < 0.001). They also reported lower diabetes-specific self-efficacy (P < 0.001) and higher emotional distress related to diabetes (P < 0.001). Difficulty following a healthy diet and emotional distress partially mediated the association between food insecurity and glycemic control.

CONCLUSIONS-Food insecurity is an independent risk factor for poor glycemic control in the safety net setting. This risk may be partially attributable to increased difficulty following a diabetes-appropriate diet and increased emotional distress regarding capacity for successful diabetes self-management. Screening patients with diabetes for food insecurity may be appropriate, particularly in the safety net setting.

C1 [Seligman, Hilary K.; Lopez, Andrea; Fernandez, Alicia] Univ Calif San Francisco, Div Gen Internal Med, San Francisco Gen Hosp, San Francisco, CA 94143 USA.

[Seligman, Hilary K.; Lopez, Andrea] Univ Calif San Francisco, San Francisco Gen Hosp, Ctr Vulnerable Populat, San Francisco, CA USA.

[Jacobs, Elizabeth A.] Univ Wisconsin, Sch Med & Publ Hlth, Div Gen Med, Madison, WI USA.

[Jacobs, Elizabeth A.] Univ Wisconsin, Sch Med & Publ Hlth, Hlth Innovat Program, Madison, WI USA.

[Tschann, Jeanne] Univ Calif San Francisco, Dept Psychiat, San Francisco, CA USA.

RP Seligman, HK (reprint author), Univ Calif San Francisco, Div Gen Internal Med, San Francisco Gen Hosp, San Francisco, CA 94143 USA.

EM hseligman@medsfgh.ucsf.edu

OI Lopez, Andrea/0000-0002-9488-4622

FU Russell Sage Foundation; The Commonwealth Fund; National Institutes of

Health (NIH)/National Center for Research Resources (NCRR)/Office of the

Director, University of California, San Francisco (UCSF)-Clinical &

Translational Science Institute (CTSI) [KL2 RR024130]; Arnold P. Gold

Foundation

FX This project was supported by funding from the Russell Sage Foundation

and The Commonwealth Fund. H.K.S. received additional support from the

National Institutes of Health (NIH)/National Center for Research

Resources (NCRR)/Office of the Director, University of California, San

Francisco (UCSF)-Clinical & Translational Science Institute (CTSI) Grant

KL2 RR024130. A.F. received additional support from an Arnold P. Gold

Foundation Professorship. The contents of the project are solely the

responsibility of the authors and do not necessarily represent the

official views of the NIH.

CR Adams EJ, 2003, J NUTR, V133, P1070

Adriaanse MC, 2008, DIABETES CARE, V31, P2268, DOI 10.2337/dc08-1074

Agardh E, 2011, INT J EPIDEMIOL, V40, P804, DOI 10.1093/ije/dyr029

ANDERSON SA, 1990, J NUTR, V120, P1559, DOI 10.1093/jn/120.suppl_11.1555

Bhattacharya J, 2004, J HEALTH ECON, V23, P839, DOI 10.1016/j.jhealeco.2003.12.008

Bickel G, 2000, GUIDE MEASURING HOUS

Coleman-Jensen A, HOUSEHOLD FOOD SECUR

Dixon LB, 2001, J NUTR, V131, P1232

Drewnowski A, 2005, J NUTR, V135, P900

Drewnowski A, 2004, AM J PUBLIC HEALTH, V94, P1555, DOI 10.2105/AJPH.94.9.1555

DREWNOWSKI A, 2005, AM J CLIN NUTR S, V82, P265

Fisher L, 2008, DIABETIC MED, V25, P1096, DOI 10.1111/j.1464-5491.2008.02533.x

Fisher L, 2010, DIABETES CARE, V33, P1034, DOI 10.2337/dc09-2175

Hager ER, 2010, PEDIATRICS, V126, pE26, DOI 10.1542/peds.2009-3146

Hurley A C, 1992, Diabetes Educ, V18, P146, DOI 10.1177/014572179201800208

Kaiser LL, 2004, AM J CLIN NUTR, V80, P1372

Karter AJ, 2002, JAMA-J AM MED ASSOC, V287, P2519, DOI 10.1001/jama.287.19.2519

Kendall A, 1996, J AM DIET ASSOC, V96, P1019, DOI 10.1016/S0002-8223(96)00271-4

Krapek K, 2004, ANN PHARMACOTHER, V38, P1357, DOI 10.1345/aph.1D612

Lee JS, 2001, J NUTR, V131, P1503

Lorig K, 1996, OUTCOME MEASURES HLT

MacKinnon DP, 2002, PSYCHOL METHODS, V7, P83, DOI 10.1037//1082-989X.7.1.83

Marjerrison S, 2011, J PEDIATR-US, V158, P607, DOI 10.1016/j.jpeds.2010.10.003

Minnesota Department of Health. Optimal diabetes care, 2011, OPT DIAB CAR 2011 DA

MORISKY DE, 1986, MED CARE, V24, P67, DOI 10.1097/00005650-198601000-00007

National Committee for Quality Assurance (NCQA), 2009, 2009 HEDIS PERF MEAS

National Quality Forum (NQF), HEM A1C MAN

Olson CM, 1999, J NUTR, V129, p521S, DOI 10.1093/jn/129.2.521S

Polonsky WH, 2005, DIABETES CARE, V28, P626, DOI 10.2337/diacare.28.3.626

Robbins JM, 2001, AM J PUBLIC HEALTH, V91, P76

Sarkar U, 2006, DIABETES CARE, V29, P823, DOI 10.2337/diacare.29.04.06.dc05-1615

Seligman HK, 2007, J GEN INTERN MED, V22, P1018, DOI 10.1007/s11606-007-0192-6

Seligman HK, 2011, ARCH INTERN MED, V171, P1204, DOI 10.1001/archinternmed.2011.287

Seligman HK, 2010, J HEALTH CARE POOR U, V21, P1227, DOI 10.1353/hpu.2010.0921

Seligman HK, 2010, NEW ENGL J MED, V363, P6, DOI 10.1056/NEJMp1000072

Seligman HK, 2010, J NUTR, V140, P304, DOI 10.3945/jn.109.112573

Tarasuk VS, 1999, J NUTR, V129, P672

Tarasuk VS, 2001, J NUTR, V131, P2670

Wilde PE, 2000, AM J AGR ECON, V82, P200, DOI 10.1111/0002-9092.00016

Wolfe WS, 1998, J AGING HEALTH, V10, P327, DOI 10.1177/089826439801000304

NR 40

TC 100

Z9 104

U1 0

U2 24

PU AMER DIABETES ASSOC

PI ALEXANDRIA

PA 1701 N BEAUREGARD ST, ALEXANDRIA, VA 22311-1717 USA

SN 0149-5992

J9 DIABETES CARE

JI Diabetes Care

PD FEB

PY 2012

VL 35

IS 2

BP 233

EP 238

DI 10.2337/dc11-1627

PG 6

WC Endocrinology & Metabolism

SC Endocrinology & Metabolism

GA 886LN

UT WOS:000299856000008

PM 22210570

OA Green Published, Other Gold

DA 2019-01-08

ER

PT J

AU Fisher, L

Hessler, DM

Polonsky, WH

Mullan, J

AF Fisher, Lawrence

Hessler, Danielle M.

Polonsky, William H.

Mullan, Joseph

TI When Is Diabetes Distress Clinically Meaningful? Establishing cut points

for the Diabetes Distress Scale

SO DIABETES CARE

LA English

DT Article

ID DEPRESSION; ADULTS; CARE

AB OBJECTIVE-To identify the pattern of relationships between the 17-item Diabetes Distress Scale (DDS17) and diabetes variables to establish scale cut points for high distress among patients with type 2 diabetes.

RESEARCH DESIGN AND METHODS-Recruited were 506 study 1 and 392 study 2 adults with type 2 diabetes from community medical groups. Multiple regression equations associated the DDS17, a 17-item scale that yields a mean-item score, with HbA(1c), diabetes self-efficacy, diet, and physical activity. Associations also were undertaken for the two-item DDS (DDS2) screener. Analyses included control variables, linear, and quadratic (curvilinear) DDS terms.

RESULTS-Significant quadratic effects occurred between the DDS17 and each diabetes variable, with increases in distress associated with poorer outcomes: study 1 HbA(1c) (P < 0.02), self-efficacy (P < 0.001), diet (P < 0.001), physical activity (P < 0.04); study 2 HbA(1c) (P < 0.03), self-efficacy (P < 0.004), diet (P < 0.04), physical activity (P = NS). Substantive curvilinear associations with all four variables in both studies began at unexpectedly low levels of DDS17: the slope increased linearly between scores 1 and 2, was more muted between 2 and 3, and reached a maximum between 3 and 4. This suggested three patient subgroups: little or no distress, <2.0; moderate distress, 2.0-2.9; high distress, >= 3.0. Parallel findings occurred for the DDS2.

CONCLUSIONS-In two samples of type 2 diabetic patients we found a consistent pattern of curvilinear relationships between the DDS and HbA(1c), diabetes self-efficacy, diet, and physical activity. The shape of these relationships suggests cut points for three patient groups: little or no, moderate, and high distress.

C1 [Fisher, Lawrence; Hessler, Danielle M.] Univ Calif San Francisco, Dept Family & Community Med, San Francisco, CA 94143 USA.

[Polonsky, William H.] Behav Diabet Inst, San Diego, CA USA.

[Mullan, Joseph] Univ Calif San Francisco, Sch Nursing, San Francisco, CA 94143 USA.

RP Fisher, L (reprint author), Univ Calif San Francisco, Dept Family & Community Med, San Francisco, CA 94143 USA.

EM fisherl@fcm.ucsf.edu

FU National Institute of Diabetes and Digestive and Kidney Diseases

[DK062732, DK061937]

FX This research was supported by grants DK062732 and DK061937 from the

National Institute of Diabetes and Digestive and Kidney Diseases.

CR Ammerman A, 2004, STARTING CONVERSATIO

Coyne JC, 1992, FAMILY HLTH PSYCHOL, P94

Fisher L, 2008, DIABETIC MED, V25, P1096, DOI 10.1111/j.1464-5491.2008.02533.x

Fisher L, 2009, DIABETIC MED, V26, P622, DOI 10.1111/j.1464-5491.2009.02730.x

Fisher L, 2000, DIABETES CARE, V23, P267, DOI 10.2337/diacare.23.3.267

Fisher L, 2008, ANN FAM MED, V6, P246, DOI 10.1370/afm.842

Fisher L, 2007, DIABETES CARE, V30, P542, DOI 10.2337/dc06-1614

Fisher L, 2010, DIABETES CARE, V33, P23, DOI 10.2337/dc09-1238

Glasgow RE, 2011, J MED INTERNET RES, V13, P70, DOI 10.2196/jmir.1391

Gonzalez JS, 2011, DIABETES CARE, V34, P236, DOI 10.2337/dc10-1970

Hessler DM, 2011, WHY DO YOUNGER ADULT

Lorig K, 1996, OUTCOME MEASURES HLT

Norman GR, 2003, MED CARE, V41, P582, DOI 10.1097/00005650-200305000-00004

O'Brien SM, 2004, BIOMETRICS, V60, P504, DOI 10.1111/j.0006-341X.2004.00196.x

Polonsky WH, 2005, DIABETES CARE, V28, P626, DOI 10.2337/diacare.28.3.626

Spitzer RL, 1999, JAMA-J AM MED ASSOC, V282, P1737, DOI 10.1001/jama.282.18.1737

Stewart AL, 2001, MED SCI SPORT EXER, V33, P1126

Toobert DJ, 2000, DIABETES CARE, V23, P943, DOI 10.2337/diacare.23.7.943

Williams B., 2006, FINDING OPTIMAL CUTP

WITTCHEN HU, 1994, J PSYCHIAT RES, V28, P57, DOI 10.1016/0022-3956(94)90036-1

NR 20

TC 146

Z9 153

U1 1

U2 28

PU AMER DIABETES ASSOC

PI ALEXANDRIA

PA 1701 N BEAUREGARD ST, ALEXANDRIA, VA 22311-1717 USA

SN 0149-5992

J9 DIABETES CARE

JI Diabetes Care

PD FEB

PY 2012

VL 35

IS 2

BP 259

EP 264

DI 10.2337/dc11-1572

PG 6

WC Endocrinology & Metabolism

SC Endocrinology & Metabolism

GA 886LN

UT WOS:000299856000013

PM 22228744

OA Other Gold, Green Published

DA 2019-01-08

ER

PT J

AU Kaas, A

Andersen, MLM

Fredheim, S

Hougaard, P

Buschard, K

Petersen, JS

de Beaufort, C

Robertson, KJ

Hansen, L

Mortensen, HB

Nielsen, LB

AF Kaas, Anne

Andersen, Marie Louise Max

Fredheim, Siri

Hougaard, Philip

Buschard, Karsten

Petersen, Jacob Steen

de Beaufort, Carine

Robertson, Kenneth J.

Hansen, Lars

Mortensen, Henrik B.

Nielsen, Lotte B.

CA Hvidoere Study Grp Childhood Diabe

TI Proinsulin, GLP-1, and glucagon are associated with partial remission in

children and adolescents with newly diagnosed type 1 diabetes

SO PEDIATRIC DIABETES

LA English

DT Article

DE GLP-1; glucagon; proinsulin; type 1 diabetes; remission phase

ID BETA-CELL FUNCTION; INSULIN AUTOANTIBODIES; GLYCEMIC CONTROL; PEPTIDE

RATIO; MELLITUS; RELEASE; NIDDM; PERSPECTIVES; ANTIBODIES; SECRETION

AB Objective: Proinsulin is a marker of beta-cell distress and dysfunction in type 2 diabetes and transplanted islets. Proinsulin levels are elevated in patients newly diagnosed with type 1 diabetes. Our aim was to assess the relationship between proinsulin, insulin dose-adjusted haemoglobin A1c (IDAA1C), glucagon-like peptide-1 (GLP-1), glucagon, and remission status the first year after diagnosis of type 1 diabetes.

Methods: Juvenile patients (n = 275) were followed 1, 6, and 12 months after diagnosis. At each visit, partial remission was defined as IDAA1C = 9%. The patients had a liquid meal test at the 1-, 6-, and 12-month visits, which included measurement of C-peptide, proinsulin, GLP-1, glucagon, and insulin antibodies (IA).

Results: Patients in remission at 6 and 12 months had significantly higher levels of proinsulin compared to non-remitting patients (p < 0.0001, p = 0.0002). An inverse association between proinsulin and IDAA1C was found at 1 and 6 months (p = 0.0008, p = 0.0022). Proinsulin was positively associated with C-peptide (p < 0.0001) and IA (p = 0.0024, p = 0.0068, p < 0.0001) at 1, 6, and 12 months. Glucagon (p < 0.0001 and p < 0.02) as well as GLP-1 (p = 0.0001 and p = 0.002) were significantly lower in remitters than in non-remitters at 6 and 12 months. Proinsulin associated positively with GLP-1 at 1 month (p = 0.004) and negatively at 6 (p = 0.002) and 12 months (p = 0.0002).

Conclusions: In type 1 diabetes, patients in partial remission have higher levels of proinsulin together with lower levels of GLP-1 and glucagon compared to patients not in remission. In new onset type 1 diabetes proinsulin level may be a sign of better residual beta-cell function.

C1 Glostrup Univ Hosp, Dept Paediat, DK-2600 Glostrup, Denmark.

[Kaas, Anne; Andersen, Marie Louise Max; Fredheim, Siri; Hansen, Lars; Mortensen, Henrik B.; Nielsen, Lotte B.] Glostrup Herlev Univ Hosp, Dept Paediat, Copenhagen, Denmark.

[Hougaard, Philip] Univ So Denmark, Dept Stat, Odense, Denmark.

[Buschard, Karsten] Rigshosp, Bartholin Inst, DK-2100 Copenhagen, Denmark.

[Petersen, Jacob Steen] Novo Nordisk AS, Malov, Denmark.

[de Beaufort, Carine] Ctr Hosp Luxembourg, Clin Pediat, Luxembourg, Luxembourg.

[Robertson, Kenneth J.] Royal Hosp Sick Children, Dept Paediat, Glasgow G3 8SJ, Lanark, Scotland.

RP Kaas, A (reprint author), Glostrup Univ Hosp, Dept Paediat, Res Pk, DK-2600 Glostrup, Denmark.

EM annekaas@dadlnet.dk

OI Mortensen, Henrik Bindesbol/0000-0001-9231-8363; Buschard,

Karsten/0000-0002-0126-7423

CR Arai T, 2010, CLIN IMMUNOL, V136, P74, DOI 10.1016/j.clim.2010.02.016

Atkinson MA, 2001, LANCET, V358, P221, DOI 10.1016/S0140-6736(01)05415-0

Blandino-Rosano M, 2008, J MOL ENDOCRINOL, V41, P35, DOI 10.1677/JME-07-0154

BOHMER K, 1991, DIABETOLOGIA, V34, P830, DOI 10.1007/BF00408359

Bosi E, 2010, J CLIN ENDOCR METAB, V95, P2607, DOI 10.1210/jc.2009-2741

Creutzfeldt WOC, 1996, DIABETES CARE, V19, P580, DOI 10.2337/diacare.19.6.580

DCCT Res Grp, 1987, J CLIN ENDOCR METAB, V65, P30

Dromey JA, 2011, J AUTOIMMUN, V36, P47, DOI 10.1016/j.jaut.2010.10.005

Forst T, 2011, DIABETES OBES METAB, V13, P542, DOI 10.1111/j.1463-1326.2011.01386.x

Holst JJ, 2006, DIABETOLOGIA, V49, P253, DOI 10.1007/s00125-005-0107-1

HOLST JJ, 1982, BIOCHEM J, V207, P381, DOI 10.1042/bj2070381

Hostens K, 1999, J CLIN INVEST, V104, P67, DOI 10.1172/JCI6438

Kaas A, 2010, CLIN EXP IMMUNOL, V161, P444, DOI 10.1111/j.1365-2249.2010.04193.x

Kahn SE, 1997, DIABETES, V46, P1725, DOI 10.2337/diabetes.46.11.1725

Kawamori D, 2009, ISLETS, V1, P276, DOI 10.4161/isl.1.3.9967

Kielgast U, 2009, CURR DIABETES REV, V5, P266, DOI 10.2174/157339909789804413

Klimek AM, 2009, AM J TRANSPLANT, V9, P2119, DOI 10.1111/j.1600-6143.2009.02740.x

LEAHY JL, 1991, DIABETES, V40, P985, DOI 10.2337/diabetes.40.8.985

LUDVIGSSON J, 1982, ACTA DIABETOL LAT, V19, P351, DOI 10.1007/BF02629258

Madsbad S, 2011, DIABETES OBES METAB, V13, P394, DOI 10.1111/j.1463-1326.2011.01357.x

Mortensen HB, 2010, PEDIATR DIABETES, V11, P218, DOI 10.1111/j.1399-5448.2009.00566.x

Mortensen HB, 2009, DIABETES CARE, V32, P1384, DOI 10.2337/dc08-1987

MULLER WA, 1971, J CLIN INVEST, V50, P1992, DOI 10.1172/JCI106691

ORSKOV C, 1994, DIABETES, V43, P535, DOI 10.2337/diabetes.43.4.535

Porksen S, 2007, J CLIN ENDOCR METAB, V92, P2910, DOI 10.1210/jc.2007-0244

PORTE D, 1989, DIABETES, V38, P1333, DOI 10.2337/diabetes.38.11.1333

Pratley RE, 2008, DIABETES OBES METAB, V10, P931, DOI 10.1111/j.1463-1326.2007.00835.x

Roder ME, 1998, J CLIN ENDOCR METAB, V83, P604, DOI 10.1210/jc.83.2.604

Scholin A, 2011, DIABETIC MED, V28, P156, DOI 10.1111/j.1464-5491.2010.03191.x

Snorgaard O, 1996, DIABETES CARE, V19, P146, DOI 10.2337/diacare.19.2.146

SPINAS GA, 1992, DIABETES CARE, V15, P632, DOI 10.2337/diacare.15.5.632

NR 31

TC 9

Z9 10

U1 0

U2 8

PU WILEY-BLACKWELL

PI HOBOKEN

PA 111 RIVER ST, HOBOKEN 07030-5774, NJ USA

SN 1399-543X

EI 1399-5448

J9 PEDIATR DIABETES

JI Pediatr. Diabetes

PD FEB

PY 2012

VL 13

IS 1

BP 51

EP 58

DI 10.1111/j.1399-5448.2011.00812.x

PG 8

WC Endocrinology & Metabolism; Pediatrics

SC Endocrinology & Metabolism; Pediatrics

GA 882FY

UT WOS:000299549900009

PM 22118630

OA Green Published

DA 2019-01-08

ER

PT J

AU Egede, LE

Dismuke, CE

AF Egede, Leonard E.

Dismuke, Clara E.

TI Serious Psychological Distress and Diabetes: A Review of the Literature

SO CURRENT PSYCHIATRY REPORTS

LA English

DT Review

DE Kessler; Psychological distress; Serious psychological distress;

Diabetes; Depression; Schizophrenia; Post-traumatic stress disorder;

PTSD; Diabetes distress; Anxiety

ID CORONARY-HEART-DISEASE; MAJOR DEPRESSION; GLYCEMIC CONTROL; MEDICATION

ADHERENCE; COMORBID DEPRESSION; GENERAL-POPULATION; PROSPECTIVE COHORT;

MENTAL-ILLNESS; HEALTH-CARE; LOW-INCOME

AB With the development of a measure of serious psychological distress (SPD) in 2002, more attention is being paid to the association of SPD with diabetes outcomes and processes of care. We review the literature on the relationship between SPD and diabetes processes of care and outcomes, as well as the literature on the relationship between specific mental health diagnoses and diabetes processes of care and outcomes during the 2010 to 2011 period. There is an extensive literature on the association of mental health diagnoses with diabetes outcomes, especially for depression. Because the Kessler scale measures a much broader range of mental health issues than any specific DSM-IV/Structured Clinical Interview for DSM Disorders diagnosis and is designed to assess SPD at the population level, additional research needs to be conducted both in clinical settings and using large administrative datasets to examine the association between SPD and diabetes outcomes and processes of care.

C1 [Egede, Leonard E.; Dismuke, Clara E.] Ralph H Johnson Vet Affairs Med Ctr, Ctr Dis Prevent & Hlth Intervent Diverse Populat, Charleston, SC 29425 USA.

[Egede, Leonard E.; Dismuke, Clara E.] Med Univ S Carolina, Ctr Hlth Dispar Res, Charleston, SC 29425 USA.

RP Egede, LE (reprint author), Ralph H Johnson Vet Affairs Med Ctr, Ctr Dis Prevent & Hlth Intervent Diverse Populat, 135 Rutledge Ave,Room 280H,POB 250593, Charleston, SC 29425 USA.

EM egedel@musc.edu

FU Center for Disease Prevention and Health Interventions for Diverse

Populations HSRD [REA 08-261]

FX The authors acknowledge and appreciate the resources provided by the

Center for Disease Prevention and Health Interventions for Diverse

Populations HSR&D program (grant REA 08-261) and the Ralph H. Johnson

Veterans Affairs Medical Center. The views expressed in this article are

those of the authors and do not necessarily represent the views of the

Department of Veterans Affairs.

CR Ali S, 2006, DIABETIC MED, V23, P1165, DOI 10.1111/j.1464-5491.2006.01943.x

Arnaud B, 2010, ALCOHOL CLIN EXP RES, V34, P1235, DOI 10.1111/j.1530-0277.2010.01201.x

Auila N, 2011, DIABETIC MED, V28, P785

Becker T, 2011, DIABETES CARE, V34, P398, DOI 10.2337/dc10-1139

Boyko EJ, 2010, DIABETES CARE, V33, P1771, DOI 10.2337/dc10-0296

Bresee L, 2011, EUR PSYCHIAT, V33, P327

Bryan C, 2010, DIABETES CARE, V33, P1034

Campayo A, 2011, CURR PSYCHIAT REP, V13, P26, DOI 10.1007/s11920-010-0165-z

Centres for Disease Control and Prevention, 2004, MMWR-MORBID MORTAL W, V53, P1089

Clouse RE, 2003, PSYCHOSOM MED, V65, P376, DOI 10.1097/01.PSY.0000041624.96580.1F

de Groot M, 2001, PSYCHOSOM MED, V63, P619, DOI 10.1097/00006842-200107000-00015

Dismuke CE, 2011, GEN HOSP PSYCHIAT, V33, P311, DOI 10.1016/j.genhosppsych.2011.03.014

Dismuke CE, 2010, GEN HOSP PSYCHIAT, V32, P484, DOI 10.1016/j.genhosppsych.2010.06.004

Dunbar JA, 2008, DIABETES CARE, V31, P2368, DOI 10.2337/dc08-0175

Egede LE, 2005, DIABETES CARE, V28, P1339, DOI 10.2337/diacare.28.6.1339

Egede LE, 2004, DIABETES CARE, V27, P1751, DOI 10.2337/diacare.27.7.1751

Egede LE, 2004, DIABETES CARE, V27, P421, DOI 10.2337/diacare.27.2.421

Egede LE, 2002, DIABETES CARE, V25, P464, DOI 10.2337/diacare.25.3.464

Egede LE, 2005, CURR DIABETES REV, V1, P235, DOI 10.2174/157339905774574356

Egede LE, 2010, GEN HOSP PSYCHIAT, V32, P563, DOI 10.1016/j.genhosppsych.2010.08.002

Egede LE, 2010, DIABETES RES CLIN PR, V87, P302, DOI 10.1016/j.diabres.2010.01.024

Egede LE, 2010, DIABETES EDUCATOR, V36, P276, DOI 10.1177/0145721710361389

Ell K, 2011, GEN HOSP PSYCHIAT

Ell K, 2010, DIABETES CARE, V33, P706, DOI 10.2337/dc09-1711

Eren I, 2008, DEPRESS ANXIETY, V25, P98, DOI 10.1002/da.20288

Finkelstein EA, 2003, DIABETES CARE, V26, P415, DOI 10.2337/diacare.26.2.415

Fisher L, 2008, ANN FAM MED, V6, P246, DOI 10.1370/afm.842

Fisher L, 2010, DIABETES CARE, V33, P1034, DOI 10.2337/dc09-2175

Fisher L, 2010, DIABETES CARE, V33, P23, DOI 10.2337/dc09-1238

Gonzalez JS, 2007, DIABETES CARE, V30, P2222, DOI 10.2337/dc07-0158

Gonzalez JS, 2008, DIABETES CARE, V31, P2398, DOI 10.2337/dc08-1341

Heckbert SR, 2010, J GEN INTERN MED, V25, P524, DOI 10.1007/s11606-010-1272-6

Hsu JH, 2011, CAN J PSYCHIAT, V56, P19, DOI 10.1177/070674371105600105

Katon W, 2008, J GEN INTERN MED, V23, P1571, DOI 10.1007/s11606-008-0731-9

Katon WJ, 2010, J GEN INTERN MED, V25, P423, DOI 10.1007/s11606-009-1248-6

Kessler R.C., 2010, INT J METHODS PSY S1, P4

Kessler RC, 2003, ARCH GEN PSYCHIAT, V60, P184, DOI 10.1001/archpsyc.60.2.184

Kessler RC, 2002, PSYCHOL MED, V32, P959, DOI 10.1017/S0033291702006074

Kessler RC, 2006, B WORLD HEALTH ORGAN, V84, P930, DOI 10.2471/BLT.06.033019

Kreyenbuhl J, 2010, SCHIZOPHRENIA BULL, V36, P428, DOI 10.1093/schbul/sbn106

Krieger N, 2011, AM J PUBLIC HEALTH, V101, P1704, DOI 10.2105/AJPH.2011.300168

Labad J, 2010, DIABETOLOGIA, V53, P467, DOI 10.1007/s00125-009-1628-9

Le Trong K, 2006, Cost Eff Resour Alloc, V4, P18, DOI 10.1186/1478-7547-4-18

Li CY, 2010, DIABETES CARE, V33, P1061, DOI 10.2337/dc09-1515

Li CY, 2009, INT J PUBLIC HEALTH, V54, P43, DOI 10.1007/s00038-009-0006-1

Lin EHB, 2004, DIABETES CARE, V27, P2154, DOI 10.2337/diacare.27.9.2154

Lin EHB, 2010, DIABETES CARE, V33, P264, DOI 10.2337/dc09-1068

Lustman PJ, 2000, DIABETES CARE, V23, P934, DOI 10.2337/diacare.23.7.934

McDade-Montez E, 2011, ANN BEHAV MED

McMillan KA, 2010, J CLIN PSYCHIAT, V71, P1168, DOI 10.4088/JCP.08m04986gry

Miller SA, 2011, GEN HOSP PSYCHIAT, V33, P116, DOI 10.1016/j.genhosppsych.2010.12.005

Mitchell CM, 2011, PSYCHOL ASSESSMENT, V23, P752, DOI 10.1037/a0023288

Nouwen A, 2011, DIABETES CARE, V34, P752, DOI 10.2337/dc10-1414

Osborn CY, 2010, DIABETES CARE, V33, P1050, DOI 10.2337/dc09-1929

Pan A, 2010, ARCH INTERN MED, V22, P1884

Pan A, 2011, ARCH GEN PSYCHIAT, V68, P42, DOI 10.1001/archgenpsychiatry.2010.176

Pirraglia PA, 2011, AM J MANAG CARE, V17, P319

Richardson LK, 2008, GEN HOSP PSYCHIAT, V30, P509, DOI 10.1016/j.genhosppsych.2008.07.001

Schoepf D, 2011, EUR PSYCHIAT

Scott-Tilley D, 2010, PERSPECT PSYCHIATR C, V46, P26, DOI 10.1111/j.1744-6163.2009.00235.x

Sorkin DH, 2011, DIABETES CARE, V34, P598, DOI 10.2337/dc10-1825

Ting RZW, 2011, DIABETES CARE, V34, P1094, DOI 10.2337/dc10-1612

Trento M, 2011, ACTA DIABETOL

Williams SL, 2010, INT J PSYCHIAT MED, V40, P233, DOI 10.2190/PM.40.3.a

ZAGARINS SE, 2011, J BEHAV MED

NR 65

TC 51

Z9 51

U1 0

U2 36

PU SPRINGER

PI NEW YORK

PA 233 SPRING ST, NEW YORK, NY 10013 USA

SN 1523-3812

J9 CURR PSYCHIAT REP

JI Curr. Psychiatry Rep.

PD FEB

PY 2012

VL 14

IS 1

BP 15

EP 22

DI 10.1007/s11920-011-0240-0

PG 8

WC Psychiatry

SC Psychiatry

GA 876FJ

UT WOS:000299092700003

PM 22002804

DA 2019-01-08

ER

PT J

AU Due-Christensen, M

Zoffmann, V

Hommel, E

Lau, M

AF Due-Christensen, M.

Zoffmann, V.

Hommel, E.

Lau, M.

TI Can sharing experiences in groups reduce the burden of living with

diabetes, regardless of glycaemic control?

SO DIABETIC MEDICINE

LA English

DT Article

DE diabetes-related distress; good and poor metabolic control;

psychosocial; support groups

ID RANDOMIZED CONTROLLED-TRIAL; OF-THE-LITERATURE; PSYCHOSOCIAL

INTERVENTIONS; TYPE-1; QUESTIONNAIRES; METAANALYSIS; DEPRESSION;

MANAGEMENT; DISTRESS; IMPROVE

AB Aims To test whether patients with Type 1 diabetes would join support groups and benefit by improving psychosocial functioning, regardless of their HbA1c levels. Methods A pre-post test with follow-up after 6 and 12 months was conducted as a concurrent mixed-method study. The convenience sample included patients with Type 1 diabetes aged = 21 years, having been diagnosed = 1 year earlier. Primary outcome was diabetes-related distress (using the Problem Areas in Diabetes scale). Secondary outcomes were psychological distress and depressive symptoms (Symptom Check List -90-R/Global Severity Index and depression subscale), well-being (World Health Organization 5) and HbA1c. Results Equal numbers of patients with HbA1c above and below 64 mmol/mol (8%) joined the support groups (n = 54). Focus group interviews revealed that major benefits were feeling less alone and being intuitively understood among peers. The patients perceived the support groups as a safe environment for sharing experiences. Problem Areas in Diabetes, Global Severity Index and depression subscale scores were significantly reduced post-intervention and maintained at 1-year follow-up. Well-being increased insignificantly. HbA1c was unchanged. Conclusions Support groups are able to reduce diabetes-related and psychological distress 1 year after the intervention for patients with both good and poor glycaemic control displaying high levels of distress. Although patients with severely high levels of diabetes-related distress might need more extensive therapeutic interventions to further reduce their level of distress. Further, interventions that target specific self-management problems are needed for patients with poor glycaemic control to help them accomplish lower levels of HbA1c. Moreover, healthcare providers must be aware that patients with good glycaemic control might have an unacknowledged psychosocial burden of living with the illness.

C1 [Due-Christensen, M.; Zoffmann, V.; Hommel, E.] Steno Diabet Ctr, DK-2820 Gentofte, Denmark.

[Lau, M.] Stolpegaard Psychotherapy Ctr, Gentofte, Denmark.

RP Due-Christensen, M (reprint author), Steno Diabet Ctr, Niels Steensens Vej 2, DK-2820 Gentofte, Denmark.

EM mtdc@steno.dk

OI Due-Christensen, Mette/0000-0003-2604-3730; Zoffmann,

Vibeke/0000-0003-0571-5331; Hommel, Eva/0000-0001-7570-5919

FU Steno Diabetes Center

FX This study was supported by grants from the Steno Diabetes Center. We

acknowledge all patients for their participation.

CR ANDERSON RM, 1995, DIABETES CARE, V18, P943, DOI 10.2337/diacare.18.7.943

Braun V., 2006, QUALITATIVE RES PSYC, V3, P77, DOI DOI 10.1191/1478088706QP0630A

Creswell J. W., 2003, RES DESIGN QUALITATI, P208

de Wit M, 2007, DIABETES CARE, V30, P2003, DOI 10.2337/dc07-0447

Derogatis L., 1994, SCL 90 R SYMPTOM CHE

DeVries JH, 2004, DIABETIC MED, V21, P1263, DOI 10.1111/j.1464-5491.2004.01386.x

Fisher EB, 2007, DIABETES EDUCATOR, V33, P1080, DOI 10.1177/0145721707309808

Grigsby AB, 2002, J PSYCHOSOM RES, V53, P1053, DOI 10.1016/S0022-3999(02)00417-8

Harkness E, 2010, DIABETES CARE, V33, P926, DOI 10.2337/dc09-1519

Hartemann-Heurtier A, 2001, DIABETES METAB, V27, P553

Heisler M., 2007, DIABETES SPECTRUM, V20, P214, DOI DOI 10.2337/DIASPECT.20.4.214

Hermanns N, 2006, DIABETOLOGIA, V49, P469, DOI 10.1007/s00125-005-0094-2

Hernandez C A, 1996, Can J Nurs Res, V28, P37

Hill-Briggs F, 2007, DIABETES EDUCATOR, V33, P1032, DOI 10.1177/0145721707308412

Kazdin A. E., 1994, HDB PSYCHOTHERAPY BE, P19

Lowe B, 2004, J AFFECT DISORDERS, V78, P131, DOI 10.1016/S0165-0327(02)00237-9

Lustman PJ, 2000, DIABETES CARE, V23, P934, DOI 10.2337/diacare.23.7.934

Olsen LR, 2004, ACTA PSYCHIAT SCAND, V110, P225, DOI 10.1111/j.1600-0447.2004.00399.x

Peyrot M, 2007, DIABETES CARE, V30, P2433, DOI 10.2337/dc07-1222

POLONSKY WH, 1995, DIABETES CARE, V18, P754, DOI 10.2337/diacare.18.6.754

Ryan RM, 2000, AM PSYCHOL, V55, P68, DOI 10.1037/0003-066X.55.1.68

Skinner TC, 2005, PSYCHOL DIABETES CAR, P141

Snoek FJ, 2011, DIABETES CARE, V34, P601, DOI 10.2337/dc10-1552

van Bastelaar KMP, 2010, DIABETIC MED, V27, P798, DOI 10.1111/j.1464-5491.2010.03025.x

van der Ven NCW, 2005, DIABETIC MED, V22, P1619

Welch GW, 1997, DIABETES CARE, V20, P760, DOI 10.2337/diacare.20.5.760

Winkley K, 2006, BRIT MED J, V333, P65, DOI 10.1136/bmj.38874.652569.55

Zoffmann V, 2006, PATIENT EDUC COUNS, V64, P78, DOI 10.1016/j.pec.2005.11.017

NR 28

TC 20

Z9 21

U1 0

U2 8

PU WILEY

PI HOBOKEN

PA 111 RIVER ST, HOBOKEN 07030-5774, NJ USA

SN 0742-3071

EI 1464-5491

J9 DIABETIC MED

JI Diabetic Med.

PD FEB

PY 2012

VL 29

IS 2

BP 251

EP 256

DI 10.1111/j.1464-5491.2011.03521.x

PG 6

WC Endocrinology & Metabolism

SC Endocrinology & Metabolism

GA 874TK

UT WOS:000298980800015

PM 22061095

DA 2019-01-08

ER

PT J

AU Karlsen, B

Oftedal, B

Bru, E

AF Karlsen, Bjorg

Oftedal, Bjorg

Bru, Edvin

TI The relationship between clinical indicators, coping styles, perceived

support and diabetes-related distress among adults with type 2 diabetes

SO JOURNAL OF ADVANCED NURSING

LA English

DT Article

DE clinical indicators; diabetes-related distress; healthcare providers;

social support; type 2 diabetes

ID PROBLEM AREAS; EMOTIONAL DISTRESS; GLYCEMIC CONTROL; DEPRESSION; SCALE;

ADJUSTMENT; VALIDITY; DUTCH

AB Aim: This article is a report of a cross-sectional study examining the degree to which clinical indicators, coping styles and perceived support from healthcare professionals and family are related to diabetes-related distress. Background: Many people with type 2 diabetes experience high levels of distress stemming from concerns and worries associated with their disease. Diabetes-related distress has predominantly been studied in relation to diabetes management and metabolic control, and to some extent in relation to coping styles and perceived social support. To date, little is known about the relative contribution of clinical indicators, coping styles and perceptions of social support to perceived distress among people with type 2 diabetes. Methods: A sample comprising 425 Norwegian adults, aged 3070, with type 2 diabetes, completed questionnaires assessing coping styles, perceived social support from health professionals and family and diabetes-related distress assessed by the Problem Areas in Diabetes Scale. Demographical and clinical data were collected by self-report. Data were collected in October 2008. Findings: Results from the regression analyses showed a greater variance in emotional distress accounted for by coping styles (21.3%) and perceived support (19.7%) than by clinical indicators (5.8%). Conclusion: Findings may indicate that healthcare providers should pay more attention to non-clinical factors such as coping styles and social support, when addressing diabetes-related distress. They should also be aware that interventions based on psychosocial approaches may primarily influence distress, and not necessarily metabolic control.

C1 [Karlsen, Bjorg; Oftedal, Bjorg] Univ Stavanger, Fac Social Sci, Dept Hlth Studies, Stavanger, Norway.

[Bru, Edvin] Univ Stavanger, Ctr Behav Res, Stavanger, Norway.

RP Karlsen, B (reprint author), Univ Stavanger, Fac Social Sci, Dept Hlth Studies, Stavanger, Norway.

EM bjorg.karlsen@uis.no

OI Bru, Edvin/0000-0003-2004-6957

FU Norwegian Diabetes Association

FX The study was supported by grants from the Norwegian Diabetes

Association.

CR Bouchard G, 2004, EUR J PERSONALITY, V18, P221, DOI 10.1002/per.512

Bru E., 2002, PSYCHOL HEALTH MED, V7, P245, DOI DOI 10.1080/13548500220139403

CARVER CS, 1989, J PERS SOC PSYCHOL, V56, P267, DOI 10.1037/0022-3514.56.2.267

Chang EC, 2007, PERS INDIV DIFFER, V43, P1149, DOI 10.1016/j.paid.2007.03.007

Costa P. T., 1996, HDB COPING THEORY RE, P44

COX DJ, 1992, J CONSULT CLIN PSYCH, V60, P628, DOI 10.1037//0022-006X.60.4.628

de Groot Mary, 2010, Diabetes Spectr, V23, P15, DOI 10.2337/diaspect.23.1.15

de Ridder D, 2001, CLIN PSYCHOL REV, V21, P205, DOI 10.1016/S0272-7358(99)00046-X

DECI EL, 1991, EDUC PSYCHOL-US, V26, P325, DOI 10.1207/s15326985ep2603&4_6

Delahanty LM, 2007, DIABETIC MED, V24, P48, DOI 10.1111/j.1464-5491.2007.02028.x

DeRidder D, 1997, PSYCHOL HEALTH, V12, P417, DOI 10.1080/08870449708406717

Fisher L, 2008, DIABETIC MED, V25, P1096, DOI 10.1111/j.1464-5491.2008.02533.x

Fisher L, 2009, DIABETIC MED, V26, P622, DOI 10.1111/j.1464-5491.2009.02730.x

Fisher L, 2001, DIABETES CARE, V24, P1751, DOI 10.2337/diacare.24.10.1751

Fisher L, 2007, DIABETES CARE, V30, P542, DOI 10.2337/dc06-1614

Fisher L, 2010, DIABETES CARE, V33, P1034, DOI 10.2337/dc09-2175

Folkman S, 2004, ANNU REV PSYCHOL, V55, P745, DOI 10.1146/annurev.psych.55.090902.141456

Folkman S., 1988, MANUAL WAYS COPING Q

Ford ME, 1998, J NATL MED ASSOC, V90, P425

Hewitt PL, 1996, HDB COPING THEORY RE, P410

Karlsen B., 2004, PSYCHOL HEALTH MED, V9, P53

Kosciulek John F., 2007, COPING CHRONIC ILLNE, P73

Lazarus R., 1991, EMOTION ADAPTATION

Lazarus R. S, 1984, STRESS APPRAISAL COP

Lloyd C, 2005, DIABETES SPECTR, V18, P121, DOI [DOI 10.2337/DIASPECT.18.2.121, 10.2337/diaspect.18.2.121]

Macrodimitris SD, 2001, HEALTH PSYCHOL, V20, P208, DOI 10.1037//0278-6133.20.3.208

Maes S., 1996, HDB COPING THEORY RE, P221

Moos RH, 2003, J CLIN PSYCHOL, V59, P1257, DOI 10.1002/jclp.10218

Papelbaum M, 2010, DIABETES RES CLIN PR, V89, P227, DOI 10.1016/j.diabres.2010.05.024

POLONSKY WH, 1995, DIABETES CARE, V18, P754, DOI 10.2337/diacare.18.6.754

Polonsky WH, 2005, DIABETES CARE, V28, P626, DOI 10.2337/diacare.28.3.626

Pouwer F, 2005, DIABETES RES CLIN PR, V70, P166, DOI 10.1016/j.diabres.2005.03.031

Sacco WP, 2006, INTERPERSONAL, COGNITIVE, AND SOCIAL NATURE OF DEPRESSION, P101

Sacco WP, 1999, INTERACTIONAL NATURE OF DEPRESSION, P329, DOI 10.1037/10311-012

SCHAFER LC, 1986, DIABETES CARE, V9, P179, DOI 10.2337/diacare.9.2.179

Sigurdardottir AK, 2008, INT J NURS STUD, V45, P526, DOI 10.1016/j.ijnurstu.2006.10.008

Snoek FJ, 2000, DIABETES CARE, V23, P1305, DOI 10.2337/diacare.23.9.1305

STEWART M, 2000, CHRONIC CONDITIONS C

Taylor S, 2006, HLTH PSYCHOL

Thorne SE, 2001, PATIENT EDUC COUNS, V42, P81, DOI 10.1016/S0738-3991(00)00095-1

Trief PM, 1998, DIABETES CARE, V21, P241, DOI 10.2337/diacare.21.2.241

Welch G, 2003, DIABETIC MED, V20, P69, DOI 10.1046/j.1464-5491.2003.00832.x

Welch GW, 1997, DIABETES CARE, V20, P760, DOI 10.2337/diacare.20.5.760

Yang J, 2009, J CLIN NURS, V18, P1295, DOI 10.1111/j.1365-2702.2008.02703.x

NR 44

TC 43

Z9 46

U1 1

U2 16

PU WILEY

PI HOBOKEN

PA 111 RIVER ST, HOBOKEN 07030-5774, NJ USA

SN 0309-2402

EI 1365-2648

J9 J ADV NURS

JI J. Adv. Nurs.

PD FEB

PY 2012

VL 68

IS 2

BP 391

EP 401

DI 10.1111/j.1365-2648.2011.05751.x

PG 11

WC Nursing

SC Nursing

GA 874UW

UT WOS:000298984600015

PM 21707728

DA 2019-01-08

ER

PT J

AU Hay, WW

AF Hay, William W., Jr.

TI Care of the Infant of the Diabetic Mother

SO CURRENT DIABETES REPORTS

LA English

DT Article

DE Gestational diabetes; Infant of diabetic mother; Large for gestational

age; Pregnancy; Hypoglycemia; Hyperbilirubinemia; Hypocalcemia;

Hypomagnesemia; Polycythemia; Hyperviscosity; Continuous glucose

monitoring; Intrauterine growth restriction; Preterm birth; Caudal

regression syndrome

ID LOW-BIRTH-WEIGHT; NEONATAL HYPOGLYCEMIA; NEWBORN-INFANTS;

IRON-DEFICIENCY; HIGH GLUCOSE; GESTATIONAL-AGE; PREGNANT-WOMEN; TERM

INFANTS; FETAL; MELLITUS

AB Gestational diabetes mellitus (GDM) from all causes of diabetes is the most common medical complication of pregnancy and is increasing in incidence, particularly as type 2 diabetes continues to increase worldwide. Despite advances in perinatal care, infants of diabetic mothers (IDMs) remain at risk for a multitude of physiologic, metabolic, and congenital complications such as preterm birth, macrosomia, asphyxia, respiratory distress, hypoglycemia, hypocalcemia, hyperbilirubinemia, polycythemia and hyperviscosity, hypertrophic cardiomyopathy, and congenital anomalies, particularly of the central nervous system. Overt type 1 diabetes around conception produces marked risk of embryopathy (neural tube defects, cardiac defects, caudal regression syndrome), whereas later in gestation, severe and unstable type 1 maternal diabetes carries a higher risk of intrauterine growth restriction, asphyxia, and fetal death. IDMs born to mothers with type 2 diabetes are more commonly obese (macrosomic) with milder conditions of the common problems found in IDMs. IDMs from all causes of GDM also are predisposed to later-life risk of obesity, diabetes, and cardiovascular disease. Care of the IDM neonate needs to focus on ensuring adequate cardiorespiratory adaptation at birth, possible birth injuries, maintenance of normal glucose metabolism, and close observation for polycythemia, hyperbilirubinemia, and feeding intolerance.

C1 Univ Colorado, Sch Med, Perinatal Res Ctr, Aurora, CO 80045 USA.

RP Hay, WW (reprint author), Univ Colorado, Sch Med, Perinatal Res Ctr, Anschutz Med Campus,F441,13243 E 23rd Ave, Aurora, CO 80045 USA.

EM bill.hay@ucdenver.edu

CR Ahlsson FSE, 2007, PEDIATRICS, V120, P958, DOI 10.1542/peds.2007-0165

Ahmed MU, 1997, BIOCHEM J, V324, P565, DOI 10.1042/bj3240565

*AM DIAB ASS, 2005, DIABETES CARE S1, V28, pS4, DOI DOI 10.2337/DIACARE

Banerjee S, 2003, MAGNESIUM RES, V16, P127

Baptiste-Roberts K, 2011, MATERN CHILD HLTH J, DOI 10.007/s10995-011-0756-2

BARD H, 1985, PEDIATRICS, V75, P1143

BARKER DJP, 1993, DIABETOLOGIA, V36, P62, DOI 10.1007/BF00399095

Barnes-Powell LL, 2007, NEONATAL NETW, V26, P283, DOI 10.1891/0730-0832.26.5.283

Beard J, 2003, J NUTR, V133, P1174

Beardsall K, 2005, ARCH DIS CHILD-FETAL, V90, pF307, DOI 10.1136/adc.2004.051979

Beardsall K, 2008, NEW ENGL J MED, V359, P1873, DOI 10.1056/NEJMoa0803725

Boluyt N, 2006, PEDIATRICS, V117, P2231, DOI 10.1542/peds.2005-1919

Bromiker R, 2006, J PEDIATR-US, V149, P640, DOI 10.1016/j.jpeds.2006.07.034

Brownlee M, 2005, DIABETES, V54, P1615, DOI 10.2337/diabetes.54.6.1615

Burguet A, 2010, DIABETES METAB, V36, P682, DOI 10.1016/j.diabet.2010.11.018

Carver TD, 1996, AM J PHYSIOL-ENDOC M, V271, pE865

CASSON F, 1997, BRIT MED J, V315, P275

Catalano PM, 2007, OBSTET GYNECOL, V109, P419, DOI 10.1097/01.AOG.0000253311.44696.85

Cetin H, 2011, J INT MED RES, V39, P815, DOI 10.1177/147323001103900314

Chu SY, 2007, DIABETES CARE, V30, P2070, DOI 10.2337/dc06-2559a

Clark RJ, 2003, J BIOL CHEM, V278, P44230, DOI 10.1074/jbc.M303810200

Coetzee E J, 2000, Semin Neonatol, V5, P221, DOI 10.1053/siny.2000.0010

Connor JR, 1996, GLIA, V17, P83, DOI 10.1002/(SICI)1098-1136(199606)17:2<83::AID-GLIA1>3.0.CO;2-7

Cordero L, 1998, ARCH PEDIAT ADOL MED, V152, P249

Cornblath M, 2000, PEDIATRICS, V105, P1141, DOI 10.1542/peds.105.5.1141

COWETT RM, 1982, PEDIATR CLIN N AM, V29, P1213

COWETT RM, 2002, NEOREVIEWS, V3, pE173, DOI DOI 10.1542/NEO.3-9-E173

Crowther CA, 2005, NEW ENGL J MED, V352, P2477, DOI 10.1056/NEJMoa042973

CRUIKSHANK DP, 1983, AM J OBSTET GYNECOL, V135, P1010

Dabelea D, 2007, DIABETES CARE, V30, pS169, DOI 10.2337/dc07-s211

De Leon DD, 2007, NAT CLIN PRACT ENDOC, V3, P57, DOI 10.1038/ncpendmet0368

DEMARINI S, 1994, OBSTET GYNECOL, V83, P918, DOI 10.1097/00006250-199406000-00003

deReginer RA, 2000, J PEDIATR-US, V137, P777, DOI 10.1067/mpd.2000.109149

DEUNGRIA M, 2003, PEDIATR RES, V133, P1174

Di Cianni G, 2005, DIABETIC MED, V22, P21, DOI 10.1111/j.1464-5491.2004.01336.x

Doblado Manuel, 2007, Curr Opin Endocrinol Diabetes Obes, V14, P488, DOI 10.1097/MED.0b013e3282f1cb92

Du XL, 2003, J CLIN INVEST, V112, P1049, DOI 10.1172/JCI200318127

Eidelman AI, 2002, SEMIN PERINATOL, V26, P232, DOI 10.1053/sper.2002.34215

ELIXHAUSER A, 1993, DIABETES CARE, V16, P1146, DOI 10.2337/diacare.16.8.1146

Ellis H, 2009, J PEDIATR SURG, V44, P2343, DOI 10.1016/j.jpedsurg.2009.07.054

GABBAY KH, 1966, SCIENCE, V151, P209, DOI 10.1126/science.151.3707.209

Gewolb IH, 1996, EXP LUNG RES, V22, P201, DOI 10.3109/01902149609050847

GEWOLB IH, 1995, LAB INVEST, V73, P59

Gewolb IH, 1997, EXP LUNG RES, V23, P245, DOI 10.3109/01902149709087370

Gustafsson J, 2009, INDIAN J MED RES, V130, P618

Harris DL, 2010, J PEDIATR-US, V157, P198, DOI 10.1016/j.jpeds.2010.02.003

Hay WW, 2010, J PEDIATR-US, V157, P180, DOI 10.1016/j.jpeds.2010.04.007

Hay WW, 2006, J PHYSIOL-LONDON, V572, P17, DOI 10.1113/jphysiol.2006.105072

HAY WW, 2008, NUTR PEDIAT BASIC SC, P311

HAY WW, 2003, TXB DIABETES PREGNAN, P201

HECK IJ, 1987, J PEDIAT, V110, P119

IKEDA H, 2011, MOL CELL BIOL, V3, P1054

Ikeda K, 1996, BIOCHEMISTRY-US, V35, P8075, DOI 10.1021/bi9530550

Jensen DM, 2004, DIABETES CARE, V27, P2819, DOI 10.2337/diacare.27.12.2819

KAISER N, 1993, DIABETES, V42, P80, DOI 10.2337/diabetes.42.1.80

Kalhan SC, 2002, NEONATAL PERINATAL M, P1357

KELLY DP, 1994, NEW ENGL J MED, V330, P913

Kitajima M, 2001, OBSTET GYNECOL, V97, P776, DOI 10.1016/S0029-7844(01)01328-X

KNOPP RH, 1992, DIABETES CARE, V15, P1605, DOI 10.2337/diacare.15.11.1605

Korshunov SS, 1997, FEBS LETT, V416, P15, DOI 10.1016/S0014-5793(97)01159-9

Koya D, 1998, DIABETES, V47, P859, DOI 10.2337/diabetes.47.6.859

LILIEN LD, 1980, J PEDIATR-US, V97, P295, DOI 10.1016/S0022-3476(80)80499-9

Lisowski LA, 2010, HERZ, V35, P19, DOI 10.1007/s00059-010-3244-3

Lozoff B, 2000, PEDIATRICS, V105, DOI 10.1542/peds.105.4.e51

MANCOJOHNSON MJ, 1994, PEDIATR RES, V36, P293, DOI 10.1203/00006450-199409000-00005

MCLELLAN AC, 1994, CLIN SCI, V87, P21, DOI 10.1042/cs0870021

Meissner T, 1997, EUR J PEDIATR, V156, P754, DOI 10.1007/s004310050706

Meur S, 2007, PAEDIAT CHILD HLTH, V17, P217, DOI DOI 10.1016/J.PAED.2007.03.004

MIMOUNI F, 1990, AM J PERINAT, V7, P203, DOI 10.1055/s-2007-999481

MIMOUNI F, 1988, J PEDIATR-US, V113, P345, DOI 10.1016/S0022-3476(88)80282-8

Mongiovi M, 2010, CURR PHARM DESIGN, V16, P2929, DOI 10.2174/138161210793176428

MORRIS MA, 1985, AM J OBSTET GYNECOL, V153, P651, DOI 10.1016/S0002-9378(85)80253-2

Nold JL, 2004, PEDIATR CLIN N AM, V51, P619, DOI 10.1016/j.pcl.2004.01.003

Ouzilleau C, 2003, CAN MED ASSOC J, V168, P403

Persson B, 2009, SEMIN FETAL NEONAT M, V14, P106, DOI 10.1016/j.siny.2009.01.003

PETRY CD, 1994, AM J PHYSIOL-ENDOC M, V267, pE507

Platas II, 2009, NEONATOLOGY, V95, P217, DOI 10.1159/000165980

Ralser Markus, 2007, J Biol, V6, P10, DOI 10.1186/jbiol61

Rao R, 1999, J NUTR, V129, P199

Rayani HH, 1999, EXP LUNG RES, V25, P69, DOI 10.1080/019021499270439

Regnault TRH, 2006, NEONATAL NUTRITION AND METABOLISM, 2ND EDITION, P32, DOI 10.1017/CBO9780511544712.004

Rolo LC, 2011, ARQ BRAS CARDIOL, V96, P386, DOI 10.1590/S0066-782X2011005000036

Rozance PJ, 2006, BIOL NEONATE, V90, P74, DOI 10.1159/000091948

Rozance PJ, 2010, EARLY HUM DEV, V86, P275, DOI 10.1016/j.earlhumdev.2010.05.002

Salhab WA, 2004, PEDIATRICS, V114, P361, DOI 10.1542/peds.114.2.361

Schaefer-Graf UM, 2008, DIABETES CARE, V31, P1858, DOI 10.2337/dc08-0039

Schwartz R, 2000, SEMIN PERINATOL, V24, P120, DOI 10.1053/sp.2000.6363

Son GH, 2010, ACTA OBSTET GYN SCAN, V89, P700, DOI 10.3109/00016341003605677

SUGAHARA K, 1994, AM J RESP CELL MOL, V11, P397, DOI 10.1165/ajrcmb.11.4.7917308

Sunehag A, 1997, PEDIATR RES, V42, P492, DOI 10.1203/00006450-199710000-00012

Thorn SR, 2011, SEMIN REPROD MED, V29, P225, DOI 10.1055/s-0031-1275516

Thorn SR, 2009, ENDOCRINOLOGY, V150, P3021, DOI 10.1210/en.2008-1789

Verhaeghe J, 2011, METABOLISM, V60, P71, DOI 10.1016/j.metabol.2010.04.013

VISSER GHA, 2008, TXB DIABETES PREGNAN

Volpe JJ, 2001, NEUROLOGY NEWBORN, P178

Wagner RK, 1999, OBSTET GYN CLIN N AM, V26, P371, DOI 10.1016/S0889-8545(05)70080-2

WARDPLATT M, 2005, SEMIN FETAL NEONAT M, V10, P341

WARSHAW JB, 2006, OSKIS PEDIAT PRINCIP, P423

Weindling MA, 2009, SEMIN FETAL NEONAT M, V14, P111

WIDNESS JA, 1981, J CLIN INVEST, V67, P637, DOI 10.1172/JCI110078

WIDNESS JA, 1989, ENDOCRINE DISORDERS, P273

Yan SF, 2003, CIRC RES, V93, P1159, DOI 10.1161/01.RES.0000103862.26506.3D

Yang J, 2006, OBSTET GYNECOL, V108, P644, DOI 10.1097/01.AOG.0000231688.08263.47

2011, DIABETES, V60, P1849

2011, PEDIATRICS, V127, P575

2007, CONFIDENTIAL ENQUIRY

NR 106

TC 49

Z9 50

U1 1

U2 23

PU CURRENT MEDICINE GROUP

PI PHILADELPHIA

PA 400 MARKET STREET, STE 700, PHILADELPHIA, PA 19106 USA

SN 1534-4827

J9 CURR DIABETES REP

JI Curr. Diabetes Rep.

PD FEB

PY 2012

VL 12

IS 1

BP 4

EP 15

DI 10.1007/s11892-011-0243-6

PG 12

WC Endocrinology & Metabolism

SC Endocrinology & Metabolism

GA 874AD

UT WOS:000298926500002

PM 22094826

DA 2019-01-08

ER

PT J

AU Curcio, R

Alexandre, NMC

Torres, HD

Lima, MHM

AF Curcio, Raquel

Costa Alexandre, Neuza Maria

Torres, Heloisa de Carvalho

Melo Lima, Maria Helena

TI Translation and adaptation of the "Diabetes Distress Scale - DDS" in

Brazilian culture

SO ACTA PAULISTA DE ENFERMAGEM

LA English

DT Article

DE Diabetes mellitus; Quality of life; Validation studies; Translating

ID QUALITY-OF-LIFE; GLYCEMIC CONTROL; SELF-CARE; HEALTH; DEPRESSION;

VALIDITY

AB Objective: To translate and adapt the Diabetes Distress Scale for the Brazilian culture. Methods: The process followed international standards for adaptation of an instrument: translation, back translation and evaluation by a panel of judges, and pretest. Results: The stages of translation and back translation were performed successfully, and the evaluation of the synthesis version by the expert panel resulted in modification of items, ensuring the equivalence between the original and translated versions. During the pretest, there were reformulations of some items, making them clearer and easier to understand. Conclusion: The Brazilian version of the Diabetes Distress Scale received satisfactory results with respect to the process of translation and cultural adaptation.

C1 [Curcio, Raquel] State Univ Campinas UNICAMP, Campinas, SP, Brazil.

[Costa Alexandre, Neuza Maria; Melo Lima, Maria Helena] State Univ Campinas UNICAMP, Fac Med Sci, Dept Nursing, Campinas, SP, Brazil.

[Torres, Heloisa de Carvalho] Univ Fed Minas Gerais, Sch Nursing, Dept Appl Nursing, Belo Horizonte, MG, Brazil.

RP Lima, MHM (reprint author), Rua Tessalia Vieira de Camargo 126,Caixa Postal 6, BR-13083887 Campinas, SP, Brazil.

EM melolima@fcm.unicamp.br

RI Lima, Maria Helena/C-5389-2018; Lima, Maria Helena/E-9595-2012

OI Lima, Maria Helena/0000-0001-6521-8324; Lima, Maria

Helena/0000-0001-6521-8324

CR Alexandre Neusa Maria Costa, 2002, Rev Panam Salud Publica, V11, P109, DOI 10.1590/S1020-49892002000200007

Beaton DE, 2000, SPINE, V25, P3186, DOI 10.1097/00007632-200012150-00014

Curcio R, 2011, REV ELETRONICA ENFER, V13, P331

El Achhab Y, 2008, DIABETES RES CLIN PR, V80, P171, DOI 10.1016/j.diabres.2007.12.020

Fisher L, 2009, DIABETIC MED, V26, P622, DOI 10.1111/j.1464-5491.2009.02730.x

Fisher L, 2008, ANN FAM MED, V6, P246, DOI 10.1370/afm.842

Fisher L, 2010, DIABETES CARE, V33, P1034, DOI 10.2337/dc09-2175

Fisher L, 2010, DIABETES CARE, V33, P23, DOI 10.2337/dc09-1238

Gonzalez JS, 2007, DIABETES CARE, V30, P2222, DOI 10.2337/dc07-0158

Graue M, 2012, INT J NURS STUD, V49, P174, DOI 10.1016/j.ijnurstu.2011.08.007

GUILLEMIN F, 1995, SCAND J RHEUMATOL, V24, P61, DOI 10.3109/03009749509099285

Herschbach P, 2005, J PSYCHOSOM RES, V58, P505, DOI 10.1016/j.jpsychores.2005.02.007

Home P, 2006, DIABETIC MED, V23, P579

Lustman PJ, 2000, DIABETES CARE, V23, P934, DOI 10.2337/diacare.23.7.934

Moreira RO, 2003, ARQ BRAS ENDOCRINOL, V47, P19

Polonsky WH, 2005, DIABETES CARE, V28, P626, DOI 10.2337/diacare.28.3.626

Ting RZW, 2011, DIABETES CARE, V34, P1094, DOI 10.2337/dc10-1612

Torres HD, 2011, ACTA PAUL ENFERM, V24, P514, DOI 10.1590/S0103-21002011000400011

Wexler DJ, 2006, DIABETOLOGIA, V49, P1489, DOI 10.1007/s00125-006-0249-9

Wynd CA, 2003, WESTERN J NURS RES, V25, P508, DOI 10.1177/0193945903252998

NR 20

TC 4

Z9 4

U1 0

U2 6

PU UNIV FED SAO PAULO, DEPT ENFERMAGEN

PI SAO PAULO

PA RUA NAPOLEAO DE BARROS, 754 VILA CLEMENTINO, SAO PAULO, CEP04024-002,

BRAZIL

SN 0103-2100

J9 ACTA PAUL ENFERM

JI Acta Paul. Enferm.

PY 2012

VL 25

IS 5

BP 762

EP 767

DI 10.1590/S0103-21002012005000025

PG 6

WC Nursing

SC Nursing

GA 170TF

UT WOS:000320875900018

OA DOAJ Gold

DA 2019-01-08

ER

PT J

AU Grzywacz, JG

Arcury, TA

Saldana, S

Kirk, JK

Bell, RA

Ip, E

Quandt, SA

AF Grzywacz, Joseph G.

Arcury, Thomas A.

Saldana, Santiago

Kirk, Julienne K.

Bell, Ronny A.

Ip, Edward

Quandt, Sara A.

TI Social Control in Older Adults' Diabetes Self Management and Well-Being

SO BEHAVIORAL MEDICINE

LA English

DT Article

DE depressive symptoms; diabetes management; ethnicity; social control

ID PSYCHOLOGICAL DISTRESS; HEALTH BEHAVIORS; MARITAL-STATUS; INTEGRATION;

GENDER; TYPE-1

AB The "dual effects" hypothesis argues that social control can be effective in promoting positive health-related behavior change, but it can also jeopardize the targeted individual's well-being. This hypothesis is tested using hemoglobin A1C as an objective indicator of behavioral compliance with diabetes self-management behavior and depressive symptoms. Differences in the effects of social control on A1C and depressive symptoms by sex and ethnicity are tested. Cross-sectional data were obtained from a multi-ethnic sample of older adults with diabetes (N = 593). Greater social control was associated with poorer rather than better odds of achieving glucose control, and with greater depressive symptoms. There was no evidence that social control has differential effects on either glucose control or depressive symptoms by sex or ethnicity. Active use of social control attempts by family members and friends, especially if they are coercive or punitive in nature, are likely counterproductive for maintaining the physical and mental health of older adults with diabetes.

C1 [Quandt, Sara A.] Wake Forest Sch Med, Dept Epidemiol & Prevent, Winston Salem, NC 27157 USA.

RP Quandt, SA (reprint author), Wake Forest Sch Med, Dept Epidemiol & Prevent, Med Ctr Blvd, Winston Salem, NC 27157 USA.

EM squandt@wakehealth.edu

OI Grzywacz, Joseph/0000-0002-2308-7781

FU National Institute of Aging [R01 AG17587]

FX This research was supported by a grant from the National Institute of

Aging (R01 AG17587).

CR Aiken L. S, 1991, MULTIPLE REGRESSION

American Diabetes Association, 2011, Diabetes Care, V34 Suppl 1, pS11, DOI 10.2337/dc11-S011

Arcury T. A., J APPL GERO IN PRESS

Arcury TA, 1999, HUM ORGAN, V58, P128, DOI 10.17730/humo.58.2.t5g838w7u1761868

August KJ, 2010, SOC SCI MED, V71, P1831, DOI 10.1016/j.socscimed.2010.08.022

Barnes LL, 2004, J GERONTOL B-PSYCHOL, V59, pS146, DOI 10.1093/geronb/59.3.S146

Berkman LF, 2000, SOC SCI MED, V51, P843, DOI 10.1016/S0277-9536(00)00065-4

Bode Bruce W, 2007, J Diabetes Sci Technol, V1, P405

de Leon CFM, 2001, J GERONTOL B-PSYCHOL, V56, pS179, DOI 10.1093/geronb/56.3.S179

HUGHES M, 1981, AM J SOCIOL, V87, P48, DOI 10.1086/227419

Kramer CK, 2011, DIABETES CARE, V34, P1238, DOI 10.2337/dc11-0079

KRAUSE N, 1993, SOC SCI MED, V36, P1429, DOI 10.1016/0277-9536(93)90385-H

Lewis MA, 1999, HEALTH PSYCHOL, V18, P63, DOI 10.1037/0278-6133.18.1.63

Lewis MA, 2004, J SOC PERS RELAT, V21, P669, DOI 10.1177/0265404045893

NATHAN DM, 1993, NEW ENGL J MED, V328, P1676, DOI 10.1056/NEJM199306103282306

Quandt SA, 2006, J GERONTOL A-BIOL, V61, P394, DOI 10.1093/gerona/61.4.394

Quandt SA, 2001, J APPL GERONTOL, V20, P356, DOI 10.1177/073346480102000307

RADLOFF L S, 1977, Applied Psychological Measurement, V1, P385, DOI 10.1177/014662167700100306

ROOK KS, 1990, PSYCHOL AGING, V5, P327, DOI 10.1037/0882-7974.5.3.327

Stephens MAP, 2010, FAM SYST HEALTH, V28, P199, DOI 10.1037/a0020513

Thorpe CT, 2008, J BEHAV MED, V31, P93, DOI 10.1007/s10865-007-9125-4

Tucker JS, 2006, HEALTH PSYCHOL, V25, P715, DOI 10.1037/0278-6133.25.6.715

Tucker JS, 2004, J GERONTOL B-PSYCHOL, V59, pP147, DOI 10.1093/geronb/59.4.P147

UMBERSON D, 1987, J HEALTH SOC BEHAV, V28, P306, DOI 10.2307/2136848

UMBERSON D, 1992, SOC SCI MED, V34, P907, DOI 10.1016/0277-9536(92)90259-S

WEISSMAN MM, 1977, AM J EPIDEMIOL, V106, P203, DOI 10.1093/oxfordjournals.aje.a112455

NR 26

TC 9

Z9 10

U1 0

U2 12

PU ROUTLEDGE JOURNALS, TAYLOR & FRANCIS LTD

PI ABINGDON

PA 2-4 PARK SQUARE, MILTON PARK, ABINGDON OX14 4RN, OXON, ENGLAND

SN 0896-4289

EI 1940-4026

J9 BEHAV MED

JI Behav. Med.

PY 2012

VL 38

IS 4

BP 115

EP 120

DI 10.1080/08964289.2012.693976

PG 6

WC Behavioral Sciences; Psychiatry

SC Behavioral Sciences; Psychiatry

GA 030ZO

UT WOS:000310608900001

PM 23121208

OA Green Accepted

DA 2019-01-08

ER

PT J

AU Al-Nsour, M

Zindah, M

Belbeisi, A

Hadaddin, R

Brown, DW

Walke, H

AF Al-Nsour, Mohannad

Zindah, Meyasser

Belbeisi, Adel

Hadaddin, Raja

Brown, David W.

Walke, Henry

TI Prevalence of Selected Chronic, Noncommunicable Disease Risk Factors in

Jordan: Results of the 2007 Jordan Behavioral Risk Factor Surveillance

Survey

SO PREVENTING CHRONIC DISEASE

LA English

DT Article

ID DIABETES-MELLITUS; WEIGHT-GAIN; PREVENTION

AB Introduction

Noncommunicable diseases (NCDs) are the leading cause of illness and death in Jordan. Since 2002, the Jordan Ministry of Health, in cooperation with the World Health Organization and the Centers for Disease Control and Prevention, established the Jordan Behavioral Risk Factor Surveillance Survey to collect information on many of the behaviors and conditions related to NCDs. The objectives of this study were to describe the prevalence of selected NCD risk factors and the relationship between body mass index and selected health conditions among a nationally representative sample of Jordanian adults aged 18 years or older.

Methods

We used a multistage sampling design to select 3,688 households, from which we randomly selected and interviewed 1 adult aged 18 years or older. A total of 3,654 adults completed the survey. We randomly selected a subsample of 889 interviewed adults and invited them to visit local health clinics for a medical evaluation; we obtained measurements, including fasting blood glucose and blood lipids, from 765 adults. Data were collected between June 1, 2007, and August 23, 2007.

Results

Nearly one-third of participants smoked cigarettes, 18% reported having been diagnosed with high blood pressure, and 10% reported frequent mental distress. Compared with survey participants who did not participate in the medical evaluation, those who participated were more likely to self-report high blood pressure, high blood cholesterol, and diabetes and report lower levels of health-related quality of life. Among participants of the medical evaluation, an estimated 11% reported they had been diagnosed with diabetes by a health professional, and 19% were diagnosed with diabetes according to laboratory testing. Approximately one-third of participants of the medical evaluation were either overweight (30%) or obese (36%). In the fully adjusted model, obese participants of the medical evaluation were nearly 3 times as likely to have high blood pressure and more than 2 times as likely to have high blood cholesterol as normal-weight participants.

Conclusion

Diabetes, high blood pressure, high cholesterol, and obesity are a public health concern in Jordan. Adequate and continuous monitoring of NCD risk factors in Jordan is needed, and the surveillance findings should be used in health promotion and disease prevention activities.

C1 [Al-Nsour, Mohannad; Zindah, Meyasser; Belbeisi, Adel; Hadaddin, Raja] Minist Hlth, Amman 11196, Jordan.

[Brown, David W.] Brown Consulting Grp, Charlotte, NC USA.

[Walke, Henry] Ctr Dis Control & Prevent, Atlanta, GA USA.

RP Al-Nsour, M (reprint author), Minist Hlth, Dahiyat Al Rasheed Alwifaq St,Bldg 94,1st Floor,O, Amman 11196, Jordan.

EM mohannadnsour973@yahoo.com

CR Belbeisi A., 2006, Morbidity and Mortality Weekly Report, V55, P653

Belbeisi A, 2009, GLOBALIZATION HEALTH, V5, DOI 10.1186/1744-8603-5-18

Belbeisi A, 2009, INT J PUBLIC HEALTH, V54, P106, DOI 10.1007/s00038-009-0014-1

Brown DW, 2009, PREV CHRONIC DIS, V6

Centers for Disease Control and Prevention, 2000, MEAS HLTH DAYS POP A

Centers for Disease Control and Prevention (CDC), 2003, MMWR Morb Mortal Wkly Rep, V52, P1042

COLDITZ GA, 1995, ANN INTERN MED, V122, P481, DOI 10.7326/0003-4819-122-7-199504010-00001

Directorate of Information Studies and Research, 2008, MORT JORD 2005

ERIKSSON KF, 1991, DIABETOLOGIA, V34, P891, DOI 10.1007/BF00400196

HANSON RL, 1995, DIABETES, V44, P261, DOI 10.2337/diabetes.44.3.261

Jordan Cancer Registry, 2009, CANC INC JORD 2006

Jordan Department of Statistics, 2007, POP FAM HLTH SURV 20

Mokdad AH, 2007, ETHN DIS S3, V17, pS3

Nelson DE, 2001, SOZ PRAVENTIV MED, V46, pS3

Ounpuu S, 2001, Chronic Dis Can, V22, P93

PISUNYER FX, 1993, ANN INTERN MED, V119, P655, DOI 10.7326/0003-4819-119-7_Part_2-199310011-00006

ROWLAND ML, 1990, AM J CLIN NUTR, V52, P1125

Tuomilehto J, 2001, NEW ENGL J MED, V344, P1343, DOI 10.1056/NEJM200105033441801

World Health Organization, PROJ MORT BURD DIS 2

Zindah M, 2008, PREV CHRONIC DIS, V5

NR 20

TC 13

Z9 13

U1 0

U2 7

PU CENTERS DISEASE CONTROL

PI ATLANTA

PA 1600 CLIFTON RD, ATLANTA, GA 30333 USA

SN 1545-1151

J9 PREV CHRONIC DIS

JI Prev. Chronic Dis.

PD JAN

PY 2012

VL 9

AR 110077

DI 10.5888/pcd9.110077

PG 9

WC Public, Environmental & Occupational Health

SC Public, Environmental & Occupational Health

GA 977GI

UT WOS:000306644300025

PM 22172192

OA Green Published

DA 2019-01-08

ER

PT J

AU Franks, MM

Hemphill, RC

Seidel, AJ

Stephens, MAP

Rook, KS

Salem, JK

AF Franks, Melissa M.

Hemphill, Rachel C.

Seidel, Amber J.

Stephens, Mary Ann Parris

Rook, Karen S.

Salem, James K.

TI Setbacks in diet adherence and emotional distress: A study of older

patients with type 2 diabetes and their spouses

SO AGING & MENTAL HEALTH

LA English

DT Article

DE chronic illness management; diabetes distress; depressive symptoms

ID DEPRESSIVE SYMPTOMS; CHRONIC ILLNESS; SOCIAL-CONTROL; LIFE-STYLE;

SELF-CARE; ADULTS; MELLITUS; SCALE; MANAGEMENT; BEHAVIORS

AB Objectives: We investigated patients' difficulties in managing their diet (i.e. diet setbacks) and associations with change in disease-specific and general emotional distress (diabetes distress and depressive symptoms) among patients with type 2 diabetes and their spouses.

Method: Data for this study were collected in couples' homes (N = 115 couples) using structured interviews and self-administered questionnaires at three time points: baseline (T1), six months after baseline (T2) and 12 months after baseline (T3).

Results: Patients' diet setbacks were associated with an increase in their diabetes distress in the shorter-term (over six months). Patients' diet setbacks were not associated with longer-term change in diabetes distress or with change in depressive symptoms at either time point (six months or one year). In contrast, spouses' perceptions of patients' diet setbacks were associated with increases in their own diabetes distress at both time points (over six months and one year), and also with an increase in their depressive symptoms in the longer-term (over one year).

Conclusion: Findings reveal detrimental consequences of patients' diet nonadherence for emotional well-being that extend to the well-being of their spouses.

C1 [Franks, Melissa M.; Seidel, Amber J.] Purdue Univ, Dept Human Dev & Family Studies, W Lafayette, IN 47907 USA.

[Hemphill, Rachel C.; Stephens, Mary Ann Parris] Kent State Univ, Dept Psychol, Kent, OH 44242 USA.

[Rook, Karen S.] Univ Calif Irvine, Dept Psychol & Social Behav, Irvine, CA USA.

[Salem, James K.] SUMMA Hlth Syst, Akron, OH USA.

RP Franks, MM (reprint author), Purdue Univ, Dept Human Dev & Family Studies, W Lafayette, IN 47907 USA.

EM mmfranks@purdue.edu

FU National Institute on Aging [R01 AG24833]

FX This research, entitled 'Mind-Body Interactions in Management of Type 2

Diabetes' was supported by grant R01 AG24833 from the National Institute

on Aging.

CR August KJ, 2010, SOC SCI MED, V71, P1831, DOI 10.1016/j.socscimed.2010.08.022

Bell RA, 2010, AM J HEALTH BEHAV, V34, P36

Berg CA, 2007, PSYCHOL BULL, V133, P920, DOI 10.1037/0033-2909.133.6.920

Beverly EA, 2008, HEALTH EDUC BEHAV, V35, P707, DOI 10.1177/1090198107299787

Center for Disease Control and Prevention, 2011, NAT DIAB FACT SHEET

Delahanty LM, 2007, DIABETIC MED, V24, P48, DOI 10.1111/j.1464-5491.2007.02028.x

Engum A, 2005, DIABETES CARE, V28, P1904, DOI 10.2337/diacare.28.8.1904

Fisher L, 2008, DIABETIC MED, V25, P1096, DOI 10.1111/j.1464-5491.2008.02533.x

Fisher L, 2002, DIABETES CARE, V25, P1564, DOI 10.2337/diacare.25.9.1564

Fisher L, 2007, DIABETES CARE, V30, P542, DOI 10.2337/dc06-1614

Franks M.M., 2010, SOCIAL SUPPORT SOCIA

Franks MM, 2010, FAM RELAT, V59, P599, DOI 10.1111/j.1741-3729.2010.00626.x

Gonder-Frederick LA, 2002, J CONSULT CLIN PSYCH, V70, P611, DOI 10.1037//0022-006X.70.3.611

Halter JB, 1999, PROCESSING OF MEDICAL INFORMATION IN AGING PATIENTS, P23

Huang ES, 2009, DIABETES CARE, V32, P2225, DOI 10.2337/dc09-0459

Huang ES, 2005, J AM GERIATR SOC, V53, P306, DOI 10.1111/j.1532-5415.2005.53119.x

Kenny D. A., 2006, DYADIC DATA ANAL

Lin EHB, 2004, DIABETES CARE, V27, P2154, DOI 10.2337/diacare.27.9.2154

Murata GH, 2004, CLIN ENDOCRINOL, V60, P451, DOI 10.1111/j.1365-2265.2004.02001.x

Nothwehr F, 2000, PREV MED, V30, P407, DOI 10.1006/pmed.2000.0658

Nouwen A, 2010, DIABETOLOGIA, V53, P2480, DOI 10.1007/s00125-010-1874-x

Penckofer S, 2007, DIABETES EDUCATOR, V33, P680, DOI 10.1177/0145721707304079

Peyrot M, 2005, DIABETIC MED, V22, P1379, DOI 10.1111/j.1464-5491.2005.01644.x

POLONSKY WH, 1995, DIABETES CARE, V18, P754, DOI 10.2337/diacare.18.6.754

Polonsky WH, 2005, DIABETES CARE, V28, P626, DOI 10.2337/diacare.28.3.626

RADLOFF L S, 1977, Applied Psychological Measurement, V1, P385, DOI 10.1177/014662167700100306

Revenson T. A., 1994, ANN BEHAVIORAL MED, V16, P122

RUBIN RR, 1991, DIABETES CARE, V14, P335, DOI 10.2337/diacare.14.4.335

Snoek FJ, 2000, DIABETES CARE, V23, P1305, DOI 10.2337/diacare.23.9.1305

Stephens MAP, 2010, FAM SYST HEALTH, V28, P199, DOI 10.1037/a0020513

Talbot F, 1999, HEALTH PSYCHOL, V18, P537

Toobert DJ, 2000, DIABETES CARE, V23, P943, DOI 10.2337/diacare.23.7.943

Travis T, 1997, DIABETES EDUCATOR, V23, P152, DOI 10.1177/014572179702300205

Trief P., 2003, FAMILIES SYSTEMS HLT, V21, P57, DOI [DOI 10.1037/H0089502, 10.1037/h0089502]

Vijan S, 2005, DIABETIC MED, V22, P32, DOI 10.1111/j.1464-5491.2004.01342.x

White P, 2007, DIABETIC MED, V24, P796, DOI 10.1111/j.1464-5491.2007.02271.x

Williams K. E., 2002, PSYCHOL HEALTH MED, V7, P127, DOI [10.1080/13548500120116076, DOI 10.1080/13548500120116076]

Yancik R, 2007, J GERONTOL A-BIOL, V62, P275, DOI 10.1093/gerona/62.3.275

NR 38

TC 10

Z9 10

U1 1

U2 12

PU ROUTLEDGE JOURNALS, TAYLOR & FRANCIS LTD

PI ABINGDON

PA 4 PARK SQUARE, MILTON PARK, ABINGDON OX14 4RN, OXFORDSHIRE, ENGLAND

SN 1360-7863

J9 AGING MENT HEALTH

JI Aging Ment. Health

PY 2012

VL 16

IS 7

BP 902

EP 910

DI 10.1080/13607863.2012.674486

PG 9

WC Geriatrics & Gerontology; Gerontology; Psychiatry

SC Geriatrics & Gerontology; Psychiatry

GA 987TI

UT WOS:000307440200012

PM 22533446

OA Green Accepted

DA 2019-01-08

ER

PT J

AU Vingilis, E

Wilk, P

AF Vingilis, Evelyn

Wilk, Piotr

TI Medical Conditions, Medication Use, and Their Relationship With

Subsequent Motor Vehicle Injuries: Examination of the Canadian National

Population Health Survey

SO TRAFFIC INJURY PREVENTION

LA English

DT Article

DE Motor vehicle injuries; Medical conditions; Medication use; Canada

ID OLDER DRIVERS; PSYCHOLOGICAL DISTRESS; RISK-FACTORS; COLLISION INJURIES;

BENZODIAZEPINE USE; SCREENING SCALES; TRAFFIC CRASHES; MENTAL-HEALTH;

INVOLVEMENT; DEPRESSION

AB Purpose: To examine the effects of various medical conditions and medications on subsequent motor vehicle injuries (MVIs).

Method: The National Population Health Survey, a large, nationally representative, longitudinal study of Canadians, included self-reported medical conditions of asthma, arthritis/rheumatism, back problems excluding arthritis, high blood pressure, migraine headaches, diabetes, heart disease and distress, and medication use during the past month for asthma, high blood pressure, diabetes, heart, codeine/pethidine (Demerol)/morphine, other pain relievers, antidepressants, tranquilizers, and sleeping medication. Path analyses were used to examine the odds of subsequent MVI for different medical conditions and medication use reported prior to the MVI (in the previous wave of the survey) while controlling for age and sex.

Results: Increased odds of subsequent MVIs were found for asthma (odds ratio [OR]: 1.864, 95% confidence interval [CI]: 1.281, 2.713), arthritis/rheumatism (OR: 1.659, 95% CI: 1.163, 2.365), back problems (OR: 2.169, 95% CI: 1.624, 2.895), and migraines (OR: 1.631, 95% CI: 1.125, 2.364) but not for high blood pressure (OR: 1.435, 95% CI: 0.944, 2.181), diabetes (OR: 1.479, 95% CI: 0.743, 2.944), heart disease (OR: 2.627, 95% CI: 0.941, 7.334) or distress (OR: 1.153, 95% CI: 0.840, 1.581). Except for migraine with codeine/pethidine/morphine, this effect persisted regardless of whether medication was used to treat the condition. Respondents who reported using certain medications, namely, codeine/pethidine/morphine (OR: 2.215, 95% CI: 1.274, 3.850), other pain medication (OR: 1.630, 95% CI: 1.242, 2.139), antidepressants (OR: 2.664. 95% CI: 1.602, 4.429), and sleeping medication (OR: 2.059, 95% CI: 1.161, 3.651), had increased odds of subsequent MVI, independent of related medical condition, whereas tranquillizers showed no increased odds of subsequent MVIs.

Conclusions: This study suggests that the relationship between medical conditions, medications, and MVIs is complex but consistent with other studies.

C1 [Vingilis, Evelyn] Univ Western Ontario, Dept Family Med, Populat & Community Hlth Unit, Schulich Sch Med & Dent, London, ON N6A 5C1, Canada.

[Vingilis, Evelyn; Wilk, Piotr] Univ Western Ontario, Dept Epidemiol & Biostat, Schulich Sch Med & Dent, London, ON N6A 5C1, Canada.

[Wilk, Piotr] Univ Western Ontario, Dept Paediat, Schulich Sch Med & Dent, London, ON N6A 5C1, Canada.

RP Vingilis, E (reprint author), Univ Western Ontario, Dept Family Med, Populat & Community Hlth Unit, Schulich Sch Med & Dent, Room 2710, London, ON N6A 5C1, Canada.

EM evingili@uwo.ca

CR Andrews G, 2001, AUST NZ J PUBL HEAL, V25, P494, DOI 10.1111/j.1467-842X.2001.tb00310.x

Begg DJ, 1999, ACCIDENT ANAL PREV, V31, P1, DOI 10.1016/S0001-4575(98)00039-6

Berger JT, 2000, J GEN INTERN MED, V15, P667, DOI 10.1046/j.1525-1497.2000.04309.x

Charlton J, 2004, 213 MON U ACC RES CT

Charlton J., 2010, 300 MON U ACC RES CT

CHIPMAN ML, 1995, ACCIDENT ANAL PREV, V27, P699, DOI 10.1016/0001-4575(95)00013-P

Cross JM, 2009, BRIT J OPHTHALMOL, V93, P400, DOI 10.1136/bjo.2008.144584

de Gier Johan J., 2009, P121

Dobbs B. M., 2005, 809690 DOT HS NAT HI

Dubois S, 2008, TRAFFIC INJ PREV, V9, P404, DOI 10.1080/15389580802161943

FOLEY DJ, 1995, J AM GERIATR SOC, V43, P776, DOI 10.1111/j.1532-5415.1995.tb07049.x

Furukawa TA, 2003, PSYCHOL MED, V33, P357, DOI 10.1017/S0033291702006700

GRATTAN E, 1968, BMJ-BRIT MED J, V1, P75, DOI 10.1136/bmj.1.5584.75

Harsch IA, 2009, DRUGS DRIVING TRAFFI, P393

Hours M, 2008, ACCIDENT ANAL PREV, V40, P1789, DOI 10.1016/j.aap.2008.06.017

Hu PS, 1998, ACCIDENT ANAL PREV, V30, P569, DOI 10.1016/S0001-4575(98)00019-0

Jenkins P, 2002, AM J IND MED, V41, P98, DOI 10.1002/ajim.10035

Kessler RC, 2002, PSYCHOL MED, V32, P959, DOI 10.1017/S0033291702006074

KOEPSEL TD, 1994, J AM GERIATR SOC, V42, P695, DOI 10.1111/j.1532-5415.1994.tb06526.x

Laflamme L, 2000, Inj Prev, V6, P293, DOI 10.1136/ip.6.4.293

Lerman Y, 1995, J TRAUMA, V39, P1058, DOI 10.1097/00005373-199512000-00007

LEVEILLE SG, 1994, EPIDEMIOLOGY, V5, P591, DOI 10.1097/00001648-199411000-00006

Lindsay VL, 2008, P 2008 AUSTR ROAD SA, P610

Lococo KH, 2006, 810681 NAT HIGHW TRA

Marshall CS, 2008, TRAFFIC INJ PREV, V9, P291

MCCLOSKEY LW, 1994, AGE AGEING, V23, P267, DOI 10.1093/ageing/23.4.267

McGwin G, 2000, AM J EPIDEMIOL, V152, P424, DOI 10.1093/aje/152.5.424

Mock C, 1999, INT J EPIDEMIOL, V28, P750, DOI 10.1093/ije/28.4.750

Muthen L. K., 2010, MPLUS USERS GUIDE

Orriols L., 2010, PLOS MED, V7, P1

PEDEN M, 2002, INJURY LEADING CAUSE

Rapoport MJ, 2009, J CLIN PSYCHIAT, V70, P663, DOI 10.4088/JCP.08m04325

Rhodes A, 2006, SOC SCI MED, V62, P828, DOI 10.1016/j.socscimed.2005.06.039

Roberts S. E., 2007, ACCIDENT ANAL PREV, V40, P559

ROBINS LN, 1988, ARCH GEN PSYCHIAT, V45, P1069

Sagberg F, 2006, ACCIDENT ANAL PREV, V38, P28, DOI 10.1016/j.aap.2005.06.018

Schwarz N, 2001, AM J EVAL, V22, P127, DOI 10.1177/109821400102200202

Sims RV, 2000, J GERONTOL A-BIOL, V55, pM22

Statistics Canada, 1996, NAT POP HLTH SURV GE

Statistics Canada, 2004, NAT POP HLTH SURV HO

Statistics Canada, 2005, INF NAT POP HLTH SUR

Statistics Canada, 2010, NAT POP HLTH SURV HO

Sudman S, 1983, ASKING QUESTIONS PRA

Thomas RE, 1998, CAN FAM PHYSICIAN, V44, P799

Transport Canada, 2011, CAN MOT VEH TRAFF CO

Vaa T., 2003, IMPAIRMENTS DIS AGE

Vernon DD, 2002, ACCIDENT ANAL PREV, V34, P237, DOI 10.1016/S0001-4575(01)00019-7

Verster J. C., 2009, DRUGS DRIVING TRAFFI

Verster JC, 2009, INT J ENV RES PUB HE, V6, P1041, DOI 10.3390/ijerph6031041

Vingilis E, 2002, TRAFFIC INJ PREV, V3, P1

Vingilis Evelyn, 2007, Traffic Inj Prev, V8, P411, DOI 10.1080/15389580701626202

Wade TJ, 1997, CAN J PUBLIC HEALTH, V88, P297

Walsh J Michael, 2004, Traffic Inj Prev, V5, P241

Wothke W, 2000, MODELING LONGITUDINAL AND MULTILEVEL DATA, P219

Zhang J, 2000, ACCIDENT ANAL PREV, V32, P117, DOI 10.1016/S0001-4575(99)00039-1

NR 55

TC 13

Z9 13

U1 0

U2 4

PU TAYLOR & FRANCIS INC

PI PHILADELPHIA

PA 325 CHESTNUT ST, SUITE 800, PHILADELPHIA, PA 19106 USA

SN 1538-9588

J9 TRAFFIC INJ PREV

JI Traffic Inj. Prev.

PY 2012

VL 13

IS 3

BP 327

EP 336

DI 10.1080/15389588.2012.654411

PG 10

WC Public, Environmental & Occupational Health; Transportation

SC Public, Environmental & Occupational Health; Transportation

GA 945MF

UT WOS:000304277200014

PM 22607256

DA 2019-01-08

ER

PT J

AU Petrofsky, J

Berk, L

Al-Nakhli, H

AF Petrofsky, Jerrold

Berk, Lee

Al-Nakhli, Hani

TI The Influence of Autonomic Dysfunction Associated with Aging and Type 2

Diabetes on Daily Life Activities

SO EXPERIMENTAL DIABETES RESEARCH

LA English

DT Review

ID SKIN BLOOD-FLOW; RESPIRATORY-DISTRESS-SYNDROME; SUBCUTANEOUS FAT

THICKNESS; VEIN ENDOTHELIAL-CELLS; HEART-RATE-VARIABILITY; PPAR-ALPHA

ACTIVATION; SWEAT GLAND DENSITY; NITRIC-OXIDE; ISOMETRIC-EXERCISE;

OXIDATIVE STRESS

AB Type 2 diabetes (T2D) and ageing have well documented effects on every organ in the body. In T2D the autonomic nervous system is impaired due to damage to neurons, sensory receptors, synapses and the blood vessels. This paper will concentrate on how autonomic impairment alters normal daily activities. Impairments include the response of the blood vessels to heat, sweating, heat transfer, whole body heating, orthostatic intolerance, balance, and gait. Because diabetes is more prevalent in older individuals, the effects of ageing will be examined. Beginning with endothelial dysfunction, blood vessels have impairment in their ability to vasodilate. With this and synaptic damage, the autonomic nervous system cannot compensate for effectors such as pressure on and heating of the skin. This and reduced ability of the heart to respond to stress, reduces autonomic orthostatic compensation. Diminished sweating causes the skin and core temperature to be high during whole body heating. Impaired orthostatic tolerance, impaired vision and vestibular sensing, causes poor balance and impaired gait. Overall, people with T2D must be made aware and counseled relative to the potential consequence of these impairments.

C1 [Petrofsky, Jerrold; Berk, Lee; Al-Nakhli, Hani] Loma Linda Univ, Sch Allied Hlth Profess, Dept Phys Therapy, Loma Linda, CA 92350 USA.

RP Petrofsky, J (reprint author), Loma Linda Univ, Sch Allied Hlth Profess, Dept Phys Therapy, Loma Linda, CA 92350 USA.

EM jpetrofsky@llu.edu

CR Al-Janabi AAHS, 2011, J GLOB INFECT DIS, V3, P133, DOI 10.4103/0974-777X.81689

Alemzadeh Ramin, 2005, Diabetes Technol Ther, V7, P587, DOI 10.1089/dia.2005.7.587

Anburajan M, 2011, IEEE ENG MED BIO, P6254, DOI 10.1109/IEMBS.2011.6091544

ANDERSON RK, 1987, J APPL PHYSIOL, V63, P1089

Azad N, 1999, J DIABETES COMPLICAT, V13, P307, DOI 10.1016/S1056-8727(99)00062-8

Baylie RL, 2011, ACTA PHYSIOL, V203, P99, DOI 10.1111/j.1748-1716.2010.02217.x

Bharara Manish, 2010, J Diabetes Sci Technol, V4, P773

Brunton S, 2006, CLIN THER, V28, P118, DOI 10.1016/j.clinthera.2006.01.011

Bulhak A, 2006, BASIC RES CARDIOL, V101, P244, DOI 10.1007/s00395-005-0580-1

Bulhak AA, 2009, AM J PHYSIOL-HEART C, V296, pH719, DOI 10.1152/ajpheart.00394.2008

Buse JB, 2007, CIRCULATION, V115, P114, DOI 10.1161/CIRCULATIONAHA.106.179294

Cameron FJ, 2010, CURR DIABETES REV, V6, P17, DOI 10.2174/157339910790442592

Campanucci V, 2010, NEURON, V66, P827, DOI 10.1016/j.neuron.2010.06.010

CAPUTI M, 1994, NUCLEIC ACIDS RES, V22, P1018, DOI 10.1093/nar/22.6.1018

Carroll SL, 2004, J NEUROPATH EXP NEUR, V63, P1144, DOI 10.1093/jnen/63.11.1144

Cui J, 2011, CLIN AUTON RES, V21, P381, DOI 10.1007/s10286-011-0126-6

da Rosa EM, 2011, ARQ BRAS CARDIOL, V97, P541, DOI 10.1590/S0066-782X2011001500014

Damon DH, 2011, AM J PHYSIOL-HEART C, V300, pH1386, DOI 10.1152/ajpheart.00300.2010

Dandona P, 1996, LANCET, V347, P444, DOI 10.1016/S0140-6736(96)90013-6

de Jongh R, 2007, NEUROUROL URODYNAM, V26, P302, DOI 10.1002/nau.20298

de Wit C, 2008, CELL COMMUN ADHES, V15, P231, DOI 10.1080/15419060802440260

DiBona GF, 2003, ACTA PHYSIOL SCAND, V177, P285, DOI 10.1046/j.1365-201X.2003.01074.x

Farage M., 2010, INFLUENCE RACE GENDE

FARAGE M. A., 2010, TXB AGING SKIN

Fedorowski A, 2010, EUR HEART J, V31, P85, DOI 10.1093/eurheartj/ehp329

Ferrari Alberto U, 2002, Am J Geriatr Cardiol, V11, P30

Fredriksson I, 2010, DIABETES, V59, P1578, DOI 10.2337/db10-0080

GATTINONI L, 1994, JAMA-J AM MED ASSOC, V271, P1772, DOI 10.1001/jama.271.22.1772

Giacco F, 2010, CIRC RES, V107, P1058, DOI 10.1161/CIRCRESAHA.110.223545

Gogg S, 2009, DIABETES, V58, P2238, DOI 10.2337/db08-0961

Grubb BP, 1997, PACE, V20, P781, DOI 10.1111/j.1540-8159.1997.tb03904.x

Grundy SM, 2006, DIABETES CARE, V29, P457, DOI 10.2337/diacare.29.02.06.dc05-1904

Gupta PK, 2008, EUR J PHARMACOL, V591, P171, DOI 10.1016/j.ejphar.2008.06.031

Harris RA, 2011, AM J HYPERTENS, V24, P557, DOI 10.1038/ajh.2011.12

Hirai DM, 2011, J APPL PHYSIOL, V111, P989, DOI 10.1152/japplphysiol.00490.2011

Hoeldtke RD, 2011, CLIN AUTON RES, V21, P19, DOI 10.1007/s10286-010-0084-4

Horowitz SH, 2006, CURR OPIN ANESTHESIO, V19, P573, DOI 10.1097/01.aco.0000245287.37905.c5

Infusino F, 2010, EUR REV MED PHARMACO, V14, P203

Inoue Y, 1996, EUR J APPL PHYSIOL O, V74, P72, DOI 10.1007/BF00376497

Jan YK, 2008, ARCH PHYS MED REHAB, V89, P137, DOI 10.1016/j.apmr.2007.07.046

Jose RM, 2005, BRIT J GEN PRACT, V55, P222

Katsilambros NL, 2011, EXP DIABETES RES, DOI 10.1155/2011/607309

Keller DM, 2011, J APPL PHYSIOL, V110, P1592, DOI 10.1152/japplphysiol.00233.2010

Kellogg DL, 1999, J APPL PHYSIOL, V86, P1185

Kellogg DL, 1998, J APPL PHYSIOL, V85, P824

KENNEY WL, 1988, MED SCI SPORT EXER, V20, P155

Kostolanska J, 2011, Vnitr Lek, V57, P533

Lawson D, 2007, MED SCI MONITOR, V13, pCR258

Lee JH, 2009, AM J PHYSIOL-CELL PH, V296, pC327, DOI 10.1152/ajpcell.00254.2008

Lin Zhong-qiu, 2011, Zhonghua Xin Xue Guan Bing Za Zhi, V39, P457

Madden KM, 2011, J CARDIOVASC PHARM, V57, P666, DOI 10.1097/FJC.0b013e31821533cc

Makrantonaki E, 2010, HAUTARZT, V61, P505, DOI 10.1007/s00105-009-1889-8

Makrantonaki E, 2010, EXP GERONTOL, V45, P801, DOI 10.1016/j.exger.2010.08.005

Maloney-Hinds C, 2009, DIABETES TECHNOL THE, V11, P39, DOI 10.1089/dia.2008.0011

Maser RE, 2003, DIABETES CARE, V26, P1895, DOI 10.2337/diacare.26.6.1895

Mayaudon H, 2010, DIABETES METAB, V36, P450, DOI 10.1016/j.diabet.2010.05.004

McLellan K, 2009, DIABETES TECHNOL THE, V11, P791, DOI 10.1089/dia.2009.0097

McLellan K, 2009, DIABETES TECHNOL THE, V11, P73, DOI 10.1089/dia.2008.0026

McLellan K, 2009, MED ENG PHYS, V31, P165, DOI 10.1016/j.medengphy.2008.08.004

MENARD S, 1994, BRIT J CANCER, V69, P1126, DOI 10.1038/bjc.1994.221

Minson CT, 2001, J APPL PHYSIOL, V91, P1619

MORELLI D, 1994, SCAND J IMMUNOL, V39, P453, DOI 10.1111/j.1365-3083.1994.tb03400.x

Murrell C, 2009, J PHYSIOL-LONDON, V587, P5959, DOI 10.1113/jphysiol.2009.179549

Nakagami G, 2010, ANN PLAS SURG, V64, P351, DOI 10.1097/SAP.0b013e3181a73078

Namer B, 2004, AUTON NEUROSCI-BASIC, V114, P72, DOI 10.1016/j.autneu.2004.06.007

Noble M, 2003, J REHABIL RES DEV, V40, P283

Nunan D, 2010, PACE, V33, P1407, DOI 10.1111/j.1540-8159.2010.02841.x

Nunan D, 2010, CLIN AUTON RES, V20, P213, DOI 10.1007/s10286-010-0073-7

Obrosova IG, 2009, NEUROTHERAPEUTICS, V6, P638, DOI 10.1016/j.nurt.2009.07.004

Oudit GY, 2006, J MOL MED, V84, P349, DOI 10.1007/s00109-005-0029-x

Oudit Gavin Y., 2007, Cardiovascular & Hematological Disorders - Drug Targets, V7, P295, DOI 10.2174/187152907782793545

PENNES HH, 1948, J APPL PHYSIOL, V1, P93

Petrofsky J, 2005, MED SCI MONITOR, V11, pCR562

Petrofsky J, 2005, MED SCI MONITOR, V11, pCR247

Petrofsky J. S., 2002, J NEUROLOGICAL ORTHO, V22, P16

Petrofsky J.S., 2005, BMC ENDOCR DISORD, V5, P4

Petrofsky J, 2007, DIABETES TECHNOL THE, V9, P377, DOI 10.1089/dia.2006.0028

Petrofsky J, 2006, MED SCI MONITOR, V12, pCR290

Petrofsky Jerrold, 2011, Journal of Medical Engineering & Technology, V35, P330, DOI 10.3109/03091902.2011.592237

Petrofsky Jerrold, 2011, Journal of Medical Engineering & Technology, V35, P262, DOI 10.3109/03091902.2011.580039

Petrofsky J, 2011, DIABETES TECHNOL THE, V13, P365, DOI 10.1089/dia.2010.0161

Petrofsky J, 2011, MED SCI MONITOR, V17, pCR1, DOI 10.12659/MSM.881315

Petrofsky J, 2010, DIABETES TECHNOL THE, V12, P1003, DOI 10.1089/dia.2010.0152

Petrofsky J, 2011, MED ENG PHYS, V33, P325, DOI 10.1016/j.medengphy.2010.10.018

Petrofsky Jerrold, 2010, Physiotherapy Theory and Practice, V26, P107, DOI 10.3109/09593980802640059

Petrofsky J, 2009, DIABETES TECHNOL THE, V11, P361, DOI 10.1089/dia.2008.0094

Petrofsky Jerrold, 2007, Physiotherapy Theory and Practice, V23, P189, DOI 10.1080/09593980701209295

Petrofsky JS, 2008, MED SCI MONITOR, V14, pCR399

Petrofsky JS, 2010, DIABETES TECHNOL THE, V12, P153, DOI 10.1089/dia.2009.0123

Petrofsky JS, 2009, MED SCI MONITOR, V15, pCR325

Petrofsky JS, 2008, DIABETES TECHNOL THE, V10, P487, DOI 10.1089/dia.2008.0009

Petrofsky JS, 2006, MED SCI MONITOR, V12, pCR273

Petrofsky Jerrold Scott, 2011, J Diabetes Sci Technol, V5, P657

Petrofsky JS, 2010, J DIABETES, V2, P41, DOI 10.1111/j.1753-0407.2009.00058.x

Petrofsky JS, 2010, DIABETES TECHNOL THE, V12, P475, DOI 10.1089/dia.2009.0125

Petrofsky JS, 2006, MED SCI MONITOR, V12, pCR21

Petrofsky JS, 2005, MED SCI MONITOR, V11, pCR515

Petrofsky JS, 2005, MED SCI MONITOR, V11, pCR470

Petrofsky JS, 2005, MED SCI MONITOR, V11, pPI43

Petrofsky JS, 2005, MED SCI MONITOR, V11, pCR153

Petrofsky JS, 2002, EUR J APPL PHYSIOL, V86, P479

PETROFSKY JS, 1975, J APPL PHYSIOL, V38, P863

PETROFSKY JS, 1992, EUR J APPL PHYSIOL O, V64, P503, DOI 10.1007/BF00843758

Philips JC, 2011, DIABETES METAB, V37, P489, DOI 10.1016/j.diabet.2011.09.004

Philips JC, 2009, DIABETES-METAB RES, V25, P442, DOI 10.1002/dmrr.969

Philips JC, 2011, AUTON NEUROSCI-BASIC, V162, P3, DOI 10.1016/j.autneu.2011.03.001

Pop-Busui R, 2009, J PERIPHER NERV SYST, V14, P1, DOI 10.1111/j.1529-8027.2009.00200.x

Potenza MA, 2009, CURR MED CHEM, V16, P94, DOI 10.2174/092986709787002853

Quagliaro L, 2005, ATHEROSCLEROSIS, V183, P259, DOI 10.1016/j.atherosclerosis.2005.03.015

Risso A, 2001, AM J PHYSIOL-ENDOC M, V281, pE924

Rizzo AM, 2010, ADV EXP MED BIOL, V698, P52, DOI 10.1007/978-1-4419-7347-4_5

ROWELL LB, 1977, J INVEST DERMATOL, V69, P154, DOI 10.1111/1523-1747.ep12497938

ROWELL LB, 1983, CIRC RES, V52, P367, DOI 10.1161/01.RES.52.4.367

ROWELL LB, 1992, INT J SPORTS MED, V13, pS25, DOI 10.1055/s-2007-1024583

Samborski P, 2011, POL ARCH MED WEWN, V121, P67

Santos-Garcia D, 2011, CEREBROVASC DIS, V32, P155, DOI 10.1159/000328651

Sato T, 2002, DIABETES, V51, P1565, DOI 10.2337/diabetes.51.5.1565

Sauerstein K, 2000, J PHYSIOL-LONDON, V529, P803, DOI 10.1111/j.1469-7793.2000.00803.x

SAVIO ML, 1994, J CLIN MICROBIOL, V32, P935

Schmidt RE, 2003, AM J PATHOL, V163, P21, DOI 10.1016/S0002-9440(10)63626-7

Singh DK, 2011, NAT REV ENDOCRINOL, V7, P176, DOI 10.1038/nrendo.2010.212

Song CH, 2011, DIABETES TECHNOL THE, V13, P803, DOI 10.1089/dia.2011.0036

Sridhar B, 2010, TOHOKU J EXP MED, V220, P107, DOI 10.1620/tjem.220.107

TAGLIABUE A, 1994, INT J OBESITY, V18, P537

Tagliabue John, 1994, N Y Times Web, pA8

TAGLIABUE M, 1994, ACTA RADIOL, V35, P230

TANKERSLEY CG, 1991, J APPL PHYSIOL, V71, P236

Triggle Chris R., 2003, Journal of Smooth Muscle Research, V39, P249, DOI 10.1540/jsmr.39.249

Trott DW, 2011, J APPL PHYSIOL, V110, P1171, DOI 10.1152/japplphysiol.01037.2010

Valko M, 2006, CHEM-BIOL INTERACT, V160, P1, DOI 10.1016/j.cbi.2005.12.009

Valko M, 2007, INT J BIOCHEM CELL B, V39, P44, DOI 10.1016/j.biocel.2006.07.001

Van Nassauw L, 2001, CELL TISSUE RES, V303, P329, DOI 10.1007/s004410000338

Vincent AM, 2004, ENDOCR REV, V25, P612, DOI 10.1210/er.2003-0019

Vinik AI, 2003, SEMIN NEUROL, V23, P365

Vinik AI, 2003, DIABETES CARE, V26, P1553, DOI 10.2337/diacare.26.5.1553

Watanabe H, 2002, J BIOL CHEM, V277, P47044, DOI 10.1074/jbc.M208277200

Wong BJ, 2003, J APPL PHYSIOL, V95, P504, DOI 10.1152/japplphysiol.00254.2003

Woodman RJ, 2006, DIABETES RES CLIN PR, V71, P59, DOI 10.1016/j.diabres.2005.05.008

Ye Xi-yun, 2010, Zhonghua Xin Xue Guan Bing Za Zhi, V38, P264

NR 139

TC 13

Z9 13

U1 0

U2 7

PU HINDAWI LTD

PI LONDON

PA ADAM HOUSE, 3RD FLR, 1 FITZROY SQ, LONDON, W1T 5HF, ENGLAND

SN 1687-5214

EI 1687-5303

J9 EXP DIABETES RES

JI Exp. Diabetes Res.

PY 2012

AR 657103

DI 10.1155/2012/657103

PG 12

WC Endocrinology & Metabolism; Medicine, Research & Experimental

SC Endocrinology & Metabolism; Research & Experimental Medicine

GA 938JI

UT WOS:000303729400001

PM 22566994

OA DOAJ Gold, Green Published

DA 2019-01-08

ER

PT J

AU Robertson, SM

Stanley, MA

Cully, JA

Naik, AD

AF Robertson, Suzanne M.

Stanley, Melinda A.

Cully, Jeffrey A.

Naik, Aanand D.

TI Positive Emotional Health and Diabetes Care: Concepts, Measurement, and

Clinical Implications

SO PSYCHOSOMATICS

LA English

DT Review

ID QUALITY-OF-LIFE; TREATMENT SATISFACTION; GLYCEMIC CONTROL; PSYCHOLOGICAL

ADJUSTMENT; CONTROLLED TRIAL; SELF-MANAGEMENT; NEGATIVE AFFECT; MOOD;

RESILIENCE; PEOPLE

AB Background: Numerous studies describe the effect of emotional problems, including diabetes-related distress and depression, on diabetes outcomes. Little attention has been devoted to positive emotional health and its potential role in facilitating patients' self-management and improved outcomes. Objective: This review describes the conceptualization and measurement of three empirically-tested aspects of positive emotional health (well-being, positive affect, resilience) in the diabetes literature and their relationship to diabetes outcomes. Method: A literature review was conducted using PubMed and the Cochrane Library databases from 1970 to January 2011 to identify studies focused on well-being, positive affect, and resilience in diabetes patients. Results: Twenty-two studies were identified based on our inclusion criteria. Well-being studies were least. likely to include conceptualizations and relied on two primary measurement instruments compared to more heterogeneous presentations found in positive affect and resilience studies. All three aspects of positive emotional health were linked to health-related outcomes and self-management. Conclusion: Positive emotional health may facilitate chronic care self-management and improved health outcomes. We present a model to guide future research and intervention development efforts designed to enhance positive emotional health. (Psychosomatics 2012; 53:1-12)

C1 [Robertson, Suzanne M.; Stanley, Melinda A.; Cully, Jeffrey A.; Naik, Aanand D.] Houston Hlth Serv Res & Dev Ctr Excellence, Michael E DeBakey Vet Affairs Med Ctr, Houston, TX USA.

[Stanley, Melinda A.; Cully, Jeffrey A.; Naik, Aanand D.] Baylor Coll Med, Menninger Dept Psychiat & Behav Sci, Houston, TX 77030 USA.

[Stanley, Melinda A.; Cully, Jeffrey A.; Naik, Aanand D.] VA S Cent Mental Illness Res Educ & Clin Ctr, Houston, TX USA.

RP Robertson, SM (reprint author), Houston VA Hlth Serv Res & Dev Ctr Excellence, Michael E DeBakey VA Med Ctr, 2002 Holcombe Blvd,MEDVAMC 152, Houston, TX 77030 USA.

EM smr1@bcm.tmc.edu

FU Houston VA Health Services Research and Development Center of Excellence

[HFP90-020]; VA Office of Academic Affiliations; National Institute of

Aging [5K23AG027144]; Doris Duke Charitable Foundation

FX This review is supported with resources and the use of facilities at the

Houston VA Health Services Research and Development Center of Excellence

(HFP90-020) at the Michael E. DeBakey VA Medical Center, Houston, TX

Suzanne M. Robertson is a VA HSR post-doctoral fellow supported by VA

Office of Academic Affiliations. Aanand D. Naik receives support from a

National Institute of Aging Career Development Award (5K23AG027144) and

a Doris Duke Charitable Foundation Clinical Scientist Development Award.

The views expressed reflect those of the authors and not necessarily

those of the Department of Veterans Affairs or Baylor College of

Medicine.

CR Anderson RM, 2010, PATIENT EDUC COUNS, V79, P277, DOI 10.1016/j.pec.2009.07.025

Bech P, 2003, INT J METH PSYCH RES, V12, P85, DOI 10.1002/mpr.145

BRADLEY C, 1990, DIABETIC MED, V7, P445, DOI 10.1111/j.1464-5491.1990.tb01421.x

Bradshaw BG, 2007, DIABETES EDUCATOR, V33, P650, DOI 10.1177/0145721707303809

Chan R, 2009, CLIN J AM SOC NEPHRO, V4, P160, DOI 10.2215/CJN.01520308

Chesney MA, 2005, INT J BEHAV MED, V12, P50, DOI 10.1207/s15327558ijbm1202_2

Ciechanowski P, 2011, CLIN DIABETES, V29, P43, DOI DOI 10.2337/DIACLIN.29.2.43

Comellas M, 2010, DIABETES EDUCATOR, V36, P141, DOI 10.1177/0145721709354606

de Ridder D, 2008, LANCET, V372, P246, DOI 10.1016/S0140-6736(08)61078-8

Denollet J, 2006, J AFFECT DISORDERS, V91, P171, DOI 10.1016/j.jad.2005.12.044

Douek IF, 2005, DIABETIC MED, V22, P634, DOI 10.1111/j.1464-5491.2005.01475.x

Folkman S, 2000, AM PSYCHOL, V55, P647, DOI 10.1037/0003-066X.55.6.647

Forlani G, 2006, J ENDOCRINOL INVEST, V29, P405, DOI 10.1007/BF03344123

Gallant MP, 2003, HEALTH EDUC BEHAV, V30, P170, DOI 10.1177/1090198102251030

Glasgow RE, 2001, DIABETES SPECTRUM, V14, P33, DOI DOI 10.2337/DIASPECT.14.1.33

Heisler M, 2010, ANN INTERN MED, V153, P507, DOI 10.7326/0003-4819-153-8-201010190-00007

Katon WJ, 2010, NEW ENGL J MED, V363, P2611, DOI 10.1056/NEJMoa1003955

Keogh KM, 2011, AM J MANAG CARE, V17, P105

Ligtenberg PC, 1998, DIABETES CARE, V21, P2196, DOI 10.2337/diacare.21.12.2196

Logtenberg SJ, 2010, DIABETES CARE, V33, P1169, DOI 10.2337/dc09-1758

Lorig KR, 2003, ANN BEHAV MED, V26, P1, DOI 10.1207/S15324796ABM2601_01

Marra Giampiero, 2004, Acta Biomed, V75, P164

Moskowitz JT, 2008, HEALTH PSYCHOL, V27, pS73, DOI 10.1037/0278-6133.27.1.S73

Naik AD, 2011, ARCH INTERN MED, V171, P453, DOI 10.1001/archinternmed.2011.70

Ong AD, 2006, J PERS SOC PSYCHOL, V91, P730, DOI 10.1037/0022-3514.91.4.730

Ozer E, 2003, PATIENT EDUC COUNS, V51, P39, DOI 10.1016/S0738-3991(02)00246-X

Papanas N, 2010, EXP CLIN ENDOCR DIAB, V118, P364, DOI 10.1055/s-0029-1243623

Pelle AJ, 2011, QUAL LIFE RES, V20, P643, DOI 10.1007/s11136-010-9792-4

Pelle AJ, 2009, QUAL LIFE RES, V18, P689, DOI 10.1007/s11136-009-9485-z

Petterson T, 1998, DIABETES CARE, V21, P930, DOI 10.2337/diacare.21.6.930

Piette JD, 2004, AM J MANAG CARE, V10, P152

Pouwer F, 2001, DIABETES CARE, V24, P1929, DOI 10.2337/diacare.24.11.1929

RYFF CD, 1995, J PERS SOC PSYCHOL, V69, P719, DOI 10.1037//0022-3514.69.4.719

Saatci E, 2010, HEALTH QUAL LIFE OUT, V8, DOI 10.1186/1477-7525-8-67

Seligman M. E. P, 2011, FLOURISH VISIONARY N

SJOBERG L, 1979, SCAND J PSYCHOL, V20, P1, DOI 10.1111/j.1467-9450.1979.tb00677.x

Skaff MM, 2009, HEALTH PSYCHOL, V28, P265, DOI 10.1037/a0014429

Spangler J G, 1993, Arch Fam Med, V2, P149, DOI 10.1001/archfami.2.2.149

Steinhardt MA, 2009, DIABETES EDUCATOR, V35, P274, DOI 10.1177/0145721708329698

Stenstrom U, 2003, DIABETES RES CLIN PR, V60, P147, DOI 10.1016/S0168-8227(03)00018-4

Stuckey HL, 2010, QUAL HEALTH RES, V20, P42, DOI 10.1177/1049732309355286

Tugade MM, 2004, J PERS, V72, P1161, DOI 10.1111/j.1467-6494.2004.00294.x

Van der Does FEE, 1998, DIABETES CARE, V21, P2085, DOI 10.2337/diacare.21.12.2085

WATSON D, 1988, J PERS SOC PSYCHOL, V54, P1063, DOI 10.1037/0022-3514.54.6.1063

Wilson M, 2004, DIABETES RES CLIN PR, V66, P263, DOI 10.1016/j.diabres.2004.04.010

Yi JP, 2008, BRIT J HEALTH PSYCH, V13, P311, DOI 10.1348/135910707X186994

Yi-Frazier JP, 2010, STRESS HEALTH, V26, P51, DOI 10.1002/smi.1258

NR 47

TC 15

Z9 15

U1 0

U2 11

PU ELSEVIER SCIENCE INC

PI NEW YORK

PA 360 PARK AVE SOUTH, NEW YORK, NY 10010-1710 USA

SN 0033-3182

J9 PSYCHOSOMATICS

JI Psychosomatics

PD JAN-FEB

PY 2012

VL 53

IS 1

BP 1

EP 12

PG 12

WC Psychiatry; Psychology

SC Psychiatry; Psychology

GA 882TB

UT WOS:000299585700001

PM 22221716

DA 2019-01-08

ER

PT J

AU Primozic, S

Tavcar, R

Avbelj, M

Dernovsek, MZ

Oblak, MR

AF Primozic, Simona

Tavcar, Rok

Avbelj, Magdalena

Dernovsek, Mojca Zvezdana

Oblak, Maja Ravnik

TI Specific cognitive abilities are associated with diabetes

self-management behavior among patients with type 2 diabetes

SO DIABETES RESEARCH AND CLINICAL PRACTICE

LA English

DT Article

DE Diabetes-self management; Cognitive functions; Executive functions;

Depression

ID BLOOD-GLUCOSE CONTROL; GLYCEMIC CONTROL; MEDICATION ADHERENCE; MAJOR

DEPRESSION; CARE; MELLITUS; DYSFUNCTION; ADULTS; DISTRESS; SYMPTOMS

AB Aim: Patients with diabetes differ in compliance to diabetes self-management which influences their long-term health. Psychological factors, namely depression and cognitive abilities, are associated with diabetes self-management behavior. The aim of the study was to identify independent association of particular cognitive functions with diabetes self-management.

Methods: In a cross sectional study 98 adults with type 2 diabetes attending Diabetes Outpatient Clinic were examined using the measures of diabetes self-management (Summary of Diabetes Self-Care Activities (SDSCA) measure), depression (Hamilton Depression Inventory (HDI)), diabetes distress (Problem Areas In Diabetes scale (PAID)), and the neuropsychological battery of tests for assessment of cognitive functions. Sociodemographic and diabetes-related data were collected. Univariate and multivariate regression analyses were used to identify and evaluate the predictors of diabetes self-management.

Results: Specific cognitive functions, namely immediate memory, visuospatial/constructional abilities, attention, and specific executive functions (planning and problem solving) were significantly associated with diabetes self-management. Among cognitive factors, planning and problem solving abilities were strongest predictors; furthermore, in a multivariate regression their association was independent from depression.

Conclusions: Specific cognitive abilities, particularly planning and problem solving, play an independent role in diabetes self-management behaviors. Assessing patients' cognitive abilities may be of value for adjusting self-management education and treatment regimen. (C) 2011 Elsevier Ireland Ltd. All rights reserved.

C1 [Primozic, Simona; Avbelj, Magdalena] Univ Childrens Hosp, Univ Med Ctr Ljubljana, Dept Paediat Endocrinol Diabet & Metab, Ljubljana 1525, Slovenia.

[Tavcar, Rok; Dernovsek, Mojca Zvezdana] Univ Psychiat Hosp Ljubljana, Ljubljana, Slovenia.

[Oblak, Maja Ravnik] Univ Med Ctr Ljubljana, Dept Endocrinol Diabet & Metab Dis, Ljubljana, Slovenia.

RP Primozic, S (reprint author), Univ Childrens Hosp, Univ Med Ctr Ljubljana, Dept Paediat Endocrinol Diabet & Metab, Bohoriceva 20, Ljubljana 1525, Slovenia.

EM simona.primozic@kclj.si

OI Avbelj Stefanija, Magdalena/0000-0002-0836-5114

FU Slovenian Research Agency [L3-6359, 1000-07-310249]; Slovene National

Research Agency [P3-0343]

FX The study was supported by the Slovenian Research Agency (grant number

L3-6359). SP was a recipient of a young researcher stipend (grant number

1000-07-310249) awarded by the Slovenian Research Agency. MA is

supported in part by the Slovene National Research Agency (grant number

P3-0343).

CR Ali S, 2006, DIABETIC MED, V23, P1165, DOI 10.1111/j.1464-5491.2006.01943.x

Awad N, 2004, J CLIN EXP NEUROPSYC, V26, P1044, DOI 10.1080/13803390490514875

Brands AMA, 2007, J INT NEUROPSYCH SOC, V13, P288, DOI 10.1017/S1355617707070312

Culbertson W. C., 2005, TOWER LONDON DREXEL

Elderkin-Thompson V, 2003, ARCH CLIN NEUROPSYCH, V18, P529, DOI 10.1016/S0887-6177(03)00022-2

Faul F, 2009, BEHAV RES METHODS, V41, P1149, DOI 10.3758/BRM.41.4.1149

Fisher L, 2010, DIABETES CARE, V33, P1034, DOI 10.2337/dc09-2175

Glasgow RE, 2007, DIABETES CARE, V30, P33, DOI 10.2337/dc06-1390

Golden C. J., 2002, STROOP COLOR WORD TE

Gonzalez JS, 2008, DIABETIC MED, V25, P1102, DOI 10.1111/j.1464-5491.2008.02535.x

Gonzalez JS, 2008, DIABETOLOGIA, V51, P1822, DOI 10.1007/s00125-008-1113-x

Gonzalez JS, 2007, DIABETES CARE, V30, P2222, DOI 10.2337/dc07-0158

Gunstad J, 2007, COMPR PSYCHIAT, V48, P57, DOI 10.1016/j.comppsych.2006.05.001

Hebert JR, 1997, AM J EPIDEMIOL, V146, P1046

Kodl CT, 2008, ENDOCR REV, V29, P494, DOI 10.1210/er.2007-0034

Landro NI, 2001, NEUROPSY NEUROPSY BE, V14, P233

Legato Marianne J, 2006, Gend Med, V3, P131, DOI 10.1016/S1550-8579(06)80202-0

Lin EHB, 2004, DIABETES CARE, V27, P2154, DOI 10.2337/diacare.27.9.2154

Manschot SM, 2006, DIABETES, V55, P1106, DOI 10.2337/diabetes.55.04.06.db05-1323

McCollum Marianne, 2005, Gend Med, V2, P246, DOI 10.1016/S1550-8579(05)80054-3

McNally K, 2010, DIABETES CARE, V33, P1159, DOI 10.2337/dc09-2116

Nguyen HT, 2010, J AM GERIATR SOC, V58, P1123, DOI 10.1111/j.1532-5415.2010.02857.x

Patel A, 2008, NEW ENGL J MED, V358, P2560, DOI 10.1056/NEJMoa0802987

Randolph C., 1998, RBANS REPEATABLE BAT

Reynolds WM, 1995, HDI HAMILTON DEPRESS

Schmitt DP, 2008, J PERS SOC PSYCHOL, V94, P168, DOI 10.1037/0022-3514.94.1.168

Sinclair AJ, 2000, DIABETES RES CLIN PR, V50, P203, DOI 10.1016/S0168-8227(00)00195-9

SPSS: An IBM Company, 2009, PASW STAT 18 0 0

Thabit H, 2009, DIABETES RES CLIN PR, V86, P208, DOI 10.1016/j.diabres.2009.09.004

Toobert DJ, 2000, DIABETES CARE, V23, P943, DOI 10.2337/diacare.23.7.943

Turner RC, 1998, LANCET, V352, P837

van den Berg E, 2010, DIABETOLOGIA, V53, P58, DOI 10.1007/s00125-009-1571-9

van Harten B, 2007, AGE AGEING, V36, P164, DOI 10.1093/ageing/afl180

Weinger K, 2005, DIABETES CARE, V28, P1346, DOI 10.2337/diacare.28.6.1346

Welch GW, 1997, DIABETES CARE, V20, P760, DOI 10.2337/diacare.20.5.760

NR 35

TC 30

Z9 33

U1 0

U2 26

PU ELSEVIER IRELAND LTD

PI CLARE

PA ELSEVIER HOUSE, BROOKVALE PLAZA, EAST PARK SHANNON, CO, CLARE, 00000,

IRELAND

SN 0168-8227

J9 DIABETES RES CLIN PR

JI Diabetes Res. Clin. Pract.

PD JAN

PY 2012

VL 95

IS 1

BP 48

EP 54

DI 10.1016/j.diabres.2011.09.004

PG 7

WC Endocrinology & Metabolism

SC Endocrinology & Metabolism

GA 866ST

UT WOS:000298403900016

PM 21963107

DA 2019-01-08

ER

PT J

AU Whisman, MA

Uebelacker, LA

AF Whisman, Mark A.

Uebelacker, Lisa A.

TI A Longitudinal Investigation of Marital Adjustment as a Risk Factor for

Metabolic Syndrome

SO HEALTH PSYCHOLOGY

LA English

DT Article

DE satisfaction; distress; discord; biomarker; metabolic syndrome

ID DEPRESSIVE SYMPTOMS; OLDER-ADULTS; WOMEN; QUALITY; ASSOCIATIONS;

METAANALYSIS; SATISFACTION; CONFLICT; DISTRESS; BEHAVIOR

AB Objective: Metabolic syndrome (MetS) is a constellation of features-central obesity, dyslipidemia, elevated blood pressure, and dysglycemia-that are associated with increased risk for cardiovascular disease, diabetes, stroke, and mortality. This longitudinal study was conducted to evaluate whether marital adjustment, which has been shown to predict a variety of health outcomes, increases risk for incidence of MetS. Methods: A population-based English sample of couples (N = 216 couples) completed a self-report measure of marital adjustment at baseline and nurse visits that included collection of blood pressure, blood samples, and anthropometric measures at baseline and 4-year follow-up. Multilevel modeling was used to evaluate the association between both partners' report of baseline marital adjustment and incidence of MetS, controlling for other baseline risk factors (demographics, current and previous smoking status, alcohol use, activity level, depressive symptoms, number of MetS criteria). Results: Gender moderated the association between marital adjustment and incidence of MetS. Follow-up analyses indicated that husbands' report of marital adjustment at baseline was significantly associated with incident MetS for wives at follow-up, controlling for baseline risk factors. Marital adjustment was not significantly associated with incident MetS for men. Conclusions: Findings suggest that MetS may be one mechanism by which poor marital adjustment increases risk for poor health outcomes in women. Improving marital adjustment may help prevent the incidence of MetS and improve health, particularly for women.

C1 [Whisman, Mark A.] Univ Colorado, Dept Psychol & Neurosci, Boulder, CO 80309 USA.

[Uebelacker, Lisa A.] Brown Univ, Warren Alpert Med Sch, Dept Psychiat & Human Behav, Providence, RI 02912 USA.

[Uebelacker, Lisa A.] Butler Hosp, Dept Psychosocial Res, Providence, RI 02906 USA.

RP Whisman, MA (reprint author), Univ Colorado, Dept Psychol & Neurosci, 345 UCB, Boulder, CO 80309 USA.

EM mark.whisman@colorado.edu

FU National Institute of Aging in the United States; consortium of U.K.

government departments

FX The data were made available through the U.K. Data Archive. The English

Longitudinal Study of Ageing (ELSA) was developed by a team of

researchers based at the National Centre for Social Research, University

College London, and the Institute for Fiscal Studies. The data were

collected by the National Centre for Social Research. The funding is

provided by the National Institute of Aging in the United States and a

consortium of U.K. government departments coordinated by the Office for

National Statistics. The developers and funders of ELSA and the U.K.

Data Archive do not bear any responsibility for the analyses or

interpretations presented here.

CR ACTIVITY & HEALTH RESEARCH, 1992, ALL DUNB NAT FITN SU

Alberti KGMM, 2009, CIRCULATION, V120, P1640, DOI 10.1161/CIRCULATIONAHA.109.192644

Baker B, 2003, J PSYCHOSOM RES, V55, P229, DOI 10.1016/S0022-3999(02)00551-2

Beach SRH, 2003, J SOC PERS RELAT, V20, P355, DOI 10.1177/0265407503020003005

Brien SE, 2006, APPL PHYSIOL NUTR ME, V31, P40, DOI 10.1139/H05-024

Cornier MA, 2008, ENDOCR REV, V29, P777, DOI 10.1210/er.2008-0024

Coyne JC, 2001, AM J CARDIOL, V88, P526, DOI 10.1016/S0002-9149(01)01731-3

Cross SE, 1997, PSYCHOL BULL, V122, P5, DOI 10.1037//0033-2909.122.1.5

Ford ES, 2008, DIABETES CARE, V31, P1898, DOI 10.2337/dc08-0423

Forthofer MS, 1996, J MARRIAGE FAM, V58, P597, DOI 10.2307/353720

Galassi A, 2006, AM J MED, V119, P812, DOI 10.1016/j.amjmed.2006.02.031

Gallo LC, 2003, PSYCHOSOM MED, V65, P952, DOI 10.1097/01.PSY.0000097350.95305.FE

Gallo LC, 2003, HEALTH PSYCHOL, V22, P453, DOI 10.1037/0278-6133.22.5.453

Gami AS, 2007, J AM COLL CARDIOL, V49, P403, DOI 10.1016/j.jacc.2006.09.032

Grundy SM, 2005, CIRCULATION, V112, P2735, DOI 10.1161/CIRCULATIONAHA.105.169404

HUSTON TL, 1991, J PERS SOC PSYCHOL, V61, P721, DOI 10.1037/0022-3514.61.5.721

Kelley H. H., 1978, INTERPERSONAL RELATI

Kenny D. A., 2006, DYADIC DATA ANAL

Kiecolt-Glaser JK, 2001, PSYCHOL BULL, V127, P472, DOI 10.1037//0033-2909.127.4.472

KIECOLTGLASER JK, 1993, PSYCHOSOM MED, V55, P395, DOI 10.1097/00006842-199309000-00001

KiecoltGlaser JK, 1997, PSYCHOSOM MED, V59, P339, DOI 10.1097/00006842-199707000-00001

Li W, 2008, CEREBROVASC DIS, V25, P539, DOI 10.1159/000131672

Marmot M., 2010, ENGLISH LONGITUDINAL

McMahon JM, 2006, COMPUT STAT DATA AN, V50, P3663, DOI 10.1016/j.csda.2005.08.008

Orth-Gomer K, 2000, JAMA-J AM MED ASSOC, V284, P3008, DOI 10.1001/jama.284.23.3008

RADLOFF L S, 1977, Applied Psychological Measurement, V1, P385, DOI 10.1177/014662167700100306

Raikkonen K, 2002, METABOLISM, V51, P1573, DOI 10.1053/meta.2002.36301

SAS Institute, 2008, SAS 9 13 WIND

Steffick D. E, 2000, DR005 HRS SURV RES C

Troxel WM, 2005, ARCH INTERN MED, V165, P1022, DOI 10.1001/archinte.165.9.1022

Walen HR, 2000, J SOC PERS RELAT, V17, P5, DOI 10.1177/0265407500171001

Whisman MA, 2005, J FAM PSYCHOL, V19, P111, DOI 10.1037/0893-3200-19.1.111

Whisman MA, 2010, J FAM PSYCHOL, V24, P367, DOI 10.1037/a0019547

Whisman MA, 2009, PSYCHOL AGING, V24, P184, DOI 10.1037/a0014759

World Health Organization, 2008, GLOB BURD DIS 2004 U

NR 35

TC 28

Z9 28

U1 0

U2 12

PU AMER PSYCHOLOGICAL ASSOC

PI WASHINGTON

PA 750 FIRST ST NE, WASHINGTON, DC 20002-4242 USA

SN 0278-6133

J9 HEALTH PSYCHOL

JI Health Psychol.

PD JAN

PY 2012

VL 31

IS 1

BP 80

EP 86

DI 10.1037/a0025671

PG 7

WC Psychology, Clinical; Psychology

SC Psychology

GA 878MM

UT WOS:000299261200012

PM 22004463

DA 2019-01-08

ER

PT J

AU Ogbera, A

Adeyemi-Doro, A

AF Ogbera, Anthonia

Adeyemi-Doro, Adekunle

TI Emotional distress is associated with poor self care in type 2 diabetes

mellitus

SO JOURNAL OF DIABETES

LA English

DT Article

DE emotional distress; glycemic control; self-care

ID PSYCHOSOCIAL FACTORS; GLYCEMIC CONTROL; PREVALENCE; ADULTS

AB Background: The aim of the present study was to evaluate possible clinical and psychosocial variables that influence diabetes self-care management in patients with type 2 diabetes mellitus (T2DM).

Methods: A total of 150 individuals with T2DM who had had diabetes for at least 6 months were recruited to this cross-sectional study. Levels of self-care and psychosocial status were determined using the Self-Care Inventory (SCI) and Problem Areas in Diabetes (PAID) scale. The PAID scores were calculated using a five-point Likert scale with options ranging from 0 (not a problem) to 4 (serious problem). Data were evaluated using non-parametric and parametric tests as appropriate.

Results: The mean age of the study participants was 69.97 perpendicular to 8.68 years. Cronbach's alpha for SCI and PAID scores was 0.85 and 0.98, respectively. People with poor glycemic control had significantly higher mean (+/- SD) total PAID scores than individuals with good glycemic control (29.5 +/- 30.9 vs 16.7 +/- 26.9, respectively; P = 0.012). There was a significant relationship between PAID scores and glycemic control (r = 0.2; P = 0.012).

Conclusions: The present study demonstrates that psychosocial factors directly influence glycemic control and diabetes self-care habits. In addition, diabetes-specific distress in study population was unrelated to the duration of diabetes, the age of the patients and anthropometric indices.

C1 [Ogbera, Anthonia; Adeyemi-Doro, Adekunle] Lagos State Univ Teaching Hosp, Dept Med, Ikeja, Lagos, Nigeria.

RP Ogbera, A (reprint author), Structured Healthcare Initiat, POB 579, Ikeja, Lagos State, Nigeria.

EM oogbera@yahoo.co.uk

CR Aikens JE, 2009, DIABETES CARE, V32, P1177, DOI 10.2337/dc09-0071

Ali S, 2006, DIABETIC MED, V23, P1165, DOI 10.1111/j.1464-5491.2006.01943.x

Delahanty LM, 2007, DIABETIC MED, V24, P48, DOI 10.1111/j.1464-5491.2007.02028.x

Farmer AJ, 2003, DIABETIC MED, V20, P996, DOI 10.1046/j.1464-5491.2003.01042.x

Greco P., 1990, DIABETES S1, V40, pA165

Lerman I, 2004, BIOMED PHARMACOTHER, V58, P566, DOI 10.1016/j.biopha.2004.09.003

Miller S. T., 2008, BMC PUBLIC HEALTH, V8, P70

Mooy JM, 2000, DIABETES CARE, V23, P197, DOI 10.2337/diacare.23.2.197

Nakahara R, 2006, PSYCHOSOMATICS, V47, P240, DOI 10.1176/appi.psy.47.3.240

Ogbera A O, 2010, West Afr J Med, V29, P158

Papelbaum M, 2010, DIABETES RES CLIN PR, V89, P227, DOI 10.1016/j.diabres.2010.05.024

POLONSKY WH, 1995, DIABETES CARE, V18, P754, DOI 10.2337/diacare.18.6.754

Snoek FJ, 2000, DIABETES CARE, V23, P1305, DOI 10.2337/diacare.23.9.1305

Spencer MS, 2006, J HEALTH CARE POOR U, V17, P88, DOI 10.1353/hpu.2006.0095

Thoolen BJ, 2006, DIABETES CARE, V29, P2257, DOI 10.2337/dc06-0617

Weinger K, 2005, DIABETES CARE, V28, P1346, DOI 10.2337/diacare.28.6.1346

NR 16

TC 26

Z9 27

U1 1

U2 7

PU WILEY-BLACKWELL

PI HOBOKEN

PA 111 RIVER ST, HOBOKEN 07030-5774, NJ USA

SN 1753-0393

J9 J DIABETES

JI J. Diabetes

PD DEC

PY 2011

VL 3

IS 4

BP 348

EP 352

DI 10.1111/j.1753-0407.2011.00156.x

PG 5

WC Endocrinology & Metabolism

SC Endocrinology & Metabolism

GA 982SJ

UT WOS:000307065400014

PM 21883978

DA 2019-01-08

ER

PT J

AU Wang, RH

Wu, LC

Hsu, HY

AF Wang, Ruey-Hsia

Wu, Li-Chu

Hsu, Hsiu-Yueh

TI A path model of health-related quality of life in Type 2 diabetic

patients: a cross-sectional study in Taiwan

SO JOURNAL OF ADVANCED NURSING

LA English

DT Article

DE health-related quality of life; nursing; path model; structural equation

modelling; Type 2 diabetes

ID METABOLIC-CONTROL; SELF-CARE; EMPOWERMENT; DEPRESSION; DISTRESS; ADULTS;

OUTCOMES; POPULATION; PROGRAM; VERSION

AB Aims. To construct a path model about relationships of perceptions of empowerment, diabetes distress, self-care behaviour and glycemic control to health-related quality of life in Taiwanese Type 2 diabetic patients, suffering from diabetes for 10 years or less. Background. Health-related quality of life is the primary end point for people with diabetes. Understanding the path model of health-related quality of life in Type 2 diabetic patients is useful for nurses to design early intervention programmes. Methods. This was a cross-sectional study. An anonymous questionnaire was used to collect data from 2007 to 2008. Glycosylated haemoglobin (HbA1c) was also collected. Data from 428 Type 2 diabetic patients were analysed with structural equation modelling to test the fit of the hypothesized path model to the data. Results. A model was produced in which self-care behaviour had a statistically significantly direct influence on satisfaction, impact and worry aspects of health-related quality of life. Glycosylated haemoglobin had a statistically significantly negative influence on satisfaction, and impact aspects of health-related quality of life. Perceptions of empowerment had a statistically significantly direct influence on satisfaction aspect of health-related quality of life. Diabetes distress had a statistically significantly direct influence on satisfaction, impact and worry aspects of health-related quality of life. Conclusions. Nurses should develop new approaches to improve various aspects of health-related quality of life. Nurses could empower patients to improve satisfaction aspect of health-related quality of life. To improve the impact and worry aspects of health-related quality of life, nurses should reduce diabetes distress felt by patients.

C1 [Wang, Ruey-Hsia] Kaohsiung Med Univ, Coll Nursing, Kaohsiung, Taiwan.

[Wu, Li-Chu] Kaohsiung Vet Gen Hosp, Kaohsiung, Taiwan.

[Hsu, Hsiu-Yueh] Meiho Inst Technol, Dept Nursing, Pingtung, Taiwan.

RP Wang, RH (reprint author), Kaohsiung Med Univ, Coll Nursing, Kaohsiung, Taiwan.

EM wrhsia@kmu.edu.tw

FU National Science Council, Taiwan [NSC 96-2314-B-037-075-MY2]

FX Thanks to the National Science Council, Taiwan for funding this study

(NSC 96-2314-B-037-075-MY2).

CR American Diabetes Association, 2010, DIAB BAS DIAB STAT

Anderson RJ, 2000, DIABETES, V49, pA64

ANDERSON RM, 1995, DIABETES CARE, V18, P943, DOI 10.2337/diacare.18.7.943

Anderson RM, 2010, PATIENT EDUC COUNS, V79, P277, DOI 10.1016/j.pec.2009.07.025

Bennett WL, 2008, HEALTH QUAL LIFE OUT, V6, DOI 10.1186/1477-7525-6-110

Black SA, 2003, DIABETES CARE, V26, P2822, DOI 10.2337/diacare.26.10.2822

Brown G.C., 2004, J DIABETES COMPLICAT, V14, P18

Bureau of Health Promotion, 2009, AD ELD HLTH

El Achhab Y, 2008, DIABETES RES CLIN PR, V80, P171, DOI 10.1016/j.diabres.2007.12.020

Fisher L, 2008, ANN FAM MED, V6, P246, DOI 10.1370/afm.842

Fisher L, 2007, DIABETES CARE, V30, P542, DOI 10.2337/dc06-1614

Forlani G, 2006, J ENDOCRINOL INVEST, V29, P405, DOI 10.1007/BF03344123

Funnell Martha Mitchell, 2009, Nursing, V39, P34, DOI 10.1097/01.NURSE.0000347073.76891.2a

Gross CC, 2007, DIABETES RES CLIN PR, V76, P455, DOI 10.1016/j.diabres.2006.09.022

Hu LT, 1999, STRUCT EQU MODELING, V6, P1, DOI 10.1080/10705519909540118

Huang IC, 2008, HEALTH QUAL LIFE OUT, V6, DOI 10.1186/1477-7525-6-87

Huang MF, 2010, INT J NURS STUD, V47, P542, DOI 10.1016/j.ijnurstu.2009.10.012

Jermendy G, 2008, HEALTH QUAL LIFE OUT, V6, DOI 10.1186/1477-7525-6-88

Karlsen B, 2004, PATIENT EDUC COUNS, V53, P299, DOI 10.1016/j.pec.2003.10.008

Li CY, 2007, DIABETES CARE, V30, P1770, DOI 10.2337/dc06-2571

Lin C. C, 2005, THESIS STATE U MICHI

Lustman PJ, 2005, J DIABETES COMPLICAT, V19, P113, DOI 10.1016/j.jdiacomp.2004.01.002

Maddigan SL, 2005, QUAL LIFE RES, V14, P1489, DOI 10.1007/s11136-005-0586-z

Nuttall F Q, 1998, J Fam Pract, V47, pS45

Papadopoulos AA, 2007, BMC PUBLIC HEALTH, V7, DOI 10.1186/1471-2458-7-186

Pibernik-Okanovic M, 2004, PATIENT EDUC COUNS, V52, P193, DOI 10.1016/S0738-3991(03)00038-7

Polit DF, 2004, NURSING RES PRINCIPL

Polonsky WH, 2005, DIABETES CARE, V28, P626, DOI 10.2337/diacare.28.3.626

Rodwell CM, 1996, J ADV NURS, V23, P305, DOI 10.1111/j.1365-2648.1996.tb02672.x

Saito I, 2006, DIABETES RES CLIN PR, V73, P51, DOI 10.1016/j.diabres.2005.11.015

Samuel-Hodge CD, 2008, DIABETES EDUCATOR, V34, P501, DOI 10.1177/0145721708316946

Schumacker R. E., 1996, BEGINNERS GUIDE STRU

Sigurthardottir AK, 2005, J CLIN NURS, V14, P301, DOI 10.1111/j.1365-2702.2004.01043.x

Tengland Per-Anders, 2007, Med Health Care Philos, V10, P197, DOI 10.1007/s11019-006-9027-1

Wang JX, 1998, J NURSING, V45, P60

Watkins KW, 2000, DIABETES CARE, V23, P1511, DOI 10.2337/diacare.23.10.1511

*WHO, 2008, DIAB

NR 37

TC 18

Z9 18

U1 2

U2 16

PU WILEY

PI HOBOKEN

PA 111 RIVER ST, HOBOKEN 07030-5774, NJ USA

SN 0309-2402

EI 1365-2648

J9 J ADV NURS

JI J. Adv. Nurs.

PD DEC

PY 2011

VL 67

IS 12

BP 2658

EP 2667

DI 10.1111/j.1365-2648.2011.05701.x

PG 10

WC Nursing

SC Nursing

GA 858XX

UT WOS:000297837700016

PM 21615464

DA 2019-01-08

ER

PT J

AU Franc, S

Daoudi, A

Mounier, S

Boucherie, B

Laroye, H

Peschard, C

Dardari, D

Juy, O

Requeda, E

Canipel, L

Charpentier, G

AF Franc, S.

Daoudi, A.

Mounier, S.

Boucherie, B.

Laroye, H.

Peschard, C.

Dardari, D.

Juy, O.

Requeda, E.

Canipel, L.

Charpentier, G.

TI Telemedicine: What more is needed for its integration in everyday life?

SO DIABETES & METABOLISM

LA English

DT Article

DE Telemedicine; Type 1 and type 2 diabetes; Medical device; Smart-phone;

Education; Organization of healthcare; Review

ID RANDOMIZED CONTROLLED-TRIAL; GLUCOSE MONITORING-SYSTEM;

DIABETIC-PATIENTS; GLYCEMIC CONTROL; INTERNET; SUPPORT; PHONE; CARE

AB The Health Authorities have huge expectations of telemedicine (TM): improved patient access to healthcare, a solution to the shortage of doctors in the face of an exponentially expanding disease, and reduced healthcare costs with improved quality.

There are a host of applications for TM in the area of diabetes. TM has been validated and has been widely used to screen for diabetic retinopathy, and a number of studies are currently underway for the follow-up of diabetic foot ulcers. However, the main indication of TM remains the follow-up and control of blood glucose. In this area, many studies have been conducted to improve glycaemic control. While most of these studies have failed to show any benefits vs. conventional care, a small number have demonstrated great efficacy of this approach with regard to glycaemia. Using these studies, we attempt to define the key qualities of a successful TM system.

How can we extend the results of these experiments beyond the framework of clinical studies and integrate them in daily practice so as to improve diabetes management?

This is the key challenge for TM, implementation of which will require reorganization of healthcare, given the evolution of medical demographics.

This reorganization will involve healthcare providers specialized in diabetes that may intervene in assigning physicians for especially distressed patients. However, such reorganization will require medico-economic evaluation before it can be implemented on a larger scale. (C) 2011 Elsevier Masson SAS. All rights reserved.

C1 [Franc, S.; Daoudi, A.; Mounier, S.; Boucherie, B.; Laroye, H.; Peschard, C.; Dardari, D.; Juy, O.; Canipel, L.; Charpentier, G.] CERITD, Corbeil Essonnes, France.

[Franc, S.; Requeda, E.; Charpentier, G.] Sud Francilien Hosp, Dept Diabet, Corbeil Essonnes, France.

RP Franc, S (reprint author), CERITD, Corbeil Essonnes, France.

EM sylvia.franc@free.fr

RI laroye, heloise/I-8082-2017

CR [Anonymous], NCT00937703

[Anonymous], NCT01192711 INS ADM

Benhamou PY, 2007, DIABETES METAB, V33, P220, DOI 10.1016/j.diabet.2007.01.002

Chabouis A, 2009, DIABETES METAB, V35, P228, DOI 10.1016/j.diabet.2008.12.001

Charpentier G, 2011, DIABETES CARE, V34, P533, DOI 10.2337/dc10-1259

Cho JH, 2006, DIABETES CARE, V29, P2625, DOI 10.2337/dc05-2371

Dardari D.FrancS.BoucherieB, 2006, DIABETES METAB S, V32, p1S73

Farmer AJ, 2005, DIABETES CARE, V28, P269

Franc S, 2009, DIABETES METAB, V35, P463, DOI 10.1016/j.diabet.2009.05.006

Franc S, 2011, DIABETES METAB 0831

Franc S, 2010, DIABETES CARE, V33, P1913, DOI 10.2337/dc10-0115

Kwon HS, 2004, DIABETES CARE, V27, P478, DOI 10.2337/diacare.27.2.478

Legmann M, TELEMEDECINE PRECONI

Lim S, 2011, DIABETES CARE, V34, P308, DOI 10.2337/dc10-1447

Massin P, 2008, DIABETES METAB, V34, P227, DOI 10.1016/j.diabet.2007.12.006

Petrovski G, 2011, INTERNET VISITS IMPR

Piette JD, 2000, AM J MED, V108, P20, DOI 10.1016/S0002-9343(99)00298-3

Quinn CC, 2008, DIABETES TECHNOL THE, V10, P160, DOI 10.1089/dia.2008.0283

Quinn CC, 2011, DIABETES CARE, V34, P1934, DOI 10.2337/dc11-0366

Rigla Mercedes, 2007, J Diabetes Sci Technol, V1, P505

Rossi MCE, 2010, DIABETES CARE, V33, P109, DOI 10.2337/dc09-1327

Rossi MCE, 2009, DIABETES TECHNOL THE, V11, P19, DOI 10.1089/dia.2008.0020

Rossi MCE, 2010, EUROPEAN ENDOCRINOLO, V6, P39

SHAMOON H, 1993, NEW ENGL J MED, V329, P977

Simon P, PLACE TELEMEDICINE O

Thompson DM, 1999, CAN MED ASSOC J, V161, P959

Verhoeven Fenne, 2010, J Diabetes Sci Technol, V4, P666

Young RJ, 2005, DIABETES CARE, V28, P278, DOI 10.2337/diacare.28.2.278

NR 28

TC 6

Z9 6

U1 2

U2 21

PU MASSON EDITEUR

PI MOULINEAUX CEDEX 9

PA 21 STREET CAMILLE DESMOULINS, ISSY, 92789 MOULINEAUX CEDEX 9, FRANCE

SN 1262-3636

J9 DIABETES METAB

JI Diabetes Metab.

PD DEC

PY 2011

VL 37

SI 4

BP S71

EP S77

DI 10.1016/S1262-3636(11)70969-7

PG 7

WC Endocrinology & Metabolism

SC Endocrinology & Metabolism

GA 872UH

UT WOS:000298834700007

PM 22208714

DA 2019-01-08

ER

PT J

AU Rzeszut, JR

AF Rzeszut, Jennifer R.

TI Children With Diabetes: The Impact of Fear of Needles

SO JOURNAL OF PEDIATRIC NURSING-NURSING CARE OF CHILDREN & FAMILIES

LA English

DT Article

ID DISTRESS

C1 Univ Penn, Philadelphia, PA 19104 USA.

RP Rzeszut, JR (reprint author), Univ Penn, Philadelphia, PA 19104 USA.

EM jeni.rzeszut@gmail.com

CR Dahlquist Lynnda M, 2002, J Pediatr Oncol Nurs, V19, P22, DOI 10.1053/jpon.2002.30009

Deacon B, 2006, J ANXIETY DISORD, V20, P946, DOI 10.1016/j.janxdis.2006.01.004

FASSLER D, 1985, AM J ORTHOPSYCHIAT, V55, P371, DOI 10.1111/j.1939-0025.1985.tb03452.x

Hanas R, 1997, PRACTICAL DIABETES, V14, P95, DOI DOI 10.1002/PDI.1960140404

Howe CJ, 2010, AM J MATERN CHILD NU, V36, P25

HUMPHREY GB, 1992, PEDIATRICS, V90, P87

MOORE KE, 1995, CLIN PEDIATR, V34, P530, DOI 10.1177/000992289503401004

RAINWATER N, 1988, CHILD FAM BEHAV THER, V10, P19

Simmons JH, 2007, DIABETES CARE, V30, P987, DOI 10.2337/dc06-1553

Sparks L, 2001, MCN Am J Matern Child Nurs, V26, P72, DOI 10.1097/00005721-200103000-00005

Walco Gary A, 2008, Pediatrics, V122 Suppl 3, pS125, DOI 10.1542/peds.2008-1055d

NR 11

TC 6

Z9 6

U1 0

U2 6

PU ELSEVIER SCIENCE INC

PI NEW YORK

PA 360 PARK AVE SOUTH, NEW YORK, NY 10010-1710 USA

SN 0882-5963

J9 J PEDIATR NURS

JI J. Pediatr. Nurs.

PD DEC

PY 2011

VL 26

IS 6

BP 589

EP 592

DI 10.1016/j.pedn.2011.09.003

PG 4

WC Nursing; Pediatrics

SC Nursing; Pediatrics

GA 045OI

UT WOS:000311705800011

PM 21925589

DA 2019-01-08

ER

PT J

AU Jaser, SS

AF Jaser, Sarah S.

TI Family Interaction in Pediatric Diabetes

SO CURRENT DIABETES REPORTS

LA English

DT Article

DE Family relations; Type 1 diabetes mellitus; Adaptation; Psychological

ID QUALITY-OF-LIFE; METABOLIC-CONTROL; GLYCEMIC CONTROL; MATERNAL

INVOLVEMENT; DEPRESSIVE SYMPTOMS; SYSTEMS THERAPY; YOUNG-CHILDREN;

RISK-FACTORS; TYPE-1; ADHERENCE

AB Adaptation to type 1 diabetes is optimized in the presence of ongoing family support and supervision. Therefore, it is particularly important to understand how family interactions influence adaptation to the illness. The purpose of this paper is to review the current literature on family interaction in youth with type 1 diabetes. Recent advancements in the literature include greater specificity of types of parental involvement, attention to the role of fathers, acknowledgment of the impact of parental distress, increased use of observational methods, and awareness of the impact of culture. Continued parental involvement-particularly monitoring-in the management of diabetes care is important as children transition into adolescence, and the best outcomes are evident when this involvement occurs in a warm, collaborative manner. Parents need support in managing their own distress to maintain this type of involvement.

C1 Yale Univ, Sch Nursing, New Haven, CT 06536 USA.

RP Jaser, SS (reprint author), Yale Univ, Sch Nursing, 100 Church St S,POB 9740, New Haven, CT 06536 USA.

EM sarah.jaser@yale.edu

FU National Institute of Diabetes and Digestive and Kidney Diseases [K23

NK088454]; National Center for Research Resources [UL1 RR024139]

FX This work was supported by awards from the National Institute of

Diabetes and Digestive and Kidney Diseases (K23 NK088454) and the

National Center for Research Resources (UL1 RR024139). The contents of

this manuscript are solely the responsibility of the author and do not

necessarily represent the official view of the US National Institutes of

Health.

CR American Diabetes Association (ADA), 2011, DIABETES CARE S1, V34, pS11, DOI DOI 10.2337/DC11-S011

Anderson BJ, 2007, DIS MANAG HEALTH OUT, V15, P101, DOI 10.2165/00115677-200715020-00005

Anderson BJ, 2002, DIABETIC MED, V19, P635, DOI 10.1046/j.1464-5491.2002.00752.x

Anderson BJ, 1999, DIABETES CARE, V22, P713, DOI 10.2337/diacare.22.5.713

Berg CA, 2008, DIABETES CARE, V31, P678, DOI 10.2337/dc07-1678

Cameron FJ, 2008, DIABETIC MED, V25, P463, DOI 10.1111/j.1464-5491.2008.02399.x

Cameron FJ, 2007, DIABETES CARE, V30, P2716, DOI 10.2337/dc07-0603

Cameron LD, 2007, J PEDIATR PSYCHOL, V32, P733, DOI 10.1093/jpepsy/jslo53

Cohen DM, 2004, J PEDIATR PSYCHOL, V29, P171, DOI 10.1093/jpepsy/jsh019

Delamater AM, 1999, DIABETES CARE, V22, P700, DOI 10.2337/diacare.22.5.700

Drotar D, 1997, J PEDIATR PSYCHOL, V22, P149

Ellis DA, 2008, J ADOLESCENT HEALTH, V42, P146, DOI 10.1016/j.jadohealth.2007.08.012

Ellis DA, 2007, J PEDIATR PSYCHOL, V32, P907, DOI 10.1093/jpepsy/jsm009

Faulkner Melissa Spezia, 2007, J Pediatr Nurs, V22, P59, DOI 10.1016/j.pedn.2006.02.008

Gale EAM, 2002, DIABETES, V51, P3353, DOI 10.2337/diabetes.51.12.3353

Gallegos-Macias Angela R, 2003, Pediatr Diabetes, V4, P19, DOI 10.1034/j.1399-5448.2003.00020.x

Grey M, 2000, J PEDIATR-US, V137, P107, DOI 10.1067/mpd.2000.106568

Grey M, 1998, DIABETES CARE, V21, P909, DOI 10.2337/diacare.21.6.909

Grey M, 2009, RES NURS HEALTH, V32, P405, DOI 10.1002/nur.20336

HEGELSON VS, 2009, J PEDIATR PSYCHOL, V34, P254

Hood KK, 2006, DIABETES CARE, V29, P1389, DOI 10.2337/dc06-0087

Horton D, 2009, J PEDIATR PSYCHOL, V34, P1008, DOI 10.1093/jpepsy/jsp022

Hsin O, 2010, J PEDIATR PSYCHOL, V35, P156, DOI 10.1093/jpepsy/jsp045

JACOBSON AM, 1994, PSYCHOSOM MED, V56, P401, DOI 10.1097/00006842-199409000-00004

Jaser SS, 2008, J PEDIATR PSYCHOL, V33, P509, DOI 10.1093/jpepsy/jsm104

Jaser SS, 2010, J PEDIATR PSYCHOL, V35, P738, DOI 10.1093/jpepsy/jsp098

Kokkonen J, 1997, NORD J PSYCHIAT, V51, P165, DOI 10.3109/08039489709109091

Kovacs M, 1997, DIABETES CARE, V20, P36, DOI 10.2337/diacare.20.1.36

Laffel LMB, 2003, DIABETES CARE, V26, P3067, DOI 10.2337/diacare.26.11.3067

Lewin AB, 2006, J PEDIATR PSYCHOL, V31, P174, DOI 10.1093/jpepsy/jsj004

Martin MT, 1998, J FAM PSYCHOL, V12, P102, DOI 10.1037/0893-3200.12.1.102

Palmer DL, 2004, J PEDIATR PSYCHOL, V29, P35, DOI 10.1093/jpepsy/jsh005

Patton SR, 2008, J CLIN PSYCHOL MED S, V15, P252, DOI 10.1007/s10880-008-9123-x

Patton SR, 2008, J PEDIATR PSYCHOL, V33, P885, DOI 10.1093/jpepsy/jsn026

Piazza-Waggoner C, 2008, J DEV BEHAV PEDIATR, V29, P101, DOI 10.1097/DBP.0b013e31815f24ce

SIEFFGEKRENKE I, 2002, J PEDIATR PSYCHOL, V27, P439

SILVERSTEIN J, 2005, DIABETES CARE, V28, P2005

Streisand R, 2005, J PEDIATR PSYCHOL, V30, P513, DOI 10.1093/jpepsy/jsi076

Swift EE, 2006, ANN BEHAV MED, V32, P39, DOI 10.1207/s15324796abm3201_5

Weinger K, 2001, J ADOLESCENT HEALTH, V29, P330, DOI 10.1016/S1054-139X(01)00270-1

Weissberg-Benchell J, 2009, J PEDIATR PSYCHOL, V34, P977, DOI 10.1093/jpepsy/jsp003

Whittemore R, 2003, DIABETES EDUCATOR, V29, P847, DOI 10.1177/014572170302900514

Whittemore R, 2004, EVIDENCE BASED PRACT

Wiebe DJ, 2005, J PEDIATR PSYCHOL, V30, P167, DOI 10.1093/jpepsy/jsi004

Williams LB, 2009, DIABETIC MED, V26, P908, DOI 10.1111/j.1464-5491.2009.02794.x

Wysocki T, 2006, J PEDIATR PSYCHOL, V31, P501, DOI 10.1093/jpepsy/jsj042

Wysocki T, 2008, BEHAV THER, V39, P33, DOI 10.1016/j.beth.2007.04.001

Wysocki T, 2007, DIABETES CARE, V30, P555, DOI 10.2337/dc06-1613

NR 48

TC 14

Z9 15

U1 0

U2 23

PU CURRENT MEDICINE GROUP

PI PHILADELPHIA

PA 400 MARKET STREET, STE 700, PHILADELPHIA, PA 19106 USA

SN 1534-4827

J9 CURR DIABETES REP

JI Curr. Diabetes Rep.

PD DEC

PY 2011

VL 11

IS 6

BP 480

EP 485

DI 10.1007/s11892-011-0222-y

PG 6

WC Endocrinology & Metabolism

SC Endocrinology & Metabolism

GA 841LA

UT WOS:000296512600005

PM 21853415

OA Green Accepted

DA 2019-01-08

ER

PT J

AU Amparo, P

Farr, SL

Dietz, PM

AF Amparo, Pamela

Farr, Sherry L.

Dietz, Patricia M.

TI Chronic Disease Risk Factors Among American Indian/Alaska Native Women

of Reproductive Age

SO PREVENTING CHRONIC DISEASE

LA English

DT Article

ID MATERNAL OBESITY; DIABETES-MELLITUS; HEALTH BEHAVIORS; PREGNANCY;

METAANALYSIS; ADULTS

AB Introduction

The magnitude of chronic conditions and risk factors among American Indian/Alaska Native women of reproductive age is unknown. The objective of our study was to estimate this magnitude.

Methods

We analyzed data for 2,821 American Indian/Alaska Native women and 105,664 non-Hispanic white women aged 18 to 44 years from the 2005 and 2007 Behavioral Risk Factor Surveillance System. We examined prevalence of high cholesterol, high blood pressure, diabetes, body mass index (kg/m(2)) >= 25.0, physical inactivity, smoking, excessive alcohol consumption, and frequent mental distress, and the cumulative number of these chronic conditions and risk factors (>= 3, 2, 1, or 0). In a multivariable, multinomial logistic regression model, we examined whether American Indian/Alaska Native race was associated with the cumulative number of chronic conditions and risk factors.

Results

American Indian/Alaska Native women, compared with white women, had significantly higher rates of high blood pressure, diabetes, obesity, smoking, and frequent mental distress. Of American Indian/Alaska Native women, 41% had 3 or more chronic conditions or risk factors compared with 27% of white women (chi(2), P < .001). After adjustment for income, education, and other demographic variables, American Indian/Alaska Native race was not associated with having either 1, 2, or 3 or more chronic conditions or risk factors.

Conclusion

Three out of every 5 American Indian/Alaska Native women aged 18 to 44 years have 3 or more chronic conditions or risk factors. Improving economic status and education for AI/AN women could help eliminate disparities in health status.

C1 [Dietz, Patricia M.] Ctr Dis Control & Prevent, Div Reprod Hlth, Natl Ctr Chron Dis Prevent & Hlth Promot, Atlanta, GA 30341 USA.

RP Dietz, PM (reprint author), Ctr Dis Control & Prevent, Div Reprod Hlth, Natl Ctr Chron Dis Prevent & Hlth Promot, MS K-22,4770 Buford Hwy NE, Atlanta, GA 30341 USA.

EM PDietz@cdc.gov

CR Arendas K, 2008, J OBSTET GYNAECOL CA, V30, P477, DOI 10.1016/S1701-2163(16)32863-8

Balsells M, 2009, J CLIN ENDOCR METAB, V94, P4284, DOI 10.1210/jc.2009-1231

Barnes PJ, 2010, PROG RESPIR RES, V39, P1

Blumberg SJ, 2009, AM J PUBLIC HEALTH, V99, P1806, DOI 10.2105/AJPH.2008.152835

Castro LC, 2002, CURR OPIN OBSTET GYN, V14, P601, DOI 10.1097/01.gco.0000045486.15021.c9

Centers for Disease Control and Prevention (CDC), 2003, MMWR Morb Mortal Wkly Rep, V52, P1148

Centers for Disease Control and Prevention (CDC), 2004, MMWR-MORBID MORTAL W, V53, P121

Centers for Disease Control and Prevention (CDC), 2003, MMWR-MORBID MORTAL W, V52, P702

Chu SY, 2007, DIABETES CARE, V30, P2070, DOI 10.2337/dc06-2559a

Doshi SR, 2006, J WOMENS HEALTH, V15, P919, DOI 10.1089/jwh.2006.15.919

Fahimi M, 2008, PREV CHRONIC DIS, V5

Frost JJ, 2008, AM J PUBLIC HEALTH, V98, P1814, DOI 10.2105/AJPH.2007.124719

Giuliano A, 1998, ANN EPIDEMIOL, V8, P160, DOI 10.1016/S1047-2797(97)00200-7

Gold RB, 2009, NEXT STEPS AM FAMILY, P14

Haskell WL, 2007, CIRCULATION, V116, P1081, DOI 10.1161/CIRCULATIONAHA.107.185649

Indian Health Service, 2011, IHS FACT SHEETS IND

Jernigan VBB, 2010, AM J PUBLIC HEALTH, V100, P677, DOI 10.2105/AJPH.2009.164285

Little J, 2004, B WORLD HEALTH ORGAN, V82, P213

Livingston J C, 2003, Minerva Ginecol, V55, P1

Lykke JA, 2009, HYPERTENSION, V53, P944, DOI 10.1161/HYPERTENSIONAHA.109.130765

NCEP Executive, 2001, JAMA-J AM MED ASSOC, V285, P2486, DOI DOI 10.1001/JAMA.285.19.2486

Nelson DE, 2003, AM J PUBLIC HEALTH, V93, P1335, DOI 10.2105/AJPH.93.8.1335

Newschaffer CJ, 1998, VALIDATION BEHAV RIS

Oken E, 2009, OBSTET GYN CLIN N AM, V36, P361, DOI 10.1016/j.ogc.2009.03.007

Rasmussen SA, 2008, AM J OBSTET GYNECOL, V198, P611, DOI 10.1016/j.ajog.2008.04.021

Taylor R, 2007, BMJ-BRIT MED J, V334, P742, DOI 10.1136/bmj.39154.700417.BE

US Department of Health and Human Services, 2004, HLTH CONS SMOK REP S

Zuckerman S, 2004, AM J PUBLIC HEALTH, V94, P53, DOI 10.2105/AJPH.94.1.53

NR 28

TC 17

Z9 17

U1 0

U2 2

PU CENTERS DISEASE CONTROL

PI ATLANTA

PA 1600 CLIFTON RD, ATLANTA, GA 30333 USA

SN 1545-1151

J9 PREV CHRONIC DIS

JI Prev. Chronic Dis.

PD NOV

PY 2011

VL 8

IS 6

AR A118

PG 10

WC Public, Environmental & Occupational Health

SC Public, Environmental & Occupational Health

GA 896JZ

UT WOS:000300563000001

PM 22005611

DA 2019-01-08

ER

PT J

AU Hessler, DM

Fisher, L

Mullan, JT

Glasgow, RE

Masharani, U

AF Hessler, Danielle M.

Fisher, Lawrence

Mullan, Joseph T.

Glasgow, Russell E.

Masharani, Umesh

TI Patient age: A neglected factor when considering disease management in

adults with type 2 diabetes

SO PATIENT EDUCATION AND COUNSELING

LA English

DT Article

DE Age; Type 2 diabetes; HbA1c; Psychosocial stressors

ID MELLITUS; SCALE; CARE

AB Objective: The average age at diagnosis for type 2 diabetes is decreasing. However, because age is most often controlled for in clinical research, little is known regarding how adult age is associated with diabetes disease-related variables.

Methods: In a community based study with type 2 diabetes patients (N = 506), after adjusting for potentially confounding variables, we examined associations between patients' age and: stress, depression, diabetes-related distress, self-efficacy, diet, exercise, and glycemic control. We then explored to what extent age interacts with these variables in their association with glycemic control.

Results: Younger age was independently associated with: greater chronic stress and negative life events, higher levels of diabetes-related distress, higher depressed affect, eating healthier foods and exercising less frequently, lower diabetes self-efficacy, and higher HbA1c. Interactions showed that younger patients with high stress and/or low self-efficacy were more likely to have higher HbA1c levels than older patients.

Conclusions: Results suggest younger adult patients with type 2 diabetes represent a unique patient subgroup with specific needs and health risks based on their developmental stage and life context.

Practice implications: Treatment programs need to target younger adult patients and may need to utilize different Media or modalities (e.g., social media) to reach this group. (C) 2011 Elsevier Ireland Ltd. All rights reserved.

C1 [Hessler, Danielle M.; Fisher, Lawrence] Univ Calif San Francisco, Dept Family & Community Med, San Francisco, CA 94143 USA.

[Mullan, Joseph T.] Univ Calif San Francisco, Dept Social & Behav Sci, Sch Nursing, San Francisco, CA 94143 USA.

[Glasgow, Russell E.] Kaiser Permanente, Inst Hlth Res, Denver, CO USA.

[Masharani, Umesh] Univ Calif San Francisco, Dept Med, San Francisco, CA 94143 USA.

RP Hessler, DM (reprint author), Univ Calif San Francisco, Dept Family & Community Med, Box 0900, San Francisco, CA 94143 USA.

EM hesslerd@fcm.ucsf.edu

FU National Institute of Diabetes, Digestive and Kidney Disease [DK062732,

DK061937]

FX This research was supported by Grants DK062732 and DK061937 from the

National Institute of Diabetes, Digestive and Kidney Disease. The

following medical groups and diabetes education centers collaborated in

this research: Alta Bates Diabetes Education Center, Brown and Toland

Medical Group, California Pacific Diabetes Education Center, Hill

Physicians Medical Group, Mann IPA, St. Luke's Diabetes Education

Center, St. Mary's Medical Center, University of California, San

Francisco Hospital and Clinics. Declaration of Competing Interests:

Nothing to declare.

CR Aiken L. S, 1991, MULTIPLE REGRESSION

Campbell JL, 2001, QUAL HEALTH CARE, V10, P90, DOI 10.1136/qhc.10.2.90

Centers for Disease Control and Prevention, 2008, DAT NAT HLTH INT SUR

CENTERS FOR DISEASE CONTROL AND PREVENTION, 2008, NAT DIAB FACT SHEET

Fisher L, 2000, DIABETES CARE, V23, P267, DOI 10.2337/diacare.23.3.267

Gilliland SS, 2002, DIABETES CARE, V25, P2178, DOI 10.2337/diacare.25.12.2178

Koopman RJ, 2005, ANN FAM MED, V3, P60, DOI 10.1370/afm.214

Li JKY, 2006, DIABETES-METAB RES, V22, P46, DOI 10.1002/dmrr.577

PALINKAS LA, 1991, DIABETIC MED, V8, P532, DOI 10.1111/j.1464-5491.1991.tb01646.x

Polonsky WH, 2005, DIABETES CARE, V28, P626, DOI 10.2337/diacare.28.3.626

RADLOFF L S, 1977, Applied Psychological Measurement, V1, P385, DOI 10.1177/014662167700100306

Scott KM, 2008, PSYCHOL MED, V38, P1659, DOI 10.1017/S0033291708003413

Sherbourne C D, 1992, Qual Life Res, V1, P235, DOI 10.1007/BF00435632

Sidell NL, 1997, HEALTH SOC WORK, V22, P5, DOI 10.1093/hsw/22.1.5

SKAFF MM, 2007, ANN M GER SOC AM SAN

SOUSA VD, 2006, ONLINE BRAZILIAN J N, P5

Toobert DJ, 2000, DIABETES CARE, V23, P943, DOI 10.2337/diacare.23.7.943

TURNER RJ, 1995, AM SOCIOL REV, V60, P104, DOI 10.2307/2096348

Zhao W, 2006, PUBLIC HEALTH, V120, P696, DOI 10.1016/j.puhe.2006.04.012

NR 19

TC 32

Z9 32

U1 0

U2 9

PU ELSEVIER IRELAND LTD

PI CLARE

PA ELSEVIER HOUSE, BROOKVALE PLAZA, EAST PARK SHANNON, CO, CLARE, 00000,

IRELAND

SN 0738-3991

J9 PATIENT EDUC COUNS

JI Patient Educ. Couns.

PD NOV

PY 2011

VL 85

IS 2

BP 154

EP 159

DI 10.1016/j.pec.2010.10.030

PG 6

WC Public, Environmental & Occupational Health; Social Sciences,

Interdisciplinary

SC Public, Environmental & Occupational Health; Social Sciences - Other

Topics

GA 850WX

UT WOS:000297231400006

PM 21112720

OA Green Accepted

DA 2019-01-08

ER

PT J

AU Hajos, TRS

Polonsky, WH

Twisk, JWR

Dain, MP

Snoek, FJ

AF Hajos, Tibor R. S.

Polonsky, William H.

Twisk, Jos W. R.

Dain, Marie-Paule

Snoek, Frank J.

TI Do physicians understand Type 2 diabetes patients' perceptions of

seriousness; the emotional impact and needs for care improvement? A

cross-national survey

SO PATIENT EDUCATION AND COUNSELING

LA English

DT Article

DE Type 2 diabetes; Patient-provider interaction; Perceptions;

Collaborative care; Concordance

ID PROVIDER PERCEPTIONS; SELF-MANAGEMENT; SATISFACTION; ATTITUDES;

DISTRESS; BARRIERS; QUALITY; DISEASE; DAWN

AB Objective: To explore across countries the extent to which physicians understand Type 2 diabetes patients' perceptions of seriousness, worries about complications, emotional distress, and needs for care improvement.

Methods: Cross-sectional data were collected in a multinational survey (SHARED). Type 2 diabetes patients (n=1609), general practitioners (n=818) and diabetes specialists (n=697) from eight countries were included. Data were gathered online and via telephone interviews. Responses from patients and professionals were compared using descriptive statistics and multilevel analyses.

Results: Patients generally perceived diabetes as a serious condition and reported moderate distress. Physicians tended to underestimate patients' perceived seriousness, while overestimating their level of distress. Physicians had difficulty estimating which diabetes complications concerned patients most, and what they needed to feel more confident about their diabetes. Patients did not wish for more consultation time, but rather active involvement, information and easy access to their physician.

Conclusion: Results of this large survey highlight the importance of patient involvement and shared decision making.

Practice implications: Further improvement of patient-provider communication as a basis for shared responsibilities and achieving optimal treatment outcomes is needed. With the growing numbers of diabetes patients worldwide, task delegation should be considered, in the framework of a multidisciplinary diabetes care model. (C) 2010 Elsevier Ireland Ltd. All rights reserved.

C1 [Hajos, Tibor R. S.; Twisk, Jos W. R.; Snoek, Frank J.] Vrije Univ Amsterdam Med Ctr, EMGO Inst, NL-1081 BT Amsterdam, Netherlands.

[Hajos, Tibor R. S.; Snoek, Frank J.] Vrije Univ Amsterdam Med Ctr, Dept Med Psychol, NL-1081 BT Amsterdam, Netherlands.

[Polonsky, William H.] Univ Calif San Diego, Behav Diabet Inst, San Diego, CA 92103 USA.

[Polonsky, William H.] Univ Calif San Diego, Dept Psychiat, San Diego, CA 92103 USA.

[Twisk, Jos W. R.] Vrije Univ Amsterdam Med Ctr, Dept Clin Epidemiol & Biostat, NL-1081 BT Amsterdam, Netherlands.

[Dain, Marie-Paule] Sanofi Aventis, Paris, France.

RP Hajos, TRS (reprint author), Vrije Univ Amsterdam Med Ctr, EMGO Inst, Van der Boechorststr 7, NL-1081 BT Amsterdam, Netherlands.

EM t.hajos@vumc.nl

FU sanofi-aventis; Eli Lilly; Roche Diagnostics; Novo Nordisk; Amylin

FX Declaration of competing interests: Tibor Hajos has no conflict of

interest. William H. Polonsky has received fees for lectures, consulting

and advisory board memberships from sanofi-aventis, Eli Lilly, Roche

Diagnostics, Novo Nordisk and Amylin and unrestricted research grant;

from Roche Diagnostics. Jos Twisk has no conflict of interest.

Marie-Paule Dain is an employee of sanofi-aventis. Frank J. Snoek has

received fees for lectures and advisory board membership from

sanofi-aventis, Eli Lilly, Roche Diagnostics and Novo Nordisk, and

unrestricted research grants from sanofi-aventis and Novo Nordisk.

CR ANDERSON RM, 1993, PATIENT EDUC COUNS, V21, P41, DOI 10.1016/0738-3991(93)90058-5

BANDURA A, 1977, PSYCHOL REV, V84, P191, DOI 10.1037/0033-295X.84.2.191

Davies M, 2006, DIABETIC MED, V23, P917, DOI 10.1111/j.1464-5491.2006.01892.x

Deutskens E, 2004, MARKET LETT, V15, P21, DOI 10.1023/B:MARK.0000021968.86465.00

Fisher L, 2008, DIABETIC MED, V25, P1096, DOI 10.1111/j.1464-5491.2008.02533.x

Fitzgerald JT, 2008, PATIENT EDUC COUNS, V70, P118, DOI 10.1016/j.pec.2007.09.005

Fitzgerald JT, 2003, DIABETES EDUCATOR, V29, P480, DOI 10.1177/014572170302900312

Holman H, 2004, PUBLIC HEALTH REP, V119, P239, DOI 10.1016/j.phr.2004.04.002

Joosten EAG, 2008, PSYCHOTHER PSYCHOSOM, V77, P219, DOI 10.1159/000126073

Loewe R, 2000, CULT MED PSYCHIAT, V24, P379, DOI 10.1023/A:1005611207687

Mead N, 2002, SOC SCI MED, V55, P283, DOI 10.1016/S0277-9536(01)00171-X

Nabi RL, 2008, HEALTH COMMUN, V23, P191, DOI 10.1080/10410230701808327

Nicholson TRJ, 2009, DIABETIC MED, V26, P447, DOI 10.1111/j.1464-5491.2009.02701.x

Peyrot M, 2006, DIABETOLOGIA, V49, P279, DOI 10.1007/s00125-005-0048-8

Peyrot M, 2005, DIABETIC MED, V22, P1379, DOI 10.1111/j.1464-5491.2005.01644.x

Shoor S, 2002, MED CARE, V40, P40

Snoek FJ, 2002, INT J CLIN PRACT, P80

Snoek FJ, 2000, DIABETES CARE, V23, P1305, DOI 10.2337/diacare.23.9.1305

Snoek FI, 2007, BMJ-BRIT MED J, V335, P458, DOI 10.1136/bmj.39315.443160.BE

Solli O, 2010, HEALTH QUAL LIFE OUT, V8, DOI 10.1186/1477-7525-8-18

Tamblyn R, 2010, ARCH INTERN MED, V170, P1064, DOI 10.1001/archinternmed.2010.167

Wagner E H, 1996, Manag Care Q, V4, P12

NR 22

TC 11

Z9 11

U1 1

U2 6

PU ELSEVIER IRELAND LTD

PI CLARE

PA ELSEVIER HOUSE, BROOKVALE PLAZA, EAST PARK SHANNON, CO, CLARE, 00000,

IRELAND

SN 0738-3991

J9 PATIENT EDUC COUNS

JI Patient Educ. Couns.

PD NOV

PY 2011

VL 85

IS 2

BP 258

EP 263

DI 10.1016/j.pec.2010.08.019

PG 6

WC Public, Environmental & Occupational Health; Social Sciences,

Interdisciplinary

SC Public, Environmental & Occupational Health; Social Sciences - Other

Topics

GA 850WX

UT WOS:000297231400022

PM 20932702

DA 2019-01-08

ER

PT J

AU Park, SW

Lee, CS

Lee, CK

Kwak, YG

Moon, C

Kim, BN

Kim, ES

Kang, JM

Oh, MD

AF Park, Sang-Won

Lee, Chang-Seop

Lee, Chi Kug

Kwak, Yee Gyung

Moon, Chisook

Kim, Baek-Nam

Kim, Eu Suk

Kang, Jae Myung

Oh, Myoung-don

TI Severity Predictors in Eschar-Positive Scrub Typhus and Role of Serum

Osteopontin

SO AMERICAN JOURNAL OF TROPICAL MEDICINE AND HYGIENE

LA English

DT Article

ID ORIENTIA TSUTSUGAMUSHI INFECTION; RESPIRATORY-DISTRESS-SYNDROME;

RICKETTSIA-TSUTSUGAMUSHI; NATURAL-RESISTANCE; DISEASE-ACTIVITY; HOST

DEFENSES; SOUTH-KOREA; GENE; MICE; CLASSIFICATION

AB We prospectively evaluated severity predictors in terms of host, microorganism, and treatment factors in 153 eschar-positive scrub typhus patients. Severity was assessed with the Acute Physiology and Chronic Health Evaluation (APACHE) II score (< 10 versus >= 10) and predefined criteria of severe complications. Genotypes of Orientia tsutsugamushi were determined. Independent risk factors for severity (APACHE II score >= 10) were old age, diabetes mellitus, serum osteopontin > 100 ng/mL, and a group of underlying diseases (congestive heart failure, cerebrovascular disease, chronic liver disease, bronchial asthma, and chronic obstructive lung diseases). Anemia (<= 10 g/dL) and C-reactive protein > 10 mg/dL were indicators of current severity. Neither the delay in antibiotics administration nor strain types (Boryong, Taguchi, or Kanda/Kawasaki) contributed to the severity. The risk factors for severe complications were similar. Serum osteopontin > 100 ng/mL had a negative predictive value of 96% for severe complications. This marker can be used to rule out severe disease status.

C1 [Moon, Chisook] Inje Univ, Coll Med, Dept Internal Med, Pusan 614735, South Korea.

Seoul Natl Univ, Coll Med, Dept Internal Med, Seoul 151, South Korea.

Boramae Med Ctr, Seoul, South Korea.

Chonbuk Natl Univ, Sch Med, Dept Internal Med, Jeonju, South Korea.

Namwon Med Ctr, Dept Internal Med, Namwon, South Korea.

Seoul Natl Univ, Bundang Hosp, Dept Internal Med, Songnam, South Korea.

Handong Global Univ, Sunlin Hosp, Dept Internal Med, Pohang, South Korea.

RP Moon, C (reprint author), Inje Univ, Coll Med, Dept Internal Med, 633-165 Gaegeum Dong, Pusan 614735, South Korea.

EM duomon@hanmail.net

RI Park, Sang Won/J-5485-2012

OI Park, Sang Won/0000-0002-0550-1897; Kim, Baek-Nam/0000-0001-5551-2132;

Kwak, Yee Gyung/0000-0002-4713-8045; Oh, Myoung-don/0000-0002-2344-7695

FU Seoul National University Hospital (SNUH) [04-2009-610]

FX This study was supported by grant 04-2009-610 from the Seoul National

University Hospital (SNUH) Research Fund.

CR Ashkar S, 2000, SCIENCE, V287, P860, DOI 10.1126/science.287.5454.860

Chang Woo-Hyun, 1995, Journal of Korean Medical Science, V10, P227

Chanta Chulapong, 2007, Journal of the Medical Association of Thailand, V90, P2366

Cho NH, 2001, INFECT IMMUN, V69, P1265, DOI 10.1128/IAI.69.3.1265-1272.2001

Chrispal A, 2010, TROP DOCT, V40, P129, DOI 10.1258/td.2010.090452

Chung DR, 2008, J INFECTION, V56, P44, DOI 10.1016/j.jinf.2007.09.009

Ewig S, 1998, AM J RESP CRIT CARE, V158, P1102, DOI 10.1164/ajrccm.158.4.9803114

GROVES MG, 1978, INFECT IMMUN, V19, P583

GROVES MG, 1980, J IMMUNOL, V125, P1395

HANSON B, 1991, INFECT IMMUN, V59, P4134

Inomata S, 2005, CYTOKINE, V30, P203, DOI 10.1016/j.cyto.2005.01.014

Iwasaki H, 1997, J CLIN MICROBIOL, V35, P3320

IWASAKI H, 1994, LANCET, V343, P1236

Iwasaki H, 2010, INT J INFECT DIS, V14, pE328, DOI 10.1016/j.ijid.2009.06.002

Kelly DJ, 2009, CLIN INFECT DIS, V48, pS203, DOI 10.1086/596576

KELLY DJ, 1990, ANN NY ACAD SCI, V590, P564, DOI 10.1111/j.1749-6632.1990.tb42267.x

Kim DM, 2010, BMC INFECT DIS, V10, DOI 10.1186/1471-2334-10-108

Kim IH, 2010, CLIN MICROBIOL INFEC, V16, P419, DOI 10.1111/j.1469-0691.2009.02825.x

Kim YS, 2004, CLIN INFECT DIS, V39, P1329, DOI 10.1086/425008

KNAUS WA, 1985, CRIT CARE MED, V13, P818, DOI 10.1097/00003246-198510000-00009

Kweon SS, 2009, EMERG INFECT DIS, V15, P1127, DOI 10.3201/eid1507.080399

Lee CS, 2010, BMC INFECT DIS, V10, DOI 10.1186/1471-2334-10-216

Liu YX, 2009, BMC INFECT DIS, V9, DOI 10.1186/1471-2334-9-82

Maeno Y, 2006, ACTA TROP, V98, P305, DOI 10.1016/j.actatropica.2006.05.001

Moron CG, 2001, MODERN PATHOL, V14, P752, DOI 10.1038/modpathol.3880385

Nau GJ, 1999, INFECT IMMUN, V67, P4223

Park JS, 2000, J KOREAN MED SCI, V15, P343, DOI 10.3346/jkms.2000.15.3.343

Park SW, 2010, AM J TROP MED HYG, V83, P930, DOI 10.4269/ajtmh.2010.09-0791

PATARCA R, 1989, J EXP MED, V170, P145, DOI 10.1084/jem.170.1.145

Raoult D, 2010, PRINCIPLES PRACTICE, P2529

Rollo EE, 1996, J LEUKOCYTE BIOL, V60, P397

Seong SY, 2001, MICROBES INFECT, V3, P11, DOI 10.1016/S1286-4579(00)01352-6

Shiga K, 1997, Kansenshogaku Zasshi, V71, P299

Sonthayanon P, 2009, J CLIN MICROBIOL, V47, P430, DOI 10.1128/JCM.01927-08

Takahashi F, 2004, LUNG, V182, P173

TAMURA A, 1995, INT J SYST BACTERIOL, V45, P589, DOI 10.1099/00207713-45-3-589

Tamura A, 2001, MICROBIOL IMMUNOL, V45, P439, DOI 10.1111/j.1348-0421.2001.tb02643.x

Thap Lon Chan, 2002, Southeast Asian Journal of Tropical Medicine and Public Health, V33, P780

Varghese GM, 2006, J INFECTION, V52, P56, DOI 10.1016/j.jinf.2005.02.001

WATT G, 1994, CLIN INFECT DIS, V18, P624, DOI 10.1093/clinids/18.4.624

Wong CK, 2005, RHEUMATOLOGY, V44, P602, DOI 10.1093/rheumatology/keh558

Yasunaga H, 2011, INTERNAL MED, V50, P37, DOI 10.2169/internalmedicine.50.4220

NR 42

TC 11

Z9 13

U1 0

U2 5

PU AMER SOC TROP MED & HYGIENE

PI MCLEAN

PA 8000 WESTPARK DR, STE 130, MCLEAN, VA 22101 USA

SN 0002-9637

EI 1476-1645

J9 AM J TROP MED HYG

JI Am. J. Trop. Med. Hyg.

PD NOV

PY 2011

VL 85

IS 5

BP 924

EP 930

DI 10.4269/ajtmh.2011.11-0134

PG 7

WC Public, Environmental & Occupational Health; Tropical Medicine

SC Public, Environmental & Occupational Health; Tropical Medicine

GA 843HC

UT WOS:000296661900025

PM 22049051

OA Green Published, Bronze

DA 2019-01-08

ER

PT J

AU Gois, C

Barbosa, A

Ferro, A

Santos, AL

Sousa, F

Akiskal, H

Akiskal, K

Figueira, ML

AF Gois, Carlos

Barbosa, Antonio

Ferro, Ana

Santos, Ana Luisa

Sousa, Filomena

Akiskal, Hagop

Akiskal, Kareen

Luisa Figueira, M.

TI The role of affective temperaments in metabolic control in patients with

type 2 diabetes

SO JOURNAL OF AFFECTIVE DISORDERS

LA English

DT Article

DE Affective temperaments; TEMPS-A; Type 2 diabetes; Diabetes psychological

adjustment; Metabolic control; Depression

ID MAJOR DEPRESSIVE DISORDER; TEMPS-A; GLYCEMIC CONTROL; ANXIETY DISORDERS;

INTERNAL CONSISTENCY; PERSONALITY; DISTRESS; VALIDATION; QUESTIONNAIRE;

ASSOCIATION

AB Background: In non-diabetic populations, anxious and depressive affective temperaments are associated with stress and distress disorders (anxious and depressive). In type 2 diabetes, distress with diabetes and distress disorders have been associated with metabolic control. There are few studies undertaken on temperament and metabolic control in patients with type 2 diabetes. The aim of our study was to examine the independent association between affective temperaments and metabolic control in patients with type 2 diabetes.

Methods: The sample included 90 patients with type 2 diabetes from two outpatient clinics aged 53.54 (SD +/- 8.05) years and with 66.7% female gender. Depression was evaluated by using the MADRS observer rating scale (mean value: 16.38 +/- 11).

Results: Patients with excessive depressive and anxious temperaments had more depressive symptoms, worse psychological adjustment to diabetes and worse metabolic control. In logistic regression (crude model), only depressive temperament was independently associated with metabolic control.

Conclusions: Affective temperaments are associated with psychological adjustment to diabetes and metabolic control in patients with type 2 diabetes. Patients above threshold on depressive temperament should be considered for greater scrutiny and psycho-education by the diabetes clinic staff. (C) 2011 Elsevier B.V. All rights reserved.

C1 [Gois, Carlos; Barbosa, Antonio; Ferro, Ana; Santos, Ana Luisa; Sousa, Filomena; Luisa Figueira, M.] Univ Lisbon, Hosp Santa Maria, Dept Psychiat, Fac Med, P-1600 Lisbon, Portugal.

[Akiskal, Hagop; Akiskal, Kareen] Univ Calif San Diego, San Diego, CA 92103 USA.

[Akiskal, Hagop; Akiskal, Kareen] Int Mood Ctr, La Jolla, CA USA.

RP Gois, C (reprint author), Univ Lisbon, Hosp Santa Maria, Dept Psychiat, Fac Med, Av Prof Egas Moniz, P-1600 Lisbon, Portugal.

EM cargois@gmail.com

OI Gois, Carlos/0000-0003-4346-4844

CR Adriaanse MC, 2008, DIABETES CARE, V31, P2268, DOI 10.2337/dc08-1074

Aikens JE, 2009, DIABETES CARE, V32, P1177, DOI 10.2337/dc09-0071

Akiskal H. S., 1995, DEPRESSION MANIA NEU, P99

Akiskal HS, 1998, J AFFECT DISORDERS, V51, P7, DOI 10.1016/S0165-0327(98)00152-9

Akiskal HS, 2005, J AFFECT DISORDERS, V85, P3, DOI 10.1016/j.jad.2004.12.001

Akiskal HS, 2005, J AFFECT DISORDERS, V85, P29, DOI 10.1016/j.jad.2003.12.009

AKISKAL HS, 1983, ARCH GEN PSYCHIAT, V40, P801

AKSIKAL HS, 2005, J AFFECT DISORDERS, V85, P45

Bloink R, 2005, J AFFECT DISORDERS, V85, P77, DOI 10.1016/S0165-0327(03)00101-0

Bryan CJ, 2008, J AFFECT DISORDERS, V108, P113, DOI 10.1016/j.jad.2007.10.003

CLARK LA, 1994, J ABNORM PSYCHOL, V103, P103, DOI 10.1037/0021-843X.103.1.103

Collins MM, 2009, DIABETIC MED, V26, P153, DOI 10.1111/j.1464-5491.2008.02648.x

COYNE JC, 1994, PSYCHOL BULL, V116, P29, DOI 10.1037/0033-2909.116.1.29

Delahanty LM, 2007, DIABETIC MED, V24, P48, DOI 10.1111/j.1464-5491.2007.02028.x

DUNN SM, 1986, DIABETES CARE, V9, P480, DOI 10.2337/diacare.9.5.480

Egede LE, 2003, DIABETES CARE, V26, P104, DOI 10.2337/diacare.26.1.104

Egede LE, 2010, DIABETES RES CLIN PR, V87, P302, DOI 10.1016/j.diabres.2010.01.024

Erfurth A, 2005, J AFFECT DISORDERS, V85, P53, DOI 10.1016/S0165-0327(03)00102-2

Figueira ML, 2008, J AFFECT DISORDERS, V111, P193, DOI 10.1016/j.jad.2008.03.001

Fisher L, 2008, DIABETIC MED, V25, P1096, DOI 10.1111/j.1464-5491.2008.02533.x

Fisher L, 2009, DIABETIC MED, V26, P622, DOI 10.1111/j.1464-5491.2009.02730.x

Fisher L, 2001, DIABETES CARE, V24, P1751, DOI 10.2337/diacare.24.10.1751

Fisher L, 2007, DIABETES CARE, V30, P542, DOI 10.2337/dc06-1614

Fisher L, 2010, DIABETES CARE, V33, P23, DOI 10.2337/dc09-1238

Gois C., 2006, PSIQUIATRIA CLIN, V27, P71

Grigsby AB, 2002, J PSYCHOSOM RES, V53, P1053, DOI 10.1016/S0022-3999(02)00417-8

Hall PA, 2009, J PSYCHOSOM RES, V67, P297, DOI 10.1016/j.jpsychores.2009.05.015

Hosmer D.W., 2000, APPL LOGISTIC REGRES

International Diabetes Federation, 2005, GLOB GUID TYP 2 DIAB

Ismail K, 2007, DIABETES CARE, V30, P1473, DOI 10.2337/dc06-2313

Katon W, 2004, DIABETES CARE, V27, P914, DOI 10.2337/diacare.27.4.914

Katon W, 2010, DEPRESSION DIABETES, P81

KENDLER KS, 1993, ARCH GEN PSYCHIAT, V50, P853

Kessler RC, 2003, JAMA-J AM MED ASSOC, V289, P3095, DOI 10.1001/jama.289.23.3095

Lane JD, 2000, DIABETES CARE, V23, P1321, DOI 10.2337/diacare.23.9.1321

Lloyd Cathy E., 2010, DEPRESSION DIABETES, P1

Lustman PJ, 1997, GEN HOSP PSYCHIAT, V19, P138, DOI 10.1016/S0163-8343(96)00170-3

LUSTMAN PJ, 1992, PSYCHOSOM MED, V54, P602, DOI 10.1097/00006842-199209000-00007

LUSTMAN PJ, 1991, PSYCHOSOM MED, V53, P305, DOI 10.1097/00006842-199105000-00004

Lustman PJ, 2000, DIABETES CARE, V23, P934, DOI 10.2337/diacare.23.7.934

Maremmani I, 2005, J AFFECT DISORDERS, V85, P17, DOI 10.1016/S0165-0327(03)00099-5

Montgomery P., 1979, BRIT J PSYCHIAT, V134, P382

Muller MJ, 2000, J AFFECT DISORDERS, V60, P137, DOI 10.1016/S0165-0327(99)00162-7

Nakahara R, 2006, PSYCHOSOMATICS, V47, P240, DOI 10.1176/appi.psy.47.3.240

Ormel J, 2004, ARCH GEN PSYCHIAT, V61, P990, DOI 10.1001/archpsyc.61.10.990

Placidi GF, 1998, J AFFECT DISORDERS, V47, P1, DOI 10.1016/S0165-0327(97)00122-5

Pouwer F, 2009, NAT REV ENDOCRINOL, V5, P665, DOI 10.1038/nrendo.2009.214

Richardson LK, 2008, GEN HOSP PSYCHIAT, V30, P509, DOI 10.1016/j.genhosppsych.2008.07.001

Rihmer A, 2009, J AFFECT DISORDERS, V116, P18, DOI 10.1016/j.jad.2008.10.024

Rihmer Z, 2010, CURR OPIN PSYCHIATR, V23, P12, DOI 10.1097/YCO.0b013e32833299d4

Rozsa S, 2008, J AFFECT DISORDERS, V106, P45, DOI 10.1016/j.jad.2007.03.016

Stratton IM, 2000, BRIT MED J, V321, P405, DOI 10.1136/bmj.321.7258.405

Tomassini A, 2009, INT J PSYCHIAT CLIN, V13, P31, DOI 10.1080/13651500802369482

Tyrer P, 2001, BRIT J PSYCHIAT, V179, P191, DOI 10.1192/bjp.179.3.191

Watson D, 2005, J RES PERS, V39, P46, DOI 10.1016/j.jrp.2004.09.006

Welch G. W., 1996, HDB PSYCHOL DIABETES, P223

Welch GW, 1997, DIABETES CARE, V20, P760, DOI 10.2337/diacare.20.5.760

NR 57

TC 22

Z9 24

U1 2

U2 8

PU ELSEVIER SCIENCE BV

PI AMSTERDAM

PA PO BOX 211, 1000 AE AMSTERDAM, NETHERLANDS

SN 0165-0327

J9 J AFFECT DISORDERS

JI J. Affect. Disord.

PD NOV

PY 2011

VL 134

IS 1-3

BP 52

EP 58

DI 10.1016/j.jad.2011.05.021

PG 7

WC Clinical Neurology; Psychiatry

SC Neurosciences & Neurology; Psychiatry

GA 831TG

UT WOS:000295753400007

PM 21641045

DA 2019-01-08

ER

PT J

AU Shles, A

Fainmesser, P

Eliakim, A

Nemet, D

AF Shles, Ayelet

Fainmesser, Pinchas

Eliakim, Alon

Nemet, Dan

TI Child abuse suspicion masquerading new onset insulin dependent diabetes

mellitus

SO JOURNAL OF PEDIATRIC ENDOCRINOLOGY & METABOLISM

LA English

DT Article

DE child maltreatment; diabetic ketoacidosis

ID NONACCIDENTAL-INJURY; MUNCHAUSEN-SYNDROME; MISTAKEN DIAGNOSIS;

PHYSICIANS; EXPERIENCE; PROXY

AB The identification and diagnosis of child abuse is a challenging task to the pediatrician. The increased awareness among both the public and medical personnel, while improving attentiveness to this important subject, can sometimes result in misdiagnosing medical conditions, thus causing distress and delay in required treatment. Numerous reports have described conditions mimicking non-accidental injuries; most of these include dermatological findings related to skin diseases, medical conditions causing pathological fractures, and rare diseases with unusual physical findings. We present a case of a 9.5-year-old child in which the workup for a suspected abusive event led to a delay in the diagnosis of insulin dependent diabetes mellitus later presented as diabetic ketoacidosis.

C1 [Eliakim, Alon; Nemet, Dan] Tel Aviv Univ, Sackler Sch Med, Endocrine Clin, Dept Pediat,Meir Med Ctr, IL-44281 Kefar Sava, Israel.

[Shles, Ayelet; Fainmesser, Pinchas] Tel Aviv Univ, Sackler Sch Med, Pediat Emergency Care Unit, Dept Pediat,Meir Med Ctr, IL-44281 Kefar Sava, Israel.

RP Nemet, D (reprint author), Tel Aviv Univ, Sackler Sch Med, Endocrine Clin, Dept Pediat,Meir Med Ctr, IL-44281 Kefar Sava, Israel.

EM dan.nemet@clalit.org.il

CR AlJasser M, 2008, EUR J PEDIATR, V167, P1221, DOI 10.1007/s00431-008-0792-0

ANH NT, 1976, JAMA-J AM MED ASSOC, V236, P2288, DOI 10.1001/jama.1976.03270210020012

BALDWIN K, 2010, CLIN ORTHOP RELAT RE

COPELAND AR, 1988, J FORENSIC SCI, V33, P1493

Flaherty EG, 2008, PEDIATRICS, V122, P611, DOI 10.1542/peds.2007-2311

HORAN PF, 1986, DIABETES CARE, V9, P302, DOI 10.2337/diacare.9.3.302

Jones R, 2008, PEDIATRICS, V122, P259, DOI 10.1542/peds.2007-2312

Karavanaki K, 2008, J ENDOCRINOL INVEST, V31, P406, DOI 10.1007/BF03346384

KIRSCHNER RH, 1985, AM J DIS CHILD, V139, P873, DOI 10.1001/archpedi.1985.02140110027022

KOVACS CS, 1993, DIABETES CARE, V16, P1294, DOI 10.2337/diacare.16.9.1294

Laskey AL, 2007, CHILD ABUSE NEGLECT, V31, P329, DOI 10.1016/j.chiabu.2006.02.017

Lindberg DM, 2008, PEDIATRICS, V121, pE945, DOI 10.1542/peds.2007-2485

MCSWEENEY JJ, 1991, DIABETES CARE, V14, P928, DOI 10.2337/diacare.14.10.928

Merrick J, 1999, Public Health Rev, V27, P279

Mimasaka S, 1998, J Clin Forensic Med, V5, P72, DOI 10.1016/S1353-1131(98)90057-8

MORRIS JL, 1985, AM J DIS CHILD, V139, P194, DOI 10.1001/archpedi.1985.02140040096037

Paterson CR, 2009, ACTA PAEDIATR, V98, P2008, DOI 10.1111/j.1651-2227.2009.01407.x

SAULSBURY FT, 1986, SOUTHERN MED J, V79, P585, DOI 10.1097/00007611-198605000-00016

Senniappan S, 2008, ACTA PAEDIATR, V97, P992

Senniappan S, 2008, ACTA PAEDIATR, V97, P836, DOI 10.1111/j.1651-2227.2008.00790.x

*US ADM CHILDR YOU, 2005, CHILD MAN 2003

Walsh J, 2008, ARCH DIS CHILD, V93, P65, DOI 10.1136/adc.2007.125443

WARDINSKY TD, 1995, MIL MED, V160, P15

WHEELER DM, 1988, BRIT MED J, V296, P1233, DOI 10.1136/bmj.296.6631.1233

NR 24

TC 0

Z9 0

U1 0

U2 1

PU WALTER DE GRUYTER & CO

PI BERLIN

PA GENTHINER STRASSE 13, D-10785 BERLIN, GERMANY

SN 0334-018X

J9 J PEDIATR ENDOCR MET

JI J. Pediatr. Endocrinol. Metab.

PD OCT

PY 2011

VL 24

IS 9-10

BP 823

EP 825

DI 10.1515/JPEM.2011.284

PG 3

WC Endocrinology & Metabolism; Pediatrics

SC Endocrinology & Metabolism; Pediatrics

GA 854BQ

UT WOS:000297470000041

PM 22145485

DA 2019-01-08

ER

PT J

AU Allen, NA

Melkus, GD

Chyun, DA

AF Allen, Nancy A.

Melkus, Gail D.

Chyun, Deborah A.

TI Physiological and Behavioral Factors Related to Physical Activity in

Black Women With Type 2 Diabetes Mellitus

SO JOURNAL OF TRANSCULTURAL NURSING

LA English

DT Article

DE women's health; methods/designs; type 2 diabetes mellitus; physical

activity; exercise; psychosocial factors; behavioral factors; Black;

African American; women

ID QUALITY-OF-LIFE; CARDIOVASCULAR-DISEASE; AFRICAN-AMERICAN; US ADULTS;

HEALTH; RISK; ASSOCIATION; DEPRESSION; EXERCISE; ANXIETY

AB Purpose: To describe relationships among physical activity (PA), physiological factors, and psychological factors in Black women with type 2 diabetes mellitus (T2DM). Methods: A cross-sectional design was used (N = 109). Data were collected on PA (activity/inactivity, TV hours, bed confinement), physiology (blood pressure, lipids, hemoglobin A1c), psychology (anxiety, emotional distress, physical functioning, bodily pain, vitality), and health care provider (HCP) support. Results: Walking was the preferred PA; TV viewing averaged 3.7 hours/day, and 24% reported confinement to bed >1 week in the last year. Inactive women had greater physiological and psychological problems than active women. Women watching TV >2 hours/day had more physiological problems than women watching TV <2 hours/day. Women reporting >1 week of confinement to bed in the last year had more physiological and psychological problems than those confined to bed <1 week. Conclusions: PA interventions in Black women with T2DM should promote walking, address TV viewing time, incorporate HCP's role of PA counseling/support, and address several psychological factors.

C1 [Allen, Nancy A.] Boston Coll, Connell Sch Nursing, Chestnut Hill, MA 02467 USA.

[Melkus, Gail D.; Chyun, Deborah A.] NYU, New York, NY USA.

RP Allen, NA (reprint author), Boston Coll, Connell Sch Nursing, 140 Commonwealth Ave, Chestnut Hill, MA 02467 USA.

EM Nancy.a.allen@bc.edu

FU National Institute of Health [R01NR05341, T32 NR 008346-06]

FX National Institute of Health Grant R01NR05341 and T32 NR 008346-06.

CR Adams-Campbell LL, 2000, PREV MED, V30, P43, DOI 10.1006/pmed.1999.0604

Allan JL, 2007, J PSYCHOSOM RES, V63, P501, DOI 10.1016/j.jpsychores.2007.08.001

[Anonymous], AM TIM US SURV

Bennett GG, 2006, AM J PUBLIC HEALTH, V96, P1681, DOI 10.2105/AJPH.2005.080580

Blanc S, 2000, J CLIN ENDOCR METAB, V85, P2223, DOI 10.1210/jc.85.6.2223

Bonnet F, 2005, ATHEROSCLEROSIS, V178, P339, DOI 10.1016/j.atgersclerosis.2004.08.035

BURGESS PM, 1987, BRIT J MED PSYCHOL, V60, P61

*CDCP, 2007, CDCS DIAB PROGR DAT

*CDCP, 2007, DIAB DAT TRENDS 2007

*CDCP, 2004, MMWR-MORBID MORTAL W, V53, P82

CROWN S, 1966, BRIT J PSYCHIAT, V112, P917, DOI 10.1192/bjp.112.490.917

De Moor MHM, 2008, ARCH GEN PSYCHIAT, V65, P897, DOI 10.1001/archpsyc.65.8.897

Duvigneaud N, 2008, J SPORT MED PHYS FIT, V48, P201

Jakes RW, 2003, EUR J CLIN NUTR, V57, P1089, DOI 10.1038/sj.ejcn.1601648

Kleefstra N, 2005, NETH J MED, V63, P215

KRISKA AM, 1990, DIABETES CARE, V13, P401, DOI 10.2337/diacare.13.4.401

Kumanyika SK, 2002, ETHNICITY DIS S3, V12, P62

Li C, 2008, DIABETIC MED, V25, P878, DOI 10.1111/j.1464-5491.2008.02477.x

Maddigan SL, 2005, QUAL LIFE RES, V14, P1489, DOI 10.1007/s11136-005-0586-z

Miller ST, 2008, BMC PUBLIC HEALTH, V8, DOI 10.1186/1471-2458-8-70

Morrato EH, 2007, DIABETES CARE, V30, P203, DOI 10.2337/dc06-1128

Myers J, 2002, NEW ENGL J MED, V346, P793, DOI 10.1056/NEJMoa011858

Nelson KM, 2002, DIABETES CARE, V25, P1722, DOI 10.2337/diacare.25.10.1722

Panagiotakos DB, 2008, ANN EPIDEMIOL, V18, P387, DOI 10.1016/j.annepidem.2007.11.002

Physical Activity Guidelines Advisory Committee, 2008, PHYS ACT GUID ADV CO

POLONSKY WH, 1995, DIABETES CARE, V18, P754, DOI 10.2337/diacare.18.6.754

Rejeski WJ, 2006, OBESITY, V14, P870, DOI 10.1038/oby.2006.101

Sharpe PA, 2008, AM J HEALTH BEHAV, V32, P701, DOI 10.5555/ajhb.2008.32.6.701

Smits JAJ, 2006, DEPRESS ANXIETY, V23, P102, DOI 10.1002/da.20146

Spencer MS, 2006, J HEALTH CARE POOR U, V17, P88, DOI 10.1353/hpu.2006.0095

Stenholm S, 2007, AGING CLIN EXP RES, V19, P277

Volpato S, 2002, DIABETES CARE, V25, P678, DOI 10.2337/diacare.25.4.678

WARE JE, 1992, MED CARE, V30, P473, DOI 10.1097/00005650-199206000-00002

Ware JE, 1997, SF 36 HLTH SURVEY MA

Wexler DJ, 2006, DIABETOLOGIA, V49, P1489, DOI 10.1007/s00125-006-0249-9

Whittemore R, 2005, J CLIN NURS, V14, P195, DOI 10.1111/j.1365-2702.2004.00937.x

WILLIAMS GC, 1998, HEALTH PSYCHOL, V17, P169

NR 37

TC 1

Z9 1

U1 0

U2 12

PU SAGE PUBLICATIONS INC

PI THOUSAND OAKS

PA 2455 TELLER RD, THOUSAND OAKS, CA 91320 USA

SN 1043-6596

J9 J TRANSCULT NURS

JI J. Transcult. Nurs.

PD OCT

PY 2011

VL 22

IS 4

BP 376

EP 385

DI 10.1177/1043659611414143

PG 10

WC Nursing

SC Nursing

GA 848TB

UT WOS:000297076700007

PM 21807961

DA 2019-01-08

ER

PT J

AU Mulvaney, SA

Hood, KK

Schlundt, DG

Osborn, CY

Johnson, KB

Rothman, RL

Wallston, KA

AF Mulvaney, Shelagh A.

Hood, Korey K.

Schlundt, David G.

Osborn, Chandra Y.

Johnson, Kevin B.

Rothman, Russell L.

Wallston, Kenneth A.

TI Development and initial validation of the barriers to diabetes adherence

measure for adolescents

SO DIABETES RESEARCH AND CLINICAL PRACTICE

LA English

DT Article

DE Adherence; Adolescents; Type 1 diabetes; Barriers; Assessment

ID METABOLIC-CONTROL; SELF-MANAGEMENT; SITUATIONAL OBSTACLES; REGIMEN

ADHERENCE; STRESS; CARE; MELLITUS; PERCEPTIONS; DISTRESS; SUPPORT

AB Aims: The purpose of this study was to develop a measure of psychosocial barriers to adherence in adolescents with type 1 diabetes (T1D) and examine relationships to patient characteristics, adherence, and hemoglobin A1C (A1C).

Methods: Barriers to diabetes adherence (BDA) items were generated by researchers, clinicians, and patients. Adolescents aged 12-17 with T1D completed the BDA and an adherence measure. Hemoglobin A1C was obtained through medical chart review.

Results: Factor analysis from 123 adolescents resulted in a 21-item, five-component solution that accounted for 64.5% of the variance. The components were stress and burnout, time pressure and planning, social support, parental autonomy support, and stigma. The BDA total and subscales were internally consistent. The BDA total and some components were associated with adherence and A1C. The BDA was the only predictor of A1C compared to demographic, clinical, and adherence variables (F 6.17, p < .05). Subjects with higher A1C (>8.5) showed a higher level of barriers (F 15.20, p < .001) and a differential profile of barriers (F 5.75, p < .05).

Conclusions: The BDA may be useful in research and clinical settings as a compliment to adherence measures and to tailor educational programs. Additional research is necessary to establish test-retest reliability and discriminant validity. (C) 2011 Elsevier Ireland Ltd. All rights reserved.

C1 [Mulvaney, Shelagh A.; Wallston, Kenneth A.] Vanderbilt Univ, Med Ctr, Sch Nursing, Nashville, TN 37240 USA.

[Mulvaney, Shelagh A.; Johnson, Kevin B.] Vanderbilt Univ, Med Ctr, Dept Biomed Informat, Nashville, TN 37240 USA.

[Mulvaney, Shelagh A.; Johnson, Kevin B.] Vanderbilt Univ, Med Ctr, Dept Pediat, Nashville, TN 37240 USA.

[Hood, Korey K.] Cincinnati Childrens Hosp Med Ctr, Div Behav Med & Clin Psychol, Cincinnati, OH USA.

[Schlundt, David G.] Vanderbilt Univ, Dept Psychol, Nashville, TN 37240 USA.

[Osborn, Chandra Y.; Rothman, Russell L.] Vanderbilt Univ, Med Ctr, Ctr Hlth Serv Res, Nashville, TN 37240 USA.

RP Mulvaney, SA (reprint author), Vanderbilt Univ, Med Ctr, Sch Nursing, 461 21st Ave S, Nashville, TN 37240 USA.

EM shelagh.mulvaney@vanderbilt.edu

RI Hanghoj, Signe/E-2440-2012

OI Osborn, Chandra/0000-0002-7668-028X

FU Vanderbilt Diabetes Research and Training Center [P60DK020593];

Vanderbilt Institute for Clinical and Translational Research, NCRR/NIH

[1 UL1 RR024975]

FX The authors wish to thank Mr. Eric Pittel and Ms. Kathleen Smith for

assistance with data collection. This research was supported by a Pilot

and Feasibility grant to Dr. Mulvaney from the Vanderbilt Diabetes

Research and Training Center (P60DK020593) and by the Vanderbilt

Institute for Clinical and Translational Research (1 UL1 RR024975 from

NCRR/NIH).

CR Admi H, 2007, QUAL HEALTH RES, V17, P1178, DOI 10.1177/1049732307307548

Auslander WF, 2010, DIABETES EDUCATOR, V36, P613, DOI 10.1177/0145721710369706

Baars R., 2005, HEALTH QUAL LIFE OUT, V3, P1

Burroughs TE, 1997, DIABETES EDUCATOR, V23, P438, DOI 10.1177/014572179702300409

Casillas J, 2010, PSYCHO-ONCOLOGY, V19, P982, DOI 10.1002/pon.1650

Delamater AM, 2000, PROMOTING ADHERENCE TO MEDICAL TREATMENT IN CHRONIC CHILDHOOD ILLNESS: CONCEPTS, METHODS, AND INTERVENTIONS, P173

DELAMATER AM, 1987, J DEV BEHAV PEDIATR, V8, P136

Delamater AM, 2006, CLIN DIABETES, V24, P71, DOI [10.2337/diaclin.24.2.71, DOI 10.2337/DIACLIN.24.2.71]

DeVellis RF, 2003, SCALE DEV THEORY APP

Di Battista AM, 2009, DIABETES EDUCATOR, V35, P465, DOI 10.1177/0145721709333492

Ellis DA, 2005, PEDIATRICS, V116, pE826, DOI 10.1542/peds.2005-0638

Farrell SP, 2004, J ADOLESCENT HEALTH, V34, P461, DOI 10.1016/S1054-139X(03)00215-5

Fisher EB, 2007, DIABETES EDUCATOR, V33, P1080, DOI 10.1177/0145721707309808

GLASGOW RE, 1986, J BEHAV MED, V9, P65, DOI 10.1007/BF00844645

GLASGOW RE, 1992, PATIENT EDUC COUNS, V19, P61, DOI 10.1016/0738-3991(92)90102-O

Glasgow RE, 2001, DIABETES SPECTRUM, V14, P33, DOI DOI 10.2337/DIASPECT.14.1.33

Greening L, 2007, J PEDIATR PSYCHOL, V32, P437, DOI 10.1093/jpepsy/jsl029

Grey M, 2000, J PEDIATR-US, V137, P107, DOI 10.1067/mpd.2000.106568

Hains AA, 2006, DIABETES CARE, V29, P818, DOI 10.2337/diacare.29.04.06.dc05-1828

Hains AA, 2000, DIABETES EDUCATOR, V26, P417, DOI 10.1177/014572170002600309

Hanna K M, 2000, Issues Compr Pediatr Nurs, V23, P165

Hanson CL, 1992, STRESS COPING CHILD, P213

Harris PA, 2009, J BIOMED INFORM, V42, P377, DOI 10.1016/j.jbi.2008.08.010

Hayes RA, 2002, PSYCHOL REP, V90, P1161, DOI 10.2466/PR0.90.4.1161-1173

Holmes CS, 2006, J PEDIATR PSYCHOL, V31, P770, DOI 10.1093/jpepsy/jsj083

Hood KK, 2009, PEDIATRICS, V124, pE1171, DOI 10.1542/peds.2009-0207

Iannotti RJ, 2006, DIABETES CARE, V29, P2263, DOI 10.2337/dc06-0685

Lancaster BM, 2010, DIABETES RES CLIN PR, V90, P243, DOI 10.1016/j.diabres.2010.07.003

Larkin ME, 2008, DIABETES EDUCATOR, V34, P511, DOI 10.1177/0145721708317869

Lenhart A., 2007, TEENS PRIVACY ONLINE, P1

Logan D, 2003, J PEDIATR PSYCHOL, V28, P383, DOI 10.1093/jpepsy/jsg028

Mulvaney SA, PATIENT ED IN PRESS

Mulvaney SA, 2008, DIABETES EDUCATOR, V34, P674, DOI 10.1177/0145721708320902

Mulvaney SA, 2010, DIABETES CARE, V33, P602, DOI 10.2337/dc09-1881

Paulhus DL, 1991, MEASURES PERSONALITY, P17, DOI DOI 10.1016/B978-0-12-590241-0.50006-X

Polonsky WH, 2005, DIABETES CARE, V28, P626, DOI 10.2337/diacare.28.3.626

Riekert KA, 2000, PROMOTING ADHERENCE TO MEDICAL TREATMENT IN CHRONIC CHILDHOOD ILLNESS: CONCEPTS, METHODS, AND INTERVENTIONS, P3

Ryan RM, 2000, AM PSYCHOL, V55, P68, DOI 10.1037/0003-066X.55.1.68

Schlundt DG, 1996, J ADOLESCENT HEALTH, V19, P282, DOI 10.1016/1054-139X(95)00197-Z

SCHLUNDT DG, 1994, DIABETES EDUCATOR, V20, P207, DOI 10.1177/014572179402000305

Siminerio LM, 2008, DIABETES EDUCATOR, V34, p18S, DOI 10.1177/0145721707313938

Simons LE, 2007, J PEDIATR PSYCHOL, V32, P831, DOI 10.1093/jpepsy/jsm030

Skinner T., 1998, J PEDIATR PSYCHOL, V25, P257

Soo H, 2009, J HEALTH PSYCHOL, V14, P933, DOI 10.1177/1359105309341146

Tabachnick B. G., 2007, USING MULTIVARIATE S

Travis T, 1997, DIABETES EDUCATOR, V23, P152, DOI 10.1177/014572179702300205

Weissberg-Benchell J, 2011, PEDIATR DIABETES, V12, P341, DOI 10.1111/j.1399-5448.2010.00720.x

Williams GC, 1998, DIABETES CARE, V21, P1644, DOI 10.2337/diacare.21.10.1644

Williams GC, 2009, ANN BEHAV MED, V37, P315, DOI 10.1007/s12160-009-9090-y

Wysocki Tim, 2006, Curr Diab Rep, V6, P117, DOI 10.1007/s11892-006-0022-y

NR 50

TC 30

Z9 30

U1 0

U2 25

PU ELSEVIER IRELAND LTD

PI CLARE

PA ELSEVIER HOUSE, BROOKVALE PLAZA, EAST PARK SHANNON, CO, CLARE, 00000,

IRELAND

SN 0168-8227

J9 DIABETES RES CLIN PR

JI Diabetes Res. Clin. Pract.

PD OCT

PY 2011

VL 94

IS 1

BP 77

EP 83

DI 10.1016/j.diabres.2011.06.010

PG 7

WC Endocrinology & Metabolism

SC Endocrinology & Metabolism

GA 841RA

UT WOS:000296529100019

PM 21737172

OA Green Accepted

DA 2019-01-08

ER

PT J

AU Siegelaar, SE

Hoekstra, JBL

DeVries, JH

AF Siegelaar, Sarah E.

Hoekstra, Joost B. L.

DeVries, J. Hans

TI Special considerations for the diabetic patient in the ICU; targets for

treatment and risks of hypoglycaemia

SO BEST PRACTICE & RESEARCH CLINICAL ENDOCRINOLOGY & METABOLISM

LA English

DT Article

DE diabetes mellitus; intensive care unit; hyperglycaemia; hypoglycaemia;

glucose variability; morbidity; mortality; intensive insulin therapy

ID CRITICALLY-ILL PATIENTS; INTENSIVE-CARE-UNIT;

RESPIRATORY-DISTRESS-SYNDROME; BLOOD-GLUCOSE CONCENTRATION; INSULIN

THERAPY; GLYCEMIC CONTROL; STRESS HYPERGLYCEMIA; INCREASED MORTALITY;

HOSPITAL MORTALITY; RANDOMIZED-TRIAL

AB Due to the diabetes pandemic the number of diabetic patients admitted to the intensive care unit (ICU) increases. Diabetic patients admitted to the ICU are more vulnerable for developing complications as compared to non-diabetic patients, but this does not directly translate into higher mortality rates. However, mortality might differ per admission diagnosis. Hyperglycaemia is common in diabetic as well as non-diabetic critically ill patients, but probably chronic hyperglycaemia is pathophysiologically different from acute hyperglycaemia. As opposed to non-diabetic patients, there is discussion about the association between hyperglycaemia and mortality in diabetic patients. They do not seem to benefit from strict glycaemic control and also glucose variability appears less harmful, although clinical trials in diabetic populations have not been performed yet. Diabetes is a risk factor for hypoglycaemia and evidence suggests that even near-normal glucose levels are associated with worse outcome. Taking this together, it is suggested to strive for moderate targets when treating hyperglycaemia in critically ill diabetic patients. (C) 2011 Elsevier Ltd. All rights reserved.

C1 [Siegelaar, Sarah E.; Hoekstra, Joost B. L.; DeVries, J. Hans] Univ Amsterdam, Acad Med Ctr, Dept Internal Med, NL-1105 AZ Amsterdam, Netherlands.

RP Siegelaar, SE (reprint author), Univ Amsterdam, Acad Med Ctr, Dept Internal Med, Meibergdreef 9,Room F4-257, NL-1105 AZ Amsterdam, Netherlands.

EM s.e.siegelaar@amc.uva.nl; j.b.hoekstra@amc.uva.nl;

j.h.devries@amc.uva.nl

OI DeVries, J. Hans/0000-0001-9196-9906

CR Ahmad R, 2007, ARCH SURG-CHICAGO, V142, P613, DOI 10.1001/archsurg.142.7.613

Al-Dorzi HM, 2010, ANAESTH INTENS CARE, V38, P695

ALEXIEWICZ JM, 1995, ANN INTERN MED, V123, P919, DOI 10.7326/0003-4819-123-12-199512150-00004

Alvarez-Lerma F, 2003, INTENS CARE MED, V29, P1069, DOI 10.1007/s00134-003-1807-y

Arabi YM, 2008, CRIT CARE MED, V36, P3190, DOI 10.1097/CCM.0b013e31818f21aa

Bochicchio GV, 2006, WORLD J SURG, V30, P114, DOI 10.1007/s00268-005-0203-x

Braithwaite SS, 2010, CRIT CARE MED, V38, P717, DOI 10.1097/CCM.0b013e3181cc8c6a

Brown JR, 2006, SEMIN THORAC CARDIOV, V18, P281, DOI 10.1053/j.semtcvs.2006.04.004

Brownlee M, 2001, NATURE, V414, P813, DOI 10.1038/414813a

Brunkhorst FM, 2008, NEW ENGL J MED, V358, P125, DOI 10.1056/NEJMoa070716

BRUNNER R, 2011, CRITICAL CARE MED

Bucerius J, 2003, THORAC CARDIOV SURG, V51, P11, DOI 10.1055/s-2003-37280

De la Rosa GD, 2008, CRIT CARE, V12, DOI 10.1186/cc7017

Dossett LA, 2008, AM SURGEON, V74, P679

Douglas SL, 2007, AM J CRIT CARE, V16, P447

Douglas SL, 2009, J CRIT CARE, V24, P302, DOI 10.1016/j.jcrc.2008.02.004

Dungan KM, 2009, LANCET, V373, P1798, DOI 10.1016/S0140-6736(09)60553-5

Durao MS, 2010, ANAESTH INTENS CARE, V38, P467

Egi M, 2011, CRITICAL CARE MED, V39, P1

Egi M, 2008, CRIT CARE MED, V36, P2249, DOI [10.1097/CCM.0b013e318181039a, 10.1097/CCM.0b013e31818039a]

Egi M, 2006, ANESTHESIOLOGY, V105, P244, DOI 10.1097/00000542-200608000-00006

Falciglia M, 2009, CRIT CARE MED, V37, P3001, DOI 10.1097/CCM.0b013e3181b083f7

Finfer S, 2009, NEW ENGL J MED, V360, P1283, DOI 10.1056/NEJMoa0810625

Finney SJ, 2003, JAMA-J AM MED ASSOC, V290, P2041, DOI 10.1001/jama.290.15.2041

Furnary Anthony P, 2006, Endocr Pract, V12 Suppl 3, P22

Furnary AP, 2003, J THORAC CARDIOV SUR, V125, P1007, DOI 10.1067/mtc.2003.181

Gandhi GY, 2007, ANN INTERN MED, V146, P233, DOI 10.7326/0003-4819-146-4-200702200-00002

Gong MN, 2005, CRIT CARE MED, V33, P1191, DOI 10.1097/01.CCM.0000165566.82925.14

Graham BB, 2010, CRIT CARE MED, V38, P16, DOI 10.1097/CCM.0b013e3181b9eaa5

Guvener M, 2002, ENDOCR J, V49, P531, DOI 10.1507/endocrj.49.531

Hermanides J, 2010, CRIT CARE MED, V38, P1430, DOI 10.1097/CCM.0b013e3181de562c

Hermanides J, 2010, CRIT CARE MED, V38, P838, DOI 10.1097/CCM.0b013e3181cc4be9

HEUSER MD, 1992, ARCH INTERN MED, V152, P1683, DOI 10.1001/archinte.152.8.1683

Hirshberg E, 2008, PEDIATR CRIT CARE ME, V9, P361, DOI 10.1097/PCC.0b013e318172d401

Holzinger U, 2010, DIABETES CARE, V33, P467, DOI 10.2337/dc09-1352

Kee Cheryl A, 2006, Can J Cardiovasc Nurs, V16, P20

Krinsley JS, 2008, CRIT CARE MED, V36, P3008, DOI 10.1097/CCM.0b013e31818b38d2

Krinsley JS, 2007, CRIT CARE MED, V35, P2262, DOI 10.1097/01.CCM.0000282073.98414.4B

Krinsley JS, 2006, SEMIN THORAC CARDIOV, V18, P317, DOI 10.1053/j.semtcvs.2006.12.003

Krinsley James Stephen, 2009, J Diabetes Sci Technol, V3, P1292

Krinsley JS, 2003, MAYO CLIN PROC, V78, P1471, DOI 10.4065/78.12.1471

Laupland KB, 2004, CRIT CARE MED, V32, P992, DOI 10.1097/01.CCM.0000119424.31648.1E

Lazar HL, 2004, CIRCULATION, V109, P1497, DOI 10.1161/01.CIR.0000121747.71054.79

Leavitt BJ, 2004, CIRCULATION, V110, pII41, DOI 10.1161/10.CIR.0000138197.07051.e7

Lemkes BA, 2010, J THROMB HAEMOST, V8, P1663, DOI 10.1111/j.1538-7836.2010.03910.x

Ligtenberg JJM, 2006, INTENS CARE MED, V32, P435, DOI 10.1007/s00134-005-0052-y

Logtenberg SJ, 2009, DIABETES TECHNOL THE, V11, P31, DOI 10.1089/dia.2008.0028

MARHOFFER W, 1992, DIABETES CARE, V15, P256, DOI 10.2337/diacare.15.2.256

MASLA M, 2010, J CARDIOTHORACIC VAS

Michalia M, 2009, INTENS CARE MED, V35, P448, DOI 10.1007/s00134-008-1288-0

Moghissi ES, 2009, DIABETES CARE, V32, P1119, DOI 10.2337/dc09-9029

Moss M, 2000, CRIT CARE MED, V28, P2187, DOI 10.1097/00003246-200007000-00001

Rabiee Atoosa, 2009, J Diabetes Sci Technol, V3, P951

Rady MY, 2005, MAYO CLIN PROC, V80, P1558, DOI 10.4065/80.12.1558

Reyes Maryrose, 2008, Dynamics, V19, P18

Ryan T, 1997, CRIT CARE MED, V25, P2009, DOI 10.1097/00003246-199712000-00018

Siegelaar SE, 2011, DIABETES CARE, V34, P855, DOI [10.2337/dc10-1684, 10.2337/dc10-1882]

Siegelaar SE, 2010, CRIT CARE, V14, DOI 10.1186/cc9369

Siegelaar SE, 2010, ENDOCR REV, V31, P171, DOI 10.1210/er.2009-0021

Skjaervold NK, 2011, ANESTHESIOLOGY, V114, P120, DOI 10.1097/ALN.0b013e3181ff4187

Slynkova K, 2006, CRIT CARE, V10, DOI 10.1186/cc5051

Stegenga ME, 2010, CRIT CARE MED, V38, P539, DOI 10.1097/CCM.0b013e3181c02726

Tissot E, 2001, EUR J CLIN MICROBIOL, V20, P260, DOI 10.1007/s10096-001-8097-5

Van den Berghe G, 2006, NEW ENGL J MED, V354, P449, DOI 10.1056/NEJMoa052521

Van den Berghe G, 2001, NEW ENGL J MED, V345, P1359, DOI 10.1056/NEJMoa011300

Van den Berghe G, 2006, DIABETES, V55, P3151, DOI 10.2337/db06-0855

Vardakas KZ, 2007, DIABETIC MED, V24, P1168, DOI 10.1111/j.1464-5491.2007.02234.x

Vincent JL, 2010, CRIT CARE, V14, DOI 10.1186/cc8866

Vriesendorp TM, 2006, CRIT CARE MED, V34, P96, DOI 10.1097/01.CCM.0000194536.89694.06

Whitcomb BW, 2005, CRIT CARE MED, V33, P2772, DOI 10.1097/01.CCM.0000189741.44071.25

NR 70

TC 6

Z9 6

U1 0

U2 3

PU ELSEVIER SCI LTD

PI OXFORD

PA THE BOULEVARD, LANGFORD LANE, KIDLINGTON, OXFORD OX5 1GB, OXON, ENGLAND

SN 1521-690X

EI 1532-1908

J9 BEST PRACT RES CL EN

JI Best Pract. Res. Clin. Endoc. Metab.

PD OCT

PY 2011

VL 25

IS 5

BP 825

EP 834

DI 10.1016/j.beem.2011.03.004

PG 10

WC Endocrinology & Metabolism

SC Endocrinology & Metabolism

GA 833CQ

UT WOS:000295859800011

PM 21925081

DA 2019-01-08

ER

PT J

AU Quinn, CC

Shardell, MD

Terrin, ML

Barr, EA

Ballew, SH

Gruber-Baldini, AL

AF Quinn, Charlene C.

Shardell, Michelle D.

Terrin, Michael L.

Barr, Erik A.

Ballew, Shoshana H.

Gruber-Baldini, Ann L.

TI Cluster-Randomized Trial of a Mobile Phone Personalized Behavioral

Intervention for Blood Glucose Control

SO DIABETES CARE

LA English

DT Article

ID DIABETES MANAGEMENT; CARE; OUTCOMES; POPULATION; DISTRESS; SIZE; US

AB OBJECTIVE-To test whether adding mobile application coaching and patient/provider web portals to community primary care compared with standard diabetes management would reduce glycated hemoglobin levels in patients with type 2 diabetes.

RESEARCH DESIGN AND METHODS-A cluster-randomized clinical trial, the Mobile Diabetes Intervention Study, randomly assigned 26 primary care practices to one of three stepped treatment groups or a control group (usual care). A total of 163 patients were enrolled and included in analysis. The primary outcome was change in glycated hemoglobin levels over a 1-year treatment period. Secondary outcomes were changes in patient-reported diabetes symptoms, diabetes distress, depression, and other clinical (blood pressure) and laboratory (lipid) values. Maximal treatment was a mobile- and web-based self-management patient coaching system and provider decision support. Patients received automated, real-time educational and behavioral messaging in response to individually analyzed blood glucose values, diabetes medications, and lifestyle behaviors communicated by mobile phone. Providers received quarterly reports summarizing patient's glycemic control, diabetes medication management, lifestyle behaviors, and evidence-based treatment options.

RESULTS-The mean declines in glycated hemoglobin were 1.9% in the maximal treatment group and 0.7% in the usual care group, a difference of 1.2% (P < 0.001) over 12 months. Appreciable differences were not observed between groups for patient-reported diabetes distress, depression, diabetes symptoms, or blood pressure and lipid levels (all P > 0.05).

CONCLUSIONS-The combination of behavioral mobile coaching with blood glucose data, lifestyle behaviors, and patient self-management data individually analyzed and presented with evidence-based guidelines to providers substantially reduced glycated hemoglobin levels over 1 year.

C1 [Quinn, Charlene C.; Shardell, Michelle D.; Terrin, Michael L.; Barr, Erik A.; Ballew, Shoshana H.; Gruber-Baldini, Ann L.] Univ Maryland, Sch Med, Dept Epidemiol & Publ Hlth, Baltimore, MD 21201 USA.

RP Quinn, CC (reprint author), Univ Maryland, Sch Med, Dept Epidemiol & Publ Hlth, Baltimore, MD 21201 USA.

EM cquinn@epi.umaryland.edu

RI Emchi, Karma/Q-1952-2016

FU University of Maryland Baltimore; WellDoc; University of Maryland

FX This research project is funded through a contract between the

University of Maryland Baltimore and WellDoc in addition to

contributions by WellDoc, CareFirst Blue Cross/Blue Shield of Maryland,

LifeScan, and Sprint. Additional funding was provided by the Maryland

Industrial Partnerships program through the University of Maryland, an

initiative of the A. James Clark School of Engineering's Maryland

Technology Enterprise Institute. No other potential conflicts of

interest relevant to this article were reported.

CR Amer Diabet Assoc, 2008, DIABETES CARE, V31, pS12, DOI 10.2337/dc08-S012

Amer Diabet Assoc, 2009, DIABETES CARE, V32, pS13, DOI 10.2337/dc09-S013

Cleland JGF, 2010, JAMA-J AM MED ASSOC, V304, P1383, DOI 10.1001/jama.2010.1387

Cowie CC, 2009, DIABETES CARE, V32, P287, DOI 10.2337/dc08-1296

Duke SAS, 2009, COCHRANE DB SYST REV, DOI 10.1002/14651858.CD005268.pub2

Fisher L, 2008, ANN FAM MED, V6, P246, DOI 10.1370/afm.842

Gaede Peter Haulund, 2003, Ugeskr Laeger, V165, P2658

Gerstein HC, 2008, NEW ENGL J MED, V358, P2545, DOI 10.1056/NEJMoa0802743

GRIFFIN S, 2000, COCHRANE DB SYST REV

Hayes RJ, 1999, INT J EPIDEMIOL, V28, P319, DOI 10.1093/ije/28.2.319

Huang ES, 2009, DIABETES CARE, V32, P2225, DOI 10.2337/dc09-0459

Knowler WC, 2002, NEW ENGL J MED, V346, P393

Kroenke K, 2001, J GEN INTERN MED, V16, P606, DOI 10.1046/j.1525-1497.2001.016009606.x

MCCOLL E, 1995, SOC SCI MED, V41, P1339, DOI 10.1016/0277-9536(95)00120-V

Peyrot M, 2005, DIABETIC MED, V22, P1379, DOI 10.1111/j.1464-5491.2005.01644.x

Polonsky WH, 2005, DIABETES CARE, V28, P626, DOI 10.2337/diacare.28.3.626

Quinn CC, 2008, DIABETES TECHNOL THE, V10, P160, DOI 10.1089/dia.2008.0283

Quinn CC, 2009, CONTEMP CLIN TRIALS, V30, P334, DOI 10.1016/j.cct.2009.02.004

Renders CM, 2001, COCHRANE DB SYST REV, V1, DOI DOI 10.1002/14651858.CD001481

ROBINS JM, 1995, J AM STAT ASSOC, V90, P122, DOI 10.2307/2291135

Saaddine JB, 2006, ANN INTERN MED, V144, P465, DOI 10.7326/0003-4819-144-7-200604040-00005

Shea S, 2006, J AM MED INFORM ASSN, V13, P40, DOI 10.1197/jamia.M1917

Solomon CG, 2003, NEW ENGL J MED, V348, P457, DOI 10.1056/NEJMe020172

VERBEKE G, 2007, REPEATED MEASUREMENT

Whitty P, 1997, QUAL LIFE RES, V6, P407, DOI 10.1023/A:1018443628933

NR 25

TC 202

Z9 208

U1 1

U2 48

PU AMER DIABETES ASSOC

PI ALEXANDRIA

PA 1701 N BEAUREGARD ST, ALEXANDRIA, VA 22311-1717 USA

SN 0149-5992

EI 1935-5548

J9 DIABETES CARE

JI Diabetes Care

PD SEP

PY 2011

VL 34

IS 9

BP 1934

EP 1942

DI 10.2337/dc11-0366

PG 9

WC Endocrinology & Metabolism

SC Endocrinology & Metabolism

GA 824IH

UT WOS:000295195100009

PM 21788632

OA Green Published, Other Gold

HC Y

HP N

DA 2019-01-08

ER

PT J

AU Welch, G

Allen, NA

Zagarins, SE

Stamp, KD

Bursell, SE

Kedziora, RJ

AF Welch, Garry

Allen, Nancy A.

Zagarins, Sofija E.

Stamp, Kelly D.

Bursell, Sven-Erik

Kedziora, Richard J.

TI Comprehensive Diabetes Management Program for Poorly Controlled Hispanic

Type 2 Patients at a Community Health Center

SO DIABETES EDUCATOR

LA English

DT Article

ID ETHNIC-DIFFERENCES; PROBLEM AREAS; CARE; RETINOPATHY; BARRIERS;

QUESTIONNAIRE; IMPROVEMENT; PREDICTORS

AB Technology and improved care coordination models can help diabetes educators and providers meet national care standards and provide culturally sensitive diabetes education that may improve diabetes outcomes. The purpose of the study was to evaluate the clinical usefulness of a nurse-led diabetes care program (Comprehensive Diabetes Management Program, CDMP) for poorly controlled Hispanic type 2 diabetes (T2DM) patients in an urban community health center setting. Patients were randomized to the intervention condition (IC; n = 21) or an attention control condition (AC; n = 18). IC and AC conditions were compared on rates of adherence to national clinical practice guidelines (blood glucose, blood pressure, foot exam, eye exam), and levels of diabetes distress, depression, and treatment satisfaction. IC patients had a significant improvement in A1C from baseline to 12-month follow-up compared with AC (-1.6% +/- 1.4% versus -0.6% +/- 1.1%; P = .01). The proportion of IC patients meeting clinical goals at follow-up tended to be higher than AC for A1c (IC = 45%; AC = 28%), systolic blood pressure (IC = 55%; AC = 28%), eye screening (IC = 91%; AC = 78%), and foot screening, (IC = 86%; AC = 72%). Diabetes distress and treatment satisfaction also showed greater improvement for IC than AC (P = .05 and P = .06, respectively), with no differences for depression. The CDMP intervention was more effective than an attention control condition in helping patients meet evidence-based guidelines for diabetes care.

C1 [Welch, Garry] Baystate Hlth Syst, Behav Med Res, Dept Behav Med Res, Springfield, MA 01105 USA.

[Allen, Nancy A.; Stamp, Kelly D.] Boston Coll, William F Connell Sch Nursing, Chestnut Hill, MA 02167 USA.

[Bursell, Sven-Erik] Univ Hawaii Manoa, Telehealth Res Inst, John A Burns Sch Med, Honolulu, HI 96822 USA.

[Kedziora, Richard J.] Estenda Solut Inc, Conshohocken, PA USA.

RP Welch, G (reprint author), Baystate Hlth Syst, Behav Med Res, Dept Behav Med Res, 140 High St,Room 2104, Springfield, MA 01105 USA.

EM garry.welch@baystatehealth.org

FU Baystate Medical Center [AA-BMC P99]

FX This study was funded by Baystate Medical Center Academic Affairs

Internal Research Grant #AA-BMC P99.

CR Abu-Qamar M, 2007, INT J EVID-BASED HEA, V5, P254, DOI 10.1111/j.1479-6988.2007.00066.x

*AM ASS DIAB ED, 2002, DIABETES ED, V28, P735

American Psychiatric Association, 2000, DIAGN STAT MAN MENT

BEASER RS, 2001, JOSLINS DIABETES DES

Bradley C, 1999, DIABETES CARE, V22, P530, DOI 10.2337/diacare.22.3.530

Bursell SE, 2001, OPHTHALMOLOGY, V108, P572, DOI 10.1016/S0161-6420(00)00604-7

Caban A, 2006, DIABETES EDUCATOR, V32, P584, DOI 10.1177/0145721706290435

Cavallerano JD, 2005, AM J OPHTHALMOL, V140, P667, DOI 10.1016/j.ajo.2005.03.075

Centers for Disease Control and Prevention, 2007, NAT DIAB FACT SHEET

Chin MH, 2001, DIABETES CARE, V24, P268, DOI 10.2337/diacare.24.2.268

Conlin PR, 2006, J REHABIL RES DEV, V43, P733, DOI 10.1682/JRRD.2005.07.0117

Dall TM, 2010, HEALTH AFFAIR, V29, DOI 10.1377/hlthaff.2009.0155

Fanning EL, 2004, DIABETES CARE, V27, P1638, DOI 10.2337/diacare.27.7.1638

Fisher L, 2010, DIABETES CARE, V33, P23, DOI 10.2337/dc09-1238

Fonda SJ, 2008, DIABETES TECHNOL THE, V10, P16, DOI 10.1089/dia.2007.0252

Goderis G, 2009, IMPLEMENT SCI, V4, DOI 10.1186/1748-5908-4-41

Hjelm-Karlsson K, 1991, West J Nurs Res, V13, P284, DOI 10.1177/019394599101300211

Khattab M, 2010, J DIABETES COMPLICAT, V24, P84, DOI 10.1016/j.jdiacomp.2008.12.008

Kroenke K, 2002, PSYCHIAT ANN, V32, P509, DOI 10.3928/0048-5713-20020901-06

Lanting LC, 2005, DIABETES CARE, V28, P2280, DOI 10.2337/diacare.28.9.2280

Latham CL, 2009, WESTERN J NURS RES, V31, P364, DOI 10.1177/0193945908328263

Lee PW, 2007, J AM BOARD FAM MED, V20, P427, DOI 10.3122/jabfm.2007.05.070045

Mensing Carole, 2007, Diabetes Care, V30 Suppl 1, pS96, DOI 10.2337/dc07-S096

Nagelkerk J, 2006, J ADV NURS, V54, P151, DOI 10.1111/j.1365-2648.2006.03799.x

*NAT ASS COMM HLTH, ON YEAR LAT HLTH CTR

Nuovo J, 2007, CURR DIABETES REV, V3, P226, DOI 10.2174/157339907782330030

Philis-Tsimikas A, 2004, DIABETES CARE, V27, P110, DOI 10.2337/diacare.27.1.110

Pleis John R, 2009, Vital Health Stat 10, P1

RUBIN R, 2000, PSYCHOL DIABETES CAR

Saaddine JB, 2006, ANN INTERN MED, V144, P465, DOI 10.7326/0003-4819-144-7-200604040-00005

Schram MT, 2009, CURR DIABETES REV, V5, P112, DOI 10.2174/157339909788166828

Spenceley SM, 2006, CAN J NURS RES, V38, P124

*US CENS BUR, 2009, AM COMM SURV B03001

*US DEP HHS, STAT APPR DIAB QUAL

Weinick RM, 2000, MED CARE RES REV, V57, P36, DOI 10.1177/107755800773743592

Welch G, 2005, DIABETES, V54, pA470

Welch G, 2003, DIABETIC MED, V20, P69, DOI 10.1046/j.1464-5491.2003.00832.x

WELCH G, 2009, J DIAB NURSING, V13, P319

Welch G, 2010, DIABETES RES CLIN PR, V88, P1, DOI 10.1016/j.diabres.2009.12.026

Welch GW, 1997, DIABETES CARE, V20, P760, DOI 10.2337/diacare.20.5.760

Whited JD, 2005, TELEMED J E-HEALTH, V11, P641, DOI 10.1089/tmj.2005.11.641

Whittemore R, 2007, J TRANSCULT NURS, V18, P157, DOI 10.1177/1043659606298615

ZAGARINS SE, 2011, J BEHAV MED

2009, DIABETES CARE S1, V32, pS6

NR 44

TC 24

Z9 25

U1 1

U2 15

PU SAGE PUBLICATIONS INC

PI THOUSAND OAKS

PA 2455 TELLER RD, THOUSAND OAKS, CA 91320 USA

SN 0145-7217

J9 DIABETES EDUCATOR

JI Diabetes Educ.

PD SEP-OCT

PY 2011

VL 37

IS 5

BP 680

EP 688

DI 10.1177/0145721711416257

PG 9

WC Endocrinology & Metabolism; Public, Environmental & Occupational Health

SC Endocrinology & Metabolism; Public, Environmental & Occupational Health

GA 819PY

UT WOS:000294841600007

PM 21918206

DA 2019-01-08

ER

PT J

AU Trief, PM

Sandberg, J

Fisher, L

Dimmock, JA

Scales, K

Hessler, DM

Weinstock, RS

AF Trief, Paula M.

Sandberg, Jonathan

Fisher, Lawrence

Dimmock, Jacqueline A.

Scales, Kasandra

Hessler, Danielle M.

Weinstock, Ruth S.

TI Challenges and lessons learned in the development and implementation of

a couples-focused telephone intervention for adults with type 2

diabetes: the Diabetes Support Project

SO TRANSLATIONAL BEHAVIORAL MEDICINE

LA English

DT Article

DE Type 2 diabetes; Glycemic control; Marital interaction; Social support;

Telephone intervention

AB Type 2 diabetes (T2DM) studies report that marital/family support relates to glycemic control, adherence, and quality of life. Yet, there are few reports on couples-focused interventions. This study aims to describe the challenges faced and lessons learned in the implementation of a theoretically based, couples intervention. Three hundred fifty couples (one partner has T2DM in poor glycemic control) are randomized to a couples intervention, individual intervention, or enhanced usual care. All contacts are by telephone to increase reach. The medical (e.g., glycemic control), psychosocial (e.g., diabetes distress), and behavioral (e.g., regimen adherence) outcomes were measured. Challenges in recruitment, assessment, and intervention with couples are described, with suggestions about how to address them. Findings concerning the efficacy and cost-effectiveness of the couples intervention, its effect on partners, and possible mechanisms of demonstrated changes, are anticipated in 2013. Interventionists need specific skills to work with couples to promote communal coping and increase the likelihood of an efficacious couples intervention.

C1 [Trief, Paula M.; Dimmock, Jacqueline A.; Scales, Kasandra; Weinstock, Ruth S.] SUNY Upstate Med Univ, Syracuse, NY 13210 USA.

[Sandberg, Jonathan] Brigham Young Univ, Provo, UT 84602 USA.

[Fisher, Lawrence; Hessler, Danielle M.] Univ Calif San Francisco, San Francisco, CA 94143 USA.

RP Trief, PM (reprint author), SUNY Upstate Med Univ, 750 E Adams St, Syracuse, NY 13210 USA.

EM triefp@upstate.edu

OI Trief, Paula/0000-0002-2827-5722

FU National Institute of Diabetes, Digestive, and Kidney Diseases of the

National Institutes of Health [1R18DK080867-01A2]

FX This study is being funded by the National Institute of Diabetes,

Digestive, and Kidney Diseases of the National Institutes of Health

(grant no. 1R18DK080867-01A2 Clinicaltrials.gov no. NCT0107523).

CR Bandura A., 1963, SOCIAL LEARNING PERS

BLACK DR, 1990, HEALTH PSYCHOL, V9, P330, DOI 10.1037//0278-6133.9.3.330

Campbell TA, 2002, EFFECTIVENESS RES MA, P311

CARDENAS L, 1987, AM J MED SCI, V293, P28, DOI 10.1097/00000441-198701000-00007

Delamater AM, 2001, DIABETES CARE, V24, P1286, DOI 10.2337/diacare.24.7.1286

DEVOOGD JN, 1993, J MUSCULOSKELET PAIN, V1, P273

DUNKELSCHETTER C, 1984, J SOC ISSUES, V40, P77, DOI 10.1111/j.1540-4560.1984.tb01108.x

Fisher L, 2000, J FAM PRACTICE, V49, P561

Fukunishi I, 1998, DIABETES RES CLIN PR, V41, P207, DOI 10.1016/S0168-8227(98)00083-7

GARAYSEVILA ME, 1995, J DIABETES COMPLICAT, V9, P81, DOI 10.1016/1056-8727(94)00021-F

GILDEN JL, 1989, J AM GERIATR SOC, V37, P1023, DOI 10.1111/j.1532-5415.1989.tb06915.x

Glasgow RE, 2006, AM J PREV MED, V30, P67, DOI 10.1016/j.amepre.2005.08.037

HERBERT TB, 1993, PSYCHOL BULL, V113, P472, DOI 10.1037//0033-2909.113.3.472

Kelley H. H., 1978, INTERPERSONAL RELATI

Kiecolt-Glaser JK, 2001, PSYCHOL BULL, V127, P472, DOI 10.1037//0033-2909.127.4.472

Lewis MA, 2006, SOC SCI MED, V62, P1369, DOI 10.1016/j.socscimed.2005.08.006

Manne S, 2007, HEALTH PSYCHOL, V26, P735, DOI 10.1037/0278-6133.26.6.735

Martire LM, 2010, ANN BEHAV MED, V40, P325, DOI 10.1007/s12160-010-9216-2

McBride CM, 2004, AM J PREV MED, V27, P232, DOI 10.1016/j.amepre.2004.06.005

Palmer C. A., 2000, PSYCHOL COUPLES ILLN, P311

PEYROT M, 1988, J MARRIAGE FAM, V50, P363, DOI 10.2307/352003

Pieper B A, 1990, Diabetes Educ, V16, P108, DOI 10.1177/014572179001600206

POLONSKY WH, 1995, DIABETES CARE, V18, P754, DOI 10.2337/diacare.18.6.754

PRIMOMO J, 1990, RES NURS HEALTH, V13, P153, DOI 10.1002/nur.4770130304

Rissman R., 1987, FAMILY SYSTEM MED, V5, P61

Robles TF, 2003, PHYSIOL BEHAV, V79, P409, DOI 10.1016/S0031-9384(03)00160-4

Rusbult CE, 2003, ANNU REV PSYCHOL, V54, P351, DOI 10.1146/annurev.psych.54.101601.145059

SCHMALING K, 2000, PSYCHOL COUPLES ILLN

SHUMAKER SA, 1998, HDB HLTH BEHAV CHANG

Siegel JE, 1996, JAMA-J AM MED ASSOC, V276, P1339, DOI 10.1001/jama.276.16.1339

Trief PM, 1998, DIABETES CARE, V21, P241, DOI 10.2337/diacare.21.2.241

Trief PM, 2004, ANN BEHAV MED, V27, P148, DOI 10.1207/s15324796abm2703_2

Trief PM, 2001, DIABETES CARE, V24, P1384, DOI 10.2337/diacare.24.8.1384

WING RR, 1991, J CONSULT CLIN PSYCH, V59, P156, DOI 10.1037/0022-006X.59.1.156

NR 34

TC 12

Z9 12

U1 0

U2 4

PU SPRINGER INTERNATIONAL PUBLISHING AG

PI CHAM

PA GEWERBESTRASSE 11, CHAM, CH-6330, SWITZERLAND

SN 1869-6716

EI 1613-9860

J9 TRANSL BEHAV MED

JI Transl. Behav. Med.

PD SEP

PY 2011

VL 1

IS 3

BP 461

EP 467

DI 10.1007/s13142-011-0057-8

PG 7

WC Public, Environmental & Occupational Health

SC Public, Environmental & Occupational Health

GA V39LI

UT WOS:000209412100016

PM 22003374

OA Green Accepted, Green Published

DA 2019-01-08

ER

PT J

AU Hajos, TRS

Pouwer, F

de Grooth, R

Holleman, F

Twisk, JWR

Diamant, M

Snoek, FJ

AF Hajos, T. R. S.

Pouwer, F.

de Grooth, R.

Holleman, F.

Twisk, J. W. R.

Diamant, M.

Snoek, F. J.

TI Initiation of insulin glargine in patients with Type 2 diabetes in

suboptimal glycaemic control positively impacts health-related quality

of life. A prospective cohort study in primary care

SO DIABETIC MEDICINE

LA English

DT Article

DE emotional well-being; insulin glargine; insulin initiation; quality of

life

ID OF-LIFE; THERAPY; SYMPTOMS; TRIAL; HYPOGLYCEMIA; SATISFACTION;

VALIDATION; MELLITUS; ANALOGS; SCALE

AB Aims To study prospectively the impact of initiating insulin glargine in suboptimally controlled insulin-naive patients with Type 2 diabetes on health-related quality of life in relation to glycaemic control.

Methods Insulin-naive Dutch patients with Type 2 diabetes in suboptimal glycaemic control (HbA(1c) > 53 mmol/mol; 7%) on maximum dose of oral glucose-lowering medications were included from 363 primary care practices (n = 911). Patients started insulin glargine and were followed up for 6 months. At baseline (start insulin therapy), 3 and 6 months, HbA(1c) was measured and patients completed self-report health-related quality of life measures, including emotional well-being (World Health Organization-5 well-being index), fear of hypoglycaemia (Hypoglycaemia Fear Survey) and diabetes symptom distress (Diabetes Symptom Checklist-revised). Data were analysed using generalized estimating equations analysis.

Results HbA(1c) (mmol/mol; %) decreased from 69 +/- 16; 8.5 +/- 1.7 to 60 +/- 11; 7.6 +/- 1.0 and 57 +/- 11; 7.3 +/- 1.0 at 3 and 6 months, respectively (P < 0.001). Pre-insulin BMI (kg/m(2)) was 30 +/- 5.7, which remained stable at 3 months (30 +/- 5.8) and increased to 31 +/- 5.9 at 6 months (P = 0.004); no significant changes in self-reported symptomatic and severe hypoglycaemia were observed, while nocturnal hypoglycaemia slightly decreased. The Hypoglycaemia Fear Survey score decreased from 14.6 +/- 16.2 to 12.1 +/- 15.2 and 10.8 +/- 14.4 at 3 and 6 months, respectively (P < 0.001). The Diabetes Symptom Checklist-revised score decreased from 15 +/- 14 to 10 +/- 12 and 10 +/- 13 (P < 0.001), with most pronounced reductions in hyperglycaemic symptoms and fatigue. The World Health Organization-5 score increased from 57 +/- 25.3 to 65 +/- 21.6 at 3-month follow-up and 67 +/- 21.8 at 6-month follow-up (P < 0.001).

Conclusions Results of this observational study demonstrate combined glycaemic and health-related quality of life benefits of initiating insulin glargine in patients with Type 2 diabetes in routine primary care.

C1 [Hajos, T. R. S.] Vrije Univ Amsterdam Med Ctr, EMGO Inst, Dept Med Psychol, NL-1081 BT Amsterdam, Netherlands.

[Hajos, T. R. S.; Twisk, J. W. R.; Diamant, M.; Snoek, F. J.] EMGO Res Inst, Amsterdam, Netherlands.

[Pouwer, F.] Tilburg Univ, Ctr Res Psychol Somat Dis CoRPS, NL-5000 LE Tilburg, Netherlands.

[de Grooth, R.] Sanofi Aventis, Gouda, Netherlands.

[Holleman, F.] Univ Amsterdam, Acad Med Ctr, Dept Internal Med, NL-1105 AZ Amsterdam, Netherlands.

[Twisk, J. W. R.] Vrije Univ Amsterdam Med Ctr, Dept Clin Epidemiol & Biostat, NL-1081 BT Amsterdam, Netherlands.

Vrije Univ Amsterdam Med Ctr, Ctr Diabet, Dept Internal Med, NL-1081 BT Amsterdam, Netherlands.

RP Hajos, TRS (reprint author), Vrije Univ Amsterdam Med Ctr, EMGO Inst, Dept Med Psychol, Van der Boechorststr 7, NL-1081 BT Amsterdam, Netherlands.

EM t.hajos@vumc.nl

FU sanofi-aventis; Amylin Pharmaceuticals Inc.; Eli Lilly and Company; Novo

Nordisk; Merck; Sharp and Dohme; Novartis; Takeda; VU Medical Centre

from sanofi-aventis

FX RdeG is an employee of sanofi-aventis. FH has acquired funds for his

activities as a principal investigator and steering committee member for

a multinational trial sponsored by sanofi-aventis. MD is a consultant

and speaker for Eli Lilly and Company, Novo Nordisk and Merck, Sharp and

Dohme, and a consultant for sanofi-aventis. Through MD, the VU

University Medical Center in Amsterdam has received research grants from

Amylin Pharmaceuticals Inc., Eli Lilly and Company, Novo Nordisk, Merck,

Sharp and Dohme, Novartis and Takeda. FJS has received a research grant

via the VU Medical Centre from sanofi-aventis for two observational

studies related to psychological outcomes of insulin glargine therapy;

he has consulted for sanofi-aventis on the topic of quality-of-life

measurement and received speakers fee from sanofi-aventis for lectures

delivered in the context of continuous medical education. There are no

other potential conflicts of interest relevant to this article.

CR Amiel SA, 2008, DIABETIC MED, V25, P245, DOI 10.1111/j.1464-5491.2007.02341.x

Bech P, 2003, INT J METH PSYCH RES, V12, P85, DOI 10.1002/mpr.145

Bouma M, 2006, Ned Tijdschr Geneeskd, V150, P2251

Boye KS, 2006, HEALTH QUAL LIFE OUT, V4, DOI 10.1186/1477-7525-4-80

Bradley C, 2008, DIABETES OBES METAB, V10, P50, DOI 10.1111/j.1463-1326.2008.00871.x

COX DJ, 1987, DIABETES CARE, V10, P617, DOI 10.2337/diacare.10.5.617

Dale J, 2010, PRIM CARE DIABETES, V4, P85, DOI 10.1016/j.pcd.2010.03.001

de Sonnaville JJJ, 1998, DIABETES CARE, V21, P919, DOI 10.2337/diacare.21.6.919

DeVries JH, 2003, DIABETES-METAB RES, V19, P357, DOI 10.1002/dmrr.405

Dluhy RG, 2008, NEW ENGL J MED, V358, P2630, DOI 10.1056/NEJMe0804182

Hartman I, 2008, CLIN MED RES, V6, P54, DOI 10.3121/cmr.2008.793

Heinemann L, 2000, DIABETES CARE, V23, P644, DOI 10.2337/diacare.23.5.644

Hirsch IB, 2005, NEW ENGL J MED, V352, P174, DOI 10.1056/NEJMra040832

Horvath K, 2007, COCHRANE DB SYST REV

Kunt T, 2009, INT J CLIN PRACT, V63, P6, DOI 10.1111/j.1742-1241.2009.02176.x

Lowe B, 2004, J AFFECT DISORDERS, V78, P131, DOI 10.1016/S0165-0327(02)00237-9

Nathan DM, 2006, DIABETES CARE, V29, P1963, DOI 10.2337/dc06-9912

Polonsky WH, 2005, DIABETES CARE, V28, P2543, DOI 10.2337/diacare.28.10.2543

Pouwer F, 2009, DIABETES-METAB RES, V25, pS4, DOI 10.1002/dmrr.981

Rubin DB, 1987, MULTIPLE IMPUTATION

Schafer JL, 2002, PSYCHOL METHODS, V7, P147, DOI 10.1037//1082-989X.7.2.147

Schreiber SA, 2008, DIABETES TECHNOL THE, V10, P121, DOI 10.1089/dia.2007.0265

Snoek FJ, 2007, HEALTH QUAL LIFE OUT, V5, DOI 10.1186/1477-7525-5-69

Swinnen SG, 2010, DIABETES CARE, V33, P1176, DOI 10.2337/dc09-2294

Testa MA, 1998, JAMA-J AM MED ASSOC, V280, P1490, DOI 10.1001/jama.280.17.1490

VanderDoes FEE, 1996, DIABETES CARE, V19, P204, DOI 10.2337/diacare.19.3.204

Vinik AI, 2007, DIABETES CARE, V30, P795, DOI 10.2337/dc06-1712

NR 27

TC 19

Z9 19

U1 0

U2 4

PU WILEY

PI HOBOKEN

PA 111 RIVER ST, HOBOKEN 07030-5774, NJ USA

SN 0742-3071

EI 1464-5491

J9 DIABETIC MED

JI Diabetic Med.

PD SEP

PY 2011

VL 28

IS 9

BP 1096

EP 1102

DI 10.1111/j.1464-5491.2011.03329.x

PG 7

WC Endocrinology & Metabolism

SC Endocrinology & Metabolism

GA 807NG

UT WOS:000293905000015

PM 21843305

DA 2019-01-08

ER

PT J

AU Castillo, O

Roig, B

Sanz, I

Herrero, R

Garay, T

Fuentes, ME

Barreales, L

Egido, JA

AF Castillo, Olga

Roig, Beatriz

Sanz, Isabel

Herrero, Rosa

Garay, Teresa

Fuentes, Manuel E.

Barreales, Laura

Egido, Jose A.

TI Agreement between information provided by stroke patients and their

relatives on psychophysical and vascular risk factors

SO INTERNATIONAL JOURNAL OF NURSING STUDIES

LA English

DT Article

DE Agreement; Psychophysical stress; Risk factors; Stroke

ID MIDDLE-AGED MEN; PROXY RESPONDENTS; PSYCHOLOGICAL STRESS;

CARDIOVASCULAR-DISEASE; ISCHEMIC-STROKE; VALIDITY; MORTALITY; DISTRESS;

VERSION; CANCER

AB Background: In studies examining vascular risk factors and the effects of stress in stroke patients, information sometimes has to be provided by a close relative.

Objectives: The present study was designed to assess agreement between the information provided by a stroke patient and his/her next-of-kin or other close relative on prior vascular risk factors and situations of psychophysical stress based on a standardized interview within 72 h after stroke.

Design: Case-control observational study.

Participants and settings: All patients with incident stroke aged 18-65 years admitted to our centre were enrolled as cases, and their coinhabiting relatives as controls. The study was conducted from January to June 2008.

Methods: A structured questionnaire about vascular risk factors and psychophysical stress factors were obtained from the participants within 72 h after stroke. Subjects were required to grade themselves or their relatives using validated Spanish versions of the Life Events Stress Scale of Holmes and Rahe, Goldberg's General Health Questionnaire GHQ-28 and the SF-12 Health Survey, as well as the Spanish Type A Behaviour Scale. The questionnaires were self-administered. Cases and controls completed the same questionnaires and which were administered with a time difference of no longer than 24 h between the two groups of subjects. Risk factors were analyzed by determining kappa statistics and interclass correlation coefficients (ICC). Bland-Altman plots were used to examine the scores obtained in graded scales.

Results: 25 patients and 25 relatives were recruited. Agreement between cases and controls was good for reported atrial fibrillation, diabetes, alcohol consumption and smoking (range 0.83-1.0). ICC were low for the questionnaires Holmes-Rahe Life Events (0.26; 95%CI: 0.08-0.57), General Health GHQ-28 (0.39; 95%CI: 0.03-0.67) and SF12 Health Survey (0.52; 95%CI: 0.16-0.76 and 0.35; 95%CI: 0.06-0.66), and good for the Type A Behaviour Scale ERCTA (0.62; 95%CI: 0.32-0.81).

Conclusions: The information obtained from family members on patient risk factors could be considered reliable, while that related to psychophysical aspects was not sufficiently reliable for research purposes. Agreement assessments could be useful to avoid misclassification biases. (C) 2011 Elsevier Ltd. All rights reserved.

C1 [Castillo, Olga; Roig, Beatriz; Sanz, Isabel; Herrero, Rosa; Garay, Teresa; Fuentes, Manuel E.; Barreales, Laura; Egido, Jose A.] Hosp Clin San Carlos, Stroke Unit, Madrid 28040, Spain.

RP Roig, B (reprint author), Hosp Clin San Carlos, Stroke Unit, C Prof Martin Lagos S-N, Madrid 28040, Spain.

EM beatrizroig@hotmail.com

RI Egido, Jose/E-3837-2015; Fuentes Ferrer, Manuel Enrique/L-1086-2017

OI Egido, Jose/0000-0003-1698-9127; Fuentes Ferrer, Manuel

Enrique/0000-0002-5177-1441

FU Ministry of Health and Social Policy (FIS); Fondo de Investigaciones

Sanitarias del Ministerio de Sanidad y Politica Social (FIS) [PI7/0124]

FX Funding. Health Research Fund of the Ministry of Health and Social

Policy (FIS).; The source of public funding of this research was "Fondo

de Investigaciones Sanitarias del Ministerio de Sanidad y Politica

Social (FIS) PI7/0124". To Dr. Fernandez and Dr.Garcia, for their help

in the manuscript elaboration.

CR ALLEN MT, 1995, BIOL PSYCHOL, V41, P1, DOI 10.1016/0301-0511(95)05123-R

ALONSO J, 1995, MED CLIN-BARCELONA, V104, P771

Boden-Albala B, 2000, Curr Atheroscler Rep, V2, P160, DOI 10.1007/s11883-000-0111-3

Carod-Artal FJ, 2006, REV NEUROLOGIA, V42, P169

DeVries AC, 2001, P NATL ACAD SCI USA, V98, P11824, DOI 10.1073/pnas.201215298

Dimsdale JE, 2008, J AM COLL CARDIOL, V51, P1237, DOI 10.1016/j.jacc.2007.12.024

Farrow D C, 1990, Epidemiology, V1, P370, DOI 10.1097/00001648-199009000-00006

Gainotti G, 1997, STROKE, V28, P2145, DOI 10.1161/01.STR.28.11.2145

Gavalda L, 1995, Gac Sanit, V9, P334

Gonzalez de Rivera J, 1984, PSIQUIS, V4, P121

Hamer M, 2008, J AM COLL CARDIOL, V52, P2156, DOI 10.1016/j.jacc.2008.08.057

Hankey GJ, 2006, STROKE, V37, P2181, DOI 10.1161/01.STR.0000229883.72010.e4

KOLONEL LN, 1977, AM J EPIDEMIOL, V106, P476, DOI 10.1093/oxfordjournals.aje.a112494

LANDIS JR, 1977, BIOMETRICS, V33, P159, DOI 10.2307/2529310

LOBO A, 1986, PSYCHOL MED, V16, P135, DOI 10.1017/S0033291700002579

LYON JL, 1992, EPIDEMIOLOGY, V3, P223, DOI 10.1097/00001648-199205000-00007

May M, 2002, STROKE, V33, P7, DOI 10.1161/hs0102.100529

*NAT I STAT, 2010, INT CLASS DIS CAUS D

NELSON LM, 1994, EPIDEMIOLOGY, V5, P204, DOI 10.1097/00001648-199403000-00011

NELSON LM, 1990, EPIDEMIOL REV, V12, P71, DOI 10.1093/oxfordjournals.epirev.a036063

Ohlin B, 2004, EUR HEART J, V25, P867, DOI 10.1016/j.ehj.2004.03.003

ROCCA WA, 1986, J CHRON DIS, V39, P907, DOI 10.1016/0021-9681(86)90039-1

ROGOT E, 1975, INT J EPIDEMIOL, V4, P51, DOI 10.1093/ije/4.1.51

ROSENGREN A, 1991, AM J CARDIOL, V68, P1171, DOI 10.1016/0002-9149(91)90189-R

SACCO RL, 2001, NEUROLOGY, V57, P31

Schneck MJ, 1997, NEUROEPIDEMIOLOGY, V16, P174, DOI 10.1159/000109684

Sharkey SW, 2005, CIRCULATION, V111, P472, DOI 10.1161/01.CIR.0000153801.51470.EB

SUTIL RC, 1997, CLIN SALUD, V8, P347

SZKLO M, 2003, LACK VALIDITY BIAS I

Tang ST, 2006, CANCER NURS, V29, P49, DOI 10.1097/00002820-200601000-00009

TEPPER A, 1993, J CLIN EPIDEMIOL, V46, P1243, DOI 10.1016/0895-4356(93)90088-I

Truelsen T, 2003, STROKE, V34, P856, DOI 10.1161/01.STR.0000062345.80774.40

Villanueva V, 2006, J ALZHEIMERS DIS, V10, P409

WEISS A, 1966, J CLIN EPIDEMIOL, V49, P1187

World Health Organization, 1998, GUID CONTR MON TOB E

NR 35

TC 1

Z9 1

U1 0

U2 9

PU PERGAMON-ELSEVIER SCIENCE LTD

PI OXFORD

PA THE BOULEVARD, LANGFORD LANE, KIDLINGTON, OXFORD OX5 1GB, ENGLAND

SN 0020-7489

J9 INT J NURS STUD

JI Int. J. Nurs. Stud.

PD AUG

PY 2011

VL 48

IS 8

BP 952

EP 958

DI 10.1016/j.ijnurstu.2011.01.003

PG 7

WC Nursing

SC Nursing

GA 826JQ

UT WOS:000295350800006

PM 21295779

DA 2019-01-08

ER

PT J

AU Swartz, JA

AF Swartz, James A.

TI Chronic Medical Conditions among Jail Detainees in Residential

Psychiatric Treatment: A Latent Class Analysis

SO JOURNAL OF URBAN HEALTH-BULLETIN OF THE NEW YORK ACADEMY OF MEDICINE

LA English

DT Article

DE Co-occurring disorders; Medical complexity; Chronic medical conditions;

Serious mental illness; Jail detainees; Latent class analysis

ID COMORBIDITY SURVEY REPLICATION; DIAGNOSTIC INTERVIEW CIDI;

MINI-MENTAL-STATE; DSM-IV DISORDERS; PSYCHOLOGICAL DISTRESS;

SUBSTANCE-ABUSE; PREVALENCE; PRISON; CARE; POPULATION

AB Studies of incarcerates with serious mental illnesses have found elevated rates of chronic medical conditions such as asthma and diabetes, and of infectious diseases such as tuberculosis compared with general population rates. This study explored the pattern of chronic medical conditions in a sample of adult detainees in psychiatric treatment in a large urban jail to develop a clinical profile encompassing the full range of medical conditions. A total of 431 male and female detainees were sampled with certainty from admissions to a residential psychiatric treatment program (overall recruitment rate = 67%). Interviews used the World Mental Health version of the Composite International Diagnostic Interview to assess psychiatric and substance use disorders per DSM-IV criteria and chronic medical conditions. Latent class analysis was conducted using 17 medical conditions as class indicators, yielding a 3-class model composed of: a latent class with a high to intermediate probability of multiple medical conditions (HMC; 12.5% of the sample); an intermediate class with a lower probability of having a smaller number of medical conditions (MMC; 43.2%); and a class with a low probability of any medical condition (44.3%). Those in the HMC class were more likely to report respiratory problems, severe headaches, musculoskeletal pain, hypertension, and arthritis, have greater functional impairment, and have a higher number of co-occurring psychiatric disorders. Being older (50+ years) and female were associated with higher odds of being in the HMC or MMC classes. The policy implications for providing medical care to incarcerates with complex mixtures of medical conditions and psychiatric disorders are considered.

C1 Univ Illinois, Jane Addams Coll Social Work, Chicago, IL 60680 USA.

RP Swartz, JA (reprint author), Univ Illinois, Jane Addams Coll Social Work, Chicago, IL 60680 USA.

EM jaswartz@uic.edu

RI Swartz, James/G-3931-2017

OI Swartz, James/0000-0003-2206-7962

FU Bureau of Justice Assistance, Office of Justice Programs, US Department

of Justice [04-DB-BX-0043]; National Institute on Drug Abuse

[5-R01-DA013943-05]

FX This study was supported by grant no. 04-DB-BX-0043 awarded to the

Illinois Criminal Justice Information Authority by the Bureau of Justice

Assistance, Office of Justice Programs, US Department of Justice and by

grant no. 5-R01-DA013943-05 from the National Institute on Drug Abuse.

Points of view or opinions contained within this document are those of

the author and do not necessarily represent the official position or

policies of the US Department of Justice, the Illinois Criminal Justice

Information Authority, or the National Institute on Drug Abuse.

CR Andrews G, 2001, AUST NZ J PUBL HEAL, V25, P494, DOI 10.1111/j.1467-842X.2001.tb00310.x

APA (American Psychiatric Association), 2002, DIAGN STAT MAN MENT

Babudieri S, 2005, J MED VIROL, V76, P311, DOI 10.1002/jmv.20375

Baillargeon J, 2004, PREV MED, V38, P607, DOI 10.1016/j.ypmed.2003.11.020

Baillargeon JG, 2008, AIDS CARE, V20, P124, DOI 10.1080/09540120701426532

Biemer Paul P., 2008, SAMPLING POPULATIONS, P489

Binswanger IA, 2009, J EPIDEMIOL COMMUN H, V63, P912, DOI 10.1136/jech.2009.090662

Binswanger IA, 2007, NEW ENGL J MED, V356, P157, DOI 10.1056/NEJMsa064115

Binswanger IA, 2010, AM J PUBLIC HEALTH, V100, P476, DOI 10.2105/AJPH.2008.149591

Callahan CM, 2002, MED CARE, V40, P771, DOI 10.1097/01.MLR.0000024610.33213.C8

Cuddeback GS, 2010, PSYCHIAT SERV, V61, P45, DOI 10.1176/appi.ps.61.1.45

Draine J, 2007, PSYCHIAT SERV, V58, P1577, DOI 10.1176/appi.ps.58.12.1577

Druss BG, 2006, GEN HOSP PSYCHIAT, V28, P145, DOI 10.1016/j.genhosppsych.2005.10.006

Fagiolini A, 2009, J CLIN PSYCHIAT, V70, P22, DOI 10.4088/JCP.7075su1c.04

Fazel S, 2006, ADDICTION, V101, P181, DOI 10.1111/j.1360-0443.2006.01316.x

Fazel S, 2002, LANCET, V359, P545, DOI 10.1016/S0140-6736(02)07740-1

FOLSTEIN MF, 1975, J PSYCHIAT RES, V12, P189, DOI 10.1016/0022-3956(75)90026-6

GUNTER TD, 2008, J AM ACAD PSYCHIAT L, V36, P35

Hennessey KA, 2009, J URBAN HEALTH, V86, P93, DOI 10.1007/s11524-008-9305-8

Kessler RC, 2005, BIOL PSYCHIAT, V58, P668, DOI 10.1016/j.biopsych.2005.04.034

Kessler RC, 2002, PSYCHOL MED, V32, P959, DOI 10.1017/S0033291702006074

Kessler RC, 2004, INT J METH PSYCH RES, V13, P93, DOI 10.1002/mpr.168

Kessler RC, 2005, ARCH GEN PSYCHIAT, V62, P617, DOI 10.1001/archpsyc.62.6.617

Kessler RC, 2005, ARCH GEN PSYCHIAT, V62, P593, DOI 10.1001/archpsyc.62.6.593

MARUSCHAK LM, 2006, US DOJ PUBLICATION N

MARUSCHAK LM, 2009, US DOJ PUBLICATION N

MURMOLA CJ, 2006, US DOJ PUBLICATION N

MUTHEN LK, 2008, MPLUS COMP PROGR VER

*NAT COMM CORR HLT, 2002, HLTH STAT SOON TO BE, V1

Nylund KL, 2007, STRUCT EQU MODELING, V14, P535, DOI 10.1080/10705510701575396

Restum ZG, 2005, AM J PUBLIC HEALTH, V95, P1689, DOI 10.2105/AJPH.2004.055053

Roberts CA, 2006, AM J PREV MED, V30, P125, DOI 10.1016/j.amepre.2005.10.018

Saitz R, 2005, ADDICTION, V100, P70, DOI 10.1111/j.1360-0443.2004.00916.x

Sheehan DV, 1996, INT CLIN PSYCHOPHARM, V11, P89, DOI 10.1097/00004850-199606003-00015

Smith KL, 2006, DRUG ALCOHOL DEPEN, V82, P231, DOI 10.1016/j.drugalcdep.2005.09.012

SOBELL LC, 1992, MEASURING ALCOHOL CONSUMPTION, P41

*SPSS INC, 2007, SPSS MAC COMP PROGR

*STAT CORP, 2009, STAT MAC COMP PROGR

Swartz J. A., 2010, STUDY COOCCURRING HL

Teplin LA, 2005, ARCH GEN PSYCHIAT, V62, P911, DOI 10.1001/archpsyc.62.8.911

Vogeli C, 2007, J GEN INTERN MED, V22, P391, DOI 10.1007/s11606-007-0322-1

*WHO, 1990, INT CLASS DIS 10 CLA

WITTCHEN HU, 1994, J PSYCHIAT RES, V28, P57, DOI 10.1016/0022-3956(94)90036-1

2009, STAT ANAL SYSTEMS CO

NR 44

TC 8

Z9 8

U1 0

U2 12

PU SPRINGER

PI NEW YORK

PA 233 SPRING ST, NEW YORK, NY 10013 USA

SN 1099-3460

J9 J URBAN HEALTH

JI J. Urban Health

PD AUG

PY 2011

VL 88

IS 4

BP 700

EP 717

DI 10.1007/s11524-011-9554-9

PG 18

WC Public, Environmental & Occupational Health; Medicine, General &

Internal

SC Public, Environmental & Occupational Health; General & Internal Medicine

GA 808XD

UT WOS:000294014400009

PM 21394659

OA Green Published

DA 2019-01-08

ER

PT J

AU Maslow, GR

Haydon, A

Mcree, AL

Ford, CA

Halpern, CT

AF Maslow, Gary R.

Haydon, Abigail

Mcree, Annie-Laurie

Ford, Carol A.

Halpern, Carolyn T.

TI Growing Up With a Chronic Illness: Social Success,

Educational/Vocational Distress

SO JOURNAL OF ADOLESCENT HEALTH

LA English

DT Article

DE Young adult; Chronic illness; Transition to adulthood; Social outcomes;

Educational outcomes; Vocational outcomes

ID CHILDHOOD-CANCER SURVIVOR; CHRONIC PHYSICAL ILLNESS;

SICKLE-CELL-DISEASE; LONG-TERM SURVIVORS; ADULT SURVIVORS; FOLLOW-UP;

LIFE; HEALTH; COHORT; TRANSITION

AB Objectives: We compared adult educational, vocational, and social outcomes among young adults with and without childhood-onset chronic illness in a nationally representative U. S. sample.

Methods: We used data from Wave IV (2008) of the National Longitudinal Study of Adolescent Health. We compared respondents who reported childhood-onset cancer, heart disease, diabetes, or epilepsy with young adults without these chronic illnesses in terms of marriage, having children, living with parents, romantic relationship quality, educational attainment, income, and employment. Multivariate models controlled for sociodemographic factors and adult-onset chronic illness.

Results: As compared with those without childhood chronic illness, respondents with childhood chronic illness had similar odds of marriage (odds ratios [OR] = .89, 95% CI: .65-1.24), having children (OR = .99, 95% CI: .70-1.42), and living with parents (OR = 1.49, 95% CI .94-2.33), and similar reports of romantic relationship quality. However, the chronic illness group had lower odds of graduating college (OR = .49, 95% CI: .31-.78) and being employed (OR = .56, 95% CI: .39-.80), and higher odds of receiving public assistance (OR = 2.13, 95% CI: 1.39-3.25), and lower mean income.

Conclusions: Young adults growing up with chronic illness succeed socially, but are at increased risk of poorer educational and vocational outcomes. (C) 2011 Society for Adolescent Health and Medicine. All rights reserved.

C1 [Maslow, Gary R.] Univ N Carolina, Cecil B Sheps Ctr Hlth Serv Res, Div Hlth Affairs, Chapel Hill, NC 27599 USA.

[Maslow, Gary R.] Duke Univ, Med Ctr, Dept Pediat, Durham, NC 27710 USA.

[Haydon, Abigail; Mcree, Annie-Laurie; Ford, Carol A.; Halpern, Carolyn T.] UNC Gillings Sch Global Hlth, Dept Maternal & Child Hlth, Chapel Hill, NC USA.

[Haydon, Abigail; Halpern, Carolyn T.] Univ N Carolina, Div Hlth Affairs, Carolina Populat Ctr, Chapel Hill, NC 27599 USA.

[Ford, Carol A.] UNC Sch Med, Dept Pediat, Chapel Hill, NC USA.

RP Maslow, GR (reprint author), Univ N Carolina, Cecil B Sheps Ctr Hlth Serv Res, Div Hlth Affairs, 725 Martin Luther King Jr Blvd,CB 7590, Chapel Hill, NC 27599 USA.

EM gmaslow@unc.edu

RI McRee, Annie/J-3077-2013

FU Eunice Kennedy Shriver National Institute of Child Health and Human

Development [P01-HD31921, R01-HD57046]

FX This research used data from Add Health, a program project directed by

Kathleen Mullan Harris and designed by J. Richard Udry, Peter S.

Bearman, and Kathleen Mullan Harris at the University of North Carolina

at Chapel Hill, and funded by grant P01-HD31921 from the Eunice Kennedy

Shriver National Institute of Child Health and Human Development, with

cooperative funding from 23 other federal agencies and foundations.

Special acknowledgment is due to Ronald R. Rindfuss and Barbara Entwisle

for assistance in the original design. Information on how to obtain the

Add Health data files is available on the Add Health Web site

(http://www.cpc.unc.edu/addhealth). No direct support was received from

grant P01-HD31921 for this analysis.; Training support for Gary Maslow,

M. D. provided by T32HP14001 from the Health Resources and Services

Administration for University of North Carolina's National Research

Service Administration Primary Care Research Fellowship. Effort by A.

Haydon and C. T. Halpern was supported by Eunice Kennedy Shriver

National Institute of Child Health and Human Development grant

R01-HD57046; C. T. Halpern, Principal Investigator. Thanks to the

National Research Service Administration Primary Care Research Fellows

for careful review of this manuscript.

CR Boman KK, 2010, CANCER-AM CANCER SOC, V116, P1385, DOI 10.1002/cncr.24840

Bussing R, 1996, ARCH PEDIAT ADOL MED, V150, P181, DOI 10.1001/archpedi.1996.02170270063009

Camfield CS, 2007, EPILEPSIA, V48, P3, DOI 10.1111/j.1528-1167.2007.01390.x

Canadian Paediatric Society, 2006, PAEDIAT CHILD HLTH, V11, P43

Ellenberg L, 2009, NEUROPSYCHOLOGY, V23, P705, DOI 10.1037/a0016674

Gerhardt CA, 2007, J ADOLESC HLTH, V40

Gledhill J, 2000, ARCH DIS CHILD, V83, P104, DOI 10.1136/adc.83.2.104

GORTMAKER SL, 1984, PEDIATR CLIN N AM, V31, P3

GORTMAKER SL, 1993, J RES ADOLESCENCE, V0003

Gurney JG, 2009, J CLIN ONCOL, V27, P2390, DOI 10.1200/JCO.2008.21.1458

Harris K. M., 2009, NATL LONGITUDINAL ST

Hartung PJ, 2005, J VOCAT BEHAV, V66, P385, DOI 10.1016/j.jvb.2004.05.006

Huurre TM, 2002, EUR CHILD ADOLES PSY, V11, P85, DOI 10.1007/s007870200015

Jacobson AM, 1997, DIABETIC MED, V14, P73, DOI 10.1002/(SICI)1096-9136(199701)14:1<73::AID-DIA294>3.0.CO;2-Q

KOKKONEN J, 1995, EUR J PEDIATR, V154, P676, DOI 10.1007/BF02079077

La Greca AM, 2002, J DEV BEHAV PEDIATR, V23, DOI 10.1097/00004703-200208000-00013

Lancashire ER, 2010, J NATL CANCER I, V102, P254, DOI 10.1093/jnci/djp498

Lipkin PH, 2007, PEDIATRICS, V119, P1218, DOI 10.1542/peds.2007-0885

Madan-Swain A, 2004, HANDBOOK OF PEDIATRIC PSYCHOLOGY IN SCHOOL SETTINGS, P637

McDonald S, 2007, SOC SCI RES, V36, P1328, DOI 10.1016/j.ssresearch.2007.01.008

Miauton L, 2003, EUR J PEDIATR, V162, P682, DOI 10.1007/s00431-003-1179-x

Mitby PA, 2003, CANCER, V97, P1115, DOI 10.1002/cncr.11117

Noll RB, 2010, PEDIATRICS, V125, pE1453, DOI 10.1542/peds.2009-2996

Pang JWY, 2008, PEDIATR BLOOD CANCER, V50, P104, DOI 10.1002/pbc.21226

PERRIN EC, 1993, PEDIATRICS, V91, P787

PLATT OS, 1994, NEW ENGL J MED, V330, P1639, DOI 10.1056/NEJM199406093302303

PLESS IB, 1989, DEV MED CHILD NEUROL, V31, P746

PLESS IB, 1971, PEDIATRICS, V47, P405

Quinn CT, 2004, BLOOD, V103, P4023, DOI 10.1182/blood-2003-11-3758

Reiss J, 2002, PEDIATRICS, V110, P1307

Roberts M. C., 2009, HDB PEDIAT PSYCHOL, P689

Shandra CL, 2008, J VOCAT REHABIL, V29, P117

Stam H, 2006, J ADOLESCENT HEALTH, V39, P4, DOI 10.1016/j.jadohealth.2005.03.011

STEIN REK, 1982, PUBLIC HEALTH REP, V97, P354

Thompson AL, 2009, PSYCHO-ONCOL, V18, P767, DOI 10.1002/pon.1471

Wakamoto H, 2000, BRAIN DEV-JPN, V22, P246, DOI 10.1016/S0387-7604(00)00121-2

Wittenburg D. C., 2002, J VOCATIONAL REHABIL, V17, P195

1990, PEDIATRICS, V86, P636

NR 38

TC 79

Z9 79

U1 0

U2 25

PU ELSEVIER SCIENCE INC

PI NEW YORK

PA 360 PARK AVE SOUTH, NEW YORK, NY 10010-1710 USA

SN 1054-139X

J9 J ADOLESCENT HEALTH

JI J. Adolesc. Health

PD AUG

PY 2011

VL 49

IS 2

BP 206

EP 212

DI 10.1016/j.jadohealth.2010.12.001

PG 7

WC Psychology, Developmental; Public, Environmental & Occupational Health;

Pediatrics

SC Psychology; Public, Environmental & Occupational Health; Pediatrics

GA 795TB

UT WOS:000292997600016

PM 21783055

OA Green Accepted

DA 2019-01-08

ER

PT J

AU Wu, FL

Juang, JH

Yeh, MC

AF Wu, Fei-Ling

Juang, Jyuhn-Huarng

Yeh, Mei Chang

TI The dilemma of diabetic patients living with hypoglycaemia

SO JOURNAL OF CLINICAL NURSING

LA English

DT Article

DE diabetes mellitus; hypoglycaemia; nurses; nursing; psychosocial factors;

self-management

ID COMPLICATIONS; EXPERIENCES; ADOLESCENT; MELLITUS; WOMEN

AB Aims. To examine the impact of the threat of hypoglycaemic episodes on people with diabetes in Taiwan.

Background. Intensive diabetes treatment in people with diabetes helps them to achieve better glycaemic control. However, it also causes more frequent hypoglycaemic episodes and has an impact on their overall quality of life. Hypoglycaemia is accompanied by various distressing symptoms which may cause excessive fear, affecting decision making in hypoglycaemic management.

Design. Purposive sampling and in-depth, face-to-face interviews were used to collect data.

Methods. Semi-structured interviews were conducted from July 2008-January 2009 with 17 individuals treated with insulin who had previous hypoglycaemic episodes. Data were analysed using qualitative content analysis.

Results. Four themes were generated from the analysis, 'inability to control fluctuations in health', challenges to interpersonal relationships', 'facing the disease alone' and 'finding a balance between competing symptoms'.

Conclusions. Hypoglycaemia is a major health issue for many people with diabetes. Understanding individuals' experiences with hypoglycaemic episodes should help practitioners become more fully involved in promoting self-management. We identified key areas that health care providers should address, including concerns about patient education and professional support for people with diabetes experiencing hypoglycaemia, to enhance problem solving skills for them and their families.

Relevance to clinical practice. We recommend that health care providers make proper use of support groups for family caregivers or other important individuals in the lives of people with diabetes to provide education, clarification, support and guidance. In addition, health care providers also need to provide clients with hypoglycaemia-related emotional support, while enhancing diabetes self-management and problem-solving skills.

C1 [Wu, Fei-Ling] Natl Taiwan Univ, Dept Nursing, Coll Med, Taipei 100, Taiwan.

[Wu, Fei-Ling] Chang Gung Inst Technol, Dept Nursing, Tao Yuan, Taiwan.

[Juang, Jyuhn-Huarng] Chang Gung Univ, Tao Yuan, Taiwan.

[Juang, Jyuhn-Huarng] Chang Gung Mem Hosp, Tao Yuan, Taiwan.

[Yeh, Mei Chang] Natl Taiwan Univ Hosp, Dept Nursing, Taipei, Taiwan.

RP Yeh, MC (reprint author), Natl Taiwan Univ, Dept Nursing, Coll Med, 1 Jen Ai Rd,Sec 1, Taipei 100, Taiwan.

EM mchang@ntu.edu.tw

OI CHANG, MEI/0000-0002-4166-3498

CR Aalto AM, 2000, EUR J PUBLIC HEALTH, V10, P68, DOI 10.1093/eurpub/10.1.68

CHAN JCN, 1996, HONG KONG PRACTITION, V18, P270

*CHIN TAIW DIAB AS, 2006, 2006 TYP 2 DIAB CAR

Ciechanowski PS, 2000, ARCH INTERN MED, V160, P3278, DOI 10.1001/archinte.160.21.3278

COX DJ, 1992, J CONSULT CLIN PSYCH, V60, P628, DOI 10.1037//0022-006X.60.4.628

*DEP HLTH EX YUAN, 2003, INTR TAIW TEL PIL PR

*DIAB CONTR COMPL, 1993, NEW ENGL J MED, V329, P977, DOI DOI 10.1056/NEJM199309303291401

Dickinson JK, 2004, DIABETES EDUCATOR, V30, P99, DOI 10.1177/014572170403000117

Dovey-Pearce G, 2007, BRIT J HEALTH PSYCH, V12, P75, DOI 10.1348/135910706X98317

Freidson E., 1970, PROFESSION MED

GONDERFREDERICK LA, 2003, PRACTICAL PSYCHOL DI, P113

Kay C, 2009, J HEALTH PSYCHOL, V14, P242, DOI 10.1177/1359105308100208

Lincoln Y. S., 1985, NATURALISTIC ENQUIRY

Luttik Marie Louise, 2005, J Cardiovasc Nurs, V20, P162

MacLean LM, 2004, QUAL HEALTH RES, V14, P113, DOI 10.1177/1049732303259804

Miles M., 1994, QUALITATIVE DATA ANA

Penckofer S, 2007, DIABETES EDUCATOR, V33, P680, DOI 10.1177/0145721707304079

POLONSKY W, 2006, DIABETES FORECAST, V59, P9

Pouwer F, 2006, PATIENT EDUC COUNS, V60, P235, DOI 10.1016/j.pec.2005.01.009

Richmond J, 1996, Br J Nurs, V5, P1054

Ritholz MD, 1998, J GEN INTERN MED, V13, P799, DOI 10.1046/j.1525-1497.1998.00243.x

Samuel-Hodge CD, 2000, DIABETES CARE, V23, P928, DOI 10.2337/diacare.23.7.928

Shiu ATY, 2002, J ADV NURS, V39, P155

UKPDS Study Group, 1998, LANCET, V352, P837, DOI DOI 10.1016/S0140-6736(98)07019-6

Weber RP, 1990, BASIC CONTENT ANAL

Wen LK, 2004, DIABETES EDUCATOR, V30, P980, DOI 10.1177/014572170403000619

Zoffmann V, 2006, PATIENT EDUC COUNS, V64, P78, DOI 10.1016/j.pec.2005.11.017

NR 27

TC 8

Z9 8

U1 0

U2 4

PU WILEY

PI HOBOKEN

PA 111 RIVER ST, HOBOKEN 07030-5774, NJ USA

SN 0962-1067

EI 1365-2702

J9 J CLIN NURS

JI J. Clin. Nurs.

PD AUG

PY 2011

VL 20

IS 15-16

BP 2277

EP 2285

DI 10.1111/j.1365-2702.2011.03725.x

PG 9

WC Nursing

SC Nursing

GA 792LB

UT WOS:000292744900018

PM 21627701

DA 2019-01-08

ER

PT J

AU Collins, RE

Lopez, LM

Marteau, TM

AF Collins, Ruth E.

Lopez, Laureen M.

Marteau, Theresa M.

TI Emotional impact of screening: a systematic review and meta-analysis

SO BMC PUBLIC HEALTH

LA English

DT Article

ID RANDOMIZED CONTROLLED-TRIAL; QUALITY-OF-LIFE; SMOKING-CESSATION

TREATMENT; ABDOMINAL AORTIC-ANEURYSM; CERVICAL SMEAR TEST; PSYCHOLOGICAL

IMPACT; BREAST-CANCER; GENETIC SUSCEPTIBILITY; GENERAL-PRACTICE;

FOLLOW-UP

AB Background: There is a widely held expectation that screening for disease has adverse emotional impacts. The aim of the current review is to estimate the short (< 4 weeks) and longer term (> 4 weeks) emotional impact of such screening.

Methods: Studies selected for inclusion were (a) randomised controlled trials in which (b) participants in one arm underwent screening and received test results, and those in a control arm did not, and (c) emotional outcomes were assessed in both arms. MEDLINE via PubMed (1950 to present), EMBASE (1980 to present), PsycINFO (1985 to present) using OVID SP, and CINAHL (1982 to present) via EBSCO were searched, using strategies developed with keywords and medical subject headings. Data were extracted on emotional outcomes, type of screening test and test results.

Results: Of the 12 studies that met the inclusion criteria, six involved screening for cancer, two for diabetes, and one each for abdominal aortic aneurysms, peptic ulcer, coronary heart disease and osteoporosis. Five studies reported data on anxiety, four on depression, two on general distress and eight on quality of life assessed between one week and 13 years after screening (median = 1.3 years). Meta-analyses revealed no significant impact of screening on longer term anxiety (pooled SMD 0.01, 95% CI -0.10, 0.11), depression (pooled SMD -0.04, 95% CI -12, 0.20), or quality of life subscales (mental and self-assessed health pooled SMDs, respectively: 0.03; -0.01, 95% CI -02, 0.04; 0.00, 95% CI -04, 0.03).

Conclusion: Screening does not appear to have adverse emotional impacts in the longer term (> 4 weeks). Too few studies assessed outcomes before four weeks to comment on the shorter term emotional impact of screening.

C1 [Collins, Ruth E.; Marteau, Theresa M.] Kings Coll London, Hlth Psychol Sect, Dept Psychol Guys, London SE1 9RT, England.

[Lopez, Laureen M.] Clin Sci FHI, Res Triangle Pk, NC 27709 USA.

RP Marteau, TM (reprint author), Kings Coll London, Hlth Psychol Sect, Dept Psychol Guys, 5th Floor Bermondsey Wing,Guys Campus, London SE1 9RT, England.

EM theresa.marteau@kcl.ac.uk

RI Collins, Ruth/A-2276-2013

OI Marteau, Theresa/0000-0003-3025-1129

FU NHS; Medical Research Council [G0500274]

FX This review was conducted as part of a grant from the NHS Heath

Technology Assessment Program (03/38 Investigations following abnormal

liver function tests). We would like to express our gratitude to

Professors McBride, Whynes and Christensen who kindly supplied data for

the analysis in this review and to those who also searched but were

unable to find raw data sets often from many years ago. We are also

grateful both to Professor Roger Jones, Dr Alison Wright and Dr Rachel

Crockett who commented on earlier drafts of the manuscript.

CR Andersen MR, 2007, PSYCHO-ONCOLOGY, V16, P814, DOI 10.1002/pon.1151

Antoni MH, 2001, HEALTH PSYCHOL, V20, P20, DOI 10.1037//0278-6133.20.1.20

Ashton HA, 2002, LANCET, V360, P1531, DOI 10.1016/S0140-6736(02)11522-4

Audrain J, 1997, ADDICT BEHAV, V22, P741, DOI 10.1016/S0306-4603(97)00060-9

Brett J, 2005, PSYCHO-ONCOLOGY, V14, P917, DOI 10.1002/pon.904

Broadstock M, 2000, EUR J HUM GENET, V8, P731, DOI 10.1038/sj.ejhg.5200532

Butow PN, 2003, MED J AUSTRALIA, V178, P77

Carver C. S, 1999, SELF SOCIAL PSYCHOL, P299

Christensen B, 2004, EUR J CARDIOV PREV R, V11, P239, DOI 10.1097/01.hjr.0000129739.30593.23

Cockburn J, 1994, J Med Screen, V1, P7

Eborall HC, 2007, BRIT MED J, V335, P486, DOI 10.1136/bmj.39303.723449.55

Eich E, 2000, COGNITION EMOTION CO

FALLOWFIELD LJ, 1990, J ROY SOC MED, V83, P547

French DP, 2006, PSYCHO-ONCOL, V15, P498, DOI 10.1002/pon.980

Goldberg D., 1978, MANUAL GEN HLTH QUES

Hansen JM, 2008, AM J GASTROENTEROL, V103, P1106, DOI 10.1111/j.1572-0241.2007.01770.x

HAYNES RB, 1978, NEW ENGL J MED, V299, P741, DOI 10.1056/NEJM197810052991403

Heshka JT, 2008, GENET MED, V10, P19, DOI 10.1097/GIM.0b013e31815f524f

Higgins J, 2008, COCHRANE HDB SYSTEMA

Holland WW, 1990, SCREENING HLTH CARE

HUNT SM, 1981, SOC SCI MED-MED SOC, V15, P221, DOI 10.1016/0271-7123(81)90005-5

Johnson James N, 2008, BMJ, V336, P863, DOI 10.1136/bmj.39514.494167.AD

Kane RA, 2009, NEW ENGL J MED, V361, P298, DOI 10.1056/NEJMe0903449

KASH KM, 1992, J NATL CANCER I, V84, P24, DOI 10.1093/jnci/84.1.24

Lerman C, 1997, HEALTH PSYCHOL, V16, P87, DOI 10.1037//0278-6133.16.1.87

LERMAN C, 1993, BREAST CANCER RES TR, V28, P145, DOI 10.1007/BF00666427

Lindholt JS, 2000, EUR J VASC ENDOVASC, V20, P79, DOI 10.1053/ejvs.1999.1087

McBride CM, 2002, CANCER EPIDEM BIOMAR, V11, P521

Meiser B, 2005, PSYCHO-ONCOL, V14, P1060, DOI 10.1002/pon.933

Michie S, 1996, BRIT J HEALTH PSYCH, V1, P3, DOI 10.1111/j.2044-8287.1996.tb00487.x

Park P, 2008, BMC PUBLIC HEALTH, V8, DOI 10.1186/1471-2458-8-350

PARKER MA, 2002, J MED SCREEN, P7

PERRY S, 1991, ARCH GEN PSYCHIAT, V48, P143

RADLOFF L S, 1977, Applied Psychological Measurement, V1, P385, DOI 10.1177/014662167700100306

Rogstad KE, 2002, BJOG-INT J OBSTET GY, V109, P364, DOI 10.1111/j.1471-0528.2002.99023.x

RUDD P, 1987, HYPERTENSION, V10, P425, DOI 10.1161/01.HYP.10.4.425

Sanderson SC, 2008, J HEALTH PSYCHOL, V13, P481, DOI 10.1177/1359105308088519

Shaw C, 1999, SOC SCI MED, V49, P1571, DOI 10.1016/S0277-9536(99)00244-0

Spielberger C. D., 1970, STAI MANUAL STATE TR

STEWART DE, 1993, OBSTET GYNECOL, V81, P280

Taylor KL, 2004, JNCI-J NATL CANCER I, V96, P1083, DOI 10.1093/jnci/djh194

Tercyak KP, 2001, HEALTH PSYCHOL, V20, P217, DOI 10.1037/0278-6133.20.3.217

Timman R, 2004, HEALTH PSYCHOL, V23, P189, DOI 10.1037/0278-6133.23.2.189

Torgerson DJ, 1997, ARCH INTERN MED, V157, P2121, DOI 10.1001/archinte.157.18.2121

Wardle J, 1999, BRIT MED J, V319, P1037, DOI 10.1136/bmj.319.7216.1037

WARE JE, 1992, MED CARE, V30, P473, DOI 10.1097/00005650-199206000-00002

WHYNES DK, 1994, QUAL LIFE RES, V3, P191, DOI 10.1007/BF00435384

WILKINSON C, 1990, BRIT MED J, V300, P440, DOI 10.1136/bmj.300.6722.440

WILSON JMG, 1971, P ROY SOC MED, V64, P1255

Yerkes RM, 1908, J COMP NEUROL PSYCHO, V18, P459, DOI 10.1002/cne.920180503

ZIGMOND AS, 1983, ACTA PSYCHIAT SCAND, V67, P361, DOI 10.1111/j.1600-0447.1983.tb09716.x

NR 51

TC 19

Z9 20

U1 0

U2 13

PU BIOMED CENTRAL LTD

PI LONDON

PA 236 GRAYS INN RD, FLOOR 6, LONDON WC1X 8HL, ENGLAND

SN 1471-2458

J9 BMC PUBLIC HEALTH

JI BMC Public Health

PD JUL 28

PY 2011

VL 11

AR 603

DI 10.1186/1471-2458-11-603

PG 10

WC Public, Environmental & Occupational Health

SC Public, Environmental & Occupational Health

GA 826TT

UT WOS:000295378400001

PM 21798046

OA DOAJ Gold, Green Published

DA 2019-01-08

ER

PT J

AU Sabourin, BC

Vallis, TM

Currie, S

AF Sabourin, Brigitte C.

Vallis, T. Michael

Currie, Shannon

TI Development and Pilot-Testing of a Brief Psychosocial Group Intervention

Protocol for Type 2 Diabetes Self-Management

SO CANADIAN JOURNAL OF DIABETES

LA English

DT Article

DE brief intervention; group intervention; knowledge translation;

psychosocial; self-management; type 2 diabetes

ID QUALITY-OF-LIFE; DISTRESS; MELLITUS; ADULTS; SCALE; DEPRESSION;

AGREEMENT; SCIENCE; CALL; CARE

AB OBJECTIVES: To develop a brief psychosocial intervention protocol for type 2 diabetes self-management and pilot-test its implementation.

METHODS: A 6-session evidence-based psychosocial intervention protocol was developed focusing on the role of behaviour modification, maintenance of motivation and emotion management in type 2 diabetes self-management. The intervention was pilot-tested with 15 individuals with type 2 diabetes recruited from participants in the Action to Control Cardiovascular Risk in Diabetes (ACCORD) study.

RESULTS: Repeated-measures analyses of variance of validated psychosocial and behavioural outcome measures revealed that following the intervention, diabetes-related distress and intake of high-fat foods decreased and exercise participation increased. Furthermore, all participants found the intervention to be helpful for diabetes management. The most helpful aspect of the group sessions, derived from inductive classical content analysis of open-ended feedback questionnaires, was the development of friendship and trust in openly sharing experiences with group members.

CONCLUSION: This promising intervention should be further tested using a randomized, controlled trial. The protocol could then be developed as a training manual so that other university-educated professionals could deliver the intervention to people with diabetes, helping to fulfill 1 of the Canadian Diabetes Association's clinical guidelines: providing psychosocial interventions as part of diabetes care.

C1 [Sabourin, Brigitte C.] Dalhousie Univ, Dept Psychol, Halifax, NS B3H 4J1, Canada.

[Vallis, T. Michael] Dalhousie Univ, QEII Diabet Management Ctr, Halifax, NS B3H 4J1, Canada.

[Currie, Shannon] Schneider & Associates Psychol Serv, Hamilton, ON, Canada.

[Currie, Shannon] Burlington Psychol & Counselling Serv, Burlington, ON, Canada.

RP Sabourin, BC (reprint author), Dalhousie Univ, Dept Psychol, 1355 Oxford St, Halifax, NS B3H 4J1, Canada.

EM brigitte.sabourin@dal.ca

OI Sabourin, Brigitte/0000-0001-7600-9974

FU Canadian Institutes of Health Research; Nova Scotia Health Research

Foundation

FX BS is supported by a doctoral fellowship from the Canadian Institutes of

Health Research and was supported through a doctoral-level scholarship

from the Nova Scotia Health Research Foundation for part of the study.

CR ACCORD STUD GRO, 2007, AM J CARDIOL, V99, P21

Beck J. S., 1995, COGNITIVE THERAPY BA

Canadian Diabetes Association Clinical Practice Guidelines Committee: Canadian, 2008, CAN J DIABETES S1, V32, pS1, DOI DOI 10.1503/CMAJ.080554

COHEN J, 1960, EDUC PSYCHOL MEAS, V20, P37, DOI 10.1177/001316446002000104

CRAIG TJ, 1978, AM J PSYCHIAT, V135, P1036

Deakin T, 2005, COCHRANE DB SYST REV, DOI 10.1002/14651858.CD003417.pub2

DIENER E, 1985, J PERS ASSESS, V49, P71, DOI 10.1207/s15327752jpa4901_13

El Achhab Y, 2008, DIABETES RES CLIN PR, V80, P171, DOI 10.1016/j.diabres.2007.12.020

Elliott TR, 2001, J CLIN PSYCHOL MED S, V8, P283, DOI 10.1023/A:1011920914079

Field A., 2009, DISCOVERING STAT USI

Fisher L, 2007, DIABETES CARE, V30, P2746, DOI 10.2337/dc07-1166

Gerstein HC, 2008, NEW ENGL J MED, V358, P2545, DOI 10.1056/NEJMoa0802743

Glasgow RE, 1999, DIABETES CARE, V22, P832, DOI 10.2337/diacare.22.5.832

Glasgow RE, 2006, CHRONIC ILLN, V2, P27, DOI 10.1179/174592006X93851

LANDIS JR, 1977, BIOMETRICS, V33, P159, DOI 10.2307/2529310

Leech NL, 2007, SCHOOL PSYCHOL QUART, V22, P557, DOI 10.1037/1045-3830.22.4.557

LUSTMAN PJ, 1992, DIABETES CARE, V15, P1631, DOI 10.2337/diacare.15.11.1631

Marcus BH, 2000, HEALTH PSYCHOL, V19, P32, DOI 10.1037/0278-6133.19.Suppl1.32

Marlatt G. A., 2005, RELAPSE PREVENTION M

Miller W. R., 2002, MOTIVATIONAL INTERVI

Nelson KM, 2002, DIABETES CARE, V25, P1722, DOI 10.2337/diacare.25.10.1722

Norris SL, 2001, DIABETES CARE, V24, P561, DOI 10.2337/diacare.24.3.561

PAVOT W, 1993, PSYCHOL ASSESSMENT, V5, P164, DOI DOI 10.1037/1040-3590.5.2.164

Peyrot M, 2005, DIABETIC MED, V22, P1379, DOI 10.1111/j.1464-5491.2005.01644.x

Polonsky WH, 2005, DIABETES CARE, V28, P626, DOI 10.2337/diacare.28.3.626

Prochaska J. O., 2008, HLTH BEHAV HLTH ED T, V4, P97

RADLOFF L S, 1977, Applied Psychological Measurement, V1, P385, DOI 10.1177/014662167700100306

Rickheim PL, 2002, DIABETES CARE, V25, P269, DOI 10.2337/diacare.25.2.269

Ruggiero LP, 1993, DIABETES SPECTRUM, V6, P22

Sultan S, 2001, J HEALTH PSYCHOL, V6, P731, DOI 10.1177/135910530100600616

SURWIT RS, 1992, DIABETES CARE, V15, P1413, DOI 10.2337/diacare.15.10.1413

Toobert DJ, 2000, DIABETES CARE, V23, P943, DOI 10.2337/diacare.23.7.943

Vallis TM, 2001, CAN J DIABETES, V25, P31

WIETKIEWITZ K, 2007, THERAPISTS GUIDE EVI, P3

Zhang XP, 2007, MED CARE, V45, P820, DOI 10.1097/MLR.0b013e3180618b55

NR 35

TC 3

Z9 4

U1 0

U2 7

PU CANADIAN DIABETES ASSOC

PI TORONTO

PA 1400-522 UNIVERSITY AVE, TORONTO, ONTARIO M5G 2R5, CANADA

SN 1499-2671

J9 CAN J DIABETES

JI Can. J. Diabetes

PD JUL

PY 2011

VL 35

IS 3

BP 287

EP 294

DI 10.1016/S1499-2671(11)53013-8

PG 8

WC Endocrinology & Metabolism

SC Endocrinology & Metabolism

GA 836AX

UT WOS:000296079800012

DA 2019-01-08

ER

PT J

AU Paradise, MB

Glozier, NS

Naismith, SL

Davenport, TA

Hickie, IB

AF Paradise, Matt B.

Glozier, Nick S.

Naismith, Sharon L.

Davenport, Tracey A.

Hickie, Ian B.

TI Subjective memory complaints, vascular risk factors and psychological

distress in the middle-aged: a cross-sectional study

SO BMC PSYCHIATRY

LA English

DT Article

ID MILD COGNITIVE IMPAIRMENT; ALZHEIMERS-DISEASE; SUBCORTICAL

HYPERINTENSITIES; SEVERE DEPRESSION; APOE GENOTYPE; OLDER-PEOPLE;

DEMENTIA; POPULATION; DISABILITY; DISORDERS

AB Background: Subjective memory complaints (SMC) are common but their significance is still unclear. It has been suggested they are a precursor of mild cognitive impairment (MCI) or dementia and an early indicator of cognitive decline. Vascular risk factors have an important role in the development of dementia and possibly MCI. We therefore aimed to test the hypothesis that vascular risk factors were associated with SMC, independent of psychological distress, in a middle-aged community-dwelling population.

Methods: A cross-sectional analysis of baseline data from the 45 and Up Study was performed. This is a cohort study of people living in New South Wales (Australia), and we explored the sample of 45, 532 participants aged between 45 and 64 years. SMC were defined as 'fair' or 'poor' on a self-reported five-point Likert scale of memory function. Vascular risk factors of obesity, diabetes, hypertension, hypercholesterolemia and smoking were identified by self- report. Psychological distress was measured by the Kessler Psychological Distress Scale. We tested the model generated from a randomly selected exploratory sample (n = 22, 766) with a confirmatory sample of equal size.

Results: 5, 479/45, 532 (12%) of respondents reported SMC. Using multivariate logistic regression, only two vascular risk factors: smoking (OR 1.18; 95% CI = 1.03 - 1.35) and hypercholesterolaemia (OR 1.19; 95% CI = 1.04 - 1.36) showed a small independent association with SMC. In contrast psychological distress was strongly associated with SMC. Those with the highest levels of psychological distress were 7.00 (95% CI = 5.41 - 9.07) times more likely to have SMC than the non-distressed. The confirmatory sample also demonstrated the strong association of SMC with psychological distress rather than vascular risk factors.

Conclusions: In a large sample of middle-aged people without any history of major affective illness or stroke, psychological distress was strongly, and vascular risk factors only weakly, associated with SMC, although we cannot discount psychological distress acting as a mediator in any association between vascular risk factors and SMC. Given this, clinicians should be vigilant regarding the presence of an affective illness when assessing middle-aged patients presenting with memory problems.

C1 [Paradise, Matt B.; Glozier, Nick S.; Naismith, Sharon L.; Hickie, Ian B.] Univ Sydney, Brain & Mind Res Inst, Camperdown, NSW 2050, Australia.

[Davenport, Tracey A.] Acad Res & Stat Consulting, W Ryde, NSW 2114, Australia.

RP Paradise, MB (reprint author), Univ Sydney, Brain & Mind Res Inst, Bldg F,94 Mallet St, Camperdown, NSW 2050, Australia.

EM matthew.paradise@sydney.edu.au

OI Glozier, Nick/0000-0002-0476-9146; Naismith, Sharon/0000-0001-9076-2778;

Paradise, Matthew/0000-0003-4002-9599

FU NHMRC Australia

FX Dr Paradise, A/Prof Naismith and Prof Hickie and are funded by an NHMRC

Australia Fellowship awarded to Prof Hickie.

CR *ABS HLTH SURV, 2001, 4817055001 ABS HLTH

Alexopoulos GS, 1997, ARCH GEN PSYCHIAT, V54, P915

Andrews G, 2001, AUST NZ J PUBL HEAL, V25, P494, DOI 10.1111/j.1467-842X.2001.tb00310.x

Banks E, 2008, INT J EPIDEMIOL, V37, P941, DOI 10.1093/ije/dym184

CHAO LL, 2008, NEUROBIOL AGING, V31, P368

Minett TSC, 2008, INT J GERIATR PSYCH, V23, P49, DOI 10.1002/gps.1836

de la Torre JC, 2004, LANCET NEUROL, V3, P184, DOI 10.1016/S1474-4422(04)00683-0

Duman RS, 2006, BIOL PSYCHIAT, V59, P1116, DOI 10.1016/j.biopsych.2006.02.013

Ferri CP, 2005, LANCET, V366, P2112, DOI 10.1016/S0140-6736(05)67889-0

Ganguli M, 2004, NEUROLOGY, V63, P115, DOI 10.1212/01.WNL.0000132523.27540.81

Geerlings MI, 1999, AM J PSYCHIAT, V156, P531

Gorelick PB, 2004, STROKE, V35, P2620, DOI 10.1161/01.STR.000143318.70292.47

HANNINEN T, 1994, J AM GERIATR SOC, V42, P1

Hickie I, 1998, PSYCHOL MED, V28, P1007, DOI 10.1017/S0033291797006090

Hickie I, 2005, BRIT J PSYCHIAT, V186, P197, DOI 10.1192/bjp.186.3.197

Hickie I, 1997, BIOL PSYCHIAT, V42, P367, DOI 10.1016/S0006-3223(96)00363-0

HICKIE I, 1995, BIOL PSYCHIAT, V37, P151, DOI 10.1016/0006-3223(94)00174-2

Hickie I.B., 2001, MED J AUSTRALIA, V175, P16

JESSEN F, ARCH GEN PSYCHIAT, V67, P414

Jonker C, 2000, INT J GERIATR PSYCH, V15, P983, DOI 10.1002/1099-1166(200011)15:11<983::AID-GPS238>3.0.CO;2-5

Jorm AF, 2004, PSYCHOL MED, V34, P1495, DOI 10.1017/S0033291704003162

Kessler RC, 2002, PSYCHOL MED, V32, P959, DOI 10.1017/S0033291702006074

Kivipelto M, 2001, NEUROLOGY, V56, P1683, DOI 10.1212/WNL.56.12.1683

Larrabee G J, 1994, Int Psychogeriatr, V6, P95, DOI 10.1017/S1041610294001663

Luchsinger JA, 2005, NEUROLOGY, V65, P545, DOI 10.1212/01.wnl.0000172914.08967.dc

Mariani E, 2007, DEMENT GERIATR COGN, V24, P448, DOI 10.1159/000110653

MEALING NM, BMC MED RES METHODOL, V10, P26

Minett TSC, 2005, AM J GERIAT PSYCHIAT, V13, P665, DOI 10.1176/appi.ajgp.13.8.665

Modrego PJ, 2004, ARCH NEUROL-CHICAGO, V61, P1290, DOI 10.1001/archneur.61.8.1290

Naismith SL, 2009, EARLY INTERV PSYCHIA, V3, P19, DOI 10.1111/j.1751-7893.2008.00102.x

Naismith SL, 2007, BMC PSYCHIATRY, V7, DOI 10.1186/1471-244X-7-32

Naismith SL, 2003, J CLIN EXP NEUROPSYC, V25, P866, DOI 10.1076/jcen.25.6.866.16472

NAISMITH SL, 2001, MED J AUSTRALIA, V175, P16

Ownby RL, 2006, ARCH GEN PSYCHIAT, V63, P530, DOI 10.1001/archpsyc.63.5.530

Purser JL, 2006, J AM GERIATR SOC, V54, P335, DOI 10.1111/j.1532-5415.2005.00589.x

Ravaglia G, 2006, DEMENT GERIATR COGN, V21, P51, DOI 10.1159/000089515

Reid LM, 2006, DEMENT GERIATR COGN, V22, P471, DOI 10.1159/000096295

Reisberg B, 2008, ALZHEIMERS DEMENT, V4, pS98, DOI 10.1016/j.jalz.2007.11.017

Saykin AJ, 2006, NEUROLOGY, V67, P834, DOI 10.1212/01.wnl.0000234032.77541.a2

Solfrizzi V, 2004, NEUROLOGY, V63, P1882, DOI 10.1212/01.WNL.0000144281.38555.E3

Stewart R, 2001, PSYCHOL MED, V31, P431

TOBIANSKY R, 1995, PSYCHOL MED, V25, P779, DOI 10.1017/S0033291700035029

World Health Organization, 2000, TECHN REP SER WHO

NR 43

TC 46

Z9 48

U1 5

U2 16

PU BIOMED CENTRAL LTD

PI LONDON

PA 236 GRAYS INN RD, FLOOR 6, LONDON WC1X 8HL, ENGLAND

SN 1471-244X

J9 BMC PSYCHIATRY

JI BMC Psychiatry

PD JUL 1

PY 2011

VL 11

AR 108

DI 10.1186/1471-244X-11-108

PG 7

WC Psychiatry

SC Psychiatry

GA 803OI

UT WOS:000293590200001

PM 21722382

OA DOAJ Gold, Green Published

DA 2019-01-08

ER

PT J

AU Pengpid, S

Peltzer, K

Van der Heever, H

AF Pengpid, Supa

Peltzer, Karl

Van der Heever, Hendry

TI Prevalence of Alcohol Use and Associated Factors in Urban Hospital

Outpatients in South Africa

SO INTERNATIONAL JOURNAL OF ENVIRONMENTAL RESEARCH AND PUBLIC HEALTH

LA English

DT Article

DE alcohol misuse; tobacco use; associated factors; hospital out-patients;

South Africa

ID IDENTIFICATION TEST AUDIT; PRIMARY-CARE; USE DISORDERS; ANXIETY

DISORDERS; PROBLEM DRINKING; PSYCHOLOGICAL DISTRESS; COLLABORATIVE

PROJECT; BRIEF INTERVENTION; SUBSTANCE USE; CONSUMPTION

AB The aim of this study was to assess the prevalence of alcohol use and associated factors among outpatients in an urban hospital in South Africa. The sample included 1,532 (56.4% men and women 43.6%) consecutively selected patients from different hospital outpatient departments. Results indicate that 41.2% of men and 18.3% of women were found to be hazardous drinkers, and 3.6% of men and 1.4% of women meet criteria for probable alcohol dependence or harmful drinking as defined by the Alcohol Use Disorder Identification Test (AUDIT). Two in five patients (40.5%) were hazardous or harmful drinkers and/or had anxiety or depression. Logistic multiple regression found that for men tobacco use and not having been diagnosed with diabetes and for women tobacco use and having been diagnosed with migraine headache was associated with hazardous and harmful drinking. Although the study is cross-sectional, it does identify groups that may be at high risk of alcohol misuse and for whom intervention is urgent. Because prevalence of hazardous and harmful alcohol use is high in this population, routine screening should be introduced in hospital out-patient settings.

C1 [Pengpid, Supa; Van der Heever, Hendry] Univ Limpopo, Dept Hlth Syst Management & Policy, ZA-0204 Pretoria, South Africa.

[Peltzer, Karl] Human Sci Res Council, HIV AIDS SIT & TB HAST, ZA-0002 Pretoria, South Africa.

[Peltzer, Karl] Univ Limpopo, Dept Psychol, ZA-0727 Sovenga, Limpopo, South Africa.

RP Pengpid, S (reprint author), Univ Limpopo, Dept Hlth Syst Management & Policy, Ga Rankuwa Campus, ZA-0204 Pretoria, South Africa.

EM Supa_Pengpid@embanet.com; Kpeltzer@hsrc.ac.za; Hendry_Heever@embanet.com

OI van der Heever, Hendry/0000-0002-2217-6504

FU University of Limpopo; FLIR-UOS

FX We thank FLIR-UOS under program collaboration with the University of

Limpopo (Project 7) for funding the study.

CR Abiodun OA, 1996, ACTA PSYCHIAT SCAND, V93, P235, DOI 10.1111/j.1600-0447.1996.tb10640.x

Allen JP, 1997, ALCOHOL CLIN EXP RES, V21, P613, DOI 10.1111/j.1530-0277.1997.tb03811.x

Andrews G, 2001, AUST NZ J PUBL HEAL, V25, P494, DOI 10.1111/j.1467-842X.2001.tb00310.x

Arch JJ, 2006, GEN HOSP PSYCHIAT, V28, P37, DOI 10.1016/j.genhosppsych.2005.08.007

Babor TF, 2001, BRIEF INTERVENTION H

Babor TF, 2001, AUDIT ALCOHOL USE DI

Bekker D., 2003, S AFRICAN FAMILY PRA, V45, P10

Brooks RT, 2006, PSYCHOL ASSESSMENT, V18, P62, DOI 10.1037/1040-3590.18.1.62

CHINYADZA E, 1993, CENT AFR J MED, V39, P26

Emmen MJ, 2005, NETH J MED, V63, P421

Emmen MJ, 2006, PATIENT EDUC COUNS, V60, P32, DOI 10.1016/j.pec.2004.11.011

Emmen MJ, 2004, BMJ-BRIT MED J, V328, P318, DOI 10.1136/bmj.37956.562130.EE

Flisher AJ, 2001, S AFR MED J, V91, P243

Freeborn DK, 2000, J BEHAV HEALTH SER R, V27, P446, DOI 10.1007/BF02287826

Goldberger BA, 2007, J ADDICT DIS, V26, P113, DOI 10.1300/J069v26S01_02

GUREJE O, 1992, DRUG ALCOHOL DEPEN, V30, P163, DOI 10.1016/0376-8716(92)90021-4

Jane-Llopis E, 2006, DRUG ALCOHOL REV, V25, P515, DOI 10.1080/09595230600944461

Johnson T, 2005, ANN EPIDEMIOL, V15, P381, DOI 10.1016/j.annepidem.2004.09.004

Kessler RC, 2003, ARCH GEN PSYCHIAT, V60, P184, DOI 10.1001/archpsyc.60.2.184

Kessler RC, 2002, PSYCHOL MED, V32, P959, DOI 10.1017/S0033291702006074

Kushner MG, 2000, CLIN PSYCHOL REV, V20, P149, DOI 10.1016/S0272-7358(99)00027-6

Liu C, 2010, BMC PUBLIC HEALTH, V10, DOI 10.1186/1471-2458-10-713

MPHI M, 1994, ADDICTION, V89, P945, DOI 10.1111/j.1360-0443.1994.tb03350.x

Newcombe DAL, 2005, DRUG ALCOHOL REV, V24, P217, DOI 10.1080/0959230500170266

Nordstrom A, 2008, NORD J PSYCHIAT, V62, P249, DOI 10.1080/08039480802141129

Panconesi A, 2008, J HEADACHE PAIN, V9, P19, DOI 10.1007/s10194-008-0006-1

Peltzer K, 2011, AFR J PSYCHIATRY, V14, P30

Peltzer K, 2006, PSYCHOL REP, V99, P176, DOI 10.2466/PR0.99.1.176-178

Reid MC, 1999, ARCH INTERN MED, V159, P1681, DOI 10.1001/archinte.159.15.1681

SAUNDERS JB, 1993, ADDICTION, V88, P791, DOI 10.1111/j.1360-0443.1993.tb02093.x

Spies G, 2009, AIDS CARE, V21, P1163, DOI 10.1080/09540120902729965

Spies G, 2009, ARCH WOMEN MENT HLTH, V12, P69, DOI 10.1007/s00737-009-0050-0

STREBEL A, 1999, ARCH WOMEN MENT HLTH, V2, P75

Swendsen JD, 2000, CLIN PSYCHOL REV, V20, P173, DOI 10.1016/S0272-7358(99)00026-4

Tsai YF, 2009, ALCOHOL CLIN EXP RES, V33, P95, DOI 10.1111/j.1530-0277.2008.00815.x

Ward CL, 2008, SUBST USE MISUSE, V43, P1395, DOI 10.1080/10826080801922744

NR 36

TC 16

Z9 16

U1 0

U2 7

PU MDPI

PI BASEL

PA ST ALBAN-ANLAGE 66, CH-4052 BASEL, SWITZERLAND

SN 1660-4601

J9 INT J ENV RES PUB HE

JI Int. J. Environ. Res. Public Health

PD JUL

PY 2011

VL 8

IS 7

BP 2629

EP 2639

DI 10.3390/ijerph8072629

PG 11

WC Environmental Sciences; Public, Environmental & Occupational Health

SC Environmental Sciences & Ecology; Public, Environmental & Occupational

Health

GA 796QS

UT WOS:000293067300004

PM 21845149

OA DOAJ Gold, Green Published

DA 2019-01-08

ER

PT J

AU van Bastelaar, K

Cuijpers, P

Pouwer, F

Riper, H

Snoek, FJ

AF van Bastelaar, Kim

Cuijpers, Pim

Pouwer, Francois

Riper, Heleen

Snoek, Frank J.

TI Development and reach of a web-based cognitive behavioural therapy

programme to reduce symptoms of depression and diabetes-specific

distress

SO PATIENT EDUCATION AND COUNSELING

LA English

DT Article

DE Depression; Diabetes; Intervention development

ID GROUP TRAINING CBGT; QUALITY-OF-LIFE; GLYCEMIC CONTROL; PSYCHOSOCIAL

ADAPTATION; COMORBID DEPRESSION; INTERNET; ADULTS; VALIDITY; ANXIETY;

METAANALYSIS

AB Objectives: There is an urgent need for more effective and efficient depression treatments in diabetes. We developed a diabetes-specific version of the Dutch web-based 'Coping with Depression' (CWD) course. Here, we report on the development, reach, patients' reasons for choosing our intervention and their characteristics.

Methods: The CWD programme was amended for use in diabetes patients with co-morbid depression. Data were collected using a telephone interview, self-report questionnaires, and medical records.

Results: Adding diabetes-specific topics to an effective web-based depression programme resulted in an 8-lesson intervention ("www.diabetergestemd.nl"), with minimal guidance by coaches. In the framework of a randomised trial, the intervention attracted serious interest of 540 patients. After screening, 255 depressed diabetes patients were enrolled. Less than half had a history of depression treatment; 80% reported the diabetes-specific approach to be an important reason for signing up.

Conclusion: We successfully developed a diabetes-specific version of the web-based CWD course, which attracted a large group of patients. Our results affirm the importance of addressing diabetes-specific issues in the context of depression treatment.

Practice implications: Our intervention could be implemented on a large scale at low costs, and may serve as a model on how to develop other illness-specific online self-help interventions. Crown Copyright (C) 2010 Published by Elsevier Ireland Ltd. All rights reserved.

C1 [van Bastelaar, Kim] Vrije Univ Amsterdam Med Ctr, Dept Med Psychol, Diabet Psychol Res Grp, NL-1081 BT Amsterdam, Netherlands.

[van Bastelaar, Kim; Cuijpers, Pim; Snoek, Frank J.] EMGO Inst Hlth & Care Res, Amsterdam, Netherlands.

[Cuijpers, Pim] Vrije Univ Amsterdam, Dept Clin Psychol, Amsterdam, Netherlands.

[Pouwer, Francois] Tilburg Univ, CoRPS Ctr Res Psychol Somat Dis, Dept Med Psychol & Neuropsychol, NL-5000 LE Tilburg, Netherlands.

[Riper, Heleen] Netherlands Inst Mental Hlth & Addict Trimbos, Utrecht, Netherlands.

RP van Bastelaar, K (reprint author), Vrije Univ Amsterdam Med Ctr, Dept Med Psychol, Diabet Psychol Res Grp, Van der Boechorststr 7, NL-1081 BT Amsterdam, Netherlands.

EM k.vanbastelaar@vumc.nl

RI Cuijpers, Pim/G-1703-2013

OI Cuijpers, Pim/0000-0001-5497-2743

FU Dutch Diabetes Research Foundation

FX This study was funded by the Dutch Diabetes Research Foundation.

CR Amer Diabet Assoc, 2009, DIABETES CARE, V32, pS13, DOI 10.2337/dc09-S013

Anderson RJ, 2001, DIABETES CARE, V24, P1069, DOI 10.2337/diacare.24.6.1069

ANDERSSON G, 2009, COGN BEHAV THER, V1

Andersson G, 2008, BRIT J PSYCHIAT, V193, P270, DOI 10.1192/bjp.bp.108.054080

Baan Caroline A, 2009, Ned Tijdschr Geneeskd, V153, pA580

BARAK A, 2009, ANN BEHAV MED

Beekman ATF, 1997, PSYCHOL MED, V27, P231, DOI 10.1017/S0033291796003510

Clarke G, 2005, J MED INTERNET RES, V7, DOI 10.2196/jmir.7.2.e16

Cuijpers P, 1998, PSYCHOL REP, V82, P735, DOI 10.2466/PR0.82.3.735-738

Cuijpers P, 2009, EVID-BASED MENT HEAL, V12, P76, DOI 10.1136/ebmh.12.3.76

Cuijpers P, 2009, CLIN PSYCHOL REV, V29, P449, DOI 10.1016/j.cpr.2009.04.005

Cuijpers P, 2008, J CLIN PSYCHIAT, V69, P1675, DOI 10.4088/JCP.v69n1102

Cuijpers P, 2008, J CONSULT CLIN PSYCH, V76, P909, DOI 10.1037/a0013075

de Graaf LE, 2009, BRIT J PSYCHIAT, V195, P73, DOI 10.1192/bjp.bp.108.054429

de Groot M, 2001, PSYCHOSOM MED, V63, P619, DOI 10.1097/00006842-200107000-00015

GEORGIADES A, 2007, CHANGES DEPRESSIVE S

Griffiths F, 2006, J MED INTERNET RES, V8, DOI 10.2196/jmir.8.2.e10

IDF, 2006, DIABETIC MED, V23, P579

Katon WJ, 2005, DIABETES CARE, V28, P2668, DOI 10.2337/diacare.28.11.2668

Kazantzis N, 2000, CLIN PSYCHOL-SCI PR, V7, P189, DOI 10.1093/clipsy/7.2.189

Lin YR, 2008, J CLIN NURS, V17, P2875, DOI 10.1111/j.1365-2702.2008.02343.x

Mosaku K, 2008, J NATL MED ASSOC, V100, P73, DOI 10.1016/S0027-9684(15)31178-0

Petrak F, 2009, CURR OPIN PSYCHIATR, V22, P211, DOI 10.1097/YCO.0b013e3283207b45

Peyrot M, 2005, DIABETIC MED, V22, P1379, DOI 10.1111/j.1464-5491.2005.01644.x

Piette JD, 2004, AM J MANAG CARE, V10, P152

Pouwer F, 2006, PATIENT EDUC COUNS, V60, P235, DOI 10.1016/j.pec.2005.01.009

RADLOFF L S, 1977, Applied Psychological Measurement, V1, P385, DOI 10.1177/014662167700100306

Rohde P, 1997, AM J PSYCHIAT, V154, P1593, DOI 10.1176/ajp.154.11.1593

Rubin Richard R, 2004, Curr Diab Rep, V4, P119, DOI 10.1007/s11892-004-0067-8

Rubin RR, 1999, DIABETES-METAB RES, V15, P205, DOI 10.1002/(SICI)1520-7560(199905/06)15:3<205::AID-DMRR29>3.0.CO;2-O

Samson A, 2008, PATIENT EDUC COUNS, V70, P426, DOI 10.1016/j.pec.2007.10.018

Snoek FJ, 2008, DIABETIC MED, V25, P1337, DOI 10.1111/j.1464-5491.2008.02595.x

Snoek FJ, 2001, PATIENT EDUC COUNS, V45, P143, DOI 10.1016/S0738-3991(01)00113-6

Snoek FJ, 2000, DIABETES CARE, V23, P1305, DOI 10.2337/diacare.23.9.1305

SNOEK FJ, 2008, PRACT DIABET INT, V23, P241

SPEK V, 2008, PSYCHOL MED, P1

Spek V, 2007, PSYCHOL MED, V37, P319, DOI 10.1017/S0033291706008944

Strecher V, 2007, ANNU REV CLIN PSYCHO, V3, P53, DOI 10.1146/annurev.clinpsy.3.022806.091428

Surwit RS, 2002, DIABETES CARE, V25, P30, DOI 10.2337/diacare.25.1.30

TATE DF, 2009, ANN BEHAV MED

TEN HM, 2001, SOC PSYCH PSYCH EPID, V36, P141

Trief PM, 1998, DIABETES CARE, V21, P241, DOI 10.2337/diacare.21.2.241

Trief PM, 2001, DIABETES CARE, V24, P1384, DOI 10.2337/diacare.24.8.1384

van Bastelaar KM, 2008, BMC PSYCHIATRY, V8, DOI 10.1186/1471-244X-8-9

van der Ven NCW, 2005, PATIENT EDUC COUNS, V56, P313, DOI 10.1016/j.pec.2004.03.005

VANMEETERENSCHR.MT, 2007, 2608010032007 RIVM

Warmerdam L, 2008, J MED INTERNET RES, V10, DOI 10.2196/jmir.1094

WITTCHEN HU, 1994, J PSYCHIAT RES, V28, P57, DOI 10.1016/0022-3956(94)90036-1

NR 48

TC 17

Z9 18

U1 0

U2 24

PU ELSEVIER IRELAND LTD

PI CLARE

PA ELSEVIER HOUSE, BROOKVALE PLAZA, EAST PARK SHANNON, CO, CLARE, 00000,

IRELAND

SN 0738-3991

J9 PATIENT EDUC COUNS

JI Patient Educ. Couns.

PD JUL

PY 2011

VL 84

IS 1

BP 49

EP 55

DI 10.1016/j.pec.2010.06.013

PG 7

WC Public, Environmental & Occupational Health; Social Sciences,

Interdisciplinary

SC Public, Environmental & Occupational Health; Social Sciences - Other

Topics

GA 791OH

UT WOS:000292674000009

PM 20619577

DA 2019-01-08

ER

PT J

AU Capuron, L

Poitou, C

Machaux-Tholliez, D

Frochot, V

Bouillot, JL

Basdevant, A

Laye, S

Clement, K

AF Capuron, L.

Poitou, C.

Machaux-Tholliez, D.

Frochot, V.

Bouillot, J. -L.

Basdevant, A.

Laye, S.

Clement, K.

TI Relationship between adiposity, emotional status and eating behaviour in

obese women: role of inflammation

SO PSYCHOLOGICAL MEDICINE

LA English

DT Article

DE Eating behaviour; inflammation; neuroticism; obesity; psychological

characteristics; weight loss

ID GENERALIZED ANXIETY DISORDER; GASTRIC BYPASS-SURGERY; LOW-GRADE

INFLAMMATION; C-REACTIVE PROTEIN; WEIGHT-LOSS; INTERFERON-ALPHA; MAJOR

DEPRESSION; MACROPHAGE INFILTRATION; INSULIN-RESISTANCE; TISSUE

AB Background. Obesity is characterized by chronic low-grade inflammation that may lead to emotional distress and behavioural symptoms. This study assessed the relationship between adiposity, low-grade inflammation, eating behaviour and emotional status in obese women awaiting gastric surgery and investigated the effects of surgery-induced weight loss on this relationship.

Method. A total of 101 women with severe or morbid obesity awaiting gastric surgery were recruited. Assessments were performed before and at 1 year post-surgery and included the measurement of neuroticism and extraversion using the revised Neuroticism-Extraversion-Openness personality inventory (NEO-PI-R) and eating behaviour using the Three-Factor Eating Questionnaire (TFEQ). Blood samples were collected for the measurement of serum inflammatory markers [interleukin-6 (IL-6), high-sensitive C-reactive protein (hsCRP)] and adipokines (leptin, adiponectin).

Results. At baseline, body mass index (BMI) was positively correlated with inflammatory markers and adipokines. Regression analyses adjusting for age and diabetes revealed that baseline concentrations of IL-6 and hsCRP were associated with the depression and anxiety facets of neuroticism, with higher inflammation predicting higher anxiety and depression. This association remained significant after adjusting for BMI. Gastric surgery induced significant weight loss, which correlated with reduced inflammation. After controlling for BMI variations, decreases in inflammatory markers, notably hsCRP, were associated with reduced anxiety and TFEQ-cognitive restraint scores.

Conclusions. These findings indicate strong associations between adiposity, inflammation and affectivity in obese subjects and show that surgery-induced weight loss is associated concomitantly with reduced inflammation and adipokines and with significant improvement in emotional status and eating behaviour. Inflammatory status appears to represent an important mediator of emotional distress and psychological characteristics of obese individuals.

C1 [Capuron, L.; Laye, S.] Univ Bordeaux 2, Lab Psychoneuroimmunol Nutr & Genet PSYNUGEN, INRA 1286, CNRS 5226, F-33076 Bordeaux, France.

[Poitou, C.; Machaux-Tholliez, D.; Basdevant, A.; Clement, K.] Hop La Pitie Salpetriere, AP HP, Nutr & Endocrinol Dept, CRNH Ile France, F-75013 Paris, France.

[Poitou, C.; Basdevant, A.; Clement, K.] INSERM, U872, Nutri Team 7, F-75006 Paris, France.

[Poitou, C.; Basdevant, A.; Clement, K.] Univ Paris 06, Ctr Rech Cordeliers, UMR S 872, F-75006 Paris, France.

[Poitou, C.; Basdevant, A.; Clement, K.] Univ Paris 05, UMR S 872, F-75006 Paris, France.

[Frochot, V.] Hop La Pitie Salpetriere, AP HP, Endocrinol & Oncol Biochem Dept, F-75013 Paris, France.

[Bouillot, J. -L.] Hop Hotel Dieu, AP HP, Dept Surg, F-75013 Paris, France.

RP Capuron, L (reprint author), Univ Bordeaux 2, Lab Psychoneuroimmunol Nutr & Genet PSYNUGEN, INRA 1286, CNRS 5226, 146 Rue Leo Saignat, F-33076 Bordeaux, France.

EM lucile.capuron@bordeaux.inra.fr

RI Clement, karine/R-1120-2017

OI Capuron, Lucile/0000-0002-2060-5918

FU Assistance Publique Hopitaux de Paris/Direction de la Recherche Clinique

[CRC P050318]; PHRC [0207]

FX This work was supported by Assistance Publique Hopitaux de

Paris/Direction de la Recherche Clinique (CRC P050318) and PHRC (0207).

We thank Dr Florence Marchelli, Madame Christine Baudoin and Madame

Patricia Ancel for their involvement in patient recruitment, data

collection and sampling at the Centre of Research on Human Nutrition

(CRNH), Ile de France.

CR Bankier B, 2008, EUR HEART J, V29, P2212, DOI 10.1093/eurheartj/ehn326

BARON RM, 1986, J PERS SOC PSYCHOL, V51, P1173, DOI 10.1037//0022-3514.51.6.1173

Bastard JP, 2006, EUR CYTOKINE NETW, V17, P4

Bastard JP, 1999, CIRCULATION, V99, P2221

Bastard JP, 2000, J CLIN ENDOCR METAB, V85, P3338, DOI 10.1210/jc.85.9.3338

Bean MK, 2008, J CLIN PSYCHOL MED S, V15, P214, DOI 10.1007/s10880-008-9124-9

Bellisle F, 2004, OBES RES, V12, P2023, DOI 10.1038/oby.2004.253

Branca F, 2007, CHALLENGE OBESITY WH

Cancello R, 2006, BJOG-INT J OBSTET GY, V113, P1141, DOI 10.1111/j.1471-0528.2006.01004.x

Cancello R, 2005, DIABETES, V54, P2277, DOI 10.2337/diabetes.54.8.2277

Canetti L, 2009, INT J EAT DISORDER, V42, P109, DOI 10.1002/eat.20592

Capuron L, 2005, BIOL PSYCHIAT, V58, P190, DOI 10.1016/j.biopsych.2005.03.033

Capuron L, 2004, BIOL PSYCHIAT, V56, P819, DOI 10.1016/j.biopsych.2004.02.009

Capuron L, 2002, NEUROPSYCHOPHARMACOL, V26, P643, DOI 10.1016/S0893-133X(01)00407-9

Capuron L, 2001, AM J PSYCHIAT, V158, P1163, DOI 10.1176/appi.ajp.158.7.1163

Capuron L, 2008, BIOL PSYCHIAT, V64, P896, DOI 10.1016/j.biopsych.2008.05.019

CHARLES SC, 1987, GASTROENTEROL CLIN N, V16, P415

Clement K, 2004, FASEB J, V18, P1657, DOI 10.1096/fj.04-2204com

Costa Jr P. T., 1985, NEO PERSONALITY INVE

Costa P. T., 1998, NEO PI R INVENTAIRE

DANTZER R, 1999, CYTOKINES STRESS DEP

de Zwaan M, 2003, COMPR PSYCHIAT, V44, P428, DOI 10.1016/S0010-440X(03)00092-0

Dixon JB, 2003, ARCH INTERN MED, V163, P2058, DOI 10.1001/archinte.163.17.2058

Evans DL, 2005, BIOL PSYCHIAT, V58, P175, DOI 10.1016/j.biopsych.2005.05.001

Faith MS, 2001, OBES RES, V9, P647, DOI 10.1038/oby.2001.86

Forsythe LKS, 2008, NUTR RES REV, V21, P117, DOI 10.1017/S0954422408138732

Gibson EL, 2006, PHYSIOL BEHAV, V89, P53, DOI 10.1016/j.physbeh.2006.01.024

Herva A, 2006, INT J OBESITY, V30, P520, DOI 10.1038/sj.ijo.0803174

Hsu LKG, 2002, INT J OBESITY, V26, P1398, DOI 10.1038/sj.ijo.0802081

Jylha P, 2006, DEPRESS ANXIETY, V23, P281, DOI 10.1002/da.20167

Karlsson J, 2000, INT J OBESITY, V24, P1715, DOI 10.1038/sj.ijo.0801442

Katz A, 2000, J CLIN ENDOCR METAB, V85, P2402, DOI 10.1210/jc.85.7.2402

Kendler KS, 2007, PSYCHOL MED, V37, P453, DOI 10.1017/S0033291706009135

Kendler KS, 2006, ARCH GEN PSYCHIAT, V63, P1113, DOI 10.1001/archpsyc.63.10.1113

Konsman JP, 2002, TRENDS NEUROSCI, V25, P154, DOI 10.1016/S0166-2236(00)02088-9

Lluch A, 1995, THESIS U H POINCARE

Lykouras L, 2008, DIGEST DIS, V26, P36, DOI 10.1159/000109384

Musselman DL, 2001, NEW ENGL J MED, V344, P961, DOI 10.1056/NEJM200103293441303

O'Rourke RW, 2009, SURGERY, V145, P255, DOI 10.1016/j.surg.2008.08.038

Plotkin SR, 1996, J NEUROIMMUNOL, V67, P41

Poitou C, 2006, OBESITY, V14, P309, DOI 10.1038/oby.2006.40

Poitou C, 2009, J CLIN ENDOCR METAB, V94, P1810, DOI 10.1210/jc.2008-2040

Provencher Veronique, 2008, Eat Behav, V9, P294, DOI 10.1016/j.eatbeh.2007.10.004

Rabe K, 2008, MOL MED, V14, P741, DOI 10.2119/2008-00058.Rabe

Raison CL, 2006, TRENDS IMMUNOL, V27, P24, DOI 10.1016/j.it.2005.11.006

Rivest S, 2000, P SOC EXP BIOL MED, V223, P22, DOI 10.1046/j.1525-1373.2000.22304.x

Roberts SB, 1999, PSYCHOL MED, V29, P1101, DOI 10.1017/S0033291799008739

ROLLAND JP, 1998, REV FRANCAISE PSYCHI, V18, P139

WATKINS LR, 1995, NEUROSCI LETT, V183, P27, DOI 10.1016/0304-3940(94)11105-R

Wellen KE, 2003, J CLIN INVEST, V112, P1785, DOI 10.1172/JCI200320514

Wilberg T, 2009, J NERV MENT DIS, V197, P187, DOI 10.1097/NMD.0b013e3181923fa0

Wolfe BL, 2006, OBES SURG, V16, P1622, DOI 10.1381/096089206779319473

NR 52

TC 46

Z9 48

U1 0

U2 19

PU CAMBRIDGE UNIV PRESS

PI NEW YORK

PA 32 AVENUE OF THE AMERICAS, NEW YORK, NY 10013-2473 USA

SN 0033-2917

J9 PSYCHOL MED

JI Psychol. Med.

PD JUL

PY 2011

VL 41

IS 7

BP 1517

EP 1528

DI 10.1017/S0033291710001984

PG 12

WC Psychology, Clinical; Psychiatry; Psychology

SC Psychology; Psychiatry

GA 780KL

UT WOS:000291854700019

PM 20961476

DA 2019-01-08

ER

PT J

AU Raja-Khan, N

Stener-Victorin, E

Wu, XK

Legro, RS

AF Raja-Khan, Nazia

Stener-Victorin, Elisabet

Wu, XiaoKe

Legro, Richard S.

TI The physiological basis of complementary and alternative medicines for

polycystic ovary syndrome

SO AMERICAN JOURNAL OF PHYSIOLOGY-ENDOCRINOLOGY AND METABOLISM

LA English

DT Review

DE acupuncture; mindfulness meditation; herbs; dietary supplements;

traditional Chinese medicine

ID RANDOMIZED CONTROLLED-TRIAL; TYPE-2 DIABETES-MELLITUS; LOW-FREQUENCY

ELECTROACUPUNCTURE; SYMPATHETIC-NERVE ACTIVITY; STRESS REDUCTION MBSR;

BLOOD-FLOW RESPONSES; QUALITY-OF-LIFE; TAI-CHI-CHUAN;

INSULIN-RESISTANCE; GREEN TEA

AB Raja-Khan N, Stener-Victorin E, Wu X, Legro RS. The physiological basis of complementary and alternative medicines for polycystic ovary syndrome. Am J Physiol Endocrinol Metab 301: E1-E10, 2011. First published April 12, 2011; doi: 10.1152/ajpendo.00667.2010.-Polycystic ovary syndrome (PCOS) is a common endocrine disorder that is characterized by chronic hyperandrogenic anovulation leading to symptoms of hirsutism, acne, irregular menses, and infertility. Multiple metabolic and cardiovascular risk factors are associated with PCOS, including insulin resistance, obesity, type 2 diabetes, hypertension, inflammation, and subclinical atherosclerosis. However, current treatments for PCOS are only moderately effective at controlling symptoms and preventing complications. This article describes how the physiological effects of major complementary and alternative medicine (CAM) treatments could reduce the severity of PCOS and its complications. Acupuncture reduces hyperandrogenism and improves menstrual frequency in PCOS. Acupuncture's clinical effects are mediated via activation of somatic afferent nerves innervating the skin and muscle, which, via modulation of the activity in the somatic and autonomic nervous system, may modulate endocrine and metabolic functions in PCOS. Chinese herbal medicines and dietary supplements may also exert beneficial physiological effects in PCOS, but there is minimal evidence that these CAM treatments are safe and effective. Mindfulness has not been investigated in PCOS, but it has been shown to reduce psychological distress and exert positive effects on the central and autonomic nervous systems, hypothalamic-pituitary-adrenal axis, and immune system, leading to reductions in blood pressure, glucose, and inflammation. In conclusion, CAM treatments may have beneficial endocrine, cardiometabolic, and reproductive effects in PCOS. However, most studies of CAM treatments for PCOS are small, nonrandomized, or uncontrolled. Future well-designed studies are needed to further evaluate the safety, effectiveness, and mechanisms of CAM treatments for PCOS.

C1 [Raja-Khan, Nazia] Penn State Univ, Milton S Hershey Med Ctr, Coll Med, Div Endocrinol Diabet & Metab, Hershey, PA 17033 USA.

[Stener-Victorin, Elisabet] Univ Gothenburg, Sahlgrenska Acad, Dept Physiol, Inst Neurosci & Physiol, Gothenburg, Sweden.

[Stener-Victorin, Elisabet; Wu, XiaoKe] Heilongjiang Univ Chinese Med, Natl Key Discipline & Clin Base, Dept Obstet & Gynecol, Harbin, Peoples R China.

[Legro, Richard S.] Penn State Univ, Milton S Hershey Med Ctr, Coll Med, Dept Obstet & Gynecol, Hershey, PA 17033 USA.

RP Raja-Khan, N (reprint author), Penn State Univ, Milton S Hershey Med Ctr, Coll Med, Div Endocrinol Diabet & Metab, 500 Univ Dr,H044, Hershey, PA 17033 USA.

EM nrajakhan@psu.edu

RI Stener-Victorin, Elisabet/W-6322-2018

OI Stener-Victorin, Elisabet/0000-0002-3424-1502; Legro,

Richard/0000-0001-9927-7584; Raja-Khan, Nazia/0000-0002-2751-9609

FU NICHD NIH HHS [K12 HD055882]

CR Ahmed MI, 2008, HUM REPROD, V23, P2564, DOI 10.1093/humrep/den273

Ainsworth BE, 2000, MED SCI SPORT EXER, V32, pS498, DOI 10.1097/00005768-200009001-00009

Anderson RA, 2004, J AGR FOOD CHEM, V52, P65, DOI 10.1021/jf034916b

Barber TM, 2006, CLIN ENDOCRINOL, V65, P137, DOI 10.1111/j.1365-2265.2006.02587.x

Barnard L, 2007, HUM REPROD, V22, P2279, DOI 10.1093/humrep/dem108

Bian Zhao-Xiang, 2006, Zhong Xi Yi Jie He Xue Bao, V4, P233

Blank SK, 2007, SEMIN REPROD MED, V25, P352, DOI 10.1055/s-2007-984741

Broadhurst CL, 2000, J AGR FOOD CHEM, V48, P849, DOI 10.1021/jf9904517

Carlson LE, 2007, BRAIN BEHAV IMMUN, V21, P1038, DOI 10.1016/j.bbi.2007.04.002

Chan CCW, 2006, J SOC GYNECOL INVEST, V13, P63, DOI 10.1016/j.jsgi.2005.10.006

Chantre P, 2002, PHYTOMEDICINE, V9, P3, DOI 10.1078/0944-7113-00078

Chen ZH, 2008, AM J CHINESE MED, V36, P939, DOI 10.1142/S0192415X08006363

Chiesa A, 2010, PSYCHOL MED, V40, P1239, DOI 10.1017/S0033291709991747

Ciampelli M, 1998, HORM RES, V49, P263, DOI 10.1159/000023185

Cussons AJ, 2009, J CLIN ENDOCR METAB, V94, P3842, DOI 10.1210/jc.2009-0870

Dasgupta A, 2006, ANN CLIN BIOCHEM, V43, P223, DOI 10.1258/000456306776865214

Davidson RJ, 2003, PSYCHOSOM MED, V65, P564, DOI 10.1097/01.PSY.0000077505.67574.E3

Deeks AA, 2010, FERTIL STERIL, V93, P2421, DOI 10.1016/j.fertnstert.2009.09.018

Dissen GA, 2009, ENDOCRINOLOGY, V150, P2906, DOI 10.1210/en.2008-1575

Dulloo AG, 1999, AM J CLIN NUTR, V70, P1040

Dvorak Z, 2011, FERTIL STERIL, V95, pE7, DOI 10.1016/j.fertnstert.2010.11.014

EISENBERG DM, 1993, NEW ENGL J MED, V328, P246, DOI 10.1056/NEJM199301283280406

Eyvazzadeh AD, 2009, FERTIL STERIL, V92, P1, DOI 10.1016/j.fertnstert.2009.05.012

Feng Y, 2009, PLOS ONE, V4, DOI 10.1371/journal.pone.0006638

Figueiroa MS, 2009, ASIAN J ANDROL, V11, P362, DOI 10.1038/aja.2009.2

Finniss DG, 2010, LANCET, V375, P686, DOI 10.1016/S0140-6736(09)61706-2

Fruzzetti F, 2002, FERTIL STERIL, V77, P936, DOI 10.1016/S0015-0282(02)02955-2

GERHARD I, 1992, GYNECOL ENDOCRINOL, V6, P171

GillingSmith C, 1997, CLIN ENDOCRINOL, V47, P93, DOI 10.1046/j.1365-2265.1997.2321049.x

Goldin PR, 2010, EMOTION, V10, P83, DOI 10.1037/a0018441

Gonzalez F, 2009, METABOLISM, V58, P954, DOI 10.1016/j.metabol.2009.02.022

Grant P, 2010, PHYTOTHER RES, V24, P186, DOI 10.1002/ptr.2900

Grant SJ, 2009, COCHRANE DB SYST REV, DOI 10.1002/14651858.CD006690.pub2

Hahn S, 2006, EXP CLIN ENDOCR DIAB, V114, P577, DOI 10.1055/s-2006-948308

Han JS, 2003, TRENDS NEUROSCI, V26, P17, DOI 10.1016/S0166-2236(02)00006-1

Heider U, 2001, FERTIL STERIL, V75, P1141, DOI 10.1016/S0015-0282(01)01805-2

Higashimura Y, 2009, AUTON NEUROSCI-BASIC, V150, P100, DOI 10.1016/j.autneu.2009.06.003

Holzel BK, 2008, SOC COGN AFFECT NEUR, V3, P55, DOI 10.1093/scan/nsm038

Holmang A, 2002, J APPL PHYSIOL, V92, P982, DOI 10.1152/japplphysiol.01194.2000

Hung JW, 2009, J REHABIL MED, V41, P924, DOI 10.2340/16501977-0445

JANSEN G, 1989, NEUROSCI LETT, V97, P305, DOI 10.1016/0304-3940(89)90615-0

Jarvill-Taylor KJ, 2001, J AM COLL NUTR, V20, P327, DOI 10.1080/07315724.2001.10719053

Jedel E, 2010, HUM REPROD, V25, P450, DOI 10.1093/humrep/dep384

Jedel E, 2011, AM J PHYSIOL-ENDOC M, V300, pE37, DOI 10.1152/ajpendo.00495.2010

JENKINS PJ, 1993, HUM REPROD, V8, P154, DOI 10.1093/humrep/8.suppl_2.154

Johansson J, 2010, AM J PHYSIOL-ENDOC M, V299, pE551, DOI 10.1152/ajpendo.00323.2010

Jonsdottir IH, 2000, IMMUNOL CELL BIOL, V78, P562, DOI 10.1046/j.1440-1711.2000.00961.x

Kagitani F, 2005, JPN J PHYSIOL, V55, P149, DOI 10.2170/jjphysiol.R2120

Kao YH, 2000, ENDOCRINOLOGY, V141, P980, DOI 10.1210/en.141.3.980

Kaptchuk TJ, 2008, BMJ-BRIT MED J, V336, P999, DOI 10.1136/bmj.39524.439618.25

Kaptchuk TJ, 2000, J CLIN EPIDEMIOL, V53, P786, DOI 10.1016/S0895-4356(00)00206-7

Kaptchuk TJ, 2002, ANN INTERN MED, V136, P817, DOI 10.7326/0003-4819-136-11-200206040-00011

Kasim-Karakas SE, 2004, J CLIN ENDOCR METAB, V89, P615, DOI 10.1210/jc.2003-030666

Kaya C, 2009, REPROD BIOMED ONLINE, V19, P721, DOI 10.1016/j.rbmo.2009.06.005

Kotsa K, 2009, FERTIL STERIL, V92, P1053, DOI 10.1016/j.fertnstert.2008.07.1757

Kubota Y, 2001, COGNITIVE BRAIN RES, V11, P281, DOI 10.1016/S0926-6410(00)00086-0

LARA HE, 1993, ENDOCRINOLOGY, V133, P2690, DOI 10.1210/en.133.6.2690

Lau CH, 2008, PHYTOTHER RES, V22, P1384, DOI 10.1002/ptr.2513

Lazar SW, 2005, NEUROREPORT, V16, P1893, DOI 10.1097/01.wnr.0000186598.66243.19

Lee MJ, 2010, J ENZYM INHIB MED CH, V25, P702, DOI 10.3109/14756360903524312

Legro RS, 1999, J CLIN ENDOCR METAB, V84, P165, DOI 10.1210/jc.84.1.165

Legro RS, 2001, AM J MED, V111, P607, DOI 10.1016/S0002-9343(01)00948-2

Liang F, 2011, EVID-BASED COMPL ALT

Lin RT, 2009, BMC COMPLEM ALTERN M, V9, DOI 10.1186/1472-6882-9-26

Lin SH, 2002, LANCET, V360, P224, DOI 10.1016/S0140-6736(02)09460-6

Linde K, 2010, FORSCH KOMPLEMENTMED, V17, P259, DOI 10.1159/000320374

Liu J. P., 2004, COCHRANE DB SYST REV

Liu X, 2010, BRIT J SPORT MED, V44, P704, DOI 10.1136/bjsm.2008.051144

LOBO RA, 1983, AM J OBSTET GYNECOL, V145, P496, DOI 10.1016/0002-9378(83)90324-1

Ludwig DS, 2008, JAMA-J AM MED ASSOC, V300, P1350, DOI 10.1001/jama.300.11.1350

Lund Irene, 2006, Acupunct Med, V24, P13

MacPherson H, 2010, PLOS MED, V7, DOI 10.1371/journal.pmed.1000261

Manneras L, 2008, ENDOCRINOLOGY, V149, P3559, DOI 10.1210/en.2008-0053

Manneras L, 2009, AM J PHYSIOL-REG I, V296, pR1124, DOI 10.1152/ajpregu.90947.2008

Manni L, 2005, REPROD BIOL ENDOCRIN, V3, DOI 10.1186/1177-7827-3-21

Matchim Y, 2010, W J NURS RES	1018

McKay DL, 2002, J AM COLL NUTR, V21, P1

Mo X, 1993, J Tradit Chin Med, V13, P115

Moffet Howard H, 2006, BMC Complement Altern Med, V6, P25, DOI 10.1186/1472-6882-6-25

Moffet HH, 2009, J ALTERN COMPLEM MED, V15, P213, DOI 10.1089/acm.2008.0356

Nagai M, 2010, J AM SOC HYPERTENS, V4, P174, DOI 10.1016/j.jash.2010.05.001

Ng EHY, 2008, SEMIN REPROD MED, V26, P14, DOI 10.1055/s-2007-992920

Ni HY, 2007, LIAONING J TRADITION, V34, P123

Norman RJ, 2007, LANCET, V370, P685, DOI 10.1016/S0140-6736(07)61345-2

Palomba S, 2010, DIABETES CARE, V33, P246, DOI 10.2337/dc09-1516

Qin BL, 2003, DIABETES RES CLIN PR, V62, P139, DOI 10.1016/S0168-8227(03)00173-6

Raja-Khan N, 2005, J CLIN OUTCOMES MANA, V12, P218

Raja-Khan N, 2011, EUR J OBSTET GYN R B, V155, P49, DOI 10.1016/j.ejogrb.2010.10.027

Rashidi B, 2009, TAIWAN J OBSTET GYNE, V48, P142, DOI 10.1016/S1028-4559(09)60275-8

Rosenzweig S, 2007, ALTERN THER HEALTH M, V13, P36

SATO A, 1992, NEUROSCI RES, V14, P242, DOI 10.1016/0168-0102(92)90071-J

Schuering Andreas N., 2008, Gynaekologisch-Geburtshilfliche Rundschau, V48, P9, DOI 10.1159/000111465

Selimoglu H, 2010, J ENDOCRINOL INVEST, V33, P234, DOI 10.3275/6560

Shapiro SL, 2009, J CLIN PSYCHOL, V65, P555, DOI 10.1002/jclp.20602

Sherman KJ, 2010, SPINE, V35, P1471, DOI 10.1097/BRS.0b013e3181c2a8d3

Sills ES, 2001, BMC WOMENS HEALTH, V1, P3, DOI DOI 10.1186/1472-6874-1-1

Stener-Victorin E, 2000, BIOL REPROD, V63, P1497, DOI 10.1095/biolreprod63.5.1497

Stener-Victorin E, 2006, J APPL PHYSIOL, V101, P84, DOI 10.1152/jappalphysiol.01593.2005

Stener-Victorin E, 2005, REPROD BIOL ENDOCRIN, V3, DOI 10.1186/1477-7827-3-44

Stener-Victorin E, 2003, AUTON NEUROSCI-BASIC, V108, P50, DOI 10.1016/j.autneu.2003.08.006

Stener-Victorin E, 2000, ACTA OBSTET GYN SCAN, V79, P180, DOI 10.1034/j.1600-0412.2000.079003180.x

Stener-Victorin E, 2009, AM J PHYSIOL-REG I, V297, pR387, DOI 10.1152/ajpregu.00197.2009

Streitberger K, 1998, LANCET, V352, P364, DOI 10.1016/S0140-6736(97)10471-8

Sun F, 2000, P SOC EXP BIOL MED, V223, P295, DOI 10.1046/j.1525-1373.2000.22342.x

Sun Guan-Cheng, 2010, Diabetes Care, V33, pe8, DOI 10.2337/dc09-1543

Sverrisdottir YB, 2008, AM J PHYSIOL-ENDOC M, V294, pE576, DOI 10.1152/ajpendo.00725.2007

Takahashi T, 2005, INT J PSYCHOPHYSIOL, V55, P199, DOI 10.1016/j.ijpsycho.2004.07.004

Teede H, 2010, BMC MED, V8, DOI 10.1186/1741-7015-8-41

Tsai JC, 2003, J ALTERN COMPLEM MED, V9, P747, DOI 10.1089/107555303322524599

Tsujiuchi T, 2002, DIABETES CARE, V25, P241, DOI 10.2337/diacare.25.1.241

Wang BQ, 2008, SHANDONG J TRADITION, V27, P138

Wang JG, 2007, FERTIL STERIL, V88, P240, DOI 10.1016/j.fertnstert.2006.11.082

Wang Jing Hao, 2008, V52, P230, DOI 10.1159/000134303

Wehr E, 2009, EUR J ENDOCRINOL, V161, P575, DOI 10.1530/EJE-09-0432

White A, 2008, ACUPUNCT MED, V26, P111, DOI 10.1136/aim.26.2.111

Witek-Janusek L, 2008, BRAIN BEHAV IMMUN, V22, P969, DOI 10.1016/j.bbi.2008.01.012

Xia W, 2011, PHYTOMEDICINE, V18, P516, DOI 10.1016/j.phymed.2010.09.007

Yang X. B., 2002, XHONGGUO SHI YIAN FA, V18, P336

Yang XM, 2011, AM J PHYSIOL-REG I, V300, pR869, DOI 10.1152/ajpregu.00334.2010

YAO T, 1982, BRAIN RES, V240, P77, DOI 10.1016/0006-8993(82)90645-X

YING CB, 1991, ACUPUNCTURE ELECTRO, V16, P1

Zhang Hongwei, 2010, Chin Med, V5, P1, DOI 10.1186/1749-8546-5-1

Zhang J, 2010, COCHRANE DB SYST REV, V9

Zhang ZJ, 2010, J AFFECT DISORDERS, V124, P9, DOI 10.1016/j.jad.2009.07.005

Zhao L, 2011, FERTIL STERIL, V95, P461, DOI 10.1016/j.fertnstert.2010.07.1090

NR 125

TC 33

Z9 35

U1 15

U2 81

PU AMER PHYSIOLOGICAL SOC

PI BETHESDA

PA 9650 ROCKVILLE PIKE, BETHESDA, MD 20814 USA

SN 0193-1849

EI 1522-1555

J9 AM J PHYSIOL-ENDOC M

JI Am. J. Physiol.-Endocrinol. Metab.

PD JUL

PY 2011

VL 301

IS 1

BP E1

EP E10

DI 10.1152/ajpendo.00667.2010

PG 10

WC Endocrinology & Metabolism; Physiology

SC Endocrinology & Metabolism; Physiology

GA 783MA

UT WOS:000292085900001

PM 21487075

OA Green Published

DA 2019-01-08

ER

PT J

AU Minet, LKR

Wagner, L

Lonvig, EM

Hjelmborg, J

Henriksen, JE

AF Minet, L. K. Rosenbek

Wagner, L.

Lonvig, E. M.

Hjelmborg, J.

Henriksen, J. E.

TI The effect of motivational interviewing on glycaemic control and

perceived competence of diabetes self-management in patients with type 1

and type 2 diabetes mellitus after attending a group education

programme: a randomised controlled trial

SO DIABETOLOGIA

LA English

DT Article

DE Clinical trial; Diabetes mellitus; Motivation; Patient education; Self

care

ID PROBLEM AREAS; METAANALYSIS; INTERVENTION; CARE; ADOLESCENTS; ATTITUDES;

DISTRESS; BEHAVIOR; SKILLS; WOMEN

AB The aim of this study was to measure the efficacy of motivational interviewing (MI) compared with usual care on changes in glycaemic control and competence of diabetes self-management in patients with diabetes mellitus.

Patients were eligible if they had type 1 or 2 diabetes mellitus, were over 18 years of age and had participated in a 4 day group education programme offered at a diabetes clinic at a university hospital in Denmark. Exclusion criteria included pregnancy, severe debilitating disease and cognitive deficit. Out of 469 patients who attended the group education programme, 349 patients were randomised to either a usual care control group or an intervention group, which received up to five individual counselling sessions in 1 year based on MI, in addition to usual care. A randomised parallel design was used and open-label allocation was done by random permuted blocks, with allocation concealment by sequentially numbered, sealed, opaque envelopes. The primary outcome was glycated haemoglobin (HbA(1c)). Analysis regarding measurements of glycated haemoglobin (HbA(1c)) and competence of self-management (using the Problem Areas in Diabetes Scale [PAID] and Perceived Competence for Diabetes Scale [PCDS]) was based on 298 participants followed for a 24 month period. Data were collected at the Department of Endocrinology at Odense University Hospital. Our hypotheses were that MI could: (1) reduce HbA(1c) levels; (2) increase self-efficacy; and (3) increase diabetes self-care, compared with usual care.

Out of the 176 included in the control group and 173 in the intervention group, 153 and 145 were analysed in the groups, respectively. When using the baseline value as covariate there were no significant differences in change score between the two study groups with regard to mean level of HbA(1c) (0.131, p = 0.221), PAID scores (-1.793, p = 0.191) or PCDS scores (0.017, p = 0.903) at the 24 month follow-up, using a mixed effects regression model. The patients in the intervention group showed significantly higher levels of perceived competence in dealing with diabetes compared with the control group (mean change score = -0.387, p = 0.002) following 1 year of intervention.

We were unable to demonstrate any benefit, over or above usual care, of MI in patients with diabetes who have just completed a diabetes education programme, and who have well-regulated diabetes.

Clinical Trials NCT00555854.

The National Board of Health, Funen County, Danish Association of Diabetes, Odense University Hospital, University of Southern Denmark and TRYG Fonden.

C1 [Minet, L. K. Rosenbek; Henriksen, J. E.] Odense Univ Hosp, Dept Endocrinol M, DK-5000 Odense, Denmark.

[Wagner, L.] Univ So Denmark, Inst Clin Res, Fac Hlth Sci, Res Unit Nursing, DK-5000 Odense C, Denmark.

[Lonvig, E. M.] Odense Univ Hosp, Dept Qual, DK-5000 Odense, Denmark.

[Minet, L. K. Rosenbek] Odense Univ Hosp, Dept Rehabil, DK-5000 Odense C, Denmark.

[Hjelmborg, J.] Univ So Denmark, Dept Biostat, DK-5000 Odense C, Denmark.

RP Minet, LKR (reprint author), Odense Univ Hosp, Dept Endocrinol M, DK-5000 Odense, Denmark.

EM lisbeth.minet@ouh.regionsyddanmark.dk

OI Rosenbek Minet, Lisbeth/0000-0001-6179-5183; Hjelmborg,

Jacob/0000-0001-9630-9149; Henriksen, Jan Erik/0000-0002-1908-7017

FU National Board of Health; Funen County; Danish Association of Diabetes;

Odense University Hospital; University of Southern Denmark; TRYG Fonden

FX The authors thank the study patients for their participation in the

study. Thanks to the team members at the Diabetes Clinic Department of

Endocrinology L. Sjoberg, I. Kamp, E. Romanczuk, I. Eggertsen, T.

Flemming Larsen, G. Kramer and B. Nielsen for their invaluable

contributions to the study. Thanks also to other staff at the Diabetes

Clinic Department of Endocrinology for co-operation and assistance

throughout the trial. Thanks also to L. Stark at the Research Unit of

General Practice for language editing of the manuscript. This study was

financially supported by the National Board of Health, Funen County,

Danish Association of Diabetes, Odense University Hospital, University

of Southern Denmark and TRYG Fonden.

CR Adolfsson ET, 2004, PATIENT EDUC COUNS, V53, P319, DOI 10.1016/j.pec.2003.07.009

Bandura A., 1997, SELF EFFICACY EXERCI

Brug J, 2007, J NUTR EDUC BEHAV, V39, P8, DOI 10.1016/j.jneb.2006.08.010

Channon S, 2003, ARCH DIS CHILD, V88, P680, DOI 10.1136/adc.88.8.680

Channon SJ, 2007, DIABETES CARE, V30, P1390, DOI 10.2337/dc06-2260

Clark M, 2001, PATIENT EDUC COUNS, V42, P247, DOI 10.1016/S0738-3991(00)00128-2

Colagiuri R, 2009, DIABETIC MED, V26, P442, DOI 10.1111/j.1464-5491.2009.02700.x

Deakin T, 2005, COCHRANE DB SYST REV, DOI 10.1002/14651858.CD003417.pub2

*DIAB CONTR COMPL, 1993, NEW ENGL J MED, V329, P977, DOI DOI 10.1056/NEJM199309303291401

Duke SAS, 2009, COCHRANE DB SYST REV, DOI 10.1002/14651858.CD005268.pub2

Fain JA, 1999, DIABETES EDUCATOR, V25, P7, DOI 10.1177/014572179902500618

FRIEDEWALD WT, 1972, CLIN CHEM, V18, P499

Funnell MM, 2004, CLIN DIABETES, V22, P123, DOI DOI 10.2337/DIACLIN.22.3.123

Gary TL, 2003, DIABETES EDUCATOR, V29, P488, DOI 10.1177/014572170302900313

Ismail K, 2004, LANCET, V363, P1589, DOI 10.1016/S0140-6736(04)16202-8

Ismail K, 2008, ANN INTERN MED, V149, P708, DOI 10.7326/0003-4819-149-10-200811180-00005

Knight KM, 2006, BRIT J HEALTH PSYCH, V11, P319, DOI 10.1348/135910705X52516

Knight KM, 2003, PSYCHOL HEALTH MED, V8, P149, DOI DOI 10.1080/1354850031000087528

Loveman E, 2003, COCHRANE DB SYST REV, DOI DOI 10.1002/14651858.CD003286

Martins RK, 2009, CLIN PSYCHOL REV, V29, P283, DOI 10.1016/j.cpr.2009.02.001

Miller W. R., 2002, MOTIVATIONAL INTERVI

Miller WR, 2009, BEHAV COGN PSYCHOTH, V37, P129, DOI 10.1017/S1352465809005128

Minet L, 2010, PATIENT EDUC COUNS, V80, P29, DOI 10.1016/j.pec.2009.09.033

Norris SL, 2002, DIABETES CARE, V25, P1159, DOI 10.2337/diacare.25.7.1159

Norris SL, 2001, DIABETES CARE, V24, P561, DOI 10.2337/diacare.24.3.561

Peyrot M, 2005, DIABETIC MED, V22, P1379, DOI 10.1111/j.1464-5491.2005.01644.x

Pill R, 1999, J ADV NURS, V29, P1492, DOI 10.1046/j.1365-2648.1999.01037.x

POLONSKY WH, 1995, DIABETES CARE, V18, P754, DOI 10.2337/diacare.18.6.754

Rollnick S., 2008, MOTIVATIONAL INTERVI

Rubak S, 2005, BRIT J GEN PRACT, V55, P305

Shojania KG, 2006, JAMA-J AM MED ASSOC, V296, P427, DOI 10.1001/jama.296.4.427

Smith DE, 1997, DIABETES CARE, V20, P52, DOI 10.2337/diacare.20.1.52

Snoek FJ, 2000, DIABETES CARE, V23, P1305, DOI 10.2337/diacare.23.9.1305

*SWED COUNC TECHN, 2009, PAT ED DIAB SYST REV

UKPDS Study Group, 1998, LANCET, V352, P837, DOI DOI 10.1016/S0140-6736(98)07019-6

Viner RM, 2003, DIABETIC MED, V20, P739, DOI 10.1046/j.1464-5491.2003.00995.x

Welch G, 2003, DIABETIC MED, V20, P69, DOI 10.1046/j.1464-5491.2003.00832.x

Welch GW, 1997, DIABETES CARE, V20, P760, DOI 10.2337/diacare.20.5.760

West DS, 2007, DIABETES CARE, V30, P1081, DOI 10.2337/dc06-1966

Williams GC, 1998, DIABETES CARE, V21, P1644, DOI 10.2337/diacare.21.10.1644

Woodcock AJ, 1999, PATIENT EDUC COUNS, V37, P65, DOI 10.1016/S0738-3991(98)00104-9

ZOFFMANN V, 2004, THESIS U AARHUS AARH

Zoffmann V, 2006, PATIENT EDUC COUNS, V64, P78, DOI 10.1016/j.pec.2005.11.017

NR 43

TC 27

Z9 27

U1 2

U2 36

PU SPRINGER

PI NEW YORK

PA 233 SPRING ST, NEW YORK, NY 10013 USA

SN 0012-186X

EI 1432-0428

J9 DIABETOLOGIA

JI Diabetologia

PD JUL

PY 2011

VL 54

IS 7

BP 1620

EP 1629

DI 10.1007/s00125-011-2120-x

PG 10

WC Endocrinology & Metabolism

SC Endocrinology & Metabolism

GA 774MZ

UT WOS:000291392000006

PM 21455729

OA Bronze

DA 2019-01-08

ER

PT J

AU O'Sullivan, EP

Avalos, G

O'Reilly, M

Dennedy, MC

Gaffney, G

Dunne, F

AF O'Sullivan, E. P.

Avalos, G.

O'Reilly, M.

Dennedy, M. C.

Gaffney, G.

Dunne, F.

CA Atlantic DIP Collaborators

TI Atlantic Diabetes in Pregnancy (DIP): the prevalence and outcomes of

gestational diabetes mellitus using new diagnostic criteria

SO DIABETOLOGIA

LA English

DT Article

DE Atlantic DIP; Gestational diabetes mellitus; Maternal and neonatal

outcomes; Pregnancy

ID INTERNATIONAL ASSOCIATION; GLUCOSE; HYPERGLYCEMIA; WOMEN

AB New diagnostic criteria for gestational diabetes mellitus (GDM) have recently been published. We wished to evaluate what impact these new criteria would have on GDM prevalence and outcomes in a predominantly European population.

The Atlantic Diabetes In Pregnancy (DIP) programme performed screening for GDM in 5,500 women with an oral glucose tolerance test at 24-28 weeks. GDM was defined according to the new International Association of Diabetes and Pregnancy Study Groups (IADPSG) criteria and compared with previous WHO criteria; maternal and neonatal adverse outcomes were prospectively recorded.

Of the participants, 12.4% and 9.4% were diagnosed with GDM using IADPSG and WHO criteria, respectively. IADPSG GDM pregnancies were associated with a statistically significant increased incidence of adverse maternal outcomes (gestational hypertension, polyhydramnios and Caesarean section) and neonatal outcomes (prematurity, large for gestational age, neonatal unit admission, neonatal hypoglycaemia and respiratory distress). The odds ratio for the development of these adverse outcomes remained significant after adjustment for maternal age, body mass index and non-European ethnicity. Those women who were classified as having normal glucose tolerance by WHO criteria but as having GDM by IADPSG criteria also had significant adverse pregnancy outcomes.

GDM prevalence is higher when using newer IADPSG, compared with WHO, criteria, and these women and their offspring experience significant adverse pregnancy outcomes. Higher rates of GDM pose a challenge to healthcare systems, but improved screening provides an opportunity to attempt to reduce the associated morbidity for mother and child.

C1 [Dunne, F.] Natl Univ Ireland, Inst Clin Sci, Sch Med, Galway, Ireland.

[O'Sullivan, E. P.; Avalos, G.; O'Reilly, M.; Dennedy, M. C.; Dunne, F.] Galway Univ Hosp, Dept Diabet, Galway, Ireland.

[Gaffney, G.] Natl Univ Ireland, Dept Obstet & Gynaecol, Galway, Ireland.

RP Dunne, F (reprint author), Natl Univ Ireland, Inst Clin Sci, Sch Med, Galway, Ireland.

EM fidelma.dunne@nuigalway.ie

OI O'Reilly, Michael/0000-0002-9108-9105; Dennedy,

Michael/0000-0001-9325-6520

FU Health Research Board of Ireland

FX Funding for the study was provided by the Health Research Board of

Ireland.

CR Agarwal MM, 2010, DIABETES CARE, V33, P2018, DOI 10.2337/dc10-0572

*AM DIAB ASS, 2010, DIABETES CARE S1, V34, pS15

Central Statistics Office, 2009, POP MIGR EST APR 200

Crowther CA, 2005, NEW ENGL J MED, V352, P2477, DOI 10.1056/NEJMoa042973

Dunne FP, 2009, DIABETES CARE, V32, P1205, DOI [10.2337/dc09-1118, 10.2337/dc09-0118]

Griffin ME, 2000, DIABETIC MED, V17, P26, DOI 10.1046/j.1464-5491.2000.00214.x

Harrington J., 2008, SLAN 2007 SURVEY LIF

HOD M, 1991, DIABETES S2, V40, pS74

Jovanovic L, 2001, JAMA-J AM MED ASSOC, V286, P2516, DOI 10.1001/jama.286.20.2516

Landon MB, 2009, NEW ENGL J MED, V361, P1339, DOI 10.1056/NEJMoa0902430

Langer O, 2000, CLIN OBSTET GYNECOL, V43, P283, DOI 10.1097/00003081-200006000-00006

Lee PA, 2003, PEDIATRICS, V111, P1253, DOI 10.1542/peds.111.6.1253

Metzger BE, 2008, NEW ENGL J MED, V358, P1991, DOI 10.1056/NEJMoa0707943

Metzger BE, 2010, DIABETES CARE, V33, P676, DOI 10.2337/dc09-1848

Ostlund I, 2003, DIABETES CARE, V26, P2107, DOI 10.2337/diacare.26.7.2107

Owens LA, 2010, DIABETES CARE, V33, P577, DOI 10.2337/dc09-0911

Rosenn B M, 2000, J Matern Fetal Med, V9, P29, DOI 10.3109/14767050009020509

Stella CL, 2008, AM J PERINAT, V25, P325, DOI 10.1055/s-2008-1078758

World Health Organization, 2006, DEF DIAGN DIAB MILL

NR 19

TC 137

Z9 140

U1 1

U2 13

PU SPRINGER

PI NEW YORK

PA 233 SPRING ST, NEW YORK, NY 10013 USA

SN 0012-186X

EI 1432-0428

J9 DIABETOLOGIA

JI Diabetologia

PD JUL

PY 2011

VL 54

IS 7

BP 1670

EP 1675

DI 10.1007/s00125-011-2150-4

PG 6

WC Endocrinology & Metabolism

SC Endocrinology & Metabolism

GA 774MZ

UT WOS:000291392000012

PM 21494772

OA Bronze

DA 2019-01-08

ER

PT J

AU Leyva, B

Zagarins, SE

Allen, NA

Welch, G

AF Leyva, Bryan

Zagarins, Sofija E.

Allen, Nancy A.

Welch, Garry

TI THE RELATIVE IMPACT OF DIABETES DISTRESS VS DEPRESSION ON GLYCEMIC

CONTROL IN HISPANIC PATIENTS FOLLOWING A DIABETES SELF-MANAGEMENT

EDUCATION INTERVENTION

SO ETHNICITY & DISEASE

LA English

DT Article

DE Diabetes Distress; Depression; Depressive Symptoms; Glycemic Control;

Type 2 Diabetes; Intervention; Hispanic; Latino

ID EMOTIONAL DISTRESS; UNITED-STATES; PROBLEM AREAS; ADULTS; CARE;

MELLITUS; GLUCOSE; ASSOCIATION; SYMPTOMS; UTILITY

AB Objectives: Studies in non-Hispanic populations have shown that depression and diabetes distress are associated with glycemic control. Although rates of depression and diabetes distress are high among Hispanics with diabetes, there is little research investigating the relationship between these factors and glycemic control in this population. The purpose of the current analysis was to examine the relative impact of change in diabetes distress and change in depressive symptoms on change in glycemic control in Hispanic patients following a diabetes self-management education (DSME) intervention.

Design: We conducted a diabetes self-management education intervention in 23 Hispanic (predominantly Puerto Rican) and 168 non-Hispanic type 2 diabetes patients and measured glycemic control (HbA1c), depressive symptoms (CES-D), and diabetes distress (PAID) at baseline and 6 months.

Results: In multiple linear regression, change in diabetes distress from baseline to six-month follow-up was significantly associated with change in HbA1c among Hispanic patients, such that a 10-point reduction on the PAID scale of diabetes distress was associated with a clinically significant reduction in HbA1c of .55 +/- .06% (P=.03). Change in depression was not associated with change in HbA1c (P=.59). Findings in non-Hispanic patients were similar.

Conclusions: Change in diabetes distress, but not change in depressive symptoms, was associated with change in HbA1c in both Hispanic and non-Hispanic patients. This analysis supports the utility of DSME in reducing diabetes distress and improving glycemic control among Hispanic patients. (Ethn Dis. 2011;21(3):322-327)

C1 [Zagarins, Sofija E.; Welch, Garry] Baystate Med Ctr, Dept Behav Med Res, Springfield, MA 01105 USA.

[Leyva, Bryan; Allen, Nancy A.] Boston Coll, William F Connell Sch Nursing, Chestnut Hill, MA 02167 USA.

RP Zagarins, SE (reprint author), Baystate Med Ctr, Dept Behav Med Res, 140 High St,Room 298, Springfield, MA 01105 USA.

EM Sofija.Zagarins@baystatehealth.org

OI Leyva, Bryan/0000-0003-1929-4910

FU National Institutes of Health [1R01DK-060076]

FX This research was supported by National Institutes of Health grant

#1R01DK-060076.

CR Adriaanse MC, 2008, DIABETES CARE, V31, P2268, DOI 10.2337/dc08-1074

AIKENS JE, 1994, J BEHAV MED, V17, P535, DOI 10.1007/BF01857596

Albright TL, 2001, FAM MED, V33, P354

Ali S, 2006, DIABETIC MED, V23, P1165, DOI 10.1111/j.1464-5491.2006.01943.x

Carter JS, 1996, ANN INTERN MED, V125, P221, DOI 10.7326/0003-4819-125-3-199608010-00011

*CDCP, 2004, MMWR-MORBID MORTAL W, V53, P941

Centers for Disease Control and Prevention, 2007, NAT DIAB FACT SHEET

Fisher L, 2008, DIABETIC MED, V25, P1096, DOI 10.1111/j.1464-5491.2008.02533.x

Fisher L, 2009, DIABETIC MED, V26, P622, DOI 10.1111/j.1464-5491.2009.02730.x

Fisher L, 2008, ANN FAM MED, V6, P246, DOI 10.1370/afm.842

Fisher L, 2010, DIABETES CARE, V33, P1034, DOI 10.2337/dc09-2175

Fisher L, 2010, DIABETES CARE, V33, P23, DOI 10.2337/dc09-1238

Glassy CM, 2010, PSYCHOL HEALTH MED, V15, P117, DOI 10.1080/13548501003615274

KAPLAN MS, 1990, SOC SCI MED, V31, P1313, DOI 10.1016/0277-9536(90)90070-9

Katon WJ, 2004, ARCH GEN PSYCHIAT, V61, P1042, DOI 10.1001/archpsyc.61.10.1042

Lewis-Fernandez R, 2005, J AM BOARD FAM PRACT, V18, P282, DOI 10.3122/jabfm.18.4.282

Lin EHB, 2006, ANN FAM MED, V4, P46, DOI 10.1370/afm.423

Narayan KMV, 2003, JAMA-J AM MED ASSOC, V290, P1884, DOI 10.1001/jama.290.14.1884

POLONSKY WH, 1995, DIABETES CARE, V18, P754, DOI 10.2337/diacare.18.6.754

RADLOFF L S, 1977, Applied Psychological Measurement, V1, P385, DOI 10.1177/014662167700100306

Shao WA, 1997, J FAM PRACTICE, V44, P161

Skaff MM, 2009, HEALTH PSYCHOL, V28, P265, DOI 10.1037/a0014429

Spencer MS, 2006, J HEALTH CARE POOR U, V17, P88, DOI 10.1353/hpu.2006.0095

Thomas JL, 2001, INT J PSYCHIAT MED, V31, P25, DOI 10.2190/FUFR-PK9F-6U10-JXRK

van Bastelaar KMP, 2010, DIABETIC MED, V27, P798, DOI 10.1111/j.1464-5491.2010.03025.x

WAUGH N, 2010, HLTH TECHNOL ASSESS, V14, P1

Weinger K, 2005, DIABETES CARE, V28, P1346, DOI 10.2337/diacare.28.6.1346

Welch G, 2003, DIABETIC MED, V20, P69, DOI 10.1046/j.1464-5491.2003.00832.x

Welch G, 2007, ETHNIC DIS, V17, P541

Welch G, 2011, DIABETES RES CLIN PR, V91, P54, DOI 10.1016/j.diabres.2010.09.036

Welch GW, 1997, DIABETES CARE, V20, P760, DOI 10.2337/diacare.20.5.760

Welch GW, 2007, TXB TYPE 2 DIABETES

NR 32

TC 13

Z9 14

U1 0

U2 12

PU INT SOC HYPERTENSION BLACKS-ISHIB

PI ATLANTA

PA 100 AUBURN AVE NE STE 401, ATLANTA, GA 30303-2527 USA

SN 1049-510X

J9 ETHNIC DIS

JI Ethn. Dis.

PD SUM

PY 2011

VL 21

IS 3

BP 322

EP 327

PG 6

WC Public, Environmental & Occupational Health

SC Public, Environmental & Occupational Health

GA 820UB

UT WOS:000294930500010

PM 21942165

DA 2019-01-08

ER

PT J

AU Weissberg-Benchell, J

Antisdel-Lomaglio, J

AF Weissberg-Benchell, Jill

Antisdel-Lomaglio, Jeanne

TI Diabetes-specific emotional distress among adolescents: feasibility,

reliability, and validity of the problem areas in diabetes-teen version

SO PEDIATRIC DIABETES

LA English

DT Article

DE adolescents; emotional distress; measure development

ID GLYCEMIC CONTROL; TYPE-1; ASSOCIATION; SYMPTOMS; CHILDREN; ANXIETY

AB Weissberg-Benchell J, Antisdel-Lomaglio J. Diabetes-specific emotional distress among adolescents: feasibility, reliability, and validity of the problem areas in diabetes-teen version.

C1 [Weissberg-Benchell, Jill; Antisdel-Lomaglio, Jeanne] Northwestern Univ, Childrens Mem Hosp, Feinberg Sch Med, Chicago, IL 60614 USA.

RP Weissberg-Benchell, J (reprint author), Northwestern Univ, Childrens Mem Hosp, Feinberg Sch Med, 2300 Childrens Plaza,POB 10, Chicago, IL 60614 USA.

EM JWBenchell@childrensmemorial.org

CR Anderson BJ, 2002, DIABETIC MED, V19, P635, DOI 10.1046/j.1464-5491.2002.00752.x

ANDERSON BJ, 1990, J PEDIATR PSYCHOL, V15, P477, DOI 10.1093/jpepsy/15.4.477

Beck AT, 1996, BECK DEPRESSION INVE

Fisher L, 2010, DIABETES CARE, V33, P23, DOI 10.2337/dc09-1238

Grey M, 1997, NURS RES, V46, P312, DOI 10.1097/00006199-199711000-00003

HANSON CL, 1987, J CONSULT CLIN PSYCH, V55, P529, DOI 10.1037/0022-006X.55.4.529

Herzer M, 2010, J PEDIATR PSYCHOL, V35, P415, DOI 10.1093/jpepsy/jsp063

Hood KK, 2006, DIABETES CARE, V29, P1389, DOI 10.2337/dc06-0087

Hood KK, 2009, PEDIATRICS, V124, pE1171, DOI 10.1542/peds.2009-0207

Ingersoll G M, 1991, Diabetes Educ, V17, P114, DOI 10.1177/014572179101700219

KOVACS M, 1985, PSYCHOPHARMACOL BULL, V21, P995

McKelvey J, 1993, Diabetes Educ, V19, P125, DOI 10.1177/014572179301900206

POLONSKY WH, 1995, DIABETES CARE, V18, P754, DOI 10.2337/diacare.18.6.754

Polonsky WH, 1996, DIABETES SPECTRUM, V9, P8

Reynolds CR, 1997, J ABNORM CHILD PSYCH, V25, P15, DOI 10.1023/A:1025751206600

SPEILBERGER C, 1973, MANUAL STATE TRAIT I

Varni JW, 2003, DIABETES CARE, V26, P631, DOI 10.2337/diacare.26.3.631

WEISSBERGBENCHELL J, 1995, DIABETES CARE, V18, P77, DOI 10.2337/diacare.18.1.77

Welch GW, 1997, DIABETES CARE, V20, P760, DOI 10.2337/diacare.20.5.760

Wysocki T, 2008, J PEDIATR PSYCHOL, V33, P875, DOI 10.1093/jpepsy/jsn024

NR 20

TC 37

Z9 38

U1 0

U2 13

PU WILEY-BLACKWELL

PI MALDEN

PA COMMERCE PLACE, 350 MAIN ST, MALDEN 02148, MA USA

SN 1399-543X

J9 PEDIATR DIABETES

JI Pediatr. Diabetes

PD JUN

PY 2011

VL 12

IS 4

BP 341

EP 344

DI 10.1111/j.1399-5448.2010.00720.x

PN 1

PG 4

WC Endocrinology & Metabolism; Pediatrics

SC Endocrinology & Metabolism; Pediatrics

GA 768UJ

UT WOS:000290963600009

PM 21443583

DA 2019-01-08

ER

PT J

AU Aida, J

Kuriyama, S

Ohmori-Matsuda, K

Hozawa, A

Osaka, K

Tsuji, I

AF Aida, J.

Kuriyama, S.

Ohmori-Matsuda, K.

Hozawa, A.

Osaka, K.

Tsuji, I.

TI The association between neighborhood social capital and self-reported

dentate status in elderly Japanese - The Ohsaki Cohort 2006 Study

SO COMMUNITY DENTISTRY AND ORAL EPIDEMIOLOGY

LA English

DT Article

DE dental status; multilevel analysis; remaining teeth; social capital;

social epidemiology

ID PSYCHOLOGICAL DISTRESS; ORAL-HEALTH; MULTILEVEL ANALYSIS; SCREENING

SCALES; INEQUALITIES; ADOLESCENTS; VALIDATION; MORTALITY; CARIES

AB Objectives: Little is known about the influence of social capital on dental health. The aim of the present cross-sectional study was to determine the association between neighborhood social capital, individual social networks and social support and the number of remaining teeth in elderly Japanese.

Methods: In December 2006, self-administered questionnaires were sent to 31 237 eligible community-dwelling individuals (response rate: 73.9%). Included in the analysis were 21 736 participants. Five neighborhood social capital variables were calculated from individual civic networks, sports and hobby networks, volunteer networks, friendship networks and social support variables. We used multilevel logistic regression models to estimate the odds ratio (OR) of having 20 or more teeth according to neighborhood social capital variables with adjustment for sex, age, individual social networks and social support, educational attainment, neighborhood educational level, dental health behavior, smoking status, history of diabetes and self-rated health.

Results: The average age of the participants was 74.9 (standard deviation; 6.6) years, and 28.5% of them had 20 or more teeth. In the univariate multilevel model, there were statistically significant associations between neighborhood sports and hobby networks, friendship networks and self-reported dentate status. In the multivariable multilevel model, compared with participants living in lowest friendship network neighborhoods, those living in highest friendship network neighborhoods had an OR 1.17 (95% confidence interval, 1.04-1.30) times higher for having 20 or more teeth.

Conclusions: There is a significant association between one network aspect of neighborhood social capital and individual dentate status regardless of individual social networks and social support.

C1 [Aida, J.] Tohoku Univ, Grad Sch Dent, Dept Int & Community Oral Hlth, Aoba Ku, Sendai, Miyagi 9808575, Japan.

[Kuriyama, S.; Ohmori-Matsuda, K.; Hozawa, A.; Tsuji, I.] Tohoku Univ, Grad Sch Dent, Dept Publ Hlth & Forens Med, Div Epidemiol, Sendai, Miyagi 9808575, Japan.

RP Aida, J (reprint author), Tohoku Univ, Grad Sch Dent, Dept Int & Community Oral Hlth, Aoba Ku, 4-1 Seiryo Machi, Sendai, Miyagi 9808575, Japan.

EM j-aida@umin.ac.jp

OI Aida, Jun/0000-0002-8405-9872

FU Ministry of Health, Labour and Welfare, Japan [H20-Junkanki

(Seishuu)-Ippan-013, H21-Choju-Ippan-001]; Japan Society for the

Promotion of Science [21390200]

FX This study was supported by Health and Labour Sciences Research Grants

(H20-Junkanki (Seishuu)-Ippan-013, H21-Choju-Ippan-001) from the

Ministry of Health, Labour and Welfare, Japan, and a Grant-in-Aid for

Scientific Research (B) (21390200) from the Japan Society for the

Promotion of Science. The authors thank Ms Yoshiko Nakata, Ms Mika

Wagatsuma, Ms Naoko Sato, Ms Yuki Takeda and Dr Toru Tsuboya for their

technical assistance.

CR Aida J, 2008, COMMUNITY DENT ORAL, V36, P149, DOI 10.1111/j.1600-0528.2007.00380.x

Aida J, 2009, SOC SCI MED, V69, P512, DOI 10.1016/j.socscimed.2009.06.003

Blakely T., 2006, METHODS SOCIAL EPIDE, P316

Boyapati L, 2007, PERIODONTOL 2000, V44, P195, DOI 10.1111/j.1600-0757.2007.00211.x

Carpiano RM, 2006, SOC SCI MED, V62, P165, DOI 10.1016/j.socscimed.2005.05.020

*FED DENT INT, 1982, INT DENT J, V32, P74

Furukawa TA, 2003, PSYCHOL MED, V33, P357, DOI 10.1017/S0033291702006700

Harpham T., 2008, SOCIAL CAPITAL HLTH, P51, DOI DOI 10.1007/978-0-387-71311-3_3

Hox J., 2002, MULTILEVEL ANAL TECH

Kawachi I, 1997, AM J PUBLIC HEALTH, V87, P1491, DOI 10.2105/AJPH.87.9.1491

Kawachi I, 2008, SOCIAL CAPITAL HLTH, V10, P1, DOI DOI 10.1007/978-0-387-71311-3_1

Kawachi Ichiro, 2000, SOCIAL EPIDEMIOLOGY, P174

Kessler RC, 2002, PSYCHOL MED, V32, P959, DOI 10.1017/S0033291702006074

Kuriyama S, 2010, J EPIDEMIOL, V20, P253, DOI 10.2188/jea.JE20090093

LANDRINE H, 1994, J BEHAV MED, V17, P331, DOI 10.1007/BF01857956

LEYLAND AH, 2001, MULTILEVEL MODELLING

Macintyre S, 2002, SOC SCI MED, V55, P125, DOI 10.1016/S0277-9536(01)00214-3

Marmot M., 1999, SOCIAL DETERMINANTS

Muraoka Y, 1996, RONEN SEISHIN IGAKU, V7, P397

Ogawa K, 2003, PUBLIC HEALTH NUTR, V6, P147, DOI 10.1079/PHN2002411

Pattussi MP, 2006, COMMUNITY DENT ORAL, V34, P344, DOI 10.1111/j.1600-0528.2006.00283.x

Pattussi MP, 2006, AM J PUBLIC HEALTH, V96, P1462, DOI 10.2105/AJPH.2005.066159

Persson GR, 2003, J CLIN PERIODONTOL, V30, P691, DOI 10.1034/j.1600-051X.2003.00360.x

Phongsavan P, 2006, SOC SCI MED, V63, P2546, DOI 10.1016/j.socscimed.2006.06.021

Pitiphat W, 2002, J PUBLIC HEALTH DENT, V62, P122, DOI 10.1111/j.1752-7325.2002.tb03432.x

Putnam Robert, 1993, MAKING DEMOCRACY WOR, P167

Rasbash J., 2009, USERS GUIDE MLWIN VE

Scheffler RM, 2008, SOC SCI MED, V66, P1603, DOI 10.1016/j.socscimed.2007.12.007

Shimizu Hiroyuki, 2002, J Epidemiol, V12, P54

Sisson KL, 2007, COMMUNITY DENT ORAL, V35, P81, DOI 10.1111/j.1600-0528.2007.00354.x

Suzuki I, 1998, J Epidemiol, V8, P152

Suzuki Naoko, 2007, Nihon Ronen Igakkai Zasshi, V44, P619, DOI 10.3143/geriatrics.44.619

Watt RG, 2007, COMMUNITY DENT ORAL, V35, P1, DOI 10.1111/j.1600-0528.2007.00348.x

Wilkinson RG, 1998, SOCIOL HEALTH ILL, V20, P578, DOI 10.1111/1467-9566.00120

NR 34

TC 22

Z9 24

U1 0

U2 16

PU WILEY-BLACKWELL

PI MALDEN

PA COMMERCE PLACE, 350 MAIN ST, MALDEN 02148, MA USA

SN 0301-5661

J9 COMMUNITY DENT ORAL

JI Community Dentist. Oral Epidemiol.

PD JUN

PY 2011

VL 39

IS 3

BP 239

EP 249

DI 10.1111/j.1600-0528.2010.00590.x

PG 11

WC Dentistry, Oral Surgery & Medicine; Public, Environmental & Occupational

Health

SC Dentistry, Oral Surgery & Medicine; Public, Environmental & Occupational

Health

GA 764JS

UT WOS:000290626400006

PM 21091754

DA 2019-01-08

ER

PT J

AU Wiltink, J

Beutel, ME

Till, Y

Ojeda, FM

Wild, PS

Munzel, T

Blankenberg, S

Michal, M

AF Wiltink, Joerg

Beutel, Manfred E.

Till, Yvonne

Ojeda, Francisco M.

Wild, Philipp S.

Muenzel, Thomas

Blankenberg, Stefan

Michal, Matthias

TI Prevalence of distress, comorbid conditions and well being in the

general population

SO JOURNAL OF AFFECTIVE DISORDERS

LA English

DT Article

DE Anxiety; Community; Depression; Somatic comorbidity; Type D; Well being

ID CORONARY-HEART-DISEASE; PATIENT HEALTH QUESTIONNAIRE; MENTAL-DISORDERS;

D PERSONALITY; MAJOR DEPRESSION; SEX-DIFFERENCES; PANIC DISORDER;

CARDIAC EVENTS; PSYCHIATRIC-DISORDERS; METABOLIC SYNDROME

AB Background: The purposes of this paper are to determine the prevalence of distress in the community, to identify its determinants and to assess its relationship to somatic conditions and subjective well being.

Methods: Distress and associated factors were investigated in a random sample of 5000 participants (35-74 years) of a community-based, prospective, observational cohort study in western Mid-Germany ("Gutenberg Heart Study") between 04/2007 and 10/2008. The sample was stratified 1:1 for gender and residence and in equal strata for decades of age. Data were assessed by self-report instruments, interviews and medical examination.

Results: We found a prevalence rate for depression of 7.2% (6.5-8.0%), social anxiety 7.0% (6.3-7.7%), panic 4.6% (4.0-5.2%), generalized anxiety 3.4% (2.9-3.9%), and Type D personality 22.1% (21.0-23.2%). Mental conditions declined by age. Depression was related to diabetes (OR = 1.99, 95% Cl = 1.26-3.15), dyslipidemia (OR = 1.35, 95% Cl = 1.02-1.79), coronary heart disease (CHD; OR = 1.88, 95% Cl = 1.04-3.39), and the history of stroke (OR = 2.43, 95% Cl = 1.02-5.76). Panic was related to the history of myocardial infarction (OR = 2.46, 95% CI = 1.15-5.25), and generalized anxiety to obesity (OR = 1.65, 95% CI = 1.11-2.44). Mental distress was unrelated to hypertension, atrial fibrillation and cancer. In ordinal logistic regression subjective physical and mental well being were associated with anxiety, depression and Type D personality.

Conclusions: While mental conditions' are highly prevalent, especially depression is associated with several somatic conditions. Mental and physical well being are strongly related to mental conditions. Future work should take into account comorbid conditions when identifying the impact of depression on CHD. (C) 2010 Elsevier B.V. All rights reserved.

C1 [Wiltink, Joerg; Beutel, Manfred E.; Till, Yvonne; Michal, Matthias] Johannes Gutenberg Univ Mainz, Clin Psychosomat Med & Psychotherapy, Univ Med Ctr, D-55131 Mainz, Germany.

[Till, Yvonne; Ojeda, Francisco M.; Wild, Philipp S.; Muenzel, Thomas; Blankenberg, Stefan] Johannes Gutenberg Univ Mainz, Clin Cardiol, Univ Med Ctr, D-55131 Mainz, Germany.

RP Wiltink, J (reprint author), Johannes Gutenberg Univ Mainz, Clin Psychosomat Med & Psychotherapy, Univ Med Ctr, Untere Zahlbacher Str 8, D-55131 Mainz, Germany.

EM joerg.wiltink@unimedizin-mainz.de

CR Akbaraly TN, 2009, DIABETES CARE, V32, P499, DOI 10.2337/dc08-1358

Alonso J, 2004, ACTA PSYCHIAT SCAND, V109, P38

ALONSO J, 2000, J CLIN PSYCHIAT S2, V68, P3

Appelros P, 2009, STROKE, V40, P1082, DOI 10.1161/STROKEAHA.108.540781

Baumeister H, 2007, INT J OBESITY, V31, P1155, DOI 10.1038/sj.ijo.0803556

Becker ES, 2001, INT J OBESITY, V25, pS5, DOI 10.1038/sj.ijo.0801688

BEEKMAN ATF, 1995, SOC PSYCH PSYCH EPID, V30, P32, DOI 10.1007/BF00784432

Beutel ME, 2002, EUR UROL, V41, P85, DOI 10.1016/S0302-2838(01)00003-3

Birmaher B, 1996, J AM ACAD CHILD PSY, V35, P1427, DOI 10.1097/00004583-199611000-00011

Carpenter KM, 2000, AM J PUBLIC HEALTH, V90, P251, DOI 10.2105/AJPH.90.2.251

Chen YH, 2009, PSYCHOSOM MED, V71, P798, DOI 10.1097/PSY.0b013e3181ad55e3

Connor KM, 2001, DEPRESS ANXIETY, V14, P137, DOI 10.1002/da.1055

Denollet J, 2006, AM J CARDIOL, V97, P970, DOI 10.1016/j.amjcard.2005.10.035

Denollet J, 2005, PSYCHOSOM MED, V67, P89, DOI 10.1097/01.psy.0000149256.81953.49

Fehm L, 2008, SOC PSYCH PSYCH EPID, V43, P257, DOI 10.1007/s00127-007-0299-4

Fleet R, 2000, J PSYCHOSOM RES, V48, P347, DOI 10.1016/S0022-3999(99)00101-4

Frasure-Smith N, 2008, ARCH GEN PSYCHIAT, V65, P62, DOI 10.1001/archgenpsychiatry.2007.4

Frasure-Smith N, 2010, POSTGRAD MED J, V86, P193, DOI 10.1136/hrt.2009.186957

Gilbody S, 2007, J GEN INTERN MED, V22, P1596, DOI 10.1007/s11606-007-0333-y

Goldbacher EM, 2007, ANN BEHAV MED, V34, P240, DOI 10.1007/BF02874549

Goldbacher EM, 2009, PSYCHOSOM MED, V71, P266, DOI 10.1097/PSY.0b013e318197a4d5

Grande G, 2004, PSYCHOTHER PSYCH MED, V54, P413, DOI 10.1088/s-2004-828376

Hach I, 2007, EUR J PUBLIC HEALTH, V17, P297, DOI 10.1093/eurpub/ckl227

Heo M, 2006, INT J OBESITY, V30, P513, DOI 10.1038/sj.ijo.0803122

Herva A, 2006, INT J OBESITY, V30, P520, DOI 10.1038/sj.ijo.0803174

Jacobi F, 2004, Bundesgesundheitsblatt Gesundheitsforschung Gesundheitsschutz, V47, P736

Jacobi F, 2004, PSYCHOL MED, V34, P597, DOI 10.1017/S0033291703001399

John U, 2005, OBES RES, V13, P101, DOI 10.1038/oby.2005.13

Katon W, 2007, GEN HOSP PSYCHIAT, V29, P147, DOI 10.1016/j.genhosppsych.2006.11.005

Katon WJ, 2003, ARCH GEN PSYCHIAT, V60, P897, DOI 10.1001/archpsyc.60.9.897

Kessler RC, 2003, J OCCUP ENVIRON MED, V45, P1257, DOI 10.1097/01.jom.0000100000.70011.bb

Kessler RC, 2005, ARCH GEN PSYCHIAT, V62, P617, DOI 10.1001/archpsyc.62.6.617

KESSLER RC, 1994, ARCH GEN PSYCHIAT, V51, P8

KNAUPER B, 1994, J PSYCHIATR RES, V28, P147, DOI 10.1016/0022-3956(94)90026-4

Kroenke K, 2007, ANN INTERN MED, V146, P317, DOI 10.7326/0003-4819-146-5-200703060-00004

Kuehner C, 2003, ACTA PSYCHIAT SCAND, V108, P163, DOI 10.1034/j.1600-0447.2003.00204.x

Ladwig KH, 2008, PSYCHIAT RES, V161, P248, DOI 10.1016/j.psychres.2008.03.011

Lamertz CM, 2002, OBES RES, V10, P1152, DOI 10.1038/oby.2002.156

Lichtman JH, 2008, CIRCULATION, V118, P1768, DOI 10.1161/CIRCULATIONAHA.108.190769

Lowe B, 2004, PSYCHOTHER PSYCHOSOM, V73, P386, DOI 10.1159/000080393

Lowe B, 2003, J PSYCHOSOM RES, V55, P515, DOI 10.1016/S0022-3999(03)00072-2

McElroy SL, 2004, J CLIN PSYCHIAT, V65, P634

McManus D, 2005, AM J CARDIOL, V96, P1076, DOI 10.1016/j.amjcard.2005.06.037

Miettola J, 2008, SCAND J PRIM HEALTH, V26, P203, DOI 10.1080/02813430802117624

Onyike CU, 2003, AM J EPIDEMIOL, V158, P1139, DOI 10.1093/aje/kwg275

Pedersen SS, 2003, EUR J CARDIOV PREV R, V10, P241, DOI 10.1097/01.hjr.0000085246.65733.06

Pedersen SS, 2006, CURR CARDIOL REV, V2, P205, DOI 10.2174/157340306778019441

PETERSON B, 1990, APPL STAT-J ROY ST C, V39, P205

Poynter B, 2009, PSYCHOSOMATICS, V50, P563, DOI 10.1176/appi.psy.50.6.563

Roberts RE, 2000, AM J EPIDEMIOL, V152, P163, DOI 10.1093/aje/152.2.163

Seeley-Wait Elizabeth, 2009, Prim Care Companion J Clin Psychiatry, V11, P231, DOI 10.4088/PCC.07m00576

Spindler H, 2009, INT J BEHAV MED, V16, P98, DOI 10.1007/s12529-009-9037-5

Spitzer RL, 1999, JAMA-J AM MED ASSOC, V282, P1737, DOI 10.1001/jama.282.18.1737

Spitzer RL, 2006, ARCH INTERN MED, V166, P1092, DOI 10.1001/archinte.166.10.1092

Stokes ME, 2000, CATEGORICAL DATA ANA

Walters K, 2008, EUR HEART J, V29, P2981, DOI 10.1093/eurheartj/ehn477

Weissman MM, 1996, JAMA-J AM MED ASSOC, V276, P293, DOI 10.1001/jama.276.4.293

Williams L, 2008, J PSYCHOSOM RES, V64, P63, DOI 10.1016/j.jpsychores.2007.06.008

Wittchen H U, 2001, BUNDESGESUNDHEITSBLA, V44, P993, DOI DOI 10.1007/S001030100269

Wittchen HU, 1998, PSYCHOL MED, V28, P109, DOI 10.1017/S0033291797005928

Wittchen HU, 2005, EUR NEUROPSYCHOPHARM, V15, P357, DOI 10.1016/j.euroneuro.2005.04.012

NR 61

TC 40

Z9 41

U1 1

U2 15

PU ELSEVIER SCIENCE BV

PI AMSTERDAM

PA PO BOX 211, 1000 AE AMSTERDAM, NETHERLANDS

SN 0165-0327

EI 1573-2517

J9 J AFFECT DISORDERS

JI J. Affect. Disord.

PD MAY

PY 2011

VL 130

IS 3

BP 429

EP 437

DI 10.1016/j.jad.2010.10.041

PG 9

WC Clinical Neurology; Psychiatry

SC Neurosciences & Neurology; Psychiatry

GA 771WT

UT WOS:000291192100009

PM 21106250

DA 2019-01-08

ER

PT J

AU Donald, M

Ware, RS

Ozolins, IZ

Begum, N

Crowther, R

Bain, C

AF Donald, Maria

Ware, Robert S.

Ozolins, Ieva Z.

Begum, Nelufa

Crowther, Ruth

Bain, Christopher

TI The role of patient activation in frequent attendance at primary care: A

population-based study of people with chronic disease

SO PATIENT EDUCATION AND COUNSELING

LA English

DT Article

DE Patient activation; Health service utilisation; Frequent attenders;

Chronic illness

ID SELF-MANAGEMENT; GENERAL-PRACTICE; HEALTH; ATTENDERS; PROGRAM; COPD; ILL

AB Objective: This study explores a range of relevant socio-demographic, physical and psychological factors in a unique examination of the risk factors for frequent attendance at primary care. The impact of patient activation for self-management on health service utilisation is of particular interest.

Methods: A population-based sample of people with chronic disease from Queensland, Australia, was interviewed using computer assisted telephone surveying. Data were collected from a random sample of 1470 people with either diabetes or a cardiovascular condition.

Results: As participants became more activated they were less likely to frequently attend their main health care provider for assistance with their chronic condition. For both conditions the association was graduated and for participants with a cardiovascular condition this association remained statistically significant even after controlling for other potentially influential factors such as disease severity, length of time since diagnosis, and psychological distress.

Conclusion: Characteristics of the individual, including patient activation and psychological functioning, as well as disease factors contribute to primary care consulting patterns among people with chronic illness.

Practical implications: Efforts to improve patient activation for self-management should remain a central element of chronic care. (C) 2010 Elsevier Ireland Ltd. All rights reserved.

C1 [Donald, Maria; Ware, Robert S.; Ozolins, Ieva Z.; Begum, Nelufa; Crowther, Ruth; Bain, Christopher] Univ Queensland, Sch Populat Hlth, Herston, Qld 4006, Australia.

RP Donald, M (reprint author), Univ Queensland, Sch Populat Hlth, Herston, Qld 4006, Australia.

EM m.donald@sph.uq.edu.au

RI Donald, Maria/G-2532-2010; Ware, Robert/B-2024-2014; Crowther,

Ruth/K-8461-2014

OI Ware, Robert/0000-0002-6129-6736; Crowther, Ruth/0000-0002-7628-5618;

Donald, Maria/0000-0002-4962-7627

FU Queensland Health

FX This research was funded by Queensland Health through the Queensland

Strategy for Chronic Disease 2005-2015.

CR Battersby MW, 2005, BRIT MED J, V330, P662, DOI 10.1136/bmj.330.7492.662

Bourbeau J, 2006, CHEST, V130, P1704, DOI 10.1378/chest.130.6.1704

Carney TA, 2001, BRIT J GEN PRACT, V51, P567

deBoer AGEM, 1997, HEALTH POLICY, V42, P101, DOI 10.1016/S0168-8510(97)00062-6

*DIAB AUSTR ROYAL, 2009, DIAB MAN GEN PRACT G

Dowrick C, 2006, MED J AUSTRALIA, V185, P61

Elzen H, 2008, J EVAL CLIN PRACT, V14, P159, DOI 10.1111/j.1365-2753.2007.00791.x

Field KS, 2001, HEALTH SOC CARE COMM, V9, P294, DOI 10.1046/j.0966-0410.2001.00303.x

Foster A, 2006, FAM PRACT, V23, P444, DOI 10.1093/fampra/cml019

Foster G, 2007, COCHRANE DATABASE SY

Furukawa TA, 2003, PSYCHOL MED, V33, P357, DOI 10.1017/S0033291702006700

Hansell A, 2003, EUR RESPIR J, V21, P279, DOI 10.1183/09031936.03.00006102

Heywood PL, 1998, FAM PRACT, V15, P198, DOI 10.1093/fampra/15.3.198

Hibbard JH, 2005, HEALTH SERV RES, V40, P1918, DOI 10.1111/j.1475-6773.2005.00438.x

Hibbard JH, 2004, HEALTH SERV RES, V39, P1005, DOI 10.1111/j.1475-6773.2004.00269.x

Kennedy A, 2007, J EPIDEMIOL COMMUN H, V61, P254, DOI 10.1136/jech.2006.053538

Kessler RC, 2003, ARCH GEN PSYCHIAT, V60, P184, DOI 10.1001/archpsyc.60.2.184

Lorig KR, 2001, MED CARE, V39, P1217, DOI 10.1097/00005650-200111000-00008

Naessens JA, 2005, ANN FAM MED, V3, P324, DOI 10.1370/afm.352

Nolte E.E., 2008, EUROPEAN OBSERVATORY

Queensland Health, 2005, QUEENSL STRAT CHRON

SCHRIRE S, 1986, FAM PRACT, V3, P272, DOI 10.1093/fampra/3.4.272

Vedsted P, 2005, PUBLIC HEALTH, V119, P118, DOI 10.1016/j.puhe.2004.03.007

Wagner E H, 1998, Eff Clin Pract, V1, P2

Wetzels R, 2005, J AM GERIATR SOC, V53, P918, DOI 10.1111/j.1532-5415.2005.53278_8.x

NR 25

TC 20

Z9 20

U1 0

U2 14

PU ELSEVIER IRELAND LTD

PI CLARE

PA ELSEVIER HOUSE, BROOKVALE PLAZA, EAST PARK SHANNON, CO, CLARE, 00000,

IRELAND

SN 0738-3991

J9 PATIENT EDUC COUNS

JI Patient Educ. Couns.

PD MAY

PY 2011

VL 83

IS 2

BP 217

EP 221

DI 10.1016/j.pec.2010.05.031

PG 5

WC Public, Environmental & Occupational Health; Social Sciences,

Interdisciplinary

SC Public, Environmental & Occupational Health; Social Sciences - Other

Topics

GA 768JC

UT WOS:000290929200014

PM 20598825

DA 2019-01-08

ER

PT J

AU Ting, RZW

Nan, H

Yu, MWM

Kong, APS

Ma, RCW

Wong, RYM

Loo, K

So, WY

Chow, CC

Ko, GTC

Wing, YK

Chan, JCN

AF Ting, Rose Z. W.

Nan, Hairong

Yu, Mandy W. M.

Kong, Alice P. S.

Ma, Ronald C. W.

Wong, Rebecca Y. M.

Loo, Kitman

So, Wing-Yee

Chow, Chun-Chung

Ko, Gary T. C.

Wing, Yun-Kwok

Chan, Juliana C. N.

TI Diabetes-Related Distress and Physical and Psychological Health in

Chinese Type 2 Diabetic Patients

SO DIABETES CARE

LA English

DT Article

ID QUALITY-OF-LIFE; SCALE; OBESITY

AB OBJECTIVE-To validate a Chinese version of the Diabetes Distress Scale (CDDS).

RESEARCH DESIGN AND METHODS-The CDDS was derived using forward-backward translation and administered in 189 Chinese type 2 diabetic patients with evaluation of its psychometric properties.

RESULTS-On the basis of principal-component analysis, three factors of the 15-item version of the CDDS (CDDS-15) accounted for 63% of the variance. The correlation coefficient between the original 17-item and 15-item scales was 0.99. The Cronbach alpha for internal consistency was 0.90, and the test-retest reliability coefficient was 0.74. The CDDS-15 score was significantly associated with glycemic control, obesity, depressive symptoms, and quality of life.

CONCLUSIONS-The CDDS-15 is a valid and reliable instrument to assess diabetes-related distress.

C1 [Ting, Rose Z. W.; Kong, Alice P. S.; Ma, Ronald C. W.; Wong, Rebecca Y. M.; Loo, Kitman; So, Wing-Yee; Chow, Chun-Chung; Ko, Gary T. C.; Chan, Juliana C. N.] Chinese Univ Hong Kong, Prince Wales Hosp, Dept Med & Therapeut, Hong Kong, Hong Kong, Peoples R China.

[Nan, Hairong; So, Wing-Yee; Ko, Gary T. C.; Chan, Juliana C. N.] Chinese Univ Hong Kong, Prince Wales Hosp, Hong Kong Inst Diabet & Obes, Hong Kong, Hong Kong, Peoples R China.

[Yu, Mandy W. M.; Wing, Yun-Kwok] Chinese Univ Hong Kong, Prince Wales Hosp, Dept Psychiat, Hong Kong, Hong Kong, Peoples R China.

RP Chan, JCN (reprint author), Chinese Univ Hong Kong, Prince Wales Hosp, Dept Med & Therapeut, Hong Kong, Hong Kong, Peoples R China.

EM jchan@cuhk.edu.hk

RI Chan, Juliana/B-7918-2016; Ma, Ronald/C-2788-2009; Kong,

Alice/P-3703-2015

OI Chan, Juliana/0000-0003-1325-1194; Ma, Ronald/0000-0002-1227-803X; Wing,

Yun kwok/0000-0002-5745-5474

CR Cheung CK, 1998, J PSYCHOL, V132, P169, DOI 10.1080/00223989809599157

Fisher L, 2009, DIABETIC MED, V26, P622, DOI 10.1111/j.1464-5491.2009.02730.x

Hlatky MA, 2010, AM HEART J, V159, DOI 10.1016/j.ahj.2009.11.004

Katz DA, 2000, J GEN INTERN MED, V15, P789, DOI 10.1046/j.1525-1497.2000.90906.x

Kong APS, 2006, CURR DIABETES REV, V2, P397, DOI 10.2174/1573399810602040397

Mullan JT, 2006, DIABETES, V55, pA426

Phillips MR, 2009, LANCET, V373, P2041, DOI 10.1016/S0140-6736(09)60660-7

Polonsky WH, 2005, DIABETES CARE, V28, P626, DOI 10.2337/diacare.28.3.626

Rabin R, 2001, ANN MED, V33, P337, DOI 10.3109/07853890109002087

Toobert DJ, 2000, DIABETES CARE, V23, P943, DOI 10.2337/diacare.23.7.943

Yang XL, 2008, CAN MED ASSOC J, V179, P427, DOI 10.1503/cmaj.071474

Yeung A, 2008, COMPR PSYCHIAT, V49, P211, DOI 10.1016/j.comppsych.2006.06.002

NR 12

TC 31

Z9 34

U1 0

U2 8

PU AMER DIABETES ASSOC

PI ALEXANDRIA

PA 1701 N BEAUREGARD ST, ALEXANDRIA, VA 22311-1717 USA

SN 0149-5992

J9 DIABETES CARE

JI Diabetes Care

PD MAY

PY 2011

VL 34

IS 5

BP 1094

EP 1096

DI 10.2337/dc10-1612

PG 3

WC Endocrinology & Metabolism

SC Endocrinology & Metabolism

GA 761RV

UT WOS:000290419200007

PM 21398526

OA Green Published, Other Gold

DA 2019-01-08

ER

PT J

AU Gonder-Frederick, LA

Schmidt, KM

Vajda, KA

Greear, ML

Singh, H

Shepard, JA

Cox, DJ

AF Gonder-Frederick, Linda A.

Schmidt, Karen M.

Vajda, Karen A.

Greear, Megan L.

Singh, Harsimran

Shepard, Jaclyn A.

Cox, Daniel J.

TI Psychometric Properties of the Hypoglycemia Fear Survey-II for Adults

With Type 1 Diabetes

SO DIABETES CARE

LA English

DT Article

ID FIT INDEXES; RELIABILITY; INVENTORY; VALIDITY; EPISODES; VERSION

AB OBJECTIVE-To perform the first comprehensive psychometric evaluation of the Hypoglycemia Fear Survey-II (HFS-II), a measure of the behavioral and affective dimensions of fear of hypoglycemia, using modern test-theory methods, including item-response theory (IRT).

RESEARCH DESIGN AND METHODS-Surveys completed in four previous studies by 777 adults with type 1 diabetes were aggregated for analysis, with 289 subjects completing both subscales of the HFS-II and 488 subjects completing only the Worry subscale. The aggregated sample (53.3% female, 44.4% using insulin pumps) had a mean age of 41.9 years, diabetes duration of 23.8 years, HbA(1c) value of 7.7%, and 1.4 severe hypoglycemic episodes in the past year. Data analysis included exploratory factor analysis using polychoric correlations and IRT. Factors were analyzed for fit, trait-level locations, point-measure correlations, and separation values.

RESULTS-Internal and test-retest reliability was good, as well as convergent validity, as demonstrated by significant correlations with other measures of psychological distress. Scores were significantly higher in subjects who had experienced severe hypoglycemia in the past year. Factor analyses validated the two subscales of the HFS-II. Item analyses showed that 12 of 15 items on the Behavior subscale, and all of the items on the Worry subscale had good-fit statistics.

CONCLUSIONS-The HFS-II is a reliable and valid measure of the fear of hypoglycemia in adults with type 1 diabetes, and actor analyses and IRT support the two separate subscales of the survey. Diabetes Care 34:801-806, 2011

C1 [Gonder-Frederick, Linda A.; Vajda, Karen A.; Greear, Megan L.; Singh, Harsimran; Shepard, Jaclyn A.; Cox, Daniel J.] Univ Virginia, Hlth Sci Ctr, Charlottesville, VA 22903 USA.

[Schmidt, Karen M.] Univ Virginia, Dept Psychol, Charlottesville, VA 22903 USA.

RP Gonder-Frederick, LA (reprint author), Univ Virginia, Hlth Sci Ctr, Charlottesville, VA 22903 USA.

EM lag3g@virginia.edu

FU National Institutes of Health, National Institute of Diabetes and

Digestive and Kidney Diseases [R21-DK-080896, R01-DK-028288,

R01-DK-051562]; LifeScan (Milpitas, CA)

FX This research was supported in part by National Institutes of Health,

National Institute of Diabetes and Digestive and Kidney Diseases Grants

R21-DK-080896, R01-DK-028288, and R01-DK-051562 and a grant from

LifeScan (Milpitas, CA). No other potential conflicts of interest

relevant to this article were reported.

CR Anderbro T, 2008, PATIENT EDUC COUNS, V73, P127, DOI 10.1016/j.pec.2008.03.022

BECK AT, 1988, J CONSULT CLIN PSYCH, V56, P893, DOI 10.1037/0022-006X.56.6.893

BECK AT, 1961, ARCH GEN PSYCHIAT, V4, P561

BENDER PM, 1980, PSYCHOL BULL, V88, P588, DOI DOI 10.1037/0033-2909.88.3.588

BROWNE MW, 1992, SOCIOL METHOD RES, V21, P230, DOI 10.1177/0049124192021002005

Clarke WL, 1998, J PEDIATR ENDOCR MET, V11, P189

COX D, 1995, DIABETES CARE, V18, P523, DOI 10.2337/diacare.18.4.523

Cox DJ, 2009, DIABETES CARE, V32, P2177, DOI 10.2337/dc08-1510

COX DJ, 1987, DIABETES CARE, V10, P617, DOI 10.2337/diacare.10.5.617

COX DJ, 1990, DIABETES CARE, V13, P458, DOI 10.2337/diacare.13.4.458

Embretson S. W., 1999, NEW RULES MEASUREMEN

Gilbert RE, 2008, CAN J DIABETES, V32, P101, DOI 10.1016/S1499-2671(08)90000-9

Gonder-Frederick LA, 2006, PEDIATR DIABETES, V7, P215, DOI 10.1111/j.1399-5448.2006.00182.x

GonderFrederick L, 1997, DIABETES CARE, V20, P1543, DOI 10.2337/diacare.20.10.1543

Hu LT, 1998, PSYCHOL METHODS, V3, P424, DOI 10.1037//1082-989X.3.4.424

Irvin A, 1994, HDB PSYCHOL DIABETES, P133

Kovatchev Boris P, 2003, Diabetes Technol Ther, V5, P817, DOI 10.1089/152091503322527021

Linacre JM, WINSTEPS RASCH MODEL

Marsh HW, 2004, STRUCT EQU MODELING, V11, P320, DOI 10.1207/s15328007sem1103_2

Muthen L. K., 1998, MPLUS USERS GUIDE VE

Shiu ATY, 2004, PUBLIC HEALTH NURS, V21, P257, DOI 10.1111/j.0737-1209.2004.21308.x

SPIELBERGER C, 1979, STATE TRAIT PERSONAL

Stargardt T, 2009, HEALTH QUAL LIFE OUT, V7, DOI 10.1186/1477-7525-7-91

Ware JE, 1996, MED CARE, V34, P220, DOI 10.1097/00005650-199603000-00003

NR 24

TC 72

Z9 75

U1 1

U2 8

PU AMER DIABETES ASSOC

PI ALEXANDRIA

PA 1701 N BEAUREGARD ST, ALEXANDRIA, VA 22311-1717 USA

SN 0149-5992

J9 DIABETES CARE

JI Diabetes Care

PD APR

PY 2011

VL 34

IS 4

BP 801

EP 806

DI 10.2337/dc10-1343

PG 6

WC Endocrinology & Metabolism

SC Endocrinology & Metabolism

GA 746DE

UT WOS:000289221800003

PM 21346182

OA Other Gold, Green Published

DA 2019-01-08

ER

PT J

AU Anderson, RT

Narayan, KMV

Feeney, P

Goff, D

Ali, MK

Simmons, DL

Sperl-Hillen, JA

Bigger, T

Cuddihy, R

O'Conner, PJ

Sood, A

Zhang, P

Sullivan, MD

AF Anderson, Roger T.

Narayan, K. M. Venkat

Feeney, Patricia

Goff, David, Jr.

Ali, Mohammed K.

Simmons, Debra L.

Sperl-Hillen, Jo-Ann

Bigger, Thomas

Cuddihy, Robert

O'Conner, Patrick J.

Sood, Ajay

Zhang, Ping

Sullivan, Mark D.

CA Action Control Cardiovasc Risk

TI Effect of Intensive Glycemic Lowering on Health-Related Quality of Life

in Type 2 Diabetes ACCORD trial

SO DIABETES CARE

LA English

DT Article

ID CONTROL CARDIOVASCULAR RISK; MELLITUS; DEPRESSION; SATISFACTION;

SEVERITY; UTILITY; DESIGN; ADULTS; CARE

AB OBJECTIVE-To compare the effect of intensive versus standard glycemic control strategies on health-related quality of life (HRQL) in a substudy of the Action to Control Cardiovascular Risk in Diabetes (ACCORD) trial.

RESEARCH DESIGN AND METHODS-A randomly selected subsample of 2,053 ACCORD participants enrolled fit the HRQL substudy was assessed at baseline and 12-, 36-, and 48-month visits. HRQL assessment included general health status (the 36-Item Short Form Health Survey [SF-36]), diabetes symptoms (the Diabetes Symptom Distress Checklist), depression (Patient Health Questionnaire [PHQ]-9), and treatment satisfaction (Diabetes Treatment Satisfaction Questionnaire [DTSQ]). Repeated-measures ANOVA models were used to estimate change in HRQL outcomes by treatment group over 48 months adjusting for model covariates. The effects of early discontinuation of the ACCORD intensive glycemic control arm on study results were explored.

RESULTS-A total of 1,956 (95%) completed the self-report HRQL instrument (s) at baseline. The intensive arm had a larger decrease in SF-36 physical health component score than the standard arm (-1.6 vs. -1.1, P = 0.0345). Treatment satisfaction (DTSQ) showed larger improvement with intensive than standard (P = 0.0004). There were no differences in mean scores of the Diabetes Symptom Checklist and PHQ-9. Effects of participant transition following discontinuation of the intensive arm on HRQL were not significant.

CONCLUSIONS-The ACCORD trial strategy of intensive glycemic control did not lead to benefits in HRQL and was associated with modest improvement in diabetes treatment satisfaction. Thus patient acceptability was apparently not compromised with intensive and complex interventions such as those used in ACCORD. Diabetes Care 34:807-812, 2011

C1 [Anderson, Roger T.] Penn State Univ, Coll Med, Dept Publ Hlth Sci, Hershey, PA USA.

[Narayan, K. M. Venkat; Ali, Mohammed K.] Emory Univ, Rollins Sch Publ Hlth, Atlanta, GA 30322 USA.

[Narayan, K. M. Venkat] Emory Univ, Sch Med, Atlanta, GA USA.

[Feeney, Patricia] Wake Forest Univ Hlth Sci, Winston Salem, NC USA.

[Goff, David, Jr.] Wake Forest Univ, Dept Epidemiol, Sch Med, Winston Salem, NC 27109 USA.

[Goff, David, Jr.] Wake Forest Univ, Dept Internal Med, Sch Med, Winston Salem, NC 27109 USA.

[Goff, David, Jr.] Wake Forest Univ, Dept Social Sci & Hlth Policy, Winston Salem, NC 27109 USA.

[Simmons, Debra L.] Cent Arkansas Vet Healthcare Syst, Little Rock, AR USA.

[Simmons, Debra L.] Univ Arkansas Med Sci, Little Rock, AR 72205 USA.

[Sperl-Hillen, Jo-Ann; O'Conner, Patrick J.] HealthPartners Res Fdn, Minneapolis, MN USA.

[Bigger, Thomas] Columbia Univ, Coll Phys & Surg, New York, NY USA.

[Sood, Ajay] Case Western Reserve Univ, Cleveland, OH 44106 USA.

[Zhang, Ping] Ctr Dis Control & Prevent, Div Diabet Translat, Natl Ctr Chron Dis Prevent & Hlth Promot, Atlanta, GA USA.

[Sullivan, Mark D.] Univ Washington, Sch Med, Seattle, WA USA.

RP Anderson, RT (reprint author), Penn State Univ, Coll Med, Dept Publ Hlth Sci, Hershey, PA USA.

EM rtanders@psu.edu

RI Narayan, K.M. Venkat/J-9819-2012

OI Narayan, K.M. Venkat/0000-0001-8621-5405

FU National Heart, Lung, and Blood Institute [N01-HC-95178, N01-HC-95179,

N01-HC-95180, N01-HC-95181, N01-HC-95182, N01-HC-95183, N01-HC-95184,

IAA-Y1-HC-9035, IAA-Y1-HC-1010]; National Institutes of Health; National

Institute of Diabetes and Digestive and Kidney Diseases; National

Institute on Aging; National Eye Institute; Centers for Disease Control

and Prevention; General Clinical Research Centers; Merck; GSK; Lilly;

Abbott Laboratories through HealthPartners Research Foundation; Amylin;

Abbott; Bayer; Daiichi Sankyo; Dexcom; Edwards Life-sciences; Eli Lilly;

Hygeia; Intarcia; Johnson and Johnson/LifeScan; MannKind; Medtronic;

Novo Nordisk; Quotient Diagnostics; ResMed; Roche; sanofi-aventis;

Schering-Plough; Takeda; Valeritas

FX The ACCORD study was supported by grants (N01-HC-95178, N01-HC-95179,

N01-HC-95180, N01-HC-95181, N01-HC-95182, N01-HC-95183, N01-HC-95184,

IAA-Y1-HC-9035, and IAA-Y1-HC-1010) from the National Heart, Lung, and

Blood Institute; by other components of the National Institutes of

Health, including the National Institute of Diabetes and Digestive and

Kidney Diseases, the National Institute on Aging, and the National Eye

Institute; by the Centers for Disease Control and Prevention; and by

General Clinical Research Centers.; The following companies provided

study medications, equipment, or supplies: Abbott Laboratories, Amylin

Pharmaceutical, AstraZeneca, Bayer Health Care, Closer Healthcare,

GlaxoSmithKline, King Pharmaceuticals, Merck, Novartis, Novo Nordisk,

Omron Healthcare, sanofi-aventis, and Schering-Plough. R.T.A. is a

consultant for Abbott Laboratories, Inc. D.G. is a consultant for Merck

Inc. and serves as a DSMB member for Takeda Inc. J.-A.S.-H. is an

investigator of clinical trials sponsored by Merck, GSK, Lilly and

Abbott Laboratories through HealthPartners Research Foundation. R.C.

serves as an investigator on clinical trials sponsored by Amylin,

Abbott, Bayer, Daiichi Sankyo, Dexcom, Edwards Life-sciences, Eli Lilly,

Hygeia, Intarcia, Johnson and Johnson/LifeScan, MannKind, Medtronic,

Merck, Novo Nordisk, Quotient Diagnostics, ResMed, Roche,

sanofi-aventis, Schering-Plough, Takeda, Valeritas; and serves as an

Advisory Board Member for Abbott, Bayer, CeQur, Eli Lilly, Novo Nordisk,

and Roche. No other potential conflicts of interest relevant to this

article were reported.

CR Anderson RT, 2009, DIABETES CARE, V32, P51, DOI 10.2337/dc08-0856

Bradley C., 1994, HDB PSYCHOL DIABETES, P111

Buse JB, 2007, AM J CARDIOL, V99, p21I, DOI 10.1016/j.amjcard.2007.03.003

Ciechanowski PS, 2003, GEN HOSP PSYCHIAT, V25, P246, DOI 10.1016/S0163-8343(03)00055-0

Currie CJ, 2006, DIABETOLOGIA, V49, P2272, DOI 10.1007/s00125-006-0380-7

Eaton WW, 1996, DIABETES CARE, V19, P1097, DOI 10.2337/diacare.19.10.1097

Egede LE, 2004, DIABETES CARE, V27, P421, DOI 10.2337/diacare.27.2.421

Gerstein HC, 2008, NEW ENGL J MED, V358, P2545, DOI 10.1056/NEJMoa0802743

Gerstein HC, 2007, AM J CARDIOL, V99, p34I, DOI 10.1016/j.amjcard.2007.03.004

Goddijn PPM, 1999, DIABETIC MED, V16, P23, DOI 10.1046/j.1464-5491.1999.00002.x

Gulliford MC, 1999, J CLIN EPIDEMIOL, V52, P773, DOI 10.1016/S0895-4356(99)00053-0

KOVACS M, 1990, J PEDIATR PSYCHOL, V15, P619, DOI 10.1093/jpepsy/15.5.619

Peyrot M, 1997, DIABETES CARE, V20, P585, DOI 10.2337/diacare.20.4.585

PEYROT M, 1994, DIABETES CARE, V17, P994, DOI 10.2337/diacare.17.9.994

Peyrot M, 2009, DIABETES CARE, V32, P1411, DOI 10.2337/dc08-2256

Riddle MC, 2010, DIABETES CARE, V33, P983, DOI 10.2337/dc09-1278

Rubin RR, 1999, DIABETES-METAB RES, V15, P205, DOI 10.1002/(SICI)1520-7560(199905/06)15:3<205::AID-DMRR29>3.0.CO;2-O

SONGER T, 1995, NIH PUBL

Spitzer RL, 1999, JAMA-J AM MED ASSOC, V282, P1737, DOI 10.1001/jama.282.18.1737

Sullivan MD, 2007, AM J CARDIOL, V99, p90I, DOI 10.1016/j.amjcard.2007.03.027

Testa MA, 1998, JAMA-J AM MED ASSOC, V280, P1490, DOI 10.1001/jama.280.17.1490

UK Prospective Diabetes Study Group, 1999, DIABETES CARE, V22, P1125, DOI DOI 10.2337/DIACARE.22.7.1125

Ware JE, 2000, SCORE VERSION 2 SF 3

Wikblad K, 1996, QUAL LIFE RES, V5, P123, DOI 10.1007/BF00435977

Zhang XP, 2007, MED CARE, V45, P820, DOI 10.1097/MLR.0b013e3180618b55

NR 25

TC 26

Z9 28

U1 0

U2 8

PU AMER DIABETES ASSOC

PI ALEXANDRIA

PA 1701 N BEAUREGARD ST, ALEXANDRIA, VA 22311-1717 USA

SN 0149-5992

J9 DIABETES CARE

JI Diabetes Care

PD APR

PY 2011

VL 34

IS 4

BP 807

EP 812

DI 10.2337/dc10-1926

PG 6

WC Endocrinology & Metabolism

SC Endocrinology & Metabolism

GA 746DE

UT WOS:000289221800004

PM 21346183

OA Green Published, Other Gold

DA 2019-01-08

ER

PT J

AU Wilfley, D

Berkowitz, R

Goebel-Fabbri, A

Hirst, K

Ievers-Landis, C

Lipman, TH

Marcus, M

Ng, D

Pham, T

Saletsky, R

Schanuel, J

Van Buren, D

AF Wilfley, Denise

Berkowitz, R.

Goebel-Fabbri, A.

Hirst, K.

Ievers-Landis, C.

Lipman, T. H.

Marcus, M.

Ng, D.

Pham, T.

Saletsky, R.

Schanuel, J.

Van Buren, D.

CA TODAY Study Grp

TI Binge Eating, Mood, and Quality of Life in Youth With Type 2 Diabetes

Baseline data from the TODAY study

SO DIABETES CARE

LA English

DT Article

ID OVERWEIGHT YOUTH; ADOLESCENTS; ATTITUDES; BEHAVIOR; OBESITY; FAMILY;

GIRLS

AB OBJECTIVE-The current study examines the prevalence of binge eating and its association with adiposity and psychosocial functioning in a large, diverse sample of youth with type 2 diabetes.

RESEARCH DESIGN AND METHODS-In the TODAY study, 678 (mean age 14.0 years; 64.9% girls) of the 704 youth randomized to the study completed a self-report measure of eating disorder symptoms and were categorized as nonovereaters, overeaters, subclinical binge eaters, or clinical binge eaters.

RESULTS-Youth with clinical (6%) and subclinical (20%) levels of binge eating had significantly higher levels and rates of extreme obesity, global eating disorder and depressive symptoms, and impaired quality of life.

CONCLUSIONS-These findings highlight the importance of evaluating youth with type 2 diabetes for the presence of binge eating. Future research is needed to determine the cumulative effects of disordered eating, obesity, and psychosocial distress on adherence to lifestyle change recommendations and longitudinal response to treatment. Diabetes Care 34:858-860, 2011

C1 [Wilfley, Denise; Van Buren, D.] Washington Univ, Sch Med, Dept Psychiat, St Louis, MO 63110 USA.

[Lipman, T. H.] Univ Penn, Sch Nursing, Philadelphia, PA 19104 USA.

[Goebel-Fabbri, A.] Harvard Univ, Sch Med, Boston, MA USA.

[Hirst, K.; Pham, T.] George Washington Univ, Ctr Biostat, Rockville, MD USA.

[Ievers-Landis, C.] Rainbow Babies & Childrens Hosp, Div Behav Pediat & Psychol, Cleveland, OH USA.

[Ievers-Landis, C.] Case Western Reserve Univ, Sch Med, Cleveland, OH 44106 USA.

[Marcus, M.] Western Psychiat Inst & Clin, Pittsburgh, PA USA.

[Ng, D.] Columbia Univ, Naomi Berry Diabet Ctr, New York, NY USA.

[Saletsky, R.] SUNY Upstate Med Univ, Syracuse, NY USA.

[Schanuel, J.] Univ Oklahoma, Hlth Sci Ctr, Dept Pediat, Oklahoma City, OK USA.

RP Wilfley, D (reprint author), Washington Univ, Sch Med, Dept Psychiat, St Louis, MO 63110 USA.

EM wilfley@psychiatry.wustl.edu

OI Zeitler, Philip/0000-0001-5756-7858

FU NIDDK/NIH [U01-DK-61212, U01-DK-61230, U01-DK-61239, U01-DK-61242,

U01-DK-61254]; National Institute of Mental Health [1K24-MH-070446-01];

National Center for Research Resources (Washington University School of

Medicine) [M01-RR-00036]; Children's Hospital Los Angeles

[M01-RR-00043-45]; University of Colorado Denver [M01-RR-00069];

Children's Hospital of Pittsburgh [M01-RR-00084, UL1-RR-024153];

Massachusetts General Hospital [M01-RR-01066, UL1-RR-025758]; Yale

University [M01-RR-00125, UL1-RR-024139]; University of Oklahoma Health

Sciences Center [M01-RR-14467]; National Center for Research Resources

Clinical and Translational Science (Children's Hospital of Philadelphia)

[UL1-RR-024134]; Washington University [UL1-RR-024992]

FX This work was completed with funding from NIDDK/NIH grant numbers

U01-DK-61212, U01-DK-61230, U01-DK-61239, U01-DK-61242, and

U01-DK-61254; from National Institute of Mental Health Grant

1K24-MH-070446-01 (to D.W.); from the National Center for Research

Resources General Clinical Research Centers Program Grants M01-RR-00036

(Washington University School of Medicine), M01-RR-00043-45 (Children's

Hospital Los Angeles), M01-RR-00069 (University of Colorado Denver),

M01-RR-00084 (Children's Hospital of Pittsburgh), M01-RR-01066

(Massachusetts General Hospital), M01-RR-00125 (Yale University), and

M01-RR-14467 (University of Oklahoma Health Sciences Center); and from

the National Center for Research Resources Clinical and Translational

Science Awards Grants UL1-RR-024134 (Children's Hospital of

Philadelphia), UL1-RR-024139 (Yale University), UL1-RR-024153

(Children's Hospital of Pittsburgh), UL1-RR-024989 (Case Western Reserve

University), UL1-RR-024992 (Washington University), UL1-RR-025758

(Massachusetts General Hospital), and UL1-RR-025780 (University of

Colorado Denver).

CR *AM DIAB ASS, 2010, DIABETES CARE S1, V33, pS11, DOI DOI 10.2337/DC10-S011

BACK AT, 1996, BECK DEPRESSION INVE

Colton P, 2004, DIABETES CARE, V27, P1654, DOI 10.2337/diacare.27.7.1654

Goldschmidt AB, 2008, OBESITY, V16, P257, DOI 10.1038/oby.2007.48

Goldschmidt AB, 2007, INT J EAT DISORDER, V40, P460, DOI 10.1002/eat.20387

Goran MI, 2003, J CLIN ENDOCR METAB, V88, P1417, DOI 10.1210/jc.2002-021442

Howe Carol J, 2008, J Am Psychiatr Nurses Assoc, V13, P376, DOI 10.1177/1078390307310154

Kovacs M., 1992, CHILDRENS DEPRESSION

Olmsted MP, 2008, DIABETES CARE, V31, P1978, DOI 10.2337/dc08-0333

Peveler RC, 2005, DIABETES CARE, V28, P84, DOI 10.2337/diacare.28.1.84

Pinhas-Hamiel O, 1999, ARCH PEDIAT ADOL MED, V153, P1063, DOI 10.1001/archpedi.153.10.1063

Tanofsky-Kraff M, 2007, OBESITY, V15, P1345, DOI 10.1038/oby.2007.162

Varni JW, 2003, DIABETES CARE, V26, P631, DOI 10.2337/diacare.26.3.631

Wildes JE, 2010, INT J OBESITY, V34, P1143, DOI 10.1038/ijo.2010.35

ZEITLER P, 2007, PEDIAT DIABETES, V8, P74

NR 15

TC 45

Z9 45

U1 0

U2 7

PU AMER DIABETES ASSOC

PI ALEXANDRIA

PA 1701 N BEAUREGARD ST, ALEXANDRIA, VA 22311-1717 USA

SN 0149-5992

EI 1935-5548

J9 DIABETES CARE

JI Diabetes Care

PD APR

PY 2011

VL 34

IS 4

BP 858

EP 860

DI 10.2337/dc10-1704

PG 3

WC Endocrinology & Metabolism

SC Endocrinology & Metabolism

GA 746DE

UT WOS:000289221800013

PM 21357794

OA Green Published, Other Gold

DA 2019-01-08

ER

PT J

AU Lamers, F

Jonkers, CCM

Bosma, H

Knottnerus, JA

van Eijk, JTM

AF Lamers, Femke

Jonkers, Catharina C. M.

Bosma, Hans

Knottnerus, J. Andre

van Eijk, Jacques Th M.

TI Treating depression in diabetes patients: does a nurse-administered

minimal psychological intervention affect diabetes-specific quality of

life and glycaemic control? A randomized controlled trial

SO JOURNAL OF ADVANCED NURSING

LA English

DT Article

DE depression; diabetes; nursing; primary care; psychological intervention;

quality of life; randomized controlled trial

ID PLACEBO-CONTROLLED TRIAL; PROBLEM AREAS; SYMPTOM CHECKLIST; ELDERLY

PERSONS; PRIMARY-CARE; DOUBLE-BLIND; TYPE-2; METAANALYSIS; OUTCOMES;

SCALE

AB P>Aims.

The aim of this study was to examine whether a nurse-administered minimal psychological intervention for depressive symptoms improves diabetes-specific quality of life and glycaemic control in older persons with diabetes.

Background.

Depression is common among persons with diabetes and may have a negative impact on diabetes. Interventions aimed at reducing depressive symptoms may positively influence diabetes-specific quality of life as well.

Methods.

A pragmatic, randomized controlled trial was carried out comparing the intervention with usual care among 208 Dutch primary care patients of >= 60 years with type 2 diabetes and co-occurring minor to moderate depression. Data on symptom distress and emotional distress were collected during 2003-2006, and haemoglobin A1c levels were obtained from general practices. Data were analysed using mixed model, repeated measures ancovas. Hba1c was collected retrospectively from general practices between December 2006-February 2007. In July 2007 we retrieved some additional HbA1c data from the medical records of the university hospital.

Results.

Only in higher-educated persons did the intervention have statistically significant effect on both emotional distress and symptom distress (DSC-R total score at 9 months P = 0 center dot 001; PAID, 9 months P = 0 center dot 03). Furthermore, we found an effect on symptom distress in men (9 months P = 0 center dot 01), and on emotional distress in persons with a shorter diabetes duration (< 7 years) (9 months P = 0 center dot 04). A significant trend over time for haemoglobin A1c was found in favour of the intervention, with a statistically significant difference between groups after 9 months (P = 0 center dot 02).

Conclusion.

The nurse-administered intervention had limited effects on diabetes-specific quality of life. As only certain subgroups benefited, ways of increasing effectiveness in other groups should be explored. The potentially beneficial effect on glycaemic control is encouraging and needs further research because of small numbers in the analysis.

C1 [Lamers, Femke; Jonkers, Catharina C. M.; Bosma, Hans; van Eijk, Jacques Th M.] Maastricht Univ, Sch Publ Hlth & Primary Care CAPHRI, Dept Social Med, Maastricht, Netherlands.

[Knottnerus, J. Andre] Maastricht Univ, Sch Publ Hlth & Primary Care CAPHRI, Dept Gen Practice, Maastricht, Netherlands.

RP Lamers, F (reprint author), Maastricht Univ, Sch Publ Hlth & Primary Care CAPHRI, Dept Social Med, Maastricht, Netherlands.

EM lamersf@mail.nih.gov

RI Knottnerus, Johannes/A-3829-2009; Lamers, Femke/G-5161-2012; Albertin,

Paloma/X-1093-2018; Lamers, F/M-9742-2018; Bosma, Hans/A-6184-2013;

Knottnerus, Andre/F-4866-2013

OI Lamers, Femke/0000-0003-4344-5766

FU Netherlands Organisation for Health Research and Development (ZonMw)

[945-03-047]

FX This study was funded by the Netherlands Organisation for Health

Research and Development (ZonMw) programme on Health Care Efficiency

Research (grant number 945-03-047).

CR Ali S, 2006, DIABETIC MED, V23, P1165, DOI 10.1111/j.1464-5491.2006.01943.x

Arbuckle RA, 2009, VALUE HEALTH, V12, P1168, DOI 10.1111/j.1524-4733.2009.00571.x

Arean PA, 2007, PSYCHIAT SERV, V58, P1057, DOI 10.1176/appi.ps.58.8.1057

Ciechanowski PS, 2000, ARCH INTERN MED, V160, P3278, DOI 10.1001/archinte.160.21.3278

Cole MG, 1999, AM J PSYCHIAT, V156, P1182

de Groot M, 2001, PSYCHOSOM MED, V63, P619, DOI 10.1097/00006842-200107000-00015

de Rekeneire N, 2003, DIABETES CARE, V26, P1986, DOI 10.2337/diacare.26.7.1986

Eigenmann CA, 2009, DIABETIC MED, V26, P425, DOI 10.1111/j.1464-5491.2009.02697.x

Gonzalez JS, 2007, DIABETES CARE, V30, P2222, DOI 10.2337/dc07-0158

GROOTENHUIS PA, 1994, DIABETIC MED, V11, P253, DOI 10.1111/j.1464-5491.1994.tb00268.x

HAMILTON M, 1960, J NEUROL NEUROSUR PS, V23, P56, DOI 10.1136/jnnp.23.1.56

Jonkers C, 2007, PATIENT EDUC COUNS, V68, P252, DOI 10.1016/j.pec.2007.06.010

Katon W, 2007, GEN HOSP PSYCHIAT, V29, P147, DOI 10.1016/j.genhosppsych.2006.11.005

Koro CE, 2004, DIABETES CARE, V27, P17, DOI 10.2337/diacare.27.1.17

Lamers F, 2006, BMC PUBLIC HEALTH, V6, DOI 10.1186/1471-2458-6-161

Lamers F, 2010, PSYCHOTHER PSYCHOSOM, V79, P217, DOI 10.1159/000313690

Larranaga I, 2005, DIABETIC MED, V22, P1047, DOI 10.1111/j.1464-5491.2005.01598.x

Lustman PJ, 1997, PSYCHOSOM MED, V59, P241, DOI 10.1097/00006842-199705000-00007

Lustman PJ, 2005, J DIABETES COMPLICAT, V19, P113, DOI 10.1016/j.jdiacomp.2004.01.002

Lustman PJ, 2000, DIABETES CARE, V23, P618, DOI 10.2337/diacare.23.5.618

Mathers CD, 2006, PLOS MED, V3, DOI 10.1371/journal.pmed.0030442

Paile-Hyvarinen M, 2007, BMC FAM PRACT, V8, DOI 10.1186/1471-2296-8-34

Richardson LK, 2008, GEN HOSP PSYCHIAT, V30, P509, DOI 10.1016/j.genhosppsych.2008.07.001

RUTTEN GEH, 2006, HUISARTS WET, V49, P137

Sheehan DV, 1997, EUR PSYCHIAT, V12, P232, DOI 10.1016/S0924-9338(97)83297-X

Snoek FJ, 2000, DIABETES CARE, V23, P1305, DOI 10.2337/diacare.23.9.1305

Spitzer RL, 1999, JAMA-J AM MED ASSOC, V282, P1737, DOI 10.1001/jama.282.18.1737

van Eijk JTM, 2004, PATIENT EDUC COUNS, V54, P87, DOI 10.1016/s0738-3991(03)00201-5

Wang MY, 2008, J CLIN NURS, V17, P2524, DOI 10.1111/j.1365-2702.2008.02301.x

Ware JE, 1993, SF 36 HLTH SURVEY MA

Welch G, 2003, DIABETIC MED, V20, P69, DOI 10.1046/j.1464-5491.2003.00832.x

Welch GW, 1997, DIABETES CARE, V20, P760, DOI 10.2337/diacare.20.5.760

Williams JW, 2004, ANN INTERN MED, V140, P1015, DOI 10.7326/0003-4819-140-12-200406150-00012

NR 33

TC 25

Z9 25

U1 0

U2 10

PU WILEY-BLACKWELL

PI HOBOKEN

PA 111 RIVER ST, HOBOKEN 07030-5774, NJ USA

SN 0309-2402

J9 J ADV NURS

JI J. Adv. Nurs.

PD APR

PY 2011

VL 67

IS 4

BP 788

EP 799

DI 10.1111/j.1365-2648.2010.05540.x

PG 12

WC Nursing

SC Nursing

GA 736OA

UT WOS:000288502800011

PM 21226754

DA 2019-01-08

ER

PT J

AU Pouwer, F

Tack, CJ

Geelhoed-Duijvestijn, PHLM

Bazelmans, E

Beekman, AT

Heine, RJ

Snoek, FJ

AF Pouwer, F.

Tack, C. J.

Geelhoed-Duijvestijn, P. H. L. M.

Bazelmans, E.

Beekman, A. T.

Heine, R. J.

Snoek, F. J.

TI Limited effect of screening for depression with written feedback in

outpatients with diabetes mellitus: a randomised controlled trial

SO DIABETOLOGIA

LA English

DT Article

DE Depression; Diabetes; Randomised controlled trial; Screening

ID CO-MORBID DEPRESSION; INTERNATIONAL DIAGNOSTIC INTERVIEW;

CORONARY-HEART-DISEASE; PRIMARY-CARE; EMOTIONAL DISTRESS; GLYCEMIC

CONTROL; PROBLEM AREAS; METAANALYSIS; PREVALENCE; VALIDITY

AB The aim of this study was to test the effectiveness of a screening procedure for depression (SCR) vs care as usual (CAU) in outpatients with diabetes. The primary outcome measured was depression score and the secondary outcomes were mental healthcare consumption, diabetes-distress and HbA(1c).

In a multicentre parallel randomised controlled trial, 223 outpatients with diabetes, who had an elevated depression score, were randomly assigned to SCR (n = 116) or CAU (n = 107), using computer generated numbers. SCR-patients were invited for a Composite International Diagnostic Interview (CIDI) to diagnose depression and/or anxiety (interviewers were not blinded for group assignment). As part of the intervention, patients and their physicians were informed of the outcome of the CIDI in a letter and provided with treatment advice. At baseline and 6 month follow-up, depression and diabetes-distress were measured using the Centre for Epidemiologic Studies Depression Scale (CES-D) and the Problem Areas in Diabetes survey (PAID). HbA(1c) levels were obtained from medical charts.

Mean CES-D depression scores decreased from baseline to 6 months in both groups (24 +/- 8 to 21 +/- 8 [CAU] and 26 +/- 7 to 22 +/- 10 [SCR] respectively [p < 0.001]), with no significant differences between groups. Neither diabetes-distress nor HbA(1c) changed significantly within and between groups. The percentage of patients receiving mental healthcare increased in the SCR group from 20% to 28%, compared with 15% to 18% in the CAU group.

Depression screening with written feedback to patient and physician does not improve depression scores and has a limited impact on mental healthcare utilisation, compared with CAU. It appears that more intensive depression management is required to improve depression outcomes in patients with diabetes.

C1 [Pouwer, F.] Tilburg Univ, Dept Med Psychol & Neuropsychol, Ctr Res Psychol Somat Dis CoRPS, NL-5000 LE Tilburg, Netherlands.

[Pouwer, F.; Snoek, F. J.] Vrije Univ Amsterdam Med Ctr, EMGO Inst, Dept Med Psychol, Diabet Psychol Res Grp, Amsterdam, Netherlands.

[Tack, C. J.] Radboud Univ Nijmegen, Med Ctr, Dept Internal Med, NL-6525 ED Nijmegen, Netherlands.

[Geelhoed-Duijvestijn, P. H. L. M.] Haaglanden Med Ctr, Dept Internal Med, The Hague, Netherlands.

[Bazelmans, E.] Radboud Univ Nijmegen, Med Ctr, Dept Med Psychol, NL-6525 ED Nijmegen, Netherlands.

[Beekman, A. T.] Vrije Univ Amsterdam Med Ctr, EMGO Inst, Dept Psychiat, Amsterdam, Netherlands.

[Heine, R. J.] Vrije Univ Amsterdam Med Ctr, Ctr Diabet, Dept Endocrinol, Amsterdam, Netherlands.

RP Pouwer, F (reprint author), Tilburg Univ, Dept Med Psychol & Neuropsychol, Ctr Res Psychol Somat Dis CoRPS, POB 90153, NL-5000 LE Tilburg, Netherlands.

EM f.pouwer@uvt.nl

RI Tack, Cees/A-2368-2014

FU Dutch Diabetes Research Foundation (Diabetesfonds) [2000.00.018]

FX Dutch Diabetes Research Foundation (Diabetesfonds), 2000.00.018.

CR Ali S, 2006, DIABETIC MED, V23, P1165, DOI 10.1111/j.1464-5491.2006.01943.x

*AM DIAB ASS, 2009, DIABETES CARE S1, V32, pS13, DOI DOI 10.2337/DC09-S013

American Psychiatric Association, 1994, DIAGN STAT MAN MENT

Andrews G, 1998, SOC PSYCH PSYCH EPID, V33, P80, DOI 10.1007/s001270050026

BAKKER K, 2004, PRACT DIAB INT, V21, P88

Barnard KD, 2006, DIABETIC MED, V23, P445, DOI 10.1111/j.1464-5491.2006.01814.x

Beekman ATF, 1997, PSYCHOL MED, V27, P231, DOI 10.1017/S0033291796003510

Bot M, 2010, DIABETIC MED, V27, P1295, DOI 10.1111/j.1464-5491.2010.03119.x

Dantzer R, 2008, NAT REV NEUROSCI, V9, P46, DOI 10.1038/nrn2297

de Groot M, 2001, PSYCHOSOM MED, V63, P619, DOI 10.1097/00006842-200107000-00015

DOWRICK C, 1995, BMJ-BRIT MED J, V311, P1274, DOI 10.1136/bmj.311.7015.1274

Egede LE, 2005, DIABETES CARE, V28, P1339, DOI 10.2337/diacare.28.6.1339

Gilbody S, 2008, CAN MED ASSOC J, V178, P997, DOI 10.1503/cmaj.070281

International Diabetes Federation, 2005, GLOB GUID TYP 2 DIAB

Ismail K, 2007, DIABETES CARE, V30, P1473, DOI 10.2337/dc06-2313

Katon W, 2010, CONTEMP CLIN TRIALS, V31, P312, DOI 10.1016/j.cct.2010.03.009

Katon WJ, 2004, ARCH GEN PSYCHIAT, V61, P1042, DOI 10.1001/archpsyc.61.10.1042

Lewis G, 1996, FAM PRACT, V13, P120, DOI 10.1093/fampra/13.2.120

Lustman PJ, 2000, DIABETES CARE, V23, P934, DOI 10.2337/diacare.23.7.934

Nicholson TRJ, 2009, DIABETIC MED, V26, P447, DOI 10.1111/j.1464-5491.2009.02701.x

Nouwen A, 2010, DIABETOLOGIA, V53, P2480, DOI 10.1007/s00125-010-1874-x

Penninx BWJH, 2007, AM J GERIAT PSYCHIAT, V15, P522, DOI 10.1097/JGP.0b013e318033ed80

Petrak F, 2010, BMC GERIATR, V10, DOI 10.1186/1471-2318-10-21

Peyrot M, 2006, DIABETES CARE, V29, P1256, DOI 10.2337/dc05-2444

Pickup JC, 2004, DIABETES CARE, V27, P813, DOI 10.2337/diacare.27.3.813

Pouwer F, 2003, DIABETOLOGIA, V46, P892, DOI 10.1007/s00125-003-1124-6

Pouwer F, 2006, PATIENT EDUC COUNS, V60, P235, DOI 10.1016/j.pec.2005.01.009

Pouwer F, 2010, DIABETIC MED, V27, P217, DOI 10.1111/j.1464-5491.2009.02903.x

Pouwer F, 2001, DIABETES CARE, V24, P1929, DOI 10.2337/diacare.24.11.1929

Pouwer F, 2009, NAT REV ENDOCRINOL, V5, P665, DOI 10.1038/nrendo.2009.214

RADLOFF L S, 1977, Applied Psychological Measurement, V1, P385, DOI 10.1177/014662167700100306

Schram MT, 2009, CURR DIABETES REV, V5, P112, DOI 10.2174/157339909788166828

Simon GE, 2007, ARCH GEN PSYCHIAT, V64, P65, DOI 10.1001/archpsyc.64.1.65

Snoek FJ, 2000, DIABETES CARE, V23, P1305, DOI 10.2337/diacare.23.9.1305

Thombs BD, 2008, JAMA-J AM MED ASSOC, V300, P2161, DOI 10.1001/jama.2008.667

Tylee A, 2007, J CLIN PSYCHIAT, V68, P27

van Bastelaar KMP, 2010, DIABETIC MED, V27, P798, DOI 10.1111/j.1464-5491.2010.03025.x

van Bastelaar KM, 2008, BMC PSYCHIATRY, V8, DOI 10.1186/1471-244X-8-9

Van der Feltz-Cornelis CM, 2010, GEN HOSP PSYCHIAT, V32, P380, DOI 10.1016/j.genhosppsych.2010.03.011

Welch GW, 1997, DIABETES CARE, V20, P760, DOI 10.2337/diacare.20.5.760

Whooley MA, 2000, J GEN INTERN MED, V15, P293, DOI 10.1046/j.1525-1497.2000.04319.x

WITTCHEN HU, 1994, J PSYCHIAT RES, V28, P57, DOI 10.1016/0022-3956(94)90036-1

NR 42

TC 29

Z9 29

U1 0

U2 4

PU SPRINGER

PI NEW YORK

PA 233 SPRING ST, NEW YORK, NY 10013 USA

SN 0012-186X

J9 DIABETOLOGIA

JI Diabetologia

PD APR

PY 2011

VL 54

IS 4

BP 741

EP 748

DI 10.1007/s00125-010-2033-0

PG 8

WC Endocrinology & Metabolism

SC Endocrinology & Metabolism

GA 732VG

UT WOS:000288217200005

PM 21221528

OA Green Published, Other Gold

DA 2019-01-08

ER

PT J

AU Stankovic, Z

Jasovic-Gasic, M

Zamaklar, M

AF Stankovic, Zana

Jasovic-Gasic, Miroslava

Zamaklar, Miroslava

TI PSYCHO-SOCIAL AND CLINICAL VARIABLES ASSOCIATED WITH DEPRESSION IN

PATIENTS WITH TYPE 2 DIABETES

SO PSYCHIATRIA DANUBINA

LA English

DT Article

DE depression; type 2 diabetes; comorbidity; risk factors; neuropathy;

diabetes related distress

ID QUALITY-OF-LIFE; GLYCEMIC CONTROL; METABOLIC SYNDROME; EUROPEAN

DEPRESSION; COMORBID DEPRESSION; RESEARCH CONSORTIUM; RISK-FACTOR;

SYMPTOMS; PREVALENCE; DISORDERS

AB Background: Type 2 diabetes (T2DM,) doubles the odds of comorbid depression. Depression is a strong predictor of developing T2DM The aim of the study was to compare depressed patients with T2DM to non-depressed ones with respect to demographic, psycho-social, clinical, anthropometric and metabolic characteristics; to examine the relationship between glycemic control and depression severity in depressed patients; to estimate the risk factors of depression.

Subjects and Methods: A group of depressed diabetic patients comprising those with a Major depressive episode, first or repeated (ICD-10; 1992) and endocrinologist-diagnosed T2DM, duration >= 5 years on oral, insulin therapy or both (N=46) and non-depressed ones (N=44) (90 in total) of both genders (< 65 years) were included in this cross-sectional study. Laboratory and non-laboratory measures were performed.. The patient Health Questionnaire (PHQ-9) and a structured interview (MINI) were used to establish diagnosis, while the Beck Depression Inventory (BDI; cut off >= 16) was used to assess the severity of depression. Scaling of Life Events (SLE) for self-assessment of life events and Problem in Areas in Diabetes (PAID) for self-assessment of diabetes distress were also performed.

Results: Statistically significant higher rates of psychiatric heredity, neuropathy, higher level of diabetes related distress and a greater number of life events in depressed patients compared to non-depressed ones were found There was a statistically significant positive correlation between BDI somatic subscore and the HbAlc level (r=0.343; p=0.020). The level of diabetes related distress (OR=1.084; p=0.000), total number of life events (OR=4.528; p=0.001) and neuropathy (OR=8.699; p=0.039) were statistically significant predictors of depression using logistic regression.

Conclusions: The results obtained showed that depression in diabetic patients was predicted by both psychological (diabetes related distress, life events) and disease-specific variables (neuropathy). The severity of self-reported somatic depressive symptoms significantly correlated with the HbAlc level in depressed diabetic patients.

C1 [Stankovic, Zana; Jasovic-Gasic, Miroslava] Univ Belgrade, Psychiat Clin, Clin Ctr Serbia, Belgrade 11000, Serbia.

[Zamaklar, Miroslava] Univ Belgrade, Inst Endocrinol Diabet & Dis Metab, Belgrade 11000, Serbia.

[Jasovic-Gasic, Miroslava; Zamaklar, Miroslava] Univ Belgrade, Sch Med, Belgrade 11000, Serbia.

RP Stankovic, Z (reprint author), Univ Belgrade, Psychiat Clin, Clin Ctr Serbia, Pasterova 2, Belgrade 11000, Serbia.

EM stankovic.zana@gmail.com

CR Abrahamian H, 2009, NEUROPSYCH DIS TREAT, V5, P261

Agbir TM, 2010, ANN AFR MED, V9, P5, DOI 10.4103/1596-3519.62617

Aikens JE, 2008, DIABETIC MED, V25, P1324, DOI 10.1111/j.1464-5491.2008.02590.x

Ali S, 2006, DIABETIC MED, V23, P1165, DOI 10.1111/j.1464-5491.2006.01943.x

Almawi W, 2008, J ENDOCRINOL INVEST, V31, P1020, DOI 10.1007/BF03345642

ALONSO J, 2004, ACTA PSYCHIAT SCAN S, V420, P21

*AM DIAB ASS, 2003, DIABETES CARE, V1, P94

Anderson RJ, 2001, DIABETES CARE, V24, P1069, DOI 10.2337/diacare.24.6.1069

[Anonymous], 1992, INT STAT CLASS DIS R

BECK AT, 1961, ARCH GEN PSYCHIAT, V4, P561

BHALLA RN, 2010, DEPRESSION

Boulton AJM, 2004, DIABETES CARE, V27, P1458, DOI 10.2337/diacare.27.6.1458

Brown LC, 2005, DIABETES CARE, V28, P1063, DOI 10.2337/diacare.28.5.1063

BROWN LC, 2006, CMAJ, V175

Collins MM, 2009, DIABETIC MED, V26, P153, DOI 10.1111/j.1464-5491.2008.02648.x

Cosgrove MP, 2008, OCCUP MED-OXFORD, V58, P7, DOI 10.1093/occmed/kqm105

Daly JM, 2009, J AM BOARD FAM MED, V22, P280, DOI 10.3122/jabfm.2009.03.080114

de Groot M, 2001, PSYCHOSOM MED, V63, P619, DOI 10.1097/00006842-200107000-00015

Despres JP, 2008, ARTERIOSCL THROM VAS, V28, P1039, DOI 10.1161/ATVBAHA.107.159228

Egede LE, 2005, PSYCHOSOM MED, V67, P46, DOI 10.1097/01.psy.0000149260.82006.fb

Egede LE, 2010, DIABETES TECHNOL THE, V12, P257, DOI 10.1089/dia.2009.0143

*EXP COMM DIAGN CL, 2003, DIABETES CARE, V26, P5, DOI DOI 10.2337/DIACARE.26.2007.S5

Filipcic I, 2010, PSYCHIAT DANUB, V22, P231

Fisher L, 2008, DIABETIC MED, V25, P1096, DOI 10.1111/j.1464-5491.2008.02533.x

Fisher L, 2001, DIABETES CARE, V24, P1751, DOI 10.2337/diacare.24.10.1751

Fisher L, 2009, DIABETES CARE, V33, P23

Fisher L, 2007, DIABETES CARE, V30, P542, DOI 10.2337/dc06-1614

FOLSTEIN MF, 1975, J PSYCHIAT RES, V12, P189, DOI 10.1016/0022-3956(75)90026-6

Georgiades A, 2007, PSYCHOSOM MED, V69, P235, DOI 10.1097/PSY.0b013e318042588d

Golden SH, 2008, JAMA-J AM MED ASSOC, V299, P2751, DOI 10.1001/jama.299.23.2751

Gonzalez JS, 2008, DIABETOLOGIA, V51, P1822, DOI 10.1007/s00125-008-1113-x

Halbreich U, 2007, J AFFECT DISORDERS, V102, P159, DOI 10.1016/j.jad.2006.09.033

Heckbert SR, 2010, J GEN INTERN MED, V25, P524, DOI 10.1007/s11606-010-1272-6

INGRAM RE, 1984, COGNITIVE THER RES, V8, P443, DOI 10.1007/BF01173284

Jakovjevic M, 2007, PSYCHIAT DANUB, V19, P76

Katon WJ, 2004, J GEN INTERN MED, V19, P1192, DOI 10.1111/j.1525-1497.2004.30405.x

Kawakami N, 1999, DIABETES CARE, V22, P1071, DOI 10.2337/diacare.22.7.1071

Knol MJ, 2006, DIABETOLOGIA, V49, P837, DOI 10.1007/s00125-006-0159-x

Kokoszka A, 2009, EUR PSYCHIAT, V24, P425, DOI 10.1016/j.eurpsy.2009.04.002

Koponen H, 2008, J CLIN PSYCHIAT, V69, P178, DOI 10.4088/JCP.v69n0202

Lecrubier Y, 1997, EUR PSYCHIAT, V12, P224, DOI 10.1016/S0924-9338(97)83296-8

Lee HJ, 2009, J AM ACAD NURSE PRAC, V21, P214, DOI 10.1111/j.1745-7599.2009.00396.x

Lin EHB, 2010, DIABETES CARE, V33, P264, DOI 10.2337/dc09-1068

Lustman PJ, 2007, DIABETES CARE, V30, P459, DOI 10.2337/dc06-1769

Lustman PJ, 1997, PSYCHOSOM MED, V59, P241, DOI 10.1097/00006842-199705000-00007

Lustman PJ, 2005, J DIABETES COMPLICAT, V19, P113, DOI 10.1016/j.jdiacomp.2004.01.002

Lustman PJ, 2000, DIABETES CARE, V23, P934, DOI 10.2337/diacare.23.7.934

LUSTMAN PJ, 2004, DIABETES SPECTRUM, V17, P160, DOI DOI 10.2337/DIASPECT.17.3.160

MCINTYRE SR, 2006, EXPERT OPINION DRUG, V5, P157

Mezuk B, 2008, AM J PUBLIC HEALTH, V98, P1480, DOI 10.2105/AJPH.2007.126441

Miyaoka Y, 1997, PSYCHIAT CLIN NEUROS, V51, P203, DOI 10.1111/j.1440-1819.1997.tb02583.x

Moreira RO, 2007, BRAZ J MED BIOL RES, V40, P269, DOI 10.1590/S0100-879X2006005000051

Musselman DL, 2003, BIOL PSYCHIAT, V54, P317, DOI 10.1016/S0006-3223(03)00569-9

*NIH, 2001, NIH PUBL

Norhammar A, 2004, J AM COLL CARDIOL, V43, P585, DOI 10.1016/j.jacc.2003.08.050

Paola LD, 2006, CURR DIABETES REV, V2, P431, DOI 10.2174/1573399810602040431

PAYKEL SE, 1971, ARCH GEN PSICHIATRY, V25, P340

Pibernik-Okanovic M, 2008, J DIABETES COMPLICAT, V22, P246, DOI 10.1016/j.jdiacomp.2007.03.002

POLONSKY WH, 1995, DIABETES CARE, V18, P754, DOI 10.2337/diacare.18.6.754

Pouwer F, 2005, DIABETES RES CLIN PR, V70, P166, DOI 10.1016/j.diabres.2005.03.031

Pouwer F, 2001, DIABETIC MED, V18, P595, DOI 10.1046/j.1464-5491.2001.00499.x

Raikkonen K, 2007, DIABETES CARE, V30, P872, DOI 10.2337/dc06-1857

Spitzer RL, 1999, JAMA-J AM MED ASSOC, V282, P1737, DOI 10.1001/jama.282.18.1737

Ustun TB, 2004, BRIT J PSYCHIAT, V184, P386, DOI 10.1192/bjp.184.5.386

van Bastelaar KMP, 2010, DIABETIC MED, V27, P798, DOI 10.1111/j.1464-5491.2010.03025.x

Van Tilburg MAL, 2001, PSYCHOSOM MED, V63, P551, DOI 10.1097/00006842-200107000-00005

Wild S, 2004, DIABETES CARE, V27, P1047, DOI 10.2337/diacare.27.5.1047

World Health Organization, 2001, WORLD HLTH REP 2001

Yoshida S, 2008, PSYCHIAT CLIN NEUROS, V63, P65

ZIMMERMAN M, 1986, J HUMAN STRESS, V4, P13

NR 70

TC 8

Z9 8

U1 1

U2 8

PU MEDICINSKA NAKLADA

PI ZAGREB

PA VLASKA 69, HR-10000 ZAGREB, CROATIA

SN 0353-5053

J9 PSYCHIAT DANUB

JI Psychiatr. Danub.

PD MAR

PY 2011

VL 23

IS 1

BP 34

EP 44

PG 11

WC Psychiatry

SC Psychiatry

GA 752OT

UT WOS:000289702700005

PM 21448095

DA 2019-01-08

ER

PT J

AU Grahovac, T

Ruzic, K

Dadic-Hero, E

Sepic-Grahovac, D

Sabljic, V

AF Grahovac, Tanja

Ruzic, Klementina

Dadic-Hero, Elizabeta

Sepic-Grahovac, Dubravka

Sabljic, Vladimir

TI WEIGHT GAIN - AS POSSIBLE PREDICTOR OF METABOLIC SYNDROME

SO PSYCHIATRIA DANUBINA

LA English

DT Article

DE metabolic syndrome; quetiapine; weight gain

ID QUETIAPINE; SCHIZOPHRENIA; DISORDER; MANIA

AB Rapid weight gain among patients with mental disorders can further compound psychological distress and negatively influence compliance. Weight gain associated with treatment with atypical ant:psychotic medication has been widely recognized as a risk factor for the development of diabetes type II and cardiovascular diseases.

This paper describes a 33-year old female patient treated for schizoaffective disorder. Within two months after introducing quetiapine the patient experienced considerable weight gain amounting to 19 kg. The replacement of antipsychotic during inpatient psychiatric care resulted in weight loss.

C1 [Grahovac, Tanja; Ruzic, Klementina; Sabljic, Vladimir] Univ Psychiat Clin Rijeka, Clin Hosp Ctr Rijeka, Rijeka 51000, Croatia.

[Dadic-Hero, Elizabeta] Sch Med, Dept Social Med & Epidemiol, Rijeka, Croatia.

[Sepic-Grahovac, Dubravka] Policlin Neurol & Psychiat Interneuron, Rijeka, Croatia.

RP Grahovac, T (reprint author), Univ Psychiat Clin Rijeka, Clin Hosp Ctr Rijeka, Cambierieva 17-7, Rijeka 51000, Croatia.

EM tanja.grahovac@ri.t-com.hr

OI Grahovac Juretic, Tanja/0000-0002-8508-6688

CR Anderson IM, 2009, J AFFECT DISORDERS, V117, P116, DOI 10.1016/j.jad.2008.12.016

Brecher M, 2007, J CLIN PSYCHIAT, V68, P597, DOI 10.4088/JCP.v68n0416

Bushe C, 2010, J PSYCHOPHARMACOL, V24, P1001, DOI 10.1177/0269881108101783

Cates ME, 2009, COMMUNITY MENT HLT J, V45, P251, DOI 10.1007/s10597-009-9200-0

Correll CU, 2007, SCHIZOPHR RES, V89, P91, DOI 10.1016/j.schres.2006.08.017

Dadic-Hero E, 2010, PSYCHIAT DANUB, V22, P381

FILAKOVIC P, 2003, PSYCHIAT DANUBINA S1, V15, pS104

FOLNEGOVICSMALC V, 2009, MEDIX, V80, P212

Grundy SM, 2004, CIRCULATION, V109, P433, DOI 10.1161/01.CIR.0000111245.75752.C6

HOTUJAC LJ, 2002, MEDICUS, V11, P207

Kozumplik O, 2010, PSYCHIAT DANUB, V22, P69

Meyer JM, 2001, J CLIN PSYCHOPHARM, V21, P369, DOI 10.1097/00004714-200108000-00003

Newcomer JW, 2007, AM J MANAG CARE, V13, pS170

Sajatovic M, 2008, BIPOLAR DISORD, V10, P662, DOI 10.1111/j.1399-5618.2008.00614.x

Stahl SM, 2008, ESSENTIAL PSYCHOPHAR

UZUN S, 2005, NUSPOJAVE PSIHOFARMA, P19

Vieta E, 2005, CURR MED RES OPIN, V21, P923, DOI 10.1185/030079905X46340

NR 17

TC 2

Z9 2

U1 0

U2 1

PU MEDICINSKA NAKLADA

PI ZAGREB

PA VLASKA 69, HR-10000 ZAGREB, CROATIA

SN 0353-5053

J9 PSYCHIAT DANUB

JI Psychiatr. Danub.

PD MAR

PY 2011

VL 23

IS 1

BP 105

EP 107

PG 3

WC Psychiatry

SC Psychiatry

GA 752OT

UT WOS:000289702700020

PM 21448110

DA 2019-01-08

ER

PT J

AU Hilliard, ME

Monaghan, M

Cogen, FR

Streisand, R

AF Hilliard, M. E.

Monaghan, M.

Cogen, F. R.

Streisand, R.

TI Parent stress and child behaviour among young children with type 1

diabetes

SO CHILD CARE HEALTH AND DEVELOPMENT

LA English

DT Article

DE behaviour; diabetes; parenting stress

ID PEDIATRIC PSYCHOLOGY; MATERNAL PERCEPTIONS; ADJUSTMENT; MOTHERS;

PRESCHOOLERS; DISCIPLINE; INVENTORY; DISTRESS; MELLITUS; YOUTH

AB Background

Parents of young children with type 1 diabetes (T1D) are responsible for executing a complex daily management regimen and are at risk for elevated levels of stress. Normative misbehaviour during the preschool years can complicate T1D management, and interpretation of behavioural concerns may vary because of child health status and parent stress. Within a paediatric transactional model framework, child characteristics (e.g. behaviour problems, metabolic control) and parent functioning (e.g. parenting stress, anxiety) likely impact one another.

Methods

Parents of 2- to 6-year-old children with T1D completed self-report measures, including the Pediatric Inventory for Parents (PIP), State-Trait Anxiety Inventory (STAI), Eyberg Child Behavior Inventory (ECBI), and 24-h Recall Interviews. Medical data were obtained by parent report and medical record review. It was hypothesized that greater parent stress and child blood glucose variability would be significantly associated with greater parent-reported child behaviour concerns.

Results

Moderate levels of parent stress and child behaviour problems were endorsed; however, parents perceived children's misbehaviour as problematic, particularly with relation to tasks relevant to diabetes management (e.g. bedtimes and mealtimes). Structural equation modelling indicated that greater general anxiety and paediatric parenting stress was associated with parent report of more problematic child behaviour. Blood glucose variability did not significantly contribute to this relationship.

Conclusions

The stress experienced by parents of young children with chronic illness appears to relate to their perception of their children's behaviour problems. Parents' experiences with developmentally normative misbehaviour may interfere with disease management and exacerbate parents' stress and the subsequent impact on well-being. Implications for supporting parents and children with T1D are discussed.

C1 [Hilliard, M. E.; Monaghan, M.; Streisand, R.] Childrens Natl Med Ctr, Dept Psychol, Washington, DC 20010 USA.

[Cogen, F. R.] Childrens Natl Med Ctr, Dept Endocrinol & Diabet, Washington, DC 20010 USA.

[Cogen, F. R.] George Washington Univ, Sch Med & Hlth Sci, Dept Pediat, Washington, DC 20052 USA.

[Streisand, R.] George Washington Univ, Sch Med & Hlth Sci, Dept Psychiat & Pediat, Washington, DC 20052 USA.

RP Streisand, R (reprint author), Childrens Natl Med Ctr, Dept Psychol, 111 Michigan Ave NW, Washington, DC 20010 USA.

EM rstreis@cnmc.org

OI Hilliard, Marisa/0000-0002-8813-629X

CR Abdul-Rasoul M, 2006, PEDIATR DIABETES, V7, P101, DOI 10.1111/j.1399-543X.2006.00155.x

Alderfer MA, 2008, J PEDIATR PSYCHOL, V33, P1046, DOI 10.1093/jpepsy/jsm083

Barry TD, 2009, CHILD FAM BEHAV THER, V31, P1, DOI 10.1080/07317100802701186

Barry TD, 2005, J AM ACAD CHILD PSY, V44, P265, DOI 10.1097/00004583-200503000-00011

Centers for Disease Control and Prevention, 2005, NAT DIAB FACT SHEET

Colvin A., 1999, RESTANDARDIZATION EY

Dabelea D, 2007, JAMA-J AM MED ASSOC, V297, P2716

EURODIAB ACE Study Group, 2000, LANCET, V355, P873

FIESE BH, 1989, J PEDIATR PSYCHOL, V14, P293, DOI 10.1093/jpepsy/14.2.293

FREUND A, 1991, RELATIONSHIP ADHEREN

Holmes CS, 2006, J PEDIATR PSYCHOL, V31, P770, DOI 10.1093/jpepsy/jsj083

Jaser SS, 2009, CHILD HEALTH CARE, V38, P91, DOI 10.1080/02739610902813229

Kline R. B., 2005, PRINCIPLES PRACTICE

KRECH KH, 1992, J CLIN CHILD PSYCHOL, V21, P115, DOI 10.1207/s15374424jccp2102_3

Lewin A. B., 2005, FAMILIES SYSTEMS HLT, V23, P56, DOI DOI 10.1037/1091-7527.23.1.56

LORENZI M, 1985, DIABETOLOGIA, V28, P734

MacCallum RC, 2000, ANNU REV PSYCHOL, V51, P201, DOI 10.1146/annurev.psych.51.1.201

MACCOBY EE, 1983, SOCIALIZATION CONTEX, V4

McDonnell CM, 2007, DIABETES CARE, V30, P2211, DOI 10.2337/dc07-0328

MIDDLEBROOK JL, 1985, BEHAV THER, V16, P494, DOI 10.1016/S0005-7894(85)80027-7

Mitchell SJ, 2009, FAM SYST HEALTH, V27, P314, DOI 10.1037/a0018191

Monaghan MC, 2009, FAM SYST HEALTH, V27, P28, DOI 10.1037/a0014770

Muthen B. O., 1998, MPLUS VERSION 6

MUTHEN LK, 2006, MPLUS VERSION 4

Patton SR, 2004, PEDIATR DIABETES, V5, P190, DOI 10.1111/j.1399-543X.2004.00058.x

Powers SW, 2002, DIABETES CARE, V25, P313, DOI 10.2337/diacare.25.2.313

Quittner AL, 2008, J PEDIATR PSYCHOL, V33, P916, DOI 10.1093/jpepsy/jsm064

Renk K, 2007, CHILD FAM BEHAV THER, V29, P37, DOI 10.1300/J019v29n01_03

ROBINSON EA, 1980, J CLIN CHILD PSYCHOL, V9, P22, DOI 10.1080/15374418009532938

SHAW DS, 1993, J ABNORMAL CHILD PSY, V21, P35

Silverstein J, 2005, DIABETES CARE, V28, P186, DOI 10.2337/diacare.28.1.186

SOKOL DK, 1996, BRAIN INJURY, V10, P493

Spielberger C. D., 1983, MANUAL STATE TRAIT A

*SPSS INC, 2007, SPSS COMP PROGR VERS

Stallwood Lynda, 2005, J Pediatr Health Care, V19, P293, DOI 10.1016/j.pedhc.2005.04.003

Streisand R, 2005, J PEDIATR PSYCHOL, V30, P513, DOI 10.1093/jpepsy/jsi076

Streisand R, 2001, J PEDIATR PSYCHOL, V26, P155, DOI 10.1093/jpepsy/26.3.155

Sullivan-Bolyai S, 2003, J Pediatr Nurs, V18, P21, DOI 10.1053/jpdn.2003.4

THOMPSON KL, 1987, J PEDIATR PSYCHOL, V12, P241, DOI 10.1093/jpepsy/12.2.241

Visconti KJ, 2002, J DEV BEHAV PEDIATR, V23, P314, DOI 10.1097/00004703-200210000-00003

WACHTEL J, 1994, J PEDIATR PSYCHOL, V19, P723, DOI 10.1093/jpepsy/19.6.723

Walker LO, 2007, J SPEC PEDIATR NURS, V12, P93, DOI 10.1111/j.1744-6155.2005.00098.x

WEBSTERSTRATTON C, 1982, J CLIN CHILD PSYCHOL, V11, P123, DOI 10.1207/s15374424jccp1102_5

WILCOX RR, 1987, ANNU REV PSYCHOL, V38, P29, DOI 10.1146/annurev.psych.38.1.29

Wilson AC, 2009, J CLIN PSYCHOL MED S, V16, P216, DOI 10.1007/s10880-009-9163-x

WYSOCKI T, 1989, DIABETES CARE, V12, P524, DOI 10.2337/diacare.12.8.524

NR 46

TC 46

Z9 46

U1 2

U2 29

PU WILEY-BLACKWELL

PI MALDEN

PA COMMERCE PLACE, 350 MAIN ST, MALDEN 02148, MA USA

SN 0305-1862

J9 CHILD CARE HLTH DEV

JI Child Care Health Dev.

PD MAR

PY 2011

VL 37

IS 2

BP 224

EP 232

DI 10.1111/j.1365-2214.2010.01162.x

PG 9

WC Psychology, Developmental; Pediatrics

SC Psychology; Pediatrics

GA 720XT

UT WOS:000287316700009

PM 21083686

DA 2019-01-08

ER

PT J

AU Engel, L

Cummins, R

AF Engel, L.

Cummins, R.

TI Impact of dose adjustment for normal eating in Australia (OzDAFNE) on

subjective wellbeing, coping resources and negative affects in adults

with type 1 diabetes: A prospective comparison study

SO DIABETES RESEARCH AND CLINICAL PRACTICE

LA English

DT Article

DE Type 1 diabetes; Structured patient education; Subjective wellbeing;

Coping; Therapeutic alliance

ID QUALITY-OF-LIFE; DEPRESSION; ANXIETY; HEALTH; MODEL

AB Aim: This prospective study examined the impact of a structured education program (OzDAFNE) on subjective wellbeing, coping resources, and negative affects in adults with type 1 diabetes. Participants completing the OzDAFNE program were compared to those using continuous subcutaneous insulin (CSII) and multiple daily injections (MDI) over the same time period.

Methods: Participants in the OzDAFNE group (N = 144) were recruited from diabetes centres throughout Australia. The comparison groups were recruited from Diabetes Australia-Victoria's membership database and comprised 383 people using MDI and 64 people using CSII. All participants completed self-report questionnaires at baseline and 12-months later. Additional assessments for OzDAFNE participants were conducted at the end of the education program and at three and six-months following the training.

Results: The results demonstrated that participants completing the OzDAFNE program experienced improved subjective wellbeing (p < .01), a greater sense of mastery and control in managing their diabetes (p < .001), and reduced diabetes-related distress (p < .001) compared to the CSII and MDI groups. However, the CSII group recorded a significant drop in self-esteem (p < .001) over the duration of the study.

Conclusions/Interpretations: The OzDAFNE program provides a powerful mastery experience for participants, positively influencing subjective wellbeing and diabetes-related distress. (C) 2010 Elsevier Ireland Ltd. All rights reserved.

C1 [Engel, L.] Hlth Psychologist MOTS Consulting, Melbourne, Vic, Australia.

[Cummins, R.] Deakin Univ, Fac Hlth Med Nursing & Behav Sci, Sch Psychol, Melbourne, Vic, Australia.

RP Engel, L (reprint author), 54 Fernhill Rd, Sandringham, Vic 3191, Australia.

EM lisaengel@netspace.net.au

FU Australian Diabetes Educators Association

FX Lisa Engel received a post-graduate grant from the Australian Diabetes

Educators Association.

CR Amiel S, 2002, BRIT MED J, V325, P746

Anderson RJ, 2001, DIABETES CARE, V24, P1069, DOI 10.2337/diacare.24.6.1069

Byrne B. M., 1996, MEASURING SELF CONCE

Cummins R. A., 2002, UNIVERSALITY SUBJECT, P7, DOI DOI 10.1007/978-94-010-0271-4_3

Cummins RA, 2000, SOC INDIC RES, V52, P55, DOI 10.1023/A:1007027822521

Cummins RA, 2003, SOC INDIC RES, V64, P225, DOI 10.1023/A:1024712527648

Cummins RA, 2003, SOC INDIC RES, V64, P159, DOI 10.1023/A:1024704320683

Cummins RA, 2010, J HAPPINESS STUD, V11, P1, DOI 10.1007/s10902-009-9167-0

Engel L., 2009, PSYCHOL IMPACT DAFNE

HARVEY D, 2008, AUSTR DIAB SOC AUSTR

HORVATH AO, 1989, J COUNS PSYCHOL, V36, P223, DOI 10.1037/0022-0167.36.2.223

Lazarus R. S, 1984, STRESS APPRAISAL COP

Leventhal H, 2008, ANNU REV PSYCHOL, V59, P477, DOI 10.1146/annurev.psych.59.103006.093643

MCINTYRE D, 2008, AUSTR DIAB SOC AUSTR

Muhlhauser I, 2002, DIABETOLOGIA, V45, P1723, DOI 10.1007/s00125-002-0987-2

Nistico H., 2002, J HAPPINESS STUD, V3, P37, DOI DOI 10.1023/A:1015678915305

Peyrot M, 1997, DIABETES CARE, V20, P585, DOI 10.2337/diacare.20.4.585

Rosenberg M., 1979, CONCEIVING SELF

SCHEIER MF, 1994, J PERS SOC PSYCHOL, V67, P1063, DOI 10.1037/0022-3514.67.6.1063

SEEMAN M, 1995, SOC SCI MED, V41, P517, DOI 10.1016/0277-9536(94)00362-W

Sheldon K. M., 2006, J HAPPINESS STUD, V7, P55, DOI DOI 10.1007/S10902-005-0868-8

SNAITH P, HOSP ANXIETY DEPRESS

Speight J, 2007, DIABETIC MED, V24, P693, DOI 10.1111/j.1464-5491.2007.02201.x

Taylor SE, 2007, ANNU REV CLIN PSYCHO, V3, P377, DOI 10.1146/annurev.clinpsy.3.022806.091520

TROCHIM W, 2006, STAT ANAL NONEQUIVAL

Welch G, 2003, DIABETIC MED, V20, P69, DOI 10.1046/j.1464-5491.2003.00832.x

NR 26

TC 7

Z9 8

U1 0

U2 5

PU ELSEVIER IRELAND LTD

PI CLARE

PA ELSEVIER HOUSE, BROOKVALE PLAZA, EAST PARK SHANNON, CO, CLARE, 00000,

IRELAND

SN 0168-8227

J9 DIABETES RES CLIN PR

JI Diabetes Res. Clin. Pract.

PD MAR

PY 2011

VL 91

IS 3

BP 271

EP 279

DI 10.1016/j.diabres.2010.11.023

PG 9

WC Endocrinology & Metabolism

SC Endocrinology & Metabolism

GA 732EW

UT WOS:000288168800008

PM 21146889

DA 2019-01-08

ER

PT J

AU Yang, Y

Salam, ZHA

Ong, BC

Yang, KS

AF Yang, Yong

Salam, Zakir-Hussain Abdul

Ong, Biauw Chi

Yang, Kok Soong

TI RESPIRATORY DYSFUNCTION IN PATIENTS WITH SEPSIS: PROTECTIVE EFFECT OF

DIABETES MELLITUS

SO AMERICAN JOURNAL OF CRITICAL CARE

LA English

DT Article

ID ACUTE LUNG INJURY; DISTRESS-SYNDROME; POLYMORPHONUCLEAR LEUKOCYTES;

UNITED-STATES; SEPTIC SHOCK; RISK-FACTORS; EPIDEMIOLOGY; NEUTROPHILS;

METABOLISM; INSULIN

AB Background Sepsis is a common complication in patients with diabetes mellitus. In a Western population, risk of respiratory dysfunction was lower in diabetic patients with sepsis.

Objective To compare organ dysfunction, particularly respiratory dysfunction, between sepsis patients with and without diabetes mellitus in an Asian population.

Method Hospital discharge data were collected for the period 2004 through 2008. Patients with sepsis, diabetes mellitus, and organ dysfunction were identified by using the International Statistical Classification of Diseases and Related Health Problems, 9th Revision, Australian Modification codes.

Results Of the 383 238 patients hospitalized during the 5 years, 2943 of the 9221 who had sepsis also had diabetes (31.9%). The most common organ dysfunctions in patients with sepsis were renal (31.5%), cardiovascular (19.2%), and respiratory (10.9%). Among patients with sepsis, respiratory dysfunction was less likely in patients with diabetes (9.4%) than in those without (11.6%; P = .002), but renal dysfunction was more likely in patients with diabetes (46.5%) than in those without (24.4%; P <.001). However, only 27.6% of patients with diabetes had a respiratory source of sepsis compared with 33.4% in patients without diabetes (P <.001). Among patients with sepsis, diabetes mellitus was a significant and independent predictor of respiratory dysfunction (odds ratio, 0.80; 95% confidence interval, 0.66-0.98) after adjustments for age, sex, ethnicity, admission to intensive care, number of comorbid conditions, and other infection sources.

Conclusion Among an Asian population, respiratory dysfunction in patients with sepsis is less likely to develop in those with diabetes than in those without diabetes. (American Journal of Critical Care. 2011;20:e41-e47)

C1 [Yang, Yong; Ong, Biauw Chi; Yang, Kok Soong] Singapore Gen Hosp, Dept Epidemiol, Singapore 169608, Singapore.

[Salam, Zakir-Hussain Abdul] Natl Univ Singapore, Singapore, Singapore.

RP Yang, Y (reprint author), Singapore Gen Hosp, Dept Epidemiol, Outram Rd, Singapore 169608, Singapore.

EM yang.yong@sgh.com.sg

CR Alba-Loureiro TC, 2007, BRAZ J MED BIOL RES, V40, P1037, DOI 10.1590/S0100-879X2006005000143

Alba-Loureiro TC, 2006, J ENDOCRINOL, V188, P295, DOI 10.1677/joe.1.06438

Alberti C, 2005, AM J RESP CRIT CARE, V171, P461, DOI 10.1164/rccm.200403-324OC

ALEXIEWICZ JM, 1995, ANN INTERN MED, V123, P919, DOI 10.7326/0003-4819-123-12-199512150-00004

ANDERSEN B, 1988, J LAB CLIN MED, V111, P275

Angus DC, 2001, CRIT CARE MED, V29, P1303, DOI 10.1097/00003246-200107000-00002

Chan JCN, 2009, JAMA-J AM MED ASSOC, V301, P2129, DOI 10.1001/jama.2009.726

Chowdhury TA, 2008, LANCET, V371, P1728, DOI 10.1016/S0140-6736(08)60738-2

Delamaire M, 1997, DIABETIC MED, V14, P29, DOI 10.1002/(SICI)1096-9136(199701)14:1<29::AID-DIA300>3.0.CO;2-V

DEYO RA, 1992, J CLIN EPIDEMIOL, V45, P613, DOI 10.1016/0895-4356(92)90133-8

Eaton S, 2002, AM J RESP CRIT CARE, V165, pA471

Ernst FR, 2006, AM J HEALTH-SYST PH, V63, P575, DOI 10.2146/ajhp050214

Esper AM, 2006, CRIT CARE MED, V34, P2576, DOI 10.1097/01.CCM.0000239114.50519.0E

Esper AM, 2009, CRIT CARE, V13, DOI 10.1186/cc7704

Esper AM, 2009, CRIT CARE, V13, DOI 10.1186/cc7717

Frank JA, 2000, CRIT CARE MED, V28, P2645, DOI 10.1097/00003246-200007000-00079

Gedeborg R, 2007, J CLIN EPIDEMIOL, V60, P155, DOI 10.1016/j.jclinepi.2006.05.013

Gong MN, 2005, CRIT CARE MED, V33, P1191, DOI 10.1097/01.CCM.0000165566.82925.14

Honiden S, 2009, CRIT CARE MED, V37, P2455, DOI 10.1097/CCM.0b013e3181a0fea5

Iscimen R, 2008, CRIT CARE MED, V36, P1518, DOI 10.1097/CCM.0b013e31816fc2c0

JUBIZ W, 1984, PROSTA LEUKOTR MED, V14, P305, DOI 10.1016/0262-1746(84)90114-8

Kornum JB, 2008, DIABETES CARE, V31, P1541, DOI 10.2337/dc08-0138

Kornum JB, 2007, DIABETES CARE, V30, P2251, DOI 10.2337/dc06-2417

Martin GS, 2003, NEW ENGL J MED, V348, P1546, DOI 10.1056/NEJMoa022139

Moss M, 2000, CRIT CARE MED, V28, P2187, DOI 10.1097/00003246-200007000-00001

Perner A, 2003, INTENS CARE MED, V29, P642, DOI 10.1007/s00134-002-1628-4

REPINE JE, 1980, J INFECT DIS, V142, P869, DOI 10.1093/infdis/142.6.869

Schoenman Julie. A, 2005, VALUE HOSP DISCHARGE

Statistics Singapore, 2012, TIM SER POP MID EST

Vanhorebeek I, 2005, LANCET, V365, P53, DOI 10.1016/S0140-6736(04)17665-4

Yang Y, 2010, J CRIT CARE, V25, P398, DOI 10.1016/j.jcrc.2009.09.001

Yilmaz M, 2007, CRIT CARE MED, V35, P1660, DOI 10.1097/01.CCM.0000269037.66955.F0

Zgibor JC, 2007, DIABETES RES CLIN PR, V75, P313, DOI 10.1016/j.diabres.2006.07.007

NR 33

TC 11

Z9 14

U1 0

U2 2

PU AMER ASSOC CRITICAL CARE NURSES

PI ALISO VIEJO

PA 101 COLUMBIA, ALISO VIEJO, CA 92656 USA

SN 1062-3264

J9 AM J CRIT CARE

JI Am. J. Crit. Care

PD MAR 1

PY 2011

VL 20

IS 2

BP E41

EP E47

DI 10.4037/ajcc2011391

PG 7

WC Critical Care Medicine; Nursing

SC General & Internal Medicine; Nursing

GA 728AO

UT WOS:000287846100002

PM 21362712

OA Bronze

DA 2019-01-08

ER

PT J

AU Storz, SM

Wylenzek, SAM

Matheis, N

Weber, MM

Kahaly, GJ

AF Storz, S. M.

Wylenzek, S. A. M.

Matheis, N.

Weber, M. M.

Kahaly, G. J.

TI Impaired psychometric testing in polyglandular autoimmunity

SO CLINICAL ENDOCRINOLOGY

LA English

DT Article

ID SUBJECTIVE HEALTH-STATUS; ADRENAL INSUFFICIENCY; HOSPITAL ANXIETY; LARGE

POPULATION; DEPRESSION SCALE; DISORDERS; REPLACEMENT; PREVALENCE;

TYPE-1; LIFE

AB P>Objective

Patients with the autoimmune polyglandular syndrome (APS) could be exposed to many limitations in daily life owing to their illness. To quantify the degree of physical and emotional distress, the psychometric profile of these patients was evaluated prospectively.

Design, patients and measurements

After a complete endocrine investigation, three international validated self-assessment questionnaires were applied in 75 patients with APS: the health-related quality of life Short-Form 36 (SF-36), the Giessen Complaint List (GBB-24) and the Hospital Anxiety and Depression Scale (HADS).

Results

Average duration of APS was 7 center dot 7 years. The most frequent disease combination was type 1 diabetes and autoimmune thyroid disease (n = 47, 62 center dot 6%). Every scale of the SF-36, GBB-24, and the HADS anxiety score demonstrated markedly impaired physical and emotional well-being, foremost in female subjects (P < 0 center dot 001). Compared to a German reference cohort, all subscales of the SF-36 were decreased (P < 0 center dot 001). Sex- and age-matched z-scores were significantly lower for physical functioning (-1 center dot 1; reference population z = 0), physical role limitations (-0 center dot 8), bodily pain (-0 center dot 7), general health perception (-1 center dot 2), vitality (-0 center dot 8), social functioning (-0 center dot 8), emotional role limitations (-1 center dot 1) and mental health (-0 center dot 5). Also, the global score of discomfort was increased in comparison with the reference population (27 center dot 27 vs 13 center dot 93, P < 0 center dot 001). Generalized anxiety (25%, P < 0 center dot 001) and depression episode (18 center dot 1%, P < 0 center dot 001) were prevalent in APS. Neither time interval between two endocrine diseases, duration of APS, age, nor autoantibody positivity influenced psychometric testing results.

Conclusion

Patients with APS have a severely impaired psychometric profile. Treatment modalities that would improve their well-being are warranted.

C1 [Storz, S. M.; Wylenzek, S. A. M.; Matheis, N.; Weber, M. M.; Kahaly, G. J.] Johannes Gutenberg Univ Mainz, Med Ctr, Dept Med 1, Endocrine Unit, D-55101 Mainz, Germany.

RP Kahaly, GJ (reprint author), Johannes Gutenberg Univ Mainz, Med Ctr, Dept Med 1, Endocrine Unit, D-55101 Mainz, Germany.

EM kahaly@ukmainz.de

CR Alonso J, 2004, ACTA PSYCHIAT SCAND, V109, P28

Bekker MHJ, 2007, GENDER MED, V4, pS178, DOI 10.1016/S1550-8579(07)80057-X

Bestor TH, 2000, HUM MOL GENET, V9, P2395, DOI 10.1093/hmg/9.16.2395

Betterle C, 2004, CLIN EXP IMMUNOL, V137, P225, DOI 10.1111/j.1365-2249.2004.02561.x

Bianchi GP, 2004, QUAL LIFE RES, V13, P45, DOI 10.1023/B:QURE.0000015315.35184.66

Bjelland I, 2002, J PSYCHOSOM RES, V52, P69, DOI 10.1016/S0022-3999(01)00296-3

Bleicken B, 2008, EUR J ENDOCRINOL, V159, P811, DOI 10.1530/EJE-08-0578

Brahler E, 2000, PSYCHOTHER PSYCH MED, V50, P14, DOI 10.1055/s-2000-13233

Brahler E., 2008, GBB 24 GIESSENER BES

Bullinger M, 1996, Rehabilitation (Stuttg), V35, pXVII

BULLINGER M, 1995, SOC SCI MED, V41, P1359, DOI 10.1016/0277-9536(95)00115-N

Bullinger M, 1996, REHABILITATION STUTT, V35, pXXVII

Bullinger M, 1998, SF 36 FRAGEBOGEN ZUM

Dekkers JC, 2008, BMC MED RES METHODOL, V8, DOI 10.1186/1471-2288-8-69

EISENBARTH GS, 2004, J MED, V350, P2068

Eley T C, 1999, Clin Child Fam Psychol Rev, V2, P21, DOI 10.1023/A:1021863324202

Engum A, 2005, J PSYCHOSOM RES, V59, P263, DOI 10.1016/j.jpychores.2005.04.002

Engum A, 2005, DIABETES CARE, V28, P1904, DOI 10.2337/diacare.28.8.1904

Engum A, 2002, ACTA PSYCHIAT SCAND, V106, P27, DOI 10.1034/j.1600-0447.2002.01250.x

Erichsen MM, 2009, J CLIN ENDOCR METAB, V94, P4882, DOI 10.1210/jc.2009-1368

Grigoriadis Sophie, 2007, Ann Clin Psychiatry, V19, P247, DOI 10.1080/10401230701653294

Gurnell EM, 2008, J CLIN ENDOCR METAB, V93, P400, DOI 10.1210/jc.2007-1134

Hahner S, 2007, J CLIN ENDOCR METAB, V92, P3912, DOI 10.1210/jc.2007-0685

Hermann-Lingen C, 2005, HADS D HOSP ANXIETY

Kahaly GJ, 2009, EUR J ENDOCRINOL, V161, P11, DOI 10.1530/EJE-09-0044

Kahaly GJ, 2005, CLIN ENDOCRINOL, V63, P395, DOI 10.1111/j.1365-2265.2005.02352.x

Kessler RC, 2005, ARCH GEN PSYCHIAT, V62, P617, DOI 10.1001/archpsyc.62.6.617

Lovas K, 2002, CLIN ENDOCRINOL, V56, P581, DOI 10.1046/j.1365-2265.2002.01466.x

Mykletun A, 2001, BRIT J PSYCHIAT, V179, P540, DOI 10.1192/bjp.179.6.540

NEUFELD M, 1980, PEDIATR ANN, V9, P43, DOI 10.3928/0090-4481-19800401-07

Obermayer-Straub P, 2000, CLIN REV ALLERG IMMU, V18, P167, DOI 10.1385/CRIAI:18:2:167

Owen CJ, 2009, ENDOCRIN METAB CLIN, V38, P419, DOI 10.1016/j.ecl.2009.01.007

Pigott TA, 2003, PSYCHIAT CLIN N AM, V26, P621, DOI 10.1016/S0193-953X(03)00040-6

Ware JE, 2000, SPINE, V25, P3130, DOI 10.1097/00007632-200012150-00008

Wilhelm KA, 2009, AUST FAM PHYSICIAN, V38, P102

ZIGMOND AS, 1983, ACTA PSYCHIAT SCAND, V67, P361, DOI 10.1111/j.1600-0447.1983.tb09716.x

NR 36

TC 5

Z9 5

U1 0

U2 2

PU WILEY

PI HOBOKEN

PA 111 RIVER ST, HOBOKEN 07030-5774, NJ USA

SN 0300-0664

EI 1365-2265

J9 CLIN ENDOCRINOL

JI Clin. Endocrinol.

PD MAR

PY 2011

VL 74

IS 3

BP 394

EP 403

DI 10.1111/j.1365-2265.2010.03952.x

PG 10

WC Endocrinology & Metabolism

SC Endocrinology & Metabolism

GA 718AY

UT WOS:000287093200018

PM 21128996

DA 2019-01-08

ER

PT J

AU Goebel-Fabbri, AE

Anderson, BJ

Fikkan, J

Franko, DL

Pearson, K

Weinger, K

AF Goebel-Fabbri, Ann E.

Anderson, Barbara J.

Fikkan, Janna

Franko, Debra L.

Pearson, Kimberly

Weinger, Katie

TI Improvement and Emergence of Insulin Restriction in Women With Type 1

Diabetes

SO DIABETES CARE

LA English

DT Article

ID CLINICAL EATING-DISORDERS; MICROVASCULAR COMPLICATIONS; MELLITUS;

BEHAVIOR; INVENTORY; OMISSION; FEMALES; ADULTS; GIRLS; IDDM

AB OBJECTIVE To determine the distinguishing characteristics of women who report stopping insulin restriction at 11 years of follow-up from those continuing to endorse insulin restriction as well as those characteristics differing in patients who continue to Use insulin appropriately from new insulin restrictors.

RESEARCH DESIGN AND METHODS This is an 11-year follow-up study of 207 women with type 1 diabetes. Insulin restriction, diabetes self-care behaviors, diabetes-specific distress, and psychiatric and eating disorder symptoms were assessed using self-report surveys.

RESULTS Of the original sample, 57% participated in the follow-up study. Mean age was 44 +/- 12 years, diabetes duration was 28 +/- 11 years, and A1C was 7.9 +/- 1.3%. At follow-up, 20 of 60 baseline insulin restrictors had stopped restriction. Women who stopped reported improved diabetes self-care and distress, fewer problems with diabetes self-management, and lower levels of psychologic distress and eating disorder symptoms. Logistic regression indicated that lower levels of fear of weight gain with improved blood glucose and fewer problems with diabetes self-management predicted stopping restriction. At follow-up, 34 women (23%) reported new restriction, and a larger proportion of new insulin restrictors, relative to nonrestrictors, endorsed fear of weight gain with improved blood glucose.

CONCLUSIONS Findings indicate that fear of weight gain associated with improved blood glucose and problems with diabetes self-care are core issues related to both the emergence and resolution of insulin restriction. Greater attention to these concerns may help treatment teams to better meet the unique treatment needs of women struggling with insulin restriction. Diabetes Care 34:545-550, 2011

C1 [Goebel-Fabbri, Ann E.; Weinger, Katie] Joslin Diabet Ctr, Boston, MA 02215 USA.

[Goebel-Fabbri, Ann E.; Pearson, Kimberly; Weinger, Katie] Harvard Univ, Sch Med, Dept Psychiat, Boston, MA 02115 USA.

[Anderson, Barbara J.] Texas Childrens Hosp, Dept Pediat, Houston, TX 77030 USA.

[Fikkan, Janna] Duke Univ, Med Ctr, Durham, NC USA.

[Franko, Debra L.] Northeastern Univ, Dept Counseling & Appl Educ Psychol, Boston, MA 02115 USA.

[Pearson, Kimberly] Massachusetts Gen Hosp, Dept Psychiat, Boston, MA 02114 USA.

RP Weinger, K (reprint author), Joslin Diabet Ctr, Boston, MA 02215 USA.

EM katie.weinger@joslin.harvard.edu

FU Center of Excellence in Women's Health; Harvard Medical School; National

Institute of Diabetes and Digestive and Kidney Diseases [R01-DK-060115,

P30-DK-36836]; National Institutes of Health; Herbert Graetz Fund

FX This project was funded by a grant from the Center of Excellence in

Women's Health, Harvard Medical School and the National Institute of

Diabetes and Digestive and Kidney Diseases (R01-DK-060115 and

P30-DK-36836). The original work was supported by grants from the

National Institutes of Health-supported Diabetes and Endocrinology

Research Center at Joslin Diabetes Center and the Herbert Graetz Fund.

CR Affenito SG, 1997, DIABETES CARE, V20, P182, DOI 10.2337/diacare.20.2.182

Bryden KS, 1999, DIABETES CARE, V22, P1956, DOI 10.2337/diacare.22.12.1956

Colton PA, 2007, DIABETES CARE, V30, P2861, DOI 10.2337/dc07-1057

COX DJ, 1987, DIABETES CARE, V10, P617, DOI 10.2337/diacare.10.5.617

DEROGATIS LR, 1983, PSYCHOL MED, V13, P595, DOI 10.1017/S0033291700048017

Goebel-Fabbri AE, 2008, DIABETES CARE, V31, P415, DOI 10.2337/dc07-2026

Greco P., 1990, DIABETES S1, V39, p165A

Jones JM, 2000, BMJ-BRIT MED J, V320, P1563, DOI 10.1136/bmj.320.7249.1563

KROLEWSKI AS, 1995, NEW ENGL J MED, V332, P1251, DOI 10.1056/NEJM199505113321902

Olmsted MP, 2008, DIABETES CARE, V31, P1978, DOI 10.2337/dc08-0333

PEVELER RC, 1990, INT J EAT DISORDER, V11, P45

POLONSKY WH, 1995, DIABETES CARE, V18, P754, DOI 10.2337/diacare.18.6.754

POLONSKY WH, 1994, DIABETES CARE, V17, P1178, DOI 10.2337/diacare.17.10.1178

Rydall AC, 1997, NEW ENGL J MED, V336, P1849, DOI 10.1056/NEJM199706263362601

SHAMOON H, 1993, NEW ENGL J MED, V329, P977

Takii M, 2008, INT J EAT DISORDER, V41, P259, DOI 10.1002/eat.20498

THELEN MH, 1991, PSYCHOL ASSESSMENT J, V3, P119, DOI DOI 10.1037/1040-3590.3.1.119

Weinger K, 2005, DIABETES CARE, V28, P1346, DOI 10.2337/diacare.28.6.1346

Welch GW, 1997, DIABETES CARE, V20, P760, DOI 10.2337/diacare.20.5.760

NR 19

TC 12

Z9 12

U1 2

U2 14

PU AMER DIABETES ASSOC

PI ALEXANDRIA

PA 1701 N BEAUREGARD ST, ALEXANDRIA, VA 22311-1717 USA

SN 0149-5992

EI 1935-5548

J9 DIABETES CARE

JI Diabetes Care

PD MAR

PY 2011

VL 34

IS 3

BP 545

EP 550

DI 10.2337/dc10-1547

PG 6

WC Endocrinology & Metabolism

SC Endocrinology & Metabolism

GA 731YA

UT WOS:000288145400003

PM 21266653

OA Green Published, Other Gold

DA 2019-01-08

ER

PT J

AU Sorkin, DH

Quyen, NM

Billimek, J

August, KJ

Greenfield, S

Kaplan, SH

AF Sorkin, Dara H.

Quyen Ngo-Metzger

Billimek, John

August, Kristin J.

Greenfield, Sheldon

Kaplan, Sherrie H.

TI Underdiagnosed and Undertreated Depression Among Racially/Ethnically

Diverse Patients With Type 2 Diabetes

SO DIABETES CARE

LA English

DT Article

ID PRIMARY-CARE PATIENTS; GLYCEMIC CONTROL; CES-D; LATINO; SERVICES;

SYMPTOMS; DISTRESS

AB OBJECTIVE-To examine racial/ethnic differences in the prevalence of depressive symptoms and in provider recognition of depression among Latino, Asian, and non-Hispanic white patients with type 2 diabetes.

RESEARCH DESIGN AND METHODS-Patients (n = 1,209) with type 2 diabetes were recruited from five university-affiliated primary care clinics for an observational study.

RESULTS-Vietnamese American (133, 59.4%) and Mexican American (351, 50.2%) patients were more likely to report symptoms consistent with clinical depression (Center for Epidemiological Studies Depression [CES-D] scale score >= 22) than non-Hispanic whites (119, 41.6%; F [2, 1206] = 8.05, P < 0.001). Despite comparable diabetes care, Vietnamese and Mexican patients with high depressive symptoms were less likely to be diagnosed and treated than non-Hispanic whites (all P values < 0.001). Minority patients who reported low levels of trust in their provider were less likely to have been diagnosed or treated for depression (adjusted odds ratio 0.65, 95% CI 0.44-0.98, P < 0.05).

CONCLUSIONS-Innovative strategies are needed to improve recognition of depressive symptoms in minority patients.

C1 [Sorkin, Dara H.] Univ Calif Irvine, Div Gen Internal Med, Irvine, CA 92717 USA.

Univ Calif Irvine, Primary Care & Hlth Policy Res Inst, Irvine, CA USA.

RP Sorkin, DH (reprint author), Univ Calif Irvine, Div Gen Internal Med, Irvine, CA 92717 USA.

EM dsorkin@uci.edu

OI Greenfield, Sheldon/0000-0003-4628-1998

FU Robert Wood Johnson Foundation [1051084, 59758]; Novo Nordisk

Foundation; National Institute of Diabetes and Digestive and Kidney

Diseases [R18DK69846, K01DK078939]

FX This work was supported by the Robert Wood Johnson Foundation (grants

1051084 and 59758), the Novo Nordisk Foundation, and the National

Institute of Diabetes and Digestive and Kidney Diseases (R18DK69846 and

K01DK078939).

CR CHENG SH, 2010, HLTH POLICY PLA 0810

Chung H, 2003, COMMUNITY MENT HLT J, V39, P33, DOI 10.1023/A:1021221806912

Ciechanowski PS, 2000, ARCH INTERN MED, V160, P3278, DOI 10.1001/archinte.160.21.3278

Fisher L, 2010, DIABETES CARE, V33, P1034, DOI 10.2337/dc09-2175

Leng JCF, 2010, J IMMIGR MINOR HEALT, V12, P234, DOI 10.1007/s10903-009-9254-7

Lustman PJ, 2000, DIABETES CARE, V23, P934, DOI 10.2337/diacare.23.7.934

*NAT DIAB QUAL IMP, 2009, PERF MEAS SET AD DIA

Pignone MP, 2002, ANN INTERN MED, V136, P765, DOI 10.7326/0003-4819-136-10-200205210-00013

*REG U CA, 2009, RED RAC DISP DIAB CO

Santor DA, 1997, PSYCHOL ASSESSMENT, V9, P233, DOI 10.1037/1040-3590.9.3.233

SIMON GE, 1995, MEDICINE, V4, P99

Ta VM, 2010, AM J PUBLIC HEALTH, V100, P115, DOI 10.2105/AJPH.2009.160762

Thom DH, 1999, ACAD MED, V74, P195, DOI 10.1097/00001888-199902000-00019

*US CENS BUR, 2000, AM FACT FIND QT P17

ZICH JM, 1990, INT J PSYCHIAT MED, V20, P259, DOI 10.2190/LYKR-7VHP-YJEM-MKM2

NR 15

TC 19

Z9 19

U1 0

U2 6

PU AMER DIABETES ASSOC

PI ALEXANDRIA

PA 1701 N BEAUREGARD ST, ALEXANDRIA, VA 22311-1717 USA

SN 0149-5992

J9 DIABETES CARE

JI Diabetes Care

PD MAR

PY 2011

VL 34

IS 3

BP 598

EP 600

DI 10.2337/dc10-1825

PG 3

WC Endocrinology & Metabolism

SC Endocrinology & Metabolism

GA 731YA

UT WOS:000288145400013

PM 21273497

OA Green Published, Other Gold

DA 2019-01-08

ER

PT J

AU Snoek, FJ

Kersch, NYA

Eldrup, E

Harman-Boehm, I

Hermanns, N

Kokoszka, A

Matthews, DR

McGuire, BE

Pibernik-Okanovic, M

Singer, J

de Wit, M

Skovlund, SE

AF Snoek, Frank J.

Kersch, Nancy Y. A.

Eldrup, Ebbe

Harman-Boehm, Ilana

Hermanns, Norbert

Kokoszka, Andrzej

Matthews, David R.

McGuire, Brian E.

Pibernik-Okanovic, Mirjana

Singer, Joelle

de Wit, Maartje

Skovlund, Soren E.

TI Monitoring of Individual Needs in Diabetes (MIND): Baseline Data From

the Cross-National Diabetes Attitudes, Wishes, and Needs (DAWN) MIND

Study

SO DIABETES CARE

LA English

DT Article

ID RANDOMIZED CONTROLLED-TRIAL; CO-MORBID DEPRESSION; VALIDITY; ADULTS;

CARE; PREVALENCE; DISTRESS; QUALITY; PEOPLE

AB OBJECTIVE-To test the feasibility and impact of implementing the computer-assisted Monitoring of Individual Needs in Diabetes (MIND) procedure, which is aimed at improving recognition and management of the psychological needs of diabetic patients in routine care.

RESEARCH DESIGN AND METHODS-The MIND study was implemented in diabetes clinics across eight countries as part of the annual review. The computerized assessment covered emotional well-being (World Health Organization 5 Well-Being Index), diabetes-related distress (Problem Areas in Diabetes), life events, and the patient's agenda. Medical data were retrieved from the charts, and agreed-upon actions were recorded.

RESULTS-Of 1,567 patients monitored using the MIND, 24.9% had either likely depression or high diabetes-related distress; 5.4% had both. Over 80% of these patients were newly identified cases, and 41% of patients with depression were referred to a mental health professional.

CONCLUSIONS-Monitoring of well-being and diabetes-related distress as part of routine diabetes care is feasible and helps to identify and discuss unmet psychosocial needs.

C1 [Snoek, Frank J.; Kersch, Nancy Y. A.; de Wit, Maartje] Vrije Univ Amsterdam, Med Ctr, Dept Med Psychol, Amsterdam, Netherlands.

[Eldrup, Ebbe] Steno Diabet Ctr, Copenhagen, Denmark.

[Harman-Boehm, Ilana] Soroka Univ Med Ctr, Beer Sheva, Israel.

[Hermanns, Norbert] Mergentheim Diabet Zentrum, Mergentheim, Germany.

[Kokoszka, Andrzej] Med Univ Warsaw & Poradnia Diabetologiczna, Dept Psychiat 2, Warsaw, Poland.

[Matthews, David R.] Oxford Ctr Diabet Endocrinol & Metab, Oxford, England.

[McGuire, Brian E.] Univ Coll Hosp, Ctr Diabet, Galway, Ireland.

[McGuire, Brian E.] Natl Univ Ireland, Sch Psychol, Galway, Ireland.

[Pibernik-Okanovic, Mirjana] Vuk Vrhovac Univ Clin, Zagreb, Croatia.

[Singer, Joelle] Rabin Med Ctr, Petah Tiqwa, Israel.

[Skovlund, Soren E.] Novo Nordisk AS, Diabet Attitudes Wishes & Needs Program, DK-2880 Bagsvaerd, Denmark.

RP Snoek, FJ (reprint author), Vrije Univ Amsterdam, Med Ctr, Dept Med Psychol, Amsterdam, Netherlands.

EM fj.snoek@vumc.nl

RI Kokoszka, Andrzej/O-7454-2018

OI Kokoszka, Andrzej/0000-0003-4518-5494

FU Novo Nordisk; National Institute for Health Research [NF-SI-0508-10272]

FX This study was supported by an unrestricted research grant from Novo

Nordisk. No other potential conflicts of interest relevant to this

article were reported.

CR Ali S, 2006, DIABETIC MED, V23, P1165, DOI 10.1111/j.1464-5491.2006.01943.x

American Diabetes Association, 2006, DIABETES CARE S1, V29, pS4

Awata S, 2007, PSYCHIAT CLIN NEUROS, V61, P112, DOI 10.1111/j.1440-1819.2007.01619.x

Barnard KD, 2006, DIABETIC MED, V23, P445, DOI 10.1111/j.1464-5491.2006.01814.x

Davies M, 2006, DIABETIC MED, V23, P917, DOI 10.1111/j.1464-5491.2006.01892.x

De Wit M, 2008, DIABETES CARE, V31, P1521, DOI 10.2337/dc08-0394

Home P, 2006, DIABETIC MED, V23, P579

Li CY, 2010, DIABETES CARE, V33, P1061, DOI 10.2337/dc09-1515

Lowe B, 2004, J AFFECT DISORDERS, V78, P131, DOI 10.1016/S0165-0327(02)00237-9

McGuire BE, 2010, DIABETOLOGIA, V53, P66, DOI 10.1007/s00125-009-1559-5

Peyrot M, 2006, DIABETES CARE, V29, P1256, DOI 10.2337/dc05-2444

Peyrot M, 2005, DIABETIC MED, V22, P1379, DOI 10.1111/j.1464-5491.2005.01644.x

Pouwer F, 2001, DIABETES CARE, V24, P1929, DOI 10.2337/diacare.24.11.1929

Simon GE, 2007, ARCH GEN PSYCHIAT, V64, P65, DOI 10.1001/archpsyc.64.1.65

Snoek FJ, 2000, DIABETES CARE, V23, P1305, DOI 10.2337/diacare.23.9.1305

NR 15

TC 57

Z9 60

U1 0

U2 14

PU AMER DIABETES ASSOC

PI ALEXANDRIA

PA 1701 N BEAUREGARD ST, ALEXANDRIA, VA 22311-1717 USA

SN 0149-5992

J9 DIABETES CARE

JI Diabetes Care

PD MAR

PY 2011

VL 34

IS 3

BP 601

EP 603

DI 10.2337/dc10-1552

PG 3

WC Endocrinology & Metabolism

SC Endocrinology & Metabolism

GA 731YA

UT WOS:000288145400014

PM 21266654

OA Green Published, Other Gold

DA 2019-01-08

ER

PT J

AU Oakley, LD

Aekwarangkoon, S

Ward, EC

AF Oakley, Linda Denise

Aekwarangkoon, Saifon

Ward, Earlise C.

TI Successful Holistic Management of Type 2 Diabetes With Depression A Very

Personal Story

SO HOLISTIC NURSING PRACTICE

LA English

DT Article

DE depression; diabetes; holistic management; illness story; narrative

ID HEALTH-CARE; STRESS; DISTRESS; RISK; STRATEGIES; DIAGNOSIS; RECOVERY;

SYMPTOMS; MELLITUS; WOMEN

AB We used illness narrative methods to evaluate the retrospective illness story of a bereaved woman who has recovered from type 2 diabetes and depression with comorbid hypertension, hyperlipidemia, vertigo, and obesity. Her spoken illness story was obtained with a single interview question: What happened? Our findings show that the patient searched for and found personal meaning in her illness. The finding of meaning in her illness appears to have supported her efforts to adopt more positive health beliefs and health behaviors. Although she received excellent health care throughout her illness, she attributes her return to good health to spiritual growth. We discuss the practice implications of this patient's illness story for clinicians who seek new insights into patients who have complex illness stories.

C1 [Oakley, Linda Denise; Ward, Earlise C.] Univ Wisconsin, Madison, WI 53792 USA.

[Aekwarangkoon, Saifon] Walailak Univ, Nakhon Si Thammarat, Thailand.

RP Oakley, LD (reprint author), Univ Wisconsin, Clin Sci Ctr Room K6-356,600 Highland Ave, Madison, WI 53792 USA.

EM ldoakley@wisc.edu

CR Aekwarangkoon S., 2006, THAI J NURSING RES, V10, P288

Anderson DHC, 2007, DIABETES SPECTRUM, V20, P10

Baldacchino D, 2001, J ADV NURS, V34, P833, DOI 10.1046/j.1365-2648.2001.01814.x

Boulos Maged N Kamel, 2003, Int J Health Geogr, V2, P2, DOI 10.1186/1476-072X-2-2

Buck Harleah G, 2006, Holist Nurs Pract, V20, P288

Clarke D, 2009, PERS INDIV DIFFER, V46, P460, DOI 10.1016/j.paid.2008.11.014

Coe CL, 2010, HDB HLTH PSYCHOL BEH, P182

Csordas TJ, 2008, J NERV MENT DIS, V196, P585, DOI [10.1097/NMD.0b013e3181812c68, 10.1097/NMD.0b013c3181812c68]

Egede LE, 2009, GEN HOSP PSYCHIAT, V31, P422, DOI 10.1016/j.genhosppsych.2009.06.007

Eriksson AK, 2008, DIABETIC MED, V25, P834, DOI 10.1111/j.1464-5491.2008.02463.x

Fisher EB, 2005, AM J PUBLIC HEALTH, V95, P1523, DOI 10.2105/AJPH.2005.066084

Gaynor SI, 2008, BEHAV MODIF, V32, P372, DOI 10.1177/0145445507309028

Golden SH, 2008, JAMA-J AM MED ASSOC, V299, P2751, DOI 10.1001/jama.299.23.2751

Guidone C, 2006, DIABETES, V55, P2025, DOI 10.2337/db06-0068

Guzman Susan, 2004, Diabetes Forecast, V57, P52

Kaslow NJ, 2007, PROF PSYCHOL-RES PR, V38, P278, DOI 10.1037/0735-7028.38.3.278

Katon W, 2004, DIABETES CARE, V27, P914, DOI 10.2337/diacare.27.4.914

Kearney MH, 2001, RES NURS HEALTH, V24, P145, DOI 10.1002/nur.1017

Kirkpatrick H, 2008, ARCH PSYCHIAT NURS, V22, P61, DOI 10.1016/j.apnu.2002.12.002

Lustman PJ, 2005, J DIABETES COMPLICAT, V19, P113, DOI 10.1016/j.jdiacomp.2004.01.002

Matthews SC, 2005, PSYCHOSOM MED, V67, P509, DOI 10.1097/01.psy.0000160467.78373.d8

McEwen BS, 2007, PHYSIOL REV, V87, P873, DOI 10.1152/physrev.00041.2006

McEwen BS, 2004, ANN NY ACAD SCI, V1032, P1, DOI 10.1193/annals.1314.001

McFarlane SI, 2001, DIABETIC MED, V18, P10, DOI 10.1046/j.1464-5491.2001.00395.x

Meyer Bonnie L, 2008, Holist Nurs Pract, V22, P81, DOI 10.1097/01.HNP.0000312656.68147.7f

Mezuk B, 2008, AM J PUBLIC HEALTH, V98, P1480, DOI 10.2105/AJPH.2007.126441

Pan A, 2011, ARCH GEN PSYCHIAT, V68, P42, DOI 10.1001/archgenpsychiatry.2010.176

Praissman S, 2008, J AM ACAD NURSE PRAC, V20, P212, DOI 10.1111/j.1745-7599.2008.00306.x

Reifsnider E, 2005, J PROF NURS, V21, P216, DOI 10.1016/j.profnurs.2005.05.006

Riley A. A., 2009, JNP-J NURSE PRACT, V5, P523, DOI DOI 10.1016/J.NURPRA.2009.03.020

Riley T, 2005, HEALTH EDUC RES, V20, P226, DOI 10.1093/her/cyg122

Rudell K, 2008, BMC PUBLIC HEALTH, V8, DOI 10.1186/1471-2458-8-207

Saha S, 2008, J NATL MED ASSOC, V100, P1275, DOI 10.1016/S0027-9684(15)31505-4

Vickers AJ, 2008, ANN INTERN MED, V149, P200, DOI 10.7326/0003-4819-149-3-200808050-00010

Whittemore Robin, 2004, J Community Health Nurs, V21, P87, DOI 10.1207/s15327655jchn2102_03

Whittemore Robin, 2004, Issues Ment Health Nurs, V25, P243, DOI 10.1080/01612840490274750

*WHO, DEPR

NR 37

TC 1

Z9 1

U1 0

U2 1

PU LIPPINCOTT WILLIAMS & WILKINS

PI PHILADELPHIA

PA 530 WALNUT ST, PHILADELPHIA, PA 19106-3621 USA

SN 0887-9311

J9 HOLIST NURS PRACT

JI Holist. Nurs. Pract.

PD MAR-APR

PY 2011

VL 25

IS 2

BP 88

EP 96

DI 10.1097/HNP.0b013e31820de840

PG 9

WC Integrative & Complementary Medicine; Nursing

SC Integrative & Complementary Medicine; Nursing

GA 785BX

UT WOS:000292203500006

PM 21325909

DA 2019-01-08

ER

PT J

AU Haugstvedt, A

Wentzel-Larsen, T

Rokne, B

Graue, M

AF Haugstvedt, Anne

Wentzel-Larsen, Tore

Rokne, Berit

Graue, Marit

TI Perceived family burden and emotional distress: similarities and

differences between mothers and fathers of children with type 1 diabetes

in a population-based study

SO PEDIATRIC DIABETES

LA English

DT Article

DE childhood diabetes; family burden; gender differences; parental

emotional distress

ID QUALITY-OF-LIFE; PATERNAL INVOLVEMENT; CHRONIC ILLNESS; MATERNAL SLEEP;

CHRONIC SORROW; PARENTS; MANAGEMENT; ASSOCIATIONS; ADOLESCENTS;

DEPRESSION

AB Background: Parenting children with diabetes entail an extra burden for the families. More information is needed about associations between perceived family burden and emotional distress in both mothers and fathers.

Objective: To analyze (i) perceived burden and emotional distress in mothers and fathers of children with type 1 diabetes and (ii) associations between parental burden and distress and factors related to the child.

Methods: Mothers (n = 103) and fathers (n = 97) of 115 children (1-15 yr) with type 1 diabetes participated in this population-based survey. The parents completed the Hopkins Symptom Checklist-25 items (HSCL-25), measuring emotional distress, and the Family Burden Scale, which includes five questions measuring perceived family burden related to the child's diabetes.

Results: Both mothers and fathers reported that the greatest burden was related to long-term health concerns. The mothers reported a significantly greater burden related to medical treatment and significantly more emotional distress than the fathers. The mothers' perceived burden was significantly correlated with emotional distress. Nighttime blood glucose measurements were significantly associated with perceived parental burden, and experienced nocturnal hypoglycemia was significantly associated with parental emotional distress.

Conclusions: The higher perceived burden related to medical treatment, the more emotional distress, and the correlations between burdens and emotional distress in mothers vs. fathers emphasize the importance of discussing both parents' roles and responsibilities in relation to the child's diabetes in follow-up. In the consultations, emphasizing nighttime caregiving and nocturnal hypoglycemia might also be important to prevent emotional distress.

C1 [Haugstvedt, Anne; Graue, Marit] Bergen Univ Coll, Fac Hlth & Social Sci, N-5020 Bergen, Norway.

[Haugstvedt, Anne; Rokne, Berit] Univ Bergen, Dept Publ Hlth & Primary Hlth Care, N-5020 Bergen, Norway.

[Haugstvedt, Anne; Graue, Marit] Haukeland Hosp, Dept Pediat, N-5021 Bergen, Norway.

[Wentzel-Larsen, Tore] Haukeland Hosp, Clin Res Ctr, N-5021 Bergen, Norway.

RP Haugstvedt, A (reprint author), Bergen Univ Coll, Fac Hlth & Social Sci, Postbox 7030, N-5020 Bergen, Norway.

EM ahau@hib.no

FU Diabetes Research Foundation of Western Norway; Norwegian Diabetes

Association; Norwegian Nurses' Association; Western Norway Regional

Health Authority; University of Bergen; Bergen University College

FX We thank all the parents and children who participated in this study.

The study was supported by the Diabetes Research Foundation of Western

Norway, the Norwegian Diabetes Association, the Norwegian Nurses'

Association, the Western Norway Regional Health Authority, the

University of Bergen, and Bergen University College.

CR Bowes S, 2009, J ADV NURS, V65, P992, DOI 10.1111/j.1365-2648.2009.04963.x

Carpentier MY, 2006, CHILD HEALTH CARE, V35, P141, DOI 10.1207/s15326888chc3502_3

Cottrell L, 2005, CLIN PEDIATR, V44, P613, DOI 10.1177/000992280504400709

Dashiff Carol, 2008, J Pediatr Nurs, V23, P101, DOI 10.1016/j.pedn.2007.08.007

Davis C. S., 2002, STAT METHODS ANAL RE

DEROGATIS LR, 1974, BEHAV SCI, V19, P1, DOI 10.1002/bs.3830190102

Gavin L, 2006, J PEDIATR PSYCHOL, V31, P481, DOI 10.1093/jpepsy/jsj043

Haugstvedt A, 2010, DIABETIC MED, V27, P72, DOI 10.1111/j.1464-5491.2009.02867.x

Hoey H, 2001, DIABETES CARE, V24, P1923, DOI 10.2337/diacare.24.11.1923

Hoey H, 2006, QUAL LIFE RES, V15, P1033, DOI 10.1007/s11136-006-0042-8

Landolt MA, 2005, J AM ACAD CHILD PSY, V44, P682, DOI 10.1097/01-chi.0000161645.98022.35

Lowes L, 2000, J ADV NURS, V32, P41, DOI 10.1046/j.1365-2648.2000.01418.x

Meltzer LJ, 2006, ARCH INTERN MED, V166, P1749, DOI 10.1001/archinte.166.16.1749

Monaghan MC, 2009, FAM SYST HEALTH, V27, P28, DOI 10.1037/a0014770

Naerde A, 2000, J AFFECT DISORDERS, V58, P181, DOI 10.1016/S0165-0327(99)00119-6

Pelchat Diane, 2007, J Child Health Care, V11, P112, DOI 10.1177/1367493507076064

Pelchat Diane, 2003, J Child Health Care, V7, P231, DOI 10.1177/13674935030074001

Polit DF, 2008, NURSING RES GENERATI

Sandanger I, 1998, SOC PSYCH PSYCH EPID, V33, P345, DOI 10.1007/s001270050064

Streisand R, 2005, J PEDIATR PSYCHOL, V30, P513, DOI 10.1093/jpepsy/jsi076

Streisand R, 2008, PATIENT EDUC COUNS, V73, P333, DOI 10.1016/j.pec.2008.06.014

Sullivan-Bolyai S, 2006, MCN-AM J MATERN-CHIL, V31, P24, DOI 10.1097/00005721-200601000-00007

Sullivan-Bolyai S, 2003, J Pediatr Nurs, V18, P21, DOI 10.1053/jpdn.2003.4

Taanila A, 1996, DEV MED CHILD NEUROL, V38, P567

WINOKUR A, 1984, BRIT J PSYCHIAT, V144, P395, DOI 10.1192/bjp.144.4.395

Wysocki T, 2006, J PEDIATR PSYCHOL, V31, P501, DOI 10.1093/jpepsy/jsj042

NR 26

TC 37

Z9 38

U1 0

U2 24

PU WILEY-BLACKWELL

PI MALDEN

PA COMMERCE PLACE, 350 MAIN ST, MALDEN 02148, MA USA

SN 1399-543X

J9 PEDIATR DIABETES

JI Pediatr. Diabetes

PD MAR

PY 2011

VL 12

IS 2

BP 107

EP 114

DI 10.1111/j.1399-5448.2010.00661.x

PG 8

WC Endocrinology & Metabolism; Pediatrics

SC Endocrinology & Metabolism; Pediatrics

GA 726DZ

UT WOS:000287700300007

PM 20522171

DA 2019-01-08

ER

PT J

AU van Son, J

Nyklicek, I

Pop, VJM

Pouwer, F

AF van Son, Jenny

Nyklicek, Ivan

Pop, Victor J. M.

Pouwer, Francois

TI Testing the effectiveness of a mindfulness-based intervention to reduce

emotional distress in outpatients with diabetes (DiaMind): design of a

randomized controlled trial

SO BMC PUBLIC HEALTH

LA English

DT Article

ID AMBULATORY BLOOD-PRESSURE; QUALITY-OF-LIFE; STRESS REDUCTION; D

PERSONALITY; SOCIAL INHIBITION; DEPRESSION SCALE; HOSPITAL ANXIETY;

GLYCEMIC CONTROL; PSYCHOMETRIC PROPERTIES; CARDIOVASCULAR-DISEASE

AB Background: Approximately 20-40% of outpatients with diabetes experience elevated levels of emotional distress, varying from disease-specific distress to general symptoms of anxiety and depression. The patient's emotional wellbeing is related to other unfavorable outcomes, like reduced quality of life, sub-optimal self-care, impaired glycemic control, higher risk of complications, and increased mortality rates. The purpose of this study is to test the effectiveness of a new diabetes-specific, mindfulness-based psychological intervention. First, with regard to reducing emotional distress; second, with respect to improving quality of life, dispositional mindfulness, and selfesteem of patients with diabetes; third, with regard to self-care and clinical outcomes; finally, a potential effect modification by clinical and personality characteristics will be explored.

Methods/Design: The Diabetes and Mindfulness study (DiaMind) is a randomized controlled trial. Patients with diabetes with low levels of emotional well-being will be recruited from outpatient diabetes clinics. Eligible patients will be randomized to an intervention group or a wait-list control group. The intervention group will receive the mindfulness program immediately, while the control group will receive the program eight months later. The primary outcome is emotional distress (anxiety, stress, depressive symptoms), for which data will be collected at baseline, four weeks, post intervention, and after six months follow-up. In addition, self-report data will be collected on quality of life, dispositional mindfulness, self-esteem, self-care, and personality, while complications and glycemic control will be assessed from medical files and blood pressure will be measured. Group differences will be analyzed with repeated measures analysis of covariance. The study is supported by grants from the Dutch Diabetes Research Foundation and Tilburg University and has been approved by a medical ethics committee.

Discussion: It is hypothesized that emotional well-being, quality of life, dispositional mindfulness, self-esteem, self-care, and blood pressure will improve significantly more in the mindfulness group compared to the control group. Results of this study can contribute to a better care for patients with diabetes with lowered levels of emotional well-being. It is expected that the first results will become available in 2012.

C1 [van Son, Jenny; Nyklicek, Ivan; Pop, Victor J. M.; Pouwer, Francois] Tilburg Univ, Ctr Res Psychol Somat Dis CoRPS, Dept Med Psychol & Neuropsychol, NL-5000 LE Tilburg, Netherlands.

RP Nyklicek, I (reprint author), Tilburg Univ, Ctr Res Psychol Somat Dis CoRPS, Dept Med Psychol & Neuropsychol, NL-5000 LE Tilburg, Netherlands.

EM I.Nyklicek@uvt.nl

FU Dutch Diabetes Research Foundation [2008.13.005]; Tilburg University

FX The study is supported by grants from the Dutch Diabetes Research

Foundation or 'Diabetesfonds' (project number 2008.13.005, awarded to Dr

I. Nyklicek) and Tilburg University. The authors wish to acknowledge and

express their special thanks to internal medicine physician Dr Marion

Blonk, for her efforts in recruiting patients at the Catharina Hospital

Eindhoven. In addition, we want to thank the internal medicine

physicians Dr Bravenboer, Dr Erdtsieck, Dr Lieverse, and Dr Spooren,

their secretaries, and the diabetes nurses of the participating

hospitals, for their continuing assistance in the recruitment of

patients for this study. Moreover, we want to thank the general

practitioners and nurse practitioners of the Health Centre Plus

Eindhoven and Vlaslant Valkenswaard, and their patients for their

participation in the pilot study.

CR Ali S, 2006, DIABETIC MED, V23, P1165, DOI 10.1111/j.1464-5491.2006.01943.x

American Diabetes Association, 2011, Diabetes Care, V34 Suppl 1, pS11, DOI 10.2337/dc11-S011

Baer RA, 2006, ASSESSMENT, V13, P27, DOI 10.1177/1073191105283504

BAER RA, 2009, COGN BEHAV THER, P1

Baer RA, 2008, ASSESSMENT, V15, P329, DOI 10.1177/1073191107313003

Barnes VA, 2004, PSYCHOSOM MED, V66, P909, DOI 10.1097/01.psy.0000145902.91749.35

Bishop SR, 2004, CLIN PSYCHOL-SCI PR, V11, P230, DOI 10.1093/clipsy/bph077

Bjelland I, 2002, J PSYCHOSOM RES, V52, P69, DOI 10.1016/S0022-3999(01)00296-3

Bogner HR, 2007, DIABETES CARE, V30, P3005, DOI 10.2337/dc07-0974

Branstrom R, 2010, ANN BEHAV MED, V39, P151, DOI 10.1007/s12160-010-9168-6

COHEN S, 1983, J HEALTH SOC BEHAV, V24, P385, DOI 10.2307/2136404

COHEN S, 1988, CLAR SYMP, P31

de Jonge P, 2007, J PSYCHOSOM RES, V63, P477, DOI 10.1016/j.jpsychores.2007.06.002

de Wit M, 2007, DIABETES CARE, V30, P2003, DOI 10.2337/dc07-0447

Denollet J, 2006, EUR HEART J, V27, P171, DOI 10.1093/eurheartj/ehi616

Denollet J, 2005, PSYCHOSOM MED, V67, P89, DOI 10.1097/01.psy.0000149256.81953.49

Egede LE, 2005, CURR DIABETES REV, V1, P235, DOI 10.2174/157339905774574356

Emons WHM, 2007, J PSYCHOSOM RES, V63, P27, DOI 10.1016/j.jphyschores.2007.03.010

Evers A, 2009, COTAN DOCUMENTATIE

Franck E, 2008, PSYCHOL BELG, V48, P25, DOI 10.5334/pb-48-1-25

Gregg JA, 2007, J CONSULT CLIN PSYCH, V75, P336, DOI 10.1037/0022-006X.75.2.336

Grigsby AB, 2002, J PSYCHOSOM RES, V53, P1053, DOI 10.1016/S0022-3999(02)00417-8

Grossman P, 2004, J PSYCHOSOM RES, V57, P35, DOI 10.1016/S0022-3999(03)00573-7

Heine RJ, 2004, HDB DIABETES MELLITU

Henry JL, 1997, PSYCHOL HEALTH MED, V2, P109

Hermanns N, 2006, DIABETOLOGIA, V49, P469, DOI 10.1007/s00125-005-0094-2

Hofmann SG, 2010, J CONSULT CLIN PSYCH, V78, P169, DOI 10.1037/a0018555

*INT DIAB FED, EP MORB

International Diabetes Federation, 2005, GLOB GUID TYP 2 DIAB

Jenkinson C, 1997, J PUBLIC HEALTH MED, V19, P179, DOI 10.1093/oxfordjournals.pubmed.a024606

Jonas BS, 2000, PSYCHOSOM MED, V62, P188, DOI 10.1097/00006842-200003000-00006

Kabat-Zinn J., 1990, FULL CATASTROPHE LIV

Lustman PJ, 2005, J DIABETES COMPLICAT, V19, P113, DOI 10.1016/j.jdiacomp.2004.01.002

Makine C, 2009, DIABETIC MED, V26, P28, DOI 10.1111/j.1464-5491.2008.02606.x

Martens EJ, 2007, J PSYCHOSOM RES, V63, P545, DOI 10.1016/j.jpsychores.2007.06.005

Morone NE, 2008, PAIN, V134, P310, DOI 10.1016/j.pain.2007.04.038

Nouwen A, 2010, DIABETOLOGIA, V53, P2480, DOI 10.1007/s00125-010-1874-x

Nyklicek I, 2008, ANN BEHAV MED, V35, P331, DOI 10.1007/s12160-008-9030-2

POLONSKY WH, 1995, DIABETES CARE, V18, P754, DOI 10.2337/diacare.18.6.754

Pouwer F, 2006, PATIENT EDUC COUNS, V60, P235, DOI 10.1016/j.pec.2005.01.009

Rosenzweig S, 2007, ALTERN THER HEALTH M, V13, P36

SANDERMAN R, 1995, METEN PERSOONLIJKHEI

Segal Z., 2002, MINDFULNESS BASED CO

Skevington SM, 2004, QUAL LIFE RES, V13, P299, DOI 10.1023/B:QURE.0000018486.91360.00

Snoek FJ, 2000, DIABETES CARE, V23, P1305, DOI 10.2337/diacare.23.9.1305

Sowers JR, 2001, HYPERTENSION, V37, P1053, DOI 10.1161/01.HYP.37.4.1053

Speca M, 2000, PSYCHOSOM MED, V62, P613, DOI 10.1097/00006842-200009000-00004

Spinhoven P, 1997, PSYCHOL MED, V27, P363, DOI 10.1017/S0033291796004382

Steed L, 2003, PATIENT EDUC COUNS, V51, P5, DOI 10.1016/S0738-3991(02)00213-6

Teasdale JD, 2000, J CONSULT CLIN PSYCH, V68, P615, DOI 10.1037/0022-006X.68.4.615

van Bastelaar KM, 2008, BMC PSYCHIATRY, V8, DOI 10.1186/1471-244X-8-9

Van der Feltz-Cornelis CM, 2010, GEN HOSP PSYCHIAT, V32, P380, DOI 10.1016/j.genhosppsych.2010.03.011

van der Ven NCW, 2005, DIABETIC MED, V22, P1619

VANBASTELAAR KM, 2011, DIABETES CARE

Wald F., 1990, NED T PSYCHOL, V45, P86

Welch GW, 1997, DIABETES CARE, V20, P760, DOI 10.2337/diacare.20.5.760

Westhoff TH, 2005, BLOOD PRESS MONIT, V10, P239, DOI 10.1097/01.mbp.0000172711.82287.7f

WICHERTS JM, 2003, NEDERLANDS TIJDSCHRI, V59, P12

World Health Organization, WHO 5 WELL BEING IND

ZIGMOND AS, 1983, ACTA PSYCHIAT SCAND, V67, P361, DOI 10.1111/j.1600-0447.1983.tb09716.x

NEEMT AANTAL MENSEN

HOE VAAK KOMT DIABET

NR 62

TC 16

Z9 16

U1 3

U2 33

PU BMC

PI LONDON

PA CAMPUS, 4 CRINAN ST, LONDON N1 9XW, ENGLAND

SN 1471-2458

J9 BMC PUBLIC HEALTH

JI BMC Public Health

PD FEB 24

PY 2011

VL 11

AR 131

DI 10.1186/1471-2458-11-131

PG 11

WC Public, Environmental & Occupational Health

SC Public, Environmental & Occupational Health

GA 732XF

UT WOS:000288222300001

PM 21349184

OA DOAJ Gold, Green Published

DA 2019-01-08

ER

PT J

AU Ireland, SE

Arthur, HM

Gunn, EA

Oczkowski, W

AF Ireland, Sandra E.

Arthur, Heather M.

Gunn, Elizabeth A.

Oczkowski, Wieslaw

TI Stroke prevention care delivery: Predictors of risk factor management

outcomes

SO INTERNATIONAL JOURNAL OF NURSING STUDIES

LA English

DT Article

DE Stroke; Prevention; Hypertension; Diabetes; Adherence

ID MINI-MENTAL-STATE; TRANSIENT ISCHEMIC ATTACK; BLOOD-PRESSURE;

SELF-EFFICACY; PSYCHOLOGICAL DISTRESS; COGNITIVE IMPAIRMENT;

PATIENT-ADHERENCE; MEDICAL-TREATMENT; HEALTH BEHAVIOR; SOCIAL SUPPORT

AB Background: Internationally, the development and implementation of stroke care guidelines have resulted in the evolution of stroke prevention outpatient clinics designed to accelerate patient access to treatment and behavioral risk reduction following transient ischemic attack or stroke.

Objectives: To examine the extent to which selected demographic, social-psychological, physiological, and adherence characteristics predicted achievement of blood pressure and glucose targets in a group of patients referred to a Canadian stroke prevention clinic with confirmed transient ischemic attack (TIA) or stroke and hypertension and/or diabetes.

Participants: A total of 313, English speaking, adult patients who were referred from family or emergency department physicians to a stroke prevention clinic provided demographic data and received social-psychological screening testing at intake. Of these, 93 participants who met criteria of confirmed TIA or stroke plus hypertension and/or diabetes were identified as the study group. Seventy-seven of study group participants completed a 6-month follow-up.

Methods: Admission screening tests included the Modified and Mini-Mental State Examinations, Trail Making Test, Clock Drawing Test, a medication self-efficacy scale, the Lubben Social Network Scale and the Geriatric Depression Scale. Family physician follow-up was ascertained 4-8 weeks after intake. At approximately 6 months after the initial screening measures, 77 study group participants completed additional measures of adherence, blood pressure and/or glycated hemoglobin.

Results: Transient ischemic attack was confirmed in 58% and stroke in 42% of the study group. Mean age was 69 years (SD = 11); 53% were male; 97% had hypertension; and 25% were diabetic; some had both. Twenty-three percent were not followed-up by family practitioners. At 6-month follow-up, 97% reported >= 80% adherence to medication; only 57% met treatment targets. A logistic regression analysis identified three independent predictors of achieving blood pressure and/or glucose targets: self-efficacy expectations (OR, 1.61; 95% Cl, 1.03-2.54; p = 0.04), Mini-Mental State (OR, 1.28; 95% Cl, 1.06-1.54; p = 0.009); and self-reported adherence (OR, 1.14; 95% Cl, 1.01-1.29; p = 0.03).

Conclusions: Three simple screening measures identified patients at increased risk of not achieving clinical risk factor management targets. (C) 2010 Elsevier Ltd. All rights reserved.

C1 [Ireland, Sandra E.] McMaster Univ, Hamilton Gen Hosp, Hamilton Hlth Sci, Hamilton, ON L8K 2X2, Canada.

[Arthur, Heather M.] McMaster Univ, Sch Nursing, Heart & Stroke Fdn Ontario, Michael G DeGroote Endowed Chair Cardiovasc Nursi, Hamilton, ON L8N 3Z5, Canada.

[Gunn, Elizabeth A.] McMaster Univ, Hamilton Gen Hosp, Cardiac Hlth & Rehabil Ctr, Hamilton, ON L8K 2X2, Canada.

[Oczkowski, Wieslaw] McMaster Univ, Hamilton Gen Hosp, Hamilton Hlth Sci Reg Stroke Program, Hamilton, ON L8L 2X2, Canada.

RP Ireland, SE (reprint author), McMaster Univ, Hamilton Gen Hosp, Hamilton Hlth Sci, Room 722,7 North,237 Barton St E, Hamilton, ON L8K 2X2, Canada.

EM irelasan@hhsc.ca

FU Hamilton Health Sciences; Heart and Stroke Foundation of Canada;

Canadian Stroke Network

FX The study was funded by a grant from the Hamilton Health Sciences New

Investigator Fund. The funding source had no involvement in the study

design, in the collection, analysis and interpretation of the data.;

Sandra Ireland was sponsored by a Nursing Research Fellowship from the

Heart and Stroke Foundation of Canada and the Canadian Stroke Network.

CR *AM HEART ASS, 2002, MULT COMPL CHALL

[Anonymous], 1994, CMAJ, V150, P899

Appelros P, 2006, EUR J NEUROL, V13, P491, DOI 10.1111/j.1468-1331.2006.01299.x

Ballard C, 2003, DEMENT GERIATR COGN, V16, P52, DOI 10.1159/000069994

Bandura A, 1998, PSYCHOL HEALTH, V13, P623, DOI 10.1080/08870449808407422

BANDURA A, 1982, AM PSYCHOL, V37, P22

Barba R, 2002, STROKE, V33, P1993, DOI 10.1161/01.STR.0000017285.73172.91

Berlowitz DR, 1998, NEW ENGL J MED, V339, P1957, DOI 10.1056/NEJM199812313392701

Black MEA, 2001, HEALTH EDUC BEHAV, V28, P200, DOI 10.1177/109019810102800206

*CAN DIAB ASS, 2006, BEST PRACT GUID

*CAN HYP ED PROGR, 2006, PERSPECTIVES CARDIOL, P28

*CAN STROK STRAT, 2008, CAN BEST PRACT REC S

Carney RA, 2002, STROKE, V33, P5, DOI 10.1161/str.33.1.5

Censori B, 1996, STROKE, V27, P1205, DOI 10.1161/01.STR.27.7.1205

Clark NM, 1999, HEALTH EDUC BEHAV, V26, P72, DOI 10.1177/109019819902600107

COLANTONIO A, 1993, J GERONTOL, V48, pS261, DOI 10.1093/geronj/48.5.S261

CRAIG HM, 1985, RES NURS HEALTH, V8, P61, DOI 10.1002/nur.4770080112

DEBUSK RF, 1994, ANN INTERN MED, V120, P721, DOI 10.7326/0003-4819-120-9-199405010-00001

DICK JPR, 1984, J NEUROL NEUROSUR PS, V47, P496, DOI 10.1136/jnnp.47.5.496

DiMatteo MR, 2000, ARCH INTERN MED, V160, P2101, DOI 10.1001/archinte.160.14.2101

DiMatteo MR, 2004, HEALTH PSYCHOL, V23, P207, DOI 10.1037/0278-6133.23.2.207

DiMatteo MR, 2004, MED CARE, V42, P200, DOI 10.1097/01.mlr.0000114908.90348.f9

Elias MF, 2004, STROKE, V35, P404, DOI 10.1161/01.STR.0000103141.82869.77

Elkins JS, 2004, NEUROLOGY, V63, P793, DOI 10.1212/01.WNL.0000137014.36689.7F

FOLSTEIN MF, 1975, J PSYCHIAT RES, V12, P189, DOI 10.1016/0022-3956(75)90026-6

Fure B, 2006, ACTA NEUROL SCAND, V114, P17, DOI 10.1111/j.1600-0404.2006.00603.x

Gladstone DJ, 2004, CAN MED ASSOC J, V170, P1099, DOI 10.1503/cmaj.1031349

Gordis L., 1979, COMPLIANCE HLTH CARE, P23

Green L. W., 1999, HLTH PROMOTION PLANN

GREMBOWSKI D, 1993, J HEALTH SOC BEHAV, V34, P89, DOI 10.2307/2137237

HAYNES RB, 2005, COCHRANE DATABASE SY, V4

HAYNES RB, 2003, COCHRANE LIB, V4

*HEART STROK FDN C, 2008, CANADIAN MED ASS J, V179, pE4

Hosemer D., 2000, APPL LOGISTIC REGRES

Jonas BS, 2000, PSYCHOSOM MED, V62, P463, DOI 10.1097/00006842-200007000-00001

Joseph LN, 1999, STROKE, V30, P16, DOI 10.1161/01.STR.30.1.16

Kernan WN, 2000, STROKE, V31, P415, DOI 10.1161/01.STR.31.2.415

Keys BA, 2000, J INT NEUROPSYCH SOC, V6, P76

Libon DJ, 1994, NEUROPSYCHOLOGY, V8, P38, DOI 10.1037/0894-4105.8.1.38

Lubben J. E., 1988, FAM COMMUNITY HEALTH, V11, P42, DOI DOI 10.1097/00003727-198811000-00008

May M, 2002, STROKE, V33, P7, DOI 10.1161/hs0102.100529

Mead GE, 2000, STROKE, V31, P714, DOI 10.1161/01.STR.31.3.714

Miller N H, 1996, J Cardiovasc Nurs, V11, P76

*MIN HLTH LONG TER, 2000, INT STROK STRAT ONT

Moses James A Jr, 2004, Arch Clin Neuropsychol, V19, P703, DOI 10.1016/j.acn.2004.02.004

Mouradian MS, 2002, STROKE, V33, P1656, DOI 10.1161/01.STR.0000017877.62543.14

Nelson MR, 2006, MED J AUSTRALIA, V185, P487

Norman G. R., 1994, BIOSTATISTICS BARE E

Pender NJ, 2002, HLTH PROMOTION NURSI

Reitan R. M, 1993, HALSTEAD REITAN NEUR

REITAN RM, 2004, COMPREHENSIVE HDB PS, V1, P385

Sacco RL, 2006, STROKE, V37, P577, DOI 10.1161/01.STR.0000199147.30016.74

SACKETT DL, 1979, COMPLIANCE HLTH CARE, P286

Samsa GP, 2000, ARCH INTERN MED, V160, P967, DOI 10.1001/archinte.160.7.967

SEEMAN TE, 1987, PSYCHOSOM MED, V49, P341, DOI 10.1097/00006842-198707000-00003

SHULMAN K, 2003, QUICK COGNITIVE SCRE

Spalletta G, 2002, J NEUROL, V249, P1541, DOI 10.1007/s00415-002-0885-z

Srikanth V, 2006, INT PSYCHOGERIATR, V18, P295, DOI 10.1017/S1041610205002711

STEPHENSON BJ, 1993, JAMA-J AM MED ASSOC, V269, P2779, DOI 10.1001/jama.269.21.2779

STRECHER VJ, 1986, HEALTH EDUC QUART, V13, P73, DOI 10.1177/109019818601300108

Suhr JA, 1999, ARCH PHYS MED REHAB, V80, P773, DOI 10.1016/S0003-9993(99)90226-5

TATEMICHI TK, 1994, J NEUROL NEUROSUR PS, V57, P202, DOI 10.1136/jnnp.57.2.202

TENG EL, 1987, J CLIN PSYCHIAT, V48, P314

TOMBAUGH TN, 1992, J AM GERIATR SOC, V40, P922, DOI 10.1111/j.1532-5415.1992.tb01992.x

Tombaugh TN, 1996, PSYCHOL ASSESSMENT, V8, P48, DOI 10.1037/1040-3590.8.1.48

Uchino BN, 1996, PSYCHOL BULL, V119, P488, DOI 10.1037/0033-2909.119.3.488

VANLATUM JD, 1995, STROKE, V26, P331

WADE DT, 1987, BRIT J PSYCHIAT, V151, P200, DOI 10.1192/bjp.151.2.200

WATSON YI, 1993, J AM GERIATR SOC, V41, P1235, DOI 10.1111/j.1532-5415.1993.tb07308.x

WHO, 2005, WHO STEPWISE APPR ST

*WORLD HEART FED, 2006, COUNTR SHOULD FORM G

*WORLD MED ASS, 1965, DECL HELS

YESAVAGE JA, 1983, J PSYCHIATR RES, V17, P37, DOI 10.1016/0022-3956(82)90033-4

NR 73

TC 1

Z9 1

U1 2

U2 9

PU PERGAMON-ELSEVIER SCIENCE LTD

PI OXFORD

PA THE BOULEVARD, LANGFORD LANE, KIDLINGTON, OXFORD OX5 1GB, ENGLAND

SN 0020-7489

J9 INT J NURS STUD

JI Int. J. Nurs. Stud.

PD FEB

PY 2011

VL 48

IS 2

BP 156

EP 164

DI 10.1016/j.ijnurstu.2010.07.003

PG 9

WC Nursing

SC Nursing

GA 725GB

UT WOS:000287632100003

PM 20673897

DA 2019-01-08

ER

PT J

AU van Bastelaar, KMP

Pouwer, F

Cuijpers, P

Riper, H

Snoek, FJ

AF van Bastelaar, Kim M. P.

Pouwer, Francois

Cuijpers, Pim

Riper, Heleen

Snoek, Frank J.

TI Web-Based Depression Treatment for Type 1 and Type 2 Diabetic Patients A

randomized, controlled trial

SO DIABETES CARE

LA English

DT Article

ID COGNITIVE-BEHAVIOR THERAPY; INTERNET-BASED TREATMENT; EMOTIONAL

DISTRESS; SYMPTOMS; ANXIETY; PSYCHOTHERAPY; METAANALYSIS; DISORDERS;

DROPOUT; ADULTS

AB OBJECTIVE-Comorbid depression is common in patients with type 1 and type 2 diabetes, adversely affecting quality of life, diabetes outcomes, and mortality. Depression can be effectively treated with cognitive behavior therapy (CBT). The Internet is a new and attractive method for delivering CBT intervention on a large scale at relatively low costs. This study evaluated the effectiveness of Web-based CBT for depression treatment in adults with type 1 or type 2 diabetes, with minimal guidance.

RESEARCH DESIGN AND METHODS-A randomized controlled trial was conducted in the Netherlands in 255 adult diabetic patients with elevated depressive symptoms. Primary outcomes were depressive symptoms. Secondary outcomes were diabetes-specific emotional distress and glycemic control. Assessments were at baseline, after treatment, and at the 1-month follow-up.

RESULTS-The Web-based CBT was effective in reducing depressive symptoms by intention-to-treat analyses (P = 0.04, d = 0.29; clinical improvement 41% vs. 24% P < 0.001) and by per-protocol analyses (P < 0.001, d = 0.70; clinical improvement, 56% vs. 24% P < 0.001). The intervention reduced diabetes-specific emotional distress (P = 0.03) but had no beneficial effect on glycemic control (P > 0.05).

CONCLUSIONS-Web-based CBT depression treatment is effective in reducing depressive symptoms in adults with type 1 and type 2 diabetes. In addition, the intervention reduces diabetes-specific emotional distress in depressed patients.

C1 [van Bastelaar, Kim M. P.; Snoek, Frank J.] Vrije Univ Amsterdam Med Ctr, Dept Med Psychol, Amsterdam, Netherlands.

[van Bastelaar, Kim M. P.; Cuijpers, Pim; Snoek, Frank J.] Vrije Univ Amsterdam Med Ctr, EMGO Inst, Inst Hlth & Care Res, Amsterdam, Netherlands.

[Pouwer, Francois] Tilburg Univ, Ctr Res Psychol Somat Dis CoRPS, Dept Med Psychol & Neuropsychol, NL-5000 LE Tilburg, Netherlands.

[Cuijpers, Pim] Vrije Univ Amsterdam, Dept Clin Psychol, Amsterdam, Netherlands.

[Riper, Heleen] Netherlands Inst Mental Hlth & Addict Trimbos, Utrecht, Netherlands.

RP van Bastelaar, KMP (reprint author), Vrije Univ Amsterdam Med Ctr, Dept Med Psychol, Amsterdam, Netherlands.

EM k.vanbastelaar@vumc.nl

RI Cuijpers, Pim/G-1703-2013

OI Cuijpers, Pim/0000-0001-5497-2743

FU Dutch Diabetes Research Foundation [2005.00.009]

FX This study was financed by the Dutch Diabetes Research Foundation (grant

number 2005.00.009).

CR *AM DIAB ASS, 2010, DIABETES CARE S1, V33, pS11, DOI DOI 10.2337/DC10-S011

Anderson RJ, 2001, DIABETES CARE, V24, P1069, DOI 10.2337/diacare.24.6.1069

Christensen H, 2004, BRIT MED J, V328, P265, DOI 10.1136/bmj.37945.566632.EE

Cohen J., 1988, STAT POWER ANAL BEHA, Vxxi

Cuijpers P, 2009, CLIN PSYCHOL REV, V29, P449, DOI 10.1016/j.cpr.2009.04.005

de Graaf LE, 2009, BRIT J PSYCHIAT, V195, P73, DOI 10.1192/bjp.bp.108.054429

Egede LE, 2010, DIABETES RES CLIN PR, V87, P302, DOI 10.1016/j.diabres.2010.01.024

Eysenbach G, 2005, J MED INTERNET RES, V7, DOI 10.2196/jmir.7.1.e11

Fisher L, 2010, DIABETES CARE, V33, P23, DOI 10.2337/dc09-1238

Hermanns N, 2006, DIABETOLOGIA, V49, P469, DOI 10.1007/s00125-005-0094-2

JACOBSON NS, 1991, J CONSULT CLIN PSYCH, V59, P12, DOI 10.1037//0022-006X.59.1.12

Kaltenthaler E, 2006, HEALTH TECHNOL ASSES, V10, P1, DOI 10.3310/hta10330

Li XM, 2006, STAT MED, V25, P2107, DOI 10.1002/sim.2343

Melville KM, 2010, BRIT J CLIN PSYCHOL, V49, P455, DOI 10.1348/014466509X472138

Peters L, 1998, PSYCHOL MED, V28, P893, DOI 10.1017/S0033291798006655

Radloff L. S., 1977, APPL PSYCHOL MEAS, V1

Simon GE, 2010, PSYCHIAT SERV, V61, P684, DOI 10.1176/ps.2010.61.7.684

Snoek FJ, 2000, DIABETES CARE, V23, P1305, DOI 10.2337/diacare.23.9.1305

Spek V, 2007, PSYCHOL MED, V37, P319, DOI 10.1017/S0033291706008944

*US BUR CENS, 2010, INT WORLD STAT

van Bastelaar KMP, 2010, DIABETIC MED, V27, P798, DOI 10.1111/j.1464-5491.2010.03025.x

van Bastelaar KM, 2008, BMC PSYCHIATRY, V8, DOI 10.1186/1471-244X-8-9

Van der Feltz-Cornelis CM, 2010, GEN HOSP PSYCHIAT, V32, P380, DOI 10.1016/j.genhosppsych.2010.03.011

VANBASTELAAR KMP, 2010, PATIENT ED COUN 0707

Warmerdam L, 2008, J MED INTERNET RES, V10, DOI 10.2196/jmir.1094

NR 25

TC 85

Z9 85

U1 0

U2 32

PU AMER DIABETES ASSOC

PI ALEXANDRIA

PA 1701 N BEAUREGARD ST, ALEXANDRIA, VA 22311-1717 USA

SN 0149-5992

J9 DIABETES CARE

JI Diabetes Care

PD FEB

PY 2011

VL 34

IS 2

BP 320

EP 325

DI 10.2337/dc10-1248

PG 6

WC Endocrinology & Metabolism

SC Endocrinology & Metabolism

GA 722LO

UT WOS:000287433900013

PM 21216855

OA Green Published, Other Gold

DA 2019-01-08

ER

PT J

AU Andreadou, E

Anagnostouli, M

Vasdekis, V

Kararizou, E

Rentzos, M

Kontaxis, T

Evdokimidis, I

AF Andreadou, Elisabeth

Anagnostouli, Maria

Vasdekis, Vassilis

Kararizou, Evangelia

Rentzos, Michael

Kontaxis, Theodoros

Evdokimidis, Ioannis

TI The impact of comorbidity and other clinical and sociodemographic

factors on health-related quality of life in Greek patients with

Parkinson's disease

SO AGING & MENTAL HEALTH

LA English

DT Article

DE comorbidity; depression; disability; sleep problems; gastrointestinal

disturbances

ID NONMOTOR SYMPTOMS; QUESTIONNAIRE; DISABILITY; DETERMINANTS; VALIDATION;

DEPRESSION; MORTALITY; DIAGNOSIS; ACCURACY; ONSET

AB Objectives: This study was designed to evaluate the impact of other common self-reported comorbid disorders (hypertension, dyslipidemia, ischemic heart disease, diabetes mellitus, minor stroke, arthritis, low back pain or osteoporosis and depression) on health-related Quality of Life (HRQoL) of Parkinson's disease (PD) patients and to explore the association of their HRQoL with various sociodemographic and clinical factors.

Methods: Data about age, gender, education, occupation, income, marital and residential status, social relations, disease duration, functional status, treatment and concomitant diseases were collected of 139 Greek patients (68 men and 71 women) with PD. Patients were consecutively recruited from the outpatient clinic of the first Neurology Department of Athens National University at Aeginition Hospital. Disease severity was assessed using the unified Parkinson's disease rating scale including Hoehn and Yahr and Schwab and England (S&E) scales. HRQoL was measured by the specific Parkinson's disease questionnaire (PDQ-39). A multivariate multiple regression model with normal errors was used for the statistical analysis.

Results: The main determinants of HRQoL were low degree of independence measured by the S&E scale (F = 35.942, p < 0.001), social isolation (F = 20.508, p < 0.001), disease duration (F = 14.983, p < 0.001), sleep (F = 6.507, p = 0.013) and gastrointestinal disturbances (F = 4.643, p = 0.035) and the presence of depression (F = 6.022, p = 0.017).

Conclusion: Among the other chronic comorbidities only depression was associated with a poor HRQoL in PD patients. Functional dependence and social isolation contributed most to worse HRQoL. Our findings suggest that adequate social support and management of depression, sleep and gastrointestinal disturbances could reduce the distress and improve HRQoL in patients with PD.

C1 [Andreadou, Elisabeth; Anagnostouli, Maria; Kararizou, Evangelia; Rentzos, Michael; Kontaxis, Theodoros; Evdokimidis, Ioannis] Univ Athens, Aeginit Hosp, Sch Med, Dept Neurol 1, Athens 11528, Greece.

[Vasdekis, Vassilis] Athens Univ Econ & Business, Dept Stat, Athens, Greece.

RP Andreadou, E (reprint author), Univ Athens, Aeginit Hosp, Sch Med, Dept Neurol 1, Athens 11528, Greece.

EM eandread@med.uoa.gr

OI Andreadou, Elisabeth/0000-0002-9634-9631

CR Alonso J, 2004, QUAL LIFE RES, V13, P283, DOI 10.1023/B:QURE.0000018472.46236.05

Aydemir O, 2005, ARCH MED RES, V36, P136, DOI 10.1016/j.arcmed.2004.12.009

Barone P, 2009, MOVEMENT DISORD, V24, P1641, DOI 10.1002/mds.22643

Caap-Ahlgren M, 2001, ARCH GERONTOL GERIAT, V32, P23, DOI 10.1016/S0167-4943(00)00087-X

Carod-Artal FJ, 2007, MOVEMENT DISORD, V22, P1408, DOI 10.1002/mds.21408

Chaudhuri KR, 2006, LANCET NEUROL, V5, P235, DOI 10.1016/S1474-4422(06)70373-8

Findley L, 2002, MOVEMENT DISORD, V17, P60

Forsaa EB, 2008, MOVEMENT DISORD, V23, P1420, DOI 10.1002/mds.22121

Fulop T, 2006, PATHOL BIOL, V54, P375, DOI 10.1016/j.patbio.2006.07.002

Gage H, 2003, J NEUROL NEUROSUR PS, V74, P163, DOI 10.1136/jnnp.74.2.163

Goetz CG, 2003, MOVEMENT DISORD, V18, P738, DOI 10.1002/mds.10473

Gulati A, 2004, MOVEMENT DISORD, V19, pS406

HARLOW SD, 1989, AM J EPIDEMIOL, V129, P233, DOI 10.1093/oxfordjournals.aje.a115129

HOEHN MM, 1967, NEUROLOGY, V17, P427, DOI 10.1212/WNL.17.5.427

HUGHES AJ, 1992, J NEUROL NEUROSUR PS, V55, P181, DOI 10.1136/jnnp.55.3.181

Karlsen KH, 2000, J NEUROL NEUROSUR PS, V69, P584, DOI 10.1136/jnnp.69.5.584

Karlsen KH, 1999, J NEUROL NEUROSUR PS, V66, P431, DOI 10.1136/jnnp.66.4.431

Katsarou Z, 2004, MOVEMENT DISORD, V19, P308, DOI 10.1002/mds.10678

Katsarou Z, 2001, QUAL LIFE RES, V10, P159, DOI 10.1023/A:1016720400862

Lyons K E, 1997, Parkinsonism Relat Disord, V3, P207, DOI 10.1016/S1353-8020(97)00021-7

Martinez-Martin P, 1998, J NEUROL, V245, pS2, DOI 10.1007/PL00007733

Martinez-Martin P, 2007, MOVEMENT DISORD, V22, P1623, DOI 10.1002/mds.21586

Menza M, 2009, MOVEMENT DISORD, V24, P1325, DOI 10.1002/mds.22586

Murray CJL, 1997, LANCET, V349, P1498, DOI 10.1016/S0140-6736(96)07492-2

Muslimovic D, 2008, NEUROLOGY, V70, P2241, DOI 10.1212/01.wnl.0000313835.33830.80

PETO V, 1995, QUAL LIFE RES, V4, P241, DOI 10.1007/BF02260863

Rahman S, 2008, MOVEMENT DISORD, V23, P1428, DOI 10.1002/mds.21667

Reuther M, 2007, PARKINSONISM RELAT D, V13, P108, DOI 10.1016/j.parkreldis.2006.07.009

Riedinger MS, 2002, AM J CRIT CARE, V11, P211

Schrag A, 2003, MOVEMENT DISORD, V18, P1250, DOI 10.1002/mds.10527

Schrag A, 2006, J NEUROL SCI, V248, P151, DOI 10.1016/j.jns.2006.05.030

SCHWAB RS, 1969, 3 S PARK DIS, P152

Sprangers MAG, 1999, SOC SCI MED, V48, P1507, DOI 10.1016/S0277-9536(99)00045-3

Tandberg E, 1998, MOVEMENT DISORD, V13, P895, DOI 10.1002/mds.870130606

Wee Hwee-Lin, 2005, Health Qual Life Outcomes, V3, P2, DOI 10.1186/1477-7525-3-2

Welsh M, 2004, NEUROL CLIN, V22, pS141, DOI 10.1016/j.ncl.2004.05.003

Winter Y, 2010, PARKINSONISM RELAT D, V16, P243, DOI 10.1016/j.parkreldis.2009.11.012

Zach M, 2004, MOVEMENT DISORD, V19, P667, DOI 10.1002/mds.10698

NR 38

TC 14

Z9 14

U1 0

U2 11

PU ROUTLEDGE JOURNALS, TAYLOR & FRANCIS LTD

PI ABINGDON

PA 4 PARK SQUARE, MILTON PARK, ABINGDON OX14 4RN, OXFORDSHIRE, ENGLAND

SN 1360-7863

J9 AGING MENT HEALTH

JI Aging Ment. Health

PY 2011

VL 15

IS 7

BP 913

EP 921

DI 10.1080/13607863.2011.569477

PG 9

WC Geriatrics & Gerontology; Gerontology; Psychiatry

SC Geriatrics & Gerontology; Psychiatry

GA 881KI

UT WOS:000299482500013

PM 21547746

DA 2019-01-08

ER

PT J

AU Rosal, MC

Borg, A

Bodenlos, JS

Tellez, T

Ockene, IS

AF Rosal, Milagros C.

Borg, Amy

Bodenlos, Jamie S.

Tellez, Trinidad

Ockene, Ira S.

TI Awareness of Diabetes Risk Factors and Prevention Strategies Among a

Sample of Low-Income Latinos With No Known Diagnosis of Diabetes

SO DIABETES EDUCATOR

LA English

DT Article

ID BREAST-CANCER RISK; SOCIOECONOMIC-STATUS; MEXICAN-AMERICANS;

CARDIOVASCULAR HEALTH; PSYCHOSOCIAL FACTORS; ETHNIC-DIFFERENCES;

PHYSICAL-ACTIVITY; PERCEIVED RISK; CARE PROVIDERS; US POPULATION

AB Purpose

This study assessed awareness of type 2 diabetes risk and severity, perceived risk factors, knowledge of diabetes prevention strategies, and challenges of and opportunities for prevention among low-income Latinos in Lawrence, Massachusetts.

Methods

Qualitative research design. Latinos with no known diagnosis of diabetes participated in 4 focus groups, conducted in Spanish, which were recorded and transcribed for systematic analysis.

Results

The sample, (N = 41) was largely female (85%) with a wide age range (22-76 years), most (71%) had an educational level of high school or less, and less than half (46%) were employed. Participants had basic knowledge of diabetes, but gaps were apparent. Many perceived family history of diabetes, poor diet, emotional distress, and stress associated with the United States as risk factors for diabetes. There was little or no awareness of risk associated with Latino ethnicity, gestational diabetes, hypertension, lipid abnormalities, or obesity. Few cited physical activity or weight loss as diabetes prevention strategies. More than half the participants perceived themselves at low risk for diabetes.

Conclusions

This Latino sample had limited knowledge of diabetes risk factors and lifestyle changes that can prevent or delay diabetes onset. Insights for intervening for diabetes prevention are offered.

C1 [Rosal, Milagros C.] Univ Massachusetts, Sch Med, Dept Med, Div Prevent & Behav Med, Worcester, MA 01655 USA.

[Bodenlos, Jamie S.] Hobart & William Smith Coll, Dept Psychol, Geneva, NY 14456 USA.

[Tellez, Trinidad] New Hampshire Dept Hlth & Human Serv, Concord, NH 03301 USA.

[Tellez, Trinidad] Greater Lawrence Family Hlth Ctr, Lawrence, MA USA.

RP Rosal, MC (reprint author), Univ Massachusetts, Sch Med, Dept Med, Div Prevent & Behav Med, 55 Lake Ave N, Worcester, MA 01655 USA.

EM Milagros.Rosal@umassmed.edu

FU National Institute of Diabetes and Digestive and Kidney diseases (NIDDK)

[R18 DK067549]

FX This project described was made possible by Grant Number R18 DK067549

from the National Institute of Diabetes and Digestive and Kidney

diseases (NIDDK). Its contents are solely the responsibility of the

authors and do not necessarily represent the official views of the

NIDDK. We thank our community partners-the Lawrence Council on Aging,

the YWCA of Greater Lawrence, and the Greater Lawrence Family Health

Center-for their contributions to the implementation of the research. We

also thank Philip Merriam, MPH, for his assistance coordinating the

recruitment of participants, and Karin Valentine Goins, MPH, for her

editorial assistance.

CR Ball K, 2006, WOMEN HEALTH, V43, P93, DOI 10.1300/J013v43n01_06

Boltri JM, 2006, DIABETES EDUCATOR, V32, P901, DOI 10.1177/0145721706295010

Brewer NT, 2004, ANN BEHAV MED, V27, P125, DOI 10.1207/s15324796abm2702_7

Byrd TL, 2004, PREV MED, V38, P192, DOI 10.1016/j.ypmed.2003.09.017

Carbone ET, 2007, PATIENT EDUC COUNS, V66, P202, DOI 10.1016/j.pec.2006.12.003

Castro FG, 2009, J BEHAV MED, V32, P89, DOI 10.1007/s10865-008-9194-z

*CDCP, 2004, MMWR-MORBID MORTAL W, V53, P935

Cohen S, 2006, PSYCHOSOM MED, V68, P414, DOI 10.1097/01.psy.0000221236.37158.b9

Coronado GD, 2004, J HEALTH CARE POOR U, V15, P576, DOI 10.1353/hpu.2004.0057

Cowie CC, 2006, DIABETES CARE, V29, P1263, DOI 10.2337/dc06-0062

Cramer JS, 2007, DIABETES EDUCATOR, V33, P503, DOI 10.1177/0145721707301680

Dalle Grave R, 2005, OBES RES, V13, P1961, DOI DOI 10.1038/OBY.2005.241

Davidson Jaime A, 2007, Endocr Pract, V13, P77

Dooley KE, 2009, LANCET INFECT DIS, V9, P737, DOI 10.1016/S1473-3099(09)70282-8

Dutton GR, 2005, DIABETES CARE, V28, P1209, DOI 10.2337/diacare.28.5.1209

Engum A, 2007, J PSYCHOSOM RES, V62, P31, DOI 10.1016/j.jpsychores.2006.07.009

Falcon LM, 2000, J GERONTOL B-PSYCHOL, V55, pS108, DOI 10.1093/geronb/55.2.S108

Ferrara A, 2002, DIABETES CARE, V25, P1625, DOI 10.2337/diacare.25.9.1625

FRANKLIN GM, 1990, AM J EPIDEMIOL, V131, P633, DOI 10.1093/oxfordjournals.aje.a115547

Gallo LC, 2003, PSYCHOL BULL, V129, P10, DOI 10.1037//0033-2909.129.1.10

George GC, 2005, J AM DIET ASSOC, V105, P916, DOI 10.1016/j.jada.2005.03.009

Getaneh A, 2008, ETHNIC DIS, V18, P342

Hannan EL, 1999, MED CARE, V37, P68, DOI 10.1097/00005650-199901000-00010

Harris MI, 1998, DIABETES CARE, V21, P1230, DOI 10.2337/diacare.21.8.1230

Helitzer DL, 2007, AM J HEALTH PROMOT, V21, P517, DOI 10.4278/0890-1171-21.6.517

Ho LS, 2008, HEALTH EDUC BEHAV, V35, P561, DOI 10.1177/1090198108315367

Hussain A, 2007, DIABETES RES CLIN PR, V76, P317, DOI 10.1016/j.diabres.2006.09.020

Jenkins JH, 2005, INT J SOC PSYCHIATR, V51, P211, DOI 10.1177/0020764005056986

Jezewski MA, 2002, WESTERN J NURS RES, V24, P840, DOI 10.1177/019394502237695

Jotkowitz AB, 2006, AM J MED, V119, P665, DOI 10.1016/j.amjmed.2006.02.010

Katapodi MC, 2004, PREV MED, V38, P388, DOI 10.1016/j.ypmed.2003.11.012

Knowler WC, 2002, NEW ENGL J MED, V346, P393

Kurian AK, 2007, ETHNIC DIS, V17, P143

La Pelle N, 2004, FIELD METHOD, V18, P85, DOI [10.1177/1525822x03259227, DOI 10.1177/1525822X03259227]

Lanting LC, 2005, DIABETES CARE, V28, P2280, DOI 10.2337/diacare.28.9.2280

Lantz PM, 2005, J HEALTH SOC BEHAV, V46, P274, DOI 10.1177/002214650504600305

Lee AJ, 2007, DIABETES CARE, V30, P878, DOI 10.2337/dc06-1816

MAINOUS AG, 2007, DIABETOLOGIA, V50, P934

Markosyan KM, 2007, AIDS BEHAV, V11, P325, DOI 10.1007/s10461-006-9147-x

Martin R, 1998, HEALTH PSYCHOL, V17, P346, DOI 10.1037//0278-6133.17.4.346

McCaul KD, 1996, HEALTH PSYCHOL, V15, P423, DOI 10.1037/0278-6133.15.6.423

Mensah GA, 2005, CIRCULATION, V111, P1233, DOI 10.1161/01.CIR.0000158136.76824.04

Mier Nelda, 2007, Prev Chronic Dis, V4, pA24

Mosca L, 2006, CIRCULATION, V113, P525, DOI 10.1161/CIRCULATIONAHA.105.588103

Mosca L, 2005, CIRCULATION, V111, P499, DOI 10.1161/01.CIR.0000154568.43333.82

NHLBI obesity Education Initiative Expert Panel, 1998, CLIN GUID ID EV TREA

Pope C., 2006, QUALITATIVE RES HLTH

Prata N, 2006, INT FAM PLAN PERSPEC, V32, P192, DOI 10.1363/3219206

Rice P, 2001, QUALITATIVE RES METH

Robb KA, 2004, CANCER EPIDEM BIOMAR, V13, P366

Rosal MC, 2004, HEALTH EDUC BEHAV, V31, P388, DOI 10.1177/1090198104263360

Rosecrans AM, 2008, HEALTH EDUC RES, V23, P272, DOI 10.1093/her/cym031

Seidel MC, 2008, DIABETES CARE, V31, P684, DOI 10.2337/dc07-1869

Taveras EM, 2006, AMBUL PEDIATR, V6, P342, DOI 10.1016/j.ambp.2006.07.004

U.S. Department of Health and Human Services Centers for Disease Control and Prevention, EARL REL SEL EST BAS

*UD DEP HHS, 2006, NAT HEALTHC DISP REP

*US BUR CENS, 2006, ANN EST POP SEX RAC

van Ryn M, 2000, SOC SCI MED, V50, P813, DOI 10.1016/S0277-9536(99)00338-X

Weinstein Neil D., 1999, Journal of the National Cancer Institute Monographs, P15

Wing RR, 1999, J CONSULT CLIN PSYCH, V67, P132, DOI 10.1037//0022-006X.67.1.132

NR 60

TC 15

Z9 15

U1 1

U2 13

PU SAGE PUBLICATIONS INC

PI THOUSAND OAKS

PA 2455 TELLER RD, THOUSAND OAKS, CA 91320 USA

SN 0145-7217

J9 DIABETES EDUCATOR

JI Diabetes Educ.

PD JAN-FEB

PY 2011

VL 37

IS 1

BP 47

EP 55

DI 10.1177/0145721710392247

PG 9

WC Endocrinology & Metabolism; Public, Environmental & Occupational Health

SC Endocrinology & Metabolism; Public, Environmental & Occupational Health

GA 715LC

UT WOS:000286885000003

PM 21220363

DA 2019-01-08

ER

PT J

AU Welch, G

Zagarins, SE

Feinberg, RG

Garb, JL

AF Welch, Garry

Zagarins, Sofija E.

Feinberg, Rebecca G.

Garb, Jane L.

TI Motivational interviewing delivered by diabetes educators: Does it

improve blood glucose control among poorly controlled type 2 diabetes

patients?

SO DIABETES RESEARCH AND CLINICAL PRACTICE

LA English

DT Article

DE Type 2 diabetes; Diabetes self management education (DSME); Motivational

interviewing; Blood glucose control; HbA1c

ID SELF-MANAGEMENT; PROGRAM; WOMEN

AB Aim: To determine whether glycemic control is improved when motivational interviewing (MI), a patient-centered behavior change strategy, is used with diabetes self management education (DSME) as compared to DSME alone.

Methods: Poorly controlled type 2 diabetes (T2DM) patients (n = 234) were randomized into 4 groups: MI + DSME or DSME alone, with or without use of a computerized summary of patient self management barriers. We compared HbA1c changes between groups at 6 months and investigated mediators of HbA1c change.

Results: Study patients attended the majority of the four intervention visits (mean 3.4), but drop-out rate was high at follow-up research visits (35%). Multiple regression showed that groups receiving MI had a mean change in HbA1c that was significantly lower (less improved) than those not receiving MI (t = 2.10; p = 0.037). Mediators of HbA1c change for the total group were diabetes self-care behaviors and diabetes distress; no between-group differences were found.

Conclusions: DSME improved blood glucose control, underlining its benefit for T2DM management. However, MI + DSME was less effective than DSME alone. Overall, weak support was found for the clinical utility of MI in the management of T2DM delivered by diabetes educators. (C) 2010 Elsevier Ireland Ltd. All rights reserved.

C1 [Welch, Garry; Zagarins, Sofija E.; Feinberg, Rebecca G.] Baystate Med Ctr, Dept Behav Med Res, Springfield, MA 01105 USA.

[Garb, Jane L.] Baystate Med Ctr, Dept Acad Affairs, Springfield, MA 01104 USA.

RP Welch, G (reprint author), Baystate Med Ctr, Dept Behav Med Res, 140 High St,Room 2104, Springfield, MA 01105 USA.

EM Garry.Welch@baystatehealth.org

RI Albertin, Paloma/X-1093-2018

FU National Institutes of Health [1R01DK060076]

FX This research was supported by National Institutes of Health grant

#1R01DK060076.

CR *AM ASS DIAB ED, 2009, DIABETES ED

*AM DIAB ASS, 1998, DIABETES CARE, V21, P2180

American Association of Diabetes Educators, 2008, DIABETES ED, V34, P445, DOI DOI 10.1177/0145721708316625

American Diabetes Association, 2008, DIABETES CARE S1, V31, pS12, DOI DOI 10.2337/DC08-S012

Barnard KD, 2007, PRACT INT DIABETES, V24, P36

Bradley C, 1999, DIABETES CARE, V22, P530, DOI 10.2337/diacare.22.3.530

Centers for Disease Control and Prevention, 2001, TEAM CAR COMPR LIF M

Funnell MM, 2005, DIABETES EDUCATOR, V31, P53, DOI 10.1177/0145721704273166

MACKINNON DP, 1993, EVALUATION REV, V17, P144, DOI 10.1177/0193841X9301700202

MACKINNON DP, 1995, MULTIVAR BEHAV RES, V30, pR2

Miller W. R., 2002, MOTIVATIONAL INTERVI

*MOT INT, 2010, MOT INT RES CLIN RES

Peyrot M, 2007, DIABETES CARE, V30, P2433, DOI 10.2337/dc07-1222

Preacher KJ, 2004, BEHAV RES METH INS C, V36, P717, DOI 10.3758/BF03206553

RADLOFF L S, 1977, Applied Psychological Measurement, V1, P385, DOI 10.1177/014662167700100306

RUBAK S, 2005, THESIS U AARHUS DENM

Smith DE, 1997, DIABETES CARE, V20, P52, DOI 10.2337/diacare.20.1.52

Weinger K, 2005, DIABETES CARE, V28, P1346, DOI 10.2337/diacare.28.6.1346

WELCH G, 2002, DIABETES SPECTRUM, V15, P203

WELCH G, 2007, DIABETES

Welch GW, 1997, DIABETES CARE, V20, P760, DOI 10.2337/diacare.20.5.760

Welch GW, 2007, TXB TYPE 2 DIABETES

West DS, 2007, DIABETES CARE, V30, P1081, DOI 10.2337/dc06-1966

Williams GC, 2005, PATIENT EDUC COUNS, V56, P28, DOI 10.1016/j.pec.2003.11.008

NR 24

TC 36

Z9 38

U1 0

U2 18

PU ELSEVIER IRELAND LTD

PI CLARE

PA ELSEVIER HOUSE, BROOKVALE PLAZA, EAST PARK SHANNON, CO, CLARE, 00000,

IRELAND

SN 0168-8227

J9 DIABETES RES CLIN PR

JI Diabetes Res. Clin. Pract.

PD JAN

PY 2011

VL 91

IS 1

BP 54

EP 60

DI 10.1016/j.diabres.2010.09.036

PG 7

WC Endocrinology & Metabolism

SC Endocrinology & Metabolism

GA 698XS

UT WOS:000285629100017

PM 21074887

OA Green Accepted

DA 2019-01-08

ER

PT J

AU Gasquoine, PG

AF Gasquoine, Philip G.

TI Cognitive impairment in common, noncentral nervous system medical

conditions of adults and the elderly

SO JOURNAL OF CLINICAL AND EXPERIMENTAL NEUROPSYCHOLOGY

LA English

DT Article

DE Cognitive impairment; Neuropsychological testing; Frontal-subcortical

brain injury; Hypoxic-ischemic encephalopathy; Cytokines

ID OBSTRUCTIVE SLEEP-APNEA; RESPIRATORY-DISTRESS-SYNDROME; BREAST-CANCER;

NEUROPSYCHOLOGICAL SEQUELAE; ADJUVANT CHEMOTHERAPY; OLDER-ADULTS;

HYPERTENSION; DISEASE; PERFORMANCE; COMPLAINTS

AB Common, noncentral nervous system medical conditions linked with cognitive impairment in adults and the elderly include: acute respiratory distress syndrome; cancer; chronic kidney disease; chronic obstructive pulmonary disease; coronary heart disease; hypertension; obesity (bariatric surgical candidates); obstructive sleep apnea; and type 2 diabetes. Cross-condition comparison of the nature and frequency of cognitive impairment is difficult as these conditions often coexist, and there exists no consensus as to the definition of cognitive impairment, nor the optimal number and type of neuropsychological tests required for evaluation. There is as yet no clear evidence for condition-specific profiles of cognitive impairment. Rather, a generalized profile consisting of subclinical levels of impairment in attention, processing speed, executive, and memory functions from bilateral frontal-subcortical ischemia fits across all conditions. This profile: occurs only in subgroups of patients; is inconsistently related to measures of illness severity; is unrelated to patient self-report or level of functional independence; is exacerbated by very high levels of emotional distress; and is reversible in some cases but can also progress to frank neurological disease (dementia) in others, especially the elderly, when multiple conditions coexist, and/or when medical condition severity progresses.

C1 Univ Texas Pan Amer, Dept Psychol & Anthropol, Edinburg, TX 78541 USA.

RP Gasquoine, PG (reprint author), Univ Texas Pan Amer, Dept Psychol & Anthropol, 1201 W Univ Dr, Edinburg, TX 78541 USA.

EM drgdrg13@yahoo.com

FU College of Social and Behavioral Sciences, University of Texas-Pan

American

FX This work was supported in part by a 2010 Summer Writing Institute Grant

from the College of Social and Behavioral Sciences, University of

Texas-Pan American.

CR AH DV, 2009, CLIN NEUROPSYCHOL, V23, P661

Aloia MS, 2004, J INT NEUROPSYCH SOC, V10, P772, DOI 10.1017/S1355617704105134

Amenta F, 2002, J NEUROL SCI, V203, P147, DOI 10.1016/S0022-510X(02)00281-2

American Psychiatric Association, 1994, DIAGN STAT MAN MENT

Anderson-Hanley C, 2003, J INT NEUROPSYCH SOC, V9, P967, DOI 10.1017/S1355617703970019

Antonelli-Incalzi R, 2008, J CLIN EXP NEUROPSYC, V30, P141, DOI 10.1080/13803390701287390

Awad N, 2004, J CLIN EXP NEUROPSYC, V26, P1044, DOI 10.1080/13803390490514875

Binder LM, 2004, J CLIN EXP NEUROPSYC, V26, P369, DOI 10.1080/13803390490510095

Boeka AG, 2008, ARCH CLIN NEUROPSYCH, V23, P467, DOI 10.1016/j.acn.2008.03.003

Bossema ER, 2006, J CLIN EXP NEUROPSYC, V28, P357, DOI 10.1080/13803390590935318

Brady CB, 2005, NEUROPSYCHOLOGY, V19, P770, DOI 10.1037/0894-4105.19.6.770

Brands AMA, 2007, J INT NEUROPSYCH SOC, V13, P288, DOI 10.1017/S1355617707070312

Brandt J, 2007, CLIN NEUROPSYCHOL, V21, P553, DOI 10.1080/13854040600770800

Castellon SA, 2004, J CLIN EXP NEUROPSYC, V26, P955, DOI 10.1080/13803390490510905

Catania A, 2009, BRAIN BEHAV IMMUN, V23, P877, DOI 10.1016/j.bbi.2009.04.006

*CDCP, A Z IND

CHELUNE GJ, 1986, INT J EAT DISORDER, V5, P701, DOI 10.1002/1098-108X(198605)5:4<701::AID-EAT2260050409>3.0.CO;2-7

Cohen RA, 2009, J CLIN EXP NEUROPSYC, V31, P96, DOI 10.1080/13803390802014594

Daurat A, 2008, J CLIN EXP NEUROPSYC, V30, P91, DOI 10.1080/13803390701236116

Delis D. C., 2000, CALIFORNIA VERBAL LE

DeLuca J, 2004, J INT NEUROPSYCH SOC, V10, P101

Denes A, 2010, BRAIN BEHAV IMMUN, V24, P708, DOI 10.1016/j.bbi.2009.09.010

Dijkstra JB, 2002, NEUROPSYCHOL REV, V12, P1, DOI 10.1023/A:1015404122161

EHRENGAFT J, 1961, J THORAC CARDIOV SUR, V42, P514

Finkelstein EA, 2005, AM J MANAG CARE, V11, P630

Gale SD, 2004, J INT NEUROPSYCH SOC, V10, P60

Gasquoine PG, 1997, NEUROPSYCHOL REV, V7, P77, DOI 10.1023/B:NERV.0000005945.58251.c0

Hopkins RO, 2004, J INT NEUROPSYCH SOC, V10, P1005, DOI 10.1017/S135561770410711X

Incalzi RA, 2003, J CLIN EXP NEUROPSYC, V25, P14, DOI 10.1076/jcen.25.1.14.13635

Jackson JC, 2009, SOUTH MED J, V102, P1150, DOI 10.1097/SMJ.0b013e3181b6a592

Kesler SR, 2009, CLIN CANCER RES, V15, P6665, DOI 10.1158/1078-0432.CCR-09-1227

Koushik NS, 2010, NEUROPSYCHOL REV, V20, P33, DOI 10.1007/s11065-009-9110-5

Larson MJ, 2007, J INT NEUROPSYCH SOC, V13, P595, DOI 10.1017/S1355617707070749

Liao DP, 1996, STROKE, V27, P2262, DOI 10.1161/01.STR.27.12.2262

Lu FP, 2009, PLOS ONE, V4, DOI 10.1371/journal.pone.0004144

Miles WR, 1922, ARCH INTERN MED, V30, P767, DOI 10.1001/archinte.1922.00110120086003

Moss M, 2005, J CLIN EXP NEUROPSYC, V27, P139, DOI 10.1080/13803390490515450

Naismith S, 2004, J CLIN EXP NEUROPSYC, V26, P43, DOI 10.1076/jcen.26.1.43.23929

Obler W, 1911, CAN MED ASSOC J, V40, P919

Ortapamuk H, 2006, ANN NUCL MED, V20, P99, DOI 10.1007/BF02985621

Ouimet LA, 2009, J CLIN EXP NEUROPSYC, V31, P73, DOI 10.1080/13803390801992725

Pedersen AD, 2009, J INT NEUROPSYCH SOC, V15, P296, DOI 10.1017/S1355617709090316

Perry VH, 2004, BRAIN BEHAV IMMUN, V18, P407, DOI 10.1016/j.bbi.2004.01.004

Prohl J, 2009, J INT NEUROPSYCH SOC, V15, P344, DOI 10.1017/S1355617709090493

Reid LM, 2006, DEMENT GERIATR COGN, V22, P471, DOI 10.1159/000096295

Rourke SB, 1999, J CLIN EXP NEUROPSYC, V21, P737, DOI 10.1076/jcen.21.6.737.863

Sanchez AI, 2004, CLIN NEUROPSYCHOL, V18, P277, DOI 10.1080/13854040490501547

Schretlen DJ, 2008, J INT NEUROPSYCH SOC, V14, P436, DOI 10.1017/S1355617708080387

Selnes OA, 2010, J INT NEUROPSYCH SOC, V16, P221, DOI 10.1017/S1355617709991196

Shilling V, 2006, BREAST CANCER RES TR, V95, P125, DOI 10.1007/s10549-005-9055-1

Stewart A, 2006, CLIN NEUROPSYCHOL, V20, P76, DOI 10.1080/138540491005875

Thornton WL, 2007, J INT NEUROPSYCH SOC, V13, P344, DOI 10.1017/S1355617707070361

Verhaeghen P, 1997, PSYCHOL BULL, V122, P231, DOI 10.1037//0033-2909.122.3.231

WALDSTEIN SR, 1991, PSYCHOL BULL, V110, P451, DOI 10.1037//0033-2909.110.3.451

Waldstein SR, 2005, ANN BEHAV MED, V29, P174, DOI 10.1207/s15324796abm2903_3

Watari K, 2006, ARCH CLIN NEUROPSYCH, V21, P787, DOI 10.1016/j.acn.2006.06.014

Wefel JS, 2008, NEUROPSYCHOL REV, V18, P121, DOI 10.1007/s11065-008-9058-x

Wefel JS, 2004, CANCER, V101, P466, DOI 10.1002/cncr.20393

Yeung SE, 2009, NEUROPSYCHOLOGY, V23, P1, DOI 10.1037/a0013849

NR 59

TC 14

Z9 14

U1 0

U2 13

PU TAYLOR & FRANCIS INC

PI PHILADELPHIA

PA 530 WALNUT STREET, STE 850, PHILADELPHIA, PA 19106 USA

SN 1380-3395

EI 1744-411X

J9 J CLIN EXP NEUROPSYC

JI J. Clin. Exp. Neuropsychol.

PY 2011

VL 33

IS 4

BP 486

EP 496

AR PII 931971666

DI 10.1080/13803395.2010.536759

PG 11

WC Psychology, Clinical; Clinical Neurology; Psychology

SC Psychology; Neurosciences & Neurology

GA 746SC

UT WOS:000289266300009

PM 21218318

DA 2019-01-08

ER

PT J

AU Hatfield, L

Schwoebel, A

Lynyak, C

AF Hatfield, Linda

Schwoebel, Ann

Lynyak, Corinne

TI Caring for the Infant of a Diabetic Mother

SO MCN-THE AMERICAN JOURNAL OF MATERNAL-CHILD NURSING

LA English

DT Article

DE Gestational diabetes; Infant; Newborn; Pregnancy in diabetics/metabolism

ID GLUCOSE-METABOLISM; PREGNANCY; FETAL; MELLITUS; OUTCOMES; NEWBORN

AB In the United States, approximately 100,000 infants are born to diabetic mothers each year. If diabetes in pregnancy is uncontrolled, the diversity of resulting health problems can have a profound effect on the embryo, the fetus, and the neonate. These infants are at risk for a multitude of physiologic, metabolic, and congenital complications such as macrosomia, asphyxia, respiratory distress, hypoglycemia, hypocalcemia, hyperbilirubinemia, polycythemia and hyperviscosity, cardiomegaly, and central nervous system disruption. Preconception control of glucose metabolism throughout the trajectory of a woman's pregnancy is a significant factor in decreasing the adverse impact of diabetes on the fetus and newborn. Meticulous attention to neonatal glucose levels, thorough physical examination, and precise diagnosis are prerequisites to appropriate care for the neonate.

C1 [Hatfield, Linda] Univ Penn, Sch Nursing, Neonatal Nurse Practitioner Program, Philadelphia, PA 19104 USA.

[Hatfield, Linda; Schwoebel, Ann; Lynyak, Corinne] Penn Hosp, Philadelphia, PA 19107 USA.

RP Hatfield, L (reprint author), Univ Penn, Sch Nursing, Neonatal Nurse Practitioner Program, Philadelphia, PA 19104 USA.

EM linda.hatfield@nursing.upenn.edu

CR [Anonymous], 2005, DIABETES CARE, V28, pS4

Armentrout Debra C, 2003, MCN Am J Matern Child Nurs, V28, P234

Barnes-Powell LL, 2007, NEONATAL NETW, V26, P283, DOI 10.1891/0730-0832.26.5.283

Bromiker R, 2006, J PEDIATR-US, V149, P640, DOI 10.1016/j.jpeds.2006.07.034

Chu SY, 2007, DIABETES CARE, V30, P2070, DOI 10.2337/dc06-2559a

Cornblath M, 2000, SEMIN PERINATOL, V24, P136, DOI 10.1053/sp.2000.6364

COWETT RM, 2002, NEOREVIEWS, V3, pE173, DOI DOI 10.1542/NEO.3-9-E173

Crowther CA, 2005, NEW ENGL J MED, V352, P2477, DOI 10.1056/NEJMoa042973

Eidelman AI, 2002, SEMIN PERINATOL, V26, P232, DOI 10.1053/sper.2002.34215

Garg M, 2006, CLIN PERINATOL, V33, P853, DOI 10.1016/j.clp.2006.10.001

Jain A, 2008, INDIAN J PEDIATR, V75, P165, DOI 10.1007/s12098-008-0067-9

Kamanu CI, 2009, ARCH GYNECOL OBSTET, V279, P857, DOI 10.1007/s00404-008-0780-7

Meur S, 2007, PAEDIAT CHILD HLTH, V17, P217, DOI DOI 10.1016/J.PAED.2007.03.004

Mitanchez D, 2007, HORM RES, V68, P265, DOI 10.1159/000104174

NAFDAY SM, 2008, AAP TXB PEDIAT CARE, P2935

Parretti E, 2001, DIABETES CARE, V24, P1319, DOI 10.2337/diacare.24.8.1319

Persson B, 2009, SEMIN FETAL NEONAT M, V14, P106, DOI 10.1016/j.siny.2009.01.003

ROHATGI PK, 2001, PRINCIPLES PRACTICE, P1848

Sasidharan P, 2004, PEDIATR CLIN N AM, V51, P999, DOI 10.1016/j.pcl.2004.03.010

Waters TP, 2009, OBSTET GYNECOL, V114, P45, DOI 10.1097/AOG.0b013e3181a9454b

Yang J, 2006, OBSTET GYNECOL, V108, P644, DOI 10.1097/01.AOG.0000231688.08263.47

NR 21

TC 6

Z9 6

U1 0

U2 4

PU LIPPINCOTT WILLIAMS & WILKINS

PI PHILADELPHIA

PA TWO COMMERCE SQ, 2001 MARKET ST, PHILADELPHIA, PA 19103 USA

SN 0361-929X

EI 1539-0683

J9 MCN-AM J MATERN-CHIL

JI MCN-Am. J. Matern.-Child Nurs.

PD JAN-FEB

PY 2011

VL 36

IS 1

BP 10

EP 16

DI 10.1097/NMC.0b013e3181fb0b4c

PG 7

WC Nursing

SC Nursing

GA 695YA

UT WOS:000285407600004

PM 21164312

DA 2019-01-08

ER

PT J

AU Howe, CJ

Ratcliffe, SJ

Tuttle, A

Dougherty, S

Lipman, TH

AF Howe, Carol J.

Ratcliffe, Sarah J.

Tuttle, Alan

Dougherty, Shayne

Lipman, Terri H.

TI Needle Anxiety in Children With Type 1 Diabetes and Their Mothers

SO MCN-THE AMERICAN JOURNAL OF MATERNAL-CHILD NURSING

LA English

DT Article

DE Blood injury phobia; Children; Diabetes; Mothers; Needle anxiety; Needle

phobia

ID INJURY PHOBIA; PAIN; FEAR; DISTRESS; BLOOD; VENIPUNCTURE; CHILDHOOD;

CANCER

AB Purpose: To report the prevalence of fear, distress, pain, and level of cooperation with insulin injections and blood glucose fingersticks in children with newly diagnosed diabetes and their mothers, and the association with diabetes control.

Study Design and Methods: Forty-six subjects (23 children and mother pairs) completed the Perceptions of Insulin Shots and Fingersticks Survey at diagnosis and 6 to 9 months later. Standard descriptive statistics, Fisher's exact tests, and Spearman correlation coefficients were used to analyze the data. Scores were analyzed for associations with age and hemoglobin A1c as a marker of diabetes control.

Results: More young children as compared to older subjects reported fear and pain with injections and fingersticks. A high percentage of mothers reported high fear and distress with needles at diagnosis. Although most improve, 13.6% of mothers continued to report high fear and distress 6 to 9 months later. Mothers' continued report of high distress correlated with the poor cooperation of children, which correlated with poorer diabetes control.

Clinical Implications: Nurses should incorporate assessment and intervention for needle anxiety in children and parents at diagnosis of diabetes through informal interview or formal survey. Nurses can effectively incorporate coping strategies into their teaching of parents and children to administer injections and fingersticks.

C1 [Howe, Carol J.; Dougherty, Shayne] Childrens Hosp Philadelphia, Div Endocrinol, Diabet Ctr Children, Philadelphia, PA 19104 USA.

[Ratcliffe, Sarah J.] Univ Penn, Sch Med, Dept Biostat & Epidemiol, Philadelphia, PA 19104 USA.

[Tuttle, Alan] Childrens Hosp Philadelphia, Div Neurol, Philadelphia, PA 19104 USA.

[Lipman, Terri H.] Childrens Hosp Philadelphia, Div Endocrinol & Diabet, Philadelphia, PA 19104 USA.

RP Howe, CJ (reprint author), Childrens Hosp Philadelphia, Div Endocrinol, Diabet Ctr Children, Philadelphia, PA 19104 USA.

EM howe@email.chop.edu

OI Ratcliffe, Sarah/0000-0002-6644-8284

CR ABUSAAD H, 1984, PAIN, V19, P163, DOI 10.1016/0304-3959(84)90836-4

Berlin I, 1997, DIABETES CARE, V20, P176, DOI 10.2337/diacare.20.2.176

Bienvenu OJ, 1998, PSYCHOL MED, V28, P1129, DOI 10.1017/S0033291798007144

Boman KK, 2004, J PEDIATR-US, V145, P373, DOI 10.1016/j.jpeds.2004.05.043

Broome M E, 1990, Oncol Nurs Forum, V17, P361

Chambers CT, 2009, CLIN THER, V31, pS77, DOI 10.1016/j.clinthera.2009.07.023

Chapman HR, 2002, BRIT DENT J, V193, P447, DOI 10.1038/sj.bdj.4801593

Cline RJW, 2006, SOC SCI MED, V63, P883, DOI 10.1016/j.socscimed.2006.03.007

DAHQUIST LM, 2002, J PEDIATR ONCOL NURS, V19, P22, DOI DOI 10.1053/JPON.2002.30009

Deacon B, 2006, J ANXIETY DISORD, V20, P946, DOI 10.1016/j.janxdis.2006.01.004

Frank RG, 1998, ARTHRIT CARE RES, V11, P166, DOI 10.1002/art.1790110304

Goodenough B, 1999, PAIN, V80, P179, DOI 10.1016/S0304-3959(98)00201-2

Hagglof B, 1994, Arctic Med Res, V53 Suppl 1, P20

HANAS R, 2001, THESIS FACULTY HLTH

Hanas R, 1997, PRACTICAL DIABETES, V14, P95, DOI DOI 10.1002/PDI.1960140404

Keck J F, 1996, J Pediatr Nurs, V11, P368, DOI 10.1016/S0882-5963(96)80081-9

MARKS I, 1988, AM J PSYCHIAT, V145, P1207

McGrath P. A., 1985, ADV PAIN RES THER, V9, P387

Simmons JH, 2007, DIABETES CARE, V30, P987, DOI 10.2337/dc06-1553

Spagrud LJ, 2008, J PAIN SYMPTOM MANAG, V36, P173, DOI 10.1016/j.jpainsymman.2007.10.009

SPARKS L, 2001, AM J MATERNAL CHILD, V26, P72, DOI DOI 10.1097/00005721/200103000-00005

ULMAN LS, 2009, COCHRANE COLLABORATI, V3, P1

Walco Gary A, 2008, Pediatrics, V122 Suppl 3, pS125, DOI 10.1542/peds.2008-1055d

WONG D, 1991, NURSING CARE INFANTS

Wright S, 2009, AUST FAM PHYSICIAN, V38, P172

Zambanini A, 1999, DIABETES RES CLIN PR, V46, P239, DOI 10.1016/S0168-8227(99)00099-6

NR 26

TC 18

Z9 18

U1 0

U2 17

PU LIPPINCOTT WILLIAMS & WILKINS

PI PHILADELPHIA

PA 530 WALNUT ST, PHILADELPHIA, PA 19106-3621 USA

SN 0361-929X

J9 MCN-AM J MATERN-CHIL

JI MCN-Am. J. Matern.-Child Nurs.

PD JAN-FEB

PY 2011

VL 36

IS 1

BP 25

EP 31

DI 10.1097/NMC.0b013e3181fc6093

PG 7

WC Nursing

SC Nursing

GA 695YA

UT WOS:000285407600006

PM 21164314

DA 2019-01-08

ER

PT J

AU Min, LC

Reuben, DB

Keeler, E

Ganz, DA

Fung, CH

Shekelle, P

Roth, CP

Wenger, NS

AF Min, Lillian C.

Reuben, David B.

Keeler, Emmett

Ganz, David A.

Fung, Constance H.

Shekelle, Paul

Roth, Carol P.

Wenger, Neil S.

TI Is Patient-Perceived Severity of a Geriatric Condition Related to Better

Quality of Care?

SO MEDICAL CARE

LA English

DT Article

DE quality of care; urinary incontinence; falls

ID CORONARY-ARTERY-DISEASE; URINARY-INCONTINENCE; ELDERLY-PATIENTS;

SEX-DIFFERENCES; BLOOD-PRESSURE; OLDER PATIENTS; DIABETES CARE; MANAGED

CARE; MEDICAL-CARE; FOLLOW-UP

AB Background: Care for falls and urinary incontinence (UI) among older patients is inadequate. One possible explanation is that physicians provide less recommended care to patients who are not as concerned about their falls and UI.

Objective: To test whether patient-reported severity for 2 geriatric conditions, falls, and UI, is associated with quality of care.

Research Design: Prospective cohort study of elders with falls and/or fear of falling (n = 384) and UI (n = 163).

Subjects: Participants in the Assessing Care of Vulnerable Elders-2 Study (2002-2003), which evaluated an intervention to improve the care for falls and UI among older (age, >= 75) ambulatory care patients with falls/fear of falling or UI. Measures: Falls Efficacy Scale (FES) and the Incontinence Quality of Life surveys measured at baseline, quality of care measured by a 13-month medical record abstraction.

Results: There was a small difference in falls quality scores across the range of FES, with greater patient-perceived falls severity associated with better odds of passing falls quality indicators (OR: 1.11 [95% CI: 1.02-1.21] per 10-point increment in FES). Greater patient-perceived UI severity (Incontinence Quality of Life score) was not associated with better quality of UI care.

Conclusions: Although older persons with greater patient-perceived falls severity receive modestly better quality of care, those with more distressing incontinence do not. For both conditions, however, even the most symptomatic patients received less than half of recommended care. Low patient-perceived severity of condition is not the basis of poor care for falls and UI.

C1 [Min, Lillian C.] Univ Michigan, Sch Med, Div Geriatr, Dept Med, Ann Arbor, MI 48109 USA.

[Reuben, David B.; Fung, Constance H.] Univ Calif Los Angeles, David Geffen Sch Med, Dept Med, Div Geriatr, Los Angeles, CA 90095 USA.

[Keeler, Emmett; Ganz, David A.; Shekelle, Paul; Wenger, Neil S.] Univ Calif Los Angeles, David Geffen Sch Med, Gen Internal Med & Hlth Serv Res, Los Angeles, CA 90095 USA.

[Shekelle, Paul; Roth, Carol P.; Wenger, Neil S.] RAND Hlth, Santa Monica, CA USA.

[Ganz, David A.; Shekelle, Paul] Greater Los Angeles Vet Affairs Healthcare Syst, Los Angeles, CA USA.

RP Min, LC (reprint author), Univ Michigan, Sch Med, Div Geriatr, Dept Med, 300 N Ingalls Bldg,Wing E,Room 966, Ann Arbor, MI 48109 USA.

EM lmin@med.umich.edu

FU AHRQ [R21 HS017621]; NIA-UCLA [K12 AG001004]; U.S. Department of

Veterans Affairs, Veterans Health Administration; VA Health Services

Research & Development (HSR&D) Service through the VA Greater Los

Angeles HSR& D Center of Excellence [VA CD2 08-012-1]; National

Institute of Aging [AG028748]

FX Dr. Min is supported by the AHRQ (R21 HS017621). Dr. Min was on the UCLA

faculty during the writing of this manuscript, supported by NIA-UCLA

(K12 AG001004). Dr. Ganz is funded by the U.S. Department of Veterans

Affairs, Veterans Health Administration, VA Health Services Research &

Development (HSR&D) Service through the VA Greater Los Angeles HSR& D

Center of Excellence (Project # VA CD2 08-012-1). This research was also

supported by the UCLA Claude Pepper Older Americans Independence Center

funded by the National Institute of Aging (AG028748).

CR [Anonymous], 2001, ANN INTERN MED, V135, P653, DOI 10.7326/0003-4819-135-8_Part_2-200110161-00004

Asch SM, 2004, INT J QUAL HEALTH C, V16, P41, DOI 10.1093/intqhc/mzh004

AYANIAN JZ, 1994, NEW ENGL J MED, V331, P1136, DOI 10.1056/NEJM199410273311707

Backer V, 2006, ANN ALLERG ASTHMA IM, V97, P490, DOI 10.1016/S1081-1206(10)60940-4

Borowsky SJ, 2000, J GEN INTERN MED, V15, P381, DOI 10.1046/j.1525-1497.2000.12088.x

Burack RC, 1998, J GEN INTERN MED, V13, P366, DOI 10.1046/j.1525-1497.1998.00116.x

Cheng EM, 2007, MOVEMENT DISORD, V22, P515, DOI 10.1002/mds.21311

Chodosh J, 2004, J AM GERIATR SOC, V52, pS189

Choudhry NK, 2005, ANN INTERN MED, V142, P260, DOI 10.7326/0003-4819-142-4-200502150-00008

Cumming RG, 2000, J GERONTOL A-BIOL, V55, pM299, DOI 10.1093/gerona/55.5.M299

Cwikel JG, 1998, DISABIL REHABIL, V20, P161, DOI 10.3109/09638289809166077

Dedier J, 2001, ARCH INTERN MED, V161, P2099, DOI 10.1001/archinte.161.17.2099

Diette GB, 2001, PEDIATRICS, V108, P432, DOI 10.1542/peds.108.2.432

Diokno AC, 2004, AM J MANAG CARE, V10, P69

Etzioni DA, 2006, DIS COLON RECTUM, V49, P1002, DOI 10.1007/s10350-006-0533-2

Fagard RH, 2003, J HUM HYPERTENS, V17, P681, DOI 10.1038/sj.jhh.1001598

Fairchild DG, 2001, J GEN INTERN MED, V16, P663, DOI 10.1111/j.1525-1497.2001.01111.x

Fine JM, 2002, ARCH INTERN MED, V162, P827, DOI 10.1001/archinte.162.7.827

Gallagher TH, 2004, AM J MANAG CARE, V10, P61

Higashi Takahiro, 2005, Health Qual Life Outcomes, V3, P75, DOI 10.1186/1477-7525-3-75

Higashi T, 2007, NEW ENGL J MED, V356, P2496, DOI 10.1056/NEJMsa066253

Humphries KH, 2001, CIRCULATION, V103, P2365

Kerr EA, 2008, ANN INTERN MED, V148, P717, DOI 10.7326/0003-4819-148-10-200805200-00004

Kinchen KS, 2003, J WOMENS HEALTH, V12, P687, DOI 10.1089/154099903322404339

Kressig RW, 2001, J AM GERIATR SOC, V49, P1456, DOI 10.1046/j.1532-5415.2001.4911237.x

Landon BE, 2004, JAMA-J AM MED ASSOC, V291, P1744, DOI 10.1001/jama.291.14.1744

LEWIS CE, 1991, ANN INTERN MED, V114, P54, DOI 10.7326/0003-4819-114-1-54

McCall DT, 2004, DIABETES CARE, V27, P1060, DOI 10.2337/diacare.27.5.1060

Mehrotra A, 2006, ANN INTERN MED, V145, P826, DOI 10.7326/0003-4819-145-11-200612050-00007

Miller M, 2000, ARCH INTERN MED, V160, P343, DOI 10.1001/archinte.160.3.343

Min LC, 2005, J AM GERIATR SOC, V53, P1705, DOI 10.1111/j.1532-5415.2005.53520.x

Min LC, 2007, MED CARE, V45, P480, DOI 10.1097/MLR.0b013e318030fff9

Patrick DL, 1999, QUALITY LIFE INSTRUM

Peters TJ, 2004, HEALTH SOC CARE COMM, V12, P53, DOI 10.1111/j.1365-2524.2004.00468.x

Pham HH, 2005, JAMA-J AM MED ASSOC, V294, P473, DOI 10.1001/jama.294.4.473

Piette JD, 2006, DIABETES CARE, V29, P725, DOI 10.2337/diacare.29.03.06.dc05-2078

POWELL LE, 1995, J GERONTOL A-BIOL, V50, pM28, DOI 10.1093/gerona/50A.1.M28

Rathore SS, 2003, JAMA-J AM MED ASSOC, V289, P2517, DOI 10.1001/jama.289.19.2517

Reuben DB, 2003, J AM GERIATR SOC, V51, P1787, DOI 10.1046/j.1532-5415.2003.51565.x

ROYSTON P, 2005, STATA J, V5, P527

Smetana GW, 2007, ARCH INTERN MED, V167, P10, DOI 10.1001/archinte.167.1.10

Solomon DH, 2003, J AM GERIATR SOC, V51, P902, DOI 10.1046/j.1365-2389.2003.513331.x

STEINGART RM, 1991, NEW ENGL J MED, V325, P226, DOI 10.1056/NEJM199107253250402

TINETTI ME, 1990, J GERONTOL, V45, pP239, DOI 10.1093/geronj/45.6.P239

Turner BJ, 2008, ANN INTERN MED, V148, P578, DOI 10.7326/0003-4819-148-8-200804150-00002

Wagner TH, 1996, UROLOGY, V47, P67, DOI 10.1016/S0090-4295(99)80384-7

Walsh JME, 1999, AM J PREV MED, V16, P307, DOI 10.1016/S0749-3797(99)00021-5

Wenger NS, 2009, J AM GERIATR SOC, V57, P547, DOI 10.1111/j.1532-5415.2008.02128.x

Wenger NS, 2003, ANN INTERN MED, V139, P740, DOI 10.7326/0003-4819-139-9-200311040-00008

Wu AW, 2001, ARCH INTERN MED, V161, P2554, DOI 10.1001/archinte.161.21.2554

NR 50

TC 2

Z9 2

U1 0

U2 4

PU LIPPINCOTT WILLIAMS & WILKINS

PI PHILADELPHIA

PA 530 WALNUT ST, PHILADELPHIA, PA 19106-3621 USA

SN 0025-7079

J9 MED CARE

JI Med. Care

PD JAN

PY 2011

VL 49

IS 1

BP 101

EP 107

DI 10.1097/MLR.0b013e3181f53523

PG 7

WC Health Care Sciences & Services; Health Policy & Services; Public,

Environmental & Occupational Health

SC Health Care Sciences & Services; Public, Environmental & Occupational

Health

GA 695XW

UT WOS:000285407100014

PM 21079526

OA Green Accepted

DA 2019-01-08

ER

PT J

AU Chen, HY

Baumgardner, DJ

Rice, JP

AF Chen, Han-Yang

Baumgardner, Dennis J.

Rice, Jessica P.

TI Health-Related Quality of Life Among Adults With Multiple Chronic

Conditions in the United States, Behavioral Risk Factor Surveillance

System, 2007

SO PREVENTING CHRONIC DISEASE

LA English

DT Article

AB Introduction

Little is known about health-related quality of life (HRQOL) among people with multiple chronic conditions. We examined the association between the number of chronic conditions and self-reported HRQOL outcomes among adults in the United States.

Methods

We used data from the Behavioral Risk Factor Surveillance System (BRFSS) in 2007 (n = 430,912) to compare 4 HRQOL measures for people with any of 8 chronic conditions. We also assessed the frequency of self-reported physical and mental distress and the number of days activity was limited because of chronic conditions. We estimated prevalence and adjusted odds ratios (AORs) and 95% confidence intervals (CIs) by using survey logistic regression analyses.

Results

People with 3 or more chronic conditions had the highest risk of reporting fair or poor health compared with respondents with no chronic conditions (AOR, 8.7; 95% CI, 8.0-9.4). People with cardiovascular conditions or diabetes had higher risk of reporting poor HRQOL outcomes than those with other chronic conditions. The odds ratios for frequent physical distress were consistently higher than those for frequent mental distress and frequent activity limitations for all conditions.

Conclusion

Strategies that help clinicians to manage their patients' chronic conditions may contribute to improved HRQOL among adults. Our findings may help to inform these strategies.

C1 [Baumgardner, Dennis J.; Rice, Jessica P.] Univ Wisconsin, Sch Med & Publ Hlth, Ctr Urban Populat Hlth, Milwaukee, WI 53233 USA.

[Baumgardner, Dennis J.] Aurora UW Med Grp, Milwaukee, WI USA.

RP Chen, HY (reprint author), Univ Wisconsin, Sch Med & Publ Hlth, Ctr Urban Populat Hlth, 1020 N 12th St,Suite 4180, Milwaukee, WI 53233 USA.

EM chen25@wisc.edu

CR Andresen EM, 1999, ARCH PHYS MED REHAB, V80, P877, DOI 10.1016/S0003-9993(99)90077-1

Andresen EM, 2003, J EPIDEMIOL COMMUN H, V57, P339, DOI 10.1136/jech.57.5.339

Bethell C., 2001, PORTRAIT CHRONICALLY

Bombard JM, 2005, AM J PREV MED, V28, P251, DOI 10.1016/j.amepre.2004.12.004

BROWNSON RC, 1994, EPIDEMIOLOGY, V5, P545

Centers for Disease Control and Prevention, CHRON DIS POW PREV C

Centers for Disease Control and Prevention, 2000, MEAS HLTH DAYS POP A

Centers for Disease Control and Prevention, 2007, BEH RISK FACT SURV S

de Visser CL, 2002, QUAL LIFE RES, V11, P249, DOI 10.1023/A:1015287825660

Dominick Kelli L, 2004, Health Qual Life Outcomes, V2, P5

Engelgau MM, 2004, ANN INTERN MED, V140, P945, DOI 10.7326/0003-4819-140-11-200406010-00035

Ford ES, 2003, CHEST, V123, P119, DOI 10.1378/chest.123.1.119

Greenlund KJ, 2002, STROKE, V33, P565, DOI 10.1161/hs0202.102882

HENNESSY CH, 1994, PUBLIC HEALTH REP, V109, P665

Heo M, 2003, OBES RES, V11, P209, DOI 10.1038/oby.2003.33

Jiang YW, 2006, HEALTH QUAL LIFE OUT, V4, DOI 10.1186/1477-7525-4-14

JUNIPER EF, 1992, THORAX, V47, P76, DOI 10.1136/thx.47.2.76

Miller W. R., 2002, MOTIVATIONAL INTERVI

Moriarty DG, 2005, PREV CHRONIC DIS, V2

Mowad L, 2004, WESTERN J NURS RES, V26, P293, DOI 10.1177/0193945903261556

Newschaffer CJ, 1998, VALIDATION BEHAV RIS

Pew Research Center for the People and the Press, 2006, CELL PHON CHALL SURV

POLONSKY WH, 1995, DIABETES CARE, V18, P754, DOI 10.2337/diacare.18.6.754

Robert SA, 2009, J GERONTOL B-PSYCHOL, V64, P378, DOI 10.1093/geronb/gbp012

Schecter S, 1999, COGNITION AGING SELF

SNOEK F., 2000, DIABETES SPECTRUM, V13, P24

Strine TW, 2005, AM J PREV MED, V28, P182, DOI 10.1016/j.ampre.2004.10.002

Thompson DR, 2002, QUAL LIFE RES, V11, P535, DOI 10.1023/A:1016354516168

Thorpe KE, 2006, HEALTH AFFAIR, V25, pW378, DOI 10.1377/hlthaff.25.w378

U. S. Department of Health and Human Services, 2000, HLTH PEOPL 2010

Valdmanis V, 2001, AM J PUBLIC HEALTH, V91, P129

Verbrugge LM, 1999, DISABIL REHABIL, V21, P295

Vogeli C, 2007, J GEN INTERN MED, V22, P391, DOI 10.1007/s11606-007-0322-1

Walker AE, 2007, CHRONIC ILLN, V3, P202, DOI 10.1177/1742395307081504

Williams LS, 1999, STROKE, V30, P1362, DOI 10.1161/01.STR.30.7.1362

NR 35

TC 30

Z9 30

U1 0

U2 5

PU CENTERS DISEASE CONTROL

PI ATLANTA

PA 1600 CLIFTON RD, ATLANTA, GA 30333 USA

SN 1545-1151

J9 PREV CHRONIC DIS

JI Prev. Chronic Dis.

PD JAN

PY 2011

VL 8

IS 1

AR UNSP A09

PG 9

WC Public, Environmental & Occupational Health

SC Public, Environmental & Occupational Health

GA V29DW

UT WOS:000208730100009

PM 21159221

DA 2019-01-08

ER

PT J

AU Manzoni, GM

Villa, V

Compare, A

Castelnuovo, G

Nibbio, F

Titon, AM

Molinari, E

Gondoni, LA

AF Manzoni, Gian Mauro

Villa, Valentina

Compare, Angelo

Castelnuovo, Gianluca

Nibbio, Ferruccio

Titon, Anna Maria

Molinari, Enrico

Gondoni, Luca Alessandro

TI Short-term effects of a multi-disciplinary cardiac rehabilitation

programme on psychological well-being, exercise capacity and weight in a

sample of obese in-patients with coronary heart disease: A

practice-level study

SO PSYCHOLOGY HEALTH & MEDICINE

LA English

DT Article

DE cardiac rehabilitation; psychological well-being; exercise capacity;

obesity; weight loss

ID QUALITY-OF-LIFE; BODY-MASS INDEX; ECHOCARDIOGRAPHIC MEASUREMENTS;

CARDIOVASCULAR-DISEASE; RISK; OVERWEIGHT; DEPRESSION; PROFILES;

OUTCOMES; PARADOX

AB Given that many patients referred to cardiac rehabilitation (CR) are obese, diet therapy, exercise training, nutritional and psychological counselling for both obesity and psychological distress should be included as important components in all CR programmes. In this practice-level, observational study we evaluated the short-term within-group effects of a four-week multi-factorial inpatient CR programme specifically addressed to weight loss, fitness improvement and psychological health increase on 176 obese in-patients with coronary heart disease (CHD). Outcome measures were exercise capacity measured with estimated metabolic equivalents (METs), body mass index (BMI) and psychological well-being (PGWBI). Results show statistically significant improvements in all the PGWBI sub-scales and total score, except in general health (p=0.393). No moderation effects were found for BMI class, age, diabetes and ejection fraction (EF). METs significantly increased by 30.3% (p0.001) and BMI decreased by 1.37 points (p0.001). Significant correlations were found between BMI and weight reductions with PGWBI anxiety and total score improvements. This multi-disciplinary CR programme including diet therapy, exercise training and psychological counselling provides indication for short-term within-group effectiveness on functional exercise capacity, BMI and PGWBI in a sample of obese in-patients with CHD. However, controlled studies are needed to corroborate the results we found.

C1 [Manzoni, Gian Mauro; Villa, Valentina; Compare, Angelo; Castelnuovo, Gianluca; Molinari, Enrico] Osped San Giuseppe, Psychol Res Lab, Ist Auxol Italiano IRCCS, Verbania, Italy.

[Manzoni, Gian Mauro; Compare, Angelo] Univ Bergamo, Dept Psychol, Bergamo, Italy.

[Castelnuovo, Gianluca; Molinari, Enrico] Catholic Univ, Dept Psychol, Milan, Italy.

[Nibbio, Ferruccio; Titon, Anna Maria; Gondoni, Luca Alessandro] Osped San Giuseppe, Unit Cardiac Rehabil, Ist Auxol Italiano IRCCS, Verbania, Italy.

RP Manzoni, GM (reprint author), Osped San Giuseppe, Psychol Res Lab, Ist Auxol Italiano IRCCS, Verbania, Italy.

EM gm.manzoni@auxologico.it

OI Castelnuovo, Gianluca/0000-0003-2633-9822; Villa,

Valentina/0000-0002-9972-1064; Manzoni, Gian Mauro/0000-0003-3384-0359;

Gondoni, Luca/0000-0002-7139-0726; Molinari, Enrico/0000-0001-8132-694X;

Compare, Angelo/0000-0002-3336-7920

CR Ades PA, 2009, CIRCULATION, V119, P2671, DOI 10.1161/CIRCULATIONAHA.108.834184

Ashley EA, 2000, LANCET, V356, P1592, DOI 10.1016/S0140-6736(00)03138-X

Bader D S, 2001, J Cardiopulm Rehabil, V21, P210, DOI 10.1097/00008483-200107000-00003

Barth J, 2009, J WOMENS HEALTH, V18, P2033, DOI 10.1089/jwh.2008.1058

Benzer W, 2007, EUR J CARDIOV PREV R, V14, P441, DOI 10.1097/HJR.0b013e32802bf7ae

Bjarnason-Wehrens B, 2007, CLIN RES CARDIOL, V96, P77, DOI 10.1007/s00392-007-0461-0

Boesch C., 2005, J CARDIOPULM REHABIL, V25, P22, DOI [10.1097/ 00008483- 200501000- 00004, DOI 10.1097/00008483-200501000-00004]

Boesch Claudia, 2005, J Cardiopulm Rehabil, V25, P14, DOI 10.1097/00008483-200501000-00004

Brochu M, 2000, J Cardiopulm Rehabil, V20, P96, DOI 10.1097/00008483-200003000-00003

Cohen J, 1988, STAT POWER ANAL BEHA

Cooper Z, 2001, BEHAV RES THER, V39, P499, DOI 10.1016/S0005-7967(00)00065-6

Davin SA, 2009, PSYCHOL HEALTH MED, V14, P716, DOI 10.1080/13548500903431501

Dela Cruz CS, 2009, CLIN CHEST MED, V30, P509, DOI 10.1016/j.ccm.2009.06.001

Dupuy HJ., 1984, ASSESSMENT QUALITY L, P170

Fabricatore AN, 2004, CLIN DERMATOL, V22, P332, DOI 10.1016/j.clindermatol.2004.01.006

Flegal KM, 2005, JAMA-J AM MED ASSOC, V293, P1861, DOI 10.1001/jama.293.15.1861

Gondoni LA, 2008, ACTA CARDIOL, V63, P153, DOI 10.2143/AC.63.2.2029521

GRAHAM I, 2007, EUR J CARDIOVASC S2, V14, P1

GROSSI E, 2002, QUESTIONARIO PSYCHOL

Gunstad John, 2007, Prev Cardiol, V10, P64, DOI 10.1111/j.1520-037X.2007.05860.x

Harvey-Berino J, 1998, CORONARY ARTERY DIS, V9, P795, DOI 10.1097/00019501-199809120-00003

Herlitz J, 2003, CORONARY ARTERY DIS, V14, P509, DOI 10.1097/00019501-200311000-00006

Jobin Jean, 2005, J Cardiopulm Rehabil, V25, P103, DOI 10.1097/00008483-200503000-00010

Kinge JM, 2010, SOC SCI MED, V71, P1864, DOI 10.1016/j.socscimed.2010.09.001

Kushner RF, 2000, NUTRITION, V16, P947, DOI 10.1016/S0899-9007(00)00404-4

Lavie Carl J, 2008, J Cardiometab Syndr, V3, P136, DOI 10.1111/j.1559-4572.2008.00002.x

Lavie CJ, 2007, AM J MED, V120, P825, DOI 10.1016/j.amjmed.2007.06.023

Lavie CJ, 2006, ARCH INTERN MED, V166, P1878, DOI 10.1001/archinte.166.17.1878

Lavie CJ, 2009, AM J MED, V122, P1106, DOI 10.1016/j.amjmed.2009.06.006

Lavie CJ, 2009, J AM COLL CARDIOL, V53, P1925, DOI 10.1016/j.jacc.2008.12.068

Lavie CJ, 1997, AM J CARDIOL, V79, P397, DOI 10.1016/S0002-9149(97)89239-9

Luppino FS, 2010, ARCH GEN PSYCHIAT, V67, P220, DOI 10.1001/archgenpsychiatry.2010.2

Michie S, 2005, PSYCHOL HEALTH MED, V10, P88, DOI DOI 10.1080/13548500512331315398

Milani RV, 1996, AM HEART J, V132, P726, DOI 10.1016/S0002-8703(96)90304-X

NIH, 1998, OBES RES S2, V6, pS1

Otterstad JE, 1997, EUR HEART J, V18, P507

Pischon T, 2008, NEW ENGL J MED, V359, P2105, DOI 10.1056/NEJMoa0801891

Rozanski A, 1999, CIRCULATION, V99, P2192, DOI 10.1161/01.CIR.99.16.2192

SAHN DJ, 1978, CIRCULATION, V58, P1072, DOI 10.1161/01.CIR.58.6.1072

Savage Patrick D, 2002, J Cardiopulm Rehabil, V22, P154, DOI 10.1097/00008483-200205000-00005

Savage PD, 2006, CORONARY ARTERY DIS, V17, P227, DOI 10.1097/00019501-200605000-00004

Sierra-Johnson J, 2005, AM J CARDIOL, V96, P211, DOI 10.1016/j.ajmcard.2005.03.046

Sommaruga Marinella, 2003, Monaldi Arch Chest Dis, V60, P40

Vanhecke TE, 2009, CLIN CARDIOL, V32, P121, DOI 10.1002/clc.20458

Whitlock G, 2009, LANCET, V373, P1083, DOI 10.1016/S0140-6736(09)60318-4

*WHO, 2006, OBES OV FACT SHEET 3

Yu CM, 2003, AM J CARDIOL, V91, P321, DOI 10.1016/S0002-9149(02)03159-4

NR 47

TC 26

Z9 26

U1 1

U2 7

PU ROUTLEDGE JOURNALS, TAYLOR & FRANCIS LTD

PI ABINGDON

PA 4 PARK SQUARE, MILTON PARK, ABINGDON OX14 4RN, OXFORDSHIRE, ENGLAND

SN 1354-8506

J9 PSYCHOL HEALTH MED

JI Psychol. Health Med.

PY 2011

VL 16

IS 2

BP 178

EP 189

AR PII 933307214

DI 10.1080/13548506.2010.542167

PG 12

WC Public, Environmental & Occupational Health

SC Public, Environmental & Occupational Health

GA 720WX

UT WOS:000287314500005

PM 21328146

DA 2019-01-08

ER

PT J

AU Chun, KM

Chesla, CA

Kwan, CML

AF Chun, Kevin M.

Chesla, Catherine A.

Kwan, Christine M. L.

TI "So We Adapt Step by Step": Acculturation experiences affecting diabetes

management and perceived health for Chinese American immigrants

SO SOCIAL SCIENCE & MEDICINE

LA English

DT Article

DE Acculturation; Diabetes management; Chinese American immigrant health;

USA

ID DISEASE MANAGEMENT; ASIAN-AMERICANS; RISK-FACTORS; PREVALENCE;

POPULATIONS; OBESITY; CARE

AB This study examines how acculturation affects type 2 diabetes management and perceived health for Chinese American immigrants in the U.S. Acculturation experiences or cultural adaptation experiences affecting diabetes management and health were solicited from an informant group of immigrant patients and their spouses (N = 40) during group, couple and individual interviews conducted from 2005 to 2008. A separate respondent group of immigrant patients and their spouses (N = 19) meeting inclusion criteria reviewed and confirmed themes generated by the informant group. Using interpretive phenomenology, three key themes in patients' and spouses' acculturation experiences were identified: a) utilizing health care, b) maintaining family relations and roles, and c) establishing community ties and groundedness in the U.S. Acculturation experiences reflecting these themes were broad in scope and not fully captured by current self-report and proxy acculturation measures. In the current study, shifting family roles and evaluations of diabetes care and physical environment in the U.S. significantly affected diabetes management and health, yet are overlooked in acculturation and health investigations. Furthermore, the salience and impact of specific acculturation experiences respective to diabetes management and perceived health varied across participants due to individual, family, developmental, and environmental factors. In regards to salience, maintaining filial and interdependent family relations in the U.S. was of particular concern for older participants and coping with inadequate health insurance in the U.S. was especially distressing for self-described lower-middle to middle-class participants. In terms of impact, family separation and relocating to ethnically similar neighborhoods in the U.S. differentially affected diabetes management and health due to participants' varied family relations and pre-migration family support levels and diverse cultural and linguistic backgrounds, respectively. Implications for expanding current conceptualizations and measures of acculturation to better comprehend its dynamic and multidimensional properties and complex effects on health are discussed. Additionally, implications for developing culturally-appropriate diabetes management recommendations for Chinese immigrants and their families are outlined. (C) 2010 Elsevier Ltd. All rights reserved.

C1 [Chun, Kevin M.] Univ San Francisco, Dept Psychol, San Francisco, CA 94117 USA.

[Chesla, Catherine A.; Kwan, Christine M. L.] Univ Calif San Francisco, Dept Family Hlth Care Nursing, San Francisco, CA 94143 USA.

RP Chun, KM (reprint author), Univ San Francisco, Dept Psychol, 2130 Fulton St, San Francisco, CA 94117 USA.

EM chunk@usfca.edu

FU National Institute of Nursing [R01-NR009111]

FX This work was supported by the National Institute of Nursing Research

Grant R01-NR009111 (Principal Investigator: Catherine A. Chesla). We

thank the following organizations and individuals: the California

Pacific Medical Center; the Chinese Community Health Resource Center;

Donaldina Cameron House; North East Medical Services; University of

California San Francisco (UCSF) Diabetes Teaching Center; UCSF Lakeshore

Family Practice; Peggy Huang; Kenneth Chang; Y.H. Cheng: Eunice Lew;

Phiona Tan; Barbara Jung, and Ivan Wu.

CR Abate N, 2003, J DIABETES COMPLICAT, V17, P39, DOI 10.1016/S1056-8727(02)00190-3

Abe-Kim J, 2001, Cultur Divers Ethnic Minor Psychol, V7, P232, DOI 10.1037/1099-9809.7.3.232

Benner P, 1994, INTERPRETATIVE PHENO

Candib LM, 2007, ANN FAM MED, V5, P547, DOI 10.1370/afm.754

Chesla CA, 2005, QUAL HEALTH RES, V15, P240, DOI 10.1177/1049732304272050

Chesla CA, 2009, DIABETES CARE, V32, P1812, DOI 10.2337/dc09-0278

Chun K. M., 2007, HDB ASIAN AM PSYCHOL, P47

Chun K. M., 2009, HDB MENTAL HLTH ACCU, P99

Chun KM, 2004, PSYCHOL HEALTH, V19, P767, DOI 10.1080/08870440410001722958

Chun KM, 2003, ACCULTURATION ADV TH

CHUN KM, 2006, ACCULTURATION PARENT, P63

Cohen MZ, 2000, HERMENEUTIC PHENOMEN

Fisher L, 2004, J FAM PSYCHOL, V18, P302, DOI 10.1037/0893-3200.18.2.302

Gomez SL, 2004, AM J PUBLIC HEALTH, V94, P1977, DOI 10.2105/AJPH.94.11.1977

Green AR, 2005, J GEN INTERN MED, V20, P1050, DOI 10.1111/j.1525-1497.2005.0223.x

Hosler AS, 2003, AM J PUBLIC HEALTH, V93, P1279, DOI 10.2105/AJPH.93.8.1279

Huang BJ, 1996, AM J EPIDEMIOL, V144, P674

Jayne RL, 2001, J NURS SCHOLARSHIP, V33, P53, DOI 10.1111/j.1547-5069.2001.00053.x

Kandula NR, 2008, DIABETES CARE, V31, P1621, DOI 10.2337/dc07-2182

LU N, 2004, J AM DIET ASSOC, V104, P771

Marin G., 2003, HDB RACIAL ETHNIC MI, P208

McDonald JT, 2005, SOC SCI MED, V61, P2469, DOI 10.1016/j.socscimed.2005.05.004

McNeely MJ, 2004, DIABETES CARE, V27, P66, DOI 10.2337/diacare.27.1.66

Miltiades HB, 2008, SOC SCI MED, V66, P704, DOI 10.1016/j.socscimed.2007.10.016

Myers H. F., 2003, ACCULTURATION ADV TH, P163, DOI DOI 10.1037/10472-011

Ngo-Metzger Q, 2003, J GEN INTERN MED, V18, P44, DOI 10.1046/j.1525-1497.2003.20205.x

PEREZESCAMILLA IT, 2007, J NUTR, V137, P860

Salant T, 2003, SOC SCI MED, V57, P71, DOI 10.1016/S0277-9536(02)00300-3

Satia JA, 2001, J AM DIET ASSOC, V101, P548, DOI 10.1016/S0002-8223(01)00137-7

Shinagawa LH, 2008, PORTRAIT CHINESE AM

Sue S., 2003, ACCULTURATION ADV TH, pxvii

SUINN RM, 1992, EDUC PSYCHOL MEAS, V52, P1041, DOI 10.1177/0013164492052004028

Trimble J. E., 2003, ACCULTURATION ADV TH

Unger JB, 2004, J COMMUN HEALTH, V29, P467, DOI 10.1007/s10900-004-3395-3

Ying YW, 1995, AM J COMMUN PSYCHOL, V23, P893, DOI 10.1007/BF02507020

ZANE N, 2003, ACCULTURATION ADV TH, P39, DOI DOI 10.1037/10472-005

NR 36

TC 34

Z9 34

U1 0

U2 18

PU PERGAMON-ELSEVIER SCIENCE LTD

PI OXFORD

PA THE BOULEVARD, LANGFORD LANE, KIDLINGTON, OXFORD OX5 1GB, ENGLAND

SN 0277-9536

J9 SOC SCI MED

JI Soc. Sci. Med.

PD JAN

PY 2011

VL 72

IS 2

BP 256

EP 264

DI 10.1016/j.socscimed.2010.11.010

PG 9

WC Public, Environmental & Occupational Health; Social Sciences, Biomedical

SC Public, Environmental & Occupational Health; Biomedical Social Sciences

GA 755EZ

UT WOS:000289911300018

PM 21147509

OA Green Accepted

DA 2019-01-08

ER

PT J

AU Osborn, CY

Mayberry, LS

Mulvaney, SA

Hess, R

AF Osborn, Chandra Y.

Mayberry, Lindsay Satterwhite

Mulvaney, Shelagh A.

Hess, Rachel

TI Patient Web Portals to Improve Diabetes Outcomes: A Systematic Review

SO CURRENT DIABETES REPORTS

LA English

DT Review

DE Patient web portal; Systematic review; Diabetes; Evaluation; Usability;

Electronic health record; Personal health record; Glycemic control;

Satisfaction; Type 1; Type 2; Providers; Caregivers; Self-care;

Behaviors; Quality of life; Diabetes distress; Management; Communication

ID RANDOMIZED CONTROLLED-TRIAL; ELECTRONIC HEALTH RECORDS;

DECISION-SUPPORT; SELF-MANAGEMENT; CARE; INTERVENTION; IMPLEMENTATION;

INFORMATION; TECHNOLOGY; PHYSICIANS

AB Patient web portals (PWPs), defined as the integration of electronic medical records and patient health records, have been related to enhanced patient outcomes. A literature review was conducted to characterize the design and evaluation of PWPs to improve health care processes and outcomes in diabetes. A summary of 26 articles revealed the positive impact PWPs have on patient outcomes, patient-provider communication, disease management, and access to and patient satisfaction with health care. Innovative and useful approaches included the evaluation of specific components of the PWPs, assessing the impact of PWPs on mediators of health behaviors, such as patient distress, identification of barriers to use, and patient willingness to pay for access. Future research should focus on relevant processes that mediate patient and provider use, impact on health care utilization, and a patient-centered approach to the design and integration of educational opportunities afforded through PWPs.

C1 [Osborn, Chandra Y.] Vanderbilt Univ, Med Ctr, Div Gen Internal Med & Publ Hlth, Ctr Hlth Serv Res, Nashville, TN 37232 USA.

[Mayberry, Lindsay Satterwhite] Vanderbilt Univ, Peabody Coll, Nashville, TN 37232 USA.

[Mulvaney, Shelagh A.] Vanderbilt Univ, Sch Nursing, Med Ctr, Nashville, TN 37232 USA.

[Hess, Rachel] Univ Pittsburgh, Dept Med, Pittsburgh, PA USA.

[Hess, Rachel] Univ Pittsburgh, Dept Epidemiol, Pittsburgh, PA 15261 USA.

RP Osborn, CY (reprint author), Vanderbilt Univ, Med Ctr, Div Gen Internal Med & Publ Hlth, Ctr Hlth Serv Res, 1215 21st Ave S,Suite 6000,MCE N Tower, Nashville, TN 37232 USA.

EM chandra.osborn@vanderbilt.edu

OI Osborn, Chandra/0000-0002-7668-028X

FU NIDDK [P60 DK020593, DK 070026]; AHRQ [R18 HS018168]; Department of

Defense

FX Dr. Chandra Y. Osborn is supported by a Diversity Supplement Award

(NIDDK P60 DK020593). Dr. Shelagh A. Mulvaney is supported by NIDDK P60

DK020593, DK 070026, and AHRQ R18 HS018168. Dr. Rachel Hess is supported

by a grant from the Department of Defense for development, evaluation,

and adaptation of a PWP.

CR Beaudoin Denise E, 2005, AMIA Annu Symp Proc, P892

Bellazzi Riccardo, 2004, Diabetes Technol Ther, V6, P567, DOI 10.1089/dia.2004.6.567

Britto MT, 2009, J AM MED INFORM ASSN, V16, P660, DOI 10.1197/jamia.M3154

Bryce CL, 2008, TELEMED J E-HEALTH, V14, P1035, DOI 10.1089/tmj.2008.0005

Burrington-Brown Jill, 2005, J AHIMA, V76, P24

Businger Alexandra, 2007, AMIA Annu Symp Proc, P887

Crosson JC, 2007, ANN FAM MED, V5, P209, DOI 10.1370/afm.696

DesRoches CM, 2008, NEW ENGL J MED, V359, P50, DOI 10.1056/NEJMsa0802005

Detmer D, 2008, BMC MED INFORM DECIS, V8, DOI 10.1186/1472-6947-8-45

Dorr D, 2007, J AM MED INFORM ASSN, V14, P156, DOI 10.1197/jamia.M2255

*EXP COMM DIAGN CL, 2002, DIABETES CARE S1, V25, pS1

Faridi Z, 2008, J EVAL CLIN PRACT, V14, P465, DOI 10.1111/j.1365-2753.2007.00881.x

Fonda SJ, 2008, DIABETES TECHNOL THE, V10, P16, DOI 10.1089/dia.2007.0252

Fonda Stephanie J, 2009, J Diabetes Sci Technol, V3, P117

Hess R, 2007, TELEMED J E-HEALTH, V13, P509, DOI 10.1089/tmj.2006.0089

Holbrook A, 2009, CAN MED ASSOC J, V181, P37, DOI 10.1503/cmaj.081272

Hsu J, 2005, J AM MED INFORM ASSN, V12, P164, DOI 10.1197/jamia.M1672

Institute of Medicine, 2001, CROSS QUAL CHASM NEW

Jha AK, 2009, NEW ENGL J MED, V360, P1628, DOI 10.1056/NEJMsa0900592

Kaufman DR, 2006, HOME HLTH CARE MAN P, V18, P223, DOI 10.1177/1084822305281949

Kim EH, 2009, J MED INTERNET RES, V11, DOI 10.2196/jmir.1256

Kim HS, 2006, J NURS CARE QUAL, V21, P266, DOI 10.1097/00001786-200607000-00012

Kittler Anne F, 2004, Inform Prim Care, V12, P129

Knecht LAD, 2006, J HOSP MED, V1, P151, DOI 10.1002/jhm.94

Kobb R, 2003, TELEMED J E-HEALTH, V9, P189, DOI 10.1089/153056203766437525

Kollmann A, 2007, J MED INTERNET RES, V9, DOI 10.2196/jmir.9.5.e36

Koonce TY, 2007, J MED LIBR ASSOC, V95, P77

Krishna Santosh, 2008, J Diabetes Sci Technol, V2, P509

Kushniruk AW, 2009, STUD HEALTH TECHNOL, V150, P883, DOI 10.3233/978-1-60750-044-5-883

Leveille SG, 2008, J GEN INTERN MED, V23, P472, DOI 10.1007/s11606-007-0443-6

Lin CT, 2005, J MED INTERNET RES, V7, P13, DOI 10.2196/jmir.7.4.e47

Lobach David F, 2006, AMIA Annu Symp Proc, P509

Ma CL, 2006, INT J MED INFORM, V75, P577, DOI 10.1016/j.ijmedinf.2005.09.001

McMahon GT, 2005, DIABETES CARE, V28, P1624, DOI 10.2337/diacare.28.7.1624

Noel HC, 2004, TELEMED J E-HEALTH, V10, P170, DOI 10.1089/1530562041641291

NORDFELDT S, J MED INTERNET RES, V12, pE17

Nordqvist C, 2009, J MED INTERNET RES, V11, DOI 10.2196/jmir.1152

Olshansky E, 2008, DIABETES EDUCATOR, V34, P1004, DOI 10.1177/0145721708327304

Quinn CC, 2008, DIABETES TECHNOL THE, V10, P160, DOI 10.1089/dia.2008.0283

Quinn CC, 2009, CONTEMP CLIN TRIALS, V30, P334, DOI 10.1016/j.cct.2009.02.004

Ralston James D, 2006, AMIA Annu Symp Proc, P1070

Ralston JD, 2009, J GEN INTERN MED, V24, P349, DOI 10.1007/s11606-008-0899-z

Ross Stephen E, 2007, AMIA Annu Symp Proc, P1097

Ross Stephen E, 2006, AMIA Annu Symp Proc, P1080

Smith Karen E, 2004, Diabetes Technol Ther, V6, P828, DOI 10.1089/dia.2004.6.828

STEAD WW, 2009, COMPUTATIONAL TECHNO

Stratton IM, 2000, BRIT MED J, V321, P405, DOI 10.1136/bmj.321.7258.405

Tang Chien Hui, 2003, AMIA Annu Symp Proc, P1026

Tang PC, 2006, J AM MED INFORM ASSN, V13, P121, DOI 10.1197/jamia.M2025

Varonen H, 2008, FAM PRACT, V25, P162, DOI 10.1093/fampra/cmn020

VERHOEVEN F, J DIABETES SCI TECHN, V4, P666

WALD JS, 2009, AMIA ANN S P, P678

Weingart SN, 2006, J AM MED INFORM ASSN, V13, P91, DOI 10.1197/jamia.M1833

Wild S, 2004, DIABETES CARE, V27, P1047, DOI 10.2337/diacare.27.5.1047

Yach D, 2006, NAT MED, V12, P62, DOI 10.1038/nm0106-62

Zickmund SL, 2008, J GEN INTERN MED, V23, P20, DOI 10.1007/s11606-007-0273-6

NR 56

TC 75

Z9 75

U1 2

U2 35

PU CURRENT MEDICINE GROUP

PI PHILADELPHIA

PA 400 MARKET STREET, STE 700, PHILADELPHIA, PA 19106 USA

SN 1534-4827

J9 CURR DIABETES REP

JI Curr. Diabetes Rep.

PD DEC

PY 2010

VL 10

IS 6

BP 422

EP 435

DI 10.1007/s11892-010-0151-1

PG 14

WC Endocrinology & Metabolism

SC Endocrinology & Metabolism

GA 736LC

UT WOS:000288495200004

PM 20890688

OA Green Accepted

DA 2019-01-08

ER

PT J

AU Mitanchez, D

AF Mitanchez, D.

TI Foetal and neonatal complications in gestational diabetes: perinatal

mortality, congenital malformations, macrosomia, shoulder dystocia,

birth injuries, neonatal complications

SO DIABETES & METABOLISM

LA English

DT Article

DE malformations; macrosomia; excessive foetal growth; hypoglycaemia;

perinatal asphyxia; birth injuries; gestational diabetes mellitus;

complications; review

ID ADVERSE PREGNANCY OUTCOMES; METABOLIC-CONTROL; GLUCOSE-LEVELS; PLASMA

ERYTHROPOIETIN; CESAREAN DELIVERY; MOTHERS; INFANTS; MELLITUS; INSULIN;

WEIGHT

AB Objective : To evaluate the risks of perinatal complications in infants born to mothers with treated or untreated gestational diabetes mellitus (GDM).

Methods :A search of the PubMed database was performed and recommendations from NICE and the French National Authority for Health were consulted.

Results : Untreated moderate or severe GDM increases the risk of foetal and neonatal complications (EL1). The risk of malformations slightly increases in newborns of mothers with GDM compared to the general population (EL2). This risk is probably associated with the presence of undiagnosed type 2 diabetes among patients with GDM (EL2). There is a linear relationship between maternal blood glucose levels and an increased birth weight (EL2). Treatment for GDM reduces the incidence of macrosomia (EL1). Although the risk of cardiomyopathy in cases of GDM cannot be accurately estimated based on the available data, severe clinical forms are rare. The risks of neonatal asphyxia and perinatal mortality are no higher in infants born to women with GDM (EL2). Birth injuries and brachial plexus injuries are rare, and no more likely to occur in cases of untreated GDM: It is difficult to assess the risk of respiratory distress, regardless of its cause. It is not possible to establish a link between GDM and neonatal respiratory problems due to insufficient data. Although the risk of neonatal hypoglycaemia is difficult to determine due to the variable definitions reported in the literature, the incidence of hypoglycaemia requiring intravenous therapy is low (EL1). The risks of hypocalcaemia (EL4) and hyperbilirubinemia (EL1) are similar to the general population.

Conclusion : Serious perinatal complications specifically associated with GDM are rare. Macrosomia has been demonstrated to be the predominant adverse outcome in cases of GDM. It is the main factor linked to reported cases of complications in GDM. Maternal obesity is an additional risk factor for complications, regardless of diabetes status. (C) 2010 Elsevier Masson SAS. All rights reserved.

C1 Univ Paris 06, Hop Armand Trousseau, AP HP, Fac Med,Serv Neonatol, F-75571 Paris 12, France.

RP Mitanchez, D (reprint author), Univ Paris 06, Hop Armand Trousseau, AP HP, Fac Med,Serv Neonatol, F-75571 Paris 12, France.

EM delphine.mitanchez@trs.aphp.fr

CR Aberg A, 2001, EARLY HUM DEV, V61, P85, DOI 10.1016/S0378-3782(00)00125-0

Allen VM, 2007, J OBSTET GYNAECOL CA, V29, P927, DOI 10.1016/S1701-2163(16)32653-6

Balsells M, 2000, DIABETES NUTR METAB, V13, P257

Cheng YW, 2008, OBSTET GYNECOL, V112, P1015, DOI 10.1097/AOG.0b013e31818b5dd9

Chu SY, 2007, AM J OBSTET GYNECOL, V197, P223, DOI 10.1016/j.ajog.2007.03.027

Cnattingius S, 1998, NEW ENGL J MED, V338, P147, DOI 10.1056/NEJM199801153380302

Cordero L, 1998, ARCH PEDIAT ADOL MED, V152, P249

Correa A, 2008, AM J OBSTET GYNECOL, V199, DOI 10.1016/j.ajog.2008.06.028

Crowther CA, 2005, NEW ENGL J MED, V352, P2477, DOI 10.1056/NEJMoa042973

Cundy T, 2000, DIABETIC MED, V17, P33, DOI 10.1046/j.1464-5491.2000.00215.x

Das S, 2009, ARCH DIS CHILD-FETAL, V94, pF419, DOI 10.1136/adc.2008.156026

Daskalakis G, 2008, ACTA OBSTET GYN SCAN, V87, P403, DOI 10.1080/00016340801908783

De Luca R, 2009, PEDIATRICS, V123, pE1064, DOI 10.1542/peds.2008-2407

DEMARINI S, 1994, OBSTET GYNECOL, V83, P918, DOI 10.1097/00006250-199406000-00003

Dudley DJ, 2007, OBSTET GYN CLIN N AM, V34, P293, DOI 10.1016/j.ogc.2007.03.001

Ehrenberg HM, 2004, AM J OBSTET GYNECOL, V191, P964, DOI 10.1016/j.ajog.2004.05.052

Esakoff TF, 2009, AM J OBSTET GYNECOL, V200, DOI 10.1016/j.ajog.2009.02.035

Garcia-Patterson A, 2004, DIABETOLOGIA, V47, P509, DOI 10.1007/s00125-004-1337-3

Gherman RB, 2006, AM J OBSTET GYNECOL, V195, P657, DOI 10.1016/j.ajog.2005.09.007

Hedderson MM, 2003, OBSTET GYNECOL, V102, P850, DOI 10.1016/S0029-7844(03)00661-6

Hollander MH, 2007, OBSTET GYNECOL SURV, V62, P125, DOI 10.1097/01.ogx.0000253303.92229.59

Horvath K, 2010, BMJ-BRIT MED J, V340, DOI 10.1136/bmj.c1395

Ilves P, 2000, ACTA PAEDIATR, V89, P680, DOI 10.1080/080352500750043990

Janssen PA, 1996, PAEDIATR PERINAT EP, V10, P52, DOI 10.1111/j.1365-3016.1996.tb00026.x

Jolly MC, 2003, EUR J OBSTET GYN R B, V111, P9, DOI 10.1016/S0301-2115(03)00154-4

Landon MB, 2009, NEW ENGL J MED, V361, P1339, DOI 10.1056/NEJMoa0902430

Langer O, 2005, AM J OBSTET GYNECOL, V192, P989, DOI 10.1016/j.ajog.2004.11.039

Langer O, 2000, CLIN OBSTET GYNECOL, V43, P283, DOI 10.1097/00003081-200006000-00006

Metzger BE, 2010, BJOG-INT J OBSTET GY, V117, P575, DOI 10.1111/j.1471-0528.2009.02486.x

Metzger BE, 2008, NEW ENGL J MED, V358, P1991, DOI 10.1056/NEJMoa0707943

Metzger BE, 2009, DIABETES, V58, P453, DOI 10.2337/db08-1112

MILLEY JR, 1984, AM J OBSTET GYNECOL, V149, P673, DOI 10.1016/0002-9378(84)90257-6

MIMOUNI F, 1990, AM J PERINAT, V7, P203, DOI 10.1055/s-2007-999481

MIMOUNI F, 1988, J PEDIATR-US, V113, P345, DOI 10.1016/S0022-3476(88)80282-8

Moore TR, 2002, AM J OBSTET GYNECOL, V186, P641, DOI 10.1067/mob.2002.122851

National Collaborating Centre for Women's and Children's Health,, 2008, DIAB PREGN MAN DIAB

Naylor CD, 1996, JAMA-J AM MED ASSOC, V275, P1165, DOI 10.1001/jama.275.15.1165

Oberhoffer R, 1997, EUR J PEDIATR, V156, P262, DOI 10.1007/s004310050596

Ong KK, 2008, DIABETES CARE, V31, P2193, DOI 10.2337/dc08-1111

Ostlund I, 2003, DIABETES CARE, V26, P2107, DOI 10.2337/diacare.26.7.2107

OSULLIVA.JB, 1973, AM J OBSTET GYNECOL, V116, P901

OSULLIVAN JB, 1966, OBSTET GYNECOL, V27, P683

OSULLIVAN JB, 1966, DIABETES, V15, P466, DOI 10.2337/diab.15.7.466

PEDERSEN J, 1954, ACTA ENDOCRINOL-COP, V16, P330, DOI 10.1530/acta.0.0160330

Piper J M, 1998, J Matern Fetal Med, V7, P148, DOI 10.1002/(SICI)1520-6661(199805/06)7:3<148::AID-MFM9>3.0.CO;2-K

Raatikainen K, 2006, OBESITY, V14, P165, DOI 10.1038/oby.2006.20

Russell NE, 2008, PEDIATR DEVEL PATHOL, V11, P10, DOI 10.2350/07-05-0277.1

SALVESEN DR, 1993, AM J OBSTET GYNECOL, V168, P88, DOI 10.1016/S0002-9378(12)90891-1

Sarkar S, 2008, SEMIN FETAL NEONAT M, V13, P248, DOI 10.1016/j.siny.2008.02.003

Schaefer-Graf UM, 2000, AM J OBSTET GYNECOL, V182, P313, DOI 10.1016/S0002-9378(00)70217-1

Schwartz R, 2000, SEMIN PERINATOL, V24, P120, DOI 10.1053/sp.2000.6363

Sheffield JS, 2002, OBSTET GYNECOL, V100, P925, DOI 10.1016/S0029-7844(02)02242-1

Silver RM, 2007, AM J OBSTET GYNECOL, V196, P433, DOI 10.1016/j.ajog.2006.11.041

SOSENKO IR, 1979, NEW ENGL J MED, V301, P859, DOI 10.1056/NEJM197910183011603

Taricco E, 2009, BJOG-INT J OBSTET GY, V116, P1729, DOI 10.1111/j.1471-0528.2009.02341.x

TERAMO K, 1983, OBSTET GYNECOL, V61, P559

TERAMO KA, 1987, OBSTET GYNECOL, V69, P710

Ullmo S, 2007, EUR HEART J, V28, P1319, DOI 10.1093/eurheartj/ehl416

VEILLE JC, 1992, OBSTET GYNECOL, V79, P51

WIDNESS JA, 1981, J CLIN INVEST, V67, P637, DOI 10.1172/JCI110078

WIDNESS JA, 1990, DIABETOLOGIA, V33, P378, DOI 10.1007/BF00404643

Yogev Yariv, 2007, Archives of Gynecology and Obstetrics, V276, P361, DOI 10.1007/s00404-007-0359-8

Zhang J, 2008, AM J OBSTET GYNECOL, V199, pS51, DOI 10.1016/j.ajog.2008.09.161

NR 63

TC 57

Z9 60

U1 1

U2 19

PU MASSON EDITEUR

PI MOULINEAUX CEDEX 9

PA 21 STREET CAMILLE DESMOULINS, ISSY, 92789 MOULINEAUX CEDEX 9, FRANCE

SN 1262-3636

EI 1878-1780

J9 DIABETES METAB

JI Diabetes Metab.

PD DEC

PY 2010

VL 36

IS 6

BP 617

EP 627

DI 10.1016/j.diabet.2010.11.013

PN 2

PG 11

WC Endocrinology & Metabolism

SC Endocrinology & Metabolism

GA 704IK

UT WOS:000286044600011

PM 21163425

DA 2019-01-08

ER

PT J

AU Thiebaugeorges, O

Guyard-Boileau, B

AF Thiebaugeorges, O.

Guyard-Boileau, B.

TI Obstetrical care in gestational diabetes and management of preterm

labour

SO DIABETES & METABOLISM

LA English

DT Article

DE gestational diabetes; obstetrical monitoring; ultrasound; threat of

preterm labour; corticosteroids; asymmetric septal hypertrophy; review

ID ARTERY DOPPLER VELOCIMETRY; UMBILICAL ARTERY; FETAL WEIGHT; HIGH-RISK;

17-ALPHA-HYDROXYPROGESTERONE CAPROATE; ULTRASOUND ESTIMATION;

RESPIRATORY-DISTRESS; METABOLIC THERAPY; MACROSOMIC FETUS;

RANDOMIZED-TRIAL

AB Aim : To investigate data enabling the development of guidelines for obstetrical monitoring and management of the threat of preterm labour in gestational diabetes.

Methodology : Systematic literature review.

Results : With gestational diabetes and the absence of other disorders or associated risk factors, there is no argument justifying a systematic clinical monitoring schedule different from other pregnancies. The relevance of ultrasound weight estimations is limited. No formula has emerged as being superior to the others or to the simple measurement of abdominal circumference for the prediction of macrosomia (EL3). The usefulness of testing for asymmetric septal hypertrophy has not been demonstrated (EL4). The routine performance of umbilical artery Doppler has no demonstrated utility in the absence of growth restriction or associated hypertension (EL4). Monthly ultrasound monitoring can be proposed for poorly-controlled diabetes or that requiring insulin. With gestational diabetes that is well-controlled with diet, foetal heart rate recording has no demonstrated use. The recording is debateable for poorly-controlled diabetes or that requiring insulin, while taking associated risk factors into consideration. Weekly recording of the foetal heart rate is often advised for type 2 diabetes diagnosed during pregnancy.

If there is a threat of preterm labour, calcium channel blockers and oxytocin receptor antagonists may be used without special precautions. The risk-benefit ratio does not appear favourable for the beta(2)-adrenergic agonists. Lung maturation with corticosteroids may be done in parallel with glycaemic testing and insulin therapy if necessary. Diabetic screening tests should be done several days after the last injection of corticosteroid. (C) 2010 Elsevier Masson SAS. All rights reserved.

C1 [Thiebaugeorges, O.] Maternite Reg Univ Nancy, Serv Obstet, F-54000 Nancy, France.

[Guyard-Boileau, B.] Hop Paule de Viguier, Serv Obstet, TSA 70034, F-31059 Toulouse 9, France.

RP Thiebaugeorges, O (reprint author), Maternite Reg Univ Nancy, Serv Obstet, 10 Rue Dr Heydenreich, F-54000 Nancy, France.

EM o.thiebaugeorges@maternite.chu-nancy.fr;

guyard-boileau.b@chu-toulouse.fr

CR Allen VM, 2007, J OBSTET GYNAECOL CA, V29, P927, DOI 10.1016/S1701-2163(16)32653-6

[Anonymous], 2001, OBSTET GYNECOL, V98, P525

[Anonymous], 2005, RAPP COM NAT TECHN E

BALLARD PL, 1975, J CLIN INVEST, V56, P1548, DOI 10.1172/JCI108236

BENACERRAF BR, 1988, AM J OBSTET GYNECOL, V159, P1118, DOI 10.1016/0002-9378(88)90425-5

BENAMI M, 1995, AM J PERINAT, V12, P437, DOI 10.1055/s-2007-994515

BOCHNER CJ, 1987, AM J OBSTET GYNECOL, V157, P703, DOI 10.1016/S0002-9378(87)80033-9

Bonomo M, 2004, DIABETES METAB, V30, P237, DOI 10.1016/S1262-3636(07)70114-3

Bracero LA, 1996, J ULTRAS MED, V15, P301

BUCHANAN TA, 1994, DIABETES CARE, V17, P275, DOI 10.2337/diacare.17.4.275

Chauhan SP, 1998, OBSTET GYNECOL, V91, P72, DOI 10.1016/S0029-7844(97)00590-5

Chauhan SP, 2000, OBSTET GYNECOL, V95, P639, DOI 10.1016/S0029-7844(99)00606-7

Colman Atalie, 2006, N Z Med J, V119, pU2146

Combs C A, 2000, J Matern Fetal Med, V9, P55, DOI 10.3109/14767050009020514

Conway DL, 2007, DIABETES CARE, V30, pS175, DOI [10.2337/dc07-s212, 10.2337/dcN/-s212]

Coomarasamy A, 2005, BJOG-INT J OBSTET GY, V112, P1461, DOI 10.1111/j.1471-0528.2005.00702.x

COOPER MJ, 1992, AM J DIS CHILD, V146, P226, DOI 10.1001/archpedi.1992.02160140092027

Dudley DJ, 2007, OBSTET GYN CLIN N AM, V34, P293, DOI 10.1016/j.ogc.2007.03.001

Dudley NJ, 2005, ULTRASOUND OBST GYN, V25, P80, DOI 10.1002/uog.1751

Engstrom J L, 1992, J Obstet Gynecol Neonatal Nurs, V21, P486, DOI 10.1111/j.1552-6909.1992.tb01769.x

FIELD NT, 1995, OBSTET GYNECOL, V86, P102, DOI 10.1016/0029-7844(95)00096-A

Fisher JE, 1997, OBSTET GYNECOL, V90, P880, DOI 10.1016/S0029-7844(97)00544-9

Gmehlin U, 1994, Zentralbl Gynakol, V116, P706

Guideline Development Group, 2008, BMJ-BRIT MED J, V336, P714, DOI 10.1136/bmj.39505.641273.AD

GUTGESELL HP, 1976, J PEDIATR-US, V89, P120, DOI 10.1016/S0022-3476(76)80945-6

Gyamfi C, 2009, AM J OBSTET GYNECOL, V201, DOI 10.1016/j.ajog.2009.06.036

HADLOCK FP, 1985, AM J OBSTET GYNECOL, V151, P333, DOI 10.1016/0002-9378(85)90298-4

HADLOCK FP, 1985, RADIOLOGY, V154, P503, DOI 10.1148/radiology.154.2.3880915

Haute Autorite de Sante ( HAS), 2007, SUIV OR FEMM ENC FON

JOHNSTONE FD, 1992, BRIT J OBSTET GYNAEC, V99, P135, DOI 10.1111/j.1471-0528.1992.tb14472.x

Kaushal K, 2003, DIABETIC MED, V20, P73, DOI 10.1046/j.1464-5491.2003.00853.x

Kehl R J, 1996, J Matern Fetal Med, V5, P273

Kernaghan D, 2007, EUR J OBSTET GYN R B, V132, P189, DOI 10.1016/j.ejogrb.2006.07.012

Kjos SL, 2007, DIABETES CARE, V30, pS200, DOI 10.2337/dc07-s216

KJOS SL, 1995, AM J OBSTET GYNECOL, V173, P1532, DOI 10.1016/0002-9378(95)90645-2

Kjos SL, 2001, DIABETES CARE, V24, P1904, DOI 10.2337/diacare.24.11.1904

KOFINAS AD, 1991, AM J PERINAT, V8, P273, DOI 10.1055/s-2007-999395

Leticee NS, 2007, C MED FOET CANN

Leung WC, 2004, ULTRASOUND OBST GYN, V24, P534, DOI 10.1002/uog.1730

Mathiesen ER, 2002, ACTA OBSTET GYN SCAN, V81, P835, DOI 10.1034/j.1600-0412.2002.810906.x

Mehdizadeh A, 2000, AM J PERINAT, V17, P233, DOI 10.1055/s-2000-10003

Metzger BE, 2007, DIABETES CARE, V30, pS251, DOI 10.2337/dc07-s225

Mulholland C, 2007, J WOMENS HEALTH, V16, P790, DOI 10.1089/jwh.2007.CDC7

NEILSON JP, 2000, COCHRANE DB SYST REV

Rebarber A, 2007, DIABETES CARE, V30, P2277, DOI 10.2337/dc07-0564

REECE EA, 1994, J ULTRAS MED, V13, P73, DOI 10.7863/jum.1994.13.2.73

RIZZO G, 1992, ULTRASOUND OBST GYN, V2, P434, DOI 10.1046/j.1469-0705.1992.02060434.x

ROSSI G, 2000, ACTA OBSTET GYN SCAN, V79, P649

Sacks DA, 2007, CLIN OBSTET GYNECOL, V50, P980, DOI 10.1097/GRF.0b013e31815a6242

SALVESEN KA, 1993, BRIT MED J, V307, P1064, DOI 10.1136/bmj.307.6911.1064-b

Schaefer-Graf UM, 2004, DIABETES CARE, V27, P297, DOI 10.2337/diacare.27.2.297

Schumacher A, 2006, Z Geburtshilfe Neonatol, V210, P184, DOI 10.1055/s-2006-951743

Serlin DC, 2009, AM FAM PHYSICIAN, V80, P57

Sherman DJ, 1998, OBSTET GYNECOL, V91, P212, DOI 10.1016/S0029-7844(97)00654-6

Sotiriadis A, 2009, COCHRANE DB SYST REV, DOI 10.1002/14651858.CD006614.pub2

Stutchfield P, 2005, BMJ-BRIT MED J, V331, P662, DOI 10.1136/bmj.38547.416493.06

Turok DK, 2003, AM FAM PHYSICIAN, V68, P1767

Vidaeff AC, 2003, OBSTET GYNECOL SURV, V58, P759, DOI 10.1097/01.OGX.0000093782.25261.AC

Waters TP, 2009, OBSTET GYNECOL, V114, P45, DOI 10.1097/AOG.0b013e3181a9454b

Westergaard HB, 2001, ULTRASOUND OBST GYN, V17, P466, DOI 10.1046/j.1469-0705.2001.00415.x

WESTIN B, 1977, ACTA OBSTET GYN SCAN, V56, P273, DOI 10.3109/00016347709154978

WIKSTROM I, 1993, GYNECOL OBSTET INVES, V35, P27, DOI 10.1159/000292658

Williams KP, 2003, AM J OBSTET GYNECOL, V188, P1366, DOI 10.1067/mob.2003.305

Wong SF, 2003, AUST NZ J OBSTET GYN, V43, P302, DOI 10.1046/j.0004-8666.2003.00094.x

ZIMMERMANN P, 1992, EUR J OBSTET GYN R B, V47, P85, DOI 10.1016/0028-2243(92)90036-X

NR 65

TC 3

Z9 4

U1 0

U2 5

PU MASSON EDITEUR

PI MOULINEAUX CEDEX 9

PA 21 STREET CAMILLE DESMOULINS, ISSY, 92789 MOULINEAUX CEDEX 9, FRANCE

SN 1262-3636

EI 1878-1780

J9 DIABETES METAB

JI Diabetes Metab.

PD DEC

PY 2010

VL 36

IS 6

BP 672

EP 681

DI 10.1016/j.diabet.2010.11.017

PN 2

PG 10

WC Endocrinology & Metabolism

SC Endocrinology & Metabolism

GA 704IK

UT WOS:000286044600015

PM 21163429

DA 2019-01-08

ER

PT J

AU Yukawa, K

Yamazaki, Y

Yonekura, Y

Togari, T

Abbott, FK

Homma, M

Park, M

Kagawa, Y

AF Yukawa, Keiko

Yamazaki, Yoshihiko

Yonekura, Yuki

Togari, Taisuke

Abbott, Fusae K.

Homma, Mieko

Park, Minjeong

Kagawa, Yumi

TI Effectiveness of Chronic Disease Self-management Program in Japan:

Preliminary report of a longitudinal study

SO NURSING & HEALTH SCIENCES

LA English

DT Article

DE chronic disease; diabetes; Japan; rheumatic disease; self-efficacy;

self-management

ID RANDOMIZED CONTROLLED-TRIAL; EDUCATION-PROGRAMS; COST-EFFECTIVENESS;

CHRONIC ILLNESS; HEALTH-STATUS; CARE; PEOPLE

AB This is the preliminary report of a study to evaluate the effectiveness of the Chronic Disease Self-management Program in Japan by comparing changes in health outcomes at the baseline and 3-month and 6-month follow-ups. The program is a patient-centered educational program for the self-management of chronic conditions. The study's participants were recruited from among the attendees of the program workshops. During the study period (August 2006 to May 2007), 18 workshops were held and 128 attendees agreed to participate in the study. The health outcomes that were measured included health status, self-management behaviors, utilization of health services, self-efficacy, satisfaction with daily living, and clinical indicators. These indicators were further analyzed by disease type: diabetes, rheumatic disease, and cardiovascular disease/dyslipidemia. The findings indicated statistically significant positive changes in health distress, coping with symptoms, stretching exercises, communication with the physician, and satisfaction with daily living. The positive changes were especially remarkable among the groups with diabetes and rheumatic disease. These findings suggest that the Chronic Disease Self-management Program can be effective for Japanese people with chronic conditions.

C1 [Yukawa, Keiko] Univ Tokyo, Grad Sch Med, Dept Hlth Sociol, Bunkyo Ku, Tokyo 1130033, Japan.

[Togari, Taisuke] Yamaguchi Univ, Grad Sch Med, Dept Hyg, Div Syst Control Med, Ube, Yamaguchi 755, Japan.

[Abbott, Fusae K.] Samuel Merritt Univ, Sch Nursing, Oakland, CA USA.

RP Yukawa, K (reprint author), Univ Tokyo, Grad Sch Med, Dept Hlth Sociol, Bunkyo Ku, 7-3-1 Hongo, Tokyo 1130033, Japan.

EM tkykeiko-tky@umin.ac.jp

FU Japanese Ministry of Health, Education and Labor

FX This study was funded by Japanese Ministry of Health, Education and

Labor research grants during the 2006 and 2007 fiscal years. The authors

appreciate all the participants in this study and the workshop leaders,

as well as everyone at the JCDSMA, the Stanford University Patient

Education Research Center, California, USA, and the Department of Health

Sociology at the University of Tokyo, Japan.

CR Bagiella E, 2000, PSYCHOPHYSIOLOGY, V37, P13, DOI 10.1017/S0048577200980648

Barlow J, 2002, PATIENT EDUC COUNS, V48, P177, DOI 10.1016/S0738-3991(02)00032-0

Bisschop MI, 2004, SOC SCI MED, V59, P721, DOI 10.1016/j.socscimed.2003.11.038

DONGBO F, 2003, B WORLD HEALTH ORGAN, V81, P174

Griffiths C, 2005, BRIT J GEN PRACT, V55, P831

Griffiths C, 2007, BRIT MED J, V334, P1254, DOI 10.1136/bmj.39227.698785.47

Higashi A, 1996, Nihon Shokakibyo Gakkai Zasshi, V93, P884

Kennedy A, 2007, J EPIDEMIOL COMMUN H, V61, P254, DOI 10.1136/jech.2006.053538

KONDO F, 2009, JPN J CLIN DIALYSIS, V25, P1513

KONDO F, 2001, LIVING HLTH LIFE CHR

KONDO F, 2005, CHRONIC DIS SELF MAN

KUSUNAGA T, 2002, JPN J HLTH MED SOCIO, V14, P1

Lorig K, 1996, OUTCOME MEASURES HLT

Lorig K, 2008, DIABETES CARE, V31, P408, DOI 10.2337/dc07-1313

Lorig KR, 1999, MED CARE, V37, P5, DOI 10.1097/00005650-199901000-00003

Lorig KR, 2003, ANN BEHAV MED, V26, P1, DOI 10.1207/S15324796ABM2601_01

Lorig KR, 2001, MED CARE, V39, P1217, DOI 10.1097/00005650-200111000-00008

Mann RJ, 2006, CHARGING AHEAD: THE GROWTH AND REGULATION OF PAYMENT CARD MARKETS, P45, DOI 10.1017/CBO9780511754227.006

MCGOWAN P, 2010, IMPLEMENTATION EVALU

*MHLW, OV PAT SURV JAP

*MHLW, OV NAT HLTH NUTR EX

Newman S, 2004, LANCET, V364, P1523, DOI 10.1016/S0140-6736(04)17277-2

Osborne RH, 2007, J RHEUMATOL, V34, P1112

Reeves D, 2008, BRIT J GEN PRACT, V58, P198, DOI 10.3399/bjgp08X277320

Richardson G, 2008, J EPIDEMIOL COMMUN H, V62, P361, DOI 10.1136/jech.2006.057430

Siu AMH, 2007, PATIENT EDUC COUNS, V65, P42, DOI 10.1016/j.pec.2006.04.013

*STANF PAT ED RES, 2010, ORG LIC OFF CHRON DI

Wagner EH, 2001, HEALTH AFFAIR, V20, P64, DOI 10.1377/hlthaff.20.6.64

Warsi A, 2004, ARCH INTERN MED, V164, P1641, DOI 10.1001/archinte.164.15.1641

Williams TR, 2008, SEMIN RADIAT ONCOL, V18, P175, DOI 10.1016/j.semradonc.2008.01.005

NR 30

TC 10

Z9 11

U1 1

U2 8

PU WILEY-BLACKWELL PUBLISHING, INC

PI MALDEN

PA COMMERCE PLACE, 350 MAIN ST, MALDEN 02148, MA USA

SN 1441-0745

J9 NURS HEALTH SCI

JI Nurs. Health Sci.

PD DEC

PY 2010

VL 12

IS 4

BP 456

EP 463

DI 10.1111/j.1442-2018.2010.00559.x

PG 8

WC Nursing

SC Nursing

GA 703SI

UT WOS:000286001200012

PM 21210924

DA 2019-01-08

ER

PT J

AU Mommersteeg, PMC

Kupper, N

Denollet, J

AF Mommersteeg, Paula M. C.

Kupper, Nina

Denollet, Johan

TI Type D personality is associated with increased metabolic syndrome

prevalence and an unhealthy lifestyle in a cross-sectional Dutch

community sample

SO BMC PUBLIC HEALTH

LA English

DT Article

ID CHRONIC HEART-FAILURE; D SCALE DS14; CARDIOVASCULAR-DISEASE; NEGATIVE

AFFECTIVITY; SOCIAL INHIBITION; CLINICAL-PRACTICE; PHYSICAL-ACTIVITY;

YOUNG-ADULTS; RISK-FACTORS; DEPRESSION

AB Background: People with Type D-Distressed-personality have a general tendency towards increased negative affectivity (NA), while at the same time inhibiting these emotions in social situations (SI). Type D personality is associated with an increased risk of adverse outcomes in patients with cardiovascular disease. Whether Type D personality is a cardiovascular risk factor in healthy populations remains to be investigated. In the present study, the relations between Type D personality and classical cardiovascular risk factors, i.e. metabolic syndrome and lifestyle were investigated in a Dutch community sample.

Methods: In a cross-sectional study 1592 participants were included, aged 20-80 years. Metabolic syndrome was defined by self-report, following the International Diabetes Federation-IDF-guidelines including an increased waist circumference, dyslipidemia, hypertension, and diabetes. In addition lifestyle factors smoking, alcohol use, exercise and dietary habits were examined. Metabolic syndrome prevalence was stratified by Type D personality (a high score on both NA and SI), lifestyle and confounders age, gender, having a partner, higher education level, cardiac history, family history of cardiovascular disease.

Results: Metabolic syndrome was more prevalent in persons with a Type D personality (13% vs. 6%). Persons with Type D personality made poorer lifestyle choices, adhered less to the physical activity norm (OR = 1.5, 95% CI = 1.1-2.0, p = .02), had a less varied diet (OR = 0.50, 95% CI = 0.40-0.70, p < .0005), and were less likely to restrict their fat intake (OR = 0.70, 95% CI = 0.50-0.90, p = .01). Type D personality was related to a twofold increased risk of metabolic syndrome (OR = 2.2, 95% CI = 1.2-4.0, p = .011), independent of lifestyle factors and confounders.

Conclusions: Type D personality is related to an increased prevalence of metabolic syndrome and unhealthy lifestyle, which suggests both behavioral and biological vulnerability for development of cardiovascular disorders

C1 [Mommersteeg, Paula M. C.; Kupper, Nina; Denollet, Johan] Tilburg Univ, Dept Med Psychol, CoRPS Ctr Res Psychol Somat Dis, Tilburg, Netherlands.

RP Mommersteeg, PMC (reprint author), Tilburg Univ, Dept Med Psychol, CoRPS Ctr Res Psychol Somat Dis, Tilburg, Netherlands.

EM P.M.C.Mommersteeg@uvt.nl

RI Kupper, Nina/B-6337-2011; Kupper, Nina/B-7045-2011

OI Kupper, Nina/0000-0001-8721-5239;

FU Netherlands Organization for Scientific Research (NWO, The Hague, The

Netherlands) [453-04-004]

FX We gratefully acknowledge our research assistants for their efforts in

data collection and data entering. This research was partly funded by a

VICI grant (#453-04-004) from the Netherlands Organization for

Scientific Research (NWO, The Hague, The Netherlands) awarded to Dr.

Johan Denollet. All authors declare that there is no conflict of

interest.

CR Ainsworth BE, 2000, MED SCI SPORT EXER, V32, pS498, DOI 10.1097/00005768-200009001-00009

Alberti KGMM, 2009, CIRCULATION, V120, P1640, DOI 10.1161/CIRCULATIONAHA.109.192644

Aquarius AE, 2009, ARCH SURG-CHICAGO, V144, P728, DOI 10.1001/archsurg.2009.75

ARSENAULT BJ, 2009, INT J OBESITY

Bigaard J, 2005, J NUTR, V135, P2263

Bindraban NR, 2008, EUR J EPIDEMIOL, V23, P37, DOI 10.1007/s10654-007-9200-8

BORKOLES E, 2009, BODY IMAGE

Cho ER, 2009, ANN EPIDEMIOL, V19, P784, DOI 10.1016/j.annepidem.2009.06.010

COHEN BE, 2009, PSYCHIAT RES

Denollet J, 2005, PSYCHOSOM MED, V67, P89, DOI 10.1097/01.psy.0000149256.81953.49

Deshmukh-Taskar PR, 2009, PUBLIC HEALTH NUTR, V12, P2493, DOI 10.1017/S1368980009991261

Djousse L, 2009, JAMA-J AM MED ASSOC, V302, P394, DOI 10.1001/jama.2009.1062

Doolan Daniel M, 2008, Prog Cardiovasc Nurs, V23, P119, DOI 10.1111/j.1751-7117.2008.00001.x

Dunbar JA, 2008, DIABETES CARE, V31, P2368, DOI 10.2337/dc08-0175

EINVIK G, 2010, EUROPEAN J CARDIOVAS

FINUCANE FM, 2010, DIABETOLOGIA

Fleming P, 2008, CAN FAM PHYSICIAN, V54, P1706

Ford ES, 2008, CARDIOVASC DIABETOL, V7, DOI 10.1186/1475-2840-7-35

Goldbacher EM, 2007, ANN BEHAV MED, V34, P240, DOI 10.1007/BF02874549

Graham I, 2007, EUR J CARDIOV PREV R, V14, pE1, DOI 10.1097/01.hjr.0000277984.31558.c4

Grande G, 2004, PSYCHOTHER PSYCH MED, V54, P413, DOI 10.1088/s-2004-828376

Hamilton MT, 2007, DIABETES, V56, P2655, DOI 10.2337/db07-0882

Hausteiner C, 2010, PSYCHOSOM MED, V72, P163, DOI 10.1097/PSY.0b013e3181cb8bae

Herva A, 2006, PSYCHOSOM MED, V68, P213, DOI 10.1097/01.psy.0000203172.02305.ea

Karlsson MR, 2007, J BEHAV MED, V30, P253, DOI 10.1007/s10865-007-9096-5

Kemper HGC, 2000, TIJDSCHRIFT SOCIALE, V78, P180

Kupper N, 2007, J PERS ASSESS, V89, P265, DOI 10.1080/00223890701629797

Kupper N, 2007, PSYCHOSOM MED, V69, P675, DOI 10.1097/PSY.0b013e318149f4a7

Lichtenstein AH, 2006, CIRCULATION, V114, P82, DOI 10.1161/CIRCULATIONAHA.106.176158

Manolio T, 2003, NEW ENGL J MED, V349, P1587, DOI 10.1056/NEJMp038136

Martens EJ, 2007, J PSYCHOSOM RES, V63, P545, DOI 10.1016/j.jpsychores.2007.06.005

Molenaar EA, 2009, DIABETES CARE, V32, P505, DOI 10.2337/dc08-1382

Molloy GJ, 2008, PSYCHOSOM MED, V70, P863, DOI 10.1097/PSY.0b013e3181842e0c

Ocke MC, 1997, INT J EPIDEMIOL, V26, pS37, DOI 10.1093/ije/26.suppl_1.S37

Pedersen SS, 2003, EUR J CARDIOV PREV R, V10, P241, DOI 10.1097/01.hjr.0000085246.65733.06

Pedersen SS, 2007, EUR J CARDIOV PREV R, V14, P135, DOI 10.1097/HJR.0b013e328045c282

Pedersen SS, 2006, CURR CARDIOL REV, V2, P205, DOI 10.2174/157340306778019441

Pedersen SS, 2009, INT J BEHAV MED, V16, P108, DOI 10.1007/s12529-008-9022-4

PELLE AJ, 2009, INT J CARDIOLOGY

Pelle AJ, 2009, J AFFECT DISORDERS, V113, P150, DOI 10.1016/j.jad.2008.05.017

SCHIFFER AA, 2009, INT J CARDIOLOGY

Schiffer AA, 2008, EUR J HEART FAIL, V10, P802, DOI 10.1016/j.ejheart.2008.06.012

Spencer EA, 2004, PUBLIC HEALTH NUTR, V7, P723, DOI 10.1079/PHN2004600

Spindler H, 2009, INT J BEHAV MED, V16, P98, DOI 10.1007/s12529-009-9037-5

Vaccarino V, 2008, PSYCHOSOM MED, V70, P40, DOI 10.1097/PSY.0b013e31815c1b85

van Dam RM, 2003, AM J CLIN NUTR, V77, P1156

van den Broek KC, 2009, J AM COLL CARDIOL, V54, P531, DOI 10.1016/j.jacc.2009.04.043

VANDENDONK M, 2009, PREVENTIVE MED

Vogelzangs N, 2009, AM J GERIAT PSYCHIAT, V17, P716, DOI 10.1097/JGP.0b013e3181aad5d7

Wendel-Vos GCW, 2009, AM J PREV MED, V37, P50, DOI 10.1016/j.amepre.2009.03.015

Williams L, 2008, J PSYCHOSOM RES, V64, P63, DOI 10.1016/j.jpsychores.2007.06.008

Yu XN, 2008, J PSYCHOSOM RES, V65, P595, DOI 10.1016/j.jpsychores.2008.06.009

NR 52

TC 53

Z9 54

U1 2

U2 16

PU BMC

PI LONDON

PA CAMPUS, 4 CRINAN ST, LONDON N1 9XW, ENGLAND

SN 1471-2458

J9 BMC PUBLIC HEALTH

JI BMC Public Health

PD NOV 19

PY 2010

VL 10

AR 714

DI 10.1186/1471-2458-10-714

PG 11

WC Public, Environmental & Occupational Health

SC Public, Environmental & Occupational Health

GA 695EC

UT WOS:000285352400001

PM 21092104

OA DOAJ Gold, Green Published

DA 2019-01-08

ER

PT J

AU Hamer, M

Stamatakis, E

Kivimaki, M

Kengne, AP

Batty, GD

AF Hamer, Mark

Stamatakis, Emmanuel

Kivimaeki, Mika

Kengne, Andre Pascal

Batty, G. David

TI Psychological Distress, Glycated Hemoglobin, and Mortality in Adults

With and Without Diabetes

SO PSYCHOSOMATIC MEDICINE

LA English

DT Article

DE epidemiology; mortality; glycated hemoglobin; psychological stress;

diabetes

ID DEPRESSIVE SYMPTOMS; GLUCOSE-METABOLISM; GLYCEMIC CONTROL; MELLITUS;

ASSOCIATION; HEALTH; COMORBIDITY; COHORT; HOORN

AB Objective: To examine associations between psychological distress, glucose metabolism, and death. There is limited information about the long-term prognosis of diabetic patients with psychological distress. Methods: In a representative cohort of 11,546 adults (6.2% with high-risk/undiagnosed diabetes and 4.8% with physician-diagnosed diabetes), we measured glycated hemoglobin A I C as an indicator of glucose metabolism and psychological distress with the 12-item General Health Questionnaire (GHQ-12). Results: During the mean follow-up of 4.7 years, there were 682 deaths (93 among diabetic, 88 in high-risk/undiagnosed diabetic [A I C >= 6.0% without physician-diagnosed diabetes], and 501 in nondiabetic participants). Psychological distress was apparent in 18.9%, 16.5%, and 13.4% of diabetic, high-risk/undiagnosed diabetic, and nondiabetic participants, respectively. In participants with diabetes, a unit increase in GHQ-12 score was associated with higher risk of death at follow-up (multivariate adjusted hazard ratio, 1.16; 95% confidence interval, 1.09-1.24). Levels of A1C were also higher in diabetic participants with distress (GHQ-12 score of >3) compared with those without (7.86% versus 7.40%; p = .008), although adjustment for A1C did alter the association between distress and mortality. In the whole sample, the coexistence of diabetes and distress was associated with an elevated risk of death, beyond that due to having either diabetes or distress alone (multivariate adjusted hazard ratio, 3.64; 95% confidence interval, 2.21-5.98). Conclusions: Psychological distress is an independent risk factor for death in diabetic patients, although impaired glucose metabolism did not explain the excess risk.

C1 [Hamer, Mark; Stamatakis, Emmanuel; Kivimaeki, Mika; Batty, G. David] UCL, Dept Epidemiol & Publ Hlth, London WC1E 6BT, England.

[Kengne, Andre Pascal] Univ Sydney, George Inst Int Hlth, Sydney, NSW 2006, Australia.

[Batty, G. David] MRC, Social & Publ Hlth Sci Unit, Glasgow, Lanark, Scotland.

[Batty, G. David] Univ Edinburgh, Ctr Cognit Ageing & Cognit Epidemiol, Edinburgh, Midlothian, Scotland.

RP Hamer, M (reprint author), UCL, Dept Epidemiol & Publ Hlth, 1-19 Torrington Pl, London WC1E 6BT, England.

EM m.hamer@ucl.ac.uk

RI Stamatakis, Emmanuel/C-4958-2009; Batty, George/Y-4401-2018; Kivimaki,

Mika/B-3607-2012; Hamer, Mark/C-1602-2008

OI Stamatakis, Emmanuel/0000-0001-7323-3225; Hamer,

Mark/0000-0002-8726-7992; Kivimaki, Mika/0000-0002-4699-5627

FU Scottish Executive; British Heart Foundation [RG 05/006]; Wellcome

Trust; National Heart, Lung, and Blood Institute [R01 HL036310];

National Institute on Aging, National Institutes of Health [R01

AG034454]; Bupa Foundation; Academy of Finland; U.K. MRC; Chief

Scientist Office at the Scottish Government Health Directorates; Medical

Research Council [MC_U130059821]

FX The Scottish Health Survey is funded, in part, by the Scottish

Executive. M.H. is supported by the British Heart Foundation (RG

05/006); E.S. is a National Institute for Health Research Fellow; G.D.B.

is a Wellcome Trust Career Development Fellow (WBS

U.1300.00.006.00012.01); M.K. is supported by Grant R01 HL036310 from

the National Heart, Lung, and Blood Institute and Grant R01 AG034454

from the National Institute on Aging, National Institutes of Health, the

Bupa Foundation, and the Academy of Finland. The Medical Research

Council (MRC) Social and Public Health Sciences Unit receives funding

from the U.K. MRC and the Chief Scientist Office at the Scottish

Government Health Directorates.

CR Adriaanse MC, 2008, DIABETIC MED, V25, P843, DOI 10.1111/j.1464-5491.2008.02464.x

Adriaanse MC, 2008, DIABETES CARE, V31, P2268, DOI 10.2337/dc08-1074

Ali S, 2006, DIABETIC MED, V23, P1165, DOI 10.1111/j.1464-5491.2006.01943.x

Aujla N, 2009, PLOS ONE, V4, DOI 10.1371/journal.pone.0007755

Batty GD, 2009, J EPIDEMIOL COMMUN H, V63, P952, DOI 10.1136/jech.2008.086892

Brotman DJ, 2007, LANCET, V370, P1089, DOI 10.1016/S0140-6736(07)61305-1

Bruce DG, 2005, DIABETOLOGIA, V48, P2532, DOI 10.1007/s00125-005-0024-3

Egede LE, 2005, DIABETES CARE, V28, P1339, DOI 10.2337/diacare.28.6.1339

Fisher L, 2010, DIABETES CARE, V33, P23, DOI 10.2337/dc09-1238

Gale CR, 2010, BIOL PSYCHIAT, V67, P189, DOI 10.1016/j.biopsych.2009.09.019

Georgiades A, 2007, PSYCHOSOM MED, V69, P235, DOI 10.1097/PSY.0b013e318042588d

Gillett Melissa J, 2009, Clin Biochem Rev, V30, P197

Gillett MJ, 2009, DIABETES CARE, V32, P1327

Goldberg DP, 1997, PSYCHOL MED, V27, P191, DOI 10.1017/S0033291796004242

Golden SH, 2008, JAMA-J AM MED ASSOC, V299, P2751, DOI 10.1001/jama.299.23.2751

Hankins Matthew, 2008, Clin Pract Epidemiol Ment Health, V4, P10, DOI 10.1186/1745-0179-4-10

Holt RIG, 2009, DIABETIC MED, V26, P641, DOI 10.1111/j.1464-5491.2009.02742.x

Icks A, 2008, DIABETIC MED, V25, P1330, DOI 10.1111/j.1464-5491.2008.02585.x

Katon WJ, 2008, AM J MED, V121, P8, DOI 10.1016/j.amjmed.2008.09.008

Katon WJ, 2005, DIABETES CARE, V28, P2668, DOI 10.2337/diacare.28.11.2668

Knol MJ, 2007, PSYCHOSOM MED, V69, P300, DOI 10.1097/PSY.0b013e31805f48b9

Ludman EJ, 2010, J GERONTOL A-BIOL, V65, P93, DOI 10.1093/gerona/glp151

Prince M, 2007, LANCET, V370, P859, DOI 10.1016/S0140-6736(07)61238-0

Rhee MK, 2008, DIABETIC MED, V25, P1361, DOI 10.1111/j.1464-5491.2008.02543.x

Richardson DB, 2009, AM J EPIDEMIOL, V169, P756, DOI 10.1093/aje/kwn411

*SCOTT GOV STAT, SCOTT HLTH SURV PUBL

Testa MA, 1998, JAMA-J AM MED ASSOC, V280, P1490, DOI 10.1001/jama.280.17.1490

NR 27

TC 20

Z9 20

U1 0

U2 10

PU LIPPINCOTT WILLIAMS & WILKINS

PI PHILADELPHIA

PA 530 WALNUT ST, PHILADELPHIA, PA 19106-3621 USA

SN 0033-3174

J9 PSYCHOSOM MED

JI Psychosom. Med.

PD NOV-DEC

PY 2010

VL 72

IS 9

BP 882

EP 886

DI 10.1097/PSY.0b013e3181f6696e

PG 5

WC Psychiatry; Psychology; Psychology, Multidisciplinary

SC Psychiatry; Psychology

GA 685ZA

UT WOS:000284665400007

PM 20884891

DA 2019-01-08

ER

PT J

AU Yokokawa, H

Goto, A

Khue, NT

Nam, TQ

Trung, TT

Khoa, VT

Nguyen, TBN

Minh, PN

Vinh, NQ

Okayama, A

Yasumura, S

AF Yokokawa, Hirohide

Goto, Aya

Nguyen Thy Khue

Tran Quang Nam

Tran The Trung

Vo Tuan Khoa

Nguyen Thi Boi Ngoc

Pham Nghiem Minh

Nguyen Quang Vinh

Okayama, Akira

Yasumura, Seiji

TI Characteristics of metabolic syndrome and its clinical components among

diabetic Vietnamese patients: A survry at two institutions in Ho Chi

Minh City

SO OBESITY RESEARCH & CLINICAL PRACTICE

LA English

DT Article

DE Metabolic syndrome; Diabetes mellitus; Disease management; Chronic

disease; Primary care

ID CHOLESTEROL EDUCATION-PROGRAM; CORONARY-HEART-DISEASE; TREATMENT PANEL

III; CARDIOVASCULAR MORTALITY; ALL-CAUSE; PREVALENCE; FEDERATION;

MELLITUS; ADULTS; AGE

AB Objective: The purpose of this study was to estimate the prevalence of metabolic syndrome among Vietnamese diabetic patients and to investigate their characteristics.

Methods: This cross-sectional study was conducted among 652 outpatients who were recruited from one public general hospital (People Hospital 115) and one private clinic (Medic Center) in Ho Chi Minh City, Vietnam. We obtained informed consent from participants, collected clinical information from medical files, and conducted patient interviews. We evaluated the collected information descriptively and diagnosed metabolic syndrome according to the International Diabetes Federation (IDF) Worldwide Definition of the Metabolic Syndrome.

Results: A total of 517 eligible patients (175 men and 342 women) were analyzed. Mean age was 54.6 years for men and 60.7 years for women. Metabolic syndrome was observed in 39.4% of men and 70.5% of women. Central obesity was more prevalent among women (72.5%) compared to men (39.4%). Participants with metabolic syndrome were less likely to endorse a sense of general well-being, and women with metabolic syndrome were likely to have less diabetes-related distress.

Conclusions: this study revealed a high prevalence of metabolic syndrome in diabetic Vietnamese patients, especially among women, and revealed central obesity as a key feature. A prospective study should be conducted in this population in order to assess impacts of metabolic syndrome on disease outcome. (C) 2010 Asian Oceanian Association for the Study of Obesity. Published by Elsevier Ltd. All rights reserved.

C1 [Yokokawa, Hirohide] Juntendo Univ, Sch Med, Dept Gen Med, Bunkyo Ku, Tokyo 1138421, Japan.

[Yokokawa, Hirohide; Goto, Aya; Yasumura, Seiji] Fukushima Med Univ, Sch Med, Dept Publ Hlth, Fukushima, Japan.

[Nguyen Thy Khue; Tran Quang Nam; Tran The Trung] Univ Med & Pharm, Dept Endocrinol, Ho Chi Minh City, Vietnam.

[Vo Tuan Khoa; Nguyen Thi Boi Ngoc] Peoples Hosp 115, Dept Endocrinol, Ho Chi Minh City, Vietnam.

[Pham Nghiem Minh] Tu Du Obstet & Gynecol Hosp, Anapathol Cytol Genet Dept, Ho Chi Minh City, Vietnam.

[Nguyen Quang Vinh] Hanh Phuc Women & Children Hosp Project, Ho Chi Minh City, Vietnam.

[Okayama, Akira] Japan AntiTB Assoc, Inst Hlth Promot & Hlth Care 1, Tokyo, Japan.

RP Yokokawa, H (reprint author), Juntendo Univ, Sch Med, Dept Gen Med, Bunkyo Ku, Hongo 2-1-1, Tokyo 1138421, Japan.

EM yokokawa@pa3.so-net.ne.jp

OI Goto, Aya/0000-0002-7927-7648

FU Health Promotion Foundation; Health Promotion Foundation, Azusawa,

Itabashi, Tokyo, Japan

FX We thank the Health Promotion Foundation for grant support, as well as

Drs. Tran Viet Thang and Do Hoang Oanh for their excellent help in data

collection.; This study was funded by a grant from the Health Promotion

Foundation, 2-8-22, Azusawa, Itabashi, Tokyo, 174-0051, Japan.

CR Alexander CM, 2003, DIABETES, V52, P1210, DOI 10.2337/diabetes.52.5.1210

[Anonymous], 2003, DIABETES ATLAS

Bruno G, 2004, DIABETES CARE, V27, P2689, DOI 10.2337/diacare.27.11.2689

Cleeman JI, 2001, JAMA-J AM MED ASSOC, V285, P2486, DOI 10.1001/jama.285.19.2486

Ford ES, 2005, DIABETES CARE, V28, P2745, DOI 10.2337/diacare.28.11.2745

FRIEDLANDER Y, 1982, ISRAEL J MED SCI, V18, P1242

Hunt KJ, 2004, CIRCULATION, V110, P1251, DOI 10.1161/01.CIR.0000140762.04598.F9

International Diabetes Federation, WORLDW DEF MET SYNDR

Kuk JL, 2005, AM J CLIN NUTR, V81, P1330

Le DSNT, 2006, J ATHEROSCLER THROMB, V13, P16

Lear SA, 2007, AM J CLIN NUTR, V86, P353

Levetan CS, 2002, DIABETES CARE, V25, P2, DOI 10.2337/diacare.25.1.2

Ministry of Health, NAT HLTH NUTR SURV 2

Mohsin Abdul, 2007, J Pak Med Assoc, V57, P235

Rubin RR, 2006, DIABETES CARE, V29, P1249, DOI 10.2337/dc05-2494

Son LNTD, 2005, DIABETES RES CLIN PR, V67, P243, DOI 10.1016/j.diabres.2004.07.014

Song SH, 2008, QJM-INT J MED, V101, P875, DOI 10.1093/qjmed/hcn109

Tong PC, 2007, DIABETES CARE, V30, P1206, DOI 10.2337/dc06-1484

WHO (Five), 5 WELL BEING IND 199

Yokokawa H, 2010, J HLTH ED, V13, P1

NR 20

TC 1

Z9 1

U1 0

U2 1

PU ELSEVIER SCI LTD

PI OXFORD

PA THE BOULEVARD, LANGFORD LANE, KIDLINGTON, OXFORD OX5 1GB, OXON, ENGLAND

SN 1871-403X

EI 1878-0318

J9 OBES RES CLIN PRACT

JI Obes. Res. Clin. Pract.

PD OCT-DEC

PY 2010

VL 4

IS 4

BP E293

EP E299

DI 10.1016/j.orcp.2010.08.006

PG 7

WC Endocrinology & Metabolism; Nutrition & Dietetics

SC Endocrinology & Metabolism; Nutrition & Dietetics

GA V24JX

UT WOS:000208407800007

PM 24345695

DA 2019-01-08

ER

PT J

AU Polonsky, WH

Fisher, L

Guzman, S

Sieber, WJ

Philis-Tsimikas, A

Edelman, SV

AF Polonsky, William H.

Fisher, Lawrence

Guzman, Susan

Sieber, William J.

Philis-Tsimikas, Athena

Edelman, Steven V.

TI Are Patients' Initial Experiences at the Diagnosis of Type 2 Diabetes

Associated With Attitudes and Self-management Over Time?

SO DIABETES EDUCATOR

LA English

DT Article

ID PERCEPTIONS; SCALE

AB Objective

The purpose of this study was to investigate patients' experiences when diagnosed with type 2 diabetes and diabetes-related distress and self-management years later.

Method

One hundred seventy-nine adults with type 2 diabetes, 1 to 5 years after diagnosis, completed a questionnaire assessing what they were told and what was recommended at diagnosis as well as current diet, physical activity, and diabetes distress.

Results

Most patients (60%) expressed confidence in their health care provider (HCP) at diagnosis, but 30% reported that a clear action plan was not communicated. Current diabetes distress was significantly lower among those who reported being reassured at diagnosis that diabetes could be managed successfully and had developed a clearer action plan with their HCP. Patients' current dietary behaviors were significantly better among those receiving a clearer action plan at diagnosis.

Conclusion

Patients' retrospective reports of reassuring HCP messages when diagnosed, along with the presentation of a clear action plan, are associated with less diabetes distress and better self-management at 1 to 5 years after diagnosis.

Practical implications

Findings suggest a long-lasting impact on patients' attitudes and behaviors when the diagnosis of type 2 diabetes is delivered clearly, a sense of hope is provided, and a specific care plan is put forward.

C1 [Polonsky, William H.] Univ Calif San Diego, Dept Psychiat, San Diego, CA 92103 USA.

[Fisher, Lawrence] Univ Calif San Diego, Dept Family & Community Med, San Diego, CA 92103 USA.

[Polonsky, William H.; Guzman, Susan] Behav Diabet Inst, San Diego, CA USA.

[Sieber, William J.] Univ Calif San Diego, Dept Family & Prevent Med, San Diego, CA 92103 USA.

[Sieber, William J.] Inner Solut Success LLC, San Diego, CA USA.

[Philis-Tsimikas, Athena] Scripps Whittier Diabet Inst, La Jolla, CA USA.

[Edelman, Steven V.] Univ Calif San Diego, Div Endocrinol & Metab, San Diego, CA 92103 USA.

[Edelman, Steven V.] Vet Affairs Med Ctr, San Diego, CA 92161 USA.

RP Polonsky, WH (reprint author), POB 2148, Del Mar, CA 92014 USA.

EM whp@behavioraldiabetes.org

FU Roche Diagnostics

FX This research was funded by an unrestricted educational grant from Roche

Diagnostics. There are no conflicts of interest for any author.

CR Adriaanse MC, 2002, DIABETIC MED, V19, P406

Beeney LJ, 1996, PATIENT EDUC COUNS, V29, P109, DOI 10.1016/0738-3991(96)00939-1

Lawson VL, 2008, DIABETIC MED, V25, P482, DOI 10.1111/j.1464-5491.2008.02394.x

Parry O, 2004, FAM PRACT, V21, P131, DOI 10.1093/fampra/cmh203

Peel E, 2004, PATIENT EDUC COUNS, V53, P269, DOI 10.1016/j.pec.2003.07.010

PibernikOkanovic M, 1996, DIABETES RES CLIN PR, V34, P99, DOI 10.1016/S0168-8227(96)01342-3

Polonsky WH, 2005, DIABETES CARE, V28, P626, DOI 10.2337/diacare.28.3.626

Thoolen BJ, 2006, DIABETES CARE, V29, P2257, DOI 10.2337/dc06-0617

Toobert DJ, 2000, DIABETES CARE, V23, P943, DOI 10.2337/diacare.23.7.943

NR 9

TC 10

Z9 10

U1 0

U2 6

PU SAGE PUBLICATIONS INC

PI THOUSAND OAKS

PA 2455 TELLER RD, THOUSAND OAKS, CA 91320 USA

SN 0145-7217

J9 DIABETES EDUCATOR

JI Diabetes Educ.

PD SEP-OCT

PY 2010

VL 36

IS 5

BP 828

EP 834

DI 10.1177/0145721710378539

PG 7

WC Endocrinology & Metabolism; Public, Environmental & Occupational Health

SC Endocrinology & Metabolism; Public, Environmental & Occupational Health

GA 682TU

UT WOS:000284426700014

PM 20729511

DA 2019-01-08

ER

PT J

AU Pena, MMEY

Barrera, VH

Cordero, XF

de Miguel, AG

Perez, MR

Andres, AL

Jimenez-Garcia, R

AF Esteban y Pena, M. M.

Hernandez Barrera, V.

Fernandez Cordero, X.

Gil de Miguel, A.

Rodriguez Perez, M.

Lopez-de Andres, A.

Jimenez-Garcia, R.

TI Self-perception of health status, mental health and quality of life

among adults with diabetes residing in a metropolitan area

SO DIABETES & METABOLISM

LA English

DT Article

DE Quality of life; Diabetes; GHQ-12; Chronic diseases; Depression

ID IQOLA PROJECT; DEPRESSION; POPULATION; DISEASE; IMPACT; SF-36;

COMORBIDITY; DISABILITY; COUNTRIES; MELLITUS

AB Aim. - The study aimed to compare the self-perception of health, physiological distress and health-related quality of life (HRQL) in subjects with and without diabetes residing in a large metropolitan area (the city of Madrid), and to identify the variables associated with the poorest HRQL among diabetes patients.

Methods. - In this case-control epidemiological study, we selected 358 patients with diabetes from the Madrid City Health Survey. For every patient, two controls without diabetes were randomly selected from the same database and matched for age, gender and health district. The resultant study population comprised 1074 subjects, who were analyzed according to their self-rated health status, with mental health assessed by the 12-item General Health Questionnaire (GHQ-12) and HRQL by the COOP/WONCA questionnaire. Independent variables included sociodemographic characteristics, lifestyle variables, associated chronic diseases and consumption of medications. Multivariate analyses were conducted using ANCOVA tests.

Results. - The prevalence of health perceived as fair or poor was 64.12% in those with diabetes vs 38.57% in those without diabetes (P<0.05). The GHQ-12 results showed that mental health was also significantly worse among diabetes sufferers, and the COOP/WONCA questionnaire results indicated significantly poorer HRQL in those with diabetes. The variables that determined a poorer perception of HRQL among diabetes sufferers were female gender, older age, obesity, lack of physical exercise, coexistence of depression, use of sleeping pills, and Alzheimer's and cerebrovascular diseases.

Conclusion. - Self-rated health and psychological well-being, and HRQL, are all considerably poorer among patients with diabetes vs those without diabetes. The poorest quality of life among those with diabetes associated with female gender, depression, lack of exercise and obesity. (C) 2010 Elsevier Masson SAS. All rights reserved.

C1 [Hernandez Barrera, V.; Gil de Miguel, A.; Lopez-de Andres, A.; Jimenez-Garcia, R.] Rey Juan Carlos Univ, Prevent Med & Publ Hlth Teaching & Res Unit, Fac Hlth Sci, Madrid 28922, Spain.

[Esteban y Pena, M. M.; Fernandez Cordero, X.] Madrid City Council, Inst Publ Hlth, Madrid 28007, Spain.

[Rodriguez Perez, M.] Madrid City Council, Dept Evaluat & Qual, Madrid 28007, Spain.

RP Jimenez-Garcia, R (reprint author), Rey Juan Carlos Univ, Prevent Med & Publ Hlth Teaching & Res Unit, Fac Hlth Sci, Av Atenas Alcorcon, Madrid 28922, Spain.

EM rodrigo.jimenez@urjc.es

FU ISCIII [ETES: PI09/90515]

FX The authors are grateful to the Public Health Institute of the Madrid

City Council (Instituto de Salud Publica - Ayuntamiento de Madrid) for

providing access to the 2005 Madrid City Health Survey database. The

present study was supported by a grant from the ISCIII (ETES:

PI09/90515). The authors also thank Patricia Santa-Olalla for

translating the abstract and title into French.

CR Aaronson N K, 1992, Qual Life Res, V1, P349, DOI 10.1007/BF00434949

Alonso J, 2004, QUAL LIFE RES, V13, P283, DOI 10.1023/B:QURE.0000018472.46236.05

Aydin IO, 2001, GEN HOSP PSYCHIAT, V23, P77

Carnes D, 2007, RHEUMATOLOGY, V46, P1168, DOI 10.1093/rheumatology/kem118

Casado J, 2001, Aten Primaria, V28, P167

de Jonge P, 2006, DIABETOLOGIA, V49, P2627, DOI 10.1007/s00125-006-0442-x

Donmez L, 2005, ARCH GERONTOL GERIAT, V40, P213, DOI 10.1016/j.archger.2004.08.006

FERNANDEZMAYORA.G, 2003, REV MULT GERONTOL, V3, P43

Glasgow RE, 1997, DIABETES CARE, V20, P562, DOI 10.2337/diacare.20.4.562

JACOBSON AM, 1994, DIABETES CARE, V17, P267, DOI 10.2337/diacare.17.4.267

Jenkinson C, 1997, AGE AGEING, V26, P7, DOI 10.1093/ageing/26.1.7

Jimenez-Garcia R, 2008, DIABETES CARE, V31, P90, DOI 10.2337/dc07-1382

Katon WJ, 2008, AM J MED, V121, P8, DOI 10.1016/j.amjmed.2008.09.008

Kempen GIJM, 1997, AM J PUBLIC HEALTH, V87, P38, DOI 10.2105/AJPH.87.1.38

Lam C. L. K., 1995, Hong Kong Medical Journal, V1, P225

Lam CLK, 2000, FAM PRACT, V17, P159, DOI 10.1093/fampra/17.2.159

Lizan Tudela L, 2000, Med Clin (Barc), V114 Suppl 3, P76

Lizan Tudela L, 1999, Aten Primaria, V24, P75

*MADR SAL, 2005, ENC SAL CIUD MADR 20

Madrid Salud, EST SAL CIUD MADR 20

Mata M, 2003, ATEN PRIM, V31, P493

Mena Martin F J, 2006, An Med Interna, V23, P357

*MIN SAN CONS INE, HLTH SPAN POP 2006

MUNOZ PE, 1979, ARCH NEUROBIOL, V42, P139

Papadopoulos AA, 2007, BMC PUBLIC HEALTH, V7, DOI 10.1186/1471-2458-7-186

PATE RR, 1995, JAMA-J AM MED ASSOC, V273, P402, DOI 10.1001/jama.273.5.402

Pena F, 2010, HIPPOCAMPUS, V20, P78, DOI 10.1002/hipo.20592

Peyrot M, 2007, DIABETES CARE, V30, P2433, DOI 10.2337/dc07-1222

Regidor E, 2001, Rev Esp Salud Publica, V75, P13

Rubin RR, 1999, DIABETES-METAB RES, V15, P205, DOI 10.1002/(SICI)1520-7560(199905/06)15:3<205::AID-DMRR29>3.0.CO;2-O

Saito I, 2006, DIABETES RES CLIN PR, V73, P51, DOI 10.1016/j.diabres.2005.11.015

Sanchez-Lopez MP, 2008, PSICOTHEMA, V20, P839

Sanchez-Lopez MP, 2008, WOMEN HEALTH, V48, P445, DOI 10.1080/03630240802575096

SCHELLEVIS FG, 1994, BRIT J GEN PRACT, V44, P259

Schlenk EA, 1998, QUAL LIFE RES, V7, P57

Shao WA, 1997, J FAM PRACTICE, V44, P161

SNEEUW KC, 1992, J CLIN EPIDEMIOL, V45, P743

Tapp RJ, 2006, DIABETES RES CLIN PR, V74, P154, DOI 10.1016/j.diabres.2006.03.012

TOMAS C, 1999, GAC SANIT, V13, P90

Van Weel C, 1993, Disabil Rehabil, V15, P96

Wagner AK, 1998, J CLIN EPIDEMIOL, V51, P925, DOI 10.1016/S0895-4356(98)00083-3

*WHO, 1983, BAS DOC

*WONCA ERGHO, 1995, MEAS FUNCT HLTH STAT

NR 43

TC 18

Z9 18

U1 0

U2 7

PU MASSON EDITEUR

PI MOULINEAUX CEDEX 9

PA 21 STREET CAMILLE DESMOULINS, ISSY, 92789 MOULINEAUX CEDEX 9, FRANCE

SN 1262-3636

J9 DIABETES METAB

JI Diabetes Metab.

PD SEP

PY 2010

VL 36

IS 4

BP 305

EP 311

DI 10.1016/j.diabet.2010.02.003

PG 7

WC Endocrinology & Metabolism

SC Endocrinology & Metabolism

GA 671BN

UT WOS:000283474800009

PM 20483647

DA 2019-01-08

ER

PT J

AU Papelbaum, M

Lemos, HM

Duchesne, M

Kupfer, R

Moreira, RO

Coutinho, WF

AF Papelbaum, M.

Lemos, H. M.

Duchesne, M.

Kupfer, R.

Moreira, R. O.

Coutinho, W. F.

TI The association between quality of life, depressive symptoms and

glycemic control in a group of type 2 diabetes patients

SO DIABETES RESEARCH AND CLINICAL PRACTICE

LA English

DT Article

DE Type 2 diabetes mellitus; Quality of life; Metabolic control; Depression

ID PROBLEM AREAS; INVENTORY; DISTRESS; SCALE

AB Aims: Several studies have demonstrated worse perception of quality of life (QoL) among patients with type 2 diabetes mellitus (T2DM). The purpose of our study was to assess QoL in a clinical sample of patients with T2DM and its association with depressive symptoms and glycemic control.

Methods: One hundred outpatients from a sequential sample underwent clinical and psychiatric evaluation. The Problem Areas of Diabetes scale (PAID) and the Beck Depression Inventory (BDI) were used to assess, respectively, QoL and the presence of overall psychopathology. The levels of glycated hemoglobin (HbA1c) were used as the main parameter of glycemic control.

Results: The perception degree of the QoL related with diabetes was associated with the severity of depressive symptoms (r = 0.503; p <0.001), but not with HbA1c levels (p = 0.117). However, the severity of general psychopathology, evaluated through the BDI scores, predicted the metabolic control, measured by HbA1c levels, among the patients in our sample (r = 0.233; p = 0.019).

Conclusions: In our study, PAID was a valuable tool for the evaluation of QoL in T2DM and the screening of depressive symptoms. However, no correlation observed between PAID scores and HbA1c levels. Self-perception evaluation of T2DM patient can help to identify susceptible subjects to current depression. (C) 2010 Elsevier Ireland Ltd. All rights reserved.

C1 [Papelbaum, M.; Duchesne, M.] Univ Fed Rio de Janeiro, Inst Psychiat, BR-22440033 Rio De Janeiro, Brazil.

[Papelbaum, M.; Lemos, H. M.; Duchesne, M.; Kupfer, R.; Moreira, R. O.; Coutinho, W. F.] State Inst Diabet & Endocrinol Rio de Janeiro, Rio De Janeiro, Brazil.

RP Papelbaum, M (reprint author), Univ Fed Rio de Janeiro, Inst Psychiat, Ave Ataulfo Paiva 204,707, BR-22440033 Rio De Janeiro, Brazil.

EM marcelo@papelbaum.com

CR Bonow RO, 2004, AM J MED, V116, P2, DOI 10.1016/j.amjmed.2003.10.014

CRRER CJ, 2008, ARQ BRAS ENDOCRINOL, V52, P515

Edelman D, 2002, DIABETES CARE, V25, P1022, DOI 10.2337/diacare.25.6.1022

Fryers T, 2003, SOC PSYCH PSYCH EPID, V38, P229, DOI 10.1007/s00127-003-0627-2

GODNEY RD, 2004, DIABETES CARE, V27, P1066

Gorenstein C, 1996, BRAZ J MED BIOL RES, V29, P453

Gregg E. W., 2009, Morbidity and Mortality Weekly Report, V58, P1259

Gross CC, 2007, DIABETES RES CLIN PR, V76, P455, DOI 10.1016/j.diabres.2006.09.022

Gusmao Ricardo M, 2005, Acta Med Port, V18, P129

Hermanns N, 2006, DIABETOLOGIA, V49, P469, DOI 10.1007/s00125-005-0094-2

Kokoszka A, 2009, EUR PSYCHIAT, V24, P425, DOI 10.1016/j.eurpsy.2009.04.002

Lin EHB, 2004, DIABETES CARE, V27, P2154, DOI 10.2337/diacare.27.9.2154

Lloyd A, 2001, Value Health, V4, P392, DOI 10.1046/j.1524-4733.2001.45029.x

Lohr KN, 1996, CLIN THER, V18, P979, DOI 10.1016/S0149-2918(96)80054-3

Lustman PJ, 1997, PSYCHOSOM MED, V59, P24, DOI 10.1097/00006842-199701000-00004

LUTSMAN PJ, 1997, PSYCHOSOM MED, V59, P24

LUTSMANN PJ, 2000, DIABETES CARE, V23, P934

Miller ST, 2008, BMC PUBLIC HEALTH, V8, DOI 10.1186/1471-2458-8-70

Peyrot M, 2010, DIABETES CARE, V33, P240, DOI 10.2337/dc09-1348

POLONSKY WH, 1995, DIABETES CARE, V18, P754, DOI 10.2337/diacare.18.6.754

Snoek FJ, 2000, DIABETES CARE, V23, P1305, DOI 10.2337/diacare.23.9.1305

Welch GW, 1997, DIABETES CARE, V20, P760, DOI 10.2337/diacare.20.5.760

Wilder RP, 2003, DIABETES CARE, V26, P1650, DOI 10.2337/diacare.26.5.1650

NR 23

TC 30

Z9 30

U1 0

U2 6

PU ELSEVIER IRELAND LTD

PI CLARE

PA ELSEVIER HOUSE, BROOKVALE PLAZA, EAST PARK SHANNON, CO, CLARE, 00000,

IRELAND

SN 0168-8227

J9 DIABETES RES CLIN PR

JI Diabetes Res. Clin. Pract.

PD SEP

PY 2010

VL 89

IS 3

BP 227

EP 230

DI 10.1016/j.diabres.2010.05.024

PG 4

WC Endocrinology & Metabolism

SC Endocrinology & Metabolism

GA 643AB

UT WOS:000281263000005

PM 20696361

DA 2019-01-08

ER

PT J

AU Barker, L

Crespo, R

Gerzoff, RB

Denham, S

Shrewsberry, M

Cornelius-Averhart, D

AF Barker, Lawrence

Crespo, Richard

Gerzoff, Robert B.

Denham, Sharon

Shrewsberry, Molly

Cornelius-Averhart, Darrlyn

TI Residence in a Distressed County in Appalachia as a Risk Factor for

Diabetes, Behavioral Risk Factor Surveillance System, 2006-2007

SO PREVENTING CHRONIC DISEASE

LA English

DT Article

AB Introduction

We compared the risk of diabetes for residents of Appalachian counties to that of residents of non-Appalachian counties after controlling for selected risk factors in states containing at least 1 Appalachian county.

Methods

We combined Behavioral Risk Factor Surveillance System data from 2006 and 2007 and conducted a logistic regression analysis, with self-reported diabetes as the dependent variable. We considered county of residence (5 classifications for Appalachian counties, based on economic development, and 1 for non-Appalachian counties), age, sex, race/ethnicity, education, household income, smoking status, physical activity level, and obesity to be independent variables. The classification "distressed" refers to counties in the worst 10%, compared with the nation as a whole, in terms of 3-year unemployment rate, per capita income, and poverty.

Results

Controlling for covariates, residents in distressed Appalachian counties had 33% higher odds (95% confidence interval, 1.10-1.60) of reporting diabetes than residents of non-Appalachian counties. We found no significant differences between other classifications of Appalachian counties and non-Appalachian counties.

Conclusions

Residents of distressed Appalachian counties are at higher risk of diabetes than are residents of other counties. States with distressed Appalachian counties should implement culturally sensitive programs to prevent diabetes.

C1 [Barker, Lawrence] Ctr Dis Control & Prevent, Div Diabet Translat, Atlanta, GA 30341 USA.

[Crespo, Richard; Shrewsberry, Molly] Marshall Univ, Huntington, WV USA.

[Denham, Sharon] Ohio Univ, Athens, OH 45701 USA.

RP Barker, L (reprint author), Ctr Dis Control & Prevent, Div Diabet Translat, 2877 Brandywine Rd,Mail Stop K-10, Atlanta, GA 30341 USA.

EM LBarker1@cdc.gov

CR [Anonymous], 1994, APP LEAD IN CANC SOW

Appalachian Regional Commission, 2016, APP REG

Birdsall S, 2008, OUTLINE AM GEOGRAPHY

Blakeney AB, 2005, ED CULTURALLY SENSIT, P161

Bovell-Benjamin AC, 2009, HEALTH PLACE, V15, P429, DOI 10.1016/j.healthplace.2008.08.001

*CDCP, 2007 NAT DIAB FACT S

Centers for Disease Control and Prevention, BEH RISK FACT SURV S

Coyne CA, 2006, PREV CHRONIC DIS, V3

Crespo R, HLTH PROMOT IN PRESS

Denham SA, 2004, FAM COMMUNITY HEALTH, V27, P170, DOI 10.1097/00003727-200404000-00010

Denham SA, 2009, DIABETES FAMILY MATT

Dinour LM, 2007, J AM DIET ASSOC, V107, P1952, DOI 10.1016/j.jada.2007.08.006

Holben DH, 2006, PREV CHRONIC DIS, V3

Holtzman D., 2003, COMMUNITY BASED HLTH, P115

Hortz B, 2009, J RURAL HEALTH, V25, P58, DOI 10.1111/j.1748-0361.2009.00199.x

Lantz PM, 1998, JAMA-J AM MED ASSOC, V279, P1703, DOI 10.1001/jama.279.21.1703

Martin KS, 2007, J NUTR EDUC BEHAV, V39, P31, DOI 10.1016/j.jneb.2006.08.021

Murasko JE, 2009, ECON HUM BIOL, V7, P376, DOI 10.1016/j.ehb.2009.04.004

Pollard Kelvin M., 2004, NEW DIVERSITY RACE E

Rizvi AA, 2007, AM J MED SCI, V333, P35, DOI 10.1097/00000441-200701000-00005

Robbins JM, 2001, AM J PUBLIC HEALTH, V91, P76

Roussos ST, 2000, ANNU REV PUBL HEALTH, V21, P369, DOI 10.1146/annurev.publhealth.21.1.369

Tessaro I, 2005, PREV CHRONIC DIS, V2

Townsend MS, 2001, J NUTR, V131, P1738

US Department of Health and Human Services Health Resources and Service Administration, FIND SHORT AR HPSA S

Wewers ME, 2000, J COMMUN HEALTH, V25, P377, DOI 10.1023/A:1005127917122

Wewers ME, 2006, PREV CHRONIC DIS, V3

Wilde PE, 2006, J NUTR, V136, P1395

Willi C, 2007, JAMA-J AM MED ASSOC, V298, P2654, DOI 10.1001/jama.298.22.2654

Williams KJ, 2008, RURAL REMOTE HEALTH, V8

NR 30

TC 14

Z9 14

U1 0

U2 4

PU CENTERS DISEASE CONTROL

PI ATLANTA

PA 1600 CLIFTON RD, ATLANTA, GA 30333 USA

SN 1545-1151

J9 PREV CHRONIC DIS

JI Prev. Chronic Dis.

PD SEP

PY 2010

VL 7

IS 5

AR A104

PG 9

WC Public, Environmental & Occupational Health

SC Public, Environmental & Occupational Health

GA V20SD

UT WOS:000208158800014

PM 20712931

DA 2019-01-08

ER

PT J

AU Ivanova, A

Nitka, D

Schmitz, N

AF Ivanova, Anna

Nitka, Danit

Schmitz, Norbert

TI Epidemiology of antidepressant medication use in the Canadian diabetes

population

SO SOCIAL PSYCHIATRY AND PSYCHIATRIC EPIDEMIOLOGY

LA English

DT Article

DE Diabetes; Depression; Antidepressant medication; Survey

ID DIAGNOSTIC INTERVIEW CIDI; PLACEBO-CONTROLLED TRIAL; PSYCHOLOGICAL

DISTRESS; MENTAL-HEALTH; DEPRESSION SYMPTOMS; GENERAL-POPULATION;

PREVENTION PROGRAM; SCREENING SCALES; DOUBLE-BLIND; MEDICINE USE

AB Depression has been shown to be a common co-morbidity in diabetes. From a public health point of view, there is a lack of population-based studies regarding the use of antidepressant medication in non-clinical samples of people with diabetes. The objective of the present study was to provide demographic and clinical information about the use of antidepressant medication in a representative community sample of people with diabetes.

The Canadian Community Health Survey 1.2 (CCHS 1.2) is a cross-sectional survey that collects information related to health status, health-care utilization and health determinants of the Canadian general population. Diabetes presence was ascertained by self-report of physician diagnosis. Depression and anxiety were assessed using a modified version of the World Mental Health Composite International Diagnostic Interview. Antidepressant use was determined through self-report.

The population prevalence of self-reported antidepressant use in the past 12 months was 8.4% (SE = 0.95) among people with diabetes (n = 1,662). People who took antidepressants had higher average body mass index (M = 32.6, SD = 6.5) than those not taking antidepressants (M = 29.2, SD = 5.7). The use of antidepressants was associated with poorer health status and higher number of co-morbid chronic conditions. Half of diabetes subjects who used antidepressant medication in the last year did not have a lifetime history of major depression.

In a community sample of people with diabetes, the prevalence of antidepressant use exceeded the prevalence of major depression. Anxiety disorders and other somatic chronic conditions were associated with the prescription of antidepressant medication in people with diabetes, but without a history of major depression.

C1 [Ivanova, Anna; Nitka, Danit; Schmitz, Norbert] McGill Univ, Douglas Mental Hlth Univ Inst, Montreal, PQ H4H 1R3, Canada.

[Schmitz, Norbert] McGill Univ, Dept Psychiat, Montreal, PQ H4H 1R3, Canada.

RP Schmitz, N (reprint author), McGill Univ, Douglas Mental Hlth Univ Inst, 6875 LaSalle Blvd, Montreal, PQ H4H 1R3, Canada.

EM norbert.schmitz@mcgill.ca

RI Schmitz, Norbert/A-5177-2010

OI Schmitz, Norbert/0000-0001-7777-6323

FU Canadian Institutes for Health Research (CIHR) [MOP-79464]; FRSQ

Chercheur-Boursier

FX This analysis is based on Statistics Canada's Canadian Community Health

Survey, Cycle 1.2 (2005), public use microdata file which contains

anonymized collected data. All computations on these microdata were

prepared by Anna Ivanova, Danit Nitka, and Norbert Schmitz, and the

responsibility for the use and interpretation of these data is entirely

that of the authors. All authors had full access to all of the data in

the study and take responsibility for the integrity of the data and the

accuracy of the data analysis. This research was funded by the Canadian

Institutes for Health Research (CIHR Grant MOP-79464) and a FRSQ

Chercheur-Boursier fellowship to Dr Schmitz.

CR Andrews G, 2001, AUST NZ J PUBL HEAL, V25, P494, DOI 10.1111/j.1467-842X.2001.tb00310.x

Beck CA, 2005, SOC PSYCH PSYCH EPID, V40, P799, DOI 10.1007/s00127-005-0968-0

Cairney J, 2007, CAN J PSYCHIAT, V52, P111, DOI 10.1177/070674370705200209

Demyttenaere K, 2008, J PSYCHOPHARMACOL, V22, P792, DOI 10.1177/0269881107083798

Deshmukh R, 2003, CLEV CLIN J MED, V70, P614, DOI 10.3949/ccjm.70.7.614

Furukawa TA, 2003, PSYCHOL MED, V33, P357, DOI 10.1017/S0033291702006700

Ghaemi SN, 2001, J CLIN PSYCHIAT, V62, P565, DOI 10.4088/JCP.v62n07a12

Gonzalez JS, 2007, DIABETES CARE, V30, P2222, DOI 10.2337/dc07-0158

Gonzalez JS, 2008, DIABETES CARE, V31, P2398, DOI 10.2337/dc08-1341

Goodnick P J, 2001, Ann Clin Psychiatry, V13, P31, DOI 10.3109/10401230109147127

Hirschfield RMA, 1997, JAMA-J AM MED ASSOC, V277, P333, DOI 10.1001/jama.277.4.333

Idler EL, 1997, J HEALTH SOC BEHAV, V38, P21, DOI 10.2307/2955359

Katon W, 2007, GEN HOSP PSYCHIAT, V29, P147, DOI 10.1016/j.genhosppsych.2006.11.005

Katon WJ, 2005, DIABETES CARE, V28, P2668, DOI 10.2337/diacare.28.11.2668

Katon WJ, 2004, ARCH GEN PSYCHIAT, V61, P1042, DOI 10.1001/archpsyc.61.10.1042

Keller MB, 2000, NEW ENGL J MED, V342, P1462, DOI 10.1056/NEJM200005183422001

Kennedy SH, 2006, EUR NEUROPSYCHOPHARM, V16, pS619, DOI 10.1016/S0924-977X(06)70007-4

Kessler RC, 2002, PSYCHOL MED, V32, P959, DOI 10.1017/S0033291702006074

Kessler RC, 2004, INT J METH PSYCH RES, V13, P93, DOI 10.1002/mpr.168

Lin EHB, 2008, J PSYCHOSOM RES, V65, P571, DOI 10.1016/j.jpsychores.2008.06.007

Lustman PJ, 1997, PSYCHOSOM MED, V59, P241, DOI 10.1097/00006842-199705000-00007

Lustman PJ, 2000, DIABETES CARE, V23, P618, DOI 10.2337/diacare.23.5.618

Lustman PJ, 1998, ANN INTERN MED, V129, P613, DOI 10.7326/0003-4819-129-8-199810150-00005

McCollum M, 2007, AM J MANAG CARE, V13, P65

McIntyre RS, 2006, EXPERT OPIN DRUG SAF, V5, P157, DOI 10.1517/14740338.5.1.157

McIntyre RS, 2006, EXPERT OPIN DRUG SAF, V5, P523, DOI 10.1517/14740338.5.4.523

Mokdad AH, 2003, JAMA-J AM MED ASSOC, V289, P76, DOI 10.1001/jama.289.1.76

*NIH, 1998, OBES RES S2, V6, P51

Ohayon MM, 1998, J CLIN EPIDEMIOL, V51, P273, DOI 10.1016/S0895-4356(97)00238-2

Okura Y, 2004, J CLIN EPIDEMIOL, V57, P1096, DOI 10.1016/j.jclinepi.2004.04.005

RADLOFF L S, 1977, Applied Psychological Measurement, V1, P385, DOI 10.1177/014662167700100306

Rubin RR, 2008, DIABETES CARE, V31, P420, DOI 10.2337/dc07-182T

Rubin RR, 2005, DIABETES CARE, V28, P830

*STAT CAN, 2002, PROF DIS CAN CAN RES

Tunks ER, 2008, CAN J PSYCHIAT, V53, P235

Williams JW, 2004, ANN INTERN MED, V140, P1015, DOI 10.7326/0003-4819-140-12-200406150-00012

WITTCHEN HU, 1994, J PSYCHIAT RES, V28, P57, DOI 10.1016/0022-3956(94)90036-1

NR 37

TC 3

Z9 4

U1 0

U2 5

PU SPRINGER HEIDELBERG

PI HEIDELBERG

PA TIERGARTENSTRASSE 17, D-69121 HEIDELBERG, GERMANY

SN 0933-7954

J9 SOC PSYCH PSYCH EPID

JI Soc. Psychiatry Psychiatr. Epidemiol.

PD SEP

PY 2010

VL 45

IS 9

BP 911

EP 919

DI 10.1007/s00127-009-0129-y

PG 9

WC Psychiatry

SC Psychiatry

GA 637VJ

UT WOS:000280846500007

PM 19730761

DA 2019-01-08

ER

PT J

AU Cusi, K

AF Cusi, Kenneth

TI The Role of Adipose Tissue and Lipotoxicity in the Pathogenesis of Type

2 Diabetes

SO CURRENT DIABETES REPORTS

LA English

DT Article

DE Adipose tissue; Lipotoxicity; NAFLD; Type 2 diabetes mellitus; Insulin

resistance

ID FREE FATTY-ACIDS; ENCODED MITOCHONDRIAL GENES; IMPAIRS

INSULIN-SECRETION; BETA-CELL DYSFUNCTION; HUMAN SKELETAL-MUSCLE;

BODY-MASS INDEX; NONALCOHOLIC STEATOHEPATITIS; HEPATIC STEATOSIS;

FAMILY-HISTORY; METABOLIC DISEASES

AB The widespread epidemics of obesity and type 2 diabetes mellitus (T2DM) suggest that both conditions are closely linked. An increasing body of evidence has shifted our view of adipose tissue from a passive energy depot to a dynamic "endocrine organ" that tightly regulates nutritional balance by means of a complex crosstalk of adipocytes with their microenvironment. Dysfunctional adipose tissue, particularly as observed in obesity, is characterized by adipocyte hypertrophy, macrophage infiltration, impaired insulin signaling, and insulin resistance. The result is the release of a host of inflammatory adipokines and excessive amounts of free fatty acids that promote ectopic fat deposition and lipotoxicity in muscle, liver, and pancreatic beta cells. This review focuses on recent work on how glucose homeostasis is profoundly altered by distressed adipose tissue. A better understanding of this relationship offers the best chance for early intervention strategies aimed at preventing the burden of T2DM.

C1 Univ Texas Hlth Sci Ctr San Antonio, Diabet Div, San Antonio, TX 78284 USA.

RP Cusi, K (reprint author), Univ Texas Hlth Sci Ctr San Antonio, Diabet Div, Room 3-380S,7703 Floyd Curl Dr, San Antonio, TX 78284 USA.

EM cusi@uthscsa.edu

FU American Diabetes Association; Burroughs Wellcome Fund; Veterans Affairs

Medical Research Fund; National Center for Research Resources [UL

1RR025767]

FX Dr. Kenneth Cusi is supported by the American Diabetes Association, the

Burroughs Wellcome Fund, the Veterans Affairs Medical Research Fund, and

by Award Number UL 1RR025767 from the National Center for Research

Resources. The content is solely the responsibility of the authors and

does not necessarily represent the official views of the National Center

for Research Resources of the National Institutes of Health.

CR Abdul-Ghani MA, 2008, CURR DIABETES REP, V8, P173, DOI 10.1007/s11892-008-0030-1

Baker JL, 2007, NEW ENGL J MED, V357, P2329, DOI 10.1056/NEJMoa072515

Belfort R, 2005, DIABETES, V54, P1640, DOI 10.2337/diabetes.54.6.1640

Belfort R, 2006, NEW ENGL J MED, V355, P2297, DOI 10.1056/NEJMoa060326

Boden G, 2002, AM J PHYSIOL-ENDOC M, V283, pE12, DOI 10.1152/ajpendo.00429.2001

Bogacka I, 2004, DIABETES CARE, V27, P1660, DOI 10.2337/diacare.27.7.1660

Brassard P, 2008, J CLIN ENDOCR METAB, V93, P837, DOI 10.1210/jc.2007-1670

Buchanan TA, 2007, DIABETES, V56, P1502, DOI 10.2337/db07-0140

Butler AE, 2003, DIABETES, V52, P102, DOI 10.2337/diabetes.52.1.102

CHAN DC, 2010, THROMB VASC BIOL, V30, P1043

Chavez AO, 2010, J CLIN ENDOCR METAB, V95, P422, DOI 10.1210/jc.2009-1387

Choi CS, 2008, P NATL ACAD SCI USA, V105, P19926, DOI 10.1073/pnas.0810339105

Choi SS, 2008, CURR OPIN LIPIDOL, V19, P295, DOI 10.1097/MOL.0b013e3282ff5e55

Civitarese AE, 2004, DIABETOLOGIA, V47, P816, DOI 10.1007/s00125-004-1359-x

Cusi K, 2000, J CLIN INVEST, V105, P311, DOI 10.1172/JCI7535

Cusi K, 2007, AM J PHYSIOL-ENDOC M, V292, pE1775, DOI 10.1152/ajpendo.00624.2006

Cusi K, 2009, CLIN LIVER DIS, V13, P545, DOI 10.1016/j.cld.2009.07.009

Cusi K, 2009, CONTEMP DIABETES, P3, DOI 10.1007/978-1-59745-260-1_1

Cusi K, 2009, CURR OPIN ENDOCRINOL, V16, P141, DOI 10.1097/MED.0b013e3283293015

Cusi K, 2009, CURR DIABETES REP, V9, P200, DOI 10.1007/s11892-009-0033-6

De Filippis E, 2008, AM J PHYSIOL-ENDOC M, V294, pE607, DOI 10.1152/ajpendo.00729.2007

DeFronzo RA, 2010, DIABETOLOGIA, V53, P435, DOI 10.1007/s00125-009-1614-2

Delghingaro-Augusto V, 2009, DIABETOLOGIA, V52, P1122, DOI 10.1007/s00125-009-1317-8

Estall JL, 2009, P NATL ACAD SCI USA, V106, P22510, DOI 10.1073/pnas.0912533106

Federici M, 2001, DIABETES, V50, P1290, DOI 10.2337/diabetes.50.6.1290

Flegal KM, 2010, JAMA-J AM MED ASSOC, V303, P235, DOI 10.1001/jama.2009.2014

Franks PW, 2010, NEW ENGL J MED, V362, P485, DOI 10.1056/NEJMoa0904130

Furuhashi M, 2008, NAT REV DRUG DISCOV, V7, P489, DOI 10.1038/nrd2589

Gastaldelli A, 2004, DIABETOLOGIA, V47, P31, DOI 10.1007/s00125-003-1263-9

Gastaldelli A, 2009, HEPATOLOGY, V50, P1087, DOI 10.1002/hep.23116

Greenfield V, 2008, CURR OPIN GASTROEN, V24, P320, DOI 10.1097/MOG.0b013e3282fbccf2

Gregor MF, 2009, DIABETES, V58, P693, DOI 10.2337/db08-1220

GULLI G, 1992, DIABETES, V41, P1575, DOI 10.2337/diabetes.41.12.1575

Han CY, 2010, DIABETES, V59, P386, DOI 10.2337/db09-0925

Handschin C, 2008, NATURE, V454, P463, DOI 10.1038/nature07206

Hojlund K, 2008, ENDOCRIN METAB CLIN, V37, P713, DOI 10.1016/j.ecl.2008.06.006

Holloszy JO, 2009, AM J CLIN NUTR, V89, p463S, DOI 10.3945/ajcn.2008.26717C

Hotamisligil GS, 2008, NAT REV IMMUNOL, V8, P923, DOI 10.1038/nri2449

Huang W, 2010, DIABETES, V59, P347, DOI 10.2337/db09-0016

Isganaitis E, 2009, DIABETES, V58, P1192, DOI 10.2337/db08-1266

Kashyap S, 2003, DIABETES, V52, P2461, DOI 10.2337/diabetes.52.10.2461

Kashyap Sangeeta R, 2008, J Cardiometab Syndr, V3, P141, DOI 10.1111/j.1559-4572.2008.00013.x

Kashyap SR, 2004, AM J PHYSIOL-ENDOC M, V287, pE537, DOI 10.1152/ajpendo.00541.2003

Khan T, 2009, MOL CELL BIOL, V29, P1575, DOI 10.1128/MCB.01300-08

Kim JY, 2007, J CLIN INVEST, V117, P2621, DOI 10.1172/JCI31021

Lefterova MI, 2009, TRENDS ENDOCRIN MET, V20, P107, DOI 10.1016/j.tem.2008.11.005

Li ZZ, 2009, J BIOL CHEM, V284, P5637, DOI 10.1074/jbc.M807616200

Liang H, 2009, AM J PHYSIOL-ENDOC M, V296, pE945, DOI 10.1152/ajpendo.90292.2008

Listenberger LL, 2003, P NATL ACAD SCI USA, V100, P3077, DOI 10.1073/pnas.0630588100

Liu L, 2009, J BIOL CHEM, V284, P36312, DOI 10.1074/jbc.M109.049817

Liu L, 2009, DIABETES, V58, P2516, DOI 10.2337/db08-1096

MATHEW M, 2010, CARDIOVASC DIABETOL, V9, P1

Mathew M, 2007, DIABETES, V56, pA674

MCGARRY JD, 1992, SCIENCE, V258, P766, DOI 10.1126/science.1439783

Minehira K, 2008, J LIPID RES, V49, P2038, DOI 10.1194/jlr.M800248-JLR200

Muhlhausler B, 2009, TRENDS ENDOCRIN MET, V20, P51, DOI 10.1016/j.tem.2008.10.006

Muniyappa R, 2008, ENDOCRIN METAB CLIN, V37, P685, DOI 10.1016/j.ecl.2008.06.001

Ogden CL, 2010, JAMA-J AM MED ASSOC, V303, P242, DOI 10.1001/jama.2009.2012

ORTIZLOPEZ C, 2010, DIABETES S, V1, P59

Patti ME, 2003, P NATL ACAD SCI USA, V100, P8466, DOI 10.1073/pnas.1032913100

Perseghin G, 1997, DIABETES, V46, P1001, DOI 10.2337/diabetes.46.6.1001

Petersen KF, 2004, NEW ENGL J MED, V350, P664, DOI 10.1056/NEJMoa031314

Poitout V, 2008, ENDOCR REV, V29, P351, DOI 10.1210/er.2007-0023

Pratipanawatr W, 2001, DIABETES, V50, P2572, DOI 10.2337/diabetes.50.11.2572

Reyna SM, 2008, DIABETES, V57, P2595, DOI 10.2337/db08-0038

Richardson DK, 2005, J BIOL CHEM, V280, P10290, DOI 10.1074/jbc.M408985200

Roden M, 2000, DIABETES, V49, P701, DOI 10.2337/diabetes.49.5.701

Rutkowski JM, 2009, FEBS J, V276, P5738, DOI 10.1111/j.1742-4658.2009.07303.x

Semple RK, 2009, J CLIN INVEST, V119, P315, DOI 10.1172/JCI37432

Shaw JE, 2009, DIABETES RES CLIN PR, V87, P4

Szendroedi J, 2009, CURR OPIN LIPIDOL, V20, P50, DOI 10.1097/MOL.0b013e328321b3a8

UNGER RH, 2001, DIABETES S1, V50, P118

Vaag A, 2007, CURR OPIN CLIN NUTR, V10, P591, DOI 10.1097/MCO.0b013e3282ab9ea6

Virkamaki A, 2001, DIABETES, V50, P2337, DOI 10.2337/diabetes.50.10.2337

Wouters K, 2008, HEPATOLOGY, V48, P474, DOI 10.1002/hep.22363

Wueest S, 2010, J CLIN INVEST, V120, P191, DOI 10.1172/JCI38388

Yang XL, 2004, BIOCHEM BIOPH RES CO, V317, P1045, DOI 10.1016/j.bbrc.2004.03.152

Ye J, 2009, INT J OBESITY, V33, P54, DOI 10.1038/ijo.2008.229

NR 78

TC 144

Z9 157

U1 1

U2 23

PU CURRENT MEDICINE GROUP

PI PHILADELPHIA

PA 400 MARKET STREET, STE 700, PHILADELPHIA, PA 19106 USA

SN 1534-4827

J9 CURR DIABETES REP

JI Curr. Diabetes Rep.

PD AUG

PY 2010

VL 10

IS 4

BP 306

EP 315

DI 10.1007/s11892-010-0122-6

PG 10

WC Endocrinology & Metabolism

SC Endocrinology & Metabolism

GA 615IT

UT WOS:000279128900008

PM 20556549

DA 2019-01-08

ER

PT J

AU Skinner, TC

Carey, ME

Cradock, S

Dallosso, HM

Daly, H

Davies, MJ

Doherty, Y

Heller, S

Khunti, K

Oliver, L

AF Skinner, T. C.

Carey, M. E.

Cradock, S.

Dallosso, H. M.

Daly, H.

Davies, M. J.

Doherty, Y.

Heller, S.

Khunti, K.

Oliver, L.

CA Desmond Collaborative

TI Depressive symptoms in the first year from diagnosis of Type 2 diabetes:

results from the DESMOND trial

SO DIABETIC MEDICINE

LA English

DT Article

DE depression; type 2 diabetes

ID RANDOMIZED CONTROLLED-TRIAL; HOSPITAL ANXIETY; METAANALYSIS; PREVALENCE;

PEOPLE; ADULTS; SCALE

AB P>Aims

To describe the course of depressive symptoms during the first year after diagnosis of Type 2 diabetes.

Methods

Post hoc analysis of data from a randomized controlled trial of self-management education for 824 individuals newly diagnosed with Type 2 diabetes. Participants completed the Depression scale of the Hospital Anxiety and Depression Scale after diagnosis and at 4, 8 and 12 months follow-up. Participants also completed the Problem Areas in Diabetes scale at 8 and 12 months follow-up. We present descriptive statistics on prevalence and persistence of depressive symptoms. Logistic regression is used to predict possible depression cases, and multiple regression to predict depressive symptomatology.

Results

The prevalence of depressive symptoms in individuals recently diagnosed with diabetes (18-22% over the year) was not significantly different from normative data for the general population (12%) in the UK. Over 20% of participants indicated some degrees of depressive symptoms over the first year of living with Type 2 diabetes; these were mostly transient episodes, with 5% (1% severe) reporting having depressive symptoms throughout the year. At 12 months post diagnosis, after controlling for baseline depressive symptoms, diabetes-specific emotional distress was predictive of depressive symptomatology.

Conclusions

The increased prevalence of depressive symptoms in diabetes is not manifest until at least 1 year post diagnosis in this cohort. However, there are a significant number of people with persistent depressive symptoms in the early stages of diabetes, and diabetes-specific distress may be contributing to subsequent development of depressive symptoms in people with Type 2 diabetes.

C1 [Skinner, T. C.] Univ Western Australia, Combined Univ Ctr Rural Hlth, Geraldton, WA 6531, Australia.

[Carey, M. E.; Dallosso, H. M.; Daly, H.] Univ Hosp Leicester NHS Trust, Diabet Res Team, Leicester, Leics, England.

[Cradock, S.] Portsmouth Diabet Ctr & Portsmouth City Primary C, Portsmouth, Hants, England.

[Davies, M. J.] Univ Leicester, Dept Cardiovasc Serv, Leicester, Leics, England.

[Heller, S.] Univ Sheffield, Sch Med, Dept Res & Dev, Sheffield, S Yorkshire, England.

[Heller, S.] Univ Sheffield, Sch Med, Acad Unit Diabet Endocrinol & Metab, Sheffield, S Yorkshire, England.

[Khunti, K.] Univ Leicester, Dept Hlth Sci, Leicester, Leics, England.

RP Skinner, TC (reprint author), Univ Western Australia, Combined Univ Ctr Rural Hlth, POB 109, Geraldton, WA 6531, Australia.

EM timskinner@cucrh.uwa.edu.au

RI Skinner, Timothy/G-2865-2010

OI Khunti, Kamlesh/0000-0003-2343-7099; Davies,

Michael/0000-0002-5196-6919; Heller, Simon/0000-0002-2425-9565; Davies,

Melanie/0000-0002-9987-9371; Skinner, Timothy/0000-0002-0018-6963

CR Ali S, 2006, DIABETIC MED, V23, P1165, DOI 10.1111/j.1464-5491.2006.01943.x

Anderson RJ, 2001, DIABETES CARE, V24, P1069, DOI 10.2337/diacare.24.6.1069

Bjelland I, 2002, J PSYCHOSOM RES, V52, P69, DOI 10.1016/S0022-3999(01)00296-3

Ciechanowski PS, 2000, ARCH INTERN MED, V160, P3278, DOI 10.1001/archinte.160.21.3278

Crawford JR, 2001, BRIT J CLIN PSYCHOL, V40, P429, DOI 10.1348/014466501163904

Davies MJ, 2008, BRIT MED J, V336, P491, DOI 10.1136/bmj.39474.922025.BE

Khunti K, 2008, DIABETIC MED, V25, P1454, DOI 10.1111/j.1464-5491.2008.02620.x

Knol MJ, 2006, DIABETOLOGIA, V49, P837, DOI 10.1007/s00125-006-0159-x

Lustman Patrick J, 2008, Curr Psychiatry Rep, V10, P495

Lustman PJ, 2000, DIABETES CARE, V23, P934, DOI 10.2337/diacare.23.7.934

Pouwer F, 2005, DIABETES RES CLIN PR, V70, P166, DOI 10.1016/j.diabres.2005.03.031

ZIGMOND AS, 1983, ACTA PSYCHIAT SCAND, V67, P361, DOI 10.1111/j.1600-0447.1983.tb09716.x

NR 12

TC 15

Z9 16

U1 0

U2 7

PU WILEY-BLACKWELL

PI MALDEN

PA COMMERCE PLACE, 350 MAIN ST, MALDEN 02148, MA USA

SN 0742-3071

J9 DIABETIC MED

JI Diabetic Med.

PD AUG

PY 2010

VL 27

IS 8

BP 965

EP 967

DI 10.1111/j.1464-5491.2010.03028.x

PG 3

WC Endocrinology & Metabolism

SC Endocrinology & Metabolism

GA 626RN

UT WOS:000279984700019

PM 20653757

DA 2019-01-08

ER

PT J

AU Ogihara, T

Mirmira, RG

AF Ogihara, Takeshi

Mirmira, Raghavendra G.

TI An islet in distress: beta cell failure in type 2 diabetes

SO JOURNAL OF DIABETES INVESTIGATION

LA English

DT Review

DE Islet; Diabetes; Insulin resistance

ID ENDOPLASMIC-RETICULUM STRESS; INSULIN GENE-TRANSCRIPTION;

UNFOLDED-PROTEIN RESPONSE; HUMAN PANCREATIC-ISLETS;

TUMOR-NECROSIS-FACTOR; FREE FATTY-ACIDS; THIOREDOXIN-INTERACTING

PROTEIN; NUCLEAR FACTOR-4-ALPHA GENE; RECEPTOR-GAMMA ACTIVATION; CHRONIC

OXIDATIVE STRESS

AB Over 200 million people worldwide suffer from diabetes, a disorder of glucose homeostasis. The majority of these individuals are diagnosed with type 2 diabetes. It has traditionally been thought that tissue resistance to the action of insulin is the primary defect in type 2 diabetes. However, recent longitudinal and genome-wide association studies have shown that insulin resistance is more likely to be a precondition, and that the failure of the pancreatic beta cell to meet the increased insulin requirements is the triggering factor in the development of type 2 diabetes. A major emphasis in diabetes research has therefore shifted to understanding the causes of beta cell failure. Collectively, these studies have implicated a complex network of triggers, which activate intersecting execution pathways leading to beta cell dysfunction and death. In the present review, we discuss these triggers (glucotoxicity, lipotoxicity, amyloid and cytokines) with respect to the pathways they activate (oxidative stress, inflammation and endoplasmic reticulum stress) and propose a model for understanding beta cell failure in type 2 diabetes. (J Diabetes Invest, doi: 10.1111/j.2040-1124.2010.00021.x, 2010)

C1 [Ogihara, Takeshi; Mirmira, Raghavendra G.] Indiana Univ Sch Med, Dept Pediat, Indianapolis, IN 46202 USA.

[Ogihara, Takeshi; Mirmira, Raghavendra G.] Indiana Univ Sch Med, Herman B Wells Ctr Pediat Res, Indianapolis, IN USA.

[Mirmira, Raghavendra G.] Indiana Univ Sch Med, Dept Med, Indianapolis, IN USA.

[Mirmira, Raghavendra G.] Indiana Univ Sch Med, Dept Cellular & Integrat Physiol, Indianapolis, IN USA.

RP Mirmira, RG (reprint author), Indiana Univ Sch Med, Dept Pediat, Indianapolis, IN 46202 USA.

EM rmirmira@iupui.edu

FU National Institutes of Health [R01 DK60581]; Ball Brothers Foundation

FX The authors were supported by grant R01 DK60581 from the National

Institutes of Health, an ADA-Takeda Mentor-based Postdoctoral

Fellowship, and a gift from the Ball Brothers Foundation (all to RGM).

CR Agostini L, 2004, IMMUNITY, V20, P319, DOI 10.1016/S1074-7613(04)00046-9

Alquier T, 2009, DIABETES, V58, P2607, DOI 10.2337/db09-0362

Amer Diabet Assoc, 2014, DIABETES CARE, V37, pS81, DOI [10.2337/dc14-S081, 10.2337/dc10-s062, 10.2337/dc11-S062, 10.2337/dc10-S062]

Anello M, 2005, DIABETOLOGIA, V48, P282, DOI 10.1007/s00125-004-1627-9

Berridge MJ, 2002, CELL CALCIUM, V32, P235, DOI 10.1016/S0143-4160(023)00182-3

Bertolotti A, 2000, NAT CELL BIOL, V2, P326

BLEICH D, 1995, ENDOCRINOLOGY, V136, P5736, DOI 10.1210/en.136.12.5736

BODEN G, 1995, DIABETES, V44, P1239, DOI 10.2337/diabetes.44.10.1239

Bouzakri K, 2009, J BIOL CHEM, V284, P27892, DOI 10.1074/jbc.M109.048058

Briaud I, 2001, DIABETES, V50, P315, DOI 10.2337/diabetes.50.2.315

Brunham LR, 2007, NAT MED, V13, P340, DOI 10.1038/nm1546

Burton PR, 2007, NATURE, V447, P661, DOI 10.1038/nature05911

Butler AE, 2003, DIABETES, V52, P102, DOI 10.2337/diabetes.52.1.102

Butler AE, 2004, DIABETES, V53, P1509, DOI 10.2337/diabetes.53.6.1509

BUTLER PC, 1990, DIABETES, V39, P752, DOI 10.2337/diabetes.39.6.752

Casas S, 2007, DIABETES, V56, P2284, DOI 10.2337/db07-0178

Cha-Molstad H, 2009, J BIOL CHEM, V284, P16898, DOI 10.1074/jbc.M109.010504

Chen J, 2008, DIABETES, V57, P938, DOI 10.2337/db07-0715

Chen M, 2005, DIABETOLOGIA, V48, P486, DOI 10.1007/s00125-005-1673-y

Chen M, 2002, ENDOCRINOLOGY, V143, P2341, DOI 10.1210/en.143.6.2341

CLARK A, 1995, DIABETES RES CLIN PR, V28, pS39, DOI 10.1016/0168-8227(95)01075-O

Cnop M, 2002, DIABETES, V51, P1005, DOI 10.2337/diabetes.51.4.1005

Cnop M, 2002, ENDOCRINOLOGY, V143, P3449, DOI 10.1210/en.2002-220273

Cnop M, 2003, DIABETOLOGIA, V46, P459, DOI 10.1007/s00125-003-1074-z

Deeney JT, 2000, J BIOL CHEM, V275, P9363, DOI 10.1074/jbc.275.13.9363

DeFronzo RA, 2010, DIABETOLOGIA, V53, P435, DOI 10.1007/s00125-009-1614-2

Dickson LM, 2004, AM J PHYSIOL-ENDOC M, V287, pE192, DOI 10.1152/ajpendo.00031.2004

EHRLICH JC, 1961, AM J PATHOL, V38, P49

Ehses JA, 2007, DIABETES, V56, P2356, DOI 10.2337/db06-1650

Eitel K, 2003, DIABETES, V52, P991, DOI 10.2337/diabetes.52.4.991

Eizirik DL, 2008, ENDOCR REV, V29, P42, DOI 10.1210/er.2007-0015

Eizirik DL, 2001, DIABETOLOGIA, V44, P2115, DOI 10.1007/s001250100021

El-Assaad W, 2003, ENDOCRINOLOGY, V144, P4154, DOI 10.1210/en.2003-0410

Elouil H, 2007, DIABETOLOGIA, V50, P1442, DOI 10.1007/s00125-007-0674-4

Emilsson V, 1997, DIABETES, V46, P313, DOI 10.2337/diabetes.46.2.313

Evans-Molina C, 2009, MOL CELL BIOL, V29, P2053, DOI 10.1128/MCB.01179-08

Festa A, 2006, DIABETES, V55, P1114, DOI 10.2337/diabetes.55.04.06.db05-1100

GRANKVIST K, 1981, BIOCHEM J, V199, P393, DOI 10.1042/bj1990393

Grant SFA, 2006, NAT GENET, V38, P320, DOI 10.1038/ng1732

Gunton JE, 2005, CELL, V122, P337, DOI 10.1016/j.cell.2005.05.027

Hagman DK, 2008, DIABETES, V57, P424, DOI 10.2337/db07-1285

Hagman DK, 2005, J BIOL CHEM, V280, P32413, DOI 10.1074/jbc.M506000200

Han X, 2002, MOL ENDOCRINOL, V16, P2145, DOI 10.1210/me.2001-0300

Harmon JS, 2009, ENDOCRINOLOGY, V150, P4855, DOI 10.1210/en.2009-0708

Henquin JC, 2004, DIABETES, V53, pS48, DOI 10.2337/diabetes.53.suppl_3.S48

HOTAMISLIGIL GS, 1995, J CLIN INVEST, V95, P2409, DOI 10.1172/JCI117936

Huang CJ, 2007, AM J PHYSIOL-ENDOC M, V293, pE1656, DOI 10.1152/ajpendo.00318.2007

Huang CJ, 2007, DIABETES, V56, P2016, DOI 10.2337/db07-0197

Hundal RS, 2002, J CLIN INVEST, V109, P1321, DOI 10.1172/JCI200214955

Ihara Y, 1999, DIABETES, V48, P927, DOI 10.2337/diabetes.48.4.927

Ishida H, 2004, METABOLISM, V53, P488, DOI 10.1016/j.metabol.2003.11.021

Jacqueminet S, 2000, METABOLISM, V49, P532, DOI 10.1016/S0026-0495(00)80021-9

Janson J, 1996, P NATL ACAD SCI USA, V93, P7283, DOI 10.1073/pnas.93.14.7283

Jiang HY, 2004, MOL CELL BIOL, V24, P1365, DOI 10.1128/MCD.24.3.1365-1377.2004

Jonas JC, 2009, DIABETES OBES METAB, V11, P65, DOI 10.1111/j.1463-1326.2009.01112.x

Jonas JC, 1999, J BIOL CHEM, V274, P14112, DOI 10.1074/jbc.274.20.14112

Joseph JW, 2004, J BIOL CHEM, V279, P51049, DOI 10.1074/jbc.M409189200

Joseph JW, 2002, DIABETES, V51, P3211, DOI 10.2337/diabetes.51.11.3211

KAHN SE, 1990, DIABETES, V39, P634, DOI 10.2337/diabetes.39.5.634

Kajimoto Y, 2004, ANN NY ACAD SCI, V1011, P168, DOI 10.1196/annals.1293.017

Kanda H, 2006, J CLIN INVEST, V116, P1494, DOI 10.1172/JC126498

Kawasaki F, 2005, AM J PHYSIOL-ENDOC M, V288, pE510, DOI 10.1152/ajpendo.00128.2004

Kelpe CL, 2003, J BIOL CHEM, V278, P30015, DOI 10.1074/jbc.M302548200

Kern PA, 2001, AM J PHYSIOL-ENDOC M, V280, pE745

Kieffer TJ, 1997, DIABETES, V46, P1087, DOI 10.2337/diabetes.46.6.1087

Kjorholt C, 2005, DIABETES, V54, P2755, DOI 10.2337/diabetes.54.9.2755

Koshkin V, 2003, J BIOL CHEM, V278, P19709, DOI 10.1074/jbc.M209709200

Kulkarni RN, 1997, J CLIN INVEST, V100, P2729, DOI 10.1172/JCI119818

KULKARNI RN, 2001, MOL BASIS PANCREAS D, P299

Kwon G, 1999, J BIOL CHEM, V274, P18702, DOI 10.1074/jbc.274.26.18702

Lameloise N, 2001, DIABETES, V50, P803, DOI 10.2337/diabetes.50.4.803

Larsen CM, 2007, NEW ENGL J MED, V356, P1517, DOI 10.1056/NEJMoa065213

Larsen CM, 2009, DIABETES CARE, V32, P1663, DOI 10.2337/dc09-0533

Laybutt DR, 2007, DIABETOLOGIA, V50, P2117, DOI 10.1007/s00125-007-0749-2

Laybutt DR, 2007, DIABETOLOGIA, V50, P752, DOI 10.1007/s00125-006-0590-z

Lee JW, 2009, CELL SIGNAL, V21, P69, DOI 10.1016/j.cellsig.2008.09.015

LEFFERT JD, 1989, P NATL ACAD SCI USA, V86, P3127, DOI 10.1073/pnas.86.9.3127

Liu M, 2007, P NATL ACAD SCI USA, V104, P15841, DOI 10.1073/pnas.0702697104

Liu SM, 2009, PLOS ONE, V4, DOI 10.1371/journal.pone.0007983

Love-Gregory LD, 2004, DIABETES, V53, P1134, DOI 10.2337/diabetes.53.4.1134

Lupi R, 2002, DIABETES, V51, P1437, DOI 10.2337/diabetes.51.5.1437

Ma K, 2010, J CLIN ENDOCR METAB, V95, P887, DOI 10.1210/jc.2009-1102

Ma YJ, 2002, J MOL BIOL, V318, P1351, DOI 10.1016/S0022-2836(02)00234-6

Maeda K, 1996, BIOCHEM BIOPH RES CO, V221, P286, DOI 10.1006/bbrc.1996.0587

Maedler K, 2003, DIABETES, V52, P726, DOI 10.2337/diabetes.52.3.726

Maedler K, 2002, J CLIN INVEST, V110, P851, DOI 10.1172/JCI200215318

Maedler K, 2002, P NATL ACAD SCI USA, V99, P8236, DOI 10.1073/pnas.122686299

Maedler K, 2001, DIABETES, V50, P69, DOI 10.2337/diabetes.50.1.69

MAFFEI M, 1995, NAT MED, V1, P1155, DOI 10.1038/nm1195-1155

Meier JJ, 2006, AM J PHYSIOL-ENDOC M, V291, pE1317, DOI 10.1152/ajpendo.00082.2006

Mohamed-Ali V, 1997, J CLIN ENDOCR METAB, V82, P4196, DOI 10.1210/jc.82.12.4196

Mokdad AH, 2003, JAMA-J AM MED ASSOC, V289, P76, DOI 10.1001/jama.289.1.76

Molina AJA, 2009, DIABETES, V58, P2303, DOI 10.2337/db07-1781

Morioka T, 2007, J CLIN INVEST, V117, P2860, DOI 10.1172/JCI30910

Nolan CJ, 2008, TRENDS ENDOCRIN MET, V19, P285, DOI 10.1016/i.tem.2008.07.006

Nolan CJ, 2006, DIABETES, V55, pS16, DOI 10.2337/db06-S003

NOVIALS A, 1993, DIABETES, V42, P1514, DOI 10.2337/diabetes.42.10.1514

Nunemaker CS, 2008, AM J PHYSIOL-ENDOC M, V295, pE1065, DOI 10.1152/ajpendo.90371.2008

Ogihara T, 2010, J BIOL CHEM, V285, P5392, DOI 10.1074/jbc.M109.064659

Ogilvie RF, 1933, J PATHOL BACTERIOL, V37, P473, DOI 10.1002/path.1700370314

OLSON LK, 1993, J CLIN INVEST, V92, P514, DOI 10.1172/JCI116596

Oyadomari S, 2002, J CLIN INVEST, V109, P525, DOI 10.1172/JC1200214550

Paz K, 1997, J BIOL CHEM, V272, P29911, DOI 10.1074/jbc.272.47.29911

Petersen KF, 2006, AM J MED, V119, p10S, DOI 10.1016/j.amjmed.2006.01.009

Pinney SE, 2010, TRENDS ENDOCRIN MET, V21, P223, DOI 10.1016/j.tem.2009.10.002

Piro S, 2002, METABOLISM, V51, P1340, DOI 10.1053/meta.2002.35200

Pirot P, 2007, DIABETES, V56, P1069, DOI 10.2337/db06-1253

Poitout V, 1996, J CLIN INVEST, V97, P1041, DOI 10.1172/JCI118496

Poitout V, 2010, BBA-MOL CELL BIOL L, V1801, P289, DOI 10.1016/j.bbalip.2009.08.006

PRENTKI M, 1992, J BIOL CHEM, V267, P5802

Richardson SJ, 2009, DIABETOLOGIA, V52, P1143, DOI 10.1007/s00125-009-1276-0

Robertson RP, 2007, CELL BIOCHEM BIOPHYS, V48, P139, DOI 10.1007/s12013-07-0026-5

Robertson RP, 2004, J BIOL CHEM, V279, P42351, DOI 10.1074/jbc.R400019200

ROCKEN C, 1992, VIRCHOWS ARCH A, V421, P339, DOI 10.1007/BF01660981

SAKO Y, 1990, ENDOCRINOLOGY, V127, P1580, DOI 10.1210/endo-127-4-1580

Sakuraba H, 2002, DIABETOLOGIA, V45, P85, DOI 10.1007/s125-002-8248-z

SCHERER PE, 1995, J BIOL CHEM, V270, P26746, DOI 10.1074/jbc.270.45.26746

Scheuner D, 2008, ENDOCR REV, V29, P317, DOI 10.1210/er.2007-0039

SHARMA A, 1995, MOL ENDOCRINOL, V9, P1127, DOI 10.1210/me.9.9.1127

Shen JS, 2002, DEV CELL, V3, P99, DOI 10.1016/S1534-5807(02)00203-4

Shimabukuro M, 1998, J BIOL CHEM, V273, P32487, DOI 10.1074/jbc.273.49.32487

Shimabukuro M, 1998, P NATL ACAD SCI USA, V95, P2498, DOI 10.1073/pnas.95.5.2498

Sigal RJ, 2004, DIABETES CARE, V27, P2518, DOI 10.2337/diacare.27.10.2518

Silander K, 2004, DIABETES, V53, P1141, DOI 10.2337/diabetes.53.4.1141

Sladek R, 2007, NATURE, V445, P881, DOI 10.1038/nature05616

Solinas G, 2006, P NATL ACAD SCI USA, V103, P16454, DOI 10.1073/pnas.0607626103

Song BB, 2008, J CLIN INVEST, V118, P3378, DOI 10.1172/JCI34587

Stein DT, 1996, J CLIN INVEST, V97, P2728, DOI 10.1172/JCI118727

Steppan CM, 2001, NATURE, V409, P307, DOI 10.1038/35053000

Stumvoll M, 2005, LANCET, V365, P1333, DOI 10.1016/S0140-6736(05)61032-X

Tabak AG, 2009, LANCET, V373, P2215, DOI 10.1016/S0140-6736(09)60619-X

Tan CP, 2008, DIABETES, V57, P2211, DOI 10.2337/db08-0130

Tanaka Y, 1999, P NATL ACAD SCI USA, V96, P10857, DOI 10.1073/pnas.96.19.10857

Tanaka Y, 2002, P NATL ACAD SCI USA, V99, P12363, DOI 10.1073/pnas.192445199

Tiedge M, 1997, DIABETES, V46, P1733, DOI 10.2337/diabetes.46.11.1733

Tomita T, 2006, DIABETOLOGIA, V49, P962, DOI 10.1007/s00125-006-0193-8

*UK PROSP DIAB STU, 1995, DIABETES, V44, P1249

Wang J, 1999, J CLIN INVEST, V103, P27, DOI 10.1172/JCI4431

Weir GC, 2004, DIABETES, V53, pS16, DOI 10.2337/diabetes.53.suppl_3.S16

Welsh N, 2005, DIABETES, V54, P3238, DOI 10.2337/diabetes.54.11.3238

WESTERMARK P, 1989, DIABETES RES CLIN PR, V7, P219, DOI 10.1016/0168-8227(89)90008-9

Weyer C, 1999, J CLIN INVEST, V104, P787, DOI 10.1172/JCI7231

Weyer C, 2001, J CLIN ENDOCR METAB, V86, P1930, DOI 10.1210/jc.86.5.1930

Wild S, 2004, DIABETES CARE, V27, P1047, DOI 10.2337/diacare.27.5.1047

Xiang AH, 2006, DIABETES, V55, P517, DOI 10.2337/diabetes.55.02.06.db05-1066

Zhang SP, 2008, DIABETES, V57, P348, DOI 10.2337/db07-0849

ZHANG YY, 1994, NATURE, V372, P425, DOI 10.1038/372425a0

Zhao AZ, 1998, J CLIN INVEST, V102, P869, DOI 10.1172/JCI3920

Zhou RB, 2010, NAT IMMUNOL, V11, P136, DOI 10.1038/ni.1831

ZHOU YP, 1994, J CLIN INVEST, V93, P870, DOI 10.1172/JCI117042

Zraika S, 2009, DIABETOLOGIA, V52, P626, DOI 10.1007/s00125-008-1255-x

NR 151

TC 17

Z9 17

U1 0

U2 9

PU WILEY

PI HOBOKEN

PA 111 RIVER ST, HOBOKEN 07030-5774, NJ USA

SN 2040-1124

J9 J DIABETES INVEST

JI J. Diabetes Investig.

PD AUG

PY 2010

VL 1

IS 4

BP 123

EP 133

DI 10.1111/j.2040-1124.2010.00021.x

PG 11

WC Endocrinology & Metabolism

SC Endocrinology & Metabolism

GA 755DI

UT WOS:000289906500002

PM 24843420

OA Green Published, Bronze

DA 2019-01-08

ER

PT J

AU Phillips, AC

Carroll, D

Thomas, GN

Gale, CR

Deary, I

Batty, GD

AF Phillips, Anna C.

Carroll, Douglas

Thomas, G. Neil

Gale, Catharine R.

Deary, Ian

Batty, G. David

TI The influence of multiple indices of socioeconomic disadvantage across

the adult life course on the metabolic syndrome: the Vietnam Experience

Study

SO METABOLISM-CLINICAL AND EXPERIMENTAL

LA English

DT Article

ID RISK-FACTORS; SOCIAL INEQUALITIES; WHITEHALL-II; COURSE ORIGINS;

ALL-CAUSE; MORTALITY; HEALTH; PREVALENCE; POSITION; POPULATION

AB Few studies have explored the relationship between individual and combined multiple indicators of socioeconomic status across the life course and the metabolic syndrome, or attempted to understand the mechanisms underlying any associations. The present study examined the associations between 4 indicators of socioeconomic status, individually and in combination, and metabolic syndrome risk in a study of male US veterans and examined the influence of health behaviors, intelligence, and psychologic distress on these associations. Participants (N = 4253) were drawn from the Vietnam Experience Study. From military service files, telephone interviews, and a medical examination, occupational, sociodemographic, health behavior, intelligence, psychologic, and health data were collected. The 4 indices of socioeconomic status were as follows: education achieved, early adulthood income, household income in midlife, and occupational prestige in midlife. Metabolic syndrome was diagnosed from the following: body mass index, fasting blood glucose or a diagnosis of diabetes, blood pressure a diagnosis of hypertension or taking antihypertensives, high-density lipoprotein cholesterol, and triglyceride levels. In models that adjusted for age, men in the lower 2 groups on the combined measure of socioeconomic status experienced a higher risk of metabolic syndrome. This association was accounted for mainly by education achieved, household income in midlife, and occupational prestige in midlife. Intelligence appeared to explain much of this association. Combined socioeconomic status measures across the life course were related to metabolic syndrome but in a threshold rather than dose-response manner. Intelligence appeared to mediate this relationship. (C) 2010 Elsevier Inc. All rights reserved.

C1 [Phillips, Anna C.; Carroll, Douglas] Univ Birmingham, Sch Sport & Exercise Sci, Birmingham B15 2TT, W Midlands, England.

[Thomas, G. Neil] Univ Birmingham, Sch Med & Dent Sci, Birmingham B15 2TT, W Midlands, England.

[Gale, Catharine R.] Univ Southampton, MRC Epidemiol Resource Ctr, Southampton SO9 5NH, Hants, England.

[Deary, Ian; Batty, G. David] Univ Edinburgh, Dept Psychol, Ctr Cognit Ageing & Cognit Epidemiol, Edinburgh EH8 9YL, Midlothian, Scotland.

[Batty, G. David] Univ Glasgow, MRC Social & Publ Hlth Sci Unit, Glasgow G12 8QQ, Lanark, Scotland.

[Batty, G. David] George Inst Int Hlth, Sydney, NSW, Australia.

RP Phillips, AC (reprint author), Univ Birmingham, Sch Sport & Exercise Sci, Birmingham B15 2TT, W Midlands, England.

EM a.c.phillips@bham.ac.uk

RI Batty, George/Y-4401-2018; Deary, Ian/C-6297-2009; Gale,

Catharine/B-1653-2012; Phillips, Anna/A-3577-2013; Thomas, G.

Neil/A-1879-2013

OI Deary, Ian/0000-0002-1733-263X; Phillips, Anna/0000-0002-5461-0598;

Thomas, G. Neil/0000-0002-2777-1847

FU UK Medical Research Council; Chief Scientist Office at the Scottish

Government Health Directorates; Biotechnology and Biological Sciences

Research Council; Engineering and Physical Sciences Research Council;

Economic and Social Research Council; Medical Research Council;

University of Edinburgh as part of the cross-council Lifelong Health and

Wellbeing initiative; Wellcome Trust [WBS U.1300.00.006.00012.01];

Medical Research Council [MC_U130059821, MC_UP_A620_1015, G0700704,

U1475000002]

FX The Medical Research Council Social and Public Health Sciences Unit

receives funding from the UK Medical Research Council and the Chief

Scientist Office at the Scottish Government Health Directorates. The

Centre for Cognitive Ageing and Cognitive Epidemiology is supported by

the Biotechnology and Biological Sciences Research Council, the

Engineering and Physical Sciences Research Council, the Economic and

Social Research Council, the Medical Research Council, and the

University of Edinburgh as part of the cross-council Lifelong Health and

Wellbeing initiative. David Batty is a Wellcome Trust Career Development

Fellow (WBS U.1300.00.006.00012.01).

CR American Psychiatric Association, 1980, DIAGN STAT MAN MENT

Batty GD, 2008, J EPIDEMIOL COMMUN H, V62, P522, DOI 10.1136/jech.2007.064881

Batty GD, 2008, DIABETOLOGIA, V51, P436, DOI 10.1007/s00125-007-0908-5

Batty GD, 2006, BRIT MED J, V332, P580, DOI 10.1136/bmj.38723.660637.AE

Blane D, 2007, REV EPIDEMIOL SANTE, V55, P31, DOI 10.1016/j.respe.2006.12.004

Blane D, 1996, BMJ-BRIT MED J, V313, P1434, DOI 10.1136/bmj.313.7070.1434

Brunner EJ, 1997, DIABETOLOGIA, V40, P1341, DOI 10.1007/s001250050830

CARROLL D, 1993, PSYCHOL HEALTH, V8, P295, DOI 10.1080/08870449308401924

Carroll D, 2009, BIOL PSYCHIAT, V66, P91, DOI 10.1016/j.biopsych.2009.02.020

Dallongeville J, 2005, DIABETES CARE, V28, P409, DOI 10.2337/diacare.28.2.409

Davey Smith G, 1994, EUROPEAN J PUBLIC HL, V4, P131

DESTEFANO F, 1988, JAMA-J AM MED ASSOC, V259, P2708

Hart CL, 1998, AM J PUBLIC HEALTH, V88, P471, DOI 10.2105/AJPH.88.3.471

Kaplan GA, 1996, BRIT MED J, V312, P999

Langenberg C, 2006, AM J PUBLIC HEALTH, V96, P2216, DOI 10.2105/AJPH.2004.049429

Lawlor DA, 2002, BRIT MED J, V325, P805, DOI 10.1136/bmj.325.7368.805

Loucks EB, 2007, ANN EPIDEMIOL, V17, P782, DOI 10.1016/j.annepidem.2007.05.003

Loucks EB, 2007, ANN EPIDEMIOL, V17, P19, DOI 10.1016/j.annepidem.2006.07.002

Paek KW, 2006, ANN EPIDEMIOL, V16, P756, DOI 10.1016/j.annepidem.2006.04.012

Park MJ, 2007, ANN EPIDEMIOL, V17, P320, DOI 10.1016/j.annepidem.2006.10.007

Perel P, 2006, DIABETES CARE, V29, P2694, DOI 10.2337/dc06-0022

Ramsay SE, 2008, DIABETES CARE, V31, P2380, DOI 10.2337/dc08-1158

Razzouk L, 2009, CURR HYPERTENS REP, V11, P127, DOI 10.1007/s11906-009-0023-8

Reppert A, 2008, AM J HEALTH PROMOT, V23, P130, DOI 10.4278/ajhp.071009105

ROBINS LN, 1987, DIAGNOSTIC INTERVIEW

Sanchez-Chaparro MA, 2008, DIABETES CARE, V31, P1884, DOI 10.2337/dc08-0431

Schooling CM, 2008, AM J EPIDEMIOL, V167, P419, DOI 10.1093/aje/kwm329

Smith GD, 2000, PUBLIC HEALTH, V114, P308, DOI 10.1016/S0033-3506(00)00353-X

Stansfeld SA, 2003, J EPIDEMIOL COMMUN H, V57, P361, DOI 10.1136/jech.57.5.361

STEVENS G, 1985, SOC SCI RES, V14, P142, DOI 10.1016/0049-089X(85)90008-0

Thomas GN, 2007, CLIN ENDOCRINOL, V66, P666, DOI 10.1111/j.1365-2265.2007.02798.x

Wamala SP, 1999, DIABETES CARE, V22, P1999, DOI 10.2337/diacare.22.12.1999

Welin L, 2008, BMC PUBLIC HEALTH, V8, DOI 10.1186/1471-2458-8-403

WILKINSON RG, 1996, UNHEALTHY SOC INEQUA

Wilkinson RG, 2003, SOCIAL DETERMINANTS

NR 35

TC 5

Z9 6

U1 0

U2 2

PU W B SAUNDERS CO-ELSEVIER INC

PI PHILADELPHIA

PA 1600 JOHN F KENNEDY BOULEVARD, STE 1800, PHILADELPHIA, PA 19103-2899 USA

SN 0026-0495

J9 METABOLISM

JI Metab.-Clin. Exp.

PD AUG

PY 2010

VL 59

IS 8

BP 1164

EP 1171

DI 10.1016/j.metabol.2009.11.009

PG 8

WC Endocrinology & Metabolism

SC Endocrinology & Metabolism

GA 635PZ

UT WOS:000280669900012

PM 20045139

DA 2019-01-08

ER

PT J

AU Melkus, GD

Chyun, D

Vorderstrasse, A

Newlin, K

Jefferson, V

Langerman, S

AF Melkus, Gail D'Eramo

Chyun, Deborah

Vorderstrasse, Allison

Newlin, Kelley

Jefferson, Vanessa

Langerman, Susan

TI The Effect of a Diabetes Education, Coping Skills Training, and Care

Intervention on Physiological and Psychosocial Outcomes in Black Women

With Type 2 Diabetes

SO BIOLOGICAL RESEARCH FOR NURSING

LA English

DT Article

DE diabetes; self-management; outcomes; Blacks; intervention; primary care

ID AFRICAN-AMERICAN WOMEN; SELF-MANAGEMENT EDUCATION; METABOLIC-CONTROL;

GLYCEMIC CONTROL; HEALTH OUTCOMES; RISK-FACTORS; ADULTS; MELLITUS;

EFFICACY; DISEASE

AB An 11-week culturally relevant group diabetes self-management training (DSMT), coping skills training (CST), and diabetes care intervention was compared to a 10-week usual diabetes education and diabetes care intervention on physiological and psychosocial outcomes in 109 Black women (aged 48 +/- 10 years) with type 2 diabetes in primary care (PC). Strong time effects for hemoglobin Alc improvement were seen in both groups from baseline to 3 months and remained similar at 12 and 24 months (p < .0001). Systolic blood pressure (p = .01) and low-density lipoprotein cholesterol levels (p = .05) improved in both groups from baseline to 24 months. Baseline quality of life ([QOL]; Medical Outcome Study Short Form-36) was low. Social function, role-emotional, and mental health domains increased initially in both groups then declined slightly, with less decline for the experimental group at 12 months. At 24 months, experimental group scores increased. General health (p = .002), vitality (p = .01), role-physical, and bodily pain (p = .02) domains increased in both groups over time. Perceived provider support for diet (p = .0001) and exercise (p = .0001) increased in both groups over time. Diabetes-related emotional distress decreased in the experimental compared to the control group (group x time, p = .01). Findings suggest that both methods of diabetes education combined with care can improve metabolic control, QOL, and perceptions of provider care. CST may further assist in long-term improvements in health outcomes. Behavioral interventions are needed in addition to routine diabetes care, particularly in PC.

C1 [Melkus, Gail D'Eramo; Chyun, Deborah; Newlin, Kelley] New York Univ, Coll Nursing, New York, NY 10003 USA.

[Vorderstrasse, Allison] Duke Univ, Sch Nursing, Durham, NC USA.

[Jefferson, Vanessa] Yale Univ, Sch Nursing, New Haven, CT 06536 USA.

[Langerman, Susan] Yale Univ Hlth Plan, New Haven, CT USA.

RP Melkus, GD (reprint author), New York Univ, Coll Nursing, 726 Broadway 1083, New York, NY 10003 USA.

EM Gail.Melkus@nyu.edu

FU NIH NINR [ROI NR 05341-01A1]

FX Research was supported by NIH NINR Funding ROI NR 05341-01A1.

CR *AM DIAB ASS, 2009, DIABETES CARE S1, V32, pS13, DOI DOI 10.2337/DC09-S013

American Association of Diabetes Educators, 2008, DIABETES ED, V34, P445, DOI DOI 10.1177/0145721708316625

Anderson-Loftin W, 2000, DIABETES EDUCATOR, V26, P821, DOI 10.1177/014572170002600511

Balukonis J, 2008, ETHNIC DIS, V18, P141

BANDURA A, 1977, PSYCHOL REV, V84, P191, DOI 10.1037/0033-295X.84.2.191

BECKLES G, 2001, DIABETES WOMEN LIFE

Brown Sharon A, 2002, Curr Diab Rep, V2, P166, DOI 10.1007/s11892-002-0077-3

Carter-Edwards L, 2004, DIABETES EDUCATOR, V30, P493, DOI 10.1177/014572170403000321

*CDCP, 2002, MMWR-MORBID MORTAL W, V51, P159

Centers for Disease Control and Prevention, 2007, NAT DIAB FACT SHEET

Centers for Disease Control and Prevention (CDC), 2002, MMWR-MORBID MORTAL W, V51, P147

CRISP AH, 1984, LANCET, V1, P616

CROWN S, 1966, BRIT J PSYCHIAT, V112, P917, DOI 10.1192/bjp.112.490.917

DERAMOMELKUS GA, 1992, DIABETES CARE, V15, P864, DOI 10.2337/diacare.15.7.864

Eakin EG, 2002, DIABETES-METAB RES, V18, P26, DOI 10.1002/dmrr.266

Eigenmann CA, 2009, DIABETIC MED, V26, P425, DOI 10.1111/j.1464-5491.2009.02697.x

Fisher EB, 2005, AM J PUBLIC HEALTH, V95, P1523, DOI 10.2105/AJPH.2005.066084

Fisher EB, 2007, DIABETES EDUCATOR, V33, p216S, DOI 10.1177/0145721707304124

Fitzgerald JT, 1996, EVAL HEALTH PROF, V19, P208, DOI 10.1177/016327879601900205

Fitzgerald JT, 1998, EVAL HEALTH PROF, V21, P52, DOI 10.1177/016327879802100103

Funnell Martha M, 2003, Curr Diab Rep, V3, P127, DOI 10.1007/s11892-003-0036-7

Garfield SA, 2003, DIABETES CARE, V26, P2670, DOI 10.2337/diacare.26.9.2670

Glasgow RE, 2006, CHRONIC ILLN, V2, P27, DOI 10.1179/174592006X93851

Glasgow RE, 2007, J MED INTERNET RES, V9, DOI 10.2196/jmir.9.2.e11

Grey M, 1998, DIABETES CARE, V21, P902, DOI 10.2337/diacare.21.6.902

Grey M, 2000, J PEDIATR-US, V137, P107, DOI 10.1067/mpd.2000.106568

HEIGHT DI, 1994, BLACK FAMILY DINNER

HERON M, 2009, NATL VITAL STAT REPO, V57

Hill-Briggs F, 2007, DIABETES EDUCATOR, V33, P1032, DOI 10.1177/0145721707308412

*HLTH PROM COUNC S, 1999, PUT FAT BACK

Howard BV, 1998, DIABETES CARE, V21, P1258, DOI 10.2337/diacare.21.8.1258

*IS INC, 1992, GLYC AFF GHB AFF ACC

KANNEL WB, 1995, MED CLIN N AM, V79, P951, DOI 10.1016/S0025-7125(16)30016-5

KAWACHI I, 1994, CIRCULATION, V89, P1992, DOI 10.1161/01.CIR.89.5.1992

Leeman J, 2008, DIABETES EDUCATOR, V34, P310, DOI 10.1177/0145721708316623

Liebman J, 2007, DIABETES EDUCATOR, V33, p132S, DOI 10.1177/0145721707304075

Maciejewski PK, 2000, BRIT J PSYCHIAT, V176, P373, DOI 10.1192/bjp.176.4.373

MCCAUL KD, 1987, MED CARE, V25, P868, DOI 10.1097/00005650-198709000-00005

MCHORNEY CA, 1993, MED CARE, V31, P247, DOI 10.1097/00005650-199303000-00006

Melkus G, 2001, DIABETES S, V50, P811

Melkus GD, 2004, APPL NURS RES, V17, P10, DOI 10.1016/j.apnr.2003.10.009

Miller CK, 2002, J AM DIET ASSOC, V102, P1069, DOI 10.1016/S0002-8223(02)90242-7

Moser DK, 2000, NURS RES, V49, P109, DOI 10.1097/00006199-200003000-00008

*NAT I DIAB DIG KI, 2007, NAT DIAB STAT 2007

Nelson KM, 2002, DIABETES CARE, V25, P1722, DOI 10.2337/diacare.25.10.1722

Newlin K, 2003, ETHNIC DIS, V13, P61

Newlin K, 2006, ETHNIC DIS, V16, P956

Norris SL, 2004, AM J MED, V117, P762, DOI 10.1016/j.amjmed.2004.05.024

Norris SL, 2002, DIABETES CARE, V25, P1159, DOI 10.2337/diacare.25.7.1159

Norris SL, 2001, DIABETES CARE, V24, P561, DOI 10.2337/diacare.24.3.561

Peyrot M, 2005, DIABETIC MED, V22, P1379, DOI 10.1111/j.1464-5491.2005.01644.x

Peyrot M, 2009, DIABETES EDUCATOR, V35, P246, DOI 10.1177/0145721708329546

Pickering TG, 2005, CIRCULATION, V111, P697, DOI [10.1161/01.CIR.0000154900.76284.F6, 10.1161/01.HYP.0000150859.47929.8e]

POLONSKY WH, 1995, DIABETES CARE, V18, P754, DOI 10.2337/diacare.18.6.754

Prochaska J O, 1984, Prog Clin Biol Res, V156, P131

PROCHASKA JO, 1992, AM PSYCHOL, V47, P1102, DOI 10.1037//0003-066X.47.9.1102

Rayman Kathleen, 2004, Health Care Women Int, V25, P900, DOI 10.1080/07399330490508604

Ruggiero L, 1998, Med Health R I, V81, P355

SHAMOON H, 1993, NEW ENGL J MED, V329, P977

SKELLY AH, 1995, DIABETES EDUCATOR, V21, P38, DOI 10.1177/014572179502100107

Smith SC, 2005, CIRCULATION, V111, P134

*STATEHEALTHFACTS, 2006, NUMB DIAB DEATHS PER

Surgenor LJ, 2000, J PSYCHOSOM RES, V49, P267, DOI 10.1016/S0022-3999(00)00181-1

Surwit RS, 2002, DIABETES CARE, V25, P30, DOI 10.2337/diacare.25.1.30

Utz SW, 2008, DIABETES EDUCATOR, V34, P854, DOI 10.1177/0145721708323642

van der Ven N., 2003, DIABETES SPECTRUM, V16, P88, DOI [DOI 10.2337/DIASPECT.16.2.88, 10.2337/diaspect.16.2.88]

WARE J, 1992, MED CARE, V30, P43

Whittemore R, 2005, J CLIN NURS, V14, P195, DOI 10.1111/j.1365-2702.2004.00937.x

Whittemore R, 2007, J TRANSCULT NURS, V18, P157, DOI 10.1177/1043659606298615

Williams GC, 1996, DIABETES, V45, p13A

NR 70

TC 19

Z9 19

U1 2

U2 10

PU SAGE PUBLICATIONS INC

PI THOUSAND OAKS

PA 2455 TELLER RD, THOUSAND OAKS, CA 91320 USA

SN 1099-8004

J9 BIOL RES NURS

JI Biol. Res. Nurs.

PD JUL

PY 2010

VL 12

IS 1

BP 7

EP 19

DI 10.1177/1099800410369825

PG 13

WC Nursing

SC Nursing

GA 617IE

UT WOS:000279273400002

PM 20484058

DA 2019-01-08

ER

PT J

AU Nefs, G

Pouwer, F

Denollet, J

Pop, VJM

AF Nefs, Giesje

Pouwer, Francois

Denollet, Johan

Pop, Victor J. M.

TI Psychological risk factors of micro- and macrovascular outcomes in

primary care patients with type 2 diabetes: rationale and design of the

DiaDDZoB Study

SO BMC PUBLIC HEALTH

LA English

DT Article

ID POSTNATAL DEPRESSION SCALE; CORONARY-HEART-DISEASE; D PERSONALITY;

CARDIOVASCULAR EVENTS; EMOTIONAL-PROBLEMS; MAJOR DEPRESSION; GLYCEMIC

CONTROL; ARTERY-DISEASE; SYMPTOMS; COMPLICATIONS

AB Background: Depression is a common psychiatric complication of diabetes, but little is known about the natural course and the consequences of depressive symptoms in primary care patients with type 2 diabetes. While depression has been related to poor glycemic control and increased risk for macrovascular disease, its association with microvascular complications remains understudied. The predictive role of other psychological risk factors such as Type D (distressed) personality and the mechanisms that possibly link depression and Type D personality with poor vascular outcomes are also still unclear.

Methods/Design: This prospective cohort study will examine: (1) the course of depressive symptoms in primary care patients with type 2 diabetes; (2) whether depressive symptoms and Type D personality are associated with the development of microvascular and/or macrovascular complications and with the risk of all-cause or vascular mortality; and (3) the behavioral and physiological mechanisms that may mediate these associations. The DiaDDZoB Study is embedded within the larger DIAZOB Primary Care Diabetes study, which covers a comprehensive cohort of type 2 diabetes patients treated by over 200 primary care physicians in South-East Brabant, The Netherlands. These patients will be followed during their lifetime and are assessed annually for demographic, clinical, lifestyle and psychosocial factors. Measurements include an interviewer-administered and self-report questionnaire, regular care laboratory tests and physical examinations, and pharmacy medication records. The DiaDDZoB Study uses data that have been collected during the original baseline assessment in 2005 (M-0; N = 2,460) and the 2007 (M-1; N = 2,225) and 2008 (M-2; N = 2,032) follow-up assessments.

Discussion: The DiaDDZoB Study is expected to contribute to the current understanding of the course of depression in primary care patients with type 2 diabetes and will also test whether depressed patients or those with Type D personality are at increased risk for (further) development of micro- andand cardiovascular disease. More knowledge about the mechanisms behind this association is needed to guide new intervention studies.

C1 [Nefs, Giesje; Pouwer, Francois; Denollet, Johan; Pop, Victor J. M.] Tilburg Univ, Dept Med Psychol & Neuropsychol, CoRPS Ctr Res Psychol Somat Dis, NL-5000 LE Tilburg, Netherlands.

RP Pouwer, F (reprint author), Tilburg Univ, Dept Med Psychol & Neuropsychol, CoRPS Ctr Res Psychol Somat Dis, NL-5000 LE Tilburg, Netherlands.

EM f.pouwer@uvt.nl

RI Nefs, Giesje/C-9992-2018

FU Netherlands Organisation for Health Research and Development [5881];

Netherlands Organisation for Scientific Research (The Hague, The

Netherlands) [453-04.004]

FX This study was supported by a ZonMW grant (call for diabetes health care

groups, application number 5881) from the Netherlands Organisation for

Health Research and Development to Victor Pop, and by Vici grant #

453-04.004 from the Netherlands Organisation for Scientific Research

(The Hague, The Netherlands) to Johan Denollet.

CR Ali S, 2006, DIABETIC MED, V23, P1165, DOI 10.1111/j.1464-5491.2006.01943.x

American Diabetes Association, 2008, DIABETES CARE, V31, P12

[Anonymous], 1996, BRIT MED J, V313, P1448

Becht MC, 2001, J AFFECT DISORDERS, V63, P209, DOI 10.1016/S0165-0327(99)00189-5

Beekman ATF, 2002, ARCH GEN PSYCHIAT, V59, P605, DOI 10.1001/archpsyc.59.7.605

Black SA, 2003, DIABETES CARE, V26, P2822, DOI 10.2337/diacare.26.10.2822

Bruce DG, 2005, DIABETOLOGIA, V48, P2532, DOI 10.1007/s00125-005-0024-3

Clouse RE, 2003, PSYCHOSOM MED, V65, P376, DOI 10.1097/01.PSY.0000041624.96580.1F

COX JL, 1987, BRIT J PSYCHIAT, V150, P782, DOI 10.1192/bjp.150.6.782

Cox JL, 1996, J AFFECT DISORDERS, V39, P185, DOI 10.1016/0165-0327(96)00008-0

de Groot M, 2001, PSYCHOSOM MED, V63, P619, DOI 10.1097/00006842-200107000-00015

Denollet J, 2005, PSYCHOSOM MED, V67, P89, DOI 10.1097/01.psy.0000149256.81953.49

Egede LE, 2005, DIABETES CARE, V28, P1339, DOI 10.2337/diacare.28.6.1339

Fisher L, 2007, DIABETES CARE, V30, P542, DOI 10.2337/dc06-1614

Hermanns N, 2006, DIABETOLOGIA, V49, P469, DOI 10.1007/s00125-005-0094-2

HERMANNS N, 2004, 40 EASD M MUN GERM

HINTZE J, 2008, PASS 2008

Katon WJ, 2005, DIABETES CARE, V28, P2668, DOI 10.2337/diacare.28.11.2668

Katon WJ, 2004, J GEN INTERN MED, V19, P1192, DOI 10.1111/j.1525-1497.2004.30405.x

Khunti K, 2000, J ROY SOC MED, V93, P183, DOI 10.1177/014107680009300407

Knol MJ, 2008, PHARMACOEPIDEM DR S, V17, P577, DOI 10.1002/pds.1608

Knol MJ, 2006, DIABETOLOGIA, V49, P837, DOI 10.1007/s00125-006-0159-x

Koopmans B, 2009, DIABETOLOGIA, V52, P2056, DOI 10.1007/s00125-009-1460-2

Koopmans B, 2009, FAM PRACT, V26, P171, DOI 10.1093/fampra/cmp016

Kop WJ, 1997, J PSYCHOSOM RES, V43, P167, DOI 10.1016/S0022-3999(97)80002-5

Kupper N, 2007, J PERS ASSESS, V89, P265, DOI 10.1080/00223890701629797

Lett HS, 2004, PSYCHOSOM MED, V66, P305, DOI 10.1097/01.psy.0000126207.43307.c0

Lin EHB, 2010, DIABETES CARE, V33, P264, DOI 10.2337/dc09-1068

Lustman PJ, 1997, GEN HOSP PSYCHIAT, V19, P138, DOI 10.1016/S0163-8343(96)00170-3

Lustman PJ, 2000, DIABETES CARE, V23, P934, DOI 10.2337/diacare.23.7.934

MacKinnon DP, 2007, ANNU REV PSYCHOL, V58, P593, DOI 10.1146/annurev.psych.58.110405.085542

Marshall SM, 2006, BRIT MED J, V333, P475, DOI 10.1136/bmj.38922.650521.80

Martens EJ, 2007, J PSYCHOSOM RES, V63, P545, DOI 10.1016/j.jpsychores.2007.06.005

Matthey S, 2006, ARCH WOMEN MENT HLTH, V9, P309, DOI 10.1007/s00737-006-0152-x

Matthey S, 2001, J AFFECT DISORDERS, V64, P175, DOI 10.1016/S0165-0327(00)00236-6

McIntyre RS, 2006, EXPERT OPIN DRUG SAF, V5, P157, DOI 10.1517/14740338.5.1.157

Mezuk B, 2008, DIABETES CARE, V31, P2383, DOI 10.2337/dc08-0985

NOUWEN A, 2009, DIABETES CARE, V32, P57

Nouwen Arie, 2009, Diabetes Care, V32, pe57, DOI 10.2337/dc09-0027

Nyklicek I, 2004, PSYCHIAT RES, V128, P111, DOI 10.1016/j.psychres.2004.05.017

OHARA MW, 1986, ARCH GEN PSYCHIAT, V43, P569

Pedersen SS, 2003, EUR J CARDIOV PREV R, V10, P241, DOI 10.1097/01.hjr.0000085246.65733.06

Pelle AJ, 2009, J AFFECT DISORDERS, V113, P150, DOI 10.1016/j.jad.2008.05.017

Peyrot M, 1999, DIABETES CARE, V22, P448, DOI 10.2337/diacare.22.3.448

Pibernik-Okanovic M, 2008, J DIABETES COMPLICAT, V22, P246, DOI 10.1016/j.jdiacomp.2007.03.002

Poortvliet MC, 2007, DIABETES NEDERLAND O

POP VJ, 1992, J AFFECT DISORDERS, V26, P105, DOI 10.1016/0165-0327(92)90041-4

Pouwer F, 2006, PATIENT EDUC COUNS, V60, P235, DOI 10.1016/j.pec.2005.01.009

Pouwer F, 2009, NAT REV ENDOCRINOL, V5, P665, DOI 10.1038/nrendo.2009.214

Rozanski A, 1999, CIRCULATION, V99, P2192, DOI 10.1161/01.CIR.99.16.2192

RUTTEN GEH, 2006, HUISARTS WET, V49, P137

Schiffer AA, 2007, HEART, V93, P814, DOI 10.1136/hrt.2006.102822

Sheeran T, 2002, PSYCHIAT RES, V109, P51, DOI 10.1016/S0165-1781(01)00364-X

Ubink-Vetmaat LJ, 2005, NETH J MED, V63, P103

UK Prospective Diabetes Study Grp, 1999, DIABETES CARE, V22, P1125

Van der Kooy K, 2007, INT J GERIATR PSYCH, V22, P613, DOI 10.1002/gps.1723

van Melle JP, 2004, PSYCHOSOM MED, V66, P814, DOI 10.1097/01.psy.0000146294.82810.9c

VANWEEL C, 2005, ANN FAM MED S1, V3, P46

Vrijhoef HJM, 2002, INT J NURS STUD, V39, P441, DOI 10.1016/S0020-7489(01)00046-3

Vuorilehto MS, 2009, PSYCHOL MED, V39, P1697, DOI 10.1017/S0033291709005182

Whooley MA, 2008, JAMA-J AM MED ASSOC, V300, P2379, DOI 10.1001/jama.2008.711

Wild S, 2004, DIABETES CARE, V27, P1047, DOI 10.2337/diacare.27.5.1047

Williams R, 2002, DIABETOLOGIA, V45, pS13, DOI 10.1007/s00125-002-0859-9

Zhang XP, 2005, AM J EPIDEMIOL, V161, P652, DOI 10.1093/aje/kwi089

Zimmet P, 2009, DIABETES RES CLIN PR, V84, P107, DOI 10.1016/j.diabres.2009.01.016

NR 65

TC 9

Z9 9

U1 0

U2 5

PU BMC

PI LONDON

PA CAMPUS, 4 CRINAN ST, LONDON N1 9XW, ENGLAND

SN 1471-2458

J9 BMC PUBLIC HEALTH

JI BMC Public Health

PD JUL 1

PY 2010

VL 10

AR 388

DI 10.1186/1471-2458-10-388

PG 11

WC Public, Environmental & Occupational Health

SC Public, Environmental & Occupational Health

GA 650OA

UT WOS:000281857700002

PM 20594337

OA DOAJ Gold, Green Published

DA 2019-01-08

ER

PT J

AU van Bastelaar, KMP

Pouwer, F

Geelhoed-Duijvestijn, PHLM

Tack, CJ

Bazelmans, E

Beekman, AT

Heine, RJ

Snoek, FJ

AF van Bastelaar, K. M. P.

Pouwer, F.

Geelhoed-Duijvestijn, P. H. L. M.

Tack, C. J.

Bazelmans, E.

Beekman, A. T.

Heine, R. J.

Snoek, F. J.

TI Diabetes-specific emotional distress mediates the association between

depressive symptoms and glycaemic control in Type 1 and Type 2 diabetes

SO DIABETIC MEDICINE

LA English

DT Article

DE depression; diabetes-specific emotional distress; glycaemic control;

type 1 diabetes; type 2 diabetes

ID RANDOMIZED CONTROLLED-TRIAL; COMORBID DEPRESSION; METAANALYSIS;

NETHERLANDS; PREVALENCE; STANDARD; VALIDITY; ADULTS; SCALE; CARE

AB P>Objectives

To investigate whether diabetes-specific emotional distress mediates the relationship between depression and glycaemic control in patients with Type 1 and Type 2 diabetes.

Research design and methods

Data were derived from the baseline assessment of a depression in diabetes screening study carried out in three tertiary diabetes clinics in the Netherlands. Most recent glycated haemoglobin (HbA(1c)) measurement was obtained from medical records. The Centre for Epidemiologic Studies Depression Scale (CES-D) and Problem Areas in Diabetes scale (PAID) were used to measure depression and diabetes-specific emotional distress respectively. Linear regression was performed to examine the mediating effect of diabetes-distress.

Results

Complete data were available for 627 outpatients with Type 1 (n = 280) and Type 2 (n = 347) diabetes. Analyses showed that diabetes-distress mediated the relation between depression and glycaemic control and not differently for both disease types. Post-hoc analyses revealed that patients depressed and distressed by their diabetes were in significantly poorer glycaemic control relative to those not depressed nor distressed (HbA(1c) 8.7 +/- 1.7 vs. 7.6 +/- 1.2% in those without depressive symptoms, 7.6 +/- 1.1% in depressed only and 7.7 +/- 1.1% in the distressed only, P < 0.001). Depressed patients without elevated diabetes-distress did not show a significantly increased risk of elevated HbA(1c).

Conclusions

In explaining the association between depression and glycaemic control, diabetes-specific emotional distress appears to be an important mediator. Addressing diabetes-specific emotional problems as part of depression treatment in diabetes patients may help improve glycaemic outcomes.

C1 [van Bastelaar, K. M. P.] Vrije Univ Amsterdam, Med Ctr, Dept Med Psychol, Diabet Psychol Res Grp,EMGO Inst Hlth & Care Res, NL-1081 BT Amsterdam, Netherlands.

[Pouwer, F.] Tilburg Univ, Dept Med Psychol & Neuropsychol, CoRPS Ctr Res Psychol Somat Dis, NL-5000 LE Tilburg, Netherlands.

[Geelhoed-Duijvestijn, P. H. L. M.] Haaglanden Med Ctr, Dept Internal Med, The Hague, Netherlands.

[Tack, C. J.] Radboud Univ Nijmegen, Med Ctr, Dept Internal Med, NL-6525 ED Nijmegen, Netherlands.

[Bazelmans, E.] Radboud Univ Nijmegen, Med Ctr, Dept Med Psychol, NL-6525 ED Nijmegen, Netherlands.

[Beekman, A. T.] Vrije Univ Amsterdam, Med Ctr, Dept Psychiat, NL-1081 BT Amsterdam, Netherlands.

[Heine, R. J.] Vrije Univ Amsterdam, Med Ctr, Dept Endocrinol, NL-1081 BT Amsterdam, Netherlands.

[Heine, R. J.] Eli Lilly & Co, Indianapolis, IN 46285 USA.

RP van Bastelaar, KMP (reprint author), Vrije Univ Amsterdam, Med Ctr, Dept Med Psychol, Diabet Psychol Res Grp,EMGO Inst Hlth & Care Res, Boechorststr 7, NL-1081 BT Amsterdam, Netherlands.

EM k.vanbastelaar@vumc.nl

RI Tack, Cees/A-2368-2014

FU Lundbeck; Eli Lilly

FX AB has received funding from Lundbeck and Eli Lilly for speaking and for

research. RJH is affiliated with Eli Lilly and Company. All other

authors declare no competing interests.

CR *ADA, 2009, DIABETES CARE S1, V32, pS13

Amsberg S, 2009, PATIENT EDUC COUNS, V77, P72, DOI 10.1016/j.pec.2009.01.015

Anderson RJ, 2001, DIABETES CARE, V24, P1069, DOI 10.2337/diacare.24.6.1069

BARON RM, 1986, J PERS SOC PSYCHOL, V51, P1173, DOI 10.1037//0022-3514.51.6.1173

Beekman ATF, 1997, PSYCHOL MED, V27, P231, DOI 10.1017/S0033291796003510

Bijl RV, 1998, SOC PSYCH PSYCH EPID, V33, P587, DOI 10.1007/s001270050098

BOUMA J, 1995, MEASURING DEPRESSION

BRYAN CJ, 2007, J AFFECT DISORDERS, P113

de Groot M, 2001, PSYCHOSOM MED, V63, P619, DOI 10.1097/00006842-200107000-00015

DeVries JH, 2004, DIABETIC MED, V21, P1263, DOI 10.1111/j.1464-5491.2004.01386.x

Fisher L, 2008, DIABETIC MED, V25, P1096, DOI 10.1111/j.1464-5491.2008.02533.x

Fisher L, 2009, DIABETES CARE, V33, P23

Fisher L, 2007, DIABETES CARE, V30, P542, DOI 10.2337/dc06-1614

Gonzalez JS, 2008, DIABETES CARE, V31, P2398, DOI 10.2337/dc08-1341

Henkel V, 2004, GEN HOSP PSYCHIAT, V26, P190, DOI 10.1016/j.genhosppsych.2004.02.003

Hermanns N, 2006, DIABETOLOGIA, V49, P469, DOI 10.1007/s00125-005-0094-2

Home P, 2006, DIABETIC MED, V23, P579

Katon WJ, 2005, DIABETES CARE, V28, P2668, DOI 10.2337/diacare.28.11.2668

Lustman PJ, 2000, DIABETES CARE, V23, P934, DOI 10.2337/diacare.23.7.934

McGuire BE, 2010, DIABETOLOGIA, V53, P66, DOI 10.1007/s00125-009-1559-5

McHale M, 2008, PSYCHOSOM MED, V70, P869, DOI 10.1097/PSY.0b013e318186dea9

Petrak F, 2009, CURR OPIN PSYCHIATR, V22, P211, DOI 10.1097/YCO.0b013e3283207b45

Pibernik-Okanovic M, 2008, DIABETIC MED, V25, P1252, DOI 10.1111/j.1464-5491.2008.02553.x

Piette JD, 2004, AM J MANAG CARE, V10, P152

Pouwer F, 2010, DIABETIC MED, V27, P217, DOI 10.1111/j.1464-5491.2009.02903.x

RADLOFF L S, 1977, Applied Psychological Measurement, V1, P385, DOI 10.1177/014662167700100306

Schram MT, 2009, CURR DIABETES REV, V5, P112, DOI 10.2174/157339909788166828

Snoek FJ, 2008, DIABETIC MED, V25, P1337, DOI 10.1111/j.1464-5491.2008.02595.x

Snoek FJ, 2000, DIABETES CARE, V23, P1305, DOI 10.2337/diacare.23.9.1305

van Bastelaar KM, 2008, BMC PSYCHIATRY, V8, DOI 10.1186/1471-244X-8-9

NR 30

TC 73

Z9 73

U1 1

U2 29

PU WILEY-BLACKWELL

PI MALDEN

PA COMMERCE PLACE, 350 MAIN ST, MALDEN 02148, MA USA

SN 0742-3071

J9 DIABETIC MED

JI Diabetic Med.

PD JUL

PY 2010

VL 27

IS 7

BP 798

EP 803

DI 10.1111/j.1464-5491.2010.03025.x

PG 6

WC Endocrinology & Metabolism

SC Endocrinology & Metabolism

GA 619OX

UT WOS:000279440600012

PM 20636961

DA 2019-01-08

ER

PT J

AU Fritschi, C

Quinn, L

AF Fritschi, Cynthia

Quinn, Laurie

TI Fatigue in patients with diabetes: A review

SO JOURNAL OF PSYCHOSOMATIC RESEARCH

LA English

DT Review

DE Diabetes; Fatigue; Review

ID AFRICAN-AMERICAN WOMEN; PHYSICAL-ACTIVITY; DEPRESSIVE SYMPTOMS;

CARDIOVASCULAR-DISEASE; OXIDATIVE STRESS; SOMATIC SYMPTOMS;

SELF-MANAGEMENT; PRIMARY-CARE; TYPE-2; EXERCISE

AB Objective: Fatigue is a common and distressing complaint among people with diabetes and likely to hinder the ability to perform daily diabetes self-management tasks. A review of the literature about diabetes-related fatigue was conducted with an eye toward creating a framework for beginning to conduct more focused studies on this subject. Methods: A literature search containing the terms diabetes, fatigue, tiredness, and symptoms was conducted to search for literature that addressed diabetes-related fatigue. Results: Diabetes presents many potential pathways for fatigue, but focused studies on this symptom are rare. Furthermore, research on diabetes-related fatigue is limited by fatigue's nonspecific symptoms and because fatigue researchers have yet to agree on standardized definition, measurement, or diagnostic criteria. Additionally, few diabetes randomized clinical trials included measurement of patient-reported outcomes, such as symptoms or health-related quality of life in their study designs, although one that did provided some meaningful finding that symptom-focused education improved self-management practices, Hb(A1c) levels, quality of life, and symptom distress. Conclusion: There is a need to standardize the definition, measurement, and diagnostic criteria of fatigue in diabetes. We present a model that can guide focused studies on fatigue in diabetes. The model capitalizes on the multidimensional phenomena (physiological, psychological, and lifestyle) associated with fatigue in diabetes. (C) 2010 Elsevier Inc. All rights reserved.

C1 [Fritschi, Cynthia; Quinn, Laurie] Univ Illinois, Coll Nursing, Dept Biobehav Hlth Sci, Chicago, IL 60612 USA.

RP Fritschi, C (reprint author), Univ Illinois, Coll Nursing MC 802, 845 S Damen Ave, Chicago, IL 60612 USA.

EM fritschi@uic.edu

OI Fritschi, Cynthia/0000-0001-5447-8315

FU National Institutes of Health/National Institute for Nursing Research

[F31 NR009751]

FX This research was funded in part by the National Institutes of

Health/National Institute for Nursing Research F31 NR009751.

CR Aaronson L S, 1999, Image J Nurs Sch, V31, P45

Aistars J, 1987, Oncol Nurs Forum, V14, P25

Amer Diabet Assoc, 2008, DIABETES CARE, V31, pS55, DOI 10.2337/dc08-S055

American Diabetes Association, DIAB STAT

American Psychiatric Association, 1994, DIAGN STAT MAN MENT

Anderson RJ, 2001, DIABETES CARE, V24, P1069, DOI 10.2337/diacare.24.6.1069

Avlund K, 2008, DAN MED BULL, V55, P147

BARTLEY SH, 1976, PSYCHOL ASPECTS PHYS, P409

Bonora E, 2001, DIABETES CARE, V24, P2023, DOI 10.2337/diacare.24.12.2023

Bonora E, 2001, DIABETOLOGIA, V44, P2107, DOI 10.1007/s001250100020

Bulpitt CJ, 1998, DIABETES CARE, V21, P111, DOI 10.2337/diacare.21.1.111

Casalenuovo GA, 2002, FATIGUE DIABETES MEL

Chasens Eileen R, 2008, Issues Ment Health Nurs, V29, P1134, DOI 10.1080/01612840802319878

Chaudhuri A, 2000, J NEUROL SCI, V179, P34, DOI 10.1016/S0022-510X(00)00411-1

Cuellar NG, 2008, J CLIN SLEEP MED, V4, P50

de Jonge P, 2006, DIABETOLOGIA, V49, P2627, DOI 10.1007/s00125-006-0442-x

de Sonnaville JJJ, 1998, DIABETES CARE, V21, P919, DOI 10.2337/diacare.21.6.919

Deray G, 2004, AM J NEPHROL, V24, P522, DOI 10.1159/000081058

Drayer RA, 2005, INT J GERIATR PSYCH, V20, P973, DOI 10.1002/gps.1389

DRIVSHOLM T, 2005, DIABETOLOGIA

FORD ES, 1995, DIABETES CARE, V18, P27, DOI 10.2337/diacare.18.1.27

Gandhi GY, 2008, JAMA-J AM MED ASSOC, V299, P2543, DOI 10.1001/jama.299.21.2543

Glass JM, 2004, J PSYCHOSOM RES, V57, P391, DOI 10.1016/j.jpsychores.2004.04.002

Golden SH, 2004, DIABETES CARE, V27, P429, DOI 10.2337/diacare.27.2.429

Golden SH, 2008, JAMA-J AM MED ASSOC, V299, P2751, DOI 10.1001/jama.299.23.2751

GROOTENHUIS PA, 1994, DIABETIC MED, V11, P253, DOI 10.1111/j.1464-5491.1994.tb00268.x

HART LK, 1982, CONCEPT CLARIFICATIO

Hays LM, 1999, DIABETES CARE, V22, P706, DOI 10.2337/diacare.22.5.706

Hill-Briggs F, 2003, DIABETES EDUCATOR, V29, P1018

*INT DIAB FED, DIAB ATL

Kant IJ, 2003, OCCUP ENVIRON MED, V60, P32

Kennedy G, 2005, FREE RADICAL BIO MED, V39, P584, DOI 10.1016/j.freeradbiomed.2005-04.020

King P, 1998, DIABETES CARE, V21, P341, DOI 10.2337/diacare.21.3.341

Koch T, 1999, J CLIN NURS, V8, P712, DOI 10.1046/j.1365-2702.1999.00320.x

Kovatchev Boris P, 2002, Diabetes Technol Ther, V4, P295, DOI 10.1089/152091502760098438

KROENKE K, 1993, ARCH INTERN MED, V153, P2474, DOI 10.1001/archinte.153.21.2474

Lavidor M, 2002, BRIT J HEALTH PSYCH, V7, P67, DOI 10.1348/135910702169367

LeMone P, 1996, DIABETES EDUCATOR, V22, P361, DOI 10.1177/014572179602200409

LEWIS G, 1992, J EPIDEMIOL COMMUN H, V46, P92, DOI 10.1136/jech.46.2.92

Lim K, 2005, PREV MED, V40, P33, DOI 10.1016/j.ypmed.2004.04.046

Lim WJ, 2005, ARCH INTERN MED, V165, P910, DOI 10.1001/archinte.165.8.910

Magnusson K, 1999, EUR J CANCER CARE, V8, P224

Maslach C, 2001, ANNU REV PSYCHOL, V52, P397, DOI 10.1146/annurev.psych.52.1.397

Mcardle W. D., 2001, EXERCISE PHYSL

McNair D M, 2003, PROFILE MOOD STATES

Miller MA, 2007, CURR VASC PHARMACOL, V5, P93, DOI 10.2174/157016107780368280

MOCK V, 2004, PSYCHOONCOLOGY

Monnier L, 2006, JAMA-J AM MED ASSOC, V295, P1681, DOI 10.1001/jama.295.14.1681

Morrato EH, 2007, DIABETES CARE, V30, P203, DOI 10.2337/dc06-1128

Morsch CM, 2006, J CLIN NURS, V15, P498, DOI 10.1111/j.1365-2702.2006.01349.x

MOSSO A, 2004, FATIGUE

*NANDA, 2001, NURS DIAGN DEF CLASS

Neuberger GB, 1997, RES NURS HEALTH, V20, P195, DOI 10.1002/(SICI)1098-240X(199706)20:3<195::AID-NUR3>3.0.CO;2-D

Nijrolder I, 2008, ANN FAM MED, V6, P519, DOI 10.1370/afm.908

O'Connor PJ, 2005, MED SCI SPORT EXER, V37, P299, DOI 10.1249/01.MMS.0000152802.89770.CF

Palinkas LA, 2004, DIABETIC MED, V21, P1185, DOI 10.1111/j.1464-5491.2004.01315.x

Petersen M, 2008, DIABETES CARE, V31, P596, DOI 10.2337/dc08-9017

Pickup JC, 2004, DIABETES CARE, V27, P813, DOI 10.2337/diacare.27.3.813

Polonsky W. H., 1999, DIABETES BURNOUT WHA

POLONSKY WH, 1995, DIABETES CARE, V18, P754, DOI 10.2337/diacare.18.6.754

Polonsky WH, 2005, DIABETES CARE, V28, P626, DOI 10.2337/diacare.28.3.626

Polonsky William H, 2002, Curr Diab Rep, V2, P153, DOI 10.1007/s11892-002-0075-5

Praet SFE, 2006, CLIN SCI, V111, P119, DOI 10.1042/CS20060041

Puetz TW, 2006, EUR J CARDIOV PREV R, V13, P886, DOI 10.1097/01.hjr.0000230102.55653.0b

Puetz TW, 2006, PSYCHOL BULL, V132, P866, DOI 10.1037/0033-2909.132.6.866

Puetz TW, 2006, SPORTS MED, V36, P767, DOI 10.2165/00007256-200636090-00004

Ream E, 1997, INT J NURS STUD, V34, P44, DOI 10.1016/S0020-7489(96)00032-6

Ream E, 1996, INT J NURS STUD, V33, P519, DOI 10.1016/0020-7489(96)00004-1

Resnick HE, 2006, J CLIN SLEEP MED, V2, P163

Rijken PM, 1998, DISABIL REHABIL, V20, P330, DOI 10.3109/09638289809166090

Rose L, 1998, J ADV NURS, V28, P295, DOI 10.1046/j.1365-2648.1998.00789.x

RUBIN RR, DEAL DIAB OV

Samuel-Hodge CD, 2000, DIABETES CARE, V23, P928, DOI 10.2337/diacare.23.7.928

Samuel-Hodge CD, 2005, ETHNIC DIS, V15, P436

Skelly AH, 2005, APPL NURS RES, V18, P213, DOI 10.1016/j.apnr.2004.08.008

Sommerfield AJ, 2004, DIABETES CARE, V27, P2335, DOI 10.2337/diacare.27.10.2335

STEWART AL, 1994, J CLIN EPIDEMIOL, V47, P719, DOI 10.1016/0895-4356(94)90169-4

Stewart ST, 2008, MED CARE, V46, P954, DOI 10.1097/MLR.0b013e318179199f

Stover JC, 2001, APPL NURS RES, V14, P72, DOI 10.1053/apnr.2001.22372

Sugahara H, 2004, PSYCHIAT RES, V128, P303, DOI 10.1016/j.psychres.2004.06.014

Theorell-Haglow J, 2006, SLEEP, V29, P751, DOI 10.1093/sleep/29.6.751

Thomas MC, 2003, DIABETES CARE, V26, P1164, DOI 10.2337/diacare.26.4.1164

Trendall J, 2000, J ADV NURS, V32, P1126, DOI 10.1046/j.1365-2648.2000.01583.x

VACCARINO AL, 2008, J AFFECT DISORD

VanderDoes FEE, 1996, DIABETES CARE, V19, P204, DOI 10.2337/diacare.19.3.204

VARNI JW, 2008, PEDIAT DIABETES

Vgontzas AN, 2006, ANN NY ACAD SCI, V1083, P329, DOI 10.1196/annals.1367.023

Von Korff M, 2005, DIABETES CARE, V28, P1326, DOI 10.2337/diacare.28.6.1326

Warren RE, 2003, DIABETES-METAB RES, V19, P408, DOI 10.1002/dmrr.396

Weijman I, 2004, J OCCUP ENVIRON MED, V46, P828, DOI 10.1097/01.jom.0000135605.62330.ca

Weijman I, 2003, OCCUP ENVIRON MED, V60, P93

Wenzel J, 2005, DIABETES EDUCATOR, V31, P98, DOI 10.1177/0145721704273242

Wessely S, 1998, Epidemiol Psichiatr Soc, V7, P10

Wijeratne C, 2007, J PSYCHOSOM RES, V62, P153, DOI 10.1016/j.jpsychores.2006.09.011

Winningham M L, 1994, Oncol Nurs Forum, V21, P23

Wood FG, 2004, J ADV NURS, V45, P190, DOI 10.1046/j.1365-2648.2003.02880.x

Zimmerman M, 2006, J NERV MENT DIS, V194, P313, DOI 10.1097/01.nmd.0000217806.16329.ff

NR 97

TC 80

Z9 81

U1 2

U2 22

PU PERGAMON-ELSEVIER SCIENCE LTD

PI OXFORD

PA THE BOULEVARD, LANGFORD LANE, KIDLINGTON, OXFORD OX5 1GB, ENGLAND

SN 0022-3999

EI 1879-1360

J9 J PSYCHOSOM RES

JI J. Psychosomat. Res.

PD JUL

PY 2010

VL 69

IS 1

BP 33

EP 41

DI 10.1016/j.jpsychores.2010.01.021

PG 9

WC Psychiatry

SC Psychiatry

GA 618NR

UT WOS:000279363100005

PM 20630261

OA Green Accepted

DA 2019-01-08

ER

PT J

AU Kerr, WC

Ye, Y

AF Kerr, William C.

Ye, Yu

TI Relationship of Life-Course Drinking Patterns to Diabetes, Heart

Problems, and Hypertension Among Those 40 and Older in the 2005 US

National Alcohol Survey

SO JOURNAL OF STUDIES ON ALCOHOL AND DRUGS

LA English

DT Article

ID PROPENSITY-SCORE; METABOLIC SYNDROME; UNITED-STATES; RISK; MORTALITY;

CONSUMPTION; DISEASE; ADULTS; METAANALYSIS; PREVALENCE

AB Objective: The goal of this study was to estimate relationships between life-course drinking patterns and the risks of self-reported diabetes, heart problems, and hypertension. Method: Respondents to the 2005 National Alcohol Survey, age 40 and older, reported ever having a doctor or health professional diagnose each of the health-problem outcomes. Retrospective earlier-life drinking patterns were characterized by lifetime abstention and the frequency of 5+ drinking days (i.e., days on which five or more drinks were consumed) in the respondent's teens, 20s, and 30s. Past-year drinking patterns were measured through intake volume and 5+ days. Potential confounders in the domains of demographics, socioeconomic resources, and other health-risk variables that is, depression, distress, sense of coherence, body mass index, tobacco use, marijuana use, childhood abuse, and family history of alcohol problems were controlled through propensity-score matching. Results: After matching, lifetime abstainers were found to be at increased risk of diabetes compared with both lifetime and current moderate drinkers. Exdrinkers were found to be at increased risk of diabetes, heart problems, and hypertension. Higher volume drinkers without monthly 5+ days were found to be at reduced risk of diabetes relative to moderate-volume current drinkers. Heavy-occasion drinkers were found to be at increased risk of hypertension. Conclusions: Regular lower quantity alcohol intake may be protective against adult onset of diabetes, but no evidence of protection from heart problems or hypertension was found. Both life course defined and past year defined drinking groups exhibit substantial clustering of confounding risk variables, indicating the need for modeling strategies like propensity-score matching. Increased risks among exdrinkers suggest a substantial "sick-quitter" effect. (J. Stud. Alcohol Drugs, 71, 515-525, 2010)

C1 [Kerr, William C.; Ye, Yu] Alcohol Res Grp, Emeryville, CA 94608 USA.

RP Kerr, WC (reprint author), Alcohol Res Grp, 6475 Christie Ave,Suite 400, Emeryville, CA 94608 USA.

EM wkerr@arg.org

FU National Institute on Alcohol Abuse and Alcoholism; National Alcohol

Research Center [P30-AA05595]

FX This research was supported by National Institute on Alcohol Abuse and

Alcoholism, National Alcohol Research Center grant P30-AA05595 awarded

to Thomas K. Greenfield (principal investigator), Alcohol Research

Group, Public Health Institute, Emeryville, CA.

CR American Association for Public Opinion Research, 2000, STAND DEF FIN DISP C

Austin PC, 2008, STAT MED, V27, P2037, DOI 10.1002/sim.3150

BRESLOW RA, 2008, ALCOHOLISM CLIN EXPT, V32, P1

Brookhart MA, 2006, AM J EPIDEMIOL, V163, P1149, DOI 10.1093/aje/kwj149

BURR ML, 1986, BRIT J NUTR, V56, P81, DOI 10.1079/BJN19860087

Caldwell TM, 2006, ALCOHOL ALCOHOLISM, V41, P650, DOI 10.1093/alcalc/agl088

Chen L, 2008, PLOS MED, V5, DOI 10.1371/journal.pmed.0050052

Corrao G, 2004, PREV MED, V38, P613, DOI 10.1016/j.ypmed.2003.11.027

Cutler JA, 2008, HYPERTENSION, V52, P818, DOI 10.1161/HYPERTENSIONAHA.108.113357

D'Agostino RB, 1998, STAT MED, V17, P2265, DOI 10.1002/(SICI)1097-0258(19981015)17:19<2265::AID-SIM918>3.0.CO;2-B

Dawson DA, 2000, ALCOHOL CLIN EXP RES, V24, P1820, DOI 10.1097/00000374-200012000-00011

Djousse L, 2004, OBES RES, V12, P1375, DOI 10.1038/oby.2004.174

Dong MX, 2004, CIRCULATION, V110, P1761, DOI 10.1161/01.CIR.0000143074.54995.7F

Dorn JM, 2007, ADDICTION, V102, P730, DOI 10.1111/j.1360-0443.2007.01765.x

Dufour MC, 2001, J NUTR, V131, p552S, DOI 10.1093/jn/131.2.552S

Emberson JR, 2005, AM J EPIDEMIOL, V161, P856, DOI 10.1093/aje/kwi111

Fan AZ, 2006, EUR J EPIDEMIOL, V21, P129, DOI 10.1007/s10654-005-5457-y

Fillmore KM, 2006, ADDICT RES THEORY, V14, P101, DOI 10.1080/16066350500497983

Gmel G, 2003, EUR J EPIDEMIOL, V18, P631, DOI 10.1023/A:1024805021504

Greenfield TK, 2000, J SUBST ABUSE, V12, P33, DOI 10.1016/S0899-3289(00)00039-0

Hodge AM, 2006, DIABETIC MED, V23, P690, DOI 10.1111/j.1464-5491.2006.01864.x

Howard AA, 2004, ANN INTERN MED, V140, P211, DOI 10.7326/0003-4819-140-6-200403160-00011

Hudson JI, 2005, EPIDEMIOLOGY, V16, P355, DOI 10.1097/01.ede.0000158224.50593.e3

Imamura F, 2009, AM J EPIDEMIOL, V170, P37, DOI 10.1093/aje/kwp096

Kerr WC, 2005, ALCOHOL CLIN EXP RES, V29, P2015, DOI 10.1097/01.alc.0000187596.92804.bd

Kerr WC, 2002, J STUD ALCOHOL, V63, P325, DOI 10.15288/jsa.2002.63.325

Kerr WC, 2008, ALCOHOL CLIN EXP RES, V32, P1623, DOI 10.1111/j.1530-0277.2008.00741.x

Kerr WC, 2009, ADDICTION, V104, P1503, DOI 10.1111/j.1360-0443.2009.02579.x

Kuper H, 2009, BMJ-BRIT MED J, V339, DOI 10.1136/bmj.b4265

Lemmens PH, 1997, CONTEMP DRUG PROBL, V24, P581

LEUVEN E, 2003, S432001 BOST COLL DE

MIDANIK LT, 2009, SUBST USE MISUSE, V44, P357

Naimi TS, 2005, AM J PREV MED, V28, P369, DOI 10.1016/j.amepre.2005.01.011

Norris SL, 2008, ANN INTERN MED, V148, P855, DOI 10.7326/0003-4819-148-11-200806030-00008

Rehm J, 2008, AM J EPIDEMIOL, V168, P866, DOI 10.1093/aje/kwn093

Rehm J, 2006, CONTEMP DRUG PROBL, V33, P205, DOI DOI 10.1177/009145090603300203

RHEM J, 2003, J CARDIOVASCULAR RIS, V10, P15

ROSENBAUM PR, 1983, BIOMETRIKA, V70, P41, DOI 10.1093/biomet/70.1.41

ROSENBAUM PR, 1985, AM STAT, V39, P33, DOI 10.2307/2683903

RUBIN DB, 1973, BIOMETRICS, V29, P185, DOI 10.2307/2529685

Rubin DB, 1997, ANN INTERN MED, V127, P757, DOI 10.7326/0003-4819-127-8_Part_2-199710151-00064

Russell M, 1997, AM J EPIDEMIOL, V146, P975

SHAPER AG, 1990, BRIT J ADDICT, V85, P837

SHAPER AG, 1988, LANCET, V2, P1267

Stewart SH, 2008, ADDICTION, V103, P1622, DOI 10.1111/j.1360-0443.2008.02317.x

Stranges S, 2004, HYPERTENSION, V44, P813, DOI 10.1161/01.HYP.0000146537.03103.f2

Valmadrid CT, 1999, JAMA-J AM MED ASSOC, V282, P239, DOI 10.1001/jama.282.3.239

Wainwright NWJ, 2008, J EPIDEMIOL COMMUN H, V62, P829, DOI 10.1136/jech.2007.066464

NR 48

TC 25

Z9 26

U1 1

U2 8

PU ALCOHOL RES DOCUMENTATION INC CENT ALCOHOL STUD RUTGERS UNIV

PI PISCATAWAY

PA C/O DEIRDRE ENGLISH, 607 ALLISON RD, PISCATAWAY, NJ 08854-8001 USA

SN 1937-1888

EI 1938-4114

J9 J STUD ALCOHOL DRUGS

JI J. Stud. Alcohol Drugs

PD JUL

PY 2010

VL 71

IS 4

BP 515

EP 525

DI 10.15288/jsad.2010.71.515

PG 11

WC Substance Abuse; Psychology

SC Substance Abuse; Psychology

GA 619EA

UT WOS:000279410800006

PM 20553659

OA Green Published

DA 2019-01-08

ER

PT J

AU McEwen, MM

Pasvogel, A

Gallegos, G

Barrera, L

AF McEwen, Marylyn Morris

Pasvogel, Alice

Gallegos, Gwen

Barrera, Lourdes

TI Type 2 Diabetes Self-Management Social Support Intervention at the

US-Mexico Border

SO PUBLIC HEALTH NURSING

LA English

DT Article

DE culturally sensitive intervention; diabetes self-management; Mexican

Americans; promotoras

ID CULTURALLY APPROPRIATE; EDUCATION; AMERICANS; CARE; QUESTIONNAIRE;

COMMUNITY; PROGRAM; RELIABILITY; DISTRESS; MELLITUS

AB Objectives

To pilot test the efficacy of a culturally tailored diabetes self-management social support intervention for Mexican American adults with Type 2 diabetes (T2DM) living in the U.S.-Mexico border region and to test the feasibility of recruiting and training promotoras to participate in intervention delivery.

Design and Sample

This study used a single-group pretest and posttest design. The convenience sample consisted of 21 Mexican American adults with T2DM. The setting for the study was a community in the Arizona-Sonora, Mexico border region.

Interventions

A bilingual, bicultural certified diabetes educator (CDE) and a nurse researcher developed the intervention to improve T2DM self-management activities for Mexican Americans. Data were collected using self-report questionnaires, glycosolated hemoglobin (HbA(1c)), and anthropometric measures.

Results

Intervention efficacy was demonstrated by an increase in participants' diabetes self-management activities and diabetes knowledge and a decrease in diabetes-related distress and sedentary behaviors. There were no significant changes in physiologic outcomes. Feasibility of recruitment and training of 2 promotoras who participated in intervention delivery was established.

Conclusions

Promotoras, in collaboration with a CDE, successfully delivered a culturally tailored diabetes self-management social support intervention for Mexican American adults with T2DM. This intervention positively affected diabetes self-management behaviors.

C1 [McEwen, Marylyn Morris; Pasvogel, Alice; Barrera, Lourdes] Univ Arizona, Tucson, AZ USA.

[Gallegos, Gwen] Carondelet Hlth Network, Tucson, AZ USA.

RP McEwen, MM (reprint author), 1305 N Martin, Tucson, AZ 85721 USA.

EM mmcewen@nursing.arizona.edu

FU NIH Center on Minority Health and Health Disparities [5P60 MD000155-04]

FX The authors wish to thank the promotoras, Josefina Meranza and Rose

Urias, and The University of Arizona College of Nursing Writer's Group.

This research was supported by the NIH Center on Minority Health and

Health Disparities 5P60 MD000155-04. PI: Cecilia Rosales, M.D., M.P.H.,

Associate Professor, MEZCOPH, University of Arizona.

CR *ADA, 2009, DIABETES CARE S1, V32, pS13

Albright TL, 2001, FAM MED, V33, P354

American Association of Diabetes Educators, 2003, DIABETES ED, V29, P818

*AZ DEP HLTH SERV, 2008, ARIZ DIAB STRAT PLAN

Brown SA, 2005, DIABETES CARE, V28, P527, DOI 10.2337/diacare.28.3.527

Brown SA, 2002, DIABETES CARE, V25, P259, DOI 10.2337/diacare.25.2.259

Campanini M, 2007, MULTIDISCIP RESP MED, V2, P100

Carranza SN, 2004, FAM MED, V36, P539

Centers for Disease Control and Prevention, 2007, BMI AD

Craig CL, 2003, MED SCI SPORT EXER, V35, P1381, DOI 10.1249/01.MSS.0000078924.61453.FB

*DIRECNET STUD GRO, 2005, COMP FING HEM A1C LE

Fisher L, 2007, DIABETES CARE, V30, P542, DOI 10.2337/dc06-1614

Garcia AA, 2001, DIABETES CARE, V24, P16, DOI 10.2337/diacare.24.1.16

Gleeson-Kreig J, 2002, PUBLIC HEALTH NURS, V19, P215, DOI 10.1046/j.0737-1209.2002.19310.x

Hertzog MA, 2008, RES NURS HEALTH, V31, P180, DOI 10.1002/nur.20247

Ingram M. G., 2005, PREV CHRONIC DIS, V2, P1

Ingram M, 2007, DIABETES EDUCATOR, V33, p172S, DOI 10.1177/0145721707304170

ISRAEL BA, 1982, PATIENT COUNS HEALTH, V4, P65, DOI 10.1016/S0190-2040(82)80002-5

Jones PS, 2001, NURS RES, V50, P300, DOI 10.1097/00006199-200109000-00008

Little RR, 2003, CLIN CHEM LAB MED, V41, P1191, DOI 10.1515/CCLM.2003.183

Lujan J, 2007, DIABETES EDUCATOR, V33, P660, DOI 10.1177/0145721707304080

Mauldon M, 2006, DIABETES EDUCATOR, V32, P751, DOI 10.1177/0145721706291999

Mensing Carole, 2007, Diabetes Care, V30 Suppl 1, pS96, DOI 10.2337/dc07-S096

McEwen MM, 2007, FAM COMMUNITY HEALTH, V30, P201, DOI 10.1097/01.FCH.0000277763.70031.0d

*NAT CTR CHRON DIS, 2005, COMM HLTH WORK PROM

*NIDDK, 2008, NAT DIAB STAT 2007 F

Norris SL, 2006, DIABETIC MED, V23, P544, DOI 10.1111/j.1464-5491.2006.01845.x

Norris SL, 2001, DIABETES CARE, V24, P561, DOI 10.2337/diacare.24.3.561

*PAN AM HLTH ORG, 2008, PAN AM HLTH ORG US M

Philis-Tsimikas A, 2004, DIABETES CARE, V27, P110, DOI 10.2337/diacare.27.1.110

Polonsky WH, 2005, DIABETES CARE, V28, P626, DOI 10.2337/diacare.28.3.626

Sixta CS, 2008, DIABETES EDUCATOR, V34, P285, DOI 10.1177/0145721708314486

Toobert DJ, 2000, DIABETES CARE, V23, P943, DOI 10.2337/diacare.23.7.943

Van de Waterbeemd Han, 2005, Expert Opin Drug Metab Toxicol, V1, P1, DOI 10.1517/17425255.1.1.1

Vincent D, 2008, NURS RES, V57, P101, DOI 10.1097/01.NNR.0000313484.18670.ab

NR 35

TC 32

Z9 34

U1 0

U2 11

PU WILEY-BLACKWELL

PI MALDEN

PA COMMERCE PLACE, 350 MAIN ST, MALDEN 02148, MA USA

SN 0737-1209

J9 PUBLIC HEALTH NURS

JI Public Health Nurs.

PD JUL-AUG

PY 2010

VL 27

IS 4

BP 310

EP 319

DI 10.1111/j.1525-1446.2010.00860.x

PG 10

WC Public, Environmental & Occupational Health; Nursing
[truncated: 1,073,502 more chars]
