# Supplementary material for: Characterizing Hotspots and Frontier Landscapes of Diabetes-Specific Distress from 2000 to 2018: A Bibliometric Study
Source: Biomed Res Int. 2020 Jan 15;2020:8691451. doi: 10.1155/2020/8691451 (PMC6985931; doi:10.1155/2020/8691451)
Supplement: Supplementary Materials — S1-1: raw citing literature and cited references. S1-2: raw citing literature and cited references (1). S1-3: raw citing literatures and cited references. S2: the number of publications by year. S3: country/region distribution. S4: institutes distribution. S5: authors distribution. S6: journals distribution. [file 8691451.f1.zip › 8691451.f1/S1-3 Raw citing literatures and cited references.doc]

FN Clarivate Analytics Web of Science

VR 1.0

PT J

AU Allgar, VL

Neal, RD

Pascoe, SW

AF Allgar, VL

Neal, RD

Pascoe, SW

TI Cancer patients consultation patterns in primary care and levels of

psychological morbidity: Findings from the health survey for England

SO PSYCHO-ONCOLOGY

LA English

DT Article

ID BREAST-CANCER; DEPRESSION; PREVALENCE; MANAGEMENT; DISTRESS

AB Aim. To determine the consultations patterns in general practice, for people with cancer and other chronic illnesses, and to assess the levels of psychological morbidity.

Methods. The following questions from the 1999 Health Survey for England were analysed: presence of a self-reported long-standing illness and its nature, numbers of contacts with general practitioner (GP) in the previous 2 weeks, contact with a GP in the previous year for anxiety/depression or a mental, nervous or emotional problem, presence of a self-reported long-standing illness of mental illness, anxiety or depression, and GHQ12 scores. For comparison purposes, data from respondents reporting having asthma, arthritis, diabetes, other long-standing illness, and no long-standing illness are presented.

Results. A third of respondents with cancer had contact with a GP in the last 2 weeks, which was slightly higher than the other illness group, however the pattern of attendances for those respondents who did consult were similar between groups. A quarter of people with cancer had spoken to a GP in the last year about being anxious/depressed, or about a mental, nervous or emotional problem. A third of cancer respondents reported high GHQ12 scores, but self-reported long-standing illness of 'mental illness/anxiety/depression' was low (4%).

Conclusion. The findings suggest that psychological morbidity may be unrecognised in some cancer patients. There is potential for these symptoms to be identified and treated in primary care, especially given the ongoing nature of the patient-doctor relationship and the easy access primary care affords. Copyright (C) 2003 John Wiley Sons, Ltd.

C1 Univ Leeds, Nuffield Inst Hlth, Ctr Res Primary Care, Leeds LS2 9PL, W Yorkshire, England.

Meanwood Grp Practice, Leeds, W Yorkshire, England.

RP Allgar, VL (reprint author), Univ Leeds, Nuffield Inst Hlth, Ctr Res Primary Care, 71-75 Clarendon Rd, Leeds LS2 9PL, W Yorkshire, England.

OI Allgar, Victoria/0000-0002-5228-2623

CR *DEP HLTH, 1999, HLTH SURV ENGL

Department of Health, 2000, NHS CANC PLAN

Fallowfield L, 2001, BRIT J CANCER, V84, P1011, DOI 10.1054/bjoc.2001.1724

Goldberg D, 1999, J CLIN PSYCHIAT, V60, P39

Goldberg D., 1988, USERS GUIDE GEN HLTH

Gulliford T, 1997, BMJ-BRIT MED J, V314, P174, DOI 10.1136/bmj.314.7075.174

Hopwood P, 2000, J CLIN ONCOL, V18, P893, DOI 10.1200/JCO.2000.18.4.893

Macleod U, 2000, BRIT MED J, V320, P1442, DOI 10.1136/bmj.320.7247.1442

Maguire P, 2000, EUR J CANCER, V36, P556

MASSIE MJ, 1990, J CLIN PSYCHIAT, V51, P12

Pascoe S, 2000, AUST NZ J PSYCHIAT, V34, P785, DOI 10.1046/j.1440-1614.2000.00817.x

Rost K, 1998, GEN HOSP PSYCHIAT, V20, P12, DOI 10.1016/S0163-8343(97)00095-9

Sellick SM, 1999, PSYCHO-ONCOL, V8, P315, DOI 10.1002/(SICI)1099-1611(199907/08)8:4<315::AID-PON391>3.0.CO;2-G

Simon GE, 1998, J CLIN PSYCHIAT, V59, P3

Zabora JR, 1997, J PSYCHOSOC ONCOL, V15, P73, DOI 10.1300/J077v15n02_05

NR 15

TC 9

Z9 9

U1 0

U2 2

PU JOHN WILEY & SONS LTD

PI CHICHESTER

PA THE ATRIUM, SOUTHERN GATE, CHICHESTER PO19 8SQ, W SUSSEX, ENGLAND

SN 1057-9249

J9 PSYCHO-ONCOL

JI Psycho-Oncol.

PD OCT-NOV

PY 2003

VL 12

IS 7

BP 736

EP 740

DI 10.1002/pon.692

PG 5

WC Oncology; Psychology; Psychology, Multidisciplinary; Social Sciences,

Biomedical

SC Oncology; Psychology; Biomedical Social Sciences

GA 727PR

UT WOS:000185667800009

PM 14502598

DA 2019-01-08

ER

PT J

AU Lapolla, A

Dalfra, MG

Masin, M

Bruttomesso, D

Piva, I

Crepaldi, C

Tortul, C

Dalla Barba, B

Fedele, D

AF Lapolla, A

Dalfra, MG

Masin, M

Bruttomesso, D

Piva, I

Crepaldi, C

Tortul, C

Dalla Barba, B

Fedele, D

TI Analysis of outcome of pregnancy in type 1 diabetics treated with

insulin pump or conventional insulin therapy

SO ACTA DIABETOLOGICA

LA English

DT Article

DE diabetes; pregnancy; insulin therapy; maternal outcome; fetal

malformations

ID FETAL MALFORMATIONS; METABOLIC-CONTROL; GLYCEMIC CONTROL; WOMEN;

INFUSION; MELLITUS; INFANTS; GLUCOSE; HEMOGLOBIN-A1C; HYPOGLYCEMIA

AB We evaluated the outcome of pregnancies followed between 1990 and 2000 in 93 women with type I diabetes, treated with conventional intensive insulin therapy (n=68) or continuous subcutaneous insulin infusion (n=25). We evaluated metabolic control (fasting and 1-hour post-prandial plasma glucose and HbA1c levels), spontaneous or induced abortions, time and mode of delivery, maternal outcome (pregnancy-induced hypertension, preeclampsia, placental insufficiency. hydramnios. hypoglycemic coma, ketoacidosis) and fetal outcome (weight, hypoglycemia, hypocalcemia, hyperbilirubinemia, fetal distress, asphyxia, hyaline membrane disease. polycythemia, shoulder dystocia, malformations). Patients treated with insulin pump more frequently had background retinopathy and clinical neuropathy. No significant differences were observed between the two groups in metabolic control and maternal outcome. Glycemic control, nonoptimal in the prepregnancy state, improved significantly during pregnancy, as shown by the progressive reduction in HbA1c levels. As regards fetal outcome, no differences were observed between the two groups in morbidity and especially in malformation rate. Patients with malformed babies did not have optimal metabolic control at conception. Thus, maternal and perinatal outcomes were comparable in patients treated with insulin pump and continuous subcutaneous insulin therapy, and depended on metabolic control. In patients in higher White's class and with more unstable glycemia, we achieved metabolic control and outcomes comparable with those of women of lower White's class and more stable glycemic values using the insulin pump. Our data suggest that insulin pump therapy is useful in problematic, complicated cases of women who want a baby.

C1 Univ Padua, Dept Med & Surg Sci, I-35137 Padua, Italy.

Univ Padua, Dept Clin & Expt Med, I-35100 Padua, Italy.

San Bortolo Hosp, Dept Metab Dis, Vicenza, Italy.

Dept Diabetol ASS2 Isontina, Monfalcone, GO, Italy.

Univ Padua, Dept Pediat, I-35100 Padua, Italy.

RP Lapolla, A (reprint author), Univ Padua, Dept Med & Surg Sci, Via E Vendramini 7, I-35137 Padua, Italy.

CR Bode BW, 1996, DIABETES CARE, V19, P324, DOI 10.2337/diacare.19.4.324

CARTA Q, 1986, DIABETES METAB, V12, P121

Casson IF, 1997, BRIT MED J, V315, P275, DOI 10.1136/bmj.315.7103.275

COUSTAN DR, 1986, JAMA-J AM MED ASSOC, V255, P631, DOI 10.1001/jama.255.5.631

Diabet Control Complications Trial Res Grp, 1996, AM J OBSTET GYNECOL, V174, P1343

FUHRMANN K, 1983, DIABETES CARE, V6, P219, DOI 10.2337/diacare.6.3.219

Gabbe SG, 2000, AM J OBSTET GYNECOL, V182, P1283, DOI 10.1067/mob.2000.106182

GOLDMAN JA, 1986, AM J OBSTET GYNECOL, V155, P293, DOI 10.1016/0002-9378(86)90812-4

HAAKENS K, 1990, J INTERN MED, V228, P457, DOI 10.1111/j.1365-2796.1990.tb00263.x

HANSON U, 1990, DIABETOLOGIA, V33, P100, DOI 10.1007/BF00401047

Holing EV, 1998, DIABETES CARE, V21, P889, DOI 10.2337/diacare.21.6.889

Home P D, 1984, Diabet Med, V1, P41

HUGGETT ASG, 1957, LANCET, V2, P368

JAYNES PK, 1985, CLIN BIOCHEM, V18, P32, DOI 10.1016/S0009-9120(85)80020-5

JORNSAY DL, 1998, DIABETES SPECTRUM, V11, P26

Kaufman FR, 1999, DIABETES-METAB RES, V15, P338, DOI 10.1002/(SICI)1520-7560(199909/10)15:5<338::AID-DMRR57>3.3.CO;2-P

KEEN H, 1963, LANCET, V2, P913

KITZMILLER JL, 1985, OBSTET GYNECOL, V66, P606

KITZMILLER JL, 1983, MANAGING OB GYN EMER, P44

KJAER K, 1992, AM J OBSTET GYNECOL, V166, P1412, DOI 10.1016/0002-9378(92)91613-F

LAURITZEN T, 1983, DIABETOLOGIA, V24, P326

Lenhard MJ, 2001, ARCH INTERN MED, V161, P2293, DOI 10.1001/archinte.161.19.2293

LINDENBAUM C, 1993, J REPROD MED, V38, P306

LUCAS MJ, 1989, AM J OBSTET GYNECOL, V161, P426, DOI 10.1016/0002-9378(89)90536-X

McElvy S S, 2000, J Matern Fetal Med, V9, P14

MELANI F, 1970, P NATL ACAD SCI USA, V67, P148, DOI 10.1073/pnas.67.1.148

MIDTHJELL K, 1994, DIABETES CARE, V17, P1235, DOI 10.2337/diacare.17.10.1235

MIODOVNIK M, 1988, DIABETES CARE, V11, P713, DOI 10.2337/diacare.11.9.713

MONTORO MN, 1993, AM J PERINAT, V10, P17, DOI 10.1055/s-2007-994692

Nordstrom L, 1998, ACTA OBSTET GYN SCAN, V77, P284, DOI 10.1034/j.1600-0412.1998.770305.x

Platt MJ, 2002, DIABETIC MED, V19, P216, DOI 10.1046/j.1464-5491.2002.00665.x

Reece EA, 2000, CLIN OBSTET GYNECOL, V43, P32, DOI 10.1097/00003081-200003000-00004

Simmons D, 2001, DIABETES CARE, V24, P2078, DOI 10.2337/diacare.24.12.2078

SKYLER JS, 1981, DIABETES CARE, V4, P311, DOI 10.2337/diacare.4.2.311

Suhonen L, 2000, DIABETOLOGIA, V43, P79, DOI 10.1007/s001250050010

TAMBORLANE WV, 1979, NEW ENGL J MED, V300, P573, DOI 10.1056/NEJM197903153001101

Vaarasmaki M, 2002, DIABETIC MED, V19, P589, DOI 10.1046/j.1464-5491.2002.00756.x

WILLHOITE MB, 1993, DIABETES CARE, V16, P450, DOI 10.2337/diacare.16.2.450

YLINEN K, 1984, BRIT MED J, V289, P345, DOI 10.1136/bmj.289.6441.345

YLINEN K, 2002, DIABETES CARE S1, V25, pS82

YLINEN K, 1998, DIABETES CARE S1, V21, pS56

NR 41

TC 34

Z9 34

U1 0

U2 1

PU SPRINGER-VERLAG ITALIA SRL

PI MILAN

PA VIA DECEMBRIO, 28, MILAN, 20137, ITALY

SN 0940-5429

EI 1432-5233

J9 ACTA DIABETOL

JI Acta Diabetol.

PD SEP

PY 2003

VL 40

IS 3

BP 143

EP 149

DI 10.1007/s00592-003-0103-1

PG 7

WC Endocrinology & Metabolism

SC Endocrinology & Metabolism

GA 737XH

UT WOS:000186257300006

PM 14605971

DA 2019-01-08

ER

PT J

AU Kalro, BN

AF Kalro, BN

TI Impaired fertility caused by endocrine dysfunction in women

SO ENDOCRINOLOGY AND METABOLISM CLINICS OF NORTH AMERICA

LA English

DT Review

ID CONGENITAL ADRENAL-HYPERPLASIA; DEPENDENT DIABETIC WOMEN; RECURRENT

PREGNANCY LOSS; LONG-TERM TREATMENT; FETAL GROWTH DELAY;

CUSHINGS-SYNDROME; ANTITHYROID ANTIBODIES; SPONTANEOUS-ABORTION;

21-HYDROXYLASE DEFICIENCY; PITUITARY-TUMORS

AB The inability to conceive is often a distressing experience for couples. This article addresses the impact of thyroid, adrenal, growth hormone disorders, and diabetes on female reproduction.

C1 Univ Pittsburgh, Sch Med, Magee Womens Hosp, Dept Obstet, Pittsburgh, PA 15213 USA.

Univ Pittsburgh, Sch Med, Magee Womens Hosp, Dept Gynecol, Pittsburgh, PA 15213 USA.

Univ Pittsburgh, Sch Med, Magee Womens Hosp, Dept Reprod Sci, Pittsburgh, PA 15213 USA.

RP Kalro, BN (reprint author), Univ Pittsburgh, Sch Med, Magee Womens Hosp, Dept Obstet, 300 Halket St, Pittsburgh, PA 15213 USA.

EM bkalro@mail.magee.edu

CR ABELOVE WA, 1954, J CLIN ENDOCR METAB, V14, P32, DOI 10.1210/jcem-14-1-32

ABOULKHAIR SA, 1964, CLIN SCI, V27, P195

AHONEN P, 1987, J CLIN ENDOCR METAB, V64, P494, DOI 10.1210/jcem-64-3-494

AKANDE EO, 1975, BRIT J OBSTET GYNAEC, V82, P552, DOI 10.1111/j.1471-0528.1975.tb00685.x

AKANDE EO, 1975, BRIT J OBSTET GYNAEC, V82, P541, DOI 10.1111/j.1471-0528.1975.tb00684.x

ANDERSON DC, 1974, CLIN ENDOCRINOL, V3, P69, DOI 10.1111/j.1365-2265.1974.tb03298.x

ANSELL JE, 1991, WERNER INGBARS THYRO, P1022

Ares S, 1997, J CLIN ENDOCR METAB, V82, P1704, DOI 10.1210/jc.82.6.1704

ARON DC, 1990, AM J OBSTET GYNECOL, V162, P244, DOI 10.1016/0002-9378(90)90859-6

ARON DC, 1987, ENDOCRIN METAB CLIN, V16, P705, DOI 10.1016/S0889-8529(18)30470-5

Atchley DW, 1933, J CLIN INVEST, V12, P297, DOI 10.1172/JCI100504

BALEN AH, 1990, BRIT J OBSTET GYNAEC, V97, P536, DOI 10.1111/j.1471-0528.1990.tb02526.x

BETTERLE C, 1993, CLIN ENDOCRINOL, V39, P35, DOI 10.1111/j.1365-2265.1993.tb01748.x

BEVAN JS, 1987, CLIN ENDOCRINOL, V27, P225, DOI 10.1111/j.1365-2265.1987.tb01148.x

BIGAZZI M, 1979, J CLIN ENDOCR METAB, V48, P9, DOI 10.1210/jcem-48-1-9

BLOCH B, 1979, S AFR MED J, V55, P57

BLUMBERG DL, 1988, NEW ENGL J MED, V319, P951

BOCCUZZI G, 1975, J CLIN ENDOCR METAB, V40, P892, DOI 10.1210/jcem-40-5-892

BOHNET HG, 1981, LANCET, V2, P1278

BRADFORD JMW, 1995, REV PSYCHIAT, V14, P755

BRAY GA, 1968, J CLIN INVEST, V47, P1640, DOI 10.1172/JCI105855

Brenner PF, 1996, AM J OBSTET GYNECOL, V175, P766, DOI 10.1016/S0002-9378(96)80082-2

BRENT F, 1950, AM J SURG, V79, P645, DOI 10.1016/0002-9610(50)90329-1

BROWN ZA, 1992, DIABETES CARE, V15, P613, DOI 10.2337/diacare.15.5.613

BUESCHER MA, 1992, OBSTET GYNECOL, V79, P130

Bussen S, 2000, HUM REPROD, V15, P545, DOI 10.1093/humrep/15.3.545

CAMARGO CA, 1989, ENDOCRINE DISORDERS, P165

CARON P, 1995, NEW ENGL J MED, V333, P601, DOI 10.1056/NEJM199508313330918

Colao A, 1997, J CLIN ENDOCR METAB, V82, P2777, DOI 10.1210/jc.82.9.2777

COUSINS L, 1983, AM J OBSTET GYNECOL, V145, P411, DOI 10.1016/0002-9378(83)90309-5

CRANE JP, 1981, FERTIL STERIL, V36, P477

Daniels GH, 1995, ENDOCR PRACT, V1, P287

DAVIS LE, 1988, OBSTET GYNECOL, V72, P108

DAVIS LE, 1989, AM J OBSTET GYNECOL, V160, P63, DOI 10.1016/0002-9378(89)90088-4

de Menis E, 1999, J CLIN ENDOCR METAB, V84, P1489, DOI 10.1210/jc.84.4.1489

DELPOZO E, 1979, OBSTET GYNECOL, V53, P282

Dendrinos S, 2000, GYNECOL ENDOCRINOL, V14, P270, DOI 10.3109/09513590009167693

DIAMOND MP, 1989, J REPROD FERTIL, V86, P1

DICKER D, 1988, AM J OBSTET GYNECOL, V158, P1161, DOI 10.1016/0002-9378(88)90245-1

DISTILLER LA, 1975, DIABETES, V24, P378, DOI 10.2337/diabetes.24.4.378

DIXIT PK, 1962, ENDOCRINOLOGY, V71, P745, DOI 10.1210/endo-71-5-745

DJURSING H, 1983, J CLIN ENDOCR METAB, V56, P1016, DOI 10.1210/jcem-56-5-1016

DRUCKER D, 1984, AM J OBSTET GYNECOL, V149, P229, DOI 10.1016/0002-9378(84)90206-0

EDEN JA, 1989, AUST NZ J OBSTET GYN, V29, P268, DOI 10.1111/j.1479-828X.1989.tb01738.x

EDWARDS CRW, 1971, BRIT MED J, V3, P462, DOI 10.1136/bmj.3.5772.462

ELIAS KA, 1987, ENDOCRINOLOGY, V120, P617, DOI 10.1210/endo-120-2-617

EZZAT S, 1992, ENDOCRIN METAB CLIN, V21, P753, DOI 10.1016/S0889-8529(18)30212-3

FEDELE L, 1980, CONTRACEPT FERTIL S, V5, P501

FELDMAN S, 1992, J CLIN ENDOCR METAB, V74, P635, DOI 10.1210/jc.74.3.635

FISCH RO, 1974, OBSTET GYNECOL, V43, P861

FITZGERALD PA, 1982, J CLIN ENDOCR METAB, V54, P413, DOI 10.1210/jcem-54-2-413

FROHMAN LA, 1991, J CLIN ENDOCR METAB, V72, P1175, DOI 10.1210/jcem-72-6-1175

FUHRMANN K, 1984, EXP CLIN ENDOCRINOL, V83, P173, DOI 10.1055/s-0029-1210327

FUHRMANN K, 1983, DIABETES CARE, V6, P219, DOI 10.2337/diacare.6.3.219

Ghazeeri GS, 2001, CURR OPIN OBSTET GYN, V13, P287, DOI 10.1097/00001703-200106000-00007

GLASS RH, 1978, FERTIL STERIL, V29, P257

GLINOER D, 1994, J CLIN ENDOCR METAB, V79, P197, DOI 10.1210/jc.79.1.197

Glinoer D, 1999, FERTIL STERIL, V72, P373

GOLDSMITH RE, 1952, J CLIN ENDOCR METAB, V12, P846, DOI 10.1210/jcem-12-7-846

GOODNER CJ, 1961, J CLIN INVEST, V40, P261, DOI 10.1172/JCI104252

GORMLEY MJJ, 1982, CLIN ENDOCRINOL, V16, P283, DOI 10.1111/j.1365-2265.1982.tb00718.x

GREENMAN GW, 1962, NEW ENGL J MED, V267, P426, DOI 10.1056/NEJM196208302670902

GREER MA, 1987, INTERNAL MED, P1918

GRIMES EM, 1973, OBSTET GYNECOL, V42, P550, DOI 10.1097/00006250-197310000-00012

Haddow JE, 1999, NEW ENGL J MED, V341, P549, DOI 10.1056/NEJM199908193410801

HANSON TH, 1974, JAMA-J AM MED ASSOC, V280, P963

HARGER JH, 1983, OBSTET GYNECOL, V62, P574

HELLEDAY J, 1993, J CLIN ENDOCR METAB, V76, P933, DOI 10.1210/jc.76.4.933

Herman-Bonert V, 1998, J CLIN ENDOCR METAB, V83, P727, DOI 10.1210/jc.83.3.727

Hierl T, 2000, CLIN ENDOCRINOL, V53, P262, DOI 10.1046/j.1365-2265.2000.01058.x

HOFFENBERG R, 1987, OXFORD TXB MED, P1030

Holdaway I M, 1999, Pituitary, V2, P29, DOI 10.1023/A:1009965803750

HOLMESWALKER DJ, 1995, CLIN ENDOCRINOL, V43, P291

HONBO KS, 1978, AM J MED, V64, P782, DOI 10.1016/0002-9343(78)90517-X

HUGHES IA, 1982, HORM RES, V16, P100, DOI 10.1159/000179489

HURLEY LS, 1976, J NUTR, V106, P1254

Irvine WJ, 1972, CLINICS ENDOCRINOLOG, V1, P549

JADRESIC A, 1982, Q J MED, V51, P189

JENSEN CC, 1954, ACTA ENDOCRINOL-COP, V15, P351, DOI 10.1530/acta.0.0150351

Joshi J V, 1993, J Postgrad Med, V39, P137

JOVANOVIC L, 1981, AM J MED, V71, P921, DOI 10.1016/0002-9343(81)90301-6

JURNEY TH, 1981, CLIN ENDOCRINOL, V14, P539, DOI 10.1111/j.1365-2265.1981.tb02963.x

Kaider AS, 1999, AM J REPROD IMMUNOL, V42, P335

KALTER H, 1987, AM J OBSTET GYNECOL, V156, P1243, DOI 10.1016/0002-9378(87)90156-6

KITZMILLER JL, 1978, AM J OBSTET GYNECOL, V131, P560, DOI 10.1016/0002-9378(78)90120-5

KJAER K, 1992, J CLIN ENDOCR METAB, V75, P524, DOI 10.1210/jc.75.2.524

KLEINBERG DL, 1977, NEW ENGL J MED, V296, P589, DOI 10.1056/NEJM197703172961103

KLINGENSMITH GJ, 1977, J PEDIATR-US, V90, P996, DOI 10.1016/S0022-3476(77)80581-7

KOERTEN JM, 1986, AM J OBSTET GYNECOL, V154, P626, DOI 10.1016/0002-9378(86)90616-2

KUHNLE U, 1995, EUR J PEDIATR, V154, P708, DOI 10.1007/BF02276713

Kutteh WH, 1999, HUM REPROD, V14, P2886, DOI 10.1093/humrep/14.11.2886

Kutteh WH, 1999, FERTIL STERIL, V71, P843, DOI 10.1016/S0015-0282(99)00091-6

la Marca A, 1999, HUM REPROD, V14, P298, DOI 10.1093/humrep/14.2.298

LAMBERTS SWJ, 1982, J CLIN ENDOCR METAB, V54, P286, DOI 10.1210/jcem-54-2-286

LANDGREN BM, 1990, INT J GYNECOL OBSTET, V32, P95, DOI 10.1016/0020-7292(90)90473-X

LANDOLT AM, 1989, NEW ENGL J MED, V320, P671

LEJEUNE B, 1993, BRIT J OBSTET GYNAEC, V100, P669, DOI 10.1111/j.1471-0528.1993.tb14236.x

Lincoln SR, 1999, J REPROD MED, V44, P455

LONGCOPE C, 1991, WERNER INGBARS THYRO, P828

LONGCOPE C, 1991, WERNER INGBARS THYRO, P1052

LOUVET JP, 1979, LANCET, V1, P1032

MACGIBBON AL, 1995, AUST NZ J OBSTET GYN, V35, P217, DOI 10.1111/j.1479-828X.1995.tb01878.x

MAGARO M, 1992, CLIN EXP RHEUMATOL, V10, P67

MAN EB, 1971, AM J OBSTET GYNECOL, V111, P905, DOI 10.1016/0002-9378(71)90945-8

MAN EB, 1975, PERINATAL THYROID PH, P117

Matalon ST, 2001, AM J REPROD IMMUNOL, V45, P72, DOI 10.1111/j.8755-8920.2001.450202.x

MCEVOY GK, 1987, DRUG INFORMATION 87, P1874

Melmed Shlomo, 1995, P413

Meyer-Bahlburg HFL, 1999, J CLIN ENDOCR METAB, V84, P1844, DOI 10.1210/jc.84.6.1844

MILLS JL, 1979, DIABETES, V28, P292, DOI 10.2337/diabetes.28.4.292

MILLS JL, 1988, NEW ENGL J MED, V318, P671, DOI 10.1056/NEJM198803173181104

MIMOUNI F, 1987, OBSTET GYNECOL, V70, P85

MIODOVNIK M, 1984, AM J OBSTET GYNECOL, V150, P372, DOI 10.1016/S0002-9378(84)80141-6

MIODOVNIK M, 1985, AM J OBSTET GYNECOL, V153, P439, DOI 10.1016/0002-9378(85)90083-3

MOLEY KH, 1991, J REPROD FERTIL, V93, P325

MOLEY KH, 1994, HUM REPROD, V9, P113, DOI 10.1093/oxfordjournals.humrep.a138298

MOLITCH ME, 1992, ENDOCRIN METAB CLIN, V21, P597, DOI 10.1016/S0889-8529(18)30204-4

MONTINI M, 1990, J ENDOCRINOL INVEST, V13, P193, DOI 10.1007/BF03349537

MONTORO M, 1981, ANN INTERN MED, V94, P31, DOI 10.7326/0003-4819-94-1-31

Mozas J, 1999, INT J GYNECOL OBSTET, V65, P71, DOI 10.1016/S0020-7292(98)00221-5

MULAIKAL RM, 1987, NEW ENGL J MED, V316, P178, DOI 10.1056/NEJM198701223160402

NADER S, 1999, MATERNAL FETAL MED, P1015

NAGAMANI M, 1980, OBSTET GYNECOL, V56, P258

NASSER M, 2002, MAYO CLIN P, V77, P297

OHARE JA, 1987, AM J MED, V83, P1080, DOI 10.1016/0002-9343(87)90945-4

PARRA A, 1966, AM J MED, V40, P961, DOI 10.1016/0002-9343(66)90208-7

PEDERSEN JF, 1981, BRIT MED J, V283, P269, DOI 10.1136/bmj.283.6286.269

PLOCKINGER U, 1990, J CLIN ENDOCR METAB, V71, P1658

PLOTZ CM, 1952, AM J MED, V13, P597, DOI 10.1016/0002-9343(52)90027-2

PONTIKIDES N, 1990, J ENDOCRINOL INVE S2, V13, P164

PRATT D, 1993, AM J OBSTET GYNECOL, V168, P837, DOI 10.1016/S0002-9378(12)90830-3

PRATT DE, 1993, FERTIL STERIL, V60, P1001

REIS RA, 1958, AM J OBSTET GYNECOL, V76, P1148, DOI 10.1016/0002-9378(58)90194-7

RODRIGUEZ S, 1981, J ENDOCRINOL INVEST, V4, P37, DOI 10.1007/BF03349411

ROSENFIELD RL, 1980, OBSTET GYNECOL, V56, P208

RZEPKA I, 1977, GINEKOL POL, V46, P157

SAKAKURA M, 1975, J CLIN ENDOCR METAB, V40, P774, DOI 10.1210/jcem-40-5-774

SEMPLE CG, 1985, BRIT J OBSTET GYNAEC, V92, P295

SHUM L, 1991, DIABETOLOGIA, V34, P289, DOI 10.1007/BF00404998

SONINO N, 1987, NEW ENGL J MED, V317, P812

SOUTH SA, 1993, J CLIN ENDOCR METAB, V76, P1048, DOI 10.1210/jc.76.4.1048

SOUTHREN AL, 1974, J CLIN ENDOCR METAB, V38, P207, DOI 10.1210/jcem-38-2-207

STAGNAROGREEN A, 1990, JAMA-J AM MED ASSOC, V264, P1422, DOI 10.1001/jama.264.11.1422

Stewart-Akers AM, 1998, AM J REPROD IMMUNOL, V39, P1

STRATFORD GA, 2000, HUM FERTIL, V3, P203

STRICKLAND DM, 1990, AM J OBSTET GYNECOL, V163, P262, DOI 10.1016/S0002-9378(11)90744-3

TACHMAN ML, 1984, ENDOCR REV, V5, P456, DOI 10.1210/edrv-5-3-456

TAFT P, 1970, AUSTRALAS ANN MED, V19, P295

Takeuchi K, 1999, OBSTET GYNECOL, V93, P848, DOI 10.1016/S0029-7844(98)00461-X

THO PT, 1979, FERTIL STERIL, V32, P389

THOMAS R, 1987, OBSTET GYNECOL, V70, P789

Tolino A, 1991, Acta Eur Fertil, V22, P275

TOYOSHIMA K, 1991, INT J FERTIL, V36, P89

TUIMALA R, 1985, ANN CHIR GYNAECOL FE, V74, P61

WHITE P, 1932, DIABETES CHILDHOOD A, P110

WILANSKY DL, 1989, AM J OBSTET GYNECOL, V160, P673, DOI 10.1016/S0002-9378(89)80056-0

WINIKOFF D, 1975, BRIT J OBSTET GYNAEC, V82, P760, DOI 10.1111/j.1471-0528.1975.tb00719.x

Winters SJ, 1997, ENDOCRINOLOGIST, V7, P167, DOI 10.1097/00019616-199707030-00006

YAMADA T, 1976, J CLIN ENDOCR METAB, V42, P87

YAP AS, 1990, AM J OBSTET GYNECOL, V163, P477, DOI 10.1016/0002-9378(90)91178-F

YESHAYA A, 1995, INT J FERTIL MENOP S, V40, P269

ZAKARIJA M, 1983, J CLIN ENDOCR METAB, V57, P1036, DOI 10.1210/jcem-57-5-1036

ZELISSEN PMJ, 1995, J AUTOIMMUN, V8, P121, DOI 10.1006/jaut.1995.0009

ZUSMAN I, 1987, TERATOLOGY, V35, P77, DOI 10.1002/tera.1420350111

NR 164

TC 12

Z9 13

U1 1

U2 2

PU W B SAUNDERS CO-ELSEVIER INC

PI PHILADELPHIA

PA 1600 JOHN F KENNEDY BOULEVARD, STE 1800, PHILADELPHIA, PA 19103-2899 USA

SN 0889-8529

EI 1558-4410

J9 ENDOCRIN METAB CLIN

JI Endocrinol. Metabol. Clin. North Amer.

PD SEP

PY 2003

VL 32

IS 3

BP 573

EP +

DI 10.1016/S0889-8529(03)00041-0

PG 21

WC Endocrinology & Metabolism

SC Endocrinology & Metabolism

GA 727DL

UT WOS:000185641300003

PM 14575026

DA 2019-01-08

ER

PT J

AU Goldacre, MJ

Duncan, ME

Cook-Mozaffari, P

Griffith, M

AF Goldacre, MJ

Duncan, ME

Cook-Mozaffari, P

Griffith, M

TI Trends in mortality rates comparing underlying-cause and multiple-cause

coding in an English population 1979-1998

SO JOURNAL OF PUBLIC HEALTH MEDICINE

LA English

DT Article

DE death certificates; multiple-cause coding; mortality

ID DISEASE

AB Until recently, national coding and analysis of routine mortality statistics in most countries included only underlying cause of death. There were changes in the rules for selection and coding of underlying cause in England in 1984 and 1993. We report on trends in mortality rates in an English region from 1979 to 1998, comparing multiple-cause and underlying-cause coded rates, for individual diseases that were affected by coding changes. Among many others, these include pneumonia, venous thromboembolism, heart failure, respiratory distress syndrome, tuberculosis, diabetes, dementia, alcohol and drug abuse, epilepsy, multiple sclerosis, stroke, asthma, peptic ulcer, appendicitis, and cancers of the breast, colon and prostate. Comparisons over time of mortality rates based on underlying cause alone will be misleading when the time-period crosses years in which rules changed for selecting underlying cause.

C1 Univ Oxford, Dept Publ Hlth, Inst Hlth Sci, Unit Hlth Care Epidemiol, Oxford OX3 7LF, England.

RP Goldacre, MJ (reprint author), Univ Oxford, Dept Publ Hlth, Inst Hlth Sci, Unit Hlth Care Epidemiol, Oxford OX3 7LF, England.

EM michael.goldacre@dphpc.ox.ac.uk

RI Duncan, Marie/A-4081-2012

CR GOLDACRE MJ, 1993, J EPIDEMIOL COMMUN H, V47, P491, DOI 10.1136/jech.47.6.491

GURALNICK L, 1966, J CHRON DIS, V19, P979, DOI 10.1016/0021-9681(66)90031-2

HARVEY I, 1991, LANCET, V338, P1407, DOI 10.1016/0140-6736(91)92291-9

HEASMAN M, 1966, STUDIES MED POPULATI, V20

*OFF NAT STAT, 1996, MORT STAT CAUS 1993, pR25

*OFF POP CENS SURV, 1985, SER OFF POP CENS SUR, pR5

Rooney C, 1996, Popul Trends, P29

World Health Organisation, 1977, MAN INT STAT CLASS D, V1, P699

NR 8

TC 50

Z9 52

U1 0

U2 1

PU OXFORD UNIV PRESS

PI OXFORD

PA GREAT CLARENDON ST, OXFORD OX2 6DP, ENGLAND

SN 0957-4832

J9 J PUBLIC HEALTH MED

JI J. Public Health Med.

PD SEP

PY 2003

VL 25

IS 3

BP 249

EP 253

DI 10.1093/pubmed/fdg058

PG 5

WC Public, Environmental & Occupational Health

SC Public, Environmental & Occupational Health

GA 723JX

UT WOS:000185430200013

PM 14575203

OA Bronze

DA 2019-01-08

ER

PT J

AU Eaton, SEM

Harris, ND

Ibrahim, S

Patel, KA

Selmi, F

Radatz, M

Ward, JD

Tesfaye, S

AF Eaton, SEM

Harris, ND

Ibrahim, S

Patel, KA

Selmi, F

Radatz, M

Ward, JD

Tesfaye, S

TI Increased sural nerve epineurial blood flow in human subjects with

painful diabetic neuropathy

SO DIABETOLOGIA

LA English

DT Article

DE diabetes mellitus; diabetic neuropathy; painful; pain; hypoxia;

epineurium; fluorescein angiography; spectrophotometry

ID CAPILLARY ABNORMALITIES; POLYNEUROPATHY; PATHOGENESIS; SEVERITY;

PATTERNS; DAMAGE

AB Aims/hypothesis. The pathogenesis of painful diabetic neuropathy remains unknown. As a consequence we still do not have any effective, rational treatments and a greater understanding of the mechanisms is urgently required. Previous studies have shown no consistent morphological differences in the nerves of patients with and without painful neuropathy. The aim of this study was to compare epineurial haemodynamics in patients with chronic painful and painless neuropathy.

Methods. The techniques of microlightguide spectrophotometry and fluorescein angiography were used to measure epineurial intravascular oxygen saturation and blood flow respectively. Eleven patients with painful and eight with painless neuropathy were studied, with the groups matched carefully in terms of severity of neuropathy and diabetes control.

Results. Intravascular oxygen saturation was higher in the painful neuropathy group compared to those without pain (median 73.8% vs 67.7%, respectively; p=0.021). Fluorescein rise time was also faster in those with painful symptoms (median 18.3 s vs 53.6 s; p=0.046) indicating higher epineurial blood flow in these subjects.

Conclusion/interpretation. These results indicate that there are distinct differences in haemodynamics within the epineurium of the sural nerve in subjects with painful and painless neuropathy. Haemodynamic factors could therefore have an important role in the pathogenesis of neuropathic pain and might offer further insight into potential treatments for this distressing condition.

C1 Royal Hallamshire Hosp, Diabet Res Unit, Sheffield S10 2JF, S Yorkshire, England.

Royal Hallamshire Hosp, Dept Phys Med, Sheffield S10 2JF, S Yorkshire, England.

Royal Hallamshire Hosp, Dept Surg & Anaesthet Sci, Sheffield S10 2JF, S Yorkshire, England.

Royal Hallamshire Hosp, Dept Neurosurg, Sheffield S10 2JF, S Yorkshire, England.

RP Tesfaye, S (reprint author), Royal Hallamshire Hosp, Diabet Res Unit, Glossop Rd, Sheffield S10 2JF, S Yorkshire, England.

OI Tesfaye, Solomon/0000-0003-1190-1472

CR ARCHER AG, 1984, DIABETOLOGIA, V27, P563, DOI 10.1007/BF00276968

ASBURY AK, 1984, NEUROLOGY, V34, P1587, DOI 10.1212/WNL.34.12.1587

BRITLAND ST, 1990, DIABETES, V39, P909, DOI 10.2337/diabetes.39.8.909

BRITLAND ST, 1990, DIABETES, V39, P898, DOI 10.2337/diabetes.39.8.898

BROWN MJ, 1976, ARCH NEUROL-CHICAGO, V33, P164, DOI 10.1001/archneur.1976.00500030020004

Cameron NE, 2001, DIABETOLOGIA, V44, P1973, DOI 10.1007/s001250100001

DYCK PJ, 1988, MUSCLE NERVE, V11, P21, DOI 10.1002/mus.880110106

Eaton S, 1999, DIABETES REV, V7, P312

FRANK KH, 1989, PHYS MED BIOL, V34, P1883, DOI 10.1088/0031-9155/34/12/011

Ibrahim S, 1999, PHYSIOL MEAS, V20, P65, DOI 10.1088/0967-3334/20/1/005

Ibrahim S, 1999, DIABETOLOGIA, V42, P737, DOI 10.1007/s001250051222

LLEWELYN JG, 1991, BRAIN, V114, P867, DOI 10.1093/brain/114.2.867

LOW PA, 1987, MUSCLE NERVE, V10, P121, DOI 10.1002/mus.880100204

MALIK RA, 1989, DIABETOLOGIA, V32, P92, DOI 10.1007/BF00505180

MALIK RA, 1993, DIABETOLOGIA, V36, P454, DOI 10.1007/BF00402283

Malik RA, 1997, DIABETES, V46, pS50, DOI 10.2337/diab.46.2.S50

NEWRICK PG, 1986, BRIT MED J, V293, P1053, DOI 10.1136/bmj.293.6554.1053

OBRIEN IAD, 1986, BRIT HEART J, V55, P348

PERBECK L, 1985, CLIN PHYSIOL, V5, P281

Tesfaye S, 1996, LANCET, V348, P1698, DOI 10.1016/S0140-6736(96)02467-1

Tesfaye S, 1996, DIABETOLOGIA, V39, P329

TESFAYE S, 1997, DIABETIC NEUROPATHY, P159

TSIGOS C, 1993, DIABETES CARE, V16, P722, DOI 10.2337/diacare.16.5.722

WATKINS PJ, 1984, BRIT MED J, V288, P168, DOI 10.1136/bmj.288.6412.168

WATKINS PJ, 1983, DIABETOLOGIA, V25, P73

Woolf CJ, 1999, LANCET, V353, P1959, DOI 10.1016/S0140-6736(99)01307-0

YOUNG RJ, 1986, DIABETES, V35, P192, DOI 10.2337/diabetes.35.2.192

YOUNG RJ, 1997, TXB DIABETES

NR 28

TC 42

Z9 42

U1 0

U2 1

PU SPRINGER-VERLAG

PI NEW YORK

PA 175 FIFTH AVE, NEW YORK, NY 10010 USA

SN 0012-186X

J9 DIABETOLOGIA

JI Diabetologia

PD JUL

PY 2003

VL 46

IS 7

BP 934

EP 939

DI 10.1007/s00125-003-1127-3

PG 6

WC Endocrinology & Metabolism

SC Endocrinology & Metabolism

GA 709VB

UT WOS:000184644300008

PM 12819899

OA Bronze

DA 2019-01-08

ER

PT J

AU Sussman, N

AF Sussman, N

TI The implications of weight changes with antipsychotic treatment

SO JOURNAL OF CLINICAL PSYCHOPHARMACOLOGY

LA English

DT Article; Proceedings Paper

CT International Meeting on Improvement Without Impairment in Psychotic and

Mood Disorders

CY JUN 20-21, 2001

CL MONTREUX, SWITZERLAND

ID DEPENDENT DIABETES-MELLITUS; CORONARY HEART-DISEASE; LIPID

ABNORMALITIES; BIPOLAR DISORDER; GLYCEMIC CONTROL; OBESE PATIENTS;

BODY-WEIGHT; GAIN; SCHIZOPHRENIA; LEPTIN

AB Patients receiving treatment with atypical antipsychotics commonly experience weight gain, which can cause considerable distress and can have deleterious effects on cardiovascular health. Because of the associated weight gain and potential direct effects on glucose metabolism, atypical antipsychotics have also been linked to the development of type II diabetes mellitus. Data on long-term treatment with these agents show that clozapine and olanzapine, followed by risperidone, were associated with the greatest degree of weight gain. A large body of data suggests that during long-term treatment, patients receiving the atypical antipsychotic quetiapine experience minimal weight gain. Data also suggest that quetiapine treatment does not increase the risk of developing type II diabetes. The use of atypical antipsychotics is increasing, as these agents are being prescribed for schizophrenia in lieu of conventional antipsychotics. Furthermore, these drugs have efficacy for treating other conditions such as bipolar disorder. Physicians prescribing atypical antipsychotics must be aware of the risk of weight gain and its associated comorbidities.

C1 NYU, Sch Med, Dept Psychiat, New York, NY 10016 USA.

RP Sussman, N (reprint author), NYU, Sch Med, Dept Psychiat, 550 1St Ave, New York, NY 10016 USA.

CR Allison DB, 1999, AM J PSYCHIAT, V156, P1686

Baile CA, 2000, ANNU REV NUTR, V20, P105, DOI 10.1146/annurev.nutr.20.1.105

Bickerdike MJ, 1999, DIABETES OBES METAB, V1, P207, DOI 10.1046/j.1463-1326.1999.00037.x

Brecher M, 2000, INT J PSYCHIAT CLIN, V4, P287, DOI 10.1080/13651500050517849

Breum L, 1995, METABOLISM, V44, P1570, DOI 10.1016/0026-0495(95)90077-2

Bustillo JR, 1996, AM J PSYCHIAT, V153, P817

Bymaster FP, 1996, NEUROPSYCHOPHARMACOL, V14, P87, DOI 10.1016/0893-133X(94)00129-N

Carnahan RM, 2001, PHARMACOTHERAPY, V21, P717, DOI 10.1592/phco.21.7.717.34575

Casey DE, 2001, INT CLIN PSYCHOPHARM, V16, pS15, DOI 10.1097/00004850-200101001-00004

Cassidy F, 1999, AM J PSYCHIAT, V156, P1417

CHAN JM, 1994, DIABETES CARE, V17, P961, DOI 10.2337/diacare.17.9.961

COLDITZ GA, 1995, ANN INTERN MED, V122, P481, DOI 10.7326/0003-4819-122-7-199504010-00001

Conley RR, 2000, J CLIN PSYCHIAT, V61, P20

Daubresse JC, 1996, OBES RES, V4, P391, DOI 10.1002/j.1550-8528.1996.tb00247.x

Dixon L, 2000, SCHIZOPHRENIA BULL, V26, P903, DOI 10.1093/oxfordjournals.schbul.a033504

Fakhoury WKH, 2001, INT CLIN PSYCHOPHARM, V16, P153, DOI 10.1097/00004850-200105000-00004

Fontaine KR, 2001, PSYCHIAT RES, V101, P277, DOI 10.1016/S0165-1781(01)00234-7

Ford ES, 1997, AM J EPIDEMIOL, V146, P214

Green AI, 2000, GEN HOSP PSYCHIAT, V22, P224, DOI 10.1016/S0163-8343(00)00081-5

Guille C, 2000, J CLIN PSYCHIAT, V61, P638, DOI 10.4088/JCP.v61n0907

Hajduch E, 1999, J BIOL CHEM, V274, P13563, DOI 10.1074/jbc.274.19.13563

HARRIS MI, 1995, NIH PUBLICATION, P1

Henderson DC, 2000, AM J PSYCHIAT, V157, P975, DOI 10.1176/appi.ajp.157.6.975

Henderson DC, 2002, CNS DRUGS, V16, P77, DOI 10.2165/00023210-200216020-00001

Houseknecht KL, 1998, J ANIM SCI, V76, P1405

HUTTUNEN M, 1995, J CLIN PSYCHOPHARM, V15, pS4, DOI 10.1097/00004714-199502001-00002

Jeanrenaud B, 2001, ANNU REV MED, V52, P339, DOI 10.1146/annurev.med.52.1.339

Kasper S, 2000, Expert Opin Pharmacother, V1, P783, DOI 10.1517/14656566.1.4.783

KENNY SJ, 1995, NIH PUBLICATION, P47

Kinon BJ, 2001, J CLIN PSYCHIAT, V62, P92, DOI 10.4088/JCP.v62n0204

Kinon BJ, 1998, J CLIN PSYCHIAT, V59, P18

Kraus T, 1999, AM J PSYCHIAT, V156, P312

Kurzthaler I, 2001, J CLIN PSYCHIAT, V62, P32

Maheux P, 1997, INT J OBESITY, V21, P97, DOI 10.1038/sj.ijo.0800372

Martin BC, 2001, SCHIZOPHR RES, V47, P281, DOI 10.1016/S0920-9964(00)00108-0

McIntyre RS, 2001, CAN J PSYCHIAT, V46, P273, DOI 10.1177/070674370104600308

Melkersson KI, 2000, J CLIN PSYCHIAT, V61, P742, DOI 10.4088/JCP.v61n1006

Melkersson KI, 1999, J CLIN PSYCHIAT, V60, P783, DOI 10.4088/JCP.v60n1112

Nemeroff CB, 2000, J CLIN PSYCHIAT, V61, P19

Newcomer JW, 2002, ARCH GEN PSYCHIAT, V59, P337, DOI 10.1001/archpsyc.59.4.337

*NHLBI OB ED IN EX, 1998, NIH PUBL

Nonogaki K, 1998, NAT MED, V4, P1152, DOI 10.1038/2647

*PFIZ INC, 2001, GEOD PRESCR INF

*PFIZ INC, 2000, BRIEF DOC ZELDOX CAP

Reinstein MJ, 1999, CLIN DRUG INVEST, V18, P99, DOI 10.2165/00044011-199918020-00002

RICHARD FJ, 1999, BIOL REPROD, V60, P10

Rosengren A, 1999, EUR HEART J, V20, P269, DOI 10.1053/euhj.1998.1235

Sernyak MJ, 2002, AM J PSYCHIAT, V159, P561, DOI 10.1176/appi.ajp.159.4.561

Sowell MO, 2002, J CLIN ENDOCR METAB, V87, P2918, DOI 10.1210/jc.87.6.2918

Sussman N, 2001, J CLIN PSYCHIAT, V62, P5

UMBRICHT DSG, 1994, J CLIN PSYCHIAT, V55, P157

Weigle DS, 1997, ANN ENDOCRINOL-PARIS, V58, P132

WILLETT WC, 1995, JAMA-J AM MED ASSOC, V273, P461, DOI 10.1001/jama.273.6.461

ZOLER ML, 1999, CLIN PSYCHIAT NEWS, V27, P20

NR 54

TC 54

Z9 57

U1 0

U2 2

PU LIPPINCOTT WILLIAMS & WILKINS

PI PHILADELPHIA

PA 530 WALNUT ST, PHILADELPHIA, PA 19106-3621 USA

SN 0271-0749

J9 J CLIN PSYCHOPHARM

JI J. Clin. Psychopharmacol.

PD JUN

PY 2003

VL 23

IS 3

SU 1

BP S21

EP S26

DI 10.1097/00004714-200306001-00005

PG 6

WC Pharmacology & Pharmacy; Psychiatry

SC Pharmacology & Pharmacy; Psychiatry

GA 699AH

UT WOS:000184033400005

PM 12832946

DA 2019-01-08

ER

PT J

AU Garcia-Patterson, A

Martin, E

Ubeda, J

Maria, MA

Adelantado, JM

Ginovart, G

de Leiva, A

Corcoy, R

AF Garcia-Patterson, A

Martin, E

Ubeda, J

Maria, MA

Adelantado, JM

Ginovart, G

de Leiva, A

Corcoy, R

TI Nurse-based management in patients with gestational diabetes

SO DIABETES CARE

LA English

DT Article

ID INTERNATIONAL WORKSHOP-CONFERENCE; PRIMARY-CARE; PRACTITIONERS;

MELLITUS; RECOMMENDATIONS

AB Objective-To compare the rate of insulin treatment and perinatal outcome in women with gestational diabetes mellitus (GDM) under endocrinologist-based versus diabetes nurse-based metabolic management.

Research Design and Methods-in a retrospective analysis, maternal characteristics, rate of insulin treatment, and perinatal outcome of patients with GDM delivering between 1 January 1995 and 30 June 1997 (n=244) receiving endocrinologist-based care were compared with those delivering between 1 July 1997 and 31 December 1999 (n=283) who received diabetes nurse-based care. The diabetes nurse's role was similar to that of an advanced practice nurse in the U.S. There were no changes in the metabolic goals and instruments or in obstetric and neonatal management. Quantitative data were compared with the Mann-Whitney U test and categorical data, with Fisher's exact test.

Results-Maternal characteristics (age, BMI, family history of diabetes, prior glucose intolerance, gestational age, and blood glucose at diagnosis of GDM) did not differ between groups treated during the two periods. Rates of insulin treatment and perinatal Outcome (hypertension, preterm delivery, cesarean section, low Apgar score, macrosomia, small- and large-for-gestational-age newborns, obstetric trauma, major malformations, hypoglycermia, hypocalcemia, polycythemia, jaundice, respiratory distress, and mortality) were also similar in both groups.

Conclusions-Comparison of periods of endocrinologist-based and diabetes nurse-based metabolic management of women with GDM showed no differences in the rate of insulin treatment and perinatal outcome. This supports a more active role of nurses in the management of women with GDM.

C1 Autonomous Univ Barcelona, Dept Endocrinol, Hosp Santa Creu & San Pau, Barcelona, Spain.

Autonomous Univ Barcelona, Dept Obstet, Hosp Santa Creu & San Pau, Barcelona, Spain.

Autonomous Univ Barcelona, Dept Pediat, Hosp Santa Creu & San Pau, Barcelona, Spain.

RP Corcoy, R (reprint author), Hosp Santa Creu & Sant Pau, Endocrinol & Nutr Dept, Avgda St Antoni Ma Claret 167, Barcelona 08025, Spain.

CR *AM DIAB ASS, 2002, DIABETES CARE S1, V25, pS94

[Anonymous], 1990, AM J OBSTET GYNECOL, V163, P1689

BANKS P, 1993, CLIN ED SERIES

Barnes L P, 1994, MCN Am J Matern Child Nurs, V19, P175

BURNIP R, 1976, AM J DIS CHILD, V130, P51, DOI 10.1001/archpedi.1976.02120020053008

CARR S, 1989, OBSTET GYNECOL, V73, P727

Corcoy R, 1988, AV DIABETOL, V1, P90

Garcia-Patterson A, 1998, AM J OBSTET GYNECOL, V179, P481, DOI 10.1016/S0002-9378(98)70383-7

Horrocks S, 2002, BRIT MED J, V324, P819, DOI 10.1136/bmj.324.7341.819

KASSIRER JP, 1994, NEW ENGL J MED, V330, P204, DOI 10.1056/NEJM199401203300310

Keohane N S, 1991, J Obstet Gynecol Neonatal Nurs, V20, P189, DOI 10.1111/j.1552-6909.1991.tb02530.x

LANGER O, 1994, AM J OBSTET GYNECOL, V170, P1036, DOI 10.1016/S0002-9378(94)70097-4

Meltzer S, 1998, CAN MED ASSOC J, V159, pS1

MERAUD B, 1985, SOINS GYNECOL OBSTET, V54, P33

METZGER BE, 1991, DIABETES, V40, P197, DOI 10.2337/diab.40.2.S197

Metzger BE, 1998, DIABETES CARE, V21, pB161

Mundinger MO, 2000, JAMA-J AM MED ASSOC, V283, P59, DOI 10.1001/jama.283.1.59

Nagusky D, 1991, Nurse Pract Forum, V2, P196

RENDERS CM, 2002, COCHRANE DATABASE SY, V1, DOI UNSP CD001481

Robinson S, 1999, LANCET, V354, P1319, DOI 10.1016/S0140-6736(99)00342-6

Simmons D, 2001, PRAC DIABETES INT, V18, P119

SMITS MW, 1995, DIABETES EDUCATOR, V21, P129, DOI 10.1177/014572179502100209

Vrijhoef HJM, 2001, J ADV NURS, V36, P546, DOI 10.1046/j.1365-2648.2001.02007.x

WEINER CP, 1987, AM J OBSTET GYNECOL, V156, P1085, DOI 10.1016/0002-9378(87)90115-3

NR 24

TC 11

Z9 11

U1 1

U2 5

PU AMER DIABETES ASSOC

PI ALEXANDRIA

PA 1701 N BEAUREGARD ST, ALEXANDRIA, VA 22311-1717 USA

SN 0149-5992

J9 DIABETES CARE

JI Diabetes Care

PD APR

PY 2003

VL 26

IS 4

BP 998

EP 1001

DI 10.2337/diacare.26.4.998

PG 4

WC Endocrinology & Metabolism

SC Endocrinology & Metabolism

GA 724UP

UT WOS:000185505300005

PM 12663563

OA Bronze

DA 2019-01-08

ER

PT J

AU Melchionda, N

Marchesini, G

Apolone, G

Cuzzolaro, M

Mannucci, E

Grossi, E

AF Melchionda, N

Marchesini, G

Apolone, G

Cuzzolaro, M

Mannucci, E

Grossi, E

CA QUOVADIS Study Grp

TI The QUOVADIS Study: Features of obese Italian patients seeking treatment

at specialist centers

SO DIABETES NUTRITION & METABOLISM

LA English

DT Article

DE obesity; anthropometry; quality of life; behavior; therapy

ID QUALITY-OF-LIFE; BODY-FAT DISTRIBUTION; PERSONS SEEKING; CHRONIC

ILLNESS; HEALTH SURVEY; SUBJECTS SOS; WEIGHT-LOSS; MASS INDEX;

QUESTIONNAIRE; OVERWEIGHT

AB Obesity is a major risk factor for several chronic diseases, but the burden associated with it also extends to psychosocial areas and to perceived health status. In 1999 an observational study on health-related quality of life in obesity was planned. The study was entirely web-based. Case Report Forms and the individual items of 7 self-administered questionnaires were directly implemented on a general database via an extranet system from 25 Italian centers. By December 2001, after enrolment had stopped, the database included anthropometric, socioeconomic and clinical data of 1944 patients (78% females). Weight-cycling was reported in over 80% of cases, overeating in 60-65%, structured physical activity in only 13-15%. Several chronic illnesses were associated. Whereas the prevalence of diabetes and hypertension was related to the degree of obesity, hyperlipidemia and coronary heart disease did not increase further with increasing obesity. A disturbed psychological mood was twice more common in females. Concern for present health was the main reason for seeking treatment in both genders; concern for body appearance was more common in females. Male subjects were more frequently assigned to dietary counseling and physical exercise, whereas in fen ales psychotherapy was more frequently considered. Various forms of behavioral approach were planned in approximately 50% of patients. Finally, very few patients were initially considered for pharmacological intervention or bariatric surgery. The study provides a comprehensive picture of Italian patients seeking treatment for obesity. Data on perceived health status, psychological well being, body image awareness, eating behavior disorders and psychopathological distress will provide clues to a comprehensive assessment of obesity, the effects of treatments and reasons for failure. (C) 2003, Editrice Kurtis.

C1 Univ Bologna, I-40126 Bologna, Italy.

Mario Negri Inst Pharmacol Res, I-20157 Milan, Italy.

Univ Roma La Sapienza, Rome, Italy.

Univ Florence, Florence, Italy.

Bracco Imaging SPA, Milan, Italy.

RP Marchesini, G (reprint author), Univ Bologna, Serv Malattie Mebab, Azienda Osped S Orsola Malpighi, Via Massarenti 9, I-40138 Bologna, Italy.

EM marchreg@med.unibo.it

RI Apolone, Giovanni/R-9799-2016

OI Apolone, Giovanni/0000-0001-5179-104X; Molinari,

Enrico/0000-0001-8132-694X; VITACOLONNA, Ester/0000-0003-1756-661X;

Compare, Angelo/0000-0002-3336-7920; Marchesini,

Giulio/0000-0003-2407-9860

CR American Psychiatric Association, 2000, DIAGN STAT MAN MENT

Apolone G, 1998, J CLIN EPIDEMIOL, V51, P1025, DOI 10.1016/S0895-4356(98)00094-8

Apolone G, 2001, PHARMACOECONOMICS, V19, P187, DOI 10.2165/00019053-200119020-00005

Astrup A, 2001, Public Health Nutr, V4, P499

BERENSON GS, 2001, PREV CARDIOL, V4, P116

Brownell K. D., 1995, EATING DISORDERS OBE

Collazo-Clavell ML, 1999, MAYO CLIN PROC, V74, P1255, DOI 10.4065/74.12.1255

DEROGATIS LR, 1976, BRIT J PSYCHIAT, V128, P280, DOI 10.1192/bjp.128.3.280

DiPietro L, 1995, Exerc Sport Sci Rev, V23, P275

DIPIETRO L, 1992, INT J OBESITY, V16, P745

Doll HA, 2000, OBES RES, V8, P160, DOI 10.1038/oby.2000.17

Dupuy HJ., 1984, ASSESSMENT QUALITY L, P170

Finkelstein MM, 2000, J FAM PRACTICE, V49, P734

Fontaine KR, 1996, J FAM PRACTICE, V43, P265

Fontaine KR, 2000, INT J EAT DISORDER, V27, P101, DOI 10.1002/(SICI)1098-108X(200001)27:1<101::AID-EAT12>3.3.CO;2-4

Fontaine KR, 1997, J NERV MENT DIS, V185, P120, DOI 10.1097/00005053-199702000-00011

Fontaine KR, 1999, QUAL LIFE RES, V8, P275, DOI 10.1023/A:1008835602894

GORMALLY J, 1982, ADDICT BEHAV, V7, P47, DOI 10.1016/0306-4603(82)90024-7

Han TS, 1998, AM J PUBLIC HEALTH, V88, P1814, DOI 10.2105/AJPH.88.12.1814

James PT, 2001, OBES RES, V9, p228S, DOI 10.1038/oby.2001.123

KARLSSON J, 1995, J CLIN EPIDEMIOL, V48, P817, DOI 10.1016/0895-4356(94)00196-W

Katz DA, 2000, J GEN INTERN MED, V15, P789, DOI 10.1046/j.1525-1497.2000.90906.x

KLESGES RC, 1990, MED SCI SPORT EXER, V22, P690, DOI 10.1249/00005768-199010000-00022

Kopelman P, 1999, J ROY COLL PHYS LOND, V33, P31

Lean MEJ, 1998, EXP CLIN ENDOCR DIAB, V106, P22, DOI 10.1055/s-0029-1212032

Mannucci E, 1999, ADDICT BEHAV, V24, P345, DOI 10.1016/S0306-4603(98)00055-0

Marchesini G, 2000, DIABETES NUTR METAB, V13, P156

Oster G, 1999, AM J PUBLIC HEALTH, V89, P1536, DOI 10.2105/AJPH.89.10.1536

Prochaska JO, 1997, AM J HEALTH PROMOT, V12, P38, DOI 10.4278/0890-1171-12.1.38

PROCHASKA JO, 1994, HEALTH PSYCHOL, V13, P47, DOI 10.1037/0278-6133.13.1.47

RAND CSW, 1990, SOUTHERN MED J, V83, P1390, DOI 10.1097/00007611-199012000-00006

Ray GT, 2000, MED CARE RES REV, V57, P92, DOI 10.1177/107755870005700106

Rosmond R, 2000, BEHAV MED, V26, P90, DOI 10.1080/08964280009595757

SOBAL J, 1991, CLIN SOCIOL REV, V9, P125

SORENSEN TIA, 1982, HUM BIOL, V54, P765

STUNKARD AJ, 1986, AM J PSYCHIAT, V143, P417

STUNKARD AJ, 1985, J PSYCHOSOM RES, V29, P71, DOI 10.1016/0022-3999(85)90010-8

STUNKARD AJ, 1992, AM J CLIN NUTR, V55, P524

SULLIVAN M, 1993, INT J OBESITY, V17, P503

Thompson D, 1999, ARCH INTERN MED, V159, P2177, DOI 10.1001/archinte.159.18.2177

WARE JE, 1992, MED CARE, V30, P473, DOI 10.1097/00005650-199206000-00002

Whitelaw DC, 2001, INT J OBESITY, V25, P1042, DOI 10.1038/sj.ijo.0801596

Williams M, 1999, ENVIRON HIST, V4, P103, DOI 10.2307/3985331

WILSON GT, 1994, ADV BEHAV RES THER, V16, P31, DOI 10.1016/0146-6402(94)90002-7

WOOLEY SC, 1994, BMJ-BRIT MED J, V309, P655

NR 45

TC 28

Z9 28

U1 0

U2 3

PU EDITRICE KURTIS S R L

PI MILAN

PA VIA LUIGI ZOJA 30, 20153 MILAN, ITALY

SN 0394-3402

J9 DIABETES NUTR METAB

JI Diabetes Nutr. Metab.

PD APR

PY 2003

VL 16

IS 2

BP 115

EP 124

PG 10

WC Endocrinology & Metabolism; Nutrition & Dietetics

SC Endocrinology & Metabolism; Nutrition & Dietetics

GA 689KW

UT WOS:000183492700006

PM 12846451

DA 2019-01-08

ER

PT J

AU van der Ven, NCW

Weinger, K

Yi, J

Pouwer, F

Ader, H

van der Ploeg, HM

Snoek, FJ

AF van der Ven, NCW

Weinger, K

Yi, J

Pouwer, F

Ader, H

van der Ploeg, HM

Snoek, FJ

TI The confidence in diabetes self-care scale - Psychometric properties of

a new measure of diabetes-specific self-efficacy in Dutch and US

patients with type 1 diabetes

SO DIABETES CARE

LA English

DT Article

ID ADOLESCENTS; ADHERENCE; MELLITUS; BEHAVIORS; ADULTS; QUESTIONNAIRE;

DEPRESSION; VARIABLES; DISTRESS; VALIDITY

AB Objective-To examine psychometric properties of the Confidence in Diabetes Self-Care (CIDS) scale, a newly developed instrument assessing diabetes-specific self-efficacy in Dutch and U.S. patients with type 1 diabetes.

Research Design and Methods-Reliability and validity of the CIDS scale were evaluated in Dutch (n=151) and U.S. (n=190) outpatients with type 1 diabetes. In addition to the CIDS scale, assessment included HbA(1c) emotional distress, fear of hypoglycemia self-esteem, anxiety, depression, and self-care behavior. The Dutch sample completed additional measures on perceived burden and importance of self-care. Test-retest reliability was established in a second Dutch sample (n=62).

Results-internal consistency (Cronbach's alpha=0.86 for Dutch patients and 0.90 U.S. patients) and test-retest reliability (Spearman's r=0.85, P<0.0001) of the CIDS scale were high. Exploratory factor analysis showed one strong general factor. Spearman's correlations between the CIDS scale and other measures were moderate and in the expected directions, and high HbA(1c) levels were associated with low CIDS scores in the U.S. sample only. Low CIDS scores were positively associated with self-care but not with glycemic control in the original samples. CIDS scores in the U.S. and Dutch samples did not show any statistically significant differences. U.S. men had higher CIDS scores than U.S. women.

Conclusions-The CIDS scale is a reliable and valid measure of diabetes-specific self-efficacy for use in patients with type 1 diabetes. High psychometric similarity allows for cross-cultural comparisons.

C1 Vrije Univ Amsterdam, Med Ctr, Dept Psychol Med, NL-1081 BT Amsterdam, Netherlands.

Vrije Univ Amsterdam, Res Inst Endocrinol Reprod & Metab, Med Ctr, Amsterdam, Netherlands.

Joslin Diabet Ctr, Behav & Mental Hlth Res Sect, Boston, MA 02215 USA.

Harvard Univ, Sch Med, Boston, MA USA.

Vrije Univ Amsterdam, Inst Res Extramural Med, Med Ctr, Amsterdam, Netherlands.

RP van der Ven, NCW (reprint author), Vrije Univ Amsterdam, Med Ctr, Dept Psychol Med, Van Boechorststr 7, NL-1081 BT Amsterdam, Netherlands.

EM ncw.van_der_ven.psychol@med.vu.nl

FU NIDDK NIH HHS [DK-42315, R01 DK042315]

CR Aalto AM, 1997, PATIENT EDUC COUNS, V30, P215, DOI 10.1016/S0738-3991(96)00963-9

Aalto AM, 1997, INT J BEHAV MED, V4, P191, DOI 10.1207/s15327558ijbm0403_1

Anderson RM, 2000, DIABETES CARE, V23, P739, DOI 10.2337/diacare.23.6.739

BANDURA A, 1977, PSYCHOL REV, V84, P191, DOI 10.1037/0033-295X.84.2.191

Bandura A, 1988, COGNITIVE PERSPECTIV, P37, DOI DOI 10.1007/978-94-009-2792-6

Bandura A., 1997, SELF EFFICACY EXERCI

Blascovich J., 1993, MEASURES PERSONALITY, P115

CONNELL CM, 1994, HEALTH PSYCHOL, V13, P263, DOI 10.1037//0278-6133.13.3.263

DEROGATIS LR, 1983, SCL90R ADM SCORING P, V2

Fournier M, 2002, PERS INDIV DIFFER, V33, P1163, DOI 10.1016/S0191-8869(02)00006-5

GLASGOW RE, 1989, HEALTH PSYCHOL, V8, P285, DOI 10.1037/0278-6133.8.3.285

GRECO P, 1990, DIABETES S1, V40, pA657

Grey M, 1998, DIABETES CARE, V21, P902, DOI 10.2337/diacare.21.6.902

Griva K, 2000, PSYCHOL HEALTH, V15, P733, DOI 10.1080/08870440008405578

GROSSMAN HY, 1987, DIABETES CARE, V10, P324, DOI 10.2337/diacare.10.3.324

Hurley A C, 1992, Diabetes Educ, V18, P146, DOI 10.1177/014572179201800208

HURLEY AC, 1989, DIABETES HLTH BELIEF

*INT RES CTR HLTH, 1991, SCOR MOS 36 IT SHORT

IRVINE A, 2004, HDB PSYCHOL DIABETES, P133

KAVANAGH DJ, 1993, J BEHAV MED, V16, P509, DOI 10.1007/BF00844820

LITTLEFIELD CH, 1992, DIABETES CARE, V15, P90, DOI 10.2337/diacare.15.1.90

LUTEIJN F, 1975, MANUAL DUTCH PERSONA

Maddux J. E., 1995, SELF EFFICACY ADAPTA, P37, DOI DOI 10.1007/978-1-4419-6868-5_2

MAIBACH E, 1995, HEALTH EDUC RES, V10, P37, DOI 10.1093/her/10.1.37

MCCAUL KD, 1987, MED CARE, V25, P868, DOI 10.1097/00005650-198709000-00005

Mollem ED, 1996, PATIENT EDUC COUNS, V29, P277, DOI 10.1016/S0738-3991(96)00926-3

Ott J, 2000, CHILD HEALTH CARE, V29, P47, DOI 10.1207/S15326888CHC2901_4

PADGETT DK, 1991, PATIENT EDUC COUNS, V18, P139, DOI 10.1016/0738-3991(91)90006-Q

POLONSKY WH, 1995, DIABETES CARE, V18, P754, DOI 10.2337/diacare.18.6.754

Pouwer F, 1999, DIABETES CARE, V22, P2004, DOI 10.2337/diacare.22.12.2004

Rosenberg M., 1979, CONCEIVING SELF

Rubin R. R., 1993, DIABETES ED, V19, P210, DOI [DOI 10.1177/014572179301900308, 10.1177/014572179301900308]

Ruggiero L, 1997, DIABETES CARE, V20, P568, DOI 10.2337/diacare.20.4.568

Schlundt DG, 1999, BEHAV MODIF, V23, P79, DOI 10.1177/0145445599231004

Senecal C, 2000, HEALTH PSYCHOL, V19, P452, DOI 10.1037//0278-6133.19.5.452

SNAITH RP, 1994, HOSPITAL ANXIETY DEP

Snoek F, 1996, GEDRAG GEZONDHEID, V24, P287

Snoek FJ, 2001, PATIENT EDUC COUNS, V45, P143, DOI 10.1016/S0738-3991(01)00113-6

Snoek FJ, 2000, DIABETES CARE, V23, P1305, DOI 10.2337/diacare.23.9.1305

Spinhoven P, 1997, PSYCHOL MED, V27, P363, DOI 10.1017/S0033291796004382

*SPSS, 2000, SPSS BAS 9 0 WIND US

Talbot F, 1997, J BEHAV MED, V20, P291, DOI 10.1023/A:1025508928696

van der Bijl J, 1999, J ADV NURS, V30, P352, DOI 10.1046/j.1365-2648.1999.01077.x

Weinger K, 2000, DIABETES, V49, pA318

NR 44

TC 90

Z9 91

U1 1

U2 16

PU AMER DIABETES ASSOC

PI ALEXANDRIA

PA 1701 N BEAUREGARD ST, ALEXANDRIA, VA 22311-1717 USA

SN 0149-5992

EI 1935-5548

J9 DIABETES CARE

JI Diabetes Care

PD MAR

PY 2003

VL 26

IS 3

BP 713

EP 718

DI 10.2337/diacare.26.3.713

PG 6

WC Endocrinology & Metabolism

SC Endocrinology & Metabolism

GA 724UM

UT WOS:000185505100027

PM 12610027

OA Bronze, Green Accepted

DA 2019-01-08

ER

PT J

AU Antai-Otong, D

AF Antai-Otong, D

TI Anxiety disorders: treatment considerations

SO NURSING CLINICS OF NORTH AMERICA

LA English

DT Article

ID POSTTRAUMATIC-STRESS-DISORDER; NATIONAL-COMORBIDITY-SURVEY; DISEASE;

CYTOKINES; BURDEN

AB Anxiety disorders are among the most widespread, recurring or chronic, and treatable mental health disorders, with a lifetime prevalence of 25% [1]. These disorders are the primary reason that people seek medical and psychiatric treatment and are often a precursor to or comorbid with mood disorders [2]. Anxiety disorders cause a considerable burden of distress and disability similar to other medical conditions, such as diabetes [3,4]. Anxiety disorders have a predictable debilitating course of anxiety and reduced productivity and interference with functioning. These disorders rarely occur as a single disorder.

C1 VA N Texas Hlth Care Syst, Employee Support Program, Mental Hlth Outpatient Clin, Dallas, TX 75216 USA.

RP Antai-Otong, D (reprint author), VA N Texas Hlth Care Syst, Employee Support Program, Mental Hlth Outpatient Clin, 4500 S Lancaster Rd, Dallas, TX 75216 USA.

EM Deborah.antai-otong@med.va.gov

CR American Psychiatric Association, 2000, DIAGN STAT MAN MENT

Andrews G, 1998, BRIT J PSYCHIAT, V173, P123, DOI 10.1192/bjp.173.2.123

Antai-Otong D, 2000, Issues Ment Health Nurs, V21, P71, DOI 10.1080/016128400248275

BECK A. T., 1985, ANXIETY DISORDER PHO

Feldmann M, 1996, ANNU REV IMMUNOL, V14, P397, DOI 10.1146/annurev.immunol.14.1.397

Greenberg PE, 1999, J CLIN PSYCHIAT, V60, P427, DOI 10.4088/JCP.v60n0702

Grossman R, 2002, PSYCHIAT CLIN N AM, V25, P317, DOI 10.1016/S0193-953X(01)00011-9

Hettema JM, 2001, AM J PSYCHIAT, V158, P1568, DOI 10.1176/appi.ajp.158.10.1568

JUDD LL, 1998, ACTA PSYCHIAT SCAN S, V393, P6

Kendler KS, 2001, ARCH GEN PSYCHIAT, V58, P1005, DOI 10.1001/archpsyc.58.11.1005

KESSLER RC, 1995, ARCH GEN PSYCHIAT, V52, P1048, DOI 10.1001/archpsyc.1995.03950240066012

KESSLER RC, 1994, ARCH GEN PSYCHIAT, V51, P8

McClane SJ, 1999, JPEN-PARENTER ENTER, V23, pS20, DOI 10.1177/014860719902300506

Noyes Jr R, 1998, ANXIETY DISORDERS

Schweizer E, 1997, J CLIN PSYCHIAT, V58, P27

Uhlenhuth E H, 1999, Depress Anxiety, V9, P107, DOI 10.1002/(SICI)1520-6394(1999)9:3<107::AID-DA2>3.0.CO;2-T

Yehuda R, 2002, PSYCHIAT CLIN N AM, V25, P341, DOI 10.1016/S0193-953X(02)00002-3

Yudkin JS, 2000, ATHEROSCLEROSIS, V148, P209, DOI 10.1016/S0021-9150(99)00463-3

NR 18

TC 0

Z9 1

U1 0

U2 2

PU W B SAUNDERS CO

PI PHILADELPHIA

PA INDEPENDENCE SQUARE WEST CURTIS CENTER, STE 300, PHILADELPHIA, PA

19106-3399 USA

SN 0029-6465

J9 NURS CLIN N AM

JI Nurs. Clin. North Am.

PD MAR

PY 2003

VL 38

IS 1

BP 35

EP +

DI 10.1016/S0029-6465(02)00059-2

PG 11

WC Nursing

SC Nursing

GA 656RX

UT WOS:000181623200005

PM 12712667

DA 2019-01-08

ER

PT J

AU Taylor, MD

Frier, BM

Gold, AE

Deary, IJ

AF Taylor, MD

Frier, BM

Gold, AE

Deary, IJ

TI Psychosocial factors and diabetes-related outcomes following diagnosis

of Type 1 diabetes in adults: The Edinburgh Prospective Diabetes Study

SO DIABETIC MEDICINE

LA English

DT Article

DE Type 1 diabetes; glycaemic control; quality of life; coping; adults

ID QUALITY-OF-LIFE; YOUNG-ADULTS; GLYCEMIC CONTROL; PSYCHIATRIC-ILLNESS;

SELF-REGULATION; MELLITUS; ADJUSTMENT; EDUCATION; ADOLESCENTS;

PERSONALITY

AB Aims To examine prospectively the relationships between psychosocial variables and diabetes-related outcomes in adults with newly diagnosed Type 1 diabetes.

Methods A total of 84 adults (48 male) with a median (range) age of 30.8 (17-51) years with newly diagnosed Type 1 diabetes were recruited for the study. Shortly after initial diagnosis each participant's personality, cognitive ability, and recent psychiatric distress were assessed. At 4 months (n = 69) and at 12 months (n = 66) after diagnosis diabetes-related outcomes were measured, including each respondent's knowledge of diabetes, satisfaction with diabetes treatment and diabetes-related quality of life. Glycated haemoglobin (HbA(1c) ) was recorded at each clinic attendance.

Results Social class (Spearman's correlation r = -0.30 and -0.28, respectively, P < 0.05) and scores on the National Adult Reading Test (r = 0.38 and 0.36, respectively, P < 0.01) were consistently associated with knowledge of diabetes at 4 months and at 12 months after diagnosis. Hierarchical regression revealed that alcohol consumption recorded at diagnosis and knowledge of diabetes at 4 months were independent predictors of glycaemic control at 12 months (adjusted r (2) = 0.16). Total scores on the Diabetes Treatment Satisfaction Questionnaire (DTSQ) at 12 months were significantly predicted by age at diagnosis (adjusted r (2) = 0.08). High neuroticism at diagnosis was consistently associated with poorer self-reported diabetes quality of life at 4 months and at 12 months after diagnosis (r s between -0.30 and -0.39, P < 0.05).

Conclusions Long-standing psychosocial factors have a significant influence on self-reported outcomes during the 12 months following diagnosis of Type 1 diabetes but may not be reliable predictors of glycaemic control. Further follow-up is necessary to determine the longer-term predictors of objective (e.g. glycaemic control) and subjective (e.g. quality of life) indicators of coping in people with diabetes.

C1 Univ Edinburgh, Sch Philosophy Psychol & Language Sci, Dept Psychol, Edinburgh EH8 9JZ, Midlothian, Scotland.

Royal Infirm, Dept Diabet, Edinburgh EH3 9YW, Midlothian, Scotland.

RP Deary, IJ (reprint author), Univ Edinburgh, Sch Philosophy Psychol & Language Sci, Dept Psychol, 7 George Sq, Edinburgh EH8 9JZ, Midlothian, Scotland.

EM Ian.Deary@ed.ac.uk

RI Deary, Ian/C-6297-2009

OI Deary, Ian/0000-0002-1733-263X

CR Ackerman P. L., 2000, J GERONTOL B-PSYCHOL, V55, P69, DOI DOI 10.1093/GERONB/55.2.P69

Ackerman PL, 1999, PSYCHOL AGING, V14, P314, DOI 10.1037//0882-7974.14.2.314

BRADLEY C, 1978, STRESS

Bradley C., 1994, HDB PSYCHOL DIABETES

Bryden KS, 2001, DIABETES CARE, V24, P1536, DOI 10.2337/diacare.24.9.1536

CLEMENT S, 1995, DIABETES CARE, V18, P1204, DOI 10.2337/diacare.18.8.1204

Cohen ST, 1997, PSYCHOSOMATICS, V38, P98, DOI 10.1016/S0033-3182(97)71477-4

COSTA PT, 1987, J PERS, V55, P299, DOI 10.1111/j.1467-6494.1987.tb00438.x

CRAWFORD JR, 1989, PERS INDIV DIFFER, V10, P585, DOI 10.1016/0191-8869(89)90043-3

DCCT Res Grp, 1988, DIABETES CARE, V11, P725

Deary IJ, 1997, BRIT J HEALTH PSYCH, V2, P35, DOI 10.1111/j.2044-8287.1997.tb00522.x

Diabet Control Complications Trial Res Grp, 1996, DIABETES CARE, V19, P195

Eysenck HJ ES., 1975, MANUAL EYSENCK PERSO

FONAGY P, 1987, ARCH DIS CHILD, V62, P1009, DOI 10.1136/adc.62.10.1009

Glasgow RE, 1999, DIABETES CARE, V22, P832, DOI 10.2337/diacare.22.5.832

Glasgow RE, 1997, DIABETES CARE, V20, P562, DOI 10.2337/diacare.20.4.562

GLASGOW RE, 1994, HDB PSYCHOL DIABETES

GOLDBERG DP, 1979, PSYCHOL MED, V9, P139, DOI 10.1017/S0033291700021644

Goldthorpe J.H., 1987, SOCIAL MOBILITY CLAS

GORDON D, 1993, DIABETIC MED, V10, P530, DOI 10.1111/j.1464-5491.1993.tb00115.x

GRAJWER LA, 1977, J PEDIATR, V9, P42

GREY M, 1995, DIABETES CARE, V18, P1330, DOI 10.2337/diacare.18.10.1330

Hampson SE, 2000, BRIT J HEALTH PSYCH, V5, P27, DOI 10.1348/135910700168748

HANSON CL, 1987, DIABETES CARE, V10, P752, DOI 10.2337/diacare.10.6.752

HEPBURN DA, 1994, DIABETIC MED, V11, P578, DOI 10.1111/j.1464-5491.1994.tb02039.x

HORN JL, 1966, J EDUC PSYCHOL, V57, P253, DOI 10.1037/h0023816

*INT RES CTR HLTH, 1991, SCOR MOS 36 IT SHORT

Jacobson AM, 1997, QUAL LIFE RES, V6, P11

JACOBSON AM, 1994, DIABETES CARE, V17, P267, DOI 10.2337/diacare.17.4.267

Jacobson AM, 1996, NEW ENGL J MED, V334, P1249, DOI 10.1056/NEJM199605093341907

Jacobson AM, 1997, DIABETIC MED, V14, P73, DOI 10.1002/(SICI)1096-9136(199701)14:1<73::AID-DIA294>3.0.CO;2-Q

KOVACS M, 1986, DIABETES CARE, V9, P472, DOI 10.2337/diacare.9.5.472

KOVACS M, 1985, J PEDIATR-US, V106, P827, DOI 10.1016/S0022-3476(85)80368-1

LANE JD, 1988, DIABETES CARE, V11, P778

LLOYD CE, 1992, DIABETIC MED, V9, P661, DOI 10.1111/j.1464-5491.1992.tb01863.x

LLOYD CE, 1991, DIABETES CARE, V15, P278

LUSTMAN PJ, 1986, J NERV MENT DIS, V174, P736, DOI 10.1097/00005053-198612000-00005

Lustman PJ, 2000, DIABETES CARE, V23, P934, DOI 10.2337/diacare.23.7.934

MADSBAD S, 1978, DIABETES, V27, P262, DOI 10.2337/diab.27.1.S262

NELSON HE, 1992, NATL ADULT READING T

Petterson T, 1998, DIABETES CARE, V21, P930, DOI 10.2337/diacare.21.6.930

Robinson DL, 1997, MED PRIN PRACT, V6, P186

RUBIN RR, 1991, DIABETES CARE, V14, P335, DOI 10.2337/diacare.14.4.335

SHAMOON H, 1993, NEW ENGL J MED, V329, P977

Skinner TC, 2002, HEALTH PSYCHOL, V21, P61, DOI 10.1037//0278-6133.21.1.61

TEBBI CK, 1990, DIABETES CARE, V13, P98, DOI 10.2337/diacare.13.2.98

Watkins KW, 2000, DIABETES CARE, V23, P1511, DOI 10.2337/diacare.23.10.1511

WATSON D, 1989, PSYCHOL REV, V96, P234, DOI 10.1037//0033-295X.96.2.234

NR 48

TC 39

Z9 41

U1 0

U2 4

PU WILEY-BLACKWELL

PI HOBOKEN

PA 111 RIVER ST, HOBOKEN 07030-5774, NJ USA

SN 0742-3071

EI 1464-5491

J9 DIABETIC MED

JI Diabetic Med.

PD FEB

PY 2003

VL 20

IS 2

BP 135

EP 146

DI 10.1046/j.1464-5491.2003.00887.x

PG 12

WC Endocrinology & Metabolism

SC Endocrinology & Metabolism

GA 645GP

UT WOS:000180968100008

PM 12581265

DA 2019-01-08

ER

PT J

AU Egede, LE

Bonadonna, RJ

AF Egede, LE

Bonadonna, RJ

TI Diabetes self-management in African Americans: An exploration of the

role of fatalism

SO DIABETES EDUCATOR

LA English

DT Article

ID FOCUS GROUPS; PSYCHOLOGICAL DISTRESS; HEALTH BELIEFS; BLACK-WOMEN;

LOCUS; CANCER; EDUCATION; RESOURCES; SYMPTOMS; CULTURE

AB PURPOSE This study was conducted to explore the concept of fatalism in relation to diabetes self-management behavior in African Americans with type 2 diabetes.

METHODS Participants (n=39) were recruited from a clinic sample of African Americans with type 2 diabetes. Seven focus groups were conducted; the sessions were recorded, transcribed, and analyzed to identify themes related to fatalism and diabetes self-management. The ISAS paradigm (individual, symbols, audience, situation), a social psychology theory, provided the theoretical framework for the study.

RESULTS Four dimensions of fatalism were identified: the meaning of diabetes, the illness experience, the individual's coping response, and the individual's religious and spiritual beliefs. For the participants in this study, fatalism seemed to characterize the nature of the interaction between the individual with diabetes and others, the meanings they attached to such interactions, and the decision to adopt an effective or ineffective diabetes self-management behavior.

CONCLUSIONS Fatalism was associated with diabetes self-management in African Americans with diabetes and was multidimensional in this population; the construct appeared to differ conceptually from the perspective of current measures.

C1 Med Univ S Carolina, Dept Med, Charleston, SC 29425 USA.

Med Univ S Carolina, Coll Nursing, Charleston, SC 29425 USA.

RP Egede, LE (reprint author), Diabet Educator, 367 W Chicago Ave, Chicago, IL 60610 USA.

FU AHRQ HHS [1K08HS11418]

CR Anderson RM, 1996, DIABETES EDUCATOR, V22, P28, DOI 10.1177/014572179602200104

ANDERSON RM, 1994, DIABETES EDUCATOR, V20, P399, DOI 10.1177/014572179402000506

ANDERSON RM, 1991, DIABETES CARE, V14, P605, DOI 10.2337/diacare.14.7.605

BASCH CE, 1987, HEALTH EDUC QUART, V14, P411, DOI 10.1177/109019818701400404

BOONE C, 1990, PSYCHOL REP, V66, P1107, DOI 10.2466/PR0.66.3.1107-1111

CALDER BJ, 1977, J MARKETING RES, V14, P353, DOI 10.2307/3150774

Chavez LR, 1997, AM J PREV MED, V13, P418

CLEMENT S, 1995, DIABETES CARE, V18, P1204, DOI 10.2337/diacare.18.8.1204

Conrad ME, 1996, ANN INTERN MED, V125, P941, DOI 10.7326/0003-4819-125-11-199612010-00026

DAVISON C, 1992, SOC SCI MED, V34, P675, DOI 10.1016/0277-9536(92)90195-V

Edelstein J, 1987, Diabetes Educ, V13, P51, DOI 10.1177/014572178701300111

Egede LE, 2002, DIABETES EDUCATOR, V28, P258, DOI 10.1177/014572170202800211

HARRIS MI, 1993, DIABETES CARE, V16, P1116, DOI 10.2337/diacare.16.8.1116

HUSSEY LC, 1989, NURS CLIN N AM, V24, P605

Joiner TE, 2001, BEHAV RES THER, V39, P887, DOI 10.1016/S0005-7967(00)00062-0

KITZINGER J, 1995, BRIT MED J, V311, P299, DOI 10.1136/bmj.311.7000.299

KNODEL J, 1993, SUCCESSFUL FOCUS GRO

Krueger R. A., 2000, FOCUS GROUPS PRACTIC

Maillet NA, 1996, DIABETES EDUCATOR, V22, P39, DOI 10.1177/014572179602200106

Mayo RM, 2001, J WOMEN AGING, V13, P57, DOI 10.1300/J074v13n01_05

*MERR WEBST INC, 1998, MERR WEBST COLL DICT

Miles M., 1994, QUALITATIVE DATA ANA

Morgan D. L., 1997, FOCUS GROUPS QUALITA

Morgan DL, 1993, SUCCESSFUL FOCUS GRO

NEFF JA, 1993, J COMMUNITY PSYCHOL, V21, P3, DOI 10.1002/1520-6629(199301)21:1<3::AID-JCOP2290210102>3.0.CO;2-9

Neighbors Harold W., 1996, Ethnicity and Disease, V6, P167

Norris SL, 2001, DIABETES CARE, V24, P561, DOI 10.2337/diacare.24.3.561

Patton M., 1990, QUALITATIVE EVALUATI

PEYROT M, 1994, DIABETES CARE, V17, P994, DOI 10.2337/diacare.17.9.994

Phillips J M, 1999, Oncol Nurs Forum, V26, P561

Powe B D, 1995, Oncol Nurs Forum, V22, P1355

Powe B D, 1999, Oncol Nurs Forum, V26, P583

POWE BD, 1995, J RELIG HEALTH, V34, P119, DOI 10.1007/BF02248767

Powe BD, 1996, NURS OUTLOOK, V44, P18, DOI 10.1016/S0029-6554(96)80020-0

POWE BD, 1995, CANCER NURS, V18, P385

*RCMAR MEAS COR, 2001, P MEAS ISS HLTH DISP

Roberts RE, 2000, PSYCHIATRY, V63, P239, DOI 10.1080/00332747.2000.11024917

ROSS CE, 1983, AM J COMMUN PSYCHOL, V11, P383, DOI 10.1007/BF00894055

ROTTER JB, 1966, PSYCHOL MONOGR, V80, P1, DOI 10.1037/h0092976

SADOWSKI CJ, 1982, J PSYCHOL, V112, P227, DOI 10.1080/00223980.1982.9915378

Schoenberg NE, 1998, DIABETES EDUCATOR, V24, P319, DOI 10.1177/014572179802400306

SCHWAB T, 1994, DIABETES EDUCATOR, V20, P221, DOI 10.1177/014572179402000308

Sofaer S, 1999, HEALTH SERV RES, V34, P1101

Talbot F, 1996, J CLIN PSYCHOL, V52, P559, DOI 10.1002/(SICI)1097-4679(199609)52:5<559::AID-JCLP11>3.0.CO;2-D

Tillotson LM, 1996, DIABETES EDUCATOR, V22, P133, DOI 10.1177/014572179602200206

*US DEP HHS, 2002, NAT I DIAB DIG KIDN

Vernon G. M., 1981, SOCIAL PSYCHOL SHARE

Vetter N J, 1991, Health Visit, V64, P191

WALLSTON BS, 1976, J CONSULT CLIN PSYCH, V44, P580, DOI 10.1037//0022-006X.44.4.580

WHEATON B, 1980, J HEALTH SOC BEHAV, V21, P100, DOI 10.2307/2136730

WHEATON B, 1983, J HEALTH SOC BEHAV, V24, P208, DOI 10.2307/2136572

NR 51

TC 65

Z9 65

U1 1

U2 3

PU AMER ASSOC DIABETES EDUCATORS

PI CHICAGO

PA STE 1240, 444 NORTH MICHIGAN AVE, CHICAGO, IL 60611-3901 USA

SN 0145-7217

J9 DIABETES EDUCATOR

JI Diabetes Educ.

PD JAN-FEB

PY 2003

VL 29

IS 1

BP 105

EP 115

DI 10.1177/014572170302900115

PG 11

WC Endocrinology & Metabolism; Public, Environmental & Occupational Health

SC Endocrinology & Metabolism; Public, Environmental & Occupational Health

GA 643UZ

UT WOS:000180880400009

PM 12632689

DA 2019-01-08

ER

PT J

AU Quattrini, C

Tesfaye, S

AF Quattrini, C

Tesfaye, S

TI Understanding the impact of painful diabetic neuropathy

SO DIABETES-METABOLISM RESEARCH AND REVIEWS

LA English

DT Article

DE diabetes; diabetic neuropathy; peripheral neuropathy; painful diabetic

neuropathy; quality of life

ID IMPAIRED GLUCOSE-TOLERANCE; QUALITY-OF-LIFE; PERIPHERAL NEUROPATHY;

GLYCEMIC CONTROL; SENSORY NEUROPATHY; NATURAL-HISTORY; PREVALENCE;

POPULATION; MELLITUS; SYMPTOMS

AB Painful neuropathy is a common and often distressing complication of diabetes, It has considerable impact on the social and psychological well-being of affected individuals. There are two distinct forms of painful neuropathy: an acute and self-limiting form that resolves within a year or a chronic form that can go on for years. There are now a number of drugs available for the treatment of neuropathic pain. However, some may fail to respond to these drugs or may have unacceptable adverse side effects. When this is the case, the patient's quality of life can be severely affected.

Health care professionals need to assess the full impact of painful neuropathy. In this article we review a number of instruments that are used to assess the severity of painful neuropathy and its impact on the quality of life. Copyright (C), 2003 John Wiley Sons, Ltd.

C1 Royal Hallamshire Hosp, Diabet Res Unit, Sheffield S10 2JF, S Yorkshire, England.

RP Tesfaye, S (reprint author), Royal Hallamshire Hosp, Diabet Res Unit, Glossop Rd, Sheffield S10 2JF, S Yorkshire, England.

OI Tesfaye, Solomon/0000-0003-1190-1472

CR Amos AF, 1997, DIABETIC MED, V14, pS7, DOI 10.1002/(SICI)1096-9136(199712)14:5+<S7::AID-DIA522>3.3.CO;2-I

ARCHER AG, 1983, J NEUROL NEUROSUR PS, V46, P491, DOI 10.1136/jnnp.46.6.491

ASBURY AK, 1970, BRAIN, V93, P555, DOI 10.1093/brain/93.3.555

Benbow SJ, 1998, QJM-MON J ASSOC PHYS, V91, P733, DOI 10.1093/qjmed/91.11.733

BENBOW SJ, 1994, DIABETIC MED, V11, P17, DOI 10.1111/j.1464-5491.1994.tb00223.x

Bennett M, 2001, PAIN, V92, P147, DOI 10.1016/S0304-3959(00)00482-6

BOULTON AJM, 1983, POSTGRAD MED J, V59, P556, DOI 10.1136/pgmj.59.695.556

Bradley C, 1999, QUAL LIFE RES, V8, P79, DOI 10.1023/A:1026485130100

Caravati C, 1933, VA MED MON, V59, P745

Carrington AL, 1996, DIABETES RES CLIN PR, V32, P19, DOI 10.1016/0168-8227(96)01198-9

CASEY EB, 1972, BMJ-BRIT MED J, V1, P656, DOI 10.1136/bmj.1.5801.656

CHAN A W, 1990, Pain Clinic, V3, P147

CHIMENES H, 1978, LANCET, V1, P883

Cleeland C. S., 1994, Annals Academy of Medicine Singapore, V23, P129

CLEELAND CS, 1990, ADV PAIN RES THER, V16, P47

Clouet F, 2001, DIABETES METAB, V27, P711

Currie CJ, 1997, DIABETIC MED, V14, P686, DOI 10.1002/(SICI)1096-9136(199708)14:8<686::AID-DIA434>3.0.CO;2-D

DUNITZ M, 2000, CHRONIC PAIN, P7

DYCK PJ, 1993, NEUROLOGY, V43, P817, DOI 10.1212/WNL.43.4.817

DYCK PJ, 1999, DIABETIC NEUROPATHY, P262

ELLENBERG M, 1974, DIABETES, V23, P418, DOI 10.2337/diab.23.5.418

Ellenberg M, 1978, Diabetes Care, V1, P10, DOI 10.2337/diacare.1.1.10

FORREST RD, 1986, DIABETIC MED, V3, P338, DOI 10.1111/j.1464-5491.1986.tb00776.x

Galer BS, 1997, NEUROLOGY, V48, P332, DOI 10.1212/WNL.48.2.332

GUY RJC, 1985, DIABETOLOGIA, V28, P131

HARRIS MI, 1987, DIABETES, V36, P523, DOI 10.2337/diabetes.36.4.523

HAYES FJ, 1994, IRISH MED J, V87, P150

KIHARA M, 1994, AM J PHYSIOL, V266, pE980

Levitt NS, 1996, DIABETES CARE, V19, P751, DOI 10.2337/diacare.19.7.751

LLEWELYN JG, 1986, ACTA NEUROPATHOL, V72, P157, DOI 10.1007/BF00685978

MASSON EA, 1989, PAIN, V38, P25, DOI 10.1016/0304-3959(89)90068-7

MELZACK R, 1975, PAIN, V1, P277, DOI 10.1016/0304-3959(75)90044-5

Meyer-Rosberg K, 2001, EUR J PAIN, V5, P391, DOI 10.1053/eujp.2001.0260

NAGHMI R, 1990, HORM METAB RES, V22, P38, DOI 10.1055/s-2007-1004844

Novella SP, 2001, MUSCLE NERVE, V24, P1229, DOI 10.1002/mus.1137

PARRY GJ, 1989, NEUROLOGY, V39, P1488, DOI 10.1212/WNL.39.11.1488

Rijken PM, 1998, DISABIL REHABIL, V20, P330, DOI 10.3109/09638289809166090

ROBINSON N, 1990, DIABETIC MED, V7, P16, DOI 10.1111/j.1464-5491.1990.tb01300.x

Russell JW, 2001, MUSCLE NERVE, V24, P1109, DOI 10.1002/mus.1122

SCOTT J, 1976, PAIN, V2, P175, DOI 10.1016/0304-3959(76)90113-5

SIMMONS D, 1991, Q J MED, V81, P1021, DOI 10.1093/qjmed/81.3.1021

Singleton JR, 2001, DIABETES CARE, V24, P1448, DOI 10.2337/diacare.24.8.1448

Singleton JR, 2001, MUSCLE NERVE, V24, P1225, DOI 10.1002/mus.1136

Snoek FJ, 2000, DIABETES CARE, V23, P1305, DOI 10.2337/diacare.23.9.1305

TENVALL GR, 2000, DIABETES COMPLICATIO, V14, P235

Tesfaye S, 1996, LANCET, V348, P1698, DOI 10.1016/S0140-6736(96)02467-1

Tesfaye S, 1996, DIABETOLOGIA, V39, P1377, DOI 10.1007/s001250050586

Tesfaye S, 1996, DIABETOLOGIA, V39, P329

TESFAYE S, 1992, DIABETOLOGIA, V35, P155, DOI 10.1007/BF00402548

TESFAYE S, 1996, DIABETIC NEUROPATHY, pCH9

THOMAS P K, 1973, Journal of the Royal College of Physicians of London, V7, P154

Vileikyte L, 1998, DIABETES, V47, pA44

Vileikyte L, 1999, DIABETES REV, V7, P387

Vinik AI, 2000, DIABETOLOGIA, V43, P957, DOI 10.1007/s001250051477

WARD JD, 1983, J ROY SOC MED, V76, P1011, DOI 10.1177/014107688307601206

WATKINS PJ, 1984, BRIT MED J, V288, P168, DOI 10.1136/bmj.288.6412.168

WATKINS PJ, 1997, TXB DIABETES, V2, P1

White Christine A, 2002, BMC Nephrol, V3, P3, DOI 10.1186/1471-2369-3-3

Wu SY, 1998, DIABETES CARE, V21, P725, DOI 10.2337/diacare.21.5.725

YOUNG MJ, 1993, DIABETOLOGIA, V36, P150, DOI 10.1007/BF00400697

Ziegler D, 1992, J Diabetes Complications, V6, P49, DOI 10.1016/1056-8727(92)90049-Q

ZIEGLER D, 1995, DIABETOLOGIA, V38, P1425, DOI 10.1007/BF00400603

ZORRILLA E, 1967, ANN INTERN MED, V67, P968, DOI 10.7326/0003-4819-67-5-968

ZRUSTOVA M, 1979, ACTA DIABETOL LAT, V16, P55, DOI 10.1007/BF02590763

NR 64

TC 74

Z9 76

U1 1

U2 6

PU WILEY

PI HOBOKEN

PA 111 RIVER ST, HOBOKEN 07030-5774, NJ USA

SN 1520-7560

J9 DIABETES-METAB RES

JI Diabetes-Metab. Res. Rev.

PD JAN-FEB

PY 2003

VL 19

SU 1

BP S2

EP S8

DI 10.1002/dmrr.360

PG 7

WC Endocrinology & Metabolism

SC Endocrinology & Metabolism

GA 651TA

UT WOS:000181337100002

PM 12577252

DA 2019-01-08

ER

PT J

AU Watkins, PJ

Buxton-Thomas, MS

Howard, ER

AF Watkins, PJ

Buxton-Thomas, MS

Howard, ER

TI Long-term outcome after gastrectomy for intractable diabetic

gastroparesis

SO DIABETIC MEDICINE

LA English

DT Article

DE diabetic autonomic neuropathy; gastroparesis; gastrectomy

ID AUTONOMIC NEUROPATHY; GASTRIC ATONY; MELLITUS; JEJUNOSTOMY; MANAGEMENT

AB Aims To examine the long-term outlook for patients with intractable vomiting from diabetic gastroparesis who underwent major gastric surgery.

Methods Of 18 patients with problems from vomiting referred to the King's Diabetes Centre during the years 1994-2000, seven were considered to suffer irreversible symptoms not alleviated by protracted periods of medical treatment. They were all Type 1 Caucasian diabetic women, mean age 32 years (range 28-37 years) with multiple symptoms of severe autonomic neuropathy. They underwent major gastric surgery comprising 70% gastric resection including pylorus and antrum, with a 60-cm Roux-en-Y loop of jejunum to prevent reflux gastritis.

Results The vomiting was relieved in six of the seven patients almost immediately after surgery and during review up to more than 6 years post-operatively. There have been no serious relapses, resulting in considerable improvement in quality of life. Unfortunately, three of the patients developed renal failure, two of them needing renal support treatments 2 and 3 years after successful gastrectomy. One patient died suddenly 5 months after successful surgery and one 3 months after starting dialysis.

Conclusions Major gastric surgery can, after careful patient selection, effectively relieve distressing vomiting from severe gastroparesis and give a greatly improved quality of life to a small group of seriously disadvantaged patients where risk of subsequent renal failure is high and where life expectancy is poor.

C1 Kings Coll Hosp London, Kings Diabet Ctr, London SE5 9RS, England.

Kings Coll Hosp London, Dept Nucl Med, London SE5 9RS, England.

Kings Coll Hosp London, Dept Surg, London SE5 9RS, England.

RP Watkins, PJ (reprint author), Kings Coll Hosp London, Kings Diabet Ctr, Denmark Hill, London SE5 9RS, England.

EM peter.watkins1@virgin.net

CR Bell DSH, 1999, DIABETES CARE, V22, P1000, DOI 10.2337/diacare.22.6.1000

Devendra D, 2000, DIABETES CARE, V23, P426, DOI 10.2337/diacare.23.3.426

DOWLING CJ, 1985, BRIT MED J, V310, P308

Ejskjaer NT, 1999, DIABETIC MED, V16, P488, DOI 10.1046/j.1464-5491.1999.00086.x

Ejskjaer NT, 1998, DIABETIC MED, V15, P723, DOI 10.1002/(SICI)1096-9136(199809)15:9<723::AID-DIA674>3.0.CO;2-7

GUY RJC, 1984, J NEUROL NEUROSUR PS, V47, P686, DOI 10.1136/jnnp.47.7.686

Horowitz M, 1996, DIABETIC MED, V13, pS16

JACOBER SJ, 1986, DIABETES CARE, V9, P217, DOI 10.2337/diacare.9.2.217

KARLSTROM L, 1989, AM J SURG, V157, P44, DOI 10.1016/0002-9610(89)90418-2

Kong MF, 1999, CLIN GERIATR MED, V15, P321

Kong MFSC, 1996, DIABETIC MED, V13, P112, DOI 10.1002/(SICI)1096-9136(199602)13:2<112::AID-DIA37>3.3.CO;2-8

LIPP RW, 1993, EUR J NUCL MED, V20, P260

Morris AD, 1997, LANCET, V350, P1505, DOI 10.1016/S0140-6736(97)06234-X

PFEIFER M, 1999, DIABETIC NEUROPATHY, P174

REARDON TM, 1989, J CLIN GASTROENTEROL, V11, P204, DOI 10.1097/00004836-198904000-00018

ROON AJ, 1972, CALIF MED, V116, P58

SAID G, 1983, BRAIN, V106, P791, DOI 10.1093/brain/106.4.791

SAMPSON MJ, 1990, Q J MED, V75, P635

Stacher G, 2001, DIABETOLOGIA, V44, P1080, DOI 10.1007/s001250100619

Winkler AS, 2000, DIABETIC MED, V17, P457, DOI 10.1046/j.1464-5491.2000.00298.x

WINKLER AS, 1999, DIABETIC MED, V16, P1

WOOTEN RL, 1961, JAMA-J AM MED ASSOC, V176, P1082, DOI 10.1001/jama.1961.03040260016004

ZANONE MM, 1993, DIABETOLOGIA, V36, P564, DOI 10.1007/BF02743275

NR 23

TC 40

Z9 41

U1 0

U2 0

PU WILEY-BLACKWELL

PI HOBOKEN

PA 111 RIVER ST, HOBOKEN 07030-5774, NJ USA

SN 0742-3071

J9 DIABETIC MED

JI Diabetic Med.

PD JAN

PY 2003

VL 20

IS 1

BP 58

EP 63

DI 10.1046/j.1464-5491.2003.00872.x

PG 6

WC Endocrinology & Metabolism

SC Endocrinology & Metabolism

GA 632XT

UT WOS:000180250700009

PM 12519321

DA 2019-01-08

ER

PT J

AU Welch, G

Weinger, K

Anderson, B

Polonsky, WH

AF Welch, G

Weinger, K

Anderson, B

Polonsky, WH

TI Responsiveness of the problem areas in diabetes (PAID) questionnaire

SO DIABETIC MEDICINE

LA English

DT Article

DE diabetes mellitus; emotional distress; PAID; validity; responsiveness

ID GLYCEMIC CONTROL; INTENSIVE TREATMENT; SCALE; INTERVENTION; VALIDATION;

EDUCATION; DISTRESS; CARE

AB Aim Responsiveness (sensitivity to change over time) is a key psychometric quality for an outcome measure. We examined the responsiveness of the Problem Areas In Diabetes (PAID) questionnaire, a measure of diabetes-specific emotional distress.

Methods PAID data were obtained from seven diabetes intervention studies following a literature search that included both published papers and conference abstracts. To estimate responsiveness we used two indices: (i) a statistical test (the dependent t -test), and (ii) a commonly used effect size index (Cohen's d).

Results Mean patient PAID scores improved from baseline to follow-up for all seven studies. Specifically, t -statistics ranged from t = 8.5 (P < 0.001) to t = 2.1 (P < 0.06). Effect size results ranged from 0.32 (i.e. small) for a disease management intervention to 0.65 (i.e. moderate) for an intensive medical/educational intervention.

Conclusions Despite the pilot nature of the studies, the pattern of findings provided strong support for the responsiveness of the PAID. Information on responsiveness helps clinical researchers select measures, accurately estimate sample size to ensure adequate statistical power, and prioritize outcomes to be assessed.

C1 Joslin Diabet Ctr, Behav & Mental Hlth Res Div, Boston, MA 02115 USA.

Univ Calif San Diego, Dept Psychiat, San Diego, CA 92103 USA.

RP Welch, G (reprint author), Joslin Diabet Ctr, Behav & Mental Hlth Res Div, Boston, MA 02115 USA.

CR Bott U, 1998, DIABETES CARE, V21, P757, DOI 10.2337/diacare.21.5.757

Clark CM, 2001, DIABETES CARE, V24, P1079, DOI 10.2337/diacare.24.6.1079

COHEN J, 1985, STAT POWER ANAL BEHA

DCCT Res Grp, 1988, DIABETES CARE, V11, P725

DELEON OA, 1995, INT J PSYCHIAT MED, V25, P81, DOI 10.2190/95FU-2TVW-UA8C-LPN8

GLASGOW RE, 1997, DIABETES SPECTRUM, V10, P12

ISHII H, 1999, DIABETES, V48, P1397

IZQUIERDO R, 2000, DIABETES, V49, P1441

JACKSON RA, 1999, DIABETES, V48, P1866

KAZIS LE, 1989, MED CARE S, V27, P178

MELKUS GD, 1999, DIABETES, V48, P158

Mollema ED, 2001, J PSYCHOSOM RES, V51, P665, DOI 10.1016/S0022-3999(01)00229-X

Nichols GA, 2000, DIABETES CARE, V23, P273, DOI 10.2337/diacare.23.3.273

POLONSKY WH, 1995, DIABETES CARE, V18, P754, DOI 10.2337/diacare.18.6.754

POLONSKY WH, 2000, DIABETES BURNOUT

RUBIN RR, 2000, J CLIN PSYCHOL, V57, P457

Snoek FJ, 2001, PATIENT EDUC COUNS, V45, P143, DOI 10.1016/S0738-3991(01)00113-6

Snoek FJ, 2000, DIABETES CARE, V23, P1305, DOI 10.2337/diacare.23.9.1305

SPILKER B, 1996, QUALITY PHARMACOECON

TODD C, 1994, HDB PSYCHOL DIABETES

Trief PM, 2001, DIABETES CARE, V24, P1384, DOI 10.2337/diacare.24.8.1384

TURRENTINE LA, 2001, DIABETES, V50

Weinger K, 2001, PATIENT EDUC COUNS, V42, P123, DOI 10.1016/S0738-3991(00)00098-7

Welch GW, 1997, DIABETES CARE, V20, P760, DOI 10.2337/diacare.20.5.760

WELCH GW, 1995, DIABETES, V44, pA958

NR 25

TC 129

Z9 133

U1 2

U2 11

PU BLACKWELL PUBLISHING LTD

PI OXFORD

PA 9600 GARSINGTON RD, OXFORD OX4 2DG, OXON, ENGLAND

SN 0742-3071

J9 DIABETIC MED

JI Diabetic Med.

PD JAN

PY 2003

VL 20

IS 1

BP 69

EP 72

DI 10.1046/j.1464-5491.2003.00832.x

PG 4

WC Endocrinology & Metabolism

SC Endocrinology & Metabolism

GA 632XT

UT WOS:000180250700011

PM 12519323

DA 2019-01-08

ER

PT J

AU Roose, SP

AF Roose, SP

TI Depression: Links with ischemic heart disease and erectile dysfunction

SO JOURNAL OF CLINICAL PSYCHIATRY

LA English

DT Article

ID NOCTURNAL PENILE TUMESCENCE; CORONARY-ARTERY DISEASE;

MYOCARDIAL-INFARCTION; RISK-FACTORS; SYMPTOMS; MEN; PROGNOSIS; MORTALITY

AB This article examines the relationships among depression, ischemic heart disease, and erectile dysfunction. Depression is an independent risk factor for the development of ischemic heart disease, and depression in the post-myocardial infarction patient is associated with increased morbidity and mortality. Ischemic heart disease and erectile dysfunction are also frequently comorbid and share many common risk factors including age, hypertension, diabetes, dyslipidemia, obesity, sedentary lifestyle, and smoking. Depression and erectile dysfunction often occur together; however, the causal relation may be difficult to determine because erectile dysfunction may be a symptom of depression, social distress accompanying erectile dysfunction may precipitate depressive symptoms, or both conditions may result from a common factor such as vascular disease.

C1 Columbia Univ Coll Phys & Surg, Dept Psychiat, New York, NY 10032 USA.

RP Roose, SP (reprint author), New York State Psychiat Inst & Hosp, 1051 Riverside Dr, New York, NY 10032 USA.

EM spr2@columbia.edu

CR Araujo AB, 1998, PSYCHOSOM MED, V60, P458, DOI 10.1097/00006842-199807000-00011

Arborelius L, 1999, J ENDOCRINOL, V160, P1, DOI 10.1677/joe.0.1600001

Ariyo AA, 2000, CIRCULATION, V102, P1773

AROMAA A, 1994, ACTA PSYCHIAT SCAND, V89, P77, DOI 10.1111/j.1600-0447.1994.tb05807.x

Burchardt M, 2000, J UROLOGY, V164, P1188, DOI 10.1016/S0022-5347(05)67138-8

Carney RM, 2002, J PSYCHOSOM RES, V53, P897, DOI 10.1016/S0022-3999(02)00311-2

Carney RM, 2001, CIRCULATION, V104, P2024, DOI 10.1161/hc4201.097834

Chung WS, 1999, EUR UROL, V36, P68, DOI 10.1159/000019929

Connerney I, 2001, LANCET, V358, P1766, DOI 10.1016/S0140-6736(01)06803-9

Derby CA, 2000, UROLOGY, V56, P302, DOI 10.1016/S0090-4295(00)00614-2

DEROGATIS LR, 1981, AM J PSYCHIAT, V138, P757

FELDMAN HA, 1994, J UROLOGY, V151, P54, DOI 10.1016/S0022-5347(17)34871-1

Ford DE, 1998, ARCH INTERN MED, V158, P1422, DOI 10.1001/archinte.158.13.1422

FRASURESMITH N, 1993, JAMA-J AM MED ASSOC, V270, P1819, DOI 10.1001/jama.270.15.1819

FRASURESMITH N, 1995, CIRCULATION, V91, P999, DOI 10.1161/01.CIR.91.4.999

Greenstein A, 1997, INT J IMPOT RES, V9, P123, DOI 10.1038/sj.ijir.3900282

KEMPER JW, 1957, ANN INTERN MED, V46, P831, DOI 10.7326/0003-4819-46-5-831

Krishnan KRR, 1997, AM J PSYCHIAT, V154, P497

LaghrissiThode F, 1997, BIOL PSYCHIAT, V42, P290, DOI 10.1016/S0006-3223(96)00345-9

Lesperance F, 2000, ARCH INTERN MED, V160, P1354, DOI 10.1001/archinte.160.9.1354

Manica A L, 1999, Arq Bras Cardiol, V73, P237, DOI 10.1590/S0066-782X1999000800012

McVary KT, 2001, J UROLOGY, V166, P1624, DOI 10.1016/S0022-5347(05)65641-8

Musselman DL, 1998, ARCH GEN PSYCHIAT, V55, P580, DOI 10.1001/archpsyc.55.7.580

O'Kane PD, 2001, INT J CLIN PRACT, V55, P219

ROOSE SP, 1991, J CLIN PSYCHIAT, V52, P34

Seidman S N, 2000, Curr Psychiatry Rep, V2, P201, DOI 10.1007/s11920-996-0008-0

Seidman SN, 2001, AM J PSYCHIAT, V158, P1623, DOI 10.1176/appi.ajp.158.10.1623

Shabsigh R, 1998, UROLOGY, V52, P848, DOI 10.1016/S0090-4295(98)00292-1

STEIGER A, 1993, ACTA PSYCHIAT SCAND, V87, P358, DOI 10.1111/j.1600-0447.1993.tb03387.x

Sullivan ME, 2001, BJU INT, V87, P838, DOI 10.1046/j.1464-410x.2001.02211.x

THASE ME, 1988, BIOL PSYCHIAT, V24, P33, DOI 10.1016/0006-3223(88)90119-9

WEI M, 1994, AM J EPIDEMIOL, V140, P930, DOI 10.1093/oxfordjournals.aje.a117181

NR 32

TC 25

Z9 25

U1 0

U2 2

PU PHYSICIANS POSTGRADUATE PRESS

PI MEMPHIS

PA P O BOX 752870, MEMPHIS, TN 38175-2870 USA

SN 0160-6689

EI 1555-2101

J9 J CLIN PSYCHIAT

JI J. Clin. Psychiatry

PY 2003

VL 64

SU 10

BP 26

EP 30

PG 5

WC Psychology, Clinical; Psychiatry

SC Psychology; Psychiatry

GA 724LL

UT WOS:000185489000005

PM 12971813

DA 2019-01-08

ER

PT J

AU Kelly, PA

Samandouras, G

Grossman, AB

Afshar, F

Besser, GM

Jenkins, PJ

AF Kelly, PA

Samandouras, G

Grossman, AB

Afshar, F

Besser, GM

Jenkins, PJ

TI Neurosurgical treatment of Nelson's syndrome

SO JOURNAL OF CLINICAL ENDOCRINOLOGY & METABOLISM

LA English

DT Article

ID CUSHINGS-DISEASE; PITUITARY IRRADIATION; ADRENALECTOMY; RADIOTHERAPY;

EXPERIENCE; MANAGEMENT; SURGERY

AB Total bilateral adrenalectomy remains the definitive procedure for cure in Cushing's disease. It is complicated by the development of Nelson's syndrome, the treatment of which remains troublesome. We report the long-term follow-up, median 17 yr (range, 8-22 yr), of 13 patients (3 males and 10 females) treated with pituitary surgery for Nelson's syndrome at a median age of 35 yr (range, 21-67 yr). The presence of a pituitary mass lesion necessitated neurosurgery in all. Pre-operatively, the median plasma ACTH level was 664 pmol/liter (range, 92-3665 pmol/liter); this fell to 29 pmol/liter (range, <2 to 1124 pmol/liter) postoperatively (P < 0.0005). Cutaneous hyperpigmentation was reduced in all and resolved in 11 patients. The pituitary tumor bulk was clearly reduced in 12 patients. There was no perioperative mortality. No patient developed a visual field defect attributable to surgery. New anterior pituitary hormone deficiency occurred in seven patients, and permanent diabetes insipidus occurred in five patients. At latest follow-up, the median plasma ACTH is 73 pmol/liter (range, <2 to 7759 pmol/liter); six patients have levels of less than 44 pmol/liter and also have a resolution of their pigmentation and no evidence of a recurrence of a pituitary mass lesion. We conclude that pituitary surgery is an efficacious treatment for mass lesions associated with Nelson's syndrome, has long-term benefit with minimal side effects, and must be considered in the management of this distressing complication.

C1 St Bartholomews & Royal London Hosp, Dept Endocrinol, London EC1A 7BE, England.

St Bartholomews & Royal London Hosp, Dept Neurosurg, London EC1A 7BE, England.

RP Jenkins, PJ (reprint author), St Bartholomews Hosp, Dept Endocrinol, London EC1A 7BE, England.

EM p.j.jenkins@qmul.ac.uk

OI Kelly, Philip/0000-0003-4338-6225

CR ATKINSON AB, 1991, CLIN ENDOCRINOL, V34, P507, DOI 10.1111/j.1365-2265.1991.tb00332.x

BARNETT AH, 1983, CLIN ENDOCRINOL OXF, V39, P91

BENNETT AH, 1973, J UROLOGY, V109, P321, DOI 10.1016/S0022-5347(17)60417-8

BURKE CW, 1990, CLIN ENDOCRINOL, V33, P525, DOI 10.1111/j.1365-2265.1990.tb03890.x

DORNHORST A, 1983, J CLIN ENDOCR METAB, V56, P985, DOI 10.1210/jcem-56-5-985

FUKUSHIMA T, 1985, NEUROSURG REV, V8, P185, DOI 10.1007/BF01815443

GANZ JC, 1993, STEREOT FUNCT NEUROS, V61, P30

GILKES JJH, 1977, BRIT MED J, V1, P996, DOI 10.1136/bmj.1.6067.996

GLENN F, 1972, ANN SURG, V175, P948, DOI 10.1097/00000658-197206010-00015

GUTHRIE FW, 1981, SURG NEUROL, V16, P316, DOI 10.1016/0090-3019(81)90262-7

HOPWOOD NJ, 1977, AM J DIS CHILD, V131, P1353, DOI 10.1001/archpedi.1977.02120250035005

HOWLETT TA, 1989, CLIN ENDOCRINOL, V31, P309, DOI 10.1111/j.1365-2265.1989.tb01255.x

JENKINS PJ, 1995, J CLIN ENDOCR METAB, V80, P165, DOI 10.1210/jc.80.1.165

KELLY WF, 1983, Q J MED, V206, P224

Kemink SAG, 2001, CLIN ENDOCRINOL, V54, P45, DOI 10.1046/j.1365-2265.2001.01187.x

LAMBERTS SWJ, 1980, J CLIN ENDOCR METAB, V51, P307, DOI 10.1210/jcem-51-2-307

LUDECKE DK, 1982, ACTA NEUROCHIR, V65, P3, DOI 10.1007/BF01405437

MANOLAS KJ, 1984, WORLD J SURG, V8, P374, DOI 10.1007/BF01655082

MCCANCE DR, 1993, CLIN ENDOCRINOL, V39, P315, DOI 10.1111/j.1365-2265.1993.tb02371.x

MELBY JC, 1988, ANN INTERN MED, V109, P445, DOI 10.7326/0003-4819-109-6-445

MercadoAsis LB, 1997, J CLIN ENDOCR METAB, V82, P514, DOI 10.1210/jc.82.2.514

MONTGOMERY DAD, 1978, BRIT J SURG, V65, P221, DOI 10.1002/bjs.1800650402

MOORE TJ, 1976, ANN INTERN MED, V85, P731, DOI 10.7326/0003-4819-85-6-731

NELSON DH, 1966, J CLIN ENDOCR METAB, V26, P722, DOI 10.1210/jcem-26-7-722

NELSON DH, 1960, ANN INTERN MED, V52, P560, DOI 10.7326/0003-4819-52-3-560

NELSON DH, 1958, NEW ENGL J MED, V259, P161, DOI 10.1056/NEJM195807242590403

Newell-Price J, 2002, J CLIN ENDOCR METAB, V87, P1640, DOI 10.1210/jc.87.4.1640

ORTH DN, 1971, NEW ENGL J MED, V285, P243, DOI 10.1056/NEJM197107292850501

Pereira MAA, 1998, CLIN ENDOCRINOL, V49, P533, DOI 10.1046/j.1365-2265.1998.00578.x

Plowman PN, 1999, CLIN ENDOCRINOL, V51, P265

PRINZ RA, 1979, ARCH SURG-CHICAGO, V114, P481

REES LH, 1971, ENDOCRINOLOGY, V89, P254, DOI 10.1210/endo-89-1-254

SCOTT HW, 1977, ANN SURG, V185, P524, DOI 10.1097/00000658-197705000-00004

SCOTT RS, 1978, CLIN ENDOCRINOL, V9, P571, DOI 10.1111/j.1365-2265.1978.tb01516.x

SPRAGUE RG, 1953, JAMA-J AM MED ASSOC, V151, P629

TRAINER PJ, 1993, CLIN ENDOCRINOL, V38, P73, DOI 10.1111/j.1365-2265.1993.tb00975.x

WELBOURN RB, 1971, BRIT J SURG, V58, P1, DOI 10.1002/bjs.1800580102

WISLAWSKI J, 1985, J NEUROSURG, V62, P68, DOI 10.3171/jns.1985.62.1.0068

Wolffenbuttel BHR, 1998, CLIN NEUROL NEUROSUR, V100, P60, DOI 10.1016/S0303-8467(97)00124-8

NR 39

TC 37

Z9 37

U1 0

U2 0

PU ENDOCRINE SOC

PI CHEVY CHASE

PA 8401 CONNECTICUT AVE, SUITE 900, CHEVY CHASE, MD 20815-5817 USA

SN 0021-972X

J9 J CLIN ENDOCR METAB

JI J. Clin. Endocrinol. Metab.

PD DEC

PY 2002

VL 87

IS 12

BP 5465

EP 5469

DI 10.1210/jc.2002-020299

PG 5

WC Endocrinology & Metabolism

SC Endocrinology & Metabolism

GA 628DC

UT WOS:000179976300019

PM 12466338

OA Bronze

DA 2019-01-08

ER

PT J

AU Fisher, L

Chesla, CA

Skaff, MM

Mullan, JT

Kanter, RA

AF Fisher, L

Chesla, CA

Skaff, MM

Mullan, JT

Kanter, RA

TI Depression and anxiety among partners of European-American and Latino

patients with type 2 diabetes

SO DIABETES CARE

LA English

DT Article

ID GENDER-DIFFERENCES; EXPRESSED EMOTION; GLYCEMIC CONTROL; SOCIAL SUPPORT;

SYMPTOMS; SPOUSES; DISTRESS; FAMILY; HEALTH; PERSPECTIVE

AB OBJECTIVE- To assess the levels of and the independent contributors to depressive affect and anxiety among partners of patients with type 2 diabetes.

RESEARCH DESIGN AND METHODS- The partners of 75 Latino and 113 European-American patients with type 2 diabetes were Assessed for level of depressive affect and anxiety and for three groups of potential stressors: demographics (age, gender, and education), patient disease status (time since diagnosis, HbA(1c)-comorbidities, And BMI), and family stress (disease impact, spouse conflict, and family closeness). Dependent variables were partner depressive affect (Center for Epidemiological Studies-Depression scale) and anxiety (Symptom Checklist [SCL-90] anxiety). Predictors of partner depressive affect and anxiety and partner-patient con-cordance rates were computed.

RESULTS- Levels of depressive affect and anxiety and rates of likely depression (21.4%) were as high for partners as they were for patients. No differences were found on depressive affect or anxiety by ethnicity, but female partner's scored higher than male partners on both measures. Partner-patient concordance rates were low. The family level variables accounted for the most variance in both depressive affect and anxiety, with demographics and disease status variables contributing modest or nonsignificant independent variance.

CONCLUSIONS- Partners of patients with type 2 diabetes experience levels of psychological distress as. high or even higher than patients, especially if the partner is female. Low levels of concordance suggest that partners can be distressed even if patients are not. Many life stresses contribute to psychological distress among. partners, not just disease-related indicators. The findings suggest the utility of evaluating both partners and patients using a life-centered rather than a disease-focused perspective.

C1 Univ Calif San Francisco, Dept Family & Community Med, San Francisco, CA 94143 USA.

No Calif Kaiser Permanente, San Francisco, CA USA.

RP Fisher, L (reprint author), Univ Calif San Francisco, Dept Family & Community Med, Box 0900, San Francisco, CA 94143 USA.

EM fisher@itsa.ucsf.edu

FU NIDDK NIH HHS [DK-49816, DK-53203]

CR Almeida DM, 1998, J PERS SOC PSYCHOL, V75, P670, DOI 10.1037/0022-3514.75.3.670

BEACH SRH, 1994, BEHAV THER, V25, P345, DOI 10.1016/S0005-7894(05)80152-2

Benazon NR, 2000, J ABNORM PSYCHOL, V109, P550, DOI 10.1037//0021-843X.109.3.550

BLOOD GW, 1994, J COMMUN DISORD, V27, P19, DOI 10.1016/0021-9924(94)90008-6

Bookwala J, 1996, PSYCHOL AGING, V11, P582, DOI 10.1037//0882-7974.11.4.582

Brummett BH, 1998, PSYCHOSOM MED, V60, P707, DOI 10.1097/00006842-199811000-00008

CAMWATH TC, 1987, BRIT MED J, V294, P409

Ciechanowski PS, 2000, ARCH INTERN MED, V160, P3278, DOI 10.1001/archinte.160.21.3278

COYNE JC, 1991, J PERS SOC PSYCHOL, V61, P404, DOI 10.1037/0022-3514.61.3.404

COYNE JC, 1987, J CONSULT CLIN PSYCH, V55, P347, DOI 10.1037//0022-006X.55.3.347

COYNE JC, 1994, PSYCHOL BULL, V116, P29, DOI 10.1037/0033-2909.116.1.29

DCCT Res Grp, 1988, DIABETES CARE, V11, P725

De Groot M, 2001, DIABETES CARE, V24, P407, DOI 10.2337/diacare.24.2.407

DEGROOT M, 1994, PSYCHOSOM MED, V56, pA176

DEROGATIS L R, 1973, Psychopharmacology Bulletin, V9, P13

DUFORT GG, 1994, PSYCHOL MED, V24, P431, DOI 10.1017/S0033291700027409

FISHER L, 1993, FAM PROCESS, V32, P69, DOI 10.1111/j.1545-5300.1993.00069.x

Fisher L, 2001, DIABETES CARE, V24, P1751, DOI 10.2337/diacare.24.10.1751

Fisher L, 2000, J FAM PRACTICE, V49, P561

Fisher L, 1998, DIABETES EDUCATOR, V24, P599, DOI 10.1177/014572179802400504

Fisher L, 2000, DIABETES CARE, V23, P267, DOI 10.2337/diacare.23.3.267

Gilligan C, 1982, DIFFERENT VOICE PSYC

HOOLEY JM, 1986, BRIT J PSYCHIAT, V148, P642, DOI 10.1192/bjp.148.6.642

HOOLEY JM, 1985, CLIN PSYCHOL REV, V5, P119, DOI 10.1016/0272-7358(85)90018-2

Kiecolt-Glaser JK, 2001, PSYCHOL BULL, V127, P472, DOI 10.1037//0033-2909.127.4.472

LEWIS FM, 1989, SOC SCI MED, V29, P1261, DOI 10.1016/0277-9536(89)90066-X

LUSTMAN PJ, 1988, DIABETES CARE, V11, P605, DOI 10.2337/diacare.11.8.605

Lustman PJ, 2000, DIABETES CARE, V23, P934, DOI 10.2337/diacare.23.7.934

MARIN G, 1991, RES HISPANIC POPULAT, V23

MCLEOD JD, 1993, J AFFECT DISORDERS, V27, P43, DOI 10.1016/0165-0327(93)90096-3

Northouse LL, 2000, SOC SCI MED, V50, P271, DOI 10.1016/S0277-9536(99)00281-6

Pearlin LI, 1981, J HEALTH SOC BEHAV, V22, P333

Piccinelli M, 2000, BRIT J PSYCHIAT, V177, P486, DOI 10.1192/bjp.177.6.486

Pieper B A, 1990, Diabetes Educ, V16, P108, DOI 10.1177/014572179001600206

Pruchno R. A., 1990, J GERONTOL, V45, P192

RADLOFF L S, 1977, Applied Psychological Measurement, V1, P385, DOI 10.1177/014662167700100306

Revenson T A, 1991, Arthritis Care Res, V4, P63, DOI 10.1002/art.1790040203

RIDEOUT EM, 1990, INT J PSYCHIAT MED, V20, P37, DOI 10.2190/YBXJ-WPDJ-QB0Y-183G

SCHWARTZ L, 1991, PAIN, V44, P61, DOI 10.1016/0304-3959(91)90148-Q

TAMBS K, 1991, J AFFECT DISORDERS, V21, P117, DOI 10.1016/0165-0327(91)90058-Z

Tower RB, 1996, PSYCHOL AGING, V11, P683, DOI 10.1037/0882-7974.11.4.683

Tschann JM, 1999, J MARRIAGE FAM, V61, P269, DOI 10.2307/353747

VanderDoes FEE, 1996, DIABETES CARE, V19, P204, DOI 10.2337/diacare.19.3.204

NR 43

TC 36

Z9 36

U1 0

U2 2

PU AMER DIABETES ASSOC

PI ALEXANDRIA

PA 1701 N BEAUREGARD ST, ALEXANDRIA, VA 22311-1717 USA

SN 0149-5992

EI 1935-5548

J9 DIABETES CARE

JI Diabetes Care

PD SEP

PY 2002

VL 25

IS 9

BP 1564

EP 1570

DI 10.2337/diacare.25.9.1564

PG 7

WC Endocrinology & Metabolism

SC Endocrinology & Metabolism

GA 724UE

UT WOS:000185504400012

PM 12196428

OA Bronze

DA 2019-01-08

ER

PT J

AU Takii, M

Uchigata, Y

Nozaki, T

Nishikata, H

Kawai, K

Komaki, G

Iwamoto, Y

Kubok, C

AF Takii, M

Uchigata, Y

Nozaki, T

Nishikata, H

Kawai, K

Komaki, G

Iwamoto, Y

Kubok, C

TI Classification of type 1 diabetic females with bulimia nervosa into

subgroups 4D according to purging behavior

SO DIABETES CARE

LA English

DT Article

ID EATING DISORDERS; INSULIN OMISSION; YOUNG-WOMEN; MELLITUS; IDDM;

DEPRESSION; INVENTORY; SCALE

AB OBJECTIVE- To classify type 1 diabetic females with bulimia nervosa (BN) by type of inappropriate compensatory behavior in order to prevent weight gain (ICB) and to investigate the group differences.

RESEACH DESIGN AND METHODS- Type I diabetic females with BN, diagnosed by structured diagnostic interview based on DSM-IV (Diagnostic and Statistical Manual of Mental Disorders, 4th ed.) criteria, were classified by type of ICB as follows: 1) only severe insulin omission as an ICB (BN-I) (n = 22), 2) both severe insulin omission and self-induced vomiting = and/or laxative abuse (BN-IP) (n = 22), or 3) no insulin omission but another ICB (BN-NI) (n = 11). The clinical characteristics of these three groups and a binge-eating disorder (BED) group (n = 24) were compared.

RESULTS- The BN-IP and BN-I groups had the highest HbA(1c) levels. The BN-IP group had the highest rates of diabetic neuropathy, retinopathy, and nephropathy. The BN-NI group had the second highest rates of neuropathy and retinopathy. The BN-IP group had the highest frequencies of diabetes- and ketoacidosis-related hospital admissions, and the BN-I group had the second highest frequencies. The BN-NI group showed the highest scores on psychological tests related to depression, anxiety, eating disorder psychopathology, and perfectionism. The had a BN-NI group had the highest rate of history of visits to a psychiatrist, and the BN-IP group the second highest history.

CONCLUSIONS- Type I diabetic females with BN seem not to be homogenous and can be classified into three distinctive subgroups by type of ICB. Individuals with BN-IP had the most severe problems with both medical and psychological/behavioral aspects. Individuals with BN-NI manifested the highest psychological distress. The BN-I group had comparatively mild distress despite having the poorest metabolic control. Each BN group manifested more severe pathology than the BED group.

C1 Kyushu Univ, Grad Sch Med Sci, Dept Psychosomat Med, Hiyashi Ku, Fukuoka 8128582, Japan.

Tokyo Womens Med Univ, Sch Med, Ctr Diabet, Tokyo, Japan.

Natl Ctr Neurol & Psychiat, NIMH, Div Psychosomat Med, Chiba, Japan.

RP Takii, M (reprint author), Kyushu Univ, Grad Sch Med Sci, Dept Psychosomat Med, Hiyashi Ku, 3-1-1 Maidashi, Fukuoka 8128582, Japan.

CR American Psychiatric Association, 1994, DIAGN STAT MAN MENT

FAIRBURN CG, 1991, BRIT MED J, V303, P17, DOI 10.1136/bmj.303.6793.17

First M. B., 1996, USERS GUIDE STRUCTUR

FROST RO, 1990, COGNITIVE THER RES, V14, P449, DOI 10.1007/BF01172967

GARNER DM, 1983, INT J EAT DISORDER, V2, P15, DOI 10.1002/1098-108X(198321)2:2<15::AID-EAT2260020203>3.0.CO;2-6

HAMILTON M, 1960, J NEUROL NEUROSUR PS, V23, P56, DOI 10.1136/jnnp.23.1.56

LAGRECA AM, 1987, DIABETES CARE, V10, P659

MARCUS MD, 1990, NEUROPSYCHOLOGICAL B, P102

POLONSKY WH, 1994, DIABETES CARE, V17, P1178, DOI 10.2337/diacare.17.10.1178

RODIN GM, 1992, DIABETES CARE, V15, P1402, DOI 10.2337/diacare.15.10.1402

RODIN GM, 1986, INT J PSYCHIAT MED, V16, P49, DOI 10.2190/HULH-CTPR-4V17-383C

RUBIN RR, 1992, DIABETES CARE, V15, P1640, DOI 10.2337/diacare.15.11.1640

Rydall AC, 1997, NEW ENGL J MED, V336, P1849, DOI 10.1056/NEJM199706263362601

SPIELBERGER CD, 1983, MANUAL STATETRAIT AN

Takii M, 1999, J PSYCHOSOM RES, V47, P221, DOI 10.1016/S0022-3999(99)00031-8

ZUNG WWK, 1972, J CLIN PSYCHOL, V28, P539, DOI 10.1002/1097-4679(197210)28:4<539::AID-JCLP2270280427>3.0.CO;2-S

NR 16

TC 15

Z9 17

U1 0

U2 0

PU AMER DIABETES ASSOC

PI ALEXANDRIA

PA 1701 N BEAUREGARD ST, ALEXANDRIA, VA 22311-1717 USA

SN 0149-5992

J9 DIABETES CARE

JI Diabetes Care

PD SEP

PY 2002

VL 25

IS 9

BP 1571

EP 1575

DI 10.2337/diacare.25.9.1571

PG 5

WC Endocrinology & Metabolism

SC Endocrinology & Metabolism

GA 724UE

UT WOS:000185504400013

PM 12196429

OA Bronze

DA 2019-01-08

ER

PT J

AU Trief, PM

Wade, MJ

Britton, KD

Weinstock, RS

AF Trief, PM

Wade, MJ

Britton, KD

Weinstock, RS

TI A prospective analysis of marital relationship factors and quality of

life in diabetes

SO DIABETES CARE

LA English

DT Article

ID HEALTH SURVEY SF-36; GLYCEMIC CONTROL; PSYCHOSOCIAL FACTORS; CHRONIC

ILLNESS; SOCIAL SUPPORT; MELLITUS; FAMILY; ADJUSTMENT; INTERVENTION;

FRAMEWORK

AB OBJECTIVE - To prospectively assess the relation between marital relationship domains (i.e., intimacy and adjustment) and health-related quality of life (HRQoL) and glycemic control of individuals with diabetes.

RESEARCH DESIGN AND METHODS - A previous cross-sectional study assessed the relationship between marital quality and adaptation to diabetes for 78 insulin-treated adults. Each participant completed two marital quality measures (Spanier Dyadic Adjustment Scale and Personal Assessment of Intimacy in Relationships Scale) at time 1 (T1). A total of 61 subjects were contacted two years later (time 2 [T2]) and re-administered four HRQoL measures (Diabetes Quality of Life Scale, Medical Outcomes Study Health Survey-SF36, Problem Areas in Diabetes Scale, and Positive and Negative Affect Schedule). Glycemic control was assessed by HbA(1c). Demographic data (age, sex, type and duration of diabetes, years married, other medical problems, family history, and years of education) were gathered from charts and questionnaires. Linear regression analyses were used to assess the ability of the two marital measures at T1 to predict HRQoL at T2, controlling for confounding HRQoL and baseline subject variables.

RESULTS - Both of the marital quality measures at T1 were prospective predictors of aspects of HRQoL at T2. Less diabetes-related distress at T2 was predicted by better marital adjustment at T1. Greater satisfaction with aspects of the diabetes care regimen at T2 was predicted by better marital adjustment and greater perceived marital intimacy at T1. Neither marital measure predicted general HRQoL or glycemic control.

CONCLUSIONS - For insulin-treated adults with diabetes, quality of marriage prospectively predicts diabetes-related quality of life. This study highlights the sensitivity of diabetes-specific measures. Future work should explore the value of interventions that target the spouse and/or couple.

C1 SUNY Upstate Med Univ, Dept Psychiat, Syracuse, NY 13210 USA.

Vet Adm Med Ctr, Syracuse, NY 13210 USA.

RP Trief, PM (reprint author), SUNY Upstate Med Univ, Dept Psychiat, 750 E Adams St, Syracuse, NY 13210 USA.

OI Trief, Paula/0000-0002-2827-5722

CR Anderson RM, 1997, DIABETES CARE, V20, P299, DOI 10.2337/diacare.20.3.299

Bowling A, 1997, MEASURING HLTH REV Q

CARDENAS L, 1987, AM J MED SCI, V293, P28, DOI 10.1097/00000441-198701000-00007

Coyne JC, 1994, J FAMILY PSYCHOL, V8, P43, DOI DOI 10.1037/0893-3200.8.1.43

*DCCT RES GROUP, 1988, DIABETES CARE, V11, P727

Fisher L, 1998, DIABETES EDUCATOR, V24, P599, DOI 10.1177/014572179802400504

GARAYSEVILA ME, 1995, J DIABETES COMPLICAT, V9, P81, DOI 10.1016/1056-8727(94)00021-F

GILDEN JL, 1989, J AM GERIATR SOC, V37, P1023, DOI 10.1111/j.1532-5415.1989.tb06915.x

HANNUM J W, 1991, Journal of Psychosocial Oncology, V9, P1, DOI 10.1300/J077v09n02_01

JACOBSON AM, 1994, DIABETES CARE, V17, P267, DOI 10.2337/diacare.17.4.267

KLAUSNER EJ, 1995, INT J MENT HEALTH, V24, P64

LLOYD CE, 1992, DIABETES CARE, V15, P166, DOI 10.2337/diacare.15.2.166

LOCKE HJ, 1959, J MARRIAGE FAMILY LI, V21, P251

Lustman PJ, 2000, DIABETES CARE, V23, P934, DOI 10.2337/diacare.23.7.934

MCHORNEY CA, 1994, MED CARE, V32, P40, DOI 10.1097/00005650-199401000-00004

Meadows K, 1996, QUAL LIFE RES, V5, P242, DOI 10.1007/BF00434746

Nouwen A, 1997, HEALTH PSYCHOL, V16, P263

PEYROT M, 1985, PSYCHOSOM MED, V47, P542, DOI 10.1097/00006842-198511000-00004

POLONSKY WH, 1995, DIABETES CARE, V18, P754, DOI 10.2337/diacare.18.6.754

Polonsky WH, 2000, DIABETES SPECTRUM, V13, P36

PRIMOMO J, 1990, RES NURS HEALTH, V13, P153, DOI 10.1002/nur.4770130304

Quittner AL, 1998, HEALTH PSYCHOL, V17, P112, DOI 10.1037/0278-6133.17.2.112

RUBIN RR, 1992, DIABETES CARE, V15, P1640, DOI 10.2337/diacare.15.11.1640

SCHAEFER MT, 1981, J MARITAL FAM THER, V7, P47, DOI 10.1111/j.1752-0606.1981.tb01351.x

SCHWARTZ LS, 1991, GEN HOSP PSYCHIAT, V13, P19, DOI 10.1016/0163-8343(91)90005-H

SPANIER GB, 1976, J MARRIAGE FAM, V38, P15, DOI 10.2307/350547

Staquet MJ, 1998, QUALITY LIFE ASSESSM

TESTA M, 1998, DIABETES CARE S3, V21, P44

THARP RG, 1966, J CONSULT PSYCHOL, V30, P426, DOI 10.1037/h0023835

Trief PM, 1998, DIABETES CARE, V21, P241, DOI 10.2337/diacare.21.2.241

TRIEF PM, 2001, DIABETES CARE, V23, P1384

WARE JE, 1992, MED CARE, V30, P473, DOI 10.1097/00005650-199206000-00002

WARE JE, 1994, SF 36 PHYSLICAL MENT

WATSON D, 1988, J PERS SOC PSYCHOL, V54, P1063, DOI 10.1037/0022-3514.54.6.1063

WEINGER K, 1998, ANN BEHAV MED, V20, pS118

Welch GW, 1997, DIABETES CARE, V20, P760, DOI 10.2337/diacare.20.5.760

WING RR, 1991, J CONSULT CLIN PSYCH, V59, P156, DOI 10.1037/0022-006X.59.1.156

NR 37

TC 73

Z9 77

U1 0

U2 8

PU AMER DIABETES ASSOC

PI ALEXANDRIA

PA 1701 N BEAUREGARD ST, ALEXANDRIA, VA 22311-1717 USA

SN 0149-5992

J9 DIABETES CARE

JI Diabetes Care

PD JUL

PY 2002

VL 25

IS 7

BP 1154

EP 1158

DI 10.2337/diacare.25.7.1154

PG 5

WC Endocrinology & Metabolism

SC Endocrinology & Metabolism

GA 724UA

UT WOS:000185504000008

PM 12087013

OA Bronze

DA 2019-01-08

ER

PT J

AU Vaarasmaki, M

Gissler, M

Ritvanen, A

Hartikainen, AL

AF Vaarasmaki, M

Gissler, M

Ritvanen, A

Hartikainen, AL

TI Congenital anomalies and first life year surveillance in Type 1 diabetic

births

SO DIABETIC MEDICINE

LA English

DT Article

DE congenital anomaly; diabetic pregnancy; infant mortality; perinatal

mortality

ID PREGNANCY; POPULATION; WOMEN; MALFORMATIONS; MORTALITY; OUTCOMES;

NEWBORN; COHORT; INFANT; TRIAL

AB Aims To evaluate the rate of congenital anomalies (CA) and the reasons for mortality from 22 weeks of gestation until 1 year of age in births by Type I diabetic mothers.

Methods Population-based cohort study using combined data from four national health registers in Finland during 1991-1995, including 954 singleton pregnancies complicated by Type 1 diabetes.

Results Sixty births (629/10 000) involved registered major CA, of which 68% (n = 41) were isolated and 22% (n = 13) multiple anomalies, and in six cases, a syndrome was diagnosed (10%). After the exclusion of syndromes, the total number of anomalies was 73. Of the malformed infants, 63% were boys. The total rate of deaths among births until 1 year of age was higher in diabetic than in non-diabetic mothers (19.9/1000 vs. 8.1/1000): especially the rates of stillborns (odds ratio 2.4; 95% confidence interval 1.2-4.7) and post-neonatal deaths (3.8; 1.6-9.2) were higher. Of perinatal mortality (PNM) from the 22nd gestational week to the age of 7 days (13.6/1000), 23% were due to CA, 23% to prematurity, and the rest were intrauterine, mostly unexplained, deaths. Respiratory distress syndrome was the main cause of death among infants.

Conclusions The rate of CAs in Type 1 diabetic pregnancies is still high, but their proportion as a cause of PNM has decreased. Though PNM rate was low, post-neonatal mortality was significantly increased, reflecting the shift of deaths from the perinatal period to a later age.

C1 Univ Oulu, Dept Obstet & Gynaecol, Oulu, Finland.

STAKES, Helsinki, Finland.

RP Vaarasmaki, M (reprint author), Univ Hosp Oulu, Dept Obstet & Gynaecol, PL 24, OYS 90029, Finland.

CR ANTTILA M, 1993, ACTA U OUL D, V273

Casson IF, 1997, BRIT MED J, V315, P275, DOI 10.1136/bmj.315.7103.275

CNATTINGIUS S, 1994, DIABETIC MED, V11, P696, DOI 10.1111/j.1464-5491.1994.tb00335.x

DAMM P, 1989, AM J OBSTET GYNECOL, V161, P1163, DOI 10.1016/0002-9378(89)90656-X

Gissler M, 1999, ACTA PAEDIATR, V88, P310, DOI 10.1080/08035259950170088

GISSLER M, 1995, SCAND J SOC MED, V23, P75, DOI 10.1177/140349489502300113

Gissler M, 1997, EUR J EPIDEMIOL, V13, P169, DOI 10.1023/A:1007379029182

HANSON U, 1986, DIABETES RES CLIN EX, V3, P71

Hawthorne G, 1997, BRIT MED J, V315, P279, DOI 10.1136/bmj.315.7103.279

Machado AF, 2001, DIABETES, V50, P1193, DOI 10.2337/diabetes.50.5.1193

REECE EA, 1994, SEMIN PERINATOL, V18, P459

RITVANEN A, 1996, EPAMUODOSTUMAREKISTE, P1

RITVANEN A, 1998, MED GENETICS

SAARIKEMPPAINEN A, 1994, J PERINAT MED, V22, P279, DOI 10.1515/jpme.1994.22.4.279

SAARIKEMPPAINEN A, 1990, LANCET, V336, P387, DOI 10.1016/0140-6736(90)91941-3

Schwartz R, 2000, SEMIN PERINATOL, V24, P120, DOI 10.1053/sp.2000.6363

Suhonen L, 2000, DIABETOLOGIA, V43, P79, DOI 10.1007/s001250050010

Vaarasmaki MS, 2000, EARLY HUM DEV, V59, P61, DOI 10.1016/S0378-3782(00)00087-6

vonKries R, 1997, EUR J PEDIATR, V156, P963, DOI 10.1007/s004310050752

NR 19

TC 19

Z9 19

U1 0

U2 3

PU BLACKWELL PUBLISHING LTD

PI OXFORD

PA 9600 GARSINGTON RD, OXFORD OX4 2DG, OXON, ENGLAND

SN 0742-3071

J9 DIABETIC MED

JI Diabetic Med.

PD JUL

PY 2002

VL 19

IS 7

BP 589

EP 593

DI 10.1046/j.1464-5491.2002.00756.x

PG 5

WC Endocrinology & Metabolism

SC Endocrinology & Metabolism

GA 568BL

UT WOS:000176522500010

PM 12099963

DA 2019-01-08

ER

PT J

AU Hultman, CM

Sparen, P

Cnattingius, S

AF Hultman, CM

Sparen, P

Cnattingius, S

TI Perinatal risk factors for infantile autism

SO EPIDEMIOLOGY

LA English

DT Article

DE infantile autism; perinatal; risk factors; maternal; intrauterine growth

ID INTRAUTERINE-GROWTH; MATERNAL SMOKING; POPULATION; PREGNANCY;

SCHIZOPHRENIA; DISORDER; DELIVERY; COMPLICATIONS; EPIDEMIOLOGY;

CHILDHOOD

AB Background. Etiologic hypotheses in infantile autism suggest a strong genetic component, as well as possible environmental risks linked to early fetal development. We evaluated the association of maternal, pregnancy, delivery, and infant characteristics and risk of infantile autism.

Methods. We conducted a case-control study nested within a population-based cohort (all Swedish children born in 1974-1993). We used prospectively recorded data from the Swedish Birth Register, which were individually linked to the Swedish Inpatient Register. Cases were 408 children (321 boys and 87 girls) discharged with a main diagnosis ion infantile autism from any hospital in Sweden before 10 years of age in the period 1987-1994, plus 2,040 matched controls. Conditional logistic regression was used to calculate odds ratios (ORs) and 95% confidence intervals (CIs).

Results. The risk of autism was associated with daily smoking in early pregnancy (OR = 1.4; CI = 1.1-1.8), maternal birth outside Europe and North America (OR - 3.0; CI = 1.7-5.2), cesarean delivery (OR = 1.6; CI = 1.1-2.3), being small for gestational age (SGA; OR = 2.1; CI = 1.1-3.0), a 5-minute Apgar score below 7 (OR = 3.2, CI = 1.2-8.2), and congenital malformations (OR = 1.8, CI = 1.1-3.1). No association was found between autism and head circumference, maternal diabetes, being a twin, or season of birth.

Conclusions. Our findings suggest that intrauterine and neonatal factors related to deviant intrauterine growth or fetal distress are important in the pathogenesis of autism.

C1 Karolinska Inst, Dept Med Epidemiol, S-17177 Stockholm, Sweden.

Univ Uppsala, Dept Neurosci, S-75105 Uppsala, Sweden.

Univ Coll S Stockholm, Stockholm Ctr Hlth Soc Transit, Huddinge, Sweden.

RP Hultman, CM (reprint author), Karolinska Inst, Dept Med Epidemiol, S-17177 Stockholm, Sweden.

CR BAILEY A, 1995, PSYCHOL MED, V25, P63, DOI 10.1017/S0033291700028099

Bolton PF, 1997, J AM ACAD CHILD PSY, V36, P272, DOI 10.1097/00004583-199702000-00018

BRESLOW NE, 1980, IARC SCI PUB, V32

BROOKE OG, 1989, BRIT MED J, V298, P795, DOI 10.1136/bmj.298.6676.795

Bryson SE, 1998, MENT RETARD DEV D R, V4, P97, DOI 10.1002/(SICI)1098-2779(1998)4:2<97::AID-MRDD6>3.3.CO;2-J

Burd L, 1999, J PERINAT MED, V27, P441, DOI 10.1515/JPM.1999.059

CEFALO RC, 1978, J REPROD MED, V21, P300

CNATTINGIUS S, 1992, J EPIDEMIOL COMMUN H, V46, P218, DOI 10.1136/jech.46.3.218

CNATTINGIUS S, 1990, SCAND J SOC MED, V18, P143, DOI 10.1177/140349489001800209

Cryan E, 1996, J AUTISM DEV DISORD, V26, P453, DOI 10.1007/BF02172829

Dalen P., 1975, SEASON BIRTH STUDY S

David AS, 1997, PSYCHOL MED, V27, P1311, DOI 10.1017/S0033291797005680

ERICSON A, 1984, ACTA PAEDIATR SCAND, V73, P69, DOI 10.1111/j.1651-2227.1984.tb09900.x

FERGUSSON DM, 1993, PEDIATRICS, V92, P815

Fergusson DM, 1998, ARCH GEN PSYCHIAT, V55, P721, DOI 10.1001/archpsyc.55.8.721

Fombonne E, 1999, PSYCHOL MED, V29, P769, DOI 10.1017/S0033291799008508

Gillberg C, 1999, DEV PSYCHOPATHOL, V11, P567, DOI 10.1017/S0954579499002217

Gillberg C, 1999, ACTA PSYCHIAT SCAND, V99, P399, DOI 10.1111/j.1600-0447.1999.tb00984.x

Gillberg IC, 1996, J INTELL DISABIL RES, V40, P24, DOI 10.1111/j.1365-2788.1996.tb00599.x

Hultman CM, 1999, BRIT MED J, V318, P421, DOI 10.1136/bmj.318.7181.421

Jedrychowski W, 1998, J EXPO ANAL ENV EPID, V8, P347

Jones PB, 1998, AM J PSYCHIAT, V155, P355, DOI 10.1176/ajp.155.3.355

Juul-Dam N, 2001, PEDIATRICS, V107, part. no., DOI 10.1542/peds.107.4.e63

Kiely J L, 1991, Paediatr Perinat Epidemiol, V5, P243, DOI 10.1111/j.1365-3016.1991.tb00707.x

KYLLERMAN M, 1982, ACTA PAEDIATR SCAND, V71, P551, DOI 10.1111/j.1651-2227.1982.tb09473.x

Lindmark G, 1991, Acta Obstet Gynecol Scand, V70, P105, DOI 10.3109/00016349109006190

LOBASCHER ME, 1970, BRIT J PSYCHIAT, V117, P525, DOI 10.1192/bjp.117.540.525

London E, 2000, ENVIRON HEALTH PERSP, V108, P401, DOI 10.1289/ehp.00108s3401

Marsal K, 1996, ACTA PAEDIATR, V85, P843, DOI 10.1111/j.1651-2227.1996.tb14164.x

Matsuishi T, 1999, J AUTISM DEV DISORD, V29, P161, DOI 10.1023/A:1023048812202

MILLBERGER S, 1996, AM J PSYCHIAT, V153, P1138

NELSON KB, 1991, PEDIATRICS, V87, P761

PALO P, 1993, AM J PERINAT, V10, P88, DOI 10.1055/s-2007-994712

Rodier PM, 1996, J COMP NEUROL, V370, P247, DOI 10.1002/(SICI)1096-9861(19960624)370:2<247::AID-CNE8>3.0.CO;2-2

SMALLEY SL, 1988, ARCH GEN PSYCHIAT, V45, P953

STROMLAND K, 1994, DEV MED CHILD NEUROL, V36, P351

THORNGRENJERNEC.K, 2001, AM COLL OBSTET GYNEC, V98, P65

TSAI LY, 1987, NEUROBIOLOGICAL ISSU, P180

WARREN RP, 1986, J AUTISM DEV DISORD, V16, P189, DOI 10.1007/BF01531729

Wyszynski DF, 1997, CLEFT PALATE-CRAN J, V34, P206, DOI 10.1597/1545-1569(1997)034<0206:MCSAOC>2.3.CO;2

NR 40

TC 320

Z9 329

U1 2

U2 41

PU LIPPINCOTT WILLIAMS & WILKINS

PI PHILADELPHIA

PA 530 WALNUT ST, PHILADELPHIA, PA 19106-3621 USA

SN 1044-3983

J9 EPIDEMIOLOGY

JI Epidemiology

PD JUL

PY 2002

VL 13

IS 4

BP 417

EP 423

DI 10.1097/01.EDE.0000016968.14007.E6

PG 7

WC Public, Environmental & Occupational Health

SC Public, Environmental & Occupational Health

GA 565PL

UT WOS:000176378600009

PM 12094096

DA 2019-01-08

ER

PT J

AU Feinstein, KW

Grunden, N

Harrison, EI

AF Feinstein, KW

Grunden, N

Harrison, EI

TI A region addresses patient safety

SO AMERICAN JOURNAL OF INFECTION CONTROL

LA English

DT Article

AB The Pittsburgh Regional Healthcare Initiative (PRHI) is a coalition of 35 hospitals, 4 major insurers, more than 30 major and small-business health care purchasers, dozens of corporate and civic leaders, organized labor, and partnerships with state and federal government all working together to deliver perfect patient care throughout Southwestern Pennsylvania. PRHI believes that in pursuing perfection, many of the challenges facing today's health care delivery system (eg, waste and error in the delivery of care, rising costs, frustration and shortage among clinicians and workers, financial distress, overcapacity, and lack of access to care) will be addressed. PRHI has identified patient safety (nosocomial infections and medication errors) and 5 clinical areas (obstetrics, orthopedic surgery, cardiac surgery, depression, and diabetes) as ideal starting points. In each of these areas of work, PRHI partners have assembled multifacility/multidisciplinary groups charged with defining perfection, establishing region-wide reporting systems, and devising and implementing recommended improvement strategies and interventions. Many design and conceptual elements of the PRHI strategy are adapted from the Toyota Production System and its Pittsburgh derivative, the Alcoa Business System. PRHI is in the proof-of-concept phase of development.

C1 Jewish Healthcare Fdn, Pittsburgh Reg Healthcare Initiat, Pittsburgh, PA 15222 USA.

RP Harrison, EI (reprint author), Jewish Healthcare Fdn, Pittsburgh Reg Healthcare Initiat, Ctr City Tower,650 Smithfield St,Suite 2330, Pittsburgh, PA 15222 USA.

CR Institute of Medicine, 1999, ERR HUM

NR 1

TC 7

Z9 7

U1 0

U2 2

PU MOSBY, INC

PI ST LOUIS

PA 11830 WESTLINE INDUSTRIAL DR, ST LOUIS, MO 63146-3318 USA

SN 0196-6553

J9 AM J INFECT CONTROL

JI Am. J. Infect. Control

PD JUN

PY 2002

VL 30

IS 4

BP 248

EP 251

AR UNSP 17/49/122645

DI 10.1067/mic.2002.122645

PG 4

WC Public, Environmental & Occupational Health; Infectious Diseases

SC Public, Environmental & Occupational Health; Infectious Diseases

GA 559VB

UT WOS:000176045100009

PM 12032502

DA 2019-01-08

ER

PT J

AU Turan, B

Osar, Z

Turan, JM

Damci, T

Ilkova, H

AF Turan, B

Osar, Z

Turan, JM

Damci, T

Ilkova, H

TI The role of coping with disease in adherence to treatment regimen and

disease control in type 1 and insulin treated type 2 diabetes mellitus

SO DIABETES & METABOLISM

LA English

DT Article

DE coping; diabetes mellitus; self-care; microangiopathic complications;

glycemic control

ID BREAST-CANCER; STRESS; STRATEGIES; DISTRESS

AB Background: Coping is defined as the behavioral and cognitive efforts used in an attempt to deal with stressful events. The objective of this study was to explore the relationship between coping with diabetes and the following outcome variables in type I and insulin treated type 2 diabetes mellitus: glycemic control, microangiopathic complications, adherence to self monitoring of blood glucose, adherence to insulin injections, and adherence to diet.

Methods: Subjects were 196 insulin treated adult diabetes patients visiting an outpatient clinic at a government university hospital in Istanbul, Turkey. Coping with disease was measured with the Turkish version of the Diabetes Coping Measure and adherence to treatment regimen was measured with a questionnaire adapted from the subscales of the Summary of Diabetes Self-Care Activities Questionnaire. Data on patients' HbA(1c) levels and severity of microangiopathic complications were obtained from their medical records.

Results: Partial correlations controlling for background variables suggested that coping was a good predictor of outcome for both type I and insulin treated type 2 diabetes mellitus. These associations were more pronounced for type I patients when compared to type 2 patients. Regressing the outcome variables on the two second-order coping factors (obtained by a factor analysis) also supported the hypothesis that coping is an important construct in explaining the outcome variables. Finally, the effect of coping on HbA(1c) was only partially mediated by adherence.

Conclusion: Coping with diabetes-related issues is an important factor in both types of diabetes, with type 1 patients showing slightly stronger associations. Therefore, training and education programs for diabetic adults might benefit from including a component that is aimed at improving coping with issues specific to diabetes.

C1 Univ Istanbul, Cerrahpasa Med Fac, Dept Internal Med, Div Metab Endocrinol & Diabet, Istanbul, Turkey.

Univ Istanbul, Inst Child Hlth, Dept Family Hlth, Istanbul, Turkey.

RP Turan, B (reprint author), Stanford Univ, Dept Psychol, Bldg 420,Jordan Hall,450 Serra Mall, Stanford, CA 94305 USA.

OI Turan, Bulent/0000-0003-2008-227X

CR ARMITAGE P, 1987, STAT METHODS MED RES

BARON RM, 1986, J PERS SOC PSYCHOL, V51, P1173, DOI 10.1037//0022-3514.51.6.1173

BILLINGS AG, 1984, J PERS SOC PSYCHOL, V46, P877, DOI 10.1037/0022-3514.46.4.877

CARVER CS, 1993, J PERS SOC PSYCHOL, V65, P375, DOI 10.1037/0022-3514.65.2.375

DAVIS MD, 1997, INT TXB DIABETES MEL, V2, P1413

DELAMATER AM, 1987, J DEV BEHAV PEDIATR, V8, P136

FEIFEL H, 1987, PSYCHOSOM MED, V49, P616, DOI 10.1097/00006842-198711000-00007

FEIFEL H, 1987, J PSYCHOSOM RES, V31, P91, DOI 10.1016/0022-3999(87)90103-6

FELTON BJ, 1984, J CONSULT CLIN PSYCH, V52, P343, DOI 10.1037/0022-006X.52.3.343

Glasgow RE, 1999, DIABETES CARE, V22, P832, DOI 10.2337/diacare.22.5.832

GOETSCH VL, 1994, BEHAV RES THER, V32, P503, DOI 10.1016/0005-7967(94)90137-6

HANSON CL, 1989, J CONSULT CLIN PSYCH, V57, P644, DOI 10.1037//0022-006X.57.5.644

Holmbeck GN, 1997, J CONSULT CLIN PSYCH, V65, P599, DOI 10.1037/0022-006X.65.4.599

JOHNSON SB, 1992, DIABETES CARE, V15, P1658, DOI 10.2337/diacare.15.11.1658

Lazarus R. S, 1984, STRESS APPRAISAL COP

Miller SM, 1989, STRESS PERSONAL CONT, P107

Peyrot M, 1999, J HEALTH SOC BEHAV, V40, P141, DOI 10.2307/2676370

Schwartz CE, 1998, BEHAV MED, V24, P41, DOI 10.1080/08964289809596380

SHAMOON H, 1993, NEW ENGL J MED, V329, P977

Smari J, 1997, PERS INDIV DIFFER, V22, P151, DOI 10.1016/S0191-8869(96)00199-7

STANTON AL, 1993, HEALTH PSYCHOL, V12, P16, DOI 10.1037//0278-6133.12.1.16

STONE AA, 1992, HOSTILITY, COPING & HEALTH, P203, DOI 10.1037/10105-014

TOOBERT DJ, 1989, HDB PSYCHOL DIABETES, P351

Turner RC, 1998, LANCET, V352, P837

Welch G., 1994, HDB PSYCHOL DIABETES, P223

Welch G, 1994, HDB PSYCHOL DIABETES, P400

Welch GW, 1997, DIABETES CARE, V20, P760, DOI 10.2337/diacare.20.5.760

*WHO, 1985, WHO TECH REP SER, V727

NR 28

TC 23

Z9 23

U1 2

U2 15

PU MASSON EDITEUR

PI PARIS 06

PA 120 BLVD SAINT-GERMAIN, 75280 PARIS 06, FRANCE

SN 1262-3636

J9 DIABETES METAB

JI Diabetes Metab.

PD JUN

PY 2002

VL 28

IS 3

BP 186

EP 193

PG 8

WC Endocrinology & Metabolism

SC Endocrinology & Metabolism

GA 576CJ

UT WOS:000176985000003

PM 12149598

DA 2019-01-08

ER

PT J

AU DeVries, JH

Eskes, SA

Snoek, FJ

Pouwer, F

van Ballegooie, E

Spijker, AJ

Kostense, PJ

Seubert, M

Heine, RJ

AF DeVries, JH

Eskes, SA

Snoek, FJ

Pouwer, F

van Ballegooie, E

Spijker, AJ

Kostense, PJ

Seubert, M

Heine, RJ

TI Continuous intraperitioneal insulin infusion in patients with 'brittle'

diabetes: favourable effects on glycaemic control and hospital stay

SO DIABETIC MEDICINE

LA English

DT Article

DE continuous intraperitoneal insulin infusion; insulin infusion systems;

quality of life

ID LONG-TERM SAFETY; IMPLANTABLE PUMPS; CLINICAL-TRIAL; DELIVERY;

COMPLICATIONS; MELLITUS; VALIDITY; DISTRESS; EFFICACY; SYSTEMS

AB Aims To evaluate the effects of continuous intraperitoneal insulin infusion (CIPII) using implantable pumps on glycaemic control and duration of hospital stay in poorly controlled 'brittle' Dutch diabetes patients, and to assess their current quality of life.

Methods Thirty-three patients were included. Glycaemic control was retrospectively assessed with HbA(1c) levels acquired before implantation, I year later and at long-term follow up of 58 months. Duration of hospital stay the year before and the year following first implantation was extracted from hospital records. Determinants of long-term glycaemic response were sought. Self-report questionnaires were administered at 58 months follow-up only, to assess current psychopathology and quality of life.

Results Mean HbA(1c) decreased from -10.0 +/- 2.3% to 9.0 +/- 1.8% (P = 0.039) 1 year after implantation and stabilized at 9.0 +/- 1.6% (P = 0.023) during long-term follow-up. Median number of hospital days in the 20 patients suffering from hospital admission before implantation decreased from 45 the year before implantation to 13 the year after (P = 0.005). Patients with a higher baseline HbA(1c) showed a larger long-term response (P < 0.001). Relatively low levels for quality of life were found, as well as a higher than expected number of patients with psychiatric symptoms.

Conclusions CIPII proved effective in complex patients with a history of poor control and hospital admission. Despite a substantial long-term improvement in glycaemic control and diminished hospital stay, normal levels of glycaemic control and quality of life were not attained.

C1 Vrije Univ Amsterdam Med Ctr, Dept Endocrinol, NL-1007 MB Amsterdam, Netherlands.

Vrije Univ Amsterdam Med Ctr, Res Inst Endocrinol Reprod & Metab, NL-1007 MB Amsterdam, Netherlands.

Vrije Univ Amsterdam Med Ctr, Dept Psychol Med, NL-1007 MB Amsterdam, Netherlands.

Isala Hosp, Zwolle, Netherlands.

Laurentius Hosp, Roermond, Netherlands.

Vrije Univ Amsterdam Med Ctr, Dept Clin Epidemiol, NL-1007 MB Amsterdam, Netherlands.

RP DeVries, JH (reprint author), Vrije Univ Amsterdam Med Ctr, Dept Endocrinol, 1 OBU 42,POB 7057, NL-1007 MB Amsterdam, Netherlands.

EM hans.devries@vumc.nl

CR Arrindell W, 1981, NEDERLANDS TIJDSCHRI, V36, P77

BROUSSOLLE C, 1994, LANCET, V343, P514, DOI 10.1016/S0140-6736(94)91462-1

DCCT Res Grp, 1988, DIABETES CARE, V11, P725

Derogatis L. R., 1977, SCL 90 ADM SCORING P

Diabet Control Complications Trial Res Grp, 1996, DIABETES CARE, V19, P195

GARDNER SB, 1989, STAT CONFIDENCE

GILL GV, 1991, BRIT MED J, V303, P285, DOI 10.1136/bmj.303.6797.285

HAARDT MJ, 1994, DIABETES CARE, V17, P847, DOI 10.2337/diacare.17.8.847

HEPP KD, 1988, LANCET, V2, P866

Irsigler K, 1990, DIABETES NUTR METAB, V3, P191

JACOBSON AM, 1990, DIABETES CARE, V13, P375, DOI 10.2337/diacare.13.4.375

Jacobson AM, 1997, QUAL LIFE RES, V6, P11

JEANDIDIER N, 1992, TRANSPLANT P, V24, P948

LLOYD CE, 1992, DIABETES CARE, V15, P166, DOI 10.2337/diacare.15.2.166

MCHORNEY CA, 1993, MED CARE, V31, P247, DOI 10.1097/00005650-199303000-00006

*MED OUTC TRUST, 1994, SCOR SF 36 HLTH SURV

OLSEN CL, 1993, INT J ARTIF ORGANS, V16, P847

Pinget M, 1998, HORM METAB RES, V30, P475, DOI 10.1055/s-2007-978916

PITT HA, 1992, ANN SURG, V216, P483, DOI 10.1097/00000658-199210000-00011

POLONSKY WH, 1995, DIABETES CARE, V18, P754, DOI 10.2337/diacare.18.6.754

Pouwer F, 1999, DIABETES CARE, V22, P2004, DOI 10.2337/diacare.22.12.2004

RENARD E, 1995, DIABETES CARE, V18, P300, DOI 10.2337/diacare.18.3.300

Renard E, 1999, DIABETES CARE, V22, P1371, DOI 10.2337/diacare.22.8.1371

Rubin RR, 1998, DIABETES SPECTRUM, V11, P81

Rydall AC, 1997, NEW ENGL J MED, V336, P1849, DOI 10.1056/NEJM199706263362601

Saudek CD, 1996, JAMA-J AM MED ASSOC, V276, P1322, DOI 10.1001/jama.276.16.1322

SAUDEK CD, 1989, NEW ENGL J MED, V321, P574, DOI 10.1056/NEJM198908313210904

SELAM JL, 1992, DIABETES CARE, V15, P877, DOI 10.2337/diacare.15.7.877

SELAM JL, 1992, DIABETES CARE, V15, P53, DOI 10.2337/diacare.15.1.53

SHAMOON H, 1993, NEW ENGL J MED, V329, P977

Snoek FJ, 2000, DIABETES CARE, V23, P1305, DOI 10.2337/diacare.23.9.1305

*SPSS, 2000, SPSS BASE 9 0 APPL G

NR 32

TC 22

Z9 22

U1 0

U2 3

PU WILEY-BLACKWELL

PI HOBOKEN

PA 111 RIVER ST, HOBOKEN 07030-5774, NJ USA

SN 0742-3071

EI 1464-5491

J9 DIABETIC MED

JI Diabetic Med.

PD JUN

PY 2002

VL 19

IS 6

BP 496

EP 501

DI 10.1046/j.1464-5491.2002.00727.x

PG 6

WC Endocrinology & Metabolism

SC Endocrinology & Metabolism

GA 559LQ

UT WOS:000176028000010

PM 12060062

OA Green Published

DA 2019-01-08

ER

PT J

AU Mestman, JH

AF Mestman, JH

TI Historical notes on diabetes and pregnancy

SO ENDOCRINOLOGIST

LA English

DT Review

ID RESPIRATORY-DISTRESS SYNDROME; METABOLIC CONTROL; AMNIOTIC-FLUID;

BLOOD-GLUCOSE; MOTHERS; MANAGEMENT; MELLITUS; INSULIN; RISK;

MALFORMATION

AB During the past two decades, as the culmination of many years of basic and clinical research in different fields of maternal-fetal-neonatal medicine, there has been a dramatic improvement in perinatal mortality and morbidity in diabetes pregnancy. Today, the chances for most women with diabetes mellitus to have a successful pregnancy are almost as good as those of a woman without diabetes mellitus, providing the health care professional and the patient adhere to the guidelines and recommendations accepted by most workers in this area. In this brief historical review, several stages in the diagnosis and management of diabetes and pregnancy are discussed, based on hallmark articles from the medical literature. After a review of the years before the discovery of insulin, the author describes each decade from 1922 until the end of 1970, [Fig. 1), in which, in his opinion, the most remarkable gains in the care of pregnant women with diabetes and neonatal health had occurred. During the past twenty years, numerous reports in the literature have reaffirmed the success of strict metabolic control, before and during pregnancy, and the proper use and interpretation of fetal well-being tests in the improvement of fetal outcome. The mechanisms of the cause of congenital malformations, the multiple factors affecting fetal growth and development, and its potential long-term effects on the offspring of diabetic mothers are the focus at the present time of intensive investigation.

C1 Univ So Calif, Keck Sch Med, Ctr Diabet & Metab Dis, Los Angeles, CA USA.

RP Mestman, JH (reprint author), 1355 San Pablo St,Suite 121, Los Angeles, CA 90033 USA.

CR Allen E, 1939, AM J OBSTET GYNECOL, V38, P982, DOI 10.1016/S0002-9378(39)90641-1

BARNES PH, 1961, CAN MED ASSOC J, V85, P681

BENNEWITZ HG, 1828, EDINBURGH MED SURG J, V30, P217

BHAGWANANI SG, 1972, LANCET, V1, P159

BLOT, 1883, COMPTES RENDUS SOC B, V5, P193

BROCARD, 1898, THESIS PARIS

BROWN JB, 1963, J OBSTET GYN BR COMM, V70, P219

CAMPBELL S, 1968, Journal of Obstetrics and Gynaecology of the British Commonwealth, V75, P568

CARRINGTON ER, 1957, OBSTET GYNECOL, V9, P664, DOI 10.1097/00006250-195706000-00008

CARRINGTON ER, 1958, JAMA-J AM MED ASSOC, V166, P245, DOI 10.1001/jama.1958.02990030043009

CARRINGTON ER, 1963, AM J OBSTET GYNECOL, V85, P669, DOI 10.1016/0002-9378(63)90314-4

CLEMENTS JA, 1972, NEW ENGL J MED, V286, P1077, DOI 10.1056/NEJM197205182862004

Coetzee EJ, 1985, DIABETES RES CLIN PR, V1, P281

COLLIER E, 1991, DIABETES, V40, P197, DOI 10.2337/diabetes.40.2.197

CONN JW, 1961, AM J MED, V31, P839, DOI 10.1016/0002-9343(61)90023-7

COUSTAN DR, 1980, AM J MED, V68, P845, DOI 10.1016/0002-9343(80)90204-1

COUSTAN DR, 1984, AM J OBSTET GYNECOL, V150, P836, DOI 10.1016/0002-9378(84)90459-9

DISTLER W, 1978, AM J OBSTET GYNECOL, V130, P424, DOI 10.1016/0002-9378(78)90284-3

DUBREUIL G, 1920, ANDREODIAS COMPT REN, V133, P1490

Duncan JM, 1882, T OBSTET SOC LONDON, V24, P256

ESSEX NL, 1973, BMJ-BRIT MED J, V4, P89, DOI 10.1136/bmj.4.5884.89

FRAINKEL N, 1980, DIABETES CARE, V3, P399

FREINKEL N, 1980, DIABETES, V29, P1023, DOI 10.2337/diabetes.29.12.1023

FUHRMANN K, 1984, EXP CLIN ENDOCRINOL, V83, P173, DOI 10.1055/s-0029-1210327

GABBE SG, 1977, AM J OBSTET GYNECOL, V129, P723, DOI 10.1016/0002-9378(77)90388-X

GABBE SG, 1977, AM J OBSTET GYNECOL, V127, P465, DOI 10.1016/0002-9378(77)90436-7

GILLMER MDG, 1975, BRIT MED J, V3, P399, DOI 10.1136/bmj.3.5980.399

GILLMER MDG, 1975, CARBOHYD METABOL, P168

GLUCK L, 1971, AM J OBSTET GYNECOL, V109, P440, DOI 10.1016/0002-9378(71)90342-5

Graham G, 1924, Proc R Soc Med, V17, P102

GREENE JW, 1963, AM J OBSTET GYNECOL, V85, P1

GREENE MF, 1989, TERATOLOGY, V39, P225, DOI 10.1002/tera.1420390303

HAGBARD L, 1960, DIABETES, V9, P296, DOI 10.2337/diab.9.4.296

HARLEY JMG, 1965, BMJ-BRIT MED J, V1, P14, DOI 10.1136/bmj.1.5426.14

HERZSTEIN J, 1946, AM J OBSTET GYNECOL, V51, P420, DOI 10.1016/S0002-9378(16)40022-0

HOET JJ, 1979, CIBA F S, V63, P283

HOET JP, 1954, DIABETES, V3, P1

HUNTINGFORD P J, 1969, Journal of Obstetrics and Gynaecology of the British Commonwealth, V76, P586

JACKSON W. P. U., 1953, S AFRICAN MED JOUR, V27, P795

JACKSON WPU, 1960, DIABETES, V9, P373, DOI 10.2337/diab.9.5.373

JONES WS, 1958, DIABETES, V7, P439, DOI 10.2337/diab.7.6.439

KARLSSON K, 1972, AM J OBSTET GYNECOL, V112, P213, DOI 10.1016/0002-9378(72)90118-4

KEMBALL ML, 1970, ARCH DIS CHILD, V45, P696, DOI 10.1136/adc.45.243.696

KOENIG RJ, 1976, DIABETES, V25, P230, DOI 10.2337/diabetes.25.3.230

KUBLI FW, 1969, FOETO PLACENTAL UNIT, P323

Lambie CG, 1926, J OBSTET GYN BR EMP, V33, P563

Langer O, 2000, NEW ENGL J MED, V343, P1134, DOI 10.1056/NEJM200010193431601

LAWRENCE RD, 1927, P ROY SOC MED, V21, P9

LECORCHE, 1885, ANN GYNEC, V24, P257

LEVER JCW, 1847, GUYS HOSP REPORT, V3

MANNING F, 1980, AM J OBSTET GYNECOL, V136, P878

MESTMAN JH, 1971, AM J OBSTET GYNECOL, V109, P41, DOI 10.1016/0002-9378(71)90831-3

MESTMAN JH, 1964, DIABETES, V13, P383, DOI 10.2337/diab.13.4.383

METZGER BE, 1980, DIABETES CARE, V3, P402, DOI 10.2337/diacare.3.3.402

METZGER BE, 1998, DIABETES CARE S2, V21, pB1

MILLER E, 1981, NEW ENGL J MED, V304, P1331, DOI 10.1056/NEJM198105283042204

MILLER HC, 1945, NEW ENGL J MED, V233, P376, DOI 10.1056/NEJM194509272331302

Miller HC, 1943, J PEDIATR-US, V23, P251, DOI 10.1016/S0022-3476(43)80001-9

MOSS JM, 1951, ANN INTERN MED, V34, P678, DOI 10.7326/0003-4819-34-3-678

NELSON DH, 1963, J CLIN ENDOCR METAB, V23, P261, DOI 10.1210/jcem-23-3-261

NELSON HB, 1953, OBSTET GYNECOL, V1, P219

OAKLEY W, 1953, BRIT MED J, V1, P1413, DOI 10.1136/bmj.1.4825.1413

OSULLIVAN J, 1961, NEW ENGL J MED, V264, P1082, DOI 10.1056/NEJM196105252642104

OSULLIVAN JB, 1964, DIABETES, V13, P278

PARTRIDGE, 1895, MED RECORD, V17, P109

PEDERSEN J, 1965, ACTA ENDOCRINOL-COP, V50, P70, DOI 10.1530/acta.0.0500070

PEDERSEN J, 1954, ACTA ENDOCRINOL-COP, V15, P282, DOI 10.1530/acta.0.0150282

PEDERSEN J, 1956, LANCET, V27, P607

PEEL J, 1962, AM J OBSTET GYNECOL, V83, P847

PEEL J, 1972, J OBSTET GYN BR COMM, V79, P385

PERSSON B, 1975, CARBOHYD METABOL, P232

PIRAT J, 1955, ACTA ENDOCRINOL-COP, V10, P192

QUINN JS, 1930, IRISH J MED SCI DUBL, P612

RAY M, 1972, AM J OBSTET GYNECOL, V114, P1

REID DD, 1955, LANCET, V2, P833

Reveno WS, 1923, J AMER MED ASSOC, V81, P2101, DOI 10.1001/jama.1923.02650250029007

RIVLIN ME, 1970, AM J OBSTET GYNECOL, V106, P875, DOI 10.1016/0002-9378(70)90482-5

ROVERSI GD, 1979, AM J OBSTET GYNECOL, V135, P567, DOI 10.1016/S0002-9378(16)32978-7

RUBIN BL, 1946, J CLIN ENDOCR METAB, V6, P347, DOI 10.1210/jcem-6-5-347

SKIPPER E, 1933, QJM, V7, P353

SMITH, 1947, J SURG, V55, P288

SONKSEN PH, 1978, LANCET, V1, P729

SUTHERLAND HW, 1973, BRIT MED J, V20, P9

TOWNER D, 1995, DIABETES CARE, V18, P1446, DOI 10.2337/diacare.18.11.1446

UNGER RH, 1958, DIABETES, V7, P455, DOI 10.2337/diab.7.6.455

VONNOORDEN C, 1917, ZUCKERKRANKHEIT

WALFORD S, 1978, LANCET, V1, P732

Walker A, 1927, P ROY SOC MED, Vxxi, P13

WHITE P, 1965, MED CLIN N AM, V49, P1015, DOI 10.1016/S0025-7125(16)33292-8

White P, 1943, J CLIN ENDOCRINOL, V3, P500, DOI 10.1210/jcem-3-9-500

WHITE P, 1949, AM J MED, V7, P609, DOI 10.1016/0002-9343(49)90382-4

Wilder RM, 1928, COLORADO MED, V25, P372

WILKERSON HLC, 1957, DIABETES, V6, P324, DOI 10.2337/diab.6.4.324

Williams JW, 1909, AM J MED SCI, V137, P1

1987, HOFMEISTER Z F, V1, P101

1985, DIABETES, V34

NR 96

TC 11

Z9 11

U1 0

U2 2

PU LIPPINCOTT WILLIAMS & WILKINS

PI PHILADELPHIA

PA 530 WALNUT ST, PHILADELPHIA, PA 19106-3621 USA

SN 1051-2144

J9 ENDOCRINOLOGIST

JI Endocrinologist

PD MAY-JUN

PY 2002

VL 12

IS 3

AR UNSP 0021-972X/2002/1203-0224

DI 10.1097/00019616-200205000-00010

PG 19

WC Endocrinology & Metabolism

SC Endocrinology & Metabolism

GA 562EB

UT WOS:000176183500009

DA 2019-01-08

ER

PT J

AU De Berardis, G

Franciosi, M

Belfiglio, M

Di Nardo, B

Greenfield, S

Kaplan, SH

Pellegrini, F

Sacco, M

Tognoni, G

Valentini, M

Nicolucci, A

AF De Berardis, G

Franciosi, M

Belfiglio, M

Di Nardo, B

Greenfield, S

Kaplan, SH

Pellegrini, F

Sacco, M

Tognoni, G

Valentini, M

Nicolucci, A

CA QuED Study Grp

TI Erectile dysfunction and quality of life in type 2 diabetic patients - A

serious problem too often overlooked

SO DIABETES CARE

LA English

DT Article

ID CORONARY HEART-DISEASE; DEPRESSIVE SYMPTOMS; OF-LIFE; ALPROSTADIL

THERAPY; SILDENAFIL CITRATE; PREVALENCE; MEN; IMPOTENCE; HEALTH; ADULTS

AB OBJECTIVE - Within the context of a large, nationwide outcomes research program in type 2 diabetes, we assess the prevalence of self-reported erectile dysfunction and evaluate its impact on quality of life.

RESEARCH DESIGN AND METHODS - The study involved 1,460 patients enrolled by 114 diabetes outpatient clinics and 112 general practitioners. Patients were asked to complete a questionnaire investigating their ability to achieve and maintain an erection. Various aspects of quality of life were also assessed depressive using the following instruments: SF-36 Health Survey, diabetes health distress, psychological adaptation to diabetes, depressive symptoms (CES-D scale), and quality of sexual life.

RESULTS - Overall, 34% of the patients reported frequent erectile problems, 24% reported occasional problems, and 42% reported no erectile problems. After adjusting for patient characteristics, erectile dysfunction was associated with higher levels of diabetes-specific health distress and worse psychological adaptation to diabetes, which were, in turn, related to worse metabolic control. Erectile problems were also associated With a dramatic increase in the prevalence of severe depressive symptoms, lower scores in the mental components of the SF-36, and a less satisfactory sexual life. A total of 63% of the patients reported that their physicians had never investigated their sexual problems.

CONCLUSIONS - Erectile dysfunction is extremely common among type 2 diabetic patients and is associated With poorer quality of life, as measured with generic and diabetes-specific instruments. Despite their relevance, sexual problems are seldom investigated by general practitioners and specialists.

C1 Ist Ric Farmacol Mario Negri, Consorzio Mario Negri Sud, Dept Clin Pharmacol & Epidemiol, I-66030 Santa Maria Imbaro, Italy.

Tufts Univ, Sch Med, Boston, MA 02111 USA.

RP Nicolucci, A (reprint author), Ist Ric Farmacol Mario Negri, Consorzio Mario Negri Sud, Dept Clin Pharmacol & Epidemiol, Via Nazl, I-66030 Santa Maria Imbaro, Italy.

EM nicolucc@cmns.mnegri.it

OI Greenfield, Sheldon/0000-0003-4628-1998; Valentini,

Miriam/0000-0002-1421-6975

CR Amato L, 1996, DIABETES METAB, V22, P314

Ansong KS, 1998, UROLOGY, V52, P834, DOI 10.1016/S0090-4295(98)00397-5

Apolone G, 1998, J CLIN EPIDEMIOL, V51, P1025, DOI 10.1016/S0895-4356(98)00094-8

Araujo AB, 1998, PSYCHOSOM MED, V60, P458, DOI 10.1097/00006842-199807000-00011

Ariyo AA, 2000, CIRCULATION, V102, P1773

BRAUNSTEIN GD, 1987, MT SINAI J MED, V54, P236

Chew KK, 2000, INT J IMPOT RES, V12, P41, DOI 10.1038/sj.ijir.3900457

COMSTOCK GW, 1976, PSYCHOL MED, V6, P551

de Leon CFM, 1998, ARCH INTERN MED, V158, P2341

DETEJADA IS, 1988, UROL CLIN N AM, V15, P17

DROLLER MJ, 1993, JAMA-J AM MED ASSOC, V270, P83

DUNN SM, 1986, DIABETES CARE, V9, P480, DOI 10.2337/diacare.9.5.480

Fedele D, 1998, DIABETES CARE, V21, P1973, DOI 10.2337/diacare.21.11.1973

Fedele D, 2000, INT J EPIDEMIOL, V29, P524, DOI 10.1093/ije/29.3.524

FELDMAN HA, 1994, J UROLOGY, V151, P54, DOI 10.1016/S0022-5347(17)34871-1

Ferketich AK, 2000, ARCH INTERN MED, V160, P1261, DOI 10.1001/archinte.160.9.1261

Franciosi M, 2001, DIABETES CARE, V24, P1870, DOI 10.2337/diacare.24.11.1870

Gary TL, 2000, DIABETES CARE, V23, P23, DOI 10.2337/diacare.23.1.23

GAVARD JA, 1993, DIABETES CARE, V16, P1167, DOI 10.2337/diacare.16.8.1167

Goldstein I, 2000, AM J CARDIOL, V86, p41F

GREENFIELD S, 1994, DIABETES CARE, V17, P32

GREENFIELD S, 1995, MED CARE, V33, pAS47

Hakim LS, 1996, ENDOCRIN METAB CLIN, V25, P379, DOI 10.1016/S0889-8529(05)70329-7

Jarow JP, 1999, J UROLOGY, V162, P722, DOI 10.1097/00005392-199909010-00024

Jonas BS, 1997, ARCH FAM MED, V6, P43, DOI 10.1001/archfami.6.1.43

Klein R, 1996, DIABETES CARE, V19, P135, DOI 10.2337/diacare.19.2.135

Langtry HD, 1999, DRUGS, V57, P967, DOI 10.2165/00003495-199957060-00015

Laumann EO, 1999, JAMA-J AM MED ASSOC, V281, P537, DOI 10.1001/jama.281.6.537

Lyness JM, 1997, ARCH INTERN MED, V157, P449, DOI 10.1001/archinte.157.4.449

MCCULLOCH DK, 1980, DIABETOLOGIA, V18, P279

McMahon CG, 2000, J UROLOGY, V164, P1192, DOI 10.1016/S0022-5347(05)67139-X

Nunnally J. C., 1978, PSYCHOMETRIC THEORY

Perttula E, 1999, POSTGRAD MED J, V75, P83, DOI 10.1136/pgmj.75.880.83

Peyrot M, 1997, DIABETES CARE, V20, P585, DOI 10.2337/diacare.20.4.585

RADLOFF L S, 1977, Applied Psychological Measurement, V1, P385, DOI 10.1177/014662167700100306

Rendell MS, 1999, JAMA-J AM MED ASSOC, V281, P421, DOI 10.1001/jama.281.5.421

RUBIN A, 1958, JAMA-J AM MED ASSOC, V168, P498, DOI 10.1001/jama.1958.03000050010003

Schein RL, 1997, INT J GERIATR PSYCH, V12, P436

Schiel R, 1999, DIABETES RES CLIN PR, V44, P115, DOI 10.1016/S0168-8227(99)00012-1

Schulz R, 2000, ARCH INTERN MED, V160, P1761, DOI 10.1001/archinte.160.12.1761

Smith KJ, 2000, ANN INTERN MED, V132, P933, DOI 10.7326/0003-4819-132-12-200006200-00002

Tan HL, 2000, PHARMACOECONOMICS, V17, P77, DOI 10.2165/00019053-200017010-00006

Ware JE, 1993, SF 36 HLTH SURVEY MA

WARE JE, 1997, MAP R WINDOWS MULTIT

WassertheilSmoller S, 1996, ARCH INTERN MED, V156, P553, DOI 10.1001/archinte.156.5.553

Whitehead E D, 1990, Clin Geriatr Med, V6, P771

Williams G, 1998, BRIT J UROL, V82, P847

Willke RJ, 1998, INT J IMPOT RES, V10, P239

Willke RJ, 1997, J UROLOGY, V157, P2124, DOI 10.1016/S0022-5347(01)64692-5

ZEMEL P, 1988, AM J CARDIOL, V61, pH27, DOI 10.1016/0002-9149(88)91102-2

NR 50

TC 157

Z9 161

U1 0

U2 4

PU AMER DIABETES ASSOC

PI ALEXANDRIA

PA 1701 N BEAUREGARD ST, ALEXANDRIA, VA 22311-1717 USA

SN 0149-5992

EI 1935-5548

J9 DIABETES CARE

JI Diabetes Care

PD FEB

PY 2002

VL 25

IS 2

BP 284

EP 291

DI 10.2337/diacare.25.2.284

PG 8

WC Endocrinology & Metabolism

SC Endocrinology & Metabolism

GA 515XU

UT WOS:000173522600005

PM 11815497

OA Other Gold

DA 2019-01-08

ER

PT J

AU Kiecolt-Glaser, JK

McGuire, L

Robles, TF

Glaser, R

AF Kiecolt-Glaser, JK

McGuire, L

Robles, TF

Glaser, R

TI Emotions, morbidity, and mortality: New perspectives from

psychoneuroimmunology

SO ANNUAL REVIEW OF PSYCHOLOGY

LA English

DT Review

DE depression; immune function; social support; interleukin 6

ID CORONARY-HEART-DISEASE; KILLER-CELL ACTIVITY;

POSTTRAUMATIC-STRESS-DISORDER; BEHAVIORAL RISK-FACTORS; TO-BRAIN

COMMUNICATION; C-REACTIVE PROTEIN; POSITIVE GAY MEN; OLDER-ADULTS;

MYOCARDIAL-INFARCTION; MARITAL CONFLICT

AB Negative emotions can intensify a variety of health threats. We provide a broad framework relating negative emotions to a range of diseases whose onset and course may be influenced by the immune system; inflammation has been linked to a spectrum of conditions associated with aging, including cardiovascular disease, osteoporosis, arthritis, type 2 diabetes, certain cancers, Alzheimer's disease, frailty and functional decline, and periodontal disease. Production of proinflammatory cytokines that influence these and other conditions can be directly stimulated by negative emotions and stressful experiences. Additionally, negative emotions also contribute to prolonged infection and delayed wound healing, processes that fuel sustained proinflammatory cytokine production. Accordingly, we argue that distress-related immune dysregulation may be one core mechanism behind a large and diverse set of health risks associated with negative emotions. Resources such as close personal relationships that diminish negative emotions enhance health in part through their positive impact on immune and endocrine regulation.

C1 Ohio State Univ, Dept Psychiat, Coll Med, Columbus, OH 43210 USA.

Ohio State Univ, Dept Mol Virol Immunol & Med Genet, Coll Med, Columbus, OH 43210 USA.

Ohio State Univ, Dept Psychol, Columbus, OH 43210 USA.

RP Kiecolt-Glaser, JK (reprint author), Ohio State Univ, Dept Psychiat, Coll Med, 1670 Upham Dr, Columbus, OH 43210 USA.

EM Kiecolt-Glaser.1@osu.edu; robles.8@osu.edu

RI foster, kylie/A-9248-2012; Kiecolt-Glaser, Janice/A-3236-2009; Glaser,

Ronald/E-3124-2011

OI Kiecolt-Glaser, Janice/0000-0003-4900-9578;

FU NIMH NIH HHS [R37 MH42096, T32 MH18831, K02 MH01467]; NIDCR NIH HHS [P50

DE17811]; NIA NIH HHS [P01 AG16321]

CR ADLER NE, 1994, AM PSYCHOL, V49, P15, DOI 10.1037//0003-066X.49.1.15

AFFLECK G, 1987, J CONSULT CLIN PSYCH, V55, P29, DOI 10.1037//0022-006X.55.1.29

ARNETZ BB, 1991, PSYCHOTHER PSYCHOSOM, V55, P76, DOI 10.1159/000288412

Aspinwall LG, 1996, PERS SOC PSYCHOL B, V22, P993, DOI 10.1177/01461672962210002

BARON RS, 1990, J PERS SOC PSYCHOL, V59, P344, DOI 10.1037/0022-3514.59.2.344

BAUM A, 1993, PSYCHOSOM MED, V55, P274, DOI 10.1097/00006842-199305000-00005

Beach SRH, 1998, CLIN PSYCHOL REV, V18, P635, DOI 10.1016/S0272-7358(98)00023-3

Bower JE, 1998, J CONSULT CLIN PSYCH, V66, P979, DOI 10.1037/0022-006X.66.6.979

BURNS EA, 1990, GERIATRIC MED, P312

BURTON R, 1893, ANATOMY MELANCHOLY, V1

CANNON JG, 1995, J GERONTOL A-BIOL, V50, P120

CARSTENSEN LL, 1992, PSYCHOL AGING, V7, P331, DOI 10.1037/0882-7974.7.3.331

CASTLE S, 1995, CLIN EXP IMMUNOL, V101, P487

Catania A, 1997, J GERONTOL A-BIOL, V52, pB93, DOI 10.1093/gerona/52A.2.B93

Christensen AJ, 1996, PSYCHOSOM MED, V58, P150, DOI 10.1097/00006842-199603000-00009

COE CL, 1993, PSYCHOSOM MED, V55, P298, DOI 10.1097/00006842-199305000-00007

Cohen HJ, 1997, J GERONTOL A-BIOL, V52, pM201, DOI 10.1093/gerona/52A.4.M201

Cohen HJ, 2000, J GERONTOL A-BIOL, V55, pM706, DOI 10.1093/gerona/55.12.M706

Cohen S, 1998, HEALTH PSYCHOL, V17, P214, DOI 10.1037/0278-6133.17.3.214

Cohen Sheldon, 1997, JAMA (Journal of the American Medical Association), V277, P1940, DOI 10.1001/jama.277.24.1940

Cole SW, 1997, J PERS SOC PSYCHOL, V72, P320, DOI 10.1037/0022-3514.72.2.320

COLE SW, 1996, PSYCHOSOM MED, V58, P1

Coplan JD, 2000, BIOL PSYCHIAT, V47, P594, DOI 10.1016/S0006-3223(00)00226-2

Costello NL, 1998, PSYCHOSOM MED, V60, P121, DOI 10.1097/00006842-199801000-00140

Croyle RT, 1998, J NATL CANCER I, V90, P1856, DOI 10.1093/jnci/90.24.1856

Davidson RJ, 2002, ANNU REV PSYCHOL, V53, P545, DOI 10.1146/annurev.psych.53.100901.135148

DeBellis MD, 1996, J TRAUMA STRESS, V9, P369, DOI 10.1002/jts.2490090218

Denollet J, 1998, CIRCULATION, V97, P167, DOI 10.1161/01.CIR.97.2.167

Denollet J, 1996, LANCET, V347, P417, DOI 10.1016/S0140-6736(96)90007-0

Dentino AN, 1999, J AM GERIATR SOC, V47, P6, DOI 10.1111/j.1532-5415.1999.tb01894.x

DeRijk R, 1997, J CLIN ENDOCR METAB, V82, P2182, DOI 10.1210/jc.82.7.2182

Dhabhar FS, 1997, BRAIN BEHAV IMMUN, V11, P286, DOI 10.1006/brbi.1997.0508

Dinan TG, 1998, PSYCHIAT CLIN N AM, V21, P325, DOI 10.1016/S0193-953X(05)70008-3

DURA JR, 1990, J ABNORM PSYCHOL, V99, P284, DOI 10.1037//0021-843X.99.3.284

EAKER ED, 1992, AM J EPIDEMIOL, V135, P854, DOI 10.1093/oxfordjournals.aje.a116381

ELLSWORTH PC, 1988, COGNITION EMOTION, V2, P301, DOI DOI 10.1080/02699938808412702

Ershler WB, 2000, ANNU REV MED, V51, P245, DOI 10.1146/annurev.med.51.1.245

ESTERLING BA, 1990, PSYCHOSOM MED, V52, P397, DOI 10.1097/00006842-199007000-00002

ESTERLING BA, 1994, HEALTH PSYCHOL, V13, P291, DOI 10.1037/0278-6133.13.4.291

Esterling BA, 1996, PSYCHOSOM MED, V58, P264, DOI 10.1097/00006842-199605000-00010

Everson SA, 1997, AM J EPIDEMIOL, V146, P142

Ferrucci L, 1999, J AM GERIATR SOC, V47, P639, DOI 10.1111/j.1532-5415.1999.tb01583.x

Fincham FD, 1999, ANNU REV PSYCHOL, V50, P47, DOI 10.1146/annurev.psych.50.1.47

Folkman S, 2000, AM PSYCHOL, V55, P647, DOI 10.1037/0003-066X.55.6.647

FRANKENHAEUSER M, 1991, PL S STRESS, P39

Frankenhaeuser M, 1986, DYNAMICS STRESS, P101, DOI DOI 10.1007/978-1-4684-5122-1_

FRASURESMITH N, 1993, JAMA-J AM MED ASSOC, V270, P1819, DOI 10.1001/jama.270.15.1819

FREDRICKSON BL, 1998, REV GEN PSYCHOL, V2, P271

Glaser R, 1999, JAMA-J AM MED ASSOC, V281, P2268, DOI 10.1001/jama.281.24.2268

Glaser R, 1999, ARCH GEN PSYCHIAT, V56, P450, DOI 10.1001/archpsyc.56.5.450

GLASER R, 1992, PSYCHOSOM MED, V54, P22, DOI 10.1097/00006842-199201000-00005

Glaser R, 2000, PSYCHOSOM MED, V62, P804, DOI 10.1097/00006842-200011000-00010

GLASER R, 1994, HDB HUMAN STRESS IMM

Glassman AH, 1998, AM J PSYCHIAT, V155, P4, DOI 10.1176/ajp.155.1.4

HAINES AP, 1987, BRIT MED J, V295, P297, DOI 10.1136/bmj.295.6593.297

Hamerman D, 1999, ANN INTERN MED, V130, P945, DOI 10.7326/0003-4819-130-11-199906010-00022

Harris TB, 1999, AM J MED, V106, P506, DOI 10.1016/S0002-9343(99)00066-2

Heim C, 2000, PSYCHONEUROENDOCRINO, V25, P1, DOI 10.1016/S0306-4530(99)00035-9

HERBERT TB, 1993, PSYCHOL BULL, V113, P472, DOI 10.1037//0033-2909.113.3.472

Herrmann C, 1998, PSYCHOSOM MED, V60, P570, DOI 10.1097/00006842-199809000-00011

Hess Evelyn V., 1994, Current Opinion in Rheumatology, V6, P474, DOI 10.1097/00002281-199409000-00004

HOLSBOER F, 1995, NEUROENDOCRINOLOGY, V62, P340, DOI 10.1159/000127023

HOUSE JS, 1988, SCIENCE, V241, P540, DOI 10.1126/science.3399889

HU DJ, 1995, J ACQ IMMUN DEF SYND, V10, P374

IRWIN M, 1991, FASEB J, V5, P3100

IRWIN M, 1987, PSYCHIAT CLIN N AM, V10, P449

Kalman B, 1999, BIOMED PHARMACOTHER, V53, P358, DOI 10.1016/S0753-3322(99)80107-3

Katon WJ, 1998, SEMIN CLIN NEUROPSYC, V3, P82

Katz IR, 1996, AM J GERIAT PSYCHIAT, V4, P1, DOI 10.1097/00019442-199624410-00001

KAWACHI I, 1994, CIRCULATION, V89, P1992, DOI 10.1161/01.CIR.89.5.1992

KAWACHI I, 1994, CIRCULATION, V90, P2225, DOI 10.1161/01.CIR.90.5.2225

Kawakami N, 1997, PSYCHOTHER PSYCHOSOM, V66, P192, DOI 10.1159/000289134

KEMENY ME, 1995, PSYCHOSOM MED, V57, P547, DOI 10.1097/00006842-199511000-00007

Kiechl S, 2001, CIRCULATION, V103, P1064, DOI 10.1161/01.CIR.103.8.1064

KIECOLT-GLASER J K, 1988, Brain Behavior and Immunity, V2, P67, DOI 10.1016/0889-1591(88)90007-4

Kiecolt-Glaser J. K., 1987, PSYCHOSOM MED, V49, P31

Kiecolt-Glaser JK, 1998, ANN NY ACAD SCI, V840, P656, DOI 10.1111/j.1749-6632.1998.tb09604.x

Kiecolt-Glaser JK, 1998, AM PSYCHOL, V53, P1209, DOI 10.1037//0003-066X.53.11.1209

KiecoltGlaser JK, 1996, J CONSULT CLIN PSYCH, V64, P324, DOI 10.1037/0022-006X.64.2.324

KIECOLTGLASER JK, 1988, PSYCHOSOM MED, V50, P213, DOI 10.1097/00006842-198805000-00001

KIECOLTGLASER JK, 1995, LANCET, V346, P1194, DOI 10.1016/S0140-6736(95)92899-5

KIECOLTGLASER JK, 1993, PSYCHOSOM MED, V55, P395, DOI 10.1097/00006842-199309000-00001

KiecoltGlaser JK, 1996, P NATL ACAD SCI USA, V93, P3043, DOI 10.1073/pnas.93.7.3043

KIECOLTGLASER JK, 1991, PSYCHOSOM MED, V53, P345, DOI 10.1097/00006842-199107000-00001

KiecoltGlaser JK, 1997, PSYCHOSOM MED, V59, P339, DOI 10.1097/00006842-199707000-00001

KIECOLTGLASER JK, 2001, IN PRESS PSYCHOL B

Koenig HG, 1997, INT J PSYCHIAT MED, V27, P233, DOI 10.2190/40NF-Q9Y2-0GG7-4WH6

Krantz DS, 2002, ANNU REV PSYCHOL, V53, P341, DOI 10.1146/annurev.psych.53.100901.135208

Kronfol Z, 2000, AM J PSYCHIAT, V157, P683, DOI 10.1176/appi.ajp.157.5.683

LEMIEUX AM, 1995, PSYCHOSOM MED, V57, P105, DOI 10.1097/00006842-199503000-00002

Leproult R, 1997, SLEEP, V20, P865

Lerman Y, 1999, PSYCHOSOM MED, V61, P828, DOI 10.1097/00006842-199911000-00017

Leventhal H, 1998, ANN REV GERONTOLOGY, V17, P138

LIEBESKIND JC, 1991, PAIN, V44, P3, DOI 10.1016/0304-3959(91)90141-J

Lutgendorf SK, 1999, J GERONTOL A-BIOL, V54, pM434, DOI 10.1093/gerona/54.9.M434

Maes M, 1997, CYTOKINE, V9, P853, DOI 10.1006/cyto.1997.0238

Maes M, 1998, CYTOKINE, V10, P313, DOI 10.1006/cyto.1997.0290

Maes M, 1999, BIOL PSYCHIAT, V45, P833, DOI 10.1016/S0006-3223(98)00131-0

Maier SF, 1998, PSYCHOL REV, V105, P83, DOI 10.1037/0033-295X.105.1.83

MALARKEY WB, 1994, PSYCHOSOM MED, V56, P41, DOI 10.1097/00006842-199401000-00006

Malarkey WB, 1996, ENDOCRINE, V5, P33, DOI 10.1007/BF02738653

Marucha PT, 1998, PSYCHOSOM MED, V60, P362, DOI 10.1097/00006842-199805000-00025

Maruta T, 2000, MAYO CLIN PROC, V75, P140

McEwen BS, 1998, NEW ENGL J MED, V338, P171, DOI 10.1056/NEJM199801153380307

Meyerowitz BE, 1998, PSYCHOL BULL, V123, P47, DOI 10.1037/0033-2909.123.1.47

Michelson D, 1996, NEW ENGL J MED, V335, P1176, DOI 10.1056/NEJM199610173351602

Miller AH, 1998, PSYCHIAT CLIN N AM, V21, P443, DOI 10.1016/S0193-953X(05)70015-0

Miller TQ, 1996, PSYCHOL BULL, V119, P322, DOI 10.1037/0033-2909.119.2.322

Mills PJ, 1999, PSYCHOSOM MED, V61, P168, DOI 10.1097/00006842-199903000-00008

Molassiotis A, 1997, J PSYCHOSOM RES, V42, P275, DOI 10.1016/S0022-3999(96)00298-X

Morag M, 1999, J PSYCHIAT RES, V33, P389, DOI 10.1016/S0022-3956(99)00010-2

Myers HF, 1995, HEALTH PSYCHOL, V14, P613, DOI 10.1037//0278-6133.14.7.613

Nolen-Hoeksema S, 1999, J PERS SOC PSYCHOL, V77, P1061, DOI 10.1037//0022-3514.77.5.1061

NOLENHOEKSEMA S, 1999, WELL BEING FDN HEDON, P330

Padgett DA, 1998, BRAIN BEHAV IMMUN, V12, P64, DOI 10.1006/brbi.1997.0512

Papanicolaou DA, 1998, ANN INTERN MED, V128, P127, DOI 10.7326/0003-4819-128-2-199801150-00009

PATRIARCA PA, 1994, JAMA-J AM MED ASSOC, V272, P1700, DOI 10.1001/jama.272.21.1700

PENNEBAKER JW, 1988, J CONSULT CLIN PSYCH, V56, P239, DOI 10.1037/0022-006X.56.2.239

Penninx BWJH, 1998, J NATL CANCER I, V90, P1888, DOI 10.1093/jnci/90.24.1888

Penninx BWJH, 1998, JAMA-J AM MED ASSOC, V279, P1720, DOI 10.1001/jama.279.21.1720

Penninx BWJH, 1999, AM J PUBLIC HEALTH, V89, P1346, DOI 10.2105/AJPH.89.9.1346

PETERSON C, 1988, J PERS SOC PSYCHOL, V55, P23, DOI 10.1037/0022-3514.55.1.23

PETRIE KJ, 1995, J CONSULT CLIN PSYCH, V63, P787, DOI 10.1037/0022-006X.63.5.787

Philibert RA, 1997, J CLIN PSYCHIAT, V58, P355, DOI 10.4088/JCP.v58n0805

Phinney JS, 1996, AM PSYCHOL, V51, P918, DOI 10.1037/0003-066X.51.9.918

Pratt LA, 1996, CIRCULATION, V94, P3123, DOI 10.1161/01.CIR.94.12.3123

Rabin B.S., 1999, STRESS IMMUNE FUNCTI

Rantanen T, 2000, J AM GERIATR SOC, V48, P613, DOI 10.1111/j.1532-5415.2000.tb04717.x

Reed GM, 1999, HEALTH PSYCHOL, V18, P354, DOI 10.1037//0278-6133.18.4.354

Ridker PM, 1997, NEW ENGL J MED, V336, P973, DOI 10.1056/NEJM199704033361401

ROJAS I, 2001, IN PRESS BRAIN BEHAV

Rozanski A, 1999, CIRCULATION, V99, P2192, DOI 10.1161/01.CIR.99.16.2192

Ryff CD, 1998, PSYCHOL INQ, V9, P1, DOI 10.1207/s15327965pli0901_1

Scheier MF, 1999, ARCH INTERN MED, V159, P829, DOI 10.1001/archinte.159.8.829

SCHEIER MF, 1992, COGNITIVE THER RES, V16, P201, DOI 10.1007/BF01173489

SCHEIER MF, 1995, PSYCHOSOM MED, V57, P255, DOI 10.1097/00006842-199505000-00007

SCHLEIFER SJ, 1989, ARCH GEN PSYCHIAT, V46, P81

SCHLEIFER SJ, 1983, JAMA-J AM MED ASSOC, V250, P374, DOI 10.1001/jama.250.3.374

Schoevers RA, 2000, BRIT J PSYCHIAT, V177, P336, DOI 10.1192/bjp.177.4.336

Schulz R, 2000, ARCH INTERN MED, V160, P1761, DOI 10.1001/archinte.160.12.1761

Seeman Teresa E., 1997, Archives of Internal Medicine, V157, P2259, DOI 10.1001/archinte.157.19.2259

Segerstrom SC, 2000, ANN BEHAV MED, V22, P180, DOI 10.1007/BF02895112

Shepperd JA, 1996, J RES PERS, V30, P517, DOI 10.1006/jrpe.1996.0038

SHERIDAN JF, 1991, J NEUROIMMUNOL, V31, P245, DOI 10.1016/0165-5728(91)90046-A

Sluzewska A, 1995, ANN NY ACAD SCI, V762, P474

Staats PS, 1999, AM FAM PHYSICIAN, V60, P42

Sternberg EM, 1997, NAT MED, V3, P264, DOI 10.1038/nm0397-264

STERNBERG EM, 1992, ANN INTERN MED, V117, P854, DOI 10.7326/0003-4819-117-10-854

STONEY CM, 1987, PSYCHOPHYSIOLOGY, V24, P127, DOI 10.1111/j.1469-8986.1987.tb00264.x

Taaffe DR, 2000, J GERONTOL A-BIOL, V55, pM709, DOI 10.1093/gerona/55.12.M709

Taylor S. E, 1989, POSITIVE ILLUSIONS C

Taylor SE, 1997, ANNU REV PSYCHOL, V48, P411, DOI 10.1146/annurev.psych.48.1.411

TAYLOR SE, 1983, AM PSYCHOL, V38, P1161, DOI 10.1037/0003-066X.38.11.1161

Taylor SE, 2000, PSYCHOL REV, V107, P411, DOI 10.1037/0033-295X.107.3.411

Taylor SE, 2000, AM PSYCHOL, V55, P99, DOI 10.1037//0003-066X.55.1.99

Turk DC, 1992, HDB PAIN ASSESSMENT, P3

Uchino BN, 1996, PSYCHOL BULL, V119, P488, DOI 10.1037/0033-2909.119.3.488

US Department of Health and Human Services, 1999, MENT HLTH REP SURG G

Vahtera J, 1997, PSYCHOL MED, V27, P693, DOI 10.1017/S0033291797004832

Vedhara K, 1999, LANCET, V353, P627, DOI 10.1016/S0140-6736(98)06098-X

Veldhuis JD, 1996, SLEEP, V19, pS221, DOI 10.1093/sleep/19.suppl_10.S221

VERBRUGGE LM, 1982, PUBLIC HEALTH REP, V97, P417

Watkins LR, 2000, ANNU REV PSYCHOL, V51, P29, DOI 10.1146/annurev.psych.51.1.29

WELLS KB, 1989, JAMA-J AM MED ASSOC, V262, P914, DOI 10.1001/jama.262.7.914

Whooley MA, 1998, ARCH INTERN MED, V158, P2129, DOI 10.1001/archinte.158.19.2129

Williams DR, 1997, ANN EPIDEMIOL, V7, P322, DOI 10.1016/S1047-2797(97)00051-3

WILLIAMS DR, 1995, ANNU REV SOCIOL, V21, P349, DOI 10.1146/annurev.so.21.080195.002025

Wolkowitz OM, 1999, PSYCHOSOM MED, V61, P698, DOI 10.1097/00006842-199909000-00011

Wu H, 1999, J GERONTOL A-BIOL, V54, pM212, DOI 10.1093/gerona/54.4.M212

Wulsin LR, 1999, PSYCHOSOM MED, V61, P6, DOI 10.1097/00006842-199901000-00003

YOSHIKAWA TT, 1983, J AM GERIATR SOC, V31, P34, DOI 10.1111/j.1532-5415.1983.tb06285.x

Zautra AJ, 1998, ARTHRIT CARE RES, V11, P271, DOI 10.1002/art.1790110408

ZHOU DH, 1993, ENDOCRINOLOGY, V133, P2523, DOI 10.1210/en.133.6.2523

NR 173

TC 554

Z9 562

U1 4

U2 111

PU ANNUAL REVIEWS

PI PALO ALTO

PA 4139 EL CAMINO WAY, PO BOX 10139, PALO ALTO, CA 94303-0139 USA

SN 0066-4308

EI 1545-2085

J9 ANNU REV PSYCHOL

JI Annu. Rev. Psychol.

PY 2002

VL 53

BP 83

EP 107

DI 10.1146/annurev.psych.53.100901.135217

PG 25

WC Psychology; Psychology, Multidisciplinary

SC Psychology

GA 524XY

UT WOS:000174039200005

PM 11752480

DA 2019-01-08

ER

PT J

AU Franciosi, M

Pellegrini, F

De Berardis, G

Belfiglio, M

Cavaliere, D

Di Nardo, B

Greenfield, S

Kaplan, SH

Sacco, M

Tognoni, G

Valentini, M

Nicolucci, A

AF Franciosi, M

Pellegrini, F

De Berardis, G

Belfiglio, M

Cavaliere, D

Di Nardo, B

Greenfield, S

Kaplan, SH

Sacco, M

Tognoni, G

Valentini, M

Nicolucci, A

CA QuED Study Group

TI The impact of blood glucose self-monitoring on metabolic control and

quality of life in type 2 diabetic patients - An urgent need for better

educational strategies

SO DIABETES CARE

LA English

DT Article; Proceedings Paper

CT Annual Meeting of the American-Diabetes-Association

CY JUN 22-26, 2001

CL PHILADELPHIA, PENNSYLVANIA

SP Amer Diabetes Assoc

AB OBJECTIVE - The role of self-monitoring of blood glucose (SMBG) in type 2 diabetes is still a matter of debate. in the framework of a nationwide outcomes research program, we investigated the frequency of SMBG and its association with metabolic control and quality of life (QoL).

RESEARCH DESIGN AND METHODS - The study involved 3,567 patients with type 2 diabetes who were recruited by 101 outpatient diabetes clinics and 103 general practitioners. Patients completed a questionnaire investigating SMBG practice and QoL (diabetes-related stress, diabetes health distress, diabetes-related worries, and Centers for Epidemiologic Studies-Depression scale).

RESULTS - Data on SMBG were available for 2,855 subjects (80% of the entire study population). Overall, 471 patients (17%) stated that they tested their blood glucose levels at home I time per day, 899 patients (31%) tested their blood glucose levels : I time per week, and 414 patients (14%) tested their blood whereas 1,071 patients (38%) stated that the never practiced SMBG. A higher frequency of SMBG was associated with better, metabolic control among subjects who were able to adjust insulin doses, whereas no relationship was found in all other patients, irrespective of the kind of treatment. Multivariate analyses showed that an SMBG Frequency ! 1 Lime per day was significantly related to higher levels of distress, worries, and depressive symptoms in non-insulin-treated patients.

CONCLUSIONS - our findings suggest that SMBG can have an important role in improving metabolic control if it is an integral part of a wider educational strategy devoted to the promotion of patient autonomy. In patients not treated with insulin, self-monitoring is associated with higher HbA(1c) levels and psychological burden. Our data do not support the extension of SMBG to this group.

C1 Ist Ric Farmacol Mario Negri, Consorzio Mario Negri Sud, Dept Clin Pharmacol & Epidemiol, I-66030 Santa Maria Imbaro, CH, Italy.

Tufts Univ, Sch Med, Boston, MA 02111 USA.

RP Nicolucci, A (reprint author), Ist Ric Farmacol Mario Negri, Consorzio Mario Negri Sud, Dept Clin Pharmacol & Epidemiol, Via Nazl, I-66030 Santa Maria Imbaro, CH, Italy.

OI Valentini, Miriam/0000-0002-1421-6975; Greenfield,

Sheldon/0000-0003-4628-1998

CR *AM DIAB ASS, 2000, DIABETES CARE S1, V24, pS80

CAMPBELL DT, 1959, PSYCHOL BULL, V56, P81, DOI 10.1037/h0046016

CRONBACH LJ, 1951, PSYCHOMETRIKA, V16, P297, DOI DOI 10.1007/BF02310555

DUNN SM, 1986, DIABETES CARE, V9, P480, DOI 10.2337/diacare.9.5.480

Evans JMM, 1999, BRIT MED J, V319, P83, DOI 10.1136/bmj.319.7202.83

Faas A, 1997, DIABETES CARE, V20, P1482, DOI 10.2337/diacare.20.9.1482

Gallichan M, 1997, BRIT MED J, V314, P964, DOI 10.1136/bmj.314.7085.964

GREENFIELD S, 1994, DIABETES CARE, V17, P32

GREENFIELD S, 1995, MED CARE, V33, pAS47

Harris MI, 2001, DIABETES CARE, V24, P979, DOI 10.2337/diacare.24.6.979

HOWARD KI, 1962, EDUC PSYCHOL MEAS, V22, P731, DOI 10.1177/001316446202200407

RADLOFF L S, 1977, Applied Psychological Measurement, V1, P385, DOI 10.1177/014662167700100306

*SAS I, 2000, SAS LANG VERS 8 1

Scorpiglione N, 1996, DIABETES RES CLIN PR, V34, P115, DOI 10.1016/S0168-8227(96)01343-5

Snijders T, 1999, MULTILEVEL ANAL INTR

SPILKER B, 1996, QUALITY LIFE PHARMAC, P681

Sullivan LM, 1999, STAT MED, V18, P855, DOI 10.1002/(SICI)1097-0258(19990415)18:7<855::AID-SIM117>3.0.CO;2-7

WARE JE, 1997, MAP R WINDOWS MULTIT

NR 18

TC 180

Z9 187

U1 0

U2 23

PU AMER DIABETES ASSOC

PI ALEXANDRIA

PA 1660 DUKE ST, ALEXANDRIA, VA 22314 USA

SN 0149-5992

J9 DIABETES CARE

JI Diabetes Care

PD NOV

PY 2001

VL 24

IS 11

BP 1870

EP 1877

DI 10.2337/diacare.24.11.1870

PG 8

WC Endocrinology & Metabolism

SC Endocrinology & Metabolism

GA 488LZ

UT WOS:000171937800004

PM 11679449

OA Bronze

DA 2019-01-08

ER

PT J

AU Mollema, ED

Snoek, FJ

Ader, HJ

Heine, RJ

van der Ploeg, HM

AF Mollema, ED

Snoek, FJ

Ader, HJ

Heine, RJ

van der Ploeg, HM

TI Insulin-treated diabetes patients with fear of self-injecting or fear of

self-testing - Psychological comorbidity and general well-being

SO JOURNAL OF PSYCHOSOMATIC RESEARCH

LA English

DT Article

DE adults; diabetes mellitus; fear of self-injecting; fear of self-testing;

psychological functioning

ID GLYCEMIC CONTROL; DEPRESSION INVENTORY; PROBLEM AREAS; QUESTIONNAIRE;

POPULATION; DISTRESS; ANXIETY; BLOOD; SCALE; EXPERIENCES

AB Objective: To examine psychological functioning and selfmanagement behaviours of Dutch adult patients with insulin-requiring diabetes mellitus suffering from extreme fear of self-injecting (FSI) and/or fear of self-testing (FST). Methods: A cross-sectional survey was performed in a sample of insulin-treated diabetes patients (n=1275; 51.1% male; age 49.7 +/- 15.8 years; 58.0% Type 1 diabetes), assessing FSI and FST. Patients completed the questionnaires concerning trait/state anxiety, depression, fear of hypoglycemia, diabetes-related distress, diabetes self-care activities, and general well-being. Comparisons were made on these measures between patients with extremely high scores on FSI and/or FST (greater than or equal to 95th percentile) and the other patients. Patients with extreme scores on FSI and/or FST were invited to take part in a second survey to assess the prevalence of major depression, common fears/phobias, and psychoneuroticism. Results: People with extreme FSI/FST scores, as compared to the other patients, reported higher levels of trait/state anxiety and depression. This group also reported more fear of hypoglycaemia. and diabetes-related distress, had lower levels of general wellbeing, and reported less frequent self-monitoring of blood glucose. The second survey showed 11.1% of patients with extreme FSI/ FST reporting scores indicating major depression. Prevalence of scores greater than or equal to the high scores on phobias (38.063.3%) and psychoneuroticism (27.8%) were consistently higher than norm group prevalences. Discussion: Extreme levels of FSI and/or FST are associated with high diabetes-related distress, poor general well-being, and psychological comorbidity, as well as poorer adherence to the diabetes treatment regimen. It is concluded that patients with extreme FSI/FST are often burdened with more than this specific phobia. (C) 2001 Elsevier Science Inc. All rights reserved.

C1 Free Univ Amsterdam, Med Ctr, Dept Psychol Med, EMGO Inst,Fac Med,Inst Res Extramural Med, NL-1081 BT Amsterdam, Netherlands.

Vrije Univ Amsterdam, Ctr Med, Dept Psychol Med, Amsterdam, Netherlands.

Vrije Univ Amsterdam, Dept Clin Epidemiol & Biostat, Med Ctr, NL-1081 HV Amsterdam, Netherlands.

Vrije Univ Amsterdam, Med Ctr, Dept Endocrinol, Amsterdam, Netherlands.

RP Mollema, ED (reprint author), Free Univ Amsterdam, Med Ctr, Dept Psychol Med, EMGO Inst,Fac Med,Inst Res Extramural Med, Van der Boechorststr 7, NL-1081 BT Amsterdam, Netherlands.

EM ed.mollema.emgo@med.vu.nl

CR ARRINDELL WA, 1980, BEHAV RES THER, V18, P229, DOI 10.1016/0005-7967(80)90080-7

ARRINDELL WA, 1986, SCL90 MANUAL MULTI D

BECK AT, 1961, ARCH GEN PSYCHIAT, V4, P561

BELL DSH, 1983, DIABETES CARE, V6, P622, DOI 10.2337/diacare.6.6.622a

Berlin I, 1997, DIABETES CARE, V20, P176, DOI 10.2337/diacare.20.2.176

Bienvenu OJ, 1998, PSYCHOL MED, V28, P1129, DOI 10.1017/S0033291798007144

Bodzin B, 1996, Home Healthc Nurse, V14, P378, DOI 10.1097/00004045-199605000-00009

BOUMAN TK, 1985, GEDRAG GEZOND, V13, P13

BOUMAN TK, 1987, MEASUREMENT DEPRESSI

BRADLEY C, 1994, HDB PSYCHOL DIABETES, P89

Derogatis L. R., 1977, SCL90 ADM SCORING PR

*EUR IDDM POL GROU, 1993, CONS GUID MAN INS DE

Evans JMM, 1999, BRIT MED J, V319, P83, DOI 10.1136/bmj.319.7202.83

Herrmann C, 1997, J PSYCHOSOM RES, V42, P17, DOI 10.1016/S0022-3999(96)00216-4

*INT DIAB FED, 1999, DIABETIC MED, V16, P1

Irvin A, 1994, HDB PSYCHOL DIABETES, P133

KOLKO DJ, 1980, J BEHAV THER EXP PSY, V11, P273, DOI 10.1016/0005-7916(80)90070-1

Lustman PJ, 1997, PSYCHOSOM MED, V59, P24, DOI 10.1097/00006842-199701000-00004

METSCH J, 1995, INT J BEHAV MED, V2, P104, DOI 10.1207/s15327558ijbm0202_2

Mollema ED, 2000, DIABETES CARE, V23, P765, DOI 10.2337/diacare.23.6.765

Morris AD, 1997, LANCET, V350, P1505, DOI 10.1016/S0140-6736(97)06234-X

Peyrot M, 1997, DIABETES CARE, V20, P585, DOI 10.2337/diacare.20.4.585

POLONSKY WH, 1995, DIABETES CARE, V18, P754, DOI 10.2337/diacare.18.6.754

Pouwer F, 1999, DIABETES CARE, V22, P2004, DOI 10.2337/diacare.22.12.2004

Ruggiero L, 1997, DIABETES CARE, V20, P568, DOI 10.2337/diacare.20.4.568

Snaith RP, 1994, HOSP ANXIETY DEPRESS

Snoek F, 1996, GEDRAG GEZONDHEID, V24, P287

Snoek FJ, 1997, DIABETIC MED, V14, P871, DOI 10.1002/(SICI)1096-9136(199710)14:10<871::AID-DIA457>3.3.CO;2-P

Snoek FJ, 2000, DIABETES CARE, V23, P1305, DOI 10.2337/diacare.23.9.1305

*SPSS, 1997, SPSS BAS 7 5 WIND US

van den Akker M, 1998, FAM PRACT, V15, P261, DOI 10.1093/fampra/15.3.261

VANDERPLOEG HM, 2000, MAN DUTCH VERS STAI

Welch GW, 1997, DIABETES CARE, V20, P760, DOI 10.2337/diacare.20.5.760

WOLPE J, 1964, BEHAV RES THER, V2, P27, DOI 10.1016/0005-7967(64)90051-8

Zambanini A, 1999, DIABETES RES CLIN PR, V46, P239, DOI 10.1016/S0168-8227(99)00099-6

Zambanini A, 1997, DIABETIC MED, V14, P321, DOI 10.1002/(SICI)1096-9136(199704)14:4<321::AID-DIA356>3.0.CO;2-H

NR 36

TC 68

Z9 70

U1 0

U2 7

PU PERGAMON-ELSEVIER SCIENCE LTD

PI OXFORD

PA THE BOULEVARD, LANGFORD LANE, KIDLINGTON, OXFORD OX5 1GB, ENGLAND

SN 0022-3999

EI 1879-1360

J9 J PSYCHOSOM RES

JI J. Psychosomat. Res.

PD NOV

PY 2001

VL 51

IS 5

BP 665

EP 672

DI 10.1016/S0022-3999(01)00229-X

PG 8

WC Psychiatry

SC Psychiatry

GA 500RL

UT WOS:000172640500006

PM 11728507

DA 2019-01-08

ER

PT J

AU Herpertz, S

Albus, C

Kielmann, R

Hagemann-Patt, H

Lichtblau, K

Kohle, K

Mann, K

Senf, W

AF Herpertz, S

Albus, C

Kielmann, R

Hagemann-Patt, H

Lichtblau, K

Kohle, K

Mann, K

Senf, W

TI Comorbidity of diabetes mellitus and eating disorders - A follow-up

study

SO JOURNAL OF PSYCHOSOMATIC RESEARCH

LA English

DT Article; Proceedings Paper

CT 23rd European Conference on Psychosomatic Research/EACLPP

CY JUN 16-21, 2000

CL OSLO, NORWAY

DE diabetes mellitus; eating disorder; follow-up

ID BECK DEPRESSION INVENTORY; ANOREXIA-NERVOSA; BULIMIA-NERVOSA; STRUCTURED

INTERVIEW; ADOLESCENT GIRLS; INSULIN OMISSION; YOUNG-ADULTS;

BODY-WEIGHT; WOMEN; BEHAVIOR

AB Objective: There is increasing evidence that the coexistence of diabetes and eating disorders (ED) leads to poor glycemic control and an increased risk of long-term complications. Methods: In a questionnaire- and interview-based study, a sample of 36 out of originally 38 (94.7%) diabetic patients with an ED (type-1: n = 13, type-2: n = 23) was assessed after a period of about 2 years in order to determine the course of EDs, body mass index (BMI), glycemic control, and psychiatric symptomatology. Results: Five patients (13.9%) of the total sample showed full remission for at least 12 consecutive weeks. Twenty-two patients (61.6%) showed no change in the diagnosis of the ED. Four patients (11.1%) shifted from subclinical to clinical EDs and five patients (13.9%) vice versa. Of the eight patients who went on to psychotherapy, only one patient (12.5%) showed full remission. Emotional distress of type-2 diabetics was considerably higher compared to type-1 diabetics, which was rather low at baseline. Except interpersonal distrust as one ED-related variable, no significant change of any psychological variable could be observed in the type-1 diabetic sample during follow-up. Of the 13 type-1 diabetic patients with an ED, five patients deliberately omitted insulin in order to lose weight. These patients showed a more serious psychopathology with regard to each measured psychological variable, a higher BMI, and worse metabolic control compared to those without insulin omission. Type-2 diabetics showed a significant increase in drive for thinness and body dissatisfaction. No considerable change could be observed with regard to BMI, glycemic control, and depressive and global psychiatric symptomatology in either diabetic subsample during follow-up. Conclusion: EDs tended to persist over time with a considerable shift within the different types of EDs. Insulin-purging in type-1 diabetics was associated with enhanced psychopathology, higher BMI, and worse metabolic control. Both mean body mass and ED-related symptoms such as ''drive for thinness'' and ''body dissatisfaction'' increased in the average obese type-2 diabetic sample, illustrating the vicious circle of low self-esteem, enhanced restraint eating, and binge eating in weight control measures, (C) 2001 Elsevier Science Inc. All rights reserved.

C1 Univ Essen Gesamthsch, Clin Psychotherapy & Psychosomat, D-45030 Essen, Germany.

Univ Cologne, Dept Psychosomat & Psychotherapy, Cologne, Germany.

Univ Essen Gesamthsch, Med Clin, Dept Endocrinol, Essen, Germany.

RP Herpertz, S (reprint author), Univ Essen Gesamthsch, Clin Psychotherapy & Psychosomat, Postfach 103043, D-45030 Essen, Germany.

RI Albus, Christian/C-1701-2015

CR Affenito SG, 1997, DIABETES CARE, V20, P182, DOI 10.2337/diacare.20.2.182

BECK AT, 1988, CLIN PSYCHOL REV, V8, P77, DOI 10.1016/0272-7358(88)90050-5

BIGGS MM, 1994, DIABETES CARE, V17, P1186, DOI 10.2337/diacare.17.10.1186

Brown SA, 1996, DIABETES CARE, V19, P613, DOI 10.2337/diacare.19.6.613

Bryden KS, 1999, DIABETES CARE, V22, P1956, DOI 10.2337/diacare.22.12.1956

Carroll P, 1999, J BEHAV MED, V22, P59, DOI 10.1023/A:1018799618864

COLAS C, 1991, DIABETOLOGIA, V34, P288, DOI 10.1007/BF00405092

COOPER A, 1997, INT J EAT DISORDER, V6, P1

COPELAND PM, 1995, HARVARD REV PSYCHIAT, V3, P46

Crow SJ, 1998, PSYCHOSOMATICS, V39, P233, DOI 10.1016/S0033-3182(98)71340-4

DEROGATIS LR, 1977, J CLIN PSYCHOL, V33, P981, DOI 10.1002/1097-4679(197710)33:4<981::AID-JCLP2270330412>3.0.CO;2-0

DEROGATIS LR, 1970, SCL90 ADM SCORING PR

Engstrom I, 1999, ACTA PAEDIATR, V88, P175, DOI 10.1080/08035259950170358

FAIRBURN CG, 1991, BRIT MED J, V303, P17, DOI 10.1136/bmj.303.6793.17

FICHTER MM, 1991, INT J EAT DISORDER, V10, P571, DOI 10.1002/1098-108X(199109)10:5<571::AID-EAT2260100510>3.0.CO;2-J

Fichter MM, 1998, INT J EAT DISORDER, V24, P227, DOI 10.1002/(SICI)1098-108X(199811)24:3<227::AID-EAT1>3.0.CO;2-O

FRIEDMAN S, 1994, ANN MED-PSYCHOL, V153, P282

GARNER DM, 1991, CLIN PSYCHOL REV, V11, P729, DOI 10.1016/0272-7358(91)90128-H

GARNER DM, 1983, INT J EAT DISORDER, V2, P15, DOI 10.1002/1098-108X(198321)2:2<15::AID-EAT2260020203>3.0.CO;2-6

GORTMAKER SL, 1993, NEW ENGL J MED, V329, P1008, DOI 10.1056/NEJM199309303291406

Hautzinger M, 1995, BECK DEPRESSIONS INV

Herpertz S, 2000, PSYCHOTHER PSYCH MED, V50, P161, DOI 10.1055/s-2000-13242

Herpertz S, 1998, DIABETES CARE, V21, P1110, DOI 10.2337/diacare.21.7.1110

Herpertz S, 1998, J PSYCHOSOM RES, V44, P503, DOI 10.1016/S0022-3999(97)00274-2

Herpertz S, 2000, INT J EAT DISORDER, V28, P68, DOI 10.1002/(SICI)1098-108X(200007)28:1<68::AID-EAT8>3.0.CO;2-R

HERPERTZ S, IN PRESS HDB EATING

Jones JM, 2000, BMJ-BRIT MED J, V320, P1563, DOI 10.1136/bmj.320.7249.1563

KENARDY J, 1994, DIABETES CARE, V17, P1197, DOI 10.2337/diacare.17.10.1197

LLOYD GG, 1987, PSYCHOTHER PSYCHOSOM, V48, P189, DOI 10.1159/000288052

Lustman PJ, 1997, PSYCHOSOM MED, V59, P24, DOI 10.1097/00006842-199701000-00004

LUSTMAN PJ, 1992, PSYCHOSOM MED, V54, P602, DOI 10.1097/00006842-199209000-00007

MULLER UA, 1996, FORDERUNGEN ST VINCE, P119

Nielsen S, 1998, EUR EAT DISORD REV, V6, P1

PEVELER RC, 1989, BEHAV RES THER, V27, P95, DOI 10.1016/0005-7967(89)90125-3

PEVELER RC, 1992, INT J EAT DISORDER, V11, P45

POLIVY J, 1995, AM PSYCHOL, V40, P191

POLLOCK M, 1995, J AM ACAD CHILD PSY, V34, P291, DOI 10.1097/00004583-199503000-00012

POLONSKY WH, 1994, DIABETES CARE, V17, P1178, DOI 10.2337/diacare.17.10.1178

POPKIN MK, 1988, ARCH GEN PSYCHIAT, V45, P64

RIEF W, 1992, PSYCHOPATHOLOGY, V25, P128, DOI 10.1159/000284763

ROBERTSON P, 1990, J PSYCHOSOM RES, V34, P535, DOI 10.1016/0022-3999(90)90028-3

ROUGHAN P, 1990, INT J OBESITY, V14, P135

Rydall AC, 1997, NEW ENGL J MED, V336, P1849, DOI 10.1056/NEJM199706263362601

STEEL JM, 1989, BRIT J PSYCHIAT, V155, P515, DOI 10.1192/bjp.155.4.515

STRIEGELMOORE RH, 1992, DIABETES CARE, V15, P1361, DOI 10.2337/diacare.15.10.1361

Takii M, 1999, J PSYCHOSOM RES, V47, P221, DOI 10.1016/S0022-3999(99)00031-8

THIEL A, 1988, Z DIFFERENTIELLE DIA, V9, P267

Vila G, 1995, EUR CHILD ADOLES PSY, V4, P270, DOI 10.1007/BF01980491

WING RR, 1989, INT J EAT DISORDER, V8, P671, DOI 10.1002/1098-108X(198911)8:6<671::AID-EAT2260080608>3.0.CO;2-5

NR 49

TC 29

Z9 30

U1 0

U2 9

PU PERGAMON-ELSEVIER SCIENCE LTD

PI OXFORD

PA THE BOULEVARD, LANGFORD LANE, KIDLINGTON, OXFORD OX5 1GB, ENGLAND

SN 0022-3999

J9 J PSYCHOSOM RES

JI J. Psychosomat. Res.

PD NOV

PY 2001

VL 51

IS 5

BP 673

EP 678

DI 10.1016/S0022-3999(01)00246-X

PG 6

WC Psychiatry

SC Psychiatry

GA 500RL

UT WOS:000172640500007

PM 11728508

DA 2019-01-08

ER

PT J

AU Elia, M

AF Elia, M

TI Obesity in the elderly

SO OBESITY RESEARCH

LA English

DT Article; Proceedings Paper

CT Symposium on Dietary Patterns in Weight Management and Health

CY APR 27-29, 2001

CL DALLAS, TEXAS

SP Slim Fast Foods Co

DE elderly; mortality; body mass index; basal metabolic rate; energy

expenditure

ID BODY-MASS INDEX; WESTERN ELECTRIC COMPANY; INTENTIONAL WEIGHT-LOSS; AGED

40-64 YEARS; PHYSICAL-ACTIVITY; RISK-FACTORS; OLDER WOMEN; MORTALITY;

MEN; HEALTH

AB In developed countries, there is a general increase in body weight and body mass index (BMI) with age, until similar to 60 years of age, when body weight and BMI begin to decline. The proportion of intra-abdominal fat, which is related to increased morbidity and mortality, progressively increases with age. There is also a progressive decline in energy intake and daily total energy expenditure (165 kcal/decade in men and 103 kcal/decade in women in developed countries), which is primarily due to a decrease in physical activity, and to a lesser extent, a decrease in basal metabolic rate. The decrease in physical activity is more pronounced in those with chronic disabilities and diseases. The BMI-mortality curves have been reported to move upward (greater overall mortality), become flatter (less effect of BMI on mortality), and in some cases shift to the right (minimum mortality occurs at a higher BMI), for a variety of possible reasons. Weight loss in the elderly has been reported to increase, decrease, or not alter mortality, but the studies are confounded by numerous methodological problems. It has been argued that there may be little benefit in encouraging weight loss in extreme old age (short life expectancy), especially when there are no obesity-related complications or biochemical risk factors and when strong resistance and distress arise from changes in lifelong habits of eating and exercise. In contrast, weight loss in the elderly can reduce morbidity from arthritis, diabetes and other conditions, reduce cardiovascular risk factors, and improve well-being. BMI also predicts morbidity in those without disease. Furthermore, increased physical activity in the elderly, which is an important component of weight management, can produce beneficial effects on muscle strength, endurance, and well-being.

C1 Univ Southampton, Southampton, Hants, England.

RP Elia, M (reprint author), Southampton Gen Hosp MP113, Inst Human Nutr, Tremona Rd, Southampton SO16 GYD, Hants, England.

CR ANDERSON JJ, 1988, AM J EPIDEMIOL, V128, P179, DOI 10.1093/oxfordjournals.aje.a114939

ANDRES R, 1985, ANN INTERN MED, V103, P1030, DOI 10.7326/0003-4819-103-6-1030

Andrews GR, 2001, BRIT MED J, V322, P728, DOI 10.1136/bmj.322.7288.728

BAUFRERE B, 2000, EUR J CLIN NUTR, V54, pS48

BAUMGARTNER RN, 1995, OBES RES, V3, P73, DOI 10.1002/j.1550-8528.1995.tb00124.x

BJORNTORP P, 1990, ARTERIOSCLEROSIS, V10, P493, DOI 10.1161/01.ATV.10.4.493

BRIERLEY EJ, 1995, QJM-MON J ASSOC PHYS, V88, P439

Burger H, 1998, AM J EPIDEMIOL, V147, P871, DOI 10.1093/oxfordjournals.aje.a009541

CAMPBELL AJ, 1990, AGE AGEING, V19, P131, DOI 10.1093/ageing/19.2.131

Campbell AJ, 1997, BRIT MED J, V315, P1065, DOI 10.1136/bmj.315.7115.1065

Dyer AR, 2000, AM J EPIDEMIOL, V152, P324, DOI 10.1093/aje/152.4.324

Elia M, 2000, EUR J CLIN NUTR, V54, pS92, DOI 10.1038/sj.ejcn.1601030

ELIA M, 1992, ENERGY METABOLISM TI, P92

ERENS B, 1999, HLTH SURVEY ENGLAND

FIATARONE MA, 1990, JAMA-J AM MED ASSOC, V263, P3029, DOI 10.1001/jama.263.22.3029

French SA, 1999, AM J EPIDEMIOL, V149, P504

GALANOS AN, 1994, J AM GERIATR SOC, V42, P368, DOI 10.1111/j.1532-5415.1994.tb07483.x

HORBER FF, 1997, NUTRITION, V13, P424

KINSELLA K, 1996, EPIDEMIOLOGY OLD AGE, P32

LACROIX AZ, 1993, AM J EPIDEMIOL, V137, P858, DOI 10.1093/oxfordjournals.aje.a116747

LAUNER LJ, 1994, JAMA-J AM MED ASSOC, V271, P1093, DOI 10.1001/jama.271.14.1093

Lehmann AB, 1996, EUR J CLIN NUTR, V50, P6

Lindsted KD, 1997, AM J EPIDEMIOL, V146, P1

MacRae PG, 1996, J AM GERIATR SOC, V44, P175, DOI 10.1111/j.1532-5415.1996.tb02435.x

Malbut-Shennan K, 1999, CORONARY ARTERY DIS, V10, P37

McMurdo MET, 2000, BRIT MED J, V321, P1149, DOI 10.1136/bmj.321.7269.1149

MORIGUTI JC, 1999, OBES RES S1, V7, P1035

*NAT RES COUNC, 1989, DIT HLTH

OSTMAN J, 1969, ACTA MED SCAND, V186, P241

PETITT DJ, 1982, AM J EPIDEMIOL, V115, P359

RISSANEN A, 1989, J CLIN EPIDEMIOL, V42, P781, DOI 10.1016/0895-4356(89)90076-0

ROBERTS SB, 1994, JAMA-J AM MED ASSOC, V272, P1601, DOI 10.1001/jama.272.20.1601

Roubenoff R, 2000, EUR J CLIN NUTR, V54, pS40, DOI 10.1038/sj.ejcn.1601024

RUUSKANEN JM, 1995, AGE AGEING, V24, P292, DOI 10.1093/ageing/24.4.292

Seidell JC, 2000, EUR J CLIN NUTR, V54, pS33, DOI 10.1038/sj.ejcn.1601023

Spataro JA, 1996, J CLIN EPIDEMIOL, V49, P849, DOI 10.1016/0895-4356(96)00067-4

Stevens J, 1998, NEW ENGL J MED, V338, P1, DOI 10.1056/NEJM199801013380101

Stevens J, 1998, OBES RES, V6, P268, DOI 10.1002/j.1550-8528.1998.tb00349.x

TAYBACK M, 1990, ARCH INTERN MED, V150, P1065, DOI 10.1001/archinte.150.5.1065

Visscher TLS, 2000, AM J EPIDEMIOL, V151, P660

Visser M, 1998, AM J CLIN NUTR, V68, P584

Waaler H T, 1988, Acta Med Scand Suppl, V723, P17

WILLERY WC, 1991, AM J CLIN NUTR, V153, P1102

Williamson DF, 1999, AM J EPIDEMIOL, V149, P491

WILLIAMSON DF, 1995, AM J EPIDEMIOL, V141, P1128, DOI 10.1093/oxfordjournals.aje.a117386

Zamboni M, 1997, AM J CLIN NUTR, V66, P111

1990, DIETARY GUIDELINES A, P261

1995, DIETARY GUIDELINES A

NR 48

TC 133

Z9 140

U1 0

U2 9

PU NORTH AMER ASSOC STUDY OBESITY

PI ROCHESTER

PA C/O DR MICHAEL JENSEN, MAYO MEDICAL CENTER, MAYO CLIN 200 FIRST ST, SW,

ROCHESTER, MN 55905 USA

SN 1071-7323

J9 OBES RES

JI Obes. Res.

PD NOV

PY 2001

VL 9

SU 4

BP 244S

EP 248S

DI 10.1038/oby.2001.126

PG 5

WC Endocrinology & Metabolism; Nutrition & Dietetics

SC Endocrinology & Metabolism; Nutrition & Dietetics

GA 496AC

UT WOS:000172371700006

PM 11707549

OA Bronze

DA 2019-01-08

ER

PT J

AU Snoek, FJ

van der Ven, NCW

Lubach, CHC

Chatrou, M

Ader, HJ

Heine, RJ

Jacobson, AM

AF Snoek, FJ

van der Ven, NCW

Lubach, CHC

Chatrou, M

Ader, HJ

Heine, RJ

Jacobson, AM

TI Effects of cognitive behavioural group training (CBGT) in adult patients

with poorly controlled insulin-dependent (type 1) diabetes: a pilot

study

SO PATIENT EDUCATION AND COUNSELING

LA English

DT Article

DE cognitive behaviour therapy; group training; type 1 diabetes; coping;

glycaemic control; well-being

ID SELF-CARE; METABOLIC CONTROL; CONTROLLED TRIAL; MELLITUS; EDUCATION;

VALIDITY; DUTCH

AB A substantial group of patients with type 1 diabetes has difficulty adhering to the treatment regimen, and as a consequence is at increased risk of developing microvascular complications. Cognitive behavioural interventions may help these patients to cope more effectively with their diabetes. We developed a 4 weeks cognitive behavioural. group training (CBGT) for patients with type I (insulin-dependent) diabetes in persistent poor glycaemic control, to help them overcome negative beliefs and attitudes towards diabetes and improve their self-care behaviours. Feasibility and efficacy of CBGT were tested in a non-randomised prospective study in 24 poorly-controlled type I diabetes patients (mean age 35.2 +/- 11.1 years; 15 female; mean HbA(1c) 9.3% (+/-1.2)), with assessments at 3 and 6 months follow-up. The program-me was delivered in small groups (n = 6-8), by a team of a diabetes nurse specialist and a psychologist. Primary outcome measures were glycosylated haemoglobin (HbA1(c)), diabetes-related emotional distress (PAID) and psychological well-being (WBQ-12). Chang diabetes self-care activities (DSCI) were documented, along with perceived barriers in diabetes questionniare (BDQ) and fear of hypoglycaemia survey (HFS). Data were analysed using repeated measures analysis of variance. The CBGT proved to be feasible in this selected group of patients and was well appreciated. Following CBGT, mean HbA(1c) dropped by 0.8% at 6 months from baseline, while emotional well-being was preserved, It is concluded that CBGT is a promising intervention that deserves further evaluation in randomised controlled trials. (C) 2001 Elsevier Science Ireland Ltd. All rights reserved.

C1 Vrije Univ Amsterdam, Med Ctr, Dept Med Psychol, Amsterdam, Netherlands.

Vrije Univ Amsterdam, Med Ctr, Dept Endocrinol, Amsterdam, Netherlands.

Vrije Univ Amsterdam, Med Ctr, Dept Clin Epidemiol & Biostat, Amsterdam, Netherlands.

Joslin Diabet Ctr, Sect Behav Res, Boston, MA 02215 USA.

Joslin Diabet Ctr, Mental Hlth Unit, Boston, MA 02215 USA.

RP Snoek, FJ (reprint author), Vrije Univ Amsterdam, Med Ctr, Dept Med Psychol, Amsterdam, Netherlands.

CR ANDERSON RM, 1995, DIABETES CARE, V18, P943, DOI 10.2337/diacare.18.7.943

Arrindell W, 1986, SCL 90 MANUAL MULTID

BECK A, 1980, COGNITIVE THERAPY DE

Becker M. H., 1974, HLTH ED MONOGRAPHS, V2, P324, DOI DOI 10.1177/109019817400200401

BROWNLEEDUFFECK M, 1987, J CONSULT CLIN PSYCH, V55, P139, DOI 10.1037//0022-006X.55.2.139

DEWEERDT I, 1990, SOC SCI MED, V30, P605, DOI 10.1016/0277-9536(90)90159-P

Ellis A., 1994, REASON EMOTION PSYCH

EMMELKAMP PMG, 1993, PSYCHOTHER PSYCHOSOM, V59, P116, DOI 10.1159/000288656

Fosbury JA, 1997, DIABETES CARE, V20, P959, DOI 10.2337/diacare.20.6.959

Glasgow RE, 1999, DIABETES CARE, V22, P832, DOI 10.2337/diacare.22.5.832

KRAAINAAR FW, 1998, BEHAV RES THER, V29, P19

Lustman PJ, 1998, ANN INTERN MED, V129, P613, DOI 10.7326/0003-4819-129-8-199810150-00005

Mollem ED, 1996, PATIENT EDUC COUNS, V29, P277, DOI 10.1016/S0738-3991(96)00926-3

Pouwer F, 1999, DIABETES CARE, V22, P2004, DOI 10.2337/diacare.22.12.2004

RUBIN RR, 1989, DIABETES CARE, V12, P673, DOI 10.2337/diacare.12.10.673

Ruggiero L, 1997, DIABETES CARE, V20, P568, DOI 10.2337/diacare.20.4.568

SALKOVSKIS PM, 1996, FRONTIERS COGNTIIVE

SHAMOON H, 1993, NEW ENGL J MED, V329, P977

Snoek F, 1996, GEDRAG GEZONDHEID, V24, P287

Snoek F. J., 1999, DIABETES SPECTRUM, V12, P147

Snoek FJ, 1999, DIABETES, V48, pA9

Snoek FJ, 2000, DIABETES CARE, V23, P1305, DOI 10.2337/diacare.23.9.1305

SPEERS MA, 1982, PAT COUNS HLTH ED, V4, P144

*SPSS INC, 2000, SPSS BAS 9 0 WIND US

VANDERVEN, 2000, PSYCHOL DIABETES CAR, P207

ZETTLER A, 1995, PSYCHOTHER PSYCHOSOM, V64, P178, DOI 10.1159/000289009

NR 26

TC 42

Z9 44

U1 0

U2 6

PU ELSEVIER SCI IRELAND LTD

PI CLARE

PA CUSTOMER RELATIONS MANAGER, BAY 15, SHANNON INDUSTRIAL ESTATE CO, CLARE,

IRELAND

SN 0738-3991

J9 PATIENT EDUC COUNS

JI Patient Educ. Couns.

PD NOV

PY 2001

VL 45

IS 2

BP 143

EP 148

DI 10.1016/S0738-3991(01)00113-6

PG 6

WC Public, Environmental & Occupational Health; Social Sciences,

Interdisciplinary

SC Public, Environmental & Occupational Health; Social Sciences - Other

Topics

GA 493YM

UT WOS:000172251100009

PM 11687328

DA 2019-01-08

ER

PT J

AU Johnson, JG

Spitzer, RL

Williams, JBW

AF Johnson, JG

Spitzer, RL

Williams, JBW

TI Health problems, impairment and illnesses associated with bulimia

nervosa and binge eating disorder among primary care and obstetric

gynaecology patients

SO PSYCHOLOGICAL MEDICINE

LA English

DT Article

ID PSYCHIATRIC COMORBIDITY; ANOREXIA-NERVOSA; LONG-TERM; MAJOR DEPRESSION;

MENTAL-DISORDERS; SUICIDE ATTEMPTS; AXIS-I; DSM-IV; PREVALENCE; SYMPTOMS

AB Background. Although psychiatric patients with eating disorders are known to be at risk for a variety of health problems, relatively little is known about eating disorders and associated health problems in other populations. An epidemiological study was conducted to investigate health problems and impairment associated with bulimia nervosa (BN) and binge eating disorder (BED) among female primary care and obstetric gynaecology patients.

Methods. Psychiatric disorders, physical illnesses, disabilities, functional status and stress were assessed among 4651 female patients (age range:18 to 99 years) at 8 primary care and 7 obstetric gynaecology clinics throughout the United States.

Results. Two hundred eighty-nine women (6(.)2 %) were diagnosed with BN or BED. The prevalence of BN was approximately 1 % among young and middle-aged women. The prevalence of BED increased steadily from early (3(.)3 %) through middle (8(.)5 %) adulthood. Anxiety disorders, mood disorders and diabetes were much more common among women with BN or BED than among women without these eating disorders. Women with BN or BED reported markedly poorer functioning and much higher levels of disability, health problems, insomnia, psychosocial stress and suicidal thoughts than did women without BN or BED, after co-occurring psychiatric disorders were controlled statistically. Yet, fewer than one of ten cases of BN or BED was recognized by the patients' physicians.

Conclusions. Patients with BN or BED often experience considerable disability, impairment, distress and co-occurring illnesses. Increased recognition of eating disorders may be a crucial step towards encouraging more patients to seek treatment for these disabling conditions.

C1 New York State Psychiat Inst & Hosp, Biometr Res Unit, New York, NY 10032 USA.

Columbia Univ, New York, NY 10027 USA.

RP Johnson, JG (reprint author), New York State Psychiat Inst & Hosp, Biometr Res Unit, Unit 60,1051 Riverside Dr, New York, NY 10032 USA.

CR American Psychiatric Association, 1994, DIAGN STAT MAN MENT

BARRETT JE, 1988, ARCH GEN PSYCHIAT, V45, P1100

BRAUN DL, 1994, PSYCHOL MED, V24, P859, DOI 10.1017/S0033291700028956

BREWERTON TD, 1995, J CLIN PSYCHIAT, V56, P77

Buekens P, 1998, EUR J OBSTET GYN R B, V81, P43, DOI 10.1016/S0301-2115(98)00134-1

Bulik CM, 1997, ACTA PSYCHIAT SCAND, V96, P101, DOI 10.1111/j.1600-0447.1997.tb09913.x

Bulik CM, 1999, ACTA PSYCHIAT SCAND, V100, P27, DOI 10.1111/j.1600-0447.1999.tb10910.x

BYRNE P, 1984, BRIT J PSYCHIAT, V144, P28, DOI 10.1192/bjp.144.1.28

Carpenter KM, 2000, AM J PUBLIC HEALTH, V90, P251, DOI 10.2105/AJPH.90.2.251

Carter JC, 1995, ADDICT BEHAV, V20, P765, DOI 10.1016/0306-4603(95)00096-8

COOPER Z, 1987, INT J EAT DISORDER, V6, P1, DOI 10.1002/1098-108X(198701)6:1<1::AID-EAT2260060102>3.0.CO;2-9

Crow SJ, 1998, PSYCHOSOMATICS, V39, P233, DOI 10.1016/S0033-3182(98)71340-4

DEEP AL, 1995, INT J EAT DISORDER, V17, P291

DEVLIN MJ, 1990, ARCH GEN PSYCHIAT, V47, P144

DEZWAAN M, 1994, HARVARD REV PSYCHIAT, V1, P310, DOI 10.3109/10673229409017098

Fairburn CG, 1997, ARCH GEN PSYCHIAT, V54, P509

FAIRBURN CG, 1985, J PSYCHIAT RES, V19, P135, DOI 10.1016/0022-3956(85)90009-3

Fava M, 1997, J NERV MENT DIS, V185, P140, DOI 10.1097/00005053-199703000-00002

Favaro A, 1997, ACTA PSYCHIAT SCAND, V95, P508, DOI 10.1111/j.1600-0447.1997.tb10139.x

FICHTER MM, 1993, INT J EAT DISORDER, V14, P1, DOI 10.1002/1098-108X(199307)14:1<1::AID-EAT2260140102>3.0.CO;2-3

FOMBONNE F, 1998, EUROPEAN ARCH PSYCHI, V248, P1

Franko DL, 2000, OBSTET GYNECOL, V95, P942, DOI 10.1016/S0029-7844(00)00792-4

Grilo CM, 1996, PSYCHIATR SERV, V47, P426

Grilo CM, 1997, PSYCHOPHARMACOL BULL, V33, P321

Heatherton TF, 1997, J ABNORM PSYCHOL, V106, P117, DOI 10.1037/0021-843X.106.1.117

Herpertz S, 1998, DIABETES CARE, V21, P1110, DOI 10.2337/diacare.21.7.1110

HOLDERNESS HC, 1994, INT J EAT DISORDER, V16, P1, DOI 10.1002/1098-108X(199407)16:1<1::AID-EAT2260160102>3.0.CO;2-T

Hsu LKG, 1996, PSYCHIAT CLIN N AM, V19, P681, DOI 10.1016/S0193-953X(05)70375-0

KAPLAN AS, 1990, CAN J PSYCHIAT, V35, P745, DOI 10.1177/070674379003500905

KAYE WH, 1989, BIOL PSYCHIAT, V25, P768, DOI 10.1016/0006-3223(89)90248-5

Keel PK, 1999, ARCH GEN PSYCHIAT, V56, P63, DOI 10.1001/archpsyc.56.1.63

Keel PK, 1997, AM J PSYCHIAT, V154, P313

KESSLER RC, 1994, ARCH GEN PSYCHIAT, V51, P8

LAESSLE RG, 1989, INT J EAT DISORDER, V8, P569, DOI 10.1002/1098-108X(198909)8:5<569::AID-EAT2260080508>3.0.CO;2-0

Miranda J, 1998, AM J OBSTET GYNECOL, V178, P212, DOI 10.1016/S0002-9378(98)80002-1

Mitchell JE, 1995, ADDICT BEHAV, V20, P725, DOI 10.1016/0306-4603(95)00095-X

NELSON E, 1983, JAMA-J AM MED ASSOC, V249, P3331, DOI 10.1001/jama.249.24.3331

PALMER EP, 1985, NEW ENGL J MED, V313, P1457, DOI 10.1056/NEJM198512053132306

READ JL, 1987, J CHRONIC DIS S1, V40, P7

ROBINS LN, 1991, PSYCHIAT DISORDERS A

SCHULBERG HC, 1988, GEN HOSP PSYCHIAT, V10, P79, DOI 10.1016/0163-8343(88)90092-8

SPITZER RL, 1993, INT J EAT DISORDER, V13, P161, DOI 10.1002/1098-108X(199303)13:2<161::AID-EAT2260130204>3.0.CO;2-R

SPITZER RL, 1993, INT J EAT DISORDER, V13, P137

SPITZER RL, 1992, INT J EAT DISORDER, V11, P191, DOI 10.1002/1098-108X(199204)11:3<191::AID-EAT2260110302>3.0.CO;2-S

SPITZER RL, 1995, JAMA-J AM MED ASSOC, V274, P1511, DOI 10.1001/jama.274.19.1511

SPITZER RL, 1994, JAMA-J AM MED ASSOC, V272, P1749, DOI 10.1001/jama.272.22.1749

Spitzer RL, 1999, JAMA-J AM MED ASSOC, V282, P1737, DOI 10.1001/jama.282.18.1737

STEWART AL, 1989, JAMA-J AM MED ASSOC, V262, P907, DOI 10.1001/jama.262.7.907

STEWART AL, 1988, MED CARE, V26, P724, DOI 10.1097/00005650-198807000-00007

Striegel-Moore RH, 1999, INT J EAT DISORDER, V25, P405, DOI 10.1002/(SICI)1098-108X(199905)25:4<405::AID-EAT5>3.0.CO;2-F

Sunday SR, 1997, PSYCHOPHARMACOL BULL, V33, P373

Telch CF, 1998, J CONSULT CLIN PSYCH, V66, P768, DOI 10.1037/0022-006X.66.5.768

THEANDER S, 1985, J PSYCHIATR RES, V19, P493, DOI 10.1016/0022-3956(85)90059-7

Wiederman MW, 1996, INT J EAT DISORDER, V20, P163, DOI 10.1002/(SICI)1098-108X(199609)20:2<163::AID-EAT6>3.0.CO;2-E

Williams M, 1999, ENVIRON HIST, V4, P103, DOI 10.2307/3985331

Wonderlich SA, 1997, PSYCHOPHARMACOL BULL, V33, P381

Yanovski SZ, 1995, ADDICT BEHAV, V20, P705, DOI 10.1016/0306-4603(96)00092-5

YANOVSKI SZ, 1993, AM J PSYCHIAT, V150, P1472

ZAIDER TI, 2000, UNPUB PROSPECTIVE LO

NR 59

TC 149

Z9 150

U1 0

U2 7

PU CAMBRIDGE UNIV PRESS

PI NEW YORK

PA 40 WEST 20TH ST, NEW YORK, NY 10011-4221 USA

SN 0033-2917

J9 PSYCHOL MED

JI Psychol. Med.

PD NOV

PY 2001

VL 31

IS 8

BP 1455

EP 1466

PG 12

WC Psychology, Clinical; Psychiatry; Psychology

SC Psychology; Psychiatry

GA 494HL

UT WOS:000172276000012

PM 11722160

DA 2019-01-08

ER

PT J

AU Fisher, L

Chesla, CA

Mullan, JT

Skaff, MM

Kanter, RA

AF Fisher, L

Chesla, CA

Mullan, JT

Skaff, MM

Kanter, RA

TI Contributors to depression in Latino and European-American patients with

type 2 diabetes

SO DIABETES CARE

LA English

DT Article

ID QUALITY-OF-LIFE; GLYCEMIC CONTROL; DISEASE MANAGEMENT;

AFRICAN-AMERICANS; METABOLIC-CONTROL; MAJOR DEPRESSION; SYMPTOMS;

ADULTS; MELLITUS; POPULATION

AB OBJECTIVE - To deter-mine the independent and cumulative contributions of diabetes and other life stresses on depression and anxiety in Latino and European-American (EA) patients with diabetes.

RESEARCH DESIGN AND METHODS - A total of 75 Latino and 113 EA patients with type 2 diabetes, recruited from managed care settings, were assessed regarding three groups of potential Stresses: demographics (age, sex, and education), disease status (functional impact, time since diagnosis, comorbidities, HbA(1c) and BMI), an family stress (financial stress, spouse conflict resolution, and family closeness). Dependent variables were depression (Center for Epidemiological Studies-Depression scale [CES-D]) and anxiety (Symptom Checklist [SCL-90]). Multiple regression equations assessed the independent contribution of each predictor on depression and anxiety.

RESULTS - For both ethnic groups, education, functional impact, and financial stress significantly and independently predicted depression; poor spouse conflict resolution was a fourth significant predictor for EA patients only. The equations accounted for a high percentage of variance (43-55%). Excluding education, the same variables predicted anxiety for both ethnic groups. The disease status and family Stress variable groups significantly predicted outcomes independently. The relationships among these variables an occurred for all patients, not only for those classified as likely depressed.

CONCLUSIONS - The findings suggest the utility of considering many life stresses, not just diabetes alone, that combine to affect depression and anxiety. We suggest that these effects are experienced cumulatively as general psychological distress for all patients with diabetes, not just those classified as likely depressed. Taken together, the findings emphasize a life-centered, patient-focused approach to the treatment of depression, rather than an exclusive disease-related perspective.

C1 Univ Calif San Francisco, Dept Family & Community Med, San Francisco, CA 94143 USA.

No Calif Kaiser Permanente, San Francisco, CA USA.

RP Fisher, L (reprint author), Univ Calif San Francisco, Dept Family & Community Med, Box 0900, San Francisco, CA 94143 USA.

EM fisher@itsa.ucsf.edu

FU NIDDK NIH HHS [DK-49816, DK-53203]

CR BETANCOURT H, 1993, AM PSYCHOL, V48, P629, DOI 10.1037/0003-066X.48.6.629

Black SA, 1998, J GERONTOL B-PSYCHOL, V53, pS198, DOI 10.1093/geronb/53B.4.S198

BLACK SA, 1999, ANN EPIDEMIOL, V9, P46

BLACK SA, 1998, J GERONTOL A-BIOL, V53, P188

CARNEY JA, 1995, SEMIN DERMATOL, V14, P90, DOI 10.1016/S1085-5629(05)80003-3

Ciechanowski PS, 2000, ARCH INTERN MED, V160, P3278, DOI 10.1001/archinte.160.21.3278

CONNELL CM, 1994, HEALTH PSYCHOL, V13, P263, DOI 10.1037//0278-6133.13.3.263

DCCT Res Grp, 1988, DIABETES CARE, V11, P725

de Groot M, 1999, J PSYCHOSOM RES, V46, P425, DOI 10.1016/S0022-3999(99)00014-8

De Groot M, 2000, DIABETES, V49, pA63

De Groot M, 2001, DIABETES CARE, V24, P407, DOI 10.2337/diacare.24.2.407

DEROGATIS L R, 1973, Psychopharmacology Bulletin, V9, P13

EVERSON SA, 2001, C DEPR MENT DIS PAT

FALICOV C, 1998, LATINO FAMILIES THER

Fisher L, 2000, DIABETES CARE, V23, P267, DOI 10.2337/diacare.23.3.267

Fisher L, 2000, FAM PROCESS, V39, P403, DOI 10.1111/j.1545-5300.2000.39402.x

Gary TL, 2000, DIABETES CARE, V23, P23, DOI 10.2337/diacare.23.1.23

GAVARD JA, 1993, DIABETES CARE, V16, P1167, DOI 10.2337/diacare.16.8.1167

Hanninen JA, 1999, DIABETES CARE, V22, P997, DOI 10.2337/diacare.22.6.997

Hodges EP, 1998, PSYCHOL REP, V83, P1081, DOI 10.2466/PR0.83.7.1081-1082

Jackson-Triche ME, 2000, J AFFECT DISORDERS, V58, P89, DOI 10.1016/S0165-0327(99)00069-5

JACOBSON AM, 1993, DIABETES CARE, V16, P1621, DOI 10.2337/diacare.16.12.1621

Johnson D., 1980, BASIC DATA DEPRESSIV

KATON W, 1990, Journal of Clinical Psychiatry, V51, P3

Kohen D, 1998, QUAL LIFE RES, V7, P197, DOI 10.1023/A:1008817812520

Kovacs M, 1997, DIABETES CARE, V20, P45, DOI 10.2337/diacare.20.1.45

LEEDOM L, 1991, PSYCHOSOMATICS, V32, P280, DOI 10.1016/S0033-3182(91)72066-5

Lewinsohn PM, 1996, J AM ACAD CHILD PSY, V35, P1120, DOI 10.1097/00004583-199609000-00009

Lipton RB, 1998, DIABETES EDUCATOR, V24, P67, DOI 10.1177/014572179802400109

Lloyd CE, 2000, DIABETIC MED, V17, P198, DOI 10.1046/j.1464-5491.2000.00260.x

Lustman PJ, 1999, DIABETES, V48, pA73

LUSTMAN PJ, 1986, J NERV MENT DIS, V174, P736, DOI 10.1097/00005053-198612000-00005

Lustman PJ, 2000, DIABETES CARE, V23, P934, DOI 10.2337/diacare.23.7.934

Lustman PJ, 2000, DIABETES CARE, V23, P618, DOI 10.2337/diacare.23.5.618

LUSTMAN PJ, 1995, DIABETES AM, P507

LYONS RA, 1994, J EPIDEMIOL COMMUN H, V48, P388, DOI 10.1136/jech.48.4.388

MARIN G, 1991, RES HISPANIC POPULAT, V23

Miranda J, 1996, J CONSULT CLIN PSYCH, V64, P868, DOI 10.1037/0022-006X.64.5.868

Miyaoka Y, 1997, PSYCHIAT CLIN NEUROS, V51, P203, DOI 10.1111/j.1440-1819.1997.tb02583.x

MOLDIN SO, 1993, PSYCHOL MED, V23, P755, DOI 10.1017/S0033291700025526

PALINKAS LA, 1991, DIABETIC MED, V8, P532, DOI 10.1111/j.1464-5491.1991.tb01646.x

Pearlin LI, 1981, J HEALTH SOC BEHAV, V22, P333

Peyrot M, 1999, DIABETES CARE, V22, P448, DOI 10.2337/diacare.22.3.448

Peyrot M, 1997, DIABETES CARE, V20, P585, DOI 10.2337/diacare.20.4.585

Polonsky WH, 2000, DIABETES, V49, pA64

RADLOFF L S, 1977, Applied Psychological Measurement, V1, P385, DOI 10.1177/014662167700100306

Rajala U, 1997, SOC PSYCH PSYCH EPID, V32, P363, DOI 10.1007/BF00805442

ROY M, 1994, COMPR PSYCHIAT, V35, P129, DOI 10.1016/0010-440X(94)90057-O

Talbot F, 1999, HEALTH PSYCHOL, V18, P537

Talbot F, 2000, DIABETES CARE, V23, P1556, DOI 10.2337/diacare.23.10.1556

Tschann JM, 1999, J MARRIAGE FAM, V61, P269, DOI 10.2307/353747

VanderDoes FEE, 1996, DIABETES CARE, V19, P204, DOI 10.2337/diacare.19.3.204

WELLS KB, 1989, JAMA-J AM MED ASSOC, V262, P914, DOI 10.1001/jama.262.7.914

WEYRER S, 1989, J PSYCHOSOM RES, V33, P633

WINOCOUR PH, 1990, DIABETES RES CLIN EX, V14, P171

Zayas L. H., 1994, FAMILY SYSTEMS MED, V12, P315, DOI 10.1037/h0089119

Zeiss AM, 1996, PSYCHOL AGING, V11, P572, DOI 10.1037/0882-7974.11.4.572

NR 57

TC 96

Z9 101

U1 0

U2 8

PU AMER DIABETES ASSOC

PI ALEXANDRIA

PA 1701 N BEAUREGARD ST, ALEXANDRIA, VA 22311-1717 USA

SN 0149-5992

EI 1935-5548

J9 DIABETES CARE

JI Diabetes Care

PD OCT

PY 2001

VL 24

IS 10

BP 1751

EP 1757

DI 10.2337/diacare.24.10.1751

PG 7

WC Endocrinology & Metabolism

SC Endocrinology & Metabolism

GA 478AW

UT WOS:000171321600009

PM 11574437

OA Bronze

DA 2019-01-08

ER

PT J

AU Trief, PM

Himes, CL

Orendorff, R

Weinstock, RS

AF Trief, PM

Himes, CL

Orendorff, R

Weinstock, RS

TI The marital relationship and psychosocial adaptation and glycemic

control of individuals with diabetes

SO DIABETES CARE

LA English

DT Article

ID HEALTH SURVEY SF-36; SOCIAL SUPPORT; CHRONIC ILLNESS; FAMILY;

ADJUSTMENT; MELLITUS; QUALITY; MANAGEMENT; FRAMEWORK; ADHERENCE

AB OBJECTIVE - To explore the relationship between marital relationship domains (i.e., intimacy and adjustment) and glycemic control and psychosocial adaptation to diabetes.

RESEARCH DESIGN AND METHODS - A total of 78 insulin-treated adults with both type 1 and type 2 diabetes were assessed on a Single occasion. They completed two marital quality measures (Spanier Dyadic Adjustment Scale and Personal Assessment of Intimacy in Relationships Scale) and four quality-of-life measures (Diabetes Quality of Life Scale, Medical Outcomes Study Health Survey, Problem Areas in Diabetes Scale, and Positive and Negative Affect Scale). Glycemic control was assessed by HbA(1c). Demographic data (age, sex, type and duration of diabetes, years married, other medical conditions, family history, disability, and years of education) were gathered from the chart and questionnaires.

RESULTS - Concerning psychosocial adaptation, both of the marital quality measures were predictors of aspects of adaptation. Better marital satisfaction was related to higher levels of diabetes-related satisfaction and less impact, as well as less diabetes-related distress and better general quality of life. Higher levels of marital intimacy were related to better diabetes-specific and general quality of life, Concerning glycemic control, there was a nonsignificant trend for marital adjustment scores to relate to HbA(1c) (P = 0.0568).

CONCLUSIONS - For insulin-treated adults with diabetes, quality of marriage is associated with adaptation to diabetes and other aspects of health-related quality of life, The suggestive finding that marital adjustment may relate to glycemic control warrants further study. Future work should also explore the impact of couples-focused interventions on adaptation, adherence, and glycemic control.

C1 SUNY Upstate Med Univ, Dept Psychiat, Syracuse, NY 13210 USA.

Syracuse Univ, Syracuse, NY USA.

Joslin Diabet Ctr, Syracuse, NY USA.

Vet Adm Med Ctr, Syracuse, NY 13210 USA.

RP Trief, PM (reprint author), SUNY Upstate Med Univ, Dept Psychiat, 750 E Adams St, Syracuse, NY 13210 USA.

OI Trief, Paula/0000-0002-2827-5722

CR BLOOM JR, 1984, SOC SCI MED, V19, P831, DOI 10.1016/0277-9536(84)90400-3

CARDENAS L, 1987, AM J MED SCI, V293, P28, DOI 10.1097/00000441-198701000-00007

DCCT Res Grp, 1988, DIABETES CARE, V11, P725

EATON WW, 1992, INT J PSYCHIAT MED, V22, P105, DOI 10.2190/DF4H-9HQW-NJEC-Q54E

Fisher L, 1998, DIABETES EDUCATOR, V24, P599, DOI 10.1177/014572179802400504

GARAYSEVILA ME, 1995, J DIABETES COMPLICAT, V9, P81, DOI 10.1016/1056-8727(94)00021-F

HAUSER ST, 1990, J PEDIATR PSYCHOL, V15, P527, DOI 10.1093/jpepsy/15.4.527

JACOBSON AM, 1994, DIABETES CARE, V17, P267, DOI 10.2337/diacare.17.4.267

KATZ AM, 1969, B MENNINGER CLIN, V33, P79

LOCKE HJ, 1959, J MARRIAGE FAMILY LI, V21, P251

Lyons R. F., 1995, RELATIONSHIPS CHRONI

MCHORNEY CA, 1994, MED CARE, V32, P40, DOI 10.1097/00005650-199401000-00004

Meadows K, 1996, QUAL LIFE RES, V5, P242, DOI 10.1007/BF00434746

PEYROT M, 1985, PSYCHOSOM MED, V47, P542, DOI 10.1097/00006842-198511000-00004

Pieper B A, 1990, Diabetes Educ, V16, P108, DOI 10.1177/014572179001600206

POLONSKY WH, 1995, DIABETES CARE, V18, P754, DOI 10.2337/diacare.18.6.754

PRIMOMO J, 1990, RES NURS HEALTH, V13, P153, DOI 10.1002/nur.4770130304

Quittner AL, 1998, HEALTH PSYCHOL, V17, P112, DOI 10.1037/0278-6133.17.2.112

Rissman R, 1987, FAMILY SYSTEMS MED, V5, P446

SCHAEFER MT, 1981, J MARITAL FAM THER, V7, P47, DOI 10.1111/j.1752-0606.1981.tb01351.x

SCHAFER LC, 1986, DIABETES CARE, V9, P179, DOI 10.2337/diacare.9.2.179

SCHWARTZ LS, 1991, GEN HOSP PSYCHIAT, V13, P19, DOI 10.1016/0163-8343(91)90005-H

SPANIER GB, 1976, J MARRIAGE FAM, V38, P15, DOI 10.2307/350547

THARP RG, 1966, J CONSULT PSYCHOL, V30, P426, DOI 10.1037/h0023835

Trief PM, 1998, DIABETES CARE, V21, P241, DOI 10.2337/diacare.21.2.241

WARE JE, 1992, MED CARE, V30, P473, DOI 10.1097/00005650-199206000-00002

WARE JE, 1994, SF 36 PHYSICAL MENTA

WATSON D, 1988, J PERS SOC PSYCHOL, V54, P1063, DOI 10.1037/0022-3514.54.6.1063

WEINGER K, 1998, ANN BEHAV MED, V20, P5118

Welch GW, 1997, DIABETES CARE, V20, P760, DOI 10.2337/diacare.20.5.760

NR 30

TC 84

Z9 88

U1 0

U2 8

PU AMER DIABETES ASSOC

PI ALEXANDRIA

PA 1660 DUKE ST, ALEXANDRIA, VA 22314 USA

SN 0149-5992

J9 DIABETES CARE

JI Diabetes Care

PD AUG

PY 2001

VL 24

IS 8

BP 1384

EP 1389

DI 10.2337/diacare.24.8.1384

PG 6

WC Endocrinology & Metabolism

SC Endocrinology & Metabolism

GA 456HE

UT WOS:000170076400013

PM 11473074

OA Bronze

DA 2019-01-08

ER

PT J

AU Mollema, ED

Snoek, FJ

Heine, RJ

van der Ploeg, HM

AF Mollema, ED

Snoek, FJ

Heine, RJ

van der Ploeg, HM

TI Phobia of self-injecting and self-testing in insulin-treated diabetes

patients: opportunities for screening

SO DIABETIC MEDICINE

LA English

DT Article

DE diabetes; phobia; self-injecting; self-testing; adult; prevalence

ID GLYCEMIC CONTROL; QUESTIONNAIRE; BLOOD; FEAR; MELLITUS; INJURY

AB Aims To define clinically relevant cut-off points for severe fear of self-injecting (FSI) and self-testing (FST) (phobia) in insulin-treated patients with diabetes, and to estimate the magnitude of these phobias in our research population.

Methods FSI and FST were assessed in a cross-sectional survey using the Diabetes Fear of Injecting and Self-testing Questionnaire (D-FISQ). A sample of 24 insulin-treated adult diabetic patients was selected from the high-scorers on FSI and/or FST (greater than or equal to 95th percentile). FSI and FST were re-assessed, after which patients participated in a behavioural avoidance test (BAT), thereby determining the current level of avoidance of either self-injecting or self-testing. FSI and FST scores were linked to the outcome of the BATs. Cut-off scores for severe FSI/FST were determined and extrapolated to the total study population (n = 1275).

Results Seven patients participated in the self-injecting BAT: two patients refused to perform an extra injection. in the self-testing BAT (n = 17) four patients declined to perform the extra blood glucose self-test. Extrapolation of FSI and FST cut-off scores to the total research population showed that 0.2-1.3% of the population scored in the severe FSI range. In FST, 0.6-0.8% of the total study population obtained scores in the cut-off range.

Conclusions Severe FSI and FST, characterized by emotional distress and avoidance behaviour, seems to occur in a small group of insulin-treated patients with diabetes. The D-FISQ can be of use to health care professionals (physicians, nurse specialists) in quickly providing valuable information on levels of FSI and FST in diabetes patients.

C1 Free Univ Amsterdam, Med Ctr, Fac Med, Dept Med Psychol,EMGO Inst, NL-1081 BT Amsterdam, Netherlands.

Free Univ Amsterdam, Med Ctr, Dept Endocrinol, NL-1081 BT Amsterdam, Netherlands.

RP Mollema, ED (reprint author), Free Univ Amsterdam, Med Ctr, Fac Med, Dept Med Psychol,EMGO Inst, Boechorststr 7, NL-1081 BT Amsterdam, Netherlands.

CR Berlin I, 1997, DIABETES CARE, V20, P176, DOI 10.2337/diacare.20.2.176

Bienvenu OJ, 1998, PSYCHOL MED, V28, P1129, DOI 10.1017/S0033291798007144

Bodzin B, 1996, Home Healthc Nurse, V14, P378, DOI 10.1097/00004045-199605000-00009

COX DJ, 1992, J CONSULT CLIN PSYCH, V60, P628, DOI 10.1037//0022-006X.60.4.628

Evans JMM, 1999, BRIT MED J, V319, P83, DOI 10.1136/bmj.319.7202.83

Glasgow RE, 1999, DIABETES CARE, V22, P832, DOI 10.2337/diacare.22.5.832

Kindt M, 1996, BEHAV RES THER, V34, P277, DOI 10.1016/0005-7967(95)00069-0

KOLKO DJ, 1980, J BEHAV THER EXP PSY, V11, P273, DOI 10.1016/0005-7916(80)90070-1

METSCH J, 1995, INT J BEHAV MED, V2, P104, DOI 10.1207/s15327558ijbm0202_2

Mollem ED, 1996, PATIENT EDUC COUNS, V29, P277, DOI 10.1016/S0738-3991(96)00926-3

Mollema ED, 2000, DIABETES CARE, V23, P765, DOI 10.2337/diacare.23.6.765

Morris AD, 1997, LANCET, V350, P1505, DOI 10.1016/S0140-6736(97)06234-X

OST LG, 1992, BEHAV THER, V23, P263, DOI 10.1016/S0005-7894(05)80385-5

RAINWATER N, 1988, CHILD FAM BEHAV THER, V10, P19

Ruggiero L, 1997, DIABETES CARE, V20, P568, DOI 10.2337/diacare.20.4.568

Snoek FJ, 1997, DIABETIC MED, V14, P871, DOI 10.1002/(SICI)1096-9136(199710)14:10<871::AID-DIA457>3.3.CO;2-P

Zambanini A, 1999, DIABETES RES CLIN PR, V46, P239, DOI 10.1016/S0168-8227(99)00099-6

Zambanini A, 1997, DIABETIC MED, V14, P321, DOI 10.1002/(SICI)1096-9136(199704)14:4<321::AID-DIA356>3.0.CO;2-H

NR 18

TC 61

Z9 61

U1 0

U2 3

PU BLACKWELL SCIENCE LTD

PI OXFORD

PA P O BOX 88, OSNEY MEAD, OXFORD OX2 0NE, OXON, ENGLAND

SN 0742-3071

J9 DIABETIC MED

JI Diabetic Med.

PD AUG

PY 2001

VL 18

IS 8

BP 671

EP 674

DI 10.1046/j.1464-5491.2001.00547.x

PG 4

WC Endocrinology & Metabolism

SC Endocrinology & Metabolism

GA 475UK

UT WOS:000171186300012

PM 11553207

DA 2019-01-08

ER

PT J

AU McIntyre, RS

McCann, SM

Kennedy, SH

AF McIntyre, RS

McCann, SM

Kennedy, SH

TI Antipsychotic metabolic effects: Weight gain, diabetes mellitus, and

lipid abnormalities

SO CANADIAN JOURNAL OF PSYCHIATRY-REVUE CANADIENNE DE PSYCHIATRIE

LA English

DT Review

DE weight gain; obesity; obesity comorbidity; diabetes mellitus;

triglyceride; atypical antipsychotics; conventional antipsychotics

ID SERUM TRIGLYCERIDE LEVELS; BIPOLAR DISORDER; BODY-WEIGHT; LEPTIN LEVELS;

PLASMA-LEVELS; FOOD-INTAKE; CLOZAPINE; OLANZAPINE; SCHIZOPHRENIA;

RECEPTORS

AB Objective: To review published and nonpublished literature describing changes in weight, glucose homeostasis, and lipid milieu with antipsychotics.

Methods: A Medline search was completed using the words weight gain, diabetes mellitus, cholesterol, triglycerides, risperidone, clozapine, olanzapine, quetiapine, ziprasidone, predictors, prolactin, obesity, and conventional antipsychotics. Publications, including original articles, review articles, letters to the editor abstracts or posters presented at professional meetings in the last 4 years, and references from published articles, were collected. Manufacturers, including Eli Lilly Canada Inc, Janssen-Ortho Inc, Pfizer Canada Inc, AstraZeneca Inc, and Novartis Pharmaceuticals, were contacted to retrieve additional medical information.

Results: The topic of antipsychotic-induced weight gain is understudied, and there ave relatively few well-controlled studies. Weight gain as a side effect has been described with both conventional and atypical antipsychotics. Moreover, some atypical antipsychotics are associated with de novo diabetes mellitus and increased serum triglyceride levels. Predictors of weight gain may be age, baseline body, mass index, appetite stimulation, previous antipsychotic exposure, and antipsychotic treatment duration.

Conclusion: Significant weight gain is reported with the existing atypical antipsychotics, The weight gain described is highly distressing to patients, may reduce treatment adherence, and may increase the relative risk for diabetes mellitus and hypertriglyceridemia. Physicians employing these agents should routinely monitor weight, fasting blood glucose, and lipid profiles.

C1 Ctr Addict & Mental Hlth, Mood & Anxiety Disorders Program, Toronto, ON M5T 1R8, Canada.

Univ Toronto, Dept Psychiat, Toronto, ON, Canada.

RP McIntyre, RS (reprint author), Ctr Addict & Mental Hlth, Mood & Anxiety Disorders Program, Clarke Site,250 Coll St, Toronto, ON M5T 1R8, Canada.

EM roger_meintyre@camh.net

CR Allison DB, 1999, J CLIN PSYCHIAT, V60, P215, DOI 10.4088/JCP.v60n0402

Allison DB, 1999, AM J PSYCHIAT, V156, P1686

AMERY W, 1997, 10 EUR COLL NEUR C S

[Anonymous], 1998, OBES RES S2, p51S, DOI DOI 10.1002/J.1550-8528.1998.TB00690.X

AULAKH CS, 1992, PSYCHOPHARMACOLOGY, V109, P444, DOI 10.1007/BF02247721

AWAD AG, 1999, 11 WORLD C PSYCH AUG

BAPTISTA T, 1990, PHARMACOL BIOCHEM BE, V37, P227, DOI 10.1016/0091-3057(90)90325-C

Baptista T, 1999, PROG NEURO-PSYCHOPH, V23, P277, DOI 10.1016/S0278-5846(98)00096-7

BAPTISTA T, 1987, PHARMACOL BIOCHEM BE, V27, P399, DOI 10.1016/0091-3057(87)90340-6

Beasley CM, 1997, J CLIN PSYCHIAT, V58, P13

BERNSTEIN JG, 1992, OBESITY

BRESCAN CM, 1997, 150 M AM PSYCH ASS M

Bromel T, 1998, MOL PSYCHIATR, V3, P76, DOI 10.1038/sj.mp.4000352

Bustillo JR, 1996, AM J PSYCHIAT, V153, P817

Bymaster FP, 1999, SCHIZOPHR RES, V37, P107, DOI 10.1016/S0920-9964(98)00146-7

CABRERA R, 1988, MOL CELL ENDOCRINOL, V58, P167, DOI 10.1016/0303-7207(88)90151-7

CALBRESE JR, 1998, ANN M AM PSYCH ASS J

CAMPFIELD LA, 1995, SCIENCE, V269, P546, DOI 10.1126/science.7624778

CHENGAPPA RKN, 1999, BIPOLAR DISORD, V1, P42

CLARK ML, 1970, CLIN PHARMACOL THER, V11, P680

Cleland R, 1998, COMMERCIAL WEIGHT LO

CONLEY RR, 1999, US PSYCH MENT HLTH C

CSERNANSKY J, 1999, BIOL PSYCHIAT S, V45, pS1

Dickson RA, 1999, SCHIZOPHR RES, V35, pS75, DOI 10.1016/S0920-9964(98)00159-5

DOSS FW, 1979, J CLIN PSYCHIAT, V40, P528

Dursun SM, 1999, J PSYCHIATR NEUROSCI, V24, P453

Farooqi IS, 1999, NEW ENGL J MED, V341, P879, DOI 10.1056/NEJM199909163411204

Frye MA, 1998, J AFFECT DISORDERS, V48, P91, DOI 10.1016/S0165-0327(97)00160-2

GALLANT DM, 1973, CURR THER RES CLIN E, V15, P915

Ganguli R, 1999, J CLIN PSYCHIAT, V60, P20

GARATTINI S, 1989, BRIT J PSYCHIAT, V155, P41, DOI 10.1192/S0007125000291757

GARDOS G, 1977, AM J PSYCHIAT, V134, P302

Gaulin BD, 1999, AM J PSYCHIAT, V156, P1270

Ghaeli P, 1996, AM J HEALTH-SYST PH, V53, P2079

Goldstein LE, 1999, PSYCHOSOMATICS, V40, P438, DOI 10.1016/S0033-3182(99)71210-7

GOODALL E, 1988, BRIT J PSYCHIAT, V153, P208, DOI 10.1192/bjp.153.2.208

GOPALASWAMY AK, 1985, ACTA PSYCHIAT SCAND, V72, P254, DOI 10.1111/j.1600-0447.1985.tb02603.x

Gordon A, 1999, AM J PSYCHIAT, V156, P968

Greenberg I, 1999, J CLIN PSYCHIAT, V60, P31

Guille C, 2000, J CLIN PSYCHIAT, V61, P638, DOI 10.4088/JCP.v61n0907

Hebebrand J, 1997, MOL PSYCHIATR, V2, P330, DOI 10.1038/sj.mp.4000282

Henderson DC, 2000, AM J PSYCHIAT, V157, P975, DOI 10.1176/appi.ajp.157.6.975

Heymsfield SB, 1999, JAMA-J AM MED ASSOC, V282, P1568, DOI 10.1001/jama.282.16.1568

HOLDEN J M C, 1970, Psychosomatics, V11, P551

Hummer M, 1995, EUR NEUROPSYCHOPHARM, V5, P437, DOI 10.1016/0924-977X(95)00012-E

HUSSAIN MZ, 1998, 48 ANN M CAN PSYCH A

ISOJARVI JIT, 1993, NEW ENGL J MED, V329, P1383, DOI 10.1056/NEJM199311043291904

JOHNSON DAW, 1979, ACTA PSYCHIAT SCAND, V59, P525, DOI 10.1111/j.1600-0447.1979.tb00252.x

JUBERT P, 1995, ANN INTERN MED, V122, P397

KAMRAN A, 1994, AM J PSYCHIAT, V151, P1395

Kelly DL, 1998, J CHILD ADOL PSYCHOP, V8, P151, DOI 10.1089/cap.1998.8.151

KHAN SA, 1987, AM FAM PHYSICIAN, V36, P107

KINON BJ, 1998, 38 ANN M NCDEU NEW C

KLETT CJ, 1960, J NEUROPSYCHIATRY, V2, P102

Koren W, 1997, ANN PHARMACOTHER, V31, P168, DOI 10.1177/106002809703100206

Kostakoglu AE, 1996, ACTA PSYCHIAT SCAND, V93, P217, DOI 10.1111/j.1600-0447.1996.tb10635.x

KOVAL MS, 1994, AM J PSYCHIAT, V151, P1520

Kraus T, 1999, AM J PSYCHIAT, V156, P312

KRYSPIN-EXNER W, 1947, Wien Klin Wochenschr, V59, P531

KUSAMAKER V, 1999, 3 INT C BIP DIS JUN

LAMBERTI JS, 1992, AM J PSYCHIAT, V149, P689

LEADBETTER R, 1992, AM J PSYCHIAT, V149, P68

Leysen JE, 1996, SEROTONIN ANTIPSYCHO, P51

Lindenmayer JP, 1999, AM J PSYCHIAT, V156, P1471

MANSON JE, 1995, NEW ENGL J MED, V333, P677, DOI 10.1056/NEJM199509143331101

Marcotte D, 1998, J AFFECT DISORDERS, V50, P245, DOI 10.1016/S0165-0327(98)00110-4

MARTIN A, 1992, AM J PSYCHIAT, V149, P714

MCELROY SL, 1998, 21 C C INT NEUR PSYC

McEvoy JP, 1999, BIOL PSYCHIAT, V46, P125, DOI 10.1016/S0006-3223(98)00377-1

MCINTYRE RS, 2000, ACTA NEUROPSYCHAITRI, V12, P42

MCINTYRE RS, 1999, PRIMARY CARE PSYCHIA, V5, P41

Melamed Y, 1998, CAN J PSYCHIAT, V43, P956

MELKERSSON KL, IN PRESS J CLIN PSYC

Meltzer S, 1998, CAN MED ASSOC J, V159, pS1

Mokdad AH, 1999, JAMA-J AM MED ASSOC, V282, P1519, DOI 10.1001/jama.282.16.1519

Montague CT, 1997, NATURE, V387, P903, DOI 10.1038/43185

MURZI E, 1991, BRAIN RES BULL, V26, P321, DOI 10.1016/0361-9230(91)90001-Z

Osser DN, 1999, J CLIN PSYCHIAT, V60, P767, DOI 10.4088/JCP.v60n1109

PARADA MA, 1988, PHARMACOL BIOCHEM BE, V30, P917, DOI 10.1016/0091-3057(88)90120-7

Peterson GA, 1996, AM J PSYCHIAT, V153, P737

PLANANSKY KAREL, 1959, JOUR CLIN AND EXPTL PSYCHOPATH, V20, P53

Popli AP, 1997, J CLIN PSYCHIAT, V58, P108, DOI 10.4088/JCP.v58n0304

Raison CL, 1997, AM J PSYCHIAT, V154, P711

Reinstein MJ, 1999, CLIN DRUG INVEST, V18, P99, DOI 10.2165/00044011-199918020-00002

Remington G, 1999, J PSYCHIATR NEUROSCI, V24, P431

Rosenbaum M, 1996, J CLIN ENDOCR METAB, V81, P3424, DOI 10.1210/jc.81.9.3424

SACHS G, 1999, 11 C PSYCH AUG HAMB

SCHOTTE A, 1996, PSYCHOPHARMACOLOGY, V17, P263

Serdula MK, 1999, JAMA-J AM MED ASSOC, V282, P1353, DOI 10.1001/jama.282.14.1353

SHANK RP, 1994, EPILEPSIA, V35, P450, DOI 10.1111/j.1528-1157.1994.tb02459.x

Sheitman BB, 1999, AM J PSYCHIAT, V156, P1471

Sobel M, 1999, J CLIN PSYCHIAT, V60, P556, DOI 10.4088/JCP.v60n0809f

STANTON JM, 1995, SCHIZOPHRENIA BULL, V21, P463, DOI 10.1093/schbul/21.3.463

TECOTT LH, 1995, NATURE, V374, P542, DOI 10.1038/374542a0

Tohen M, 1998, J CLIN PSYCHIAT, V59, P38

Tran PV, 1997, J CLIN PSYCHOPHARM, V17, P407, DOI 10.1097/00004714-199710000-00010

UMBRICHT DSG, 1994, J CLIN PSYCHIAT, V55, P157

UvnasMoberg K, 1996, NEUROENDOCRINOLOGY, V63, P269, DOI 10.1159/000126970

VAMPINI C, 1994, NEUROPSYCHOPHARMACOL, V10, pS249

Verrotti A, 1999, NEUROLOGY, V53, P230, DOI 10.1212/WNL.53.1.230

WADE GN, 1992, NEUROSCI BIOBEHAV R, V16, P235, DOI 10.1016/S0149-7634(05)80183-6

WALSH AES, 1994, PSYCHOPHARMACOLOGY, V116, P120, DOI 10.1007/BF02244883

WANG DY, 1987, EUR J CANCER CLIN ON, V23, P1541, DOI 10.1016/0277-5379(87)90098-8

Wetterling T, 1999, J CLIN PSYCHOPHARM, V19, P316, DOI 10.1097/00004714-199908000-00006

*WHO, 1997, WHONUTNCD981

Wirshing DA, 1998, BIOL PSYCHIAT, V44, P778, DOI 10.1016/S0006-3223(98)00100-0

Wirshing DA, 1999, J CLIN PSYCHIAT, V60, P358, DOI 10.4088/JCP.v60n0602

YAMADA J, 1995, LIFE SCI, V57, P819, DOI 10.1016/0024-3205(95)02010-G

Yanovski JA, 1999, JAMA-J AM MED ASSOC, V282, P1504, DOI 10.1001/jama.282.16.1504

ZOLER ML, 1999, CLIN PSYCHIAT NEWS, V27, P20

ZORN SH, 1998, INTERACTIVE MONOAMIN, V4, P377

NR 111

TC 195

Z9 199

U1 0

U2 9

PU SAGE PUBLICATIONS INC

PI THOUSAND OAKS

PA 2455 TELLER RD, THOUSAND OAKS, CA 91320 USA

SN 0706-7437

EI 1497-0015

J9 CAN J PSYCHIAT

JI Can. J. Psychiat.-Rev. Can. Psychiat.

PD APR

PY 2001

VL 46

IS 3

BP 273

EP 281

DI 10.1177/070674370104600308

PG 9

WC Psychiatry

SC Psychiatry

GA 421UR

UT WOS:000168082400009

PM 11320682

DA 2019-01-08

ER

PT J

AU Sarici, SU

Alpay, F

Dundaroz, MR

Gokcay, E

AF Sarici, SU

Alpay, F

Dundaroz, MR

Gokcay, E

TI Neonatal diabetes mellitus: Patient report and review of the literature

SO JOURNAL OF PEDIATRIC ENDOCRINOLOGY & METABOLISM

LA English

DT Article

ID PATERNAL UNIPARENTAL DISOMY; TRANSIENT; CHROMOSOME-6; ISODISOMY; 6Q

AB A female infant born at 33 weeks gestation to a gestationally diabetic mother developed apnea and respiratory distress at 6 hours of age. Laboratory investigation demonstrated persistent hyperglycemia, and the patient was treated with continuous intravenous and subsequent subcutaneous insulin therapy, Detailed laboratory investigation to reveal the etiology of hyperglycemia and further endocrine evaluation were not significant. The baby's insulin requirement has continued thereafter, and she is being followed up in an outpatient clinic still under insulin therapy at 18 months of age. Neonatal diabetes mellitus should be considered in the differential diagnosis of neonatal hyperglycemia, and it may develop in newborns born to diabetic mothers, as well as neonatal hypoglycemia. Insulin treatment with close blood glucose monitoring is essential as long as hyperglycemia persists since neonatal diabetes mellitus may be either transient or permanent and it is not possible to differentiate these two outcomes before 18 months of age.

C1 Gulhane Mil Med Acad, Dept Pediat, Div Newborn Med, TR-06018 Ankara, Turkey.

RP Sarici, SU (reprint author), Gulhane Mil Med Acad, Dept Pediat, Div Newborn Med, TR-06018 Ankara, Turkey.

CR ABRAMOWICZ MJ, 1994, J CLIN INVEST, V94, P418, DOI 10.1172/JCI117339

Arthur EI, 1997, EUR J HUM GENET, V5, P417

Bappal B, 1999, ARCH DIS CHILD, V80, pF209, DOI 10.1136/fn.80.3.F209

Battin M, 1996, J Perinatol, V16, P288

Cave H, 2000, DIABETES, V49, P108, DOI 10.2337/diabetes.49.1.108

Christian SL, 1999, J PEDIATR-US, V134, P42, DOI 10.1016/S0022-3476(99)70370-7

Gardner RJ, 1998, CLIN GENET, V54, P522

Gardner RJ, 1999, J MED GENET, V36, P192

GOTTSCHALK ME, 1992, DIABETES CARE, V15, P1273, DOI 10.2337/diacare.15.10.1273

Hermann R, 2000, PEDIATRICS, V105, P49, DOI 10.1542/peds.105.1.49

Hermann R, 1997, EUR J PEDIATR, V156, P740

HUME R, 1998, FORFAR ARNEILLS TXB, P298

KALKAN SC, 1997, NEONATAL PERINATAL M, P1462

Kruger C, 1997, EUR J PEDIATR, V156, P1

Marquis E, 2000, EUR J HUM GENET, V8, P137, DOI 10.1038/sj.ejhg.5200401

NIELSEN F, 1989, ACTA PAEDIATR SCAND, V78, P469, DOI 10.1111/j.1651-2227.1989.tb11114.x

POLK DH, 1998, AVERYS DIS NEWBORN, P1240

SALERNO M, 1994, J PEDIATR ENDOCRINOL, V7, P47

Shehadeh N, 1996, ACTA PAEDIATR, V85, P1415

Shehadeh N, 1998, J PEDIATR ENDOCR MET, V11, P575

SHIELD JPH, 1995, ARCH DIS CHILD, V72, P56, DOI 10.1136/adc.72.1.56

TEMPLE IK, 1995, NAT GENET, V9, P110, DOI 10.1038/ng0295-110

VONMUHLENDAHL E, 1995, NEW ENGL J MED, V333, P704, DOI 10.1056/NEJM199509143331105

WEIMERSKIRCH D, 1993, J PEDIATR-US, V122, P598, DOI 10.1016/S0022-3476(05)83544-9

Whiteford ML, 1997, J MED GENET, V34, P167, DOI 10.1136/jmg.34.2.167

NR 25

TC 7

Z9 8

U1 0

U2 1

PU WALTER DE GRUYTER GMBH

PI BERLIN

PA GENTHINER STRASSE 13, D-10785 BERLIN, GERMANY

SN 0334-018X

EI 2191-0251

J9 J PEDIATR ENDOCR MET

JI J. Pediatr. Endocrinol. Metab.

PD APR

PY 2001

VL 14

IS 4

BP 451

EP 454

PG 4

WC Endocrinology & Metabolism; Pediatrics

SC Endocrinology & Metabolism; Pediatrics

GA 421XJ

UT WOS:000168088600013

PM 11327380

DA 2019-01-08

ER

PT J

AU Weinger, K

Jacobson, AM

AF Weinger, K

Jacobson, AM

TI Psychosocial and quality of life correlates of glycemic control during

intensive treatment of type 1 diabetes

SO PATIENT EDUCATION AND COUNSELING

LA English

DT Article

DE type 1 diabetes; quality of life; emotional distress; barriers

ID BLOOD-GLUCOSE CONTROL; METABOLIC CONTROL; SELF-CARE; MELLITUS; THERAPY;

HYPOGLYCEMIA; MANAGEMENT; DEPRESSION; BEHAVIORS; EDUCATION

AB To identify emotional and attitudinal barriers to improved glycemic control (HbA(1c)) during intensive diabetes treatment, 55 patients attending a 4-5 month intensive diabetes medical/education clinic were followed. Subjects completed a battery of psychological surveys, had HbA(1c) and body mass index measured, and rated their attitude toward weight gain and the extent of problems with specific self-management behaviors before and after the medical intervention. Although HbA(1c) improved on average, 29% had only modest improvement and 16% showed no improvement. The number of diabetes-related annoyances, worry about hypoglycemia, and diabetes-related emotional distress diminished. Only the satisfaction subscale of the Diabetes Quality of Life survey, diabetes-related emotional distress, and problems with self-management behaviors correlated with HbA(1c). Treatment-related frustration and emotional distress may initially act as motivators to improve glycemia but can later become barriers to that goal. Interventions designed to help patients overcome attitudinal barriers should be incorporated into medical programs geared toward improving glycemia. (C) 2001 Elsevier Science Ireland Ltd. All rights reserved.

C1 Harvard Univ, Sch Med, Joslin Diabet Ctr, Sect Behav & Mental Hlth Res, Boston, MA 02215 USA.

RP Weinger, K (reprint author), Harvard Univ, Sch Med, Joslin Diabet Ctr, Sect Behav & Mental Hlth Res, Boston, MA 02215 USA.

FU NINR NIH HHS [F32 NR007157-03, F32 NR007157-01A1, F32 NR007157-02,

NR07157]; NIDDK NIH HHS [R01 DK042315-08, NIDDK42315]

CR Anderson B, 1997, J PEDIATR-US, V130, P257, DOI 10.1016/S0022-3476(97)70352-4

Beck A. T., 1976, COGNITIVE THERAPY EM

Bradley C, 1994, HDB PSYCHOL DIABETES, P65

COX DJ, 1984, HEALTH PSYCHOL, V3, P63, DOI 10.1037/0278-6133.3.1.63

COX DJ, 1987, DIABETES CARE, V10, P617, DOI 10.2337/diacare.10.5.617

Day JL, 1996, DIABETIC MED, V13, P564, DOI 10.1002/(SICI)1096-9136(199606)13:6<564::AID-DIA127>3.0.CO;2-0

Diabet Control Complications Trial Res Grp, 1996, DIABETES CARE, V19, P195

Diabet Control Complications Trial Res Grp, 1995, DIABETES CARE, V18, P1415

GLASGOW RE, 1987, J CHRON DIS, V40, P399, DOI 10.1016/0021-9681(87)90173-1

Grey M, 1999, APPL NURS RES, V12, P3, DOI 10.1016/S0897-1897(99)80123-2

JACOBSON AM, 1990, DIABETES CARE, V13, P375, DOI 10.2337/diacare.13.4.375

JACOBSON AM, 1994, DIABETES CARE, V17, P267, DOI 10.2337/diacare.17.4.267

Jacobson AM, 1998, ANN INTERN MED, V129, P656, DOI 10.7326/0003-4819-129-8-199810150-00016

Jacobson AM, 1996, NEW ENGL J MED, V334, P1249, DOI 10.1056/NEJM199605093341907

Keefe F J, 1993, Arthritis Care Res, V6, P213, DOI 10.1002/art.1790060408

Kinsley BT, 1999, DIABETES CARE, V22, P1022, DOI 10.2337/diacare.22.7.1022

LOCKINGTON TJ, 1989, DIABETIC MED, V6, P309, DOI 10.1111/j.1464-5491.1989.tb01170.x

LUSTMAN PJ, 1986, J NERV MENT DIS, V174, P736, DOI 10.1097/00005053-198612000-00005

Lustman PJ, 1998, ANN INTERN MED, V129, P613, DOI 10.7326/0003-4819-129-8-199810150-00005

MAZZE RS, 1984, DIABETES CARE, V7, P360, DOI 10.2337/diacare.7.4.360

*MED OUTC TRUST, 1993, SCOR SF 36 HLTH SURV, P19

PEYROT M, 1985, PSYCHOSOM MED, V47, P542, DOI 10.1097/00006842-198511000-00004

POLONSKY WH, 1995, DIABETES CARE, V18, P754, DOI 10.2337/diacare.18.6.754

POLONSKY WH, 1992, HEALTH PSYCHOL, V11, P199, DOI 10.1037/0278-6133.11.3.199

REICHARD P, 1993, NEW ENGL J MED, V329, P304, DOI 10.1056/NEJM199307293290502

RUBIN RR, 1989, DIABETES CARE, V12, P673, DOI 10.2337/diacare.12.10.673

RUBIN RR, 1991, DIABETES CARE, V14, P335, DOI 10.2337/diacare.14.4.335

SHAMOON H, 1993, NEW ENGL J MED, V329, P977

vanDulmen AM, 1996, PSYCHOSOM MED, V58, P508, DOI 10.1097/00006842-199609000-00013

WEINBERG JS, 1987, COGNITIVE THERAPY AP, P213

NR 30

TC 49

Z9 51

U1 0

U2 1

PU ELSEVIER SCI IRELAND LTD

PI CLARE

PA CUSTOMER RELATIONS MANAGER, BAY 15, SHANNON INDUSTRIAL ESTATE CO, CLARE,

IRELAND

SN 0738-3991

J9 PATIENT EDUC COUNS

JI Patient Educ. Couns.

PD FEB

PY 2001

VL 42

IS 2

BP 123

EP 131

DI 10.1016/S0738-3991(00)00098-7

PG 9

WC Public, Environmental & Occupational Health; Social Sciences,

Interdisciplinary

SC Public, Environmental & Occupational Health; Social Sciences - Other

Topics

GA 392XW

UT WOS:000166438700004

PM 11118778

DA 2019-01-08

ER

PT J

AU Magee, LA

AF Magee, LA

TI Treating hypertension in women of child-bearing age and during pregnancy

SO DRUG SAFETY

LA English

DT Review

ID FOR-GESTATIONAL-AGE; CONVERTING-ENZYME-INHIBITORS;

SOCIETY-CONSENSUS-CONFERENCE; BLOOD-PRESSURE MEASUREMENT; MILD CHRONIC

HYPERTENSION; FETAL GROWTH-RETARDATION; CORONARY HEART-DISEASE; MEAN

ARTERIAL-PRESSURE; BIRTH-WEIGHT INFANTS; LOW-DOSE ASPIRIN

AB Hypertension is found among 1 to 6% of young women, Treatment aims to decrease cardiovascular risk, the magnitude of which is less dependent on the absolute level of blood pressure (BP) than on associated cardiovascular risk factors, hypertension-related target organ damage and/or concomitant disease. Lifestyle modifications are recommended for all hypertensive individuals. The threshold of BP at which antihypertensive therapy should be initiated is based on absolute cardiovascular risk. Most young women are at low risk and not in need of anti-hypertensive therapy. All antihypertensive agents appear to be equally efficacious: choice depends on personal preference, social circumstances and an agent's effect on cardiovascular risk factors, target organ damage and/or concomitant disease.

Although most agents are appropriate for, and tolerated well by, young women, another consideration remains that of pregnancy, 50% of which are unplanned. A clinician must be aware of a woman's method of contraception and the potential of an antihypertensive agent to cause birth defects following inadvertent exposure in earl pregnancy. Conversely, if an oral contraceptive is effective and well tolerated, but the woman's BP becomes mildly elevated, continuing the contraceptive and initiating antihypertensive treatment mat; not be contraindicated, especially if the ability to plan pregnancy is important (e.g, in type 1 diabetes mellitus). No commonly used antihypertensive is known to he teratogenic, although ACE inhibitors and angiotensin receptor antagonists should be discontinued, and any antihypertensive drugs should be continued in pregnancy only if anticipated benefits out-weigh potential reproductive risk(s).

The hypertensive disorders of pregnancy complicate 5 to 10% of pregnancies and are a leading cause of maternal and perinatal mortality and morbidity. Treatment aims to improve pregnancy outcome. There is consensus that severe maternal hypertension (systolic BP greater than or equal to 170mm Hg and/or diastolic BP greater than or equal to 110mm Hg) should be treated immediately to avoid maternal stroke, death and, possibly, eclampsia. Parenteral hydralazine may he associated with a higher risk of maternal hypotension, and intravenous labetalol with neonatal bradycardia. There is no consensus as to whether mild-to-moderate hyper tension in pregnancy should be treated: the risks of transient severe hypertension, antenatal hospitalisation, proteinuria at delivery and neonatal respiratory distress syndrome, may be decreased by therapy, but intrauterine fetal grow th may also be impaired, particularly by atenolol. Methyldopa and other beta -blockers have been used most extensively. Reporting bias and the uncertainty of outcomes as defined warrant cautious interpretation of these findings and preclude treatment recommendations.

C1 Univ British Columbia, Childrens & Womens Hlth Ctr British Columbia, Vancouver, BC V6H 3N1, Canada.

RP Magee, LA (reprint author), Univ British Columbia, Childrens & Womens Hlth Ctr British Columbia, 1U59 4500 Oak St, Vancouver, BC V6H 3N1, Canada.

EM lmagee@cw.bc.ca

CR ALES K, 1990, AM J OBSTET GYNECOL, V162, P288, DOI 10.1016/0002-9378(90)90867-7

*AM AC PED COMM DR, 1994, PEDIATRICS, V93, P137

ANDERSON KM, 1991, CIRCULATION, V83, P356, DOI 10.1161/01.CIR.83.1.356

[Anonymous], 1990, AM J OBSTET GYNECOL, V163, P1689

AUGUST P, 1990, AM J OBSTET GYNECOL, V163, P1612, DOI 10.1016/0002-9378(90)90639-O

Badawi N, 1998, BRIT MED J, V317, P1549, DOI 10.1136/bmj.317.7172.1549

Barker DJP, 1997, CLIN OBSTET GYNECOL, V40, P853, DOI 10.1097/00003081-199712000-00019

BARR M, 1994, TERATOLOGY, V50, P399, DOI 10.1002/tera.1420500606

Barton J. R., 1999, American Journal of Obstetrics and Gynecology, V180, pS48

Bernstein IM, 2000, AM J OBSTET GYNECOL, V182, P198, DOI 10.1016/S0002-9378(00)70513-8

Briggs GG, 1994, DRUGS PREGNANCY LACT

BROUWER RM, 1985, CARDIOVASC PHARM, V7, pS88

Brown MA, 1996, MED J AUSTRALIA, V165, P360

Brown MA, 2000, AUST NZ J OBSTET GYN, V40, P133, DOI 10.1111/j.1479-828X.2000.tb01136.x

Brown MA, 1997, MED J AUSTRALIA, V166, P640

BURROWS RF, 1995, AM J OBSTET GYNECOL, V173, P929, DOI 10.1016/0002-9378(95)90368-2

Burrows RF, 1998, AUST NZ J OBSTET GYN, V38, P306, DOI 10.1111/j.1479-828X.1998.tb03072.x

Burrows RF, 1996, AM J OBSTET GYNECOL, V174, P800, DOI 10.1016/S0002-9378(96)70477-5

BUTTERS L, 1990, BMJ-BRIT MED J, V301, P587, DOI 10.1136/bmj.301.6752.587

Chappell LC, 1999, LANCET, V354, P810, DOI 10.1016/S0140-6736(99)80010-5

Coetzee EJ, 1998, BRIT J OBSTET GYNAEC, V105, P300, DOI 10.1111/j.1471-0528.1998.tb10090.x

COLLINS R, 1985, BMJ-BRIT MED J, V290, P17, DOI 10.1136/bmj.290.6461.17

DAVEY DA, 1986, CLIN EXP HYPERTENS B, V5, P97, DOI 10.3109/10641958609023478

*DEP HLTH, 1989, REP CONF ENQ MAT DEA

Department of Health, 1998, WHY MOTH DIE REP CON

Department of Health Welsh Office Scottish Office Department of Health Department of Health and Social Services Northern Ireland, 1996, REP CONF ENQ MAT DEA

DOUGLAS KA, 1994, BMJ-BRIT MED J, V309, P1395, DOI 10.1136/bmj.309.6966.1395

DUCEY JP, 1992, ANESTHESIOLOGY, V77, P829, DOI 10.1097/00000542-199210000-00034

Dwyer T, 1999, BRIT MED J, V319, P1325, DOI 10.1136/bmj.319.7221.1325

Easterling TR, 1999, OBSTET GYNECOL, V93, P725, DOI 10.1016/S0029-7844(98)00522-5

*ECL TRIAL COLL GR, 1995, LANCET, V345, P1455

Eriksson JG, 1999, BMJ-BRIT MED J, V318, P427, DOI 10.1136/bmj.318.7181.427

ESKENAZI B, 1993, AM J OBSTET GYNECOL, V169, P1112, DOI 10.1016/0002-9378(93)90265-K

Feldman RD, 1999, CAN MED ASSOC J, V161, pS1

FERRAZZANI S, 1990, AM J OBSTET GYNECOL, V162, P366, DOI 10.1016/0002-9378(90)90387-M

Ferrer RL, 2000, OBSTET GYNECOL, V96, P849, DOI 10.1016/S0029-7844(00)00938-8

Forsen T, 1999, BRIT MED J, V319, P1403, DOI 10.1136/bmj.319.7222.1403

FORSEN T, 1999, BMJ-BRIT MED J, V315, P837

Friedman E A, 1976, Perspect Nephrol Hypertens, V5, P13

Friedman SA, 1999, CHESLEYS HYPERTENSIV, P201

GALLERY EDM, 1979, Q J MED, V48, P593

GALLERY EDM, 1999, CHESLEYS HYPERTENSIV, P327

Gifford RW, 2000, AM J OBSTET GYNECOL, V183, pS1, DOI 10.1067/mob.2000.107928

GILSTRAP LC, 1978, SEMIN PERINATOL, V2, P73

Greer IA, 1999, LANCET, V353, P1258, DOI 10.1016/S0140-6736(98)10265-9

Gress TW, 2000, NEW ENGL J MED, V342, P905, DOI 10.1056/NEJM200003303421301

GUYTON AC, 1991, SCIENCE, V252, P1813, DOI 10.1126/science.2063193

HACK M, 1995, FUTURE CHILD, V5, P176, DOI 10.2307/1602514

Hales CN, 1997, BRIT MED BULL, V53, P109

Hansson L, 1998, LANCET, V351, P1755, DOI 10.1016/S0140-6736(98)04311-6

Hauth JC, 2000, OBSTET GYNECOL, V95, P24, DOI 10.1016/S0029-7844(99)00462-7

Helewa ME, 1997, CAN MED ASSOC J, V157, P715

Heyborne KD, 2000, AM J OBSTET GYNECOL, V183, P523, DOI 10.1067/mob.2000.106757

Horsager R, 1995, 15 ANN M SOC PER OBS

HURST JA, 1995, CLIN DYSMORPHOL, V4, P359, DOI 10.1097/00019605-199510000-00013

IMPEY L, 1993, BRIT J OBSTET GYNAEC, V100, P959, DOI 10.1111/j.1471-0528.1993.tb15120.x

JACKSON R, 1993, BRIT MED J, V307, P107, DOI 10.1136/bmj.307.6896.107

JOFFRES MR, 1992, CAN MED ASSOC J S, V146, P28

*JOINT NAT COMM PR, 1997, ARCH INTERN MED, V157, P2413, DOI DOI 10.1001/ARCHINTE.157.21.2413

KHONG TY, 1986, BRIT J OBSTET GYNAEC, V93, P1049, DOI 10.1111/j.1471-0528.1986.tb07830.x

Kim CR, 1996, ARCH PEDIAT ADOL MED, V150, P686, DOI 10.1001/archpedi.1996.02170320032005

Kok JH, 1998, BRIT J OBSTET GYNAEC, V105, P162, DOI 10.1111/j.1471-0528.1998.tb10046.x

Lapillonne A, 1997, ACTA PAEDIATR, V86, P173

LAW CM, 1993, BRIT MED J, V306, P24, DOI 10.1136/bmj.306.6869.24

Leon DA, 1998, BMJ-BRIT MED J, V317, P241, DOI 10.1136/bmj.317.7153.241

Lindberg B S, 1992, Int J Technol Assess Health Care, V8 Suppl 1, P57

Lip GYH, 1997, AM J CARDIOL, V79, P1436, DOI 10.1016/S0002-9149(97)00163-X

Lip GYH, 1997, LANCET, V350, P1446, DOI 10.1016/S0140-6736(05)64207-9

Liu LS, 1998, J HYPERTENS, V16, P1823, DOI 10.1097/00004872-199816120-00016

LONG PA, 1980, BRIT J OBSTET GYNAEC, V87, P13, DOI 10.1111/j.1471-0528.1980.tb04419.x

LUCAS MJ, 1995, NEW ENGL J MED, V333, P201, DOI 10.1056/NEJM199507273330401

Lydakis C, 1999, AM J HYPERTENS, V12, P541, DOI 10.1016/S0895-7061(99)00031-X

MABIE WC, 1986, OBSTET GYNECOL, V67, P197, DOI 10.1097/00006250-198602000-00008

MacMahon S, 1993, J VASC MED BIOL, V4, P265

Magee L, 2000, LANCET, V355, P1367, DOI 10.1016/S0140-6736(05)72600-3

Magee LA, 1999, BRIT MED J, V318, P1332, DOI 10.1136/bmj.318.7194.1332

Magee LA, 1996, AM J OBSTET GYNECOL, V174, P823, DOI 10.1016/S0002-9378(96)70307-1

Magee LA, 2000, EUR J OBSTET GYN R B, V88, P15, DOI 10.1016/S0301-2115(99)00113-X

MAGEE LA, 1996, THESIS U TORONTO TOR

MAGEE LA, 1997, EMBRYONIC MED THERAP, P390

MATERSON BJ, 1993, NEW ENGL J MED, V328, P914, DOI 10.1056/NEJM199304013281303

McIntire DD, 1999, NEW ENGL J MED, V340, P1234, DOI 10.1056/NEJM199904223401603

Misra DP, 1996, PAEDIATR PERINAT EP, V10, P244, DOI 10.1111/j.1365-3016.1996.tb00048.x

MOORE MP, 1983, BRIT MED J, V287, P580, DOI 10.1136/bmj.287.6392.580

Moutquin JM, 1997, CAN MED ASSOC J, V157, P907

NAEYE RL, 1987, AM J DIS CHILD, V141, P50, DOI 10.1001/archpedi.1987.04460010050022

NAEYE RL, 1979, AM J OBSTET GYNECOL, V133, P8, DOI 10.1016/0002-9378(79)90402-2

NIMROD CA, 1999, MED FETUS MOTHER, P267

ORNSTEIN M, IN PRESS HDB HYPERTE

OSMOND C, 1993, BRIT MED J, V307, P1519, DOI 10.1136/bmj.307.6918.1519

PAGE EW, 1976, AM J OBSTET GYNECOL, V126, P821, DOI 10.1016/0002-9378(76)90671-2

PAGE EW, 1976, AM J OBSTET GYNECOL, V125, P740, DOI 10.1016/0002-9378(76)90839-5

Peek M, 1996, OBSTET GYNECOL, V88, P1030, DOI 10.1016/S0029-7844(96)00350-X

Penny JA, 1998, AM J OBSTET GYNECOL, V178, P521, DOI 10.1016/S0002-9378(98)70432-6

PIETRANTONI M, 1994, CLIN EXP HYPERTENS, V16, P479, DOI 10.3109/10641969409067957

Poppas A, 1997, CIRCULATION, V95, P2407, DOI 10.1161/01.CIR.95.10.2407

Psaty BM, 1997, JAMA-J AM MED ASSOC, V277, P739, DOI 10.1001/jama.277.9.739

Ramsay LE, 1999, BRIT MED J, V319, P630, DOI 10.1136/bmj.319.7210.630

Rey E, 1997, CAN MED ASSOC J, V157, P1245

REY E, 1994, AM J OBSTET GYNECOL, V171, P410, DOI 10.1016/0002-9378(94)90276-3

RichEdwards JW, 1997, BRIT MED J, V315, P396, DOI 10.1136/bmj.315.7105.396

ROBERTS JM, 1993, LANCET, V341, P1447, DOI 10.1016/0140-6736(93)90889-O

SAFTLAS AF, 1990, AM J OBSTET GYNECOL, V163, P460, DOI 10.1016/0002-9378(90)91176-D

Saudan P, 1998, BRIT J OBSTET GYNAEC, V105, P1177, DOI 10.1111/j.1471-0528.1998.tb09971.x

SHEAR R, 1999, CAN J CME	FEB, P69

Shennan A, 1996, LANCET, V347, P139, DOI 10.1016/S0140-6736(96)90338-4

SIBAI BM, 1987, OBSTET GYNECOL, V70, P323

SIBAI BM, 1983, OBSTET GYNECOL, V61, P571

SIBAI BM, 1986, OBSTET GYNECOL, V67, P517

SIBAI BM, 1984, OBSTET GYNECOL, V64, P319

Sibai BM, 1998, NEW ENGL J MED, V339, P667, DOI 10.1056/NEJM199809033391004

SIBAI BM, 1992, AM J OBSTET GYNECOL, V167, P879, DOI 10.1016/S0002-9378(12)80005-6

SKRABANEK P, 1992, LANCET, V340, P1208, DOI 10.1016/0140-6736(92)92902-R

Staessen JA, 1997, LANCET, V350, P757, DOI 10.1016/S0140-6736(97)05381-6

Stanley CA, 1999, NEW ENGL J MED, V340, P1200, DOI 10.1056/NEJM199904153401510

Strauss RS, 2000, JAMA-J AM MED ASSOC, V283, P625, DOI 10.1001/jama.283.5.625

*TER SOC PUBL AFF, 1994, TERATOLOGY, V49, P446

TYSON JE, 1995, PEDIATRICS, V95, P534

UMANS JG, 1999, CHESLEYS HYPERTENSIV, P581

United States Food and Drug Administration, 1979, FED REGISTER, V44, P37434

van Pampus MG, 1998, EUR J OBSTET GYN R B, V76, P31, DOI 10.1016/S0301-2115(97)00146-2

Visser W, 1995, EUR J OBSTET GYN R B, V63, P147, DOI 10.1016/0301-2115(95)02260-0

von Dadelszen P, 2000, LANCET, V355, P87, DOI 10.1016/S0140-6736(98)08049-0

WAISMAN GD, 1988, AM J OBSTET GYNECOL, V159, P308, DOI 10.1016/S0002-9378(88)80072-3

*WHO EXP COMM, 1996, 862 WHO EXP COMM

WHO. World Health Organization-International Society of Hypertension Guideline for the Management of Hypertension (Guideline Sub-Committee), 1999, J HYPERTENS, P151

WITTMANN BK, 1988, CAN MED ASSOC J, V139, P37

Woodall SM, 1996, PEDIATR RES, V40, P438, DOI 10.1203/00006450-199609000-00012

YEMINI M, 1989, EUR J OBSTET GYN R B, V30, P193, DOI 10.1016/0028-2243(89)90068-3

1997, MMWR MORB MORTAL WKL, V46, P240

NR 130

TC 40

Z9 43

U1 0

U2 8

PU ADIS INT LTD

PI NORTHCOTE

PA 5 THE WAREHOUSE WAY, NORTHCOTE 0627, AUCKLAND, NEW ZEALAND

SN 0114-5916

EI 1179-1942

J9 DRUG SAFETY

JI Drug Saf.

PY 2001

VL 24

IS 6

BP 457

EP 474

DI 10.2165/00002018-200124060-00004

PG 18

WC Public, Environmental & Occupational Health; Pharmacology & Pharmacy;

Toxicology

SC Public, Environmental & Occupational Health; Pharmacology & Pharmacy;

Toxicology

GA 432BJ

UT WOS:000168666600004

PM 11368252

DA 2019-01-08

ER

PT J

AU Snoek, FJ

Pouwer, F

Welch, GW

Polonsky, WH

AF Snoek, FJ

Pouwer, F

Welch, GW

Polonsky, WH

TI Diabetes-related emotional distress in Dutch and US diabetic patients -

Cross-cultural validity of the problem areas in diabetes scale

SO DIABETES CARE

LA English

DT Article

ID QUESTIONNAIRE; PREVALENCE; MELLITUS; STRESS; PEOPLE

AB OBJECTIVE - To examine the cross-cultural validity of the Problem Areas in Diabetes Scale (PAID) in Dutch and U.S. diabetic patients.

RESEARCH DESIGN AND METHODS - A total of 1,472 Dutch people with diabetes completed the PAID along with other self-report measures of affect. Statistics covered Cronbach's or, exploratory factor analysis (EFA), and confirmatory factor analysis (CFA), Pearson's product-moment correlation, and t tests. Psychometric properties of PAID were compared for Dutch and U.S. diabetic patients.

RESULTS - Internal consistency of the Dutch PAID was high and stable across sex and type of diabetes. Test-retest reliability was high. Principal component analyses confirmed 1 general 20-item factor, whereas EFA identified 4 new subdimensions: negative emotions, treatment problems, food-related problems, and lack of social support. These dimensions were confirmed with CFA and were replicated in the U.S. sample. The PAID and its subscales demonstrated moderate to high associations in the expected direction with other measures of affect. Dutch and U.S. subjects reported having the same problem areas, with U.S. patients reporting higher emotional distress levels both in type 1 and type 2 diabetes.

CONCLUSIONS - The Dutch and U.S. 20-item PAID appeared to be psychometrically equivalent, which allowed for cross-cultural comparisons.

C1 Free Univ Amsterdam, Med Ctr, Dept Med Psychol, NL-1081 BT Amsterdam, Netherlands.

Joslin Diabet Ctr, Behav Res Sect, Boston, MA 02215 USA.

Univ Calif San Diego, Dept Psychiat, La Jolla, CA 92093 USA.

RP Snoek, FJ (reprint author), Free Univ Amsterdam, Med Ctr, Dept Med Psychol, Van der Boechorststr 7, NL-1081 BT Amsterdam, Netherlands.

CR AIKENS JE, 1992, J CONSULT CLIN PSYCH, V60, P113, DOI 10.1037/0022-006X.60.1.113

Bentler P.M., 1995, EQS WINDOWS USERS GU

Bijl RV, 1998, SOC PSYCH PSYCH EPID, V33, P587, DOI 10.1007/s001270050098

BRADLEY C, 1990, DIABETIC MED, V7, P445, DOI 10.1111/j.1464-5491.1990.tb01421.x

COX DJ, 1987, DIABETES CARE, V10, P617, DOI 10.2337/diacare.10.5.617

COX DJ, 1992, J CONSULT CLIN PSYCH, V60, P628, DOI 10.1037//0022-006X.60.4.628

Floyd FJ, 1995, PSYCHOL ASSESSMENT, V7, P286, DOI 10.1037/1040-3590.7.3.286

GAFVELS C, 1993, DIABETIC MED, V10, P768, DOI 10.1111/j.1464-5491.1993.tb00162.x

Glasgow RE, 1999, DIABETES CARE, V22, P832, DOI 10.2337/diacare.22.5.832

GLASGOW RE, 1997, DIABETES SPECTRUM, V10, P12

Herschbach P, 1997, HEALTH PSYCHOL, V16, P171, DOI 10.1037/0278-6133.16.2.171

Ishii H. W. G., 1999, DIABETES S1, V48, pA139

Jacobson AM, 1996, NEW ENGL J MED, V334, P1249, DOI 10.1056/NEJM199605093341907

KESSLER RC, 1994, ARCH GEN PSYCHIAT, V51, P8

MAZZE RS, 1984, DIABETES CARE, V7, P360, DOI 10.2337/diacare.7.4.360

Meadows K, 1996, QUAL LIFE RES, V5, P242, DOI 10.1007/BF00434746

METSCH J, 1995, INT J BEHAV MED, V2, P104, DOI 10.1207/s15327558ijbm0202_2

Nichols GA, 2000, DIABETES CARE, V23, P273, DOI 10.2337/diacare.23.3.273

Nouwen A, 1997, HEALTH PSYCHOL, V16, P263

Plowright R, 1999, QUAL LIFE RES, V8, P650

POLONSKY WH, 1995, DIABETES CARE, V18, P754, DOI 10.2337/diacare.18.6.754

Pouwer F, 1999, DIABETES CARE, V22, P2004, DOI 10.2337/diacare.22.12.2004

RUBIN RR, 1992, DIABETES CARE, V15, P1640, DOI 10.2337/diacare.15.11.1640

Snoek F, 1996, GEDRAG GEZONDHEID, V24, P287

SPIELBERGER CD, 1983, MANUAL STATE TRAIT A

SPSS, 1997, SPSS BASE 7 5 APPL G

Van der Ploeg H. M., 1999, HANDLEIDING BIJ ZELF

WELBORN TA, 1983, DIABETES CARE, V6, P315, DOI 10.2337/diacare.6.3.315

Welch GW, 1997, DIABETES CARE, V20, P760, DOI 10.2337/diacare.20.5.760

West S. G., 1995, STRUCTURAL EQUATION

NR 30

TC 225

Z9 229

U1 5

U2 24

PU AMER DIABETES ASSOC

PI ALEXANDRIA

PA 1660 DUKE ST, ALEXANDRIA, VA 22314 USA

SN 0149-5992

J9 DIABETES CARE

JI Diabetes Care

PD SEP

PY 2000

VL 23

IS 9

BP 1305

EP 1309

DI 10.2337/diacare.23.9.1305

PG 5

WC Endocrinology & Metabolism

SC Endocrinology & Metabolism

GA 348UT

UT WOS:000089005400018

PM 10977023

OA Bronze

DA 2019-01-08

ER

PT J

AU Meadows, KA

Abrams, C

Sandbaek, A

AF Meadows, KA

Abrams, C

Sandbaek, A

TI Adaptation of the Diabetes Health Profile (DHP-1) for use with patients

with Type 2 diabetes mellitus: psychometric evaluation and

cross-cultural comparison

SO DIABETIC MEDICINE

LA English

DT Article

DE cross-cultural adaptation; diabetes mellitus; diabetes-related

psychological distress and behaviour; reliability; validity

ID QUALITY-OF-LIFE; INSTRUMENTS; RELIABILITY

AB Aims To adapt the Diabetes Health Profile (DHP-1) for use with English speaking patients with Type 2 diabetes mellitus and to evaluate the psychometric properties of the adapted measure in a UK; and Danish sample of insulin, tablet and diet-treated patients with Type 2 diabetes.

Methods Following linguistic adaptation using the forward-backward translation procedure, the 32-item DHP-1 was sent to 650 and 800 consecutively selected UK and Danish patients with Type 2 diabetes. Construct validity was assessed using principal axis factoring. Factor stability was assessed across language groups using the coefficient of congruence. Reliability was evaluated using Cronbach's alpha and multi-trait analysis, including item convergent/discriminant validity. Subscale discriminant validity was assessed through known groups with one-way ANOVA and post hoc Scheffe tests for multiple comparisons.

Results Eighteen items (56.25%) were retained following initial item analysis. A three-factor solution accounting for 45.6% and 40.3% of the total explained variance was identified in the UK and Danish samples, respectively. Factors were interpreted as psychological distress (PD), barriers to activity (BA and disinhibited eating (DE). Factor congruence between language groups ranged from 0.98 to 0.99 and Cronbach's alpha ranged between 0.70 and 0.88. Item scaling success for both language versions was 88.9%. BA scores discriminated between treatment groups in both language groups (F = 24.24, P < 0.001; F = 7.68, P < 0.001) and PD scores in the UK sample (F = 20.97, P < 0.001).

Conclusions The DHP-18 developed for use with patients with Type 2 diabetes has been shown to have satisfactory internal reliability and validity and measurement equivalence across language groups.

C1 Univ Hull, Appl Stat Ctr, Hlth & Survey Res Unit, Kingston Upon Hull HU6 7RX, N Humberside, England.

RP Meadows, KA (reprint author), Univ Hull, Appl Stat Ctr, Hlth & Survey Res Unit, Kingston Upon Hull HU6 7RX, N Humberside, England.

EM k.a.meadows@maths.hull.ac.uk

CR BRADLEY C, 1990, DIABETIC MED, V7, P445, DOI 10.1111/j.1464-5491.1990.tb01421.x

Brislin RW, 1973, CROSS CULTURAL RES M

BUCQUET D, 1990, SOC SCI MED, V30, P829, DOI 10.1016/0277-9536(90)90207-9

BULLINGER M, 1993, QUAL LIFE RES, V2, P451, DOI 10.1007/BF00422219

CATTELL RB, 1966, HDB MULTIVARIATE EXP

Child D., 1970, ESSENTIALS FACTOR AN

CRONBACH LJ, 1951, PSYCHOMETRIKA, V16, P297, DOI DOI 10.1007/BF02310555

DCCT Res Grp, 1988, DIABETES CARE, V11, P725

DUNTEMAN GH, 1994, FACTOR ANAL RELATED, P157

FILLENBAUM GG, 1984, J HLTH ILLNESS, V20, P45

Goddijn P, 1996, QUAL LIFE RES, V5, P433, DOI 10.1007/BF00449918

Goddijn PPM, 1999, DIABETIC MED, V16, P23, DOI 10.1046/j.1464-5491.1999.00002.x

GUILLEMIN F, 1993, J CLIN EPIDEMIOL, V46, P1417, DOI 10.1016/0895-4356(93)90142-N

Harman HH, 1960, MODERN FACTOR ANAL

HAYS RD, 1990, BEHAV RES METH INSTR, V22, P167, DOI 10.3758/BF03203140

HAYS RD, 1993, QUAL LIFE RES, V2, P441, DOI 10.1007/BF00422218

Herdman M, 1997, QUAL LIFE RES, V6, P237

HUI CH, 1985, J CROSS CULT PSYCHOL, V16, P131, DOI 10.1177/0022002185016002001

MEADOWS K, 1994, QUAL LIFE RES, V3, P67

Meadows K, 1996, QUAL LIFE RES, V5, P242, DOI 10.1007/BF00434746

Nunnally JC, 1994, PSYCHOMETRIC THEORY, P752

POLONSKY WH, 1995, DIABETES CARE, V18, P754, DOI 10.2337/diacare.18.6.754

Rasch G, 1980, PROBABILISTIC MODELS

*SPSS INC, 1998, SPSS WIND VERS 8 0 2

TABACHNIK C, 1983, USING MULTIVARIATE S

Ware JE, 1998, QUAL LIFE RES, V7, P235, DOI 10.1023/A:1024946316424

WARE JEJ, 1993, SF36 HLTH SURVEY MAN

WILSON AE, 1993, DIABETIC MED, V10, P378, DOI 10.1111/j.1464-5491.1993.tb00084.x

YOUNGMAN MB, 1980, ANAL SOCIAL ED RES D

NR 29

TC 47

Z9 47

U1 0

U2 4

PU WILEY

PI HOBOKEN

PA 111 RIVER ST, HOBOKEN 07030-5774, NJ USA

SN 0742-3071

EI 1464-5491

J9 DIABETIC MED

JI Diabetic Med.

PD AUG

PY 2000

VL 17

IS 8

BP 572

EP 580

DI 10.1046/j.1464-5491.2000.00322.x

PG 9

WC Endocrinology & Metabolism

SC Endocrinology & Metabolism

GA 363CE

UT WOS:000089816100003

PM 11073178

DA 2019-01-08

ER

PT J

AU Herpertz, S

Albus, C

Lichtblau, K

Kohle, K

Mann, K

Senf, W

AF Herpertz, S

Albus, C

Lichtblau, K

Kohle, K

Mann, K

Senf, W

TI Relationship of weight and eating disorders in type 2 diabetic patients:

A multicenter study

SO INTERNATIONAL JOURNAL OF EATING DISORDERS

LA English

DT Article

DE diabetes mellitus; eating disorder; obesity; glycemic control

ID OBESE PATIENTS; BINGE; VALIDATION; PSYCHOPATHOLOGY; OVERWEIGHT; FEMALES

AB Objective: Previous work suggested that the degree of psychiatric symptomatology evidenced in overweight individuals was related to the severity of binge eating problems and not related to the severity of overweight. In a multicenter study, we investigated the relationship between weight and eating disorders (EDs) in a sample of type 2 diabetic patients. Methods: Three hundred twenty-two patients with type 2 diabetes were stratified to various weight categories. Glycemic control, eating and body-related psychological problems, self-esteem, depressive, and general psychopathology of diabetic patients with and without an ED were compared. Results: Eighty-one percent of all type 2 diabetic patients were overweight or obese. Prevalence rates of EDs ranged from 6.5% to 9.0%. Binge eating disorder was the most diagnosed ED. There was a strong relationship between body mass index (BMI) and eating disturbance-related variables and a weak or no relationship between BMI and depression or general psychopathologic variables. Patients with an ED showed a greater psychopathology compared to patients without an ED. The diagnosis of an ED did not seem to have a specific influence on glycemic control. Conclusions: Our results in a type 2 diabetic sample indicate that weight might have an impact on body and eating- related psychological distress in type 2 diabetic patients, but is of minor or no importance for depressive symptomatology, lower self-esteem, and general psychiatric symptomatology. Type 2 diabetic patients with an ED, however, suffer from considerable psychiatric symptomatology. (C) 2000 by John Wiley & Sons, Inc.

C1 Univ Essen Gesamthsch, Clin Psychotherapy & Psychosomat, D-45030 Essen, Germany.

Univ Cologne, Dept Psychosomat & Psychotherapy, Cologne, Germany.

Univ Essen Gesamthsch, Med Clin, Dept Endocrinol, Essen, Germany.

RP Herpertz, S (reprint author), Univ Essen Gesamthsch, Clin Psychotherapy & Psychosomat, Postfach 103043, D-45030 Essen, Germany.

RI Albus, Christian/C-1701-2015

CR American Psychiatric Association, 1994, DIAGN STAT MAN MENT

BERGER M, 1996, FORDERUNGEN STVINCEN

BRUCE B, 1992, INT J EAT DISORDER, V12, P365, DOI 10.1002/1098-108X(199212)12:4<365::AID-EAT2260120404>3.0.CO;2-M

COOPER PJ, 1987, INT J EAT DISORDER, V6, P485, DOI 10.1002/1098-108X(198707)6:4<485::AID-EAT2260060405>3.0.CO;2-O

DEROGATIS LR, 1977, J CLIN PSYCHOL, V33, P981, DOI 10.1002/1097-4679(197710)33:4<981::AID-JCLP2270330412>3.0.CO;2-0

DEZWAAN M, 1994, INT J EAT DISORDER, V15, P43, DOI 10.1002/1098-108X(199401)15:1<43::AID-EAT2260150106>3.0.CO;2-6

*DTSCH AD GES, 1995, ADIPOSITAS, V5, P6

Fichter MM, 1998, INT J EAT DISORDER, V24, P227, DOI 10.1002/(SICI)1098-108X(199811)24:3<227::AID-EAT1>3.0.CO;2-O

GARNER DM, 1983, INT J EAT DISORDER, V2, P15, DOI 10.1002/1098-108X(198321)2:2<15::AID-EAT2260020203>3.0.CO;2-6

GARROW JS, 1985, INT J OBESITY, V9, P147

GORTMAKER SL, 1993, NEW ENGL J MED, V329, P1008, DOI 10.1056/NEJM199309303291406

Hautzinger M, 1995, BECK DEPRESSIONS INV

Herpertz S, 1998, J PSYCHOSOM RES, V44, P503, DOI 10.1016/S0022-3999(97)00274-2

KENARDY J, 1994, DIABETES CARE, V17, P1197, DOI 10.2337/diacare.17.10.1197

MARCUS MD, 1992, DIABETES CARE, V15, P253, DOI 10.2337/diacare.15.2.253

MARCUS MD, 1985, ADDICT BEHAV, V10, P163, DOI 10.1016/0306-4603(85)90022-X

MARCUS MD, 1990, INT J EAT DISORDER, V9, P68

MEHNERT H, 1984, DIABETOLOGIE KLIN PR, P186

Nielsen S, 1998, EUR EAT DISORD REV, V6, P1

POLIVY J, 1985, AM PSYCHOL, V40, P191

SOREMBE V, 1979, SKALA ERFASSUNG SELB

SPITZER RL, 1993, INT J EAT DISORDER, V13, P137

SPITZER RL, 1992, INT J EAT DISORDER, V11, P191, DOI 10.1002/1098-108X(199204)11:3<191::AID-EAT2260110302>3.0.CO;2-S

STEEL JM, 1987, BRIT MED J, V294, P859, DOI 10.1136/bmj.294.6576.859

STEWART AL, 1983, AM J PUBLIC HEALTH, V73, P171, DOI 10.2105/AJPH.73.2.171

STHERPERTZ, 1998, DIABETES CARE, V21, P1

TELCH CF, 1994, INT J EAT DISORDER, V15, P53, DOI 10.1002/1098-108X(199401)15:1<53::AID-EAT2260150107>3.0.CO;2-0

TELCH CF, 1988, INT J EAT DISORDER, V7, P115, DOI 10.1002/1098-108X(198801)7:1<115::AID-EAT2260070112>3.0.CO;2-W

THIEL A, 1988, Z DIFFERENTIELLE DIA, V9, P267

Waadt S., 1992, BULIMIE

WADDEN TA, 1984, J CONSULT CLIN PSYCH, V52, P1104, DOI 10.1037//0022-006X.52.6.1104

WING RR, 1990, DIABETES CARE, V13, P170, DOI 10.2337/diacare.13.2.170

WING RR, 1989, INT J EAT DISORDER, V8, P671, DOI 10.1002/1098-108X(198911)8:6<671::AID-EAT2260080608>3.0.CO;2-5

NR 33

TC 38

Z9 41

U1 0

U2 5

PU JOHN WILEY & SONS INC

PI NEW YORK

PA 605 THIRD AVE, NEW YORK, NY 10158-0012 USA

SN 0276-3478

J9 INT J EAT DISORDER

JI Int. J. Eating Disord.

PD JUL

PY 2000

VL 28

IS 1

BP 68

EP 77

DI 10.1002/(SICI)1098-108X(200007)28:1<68::AID-EAT8>3.0.CO;2-R

PG 10

WC Psychology, Clinical; Nutrition & Dietetics; Psychiatry; Psychology

SC Psychology; Nutrition & Dietetics; Psychiatry

GA 313GH

UT WOS:000086991600008

PM 10800015

DA 2019-01-08

ER

PT J

AU Fisher, L

Chesla, CA

Skaff, MA

Gilliss, C

Kanter, RA

Lutz, CP

Bartz, RJ

AF Fisher, L

Chesla, CA

Skaff, MA

Gilliss, C

Kanter, RA

Lutz, CP

Bartz, RJ

TI Disease management status: A typology of Latino and Euro-American

patients with type 2 diabetes

SO BEHAVIORAL MEDICINE

LA English

DT Article

DE chronic disease management; type 2 diabetes; typology

ID QUALITY-OF-LIFE; PHYSICAL-ACTIVITY; HEALTH; PSYCHOLOGY; CARE

AB The number of indicators of chronic disease management is large and no practical framework is available to guide selection for use in intervention programs. In addition, it is often difficult to integrate multiple disease management indicators into a comprehensive picture of patient functioning The authors present a heuristic framework for creating a profile of disease management and an empirically based descriptive typology of disease manage ment behavior: From the literature, they identified 5 domains of disease management indicators: biologic, general health and functional status, emotional tone, quality of life, and behavioral. They selected II scales and assessed 187 patients with type 2 diabetes. Five statistically replicable patient disease management types were derived from exploratory and confirmatory cluster analyses and a descriptive narrative was created for each: balanced (33%), problematic (6%), coasters (34%), discouraged (16%), and distressed (11%). The typology describes different presentations of disease management that can be linked with patient-tailored interventions for patients with type 2 diabetes.

C1 Univ Calif San Francisco, Dept Family & Community Med, San Francisco, CA 94143 USA.

Yale Univ, New Haven, CT 06520 USA.

Kaiser Permanente, Oakland, CA 94612 USA.

RP Fisher, L (reprint author), Univ Calif San Francisco, Dept Family & Community Med, Box 0900, San Francisco, CA 94143 USA.

EM fisher@itsa.ucsf.edu

FU NIDDK NIH HHS [DK-49816]

CR ADA (American Diabetics Association), 1994, DIABETES CARE, V17, P519

Anderson RM, 1997, DIABETES CARE, V20, P299, DOI 10.2337/diacare.20.3.299

ANDERSON RM, 1990, PATIENT EDUC COUNS, V16, P231, DOI 10.1016/0738-3991(90)90072-S

BORGEN FH, 1987, J COUNS PSYCHOL, V34, P456, DOI 10.1037/0022-0167.34.4.456

Boyer JG, 1997, MED CARE, V35, P440, DOI 10.1097/00005650-199705000-00003

CATTELL RB, 1966, HDB MULTIVARIATE EXP

Coyne JC, 1994, J FAMILY PSYCHOL, V8, P43, DOI DOI 10.1037/0893-3200.8.1.43

DCCT Res Grp, 1988, DIABETES CARE, V11, P725

DEROGATIS L R, 1973, Psychopharmacology Bulletin, V9, P13

DORGAN JF, 1994, AM J EPIDEMIOL, V139, P662, DOI 10.1093/oxfordjournals.aje.a117056

ELLWOOD PM, 1988, NEW ENGL J MED, V318, P1549, DOI 10.1056/NEJM198806093182329

FISHER L, 1995, FAM PROCESS, V34, P161, DOI 10.1111/j.1545-5300.1995.00161.x

Fisher L, 1996, J FAM PSYCHOL, V10, P180

FISHER L, 1992, FAM PROCESS, V32, P399

FISHER L, 1998, HLTH PSYCHOL, V17, P1

Frankenfield DC, 1998, J AM DIET ASSOC, V98, P439, DOI 10.1016/S0002-8223(98)00100-X

GAVARD JA, 1993, DIABETES CARE, V16, P1167, DOI 10.2337/diacare.16.8.1167

GLASGOW RE, 1995, DIABETES CARE, V18, P117, DOI 10.2337/diacare.18.1.117

GLASGOW RE, IN PRESS ANN BEHAV M

GOODALL T, 1991, HLTH PSYCHOL, V11, P1

Harris J, 1919, BIOMETRIC STUDY BASA

Heyman R. E., 1994, J FAMILY PSYCHOL, V8, P432, DOI DOI 10.1037/0893-3200.8.4.432

JACOBSON AM, 1994, DIABETES CARE, V17, P267, DOI 10.2337/diacare.17.4.267

Johnson D., 1980, BASIC DATA DEPRESSIV

JOHNSON SB, 1992, DIABETES CARE, V15, P1658, DOI 10.2337/diacare.15.11.1658

KAPLAN RM, 1994, HEALTH PSYCHOL, V13, P451, DOI 10.1037//0278-6133.13.6.451

KAPLAN RM, 1990, AM PSYCHOL, V45, P1211, DOI 10.1037/0003-066X.45.11.1211

KLEINMAN A, 1978, ANN INTERN MED, V88, P251, DOI 10.7326/0003-4819-88-2-251

Kleinman A., 1980, PATIENTS HEALERS CON

LAPORTE RE, 1983, J GERONTOL, V38, P394, DOI 10.1093/geronj/38.4.394

Lorr M, 1983, CLUSTER ANAL SOCIAL

MARIN G, 1991, APPL SOCIAL RES METH

MILLIGAN GW, 1980, PSYCHOMETRIKA, V45, P325, DOI 10.1007/BF02293907

Myers L, 1998, ADHERENCE TREATMENT, P1

PAFFENBARGER RS, 1993, MED SCI SPORT EXER, V25, P60, DOI 10.1249/00005768-199301000-00010

PEARLIN LI, 1980, J HEALTH SOC BEHAV, V22, P333

TOOBERT DJ, 1994, HDB PSYCHOL DIABETES, P351

VanderDoes FEE, 1996, DIABETES CARE, V19, P204, DOI 10.2337/diacare.19.3.204

WARE JE, 1992, MED CARE, V30, P473, DOI 10.1097/00005650-199206000-00002

1992, VITAL STAT US

NR 40

TC 17

Z9 17

U1 0

U2 5

PU ROUTLEDGE JOURNALS, TAYLOR & FRANCIS LTD

PI ABINGDON

PA 4 PARK SQUARE, MILTON PARK, ABINGDON OX14 4RN, OXFORDSHIRE, ENGLAND

SN 0896-4289

EI 1940-4026

J9 BEHAV MED

JI Behav. Med.

PD SUM

PY 2000

VL 26

IS 2

BP 53

EP 66

DI 10.1080/08964280009595752

PG 14

WC Behavioral Sciences; Psychiatry

SC Behavioral Sciences; Psychiatry

GA 384JC

UT WOS:000165938200001

PM 11147290

DA 2019-01-08

ER

PT J

AU Mollema, ED

Snoek, FJ

Pouwer, F

Heine, RJ

van der Ploeg, HM

AF Mollema, ED

Snoek, FJ

Pouwer, F

Heine, RJ

van der Ploeg, HM

TI Diabetes fear of injecting and self-testing questionnaire - A

psychometric evaluation

SO DIABETES CARE

LA English

DT Article

ID PHOBIA; VALIDATION; DISTRESS; SCALE

AB OBJECTIVE - To study the psychometric properties of the Diabetes Fear of Injecting and Self-Testing Questionnaire (D-FISQ).

RESEARCH DESIGN AND METHODS - Two groups of patients were studied. Sample A consisted of 252 insulin-treated diabetes patients. Sample B incorporated 24 insulin-treated patients with high scores (greater than or equal to 95th percentile) on the D-FISQ. Test-retest correlations were assessed in both samples. Discriminant and convergent validity of the D-FISQ were assessed with questionnaires concerning fear of hypoglycemia, trait anxiety, and fear of bodily injury, illness, or death. To evaluate criterion-related validity, sample B participated in a behavioral avoidance test (BAT), in which the current level of avoidance of either self-injecting or self-testing was determined. Exploratory factor analysis (EM) was performed to study whether 2 factors (fear of self-injecting [FSI] and fear of self-testing [FST]) could be detected.

RESULTS- Test-retest correlations ranged from 0.50 to 0.68 (P < 0.001). Correlations between D-FISQ and fear of hypoglycemia, trait anxiety, and fear of bodily injury, illness, or death ranged from 0.28 to 0.45 (P < 0.001). Patients who refused to do a BA? for self-injecting or self-testing had higher scores on FSI (P = 0.095) and FST (P = 0.01). EFA yielded 2 separate factors, FSI and FST.

CONCLUSIONS - Results from this study support reliability and validity of the D-FISQ, a self-report instrument that can be used for both clinical and research purposes.

C1 Free Univ Amsterdam, Fac Med, EMGO Inst, NL-1081 BT Amsterdam, Netherlands.

Free Univ Amsterdam, Dept Med Psychol, NL-1081 BT Amsterdam, Netherlands.

Free Univ Amsterdam, Res Inst Endocrinol Reprod & Metab, NL-1081 BT Amsterdam, Netherlands.

RP Mollema, ED (reprint author), Free Univ Amsterdam, Fac Med, EMGO Inst, Boechorststr 7, NL-1081 BT Amsterdam, Netherlands.

CR ARRINDELL WA, 1980, BEHAV RES THER, V18, P229, DOI 10.1016/0005-7967(80)90080-7

ARRINDELL WA, 1986, SCL90 HANDLEIDING EE

Berlin I, 1997, DIABETES CARE, V20, P176, DOI 10.2337/diacare.20.2.176

Bodzin B, 1996, Home Healthc Nurse, V14, P378, DOI 10.1097/00004045-199605000-00009

BOUMAN TK, 1987, THESIS U GRONINGEN

BRADLEY C, 1994, HDB PSYCHOL DIABETES, P89

Floyd FJ, 1995, PSYCHOL ASSESSMENT, V7, P286, DOI 10.1037/1040-3590.7.3.286

Herrmann C, 1997, J PSYCHOSOM RES, V42, P17, DOI 10.1016/S0022-3999(96)00216-4

Irvin A, 1994, HDB PSYCHOL DIABETES, P133

JACOBSON AM, 1994, DIABETES CARE, V17, P267, DOI 10.2337/diacare.17.4.267

Kindt M, 1996, BEHAV RES THER, V34, P277, DOI 10.1016/0005-7967(95)00069-0

KOLKO DJ, 1980, J BEHAV THER EXP PSY, V11, P273, DOI 10.1016/0005-7916(80)90070-1

LUTEIJN F, 1979, HANDLEIDING NPV MANU

METSCH J, 1995, INT J BEHAV MED, V2, P104, DOI 10.1207/s15327558ijbm0202_2

Mollema E. D., 1997, Diabetologia, V40, pA637

MOORE KE, 1995, CLIN PEDIATR, V34, P530, DOI 10.1177/000992289503401004

OST LG, 1992, BEHAV THER, V23, P263, DOI 10.1016/S0005-7894(05)80385-5

Pouwer F, 1999, DIABETES CARE, V22, P2004, DOI 10.2337/diacare.22.12.2004

RAINWATER N, 1988, CHILD FAM BEHAV THER, V10, P19

Snoek F, 1996, GEDRAG GEZONDHEID, V24, P287

Snoek FJ, 1997, DIABETIC MED, V14, P871, DOI 10.1002/(SICI)1096-9136(199710)14:10<871::AID-DIA457>3.3.CO;2-P

SPIELBERGER CD, 1983, MANUAL STATE TRAIT A

*SPSS, 1997, SPSS BAS 7 5 WIND US

Streiner DL, 1995, HLTH MEASUREMENT SCA

VANDERPLOEG HM, 1980, HANDLEIDING ZELF BEO

Welch G, 1994, HDB PSYCHOL DIABETES, P400

Welch GW, 1997, DIABETES CARE, V20, P760, DOI 10.2337/diacare.20.5.760

Zambanini A, 1997, DIABETIC MED, V14, P321, DOI 10.1002/(SICI)1096-9136(199704)14:4<321::AID-DIA356>3.0.CO;2-H

ZAMBANINI A, 1999, DIABETES RES CLIN PR, V46, P289

NR 29

TC 51

Z9 52

U1 0

U2 2

PU AMER DIABETES ASSOC

PI ALEXANDRIA

PA 1660 DUKE ST, ALEXANDRIA, VA 22314 USA

SN 0149-5992

J9 DIABETES CARE

JI Diabetes Care

PD JUN

PY 2000

VL 23

IS 6

BP 765

EP 769

DI 10.2337/diacare.23.6.765

PG 5

WC Endocrinology & Metabolism

SC Endocrinology & Metabolism

GA 318ZF

UT WOS:000087314000011

PM 10840993

OA Bronze

DA 2019-01-08

ER

PT J

AU Janssen, MMJ

Snoek, FJ

de Jongh, RT

Casteleijn, S

Deville, W

Heine, RJ

AF Janssen, MMJ

Snoek, FJ

de Jongh, RT

Casteleijn, S

Deville, W

Heine, RJ

TI Biological and behavioural determinants of the frequency of mild,

biochemical hypoglycaemia in patients with Type 1 diabetes on multiple

insulin injection therapy

SO DIABETES-METABOLISM RESEARCH AND REVIEWS

LA English

DT Article

DE hypoglycaemia; Type 1 diabetes mellitus; multiple insulin injection

therapy

ID SEVERE HYPOGLYCEMIA; IDDM; AWARENESS; ADULTS; RISK; UNAWARENESS;

MELLITUS

AB Background Severe hypoglycaemic episodes are an important source of morbidity in people with Type 1 diabetes. The occurrence of severe hypoglycaemia is strongly related to the frequency of low blood glucose readings. The aim of this exploratory study was to identify determinants of the frequency of mild, biochemical hypoglycaemia in patients with Type I diabetes treated with multiple insulin injection therapy.

Methods We studied 31 patients with Type 1 diabetes in reasonable glycaemic control (HbA(1c)less than or equal to 8.3%) during multiple injection therapy. The study had a prospective, observational design. We used standardised home blood glucose monitoring (HBGM) diaries to assess the frequency of hypoglycaemia (HBGM readings<3.5 mmol/L) over a period of 6 weeks. Potential determinants studied included biological factors, self-management factors (including weekly total physical activity and vigorous physical activity), psychological factors (including psychological distress) and mediating factors [average and standard deviation (SD) of the HBGM readings and self-reported hypoglycaemia awareness].

Results Determinants of mild hypoglycaemia frequency identified in univariate regression analyses were: SD and mean of HBGM (beta 0.6, p = 0.001 and beta -0.6, p = 0.001), diabetes duration (beta 0.5, p = 0.008) and self-reported hypoglycaemia unawareness (beta -0.5, p = 0.003). A trend was observed for performance of vigorous physical activities (beta 0.3, p = 0.06) and external eating behaviour (beta -0.3, p = 0.1). These relations were confirmed in multivariate analyses.

Conclusions Patients with Type I diabetes who have a high blood glucose variability and low average blood glucose concentration, diabetes of long duration, low body mass index, self-reported impaired awareness of hypoglycaemia and those participating in vigorous physical activities, specifically require interventions aimed at preventing hypoglycaemia. Copyright (C) 2000 John Wiley & Sons, Ltd.

C1 Free Univ Amsterdam Hosp, Dept Endocrinol, Res Inst Endocrinol Reprod & Metab, NL-1007 MB Amsterdam, Netherlands.

Free Univ Amsterdam, Fac Med, Dept Epidemiol & Biostat, NL-1081 BT Amsterdam, Netherlands.

RP Heine, RJ (reprint author), Free Univ Amsterdam Hosp, Dept Endocrinol, Res Inst Endocrinol Reprod & Metab, POB 7057, NL-1007 MB Amsterdam, Netherlands.

RI Deville, Walter/B-5160-2013

CR ARRINDELL WA, 1975, SCL 90 HANDLEIDING M

Boyle PJ, 1995, NEW ENGL J MED, V333, P1726, DOI 10.1056/NEJM199512283332602

Cameron M E, 1988, MANUAL METHODOLOGY F

Clarke WL, 1997, DIABETES EDUCATOR, V23, P55, DOI 10.1177/014572179702300107

Clarke WL, 1999, DIABETES CARE, V22, P580, DOI 10.2337/diacare.22.4.580

CLARKE WL, 1995, DIABETES CARE, V18, P517, DOI 10.2337/diacare.18.4.517

CRYER PE, 1997, HYPOGLYCEMIA PATHOPH, P91

*DIAB CONTR COMPL, 1997, DIABETES, V46, P271, DOI DOI 10.2337/DIABETES.46.2.271

Diabet Control Complications Trial Res Grp, 1996, DIABETES CARE, V19, P195

Diabet Control Complications Trial Res Grp, 1995, DIABETES CARE, V18, P1415

Ferrannini E, 1997, J CLIN INVEST, V100, P1166, DOI 10.1172/JCI119628

GOLD AE, 1994, DIABETES CARE, V17, P697, DOI 10.2337/diacare.17.7.697

GONDERFREDERICK LA, 1990, HEALTH PSYCHOL, V9, P503, DOI 10.1037//0278-6133.9.5.503

HELLER SR, 1991, DIABETES, V40, P223, DOI 10.2337/diabetes.40.2.223

Kovatchev BP, 1998, DIABETES CARE, V21, P1870, DOI 10.2337/diacare.21.11.1870

MAYER EJ, 1991, AM J CLIN NUTR, V53, P812

METSCH J, 1995, INT J BEHAV MED, V2, P104, DOI 10.1207/s15327558ijbm0202_2

MONTOYE H, 1996, MEASURING PHYSICAL A

Pampanelli S, 1996, DIABETOLOGIA, V39, P677

PRAMMING S, 1990, J INTERN MED, V228, P641, DOI 10.1111/j.1365-2796.1990.tb00292.x

SALLIS JF, 1985, AM J EPIDEMIOL, V121, P91, DOI 10.1093/oxfordjournals.aje.a113987

THORSTEINSSON B, 1986, DIABETIC MED, V3, P147, DOI 10.1111/j.1464-5491.1986.tb00726.x

VANSTRIEN T, 1984, 01186 AGR U DEP HUM

VENEMAN T, 1993, DIABETES, V42, P1233, DOI 10.2337/diabetes.42.9.1233

WREDLING RAM, 1992, DIABETES CARE, V15, P518, DOI 10.2337/diacare.15.4.518

1996, NEVO TABEL NEDERLAND

NR 26

TC 20

Z9 20

U1 0

U2 3

PU JOHN WILEY & SONS LTD

PI W SUSSEX

PA BAFFINS LANE CHICHESTER, W SUSSEX PO19 1UD, ENGLAND

SN 1520-7552

J9 DIABETES METAB RES

JI Diabetes-Metab. Res. Rev.

PD MAY-JUN

PY 2000

VL 16

IS 3

BP 157

EP 163

DI 10.1002/1520-7560(0000)9999:9999<::AID-DMRR104>3.0.CO;2-U

PG 7

WC Endocrinology & Metabolism

SC Endocrinology & Metabolism

GA 328DR

UT WOS:000087834000002

PM 10867714

DA 2019-01-08

ER

PT J

AU Ladwig, KH

Muhlberger, N

Walter, H

Schumacher, K

Popp, K

Holle, R

Zitzmann-Roth, E

Schomig, A

AF Ladwig, KH

Muhlberger, N

Walter, H

Schumacher, K

Popp, K

Holle, R

Zitzmann-Roth, E

Schomig, A

TI Gender differences in emotional disability and negative health

perception in cardiac patients 6 months after stent implantation

SO JOURNAL OF PSYCHOSOMATIC RESEARCH

LA English

DT Article

DE gender differences; emotional disability; health perception; stent

implantation; coronary artery disease

ID ACUTE MYOCARDIAL-INFARCTION; CORONARY-ARTERY DISEASE; REGIMEN ISAR

TRIAL; HOSPITAL ANXIETY; FOLLOW-UP; THROMBOLYTIC THERAPY; DEPRESSION

SCALE; WOMEN; MORTALITY; RISK

AB Objectives: In this study we evaluate gender differences in affective adaptation and health perception in patients 6 months after stent implantation. Background: Assessment of gender-specific behavioral strategies to cope with serious cardiac disease conditions has not been given much attention until now. preliminary data suggest greater impairments in female patients, which might be of clinical relevance. Methods: Three hundred seventeen patients were eligible for the 6-month follow-up investigation, 78 (24.6%) of whom were women. The women were significantly older but did not differ from men in their cardiac risk features and treatment procedures. There were no gender differences in prevalence of hypertension, hyper-cholesterolemia, and family history. Men had a significantly higher prevalence of smoking than women, whereas women had a significantly higher prevalence of diabetes than men. A structured interview and a standardized psychodiagnostic assessment was carried out, which covered domains of affective dysfunction (depression, anxiety, intrusion, and avoidance), vegetative symptoms (sleeping disorders), and parameters of negative health perception. Results: There were no significant gender differences in the prevalence of depressive symptoms. Women exhibited higher mean values of anxiety than men, which did not reach significance. Sleeping disorders were significantly more prevalent ill women. The absolute level of being distressed by intrusive thoughts and avoidance behavior related to the severe underlying disease process was low in the total group of patients examined. Measurable gender differences did not emerge. Fifty-one (16.5%) patients exhibited pessimistic anticipation of dire consequences and severe signs of negative health perception (NHP group). There was a trend, although not statistically significant, toward more women being in the NHP group. The distribution of cardiac risk factors, however, was completely balanced in the NHP+ and NHP- patient groups. Objective somatic cardiac disease parameters did not account for the negative health perception. NHP was. however, associated with significantly more prestent angina pectoris (p < 0.040) and poststent angina pectoris (p < 0.0001). High levels of anxiety, depression. and of disturbed sleep also led to a sharp separation between patients with high degrees of an anticipated incapacitation due to the disease process. Univariate regression analysis suggested an effect of female gender on the occurrence of NHP (odds ratio 1.70; 95% CI 0.88 to 3.25), which was of borderline significance. Control for confounders in a multiple regression model, however, eliminated the gender effect (odds ratio 1.04, 95% CI 0.48 to 2.23). Poststent chest pain (odds ratio 7.75, 95% CI 3.28 to 18.32) and sleeping disorders (odds ratio 1.32, 95% CI 1.16 to 1.51) were identified as the most powerful confounders of the gender-NHP association. Conclusion: Contrary to expectation. women were not per se more distressed than men in all areas of adaptation of the midterm course after stent implantation, although the higher levels of anxiety and sleeping disorders in women deserve attention. A considerable proportion of patients exhibited a pessimistic disease perspective independent of their somatic status. which was associated with affective morbidity. The tendency toward more negative health perception in women may be due to their more frequent occurrence of chest pain and sleeping disorders. (C) 2000 Elsevier Science Inc. All rights reserved.

C1 Tech Univ Munich, Klinikum Rechts Isar, Inst & Poliklin Psychosomat Med Med Psychol & Psy, D-81675 Munich, Germany.

GSF, Natl Res Ctr Environm & Hlth, MEDIS Inst Med Informat & Hlth Serv Res, Munich, Germany.

Tech Univ Munich, Klinikum Rechts Isar, Med Klin 1, D-8000 Munich, Germany.

RP Ladwig, KH (reprint author), Tech Univ Munich, Klinikum Rechts Isar, Inst & Poliklin Psychosomat Med Med Psychol & Psy, Langerstraae 3, D-81675 Munich, Germany.

RI Ladwig, Karl-Heinz/B-5351-2014; Holle, Rolf/D-9333-2013

OI Ladwig, Karl-Heinz/0000-0003-0710-1720; Holle, Rolf/0000-0001-5395-2695

CR APPELS A, 1988, EUR HEART J, V9, P758, DOI 10.1093/eurheartj/9.7.758

Asplund R, 1998, J INTERN MED, V243, P209

AYANIAN JZ, 1991, NEW ENGL J MED, V325, P221, DOI 10.1056/NEJM199107253250401

Barefoot JC, 1996, AM J CARDIOL, V78, P613, DOI 10.1016/S0002-9149(96)00380-3

BERGELSON BA, 1995, CHEST, V108, P1510, DOI 10.1378/chest.108.6.1510

Billing E, 1997, EUR HEART J, V18, P911

Boogard M, 1985, J CARDIOPULMONARY RE, V5, P379

BUENO H, 1995, CIRCULATION, V92, P1133, DOI 10.1161/01.CIR.92.5.1133

Collijn DH, 1996, J PSYCHOSOM RES, V40, P271, DOI 10.1016/0022-3999(95)00547-1

CONN VS, 1991, J ADV NURS, V16, P1026, DOI 10.1111/j.1365-2648.1991.tb03362.x

Crowe JM, 1996, HEART LUNG, V25, P98, DOI 10.1016/S0147-9563(96)80111-7

DITTRICH H, 1988, AM J CARDIOL, V62, P1, DOI 10.1016/0002-9149(88)91356-2

EAKER ED, 1992, AM J EPIDEMIOL, V135, P854, DOI 10.1093/oxfordjournals.aje.a116381

Frasure-Smith N, 1999, PSYCHOSOM MED, V61, P26, DOI 10.1097/00006842-199901000-00006

FRASURESMITH N, 1993, JAMA-J AM MED ASSOC, V270, P1819, DOI 10.1001/jama.270.15.1819

Friedman BH, 1998, BIOL PSYCHOL, V47, P243, DOI 10.1016/S0301-0511(97)00027-6

HEALY B, 1991, NEW ENGL J MED, V325, P274, DOI 10.1056/NEJM199107253250408

HERRMANN C, 1991, PSYCHOTHER PSYCH MED, V41, P83

HOROWITZ M, 1979, PSYCHOSOM MED, V41, P209, DOI 10.1097/00006842-197905000-00004

Irvine J, 1998, CLIN PSYCHOL PSYCHOT, V5, P86

KAPLAN GA, 1983, AM J EPIDEMIOL, V117, P292, DOI 10.1093/oxfordjournals.aje.a113541

Kastrati A, 1997, CIRCULATION, V96, P462

KOP WJ, 1993, INT J CARDIOL, V42, P269, DOI 10.1016/0167-5273(93)90059-P

Ladwig KH, 1997, EUR HEART J, V18, P582

LADWIG KH, 1991, EUR HEART J, V12, P959

LADWIG KH, 1994, LANCET, V343, P20, DOI 10.1016/S0140-6736(94)90877-X

Lesperance F, 1996, PSYCHOSOM MED, V58, P99, DOI 10.1097/00006842-199603000-00001

MAYNARD C, 1991, AM J CARDIOL, V68, P529, DOI 10.1016/0002-9149(91)90791-I

MURPHY JM, 1986, ACTA PSYCHIAT SCAND, V73, P113, DOI 10.1111/j.1600-0447.1986.tb10576.x

Oka RK, 1996, AM J CARDIOL, V78, P861, DOI 10.1016/S0002-9149(96)00457-2

Perkins S, 1998, HEART LUNG, V27, P37, DOI 10.1016/S0147-9563(98)90067-X

Petrie KJ, 1996, BRIT MED J, V312, P1191

RIEGEL B, 1995, HEART LUNG, V24, P457, DOI 10.1016/S0147-9563(95)80023-9

Schiaffino KM, 1998, HEALTH PSYCHOL, V17, P262, DOI 10.1037/0278-6133.17.3.262

SCHLEIFER SJ, 1989, ARCH INTERN MED, V149, P1785, DOI 10.1001/archinte.149.8.1785

SCHRON EB, 1991, CIRCULATION S2, V84, P245

Schuhlen H, 1997, CIRCULATION, V95, P2015, DOI 10.1161/01.CIR.95.8.2015

SILVERSTONE PH, 1994, J PSYCHOSOM RES, V38, P441, DOI 10.1016/0022-3999(94)90105-8

*STAT BUND, 1998, GES STAT BUND, V12

STERN MJ, 1976, J CHRON DIS, V29, P513, DOI 10.1016/0021-9681(76)90015-1

VERMURA K, 1988, WORLD HLTH STAT Q, V41, P155

Weaver WD, 1996, JAMA-J AM MED ASSOC, V275, P777, DOI 10.1001/jama.275.10.777

WENGER NK, 1993, NEW ENGL J MED, V329, P247, DOI 10.1056/NEJM199307223290406

WIKLUND I, 1989, EUR HEART J, V10, P464, DOI 10.1093/oxfordjournals.eurheartj.a059511

WIKLUND I, 1993, EUR HEART J, V14, P1315, DOI 10.1093/eurheartj/14.10.1315

ZIGMOND AS, 1983, ACTA PSYCHIAT SCAND, V67, P361, DOI 10.1111/j.1600-0447.1983.tb09716.x

NR 46

TC 17

Z9 17

U1 0

U2 4

PU PERGAMON-ELSEVIER SCIENCE LTD

PI OXFORD

PA THE BOULEVARD, LANGFORD LANE, KIDLINGTON, OXFORD OX5 1GB, ENGLAND

SN 0022-3999

J9 J PSYCHOSOM RES

JI J. Psychosomat. Res.

PD APR-MAY

PY 2000

VL 48

IS 4-5

BP 501

EP 508

DI 10.1016/S0022-3999(99)00111-7

PG 8

WC Psychiatry

SC Psychiatry

GA 329HA

UT WOS:000087900500022

PM 10880672

DA 2019-01-08

ER

PT J

AU Nichols, GA

Hillier, TA

Javor, K

Brown, JB

AF Nichols, GA

Hillier, TA

Javor, K

Brown, JB

TI Predictors of glycemic control in insulin-using adults with type 2

diabetes

SO DIABETES CARE

LA English

DT Article

ID FORM HEALTH SURVEY; METABOLIC CONTROL; WEIGHT CHANGE; MELLITUS; DISEASE;

RETINOPATHY; PROGRESSION; CHILDREN; GLUCOSE; CARE

AB OBJECTIVE - To determine the characteristics that influence glycemic control among insulin-using adults with type 2 diabetes.

RESEARCH DESIGN AND METHODS - We studied all 1,333 eligible members of a large not-for-profit health maintenance organization who responded to a 1997 survey We tested associations among demographic, treatment, and psychometric variables with mean 1997 HbA(1c) values. The Problem Areas in Diabetes (PAID) instrument was used to assess the emotional effect of living with diabetes, and the Short Form 12 Physical Function Scale was used to assess the effect of physical limitations on daily activities. Based on differences between and within treatment groups, we built models to predict glycemic control for subgroups of subjects who were using insulin alone and those who were using insulin in combination with an oral hypoglycemic agent.

RESULTS - Younger age, lower BMI, and increased emotional distress about diabetes (according to the PAID scale) were all significant predictors (P < 0.05) of worse glycemic control. However, except among individuals with an HbA(1c) level of >8.0 who were receiving combination therapy, only similar to 100% of the variance in glycemic control could be predicted by demographic, treatment, or psychometric characteristics.

CONCLUSIONS - Personal characteristics explain little of the variation in glycemic control in insulin-using adults with type 2 diabetes. Possible explanations are that the reduced complexity of control in type 2 diabetes makes the disease less sensitive to personal factors than control in type 1 diabetes, that health-related behavior is less driven by personal and environmental characteristics among older individuals, or that, in populations exposed to aggressive glycemic control with oral hypoglycemic agents and nurse care managers, personal differences become largely irrelevant.

C1 Kaiser Permanente, Ctr Hlth Res, NW Div, Portland, OR 97227 USA.

Eli Lilly & Co, Indianapolis, IN 46285 USA.

RP Nichols, GA (reprint author), Kaiser Permanente, Ctr Hlth Res, NW Div, 3800 N Kaiser Ctr Dr, Portland, OR 97227 USA.

CR *AM DIAB ASS, 1999, DIABETES CARE S1, V22, pS27

*AM DIAB ASS, 1997, DIABETES CARE, V20, P23

ANDERSON BJ, 1981, DIABETES CARE, V4, P586, DOI 10.2337/diacare.4.6.586

Brown JB, 1999, DIABETES CARE, V22, P1116, DOI 10.2337/diacare.22.7.1116

Cowie CC, 1995, DIABETES AM, V2, P85

Davidson MB, 1998, DIABETES CARE, V21, P2152, DOI 10.2337/diacare.21.12.2152

DAVIS WK, 1987, HEALTH PSYCHOL, V6, P1

Edelman SV, 1998, MED CLIN N AM, V82, P665, DOI 10.1016/S0025-7125(05)70019-5

Gandek B, 1998, J CLIN EPIDEMIOL, V51, P1171, DOI 10.1016/S0895-4356(98)00109-7

GREENLICK M, 1998, HLTH CARE RES HMO 2

Harris MI, 1999, DIABETES CARE, V22, P403, DOI 10.2337/diacare.22.3.403

HENSRUD DD, 1995, OBES RES, V3, pS217, DOI 10.1002/j.1550-8528.1995.tb00467.x

HEYDEN S, 1985, J CHRON DIS, V38, P281, DOI 10.1016/0021-9681(85)90073-6

Jenkinson C, 1997, J PUBLIC HEALTH MED, V19, P179, DOI 10.1093/oxfordjournals.pubmed.a024606

Kannel William B., 1996, American Journal of Clinical Nutrition, V63, p419S, DOI 10.1093/ajcn/87.6.1602

KENNY SJ, 1995, DIABETES AM, P47

Klein BEK, 1998, DIABETES CARE, V21, P236, DOI 10.2337/diacare.21.2.236

KLEIN R, 1994, ARCH OPHTHALMOL-CHIC, V112, P1217, DOI 10.1001/archopht.1994.01090210105023

KOVACS M, 1989, DIABETES CARE, V12, P409, DOI 10.2337/diacare.12.6.409

Mayfield Jennifer, 1998, Clinical Diabetes, V16, P161

Meigs JB, 1997, AM J MED, V102, P38, DOI 10.1016/S0002-9343(96)00383-X

*NAT COMM QUAL ASS, 1999, HEDIS 2000, V2, P91

NOPPA H, 1980, AM J EPIDEMIOL, V111, P693, DOI 10.1093/oxfordjournals.aje.a112947

OHKUBO Y, 1995, DIABETES RES CLIN PR, V28, P103, DOI 10.1016/0168-8227(95)01064-K

POLONSKY WH, 1995, DIABETES CARE, V18, P754, DOI 10.2337/diacare.18.6.754

Rosilio M, 1998, DIABETES CARE, V21, P1146, DOI 10.2337/diacare.21.7.1146

Scanlon D P, 1998, Jt Comm J Qual Improv, V24, P5

SHAMOON H, 1993, NEW ENGL J MED, V329, P977

Testa MA, 1998, JAMA-J AM MED ASSOC, V280, P1490, DOI 10.1001/jama.280.17.1490

Trief PM, 1998, DIABETES CARE, V21, P241, DOI 10.2337/diacare.21.2.241

Turner RC, 1998, LANCET, V352, P837

Ware JE, 1996, MED CARE, V34, P220, DOI 10.1097/00005650-199603000-00003

Welch GW, 1997, DIABETES CARE, V20, P760, DOI 10.2337/diacare.20.5.760

WILSON W, 1986, DIABETES CARE, V9, P614, DOI 10.2337/diacare.9.6.614

NR 34

TC 79

Z9 81

U1 0

U2 6

PU AMER DIABETES ASSOC

PI ALEXANDRIA

PA 1660 DUKE ST, ALEXANDRIA, VA 22314 USA

SN 0149-5992

J9 DIABETES CARE

JI Diabetes Care

PD MAR

PY 2000

VL 23

IS 3

BP 273

EP 277

DI 10.2337/diacare.23.3.273

PG 5

WC Endocrinology & Metabolism

SC Endocrinology & Metabolism

GA 289GA

UT WOS:000085611100003

PM 10868850

OA Bronze

DA 2019-01-08

ER

PT J

AU Aalto, AM

Uutela, A

Aro, AR

AF Aalto, AM

Uutela, A

Aro, AR

TI Disease-related distress among insulin-treated diabetic patients -

Associations with health status, psychosocial factors and self-care

practices

SO EUROPEAN JOURNAL OF PUBLIC HEALTH

LA English

DT Article

DE diabetes cognitions; diabetes distress; health status; insulin-treated

diabetes; social support

ID QUALITY-OF-LIFE; LONG-TERM RECOVERY; SOCIAL SUPPORT; METABOLIC CONTROL;

GLYCOSYLATED HEMOGLOBIN; EMOTIONAL ADJUSTMENT; MELLITUS; COMPLICATIONS;

EFFICACY; SCALES

AB Background: Diabetes is a chronic illness that affects many domains of life. This cross sectional study examined disease-related distress and its associations with health and psychosocial factors among insulin treated diabetic persons. Method: The sample (n=423) was drawn from the Social Insurance Institution's drug registry. Health indicators were duration of diabetes, complications, and glycaemic control. Psychosocial measures included general and diabetes-specific social support, diabetes locus of control (DLC), self-efficacy, diabetes health beliefs and self-care practices and subjective health. Results: Most respondents reported moderate diabetes-related distress. In multivariate analysis, severe diabetes distress was related to lower net benefits of regimen (beta = -0.29, and p<0.001), poorer perceived health (beta = -0.25, and p<0.001), higher perceived threat of complications (beta = 0.19, and p<0.001), less adequate social support (beta = -0.16, and p<0.01), stronger beliefs in chance (beta = 0.15, and p<0.01) and internal DLC (beta = 0.11, and p<0.05) and more frequent exercising (beta = 0.10, and p<0.05). Conclusions: The results suggest that emotional adjustment to diabetes is predominantly determined by diabetes related cognitions and subjective health and to a lesser degree by objective diabetes indicators, which underlines the importance of assisting the patient with adjustment with the disease in health care centres.

C1 Natl Res & Dev Ctr Welf & Hlth, FIN-00531 Helsinki, Finland.

Natl Publ Hlth Inst, Helsinki, Finland.

RP Aalto, AM (reprint author), Natl Res & Dev Ctr Welf & Hlth, POB 220, FIN-00531 Helsinki, Finland.

CR Aalto AM, 1997, PATIENT EDUC COUNS, V30, P215, DOI 10.1016/S0738-3991(96)00963-9

AALTO AM, 1993, SOSIAALILAAKETIETEEL, V30, P328

ARY DV, 1986, DIABETES CARE, V9, P168, DOI 10.2337/diacare.9.2.168

BANDURA A, 1977, PSYCHOL REV, V84, P191, DOI 10.1037/0033-295X.84.2.191

BECKER MH, 1975, MED CARE, V13, P10, DOI 10.1097/00005650-197501000-00002

CHASE HP, 1989, JAMA-J AM MED ASSOC, V261, P1155, DOI 10.1001/jama.261.8.1155

CONNELL CM, 1994, HEALTH PSYCHOL, V13, P263, DOI 10.1037//0278-6133.13.3.263

CRONENWETT LR, 1985, NURS RES, V34, P93

CUNNINGHAM AJ, 1991, PATIENT EDUC COUNS, V17, P71, DOI 10.1016/0738-3991(91)90052-7

DUNN SM, 1986, DIABETES CARE, V9, P480, DOI 10.2337/diacare.9.5.480

FERRARO LA, 1987, PSYCHOL REP, V61, P763, DOI 10.2466/pr0.1987.61.3.763

GAFVELS C, 1993, DIABETIC MED, V10, P768, DOI 10.1111/j.1464-5491.1993.tb00162.x

GIVEN CW, 1983, RES NURS HEALTH, V6, P127, DOI 10.1002/nur.4770060306

GROSSMAN HY, 1987, DIABETES CARE, V10, P324, DOI 10.2337/diacare.10.3.324

HANESTAD BR, 1991, SCAND J SOC MED, V19, P57, DOI 10.1177/140349489101900111

Harris R, 1987, Diabetes Educ, V13, P292, DOI 10.1177/014572178701300310

House J. S., 1981, WORK STRESS SOCIAL S

KANGAS T, 1995, 58 GUMM KIRJ OY

KING KB, 1993, HEALTH PSYCHOL, V12, P56, DOI 10.1037/0278-6133.12.1.56

KLEIN R, 1988, JAMA-J AM MED ASSOC, V260, P2864, DOI 10.1001/jama.260.19.2864

KUGLER J, 1994, J PSYCHOSOM RES, V38, P403, DOI 10.1016/0022-3999(94)90101-5

Lasar M, 1993, Schweiz Arch Neurol Psychiatr (1985), V144, P147

LENNON MC, 1990, SOC SCI MED, V31, P477, DOI 10.1016/0277-9536(90)90043-R

LEWIS KS, 1990, DIABETIC MED, V7, P148, DOI 10.1111/j.1464-5491.1990.tb01350.x

LITTLEFIELD CH, 1990, HEALTH PSYCHOL, V9, P737, DOI 10.1037//0278-6133.9.6.737

LLOYD CE, 1992, DIABETES CARE, V15, P166, DOI 10.2337/diacare.15.2.166

LUNDMAN B, 1990, RES NURS HEALTH, V13, P255, DOI 10.1002/nur.4770130408

MANNE SL, 1989, J PERS SOC PSYCHOL, V56, P608, DOI 10.1037//0022-3514.56.4.608

MASON C, 1985, SOC SCI MED, V21, P1329, DOI 10.1016/0277-9536(85)90436-8

NAESS S, 1995, SCAND J SOC MED, V23, P179

PADGETT DK, 1993, J NERV MENT DIS, V181, P123, DOI 10.1097/00005053-199302000-00008

PASTOR MA, 1993, BRIT J RHEUMATOL, V32, P484

PEACOCK I, 1984, J CLIN PATHOL, V37, P841, DOI 10.1136/jcp.37.8.841

PRAMMING S, 1991, DIABETIC MED, V8, P217, DOI 10.1111/j.1464-5491.1991.tb01575.x

PRIMOMO J, 1990, RES NURS HEALTH, V13, P153, DOI 10.1002/nur.4770130304

REID DW, 1984, RES LOCUS CONTROL CO, V3, P361

RUBIN RR, 1991, DIABETES CARE, V14, P335, DOI 10.2337/diacare.14.4.335

SCHAFER LC, 1986, DIABETES CARE, V9, P179, DOI 10.2337/diacare.9.2.179

STEWART AL, 1988, MED CARE, V26, P724, DOI 10.1097/00005650-198807000-00007

WALLSTON BS, 1978, HEALTH EDUC QUART, V6, P107, DOI 10.1177/109019817800600107

WEINBERGER M, 1994, MED CARE, V32, P1173, DOI 10.1097/00005650-199412000-00002

WIKBLAD KF, 1990, J ADV NURS, V15, P1083, DOI 10.1111/j.1365-2648.1990.tb01990.x

WIKBY A, 1993, QUAL LIFE RES, V2, P281, DOI 10.1007/BF00434799

YATES BC, 1995, RES NURS HEALTH, V18, P193, DOI 10.1002/nur.4770180303

NR 44

TC 5

Z9 5

U1 0

U2 5

PU OXFORD UNIV PRESS

PI OXFORD

PA GREAT CLARENDON ST, OXFORD OX2 6DP, ENGLAND

SN 1101-1262

J9 EUR J PUBLIC HEALTH

JI Eur. J. Public Health

PD MAR

PY 2000

VL 10

IS 1

BP 68

EP 74

DI 10.1093/eurpub/10.1.68

PG 7

WC Public, Environmental & Occupational Health

SC Public, Environmental & Occupational Health

GA 302ED

UT WOS:000086351700015

OA Bronze

DA 2019-01-08

ER

PT J

AU Phillips, KA

AF Phillips, KA

TI Quality of life for patients with body dysmorphic disorder

SO JOURNAL OF NERVOUS AND MENTAL DISEASE

LA English

DT Article; Proceedings Paper

CT 152nd Annual Meeting of the American-Psychiatric-Association

CY MAY 15-20, 1999

CL WASHINGTON, D.C.

SP Amer Psychiat Assoc

ID OBSESSIVE-COMPULSIVE SCALE; STRUCTURED CLINICAL INTERVIEW; FORM HEALTH

SURVEY; OF-LIFE; IMAGINED UGLINESS; RELIABILITY; VALIDITY; PREVALENCE;

DEPRESSION; FEATURES

AB Body dysmorphic disorder (BDD) is a relatively common, distressing and impairing disorder. Quality of life in BDD, however, has not been investigated. in this study, 62 consecutive outpatients with BDD were evaluated with the self-report Medical Out-comes Study 36-Item Short-Form Health Survey (SF-36) and other scales. SF-36 scores were descriptively compared to published norms fbr several populations. Physical health-related quality of life scares were generally worse than general U.S. population norms and better than norms for outpatients with a medical illness or depression. However, in all mental health domains, BDD subjects' scores were notably worse than norms for the general U.S. population and for patients with depression, diabetes, or a recent myocardial infarction. More severe BDD symptoms and greater delusionality were associated with poorer mental health-related quality of life. These results indicate that patients with BDD have notably poor mental health status and mental health-related quality of life.

C1 Butler Univ, Providence, RI 02906 USA.

Brown Univ, Sch Med, Dept Psychiat & Human Behav, Providence, RI 02906 USA.

RP Phillips, KA (reprint author), Butler Univ, 345 Blackstone Blvd, Providence, RI 02906 USA.

FU NIMH NIH HHS [R-29MH54841]

CR American Psychiatric Association, 1994, DIAGN STAT MAN MENT

Awad AG, 1997, J PSYCHIATR NEUROSCI, V22, P229

Cotterill JA, 1997, BRIT J DERMATOL, V137, P246, DOI 10.1046/j.1365-2133.1997.18131897.x

COTTERILL JA, 1981, BRIT J DERMATOL, V104, P611, DOI 10.1111/j.1365-2133.1981.tb00746.x

Cotterill JA, 1996, DERMATOL CLIN, V14, P457, DOI 10.1016/S0733-8635(05)70373-9

DeMarco LM, 1998, J NERV MENT DIS, V186, P724, DOI 10.1097/00005053-199811000-00011

DENCKER SJ, 1992, NORD J PSYCHIAT, V46, P91

Eisen JL, 1998, AM J PSYCHIAT, V155, P102, DOI 10.1176/ajp.155.1.102

GOODMAN WK, 1989, ARCH GEN PSYCHIAT, V46, P1006

GOODMAN WK, 1989, ARCH GEN PSYCHIAT, V46, P1012

HAMILTON M, 1960, J NEUROL NEUROSUR PS, V23, P56, DOI 10.1136/jnnp.23.1.56

HAY GG, 1970, BRIT J PSYCHIAT, V116, P399, DOI 10.1192/bjp.116.533.399

KOBLENZER CS, 1985, ARCH DERMATOL, V121, P780

Koran LM, 1996, AM J PSYCHIAT, V153, P783

MASSION AO, 1993, AM J PSYCHIAT, V150, P600

MCHORNEY CA, 1993, MED CARE, V31, P247, DOI 10.1097/00005650-199303000-00006

Perugi G, 1998, COMPR PSYCHIAT, V39, P63, DOI 10.1016/S0010-440X(98)90080-3

Perugi G, 1997, Int J Psychiatry Clin Pract, V1, P77, DOI 10.3109/13651509709024707

PHILLIPS KA, 1994, PSYCHOPHARMACOL BULL, V30, P179

Phillips KA, 1996, J NERV MENT DIS, V184, P125, DOI 10.1097/00005053-199602000-00012

PHILLIPS KA, 1993, AM J PSYCHIAT, V150, P302

PHILLIPS KA, 1991, AM J PSYCHIAT, V148, P1138

Phillips KA, 1997, PSYCHOPHARMACOL BULL, V33, P17

Phillips KA, 1995, 148 ANN M AM PSYCH A, P157

PHILLIPS KA, 1996, BROKEN MIRROR UNDERS

PHILLIPS KA, 1996, AM J PSYCHIAT

Revicki DA, 1997, HARVARD REV PSYCHIAT, V5, P75, DOI 10.3109/10673229709034730

Russo J, 1998, MED CARE, V36, P752, DOI 10.1097/00005650-199805000-00015

SIMEON D, 1995, AM J PSYCHIAT, V152, P1207

SPITZER RL, 1992, ARCH GEN PSYCHIAT, V49, P624

Veale D, 1996, BRIT J PSYCHIAT, V169, P196, DOI 10.1192/bjp.169.2.196

Ware JE, 1993, SF 36 HLTH SURVEY MA

WELLS KB, 1989, JAMA-J AM MED ASSOC, V262, P914, DOI 10.1001/jama.262.7.914

WILLIAMS JBW, 1992, ARCH GEN PSYCHIAT, V49, P630

NR 34

TC 90

Z9 90

U1 1

U2 4

PU LIPPINCOTT WILLIAMS & WILKINS

PI PHILADELPHIA

PA 530 WALNUT ST, PHILADELPHIA, PA 19106-3621 USA

SN 0022-3018

J9 J NERV MENT DIS

JI J. Nerv. Ment. Dis.

PD MAR

PY 2000

VL 188

IS 3

BP 170

EP 175

DI 10.1097/00005053-200003000-00007

PG 6

WC Clinical Neurology; Psychiatry

SC Neurosciences & Neurology; Psychiatry

GA 294QV

UT WOS:000085922600007

PM 10749282

DA 2019-01-08

ER

EF
